# Mechanism of *ERBB2* gene overexpression by the formation of super-enhancer with genomic structural abnormalities in lung adenocarcinoma without clinically actionable genetic alterations

**DOI:** 10.1186/s12943-024-02035-6

**Published:** 2024-06-11

**Authors:** Syuzo Kaneko, Ken Takasawa, Ken Asada, Kouya Shiraishi, Noriko Ikawa, Hidenori Machino, Norio Shinkai, Maiko Matsuda, Mari Masuda, Shungo Adachi, Satoshi Takahashi, Kazuma Kobayashi, Nobuji Kouno, Amina Bolatkan, Masaaki Komatsu, Masayoshi Yamada, Mototaka Miyake, Hirokazu Watanabe, Akiko Tateishi, Takaaki Mizuno, Yu Okubo, Masami Mukai, Tatsuya Yoshida, Yukihiro Yoshida, Hidehito Horinouchi, Shun-Ichi Watanabe, Yuichiro Ohe, Yasushi Yatabe, Vassiliki Saloura, Takashi Kohno, Ryuji Hamamoto

**Affiliations:** 1 Division of Medical AI Research and Development, National Cancer Center Research Institute, 5-1-1 Tsukiji, Chuo-Ku, Tokyo, 104-0045 Japan; 2 Cancer Translational Research Team, RIKEN Center for Advanced Intelligence Project, Tokyo, 103-0027 Japan; 3 Division of Genome Biology, National Cancer Center Research Institute, Tokyo, 104-0045 Japan; 4 Department of Proteomics, National Cancer Center Research Institute, Tokyo, 104-0045 Japan; 5 Endoscopy Division, National Cancer Center Hospital, Tokyo, 104-0045 Japan; 6 Department of Diagnostic Radiology, National Cancer Center Hospital, Tokyo, 104-0045 Japan; 7 Department of Thoracic Oncology, National Cancer Center Hospital, Tokyo, 104-0045 Japan; 8 Department of Experimental Therapeutics, National Cancer Center Hospital, Tokyo, 104-0045 Japan; 9 Department of Thoracic Surgery, National Cancer Center Hospital, Tokyo, 104-0045 Japan; 10 Division of Medical Informatics, National Cancer Center Hospital, Tokyo, 104-0045 Japan; 11 Department of Diagnostic Pathology, National Cancer Center Hospital, Tokyo, 104-0045 Japan; 12 Center for Cancer Research, National Cancer Institute, Bethesda, MD 20892 USA

**Keywords:** Lung adenocarcinoma, Super-enhancers, Structural variations, Targeted therapy, Driver mutations, Integrated analysis, Precision medicine

## Abstract

**Background:**

In an extensive genomic analysis of lung adenocarcinomas (LUADs), driver mutations have been recognized as potential targets for molecular therapy. However, there remain cases where target genes are not identified. Super-enhancers and structural variants are frequently identified in several hundred loci per case. Despite this, most cancer research has approached the analysis of these data sets separately, without merging and comparing the data, and there are no examples of integrated analysis in LUAD.

**Methods:**

We performed an integrated analysis of super-enhancers and structural variants in a cohort of 174 LUAD cases that lacked clinically actionable genetic alterations. To achieve this, we conducted both WGS and H3K27Ac ChIP-seq analyses using samples with driver gene mutations and those without, allowing for a comprehensive investigation of the potential roles of super-enhancer in LUAD cases.

**Results:**

We demonstrate that most genes situated in these overlapped regions were associated with known and previously unknown driver genes and aberrant expression resulting from the formation of super-enhancers accompanied by genomic structural abnormalities. Hi-C and long-read sequencing data further corroborated this insight. When we employed CRISPR-Cas9 to induce structural abnormalities that mimicked cases with outlier *ERBB2* gene expression, we observed an elevation in *ERBB2* expression. These abnormalities are associated with a higher risk of recurrence after surgery, irrespective of the presence or absence of driver mutations.

**Conclusions:**

Our findings suggest that aberrant gene expression linked to structural polymorphisms can significantly impact personalized cancer treatment by facilitating the identification of driver mutations and prognostic factors, contributing to a more comprehensive understanding of LUAD pathogenesis.

**Supplementary Information:**

The online version contains supplementary material available at 10.1186/s12943-024-02035-6.

## Introduction

Lung adenocarcinoma (LUAD) is a major subtype of non-small cell lung cancer (NSCLC), with *ALK, EGFR,* and *KRAS* gene mutations being the most common driver gene mutations [1]. These mutations are critical for selecting targeted therapies and determining treatment strategies, with specific molecularly targeted therapies available for patients carrying these gene mutations [1]. Driver gene mutations are detected in approximately 50 ~ 70% of patients, though the exact percentage may vary depending on the study or patient population [2–4]. Despite the prevalence of identifiable driver mutations in a significant portion of the patient population, a considerable number of lung adenocarcinoma (LUAD) patients lack these specific somatic mutations, presenting challenges in both diagnosis and treatment planning.

Advancements in whole-genome sequencing (WGS) technology have made it possible to investigate novel lung cancer-related mutations and complex structural variants. structural variants have emerged as key events in causing copy number alterations (CNAs), generating gene fusions, and dysregulating gene expression through super-enhancer hijacking and the disruption of 3D genomic structure [5]. However, determining structural variant events related to super-enhancer formation using WGS alone remains challenging [6–8]. Furthermore, it is unclear whether these events can serve as druggable targets as driver mutations [9].

The super-enhancers span extensive genomic regions, with median sizes remarkably larger than those of typical enhancers. From a molecular biology perspective, it has been discovered that the super-enhancer region encompasses numerous factors related to enhancer activity, including RNA polymerase II (RNA Pol II), RNA from transcribed enhancer loci (eRNA), histone acetyltransferases p300 and CBP, chromatin factors such as cohesin, and histone modifications (histone H3 lysine 27 acetylation (H3K27Ac), H3 lysine 4 di-methylation (H3K4me2), H3 lysine 4 mono-methylation (H3K4me1). Additionally, increased chromatin accessibility has been identified within these regions. Abnormalities in the function of super-enhancers have been reported to be associated with cancer, type 1 diabetes, and Alzheimer’s disease [10, 11]. Particularly in cancer, super-enhancers may play a crucial role in the dysregulation of gene expression. For instance, during tumorigenesis, malignant cells acquire super-enhancers in key oncogenes, and higher levels of transcription of these genes have been reported compared to normal cells [12, 13]. However, it remains unclear whether these phenomena are genuinely attributable to epigenetic abnormalities or result from genomic alterations [10, 14]. Recent studies have shed light on the role of extrachromosomal DNA (ecDNA) in connection with structural variants. Not merely isolated circular DNAs, these ecDNAs form substantial clusters that potentially catalyze the emergence of super-enhancers [15].

ERBB2, also known as HER2 (human epidermal growth factor receptor 2), is a receptor tyrosine kinase that belongs to the epidermal growth factor receptor (EGFR) family [16, 17]. It plays a crucial role in cell growth, differentiation, and survival. Overexpression or amplification of *ERBB2* has been reported in various cancers, including breast cancer and NSCLC, and is associated with aggressive disease and poor prognosis [18]. As a druggable target, ERBB2 has been the focus of several targeted therapies. In breast cancer, the monoclonal antibody trastuzumab has been successfully used to treat patients with HER2-positive tumors [19]. Other HER2-targeted therapies include pertuzumab (another monoclonal antibody), ado-trastuzumab emtansine (an antibody–drug conjugate), and small molecule tyrosine kinase inhibitors such as lapatinib and neratinib [20–23]. In the context of NSCLC, ERBB2-targeted therapies have shown promise in clinical trials, particularly for patients with *ERBB2* mutations or amplifications [24].

In this study, we aimed to identify genomic alterations accompanied by the formation of super-enhancers. To achieve this, we conducted both WGS and H3K27Ac chromatin immunoprecipitation sequencing (ChIP-seq) analyses using cases with driver gene mutations and those without, allowing for a comprehensive investigation of the potential roles of super-enhancers in the context of these genetic alterations. Specifically, the super-enhancer formation surrounding the *ERBB2* gene locus is associated with exceptionally high gene expression and involves structural variant events, as revealed by Hi-C and long-read sequencing. We provide evidence that an increase in *ERBB2* gene expression occurred when one of the structural variant events, specifically an inversion, brought the *ERBB2* genomic region near the *HNF1β* gene locus. Finally, 23 genes displaying significantly aberrant expression patterns were identified as potential indicators of driver mutations in LUAD. These genes were associated with decreased recurrent-free survival in patients, suggesting their clinical relevance as prognostic factors for postoperative outcomes.

## Materials and methods

### Ethical considerations and clinical materials

All methods used in this study adhered to the ethical guidelines for medical and health research involving human subjects. Informed consent was obtained from all participating patients. The institutional review board of the National Cancer Center (NCC) approved the study (2005–109, 2016–496, 2019–018), which was conducted in accordance with the Declaration of Helsinki.

Patient samples and clinical records were collected based on the Public/Private R&D Investment Strategic Expansion PrograM (PRISM), an in-house lung cancer database of the NCC Japan, containing clinical information (*n* = 1,714), whole-exome sequencing (WES, *n* = 1,599) and RNA sequencing (RNA-seq, *n* = 1,682). In addition, DNA methylation data (*n* = 402) and H3K27Ac ChIP-seq data (*n* = 222) were collected as of April 15, 2023.

Tumor samples were collected from individuals who underwent either surgery or medical treatment at the NCC hospital in Tokyo, Japan, between 1997 and 2019. Data for the analysis was retrospectively gathered from electronic medical records. Tumor diagnoses were made through cytological and/or histological evaluations, following the World Health Organization classification guidelines. Freshly frozen tissue samples from surgical specimens were obtained from the NCC Biobank.

### WGS

We used the AllPrep DNA/RNA mini kit to extract genomic DNA from fresh frozen samples. Sequencing was performed on the Illumina HiSeq 2500 or Illumina NovaSeq 6000 platforms. To identify somatic mutations of tumor samples, we have analyzed the tumor tissues at a coverage of 100X, and the peripheral blood lymphocytes from the same cases at a coverage of 30X in WGS. The raw sequencing data was then processed using the NVIDIA Clara Parabricks, a GPU-based framework for genomic sequence analysis. For structural variant calling, we utilized Manta, a specialized tool. To consolidate and screen the detected variants, we applied SURVIVOR, a tool that aids in eliminating potential false positives and enhancing the precision and trustworthiness of the resultant structural variant dataset. More comprehensive methods are available in supplementary methods.

### Identification of LUAD without clinically actionable genetic alterations (CAGAs)

To investigate the underlying mechanism of non-CAGAs LUAD-specific cancer pathogenesis, we filtered out the cases with mutations in specific genes by identifying the driver mutations. These genes were annotated as pathogenic or likely pathogenic in the ClinVar database, or as oncogenic or likely oncogenic in the OncoKB database [25, 26]. Specifically, the genes analyzed included *EGFR, KRAS, BRAF, ERBB2, MET* skipping, as well as fusion genes of *ALK, ROS1, NRG1, RET, NTRK,* and *FGFR,* which were considered as CAGAs. We identified these gene mutations using both WES and RNA-seq datasets.

### ChIP-seq

The ChIP-seq procedure used in the study has been previously described using semi-automated dual-arm robot [27]. The full method for ChIP-seq analysis is available in supplementary methods.

### Overlap analysis of super-enhancers and structural variants

To investigate potential functional relationships or co-regulation between genomic regions, we examined the genomic coordinates of the peaks to ascertain whether their ranges intersect. This overlap can manifest as partial, wherein only a segment of one peak intersects with the other, or complete, where one peak is entirely subsumed by the other. Specifically, for the overlap analysis of super-enhancer and structural variant regions, we employed the findOverlappingPeaks function from the “ChIPpeakAnno” R package. Recognizing that structural variant events are characterized by extensive disruptions involving the 3D genomic structure, we deem an overlap between a 20 kb region surrounding the genomic breakpoint and super-enhancer region to be significant.

### Super-enhancer (SE)-to-gene links analysis

In light of a noticeable bias inherent in differing RNA-seq methodologies, we used samples processed through polyA RNA-seq for the following SE-to-gene links analysis (*n* = 142). Given that super-enhancer regions are often annotated over large areas encompassing multiple gene clusters, we first examined the correlation between H3K27Ac peaks and gene expression, referred to as peak-to-gene links. We then extracted the genes that were annotated as super-enhancer regions by the method of rank ordering of super-enhancers (ROSE) and as structural variants by Manta. The comprehensive methodology for the SE-to-gene links analysis can be found in supplementary methods.

### Hi-C

The high-throughput chromosome conformation capture (Hi-C) procedure has been previously described in the study by Rao et al. [28]. The full method for the Hi-C analysis is described in supplementary methods.

### Long-read sequencing

The complete methodology for acquiring long-read sequencing data using the PacBio Sequel II system is detailed in supplementary methods. For de novo assembly to obtain contiguous assemblies, we employed hifiasm (v0.16.1-r375) in combination with the Hi-C dataset and option -t 86. Note that this is particularly beneficial when assembling complex genomes or resolving repetitive regions, which are often difficult to decipher with short-read sequencing data. As the obtained genomic data is too large in size, visualizing the entire genome region is challenging. Therefore, we used the Bandage’s reduce command and options --scope aroundblast --evfilter 1e-100 --distance 2 to extract the *ERBB2* cDNA sequence as a query in the assembly graph, along with adjacent nodes. To query sequences and visualize *ERBB2* and *HNF1β* genes, we locally performed a BLAST search (v2.9.0) with filter parameters e-value 1e-100 and bit score 10,000 to identify genomic regions encompassing GRCh38: chr17:37,686,431–37,745,059 and GRCh38: chr17: 39,687,914 – 39,730,426 within Bandage. The continuity of genomes assembled with PacBio long reads is crucial due to its capacity for improved structural variant detection and its ability to resolve complex regions. To determine the continuity of the genome sequence according to Bandage’s rule, the following conditions were followed: one of the edges connected to node A uniquely leads to node B in all possible paths, or one of the edges connected to node B uniquely leads to node A in all possible paths.

### Targeted chromosomal rearrangements

The generation of inducible Cas9 expression in cell lines is detailed in supplementary methods. To design highly specific single guide RNAs (sgRNAs) targeting the genomic regions near the cleavage sites that cause structural variants identified from the WGS of LUAD, we used crispRdesignR (v1.1.6) package and further verified selected sgRNAs using CRISPR-Cas9 guide RNA design checker (Integrated DNA Technologies). The sgRNAs were then synthesized with the molecules comprising both crRNA and tracrRNA sequences with chemical modifications for a high level of functional stability (Integrated DNA Technologies). The targeted sequences for sgRNA were as follows:

- gRNA #1: 5'-GTT ATG AAC ATT GGC AAT GT-3',
- gRNA #2: 5'-GTC ACC TAG ATG CCC ATC CA-3',
- gRNA #3: 5'-GAG ACT GGC GTG CAG CGC GA-3',
- gRNA #4: 5'-GCC TAG GAG ATC AAA ATC TG-3'.

We then transfected Cas9-inducible HBEC3-KT and HSAEC1-KT cells with single guide RNAs (sgRNAs) using Lipofectamine RNAiMax transfection reagent (ThermoFisher Scientific, 13778–150) according to the manufacturer’s instructions to achieve targeted chromosomal rearrangements. To screen for the presence of mutations or small insertions/deletions (indels) in the specific DNA region of interest, we performed T7 endonuclease I (T7EI) mismatch detection assays using the Alt-R Genome Editing Detection Kit (Integrated DNA Technologies, 1075932) according to the manufacturer’s instructions. Genomic inversion of *HNF1β-ERBB2* region was confirmed by PCR and sequencing.

### FACS

Forty-eight hours post-transfection, cells were subjected to analysis. The cells were resuspended in 50 µL of Stain buffer (BD, 554656) and treated with 5 µL (2.5 µg) of Human BD Fc Block (BD, 564219) per 10^6^ cells, followed by a 10-min incubation. Then, we added either Anti-Her2/neu (BD, 340552) or Mouse IgG1 (20 µL, 0.1 µg/20 µL) and incubated at 4 °C for at least 30 min. We obtained the data from 50,000 individual cells. The detailed FACS analysis method is available in supplementary methods.

### Recurrence-free survival (RFS) analysis

We utilized the most comprehensive RNA-seq dataset available for LUAD (*n* = 1,115). To identify LUAD cases exhibiting outlier gene expression, we calculated the quartiles for each gene expression dataset and ascertained the interquartile range (IQR). We then computed the upper bound for the outliers in the data, which was specifically defined as the third quartile plus 1.5 times the interquartile range. This approach is considered robust for detecting outliers and is applicable across polyA RNA-seq, Ribo-Zero RNA-seq, and SMART-seq methods, irrespective of the differences in these techniques. Outlier genes used for RFS analysis were described in Table 1. RFS curves for cases with and without outlier gene expression were estimated using the Kaplan–Meier method. Differences in RFS, including postoperative recurrence, were assessed using the log-rank test. GraphPad Prism (GraphPad Software, v9) was employed for statistical analyses.

**Table 1:**
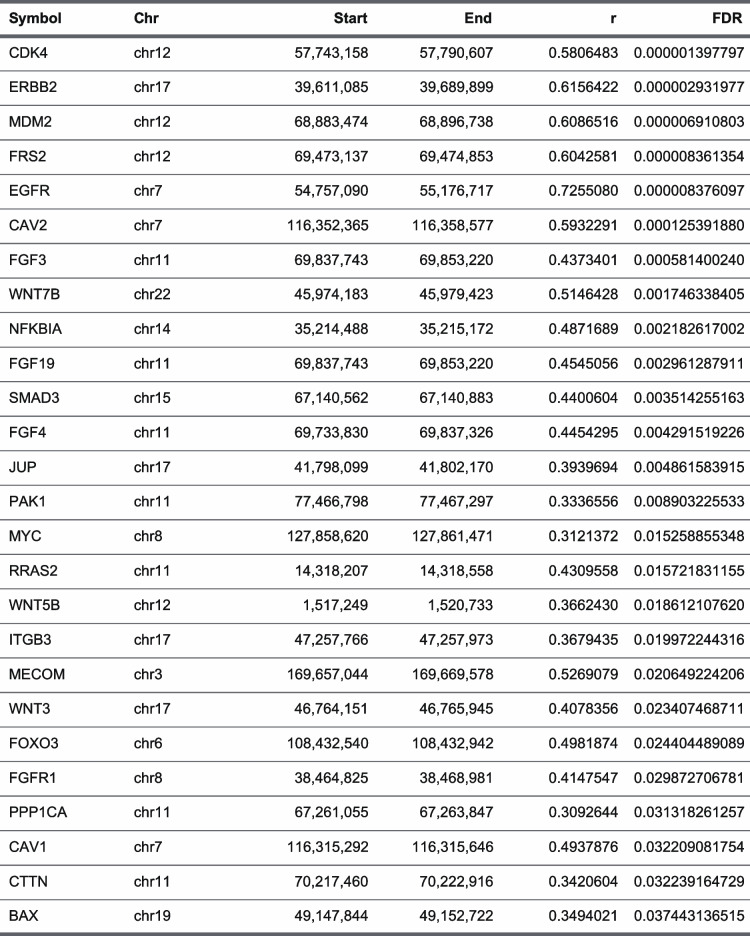
A ranked list of genes according to the SE-to-gene links analysis. The peak-to-gene links analysis was conducted on the non-CAGAs LUAD cohort. The peaks annotated as SE regions with FDR less than 0.05 were extracted. The gene symbol (Symbol), chromosome number (Chr), Start and End positions, r as correlation coefficient, and FDR are displayed and ranked based on FDR scores

### Bioinformatic analysis

The complete methods are available in supplementary methods.

### Statistical analysis

Comparisons between group means were performed using a two-tailed student’s t-test as indicated. *P*-value of less than 0.05 was considered statistically significant.

## Results

### Identification of driver mutations driven by super-enhancer formation with structural variants

Given the presence of somatic mutations relevant to cancer in a subset of patients with lung adenocarcinoma (LUAD), we often encounter significant challenges when attempting to apply targeted therapies. To broaden the scope of precision medicine to include patients with clinically actionable genetic alterations (CAGAs), we used a comprehensive strategy to classify patients with LUAD. Our initial classification scheme emphasized the identification of primary mutations in essential oncogenes, such as *EGFR*, *KRAS*, *BRAF*, *ERBB2*, and *MET* (exon skipping), as well as in oncogenic fusion genes, including *ALK*, *ROS1*, *NRG1*, *RET*, *NTRK*, and *FGFR*. Identifying these mutations is particularly important for cancer therapy because targeted treatments specifically designed for these mutations have demonstrated significant therapeutic benefits [1]. Therefore, we selected LUAD cases from 938 patients using WES and poly(A) RNA-seq dataset. From the subset that did not possess these CAGAs (*n* = 420, termed non-CAGAs), we selected 174 cases for WGS and H3K27Ac ChIP-seq analyses (Fig. S1A). Of note, driver mutations in genes including *EGFR*, *KRAS*, *BRAF*, and *ERBB2* were identified in 476 cases, representing 50.7% of the entire cohort (Fig. S1B). Importantly, a higher frequency of mutations was observed in the non-CAGA cohort (Fig. S2A, and the oncoprints shown in Fig. S2B and C), suggesting that these variants may not serve as specific markers for non-CAGA cases but rather indicate a general elevation in mutation frequency. Furthermore, the CNV and SV landscapes revealed hotspots associated with the *CDK4/MDM2* loci, where copy number amplification was observed. This may be partly explained by complex chromothripsis events characterized by extensive copy number amplification (Fig. S3).

To elucidate the distinct characteristics between normal and tumor tissues, we conducted H3K27Ac ChIP-seq analysis of seven non-CAGA LUADs. Adjacent matched tissues were used as normal controls for comparison. The PCA results indicated that, while the adjacent tissues manifested homogenously, the lung adenocarcinoma samples exhibited diverse features (Fig. S4). Subsequently, we performed WGS and H3K27Ac ChIP-seq in and 174 patients without CAGAs (non-CAGAs, see Fig. S2B and C and Table S1) and 45 patients with CAGAs to comprehensively investigate the potential roles of super-enhancers in our LUAD cohort (QC data are summarized in Fig. S5 and Dataset S1).

**Fig. 1:**
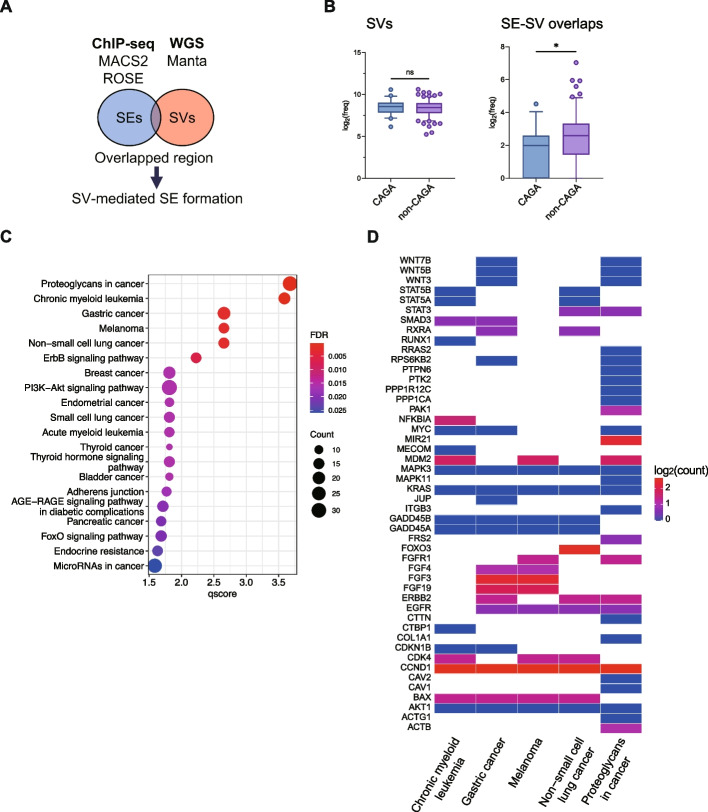
Intersection of SVs with SEs in non-CAGAs LUAD. **A** Bioinformatics methodology employed to detect SEs that are potentially regulated by SV events. Given the extensive perturbations to the 3D genomic architecture associated with SV events, a significant association is inferred when a 20-kb region surrounding the genomic breakpoint exhibits a substantial overlap with an SE region. A comprehensive description of the methods can be found in the method section. **B** Comparison of log_2_ frequency of SV events per sample (left side) and SE-SV overlaps per sample (right side) between CAGAs LUAD (*n* = 45) and non-CAGAs LUAD (*n* = 174). Statistical analysis was conducted using a two-sided t-test. * *P* < 0.05, ns: not significant. **C** KEGG pathway enrichment analysis on gene clusters annotated as SE regions with concurrent SVs in non-CAGAs LUAD samples. The statistical significance of the enriched pathways was determined using the enrichKEGG function from the clusterProfiler R package. The background gene set was defined as genes annotated with the SV regions alone. Comparable results were observed when using genes annotated with SE regions alone as the background (data not shown). False discovery rate (FDR) was calculated using the Bonferroni correction method, and the q-scores (qscore) were represented as -log_10_(FDR). The 20 enriched pathways are displayed (FDR < 0.05). **D** The gene clusters obtained from the top 5 enriched pathways in non-CAGAs LUAD. The counts of individual genes annotated in regions where SE and SV overlaps were provided

To explore the direct correlation between the formation of super-enhancers and genomic structural variants, and to better understand their molecular interplay in disease mechanisms, we employed Manta analysis to identify genomic breakpoints from WGS and ROSE analysis to identify super-enhancer regions from ChIP-seq and obtained the genomic loci where these two sets of data overlapped (Fig. 1A, super-enhancer and structural variant regions summarized in Dataset S2-3, 4–5, respectively). Although the total number of loci in the entire dataset was 67,349 and 69,991, we found that only a small fraction, 700 (~ 1%), showed overlapping regions (Fig. S6, genome coordinates listed in Dataset S6-7), suggesting that structural variants play a confined role in specific regions as direct triggers for the formation of super-enhancers in non-CAGAs LUAD. A noteworthy finding was that when focusing on regions where super-enhancers and structural variants overlapped, the frequency of overlaps per patient in non-CAGA was substantially higher than that in CAGAs LUAD (Fig. 1B). This suggest that in some instances, the concurrent presence of super-enhancers and structural polymorphisms may act as discriminating factors for non-CAGA LUAD. Furthermore, all pathways were significantly associated with cancer-related processes in the non-CAGA LUAD group (Fig. 1C). Conversely, cancer-related pathways were not consistently observed for gene groups located near the super-enhancers and structural variant regions alone (Fig. S7). Finally, we confirmed the formation of super-enhancers accompanied by structural variants in genes such as *BAX, CCND1, CDK4, EGFR, ERBB2, FOXO3, RXRA,* and *STAT3,* which are all frequently related to NSCLC (Fig. 1D, Fig. S8).

To explore the potential impact of structural variants on gene expression in our dataset, we conducted an integrated analysis of RNA sequencing data and structural variants. Most genes showed no significant changes in expression levels (black dotted line in Fig. S9A). However, a subset of genes (*n* = 632) exhibited elevated expression levels, which may be associated with the presence of structural variants (red dotted line). Conversely, 170 genes exhibited decreased expression levels (blue dotted line). Notably, no significant differences in outliner gene expression (red dots, *n* = 20) were observed between the non-CAGA and CAGA cohorts, suggesting that structural variants alone cannot be used to distinguish between non-CAGA and CAGA cases (Fig. S9B). Hence, identifying the genomic regions where both super-enhancers and structural variant coexist might offer insights into the cancer-related attributes of non-CAGAs LUAD. These findings suggest that the genes identified in these regions have potential therapeutic implications.

### Impact of gene expression on super-enhancer formation accompanied by structural variants in non-CAGAs LUAD

**Fig. 2:**
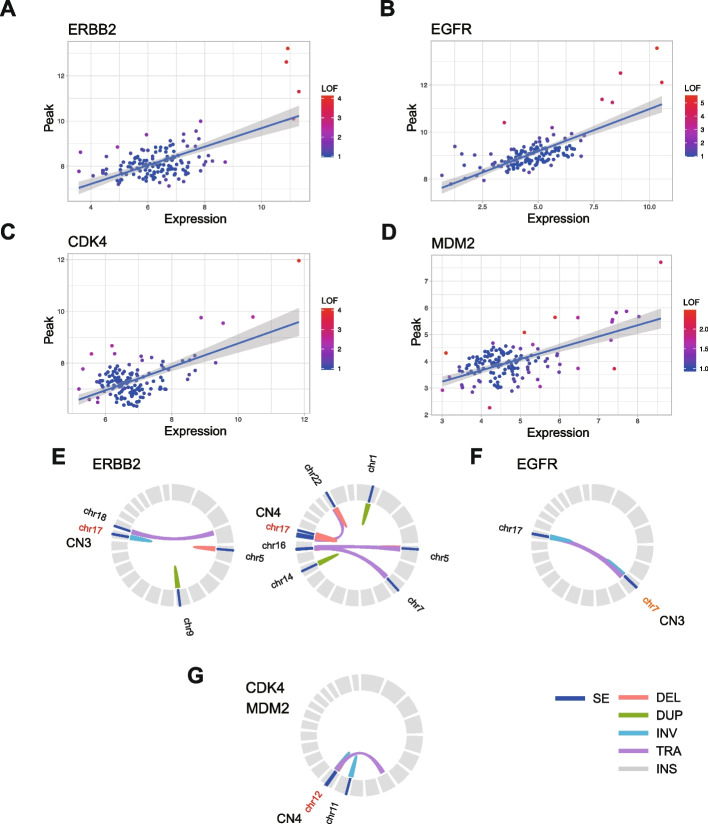
Gene expression on SE formation accompanied by SVs in non-CAGAs LUAD. **A**-**D** Correlation of H3K27Ac peaks with the expression of the genes (*n* = 142). The genes annotated as both SE regions and SVs were extracted (SE-to-gene links analysis). The straight line (blue) is to represent the best fit for the data points based on the least squares method. A linear model was used for the smoothing. To measure outliers, the local outlier factor (LOF) method was employed. For each data point, the 10 nearest data points were identified. A comprehensive description of the methods can be found in the supplementary methods. Each data point is represented in a heatmap according to LOF scores. **E–G** Circos plots of individual non-CAGAs LUAD samples. Representative cases detected by the LOF method are shown. SE regions are indicated by blue bands. The chromosomal number of the origin region, where the SE and SV overlap, is denoted in red. DEL: deletion, DUP: duplication, INV: inversion, TRA: translocation, INS: insertion, SE: super-enhancer. The absolute CNV calls were indicated in an outer ring of the Circos plots

To investigate distinct cellular or tissue signatures within LUAD through transcriptional profiling, we performed a clustering analysis on the entire RNA-seq dataset comprising 938 cases. This analysis revealed that a specific subset of non-CAGA LUAD cases exhibited prominent characteristics similar to those of limbal and corneal epithelial stem cells. In contrast, the EGFR mutation-positive group was markedly enriched in Type II pneumocytes and epithelial progenitor cells as shown in group 1 and 3, respectively (Fig. S10 and Table S2). To decipher the super-enhancer and structural variant landscape in our non-CAGAs LUAD cohort and better understand its impact on gene expression, we performed a peak-to-gene links analysis [29], by correlating H3K27Ac peaks within 0.5 M bp of the gene promoter with the expression of the gene (*n* = 142). In this analysis, 10,683 genes were identified to have a significant quantitative correlation with H3K27Ac peaks (FDR < 0.05, top 1,000 lists summarized in Dataset S8). A notable observation from our data suggests a positive correlation among gene clusters annotated as super-enhancer regions that also have accompanying structural variants (Fig. S11). Strikingly, genes such as *ERBB2* and *EGFR*, which are recognized as representative driver genes in LUAD, ranked prominently in this assessment (Fig. 2A, B, Table 1). Moreover, although *CDK4* and *MDM2* have been demonstrated to be involved in lung cancer, their roles as therapeutic targets have not yet been firmly established [30]. Regardless, they were ranked the most prominent in this assessment (Fig. 2C, D, Table 1). In a limited number of non-CAGA LUAD cases involving the *ERBB2, EGFR, CDK4,* and *MDM2* genes, we identified events where gene expression was induced to a considerable extent that they were deemed outliers (Fig. 2A-D). We confirmed that the super-enhancer and structural variants overlaps, which served as the origin of the genomic rearrangements, were present in all these cases (Fig. 2E-G). Importantly, structural variations associated with super-enhancers did not exhibit extensive copy number amplification, albeit with a moderate gain in copy number (Fig. 2E-G). Finally, to elucidate the differences in gene expression patterns and pathway engagements, particularly between those with super-enhancers and structural variants in genes including *ERBB2*, *EGFR*, *KRAS*, *CCND1*, *MDM2*, and those primarily displaying copy number alterations (CNAs), we conducted a comparative expression analysis. This analysis distinctly identified the chemokine activity pathway as significantly involved in cases with super-enhancers and structural variants, as highlighted in Group 4 (Fig. S12, Table S3). These findings indicate that H3K27Ac peaks provide a more explicit marker for gene expression amplification associated with the formation of super-enhancers concomitant with structural variants. Therefore, our analysis of the super-enhancer and structural variant landscape successfully identified gene clusters with strong correlations to expression levels. However, in instances where super-enhancer and structural variant overlaps were present, we observed an exceptionally aberrant elevation in gene expression.

### Candidates of driver mutations driven by exceptionally aberrant elevation in gene expression

Our SE-to-gene link analysis revealed a group of genes displaying remarkably aberrant expression that are compelling candidates for driver mutations driven by both super-enhancers and structural variants. Therefore, additional driver genes may need to be identified. Indeed, genes such as *FRS2* and *CAV2* may emerge as candidates (Fig. S13, the peak-to-gene link analysis for all other candidate genes, as shown in Fig. S14 and the Circos plots shown in Fig. S15). *FRS2* (Fibroblast Growth Factor Receptor Substrate 2) plays a critical role in activating the MAPK and PI3K signaling pathways, which are essential for cell proliferation, migration, and survival [31]. It has been identified as oncogenic and is amplified in high-grade serous ovarian cancer, highlighting its potential as a driver gene in oncogenesis [32]. Similarly, *CAV2* (Caveolin 2) is implicated in cancer progression; genetic variants leading to high CAV2 expression have been shown to promote pancreatic cancer progression and are associated with poor prognosis [33]. Furthermore, CAV2 influences focal adhesion and extracellular matrix organization pathways, underscoring its role in tumor development and metastasis [33]. In summary, this analysis suggests that *FRS2* and *CAV2* are involved in the molecular dynamics of non-CAGA LUAD. A deeper understanding of their molecular mechanisms may provide insights into potential therapeutic strategies.

### Chromosomal structure of super-enhancer and structural variant overlapped *ERBB2* gene locus

**Fig. 3:**
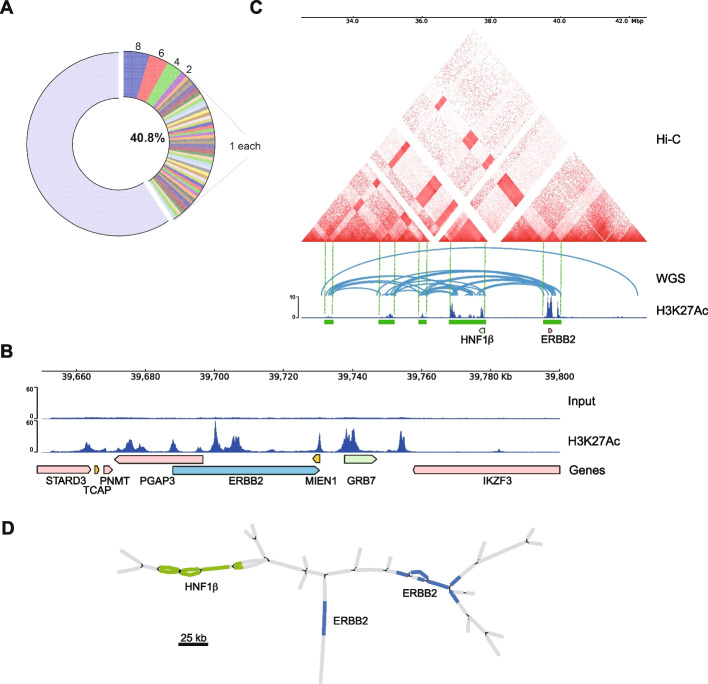
Extensive chromosomal rearrangement coincident with SE formation in the *ERBB2* locus. **A** Pie chart illustrating the proportion of non-CAGAs LUAD patients (*n* = 174) with SE formation associated with SV events. The frequency of the relevant cases displayed as numbers around the outer edge of the pie chart are shown. We counted the number of cases that overlapped with genes extracted through KEGG pathway enrichment analysis (FDR < 0.05). **B** Genome browser view of H3K27Ac ChIP-seq tracks for PDX model in LUAD. The region on chromosome 17: 39,650,000 to 39,800,000, with a center on the *ERBB2* gene is shown. **C** Integrative visualization of Hi-C, WGS, and ChIP-seq data in a sample where *ERBB2* expression was identified as an outlier. The figure presents a broad region on chromosome 17, spanning from 32,500,000 to 42,500,000. In this Hi-C analysis with a triangular view, chromosomal rearrangements are mainly represented as heatmaps, which provide a visual representation of the frequency of interactions between separate genomic regions within the cell nucleus. A missing region in the heatmap represents areas that have not been annotated in the reference genome. In the WGS track, connected breakpoints detected by Manta are linked with light blue lines. The genome browser view of H3K27Ac ChIP-seq tracks was shown at the bottom. A region where high contact frequency (Hi-C), clustering of genomic breakpoints (WGS), and peaks in H3K27Ac signal (ChIP-seq) were observed in distal genomic regions was marked with a light green bar at the bottom. **D** The assembly graph as a single node style demonstrates the de novo assembly of chromosomal rearrangement around the *HNF1β-ERBB2* loci. Two distinct paths show the connection between the *HNF1β* and *ERBB2* genes. The *HNF1β* gene is represented in green, while the *ERBB2* gene is displayed in blue. A 25 kb genome size bar is shown for reference

Considering the notably aberrant increase in gene expression driven by super-enhancer formation associated with SV events, it is noteworthy that such super-enhancer and structural variant overlapping cases were observed in 40.8% of patients with non-CAGA LUAD (Fig. 3A, gene clusters on KEGG pathway enrichment analysis: FDR < 0.05). Although a small patient group with super-enhancer and structural variant formation was observed for the *ZFP36L1*, *DDIT4*, and *MIR21*, unique super-enhancer and structural variant formations were observed in individual patients (Table S4). Among these, we focused on non-CAGAs LUAD cases displaying super-enhancer formation around ERBB2, comprising 1.15% of non-CAGA LUAD patients (Table S4). To further evaluate the validity of *ERBB2* as a potential drug target in non-CAGA LUAD cases, we conducted H3K27Ac ChIP-seq analysis in HER2-overexpressing LUAD cases verified by IHC and RNA-seq; however, its relationship with genomic amplification remains unclear [34]. These analyses were performed using patient-derived xenograft (PDX) models established at the NCC Japan [34, 35]. Extensive super-enhancer formation in the *HER2* region was indeed observed (Fig. 3B, Fig. S16A). This super-enhancer formation led to marked overexpression of HER2, as evidenced by both transcripts (Fig. S16B), and protein levels (Fig. S16C). To investigate the activation mechanisms of overexpressed ERBB2, we analyzed the same PDX samples as previously mentioned: one harboring an EGFR activating mutation L858R, sample #1, and the other exhibiting ERBB2 overexpression, sample #2. This analysis was performed utilizing both mass spectrometry and reverse-phase protein array methodologies. Although we confirmed ERBB2 overexpression (Fig. S17A), we did not observe a significant increase in phosphorylated ERBB2 at Y1248—a well-established marker of ERBB2 activation (Fig. S17B). However, we found that phosphorylation levels of ERK1/2 and the S6 ribosomal protein within the PI3K-AKT-mTOR pathway were found to be comparable in both PDX samples (Fig. S17C). Despite the limited number of samples, this suggests that there are common activation mechanisms in ERBB2 overexpressed cases that do not depend on its Tyr-1248 phosphorylation, indicating alternative pathways could be involved in ERBB2-driven signaling [36]. Importantly, drug testing using the pan-HER inhibitor, poziotinib exhibited a significantly promising effect, whereas afatinib showed no antitumor effect. In contrast, trastuzumab deruxtecan (T-DXd) induces significant tumor shrinkage in a dose-dependent manner [34]. These findings suggest that ERBB2-targeting therapies, particularly poziotinib and T-DXd, could be effective therapeutic options for LUAD with super-enhancer formation around *ERBB2*.

To delve deeper into large-scale chromosomal structural changes and interactions, we conducted Hi-C analysis of cases exhibiting extensive super-enhancer formation surrounding the *ERBB2* gene. Genomic alterations coinciding with H3K27Ac peaks were corroborated by the Hi-C results, as demonstrated by altered genomic organization (Fig. 3C). To directly identify the bona fide structural variants, we conducted de novo assembly using long-read sequencing with the PacBio Sequel II platform in conjunction with Hi-C data obtained from the same specimen (Fig. S18A, referring to Materials and methods). Upon analysis, we observed that the *ERBB2* gene loci were situated in closer proximity (~ 125 kb) to the *HNF1β* gene loci compared to their respective positions in the standard GRCh38 reference genome (~ 1.9 Mb apart) (Fig. 3D) while preserving contiguity (Fig. S18B). This observation suggested that a structural variant event was responsible for the rearrangement of the *ERBB2-HNF1β* gene loci (Fig. 3D). This comprehensive analysis not only elucidates the complex genomic landscape of non-CAGA LUAD, but also highlights the potential of ERBB2-targeting therapies for a subset of patients with specific super-enhancer formations.

### Targeted chromosomal rearrangements between *ERBB2* and *HNF1β* loci in cultured cells

**Fig. 4:**
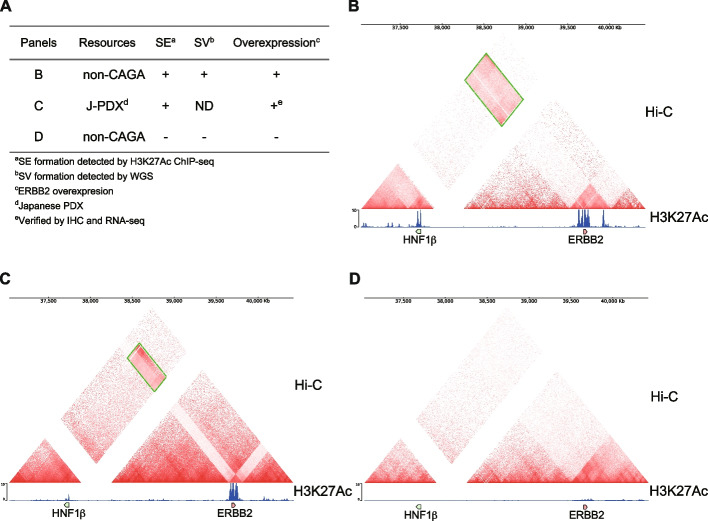
Integrated visualization of Hi-C and ChIP-seq data. **A** Table summary of the case used in the integrated analysis of Hi-C and ChIP-seq. **B**-**D** The displayed region on chromosome 17, which covers positions 37,034,000 to 40,422,000, lies between the *HNF1β* and *ERBB2* gene loci. The region enclosed by the green dashed line indicates the proximity of the *HNF1β* and *ERBB2* gene loci. The case shown in panel D was used as a negative control in which ERBB2 is not overexpressed. ^d^Japanese PDX [35]. ^e^Verified by IHC and RNA-seq [34]

**Fig. 5:**
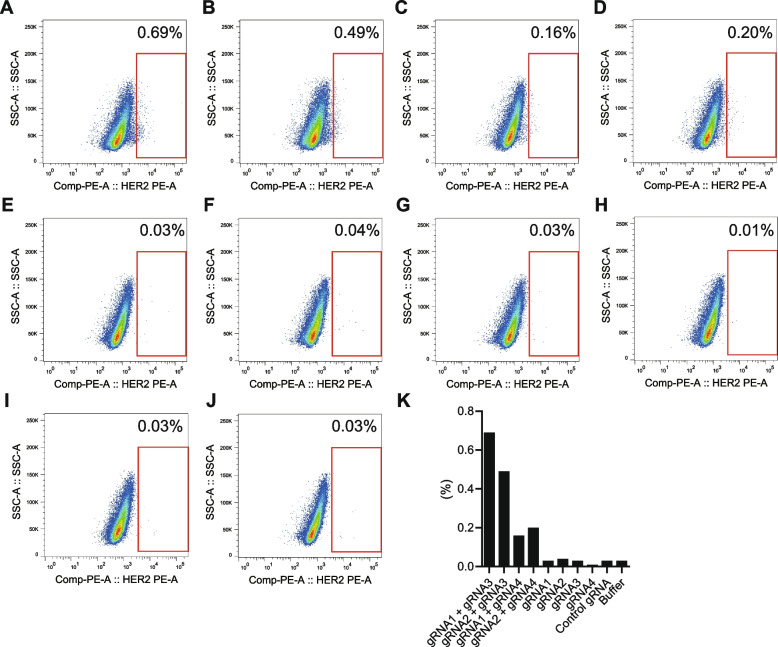
Genomic inversion induced by CRISPR-Cas9 mediated double-stranded break at *HNF1β* and *ERBB2* gene loci. Cas9-inducible HBEC3-KT cells were transfected with the combinations of guide RNAs, **A** gRNAs #1 and #2, **B** gRNAs #2 and #3, **C** gRNAs #1 and #4, **D** gRNAs #2 and #4, **E** gRNA #1, **F** gRNA #2, **G** gRNA #3, **H** gRNA #4, **I** Control gRNA, **J** Buffer. Cells within the red-framed region were designated as HER2 overexpressed cells. **K** In each experimental condition, the proportion of HER2 overexpressed cells (%) within the red-framed region was summarized

To directly determine whether the characteristic structural abnormalities obtained from the aforementioned results led to aberrant gene expression, we induced genomic structural abnormalities in cultured cells using the CRISPR-Cas9 system. WGS and Hi-C analyses revealed highly complex structural abnormalities in the *ERBB2* region. Meanwhile, from the Hi-C analysis results (Fig. 4A), we identified common chromosomal inversions in the *ERBB2* and *HNF1β* gene loci, respectively (Fig. 4B-D). Therefore, we designed gRNAs targeting the regions adjacent to these two breakpoints and attempted to induce chromosomal inversions in HBEC3-KT and HSAEC1-KT cells (Fig. S19). These cell lines, immortalized with CDK4 and hTERT, represent human bronchial and small airway epithelial cells, respectively, and neither form colonies on soft agar nor initiate tumor growth in mice [37, 38]. No oncogenic mutations in *EGFR* have been detected in HBEC3-KT using WES [39]. To confirm specific inversions, we employed T7EI assays and sequencing techniques (Fig. S20-21). Chromosomal inversions require simultaneous double-strand breaks at two distinct locations. When double-strand breaks were simultaneously induced, approximately 0.20–0.69% of the cells displayed an increase in HER2 expression, as confirmed by FACS (Fig. 5A-B, Fig. S22A-B) and RT-PCR (Fig. S23). This is comparable to the reported frequency of chromosomal inversions of approximately 1–8% [40, 41]. This increase was also observed with gRNAs targeting different sequences, albeit in the proximate regions (Fig. 5C-D, Fig. S22C-D). Conversely, upon inducing a break at only one site, we observed no significant difference in HER2 expression compared to baseline, with an approximate frequency of 0.01–0.04% (Fig. 5E-J, Fig. S22E-J, summarized in Fig. 5K, Fig. S22K). These results indicate that an increase in HER2 expression occurs only when double-strand breaks are induced in both *ERBB2* and *HNF1β* genomic regions, strongly suggesting that the observed genomic structural abnormalities directly impact HER2 expression.

### Significance of outlier genes in clinical outcomes

**Fig. 6:**
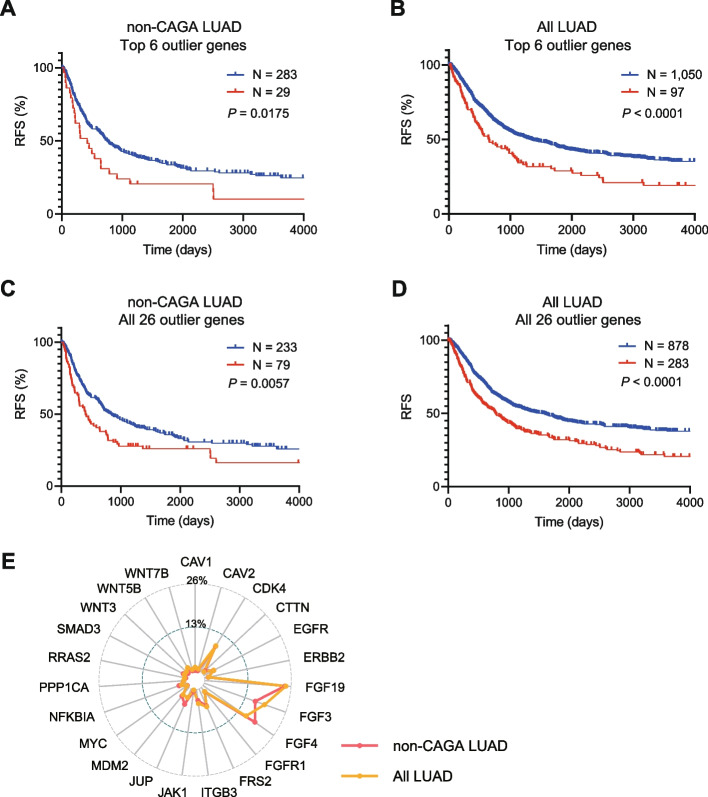
Aberrant gene expression is associated with an increased risk of postoperative recurrence in LUAD. Kaplan–Meier analysis was performed for LUAD patients with identified outlier genes, focusing on RFS in cases exhibiting abnormal gene expression as outliers (refer to Materials and methods). **A** non-CAGAs LUAD cases stratified by the top 6 outlier genes. **B** all LUAD cases stratified by the top 6 outlier genes. **C** non-CAGAs LUAD cases stratified by all 26 outlier genes. **D** all LUAD cases stratified by all 26 outlier genes. Red lines: LUAD cases with aberrant outlier gene expression. Blue lines: LUAD cases without aberrant outlier gene expression. **E** A radar chart illustrating the prevalence of the 23 genes in cases containing at least one outlier gene. Red line: non-CAGAs LUAD cases; Orange line: all LUAD cases

Our SE-to-gene link analysis, prioritized by the top six genes (*CDK4, ERBB2, MDM2, FRS2, EGFR, CAV2*), identified a set of genes displaying markedly aberrant expression patterns, indicative of potential driver mutations (Table 1). To evaluate the clinical implications of gene overexpression in the absence of somatic mutations, we analyzed its correlation with recurrence-related clinical outcomes. In patients with non-CAGA LUAD (*n* = 312), the presence of pronounced aberrant gene expression elevation, as ascertained by gene expression outlier analysis (refer to Materials and methods), was associated with significantly decreased RFS compared to those without such elevation (Fig. 6A). Moreover, these results were observed irrespective of driver mutations in the LUAD cohort (*n* = 1,147, Fig. 6B). Additionally, comparable results were obtained when the entire set of 26 genes extracted from the SE-to-gene link analysis (Table 1) was considered as the target group (Fig. 6C-D). Among the 26 genes, all LUAD cases with outliers in the 23 gene groups exhibited an increased risk of recurrence, particularly with *FGF3, FGF4* and *FGF19*, which are involved in recurrence risk (Fig. 6E). These findings underscore the robustness of the gene set derived from the super-enhancer and structural variant landscape analyses and imply that regardless of the presence or absence of driver gene mutations, such as CAGAs, the identified genes possess clinical significance as prognostic factors for predicting postoperative outcomes in LUAD.

## Discussion

Although super-enhancers and structural variants are often detected in the range of several hundred spots per case, most cancer research conducted thus far has analyzed these datasets independently [42], and there are no examples of integrated analysis in LUAD. In this study, we focused on understanding the interplay between super-enhancers and structural variants in the regulation of gene expression in non-CAGA LUAD. We found that the co-localization of super-enhancers and structural variants was limited, accounting for approximately 1% of the overall spots detected using our methodology. However, this co-localization was observed in approximately 40% of non-CAGA LUAD cases. Importantly, genes such as *ERBB2, EGFR, CDK4,* and *MDM2,* all with established links to NSCLC, demonstrated increased expression due to super-enhancer and structural variant overlap without extensive copy number amplifications. Furthermore, we identified clusters of genes that form super-enhancers linked to structural variations. This indicates that adjacent genes, including *FRS2*, *CAV2*, *FGF3*, *FGF4*, and *FGF19*, may also serve as driver genes besides well-established driver genes [32, 43–45]. Although further investigation is required to determine whether these genes are drivers, our analysis lies in the extension of the driver mutation concept from solely somatic mutations to include driver changes due to overexpression in wild-type genes [46–49].

Therapies targeting HER2, such as poziotinib and T-DXd, have shown significant efficacy in treating PDX models of LUAD with super-enhancer formation in the vicinity of the *ERBB2* gene. To further elucidate the influence of genomic structure on gene expression, we utilized the CRISPR-Cas9 system to induce chromosomal translocation between the *ERBB2* and *HNF1β* loci within a cell culture system. Our results revealed that an increase in HER2 expression was observed only when double-strand breaks occurred concurrently at both loci. Although this observation strongly reinforces the hypothesis that structural abnormalities within the gene directly influence ERBB2 expression, the structural variant event alone seems insufficient for full ERBB2 activation and subsequent cellular transformation. This suggests a potential need for other genetic or epigenetic alterations. In line with this, it would be intriguing to explore how EGF influences ERBB2 expression mediated by super-enhancers and structural variant formation. Thus, our culture conditions may unmask the complete array of genetic and epigenetic modifications necessary for cellular transformation. Overall, these findings underscore the pivotal role of genomic structures, such as super-enhancers and structural variants, in modulating gene expression in non-CAGA LUAD.

One of the most recent and ambitious efforts in this field is TRACERx, which was designed to trace genetic alterations in cancer, providing a profound understanding of how these driver genes contribute to disease progression and treatment responses [50, 51]. Such mutations often have considerable implications for the function or regulation of associated proteins, and when present, these mutations can lead to disease states such as cancer. However, when these mutations are absent, it becomes notably challenging to categorize a gene as a “driver” gene. In the context of our research, we propose a promising alternative approach for instances in which mutations in driver genes are not detected. In addition, the identification of super-enhancers and structural variants is a qualitative process that is less burdened by the complexities associated with quantitative analysis such as RNA-seq. Therefore, our approach presents an alternative pathway for identifying potential driver events and provides a new direction for research in cases where conventional methods fail to identify somatic mutations within the protein-coding regions of driver genes.

Copy number amplification is a significant event in cancer that often results in the overexpression of oncogenes and promotes tumor development and progression [52, 53]. It is plausible that regions of the genome with amplified copy numbers also coincide with areas where super-enhancers and structural variants overlap, leading to further enhancement of gene expression. Indeed, within our non-CAGA cohort, specific cases demonstrated complex chromothripsis events characterized by extensive copy number amplification around the *CDK4/MDM2* loci (Fig. S3). Since chromothripsis inherently involves complex structural variations, further investigations are required to determine whether the analyses of super-enhancers associated with structural variations indicate chromothripsis events [54]. However, it is important to note that while copy number amplification often leads to the overexpression of genes, gene expression is also regulated by other factors, including epigenetic changes and transcription factor binding [8, 55]. Therefore, an understanding of genomic-epigenetic configurations could potentially aid in the accurate identification of target genes for therapeutic interventions.

Translating our findings from WGS and ChIP-seq analyses for clinical applications requires prospective trials. By applying our method, we identified a preponderance of probable driver genes, some of which are currently under clinical investigation [30, 56–59]. This approach offers significant benefits for patient selection and potentially improves the efficacy of clinical trials by targeting individuals with relevant genetic profile. This may lead to more personalized treatment strategies, enhanced therapeutic outcomes, and better patient prognoses. However, this study has some limitations must be acknowledged. For example, the size of the obtained clinical samples may impose constraints on the scope and depth of the analyses that can be performed. Furthermore, the quality and quantity of genomic and epigenomic data may have been affected by the small sample size, potentially influencing the statistical power and reliability of the study outcomes. Despite these limitations, we previously reported that automated techniques using a dual-arm robot [27] can partially mitigate these challenges, enabling more efficient and accurate data collection and analysis.

In summary, our study provides valuable insights into the interplay between genomic and epigenetic configurations in non-CAGAs LUAD. We envision that our findings will contribute to the development of novel therapeutic strategies for patients with non-CAGAs LUAD by identifying potential therapeutic targets. Our work paves the way for further research to verify and expand upon these findings, aiming to improve patient outcomes in LUAD.

## Conclusions

Our study elucidated the intricate interplay between super-enhancers and structural variants in non-CAGA LUAD, underscoring their significant contribution to the modulation of gene expression. The methodology employed facilitated the identification of a substantial number of putative driver genes, thereby enabling a more precise selection of patients for clinical trials, potentially augmenting the effectiveness of personalized therapeutic approaches and improving patient prognoses.

### Supplementary Information

Additional file 1. Includes Figs. S1 – 23 and Tables S1 – 4.

Additional file 2. Contains supplementary methods.

Additional file 3. Represents Dataset S1 for the multiple QC reports generated by the nf-core/chipseq analysis pipeline.

Additional file 4. Provides Dataset S2 for the list of super-enhancer regions of each non-CAGAs LUAD analyzed by ROSE.

Additional file 5. Includes Dataset S3 for the list of super-enhancer regions of each CAGAs LUAD analyzed by ROSE.

Additional file 6. Includes Dataset S4 for the list of structural variant regions of each non-CAGAs LUAD analyzed by Manta.

Additional file 7. Includes Dataset S5 for the list of structural variant regions of each CAGAs LUAD analyzed by Manta.

Additional file 8. Provides Dataset S6 for the list of genome coordinates for regions where super-enhancer and structural variant overlap in non-CAGAs LUAD.

Additional file 9. Includes Dataset S7 for the list of genome coordinates for regions where super-enhancer and structural variant overlap in CAGAs LUAD.

Additional file 10. Provides Dataset S8 for the results of the peak-to-gene links analysis showing a scatter plot of the top 1,000 expression values and peak values, their correlation coefficient, and the null distribution of the correlation coefficient.

Additional file 11. The uncropped images include uncropped immunoblot data shown in Fig. S16C.

## Data Availability

The dataset for exploring genomic-epigenetic configurations in lung adenocarcinoma without clinically actionable genetic alterations is available at 10.6084/m9.figshare.22826636. The dataset encompasses the following files: 1) complete_p2gl_dataset.tsv contains a comprehensive array of peak-to-gene links analysis in non-CAGAs LUAD. 2) LUAD_56.bigWig, LUAD_JPDX0057.bigWig, and LUAD_222.bigWig include H3K27Ac ChIP-seq data for genome-wide visualization and analysis, as shown in Fig. 4B-D, respectively. 3) LUAD_56.mcool, LUAD_JPDX0057.mcool, and LUAD_222.mcool represent Hi-C data for genome-wide visual representation and analysis, as demonstrated in Fig. 4B-D, respectively. Raw sequence data, including WGS, Hi-C, RNA-seq and ChIP-seq, are not currently accessible to the public due to reasons of sensitivity. However, these can be procured from the corresponding author, provided a reasonable request is submitted. Each request will be evaluated for appropriateness and necessitate an appropriate data access agreement that aligns with the relevant ethical approvals. This procedure is coordinated through our platform, Mine (https://www.nibiohn.go.jp/mine/). Mine publicly showcases the outcomes of the inter-agency research project “Development of artificial intelligence to accelerate drug discovery”, which operates under the framework of the PRISM project in Japan. One of the projects focuses on drug development targeting patients with a condition termed “Pan-negative” lung cancer, where no specific therapeutic targets have been identified.

## Abbreviations

ALK: Anaplastic lymphoma kinase
BAX: BCL2 associated X
BLAST: Basic local alignment search tool
BRAF: B-Raf proto-oncogene
CAGAs: Clinically actionable genetic alterations
CAV2: Caveolin 2
CCND1: Cyclin D1
CDK4: Cyclin-dependent kinase 4
ChIP-seq: Chromatin immunoprecipitation sequencing
CNAs: Copy number alterations
DDIT4: DNA damage-inducible transcript 4
EGFR: Epidermal growth factor receptor
ERBB2/HER2: Human epidermal growth factor receptor 2
FGF3: Fibroblast growth factor 3
FGF4: Fibroblast growth factor 4
FGF19: Fibroblast growth factor 19
FGFR: Fibroblast growth factor receptor
FOXO3: Forkhead box O3
FRS2: Fibroblast growth factor receptor substrate 2
FDR: False discovery rate
H3K27Ac: Histone H3 lysine 27 acetylation
HNF1β: Hepatocyte nuclear factor 1 beta
Hi-C: High-throughput chromosome conformation capture
KEGG: Kyoto encyclopedia of genes and genomes
KRAS: Kirsten rat sarcoma
LUADs: Lung adenocarcinomas
MDM2: MDM2 proto-oncogene
MET: MET proto-oncogene
MIR21: MicroRNA 21
NCC: National cancer center
NRG1: Neuregulin 1
NSCLC: Non-small cell lung cancer
NTRK: Neurotrophic tyrosine kinase
PDX: Patient-derived xenograft
polyA RNA-seq: Polyadenylated RNA sequencing
PRISM: Public/Private R&D Investment Strategic Expansion PrograM
RET: Ret proto-oncogene
RNA-seq: RNA sequencing
ROSE: Rank ordering of super-enhancers
ROS1: ROS proto-oncogene 1
Ribo-Zero RNA-seq: Ribosomal RNA depletion sequencing
RXRA: Retinoid X receptor alpha
SE: Super-enhancer
SMART-seq: Switching mechanism at 5' end of RNA template sequencing
STAT3: Signal transducer and activator of transcription 3
SVs: Structural variants
T-DXd: Trastuzumab deruxtecan
T7EI: T7 endonuclease I
TRACERx: Tracking cancer evolution through therapy
WES: Whole-exome sequencing
WGS: Whole-genome sequencing
ZFP36L1: ZFP36 ring finger protein-like 1
