# Supplementary material for: Mechanism of ERBB2 gene overexpression by the formation of super-enhancer with genomic structural abnormalities in lung adenocarcinoma without clinically actionable genetic alterations
Source: Mol Cancer. 2024 Jun 11;23:126. doi: 10.1186/s12943-024-02035-6 (PMC11165761; doi:10.1186/s12943-024-02035-6)
Supplement: Supplementary file 1 — Additional file 1. Includes Figs. S1 – 23 and Tables S1 – 4. [file 12943_2024_2035_MOESM1_ESM.docx]

**Molecular Cancer *(Research*** ***Articles)***

**Supplementary information**

**Additional file 1**

**Mechanism of *ERBB2* gene overexpression by the formation of super-enhancer with genomic structural abnormalities in lung adenocarcinoma without clinically actionable genetic alterations**

Syuzo Kaneko et al.

**It includes Figs. S1 – 23 and Tables S1 – 4.**

**Fig. S1** Sample selection and driver mutations in this cohort. **A** We conducted WES and poly(A) RNA-seq on 938 samples. From the samples that did not have CAGAs, termed non-CAGAs (*n* = 420), we further performed WGS and ChIP-seq analyses on 174 cases. **B** Pie chart showing the number of cases for each driver mutation. Gene fusion include *ALK*, *ROS1*, *NRG1*, *RET*, *NTRK*, and *FGFR* (see Materials and methods).

**Fig. S2 A** Mutation frequency of non-CAGA and CAGA LUAD. The mutation frequency was calculated as the number of mutations per megabase of the entire genomic region. Mutational landscapes of non-CAGA (**B**) and CAGA LUAD (**C**). We considered CAGA cases to have clinically actionable genetic alterations when mutations in genes such as *EGFR, KRAS, BRAF, ERBB2,* and *MET* skipping, as well as fusion genes of *ALK, ROS1, NRG1, RET, NTRK,* and *FGFR,* were annotated as pathogenic or likely pathogenic. The 21 key oncogenes are illustrated. Genes with CN>4 and CN>2 are shown as red dots of different sizes. Cases marked with an asterisk have not undergone RNA-seq.

**Fig. S3** **A** CNV and SV landscape in non-CAGA LUAD. The bin size for CNV and SV analysis was set to 5 Mb each. We displayed the averaged copy-number and SV frequency in a heatmap. SV hotspot regions are indicated with dashed arrows. **B** Representative cases harboring focal amplifications in the *CDK4/MDM2* genomic region within the non-CAGA cohort. To reconstruct and analyze complex DNA amplifications involving structures such as amplicons, we utilized AmpliconArchitect.

**Fig. S4** PCA analysis of H3K27Ac ChIP-seq of lung adenocarcinoma tissues for non-CAGAs and matched adjacent tissues. N: matched adjacent normal tissues, T: non-CAGAs lung adenocarcinoma tissue.

**Fig. S5** The quality control (QC) reports for the ChIP-seq dataset (*n* = 221) used in this study. The report was formulated using Picard within the nf-core/chipseq analytical pipeline. PF_READS: the number of reads that passed Illumina's quality filter, referred to as "Pass Filter" reads. PF_READS_ALINED: the count of those PF reads that were successfully aligned to a reference sequence. PF_HQ_ALINED_READS: the count of those PF reads that were aligned to the reference sequence with a mapping quality of Q20 or higher. PF_READS_IMPROPER_PAIRS: the number of primary reads that, although aligned, didn't align appropriately in pairs.

**Fig. S6** A bar graph depicting the total count of structural variations (SVs) and super-enhancers (SEs) in each cohort, including non-CAGAs LUAD cases (*n* = 174) (**A**) and CAGA LUAD cases (*n* = 45) (**B**). The number of regions where SEs and SVs overlap are also displayed.

**Fig. S7** KEGG pathway enrichment analysis on gene clusters annotated as super-enhancers (**A**) or structural variants (**B**) regions in non-CAGA LUAD cases. The analysis of these results was conducted utilizing the same methodology as employed in the examination of the data presented in **Fig. 1C**. The top 20 enriched pathways are displayed. The analysis of these results was conducted utilizing the same methodology as employed in the examination of the data presented in Fig. 1C. The top 20 enriched pathways are displayed. The analysis indicated that cancer-related pathways were not consistently observed for gene groups located near super-enhancers or structural variant regions alone. However, when analyzing the overlap between super-enhancers and structural variants, we observed a different trend. Specifically, all pathways identified in this overlap analysis were significantly associated with cancer-related processes within the non-CAGA LUAD group, as illustrated in **Fig. 1C**. This finding underscores the potential synergistic effect of super-enhancer and structural variant co-localization in driving cancer-related gene expression changes, particularly in non-CAGA LUAD cases, highlighting the interplay between genomic features in the cancer landscape.

**Fig. S8** The entire gene clusters obtained from all 20 enriched pathways in non-CAGA LUAD (FDR < 0.05). The counts of individual genes annotated in regions where super-enhancer and structural variant overlaps were provided.


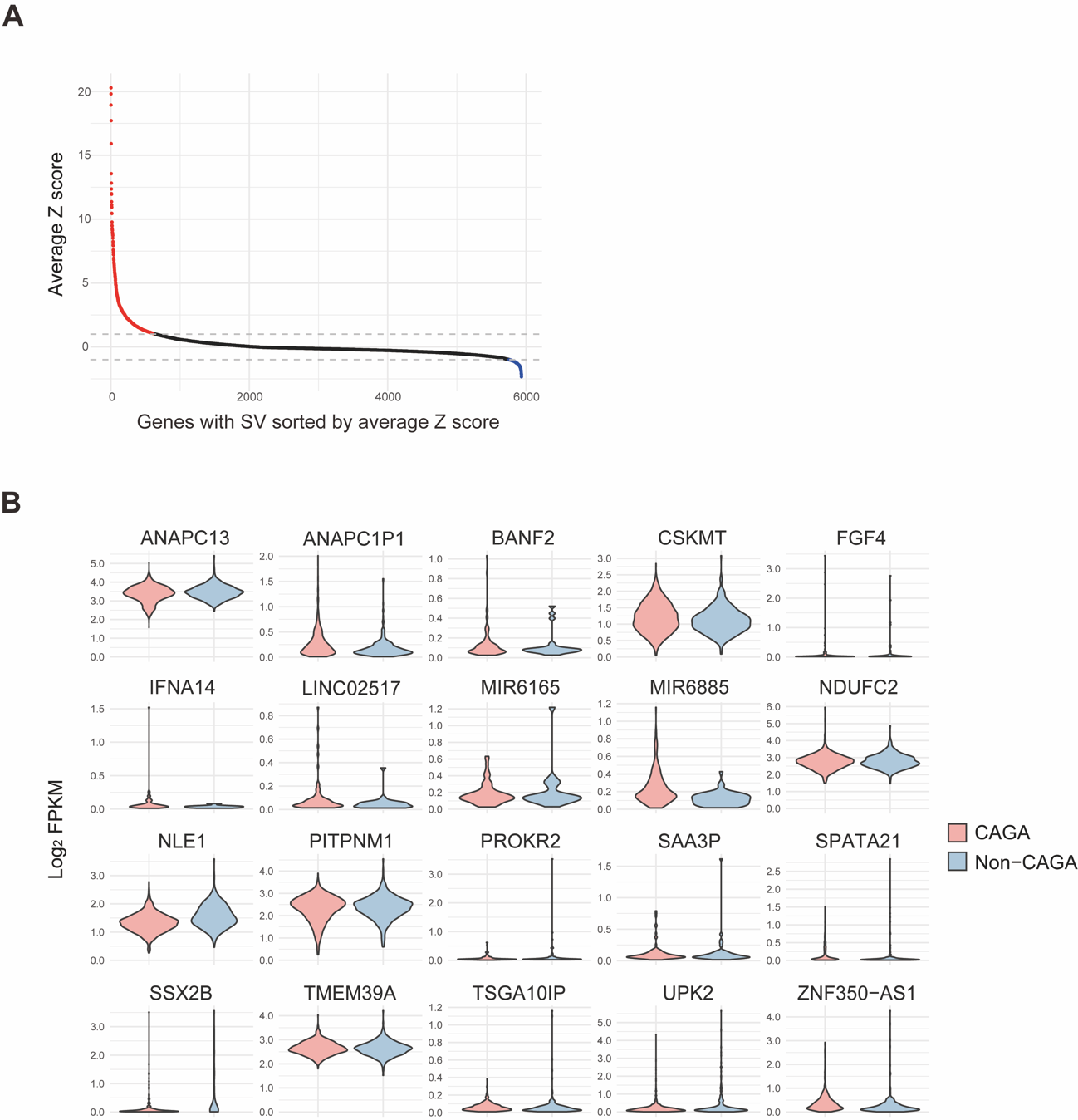


**Fig. S9** Gene expression in non-CAGAs LUAD where structural variants are found within gene body region. (**A**) We extracted expression information in the form of z-scores for genes where a structural variant intersects with the gene body region in our cohort (*n* = 174). The red dotted line: z-score > 1, the blue dotted line: z-score < －1, the black dotted line: z-score < |1|. (**B**) Violin plots showing a comparative expression analysis between the non-CAGA and CAGA cohorts in the top red dotted outlier genes (*n* = 20). These findings elucidate the differential gene expression patterns linked to the presence of structural variants, specifically highlighting the 20 genes that exhibit the most substantial expression elevation for a closer examination of variations between CAGA and non-CAGA groups. The objective of this analytical approach is to underscore that structural variants in isolation do not serve as discriminators between these groups.


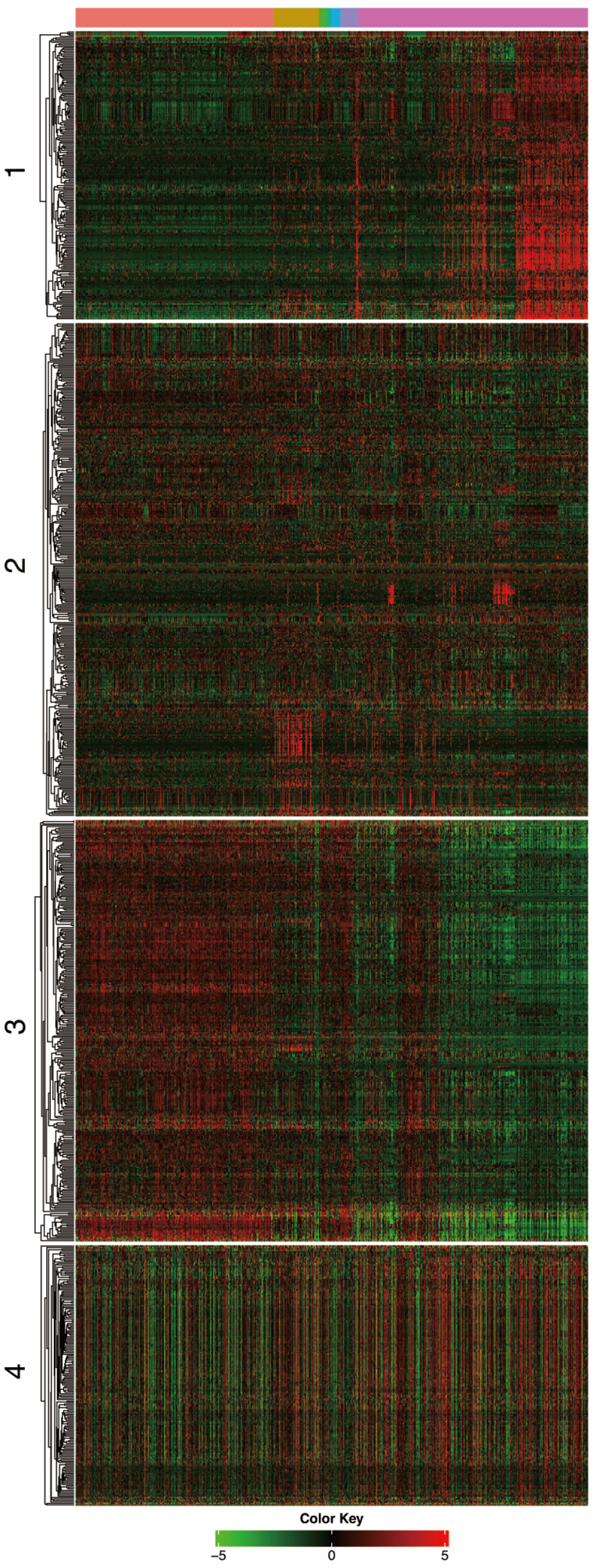


**Fig. S10** K-means clustering analysis of the poly(A) RNA-seq datasets. We juxtaposed cases with specific mutations, including EGFR (orange, *n* = 361), KRAS (brown, *n* = 85), BRAF (green, *n* = 14), MET skipping (light green, *n* = 9), ERBB2 (blue, *n* = 16), and gene fusion (purple, *n* = 33) against the non-CAGA cases (pink, *n* = 420), which are highlighted at the top. Relying on the within-group sum of squares plot, we organized the top 1,000 genes into four discernible clusters (k = 4). We subsequently conducted an enrichment analysis of these gene groupings, referencing the “CellMaker Augmented 2021” in Enrichr database. The enriched pathways derived from this analysis are detailed in Table S2.

**Fig. S11** Dot-plot for comparing the associations of H3K27Ac peak to gene expression between all annotated genes (*n* = 10,683) and the genes overlapping with super-enhancer and structural variant regions (*n* = 26, refer to as super-enhancer and structural variant landscape (SE-SV landscape)). The association scores are derived from the correlation coefficients (r) determined by the peak-to-gene links analysis (see methods). Statistical analysis was performed using a two-sided t-test. A significance level of **P* < 0.05 was applied. Red lines indicate the median.

**Fig. S12** K-means clustering analysis of the poly(A) RNA-seq datasets. We juxtaposed cases with specific copy number amplifications (CNLR_MEDIAN >= 1), including *ERBB2, EGFR, KRAS, CCND1, MDM2* genes (green, *n* = 31) against those genes with super-enhancer and structural variant (red, *n* = 9), which are highlighted at the top. Relying on the within-group sum of squares plot, we organized the top 1,000 genes into four discernible clusters (k = 4). We subsequently conducted an enrichment analysis of these gene groupings, referencing the “GO Molecular Function” in Enrichr database. The enriched pathways derived from this analysis are detailed in Table S3.

**Fig. S13** Examples of gene expression on super-enhancer formation accompanied by structural variants in non-CAGA LUAD. **A-B** SE-to-gene links analysis in Fig. 2 but showing other top-ranked genes, *FRS2* and *CAV2* (Table 1). Each data point is represented in a heatmap according to LOF scores. **C-D** Circos plots of individual non-CAGA LUAD samples extracted by LOF methods as shown in Fig. 2. The chromosomal number of the origin region, where the super-enhancer and structural variant overlap, is denoted in red. DEL: deletion, DUP: duplication, INV: inversion, TRA: translocation, INS: insertion, SE: super-enhancer.


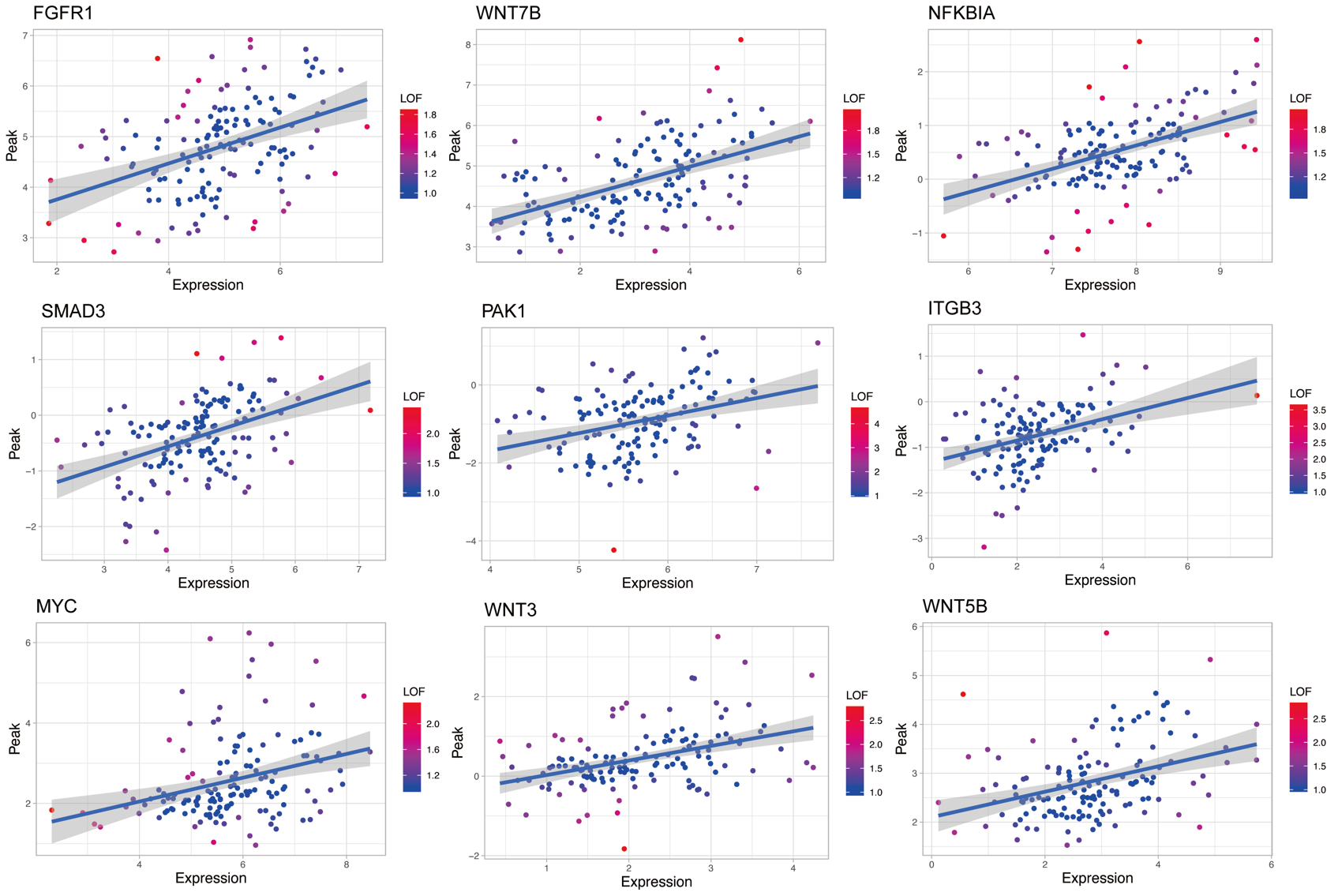


**Fig. S14** SE-to-gene links analysis for genes listed in Table 1.

**Fig. S15** Circos plots of representative non-CAGA LUAD samples for genes listed in Table 1. The absolute CNV calls were indicated in an outer ring of the Circos plots.

**Fig. S16** Overexpression of ERBB2 in PDX samples. **A** Comparison of H3K27Ac ChIP-seq peaks in the PDX samples (#1 and #2) obtained from LUAD patients. **B** RT-PCR analysis conducted on PDX samples #1 and #2. **C** Examination of ERBB2 protein levels using whole-cell extracts derived from PDX samples. On the left side, the protein size marker is displayed. GAPDH serves as a loading control.

**Fig. S17** Phosphorylation status of ERBB2-overexpressed PDX samples. Mass spectrometry (MS) and reverse-phase protein array (RPPA) analyses were conducted on two PDX samples; sample #1, featuring an EGFR activating mutation L858R, and sample #2, demonstrating ERBB2 overexpression. **A** Bar graph shows total ion intensity corresponding to the ERBB2 protein as detected by MS analysis with *n* = 2 as technical replicates. Dot-plot shows relative signal intensities of total ERBB2 protein detected by RPPA with *n* = 12 as technical replicates. **B** Dot-plots showing relative signal intensities of the phosphorylated ERBB2 (Y1221/1222 and Y1248) detected by RPPA with *n* = 12 as technical replicates. **C** Bar graph shows total ion intensity corresponding to the phosphorylated S6 ribosomal protein, as identified by MS analysis with *n* = 2 as technical replicates. Dot-plots show relative signal intensities of the phosphorylated ERK1/2 (T202/Y204), S6RP (S235/236) and S6RP (S240/244) detected by RPPA with *n* = 12 as technical replicates. Statistical analysis was performed using a two-sided t-test. A significance level of ****P < 0.0001 was applied. Red lines on dot-blot indicate the median. ns: not significant. Note that the antibodies used in the RPPA experiment, specifically ERBB2/HER2 pY1211/1222 and pY1248, have been validated through an experiment involving serum starvation overnight followed by a 30-minute treatment of A431 cells with EGF (250 ng/mL).

**Fig. S18** **A** The whole assembly graph to demonstrate de novo assembly of long-read sequencing. The raw assembled genomic data is too large in size, making it challenging to visualize the entire genome region. Therefore, we used Bandage's reduce command (see methods) to extract the *ERBB2* cDNA sequence as a query in the assembly graph, along with adjacent nodes. The *HNF1β* gene is represented in green, while the *ERBB2* gene is displayed in blue. A single node style is shown. **B** The continuity of the genome sequence is demonstrated according to Bandage's rule (See Methods), with bright green indicating the starting node, dark green representing contiguous nodes, light green for possibly contiguous nodes, and grey for nodes that are not contiguous.


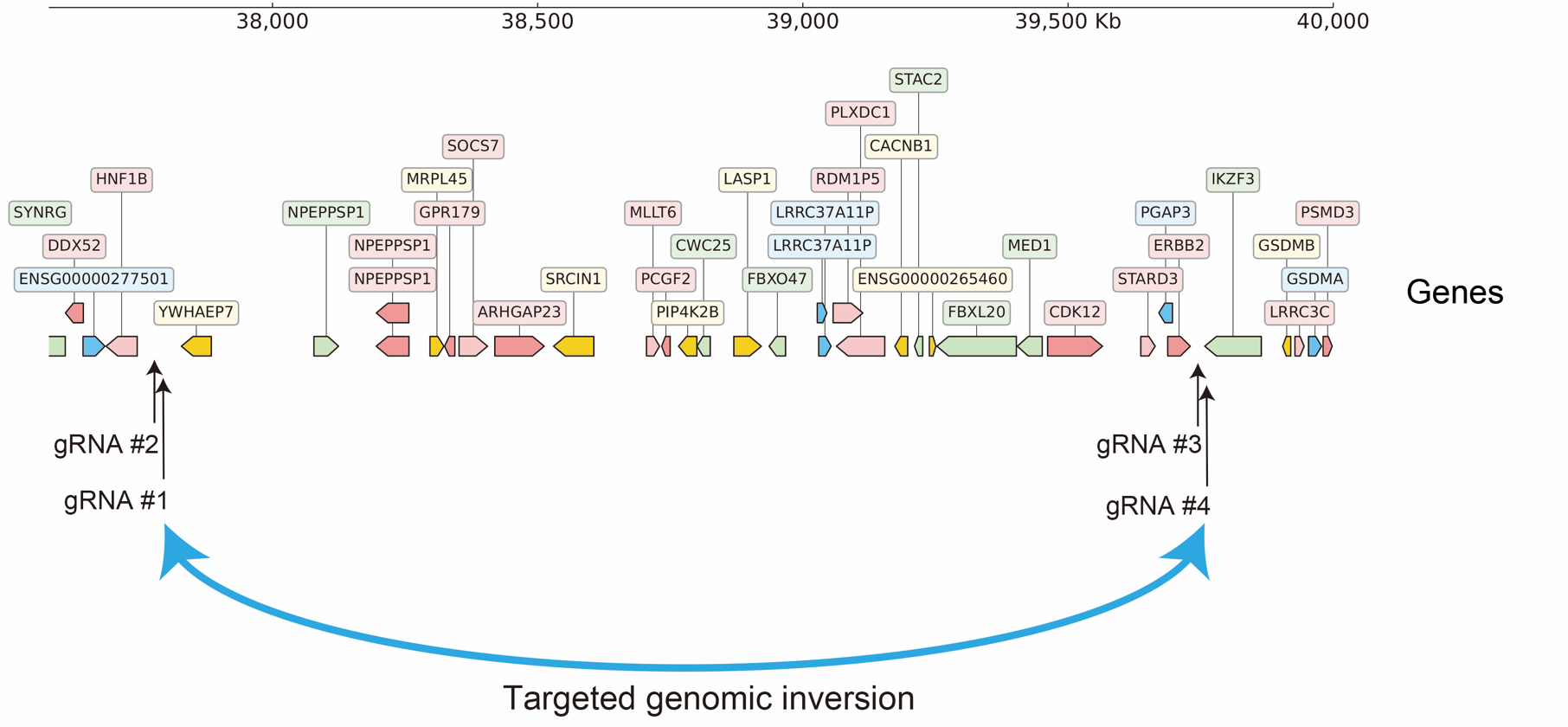


**Fig. S19** Strategy of targeted chromosomal rearrangements. The positions of the guide RNAs (gRNAs) are indicated by arrows, along with the corresponding gene tracks to provide context for the genomic locations.

**Fig. S20 A** Gel images representing DNA electrophoresis. T7EI assay (See Methods) corresponding to each gRNA used in Fig. 4A-D. The presence or absence of T7EI and lane numbers are shown below the gel images. A 100 bp DNA ladder size marker is displayed on the left side of the gel images. **B** Expected DNA fragment sizes in each lane. Size1, Size2, and Size3 are indicated in descending order of DNA size.

**Fig. S21** Chromatograms obtained from capillary sequencing are presented to demonstrate the presence of genomic inversions. PCR was performed using primers designed to flank the double-strand break region induced by CRISPR-Cas9. The sequence of the resulting PCR product was subsequently determined. The results correspond to the respective gRNA combinations: gRNAs #1 and #2 (**A**), gRNAs #2 and #3 (**B**), gRNAs #1 and #4 (**C**), and gRNAs #2 and #4 (**D**).

**Fig. S22** HER2 expression analyzed by FACS. Cas9-inducible HSAEC1-KT cells were transfected with guide RNAs. The combinations of gRNAs and the analysis methodology employed are the same as those described in **Fig. 5**.

**Fig. S23** Verification of ERBB2 expression levels. **A** Cells sorted via FACS. The sorted population is highlighted with large blue dots. **B** The isolated cells were cultured clonally and subsequently analyzed through RT-PCR. NC represents non-treated bulk cells serving as control.

**Table S1** The composition of lung adenocarcinoma cases investigated in this study is delineated based on the specific driver mutations present. The numbers enclosed in parentheses signify the count of cases associated with each respective mutation: *ALK* fusion, *EGFR, KRAS, MET* skipping, *NRG1* fusion, *RET* fusion, *ROS1* fusion. The identification of non-CAGA LUAD is described in the methods.

**Table S2** List of enriched pathways on each group. This table corresponds to the K-means clustering analysis in Fig. S10.

**Table S3** List of enriched pathways on each group. This table corresponds to the K-means clustering analysis in Fig. S12.

**Table S4** List of genes overlapped with super-enhancer formation associated with structural variant events. This table corresponds to the pie chart in Fig. 3A. Note that *CAV2* forms a super-enhancer independently, without overlap with *MET*. Our comprehensive evaluation also identified extensive super-enhancers associated with structural variants affecting both *FRS2* and *MDM2*, or *MDM2* alone.
