# Supplementary material for: Mechanism of ERBB2 gene overexpression by the formation of super-enhancer with genomic structural abnormalities in lung adenocarcinoma without clinically actionable genetic alterations
Source: Mol Cancer. 2024 Jun 11;23:126. doi: 10.1186/s12943-024-02035-6 (PMC11165761; doi:10.1186/s12943-024-02035-6)
Supplement: Supplementary file 2 — Additional file 2. Contains supplementary methods. [file 12943_2024_2035_MOESM2_ESM.docx]

**Molecular Cancer *(Research*** ***Articles)***

**Supplementary information**

**Additional file 2**

**Mechanism of *ERBB2* gene overexpression by the formation of super-enhancer with genomic structural abnormalities in lung adenocarcinoma without clinically actionable genetic alterations**

Syuzo Kaneko et al.

**It includes supplementary methods.**

**WGS**

Genomic DNA was extracted from fresh frozen samples using AllPrep DNA/RNA mini kit (Qiagen, 80204). DNA was sequenced on an Illumina HiSeq 2500 or Illumina NovaSeq 6000. To identify somatic mutations of tumor samples, we have analyzed the tumor tissues at a coverage of 100X, and the peripheral blood lymphocytes from the same cases at a coverage of 30X in WGS. As a quality standard for WGS, in all cases, we have set the criteria that the proportion of bases with QV30/20 or higher should be 75%/90% or more. After removing duplicate reads, we ensure the acquisition of data with at least N: 90G bases and T: 300G bases as a standardized methodology. The raw data were analyzed by Clara Parabricks (NVIDIA), GPU-based genome sequencing analysis framework. Briefly, the resulting NGS reads were mapped to the hg38 reference genome using BWA-MEM. The quality score recalibration, indel realignment, duplicate removal, single nucleotide variant (SNV), and indel discovery based on genome analysis toolkit (GATK version 4.1) [1] were conducted using standard hard filtering parameters according to the GATK Best Practices recommendations [2, 3]. Mutation calling to generate a somatic variant call format (VCF) file was performed using Mutect2 of GATK 4.1.

**Structural variant (SV) calling**

We employed Manta, a tool developed and maintained by Illumina, to identify somatic variations in tumor/normal pairs derived from WGS data [4]. Manta detects DNA rearrangements characterized by one or more breakpoints. These rearrangements can manifest as insertions where a specific DNA region is introduced, deletions where a particular segment is removed, inversions where a DNA segment undergoes flipping or reversal, and translocations where a segment is excised and then reintroduced into a different genomic location. To merge and filter the identified variants, we employed SURVIVOR, which removes potential false positives and improve the accuracy and reliability of the final SV set [5]. Lastly, we used VCFanno to annotate the filtered SVs with relevant genomic features, allowing for a comprehensive assessment of the potential functional consequences of the detected SVs [6].

**Copy number variation analysis**

For the assessment of copy number variations and the architecture of the amplicon, we employed the FACETS and AmpliconArchitect algorithm. This tool is designed to extract both fraction and allele-specific copy number estimates from paired tumor and normal WGS data. [7, 8].

**ChIP-seq analysis**

Archived frozen tissues from the NCC biobank were crosslinked using 1% formaldehyde (Sigma-Aldrich, 252549) in 1× PBS (FUJIFILM Wako, 293-72601) for 10 minutes at room temperature. The crosslinking reaction was quenched by adding 10x glycine solution (Cell Signaling Technology, #7005) and allowing it to react for 5 minutes at room temperature. Crosslinked tissues were then washed with ice-cold 1× PBS and resuspended in 1 mL of 1× Buffer A (Cell Signaling Technology, #7006) containing 1 mM PMSF and 1× PIC, followed by incubation on ice for 10 minutes. After centrifugation, the resulting nuclei pellets were resuspended in 1 mL of Buffer B (Cell Signaling Technology, #7007) containing 0.5 mM dithiothreitol (DTT; Cell Signaling Technology, #7016). Another round of centrifugation was performed and the pellets were then resuspended in 100 µL of Buffer B with 0.5 mM DTT. Micrococcal nuclease was added to the nuclei mixture and incubated for 20 minutes at 37 °C with frequent agitation (800 rpm). The digestion of DNA was halted by adding 20 µL of 0.5 M EDTA (pH 8.0). After another centrifugation, the resulting nuclei pellets were resuspended in 200 µL of ChIP buffer as described in reference [61]. The samples were then sonicated using a Bioruptor II (BM Equipment, BR2006A) for 30 minutes with high 30-second ON and 30-second OFF cycles to generate DNA fragments of approximately 300 base pairs. H3K27Ac antibody (2 µg, Abcam ab4729, lot GR3305164-1) was added to the solubilized chromatin along with 1% SDS, mixed briefly, and then placed in an ultrasonic water bath for 40 minutes with low 90-second ON and 30-second OFF cycles. The supernatants, after centrifugation, were incubated with 2 μl of FG Beads HM Protein G for 30 minutes at 4°C with rotation. The beads were washed twice with 800 μl of ChIP buffer, once with 800 μl of Wash buffer, and once with 800 μl of LiCl buffer [9]. Chromatin was eluted from the beads using 150 μl of ChIP elution buffer (Cell Signaling Technology, #7009) for 30 minutes at 65°C. The crosslinks were then reversed by adding 6 μl of 5M NaCl and 2 μl of Proteinase K (New England Biolab, P8107S). This mixture was incubated for approximately 16 hours at 65°C to ensure complete reversal of the crosslinks. DNA libraries were then prepared using the KAPA HyperPlus library preparation kit (Kapa Biosystems, KK8514), following the manufacturer's instructions. The DNA libraries were amplified using a 10-cycle PCR process. Finally, the DNA libraries were sequenced on an Illumina NovaSeq 6000 platform.

**Super-enhancer (SE) analysis**

We used nf-core/chipseq (v1.2.2) [10] bioinformatics pipeline to perform quality control on raw data. The ChIP-seq reads were then mapped to the human genome hg38 using Bowtie (v2.2.9) with parameters -local. Duplicate reads were removed with Samtools (v1.3.1). Enriched regions (ERs) were identified using MACS2 with the corresponding input as a control [11]. Inputs were prepared separately for male and female subjects, encompassing a pooled sample of 9 or 7 LUAD cases for each group. To identify SE regions, we performed rank ordering of super-enhancers (ROSE) analysis [12, 13]. First, we merged ERs that were within 12.5 kb of each other. Then, we ranked the merged regions by their H3K27ac ChIP-seq signal intensity, and identified regions as super-enhancers.

**RNA-seq clustering analysis**

For differential expression and pathway analysis of RNA-seq data, we employed iDEP, a web-based bioinformatics analysis pipeline [14]. We uploaded the FPKM (fragments per kilobase of exon per million reads mapped) values, which were then pre-processed and clustered using the built-in functions with default parameters. For pathway enrichment analysis, we utilized Enrichr, which offers a comprehensive collection of diverse gene set libraries [15].

**SE-to-gene links analysis**

To obtain gene expression dataset, total RNA from tissue samples was extracted employing the same kit as used for genomic analysis described above, with adaptations to acquire both DNA and RNA. RNA-seq was conducted utilizing polyA RNA-seq, Ribo-Zero RNA-seq, or SMART-seq [16], contingent upon the sample conditions. Nonetheless, due to a discernible bias in the sample preparation method as revealed by principal component analysis (PCA), solely samples processed using polyA RNA-seq were employed for the subsequent SE-to-gene links analysis (N = 142). Paired-end reads were acquired, and read mapping was executed using STAR (v 2.4.2a) [17], employing human genome and transcriptome data (GENCODE v31) as reference datasets. Transcripts per million (TPM) were computed utilizing the StringTie (v2.0.4) [18].

As SE regions are typically annotated over extensive areas spanning multiple gene clusters, we initially investigated the correlation between H3K27Ac peaks and gene expression (Peak-to-gene links). Concisely, the lower 25% of gene expression data and H3K27Ac peak data were eliminated, and all possible interactions between ChIP-seq peaks and genes within a 0.5 M bp range were identified. Pearson correlation was calculated between ChIP-seq peak (log_2_(CPM)) and gene expression (log_2_(TPM + 1)) to ascertain the significance of these correlations by constructing a random model that accounts for incidental associations. For each chromosome, 10,000 random peaks on distinct chromosomes and within 0.5 M bp from transcription start sites (TSSs) were correlated with the expression of every gene on the chromosome. Subsequently, the mean and standard deviation representing these non-specific correlations were computed. This methodology facilitated the calculation of p-values for each correlation and adjustment for multiple hypotheses using the Benjamini-Hochberg procedure (FDR). Thereafter, all correlations with an FDR below 0.05 were selected. Lastly, the genes annotated as SE regions by ROSE and as SVs by Manta were extracted.

**Hi-C**

The Hi-C procedure has been previously described in the study by Rao et al [19].　Briefly, we collected a fixed tissue sample, equivalent to less than 10 µg of DNA. We added 300 µl of complete Hi-C lysis buffer (10mM Tris-HCl [pH 8.0], 10mM NaCl, 0.2% Igepal CA630 and Protease inhibitor cocktail (PIC, Sigma, P8340)) to the crosslinked cell pellet and gently resuspended the cells. The mixture was then incubated on ice for 30 minutes. After incubation, we centrifuged the sample at 2500 ×g, 4°C for 5 minutes and discarded the supernatant. Next, we added 500 µl of Hi-C lysis buffer without PIC and briefly mixed the sample. We then centrifuged it again at 2500 ×g, 25°C for 5 minutes and discarded the supernatant. To permeabilize the nuclear membrane, we gently resuspended the pellet in 50 µl of 0.5% SDS and incubated it at 62°C for 10 minutes without agitation. We quickly spun the sample, then added 145 µl of MilliQ and 25 µl of 10% Triton X-100 to quench the SDS. We mixed the sample well by pipetting, while being careful to avoid foaming. The sample was then incubated at 37°C for 15 minutes at 650 rpm. For MboI digestion, we added 25 µl of 10× NEBuffer 2 (NEB, B7002S) and 100U of MboI (NEB, R0147M) to the sample. We gently resuspended the mixture and digested the chromatin for approximately 16 hours at 37°C with a gentle rotation. We incubated the sample at 62°C for 20 minutes and then cooled it to 25°C for 5 minutes. We then added 50 µl of fill-in master mix [19], mixed the sample well by pipetting, and incubated it at 37°C for 90 minutes with rotation. Next, we added 900 µl of ligation master mix [19], mixed the sample by inverting, and incubated it at 20°C for 4 hours with slow rotation. After incubation, we centrifuged the sample at 2500 ×g, 25°C for 5 minutes and discarded the supernatant, being careful not to disturb the loose pellet. We then resuspended the pelleted nuclei in 300 µl of crosslink reversal solution (10mM Tris-HCl [pH 8.0], 0.5M NaCl, 1% SDS) and added 50 µl of Proteinase K (800U/ml, NEB, P8107S). The sample was incubated at 55°C for 30 minutes, and then the temperature was raised to 68°C. The sample was incubated for approximately 16 hours at 1000 rpm to reverse-crosslink the sample. To extract DNA from the sample, we cooled it to room temperature, added 1 µl of Glycogen (Nacalai, 17110-11), and mixed it well. Then, we added an equal volume of phenol-chloroform-isoamyl alcohol mixture (25:24:1), vortexed it well, and centrifuged it for 5 minutes at 17,000 ×g. We carefully transferred the supernatant to a fresh tube and added 100% ethanol, incubated it at $-$80°C for 15 minutes, and centrifuged it at maximum speed for 15 minutes at 2°C. We then added 70% ethanol, centrifuged the sample at maximum speed for 5 minutes, and discarded the supernatant. After evaporating the ethanol, we dissolved the DNA pellet in 1× Tris buffer (10 mM Tris-HCl [pH 8.0]) and incubated it at 37°C for 15 minutes to fully dissolve the DNA. To make the library suitable for Illumina high-throughput sequencing, we subjected the DNA to shearing using a Bioruptor II (BM Equipment, BR2006A) for 20 minutes with low power settings of 30 seconds ON and 90 seconds OFF cycles. After shearing the DNA, we verified its size distribution to ensure successful sonication. We used the Agilent TapeStation High Sensitivity DNA (D5000) system to assess the DNA fragment size. If necessary, we repeated the sonication process until the DNA was optimally sized. Finally, we filled up the samples to 300 µl with 1× Tris buffer. To prepare the DNA library for sequencing, we mixed 10 mg/ml Dynabeads MyOne Streptavidin T1 beads (ThermoFisher Scientific, 65601) and aliquoted 150 µl into a low-binding 1.5ml tube. After washing the beads, we resuspended them in 300 µl of 2× BB (2 M NaCl, 10mM Tris-HCl [pH 7.5], 1mM EDTA) and incubated them with biotinylated DNA at room temperature for 15 minutes to bind. We then washed the beads sequentially in three different buffers as described [19] and removed unbound DNA by separating the beads on a magnet and discarding the supernatant. Next, we repaired the ends of the sheared DNA and removed biotin from unligated "dangling ends" by incubating the beads in an end repair master mix and a subsequent wash in two different buffers as described [19]. We then added an A-tailing master mix to the beads, incubated them, and rewashed them. After adding a Quick ligation reaction buffer (NEB, B2200S), Quick T4 DNA ligase (NEB, M2200L), and xGen UDI-UMI Adapters (IDT, 10005903, full-length), we incubated the beads and washed them twice with 1× TWB (1× BB, 0.05% Tween-20) at 55°C and once with 1× Tris buffer at 25°C. Finally, we detached DNA from the T1 beads and quantified DNA concentration using the Next Library Quant Kit for Illumina (NEB, E7630L). To perform PCR amplification, we used a reaction mix consisting of 47.5 µL eluted DNA library, 50 µL of 2X KAPA HiFi HotStart ReadyMix (Kapa Biosystems, KK2602), and 2.5 µL of 20 µM xGen Library Amplification Primer Mix (IDT, 1077677). The PCR cycle conditions were as follows: initial denaturation at 95°C for 45 seconds, denaturation at 98°C for 15 seconds, annealing at 60°C for 30 seconds, extension at 72°C for 30 seconds, and final extension at 72°C for 1 minute. After amplification, we performed AMPure XP beads (Beckman Coulter, A63881) size selection by adding pre-warmed beads at a 1:1 volume ratio, incubating for 5 minutes at room temperature, separating on a magnet, washing twice with 500 µl of 80% ethanol, and resuspending in 20 µl of EB buffer (Qiagen, 19086). We verified the successful size selection using the Agilent's TapeStation High Sensitivity DNA (D5000) system. Finally, we sequenced the library on an Illumina platform (NovaSeq 6000) with paired-end 2 × 150 bp reads.

We utilized the nf-core/hic (v1.3.0) bioinformatics pipeline [10], primarily based on HiC-Pro data processing, to process and analyze Hi-C data following best-practice analysis methods. This pipeline includes a two-step strategy for mapping reads spanning ligation sites utilizing GRCh38 human genome reference sequence, detection of valid interaction products, duplicate removal, and generation of raw and normalized contact maps [20]. Subsequently, for exploratory analysis, we employed the HiGlass package [21], and CoolBox was used for visualizing contact maps [22].

**Long-read sequencing**

The genomic DNA provided was fragmented to approximately 10 kb using the Megaruptor3 Shearing Kit (Diagenode). After smoothing the ends of the fragmented DNA, 3'-dA tailing was performed, and the adapter was ligated to create a template DNA for PCR amplification using the SMRTbell gDNA Sample Amplification Kit (Pacific Biosciences). PCR was performed under two different enzyme and primer conditions, and the resulting amplification products were mixed. The mixed PCR products were then subjected to end smoothing, 3'-dA tailing, and adapter ligation to create a sequencing library for use as a template for sequencing. The size distribution of the prepared sequencing library was confirmed using pulse-field gel electrophoresis, and size selection was performed using BluePippin (Sage Science). Sequencing templates were created by binding DNA polymerase and sequencing primers complementary to the SMRTbell adapters located at both ends of the sequencing library. These templates were then applied to SMRT Cells and immobilized in wells, known as Zero-mode waveguides (ZMW), for sequencing reactions. The PacBio Sequel II system was then used to obtain base sequences with approximately 20X coverage. Finally, highly accurate base sequence data with a quality value of 20 or greater (≧QV20) were obtained in the form of HiFi reads, which were created in the CCS (Circular Consensus Sequencing) mode of the PacBio Sequel II system. For the PacBio sequencing data analysis, the PacBio tool was utilized, which is available on the GitHub repository (https://github.com/PacificBiosciences/). Briefly, we removed duplicate reads of an amplified library using pbmarkdup. We utilized the pbmm2 wrapper, which is a minimap2 frontend for PacBio native data formats, to generate bam output. SV calling and analysis tools pbsv are used to detect and analyze SVs.

**Inducible Cas9 expression in cell line**

HBEC3-KT cells (#CRL-4051, lot #70016527) and HSAEC1-KT cells (#CRL-4050, lot #70023546) were purchased from American Type Culture Collection (ATCC). Cell culture experiments were conducted within 10 passages after receipt of the cells. Cell culture conditions were performed according to the manufacturer's instructions. Briefly, HBEC3-KT cells were cultured in airway epithelial cell basal medium (ATCC, PCS-300-030) supplemented with bronchial epithelial cell growth kit (ATCC, PCS-300-040) and penicillin-streptomycin solution (FUJIFILM-WAKO, 168-23191). HSAEC1-KT cells were cultured in SABM basal medium (Lonza, CC-3119) supplemented with SAGM SingleQuots kit (Lonza, CC-4124) excluding GA-1000 and 1% penicillin-streptomycin solution (FUJIFILM-WAKO, 168-23191). 293T cells were purchased from the ATCC and cultured in D-MEM (high glucose) with L-glutamine and phenol red (FUJIFILM-WAKO, 044-29765) supplemented with 10% fetal bovine serum (FBS) (Gibco, #10270106, lot.42Q2366K) and 1% antibiotic-antimycotic solution (FUJIFILM-WAKO, 161-23181). All cultures were incubated at 37°C at 5% CO2. All cell lines were routinely tested for mycoplasma contamination using an e-Myco mycoplasma PCR detection kit (iNtRON Biotechnology, #25235). The results of the STI analysis are available on the ATCC website (<https://www.atcc.org/>).

HEK293T cells were seeded in 6-well dishes at a density of 1.0 × 10^6^ cells per well in 2 mL of Opti-MEM I Reduced Serum Medium (Thermo Fisher Scientific, 31985070) supplemented with 5% FBS and 1% antibiotic-antimycotic solution. The viral vector is as follows: Edit-R Inducible Lentiviral hEF1a-Blast-Cas9 Nuclease Plasmid DNA (GE Healthcare, CAS11229). The lentiviral packaging plasmids, pMD2.G and psPAX2 were obtained from Addgene (#12259 and #12260). To produce lentiviruses, viral vector and packaging plasmids described above were co-transfected into the HEK293T using Lipofectamine 3000 (Thermo Fisher Scientific, L3000-008), according to the manufacturer’s instructions. The medium was replaced with fresh medium 6 hours after transfection. After 24 h of transfection, the cell culture medium was collected and 2 ml of fresh medium was added again. 24 hours later, the cell culture medium containing the viral fluid was collected together and filtered through a 0.45 μm filter (Sartorius, S7598FXOSK). The resulting lentiviruses were concentrated by Lenti-X Concentrator (Takara Bio, 631231). The titer of recombinant lentiviruses was determined by Lenti-X qRT-PCR Titration kit (Takara Bio, 631235).

A lentivirus transduction system was used to generate a conditional expression [23]. HBEC3-KT and HSAEC1-KT cells were seeded in 6-well dishes at a density of 3.75 × 10^4^ cells and 7.5 × 10^4^ cells per well, respectively. The following day, each virus at the described MOI was added to the cultured cells without polybrene. After 48 h of infection, the culture was replaced with fresh medium and continued for 3 days. Cas9 conditional expression cells were selected with blasticidin S (10 μg/ml) (FUJIFILM Wako, 029-18701). The Cas9 protein was induced by the administration of doxycycline (1 μg/ml).

**FACS**

Cells were analyzed 48 hours after transfection treatment. After trypsin treatment, cells were neutralized with 2% FBS/PBS (2 mL) and centrifuged at 1200 g for 4 minutes at 4°C. The supernatant was removed and cells were washed with PBS by centrifugation and removal of the supernatant. Cells were suspended in 50 µL of Stain buffer (BD, 554656) and incubated at room temperature for 10 minutes with 5 µL (2.5 µg) of Human BD Fc Block (BD, 564219) per 10^6^ cells. Anti-Her2/neu (BD, 340552) or Mouse IgG1 (20 µL, 0.1 µg/20 µL) was added and incubated at 4°C for at least 30 minutes. Cells were washed twice with 1 mL of Stain buffer and suspended in 500 µL of Stain buffer. The cell suspension was placed on ice and passed through a cell strainer (Falcon, 352235) just before measurement on the FACS Melody (BD). We imported data from 50,000 isolated cells into FlowJo software (BD) and visualized the data, set gates, and measured fluorescence intensity.

**RT-PCR**

Total RNA was extracted using QIAzol Lysis Reagent and RNeasy Plus Mini Kit (Qiagen, 73404), and cDNA was synthesized using the PrimeScript RT Reagent Kit (TaKaRa Bio, RR037A), according to the manufacturer’s instructions. Real-time PCR reactions were performed using TB Green Premix Ex Taq II (TaKaRa Bio, RR820A) and the CFX96 Touch system (Bio-Rad). mRNA levels were normalized to that of GAPDH (used as an internal control) using the ΔCq method. For quantitative real-time PCR, we used the following primers: ERBB2_Forward: 5'- ATC TTT GGG AGC CTG GCA TTT C -3'; ERBB2_Reverse, 5'- TCA AAC ACT TGG AGC TGC TCT G -3 '; EGFR_Forward: 5'- AAC ACC CTG GTC TGG AAG TAC G -3 '; EGFR_Reverse: 5'- TCG TTG GAC AGC CTT CAA GAC C -3 '; KRAS_Forward: 5'- CAG TAG ACA CAA AAC AGG CTC AG -3 '; KRAS_Reverse: 5'- TGT CGG ATC TCC CTC ACC AAT G -3 '; GAPDH_Forward: 5'- GCA AAT TCC ATG GCA CCG TC-3'; GAPDH_Reverse: 5'- TCG CCC CAC TTG ATT TTG G-3 '.

**Western blotting**

PDX tumor samples were directly lysed with CelLyticM cell lysis reagent (Sigma-Aldrich, C2978) containing protease inhibitors. Whole-cell lysates were passed through a 25-gauge needle ten times prior to centrifugation. Total protein concentration was measured using the Pierce 660 nm Protein Assay Reagent (Thermo Fisher Scientific, 22660). Whole-cell lysates mixed with Pierce Lane Marker Reducing Sample Buffer (Thermo Fisher Scientific, 39000) were boiled at 95 °C for 5 min, loaded into separate lanes on a sodium dodecyl sulfate (SDS) polyacrylamide gel (Bio-Rad, 456-9034), and then transferred to a nitrocellulose membrane (GE Healthcare, 10600012) following electrophoresis. After blocking with 5% skimmed milk (Fujifilm Wako Pure Chemical Co., 190-12865), the membranes were incubated with primary antibodies at 4 °C overnight. Protein bands were labeled with horseradish peroxidase (HRP)-conjugated secondary antibodies and visualized using ECL Prime western blotting detection reagent (GE Healthcare, RPN2236) and ImageQuant LAS 4000 (GE Healthcare). The primary antibodies used were anti-HER2/ErbB2 (Cell Signaling Technology, #2165; 1:1000) and GAPDH (Cell Signaling Technology, #2118; 1:1000). The secondary HRP-conjugated antibodies used were anti-mouse IgG (GE Healthcare, NA931; 1:5000) and anti-rabbit IgG (GE Healthcare, NA934; 1:5000).

**Mass spectrometry analysis**

For the sample preparation used in mass spectrometry analysis, we slightly modified the protocol provided by the Mass Spec Sample Prep Kit for Cultured Cells (Thermo Scientific). Specifically, we homogenized the PDX samples in lysis buffer, supplemented with benzonase to degrade nucleic acids, and precipitated the proteins using acetone. The precipitated proteins were then re-dissolved in guanidine hydrochloride, reduced with TCEP, alkylated with iodoacetamide, and digested with lysyl endopeptidase and trypsin. For phosphopeptide analysis, we utilized the TiO2 phosphopeptide enrichment kits (Thermo Scientific), preparing the samples according to the instructions provided. The resulting peptides were analyzed using an Evosep One LC system (EVOSEP) coupled to a Q-Exactive HF-X mass spectrometer (Thermo Scientific). The mobile phases consisted of 0.1% formic acid in water as solution A and 0.1% formic acid in 99.9% acetonitrile as solution B. The analysis was conducted in data-dependent acquisition mode, selecting the top 25 mass spectrometry spectra between 380 and 1500 m/z for recording. All MS/MS spectra were searched against the protein sequences from the mouse and human Swiss-Prot database using Proteome Discoverer 2.5 with the SEQUEST search engine. The false discovery rate (FDR) for peptide spectrum matches was set to 1%. For quantification, data were used after excluding quantification values derived from mouse-specific peptides.

**Reverse-phase protein array (RPPA)**

To conduct quantitative proteomic analysis and profile the expression and modification of signaling proteins, we utilized the reverse-phase protein array (RPPA), an antibody-based technique, for the analysis of cellular proteins as previously described [24, 25]. Briefly, cell lysates were prepared using RIPA buffer (Thermo Fisher Scientific) with both phosphatase and protease inhibitor cocktails (Thermo Fisher Scientific). After the protein concentration of the lysates was determined using Direct Detect (Merck), SDS sample buffer (Thermo Fisher Scientific) and DTT were added to the lysates for denaturation at 100°C for 10 minutes. Four-step twofold serial dilutions of the denatured lysates were printed in quadruplicate onto ONCYTE SuperNOVA nitrocellulose film slides (Grace Bio-Labs) using a robotic spotter (Genex Arrayer, Kaken Geneqs Inc.). The array slides were blocked with SuperG blocking buffer (Grace Bio-Labs) at room temperature for 1 hour and probed with primary antibodies at 4°C overnight. Following tyramide signal amplification (Dako), IRDye 680RD-streptavidin conjugate (LI-COR) was applied to the slides. Near-infrared (NIR) fluorescence images were captured by an InnoScan 710AL microarray scanner (Innopsys) and quantified using Mapix software (Innopsys). The fluorescence intensity of each spot was normalized to that of the protein load for the corresponding spot. Antibodies purchased from Cell Signaling Technology (CST) used in the RPPA were HER2/ErbB2 (29D8) rabbit mAb, catalog #2165; p-HER2/ErbB2 (Tyr-1221/1222) (6B12) rabbit mAb, catalog #2243; p-HER2/ErbB2 (Tyr-1248) antibody, catalog #2247; P-44/42 MAPK (Erk1/2) (Thr202/Tyr204) (D13.14.4E) XP Rabbit mAb, catalog #4370; P-S6 Ribosomal Protein (Ser235/236) D57.2.2E XP Rabbit mAb, catalog #4858; P-S6 Ribosomal Protein (Ser240/244) (D68F8) XP Rabbit mAb, catalog #5364.

**Bioinformatic analysis**

The bioinformatics analysis packages used for this study are as follows, including the available links and purposes:

[Bandage](https://rrwick.github.io/Bandage/): visualizing and analyzing de novo assembly graphs.

[BLAST search (v2.9.0)](https://ftp.ncbi.nlm.nih.gov/blast/executables/blast+/): comparing primary biological sequence information.

[Bowtie (v2.2.9)](http://bowtie-bio.sourceforge.net/index.shtml): aligning sequencing reads to long reference sequences.

[BWA-MEM](https://github.com/lh3/bwa): mapping low-divergent sequences against a large reference genome.

[Clara Parabricks (NVIDIA)](https://www.nvidia.com/en-us/clara/genomics/): accelerating analysis of next-generation sequencing data.

[CoolBox](https://github.com/GangCaoLab/CoolBox): interactive genomic data visualization and analysis.

[GATK version 4.1](https://gatk.broadinstitute.org/hc/en-us): identifying SNPs and indels in genomic sequencing data.

[GraphPad Prism (v9)](https://www.graphpad.com/scientific-software/prism/): scientific graphing, curve fitting, statistics, and data organization.

[hifiasm (v0.16.1-r375)](https://github.com/chhylp123/hifiasm): assembling high-fidelity reads, like PacBio HiFi reads.

[HiGlass](https://higlass.io/): interactive, multi-scale, and multi-modal visualization of genomic data.

[MACS2](https://github.com/taoliu/MACS): identifying transcription factor binding sites.

[Manta](https://github.com/Illumina/manta): discovering structural variants and indels in genomic data.

[nf-core/chipseq (v1.2.2)](https://nf-co.re/chipseq): ChIP-seq data analysis.

[nf-core/hic (v1.3.0)](https://nf-co.re/hic): processing and analyzing Hi-C data.

[PacBio tool](https://github.com/PacificBiosciences/pbbioconda): analyzing Pacific Biosciences (PacBio) long-read sequencing data.

Peak-to-gene links (in-house code): linking identified genomic peaks to associated genes.

[ROSE](https://github.com/younglab/ROSE): ranking super-enhancers based on their control over associated transcription.

[Samtools (v1.3.1)](http://www.htslib.org/download/): manipulating alignments in the SAM format.

[STAR (v 2.4.2a)](https://github.com/alexdobin/STAR): aligning RNA-seq reads to a reference genome.

[StringTie (v2.0.4)](https://ccb.jhu.edu/software/stringtie/): assembling RNA-Seq alignments into potential transcripts.

Bioconductor libraries [26, 27] used for bioinformatics analysis using R language (v4.2.2) in our study are as follows: BRGenomics (v1.10.0), ChIPpeakAnno (v3.32.0), clusterProfiler (v4.6.2), crispRdesignR (v1.1.6), data.table (v1.14.8), DOSE (v3.24.2), dplyr (v1.1.1), EnsDb.Hsapiens.v86 (v2.99.0), enrichplot (v1.18.3), flextable (v0.9.0), GenomicDistributions (v1.6.0), ggplot2 (v3.4.1), ggsci (v3.0.0), ggbio (v1.46.0), ggradar (v0.2), gridExtra (v2.3), gtsummary (v1.7.0), lubridate (v1.9.2), magrittr (v2.0.3), motifStack (v1.42.0), org.Hs.eg.db (v3.16.0), pipeR (v0.6.1.3), pzfx (v0.3.0) , RColorBrewer (v1.1.3), reshape2 (v1.4.4), rlist (v0.4.6.2), rmdformats (v1.0.4), stringr (v1.5.0), tidyverse (v2.0.0), TxDb.Hsapiens.UCSC.hg38.knownGene (v3.16.0), VariantAnnotation (v1.44.1), webshot (v0.5.4), yaml (v2.3.7). GitHub or Bioconductor repository sites for these R packages were listed as follows:

The circos plots were drawn using an in-house python script. For the KEGG pathway analysis, we employed the clusterProfiler package. The results were considered statistically significant when the *P*-value was less than 0.05.

**Supplementary references**

1. McKenna A, Hanna M, Banks E, Sivachenko A, Cibulskis K, Kernytsky A, et al. The Genome Analysis Toolkit: a MapReduce framework for analyzing next-generation DNA sequencing data. Genome Res 2010, 20**:**1297-1303.

2. DePristo MA, Banks E, Poplin R, Garimella KV, Maguire JR, Hartl C, et al. A framework for variation discovery and genotyping using next-generation DNA sequencing data. Nat Genet 2011, 43**:**491-498.

3. Van der Auwera GA, Carneiro MO, Hartl C, Poplin R, Del Angel G, Levy-Moonshine A, et al. From FastQ data to high confidence variant calls: the Genome Analysis Toolkit best practices pipeline. Curr Protoc Bioinformatics 2013, 43**:**11 10 11-11 10 33.

4. Chen X, Schulz-Trieglaff O, Shaw R, Barnes B, Schlesinger F, Kallberg M, et al. Manta: rapid detection of structural variants and indels for germline and cancer sequencing applications. Bioinformatics 2016, 32**:**1220-1222.

5. Jeffares DC, Jolly C, Hoti M, Speed D, Shaw L, Rallis C, et al. Transient structural variations have strong effects on quantitative traits and reproductive isolation in fission yeast. Nature Communications 2017, 8.

6. Pedersen BS, Layer RM, Quinlan AR. Vcfanno: fast, flexible annotation of genetic variants. Genome Biol 2016, 17**:**118.

7. Shen R, Seshan VE. FACETS: allele-specific copy number and clonal heterogeneity analysis tool for high-throughput DNA sequencing. Nucleic Acids Res 2016, 44**:**e131.

8. Deshpande V, Luebeck J, Nguyen ND, Bakhtiari M, Turner KM, Schwab R, et al. Exploring the landscape of focal amplifications in cancer using AmpliconArchitect. Nat Commun 2019, 10**:**392.

9. Kaneko S, Mitsuyama T, Shiraishi K, Ikawa N, Shozu K, Dozen A, et al. Genome-Wide Chromatin Analysis of FFPE Tissues Using a Dual-Arm Robot with Clinical Potential. Cancers (Basel) 2021, 13.

10. Ewels PA, Peltzer A, Fillinger S, Patel H, Alneberg J, Wilm A, et al. The nf-core framework for community-curated bioinformatics pipelines. Nat Biotechnol 2020, 38**:**276-278.

11. Zhang Y, Liu T, Meyer CA, Eeckhoute J, Johnson DS, Bernstein BE, et al. Model-based analysis of ChIP-Seq (MACS). Genome Biol 2008, 9**:**R137.

12. Whyte WA, Orlando DA, Hnisz D, Abraham BJ, Lin CY, Kagey MH, et al. Master transcription factors and mediator establish super-enhancers at key cell identity genes. Cell 2013, 153**:**307-319.

13. Loven J, Hoke HA, Lin CY, Lau A, Orlando DA, Vakoc CR, et al. Selective inhibition of tumor oncogenes by disruption of super-enhancers. Cell 2013, 153**:**320-334.

14. Ge SX, Son EW, Yao R. iDEP: an integrated web application for differential expression and pathway analysis of RNA-Seq data. BMC Bioinformatics 2018, 19**:**534.

15. Kuleshov MV, Jones MR, Rouillard AD, Fernandez NF, Duan Q, Wang Z, et al. Enrichr: a comprehensive gene set enrichment analysis web server 2016 update. Nucleic Acids Res 2016, 44**:**W90-97.

16. Adiconis X, Borges-Rivera D, Satija R, DeLuca DS, Busby MA, Berlin AM, et al. Comparative analysis of RNA sequencing methods for degraded or low-input samples. Nat Methods 2013, 10**:**623-629.

17. Dobin A, Davis CA, Schlesinger F, Drenkow J, Zaleski C, Jha S, et al. STAR: ultrafast universal RNA-seq aligner. Bioinformatics 2013, 29**:**15-21.

18. Pertea M, Pertea GM, Antonescu CM, Chang TC, Mendell JT, Salzberg SL. StringTie enables improved reconstruction of a transcriptome from RNA-seq reads. Nat Biotechnol 2015, 33**:**290-295.

19. Rao SS, Huntley MH, Durand NC, Stamenova EK, Bochkov ID, Robinson JT, et al. A 3D map of the human genome at kilobase resolution reveals principles of chromatin looping. Cell 2014, 159**:**1665-1680.

20. Servant N, Varoquaux N, Lajoie BR, Viara E, Chen CJ, Vert JP, et al. HiC-Pro: an optimized and flexible pipeline for Hi-C data processing. Genome Biol 2015, 16**:**259.

21. Kerpedjiev P, Abdennur N, Lekschas F, McCallum C, Dinkla K, Strobelt H, et al. HiGlass: web-based visual exploration and analysis of genome interaction maps. Genome Biol 2018, 19**:**125.

22. Xu W, Zhong Q, Lin D, Zuo Y, Dai J, Li G, et al. CoolBox: a flexible toolkit for visual analysis of genomics data. BMC Bioinformatics 2021, 22**:**489.

23. Machino H, Kaneko S, Komatsu M, Ikawa N, Asada K, Nakato R, et al. The metabolic stress-activated checkpoint LKB1-MARK3 axis acts as a tumor suppressor in high-grade serous ovarian carcinoma. Commun Biol 2022, 5**:**39.

24. Masuda M, Chen WY, Miyanaga A, Nakamura Y, Kawasaki K, Sakuma T, et al. Alternative mammalian target of rapamycin (mTOR) signal activation in sorafenib-resistant hepatocellular carcinoma cells revealed by array-based pathway profiling. Mol Cell Proteomics 2014, 13**:**1429-1438.

25. Masuda M, Yamada T. Signaling pathway profiling using reverse-phase protein array and its clinical applications. Expert Rev Proteomics 2017, 14**:**607-615.

26. Huber W, Carey VJ, Gentleman R, Anders S, Carlson M, Carvalho BS, et al. Orchestrating high-throughput genomic analysis with Bioconductor. Nat Methods 2015, 12**:**115-121.

27. Gentleman RC, Carey VJ, Bates DM, Bolstad B, Dettling M, Dudoit S, et al. Bioconductor: open software development for computational biology and bioinformatics. Genome Biol 2004, 5**:**R80.
