# Supplementary material for: Mechanism of ERBB2 gene overexpression by the formation of super-enhancer with genomic structural abnormalities in lung adenocarcinoma without clinically actionable genetic alterations
Source: Mol Cancer. 2024 Jun 11;23:126. doi: 10.1186/s12943-024-02035-6 (PMC11165761; doi:10.1186/s12943-024-02035-6)
Supplement: Supplementary file 3 — Additional file 3. Represents Dataset S1 for the multiple QC reports generated by the nf-core/chipseq analysis pipeline. [file 12943_2024_2035_MOESM3_ESM.html]

MultiQC Report


# Toggle navigation v1.9

Loading report..

- LIB: FastQC (raw)
  - Sequence Counts
  - Sequence Quality Histograms
  - Per Sequence Quality Scores
  - Per Base Sequence Content
  - Per Sequence GC Content
  - Per Base N Content
  - Sequence Length Distribution
  - Sequence Duplication Levels
  - Overrepresented sequences
  - Adapter Content
  - Status Checks
- LIB: cutadapt (trimmed)
  - Filtered Reads
  - Trimmed Sequence Lengths
- LIB: FastQC (trimmed)
  - Sequence Counts
  - Sequence Quality Histograms
  - Per Sequence Quality Scores
  - Per Base Sequence Content
  - Per Sequence GC Content
  - Per Base N Content
  - Sequence Length Distribution
  - Sequence Duplication Levels
  - Overrepresented sequences
  - Adapter Content
  - Status Checks
- LIB: SAMTools
  - Percent Mapped
  - Alignment metrics
  - Samtools Flagstat
  - XY counts
  - Mapped reads per contig
- MERGED LIB: SAMTools (unfiltered)
  - Percent Mapped
  - Alignment metrics
  - Samtools Flagstat
  - XY counts
  - Mapped reads per contig
- MERGED LIB: Preseq (unfiltered)
- MERGED LIB: SAMTools (filtered)
  - Percent Mapped
  - Alignment metrics
  - Samtools Flagstat
  - XY counts
  - Mapped reads per contig
- MERGED LIB: Picard
  - Alignment Summary
  - Base Distribution
  - Insert Size
  - Mark Duplicates
  - Mean Base Quality by Cycle
  - Base Quality Distribution
- MERGED LIB: deepTools
  - Fingerprint plot
  - Read Distribution Profile after Annotation
- MERGED LIB: MACS2 peak count
- MERGED LIB: MACS2 FRiP score
- MERGED LIB: HOMER peak annotation
- MERGED LIB: spp strand-shift correlation
- MERGED LIB: spp NSC coefficient
- MERGED LIB: spp RSC coefficient
- MERGED LIB: featureCounts
- nf-core/chipseq Software Versions
- nf-core/chipseq Workflow Summary

Toolbox

### MultiQC Toolbox

#### Apply Highlight Samples

This report has flat image plots that won't be highlighted.

+

Regex mode off
help
 Clear

#### Apply Rename Samples

This report has flat image plots that won't be renamed.  
See the documentation
for help.

+

Click here for bulk input.

Paste two columns of a tab-delimited table here (eg. from Excel).

First column should be the old name, second column the new name.

Add

Regex mode off
help
 Clear

#### Apply Show / Hide Samples

This report has flat image plots that won't be hidden.  
See the documentation
for help.

Hide matching samples

Show only matching samples

+

Warning! This can take a few seconds.

Regex mode off
help
 Clear

#### Export Plots

- Images
- Data

px

px

Aspect ratio

PNG
JPEG
SVG

Plot scaling

X

Download the raw data used to create the plots in this report below:

Format:

Tab-separated
Comma-separated
JSON

Note that additional data was saved in `multiqc_data` when this report was generated.

---

##### Choose Plots

 All
 None

---


   Download Plot Images

If you use plots from MultiQC in a publication or presentation, please cite:

> **MultiQC: Summarize analysis results for multiple tools and samples in a single report**  
> *Philip Ewels, Måns Magnusson, Sverker Lundin and Max Käller*  
> Bioinformatics (2016)  
> doi: 10.1093/bioinformatics/btw354  
> PMID: 27312411

#### Save Settings

You can save the toolbox settings for this report to the browser.

 Save


---

#### Load Settings

Choose a saved report profile from the dropdown box below:

[ select ]

Load
 Delete
 Set default
 Clear default

#### About MultiQC

This report was generated using MultiQC, version 1.9

You can see a YouTube video describing how to use MultiQC reports here:
https://youtu.be/qPbIlO\_KWN0

For more information about MultiQC, including other videos and
extensive documentation, please visit http://multiqc.info

You can report bugs, suggest improvements and find the source code for MultiQC on GitHub:
https://github.com/ewels/MultiQC

MultiQC is published in Bioinformatics:

> **MultiQC: Summarize analysis results for multiple tools and samples in a single report**  
> *Philip Ewels, Måns Magnusson, Sverker Lundin and Max Käller*  
> Bioinformatics (2016)  
> doi: 10.1093/bioinformatics/btw354  
> PMID: 27312411

# 

A modular tool to aggregate results from bioinformatics analyses across many samples into a single report.

> This report has been generated by the nf-core/chipseq analysis pipeline. For information about how to interpret these results, please see the documentation.

#### JavaScript Disabled

MultiQC reports use JavaScript for plots and toolbox functions. It looks like
you have JavaScript disabled in your web browser. Please note that many of the report
functions will not work as intended.

Loading report..

Report
generated on 2022-03-20, 16:45
based on data in:
`/media/rc/My_Book_Duo/nf_core_chipseq/files/work/ec/e3d7626a1f84dbd2f7ecfe8fe6f45a`

---

×
don't show again

**Welcome!** Not sure where to start?  
Watch a tutorial video
  *(6:06)*

## LIB: FastQC (raw)

LIB: FastQC (raw) This section of the report shows FastQC results before adapter trimming for individual libraries.

### Sequence Counts Help

Sequence counts for each sample. Duplicate read counts are an estimate only.

This plot show the total number of reads, broken down into unique and duplicate
if possible (only more recent versions of FastQC give duplicate info).

You can read more about duplicate calculation in the
FastQC documentation.
A small part has been copied here for convenience:

*Only sequences which first appear in the first 100,000 sequences
in each file are analysed. This should be enough to get a good impression
for the duplication levels in the whole file. Each sequence is tracked to
the end of the file to give a representative count of the overall duplication level.*

*The duplication detection requires an exact sequence match over the whole length of
the sequence. Any reads over 75bp in length are truncated to 50bp for this analysis.*

Flat image plot. Toolbox functions such as highlighting / hiding samples will not work (see the docs).

Number of reads
Percentages

---

### Sequence Quality Histograms Help

The mean quality value across each base position in the read.

To enable multiple samples to be plotted on the same graph, only the mean quality
scores are plotted (unlike the box plots seen in FastQC reports).

Taken from the FastQC help:

*The y-axis on the graph shows the quality scores. The higher the score, the better
the base call. The background of the graph divides the y axis into very good quality
calls (green), calls of reasonable quality (orange), and calls of poor quality (red).
The quality of calls on most platforms will degrade as the run progresses, so it is
common to see base calls falling into the orange area towards the end of a read.*

Flat image plot. Toolbox functions such as highlighting / hiding samples will not work (see the docs).

---

### Per Sequence Quality Scores Help

The number of reads with average quality scores. Shows if a subset of reads has poor quality.

From the FastQC help:

*The per sequence quality score report allows you to see if a subset of your
sequences have universally low quality values. It is often the case that a
subset of sequences will have universally poor quality, however these should
represent only a small percentage of the total sequences.*

Flat image plot. Toolbox functions such as highlighting / hiding samples will not work (see the docs).

---

### Per Base Sequence Content Help

The proportion of each base position for which each of the four normal DNA bases has been called.

To enable multiple samples to be shown in a single plot, the base composition data
is shown as a heatmap. The colours represent the balance between the four bases:
an even distribution should give an even muddy brown colour. Hover over the plot
to see the percentage of the four bases under the cursor.

**To see the data as a line plot, as in the original FastQC graph, click on a sample track.**

From the FastQC help:

*Per Base Sequence Content plots out the proportion of each base position in a
file for which each of the four normal DNA bases has been called.*

*In a random library you would expect that there would be little to no difference
between the different bases of a sequence run, so the lines in this plot should
run parallel with each other. The relative amount of each base should reflect
the overall amount of these bases in your genome, but in any case they should
not be hugely imbalanced from each other.*

*It's worth noting that some types of library will always produce biased sequence
composition, normally at the start of the read. Libraries produced by priming
using random hexamers (including nearly all RNA-Seq libraries) and those which
were fragmented using transposases inherit an intrinsic bias in the positions
at which reads start. This bias does not concern an absolute sequence, but instead
provides enrichement of a number of different K-mers at the 5' end of the reads.
Whilst this is a true technical bias, it isn't something which can be corrected
by trimming and in most cases doesn't seem to adversely affect the downstream
analysis.*

Click a sample row to see a line plot for that dataset.

##### Rollover for sample name

 Export Plot

Position: -

%T: -

%C: -

%A: -

%G: -

---

### Per Sequence GC Content Help

The average GC content of reads. Normal random library typically have a
roughly normal distribution of GC content.

From the FastQC help:

*This module measures the GC content across the whole length of each sequence
in a file and compares it to a modelled normal distribution of GC content.*

*In a normal random library you would expect to see a roughly normal distribution
of GC content where the central peak corresponds to the overall GC content of
the underlying genome. Since we don't know the the GC content of the genome the
modal GC content is calculated from the observed data and used to build a
reference distribution.*

*An unusually shaped distribution could indicate a contaminated library or
some other kinds of biased subset. A normal distribution which is shifted
indicates some systematic bias which is independent of base position. If there
is a systematic bias which creates a shifted normal distribution then this won't
be flagged as an error by the module since it doesn't know what your genome's
GC content should be.*

Flat image plot. Toolbox functions such as highlighting / hiding samples will not work (see the docs).

Percentages
Counts

---

### Per Base N Content Help

The percentage of base calls at each position for which an `N` was called.

From the FastQC help:

*If a sequencer is unable to make a base call with sufficient confidence then it will
normally substitute an `N` rather than a conventional base call. This graph shows the
percentage of base calls at each position for which an `N` was called.*

*It's not unusual to see a very low proportion of Ns appearing in a sequence, especially
nearer the end of a sequence. However, if this proportion rises above a few percent
it suggests that the analysis pipeline was unable to interpret the data well enough to
make valid base calls.*

Flat image plot. Toolbox functions such as highlighting / hiding samples will not work (see the docs).

---

### Sequence Length Distribution

The distribution of fragment sizes (read lengths) found.
See the FastQC help

Flat image plot. Toolbox functions such as highlighting / hiding samples will not work (see the docs).

---

### Sequence Duplication Levels Help

The relative level of duplication found for every sequence.

From the FastQC Help:

*In a diverse library most sequences will occur only once in the final set.
A low level of duplication may indicate a very high level of coverage of the
target sequence, but a high level of duplication is more likely to indicate
some kind of enrichment bias (eg PCR over amplification). This graph shows
the degree of duplication for every sequence in a library: the relative
number of sequences with different degrees of duplication.*

*Only sequences which first appear in the first 100,000 sequences
in each file are analysed. This should be enough to get a good impression
for the duplication levels in the whole file. Each sequence is tracked to
the end of the file to give a representative count of the overall duplication level.*

*The duplication detection requires an exact sequence match over the whole length of
the sequence. Any reads over 75bp in length are truncated to 50bp for this analysis.*

*In a properly diverse library most sequences should fall into the far left of the
plot in both the red and blue lines. A general level of enrichment, indicating broad
oversequencing in the library will tend to flatten the lines, lowering the low end
and generally raising other categories. More specific enrichments of subsets, or
the presence of low complexity contaminants will tend to produce spikes towards the
right of the plot.*

Flat image plot. Toolbox functions such as highlighting / hiding samples will not work (see the docs).

---

### Overrepresented sequences Help

The total amount of overrepresented sequences found in each library.

FastQC calculates and lists overrepresented sequences in FastQ files. It would not be
possible to show this for all samples in a MultiQC report, so instead this plot shows
the *number of sequences* categorized as over represented.

Sometimes, a single sequence may account for a large number of reads in a dataset.
To show this, the bars are split into two: the first shows the overrepresented reads
that come from the single most common sequence. The second shows the total count
from all remaining overrepresented sequences.

From the FastQC Help:

*A normal high-throughput library will contain a diverse set of sequences, with no
individual sequence making up a tiny fraction of the whole. Finding that a single
sequence is very overrepresented in the set either means that it is highly biologically
significant, or indicates that the library is contaminated, or not as diverse as you expected.*

*FastQC lists all of the sequences which make up more than 0.1% of the total.
To conserve memory only sequences which appear in the first 100,000 sequences are tracked
to the end of the file. It is therefore possible that a sequence which is overrepresented
but doesn't appear at the start of the file for some reason could be missed by this module.*

Flat image plot. Toolbox functions such as highlighting / hiding samples will not work (see the docs).

---

### Adapter Content Help

The cumulative percentage count of the proportion of your
library which has seen each of the adapter sequences at each position.

Note that only samples with ≥ 0.1% adapter contamination are shown.

There may be several lines per sample, as one is shown for each adapter
detected in the file.

From the FastQC Help:

*The plot shows a cumulative percentage count of the proportion
of your library which has seen each of the adapter sequences at each position.
Once a sequence has been seen in a read it is counted as being present
right through to the end of the read so the percentages you see will only
increase as the read length goes on.*

No samples found with any adapter contamination > 0.1%

---

### Status Checks Help

Status for each FastQC section showing whether results seem entirely normal (green),
slightly abnormal (orange) or very unusual (red).

FastQC assigns a status for each section of the report.
These give a quick evaluation of whether the results of the analysis seem
entirely normal (green), slightly abnormal (orange) or very unusual (red).

It is important to stress that although the analysis results appear to give a pass/fail result,
these evaluations must be taken in the context of what you expect from your library.
A 'normal' sample as far as FastQC is concerned is random and diverse.
Some experiments may be expected to produce libraries which are biased in particular ways.
You should treat the summary evaluations therefore as pointers to where you should concentrate
your attention and understand why your library may not look random and diverse.

Specific guidance on how to interpret the output of each module can be found in the relevant
report section, or in the FastQC help.

In this heatmap, we summarise all of these into a single heatmap for a quick overview.
Note that not all FastQC sections have plots in MultiQC reports, but all status checks
are shown in this heatmap.

Sort by highlight

loading..

---

## LIB: cutadapt (trimmed)

LIB: cutadapt (trimmed) This section of the report shows the length of trimmed reads by cutadapt for individual libraries.

### Filtered Reads

This plot shows the number of reads (SE) / pairs (PE) removed by Cutadapt.

Flat image plot. Toolbox functions such as highlighting / hiding samples will not work (see the docs).

Counts
Percentages

---

### Trimmed Sequence Lengths Help

This plot shows the number of reads with certain lengths of adapter trimmed.

Obs/Exp shows the raw counts divided by the number expected due to sequencing errors.
A defined peak may be related to adapter length.

See the cutadapt documentation
for more information on how these numbers are generated.

Flat image plot. Toolbox functions such as highlighting / hiding samples will not work (see the docs).

Counts
Obs/Exp

---

## LIB: FastQC (trimmed)

LIB: FastQC (trimmed) This section of the report shows FastQC results after adapter trimming for individual libraries.

### Sequence Counts Help

Sequence counts for each sample. Duplicate read counts are an estimate only.

This plot show the total number of reads, broken down into unique and duplicate
if possible (only more recent versions of FastQC give duplicate info).

You can read more about duplicate calculation in the
FastQC documentation.
A small part has been copied here for convenience:

*Only sequences which first appear in the first 100,000 sequences
in each file are analysed. This should be enough to get a good impression
for the duplication levels in the whole file. Each sequence is tracked to
the end of the file to give a representative count of the overall duplication level.*

*The duplication detection requires an exact sequence match over the whole length of
the sequence. Any reads over 75bp in length are truncated to 50bp for this analysis.*

Flat image plot. Toolbox functions such as highlighting / hiding samples will not work (see the docs).

Number of reads
Percentages

---

### Sequence Quality Histograms Help

The mean quality value across each base position in the read.

To enable multiple samples to be plotted on the same graph, only the mean quality
scores are plotted (unlike the box plots seen in FastQC reports).

Taken from the FastQC help:

*The y-axis on the graph shows the quality scores. The higher the score, the better
the base call. The background of the graph divides the y axis into very good quality
calls (green), calls of reasonable quality (orange), and calls of poor quality (red).
The quality of calls on most platforms will degrade as the run progresses, so it is
common to see base calls falling into the orange area towards the end of a read.*

Flat image plot. Toolbox functions such as highlighting / hiding samples will not work (see the docs).

---

### Per Sequence Quality Scores Help

The number of reads with average quality scores. Shows if a subset of reads has poor quality.

From the FastQC help:

*The per sequence quality score report allows you to see if a subset of your
sequences have universally low quality values. It is often the case that a
subset of sequences will have universally poor quality, however these should
represent only a small percentage of the total sequences.*

Flat image plot. Toolbox functions such as highlighting / hiding samples will not work (see the docs).

---

### Per Base Sequence Content Help

The proportion of each base position for which each of the four normal DNA bases has been called.

To enable multiple samples to be shown in a single plot, the base composition data
is shown as a heatmap. The colours represent the balance between the four bases:
an even distribution should give an even muddy brown colour. Hover over the plot
to see the percentage of the four bases under the cursor.

**To see the data as a line plot, as in the original FastQC graph, click on a sample track.**

From the FastQC help:

*Per Base Sequence Content plots out the proportion of each base position in a
file for which each of the four normal DNA bases has been called.*

*In a random library you would expect that there would be little to no difference
between the different bases of a sequence run, so the lines in this plot should
run parallel with each other. The relative amount of each base should reflect
the overall amount of these bases in your genome, but in any case they should
not be hugely imbalanced from each other.*

*It's worth noting that some types of library will always produce biased sequence
composition, normally at the start of the read. Libraries produced by priming
using random hexamers (including nearly all RNA-Seq libraries) and those which
were fragmented using transposases inherit an intrinsic bias in the positions
at which reads start. This bias does not concern an absolute sequence, but instead
provides enrichement of a number of different K-mers at the 5' end of the reads.
Whilst this is a true technical bias, it isn't something which can be corrected
by trimming and in most cases doesn't seem to adversely affect the downstream
analysis.*

Click a sample row to see a line plot for that dataset.

##### Rollover for sample name

 Export Plot

Position: -

%T: -

%C: -

%A: -

%G: -

---

### Per Sequence GC Content Help

The average GC content of reads. Normal random library typically have a
roughly normal distribution of GC content.

From the FastQC help:

*This module measures the GC content across the whole length of each sequence
in a file and compares it to a modelled normal distribution of GC content.*

*In a normal random library you would expect to see a roughly normal distribution
of GC content where the central peak corresponds to the overall GC content of
the underlying genome. Since we don't know the the GC content of the genome the
modal GC content is calculated from the observed data and used to build a
reference distribution.*

*An unusually shaped distribution could indicate a contaminated library or
some other kinds of biased subset. A normal distribution which is shifted
indicates some systematic bias which is independent of base position. If there
is a systematic bias which creates a shifted normal distribution then this won't
be flagged as an error by the module since it doesn't know what your genome's
GC content should be.*

Flat image plot. Toolbox functions such as highlighting / hiding samples will not work (see the docs).

Percentages
Counts

---

### Per Base N Content Help

The percentage of base calls at each position for which an `N` was called.

From the FastQC help:

*If a sequencer is unable to make a base call with sufficient confidence then it will
normally substitute an `N` rather than a conventional base call. This graph shows the
percentage of base calls at each position for which an `N` was called.*

*It's not unusual to see a very low proportion of Ns appearing in a sequence, especially
nearer the end of a sequence. However, if this proportion rises above a few percent
it suggests that the analysis pipeline was unable to interpret the data well enough to
make valid base calls.*

Flat image plot. Toolbox functions such as highlighting / hiding samples will not work (see the docs).

---

### Sequence Length Distribution

The distribution of fragment sizes (read lengths) found.
See the FastQC help

Flat image plot. Toolbox functions such as highlighting / hiding samples will not work (see the docs).

---

### Sequence Duplication Levels Help

The relative level of duplication found for every sequence.

From the FastQC Help:

*In a diverse library most sequences will occur only once in the final set.
A low level of duplication may indicate a very high level of coverage of the
target sequence, but a high level of duplication is more likely to indicate
some kind of enrichment bias (eg PCR over amplification). This graph shows
the degree of duplication for every sequence in a library: the relative
number of sequences with different degrees of duplication.*

*Only sequences which first appear in the first 100,000 sequences
in each file are analysed. This should be enough to get a good impression
for the duplication levels in the whole file. Each sequence is tracked to
the end of the file to give a representative count of the overall duplication level.*

*The duplication detection requires an exact sequence match over the whole length of
the sequence. Any reads over 75bp in length are truncated to 50bp for this analysis.*

*In a properly diverse library most sequences should fall into the far left of the
plot in both the red and blue lines. A general level of enrichment, indicating broad
oversequencing in the library will tend to flatten the lines, lowering the low end
and generally raising other categories. More specific enrichments of subsets, or
the presence of low complexity contaminants will tend to produce spikes towards the
right of the plot.*

Flat image plot. Toolbox functions such as highlighting / hiding samples will not work (see the docs).

---

### Overrepresented sequences Help

The total amount of overrepresented sequences found in each library.

FastQC calculates and lists overrepresented sequences in FastQ files. It would not be
possible to show this for all samples in a MultiQC report, so instead this plot shows
the *number of sequences* categorized as over represented.

Sometimes, a single sequence may account for a large number of reads in a dataset.
To show this, the bars are split into two: the first shows the overrepresented reads
that come from the single most common sequence. The second shows the total count
from all remaining overrepresented sequences.

From the FastQC Help:

*A normal high-throughput library will contain a diverse set of sequences, with no
individual sequence making up a tiny fraction of the whole. Finding that a single
sequence is very overrepresented in the set either means that it is highly biologically
significant, or indicates that the library is contaminated, or not as diverse as you expected.*

*FastQC lists all of the sequences which make up more than 0.1% of the total.
To conserve memory only sequences which appear in the first 100,000 sequences are tracked
to the end of the file. It is therefore possible that a sequence which is overrepresented
but doesn't appear at the start of the file for some reason could be missed by this module.*

660 samples had less than 1% of reads made up of overrepresented sequences

---

### Adapter Content Help

The cumulative percentage count of the proportion of your
library which has seen each of the adapter sequences at each position.

Note that only samples with ≥ 0.1% adapter contamination are shown.

There may be several lines per sample, as one is shown for each adapter
detected in the file.

From the FastQC Help:

*The plot shows a cumulative percentage count of the proportion
of your library which has seen each of the adapter sequences at each position.
Once a sequence has been seen in a read it is counted as being present
right through to the end of the read so the percentages you see will only
increase as the read length goes on.*

No samples found with any adapter contamination > 0.1%

---

### Status Checks Help

Status for each FastQC section showing whether results seem entirely normal (green),
slightly abnormal (orange) or very unusual (red).

FastQC assigns a status for each section of the report.
These give a quick evaluation of whether the results of the analysis seem
entirely normal (green), slightly abnormal (orange) or very unusual (red).

It is important to stress that although the analysis results appear to give a pass/fail result,
these evaluations must be taken in the context of what you expect from your library.
A 'normal' sample as far as FastQC is concerned is random and diverse.
Some experiments may be expected to produce libraries which are biased in particular ways.
You should treat the summary evaluations therefore as pointers to where you should concentrate
your attention and understand why your library may not look random and diverse.

Specific guidance on how to interpret the output of each module can be found in the relevant
report section, or in the FastQC help.

In this heatmap, we summarise all of these into a single heatmap for a quick overview.
Note that not all FastQC sections have plots in MultiQC reports, but all status checks
are shown in this heatmap.

Sort by highlight

loading..

---

## LIB: SAMTools

Samtools This section of the report shows SAMTools results for individual libraries.

### Percent Mapped Help

Alignment metrics from `samtools stats`; mapped vs. unmapped reads.

For a set of samples that have come from the same multiplexed library,
similar numbers of reads for each sample are expected. Large differences in numbers might
indicate issues during the library preparation process. Whilst large differences in read
numbers may be controlled for in downstream processings (e.g. read count normalisation),
you may wish to consider whether the read depths achieved have fallen below recommended
levels depending on the applications.

Low alignment rates could indicate contamination of samples (e.g. adapter sequences),
low sequencing quality or other artefacts. These can be further investigated in the
sequence level QC (e.g. from FastQC).

Flat image plot. Toolbox functions such as highlighting / hiding samples will not work (see the docs).

Number of Reads
Percentages

---

### Alignment metrics

This module parses the output from `samtools stats`. All numbers in millions.

loading..

---

### Samtools Flagstat

This module parses the output from `samtools flagstat`. All numbers in millions.

loading..

---

### XY counts

Flat image plot. Toolbox functions such as highlighting / hiding samples will not work (see the docs).

Number of Reads
Percent of X+Y Reads

---

### Mapped reads per contig

The `samtools idxstats` tool counts the number of mapped reads per chromosome / contig. Chromosomes with < 0.1% of the total aligned reads are omitted from this plot.

Flat image plot. Toolbox functions such as highlighting / hiding samples will not work (see the docs).

Normalised Counts
Observed over Expected Counts
Raw Counts

---

## MERGED LIB: SAMTools (unfiltered)

Samtools This section of the report shows SAMTools results after merging libraries and before filtering.

### Percent Mapped Help

Alignment metrics from `samtools stats`; mapped vs. unmapped reads.

For a set of samples that have come from the same multiplexed library,
similar numbers of reads for each sample are expected. Large differences in numbers might
indicate issues during the library preparation process. Whilst large differences in read
numbers may be controlled for in downstream processings (e.g. read count normalisation),
you may wish to consider whether the read depths achieved have fallen below recommended
levels depending on the applications.

Low alignment rates could indicate contamination of samples (e.g. adapter sequences),
low sequencing quality or other artefacts. These can be further investigated in the
sequence level QC (e.g. from FastQC).

Flat image plot. Toolbox functions such as highlighting / hiding samples will not work (see the docs).

Number of Reads
Percentages

---

### Alignment metrics

This module parses the output from `samtools stats`. All numbers in millions.

loading..

---

### Samtools Flagstat

This module parses the output from `samtools flagstat`. All numbers in millions.

loading..

---

### XY counts

Flat image plot. Toolbox functions such as highlighting / hiding samples will not work (see the docs).

Number of Reads
Percent of X+Y Reads

---

### Mapped reads per contig

The `samtools idxstats` tool counts the number of mapped reads per chromosome / contig. Chromosomes with < 0.1% of the total aligned reads are omitted from this plot.

Flat image plot. Toolbox functions such as highlighting / hiding samples will not work (see the docs).

Normalised Counts
Observed over Expected Counts
Raw Counts

---

## MERGED LIB: Preseq (unfiltered)

MERGED LIB: Preseq (unfiltered) This section of the report shows Preseq results after merging libraries and before filtering.

### Complexity curve

Note that the x axis is trimmed at the point where all the datasets show 80% of their maximum y-value, to avoid ridiculous scales.

Flat image plot. Toolbox functions such as highlighting / hiding samples will not work (see the docs).

---

## MERGED LIB: SAMTools (filtered)

Samtools This section of the report shows SAMTools results after merging libraries and after filtering.

### Percent Mapped Help

Alignment metrics from `samtools stats`; mapped vs. unmapped reads.

For a set of samples that have come from the same multiplexed library,
similar numbers of reads for each sample are expected. Large differences in numbers might
indicate issues during the library preparation process. Whilst large differences in read
numbers may be controlled for in downstream processings (e.g. read count normalisation),
you may wish to consider whether the read depths achieved have fallen below recommended
levels depending on the applications.

Low alignment rates could indicate contamination of samples (e.g. adapter sequences),
low sequencing quality or other artefacts. These can be further investigated in the
sequence level QC (e.g. from FastQC).

Flat image plot. Toolbox functions such as highlighting / hiding samples will not work (see the docs).

Number of Reads
Percentages

---

### Alignment metrics

This module parses the output from `samtools stats`. All numbers in millions.

loading..

---

### Samtools Flagstat

This module parses the output from `samtools flagstat`. All numbers in millions.

loading..

---

### XY counts

Flat image plot. Toolbox functions such as highlighting / hiding samples will not work (see the docs).

Number of Reads
Percent of X+Y Reads

---

### Mapped reads per contig

The `samtools idxstats` tool counts the number of mapped reads per chromosome / contig. Chromosomes with < 0.1% of the total aligned reads are omitted from this plot.

Flat image plot. Toolbox functions such as highlighting / hiding samples will not work (see the docs).

Normalised Counts
Observed over Expected Counts
Raw Counts

---

## MERGED LIB: Picard

MERGED LIB: Picard This section of the report shows picard results after merging libraries and after filtering.

### Alignment Summary

Please note that Picard's read counts are divided by two for paired-end data.

Flat image plot. Toolbox functions such as highlighting / hiding samples will not work (see the docs).

Number of Reads
Percentages

---

### Base Distribution

Plot shows the distribution of bases by cycle.

Flat image plot. Toolbox functions such as highlighting / hiding samples will not work (see the docs).

% Adenine
% Cytosine
% Guanine
% Thymine
% Undetermined

---

### Insert Size

Plot shows the number of reads at a given insert size. Reads with different orientations are summed.

Flat image plot. Toolbox functions such as highlighting / hiding samples will not work (see the docs).

Counts
Percentages

---

### Mark Duplicates Help

Number of reads, categorised by duplication state. **Pair counts are doubled** - see help text for details.

The table in the Picard metrics file contains some columns referring
read pairs and some referring to single reads.

To make the numbers in this plot sum correctly, values referring to pairs are doubled
according to the scheme below:

- `READS_IN_DUPLICATE_PAIRS = 2 * READ_PAIR_DUPLICATES`
- `READS_IN_UNIQUE_PAIRS = 2 * (READ_PAIRS_EXAMINED - READ_PAIR_DUPLICATES)`
- `READS_IN_UNIQUE_UNPAIRED = UNPAIRED_READS_EXAMINED - UNPAIRED_READ_DUPLICATES`
- `READS_IN_DUPLICATE_PAIRS_OPTICAL = 2 * READ_PAIR_OPTICAL_DUPLICATES`
- `READS_IN_DUPLICATE_PAIRS_NONOPTICAL = READS_IN_DUPLICATE_PAIRS - READS_IN_DUPLICATE_PAIRS_OPTICAL`
- `READS_IN_DUPLICATE_UNPAIRED = UNPAIRED_READ_DUPLICATES`
- `READS_UNMAPPED = UNMAPPED_READS`

Flat image plot. Toolbox functions such as highlighting / hiding samples will not work (see the docs).

Number of Reads
Percentages

---

### Mean Base Quality by Cycle Help

Plot shows the mean base quality by cycle.

This metric gives an overall snapshot of sequencing machine performance.
For most types of sequencing data, the output is expected to show a slight
reduction in overall base quality scores towards the end of each read.

Spikes in quality within reads are not expected and may indicate that technical
problems occurred during sequencing.

Flat image plot. Toolbox functions such as highlighting / hiding samples will not work (see the docs).

---

### Base Quality Distribution

Plot shows the count of each base quality score.

Flat image plot. Toolbox functions such as highlighting / hiding samples will not work (see the docs).

---

## MERGED LIB: deepTools

deepTools This section of the report shows ChIP-seq QC plots generated by deepTools.

### Fingerprint plot

Signal fingerprint according to plotFingerprint

Flat image plot. Toolbox functions such as highlighting / hiding samples will not work (see the docs).

---

### Read Distribution Profile after Annotation

Accumulated view of the distribution of sequence reads related to the closest annotated gene.
All annotated genes have been normalized to the same size.

- Green: -3.0Kb upstream of gene to TSS
- Yellow: TSS to TES
- Pink: TES to 3.0Kb downstream of gene

Flat image plot. Toolbox functions such as highlighting / hiding samples will not work (see the docs).

---

## MERGED LIB: MACS2 peak count

is calculated from total number of peaks called by MACS2

Flat image plot. Toolbox functions such as highlighting / hiding samples will not work (see the docs).

Counts
Percentages

---

## MERGED LIB: MACS2 FRiP score

is generated by calculating the fraction of all mapped reads that fall into the MACS2 called peak regions. A read must overlap a peak by at least 20% to be counted. See FRiP score.

Flat image plot. Toolbox functions such as highlighting / hiding samples will not work (see the docs).

Counts
Percentages

---

## MERGED LIB: HOMER peak annotation

is generated by calculating the proportion of peaks assigned to genomic features by HOMER annotatePeaks.pl.

Flat image plot. Toolbox functions such as highlighting / hiding samples will not work (see the docs).

Counts
Percentages

---

## MERGED LIB: spp strand-shift correlation

generated using run\_spp.R script from phantompeakqualtools.

Flat image plot. Toolbox functions such as highlighting / hiding samples will not work (see the docs).

---

## MERGED LIB: spp NSC coefficient

generated using run\_spp.R script from phantompeakqualtools.

Flat image plot. Toolbox functions such as highlighting / hiding samples will not work (see the docs).

Counts
Percentages

---

## MERGED LIB: spp RSC coefficient

generated using run\_spp.R script from phantompeakqualtools.

Flat image plot. Toolbox functions such as highlighting / hiding samples will not work (see the docs).

Counts
Percentages

---

## MERGED LIB: featureCounts

Subread featureCounts This section of the report shows featureCounts results for the number of reads assigned to merged library consensus peaks.

Flat image plot. Toolbox functions such as highlighting / hiding samples will not work (see the docs).

Number of Reads
Percentages

---

## nf-core/chipseq Software Versions

are collected at run time from the software output.

nf-core/chipseq
:   `v1.2.2`

Nextflow
:   `v21.10.6`

FastQC
:   `v0.11.9`

Trim Galore!
:   `v0.6.4_dev`

BWA
:   `v0.7.17-r1188`

Samtools
:   `v1.10`

BEDTools
:   `v2.29.2`

BamTools
:   `v2.5.1`

deepTools
:   `v3.4.3`

Picard
:   `v2.23.1`

R
:   `v3.6.3`

Pysam
:   `v0.15.3`

MACS2
:   `v2.2.7.1`

featureCounts
:   `v2.0.1`

Preseq
:   `v2.0.3`

MultiQC
:   `v1.9`

---

## nf-core/chipseq Workflow Summary

- this information is collected when the pipeline is started.

Run Name
:   `soggy_mendel`

Data Type
:   `Paired-End`

Design File
:   `/media/rc/My_Book_Duo/nf_core_chipseq/files/all_nf_core_ChIP_seq_sample_info.csv`

Genome
:   `GRCh38`

Fasta File
:   `/home/rc/.nextflow/igenomes/Homo_sapiens/NCBI/GRCh38/Sequence/WholeGenomeFasta/genome.fa`

GTF File
:   `/home/rc/.nextflow/igenomes/Homo_sapiens/NCBI/GRCh38/Annotation/Genes/genes.gtf`

Gene BED File
:   `/home/rc/.nextflow/igenomes/Homo_sapiens/NCBI/GRCh38/Annotation/Genes/genes.bed`

BWA Index
:   `/home/rc/.nextflow/igenomes/Homo_sapiens/NCBI/GRCh38/Sequence/BWAIndex/genome.fa`

Blacklist BED
:   `/home/rc/.nextflow/assets/nf-core/chipseq/assets/blacklists/hg38-blacklist.bed`

MACS2 Genome Size
:   `2.7e9`

Min Consensus Reps
:   `1`

MACS2 Narrow Peaks
:   `No`

MACS2 Broad Cutoff
:   `0.1`

Trim R1
:   `0 bp`

Trim R2
:   `0 bp`

Trim 3' R1
:   `0 bp`

Trim 3' R2
:   `0 bp`

NextSeq Trim
:   `0 bp`

Fingerprint Bins
:   `500000`

Save Genome Index
:   `No`

Max Resources
:   `750 GB memory, 46 cpus, 10d time per job`

Container
:   `singularity - nfcore/chipseq:1.2.2`

Output Dir
:   `/media/rc/My_Book_Duo/nf_core_chipseq/all_result2`

Launch Dir
:   `/media/rc/My_Book_Duo/nf_core_chipseq/files`

Working Dir
:   `/media/rc/My_Book_Duo/nf_core_chipseq/files/work`

Script Dir
:   `/home/rc/.nextflow/assets/nf-core/chipseq`

User
:   `rc`

Config Profile
:   `singularity`

**MultiQC v1.9**
- Written by Phil Ewels,
available on GitHub.

This report uses HighCharts,
jQuery,
jQuery UI,
Bootstrap,
FileSaver.js and
clipboard.js.

×

### Plot Table Data

Select Column

Select Column

Please select two table columns.

Close

×

### Regex Help

Toolbox search strings can behave as regular expressions (regexes). Click a button below to see an example of it in action. Try modifying them yourself in the text box.

`^` (start of string)
`$` (end of string)
`[]` (character choice)
`\d` (shorthand for `[0-9]`)
`\w` (shorthand for `[0-9a-zA-Z_]`)
`.` (any character)
`\.` (literal full stop)
`()` `|` (group / separator)
`*` (prev char 0 or more)
`+` (prev char 1 or more)
`?` (prev char 0 or 1)
`{}` (char num times)
`{,}` (count range)

```
samp_1
samp_1_edited
samp_2
samp_2_edited
samp_3
samp_3_edited
prepended_samp_1
tmp_samp_1_edited
tmpp_samp_1_edited
tmppp_samp_1_edited
#samp_1_edited.tmp
samp_11
samp_11111
```

See regex101.com for a more heavy duty testing suite.

Close
