# Supplementary material for: Mechanism of ERBB2 gene overexpression by the formation of super-enhancer with genomic structural abnormalities in lung adenocarcinoma without clinically actionable genetic alterations
Source: Mol Cancer. 2024 Jun 11;23:126. doi: 10.1186/s12943-024-02035-6 (PMC11165761; doi:10.1186/s12943-024-02035-6)
Supplement: Supplementary file 11 — Additional file 11. The uncropped images include uncropped immunoblot data shown in Fig. S16C. [file 12943_2024_2035_MOESM11_ESM.docx]

**Molecular Cancer *(Research*** ***Articles)***

**Supplementary information**

**Mechanism of *ERBB2* gene overexpression by the formation of super-enhancer with genomic structural abnormalities in lung adenocarcinoma without clinically actionable genetic alterations**

Syuzo Kaneko et al.

**It includes the uncropped images.**

Uncropped immunoblot data shown in Fig. S16C.
