# Supplementary material for: Mechanism of ERBB2 gene overexpression by the formation of super-enhancer with genomic structural abnormalities in lung adenocarcinoma without clinically actionable genetic alterations
Source: Mol Cancer. 2024 Jun 11;23:126. doi: 10.1186/s12943-024-02035-6 (PMC11165761; doi:10.1186/s12943-024-02035-6)
Supplement: Supplementary file 10 — Additional file 10. Provides Dataset S8 for the results of the peak-to-gene links analysis showing a scatter plot of the top 1,000 expression values and peak values, their correlation coefficient, and the null distribution of the correlation coefficient. [file 12943_2024_2035_MOESM10_ESM.pdf]

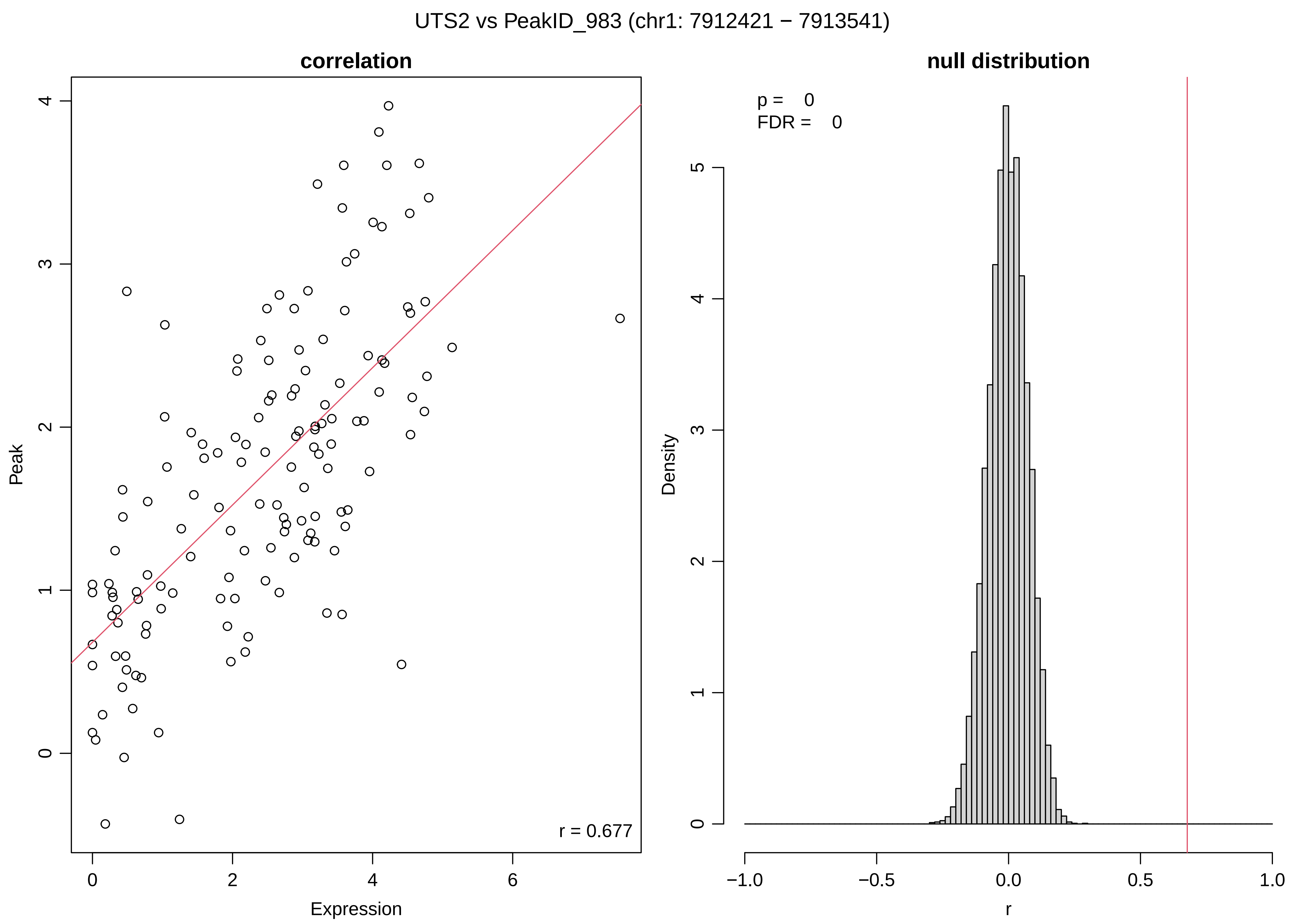
UTS2 vs PeakID_983 (chr1: 7912421 − 7913541)
correlation
Peak
Expression
r = 0.677
null distribution
p = 0
FDR = 0
Density
r

DMRTA2 vs PeakID_6850 (chr1: 50422496 − 50424279)

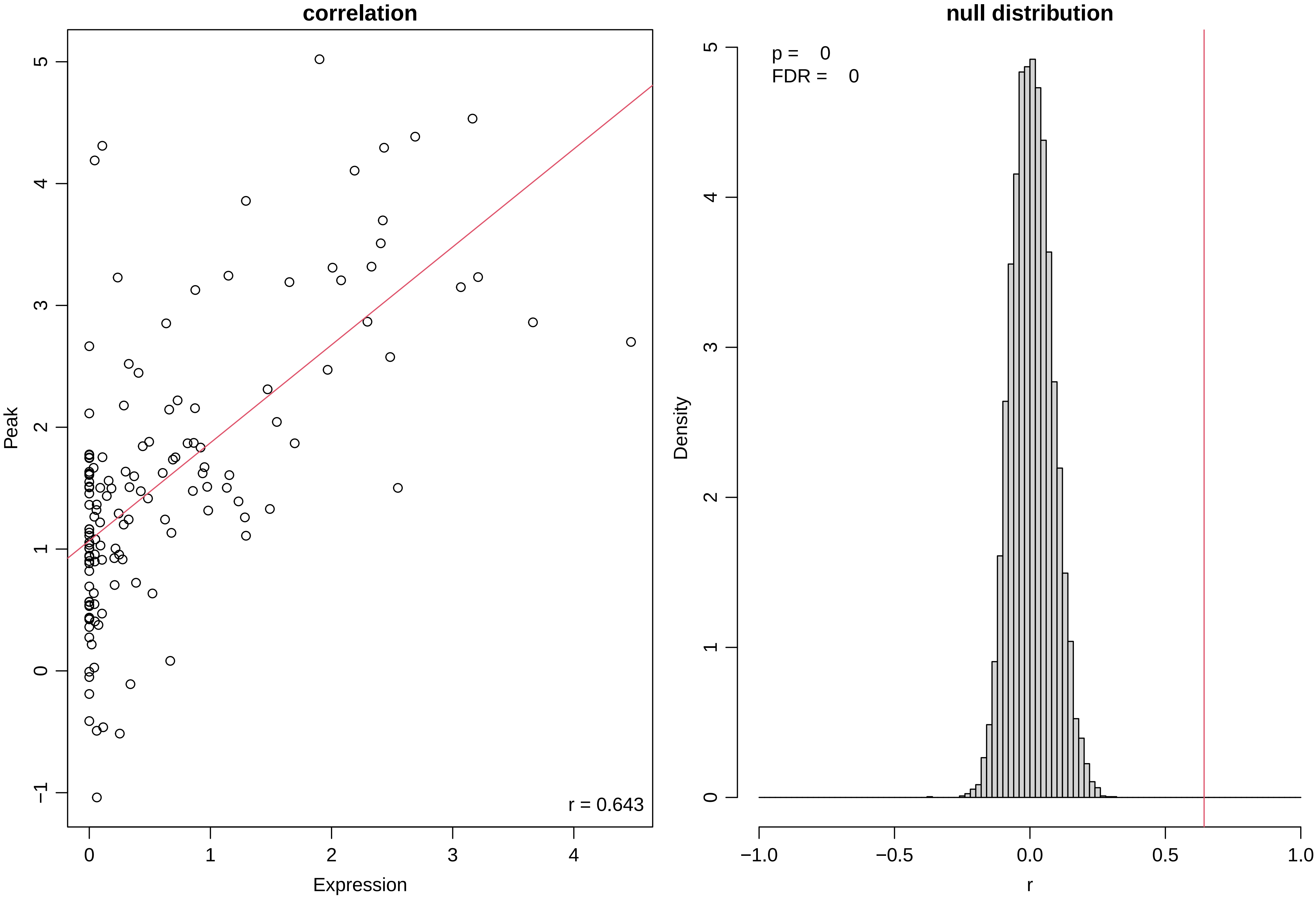

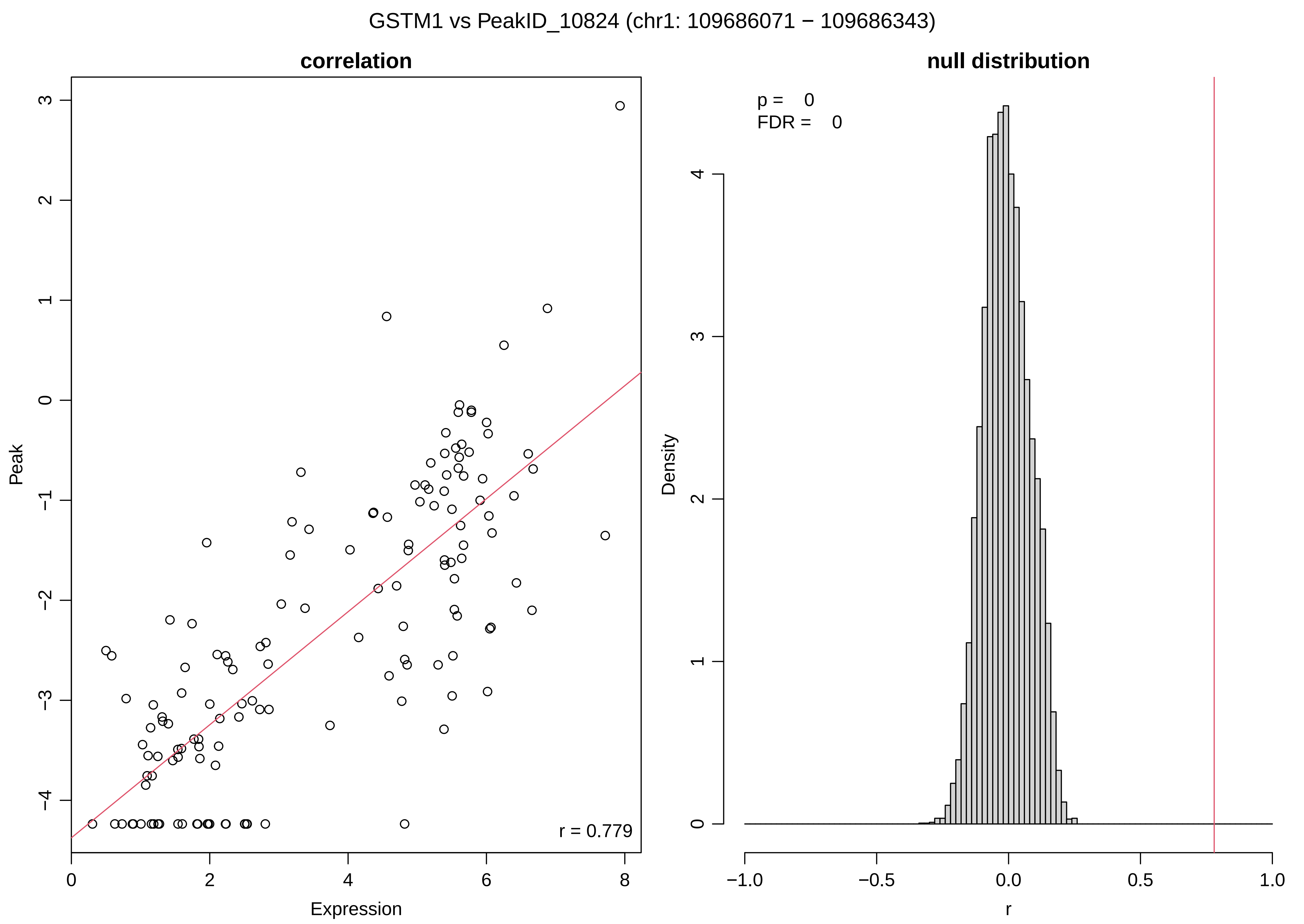
GSTM1 vs PeakID_10824 (chr1: 109686071 − 109686343)
correlation
Peak
Expression
r = 0.779
null distribution
p = 0
FDR = 0
Density
r

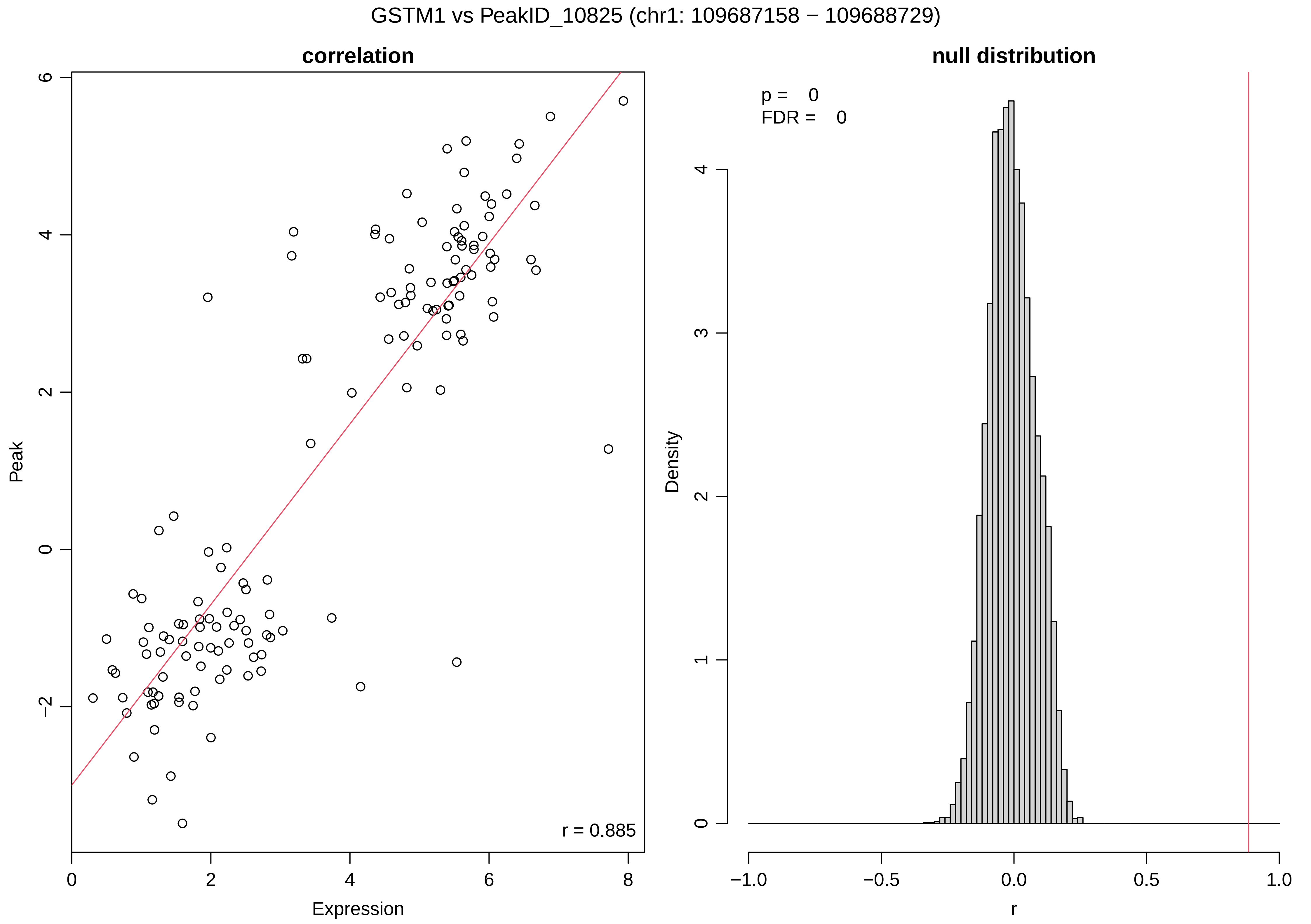
GSTM1 vs PeakID_10825 (chr1: 109687158 − 109688729)
correlation
Peak
Expression
r = 0.885
null distribution
p = 0
FDR = 0
Density
r

C5orf17 vs PeakID_188769 (chr5: 23951040 − 23952588)

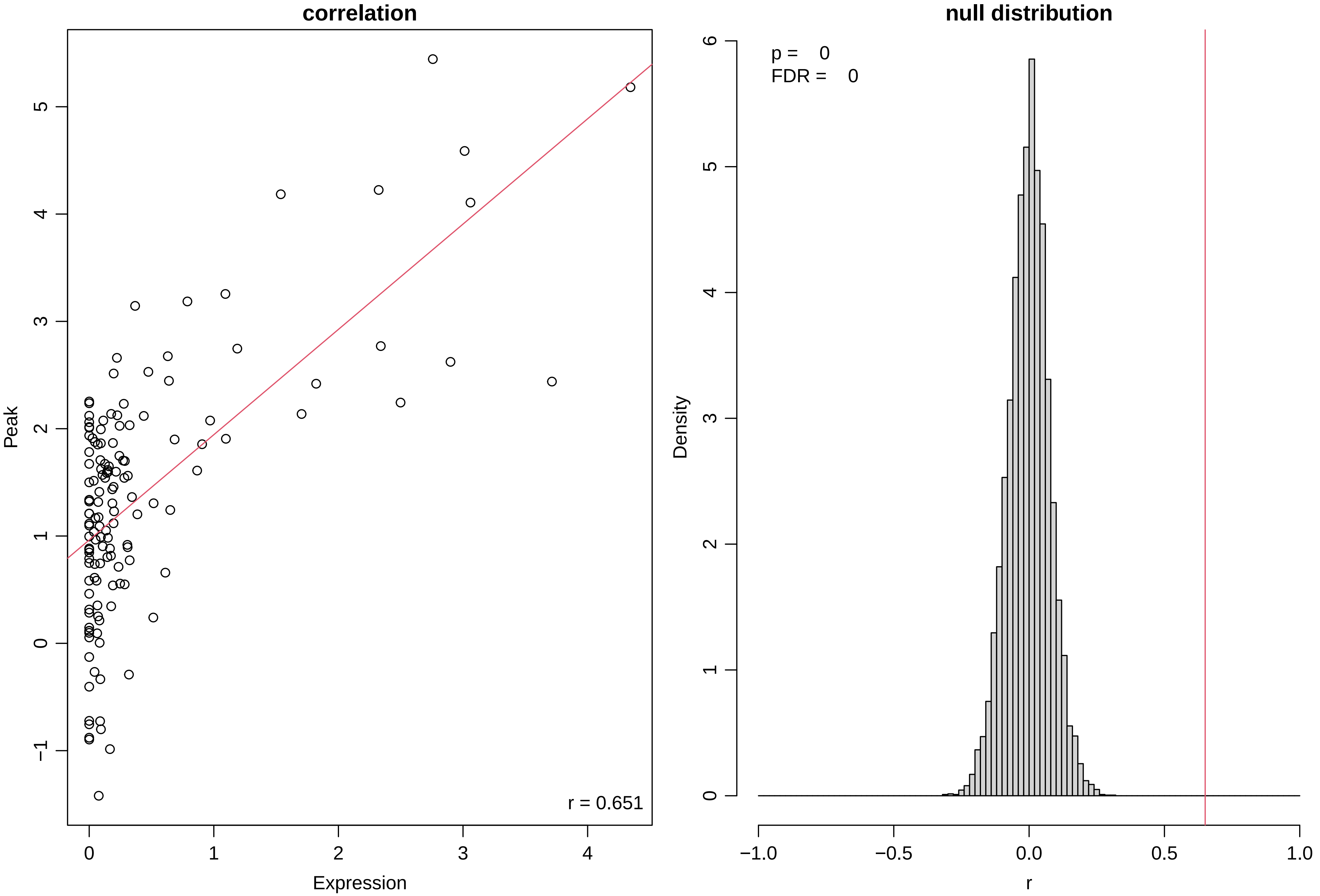

ISL1 vs PeakID_191099 (chr5: 51382527 − 51384472)

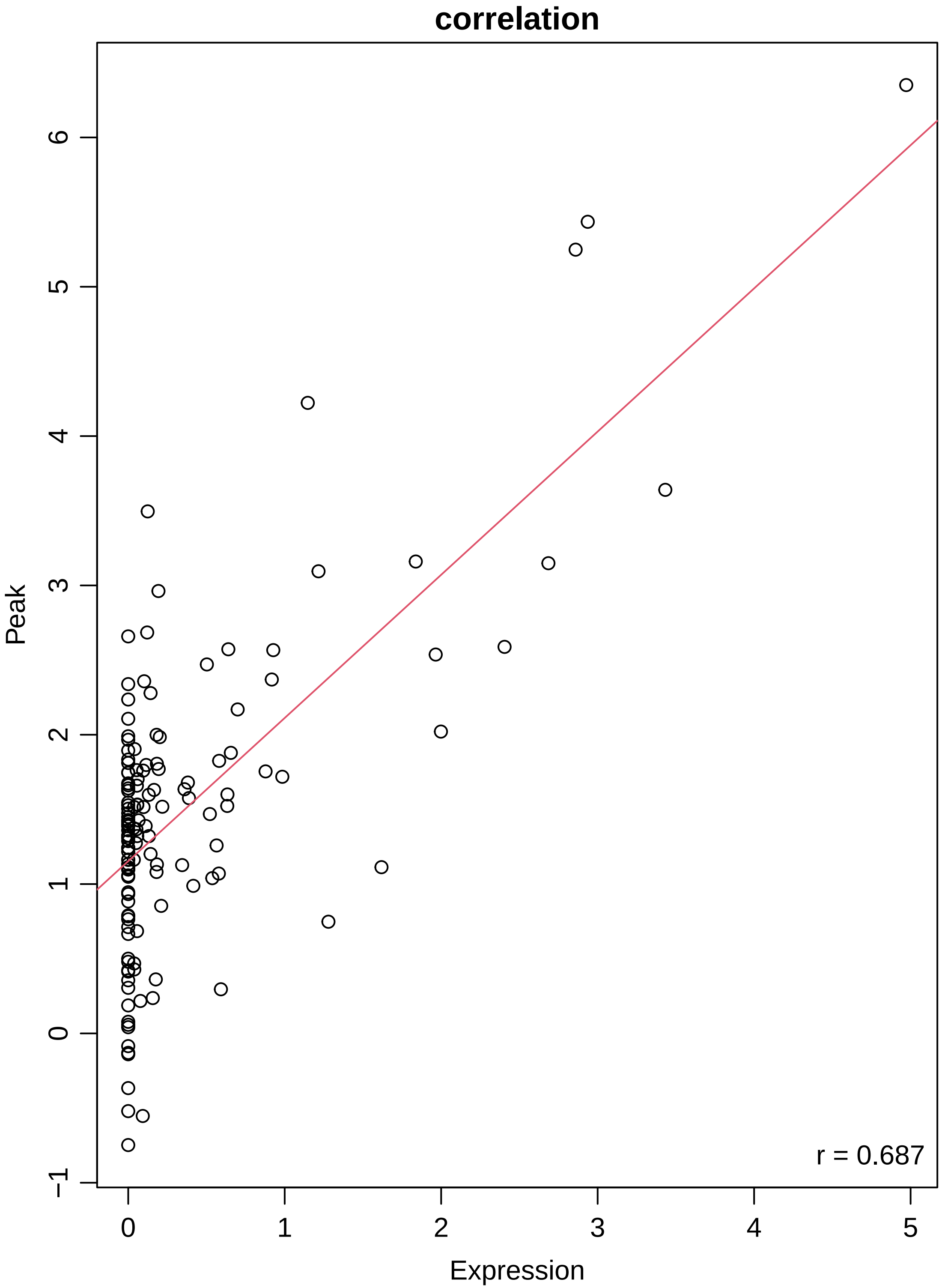

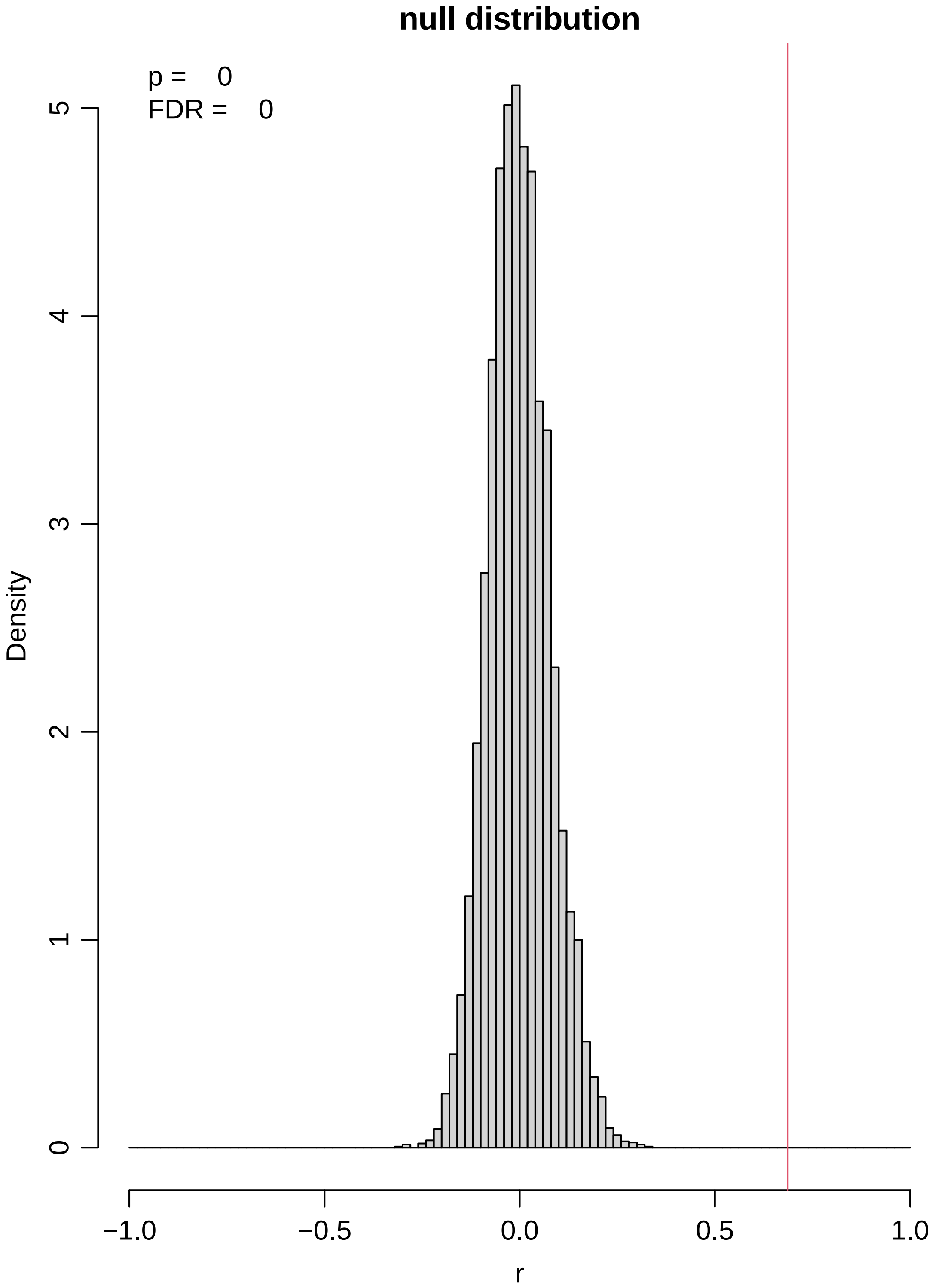

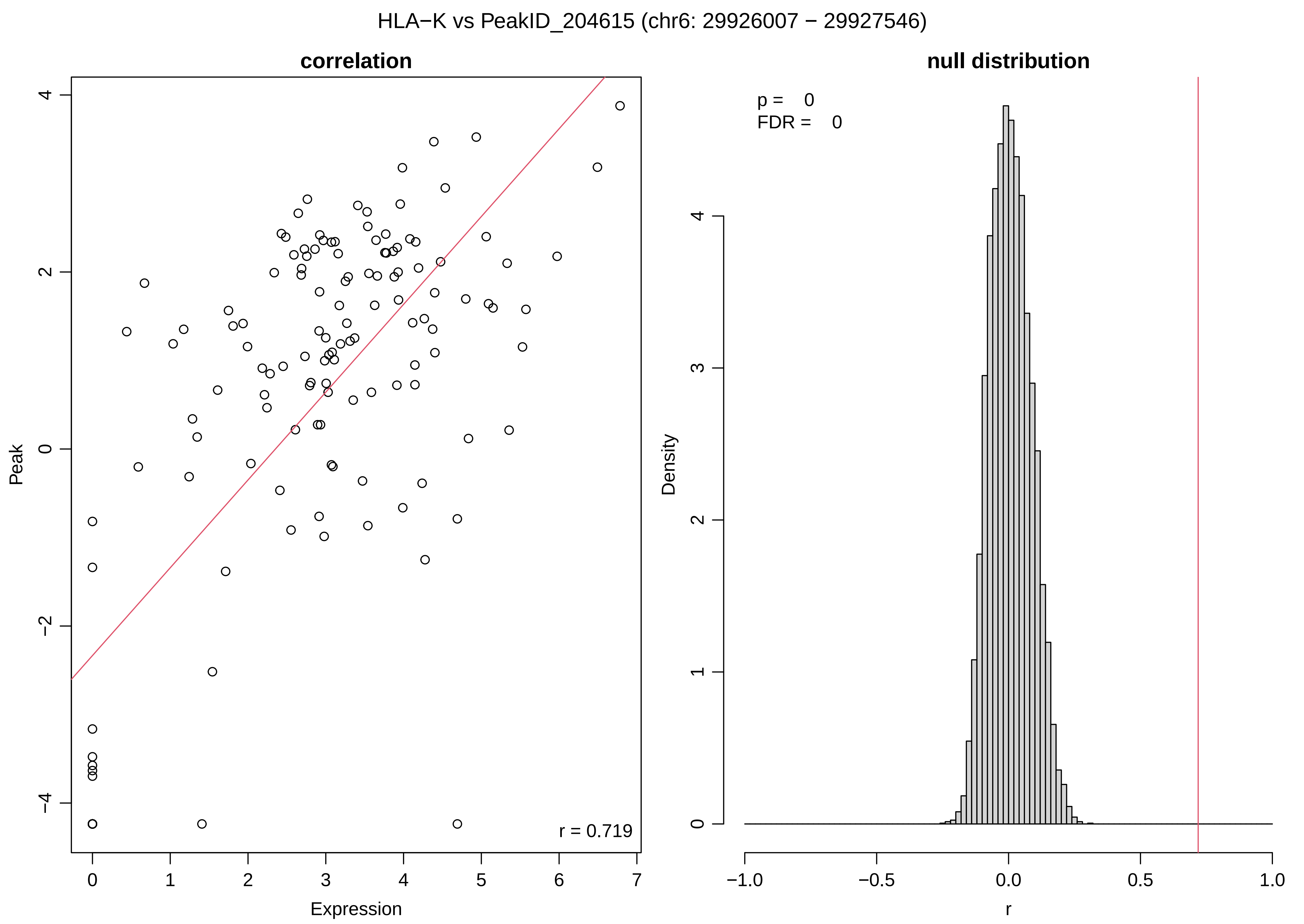

HLA−K vs PeakID_204615 (chr6: 29926007 − 29927546)
correlation
Peak
Expression
r = 0.719
null distribution
p = 0
FDR = 0
Density
r

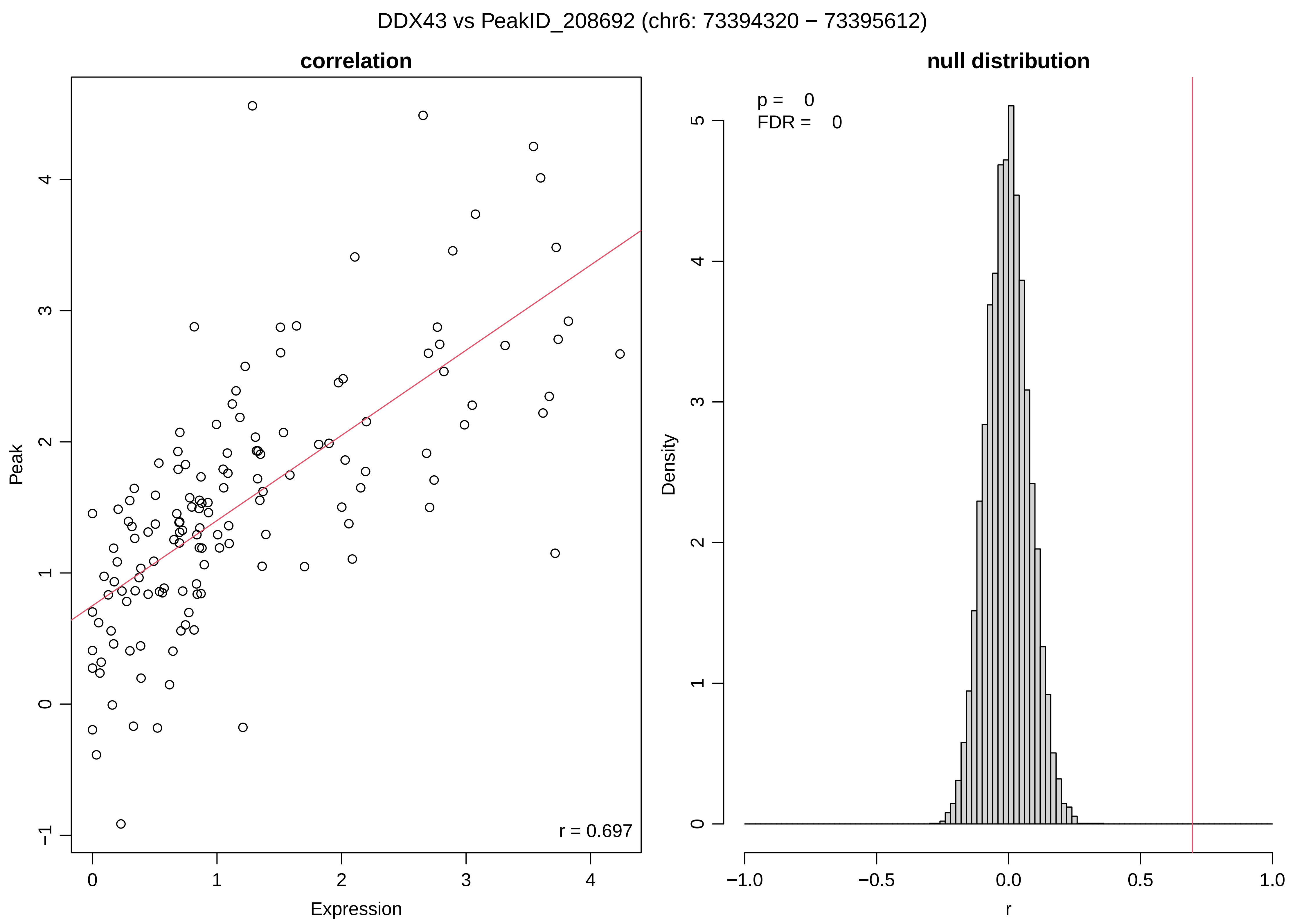

DDX43 vs PeakID_208692 (chr6: 73394320 − 73395612)
correlation
Peak
Expression
r = 0.697
null distribution
p = 0
FDR = 0
Density
r

AC074351.1 vs PeakID_224247 (chr7: 54757090 − 55176717)

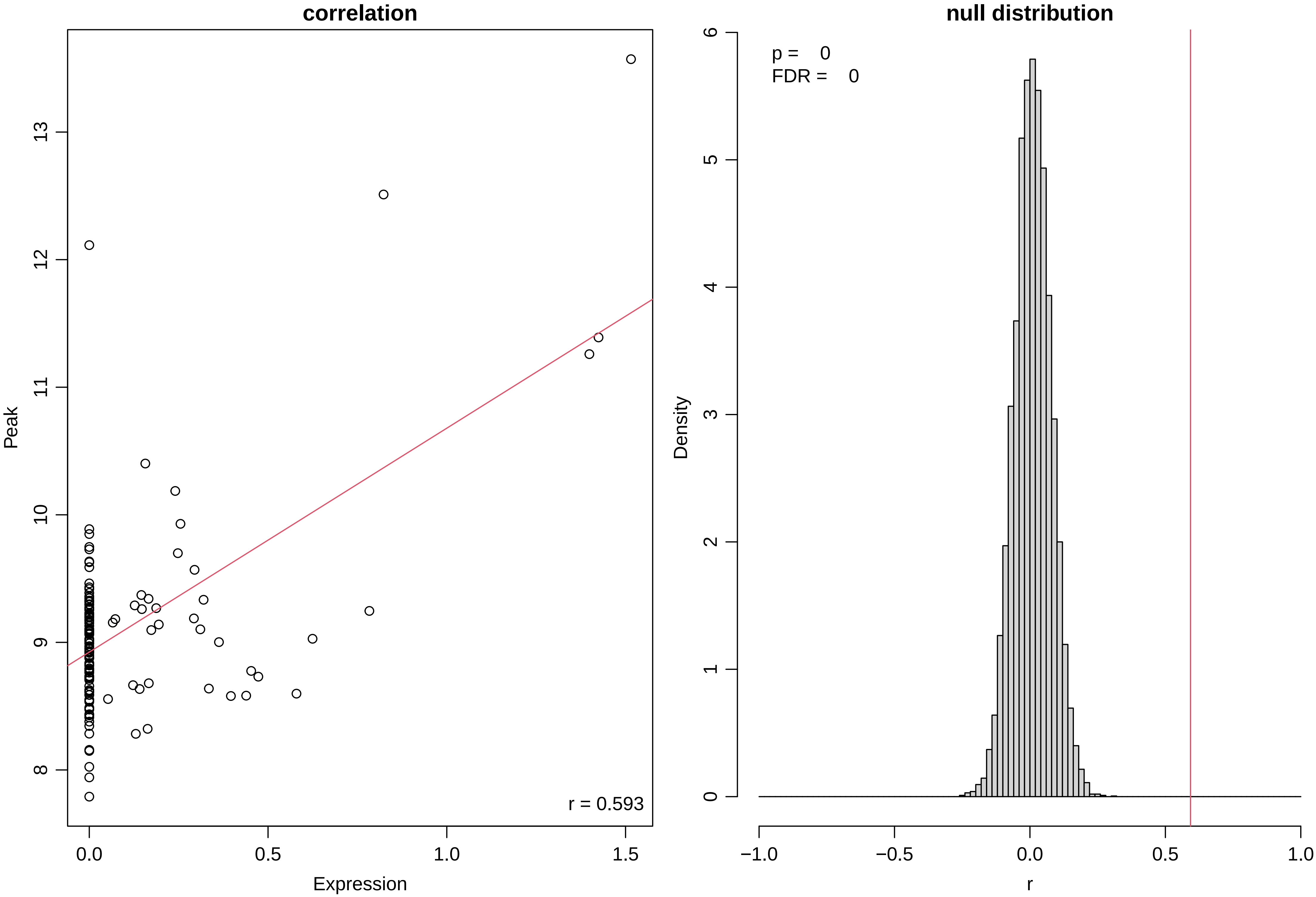

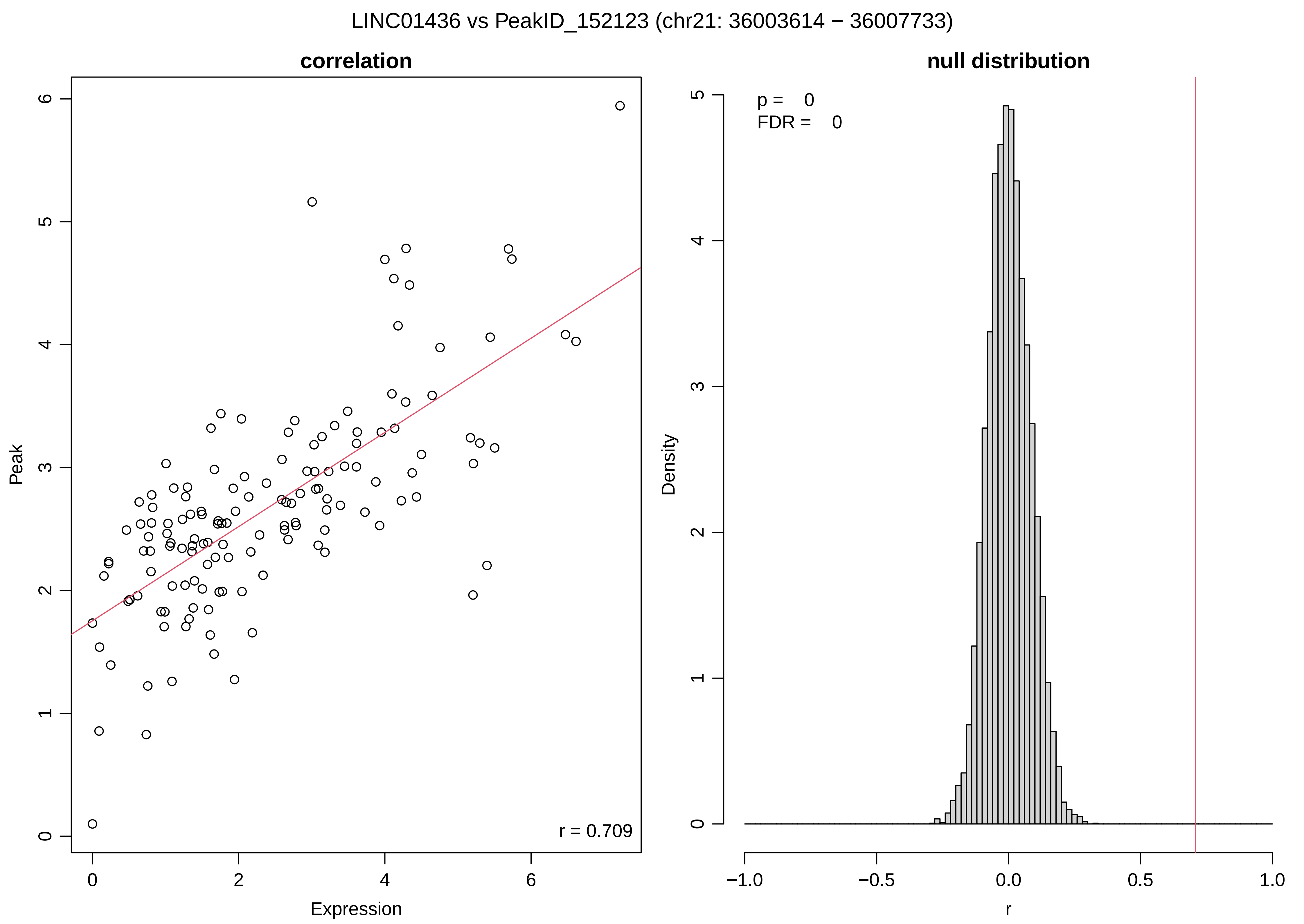

LINC01436 vs PeakID_152123 (chr21: 36003614 − 36007733)
correlation
null distribution
Peak
Expression
r = 0.709
p = 0
FDR = 0
Density
r

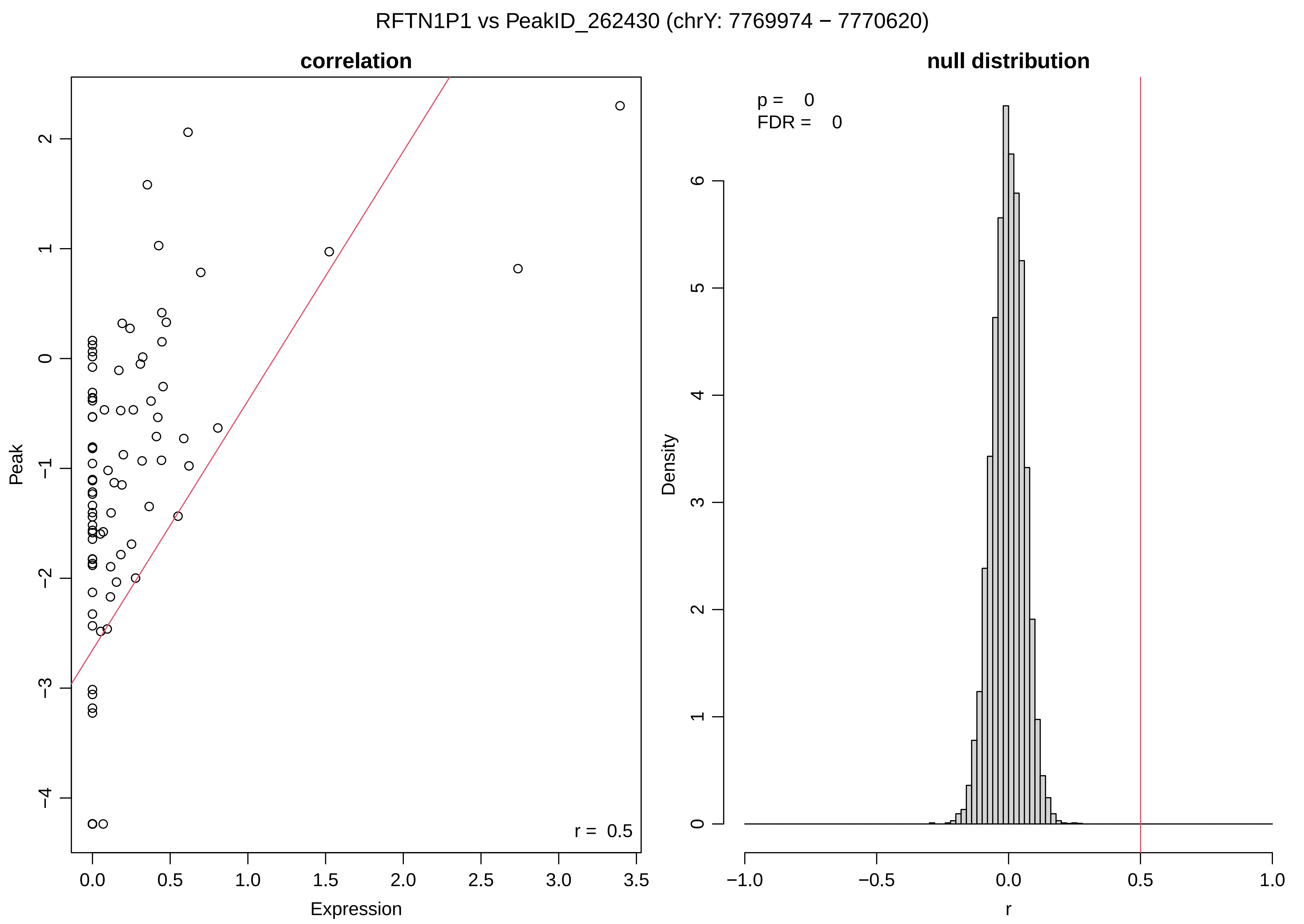
RFTN1P1 vs PeakID_262430 (chrY: 7769974 − 7770620)
correlation
Peak
Expression
r = 0.5
null distribution
p = 0
FDR = 0
Density
r

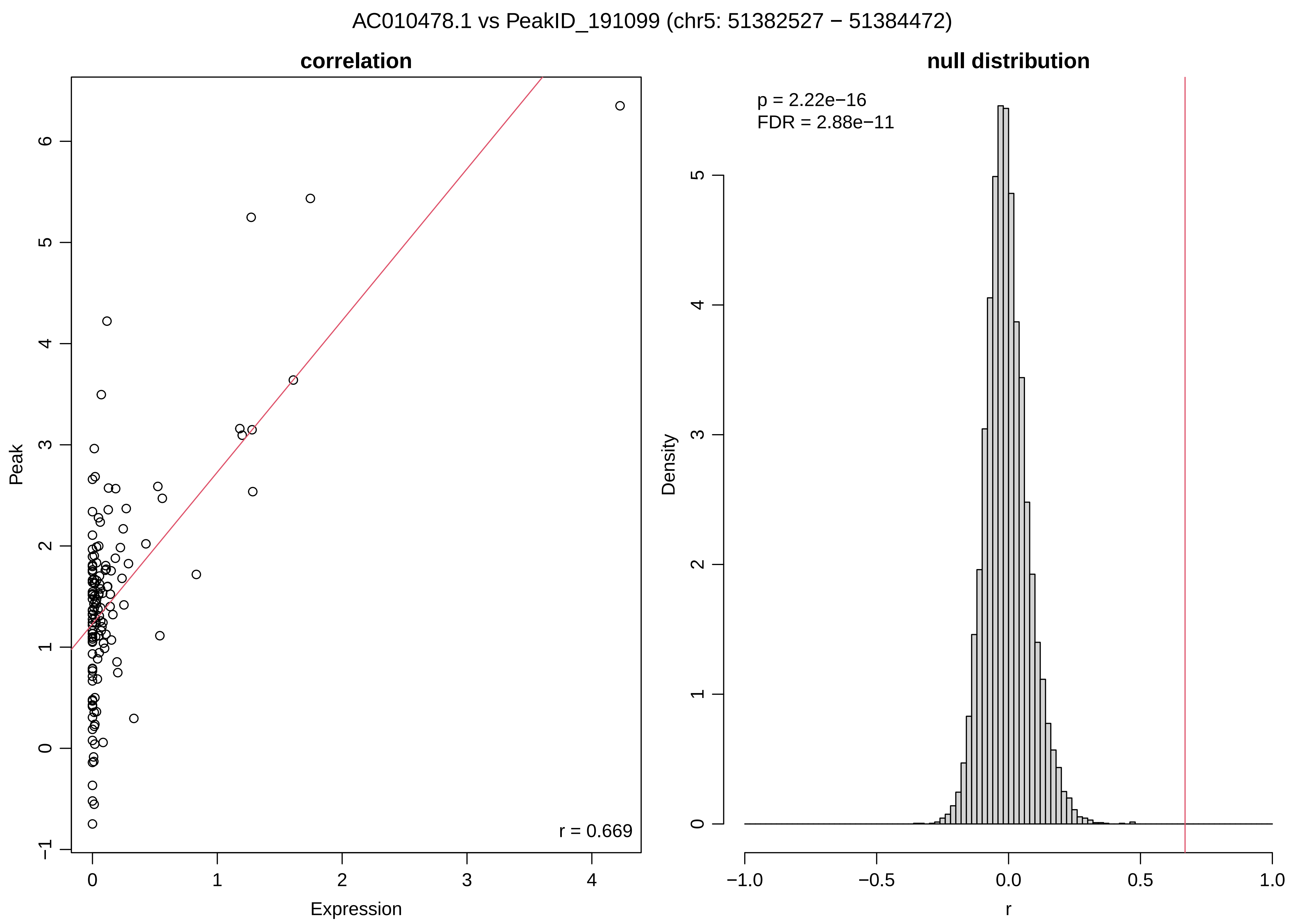
AC010478.1 vs PeakID_191099 (chr5: 51382527 − 51384472)
correlation
Peak
Expression
r = 0.669
null distribution
p = 2.22e−16
FDR = 2.88e−11
Density
r

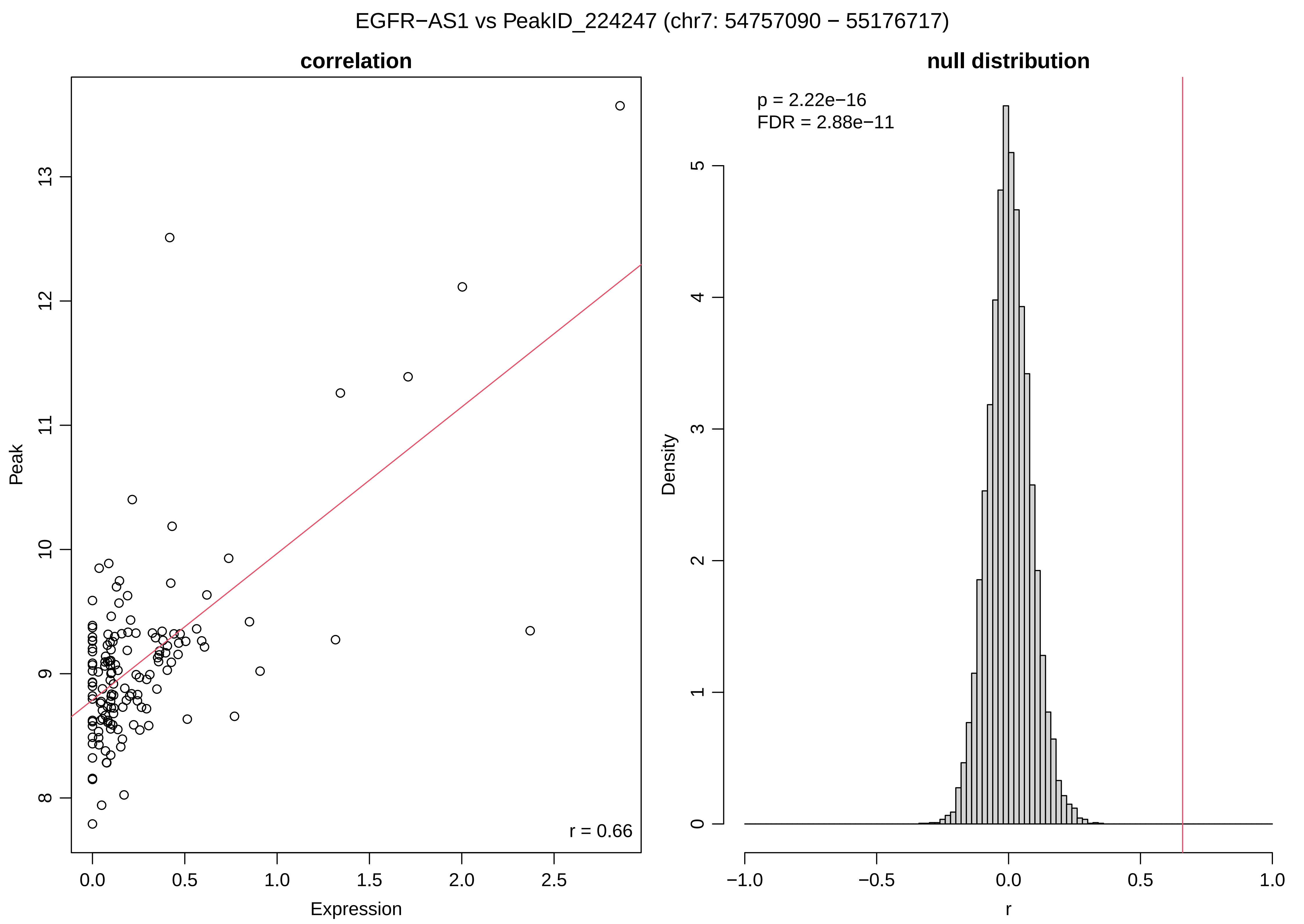
EGFR−AS1 vs PeakID_224247 (chr7: 54757090 − 55176717)
correlation
Peak
Expression
r = 0.66
null distribution
p = 2.22e−16
FDR = 2.88e−11
Density
r

EGFR−AS1 vs PeakID_224248 (chr7: 55176994 − 55203836)

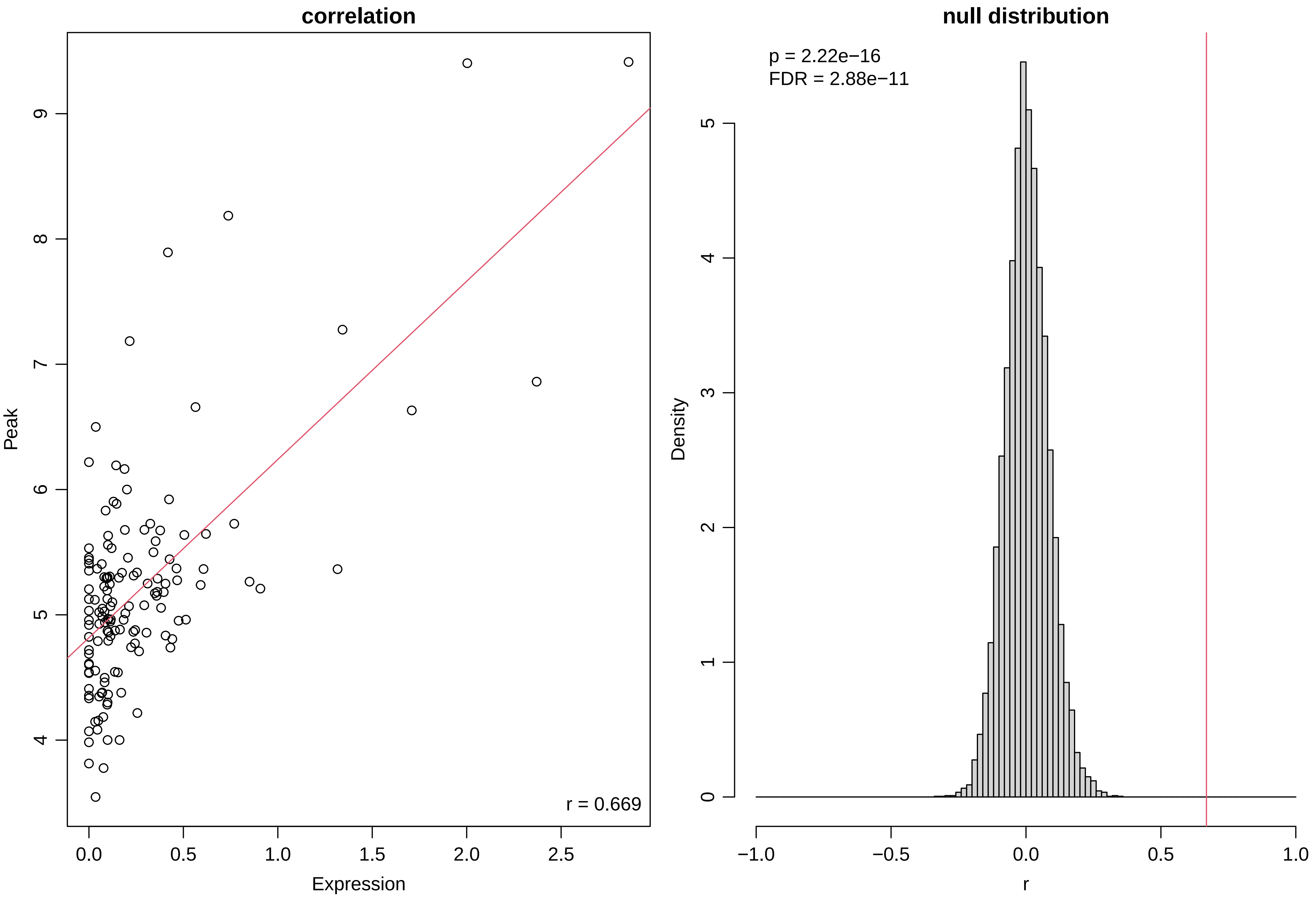

ZBED9 vs PeakID_204572 (chr6: 28586320 − 28587598)

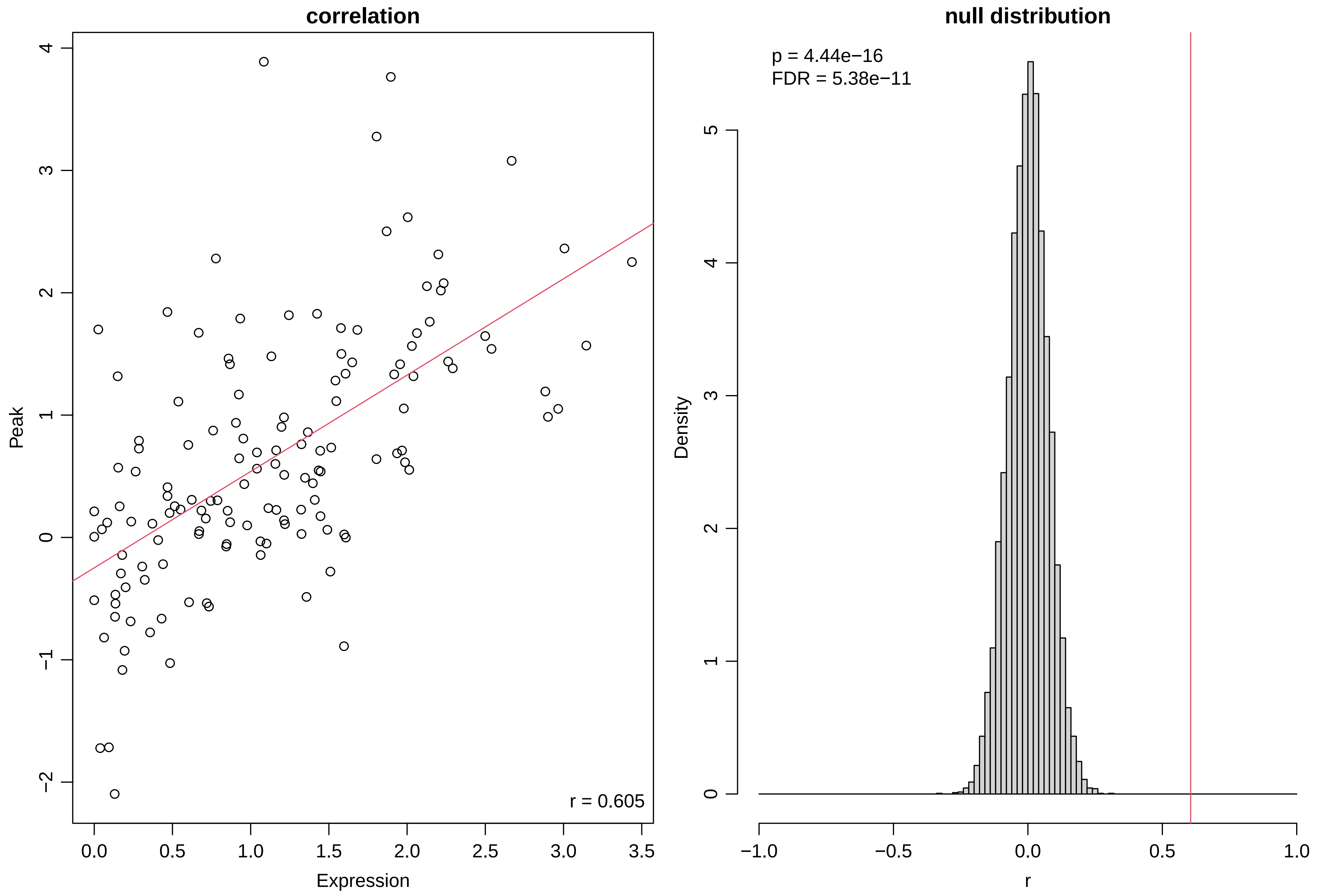

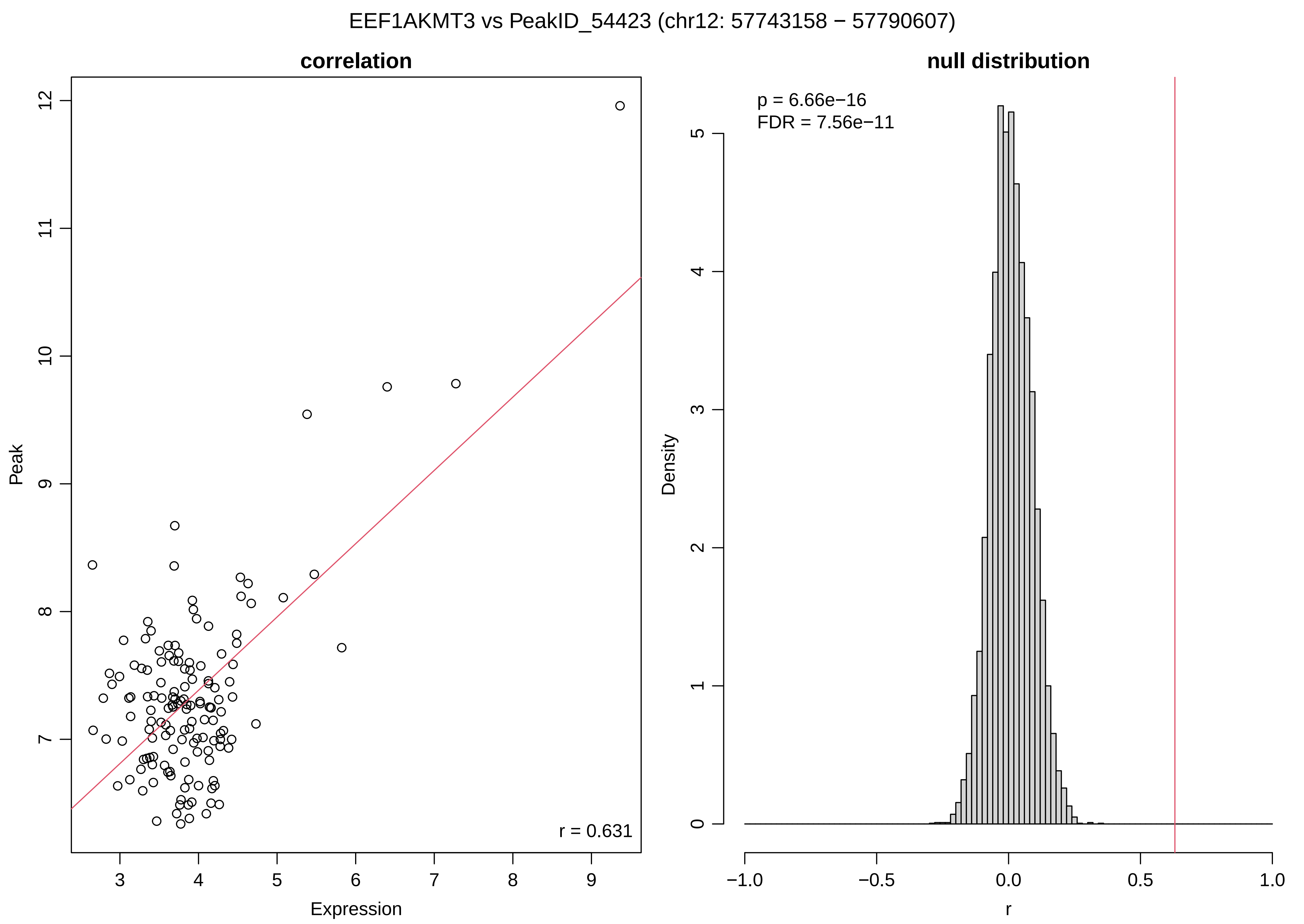
EEF1AKMT3 vs PeakID_54423 (chr12: 57743158 − 57790607)
correlation
Peak
Expression
r = 0.631
null distribution
p = 6.66e−16
FDR = 7.56e−11
Density
r

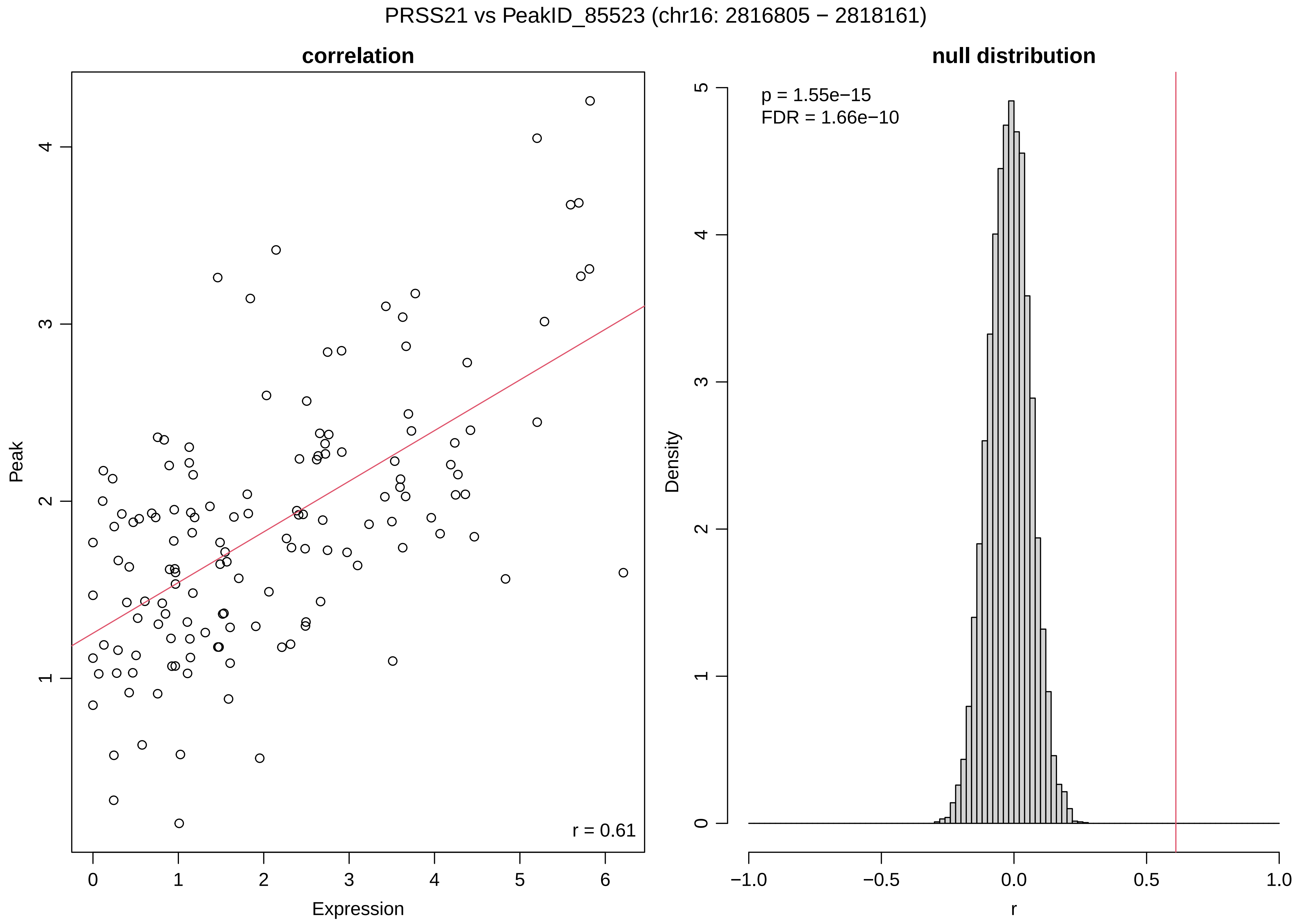
PRSS21 vs PeakID_85523 (chr16: 2816805 − 2818161)
correlation
Peak
Expression
r = 0.61
0
1
2
3
4
5
6
1
2
3
4
null distribution
Density
r
p = 1.55e−15
FDR = 1.66e−10
−1.0
−0.5
0.0
0.5
1.0
0
1
2
3
4
5

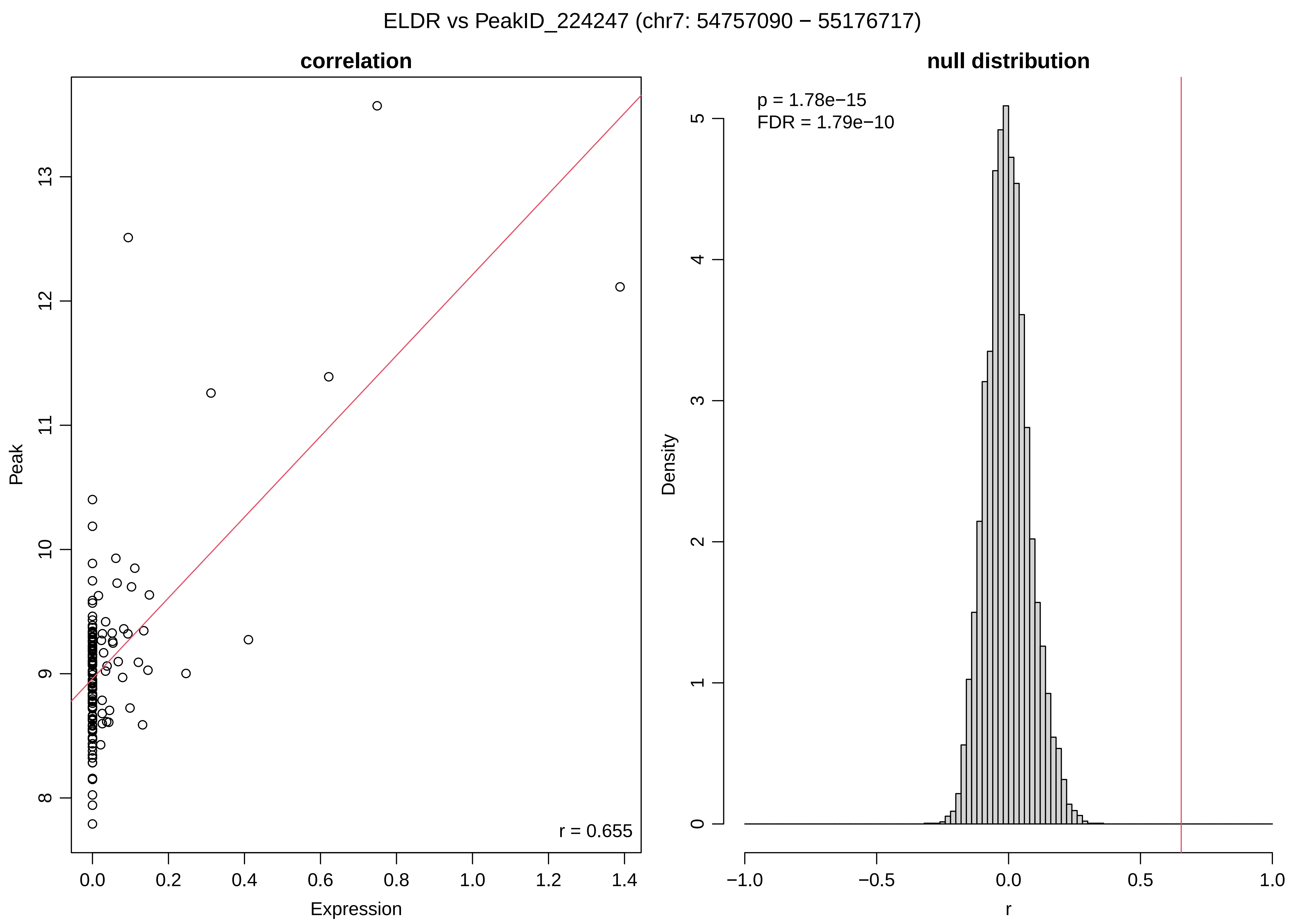
ELDR vs PeakID_224247 (chr7: 54757090 − 55176717)
correlation
Peak
Expression
r = 0.655
13
12
11
10
9
8
0.0
0.2
0.4
0.6
0.8
1.0
1.2
1.4
null distribution
p = 1.78e−15
FDR = 1.79e−10
Density
r
5
4
3
2
1
0
−1.0
−0.5
0.0
0.5
1.0

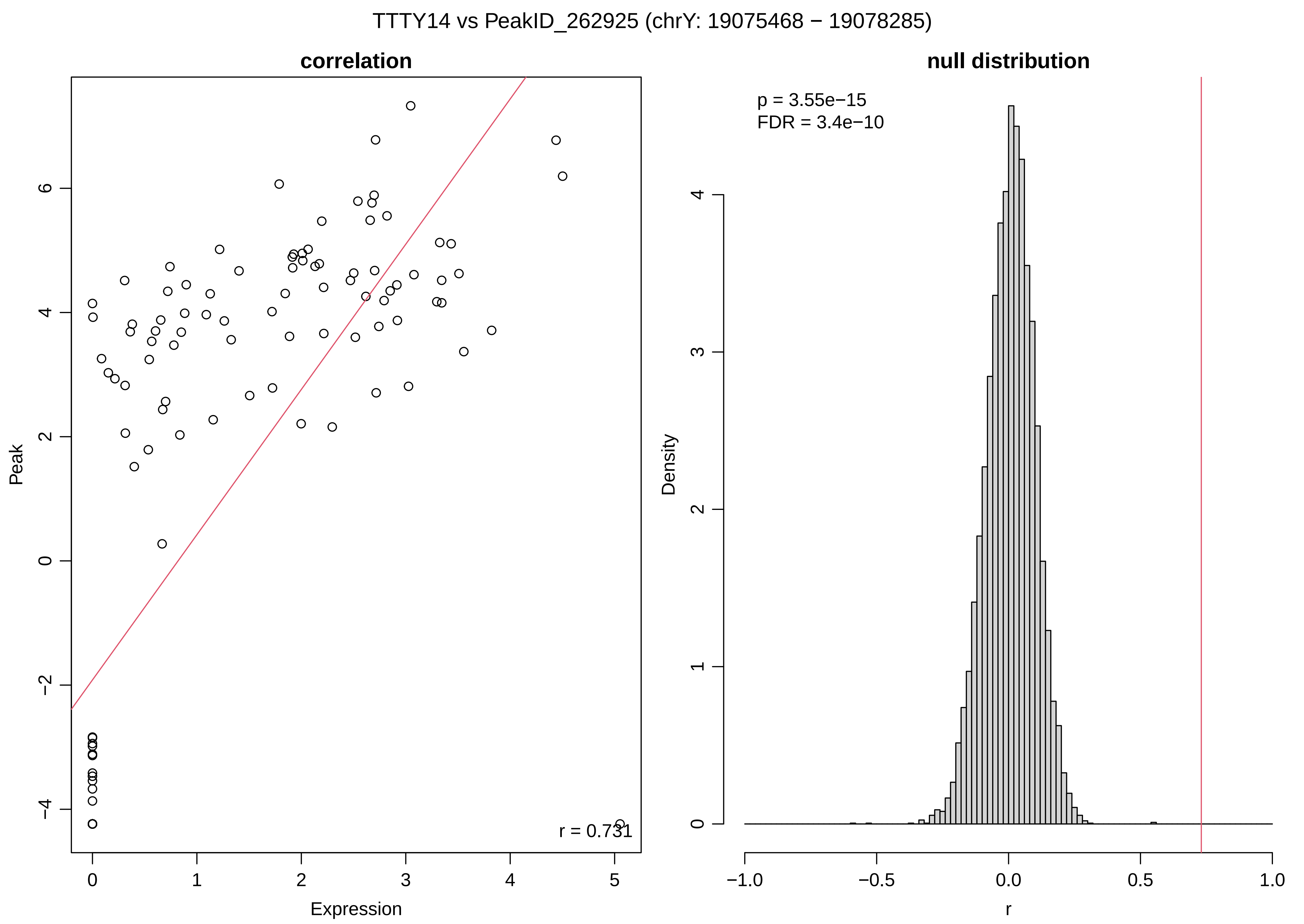
TTTY14 vs PeakID_262925 (chrY: 19075468 − 19078285)
correlation
Peak
Expression
r = 0.731
null distribution
p = 3.55e−15
FDR = 3.4e−10
Density
r

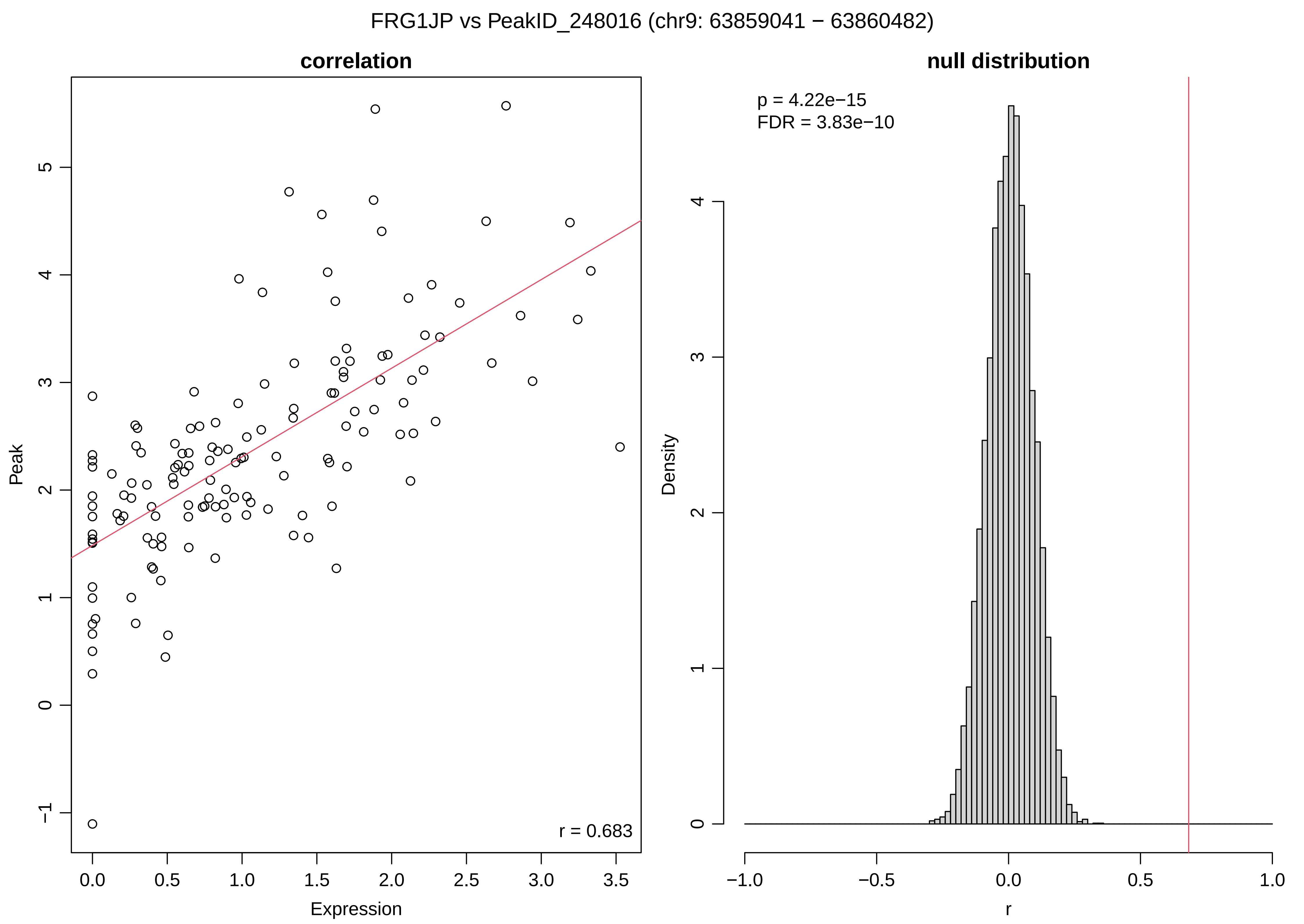
FRG1JP vs PeakID_248016 (chr9: 63859041 − 63860482)
correlation
null distribution
Peak
Expression
r = 0.683
p = 4.22e−15
FDR = 3.83e−10
Density
r

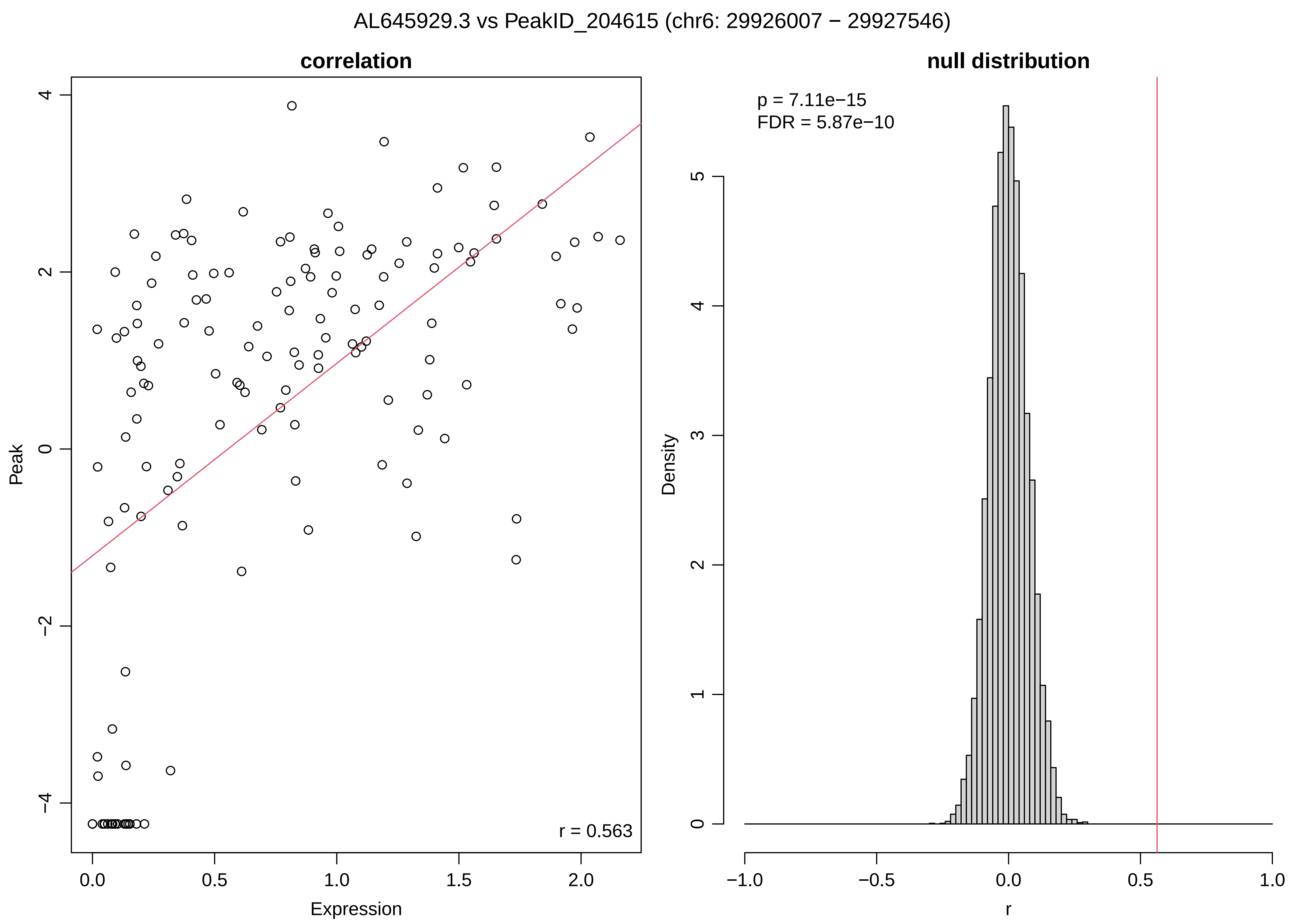
AL645929.3 vs PeakID_204615 (chr6: 29926007 − 29927546)
correlation
Peak
Expression
r = 0.563
null distribution
p = 7.11e−15
FDR = 5.87e−10
Density
r

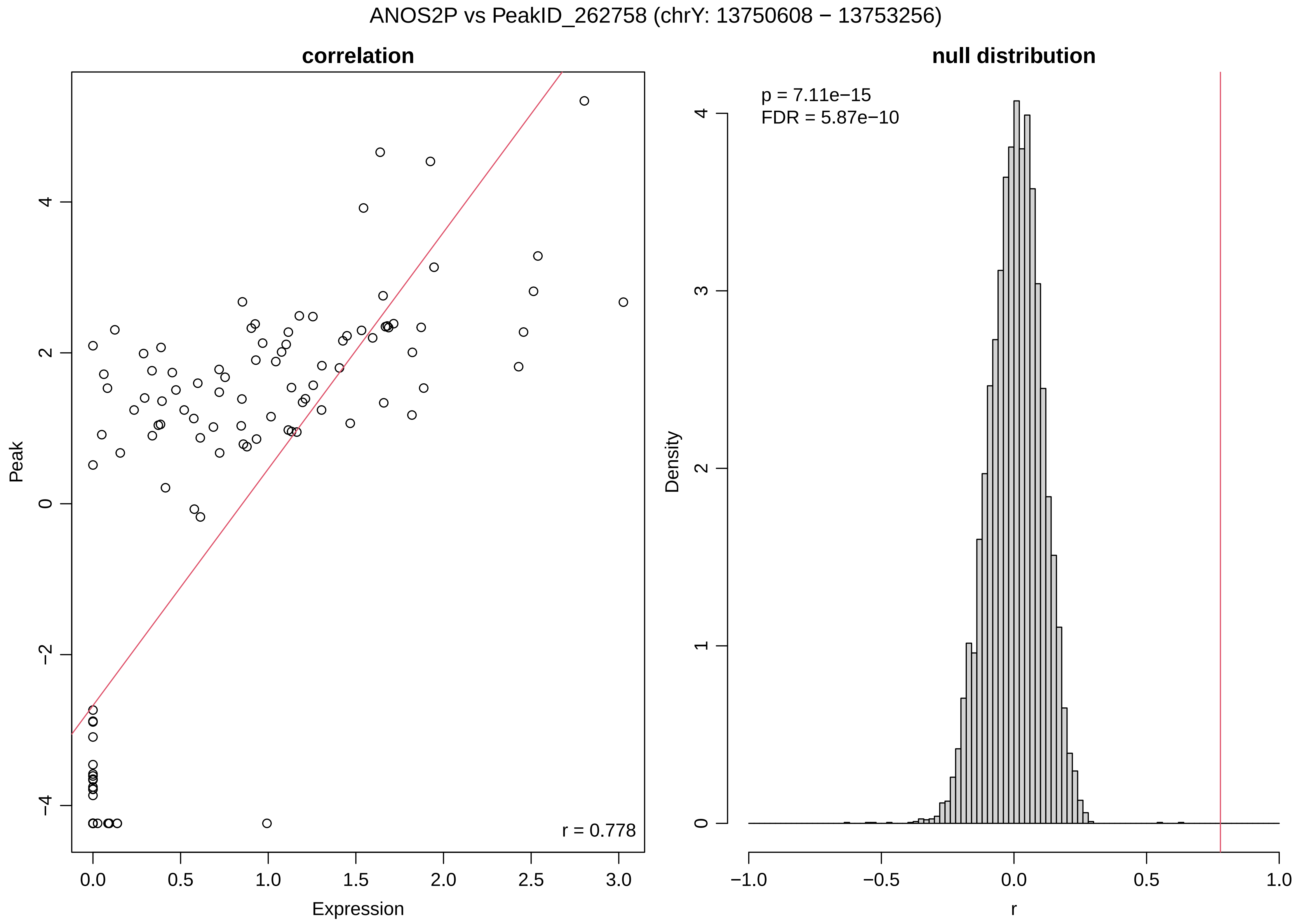

ANOS2P vs PeakID_262758 (chrY: 13750608 − 13753256)
correlation
Peak
Expression
r = 0.778
null distribution
p = 7.11e−15
FDR = 5.87e−10
Density
r

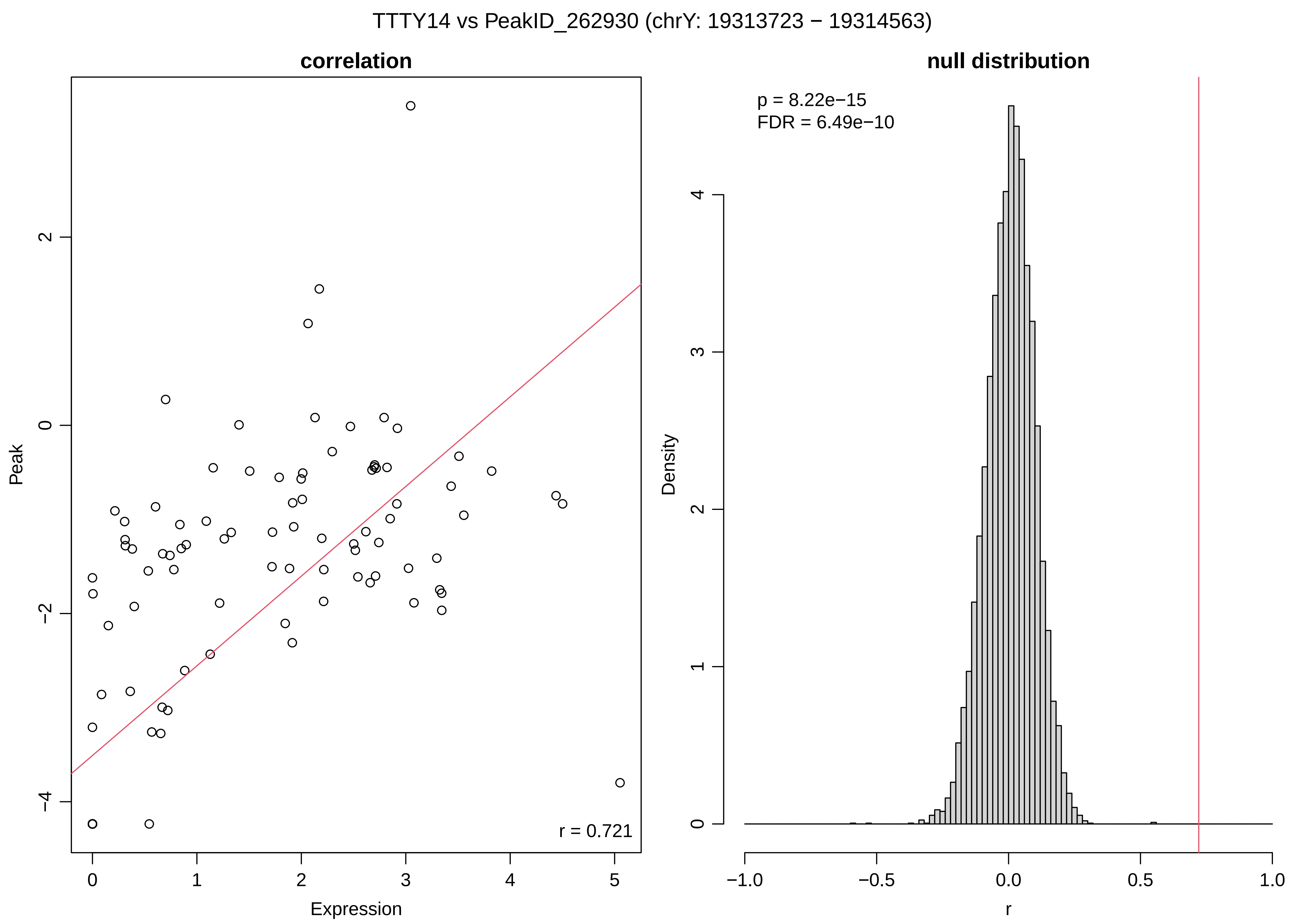
TTTY14 vs PeakID_262930 (chrY: 19313723 − 19314563)
correlation
Peak
Expression
r = 0.721
null distribution
p = 8.22e−15
FDR = 6.49e−10
Density
r

AC013652.1 vs PeakID_77590 (chr15: 39419893 − 39421375)

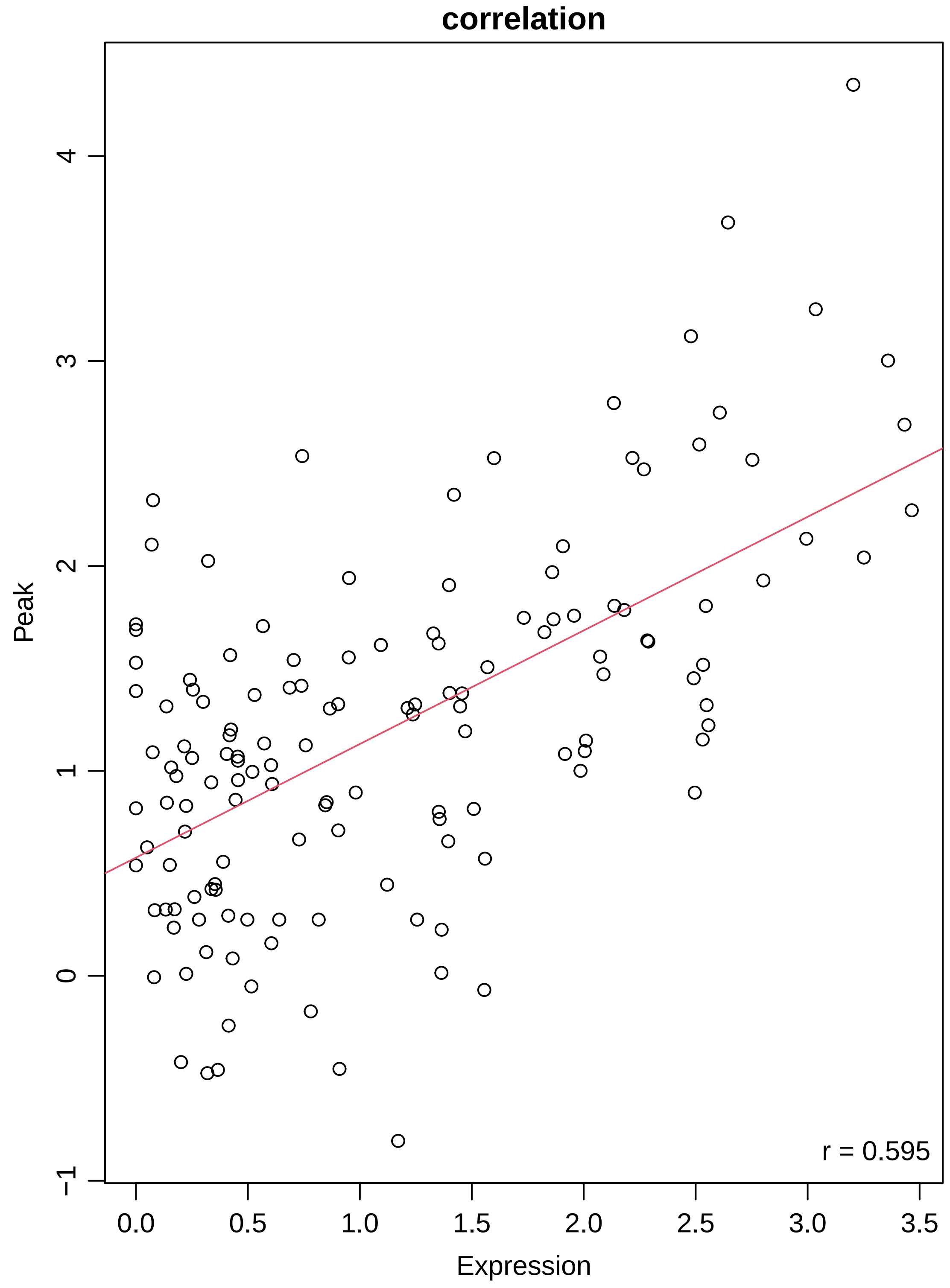

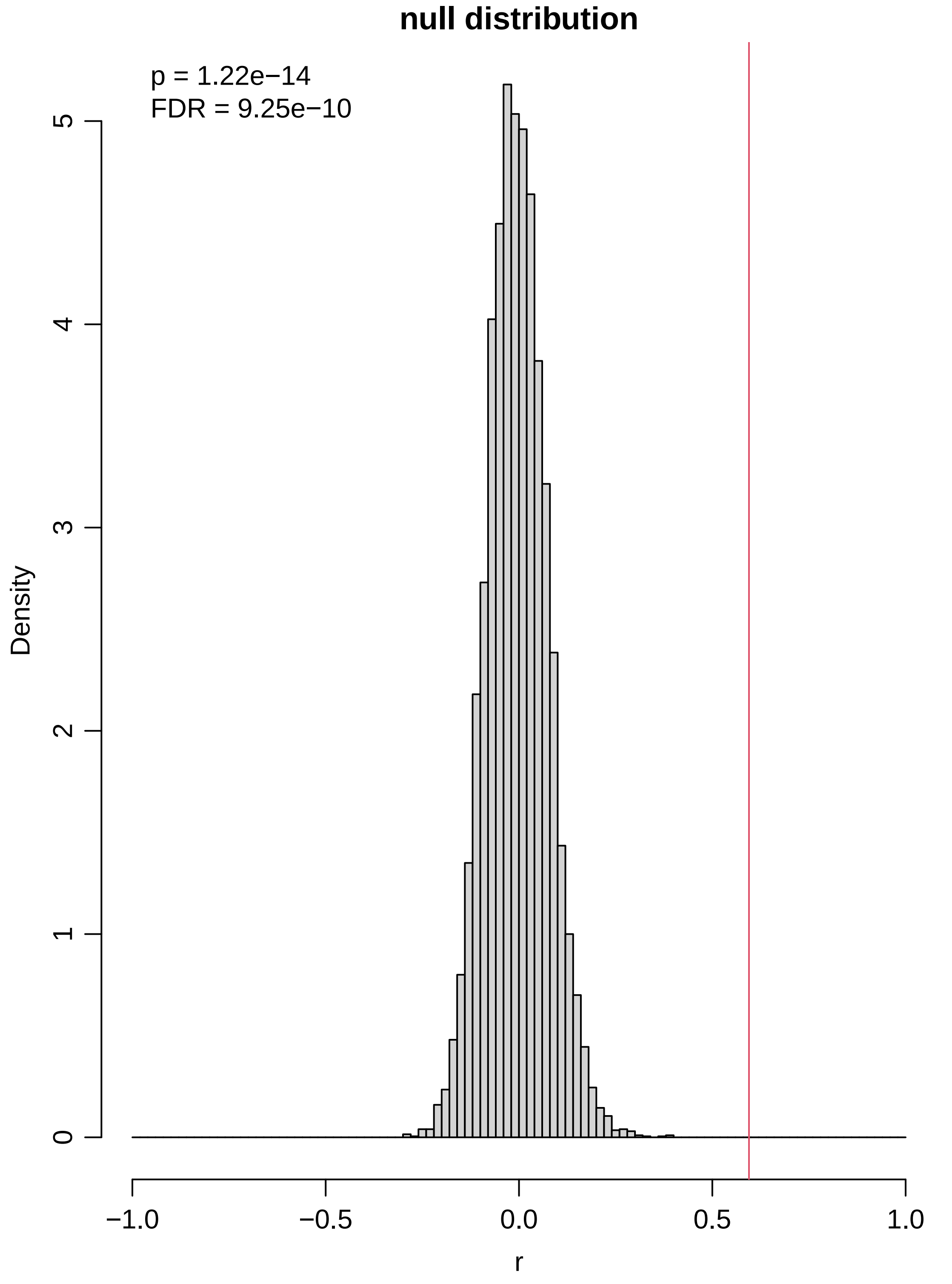

ALOX12P2 vs PeakID_95030 (chr17: 6853673 − 6854992)

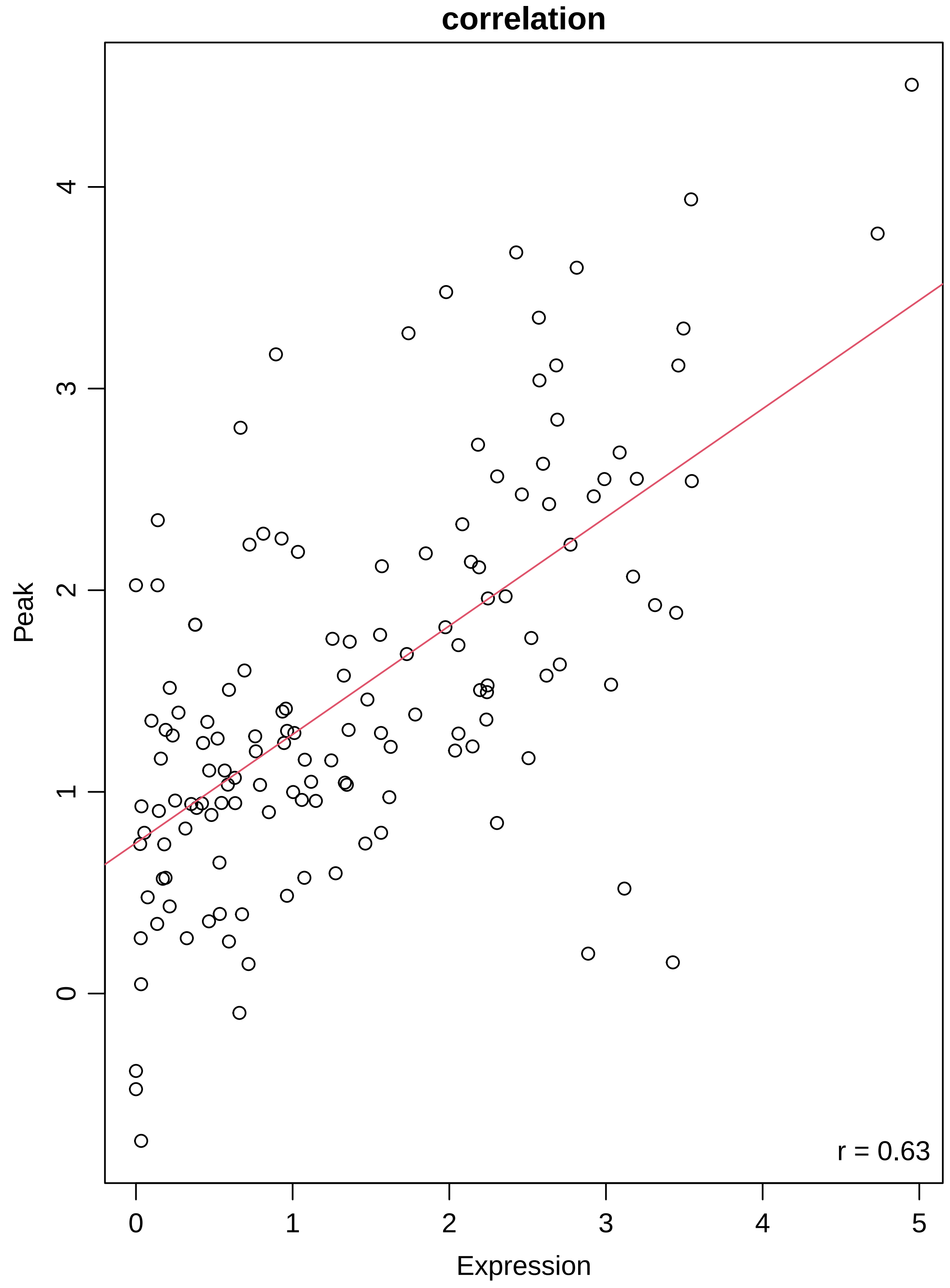

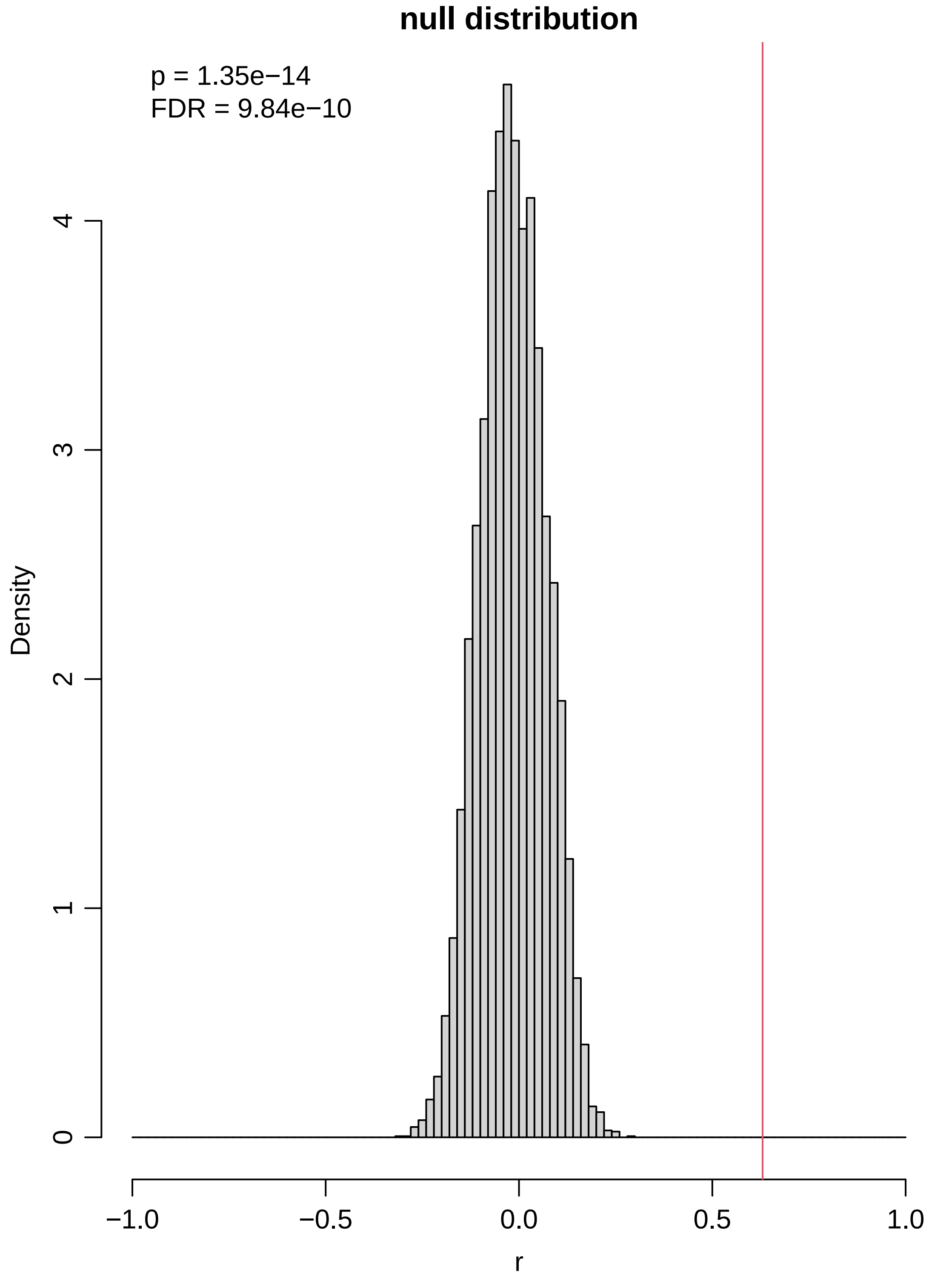

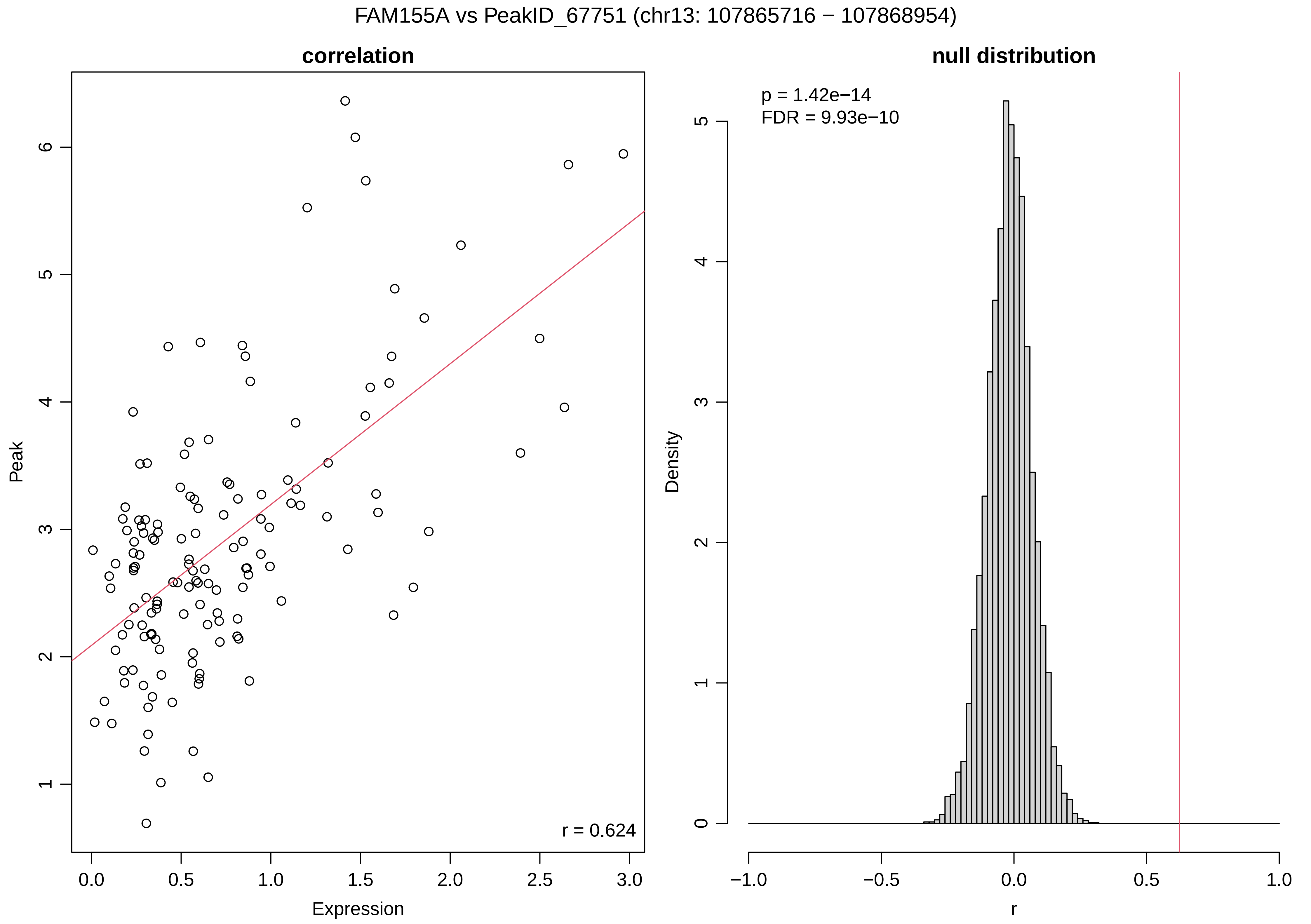

FAM155A vs PeakID_67751 (chr13: 107865716 − 107868954)
correlation
Peak
Expression
r = 0.624
null distribution
p = 1.42e−14
FDR = 9.93e−10
Density
r

AC068134.2 vs PeakID_140746 (chr2: 232416152 − 232421999)

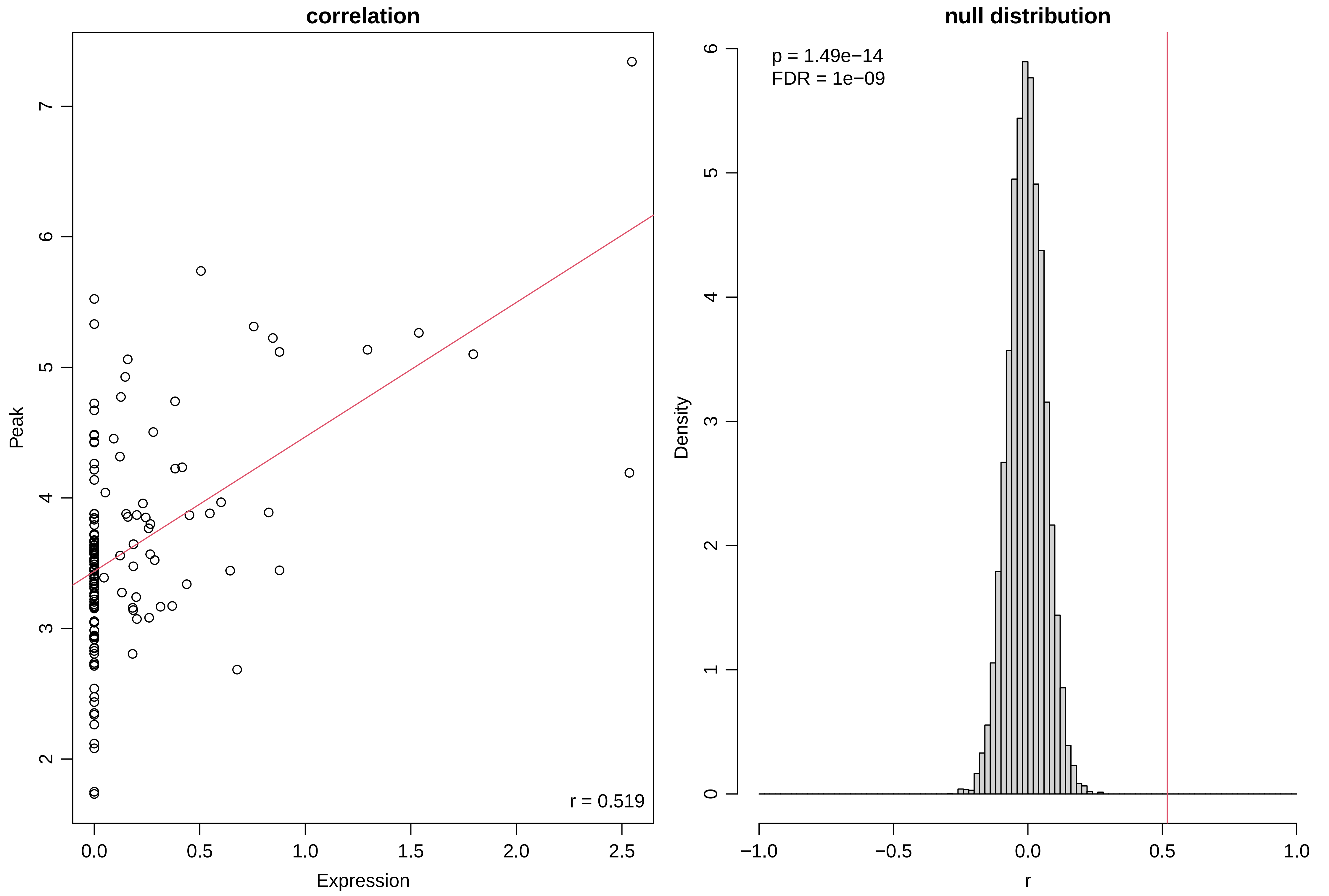

RNU4−20P vs PeakID_54828 (chr12: 59570193 − 59572647)

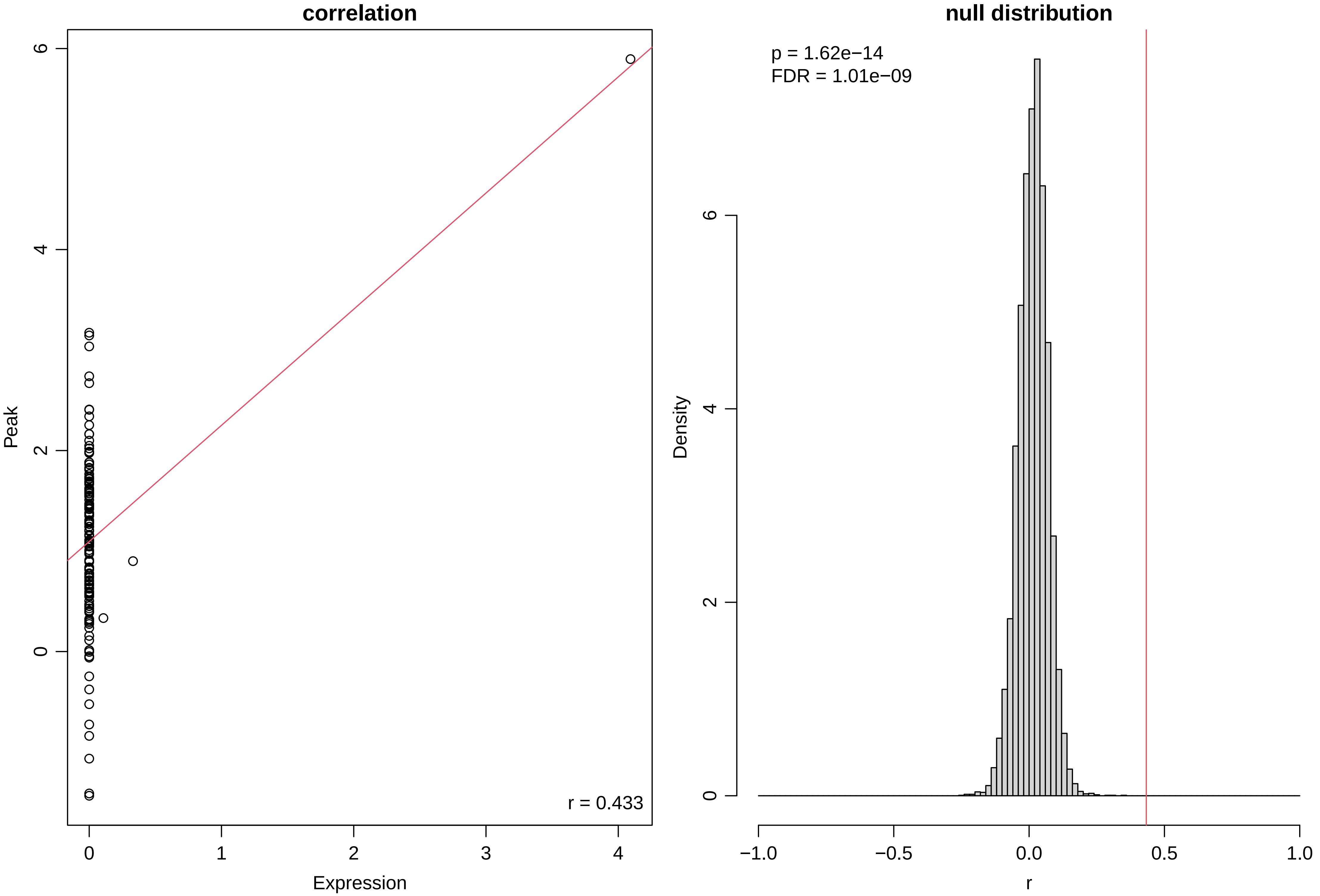

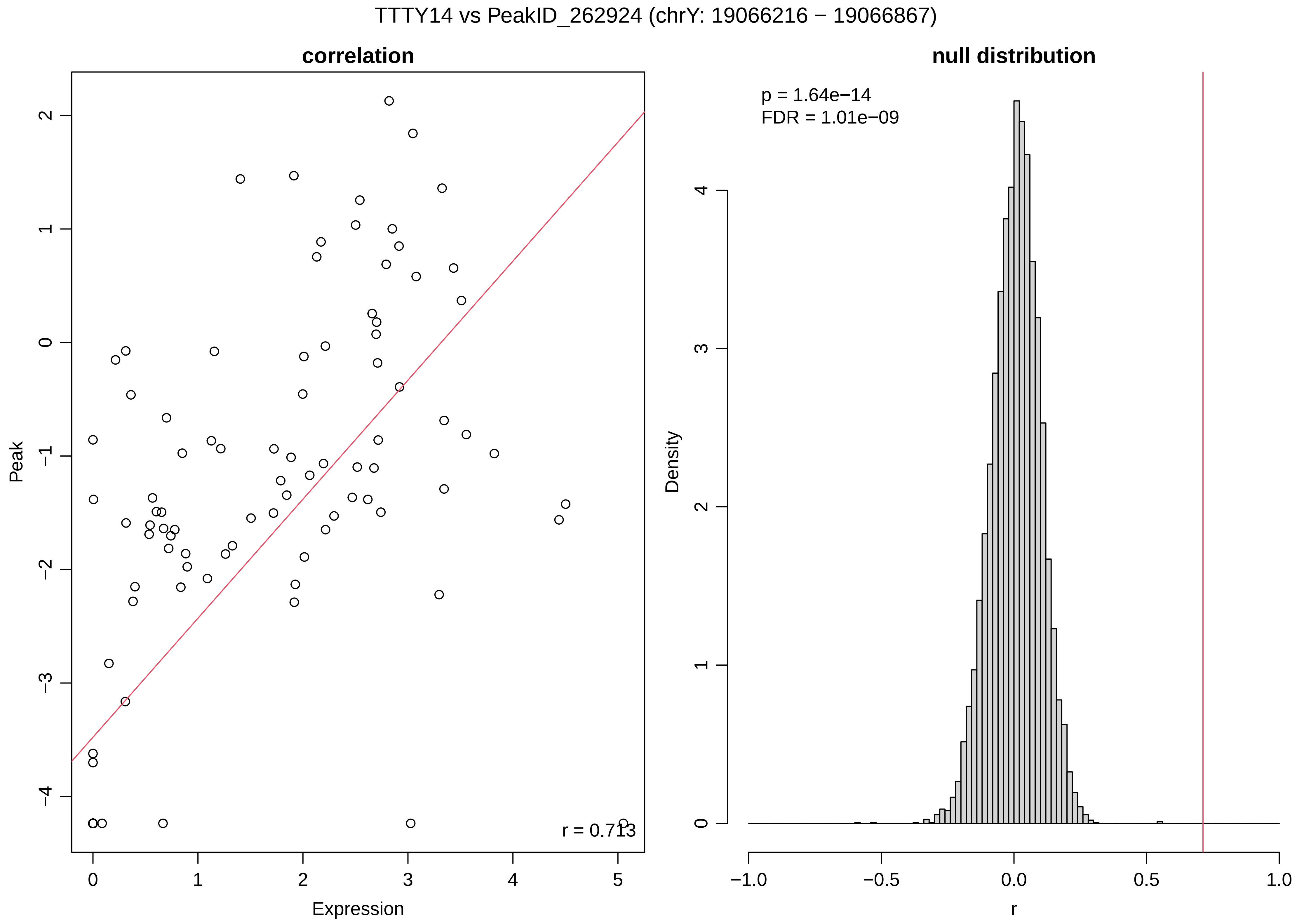

TTTY14 vs PeakID_262924 (chrY: 19066216 − 19066867)
correlation
Peak
Expression
r = 0.713
null distribution
p = 1.64e−14
FDR = 1.01e−09
Density
r

TTTY14 vs PeakID_262928 (chrY: 19268054 − 19269133)

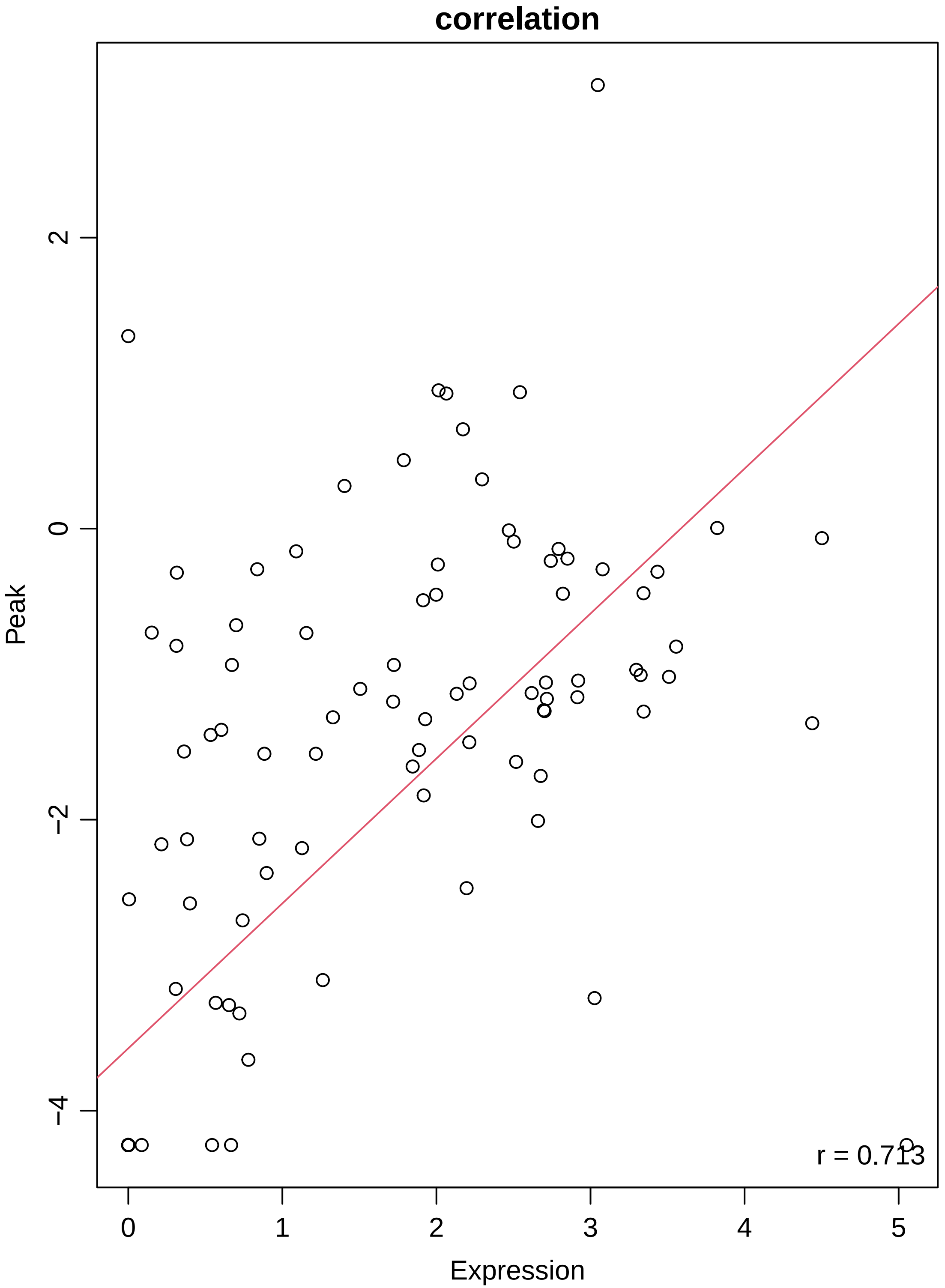

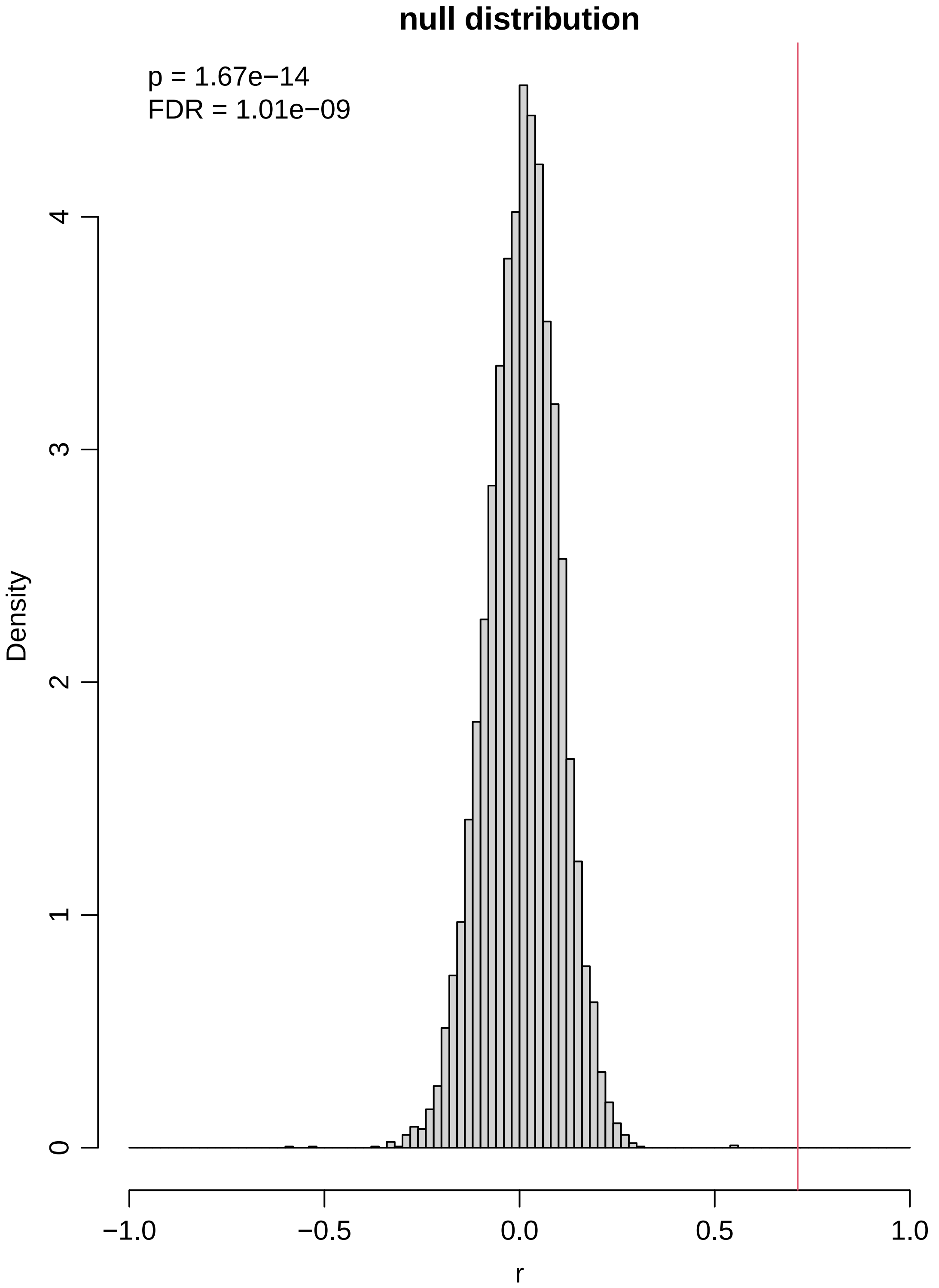

TTTY14 vs PeakID_262931 (chrY: 19320986 − 19323286)

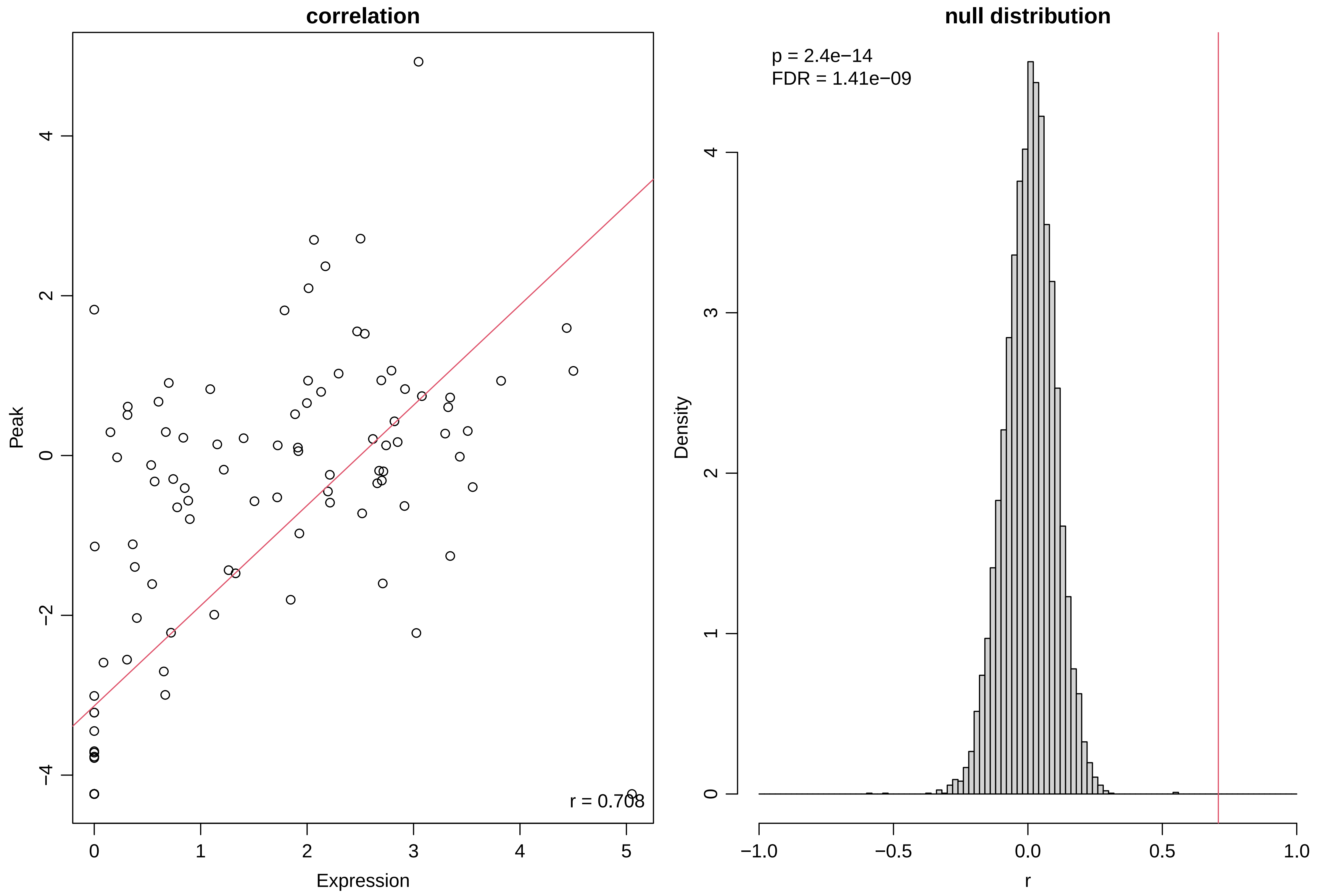

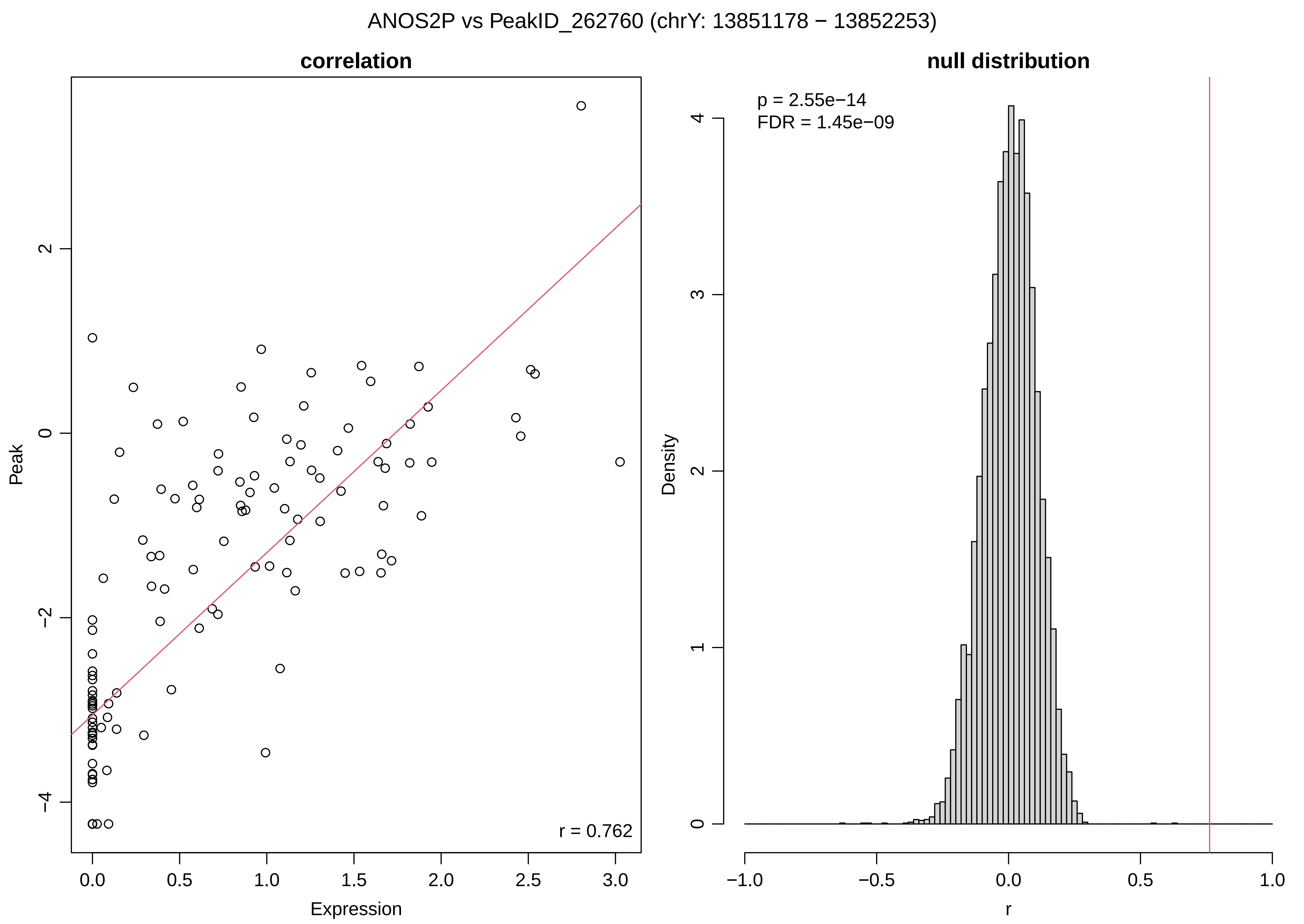

ANOS2P vs PeakID_262760 (chrY: 13851178 − 13852253)
correlation
Peak
Expression
r = 0.762
null distribution
p = 2.55e−14
FDR = 1.45e−09
Density
r

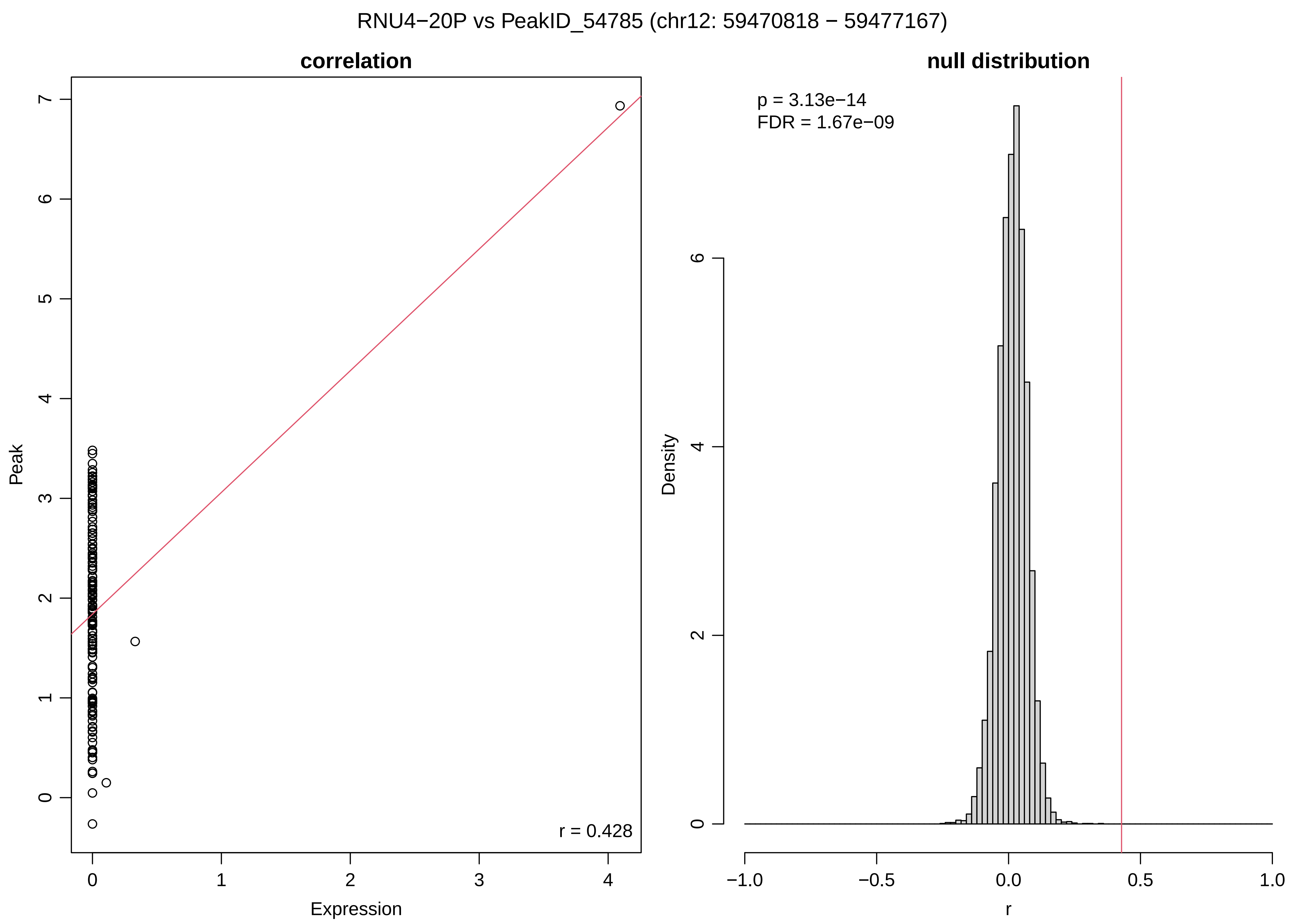

RNU4−20P vs PeakID_54785 (chr12: 59470818 − 59477167)
correlation
Peak
Expression
r = 0.428
null distribution
p = 3.13e−14
FDR = 1.67e−09
Density
r

RNU4−20P vs PeakID_54835 (chr12: 59583751 − 59588494)

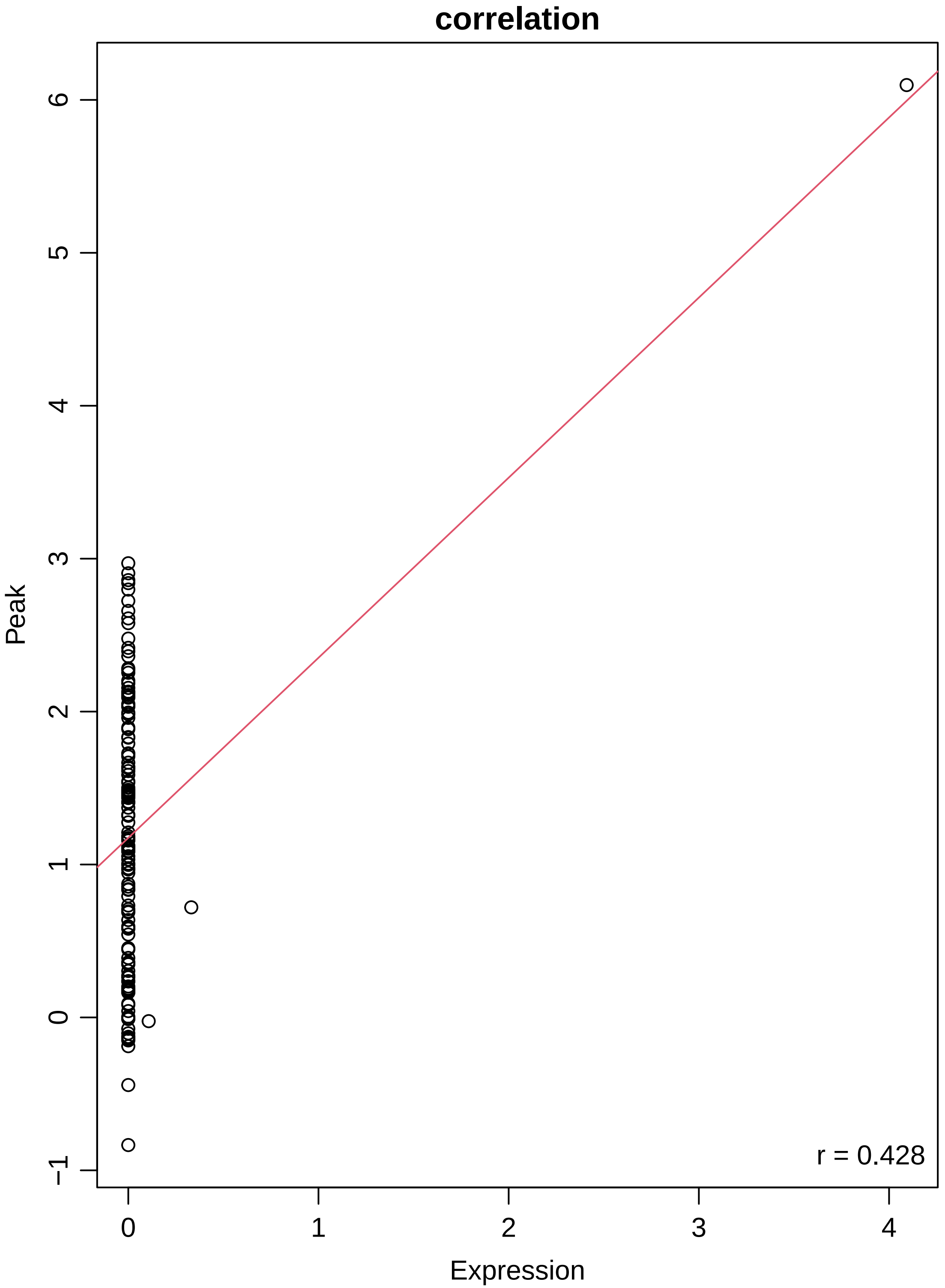

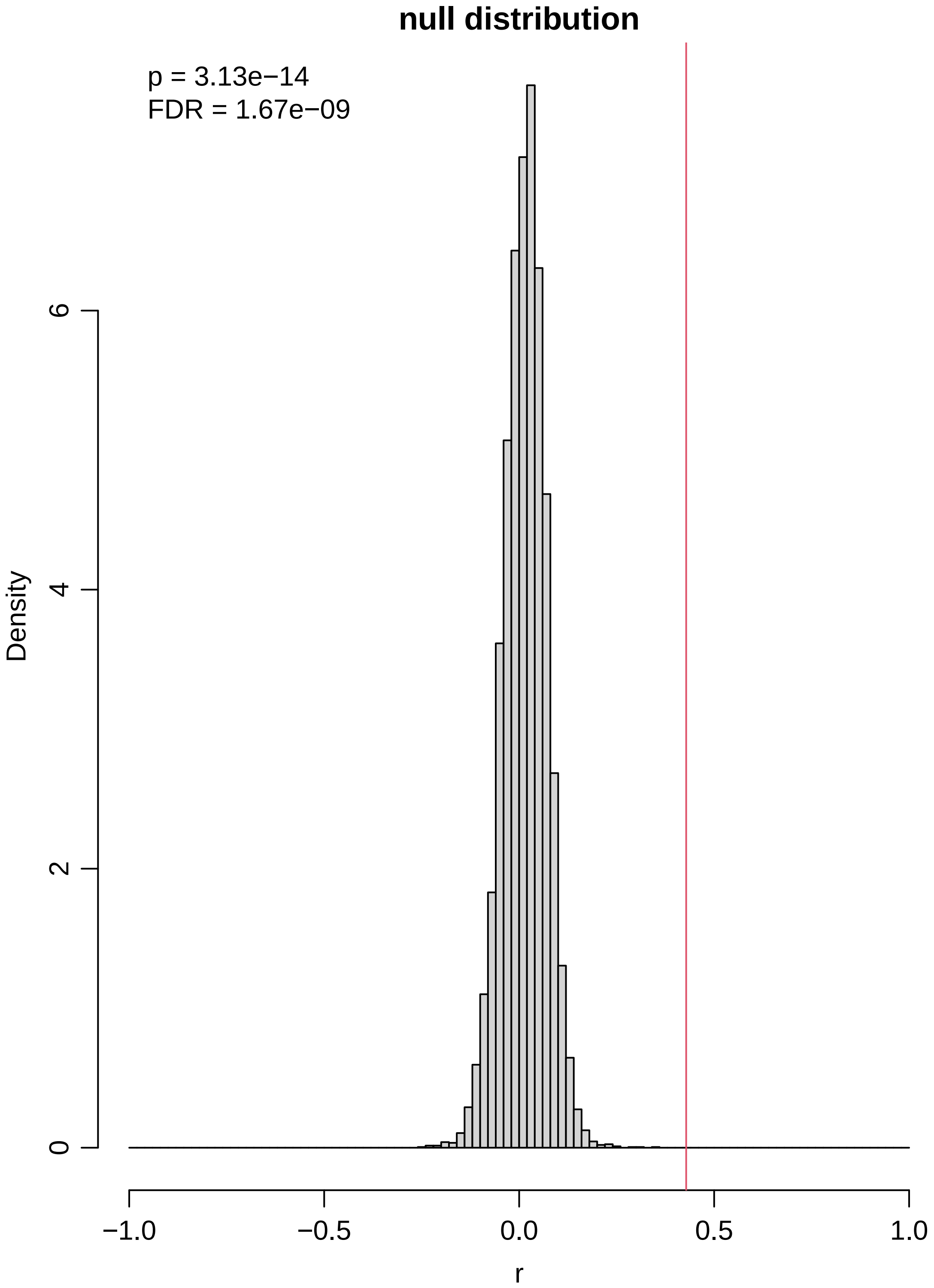

TTTY14 vs PeakID_262927 (chrY: 19265744 − 19266376)

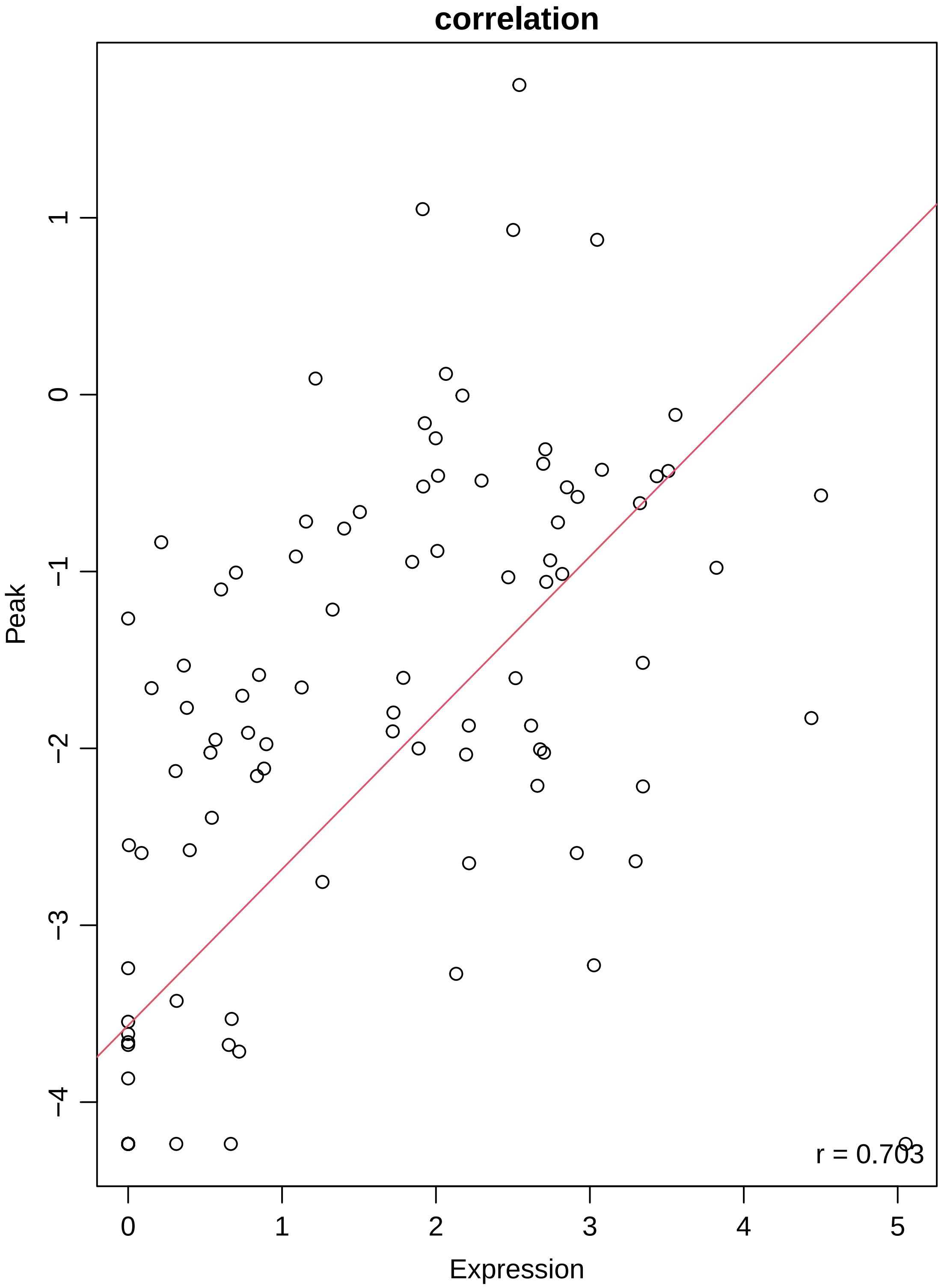

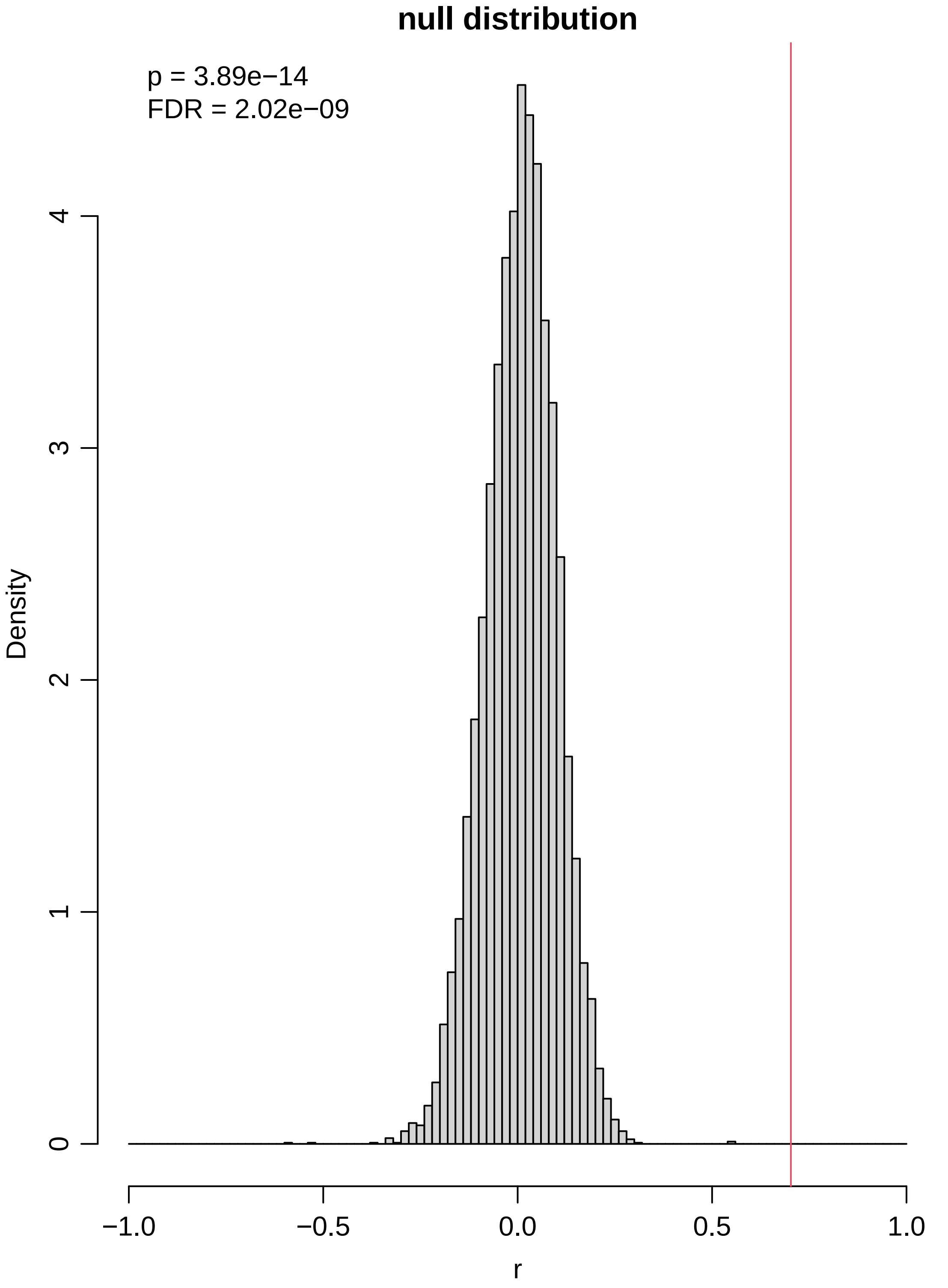

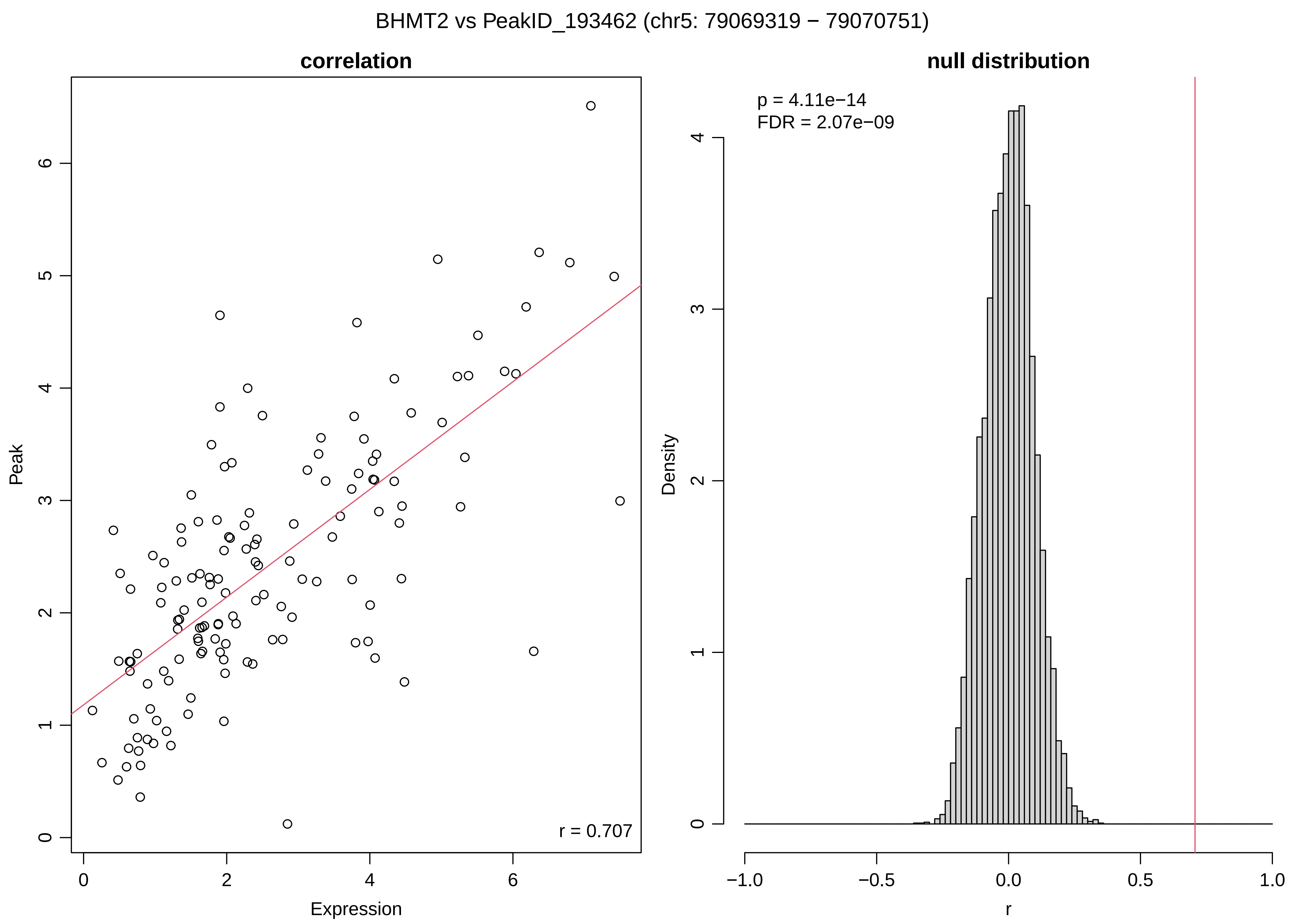
BHMT2 vs PeakID_193462 (chr5: 79069319 − 79070751)
correlation
Peak
Expression
r = 0.707
null distribution
p = 4.11e−14
FDR = 2.07e−09
Density
r

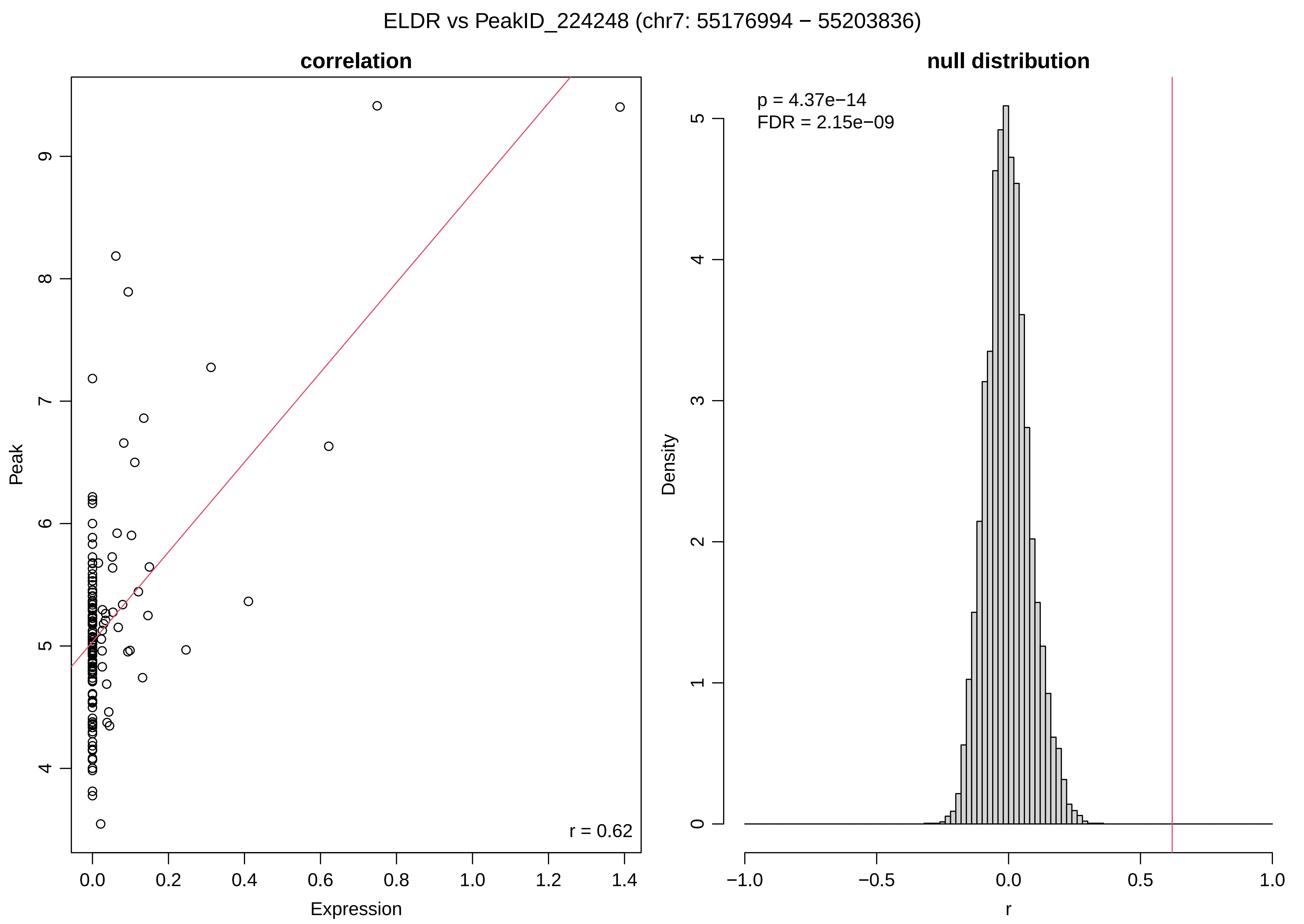
ELDR vs PeakID_224248 (chr7: 55176994 − 55203836)
correlation
Peak
Expression
r = 0.62
0.0
0.2
0.4
0.6
0.8
1.0
1.2
1.4
4
5
6
7
8
9
null distribution
p = 4.37e−14
FDR = 2.15e−09
Density
r
−1.0
−0.5
0.0
0.5
1.0
0
1
2
3
4
5

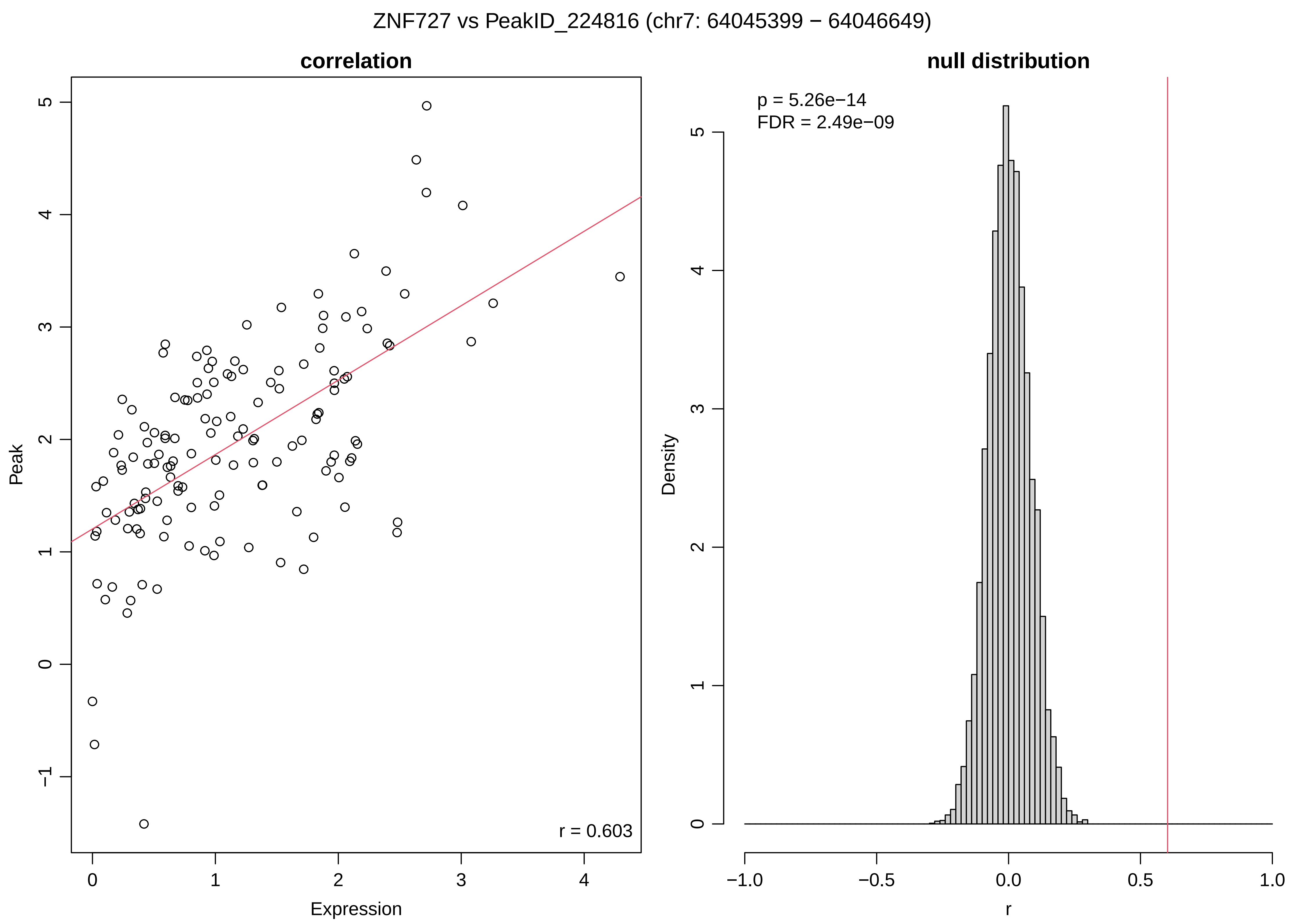

ZNF727 vs PeakID_224816 (chr7: 64045399 − 64046649)
correlation
null distribution
Peak
Expression
r = 0.603
p = 5.26e−14
FDR = 2.49e−09
Density
r

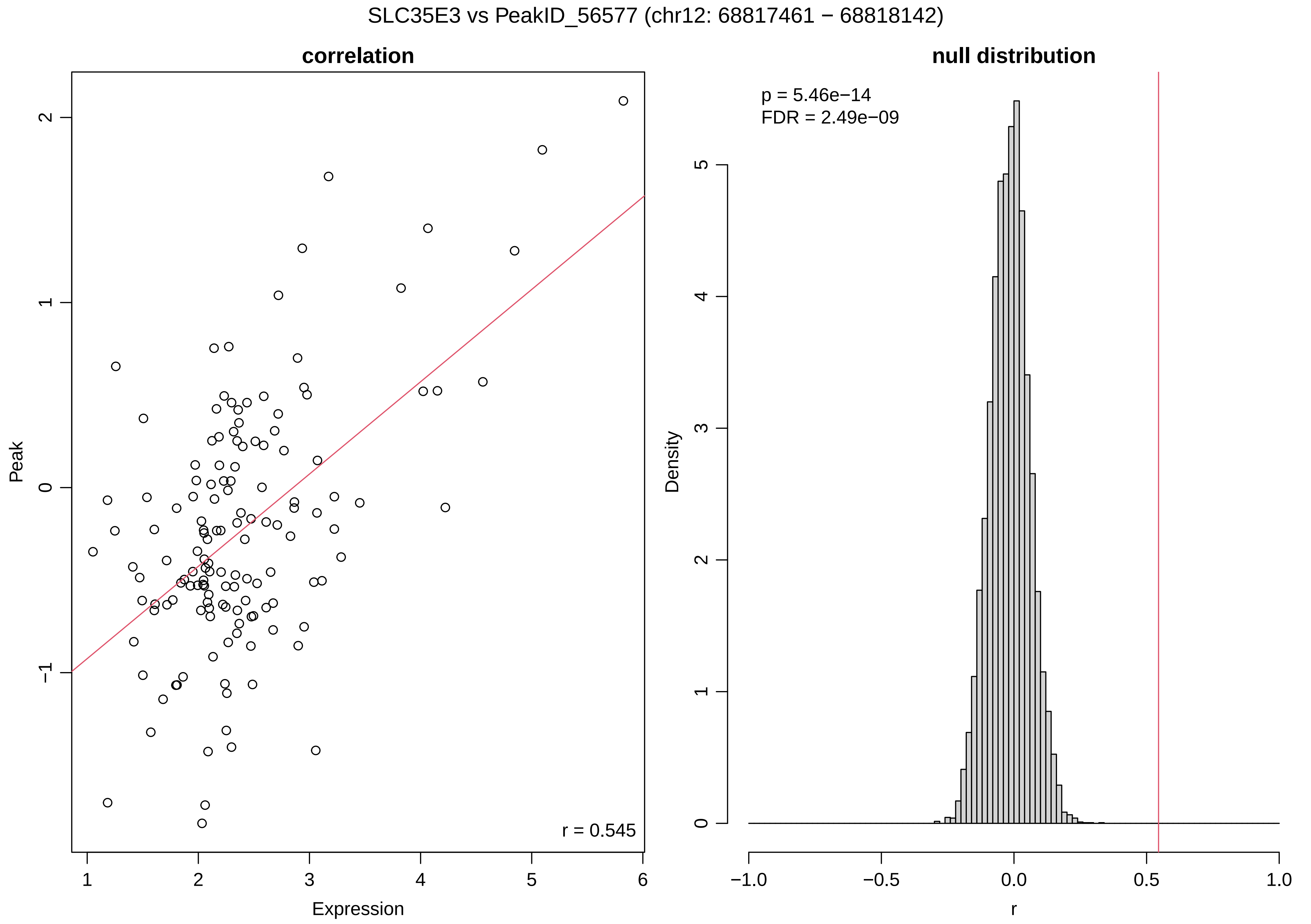
SLC35E3 vs PeakID_56577 (chr12: 68817461 − 68818142)
correlation
Peak
Expression
r = 0.545
null distribution
p = 5.46e−14
FDR = 2.49e−09
Density
r

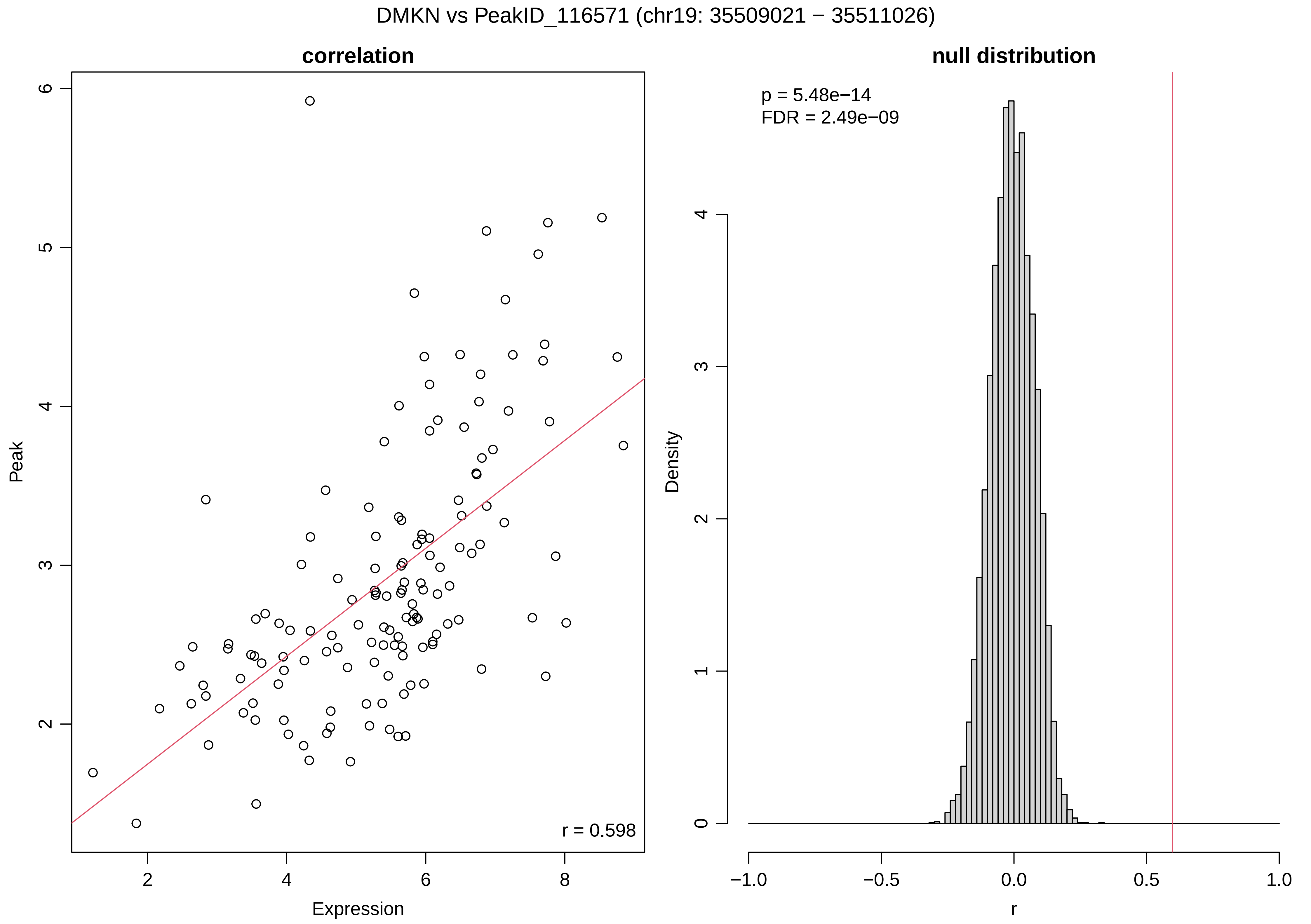
DMKN vs PeakID_116571 (chr19: 35509021 − 35511026)
correlation
Peak
Expression
r = 0.598
null distribution
p = 5.48e−14
FDR = 2.49e−09
Density
r

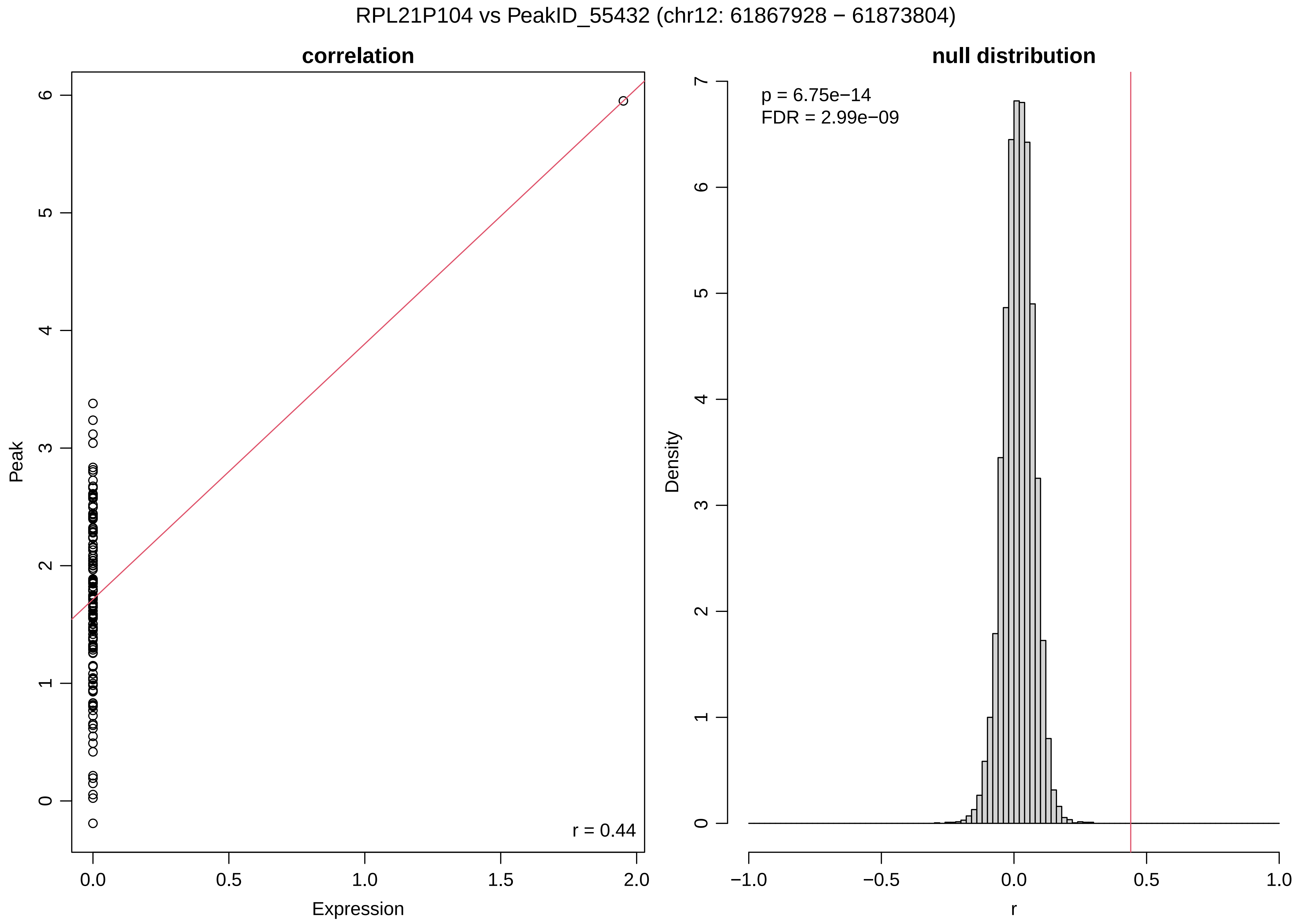
RPL21P104 vs PeakID_55432 (chr12: 61867928 − 61873804)
correlation
Peak
Expression
r = 0.44
0.0
0.5
1.0
1.5
2.0
0
1
2
3
4
5
6
null distribution
p = 6.75e−14
FDR = 2.99e−09
Density
r
−1.0
−0.5
0.0
0.5
1.0
0
1
2
3
4
5
6
7

AC092506.1 vs PeakID_10535 (chr1: 104072996 − 104073837)

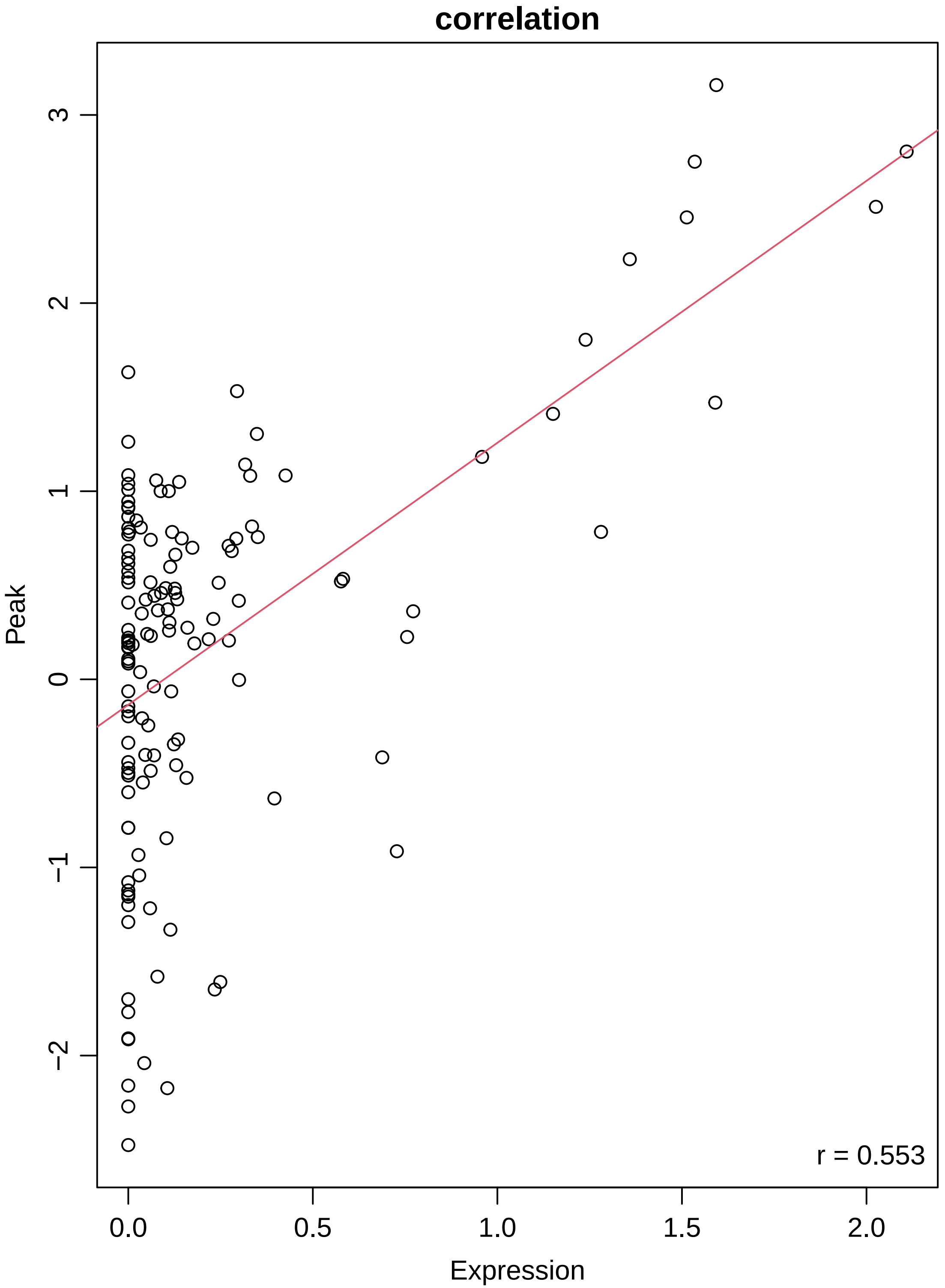

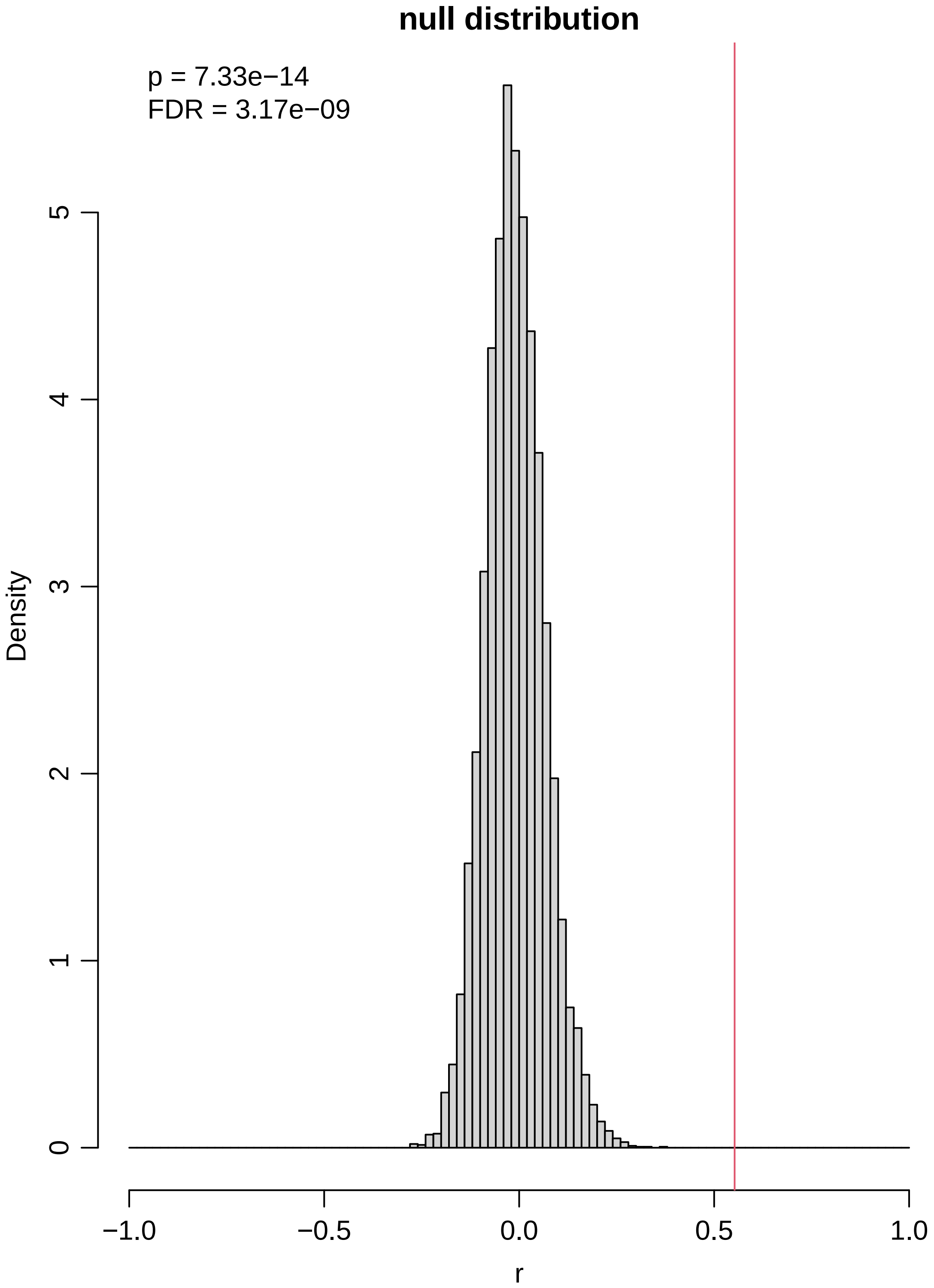

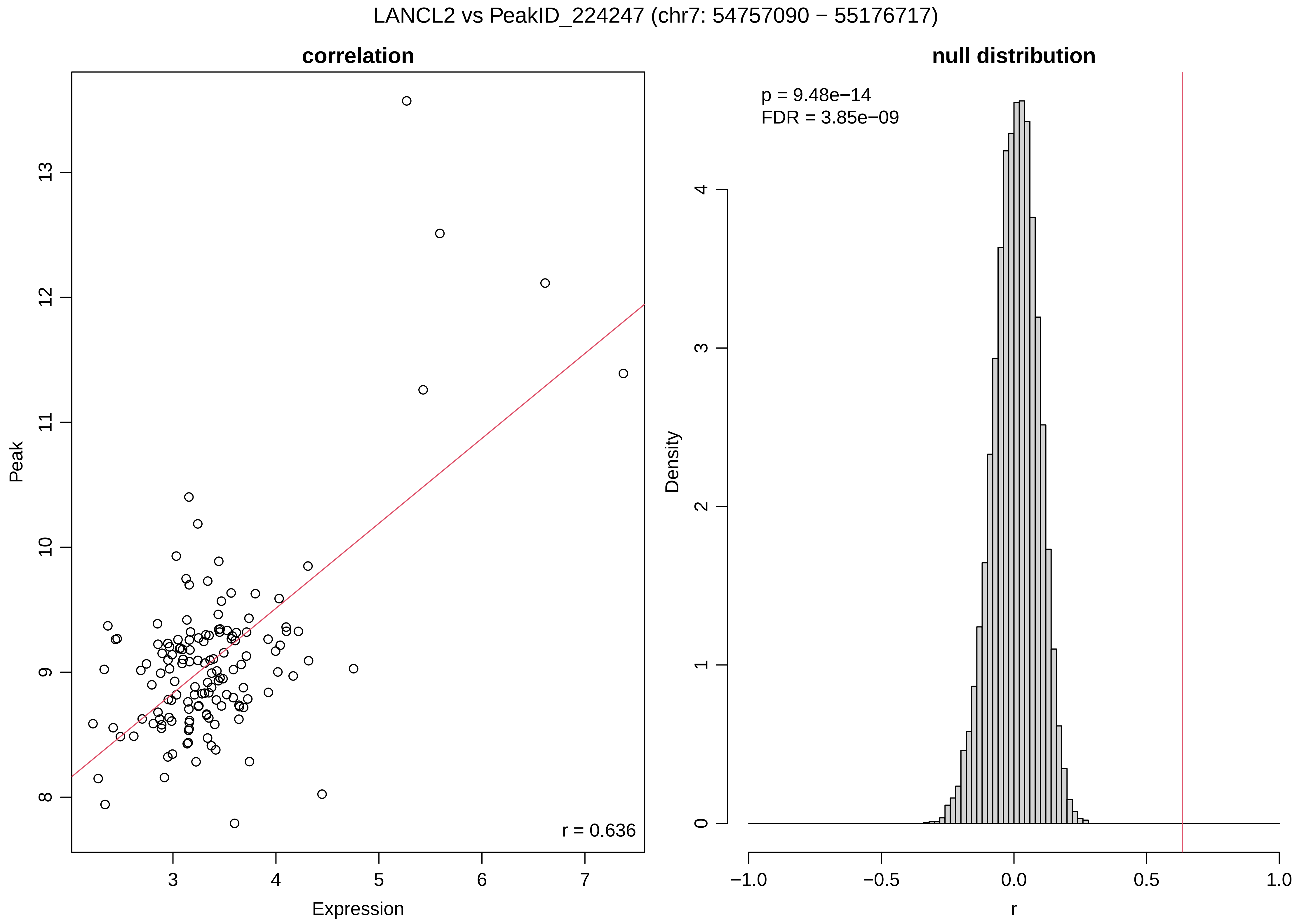
LANCL2 vs PeakID_224247 (chr7: 54757090 − 55176717)
correlation
null distribution
p = 9.48e−14
FDR = 3.85e−09
r = 0.636
Peak
Expression
Density
r

OOEP vs PeakID_208692 (chr6: 73394320 − 73395612)

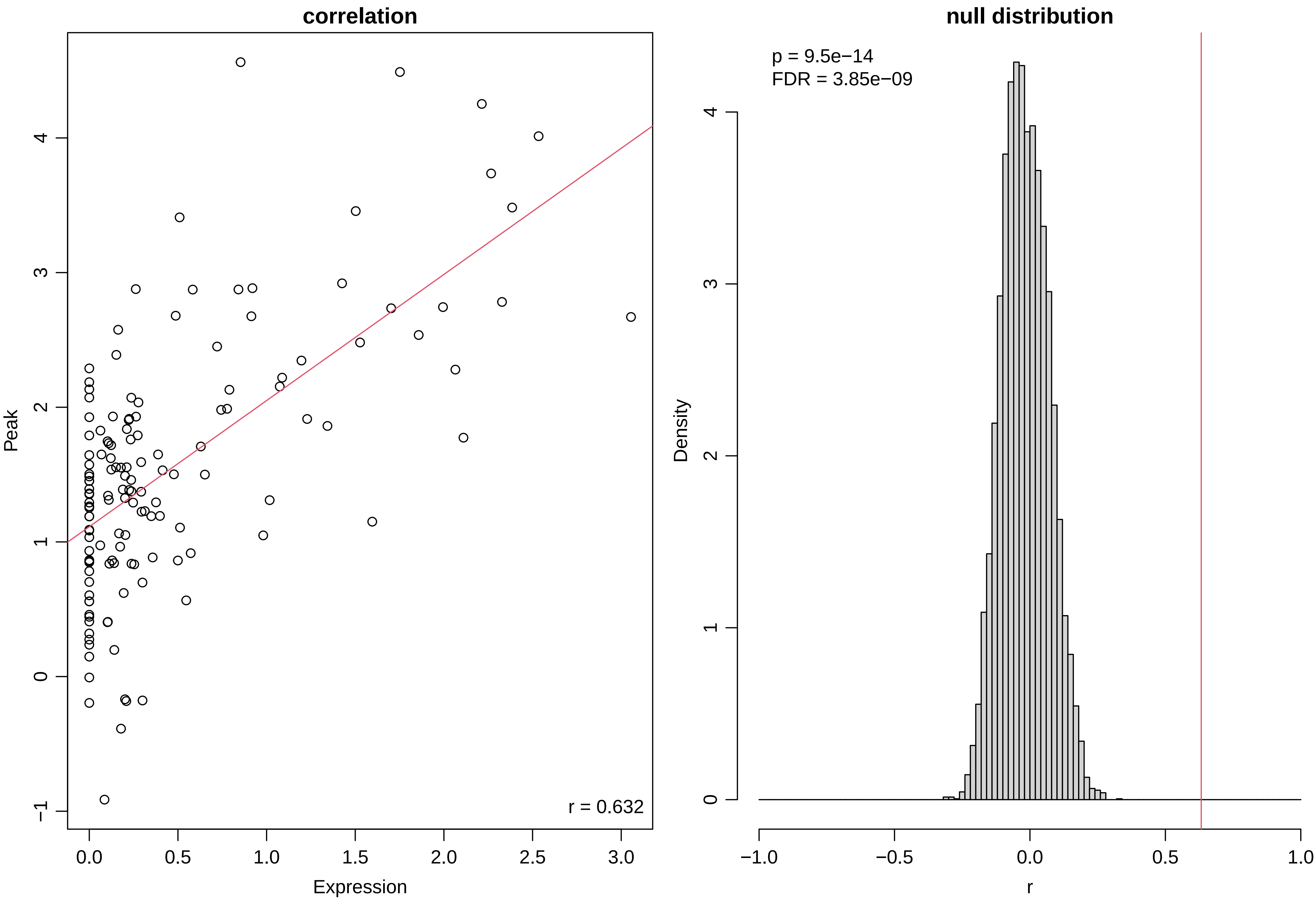

GJB7 vs PeakID_209234 (chr6: 87328463 − 87329601)

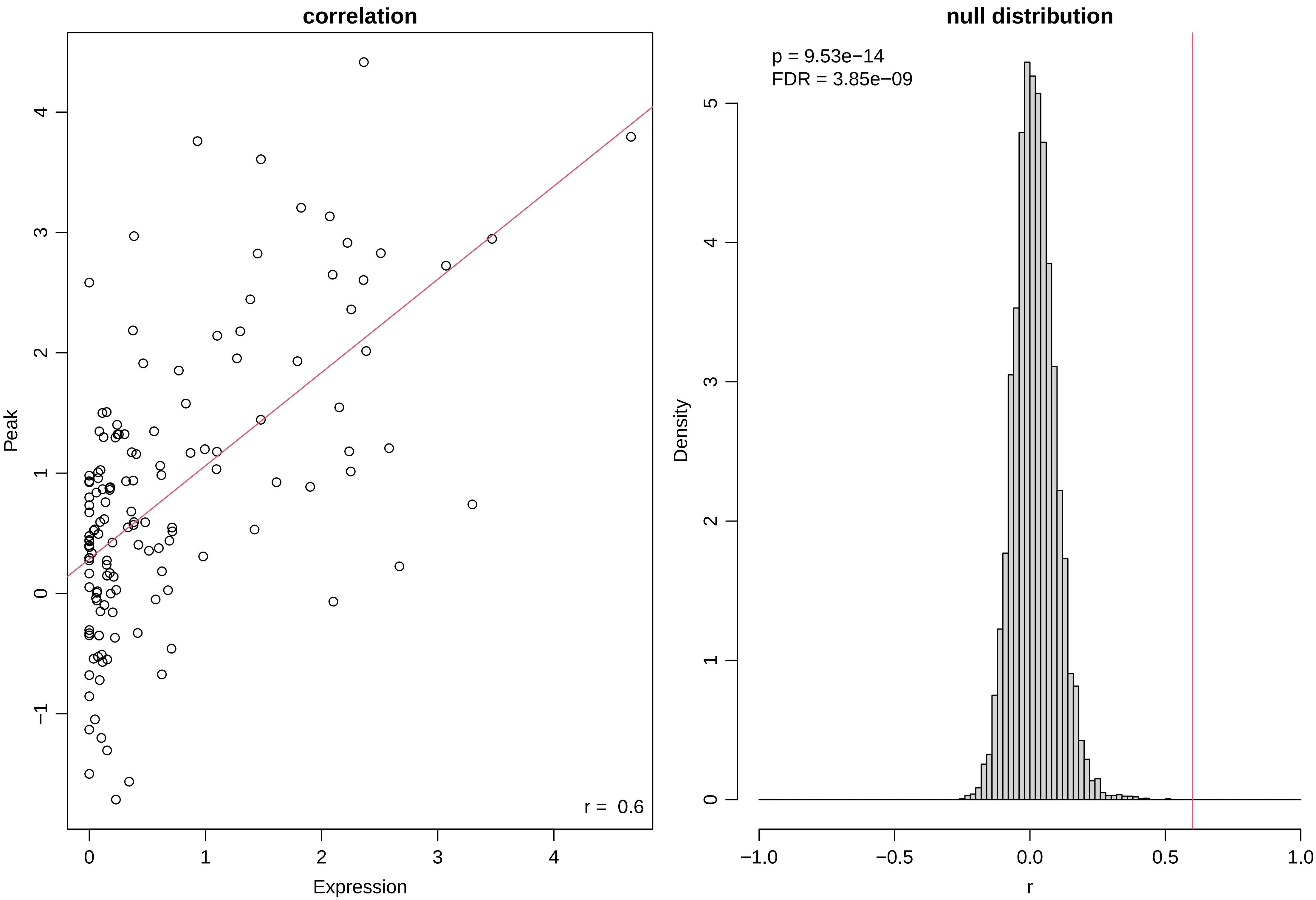

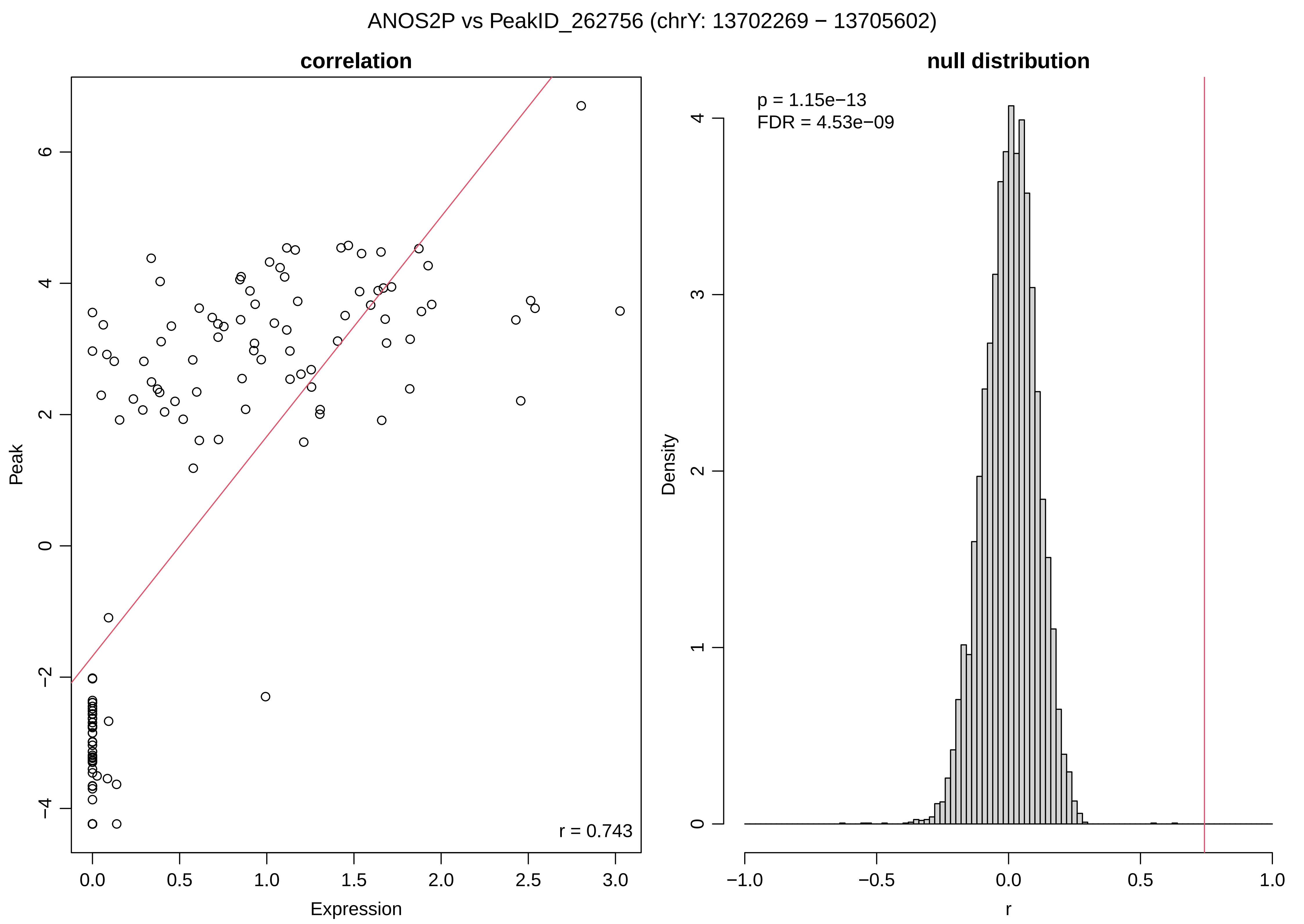

ANOS2P vs PeakID_262756 (chrY: 13702269 − 13705602)
correlation
Peak
Expression
r = 0.743
null distribution
p = 1.15e−13
FDR = 4.53e−09
Density
r

YEATS4 vs PeakID_56878 (chr12: 69552001 − 69556644)

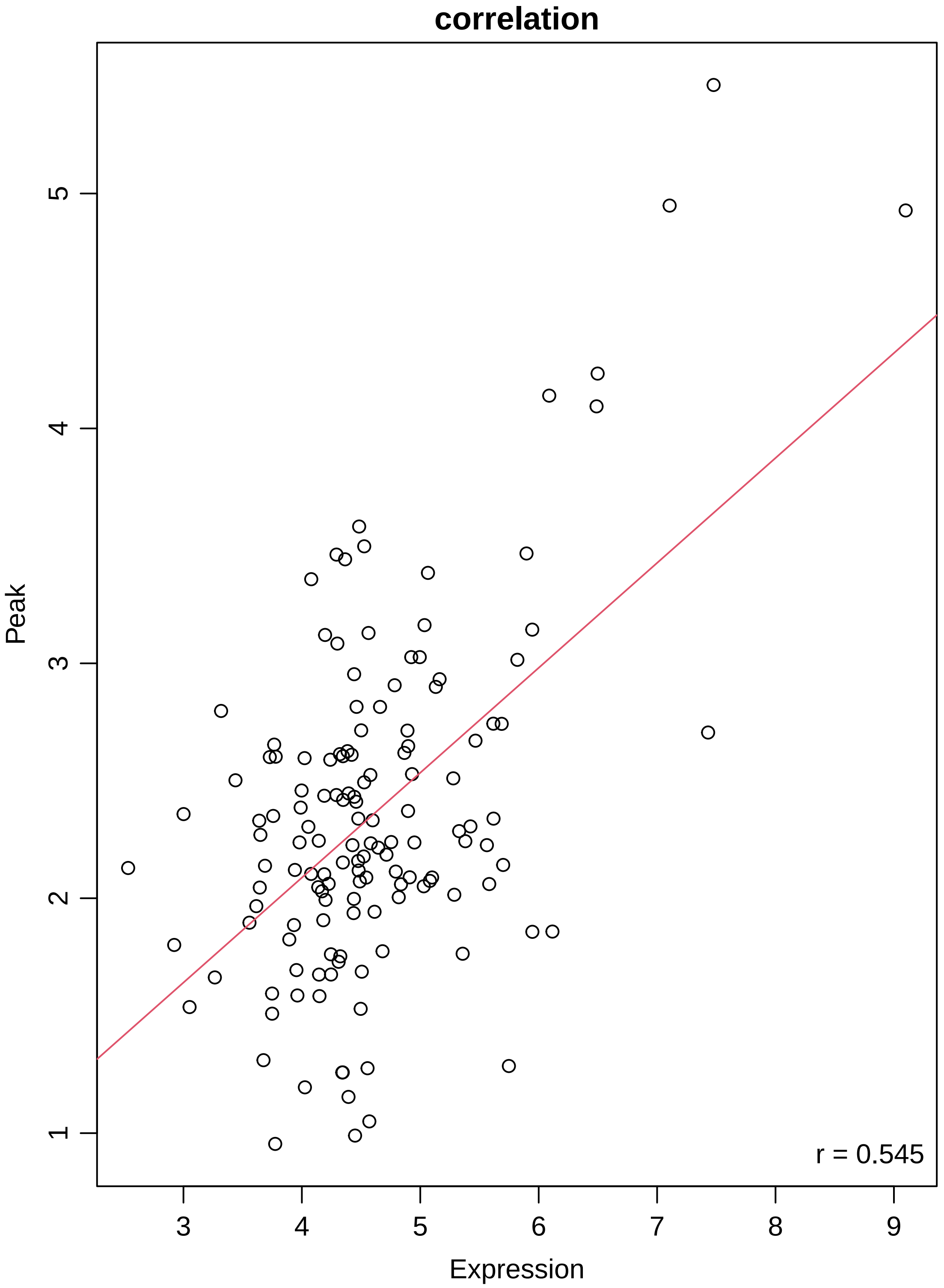

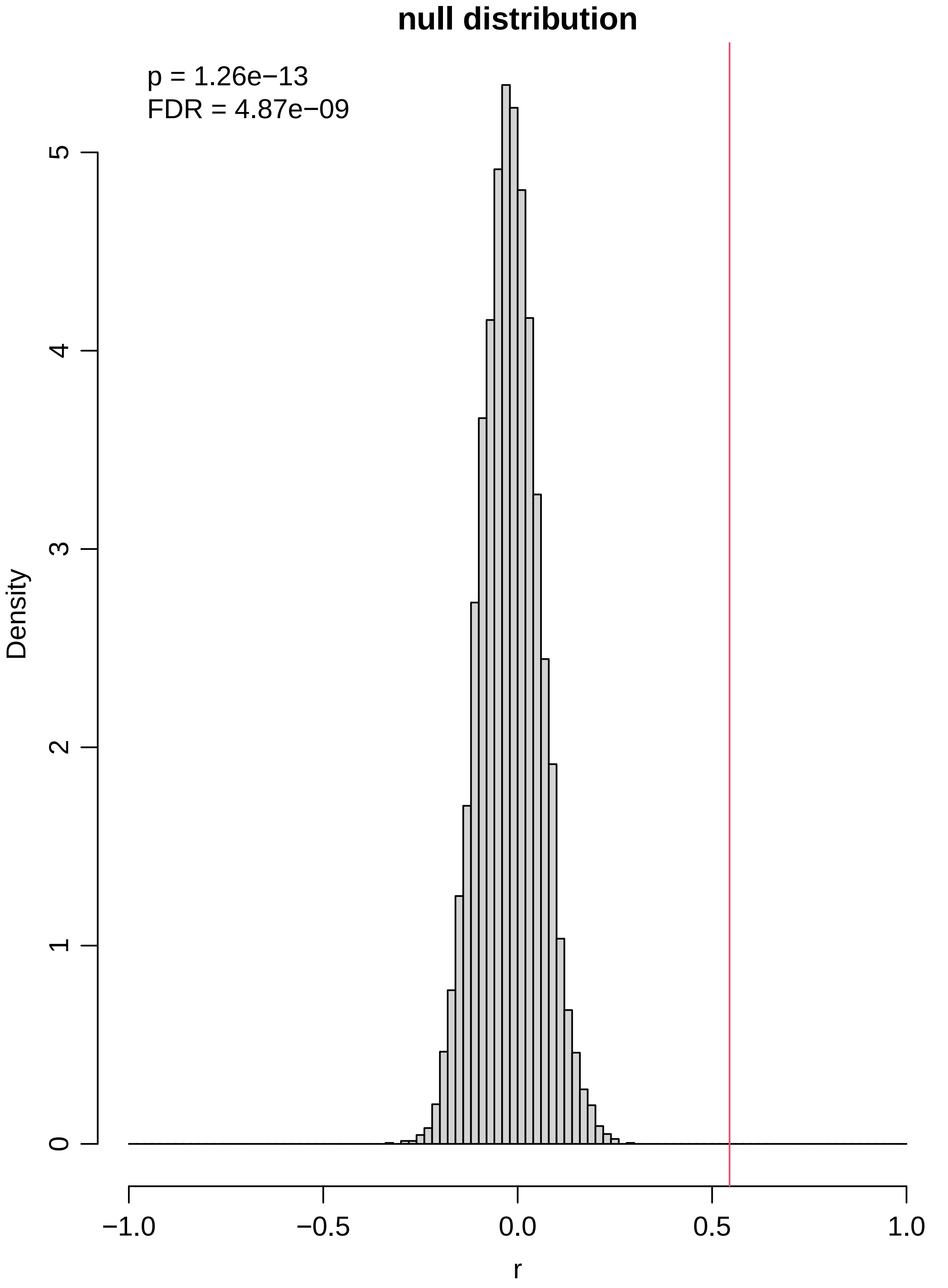

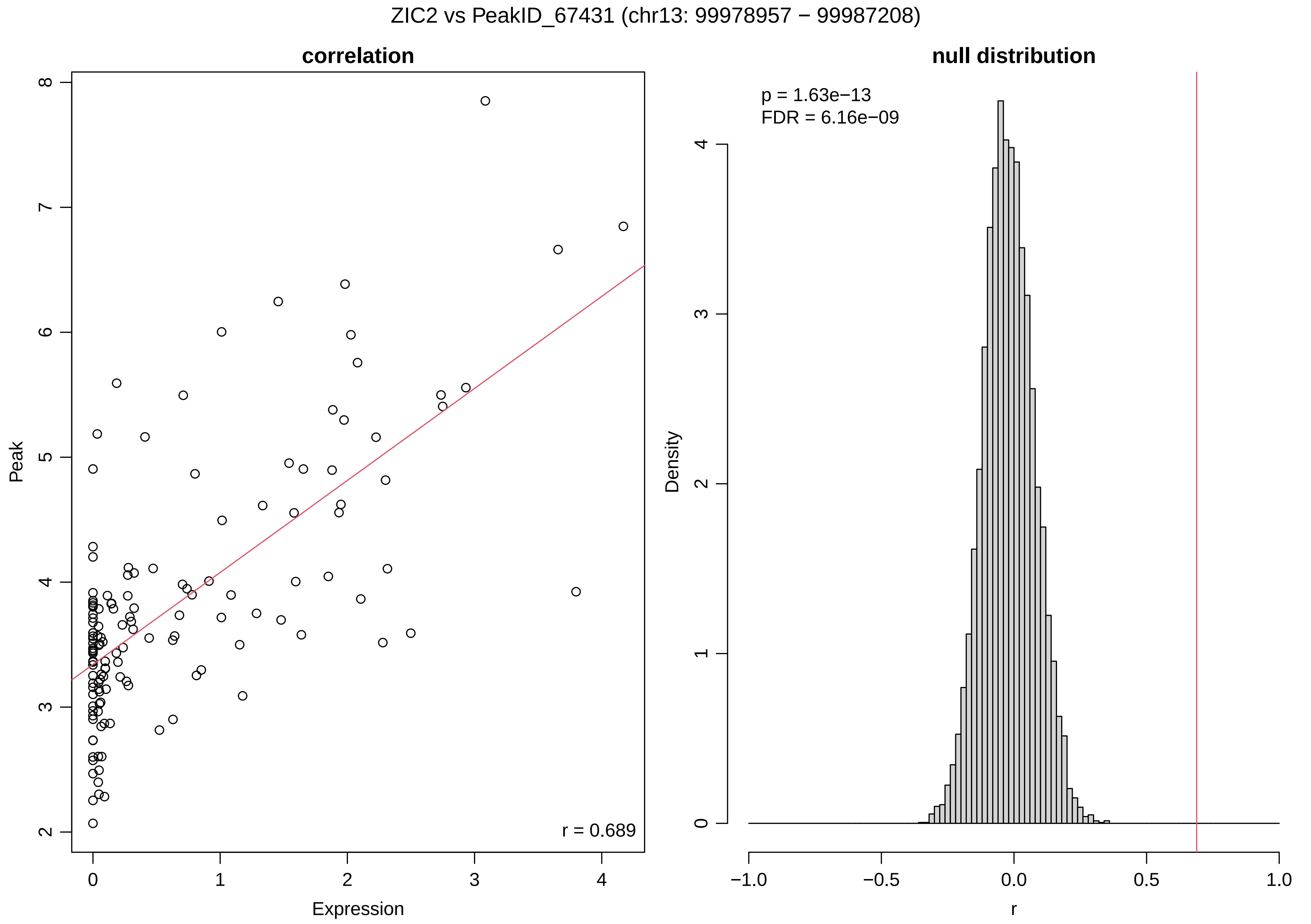

ZIC2 vs PeakID_67431 (chr13: 99978957 − 99987208)
correlation
Peak
Expression
r = 0.689
null distribution
p = 1.63e−13
FDR = 6.16e−09
Density
r

MET vs PeakID_229010 (chr7: 116670297 − 116718405)

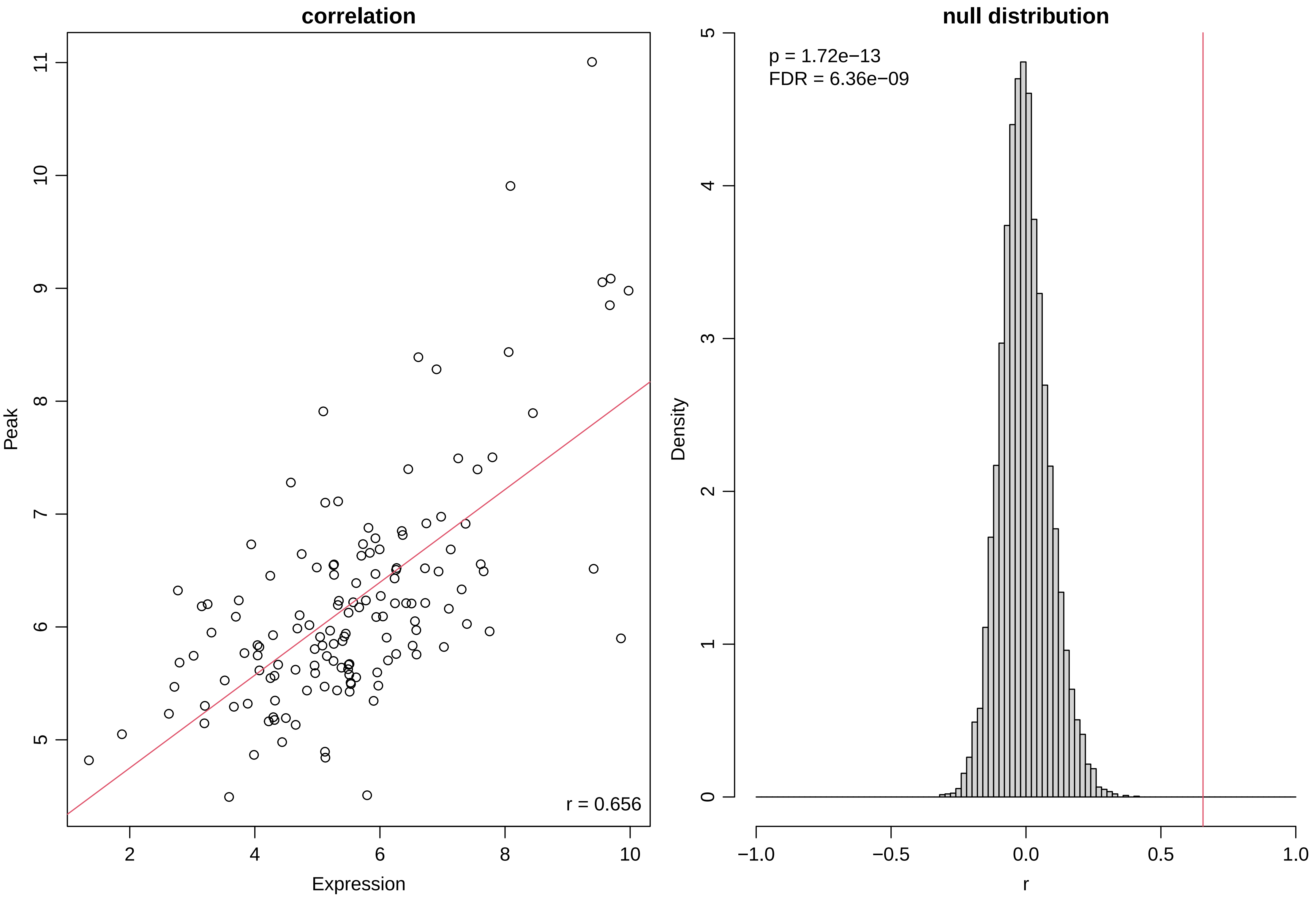

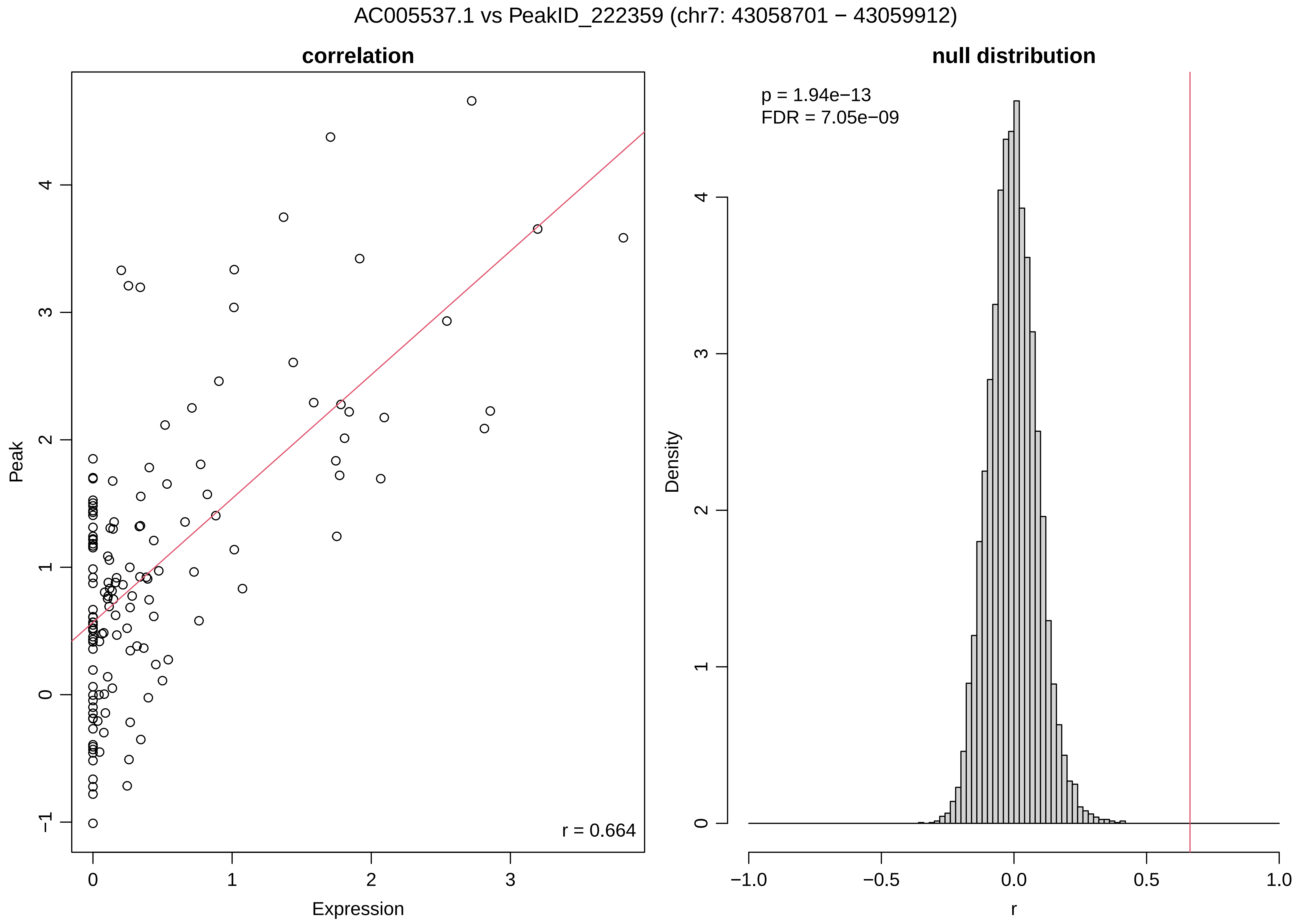
AC005537.1 vs PeakID_222359 (chr7: 43058701 − 43059912)
correlation
Peak
Expression
r = 0.664
null distribution
p = 1.94e−13
FDR = 7.05e−09
Density
r

SLC35E3 vs PeakID_56599 (chr12: 68849192 − 68850562)

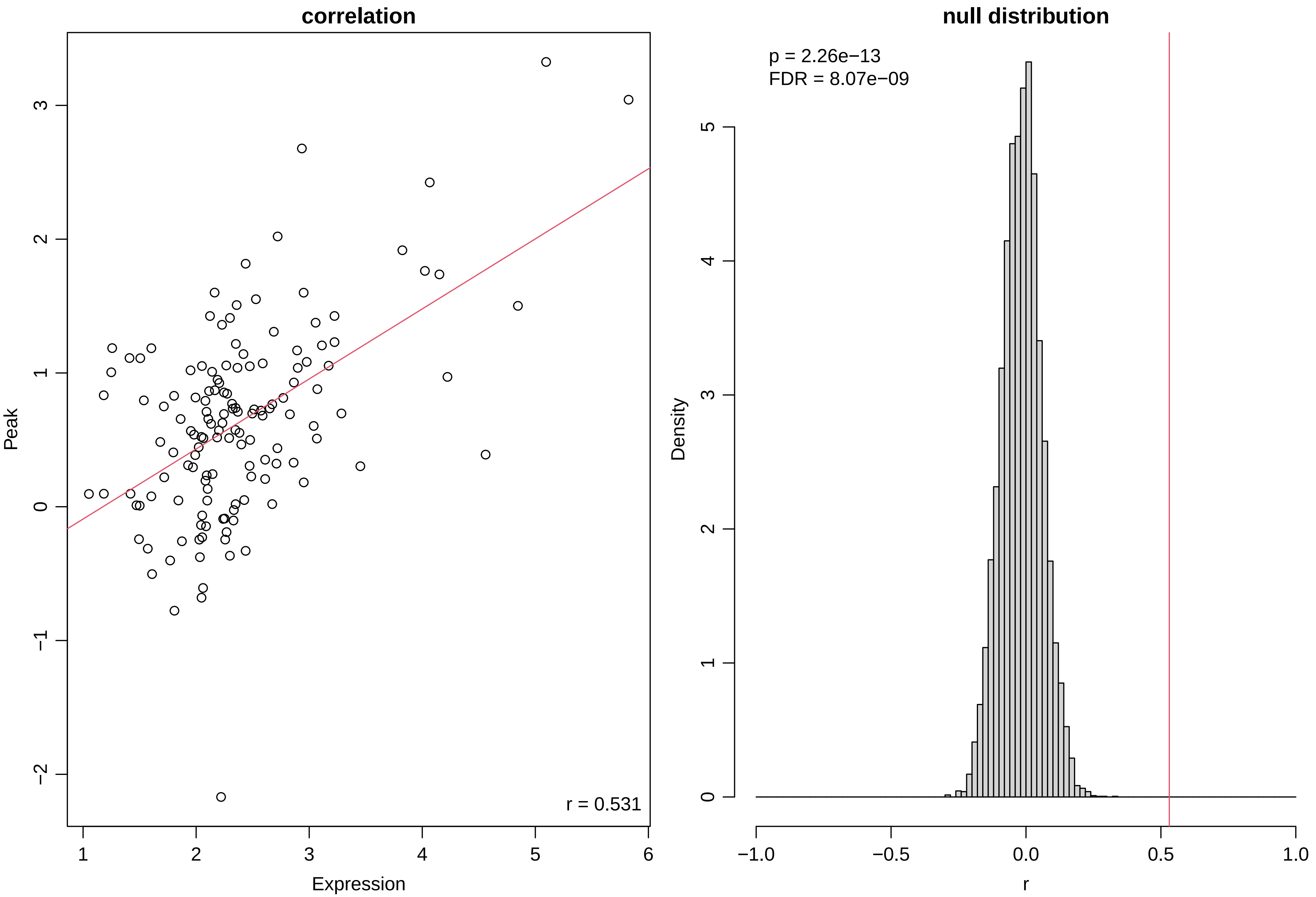

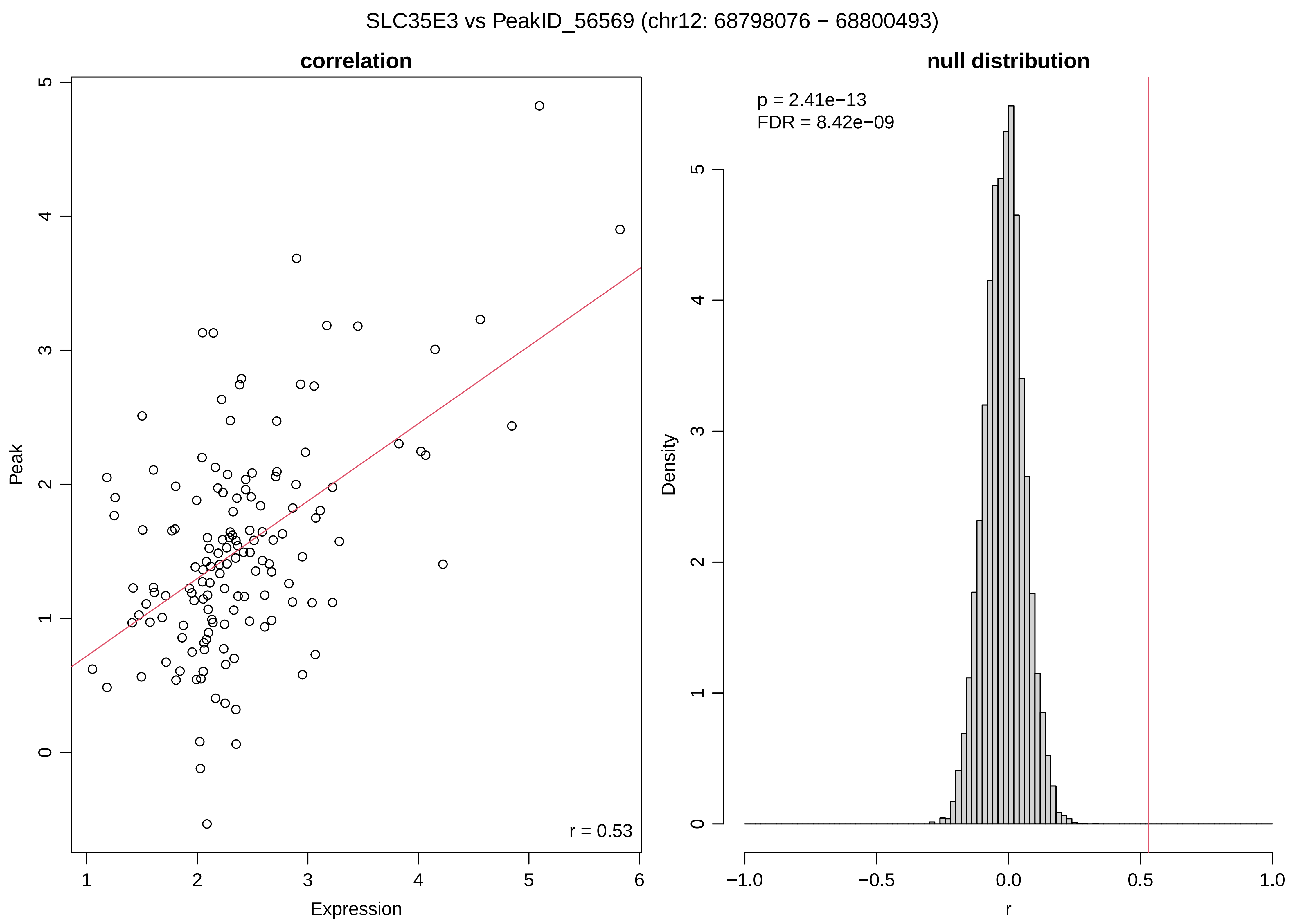
SLC35E3 vs PeakID_56569 (chr12: 68798076 − 68800493)
correlation
Peak
Expression
r = 0.53
null distribution
p = 2.41e−13
FDR = 8.42e−09
Density
r

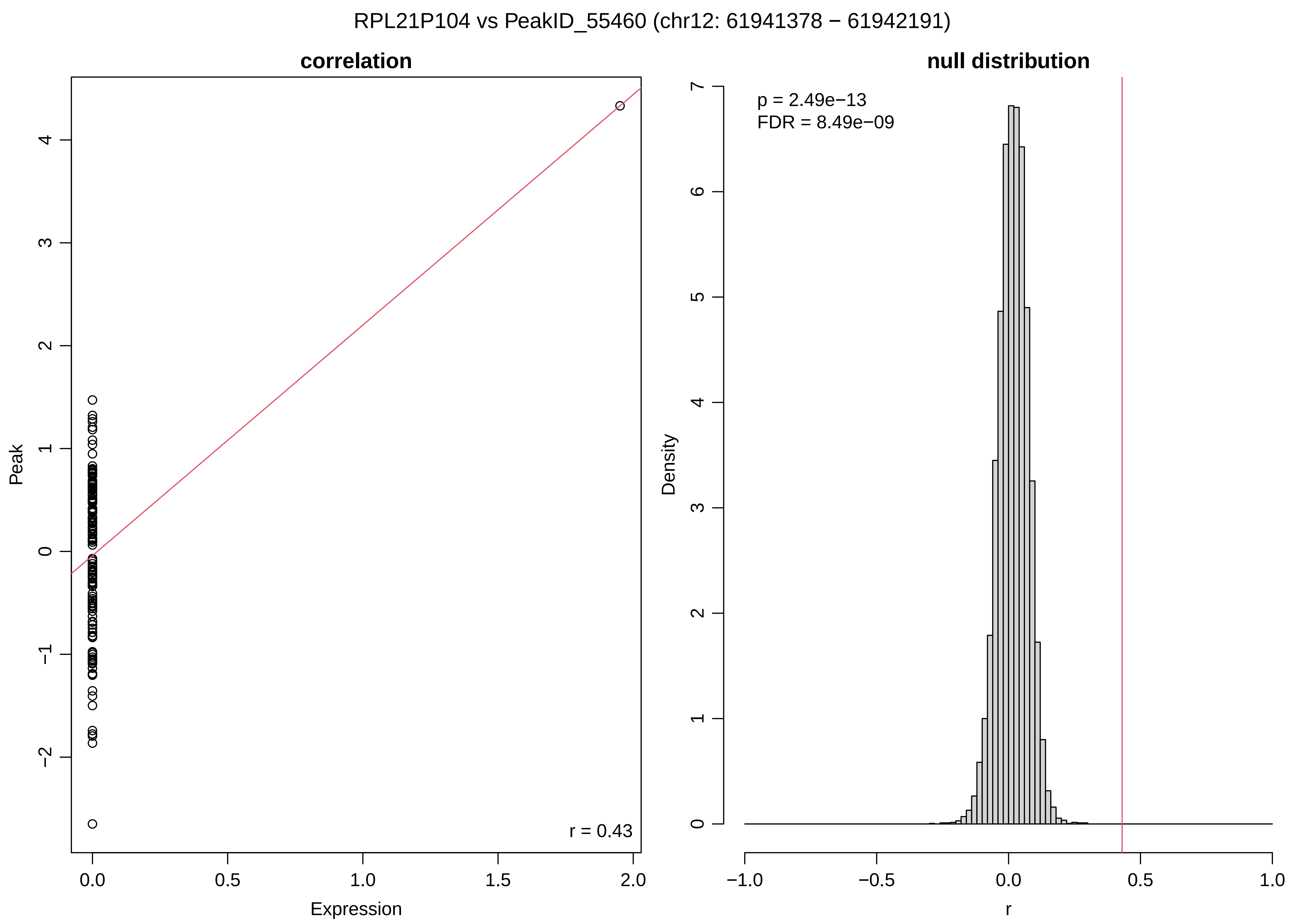
RPL21P104 vs PeakID_55460 (chr12: 61941378 − 61942191)
correlation
null distribution
p = 2.49e−13
FDR = 8.49e−09
r = 0.43
Peak
Expression
Density
r

RNU4−20P vs PeakID_54829 (chr12: 59573177 − 59578166)

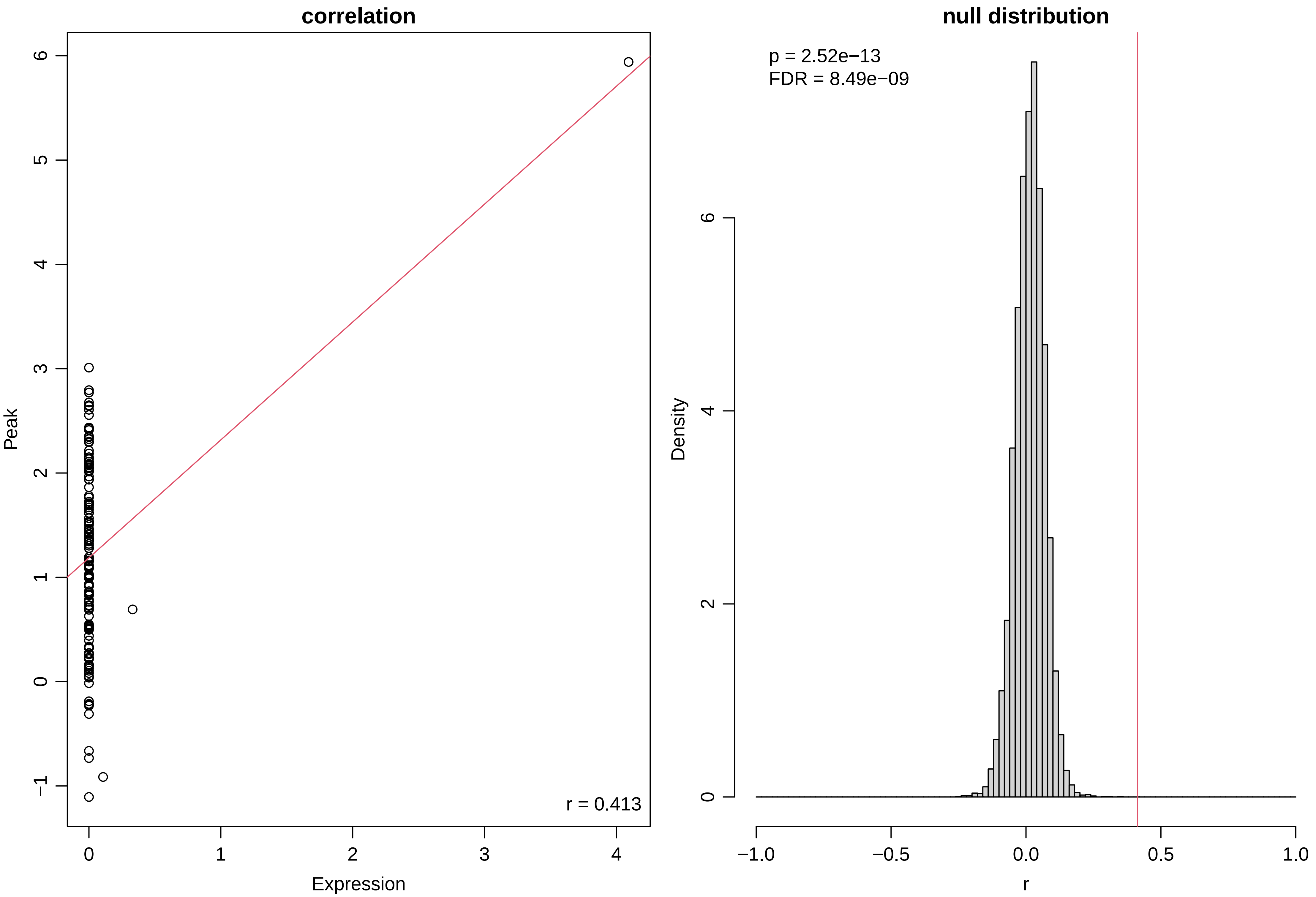

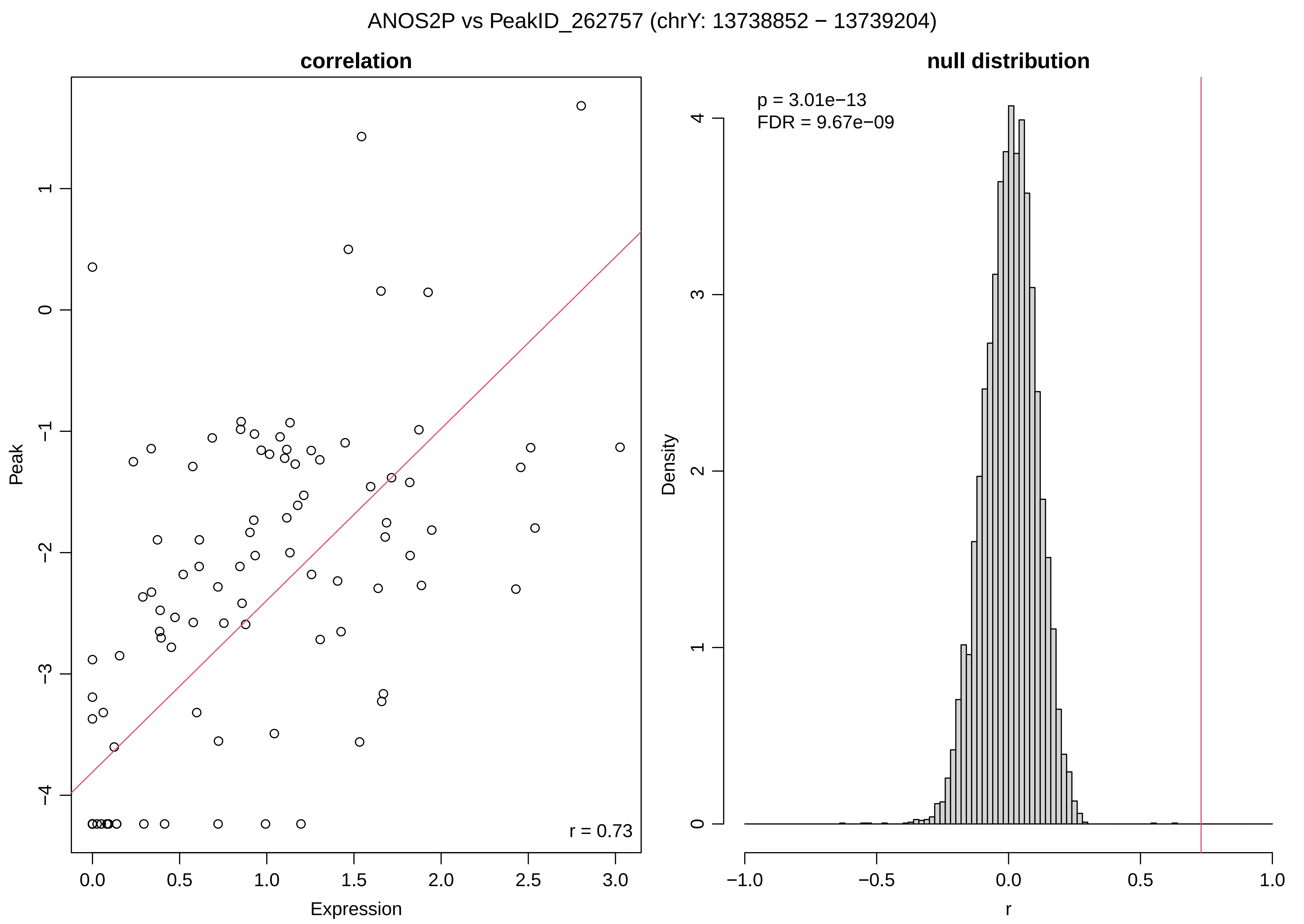
ANOS2P vs PeakID_262757 (chrY: 13738852 − 13739204)
correlation
Peak
Expression
r = 0.73
null distribution
p = 3.01e−13
FDR = 9.67e−09
Density
r

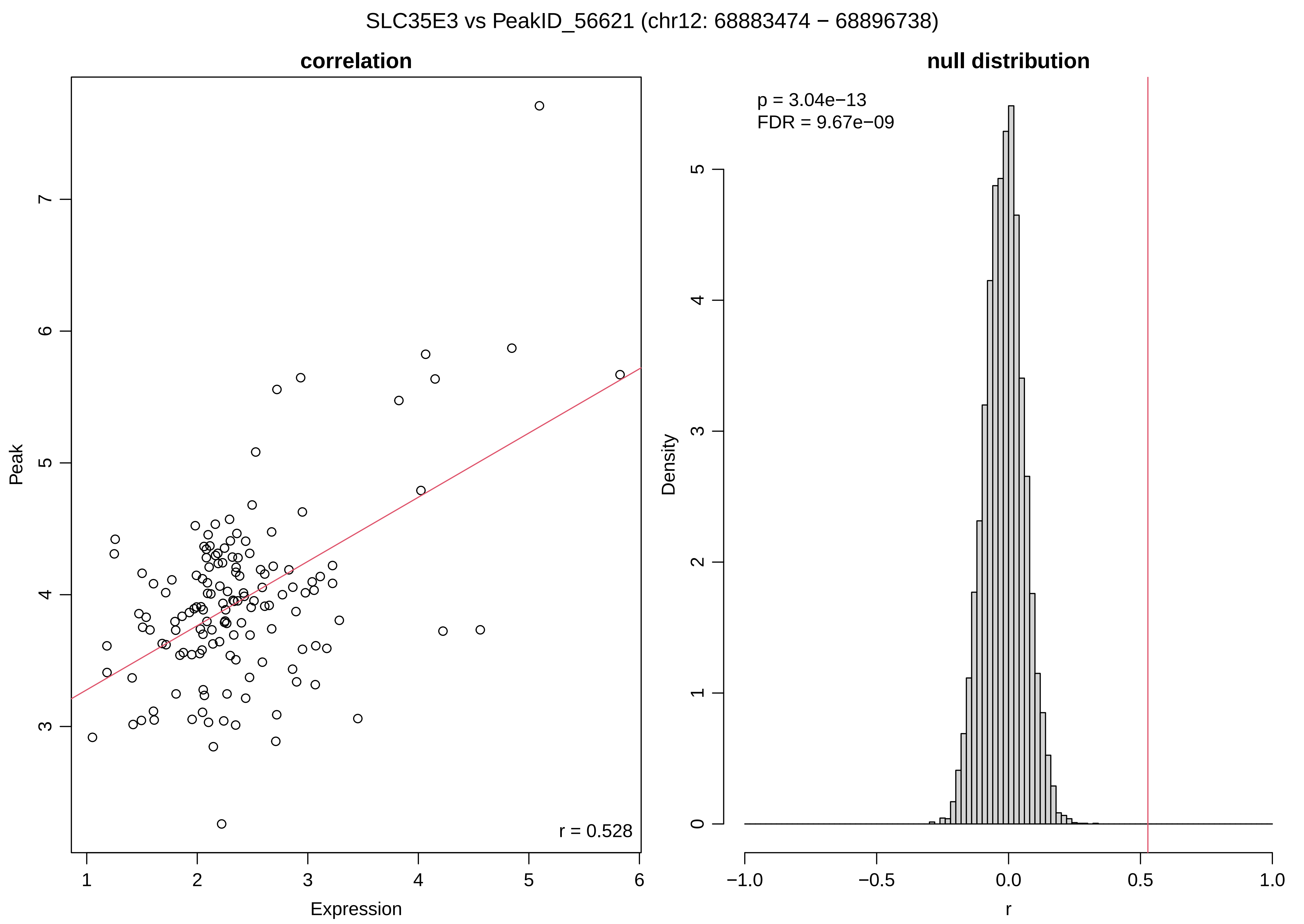
SLC35E3 vs PeakID_56621 (chr12: 68883474 − 68896738)
correlation
null distribution
p = 3.04e−13
FDR = 9.67e−09
Peak
Expression
r = 0.528
Density
r

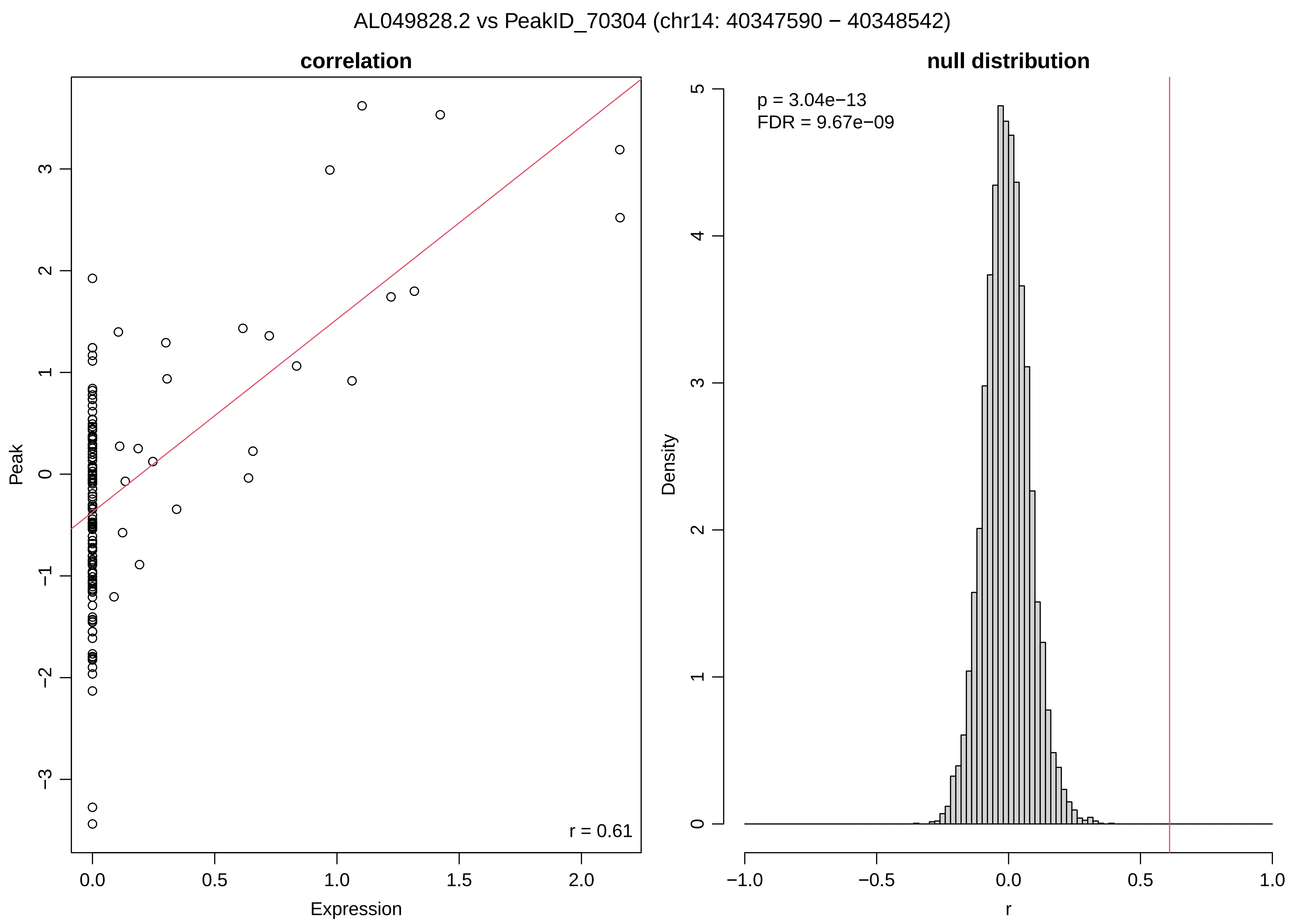
AL049828.2 vs PeakID_70304 (chr14: 40347590 − 40348542)
correlation
Peak
Expression
r = 0.61
null distribution
Density
r
p = 3.04e−13
FDR = 9.67e−09

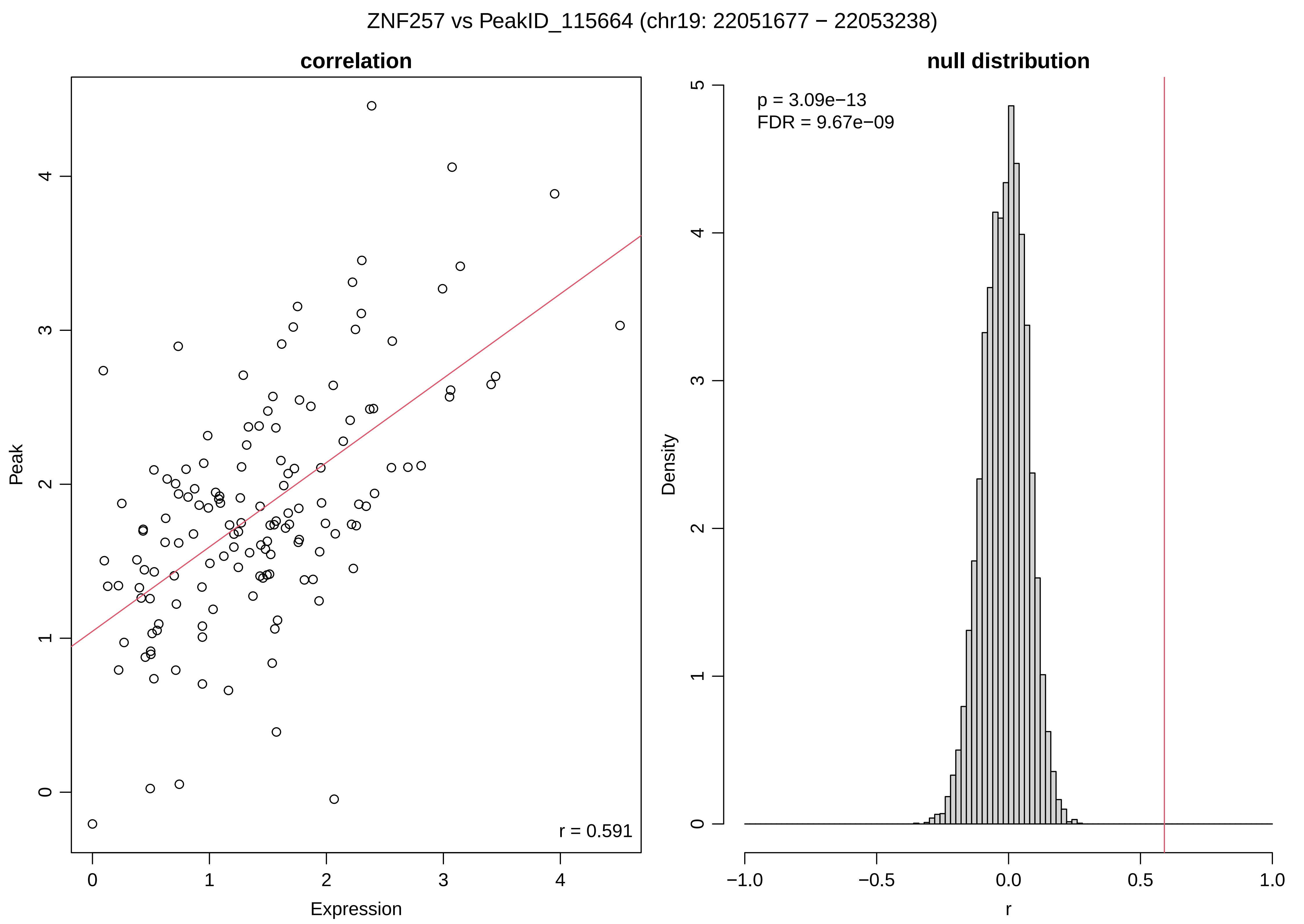
ZNF257 vs PeakID_115664 (chr19: 22051677 − 22053238)
correlation
null distribution
p = 3.09e−13
FDR = 9.67e−09
r = 0.591
Peak
Expression
Density
r
0
1
2
3
4
5
−1.0
−0.5
0.0
0.5
1.0

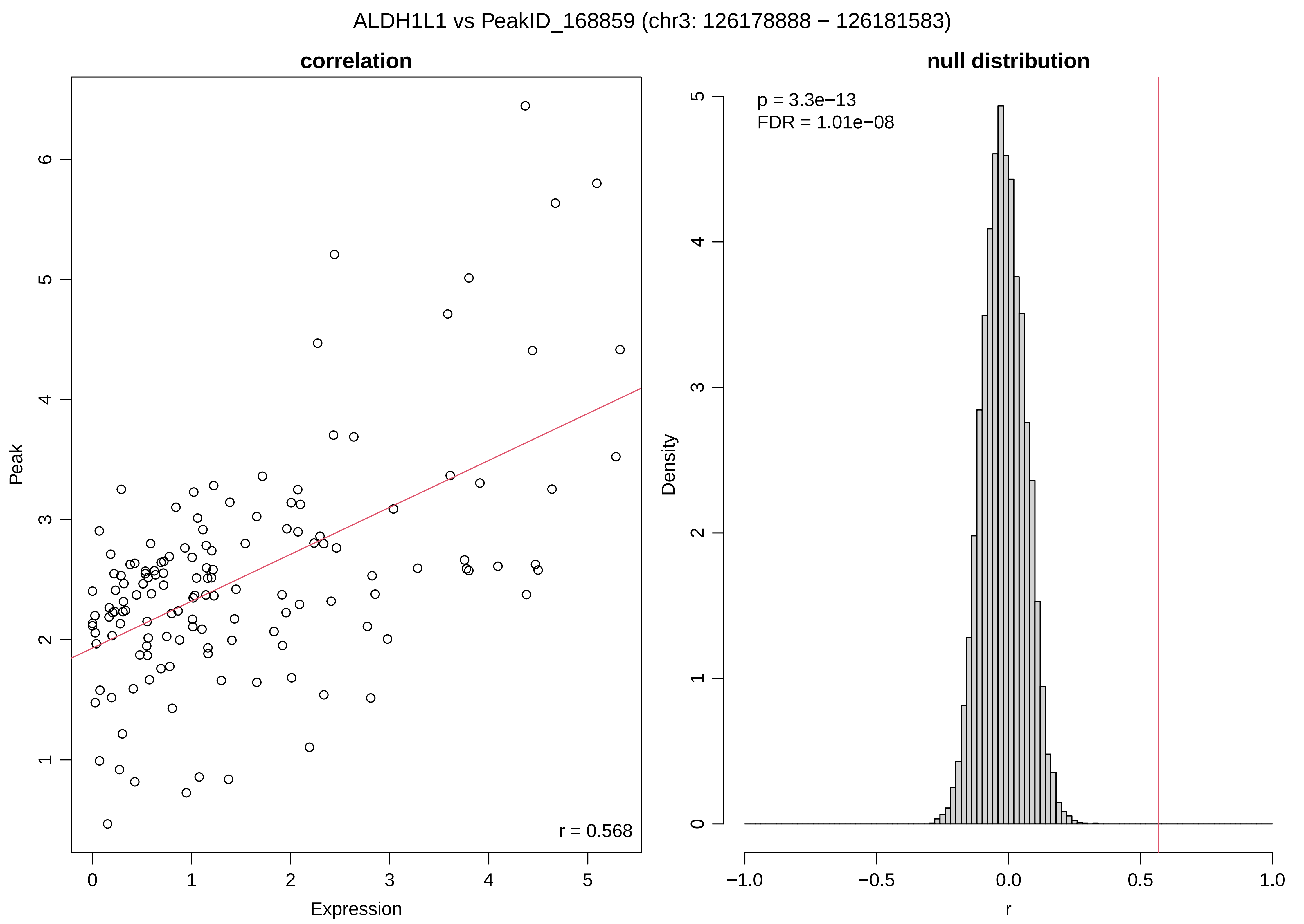

ALDH1L1 vs PeakID_168859 (chr3: 126178888 − 126181583)
correlation
Peak
Expression
r = 0.568
null distribution
p = 3.3e−13
FDR = 1.01e−08
Density
r

RNU4−20P vs PeakID_54801 (chr12: 59506184 − 59507297)

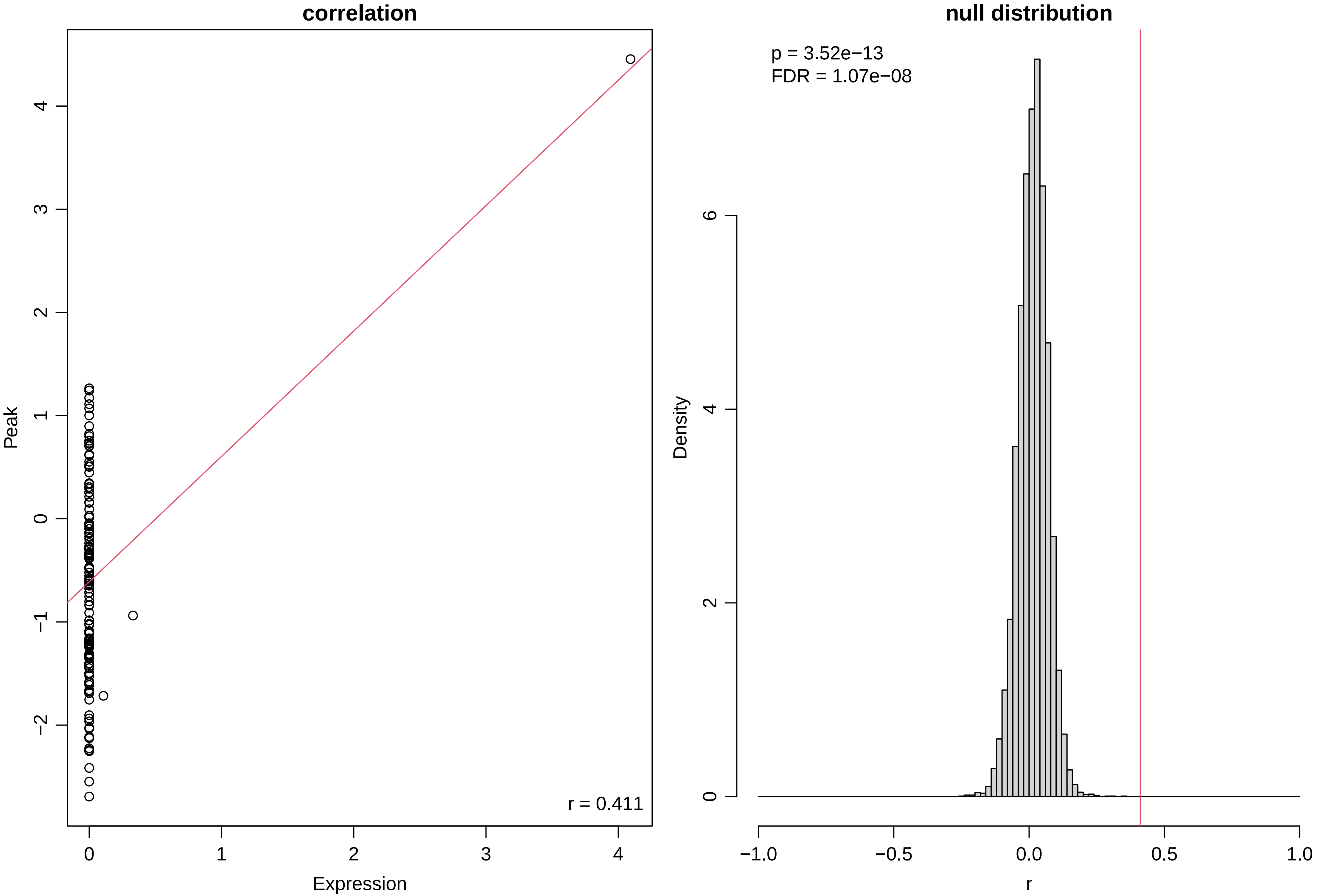

PAX8−AS1 vs PeakID_132258 (chr2: 113233800 − 113243770)

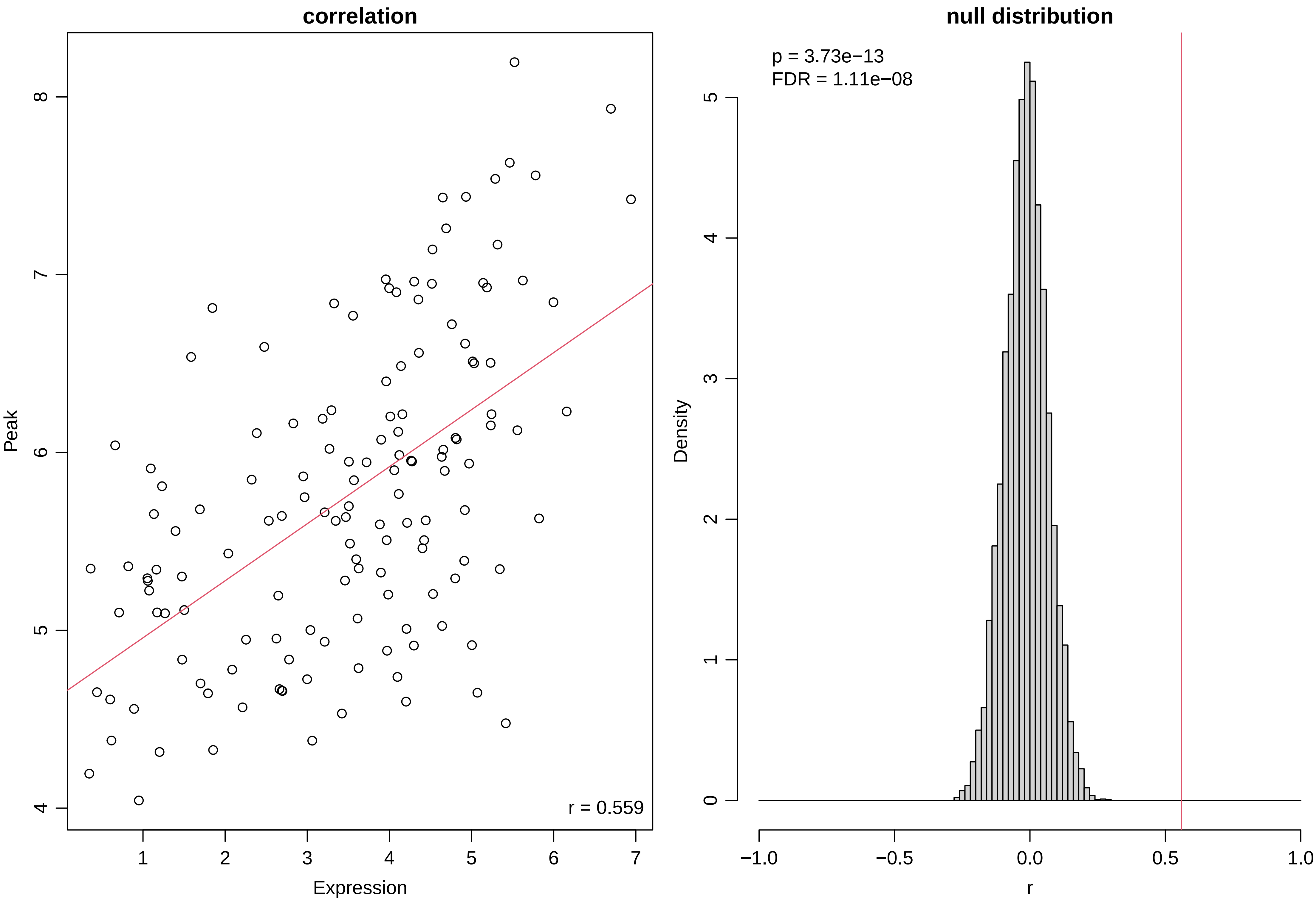

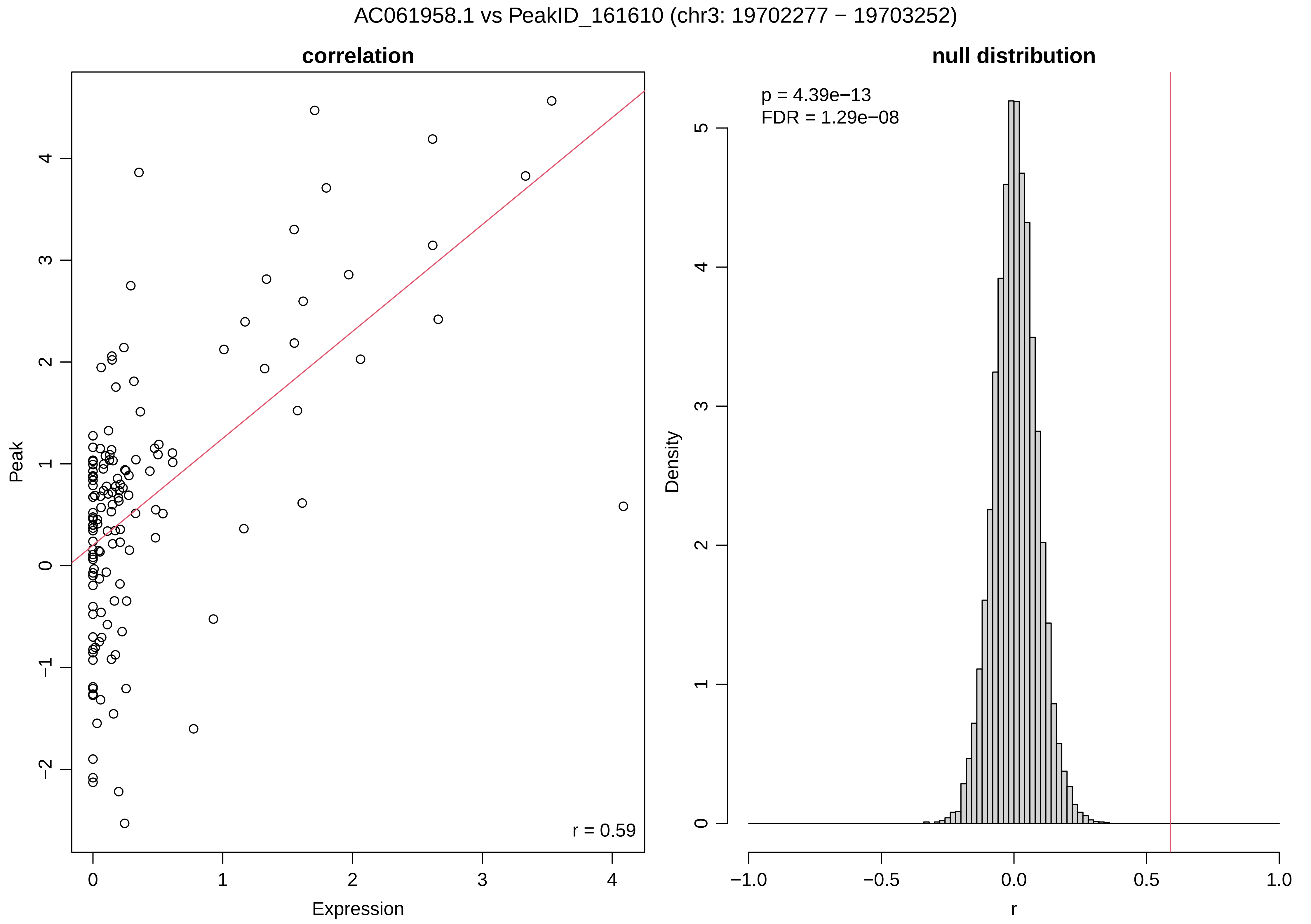
AC061958.1 vs PeakID_161610 (chr3: 19702277 − 19703252)
correlation
Peak
Expression
r = 0.59
null distribution
p = 4.39e−13
FDR = 1.29e−08
Density
r

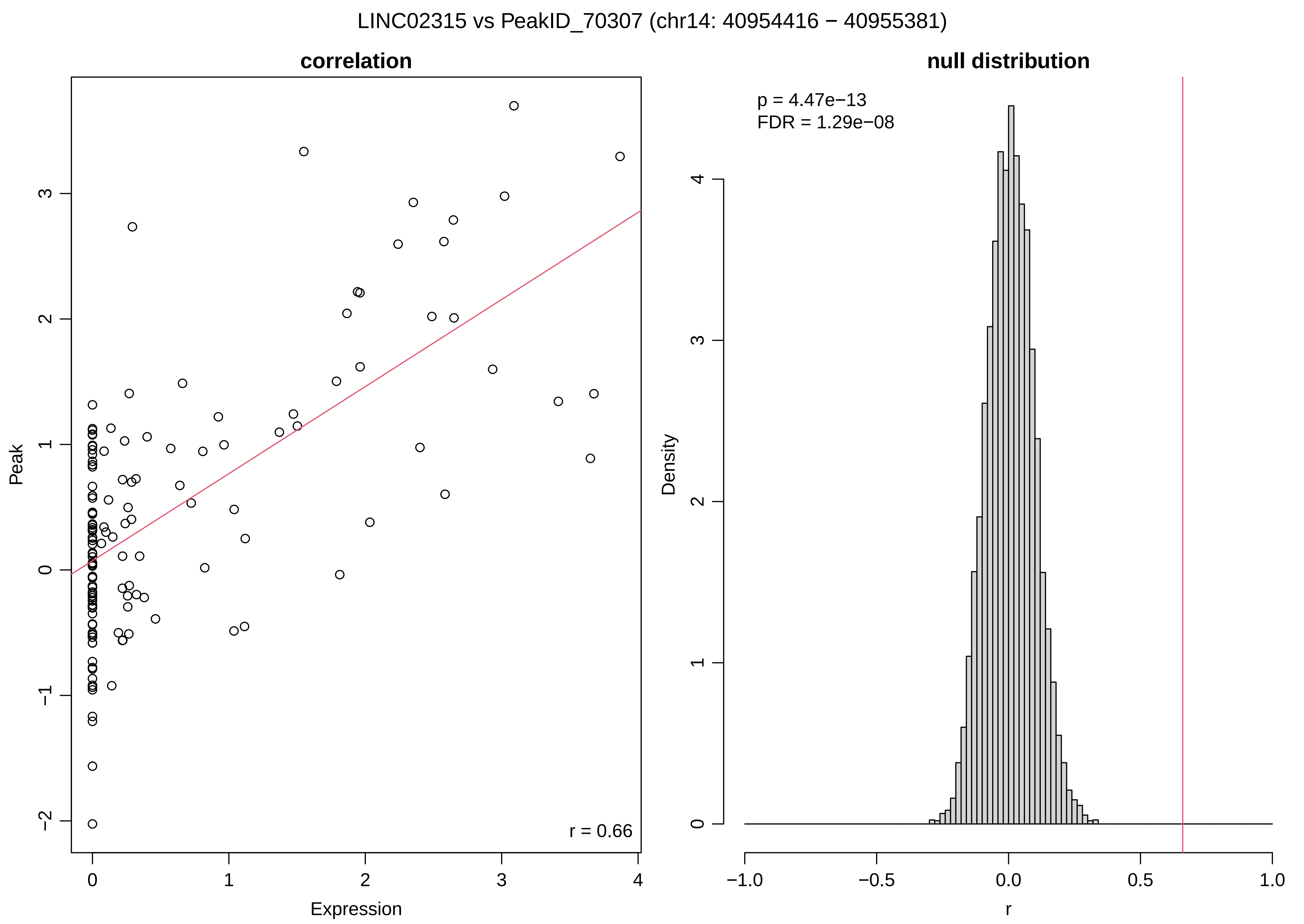

LINC02315 vs PeakID_70307 (chr14: 40954416 − 40955381)
correlation
Peak
Expression
r = 0.66
null distribution
p = 4.47e−13
FDR = 1.29e−08
Density
r

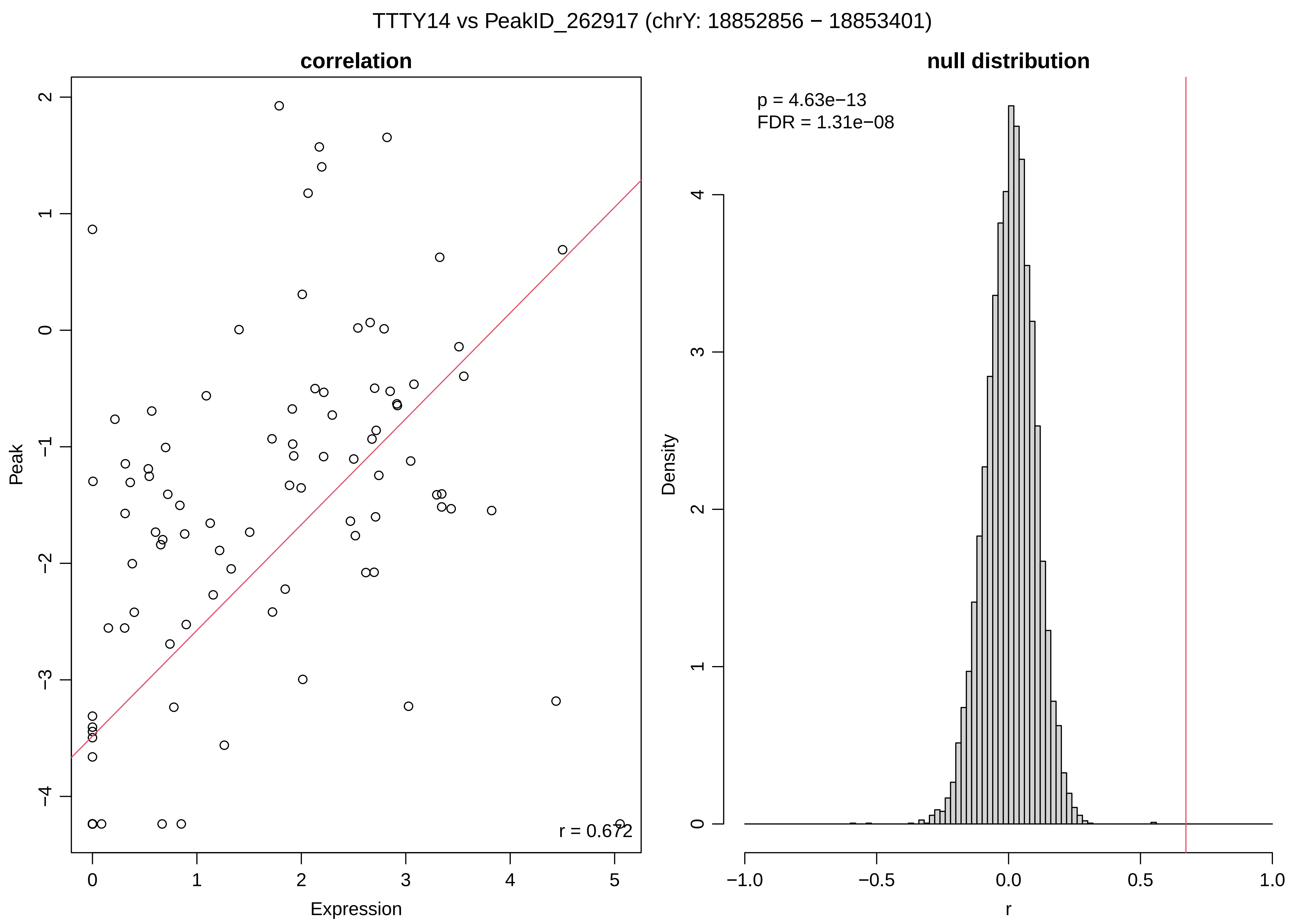

TTTY14 vs PeakID_262917 (chrY: 18852856 − 18853401)
correlation
Peak
Expression
r = 0.672
null distribution
p = 4.63e−13
FDR = 1.31e−08
Density
r

EGFR−AS1 vs PeakID_224252 (chr7: 55221408 − 55290386)

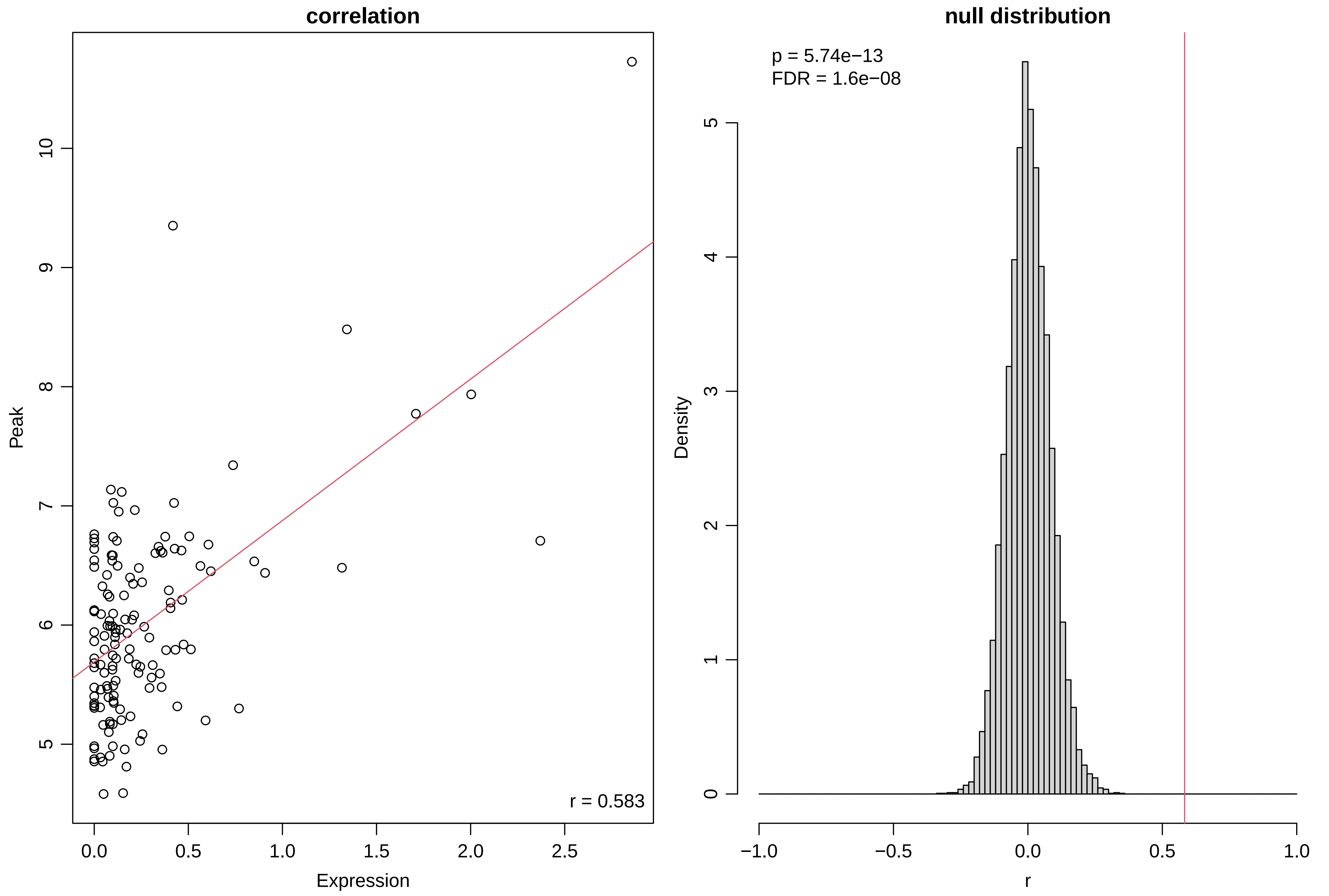

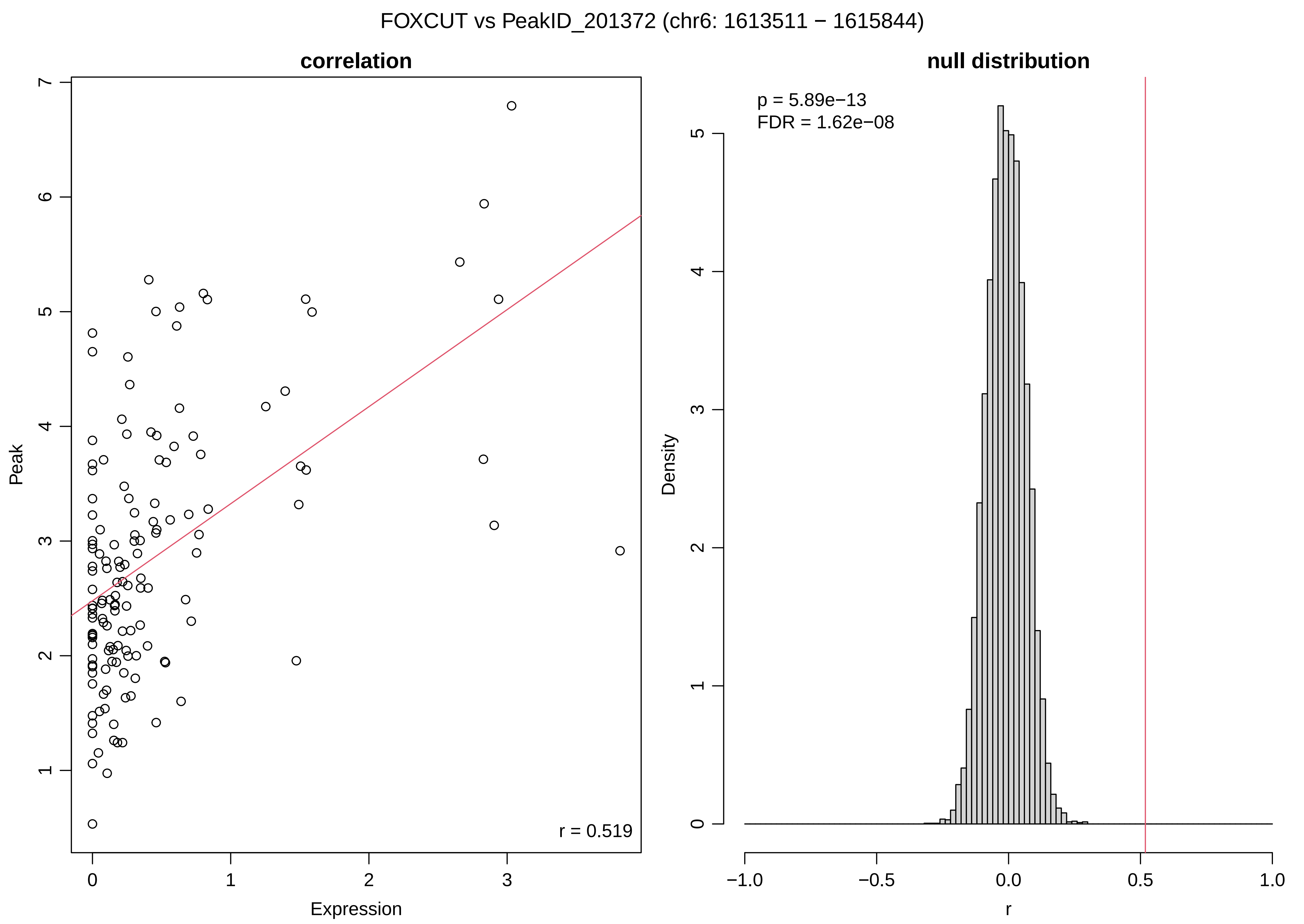
FOXCUT vs PeakID_201372 (chr6: 1613511 − 1615844)
correlation
Peak
Expression
r = 0.519
null distribution
p = 5.89e−13
FDR = 1.62e−08
Density
r

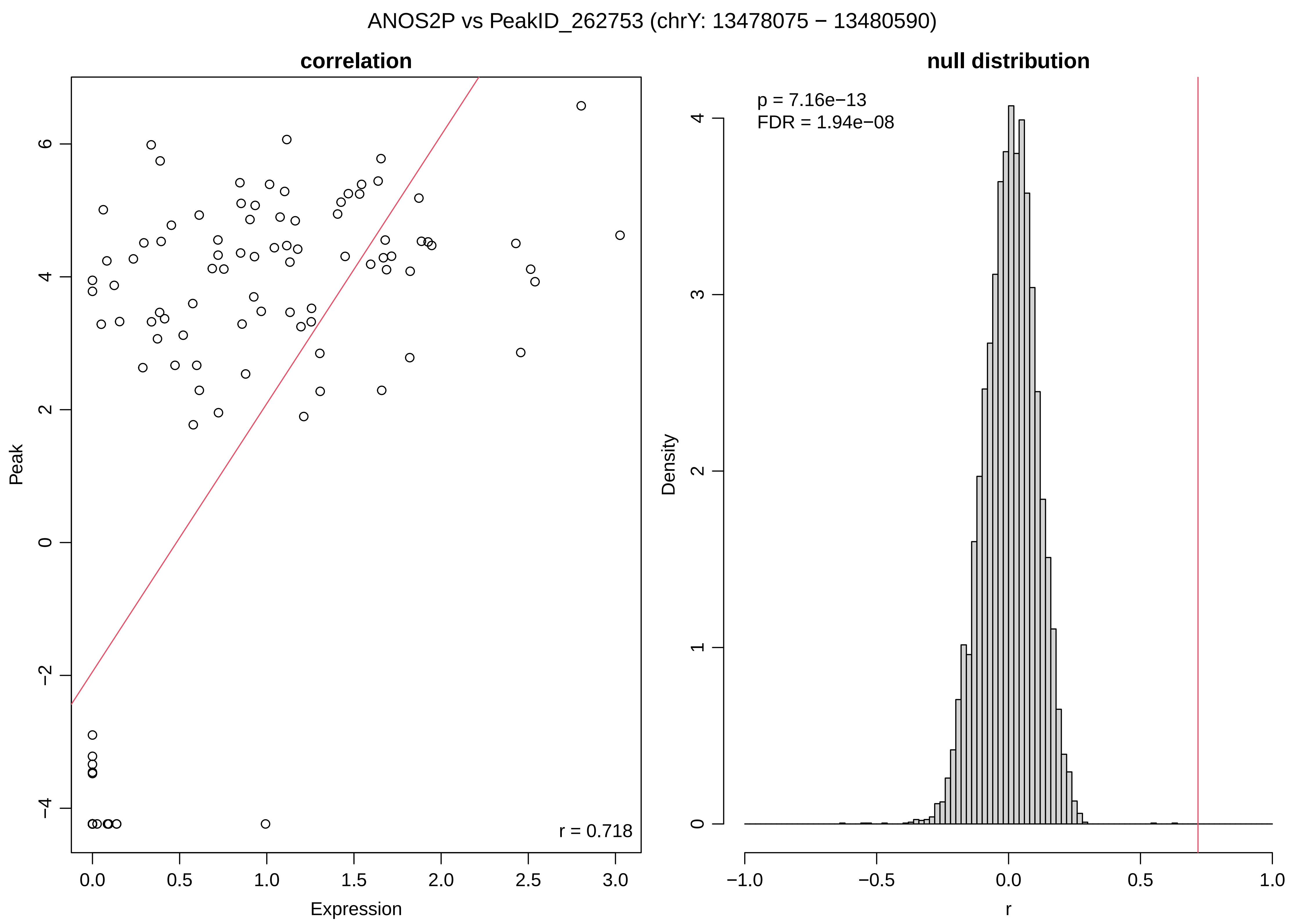
ANOS2P vs PeakID_262753 (chrY: 13478075 − 13480590)
correlation
null distribution
Peak
Expression
r = 0.718
p = 7.16e−13
FDR = 1.94e−08
Density
r

AC087491.1 vs PeakID_99043 (chr17: 39611085 − 39689899)

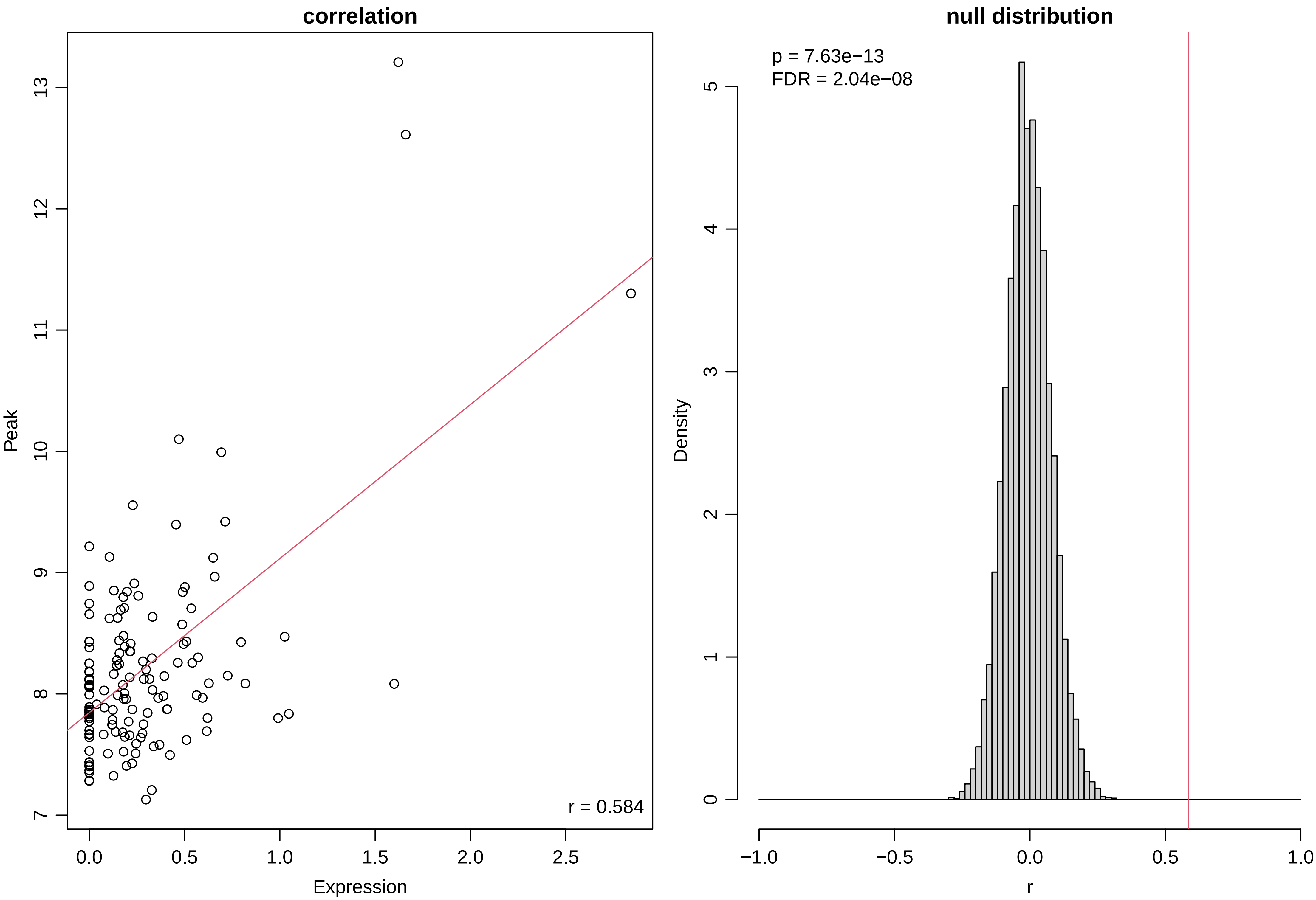

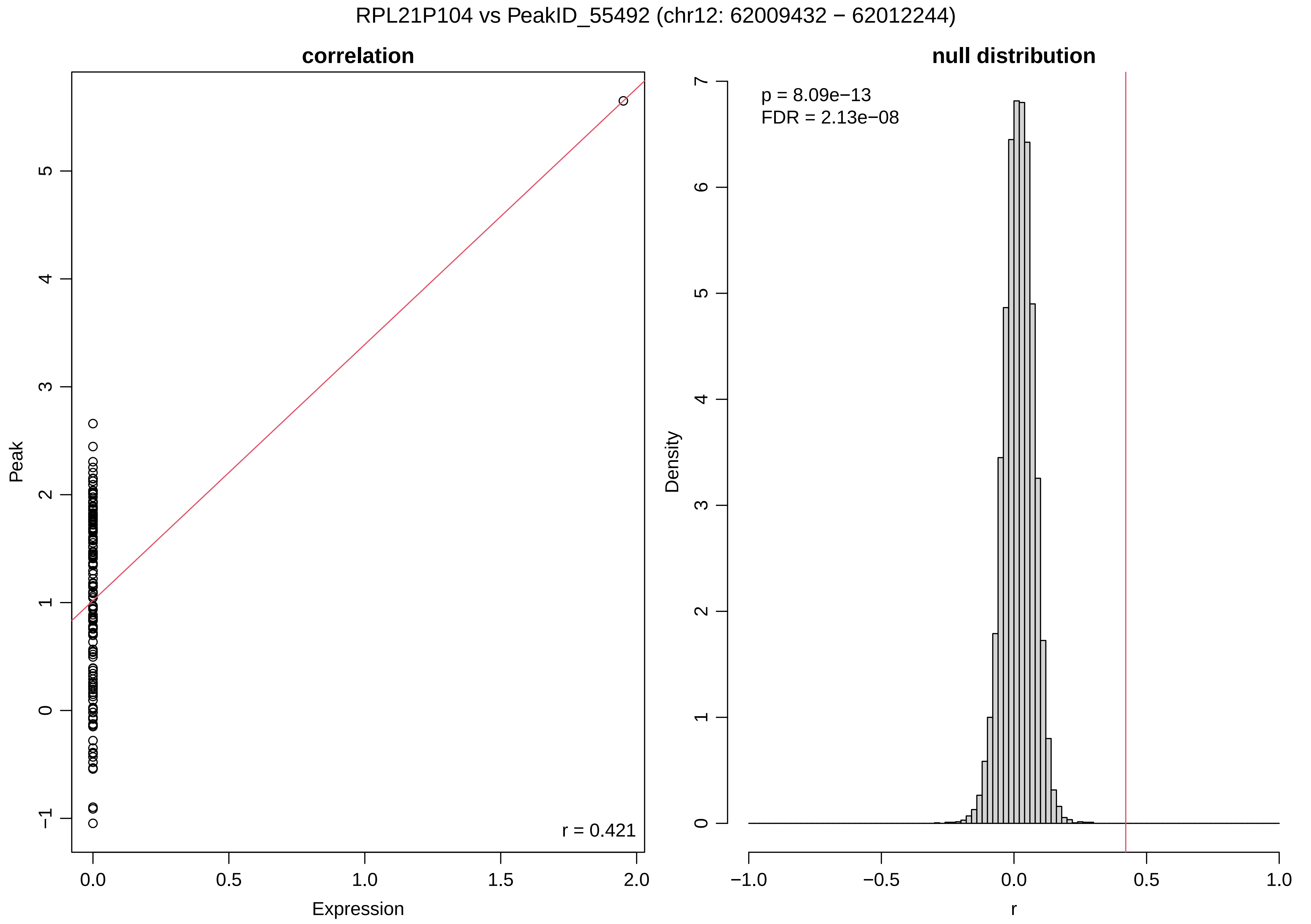

RPL21P104 vs PeakID_55492 (chr12: 62009432 − 62012244)
correlation
Peak
Expression
r = 0.421
null distribution
Density
r
p = 8.09e−13
FDR = 2.13e−08

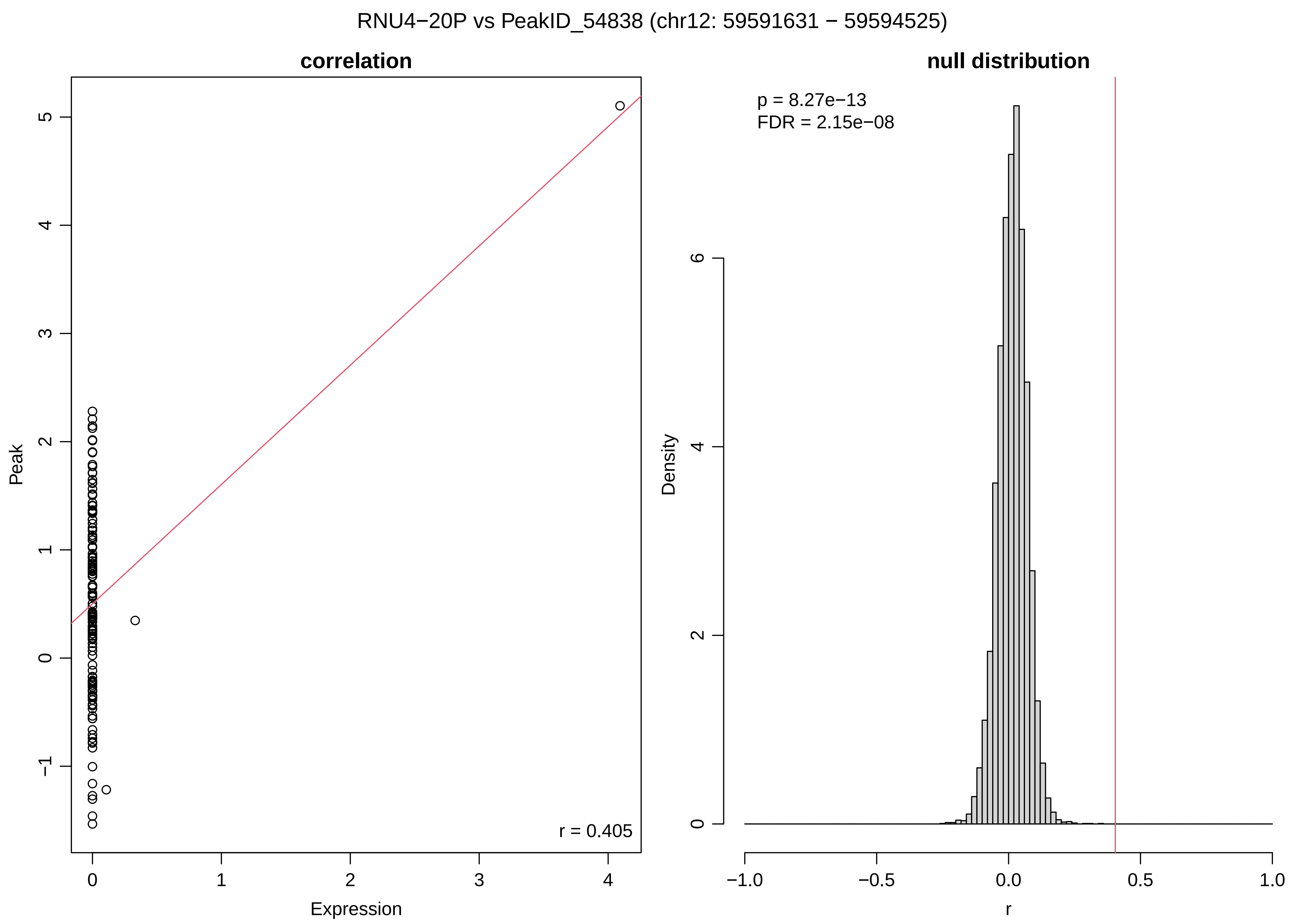
RNU4−20P vs PeakID_54838 (chr12: 59591631 − 59594525)
correlation
Peak
Expression
r = 0.405
null distribution
p = 8.27e−13
FDR = 2.15e−08
Density
r

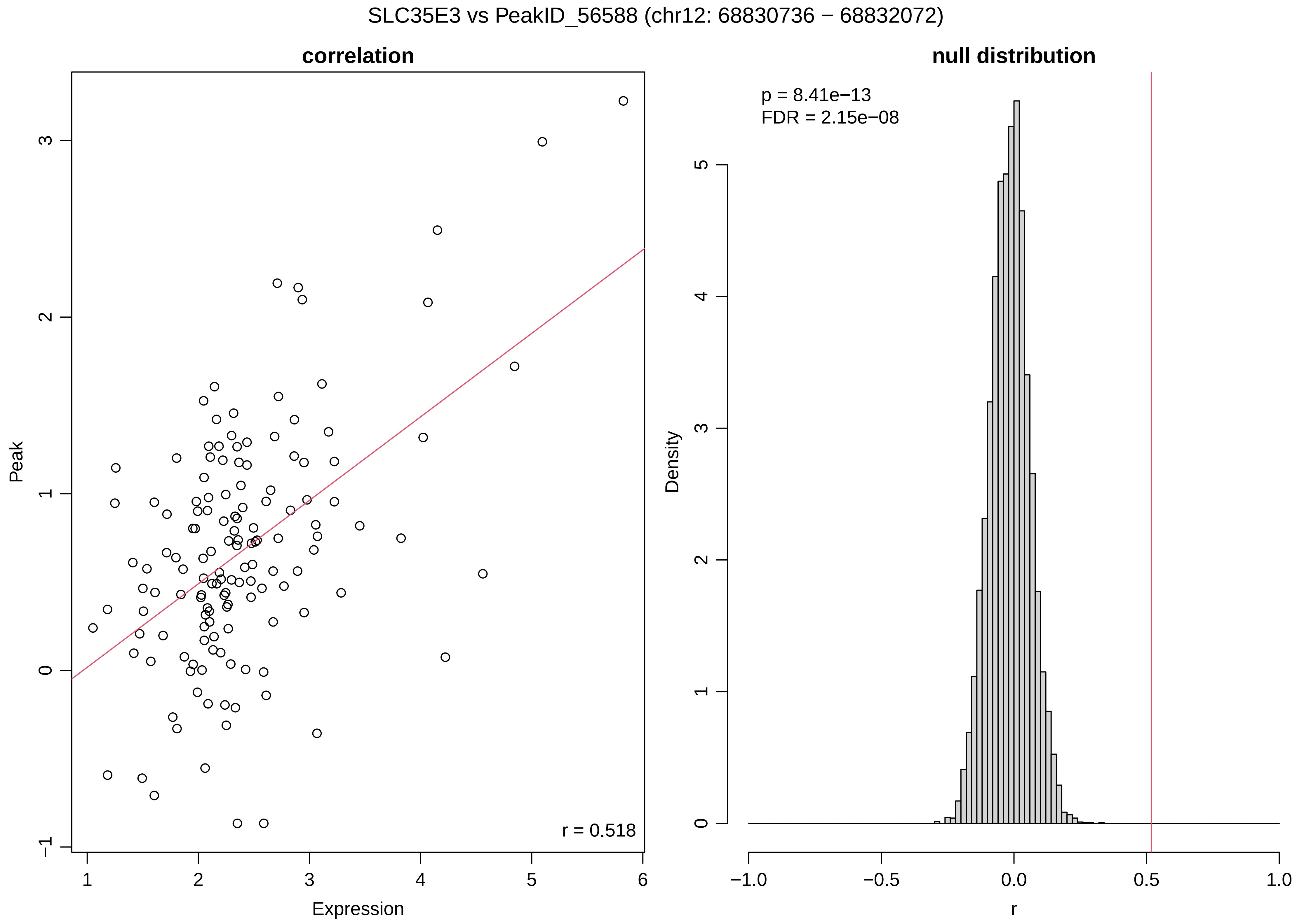
SLC35E3 vs PeakID_56588 (chr12: 68830736 − 68832072)
correlation
null distribution
p = 8.41e−13
FDR = 2.15e−08
r = 0.518
Peak
Expression
Density
r

LHFPL4 vs PeakID_160491 (chr3: 9553289 − 9554310)

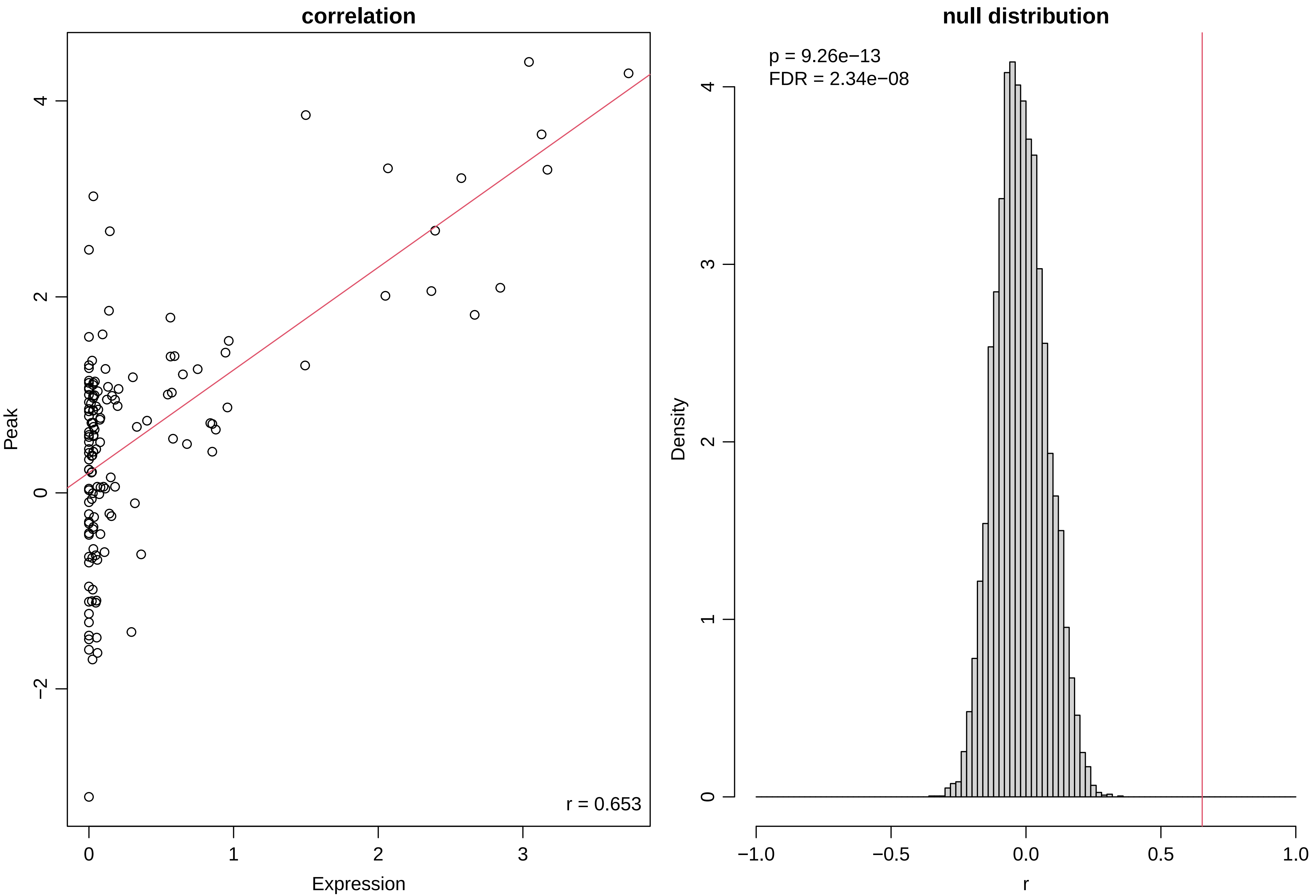

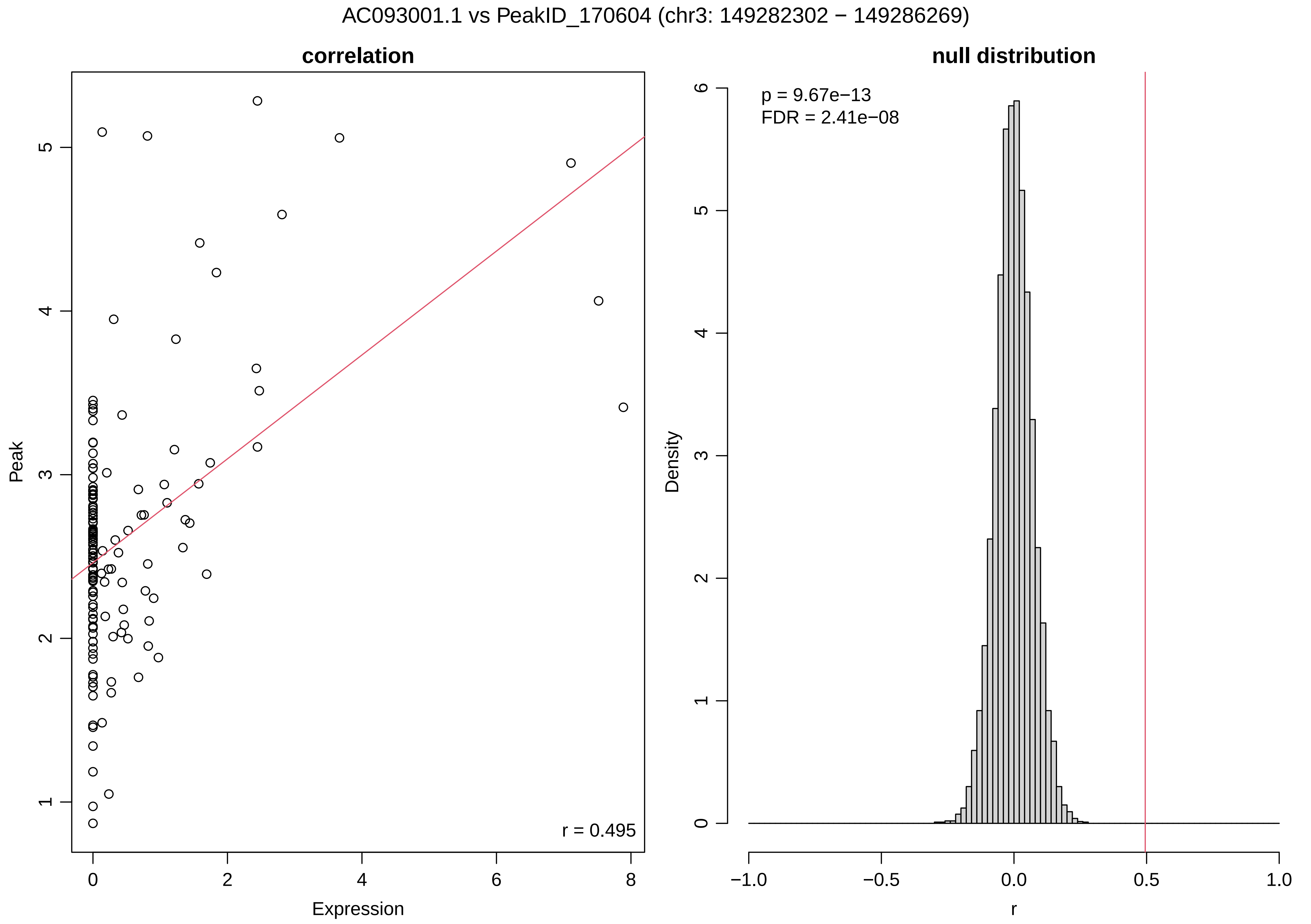

AC093001.1 vs PeakID_170604 (chr3: 149282302 − 149286269)
correlation
null distribution
Peak
Expression
r = 0.495
Density
r
p = 9.67e−13
FDR = 2.41e−08

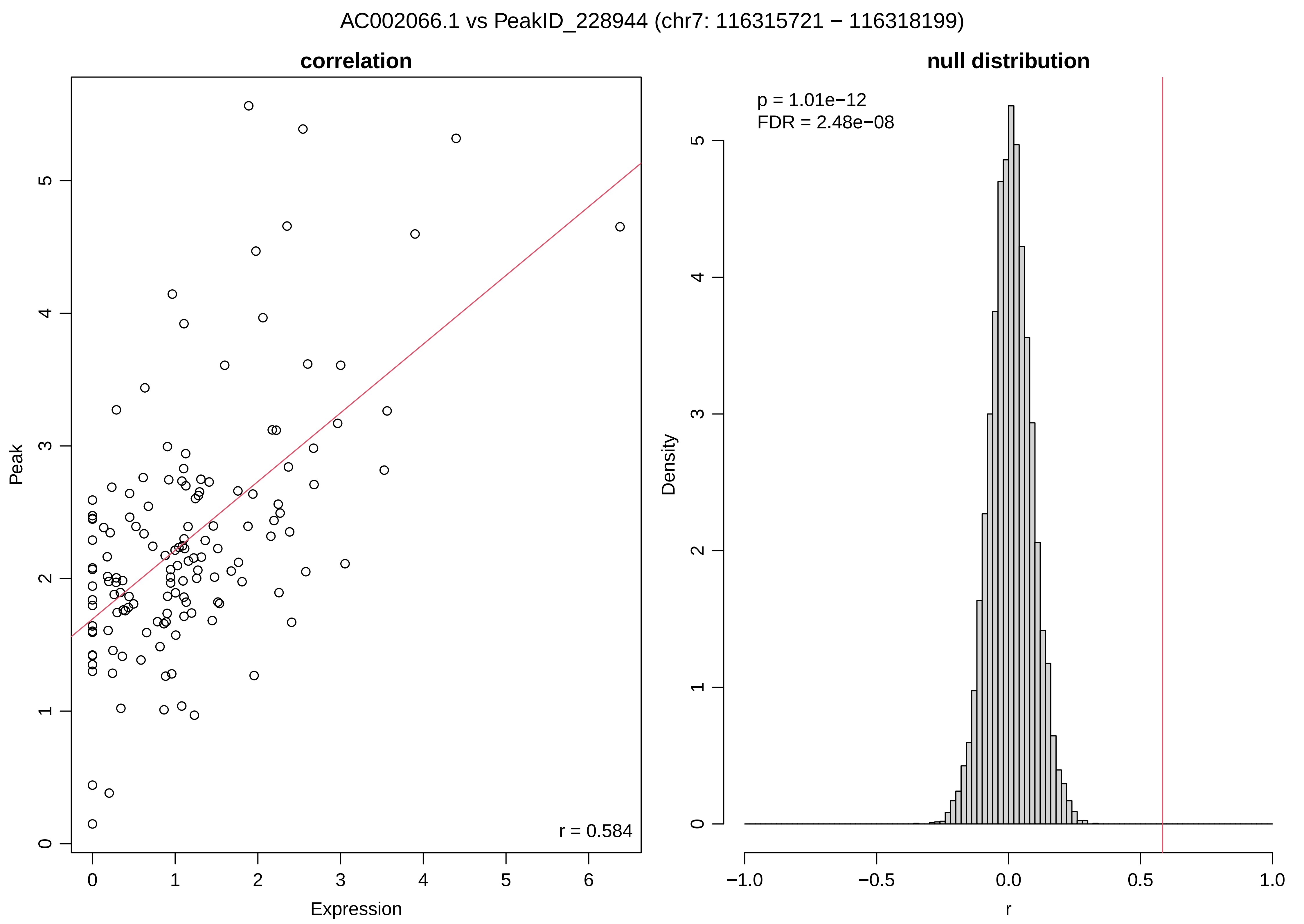

AC002066.1 vs PeakID_228944 (chr7: 116315721 − 116318199)
correlation
Peak
Expression
r = 0.584
null distribution
p = 1.01e−12
FDR = 2.48e−08
Density
r

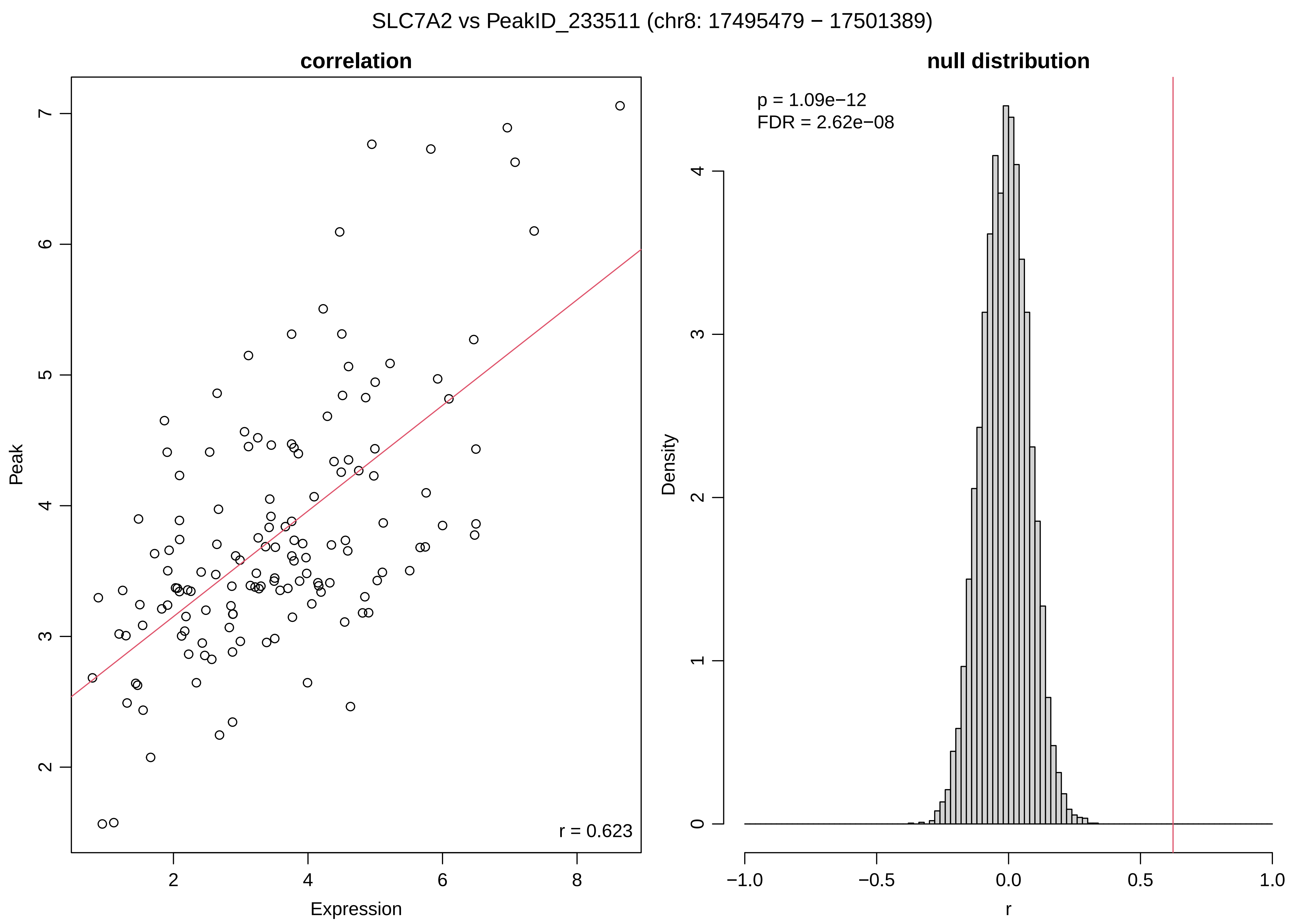
SLC7A2 vs PeakID_233511 (chr8: 17495479 − 17501389)
correlation
Peak
Expression
r = 0.623
null distribution
p = 1.09e−12
FDR = 2.62e−08
Density
r

AC090061.1 vs PeakID_56621 (chr12: 68883474 − 68896738)

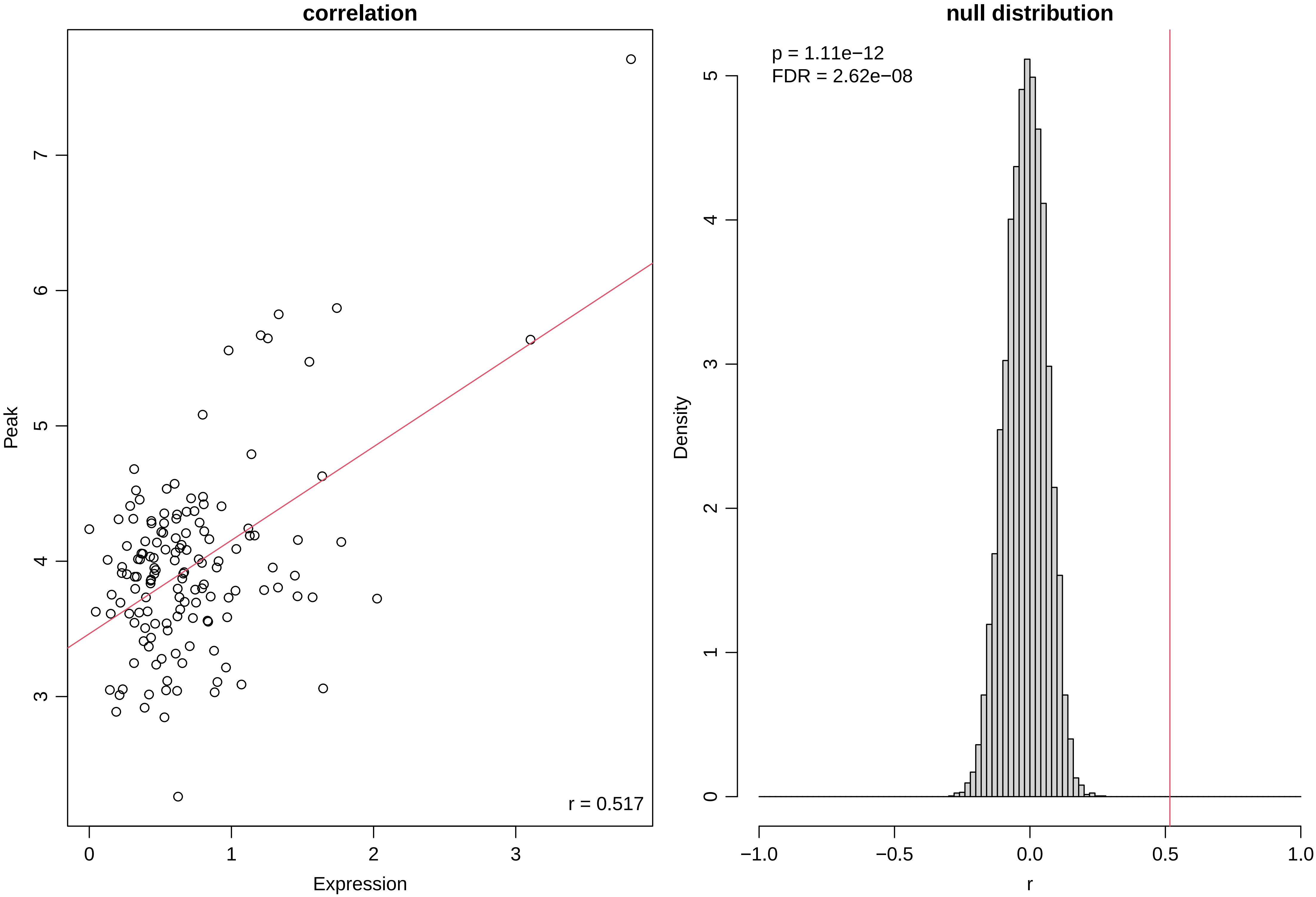

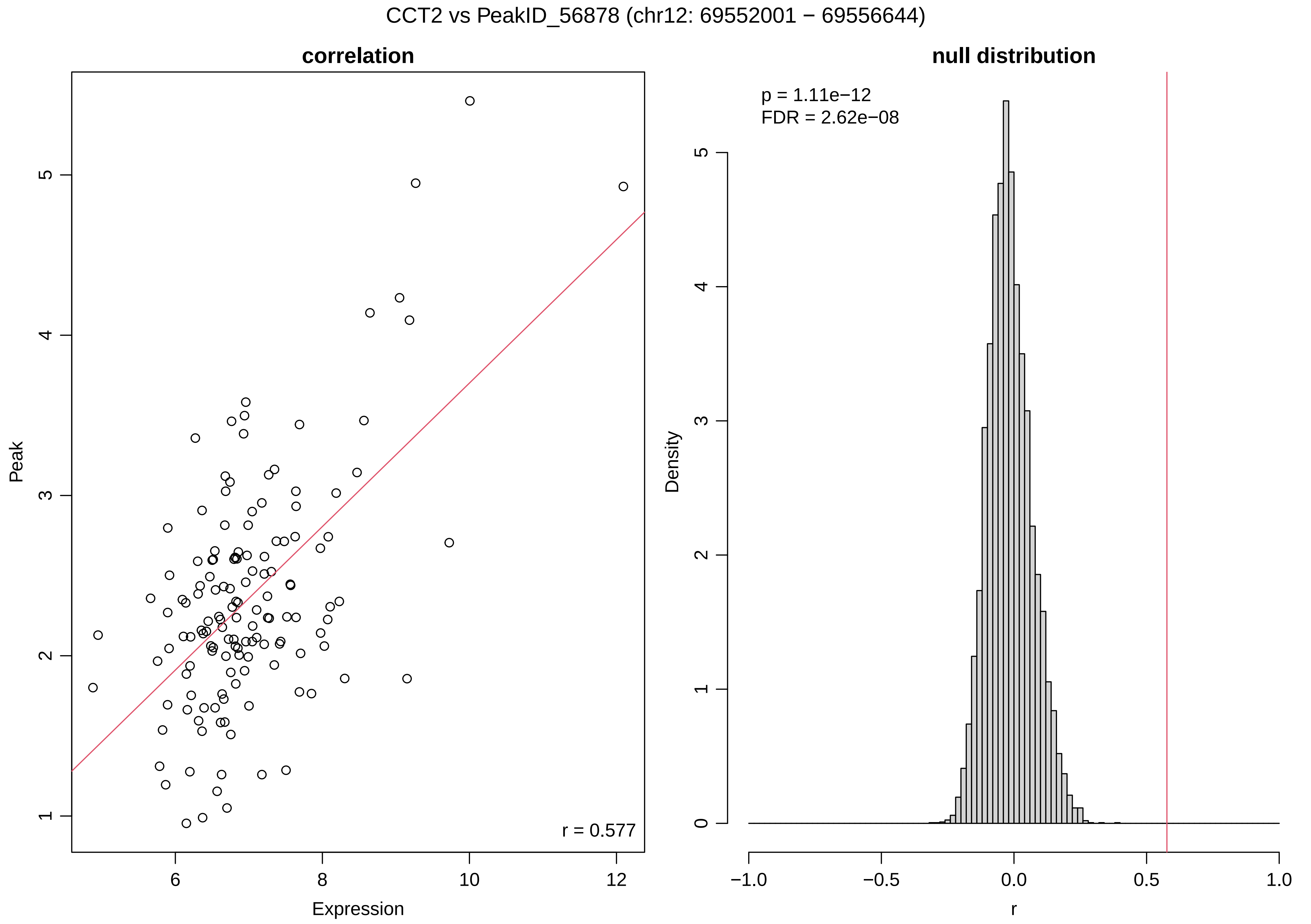

CCT2 vs PeakID_56878 (chr12: 69552001 − 69556644)
correlation
Peak
Expression
r = 0.577
null distribution
p = 1.11e−12
FDR = 2.62e−08
Density
r

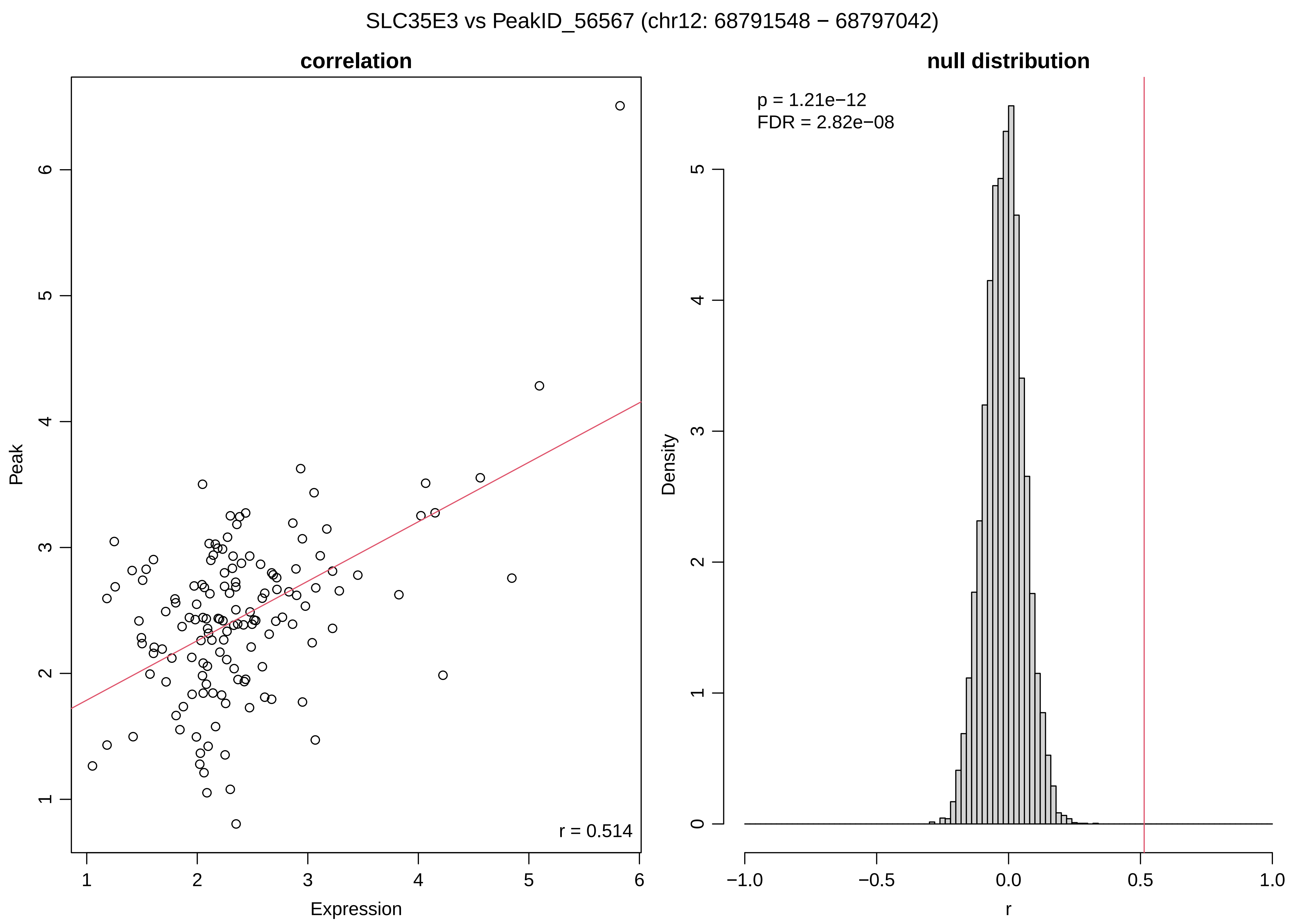
SLC35E3 vs PeakID_56567 (chr12: 68791548 − 68797042)
correlation
Peak
Expression
r = 0.514
1
2
3
4
5
6
null distribution
p = 1.21e−12
FDR = 2.82e−08
Density
r
0
1
2
3
4
5
−1.0
−0.5
0.0
0.5
1.0

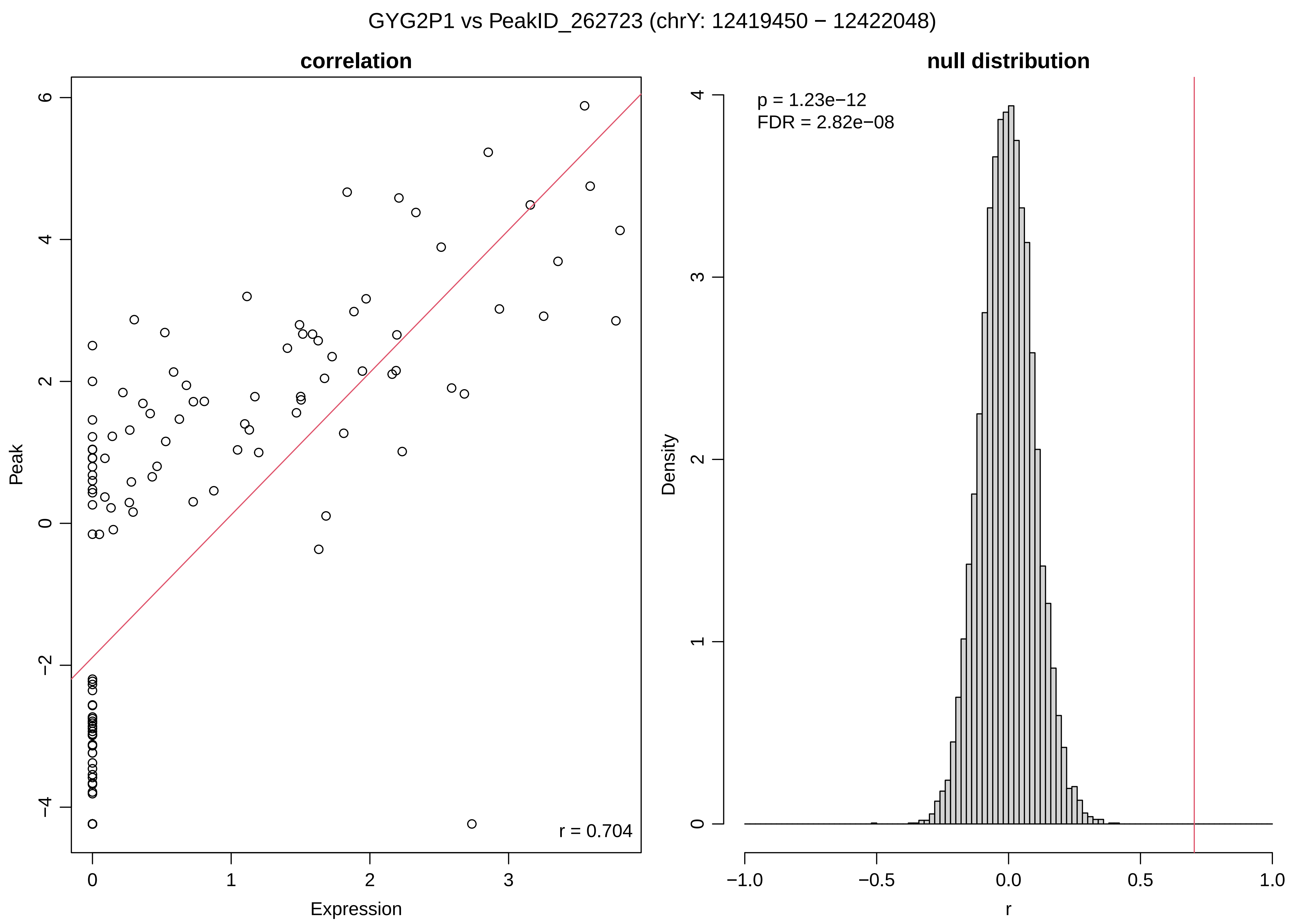

GYG2P1 vs PeakID_262723 (chrY: 12419450 − 12422048)
correlation
Peak
Expression
r = 0.704
null distribution
Density
r
p = 1.23e−12
FDR = 2.82e−08

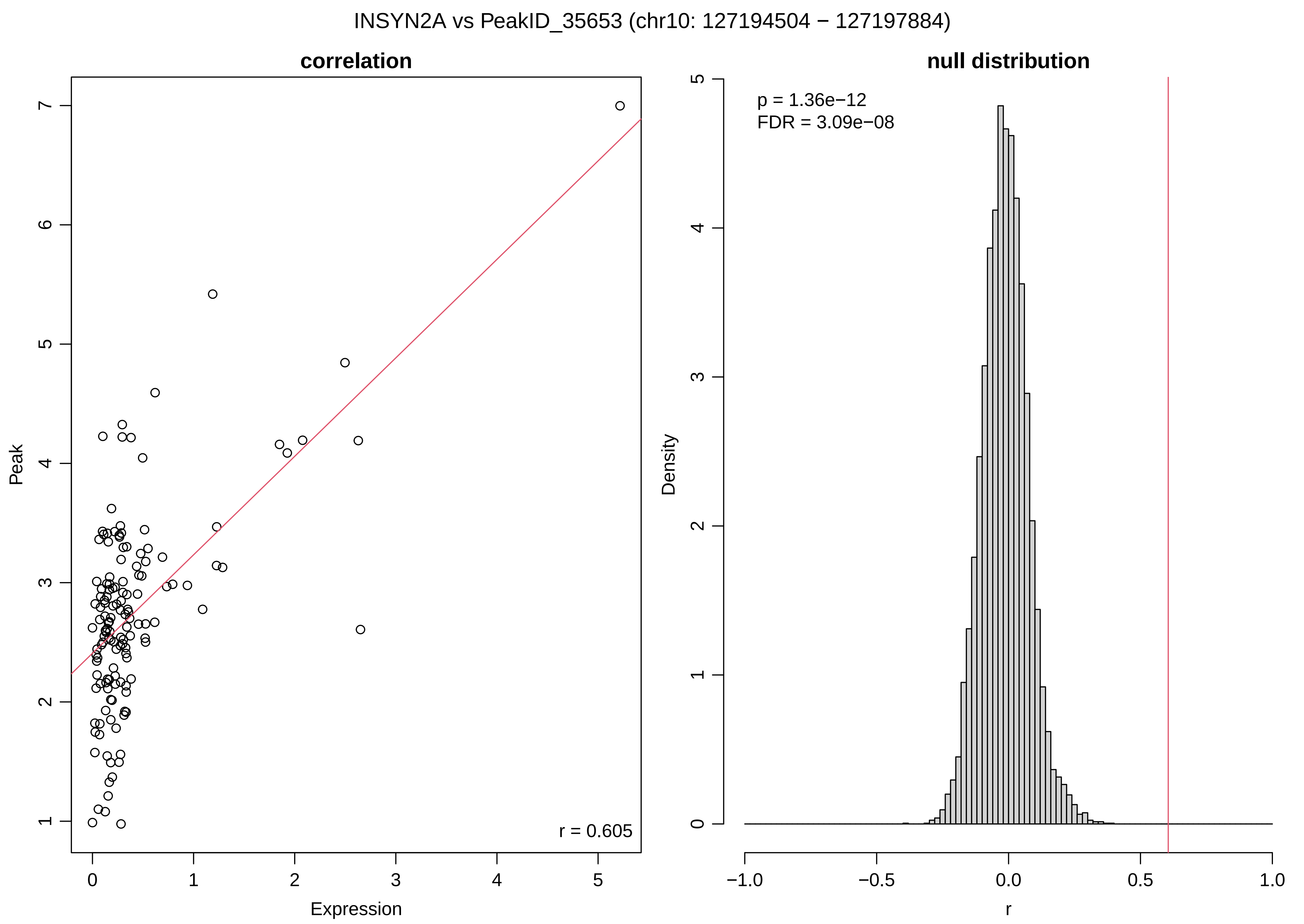
INSYN2A vs PeakID_35653 (chr10: 127194504 − 127197884)
correlation
Peak
Expression
r = 0.605
null distribution
p = 1.36e−12
FDR = 3.09e−08
Density
r

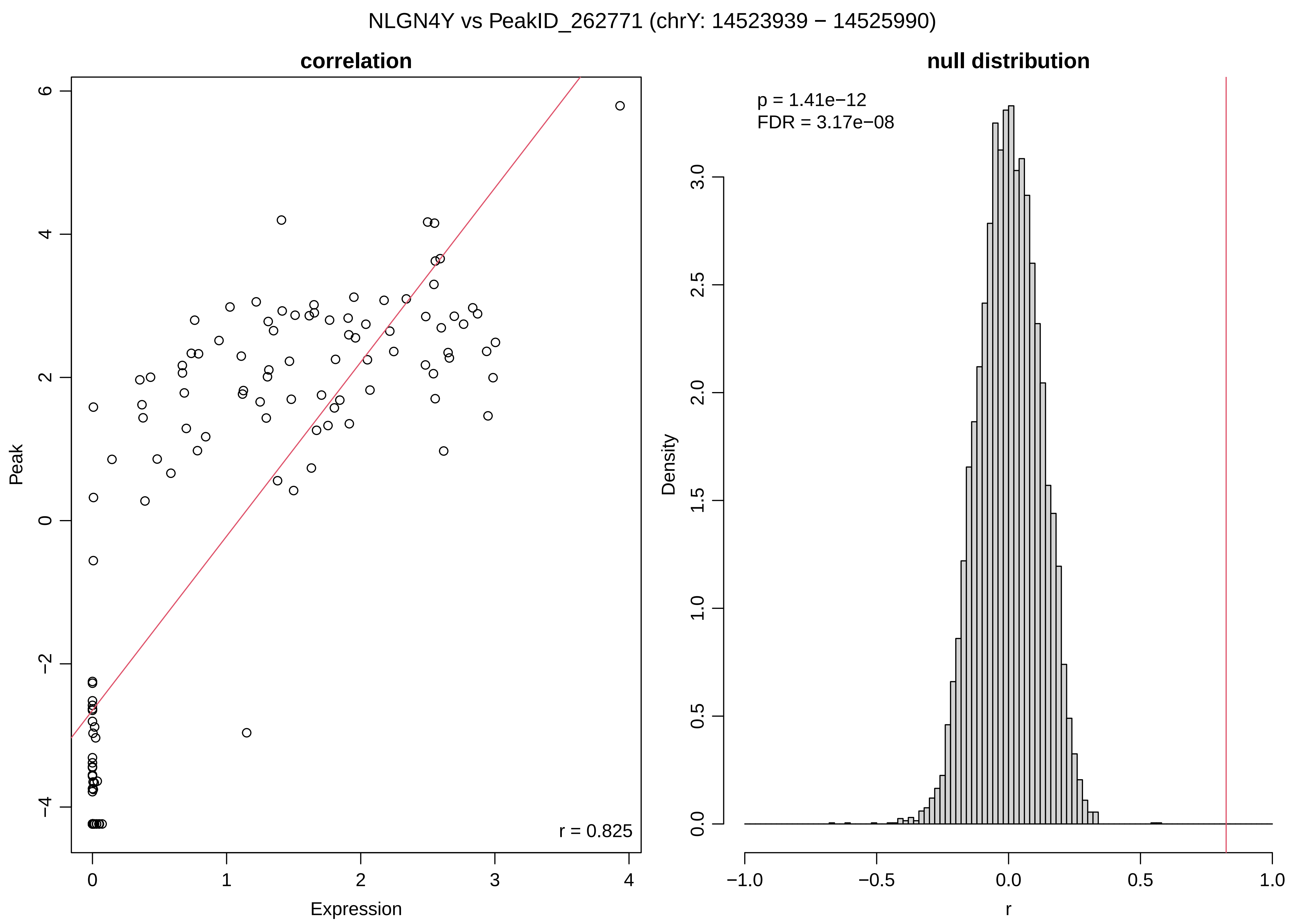
NLGN4Y vs PeakID_262771 (chrY: 14523939 − 14525990)
correlation
Peak
Expression
r = 0.825
null distribution
p = 1.41e−12
FDR = 3.17e−08
Density
r

AP000547.3 vs PeakID_154332 (chr22: 16601014 − 16603507)

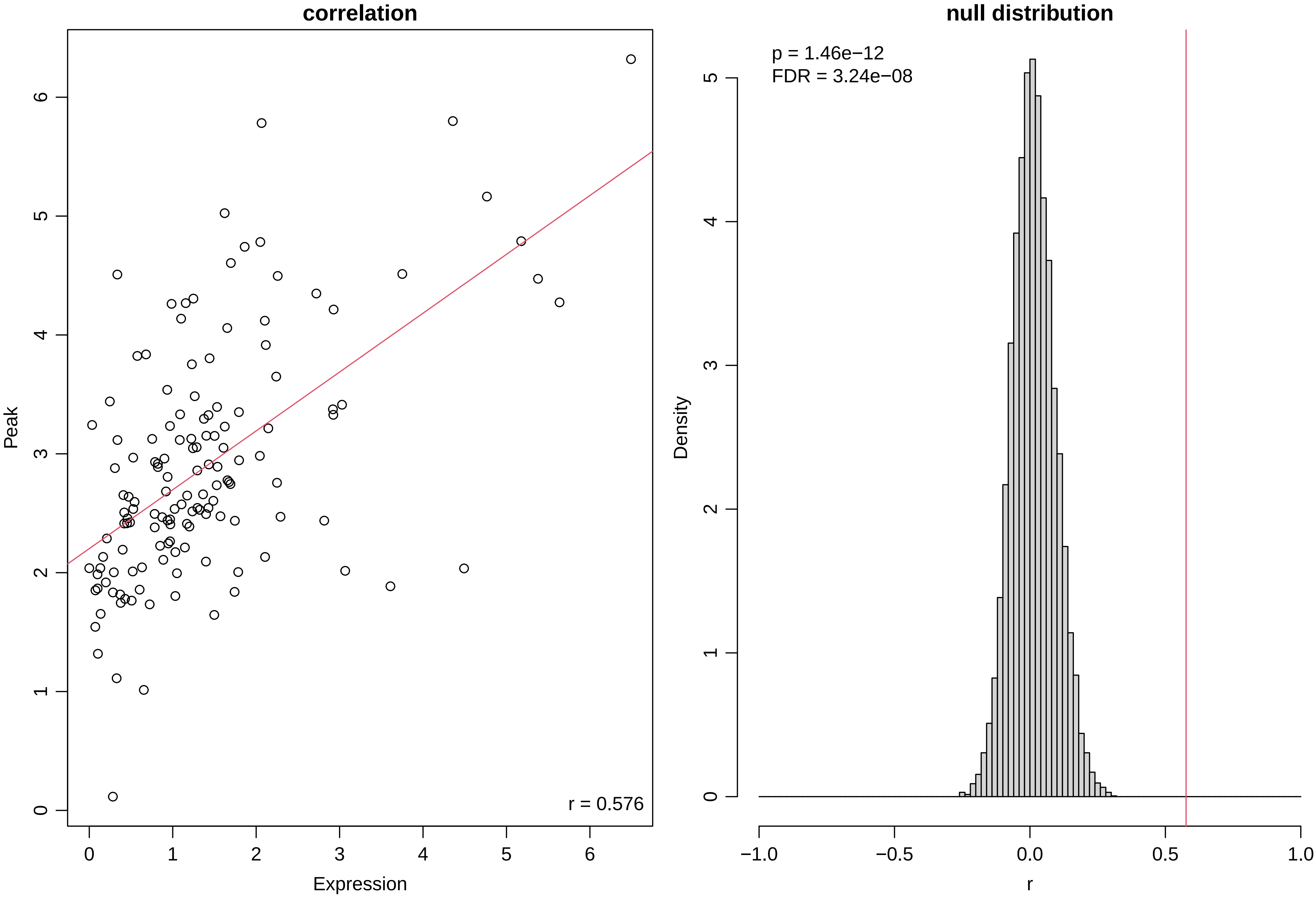

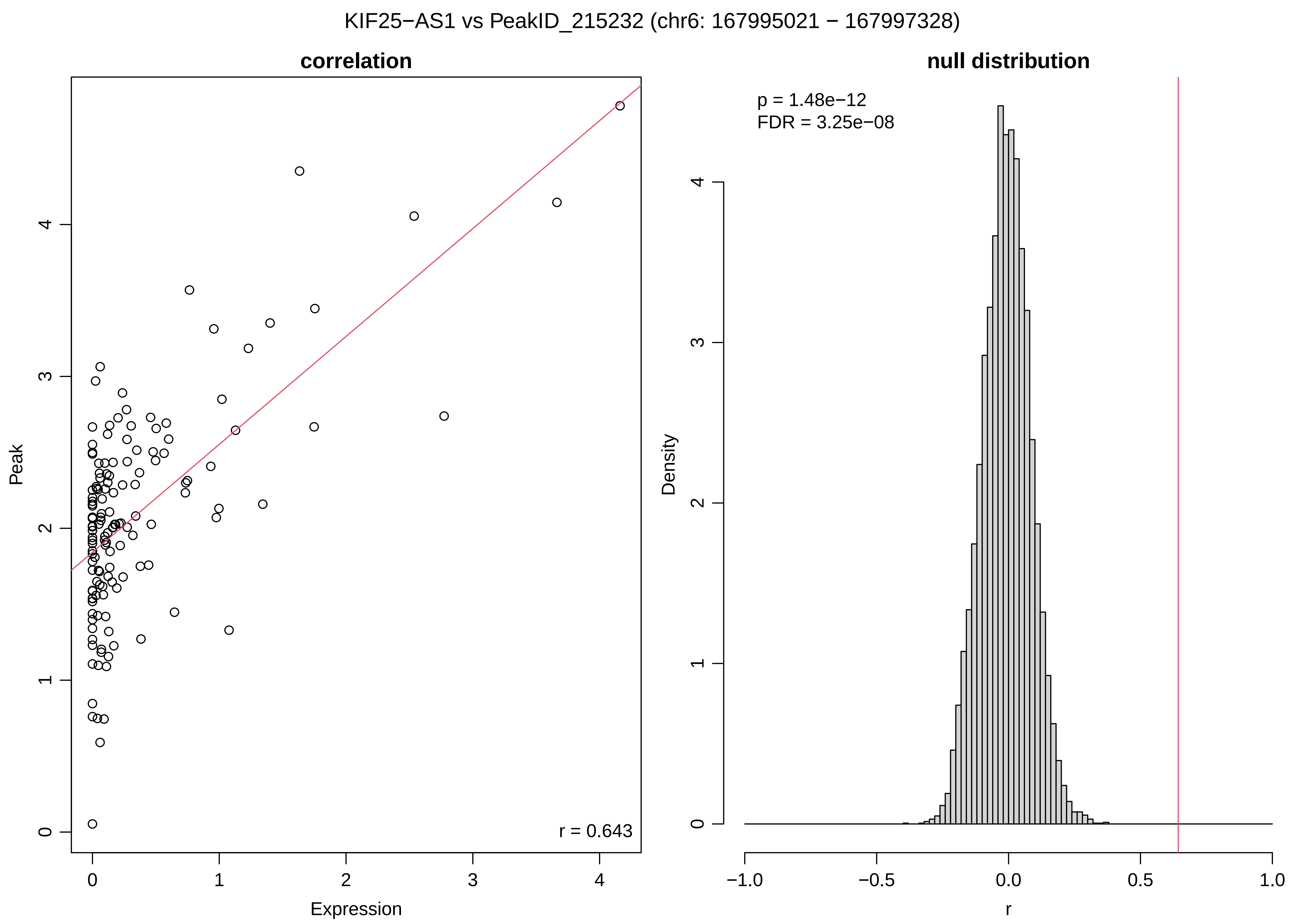
KIF25−AS1 vs PeakID_215232 (chr6: 167995021 − 167997328)
correlation
Peak
Expression
r = 0.643
null distribution
p = 1.48e−12
FDR = 3.25e−08
Density
r

AL591926.2 vs PeakID_247813 (chr9: 41653080 − 41655722)

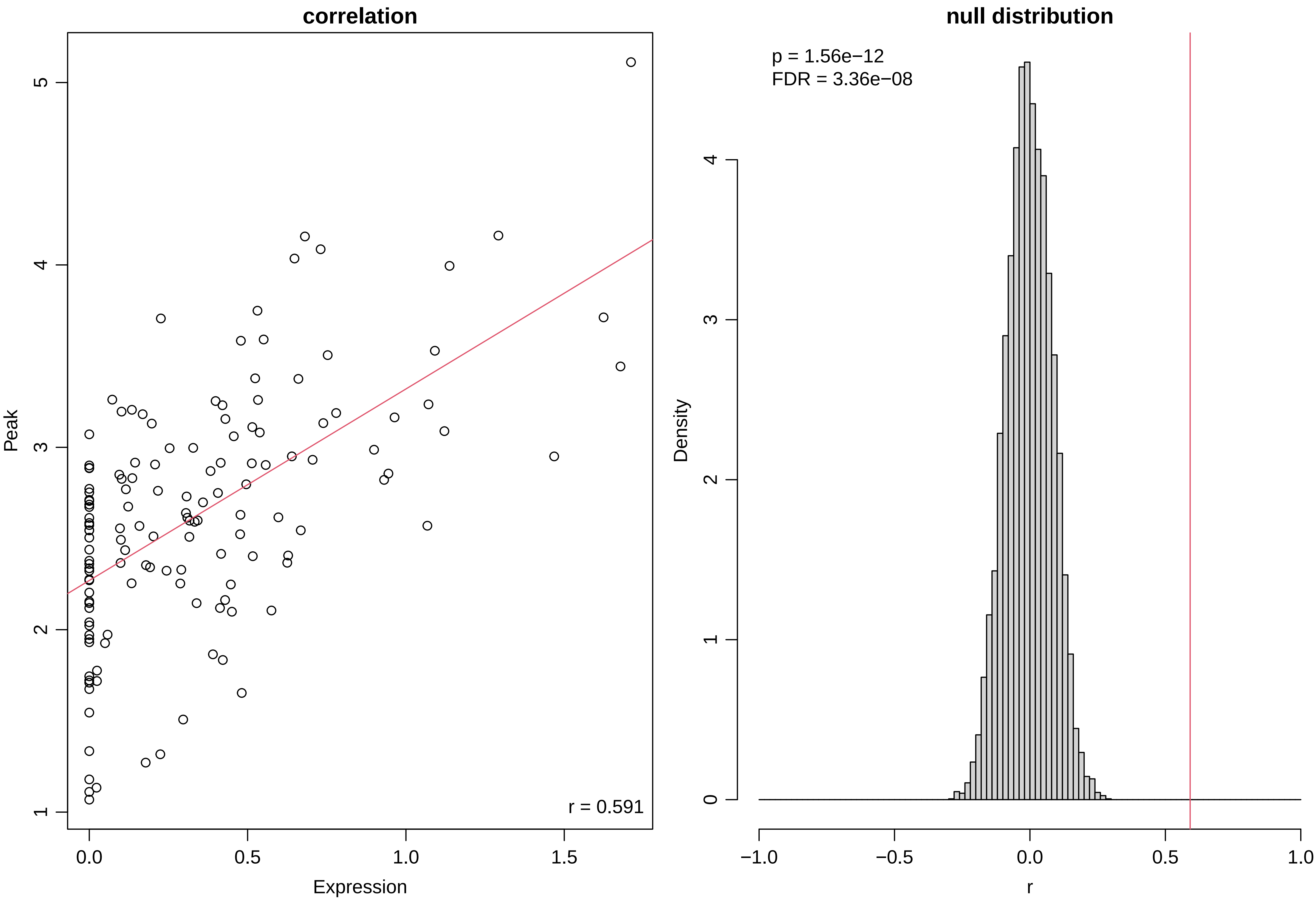

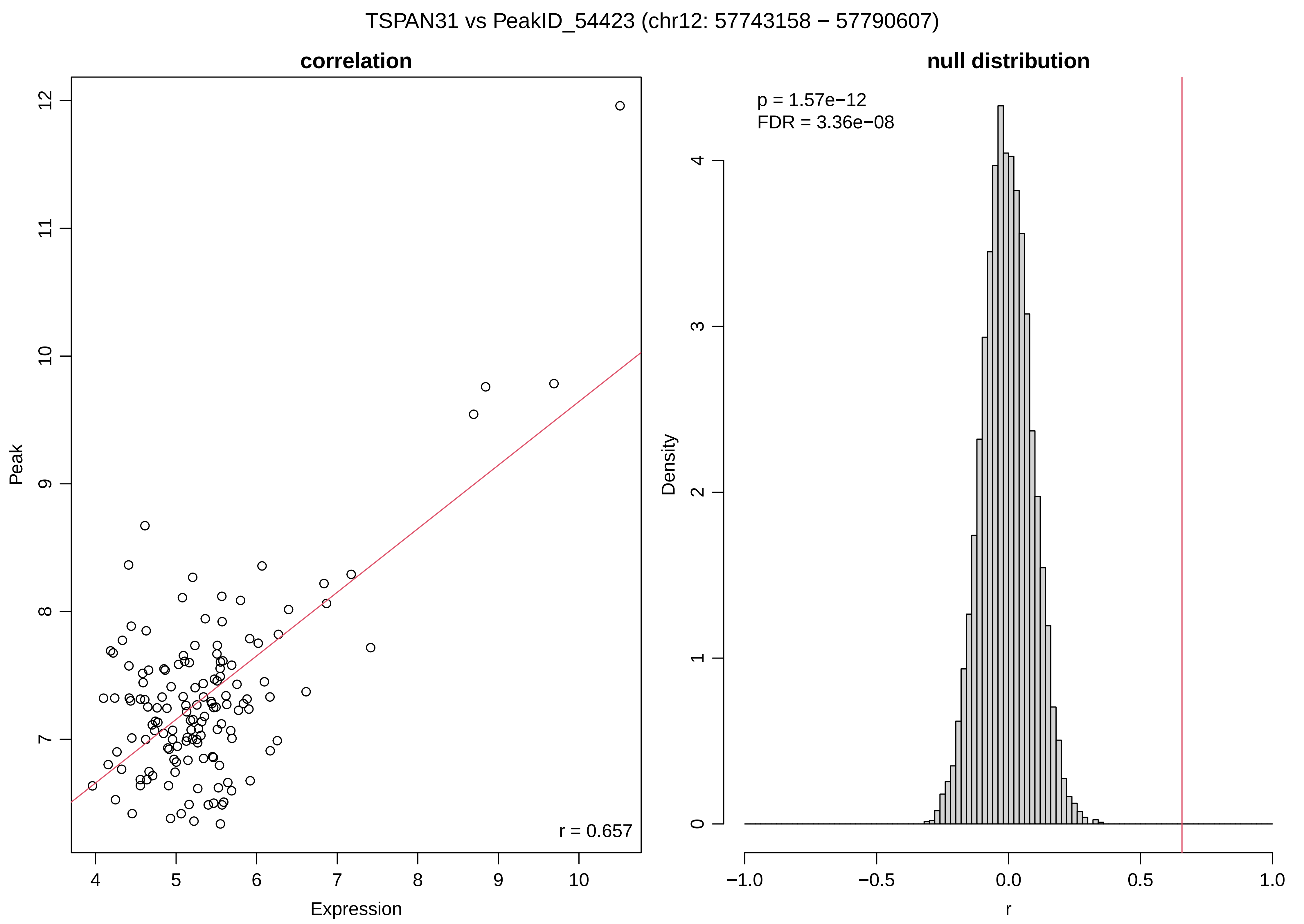
TSPAN31 vs PeakID_54423 (chr12: 57743158 − 57790607)
correlation
Peak
Expression
r = 0.657
null distribution
p = 1.57e−12
FDR = 3.36e−08
Density
r

YEATS4 vs PeakID_56860 (chr12: 69518095 − 69519979)

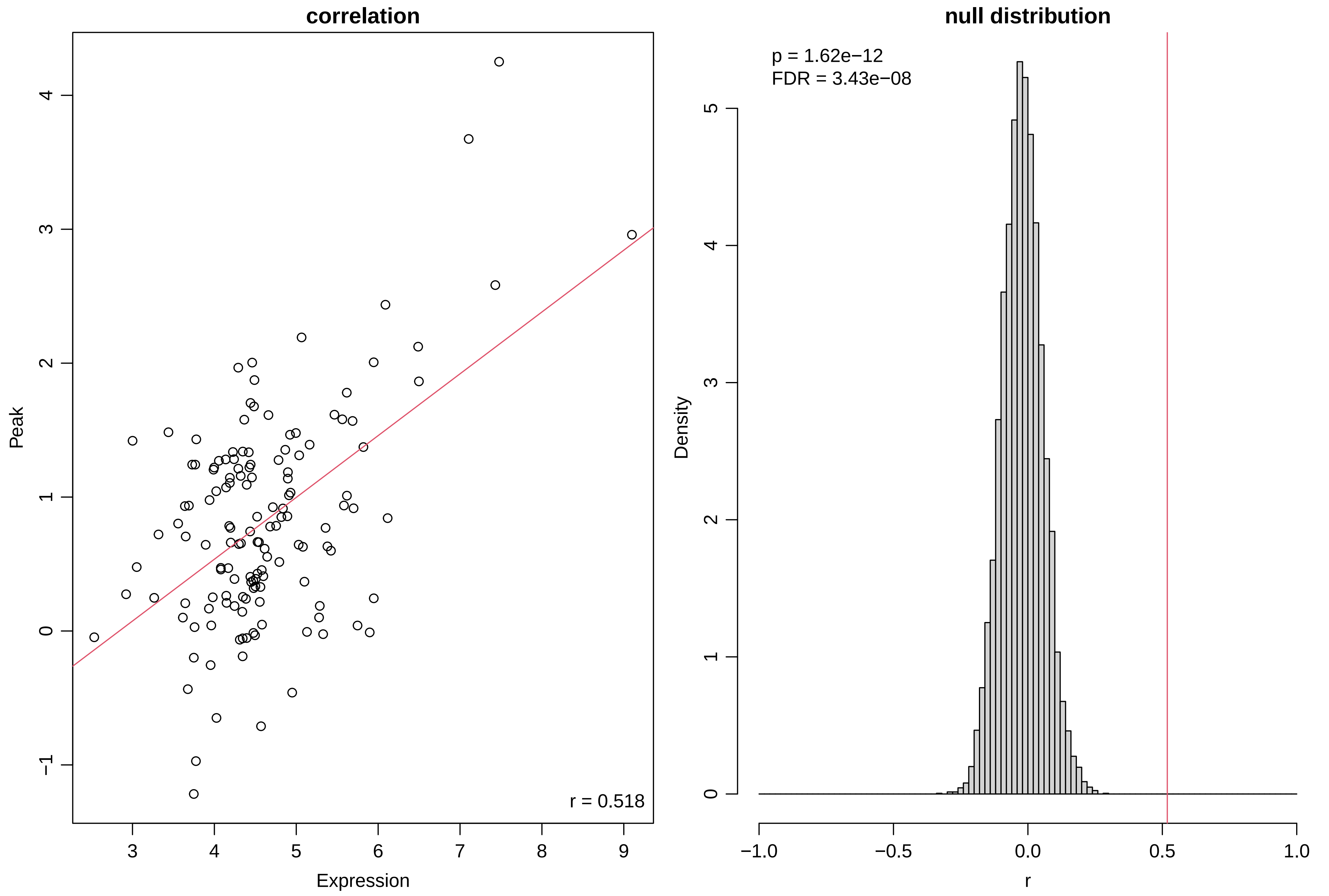

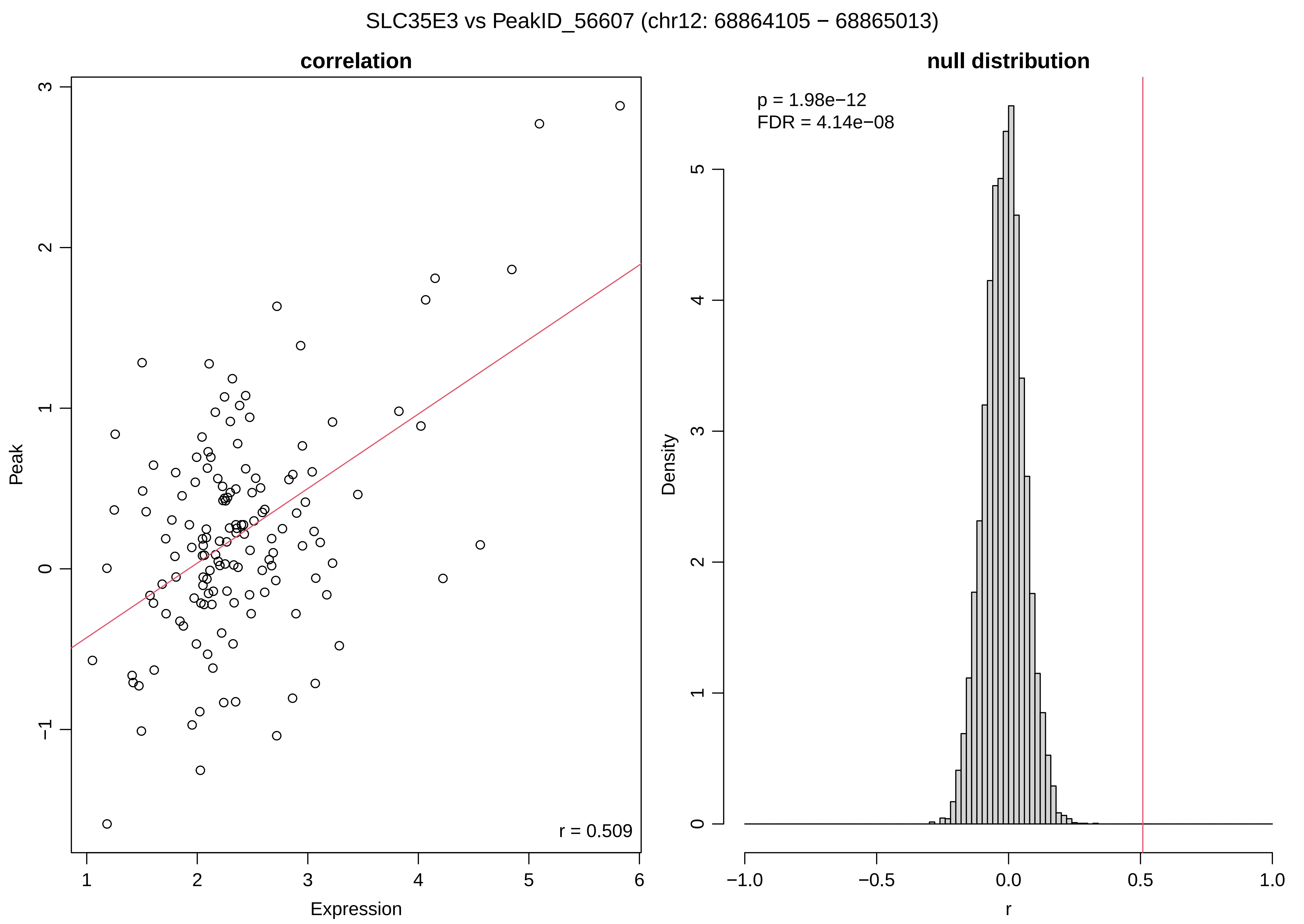

SLC35E3 vs PeakID_56607 (chr12: 68864105 − 68865013)
correlation
Peak
Expression
r = 0.509
null distribution
p = 1.98e−12
FDR = 4.14e−08
Density
r

LINC00524 vs PeakID_75250 (chr14: 101456859 − 101459715)

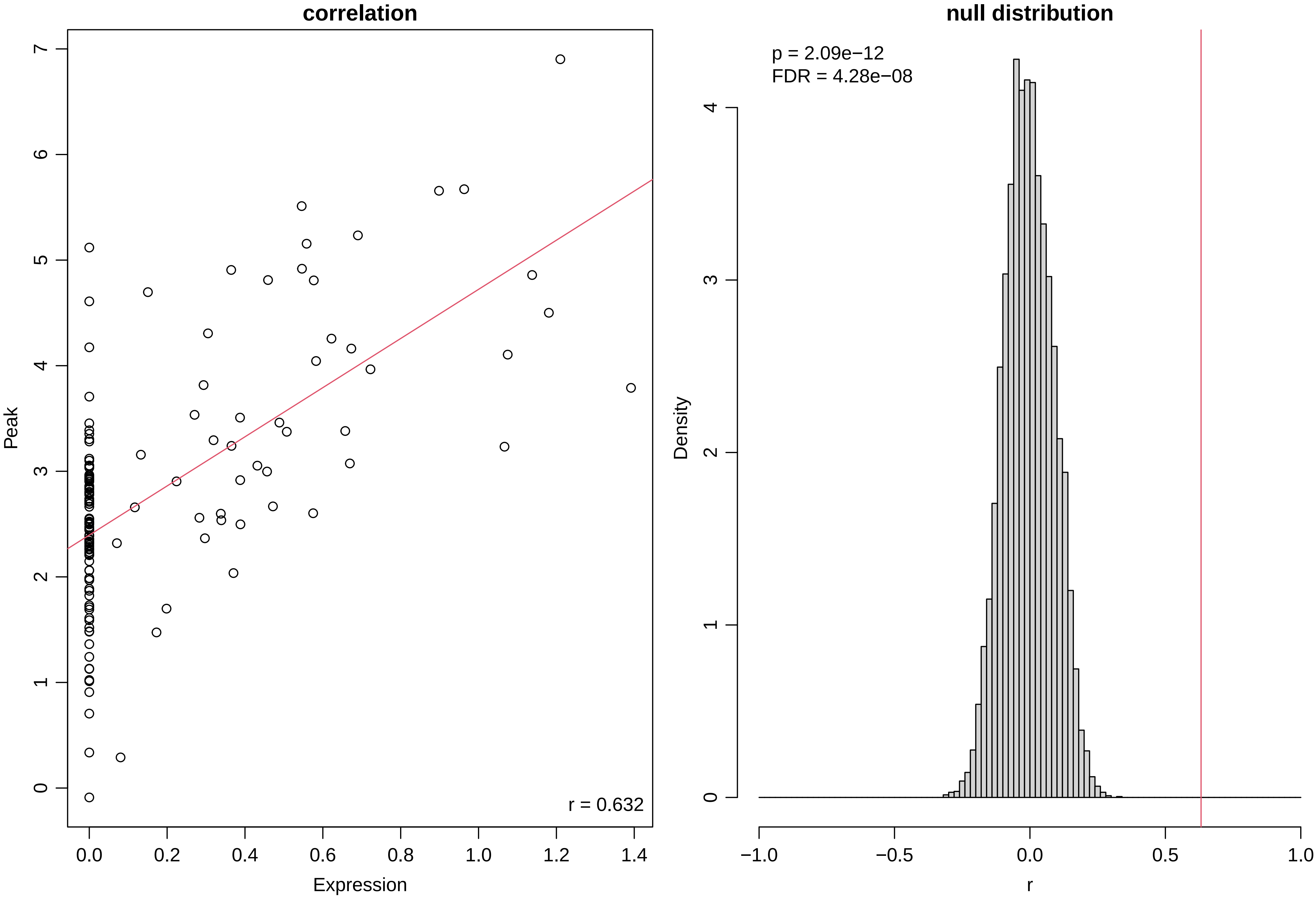

GYG2P1 vs PeakID_262720 (chrY: 12390568 − 12394593)

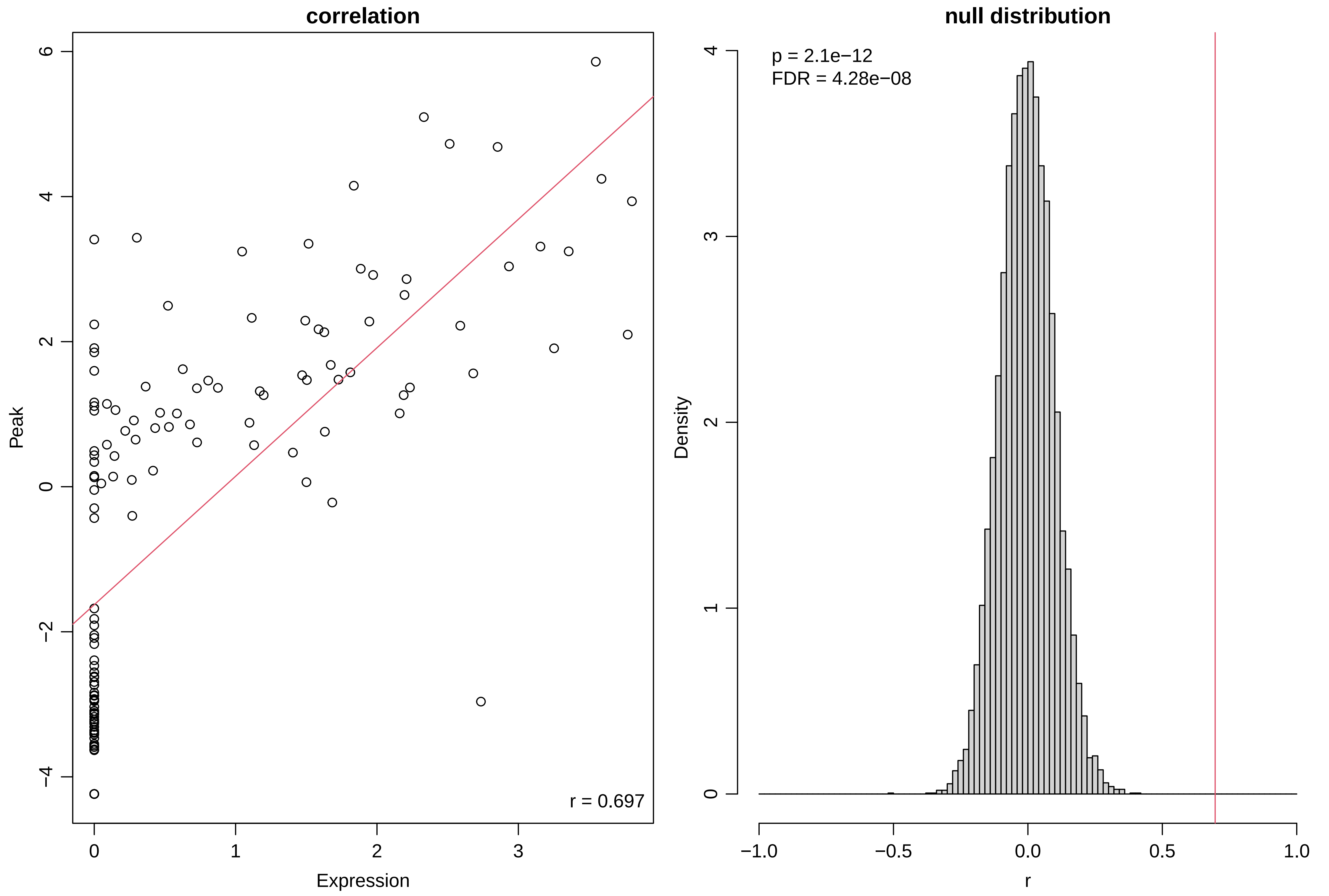

AC107881.1 vs PeakID_37843 (chr11: 12450122 − 12451382)

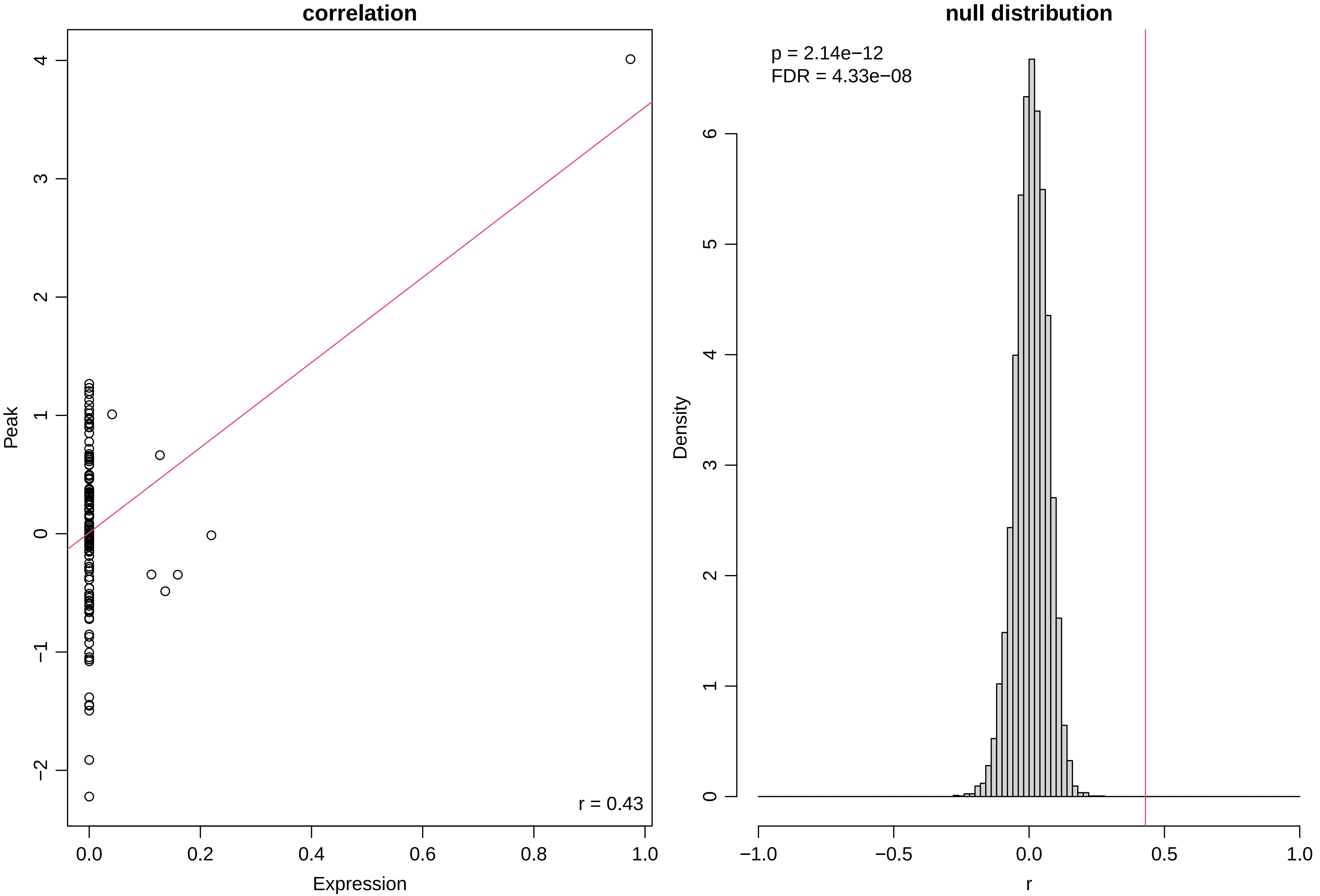

AC107881.1 vs PeakID_37896 (chr11: 12536341 − 12537262)

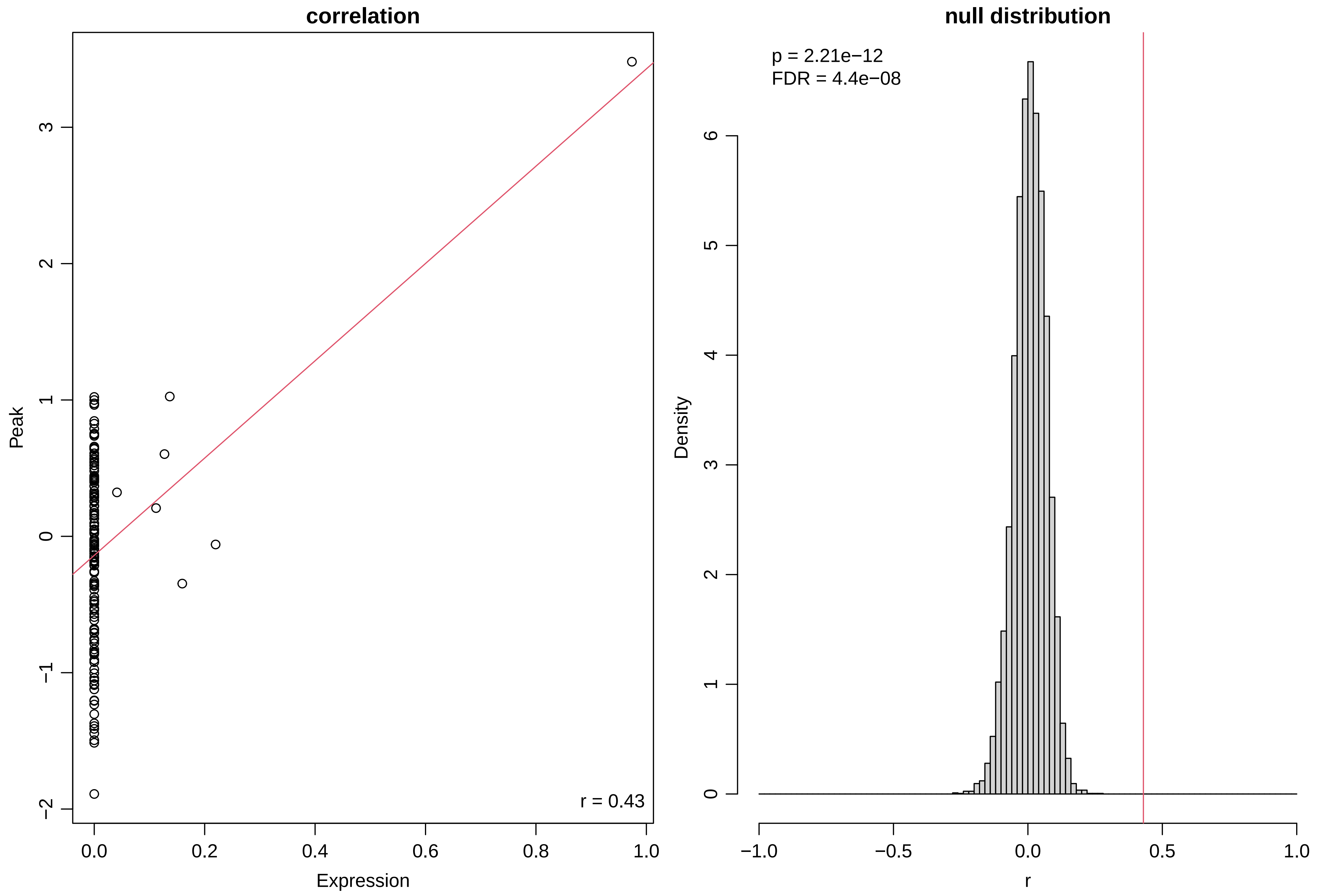

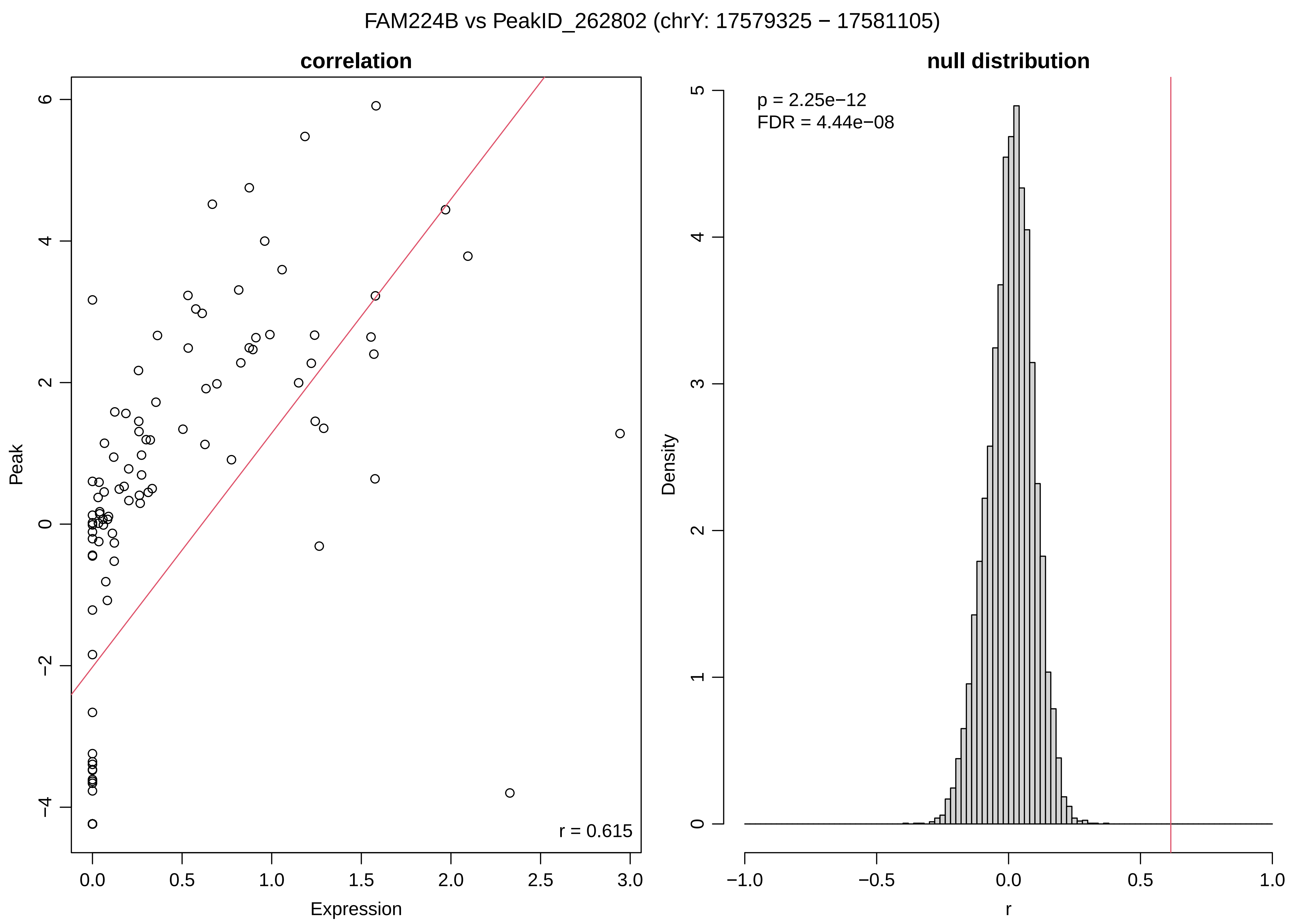

FAM224B vs PeakID_262802 (chrY: 17579325 − 17581105)
correlation
Peak
Expression
r = 0.615
null distribution
Density
r
p = 2.25e−12
FDR = 4.44e−08

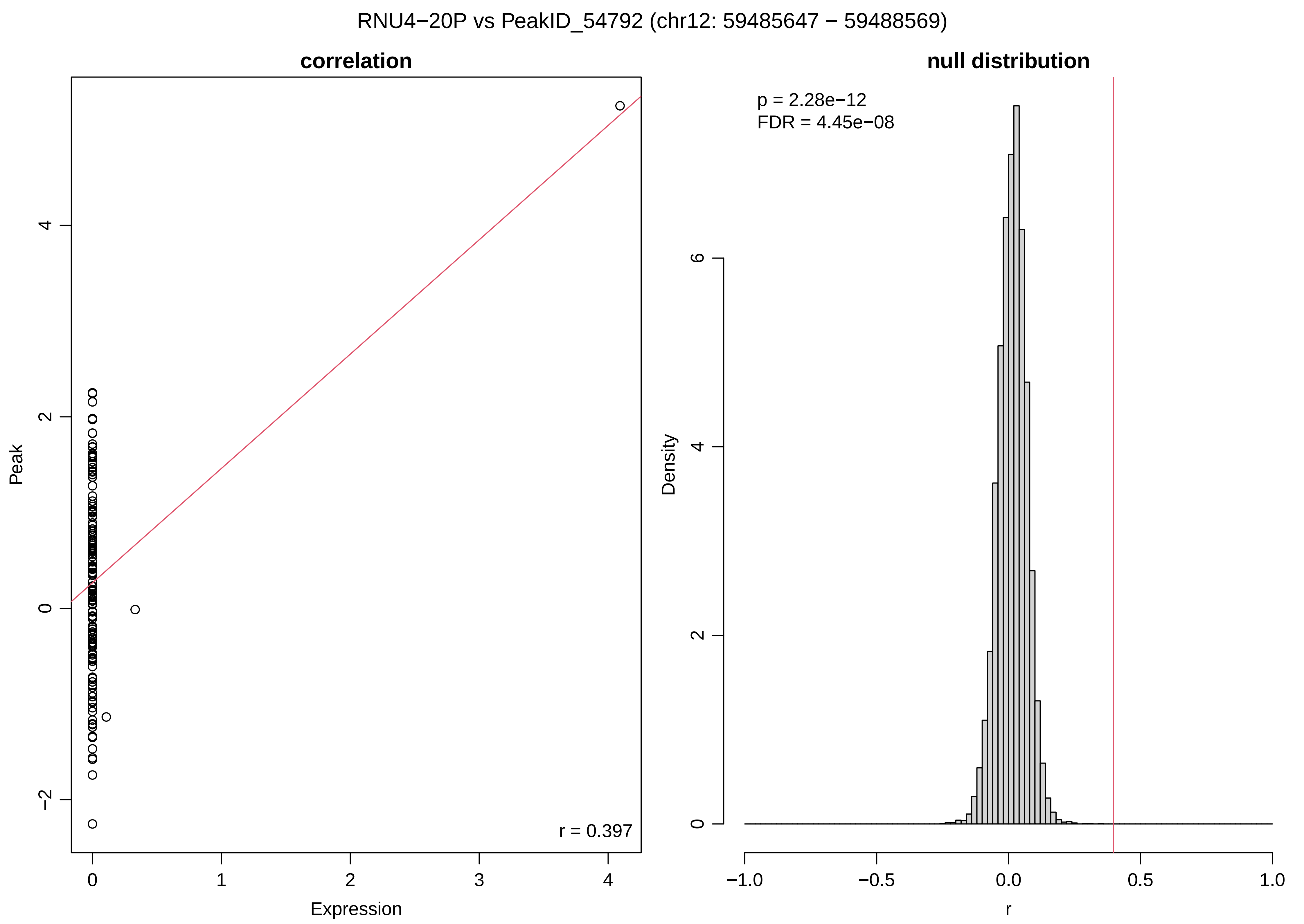

RNU4−20P vs PeakID_54792 (chr12: 59485647 − 59488569)
correlation
Peak
Expression
r = 0.397
null distribution
p = 2.28e−12
FDR = 4.45e−08
Density
r

RNU4−20P vs PeakID_54811 (chr12: 59523087 − 59527201)

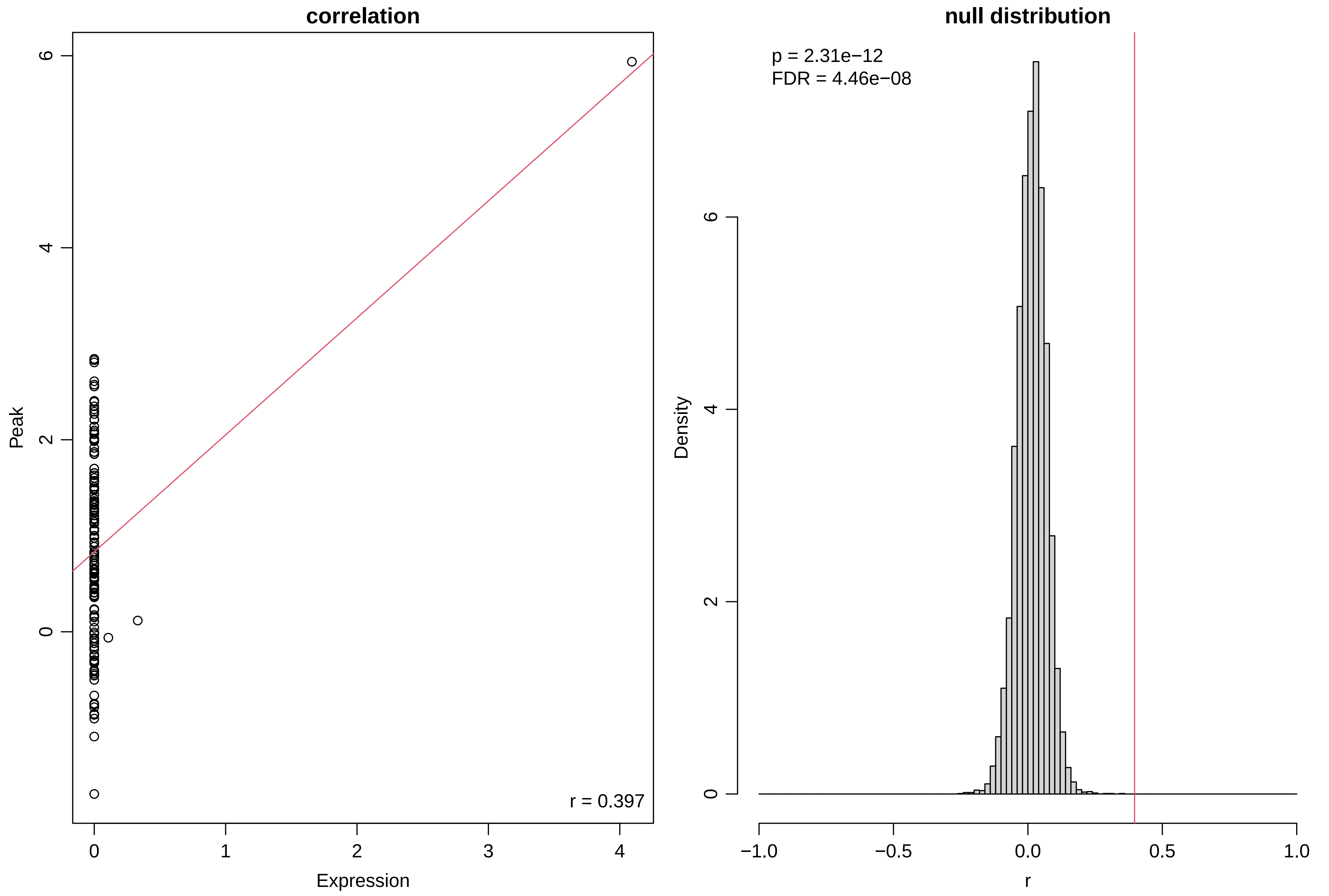

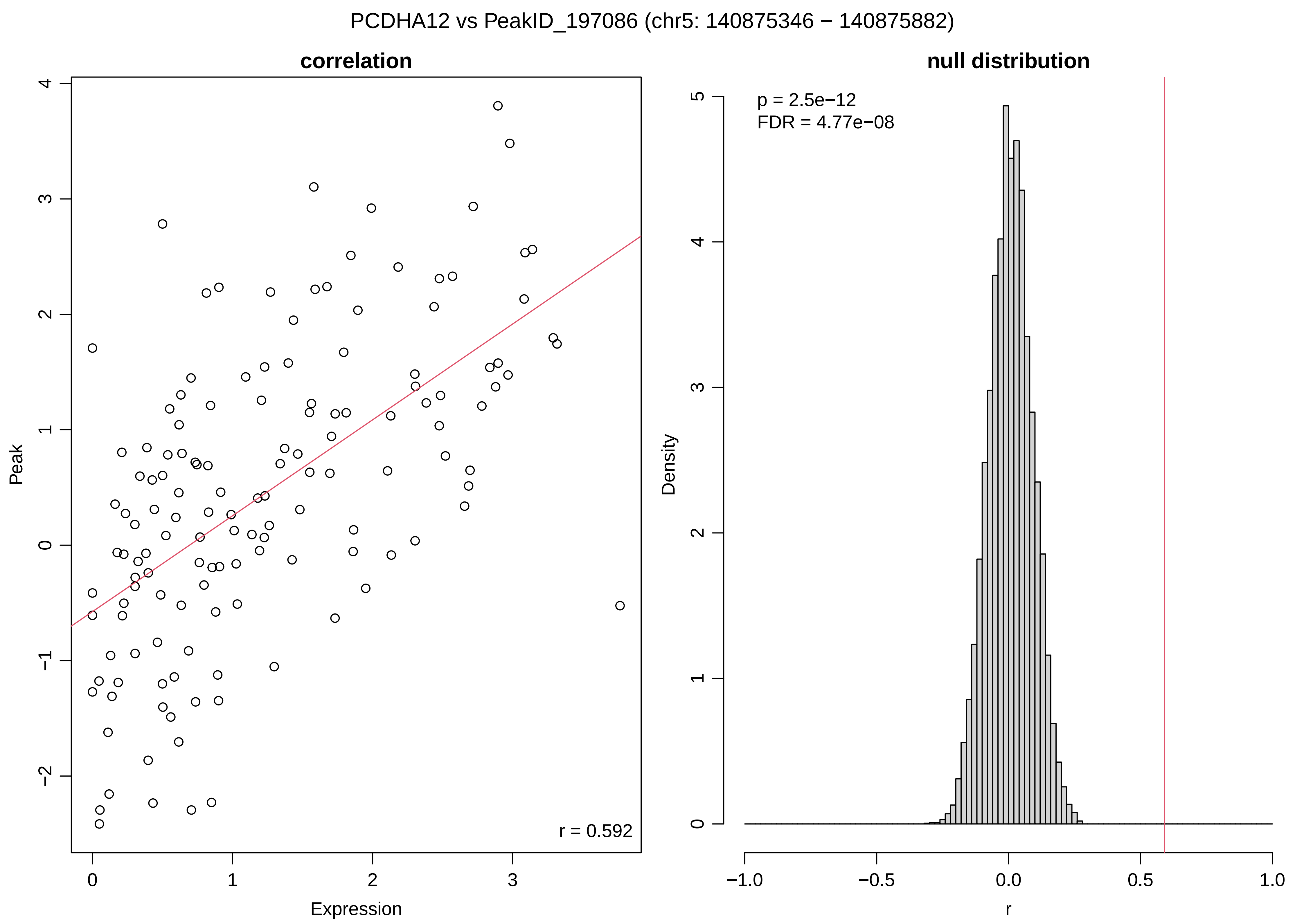
PCDHA12 vs PeakID_197086 (chr5: 140875346 − 140875882)
correlation
Peak
Expression
r = 0.592
null distribution
p = 2.5e−12
FDR = 4.77e−08
Density
r

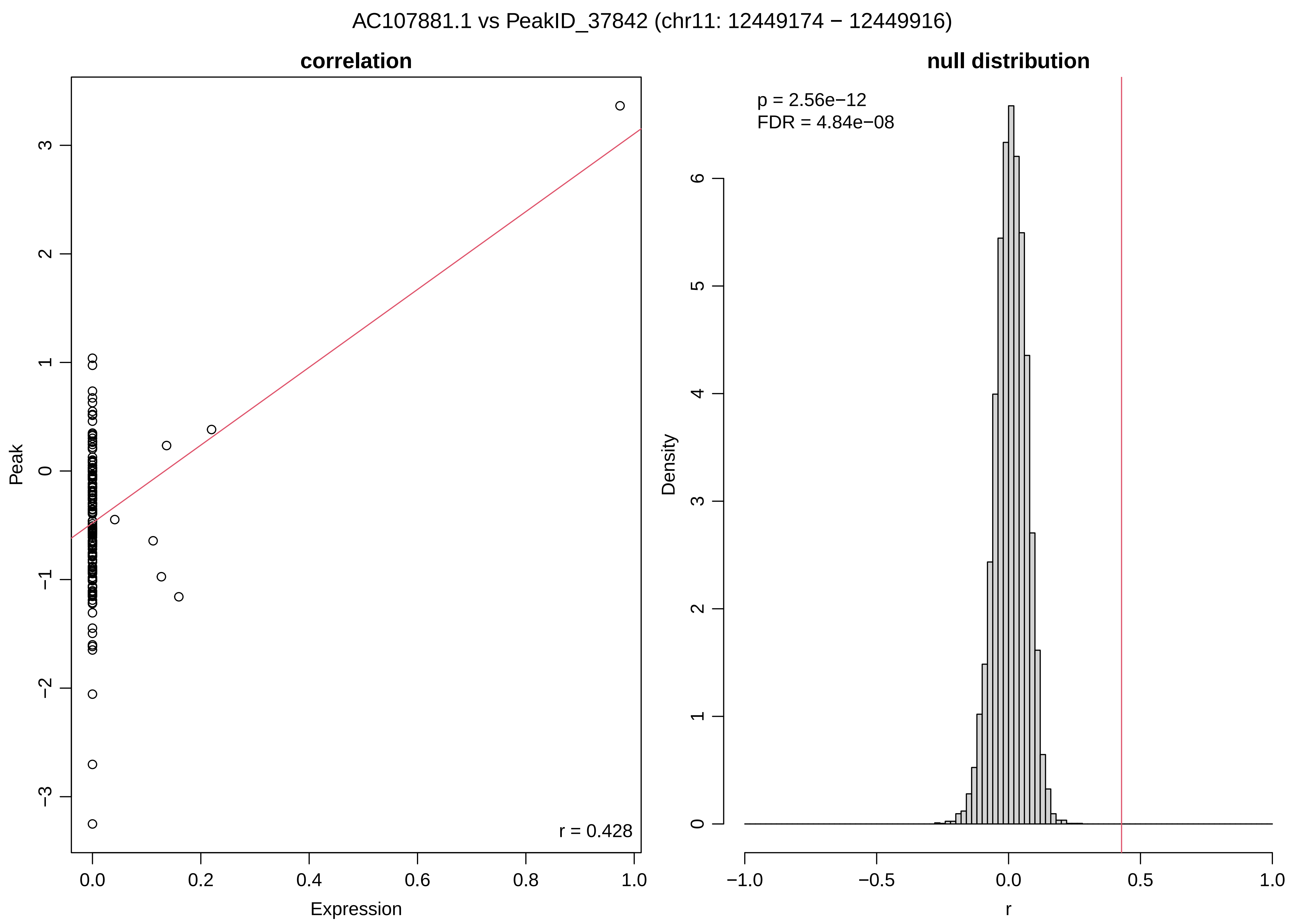
AC107881.1 vs PeakID_37842 (chr11: 12449174 − 12449916)
correlation
Peak
Expression
r = 0.428
null distribution
p = 2.56e−12
FDR = 4.84e−08
Density
r

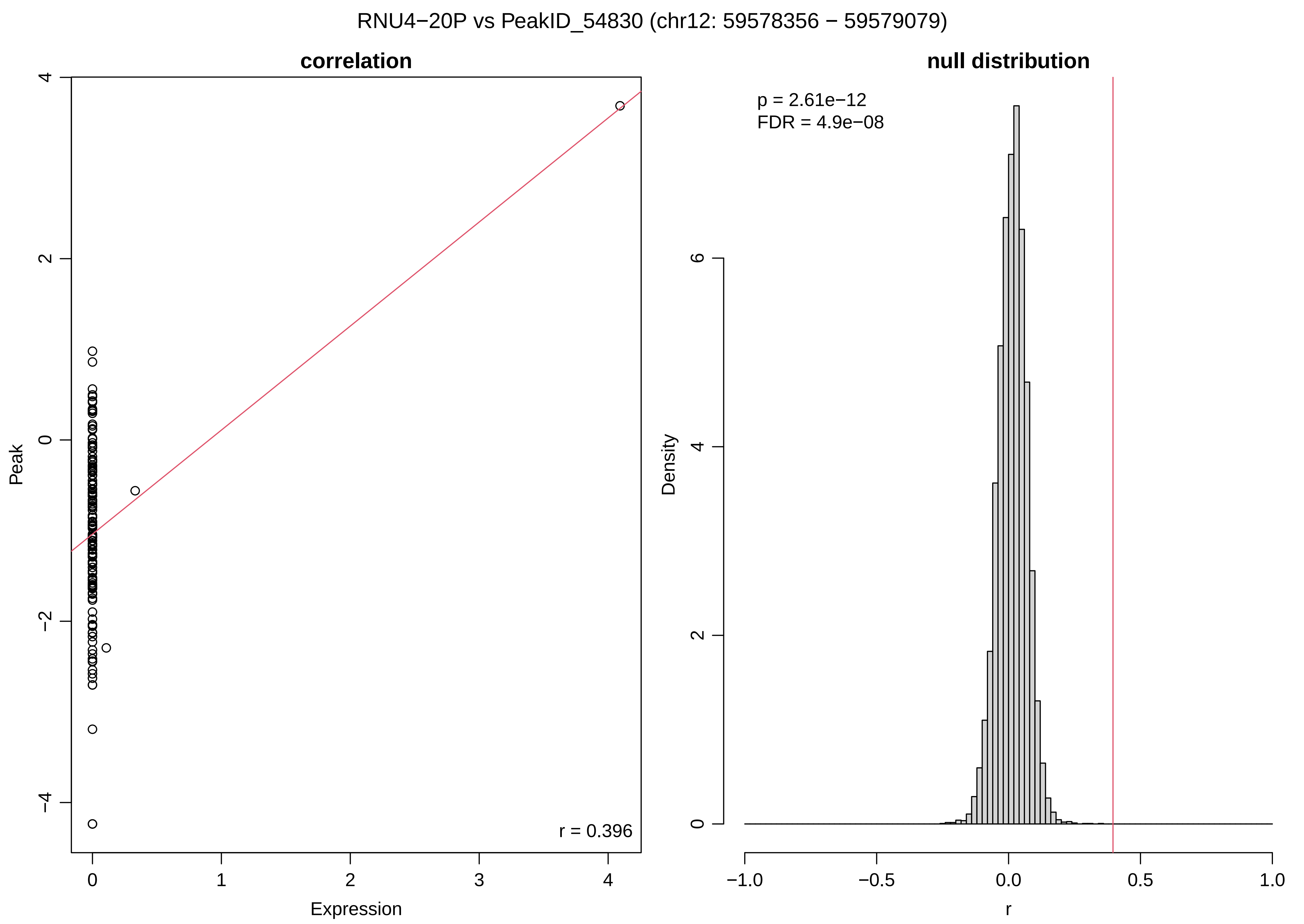
RNU4−20P vs PeakID_54830 (chr12: 59578356 − 59579079)
correlation
Peak
Expression
r = 0.396
4
2
0
−2
−4
0
1
2
3
4
null distribution
p = 2.61e−12
FDR = 4.9e−08
Density
r
6
4
2
0
−1.0
−0.5
0.0
0.5
1.0

SLC35E3 vs PeakID_56596 (chr12: 68845709 − 68846772)

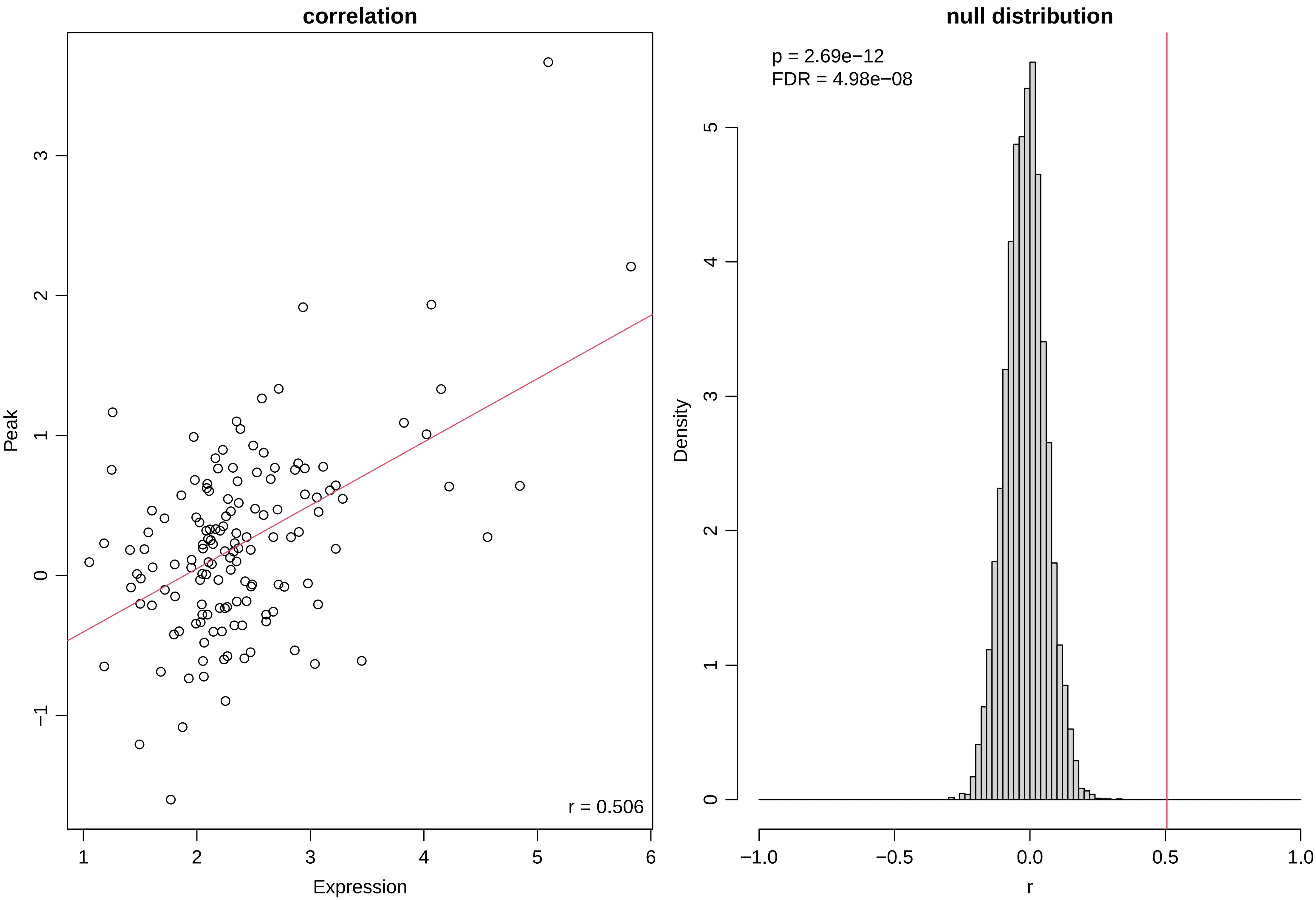

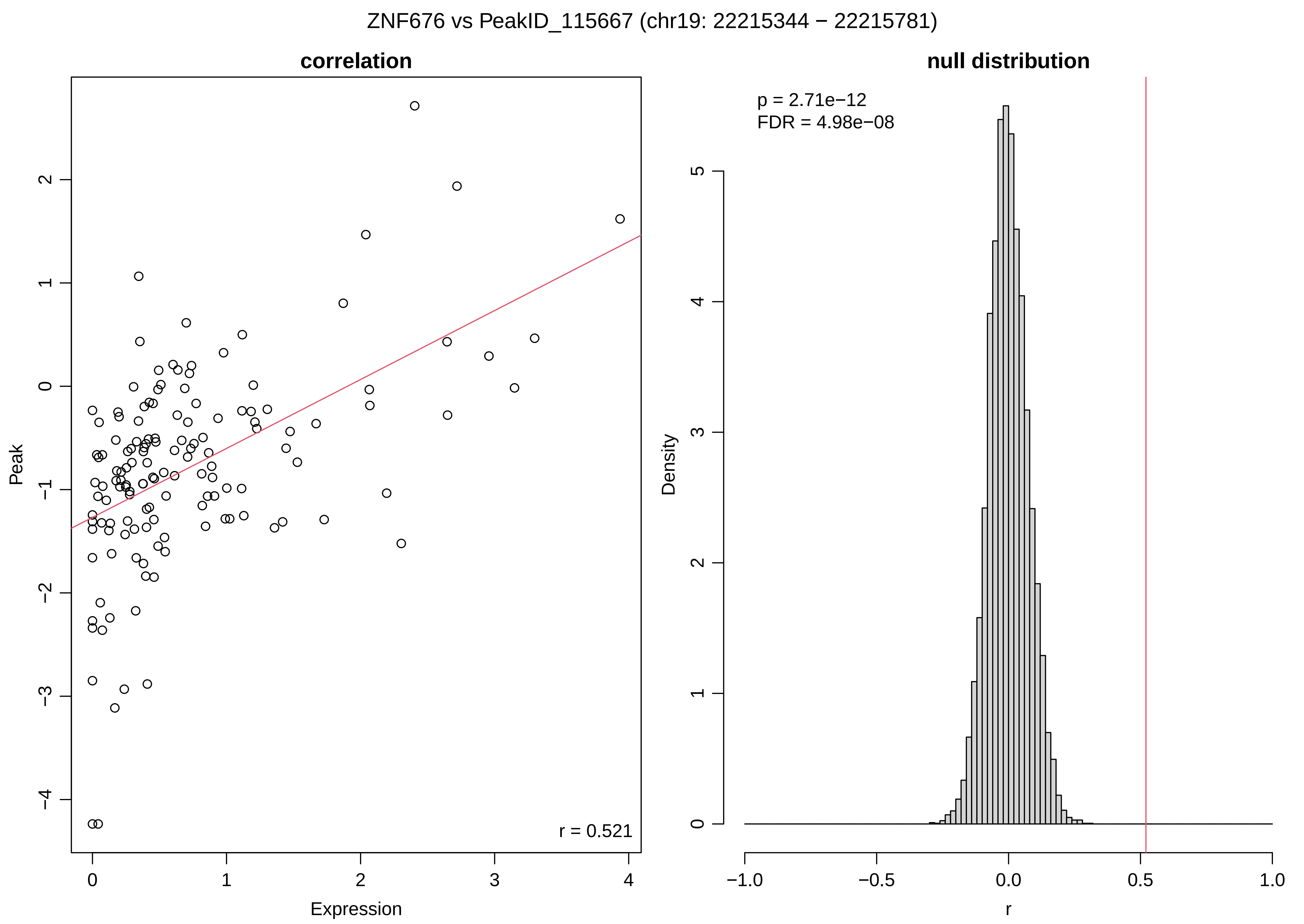

ZNF676 vs PeakID_115667 (chr19: 22215344 − 22215781)
correlation
Peak
Expression
r = 0.521
null distribution
p = 2.71e−12
FDR = 4.98e−08
Density
r

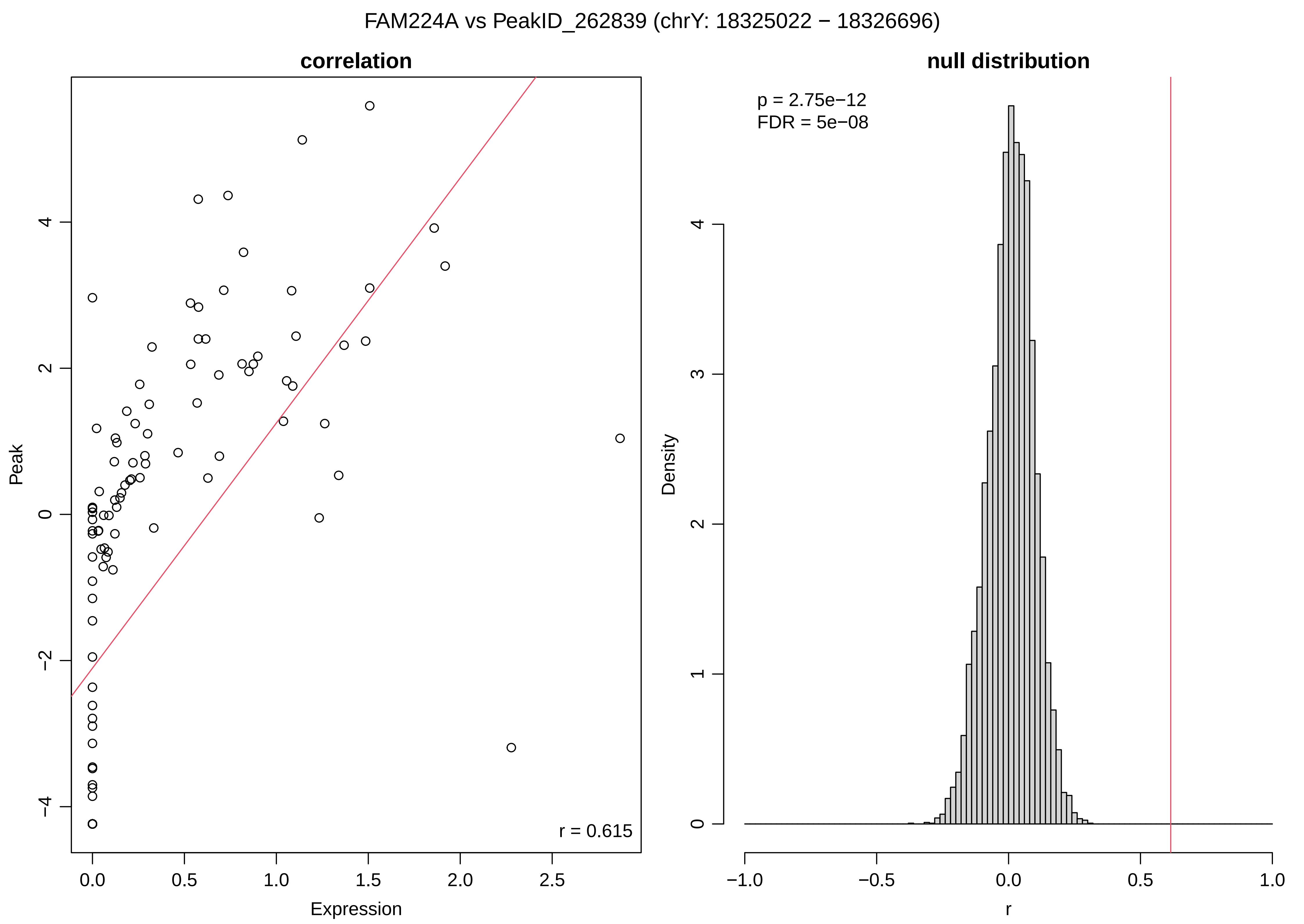
FAM224A vs PeakID_262839 (chrY: 18325022 − 18326696)
correlation
Peak
Expression
r = 0.615
null distribution
p = 2.75e−12
FDR = 5e−08
Density
r

TTTY14 vs PeakID_262919 (chrY: 18893923 − 18894704)

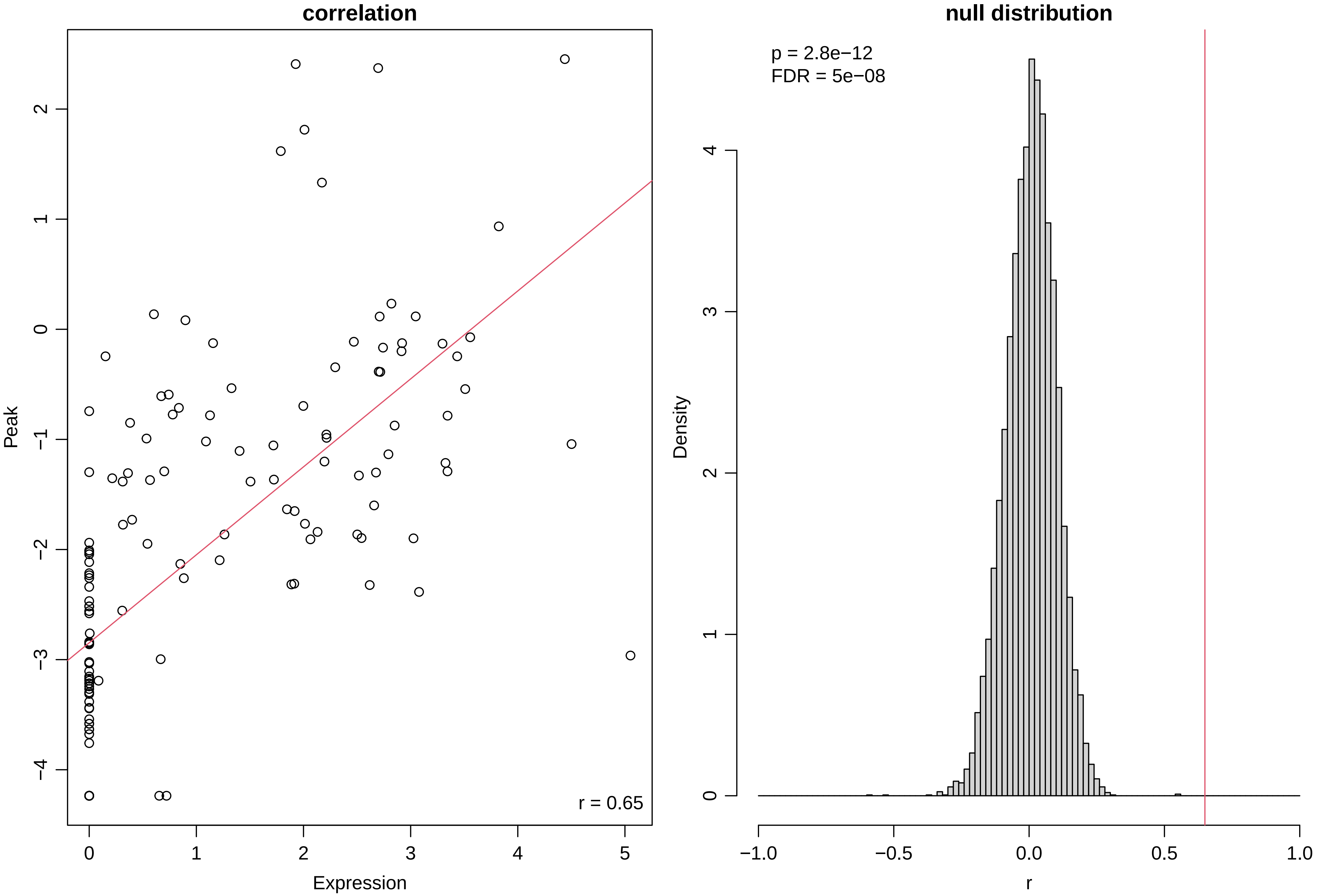

LCAL1 vs PeakID_208955 (chr6: 79307149 − 79313380)

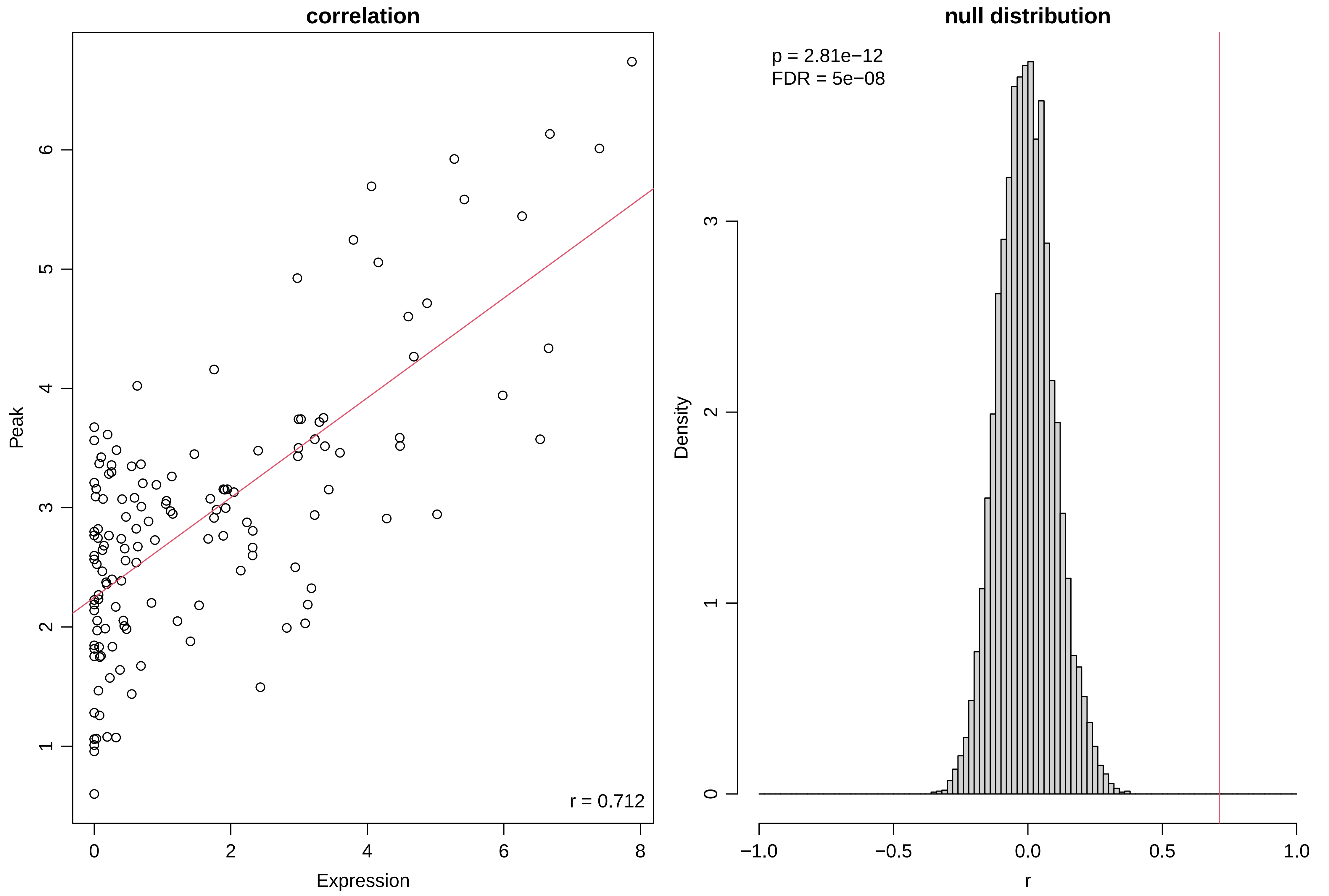

HMGA2 vs PeakID_56092 (chr12: 65822504 − 65838533)

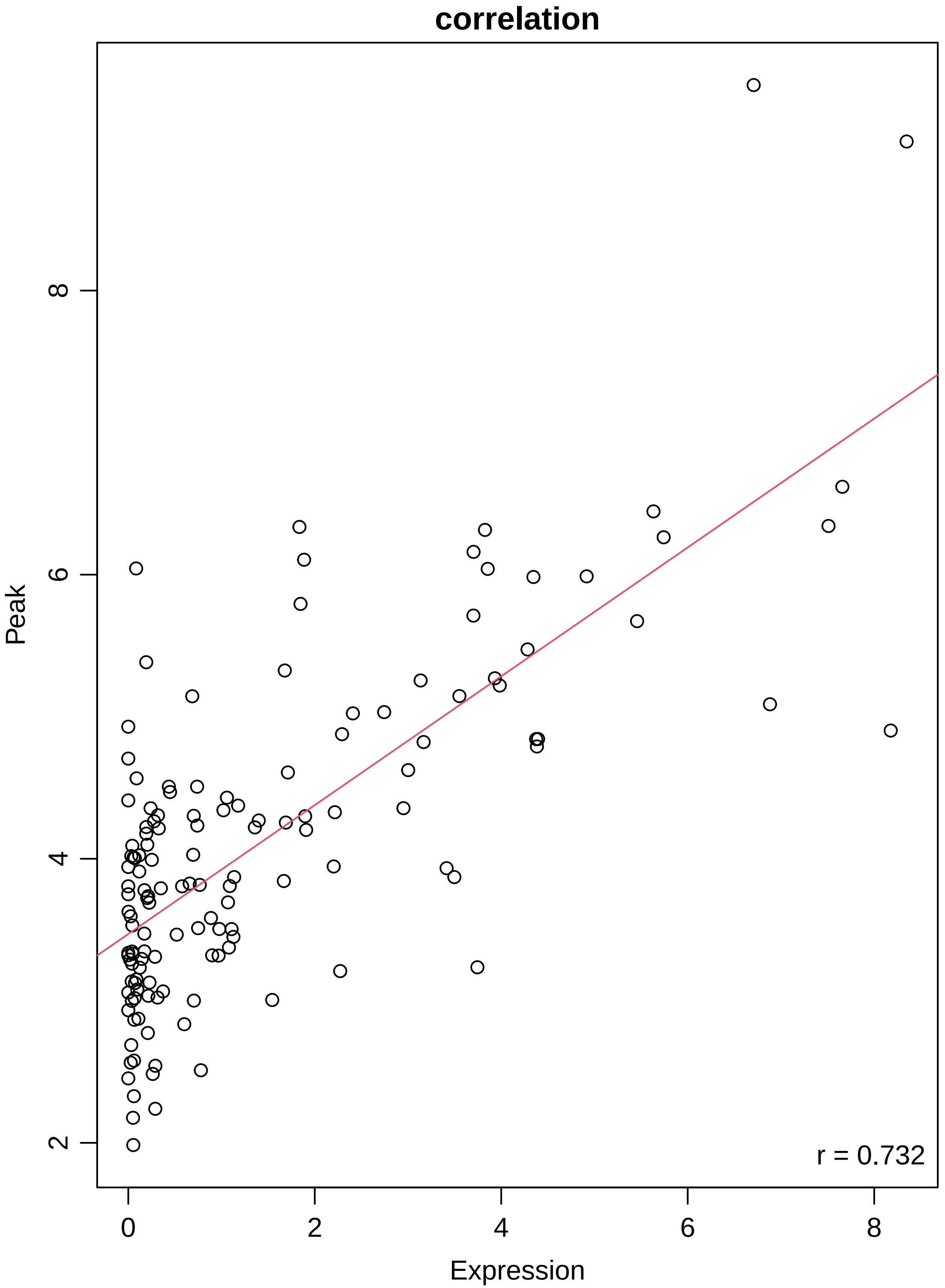

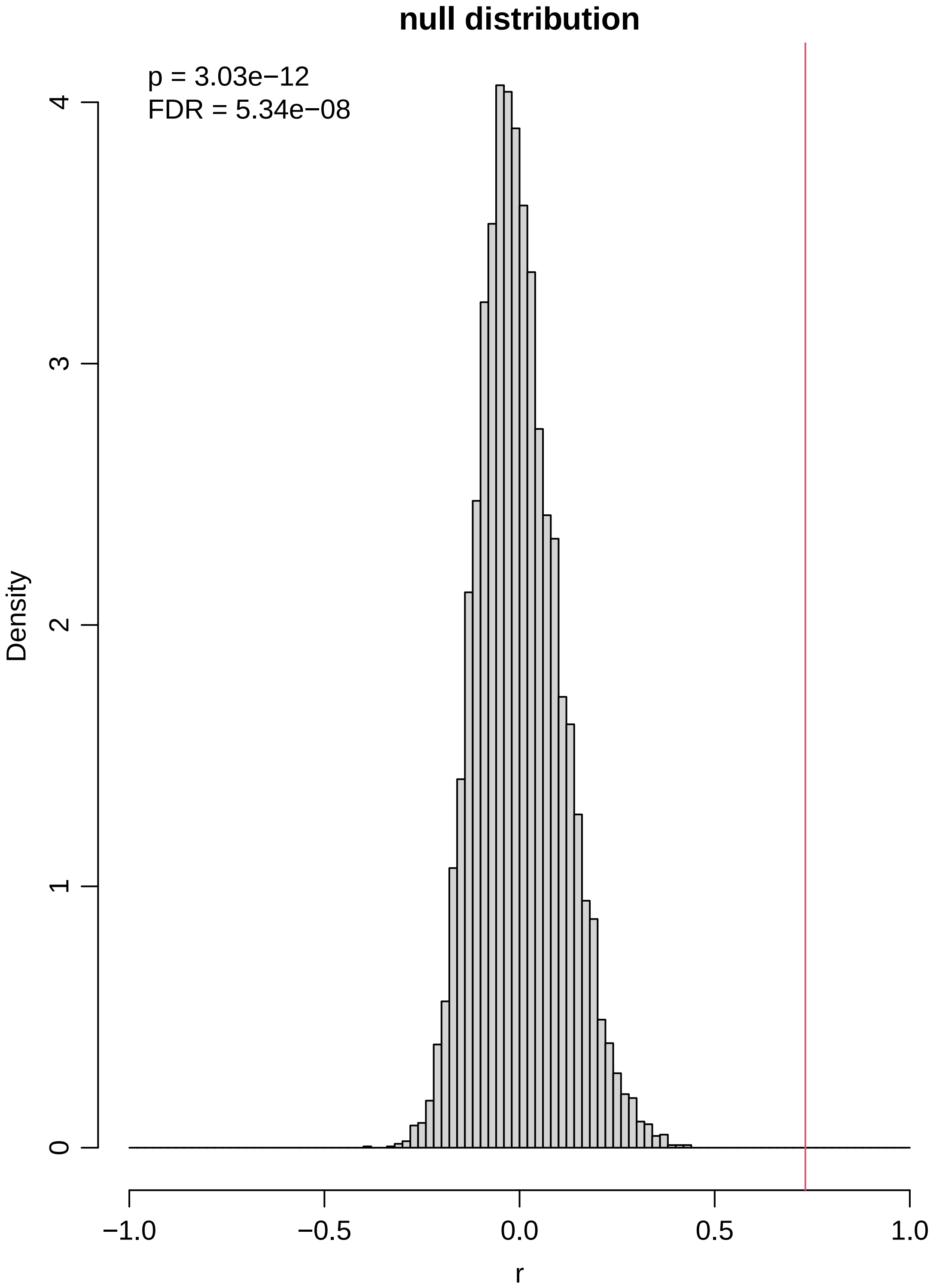

MYCN vs PeakID_123406 (chr2: 15939482 − 15944165)

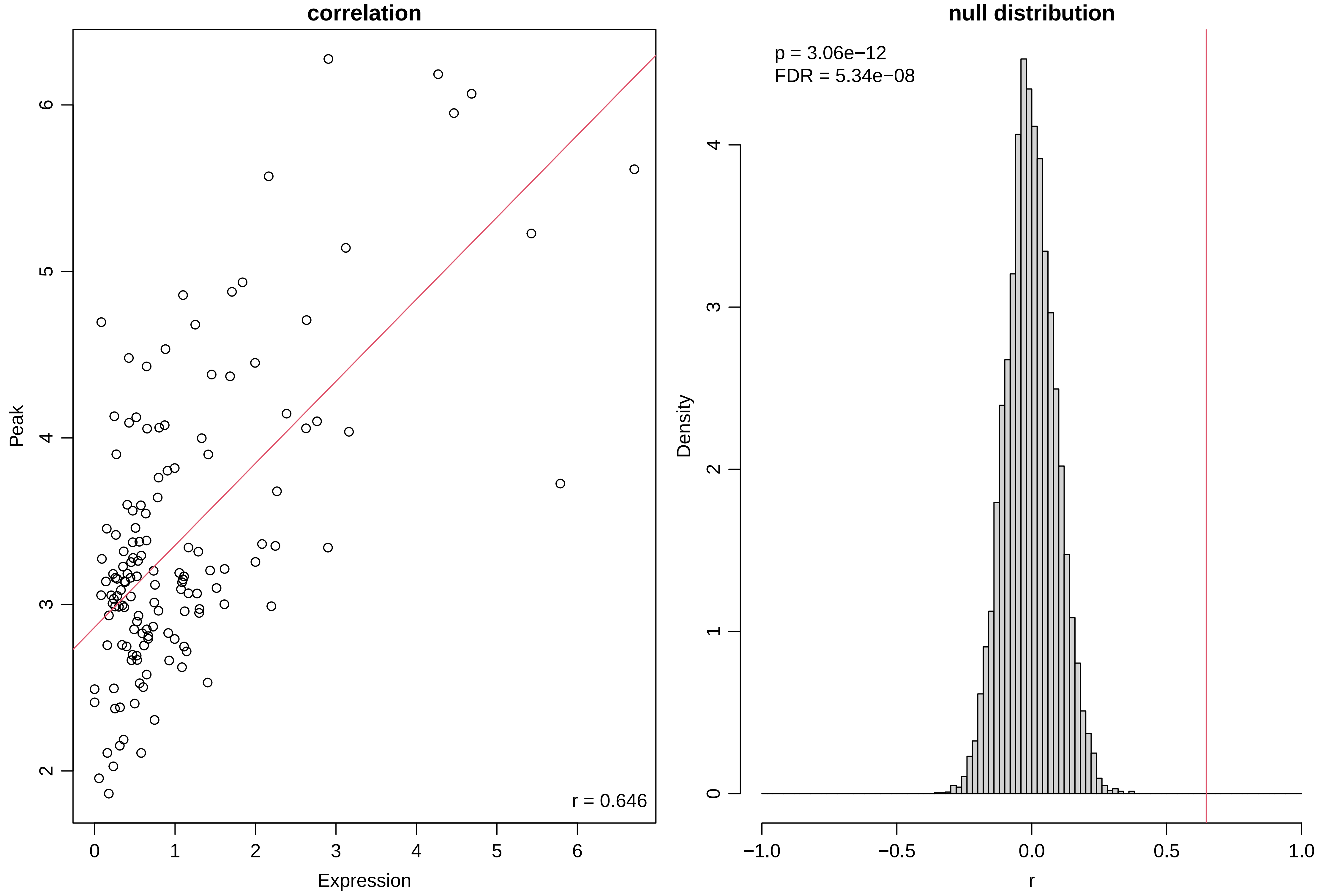

GSTM1 vs PeakID_10828 (chr1: 109711704 − 109712417)

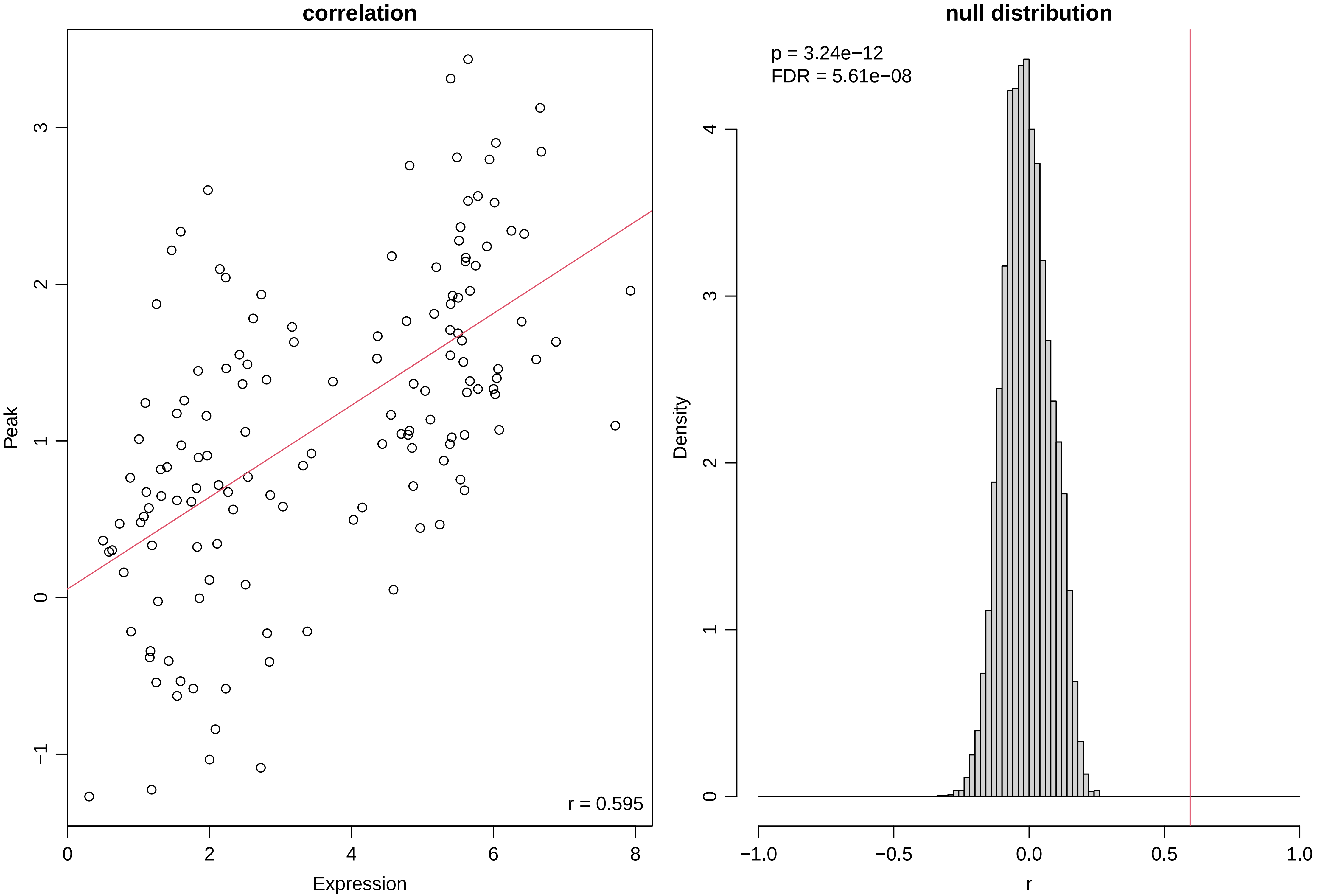

ZFY−AS1 vs PeakID_262337 (chrY: 3000907 − 3001416)

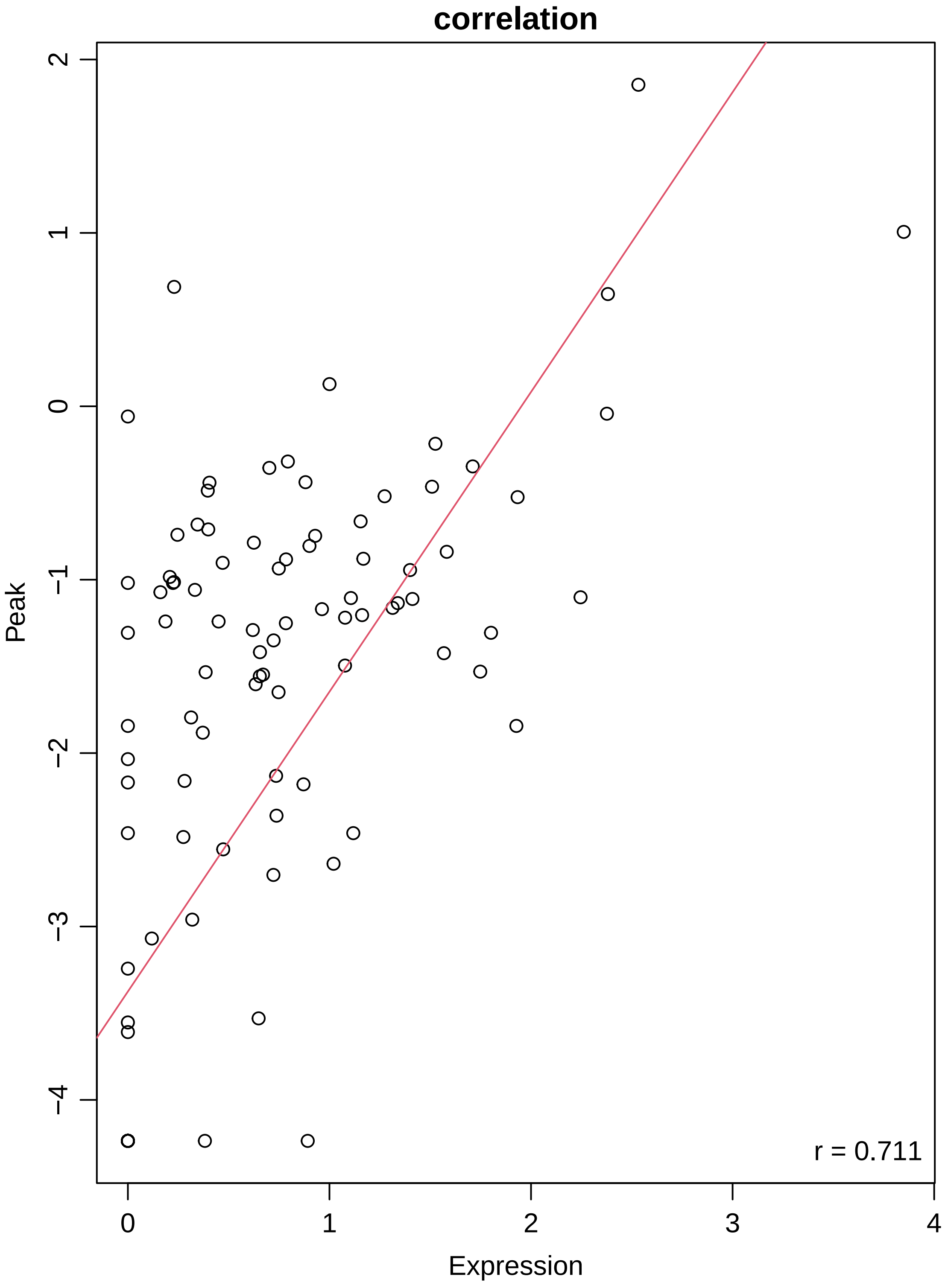

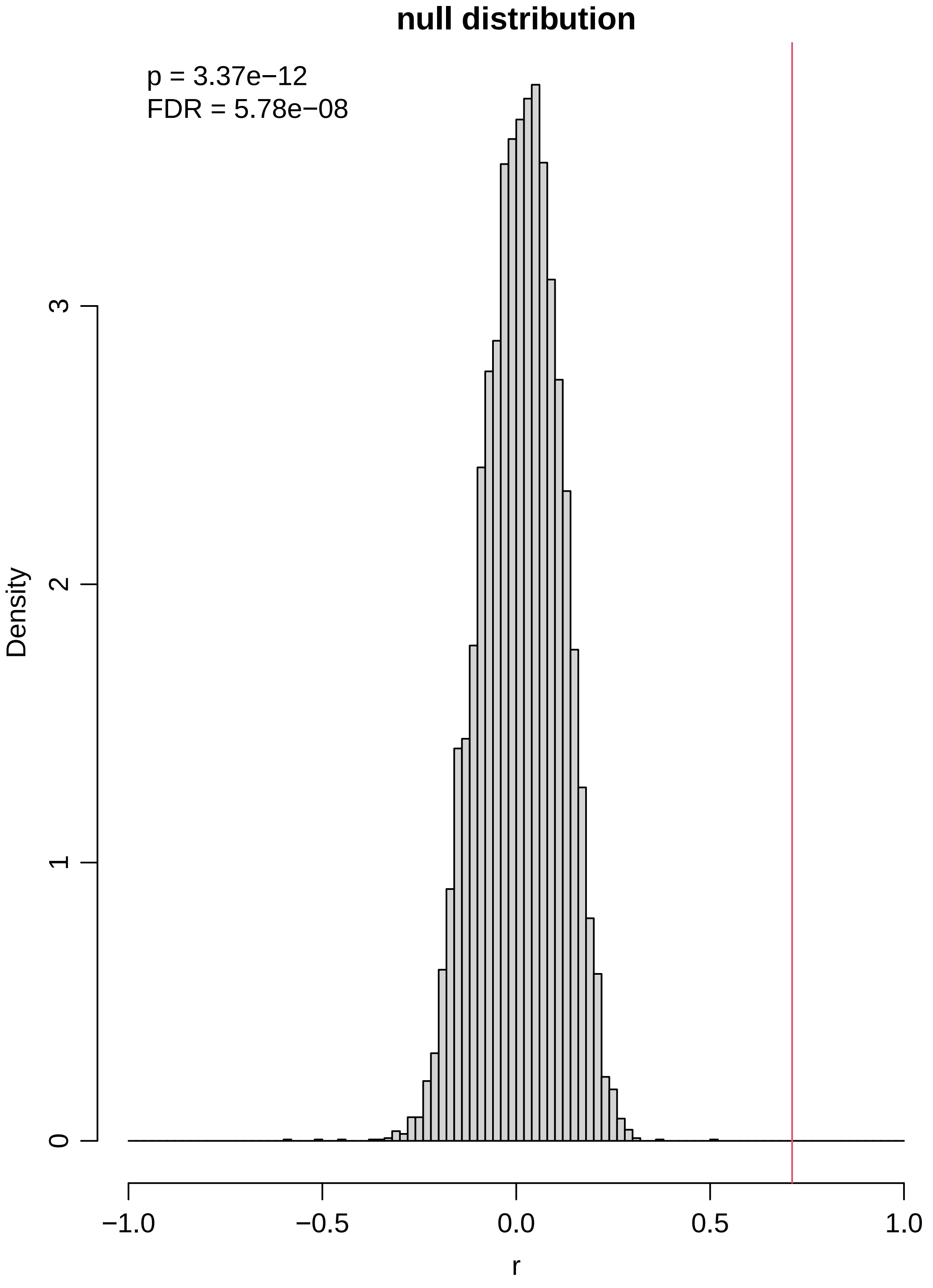

NECTIN1 vs PeakID_46771 (chr11: 119680352 − 119703740)

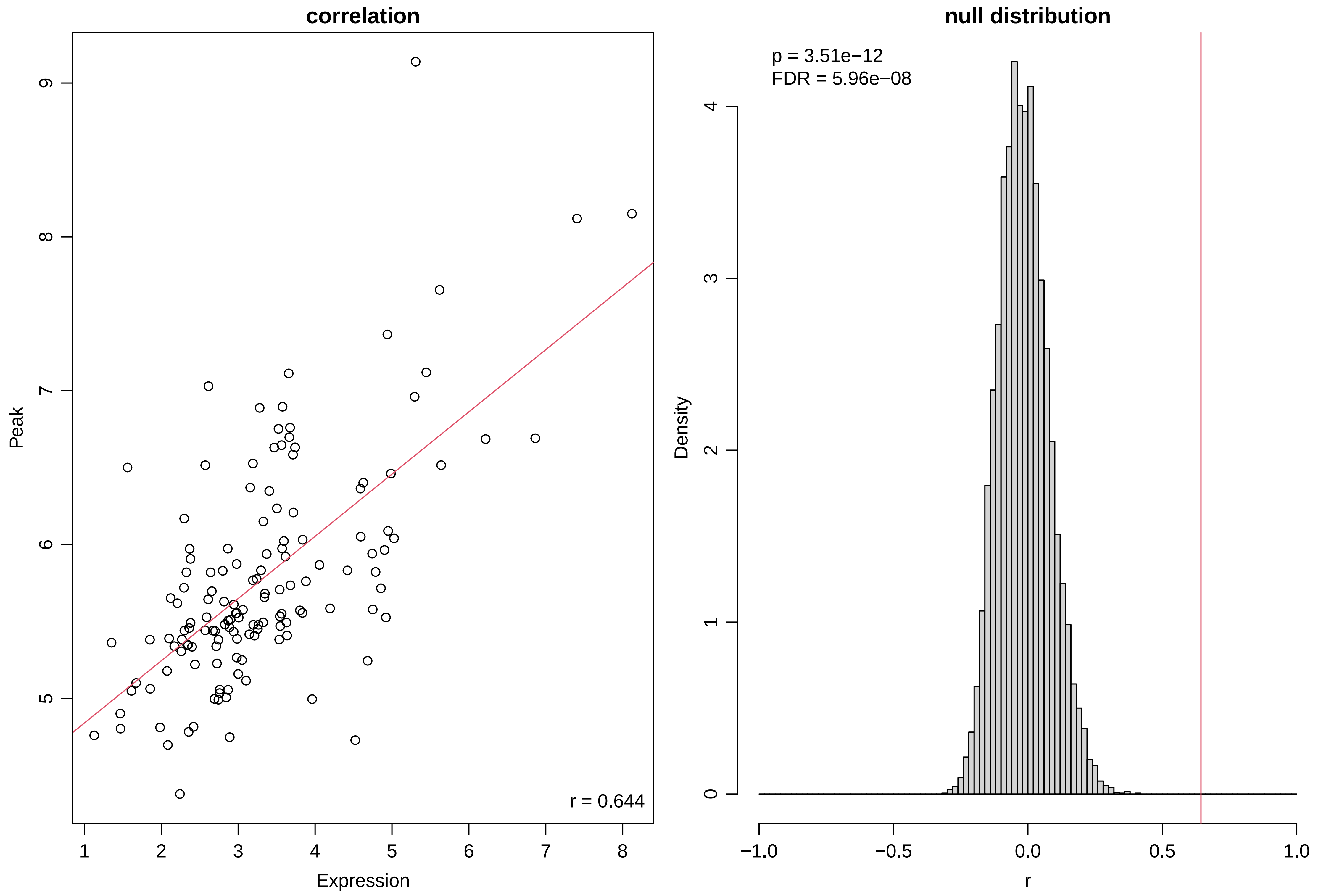

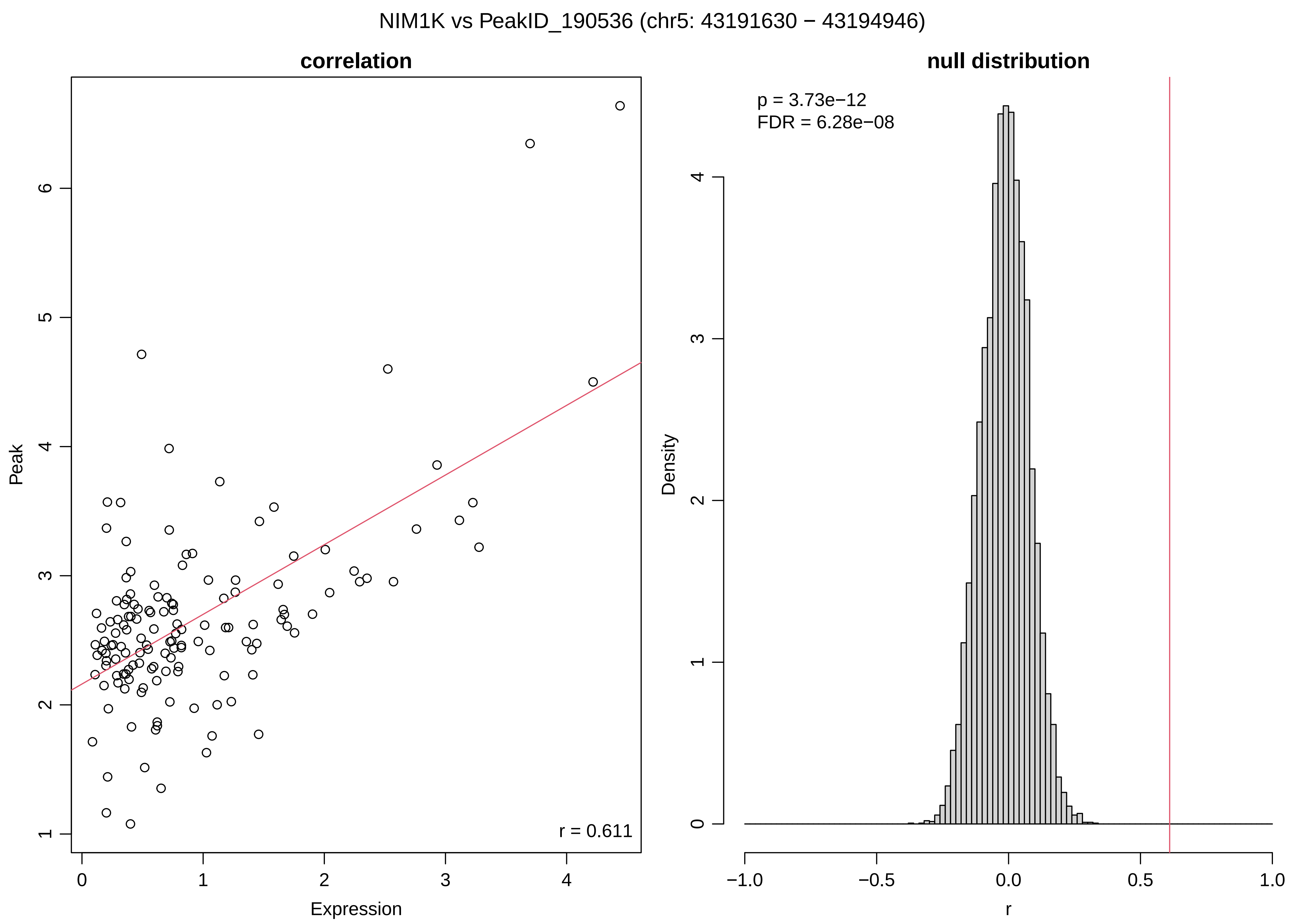
NIM1K vs PeakID_190536 (chr5: 43191630 − 43194946)
correlation
Peak
Expression
r = 0.611
null distribution
p = 3.73e−12
FDR = 6.28e−08
Density
r

SMOC1 vs PeakID_72626 (chr14: 69997549 − 69998004)

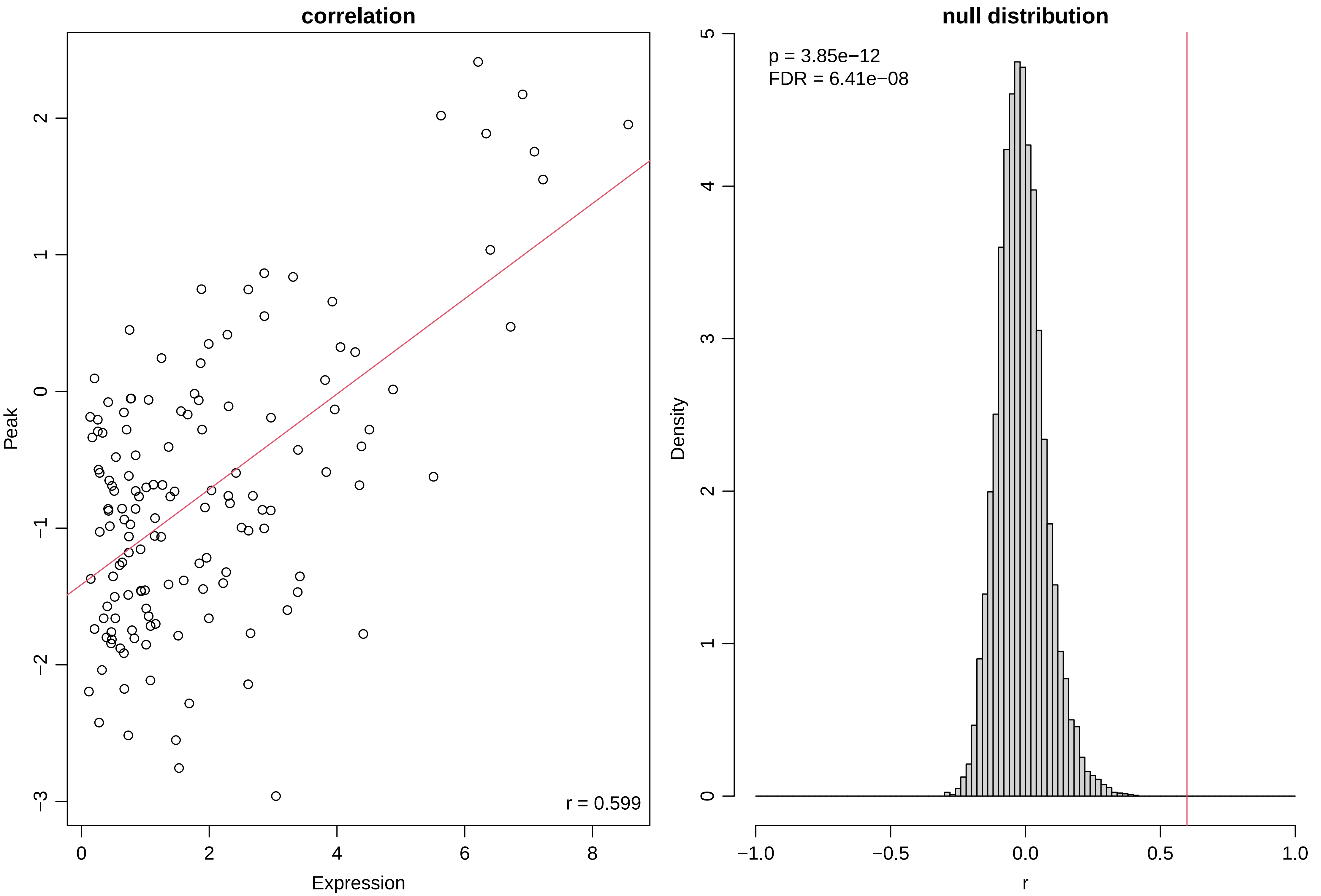

HCG4B vs PeakID_204615 (chr6: 29926007 − 29927546)

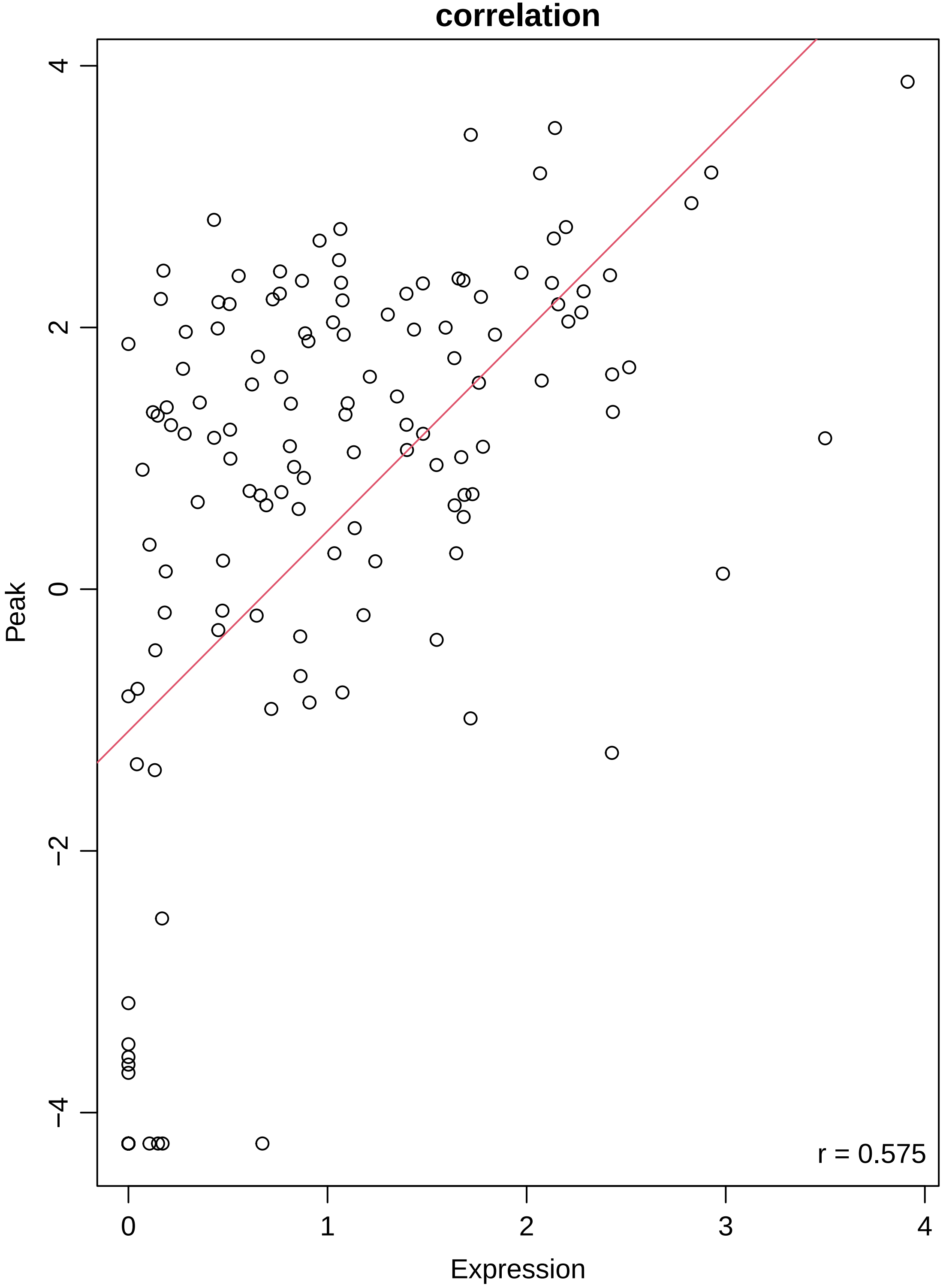

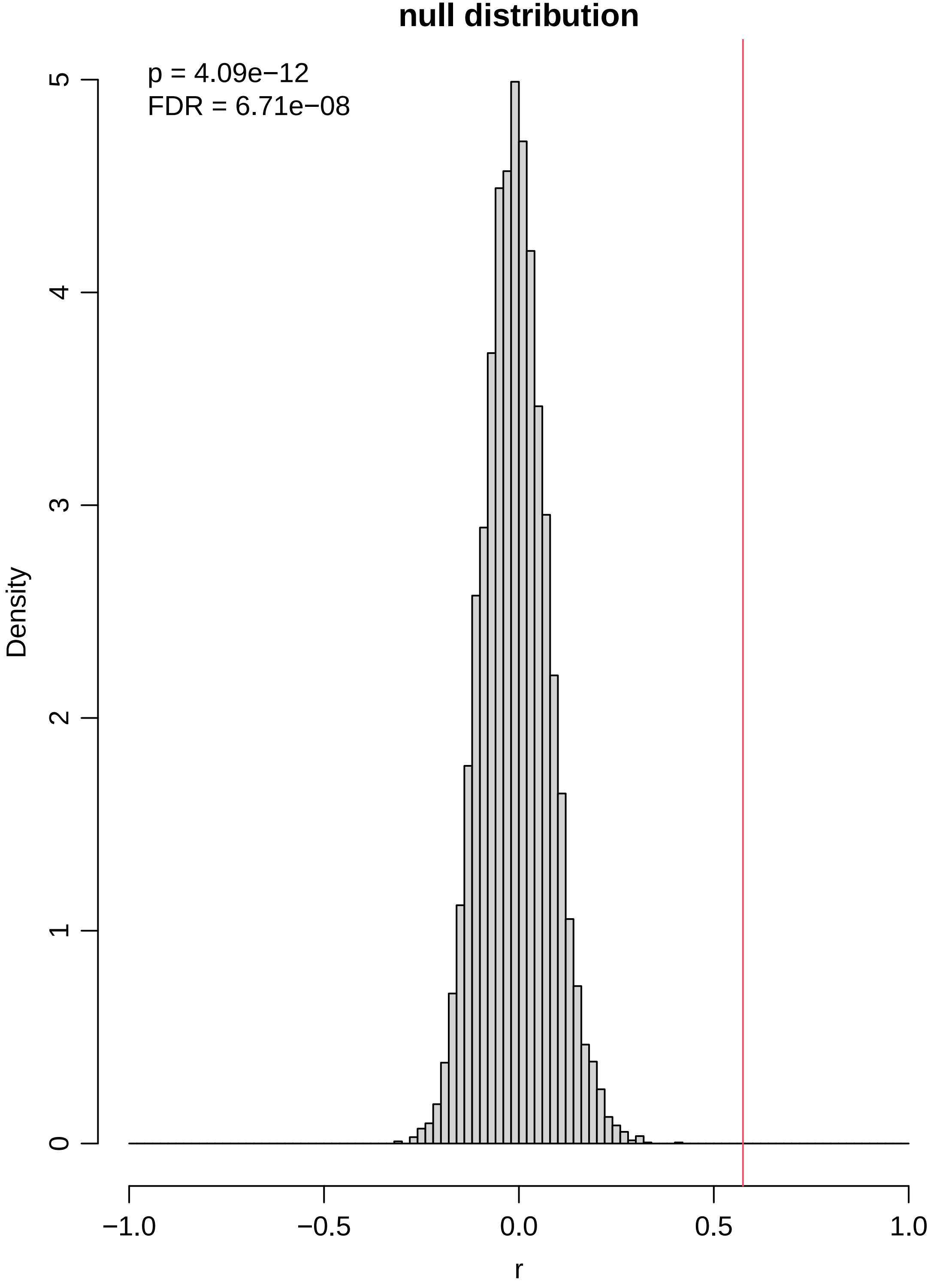

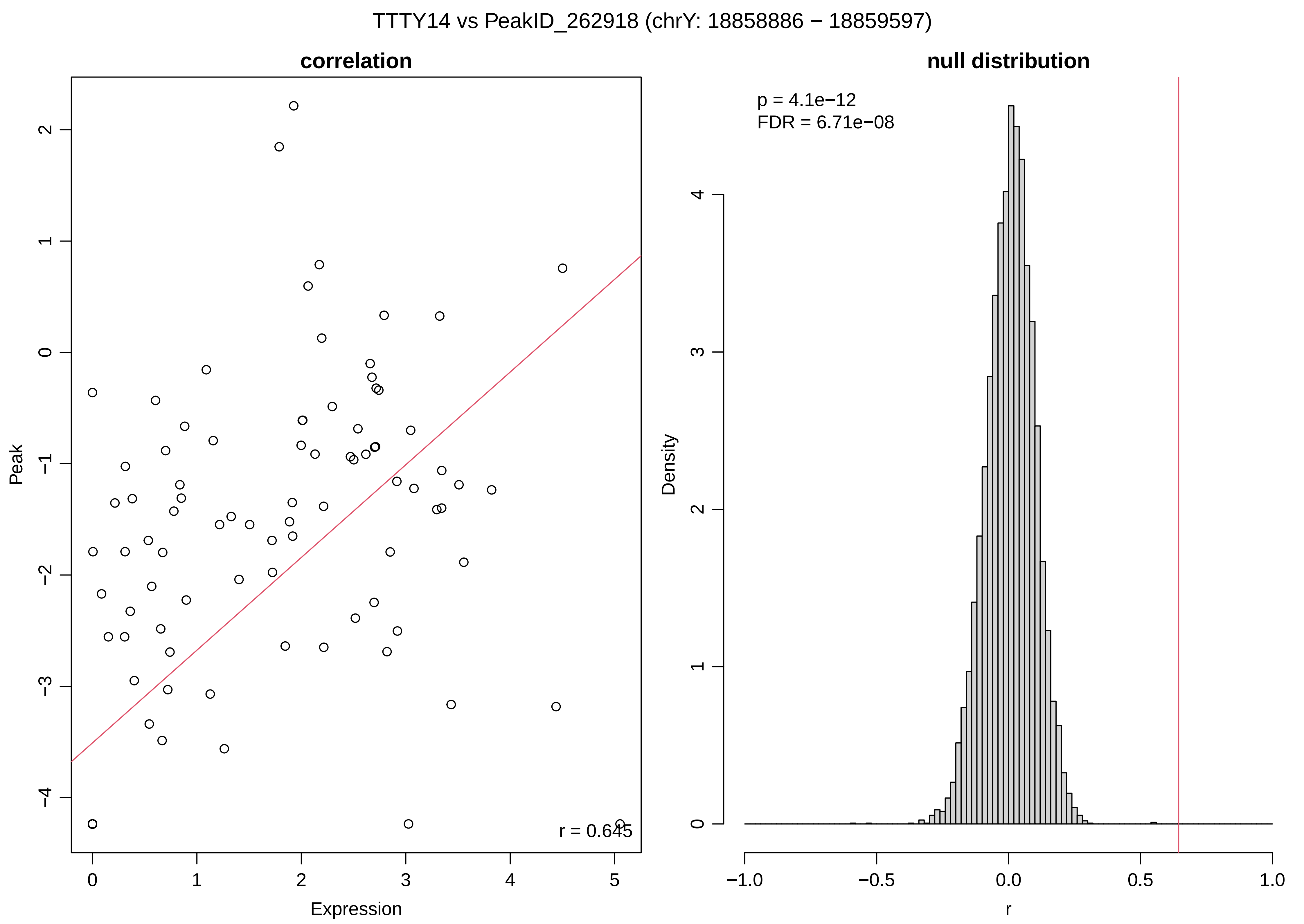

TTTY14 vs PeakID_262918 (chrY: 18858886 − 18859597)
correlation
Peak
Expression
r = 0.645
null distribution
p = 4.1e−12
FDR = 6.71e−08
Density
r

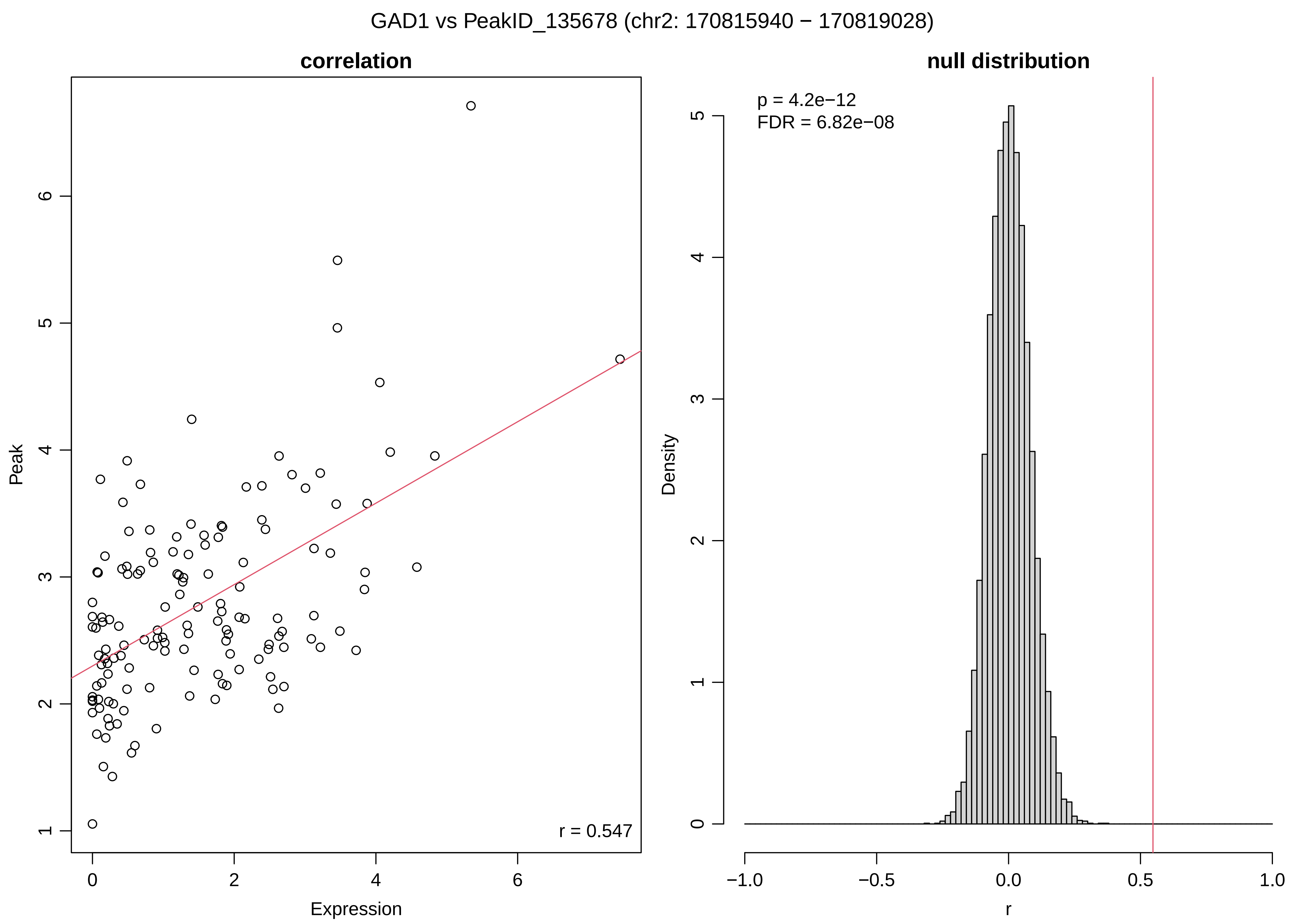
GAD1 vs PeakID_135678 (chr2: 170815940 − 170819028)
correlation
Peak
Expression
r = 0.547
0
2
4
6
1
2
3
4
5
6
null distribution
p = 4.2e−12
FDR = 6.82e−08
Density
r
−1.0
−0.5
0.0
0.5
1.0
0
1
2
3
4
5

CCT2 vs PeakID_56860 (chr12: 69518095 − 69519979)

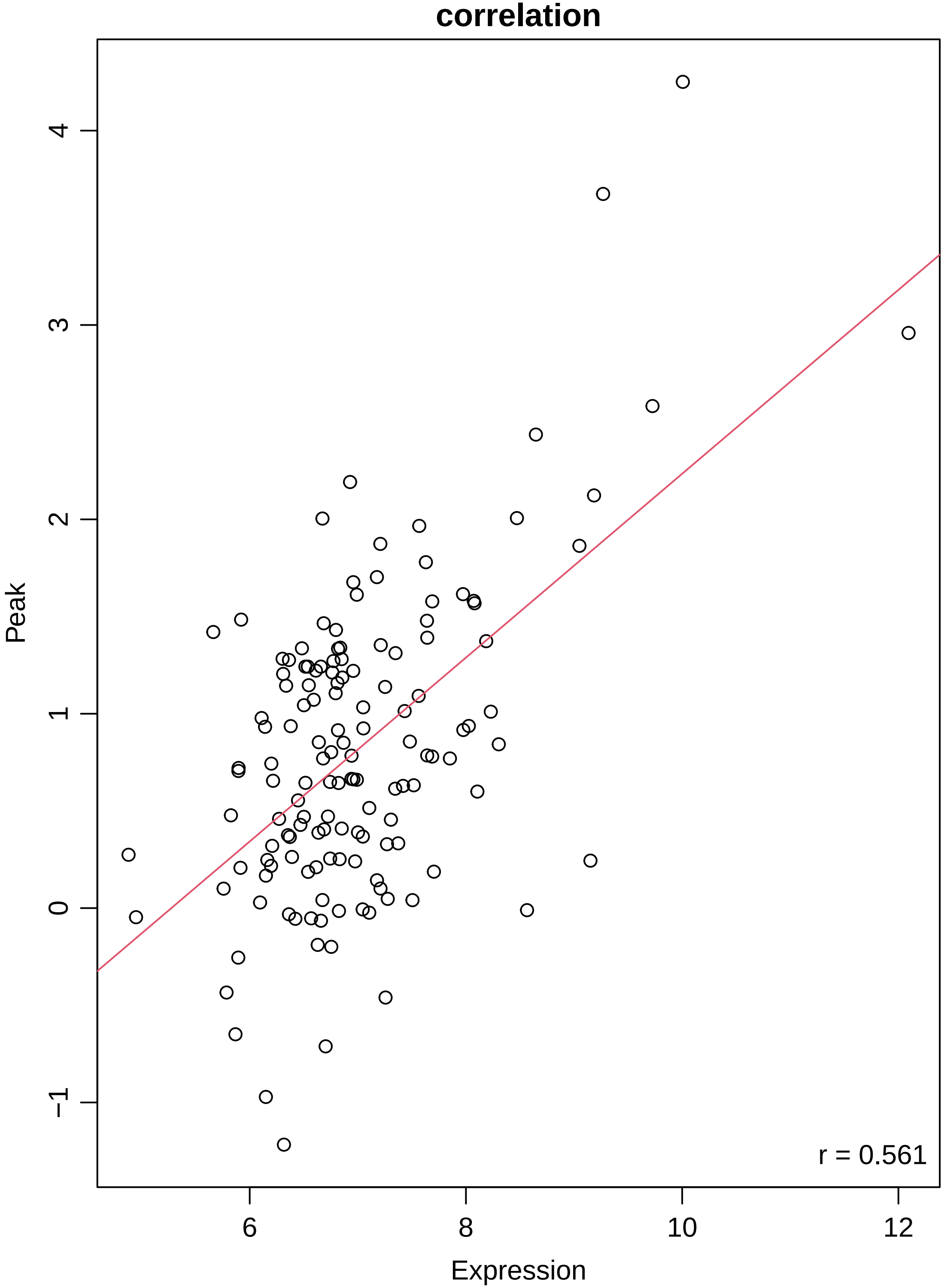

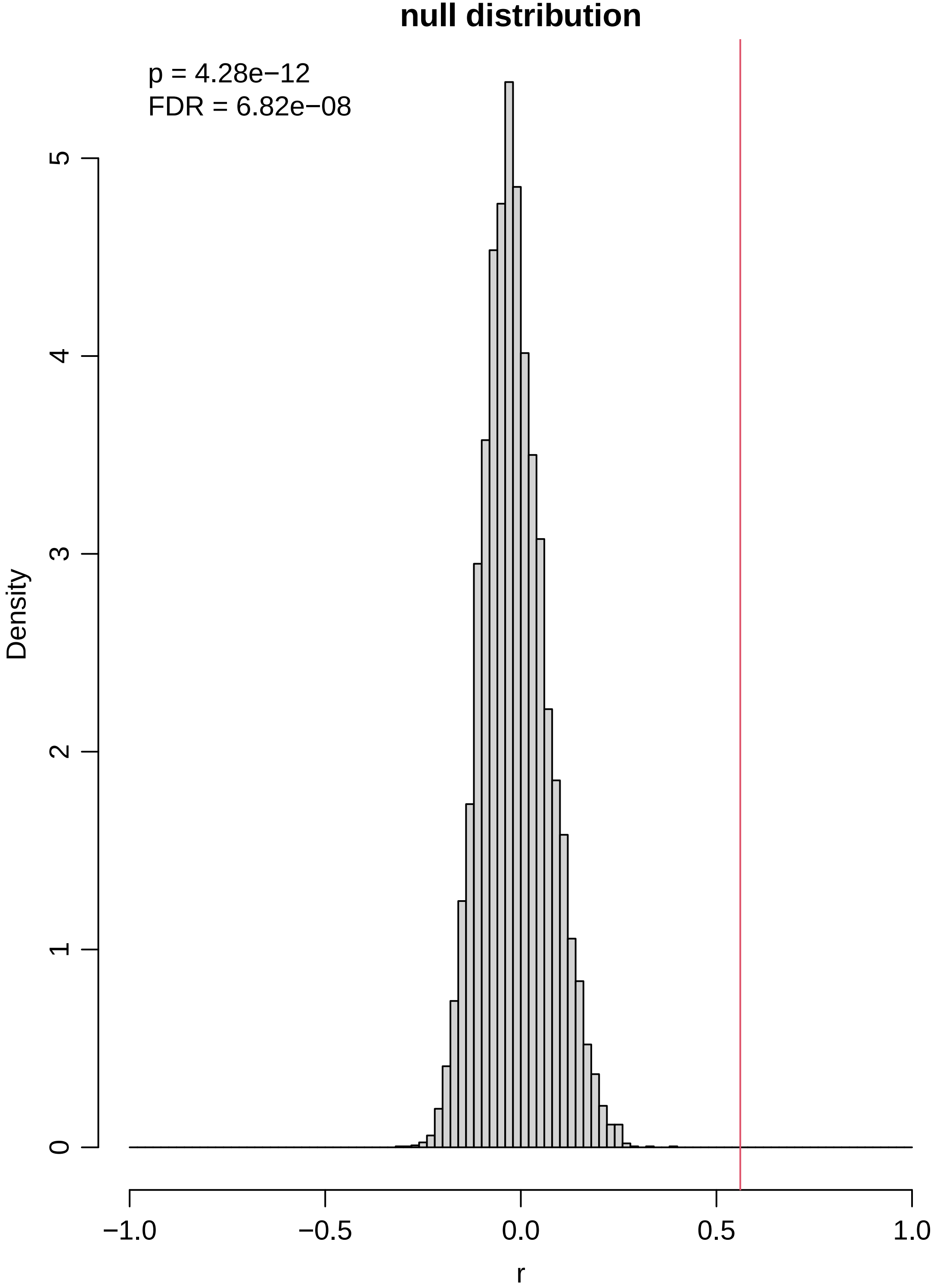

OR8G5 vs PeakID_47244 (chr11: 124170375 − 124171652)

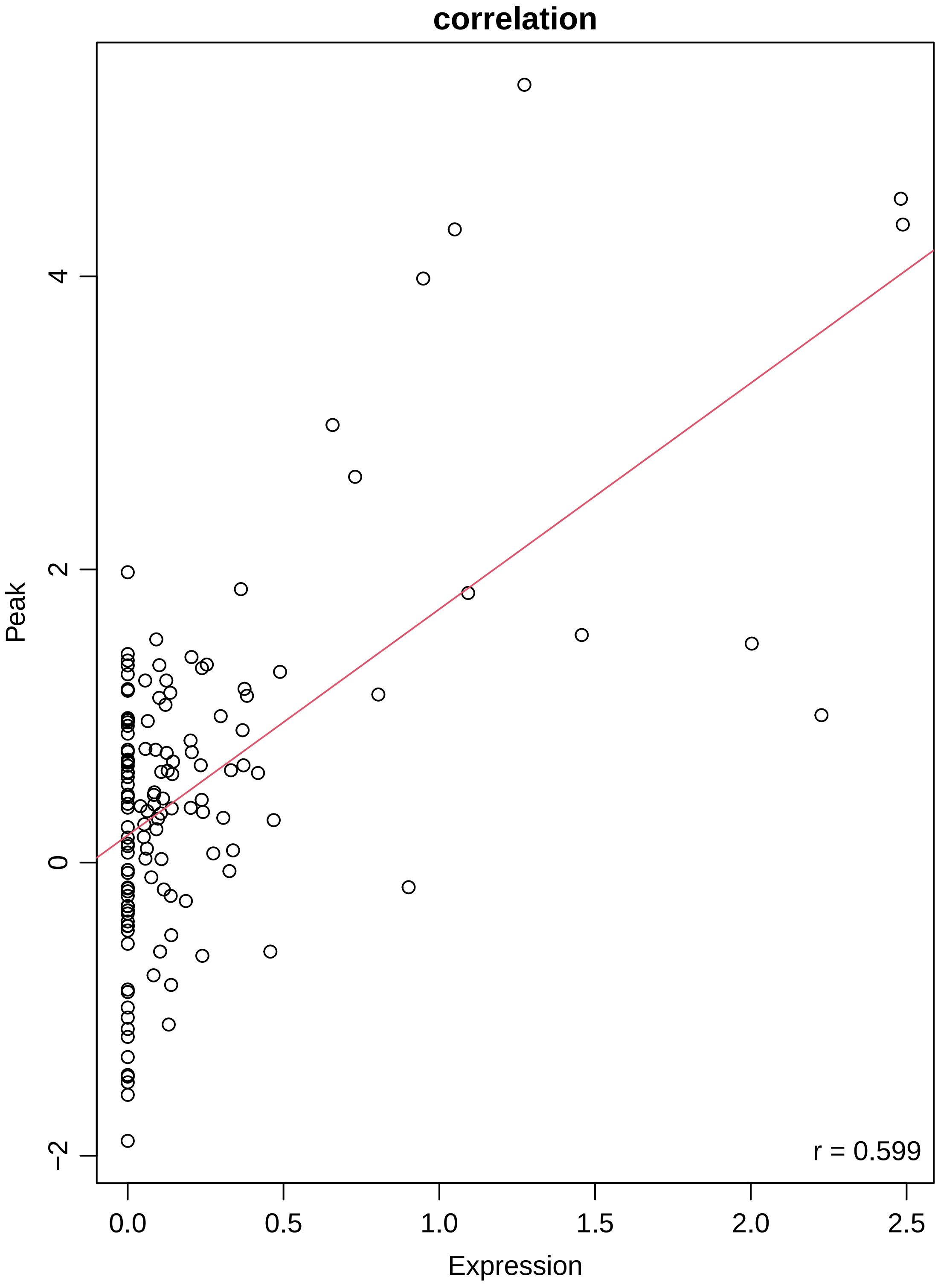

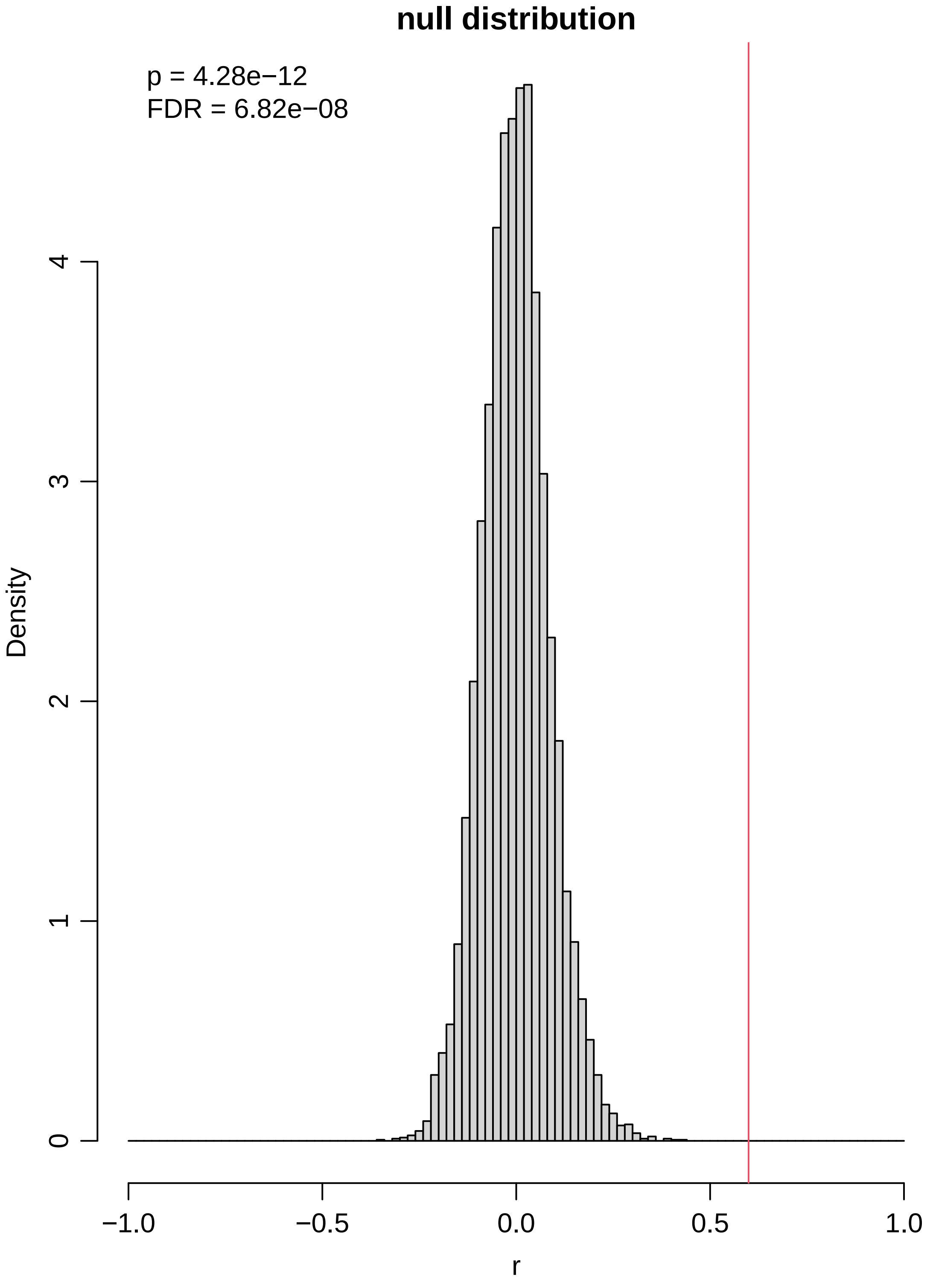

SLC35E3 vs PeakID_56587 (chr12: 68829484 − 68829764)

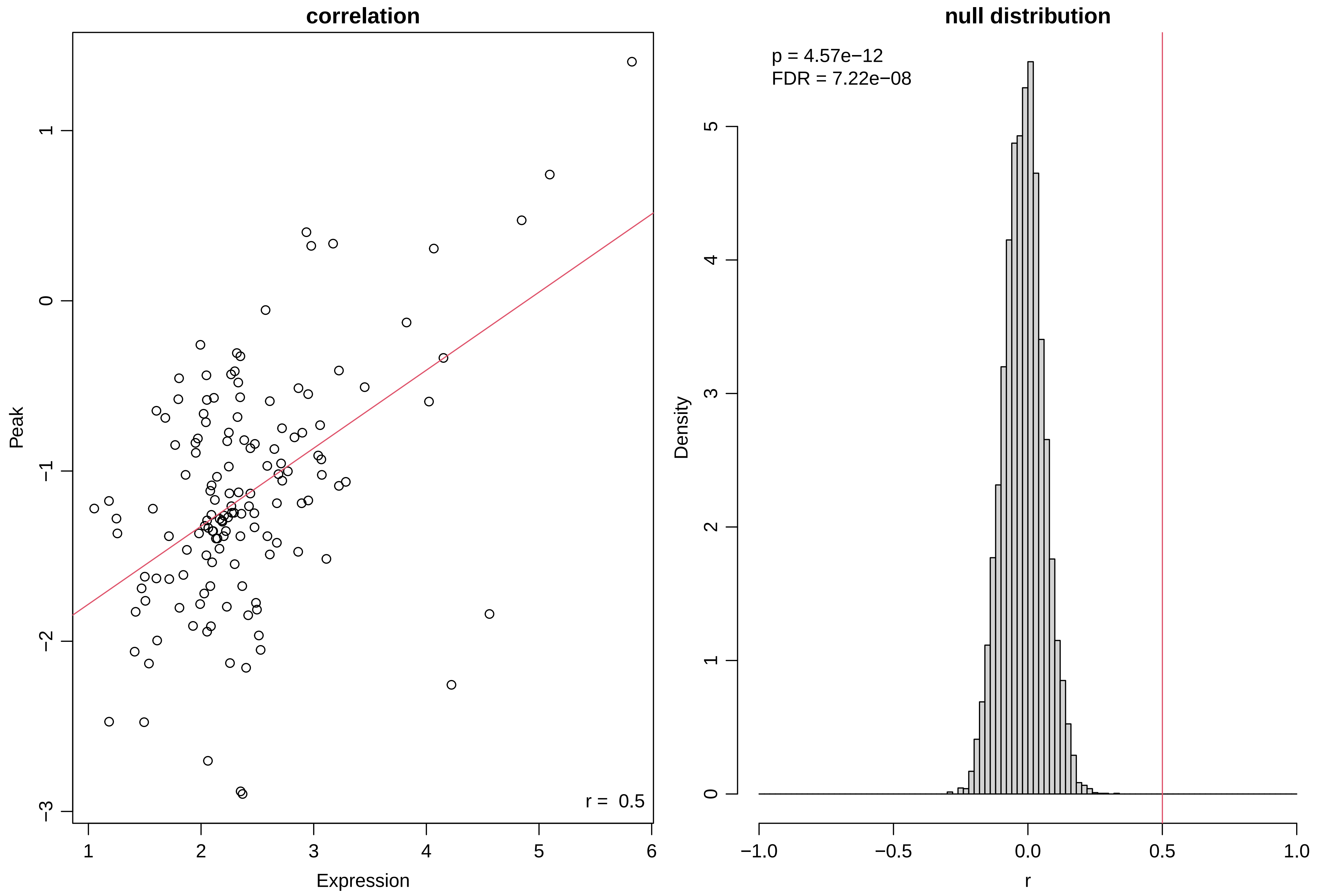

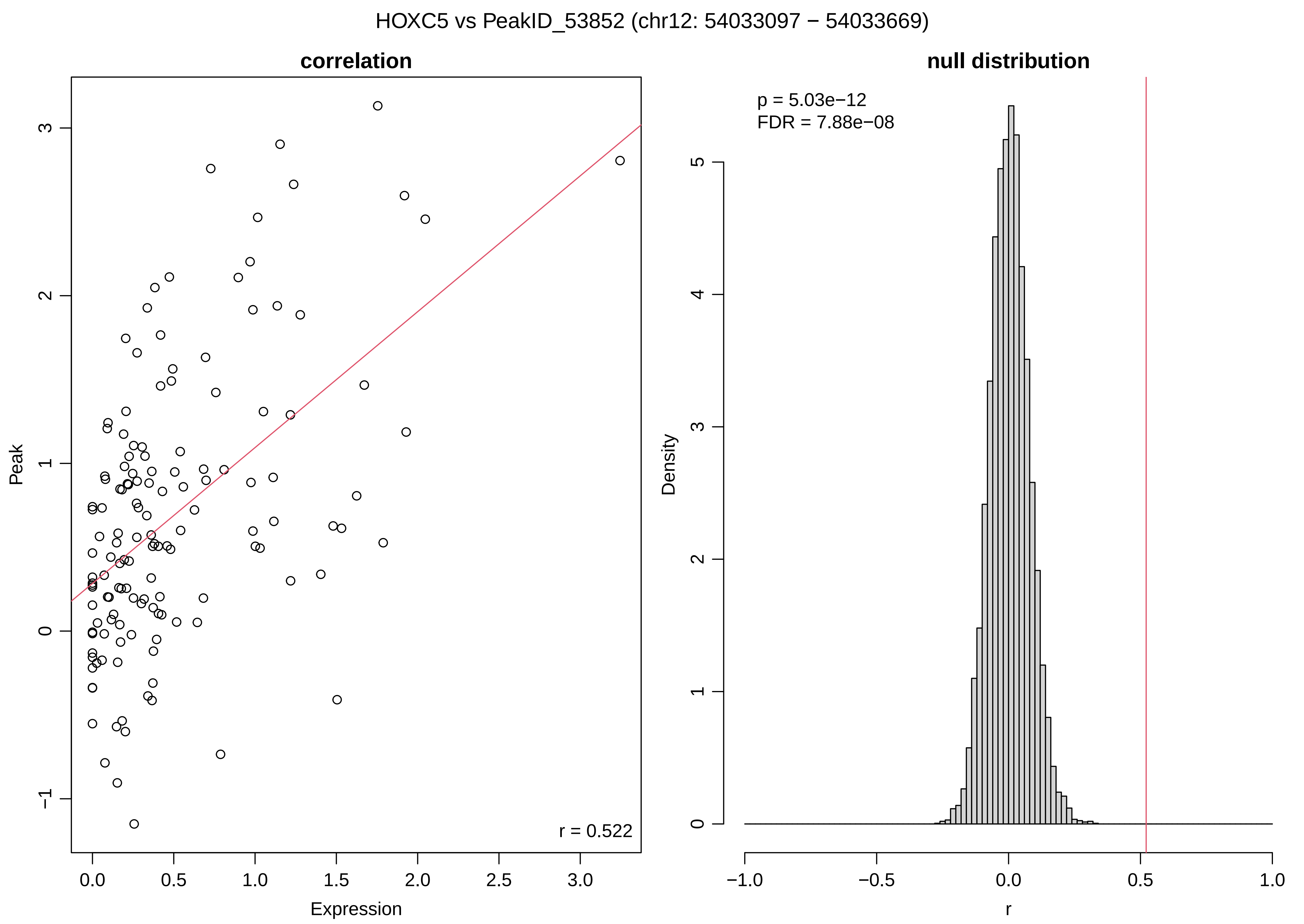
HOXC5 vs PeakID_53852 (chr12: 54033097 − 54033669)
correlation
Peak
Expression
r = 0.522
null distribution
p = 5.03e−12
FDR = 7.88e−08
Density
r

LINC00960 vs PeakID_166127 (chr3: 75671790 − 75672816)

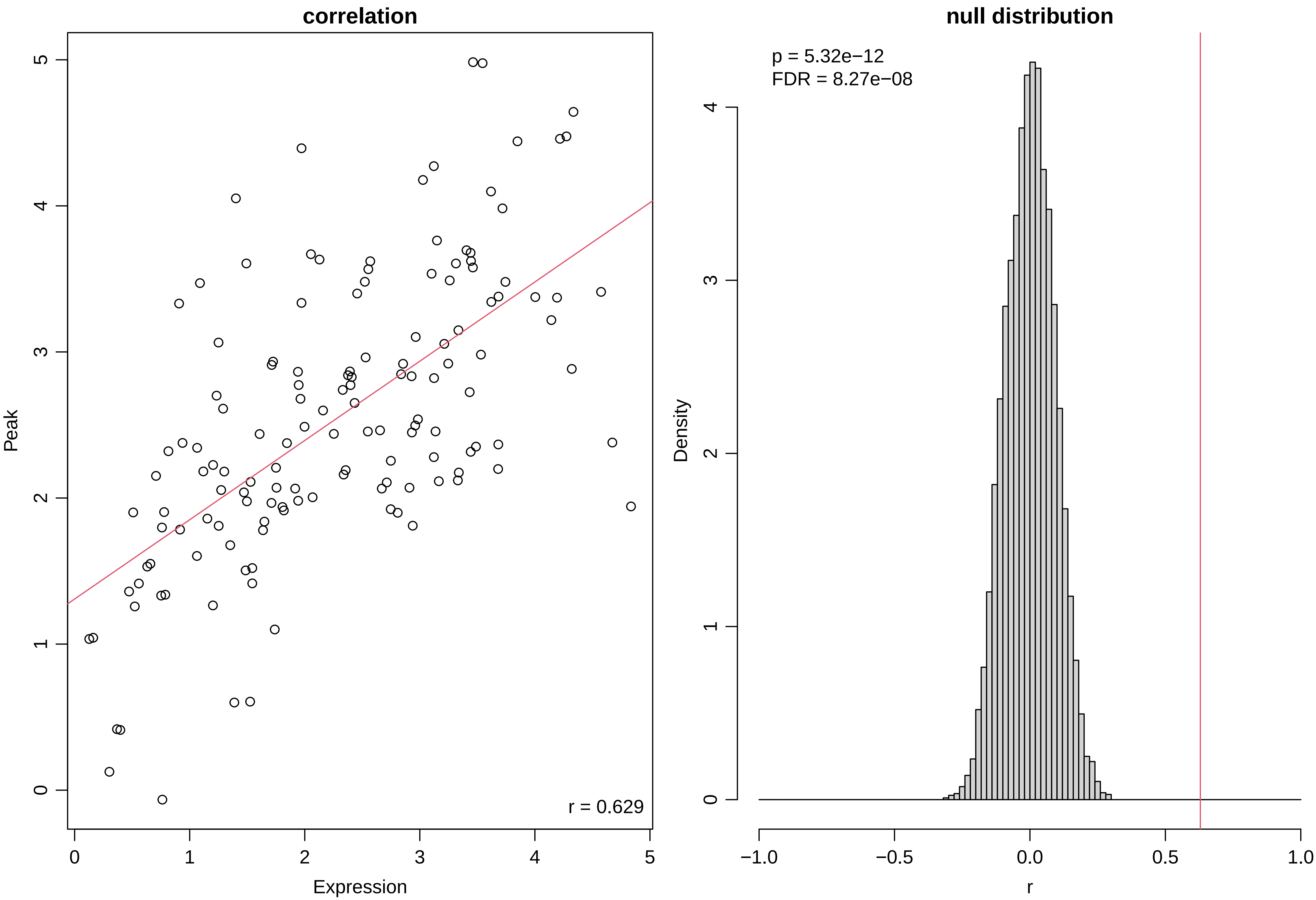

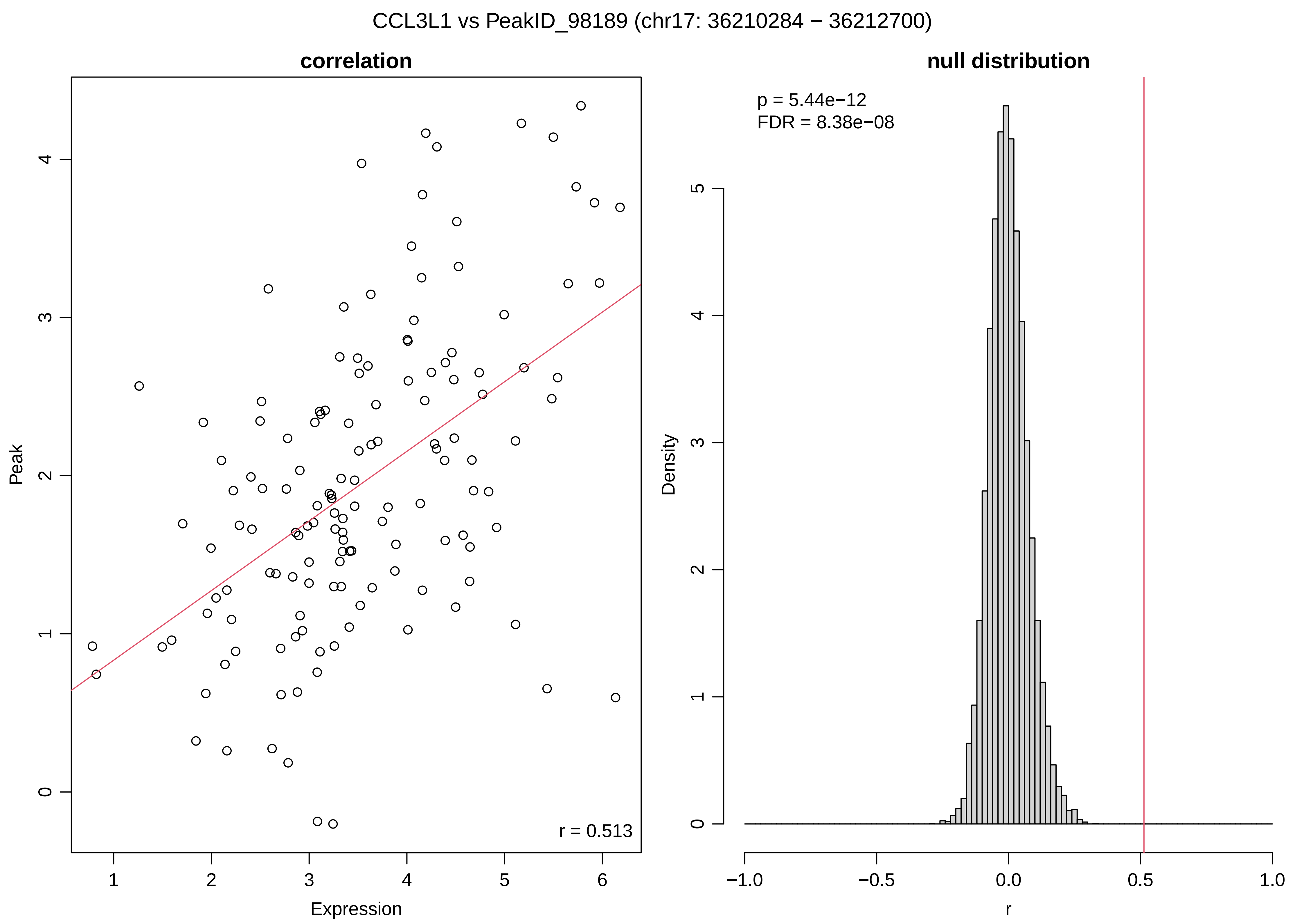

CCL3L1 vs PeakID_98189 (chr17: 36210284 − 36212700)
correlation
Peak
Expression
r = 0.513
0
1
2
3
4
1
2
3
4
5
6
null distribution
p = 5.44e−12
FDR = 8.38e−08
Density
r
0
1
2
3
4
5
−1.0
−0.5
0.0
0.5
1.0

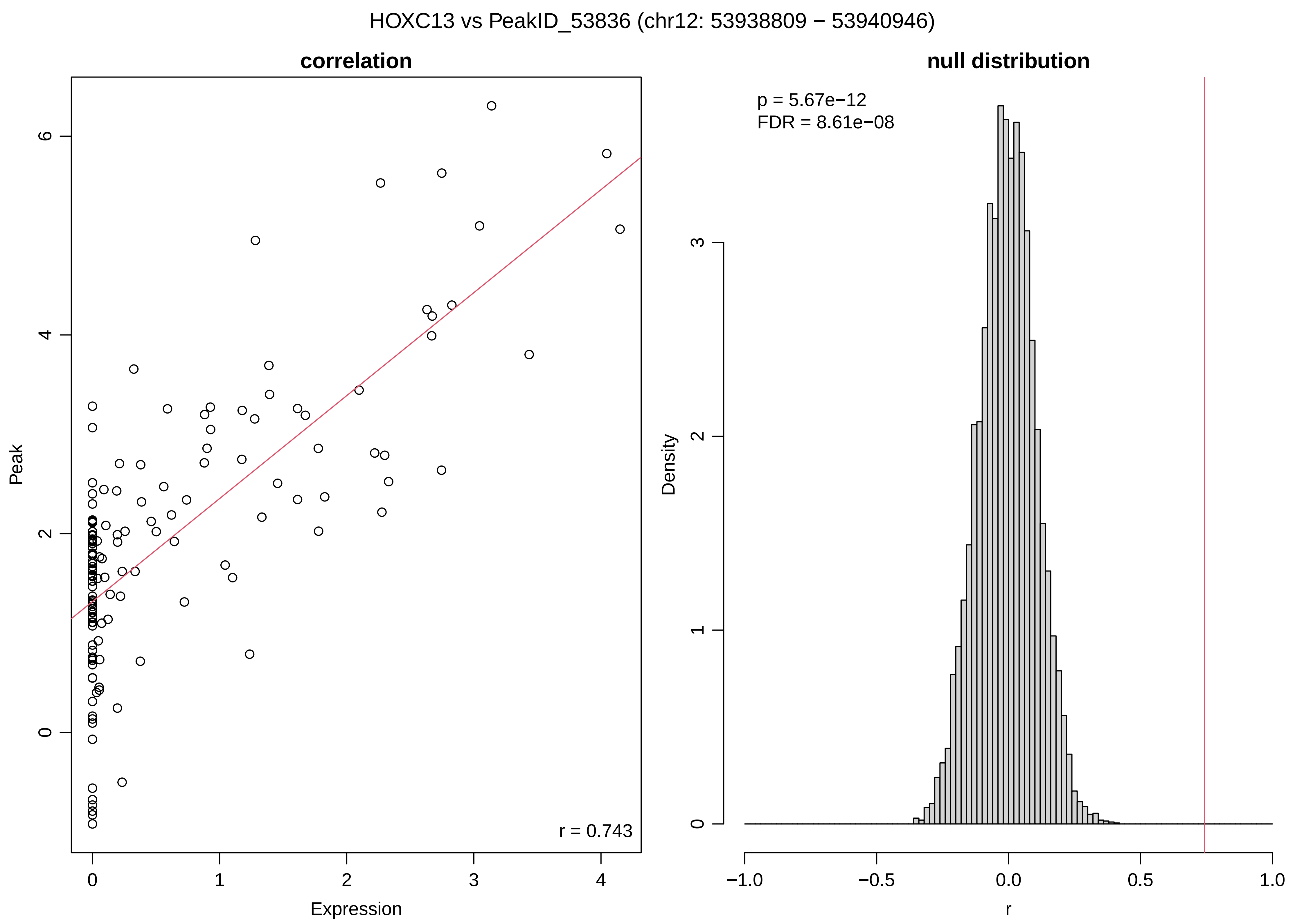
HOXC13 vs PeakID_53836 (chr12: 53938809 − 53940946)
correlation
Peak
Expression
r = 0.743
0
1
2
3
4
6
null distribution
p = 5.67e−12
FDR = 8.61e−08
Density
r
−1.0
−0.5
0.0
0.5
1.0

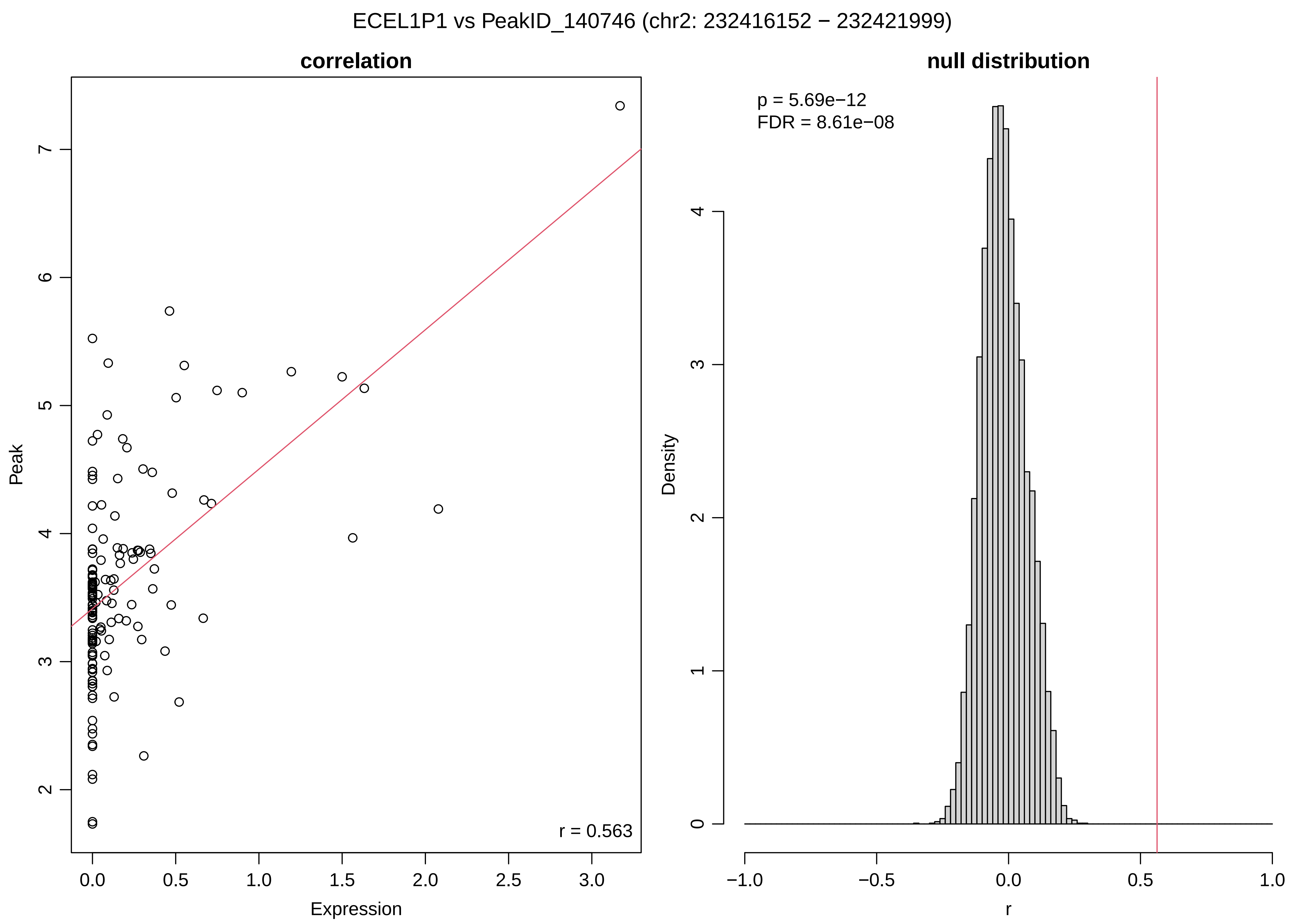

ECEL1P1 vs PeakID_140746 (chr2: 232416152 − 232421999)
correlation
Peak
Expression
r = 0.563
null distribution
p = 5.69e−12
FDR = 8.61e−08
Density
r

RNU4−20P vs PeakID_54796 (chr12: 59496253 − 59502103)

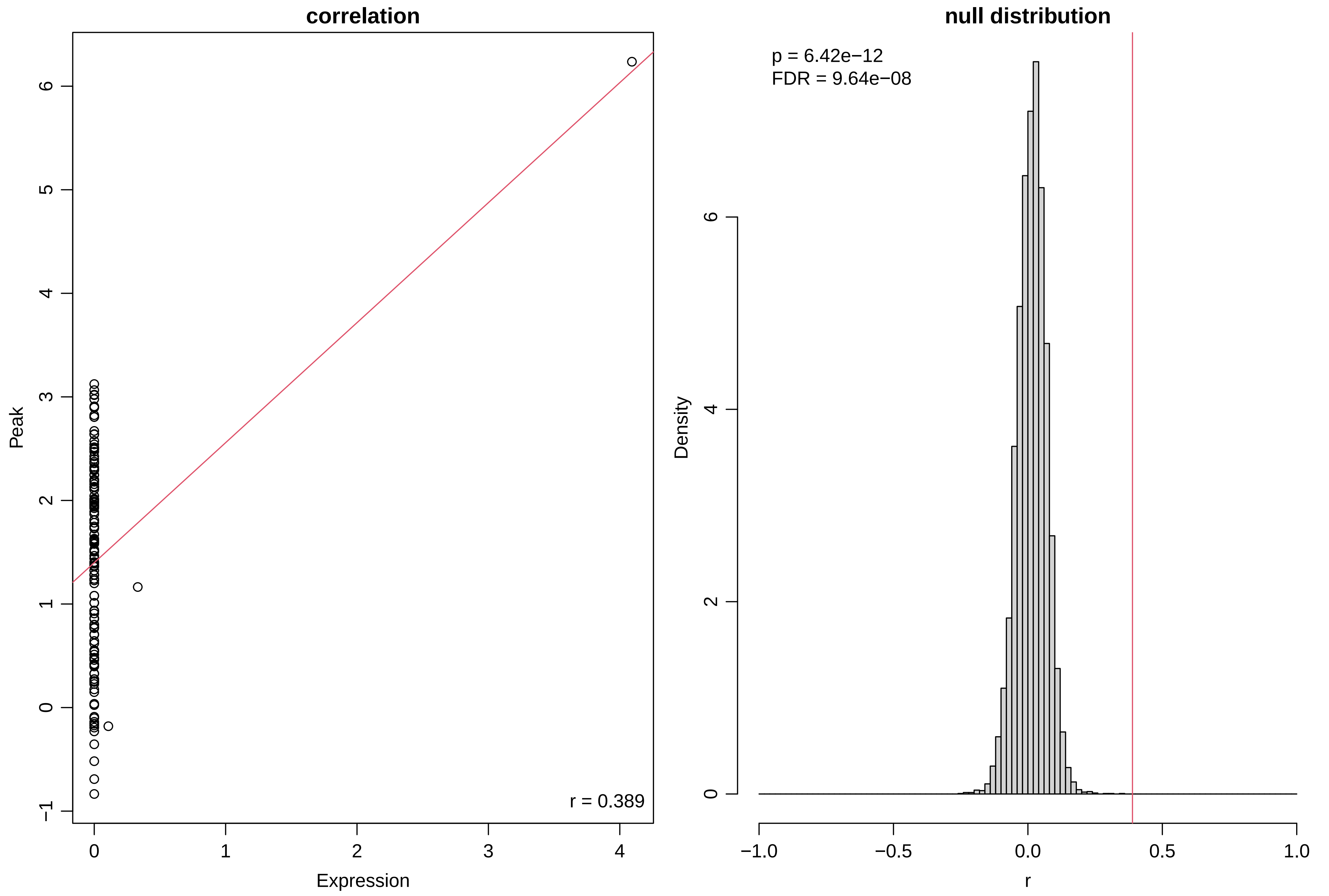

TUSC8 vs PeakID_65000 (chr13: 44401827 − 44407104)

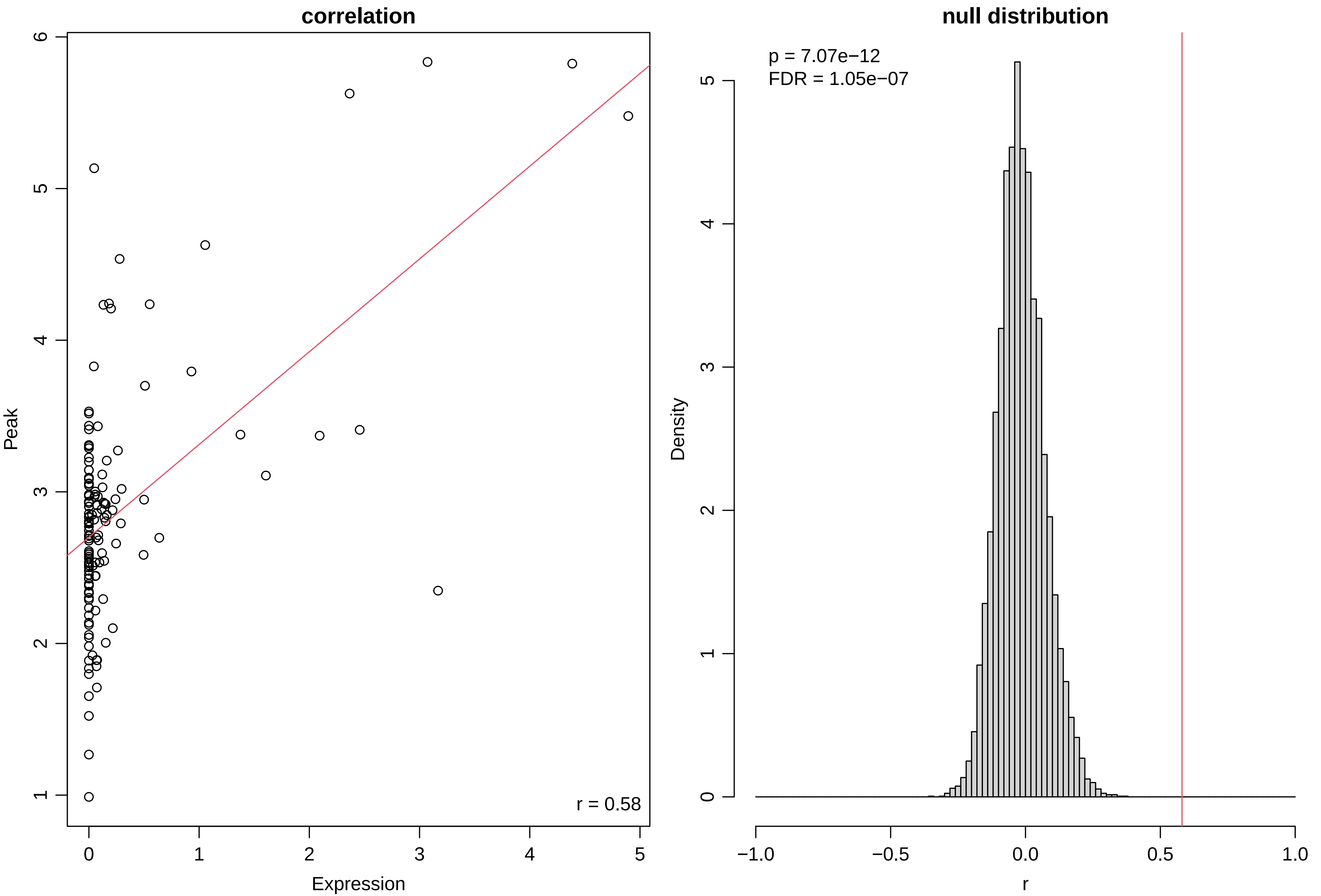

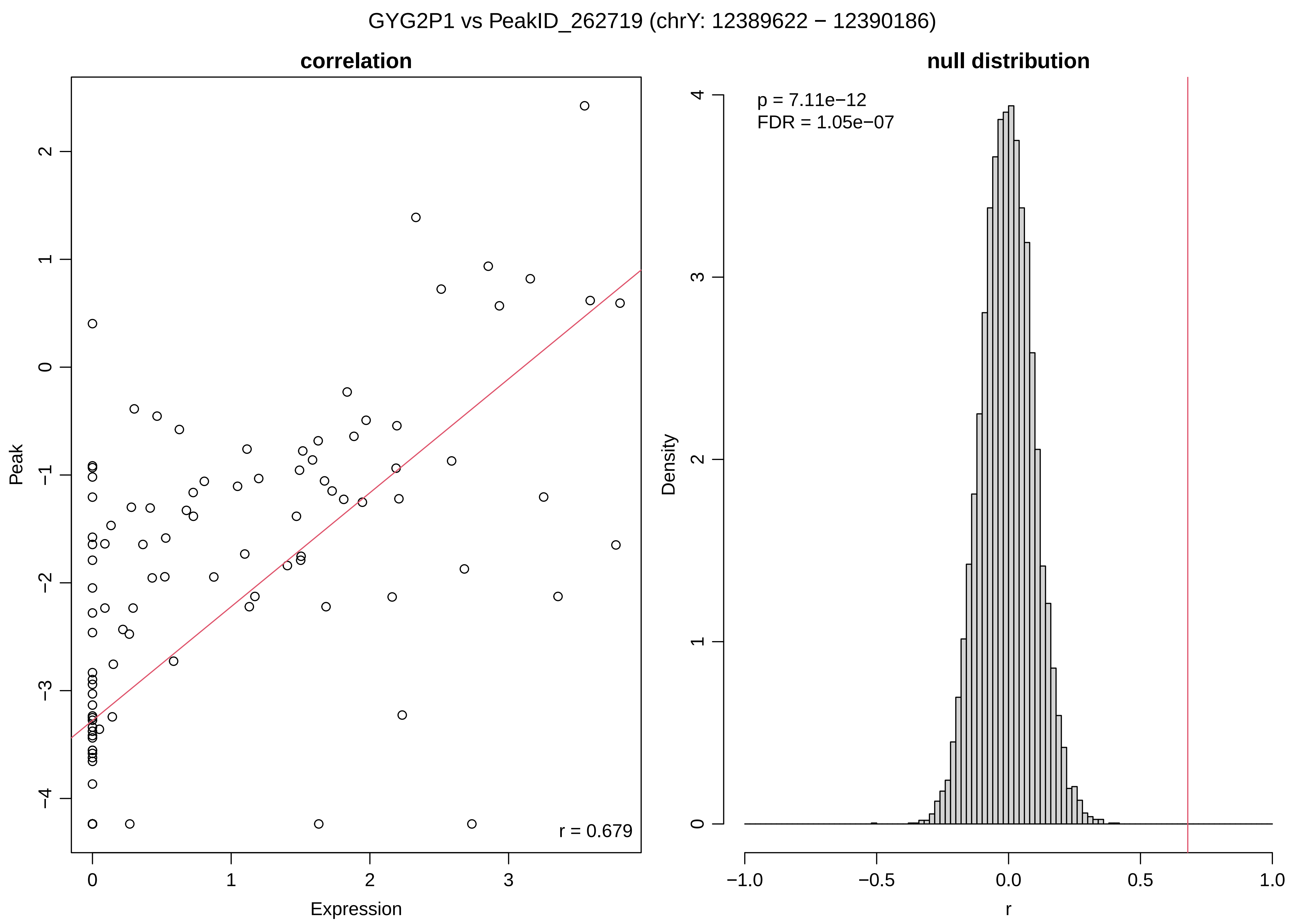

GYG2P1 vs PeakID_262719 (chrY: 12389622 − 12390186)
correlation
Peak
Expression
r = 0.679
null distribution
p = 7.11e−12
FDR = 1.05e−07
Density
r

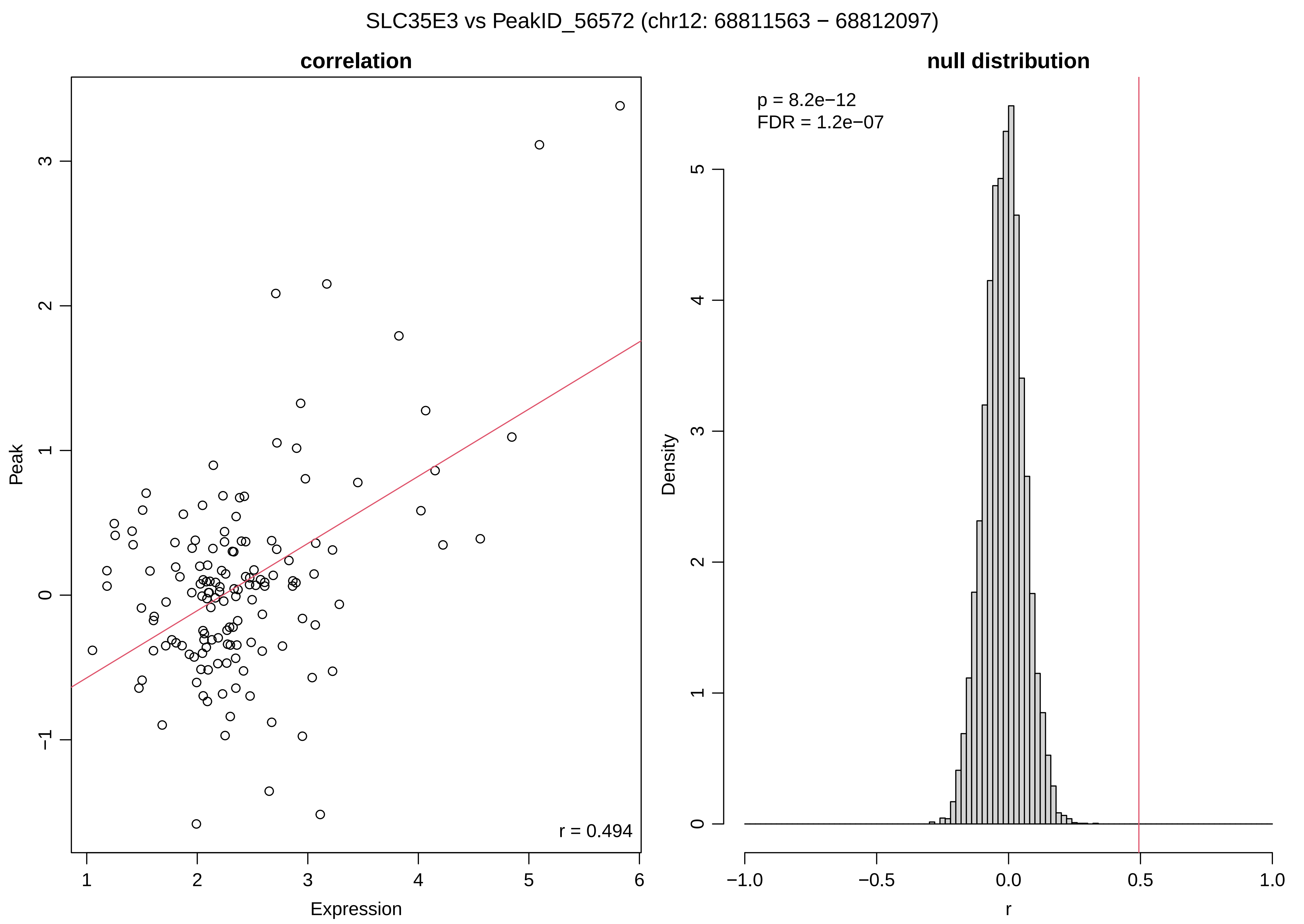
SLC35E3 vs PeakID_56572 (chr12: 68811563 − 68812097)
correlation
Peak
Expression
r = 0.494
null distribution
p = 8.2e−12
FDR = 1.2e−07
Density
r

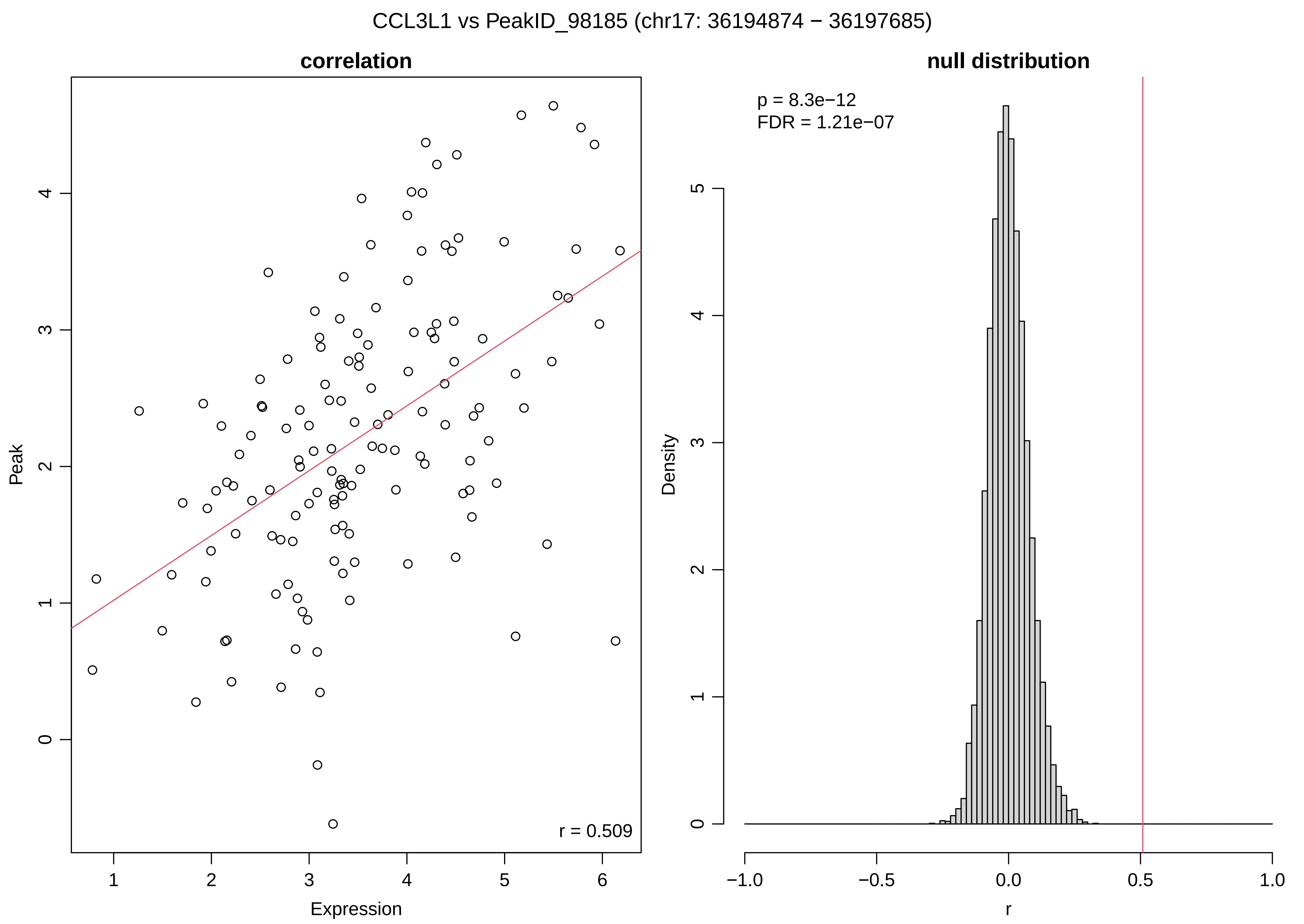
CCL3L1 vs PeakID_98185 (chr17: 36194874 − 36197685)
correlation
Peak
Expression
r = 0.509
null distribution
p = 8.3e−12
FDR = 1.21e−07
Density
r

LINC00621 vs PeakID_63215 (chr13: 22914898 − 22916742)

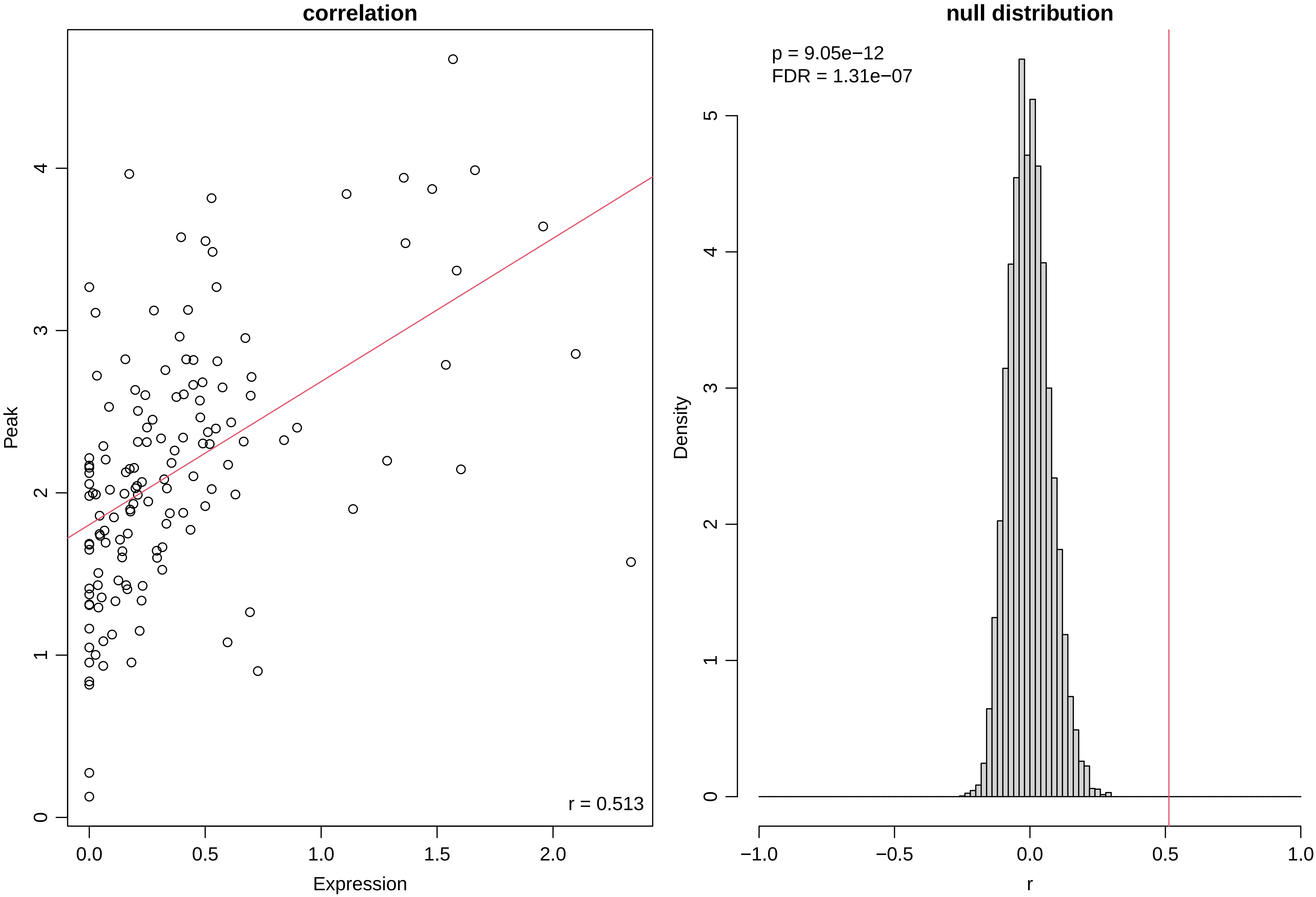

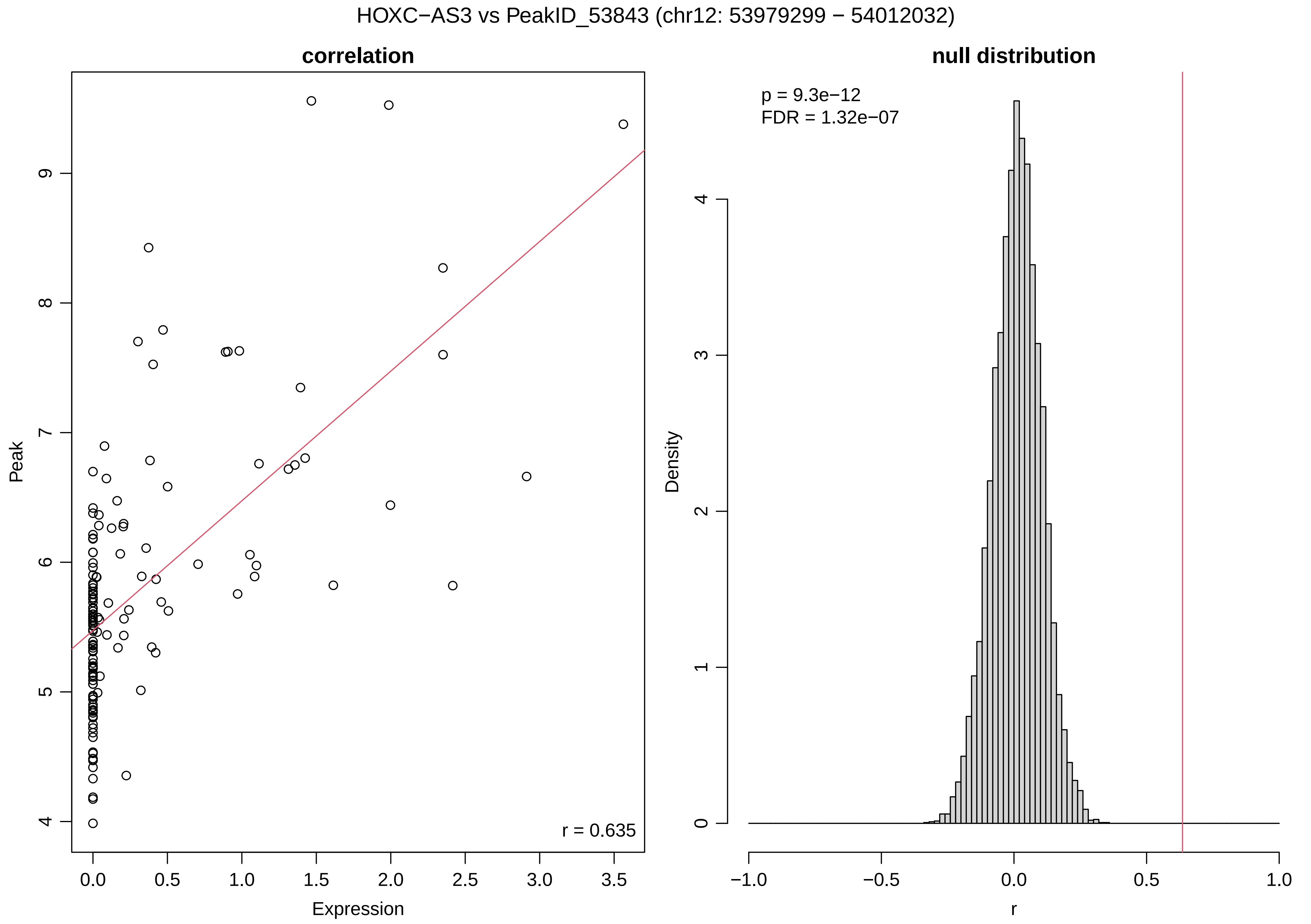
HOXC−AS3 vs PeakID_53843 (chr12: 53979299 − 54012032)
correlation
Peak
Expression
r = 0.635
4
5
6
7
8
9
0.0
0.5
1.0
1.5
2.0
2.5
3.0
3.5
null distribution
p = 9.3e−12
FDR = 1.32e−07
Density
r
0
1
2
3
4
−1.0
−0.5
0.0
0.5
1.0

RPL23AP3 vs PeakID_152123 (chr21: 36003614 − 36007733)

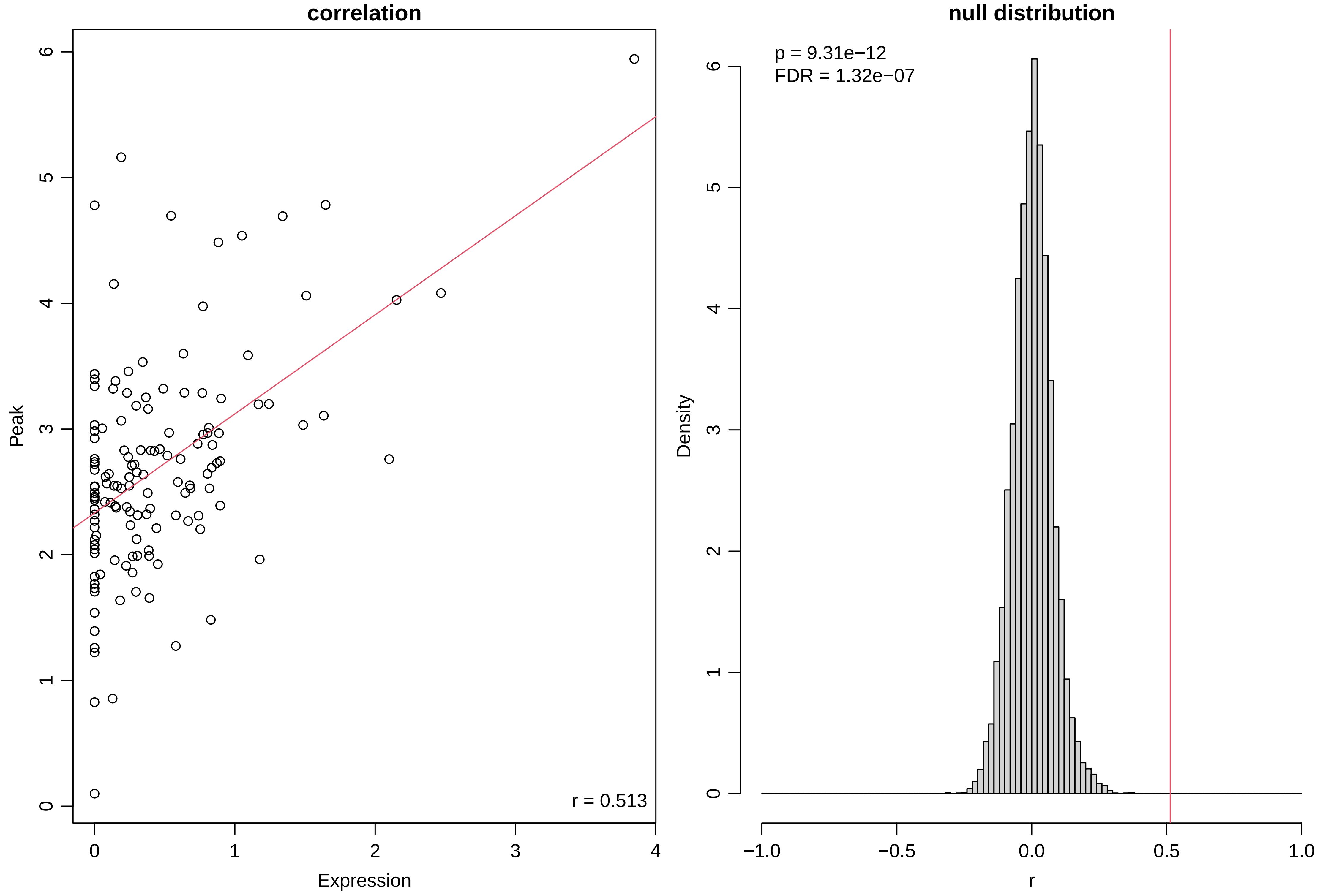

SLC35E3 vs PeakID_56662 (chr12: 68979947 − 68982175)

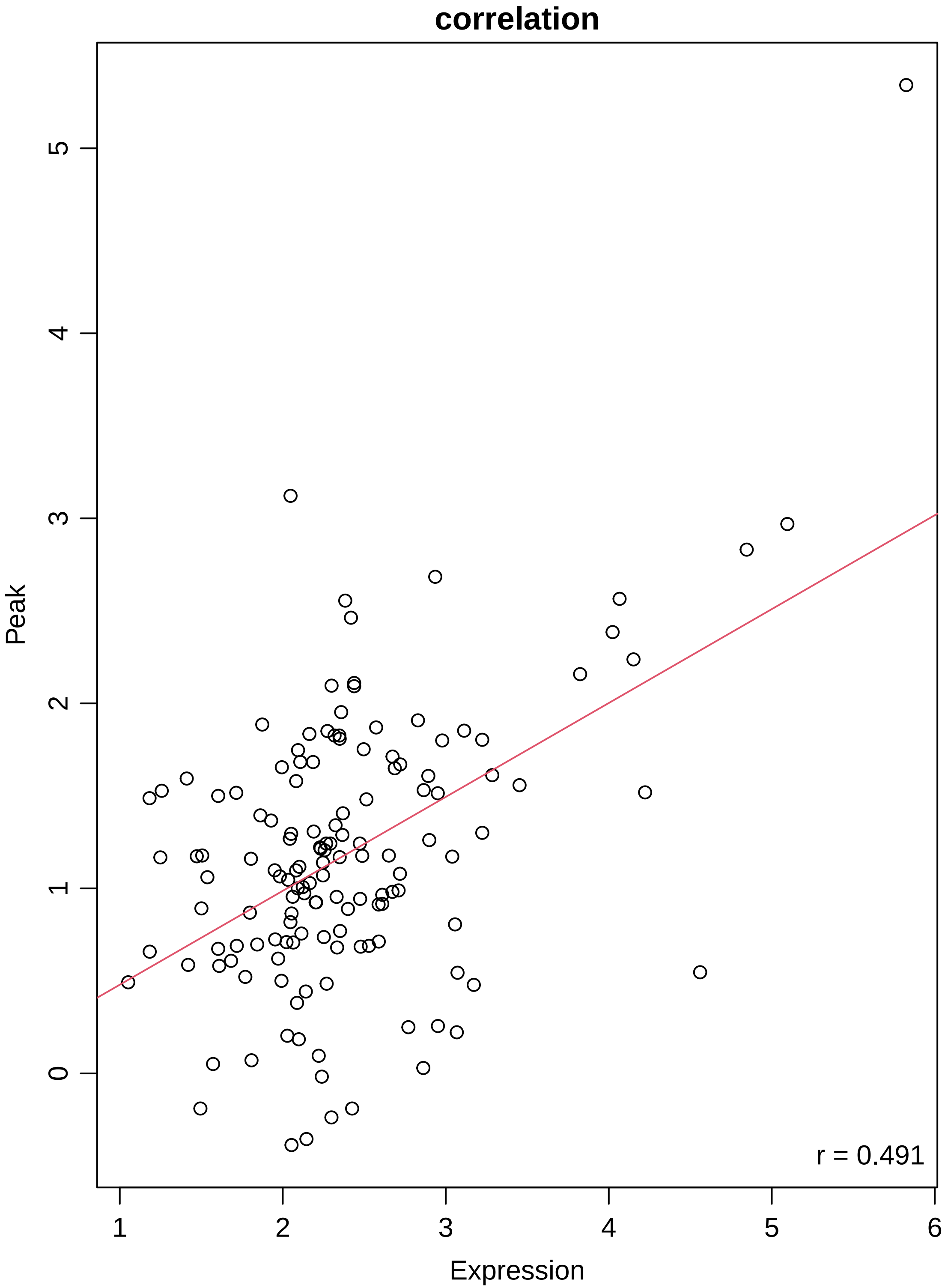

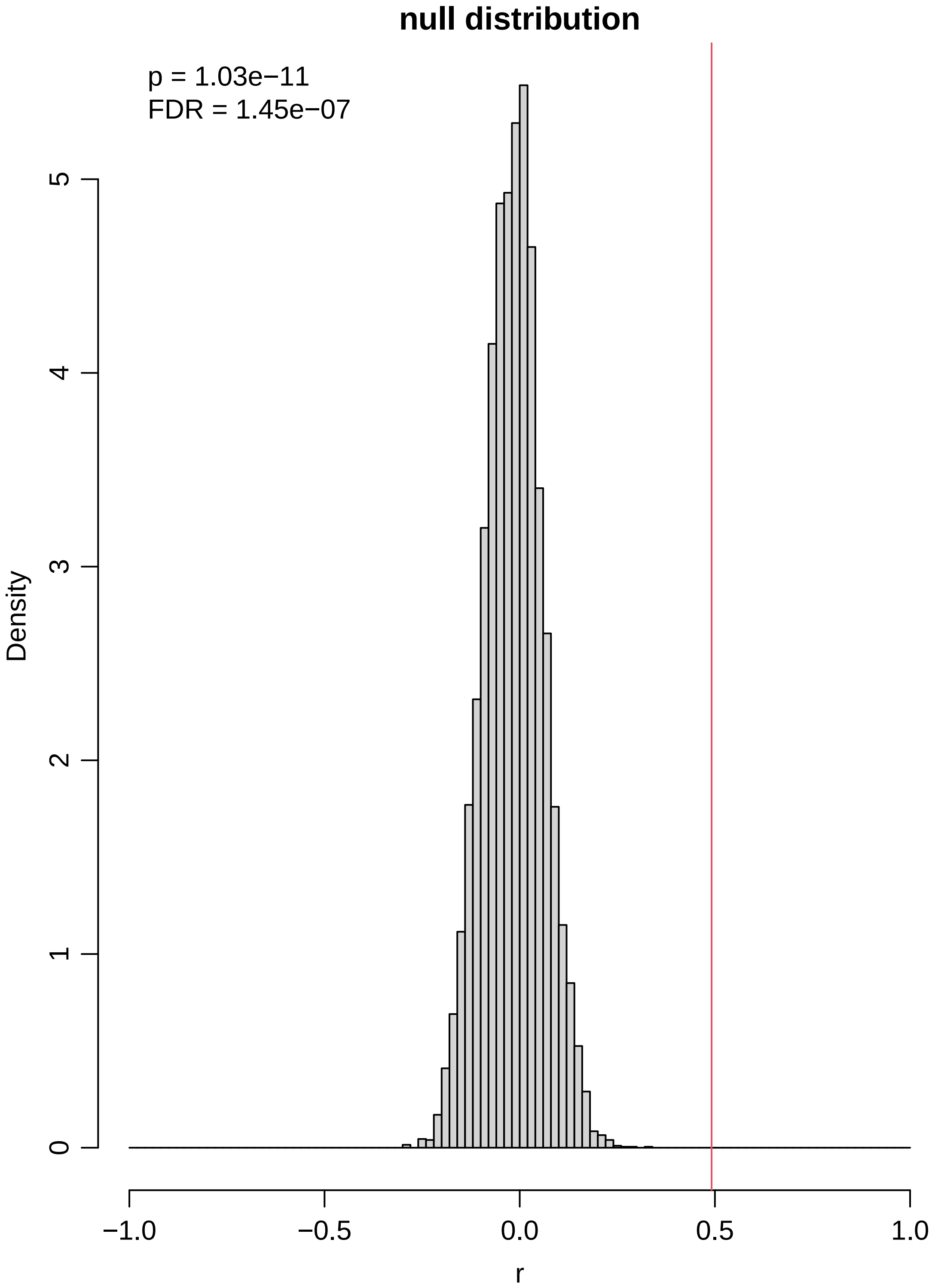

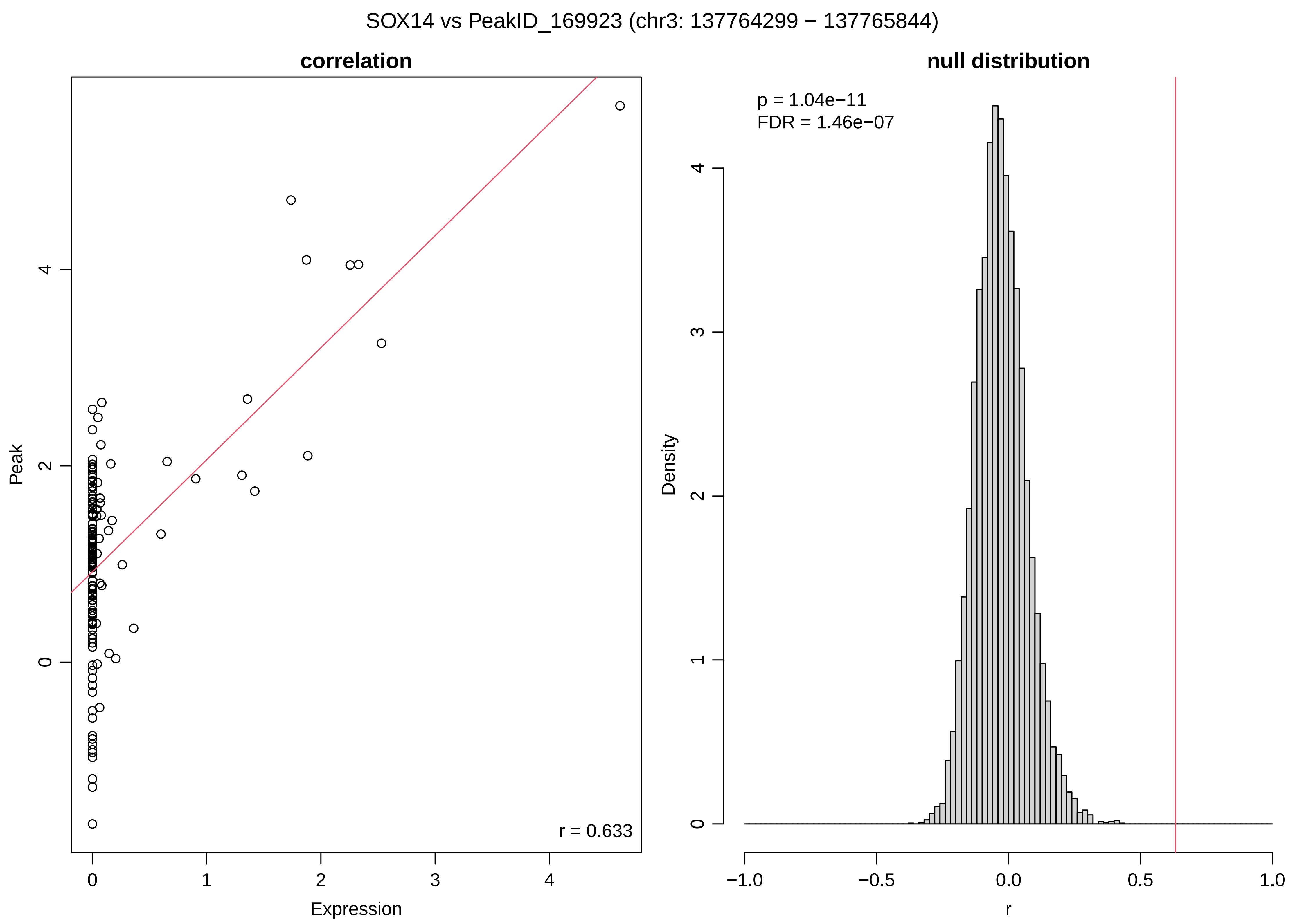
SOX14 vs PeakID_169923 (chr3: 137764299 − 137765844)
correlation
null distribution
p = 1.04e−11
FDR = 1.46e−07
r = 0.633
Peak
Expression
Density
r
0
2
4
0
1
2
3
4
−1.0
−0.5
0.0
0.5
1.0

STARD3 vs PeakID_99043 (chr17: 39611085 − 39689899)

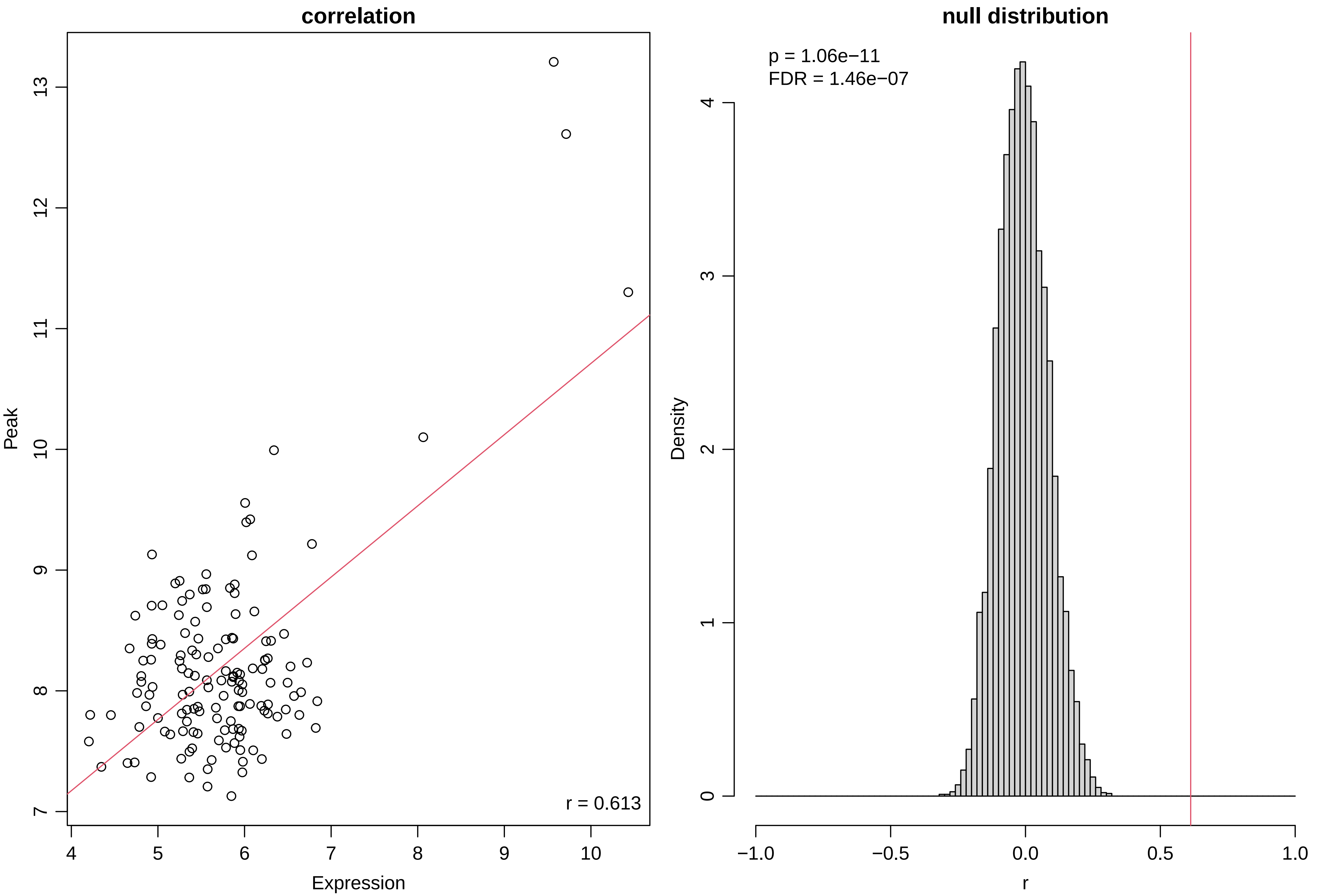

RNU4−20P vs PeakID_54831 (chr12: 59579380 − 59580633)

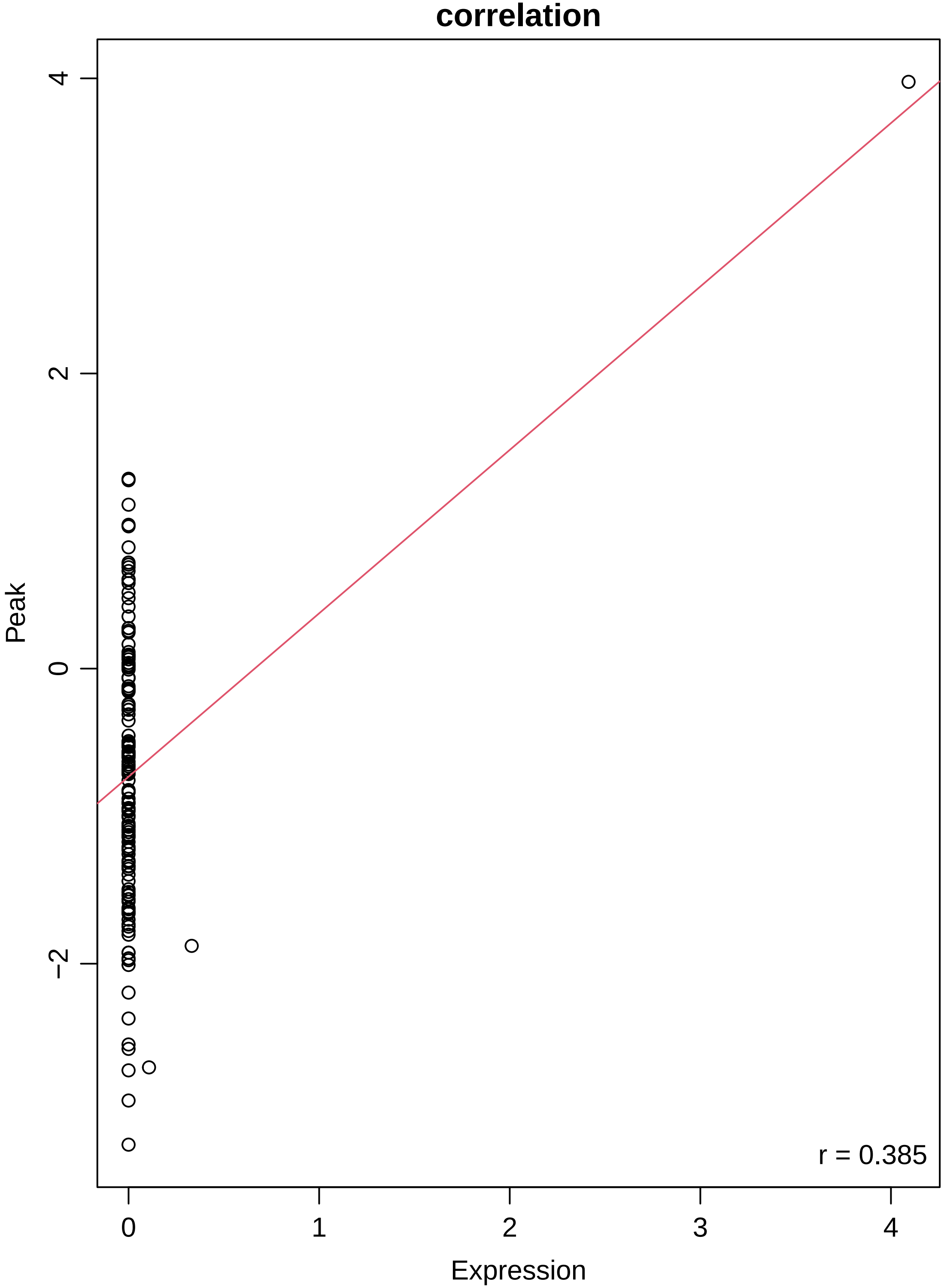

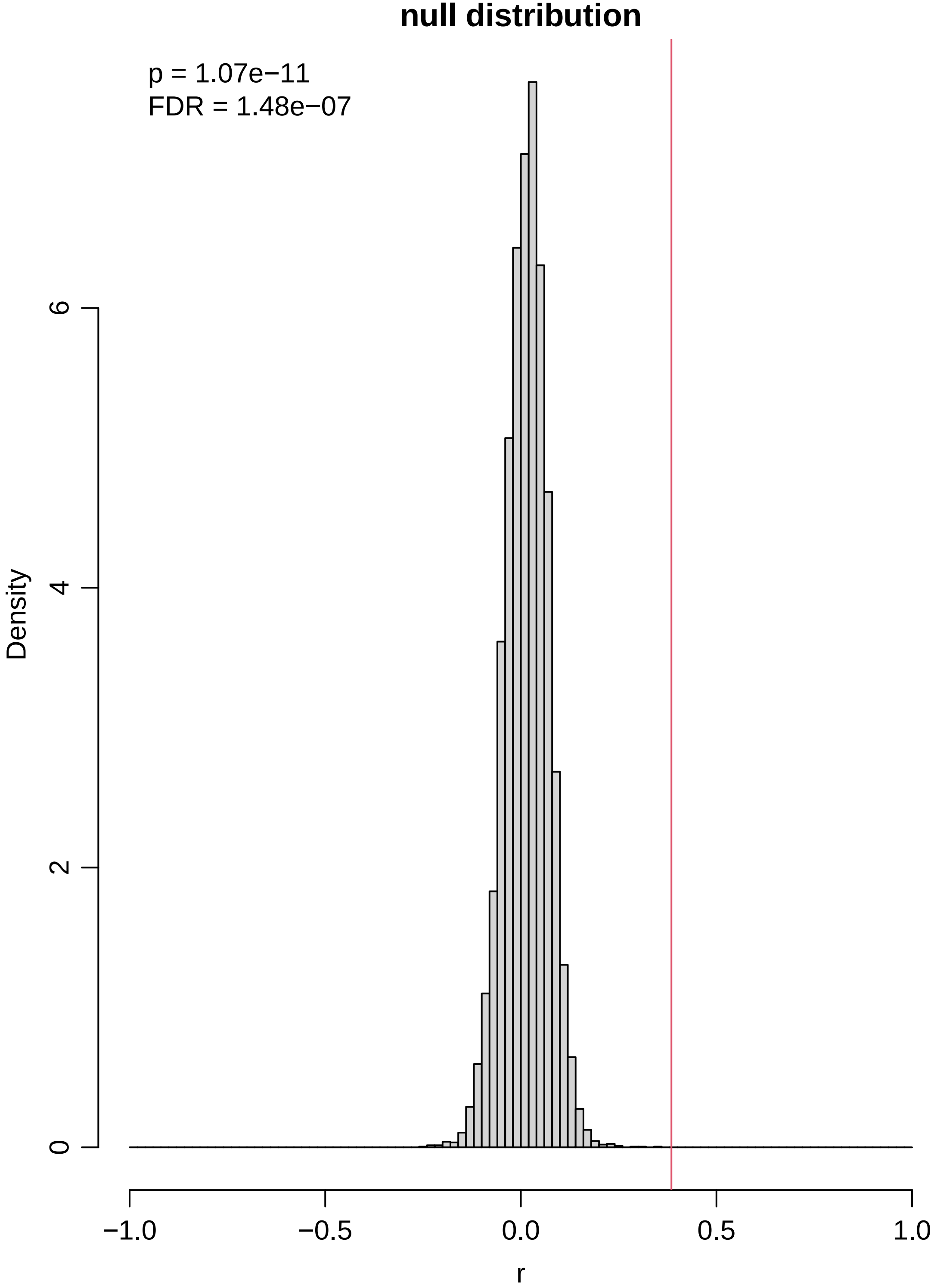

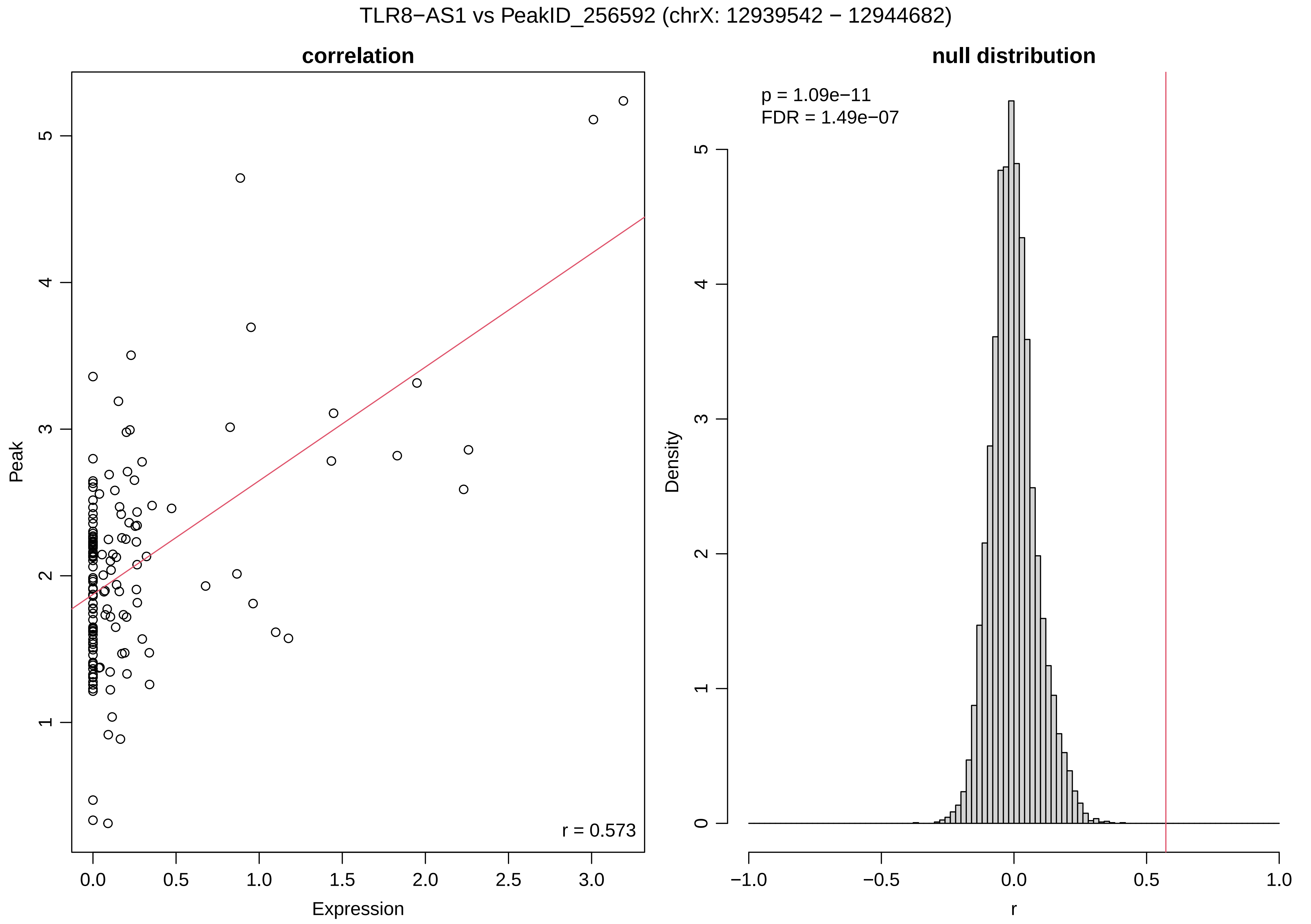
TLR8−AS1 vs PeakID_256592 (chrX: 12939542 − 12944682)
correlation
null distribution
Peak
Expression
r = 0.573
p = 1.09e−11
FDR = 1.49e−07
Density
r
0.0
0.5
1.0
1.5
2.0
2.5
3.0
1
2
3
4
5
−1.0
−0.5
0.0
0.5
1.0
0
1
2
3
4
5

RPS4Y1 vs PeakID_262338 (chrY: 3001791 − 3005953)

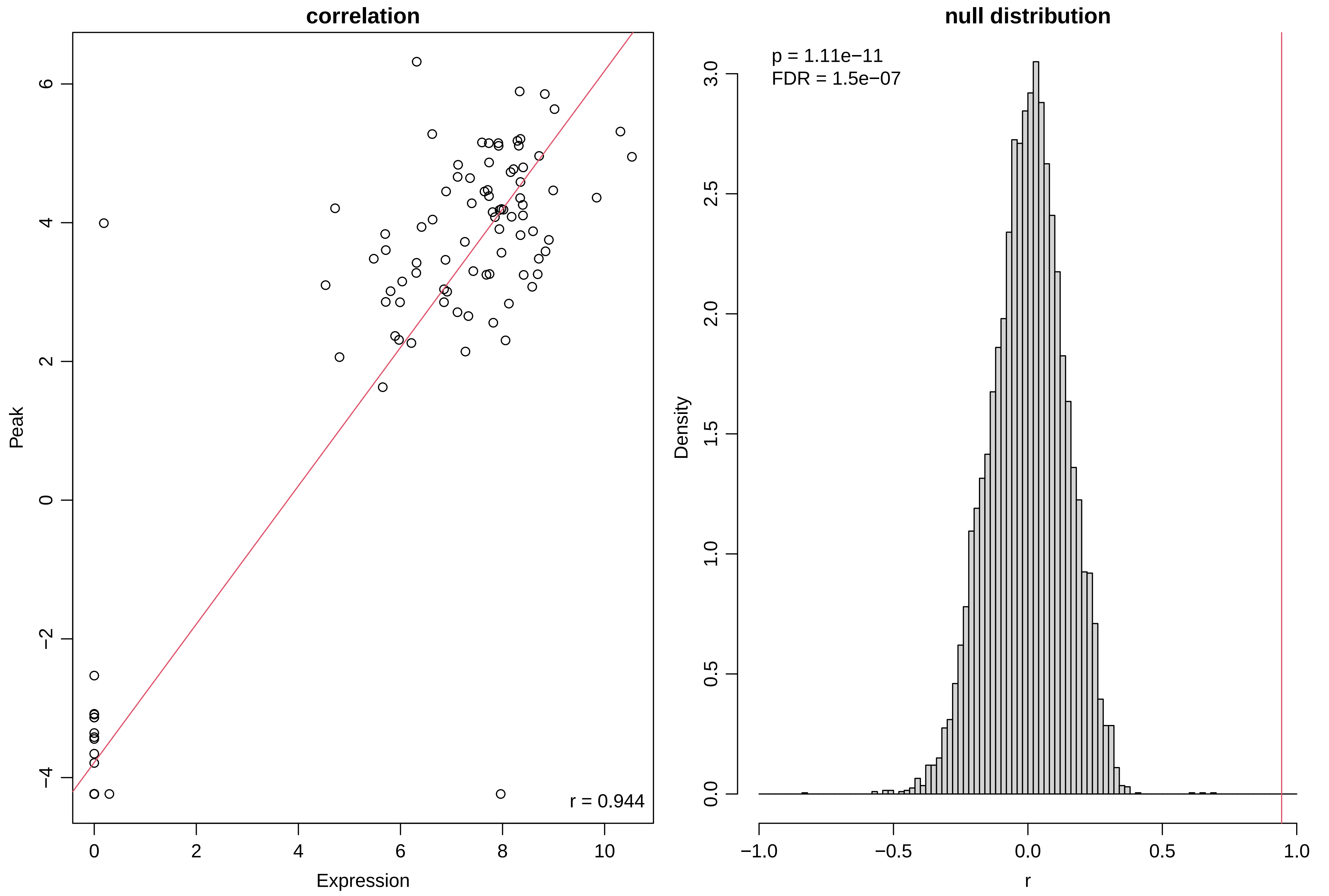

HGC6.3 vs PeakID_215232 (chr6: 167995021 − 167997328)

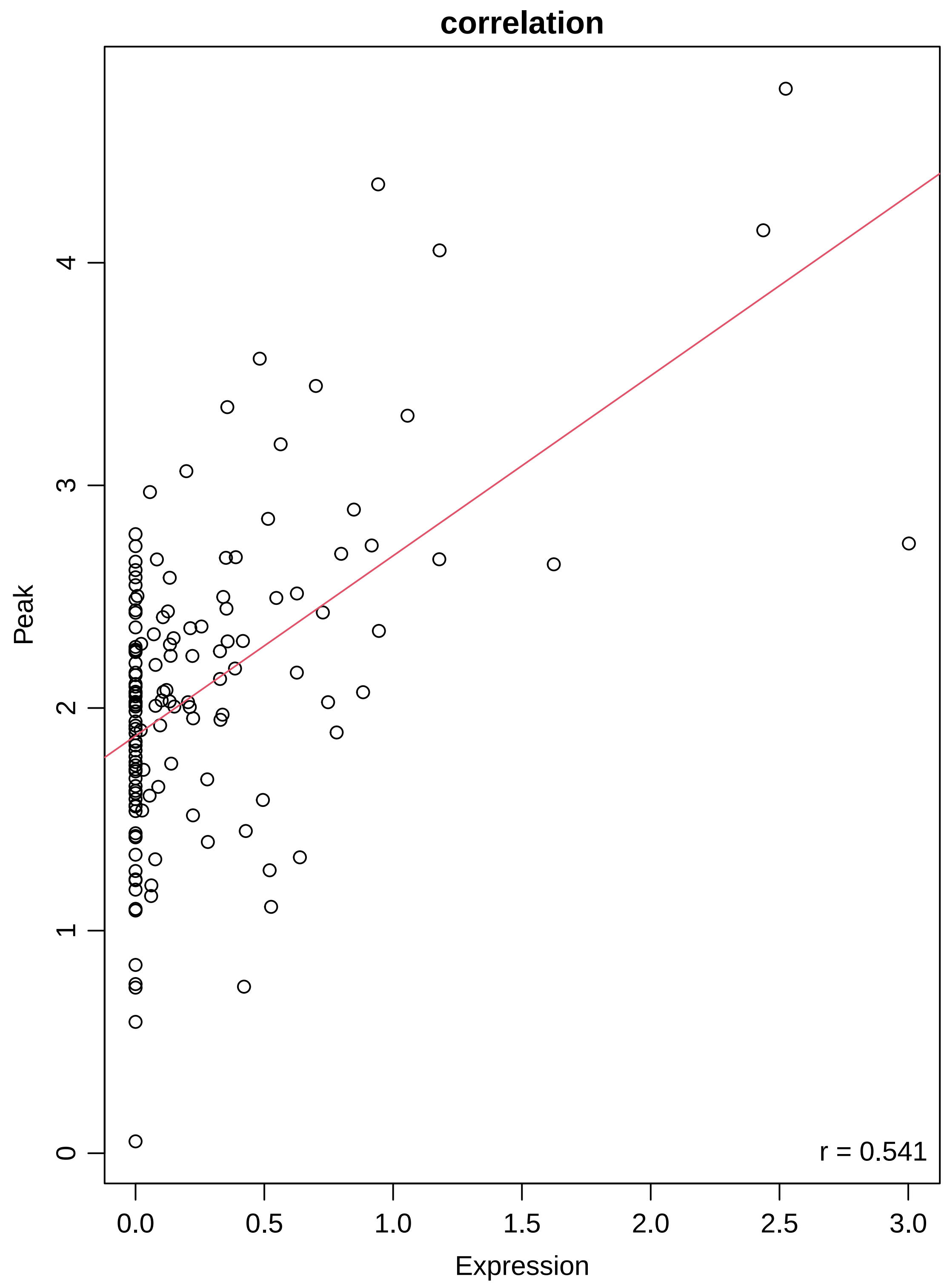

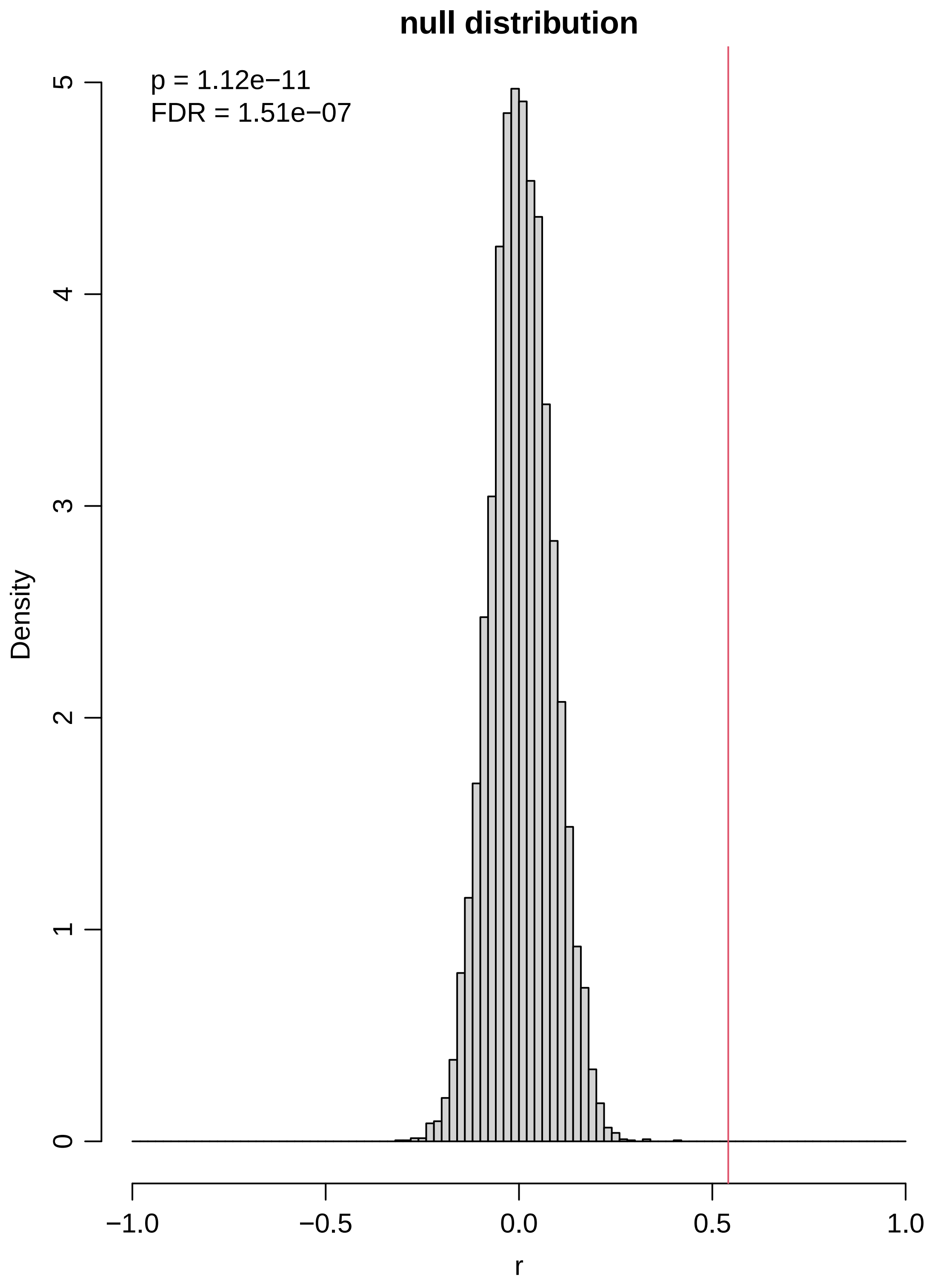

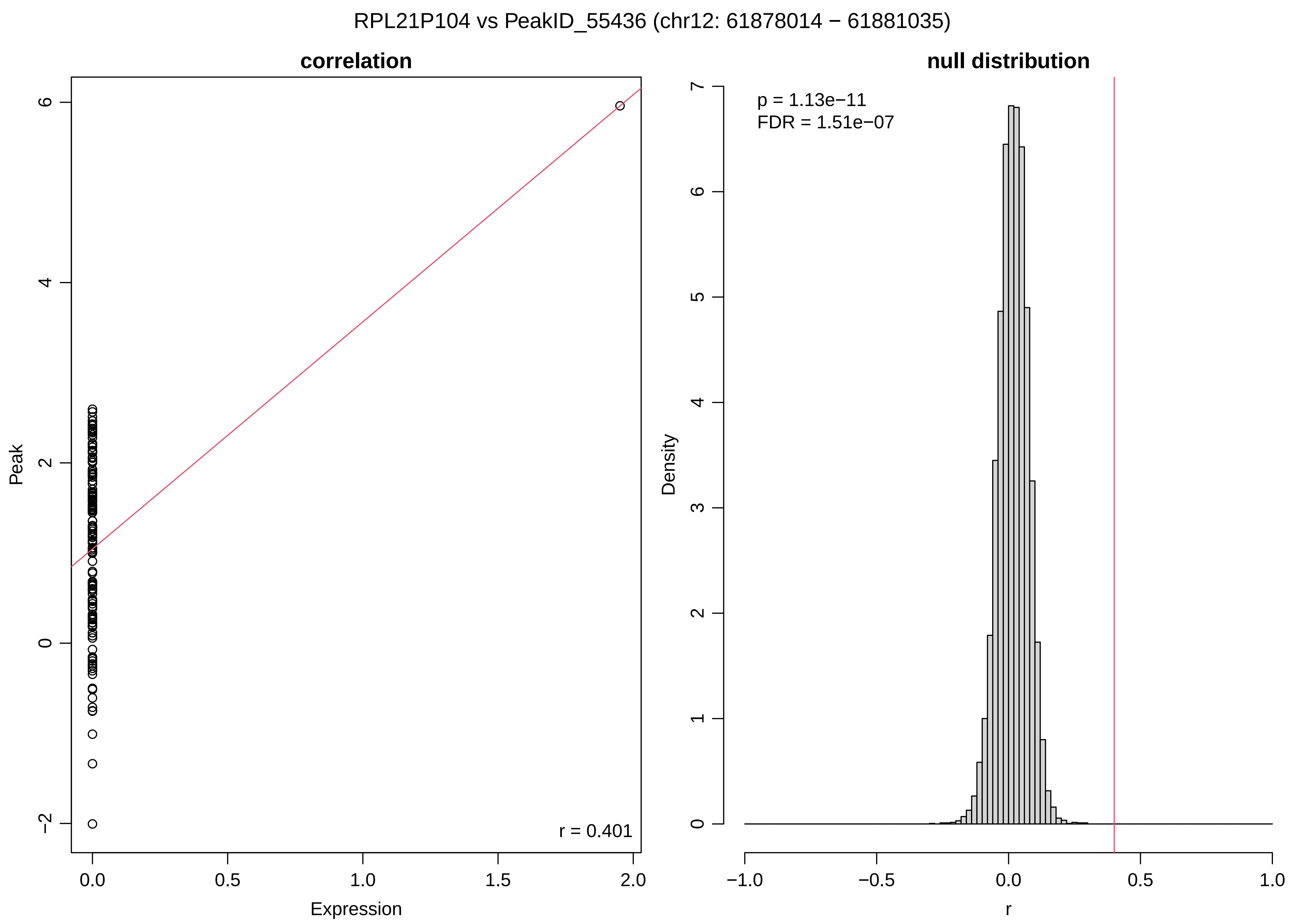

RPL21P104 vs PeakID_55436 (chr12: 61878014 − 61881035)
correlation
Peak
Expression
r = 0.401
−2
0
2
4
6
0.0
0.5
1.0
1.5
2.0
null distribution
p = 1.13e−11
FDR = 1.51e−07
Density
r
0
1
2
3
4
5
6
7
−1.0
−0.5
0.0
0.5
1.0

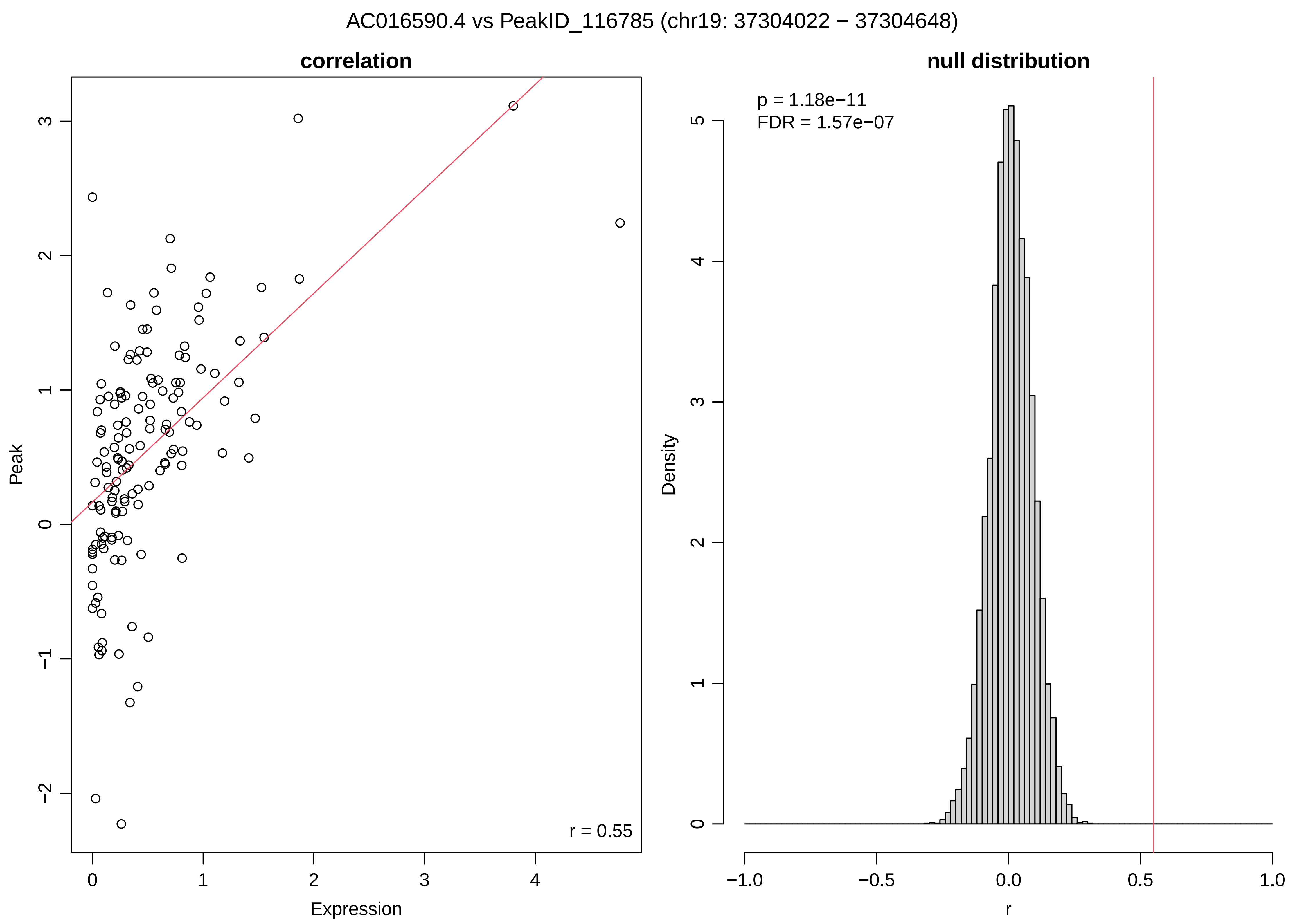
AC016590.4 vs PeakID_116785 (chr19: 37304022 − 37304648)
correlation
null distribution
Peak
Expression
r = 0.55
p = 1.18e−11
FDR = 1.57e−07
Density
r
−2
−1
0
1
2
3
4
−1.0
−0.5
0.0
0.5
1.0
5

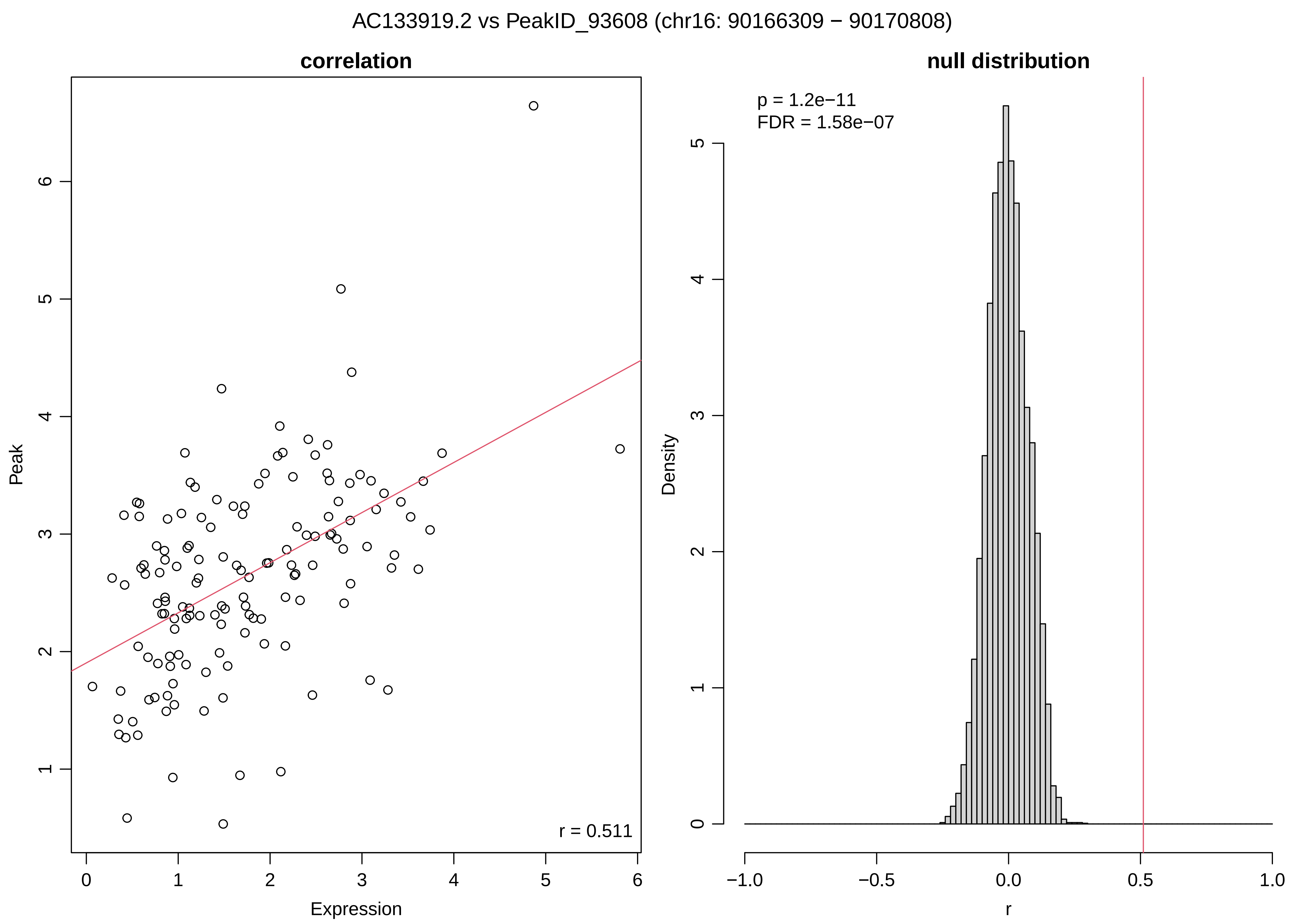

AC133919.2 vs PeakID_93608 (chr16: 90166309 − 90170808)
correlation
Peak
Expression
r = 0.511
0
1
2
3
4
5
6
null distribution
p = 1.2e−11
FDR = 1.58e−07
Density
r
−1.0
−0.5
0.0
0.5
1.0

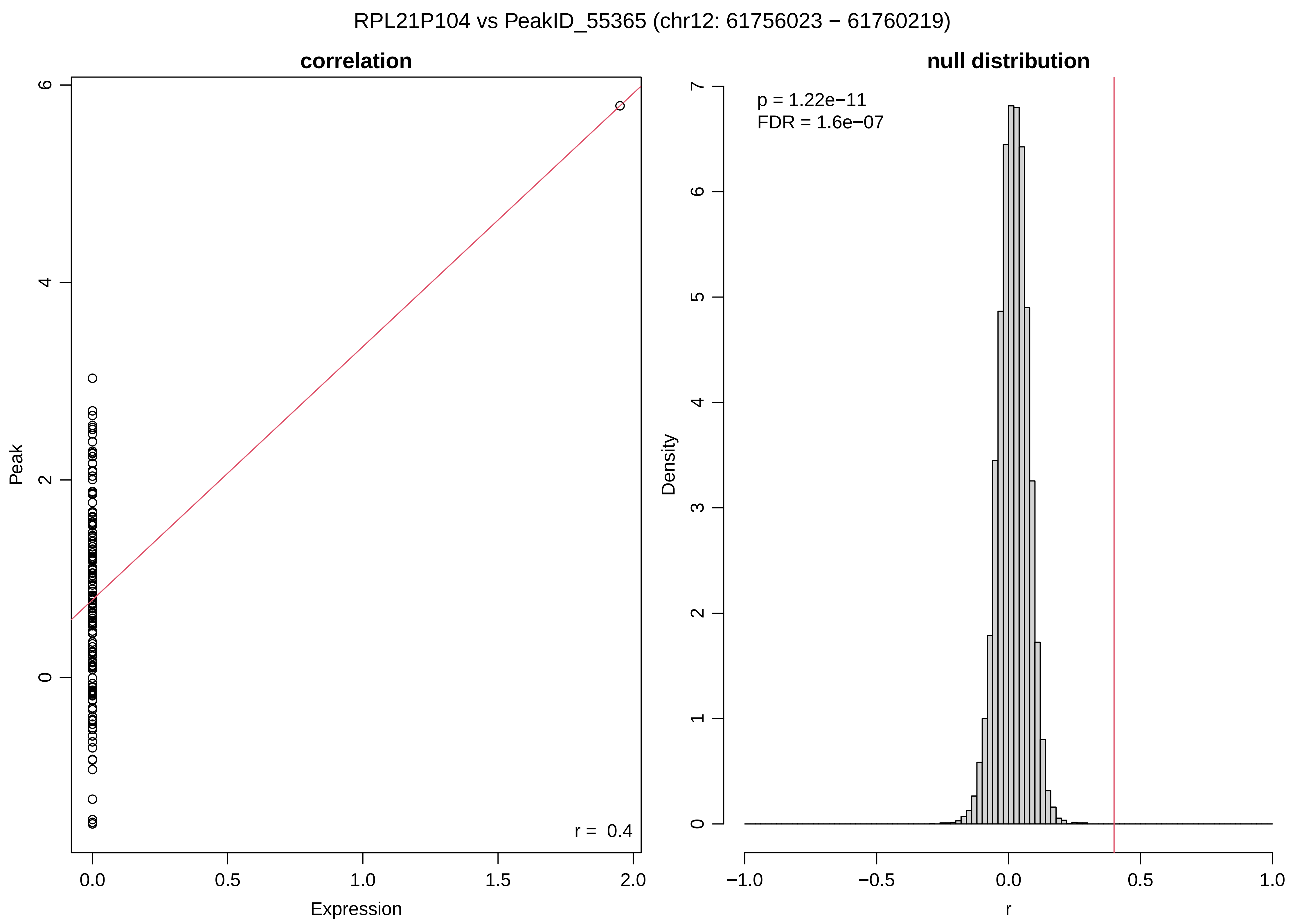

RPL21P104 vs PeakID_55365 (chr12: 61756023 − 61760219)
correlation
Peak
Expression
r = 0.4
0
2
4
6
0.0
0.5
1.0
1.5
2.0
null distribution
p = 1.22e−11
FDR = 1.6e−07
Density
r
0
1
2
3
4
5
6
7
−1.0
−0.5
0.0
0.5
1.0

NLGN4Y vs PeakID_262768 (chrY: 14329172 − 14329923)

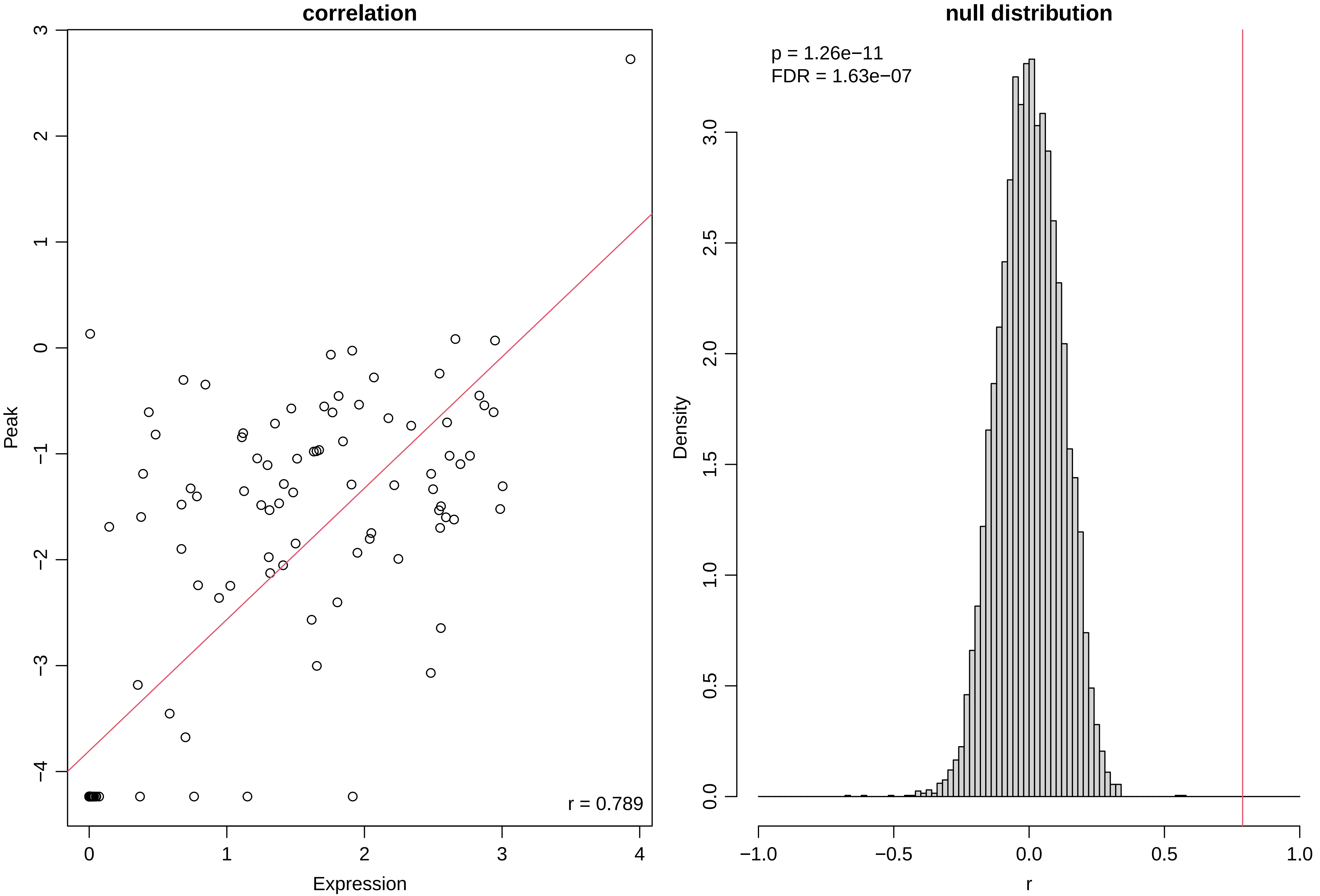

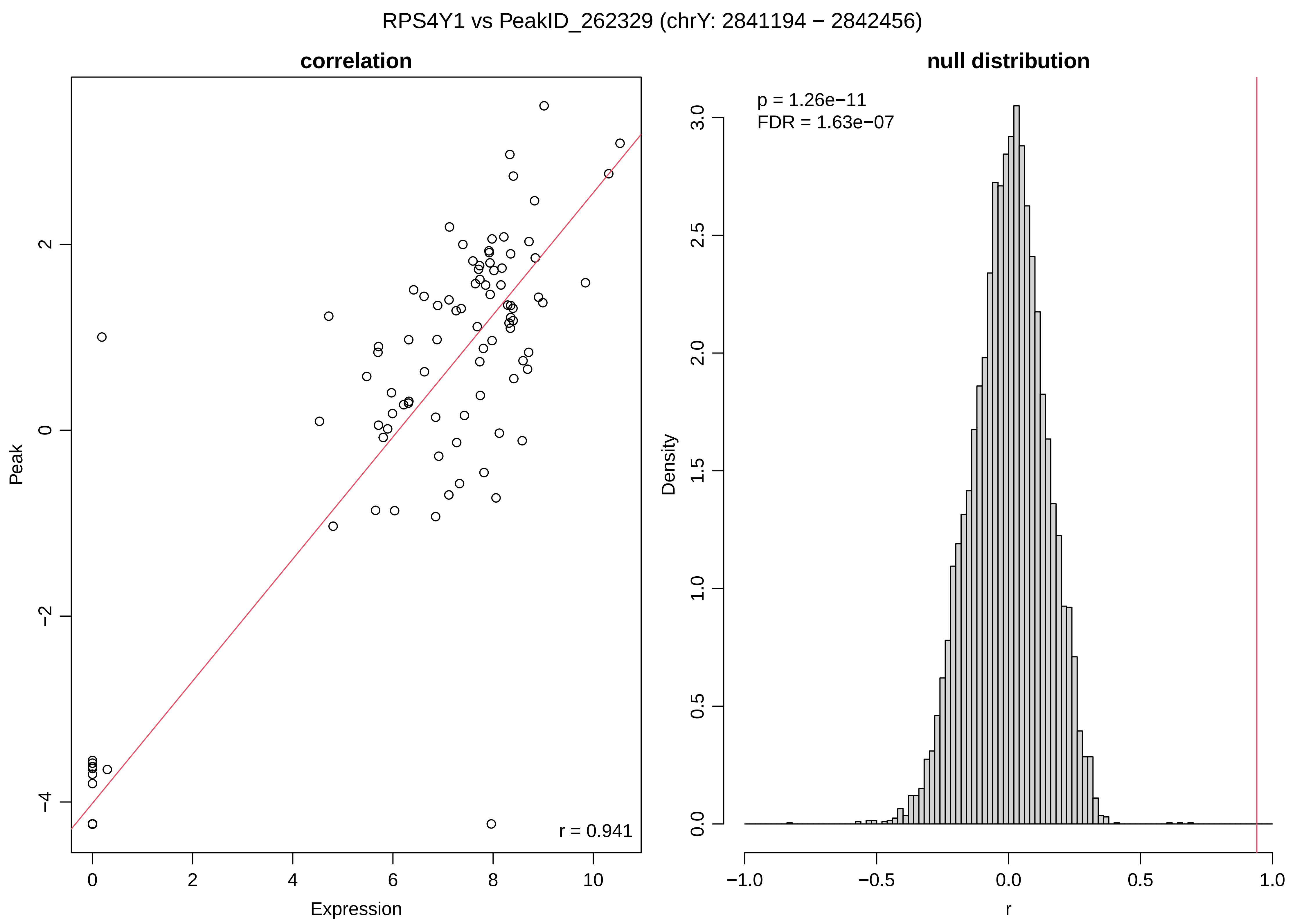
RPS4Y1 vs PeakID_262329 (chrY: 2841194 − 2842456)
correlation
Peak
Expression
r = 0.941
null distribution
p = 1.26e−11
FDR = 1.63e−07
Density
r

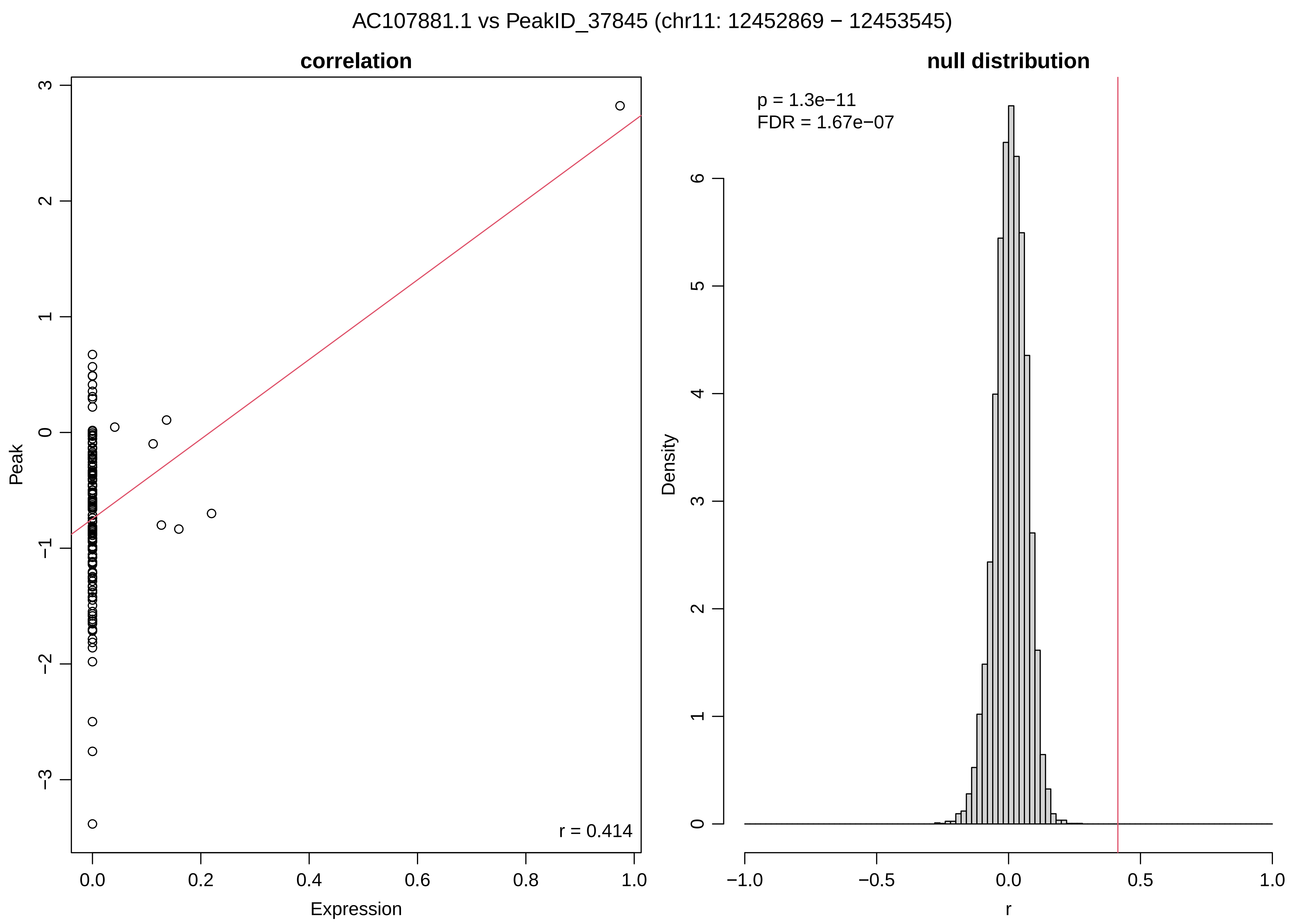
AC107881.1 vs PeakID_37845 (chr11: 12452869 − 12453545)
correlation
Peak
Expression
r = 0.414
3
2
1
0
−1
−2
−3
0.0
0.2
0.4
0.6
0.8
1.0
null distribution
p = 1.3e−11
FDR = 1.67e−07
Density
r
6
5
4
3
2
1
0
−1.0
−0.5
0.0
0.5
1.0

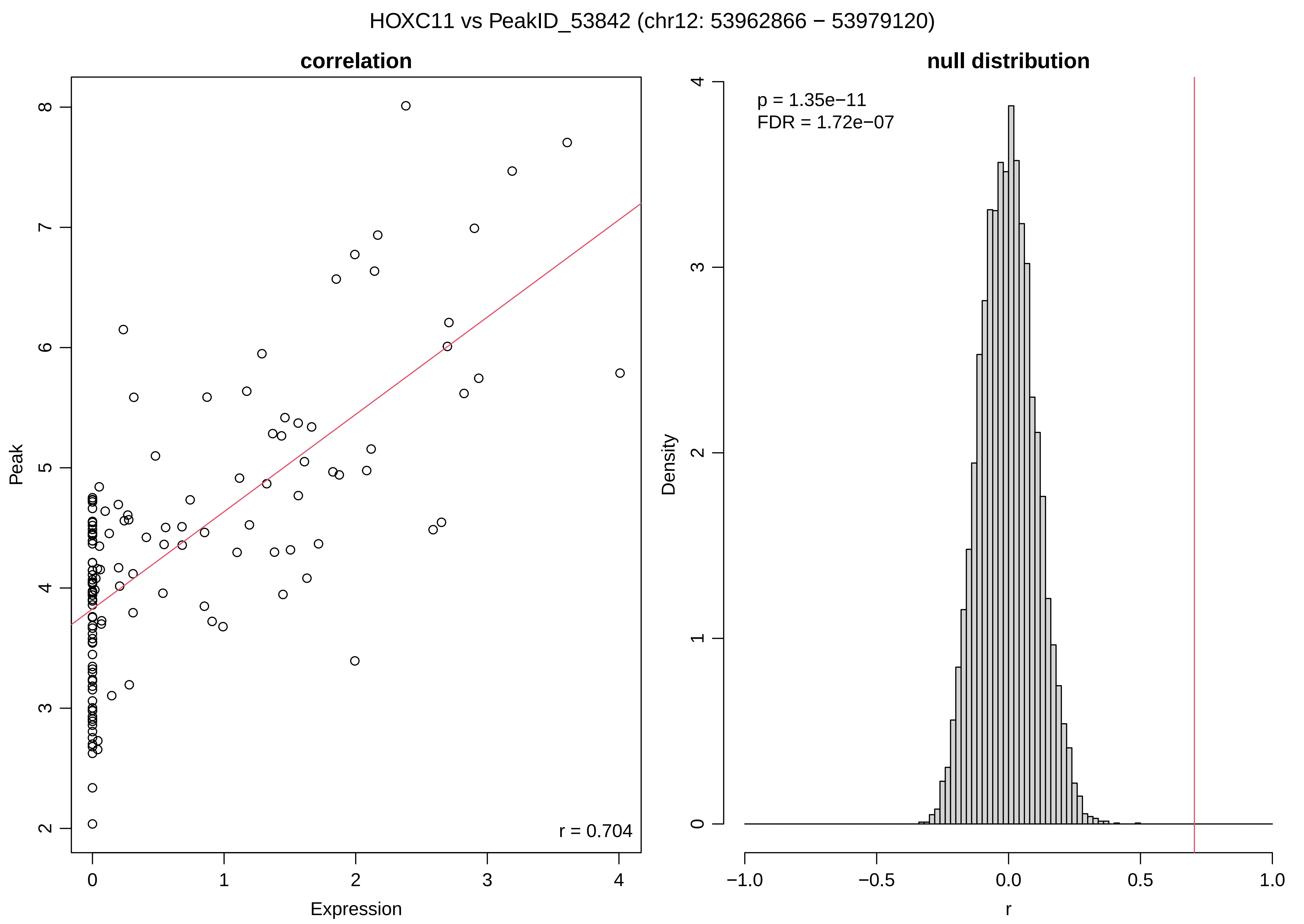
HOXC11 vs PeakID_53842 (chr12: 53962866 − 53979120)
correlation
Peak
Expression
r = 0.704
8
7
6
5
4
3
2
0
1
2
3
4
null distribution
p = 1.35e−11
FDR = 1.72e−07
Density
r
4
3
2
1
0
−1.0
−0.5
0.0
0.5
1.0

AC002066.1 vs PeakID_228943 (chr7: 116315292 − 116315646)

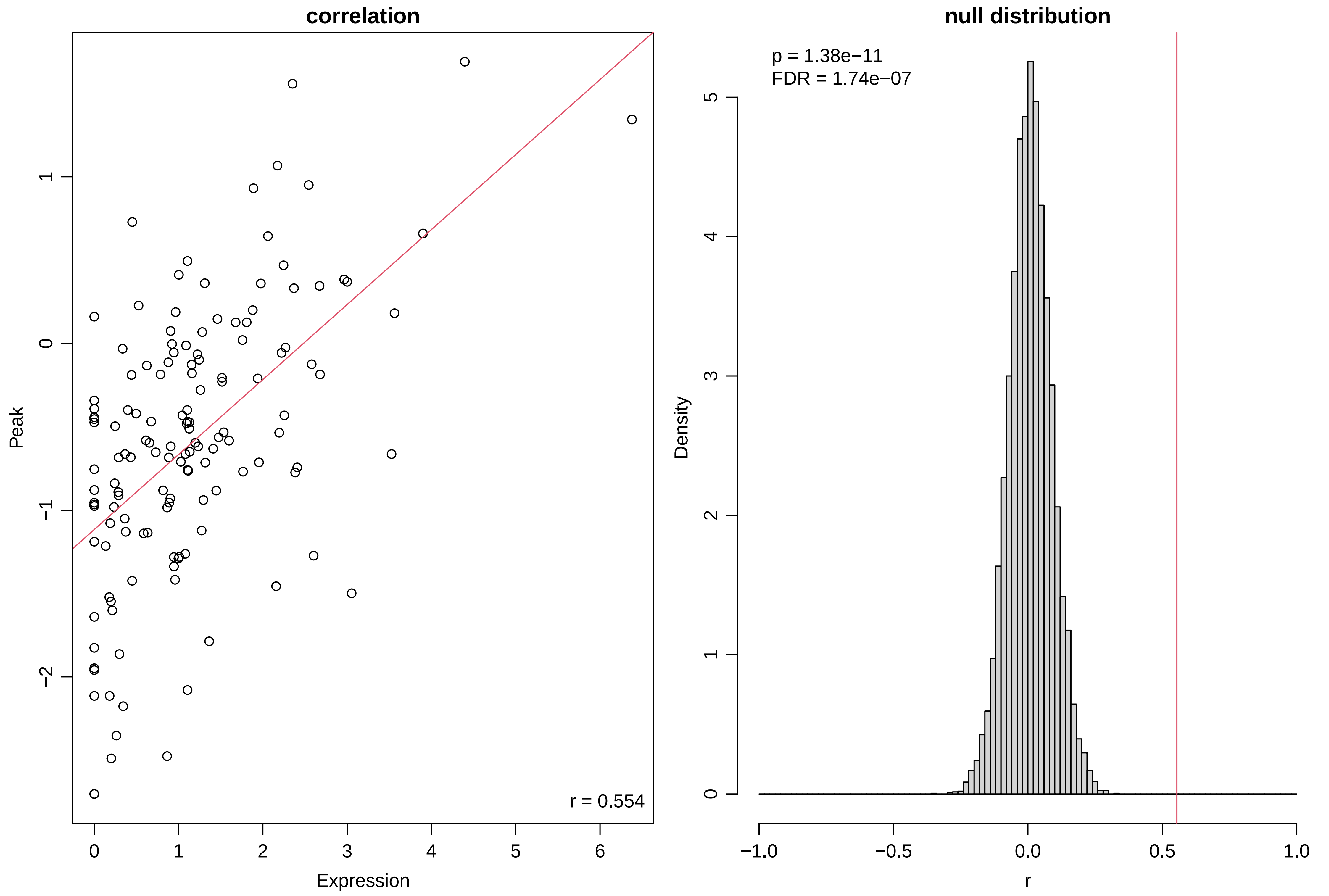

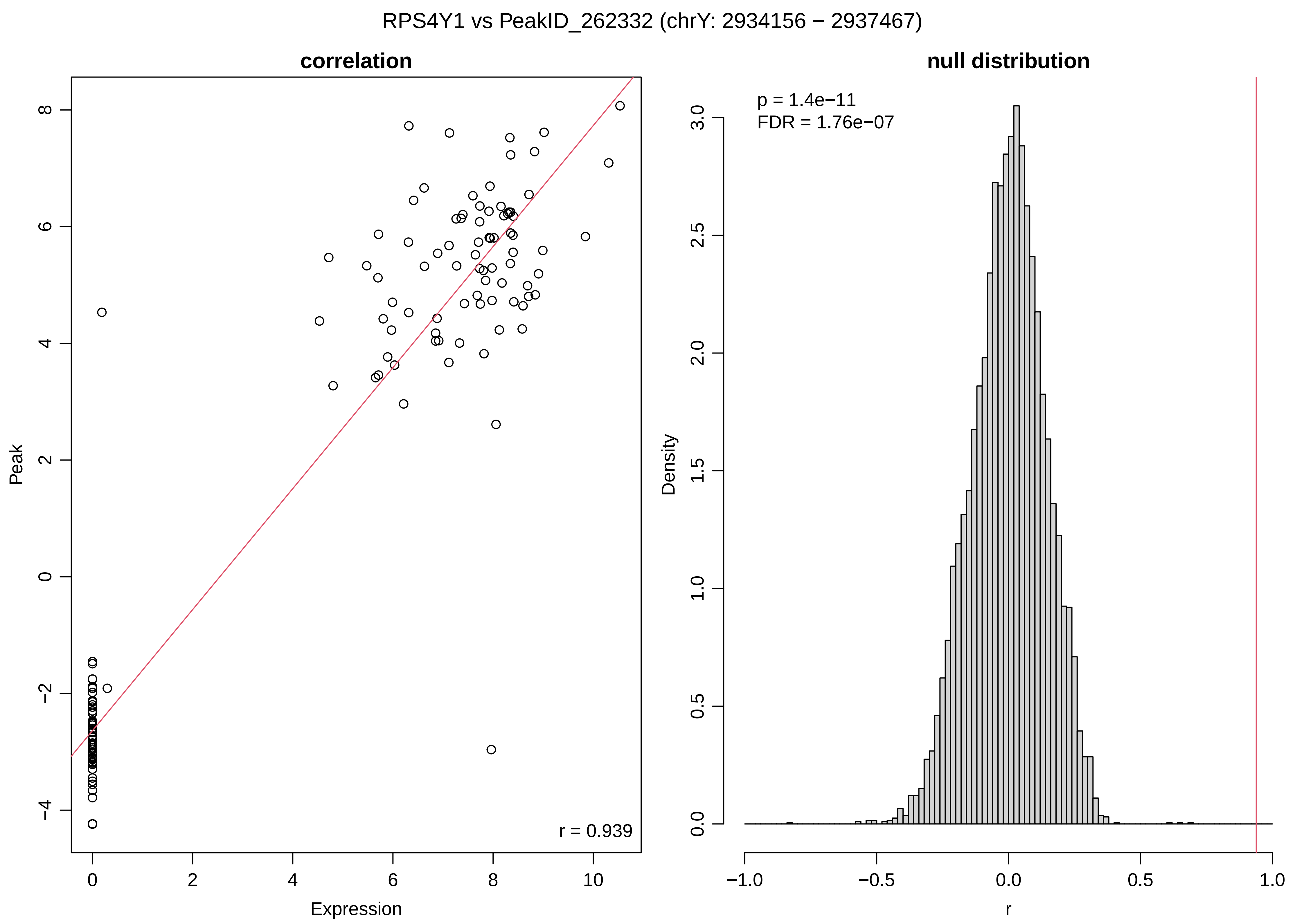
RPS4Y1 vs PeakID_262332 (chrY: 2934156 − 2937467)
correlation
Peak
Expression
r = 0.939
null distribution
p = 1.4e−11
FDR = 1.76e−07
Density
r

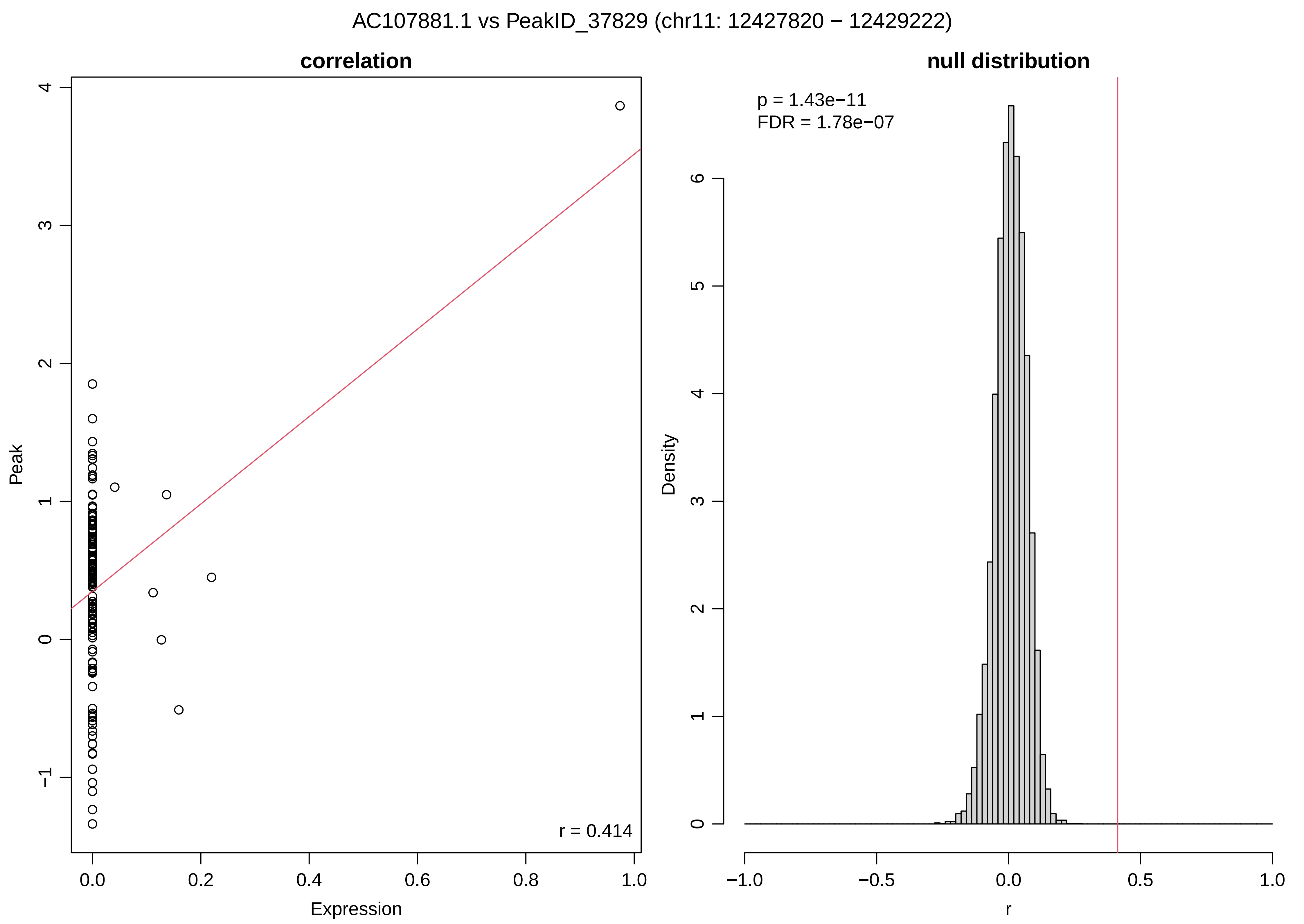

AC107881.1 vs PeakID_37829 (chr11: 12427820 − 12429222)
correlation
Peak
Expression
r = 0.414
null distribution
p = 1.43e−11
FDR = 1.78e−07
Density
r

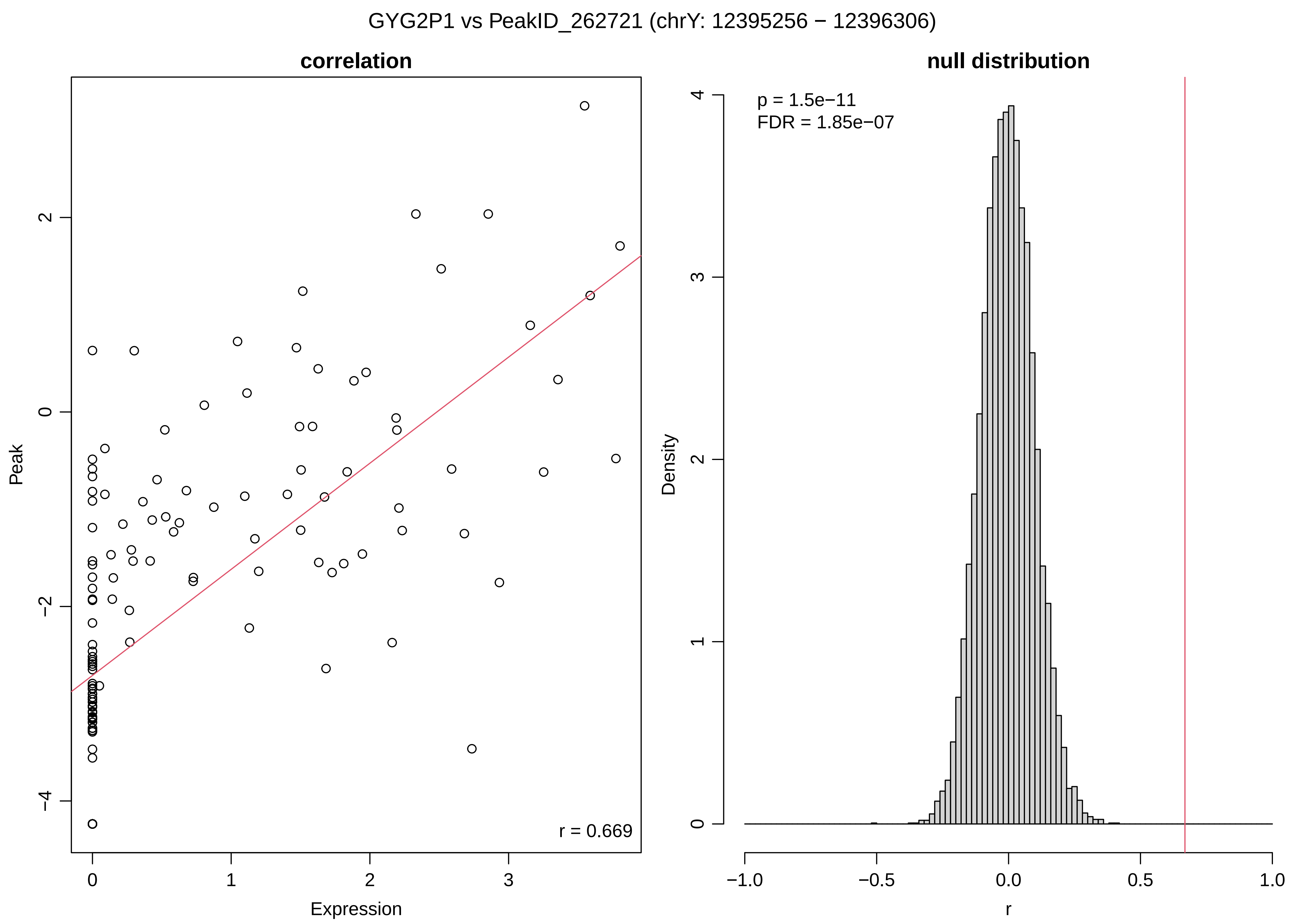
GYG2P1 vs PeakID_262721 (chrY: 12395256 − 12396306)
correlation
Peak
Expression
r = 0.669
null distribution
p = 1.5e−11
FDR = 1.85e−07
Density
r

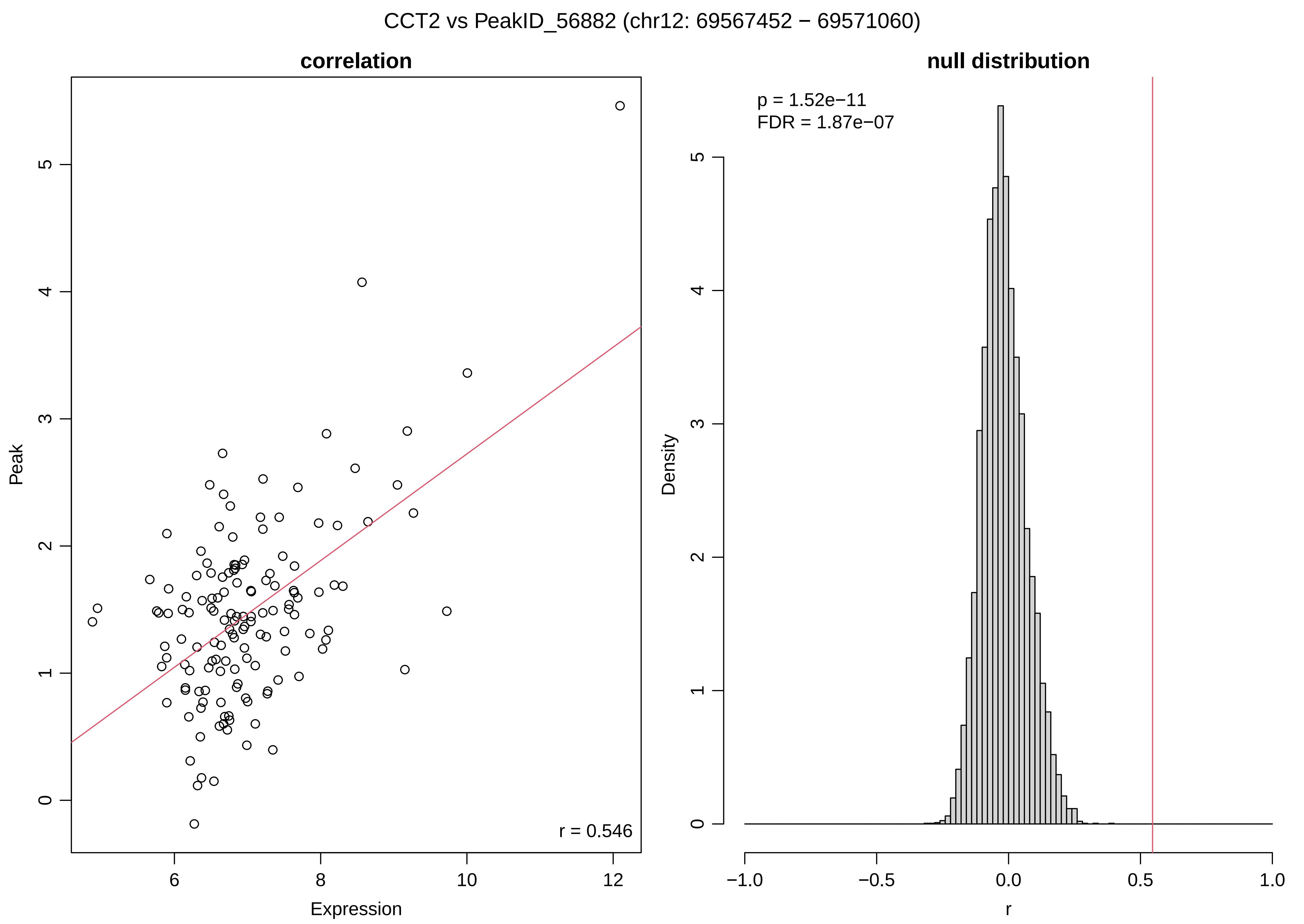
CCT2 vs PeakID_56882 (chr12: 69567452 − 69571060)
correlation
Peak
Expression
r = 0.546
null distribution
p = 1.52e−11
FDR = 1.87e−07
Density
r

MIR4713HG vs PeakID_78750 (chr15: 51036873 − 51039197)

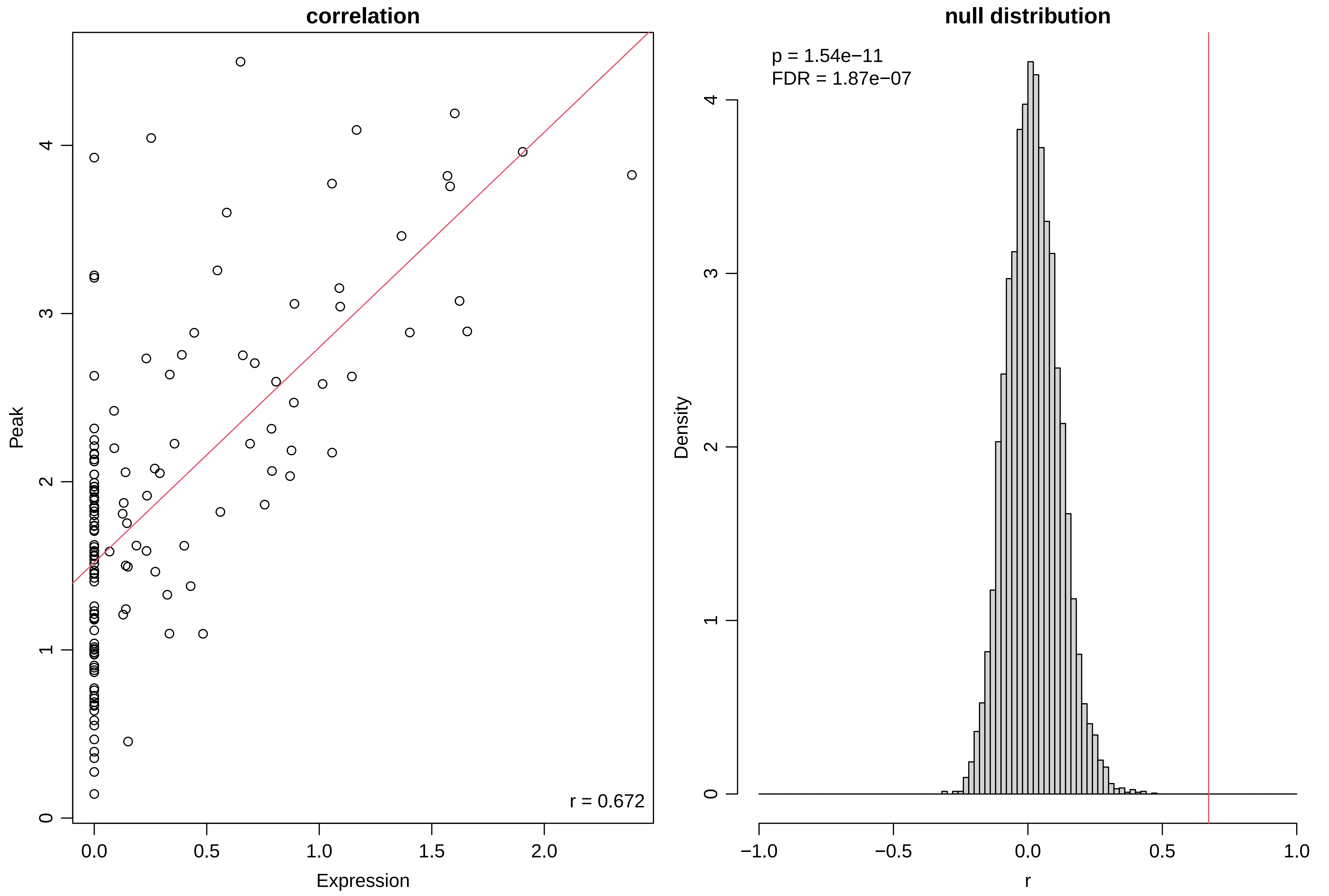

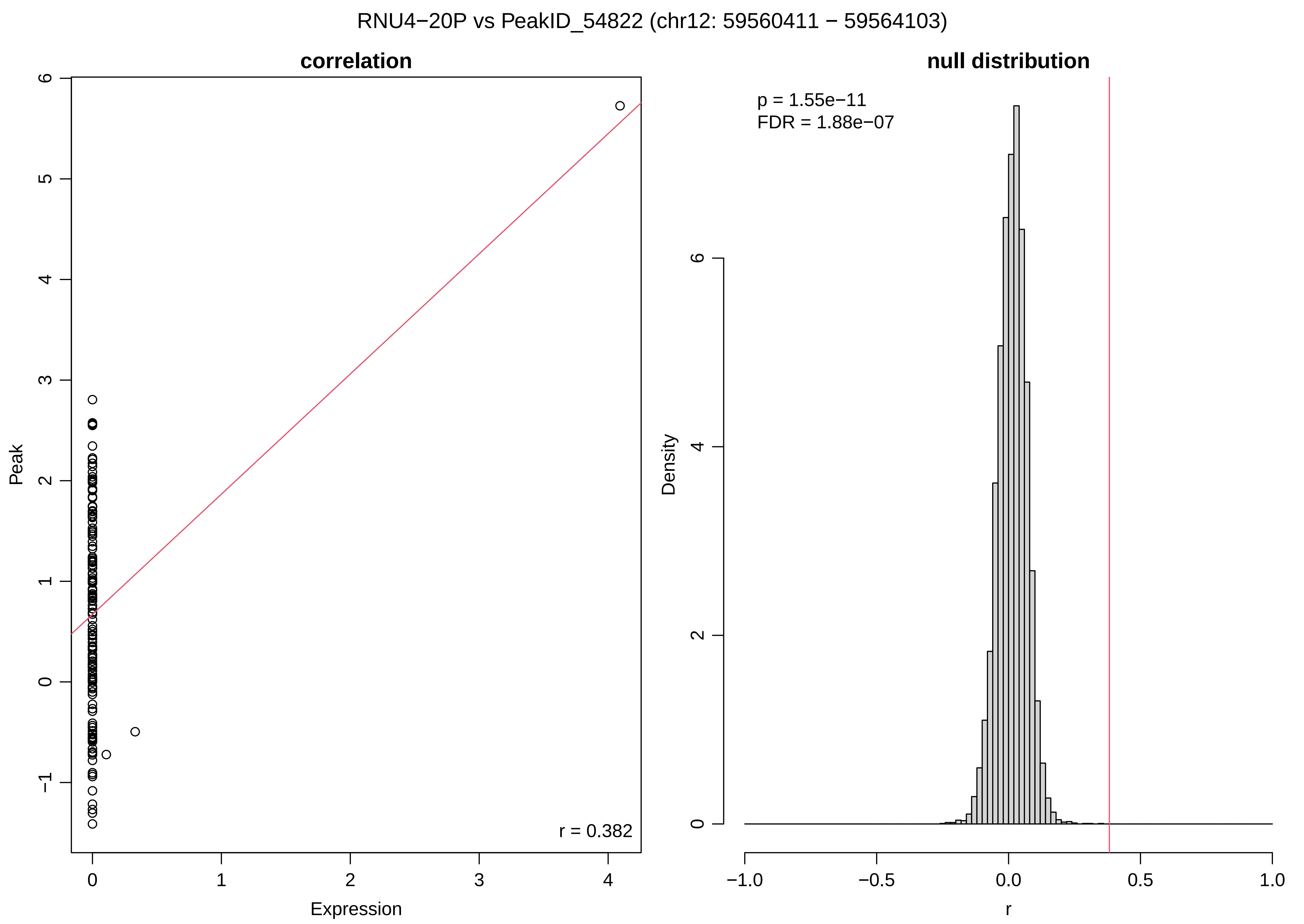
RNU4−20P vs PeakID_54822 (chr12: 59560411 − 59564103)
correlation
Peak
Expression
r = 0.382
0
1
2
3
4
−1
5
6
null distribution
p = 1.55e−11
FDR = 1.88e−07
Density
r
−1.0
−0.5
0.0
0.5
1.0

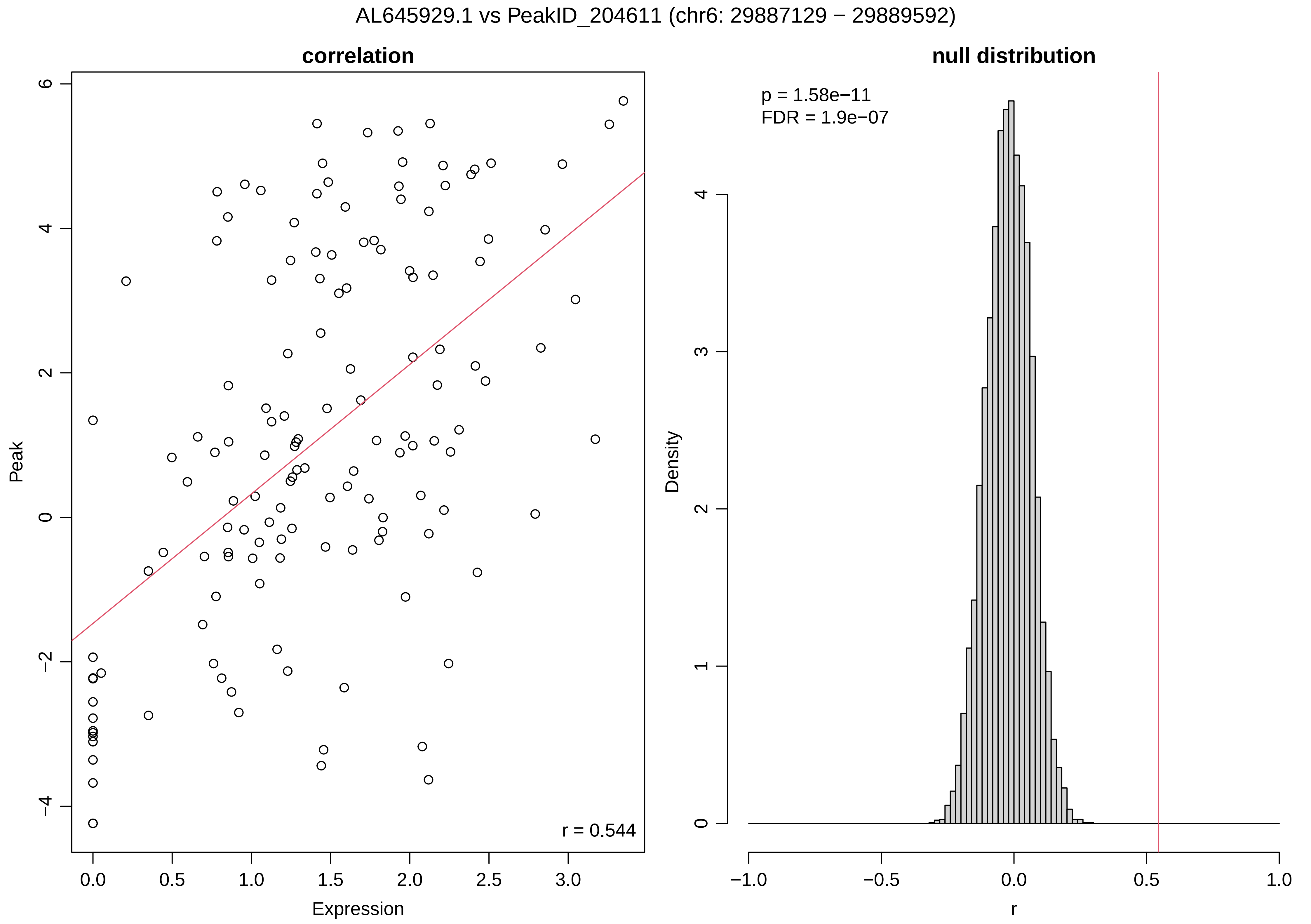
AL645929.1 vs PeakID_204611 (chr6: 29887129 − 29889592)
correlation
Peak
Expression
r = 0.544
null distribution
p = 1.58e−11
FDR = 1.9e−07
Density
r

RPL21P104 vs PeakID_55481 (chr12: 61991705 − 61995844)

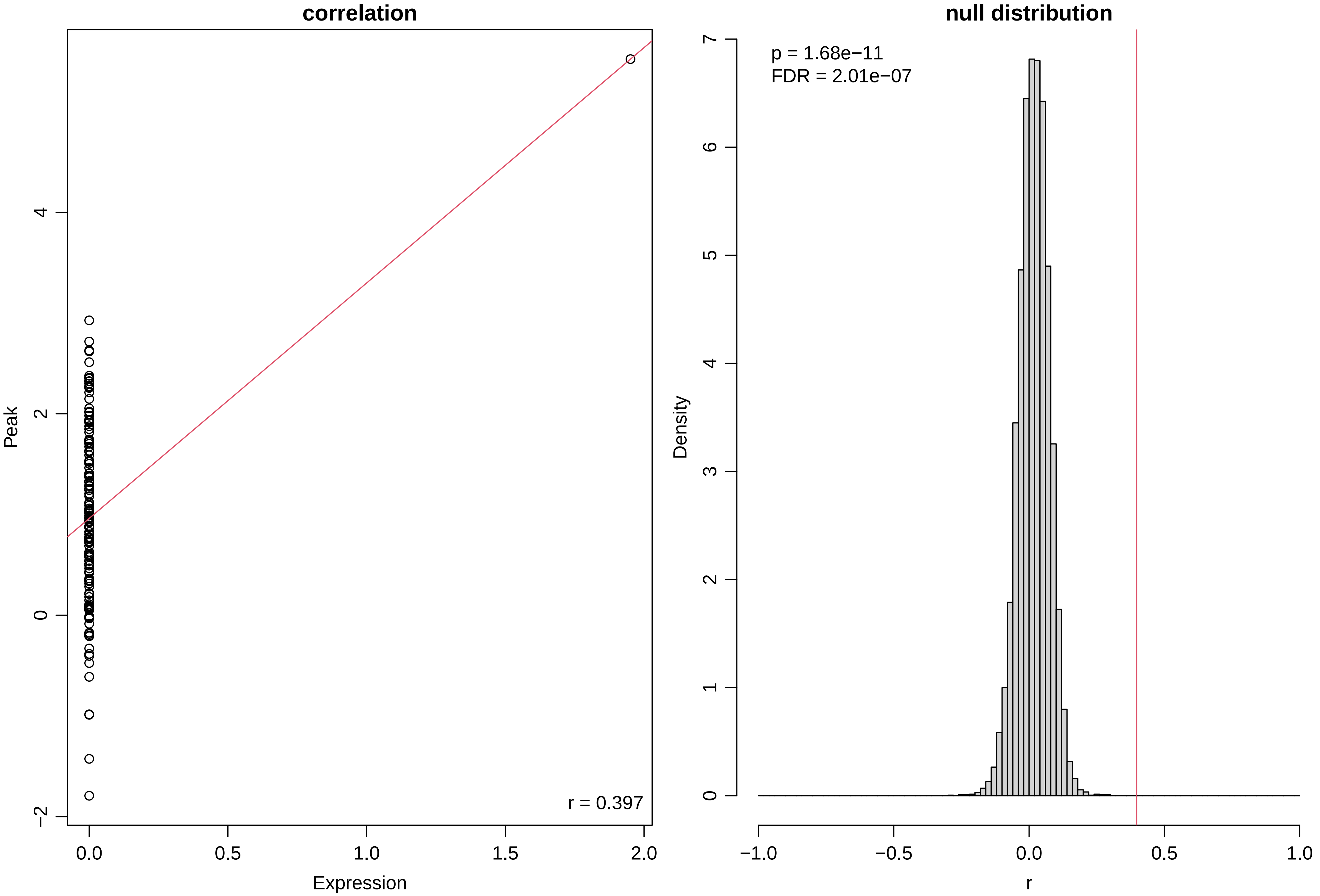

BTBD7P1 vs PeakID_25153 (chr10: 13157739 − 13159246)

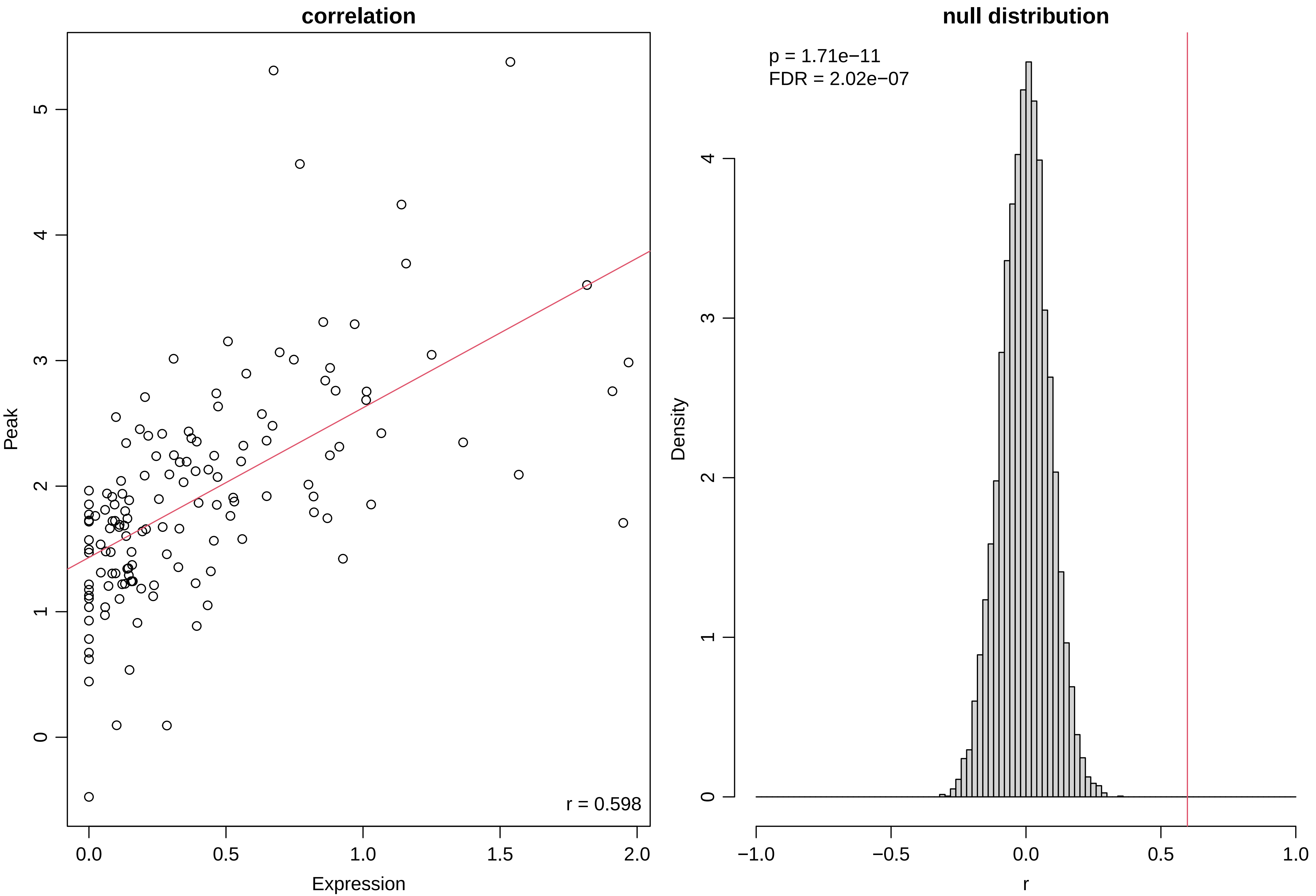

DACT2 vs PeakID_215314 (chr6: 168317944 − 168321556)

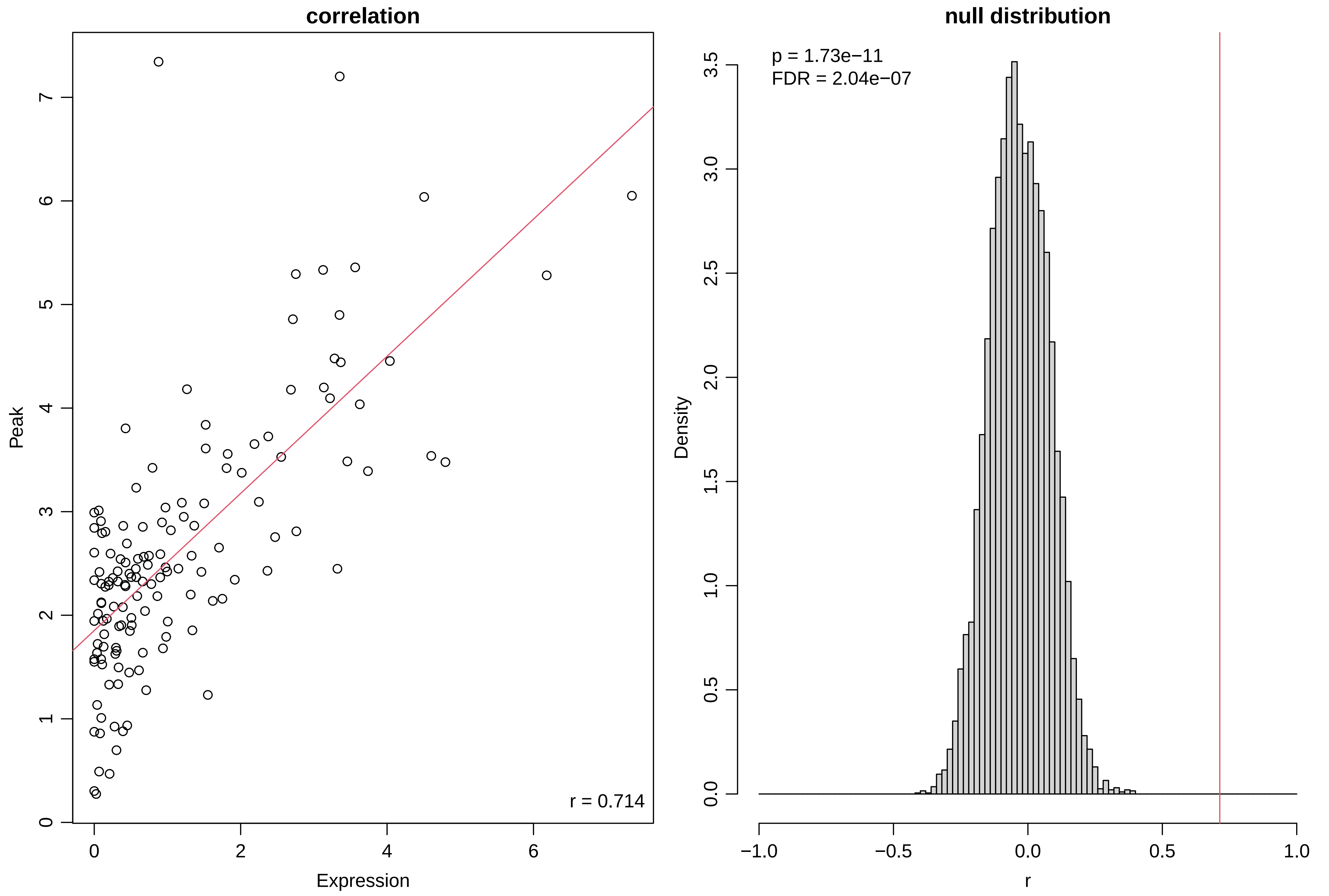

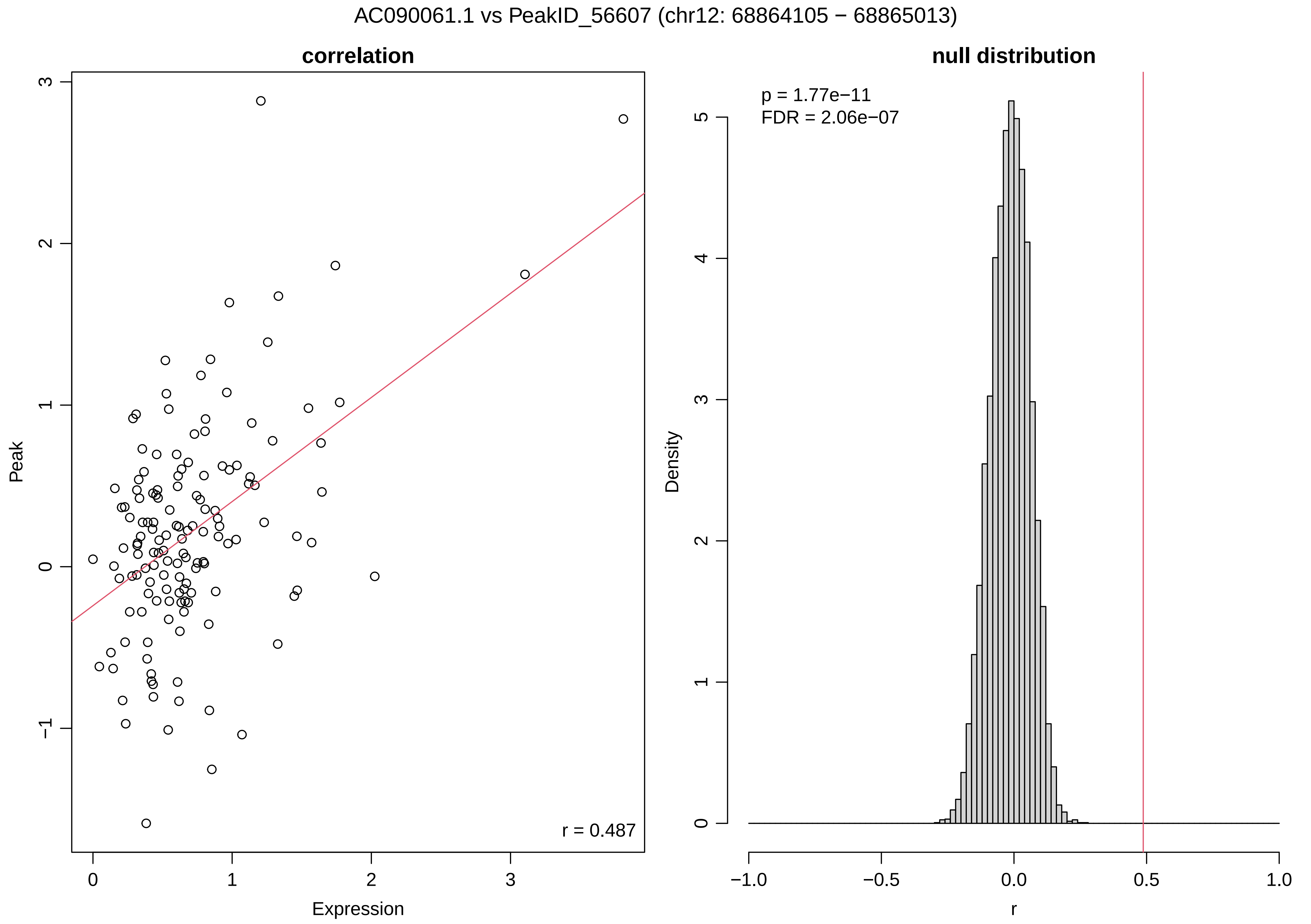
AC090061.1 vs PeakID_56607 (chr12: 68864105 − 68865013)
correlation
Peak
Expression
r = 0.487
null distribution
p = 1.77e−11
FDR = 2.06e−07
Density
r

FOXCUT vs PeakID_201369 (chr6: 1606378 − 1607350)

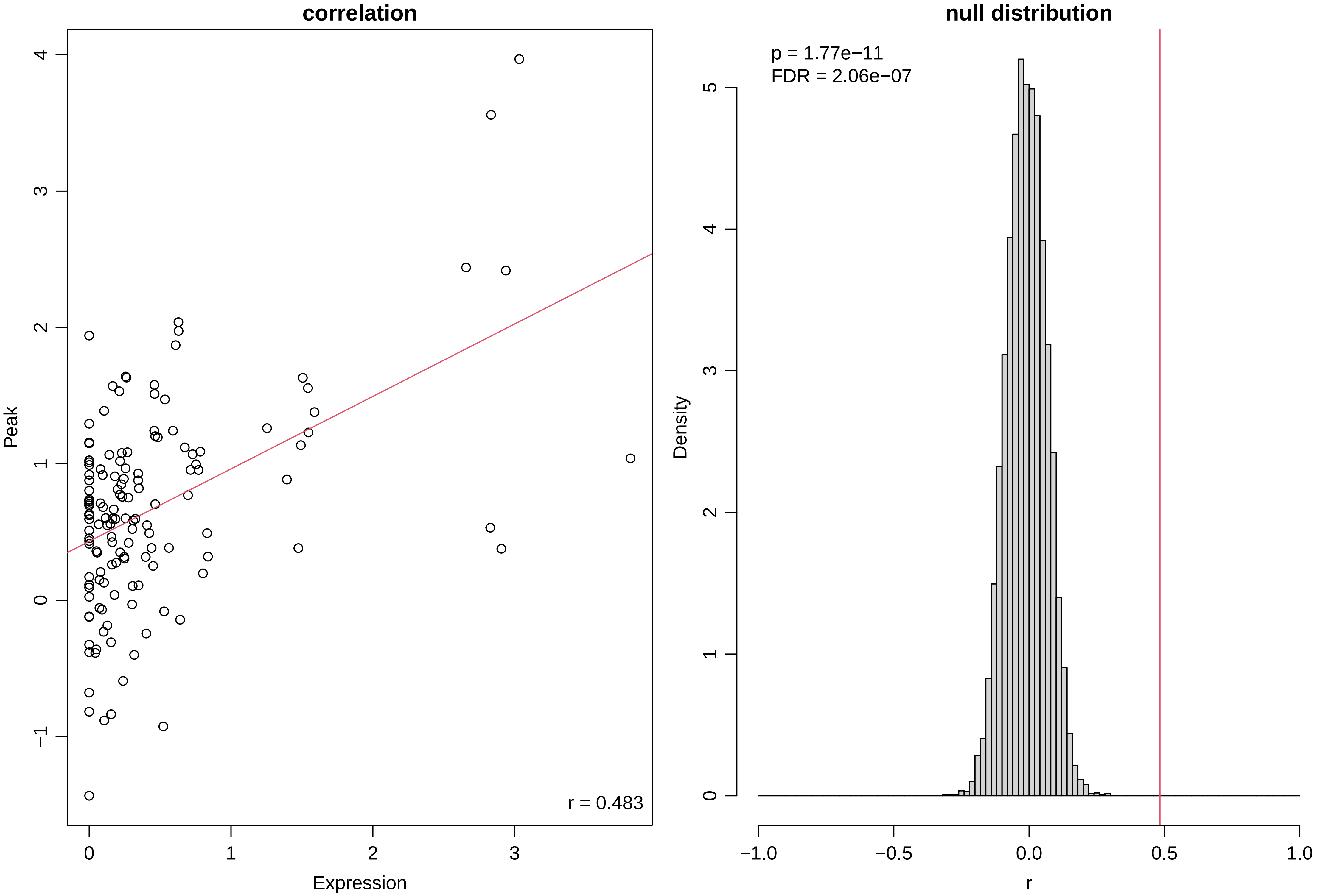

RNU4−20P vs PeakID_54787 (chr12: 59478510 − 59478905)

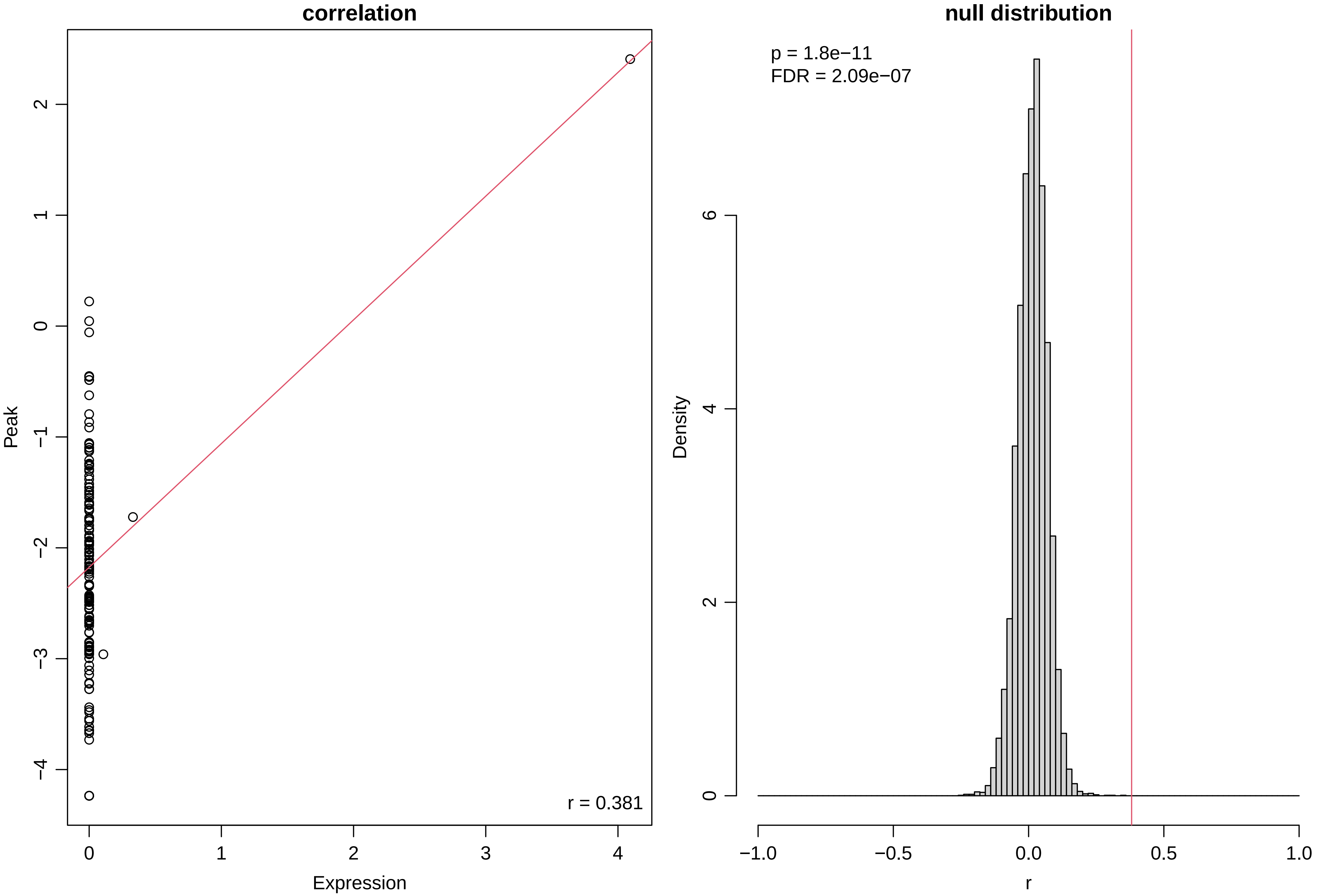

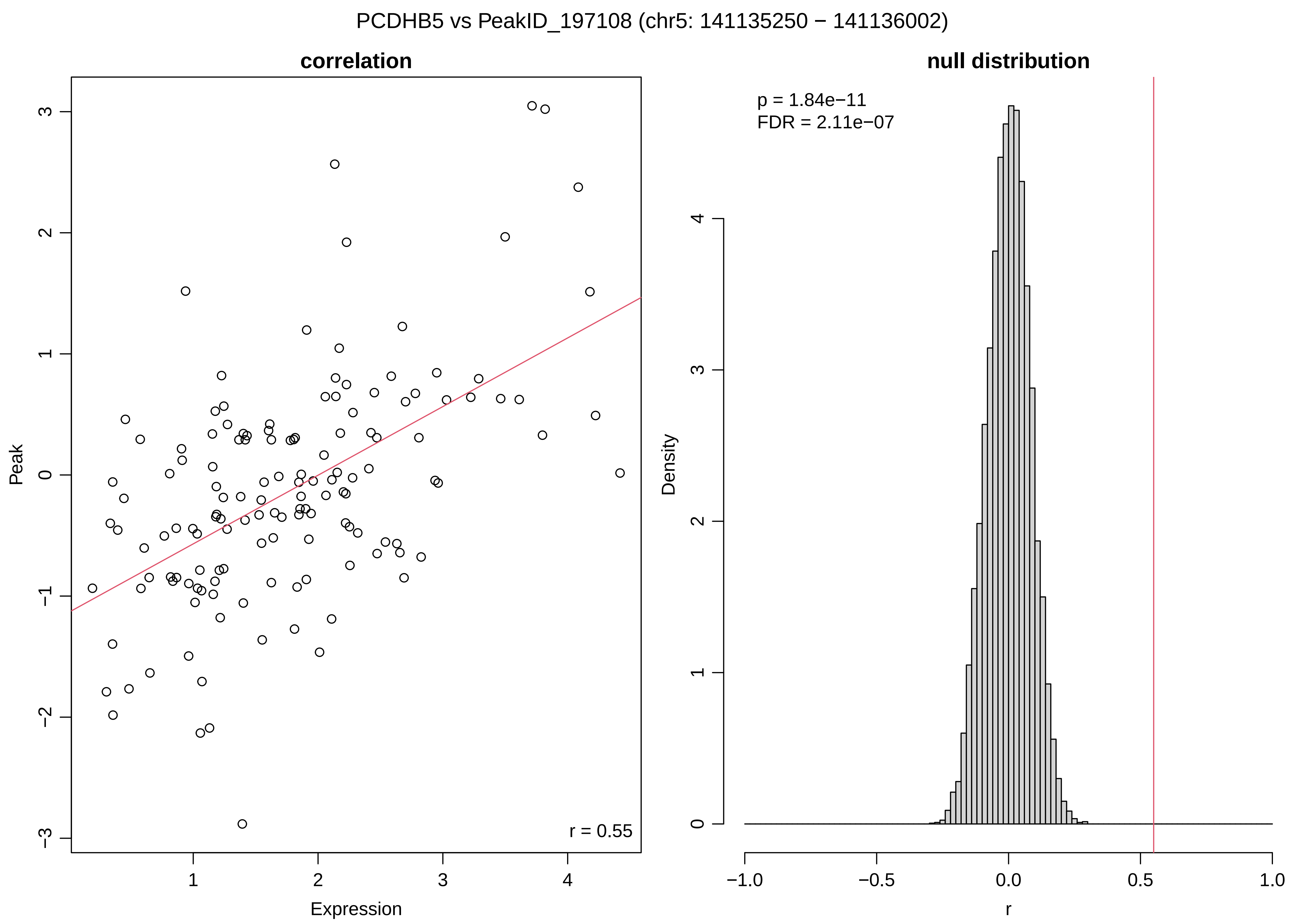
PCDHB5 vs PeakID_197108 (chr5: 141135250 − 141136002)
correlation
Peak
Expression
r = 0.55
null distribution
p = 1.84e−11
FDR = 2.11e−07
Density
r

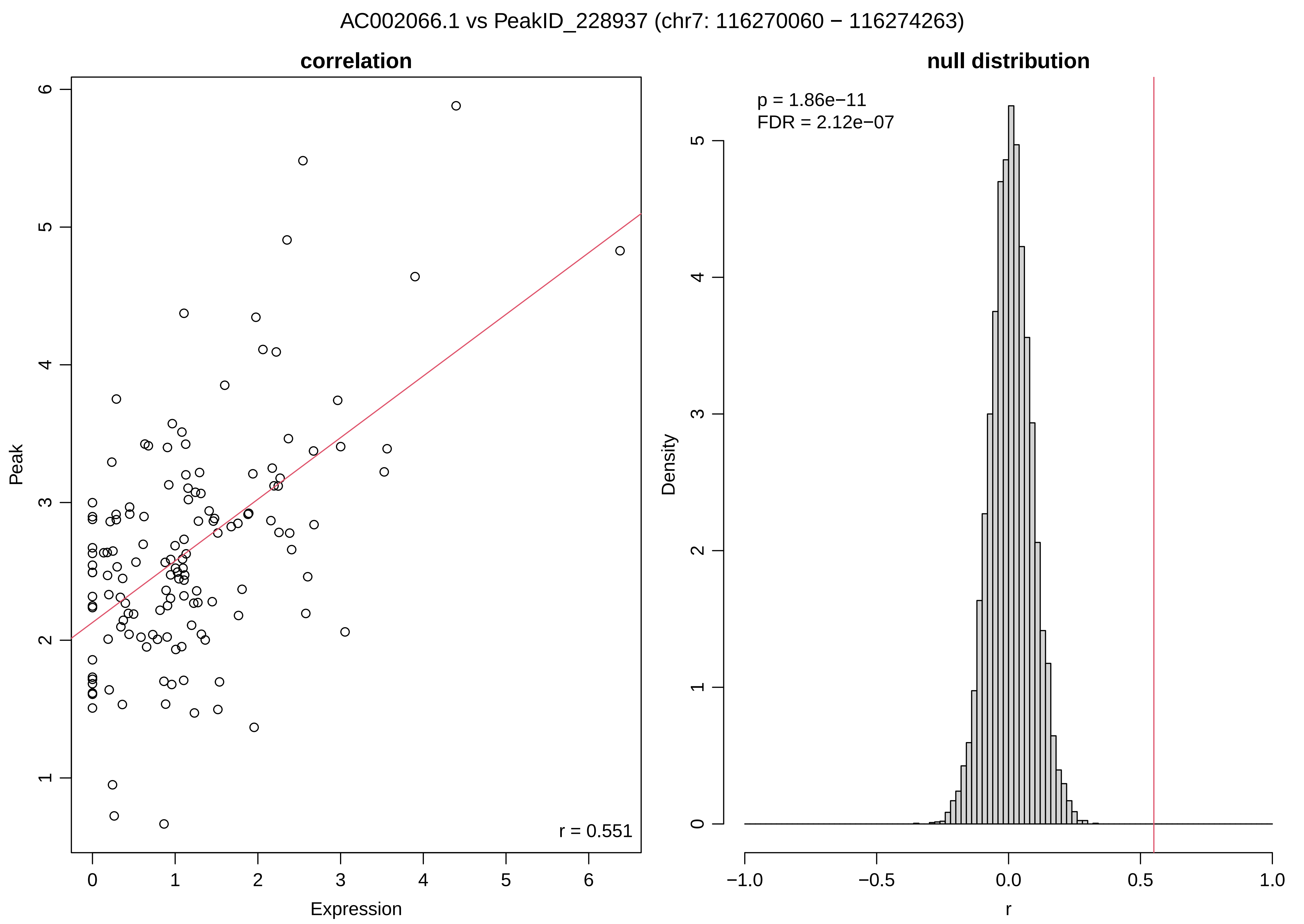

AC002066.1 vs PeakID_228937 (chr7: 116270060 − 116274263)
correlation
Peak
Expression
r = 0.551
null distribution
p = 1.86e−11
FDR = 2.12e−07
Density
r

AC002066.1 vs PeakID_228970 (chr7: 116440236 − 116444787)

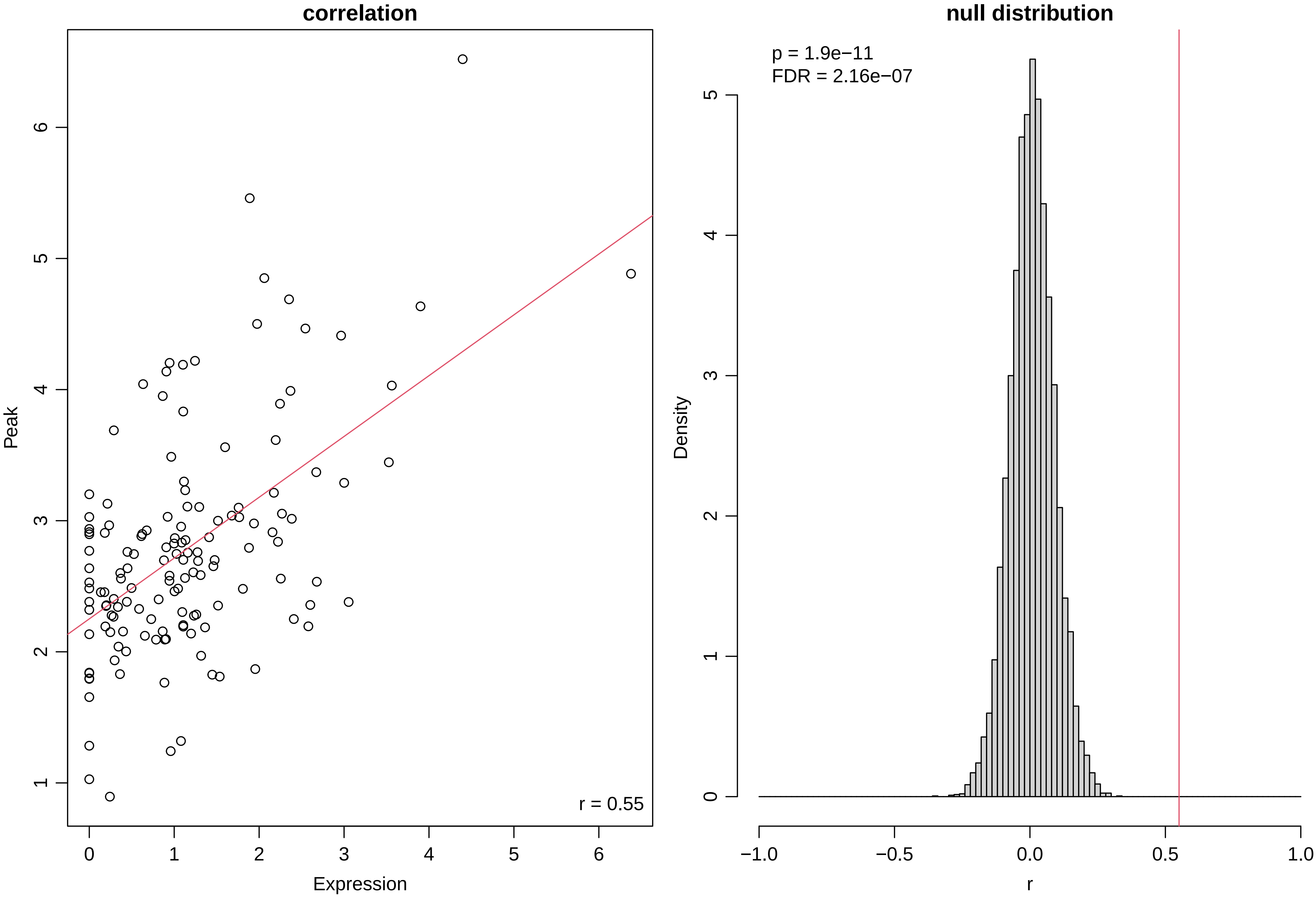

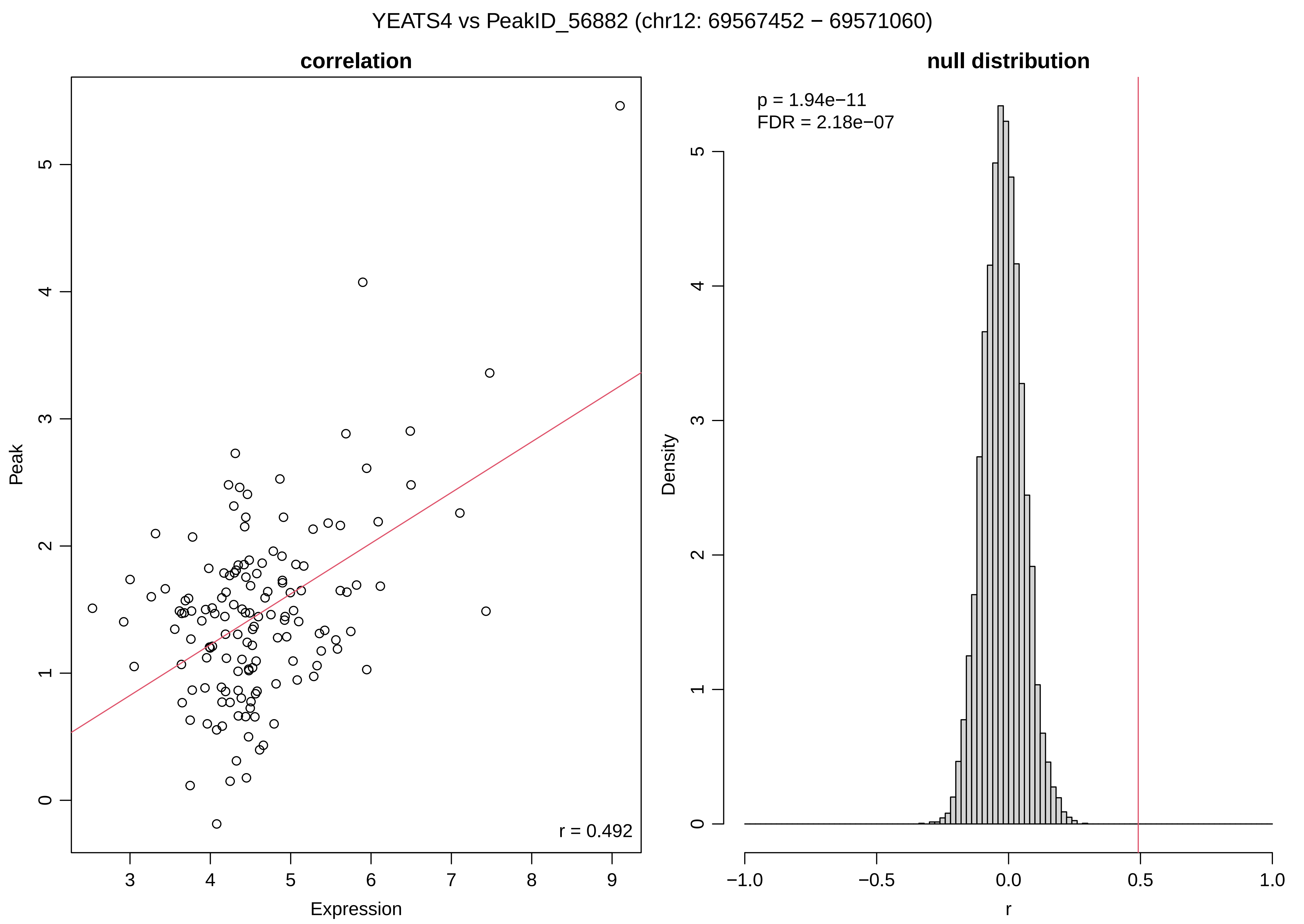

YEATS4 vs PeakID_56882 (chr12: 69567452 − 69571060)
correlation
Peak
Expression
r = 0.492
null distribution
p = 1.94e−11
FDR = 2.18e−07
Density
r

ZNF444P1 vs PeakID_79471 (chr15: 58864018 − 58867538)

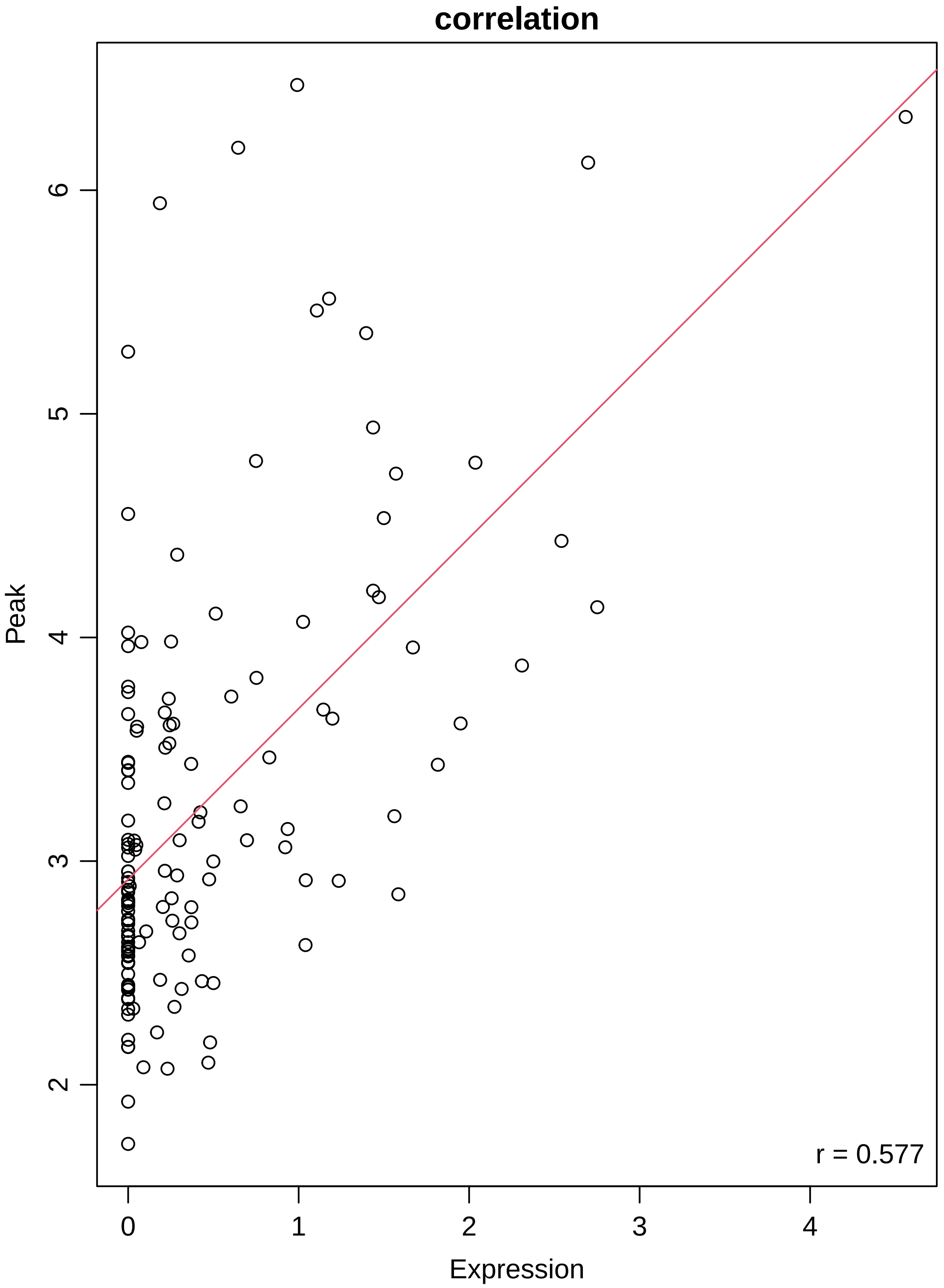

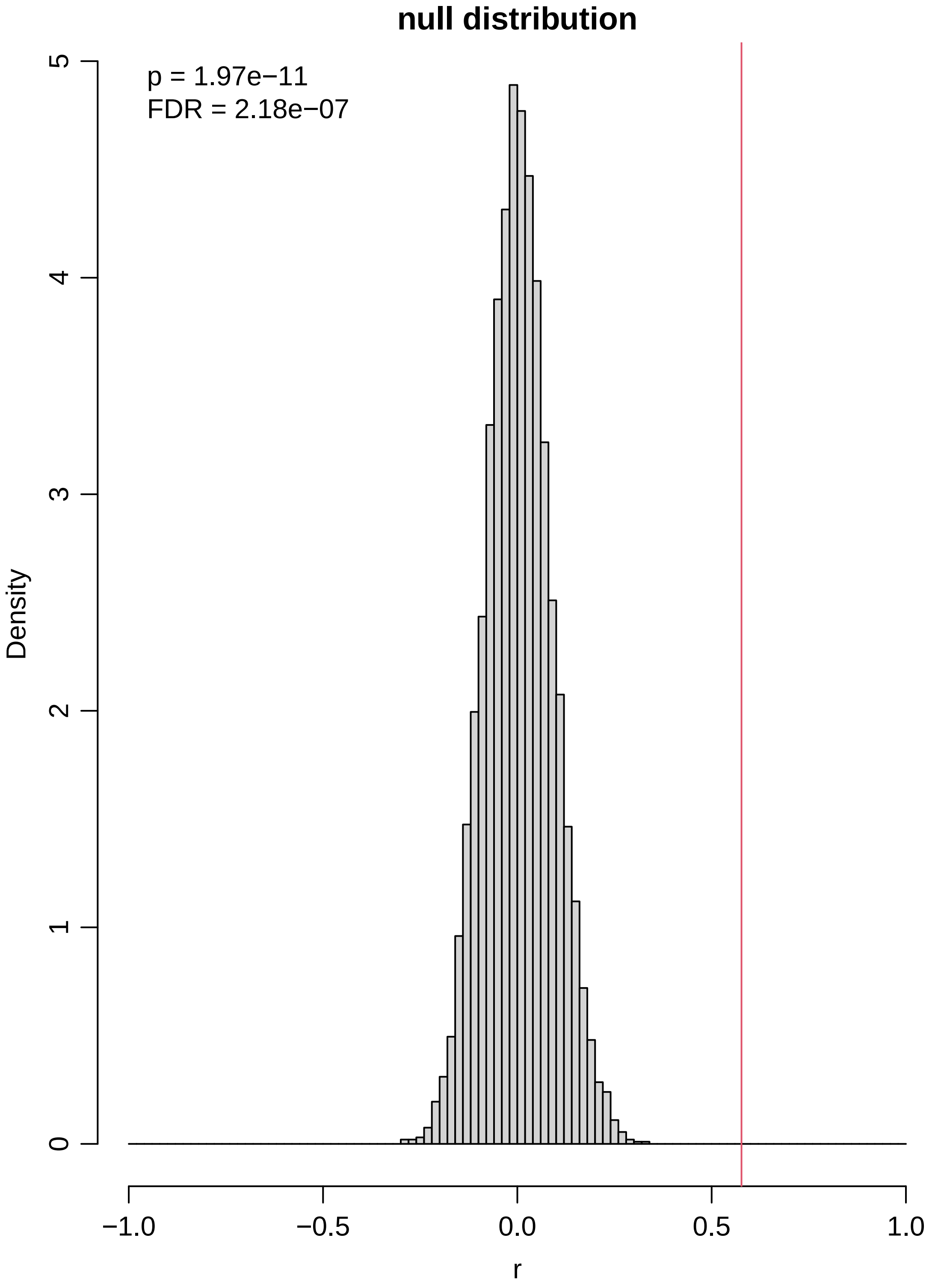

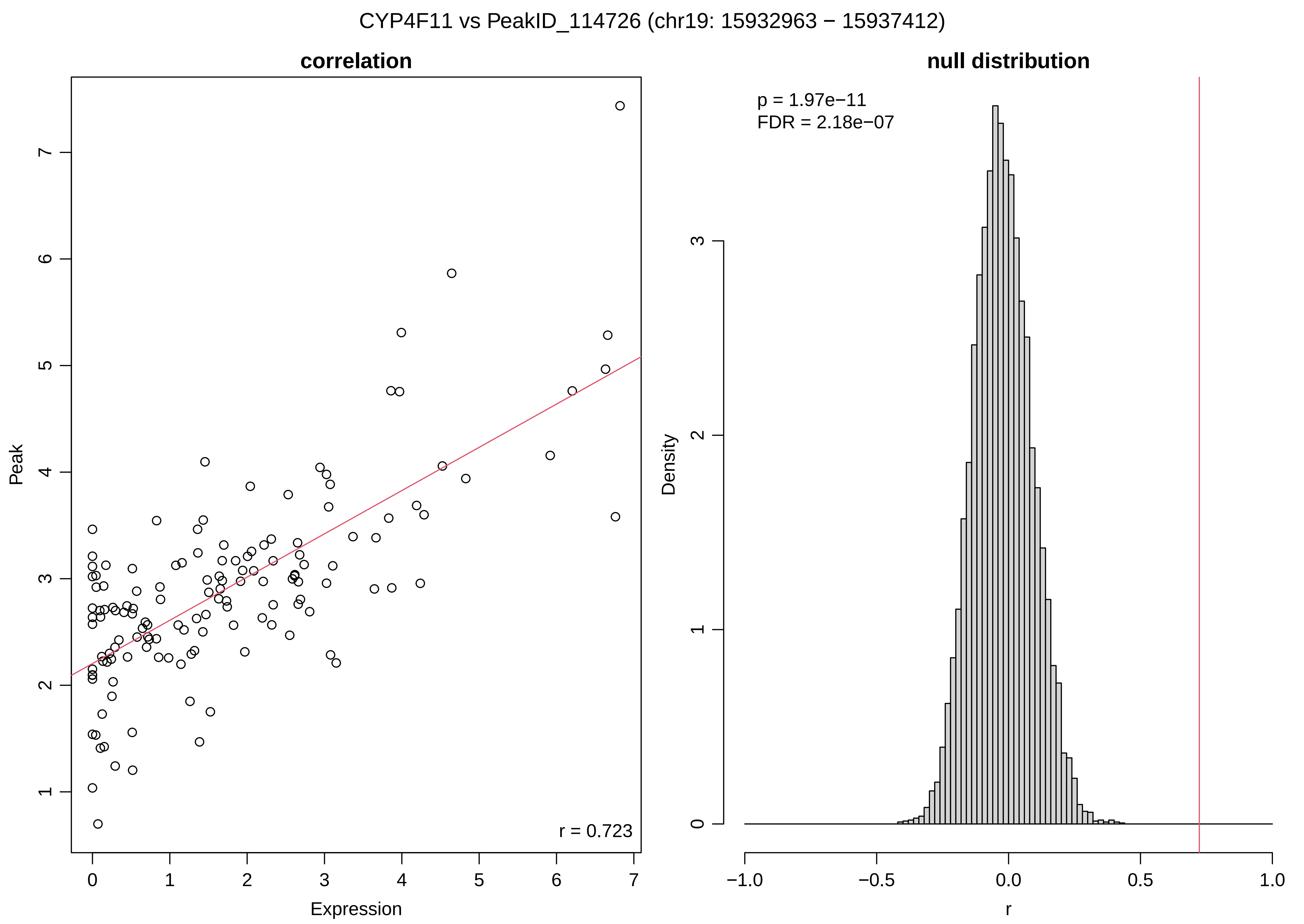

CYP4F11 vs PeakID_114726 (chr19: 15932963 − 15937412)
correlation
Peak
Expression
r = 0.723
null distribution
p = 1.97e−11
FDR = 2.18e−07
Density
r

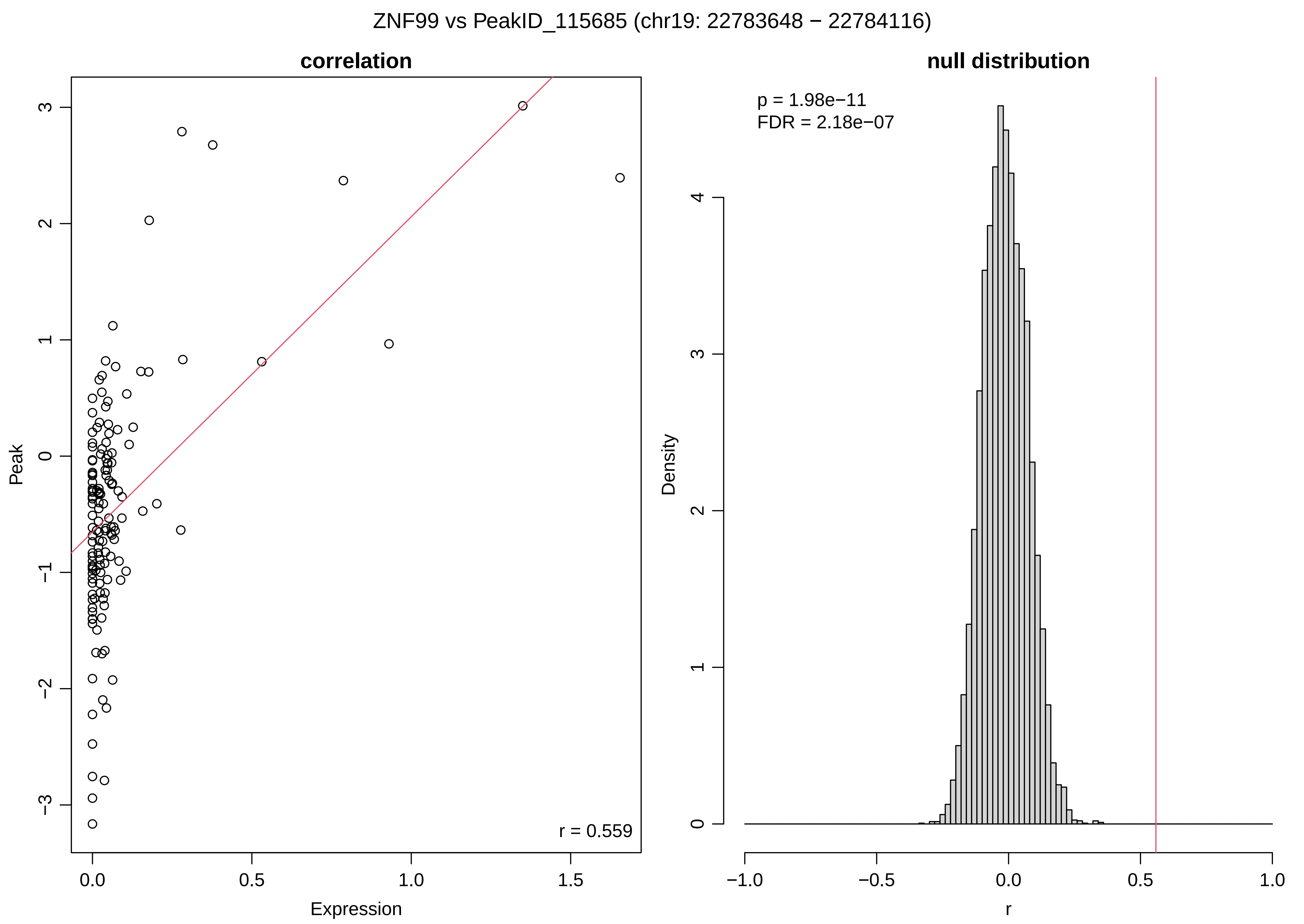
ZNF99 vs PeakID_115685 (chr19: 22783648 − 22784116)
correlation
Peak
Expression
r = 0.559
null distribution
p = 1.98e−11
FDR = 2.18e−07
Density
r

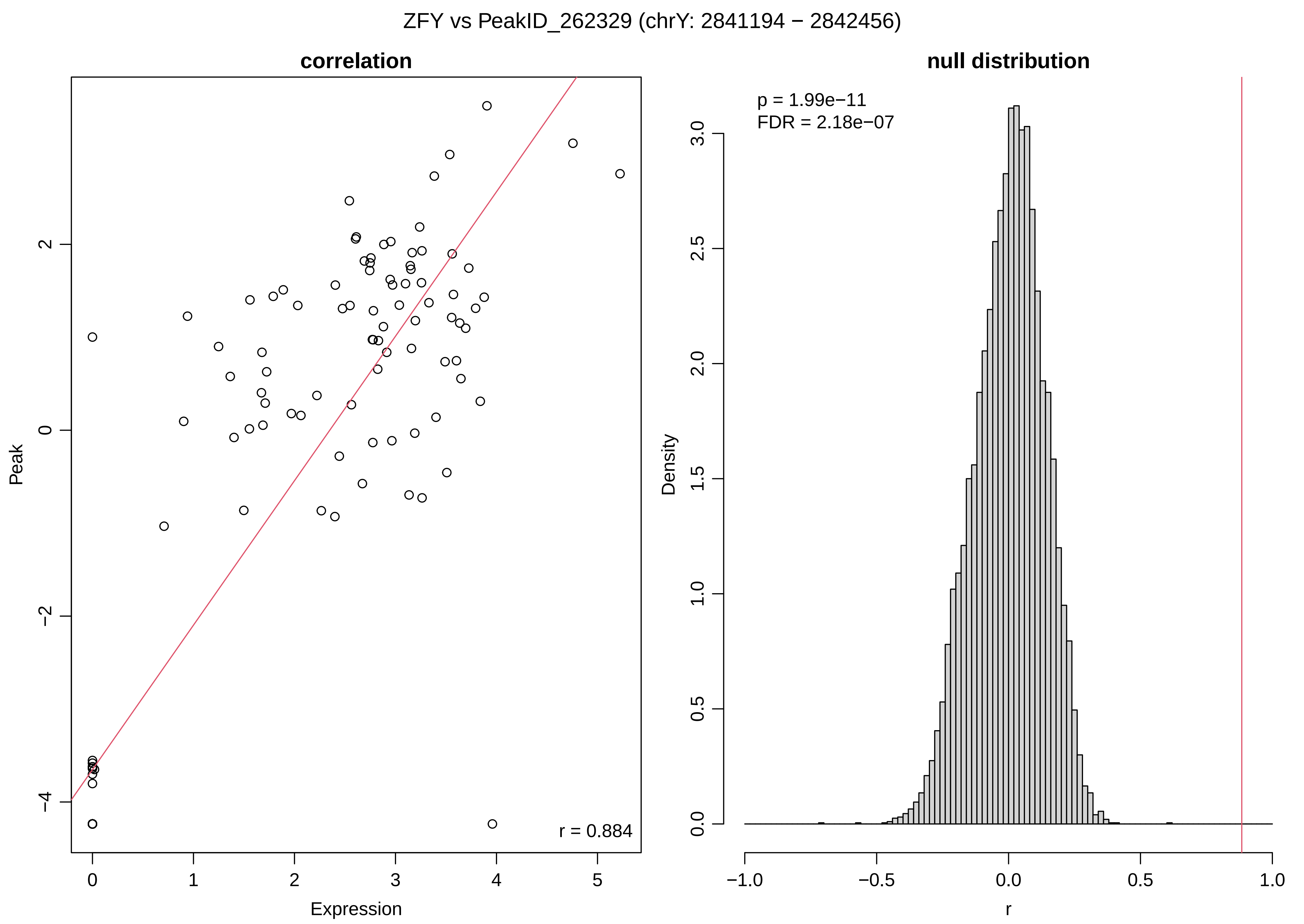
ZFY vs PeakID_262329 (chrY: 2841194 − 2842456)
correlation
Peak
Expression
r = 0.884
null distribution
p = 1.99e−11
FDR = 2.18e−07
Density
r

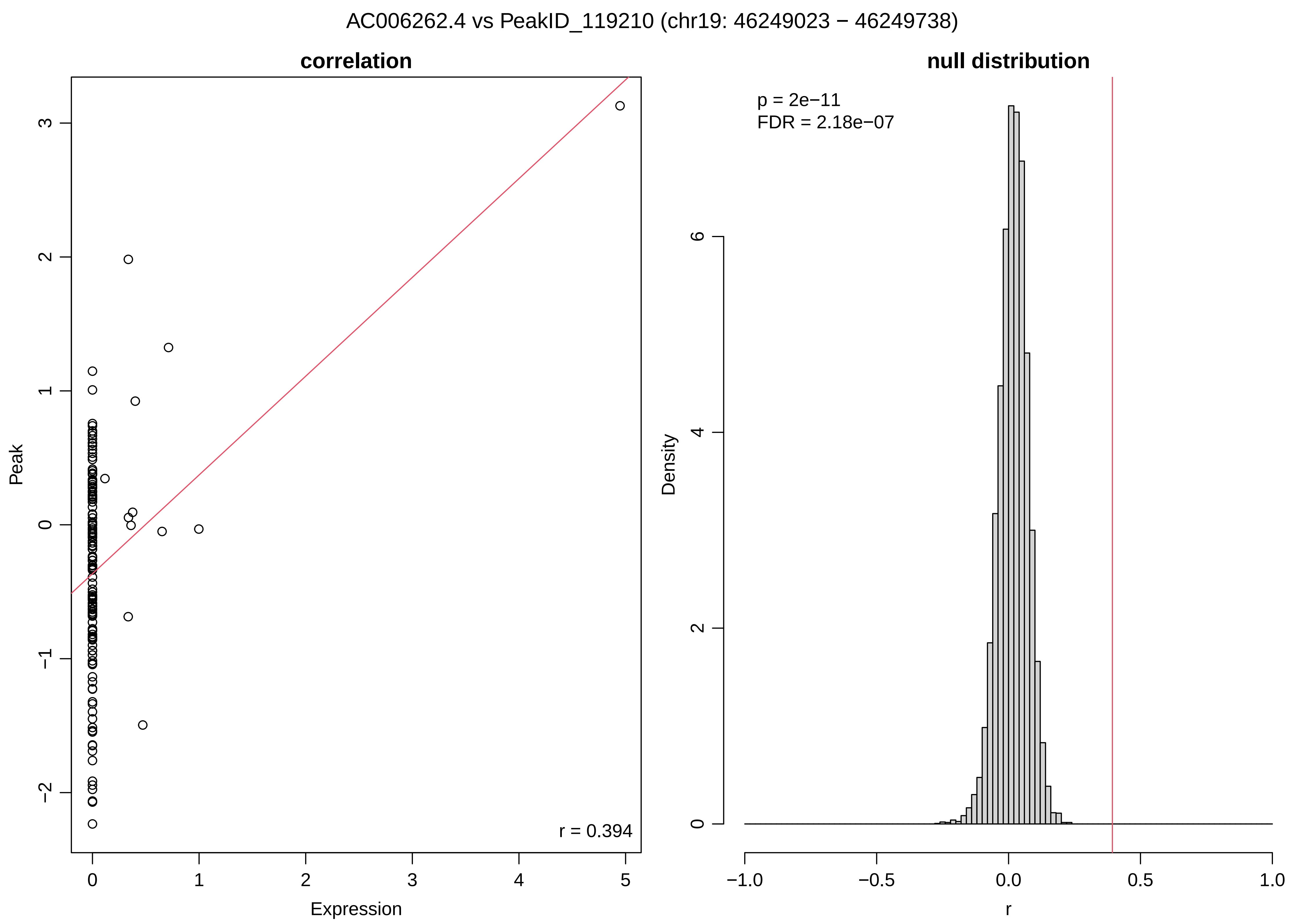

AC006262.4 vs PeakID_119210 (chr19: 46249023 − 46249738)
correlation
Peak
Expression
r = 0.394
null distribution
p = 2e−11
FDR = 2.18e−07
Density
r

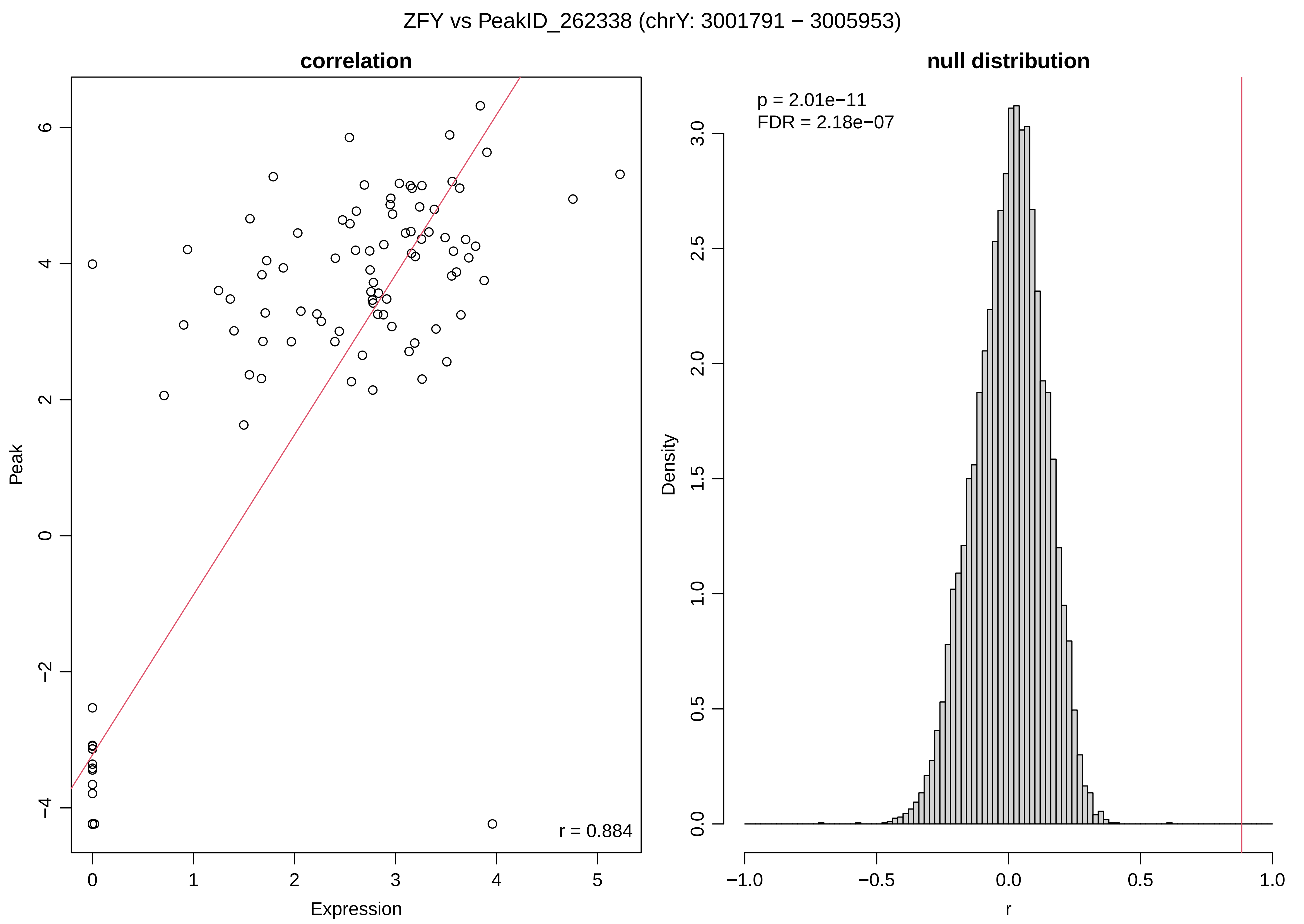
ZFY vs PeakID_262338 (chrY: 3001791 − 3005953)
correlation
Peak
Expression
r = 0.884
0
1
2
3
4
5
−4
−2
0
2
4
6
null distribution
p = 2.01e−11
FDR = 2.18e−07
Density
r
−1.0
−0.5
0.0
0.5
1.0
0.0
0.5
1.0
1.5
2.0
2.5
3.0

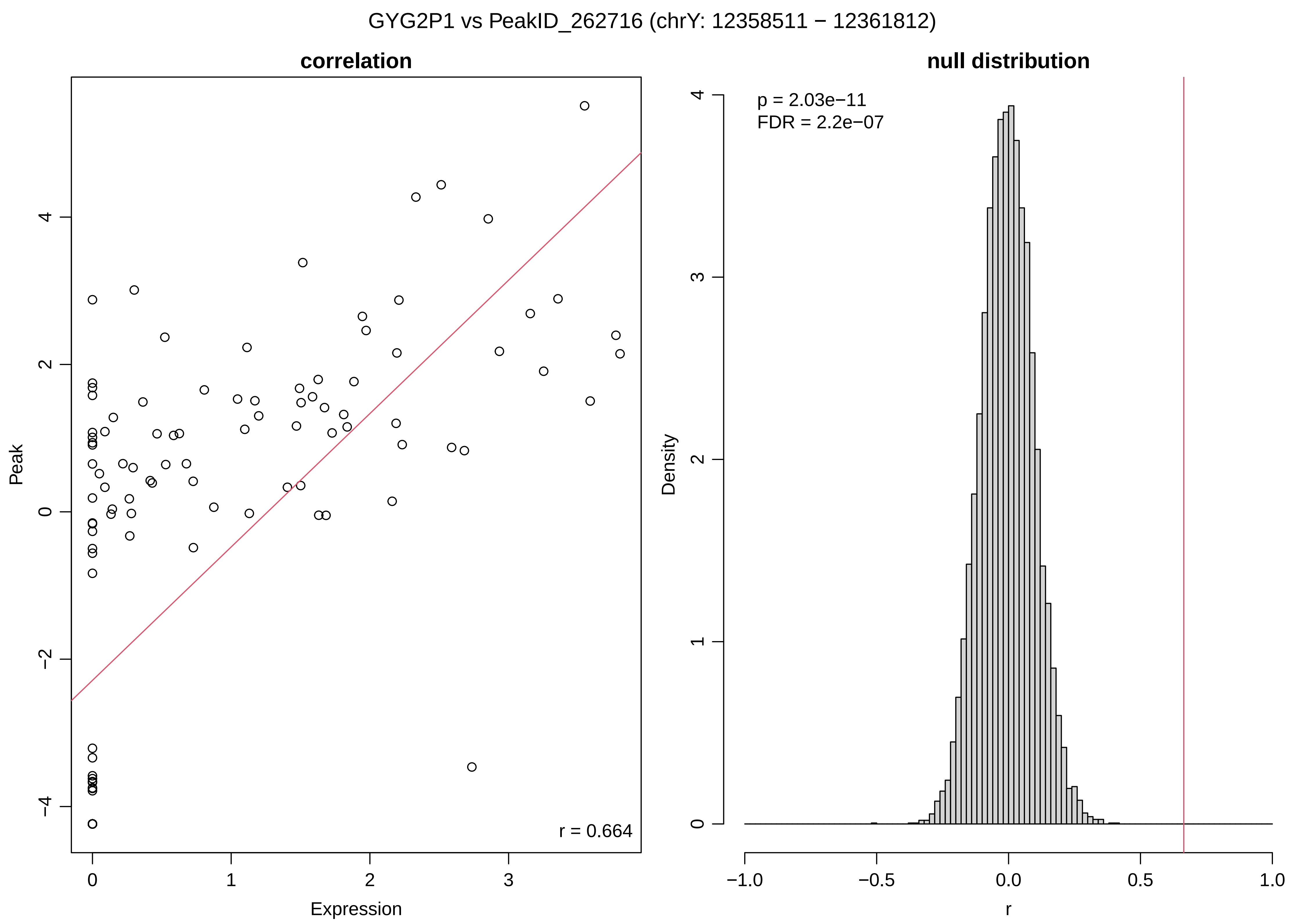

GYG2P1 vs PeakID_262716 (chrY: 12358511 − 12361812)
correlation
Peak
Expression
r = 0.664
null distribution
p = 2.03e−11
FDR = 2.2e−07
Density
r

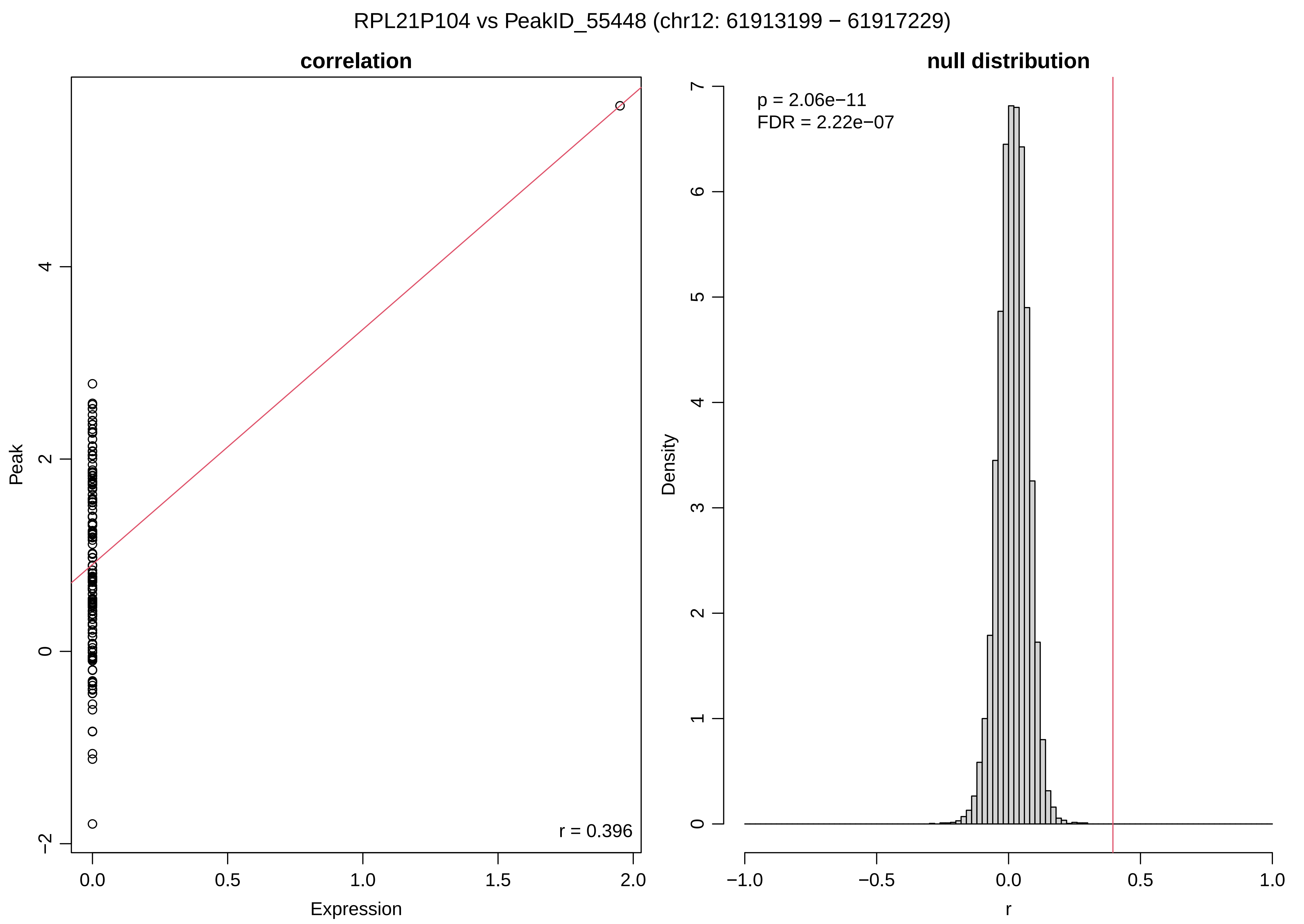
RPL21P104 vs PeakID_55448 (chr12: 61913199 − 61917229)
correlation
null distribution
Peak
Expression
r = 0.396
p = 2.06e−11
FDR = 2.22e−07
Density
r

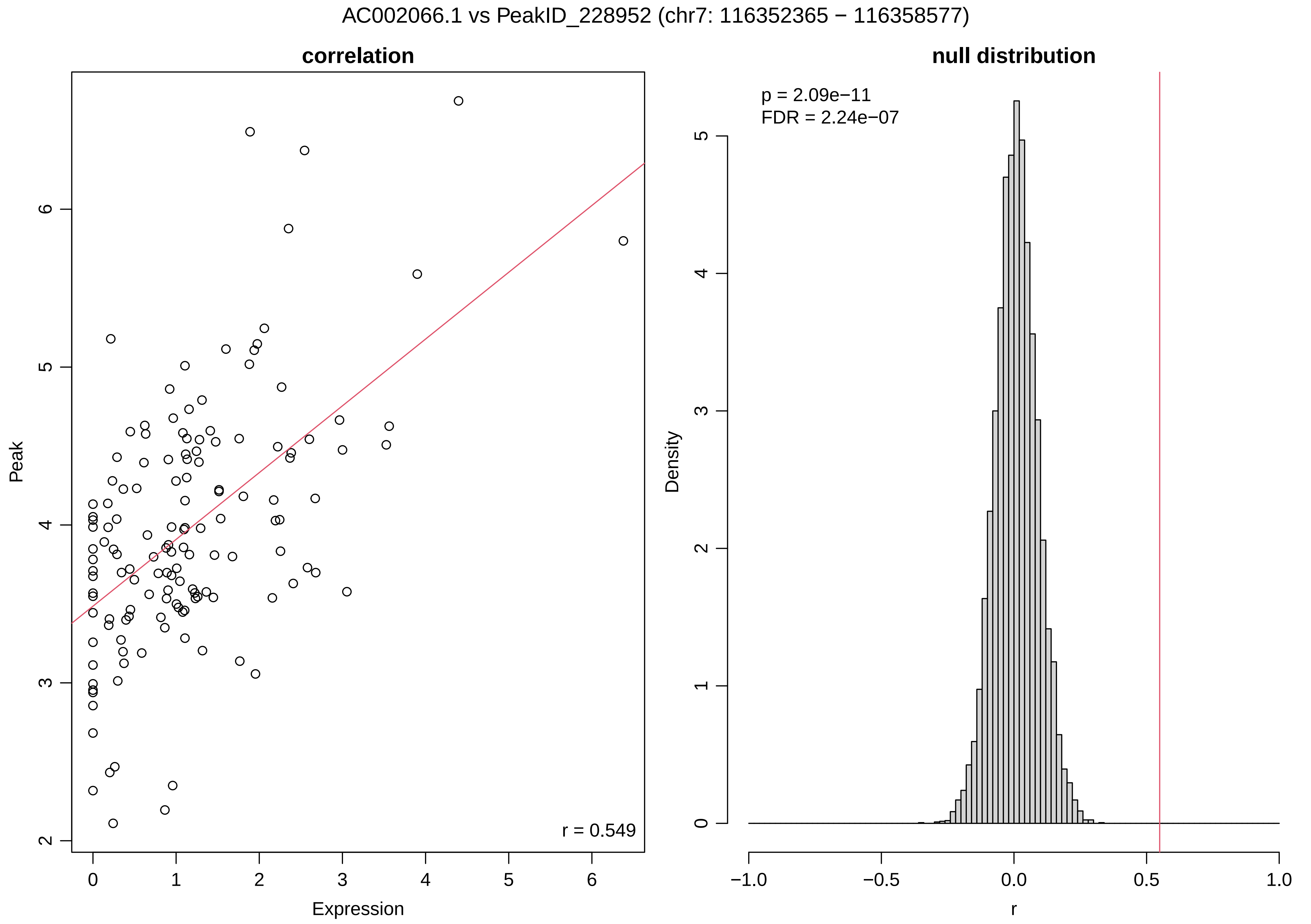
AC002066.1 vs PeakID_228952 (chr7: 116352365 − 116358577)
correlation
Peak
Expression
r = 0.549
null distribution
p = 2.09e−11
FDR = 2.24e−07
Density
r

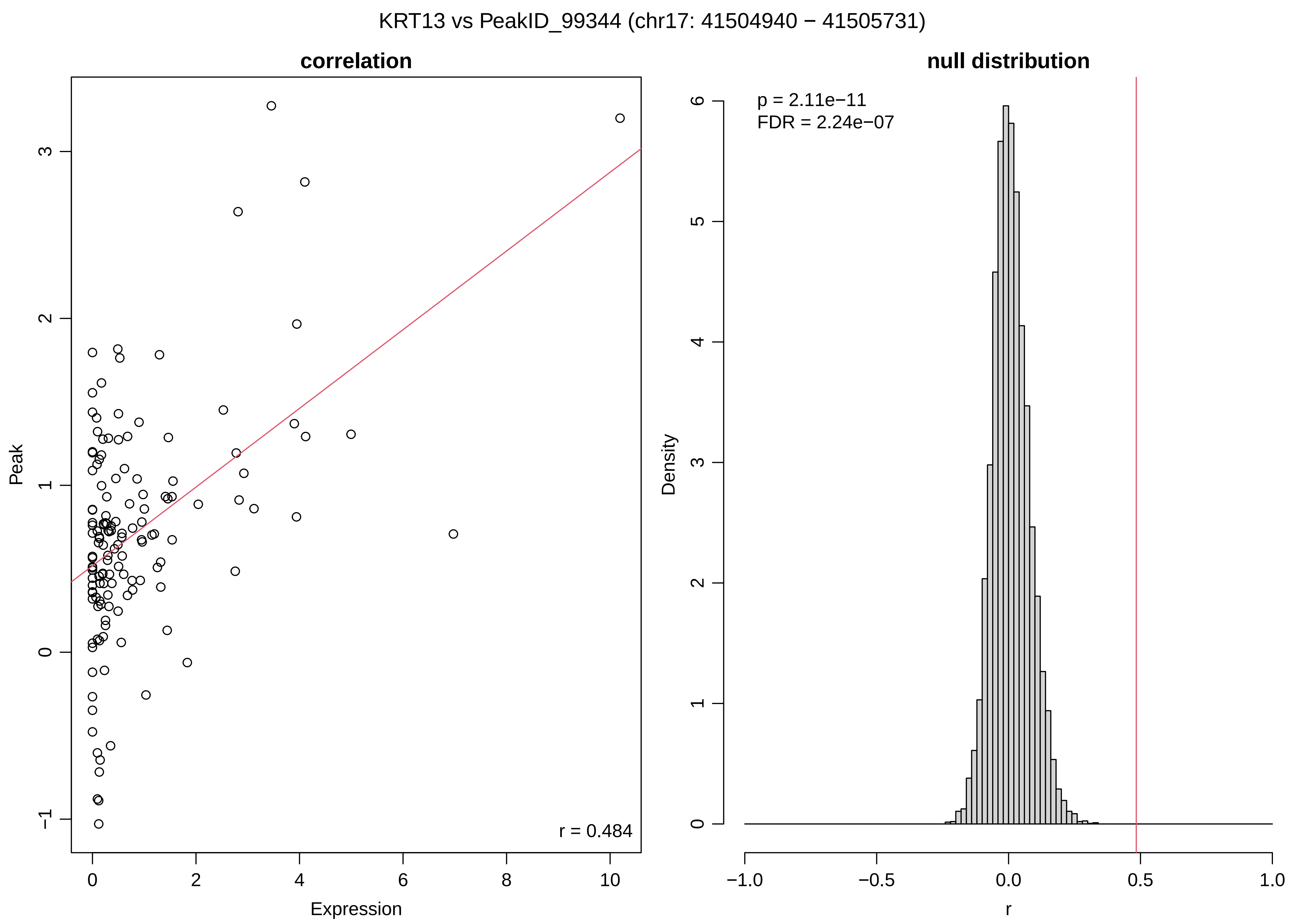
KRT13 vs PeakID_99344 (chr17: 41504940 − 41505731)
correlation
Peak
Expression
r = 0.484
null distribution
p = 2.11e−11
FDR = 2.24e−07
Density
r

AC026784.1 vs PeakID_188769 (chr5: 23951040 − 23952588)

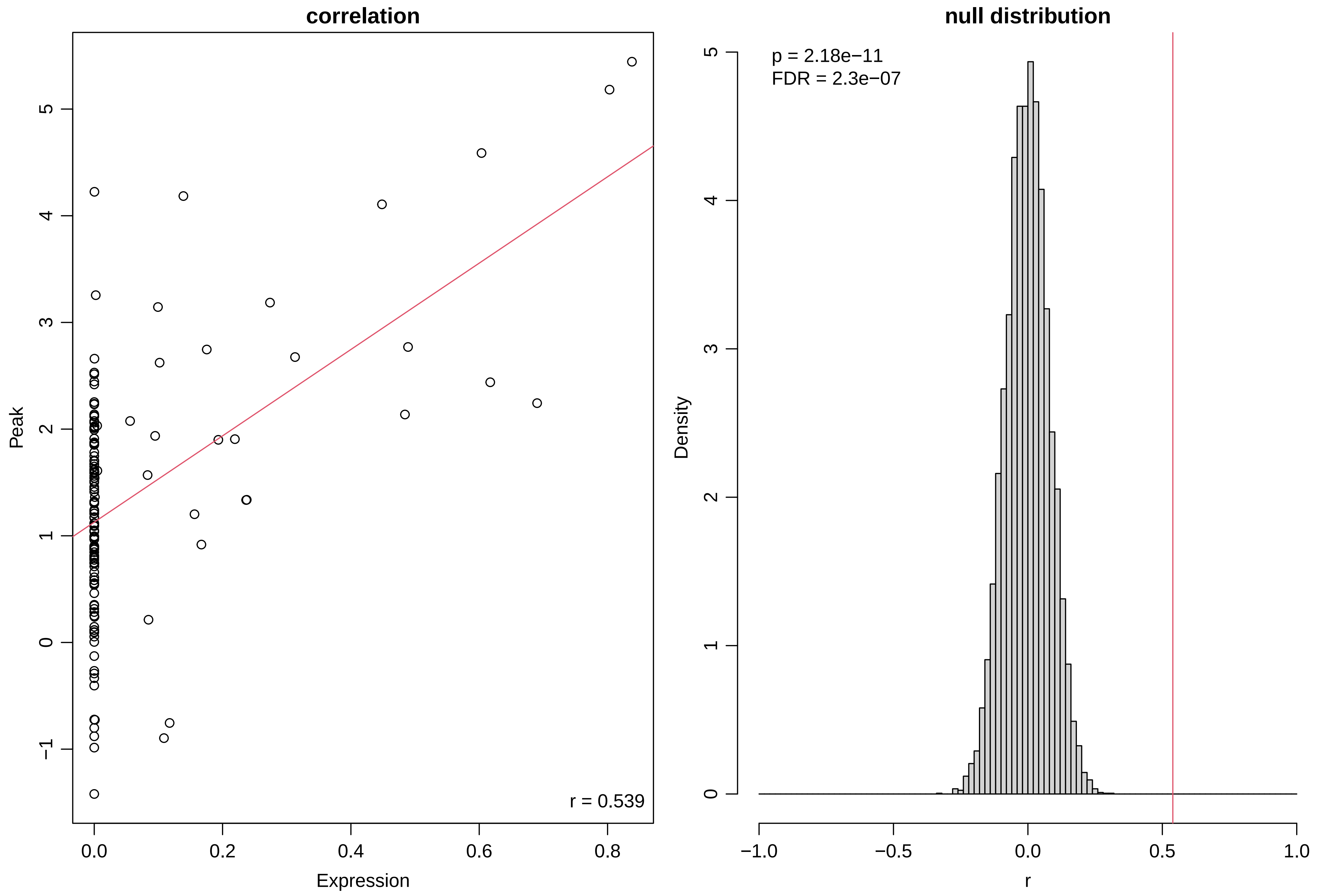

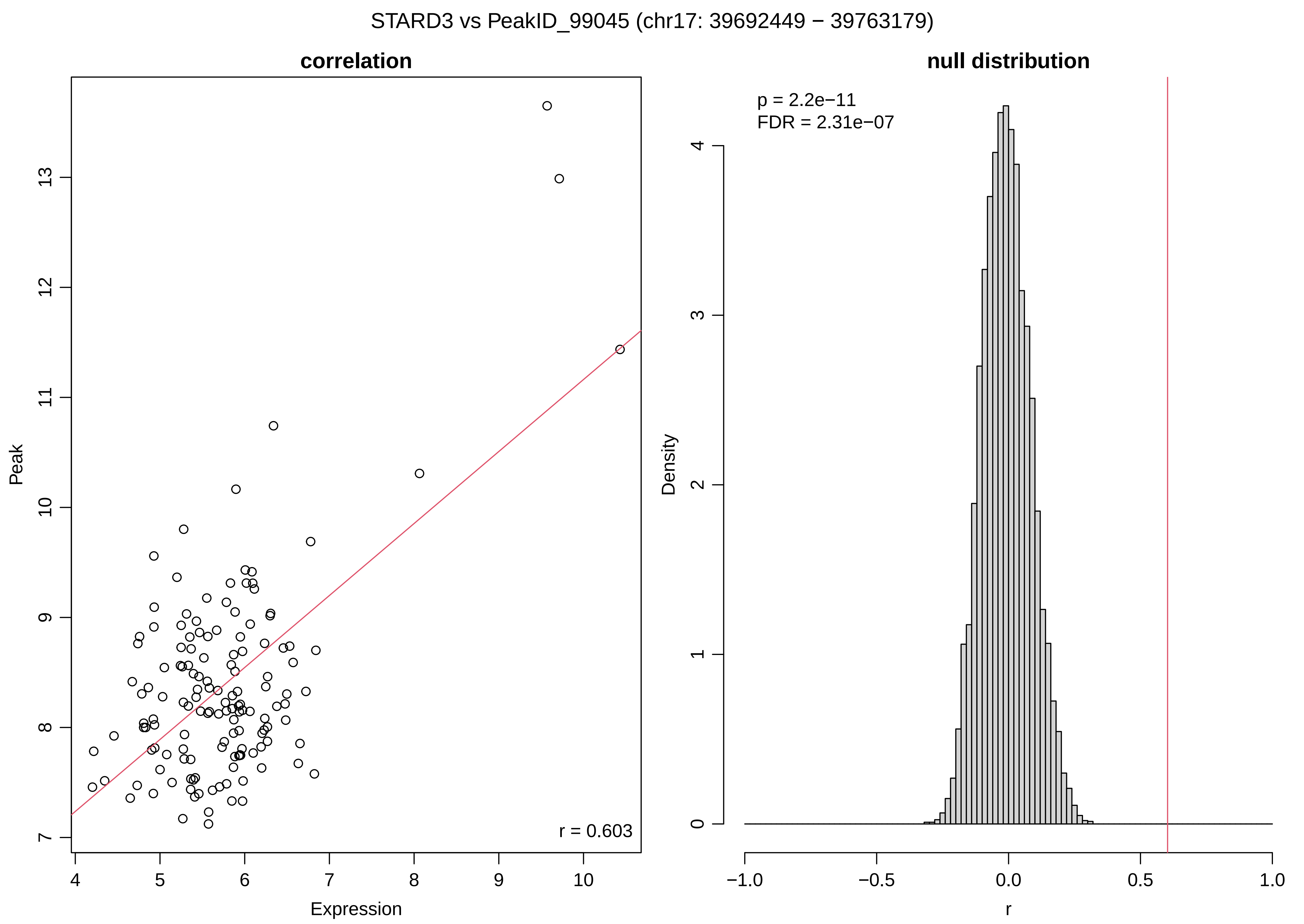

STARD3 vs PeakID_99045 (chr17: 39692449 − 39763179)
correlation
Peak
Expression
r = 0.603
13
12
11
10
9
8
7
4
5
6
7
8
9
10
null distribution
p = 2.2e−11
FDR = 2.31e−07
Density
r
4
3
2
1
0
−1.0
−0.5
0.0
0.5
1.0

ANOS2P vs PeakID_262759 (chrY: 13775925 − 13776735)

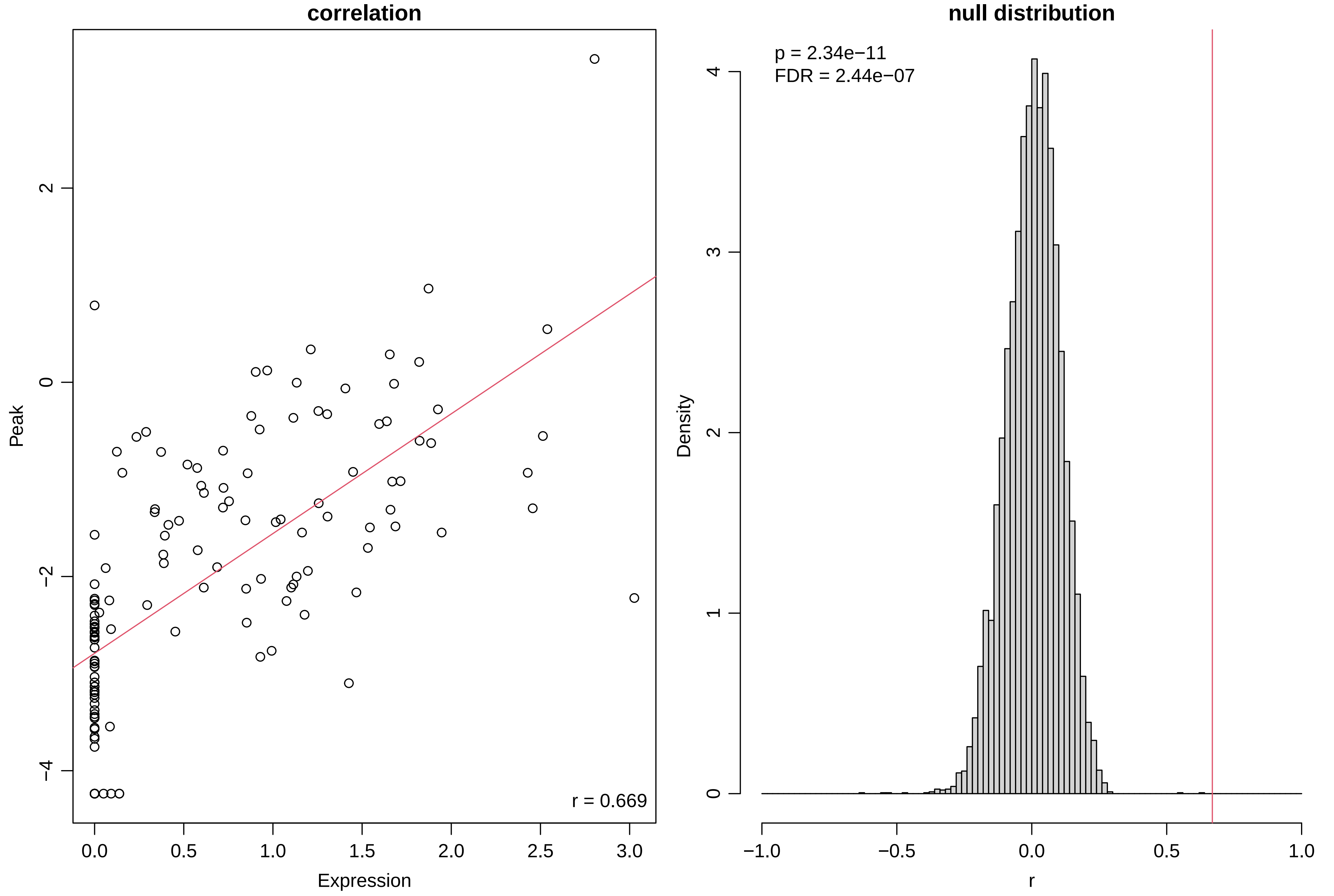

ZNF98 vs PeakID_115669 (chr19: 22421848 − 22422335)

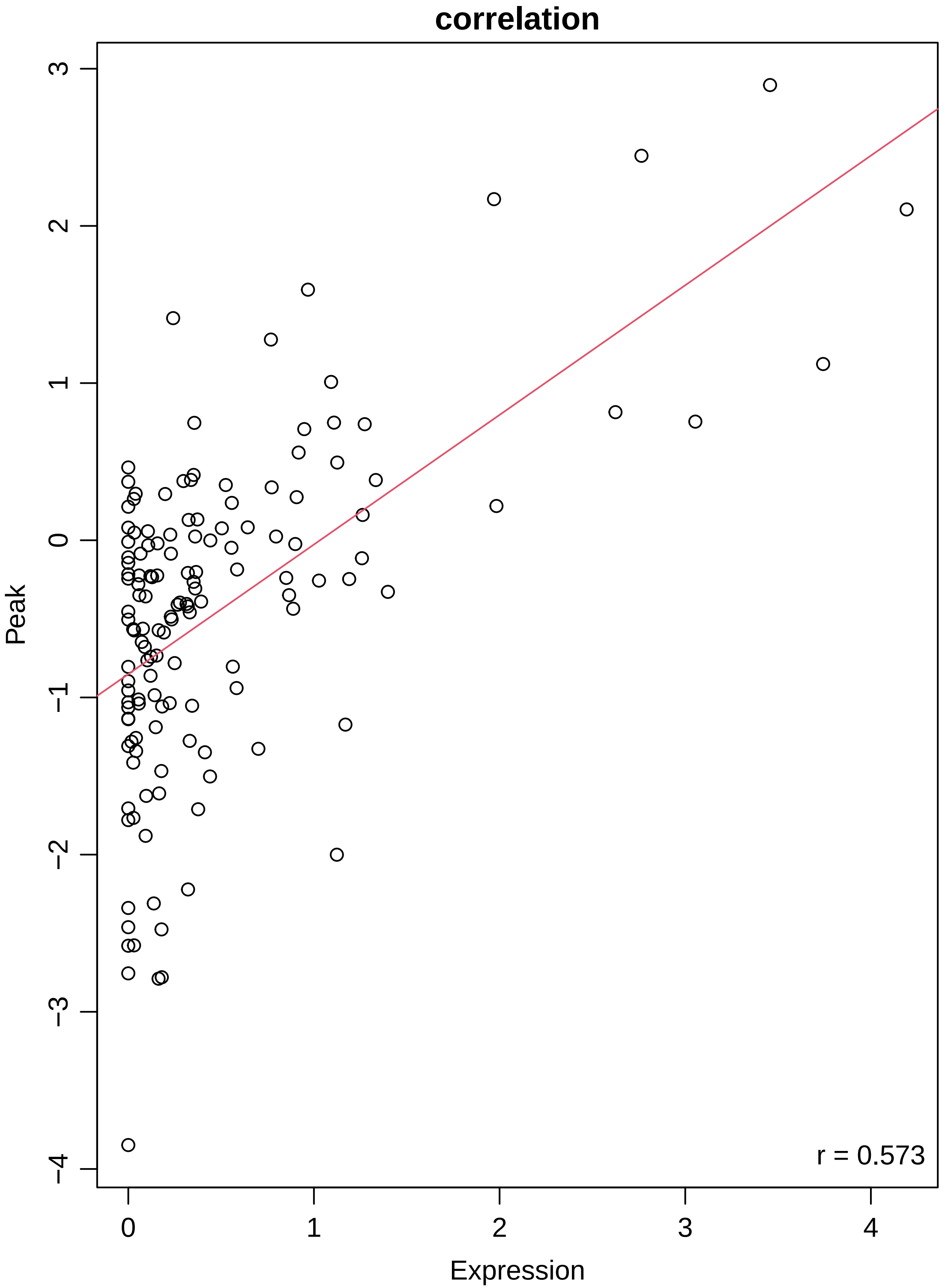

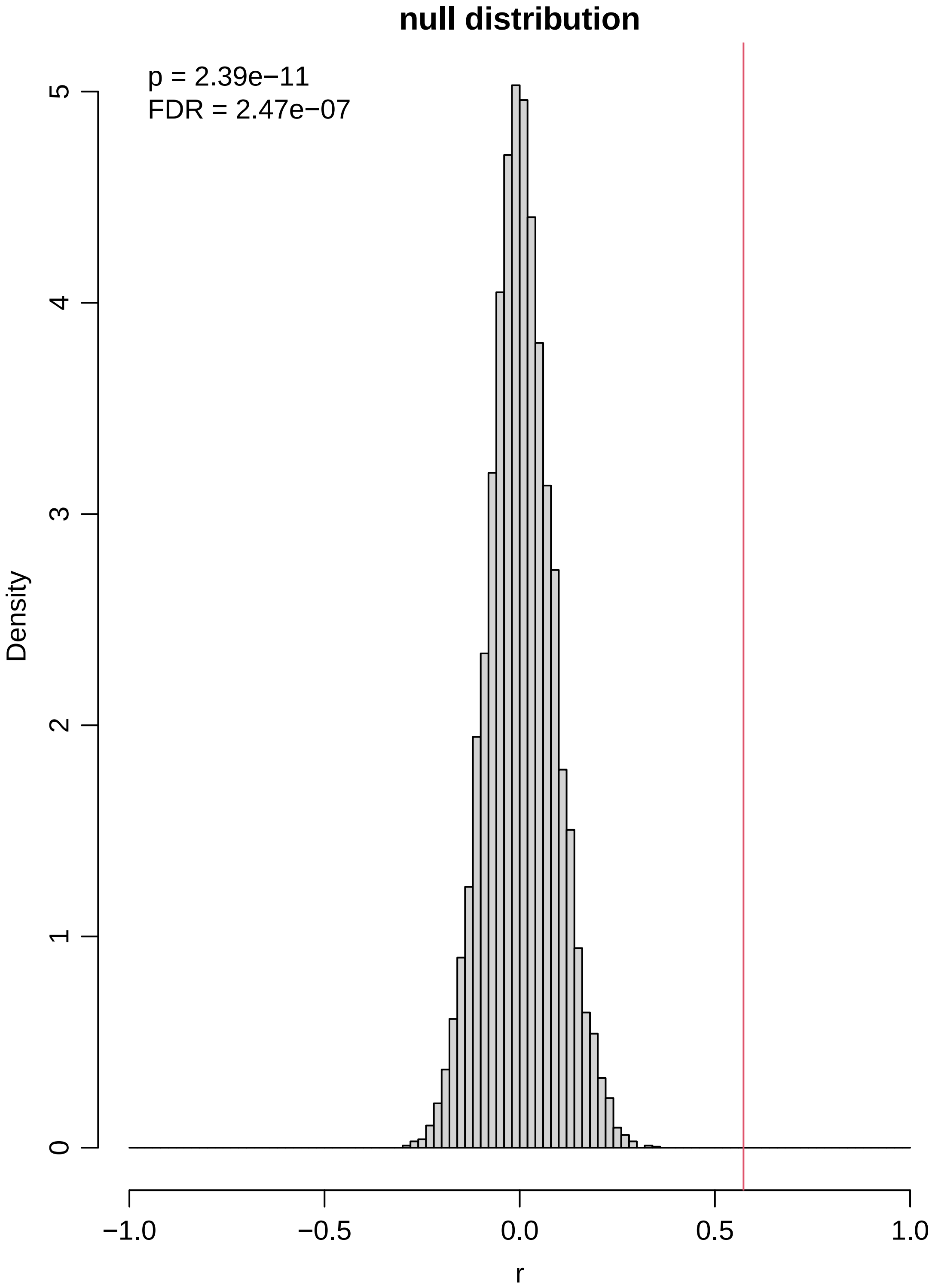

CCT2 vs PeakID_56923 (chr12: 69652828 − 69659651)

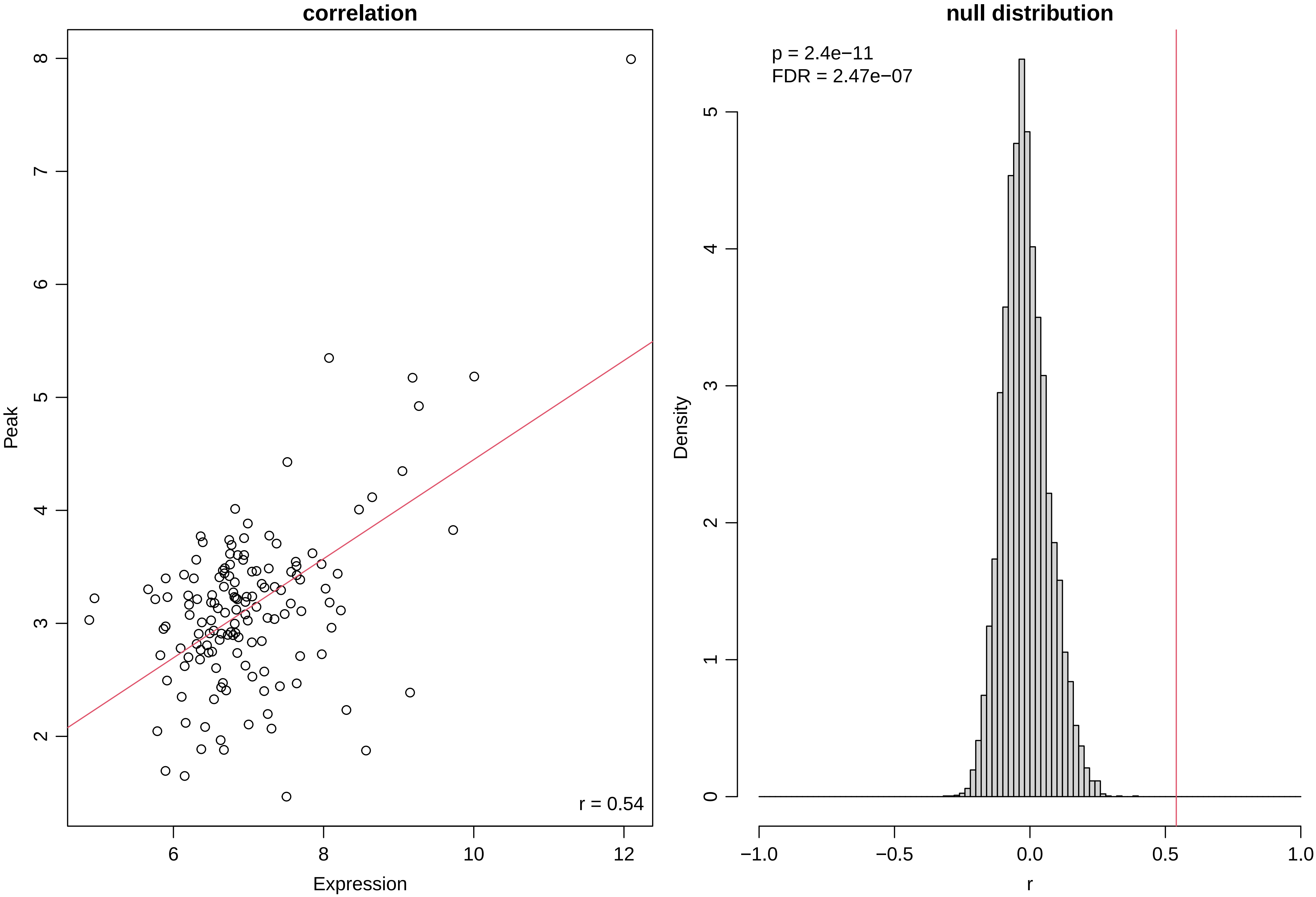

RPL7AP28 vs PeakID_183251 (chr4: 131974949 − 131976849)

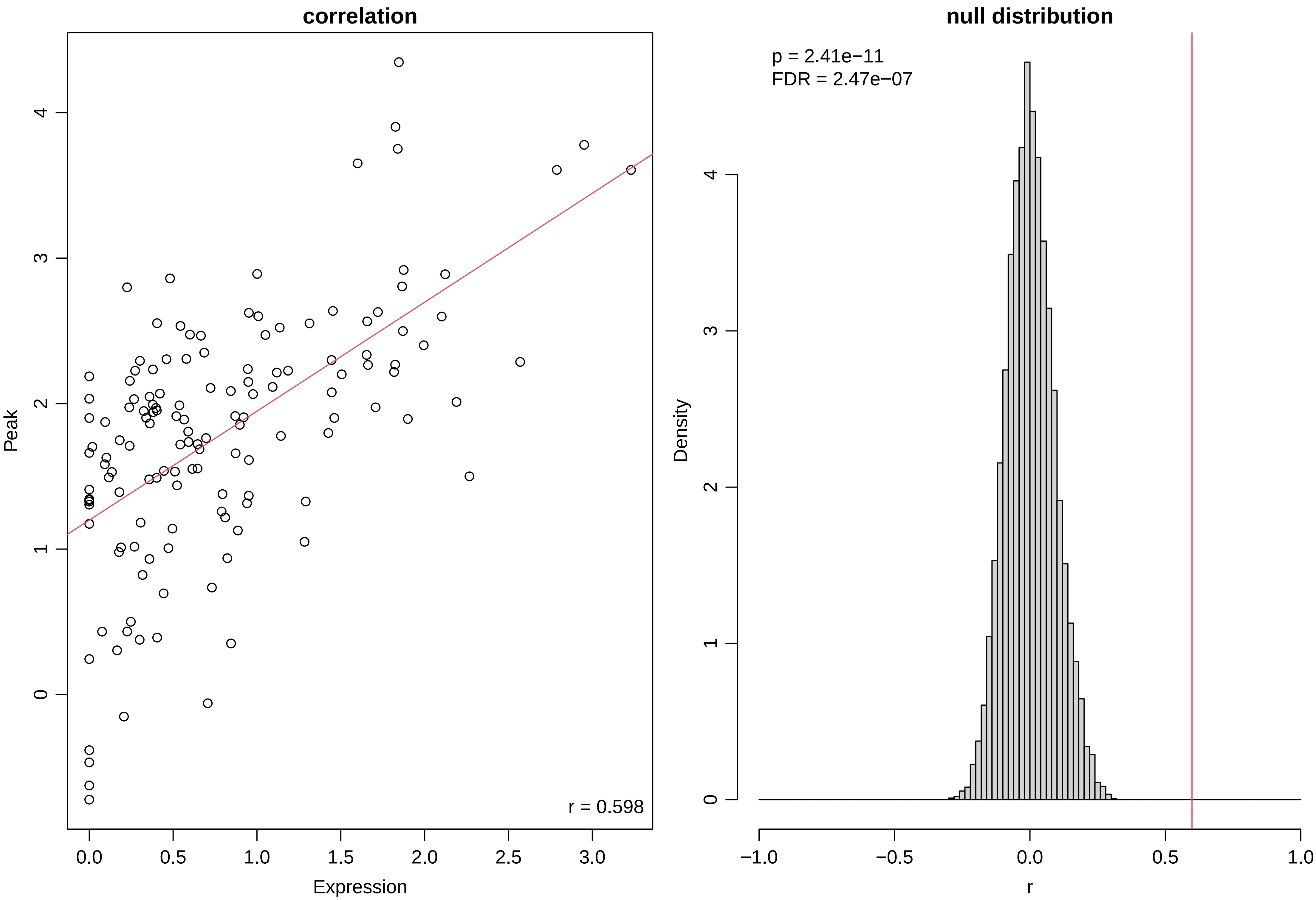

AC104383.2 vs PeakID_37617 (chr11: 11810675 − 11812278)

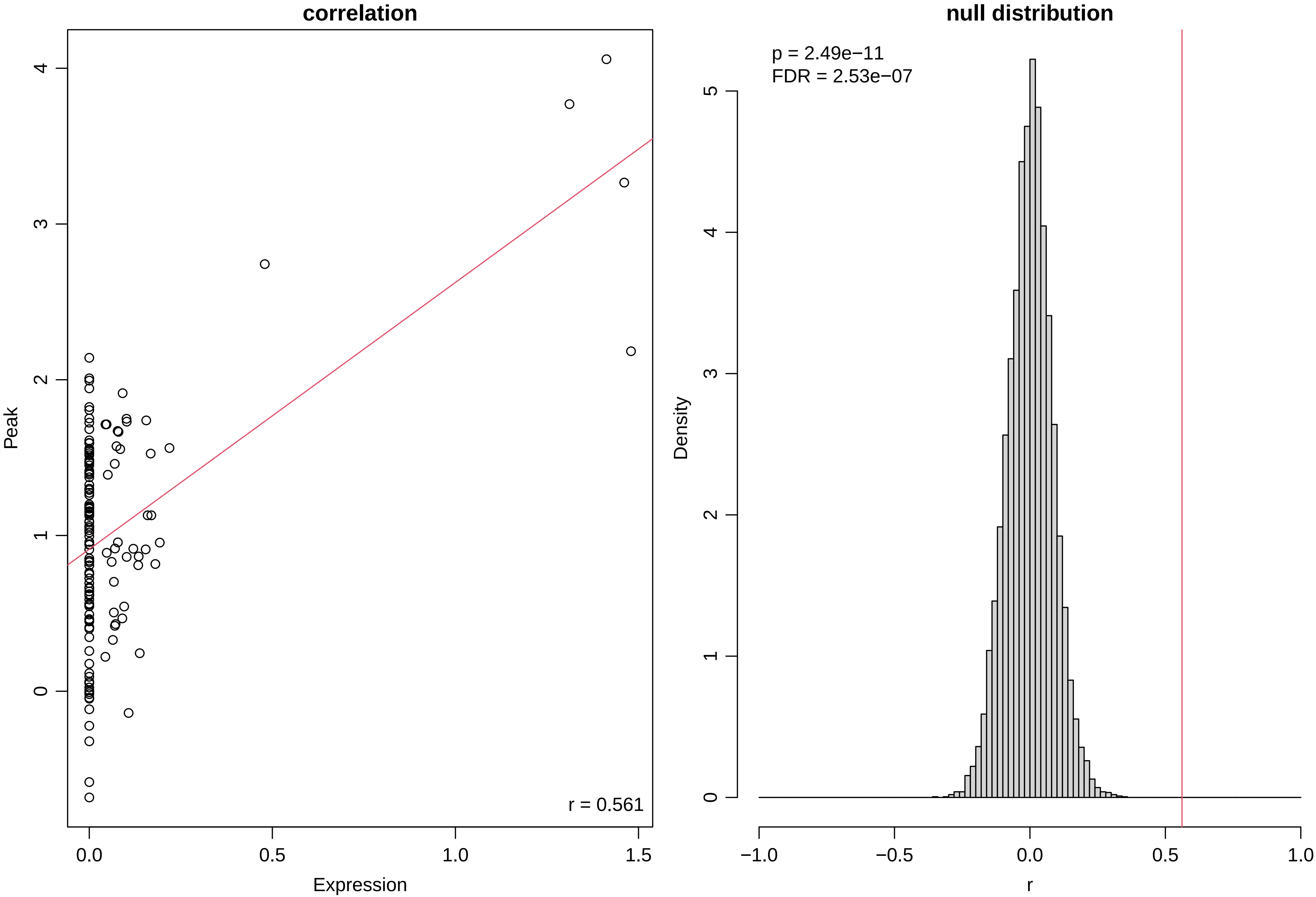

RPL21P104 vs PeakID_55424 (chr12: 61857508 − 61858399)

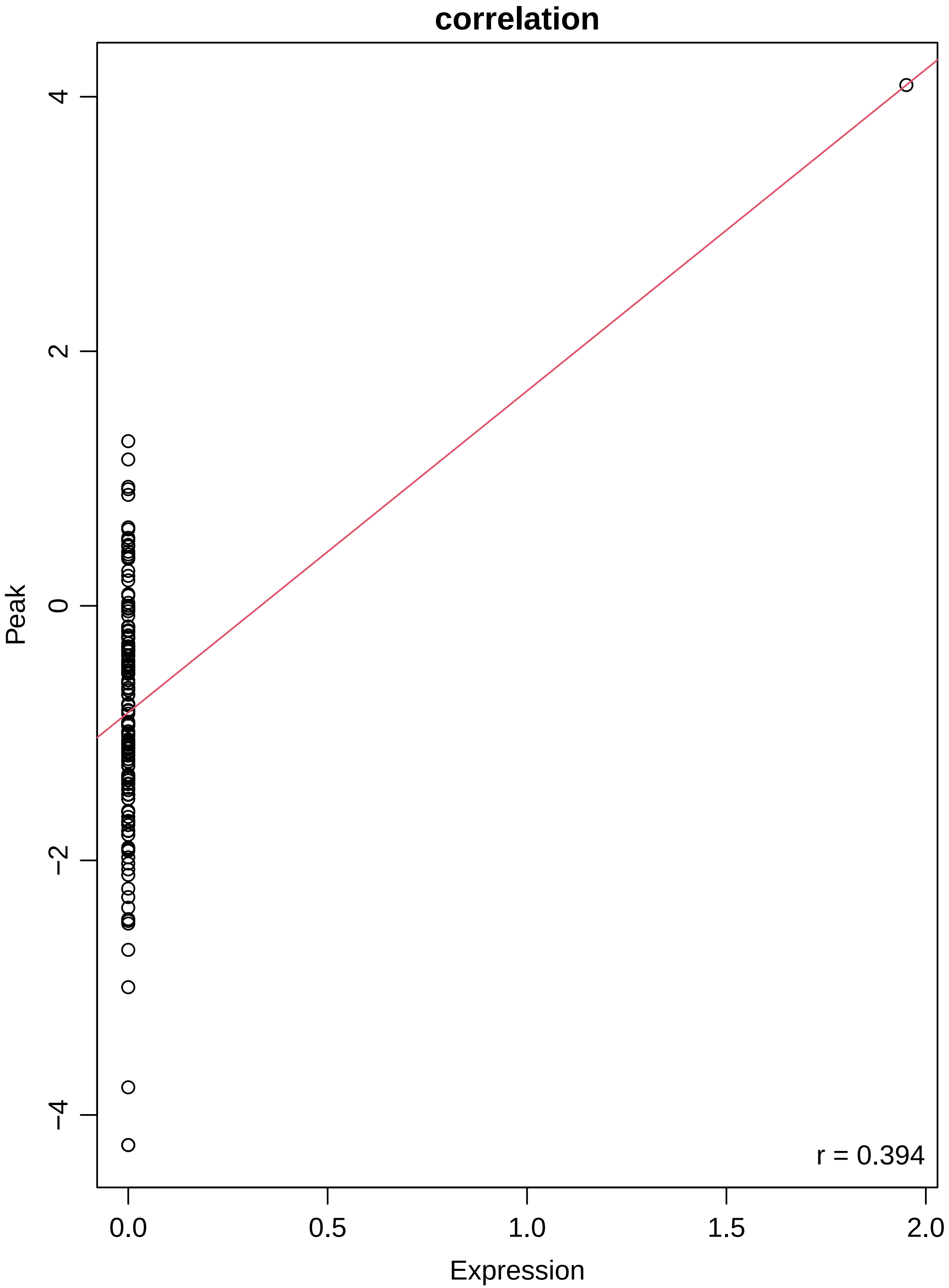

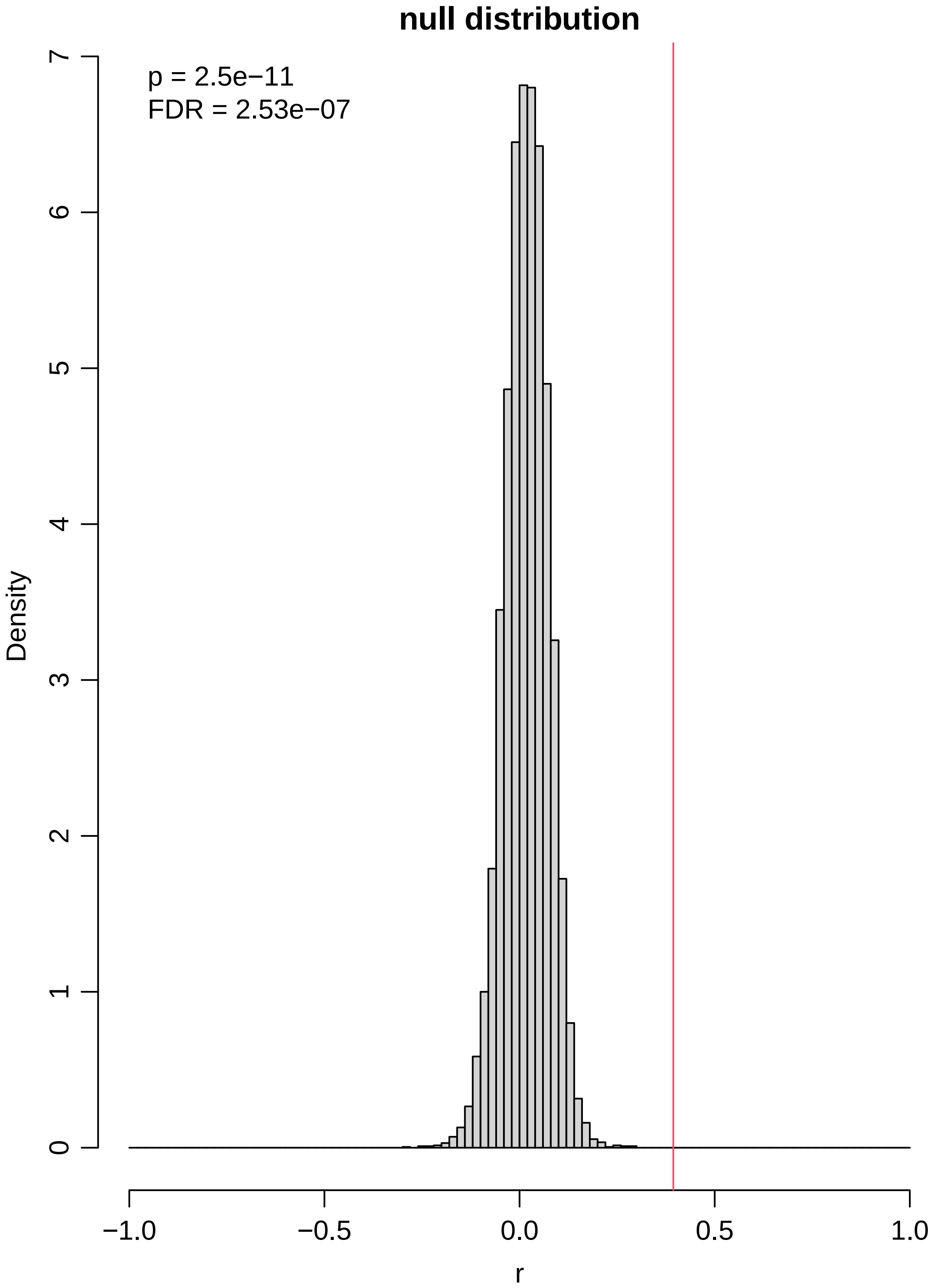

EGFR−AS1 vs PeakID_224249 (chr7: 55204101 − 55218995)

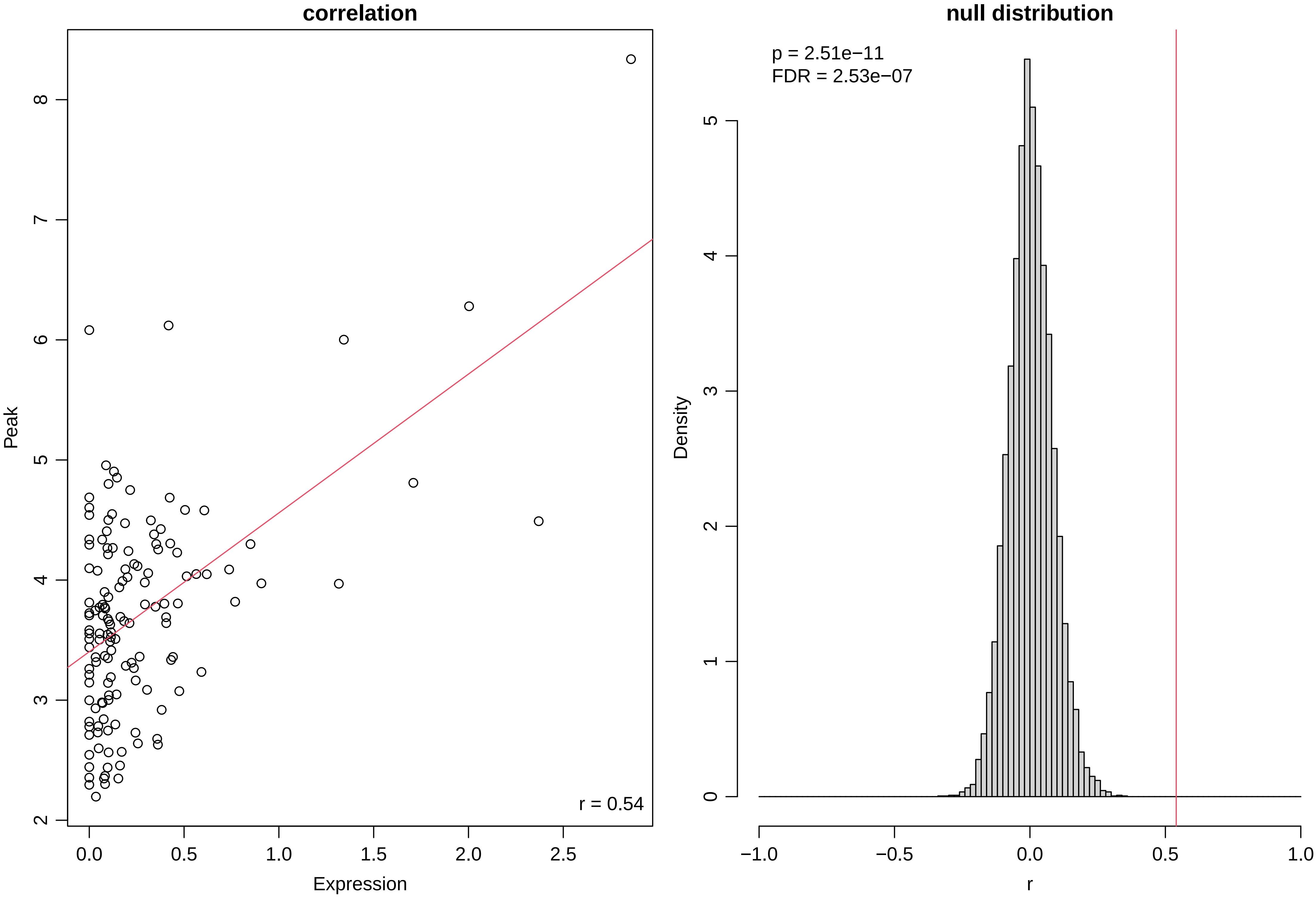

AC025811.1 vs PeakID_115672 (chr19: 22427123 − 22428839)

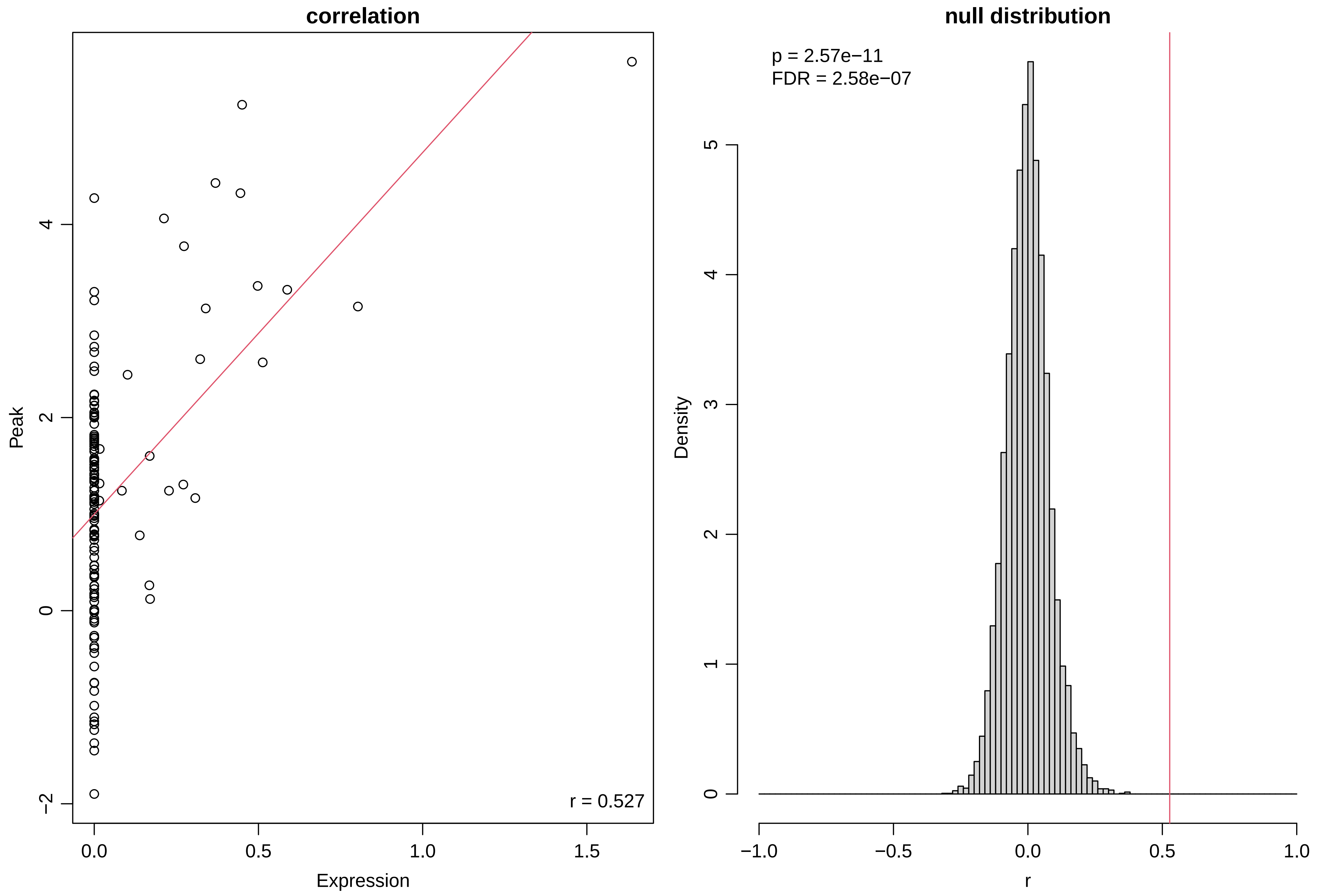

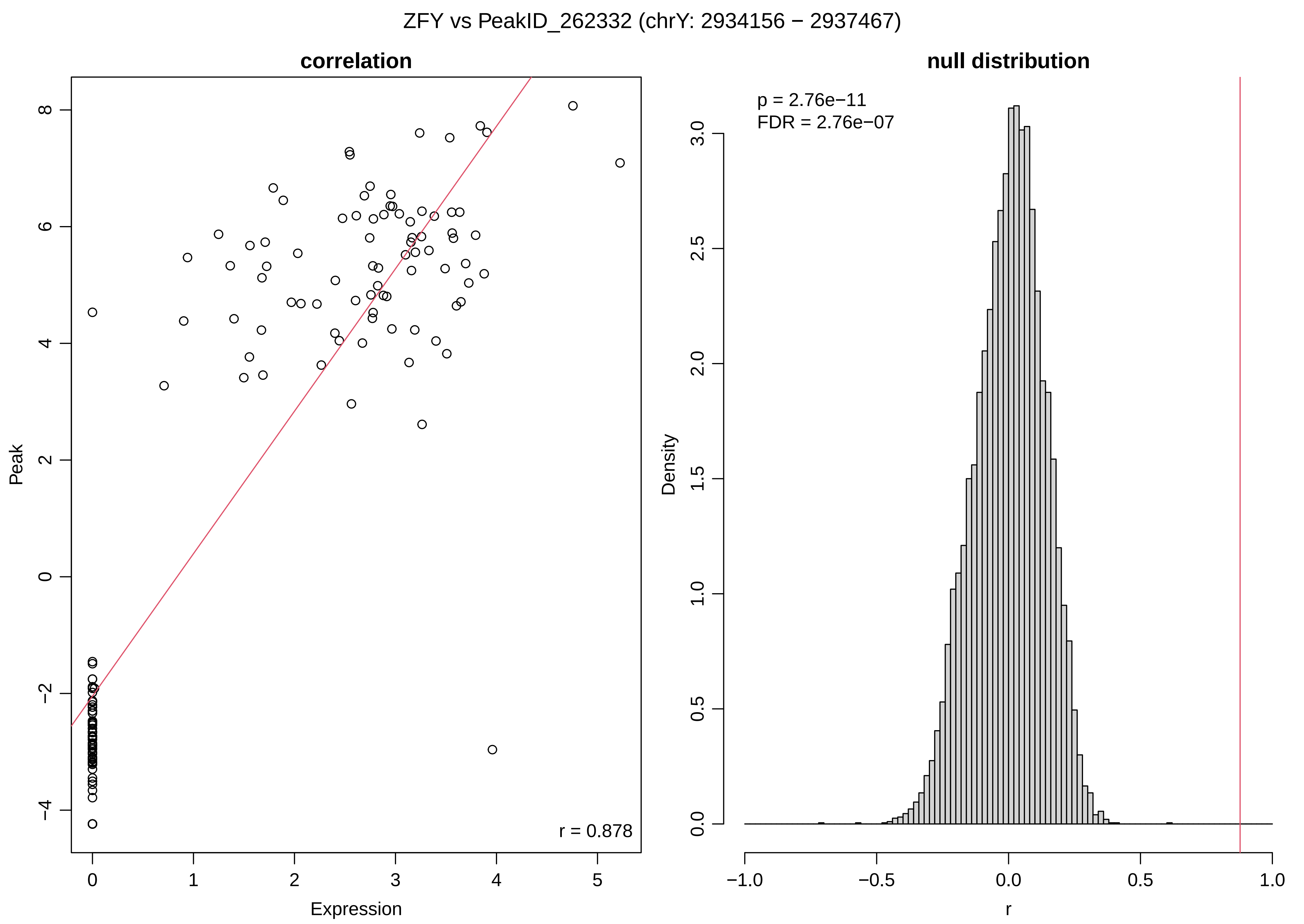
ZFY vs PeakID_262332 (chrY: 2934156 − 2937467)
correlation
Peak
Expression
r = 0.878
null distribution
p = 2.76e−11
FDR = 2.76e−07
Density
r

AC090061.1 vs PeakID_56596 (chr12: 68845709 − 68846772)

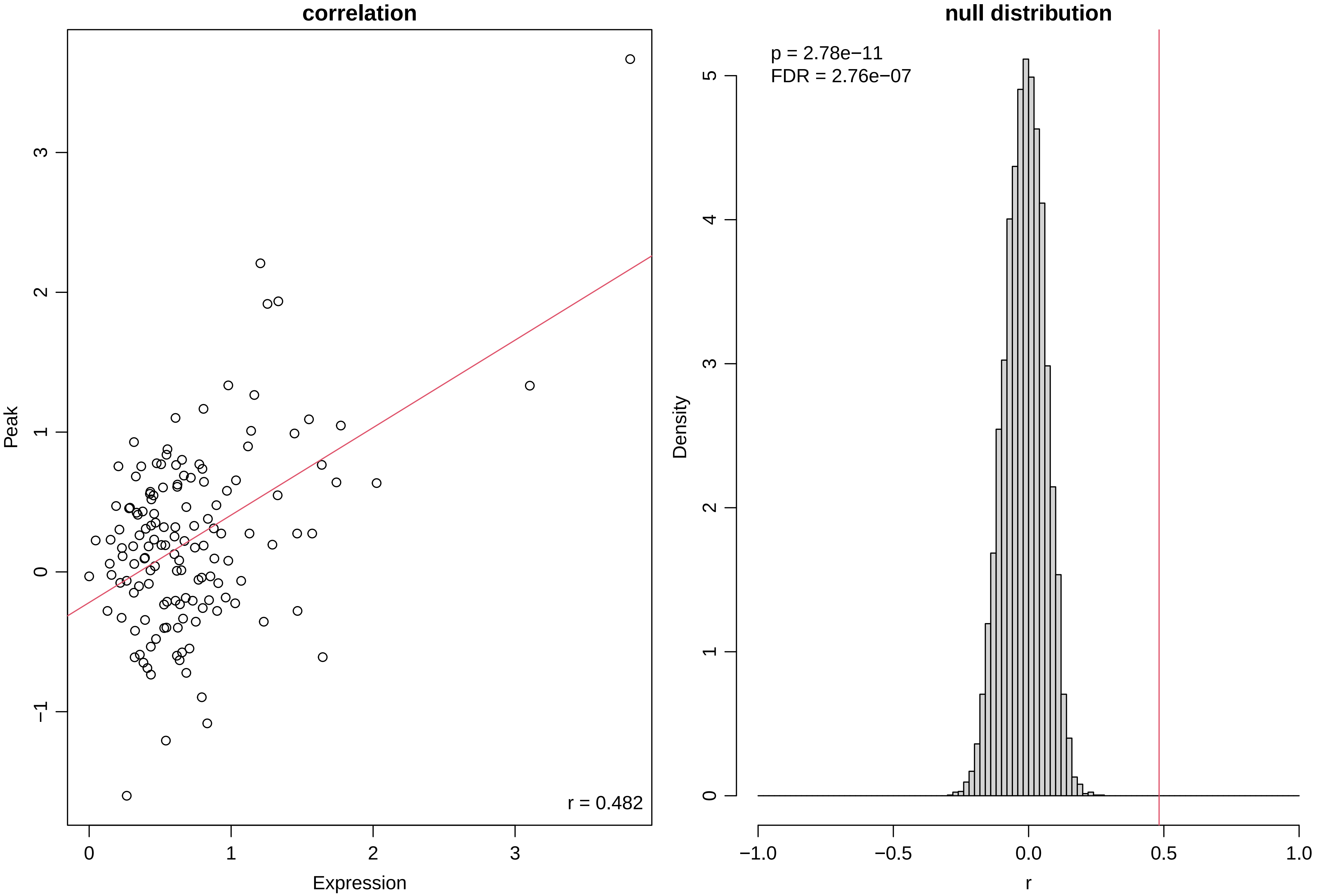

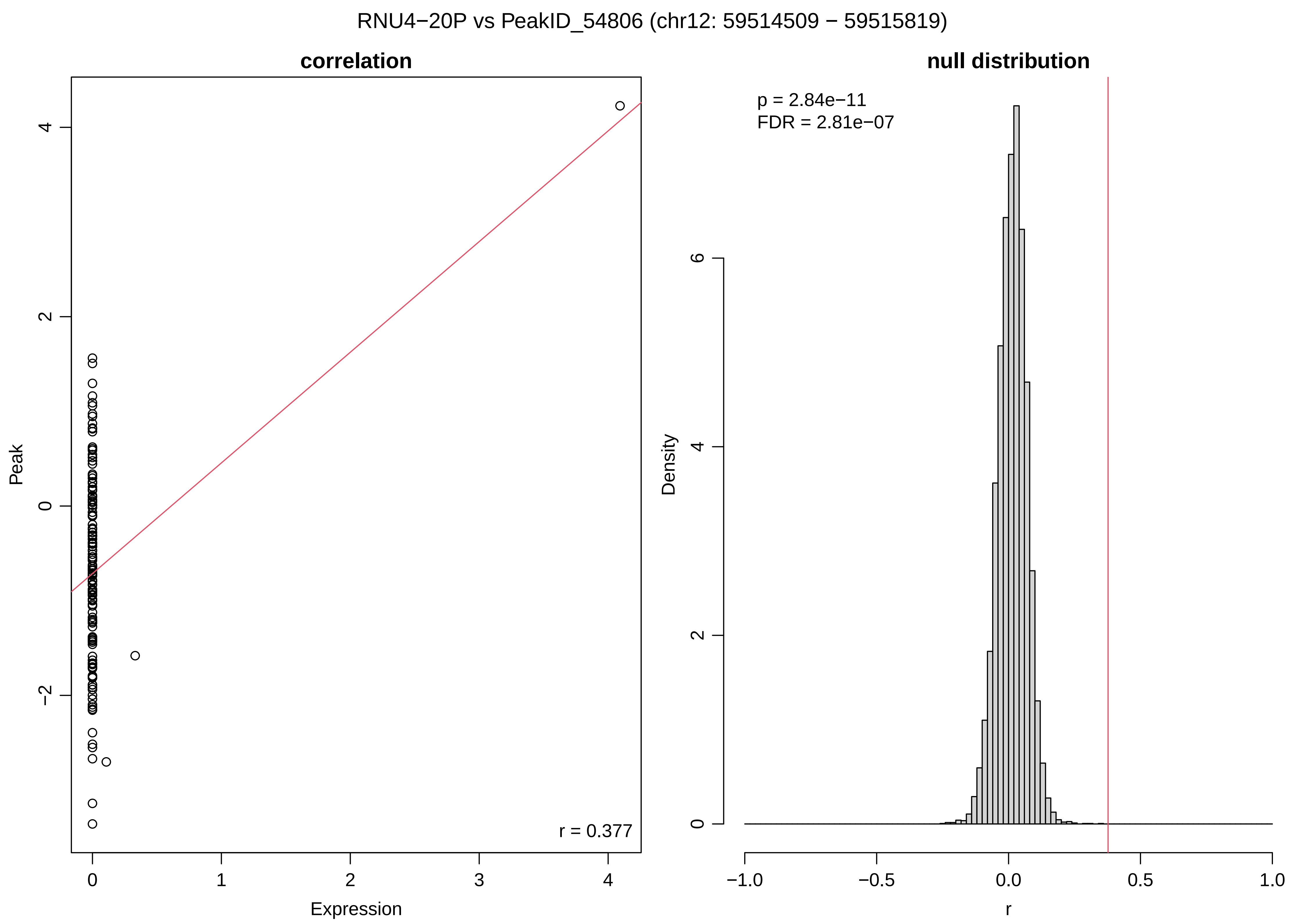
RNU4−20P vs PeakID_54806 (chr12: 59514509 − 59515819)
correlation
Peak
Expression
r = 0.377
4
2
0
−2
0
1
2
3
4
null distribution
p = 2.84e−11
FDR = 2.81e−07
Density
r
6
4
2
0
−1.0
−0.5
0.0
0.5
1.0

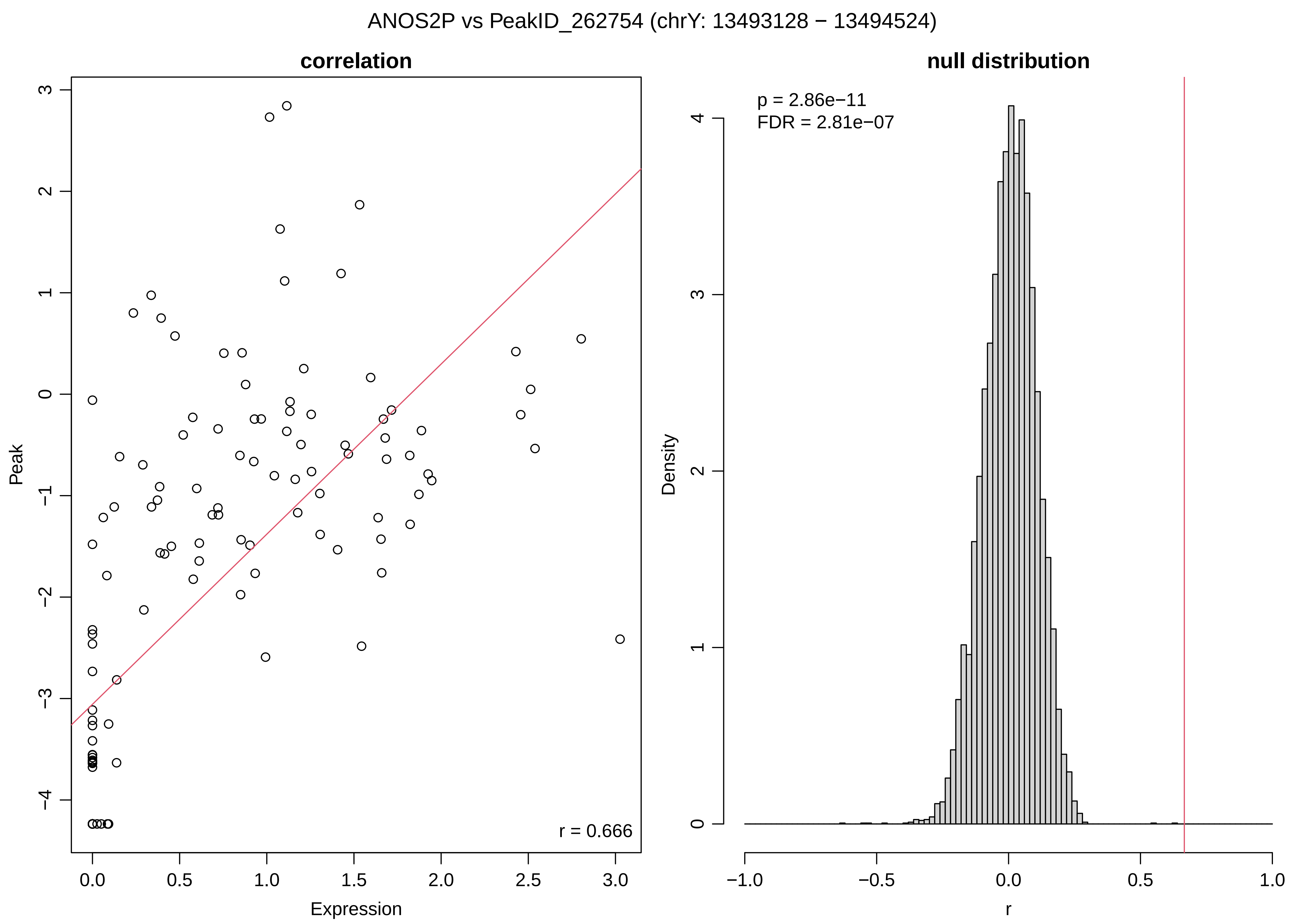

ANOS2P vs PeakID_262754 (chrY: 13493128 − 13494524)
correlation
Peak
Expression
r = 0.666
null distribution
p = 2.86e−11
FDR = 2.81e−07
Density
r

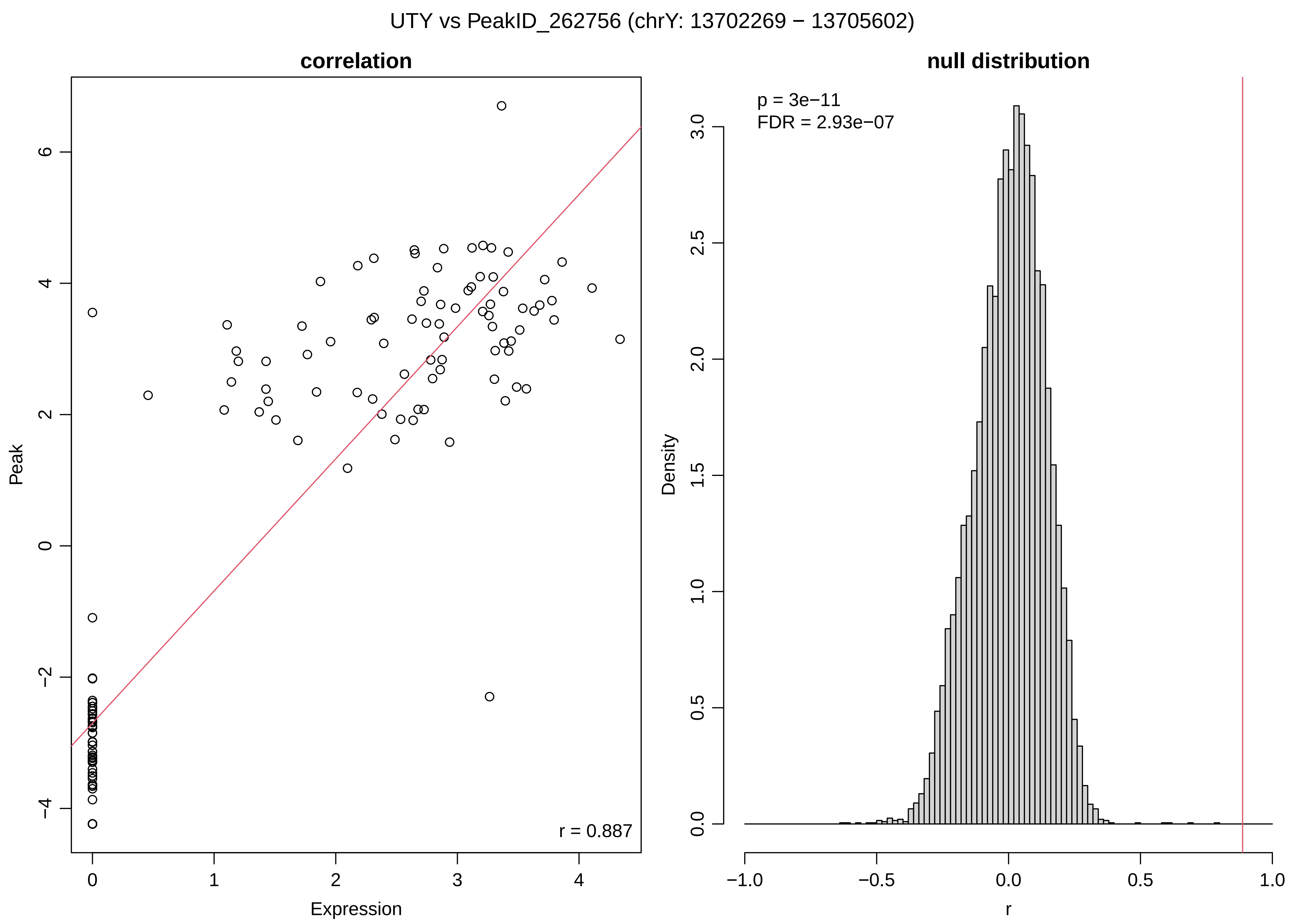
UTY vs PeakID_262756 (chrY: 13702269 − 13705602)
correlation
Peak
Expression
r = 0.887
null distribution
p = 3e−11
FDR = 2.93e−07
Density
r

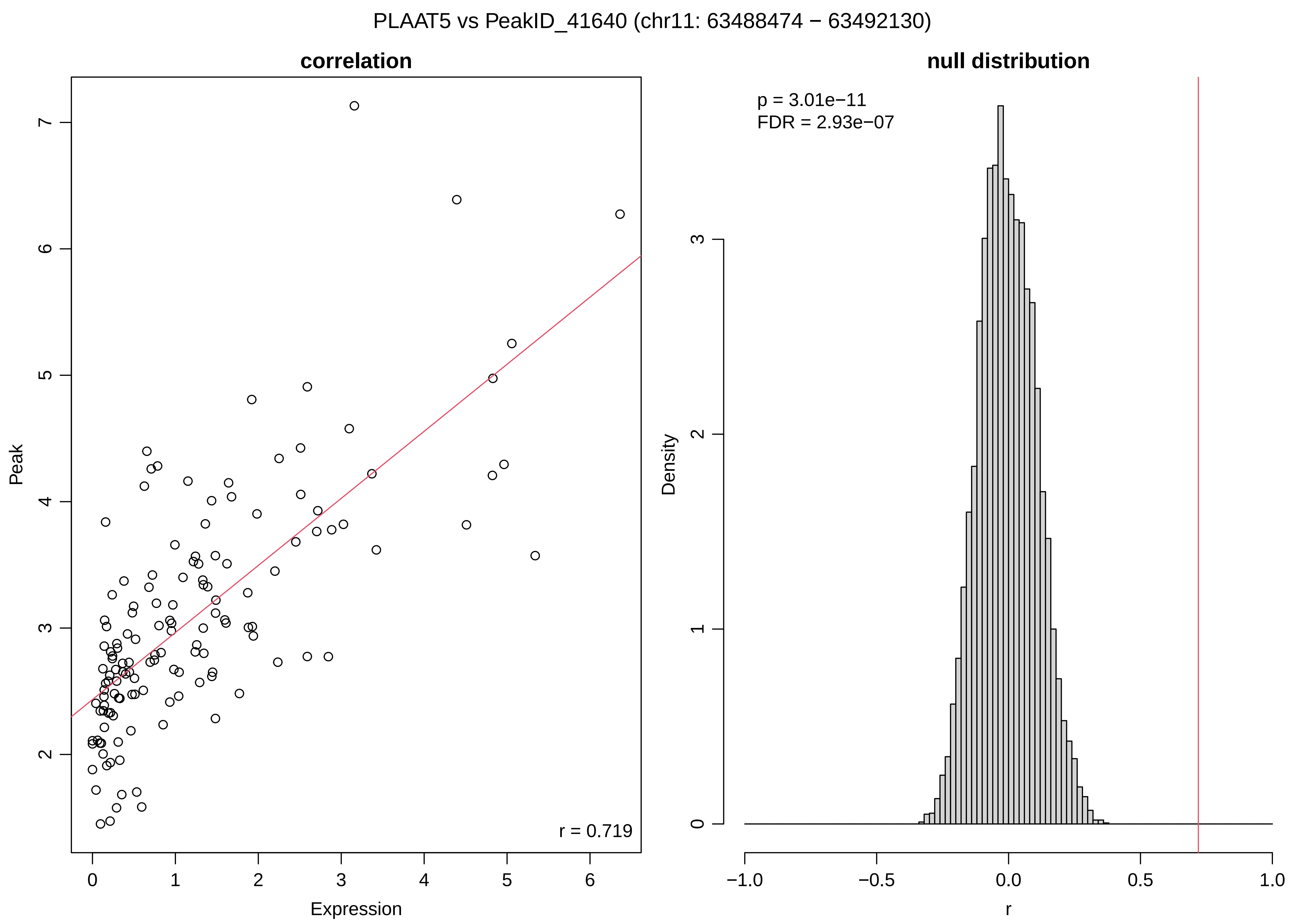
PLAAT5 vs PeakID_41640 (chr11: 63488474 − 63492130)
correlation
Peak
Expression
r = 0.719
null distribution
p = 3.01e−11
FDR = 2.93e−07
Density
r

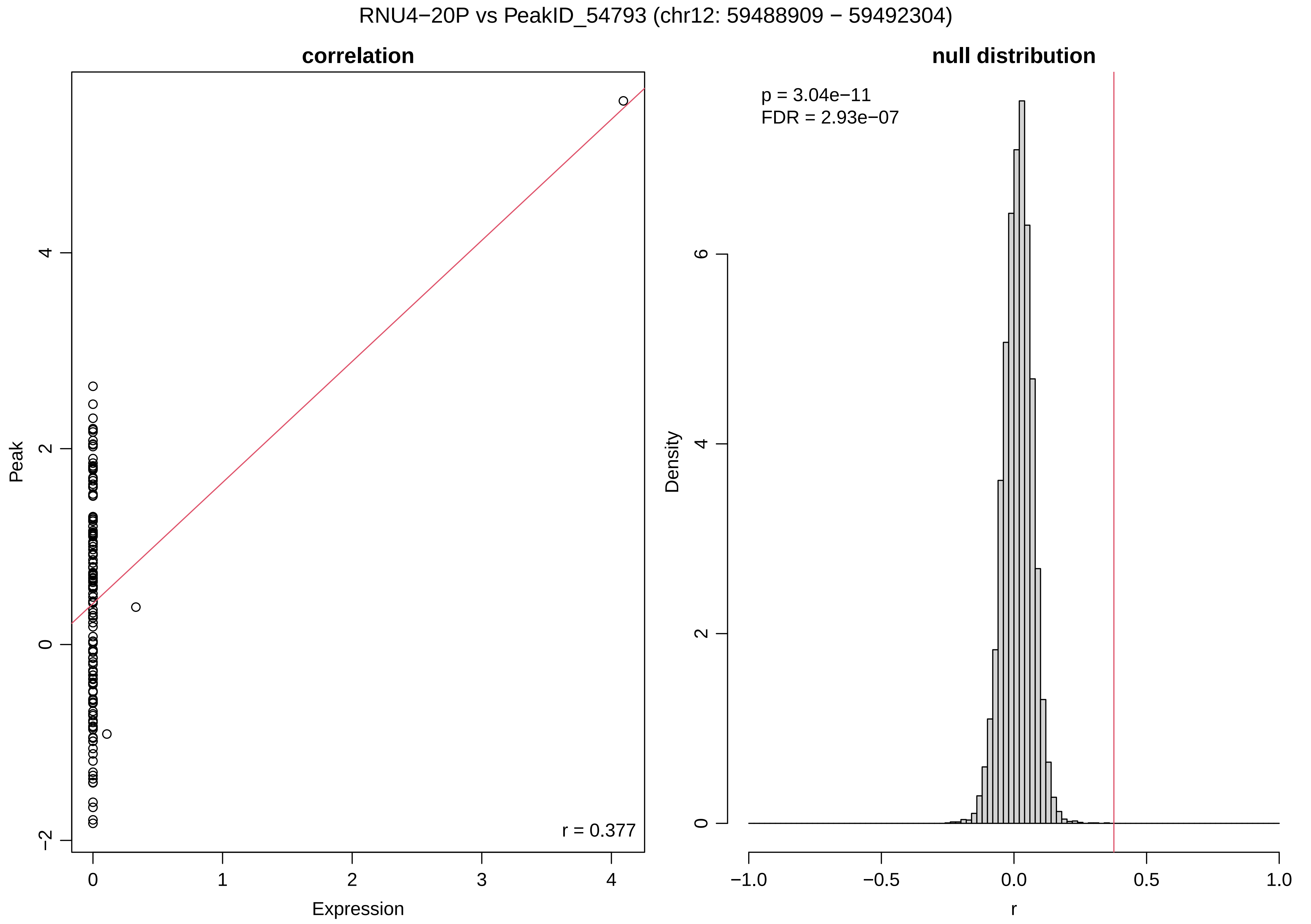

RNU4−20P vs PeakID_54793 (chr12: 59488909 − 59492304)
correlation
Peak
Expression
r = 0.377
null distribution
p = 3.04e−11
FDR = 2.93e−07
Density
r

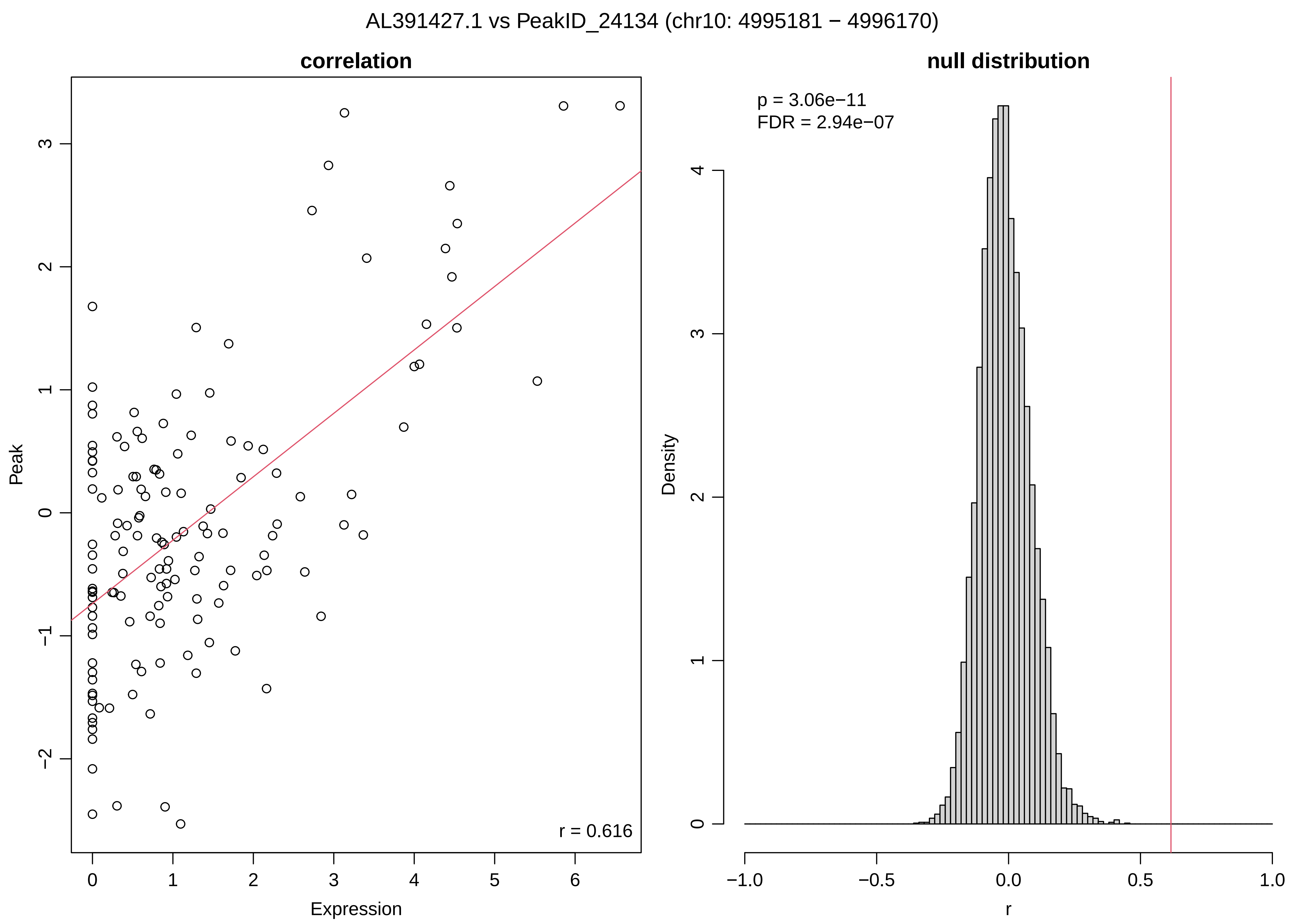
AL391427.1 vs PeakID_24134 (chr10: 4995181 − 4996170)
correlation
Peak
Expression
r = 0.616
null distribution
p = 3.06e−11
FDR = 2.94e−07
Density
r

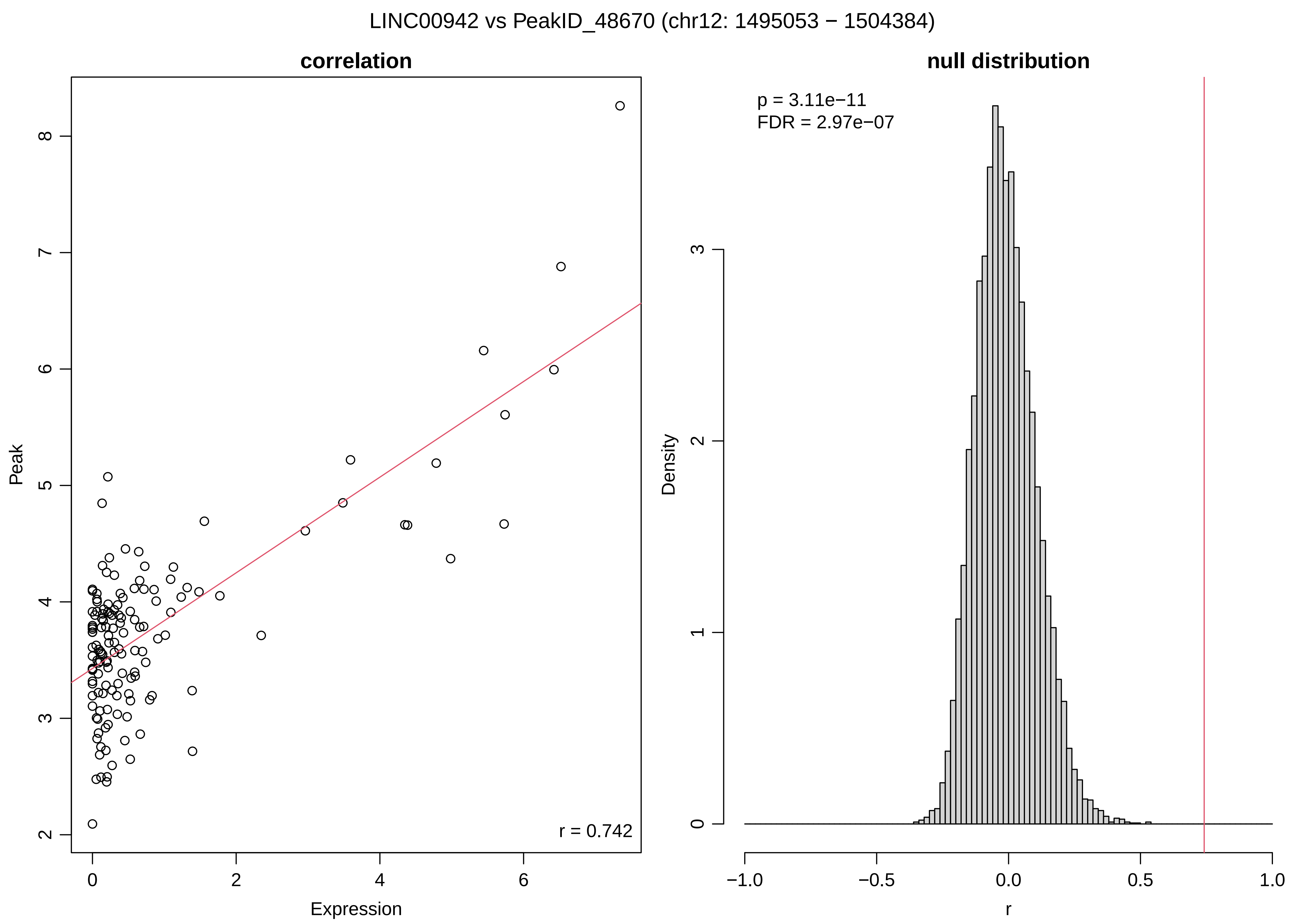
LINC00942 vs PeakID_48670 (chr12: 1495053 − 1504384)
correlation
Peak
Expression
r = 0.742
null distribution
p = 3.11e−11
FDR = 2.97e−07
Density
r

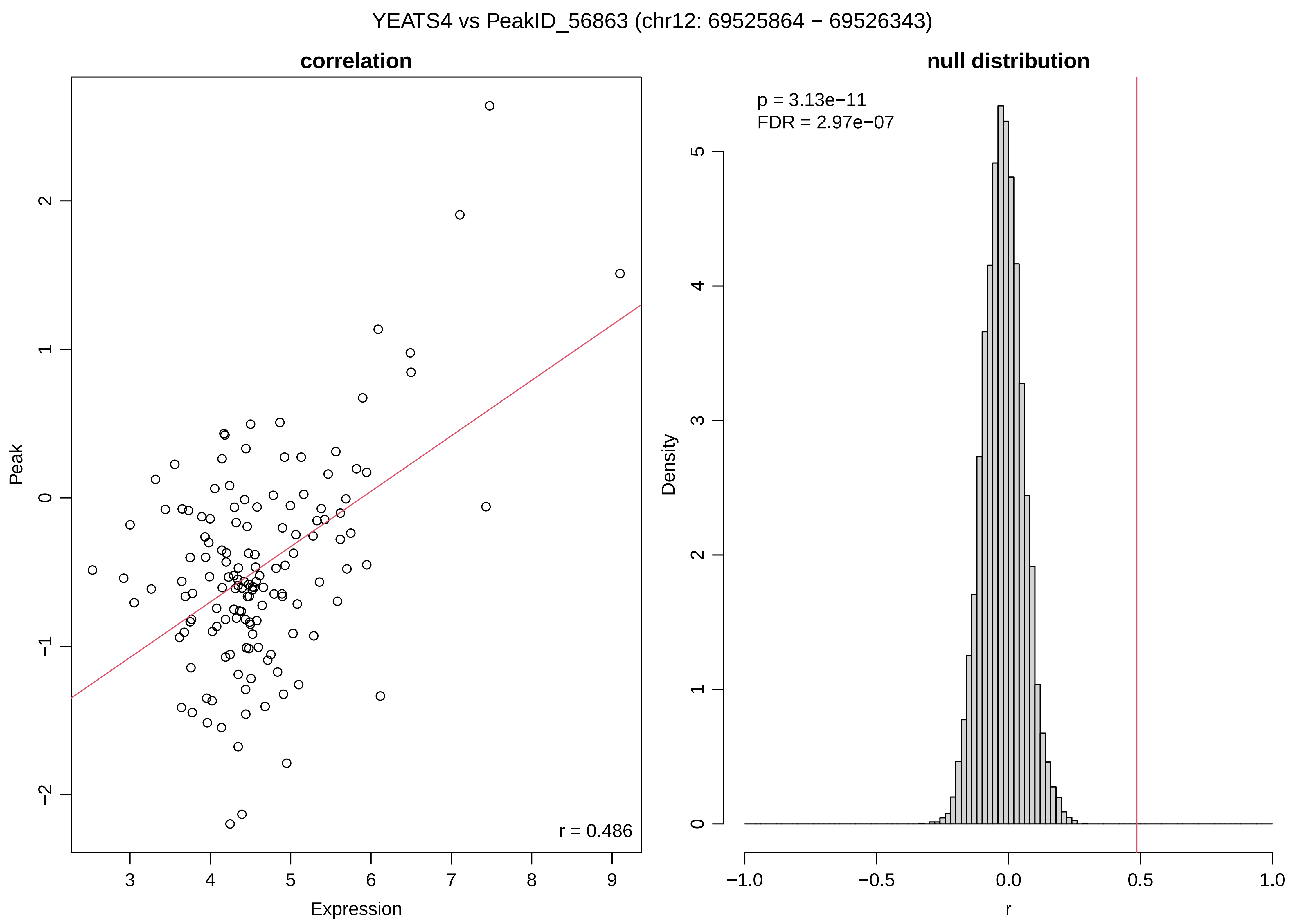
YEATS4 vs PeakID_56863 (chr12: 69525864 − 69526343)
correlation
Peak
Expression
r = 0.486
null distribution
p = 3.13e−11
FDR = 2.97e−07
Density
r

KCNMB4 vs PeakID_57014 (chr12: 70365064 − 70368222)

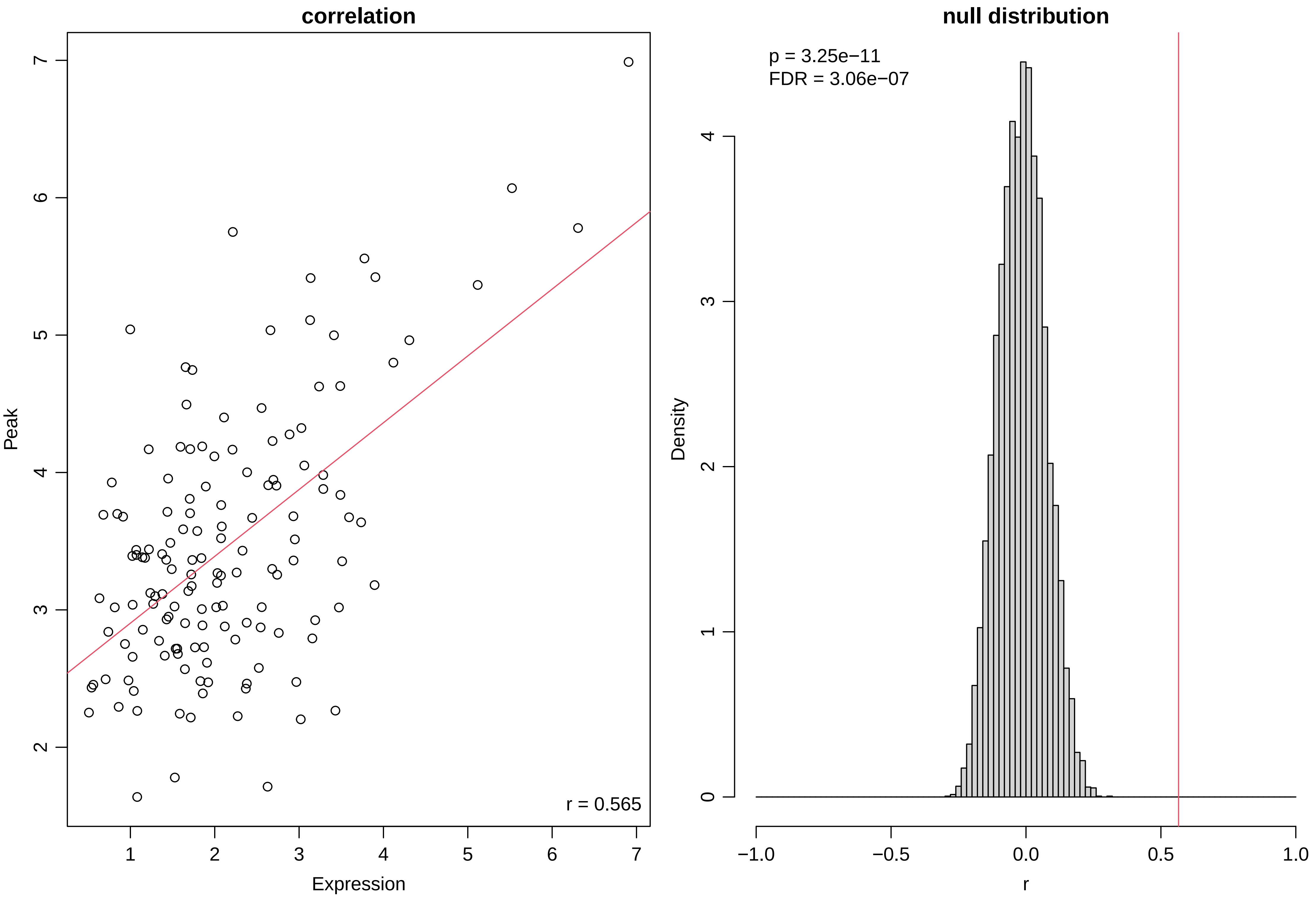

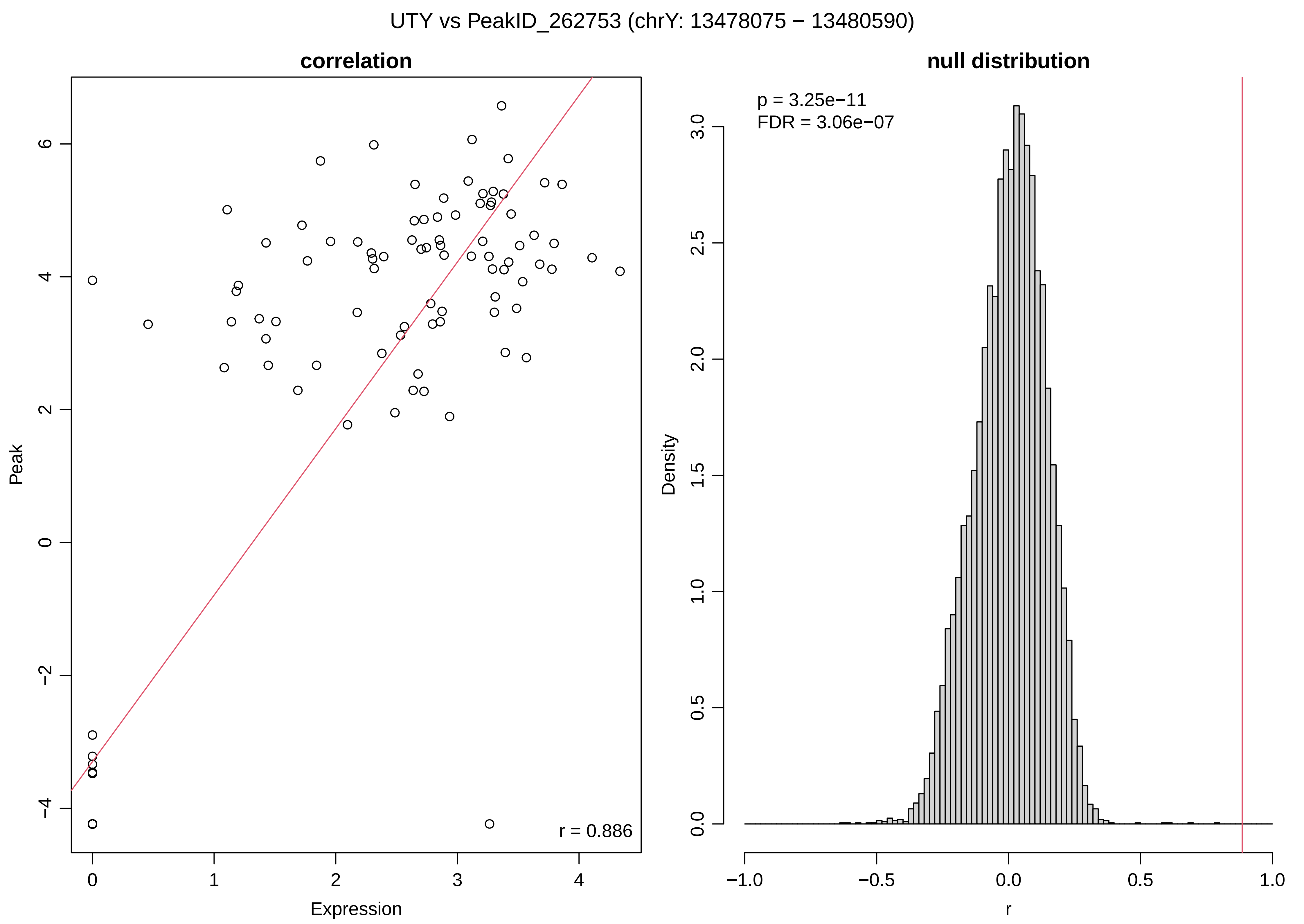

UTY vs PeakID_262753 (chrY: 13478075 − 13480590)
correlation
null distribution
p = 3.25e−11
FDR = 3.06e−07
r = 0.886
Peak
Expression
Density
r

SMOC1 vs PeakID_72628 (chr14: 69999586 − 70000349)

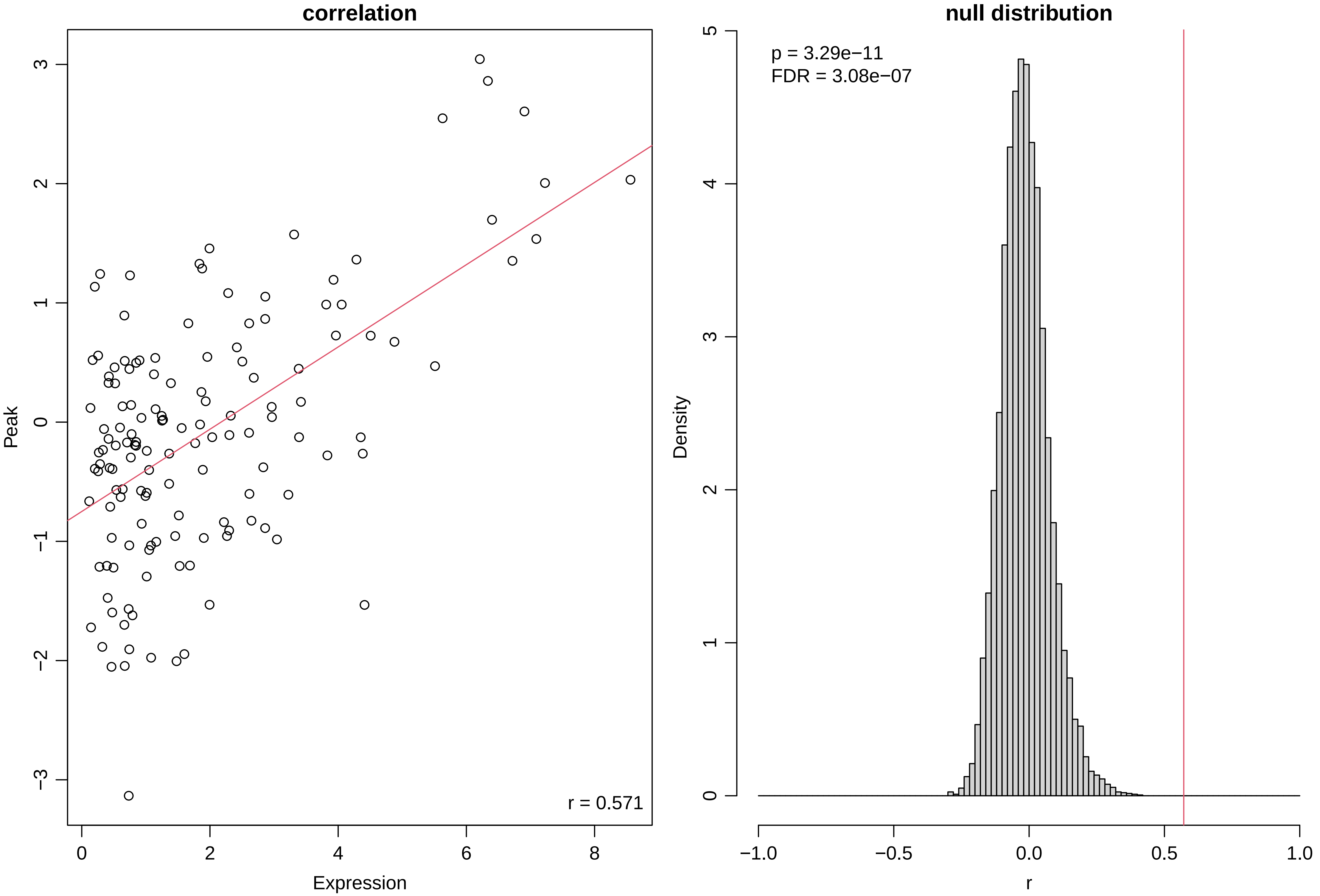

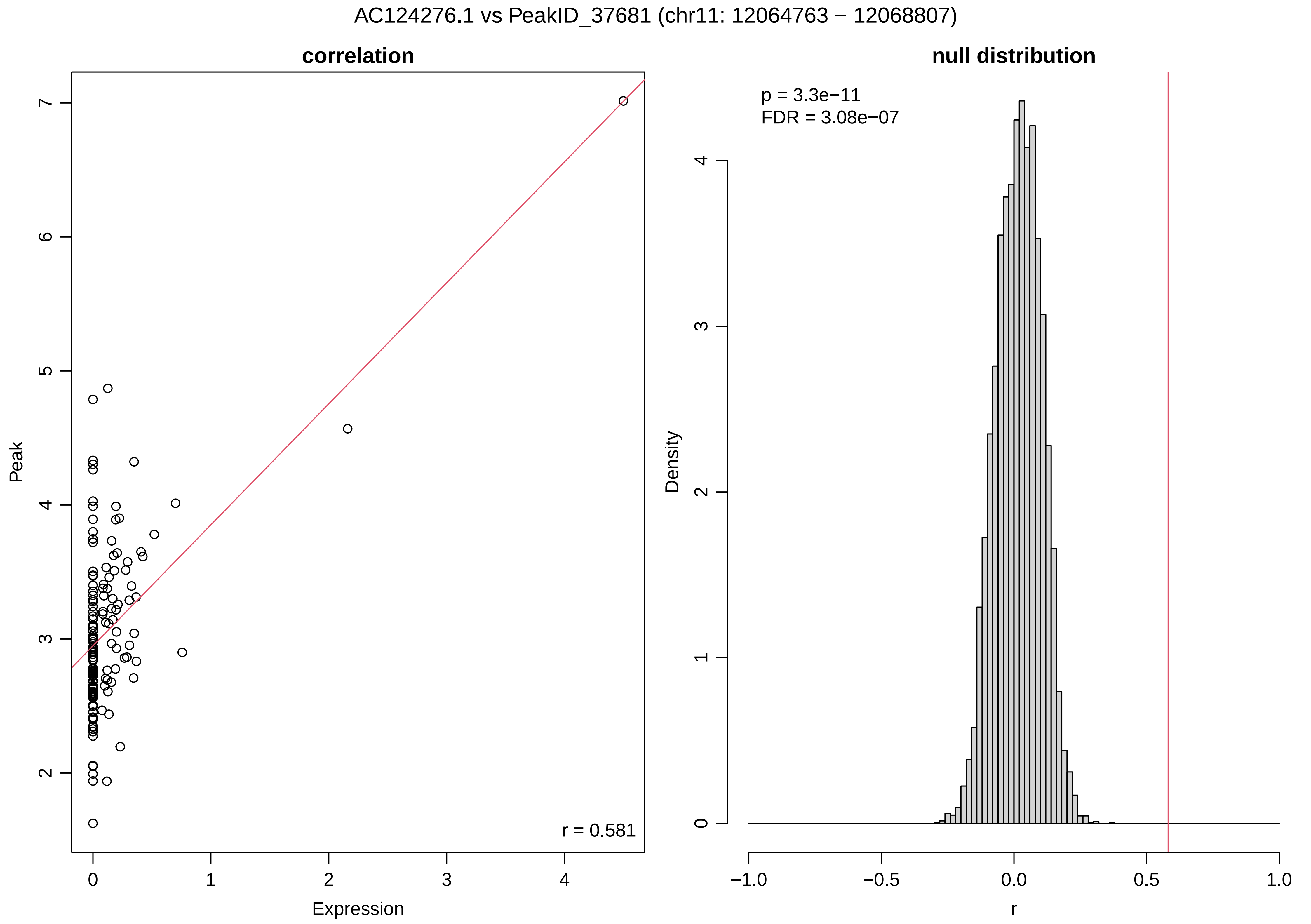

AC124276.1 vs PeakID_37681 (chr11: 12064763 − 12068807)
correlation
Peak
Expression
r = 0.581
null distribution
p = 3.3e−11
FDR = 3.08e−07
Density
r

RPL21P104 vs PeakID_55425 (chr12: 61859035 − 61860050)

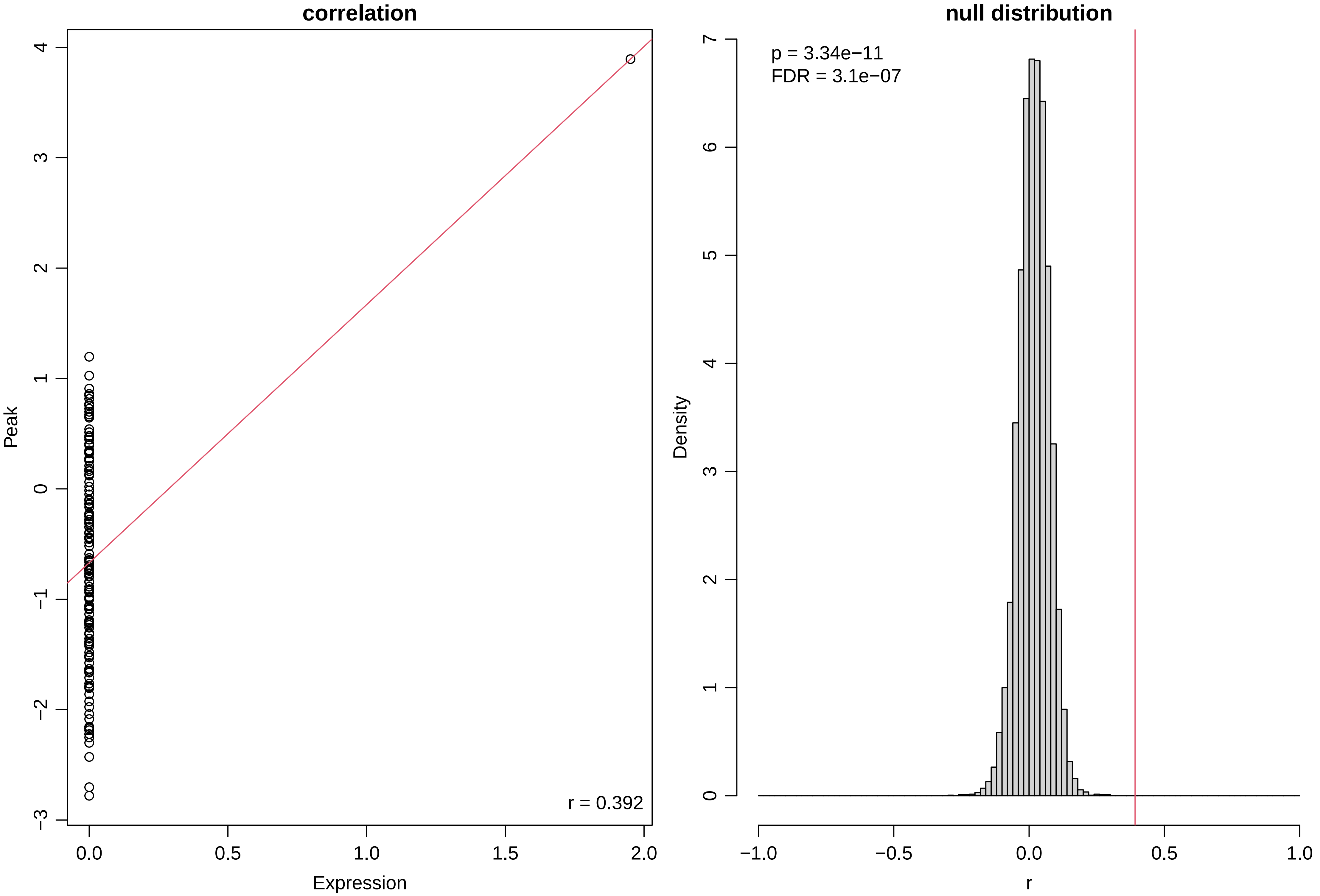

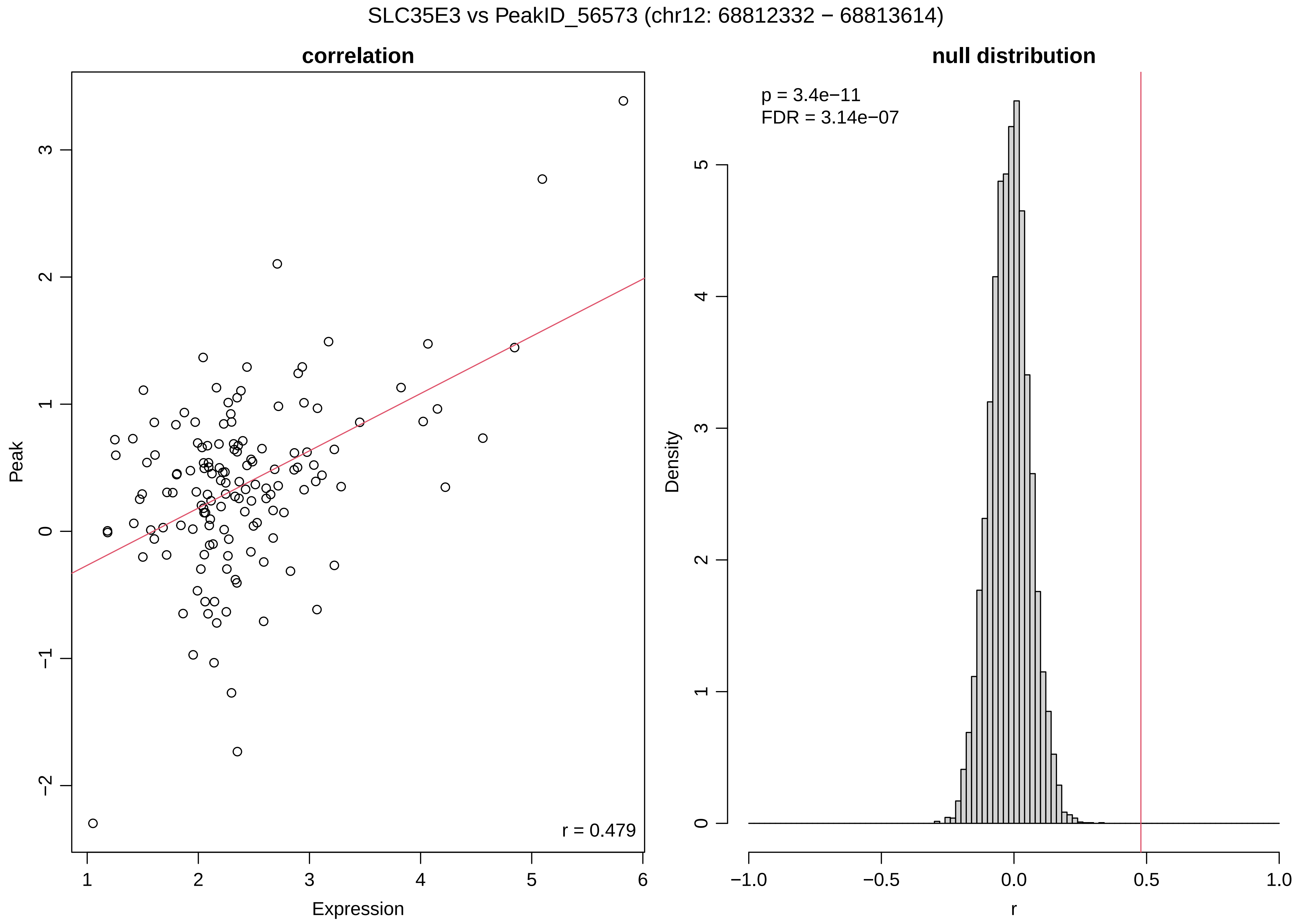

SLC35E3 vs PeakID_56573 (chr12: 68812332 − 68813614)
correlation
Peak
Expression
r = 0.479
null distribution
p = 3.4e−11
FDR = 3.14e−07
Density
r

DPP10−AS1 vs PeakID_132376 (chr2: 115160758 − 115162434)

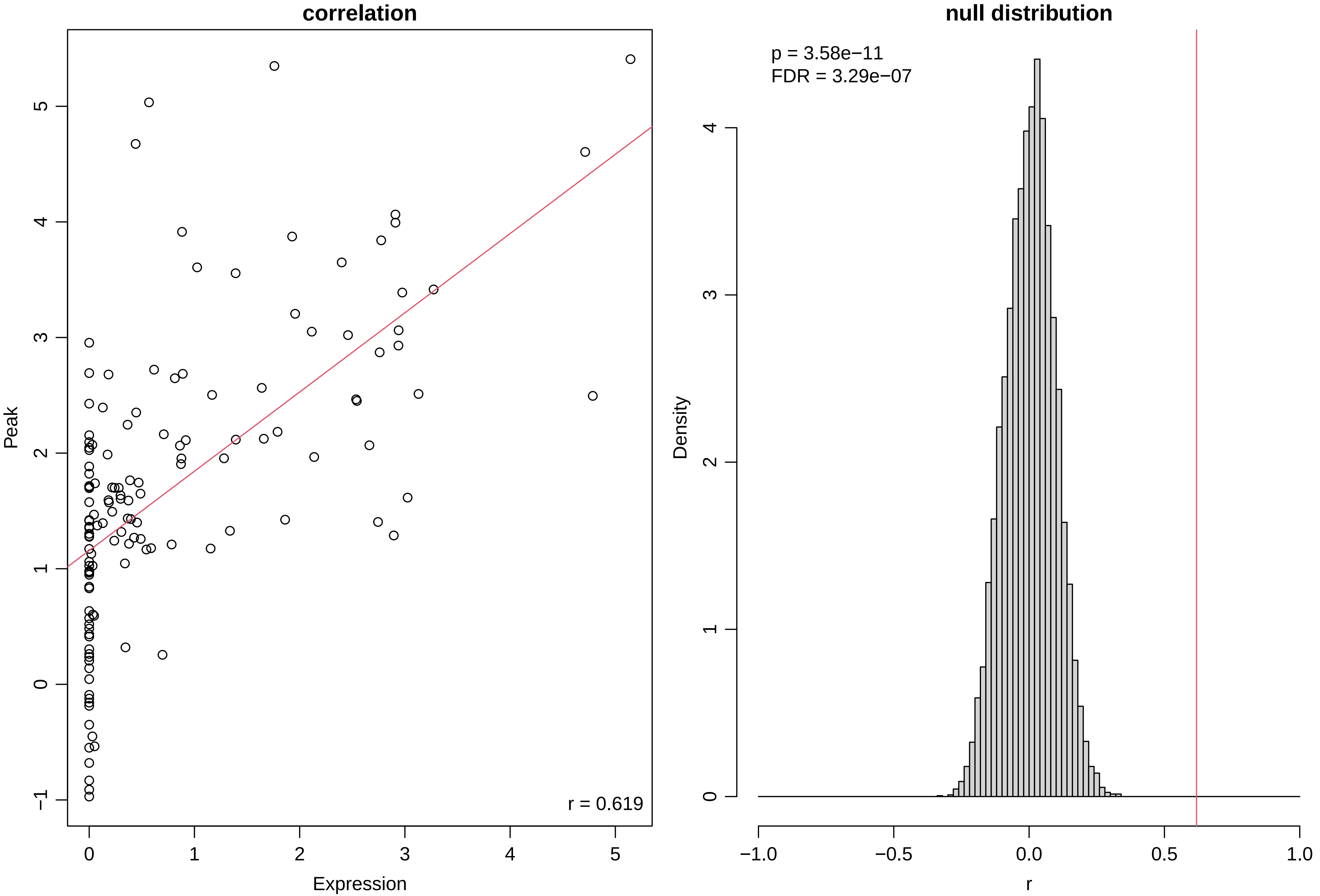

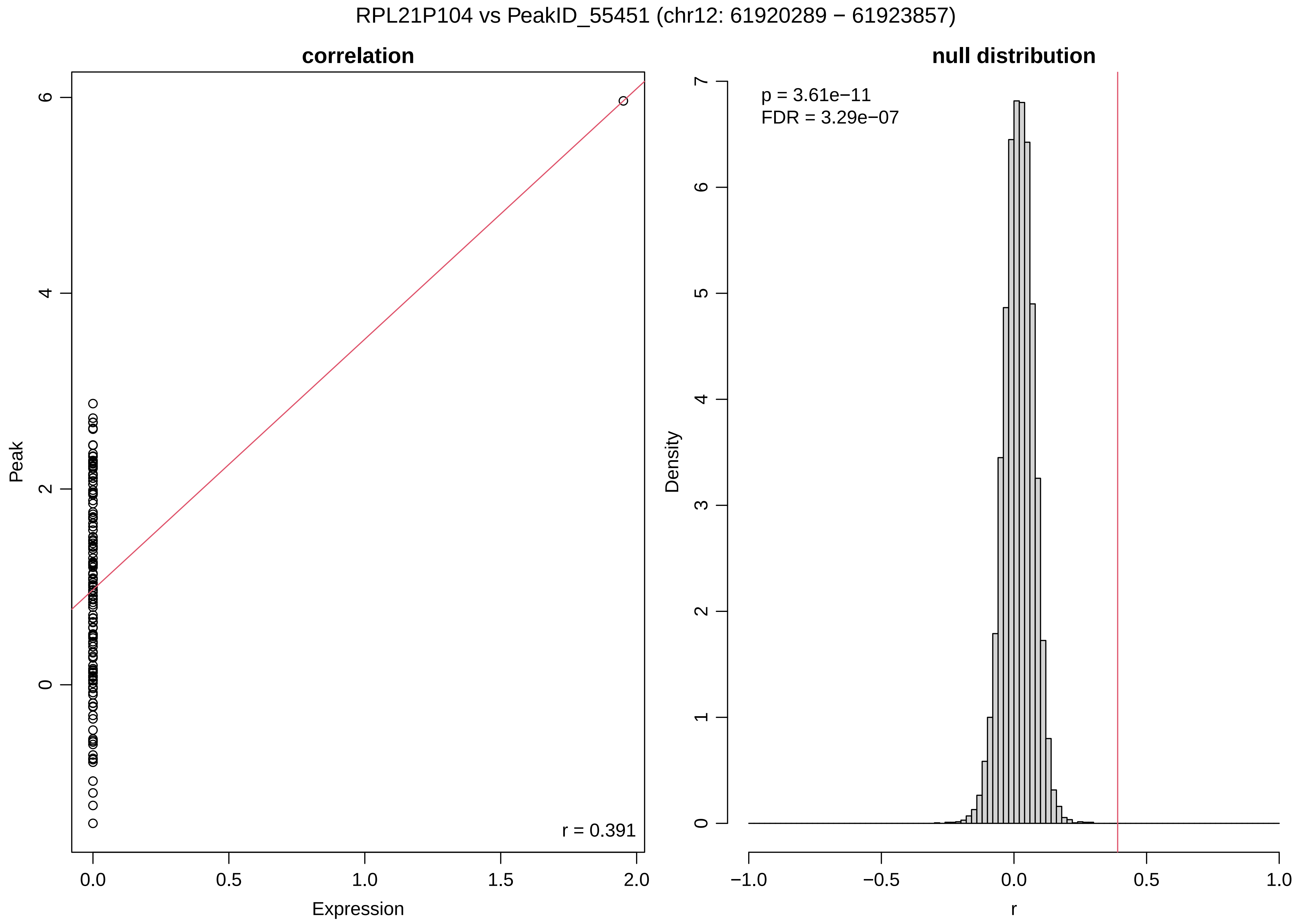
RPL21P104 vs PeakID_55451 (chr12: 61920289 − 61923857)
correlation
Peak
Expression
r = 0.391
null distribution
Density
r
p = 3.61e−11
FDR = 3.29e−07

AC131206.1 vs PeakID_49419 (chr12: 7438549 − 7440500)

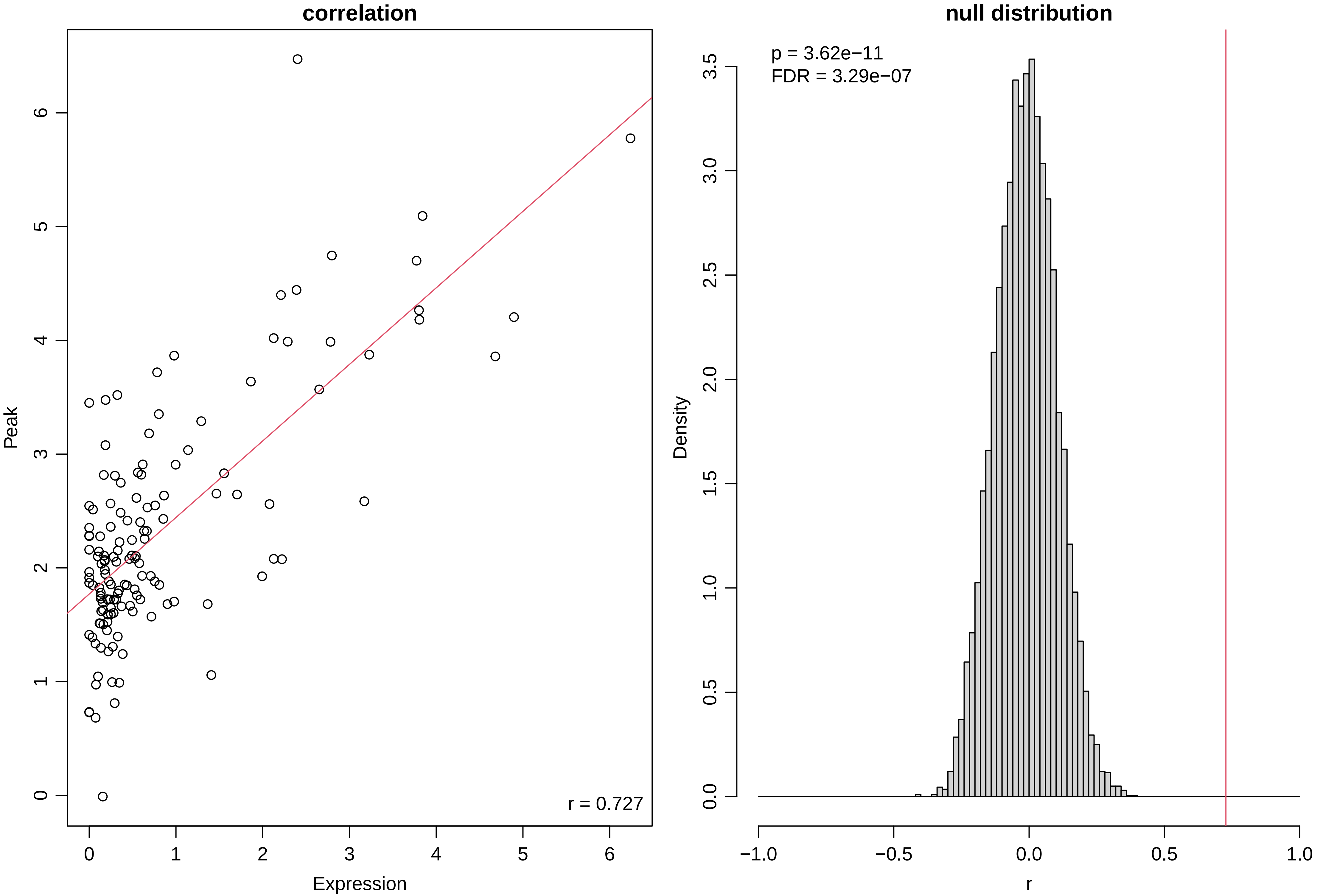

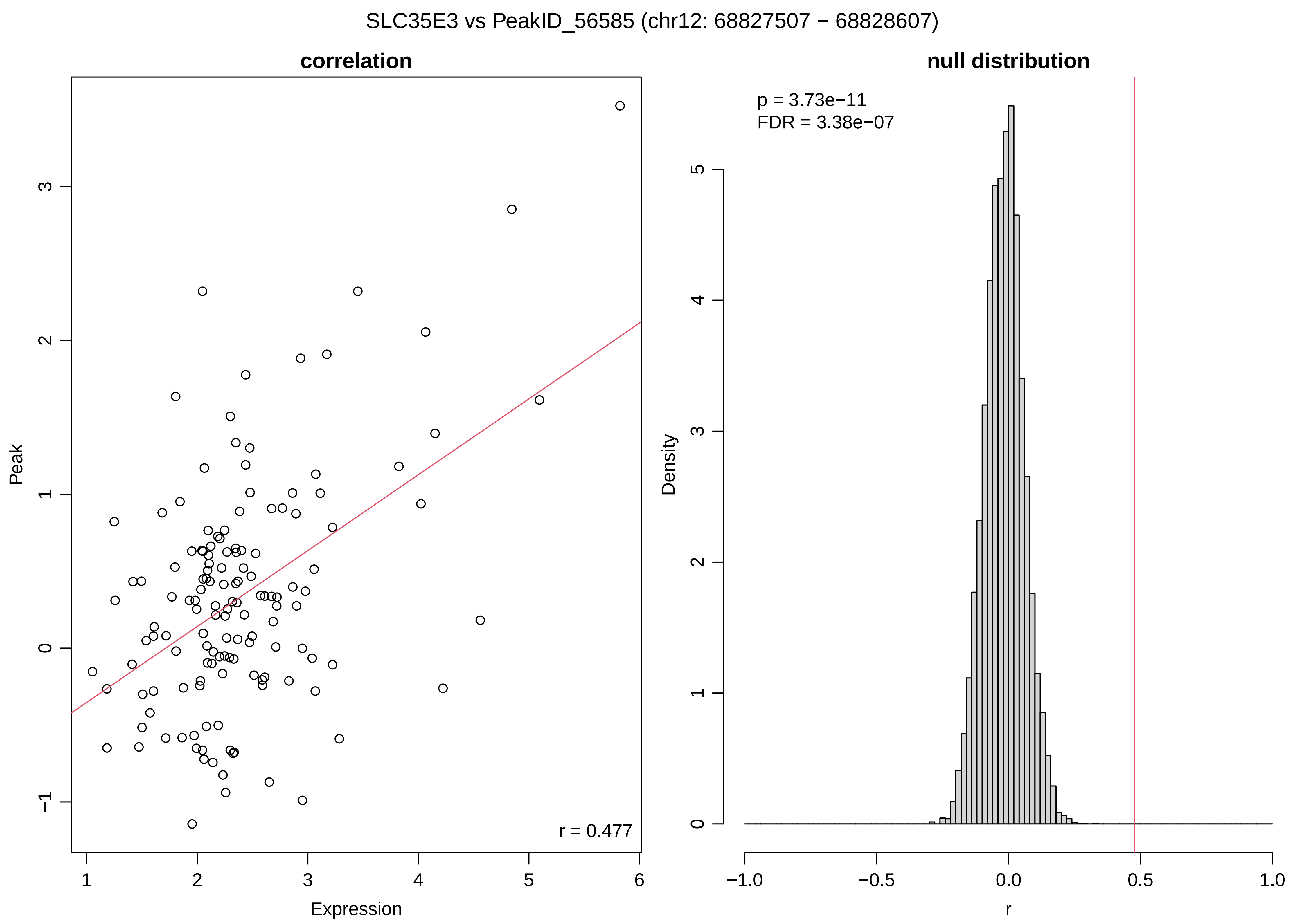
SLC35E3 vs PeakID_56585 (chr12: 68827507 − 68828607)
correlation
Peak
Expression
r = 0.477
null distribution
p = 3.73e−11
FDR = 3.38e−07
Density
r

ZNF98 vs PeakID_115672 (chr19: 22427123 − 22428839)

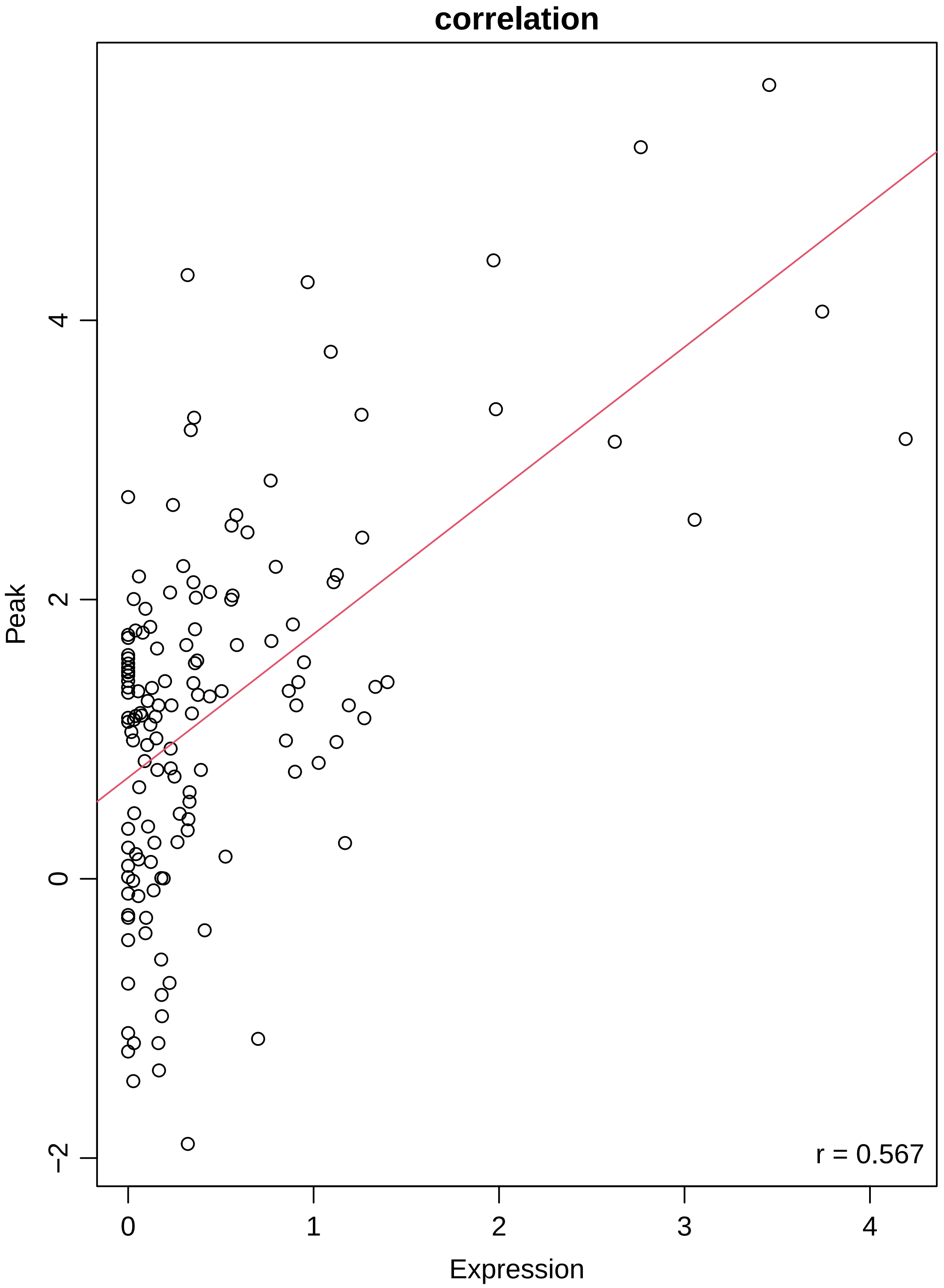

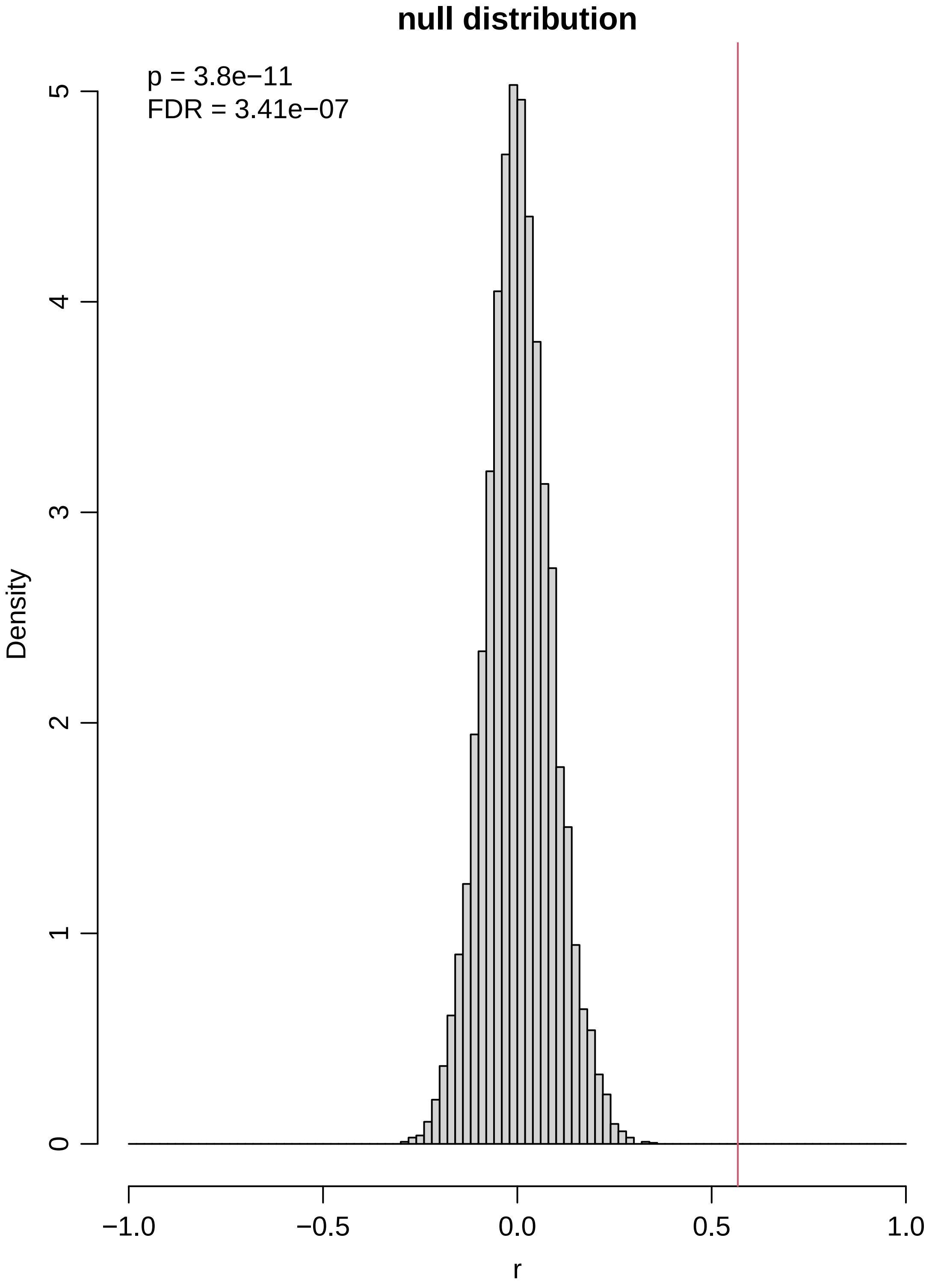

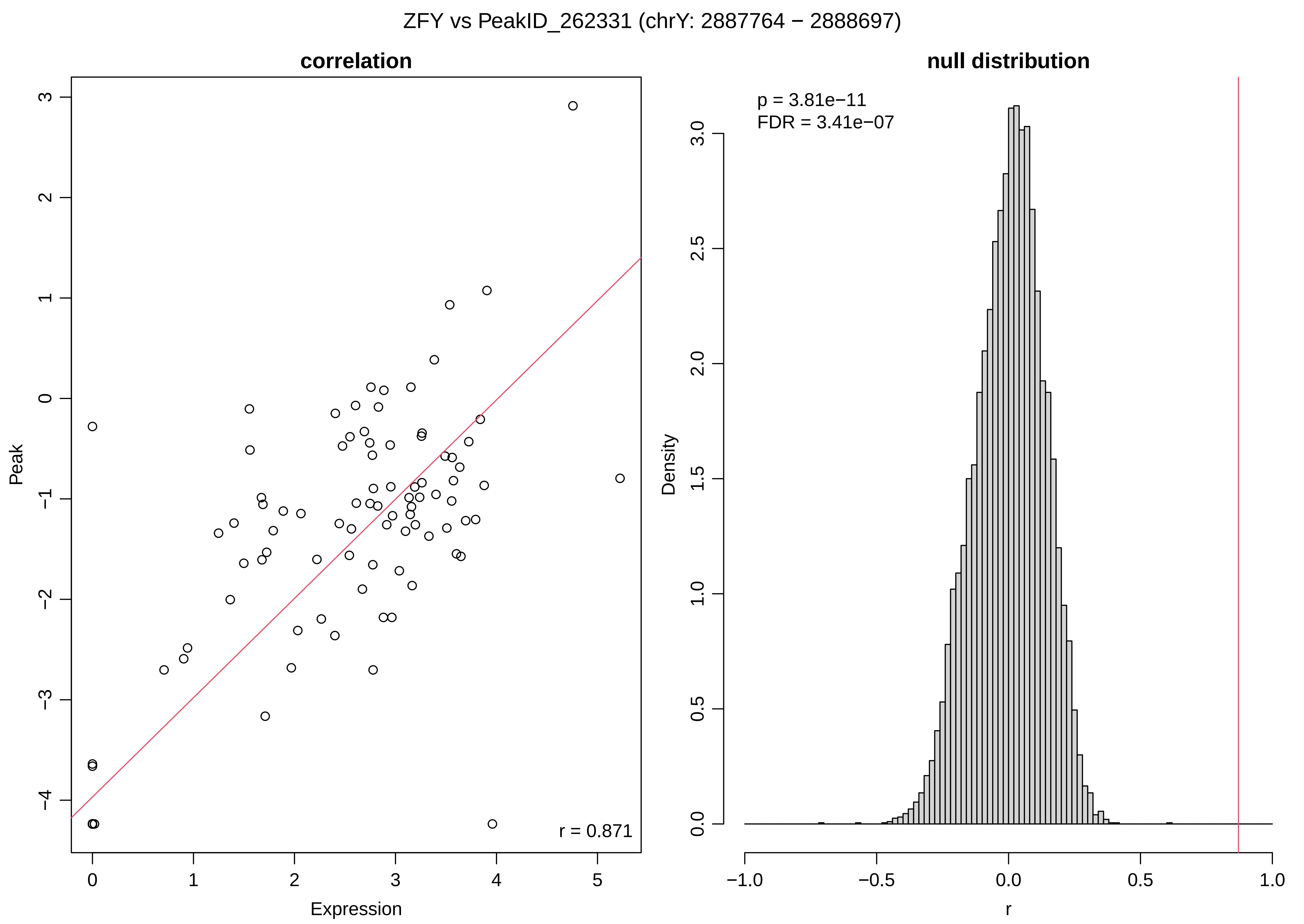
ZFY vs PeakID_262331 (chrY: 2887764 − 2888697)
correlation
Peak
Expression
r = 0.871
null distribution
p = 3.81e−11
FDR = 3.41e−07
Density
r

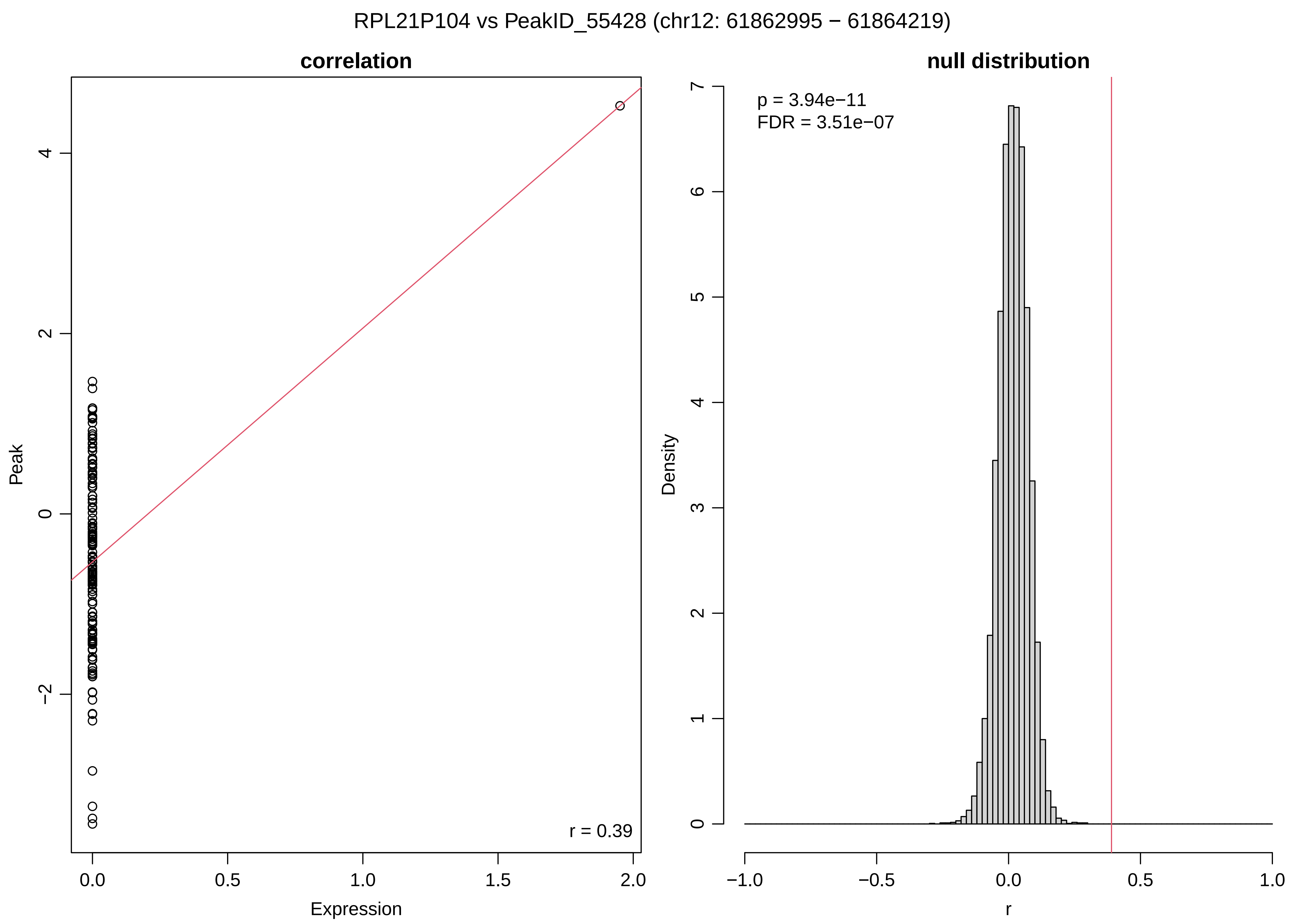
RPL21P104 vs PeakID_55428 (chr12: 61862995 − 61864219)
correlation
Peak
Expression
r = 0.39
null distribution
p = 3.94e−11
FDR = 3.51e−07
Density
r

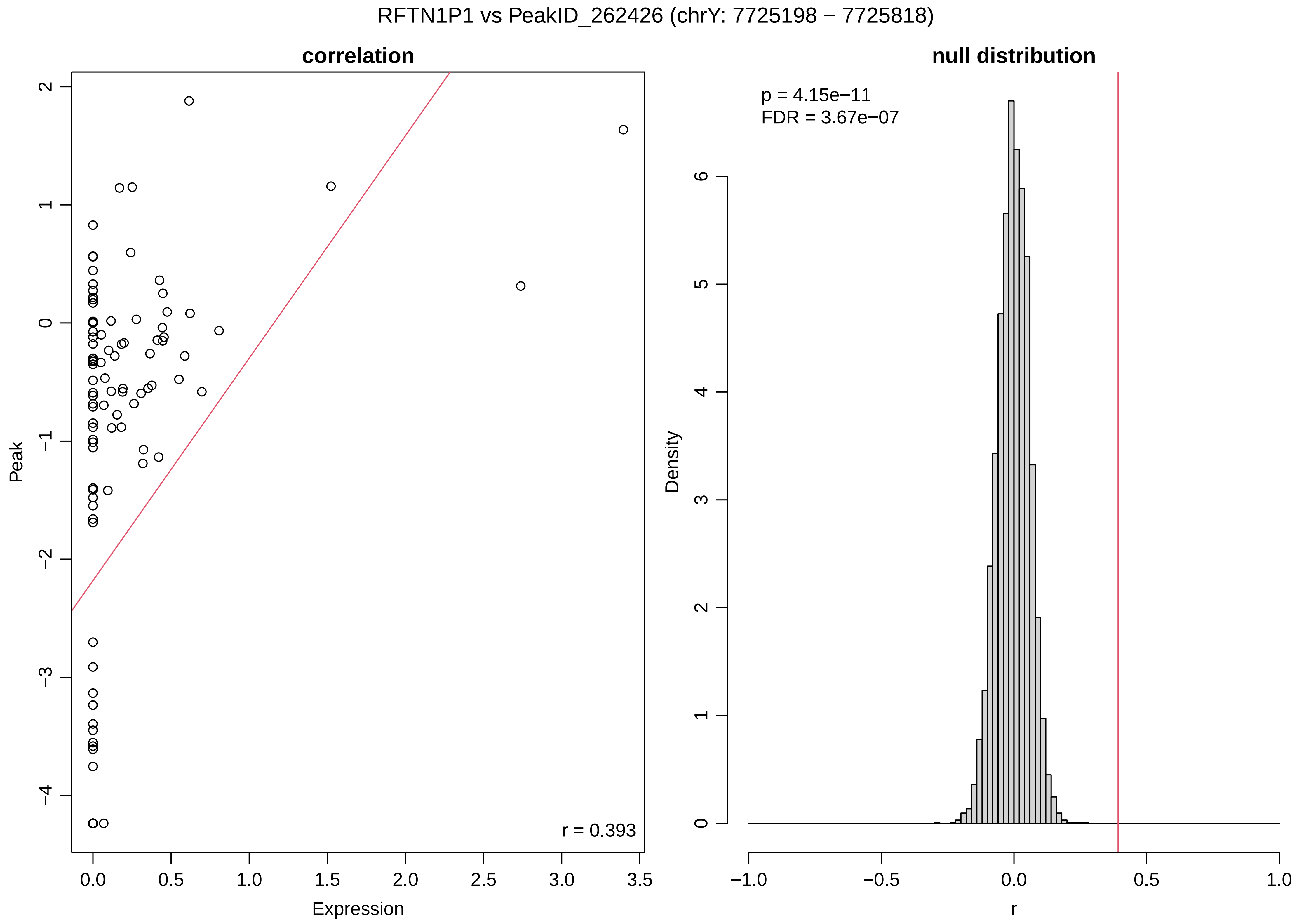

RFTN1P1 vs PeakID_262426 (chrY: 7725198 − 7725818)
correlation
Peak
Expression
r = 0.393
null distribution
p = 4.15e−11
FDR = 3.67e−07
Density
r

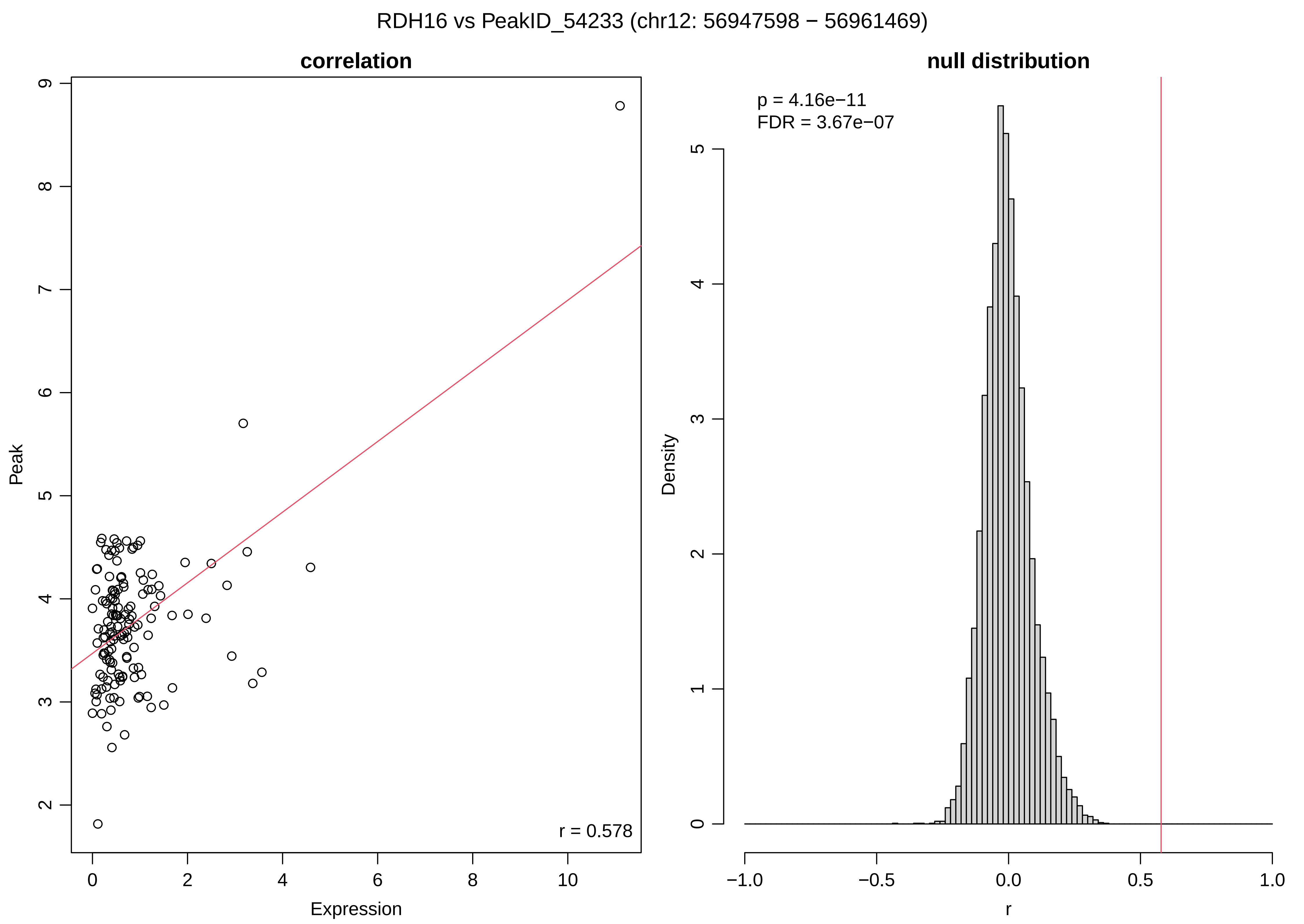

RDH16 vs PeakID_54233 (chr12: 56947598 − 56961469)
correlation
Peak
Expression
r = 0.578
null distribution
p = 4.16e−11
FDR = 3.67e−07
Density
r

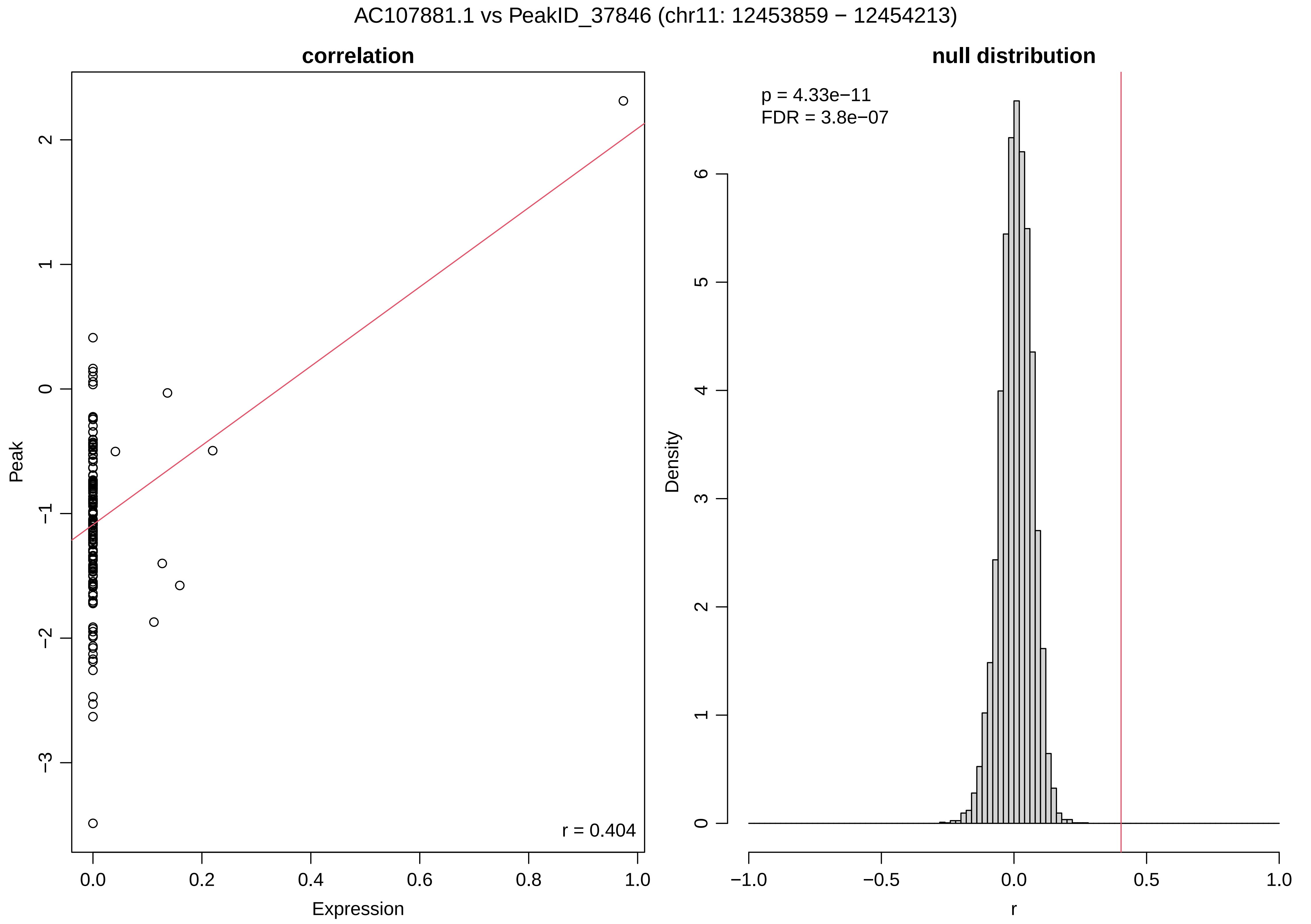
AC107881.1 vs PeakID_37846 (chr11: 12453859 − 12454213)
correlation
Peak
Expression
r = 0.404
null distribution
p = 4.33e−11
FDR = 3.8e−07
Density
r

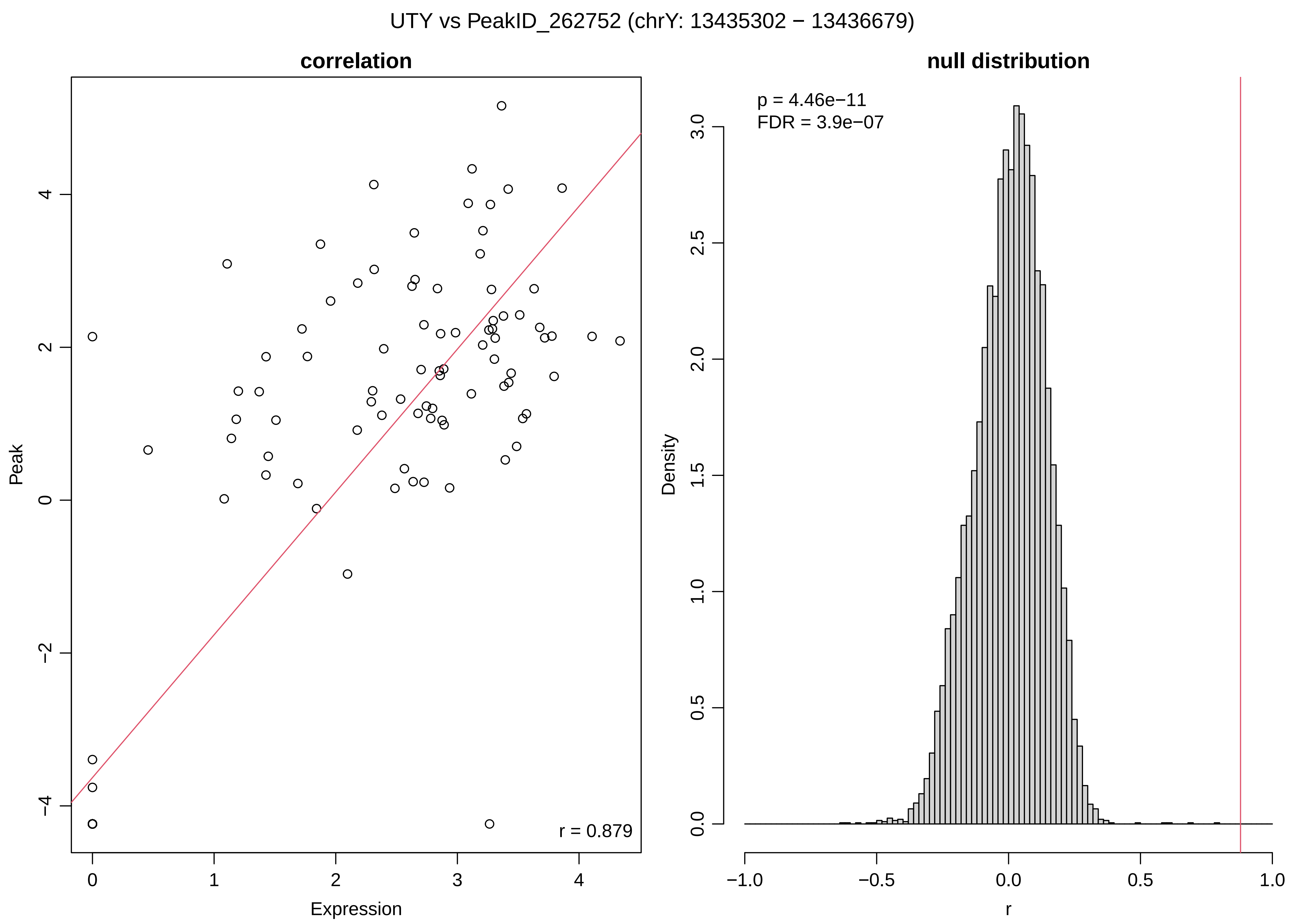

UTY vs PeakID_262752 (chrY: 13435302 − 13436679)
correlation
Peak
Expression
r = 0.879
null distribution
p = 4.46e−11
FDR = 3.9e−07
Density
r

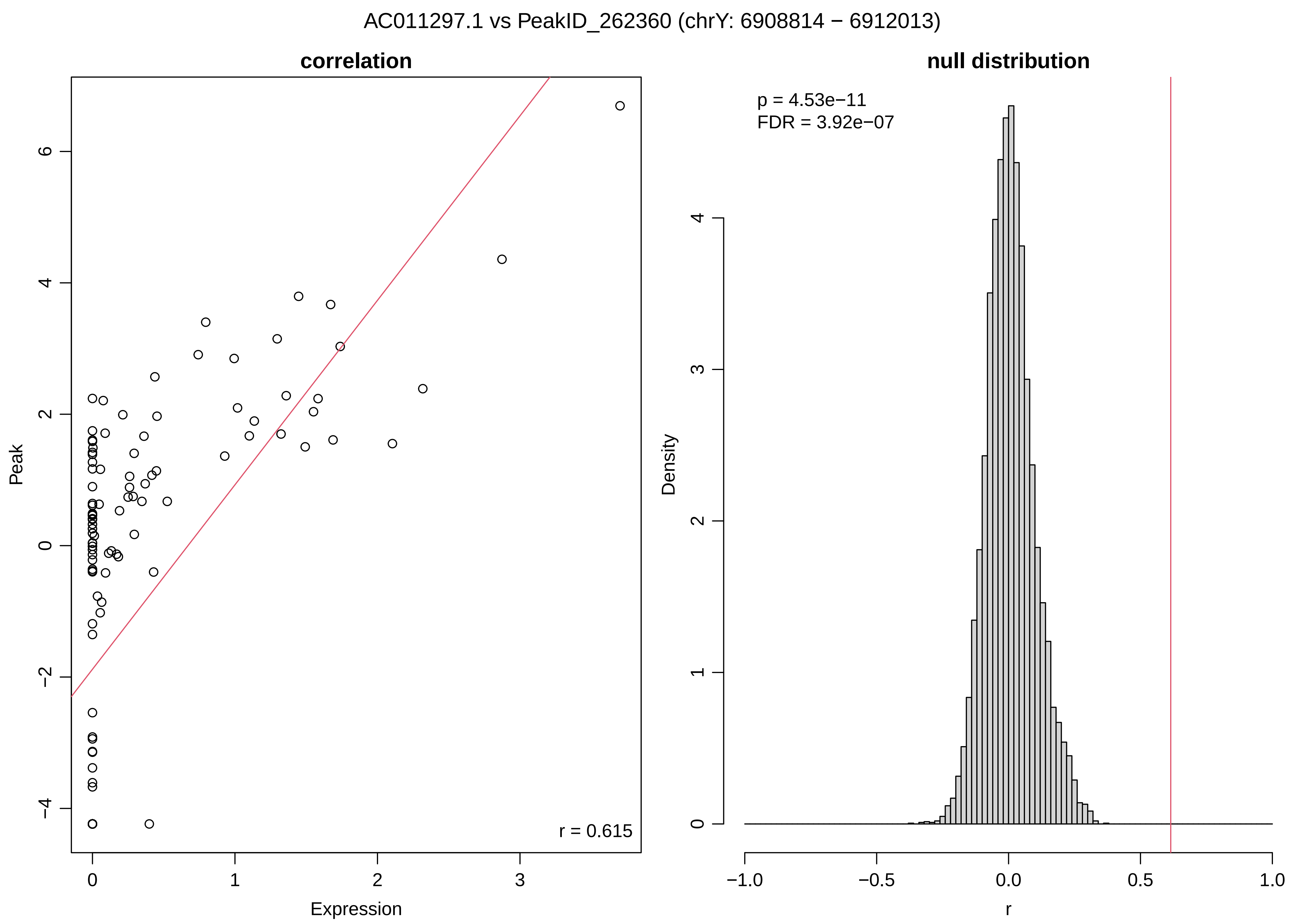
AC011297.1 vs PeakID_262360 (chrY: 6908814 − 6912013)
correlation
Peak
Expression
r = 0.615
6
4
2
0
−2
−4
0
1
2
3
null distribution
p = 4.53e−11
FDR = 3.92e−07
Density
r
4
3
2
1
0
−1.0
−0.5
0.0
0.5
1.0

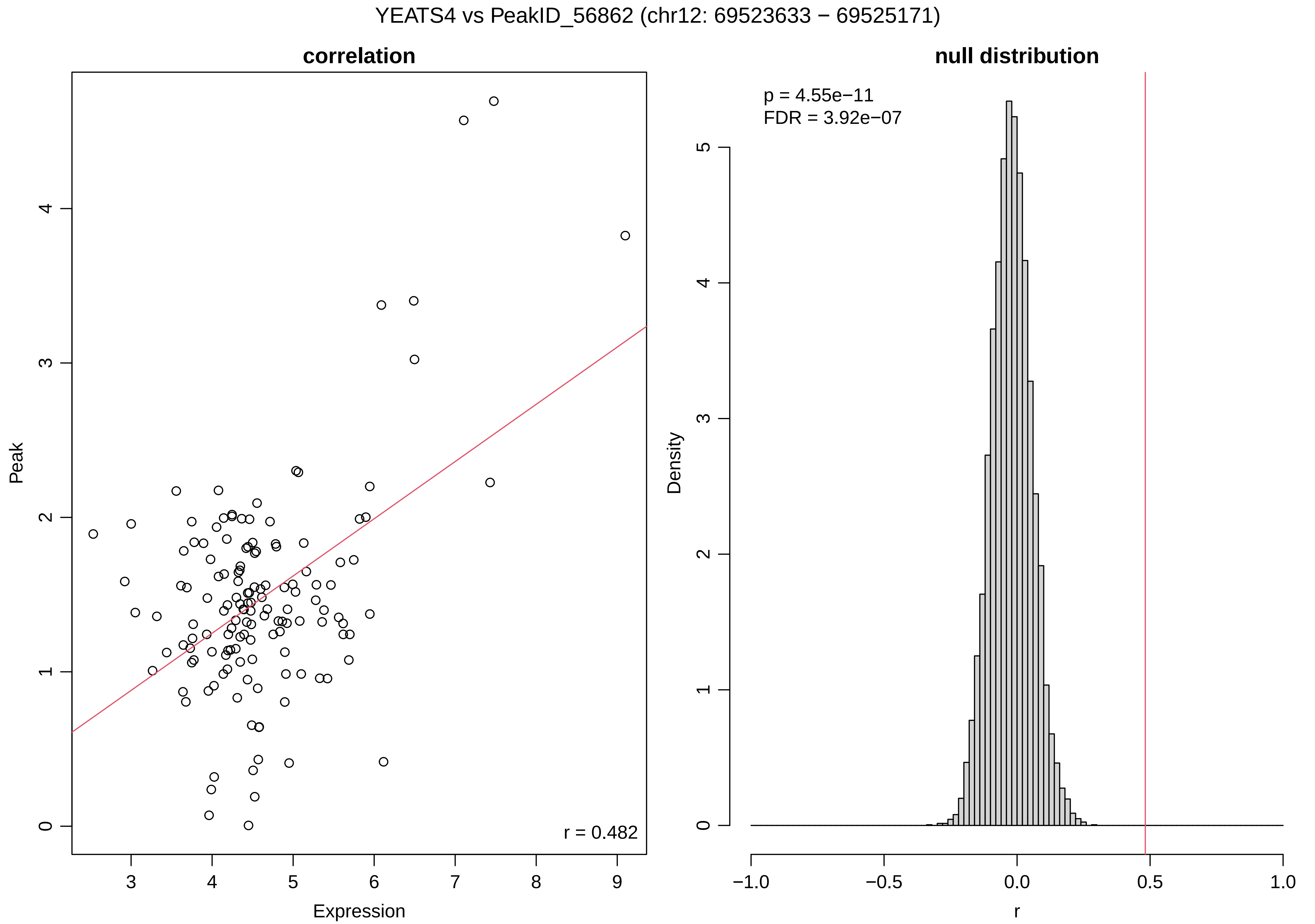

YEATS4 vs PeakID_56862 (chr12: 69523633 − 69525171)
correlation
Peak
Expression
r = 0.482
null distribution
p = 4.55e−11
FDR = 3.92e−07
Density
r

UTY vs PeakID_262747 (chrY: 13282506 − 13283712)

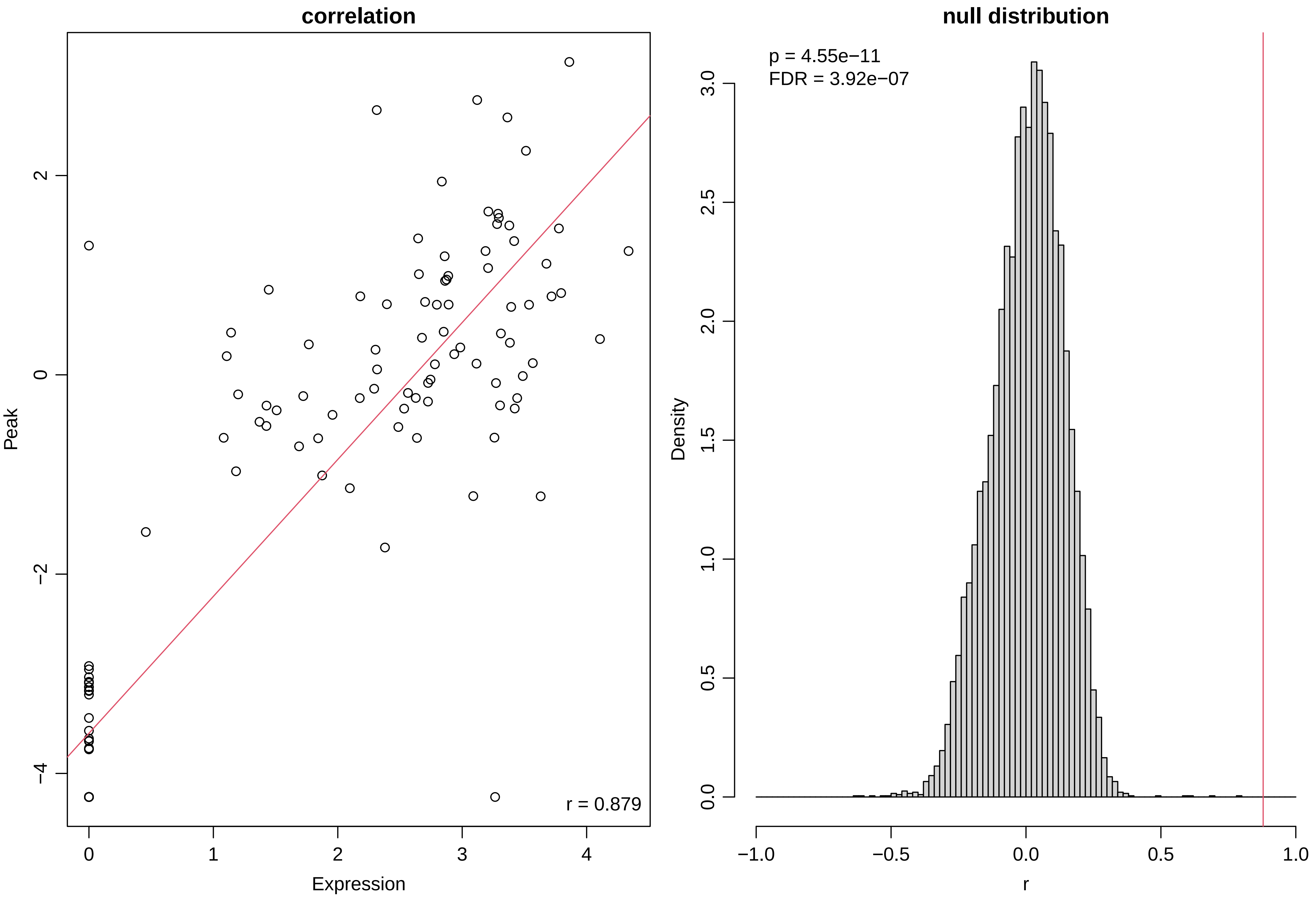

LINC00923 vs PeakID_83930 (chr15: 97875454 − 97875958)

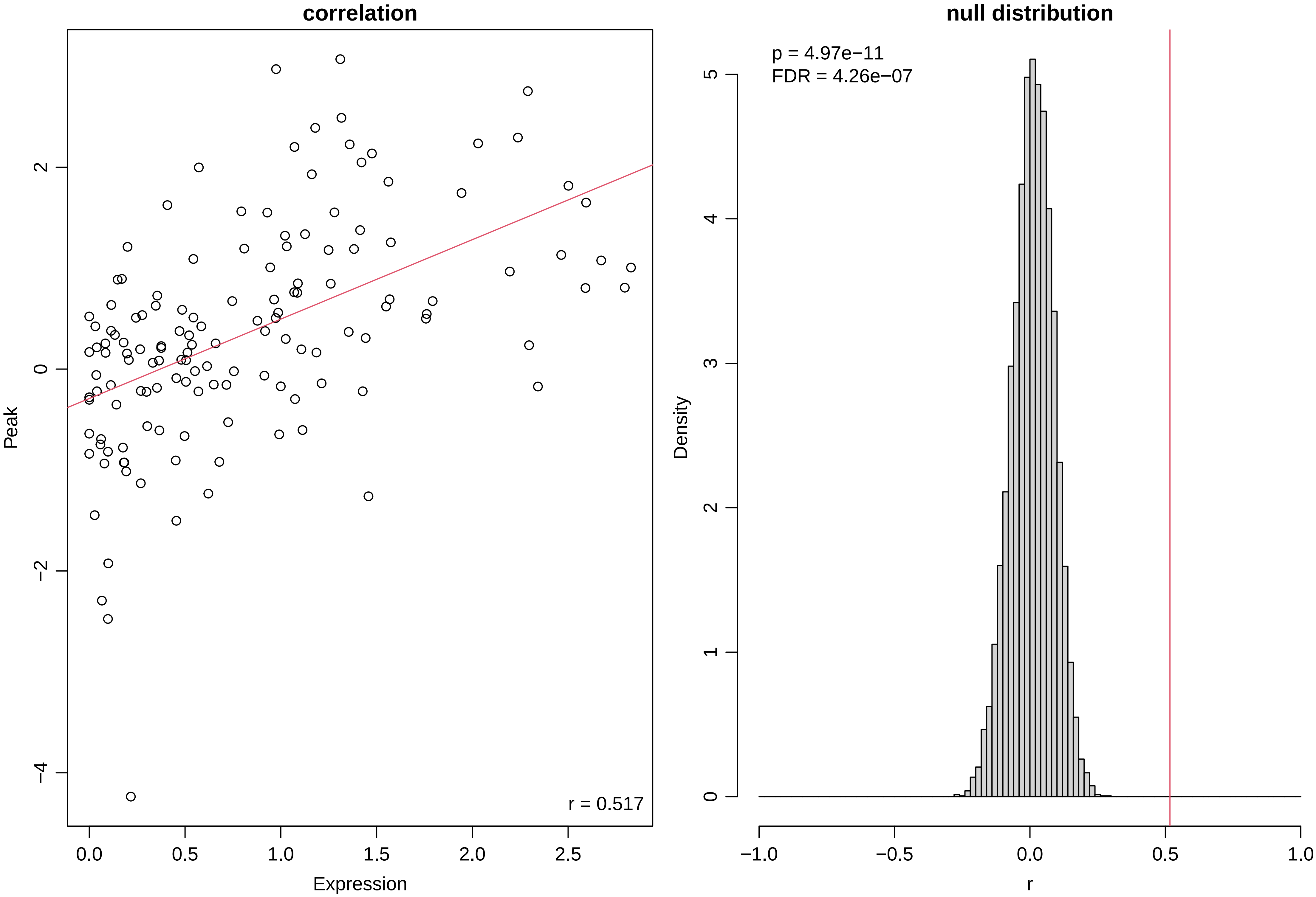

ESPNL vs PeakID_141709 (chr2: 238127350 − 238128193)

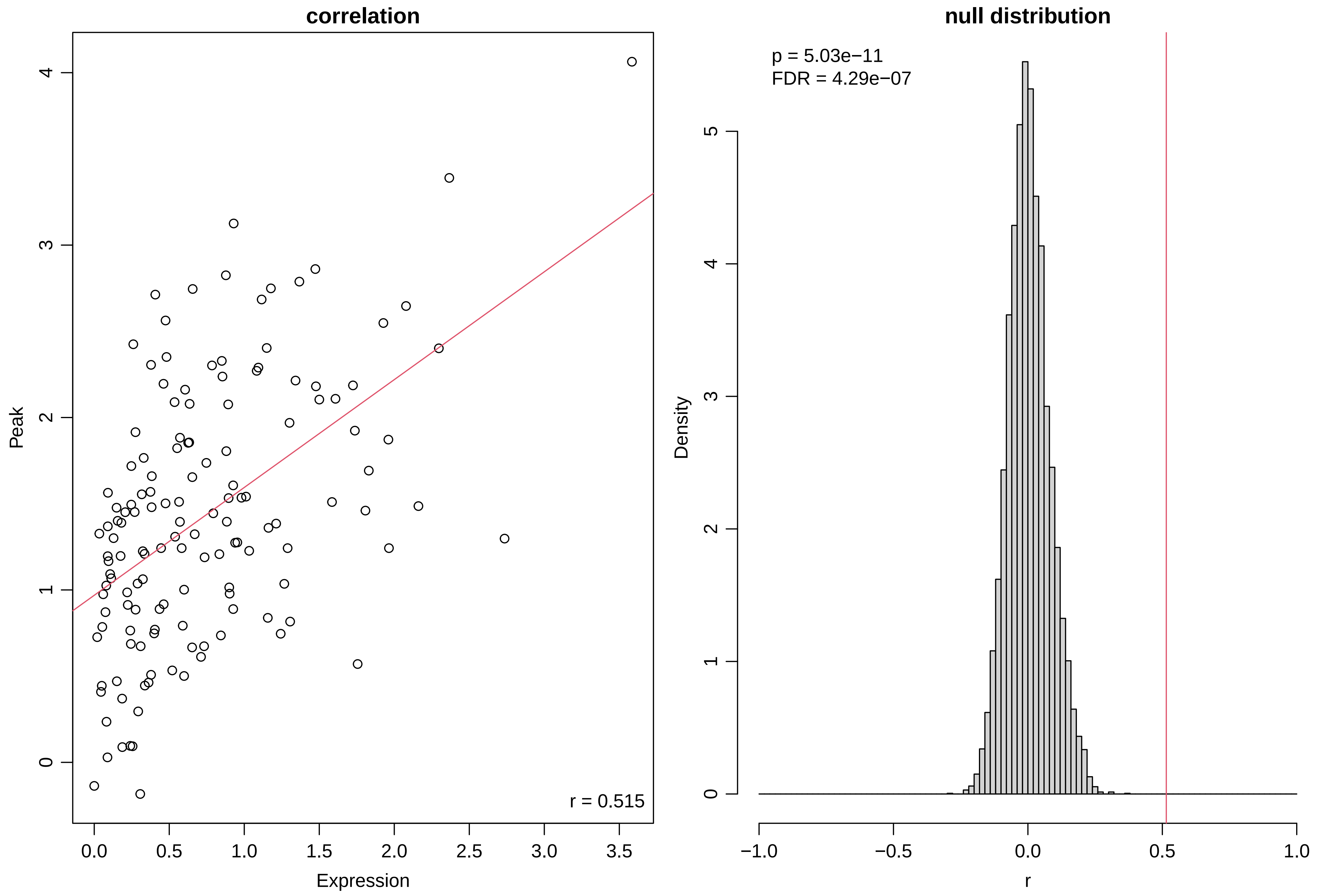

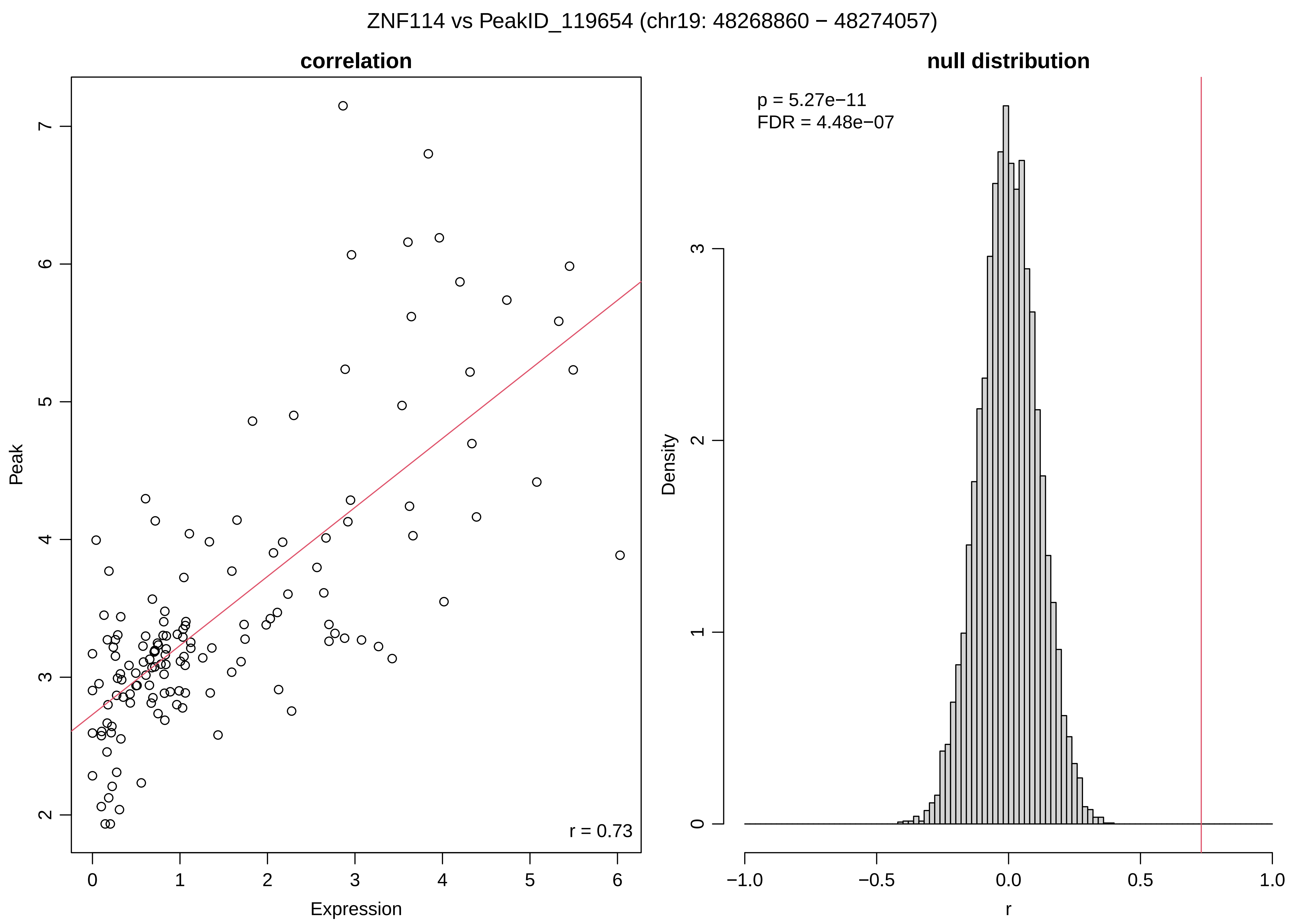

ZNF114 vs PeakID_119654 (chr19: 48268860 − 48274057)
correlation
null distribution
p = 5.27e−11
FDR = 4.48e−07
r = 0.73
Peak
Expression
Density
r

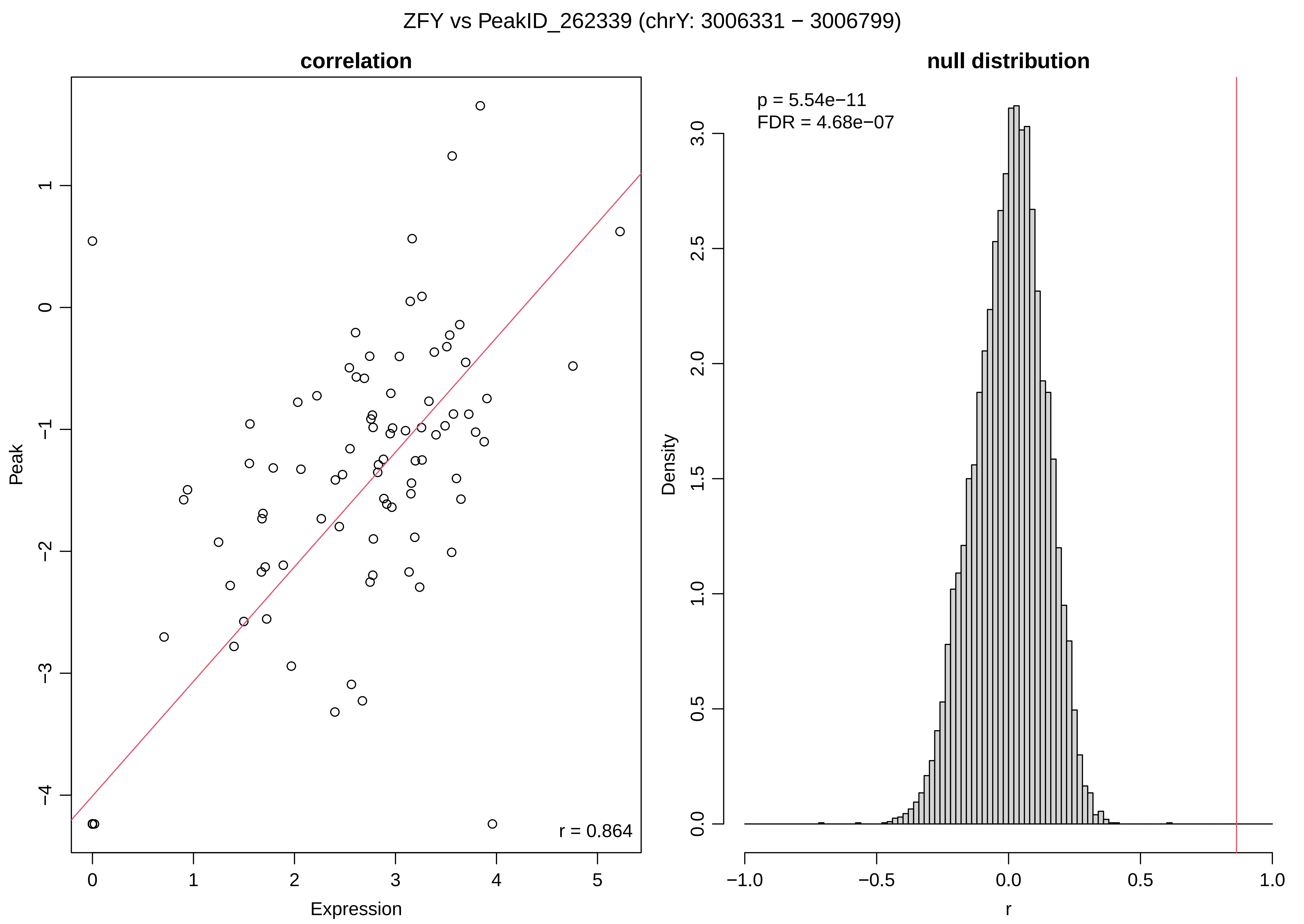
ZFY vs PeakID_262339 (chrY: 3006331 − 3006799)
correlation
Peak
Expression
r = 0.864
null distribution
p = 5.54e−11
FDR = 4.68e−07
Density
r

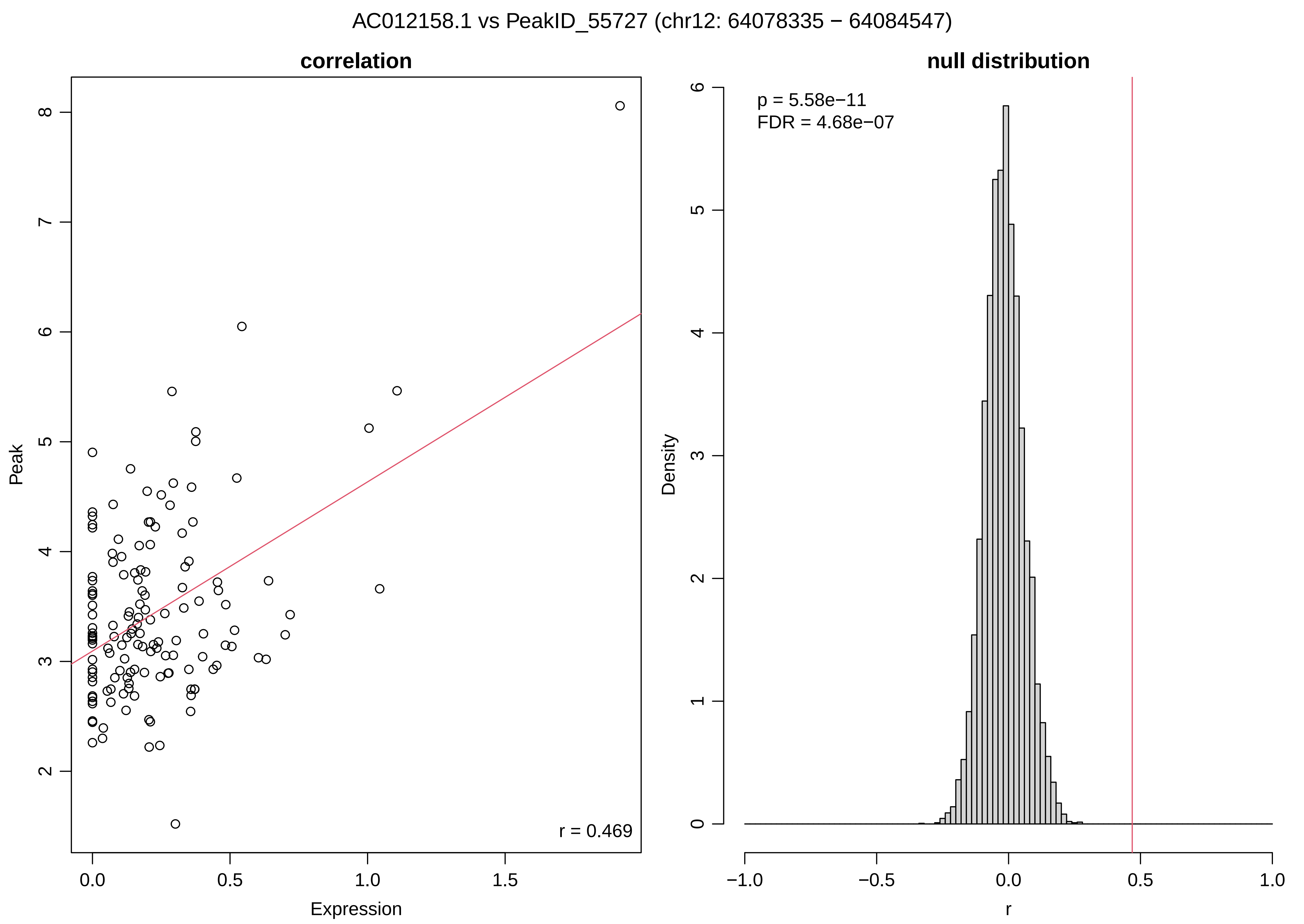
AC012158.1 vs PeakID_55727 (chr12: 64078335 − 64084547)
correlation
Peak
Expression
r = 0.469
null distribution
p = 5.58e−11
FDR = 4.68e−07
Density
r

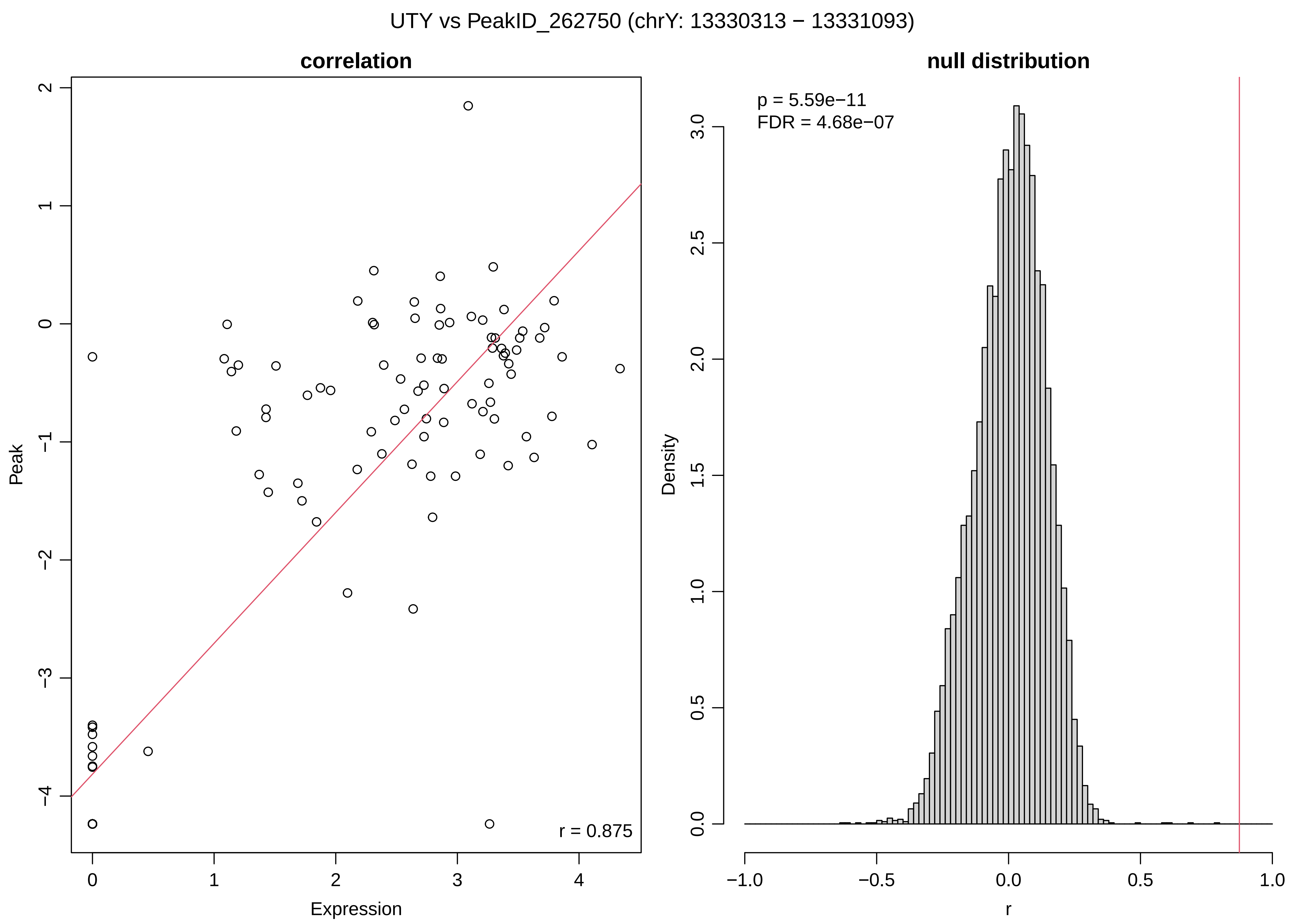

UTY vs PeakID_262750 (chrY: 13330313 − 13331093)
correlation
Peak
Expression
r = 0.875
2
1
0
−1
−2
−3
−4
0
1
2
3
4
null distribution
p = 5.59e−11
FDR = 4.68e−07
Density
r
0.0
0.5
1.0
1.5
2.0
2.5
3.0
−1.0
−0.5
0.0
0.5
1.0

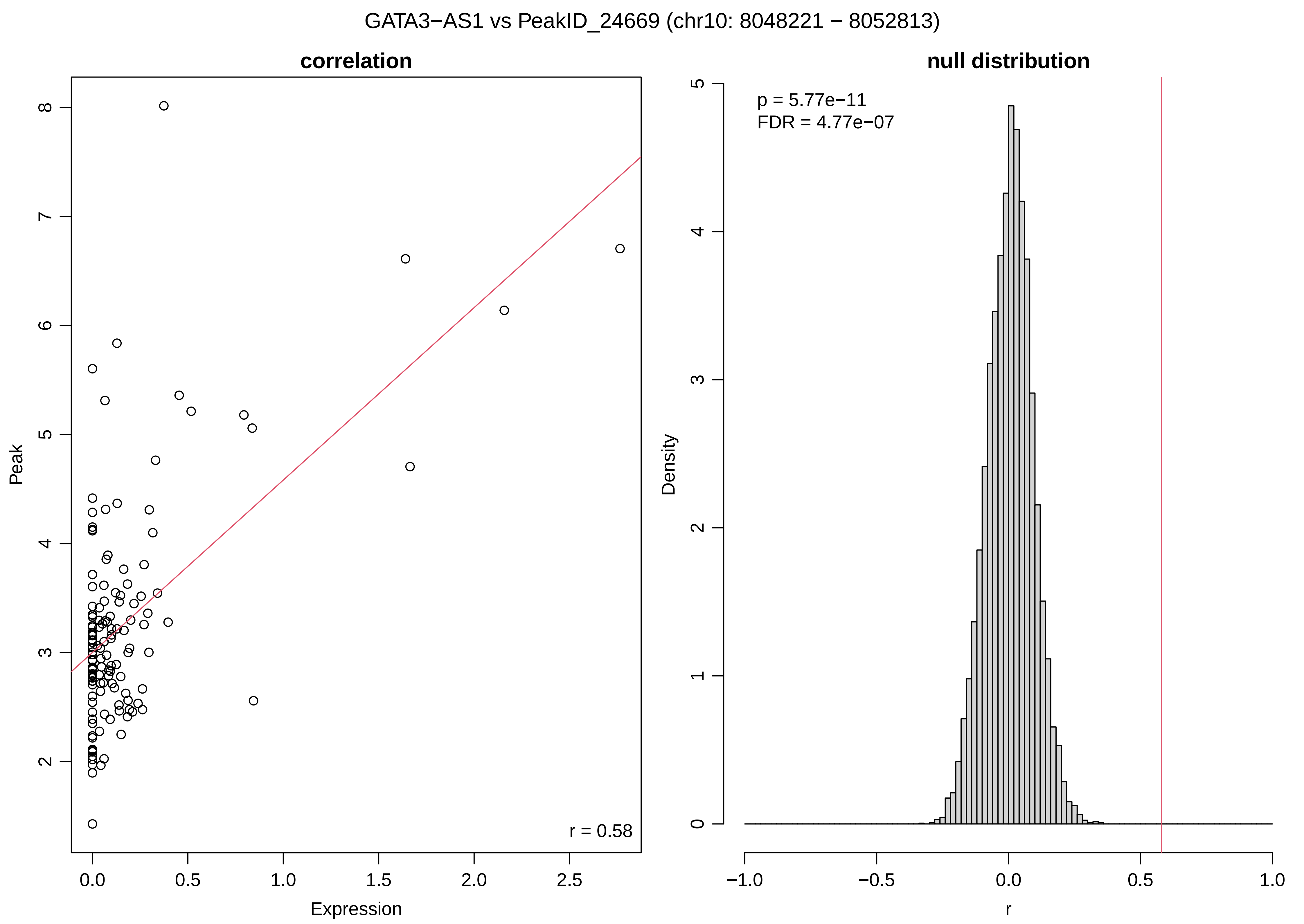

GATA3−AS1 vs PeakID_24669 (chr10: 8048221 − 8052813)
correlation
Peak
Expression
r = 0.58
null distribution
Density
r
p = 5.77e−11
FDR = 4.77e−07

EIF1AY vs PeakID_262957 (chrY: 20574956 − 20576767)

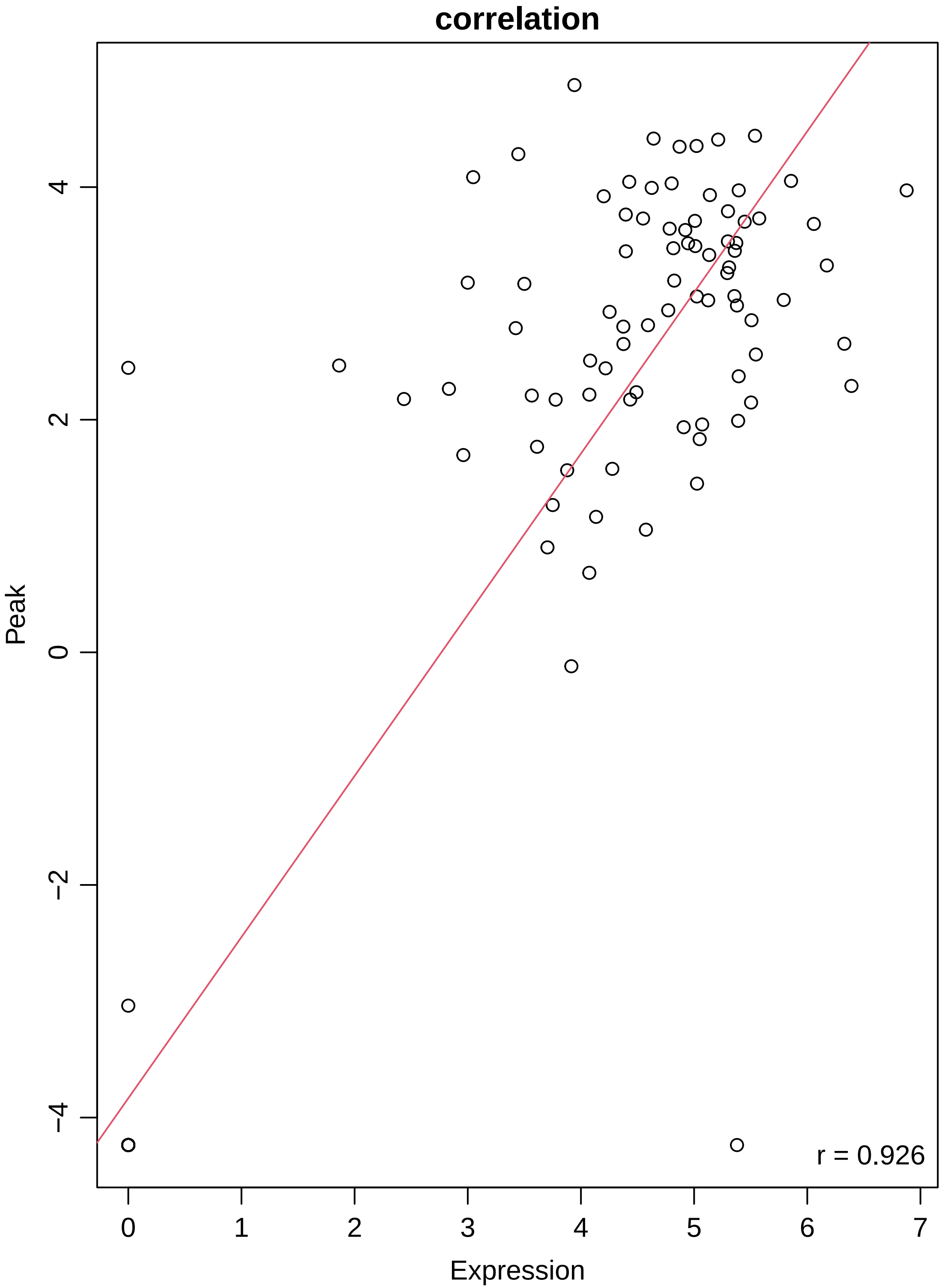

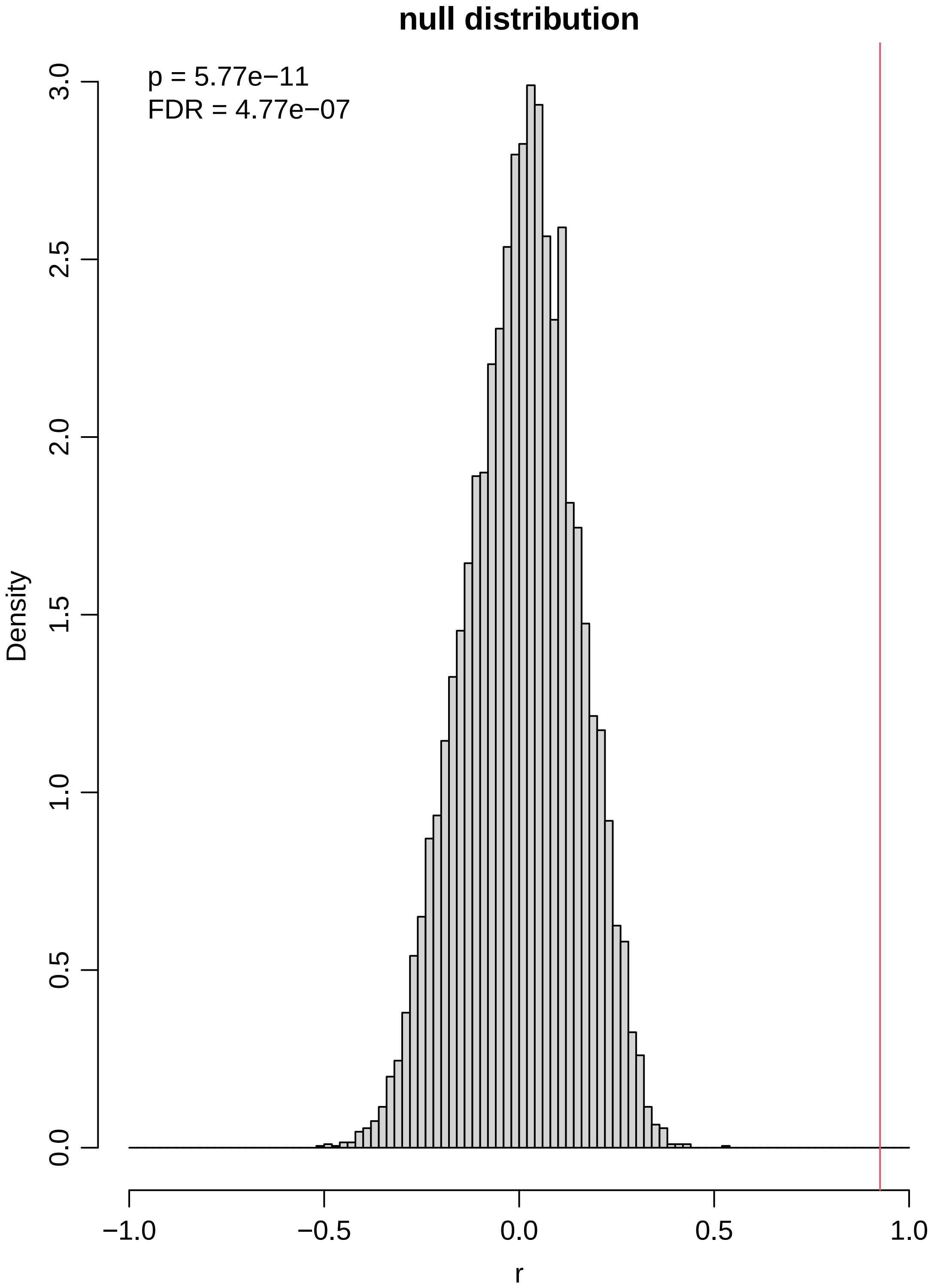

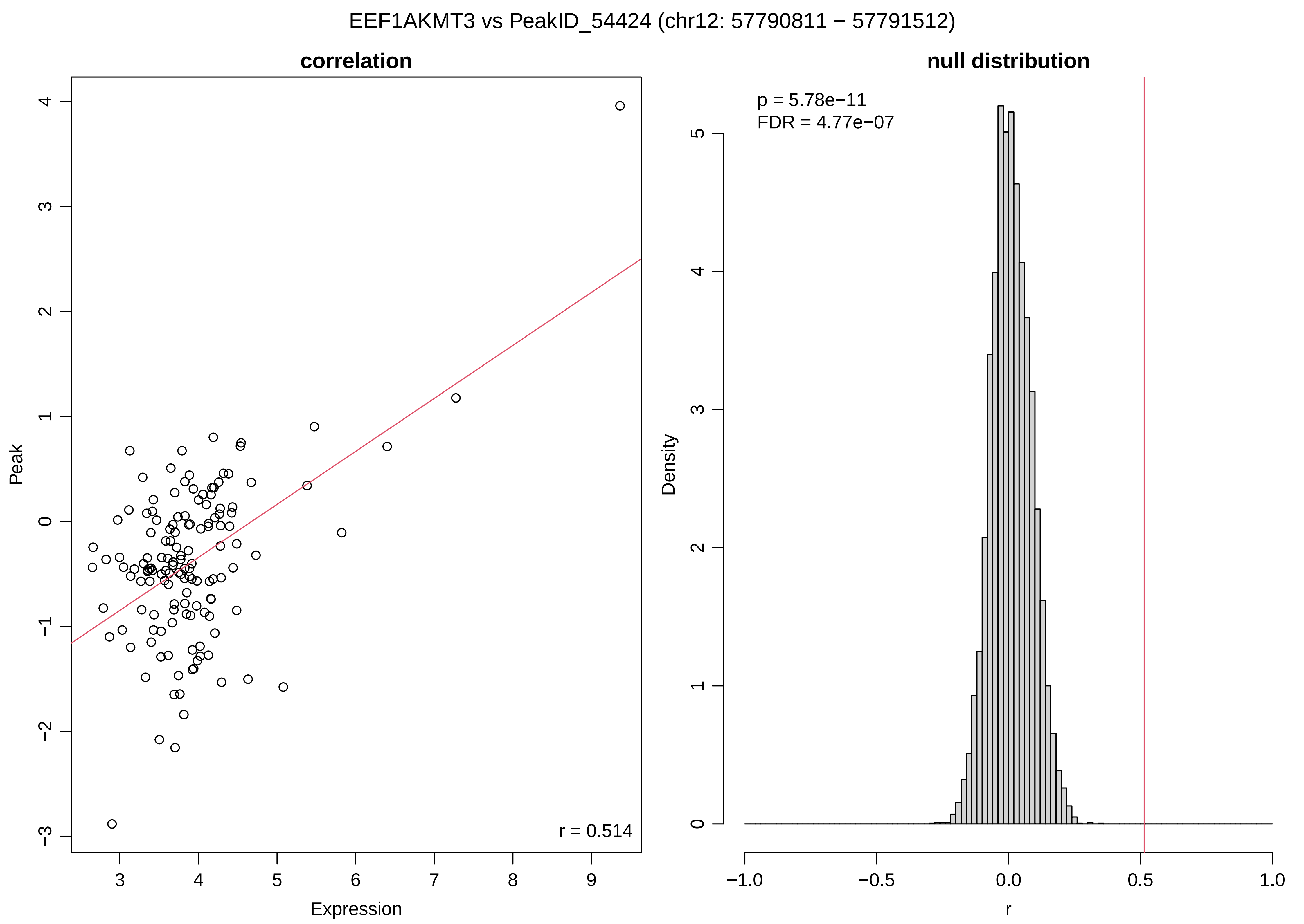

EEF1AKMT3 vs PeakID_54424 (chr12: 57790811 − 57791512)
correlation
Peak
Expression
r = 0.514
null distribution
p = 5.78e−11
FDR = 4.77e−07
Density
r

RNU4−20P vs PeakID_54836 (chr12: 59588854 − 59590180)

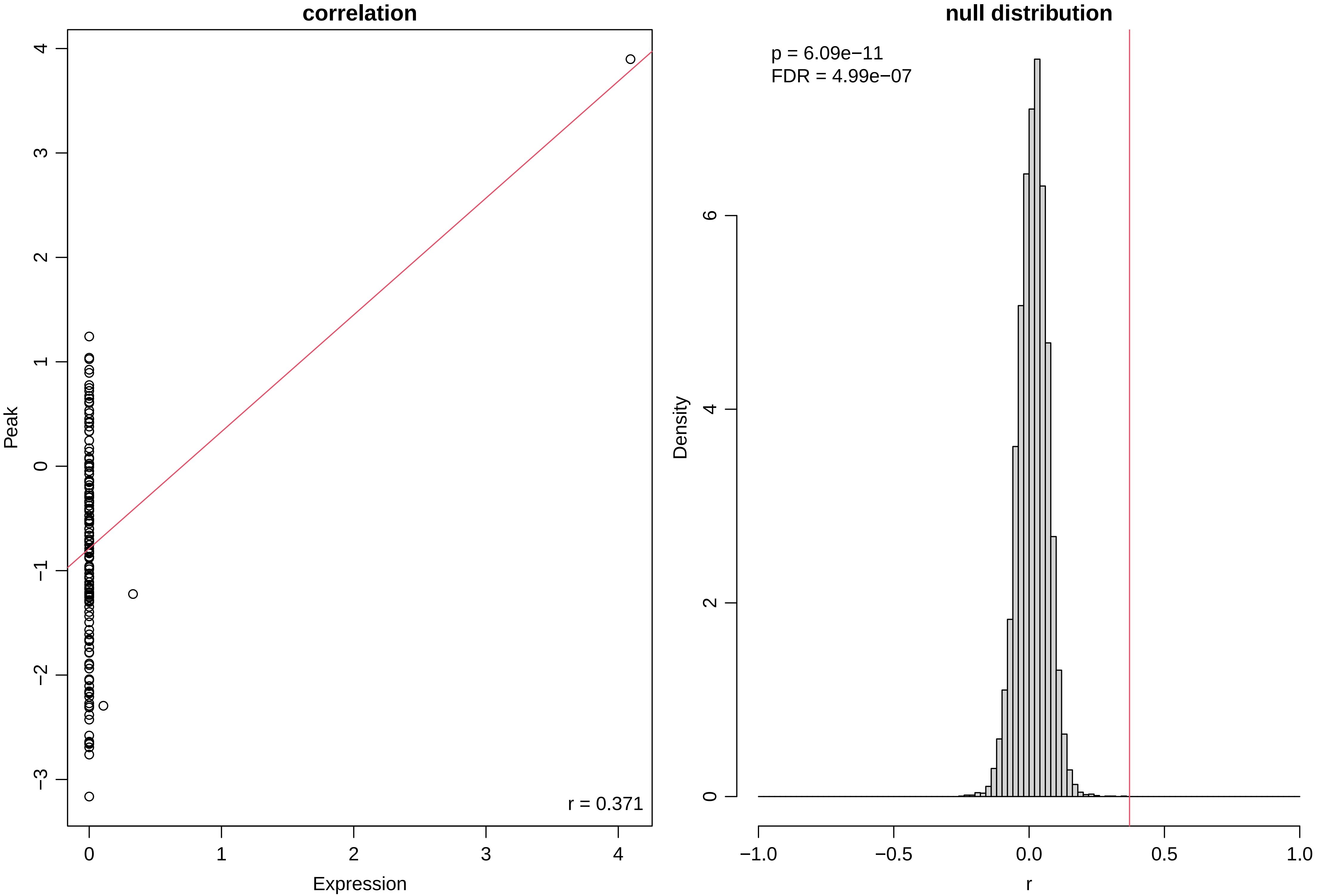

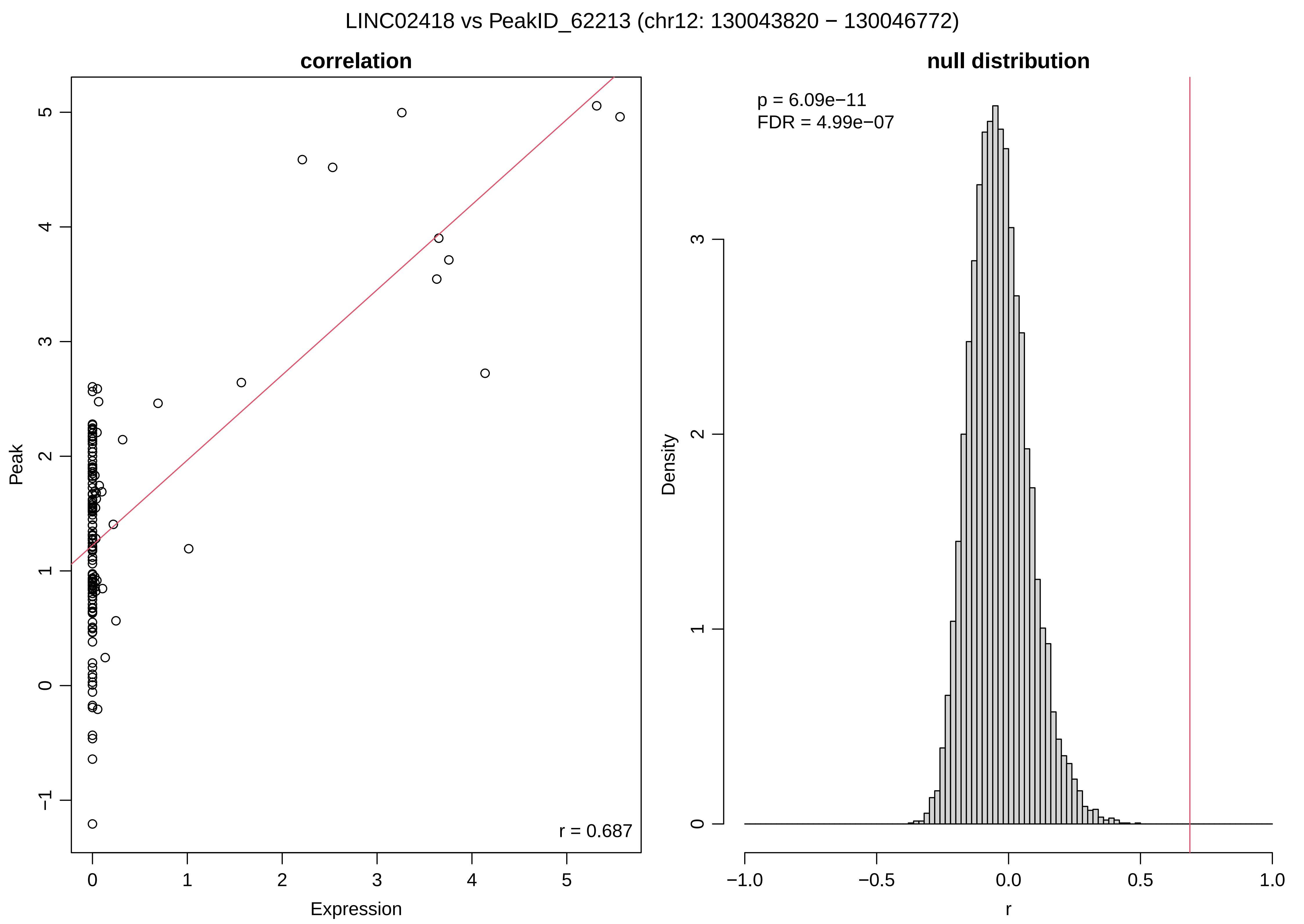

LINC02418 vs PeakID_62213 (chr12: 130043820 − 130046772)
correlation
Peak
Expression
r = 0.687
null distribution
p = 6.09e−11
FDR = 4.99e−07
Density
r

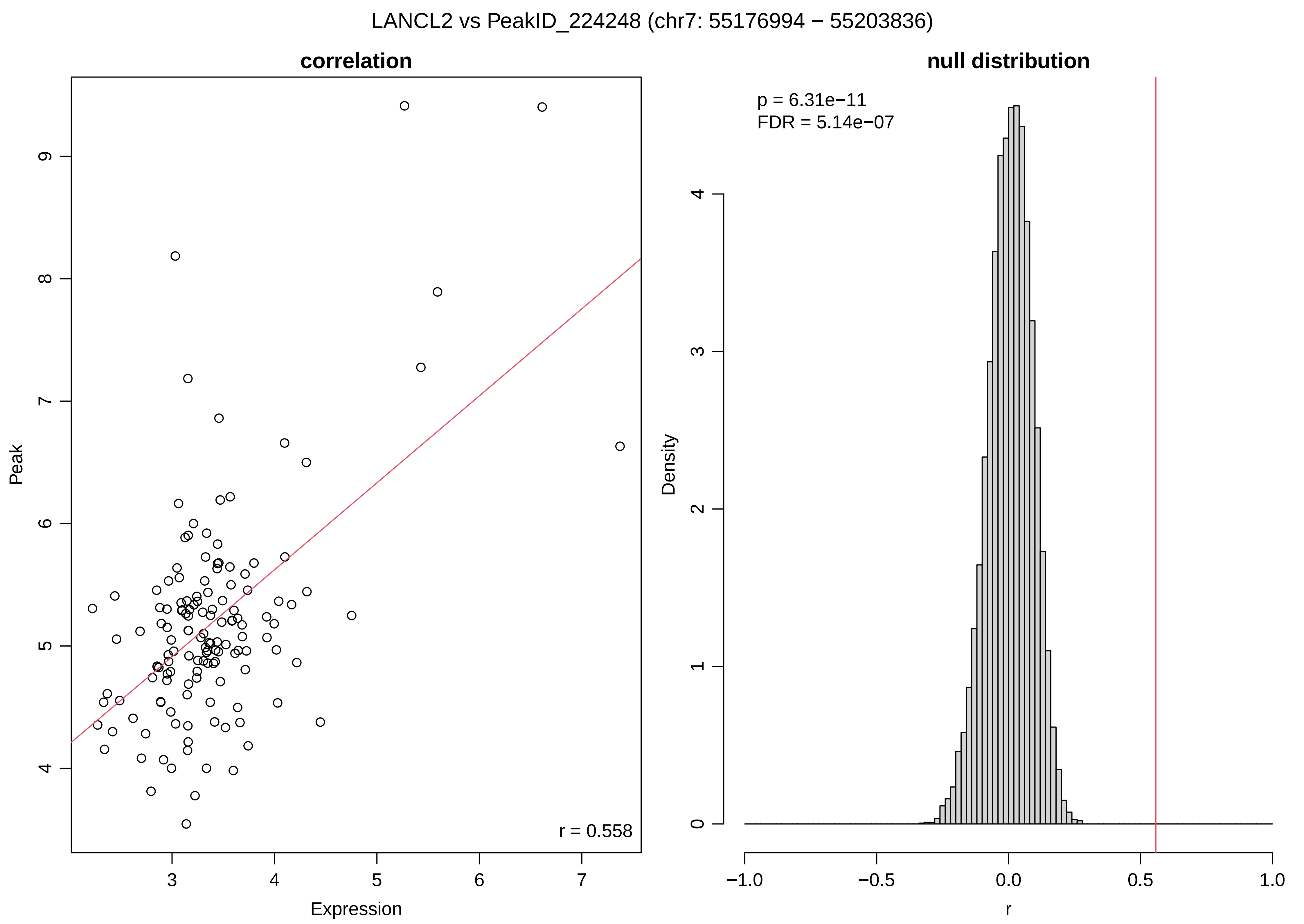
LANCL2 vs PeakID_224248 (chr7: 55176994 − 55203836)
correlation
Peak
Expression
r = 0.558
null distribution
p = 6.31e−11
FDR = 5.14e−07
Density
r

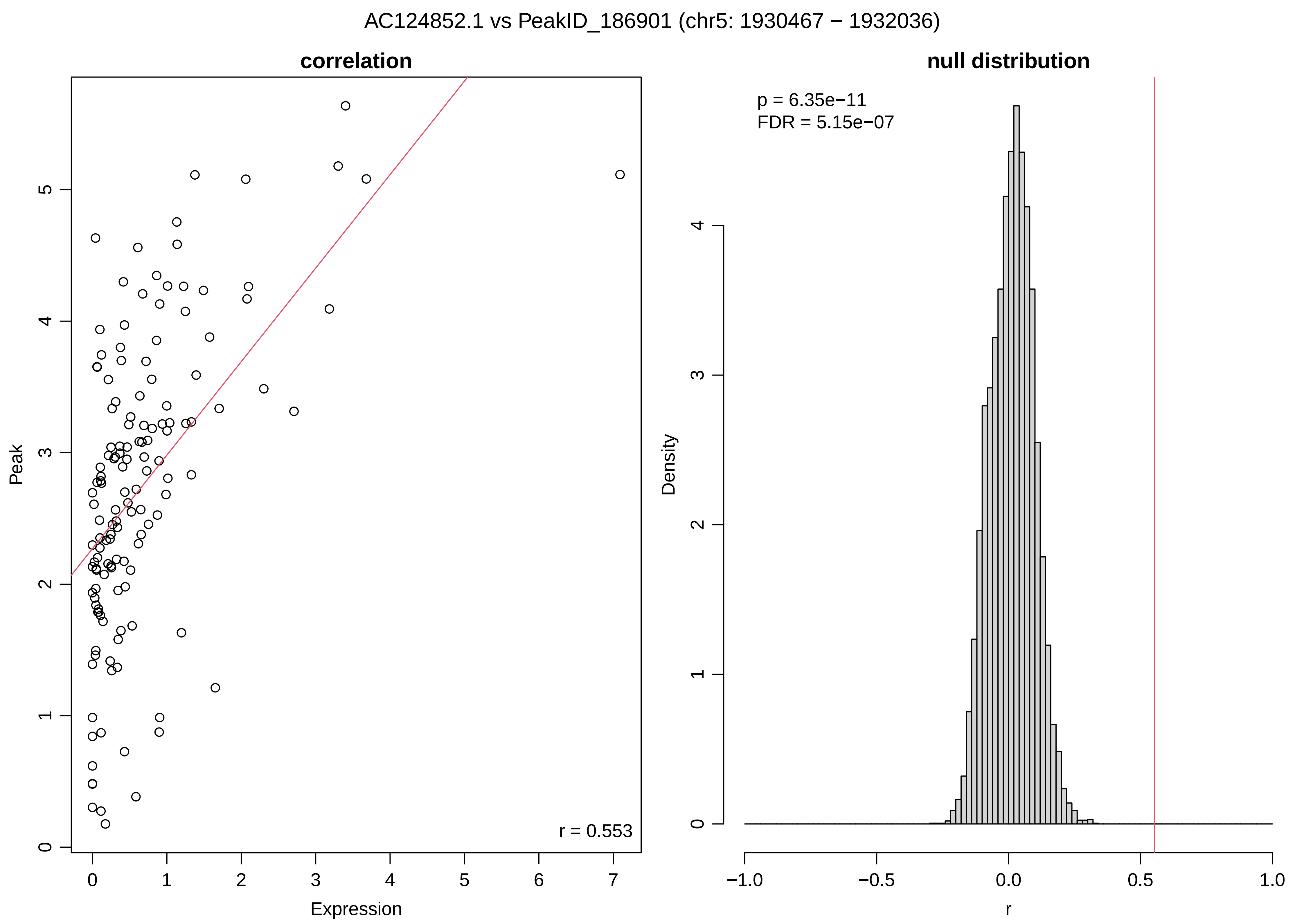
AC124852.1 vs PeakID_186901 (chr5: 1930467 − 1932036)
correlation
Peak
Expression
r = 0.553
null distribution
p = 6.35e−11
FDR = 5.15e−07
Density
r

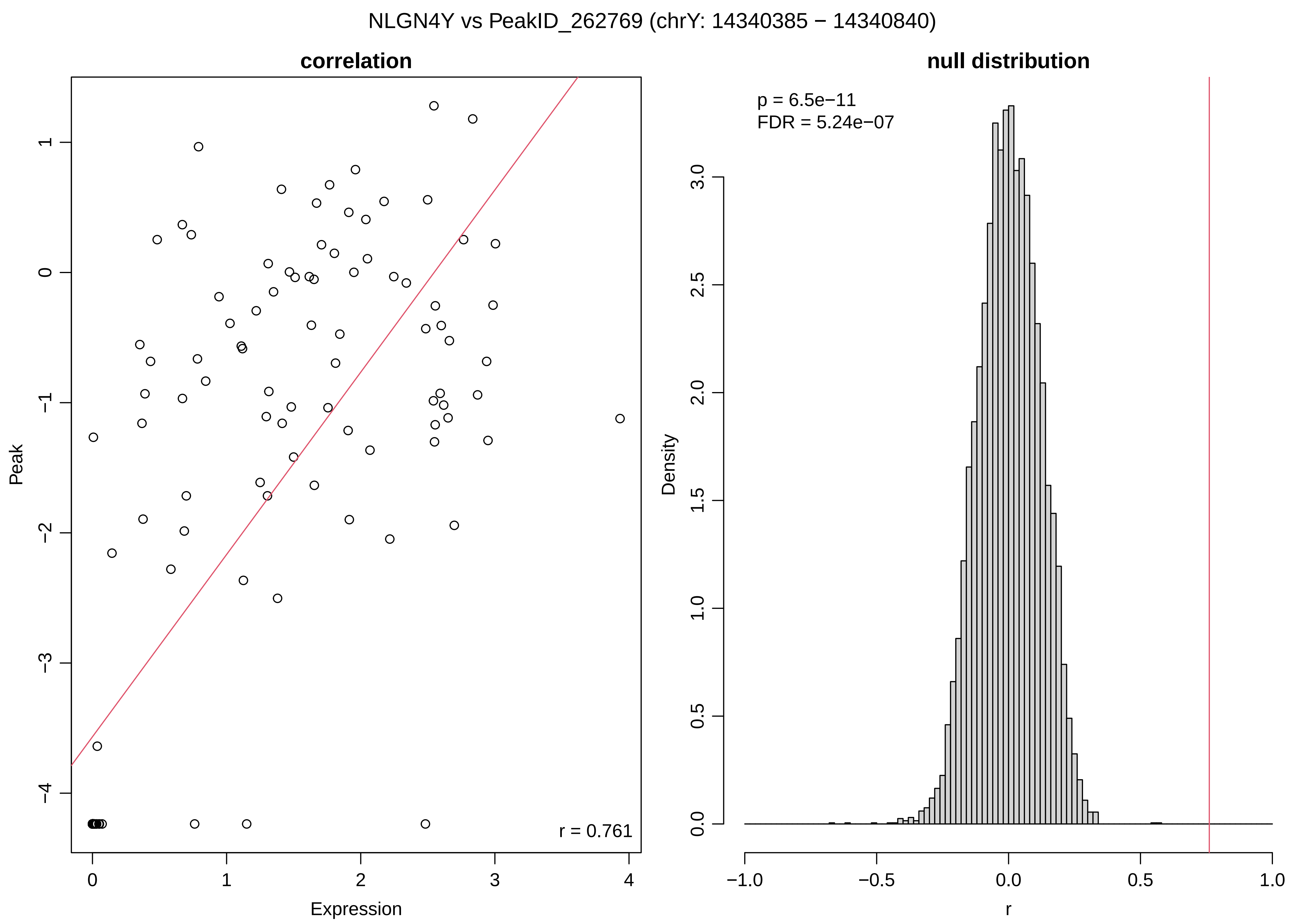

NLGN4Y vs PeakID_262769 (chrY: 14340385 − 14340840)
correlation
Peak
Expression
r = 0.761
null distribution
p = 6.5e−11
FDR = 5.24e−07
Density
r

LINC02403 vs PeakID_54572 (chr12: 58336810 − 58346102)

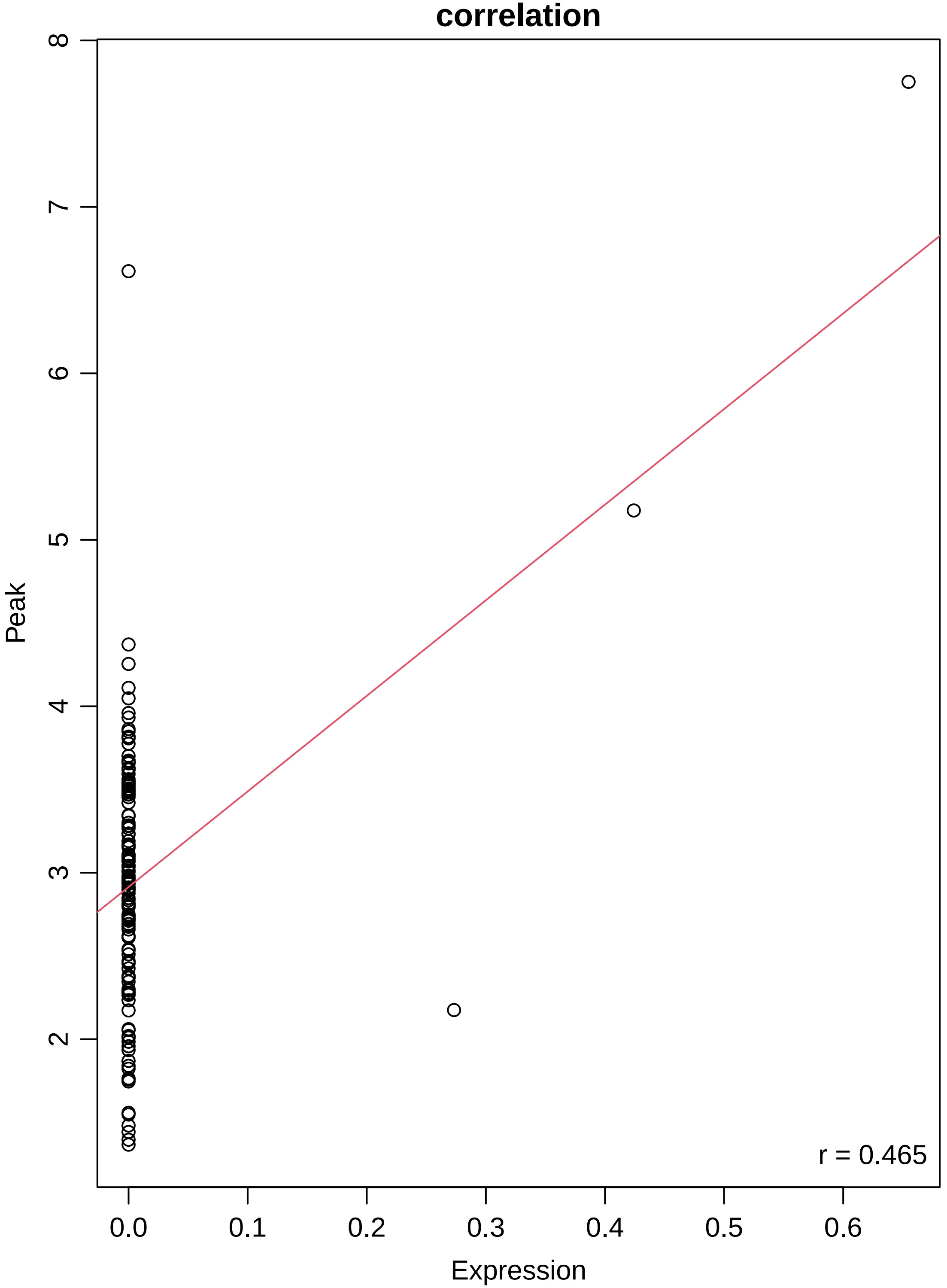

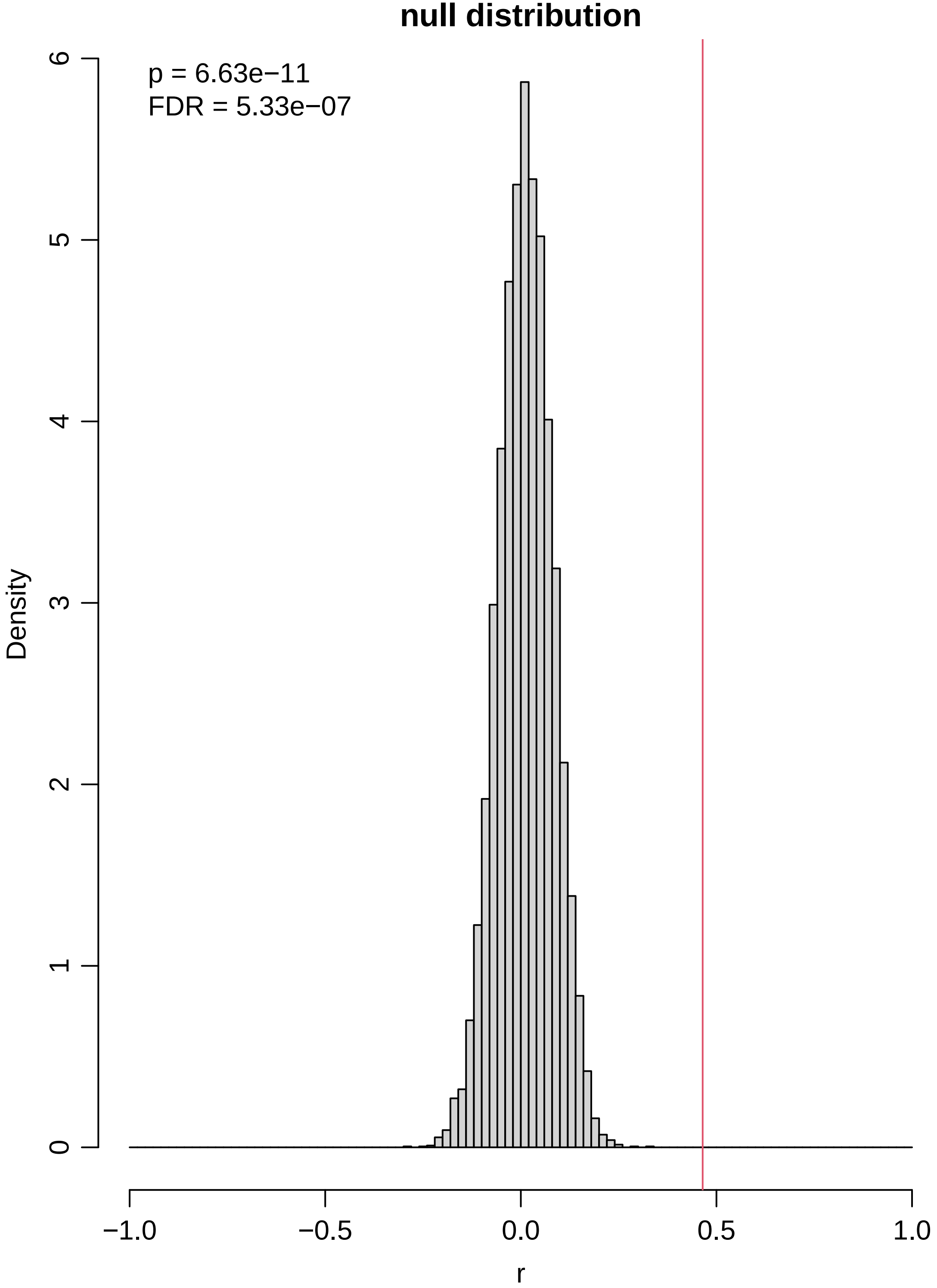

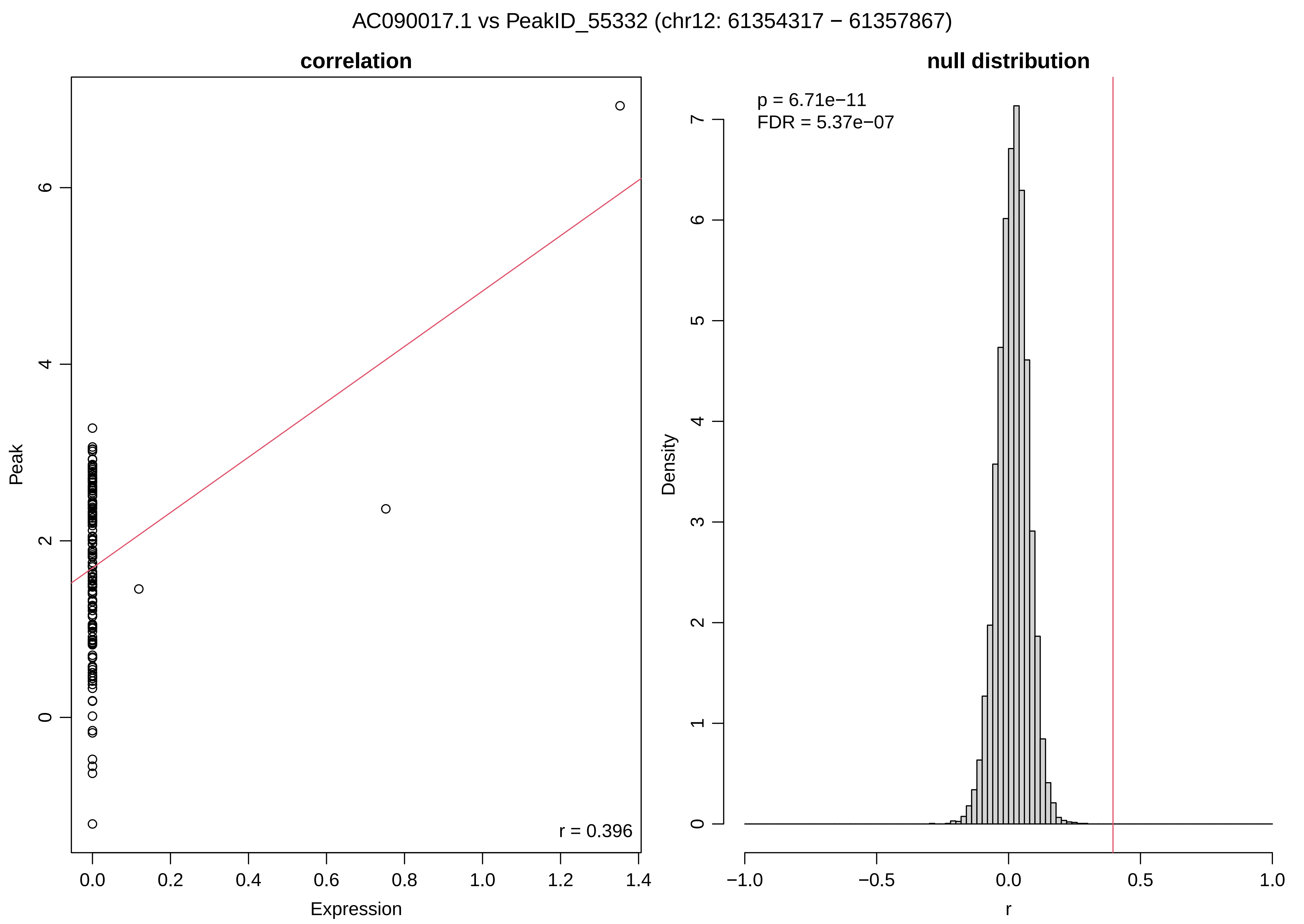
AC090017.1 vs PeakID_55332 (chr12: 61354317 − 61357867)
correlation
null distribution
p = 6.71e−11
FDR = 5.37e−07
r = 0.396
Peak
Expression
Density
r

METTL1 vs PeakID_54423 (chr12: 57743158 − 57790607)

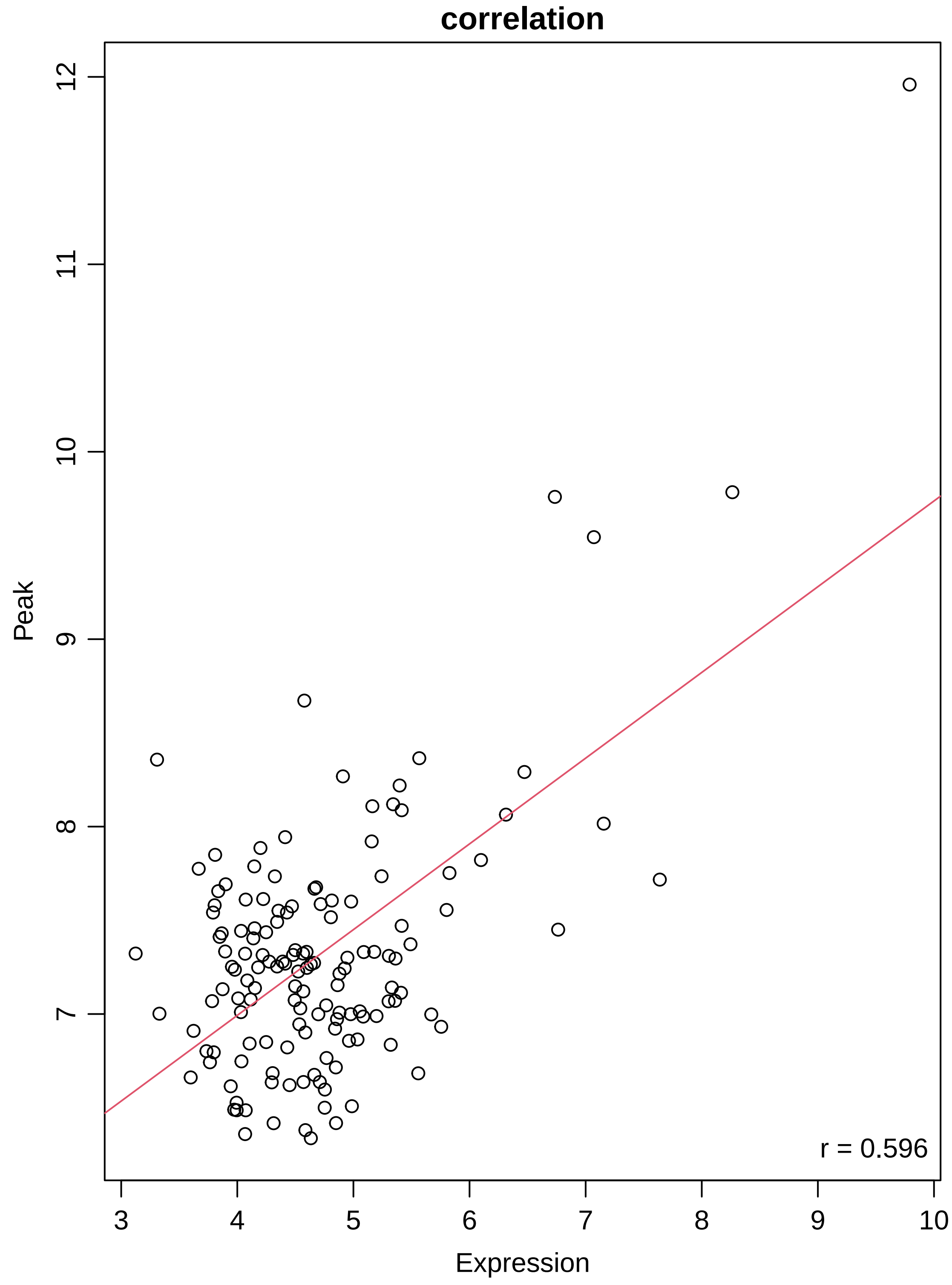

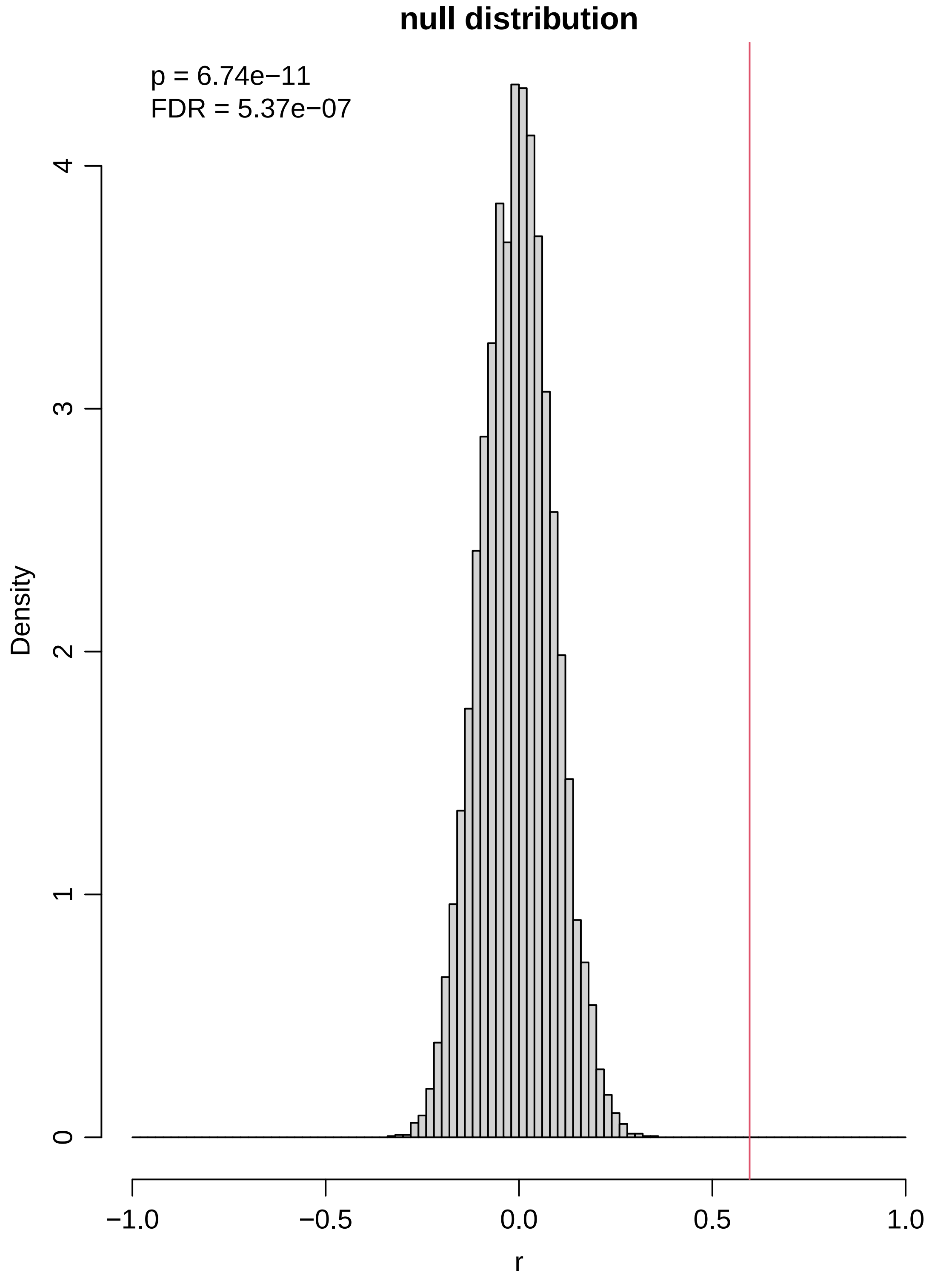

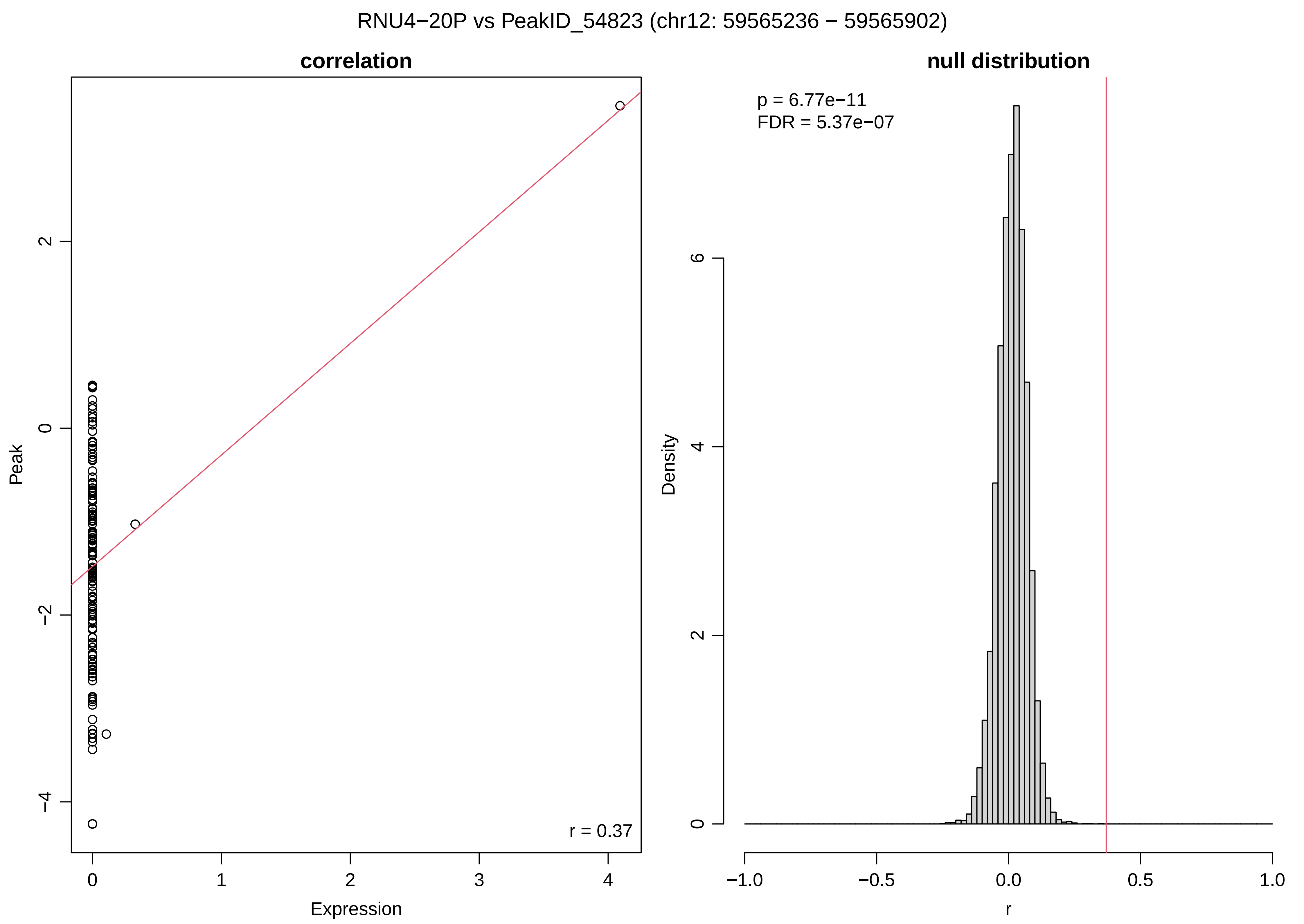

RNU4−20P vs PeakID_54823 (chr12: 59565236 − 59565902)
correlation
null distribution
p = 6.77e−11
FDR = 5.37e−07
r = 0.37
Peak
Expression
Density
r
2
0
−2
−4
0
1
2
3
4
6
4
2
0
−1.0
−0.5
0.0
0.5
1.0

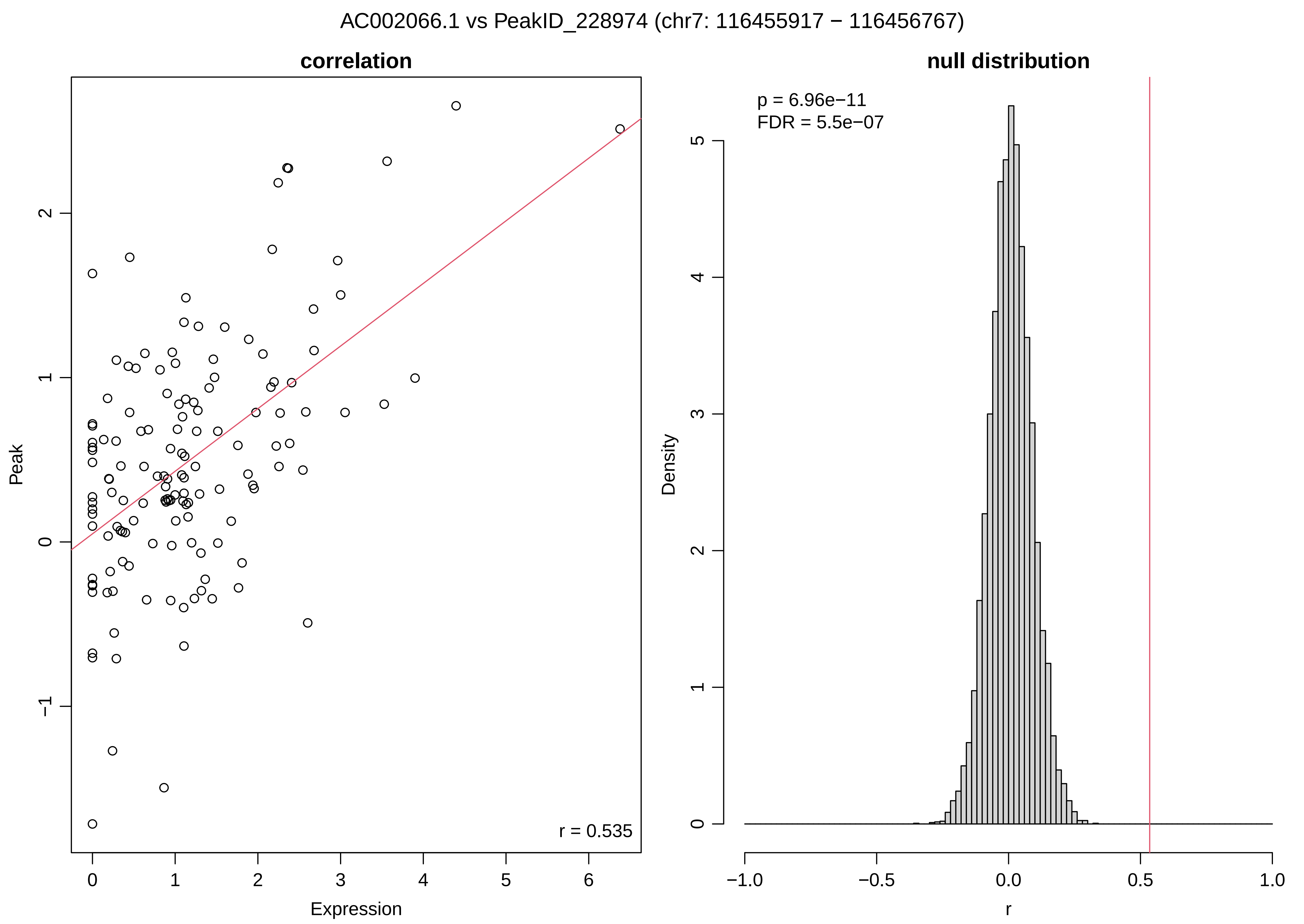
AC002066.1 vs PeakID_228974 (chr7: 116455917 − 116456767)
correlation
null distribution
p = 6.96e−11
FDR = 5.5e−07
r = 0.535
Peak
Expression
Density
r

AC010605.1 vs PeakID_117161 (chr19: 39030648 − 39033625)

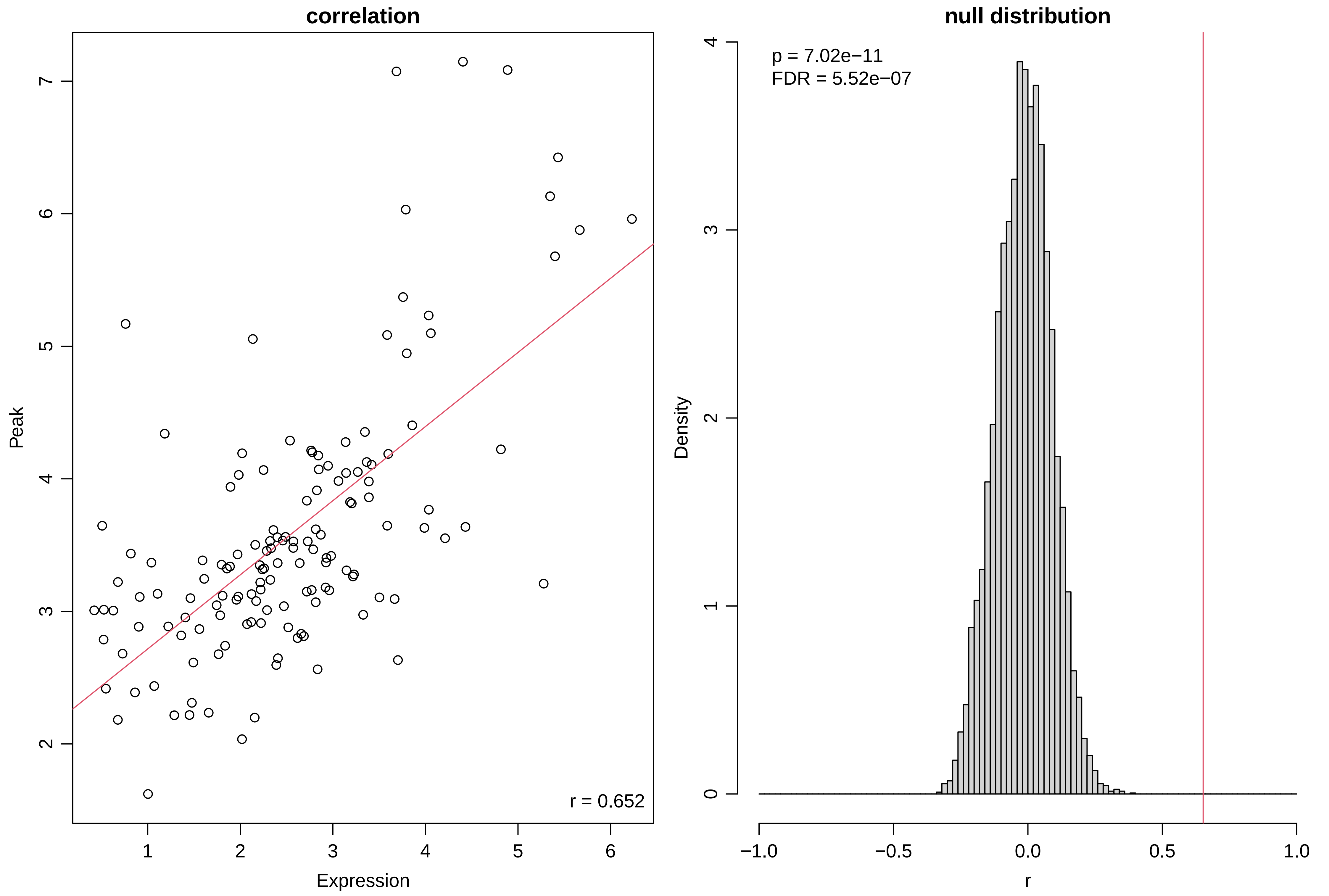

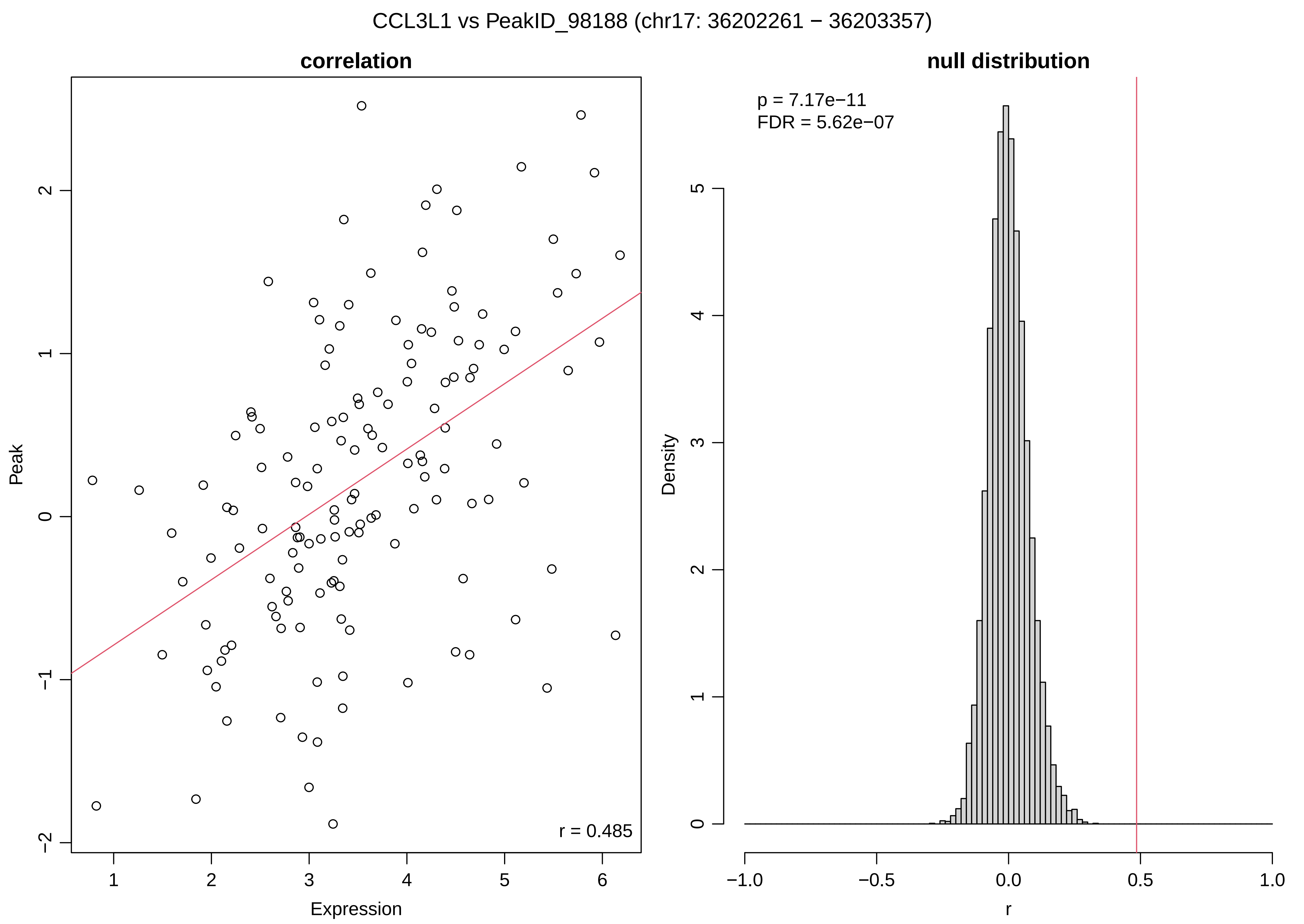
CCL3L1 vs PeakID_98188 (chr17: 36202261 − 36203357)
correlation
Peak
Expression
r = 0.485
null distribution
p = 7.17e−11
FDR = 5.62e−07
Density
r

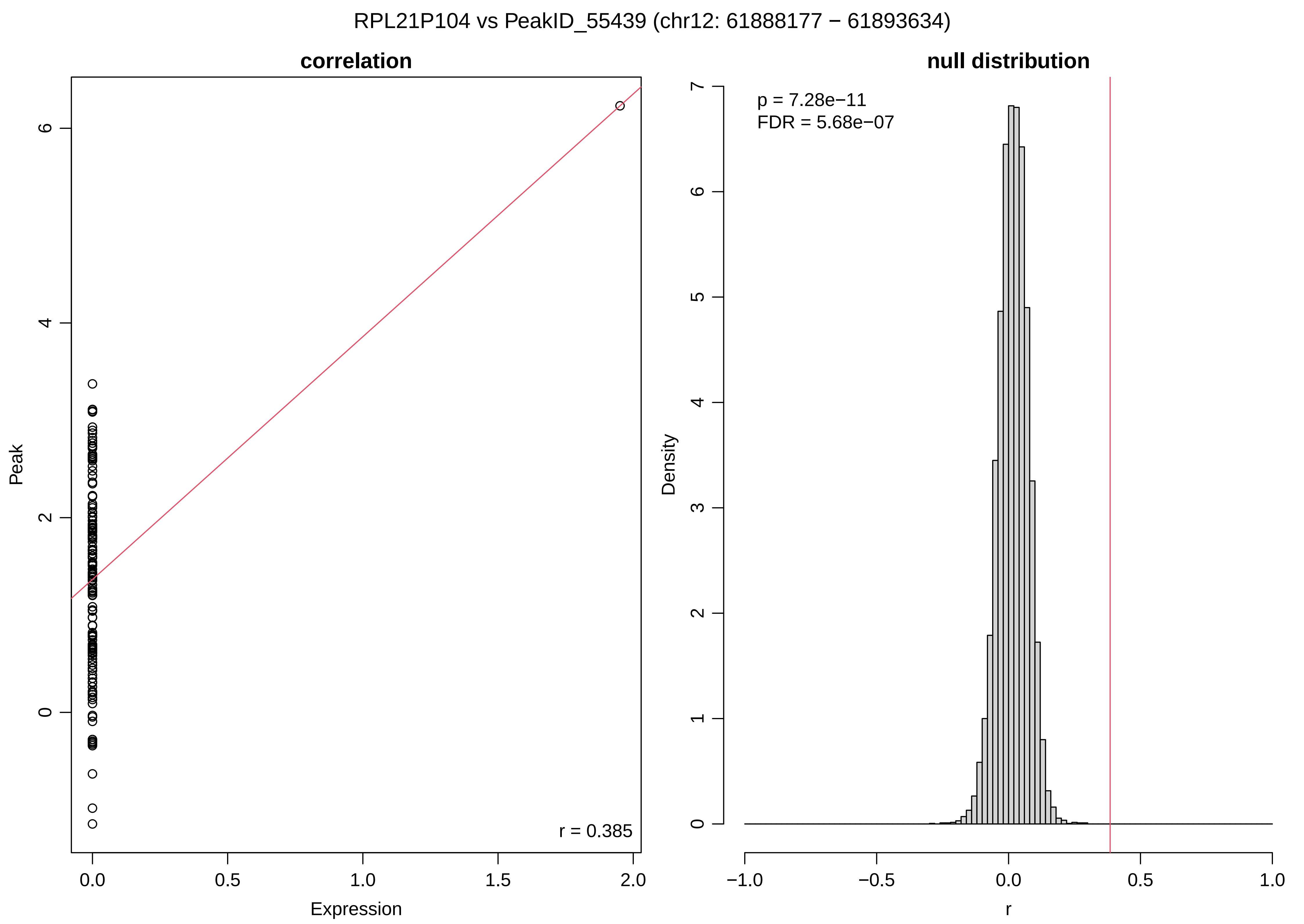
RPL21P104 vs PeakID_55439 (chr12: 61888177 − 61893634)
correlation
null distribution
p = 7.28e−11
FDR = 5.68e−07
r = 0.385
Peak
Expression
Density
r
0.0
0.5
1.0
1.5
2.0
−1.0
−0.5
0.0
0.5
1.0
0
2
4
6
0
1
2
3
4
5
6
7

KBTBD12 vs PeakID_169075 (chr3: 127915063 − 127916307)

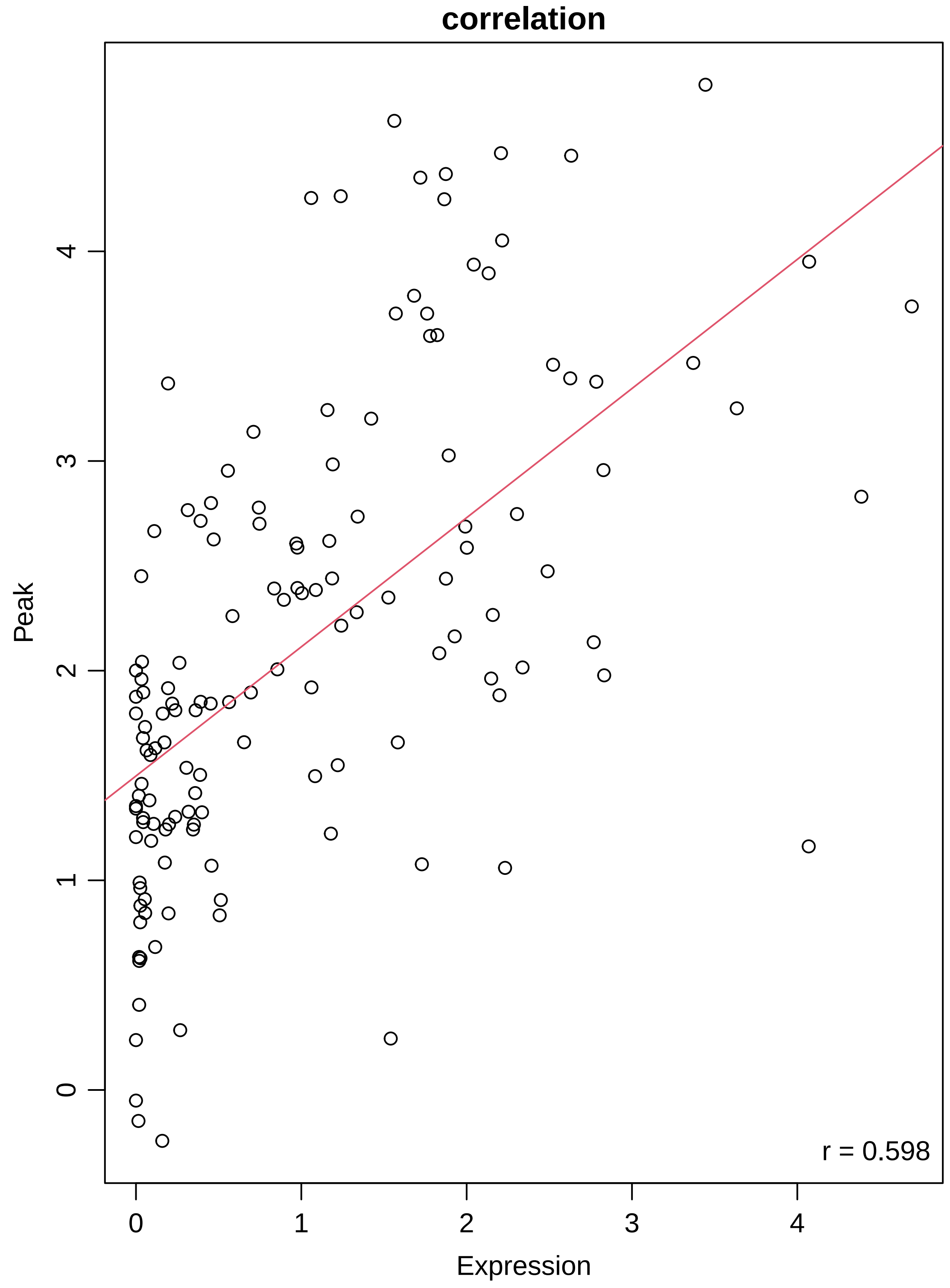

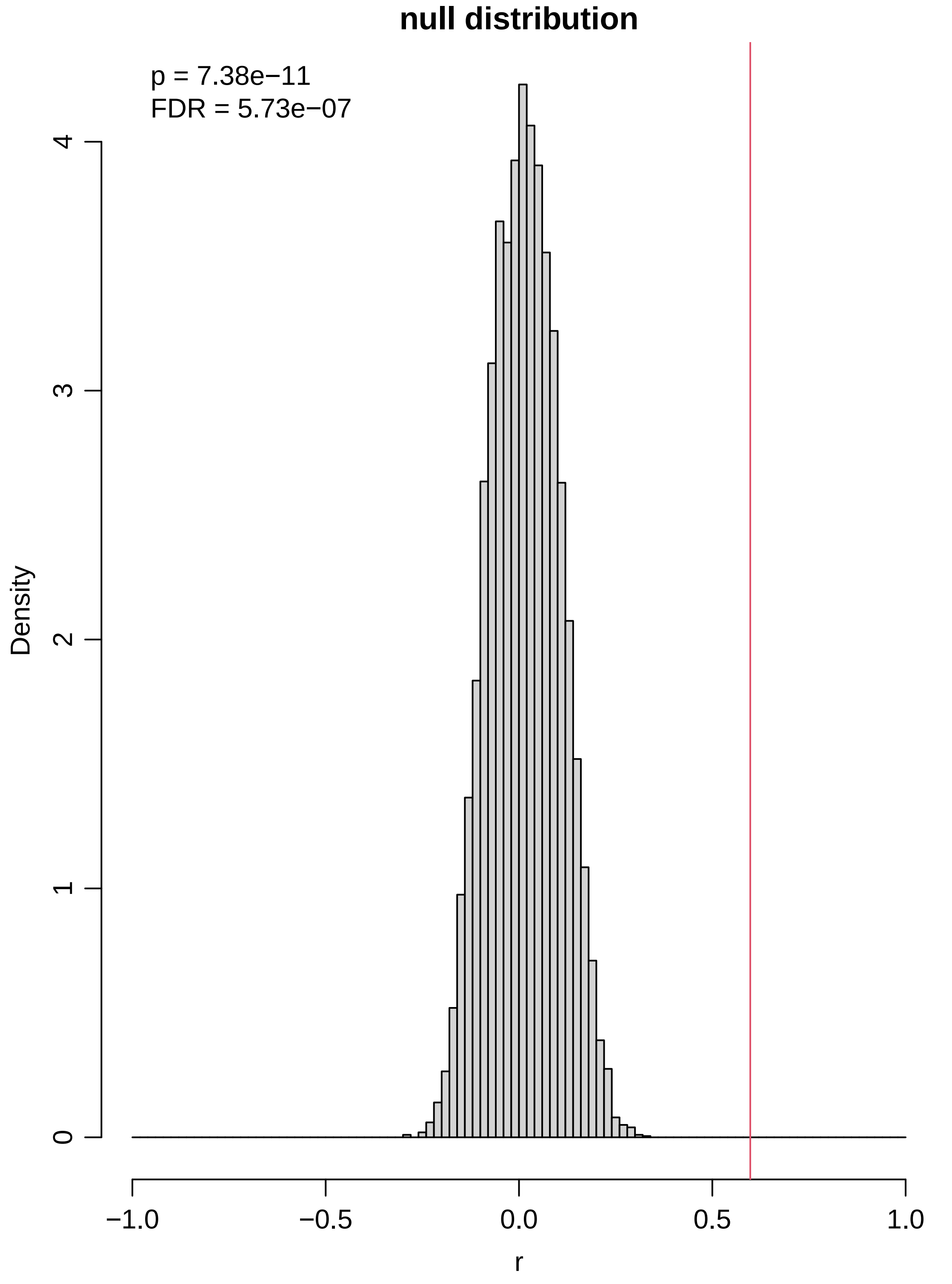

RNU4−20P vs PeakID_54821 (chr12: 59557490 − 59560012)

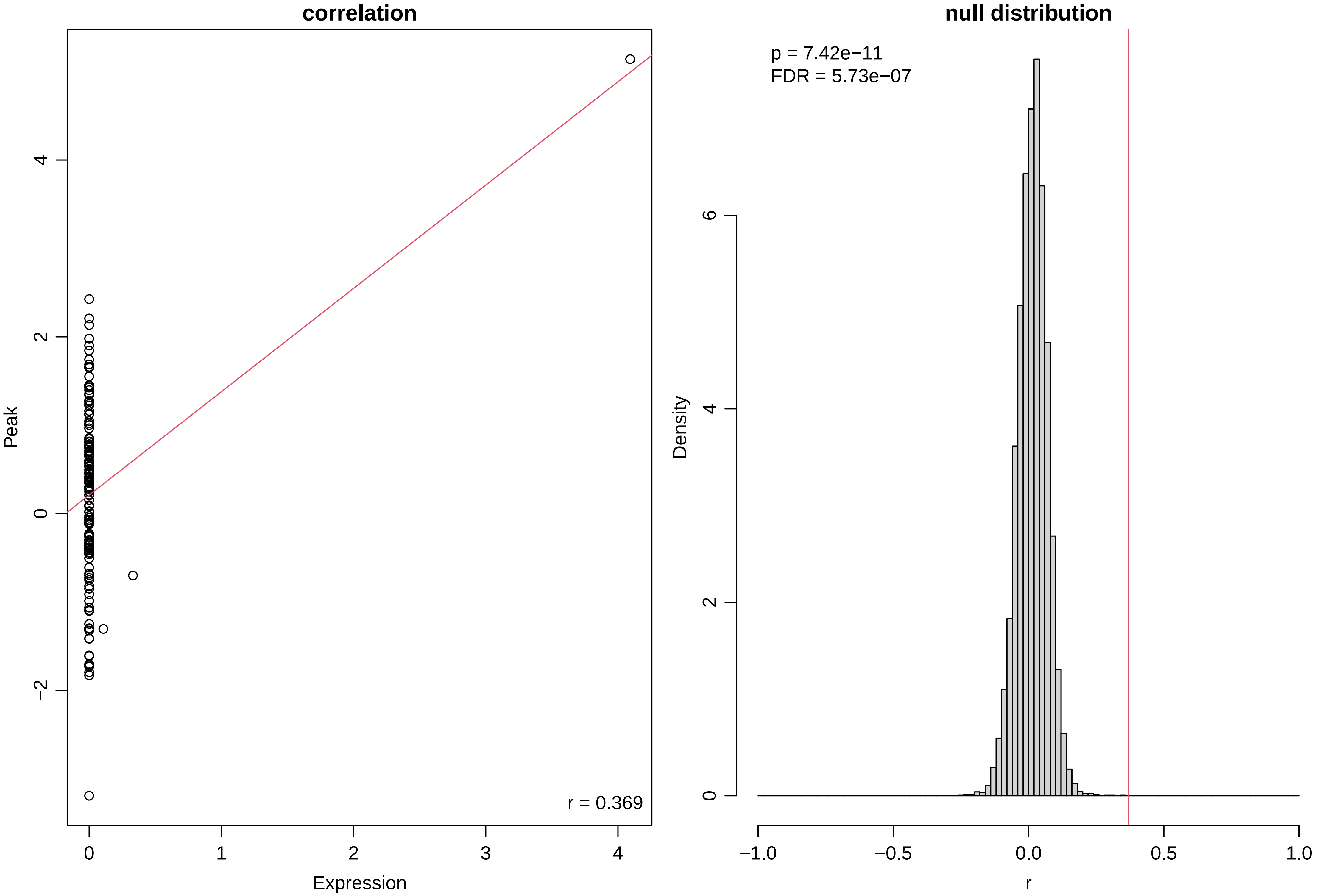

SLC35E3 vs PeakID_56658 (chr12: 68973555 − 68974449)

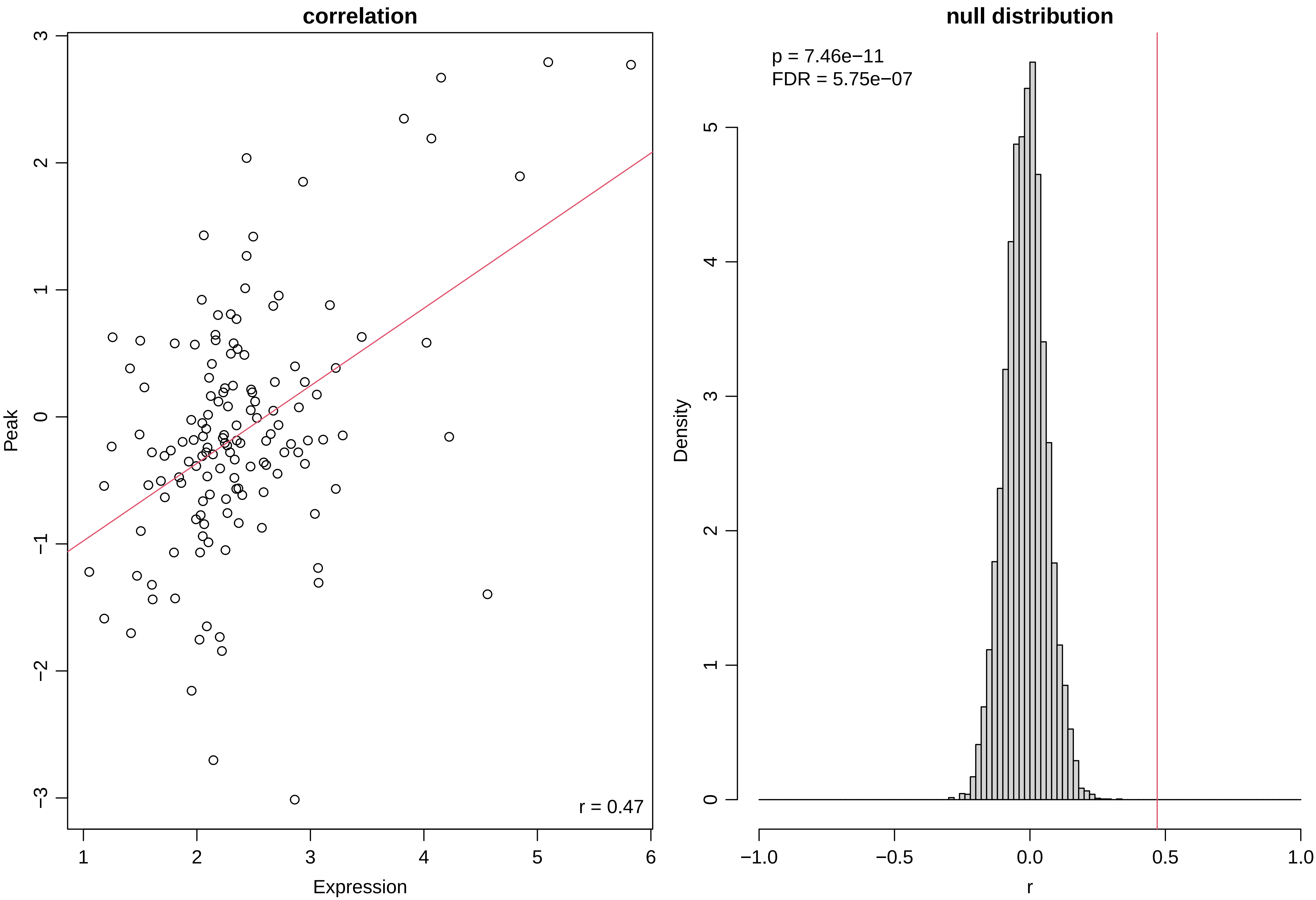

RPS4Y1 vs PeakID_262331 (chrY: 2887764 − 2888697)

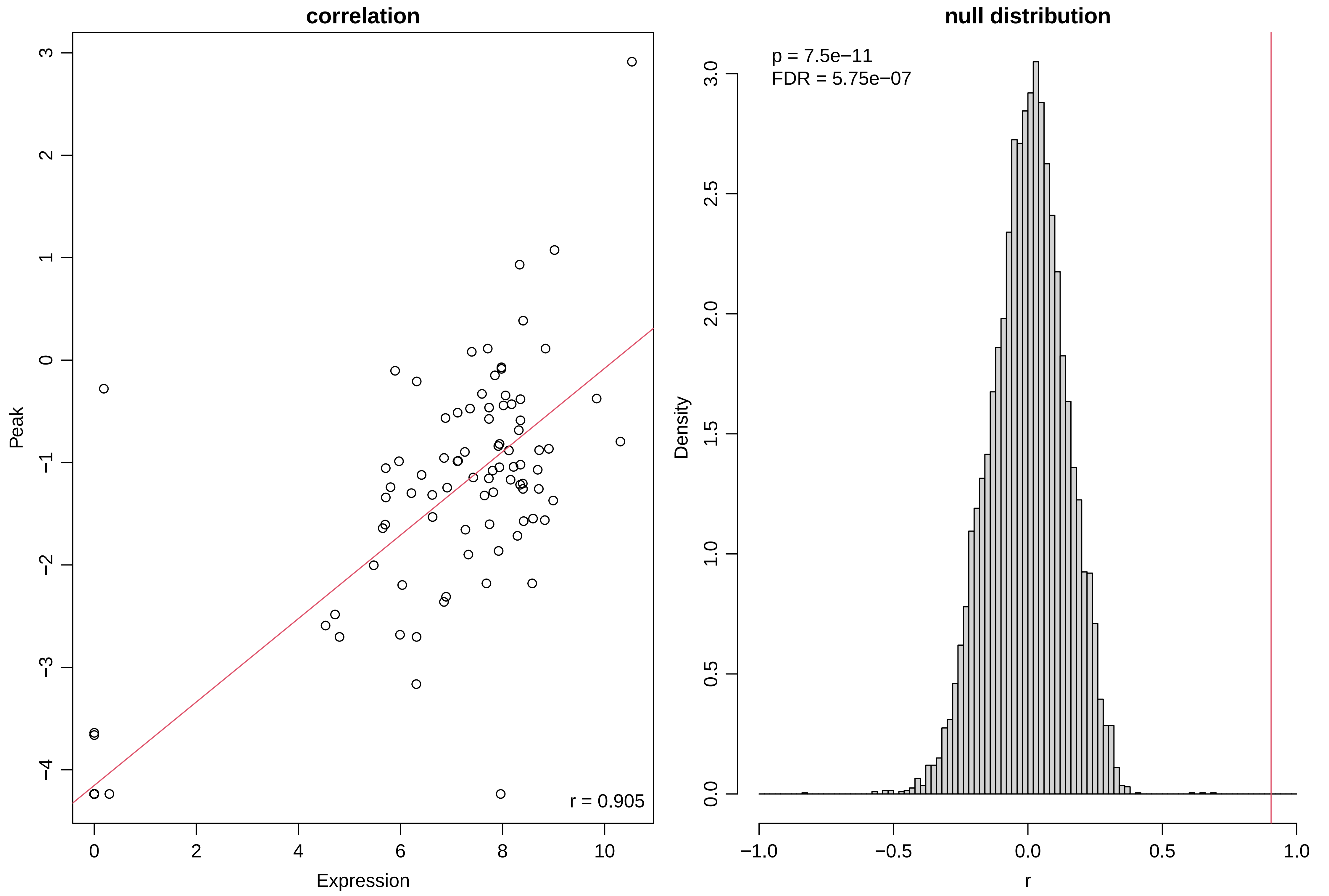

CLDN6 vs PeakID_85568 (chr16: 3017037 − 3018452)

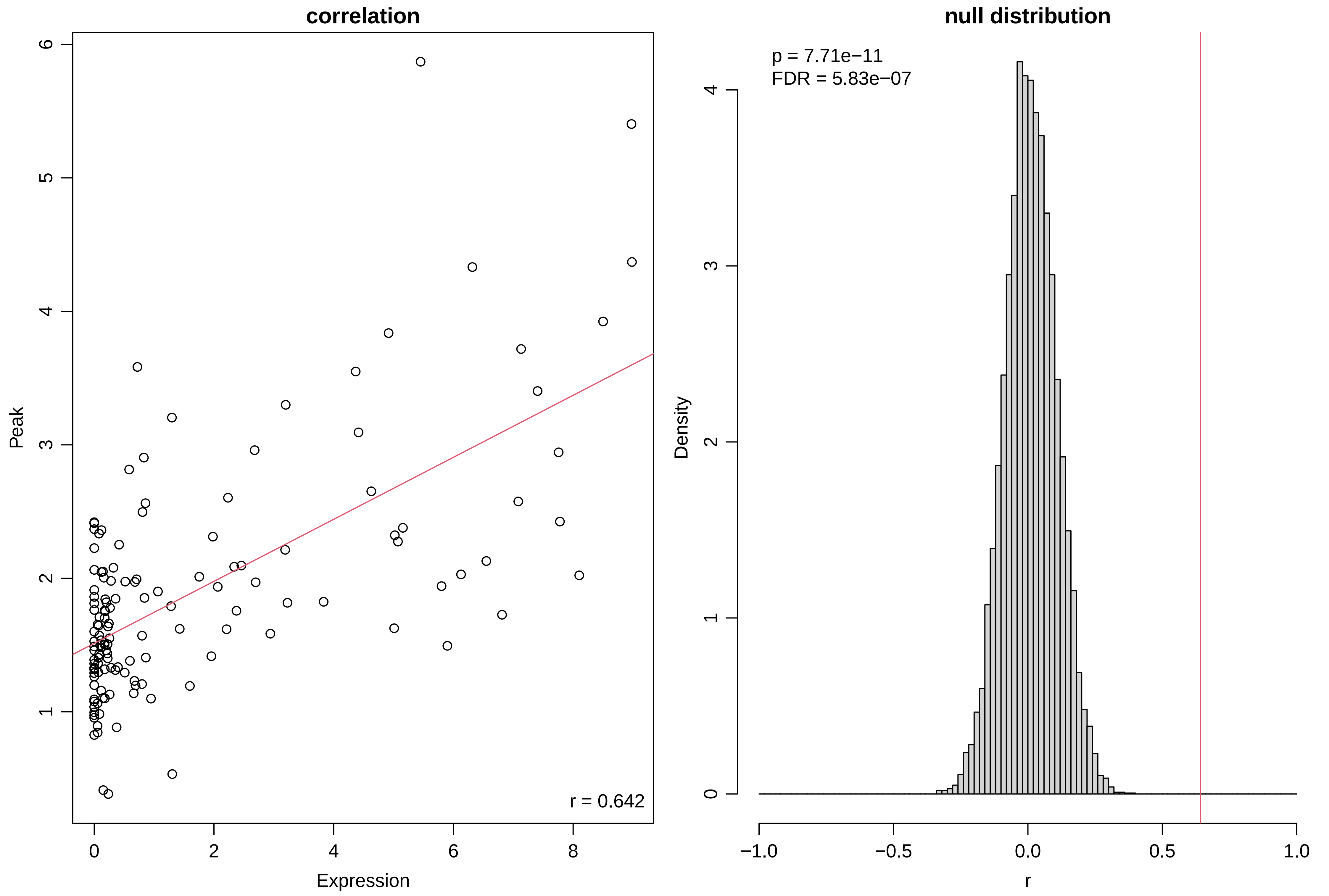

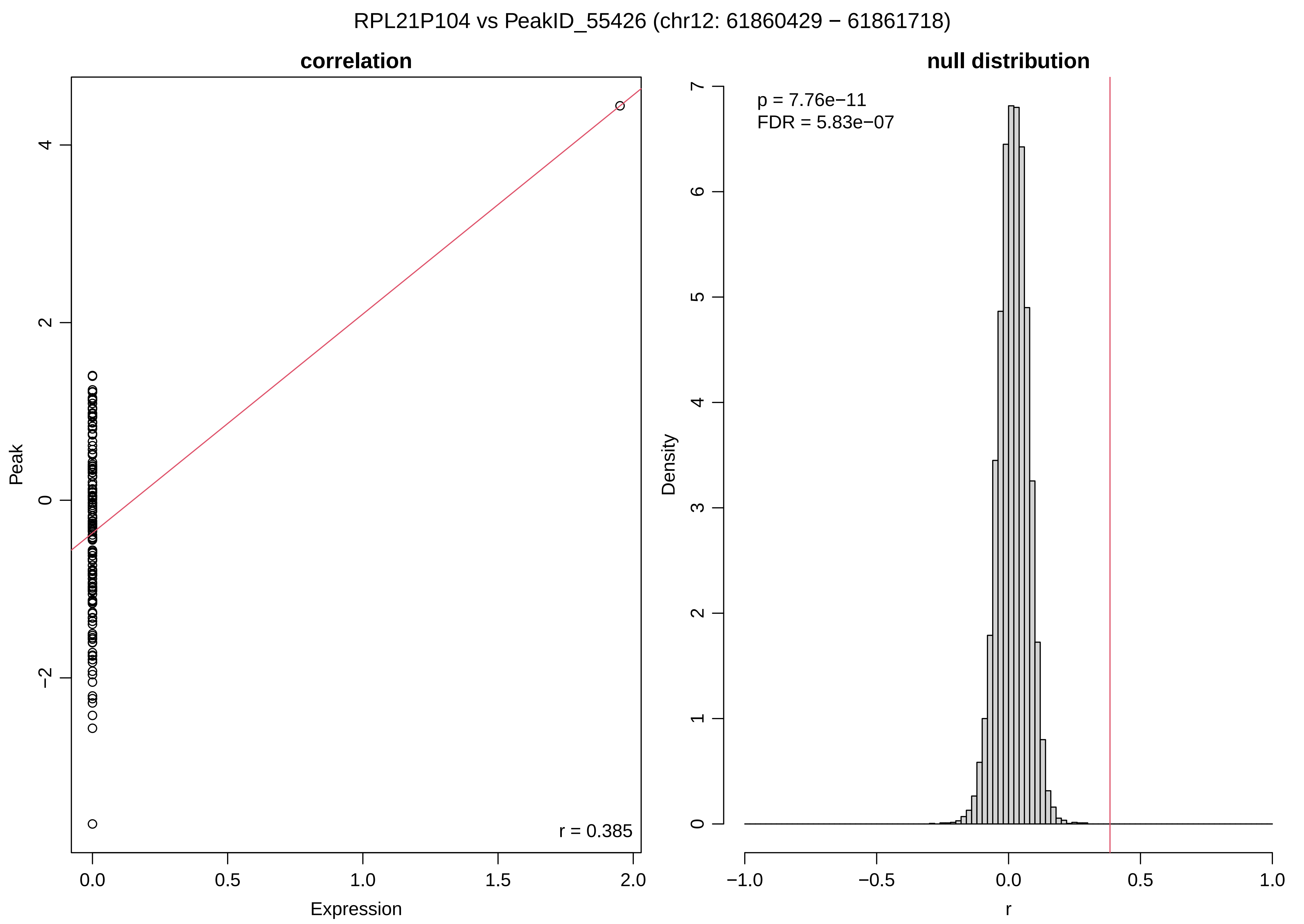
RPL21P104 vs PeakID_55426 (chr12: 61860429 − 61861718)
correlation
null distribution
p = 7.76e−11
FDR = 5.83e−07
r = 0.385
Peak
Expression
Density
r
0.0
0.5
1.0
1.5
2.0
−2
0
2
4
−1.0
−0.5
0.0
0.5
1.0
0
1
2
3
4
5
6
7

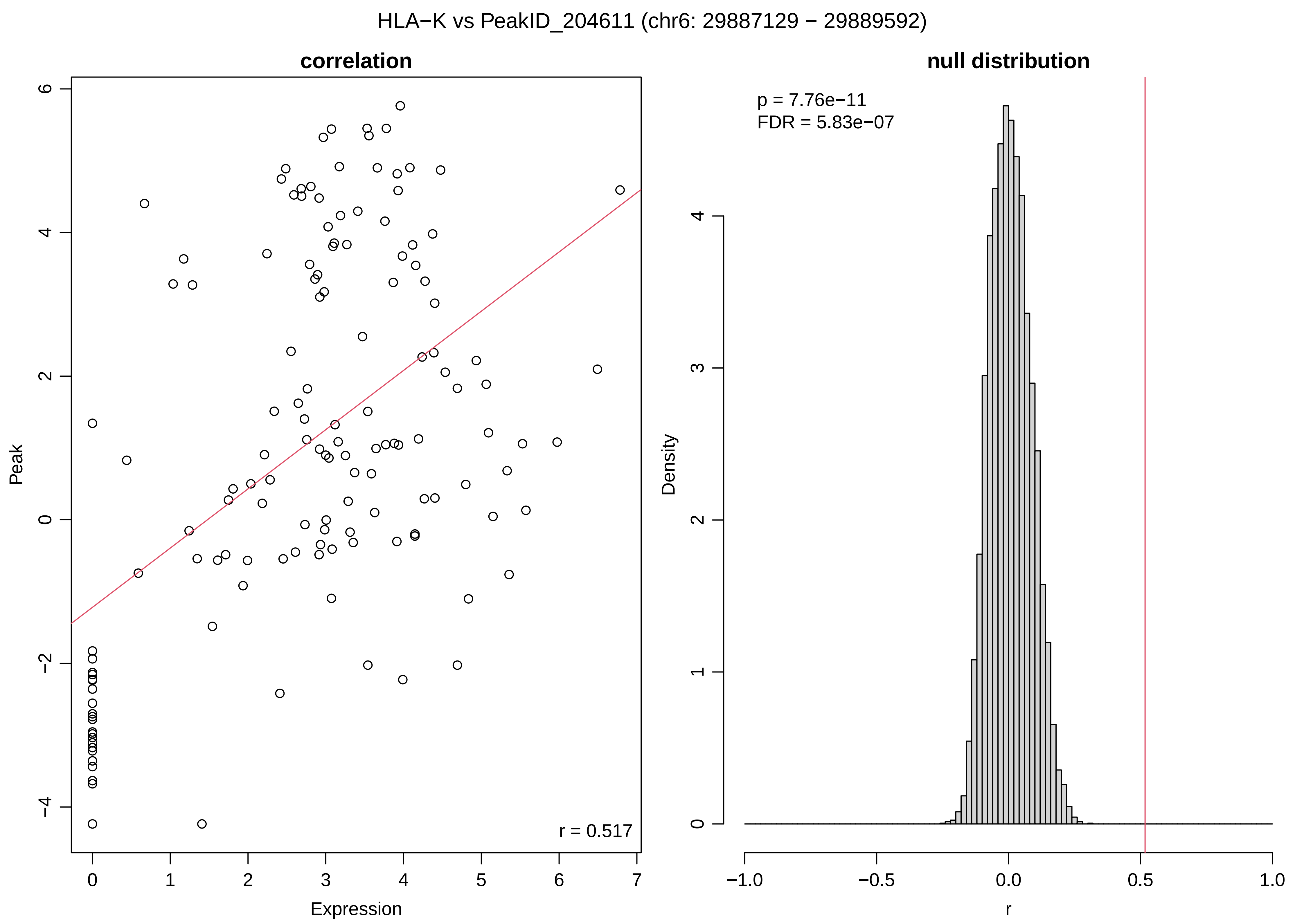
HLA−K vs PeakID_204611 (chr6: 29887129 − 29889592)
correlation
Peak
Expression
r = 0.517
null distribution
p = 7.76e−11
FDR = 5.83e−07
Density
r

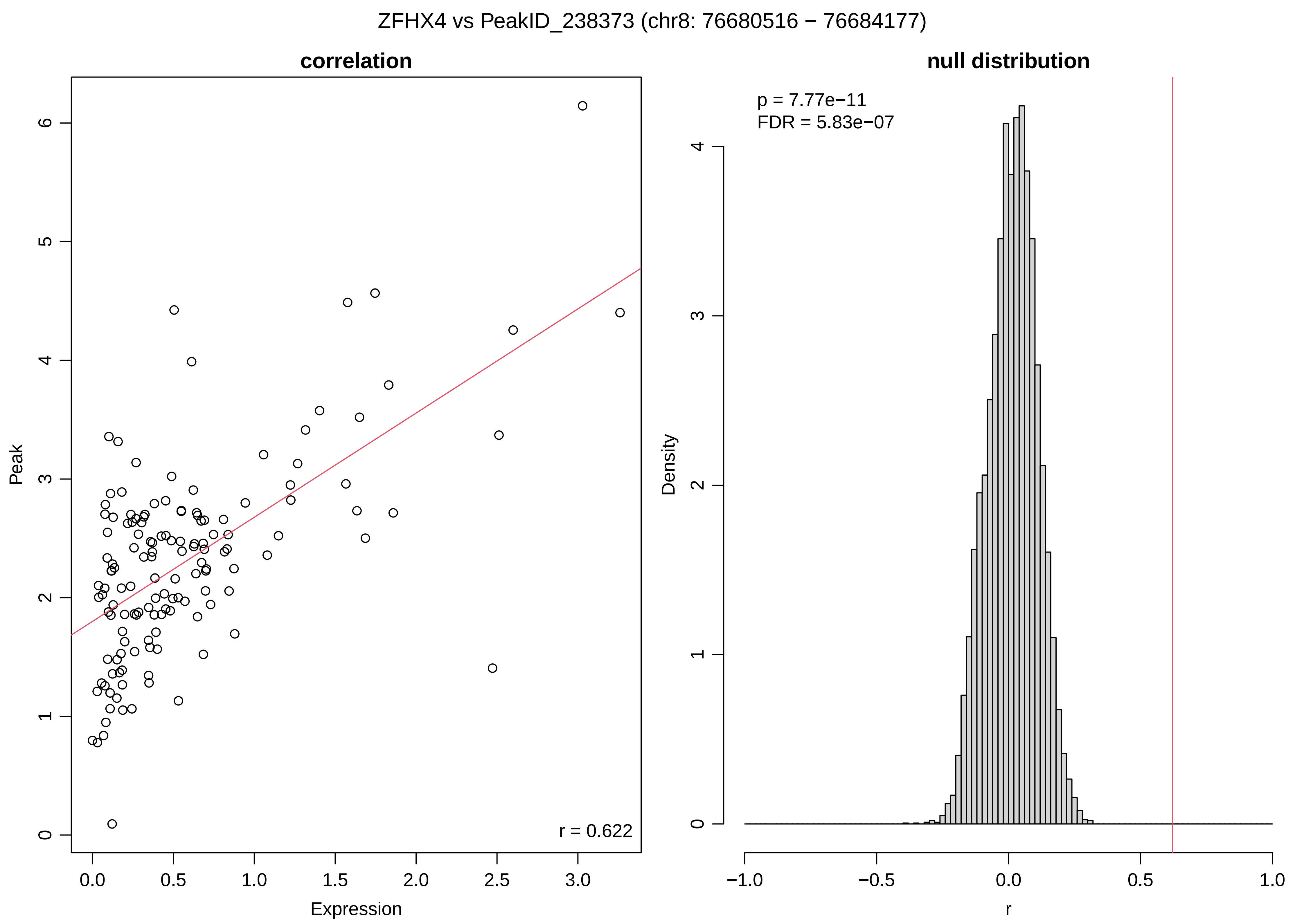
ZFHX4 vs PeakID_238373 (chr8: 76680516 − 76684177)
correlation
Peak
Expression
r = 0.622
null distribution
p = 7.77e−11
FDR = 5.83e−07
Density
r

YEATS4 vs PeakID_56864 (chr12: 69526699 − 69530100)

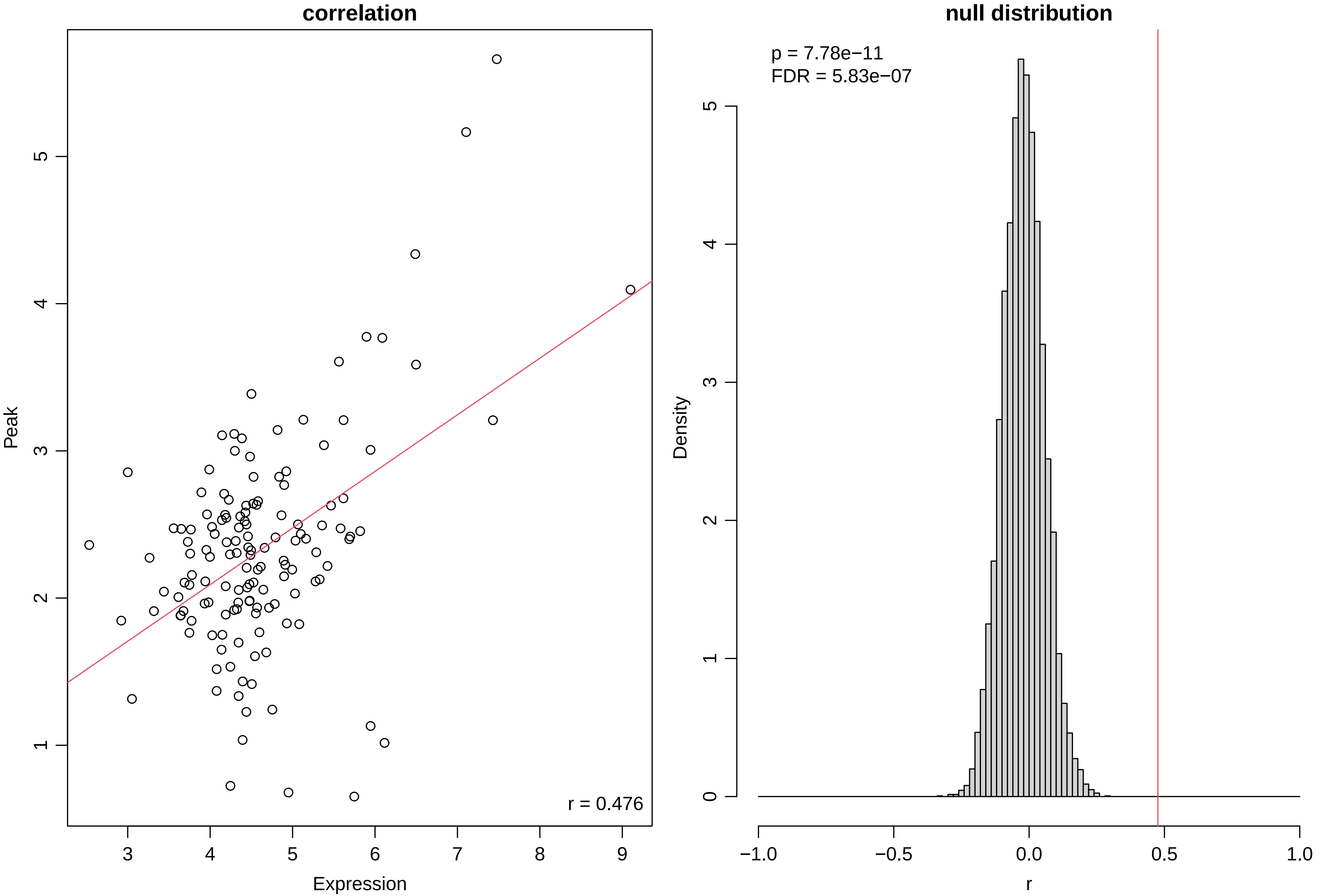

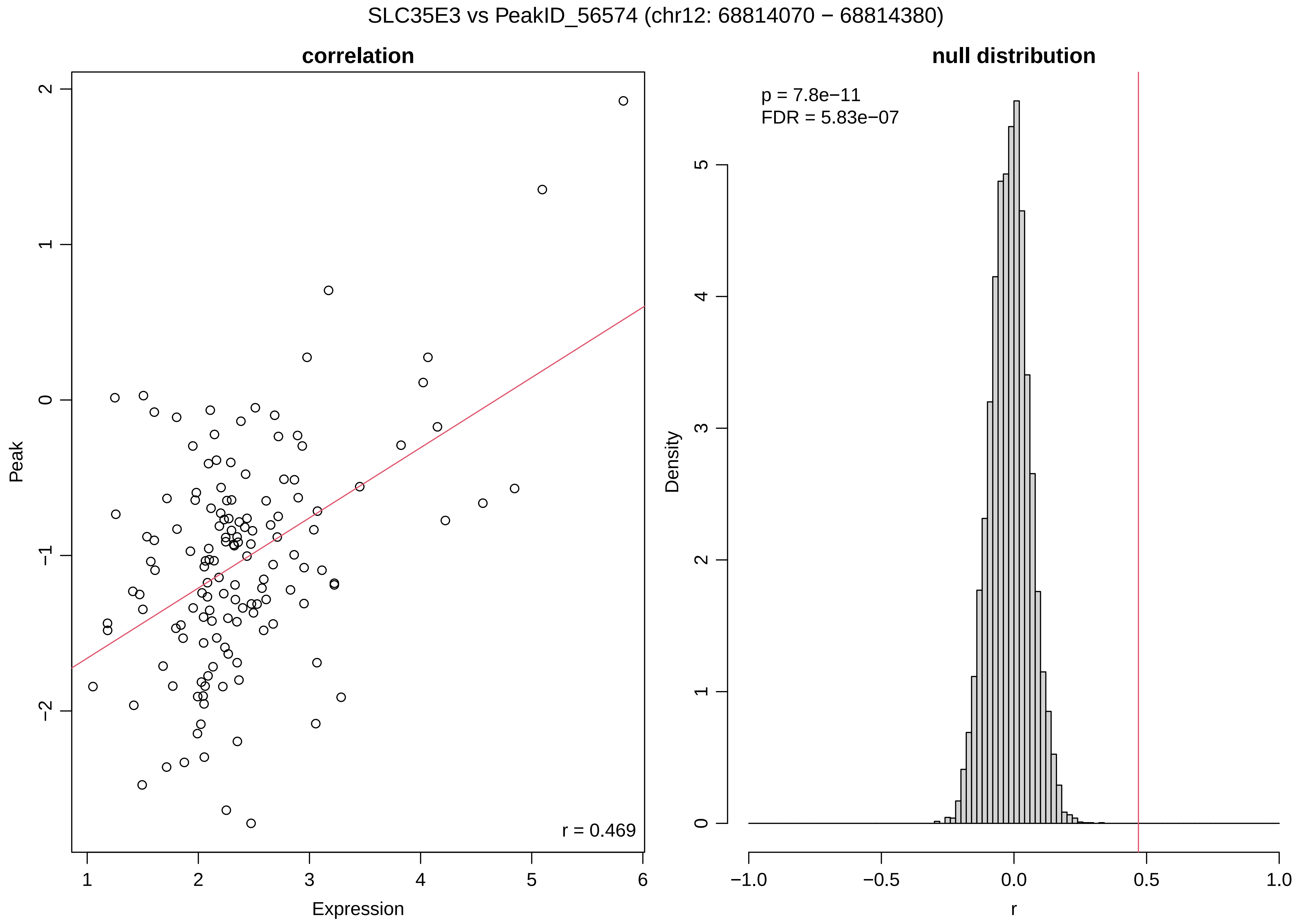
SLC35E3 vs PeakID_56574 (chr12: 68814070 − 68814380)
correlation
Peak
Expression
r = 0.469
null distribution
p = 7.8e−11
FDR = 5.83e−07
Density
r

MYO3B vs PeakID_135624 (chr2: 170178063 − 170179824)

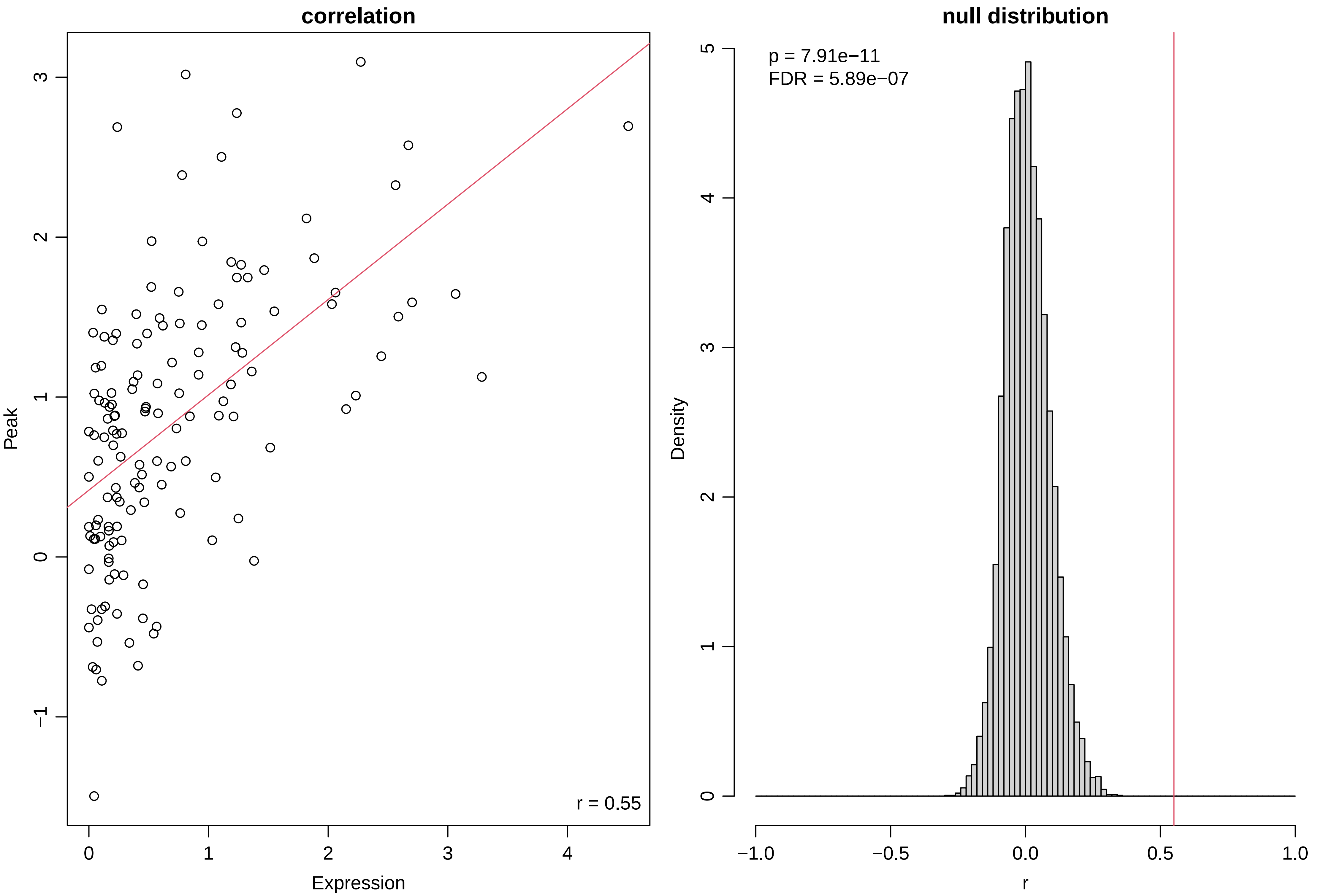

AC092821.1 vs PeakID_49720 (chr12: 9509261 − 9511009)

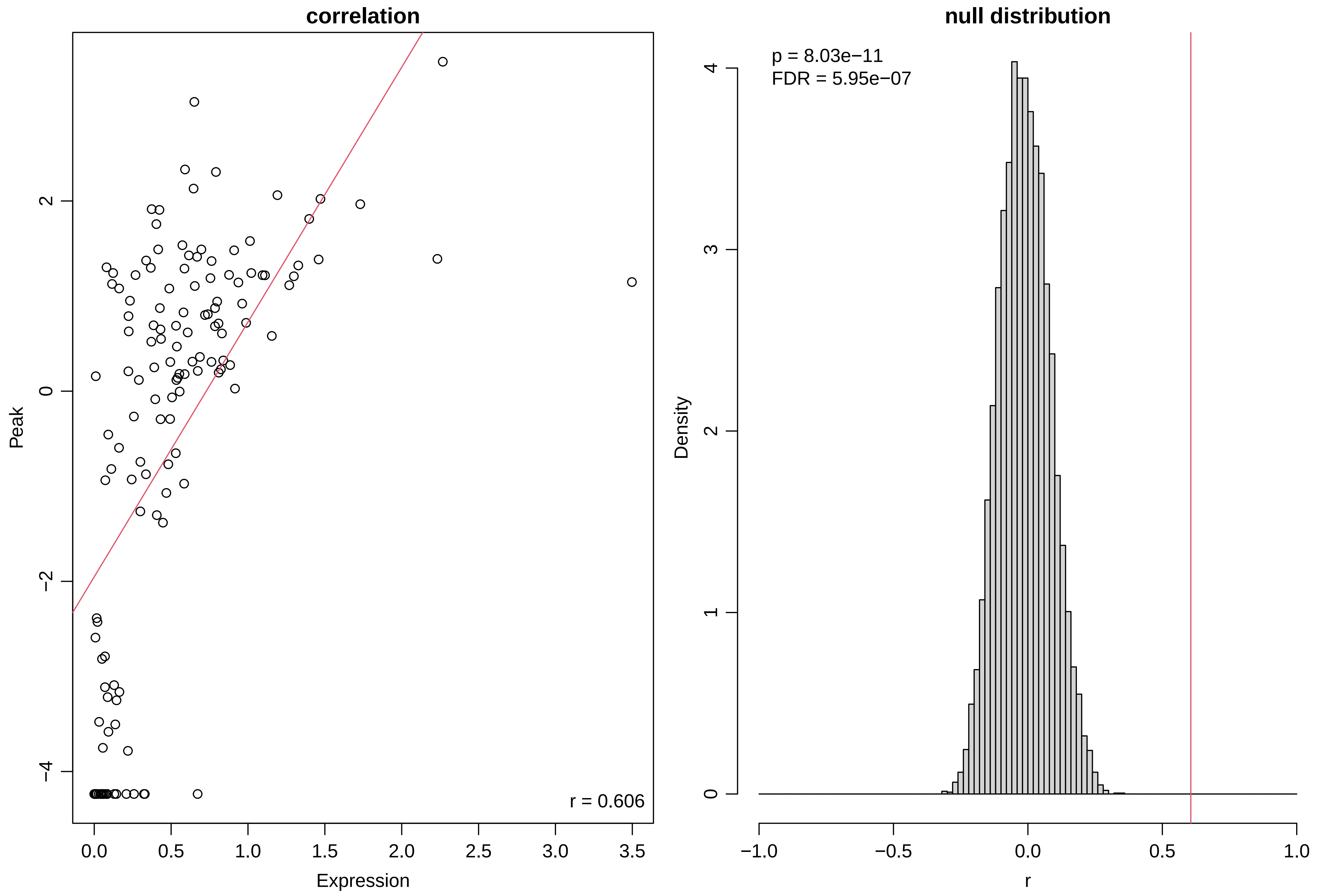

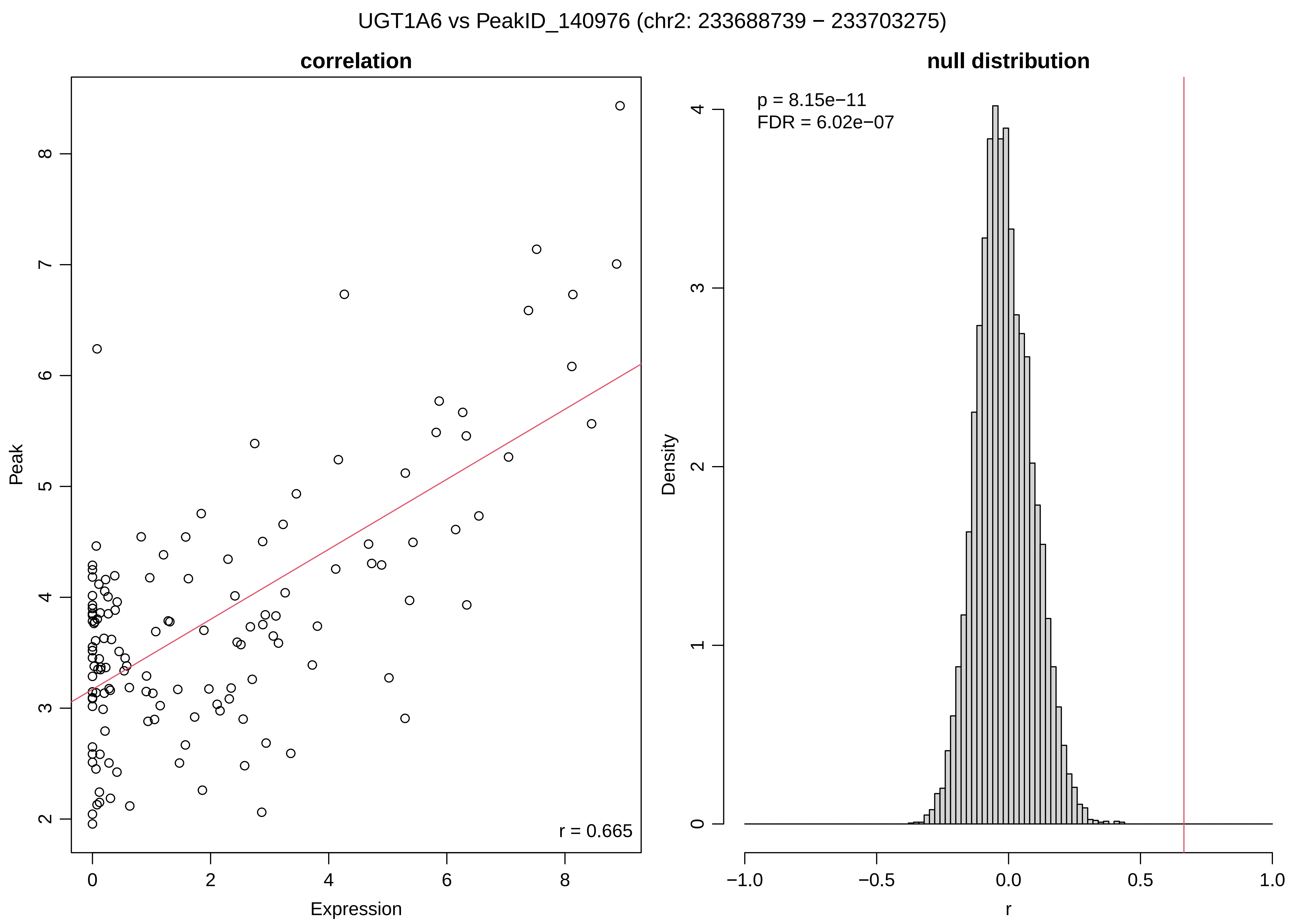

UGT1A6 vs PeakID_140976 (chr2: 233688739 − 233703275)
correlation
Peak
Expression
r = 0.665
null distribution
p = 8.15e−11
FDR = 6.02e−07
Density
r

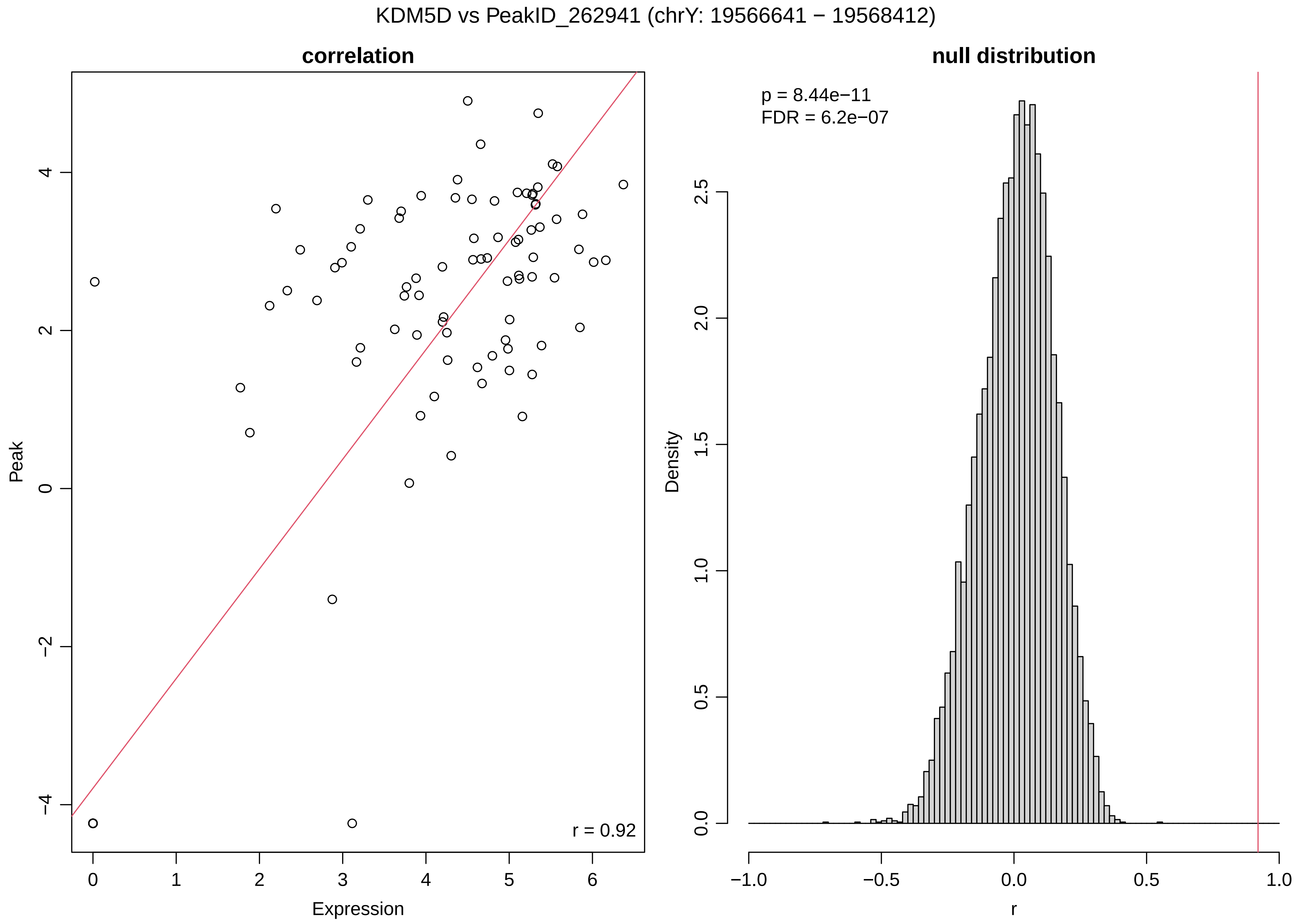
KDM5D vs PeakID_262941 (chrY: 19566641 − 19568412)
correlation
Peak
Expression
r = 0.92
null distribution
p = 8.44e−11
FDR = 6.2e−07
Density
r

YEATS4 vs PeakID_56837 (chr12: 69475065 − 69478102)

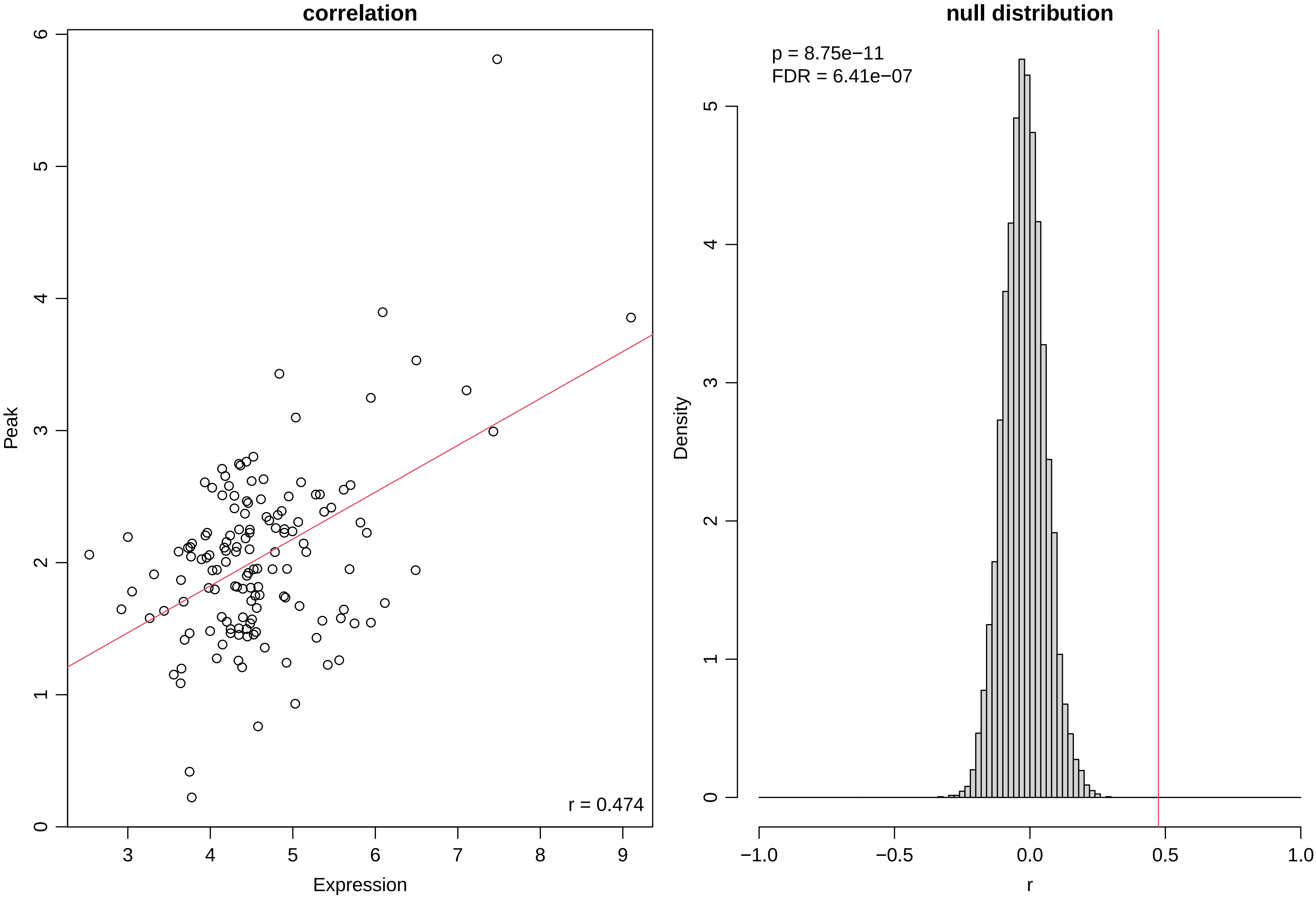

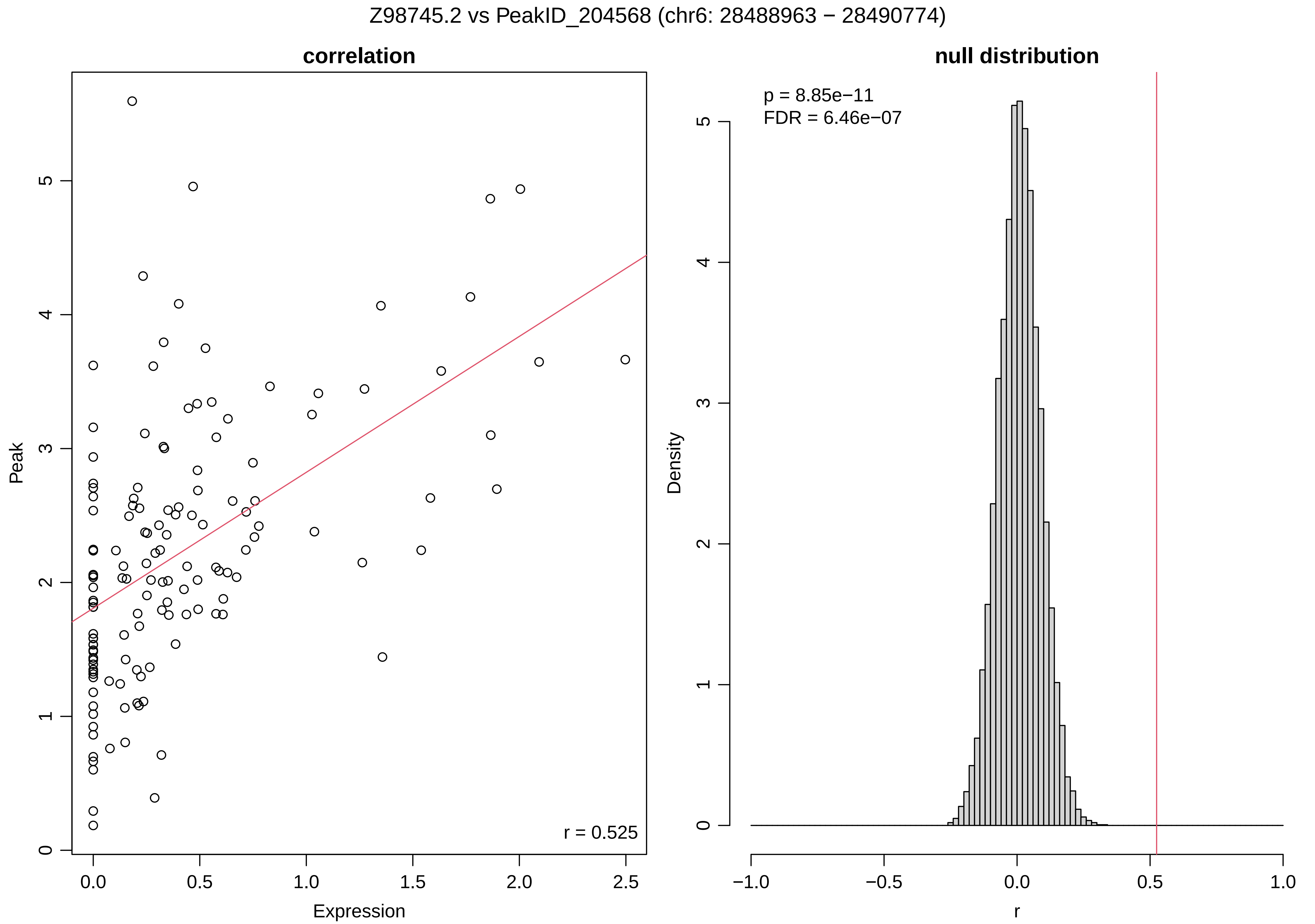

Z98745.2 vs PeakID_204568 (chr6: 28488963 − 28490774)
correlation
Peak
Expression
r = 0.525
0
1
2
3
4
5
0.0
0.5
1.0
1.5
2.0
2.5
null distribution
p = 8.85e−11
FDR = 6.46e−07
Density
r
0
1
2
3
4
5
−1.0
−0.5
0.0
0.5
1.0

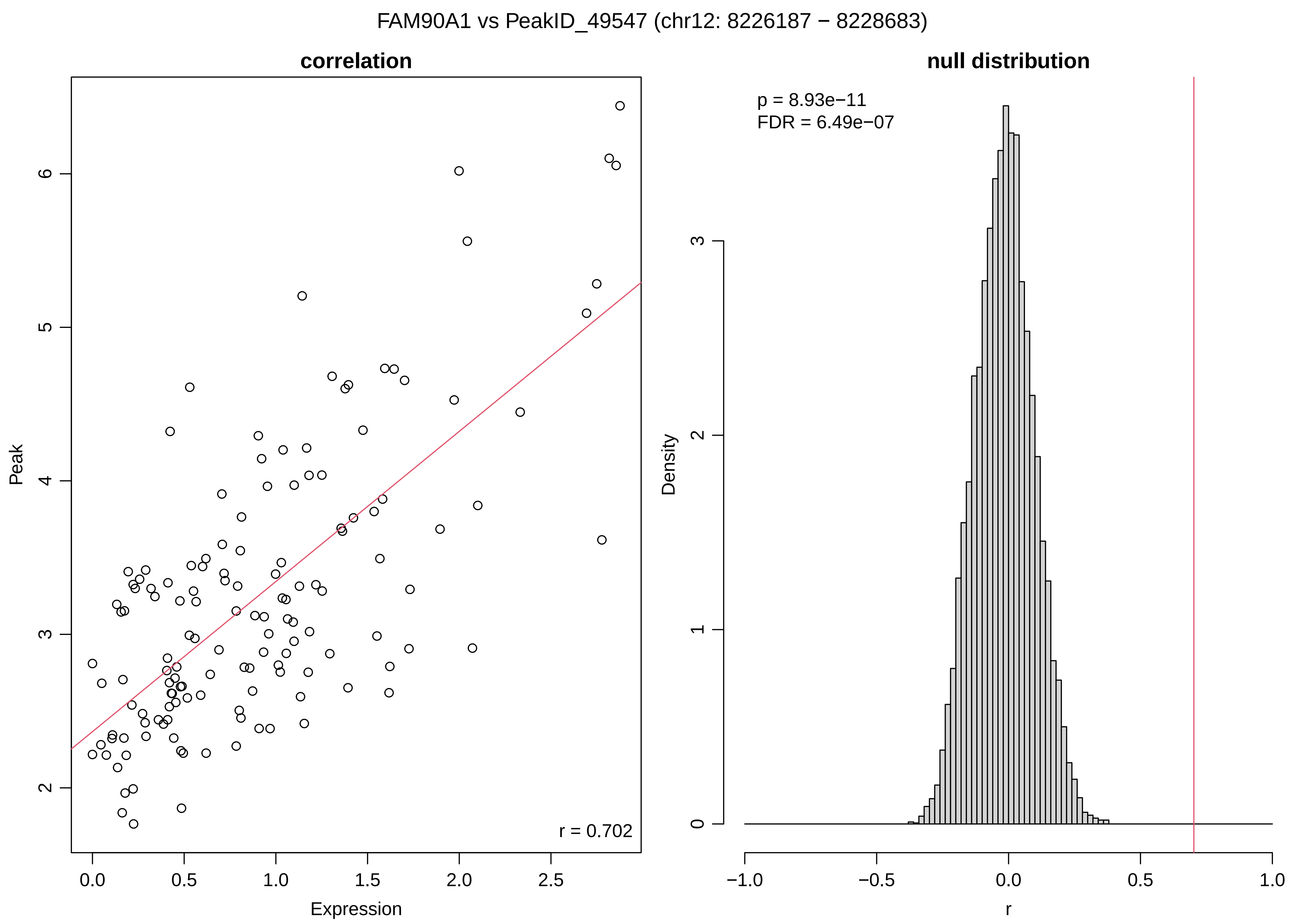

FAM90A1 vs PeakID_49547 (chr12: 8226187 − 8228683)
correlation
Peak
Expression
r = 0.702
null distribution
p = 8.93e−11
FDR = 6.49e−07
Density
r

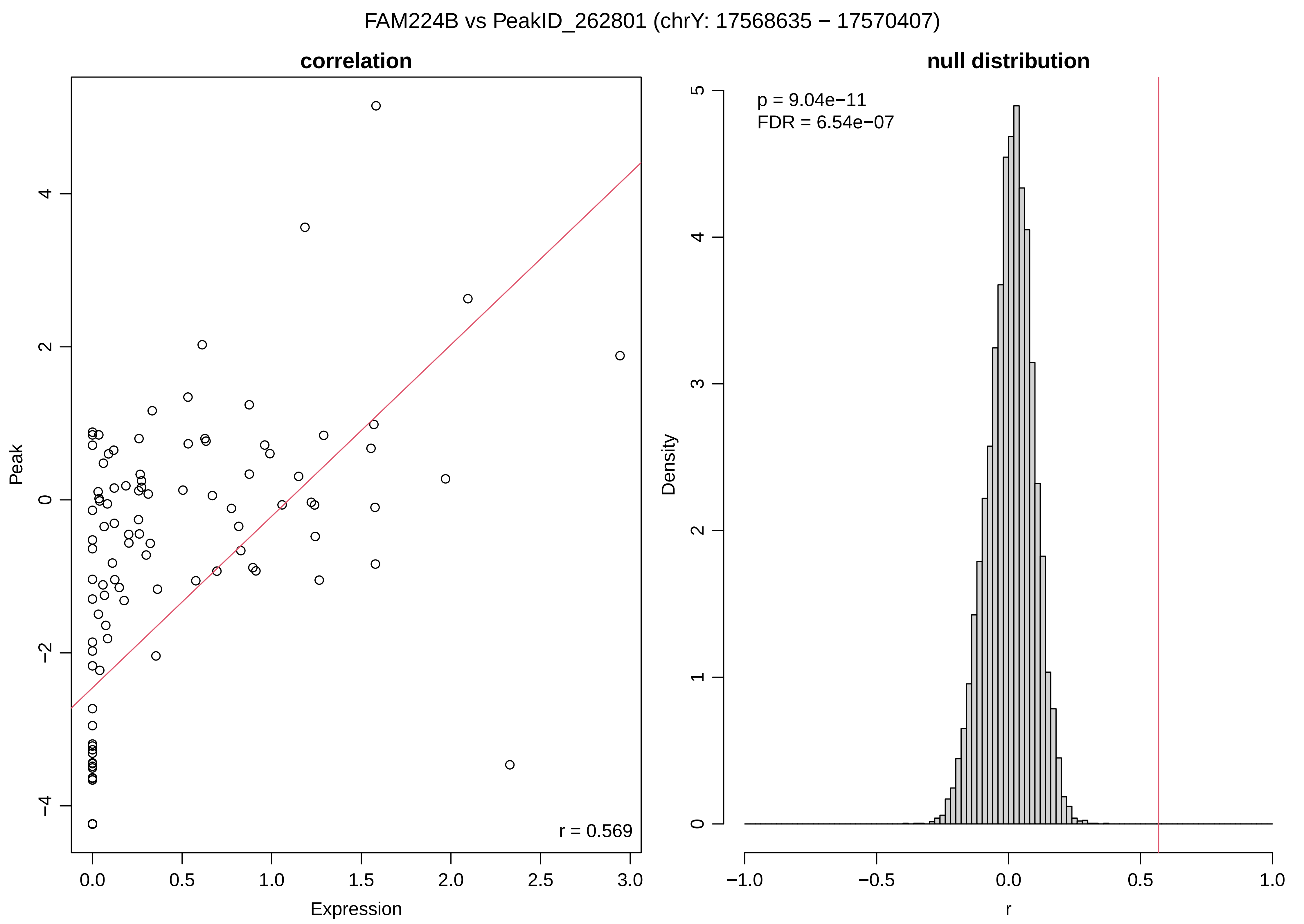
FAM224B vs PeakID_262801 (chrY: 17568635 − 17570407)
correlation
Peak
Expression
r = 0.569
null distribution
p = 9.04e−11
FDR = 6.54e−07
Density
r

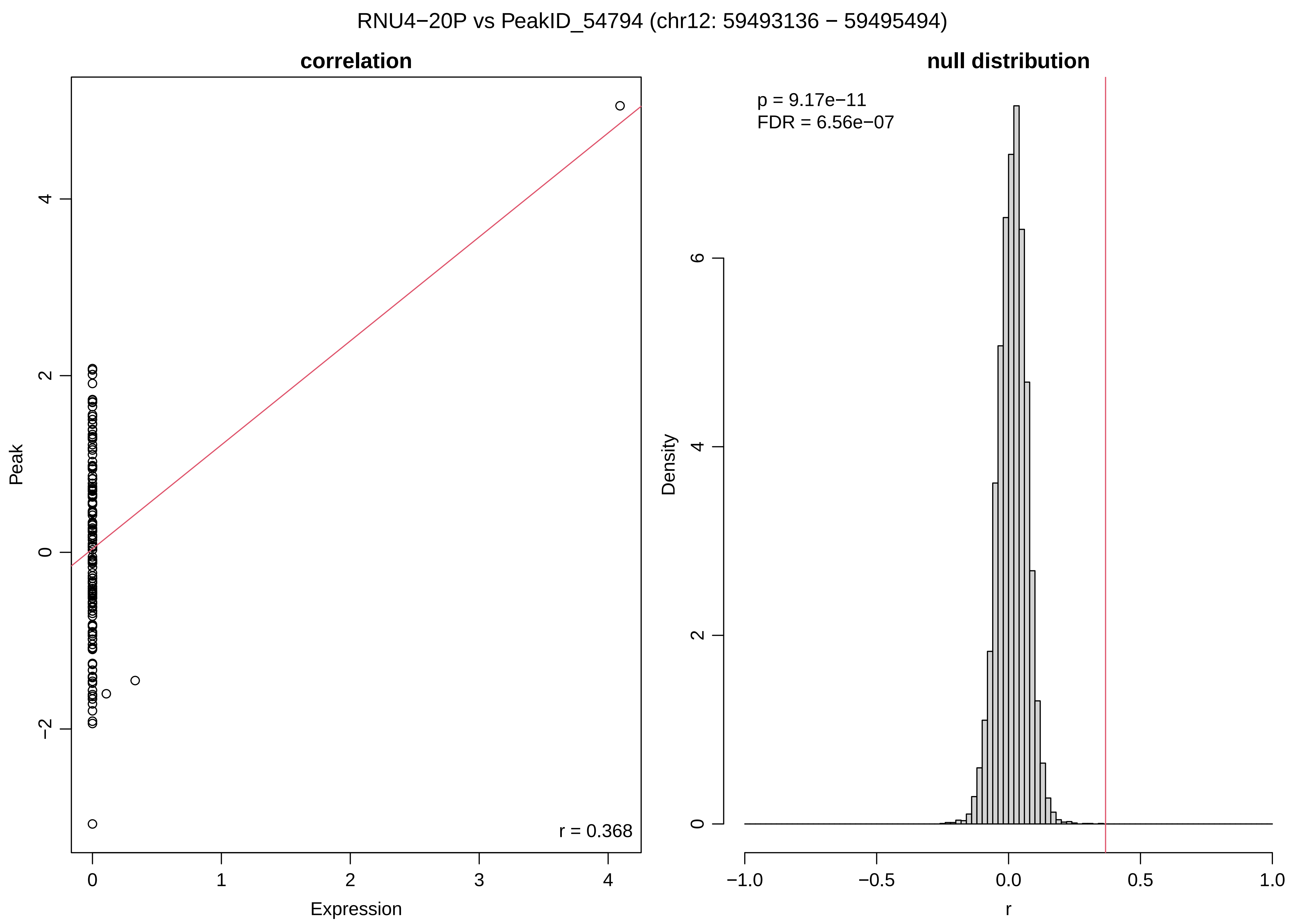
RNU4−20P vs PeakID_54794 (chr12: 59493136 − 59495494)
correlation
Peak
Expression
r = 0.368
null distribution
p = 9.17e−11
FDR = 6.56e−07
Density
r

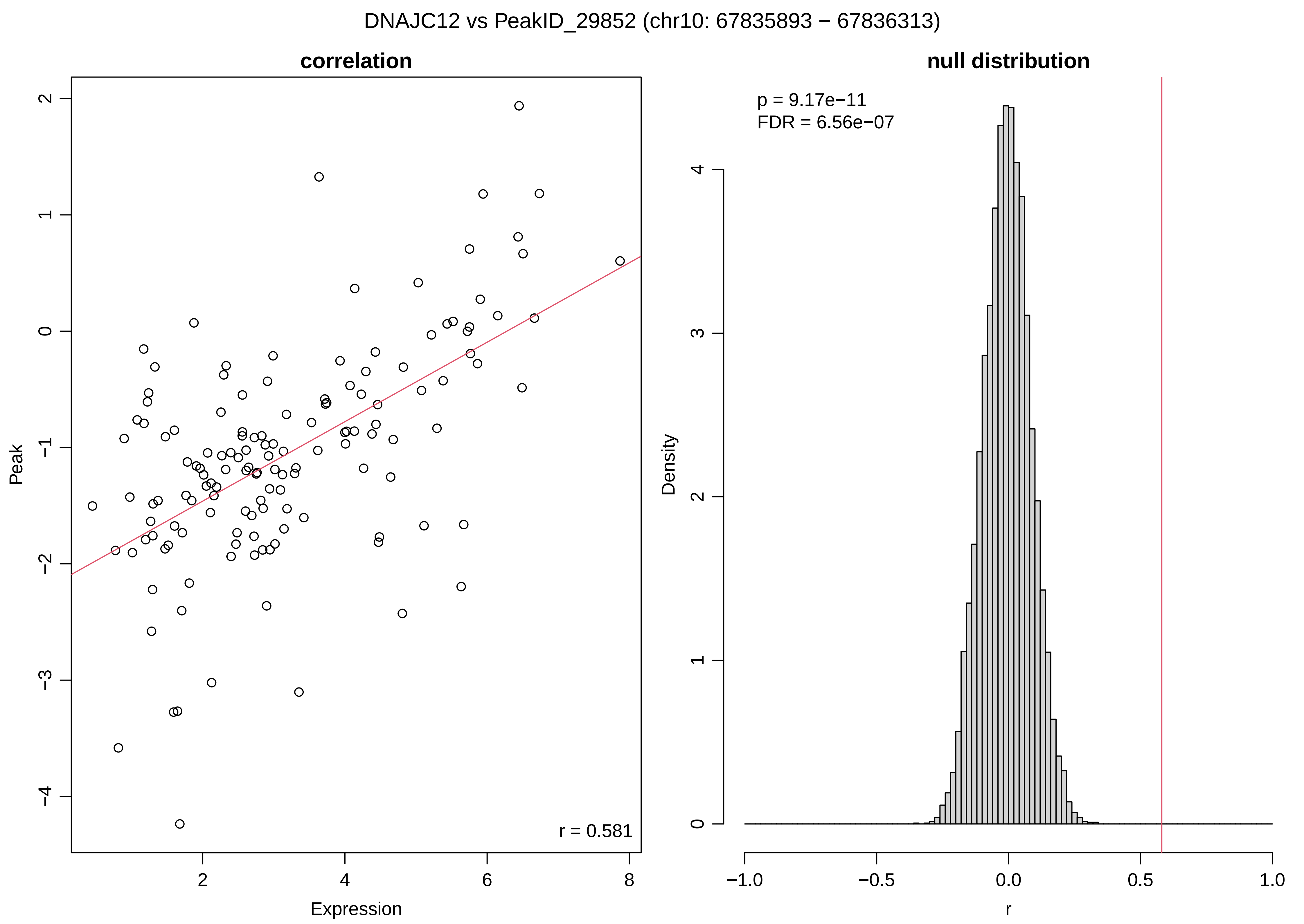
DNAJC12 vs PeakID_29852 (chr10: 67835893 − 67836313)
correlation
Peak
Expression
r = 0.581
null distribution
p = 9.17e−11
FDR = 6.56e−07
Density
r

SMOC1 vs PeakID_72627 (chr14: 69998239 − 69999337)

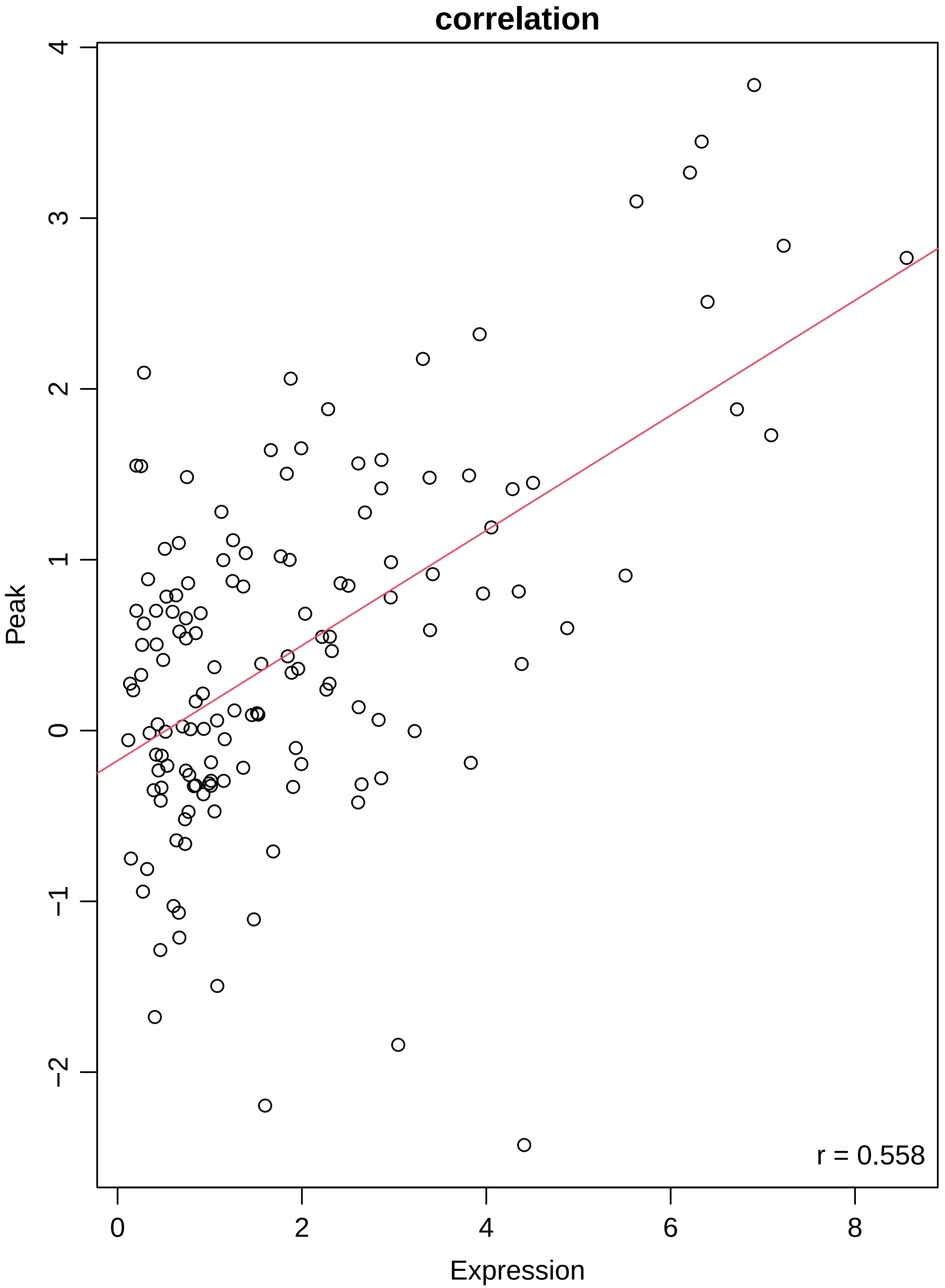

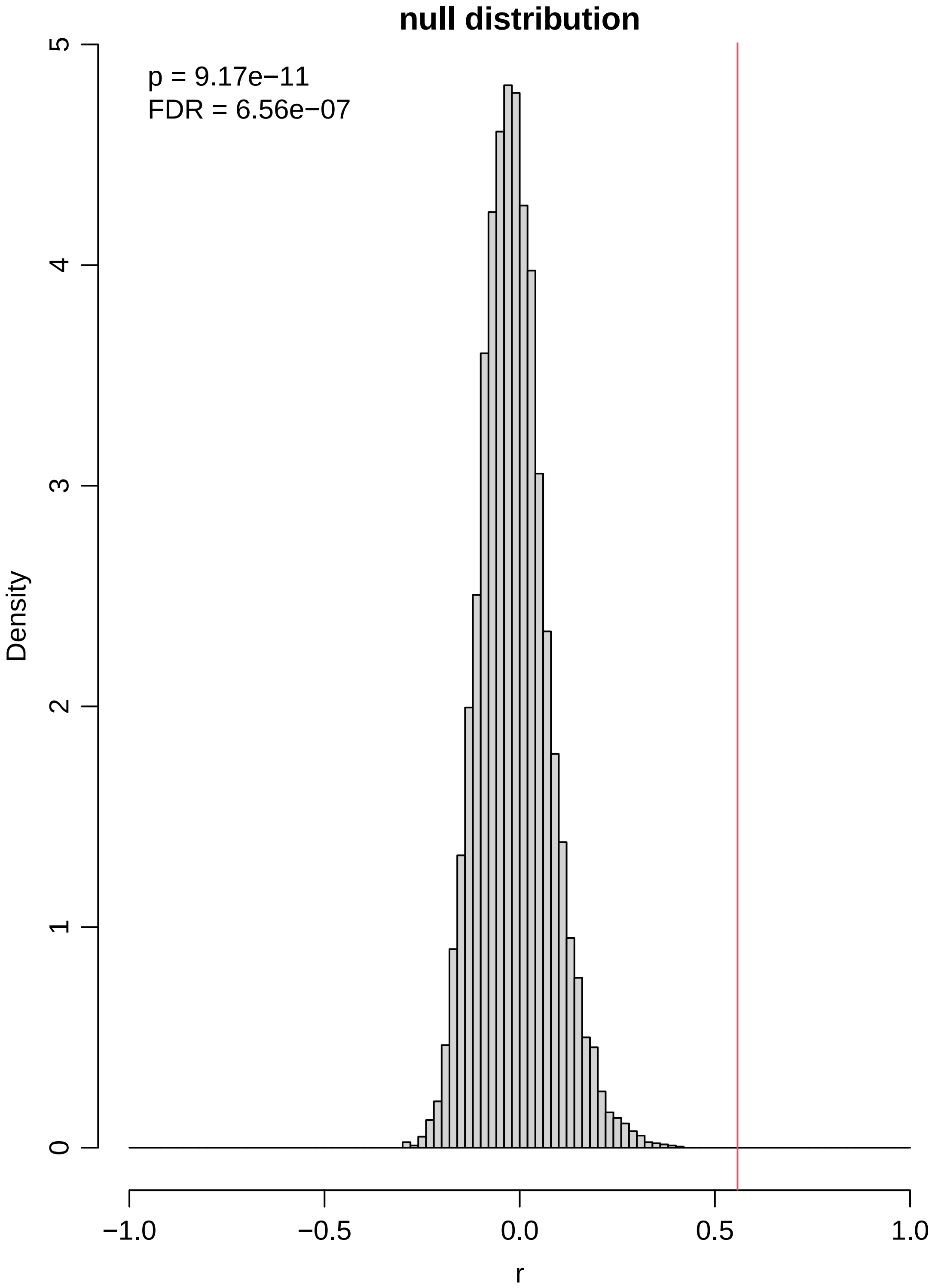

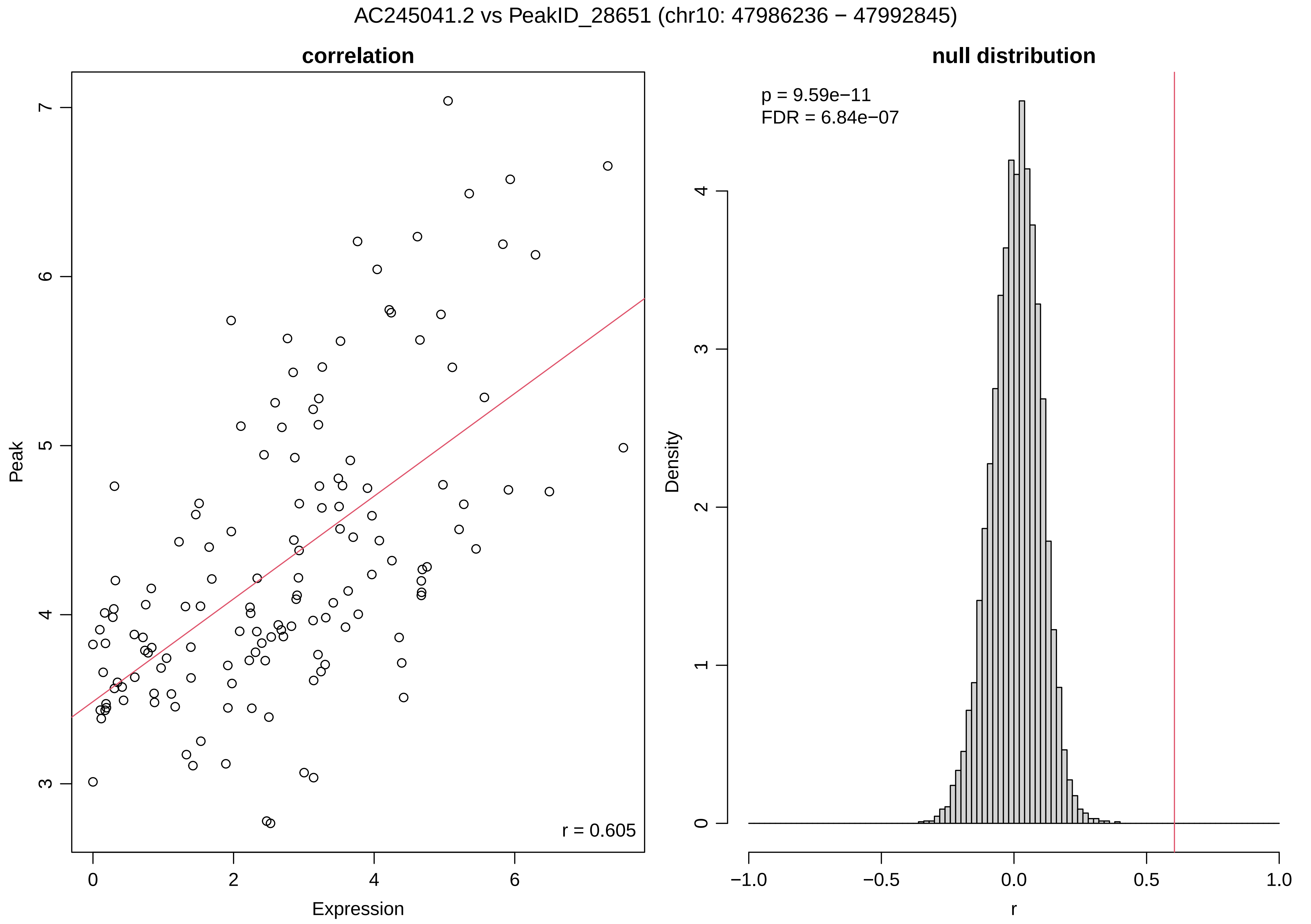

AC245041.2 vs PeakID_28651 (chr10: 47986236 − 47992845)
correlation
Peak
Expression
r = 0.605
null distribution
p = 9.59e−11
FDR = 6.84e−07
Density
r

TDRD9 vs PeakID_75739 (chr14: 103928102 − 103929892)

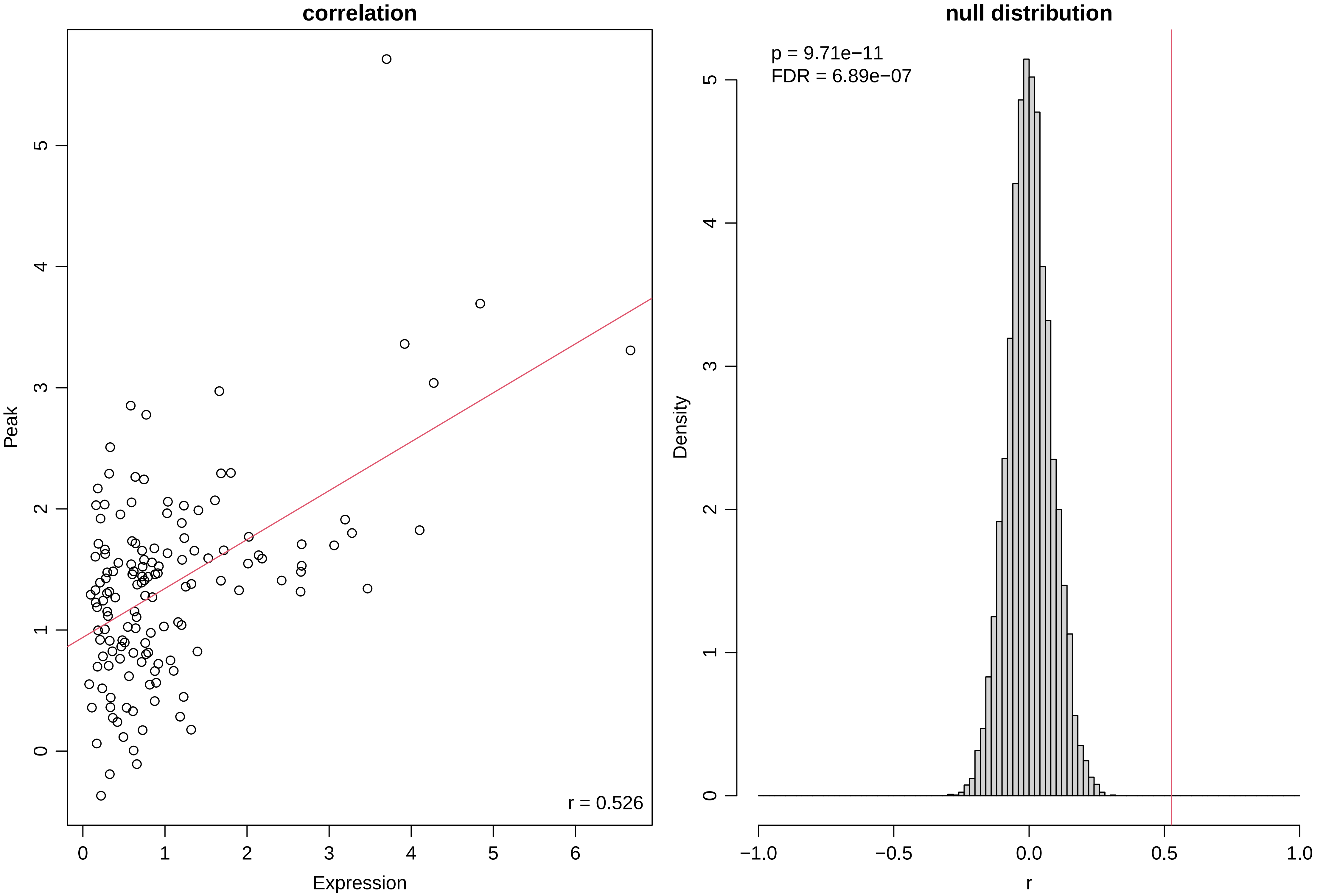

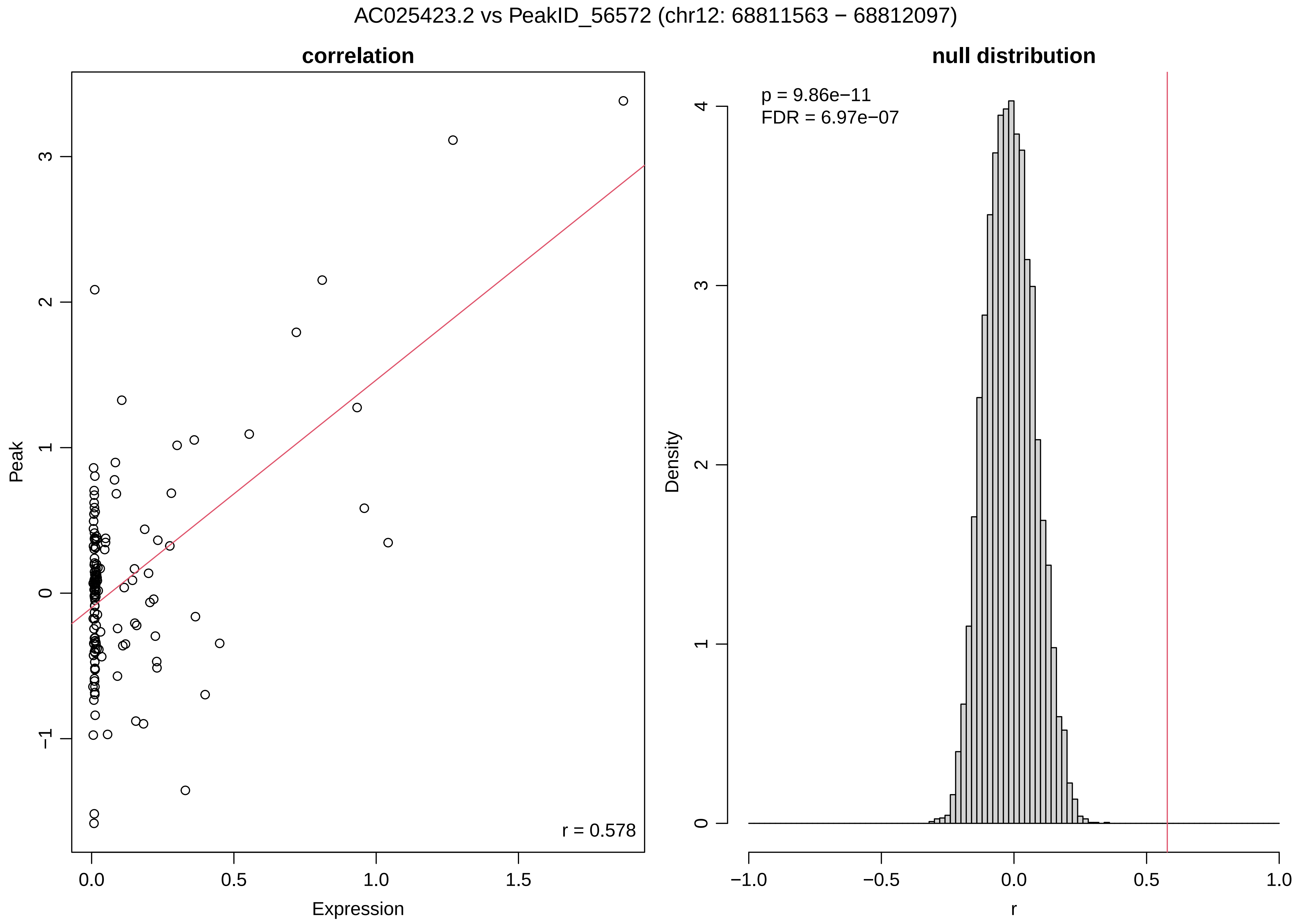
AC025423.2 vs PeakID_56572 (chr12: 68811563 − 68812097)
correlation
Peak
Expression
r = 0.578
null distribution
p = 9.86e−11
FDR = 6.97e−07
Density
r

FBXL13 vs PeakID_228112 (chr7: 102972919 − 102979011)

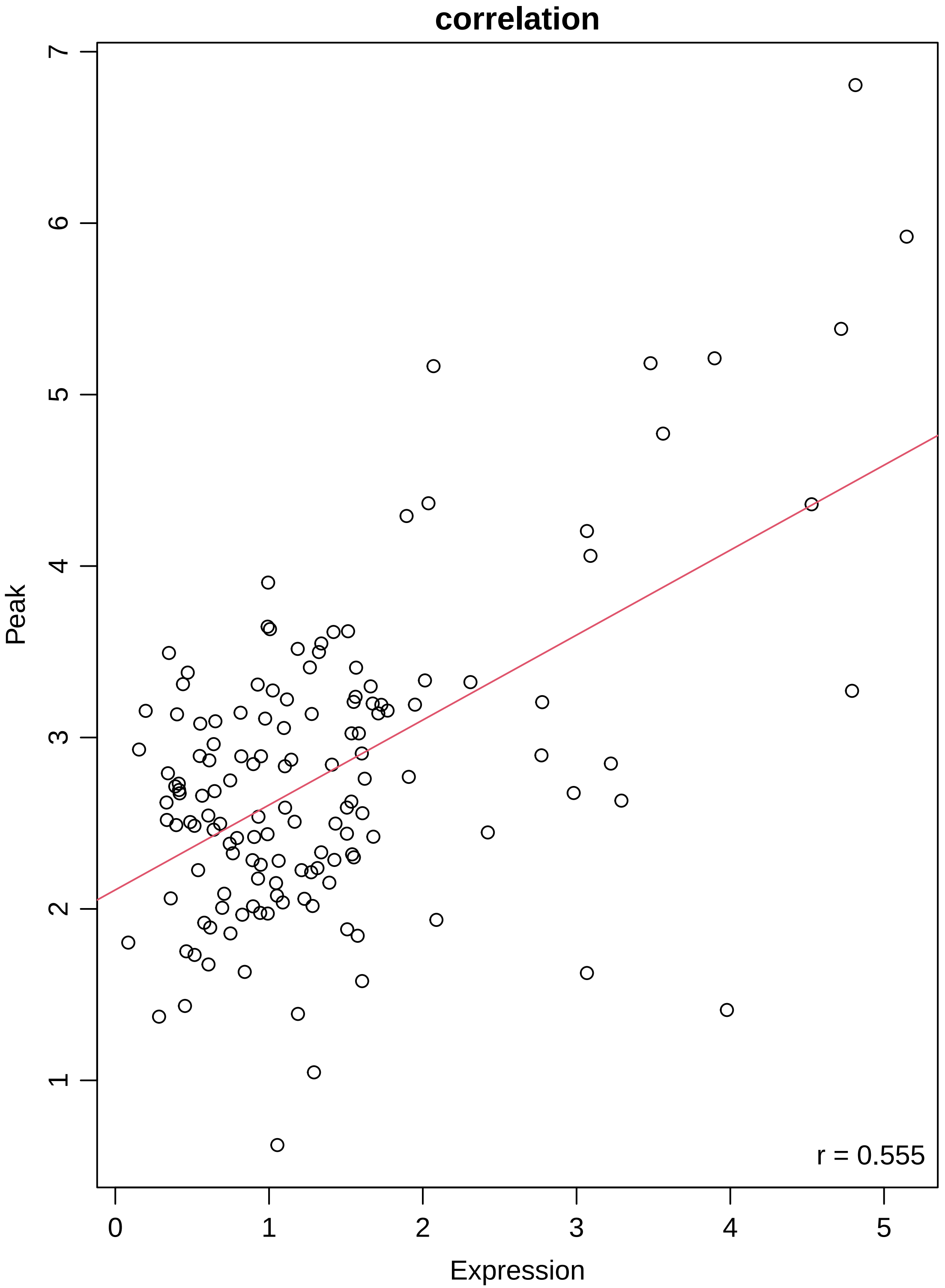

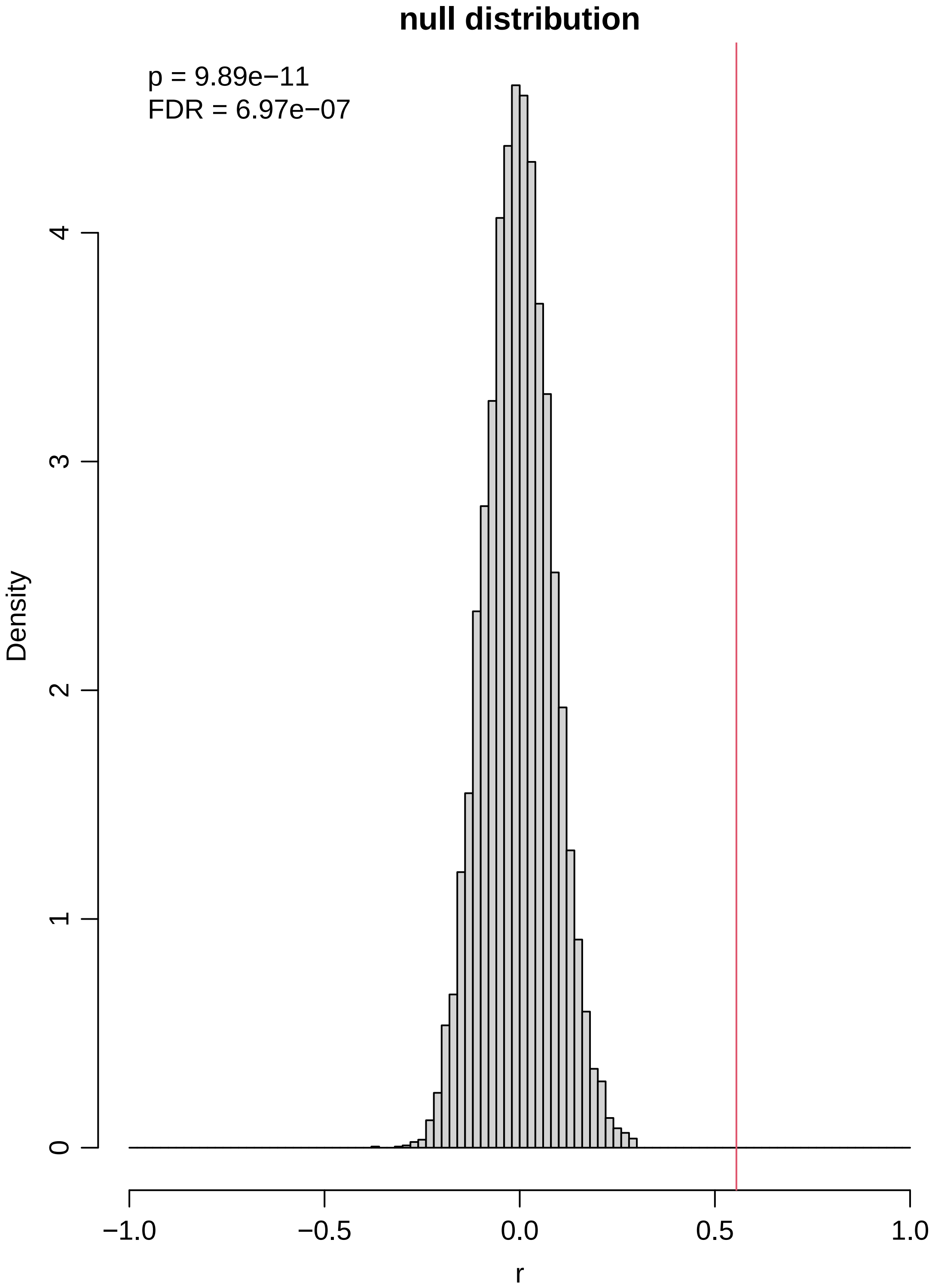

FAM224B vs PeakID_262810 (chrY: 17754804 − 17755090)

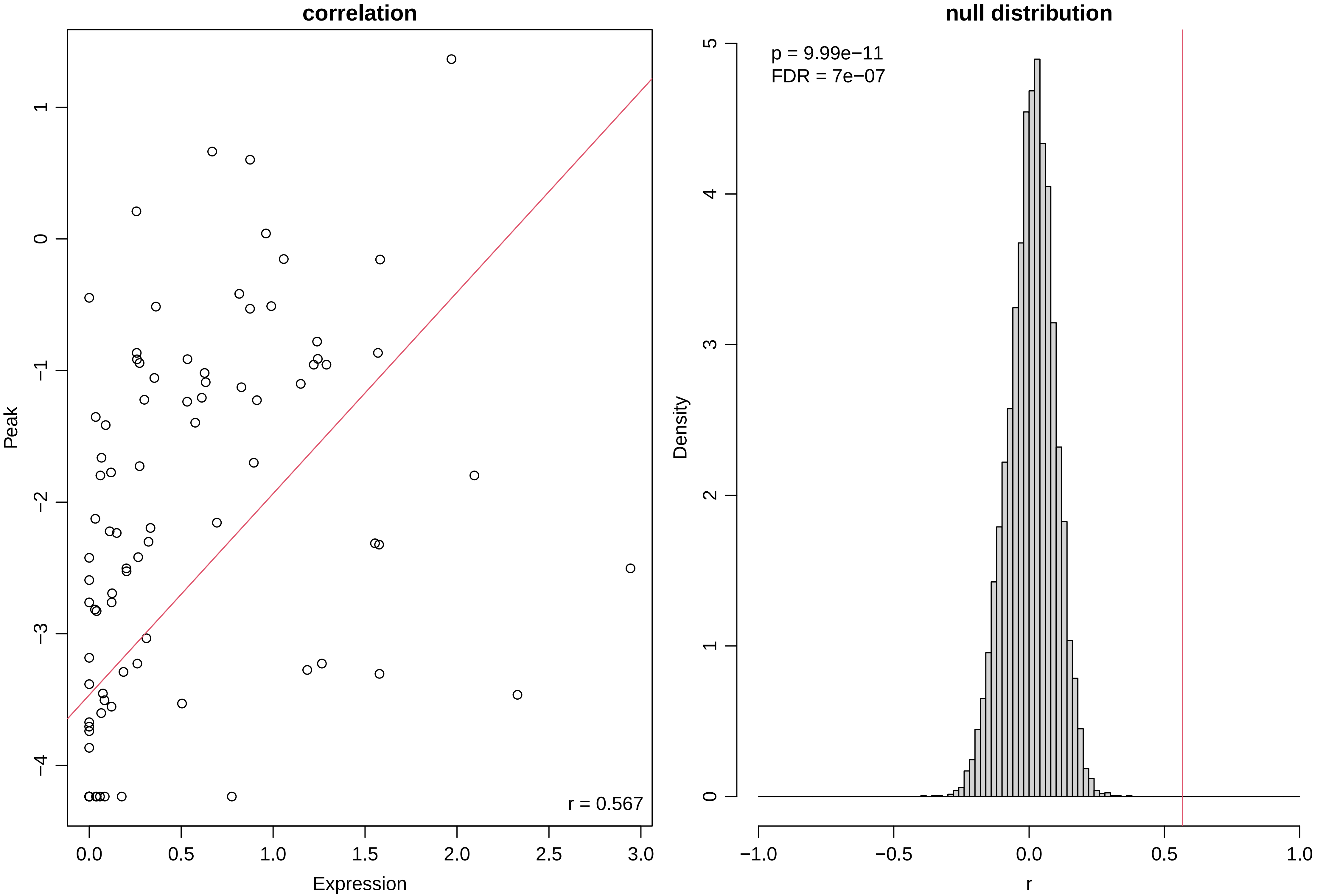

SMOC1 vs PeakID_72616 (chr14: 69878755 − 69880594)

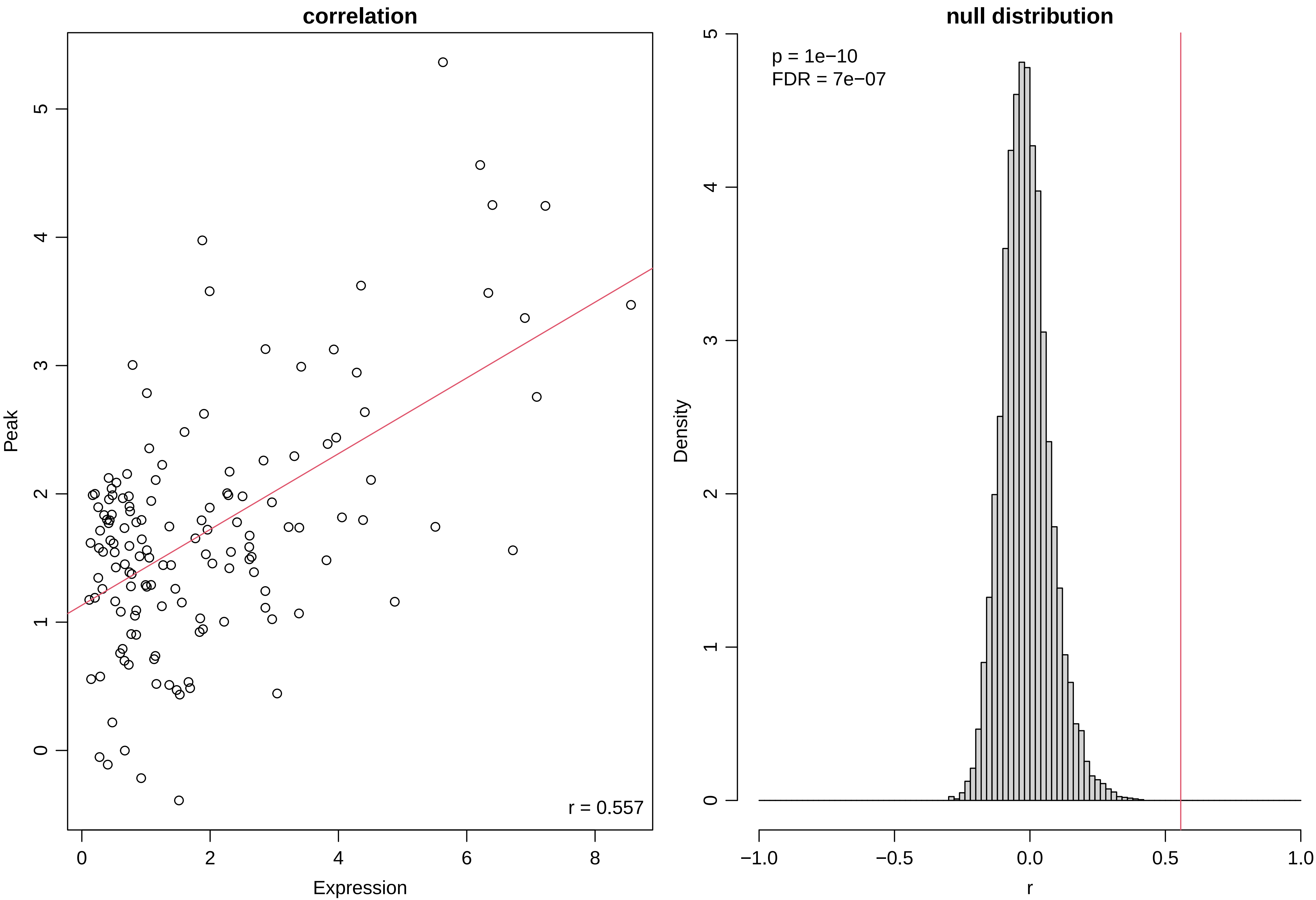

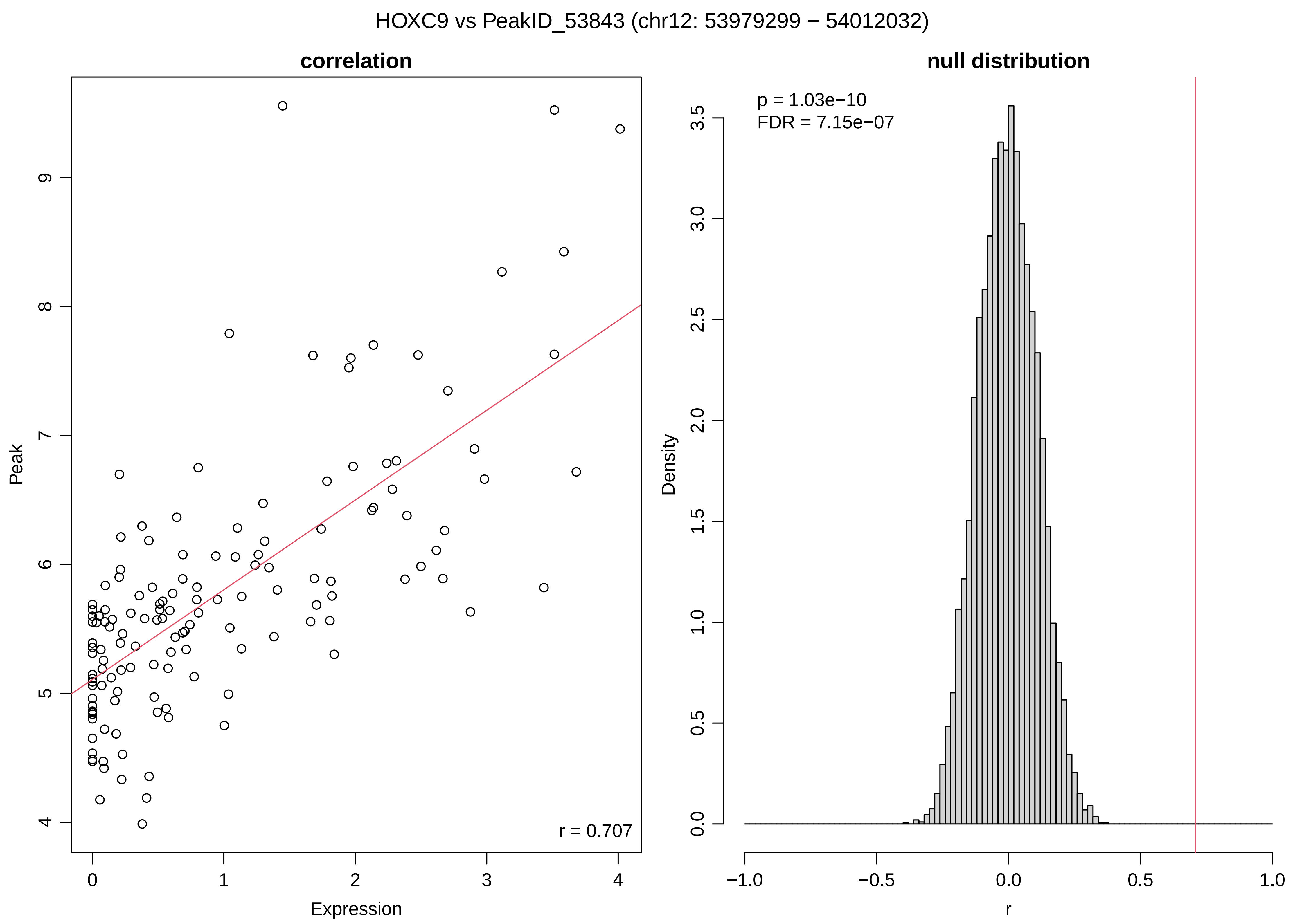
HOXC9 vs PeakID_53843 (chr12: 53979299 − 54012032)
correlation
null distribution
p = 1.03e−10
FDR = 7.15e−07
r = 0.707
Peak
Expression
Density
r
4
5
6
7
8
9
0
1
2
3
4
0.0
0.5
1.0
1.5
2.0
2.5
3.0
3.5
−1.0
−0.5
0.0
0.5
1.0

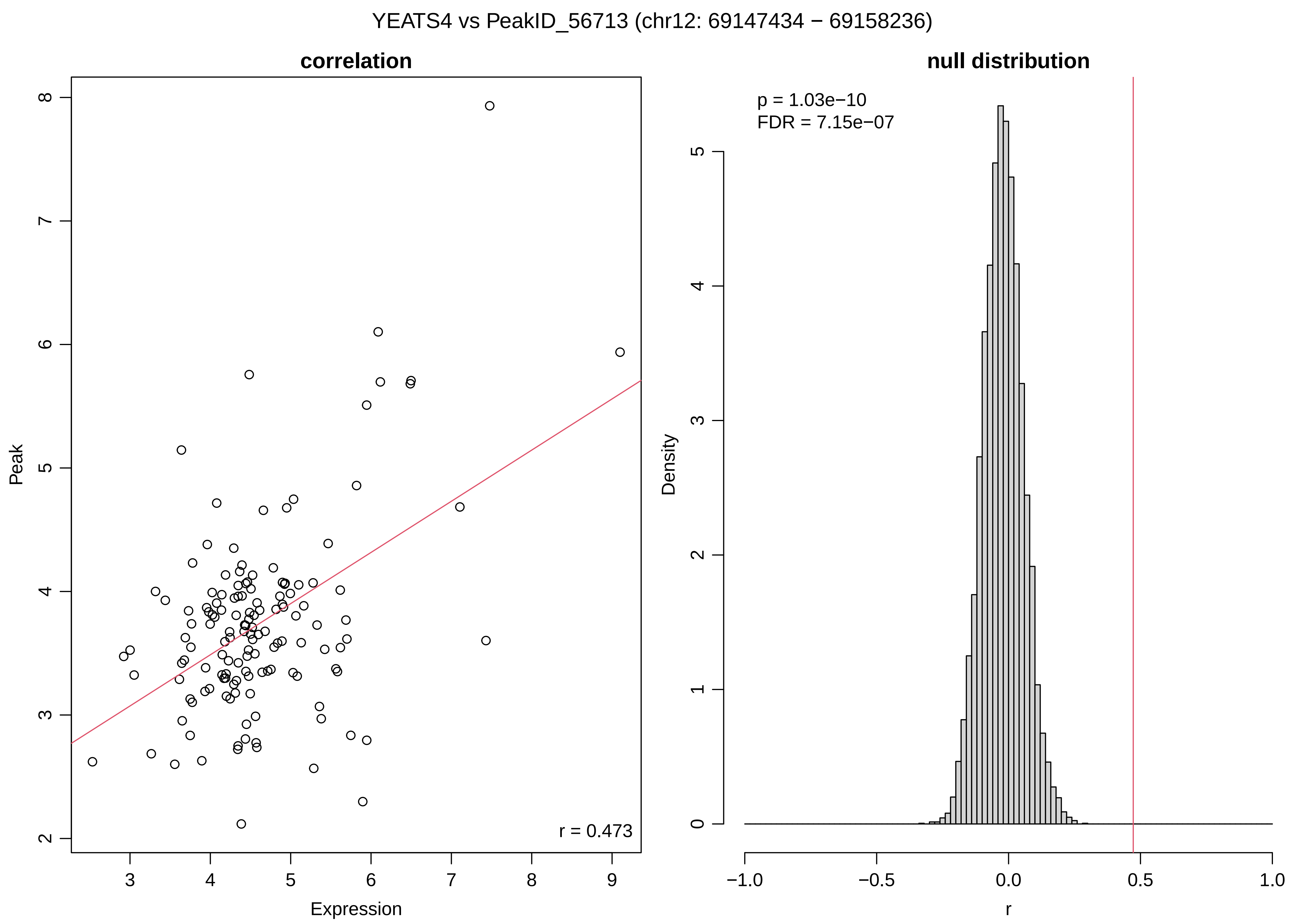
YEATS4 vs PeakID_56713 (chr12: 69147434 − 69158236)
correlation
Peak
Expression
r = 0.473
null distribution
p = 1.03e−10
FDR = 7.15e−07
Density
r

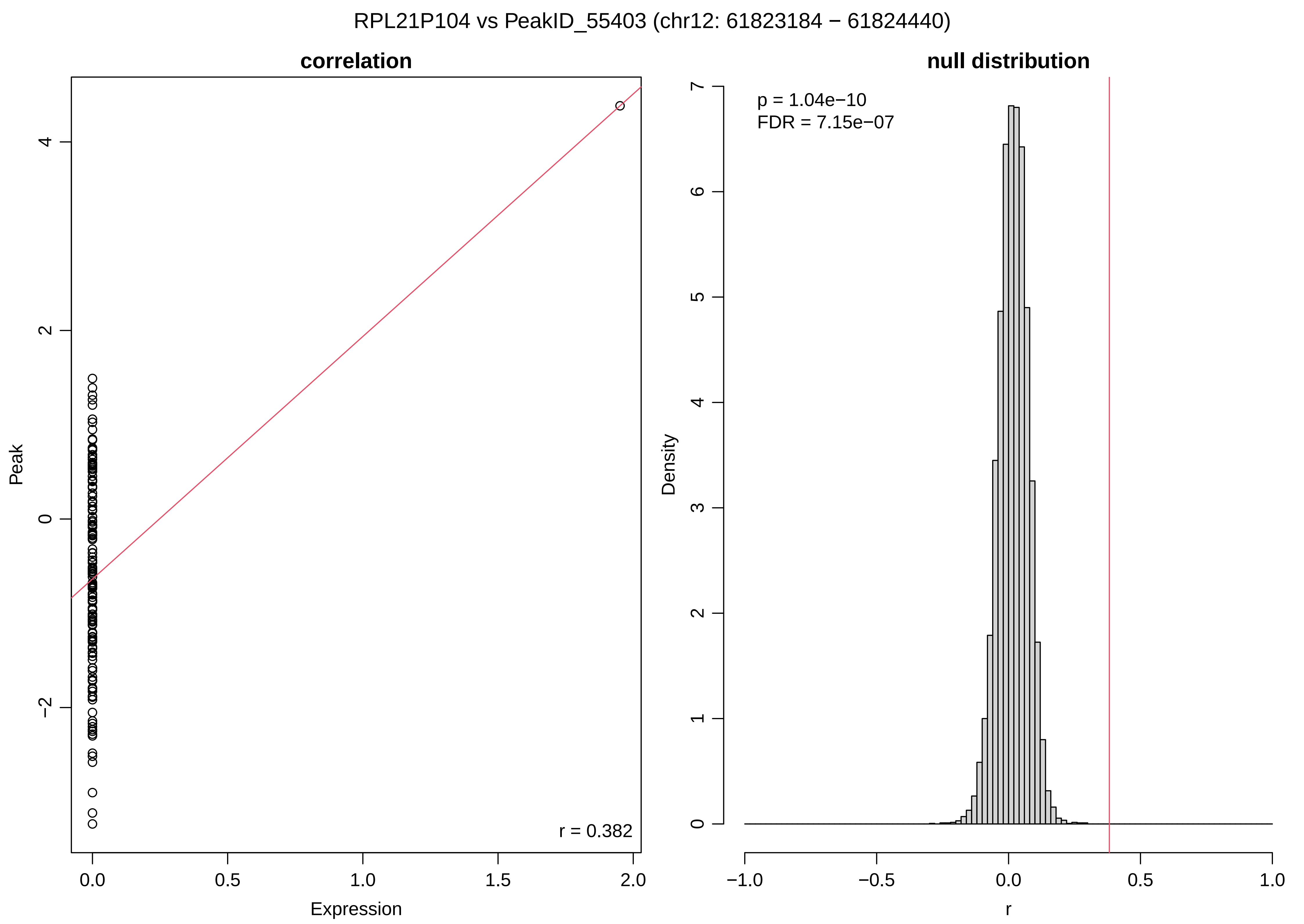
RPL21P104 vs PeakID_55403 (chr12: 61823184 − 61824440)
correlation
Peak
Expression
r = 0.382
null distribution
p = 1.04e−10
FDR = 7.15e−07
Density
r

ARX vs PeakID_257365 (chrX: 25010809 − 25014036)

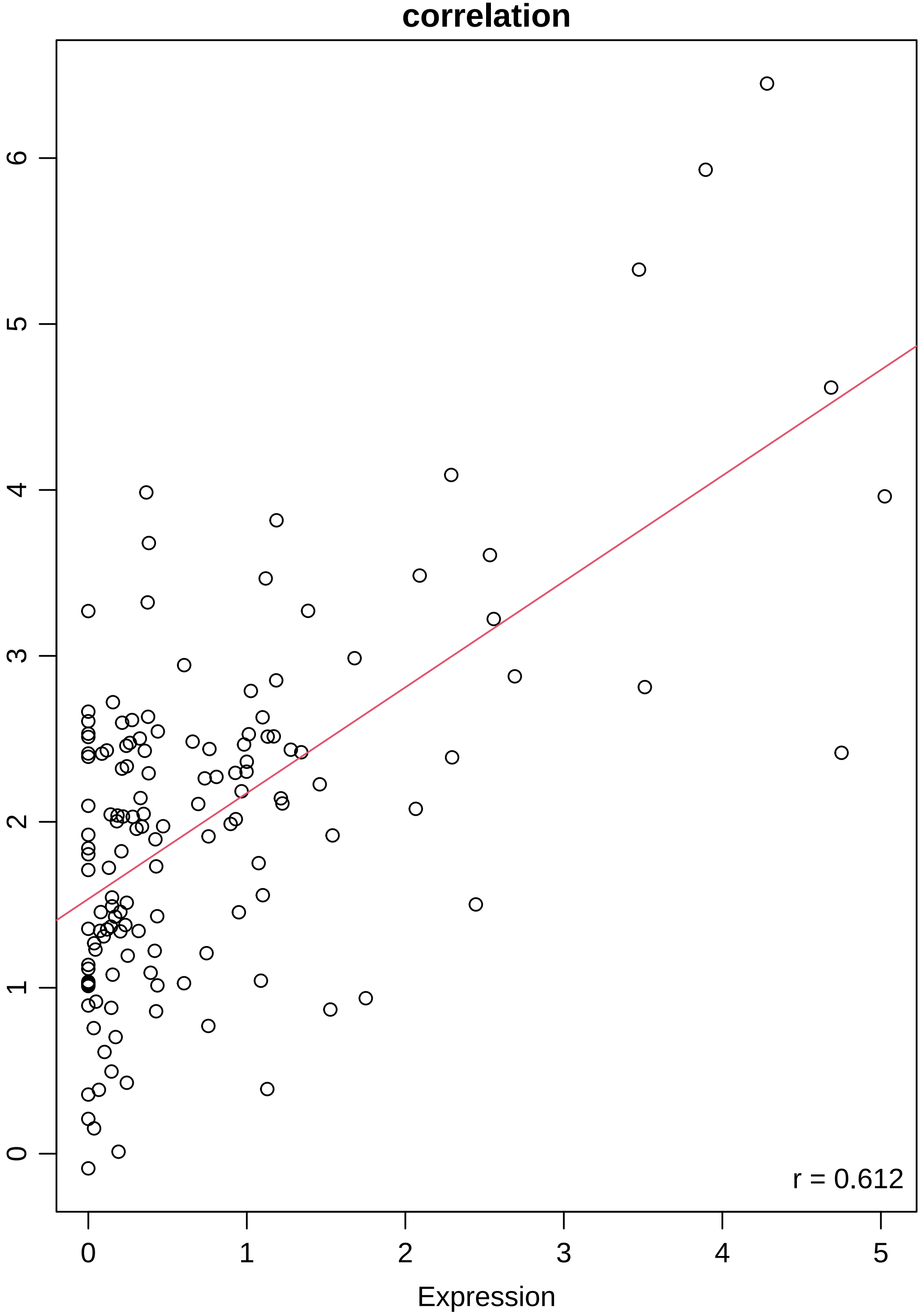

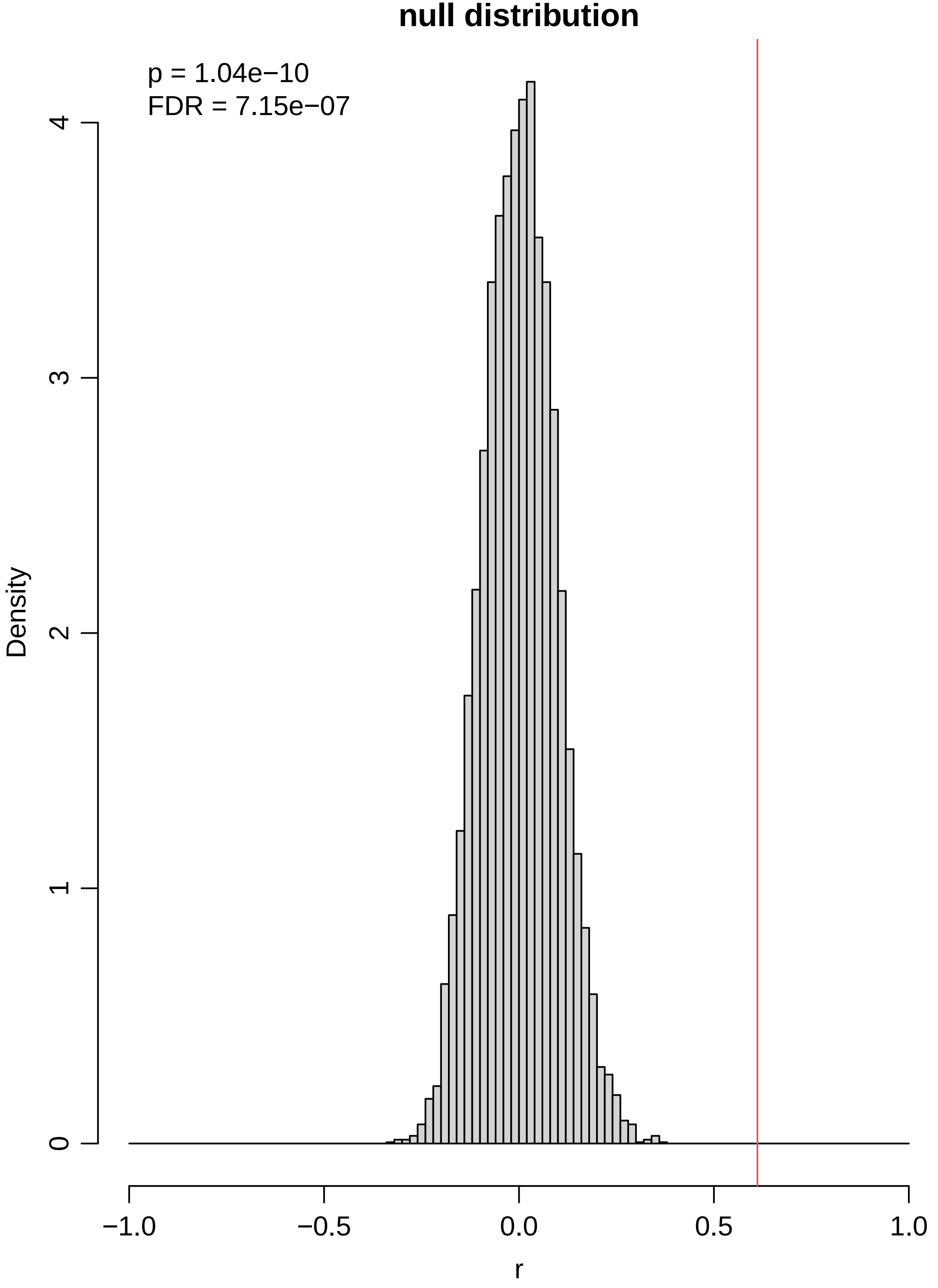

APOBEC3B vs PeakID_157636 (chr22: 38980677 − 38987686)

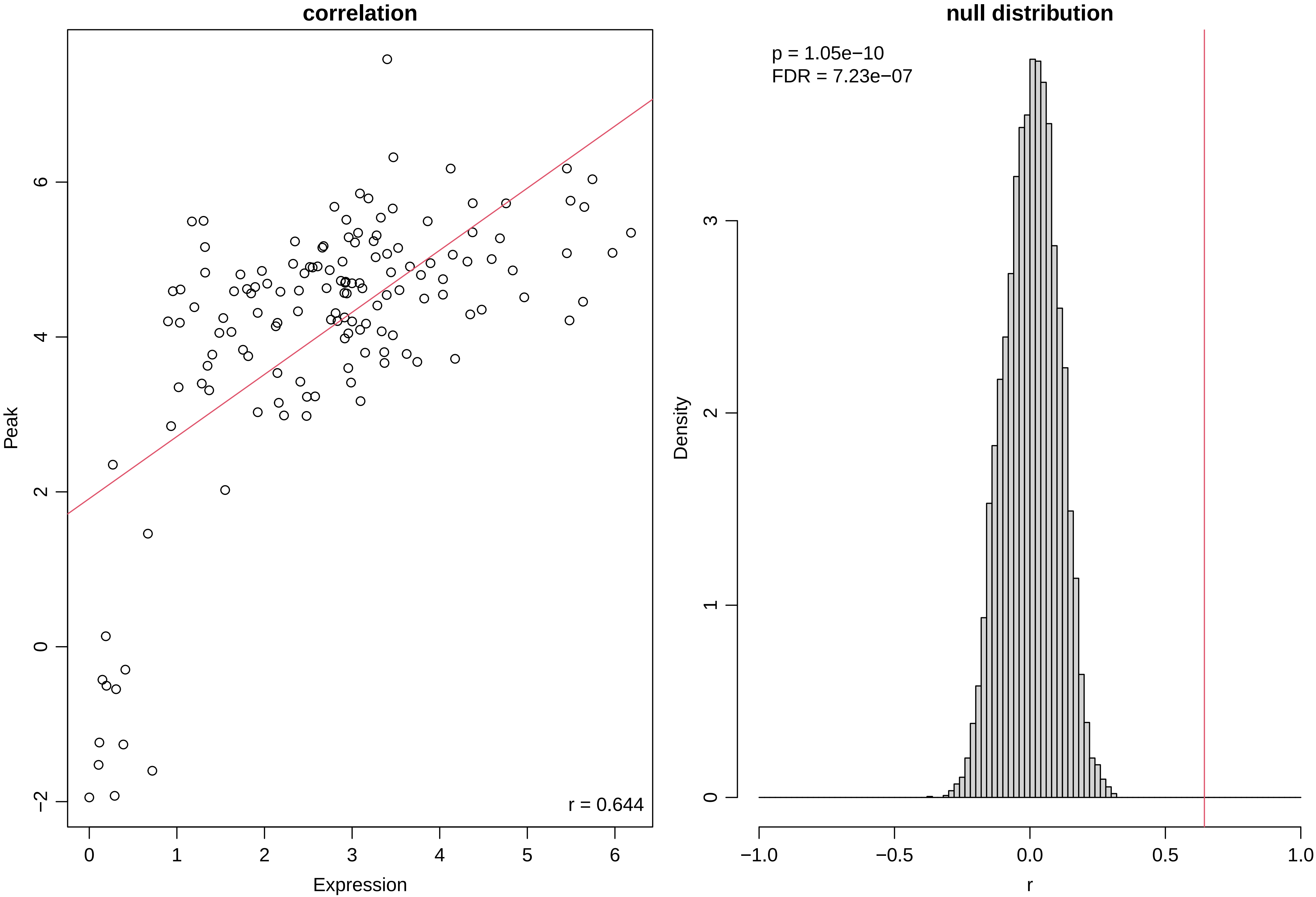

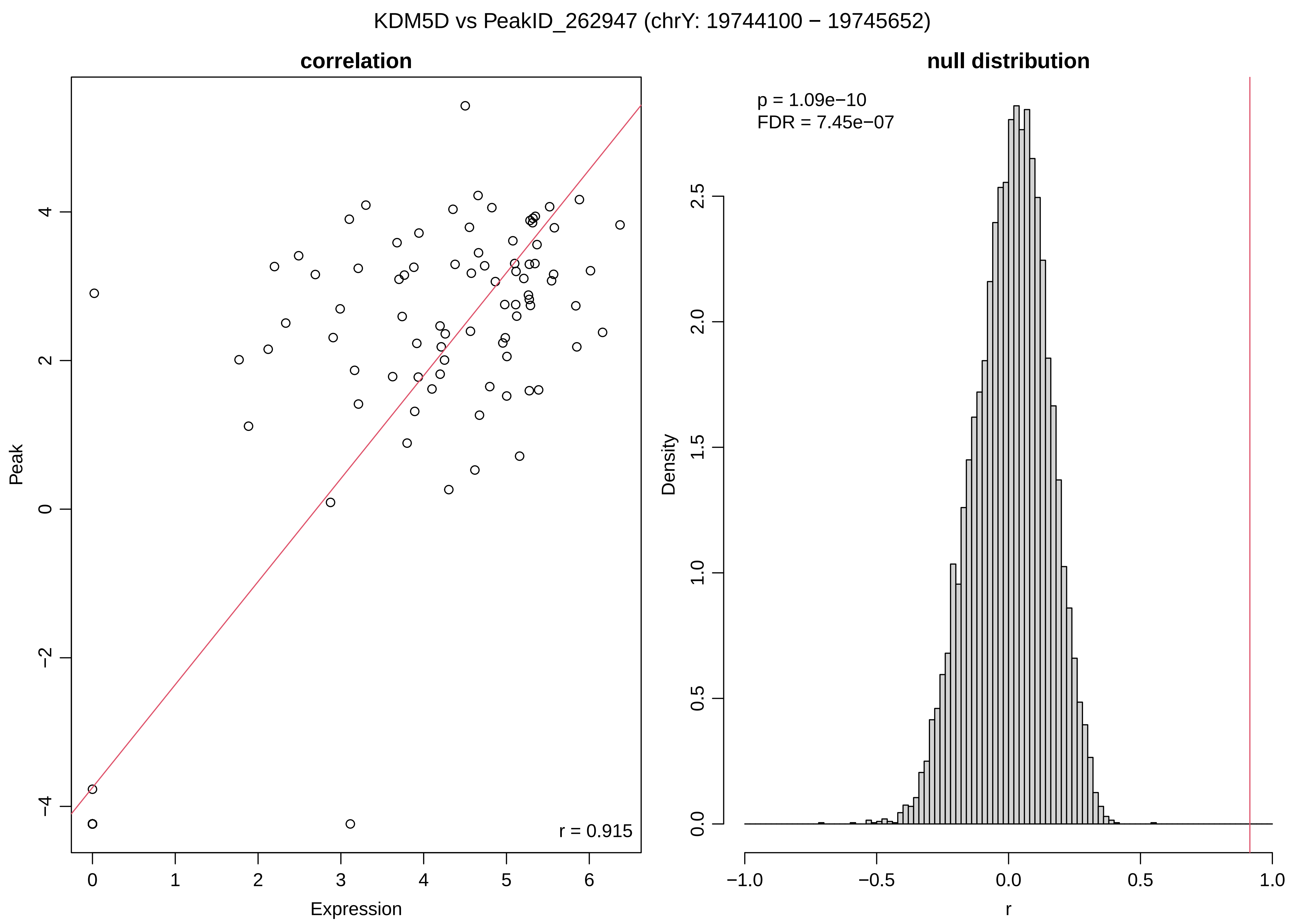

KDM5D vs PeakID_262947 (chrY: 19744100 − 19745652)
correlation
Peak
Expression
r = 0.915
null distribution
p = 1.09e−10
FDR = 7.45e−07
Density
r

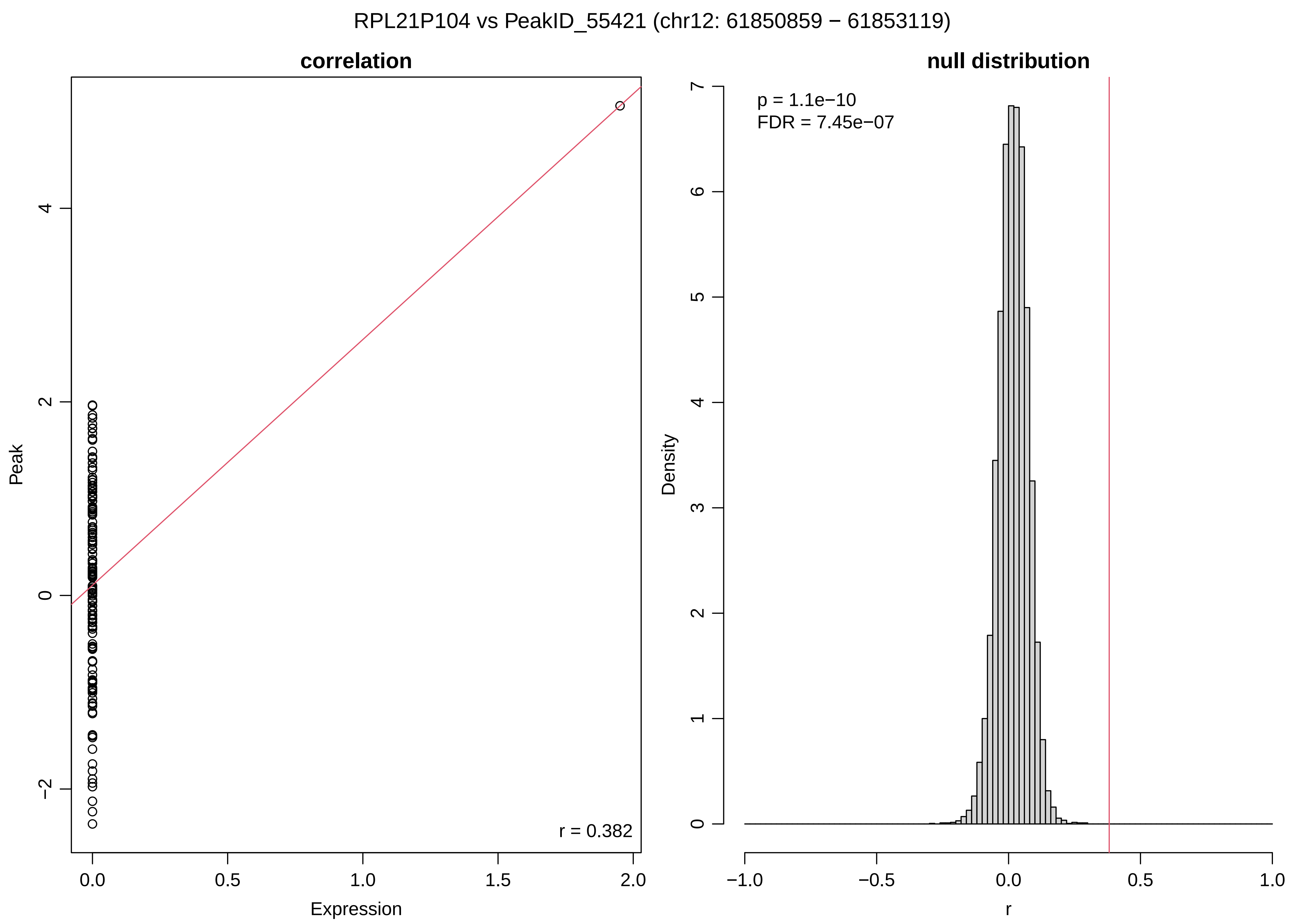
RPL21P104 vs PeakID_55421 (chr12: 61850859 − 61853119)
correlation
Peak
Expression
r = 0.382
null distribution
Density
r
p = 1.1e−10
FDR = 7.45e−07

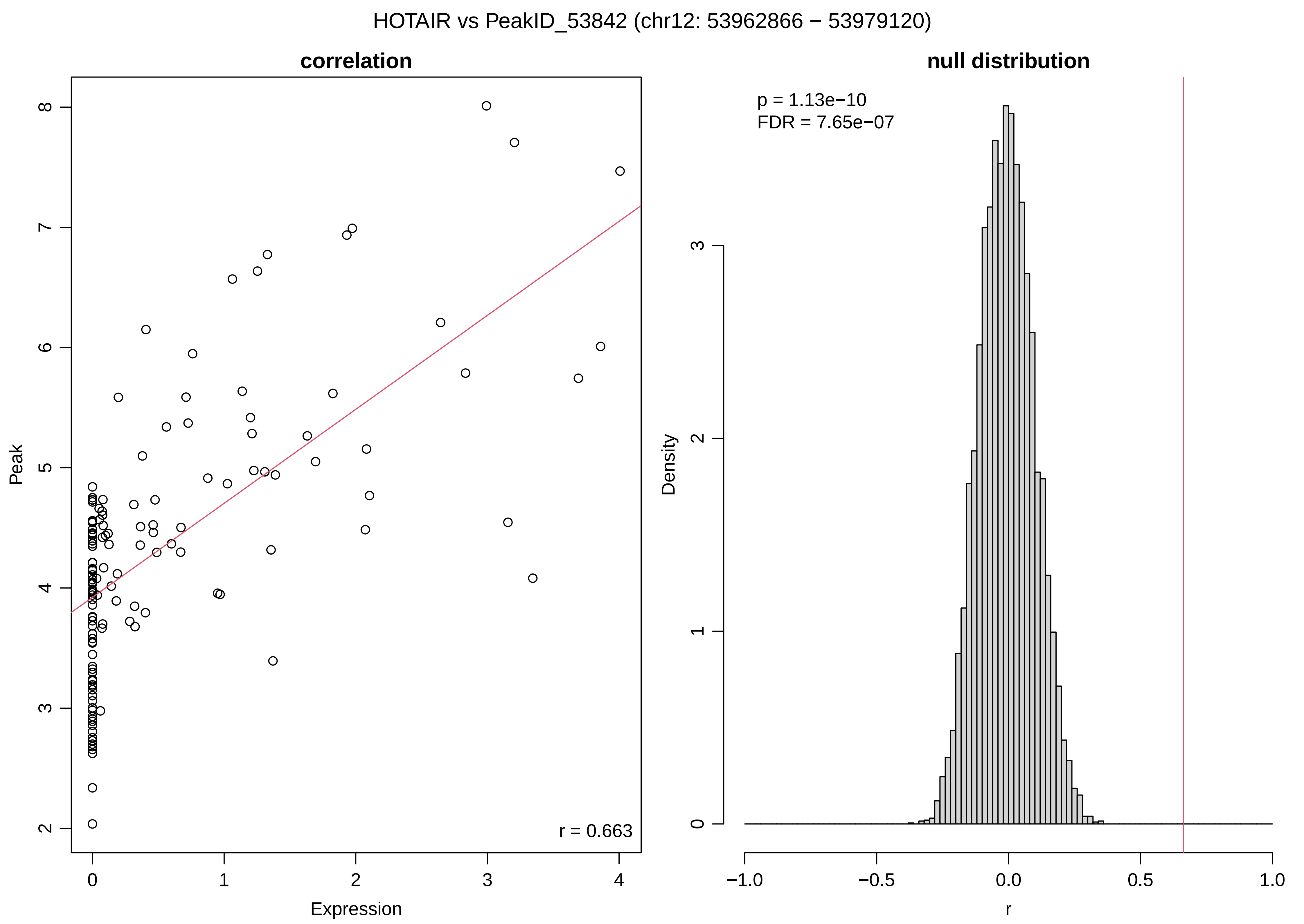

HOTAIR vs PeakID_53842 (chr12: 53962866 − 53979120)
correlation
Peak
Expression
r = 0.663
null distribution
p = 1.13e−10
FDR = 7.65e−07
Density
r

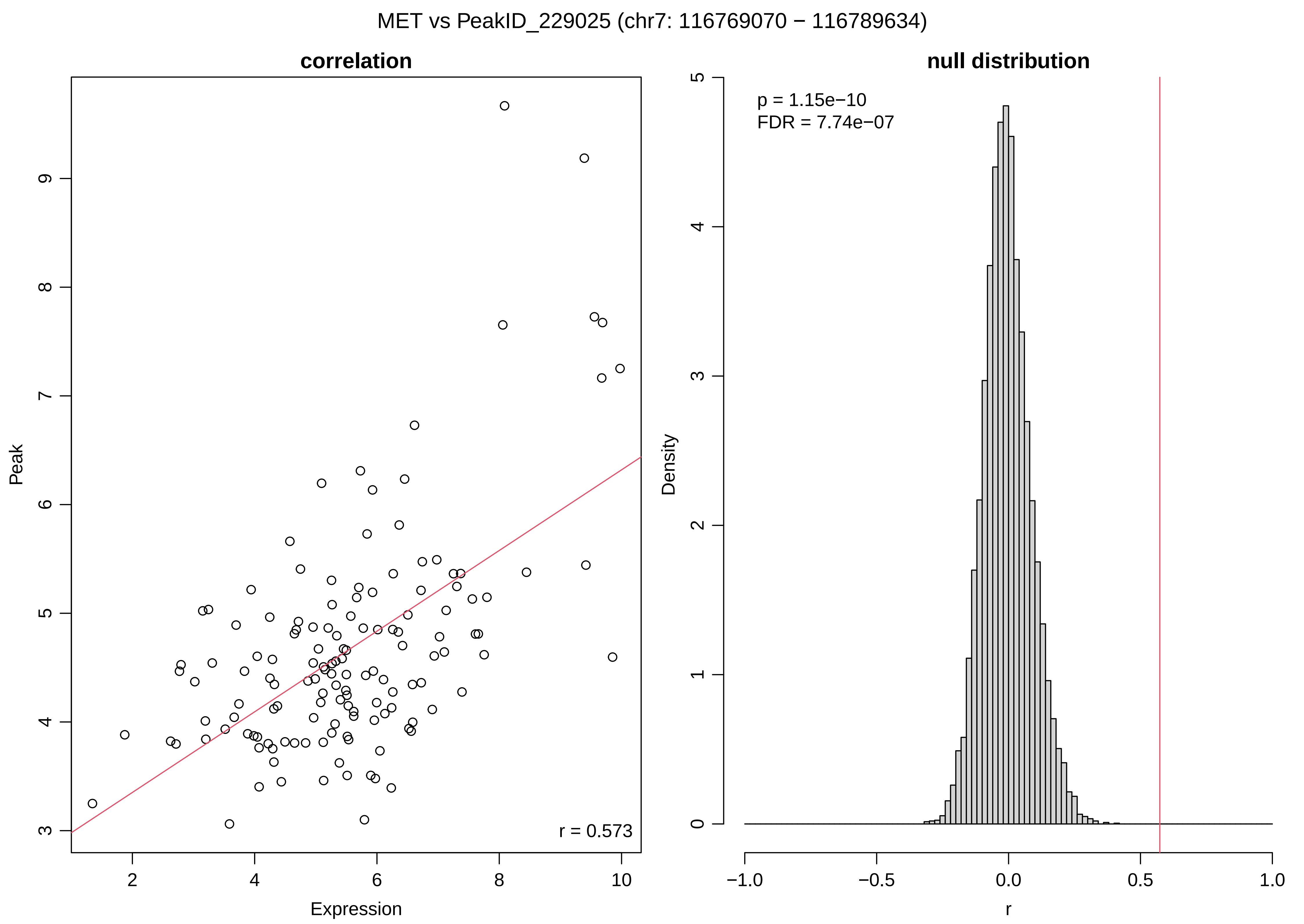
MET vs PeakID_229025 (chr7: 116769070 − 116789634)
correlation
Peak
Expression
r = 0.573
null distribution
Density
r
p = 1.15e−10
FDR = 7.74e−07

LINC01515 vs PeakID_29806 (chr10: 65570265 − 65571743)

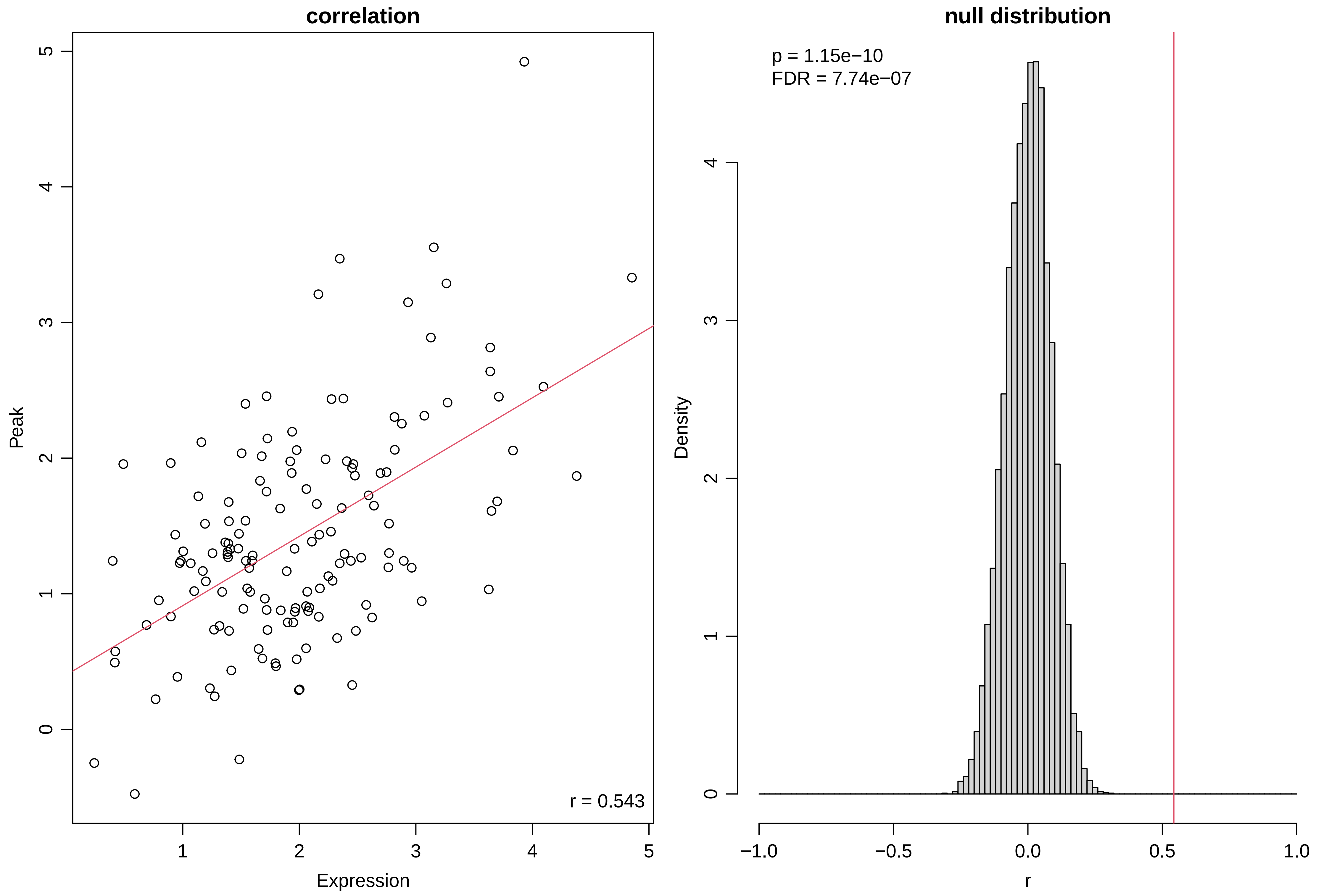

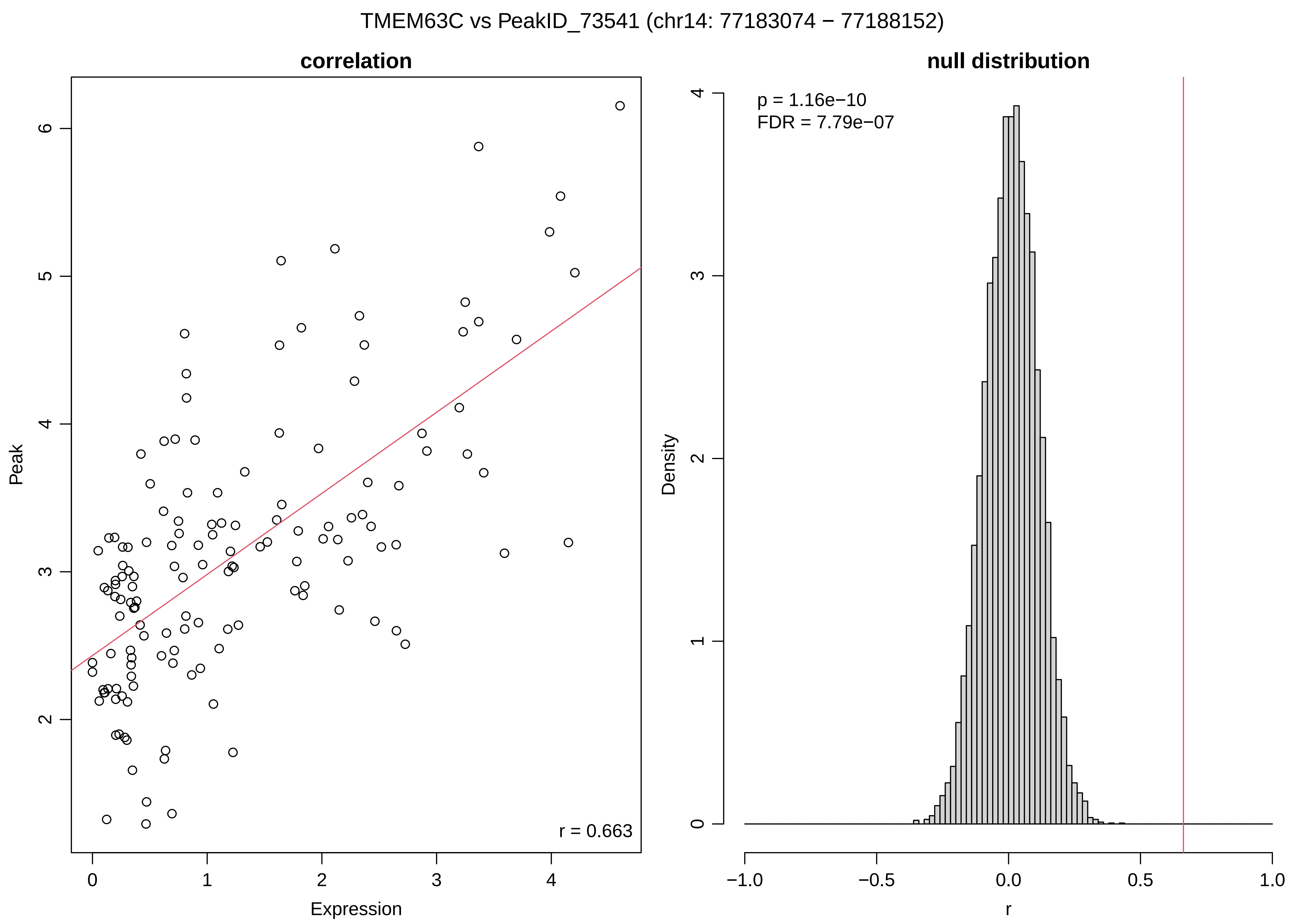

TMEM63C vs PeakID_73541 (chr14: 77183074 − 77188152)
correlation
Peak
Expression
r = 0.663
null distribution
p = 1.16e−10
FDR = 7.79e−07
Density
r

AC000032.1 vs PeakID_10825 (chr1: 109687158 − 109688729)

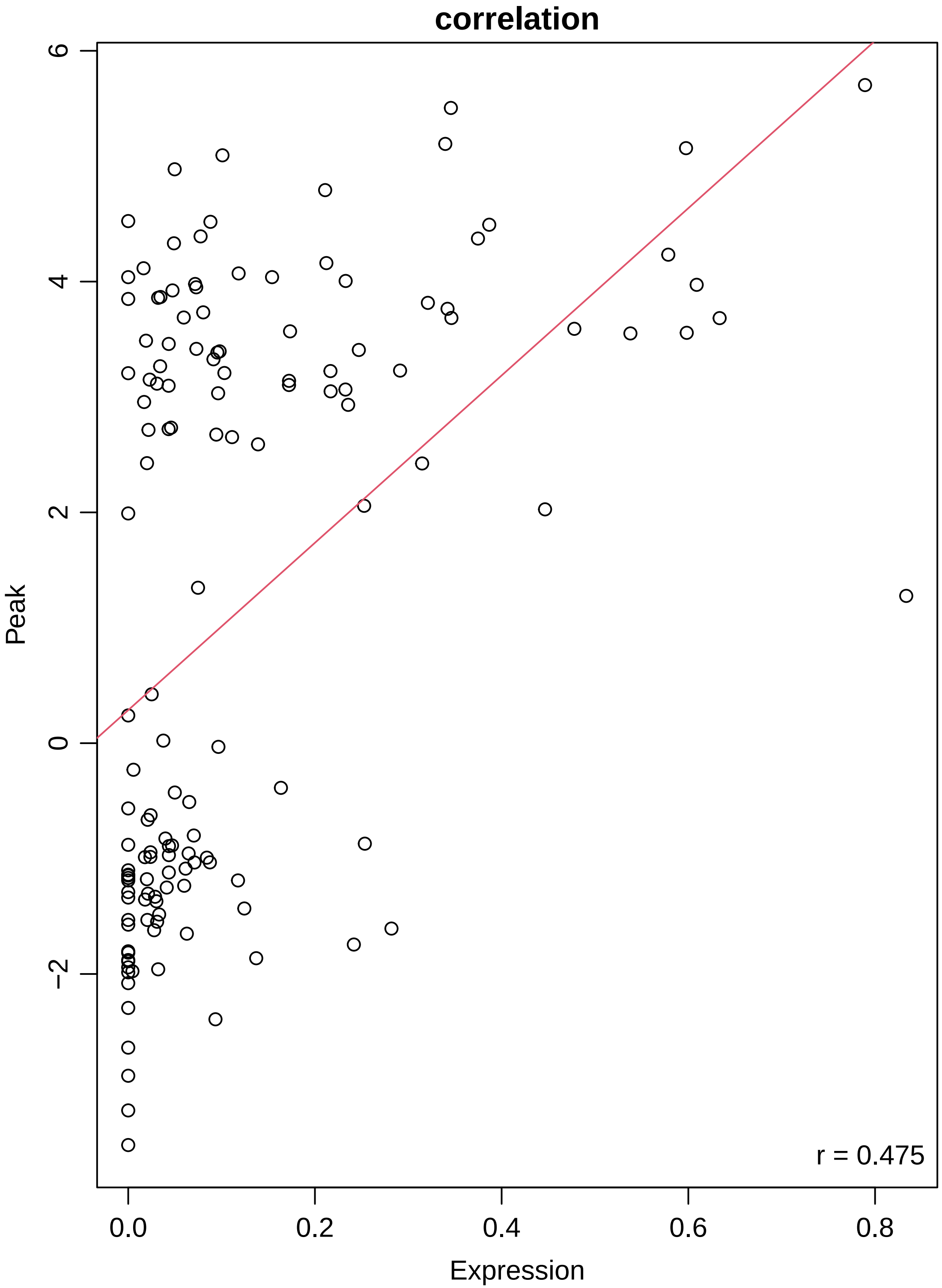

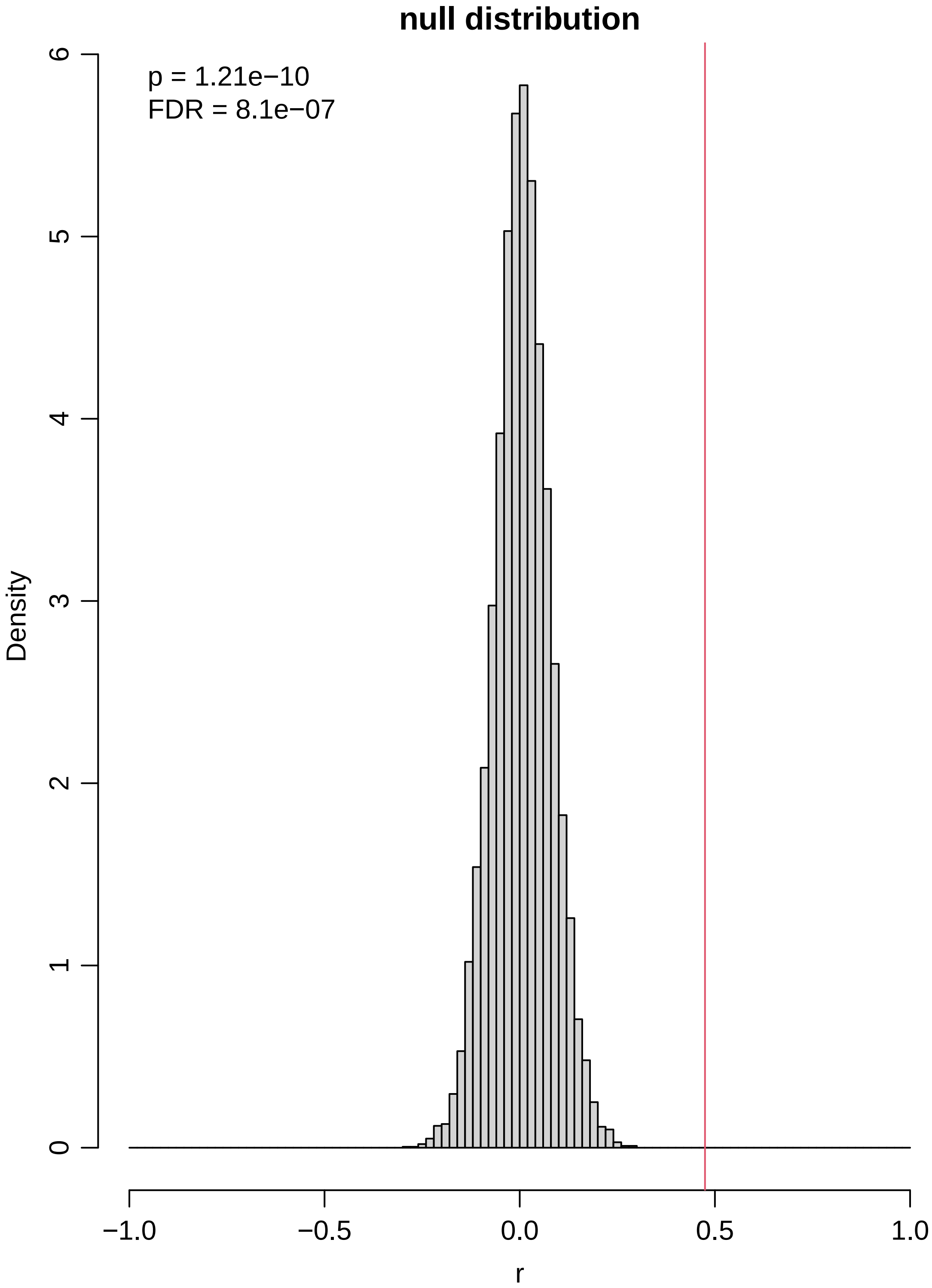

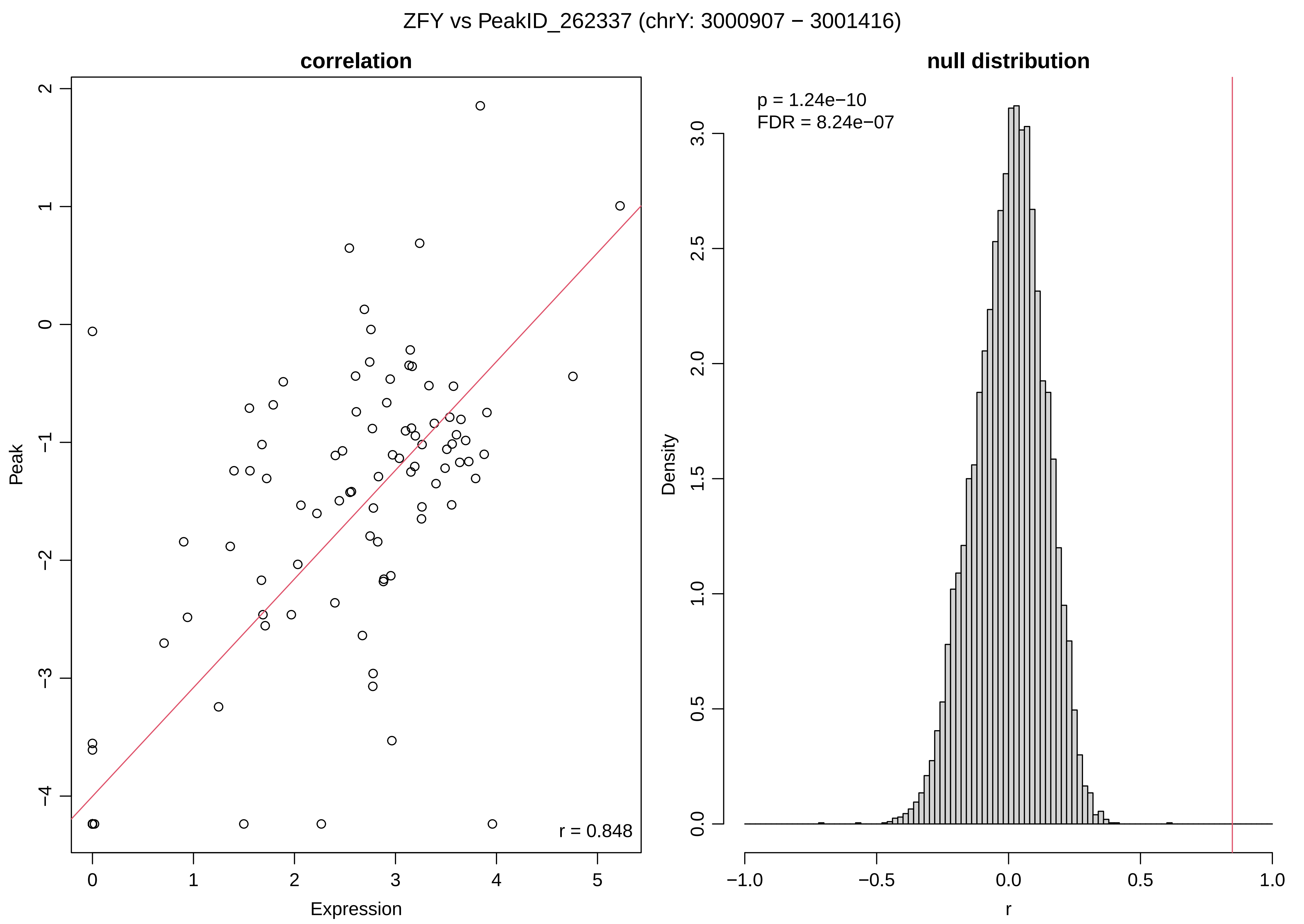
ZFY vs PeakID_262337 (chrY: 3000907 − 3001416)
correlation
null distribution
Peak
Expression
r = 0.848
p = 1.24e−10
FDR = 8.24e−07
Density
r

FRG1BP vs PeakID_145127 (chr20: 30376508 − 30377819)

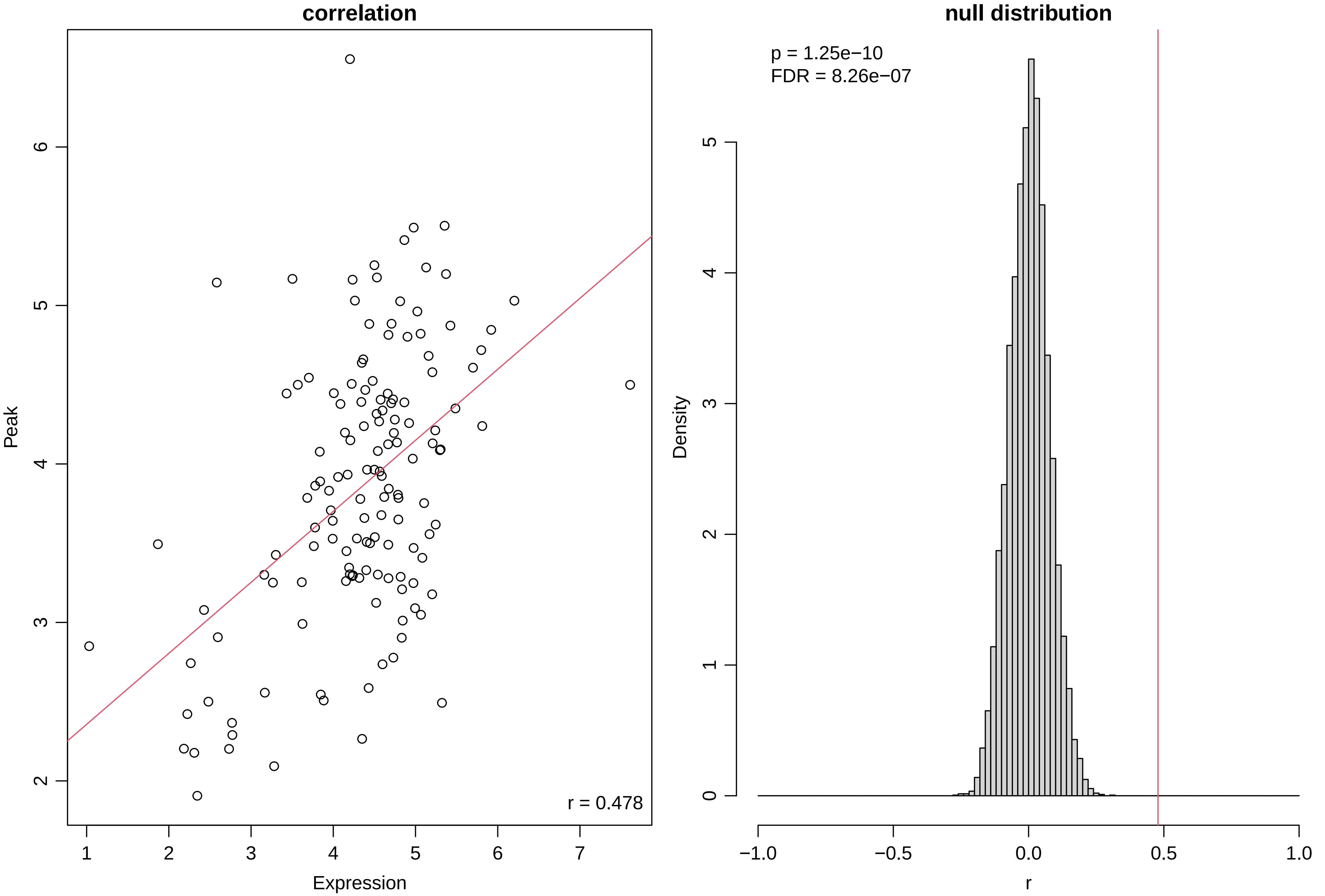

SLC35E3 vs PeakID_56608 (chr12: 68865287 − 68866701)

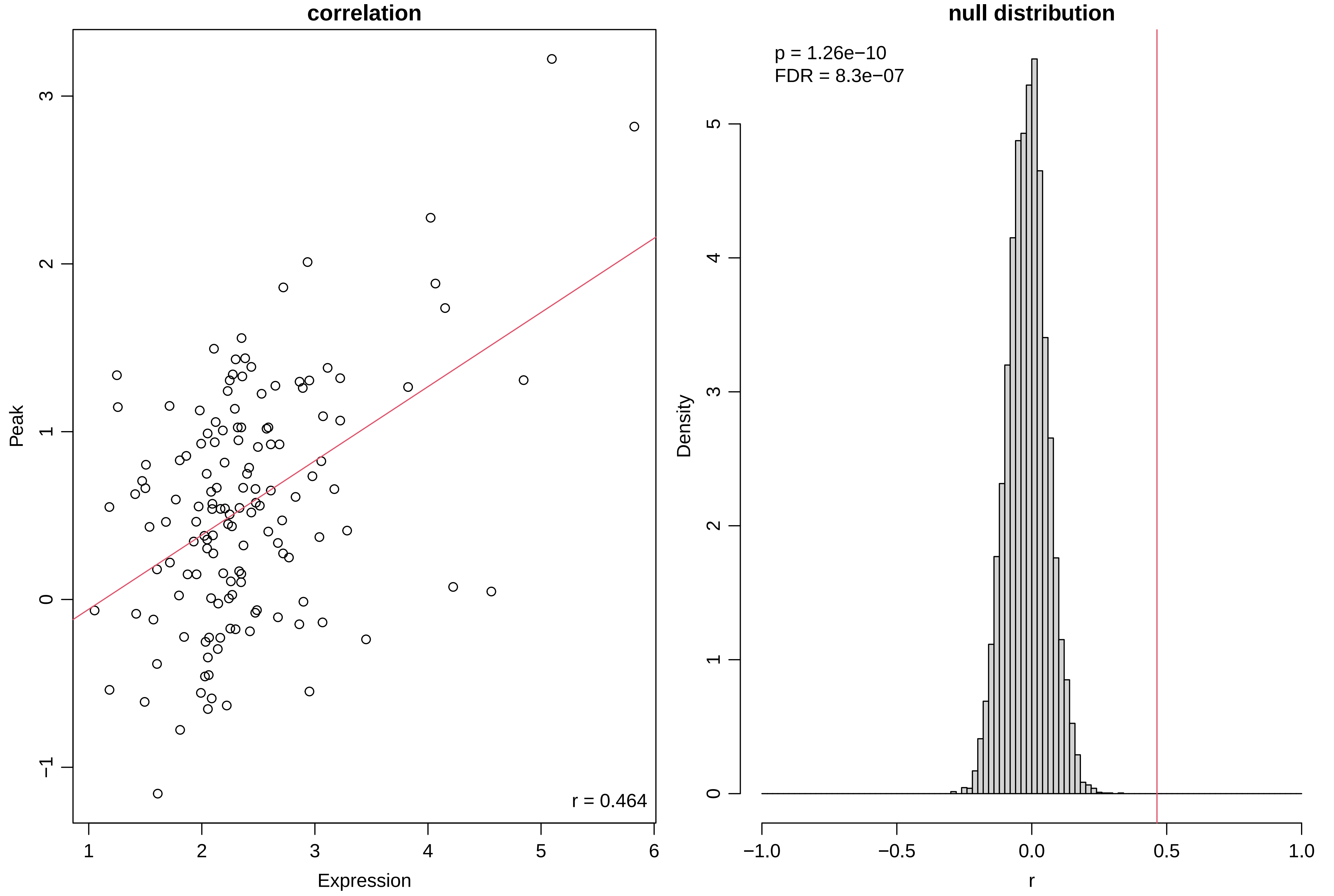

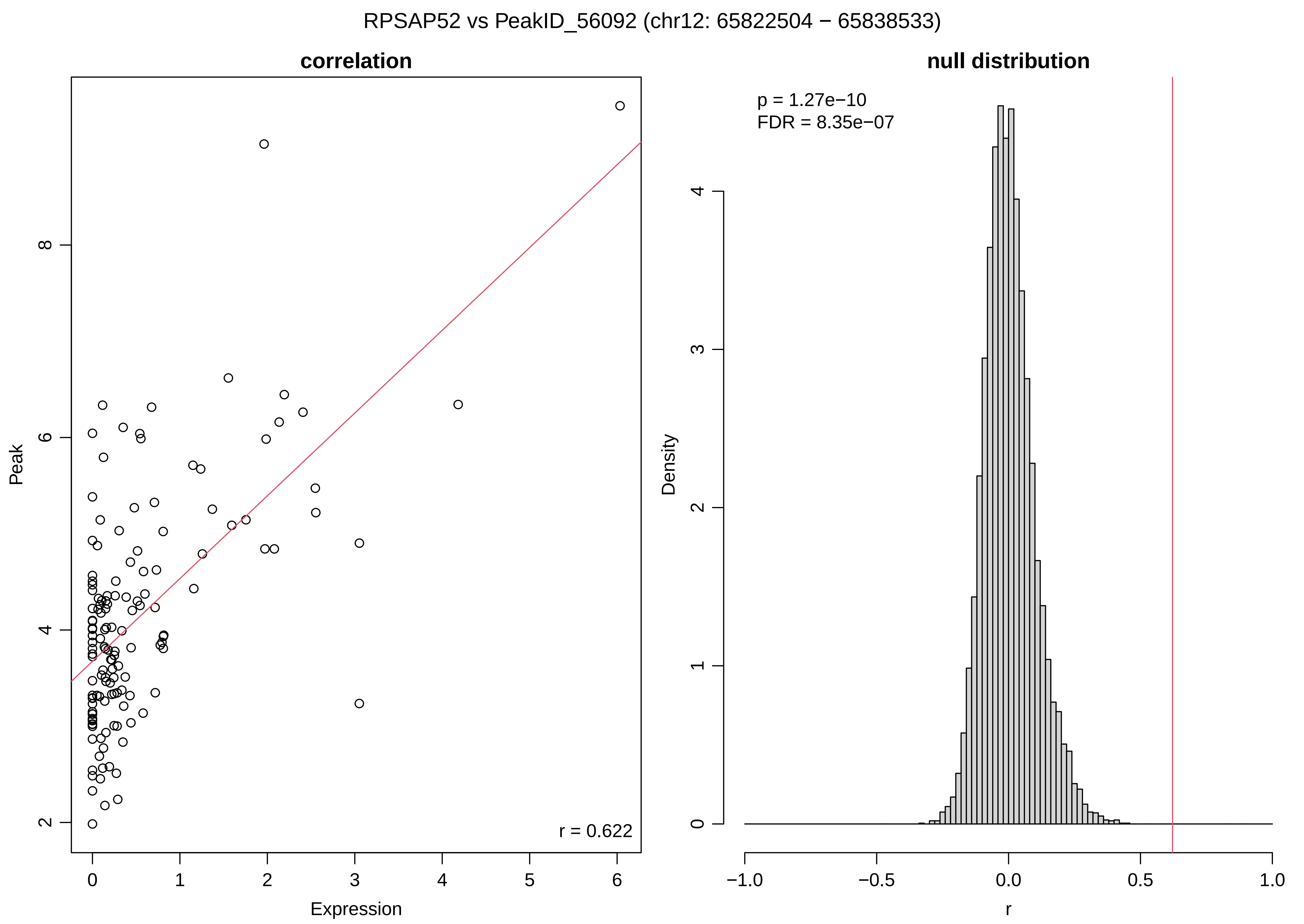
RPSAP52 vs PeakID_56092 (chr12: 65822504 − 65838533)
correlation
Peak
Expression
r = 0.622
null distribution
p = 1.27e−10
FDR = 8.35e−07
Density
r

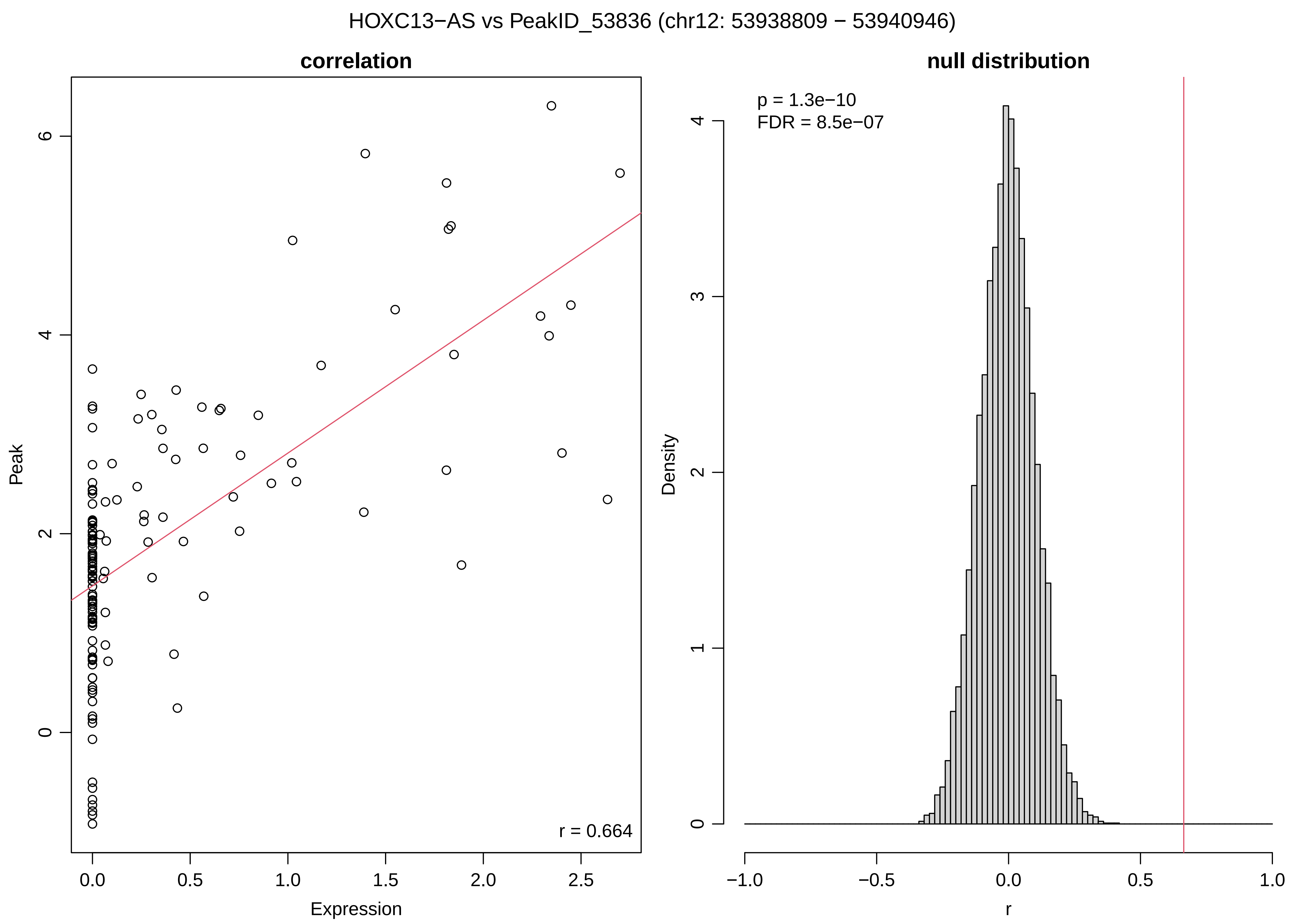
HOXC13−AS vs PeakID_53836 (chr12: 53938809 − 53940946)
correlation
Peak
Expression
r = 0.664
null distribution
p = 1.3e−10
FDR = 8.5e−07
Density
r

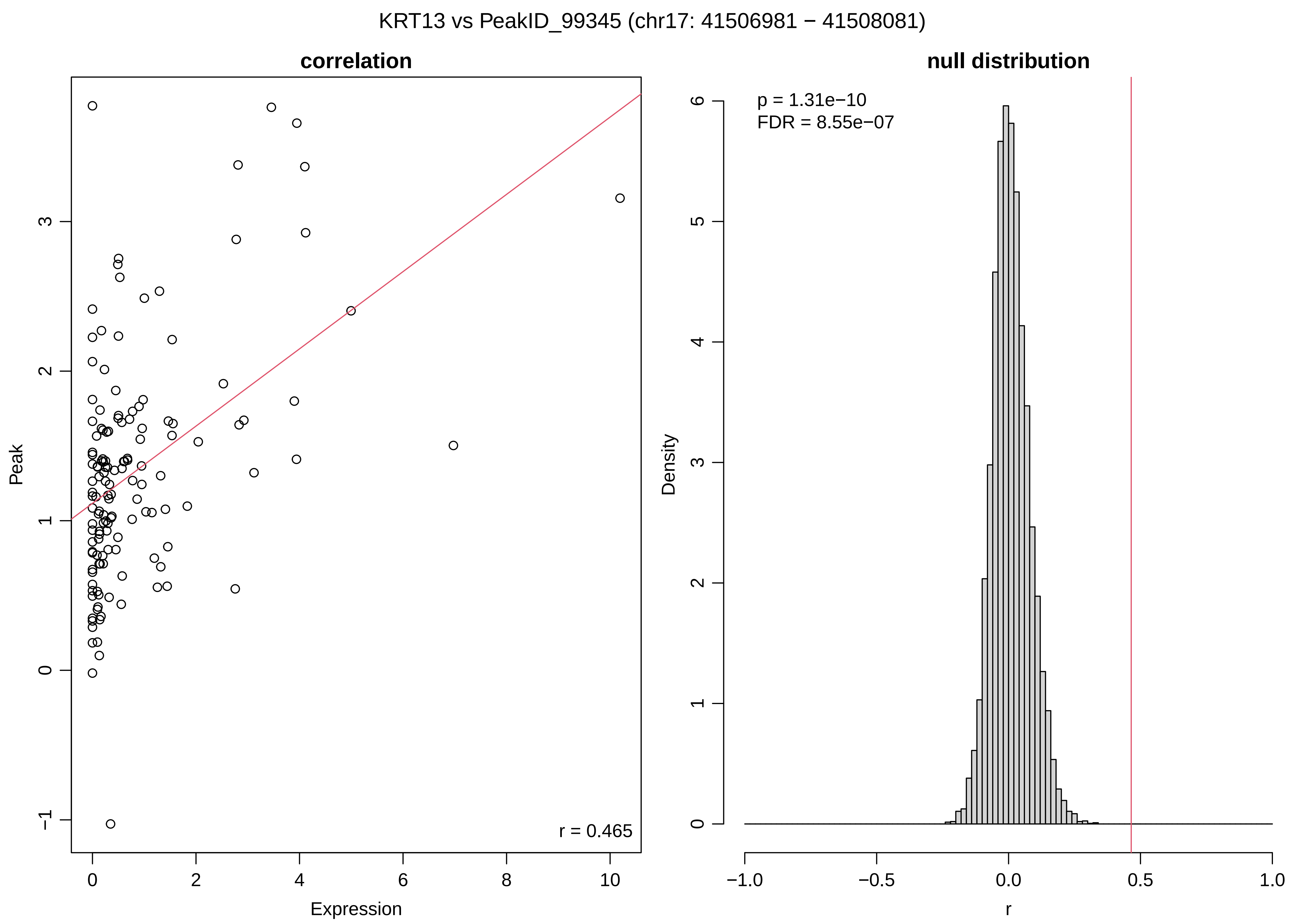
KRT13 vs PeakID_99345 (chr17: 41506981 − 41508081)
correlation
null distribution
Peak
Expression
r = 0.465
p = 1.31e−10
FDR = 8.55e−07
Density
r

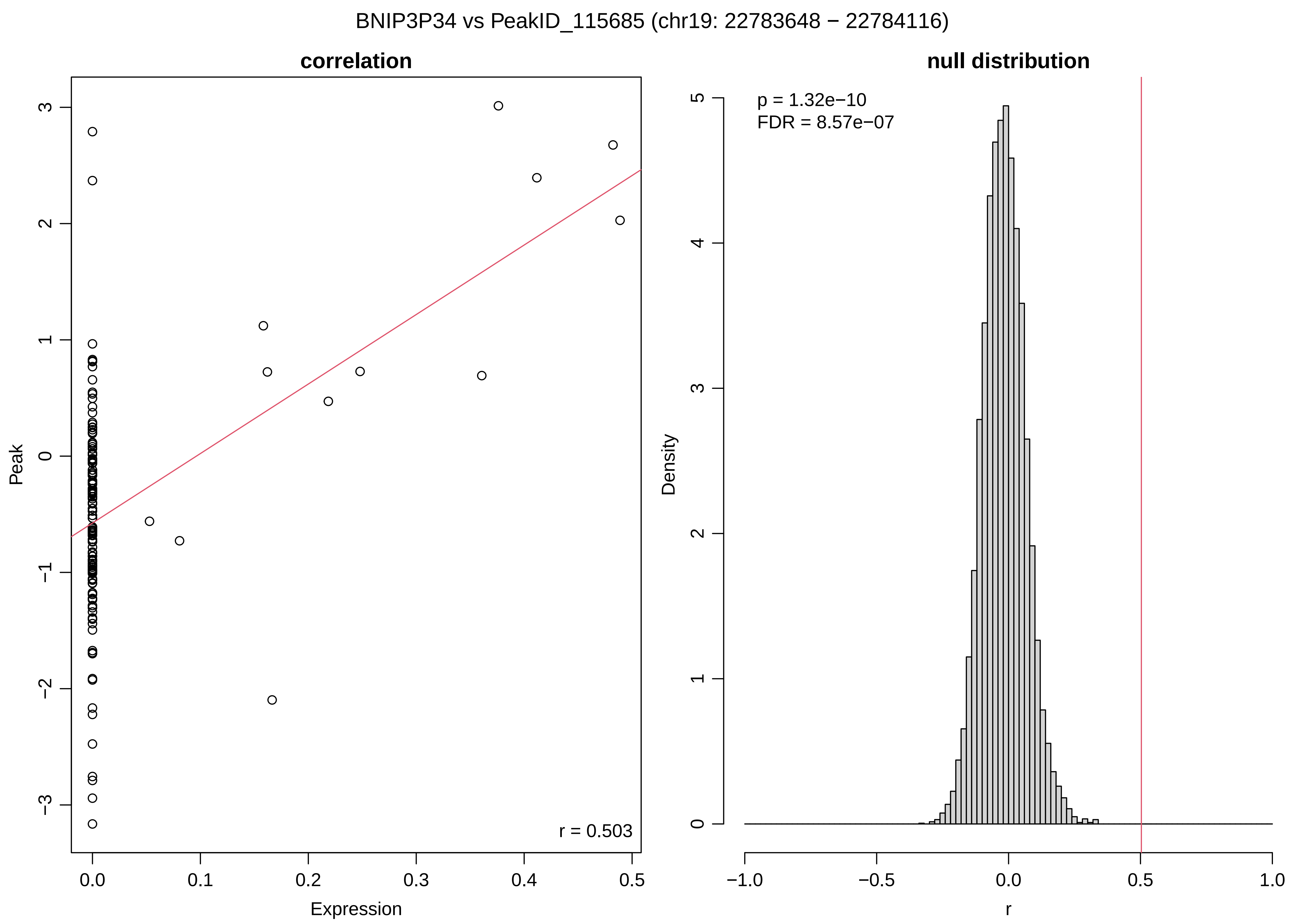
BNIP3P34 vs PeakID_115685 (chr19: 22783648 − 22784116)
correlation
Peak
Expression
r = 0.503
null distribution
p = 1.32e−10
FDR = 8.57e−07
Density
r
3
2
1
0
−1
−2
−3
0.0
0.1
0.2
0.3
0.4
0.5
5
4
3
2
1
0
−1.0
−0.5
0.0
0.5
1.0

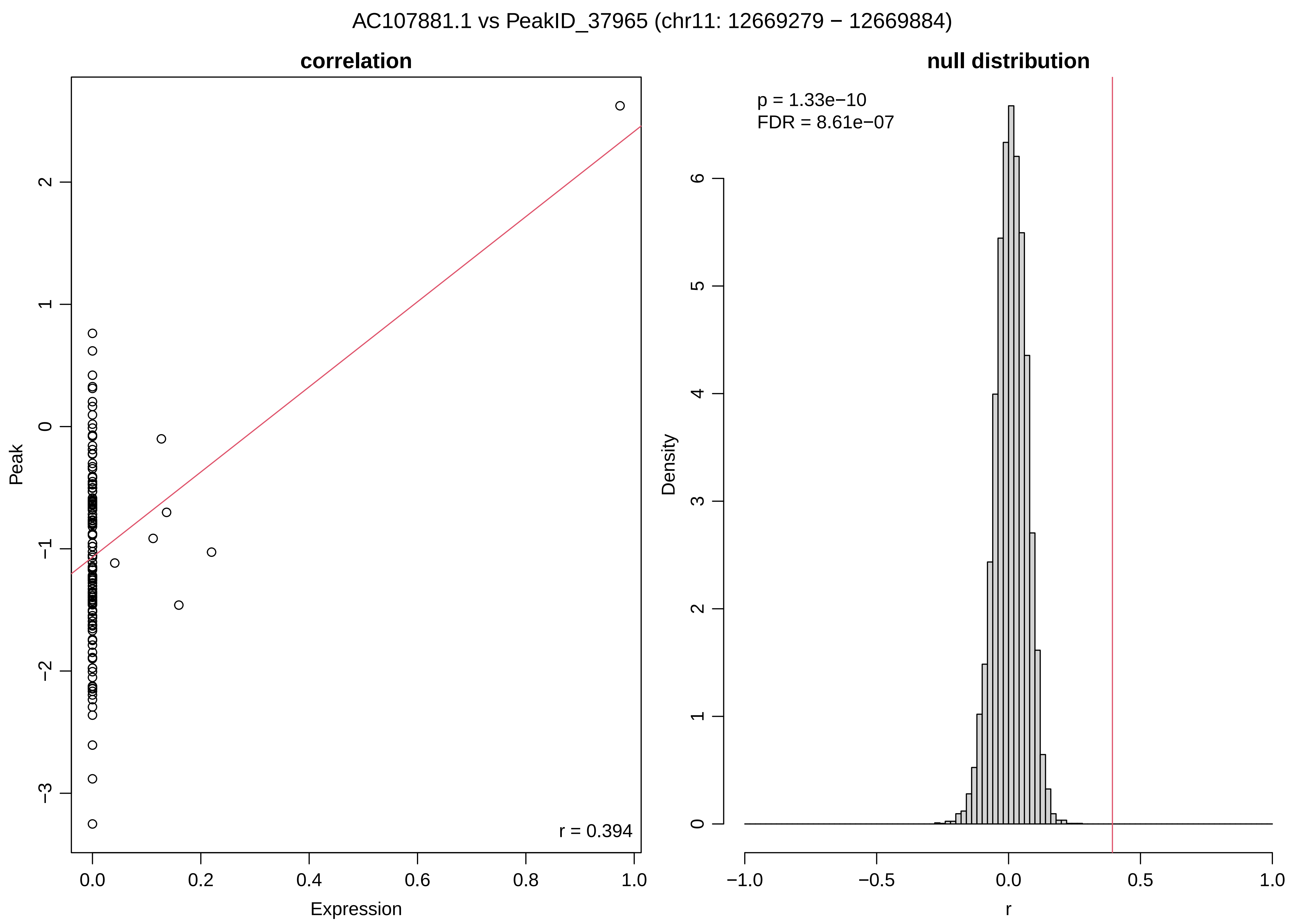

AC107881.1 vs PeakID_37965 (chr11: 12669279 − 12669884)
correlation
Peak
Expression
r = 0.394
null distribution
p = 1.33e−10
FDR = 8.61e−07
Density
r

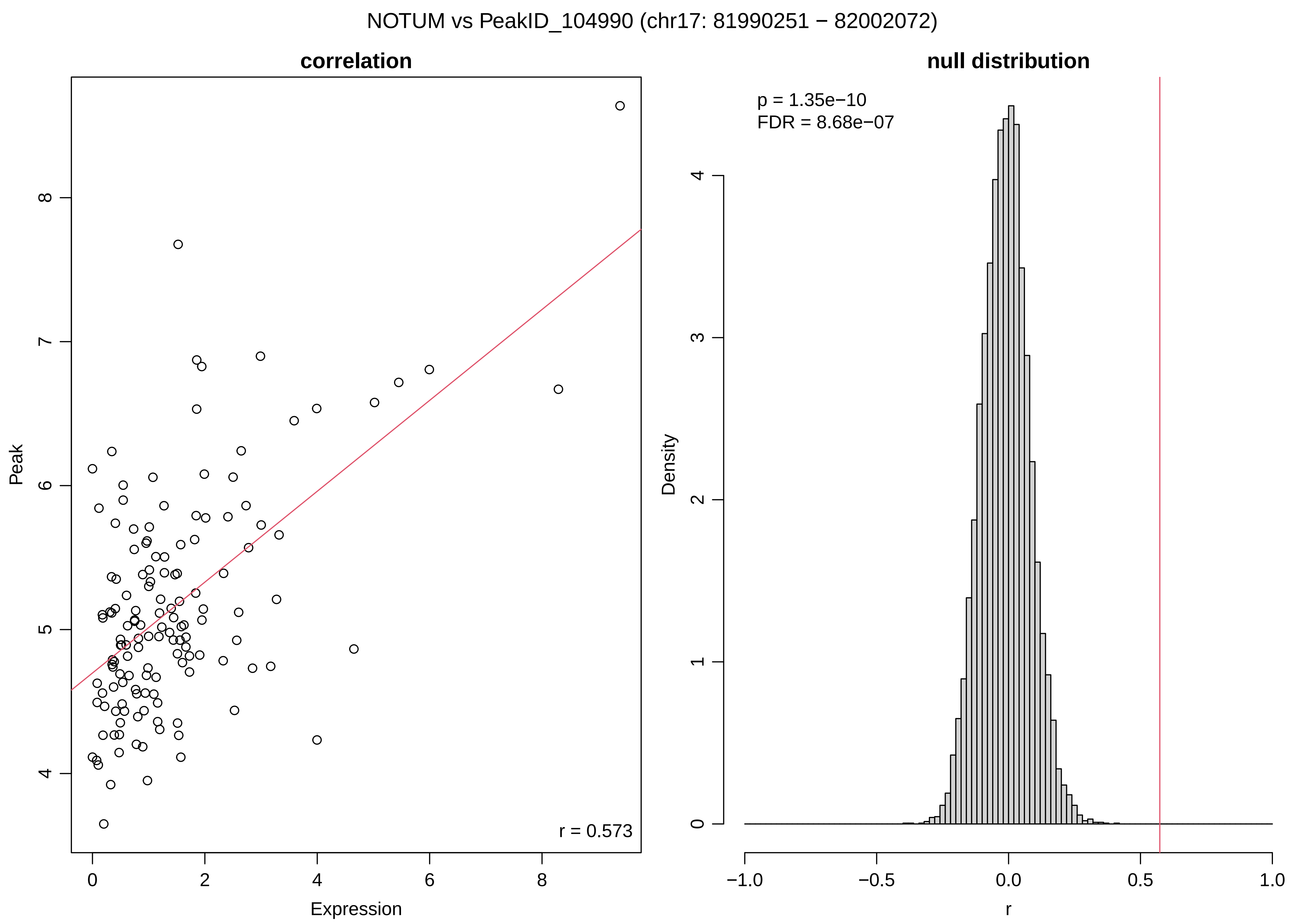
NOTUM vs PeakID_104990 (chr17: 81990251 − 82002072)
correlation
null distribution
p = 1.35e−10
FDR = 8.68e−07
r = 0.573
Peak
Expression
Density
r

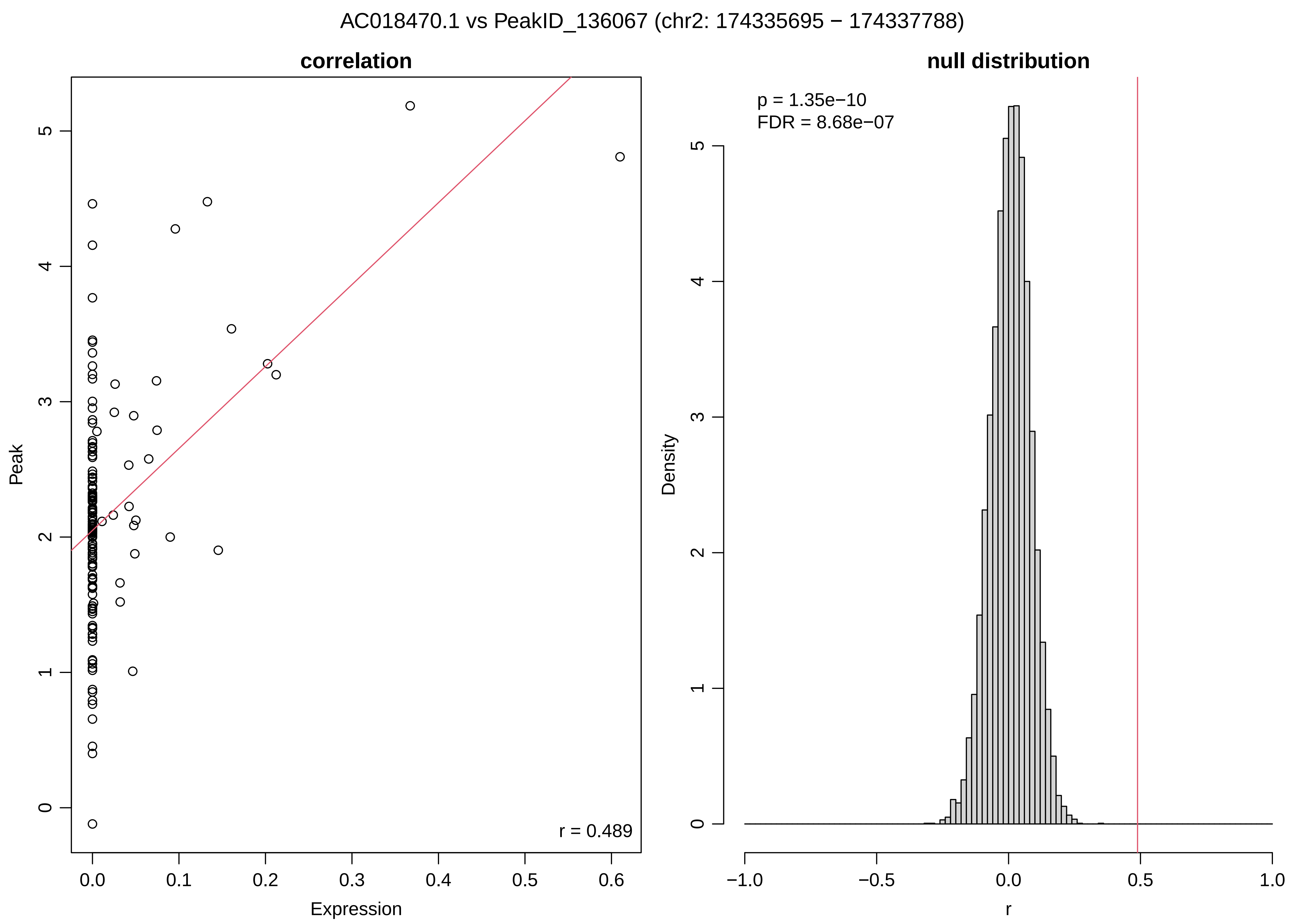

AC018470.1 vs PeakID_136067 (chr2: 174335695 − 174337788)
correlation
Peak
Expression
r = 0.489
null distribution
p = 1.35e−10
FDR = 8.68e−07
Density
r

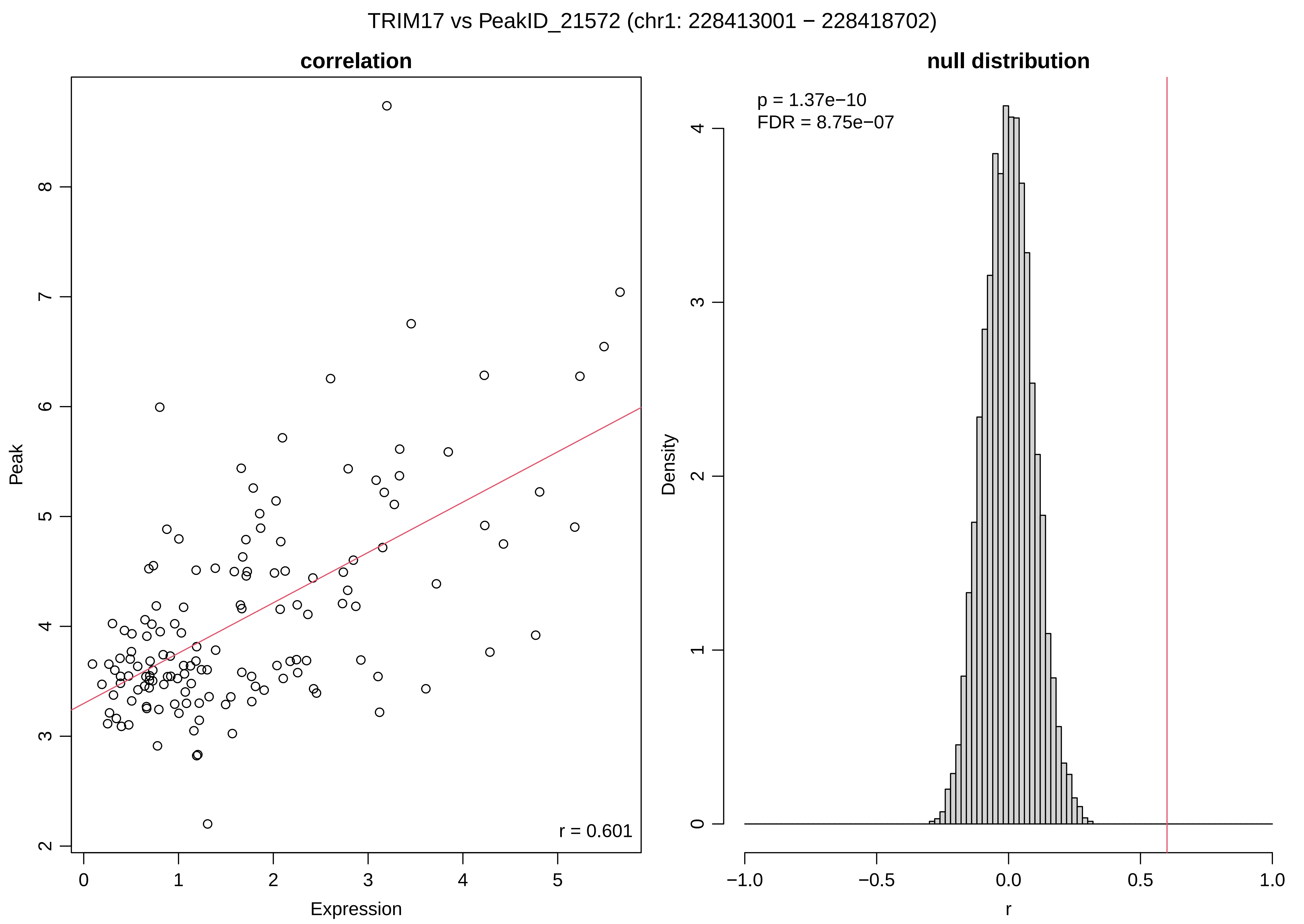

TRIM17 vs PeakID_21572 (chr1: 228413001 − 228418702)
correlation
Peak
Expression
r = 0.601
null distribution
p = 1.37e−10
FDR = 8.75e−07
Density
r

LINC00824 vs PeakID_243186 (chr8: 128611550 − 128617836)

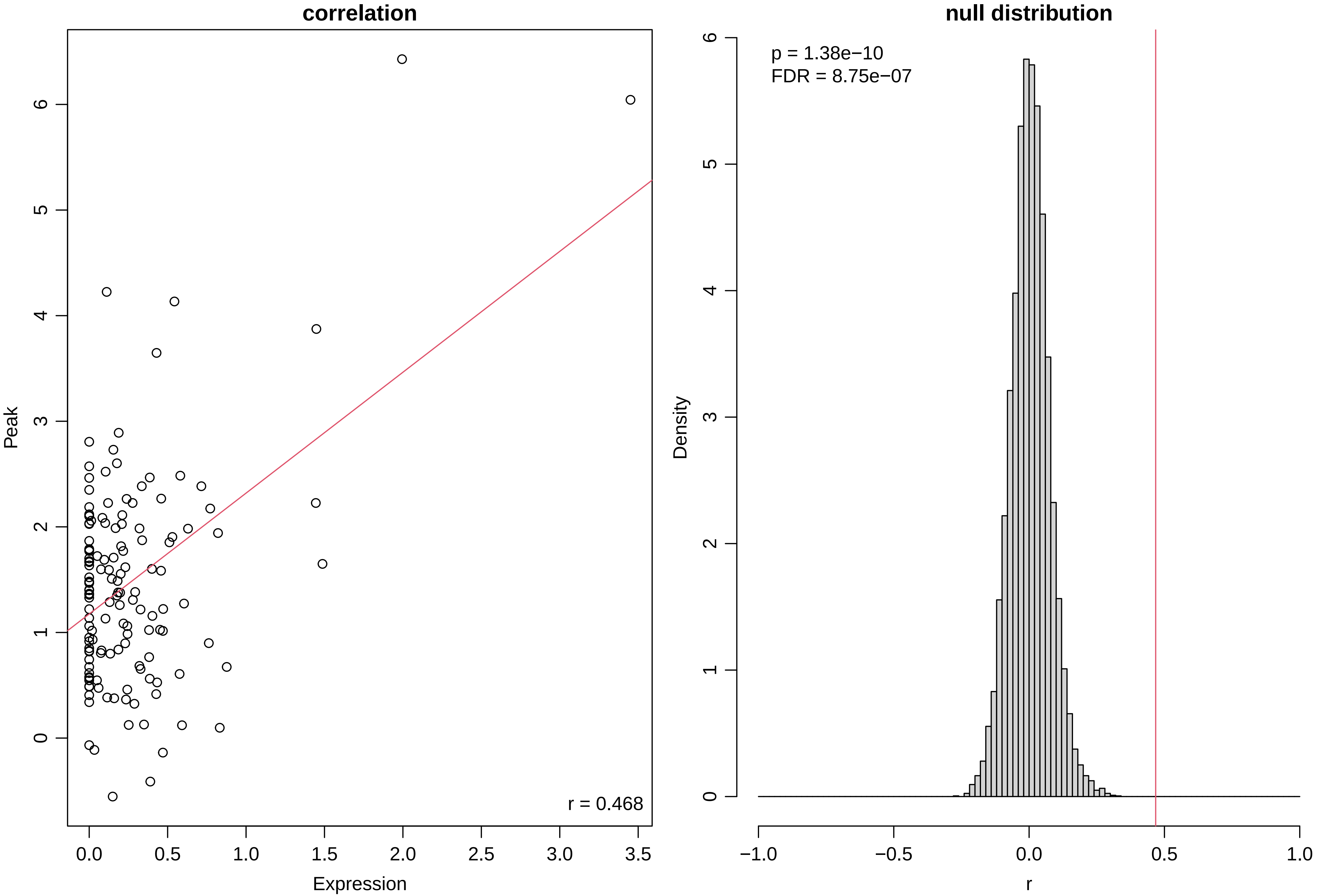

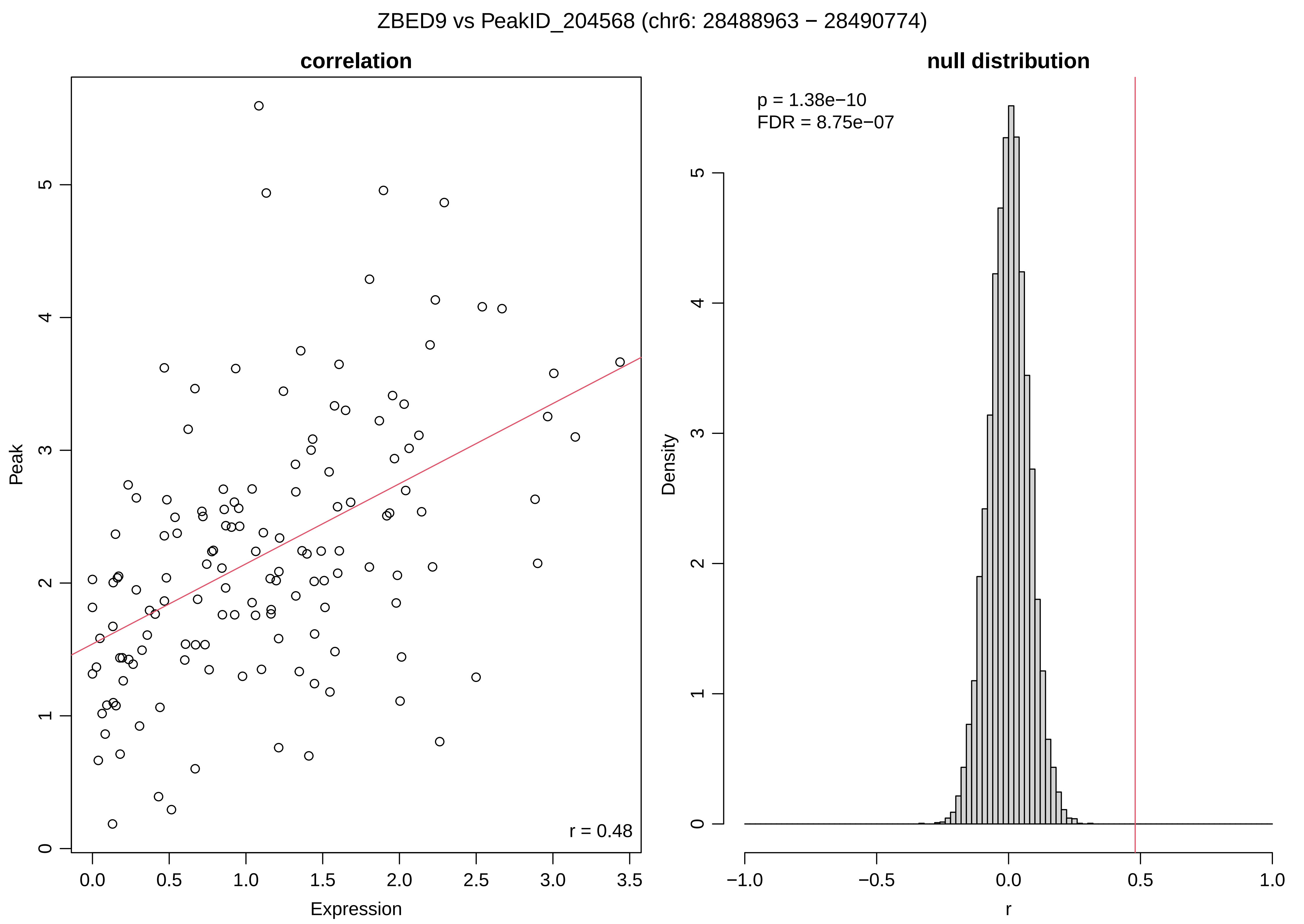
ZBED9 vs PeakID_204568 (chr6: 28488963 − 28490774)
correlation
null distribution
Peak
Expression
r = 0.48
p = 1.38e−10
FDR = 8.75e−07
Density
r

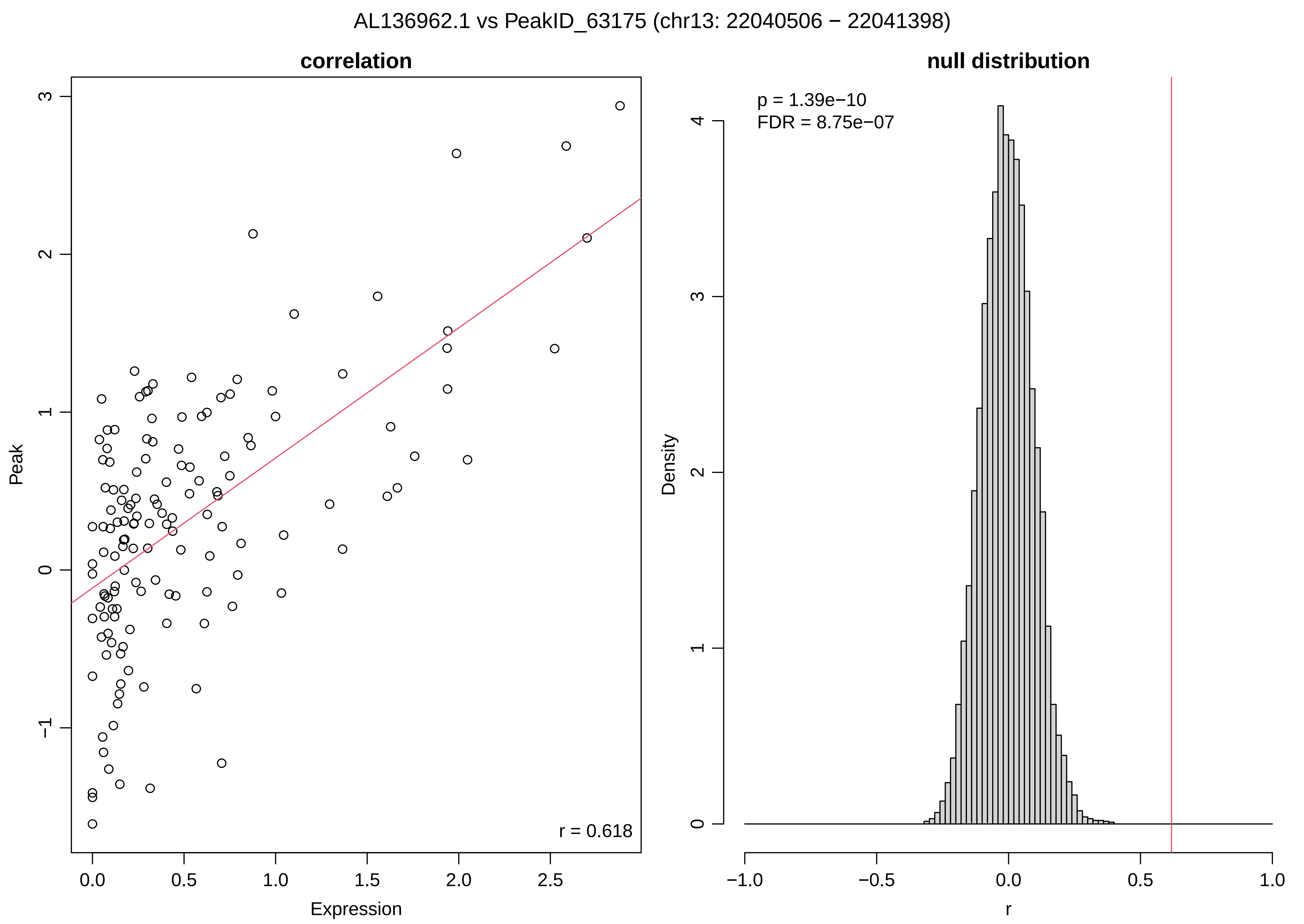
AL136962.1 vs PeakID_63175 (chr13: 22040506 − 22041398)
correlation
Peak
Expression
r = 0.618
null distribution
p = 1.39e−10
FDR = 8.75e−07
Density
r

SLC25A24P1 vs PeakID_10648 (chr1: 108272393 − 108274114)

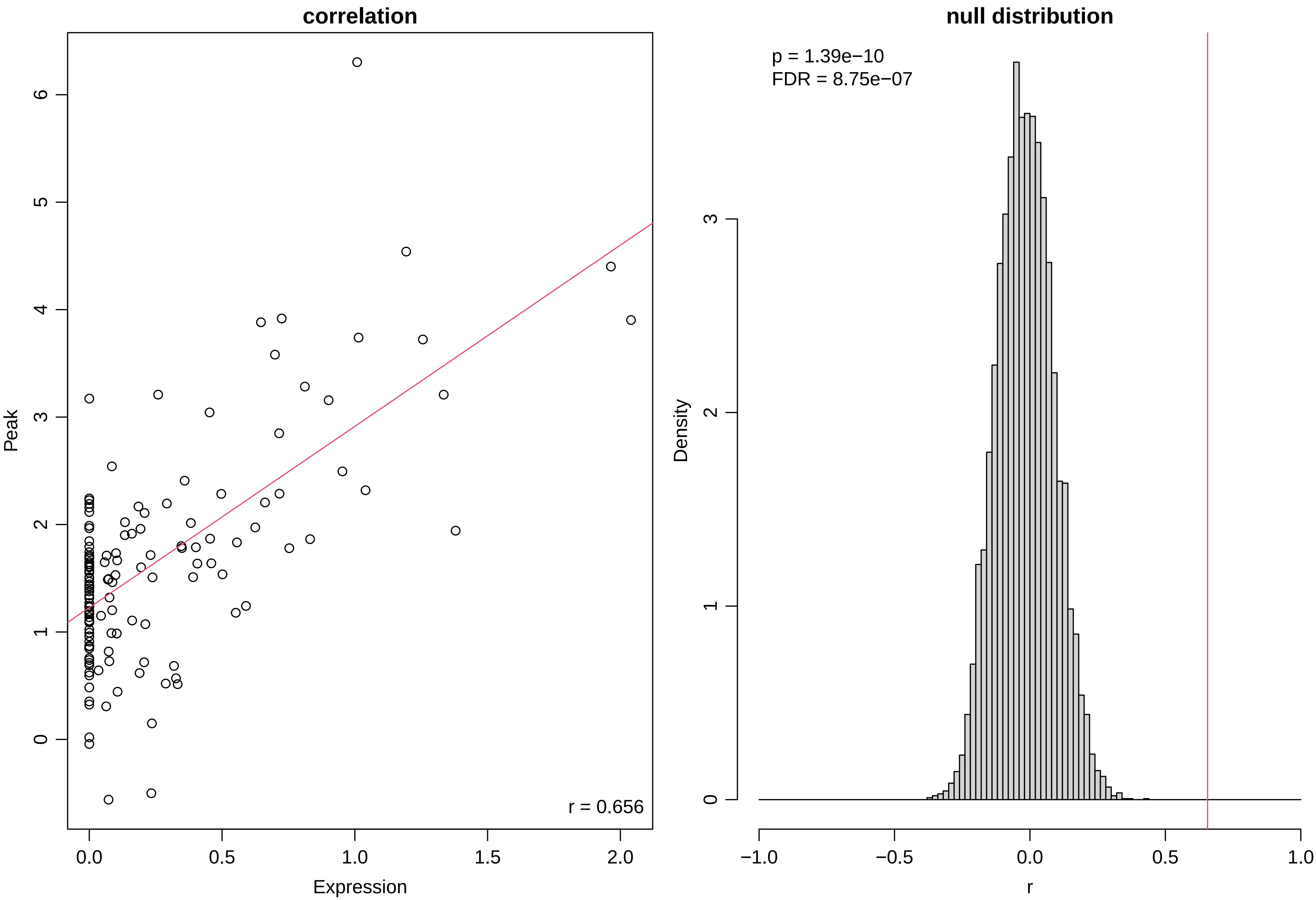

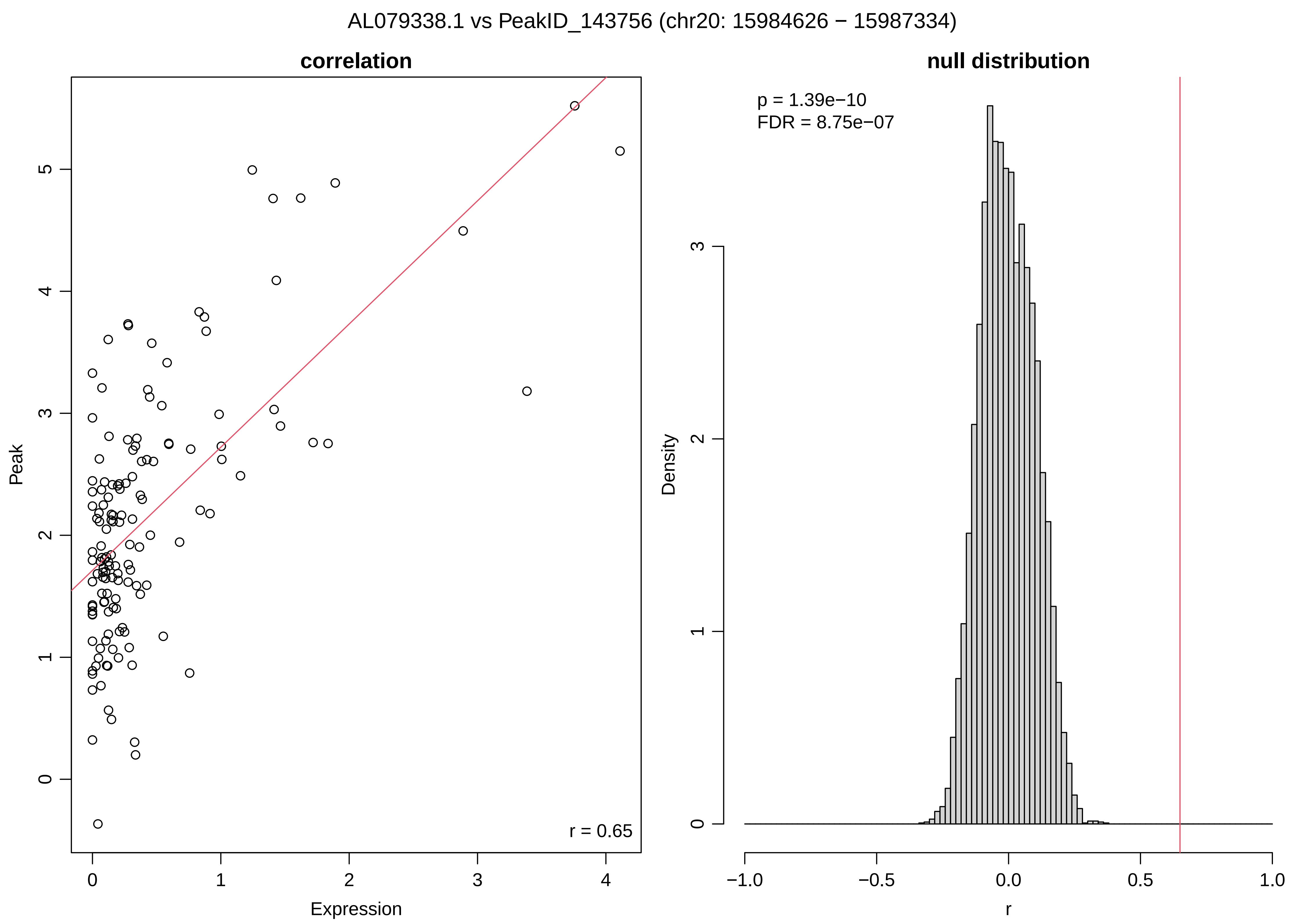

AL079338.1 vs PeakID_143756 (chr20: 15984626 − 15987334)
correlation
Peak
Expression
r = 0.65
0
1
2
3
4
5
null distribution
p = 1.39e−10
FDR = 8.75e−07
Density
r
−1.0
−0.5
0.0
0.5
1.0

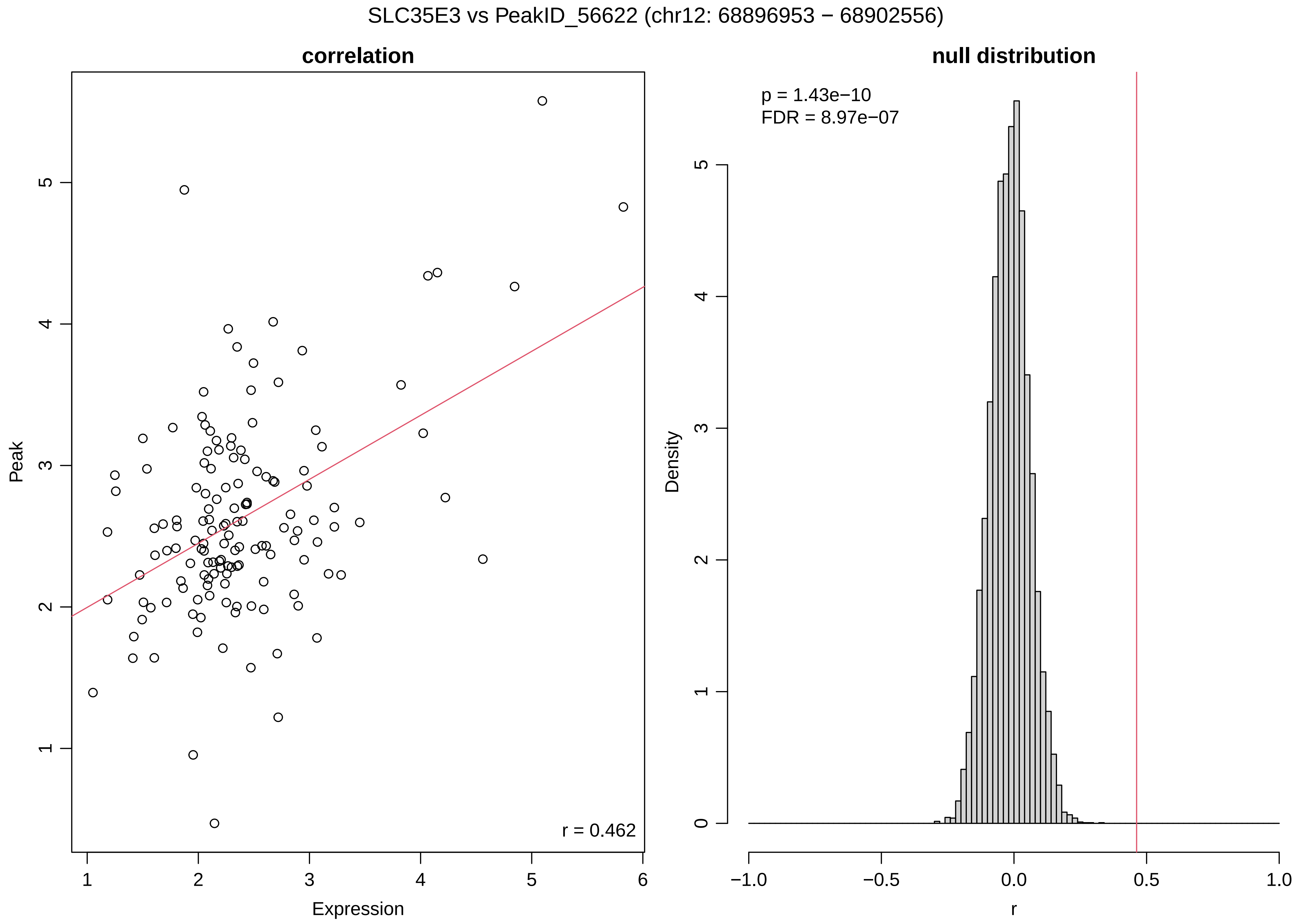
SLC35E3 vs PeakID_56622 (chr12: 68896953 − 68902556)
correlation
Peak
Expression
r = 0.462
null distribution
p = 1.43e−10
FDR = 8.97e−07
Density
r

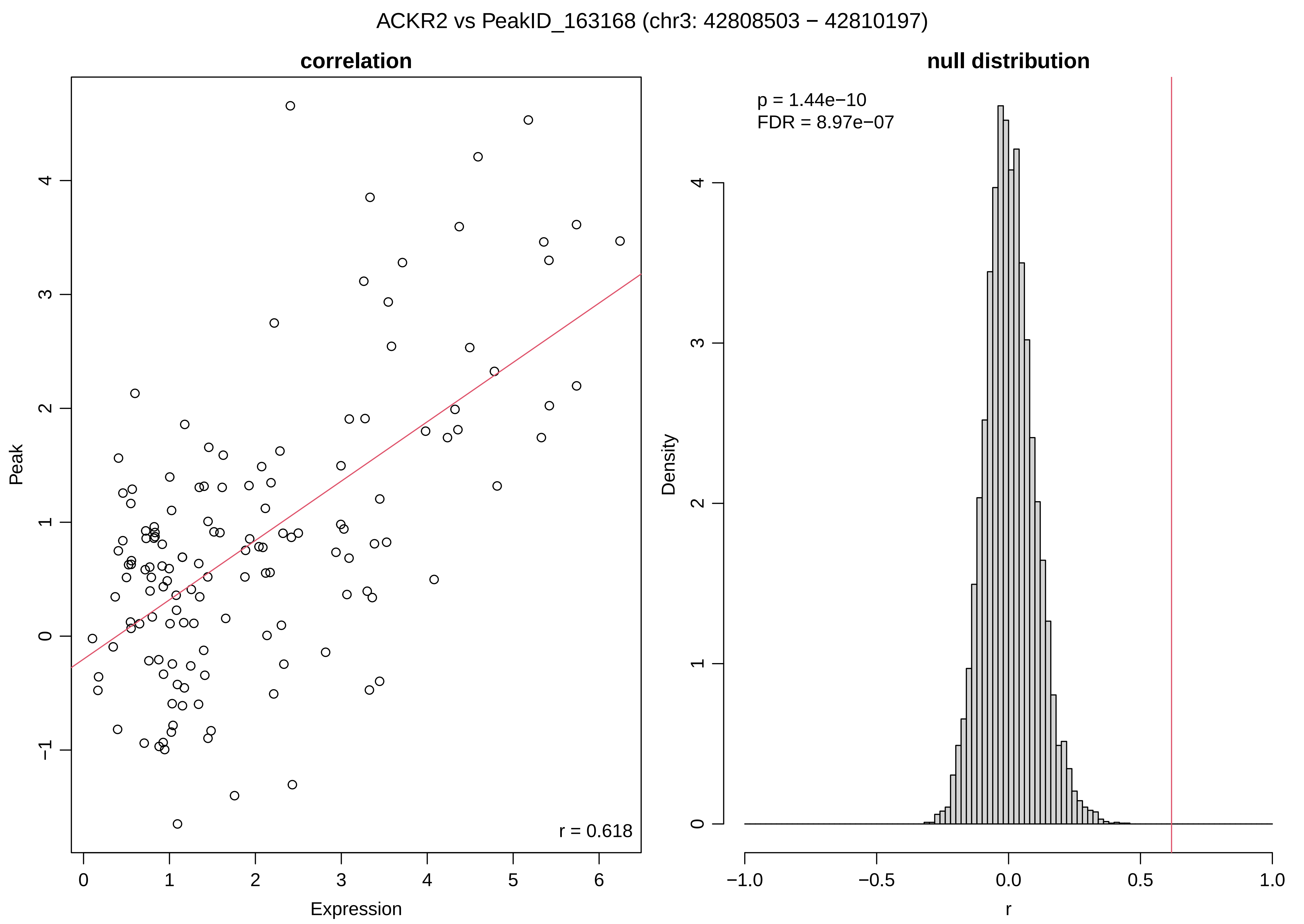

ACKR2 vs PeakID_163168 (chr3: 42808503 − 42810197)
correlation
Peak
Expression
r = 0.618
null distribution
p = 1.44e−10
FDR = 8.97e−07
Density
r

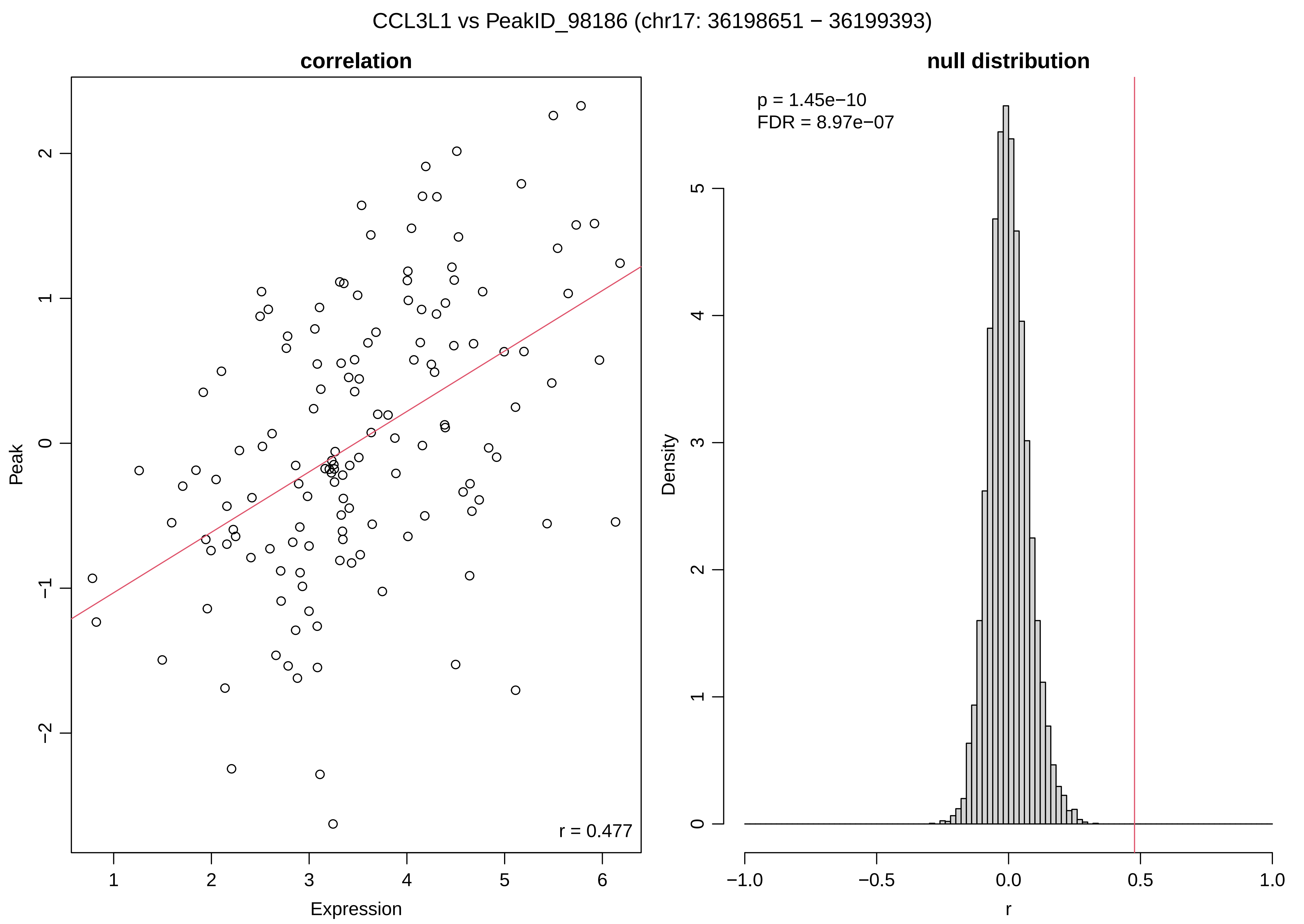

CCL3L1 vs PeakID_98186 (chr17: 36198651 − 36199393)
correlation
null distribution
Peak
Expression
r = 0.477
p = 1.45e−10
FDR = 8.97e−07
Density
r

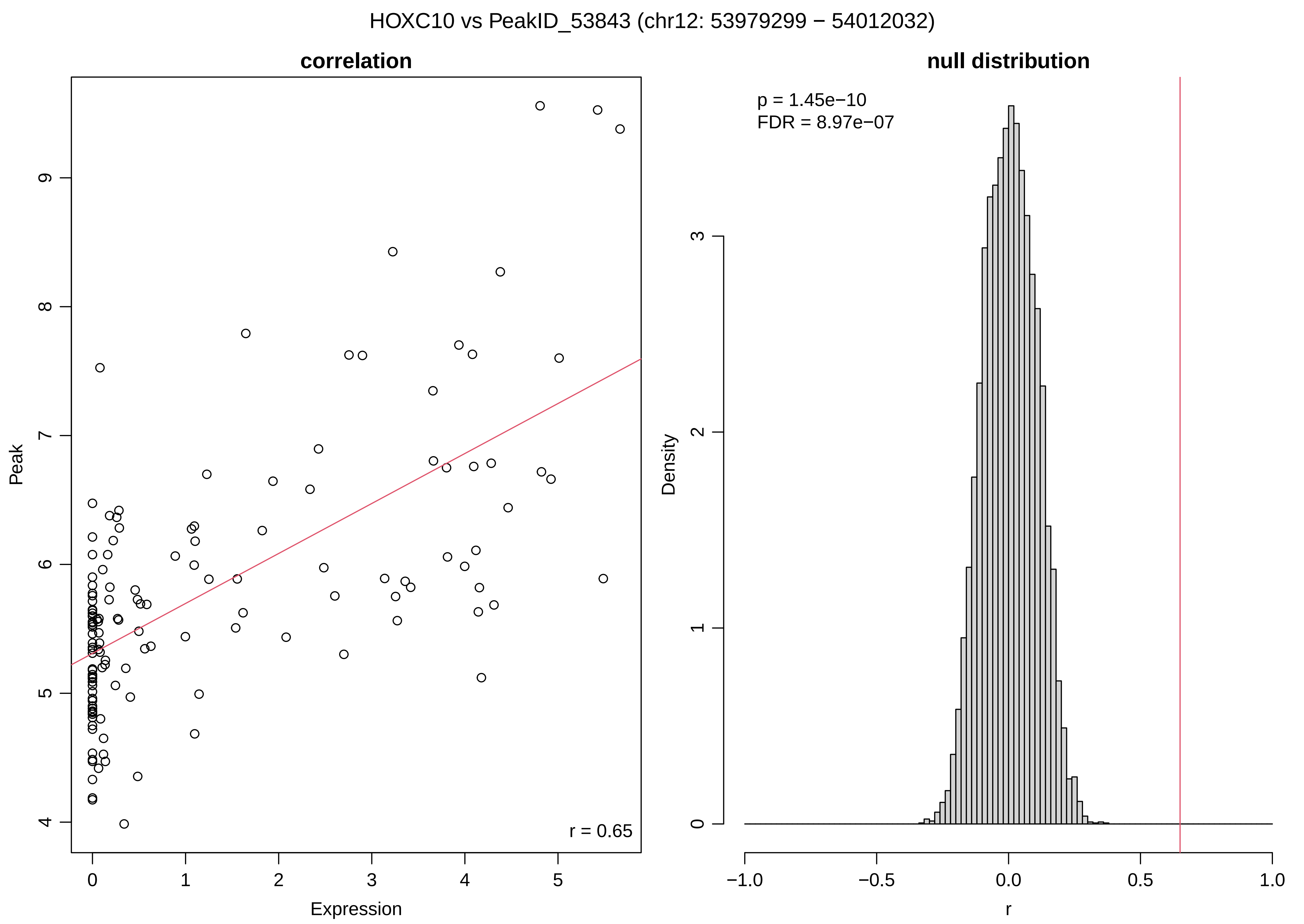
HOXC10 vs PeakID_53843 (chr12: 53979299 − 54012032)
correlation
Peak
Expression
r = 0.65
null distribution
p = 1.45e−10
FDR = 8.97e−07
Density
r
4
5
6
7
8
9
0
1
2
3
4
5
0
1
2
3
−1.0
−0.5
0.0
0.5
1.0

FZD2 vs PeakID_100047 (chr17: 44541825 − 44542567)

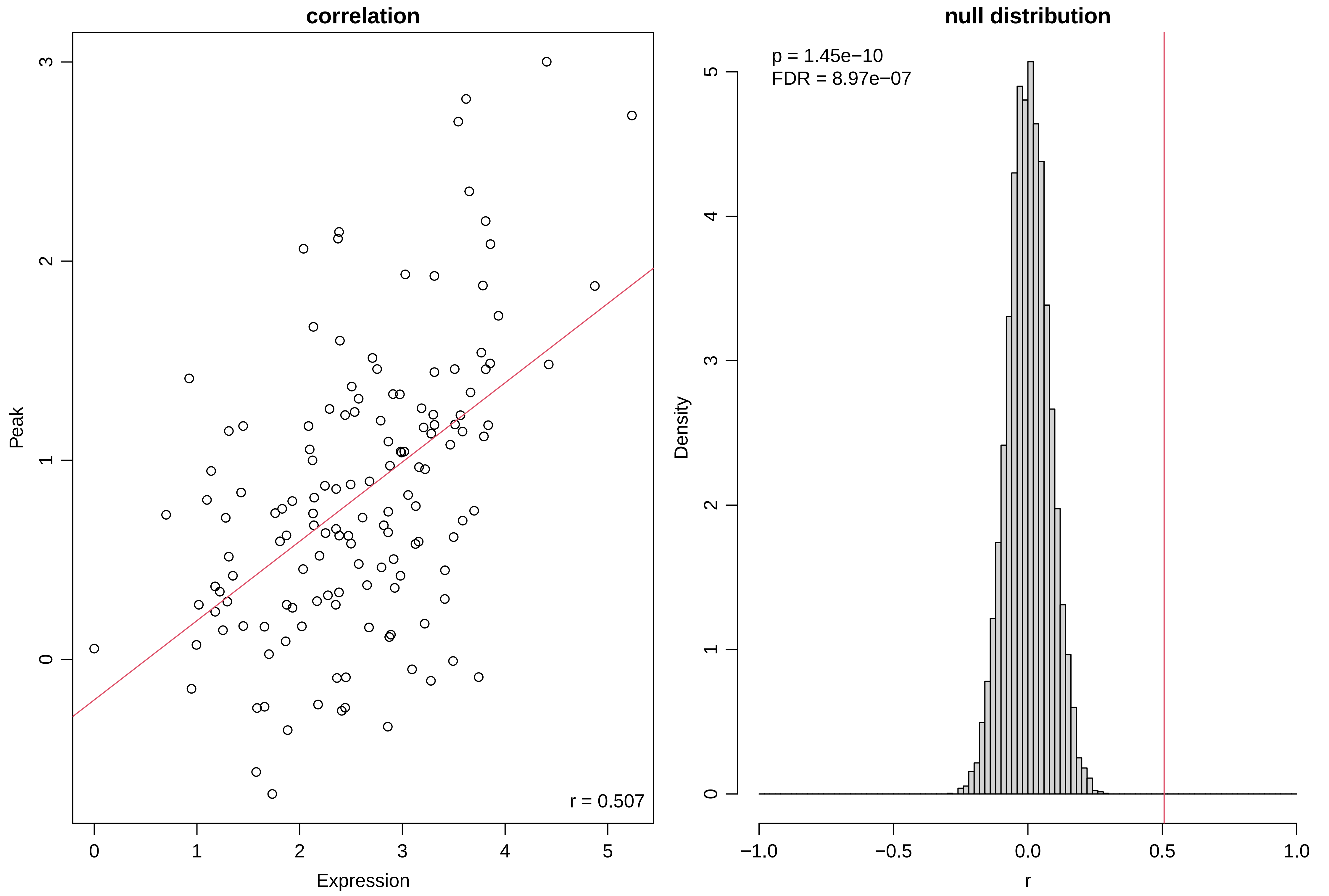

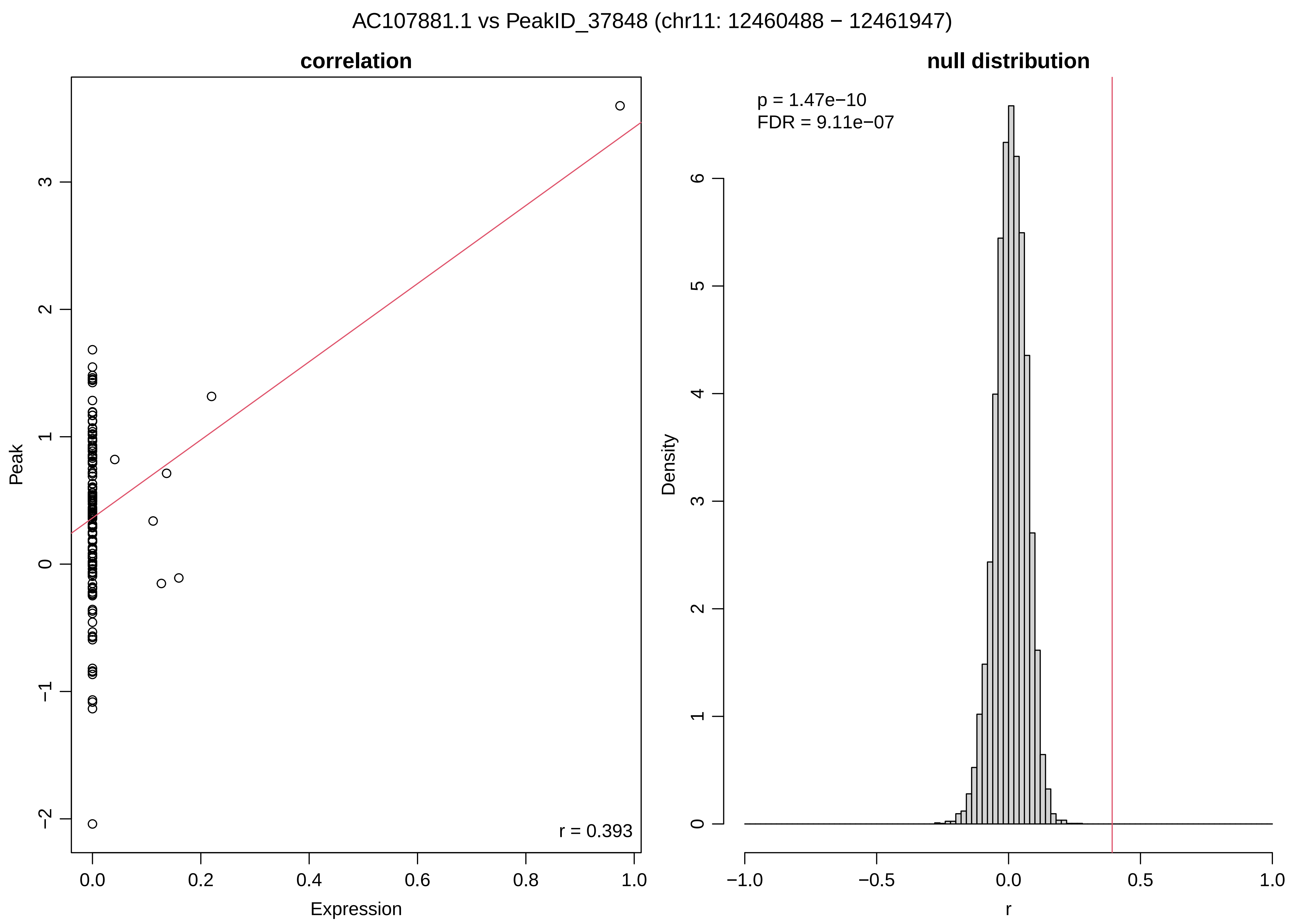
AC107881.1 vs PeakID_37848 (chr11: 12460488 − 12461947)
correlation
Peak
Expression
r = 0.393
null distribution
p = 1.47e−10
FDR = 9.11e−07
Density
r

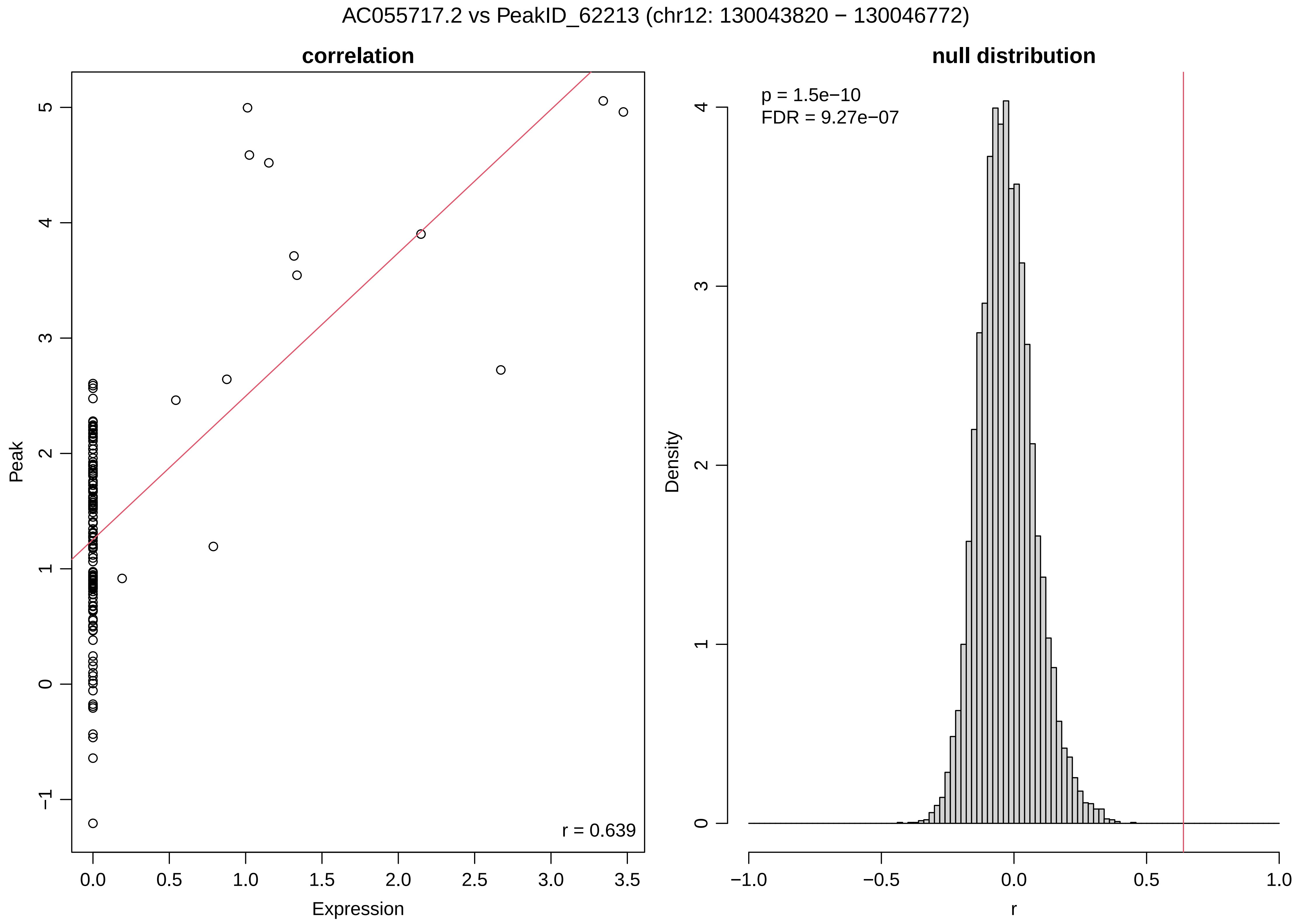
AC055717.2 vs PeakID_62213 (chr12: 130043820 − 130046772)
correlation
null distribution
Peak
Expression
r = 0.639
p = 1.5e−10
FDR = 9.27e−07
Density
r

CALML5 vs PeakID_24234 (chr10: 5492139 − 5501783)

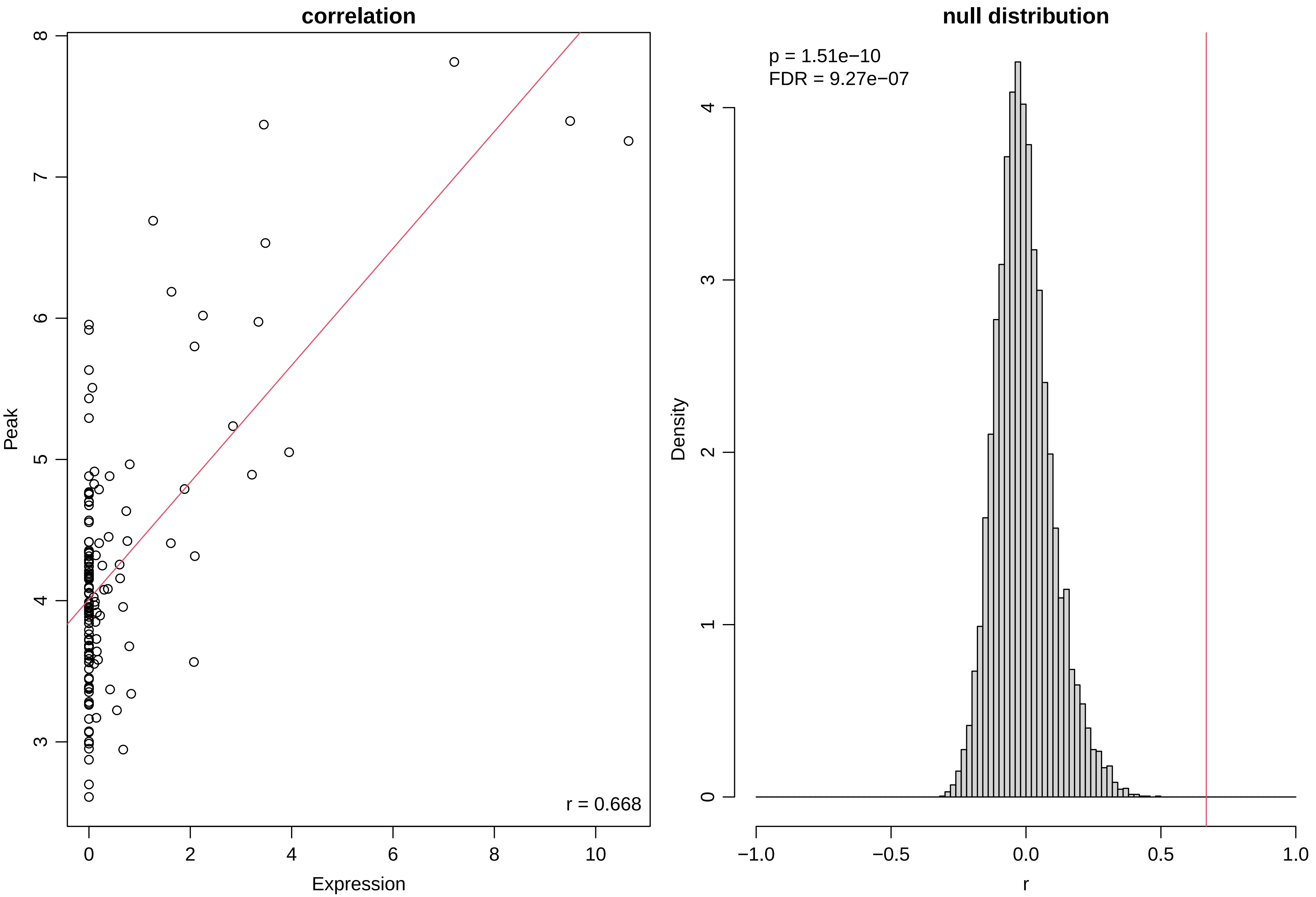

TXLNGY vs PeakID_262941 (chrY: 19566641 − 19568412)

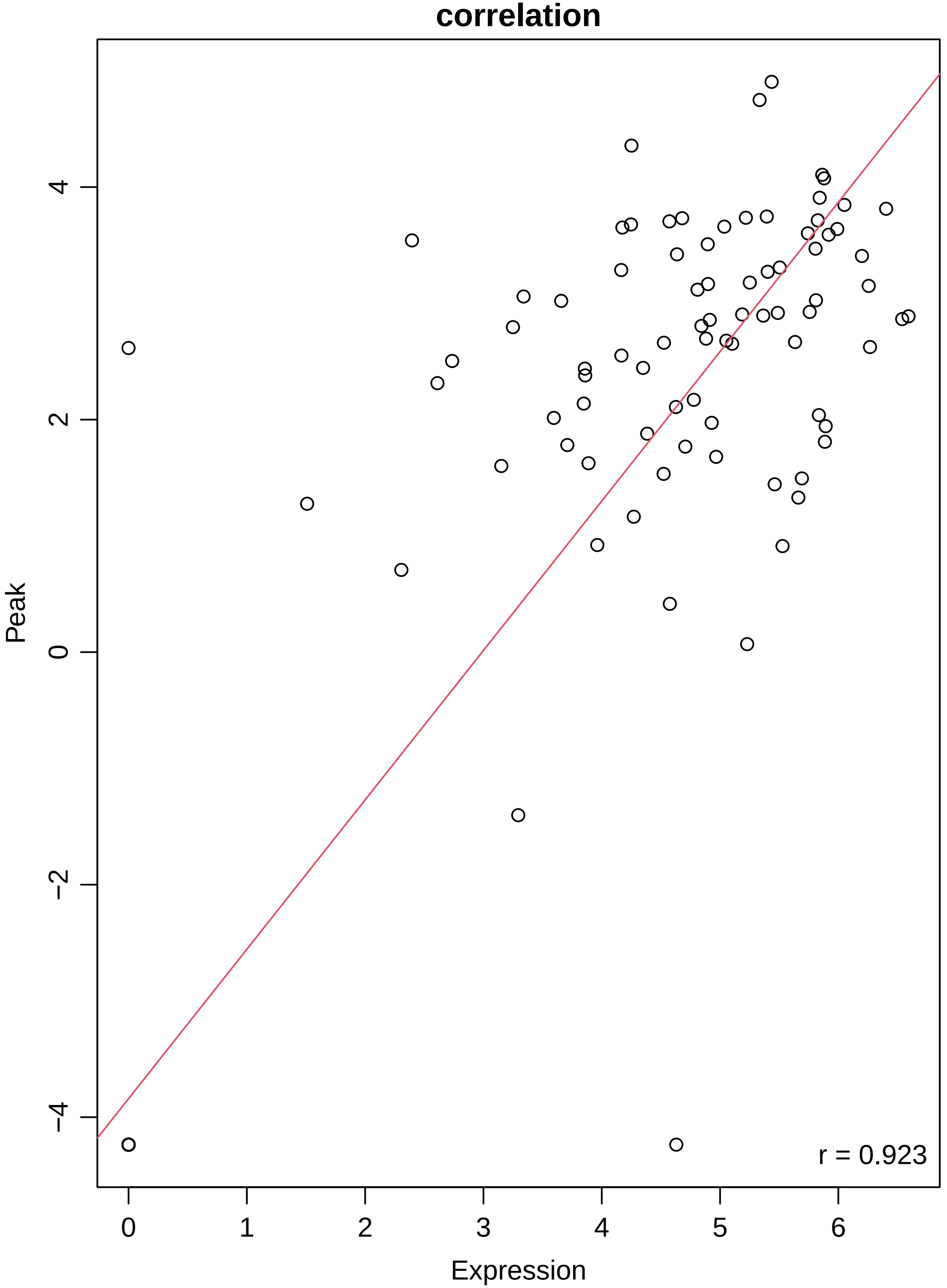

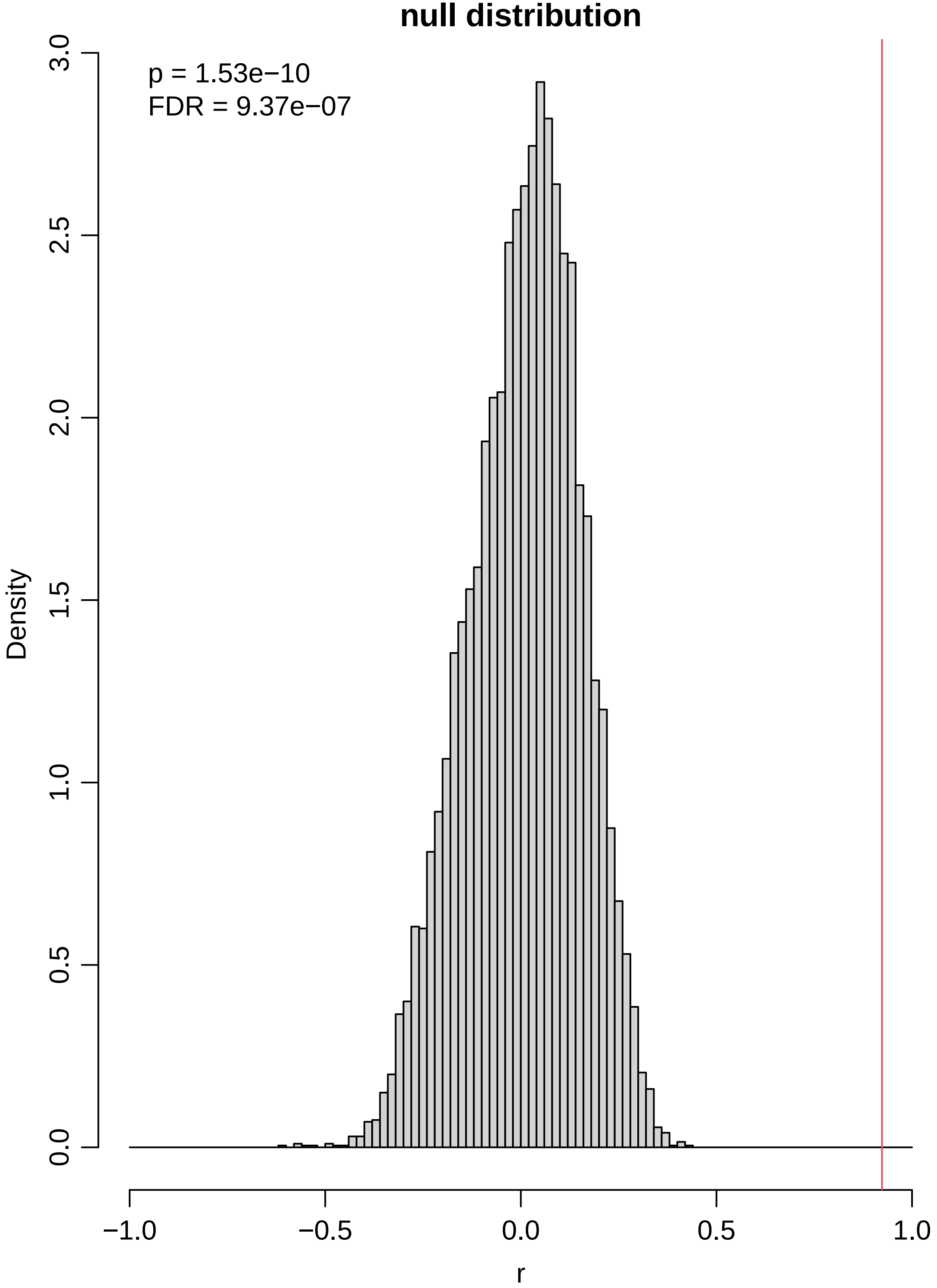

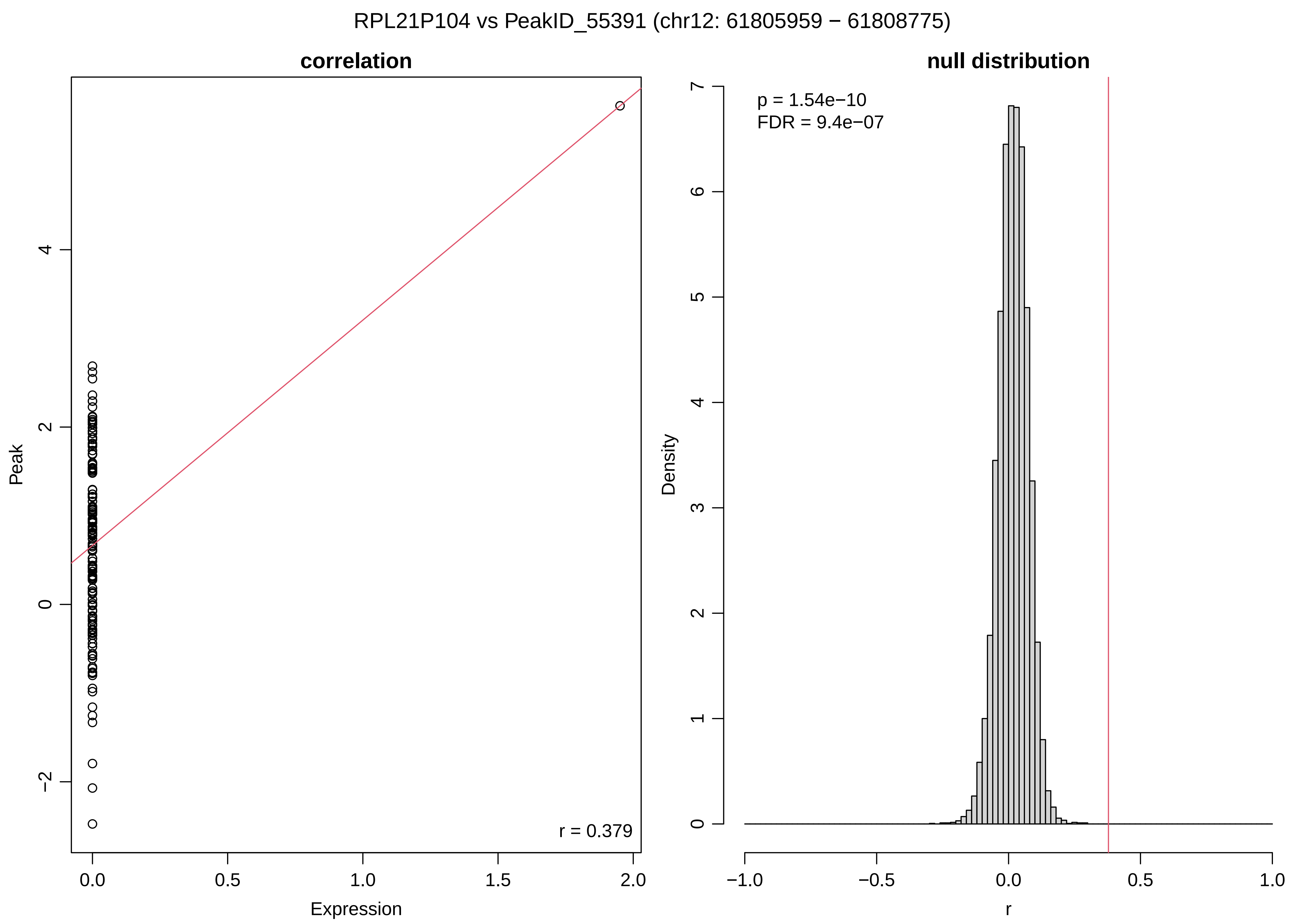

RPL21P104 vs PeakID_55391 (chr12: 61805959 − 61808775)
correlation
Peak
Expression
r = 0.379
null distribution
p = 1.54e−10
FDR = 9.4e−07
Density
r

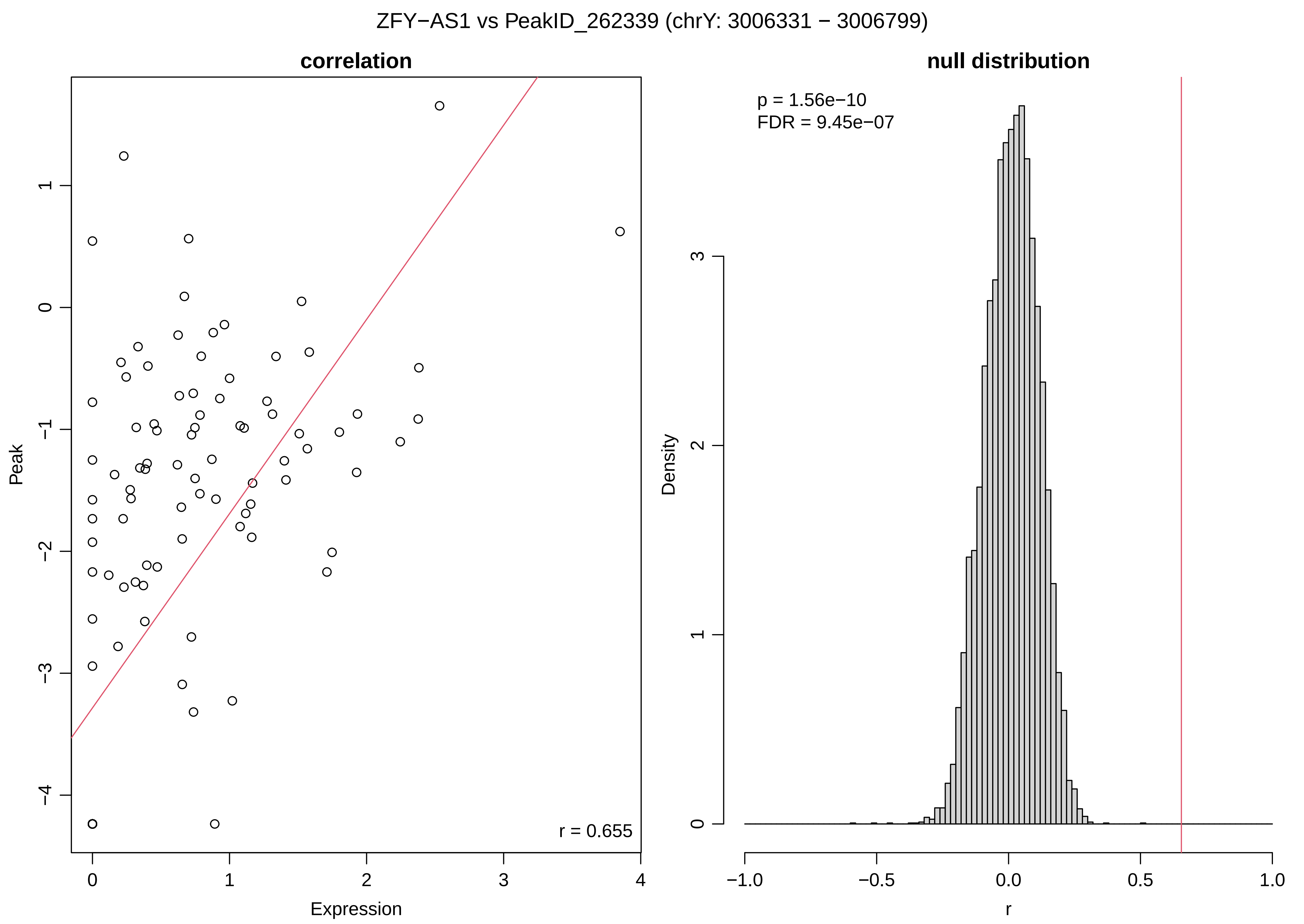
ZFY−AS1 vs PeakID_262339 (chrY: 3006331 − 3006799)
correlation
Peak
Expression
r = 0.655
null distribution
p = 1.56e−10
FDR = 9.45e−07
Density
r

AC007405.2 vs PeakID_135678 (chr2: 170815940 − 170819028)

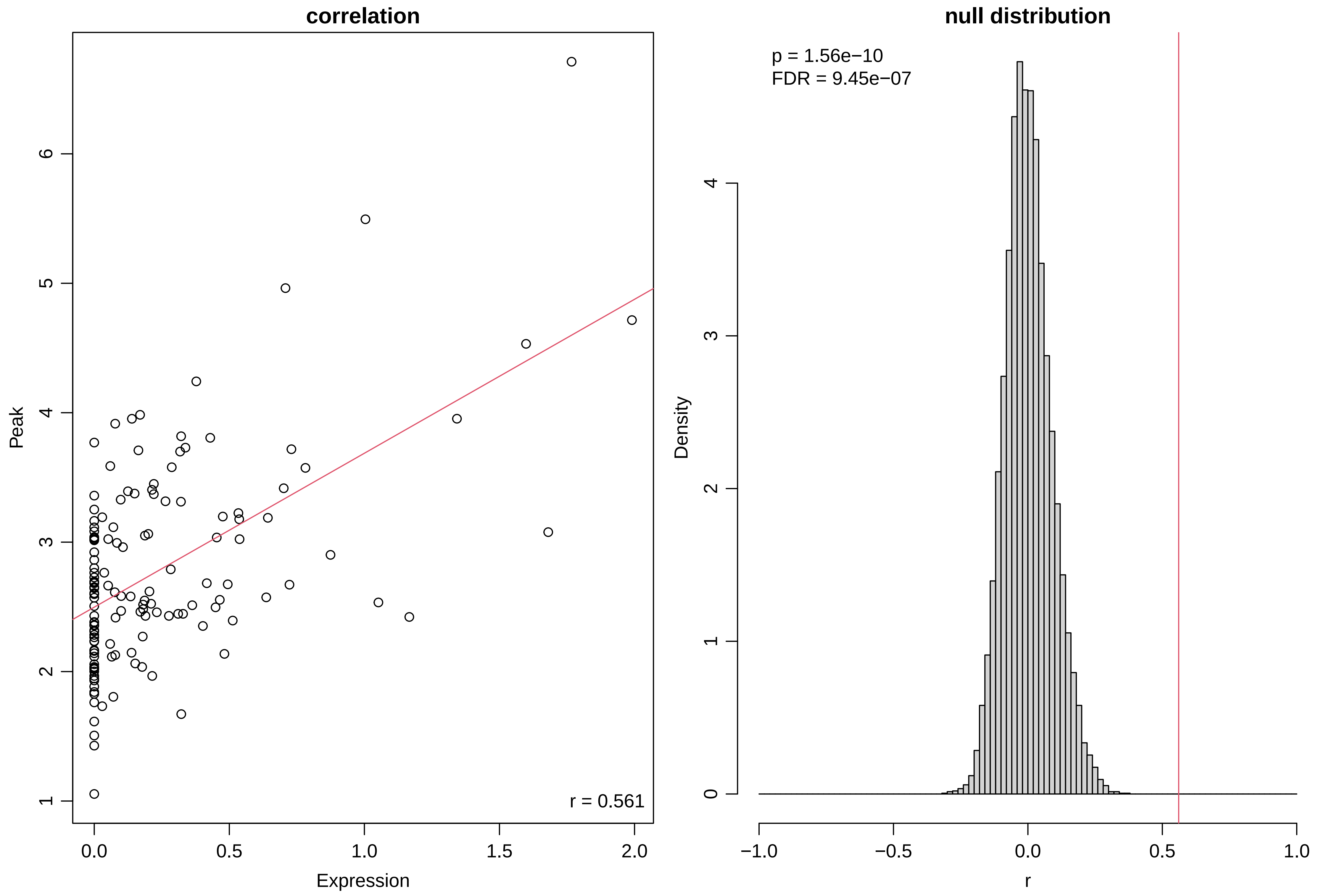

AC025569.1 vs PeakID_57025 (chr12: 70468128 − 70468616)

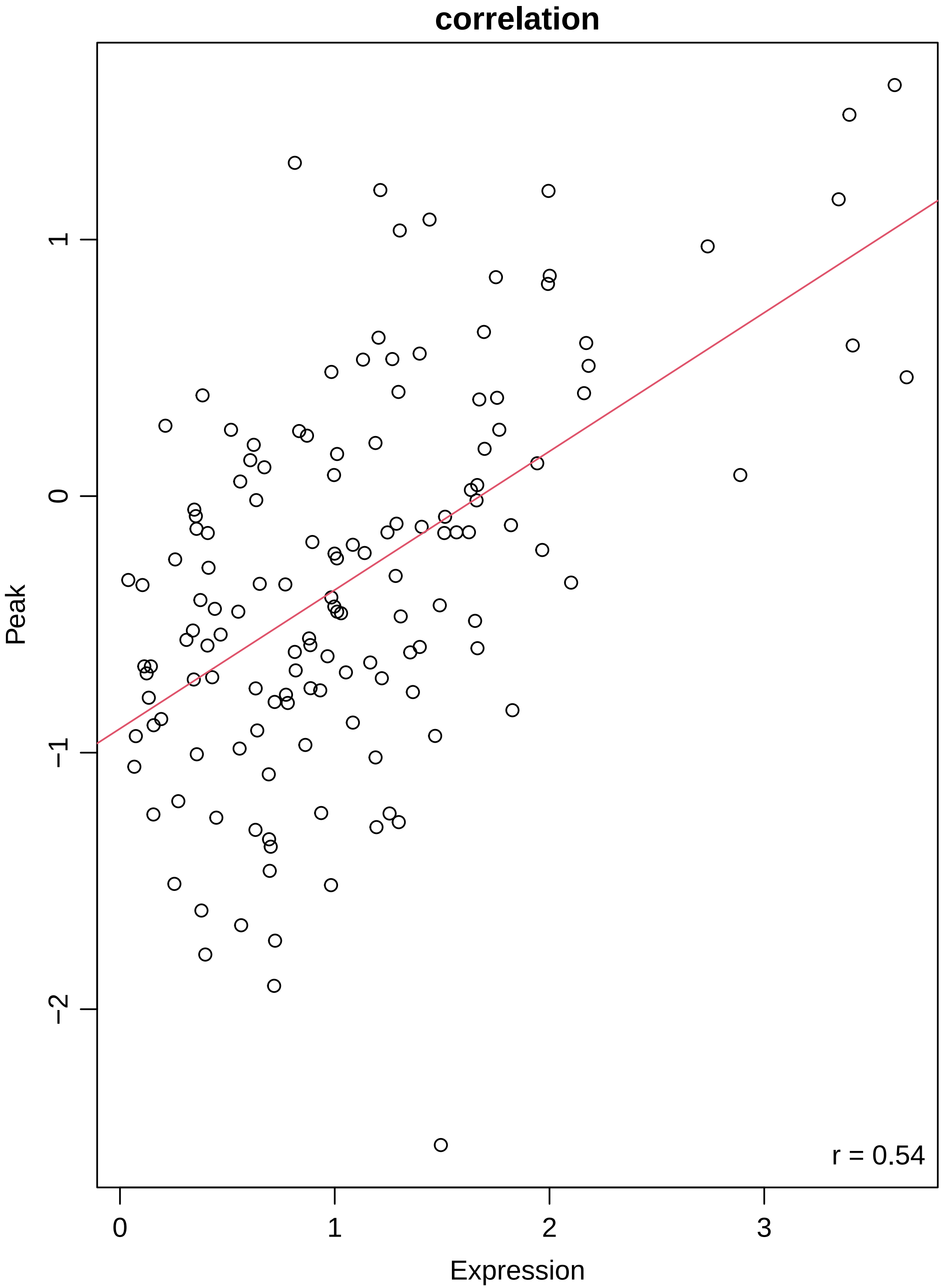

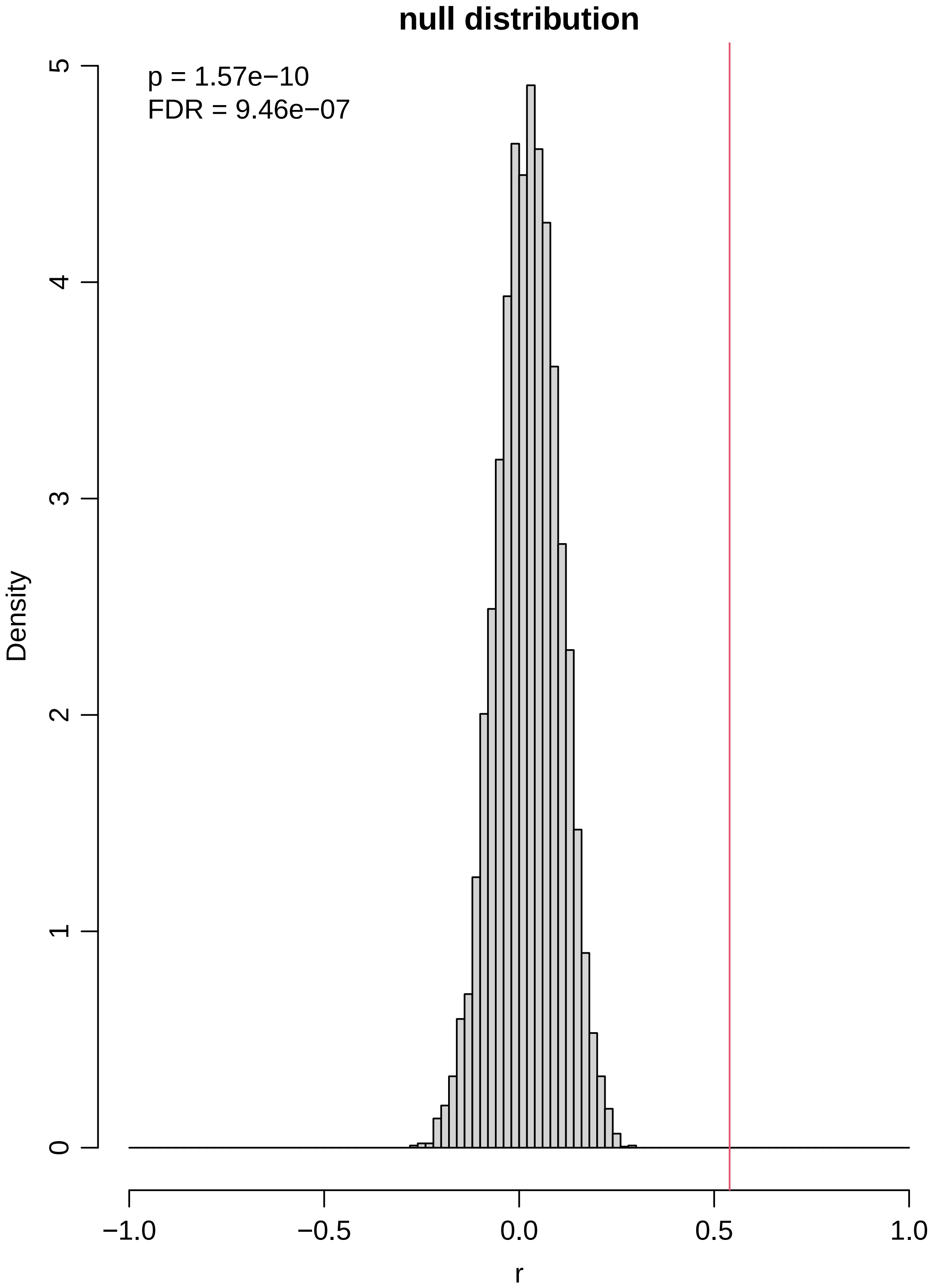

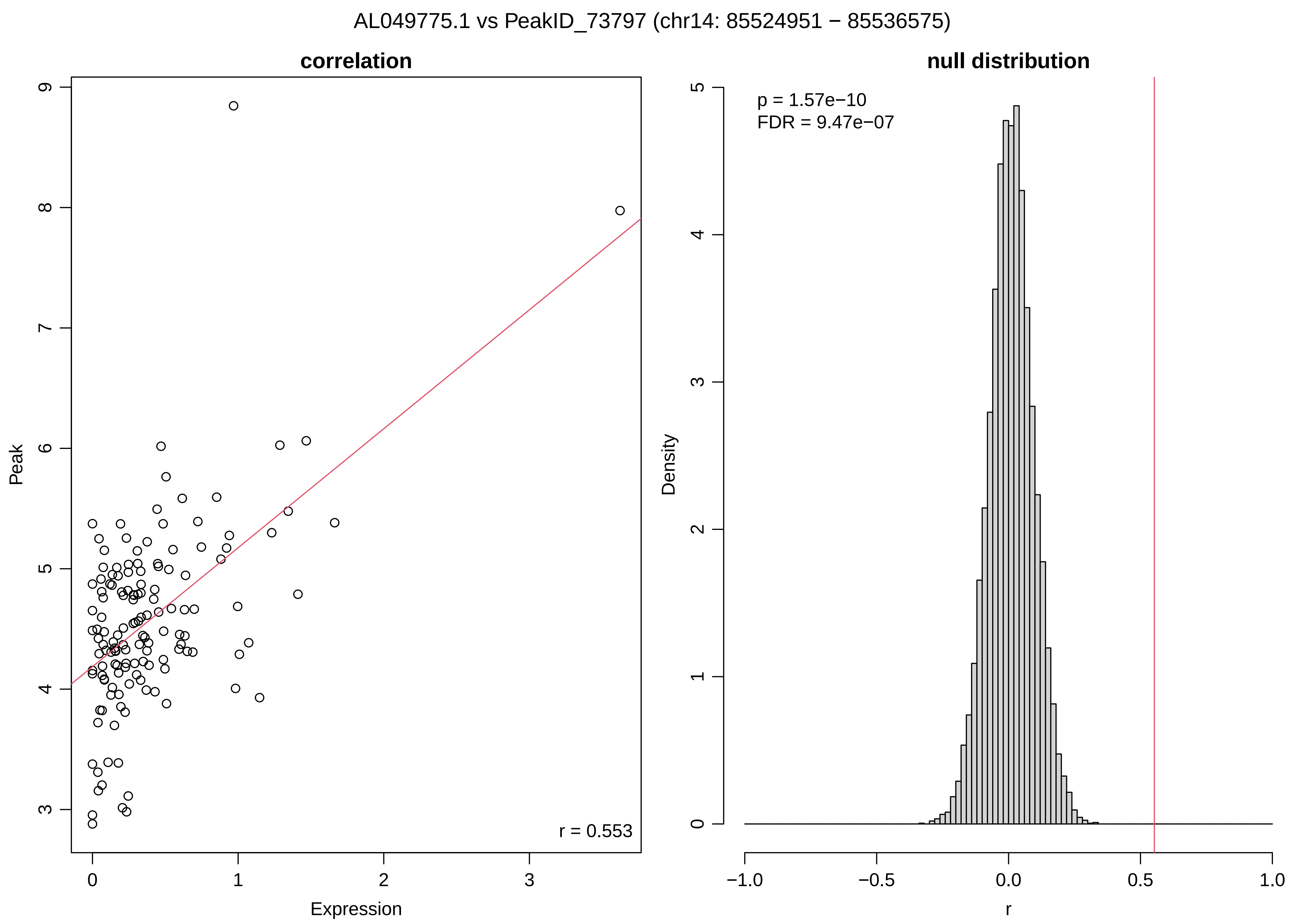
AL049775.1 vs PeakID_73797 (chr14: 85524951 − 85536575)
correlation
null distribution
Peak
Expression
r = 0.553
Density
r
p = 1.57e−10
FDR = 9.47e−07

DDX3Y vs PeakID_262742 (chrY: 12904139 − 12906462)

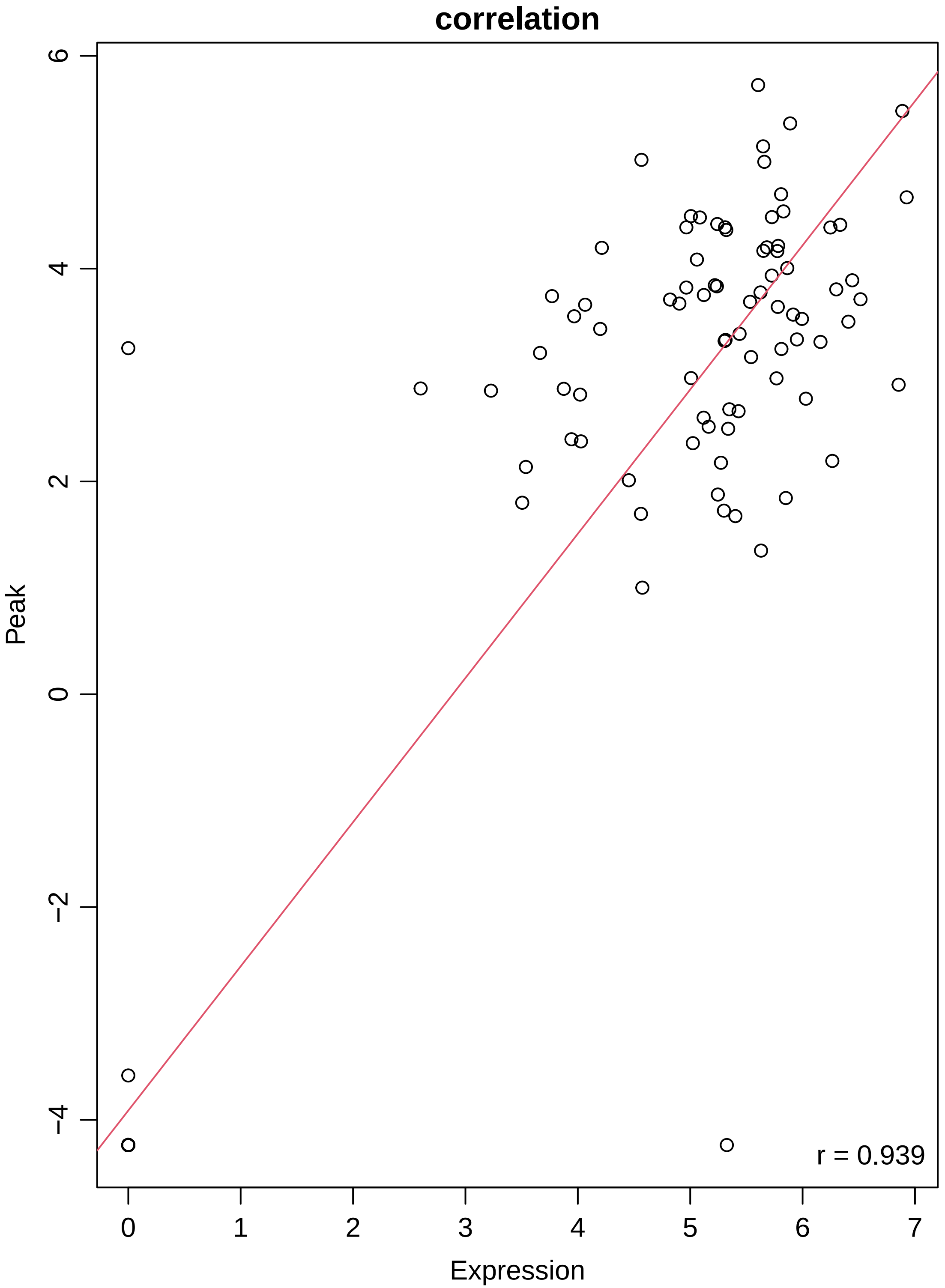

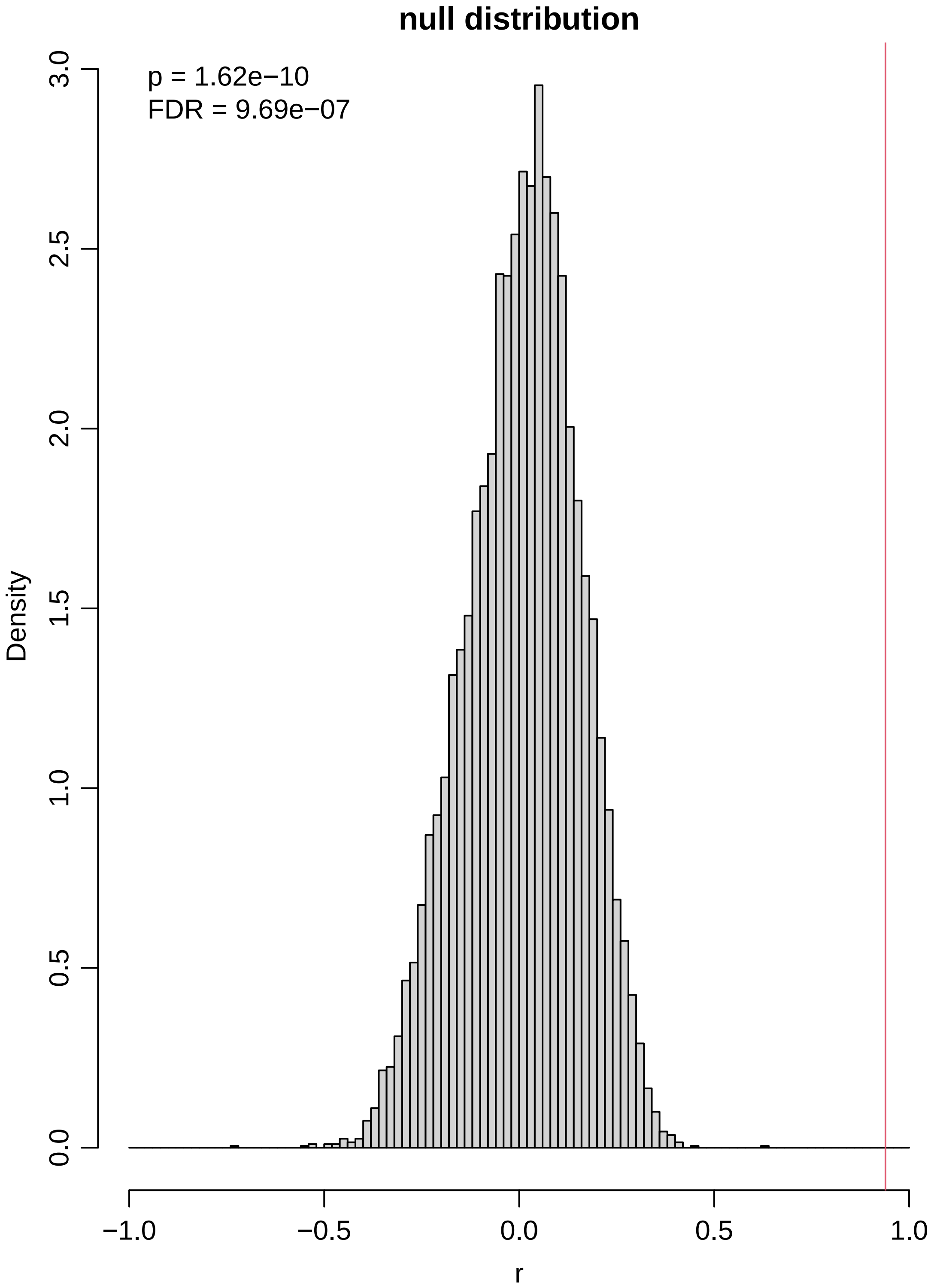

AC092881.1 vs PeakID_57004 (chr12: 70054354 − 70054749)

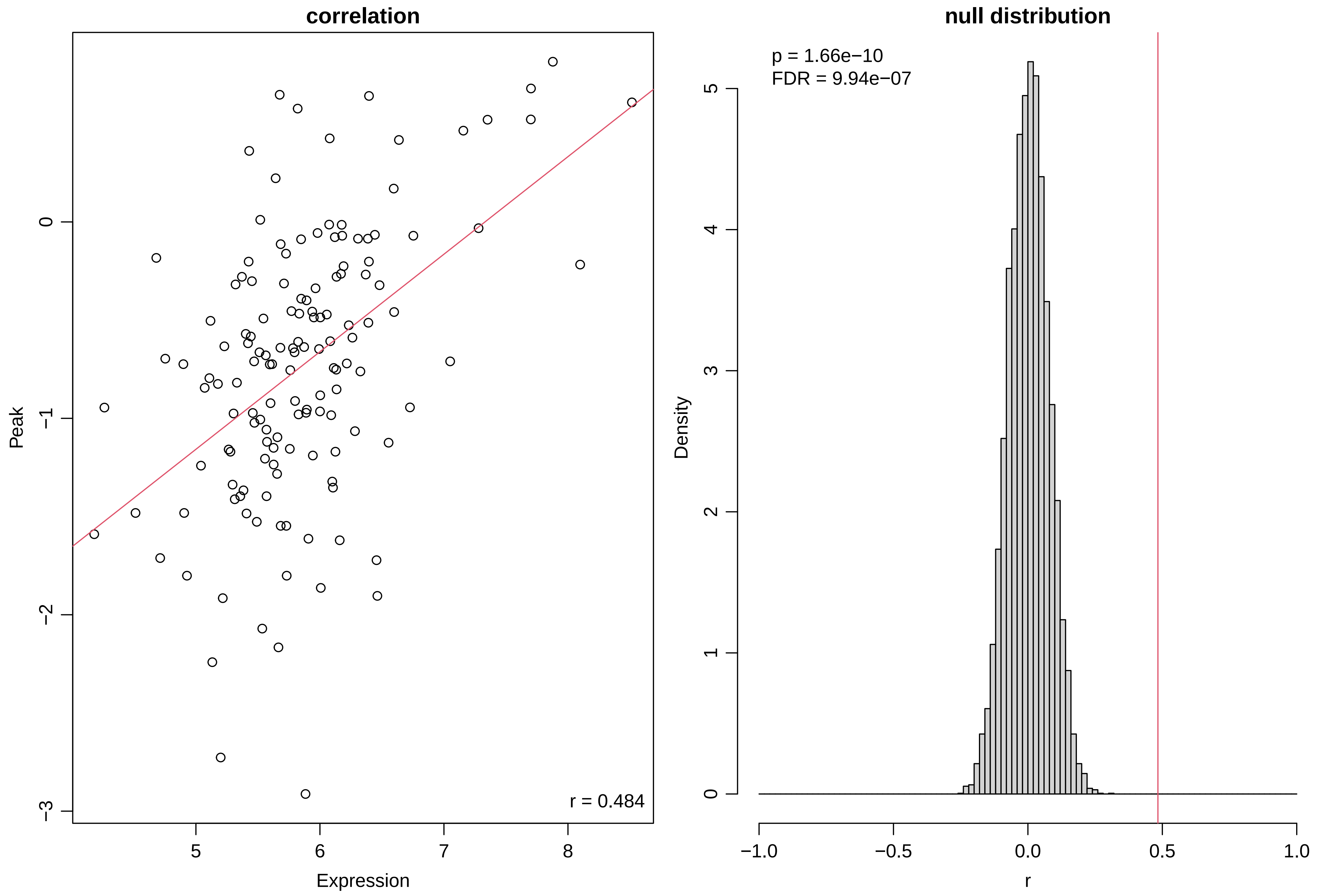

AC106754.1 vs PeakID_188789 (chr5: 25189848 − 25191022)

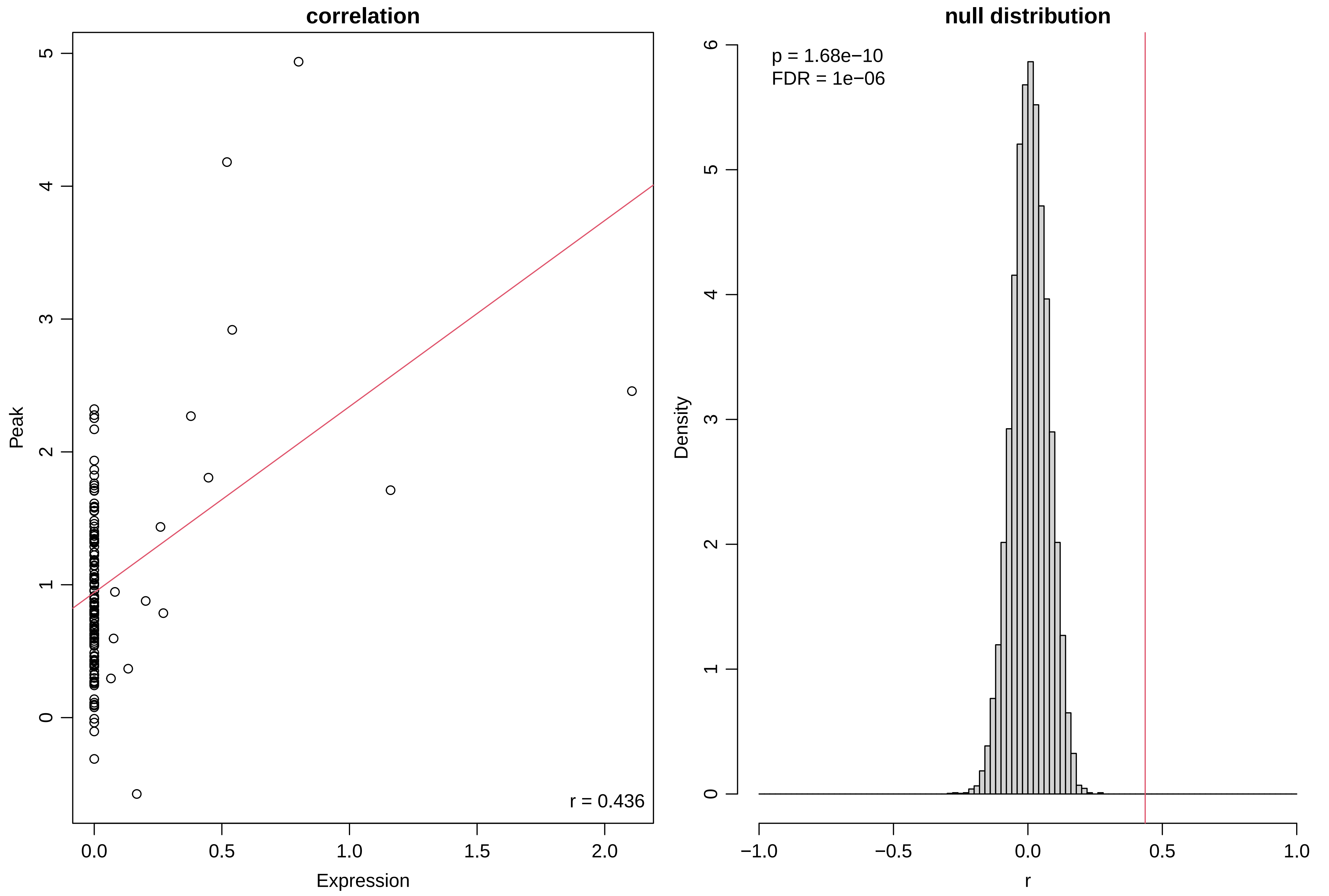

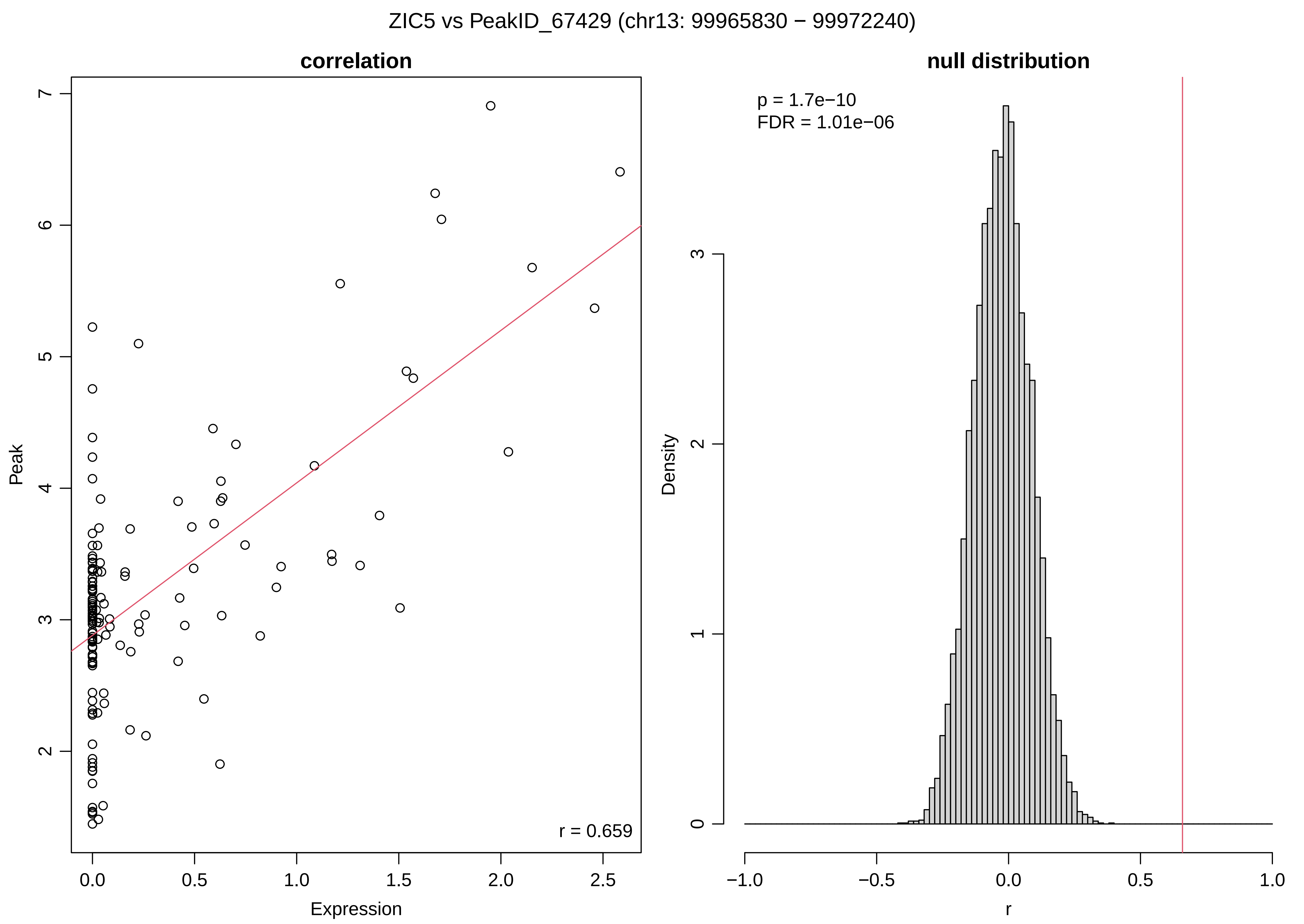

ZIC5 vs PeakID_67429 (chr13: 99965830 − 99972240)
correlation
Peak
Expression
r = 0.659
null distribution
p = 1.7e−10
FDR = 1.01e−06
Density
r

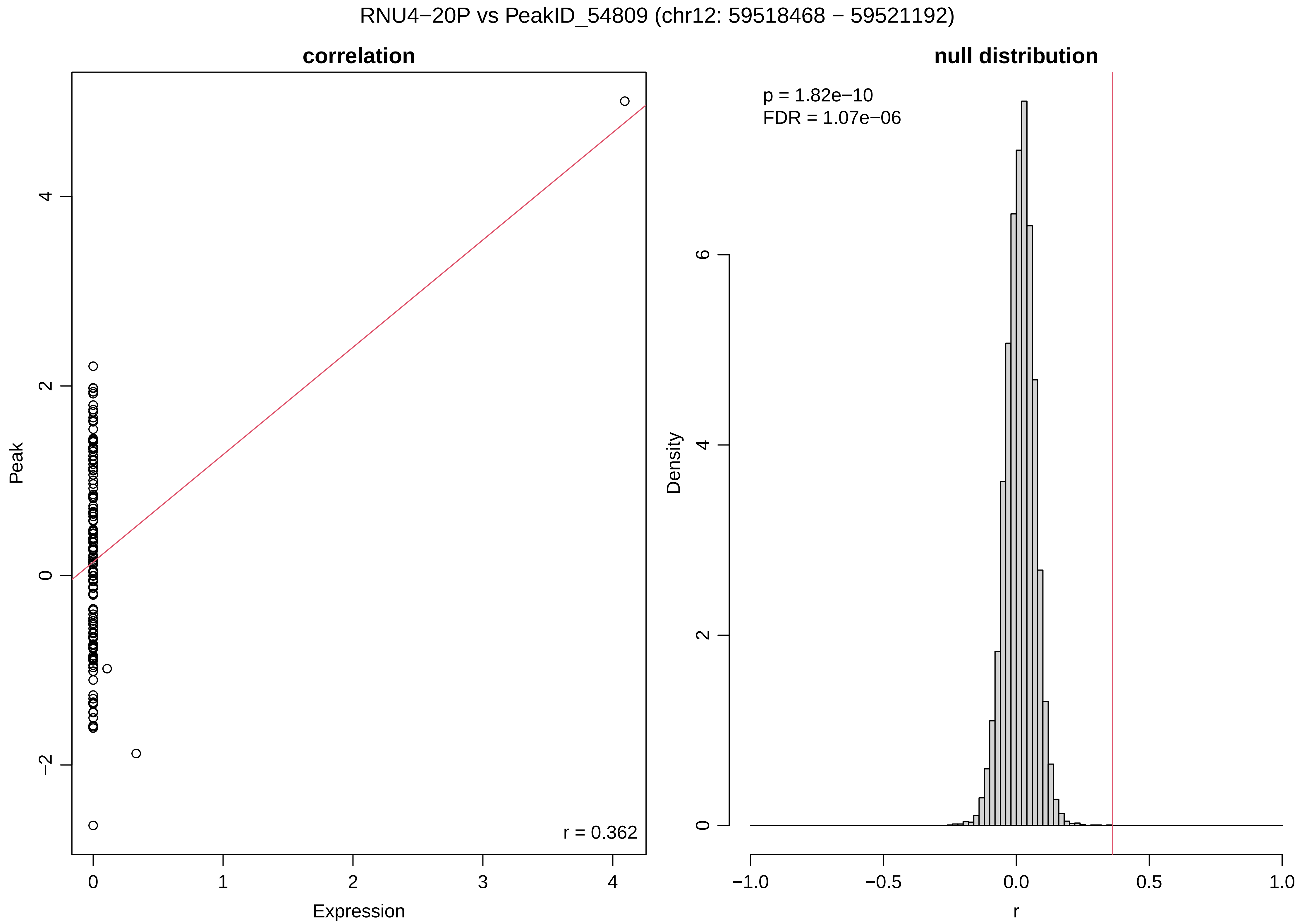
RNU4−20P vs PeakID_54809 (chr12: 59518468 − 59521192)
correlation
Peak
Expression
r = 0.362
null distribution
p = 1.82e−10
FDR = 1.07e−06
Density
r

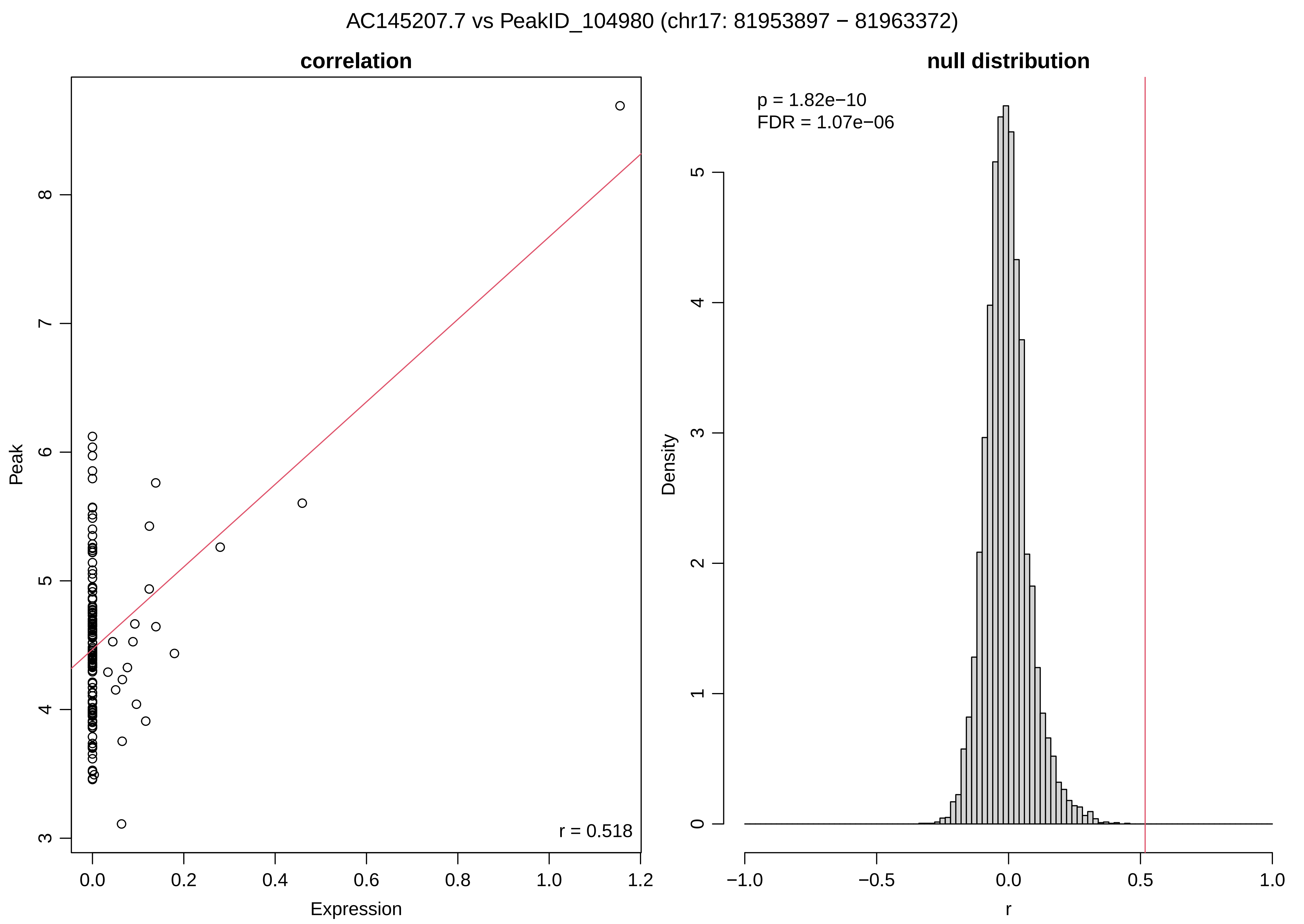

AC145207.7 vs PeakID_104980 (chr17: 81953897 − 81963372)
correlation
Peak
Expression
r = 0.518
null distribution
p = 1.82e−10
FDR = 1.07e−06
Density
r

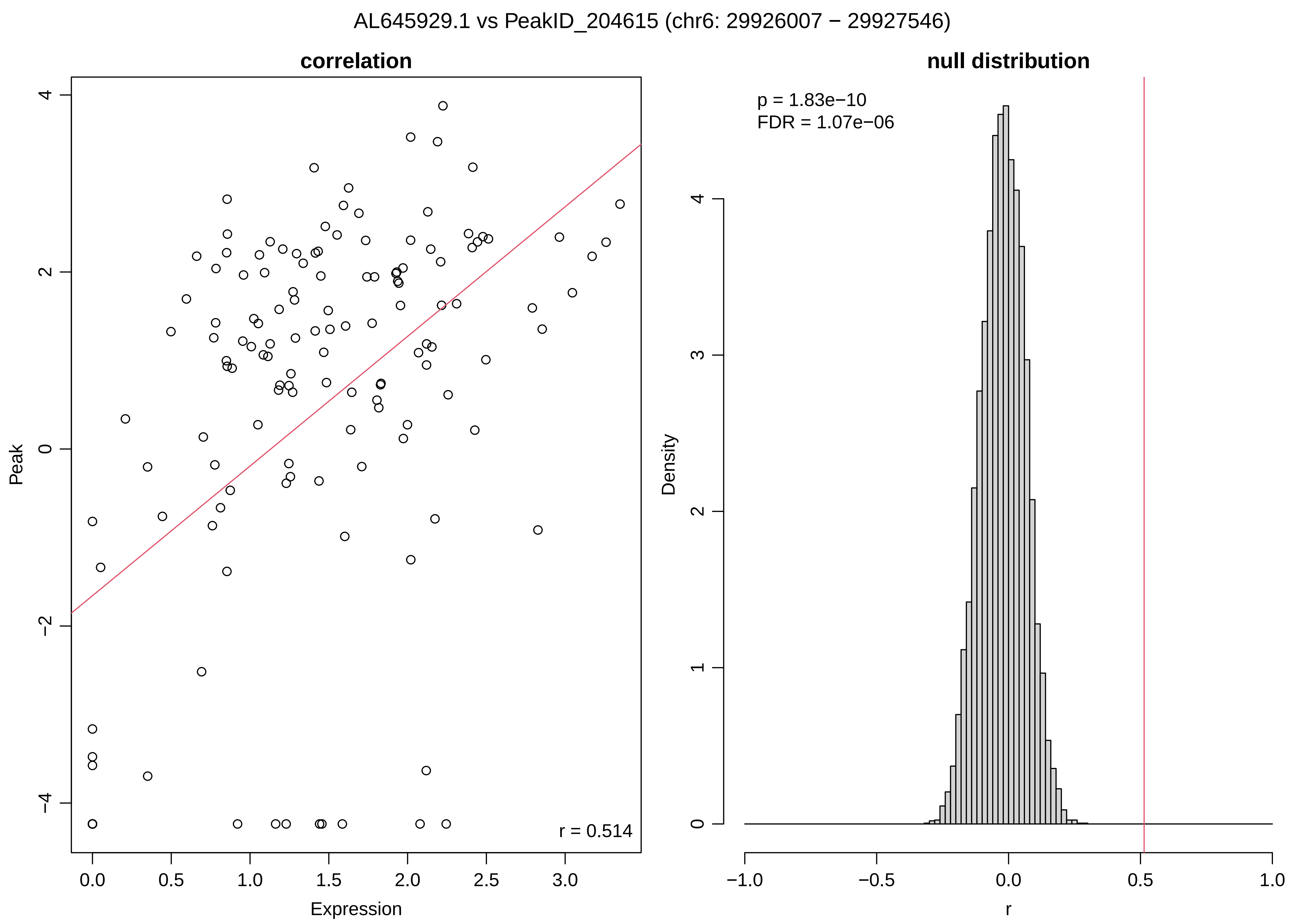
AL645929.1 vs PeakID_204615 (chr6: 29926007 − 29927546)
correlation
Peak
Expression
r = 0.514
4
2
0
−2
−4
0.0
0.5
1.0
1.5
2.0
2.5
3.0
null distribution
p = 1.83e−10
FDR = 1.07e−06
Density
r
0
1
2
3
4
−1.0
−0.5
0.0
0.5
1.0

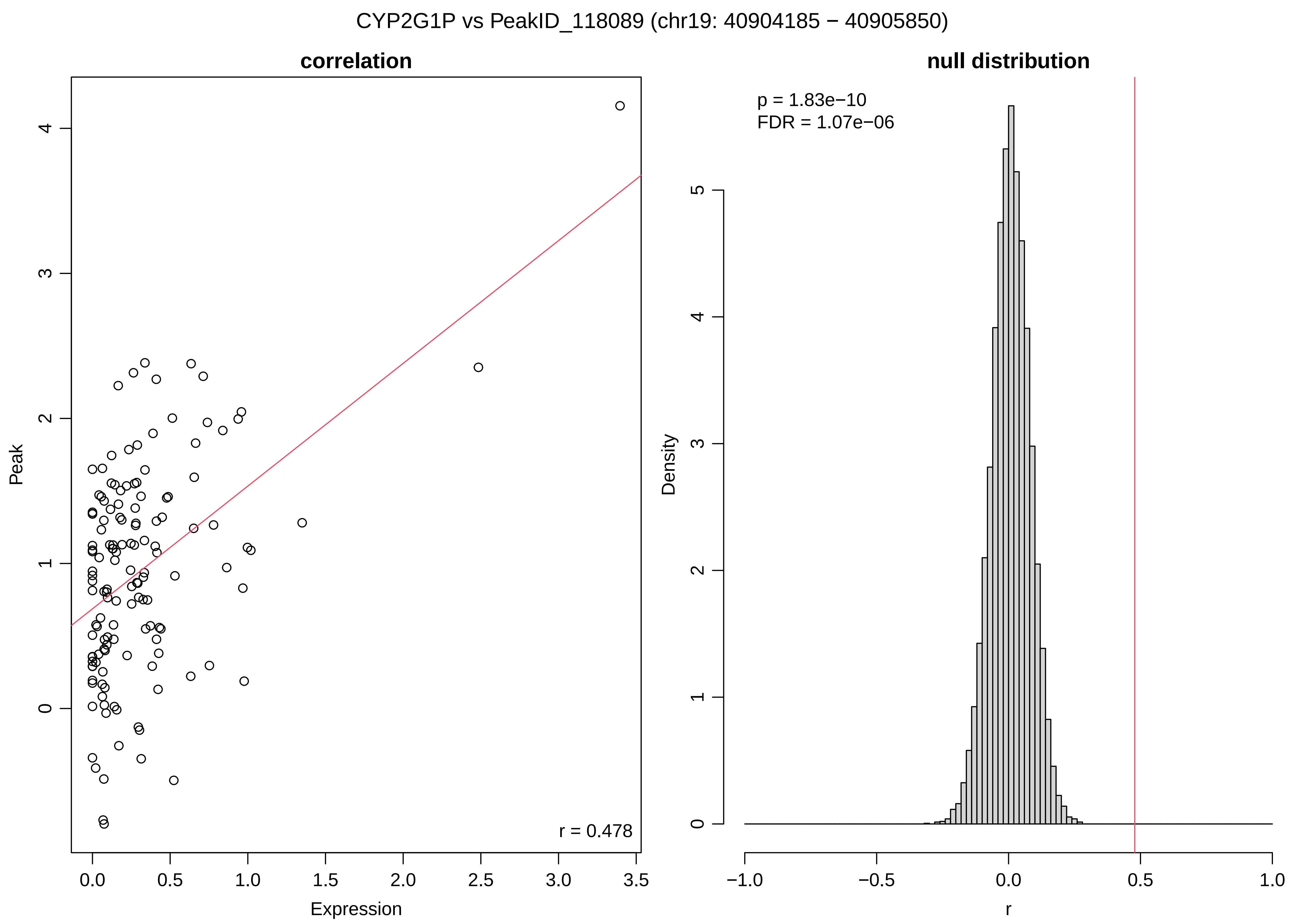
CYP2G1P vs PeakID_118089 (chr19: 40904185 − 40905850)
correlation
Peak
Expression
r = 0.478
null distribution
p = 1.83e−10
FDR = 1.07e−06
Density
r

TBX18 vs PeakID_209164 (chr6: 84761987 − 84764664)

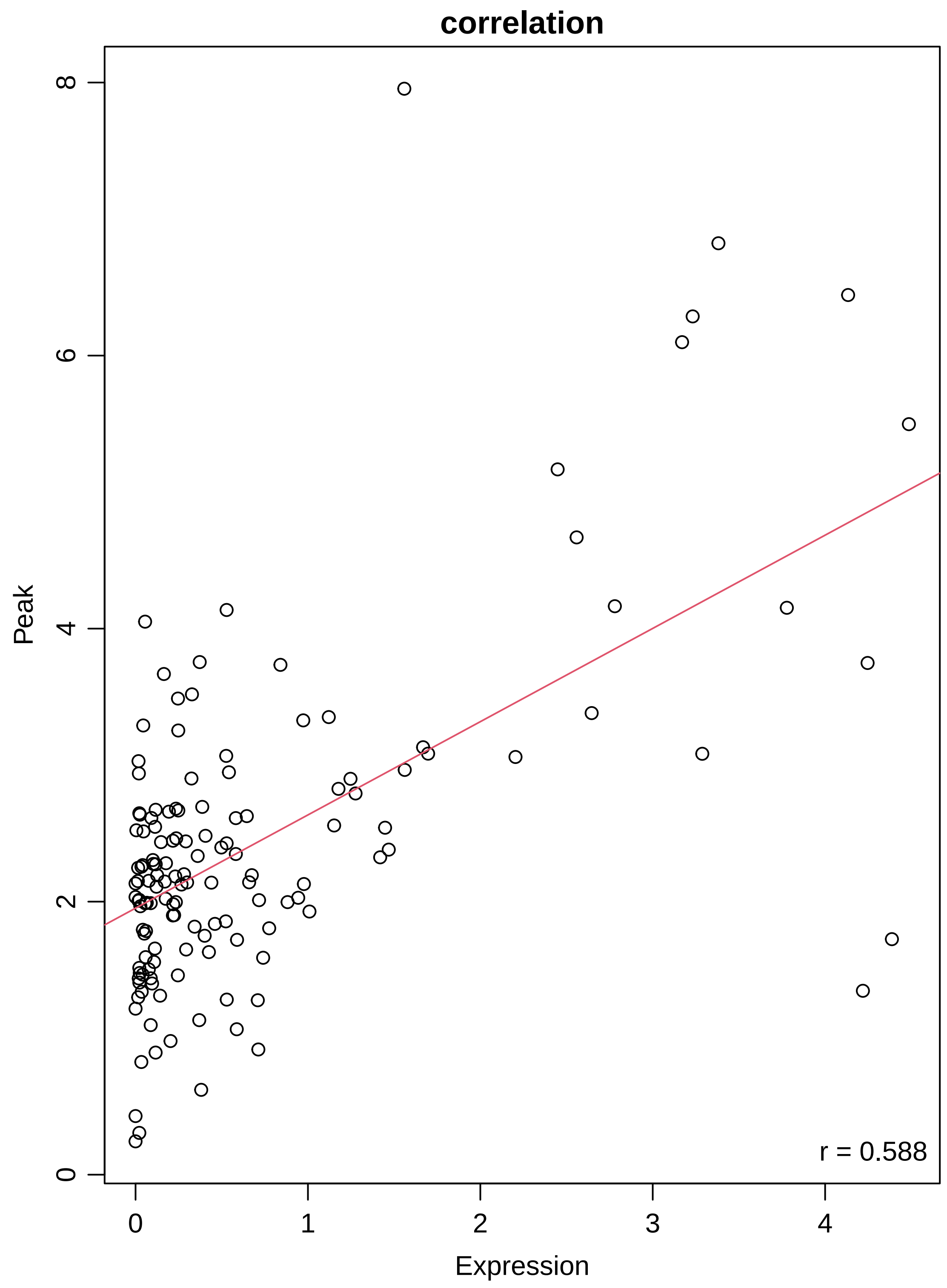

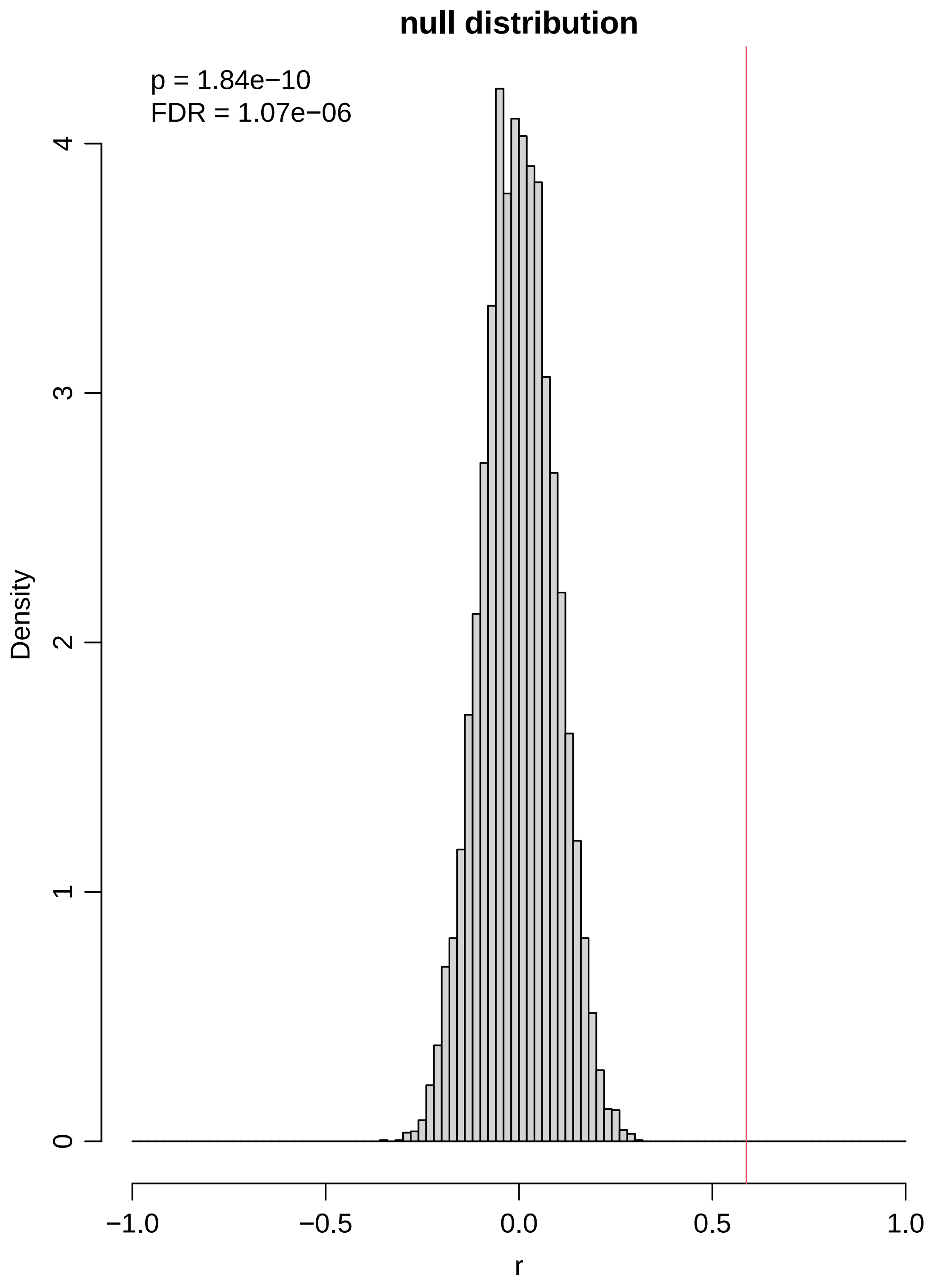

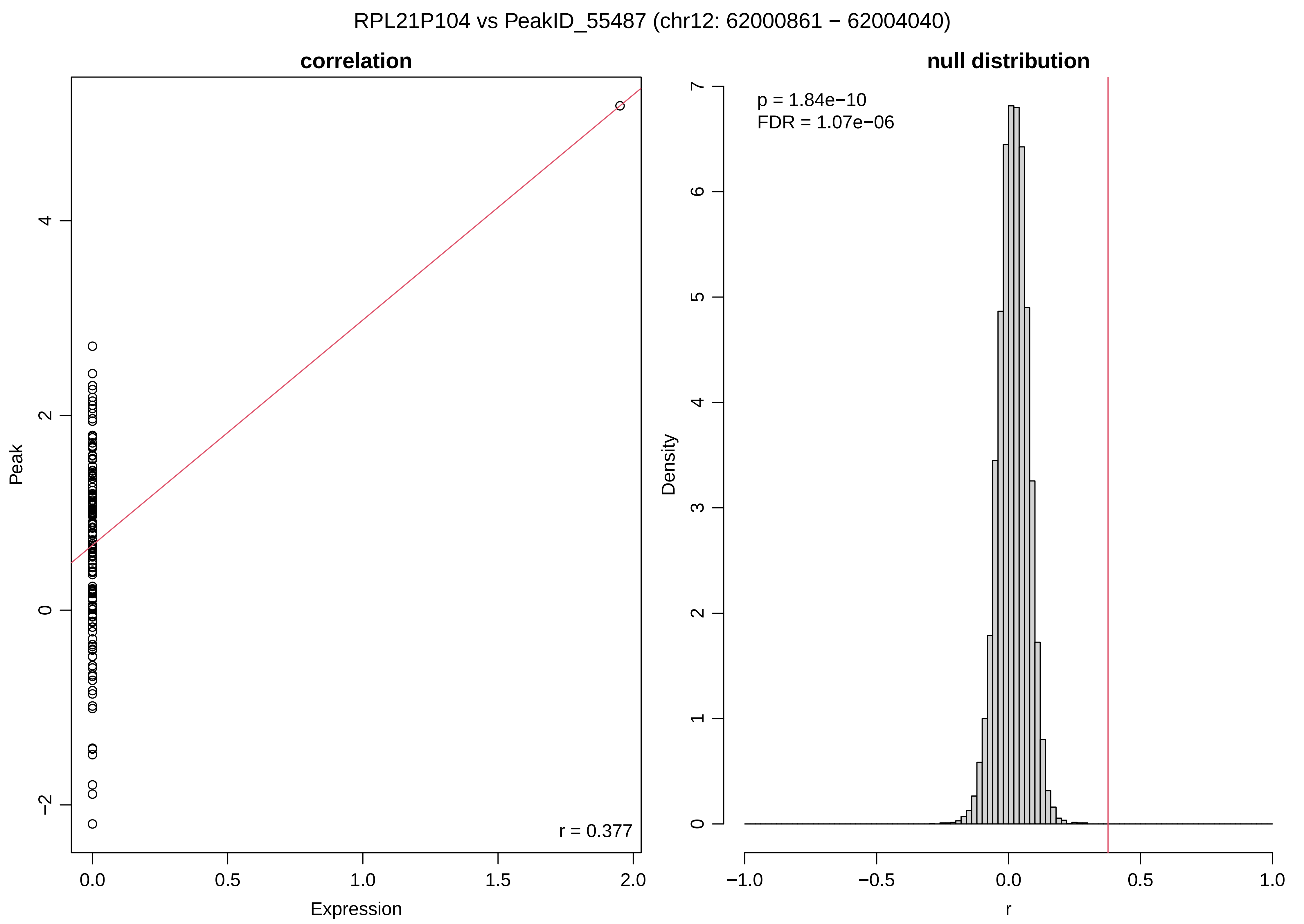

RPL21P104 vs PeakID_55487 (chr12: 62000861 − 62004040)
correlation
Peak
Expression
r = 0.377
−2
0
2
4
0.0
0.5
1.0
1.5
2.0
null distribution
Density
r
p = 1.84e−10
FDR = 1.07e−06
0
1
2
3
4
5
6
7
−1.0
−0.5
0.0
0.5
1.0

AL391427.1 vs PeakID_24133 (chr10: 4994379 − 4994866)

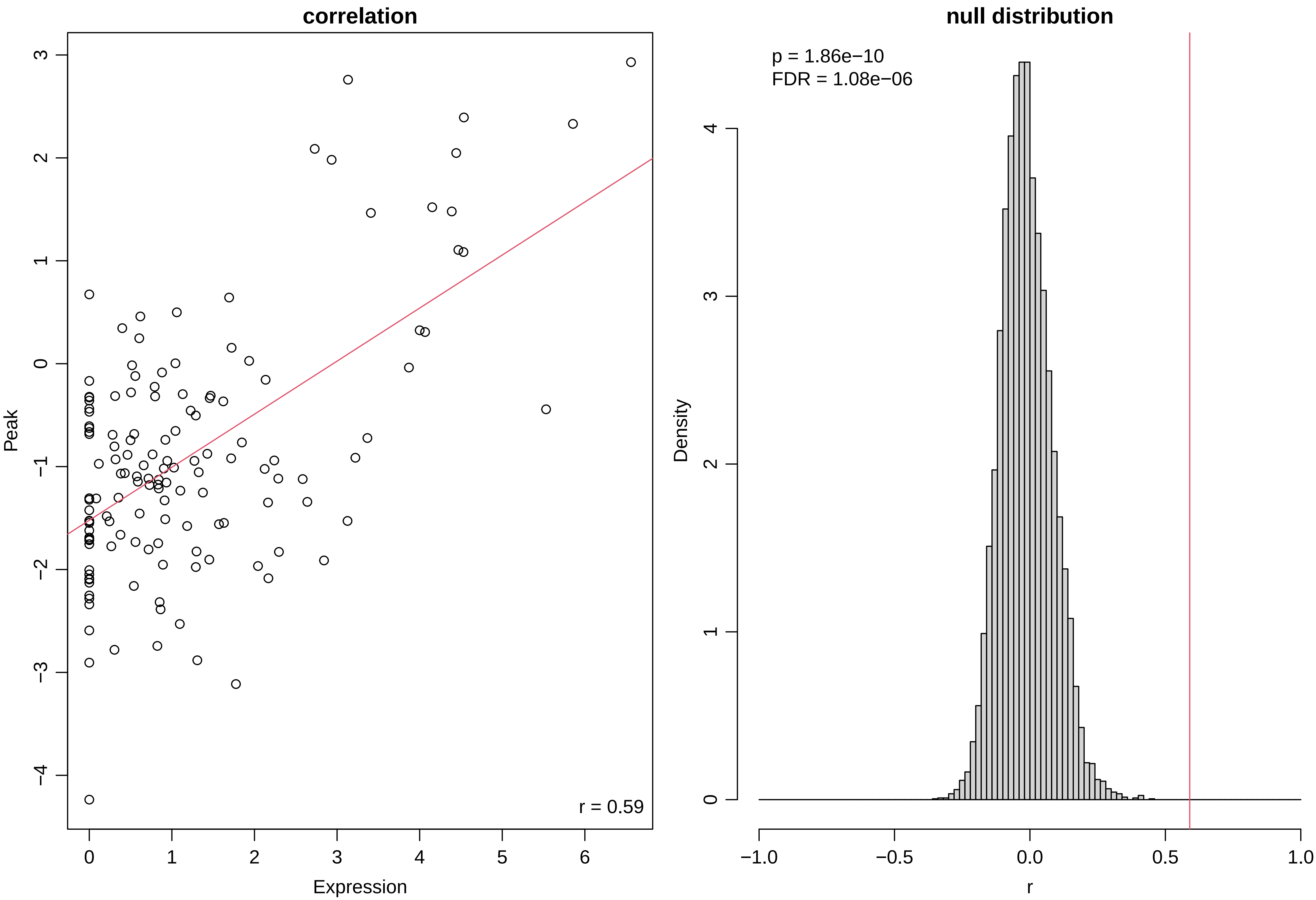

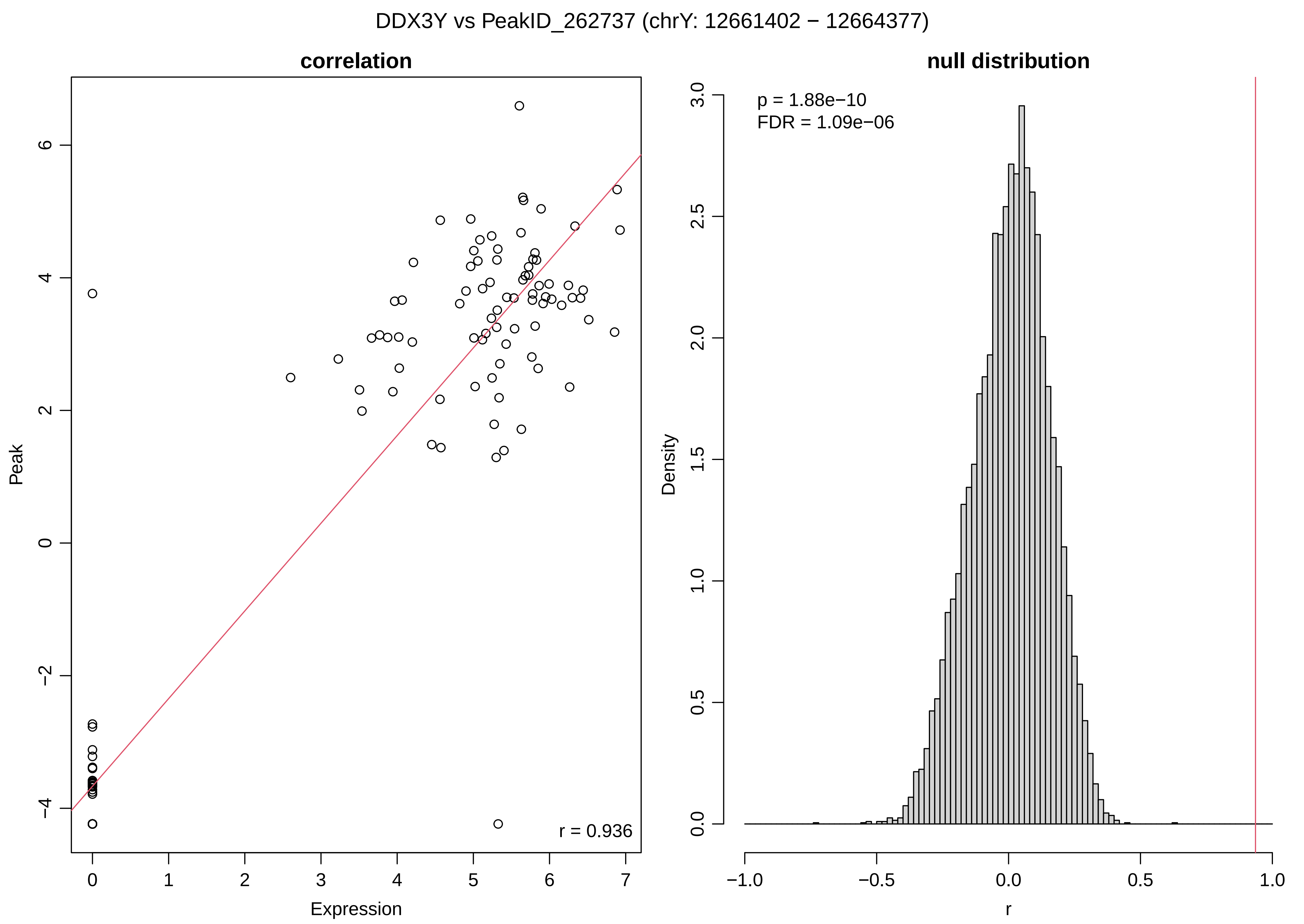
DDX3Y vs PeakID_262737 (chrY: 12661402 − 12664377)
correlation
Peak
Expression
r = 0.936
null distribution
Density
r
p = 1.88e−10
FDR = 1.09e−06
−4
−2
0
2
4
6
0
1
2
3
4
5
6
7
0.0
0.5
1.0
1.5
2.0
2.5
3.0
−1.0
−0.5
0.0
0.5
1.0

RNU4−20P vs PeakID_54837 (chr12: 59590507 − 59590921)

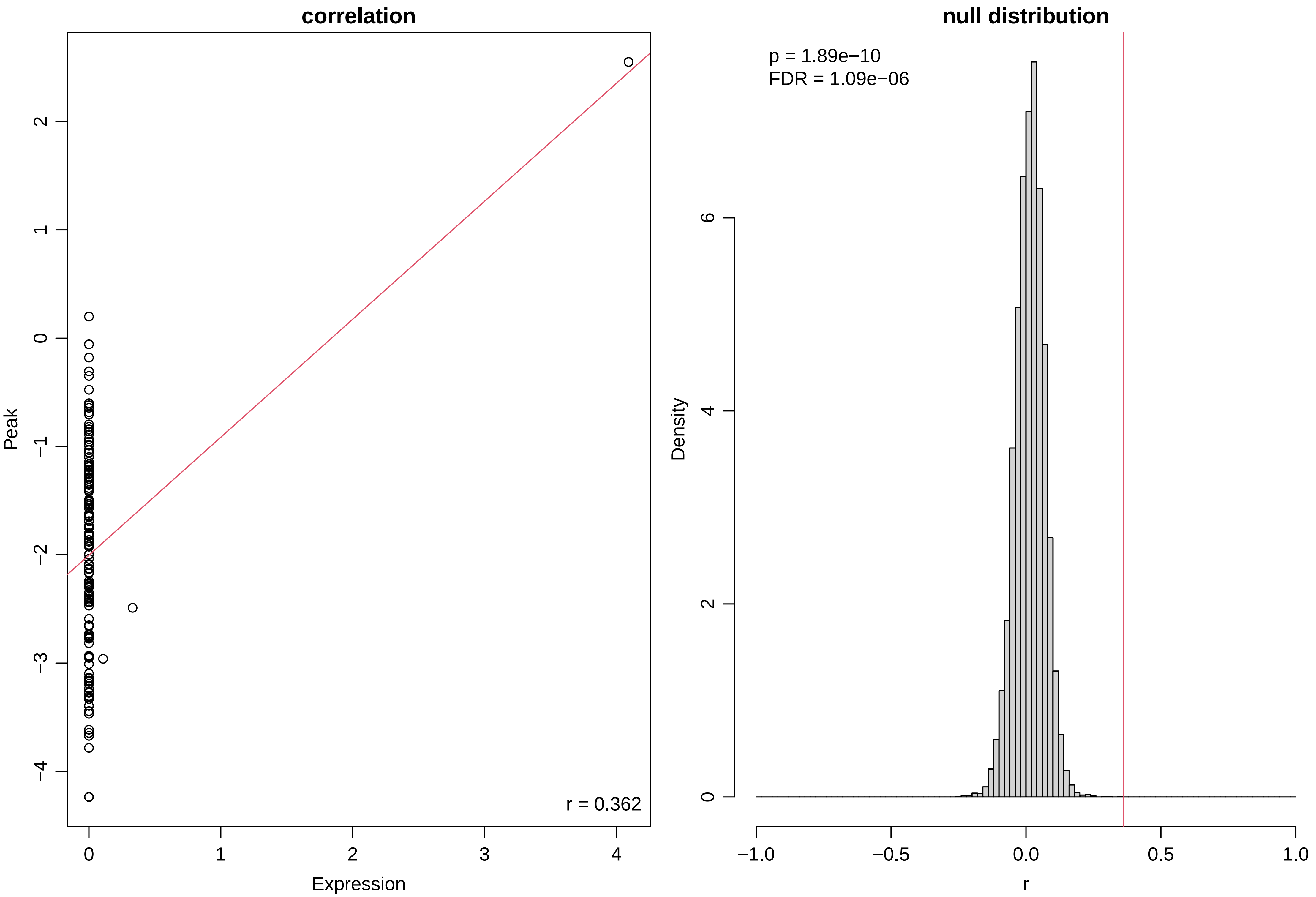

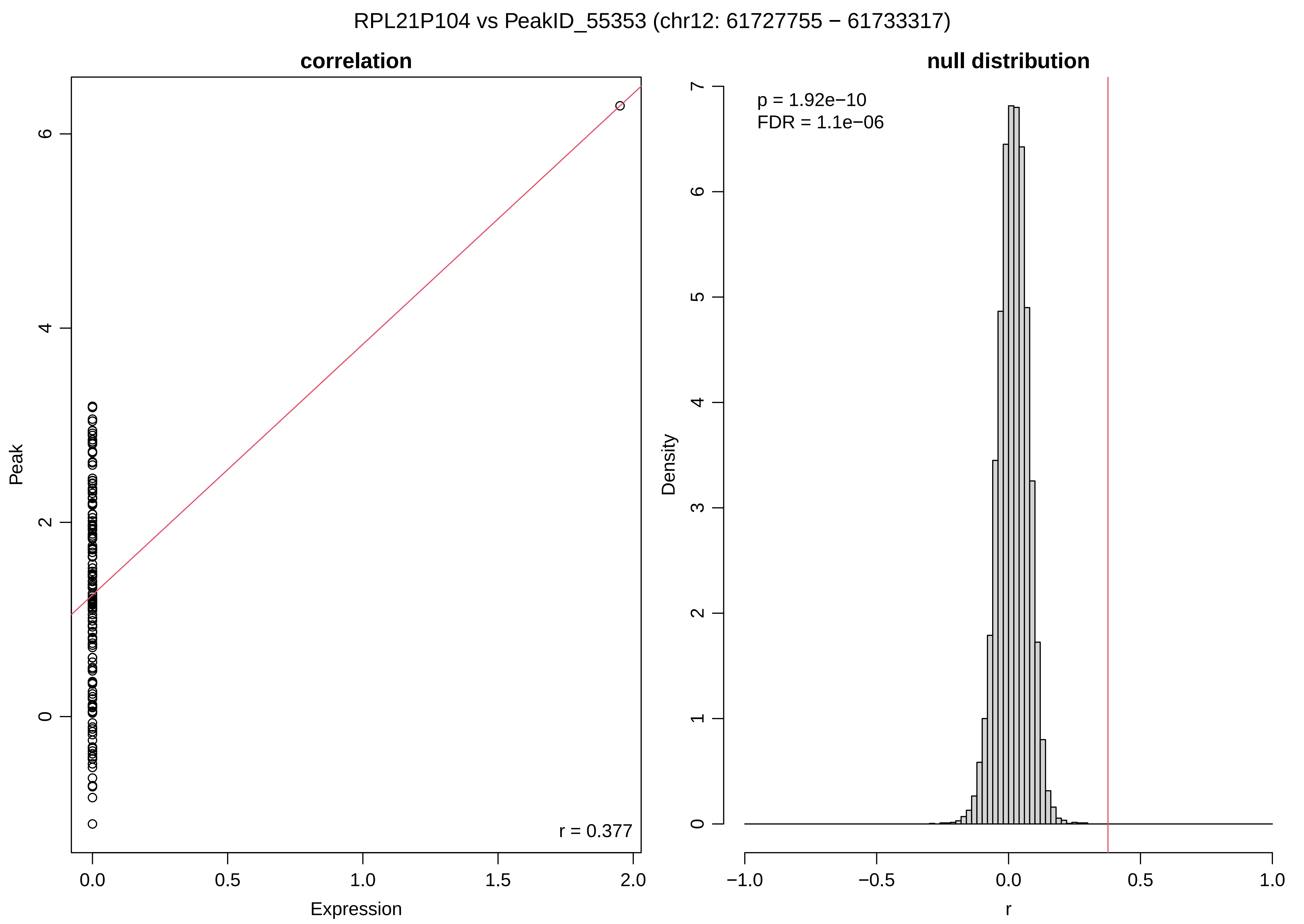
RPL21P104 vs PeakID_55353 (chr12: 61727755 − 61733317)
correlation
Peak
Expression
r = 0.377
0
2
4
6
0.0
0.5
1.0
1.5
2.0
null distribution
p = 1.92e−10
FDR = 1.1e−06
Density
r
0
1
2
3
4
5
6
7
−1.0
−0.5
0.0
0.5
1.0

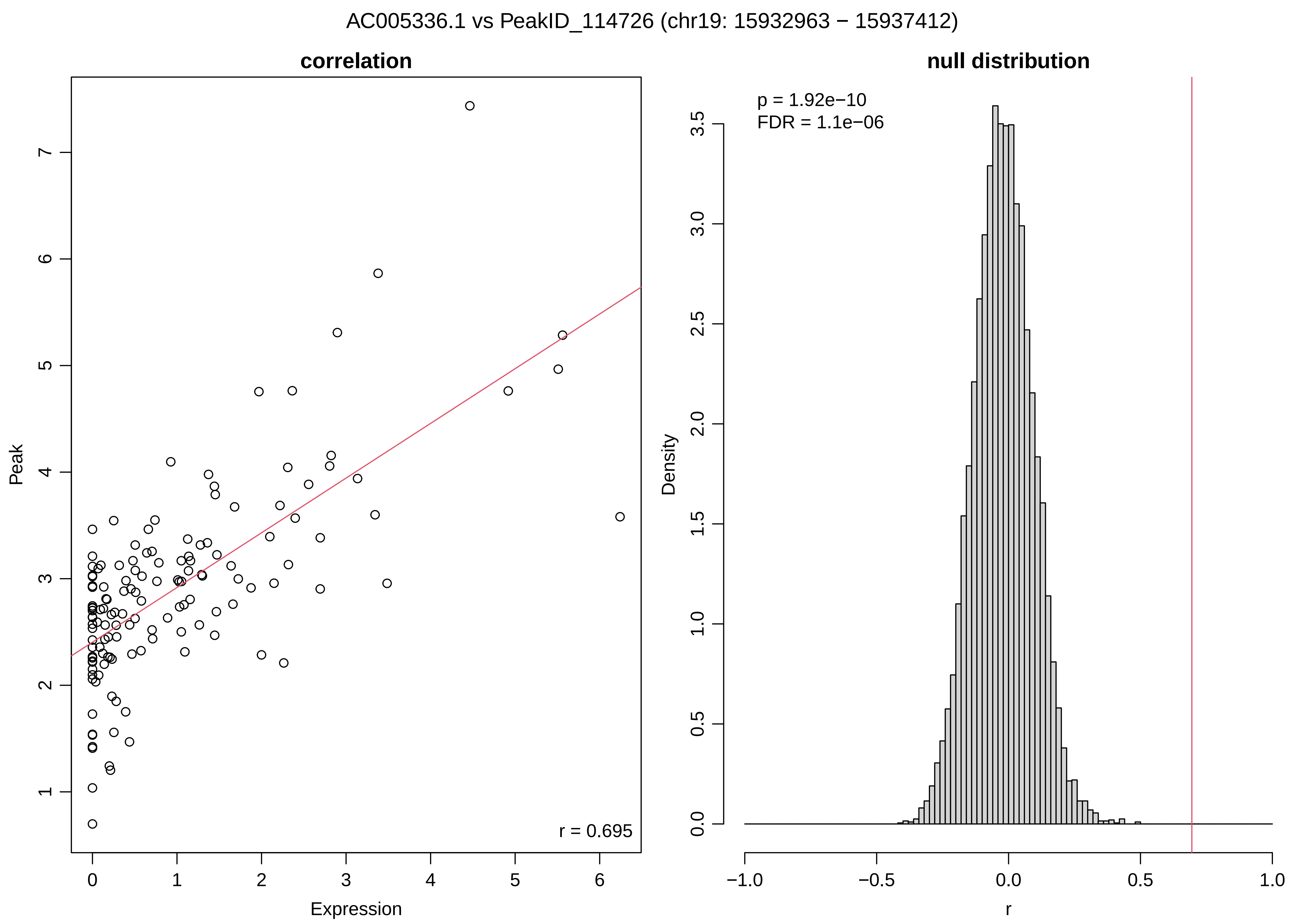
AC005336.1 vs PeakID_114726 (chr19: 15932963 − 15937412)
correlation
null distribution
Peak
Expression
r = 0.695
p = 1.92e−10
FDR = 1.1e−06
Density
r

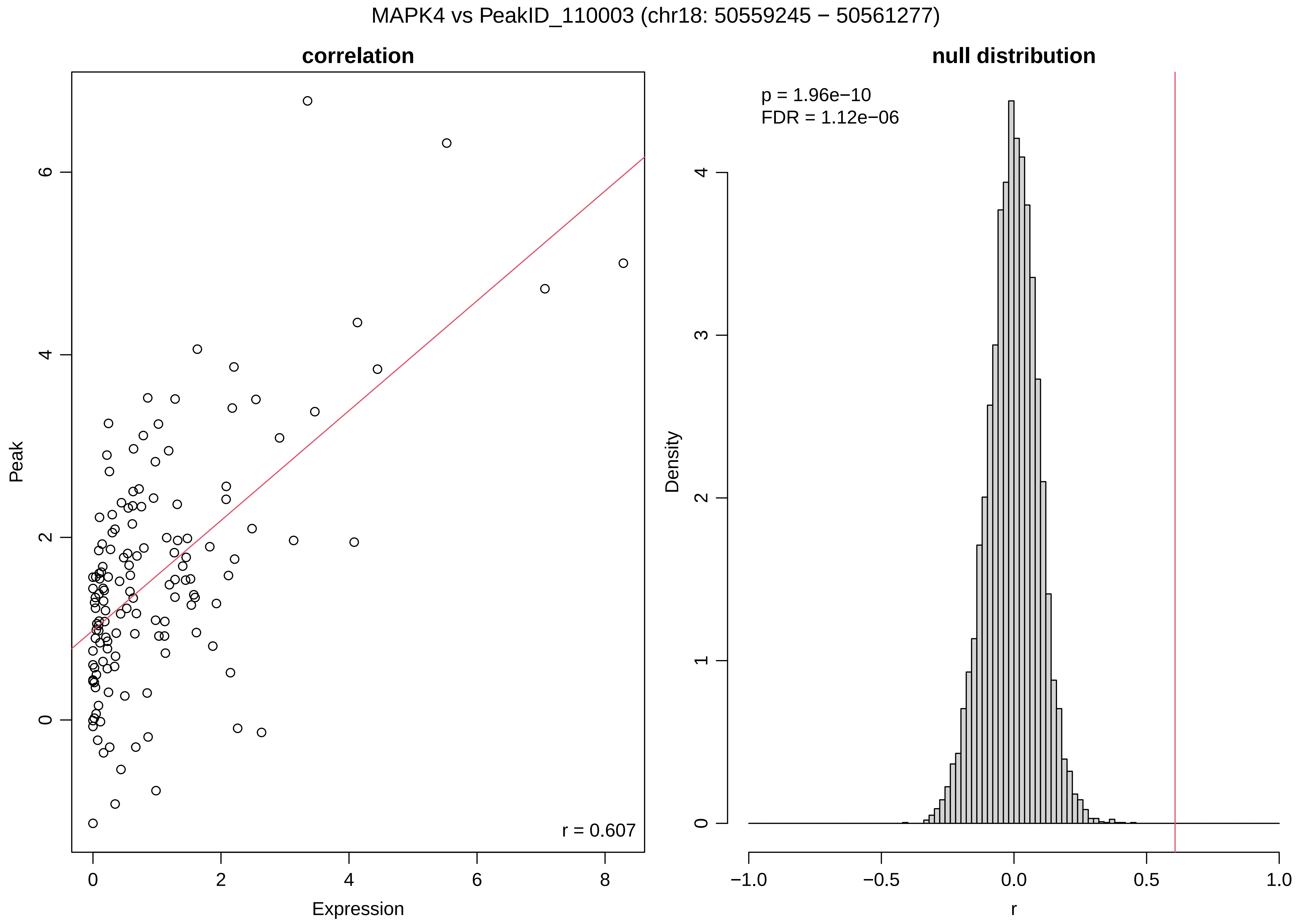
MAPK4 vs PeakID_110003 (chr18: 50559245 − 50561277)
correlation
Peak
Expression
r = 0.607
0
2
4
6
8
null distribution
p = 1.96e−10
FDR = 1.12e−06
Density
r
−1.0
−0.5
0.0
0.5
1.0

RFTN1P1 vs PeakID_262428 (chrY: 7729105 − 7730696)

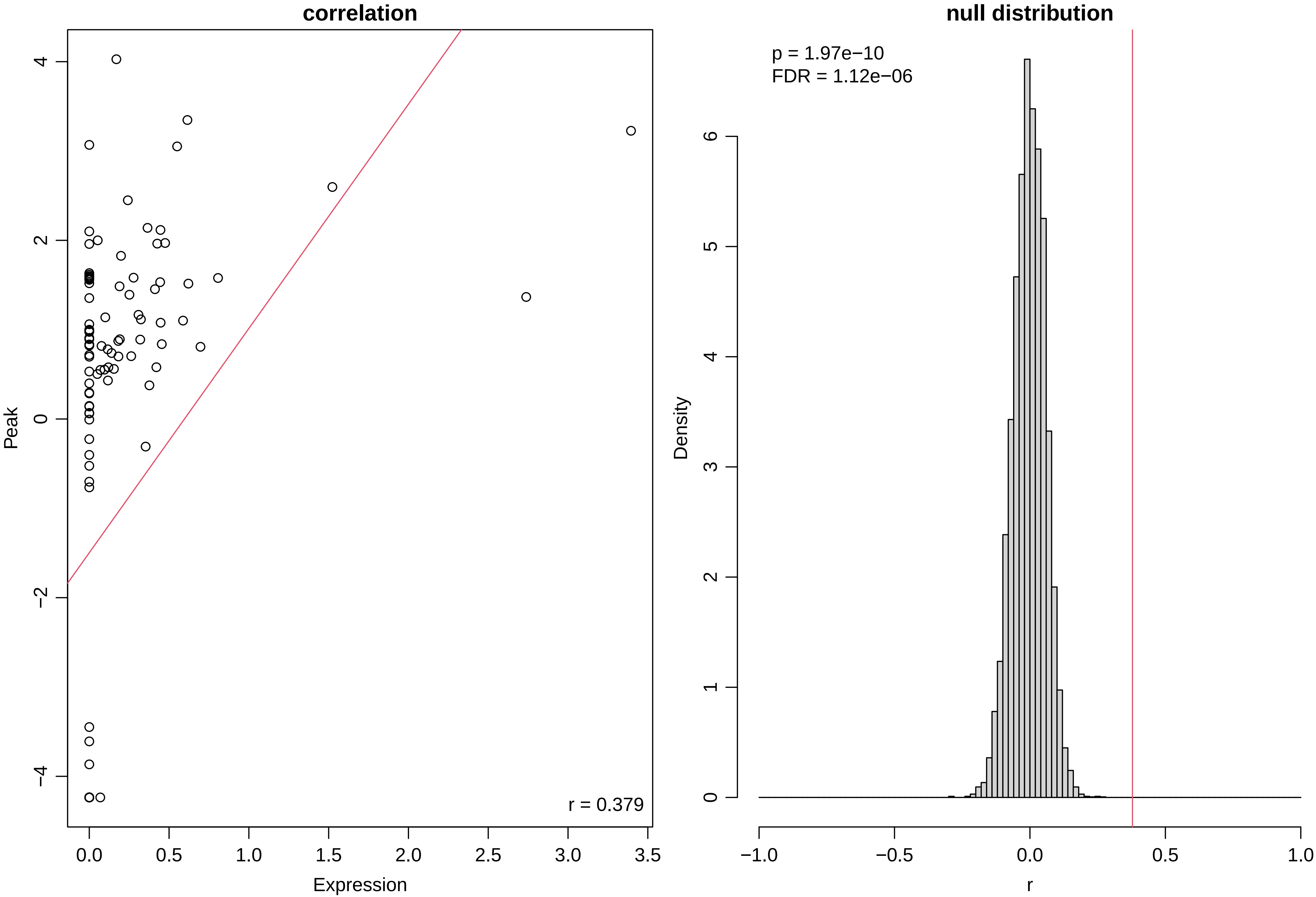

AC107881.1 vs PeakID_37819 (chr11: 12413607 − 12414135)

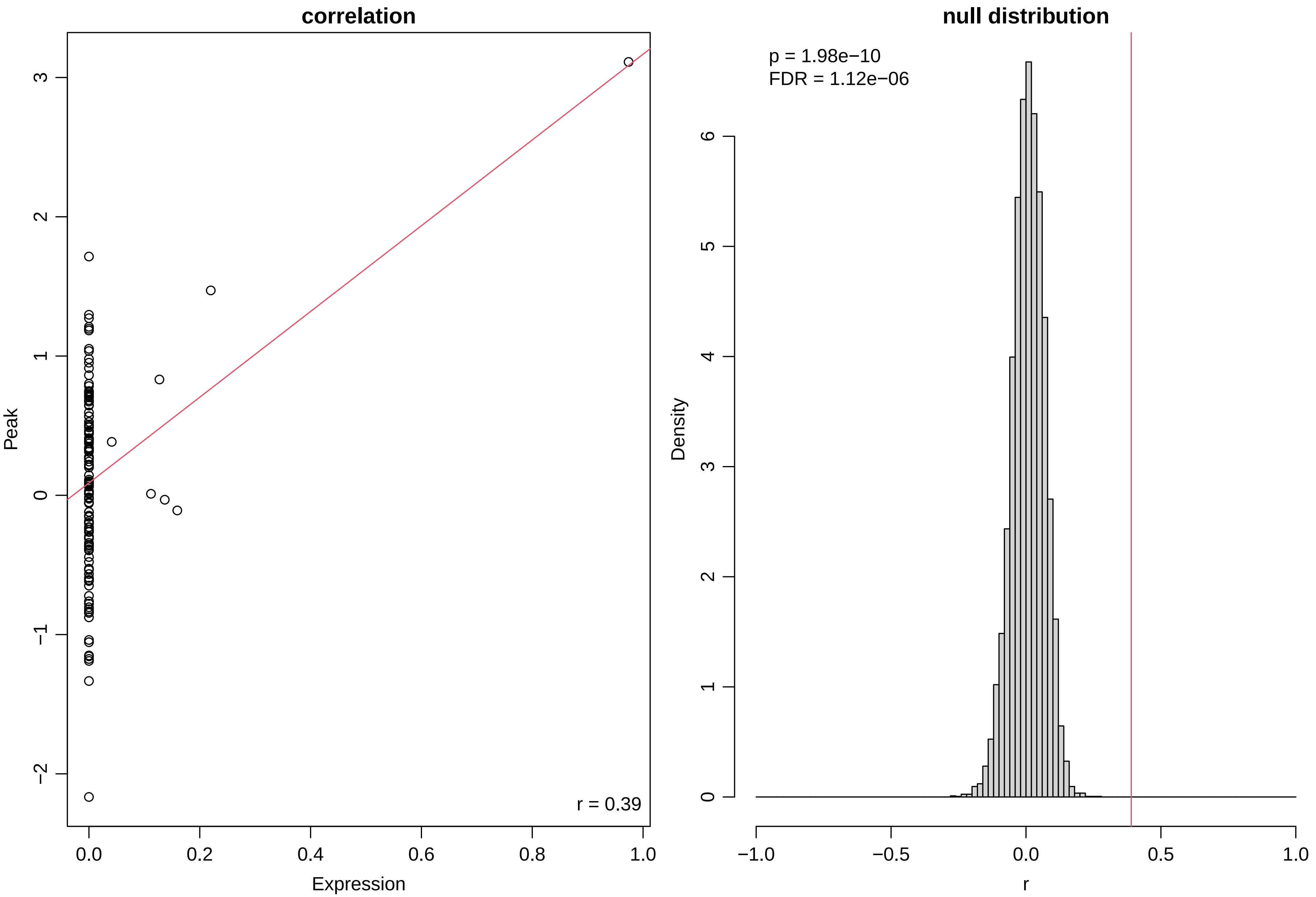

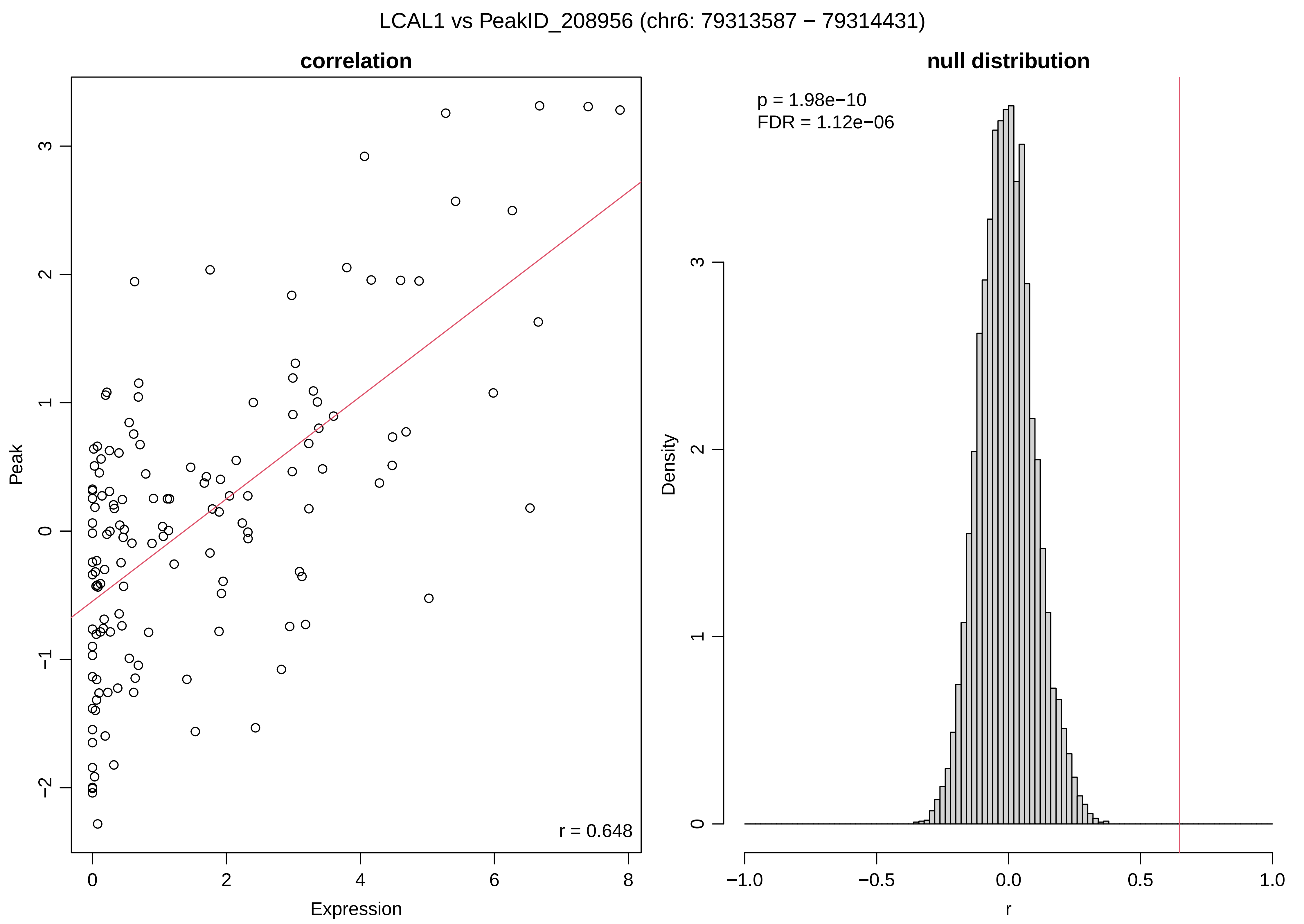

LCAL1 vs PeakID_208956 (chr6: 79313587 − 79314431)
correlation
Peak
Expression
r = 0.648
null distribution
p = 1.98e−10
FDR = 1.12e−06
Density
r

AC107881.1 vs PeakID_37988 (chr11: 12710635 − 12711996)

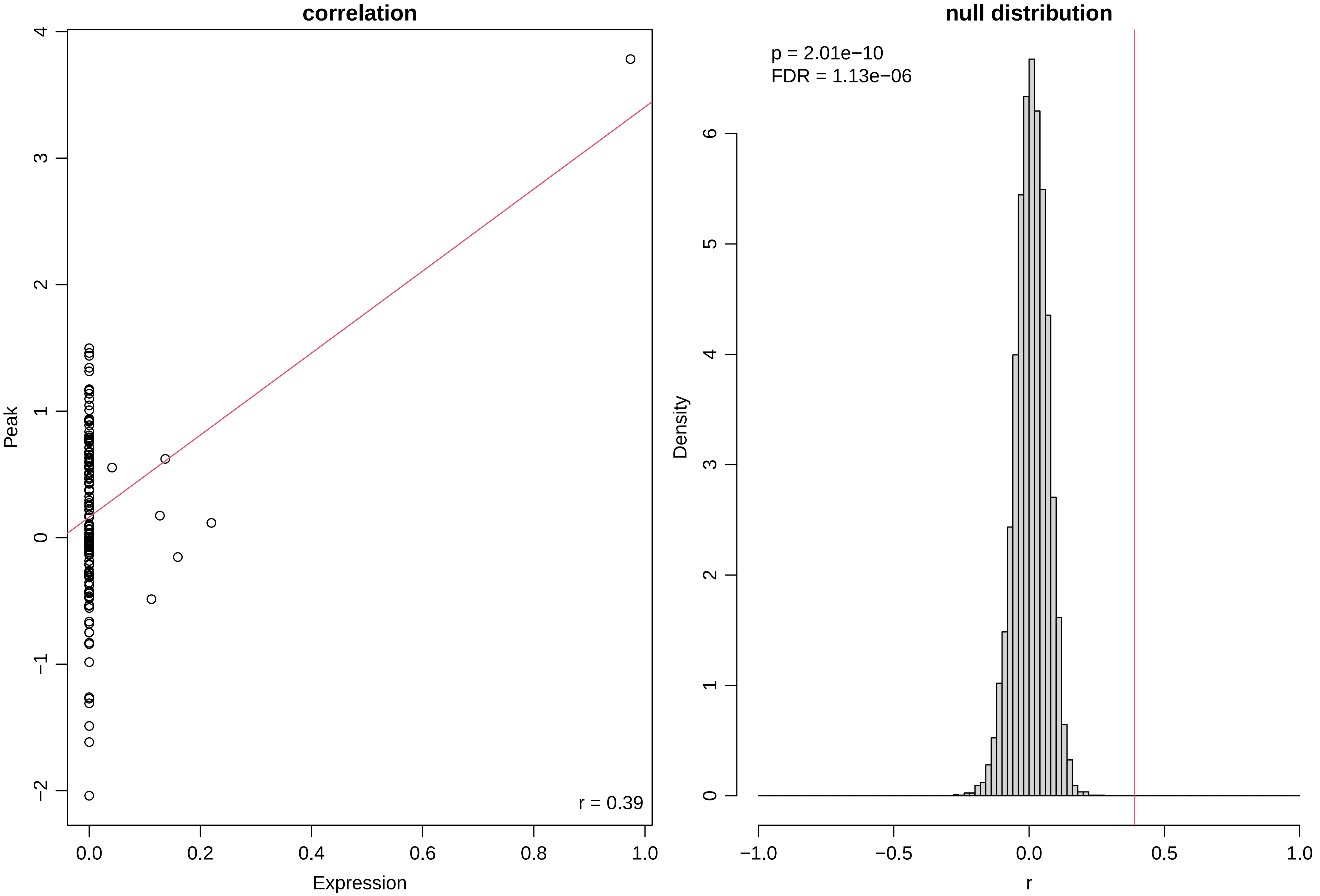

AC090061.1 vs PeakID_56569 (chr12: 68798076 − 68800493)

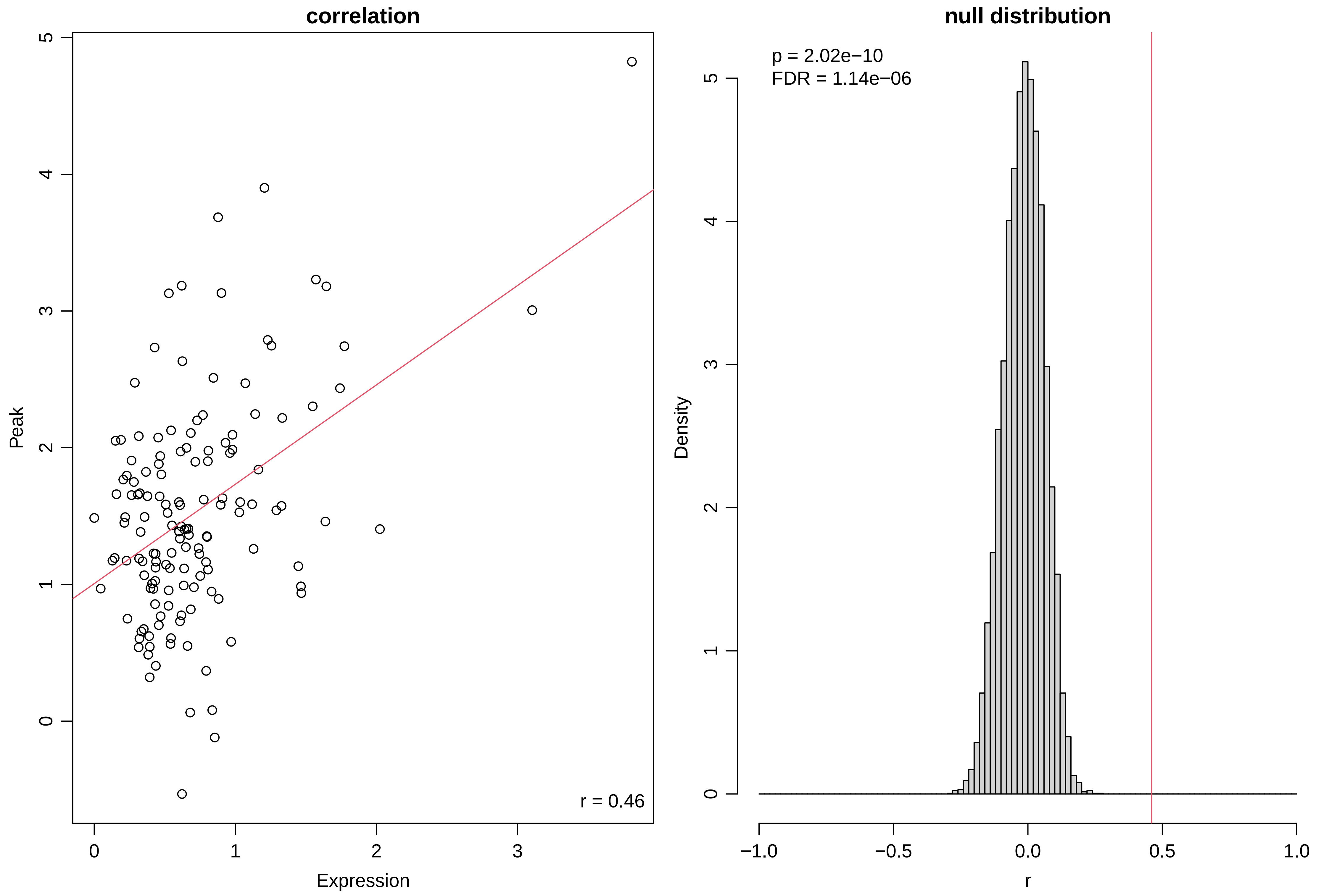

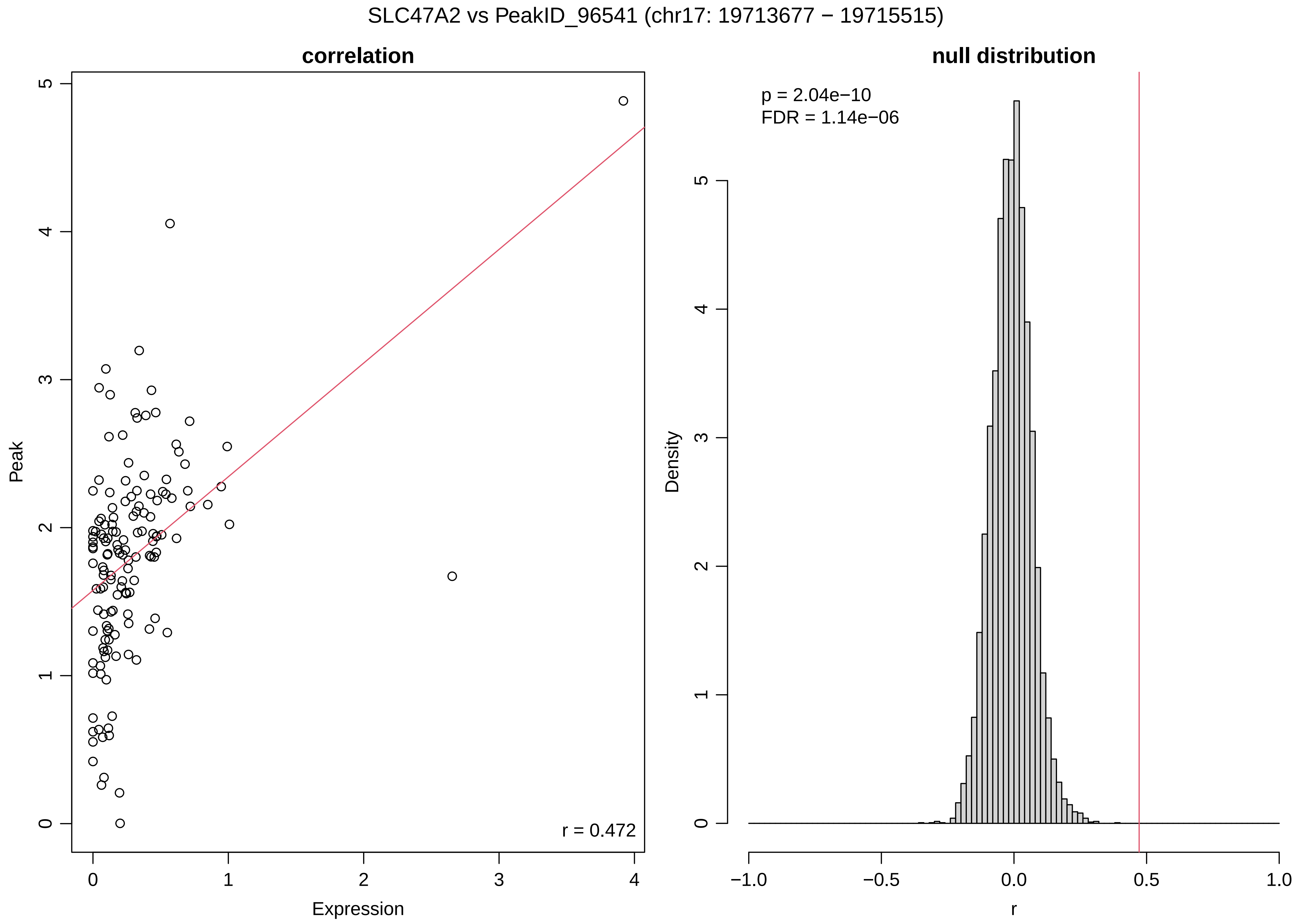
SLC47A2 vs PeakID_96541 (chr17: 19713677 − 19715515)
correlation
null distribution
Peak
Expression
r = 0.472
p = 2.04e−10
FDR = 1.14e−06
Density
r

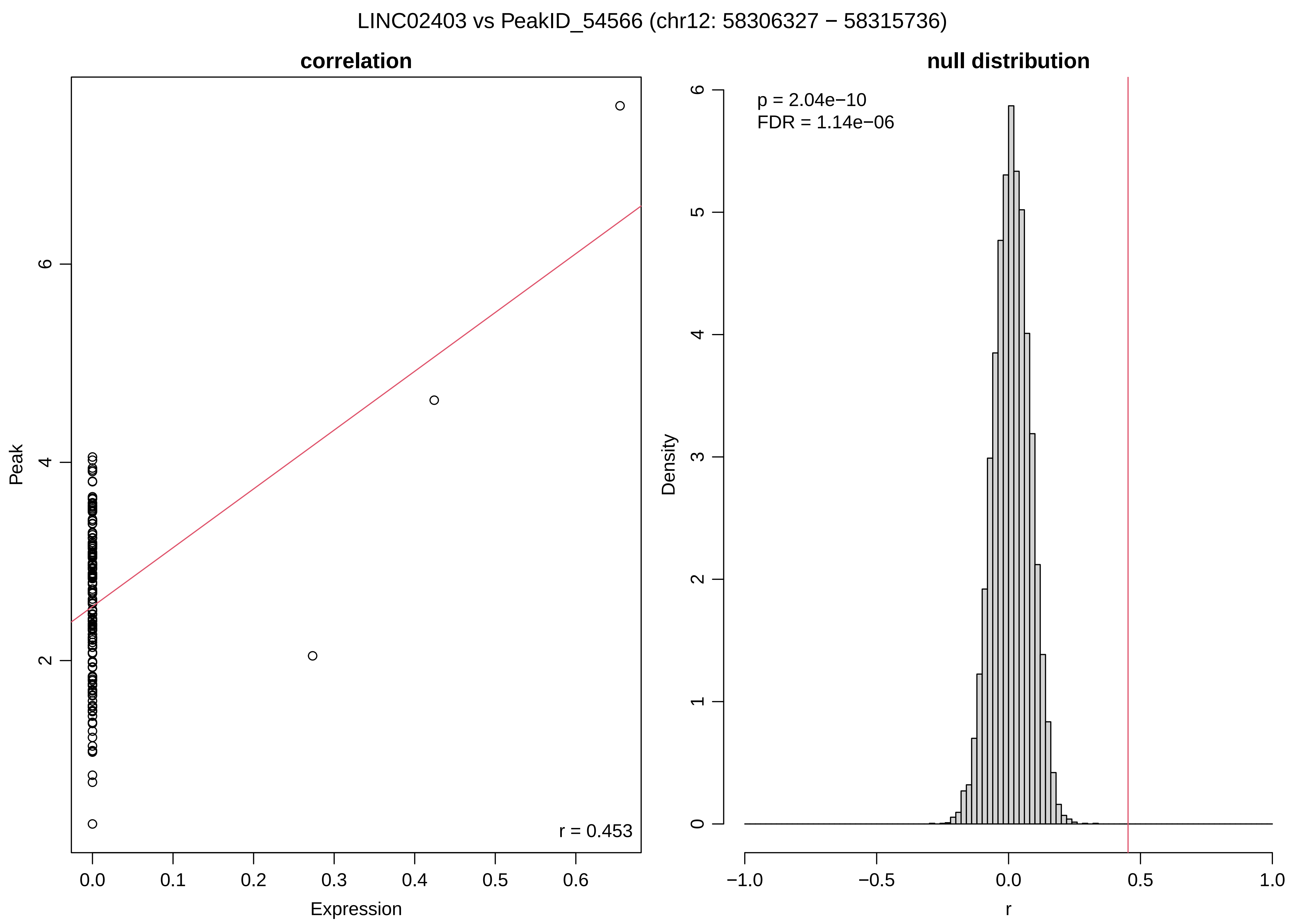
LINC02403 vs PeakID_54566 (chr12: 58306327 − 58315736)
correlation
Peak
Expression
r = 0.453
null distribution
Density
r
p = 2.04e−10
FDR = 1.14e−06

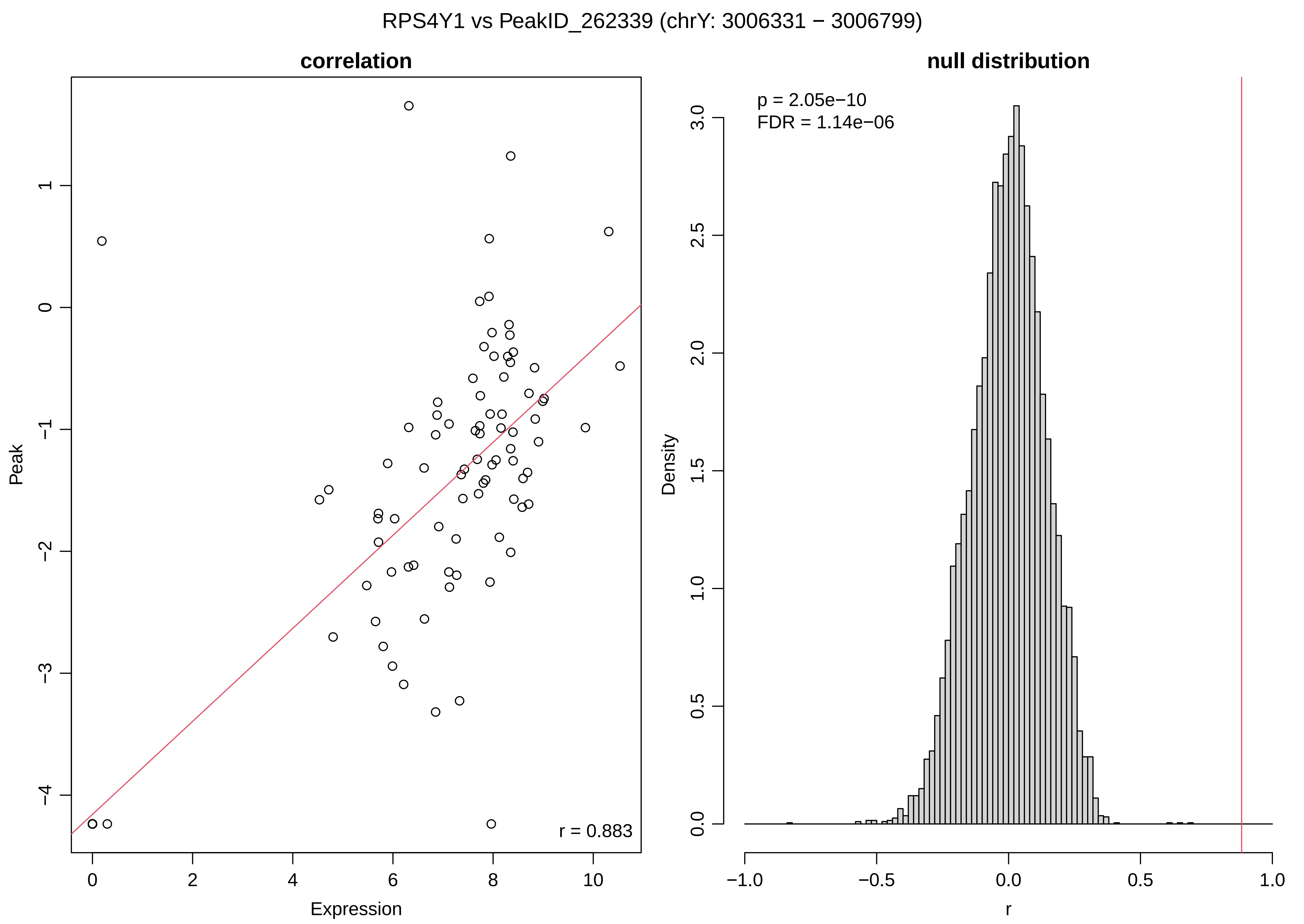
RPS4Y1 vs PeakID_262339 (chrY: 3006331 − 3006799)
correlation
Peak
Expression
r = 0.883
null distribution
p = 2.05e−10
FDR = 1.14e−06
Density
r

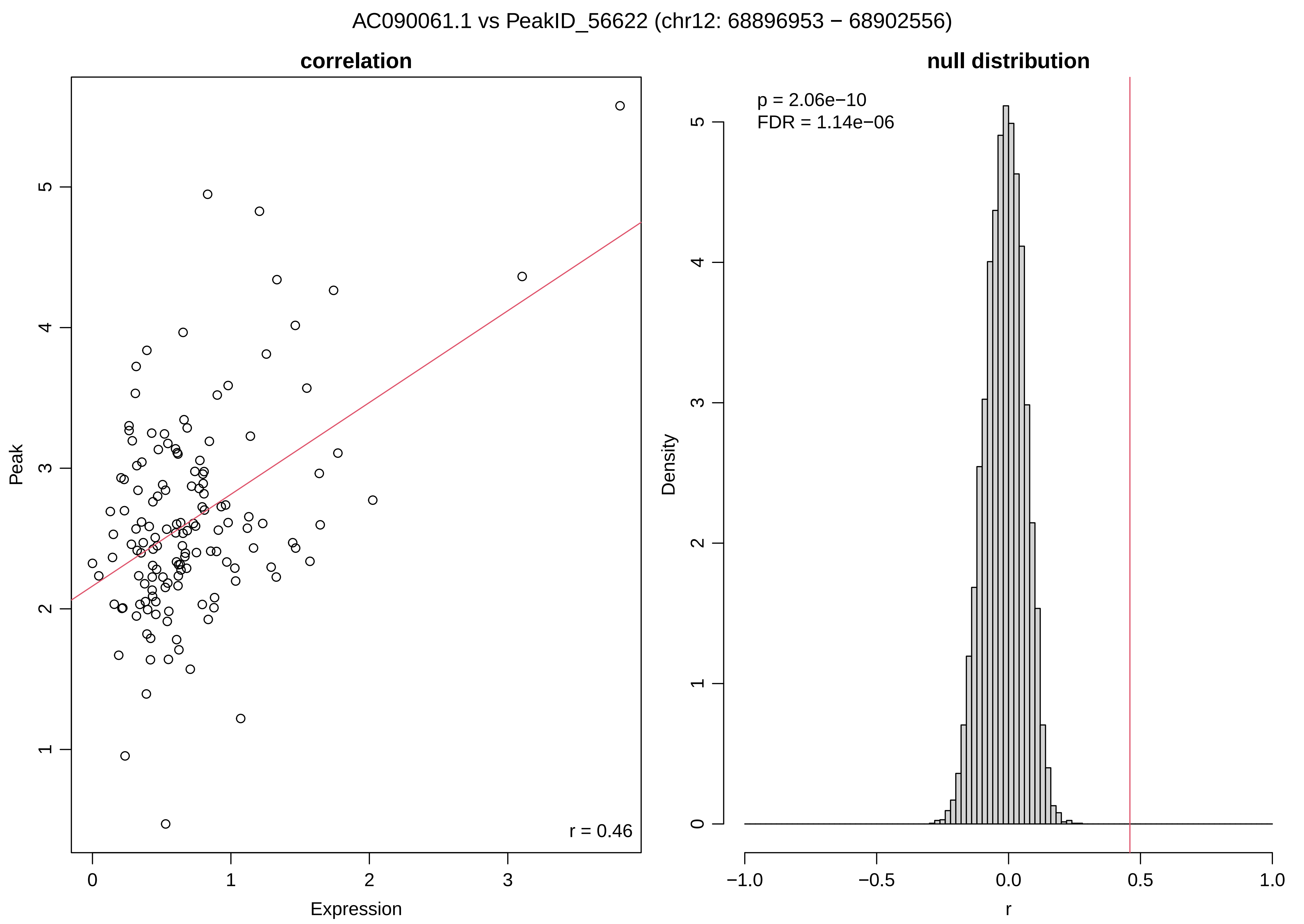
AC090061.1 vs PeakID_56622 (chr12: 68896953 − 68902556)
correlation
Peak
Expression
r = 0.46
null distribution
p = 2.06e−10
FDR = 1.14e−06
Density
r

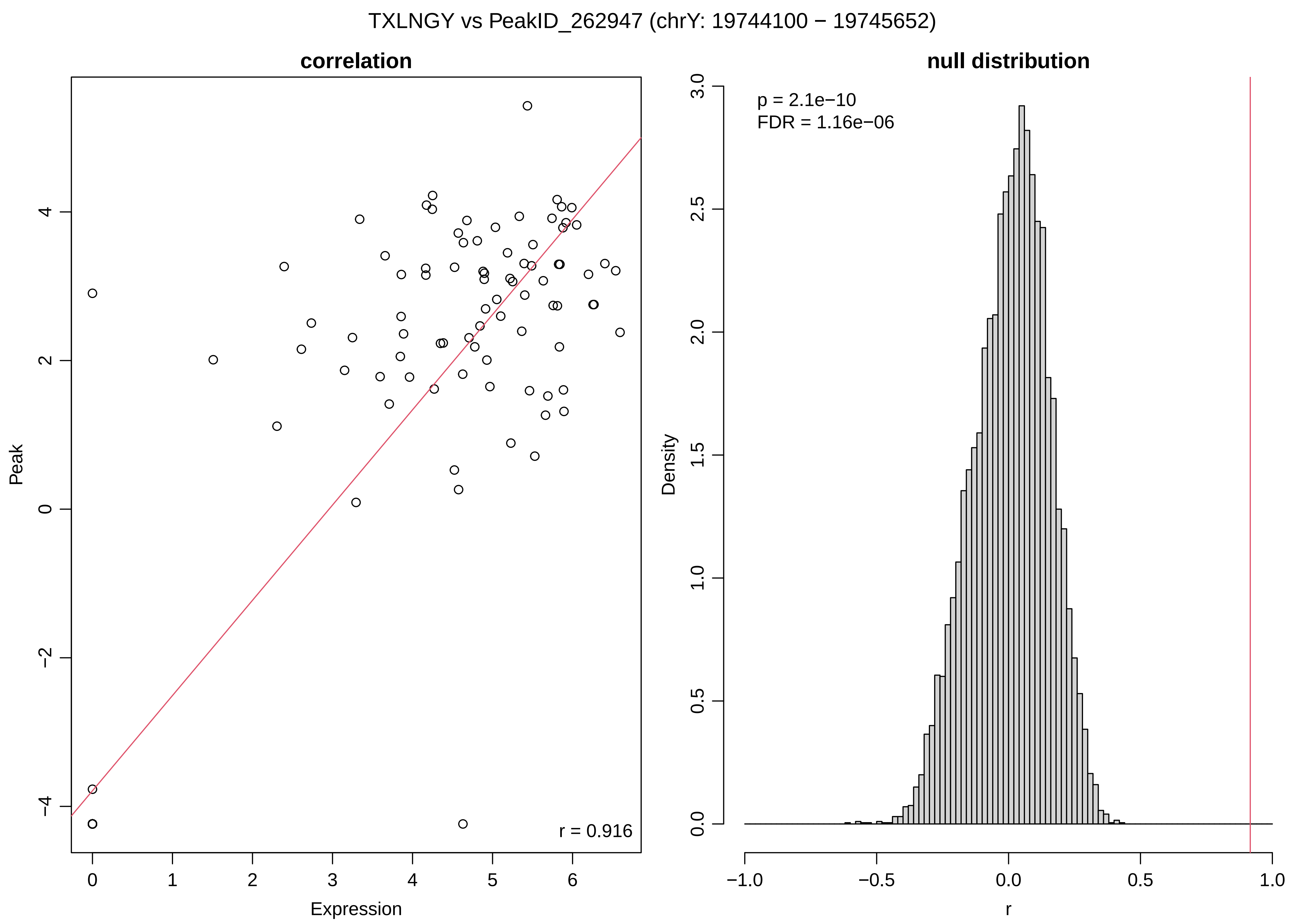
TXLNGY vs PeakID_262947 (chrY: 19744100 − 19745652)
correlation
Peak
Expression
r = 0.916
null distribution
Density
r
p = 2.1e−10
FDR = 1.16e−06

BBOX1−AS1 vs PeakID_39116 (chr11: 27217813 − 27220455)

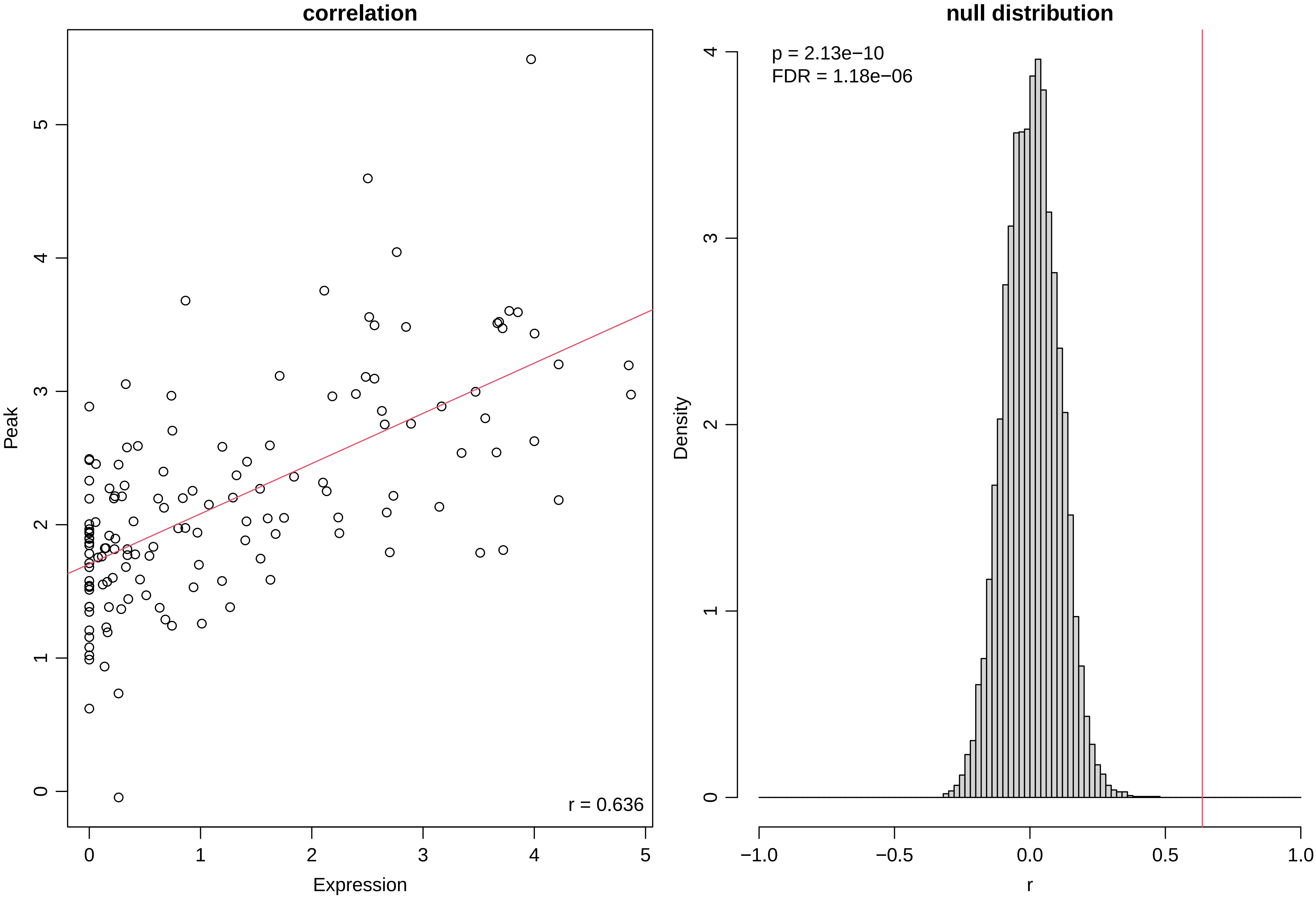

KRT16P3 vs PeakID_96655 (chr17: 20503283 − 20504569)

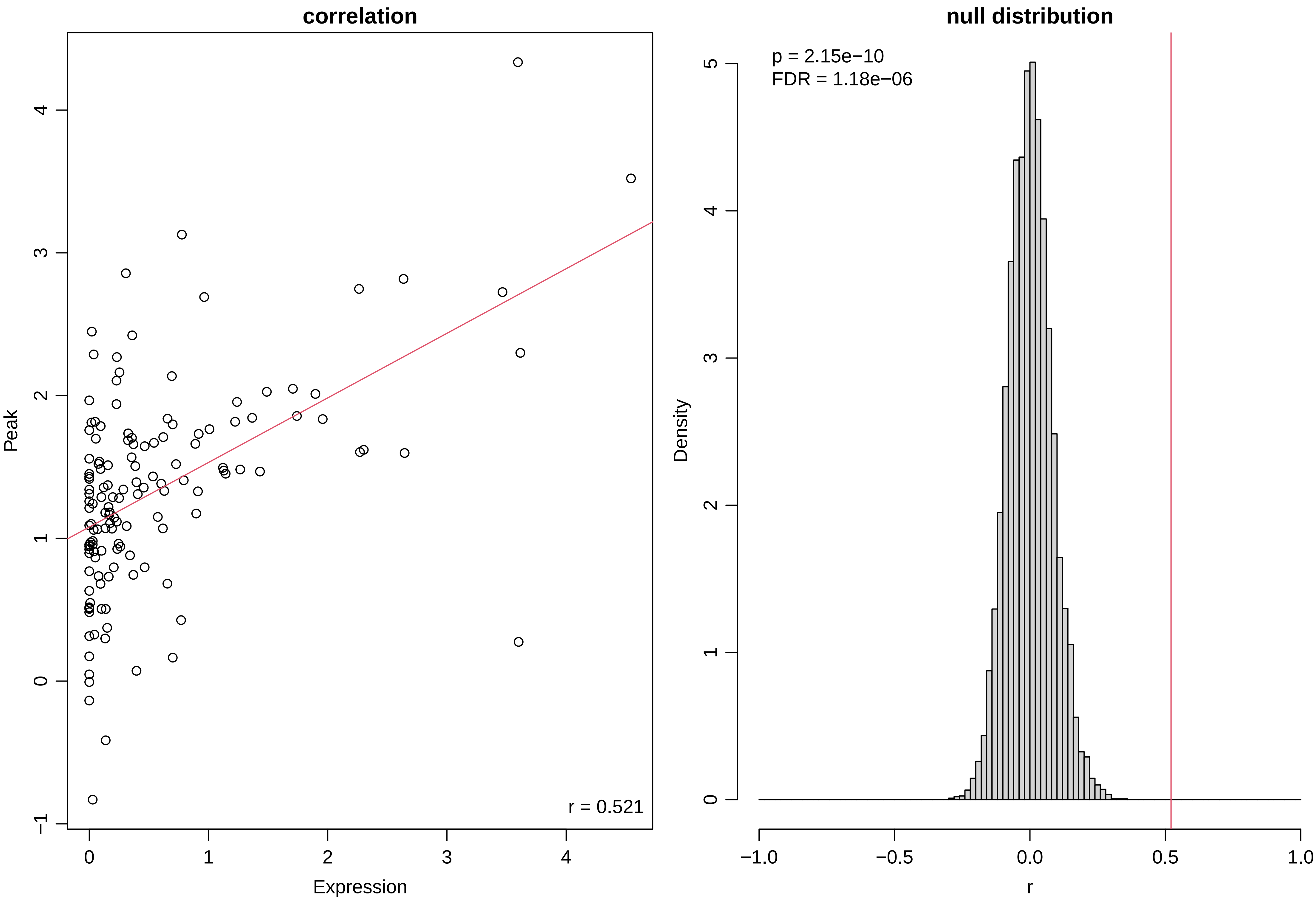

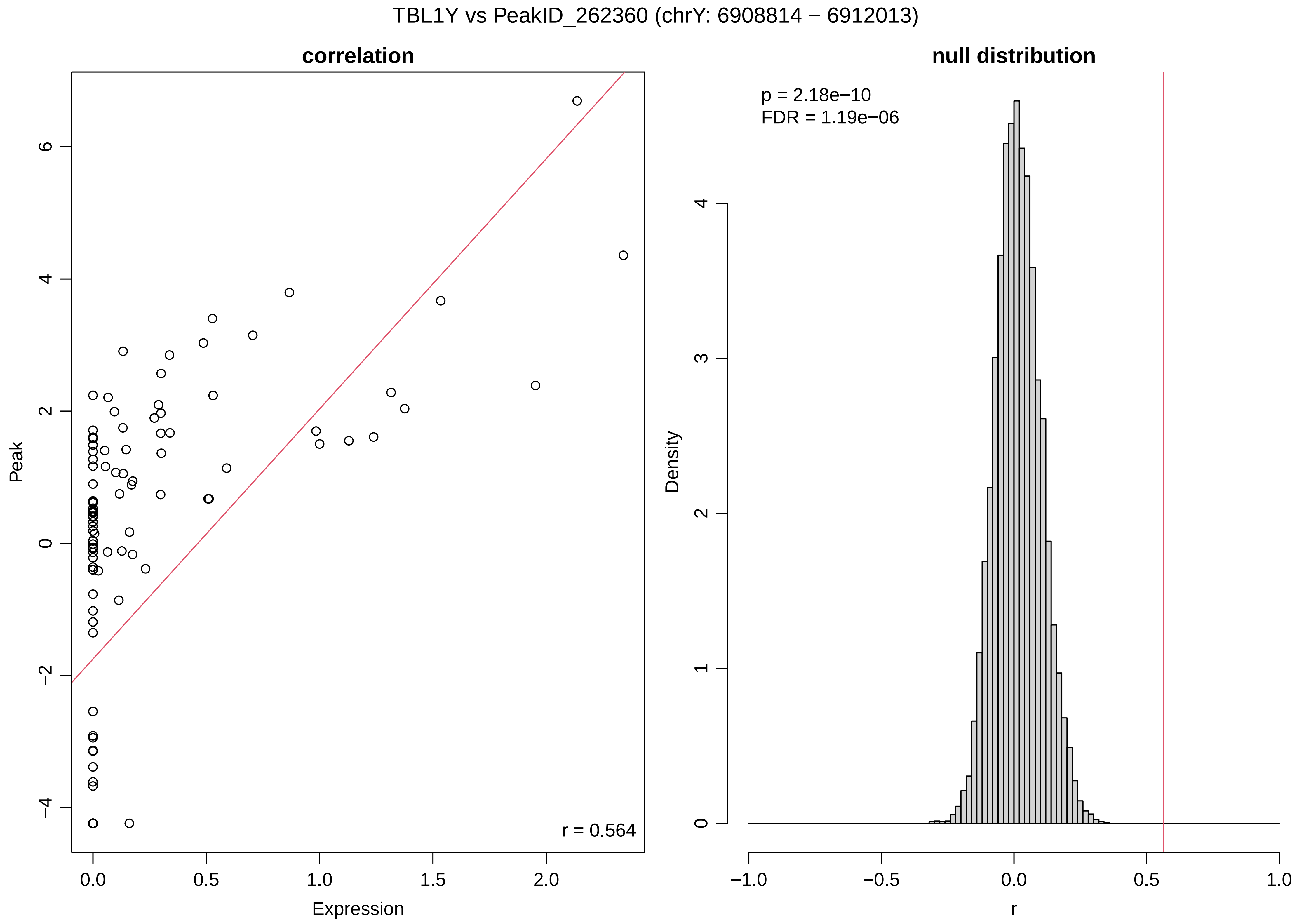

TBL1Y vs PeakID_262360 (chrY: 6908814 − 6912013)
correlation
Peak
Expression
r = 0.564
null distribution
p = 2.18e−10
FDR = 1.19e−06
Density
r

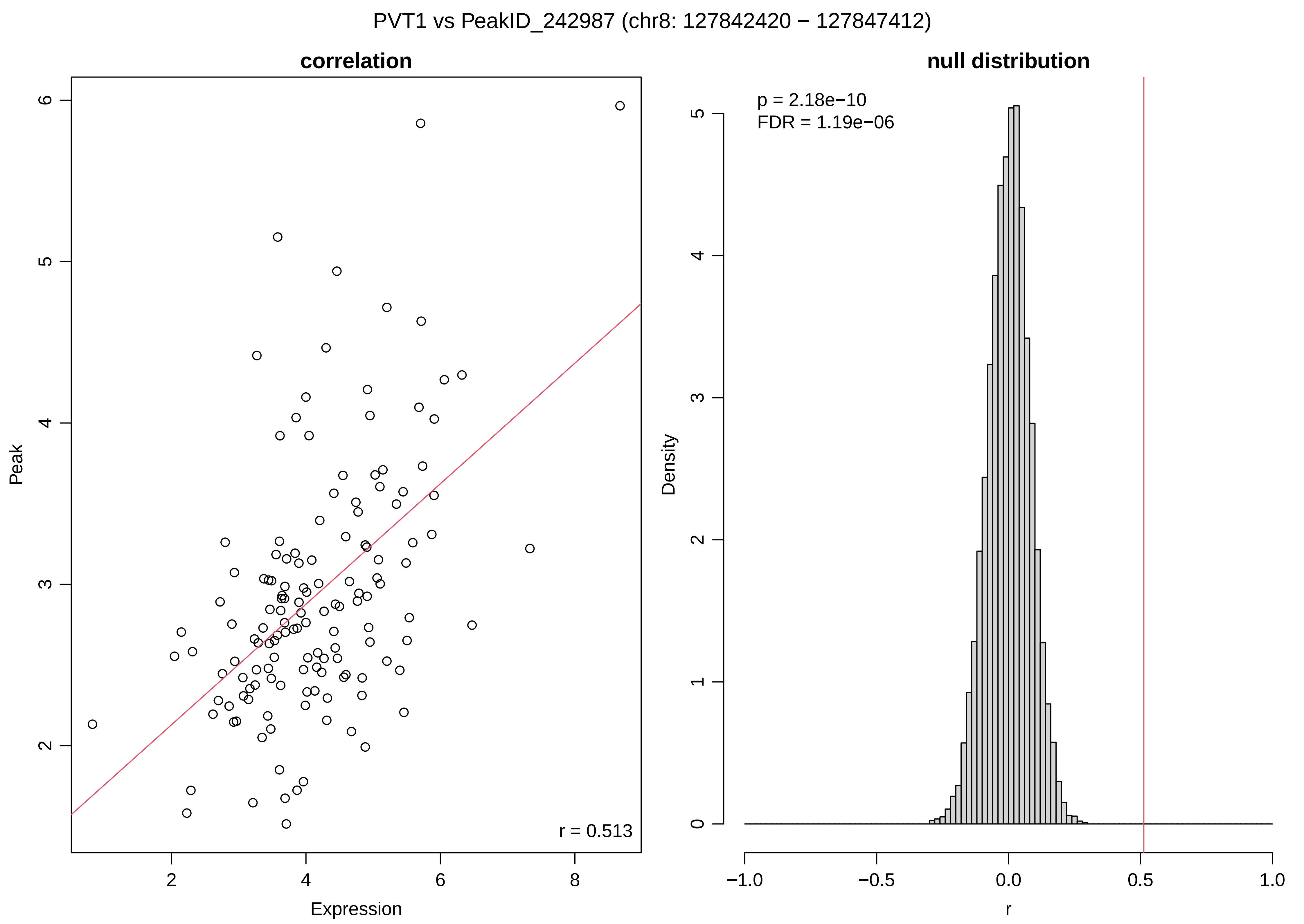

PVT1 vs PeakID_242987 (chr8: 127842420 − 127847412)
correlation
Peak
Expression
r = 0.513
null distribution
p = 2.18e−10
FDR = 1.19e−06
Density
r

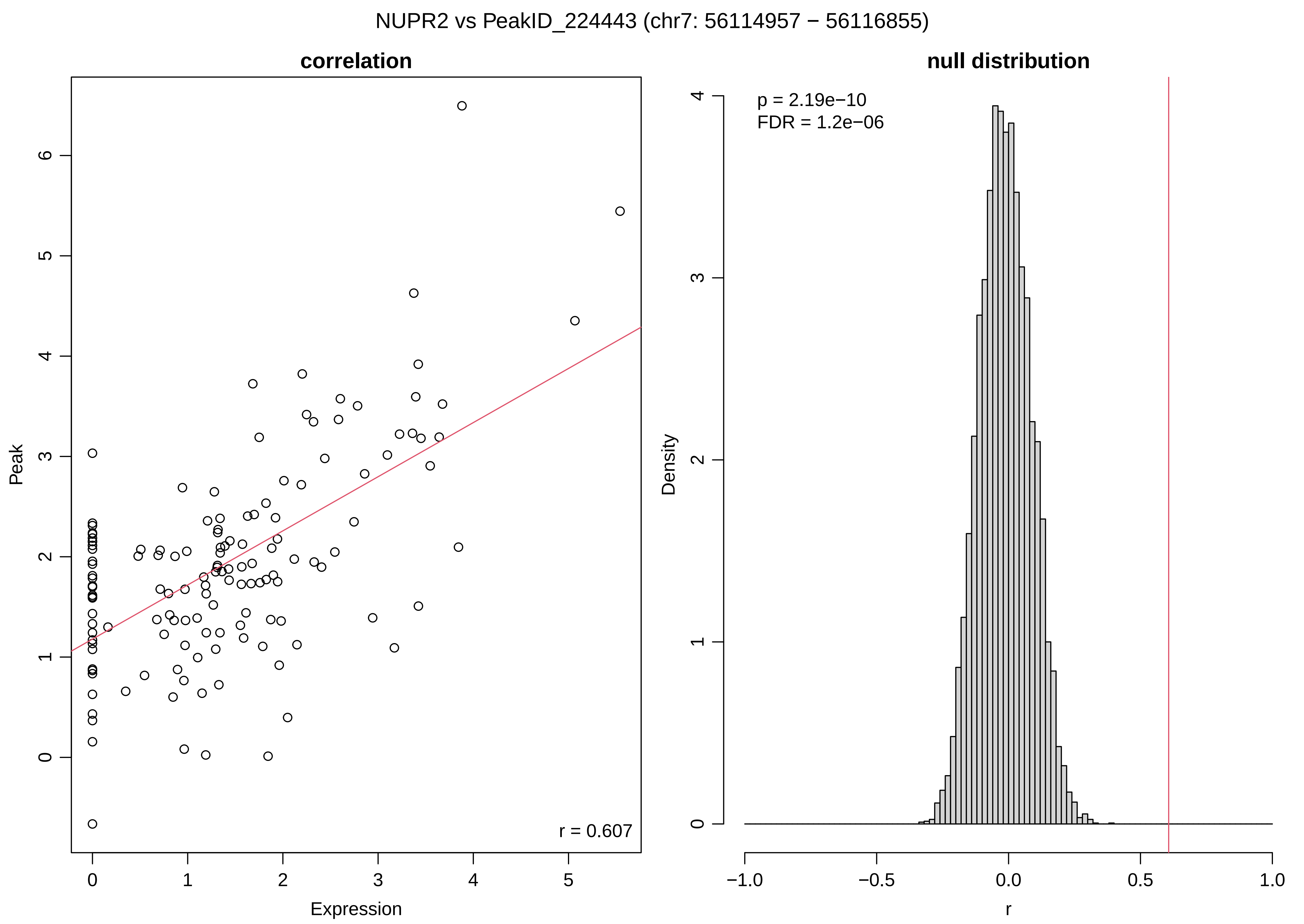
NUPR2 vs PeakID_224443 (chr7: 56114957 − 56116855)
correlation
null distribution
Peak
Expression
r = 0.607
p = 2.19e−10
FDR = 1.2e−06
Density
r

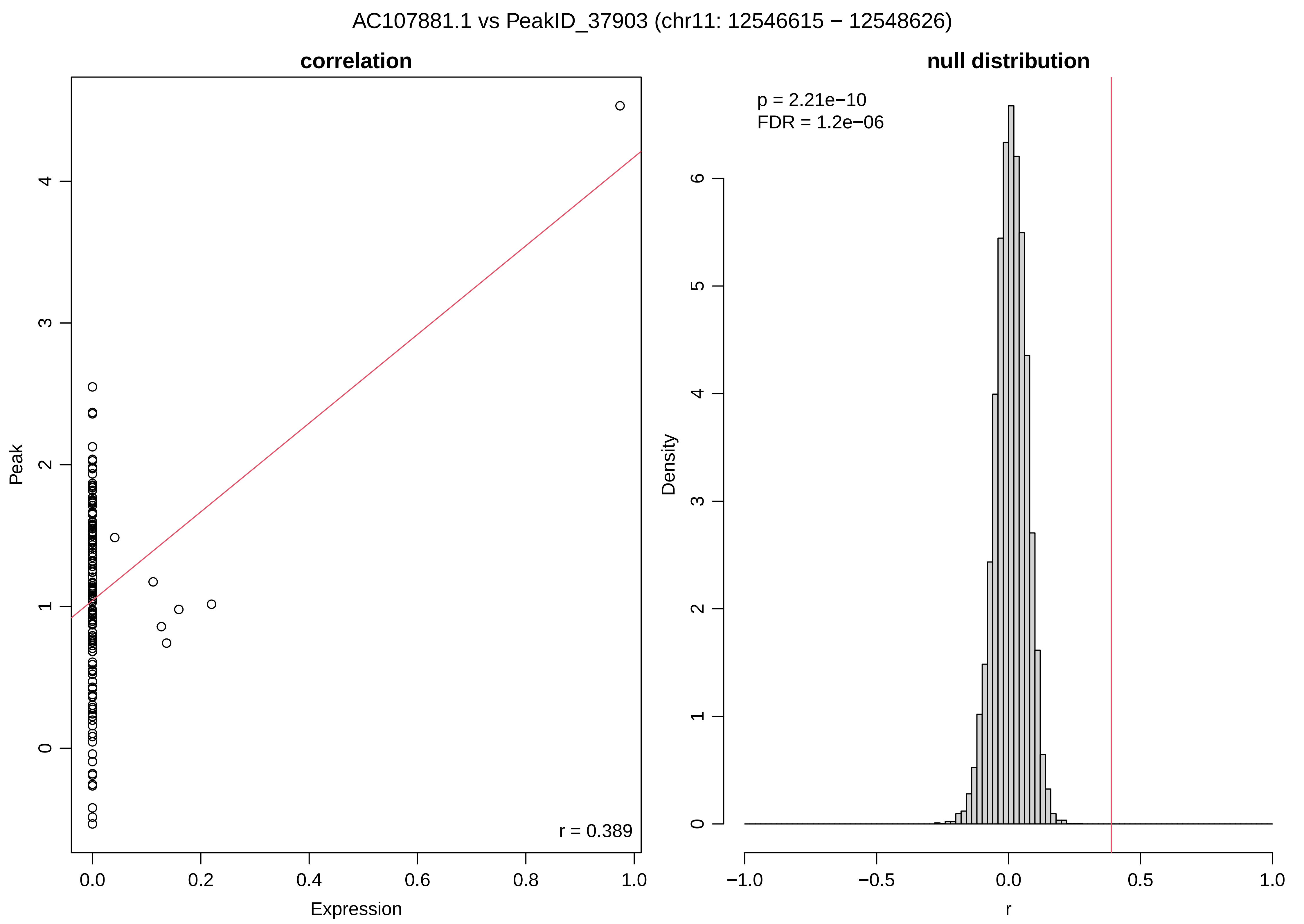
AC107881.1 vs PeakID_37903 (chr11: 12546615 − 12548626)
correlation
null distribution
Peak
Expression
r = 0.389
p = 2.21e−10
FDR = 1.2e−06
Density
r
0.0
0.2
0.4
0.6
0.8
1.0
−1.0
−0.5
0.5
0
1
2
3
4
5
6

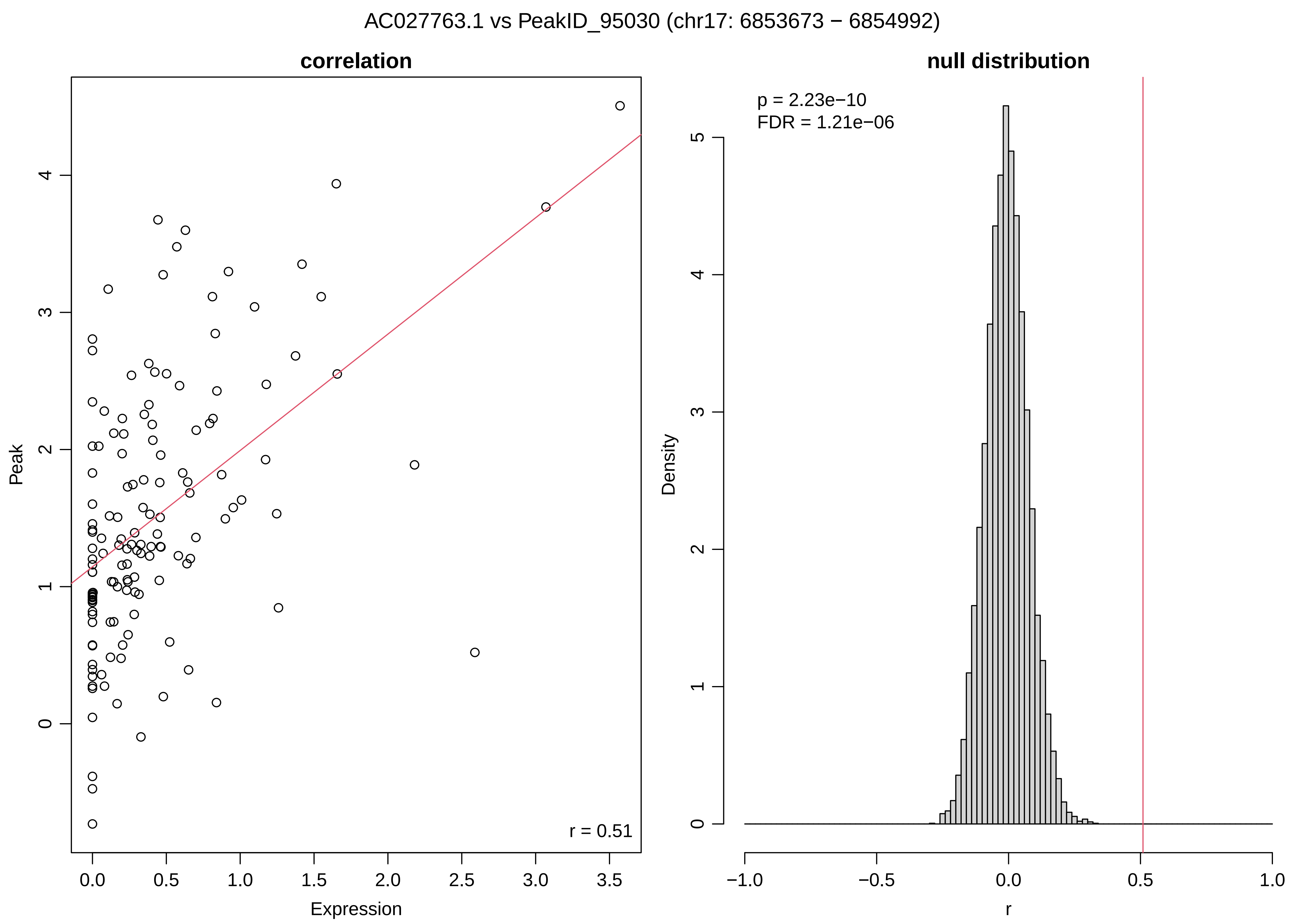

AC027763.1 vs PeakID_95030 (chr17: 6853673 − 6854992)
correlation
Peak
Expression
r = 0.51
null distribution
p = 2.23e−10
FDR = 1.21e−06
Density
r

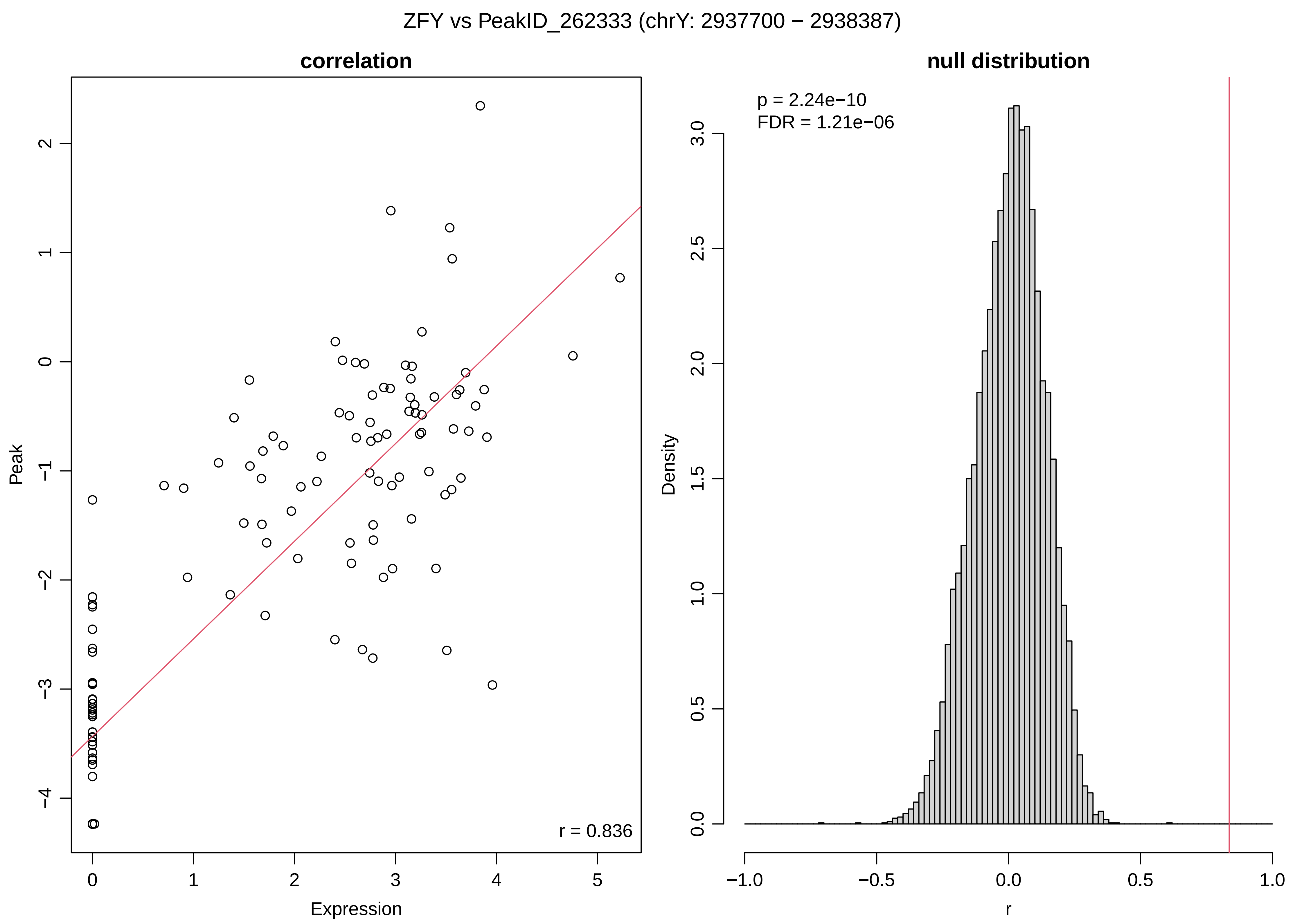

ZFY vs PeakID_262333 (chrY: 2937700 − 2938387)
correlation
Peak
Expression
r = 0.836
null distribution
p = 2.24e−10
FDR = 1.21e−06
Density
r

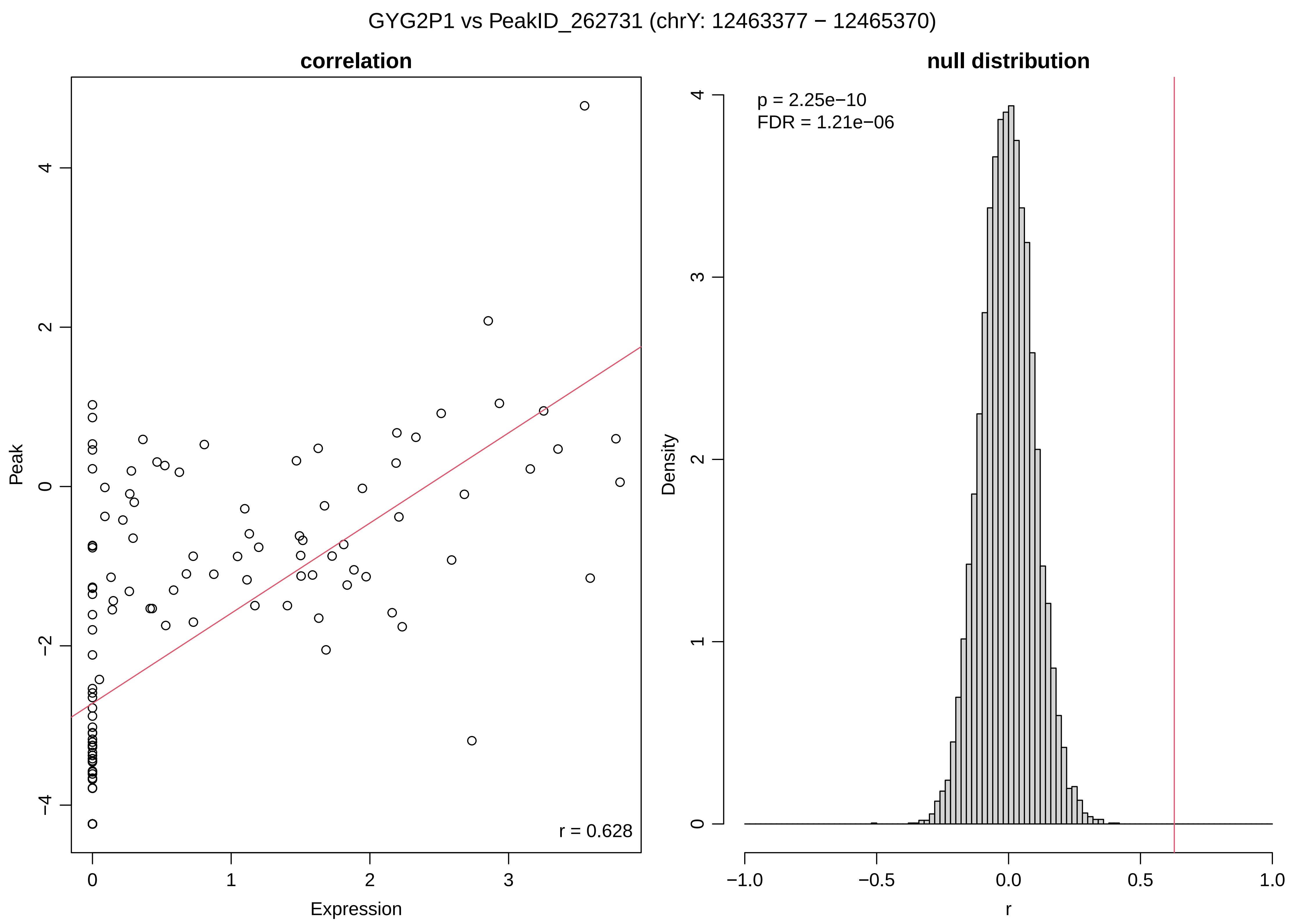
GYG2P1 vs PeakID_262731 (chrY: 12463377 − 12465370)
correlation
Peak
Expression
r = 0.628
null distribution
Density
r
p = 2.25e−10
FDR = 1.21e−06

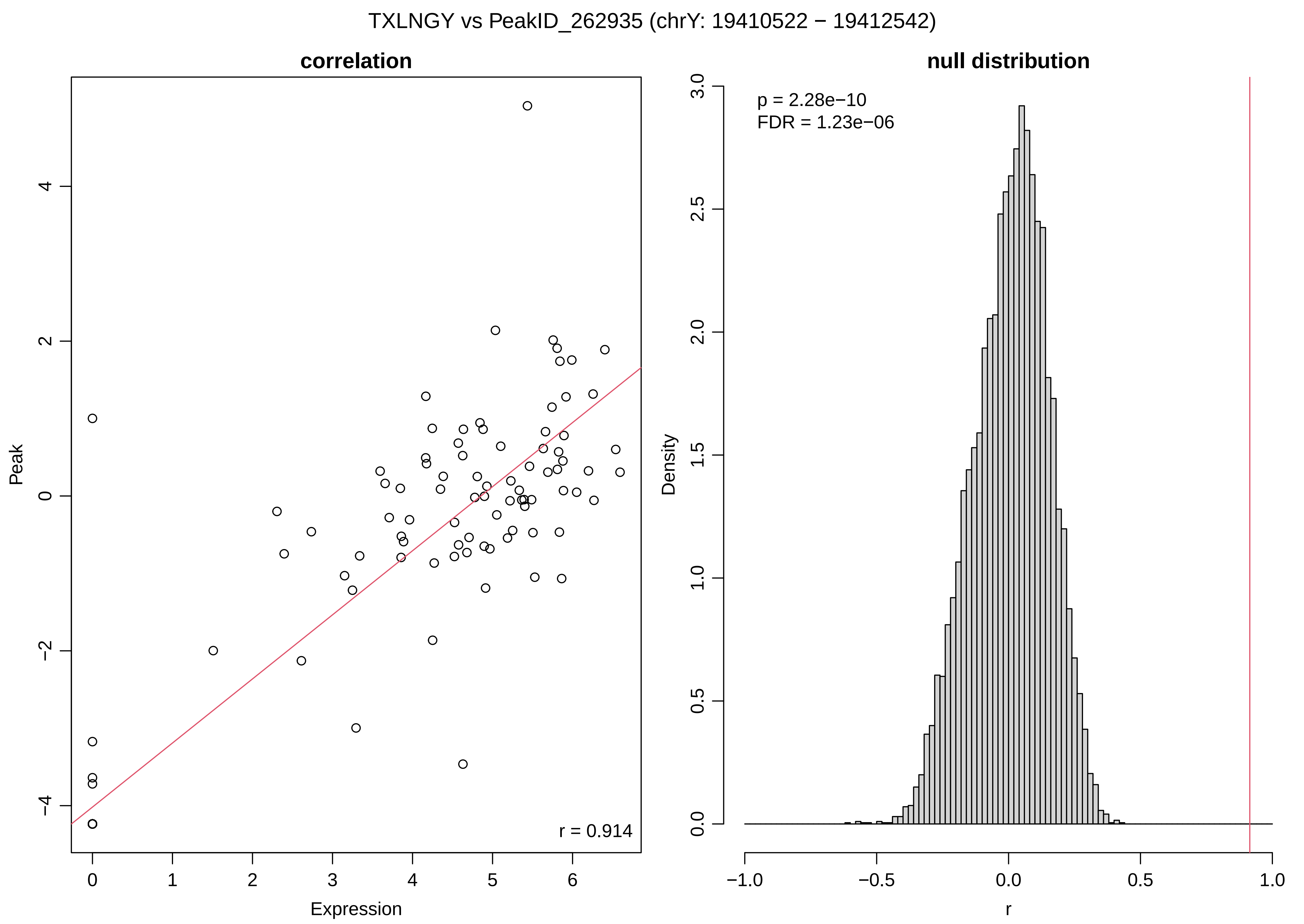

TXLNGY vs PeakID_262935 (chrY: 19410522 − 19412542)
correlation
Peak
Expression
r = 0.914
null distribution
Density
r
p = 2.28e−10
FDR = 1.23e−06

RNU4−20P vs PeakID_54812 (chr12: 59527588 − 59533679)

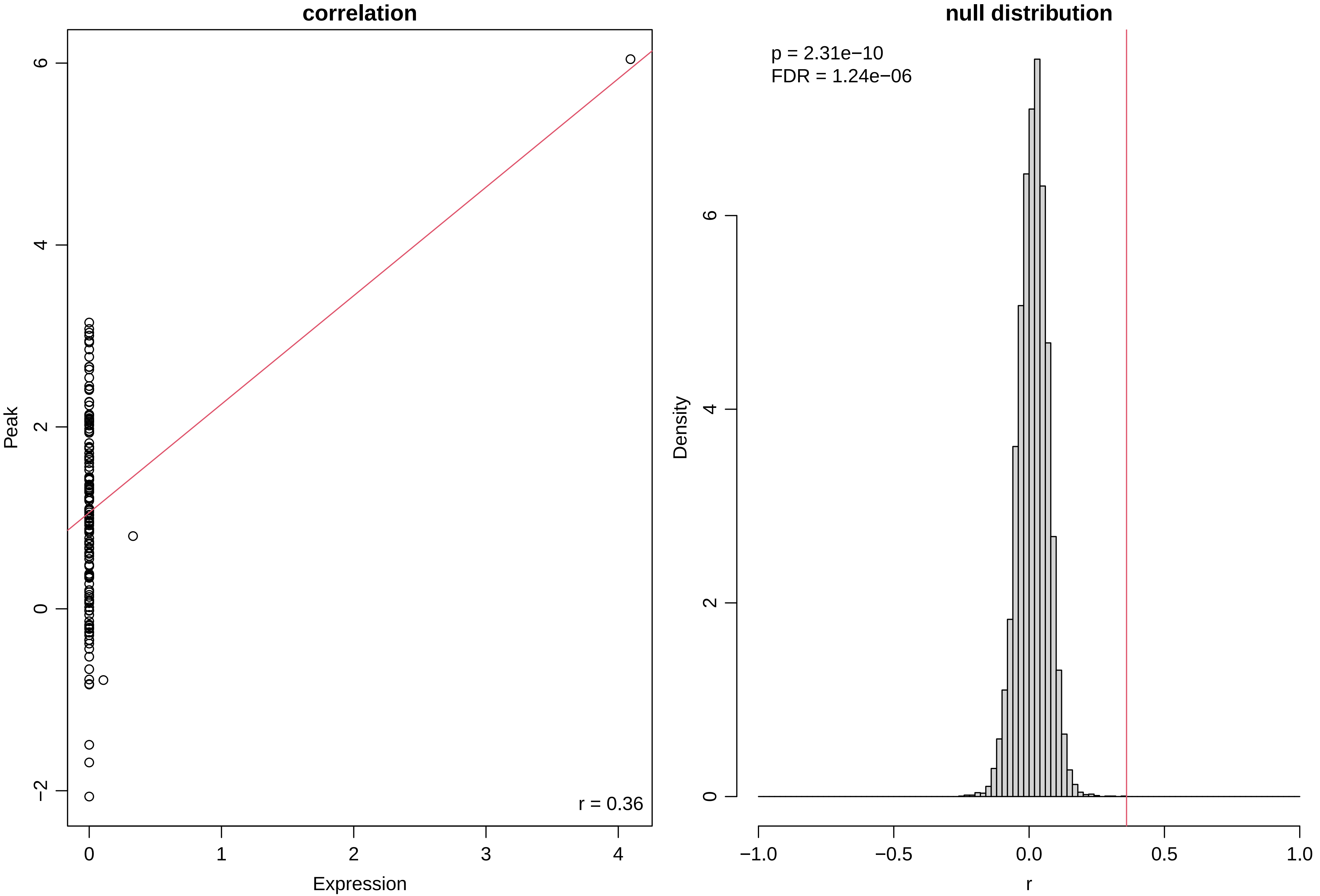

SLC35E3 vs PeakID_56591 (chr12: 68835569 − 68835796)

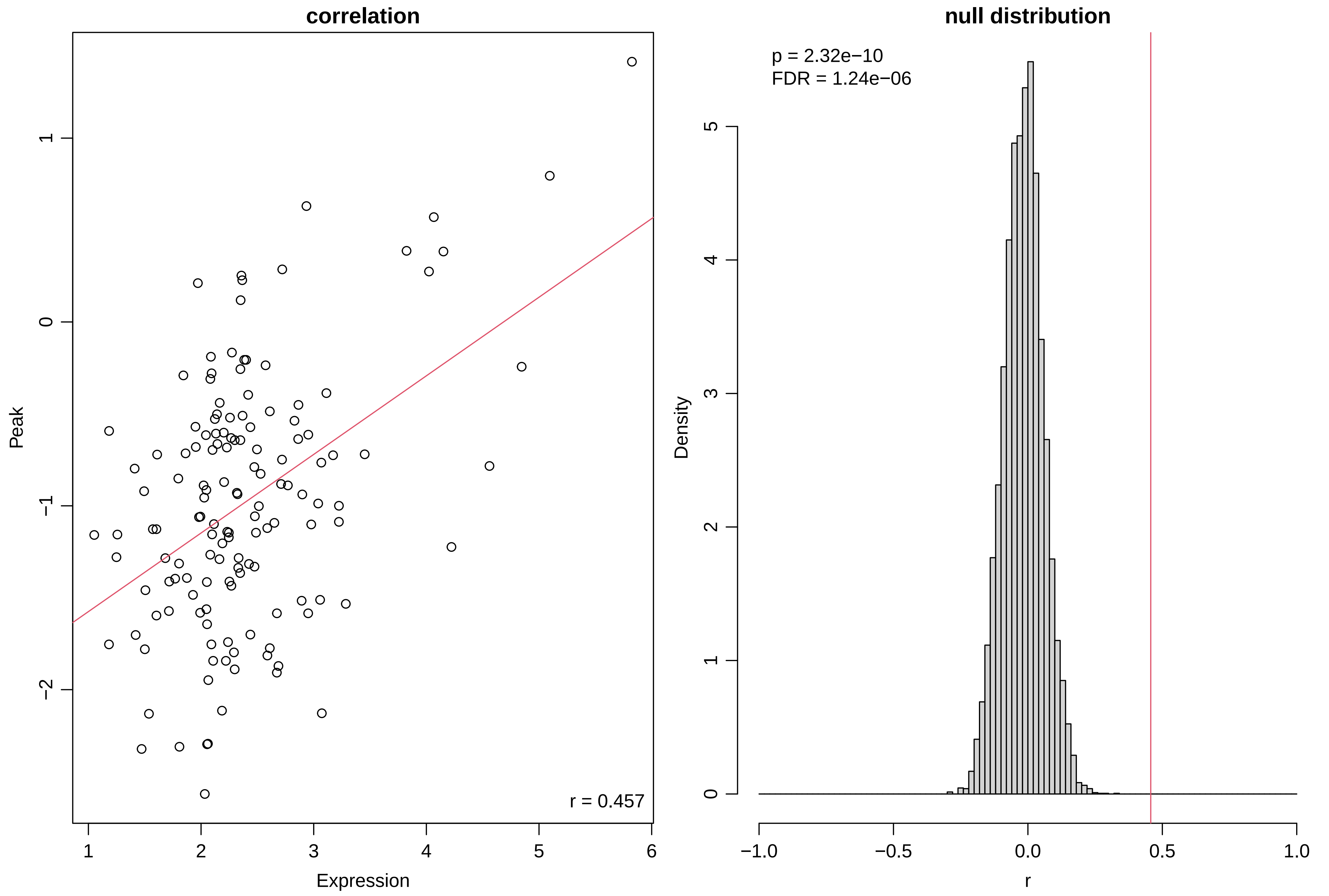

RPL21P104 vs PeakID_55446 (chr12: 61905621 − 61912279)

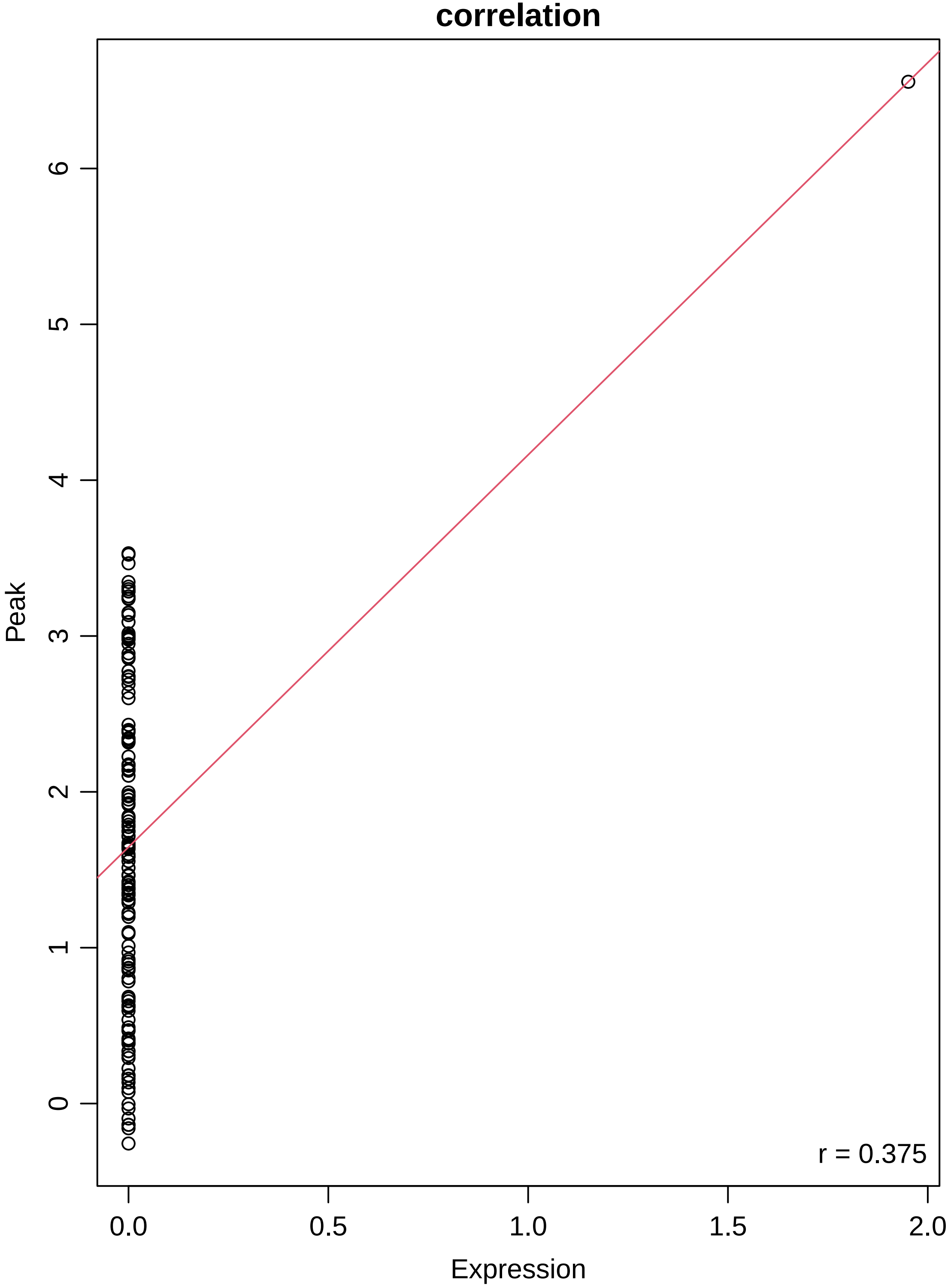

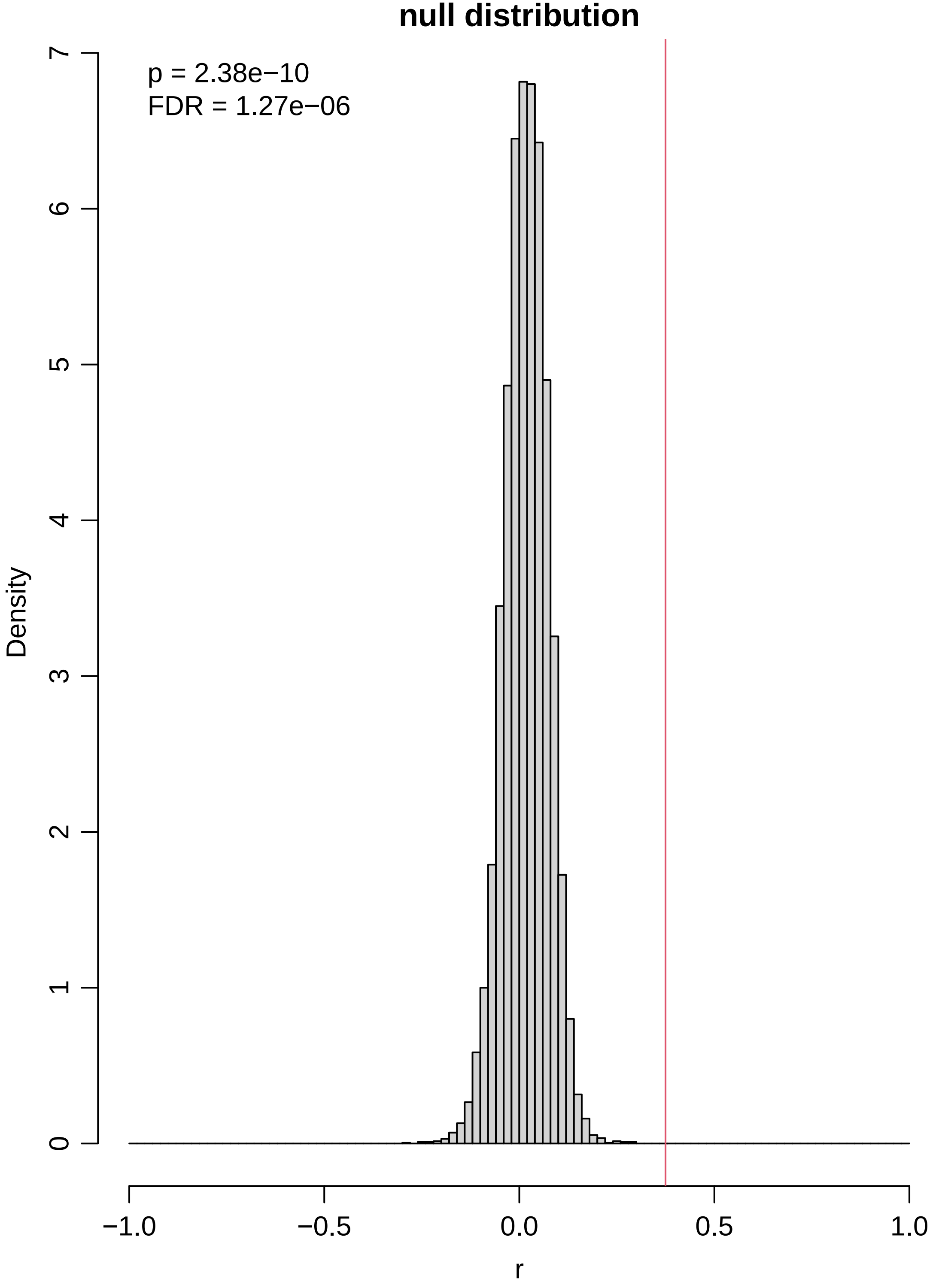

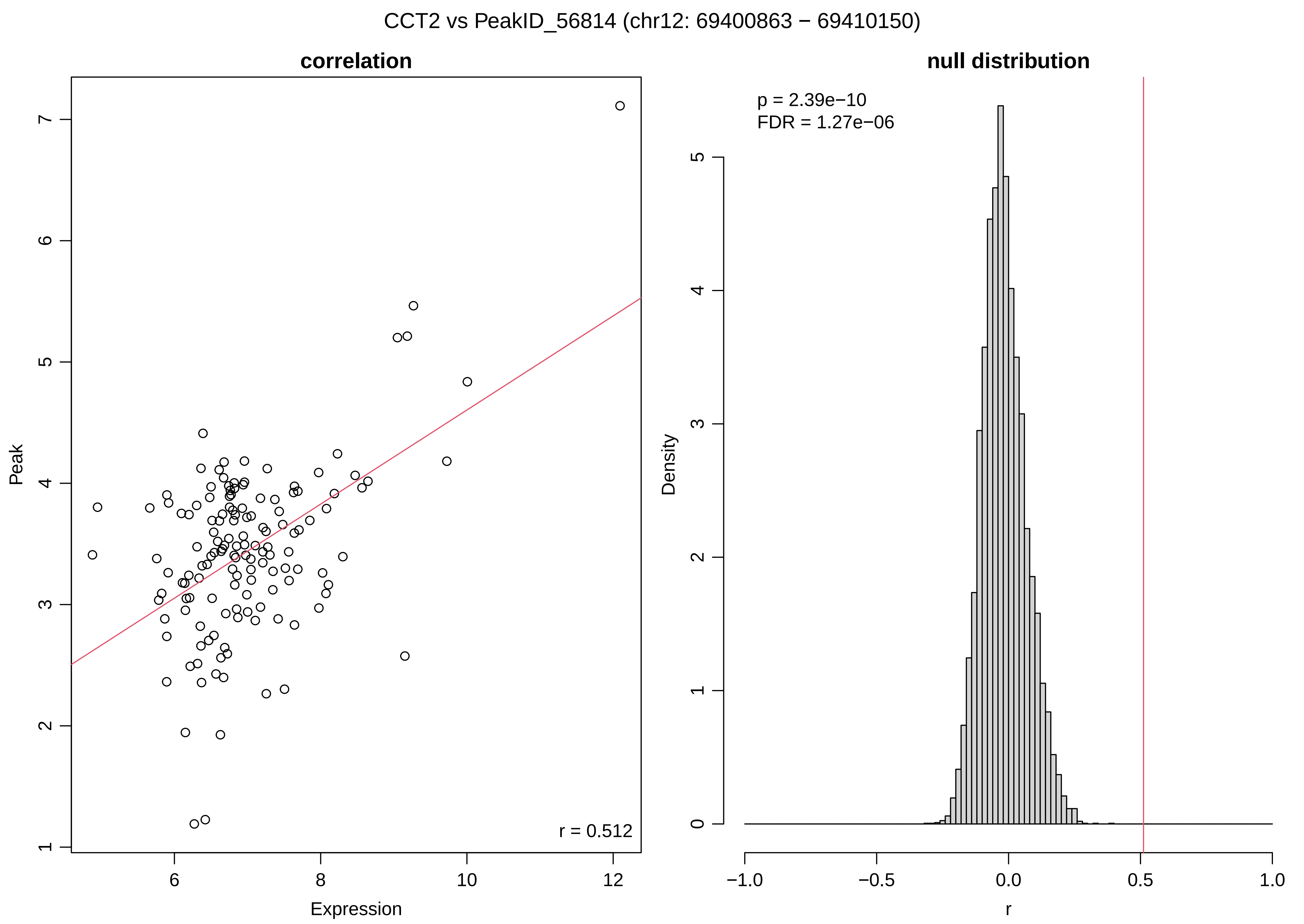

CCT2 vs PeakID_56814 (chr12: 69400863 − 69410150)
correlation
Peak
Expression
r = 0.512
null distribution
p = 2.39e−10
FDR = 1.27e−06
Density
r

AP000829.1 vs PeakID_106749 (chr18: 976333 − 977098)

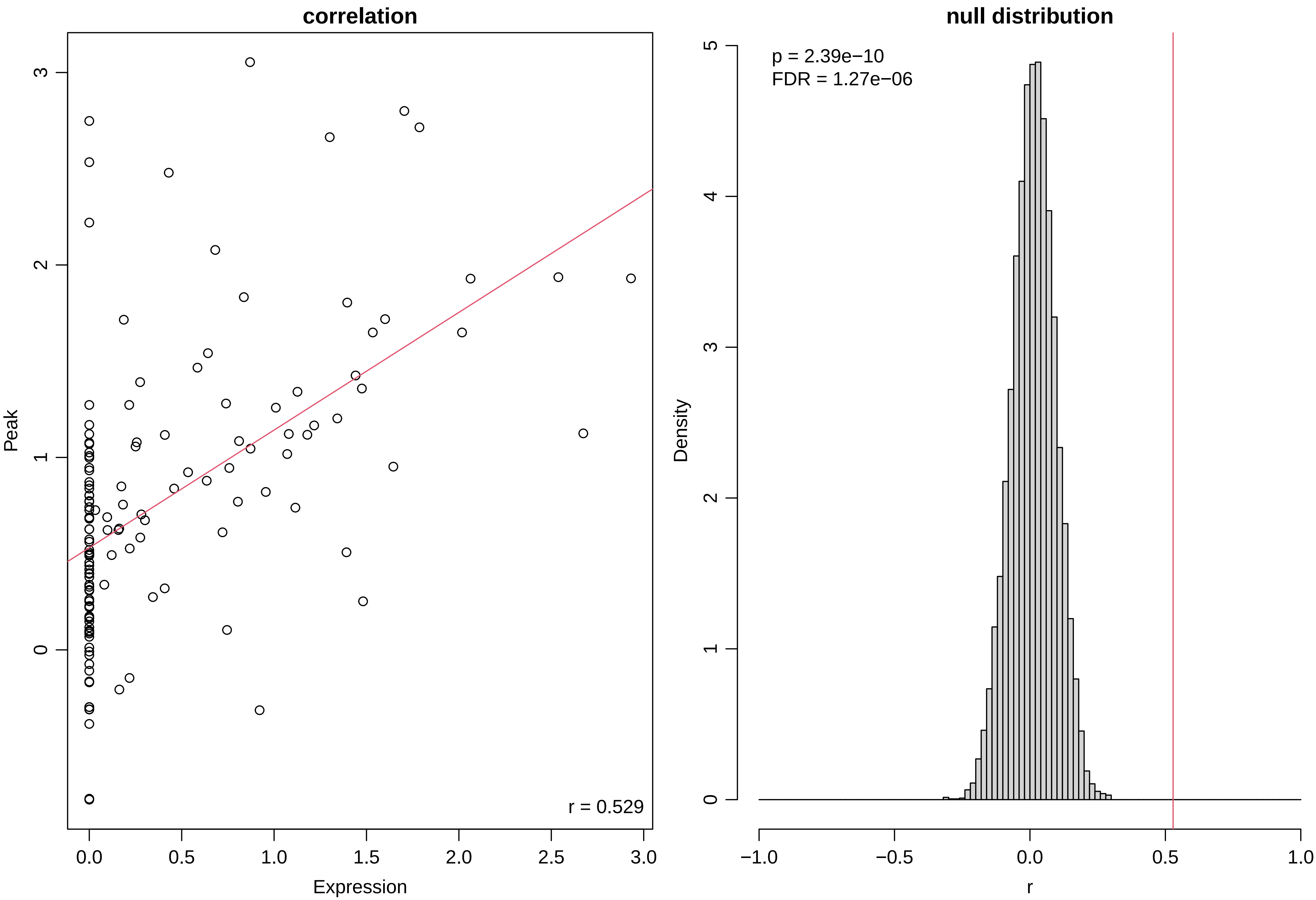

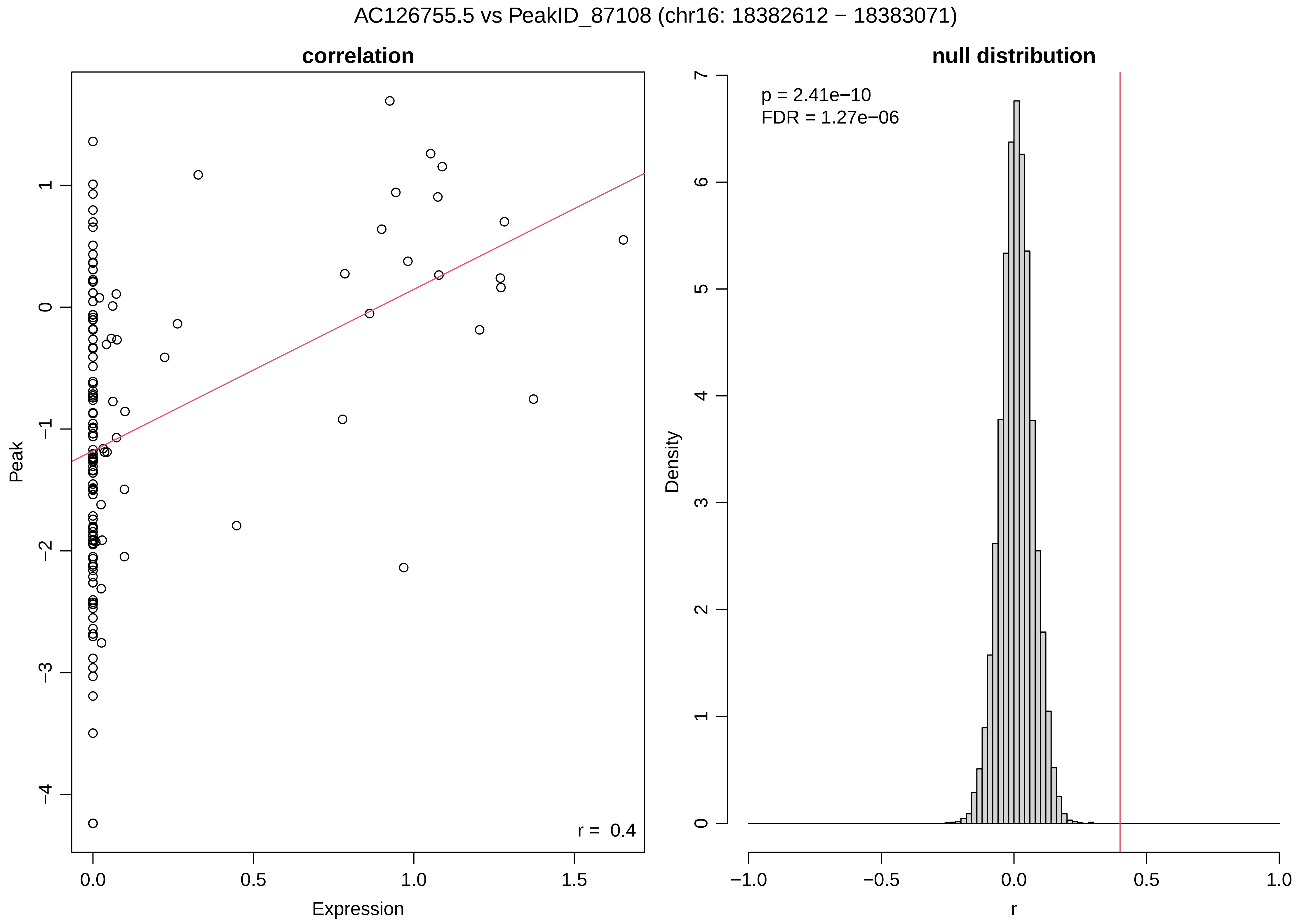
AC126755.5 vs PeakID_87108 (chr16: 18382612 − 18383071)
correlation
Peak
Expression
r = 0.4
null distribution
p = 2.41e−10
FDR = 1.27e−06
Density
r

LINC01167 vs PeakID_36057 (chr10: 132964229 − 132965794)

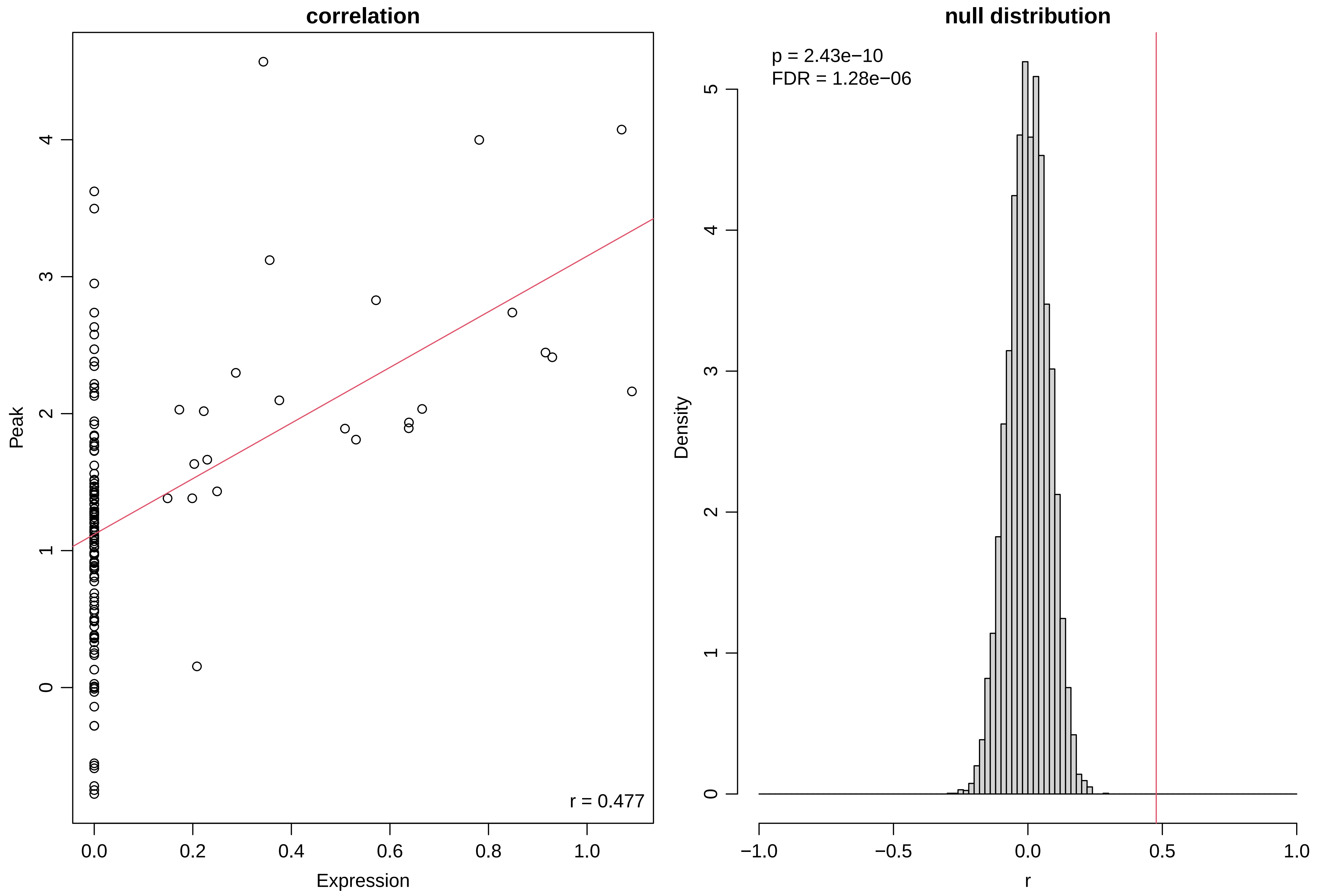

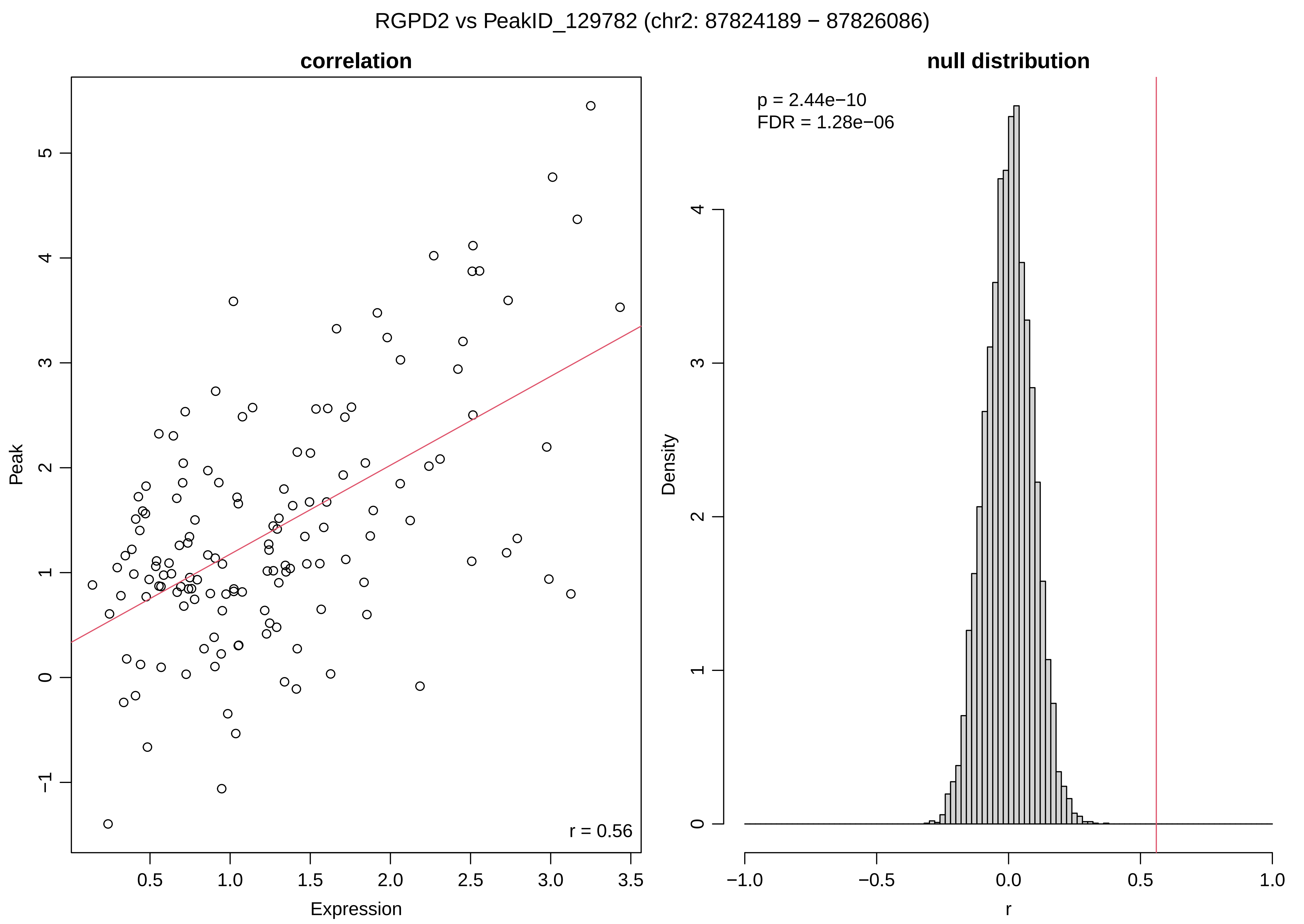
RGPD2 vs PeakID_129782 (chr2: 87824189 − 87826086)
correlation
null distribution
p = 2.44e−10
FDR = 1.28e−06
r = 0.56
Peak
Expression
Density
r
−1
0
1
2
3
4
5
0.5
1.0
1.5
2.0
2.5
3.0
3.5
−1.0
−0.5
0.0
0.5
1.0

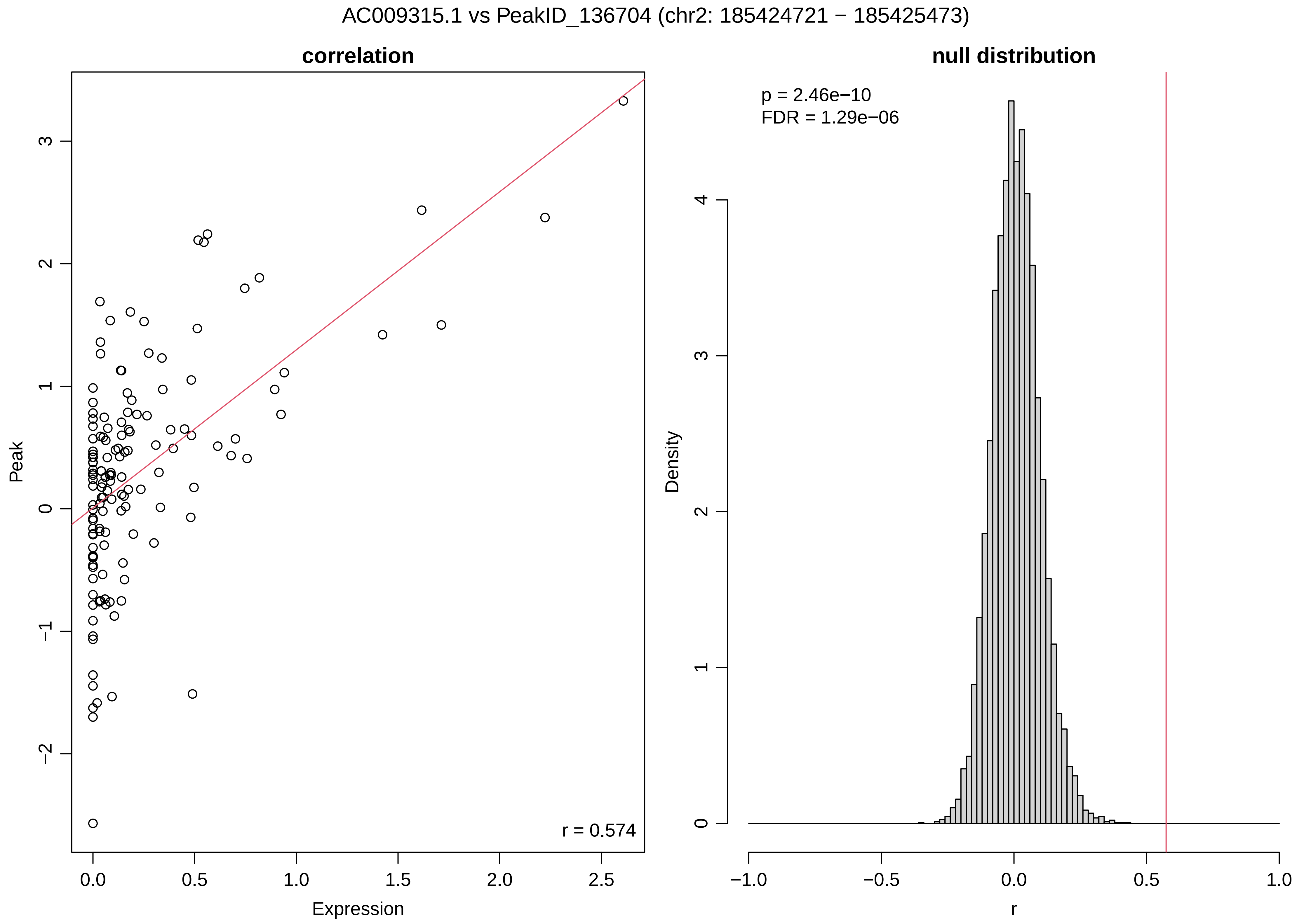
AC009315.1 vs PeakID_136704 (chr2: 185424721 − 185425473)
correlation
Peak
Expression
r = 0.574
null distribution
p = 2.46e−10
FDR = 1.29e−06
Density
r

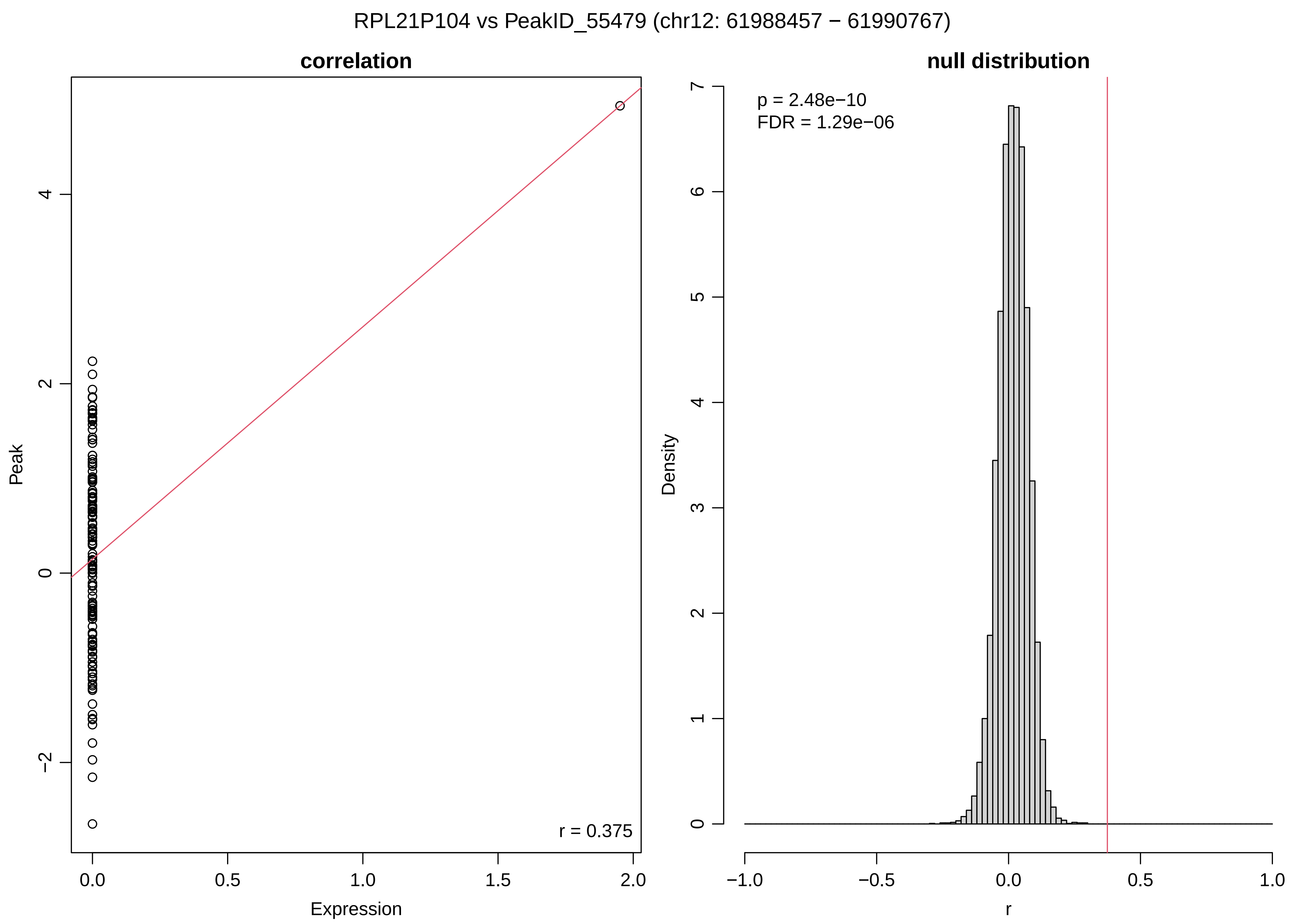
RPL21P104 vs PeakID_55479 (chr12: 61988457 − 61990767)
correlation
null distribution
p = 2.48e−10
FDR = 1.29e−06
r = 0.375
Peak
Expression
Density
r
−2
0
2
4
0.0
0.5
1.0
1.5
2.0
0
1
2
3
4
5
6
7
−1.0
−0.5
0.0
0.5
1.0

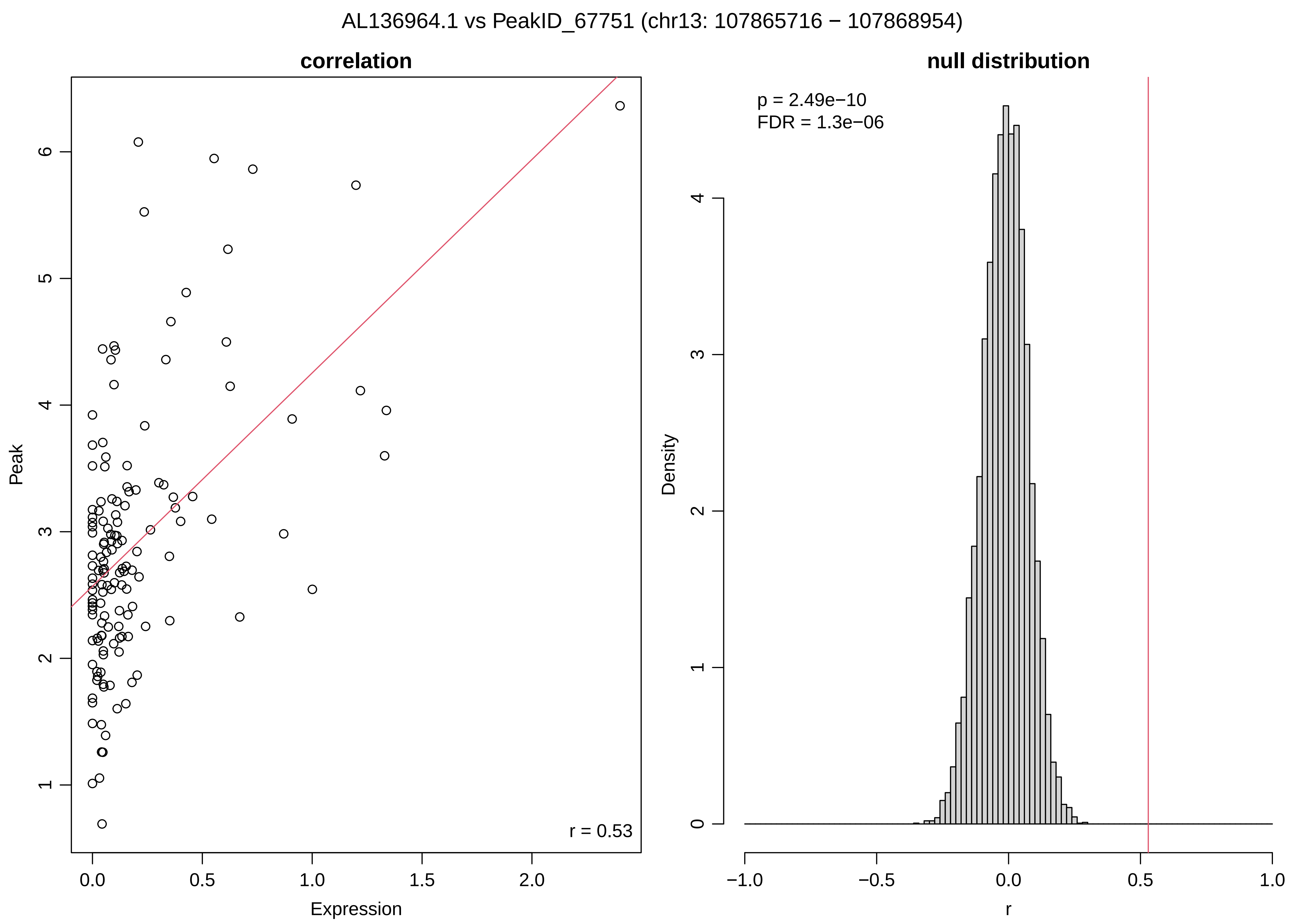
AL136964.1 vs PeakID_67751 (chr13: 107865716 − 107868954)
correlation
null distribution
p = 2.49e−10
FDR = 1.3e−06
r = 0.53
Peak
Expression
Density
r

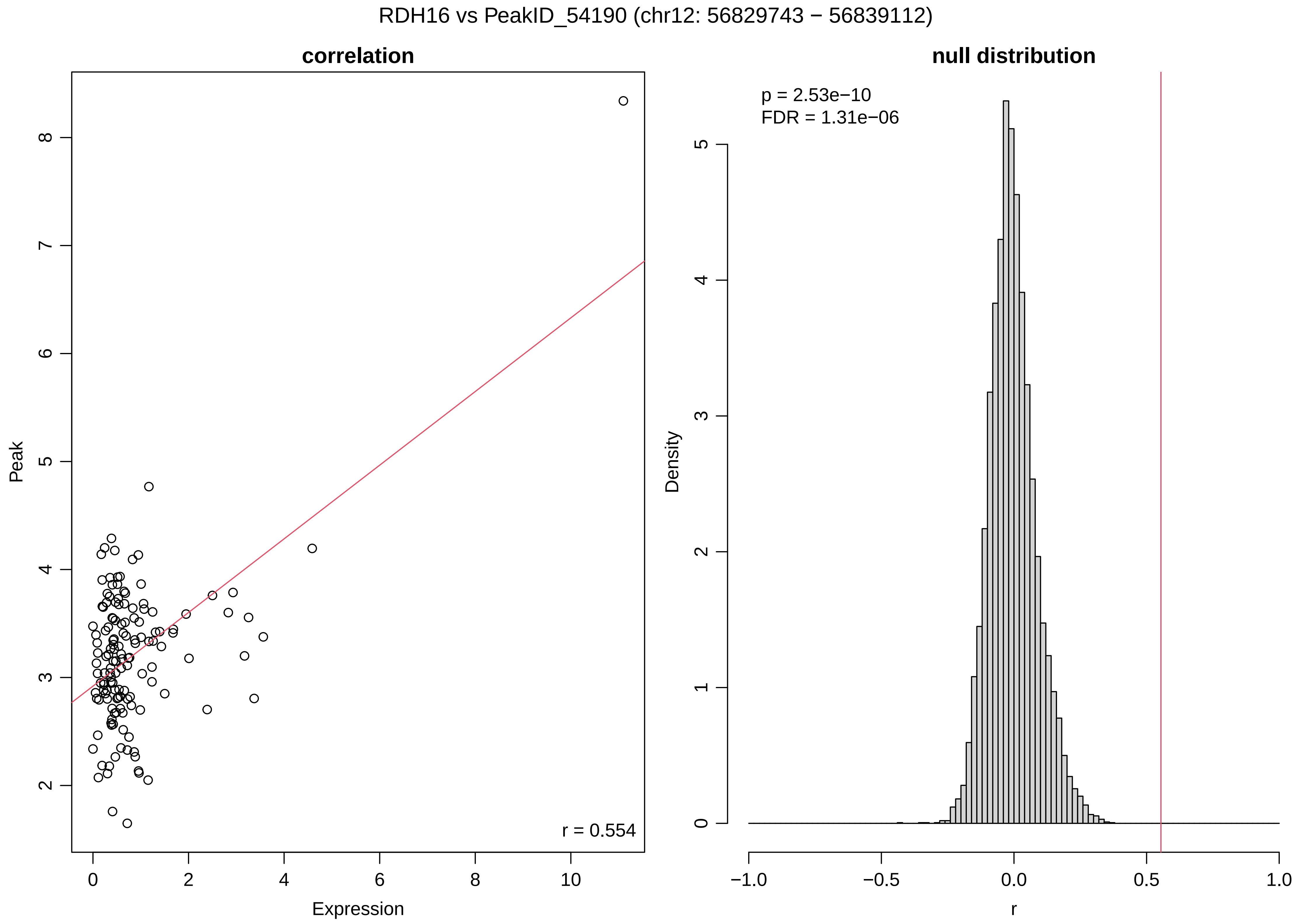

RDH16 vs PeakID_54190 (chr12: 56829743 − 56839112)
correlation
Peak
Expression
r = 0.554
null distribution
p = 2.53e−10
FDR = 1.31e−06
Density
r

AC092634.4 vs PeakID_224811 (chr7: 63925076 − 63927554)

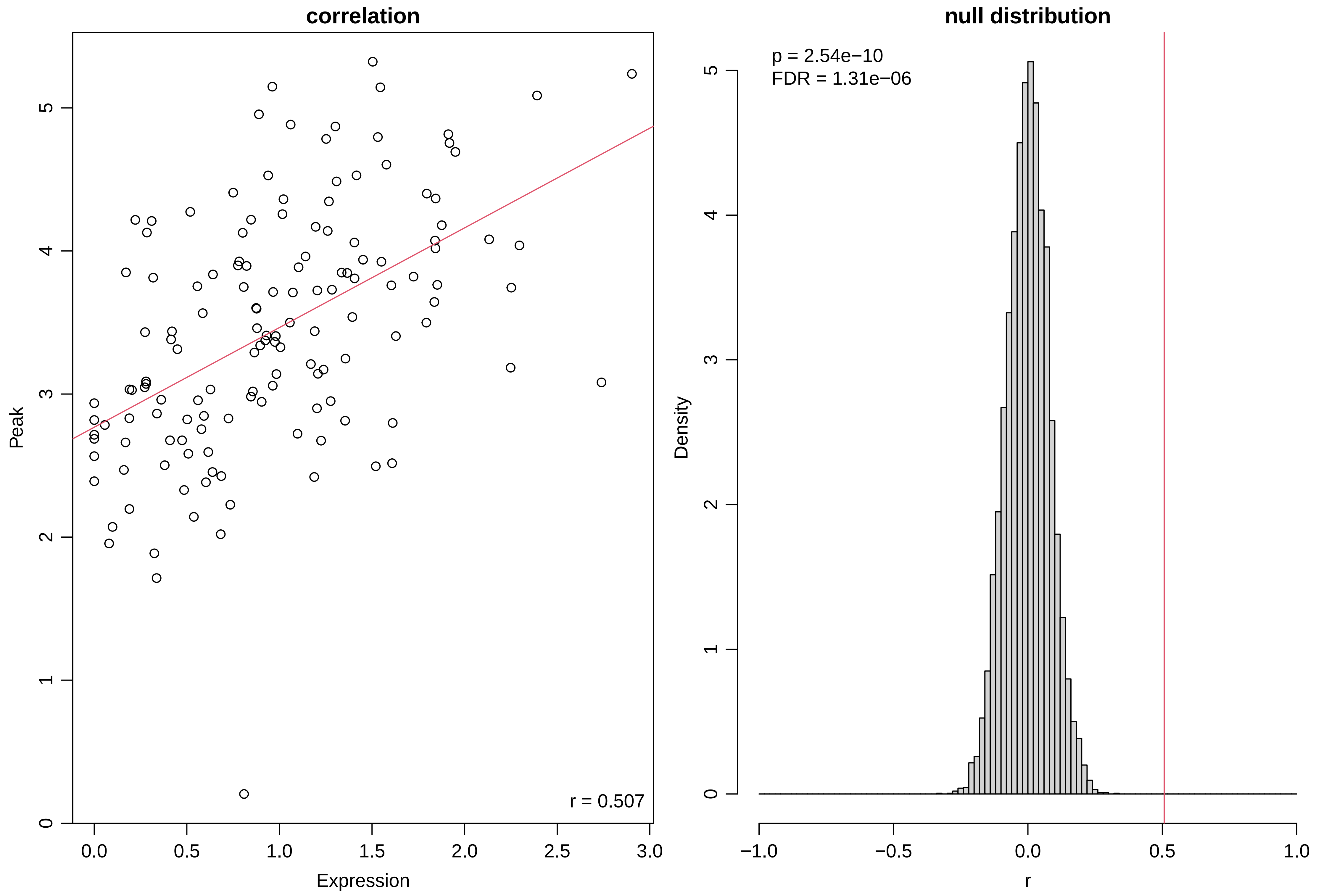

SYCP2L vs PeakID_202485 (chr6: 10886089 − 10887804)

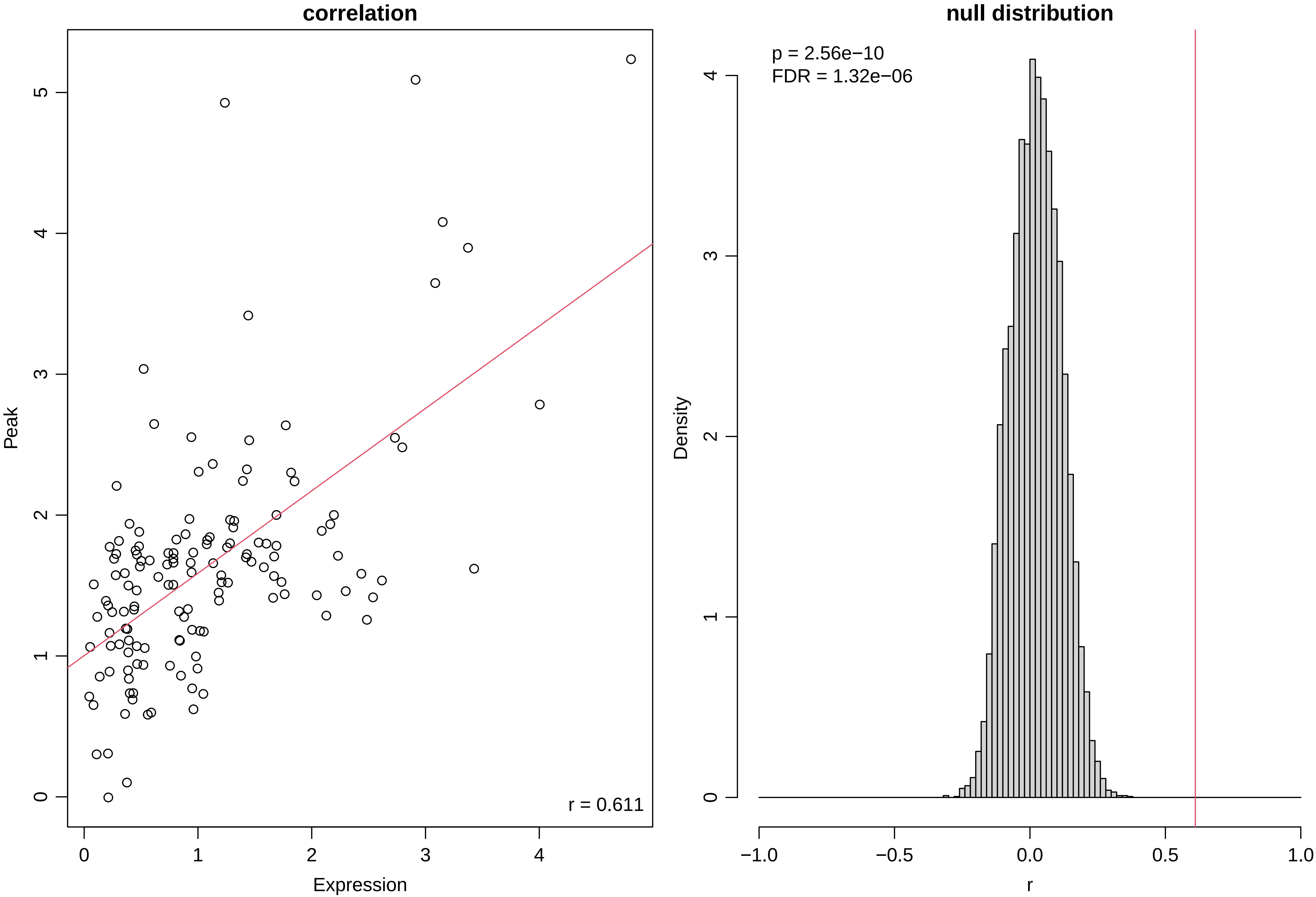

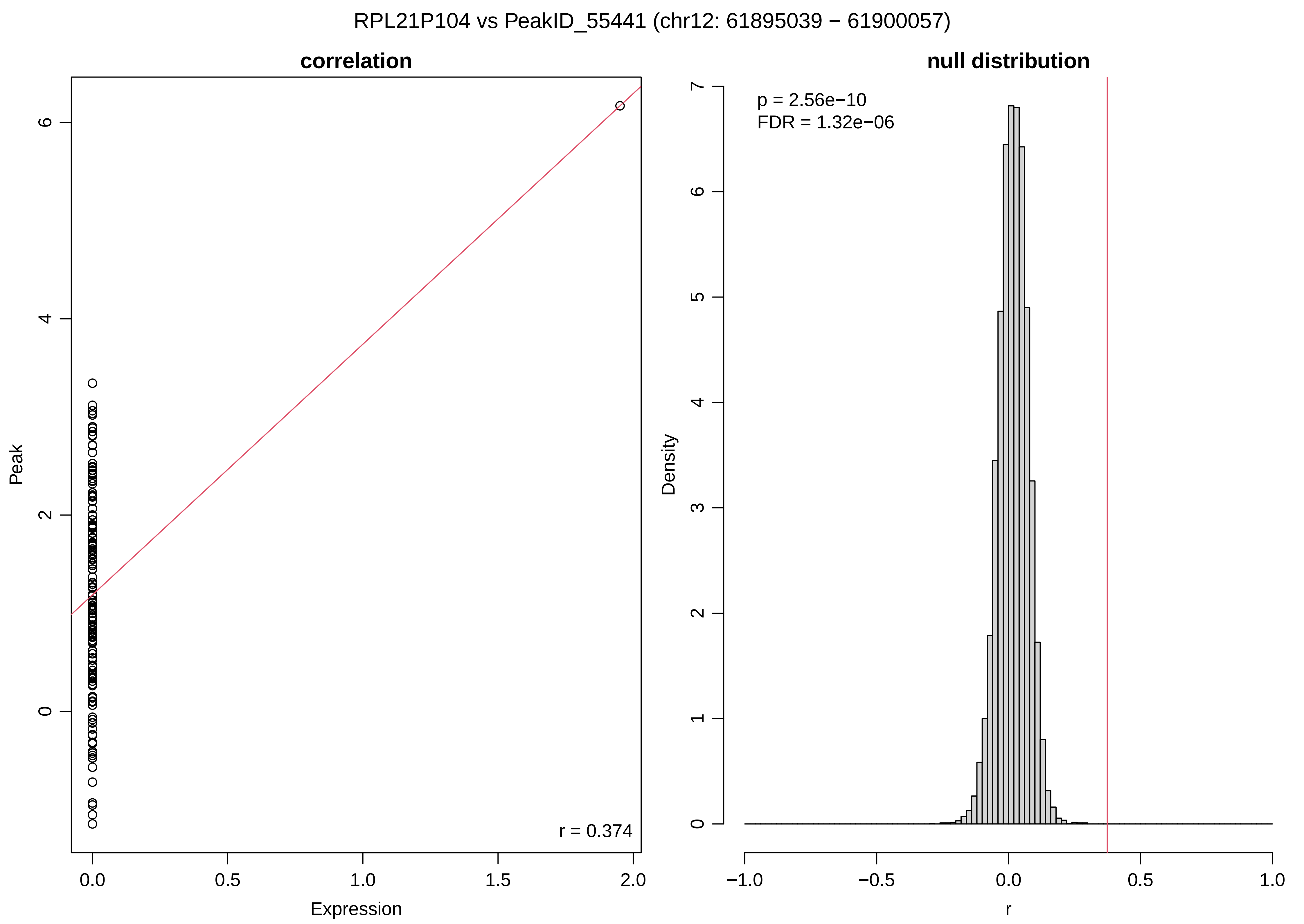

RPL21P104 vs PeakID_55441 (chr12: 61895039 − 61900057)
correlation
Peak
Expression
r = 0.374
0.0
0.5
1.0
1.5
2.0
0
2
4
6
null distribution
p = 2.56e−10
FDR = 1.32e−06
Density
r
−1.0
−0.5
0.0
0.5
1.0
0
1
2
3
4
5
6
7

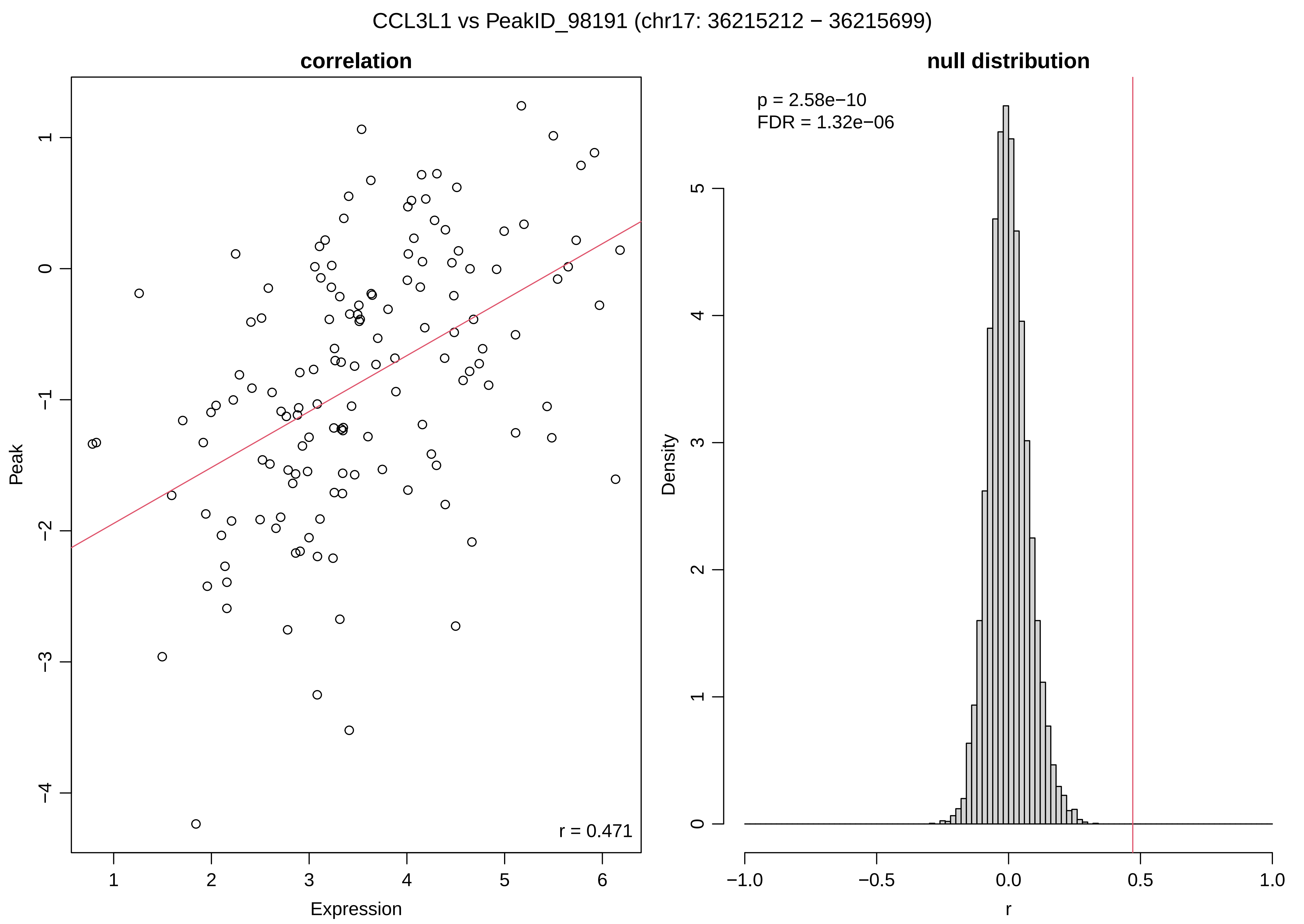
CCL3L1 vs PeakID_98191 (chr17: 36215212 − 36215699)
correlation
Peak
Expression
r = 0.471
null distribution
p = 2.58e−10
FDR = 1.32e−06
Density
r

UTY vs PeakID_262754 (chrY: 13493128 − 13494524)

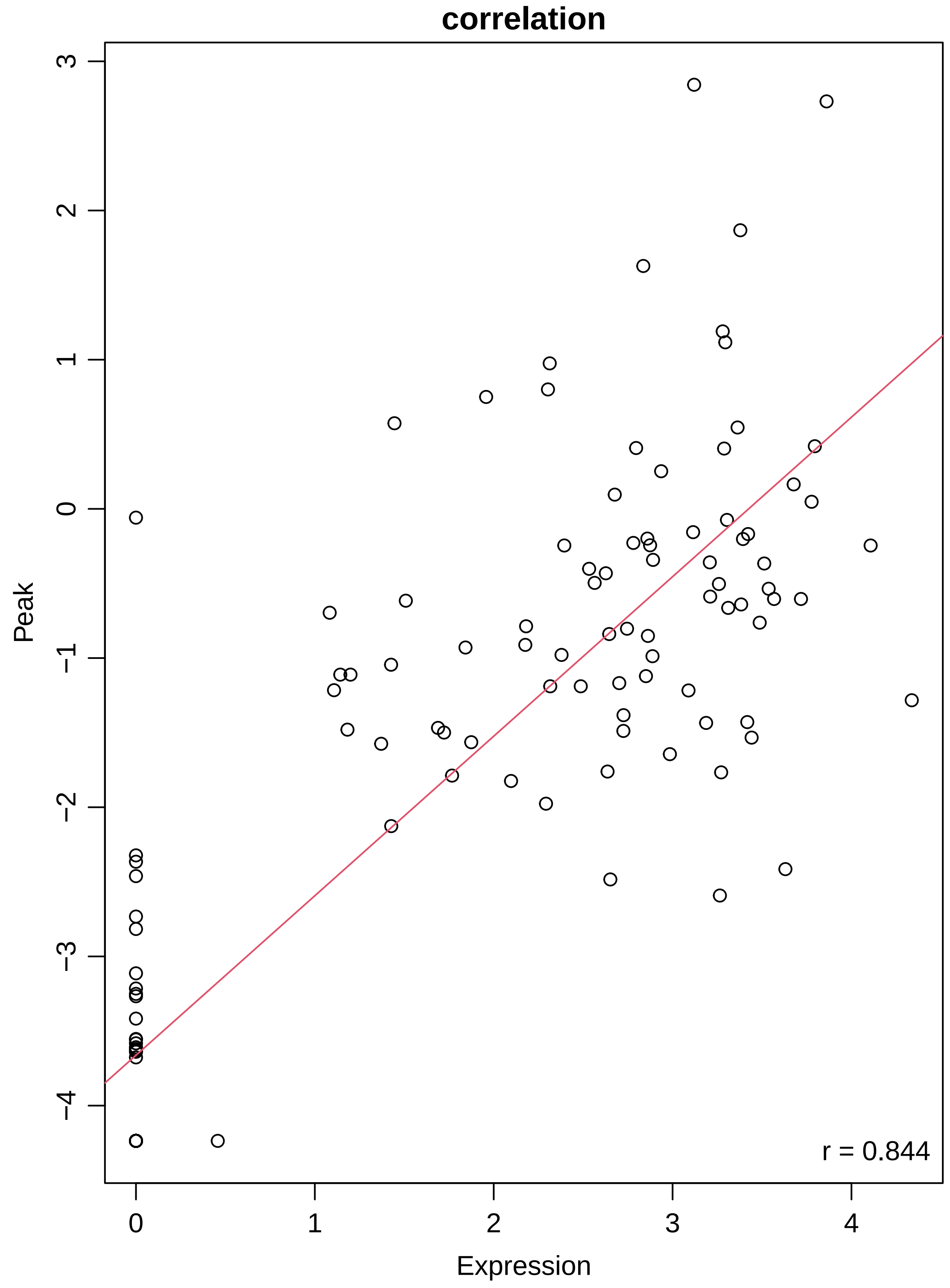

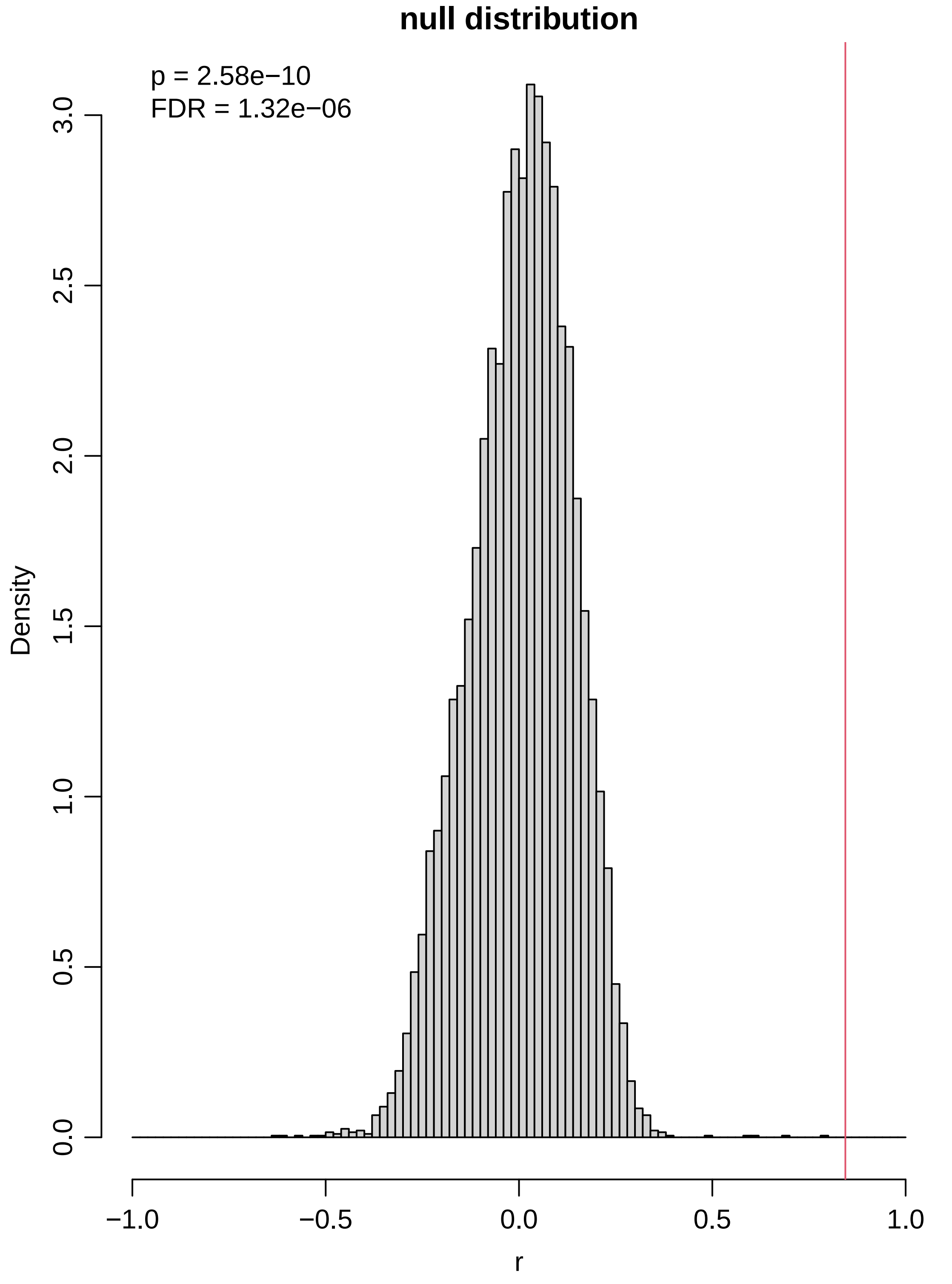

AC002066.1 vs PeakID_228963 (chr7: 116425738 − 116427739)

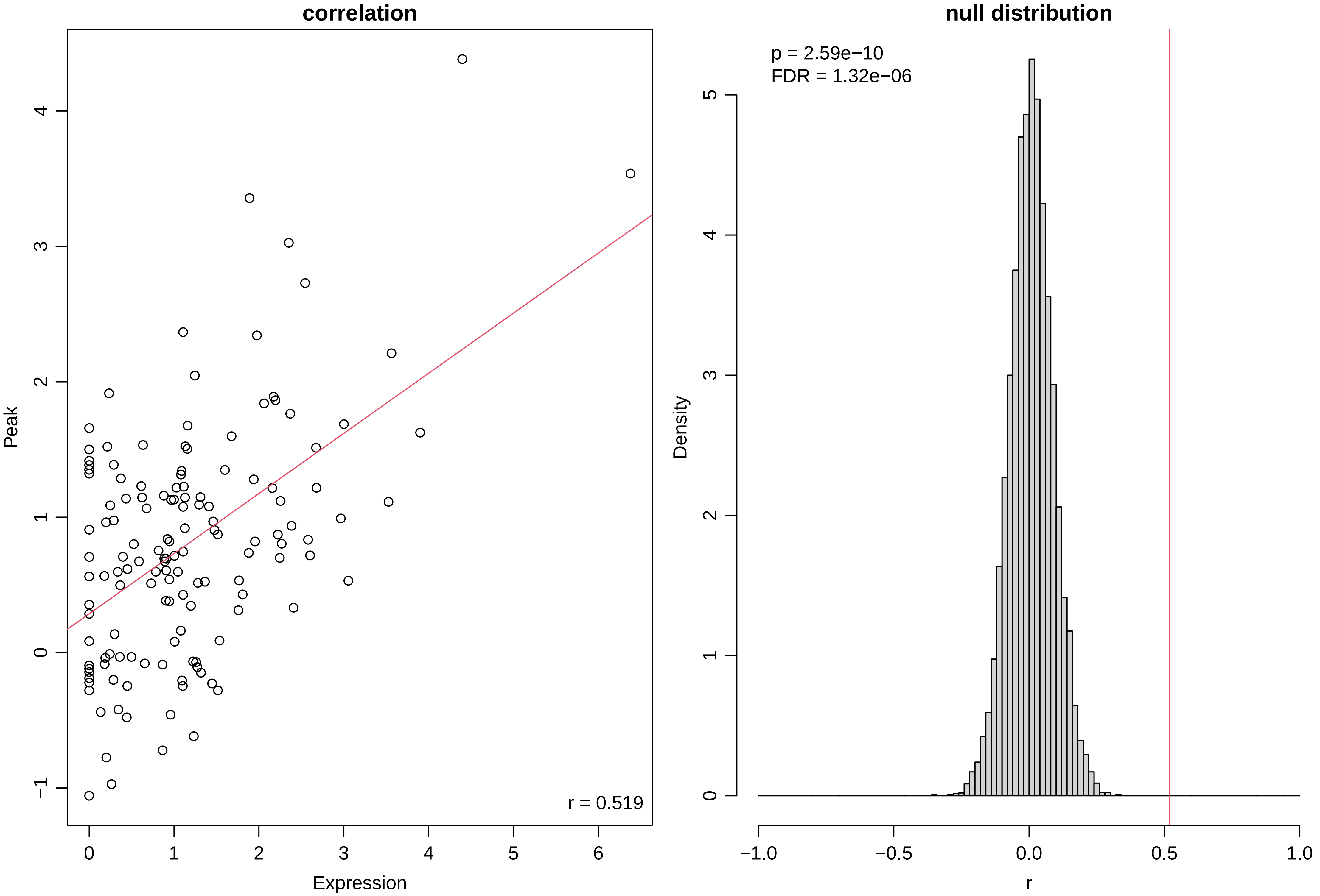

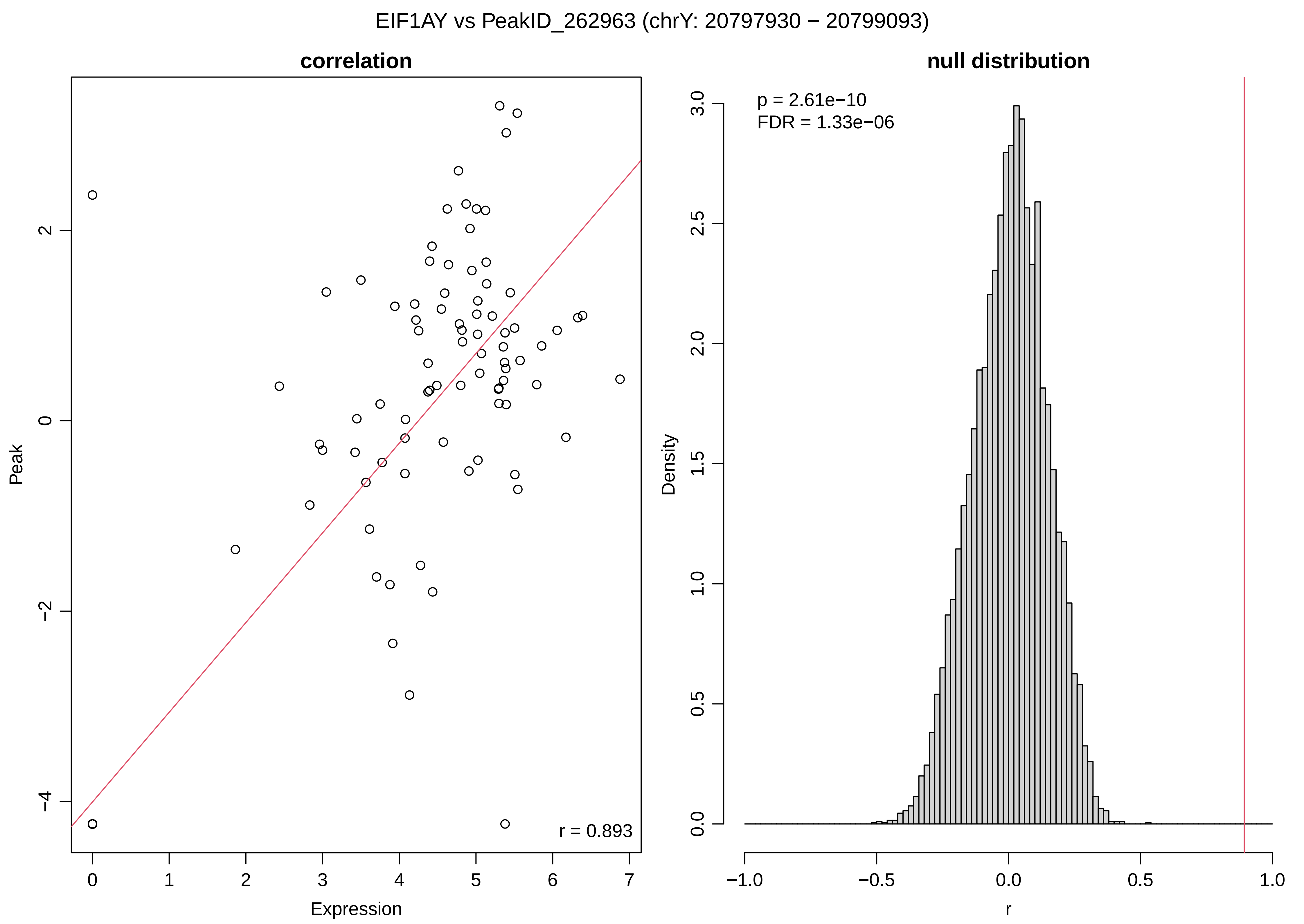
EIF1AY vs PeakID_262963 (chrY: 20797930 − 20799093)
correlation
null distribution
p = 2.61e−10
FDR = 1.33e−06
r = 0.893
Peak
Expression
Density
r

RIMKLB vs PeakID_49602 (chr12: 8679813 − 8685283)

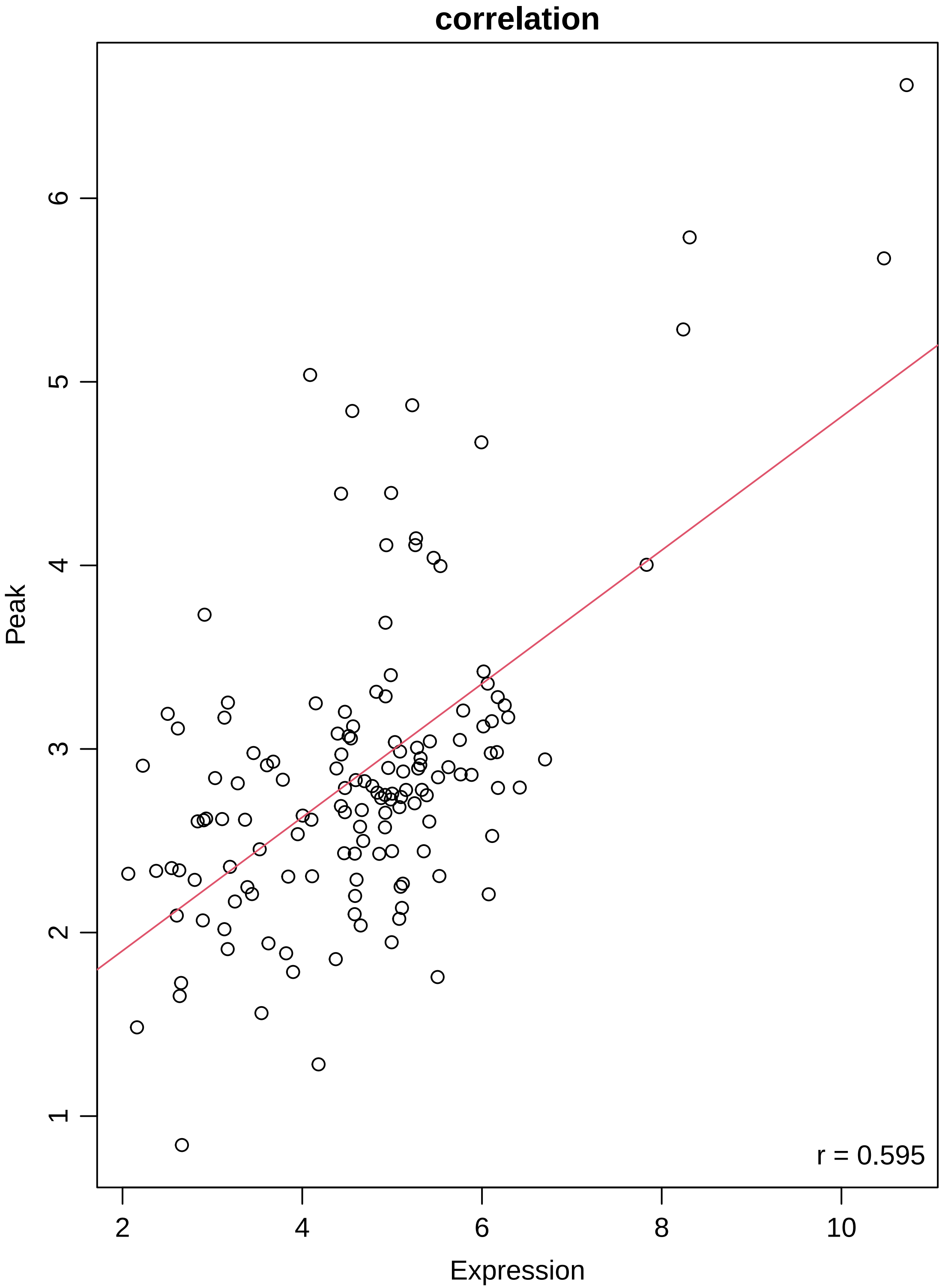

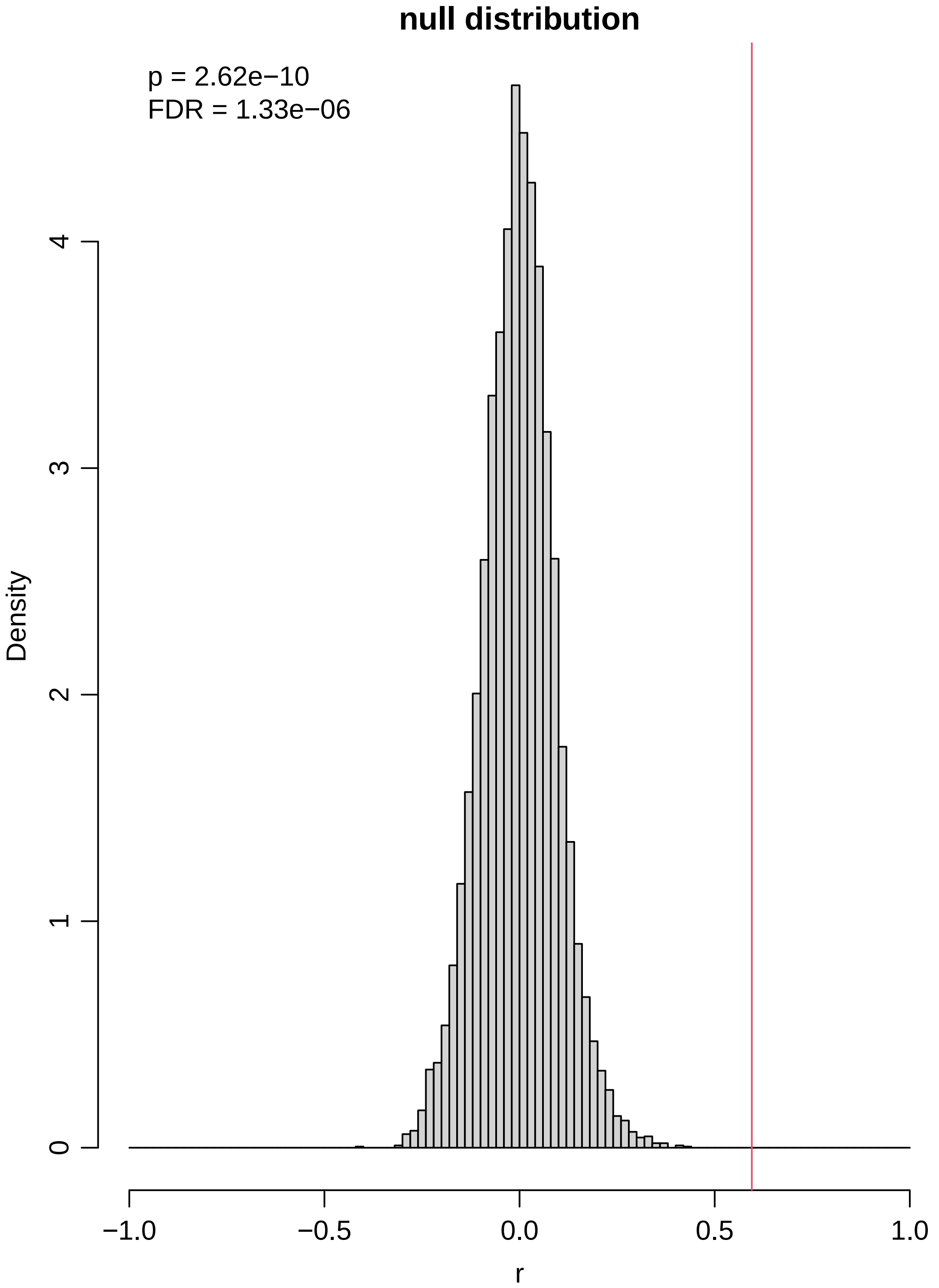

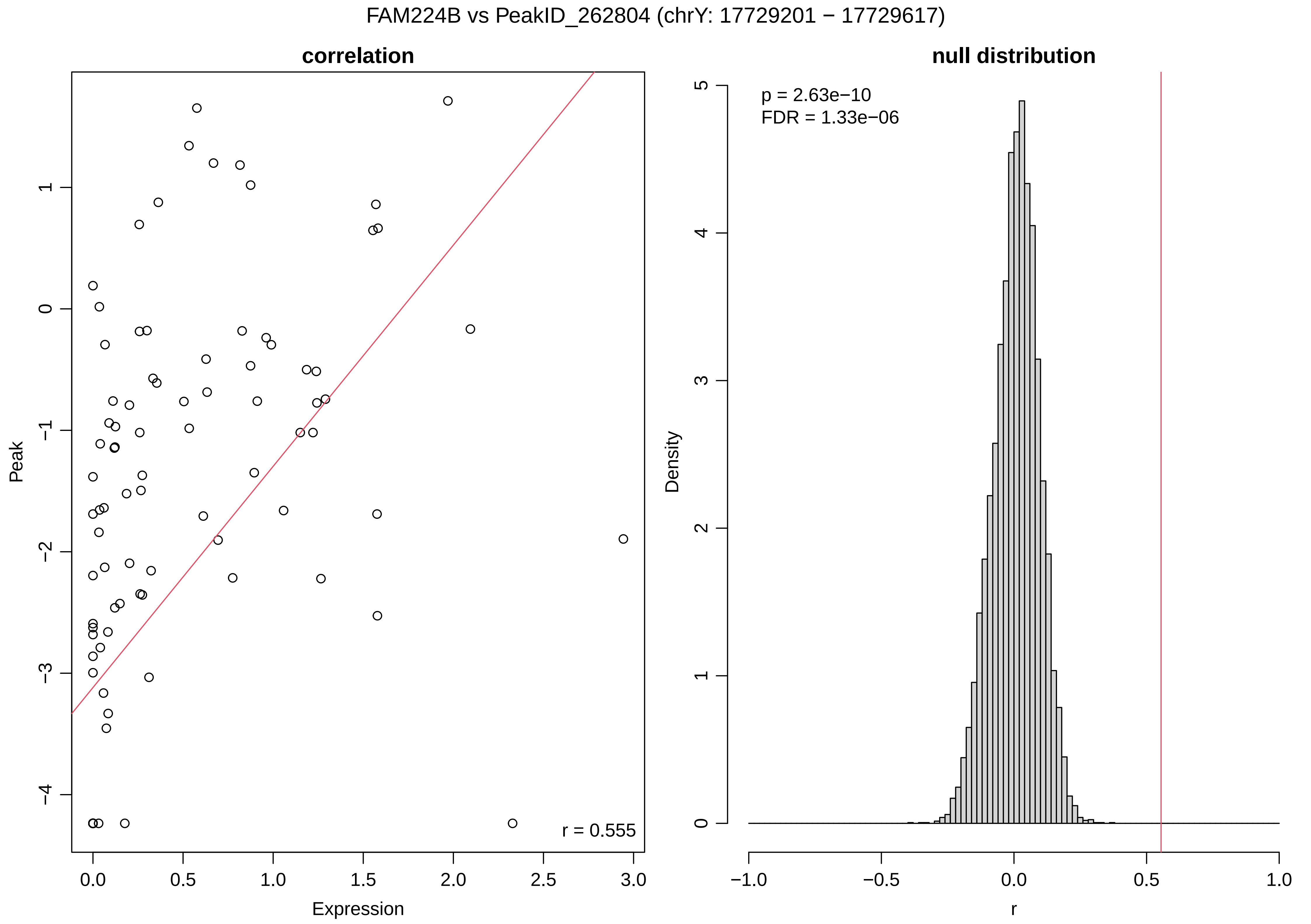
FAM224B vs PeakID_262804 (chrY: 17729201 − 17729617)
correlation
Peak
Expression
r = 0.555
null distribution
Density
r
p = 2.63e−10
FDR = 1.33e−06

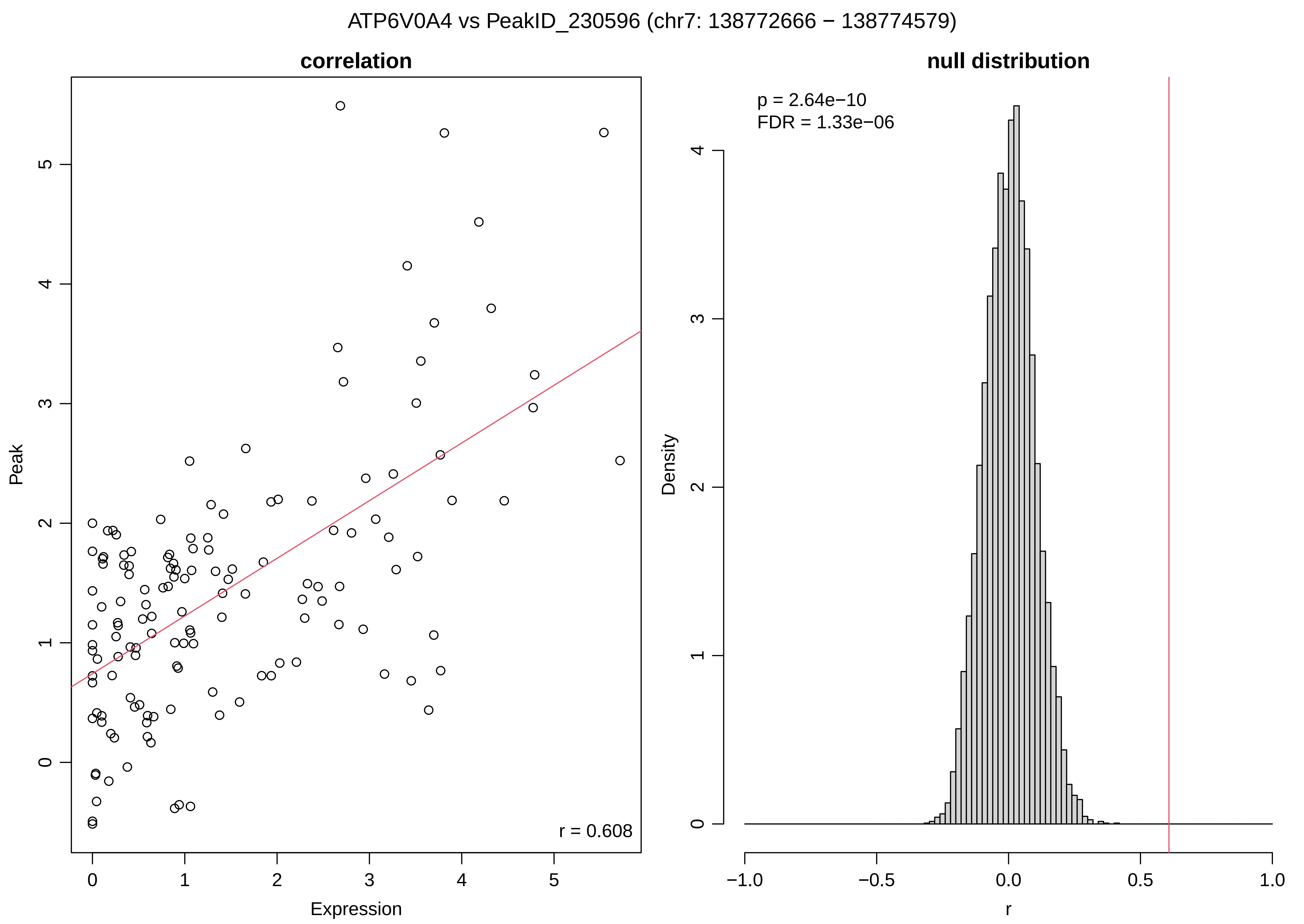
ATP6V0A4 vs PeakID_230596 (chr7: 138772666 − 138774579)
correlation
Peak
Expression
r = 0.608
null distribution
p = 2.64e−10
FDR = 1.33e−06
Density
r

AC104041.1 vs PeakID_82505 (chr15: 81995159 − 81995973)

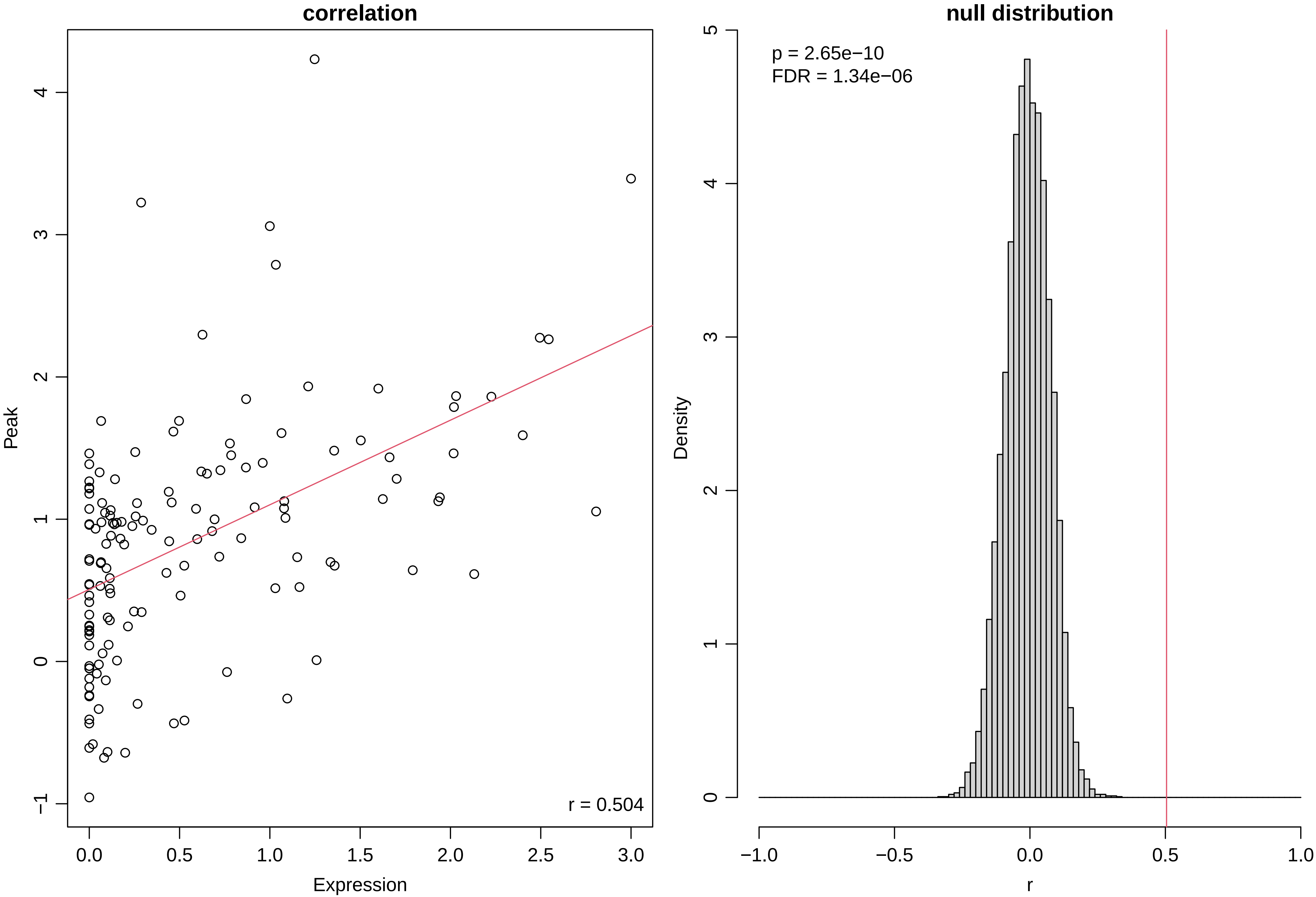

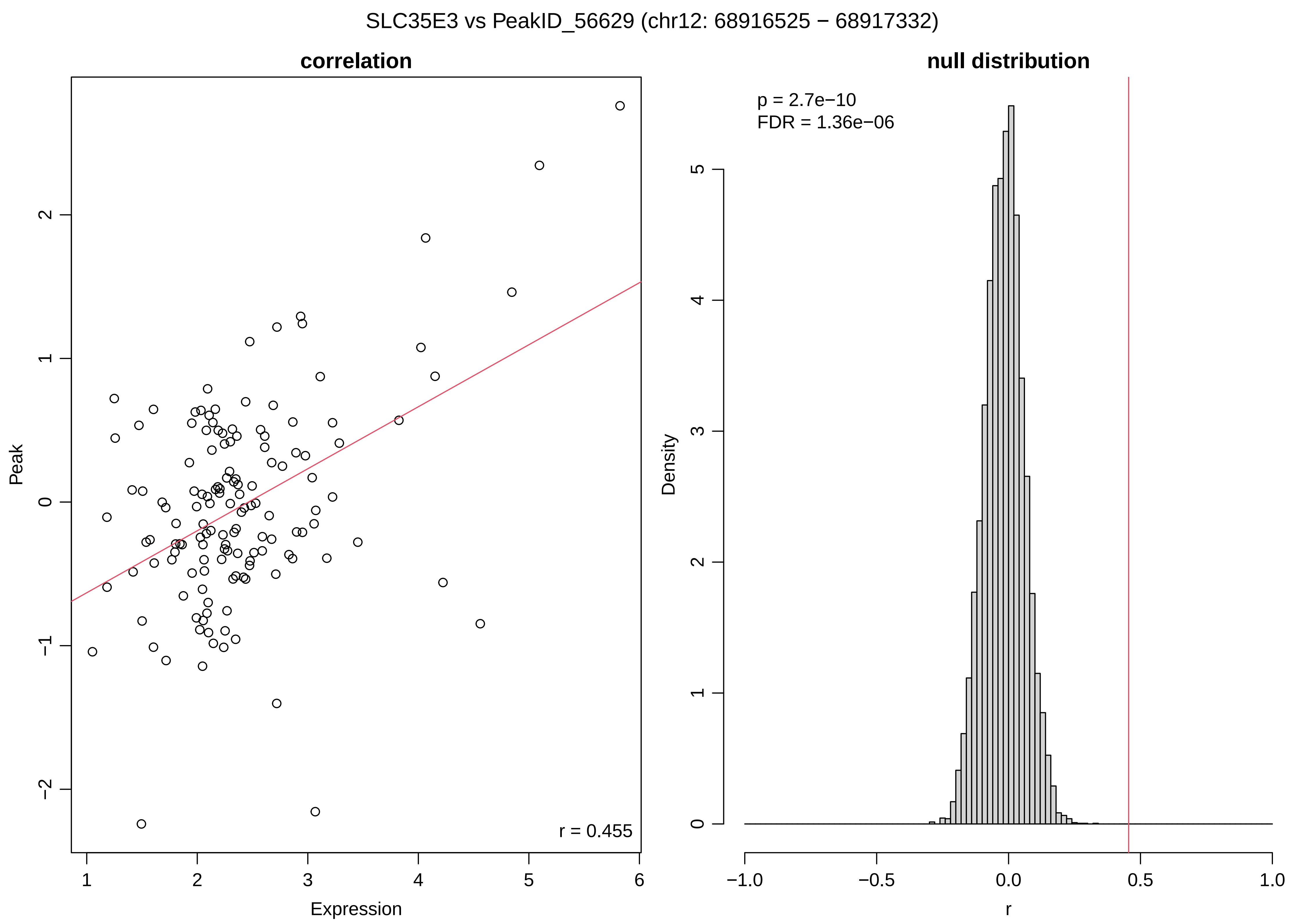
SLC35E3 vs PeakID_56629 (chr12: 68916525 − 68917332)
correlation
null distribution
Peak
Expression
r = 0.455
Density
r
p = 2.7e−10
FDR = 1.36e−06
−2
−1
0
1
2
3
4
5
6
−1.0
−0.5
0.0
0.5
1.0

SLC35E3 vs PeakID_56641 (chr12: 68937240 − 68937527)

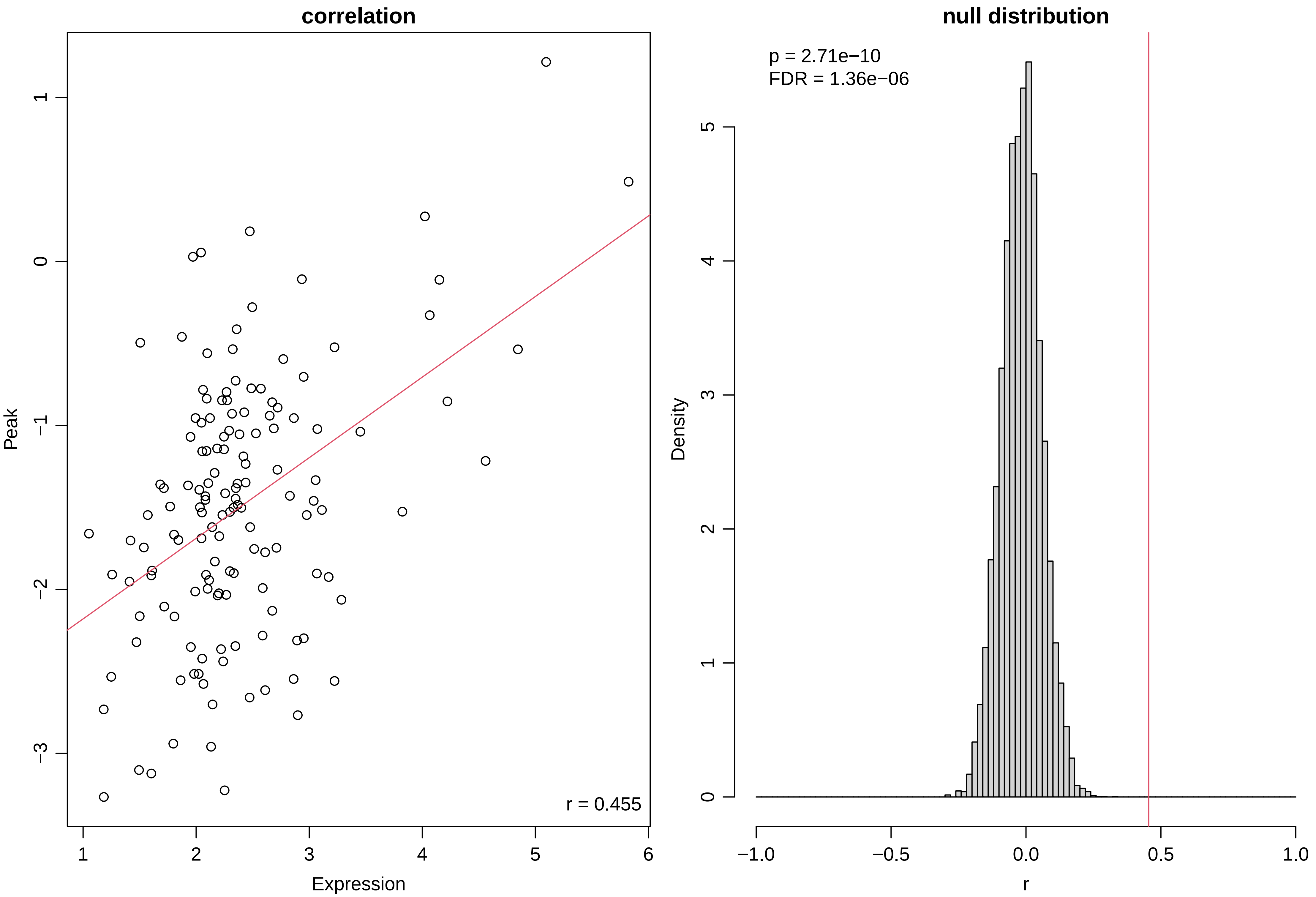

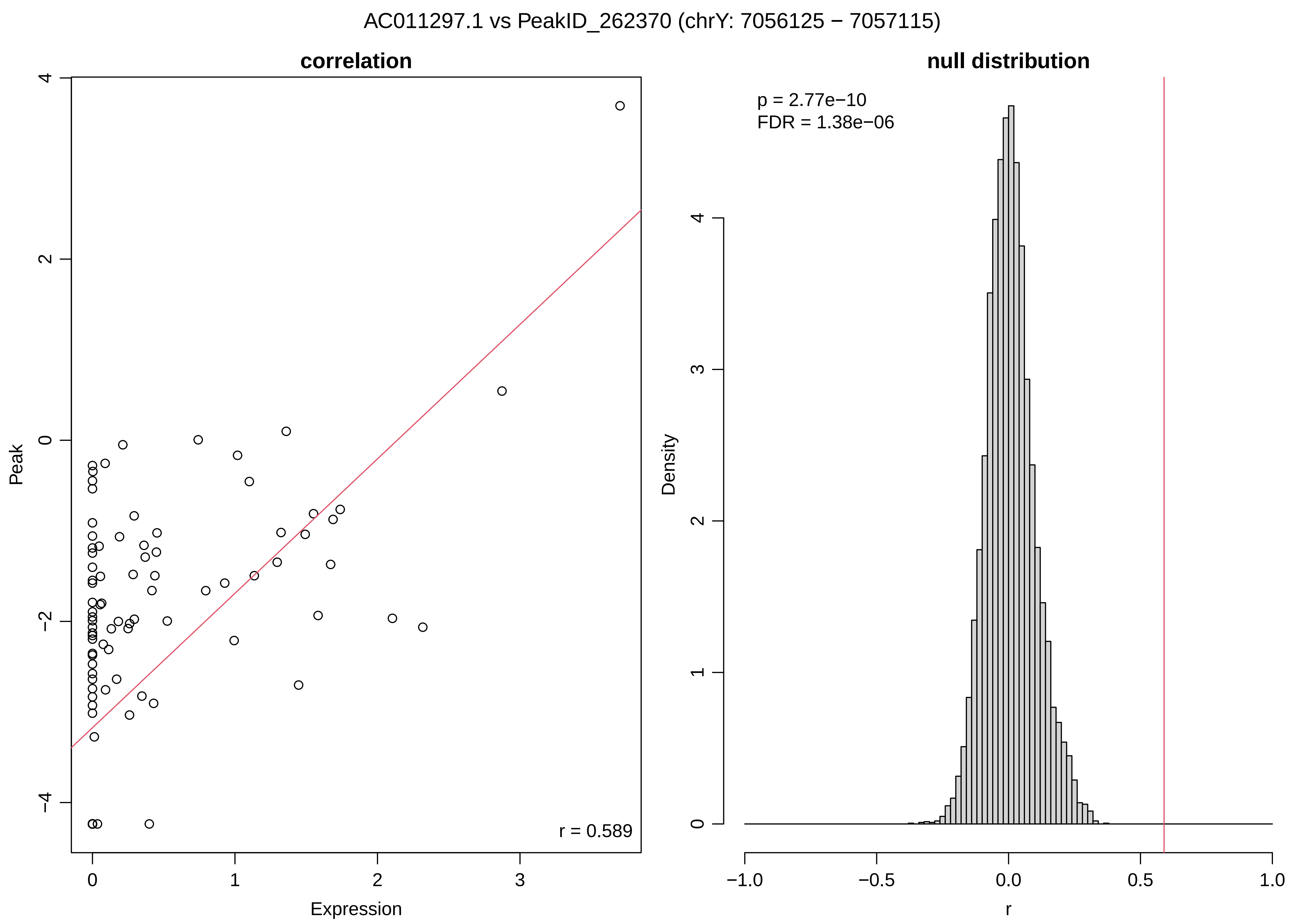
AC011297.1 vs PeakID_262370 (chrY: 7056125 − 7057115)
correlation
Peak
Expression
r = 0.589
null distribution
p = 2.77e−10
FDR = 1.38e−06
Density
r

CDK4 vs PeakID_54423 (chr12: 57743158 − 57790607)

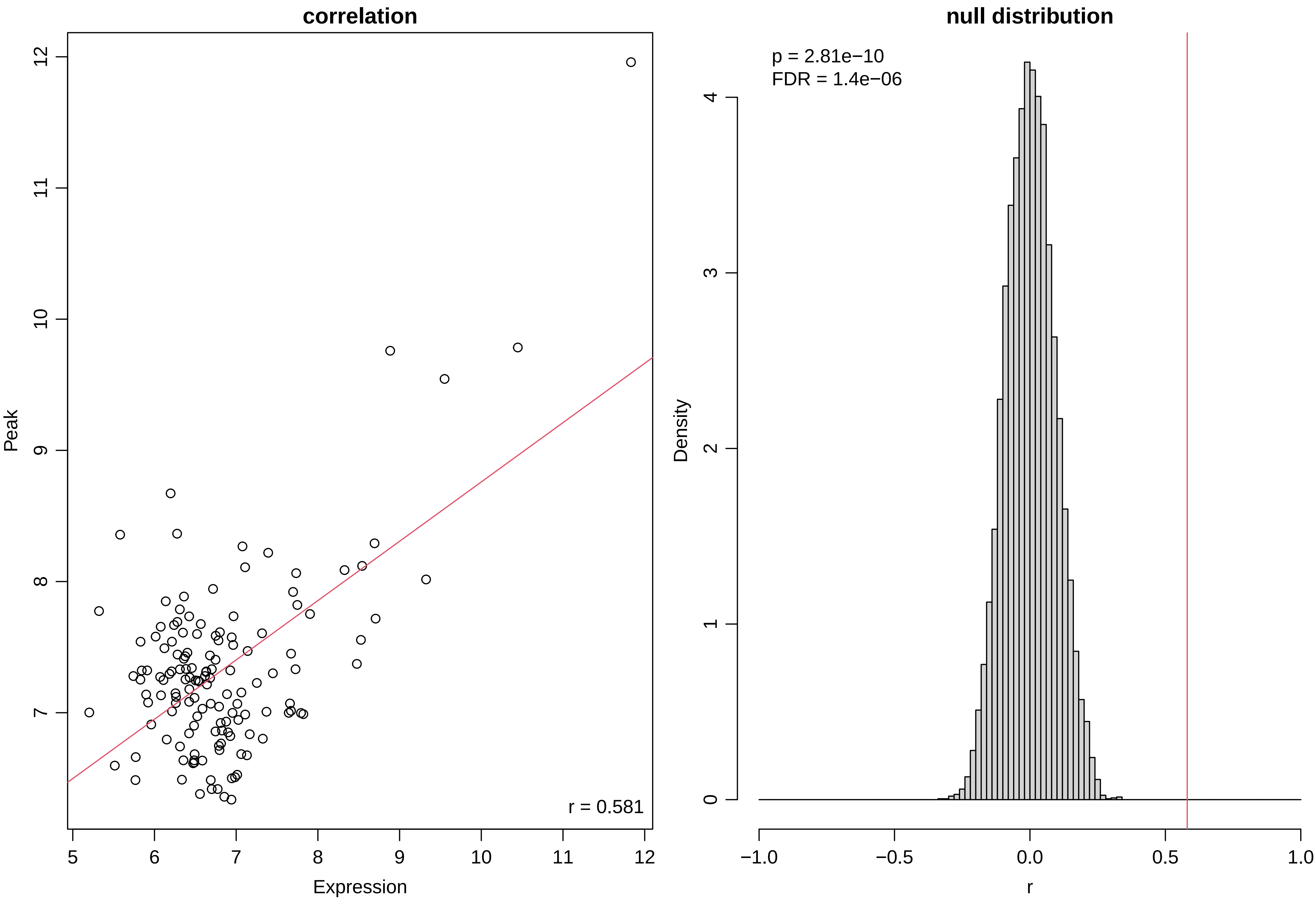

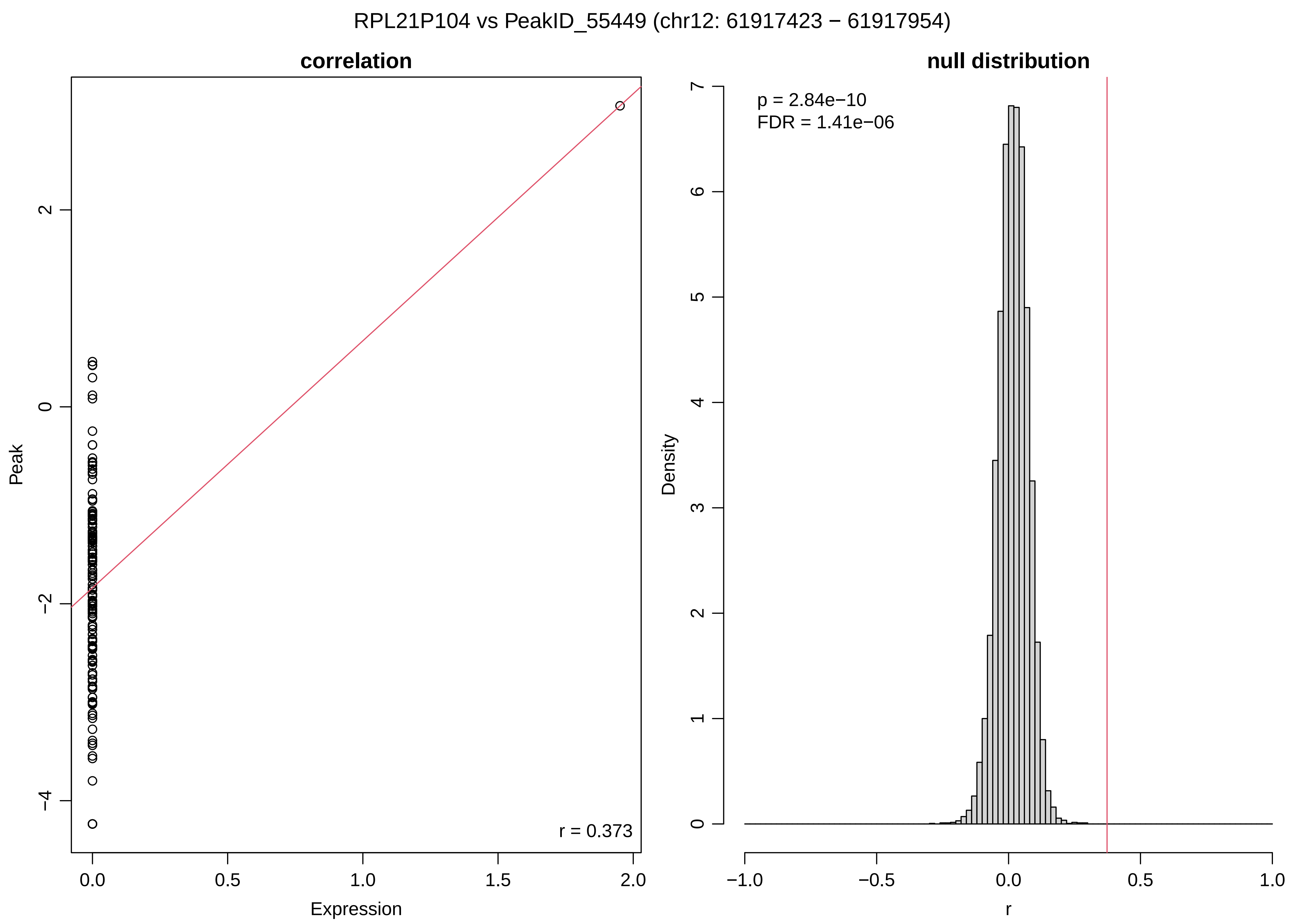

RPL21P104 vs PeakID_55449 (chr12: 61917423 − 61917954)
correlation
Peak
Expression
r = 0.373
null distribution
p = 2.84e−10
FDR = 1.41e−06
Density
r

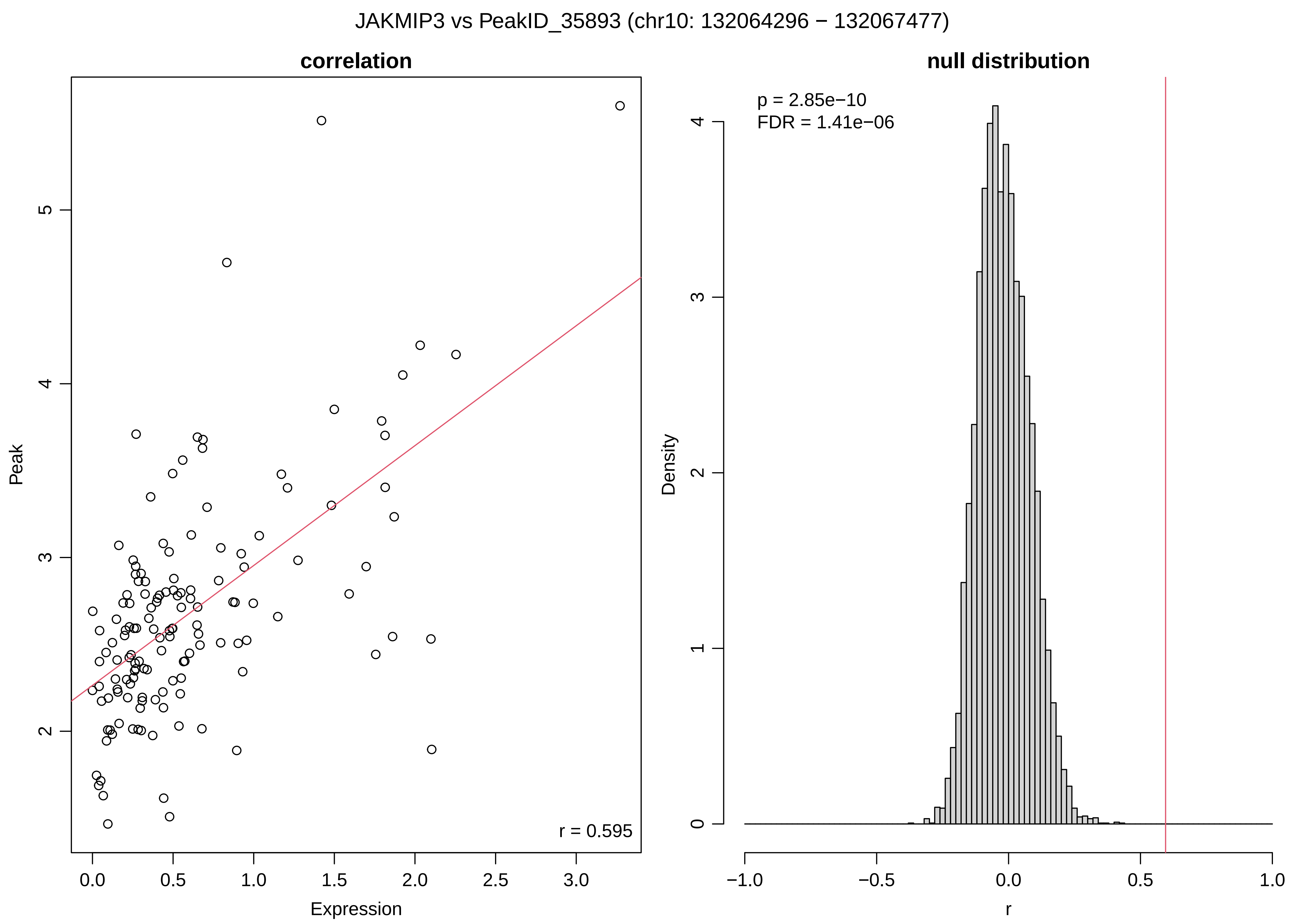

JAKMIP3 vs PeakID_35893 (chr10: 132064296 − 132067477)
correlation
Peak
Expression
r = 0.595
null distribution
p = 2.85e−10
FDR = 1.41e−06
Density
r

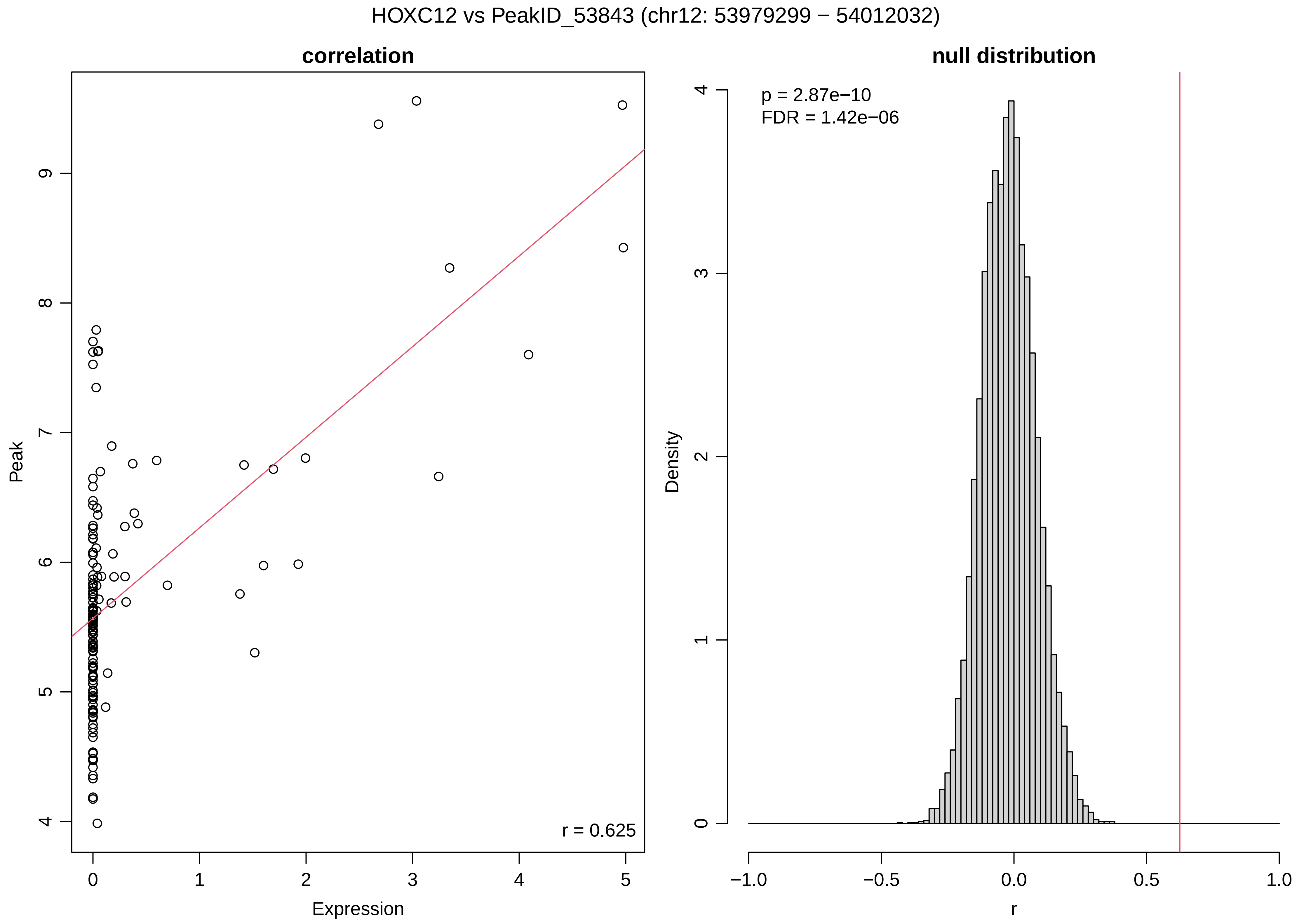
HOXC12 vs PeakID_53843 (chr12: 53979299 − 54012032)
correlation
Peak
Expression
r = 0.625
null distribution
Density
r
p = 2.87e−10
FDR = 1.42e−06

AC107881.1 vs PeakID_38020 (chr11: 12766842 − 12767370)

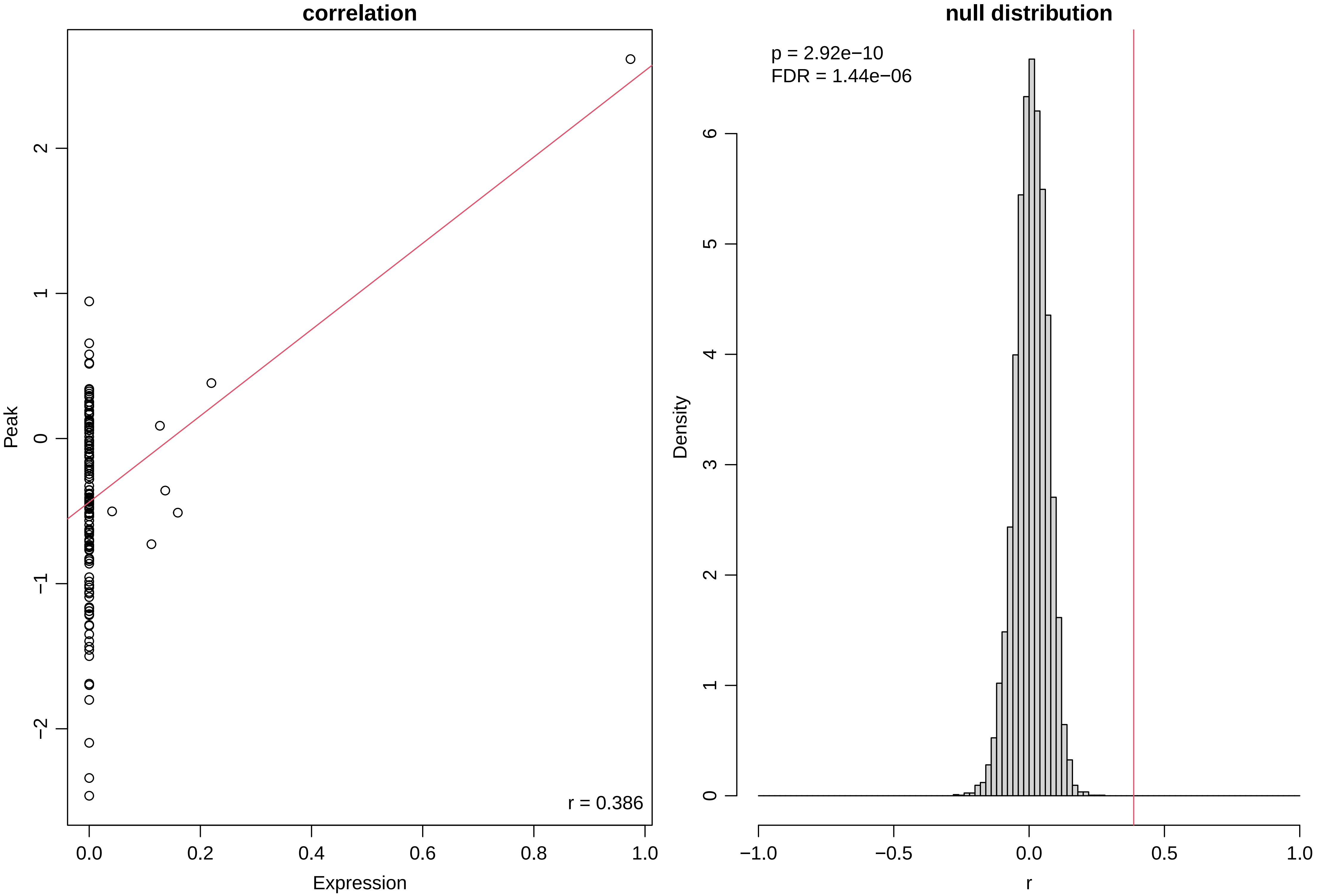

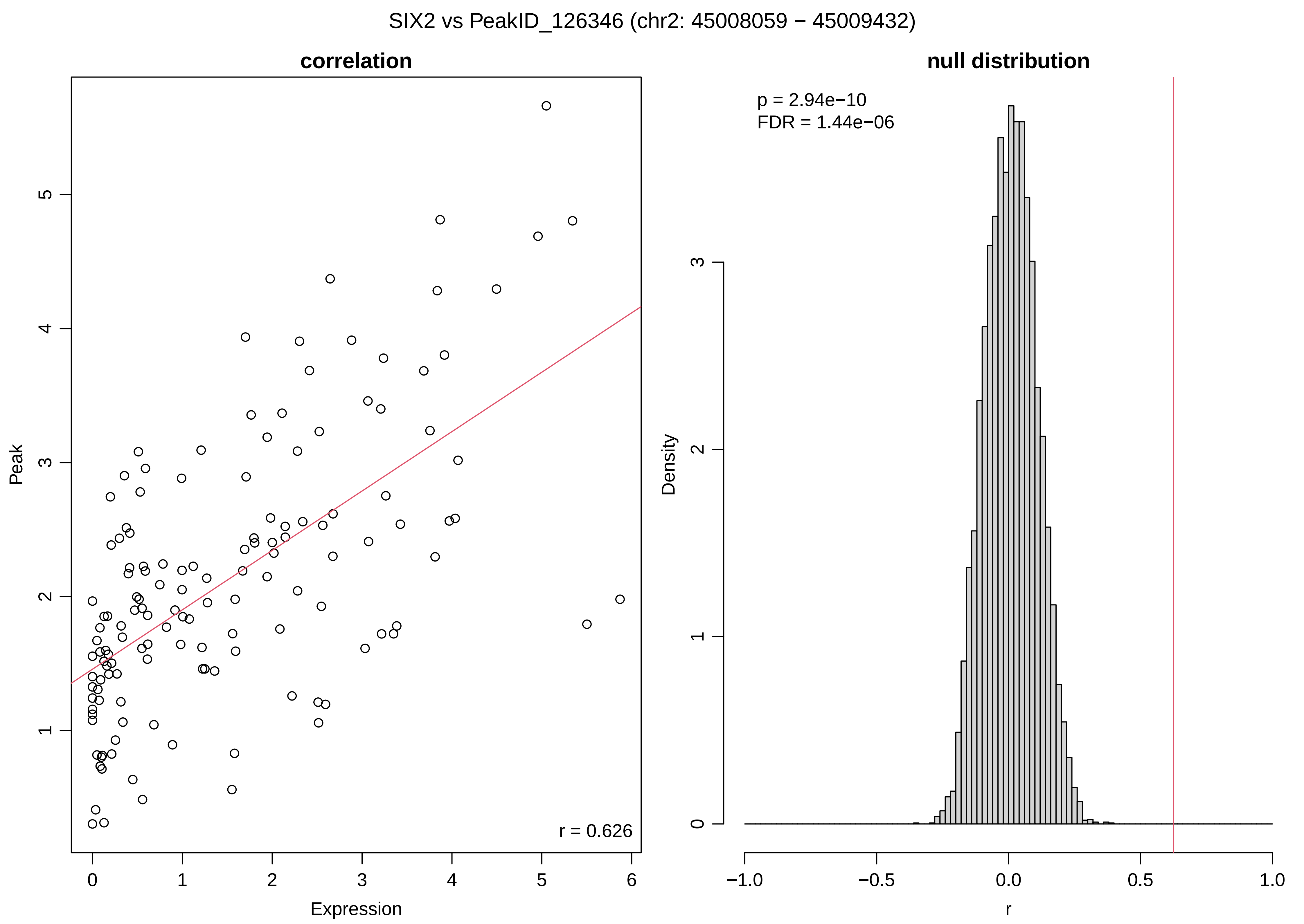
SIX2 vs PeakID_126346 (chr2: 45008059 − 45009432)
correlation
null distribution
p = 2.94e−10
FDR = 1.44e−06
r = 0.626
Peak
Expression
Density
r
0
1
2
3
4
5
6
−1.0
−0.5
0.0
0.5
1.0

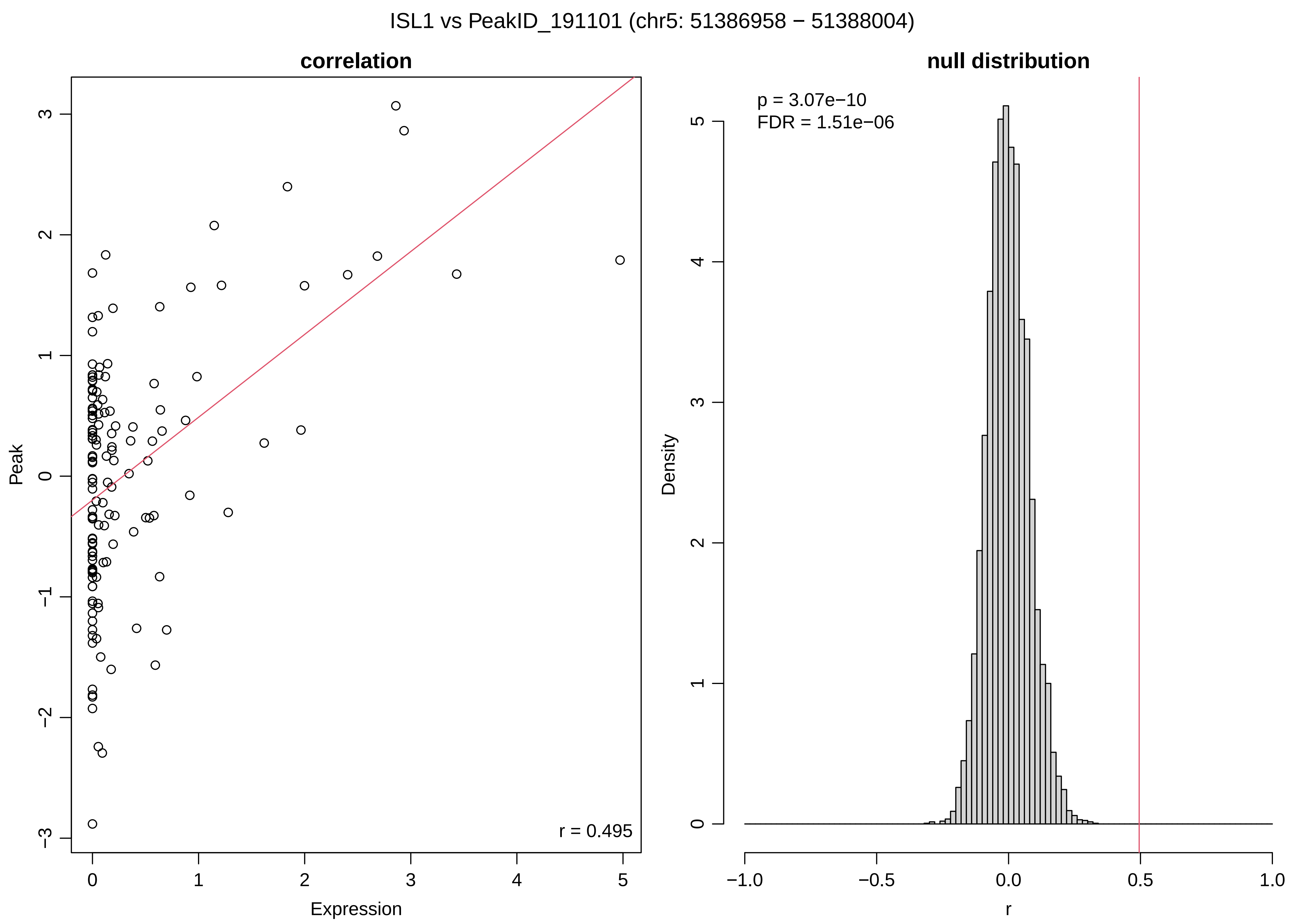
ISL1 vs PeakID_191101 (chr5: 51386958 − 51388004)
correlation
Peak
Expression
r = 0.495
null distribution
p = 3.07e−10
FDR = 1.51e−06
Density
r

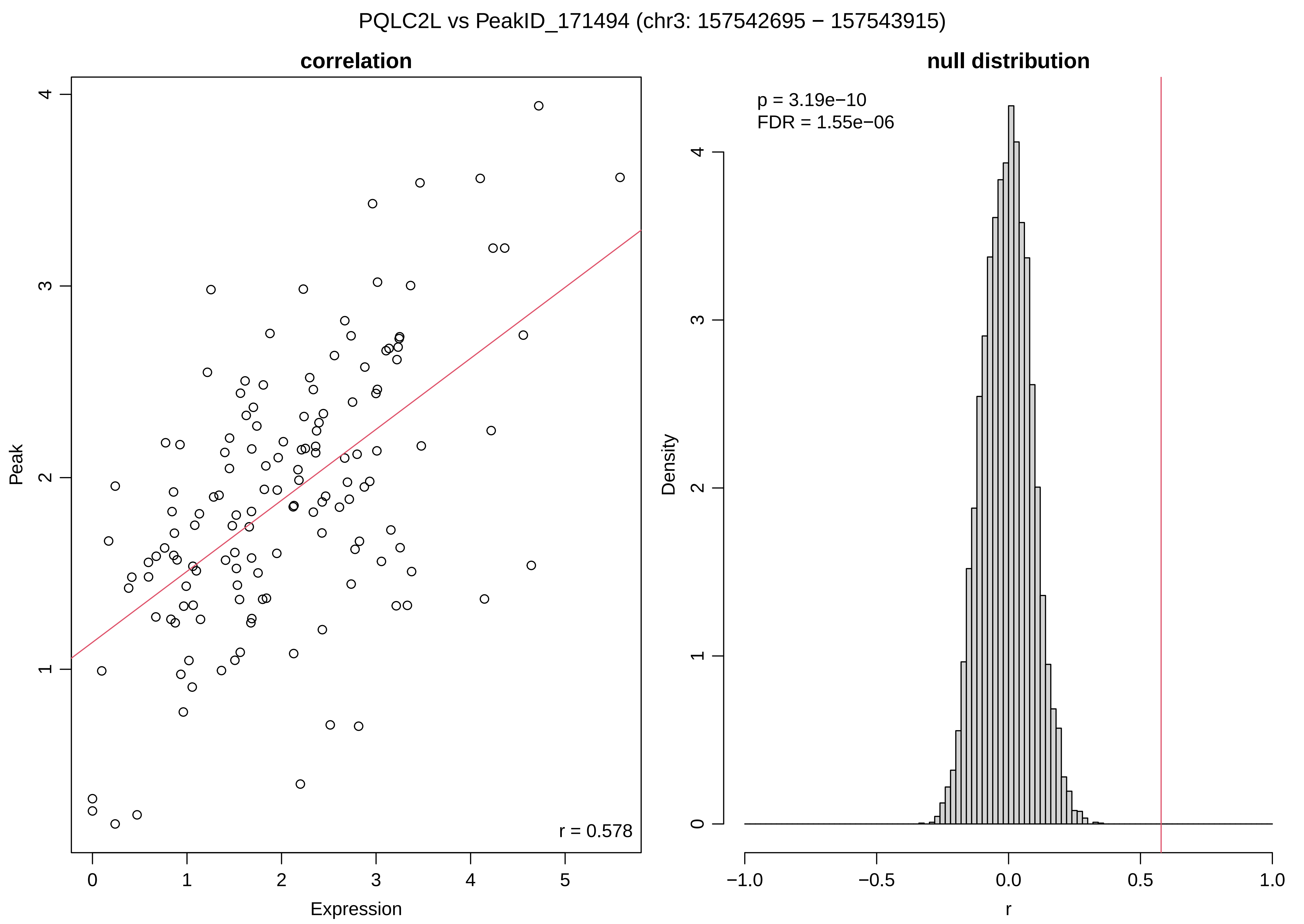
PQLC2L vs PeakID_171494 (chr3: 157542695 − 157543915)
correlation
Peak
Expression
r = 0.578
null distribution
p = 3.19e−10
FDR = 1.55e−06
Density
r

SPTSSB vs PeakID_171805 (chr3: 161370790 − 161373956)

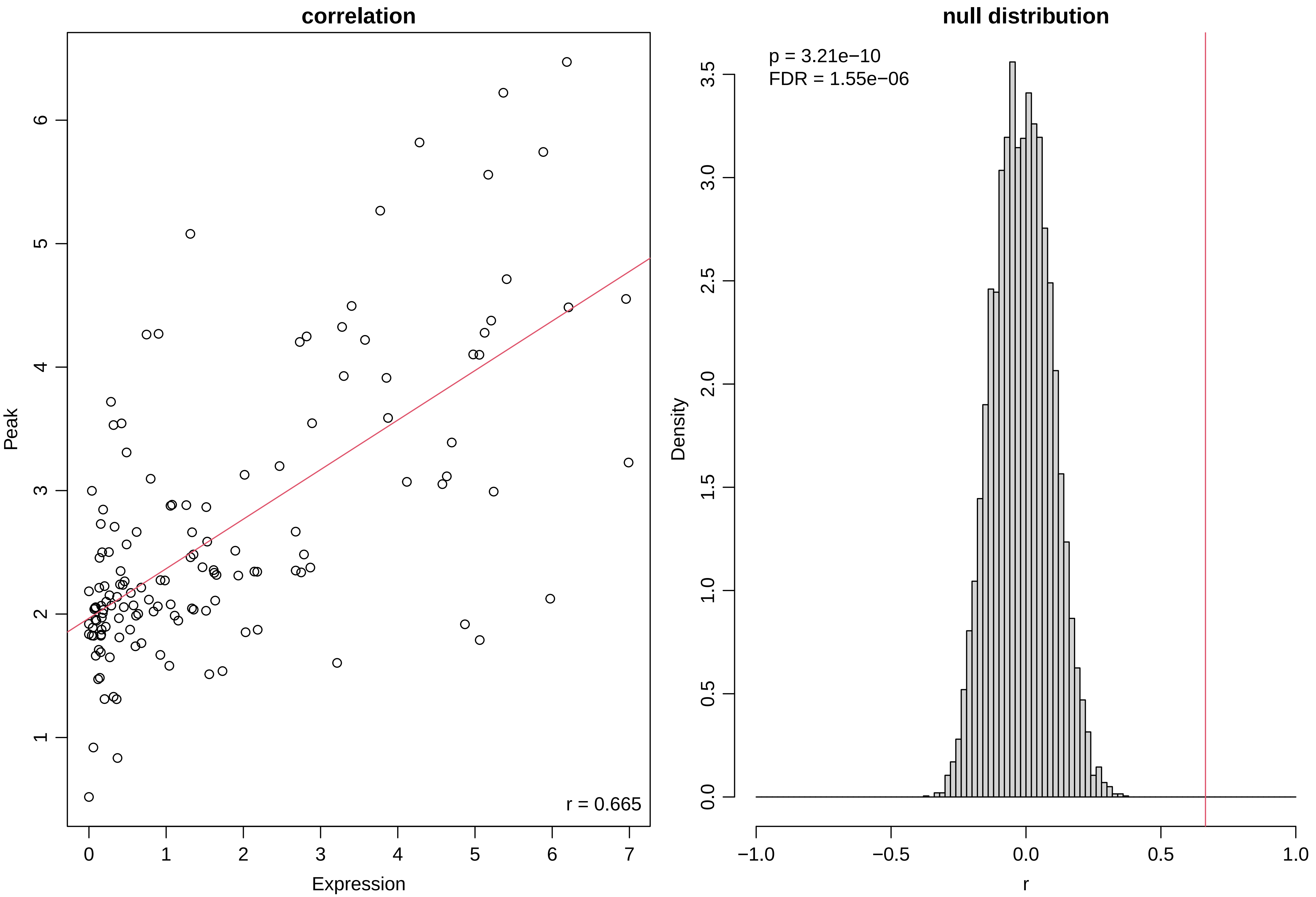

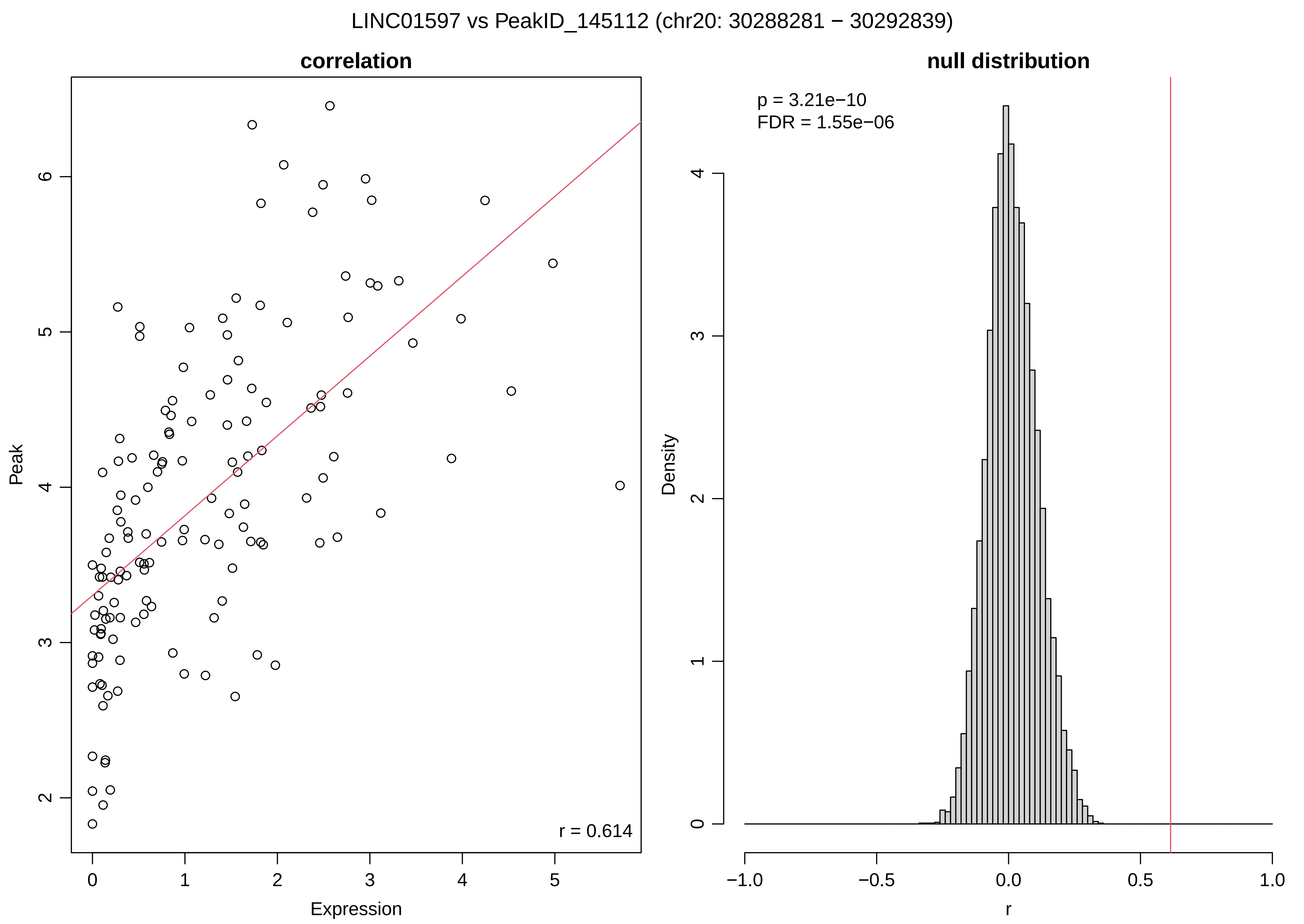

LINC01597 vs PeakID_145112 (chr20: 30288281 − 30292839)
correlation
Peak
Expression
r = 0.614
null distribution
p = 3.21e−10
FDR = 1.55e−06
Density
r

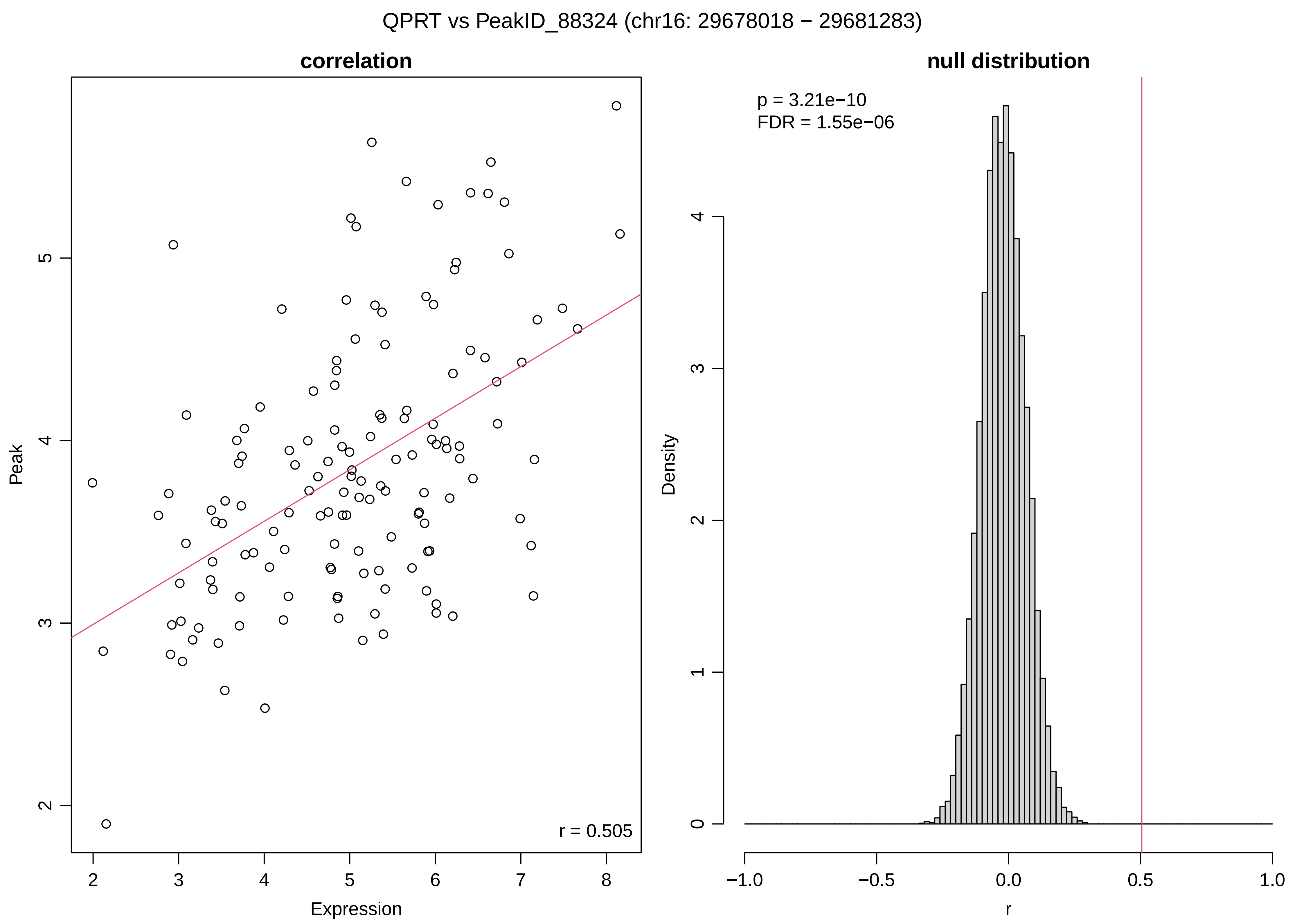

QPRT vs PeakID_88324 (chr16: 29678018 − 29681283)
correlation
null distribution
p = 3.21e−10
FDR = 1.55e−06
r = 0.505
Peak
Expression
Density
r
2
3
4
5
6
7
8
−1.0
−0.5
0.0
0.5
1.0
0
1
2
3
4

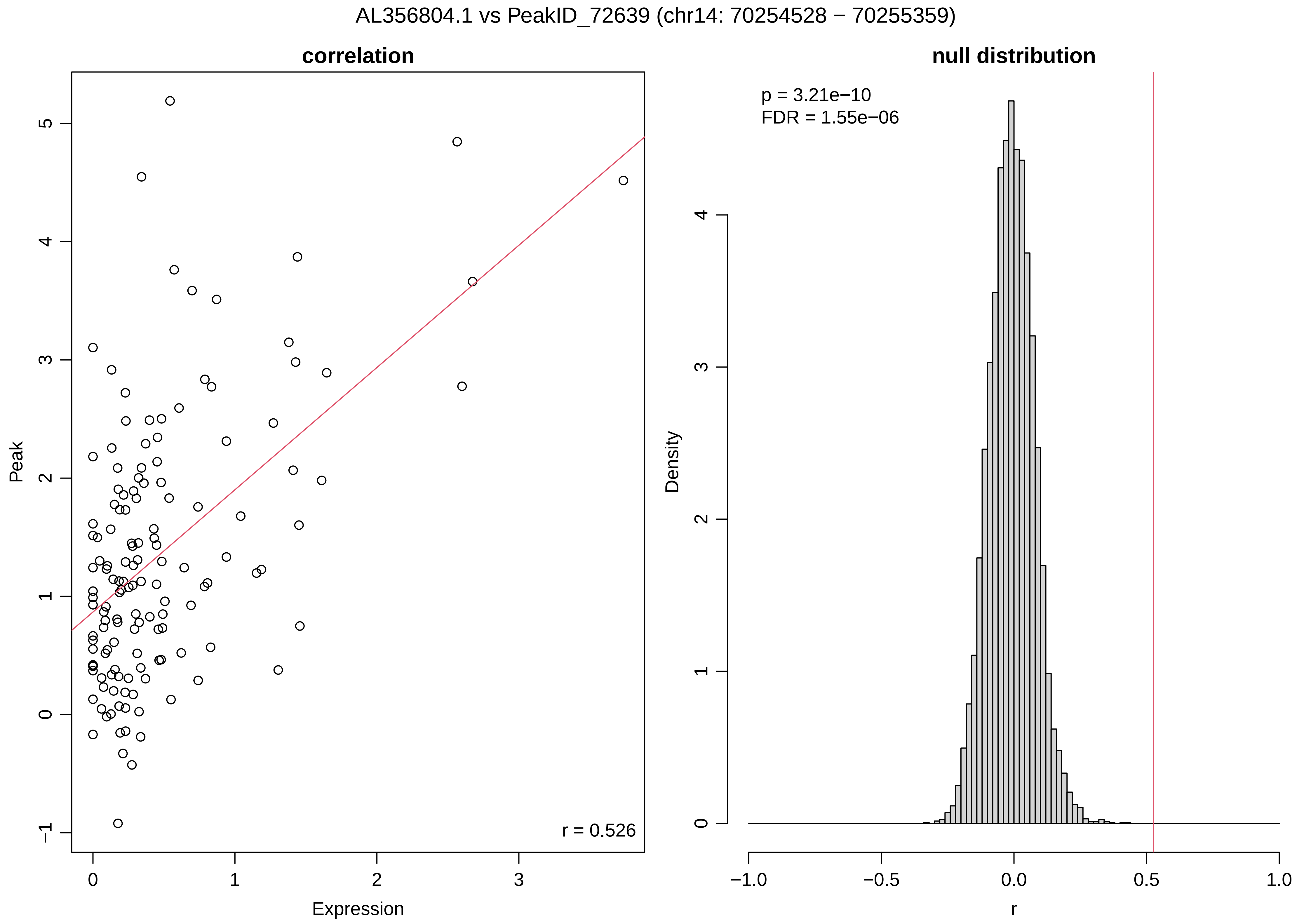

AL356804.1 vs PeakID_72639 (chr14: 70254528 − 70255359)
correlation
Peak
Expression
r = 0.526
null distribution
p = 3.21e−10
FDR = 1.55e−06
Density
r

RPL21P104 vs PeakID_55482 (chr12: 61996234 − 61997035)

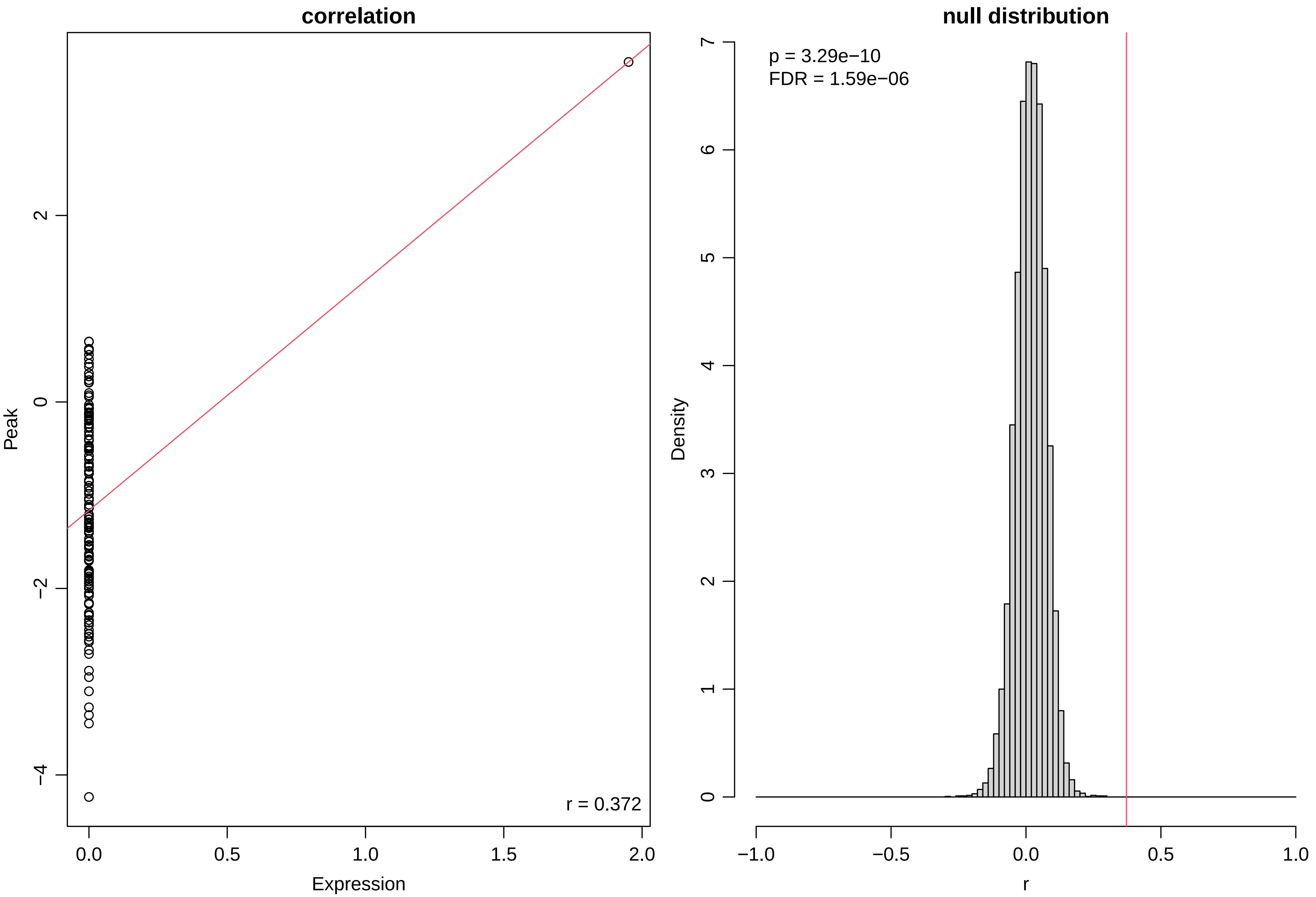

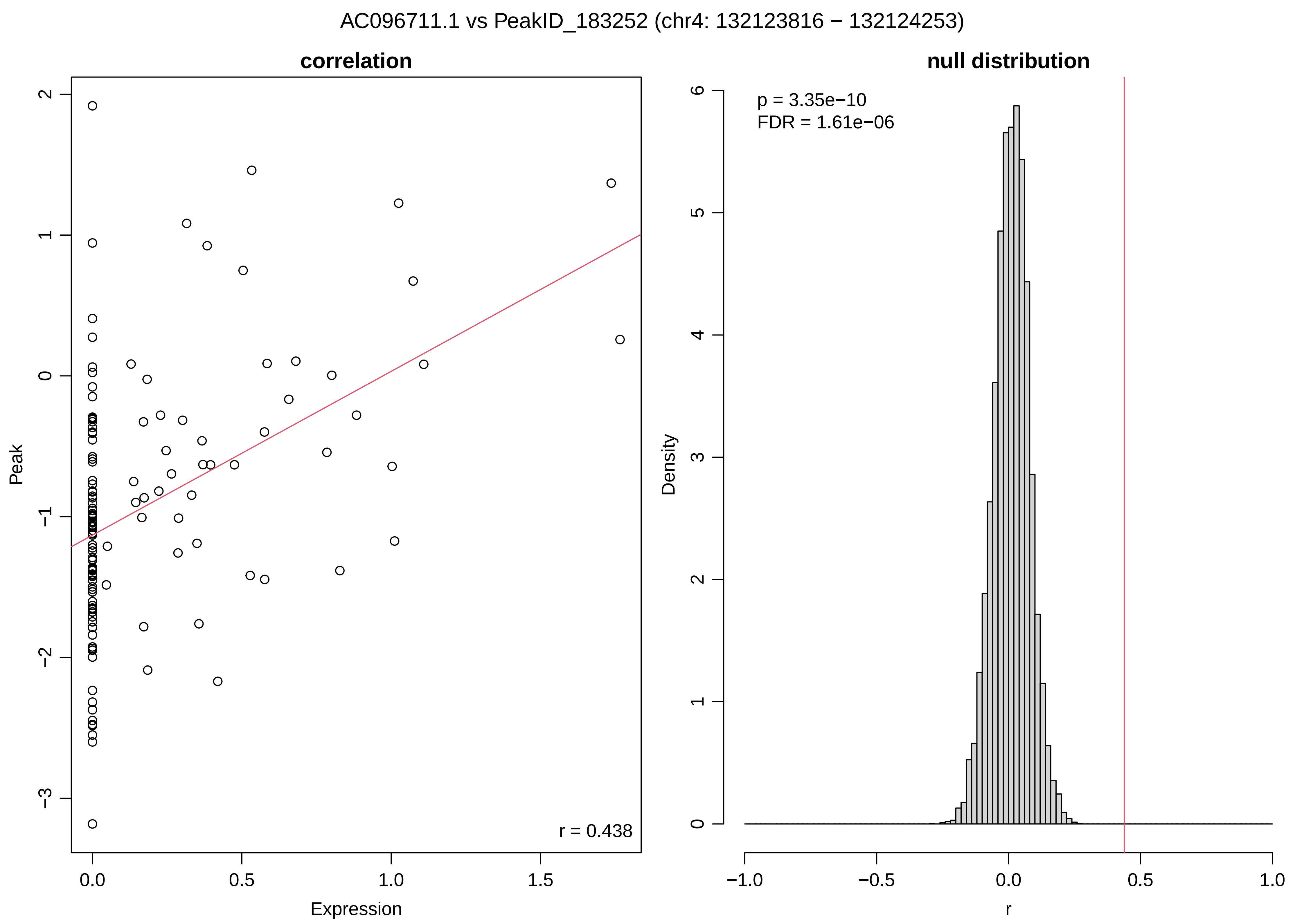

AC096711.1 vs PeakID_183252 (chr4: 132123816 − 132124253)
correlation
null distribution
Peak
Expression
r = 0.438
Density
r
p = 3.35e−10
FDR = 1.61e−06

AC107881.1 vs PeakID_37994 (chr11: 12721170 − 12721407)

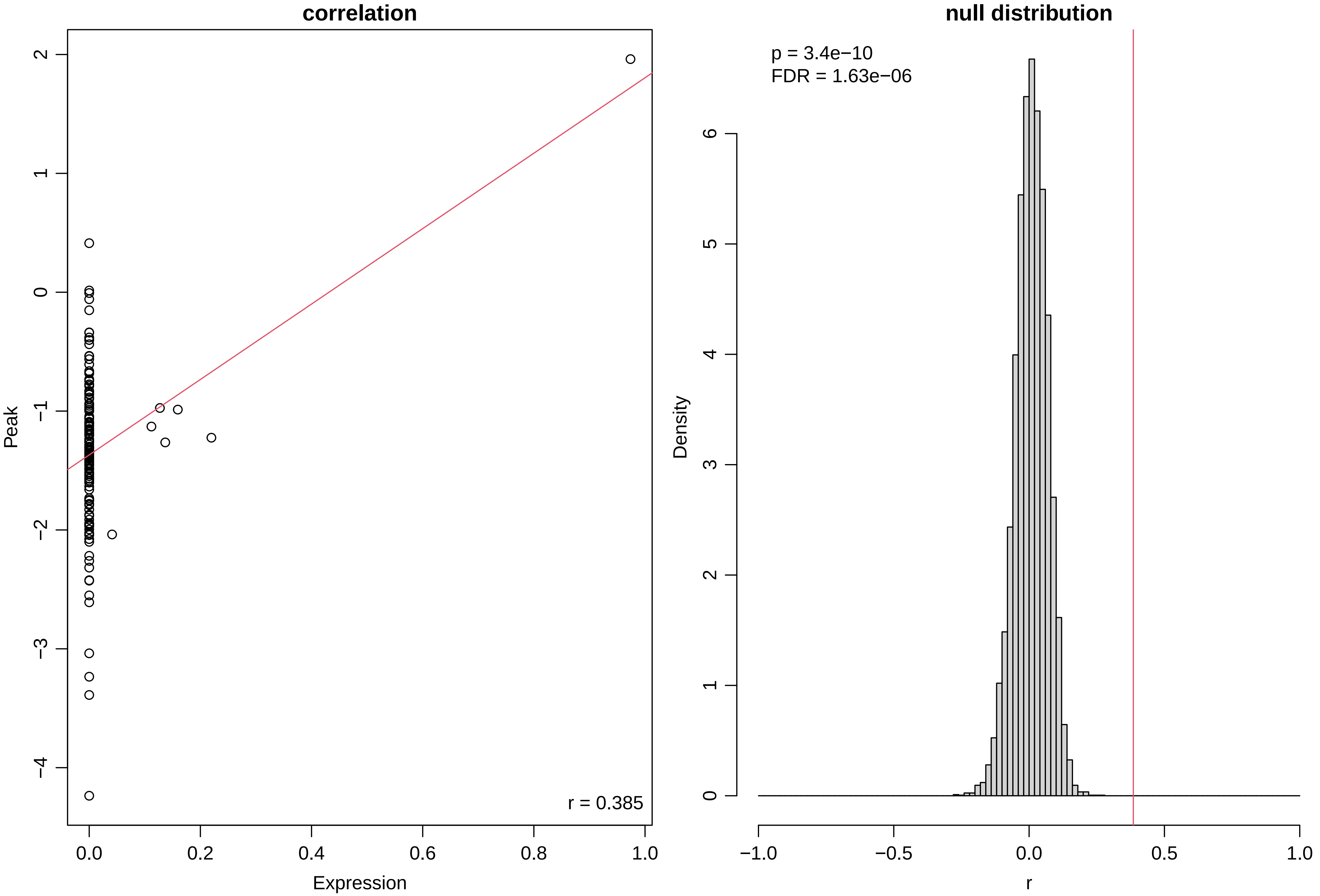

AC020907.1 vs PeakID_116494 (chr19: 35089789 − 35091640)

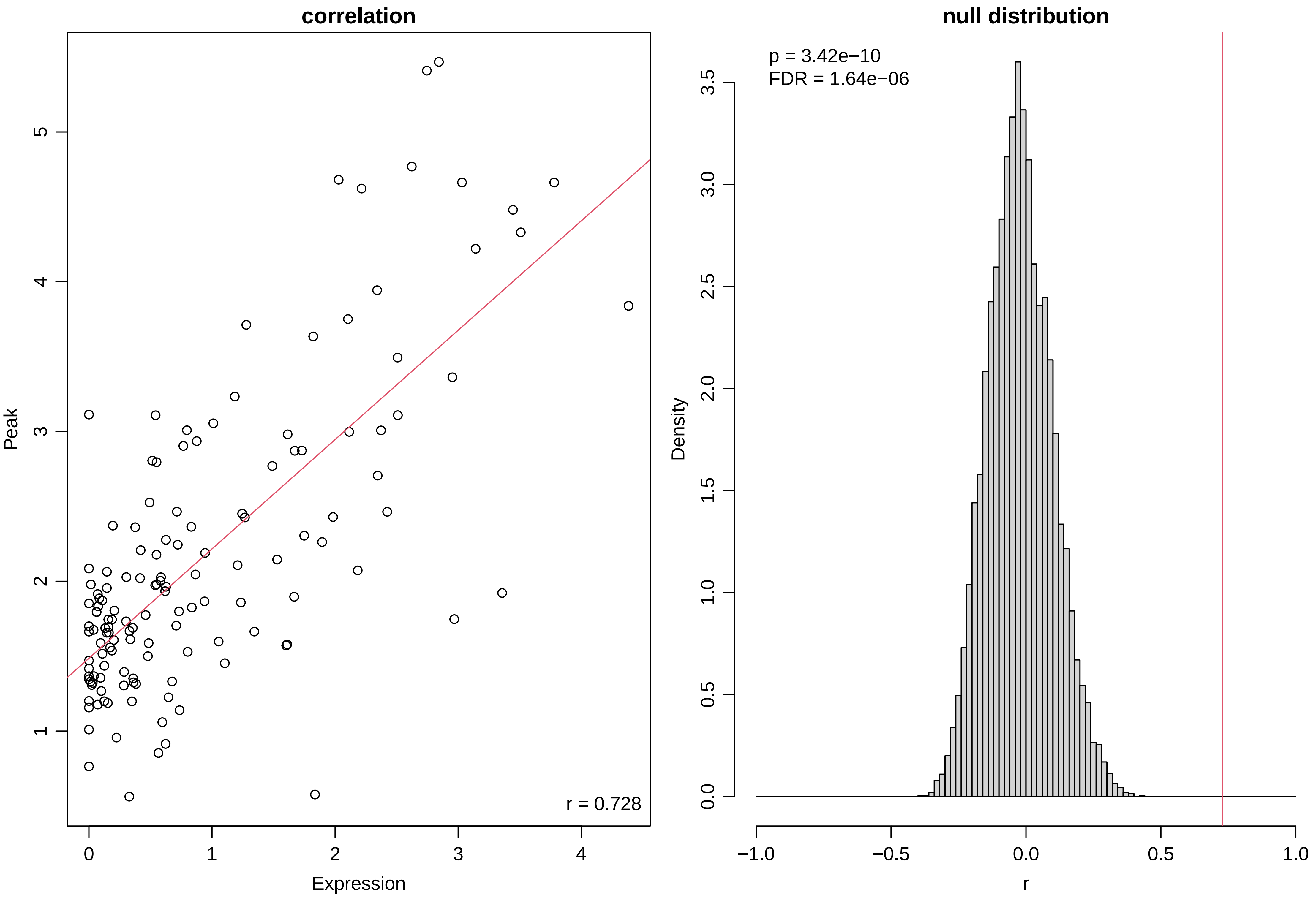

CLDN10−AS1 vs PeakID_66916 (chr13: 95530456 − 95534155)

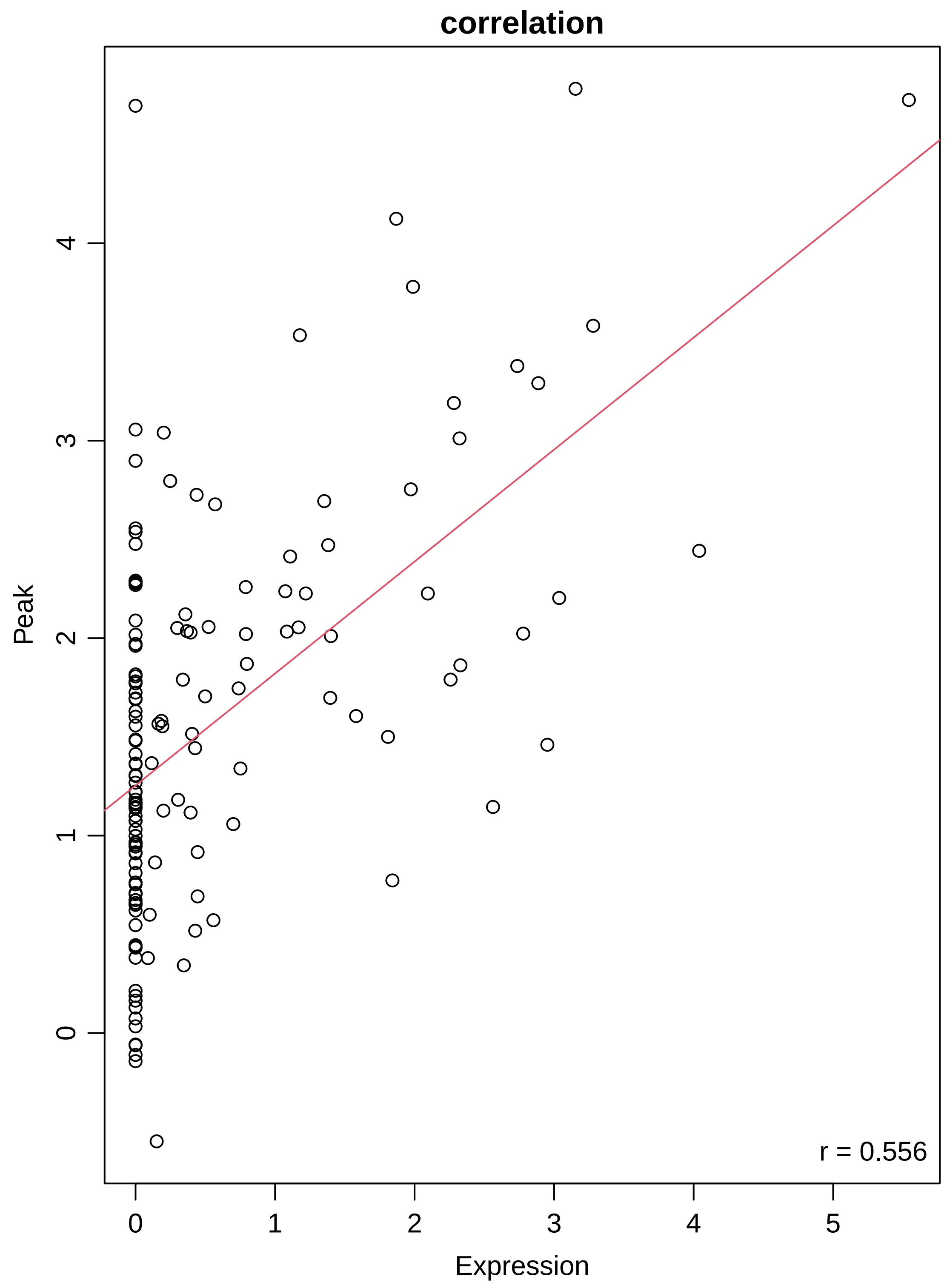

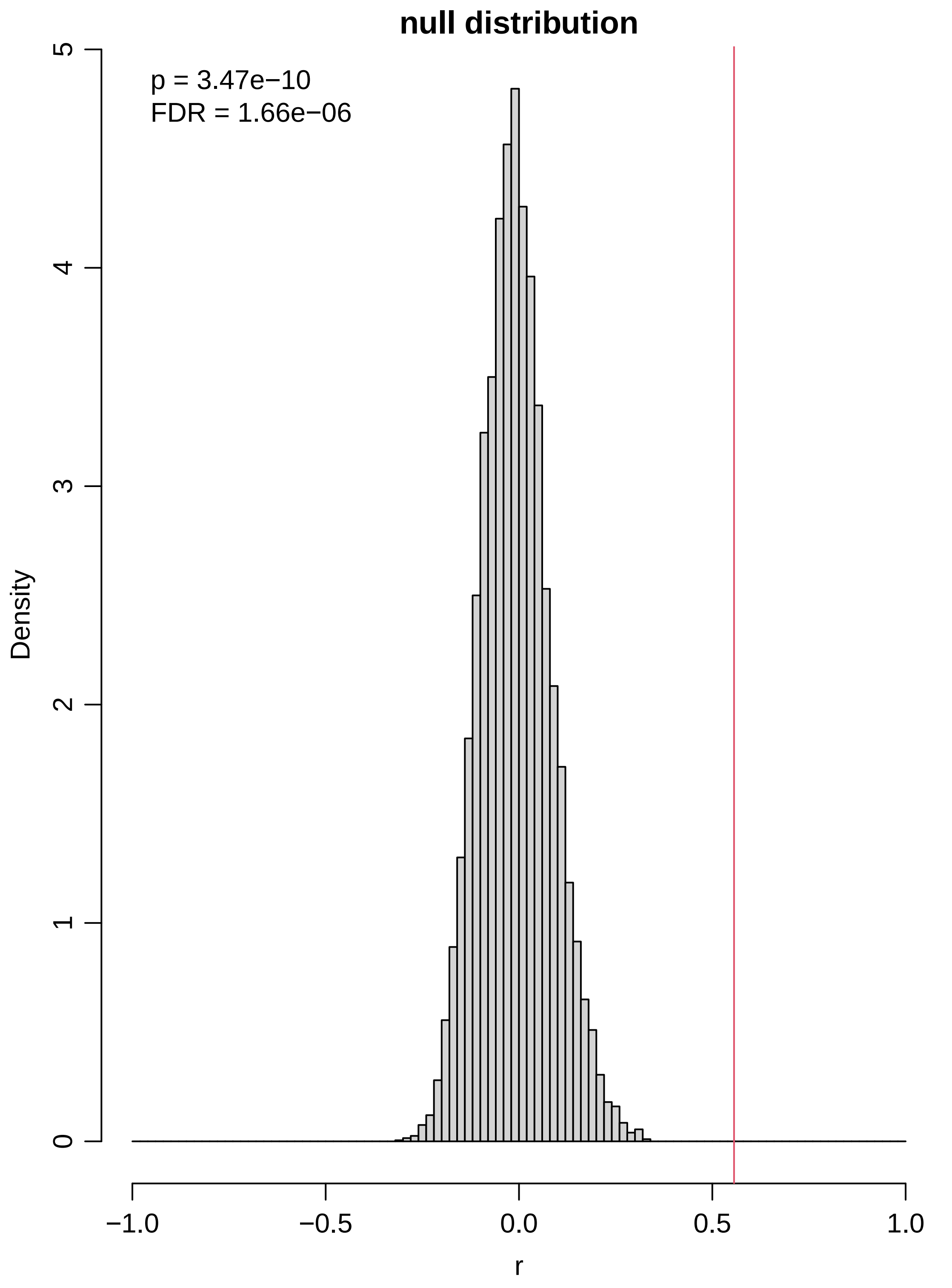

NOTUM vs PeakID_104980 (chr17: 81953897 − 81963372)

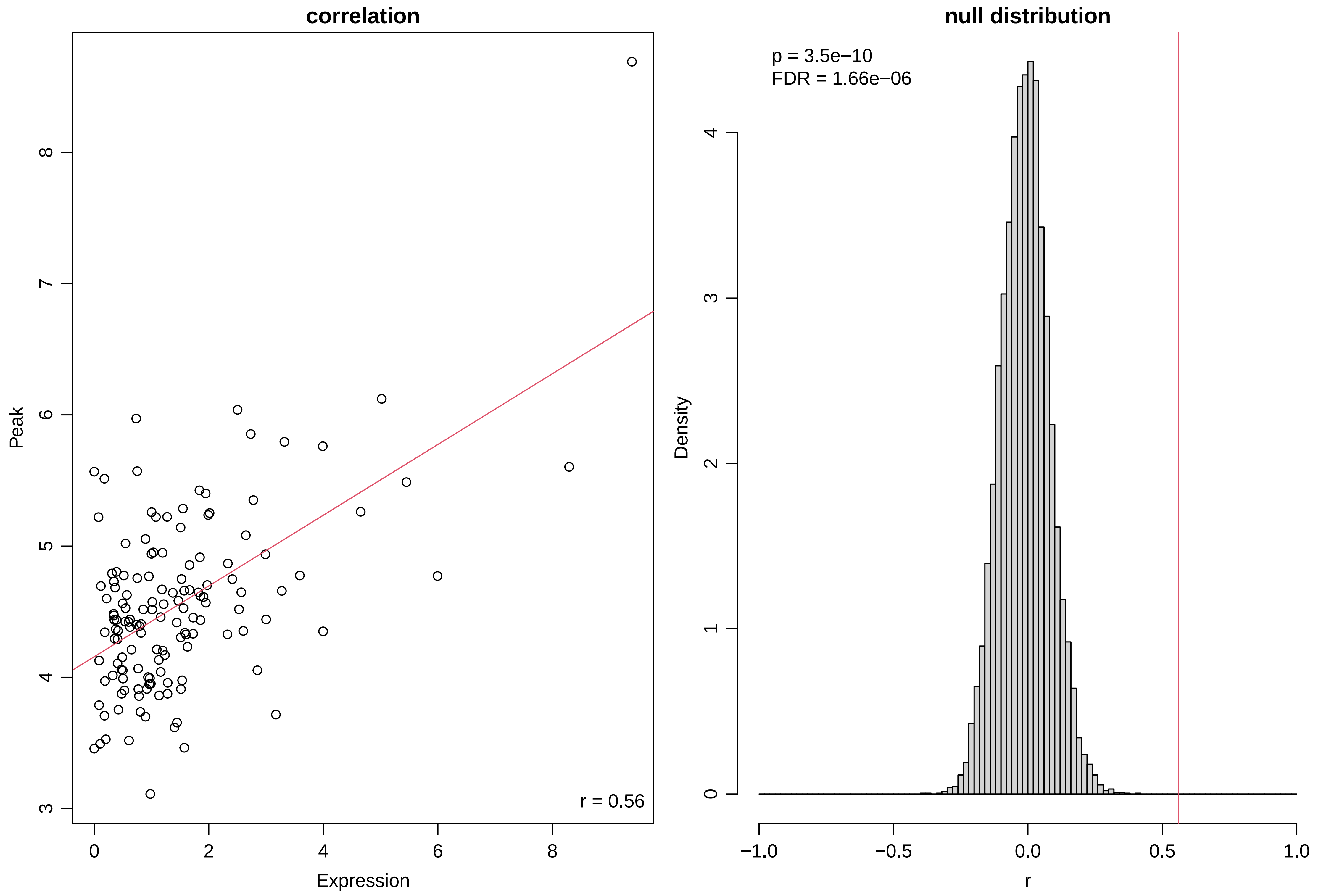

DUX4L27 vs PeakID_52228 (chr12: 34218314 − 34219252)

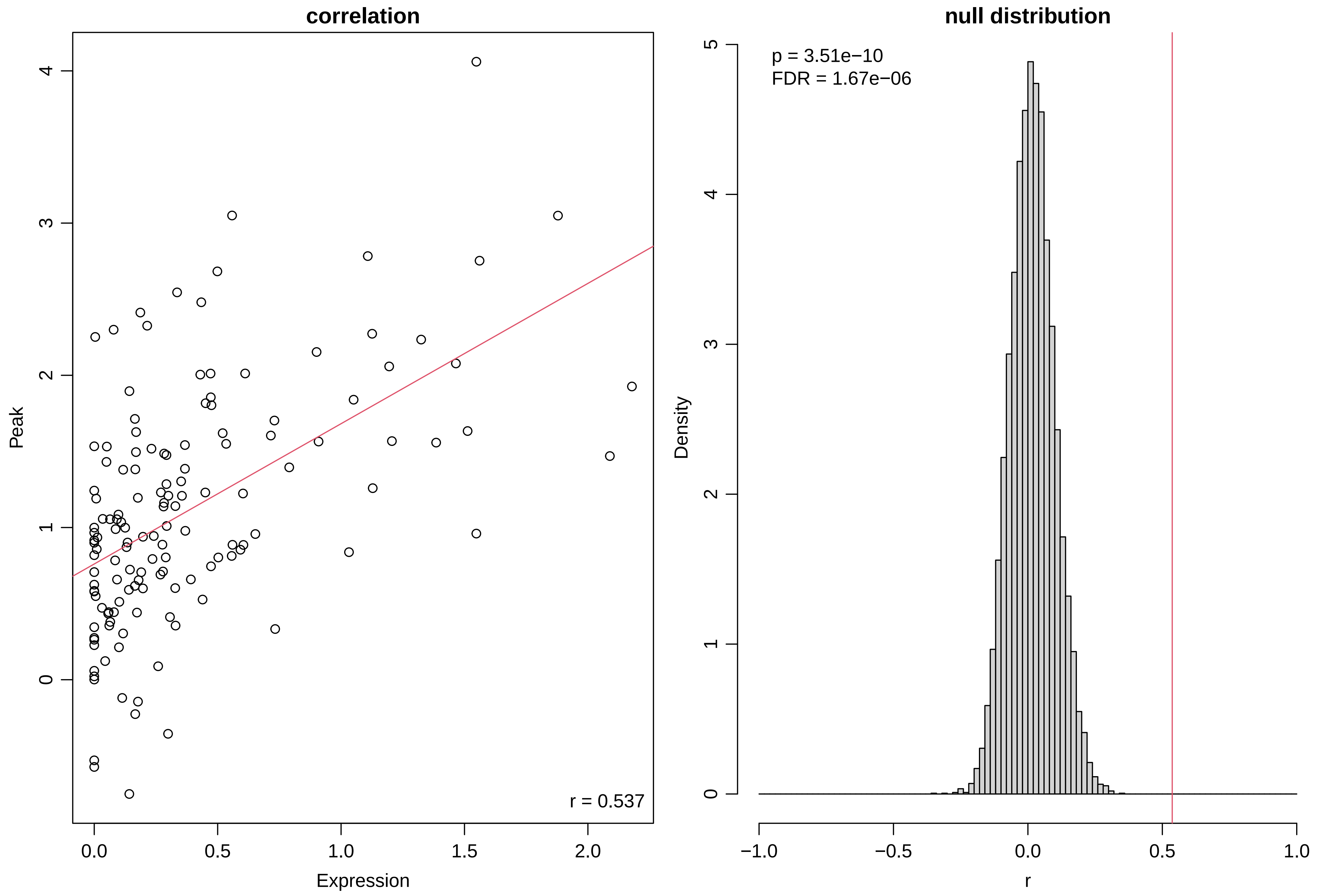

AC009271.1 vs PeakID_110299 (chr18: 55892936 − 55893961)

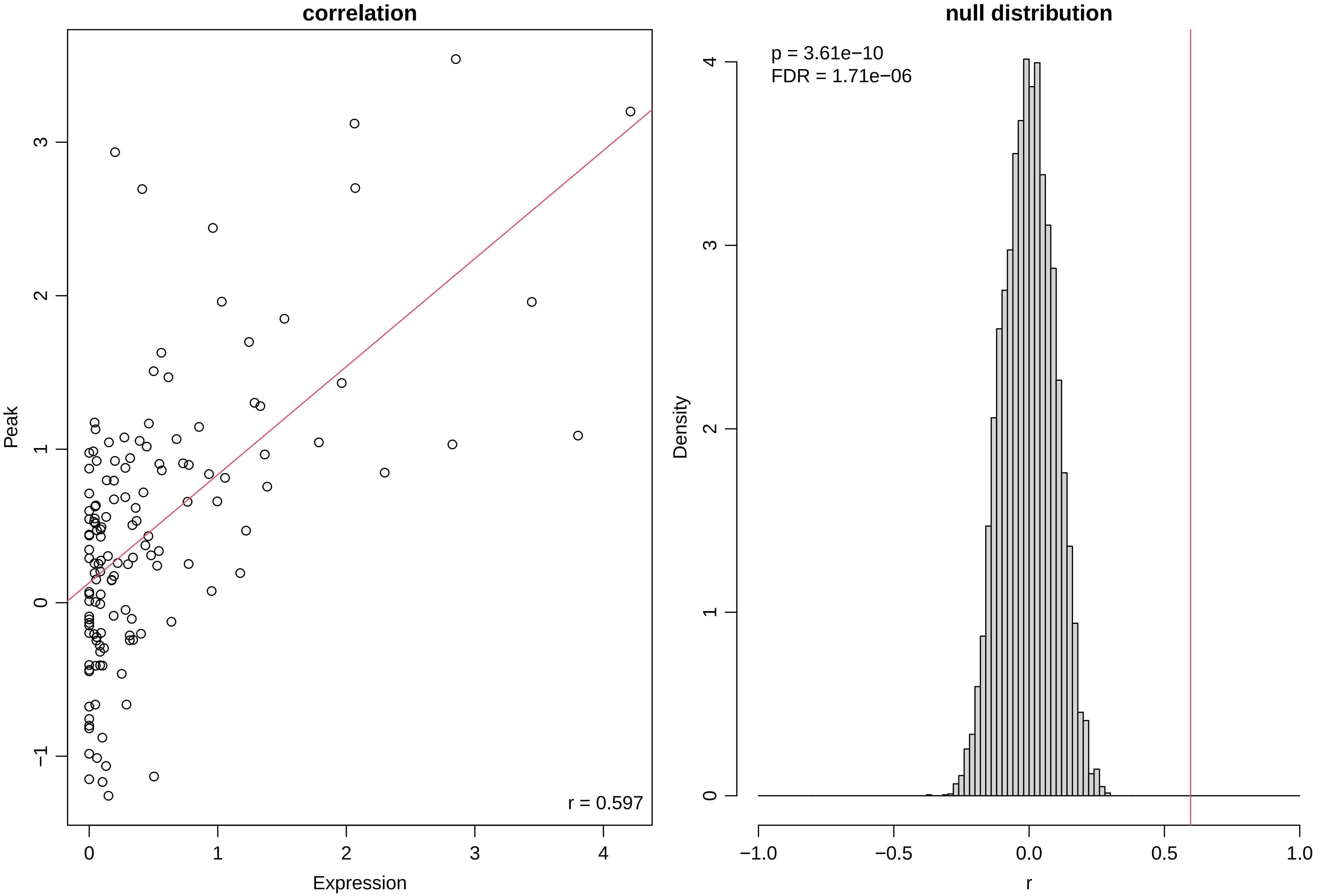

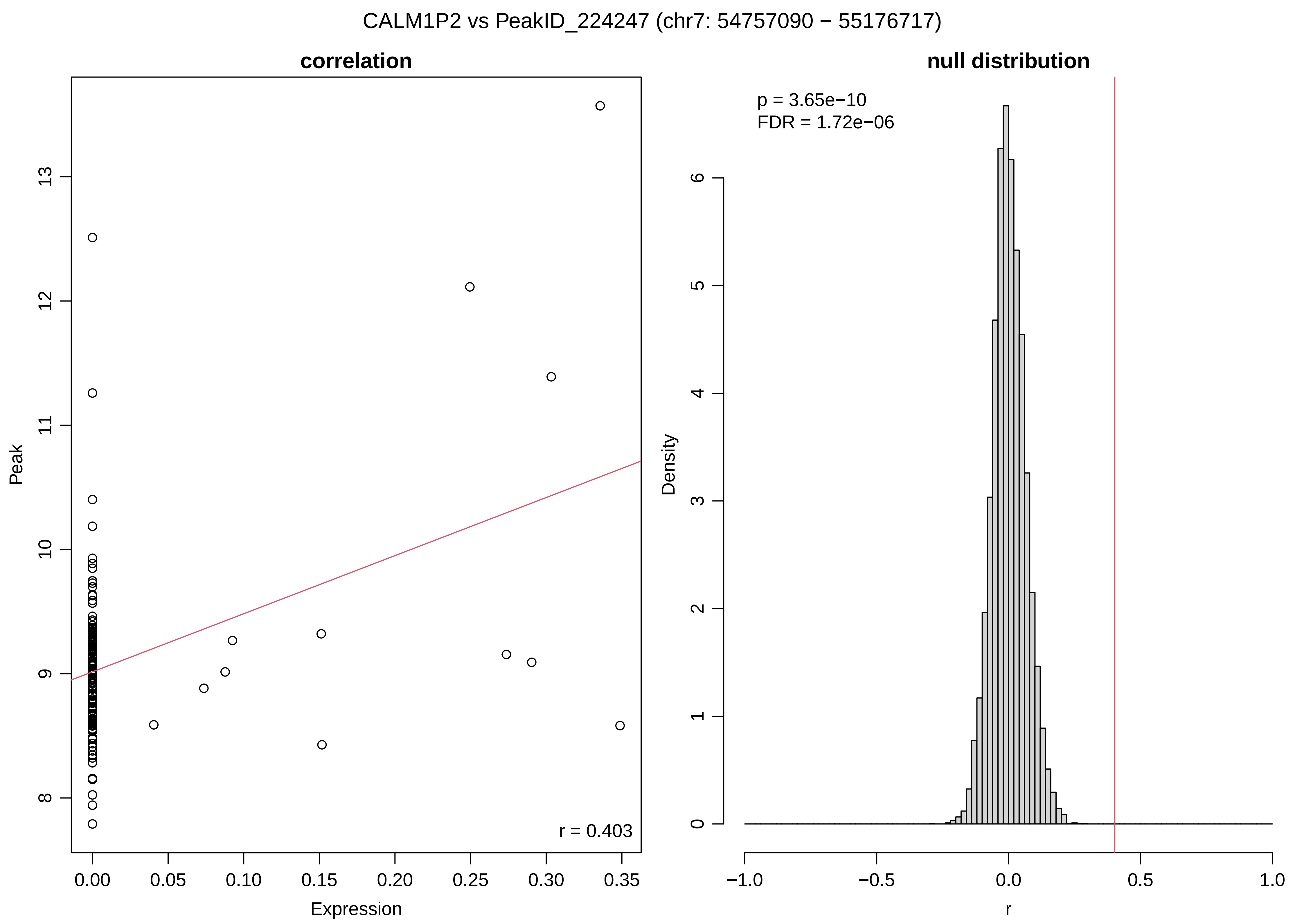

CALM1P2 vs PeakID_224247 (chr7: 54757090 − 55176717)
correlation
Peak
Expression
r = 0.403
0.00
0.05
0.10
0.15
0.20
0.25
0.30
0.35
8
9
10
11
12
13
null distribution
p = 3.65e−10
FDR = 1.72e−06
Density
r
−1.0
−0.5
0.0
0.5
1.0
0
1
2
3
4
5
6

AP000844.2 vs PeakID_48060 (chr11: 131896522 − 131897340)

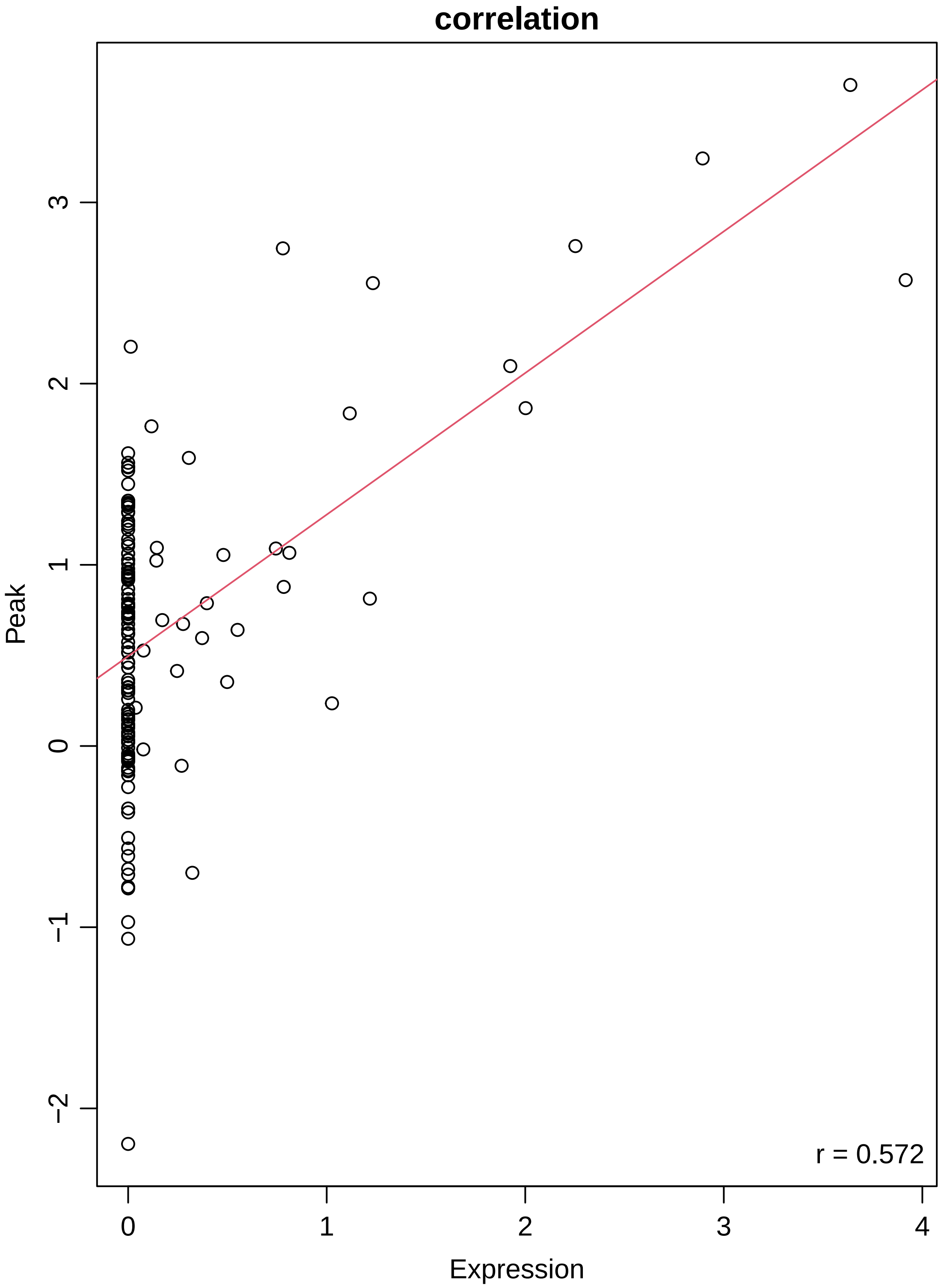

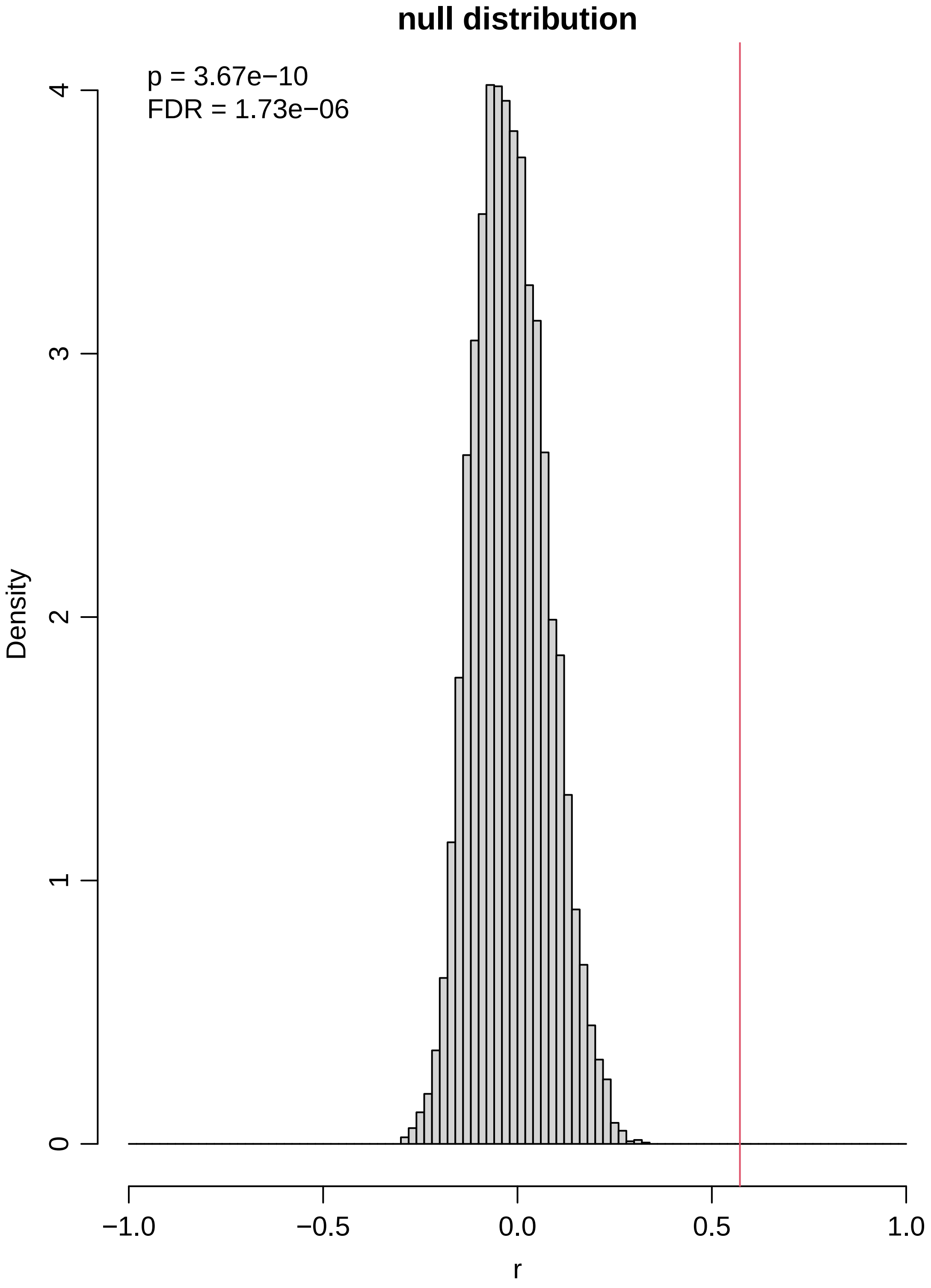

AC126755.5 vs PeakID_87109 (chr16: 18383559 − 18384167)

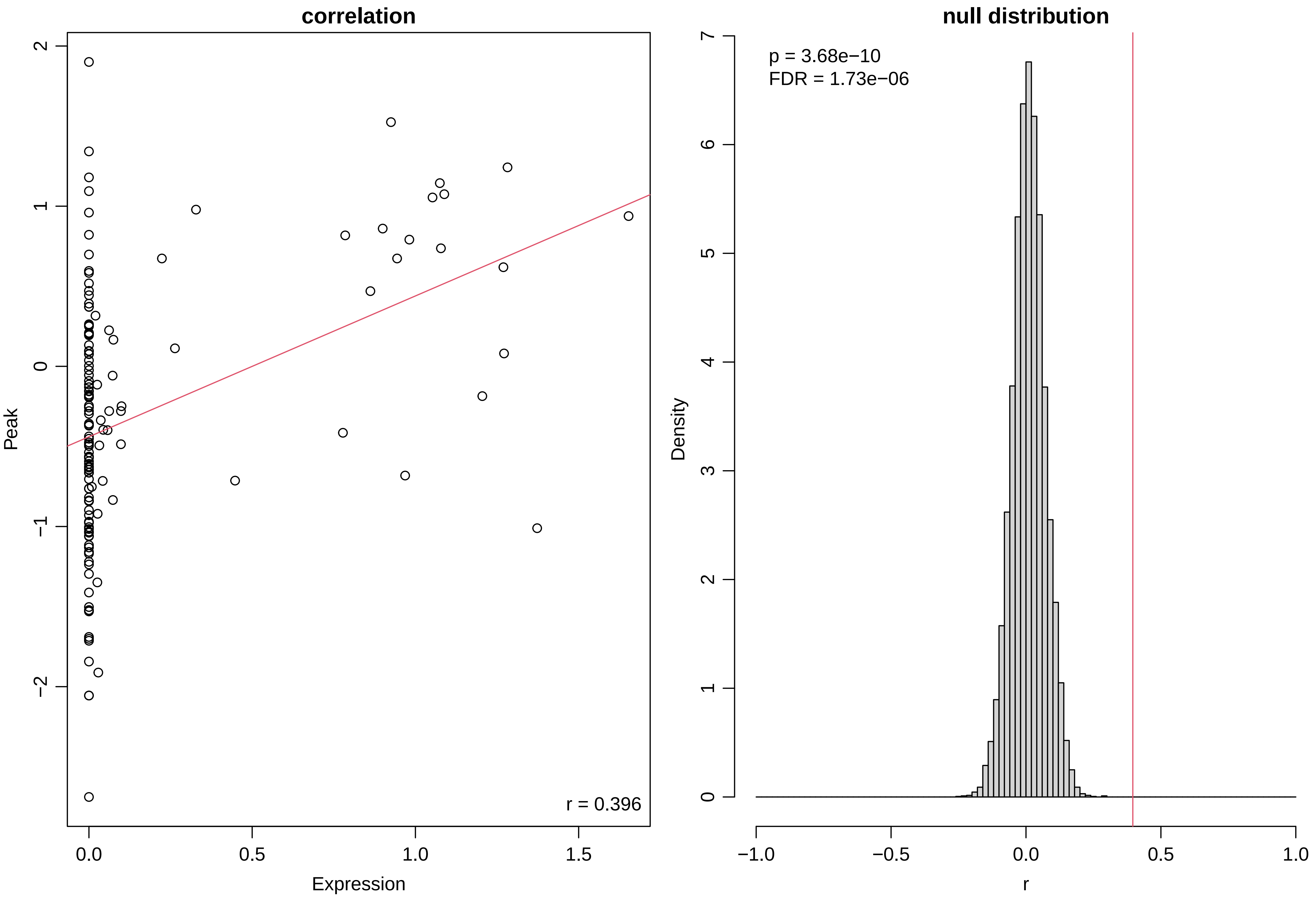

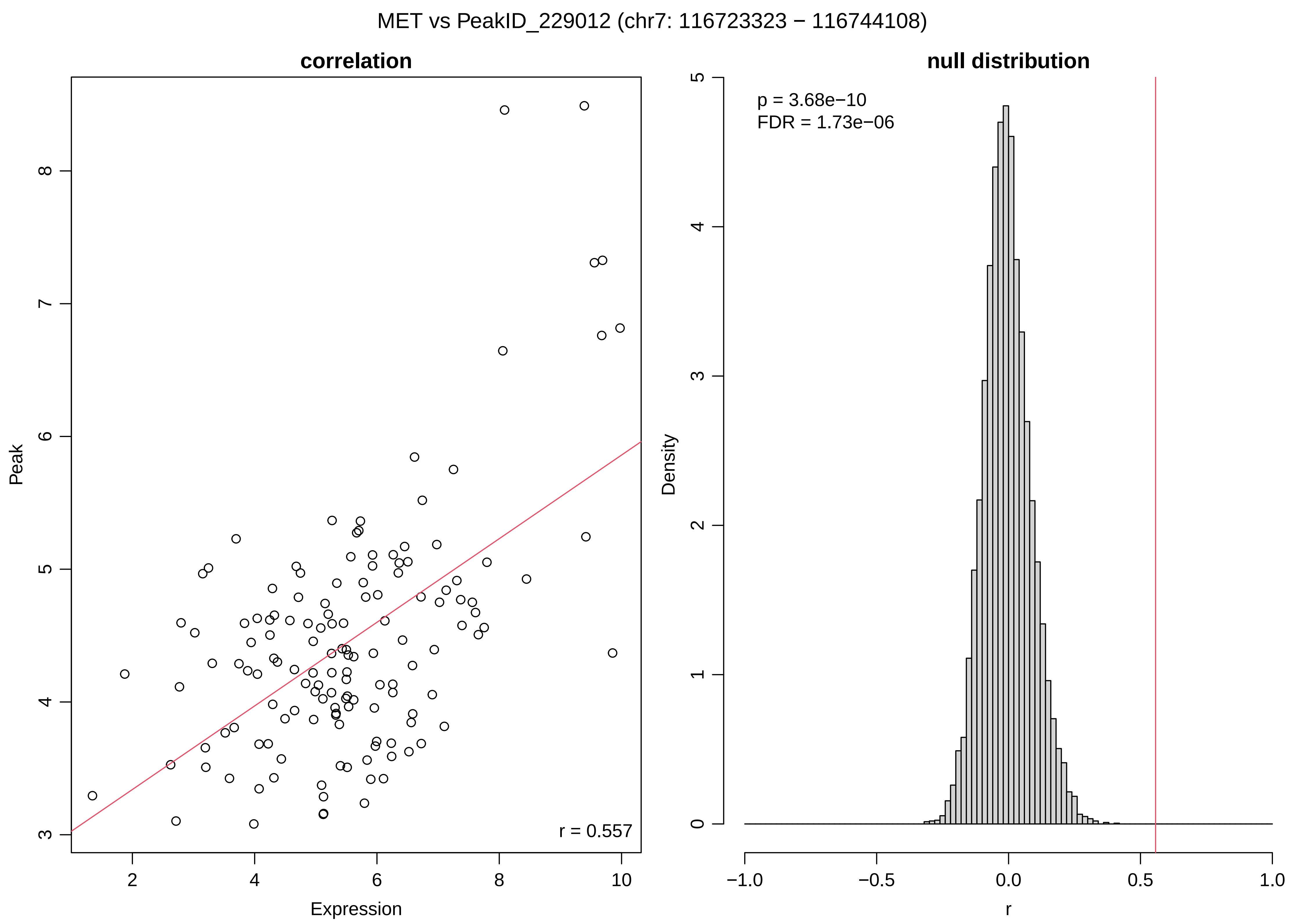

MET vs PeakID_229012 (chr7: 116723323 − 116744108)
correlation
Peak
Expression
r = 0.557
null distribution
p = 3.68e−10
FDR = 1.73e−06
Density
r

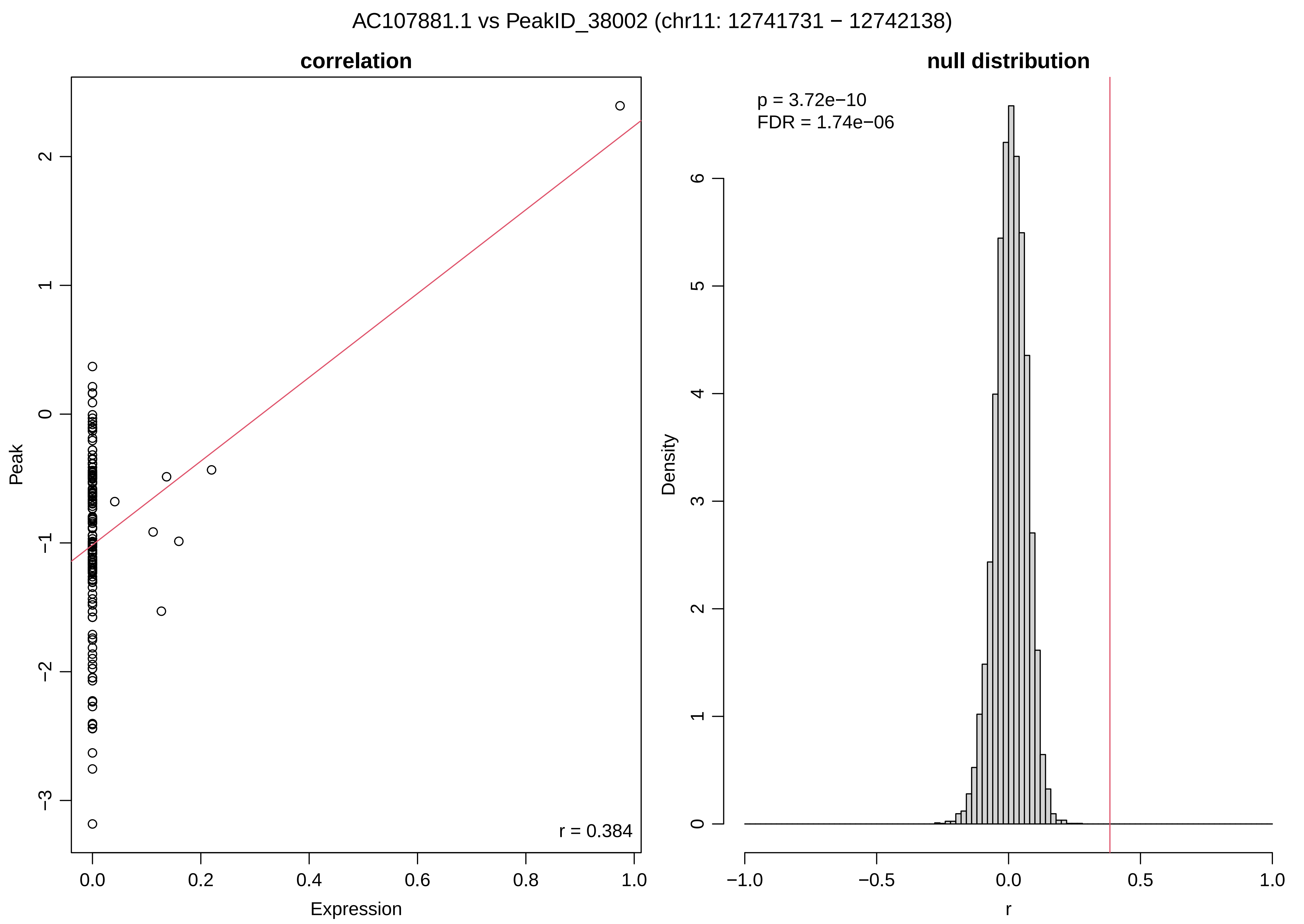
AC107881.1 vs PeakID_38002 (chr11: 12741731 − 12742138)
correlation
Peak
Expression
r = 0.384
null distribution
p = 3.72e−10
FDR = 1.74e−06
Density
r

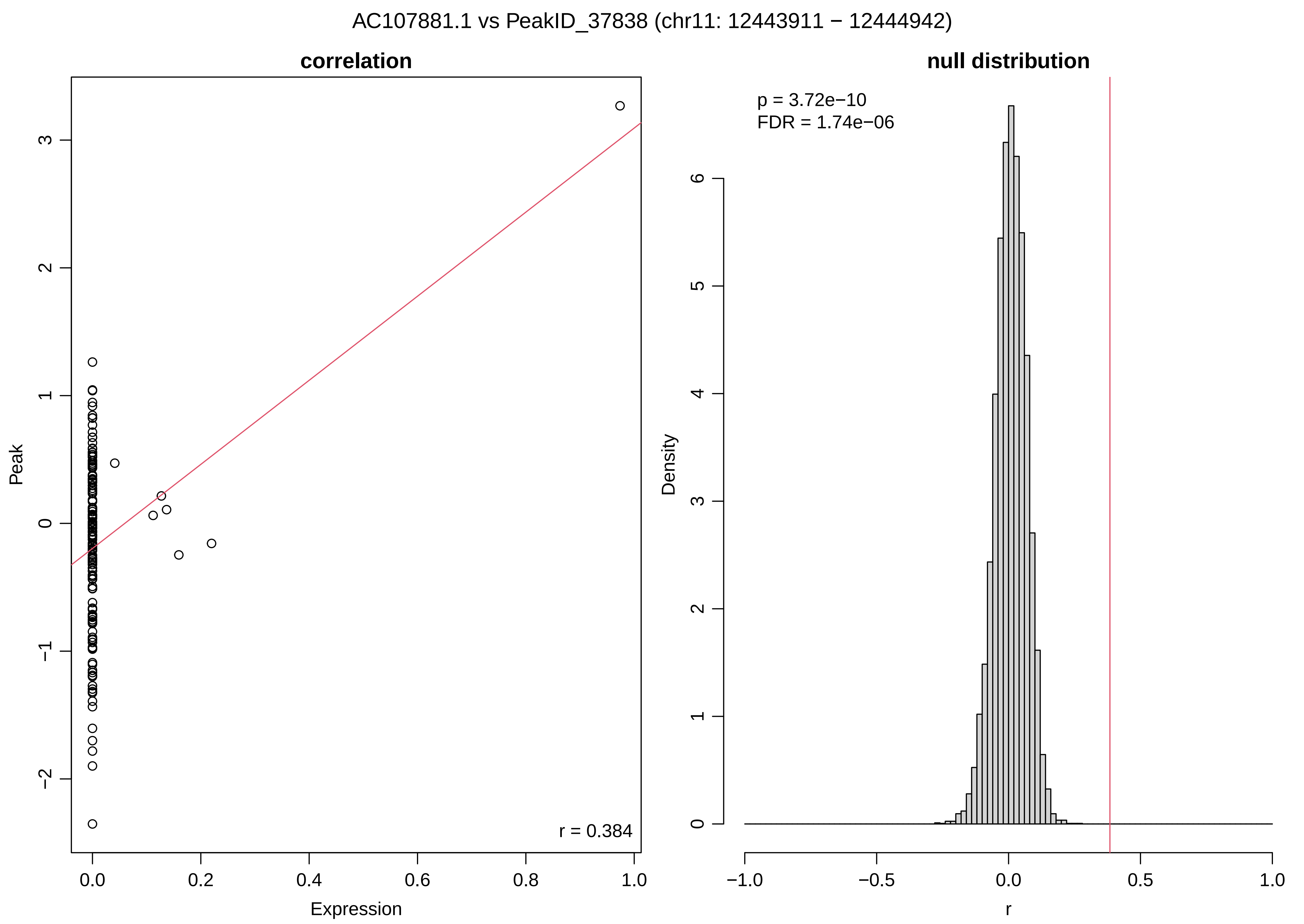
AC107881.1 vs PeakID_37838 (chr11: 12443911 − 12444942)
correlation
null distribution
p = 3.72e−10
FDR = 1.74e−06
r = 0.384
Peak
Expression
Density
r
0.0
0.2
0.4
0.6
0.8
1.0
−2
−1
0
1
2
3
−1.0
−0.5
0.0
0.5
1.0
0
1
2
3
4
5
6

FAM224B vs PeakID_262812 (chrY: 17806876 − 17807776)

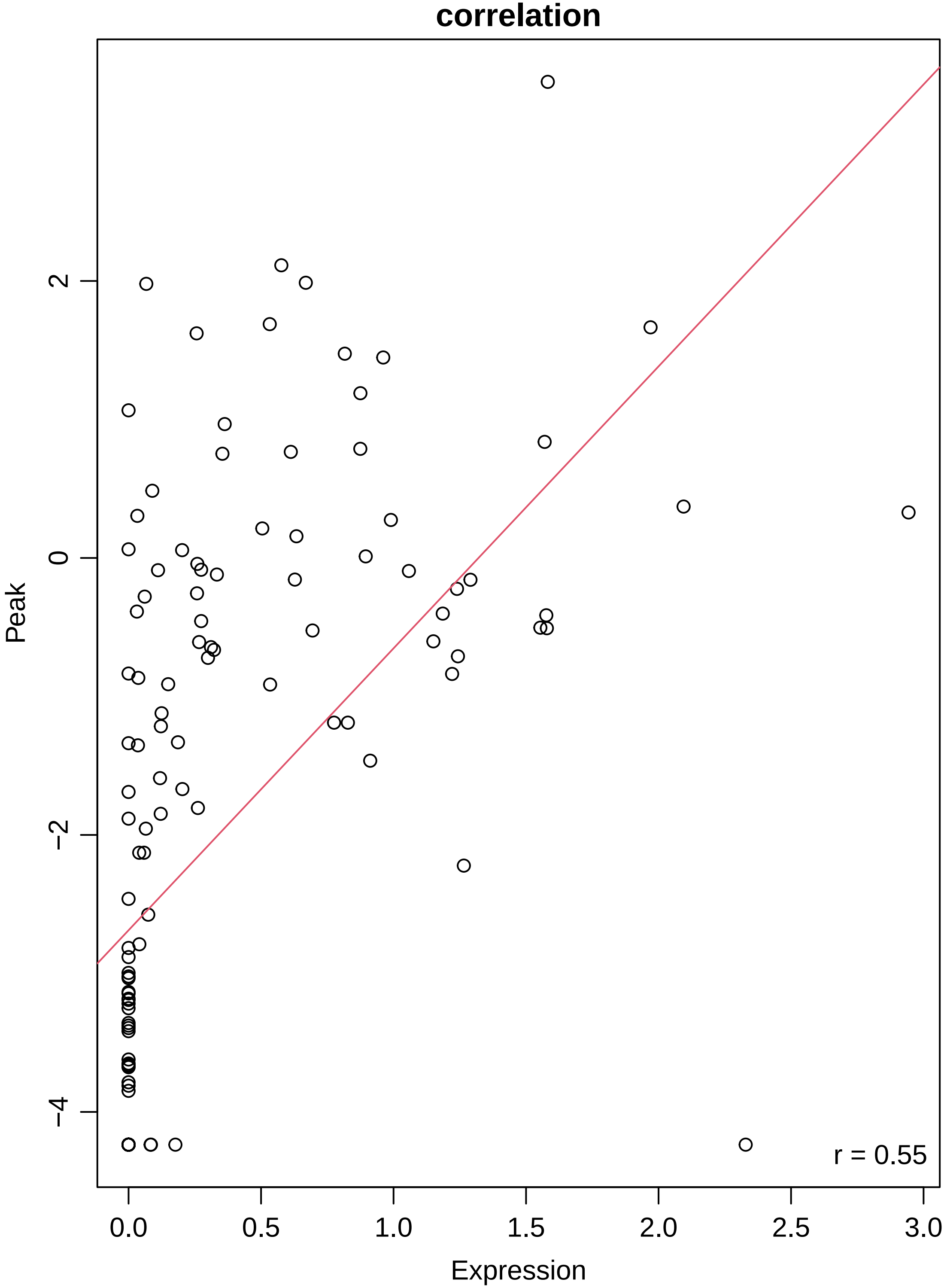

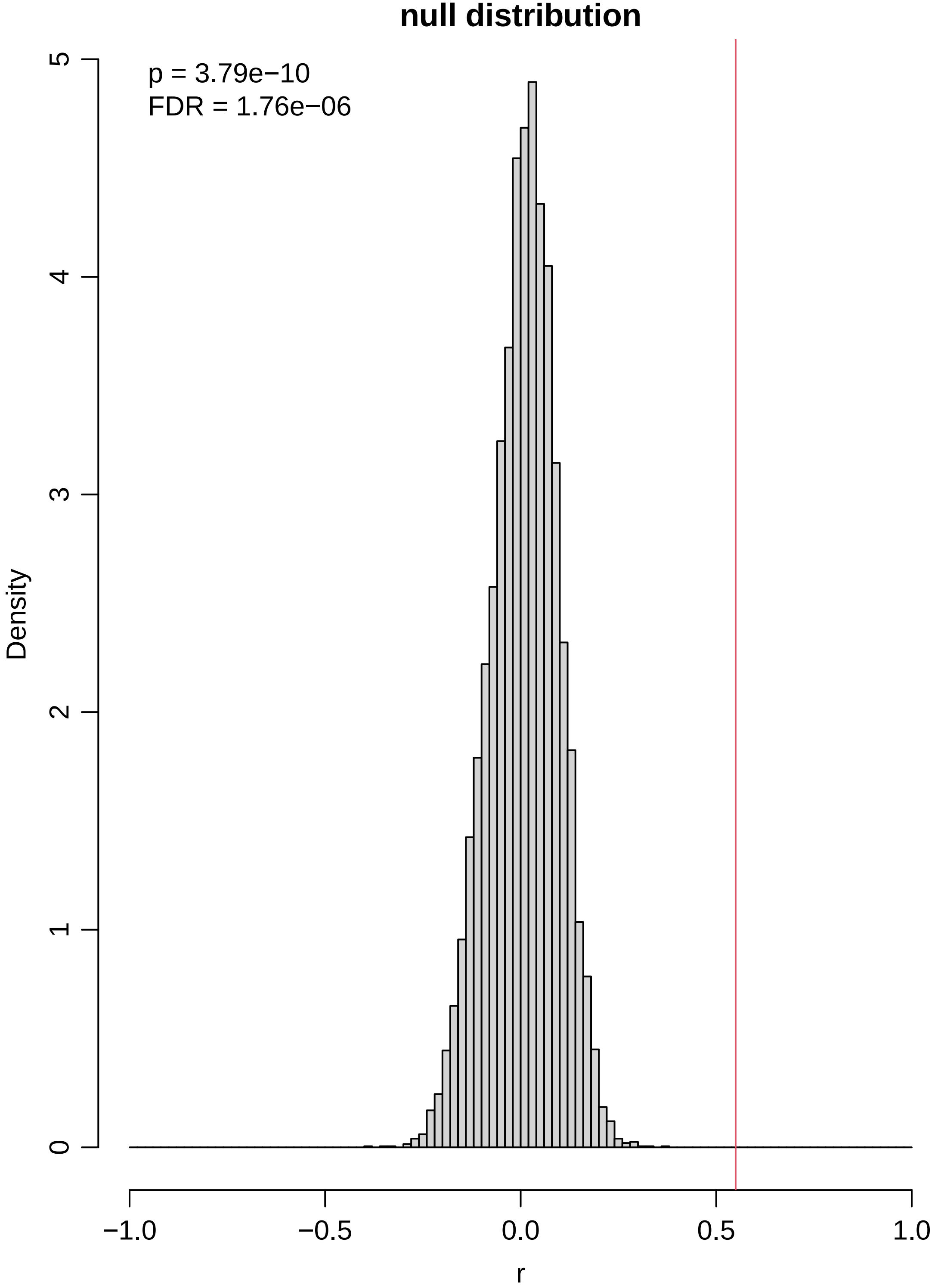

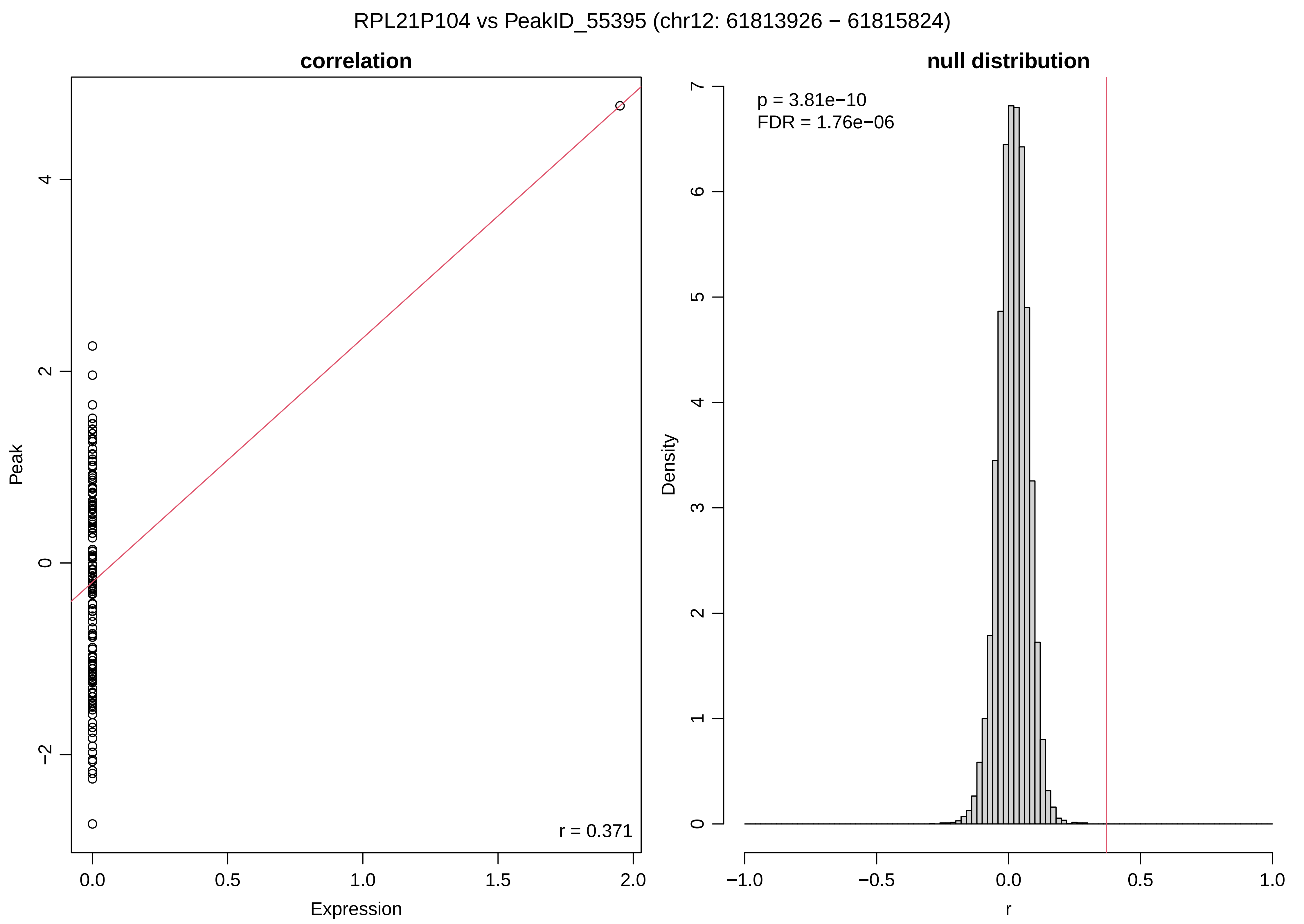

RPL21P104 vs PeakID_55395 (chr12: 61813926 − 61815824)
correlation
Peak
Expression
r = 0.371
null distribution
Density
r
p = 3.81e−10
FDR = 1.76e−06

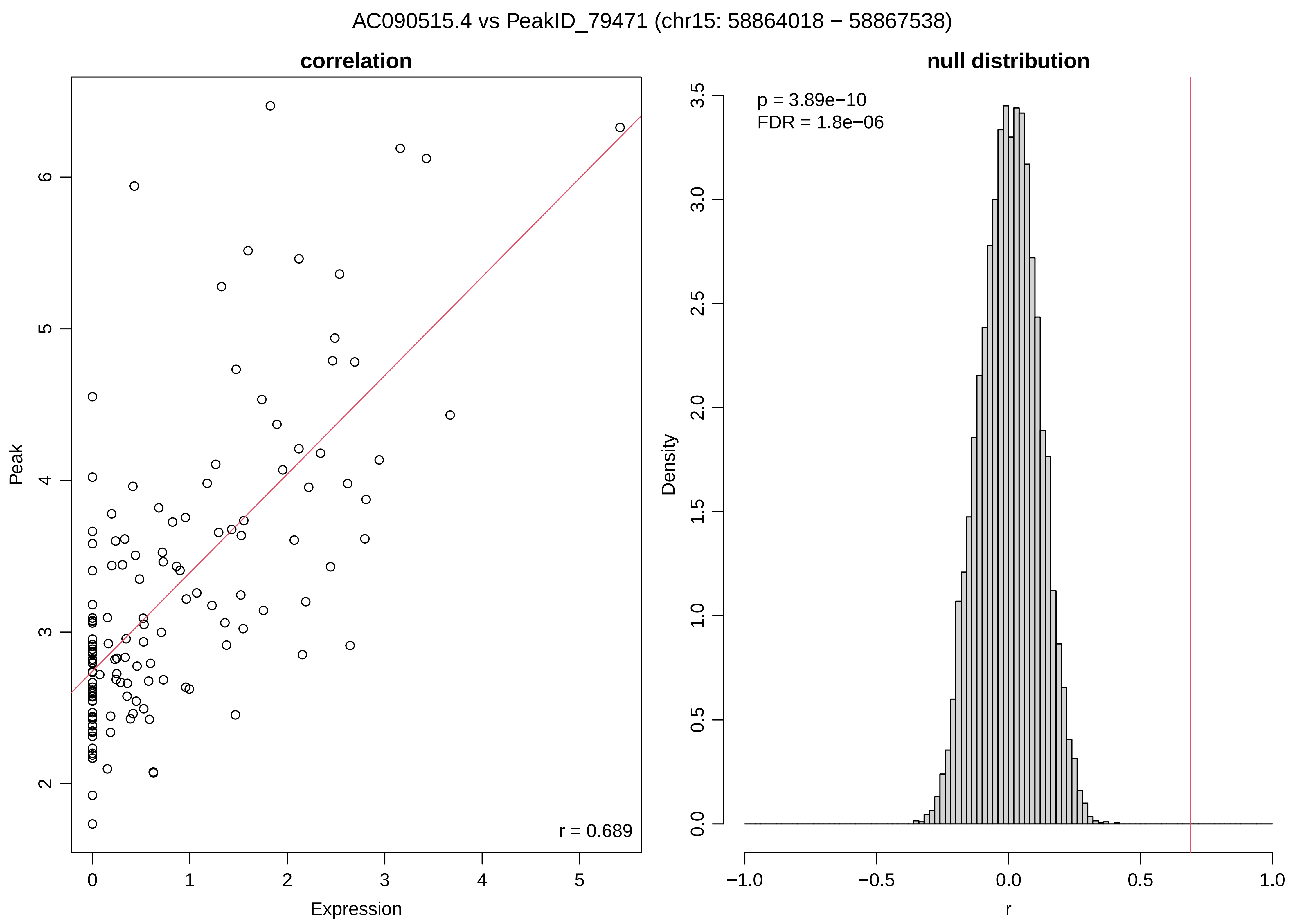
AC090515.4 vs PeakID_79471 (chr15: 58864018 − 58867538)
correlation
Peak
Expression
r = 0.689
null distribution
p = 3.89e−10
FDR = 1.8e−06
Density
r

SLC35E3 vs PeakID_56609 (chr12: 68867556 − 68868890)

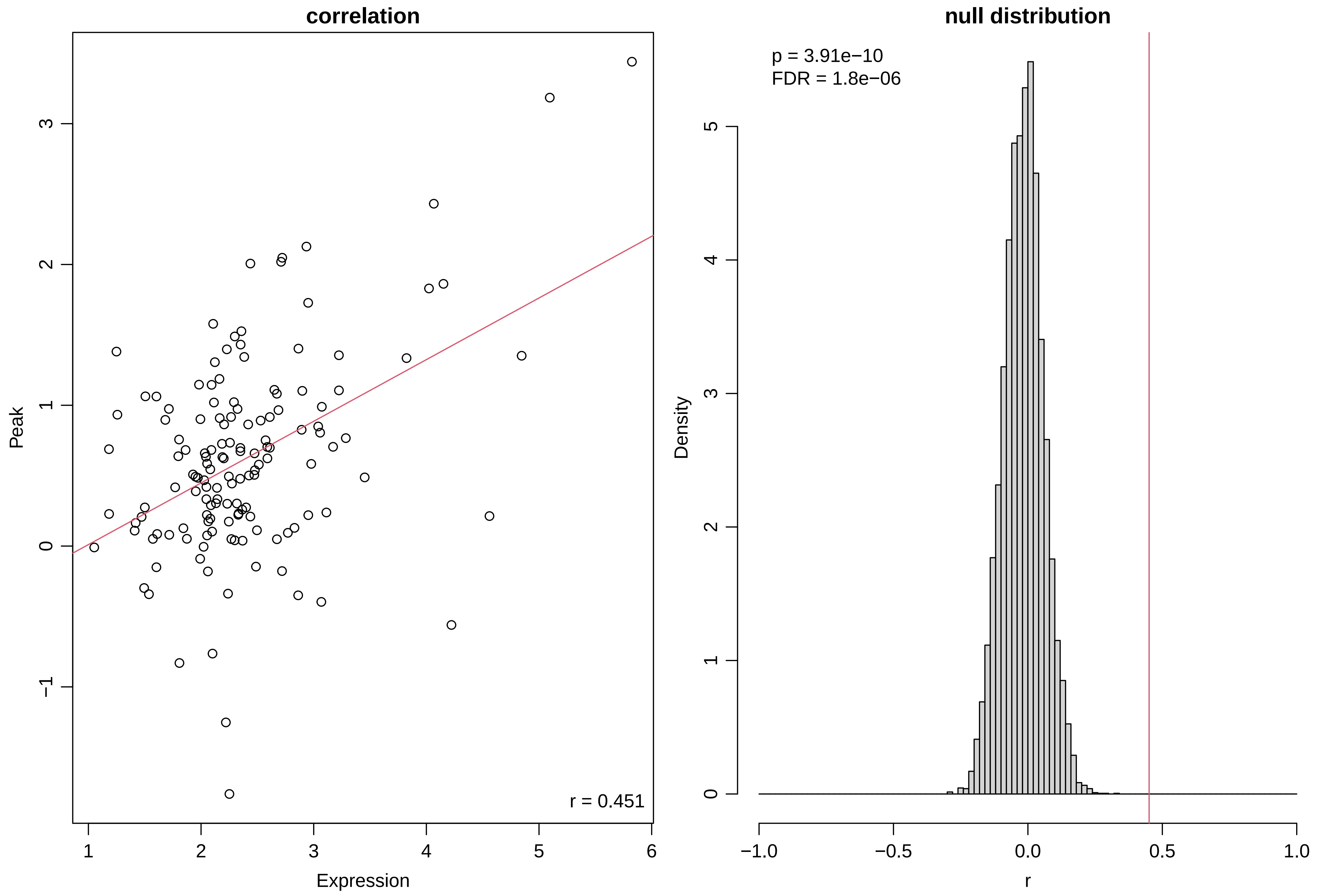

PVT1 vs PeakID_242988 (chr8: 127848279 − 127849453)

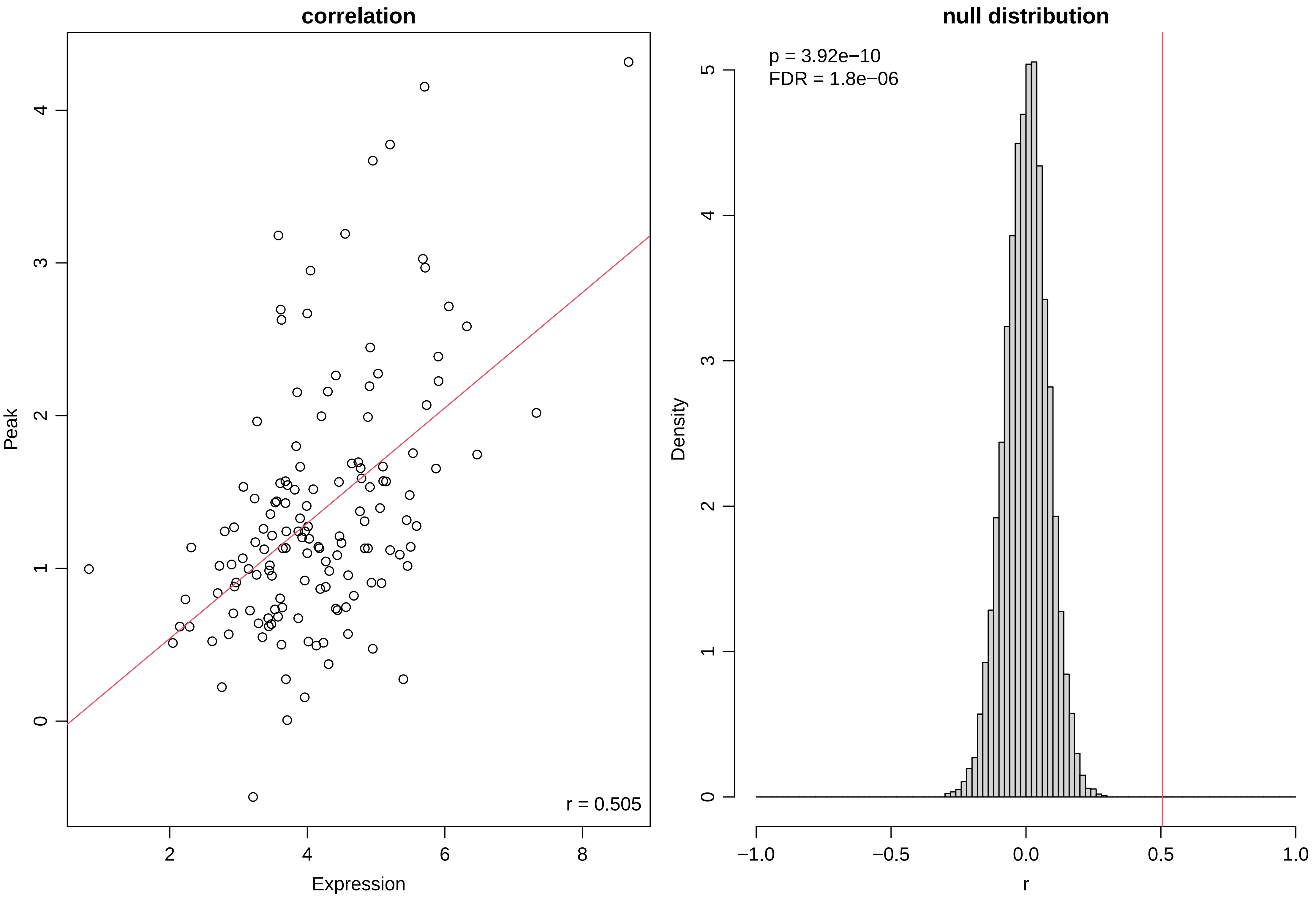

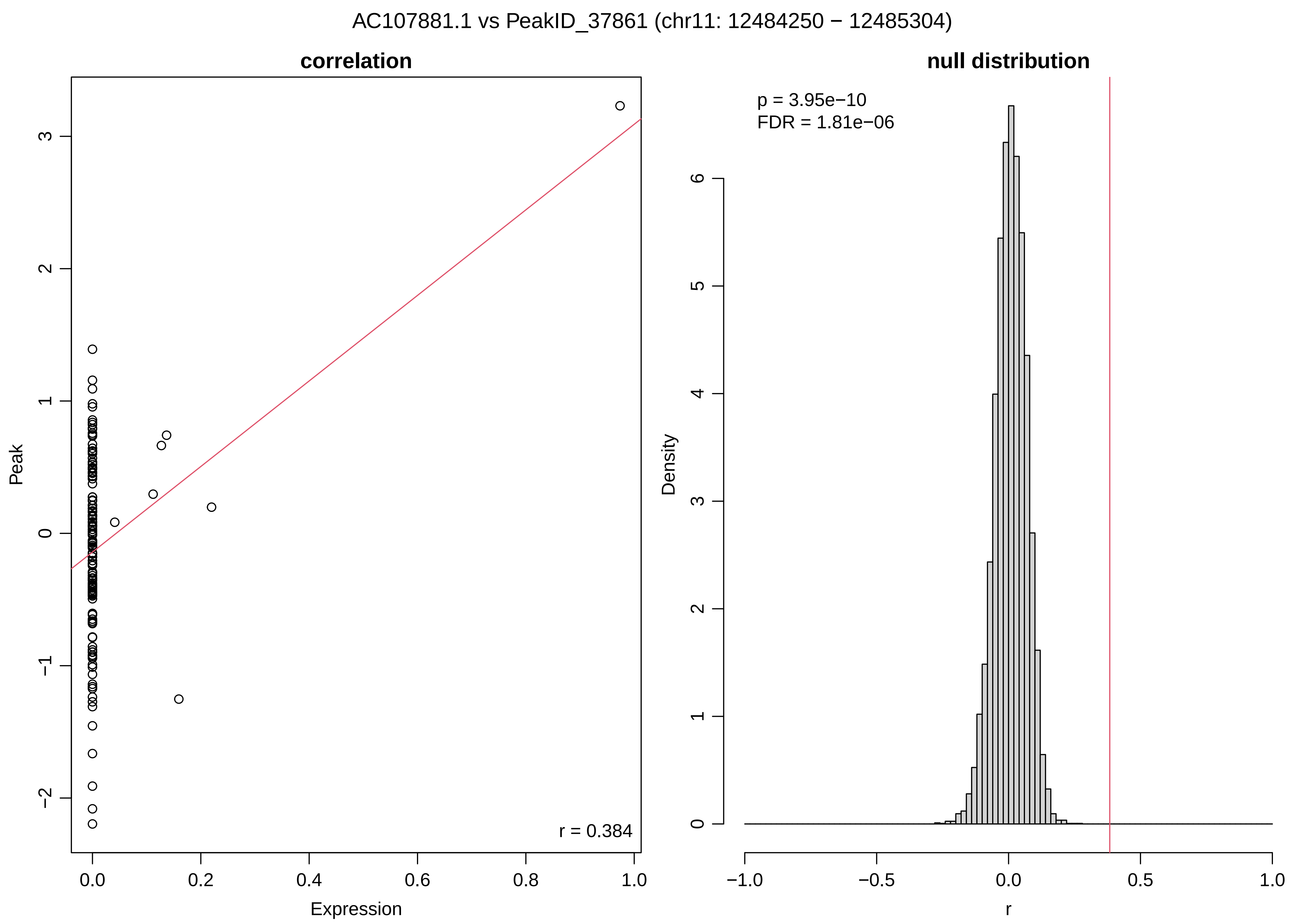
AC107881.1 vs PeakID_37861 (chr11: 12484250 − 12485304)
correlation
null distribution
p = 3.95e−10
FDR = 1.81e−06
r = 0.384
Peak
Expression
Density
r

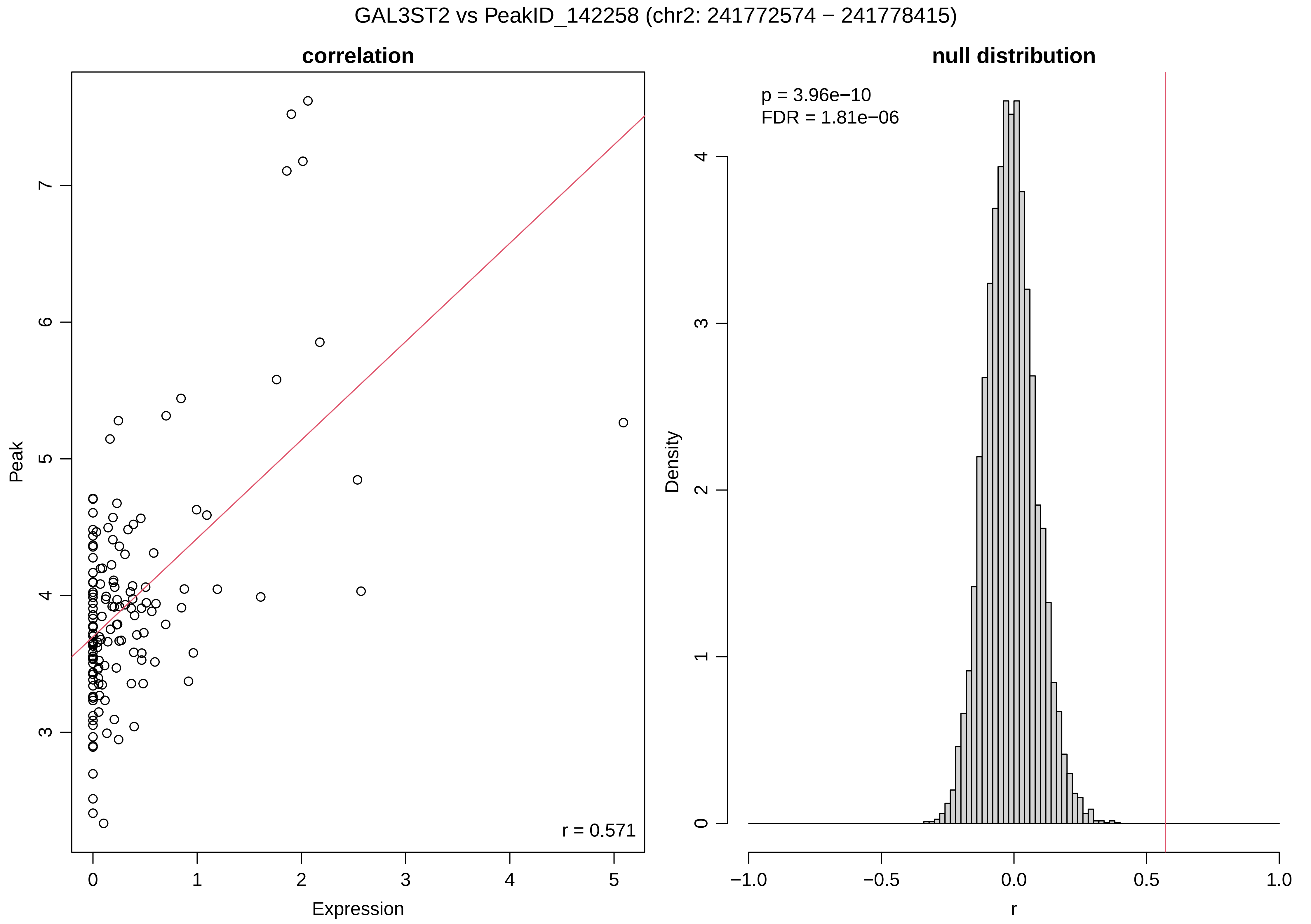
GAL3ST2 vs PeakID_142258 (chr2: 241772574 − 241778415)
correlation
Peak
Expression
r = 0.571
null distribution
p = 3.96e−10
FDR = 1.81e−06
Density
r

AKR1C1 vs PeakID_24120 (chr10: 4930960 − 4949938)

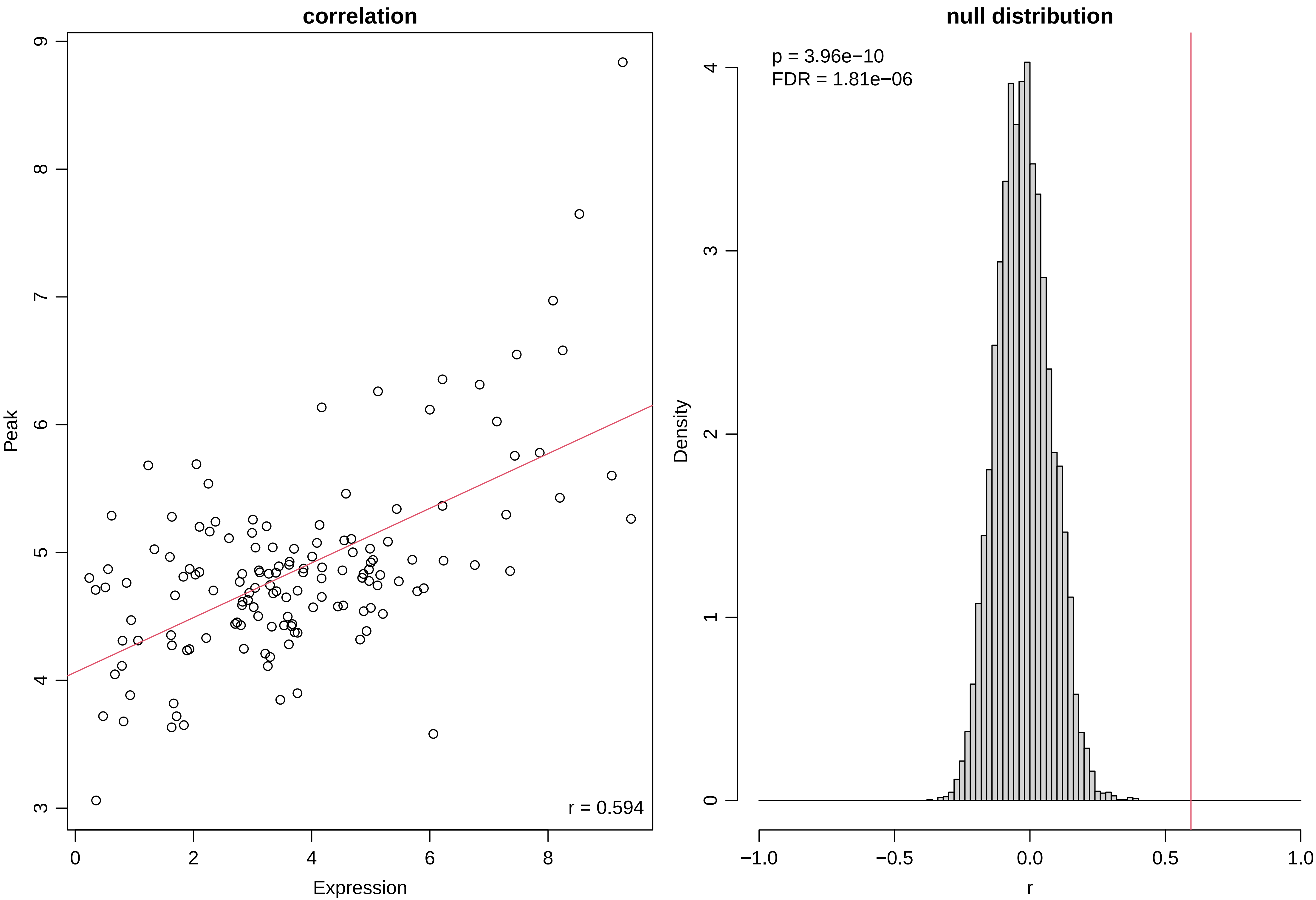

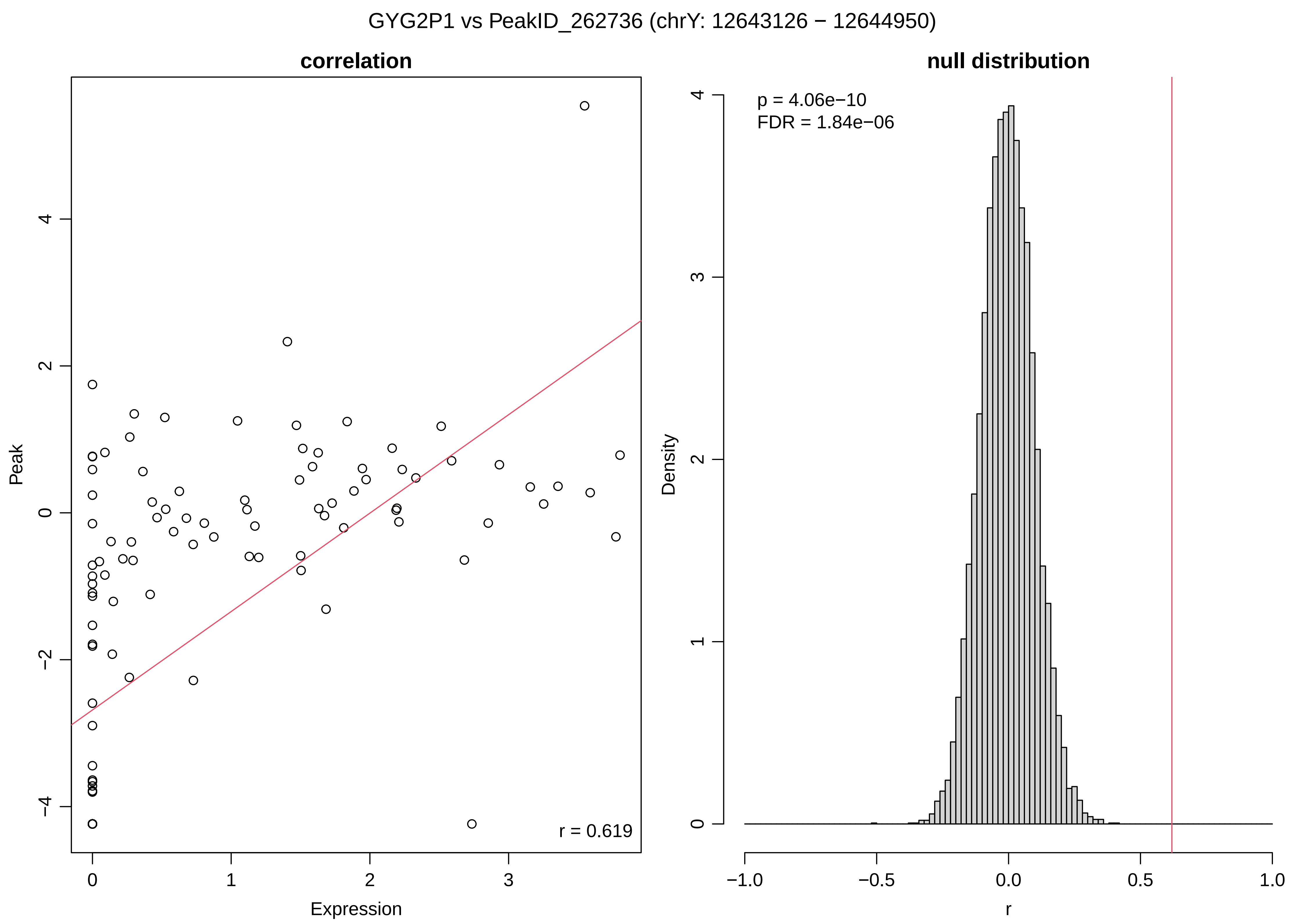
GYG2P1 vs PeakID_262736 (chrY: 12643126 − 12644950)
correlation
Peak
Expression
r = 0.619
null distribution
p = 4.06e−10
FDR = 1.84e−06
Density
r

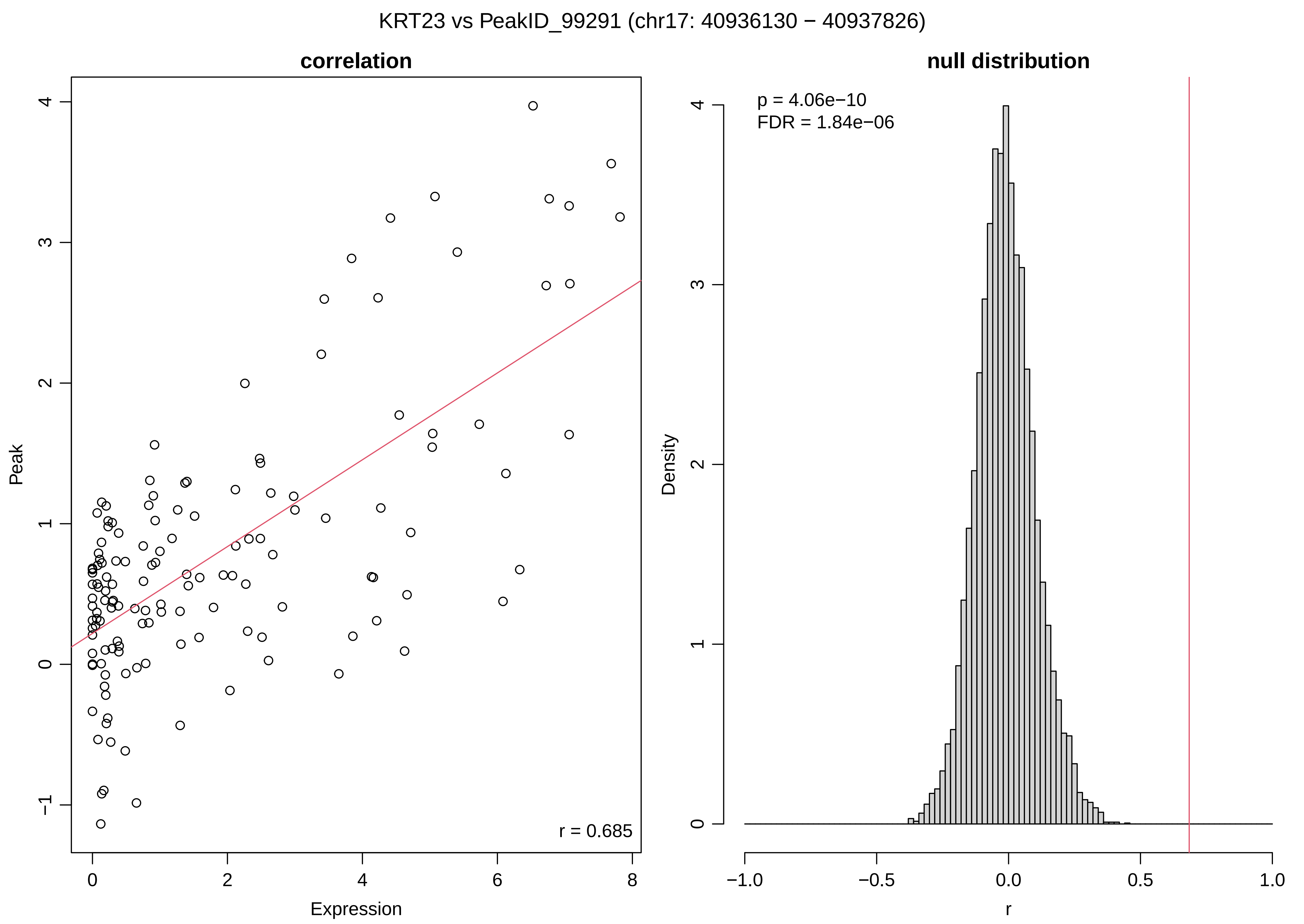
KRT23 vs PeakID_99291 (chr17: 40936130 − 40937826)
correlation
Peak
Expression
r = 0.685
null distribution
Density
r
p = 4.06e−10
FDR = 1.84e−06

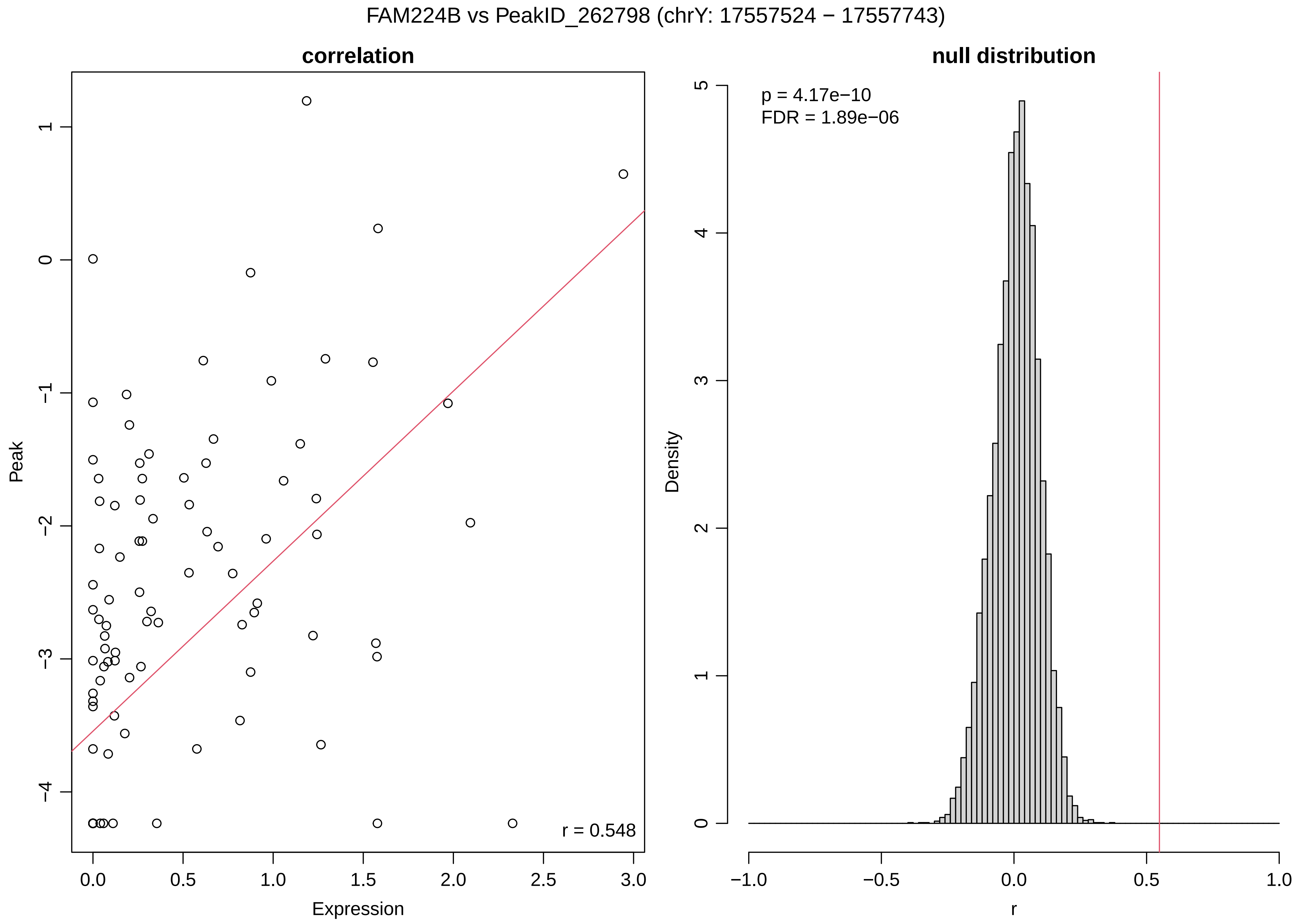

FAM224B vs PeakID_262798 (chrY: 17557524 − 17557743)
correlation
Peak
Expression
r = 0.548
null distribution
p = 4.17e−10
FDR = 1.89e−06
Density
r

AC002066.1 vs PeakID_228982 (chr7: 116498321 − 116506138)

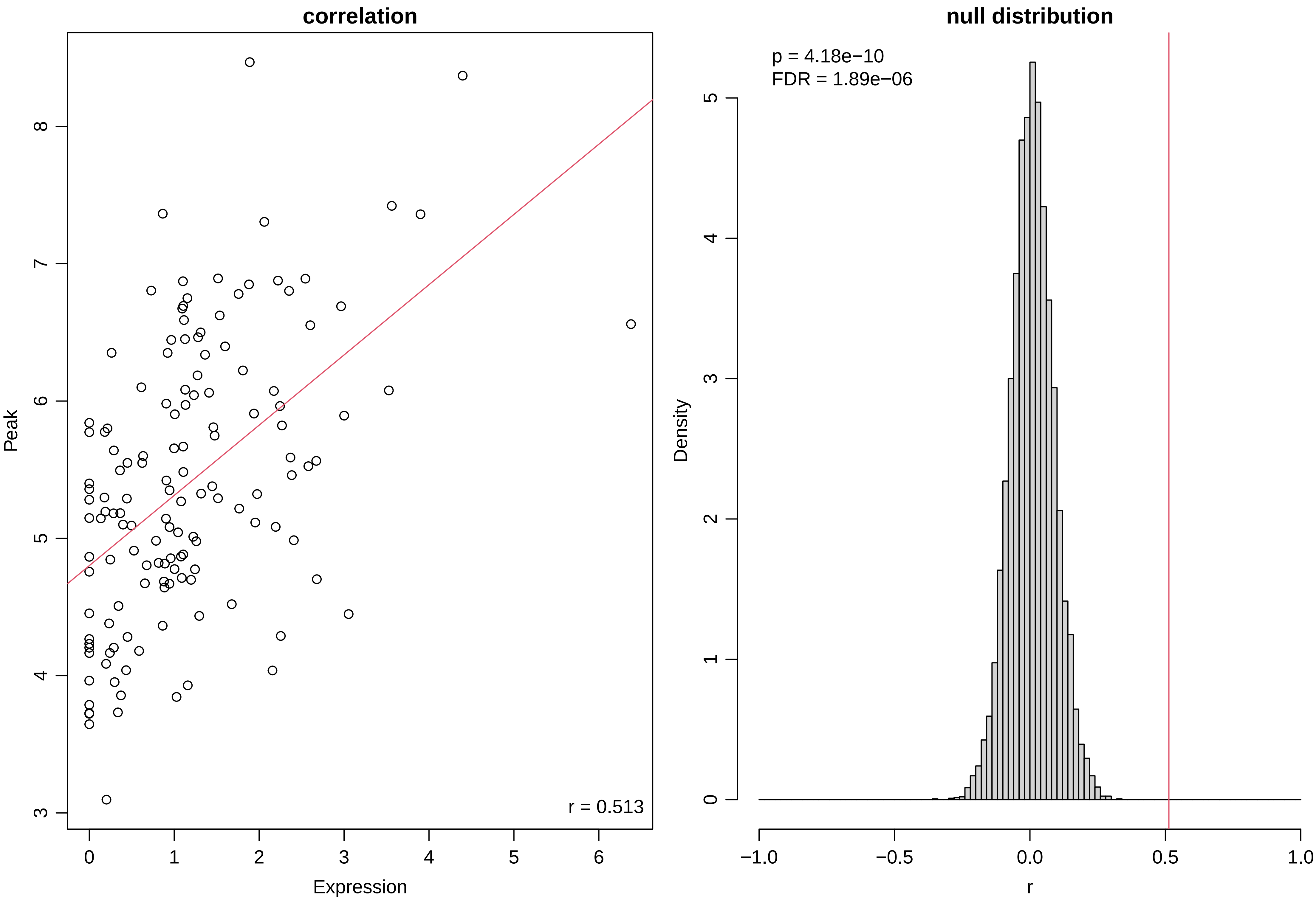

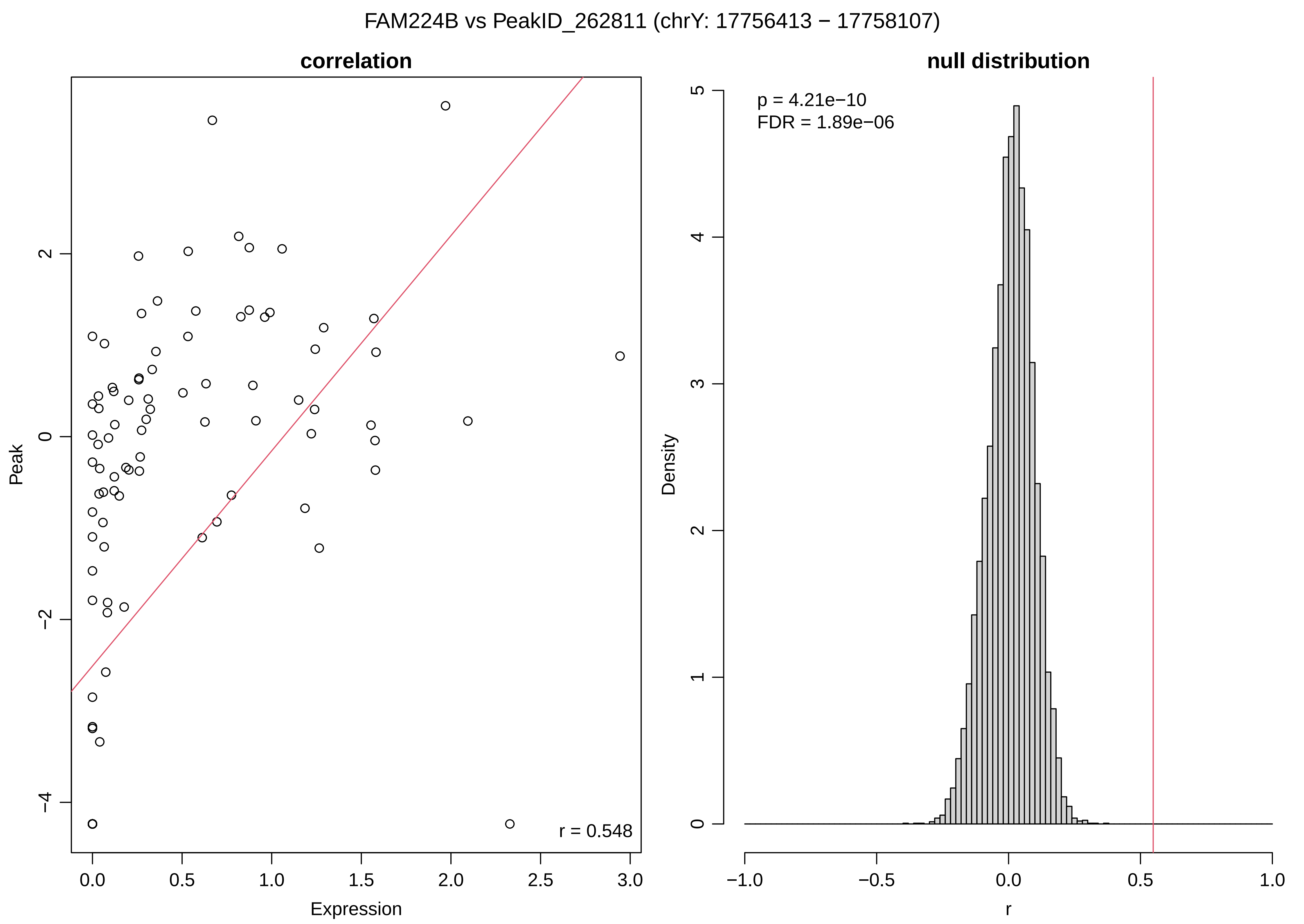
FAM224B vs PeakID_262811 (chrY: 17756413 − 17758107)
correlation
Peak
Expression
r = 0.548
null distribution
p = 4.21e−10
FDR = 1.89e−06
Density
r

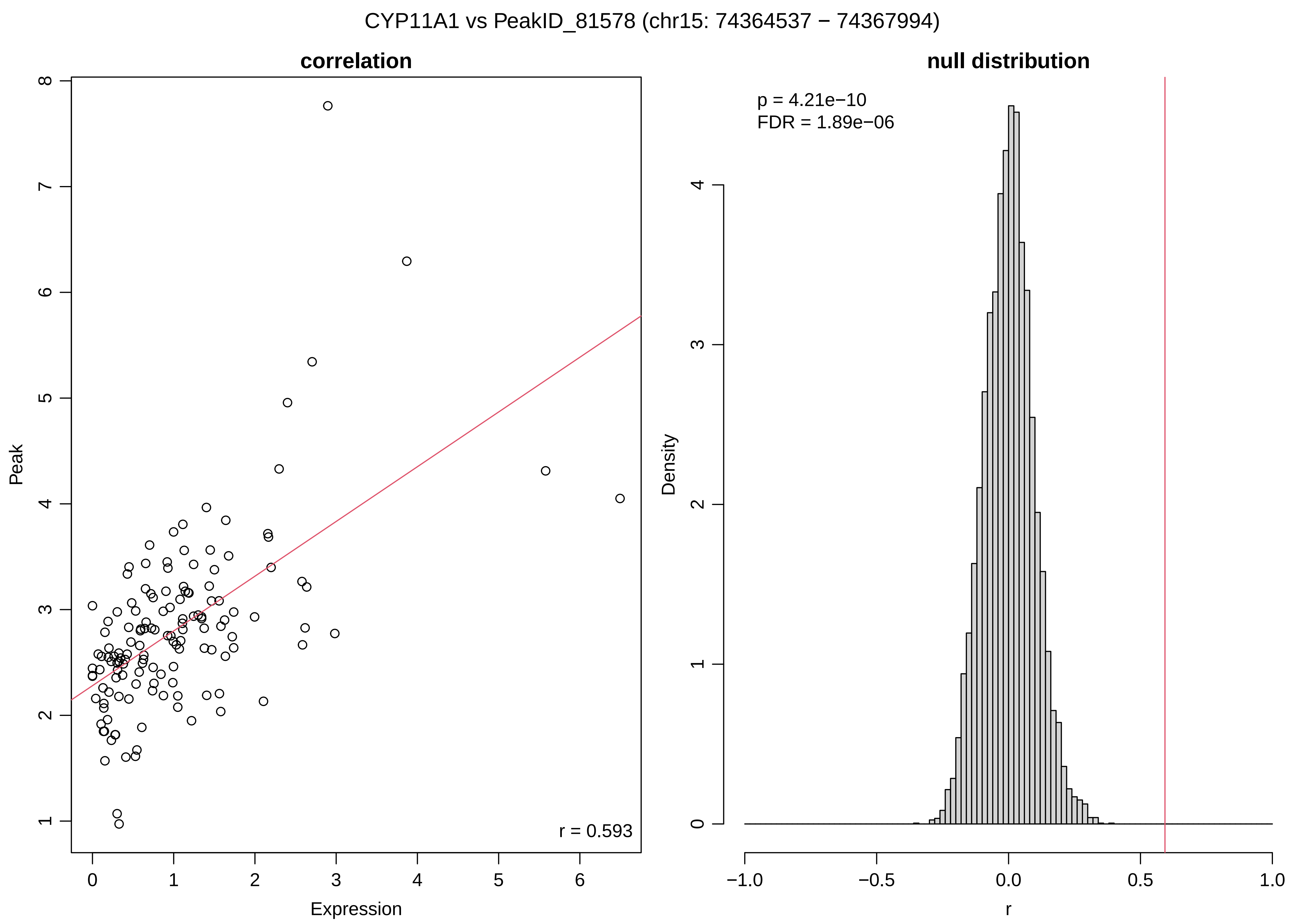
CYP11A1 vs PeakID_81578 (chr15: 74364537 − 74367994)
correlation
Peak
Expression
r = 0.593
null distribution
p = 4.21e−10
FDR = 1.89e−06
Density
r

TTTY10 vs PeakID_262957 (chrY: 20574956 − 20576767)

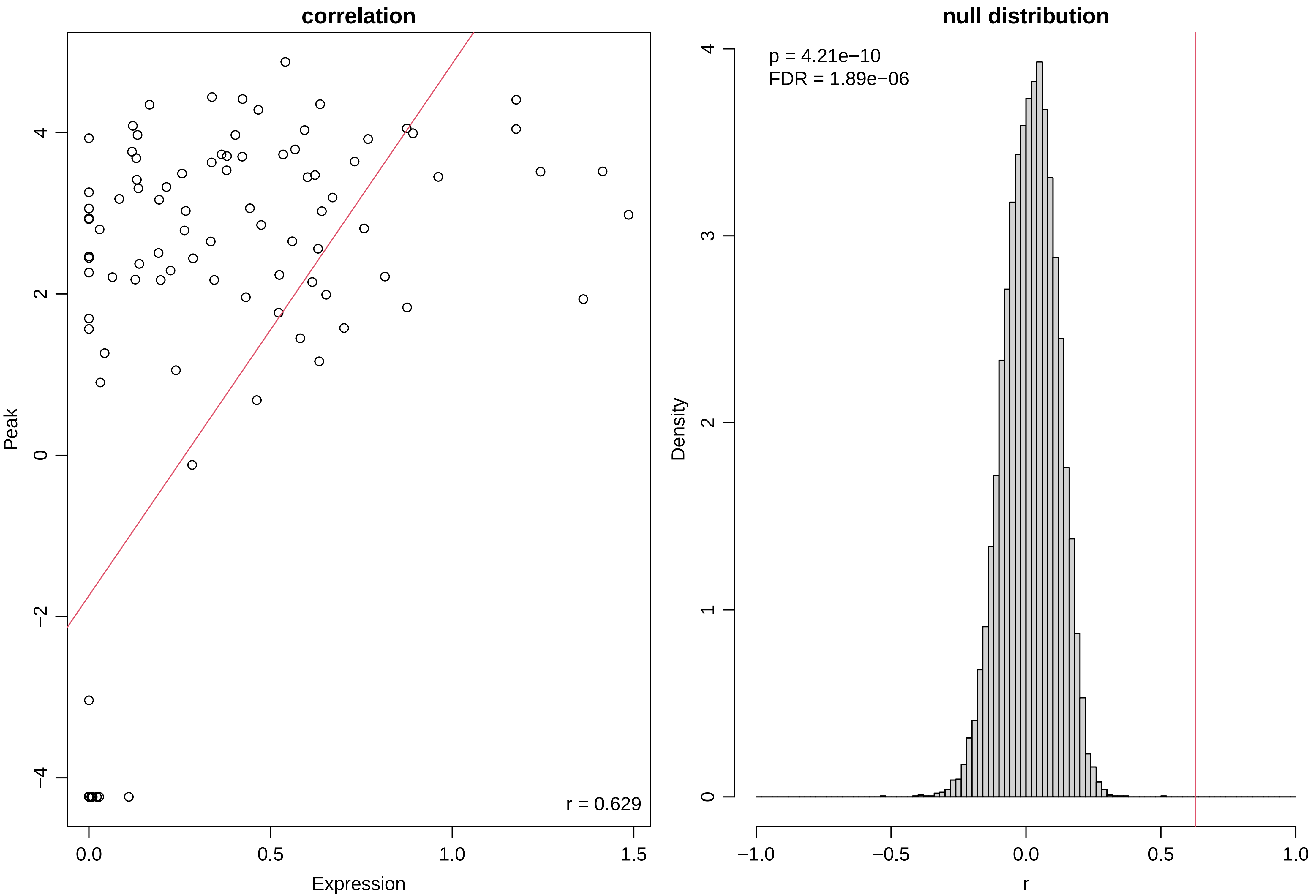

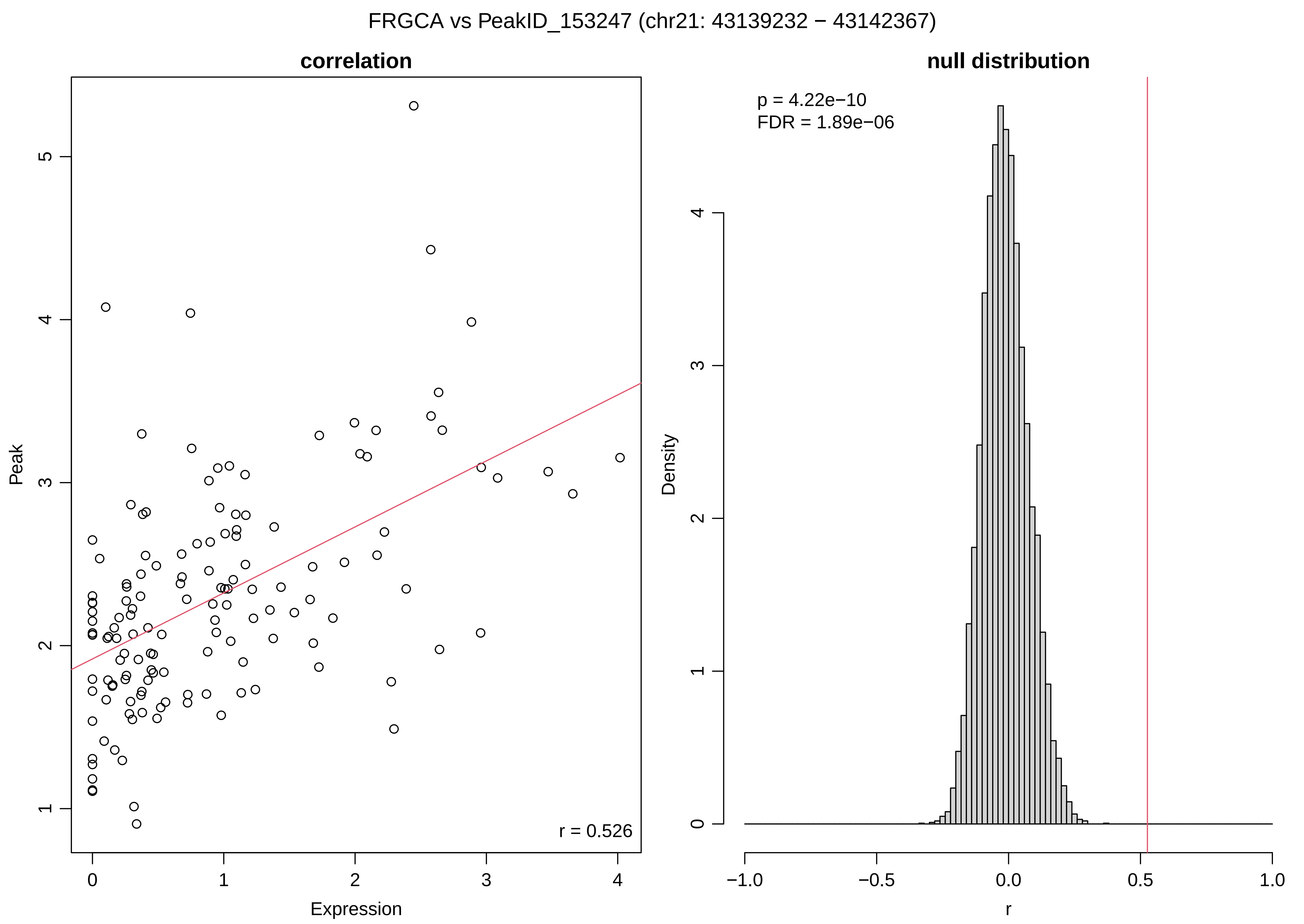

FRGCA vs PeakID_153247 (chr21: 43139232 − 43142367)
correlation
Peak
Expression
r = 0.526
null distribution
p = 4.22e−10
FDR = 1.89e−06
Density
r

RNF212 vs PeakID_175456 (chr4: 1112036 − 1115038)

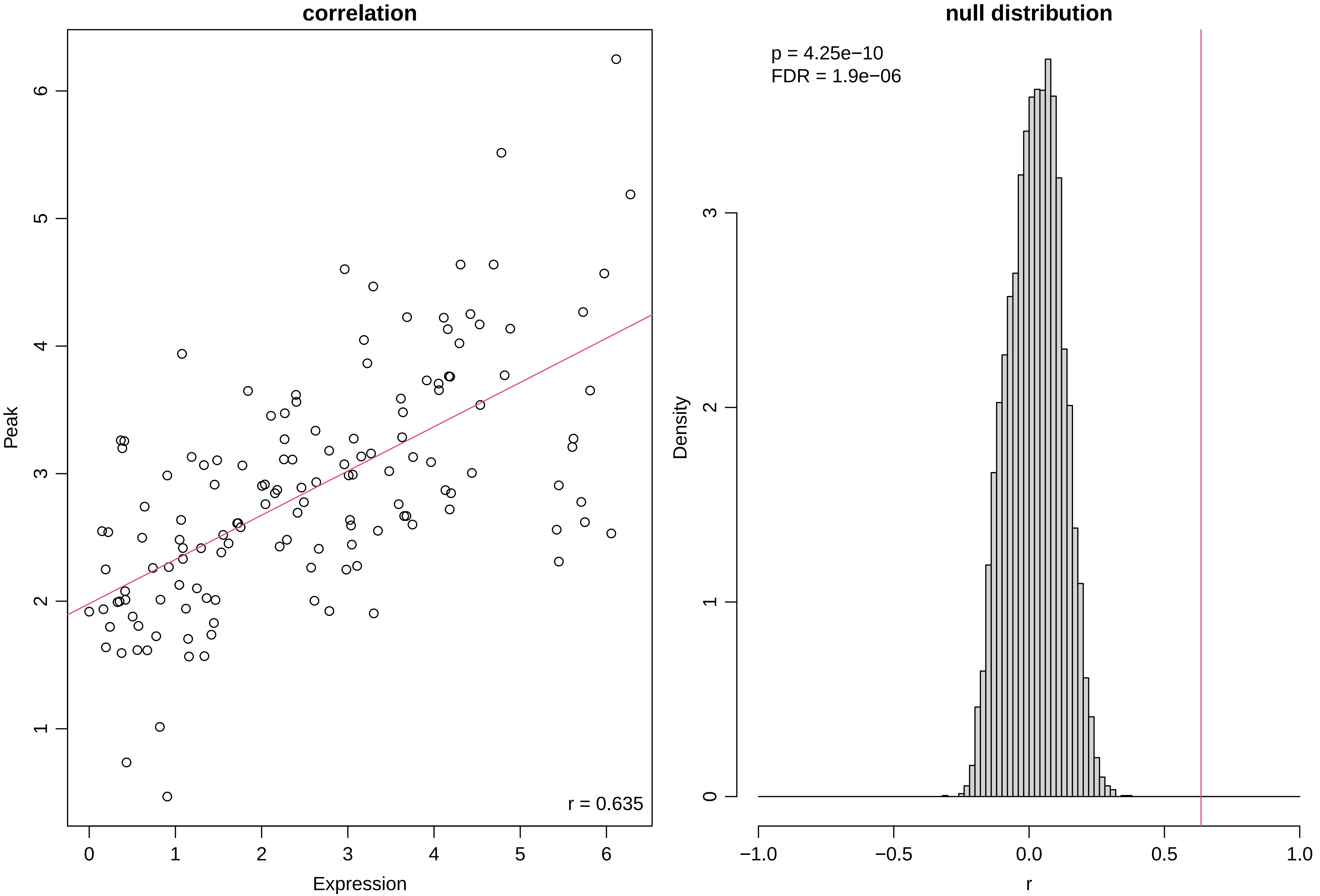

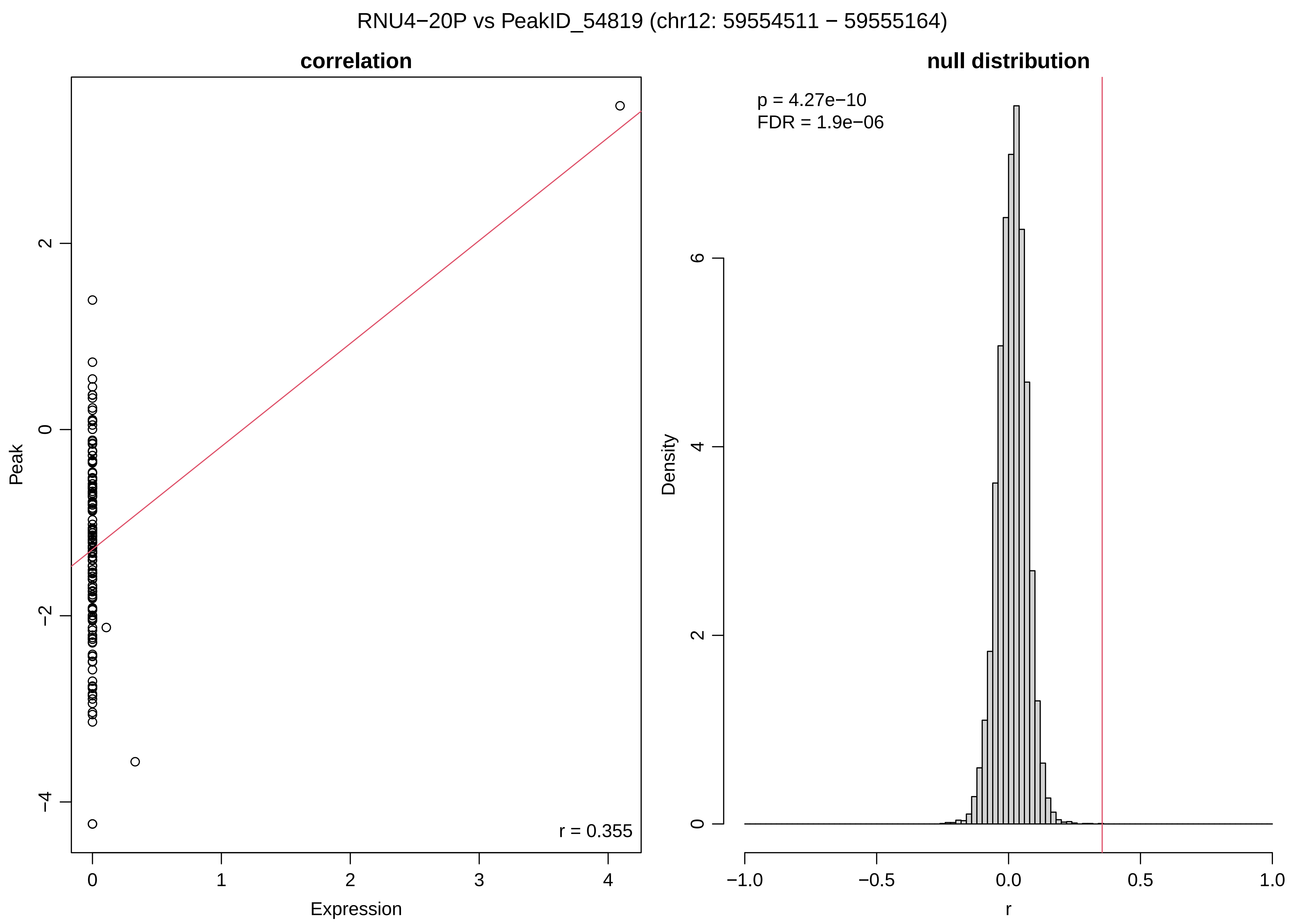
RNU4−20P vs PeakID_54819 (chr12: 59554511 − 59555164)
correlation
Peak
Expression
r = 0.355
null distribution
p = 4.27e−10
FDR = 1.9e−06
Density
r

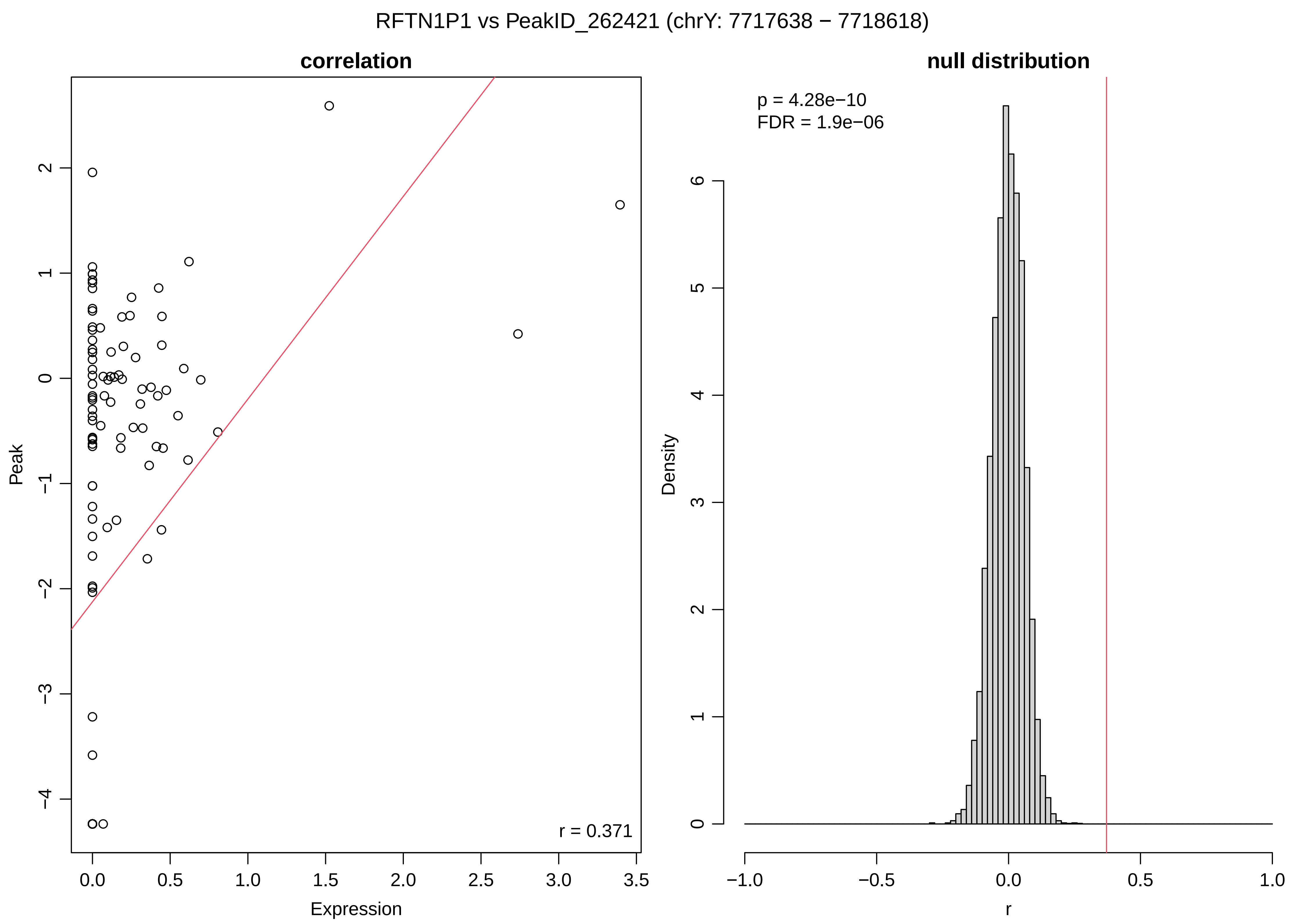

RFTN1P1 vs PeakID_262421 (chrY: 7717638 − 7718618)
correlation
Peak
Expression
r = 0.371
null distribution
p = 4.28e−10
FDR = 1.9e−06
Density
r

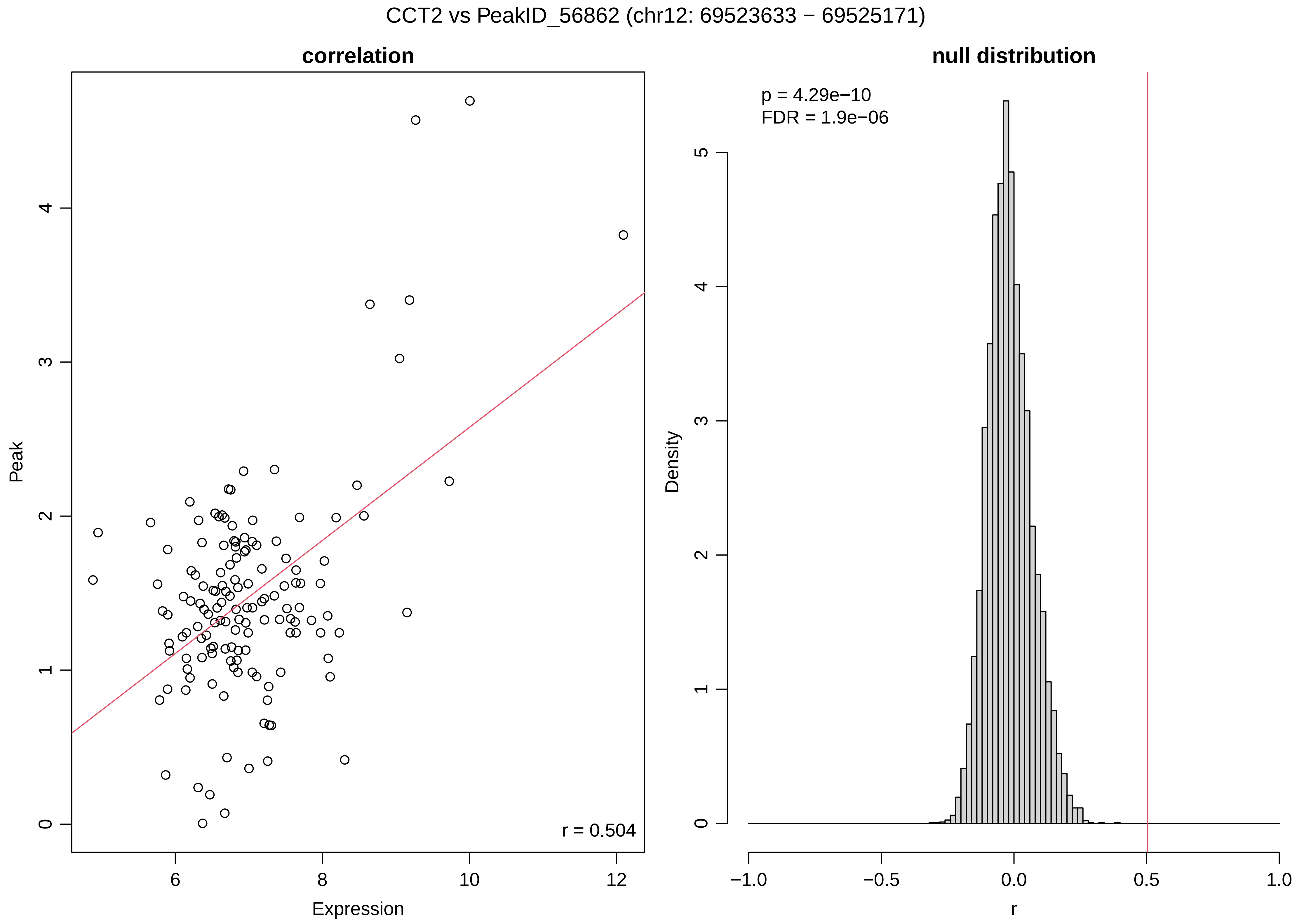
CCT2 vs PeakID_56862 (chr12: 69523633 − 69525171)
correlation
Peak
Expression
r = 0.504
null distribution
p = 4.29e−10
FDR = 1.9e−06
Density
r

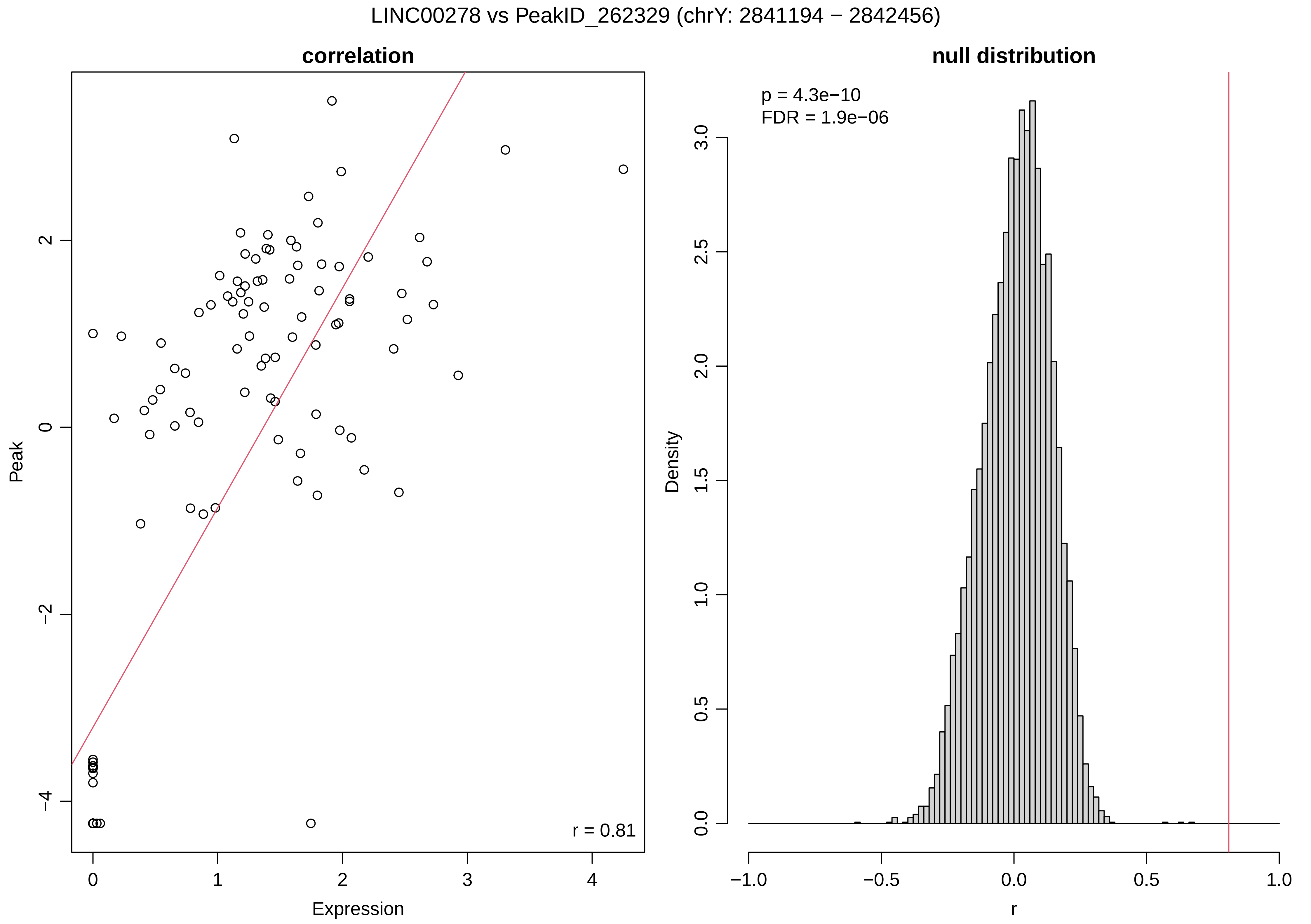
LINC00278 vs PeakID_262329 (chrY: 2841194 − 2842456)
correlation
Peak
Expression
r = 0.81
null distribution
p = 4.3e−10
FDR = 1.9e−06
Density
r

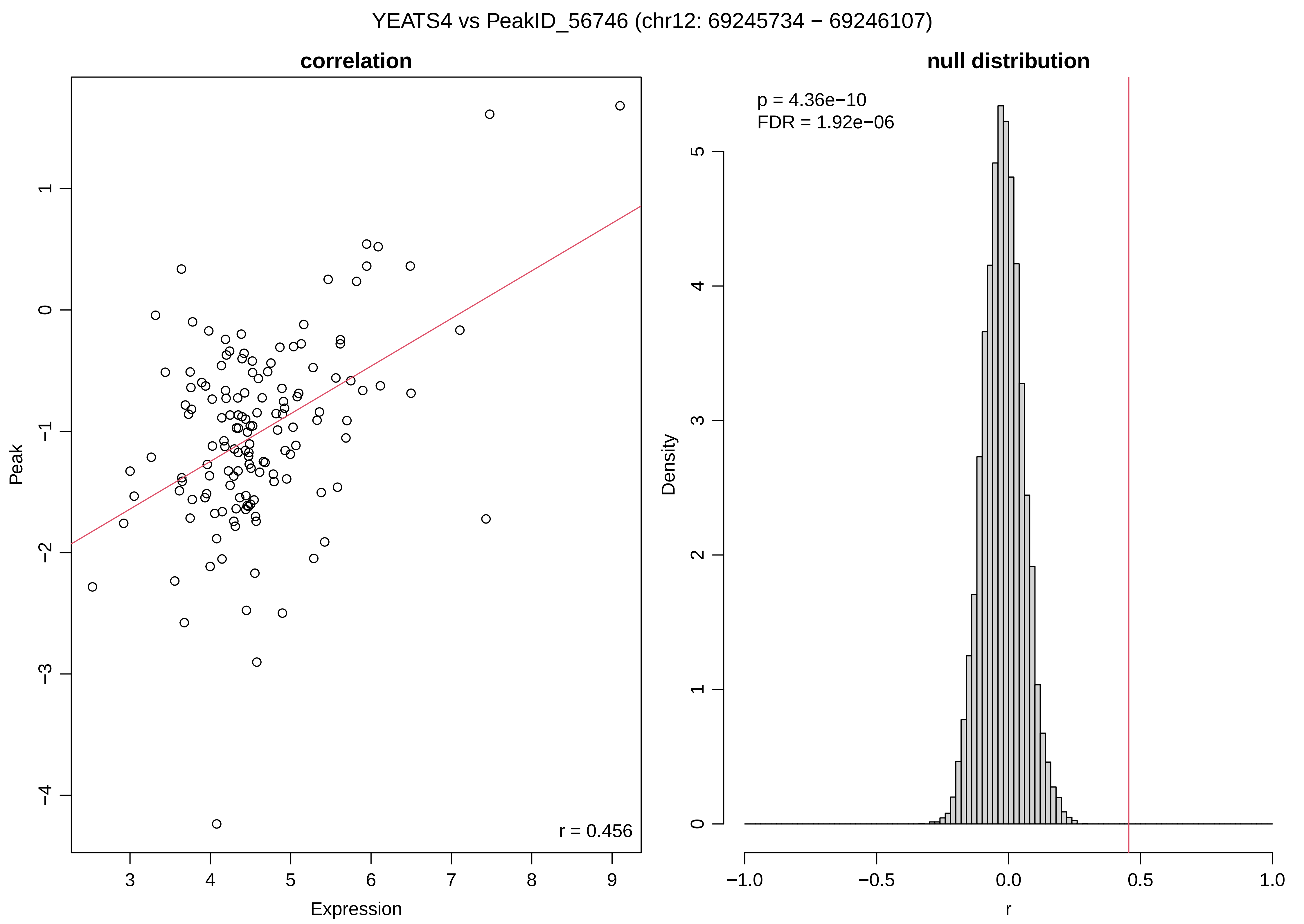
YEATS4 vs PeakID_56746 (chr12: 69245734 − 69246107)
correlation
null distribution
Peak
Expression
r = 0.456
p = 4.36e−10
FDR = 1.92e−06
Density
r

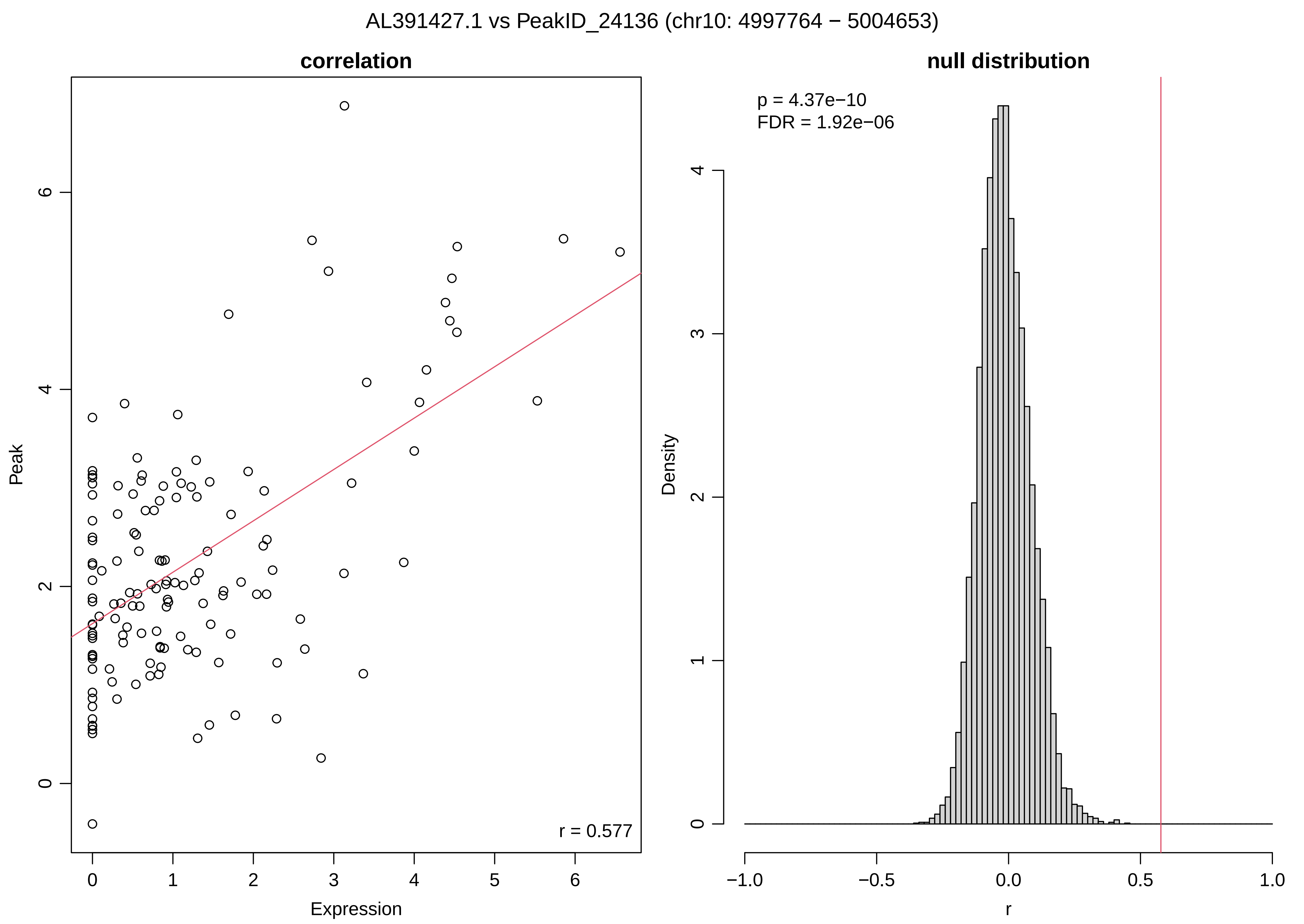
AL391427.1 vs PeakID_24136 (chr10: 4997764 − 5004653)
correlation
Peak
Expression
0
2
4
6
0
1
2
3
4
5
6
r = 0.577
null distribution
p = 4.37e−10
FDR = 1.92e−06
Density
r
0
1
2
3
4
−1.0
−0.5
0.0
0.5
1.0

FAM224A vs PeakID_262841 (chrY: 18335643 − 18337356)

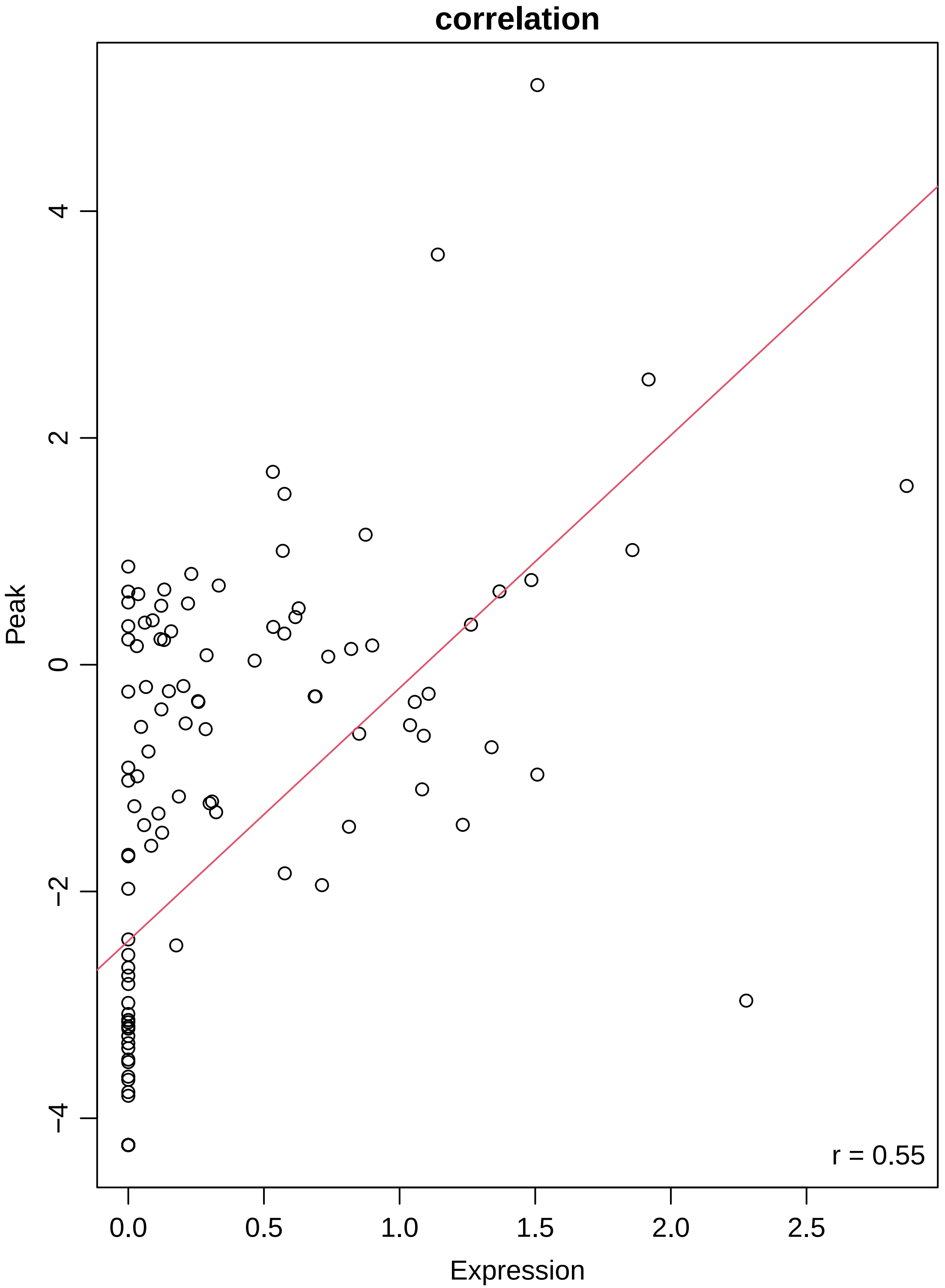

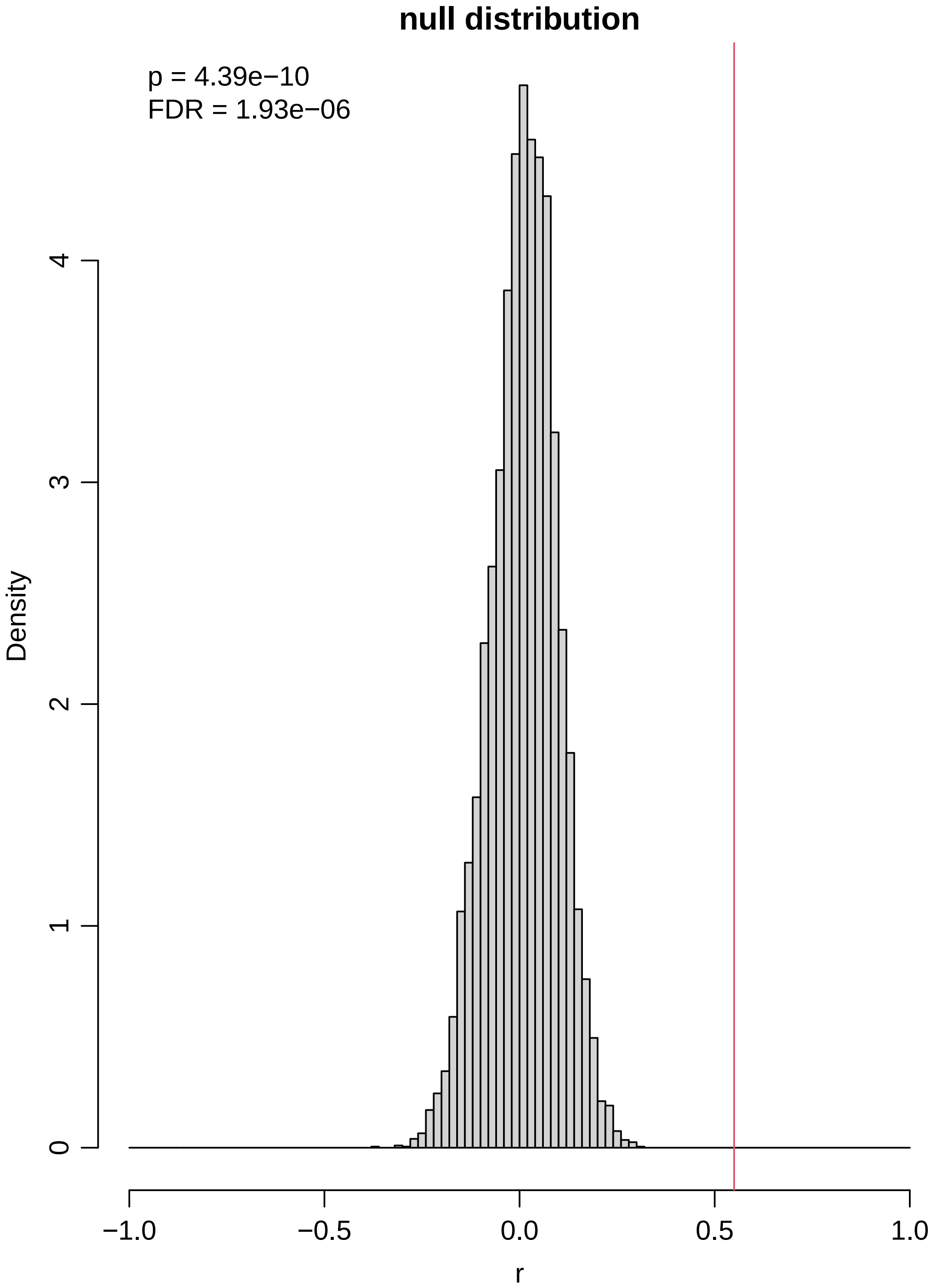

LINC02403 vs PeakID_54567 (chr12: 58316002 − 58320067)

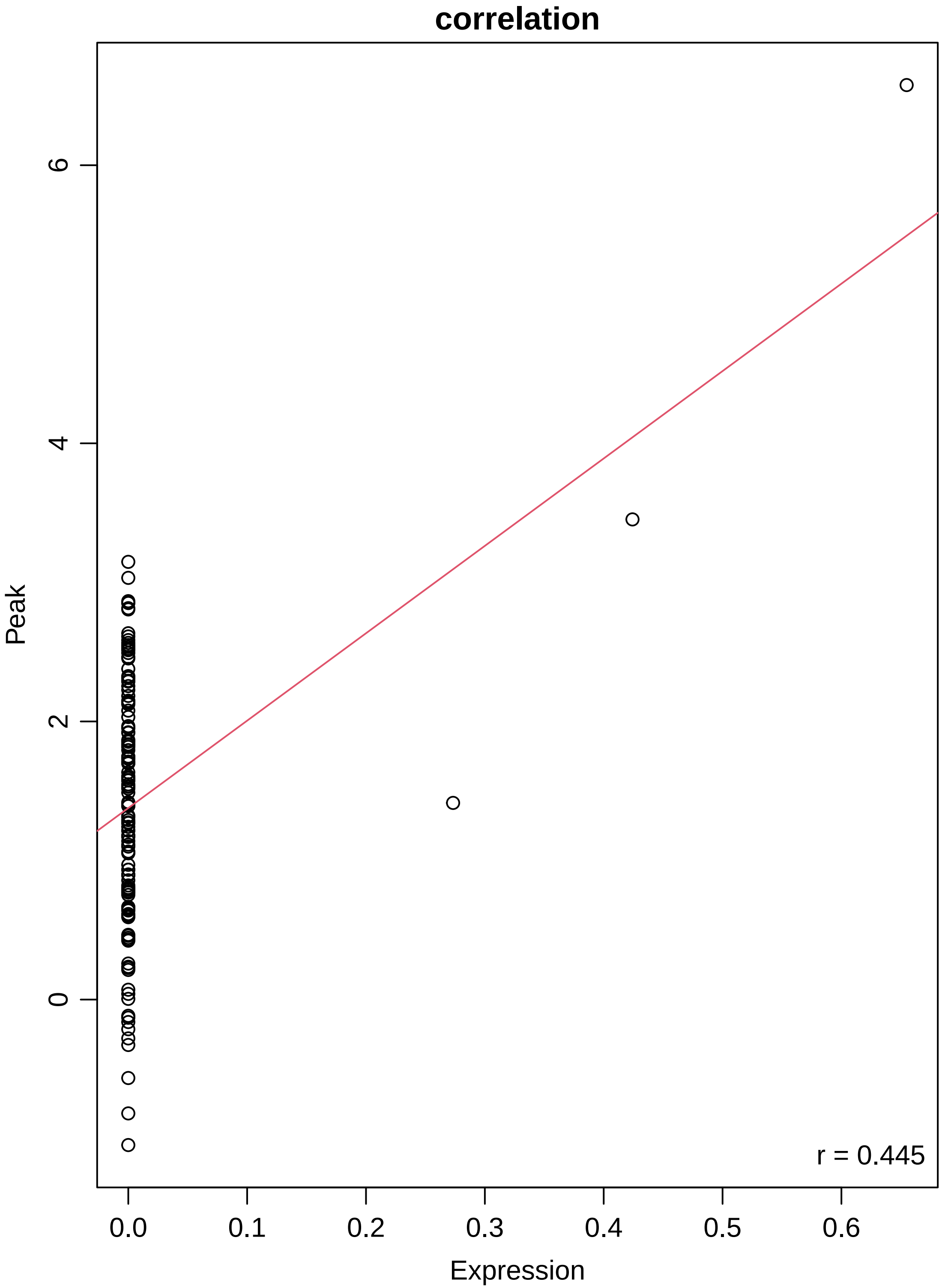

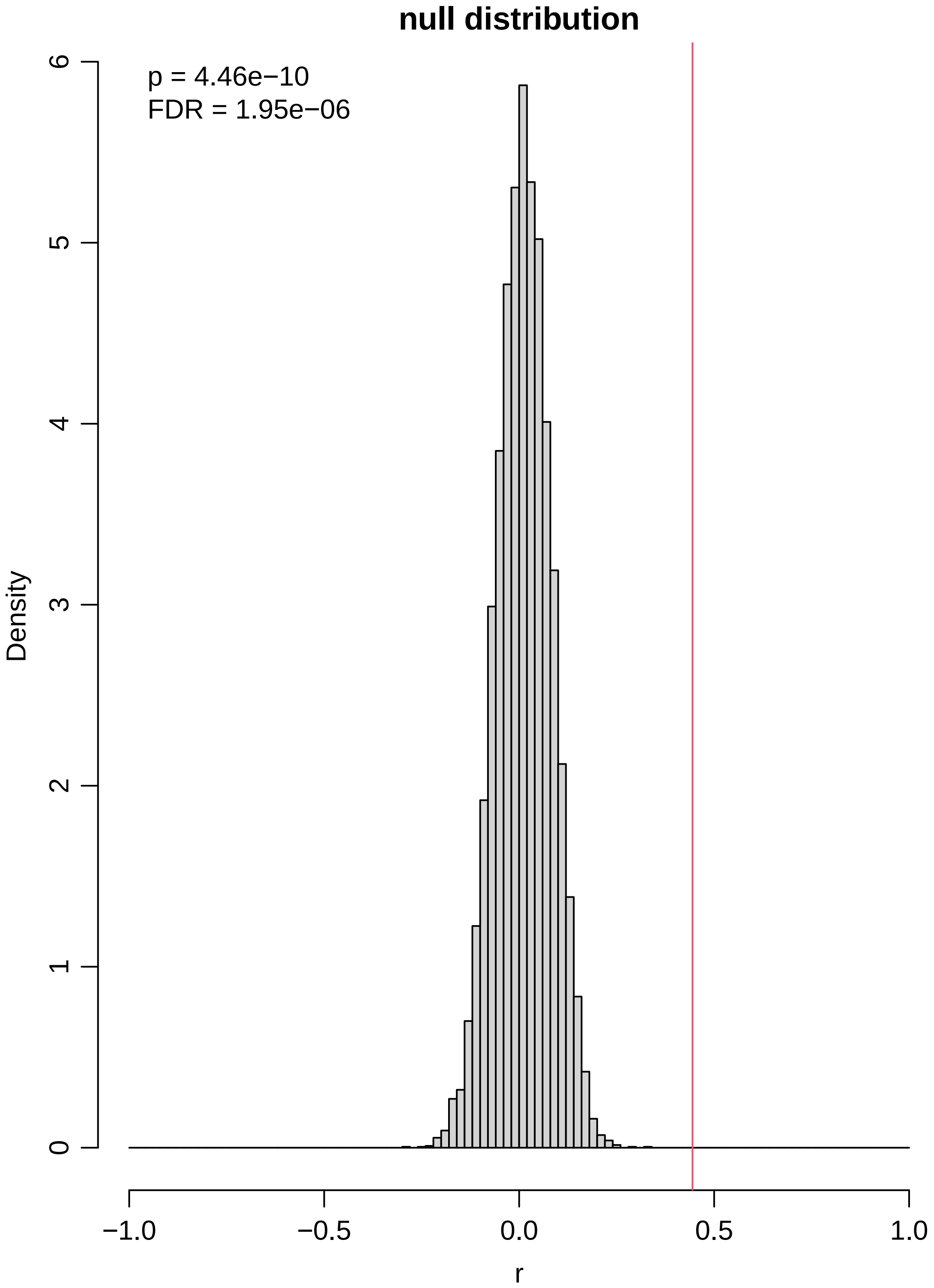

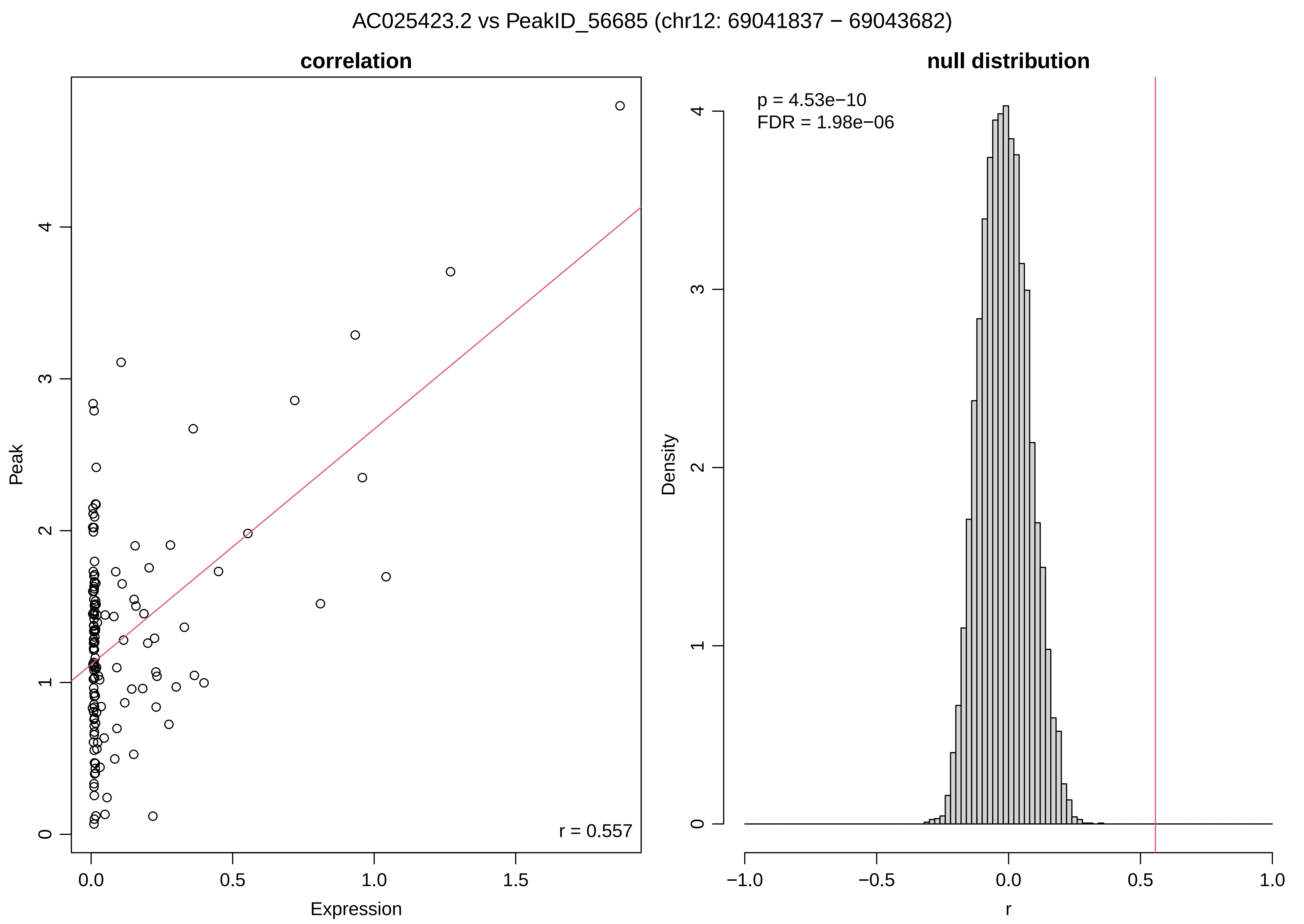
AC025423.2 vs PeakID_56685 (chr12: 69041837 − 69043682)
correlation
Peak
Expression
r = 0.557
null distribution
p = 4.53e−10
FDR = 1.98e−06
Density
r

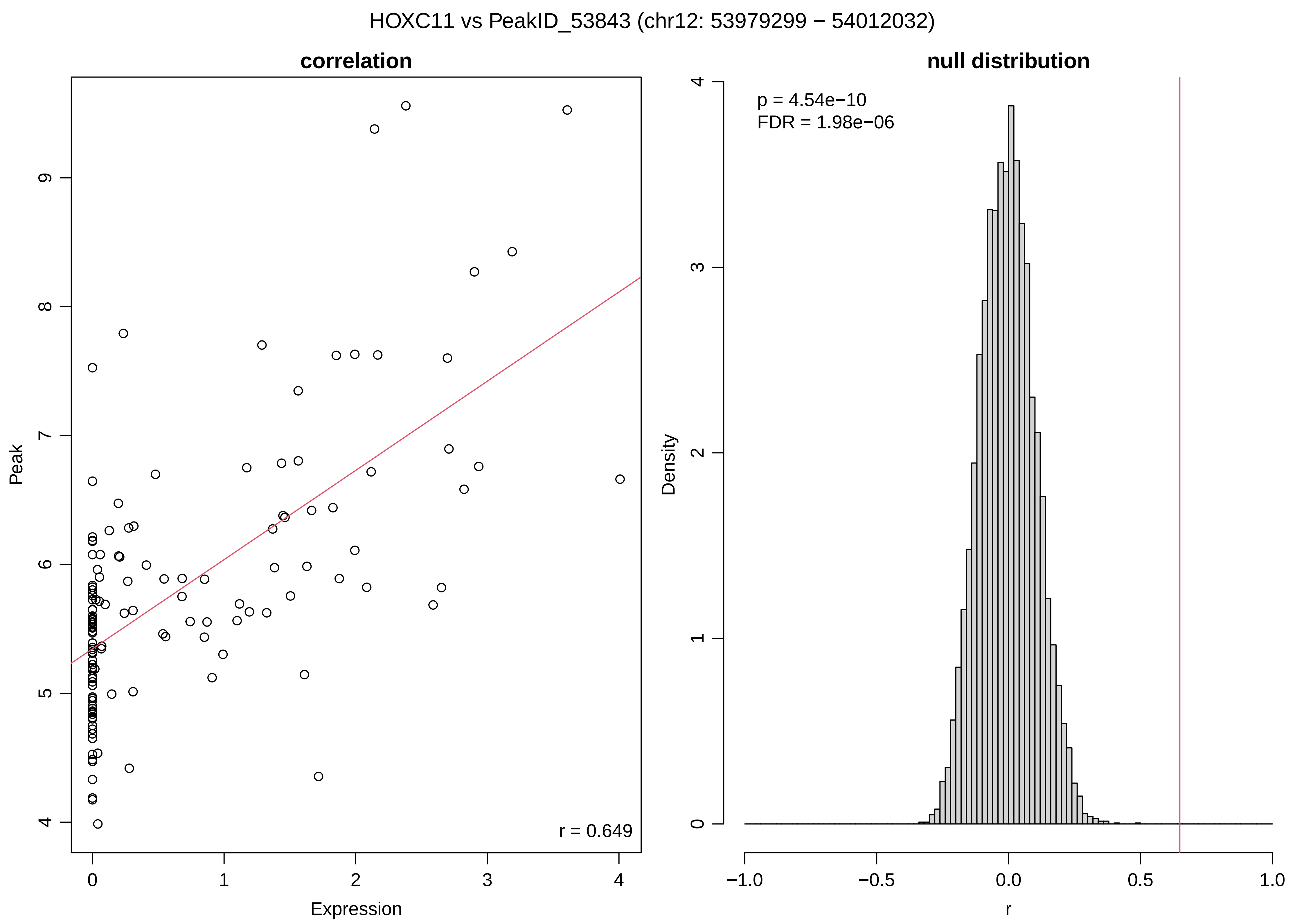
HOXC11 vs PeakID_53843 (chr12: 53979299 − 54012032)
correlation
null distribution
Peak
Expression
r = 0.649
Density
r
p = 4.54e−10
FDR = 1.98e−06
0
1
2
3
4
5
6
7
8
9
−1.0
−0.5
0.0
0.5
1.0

NR0B1 vs PeakID_257393 (chrX: 30305663 − 30309362)

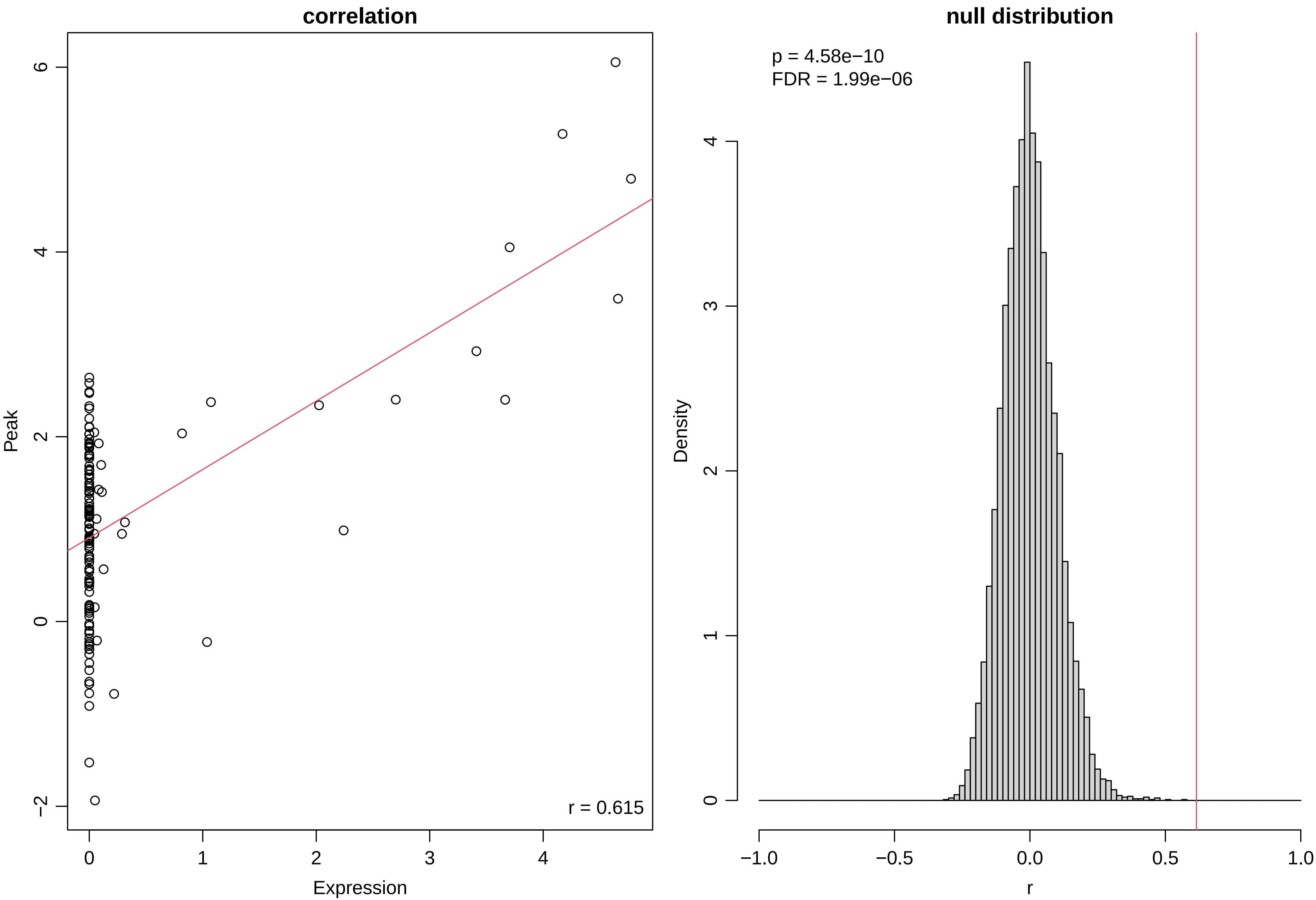

RFTN1P1 vs PeakID_262422 (chrY: 7718899 − 7719345)

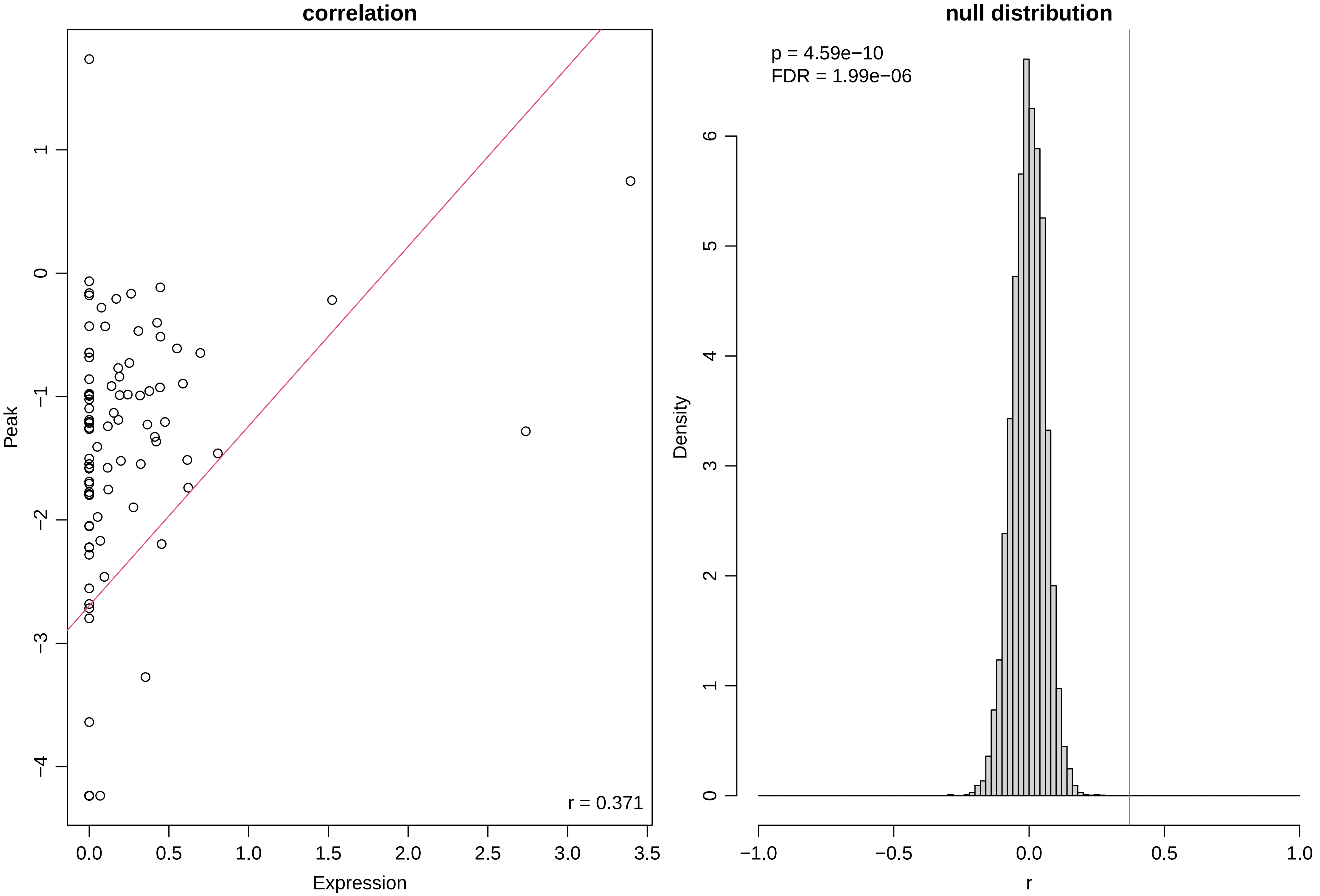

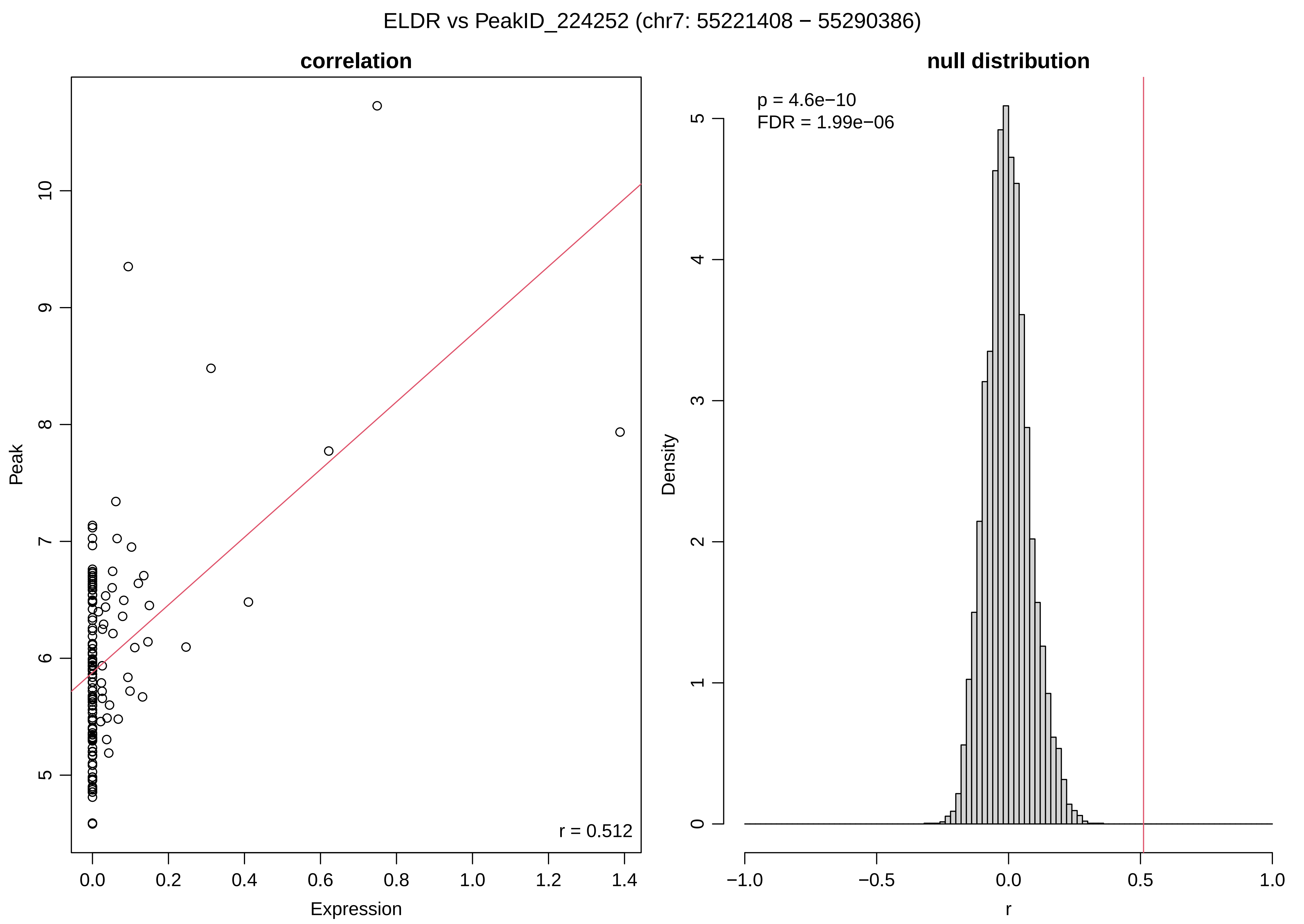
ELDR vs PeakID_224252 (chr7: 55221408 − 55290386)
correlation
Peak
Expression
r = 0.512
null distribution
p = 4.6e−10
FDR = 1.99e−06
Density
r

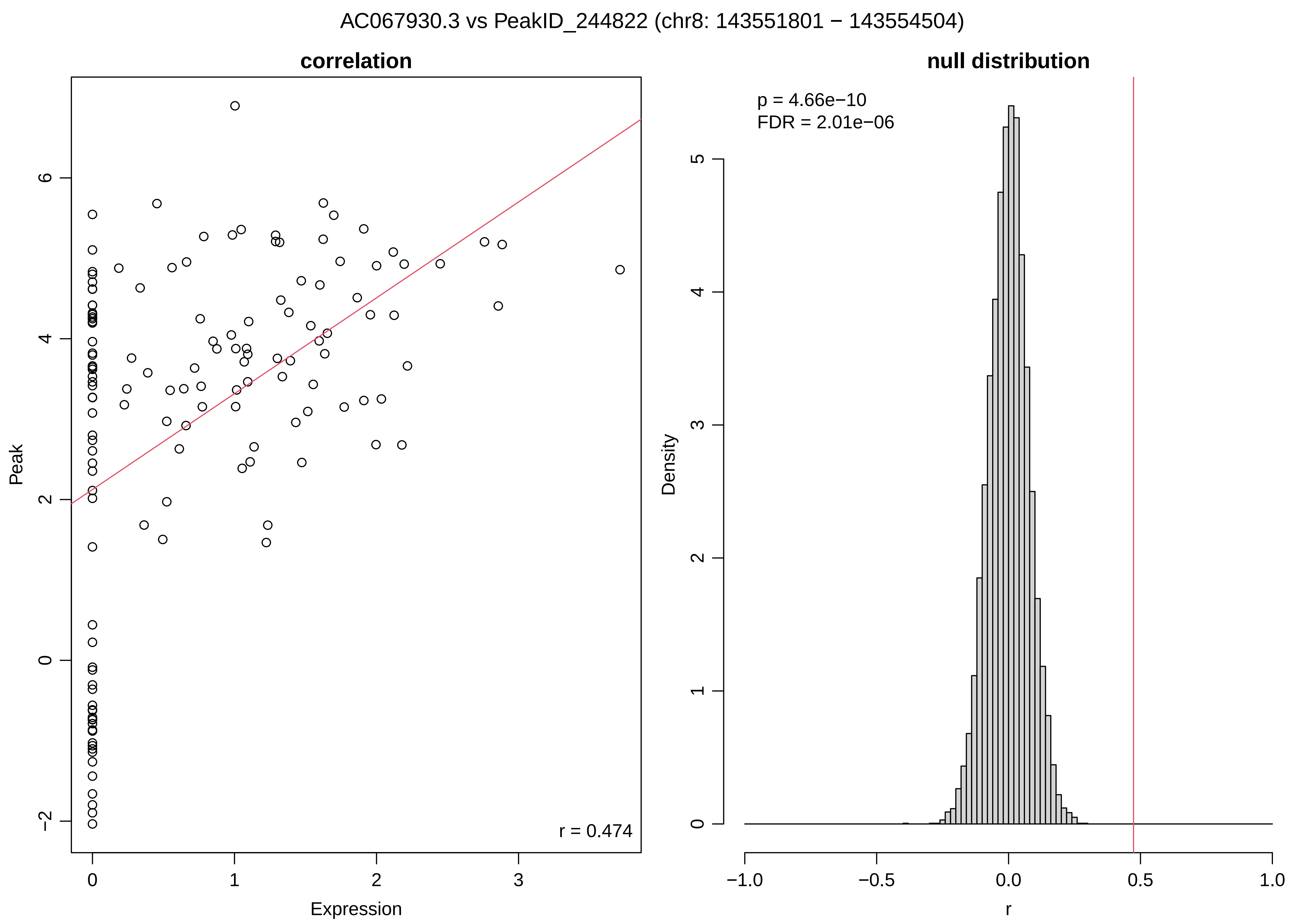
AC067930.3 vs PeakID_244822 (chr8: 143551801 − 143554504)
correlation
Peak
Expression
r = 0.474
null distribution
p = 4.66e−10
FDR = 2.01e−06
Density
r

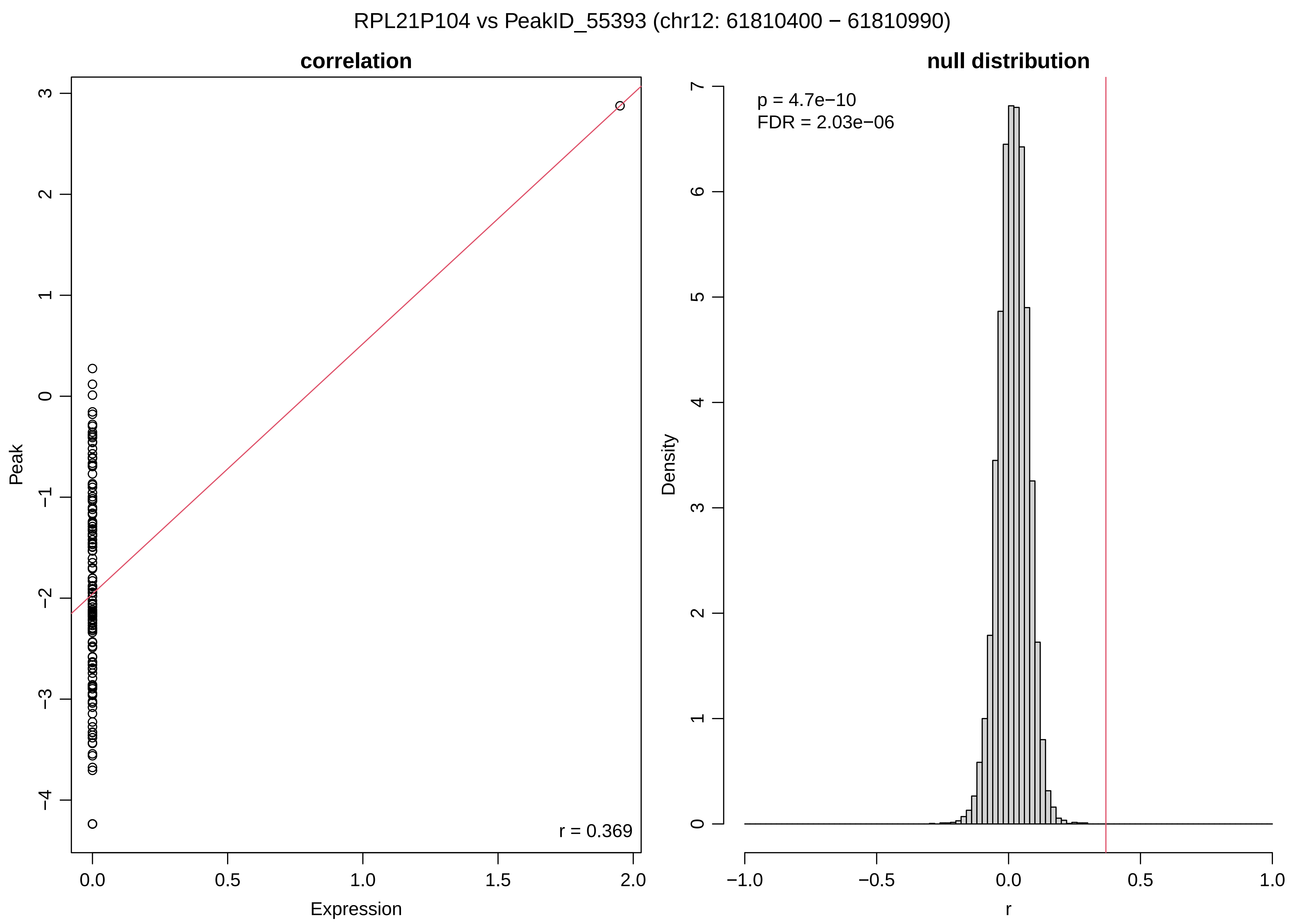

RPL21P104 vs PeakID_55393 (chr12: 61810400 − 61810990)
correlation
Peak
Expression
r = 0.369
null distribution
p = 4.7e−10
FDR = 2.03e−06
Density
r

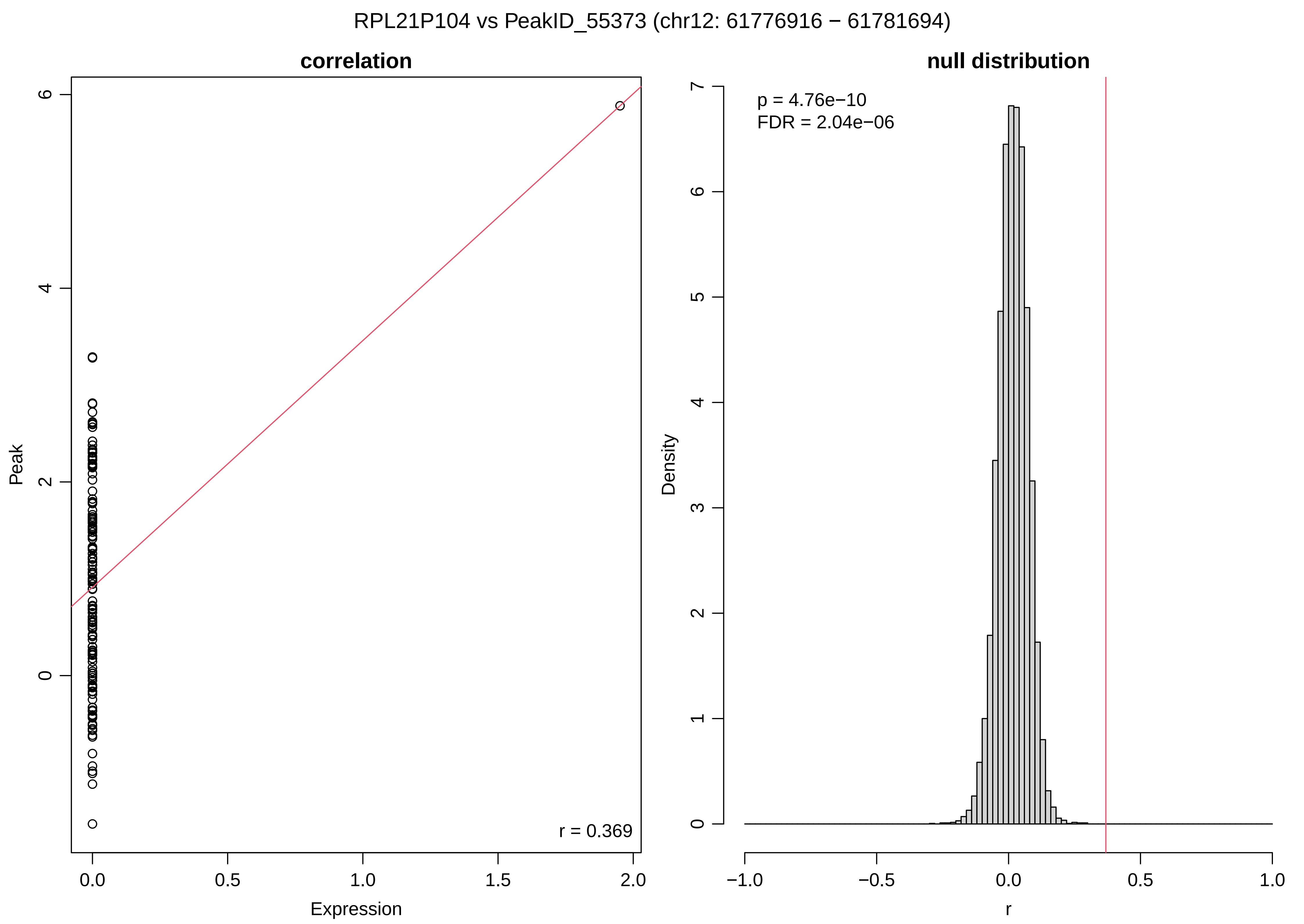
RPL21P104 vs PeakID_55373 (chr12: 61776916 − 61781694)
correlation
Peak
Expression
r = 0.369
0.0
0.5
1.0
1.5
2.0
0
2
4
6
null distribution
p = 4.76e−10
FDR = 2.04e−06
Density
r
−1.0
−0.5
0.0
0.5
1.0
0
1
2
3
4
5
6
7

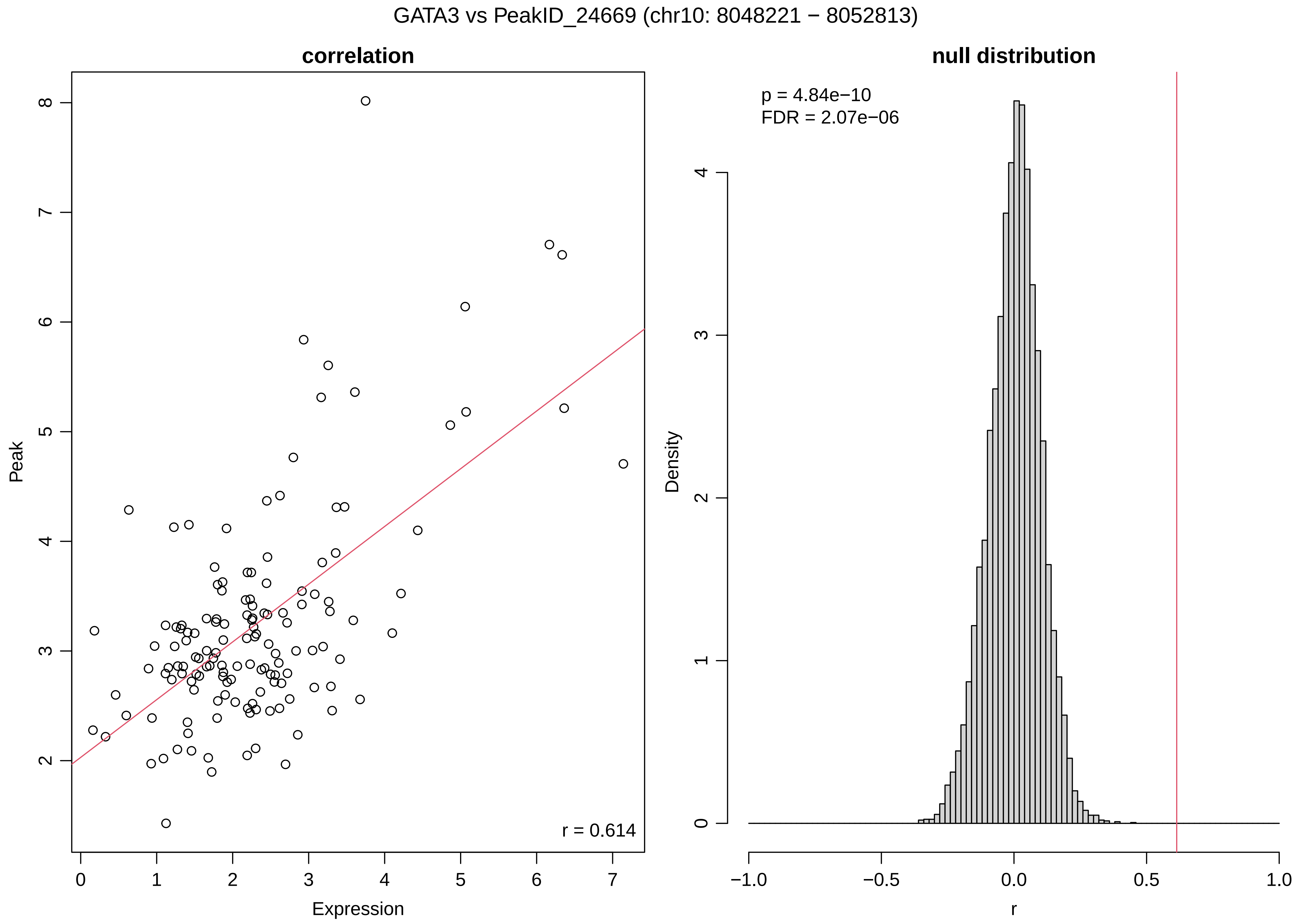
GATA3 vs PeakID_24669 (chr10: 8048221 − 8052813)
correlation
Peak
Expression
r = 0.614
null distribution
p = 4.84e−10
FDR = 2.07e−06
Density
r

BMP7 vs PeakID_149015 (chr20: 57261890 − 57267904)

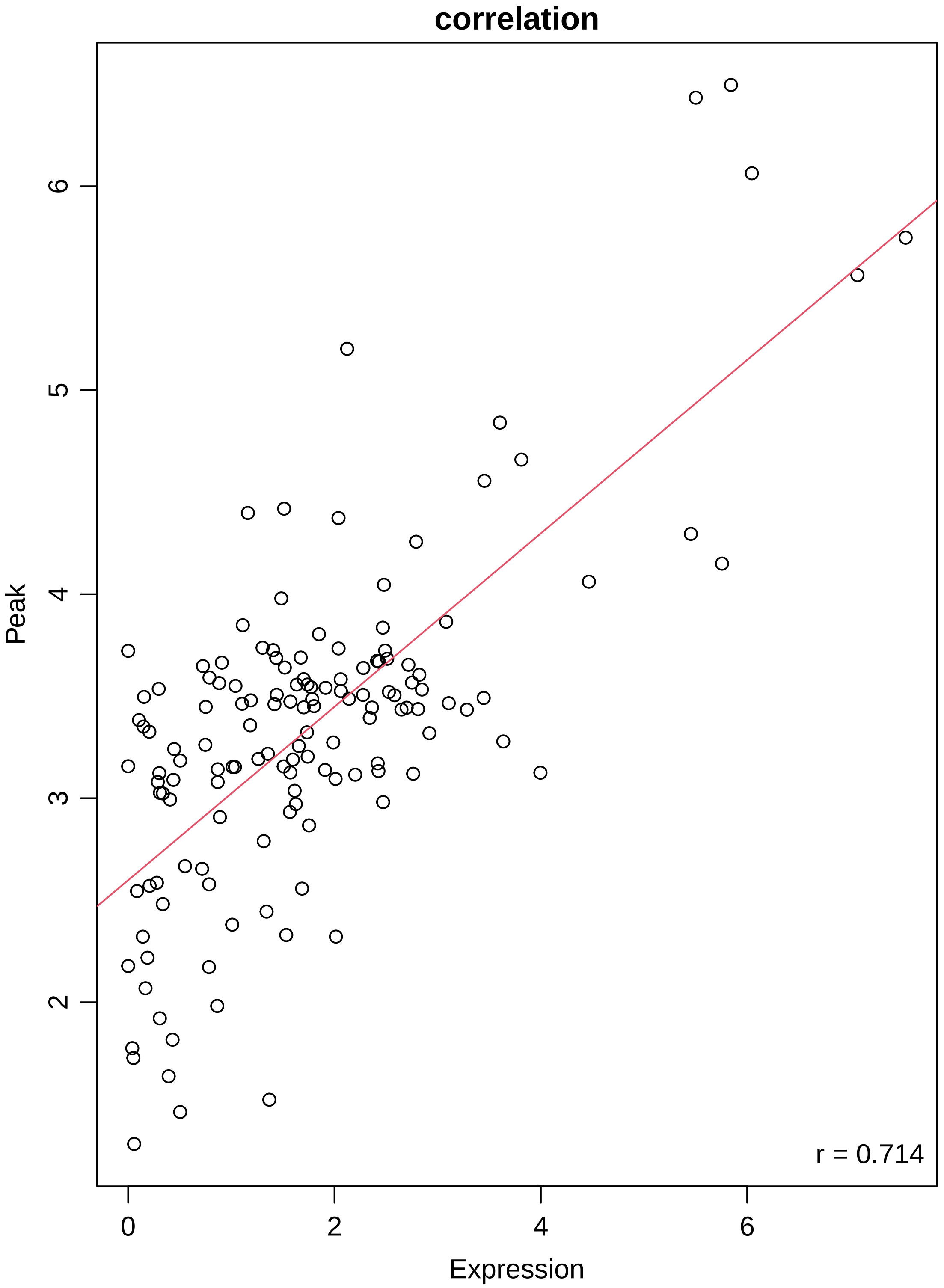

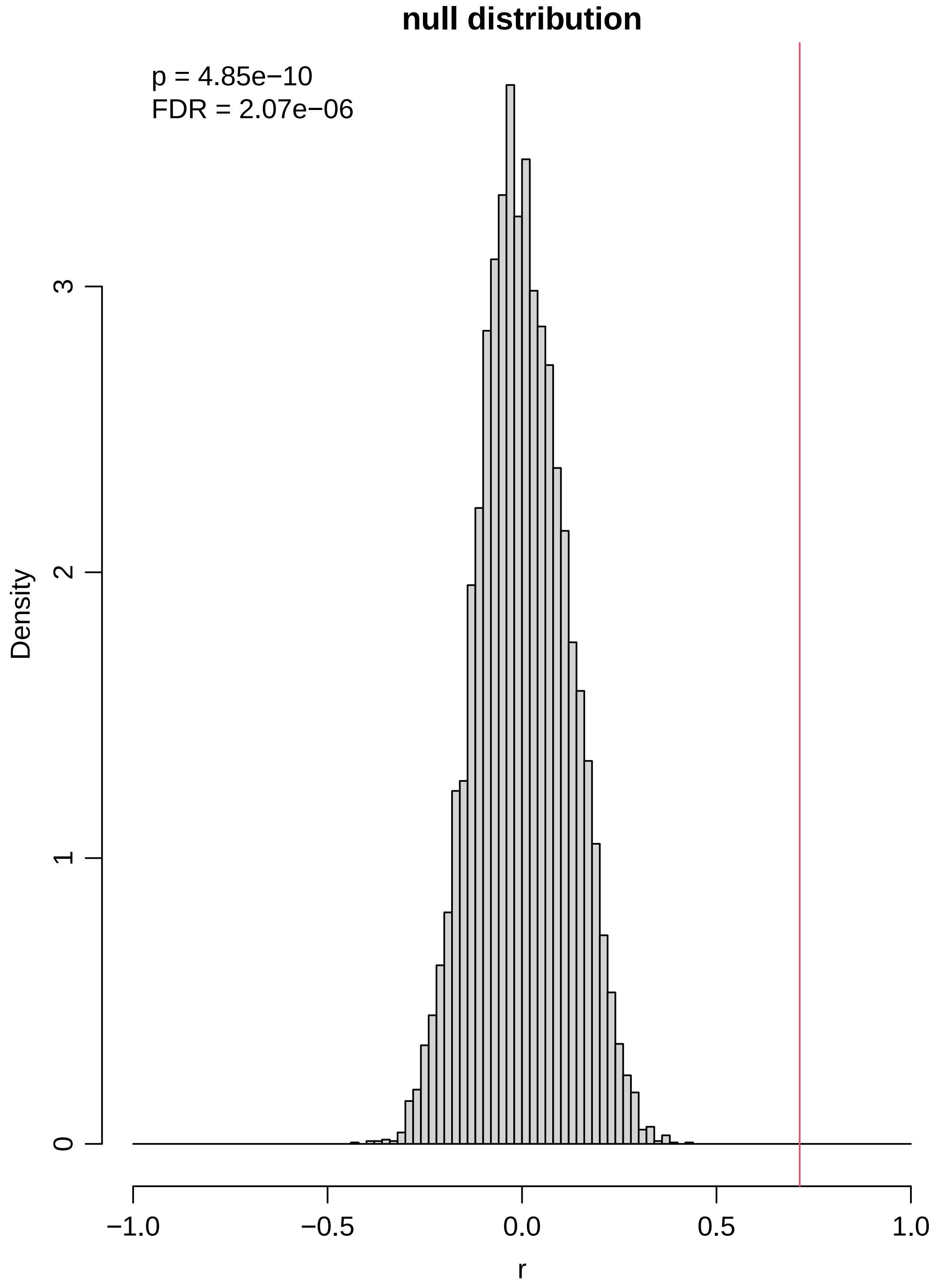

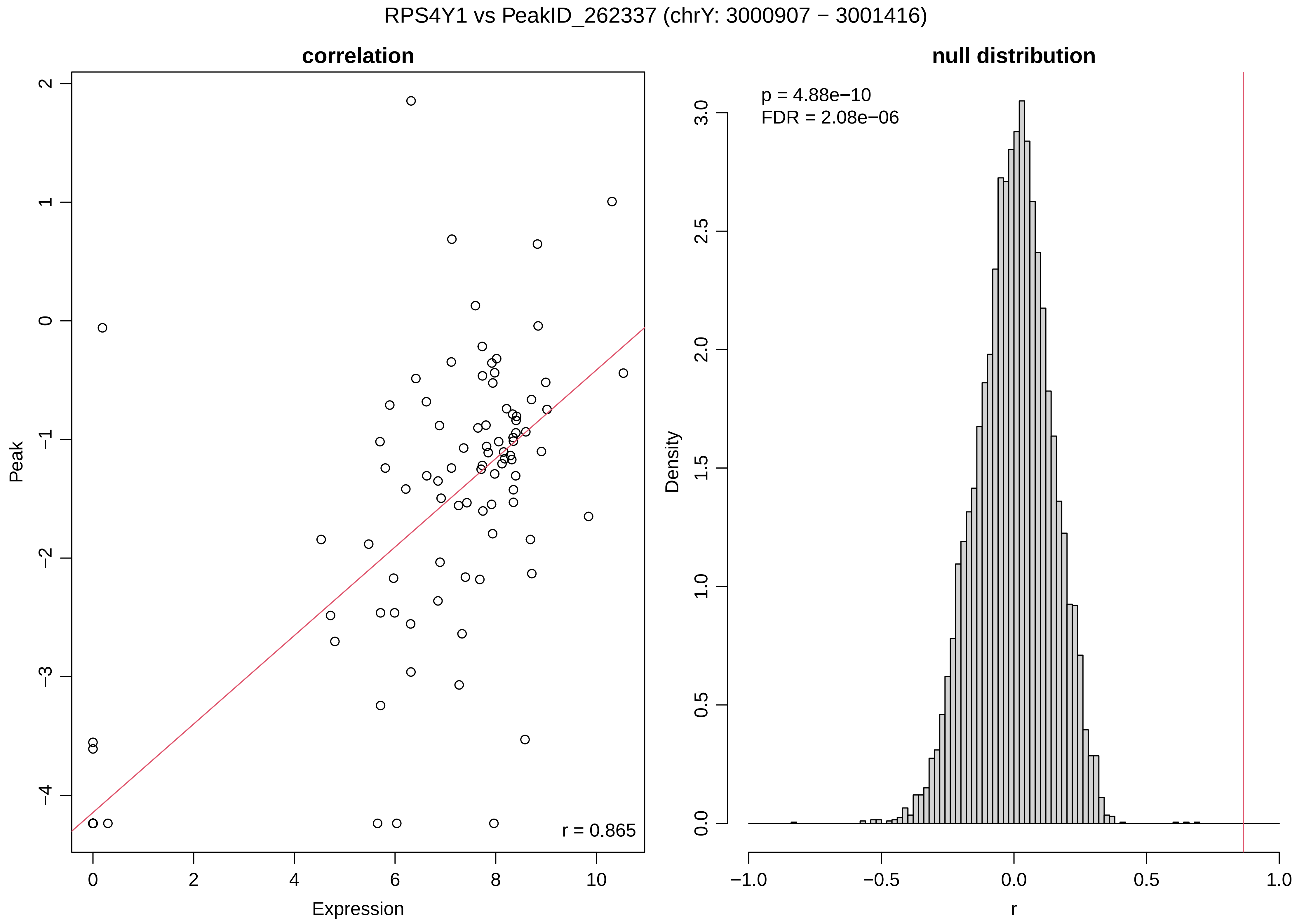

RPS4Y1 vs PeakID_262337 (chrY: 3000907 − 3001416)
correlation
Peak
Expression
r = 0.865
null distribution
p = 4.88e−10
FDR = 2.08e−06
Density
r

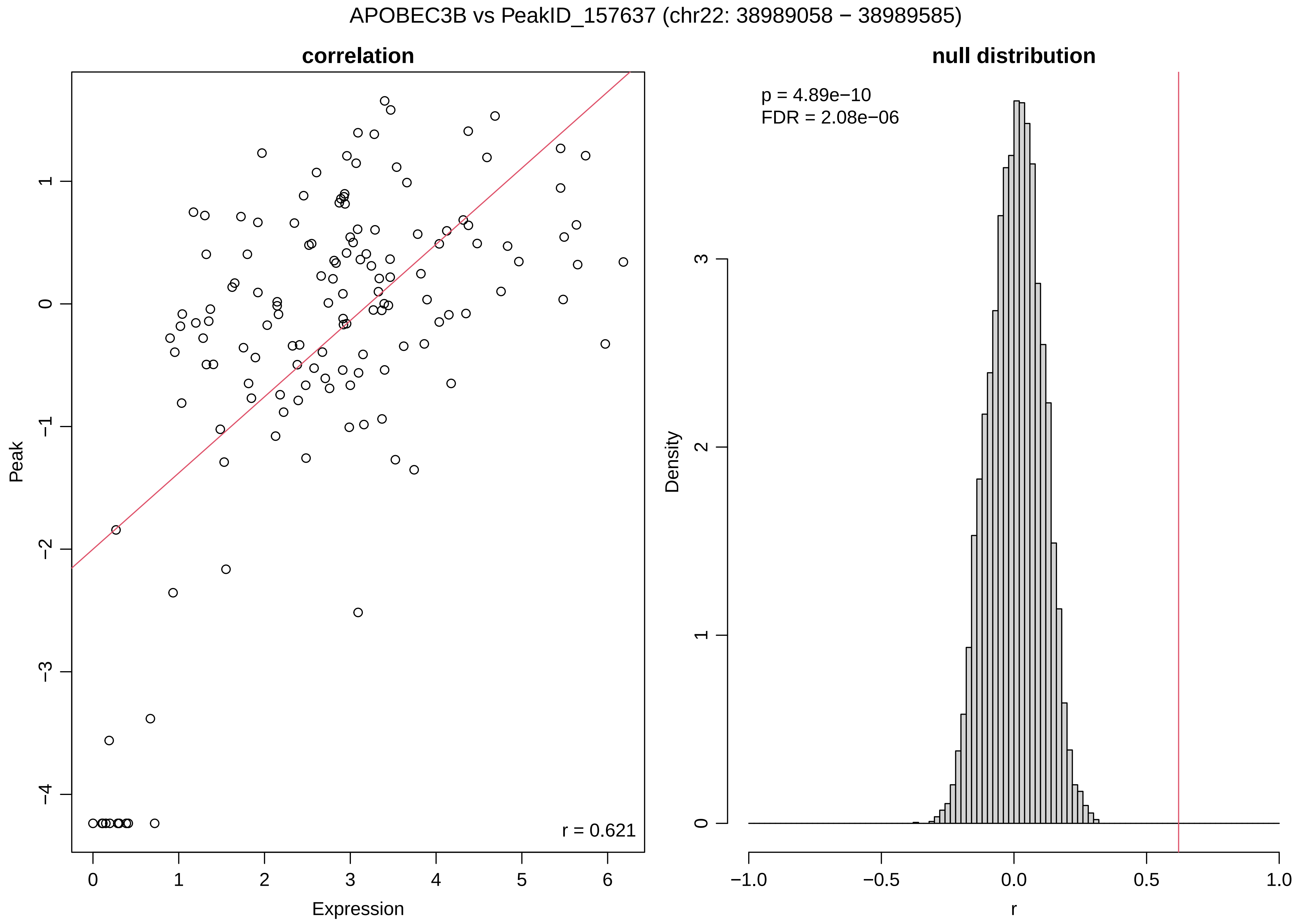

APOBEC3B vs PeakID_157637 (chr22: 38989058 − 38989585)
correlation
Peak
Expression
r = 0.621
null distribution
p = 4.89e−10
FDR = 2.08e−06
Density
r

CCL28 vs PeakID_190606 (chr5: 43407830 − 43413285)

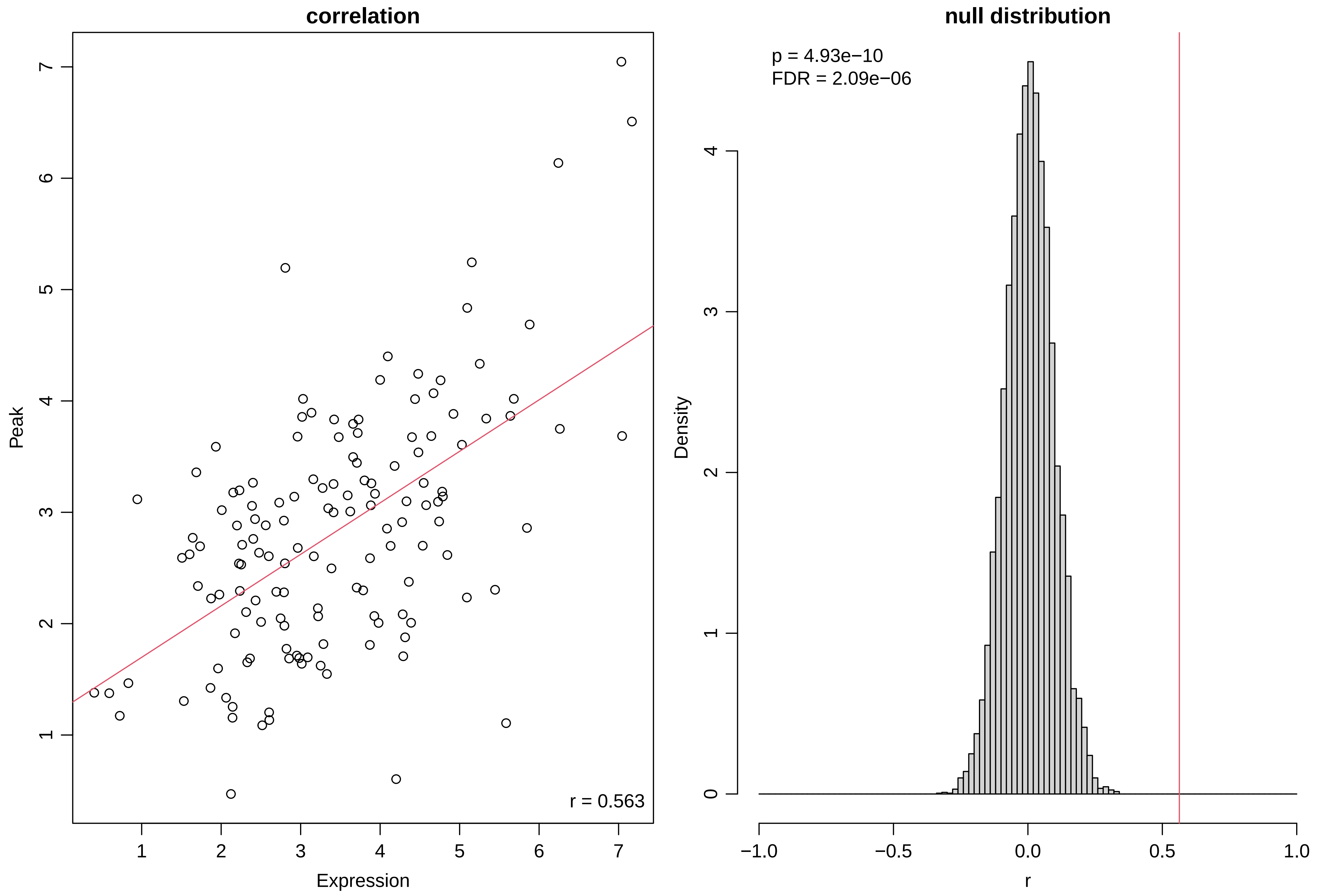

ZFY−AS1 vs PeakID_262338 (chrY: 3001791 − 3005953)

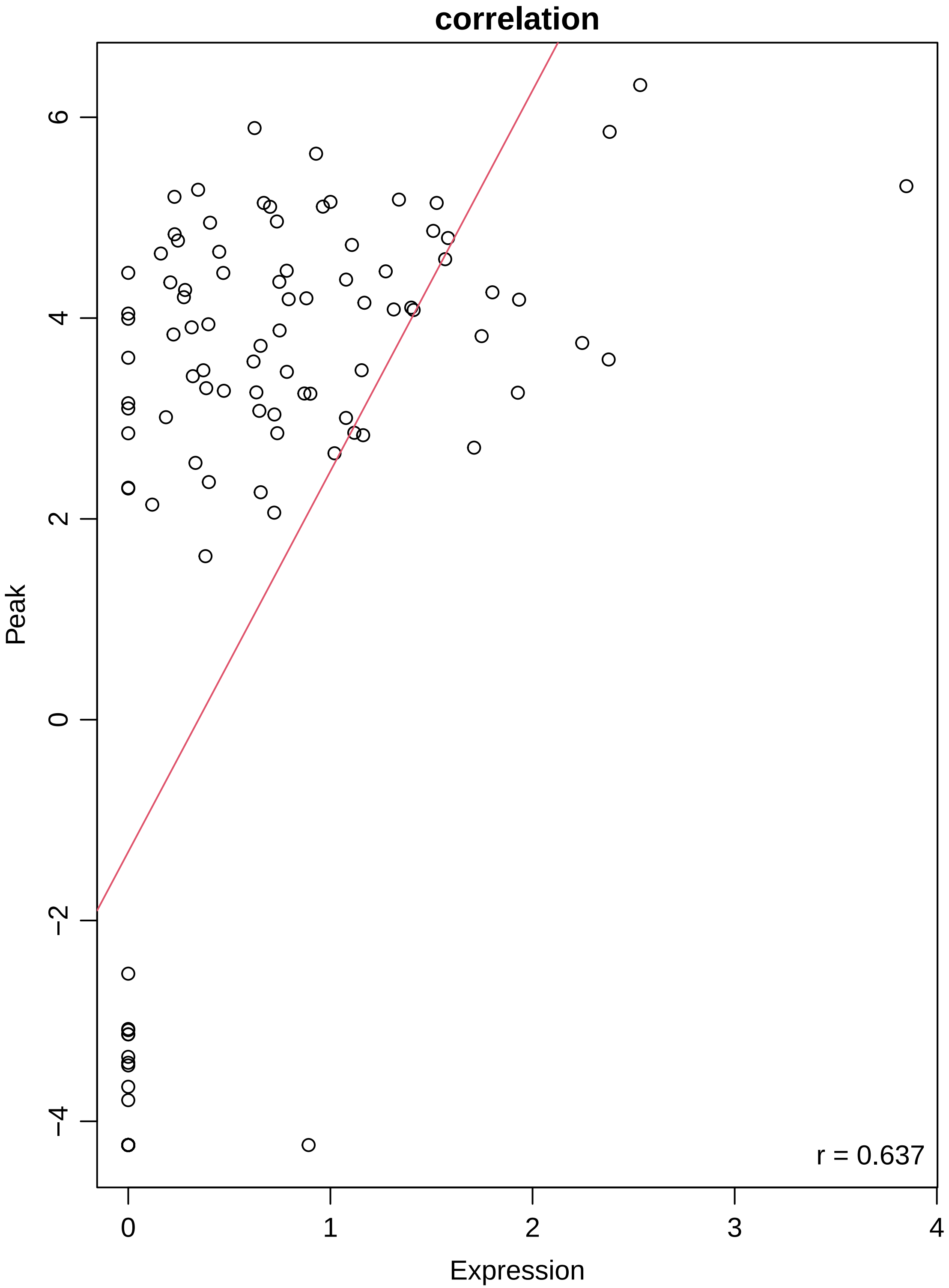

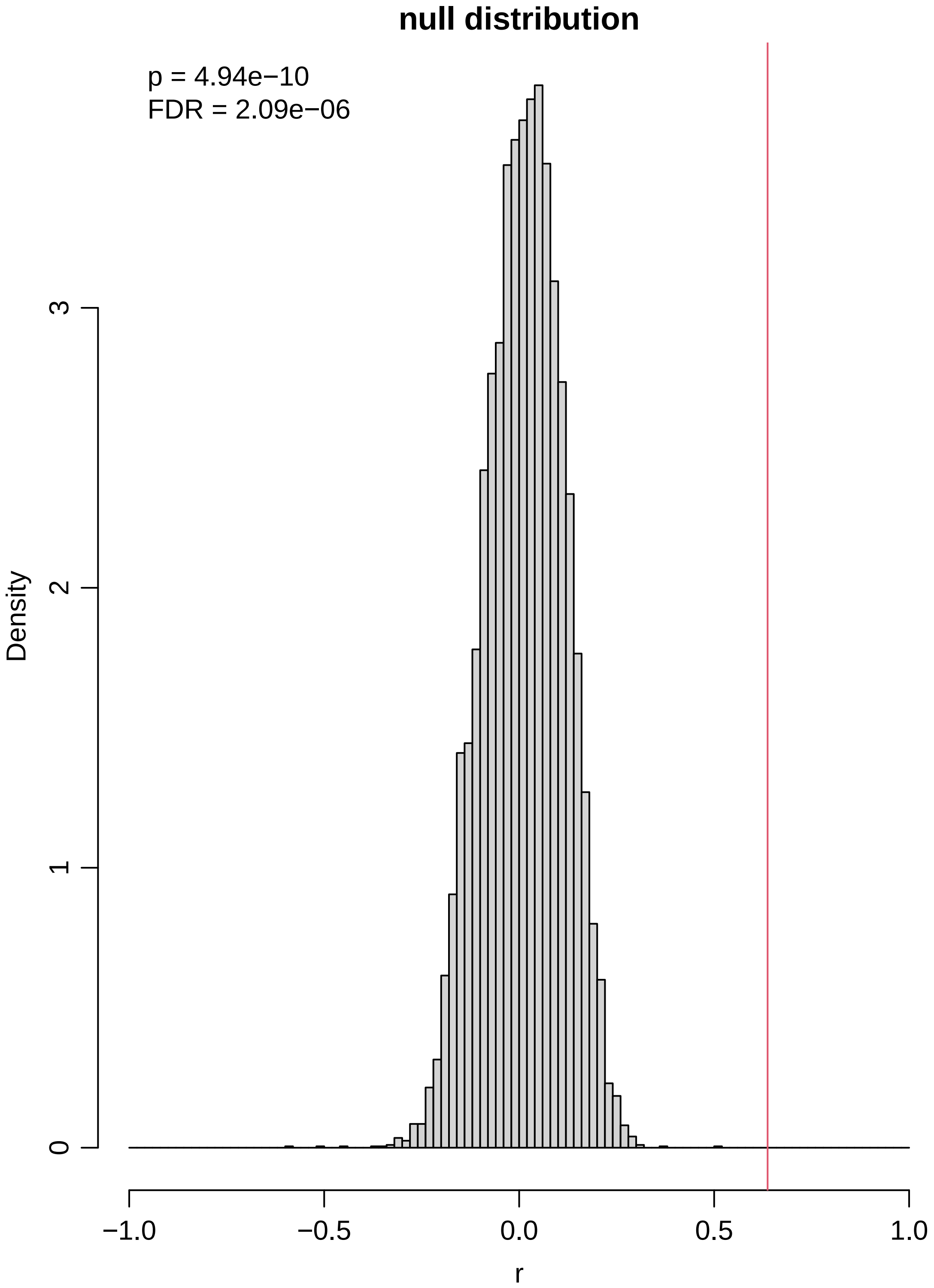

LINC00278 vs PeakID_262338 (chrY: 3001791 − 3005953)

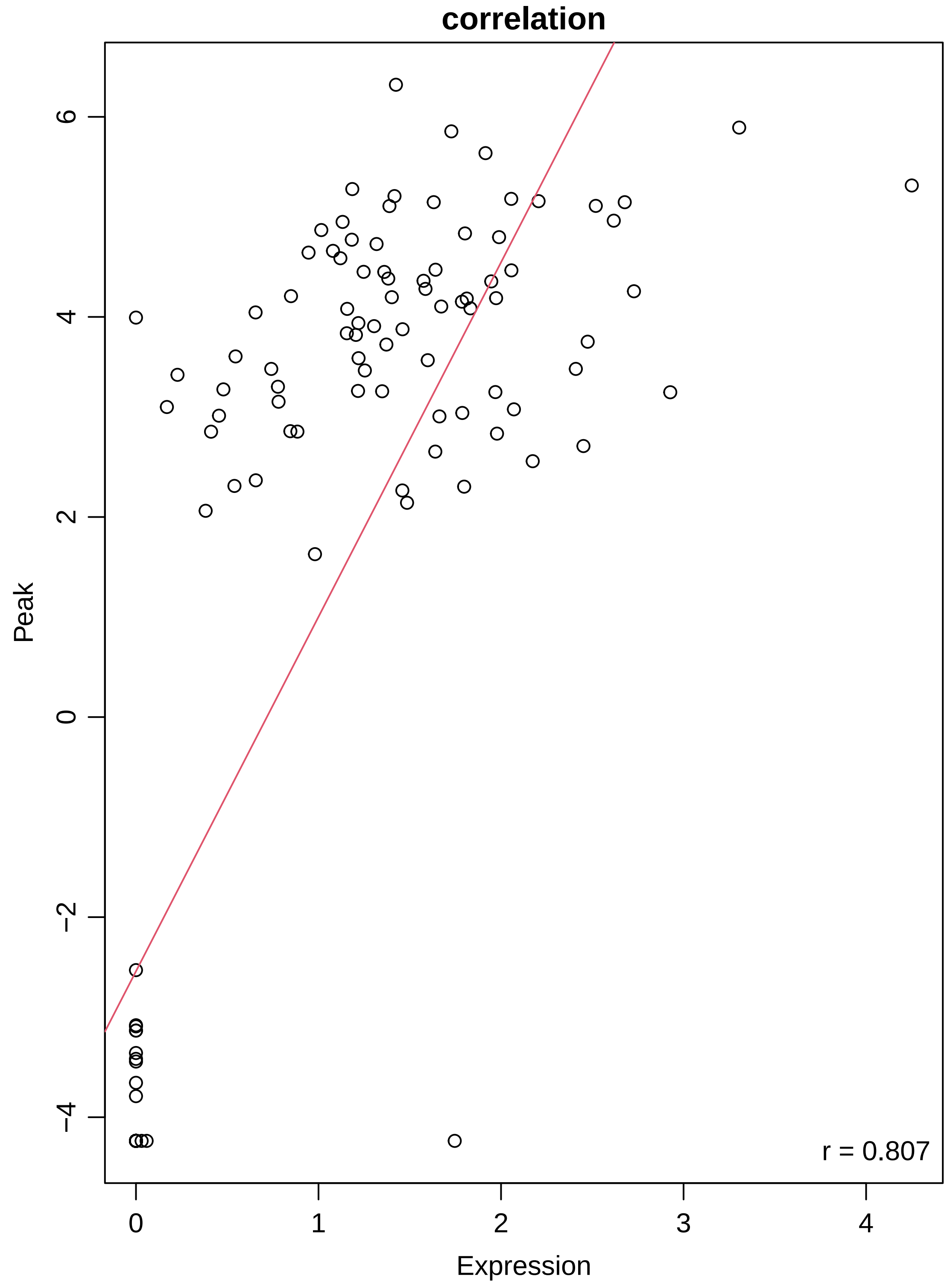

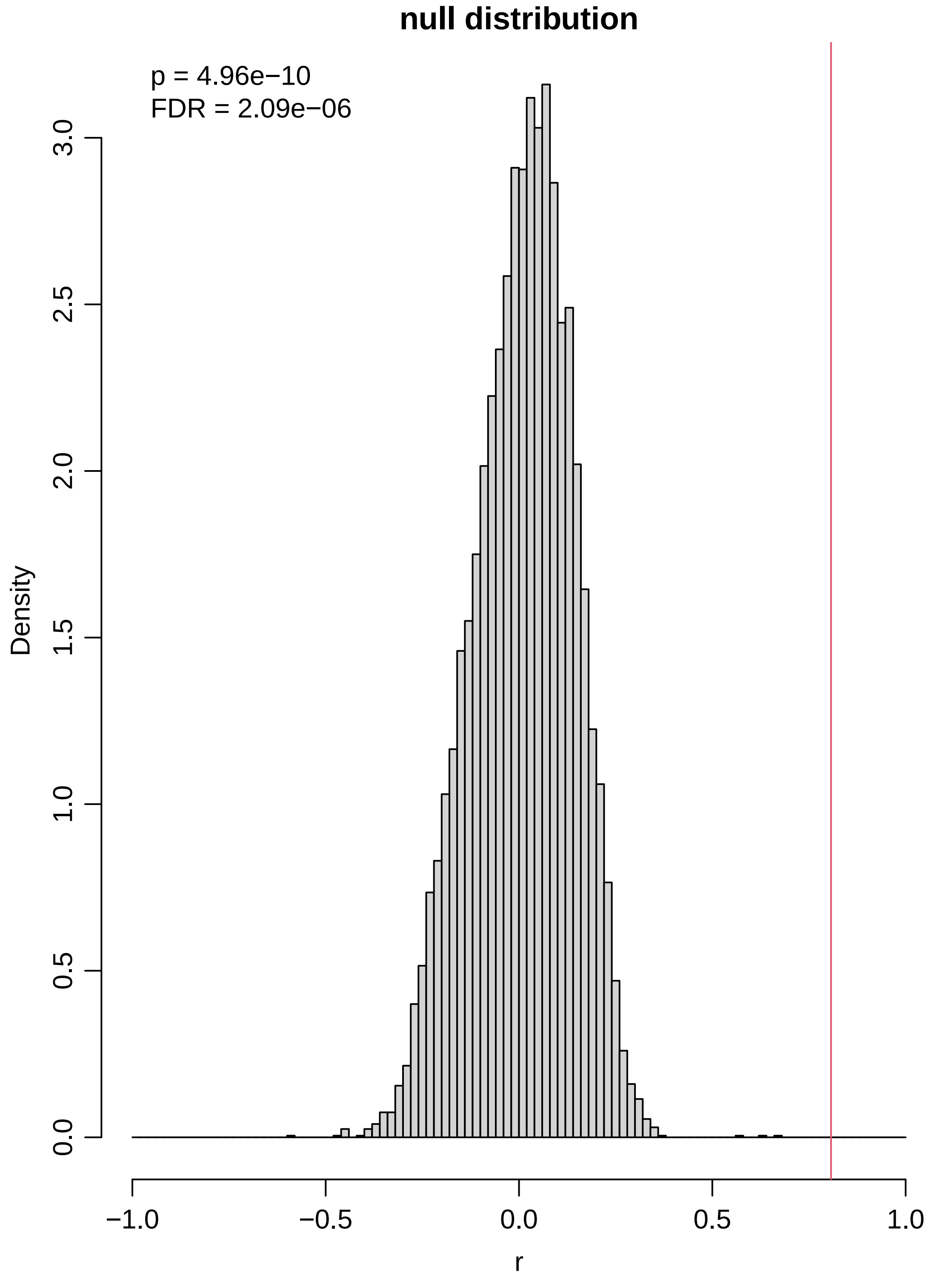

AC002066.1 vs PeakID_228984 (chr7: 116521335 − 116529136)

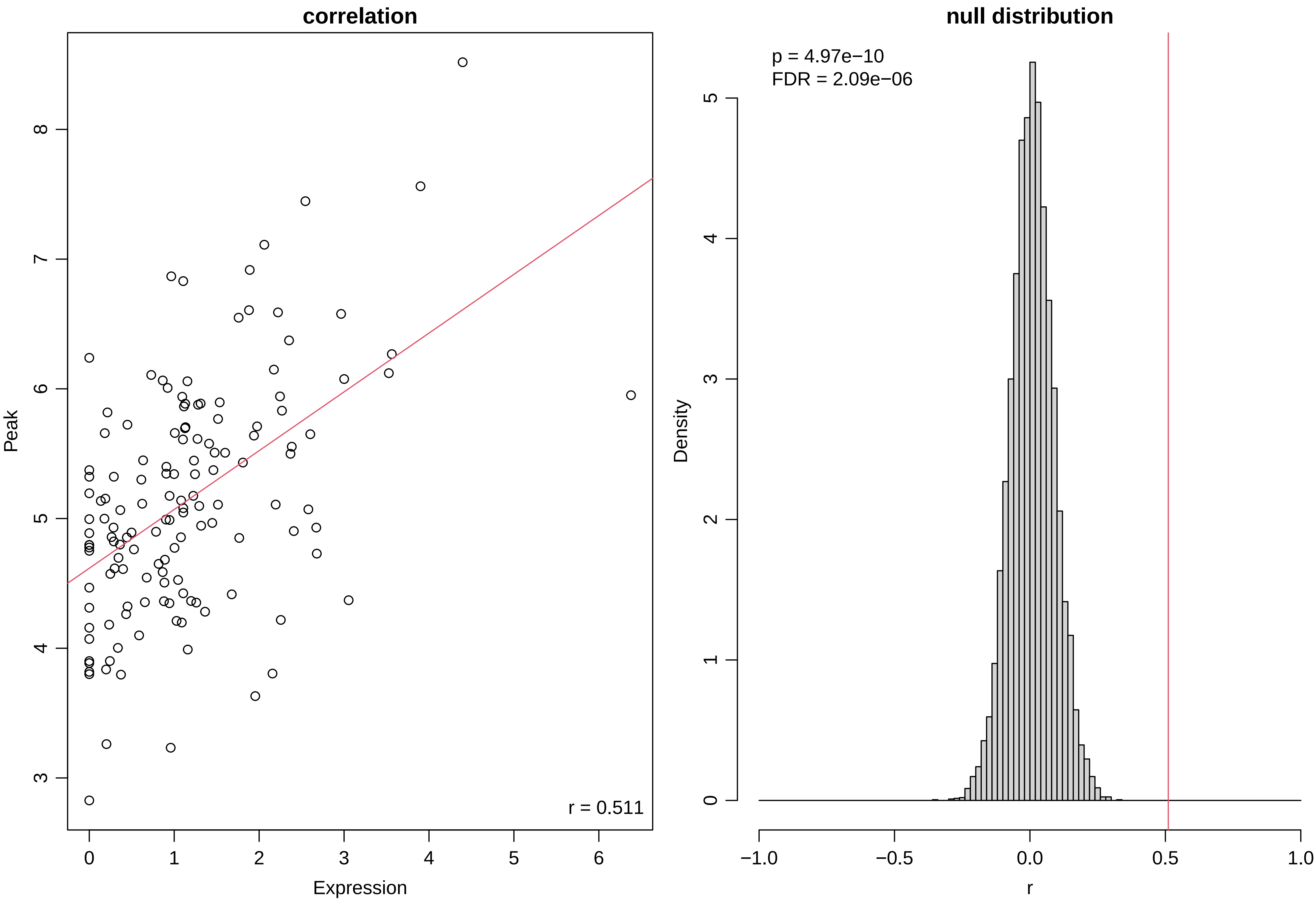

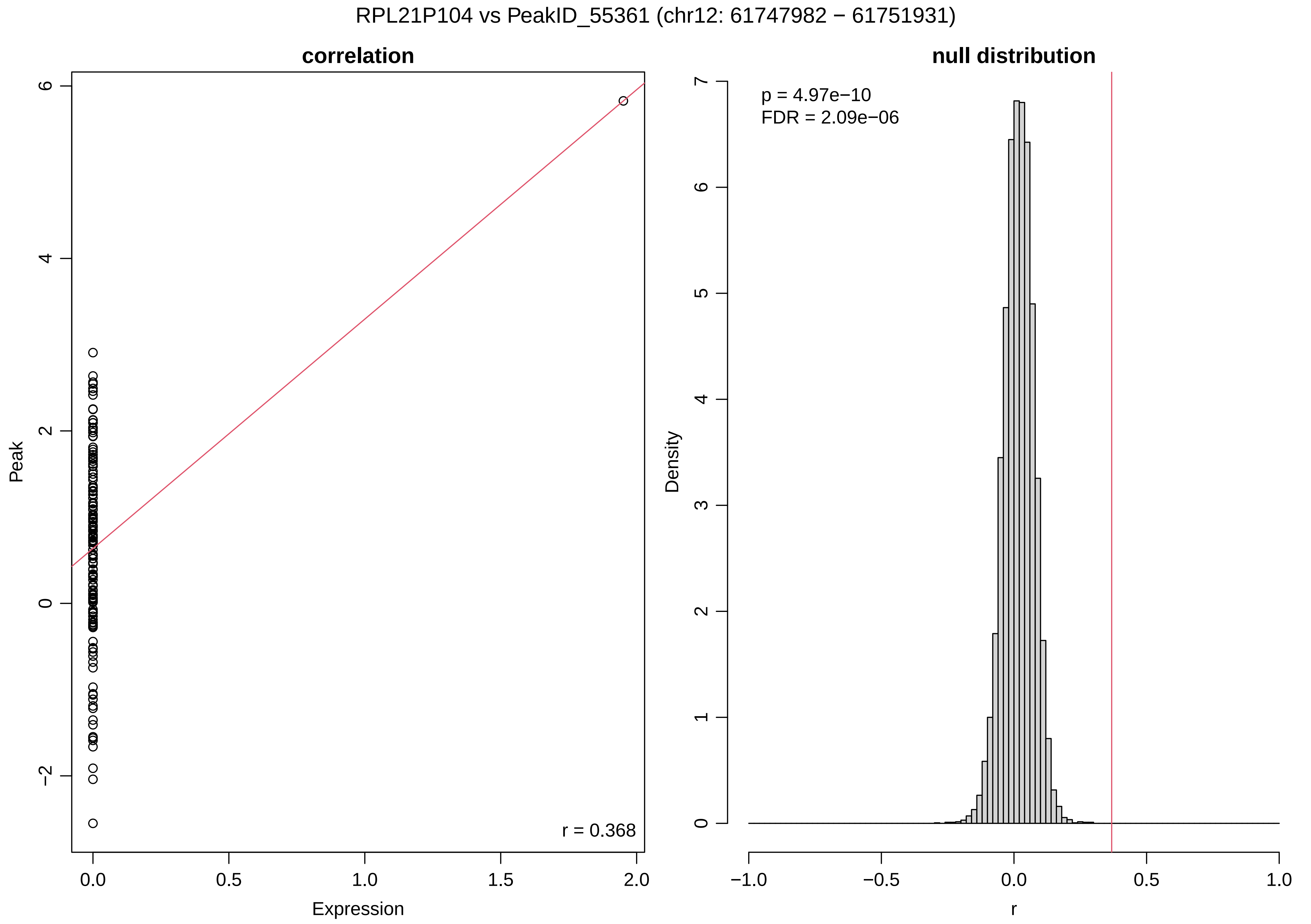
RPL21P104 vs PeakID_55361 (chr12: 61747982 − 61751931)
correlation
Peak
Expression
r = 0.368
null distribution
Density
r
p = 4.97e−10
FDR = 2.09e−06

CHRNB4 vs PeakID_82141 (chr15: 78719474 − 78720613)

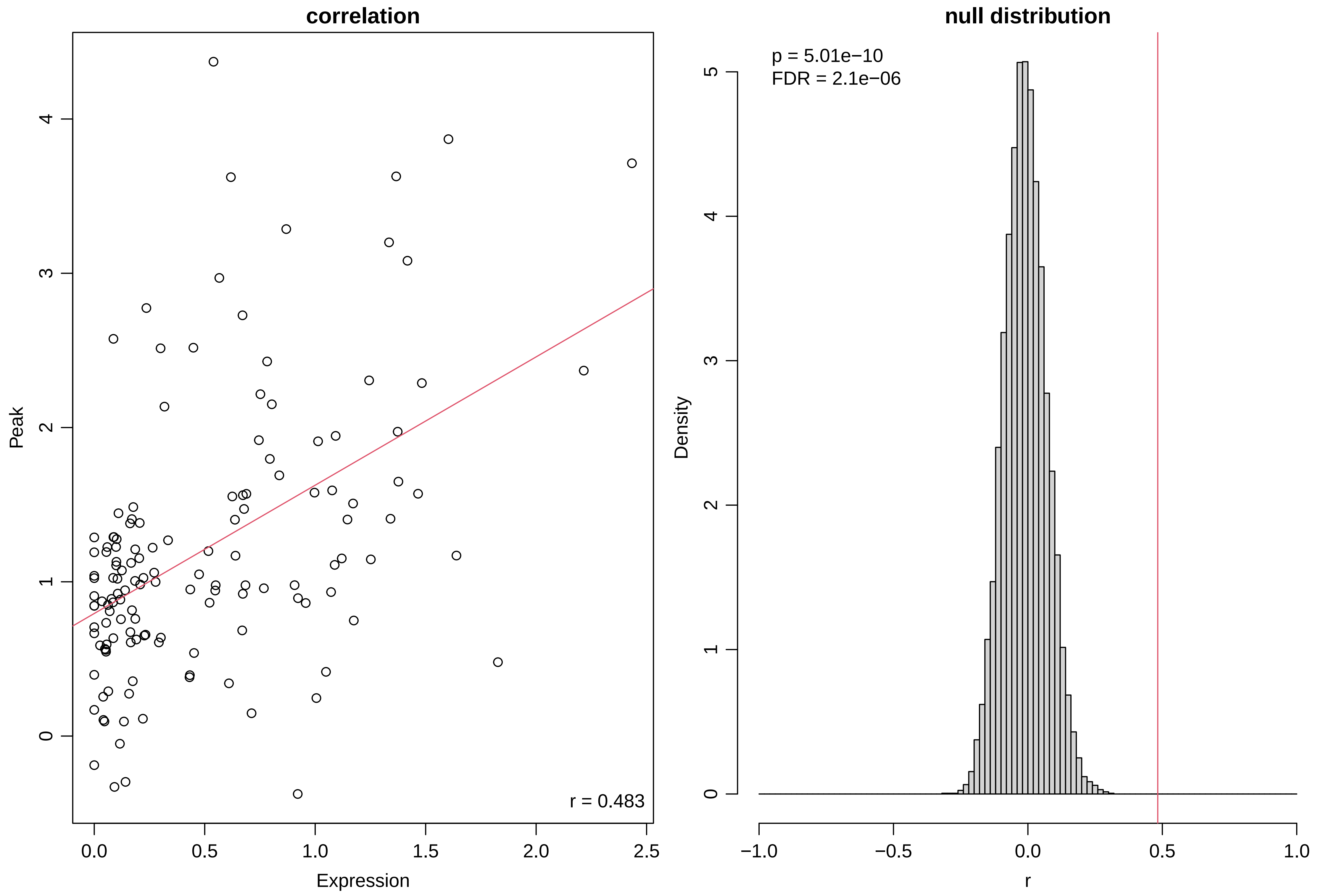

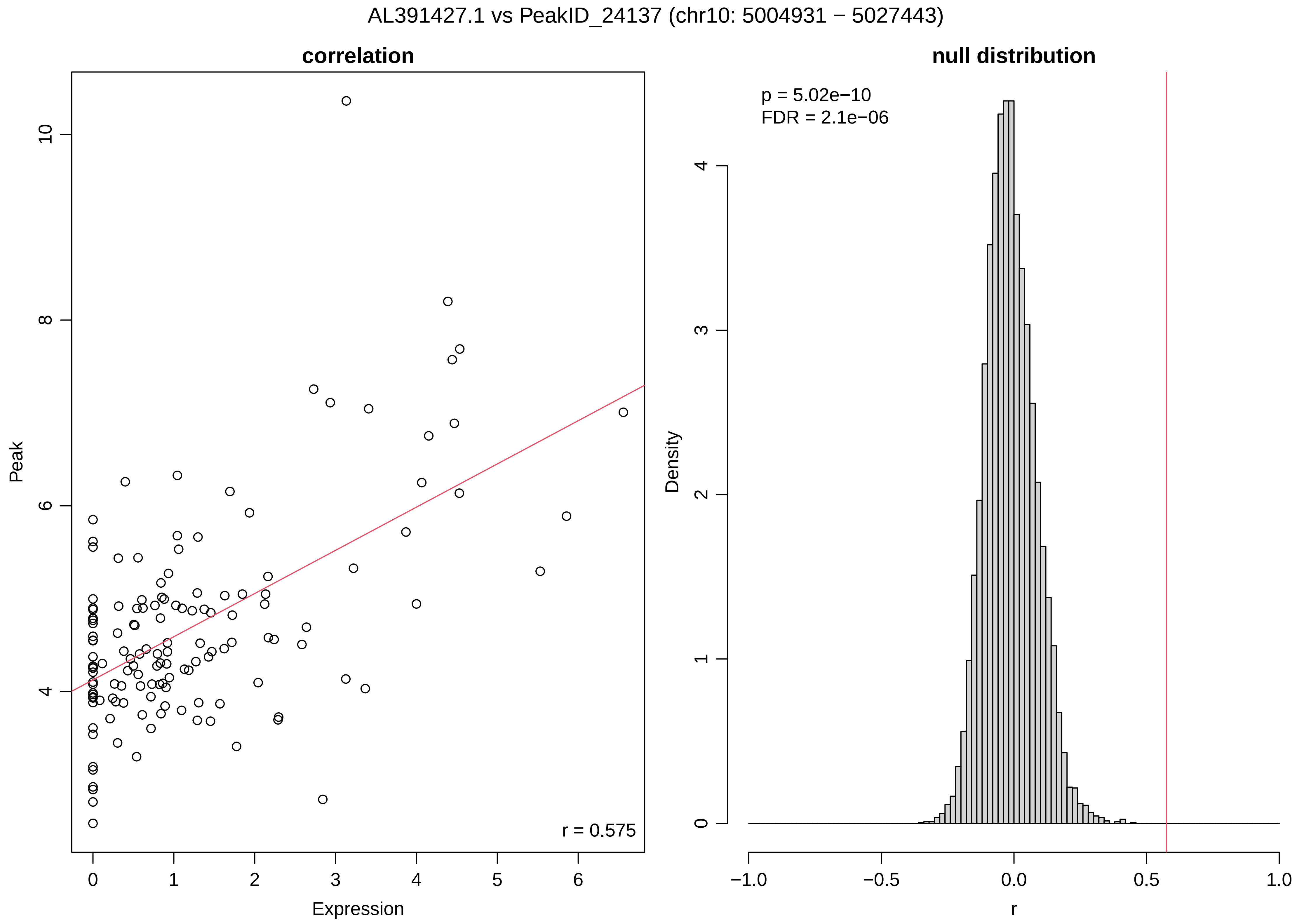

AL391427.1 vs PeakID_24137 (chr10: 5004931 − 5027443)
correlation
Peak
Expression
r = 0.575
null distribution
p = 5.02e−10
FDR = 2.1e−06
Density
r

FOXL2 vs PeakID_170059 (chr3: 138944870 − 138947729)

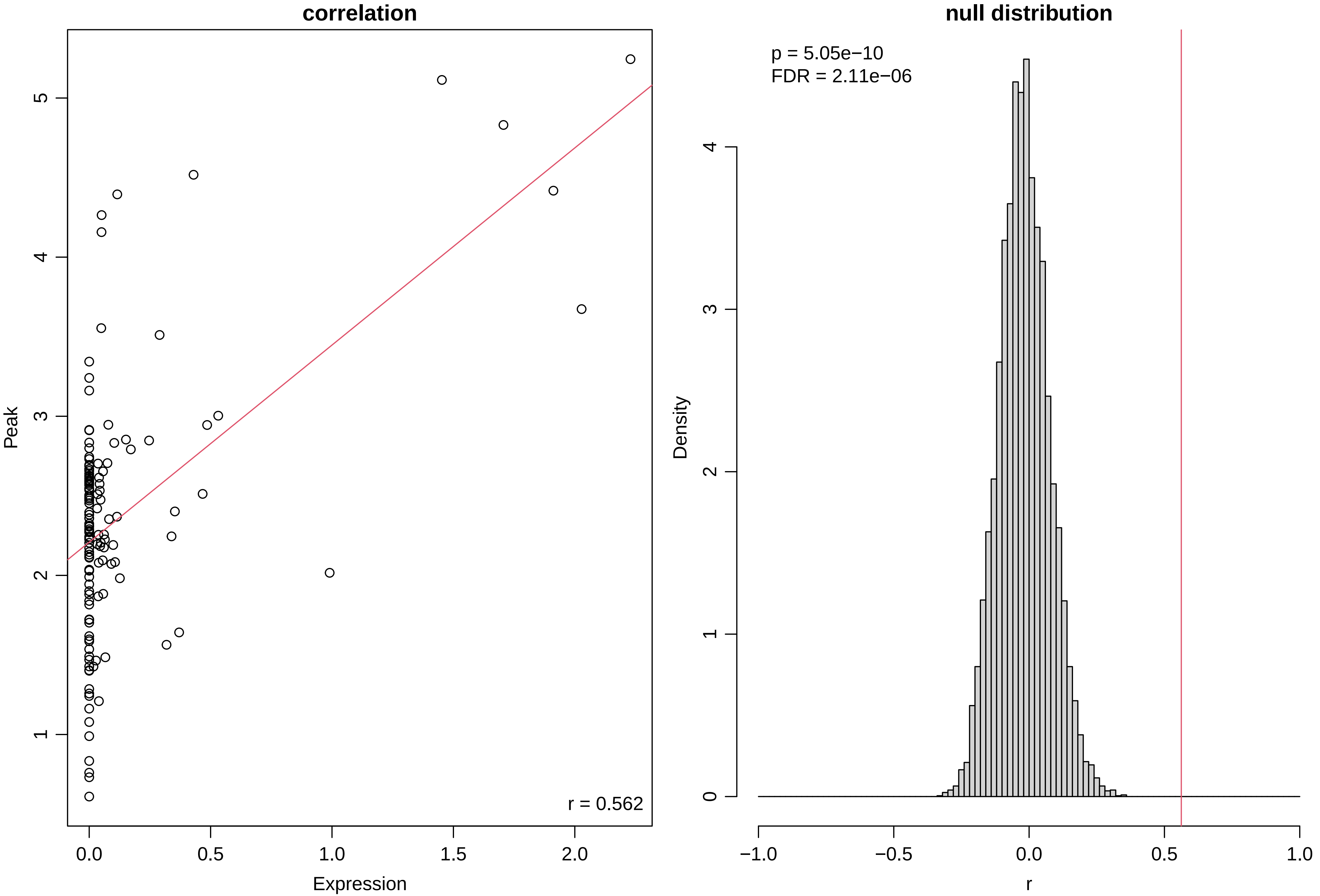

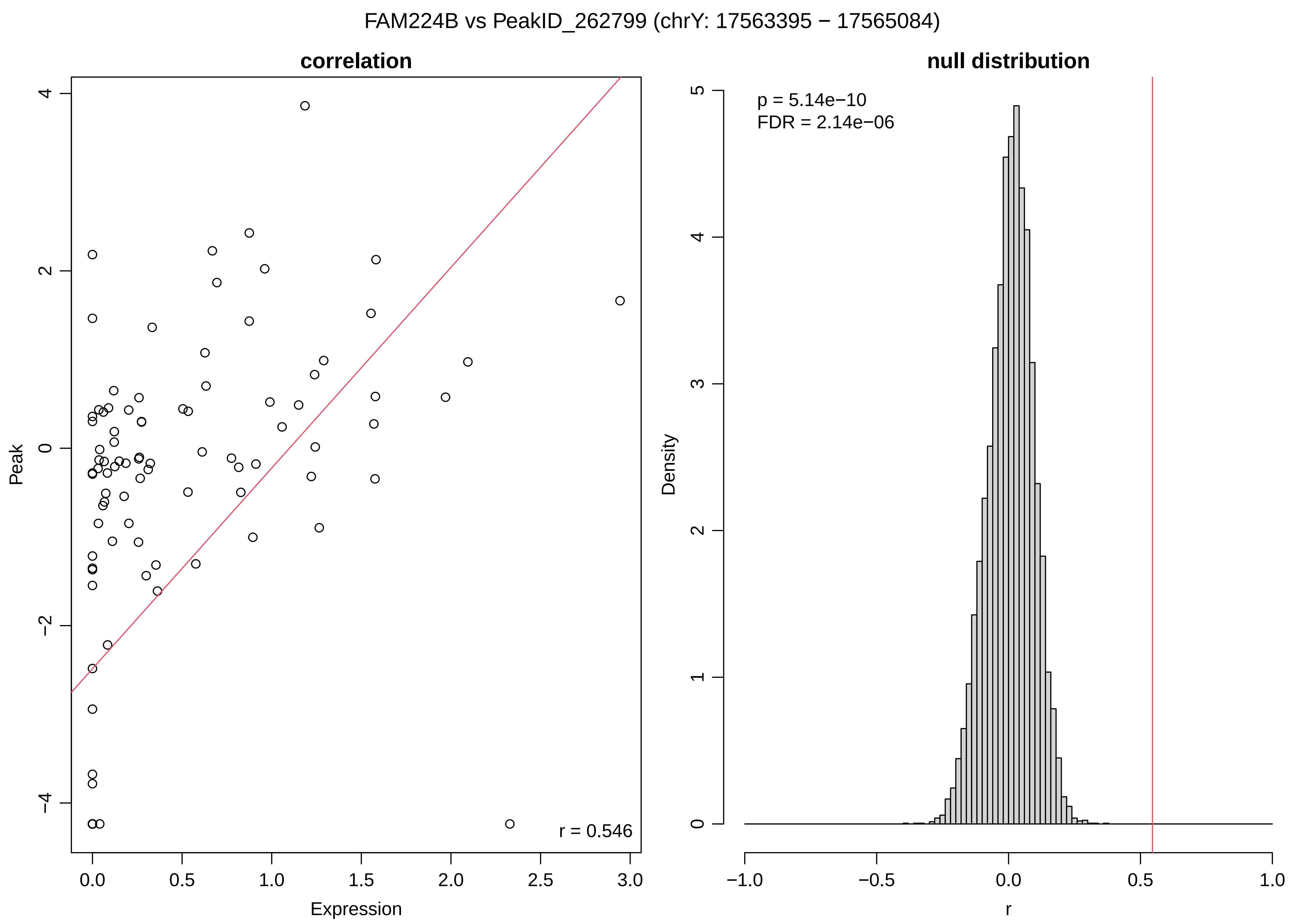
FAM224B vs PeakID_262799 (chrY: 17563395 − 17565084)
correlation
Peak
Expression
r = 0.546
null distribution
p = 5.14e−10
FDR = 2.14e−06
Density
r

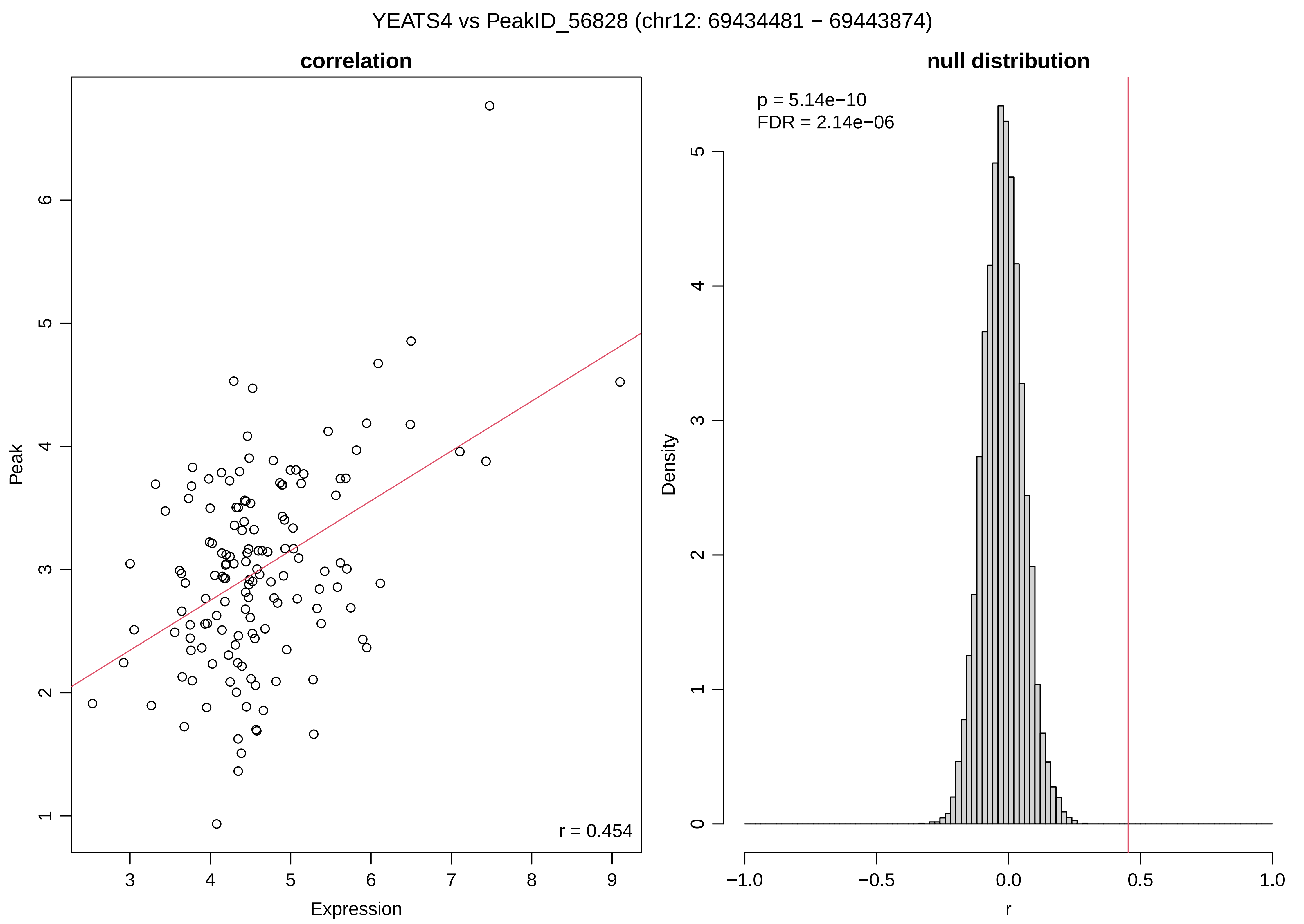

YEATS4 vs PeakID_56828 (chr12: 69434481 − 69443874)
correlation
Peak
Expression
r = 0.454
null distribution
p = 5.14e−10
FDR = 2.14e−06
Density
r

AC107881.1 vs PeakID_37984 (chr11: 12704158 − 12704556)

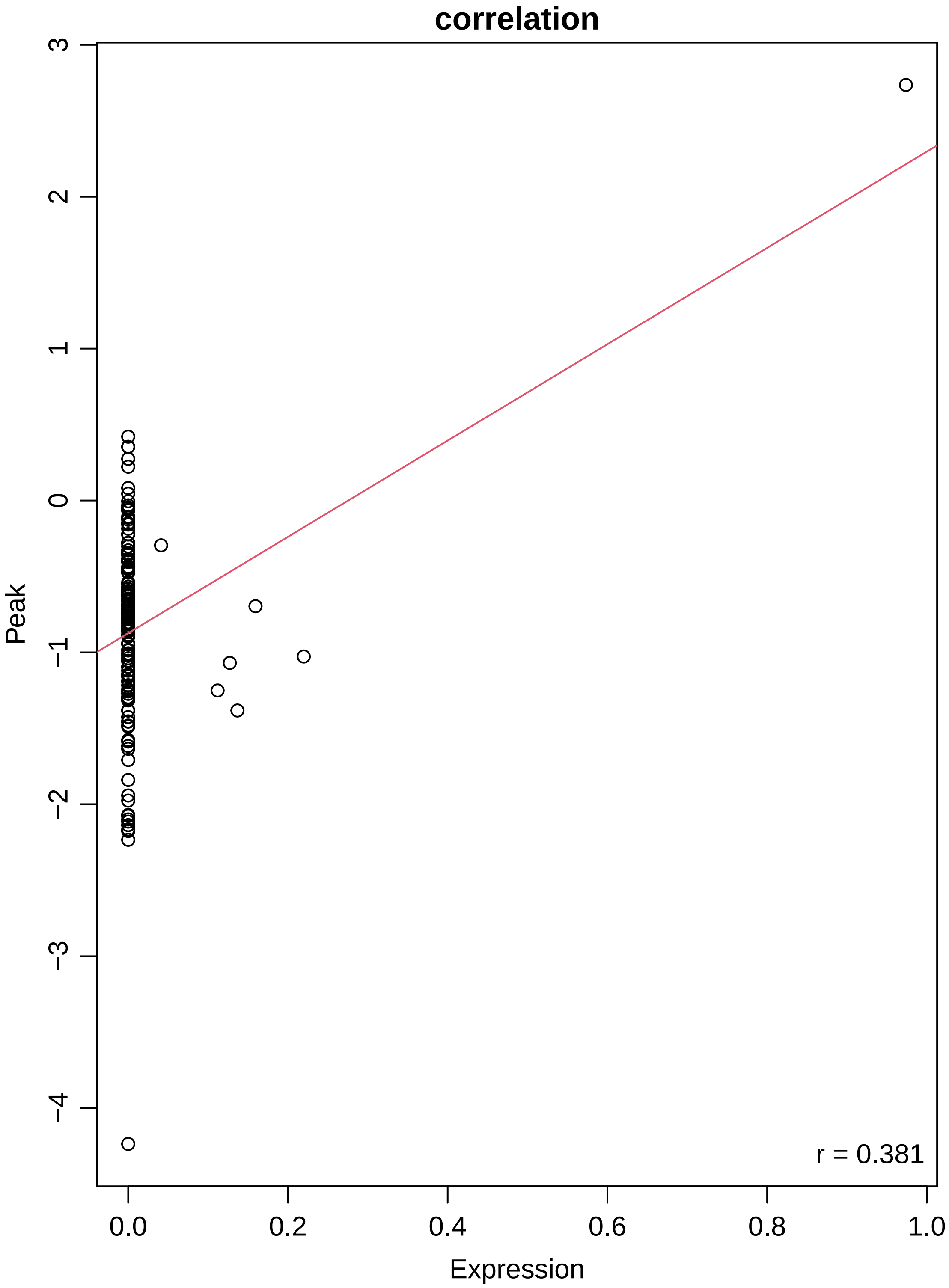

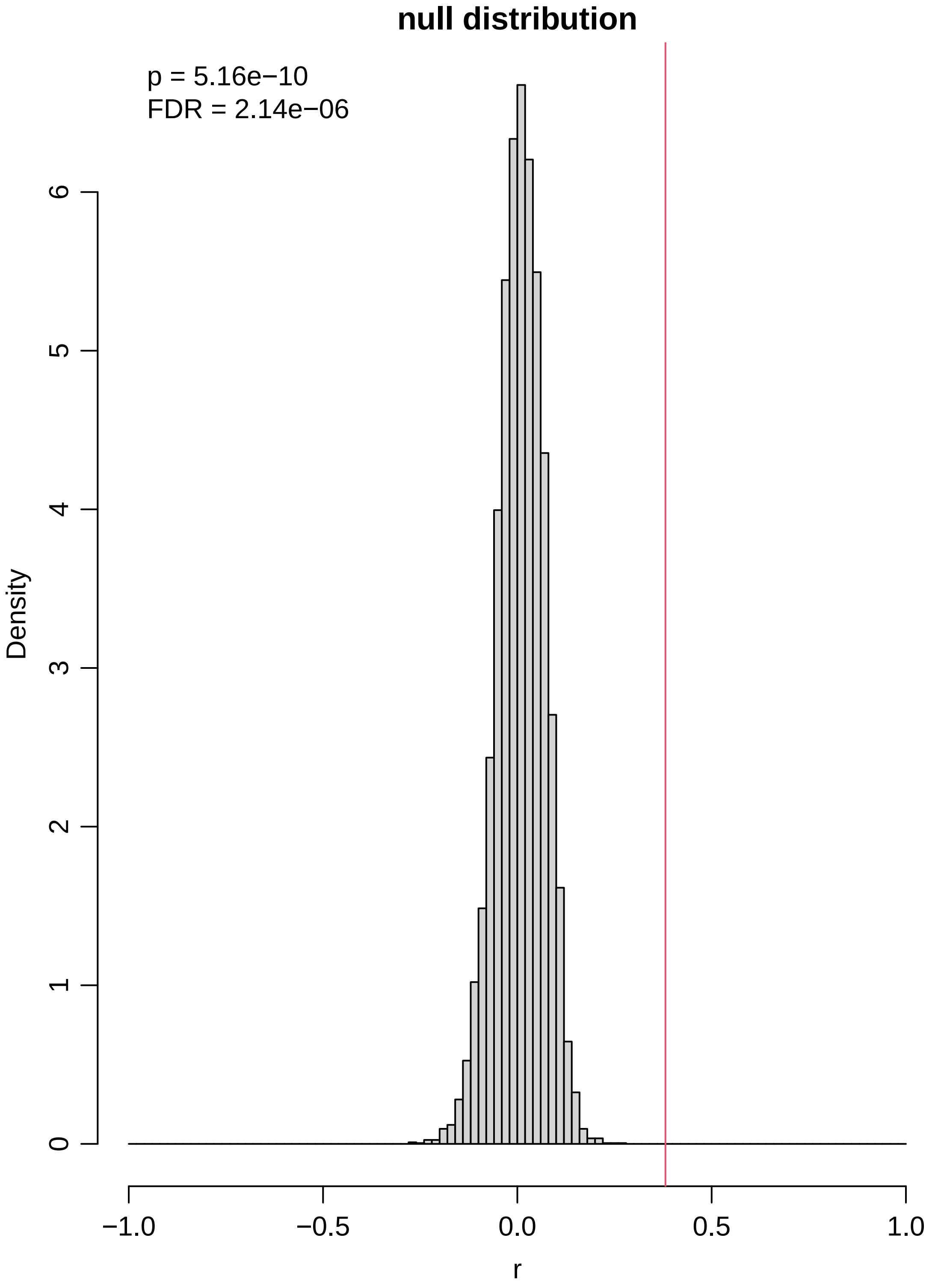

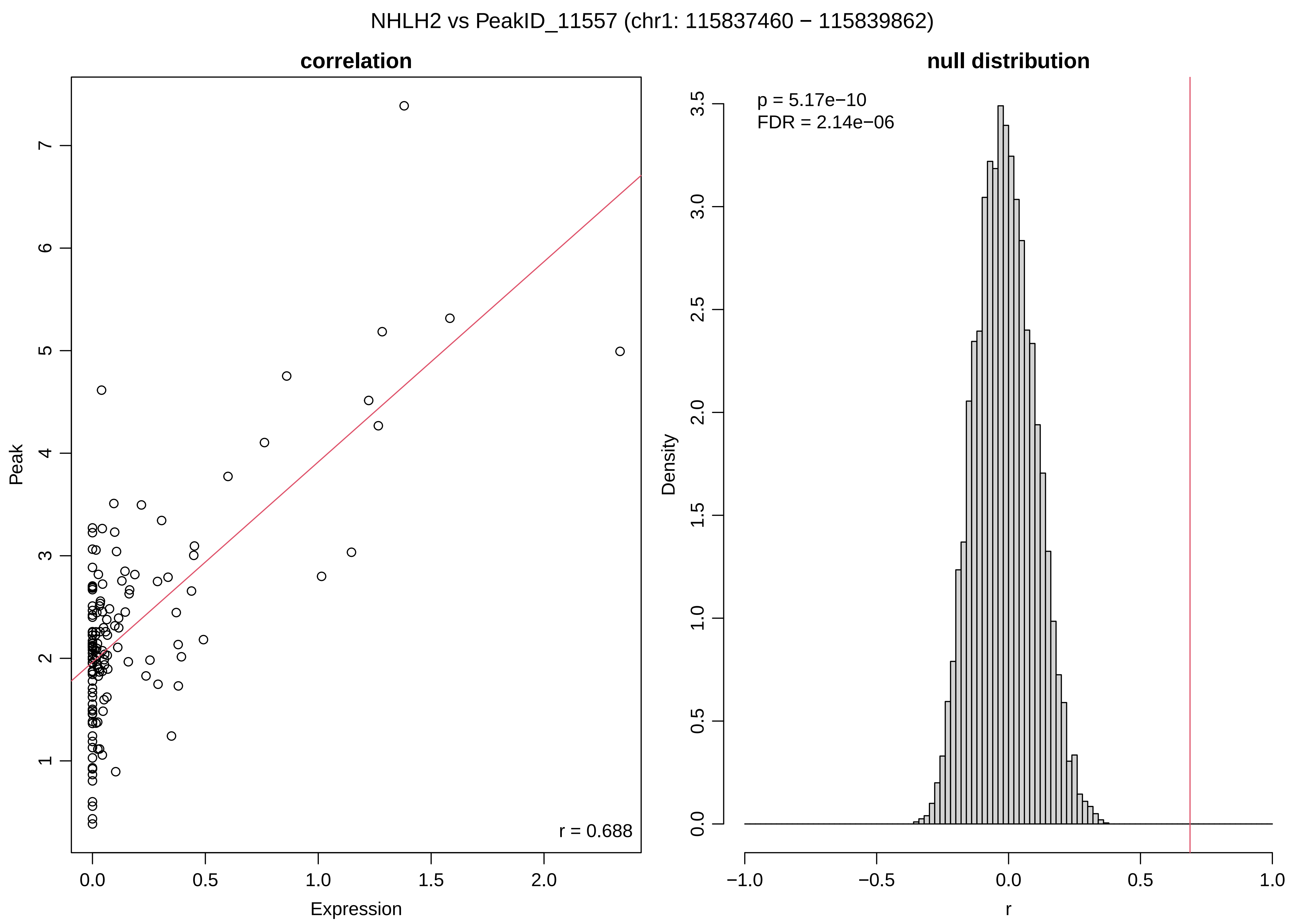
NHLH2 vs PeakID_11557 (chr1: 115837460 − 115839862)
correlation
Peak
Expression
r = 0.688
null distribution
p = 5.17e−10
FDR = 2.14e−06
Density
r

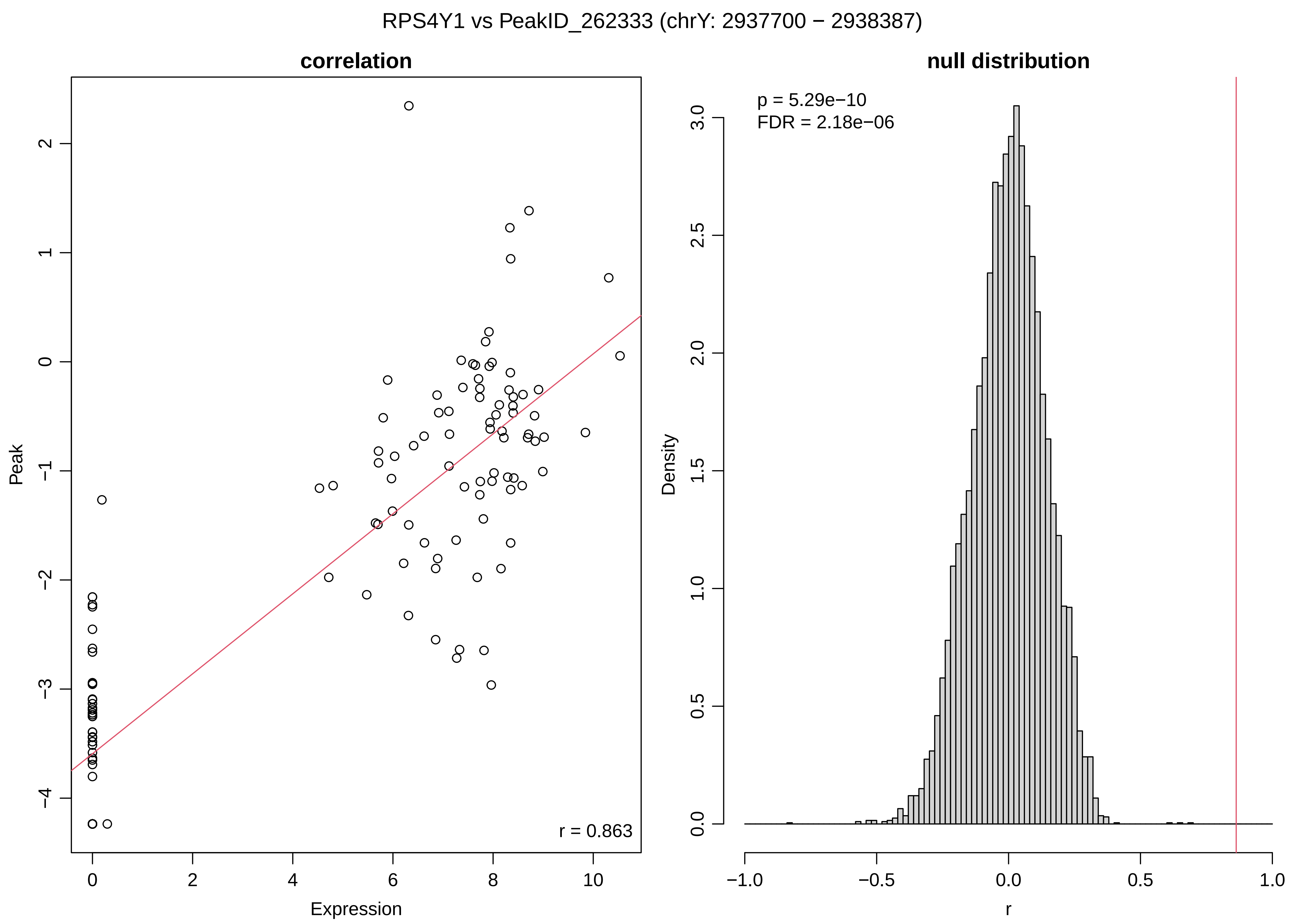
RPS4Y1 vs PeakID_262333 (chrY: 2937700 − 2938387)
correlation
Peak
Expression
r = 0.863
null distribution
p = 5.29e−10
FDR = 2.18e−06
Density
r

AC012317.2 vs PeakID_87735 (chr16: 23753956 − 23755743)

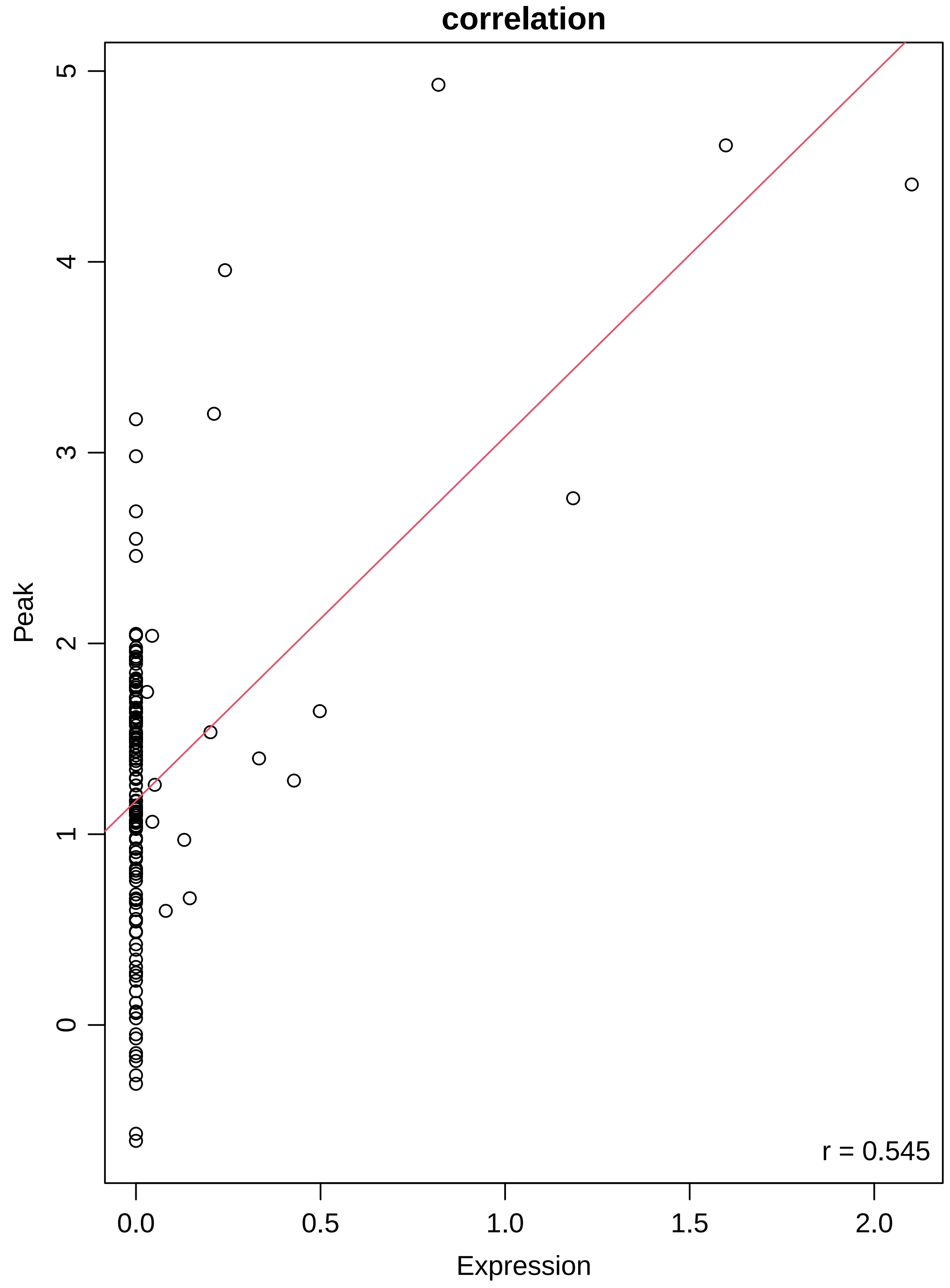

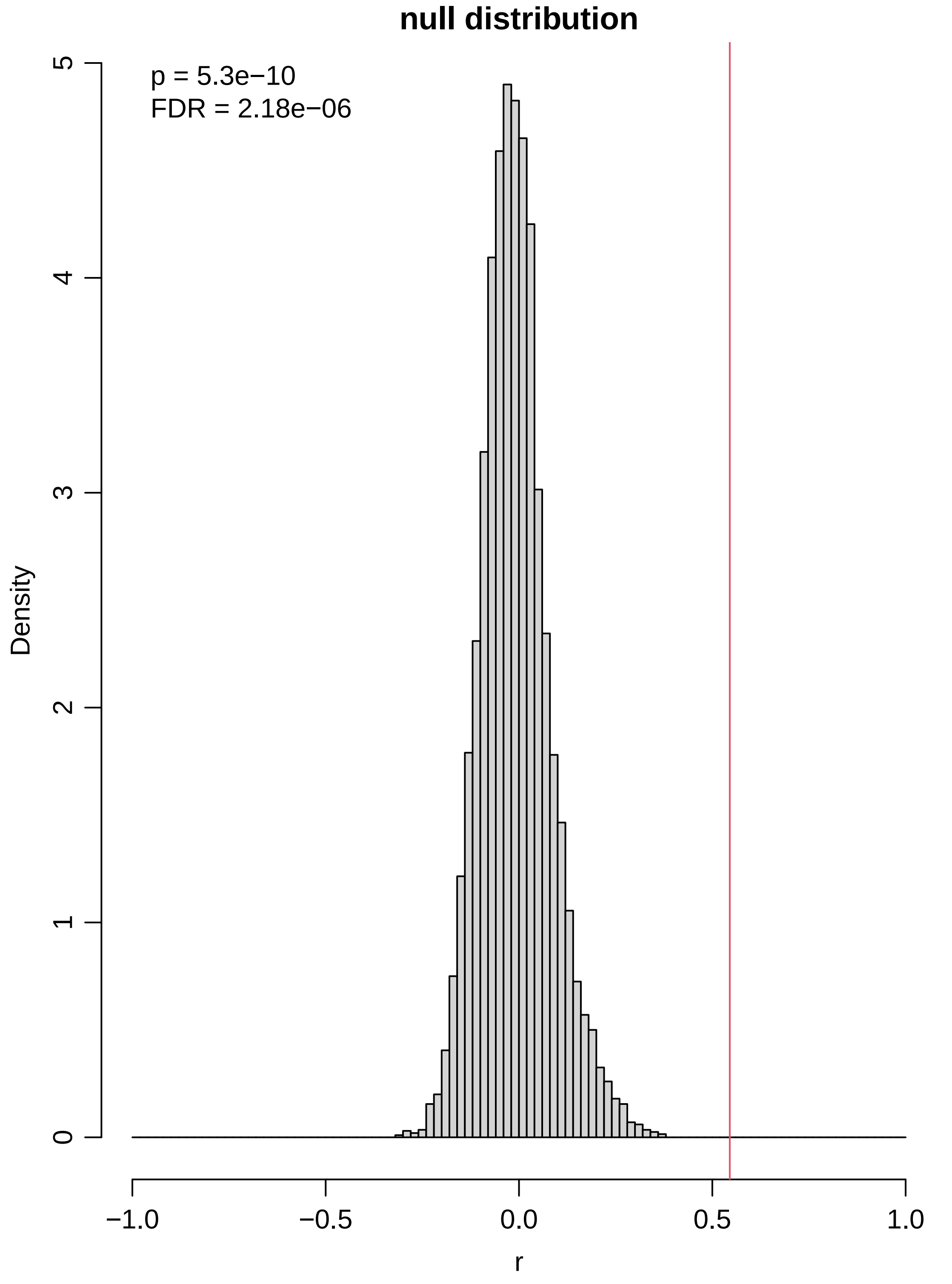

EFNA5 vs PeakID_194775 (chr5: 107668165 − 107673772)

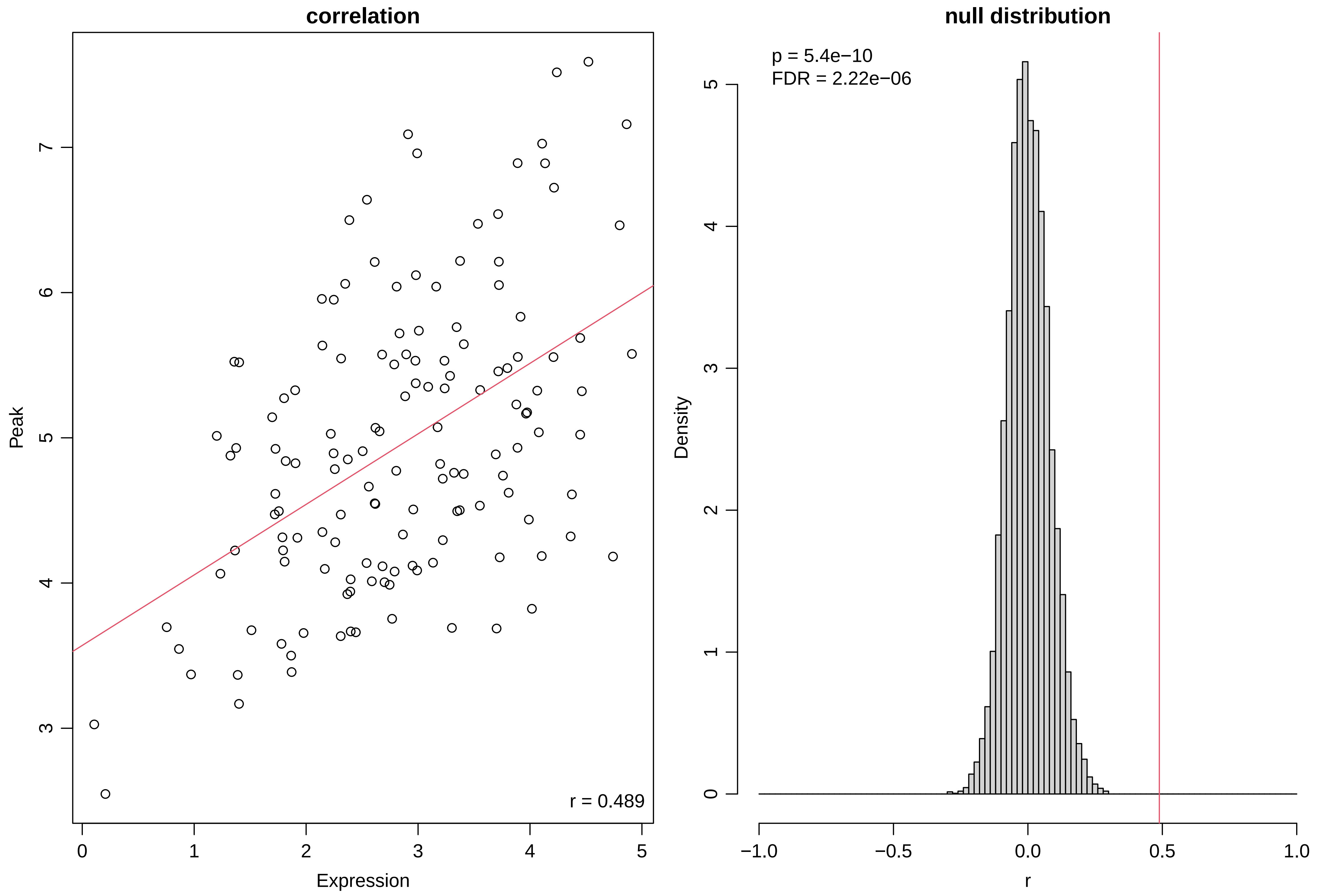

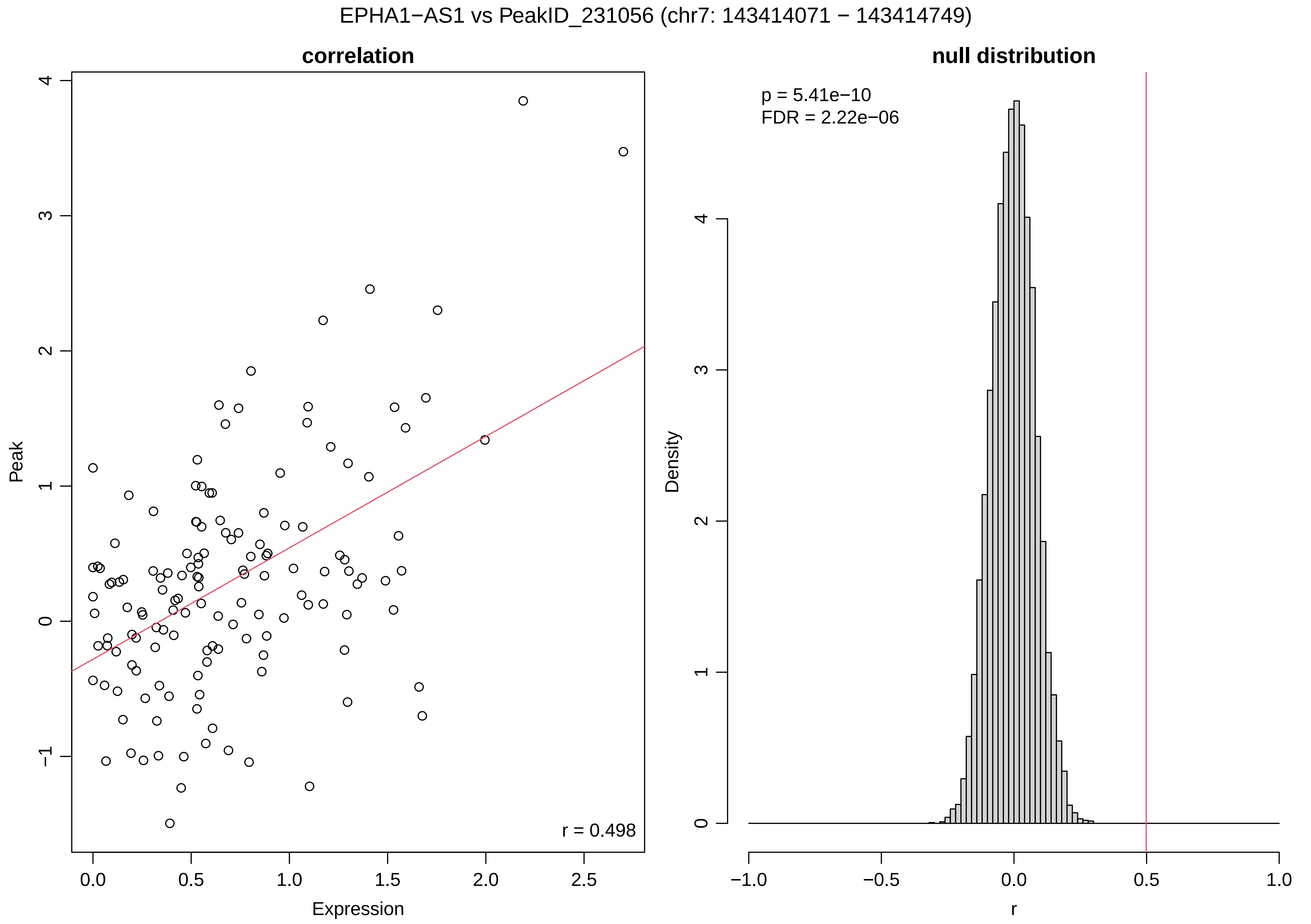

EPHA1−AS1 vs PeakID_231056 (chr7: 143414071 − 143414749)
correlation
Peak
Expression
r = 0.498
null distribution
p = 5.41e−10
FDR = 2.22e−06
Density
r

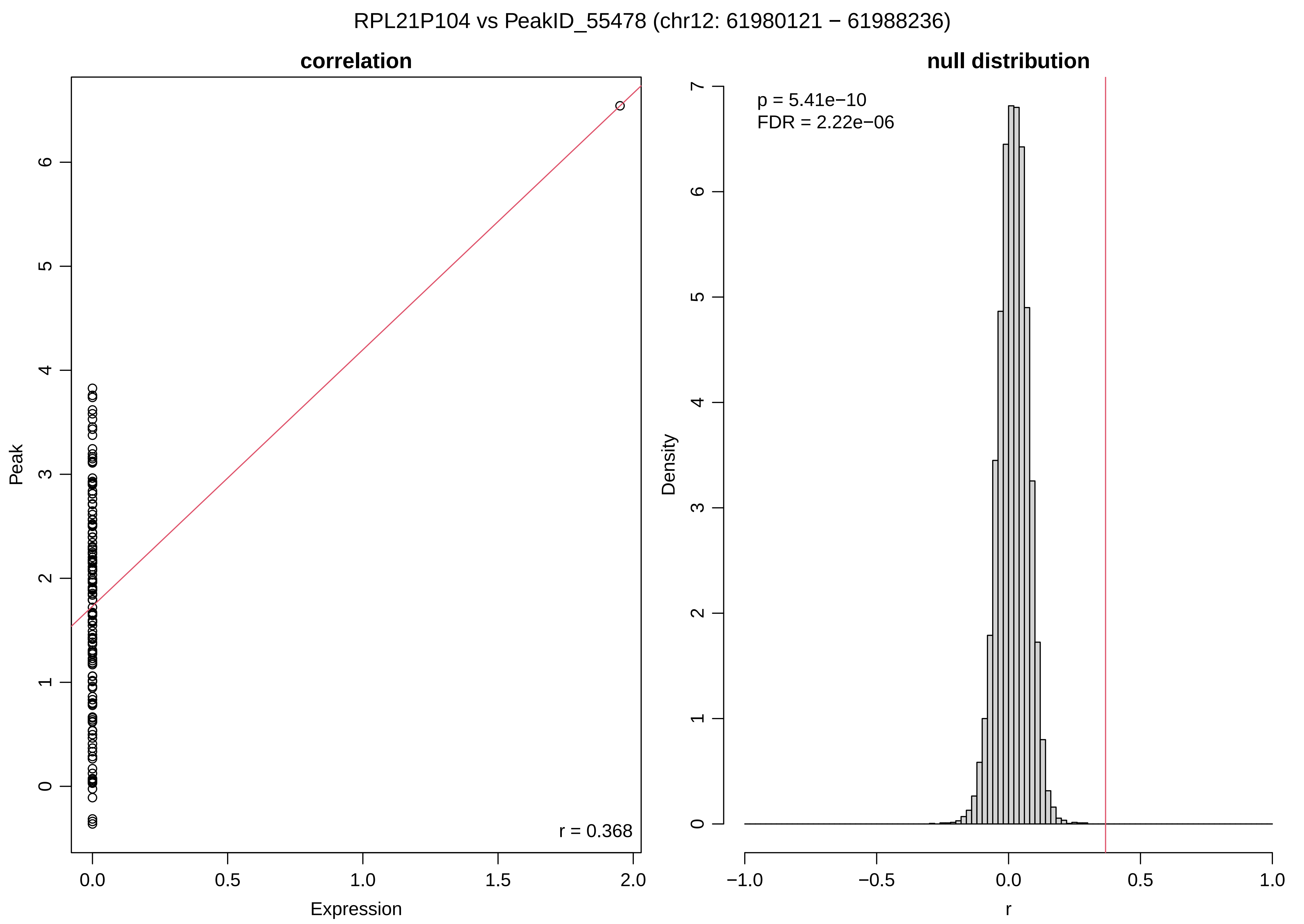
RPL21P104 vs PeakID_55478 (chr12: 61980121 − 61988236)
correlation
Peak
Expression
r = 0.368
null distribution
p = 5.41e−10
FDR = 2.22e−06
Density
r

AC107881.1 vs PeakID_37878 (chr11: 12508804 − 12510301)

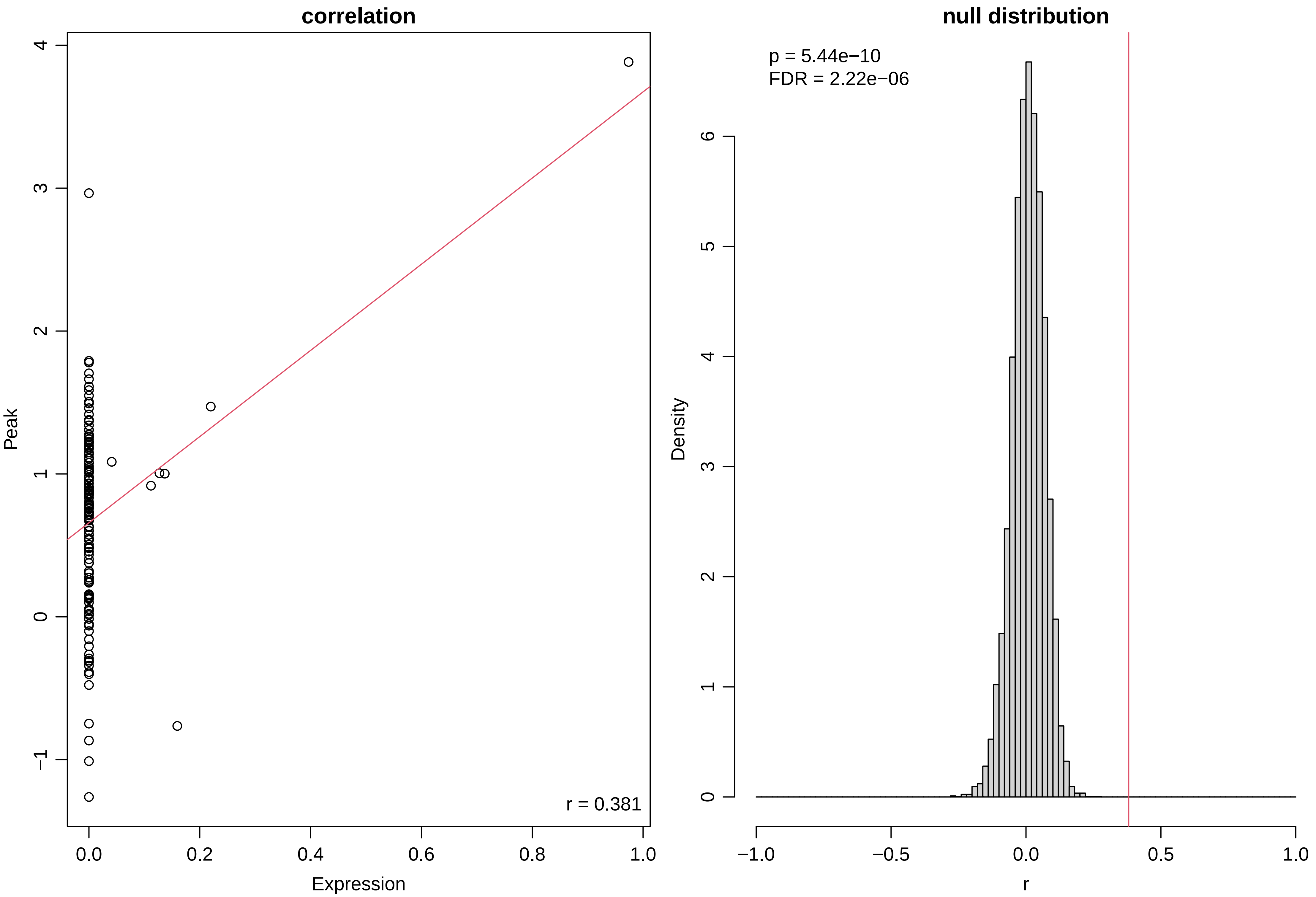

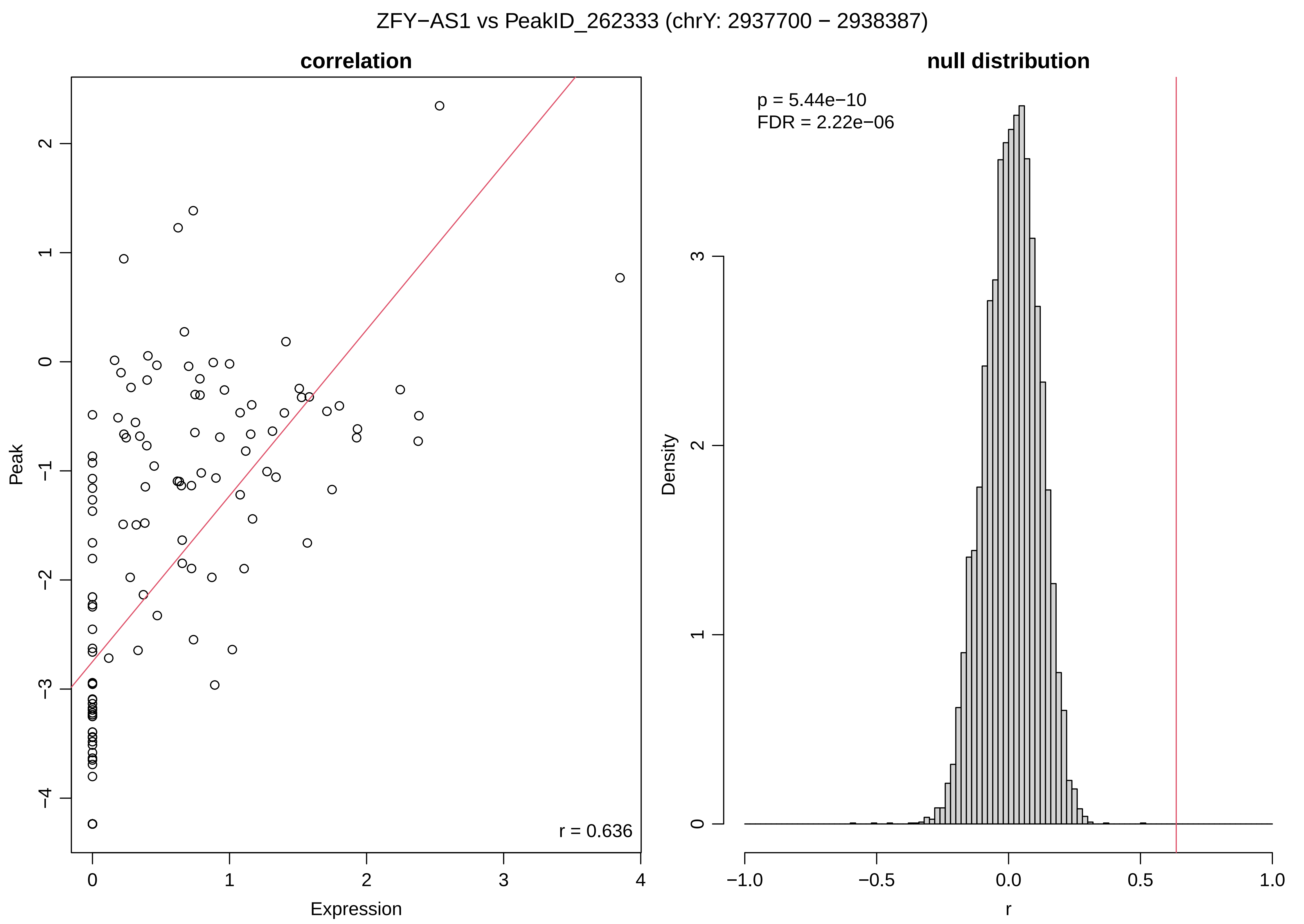
ZFY−AS1 vs PeakID_262333 (chrY: 2937700 − 2938387)
correlation
null distribution
p = 5.44e−10
FDR = 2.22e−06
r = 0.636
Peak
Expression
Density
r
2
1
0
−1
−2
−3
−4
0
1
2
3
4
−1.0
−0.5
0.0
0.5
1.0

ZFY−AS1 vs PeakID_262329 (chrY: 2841194 − 2842456)

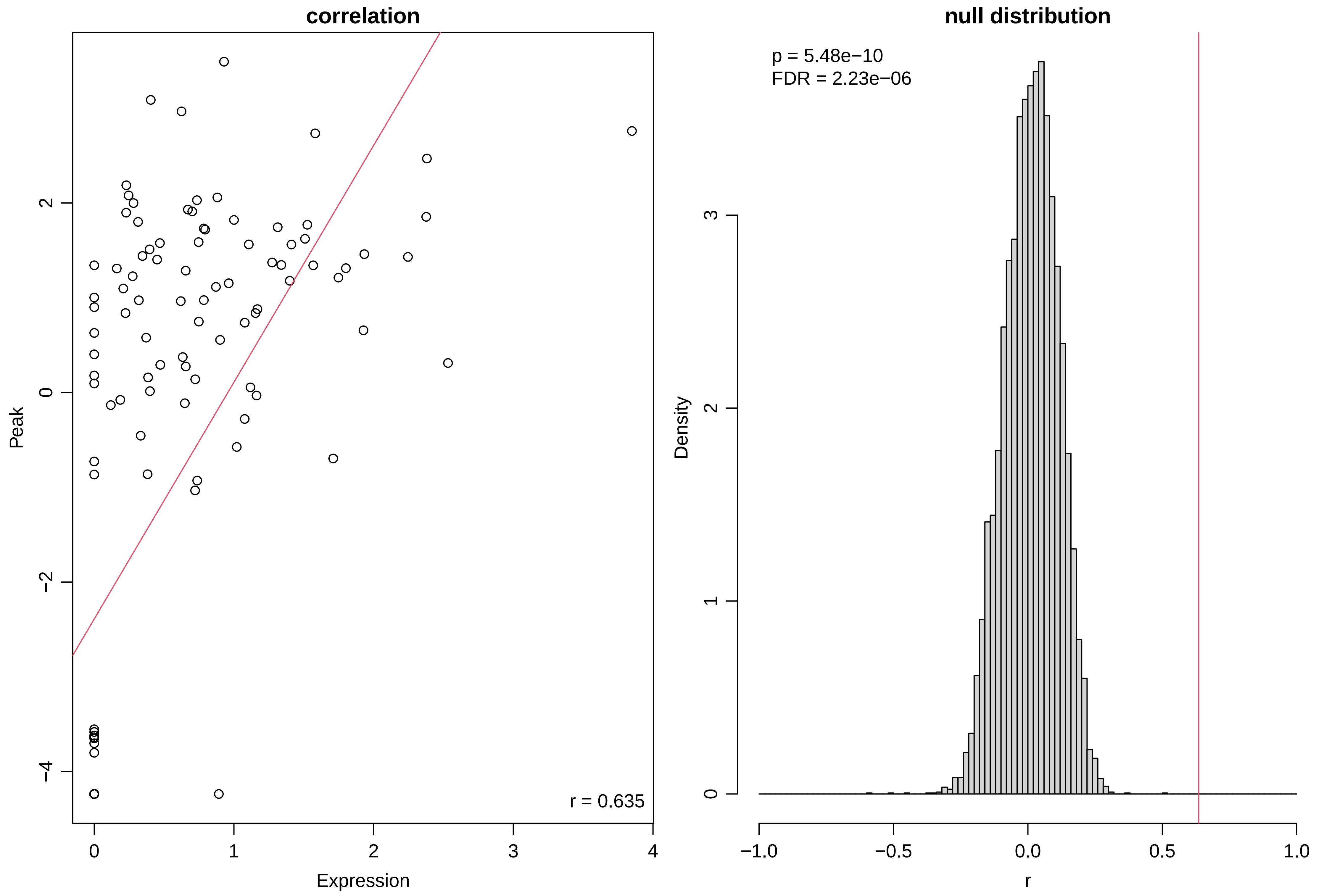

VGF vs PeakID_227821 (chr7: 101161314 − 101165765)

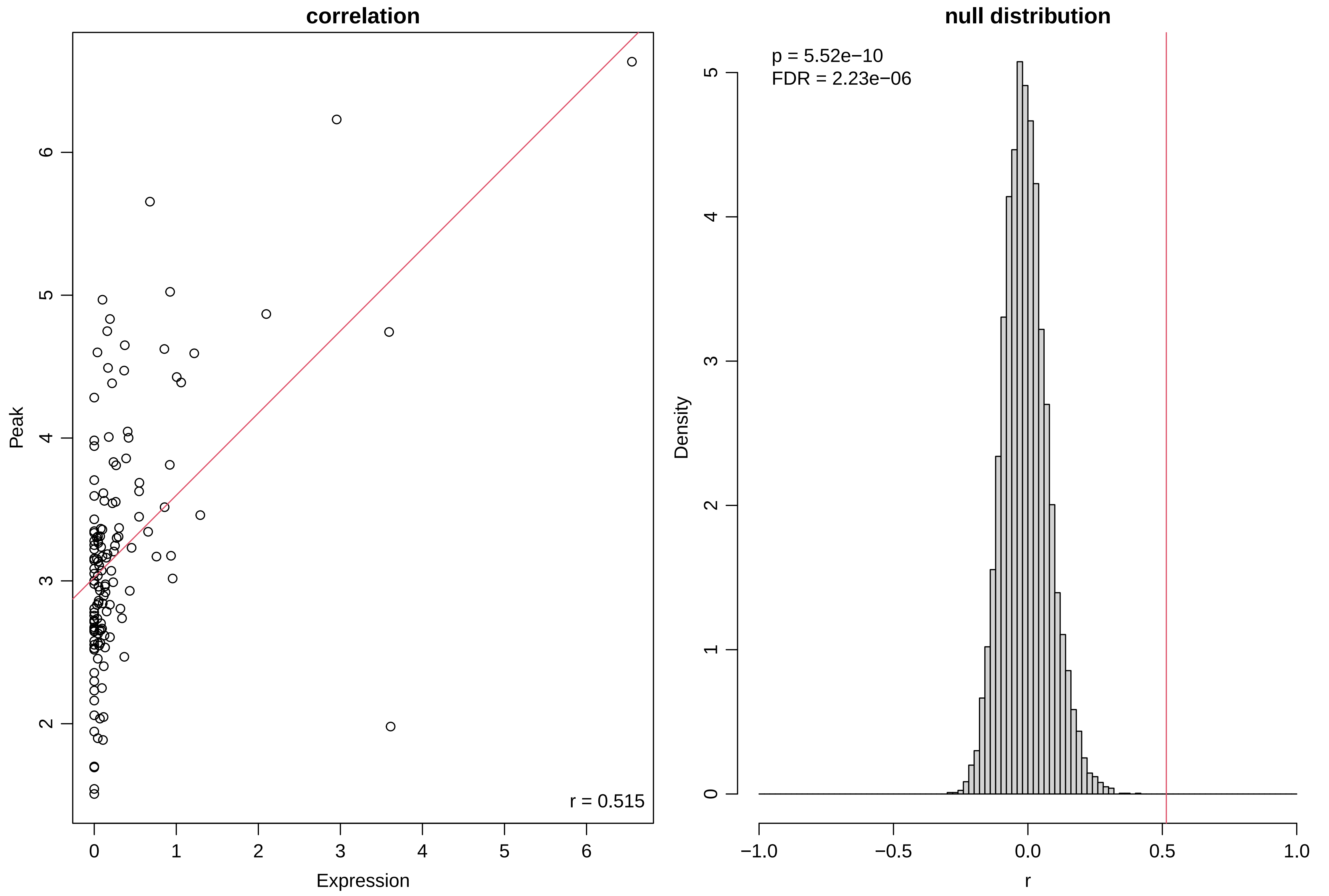

GTSF1 vs PeakID_53943 (chr12: 54473026 − 54474461)

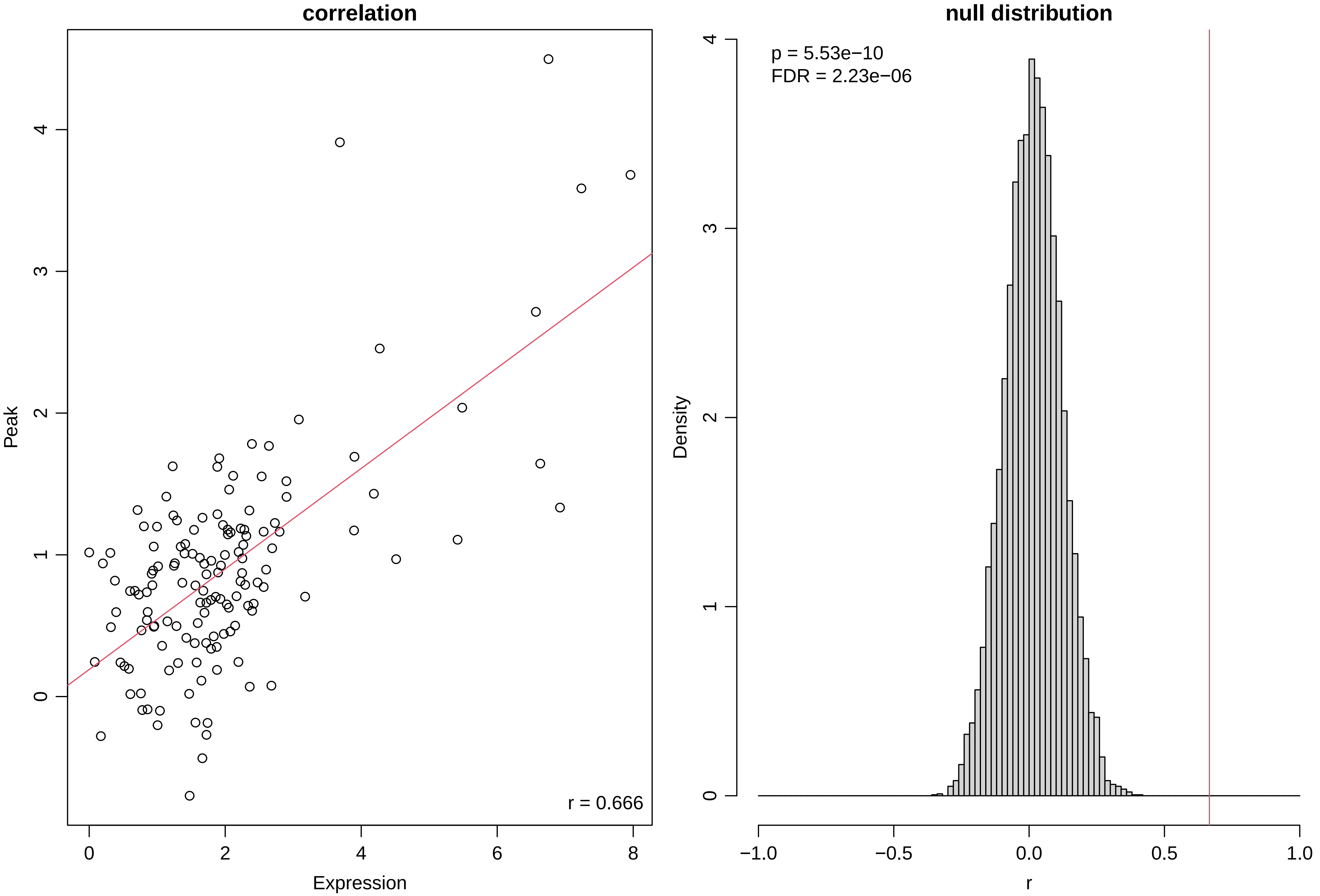

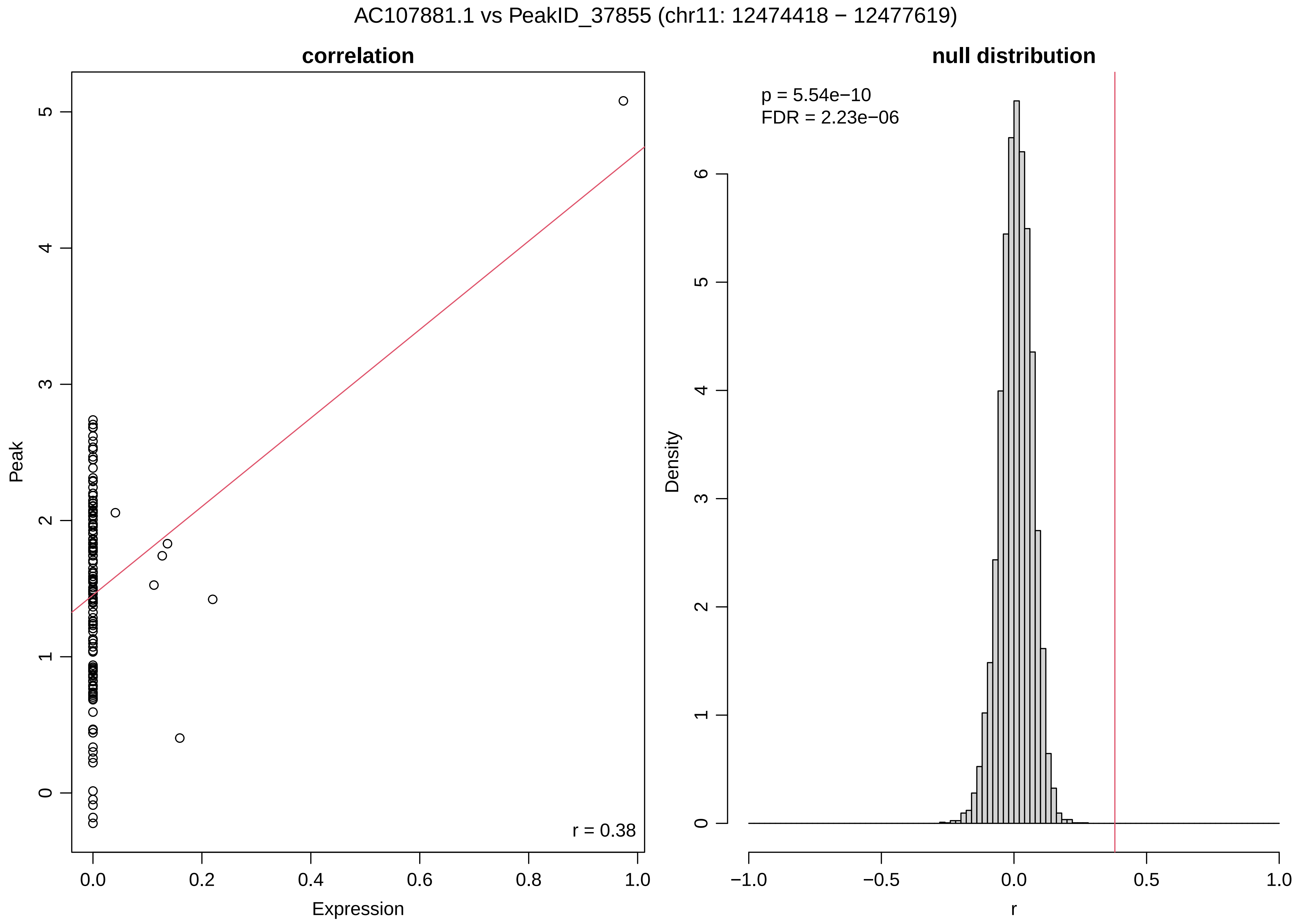

AC107881.1 vs PeakID_37855 (chr11: 12474418 − 12477619)
correlation
Peak
Expression
r = 0.38
0.0
0.2
0.4
0.6
0.8
1.0
0
1
2
3
4
5
null distribution
p = 5.54e−10
FDR = 2.23e−06
Density
r
−1.0
−0.5
0.0
0.5
1.0
0
1
2
3
4
5
6

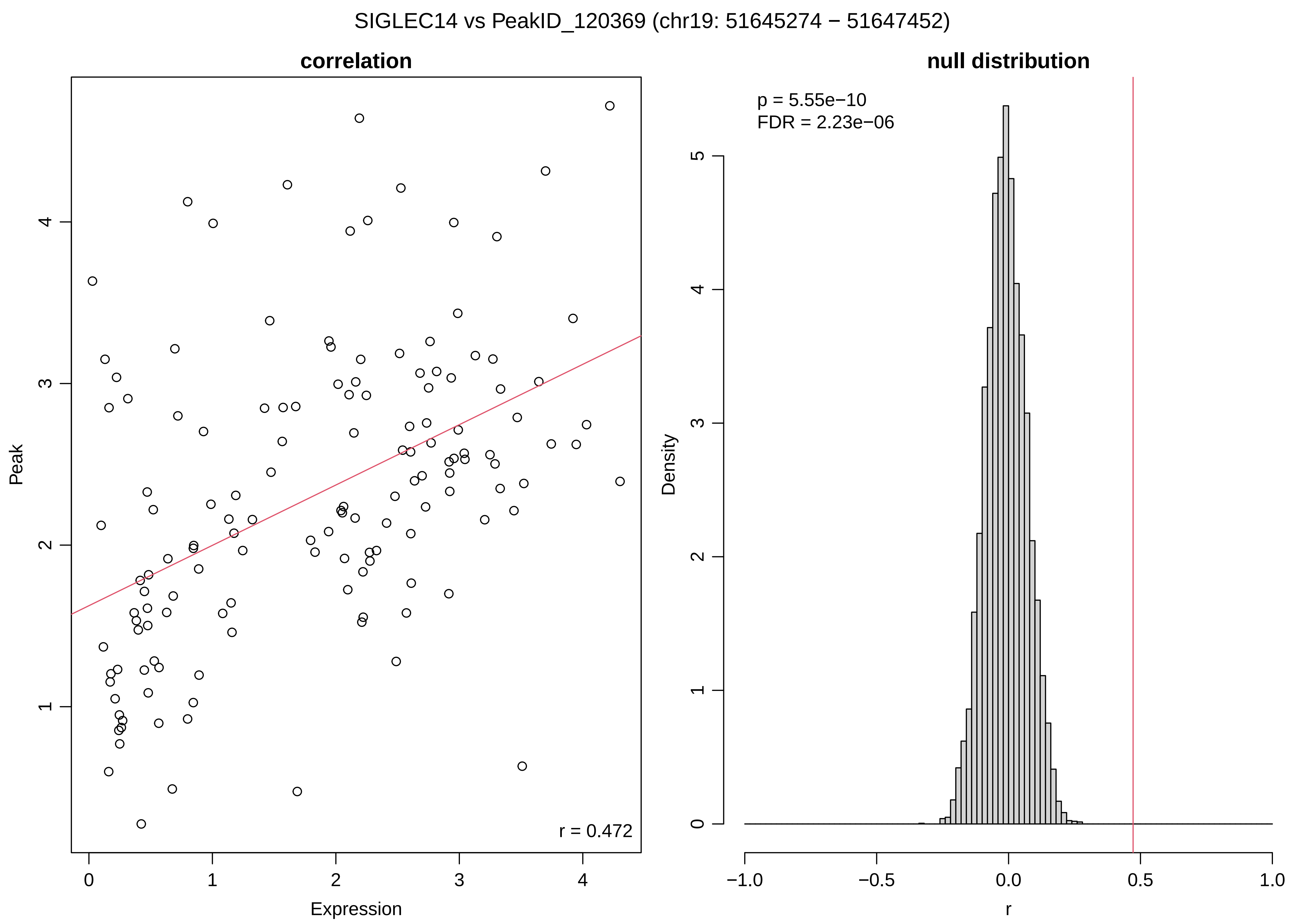

SIGLEC14 vs PeakID_120369 (chr19: 51645274 − 51647452)
correlation
Peak
Expression
r = 0.472
0
1
2
3
4
null distribution
p = 5.55e−10
FDR = 2.23e−06
Density
r
0
1
2
3
4
5
−1.0
−0.5
0.0
0.5
1.0

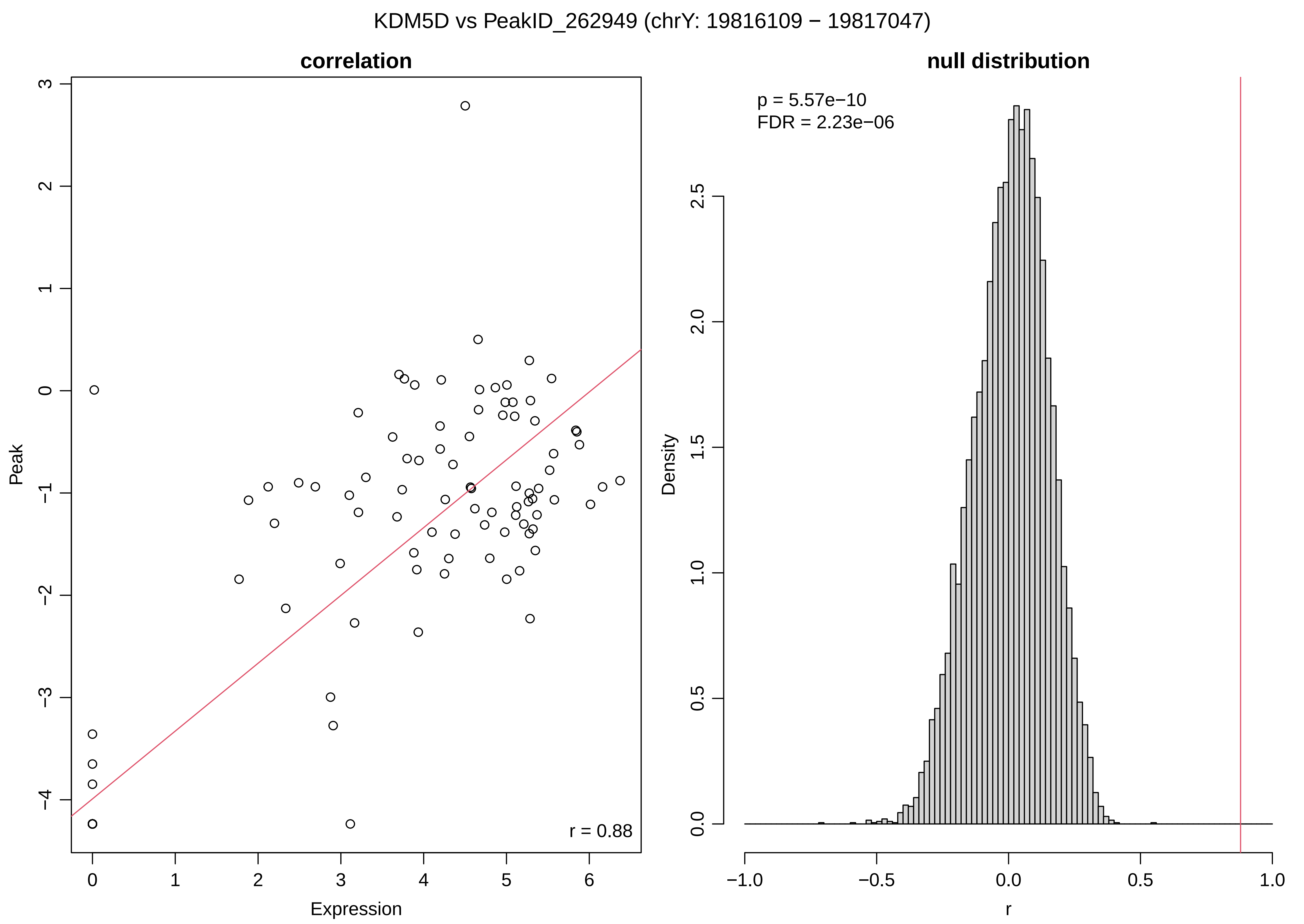
KDM5D vs PeakID_262949 (chrY: 19816109 − 19817047)
correlation
Peak
Expression
r = 0.88
null distribution
p = 5.57e−10
FDR = 2.23e−06
Density
r

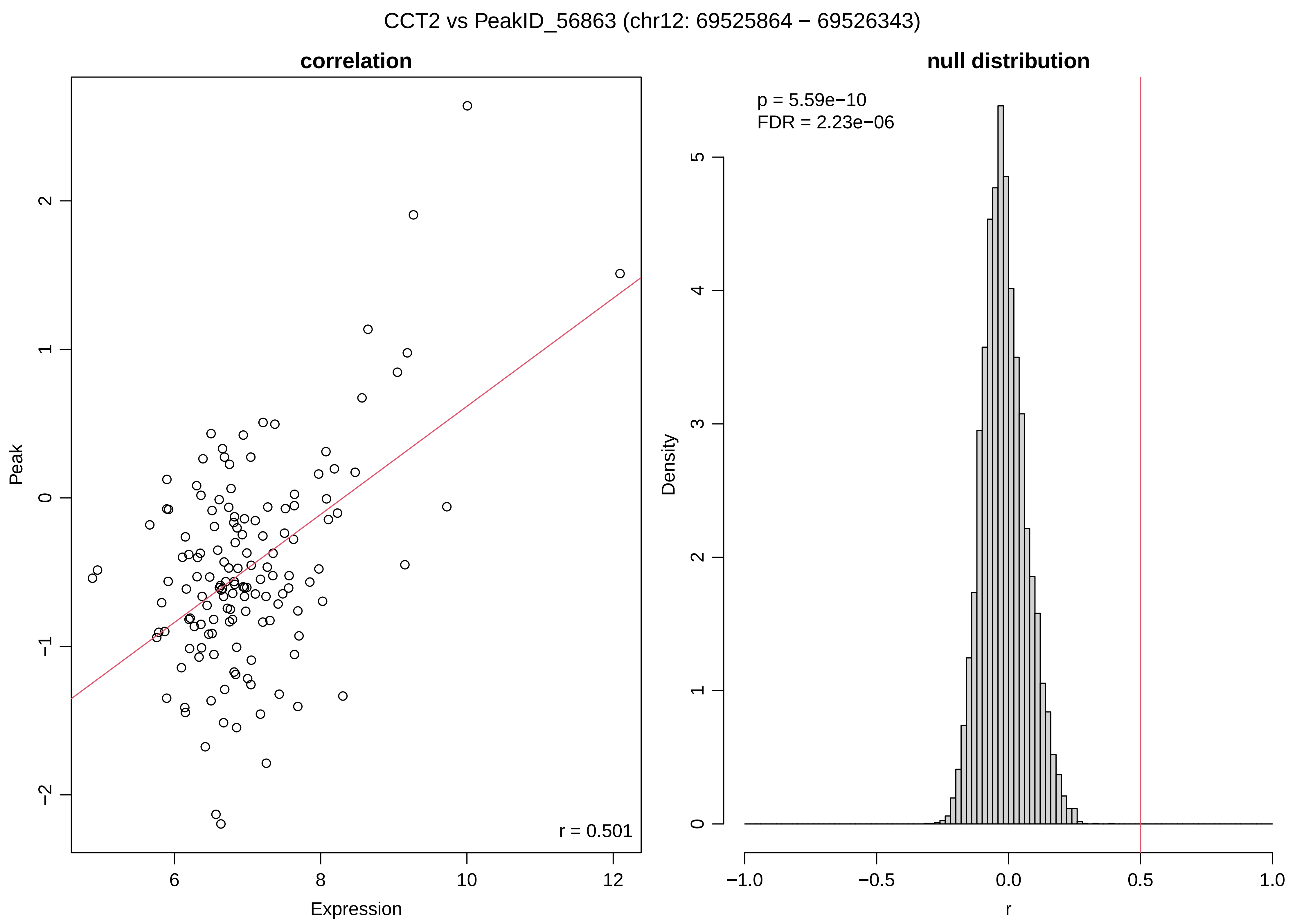

CCT2 vs PeakID_56863 (chr12: 69525864 − 69526343)
correlation
null distribution
Peak
Expression
r = 0.501
p = 5.59e−10
FDR = 2.23e−06
Density
r

SLC25A24P1 vs PeakID_10653 (chr1: 108420223 − 108421911)

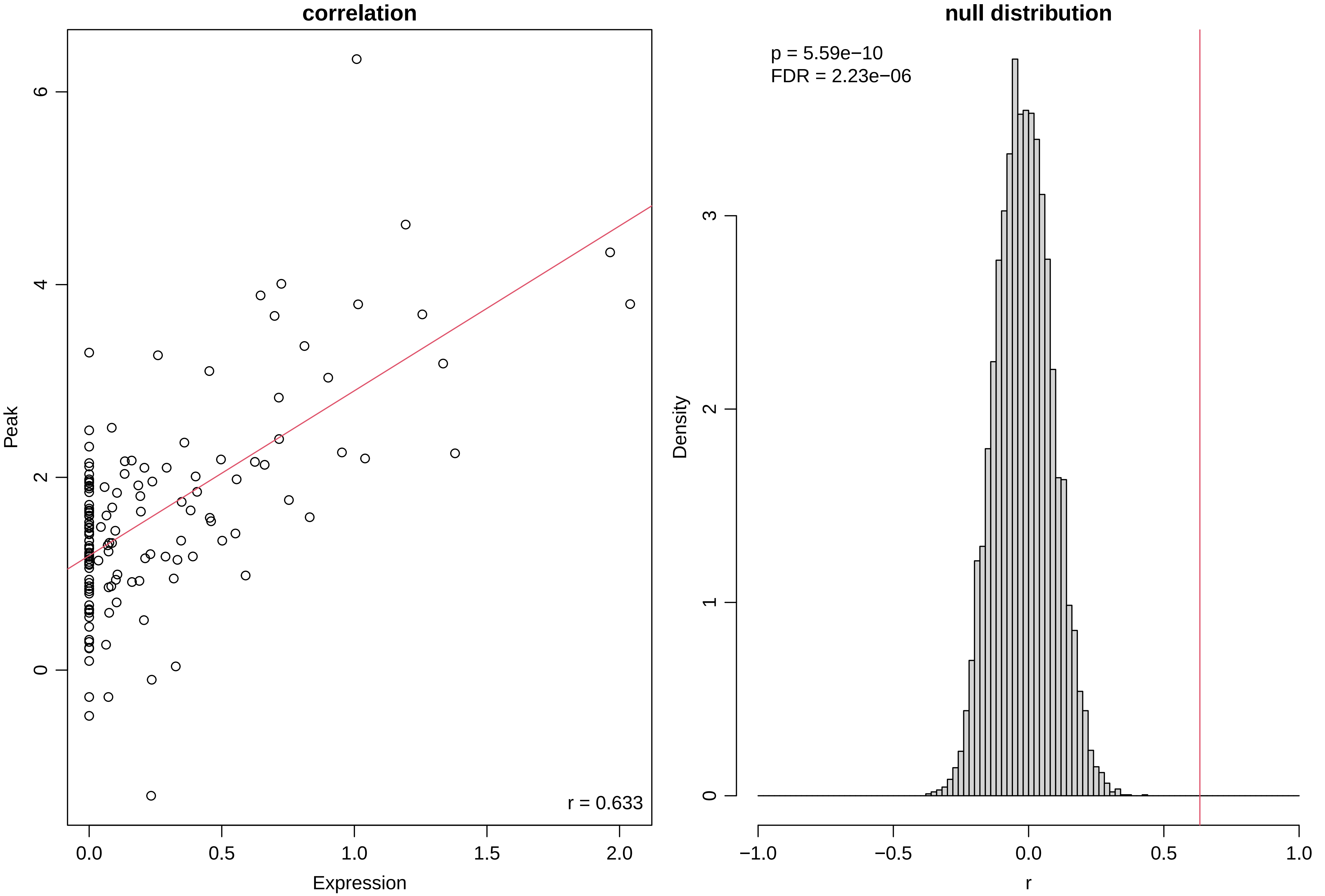

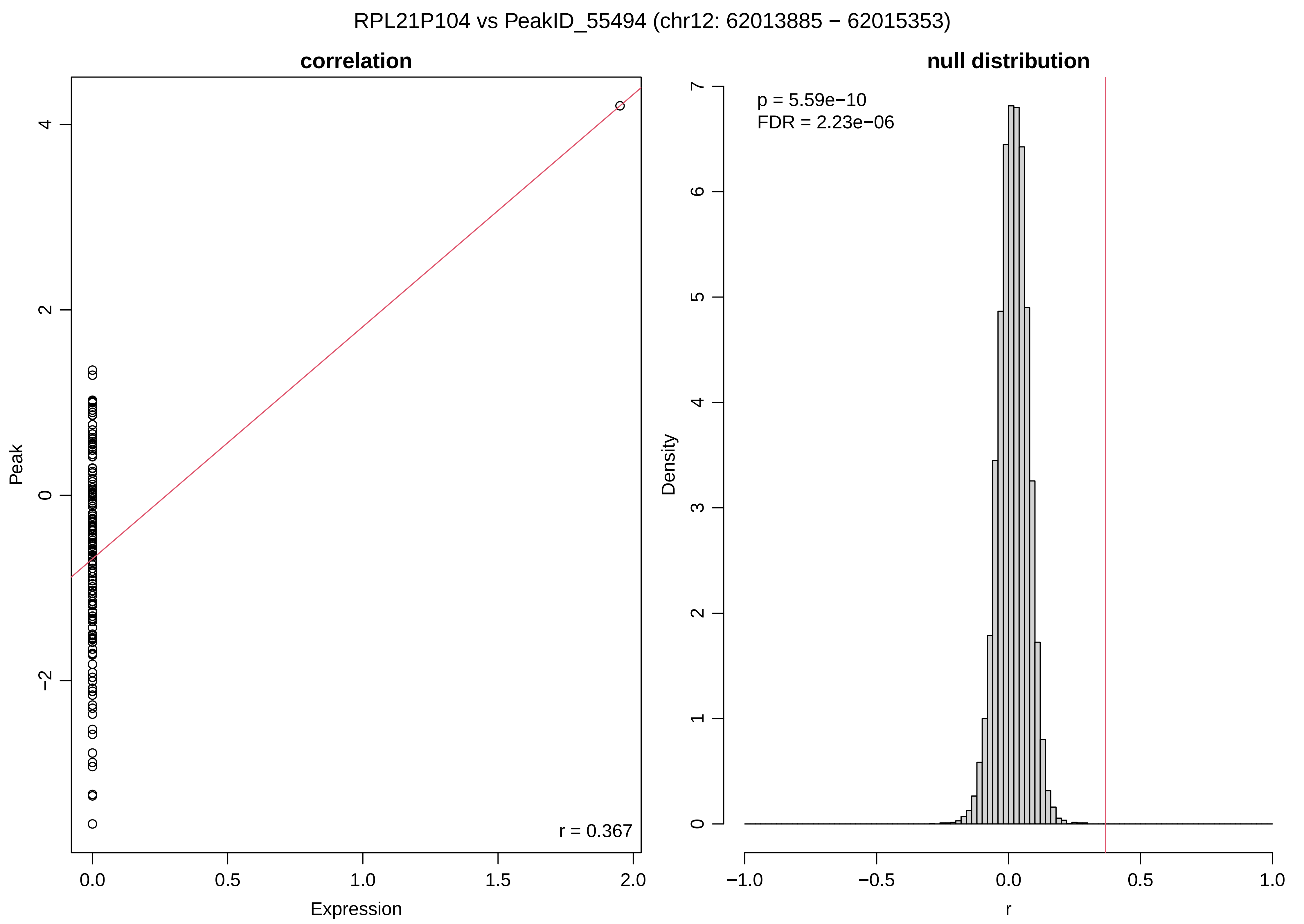
RPL21P104 vs PeakID_55494 (chr12: 62013885 − 62015353)
correlation
Peak
Expression
r = 0.367
4
2
0
−2
0.0
0.5
1.0
1.5
2.0
null distribution
p = 5.59e−10
FDR = 2.23e−06
Density
r
0
1
2
3
4
5
6
7
−1.0
−0.5
0.0
0.5
1.0

ZNF300 vs PeakID_198356 (chr5: 150904556 − 150905478)

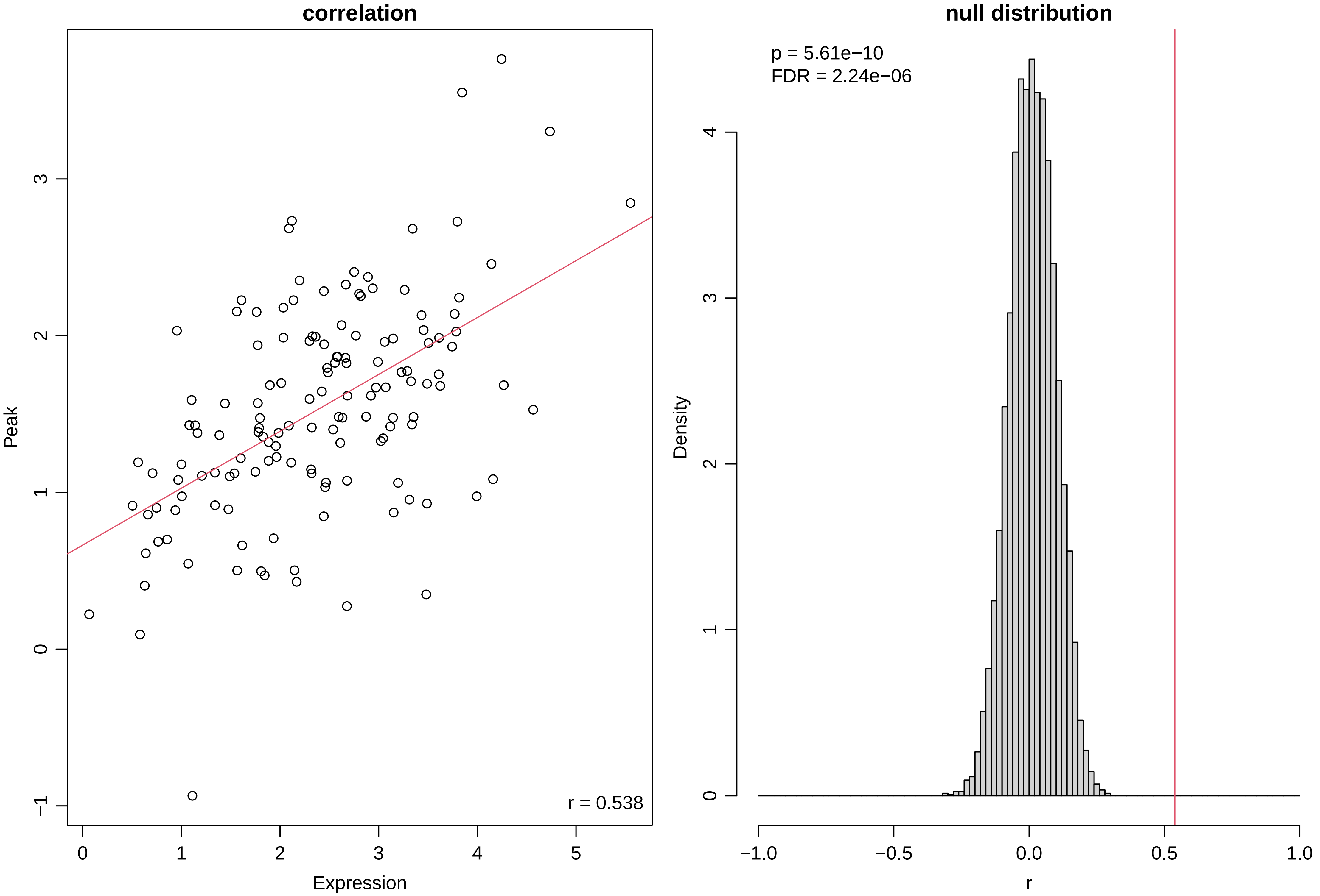

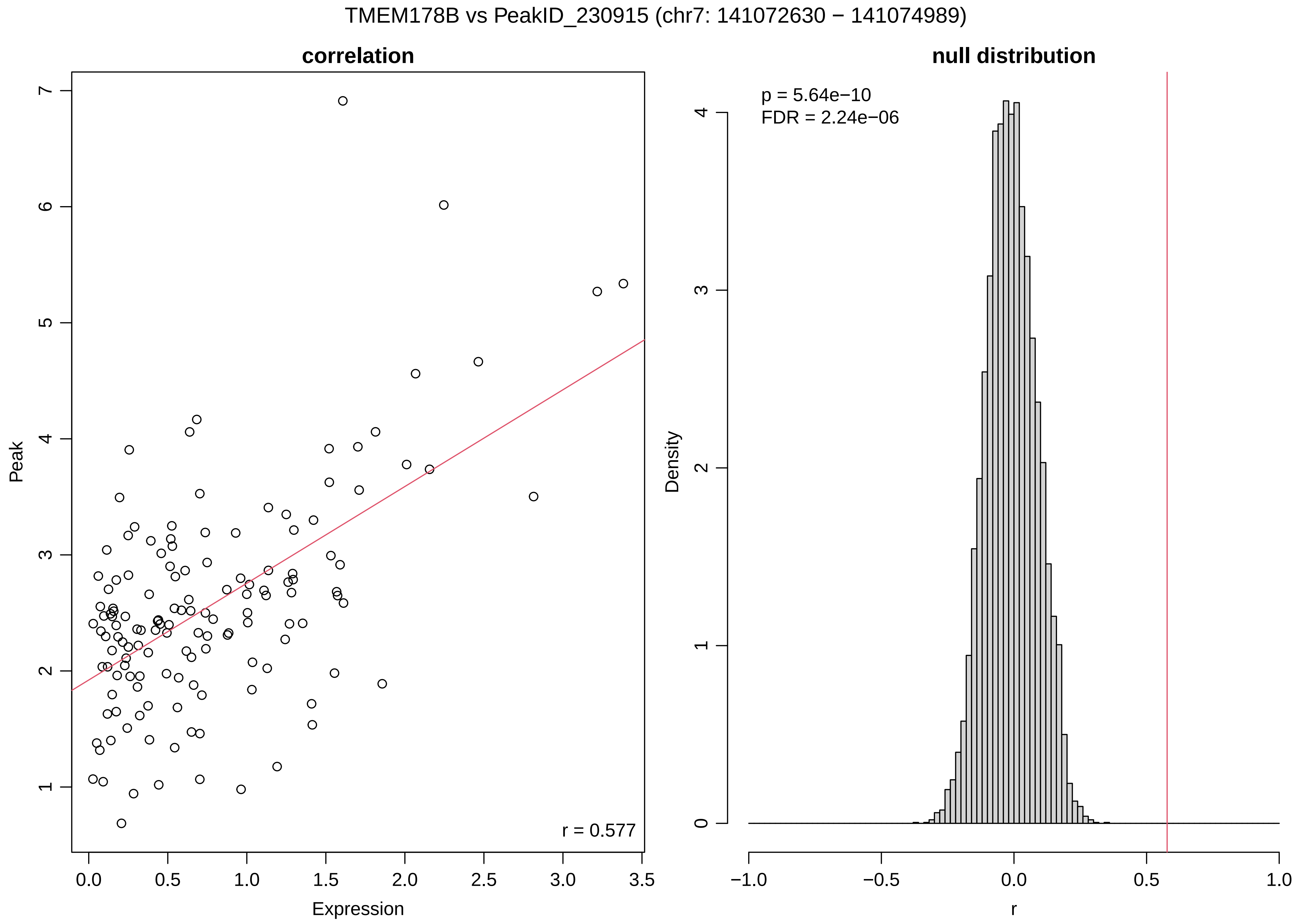
TMEM178B vs PeakID_230915 (chr7: 141072630 − 141074989)
correlation
Peak
Expression
r = 0.577
null distribution
p = 5.64e−10
FDR = 2.24e−06
Density
r

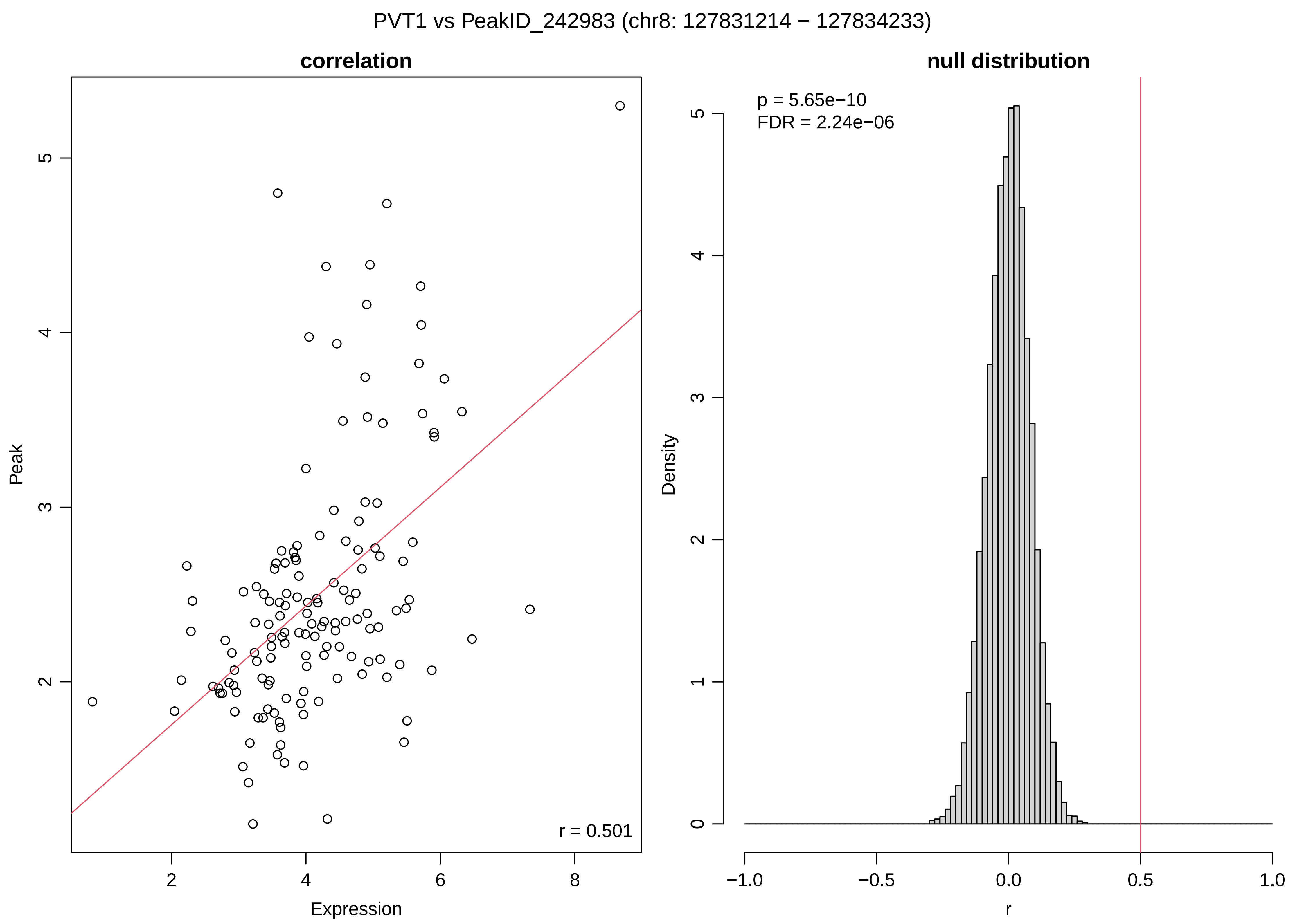

PVT1 vs PeakID_242983 (chr8: 127831214 − 127834233)
correlation
Peak
Expression
r = 0.501
null distribution
p = 5.65e−10
FDR = 2.24e−06
Density
r

RRP7A vs PeakID_158256 (chr22: 42528705 − 42530770)

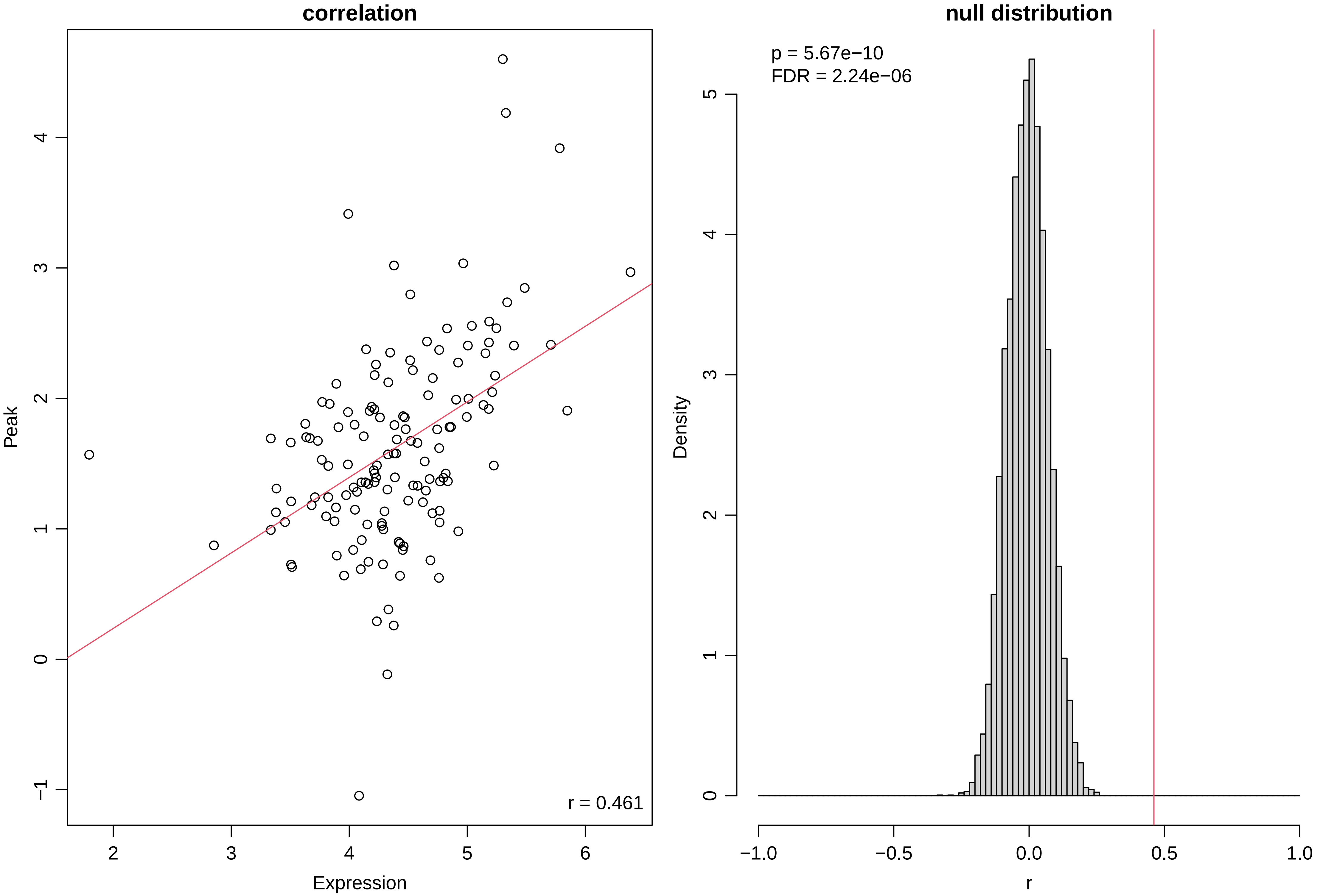

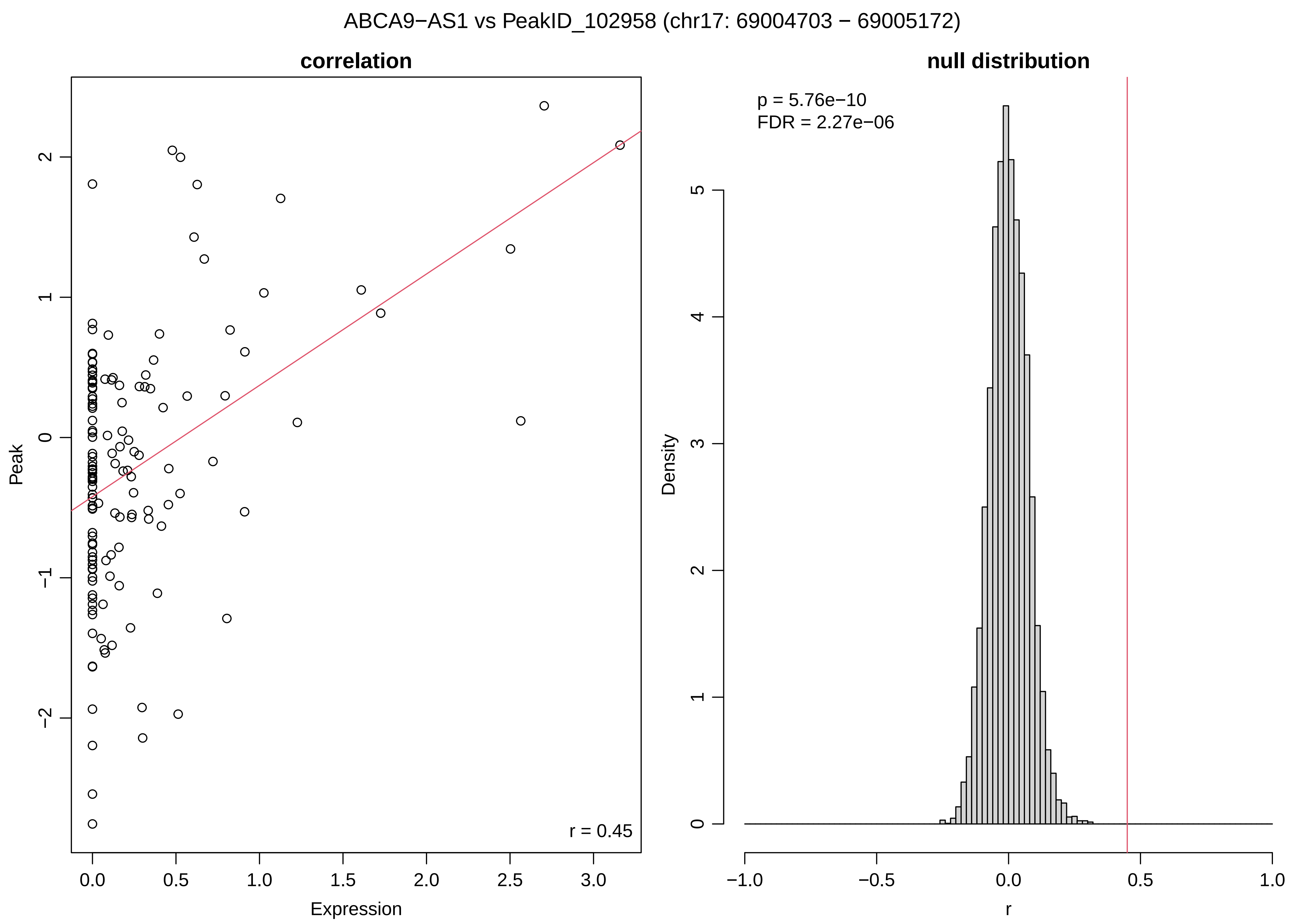
ABCA9−AS1 vs PeakID_102958 (chr17: 69004703 − 69005172)
correlation
Peak
Expression
r = 0.45
null distribution
p = 5.76e−10
FDR = 2.27e−06
Density
r

DMGDH vs PeakID_193462 (chr5: 79069319 − 79070751)

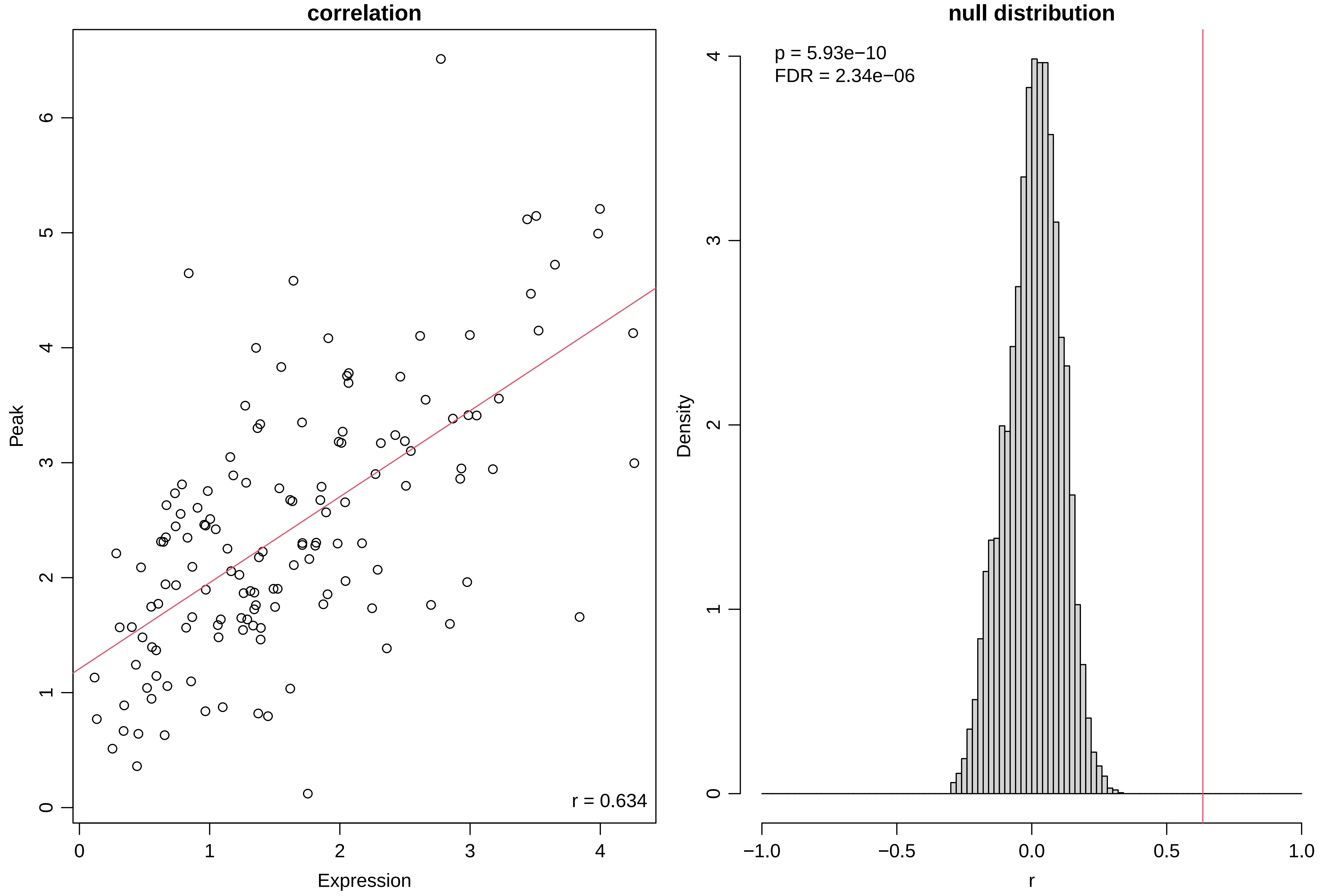

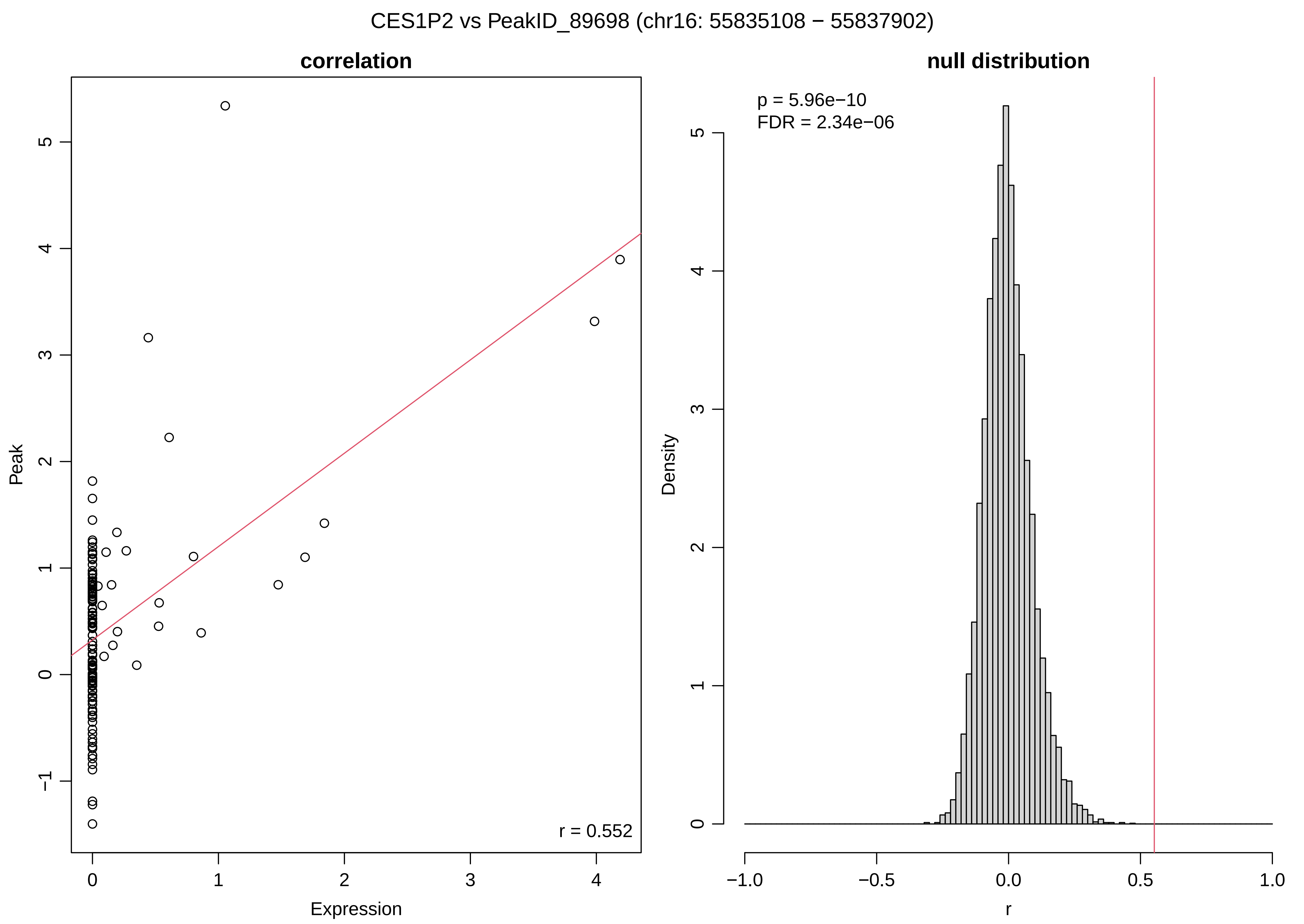

CES1P2 vs PeakID_89698 (chr16: 55835108 − 55837902)
correlation
Peak
Expression
r = 0.552
null distribution
p = 5.96e−10
FDR = 2.34e−06
Density
r

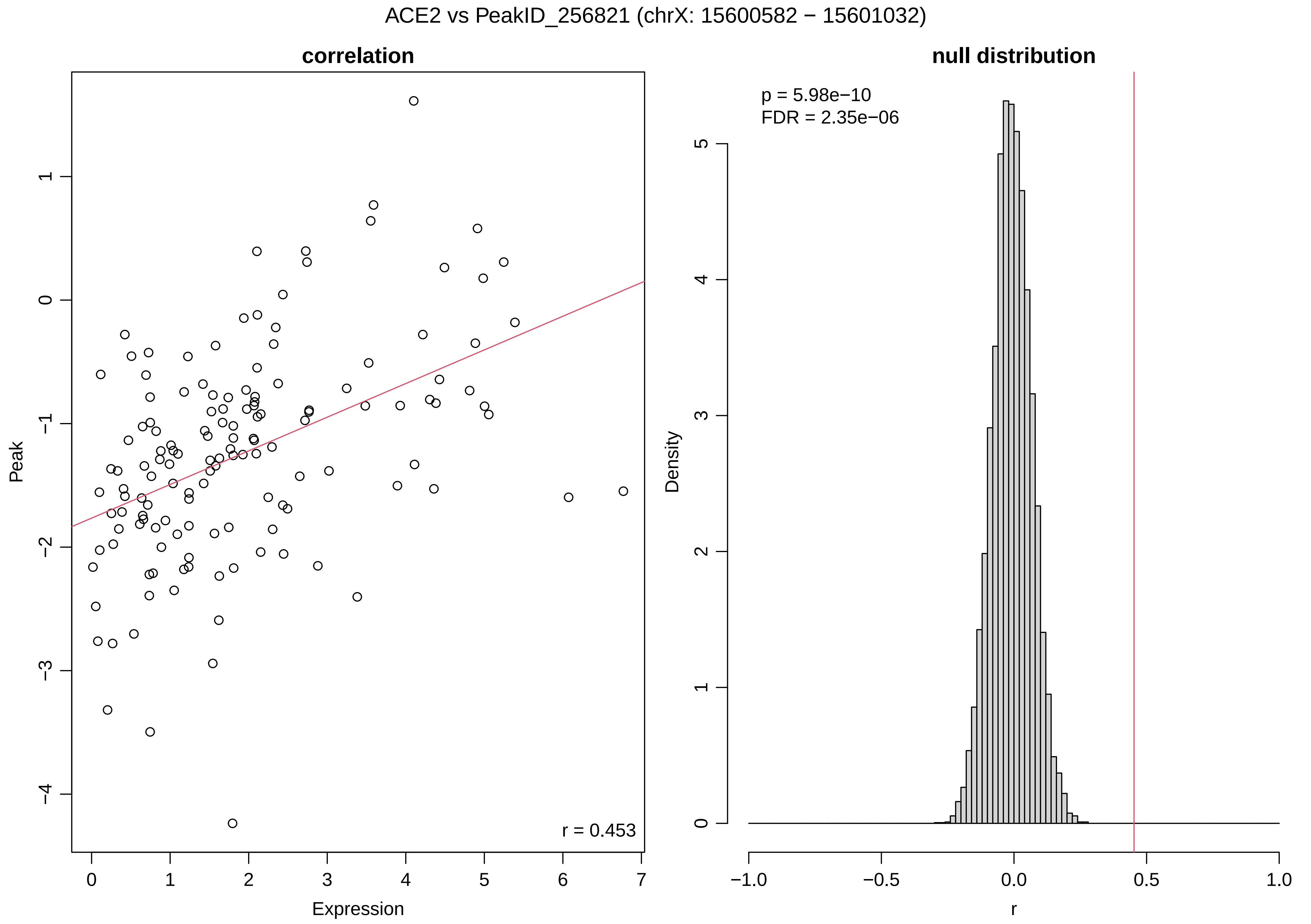
ACE2 vs PeakID_256821 (chrX: 15600582 − 15601032)
correlation
null distribution
p = 5.98e−10
FDR = 2.35e−06
r = 0.453
Peak
Expression
Density
r
1
0
−1
−2
−3
−4
0
1
2
3
4
5
6
7
−1.0
−0.5
0.0
0.5
1.0

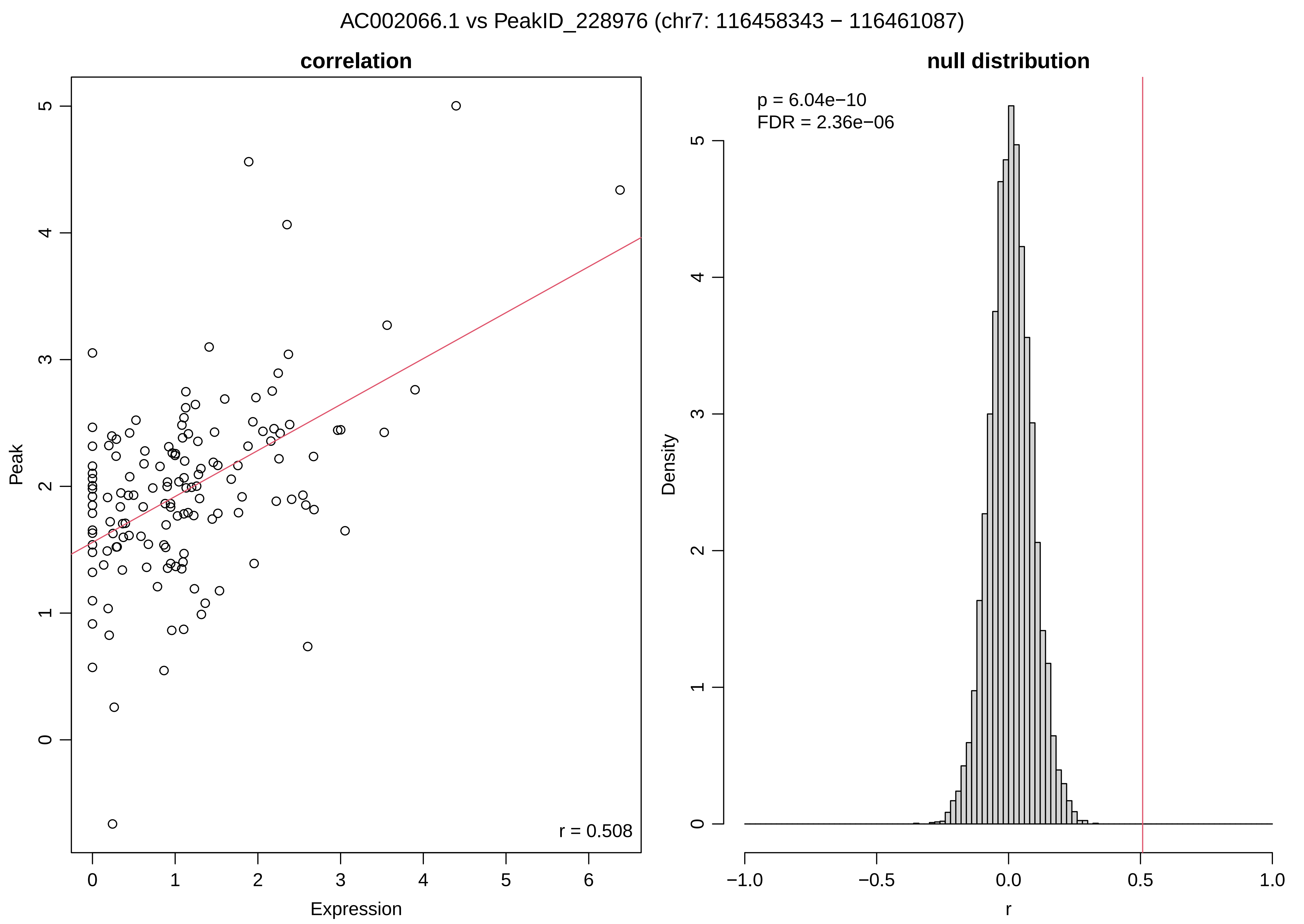
AC002066.1 vs PeakID_228976 (chr7: 116458343 − 116461087)
correlation
Peak
Expression
r = 0.508
null distribution
p = 6.04e−10
FDR = 2.36e−06
Density
r

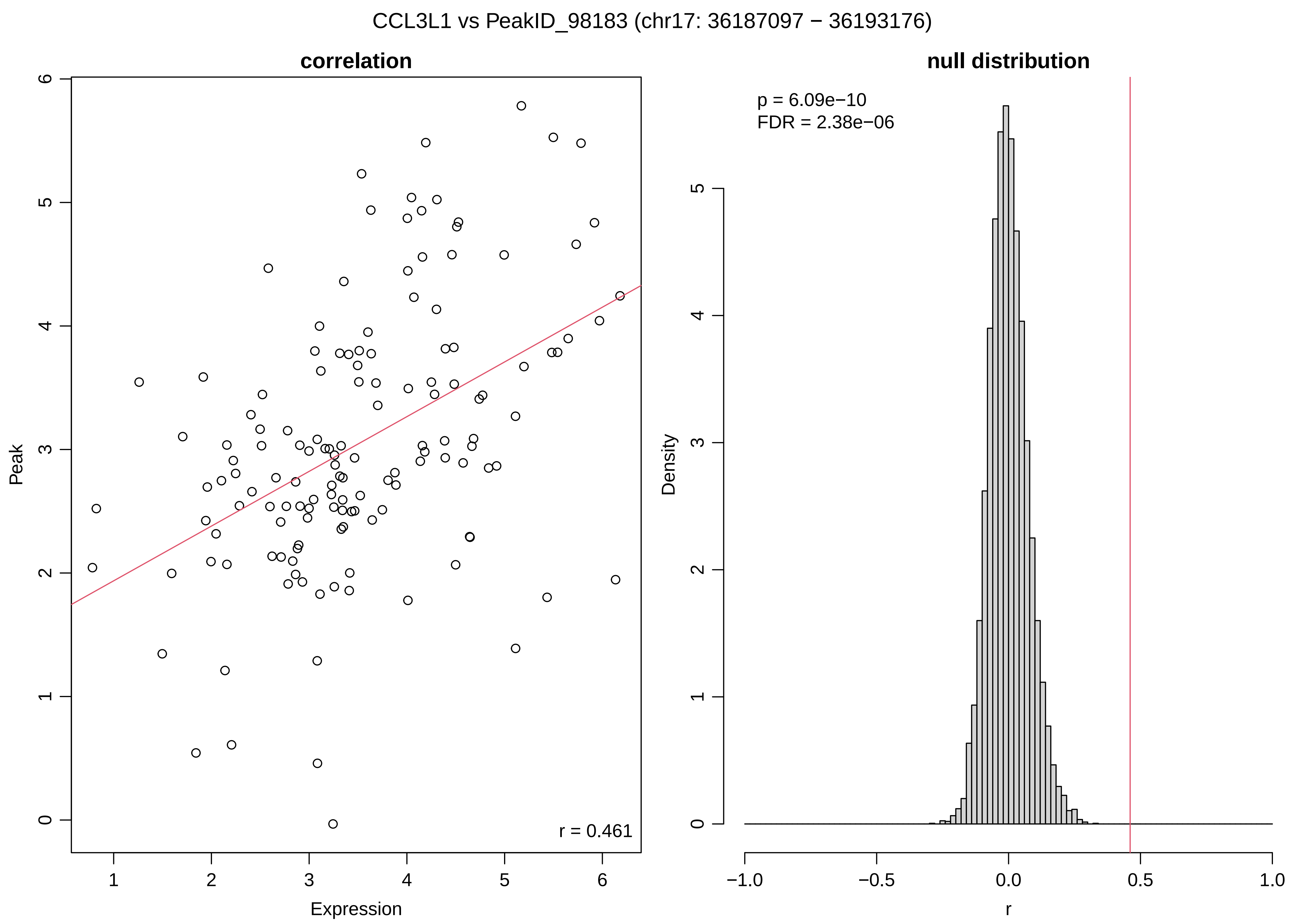

CCL3L1 vs PeakID_98183 (chr17: 36187097 − 36193176)
correlation
Peak
Expression
r = 0.461
null distribution
p = 6.09e−10
FDR = 2.38e−06
Density
r

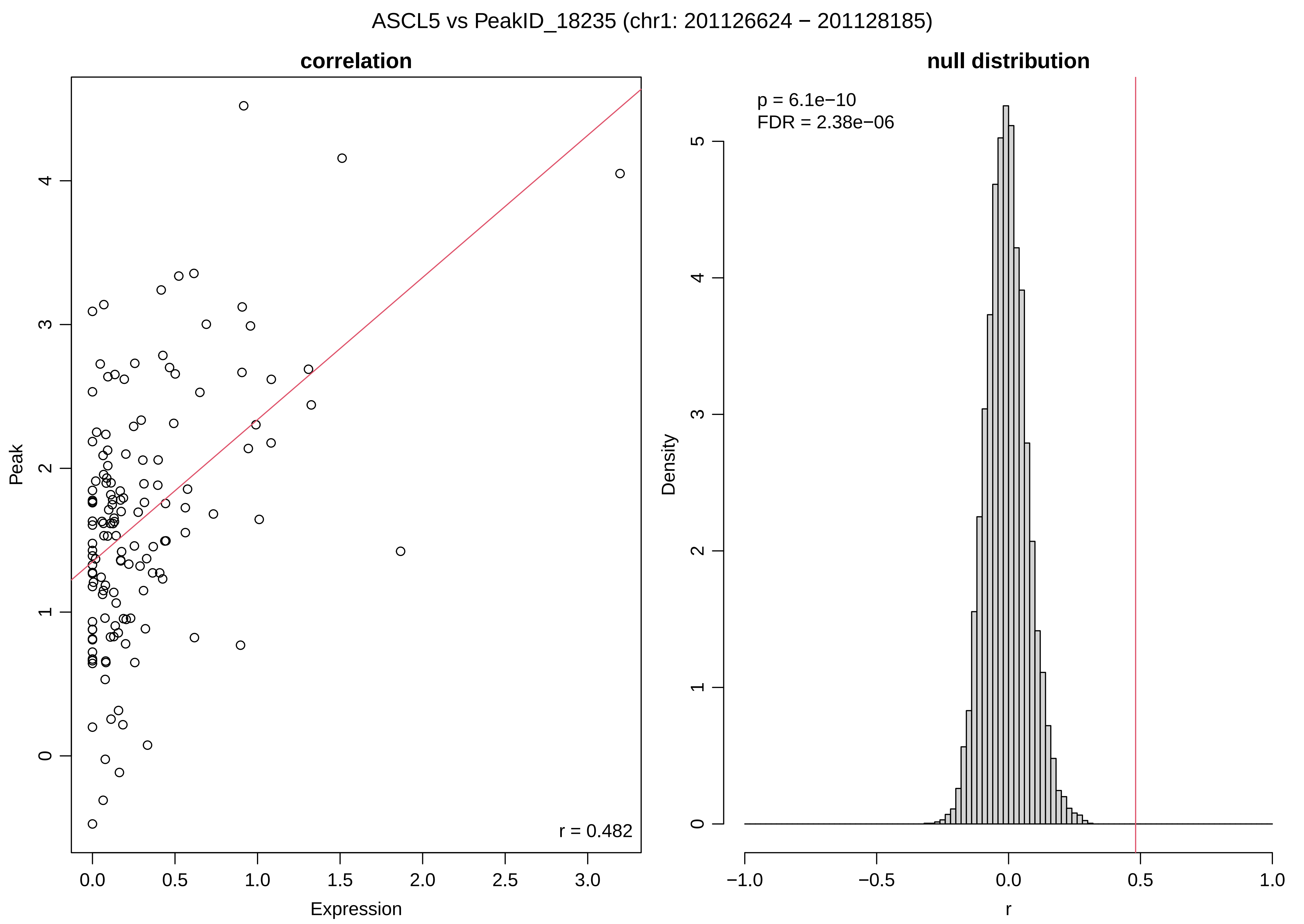
ASCL5 vs PeakID_18235 (chr1: 201126624 − 201128185)
correlation
Peak
Expression
r = 0.482
null distribution
p = 6.1e−10
FDR = 2.38e−06
Density
r

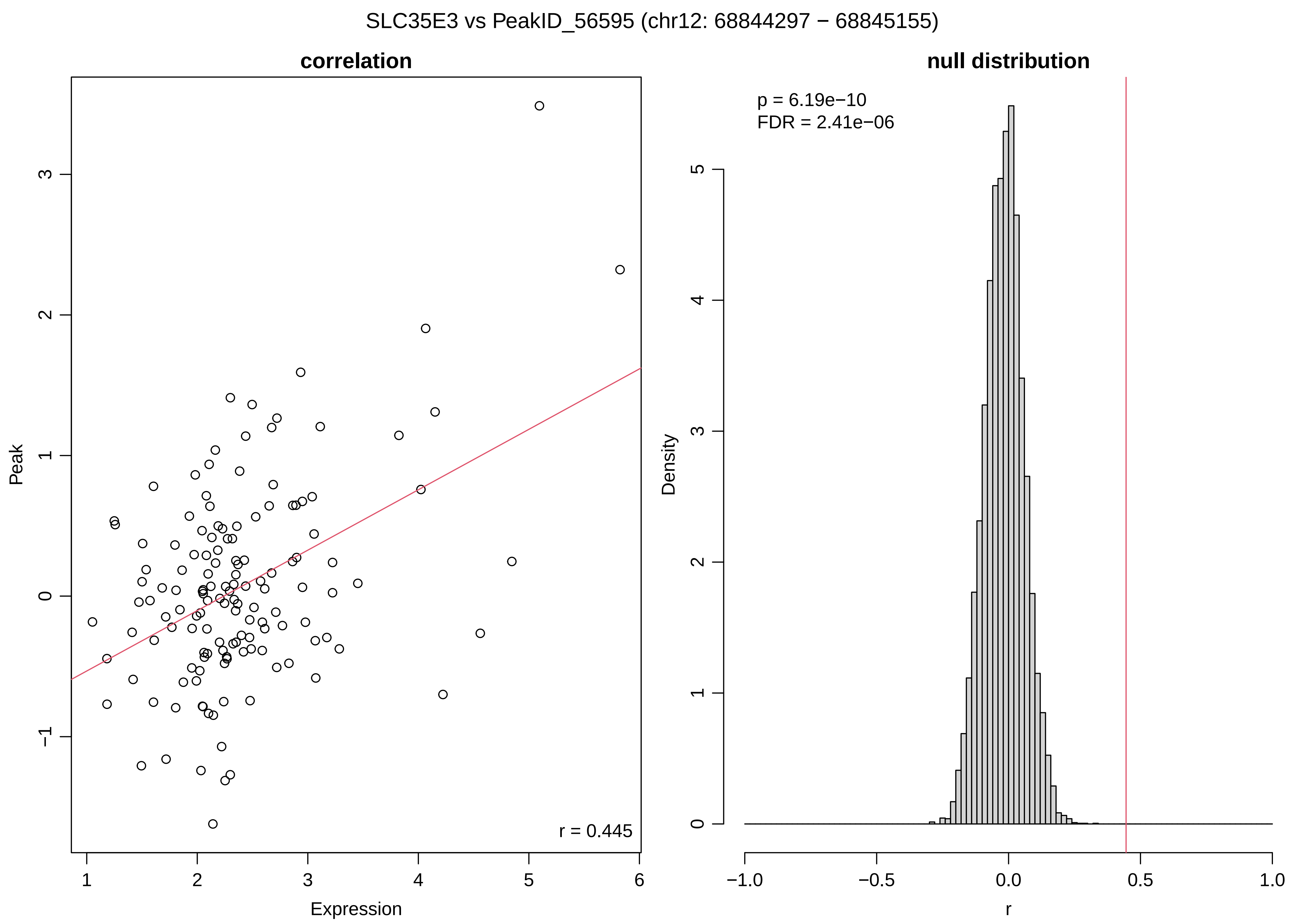

SLC35E3 vs PeakID_56595 (chr12: 68844297 − 68845155)
correlation
null distribution
Peak
Expression
r = 0.445
p = 6.19e−10
FDR = 2.41e−06
Density
r

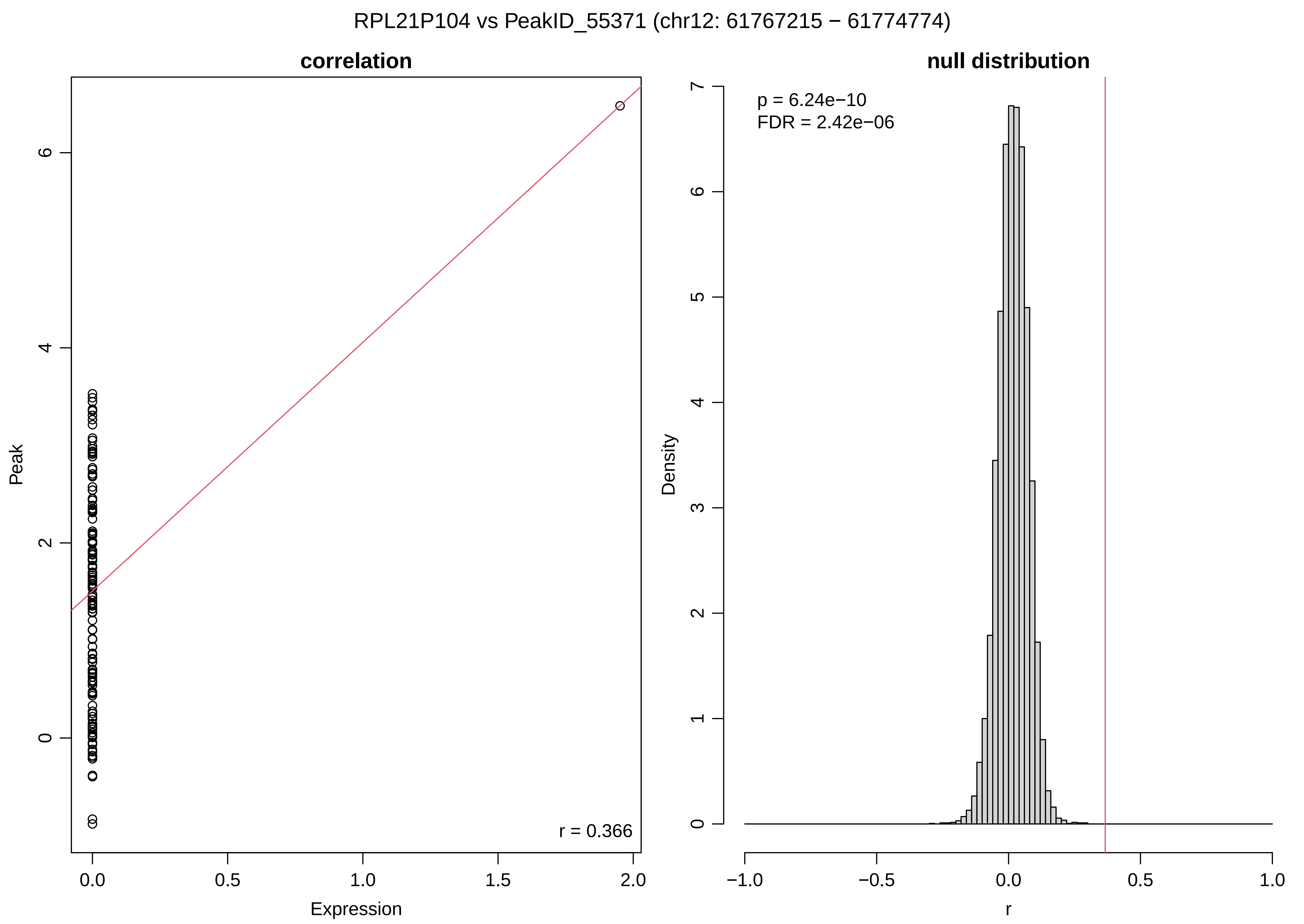

RPL21P104 vs PeakID_55371 (chr12: 61767215 − 61774774)
correlation
null distribution
Peak
Expression
r = 0.366
Density
r
p = 6.24e−10
FDR = 2.42e−06
0.0
0.5
1.0
1.5
2.0
0
2
4
6
−1.0
−0.5
0.0
0.5
1.0
0
1
2
3
4
5
6
7

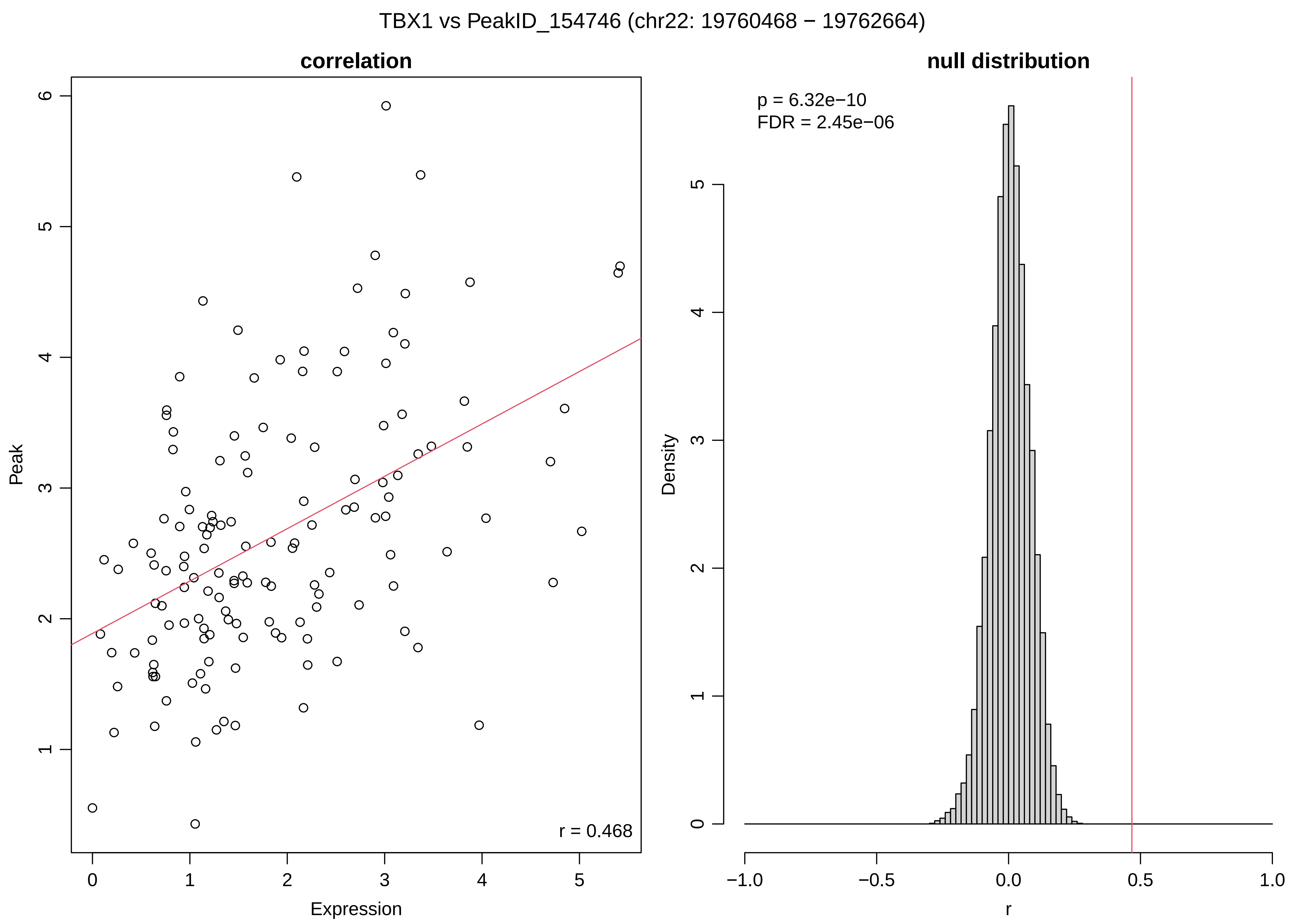
TBX1 vs PeakID_154746 (chr22: 19760468 − 19762664)
correlation
Peak
Expression
r = 0.468
null distribution
p = 6.32e−10
FDR = 2.45e−06
Density
r

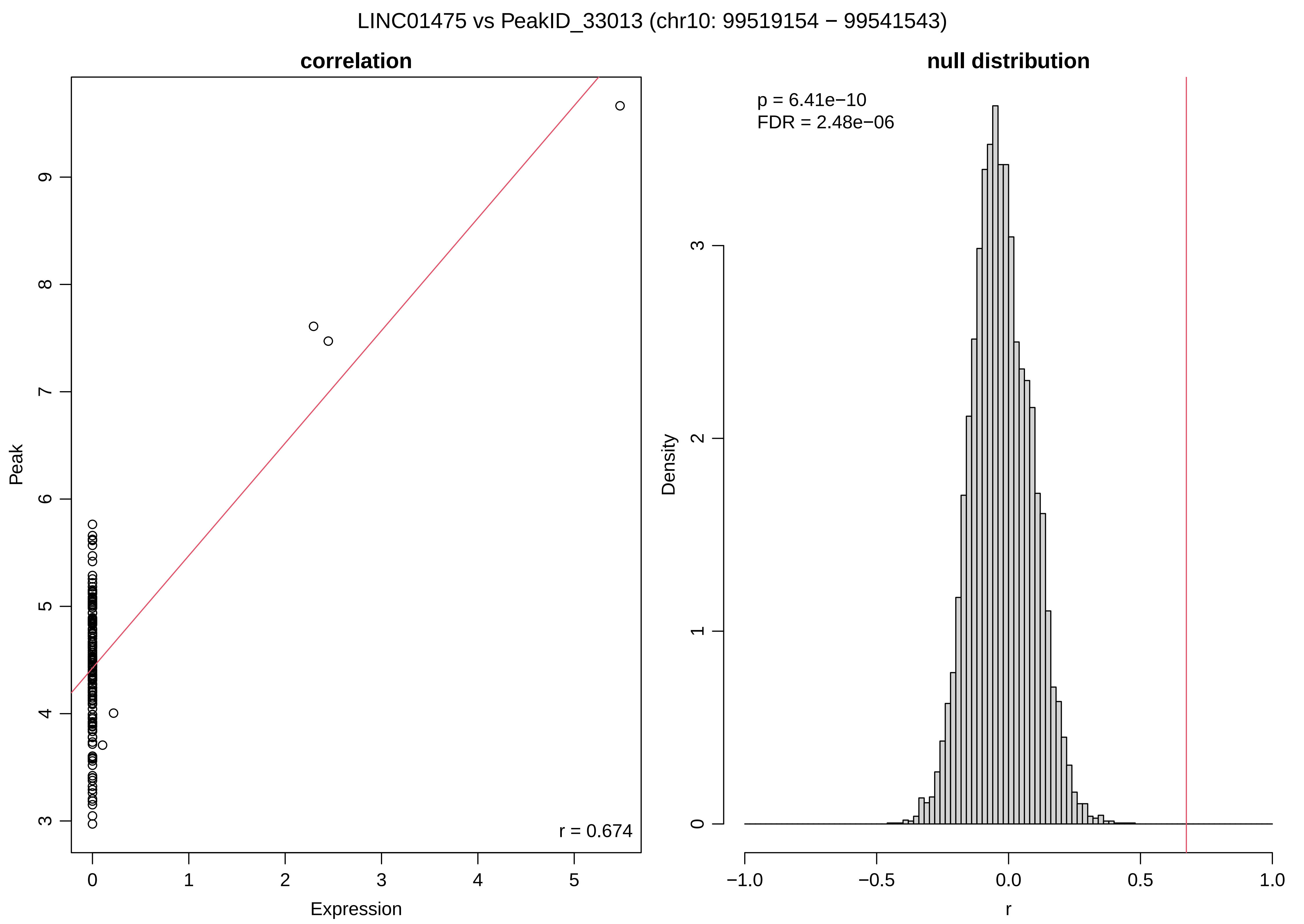

LINC01475 vs PeakID_33013 (chr10: 99519154 − 99541543)
correlation
Peak
Expression
r = 0.674
null distribution
p = 6.41e−10
FDR = 2.48e−06
Density
r

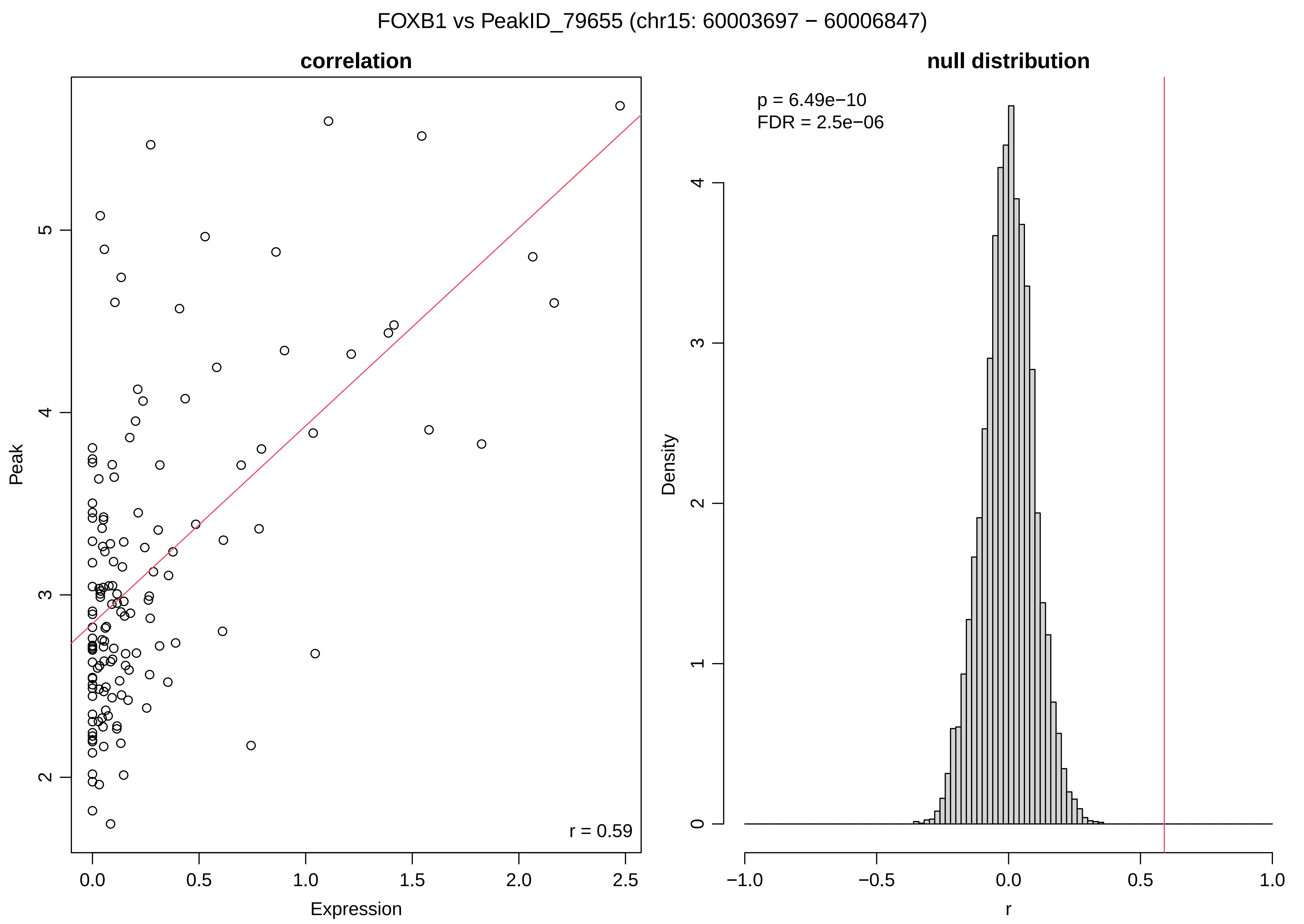
FOXB1 vs PeakID_79655 (chr15: 60003697 − 60006847)
correlation
Peak
Expression
r = 0.59
null distribution
p = 6.49e−10
FDR = 2.5e−06
Density
r
0.0
0.5
1.0
1.5
2.0
2.5
2
3
4
5
−1.0
−0.5
0.0
0.5
1.0
0
1
2
3
4

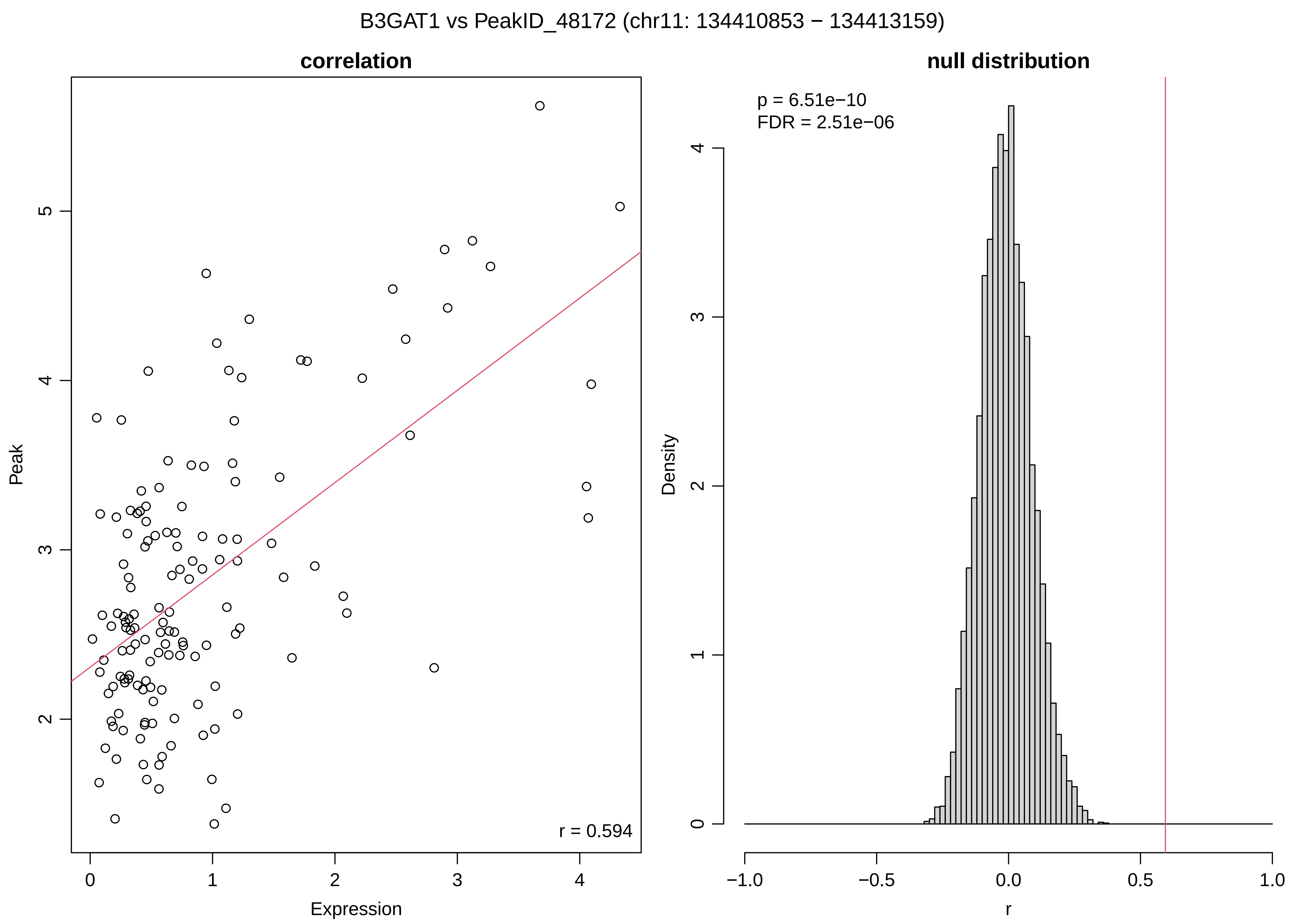
B3GAT1 vs PeakID_48172 (chr11: 134410853 − 134413159)
correlation
Peak
Expression
r = 0.594
0
1
2
3
4
5
null distribution
p = 6.51e−10
FDR = 2.51e−06
Density
r
−1.0
−0.5
0.0
0.5
1.0

LINC00470 vs PeakID_106755 (chr18: 1406910 − 1407264)

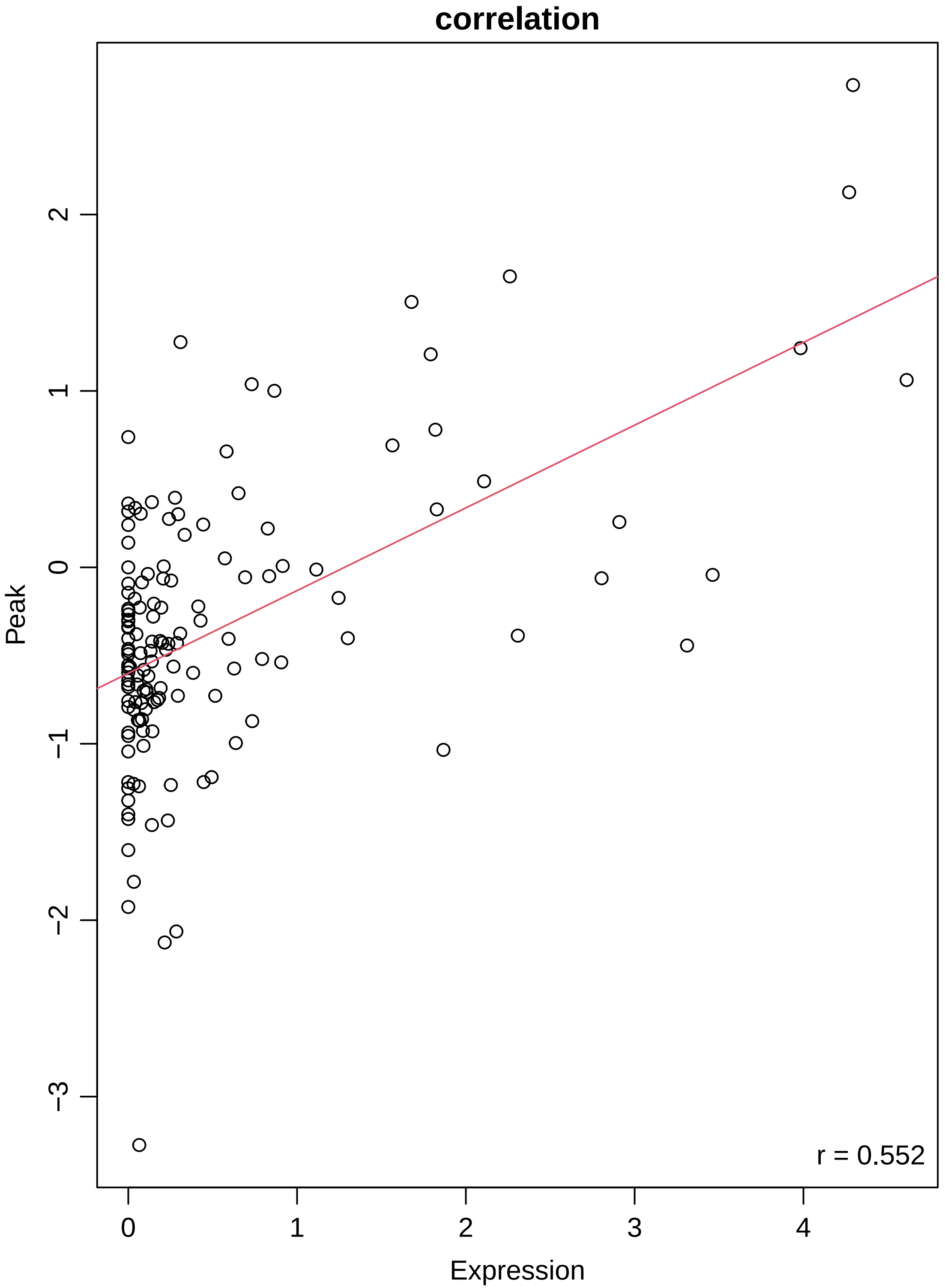

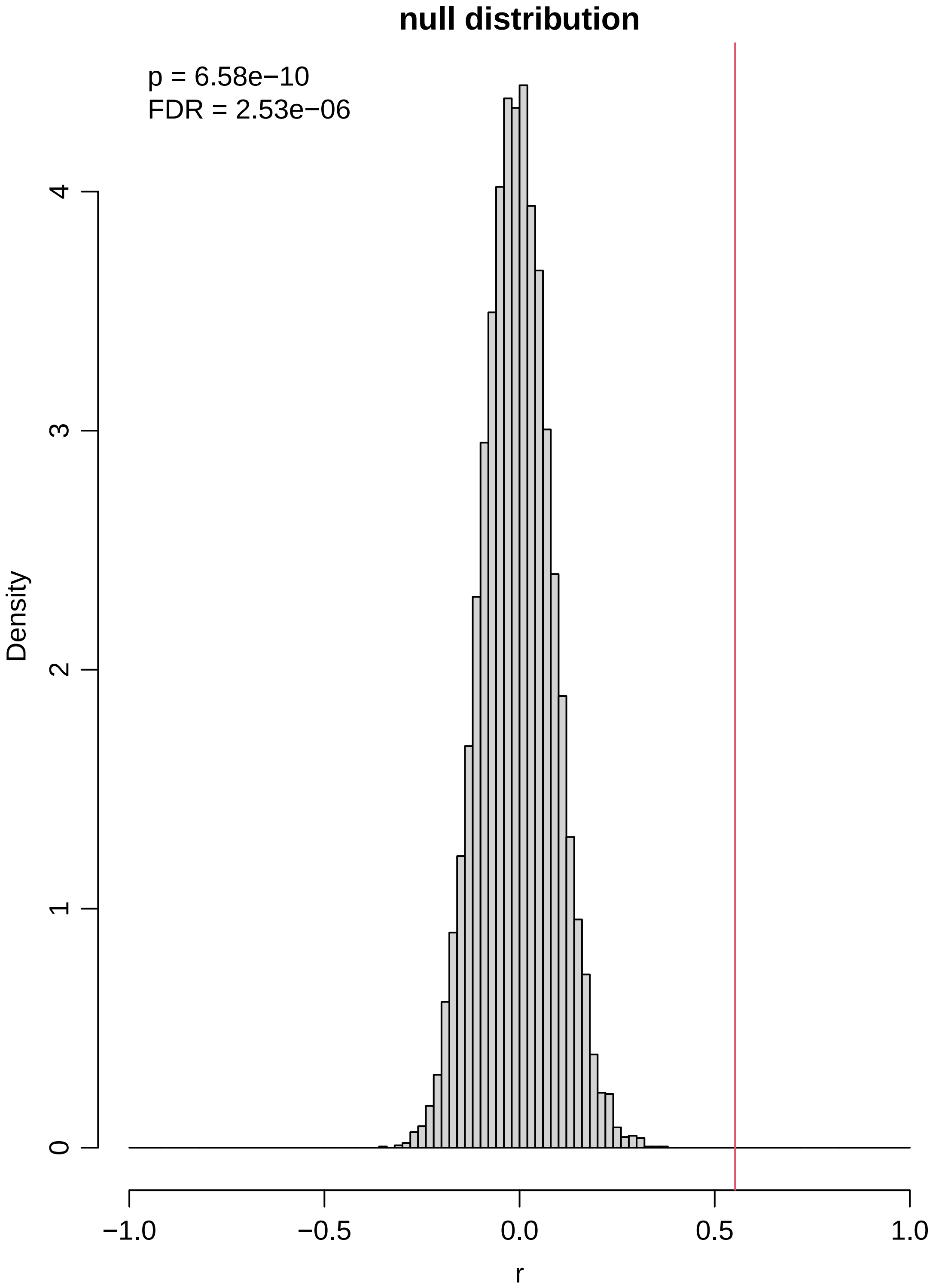

AC107881.1 vs PeakID_37881 (chr11: 12513236 − 12514195)

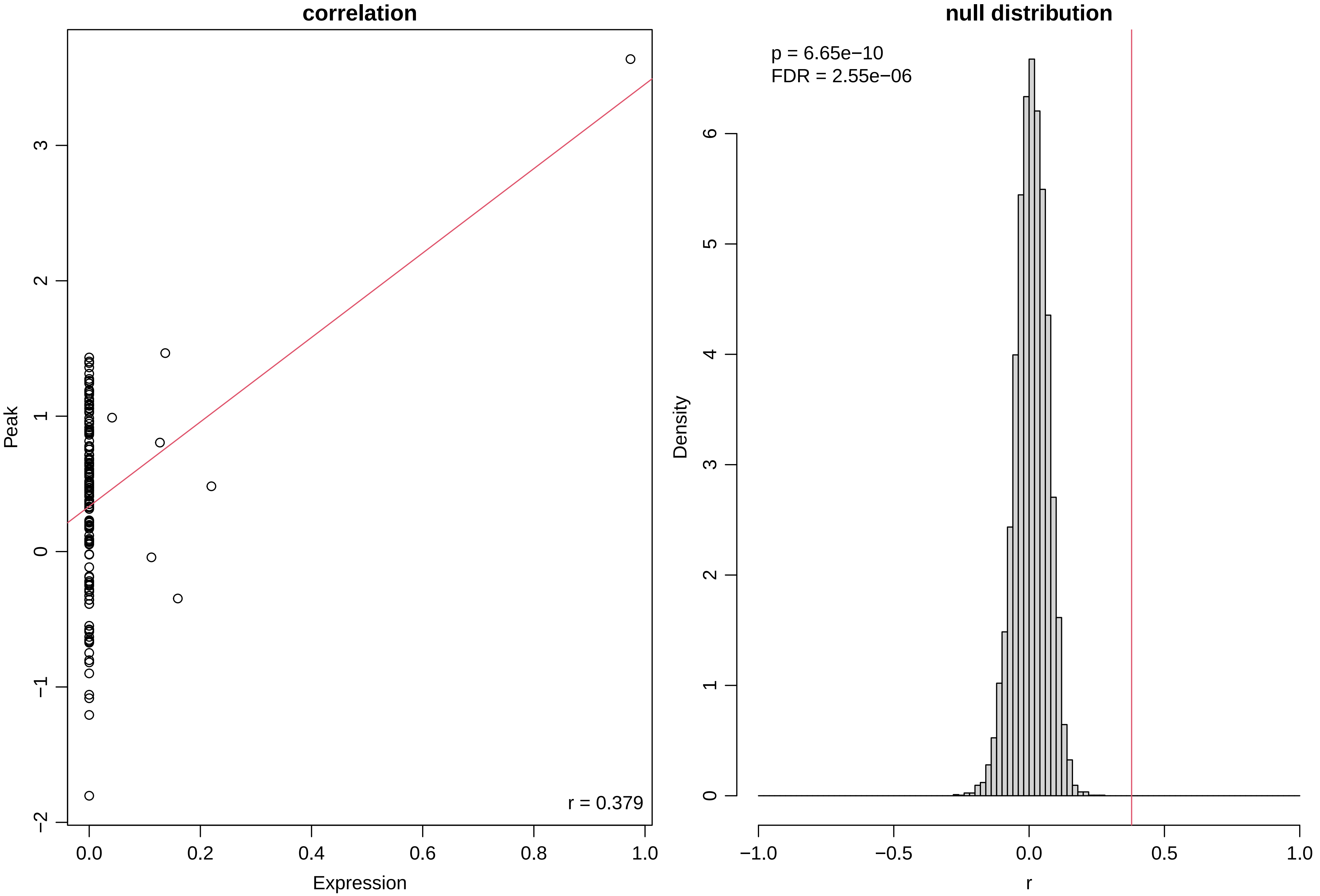

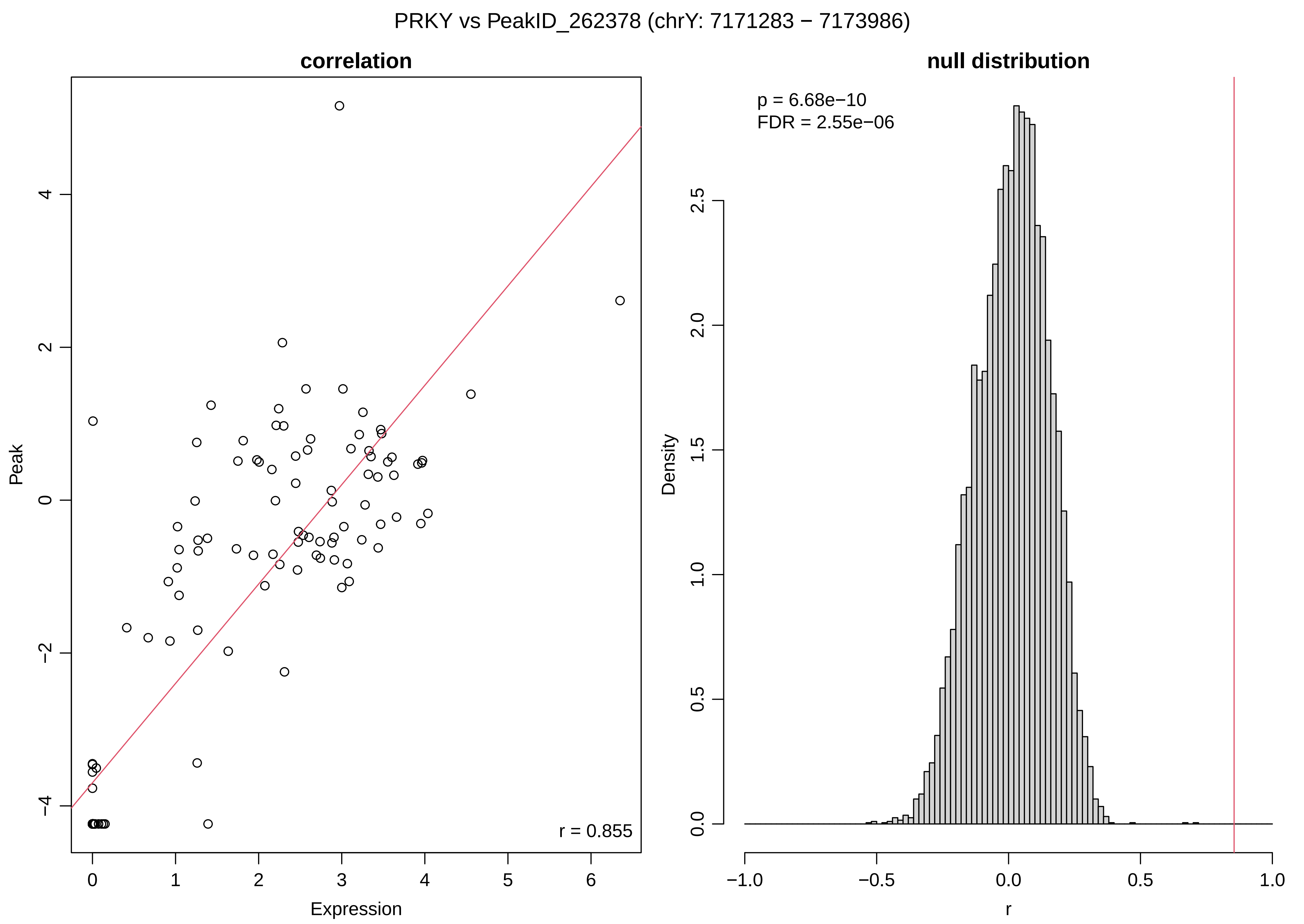

PRKY vs PeakID_262378 (chrY: 7171283 − 7173986)
correlation
Peak
Expression
r = 0.855
null distribution
p = 6.68e−10
FDR = 2.55e−06
Density
r

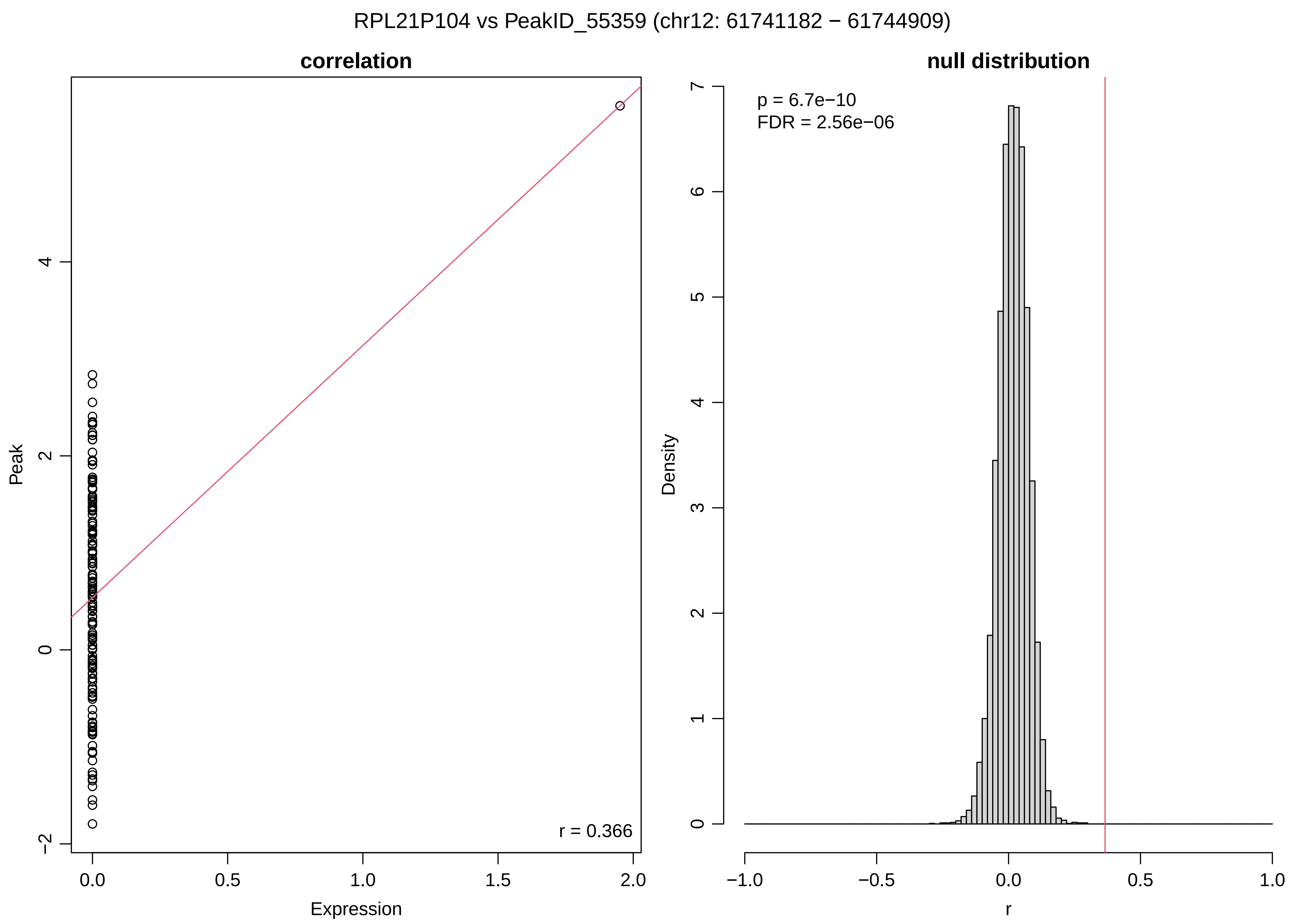
RPL21P104 vs PeakID_55359 (chr12: 61741182 − 61744909)
correlation
Peak
Expression
r = 0.366
null distribution
p = 6.7e−10
FDR = 2.56e−06
Density
r

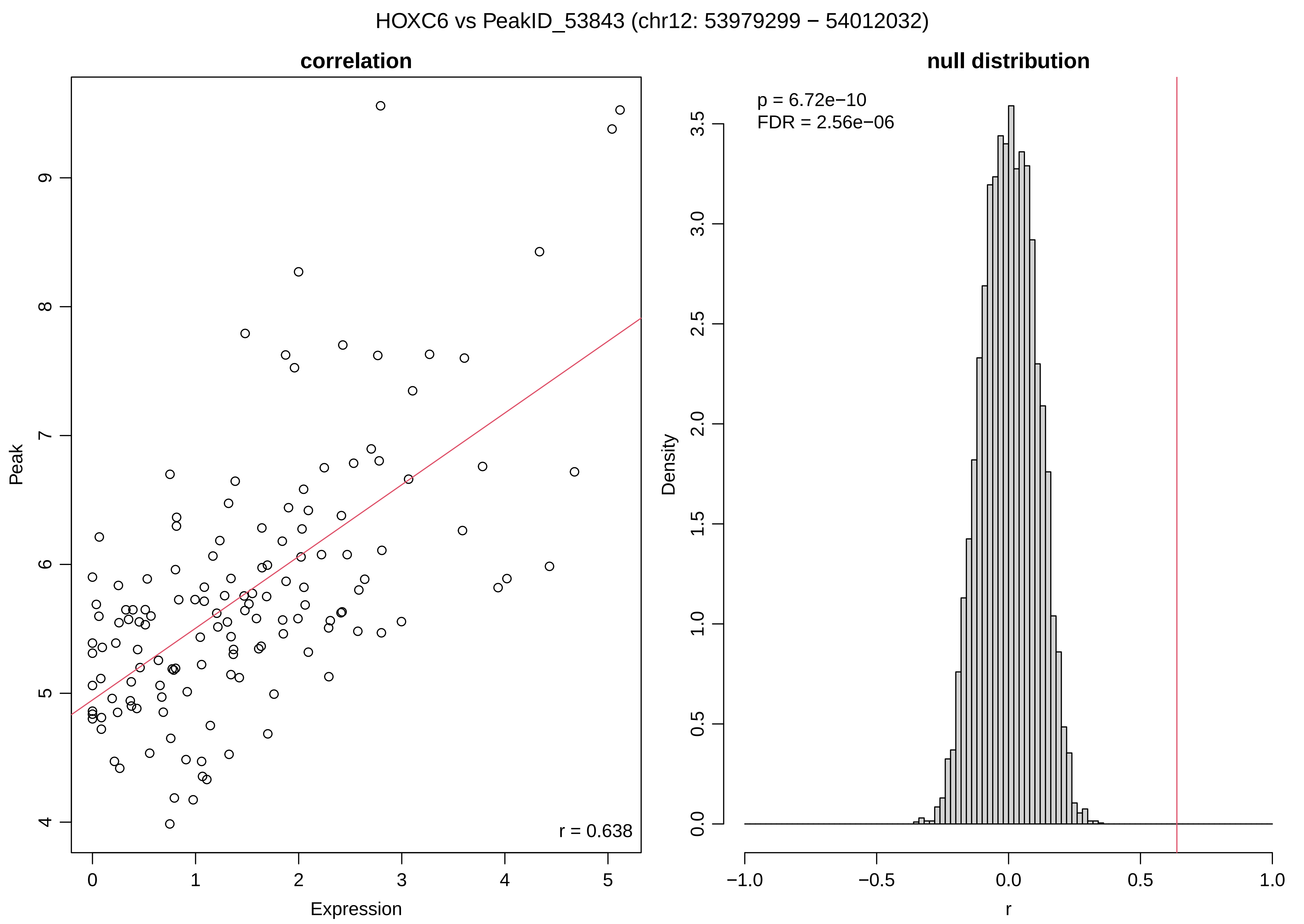

HOXC6 vs PeakID_53843 (chr12: 53979299 − 54012032)
correlation
Peak
Expression
r = 0.638
null distribution
p = 6.72e−10
FDR = 2.56e−06
Density
r

CASC9 vs PeakID_238274 (chr8: 75323473 − 75325065)

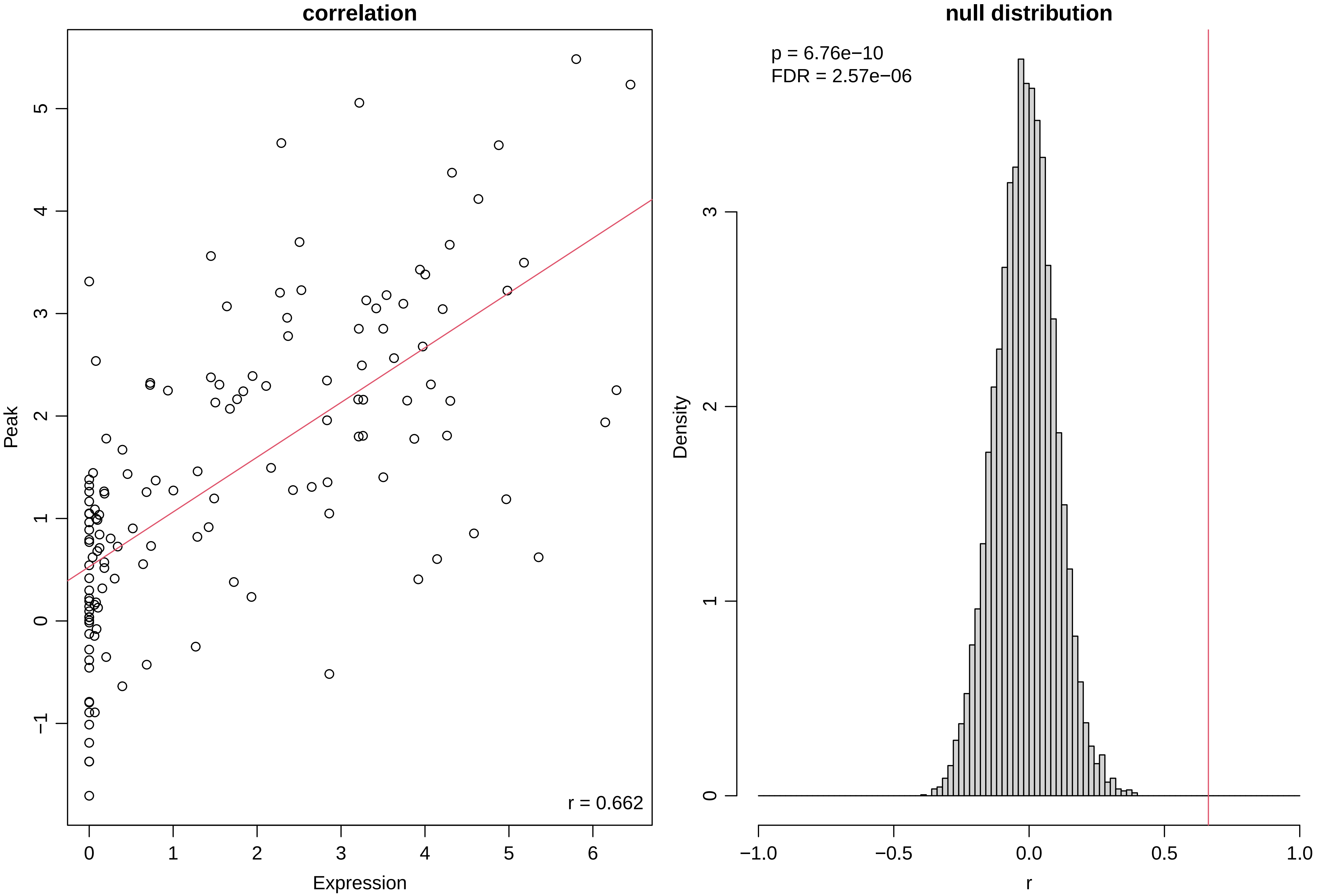

RPL21P104 vs PeakID_55495 (chr12: 62015602 − 62017275)

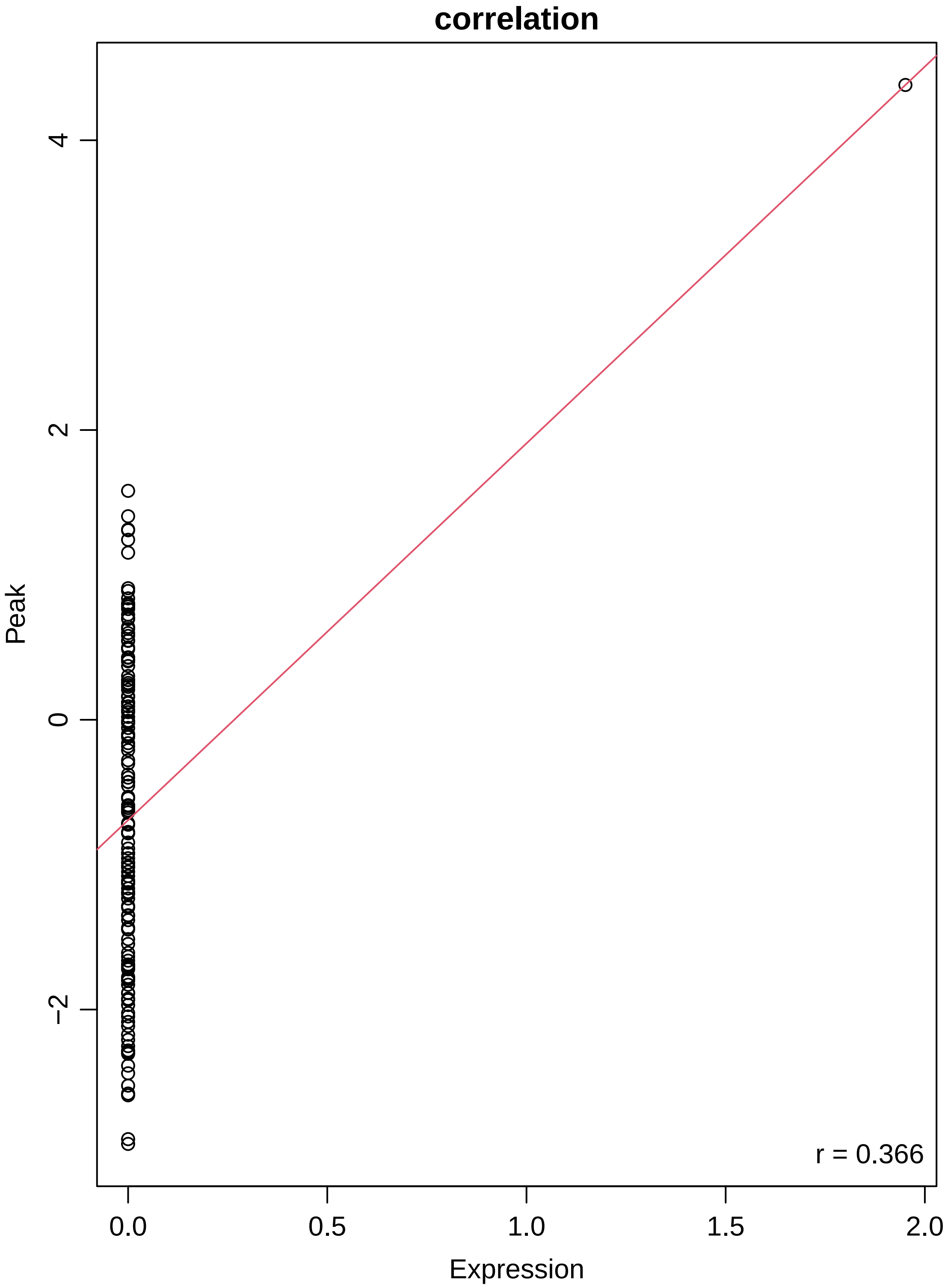

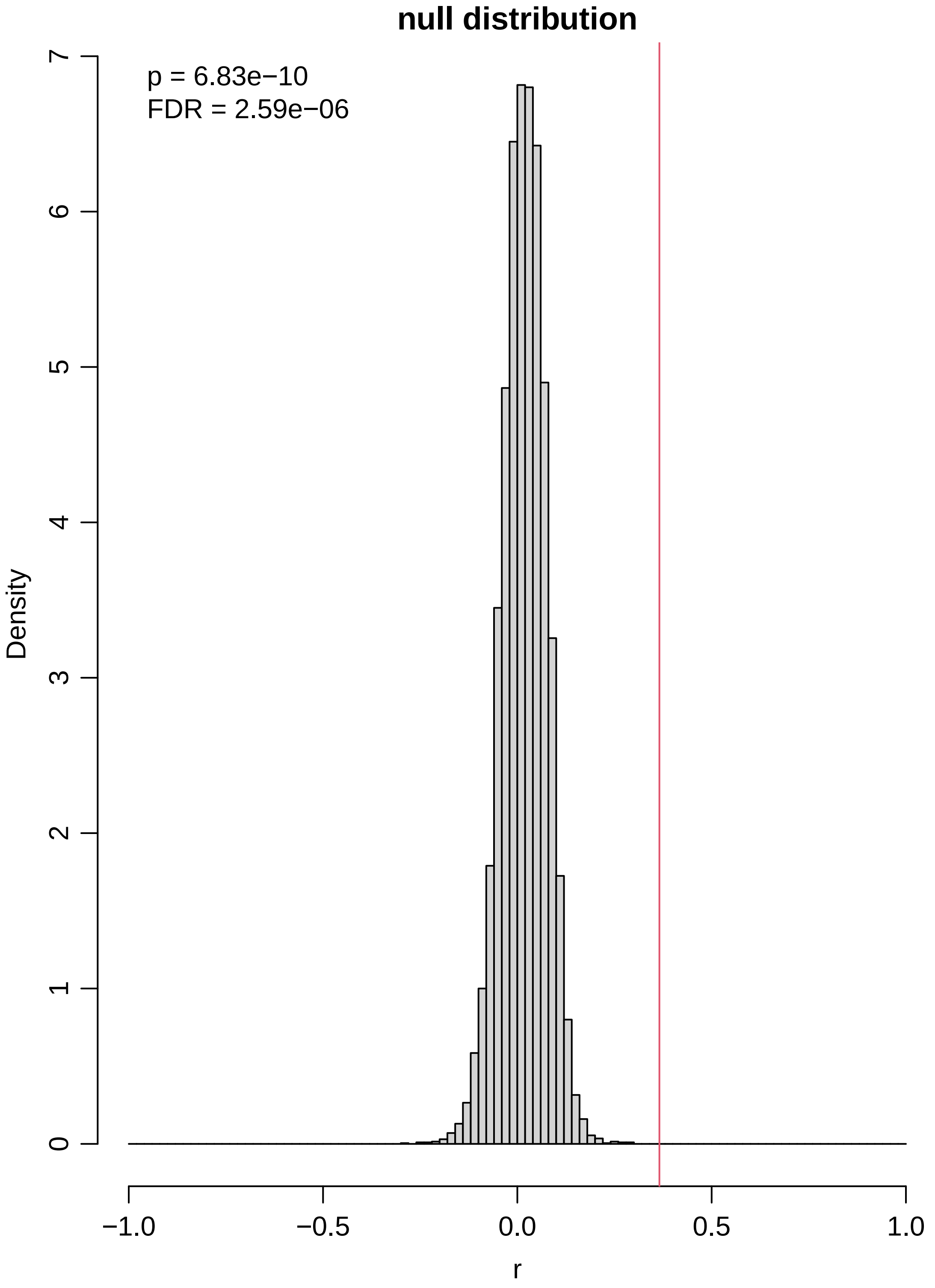

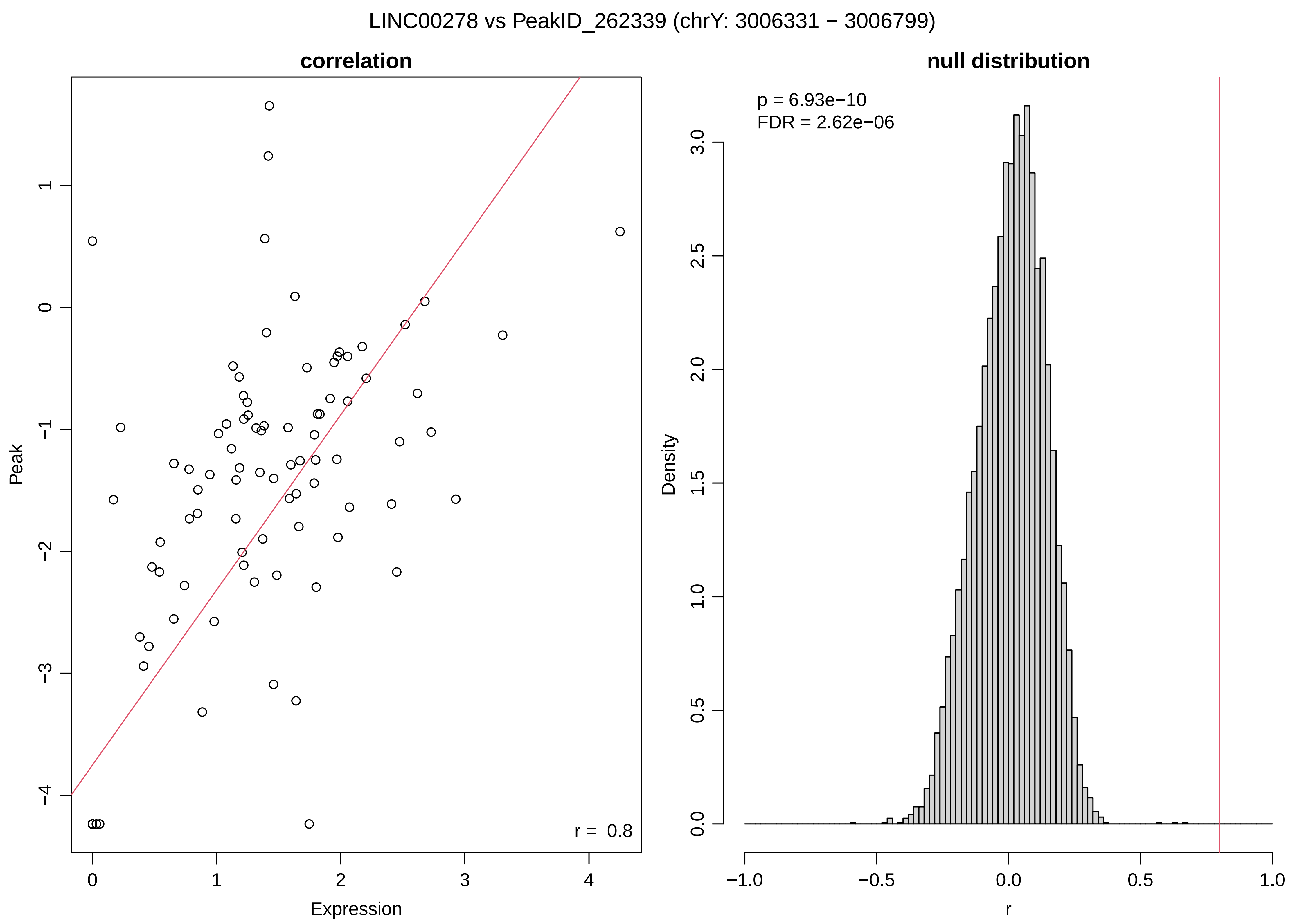

LINC00278 vs PeakID_262339 (chrY: 3006331 − 3006799)
correlation
Peak
Expression
r = 0.8
null distribution
p = 6.93e−10
FDR = 2.62e−06
Density
r

RPL21P104 vs PeakID_55499 (chr12: 62020050 − 62022315)

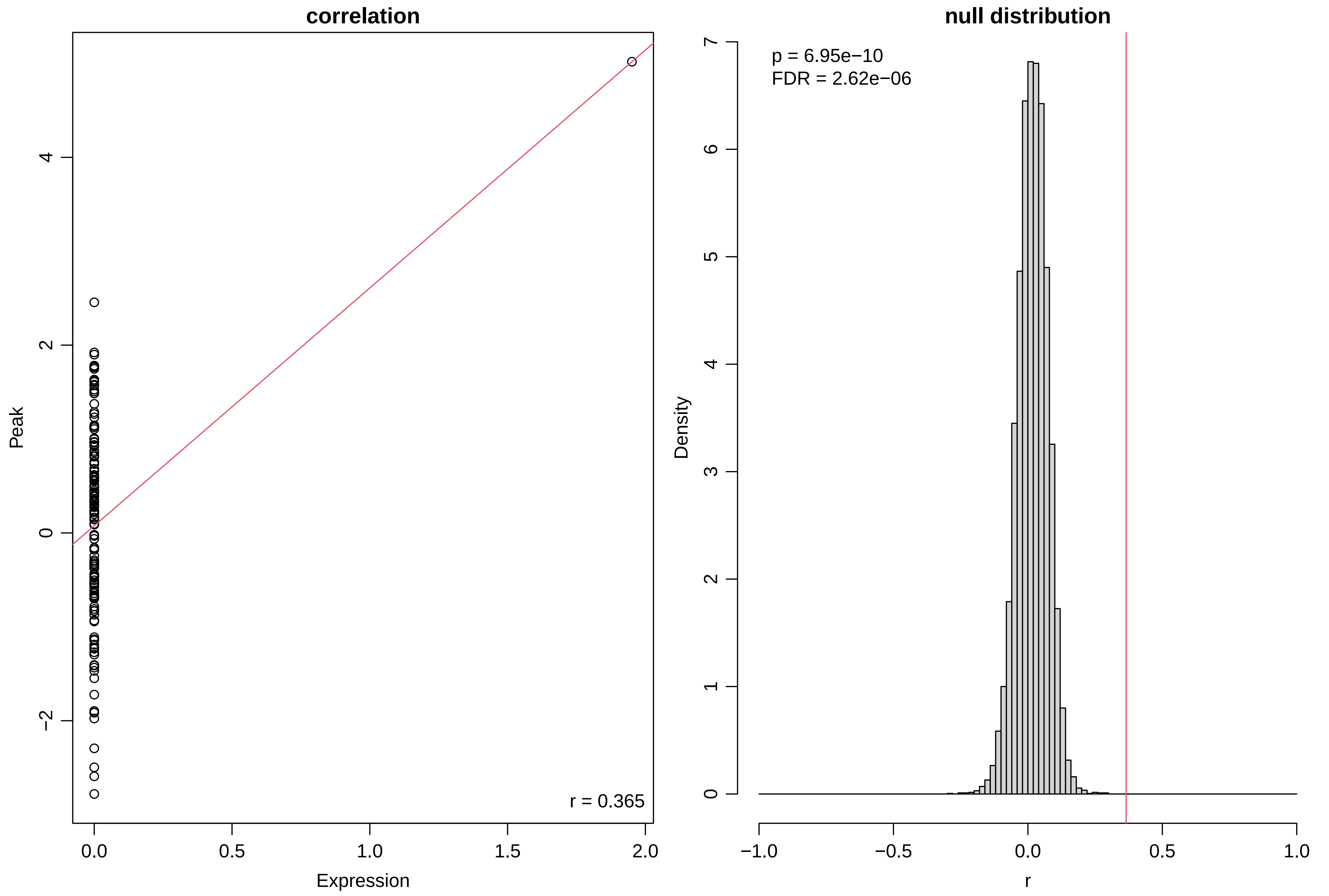

TKTL1 vs PeakID_262036 (chrX: 154295922 − 154296389)

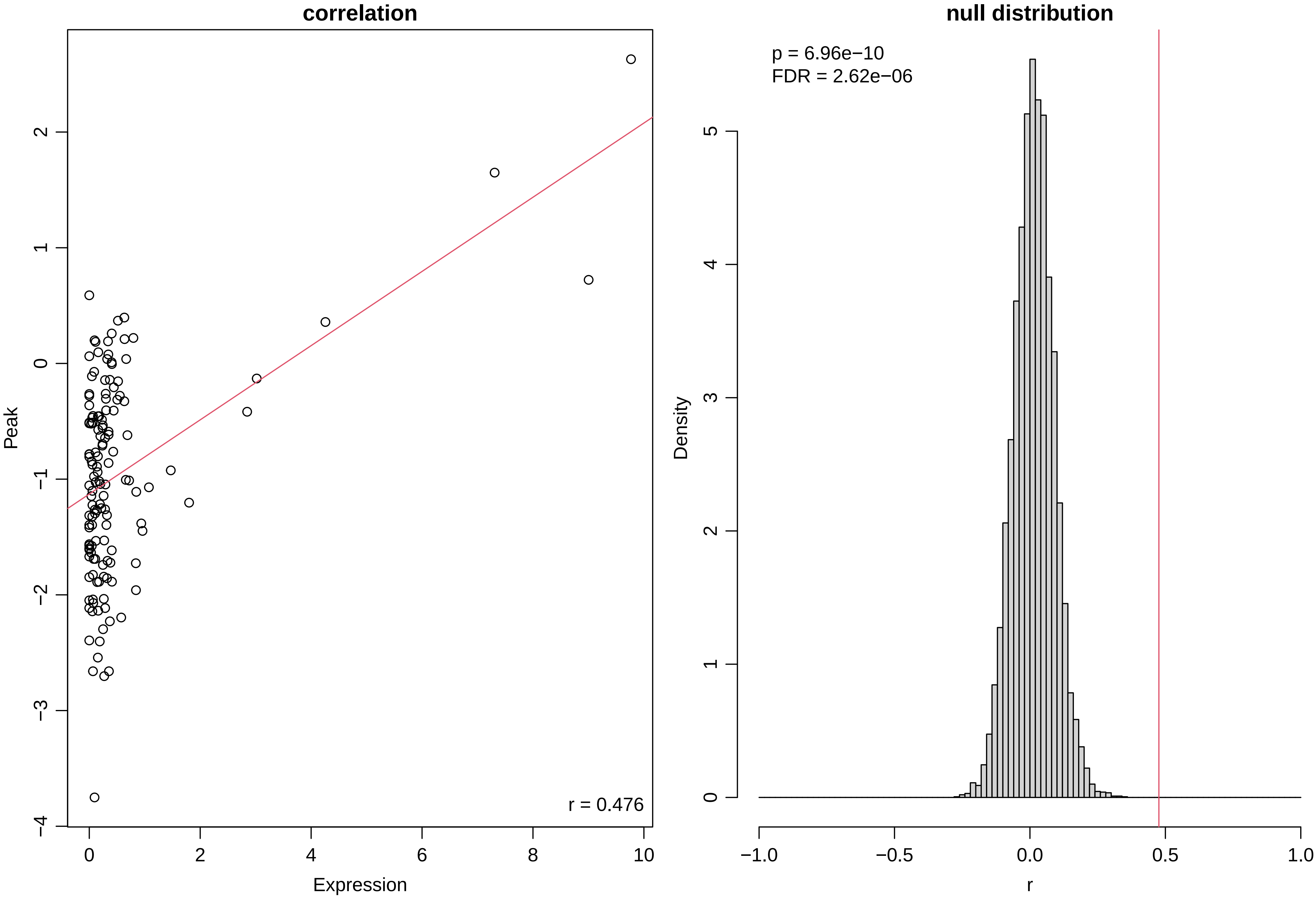

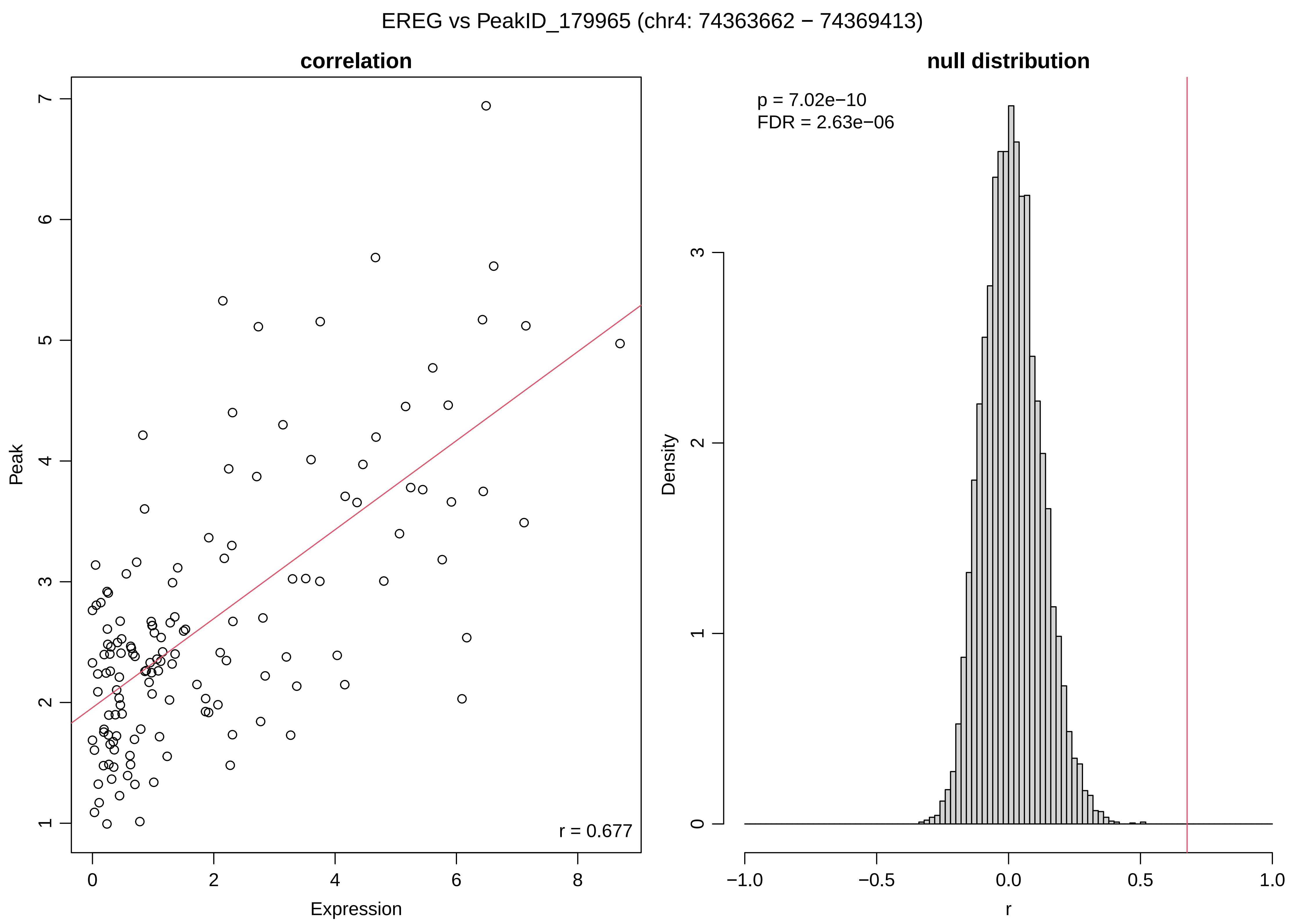
EREG vs PeakID_179965 (chr4: 74363662 − 74369413)
correlation
Peak
Expression
r = 0.677
null distribution
p = 7.02e−10
FDR = 2.63e−06
Density
r

RPL21P104 vs PeakID_55364 (chr12: 61754702 − 61755789)

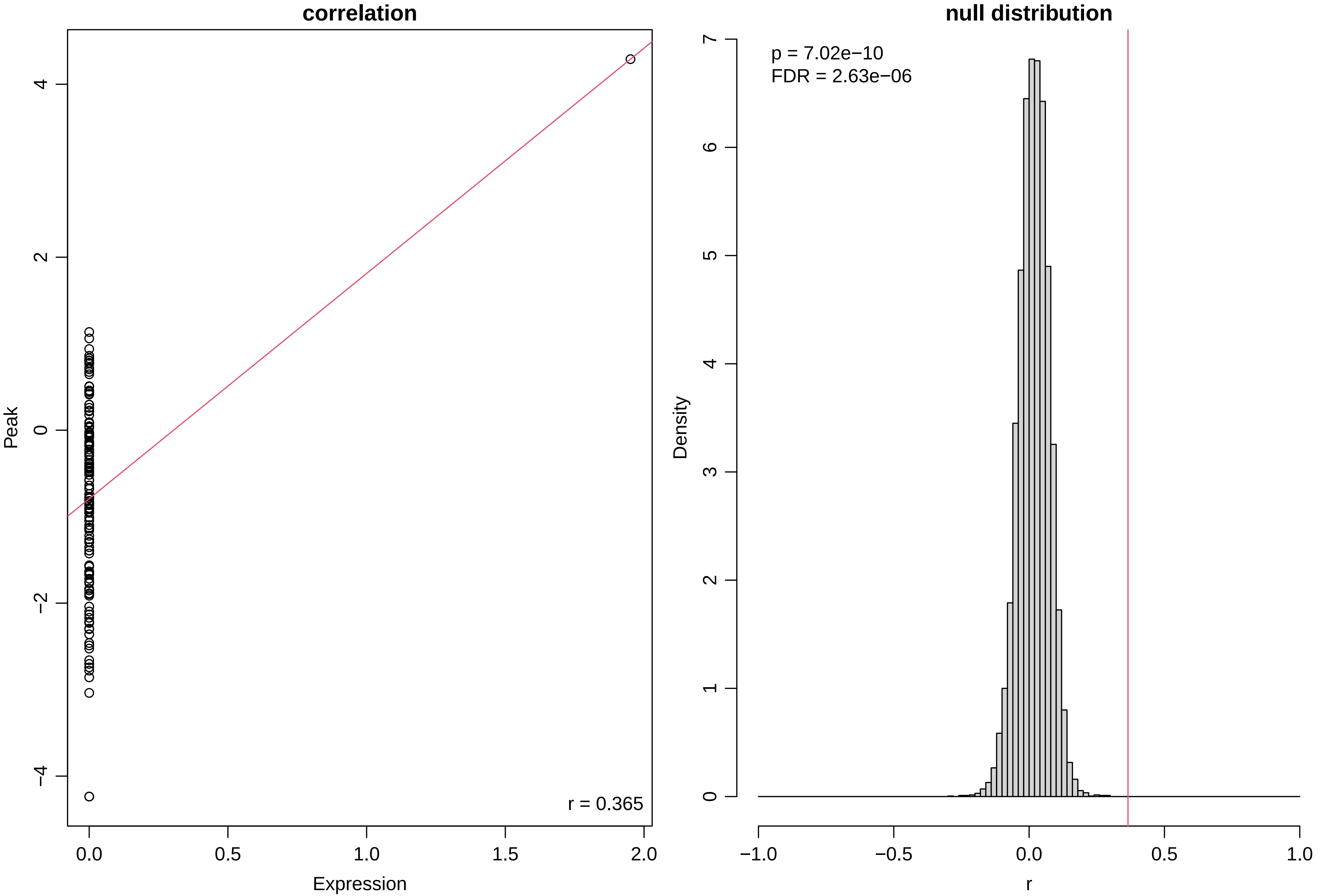

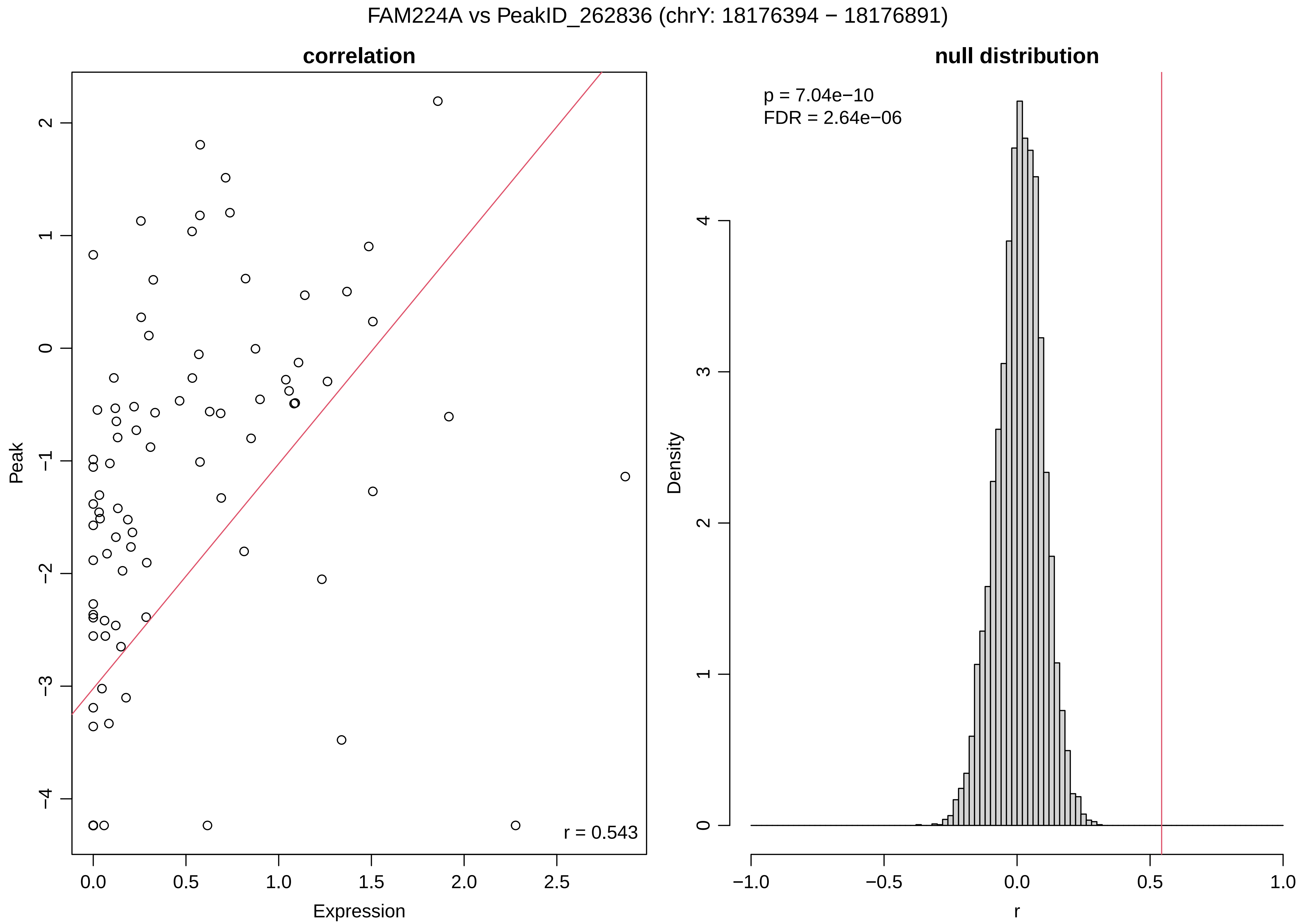
FAM224A vs PeakID_262836 (chrY: 18176394 − 18176891)
correlation
Peak
Expression
r = 0.543
null distribution
p = 7.04e−10
FDR = 2.64e−06
Density
r

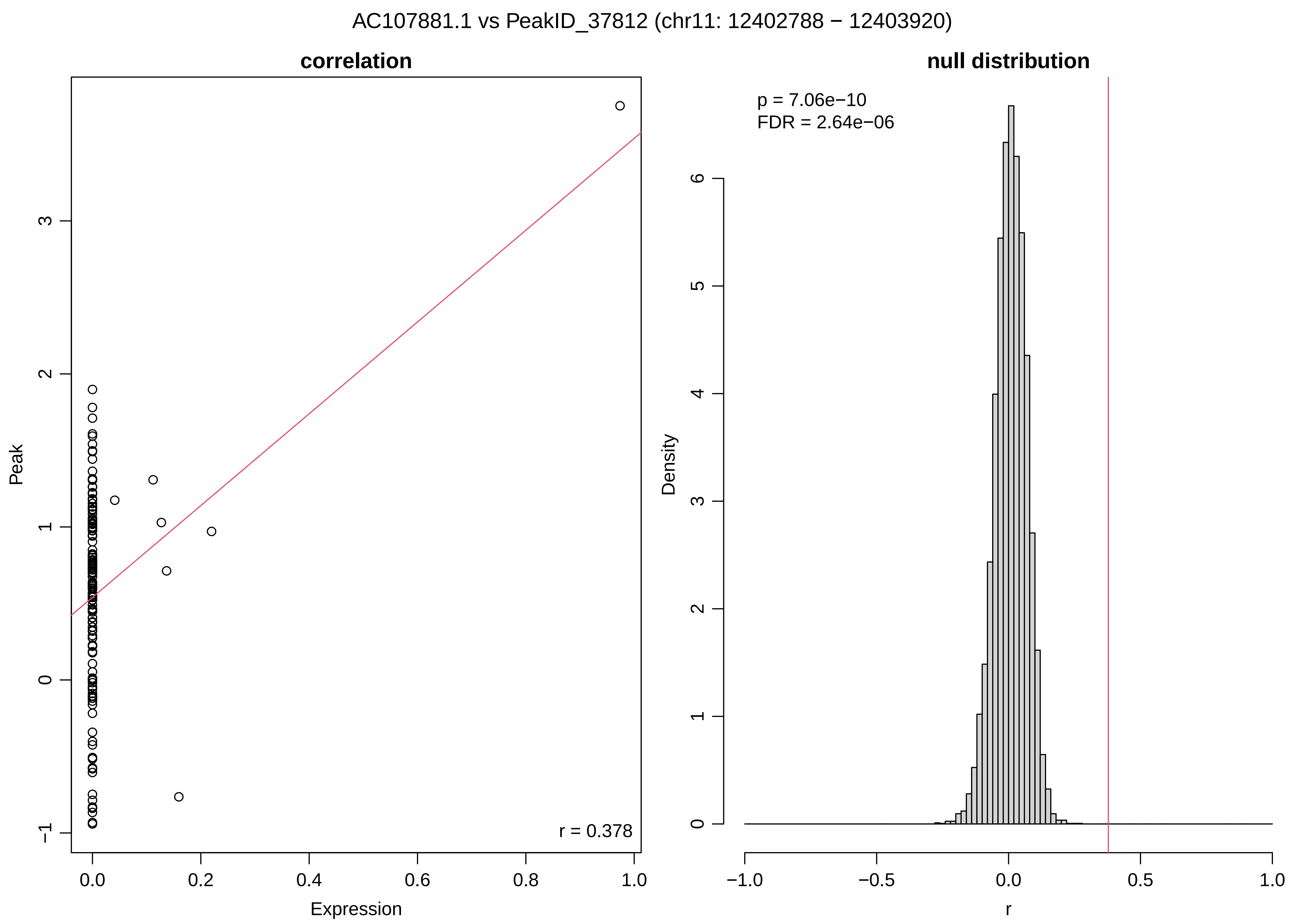

AC107881.1 vs PeakID_37812 (chr11: 12402788 − 12403920)
correlation
Peak
Expression
r = 0.378
0.0
0.2
0.4
0.6
0.8
1.0
−1
0
1
2
3
null distribution
p = 7.06e−10
FDR = 2.64e−06
Density
r
−1.0
−0.5
0.0
0.5
1.0
0
1
2
3
4
5
6

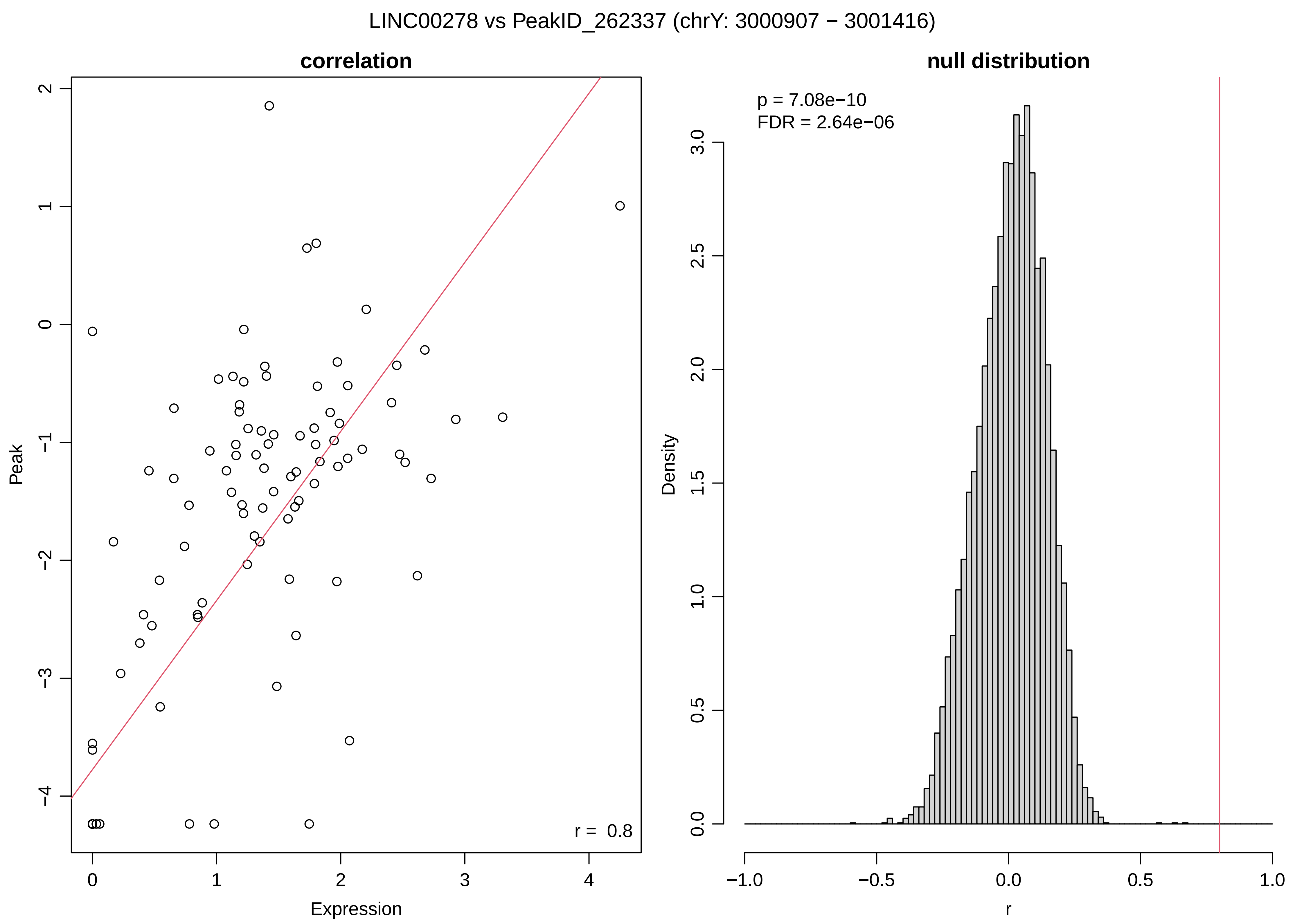

LINC00278 vs PeakID_262337 (chrY: 3000907 − 3001416)
correlation
Peak
Expression
r = 0.8
null distribution
p = 7.08e−10
FDR = 2.64e−06
Density
r

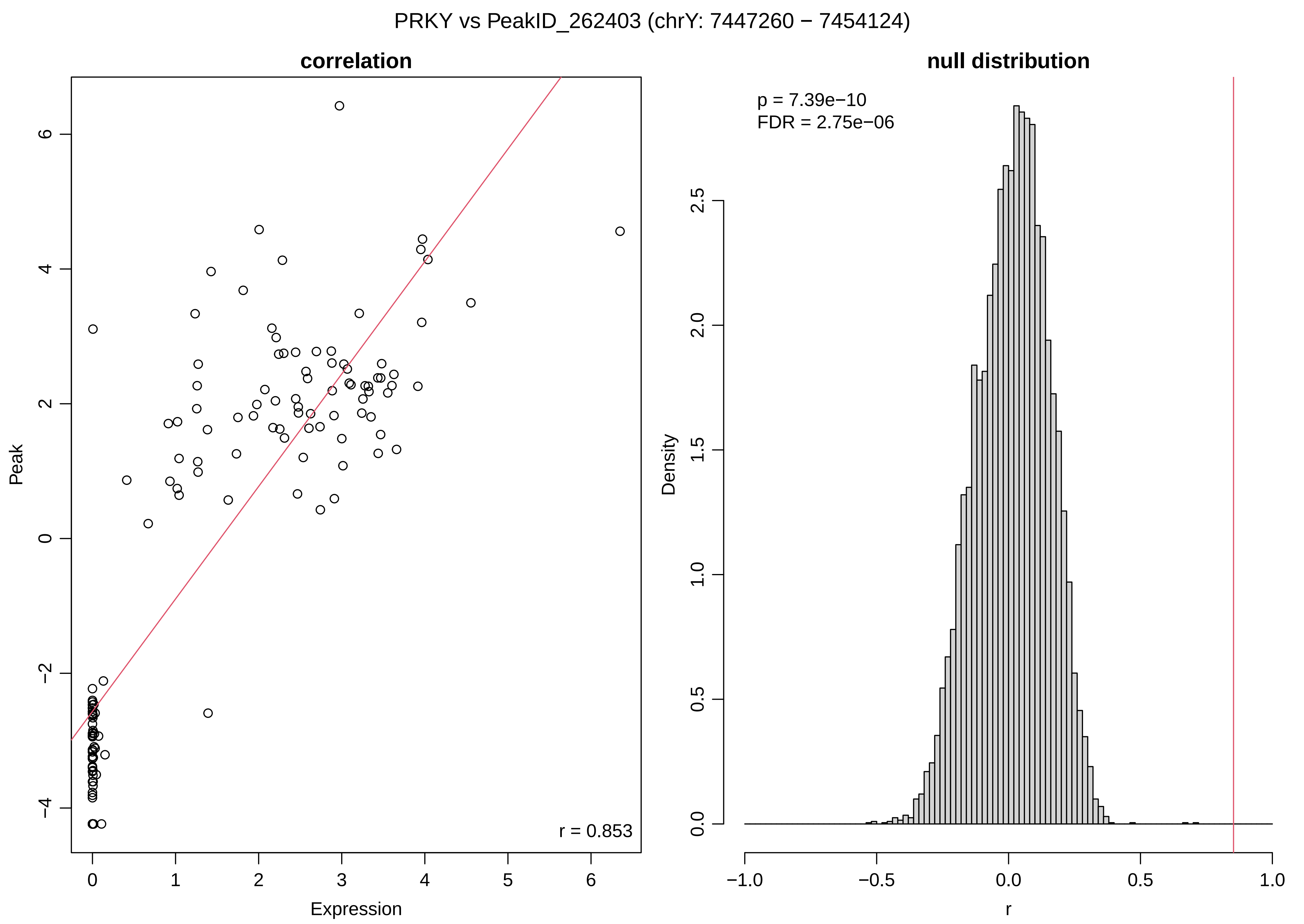
PRKY vs PeakID_262403 (chrY: 7447260 − 7454124)
correlation
null distribution
p = 7.39e−10
FDR = 2.75e−06
r = 0.853
Peak
Expression
Density
r
6
4
2
0
−2
−4
0
1
2
3
4
5
6
2.5
2.0
1.5
1.0
0.5
0.0
−1.0
−0.5
0.0
0.5
1.0

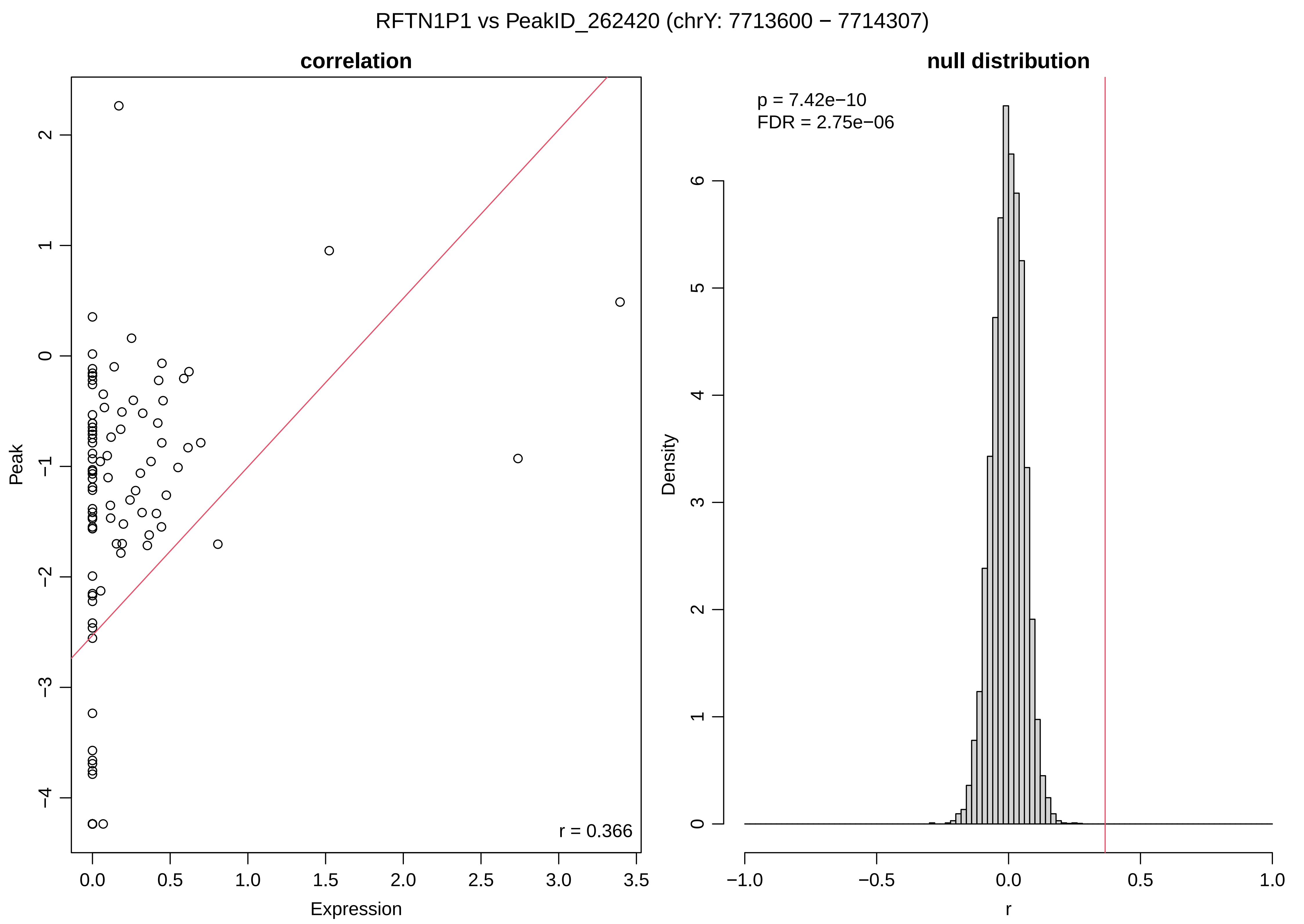
RFTN1P1 vs PeakID_262420 (chrY: 7713600 − 7714307)
correlation
Peak
Expression
r = 0.366
null distribution
p = 7.42e−10
FDR = 2.75e−06
Density
r

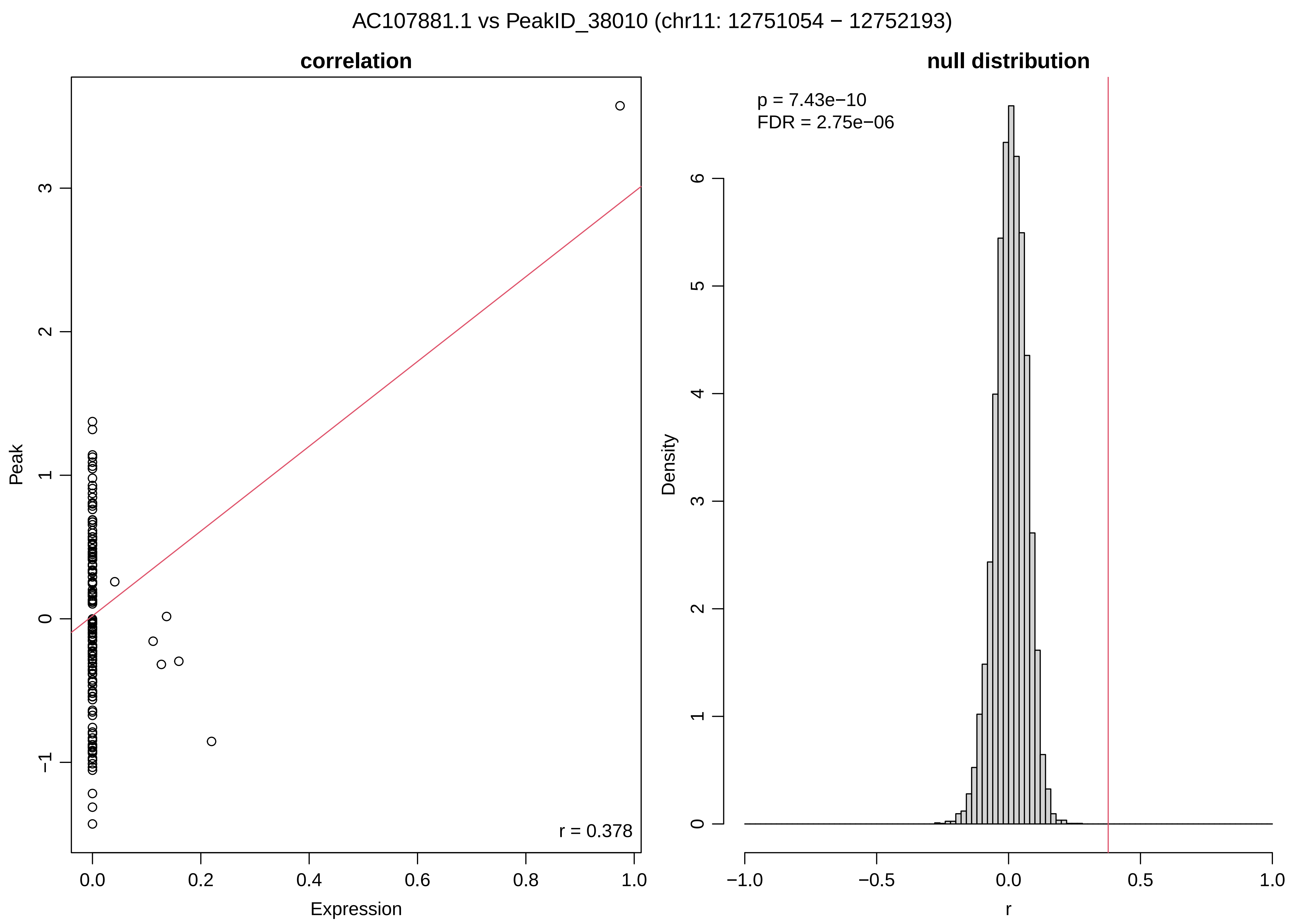
AC107881.1 vs PeakID_38010 (chr11: 12751054 − 12752193)
correlation
Peak
Expression
r = 0.378
null distribution
p = 7.43e−10
FDR = 2.75e−06
Density
r

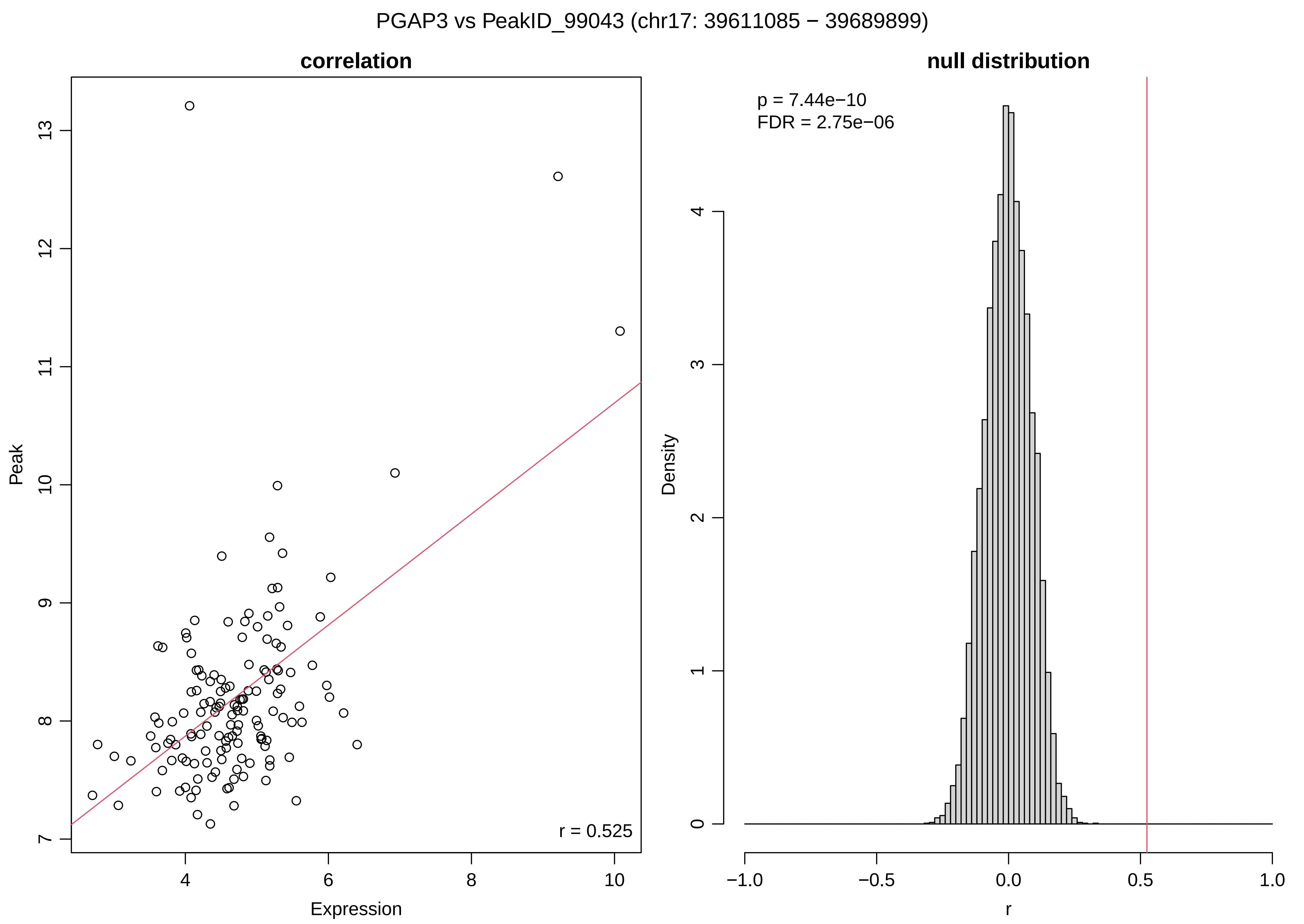

PGAP3 vs PeakID_99043 (chr17: 39611085 − 39689899)
correlation
Peak
Expression
r = 0.525
7
8
9
10
11
12
13
4
6
8
10
null distribution
p = 7.44e−10
FDR = 2.75e−06
Density
r
0
1
2
3
4
−1.0
−0.5
0.0
0.5
1.0

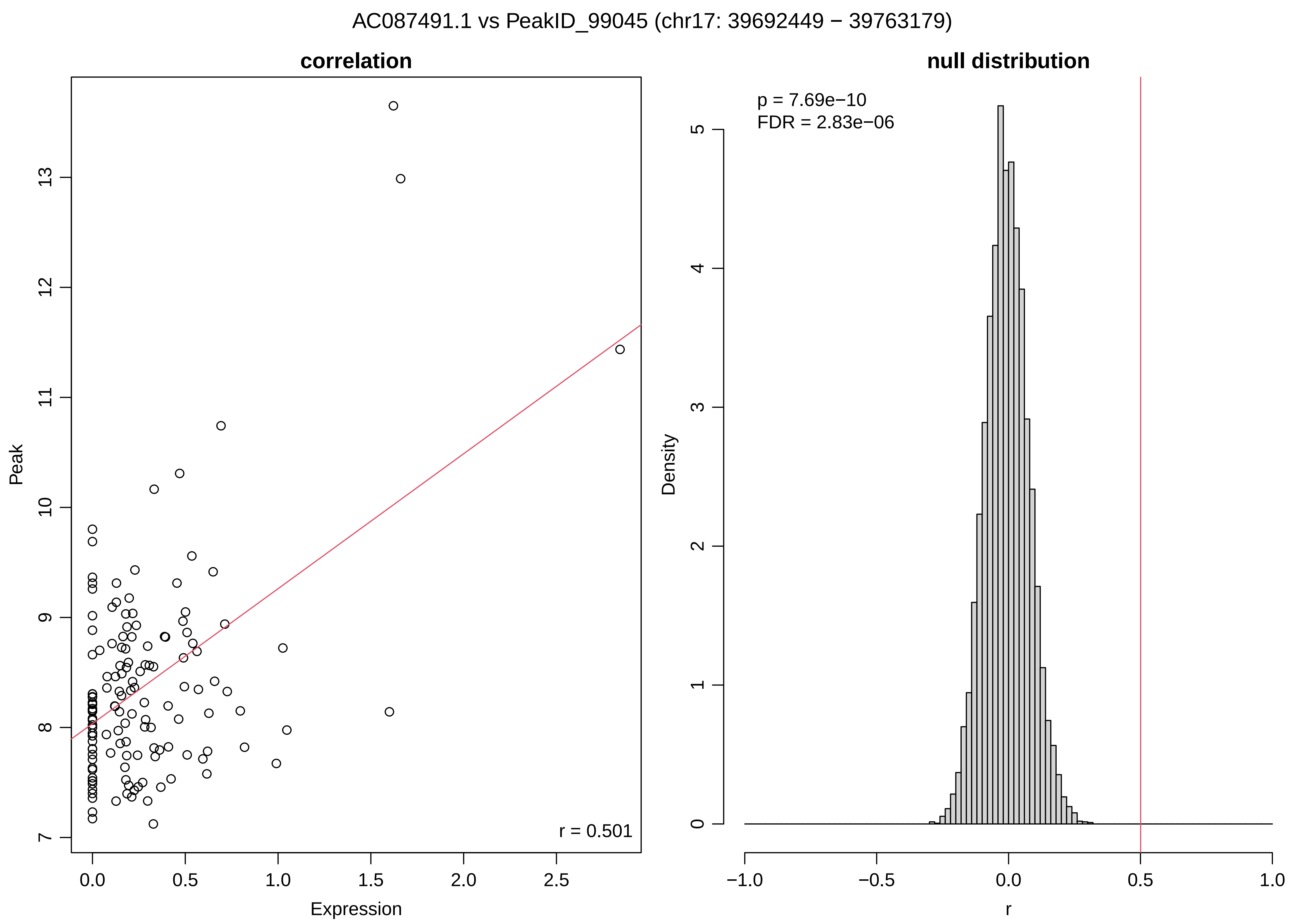
AC087491.1 vs PeakID_99045 (chr17: 39692449 − 39763179)
correlation
Peak
Expression
r = 0.501
null distribution
p = 7.69e−10
FDR = 2.83e−06
Density
r

AC011447.7 vs PeakID_115527 (chr19: 20234933 − 20238701)

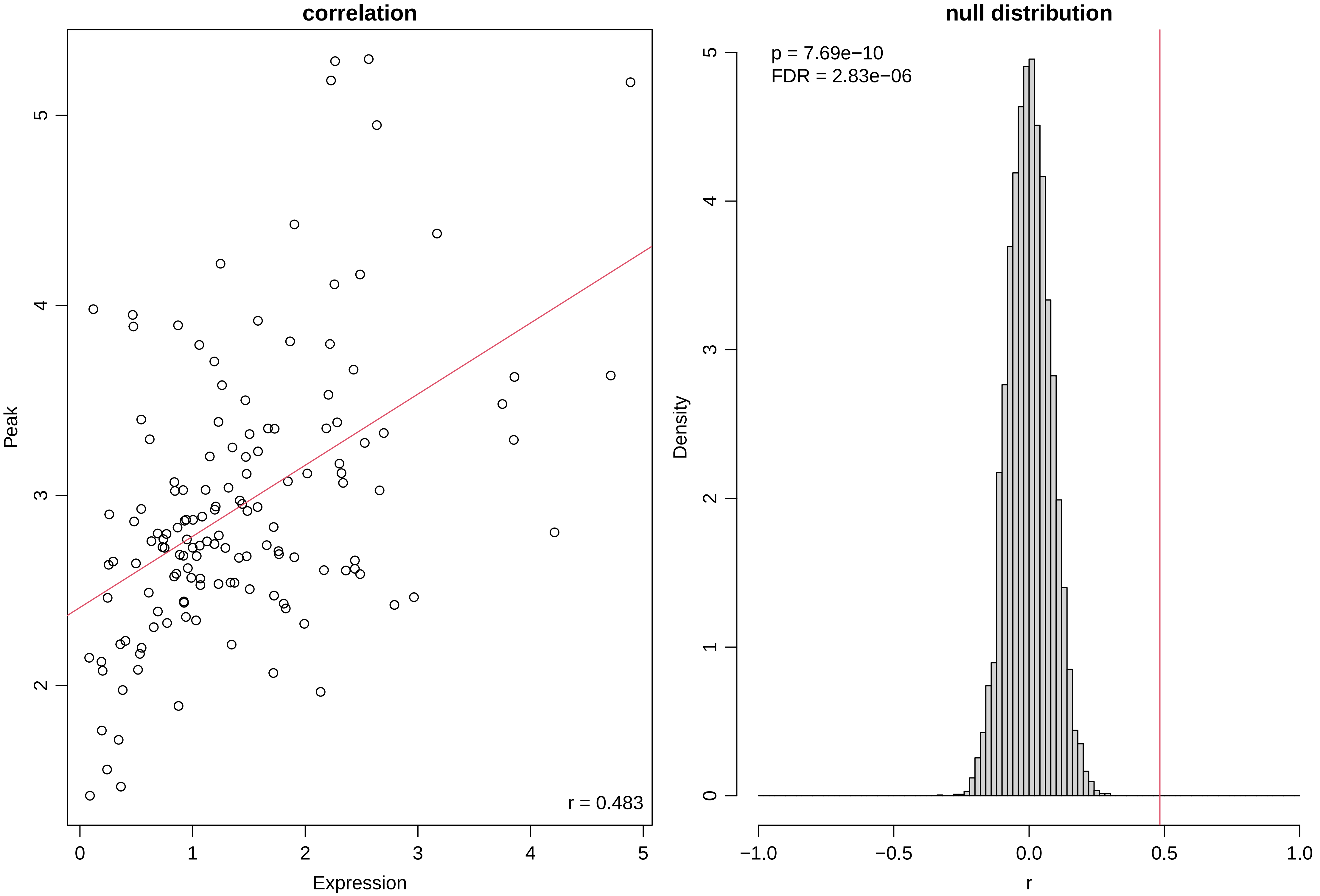

SLC35E3 vs PeakID_56584 (chr12: 68826116 − 68827241)

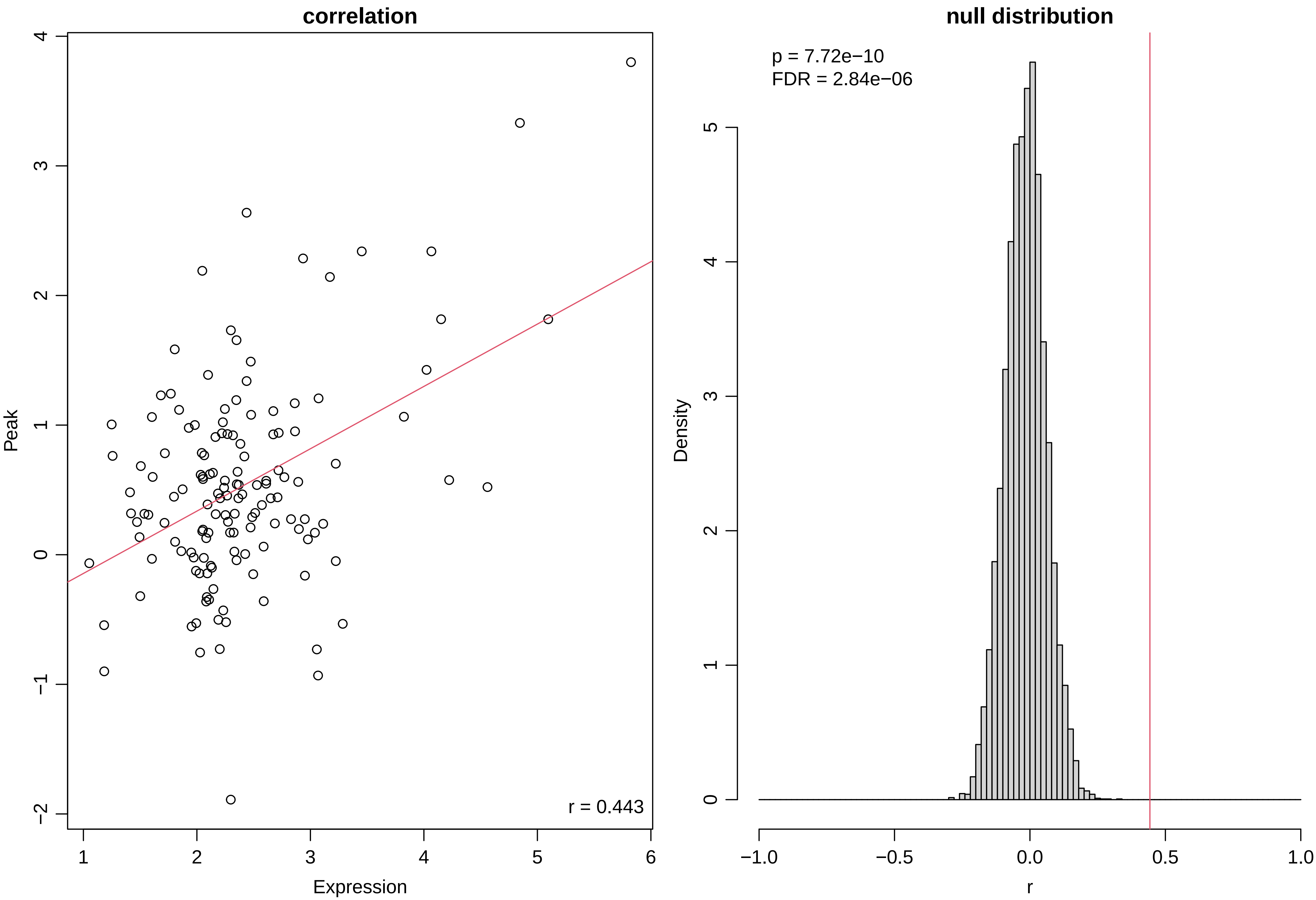

AL645929.3 vs PeakID_204611 (chr6: 29887129 − 29889592)

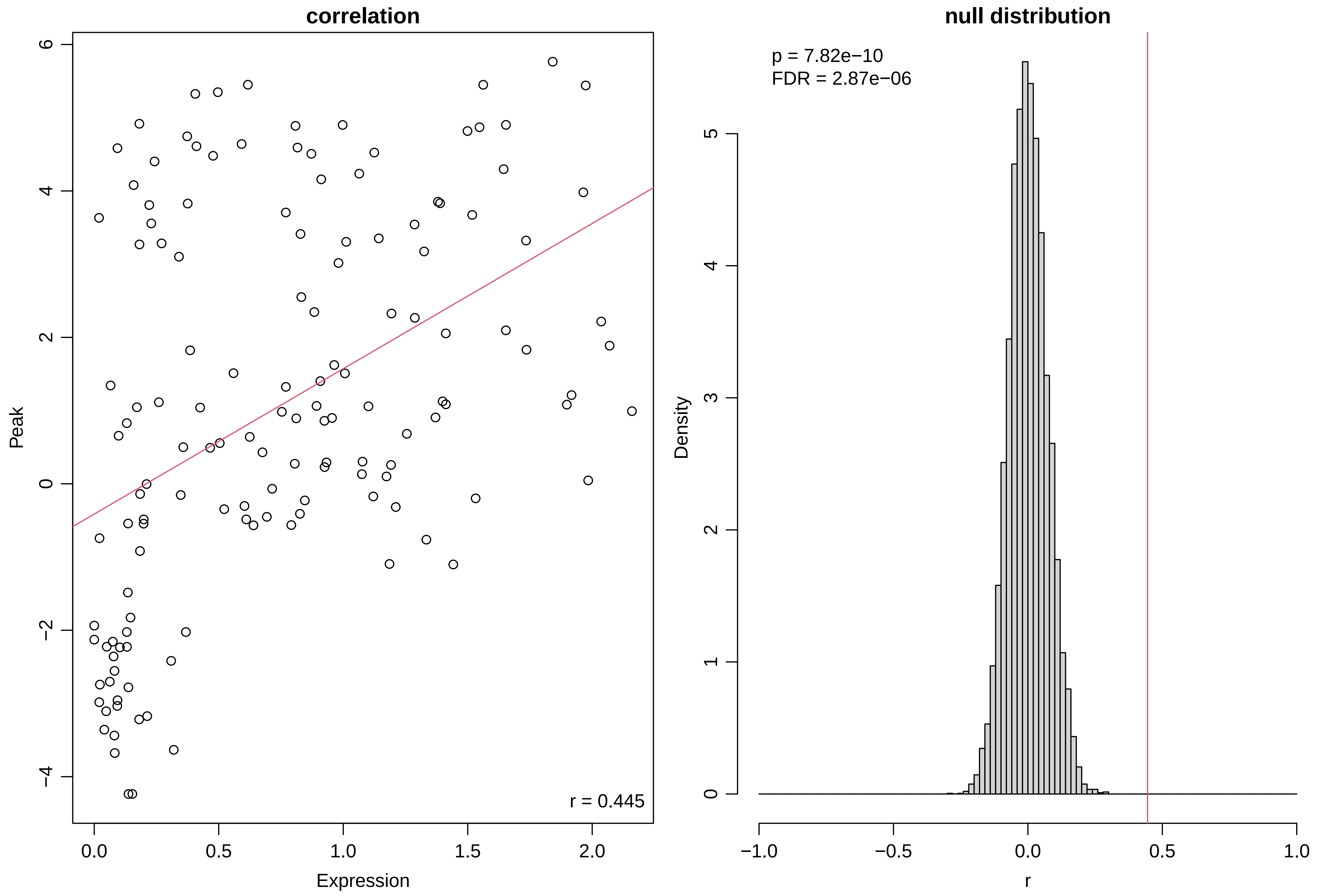

AC138649.5 vs PeakID_76722 (chr15: 22713992 − 22716520)

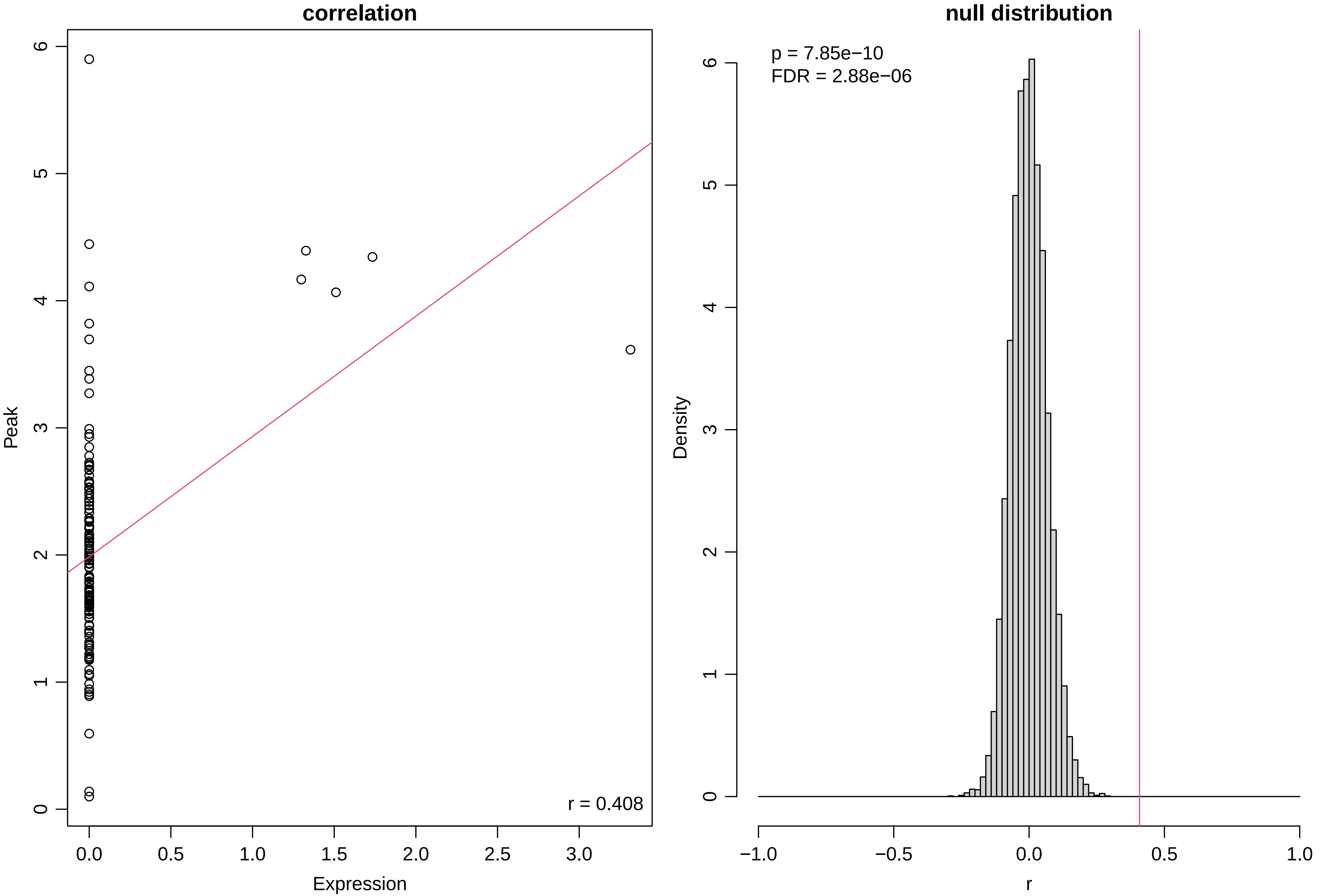

AC018755.4 vs PeakID_120369 (chr19: 51645274 − 51647452)

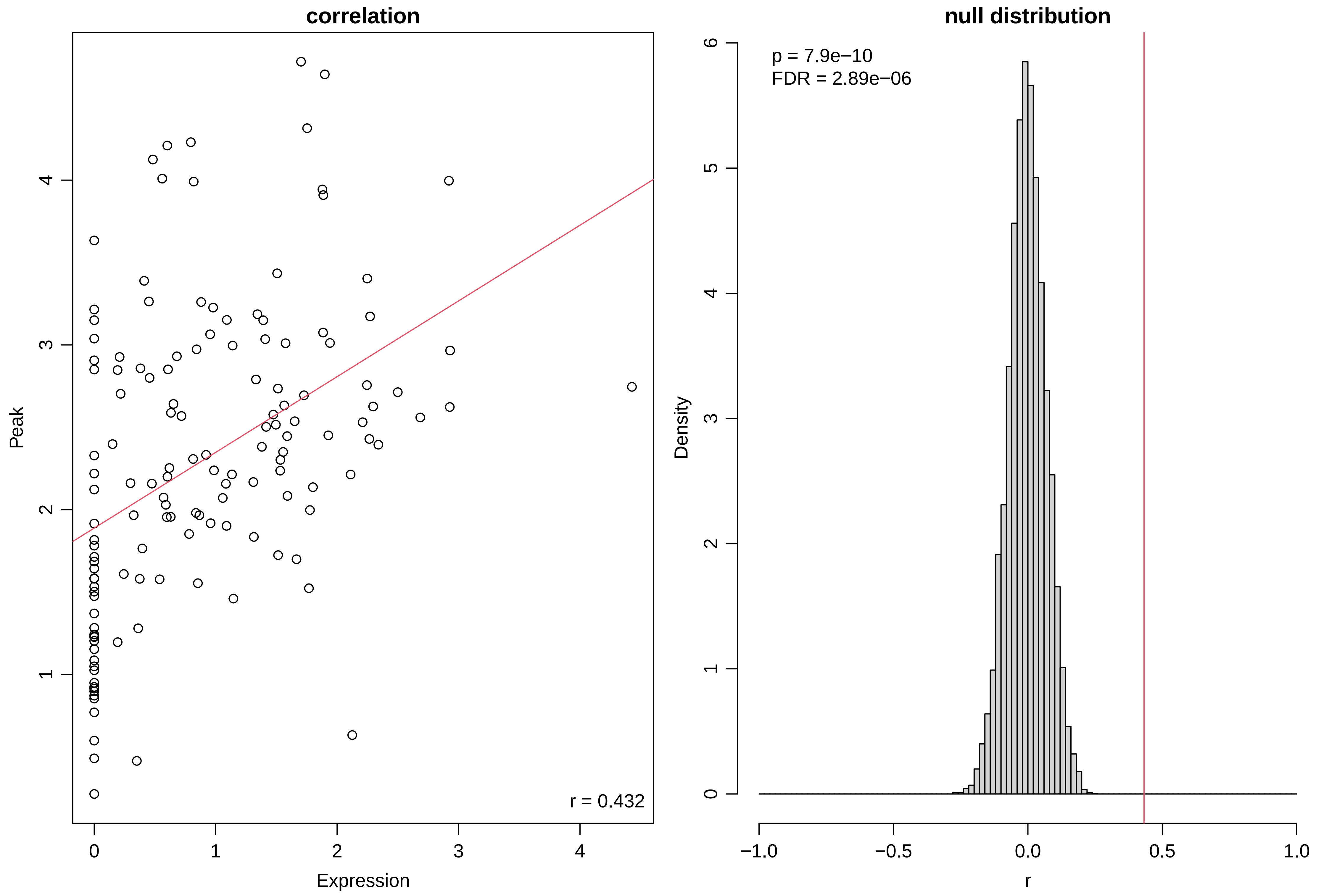

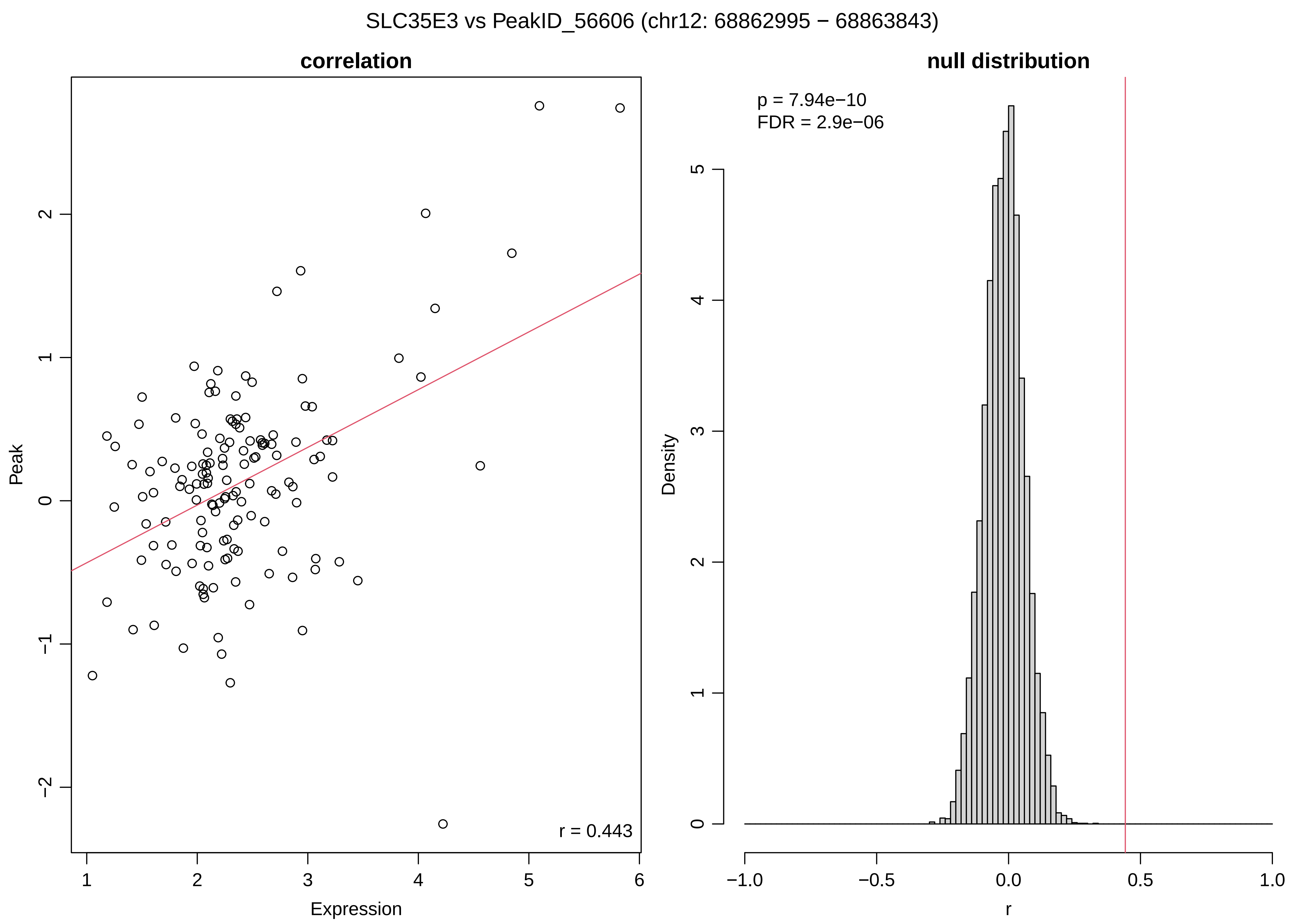
SLC35E3 vs PeakID_56606 (chr12: 68862995 − 68863843)
correlation
null distribution
p = 7.94e−10
FDR = 2.9e−06
r = 0.443
Peak
Expression
Density
r
−2
−1
0
1
2
1
2
3
4
5
6
0
1
2
3
4
5
−1.0
−0.5
0.0
0.5
1.0

RPL21P104 vs PeakID_55438 (chr12: 61885058 − 61887335)

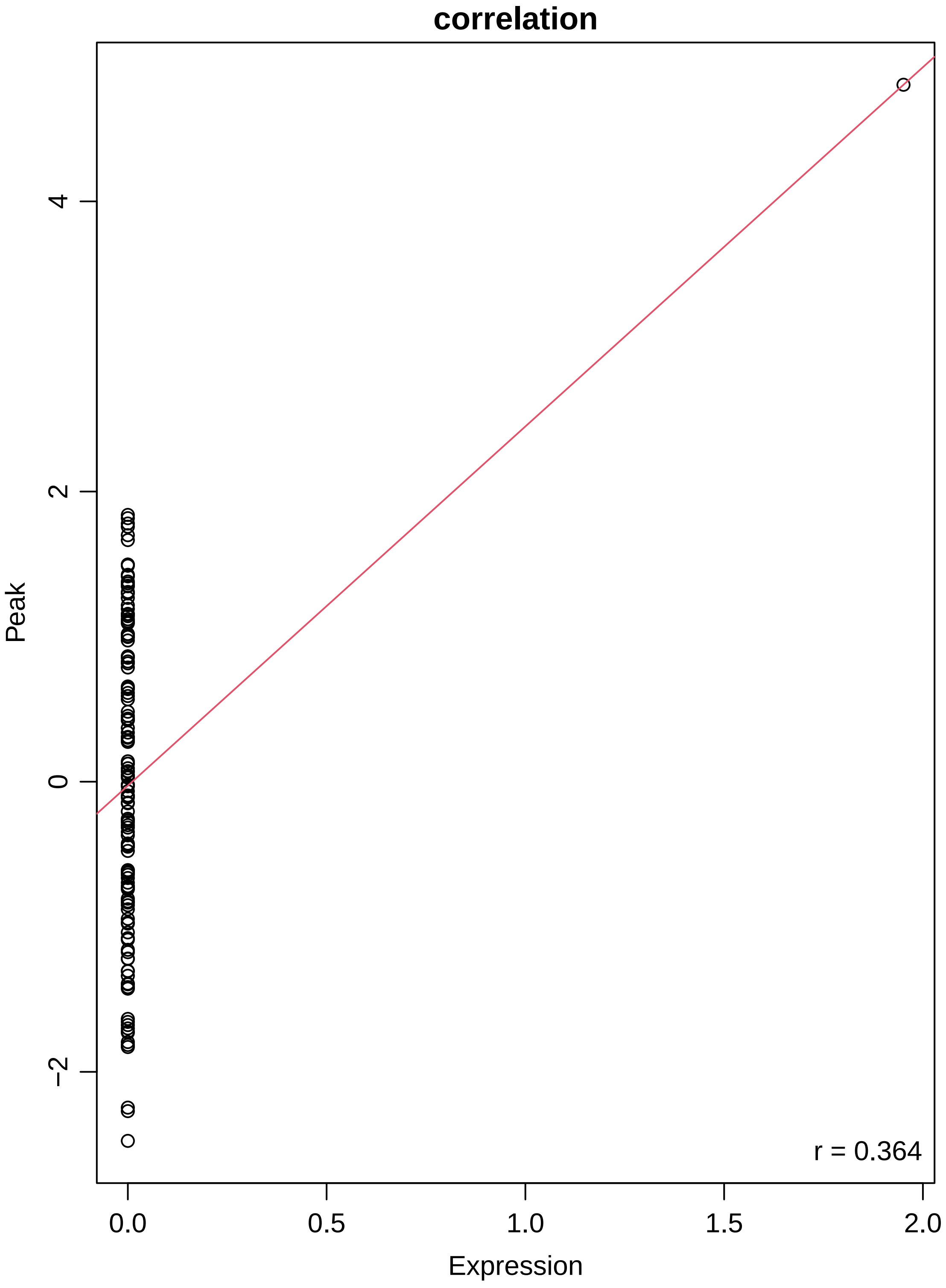

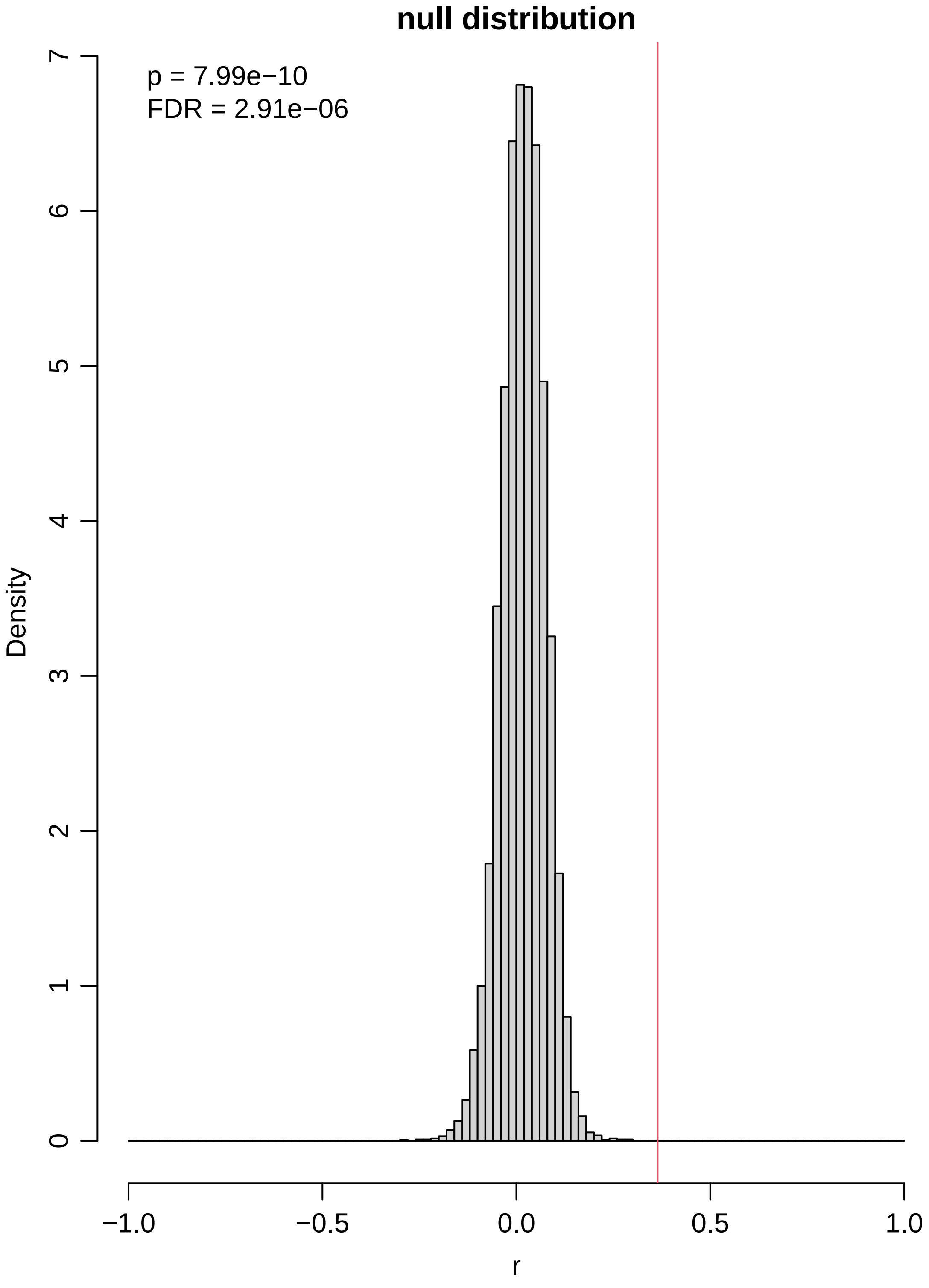

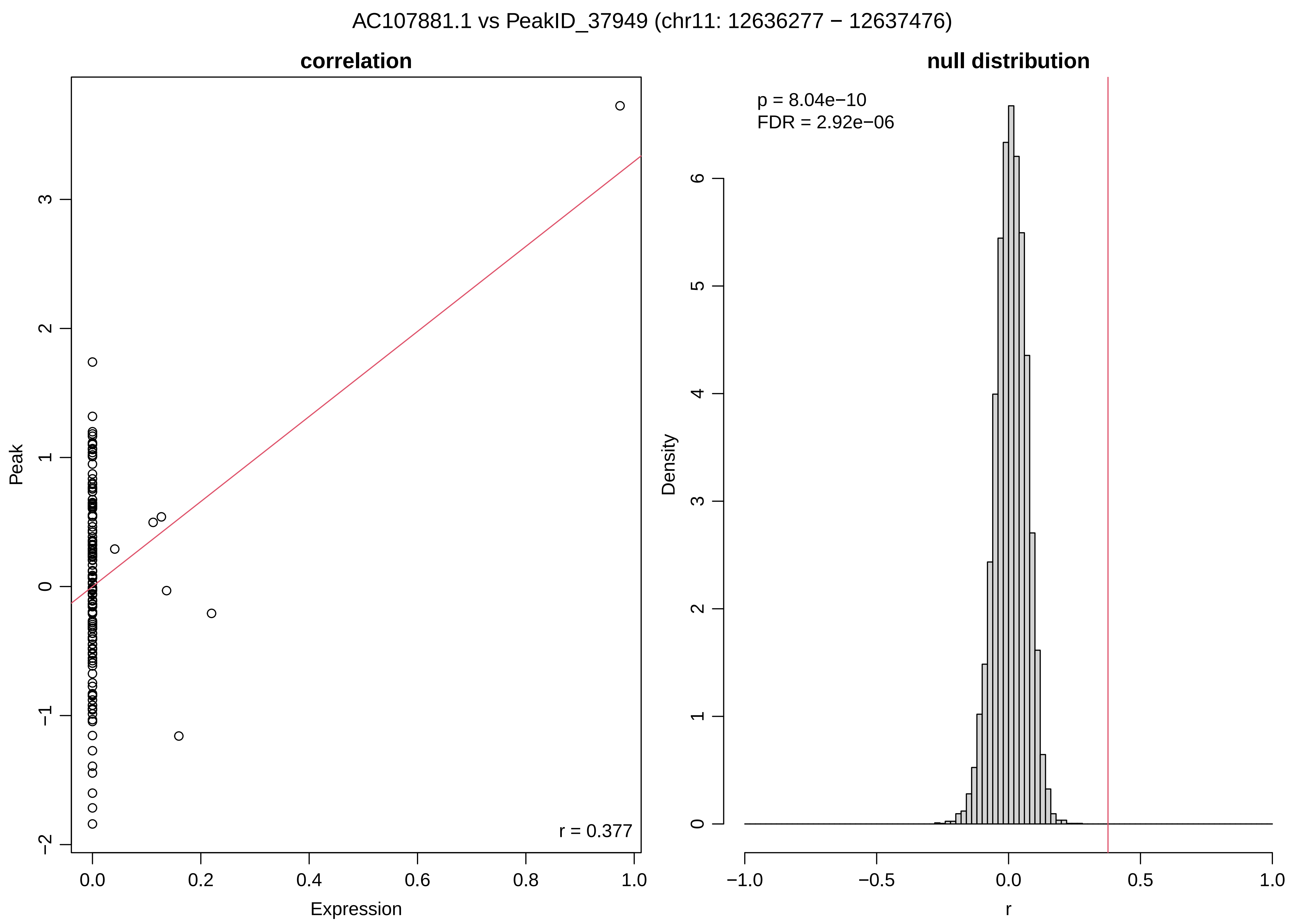
AC107881.1 vs PeakID_37949 (chr11: 12636277 − 12637476)
correlation
Peak
Expression
r = 0.377
3
2
1
0
−1
−2
0.0
0.2
0.4
0.6
0.8
1.0
null distribution
p = 8.04e−10
FDR = 2.92e−06
Density
r
6
5
4
3
2
1
0
−1.0
−0.5
0.0
0.5
1.0

AL049870.2 vs PeakID_70362 (chr14: 44913087 − 44913775)

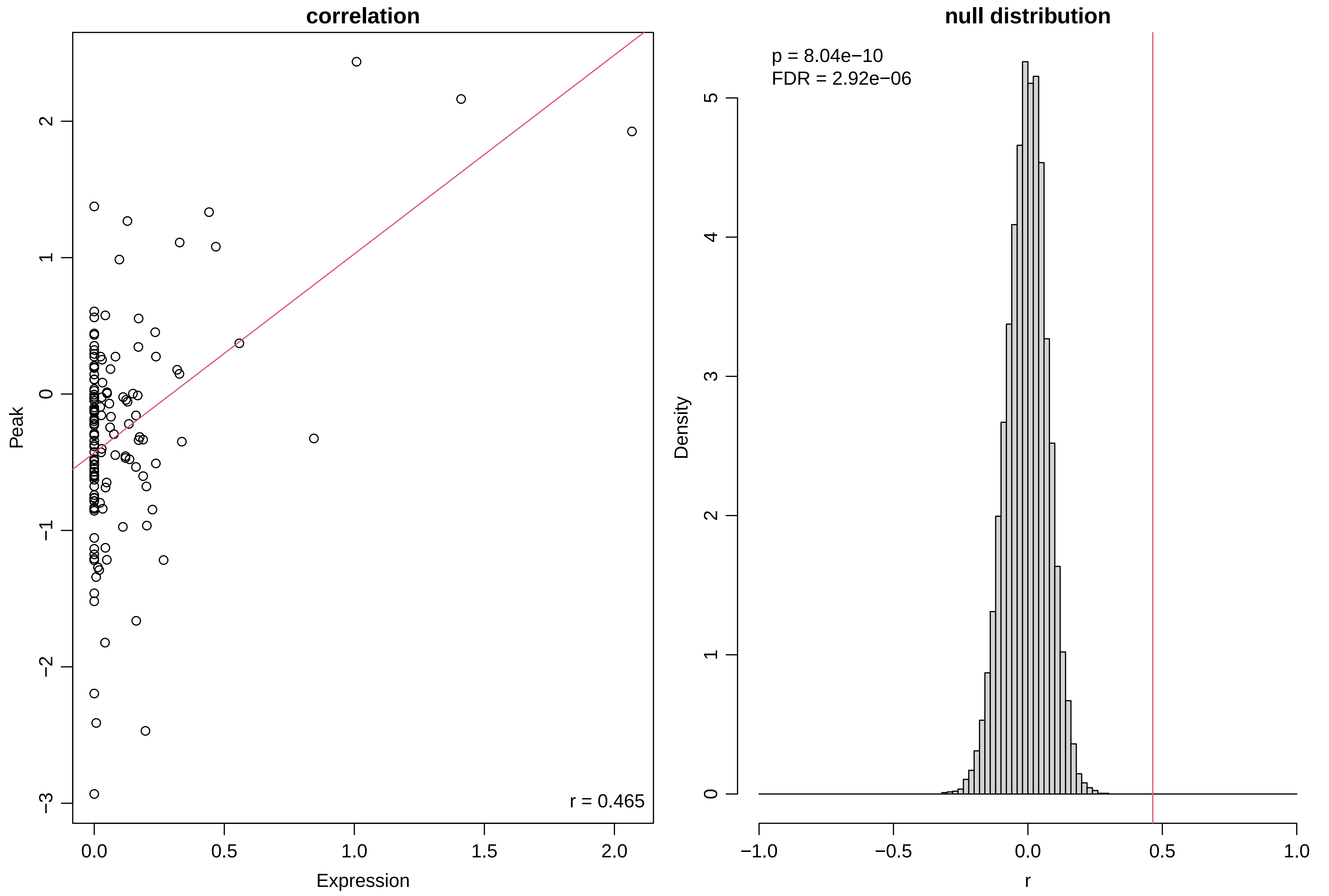

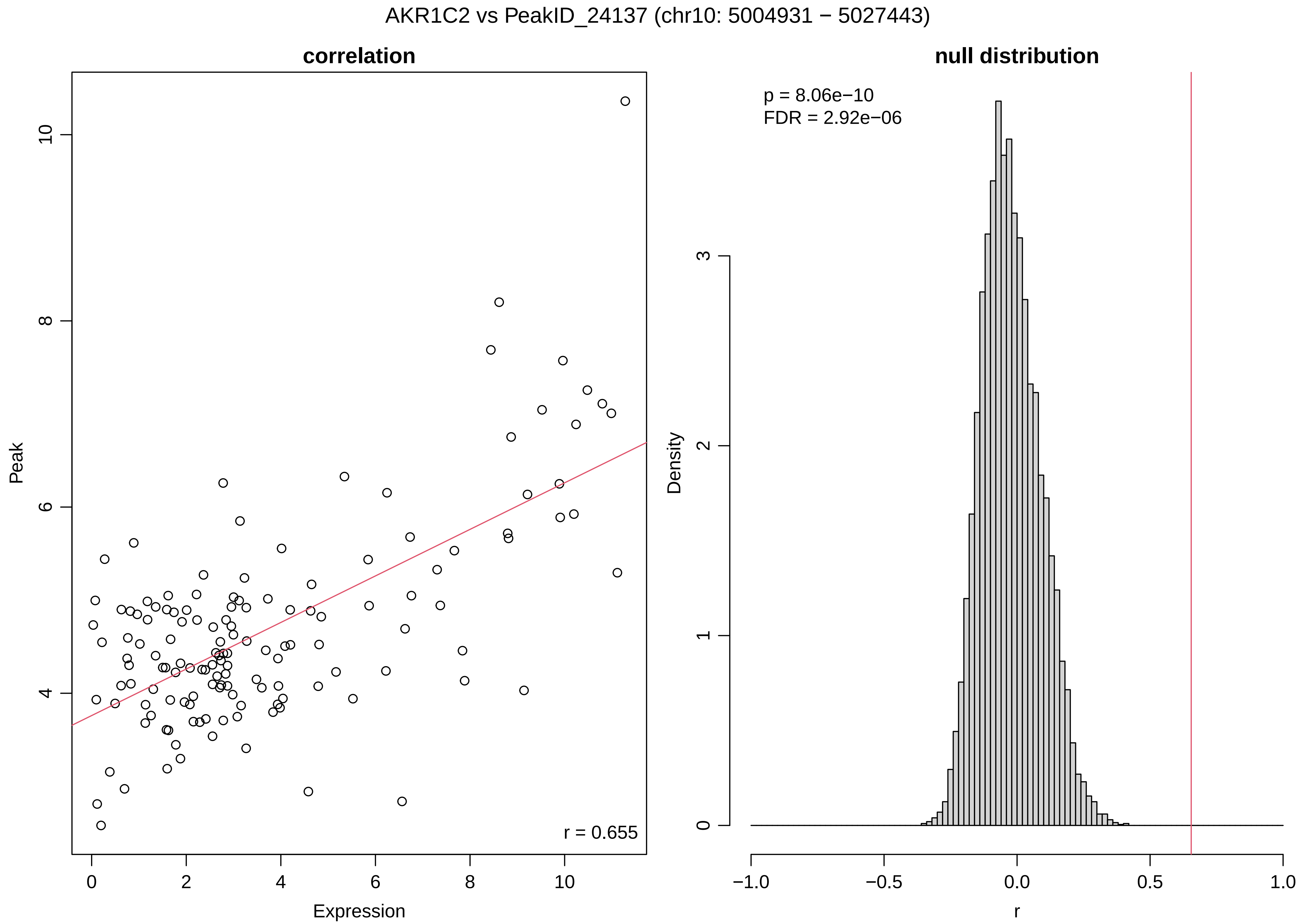

AKR1C2 vs PeakID_24137 (chr10: 5004931 − 5027443)
correlation
Peak
Expression
r = 0.655
null distribution
p = 8.06e−10
FDR = 2.92e−06
Density
r

AC016582.3 vs PeakID_116818 (chr19: 37816688 − 37818104)

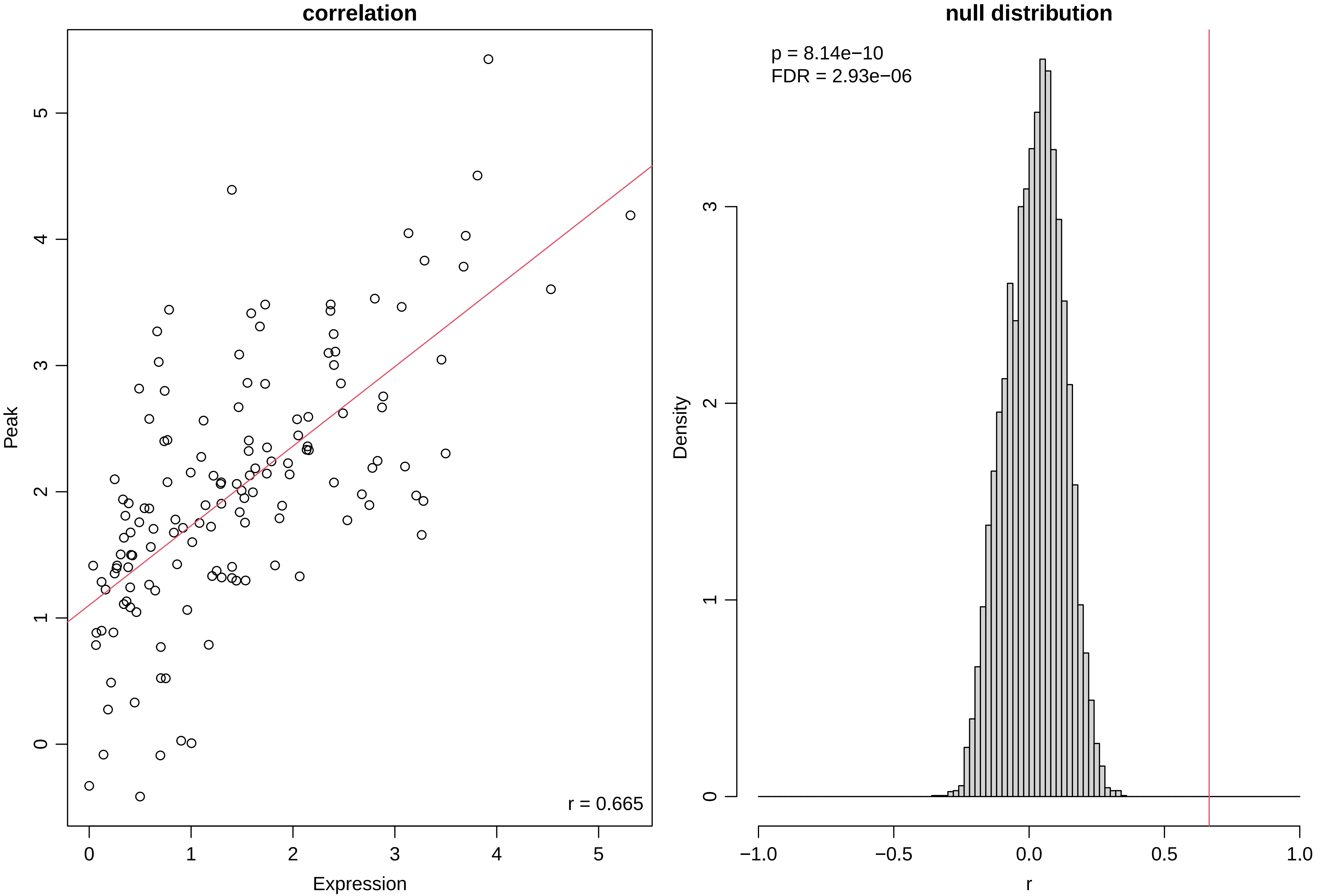

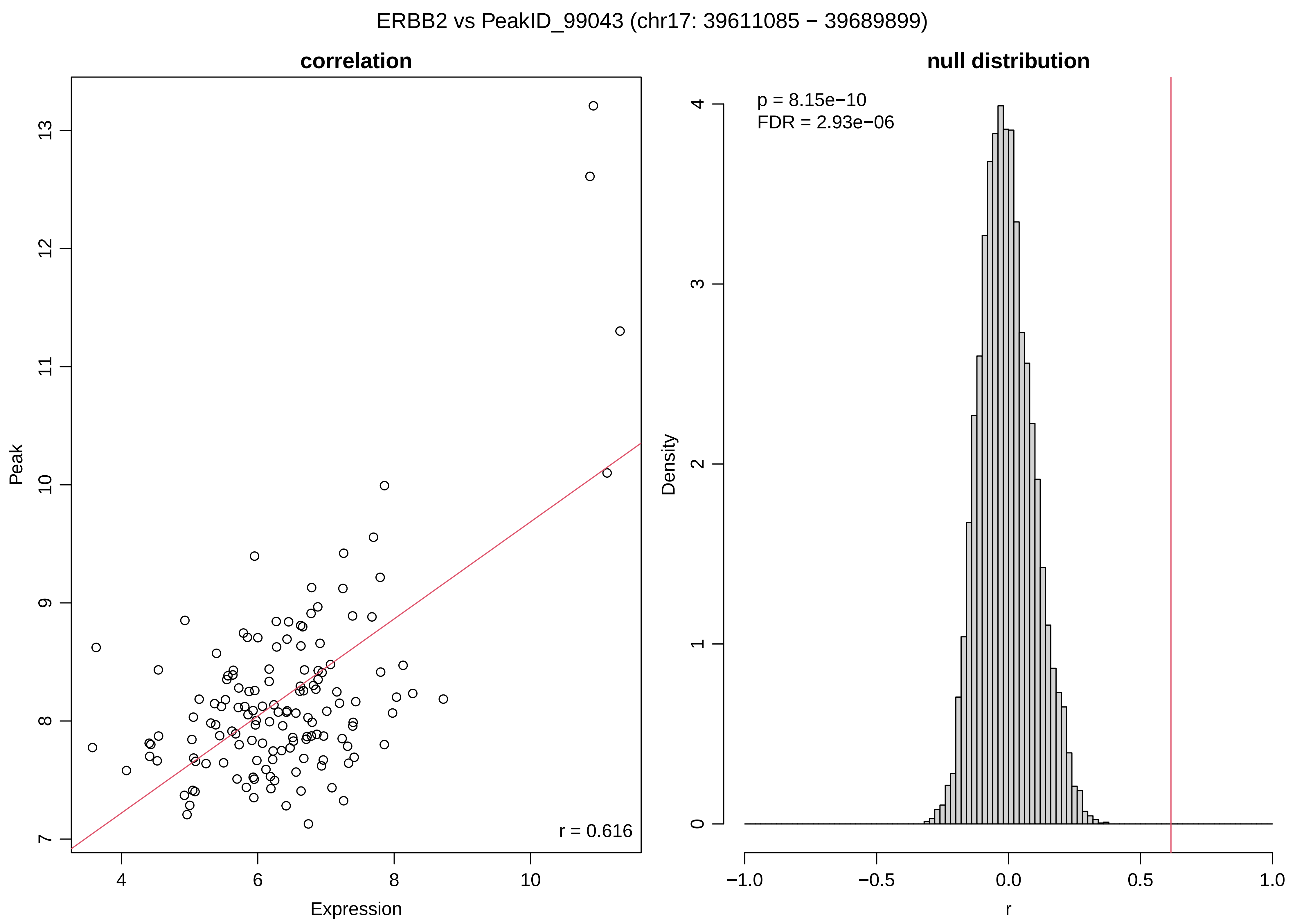

ERBB2 vs PeakID_99043 (chr17: 39611085 − 39689899)
correlation
Peak
Expression
r = 0.616
7
8
9
10
11
12
13
4
6
8
10
null distribution
p = 8.15e−10
FDR = 2.93e−06
Density
r
0
1
2
3
4
−1.0
−0.5
0.0
0.5
1.0

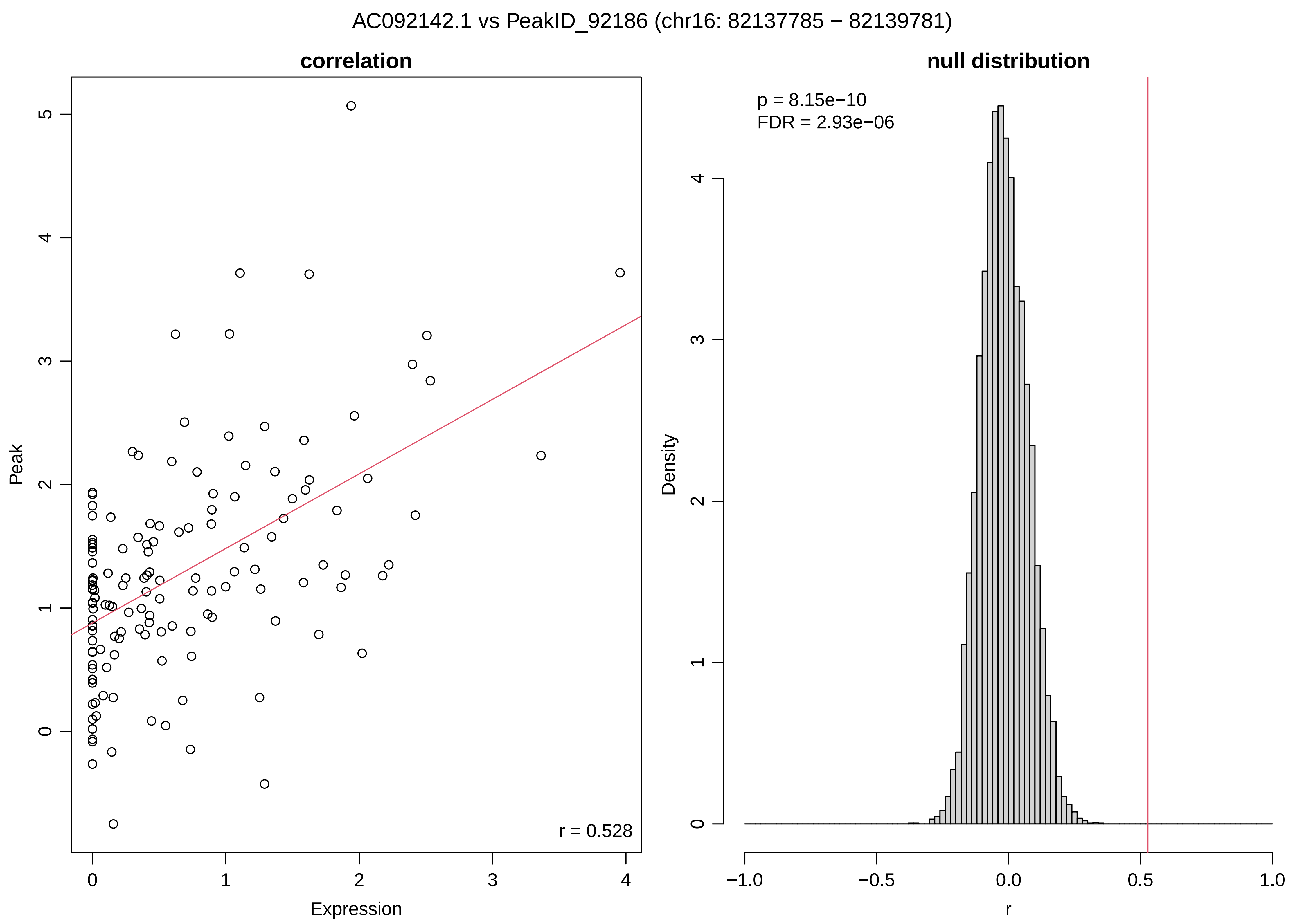

AC092142.1 vs PeakID_92186 (chr16: 82137785 − 82139781)
correlation
Peak
Expression
r = 0.528
null distribution
p = 8.15e−10
FDR = 2.93e−06
Density
r

LINC02403 vs PeakID_54451 (chr12: 58026095 − 58027855)

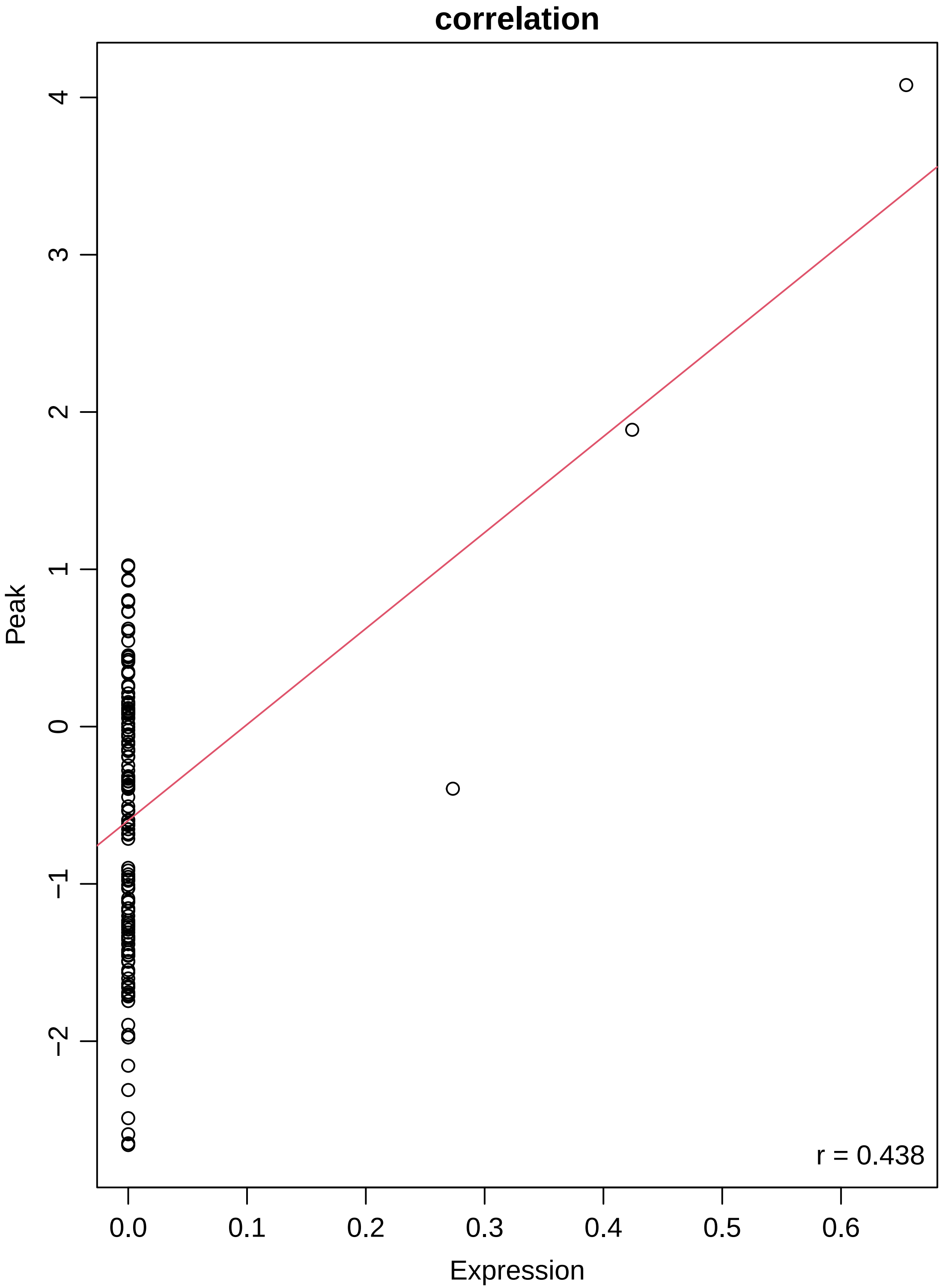

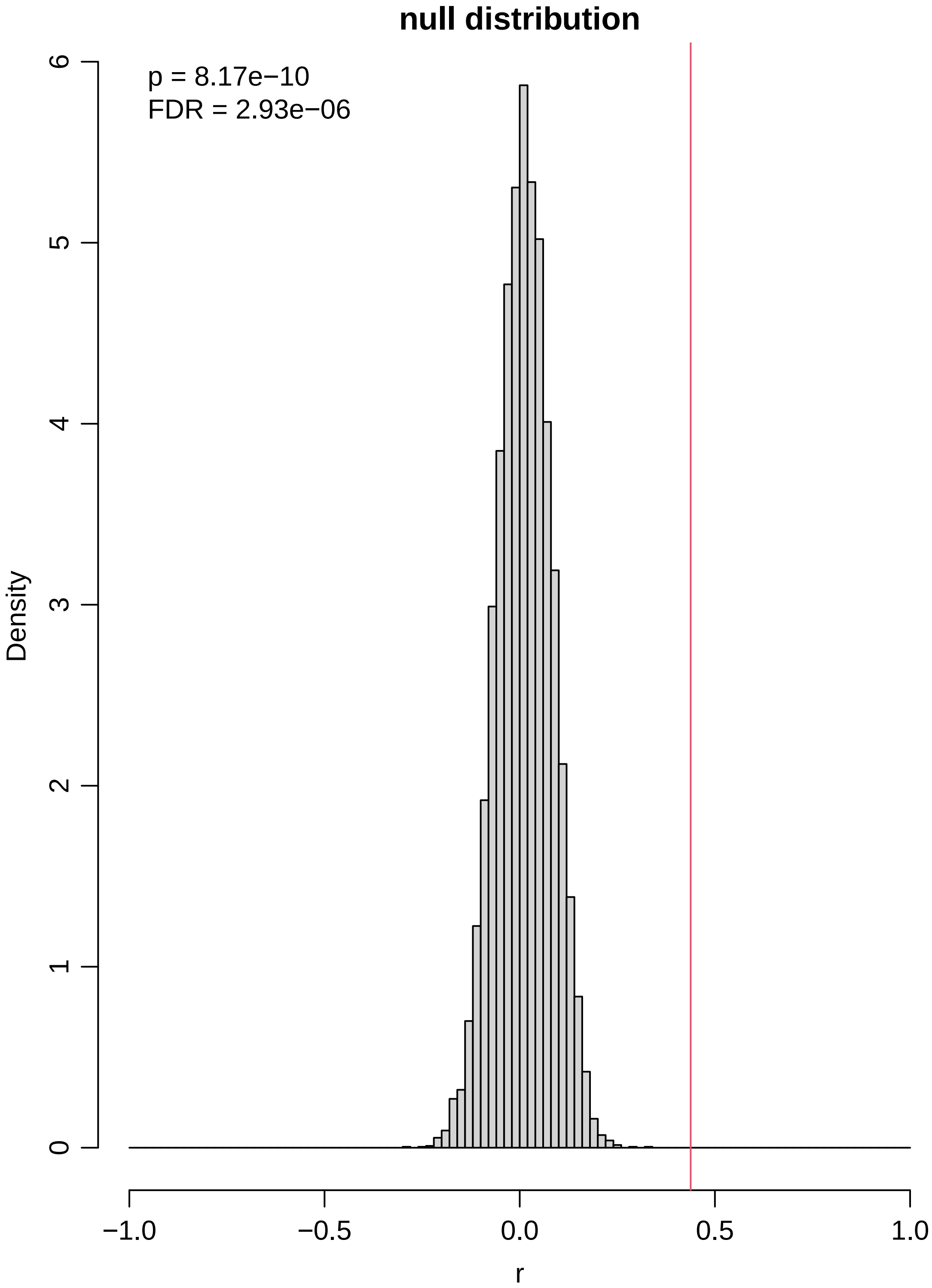

AL390800.1 vs PeakID_70306 (chr14: 40628732 − 40630236)

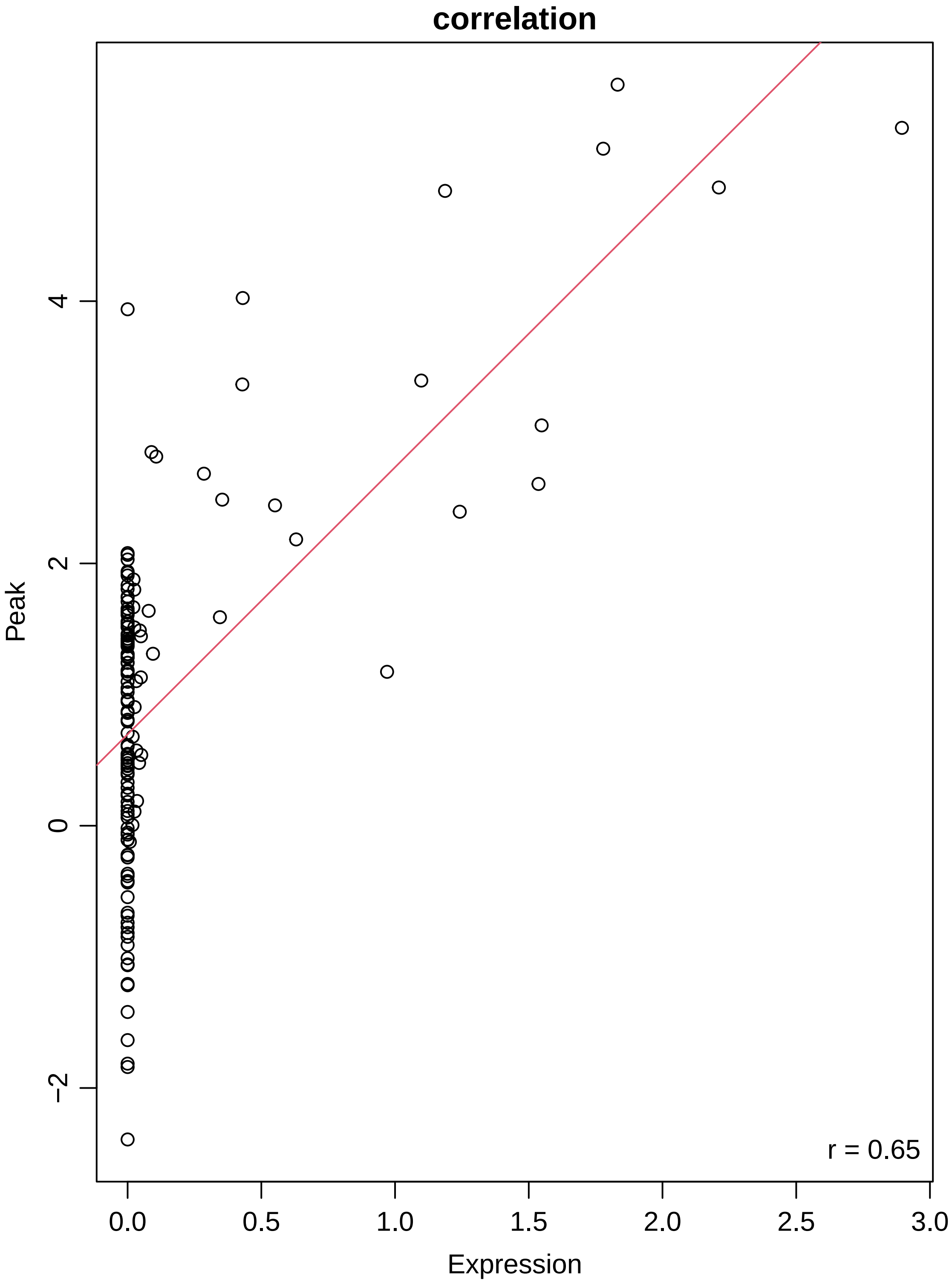

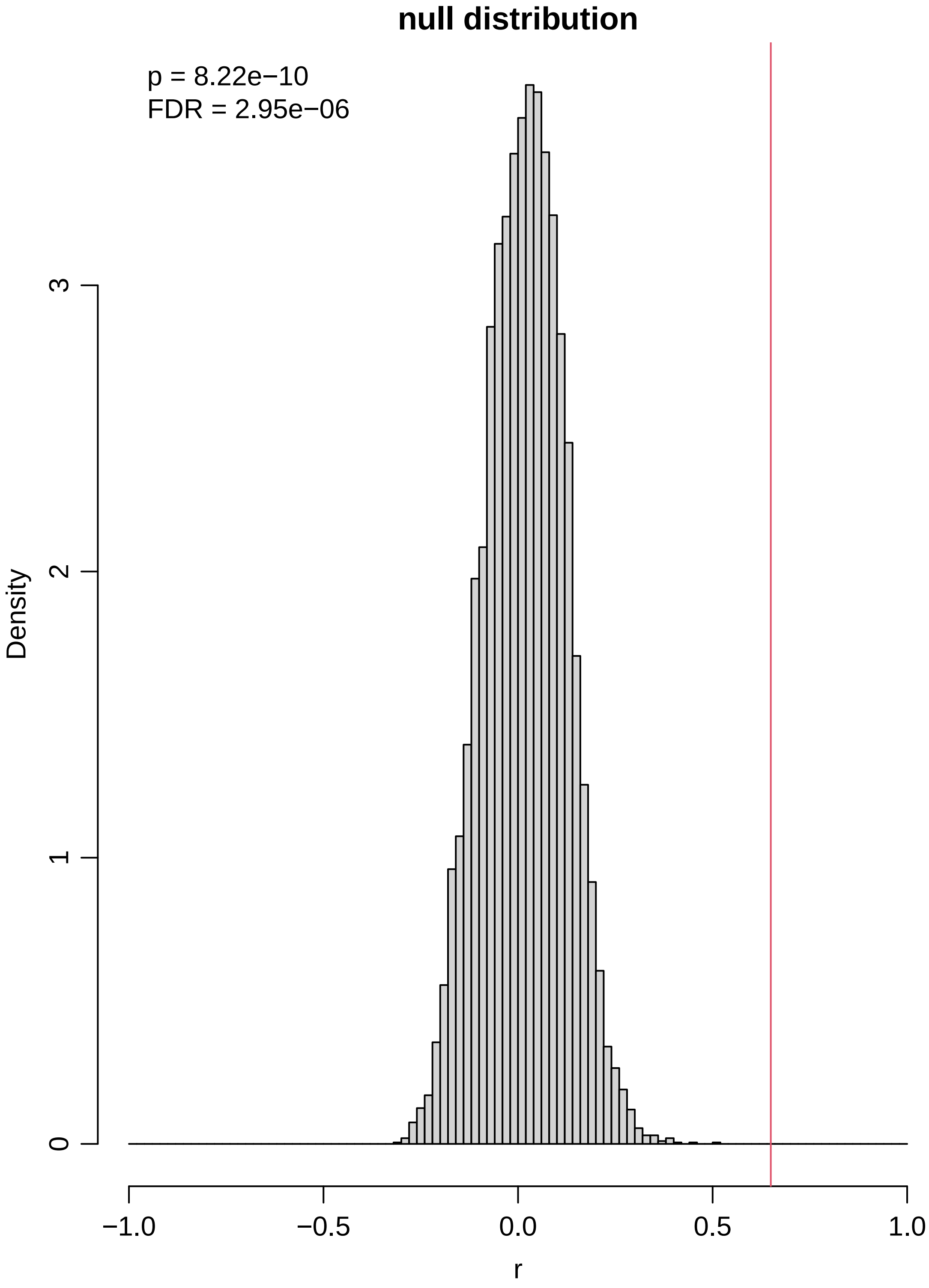

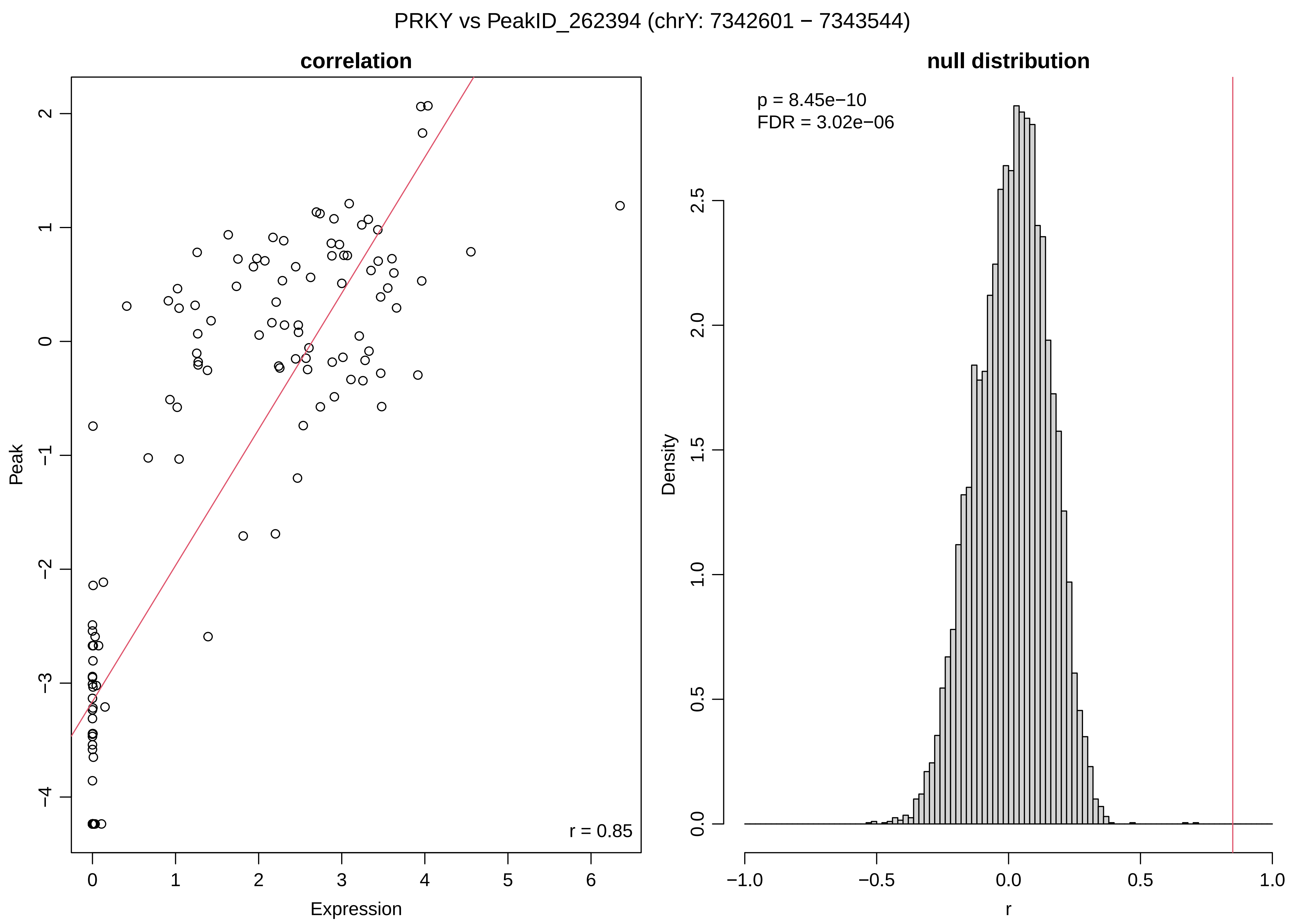
PRKY vs PeakID_262394 (chrY: 7342601 − 7343544)
correlation
Peak
Expression
r = 0.85
null distribution
p = 8.45e−10
FDR = 3.02e−06
Density
r

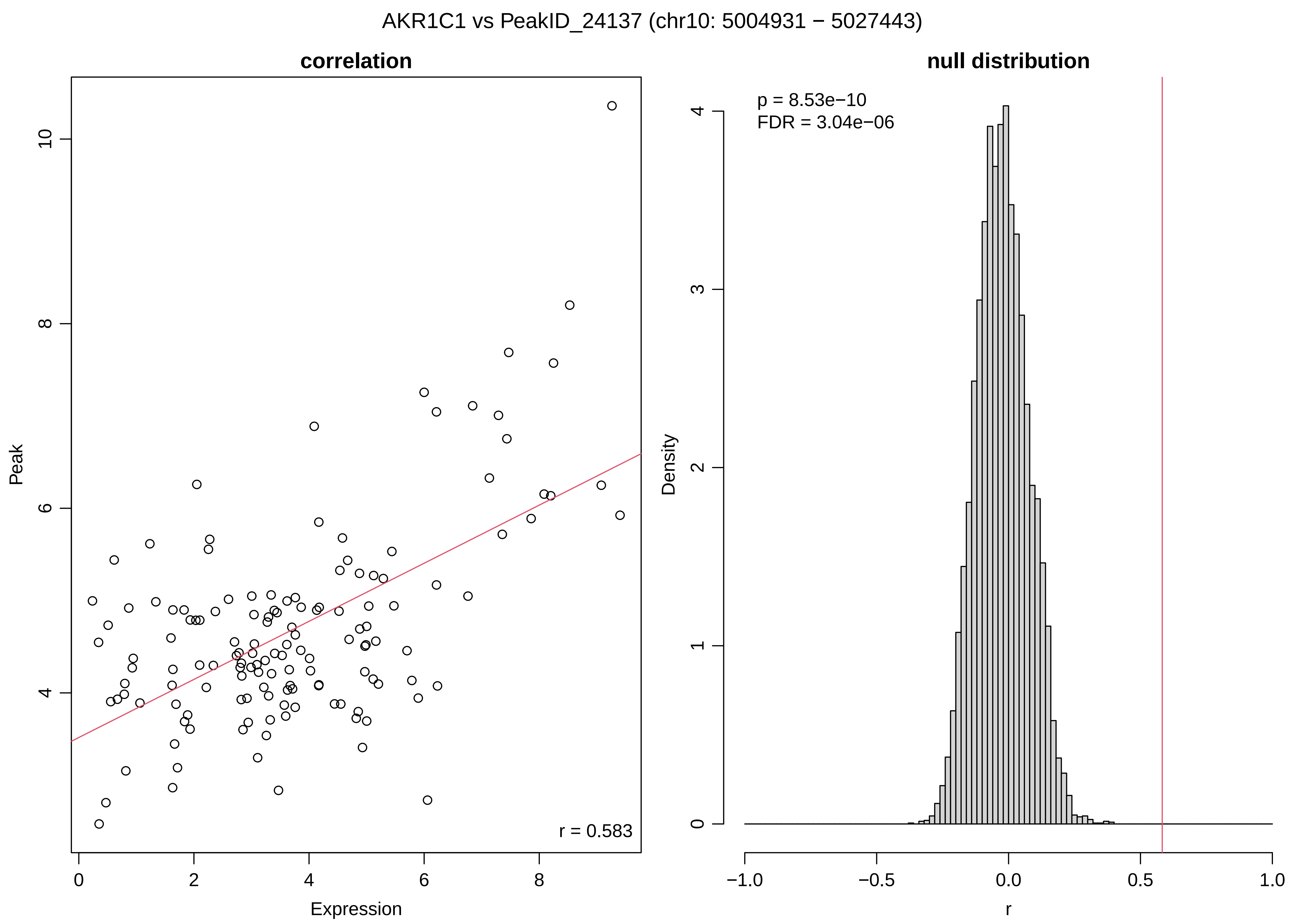

AKR1C1 vs PeakID_24137 (chr10: 5004931 − 5027443)
correlation
Peak
Expression
r = 0.583
0
2
4
6
8
10
null distribution
p = 8.53e−10
FDR = 3.04e−06
Density
r
−1.0
−0.5
0.0
0.5
1.0
1
3

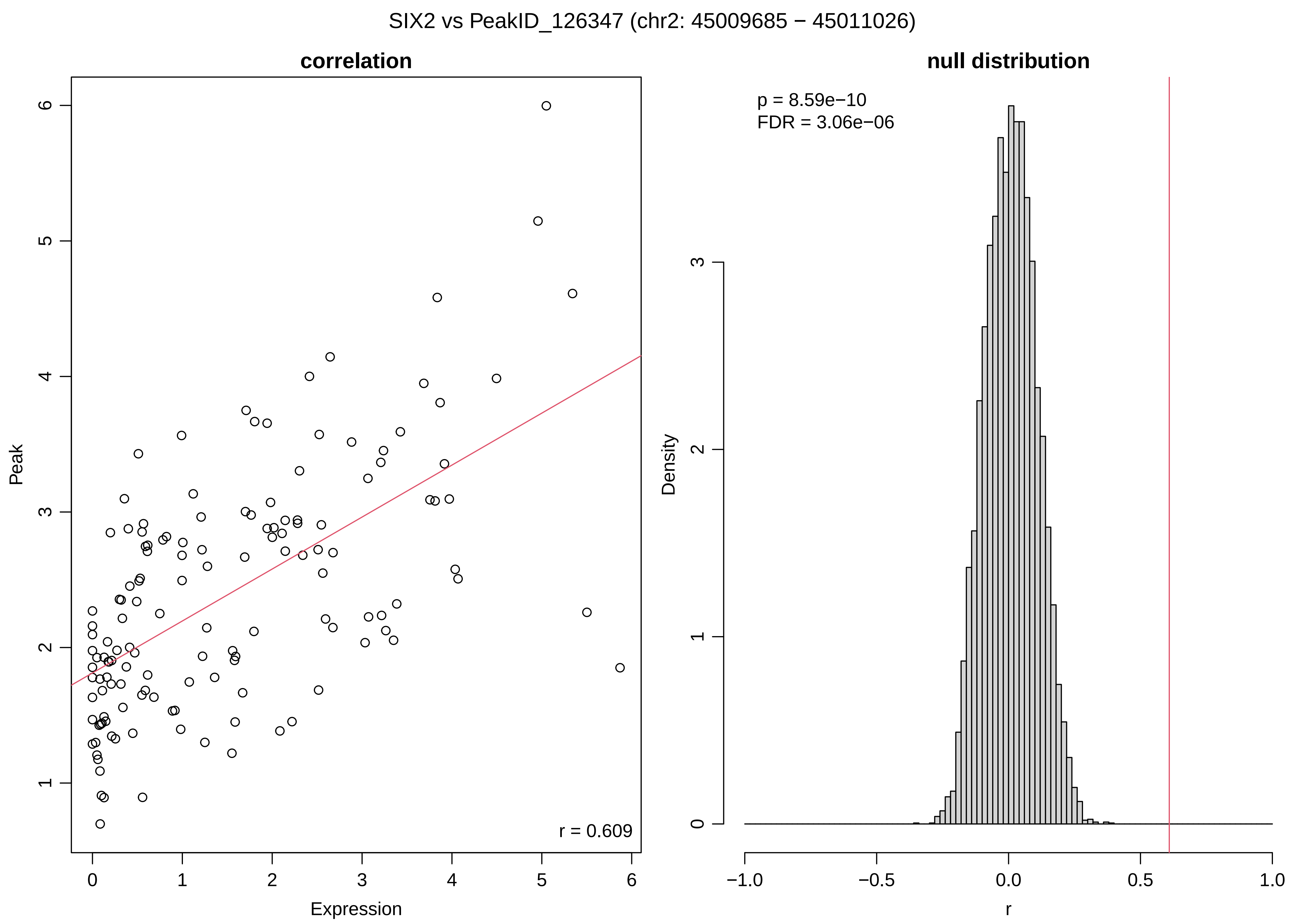
SIX2 vs PeakID_126347 (chr2: 45009685 − 45011026)
correlation
Peak
Expression
r = 0.609
null distribution
p = 8.59e−10
FDR = 3.06e−06
Density
r

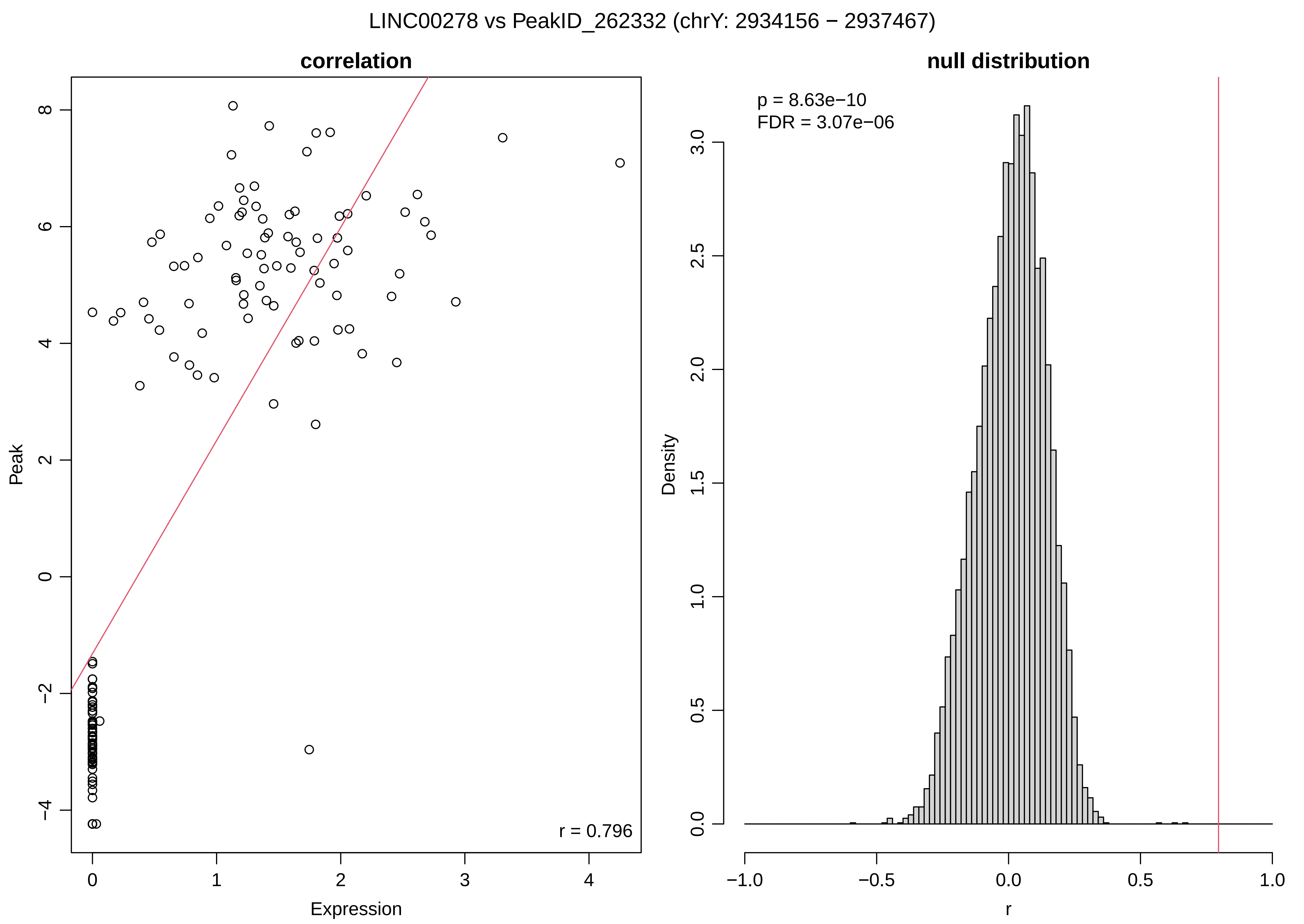

LINC00278 vs PeakID_262332 (chrY: 2934156 − 2937467)
correlation
r = 0.796
Peak
Expression
null distribution
p = 8.63e−10
FDR = 3.07e−06
Density
r

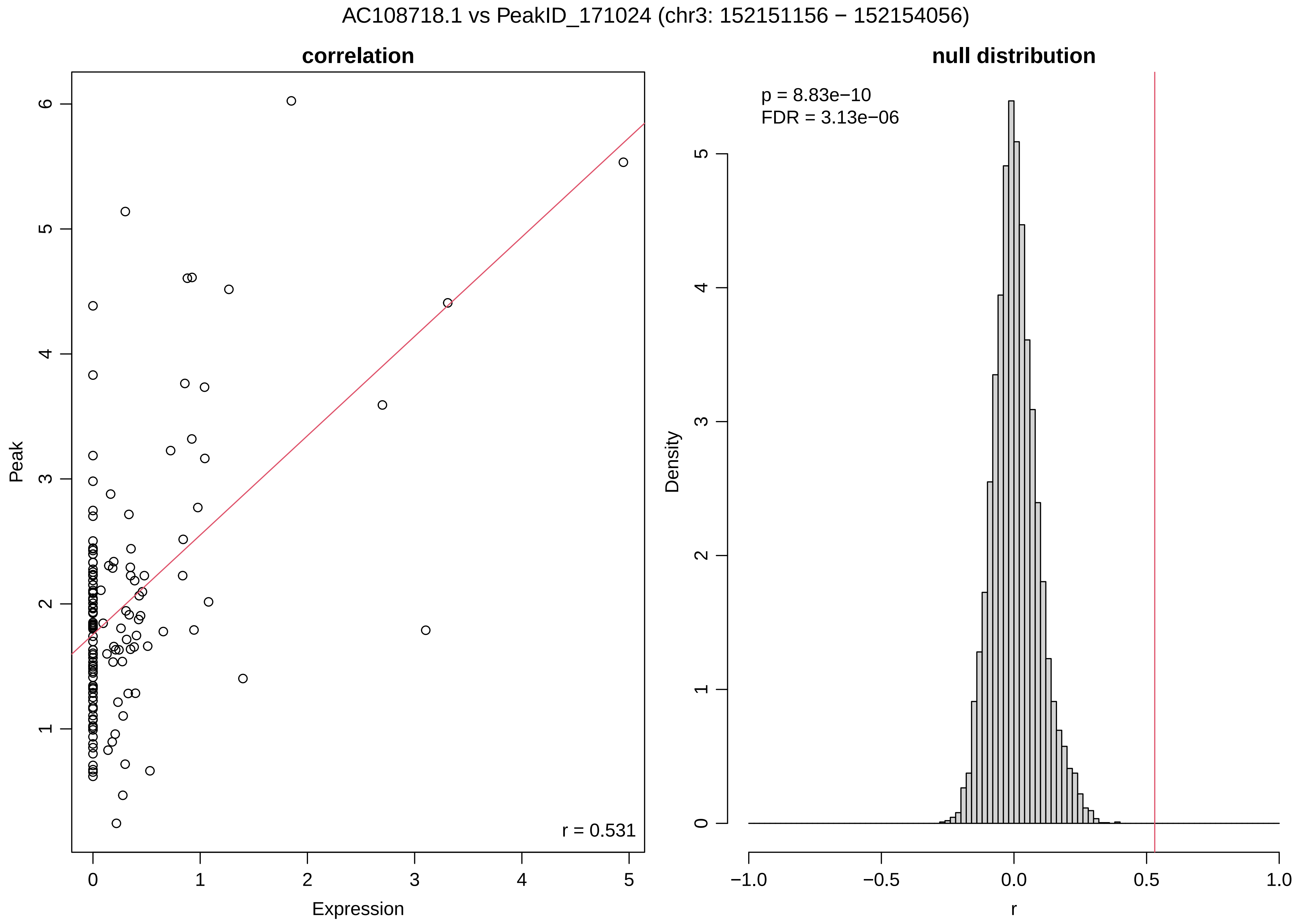

AC108718.1 vs PeakID_171024 (chr3: 152151156 − 152154056)
correlation
Peak
Expression
r = 0.531
null distribution
p = 8.83e−10
FDR = 3.13e−06
Density
r

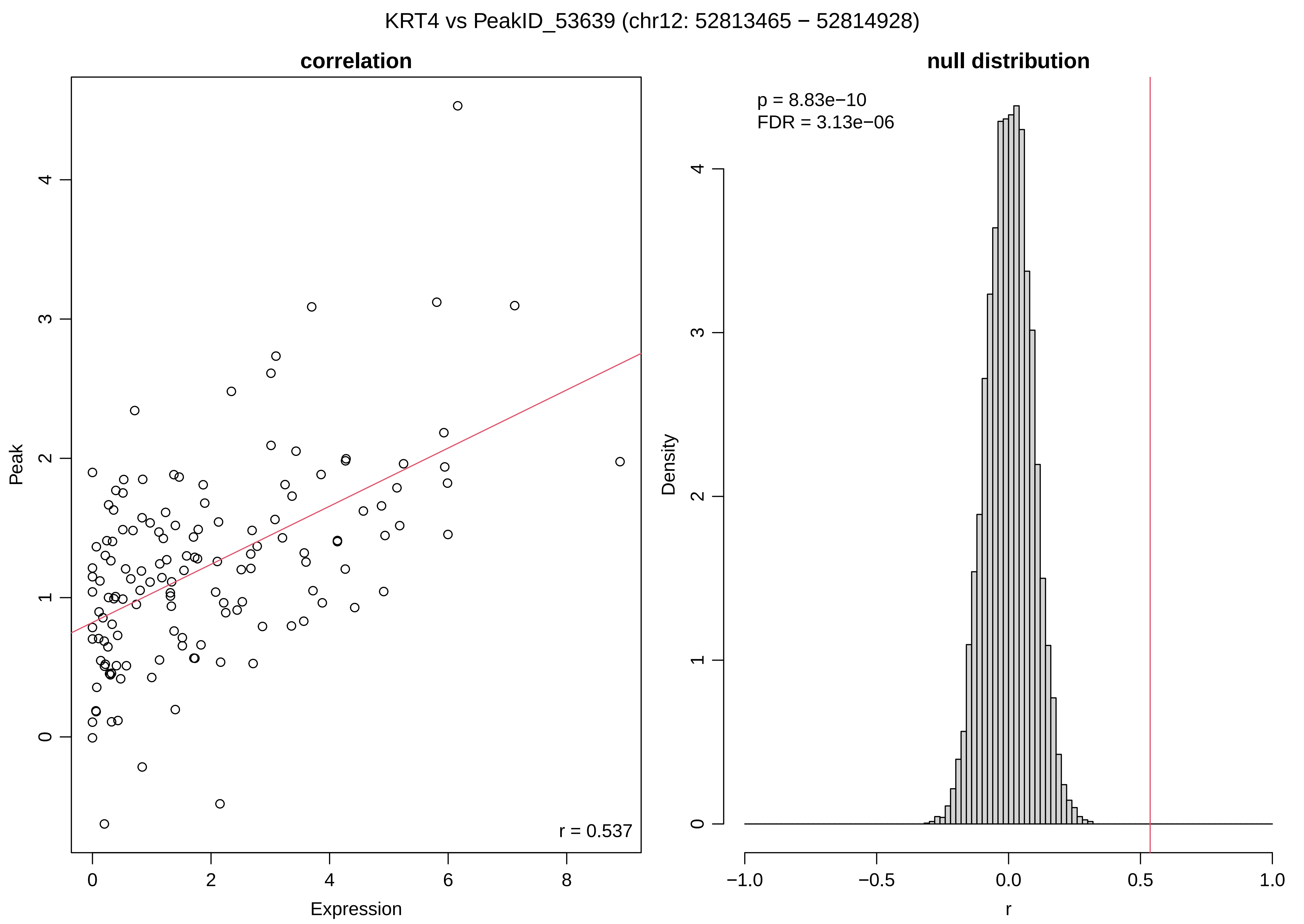
KRT4 vs PeakID_53639 (chr12: 52813465 − 52814928)
correlation
Peak
Expression
r = 0.537
0
1
2
3
4
0
2
4
6
8
null distribution
p = 8.83e−10
FDR = 3.13e−06
Density
r
0
1
2
3
4
−1.0
−0.5
0.0
0.5
1.0

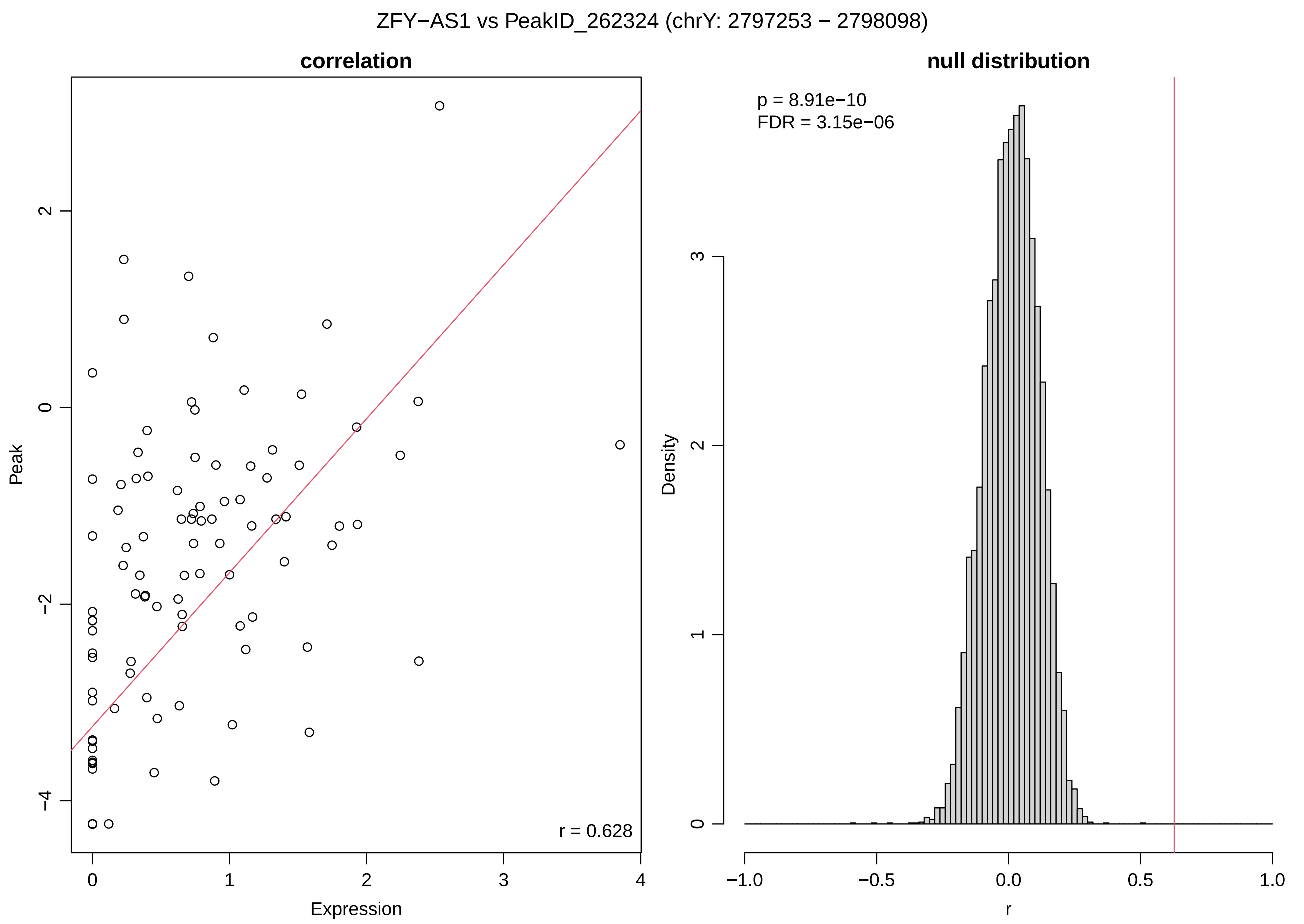
ZFY−AS1 vs PeakID_262324 (chrY: 2797253 − 2798098)
correlation
null distribution
p = 8.91e−10
FDR = 3.15e−06
r = 0.628
Peak
Expression
Density
r

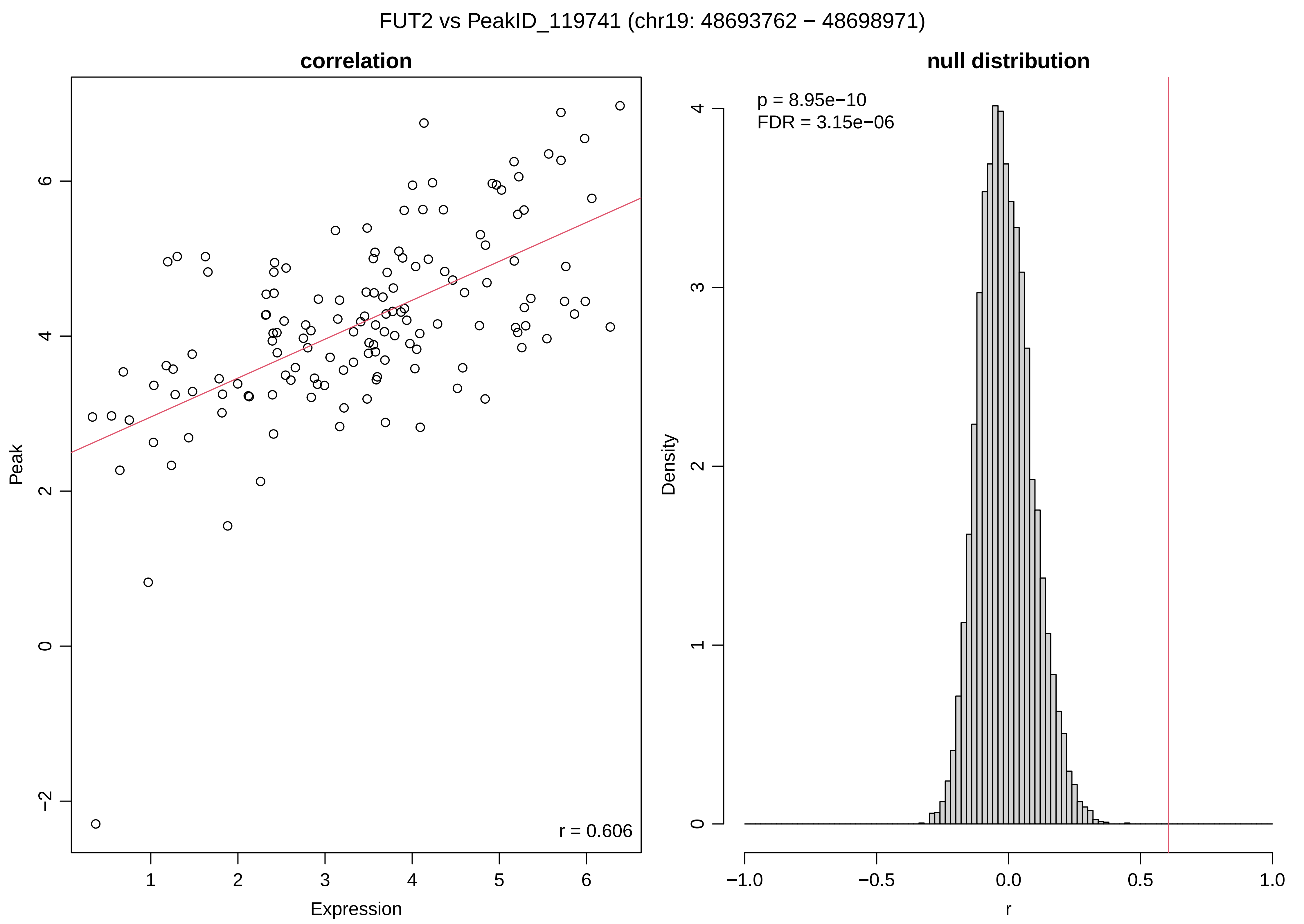
FUT2 vs PeakID_119741 (chr19: 48693762 − 48698971)
correlation
null distribution
Peak
Expression
r = 0.606
Density
r
p = 8.95e−10
FDR = 3.15e−06
−2
0
2
4
6
1
2
3
4
5
6
−1.0
−0.5
0.0
0.5
1.0

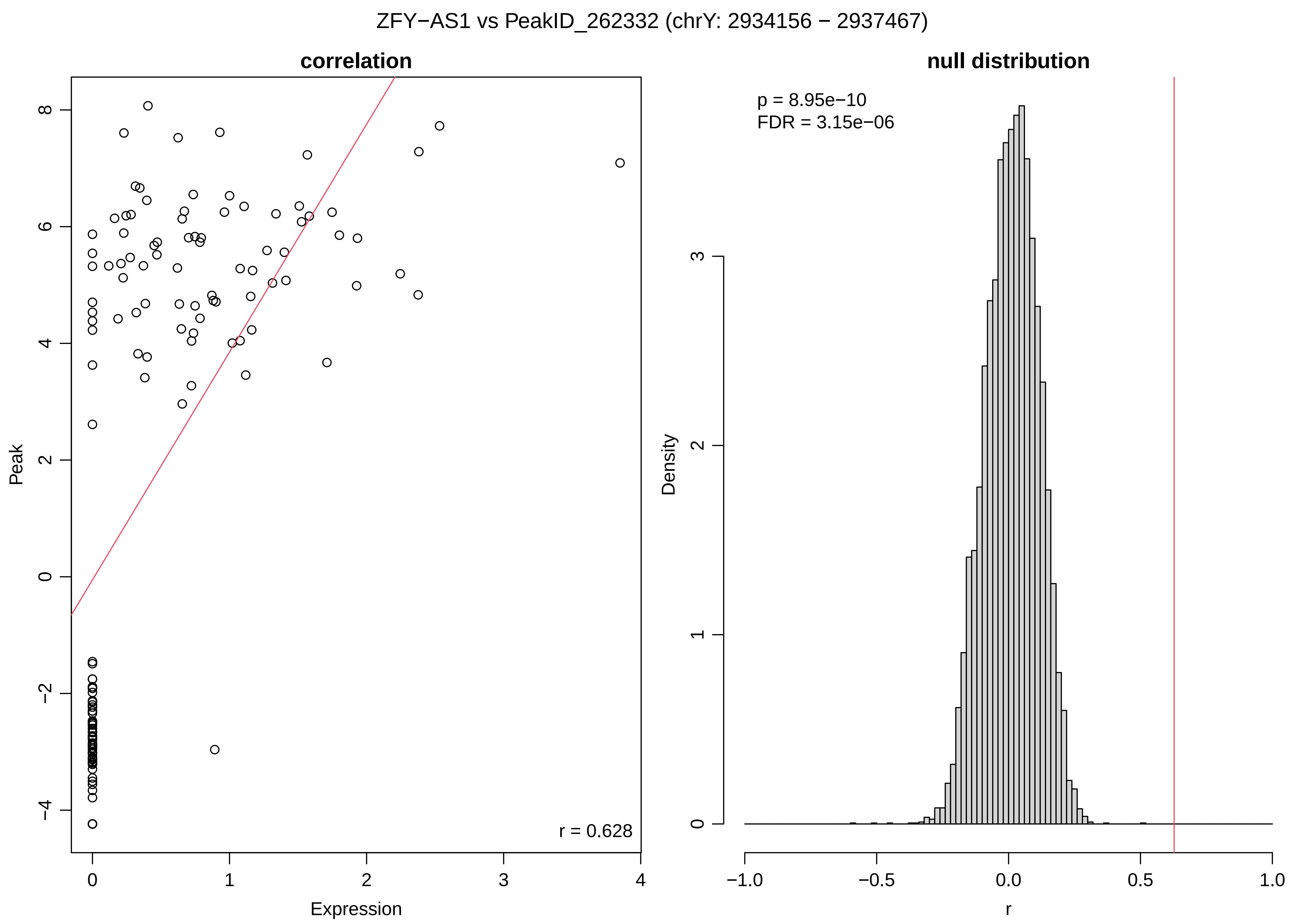

ZFY−AS1 vs PeakID_262332 (chrY: 2934156 − 2937467)
correlation
Peak
Expression
r = 0.628
8
6
4
2
0
−2
−4
0
1
2
3
4
null distribution
p = 8.95e−10
FDR = 3.15e−06
Density
r
3
2
1
0
−1.0
−0.5
0.0
0.5
1.0

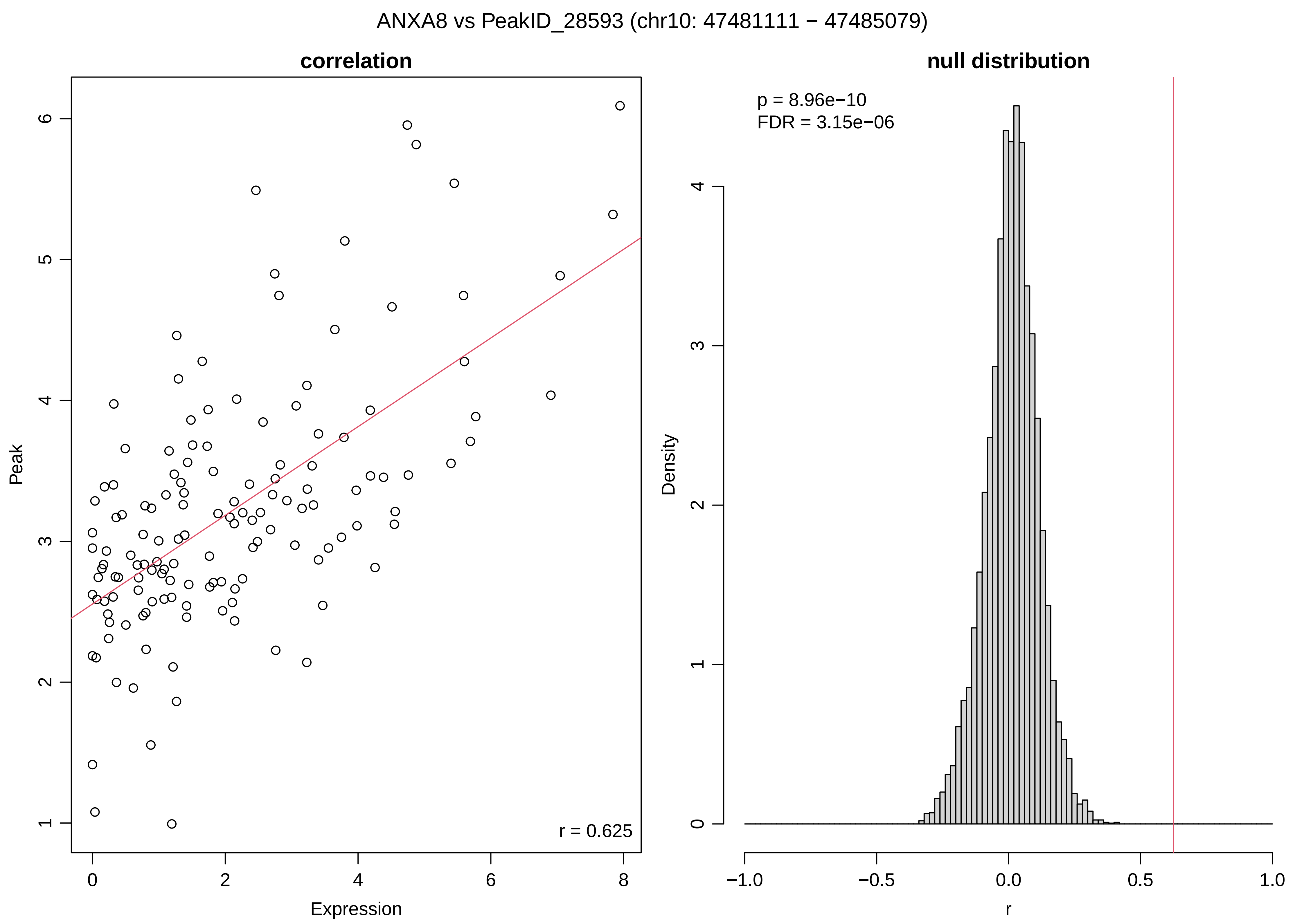
ANXA8 vs PeakID_28593 (chr10: 47481111 − 47485079)
correlation
Peak
Expression
r = 0.625
null distribution
p = 8.96e−10
FDR = 3.15e−06
Density
r

AC107881.1 vs PeakID_37993 (chr11: 12720546 − 12720892)

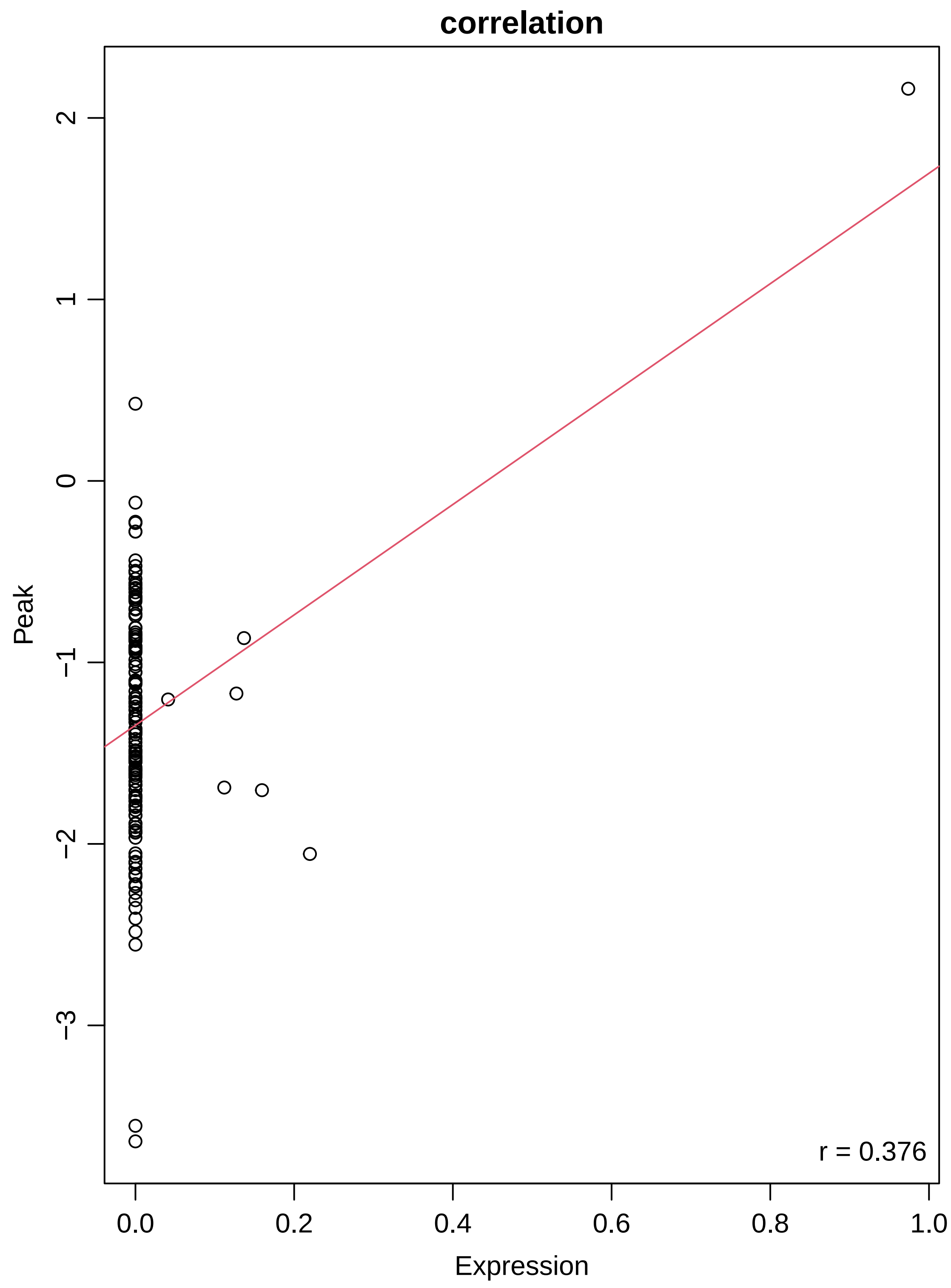

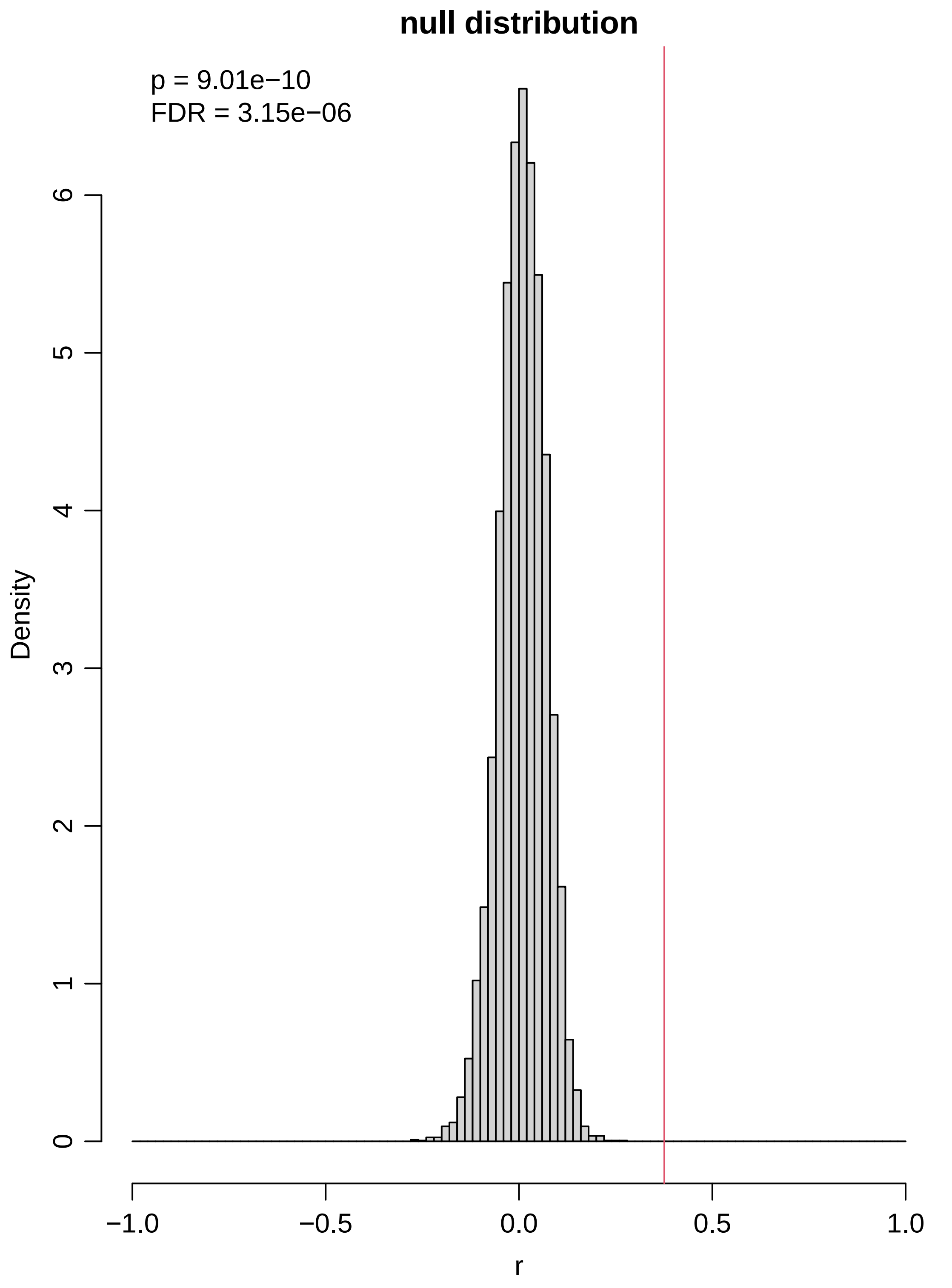

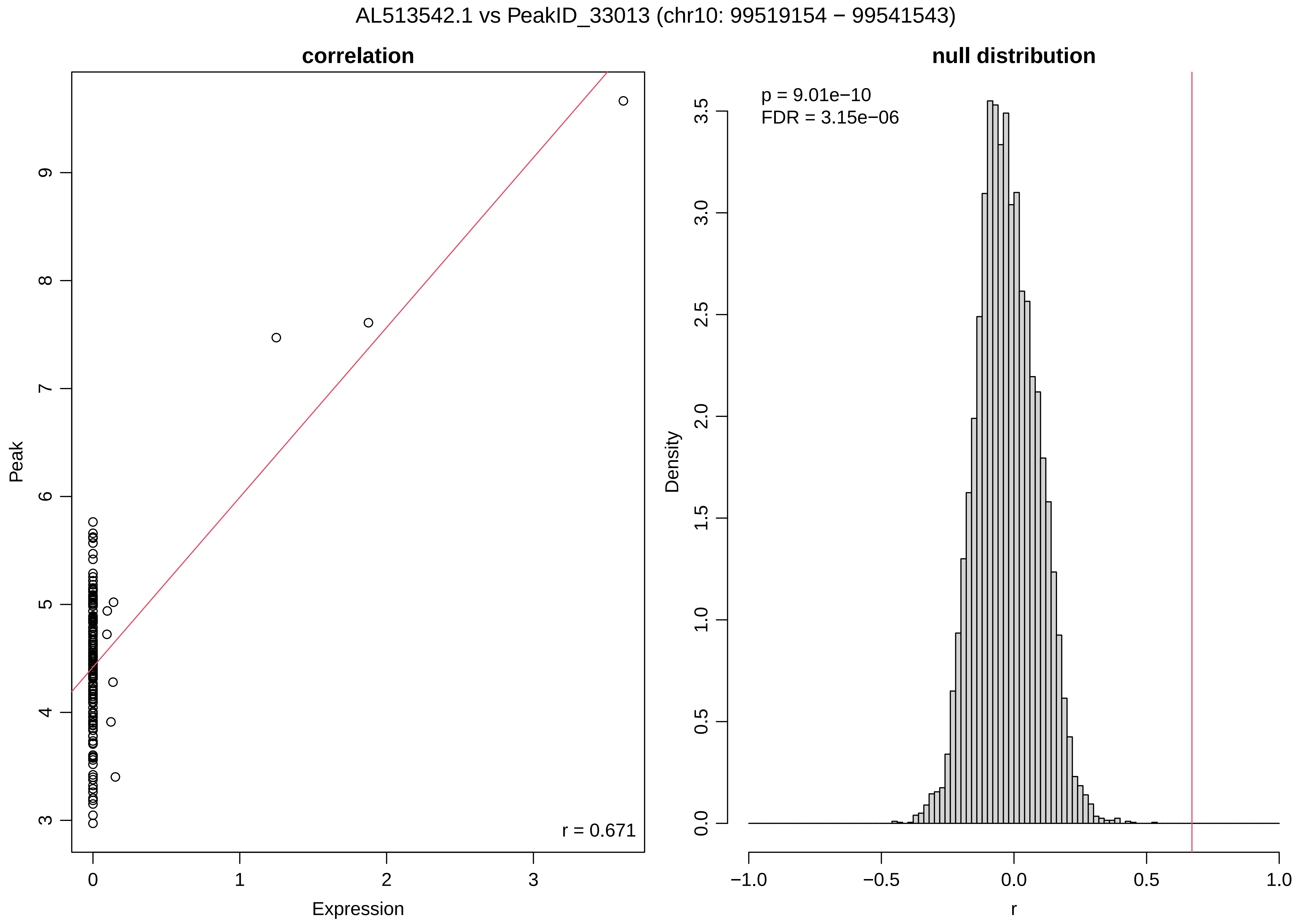
AL513542.1 vs PeakID_33013 (chr10: 99519154 − 99541543)
correlation
Peak
Expression
r = 0.671
null distribution
p = 9.01e−10
FDR = 3.15e−06
Density
r

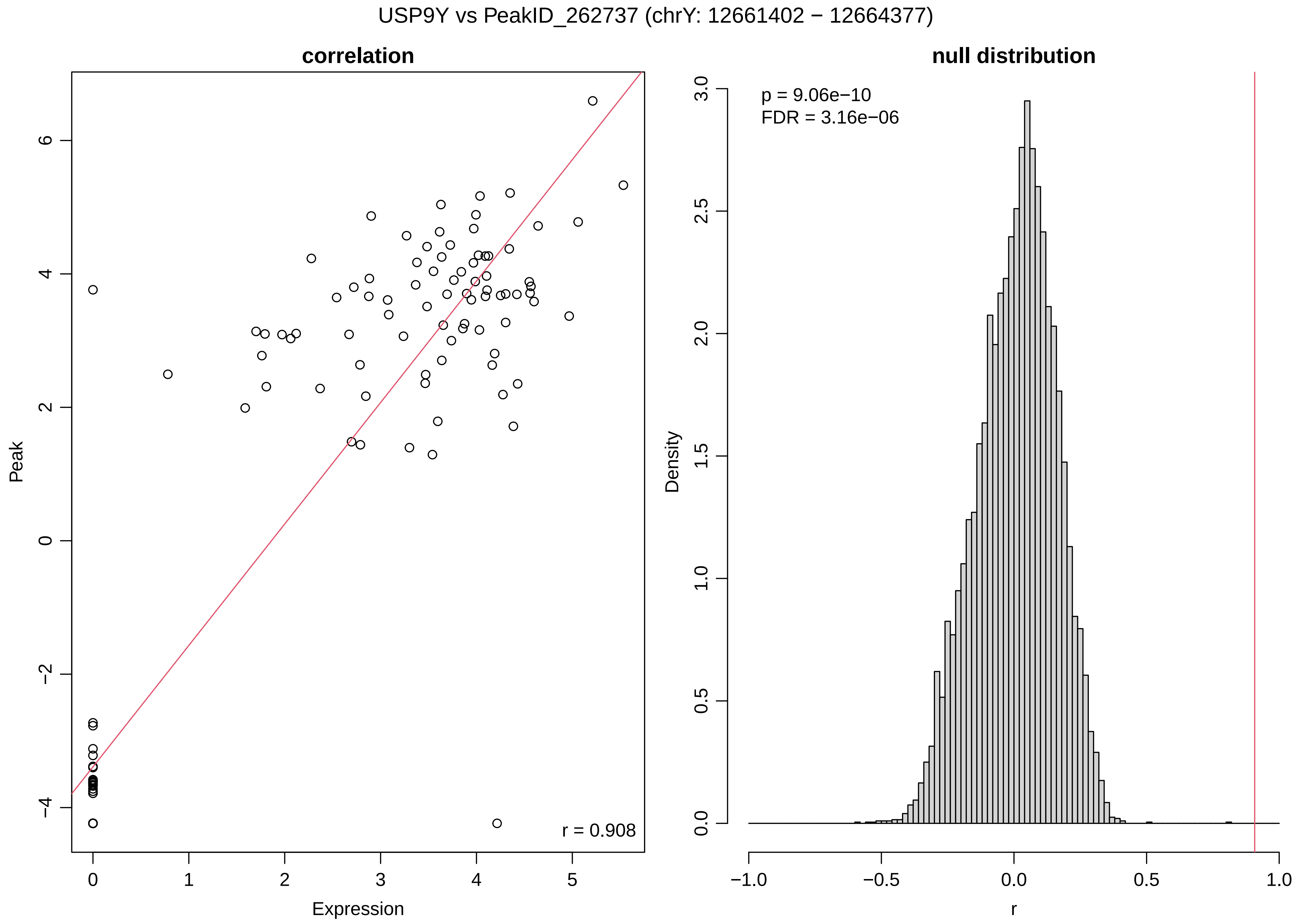
USP9Y vs PeakID_262737 (chrY: 12661402 − 12664377)
correlation
Peak
Expression
r = 0.908
null distribution
p = 9.06e−10
FDR = 3.16e−06
Density
r

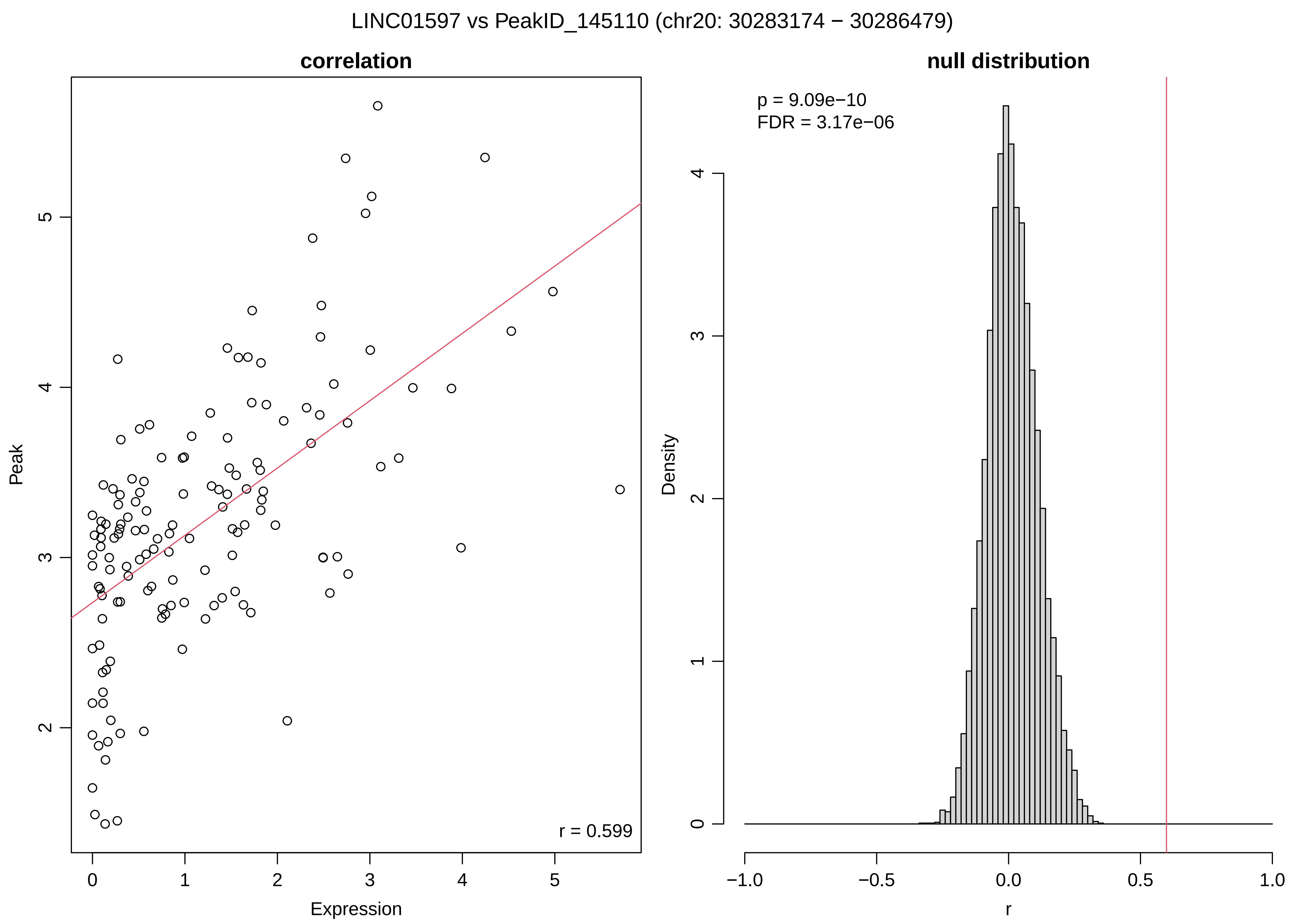
LINC01597 vs PeakID_145110 (chr20: 30283174 − 30286479)
correlation
Peak
Expression
r = 0.599
null distribution
p = 9.09e−10
FDR = 3.17e−06
Density
r

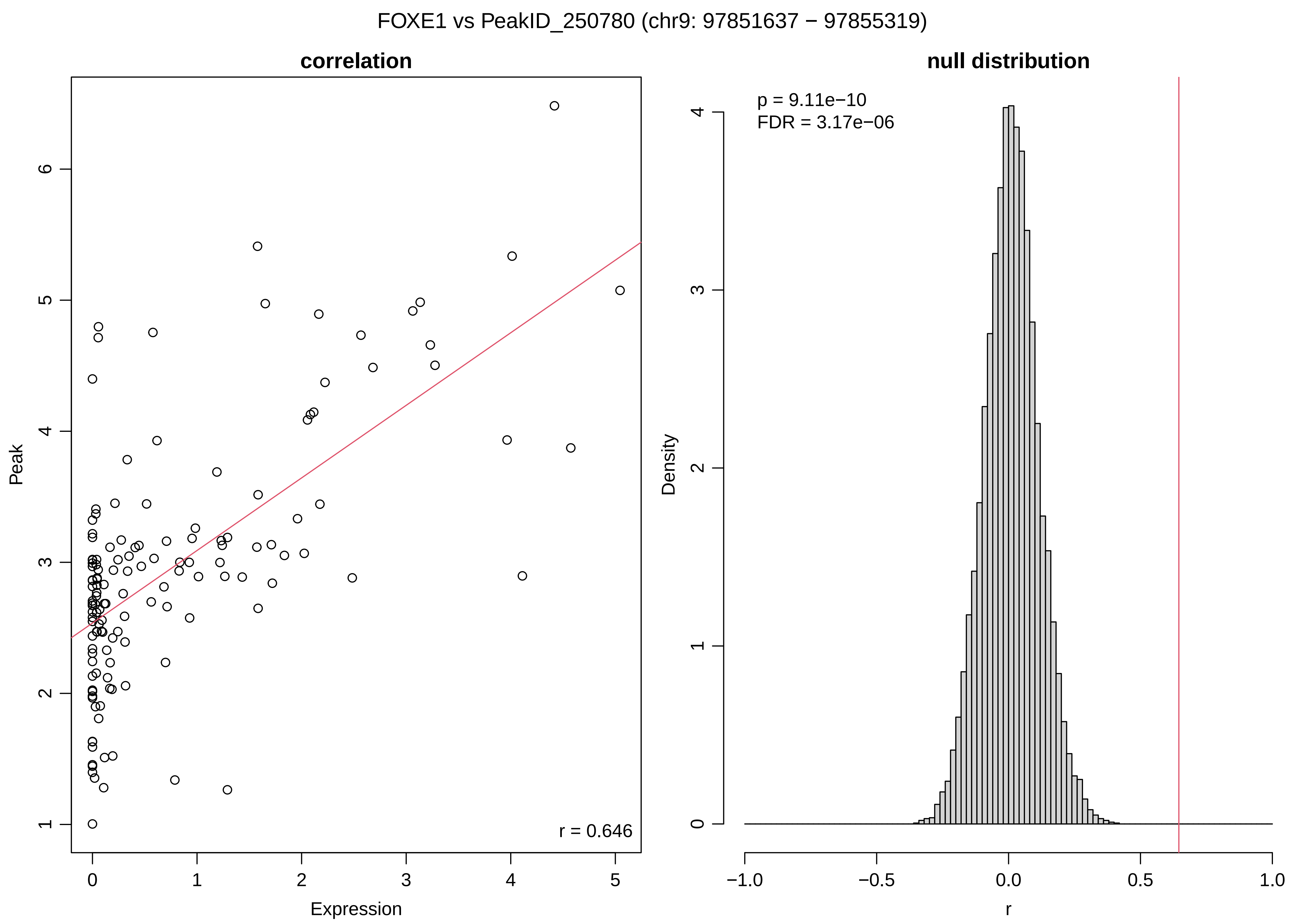

FOXE1 vs PeakID_250780 (chr9: 97851637 − 97855319)
correlation
Peak
Expression
r = 0.646
null distribution
p = 9.11e−10
FDR = 3.17e−06
Density
r

LINC01108 vs PeakID_203036 (chr6: 14299828 − 14300716)

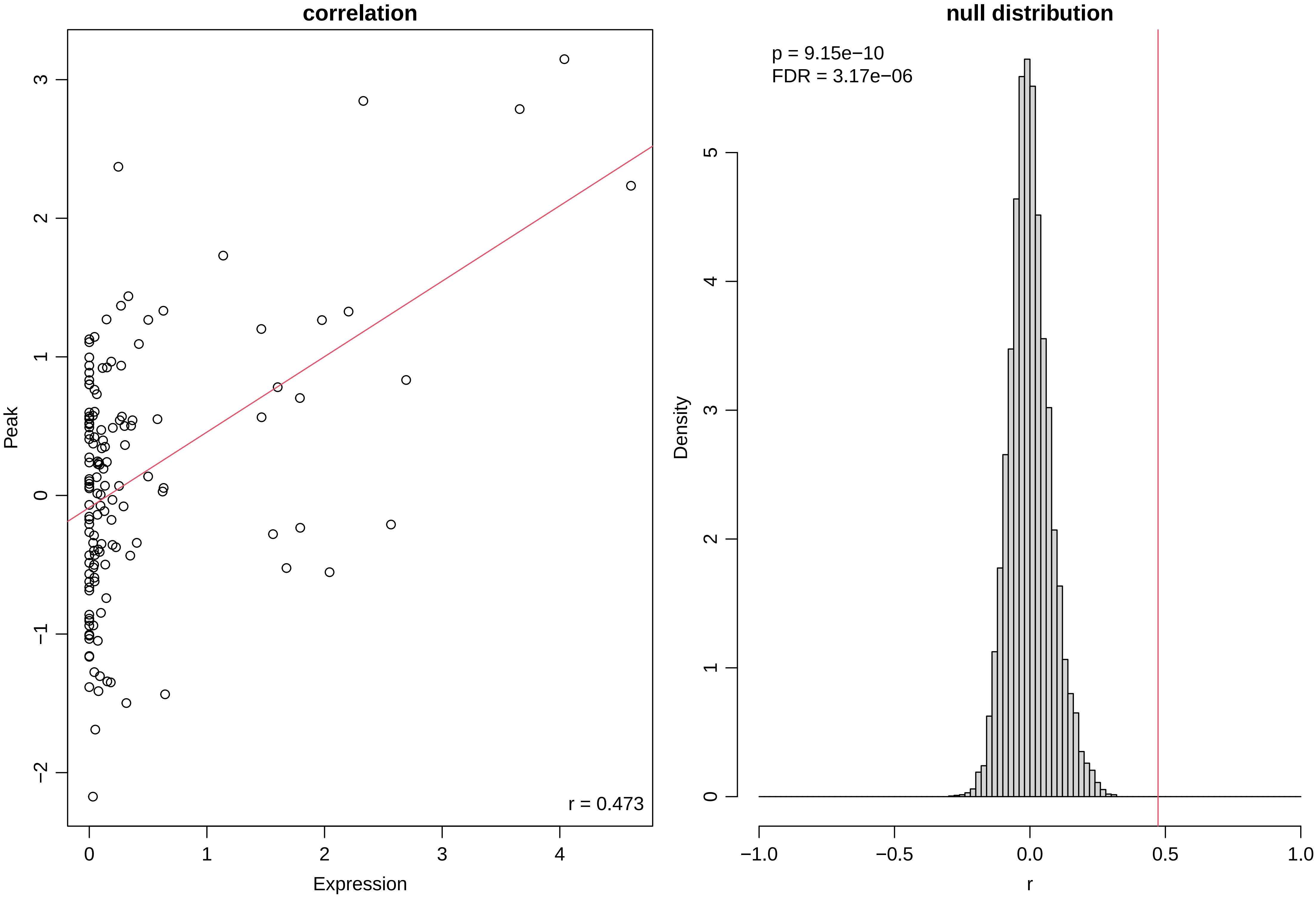

SLC35E3 vs PeakID_56613 (chr12: 68873064 − 68874900)

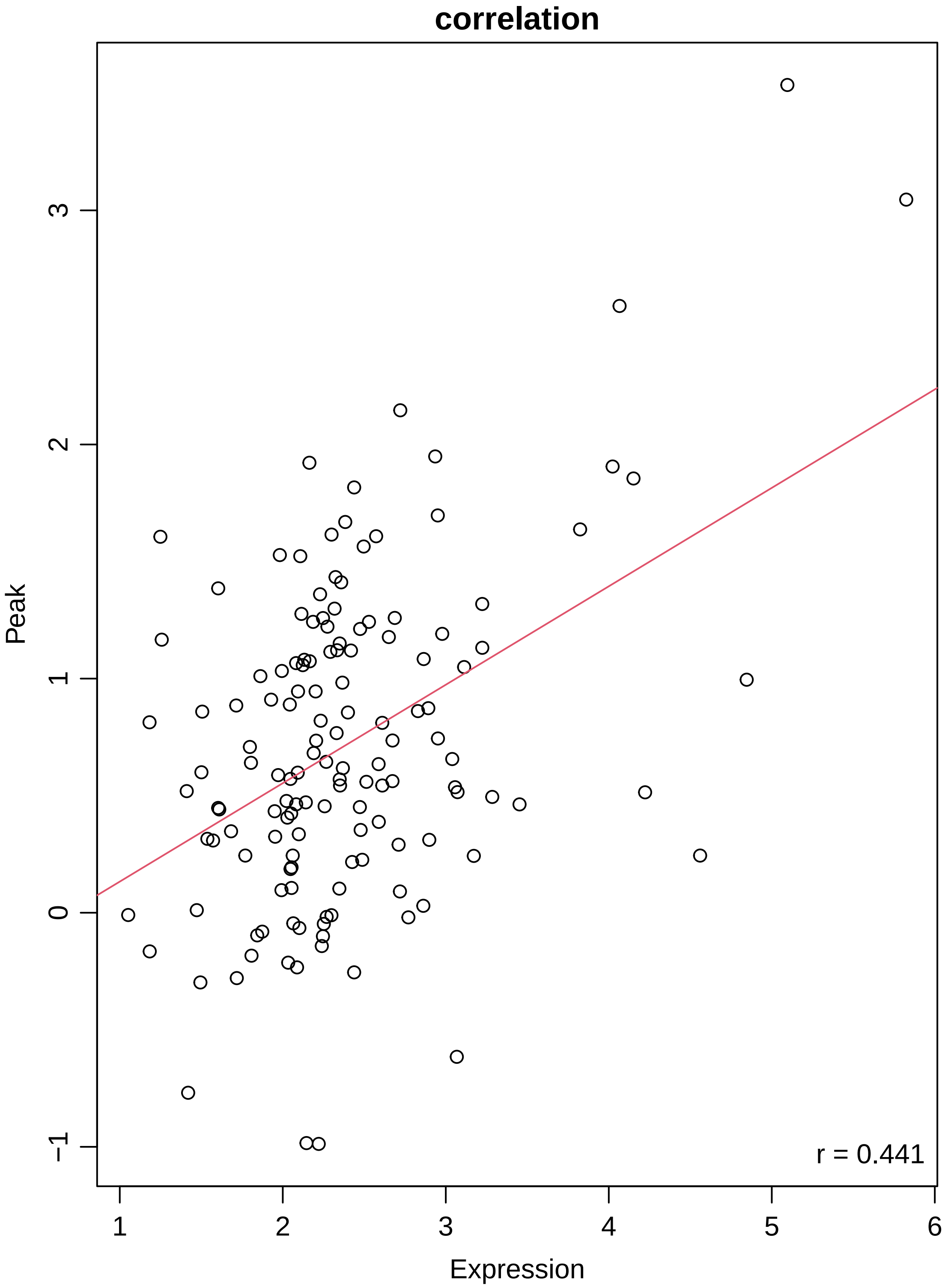

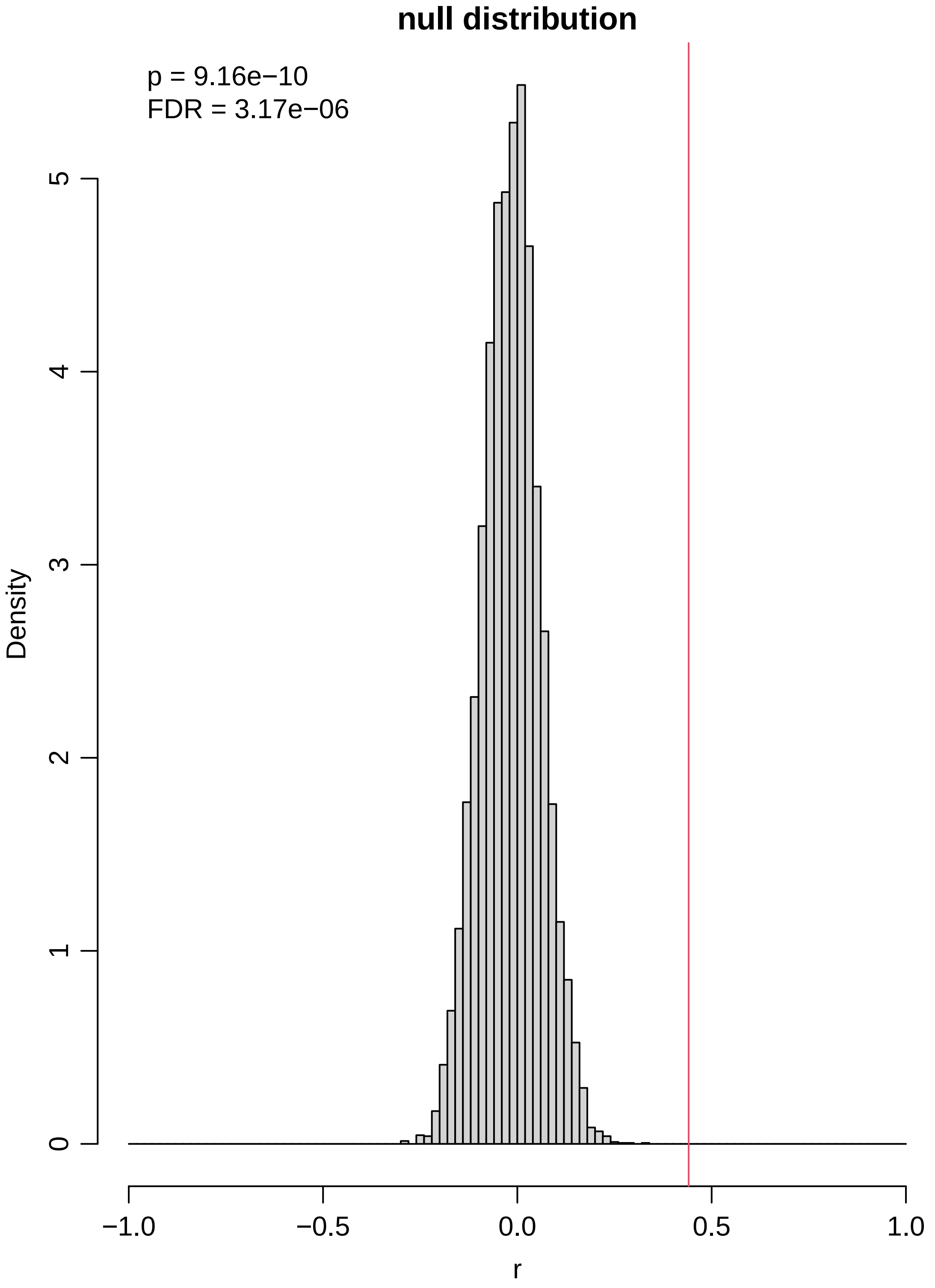

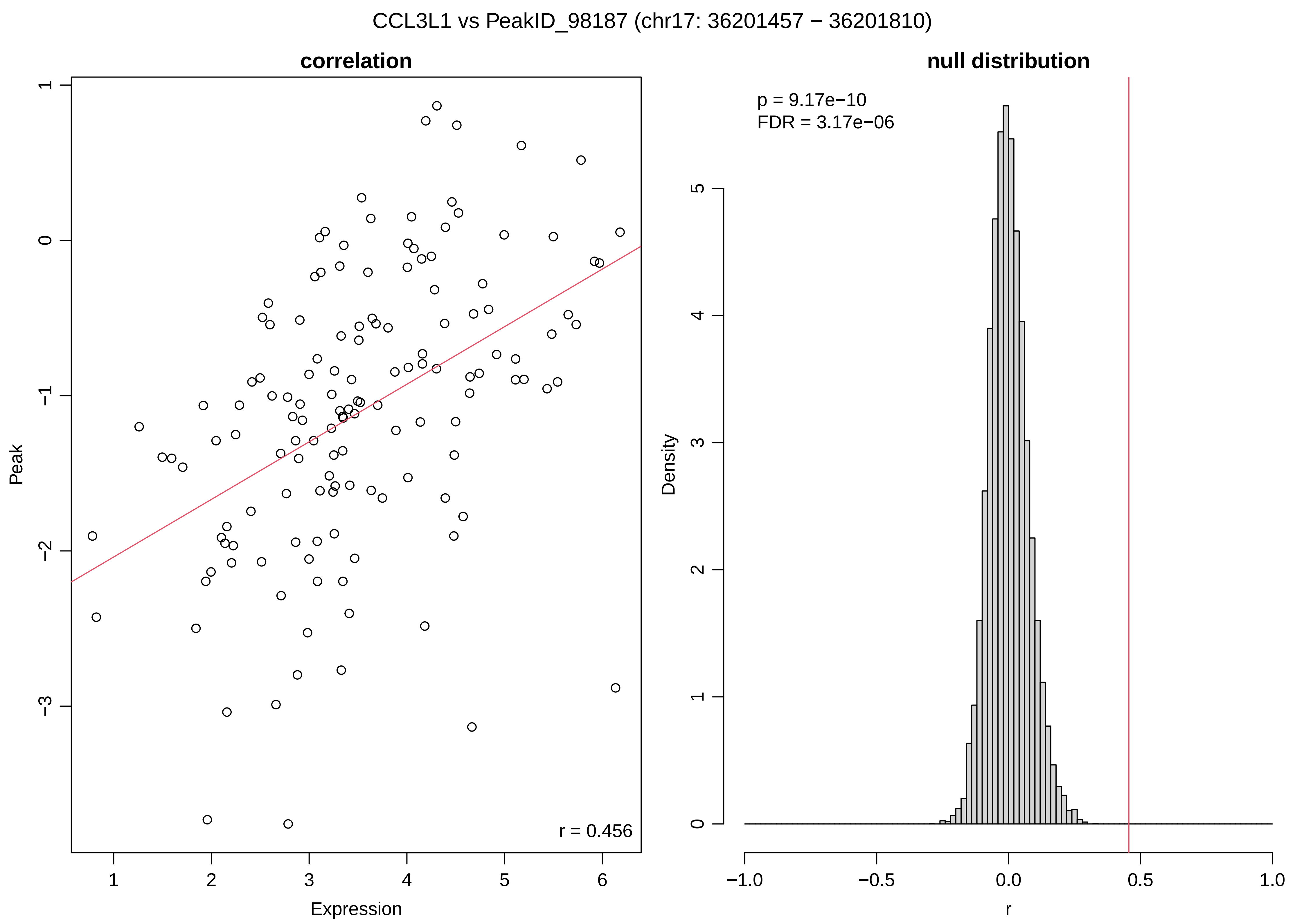
CCL3L1 vs PeakID_98187 (chr17: 36201457 − 36201810)
correlation
Peak
Expression
r = 0.456
null distribution
p = 9.17e−10
FDR = 3.17e−06
Density
r

RDH16 vs PeakID_54191 (chr12: 56839356 − 56844533)

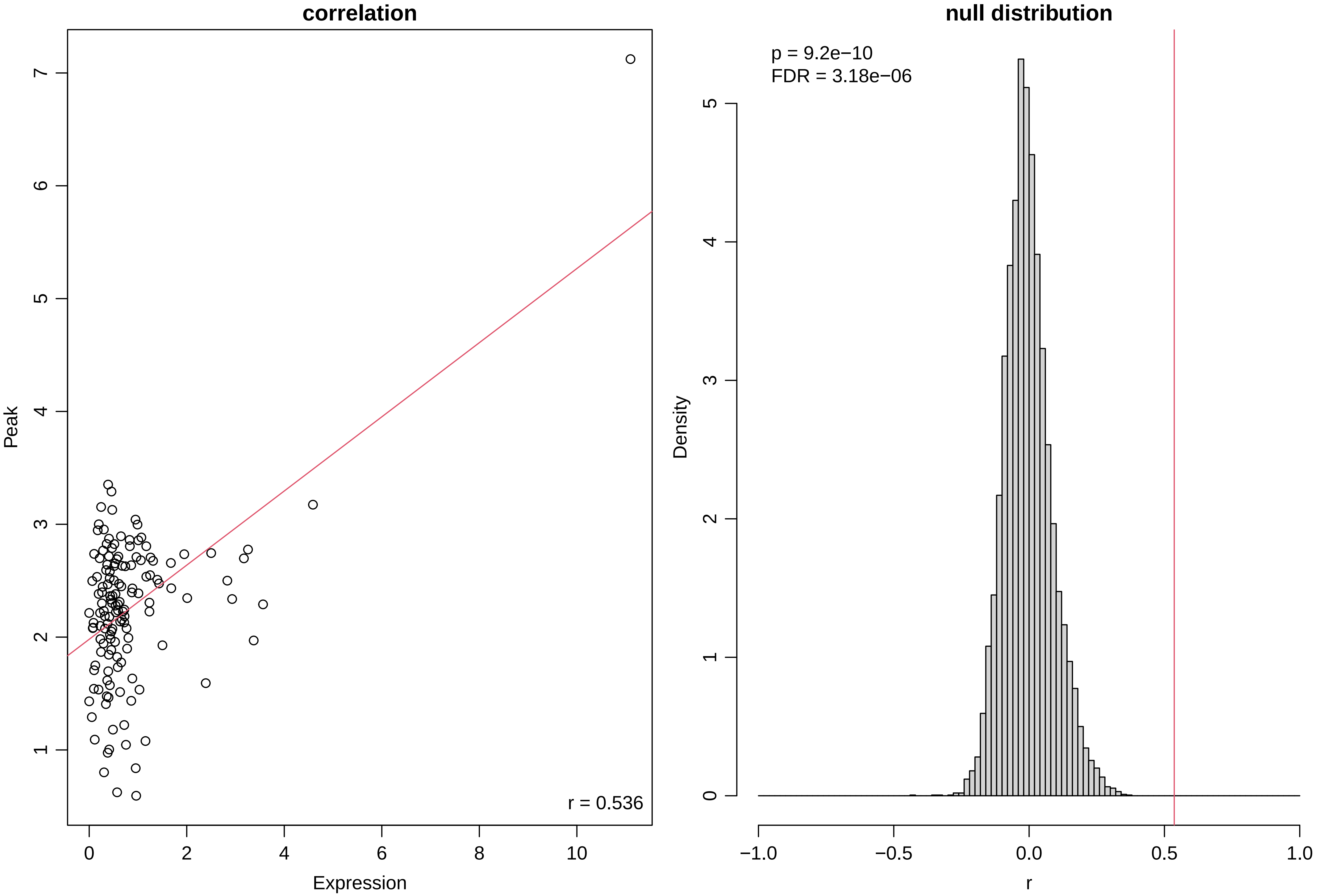

LINC01060 vs PeakID_186372 (chr4: 188444744 − 188445635)

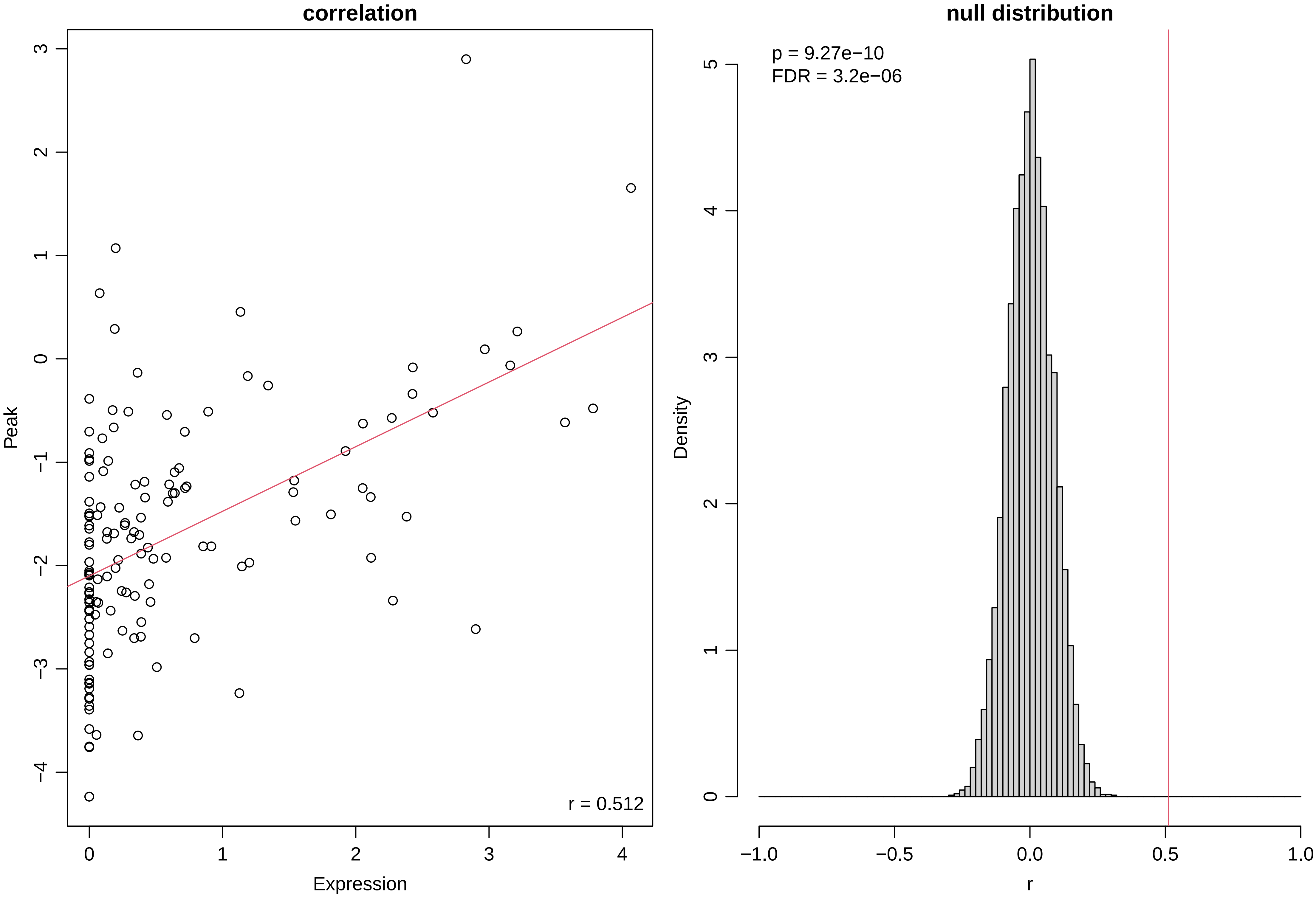

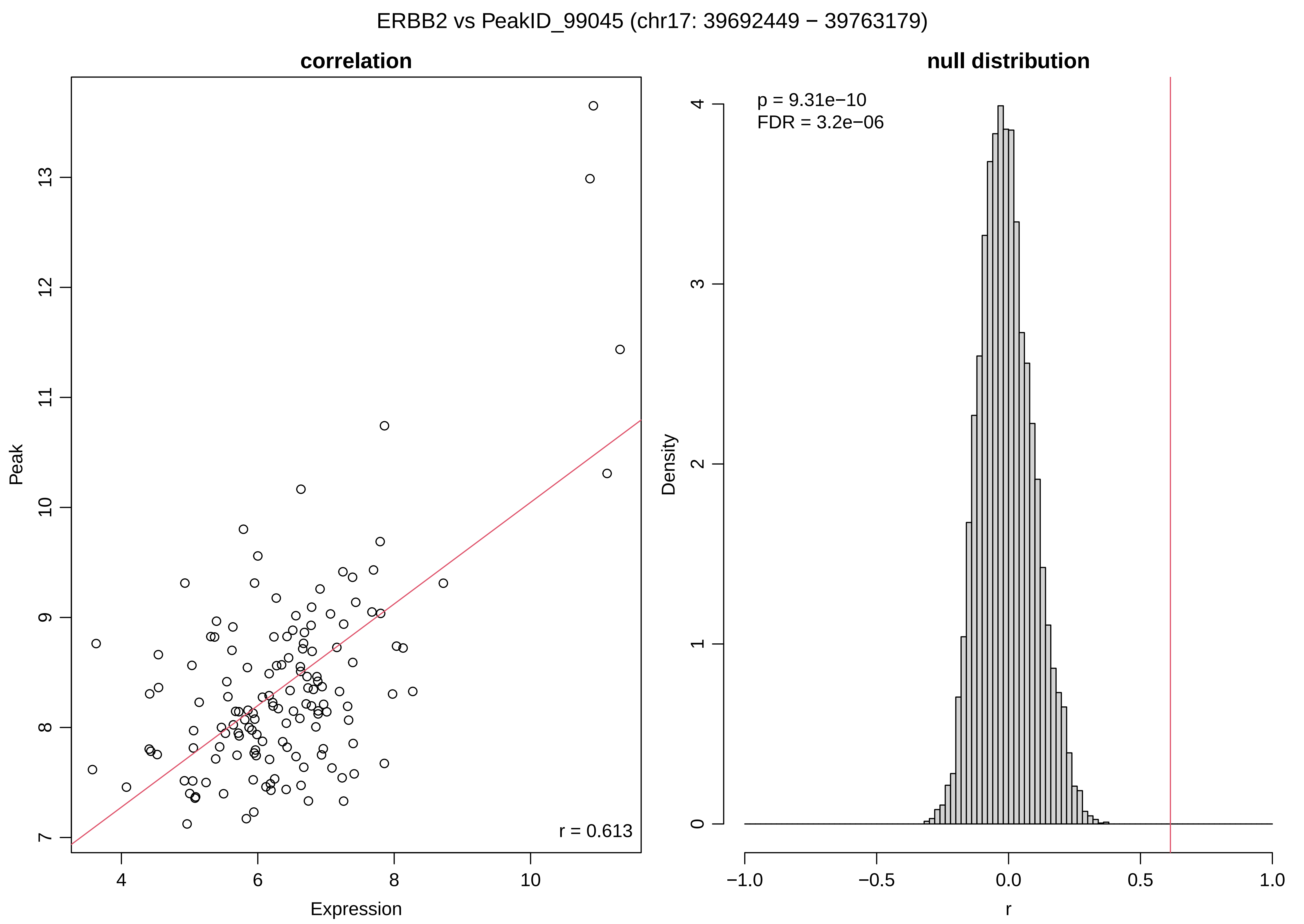
ERBB2 vs PeakID_99045 (chr17: 39692449 − 39763179)
correlation
Peak
Expression
r = 0.613
null distribution
p = 9.31e−10
FDR = 3.2e−06
Density
r

TXLNGY vs PeakID_262949 (chrY: 19816109 − 19817047)

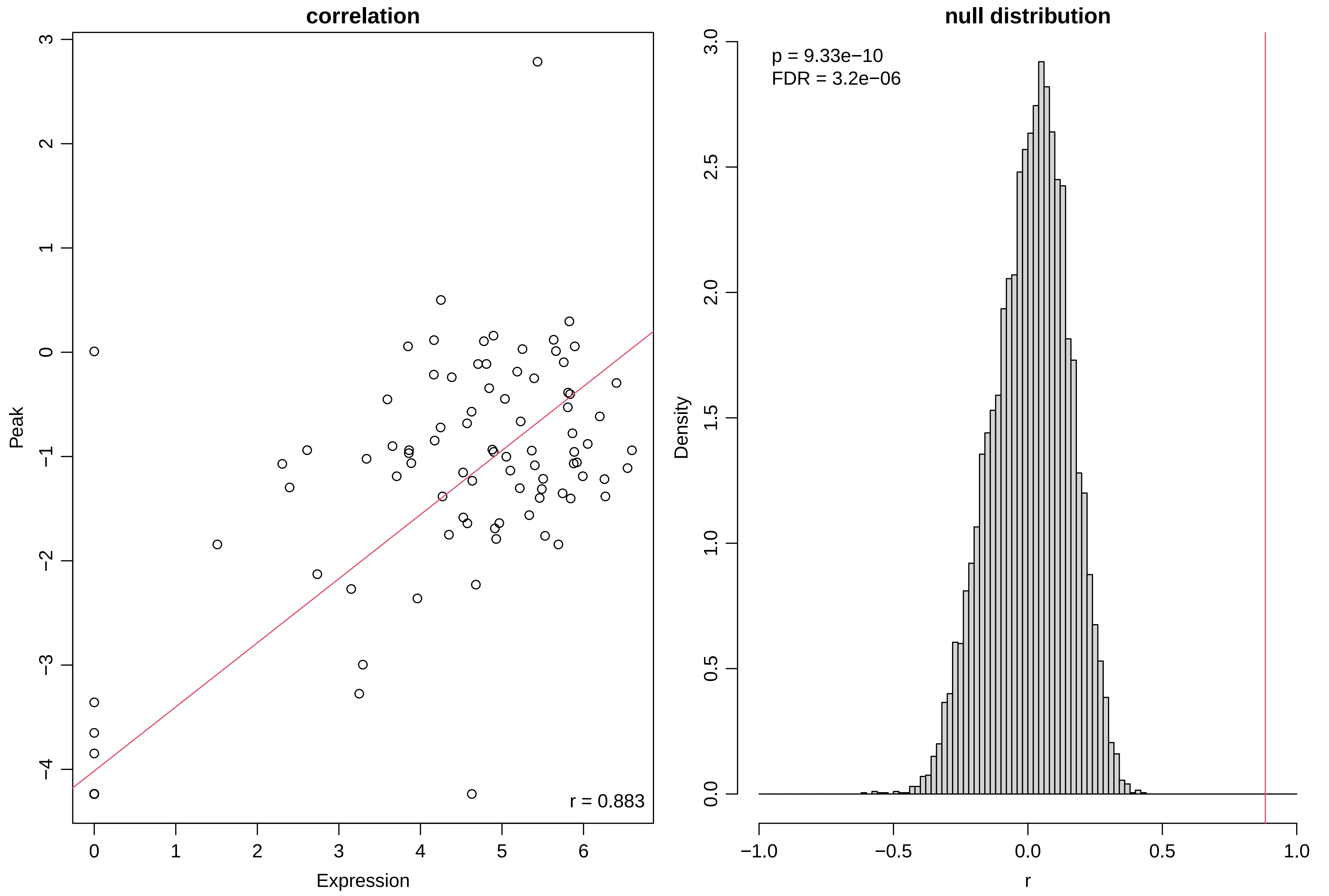

AL161630.1 vs PeakID_252634 (chr9: 117642402 − 117643930)

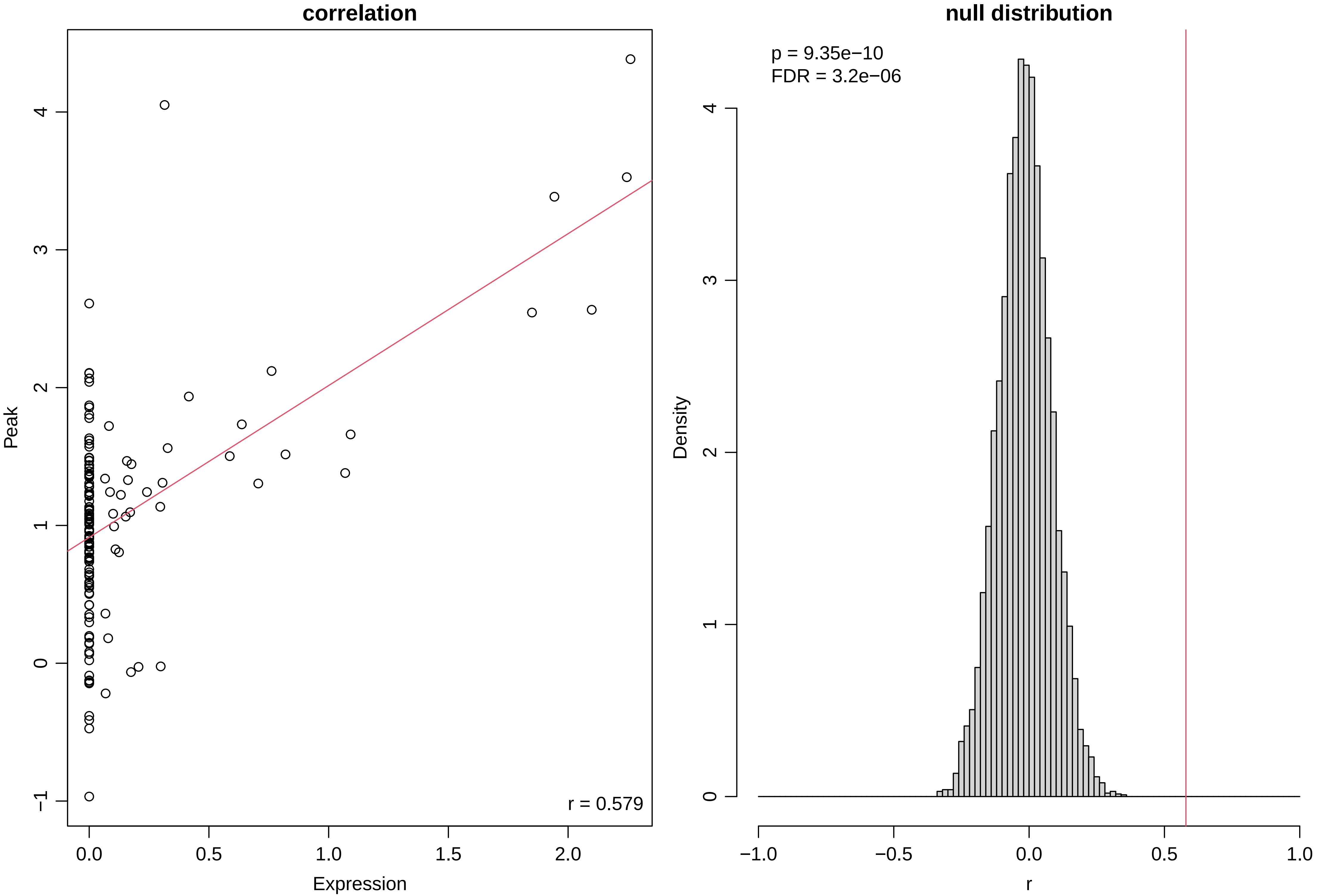

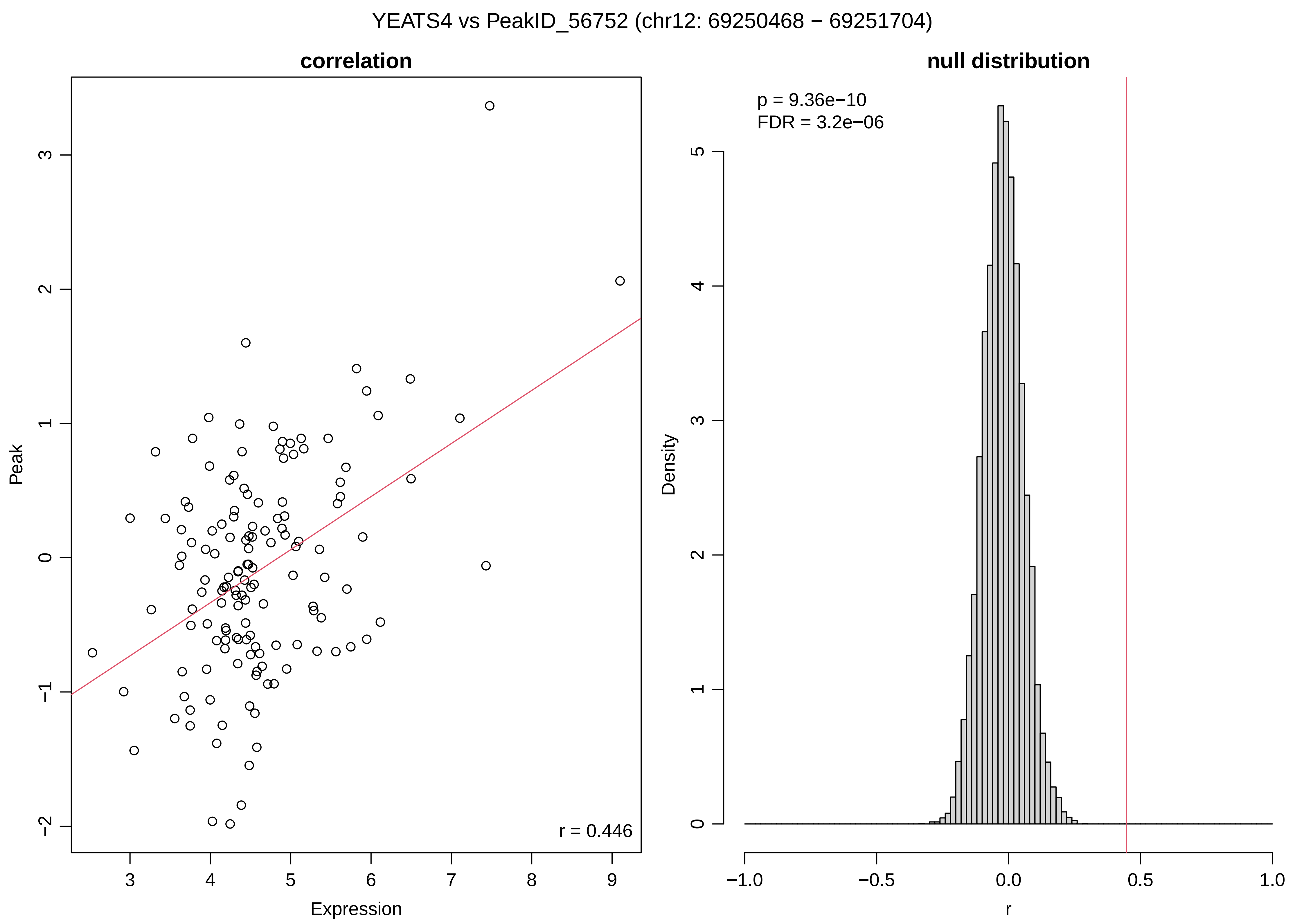
YEATS4 vs PeakID_56752 (chr12: 69250468 − 69251704)
correlation
Peak
Expression
r = 0.446
null distribution
p = 9.36e−10
FDR = 3.2e−06
Density
r

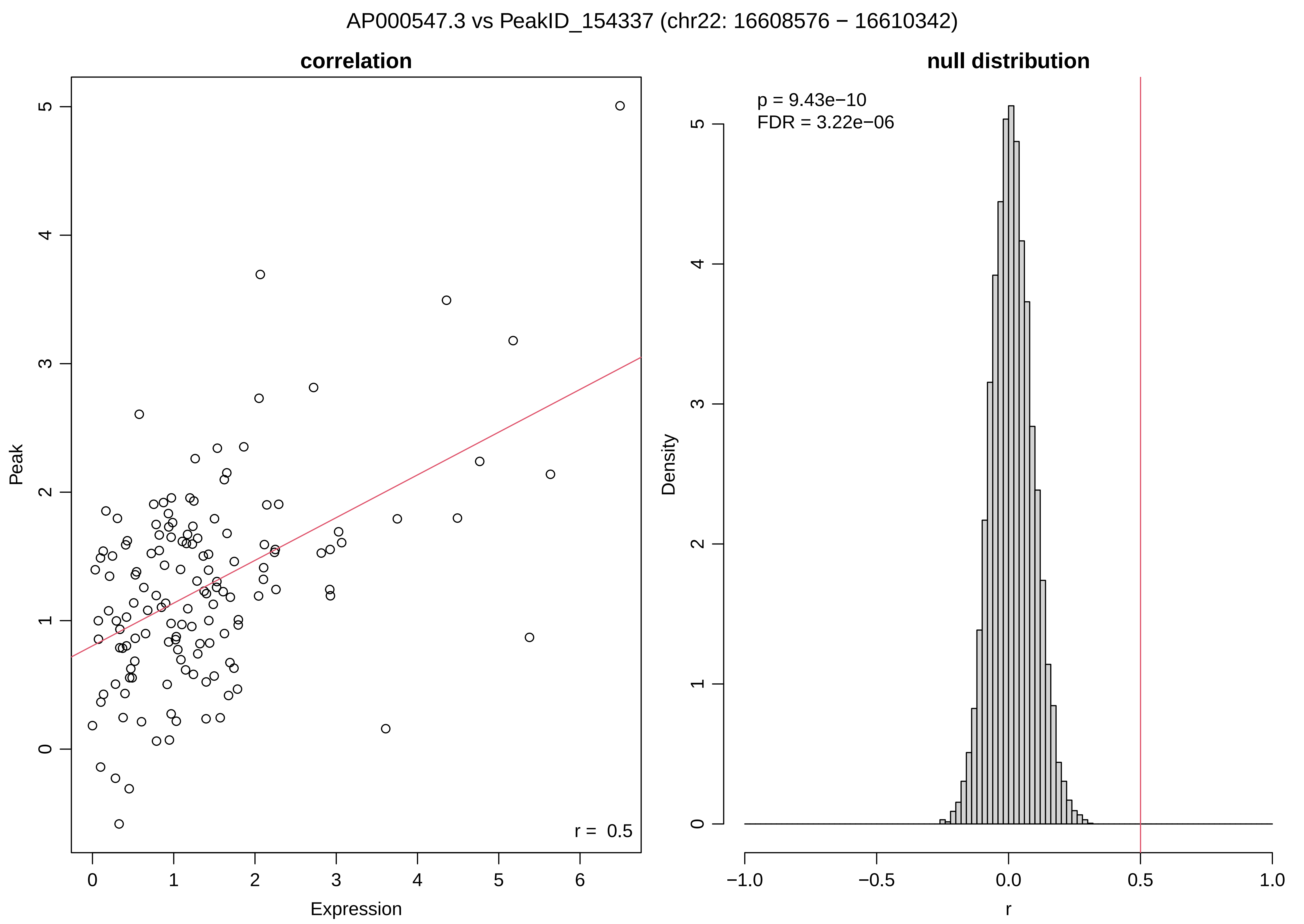

AP000547.3 vs PeakID_154337 (chr22: 16608576 − 16610342)
correlation
null distribution
p = 9.43e−10
FDR = 3.22e−06
r = 0.5
Peak
Expression
Density
r

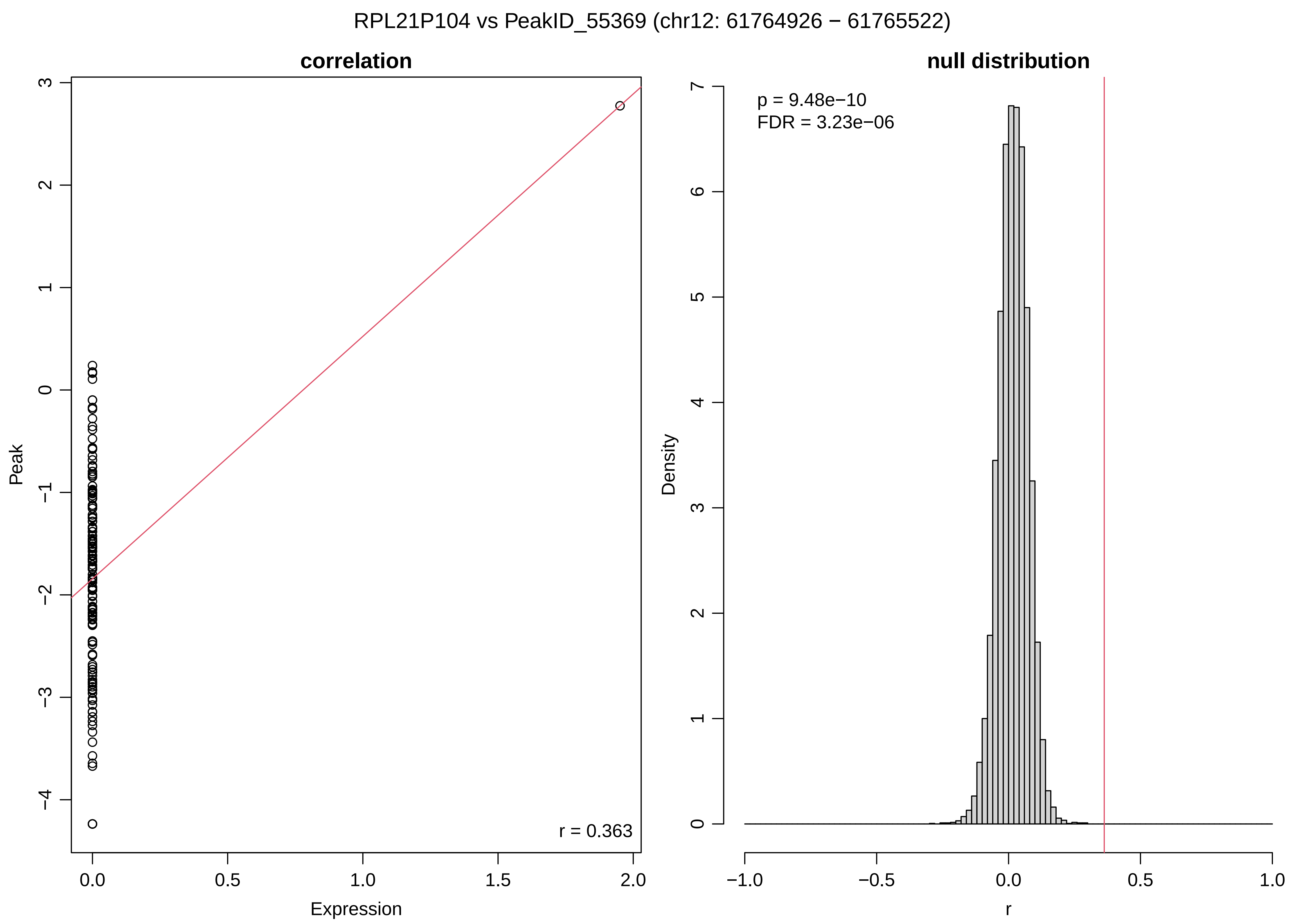

RPL21P104 vs PeakID_55369 (chr12: 61764926 − 61765522)
correlation
Peak
Expression
r = 0.363
null distribution
p = 9.48e−10
FDR = 3.23e−06
Density
r

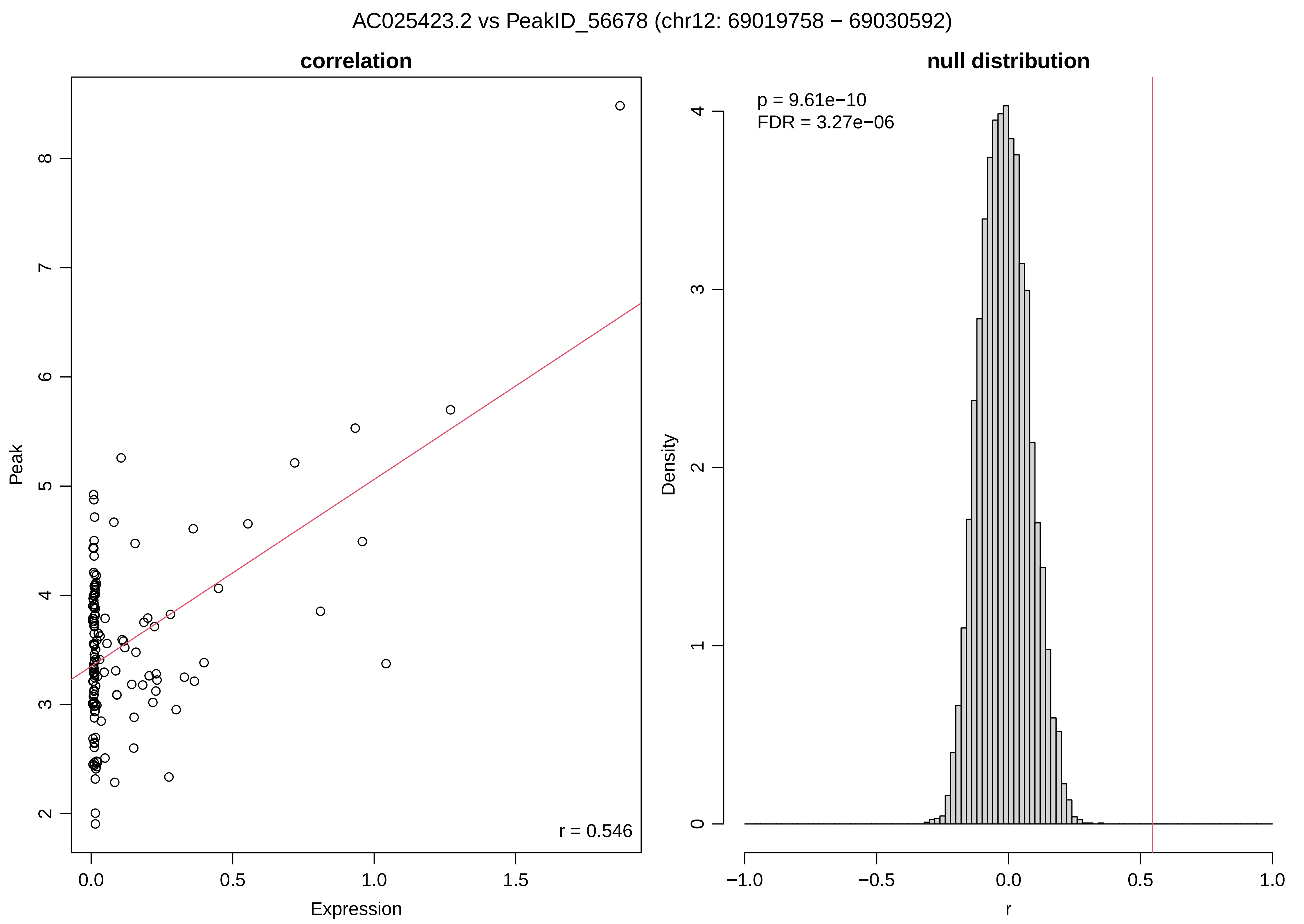
AC025423.2 vs PeakID_56678 (chr12: 69019758 − 69030592)
correlation
Peak
Expression
r = 0.546
null distribution
p = 9.61e−10
FDR = 3.27e−06
Density
r

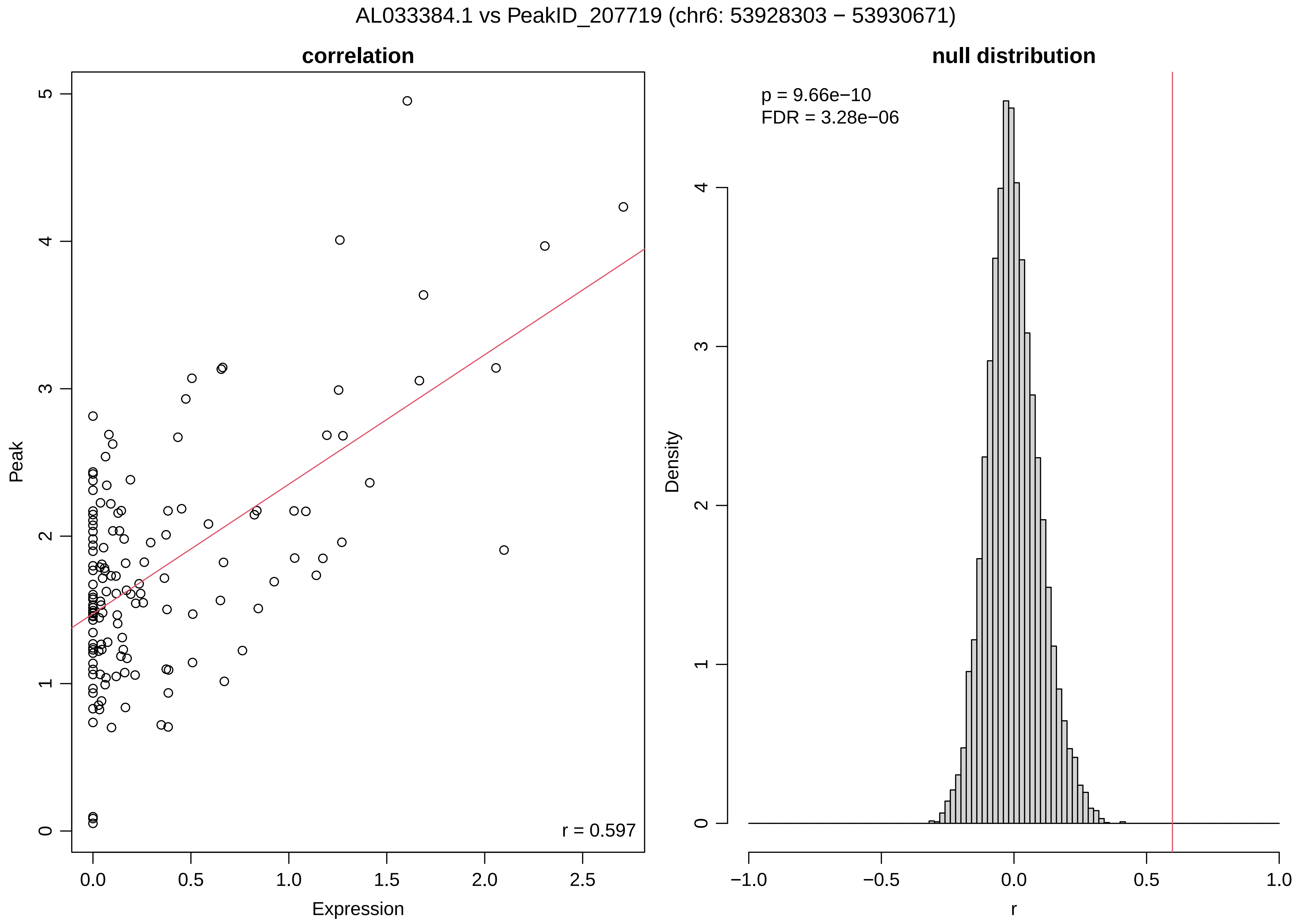
AL033384.1 vs PeakID_207719 (chr6: 53928303 − 53930671)
correlation
Peak
Expression
r = 0.597
0.0
0.5
1.0
1.5
2.0
2.5
0
1
2
3
4
5
null distribution
p = 9.66e−10
FDR = 3.28e−06
Density
r
−1.0
−0.5
0.0
0.5
1.0
0
1
2
3
4

HOTAIR vs PeakID_53843 (chr12: 53979299 − 54012032)

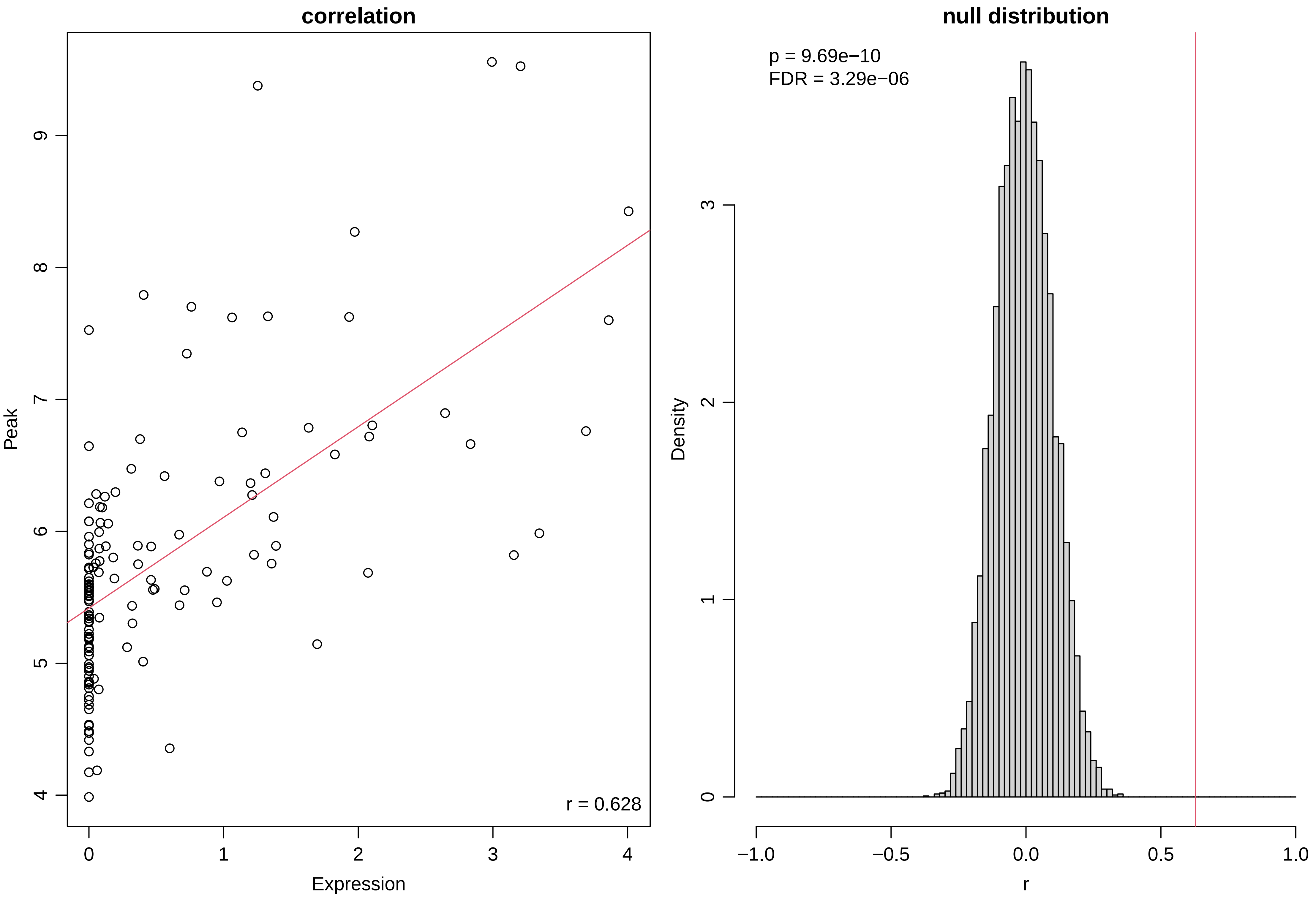

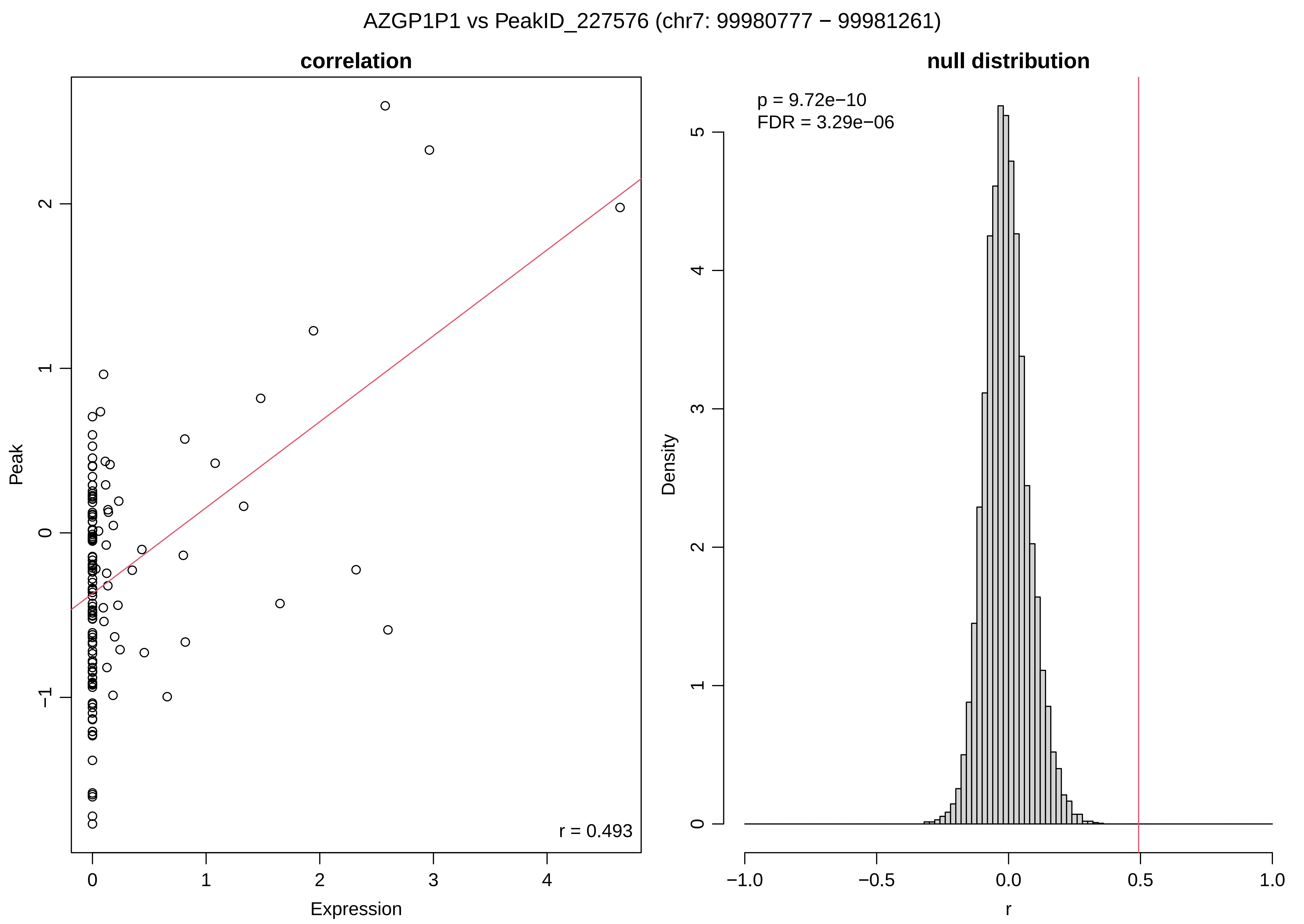
AZGP1P1 vs PeakID_227576 (chr7: 99980777 − 99981261)
correlation
Peak
Expression
r = 0.493
0
1
2
3
4
−1
null distribution
p = 9.72e−10
FDR = 3.29e−06
Density
r
−1.0
−0.5
0.0
0.5
1.0
5

RNU4−20P vs PeakID_54832 (chr12: 59580991 − 59581935)

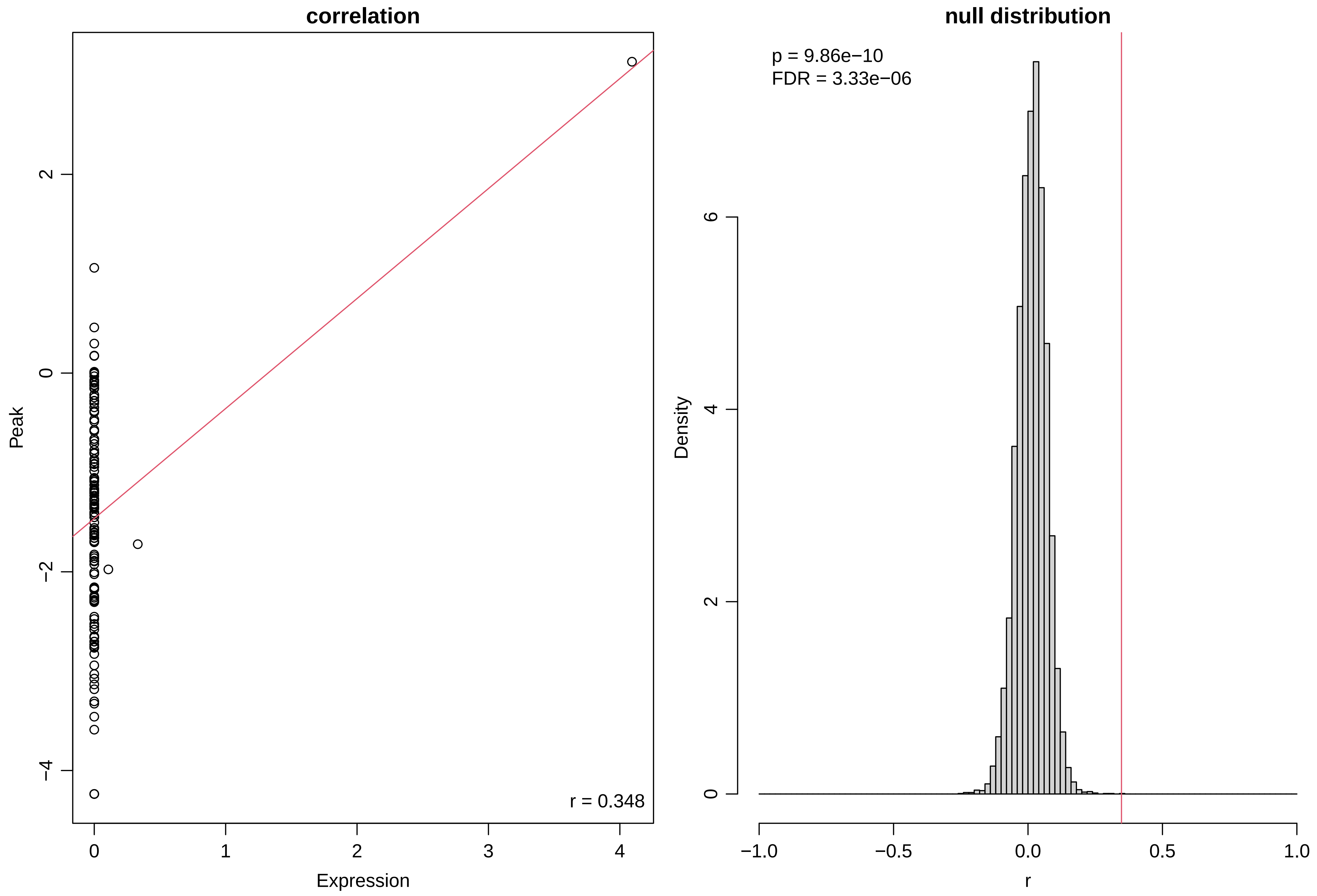

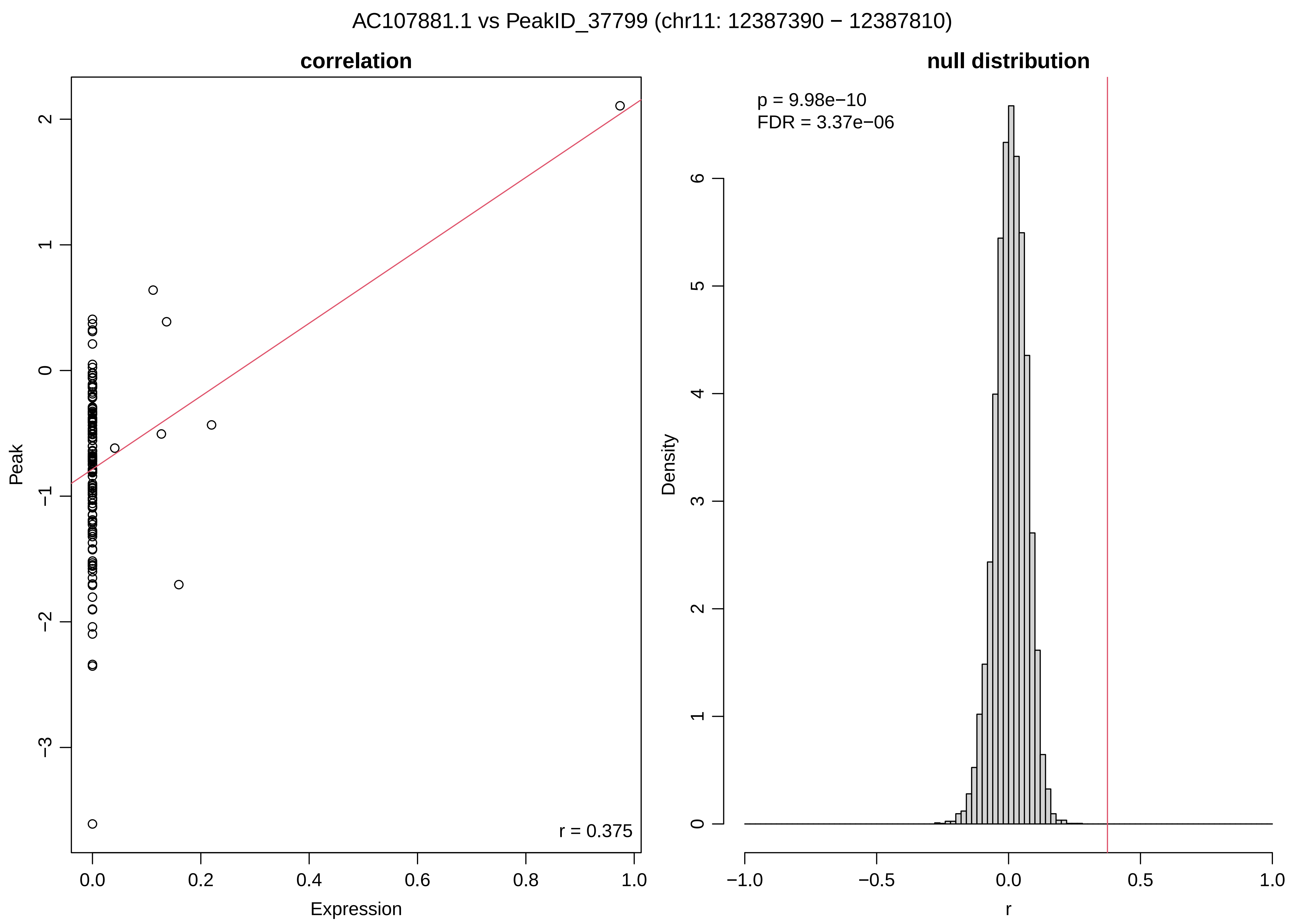

AC107881.1 vs PeakID_37799 (chr11: 12387390 − 12387810)
correlation
Peak
Expression
r = 0.375
2
1
0
−1
−2
−3
0.0
0.2
0.4
0.6
0.8
1.0
null distribution
p = 9.98e−10
FDR = 3.37e−06
Density
r
6
5
4
3
2
1
0
−1.0
−0.5
0.0
0.5
1.0

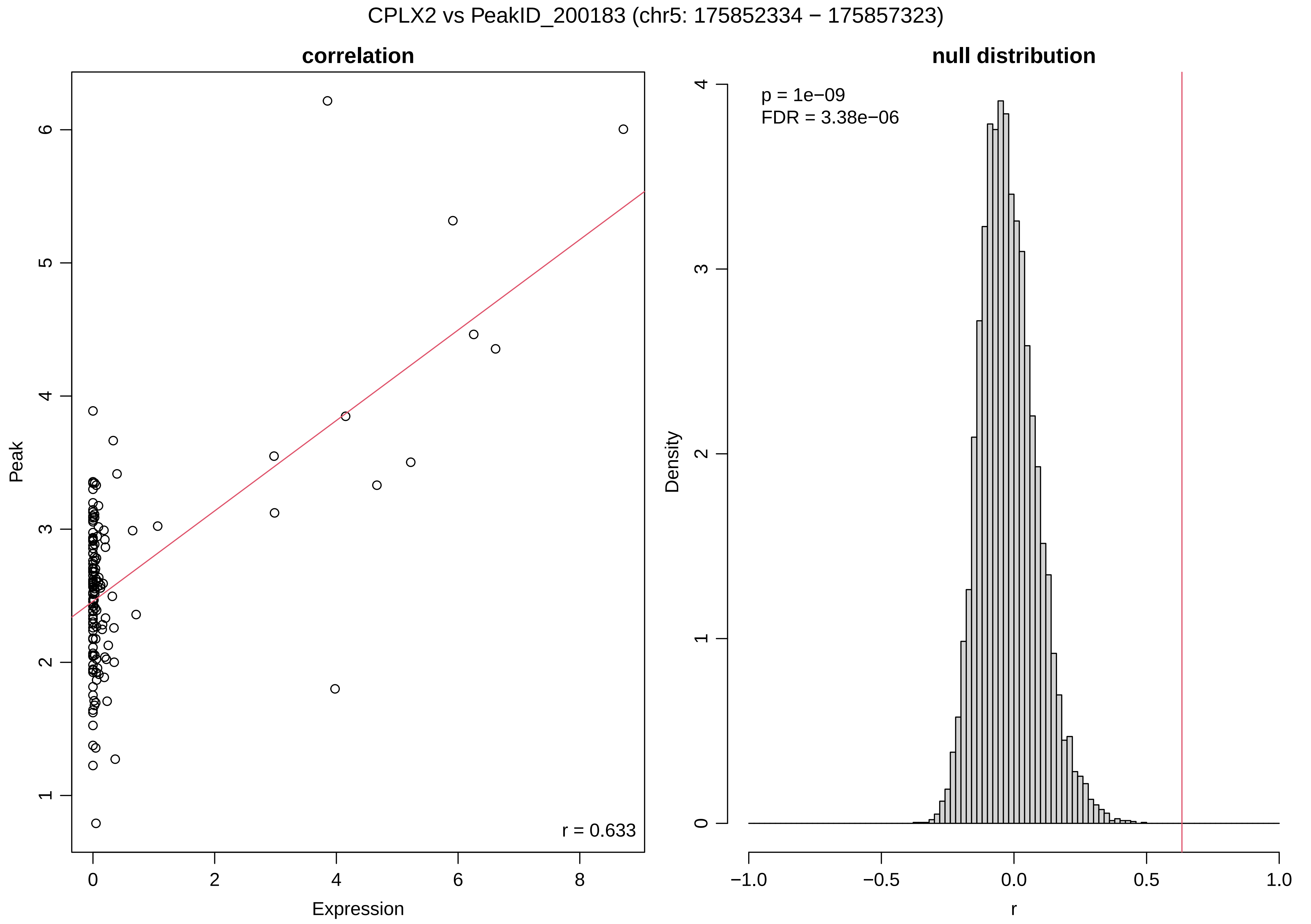
CPLX2 vs PeakID_200183 (chr5: 175852334 − 175857323)
correlation
Peak
Expression
r = 0.633
0
2
4
6
8
1
2
3
4
5
6
null distribution
p = 1e−09
FDR = 3.38e−06
Density
r
−1.0
−0.5
0.0
0.5
1.0
0
1
2
3
4

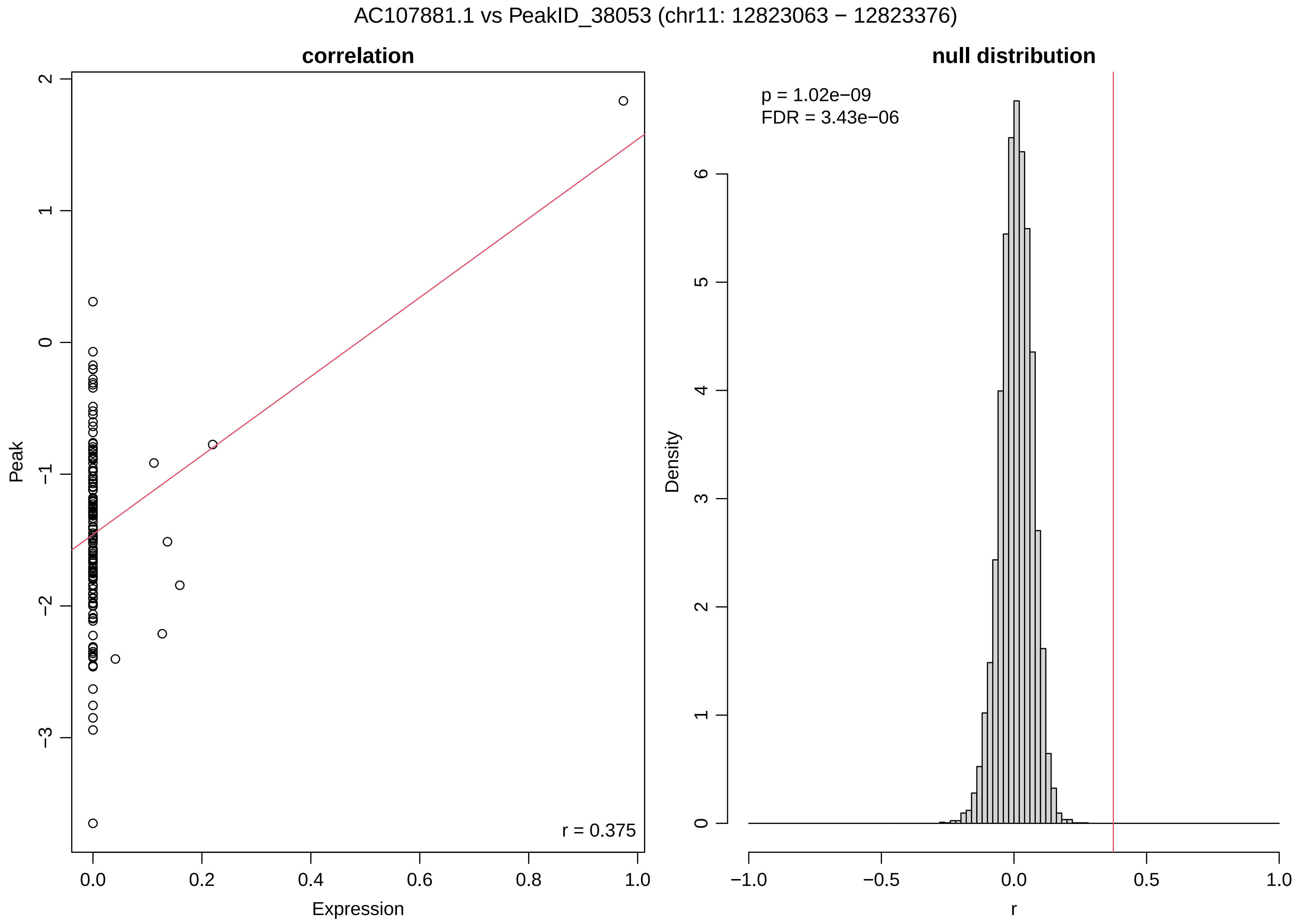
AC107881.1 vs PeakID_38053 (chr11: 12823063 − 12823376)
correlation
Peak
Expression
r = 0.375
null distribution
p = 1.02e−09
FDR = 3.43e−06
Density
r

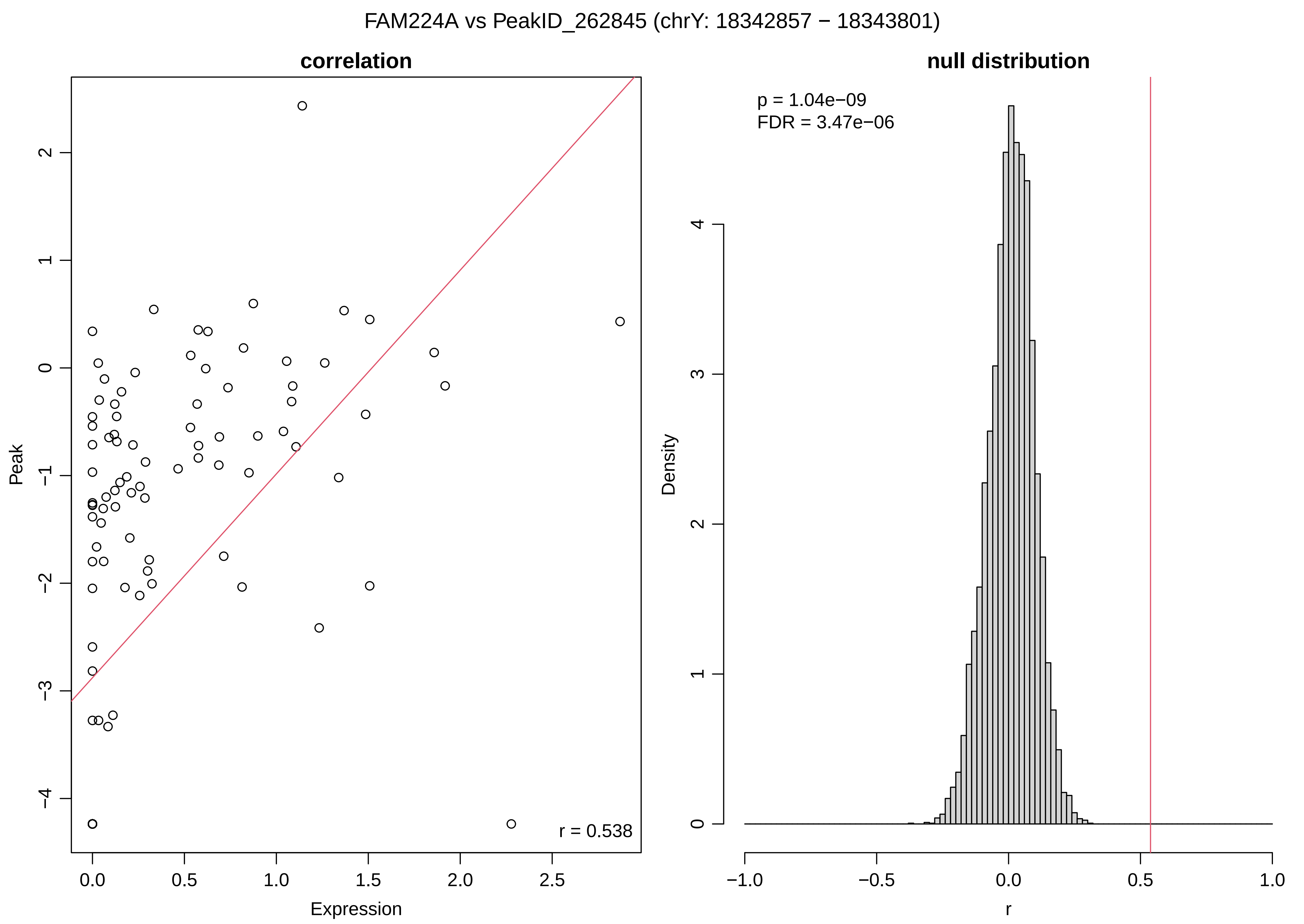

FAM224A vs PeakID_262845 (chrY: 18342857 − 18343801)
correlation
Peak
Expression
r = 0.538
null distribution
p = 1.04e−09
FDR = 3.47e−06
Density
r

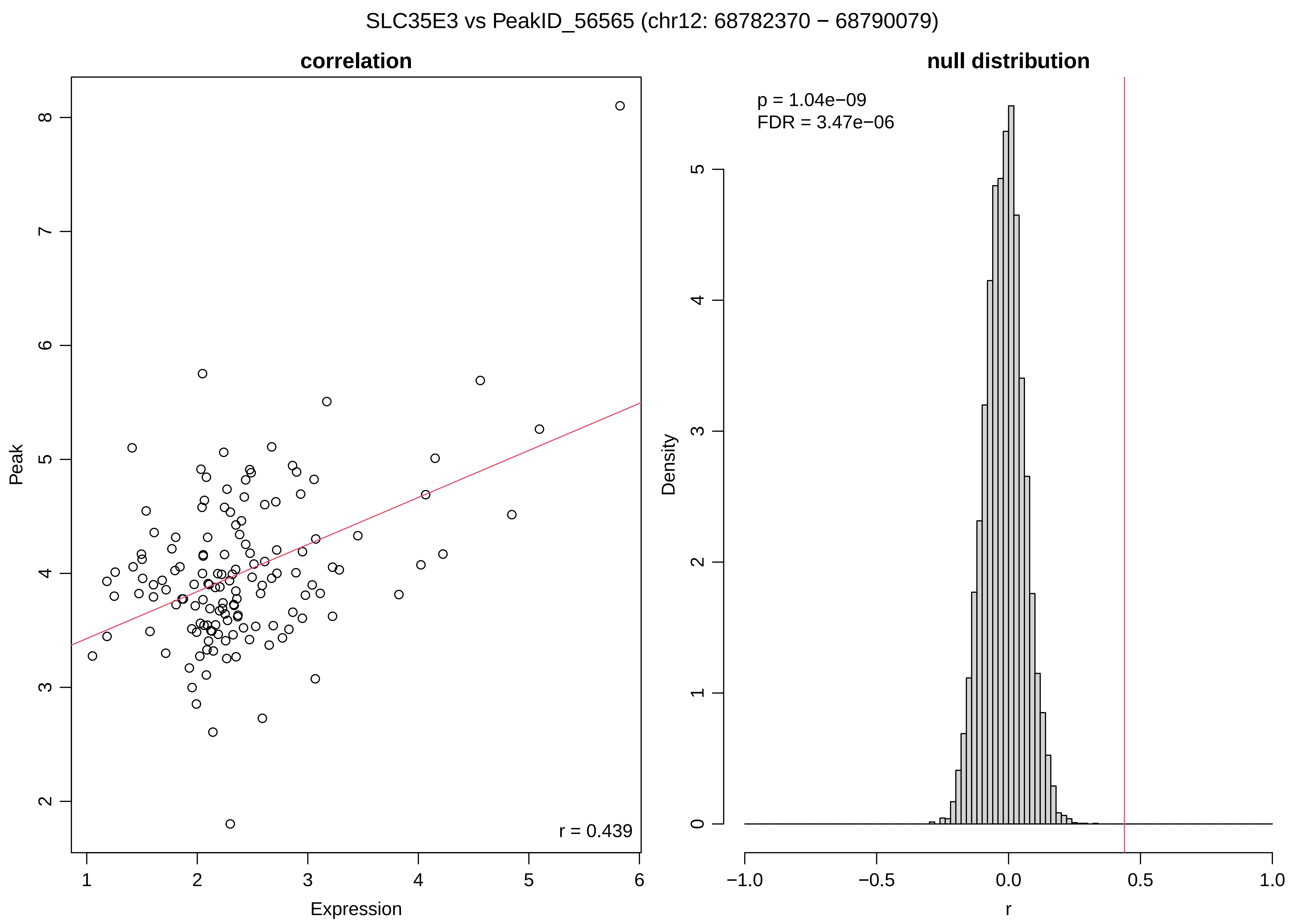
SLC35E3 vs PeakID_56565 (chr12: 68782370 − 68790079)
correlation
Peak
Expression
r = 0.439
null distribution
p = 1.04e−09
FDR = 3.47e−06
Density
r

LRP1B vs PeakID_133852 (chr2: 142129848 − 142133276)

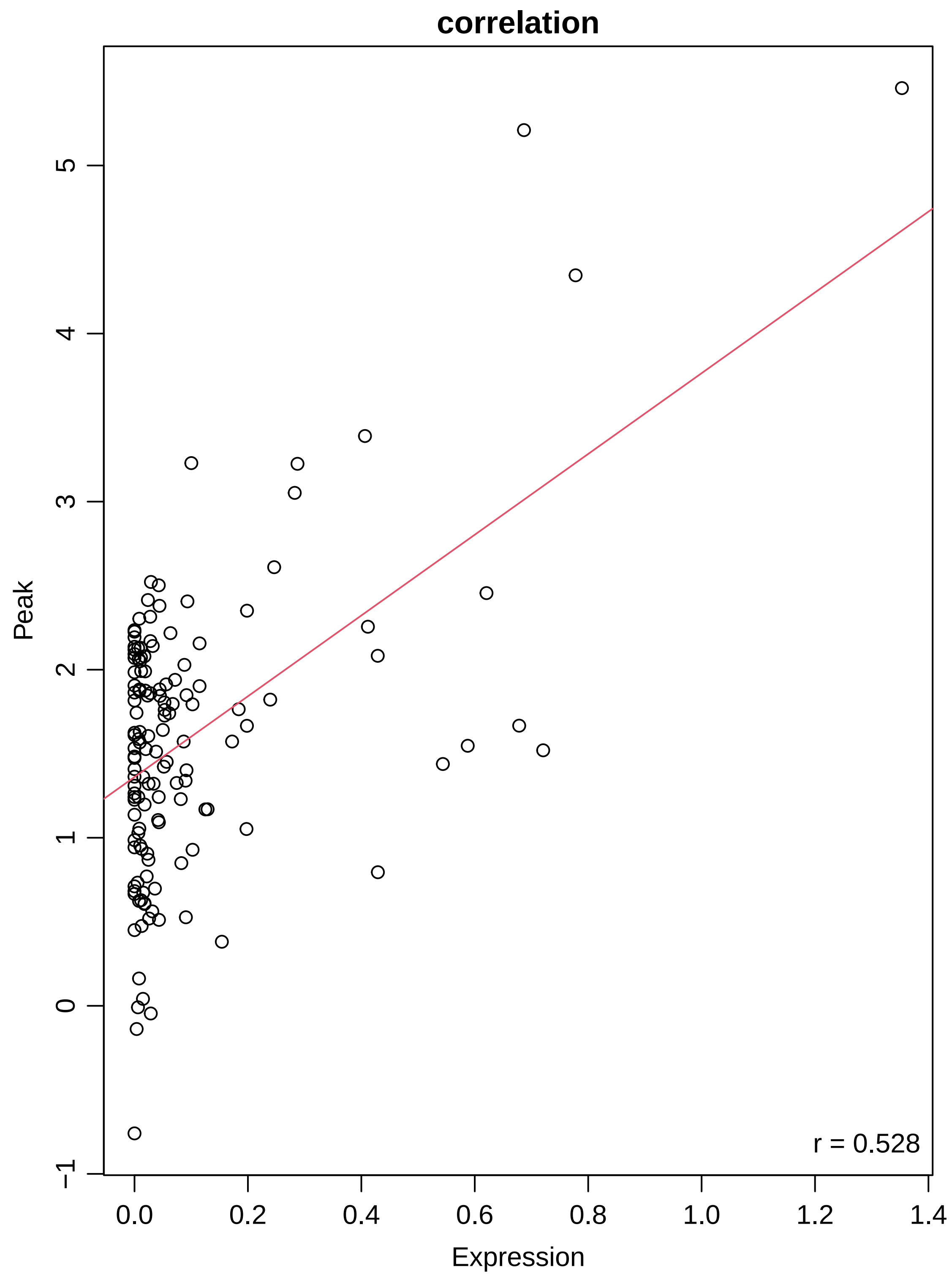

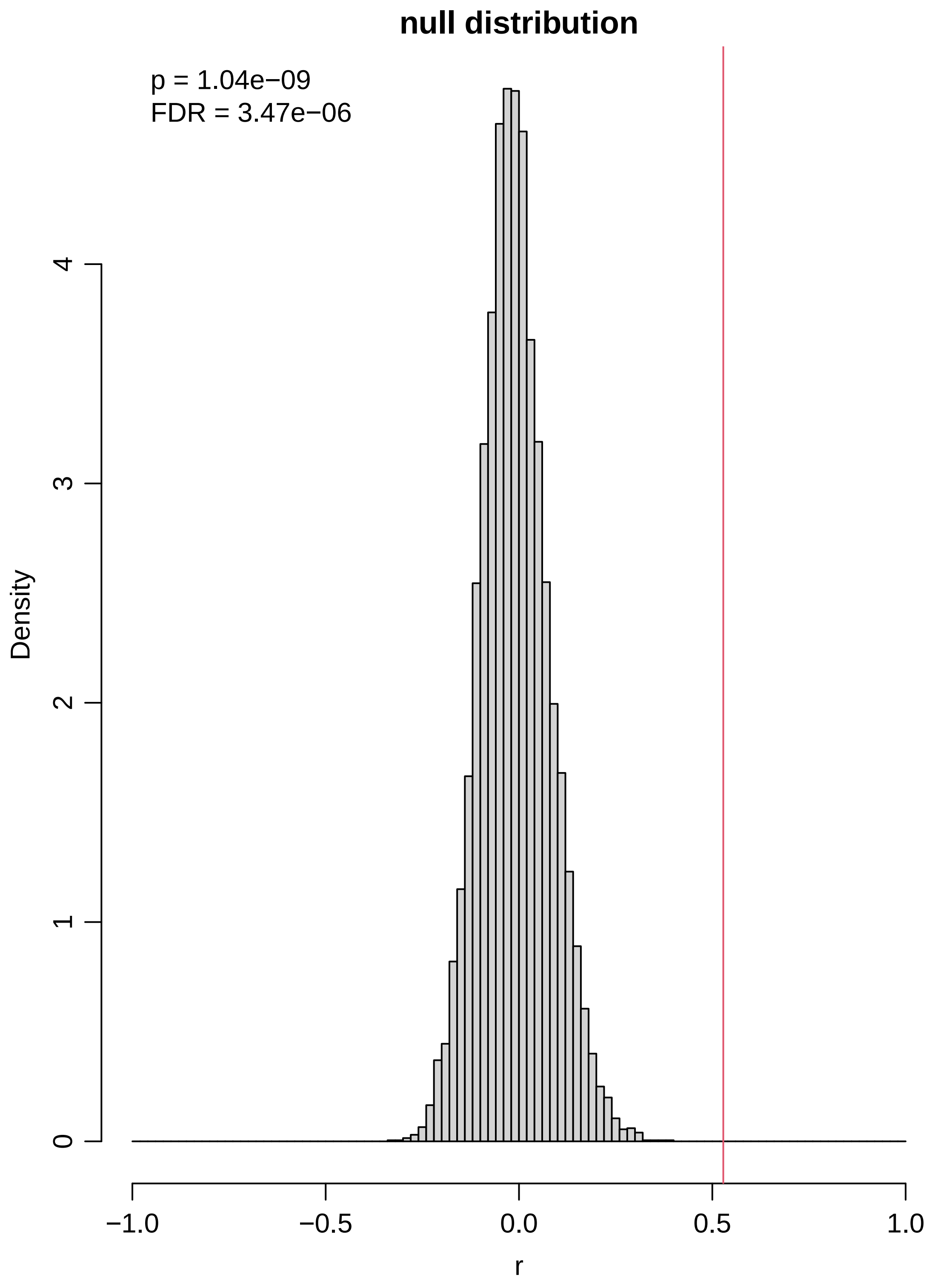

ALG1L vs PeakID_168840 (chr3: 125935528 − 125937087)

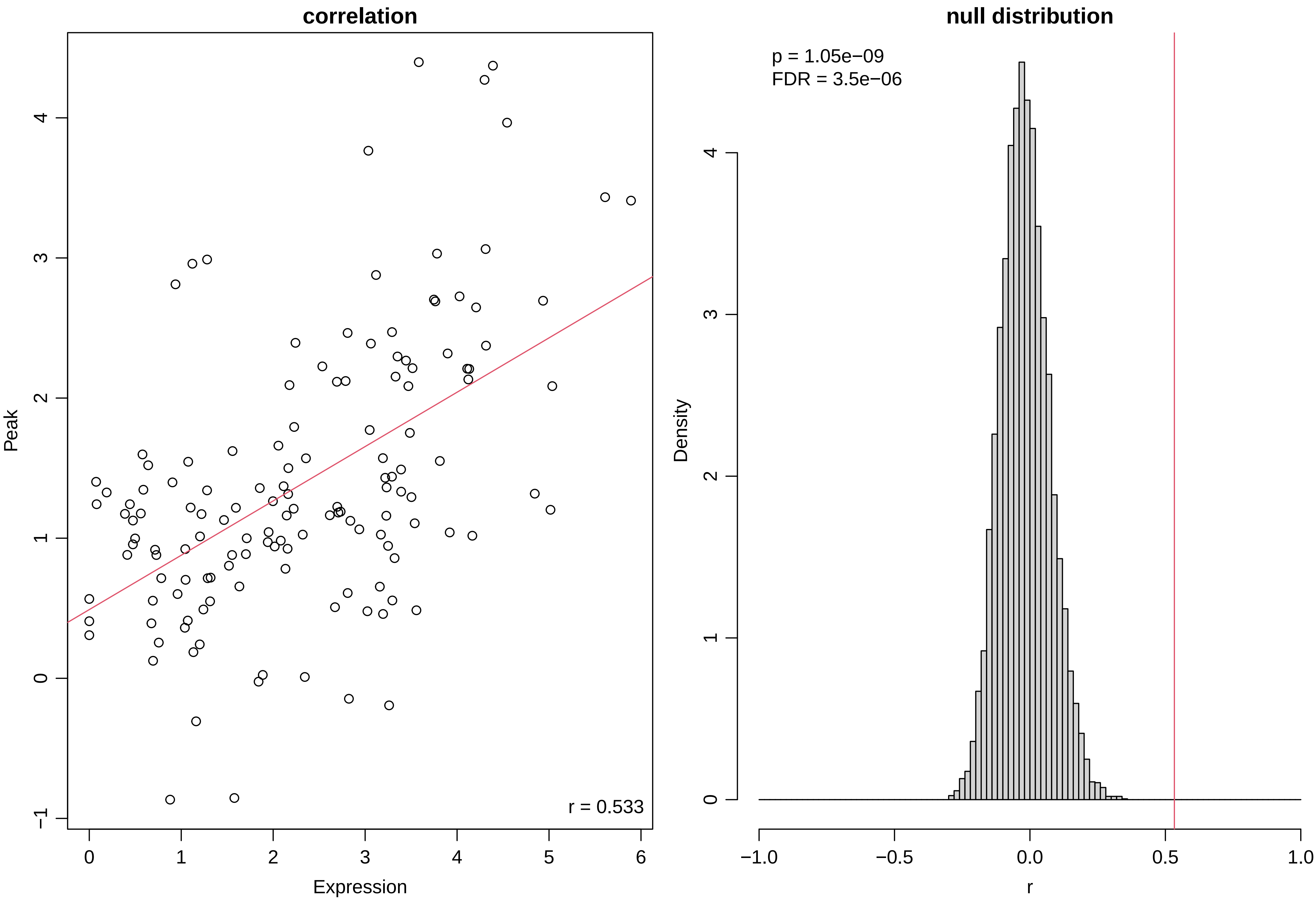

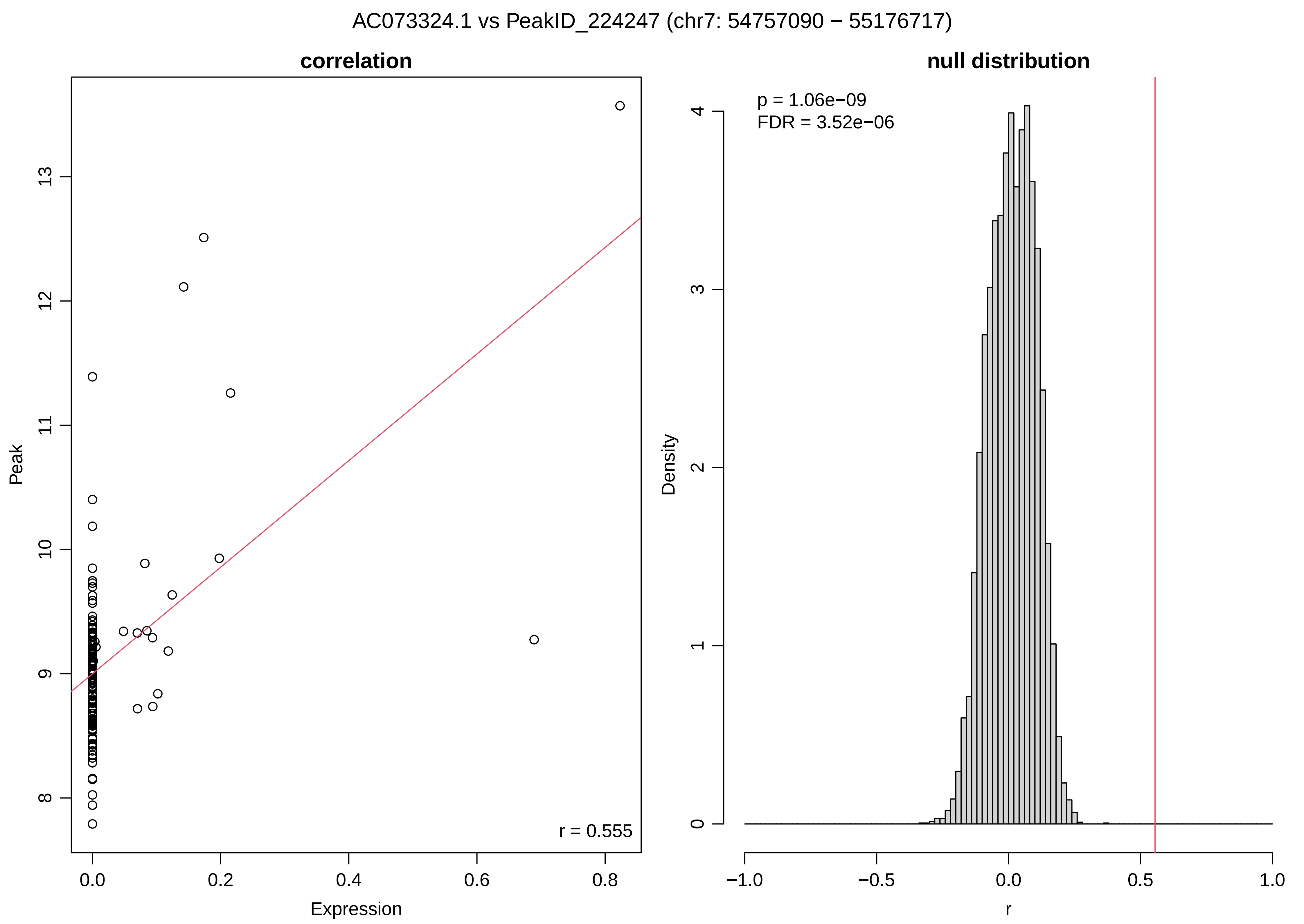

AC073324.1 vs PeakID_224247 (chr7: 54757090 − 55176717)
correlation
Peak
Expression
r = 0.555
null distribution
p = 1.06e−09
FDR = 3.52e−06
Density
r

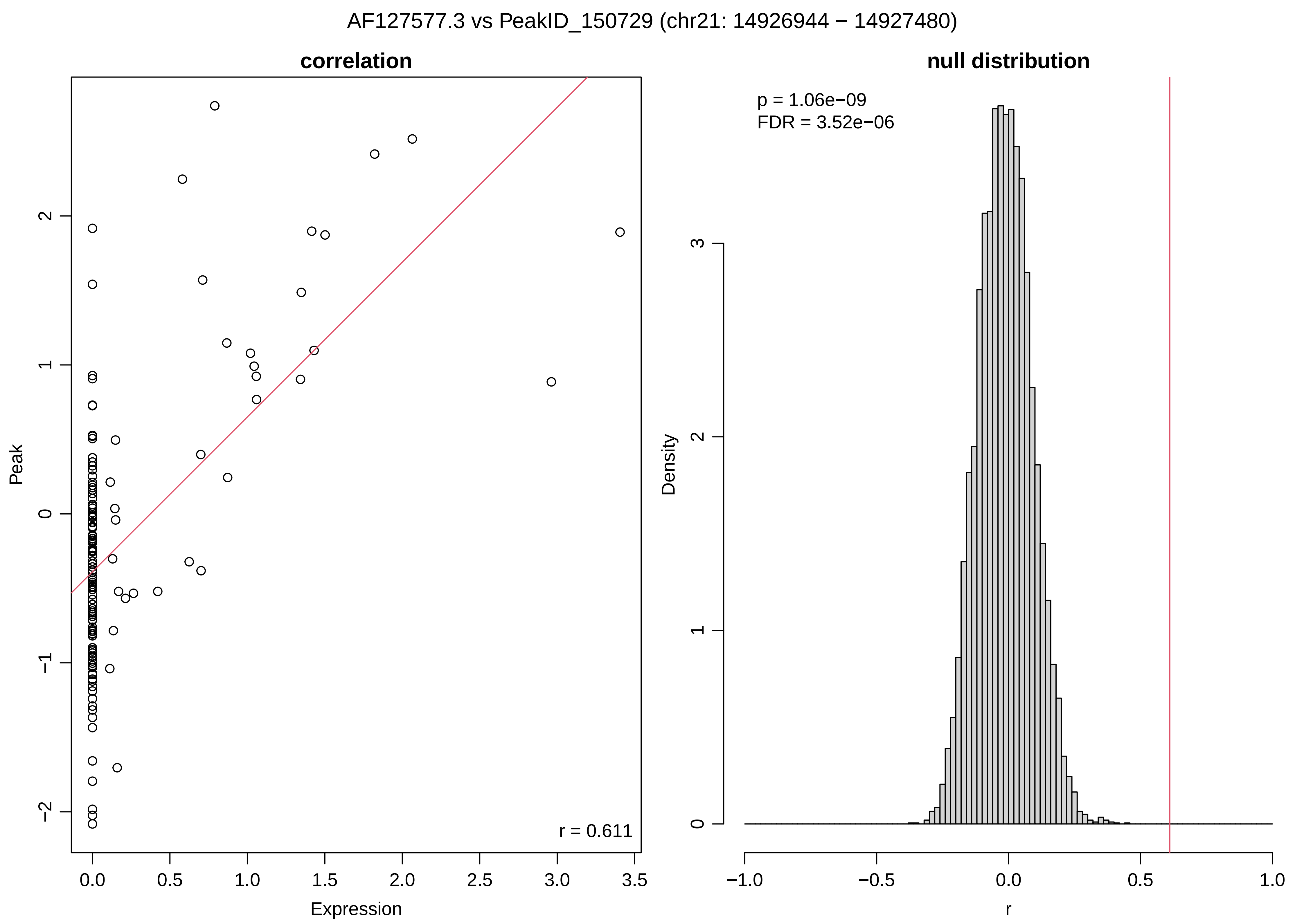
AF127577.3 vs PeakID_150729 (chr21: 14926944 − 14927480)
correlation
Peak
Expression
r = 0.611
null distribution
p = 1.06e−09
FDR = 3.52e−06
Density
r

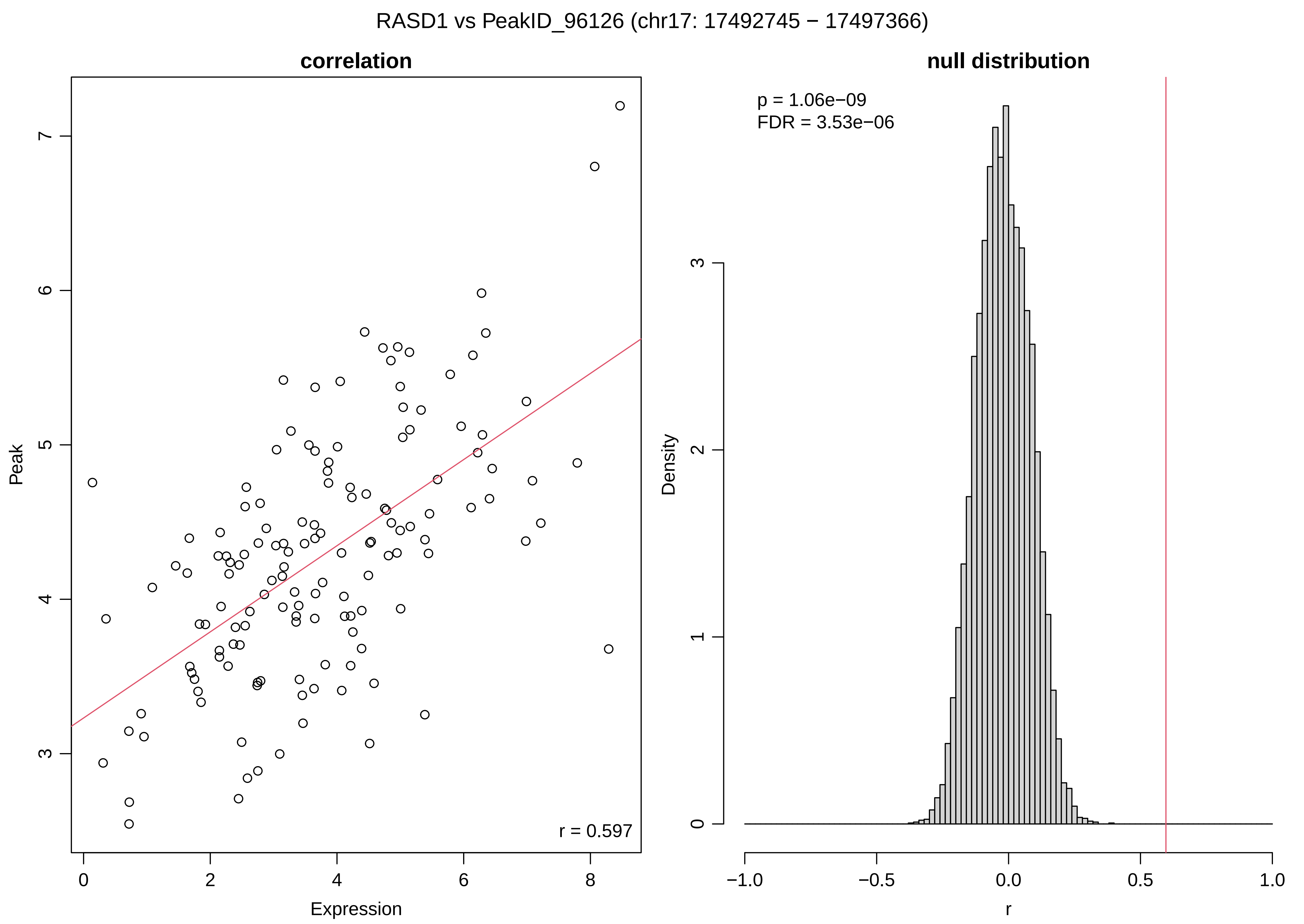

RASD1 vs PeakID_96126 (chr17: 17492745 − 17497366)
correlation
Peak
Expression
r = 0.597
null distribution
p = 1.06e−09
FDR = 3.53e−06
Density
r

ASPH vs PeakID_237334 (chr8: 61756414 − 61757081)

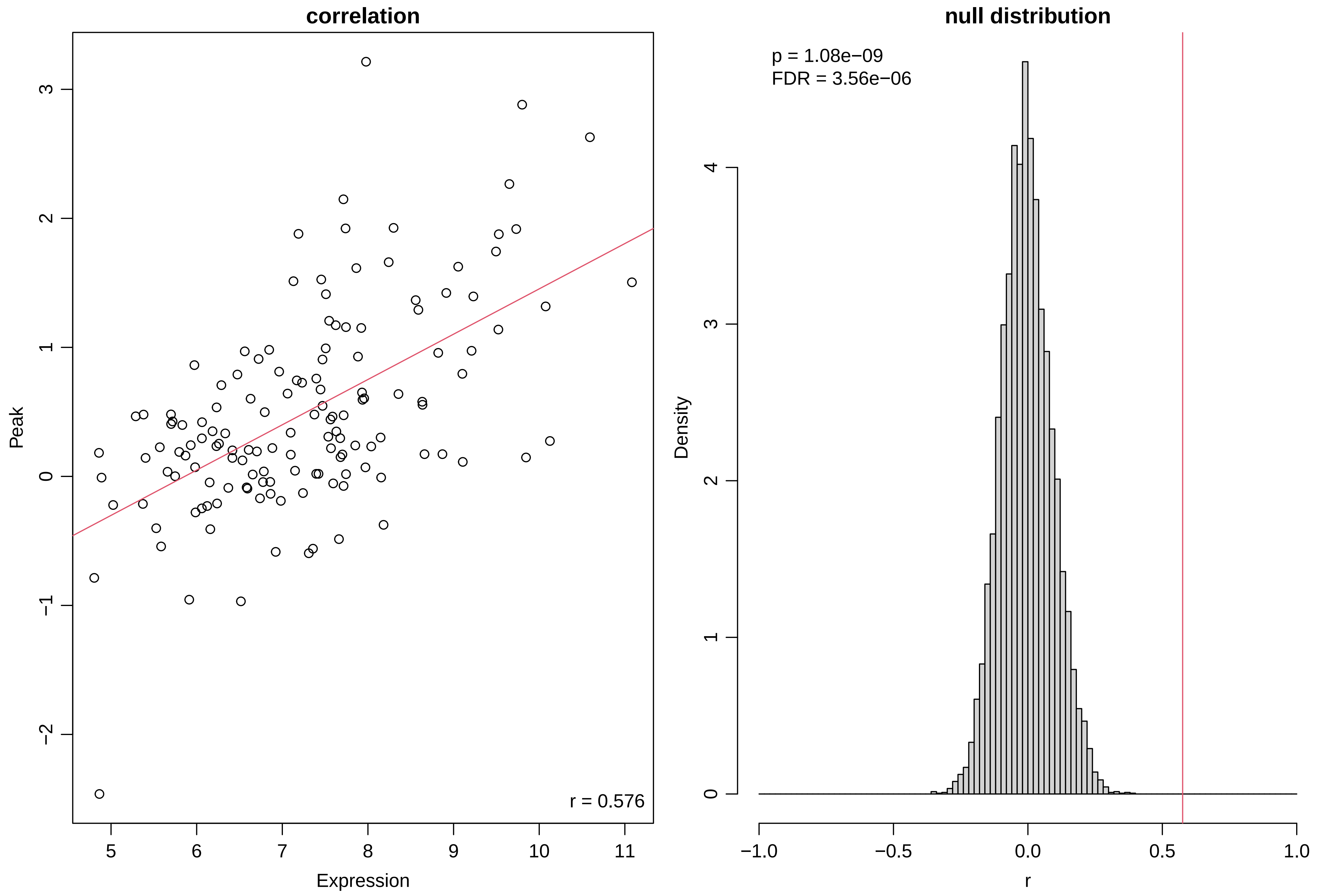

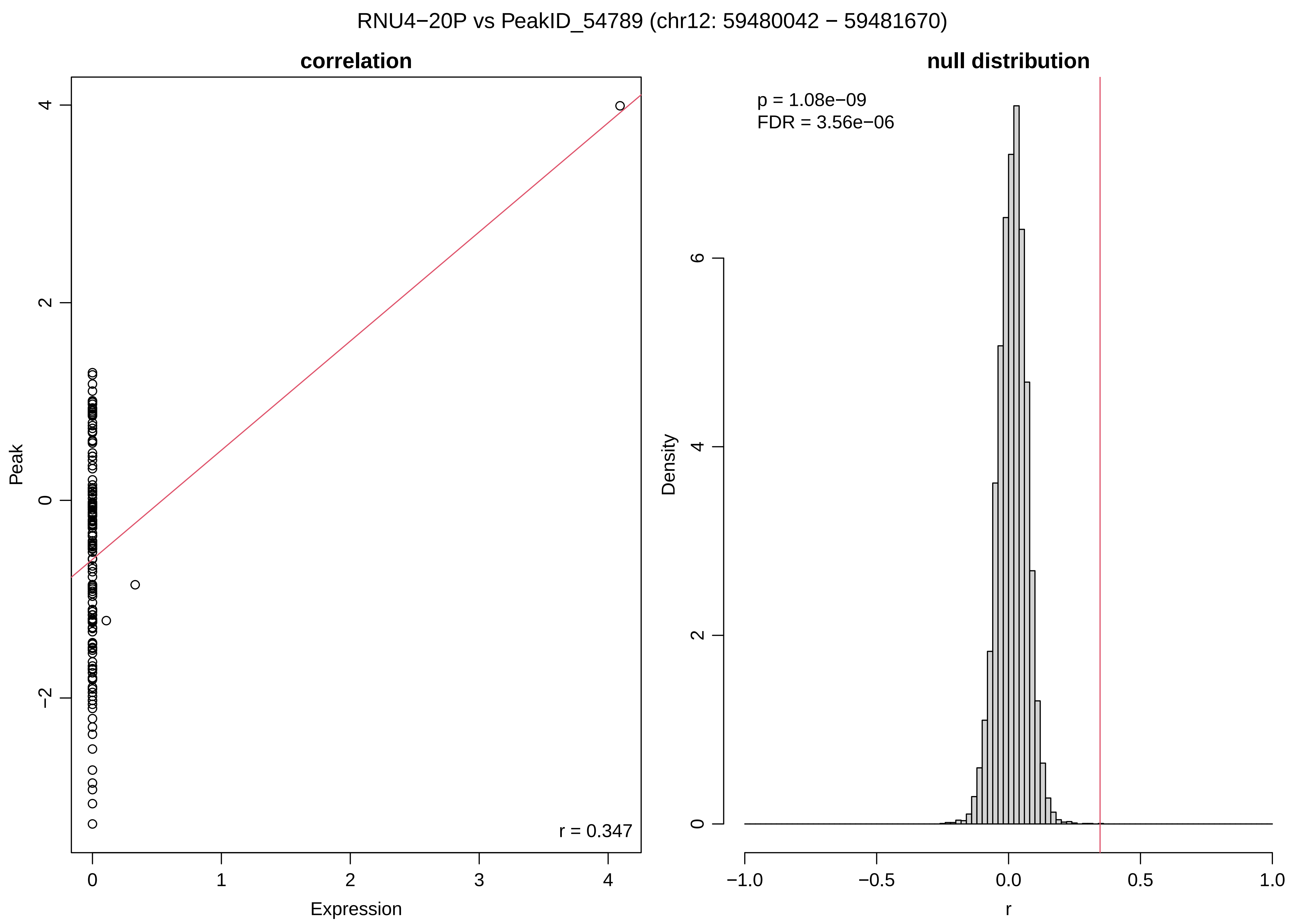

RNU4−20P vs PeakID_54789 (chr12: 59480042 − 59481670)
correlation
Peak
Expression
r = 0.347
null distribution
p = 1.08e−09
FDR = 3.56e−06
Density
r

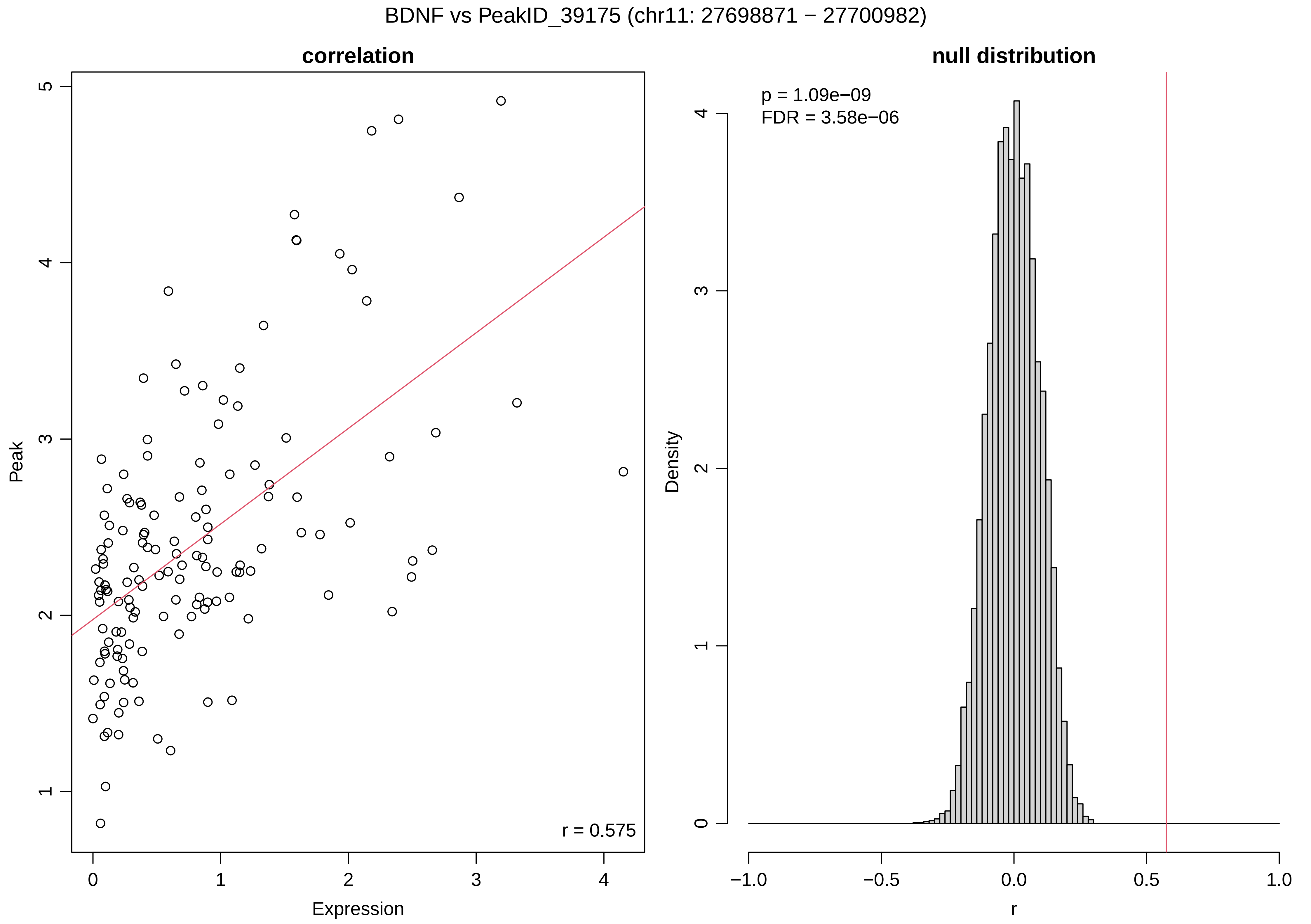

BDNF vs PeakID_39175 (chr11: 27698871 − 27700982)
correlation
Peak
Expression
r = 0.575
null distribution
p = 1.09e−09
FDR = 3.58e−06
Density
r

TSFM vs PeakID_54423 (chr12: 57743158 − 57790607)

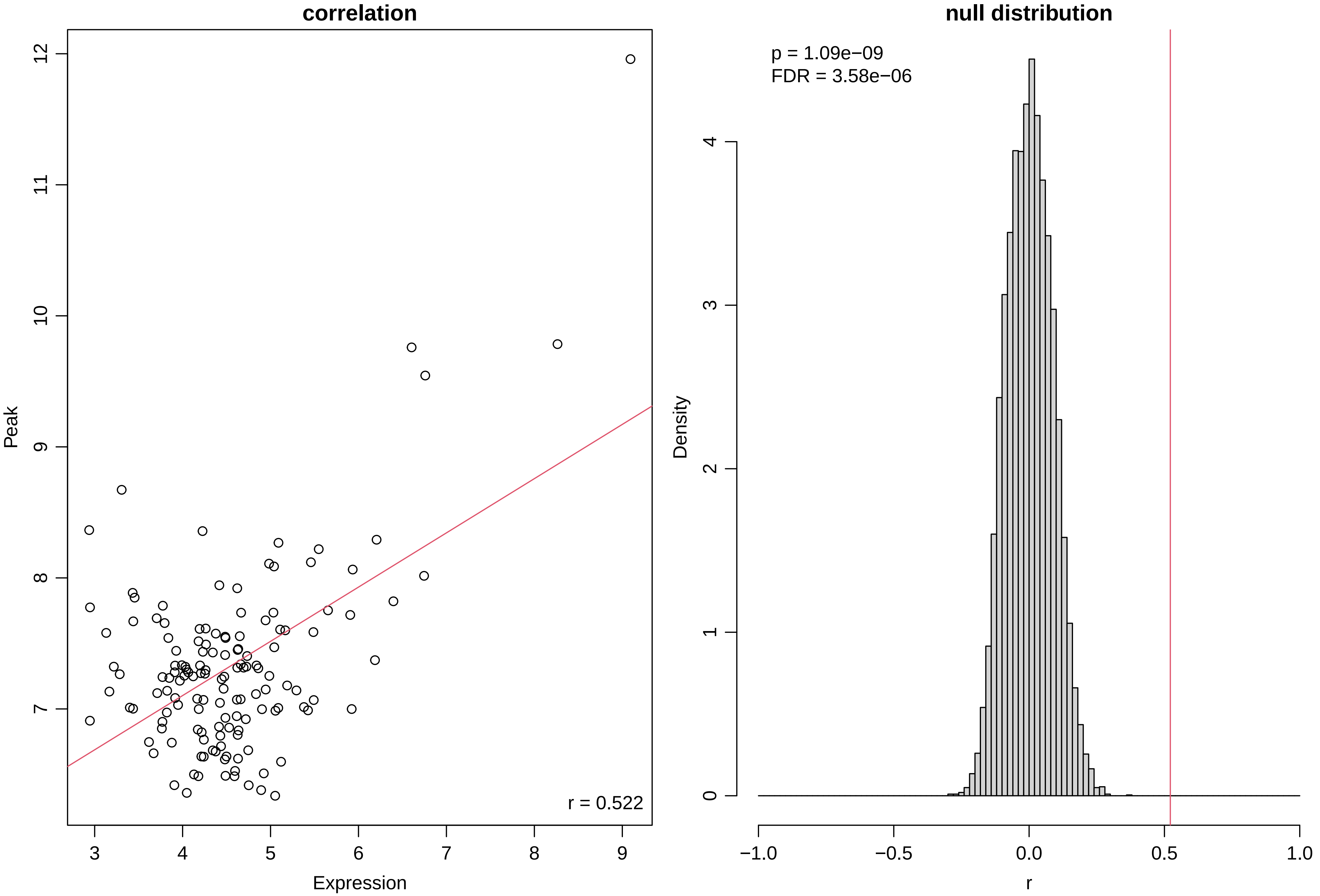

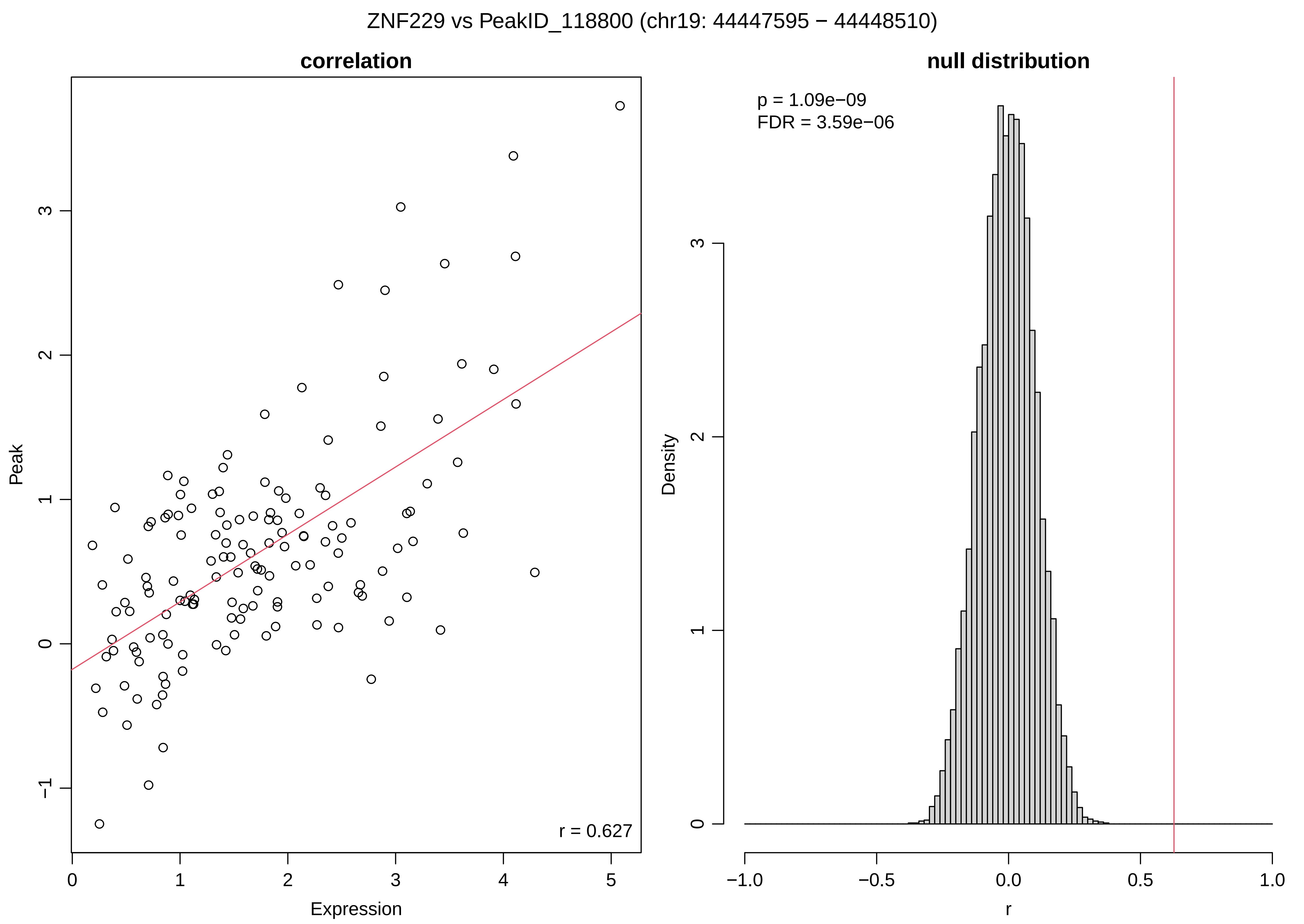
ZNF229 vs PeakID_118800 (chr19: 44447595 − 44448510)
correlation
Peak
Expression
r = 0.627
null distribution
p = 1.09e−09
FDR = 3.59e−06
Density
r

PAH vs PeakID_59236 (chr12: 102916005 − 102918488)

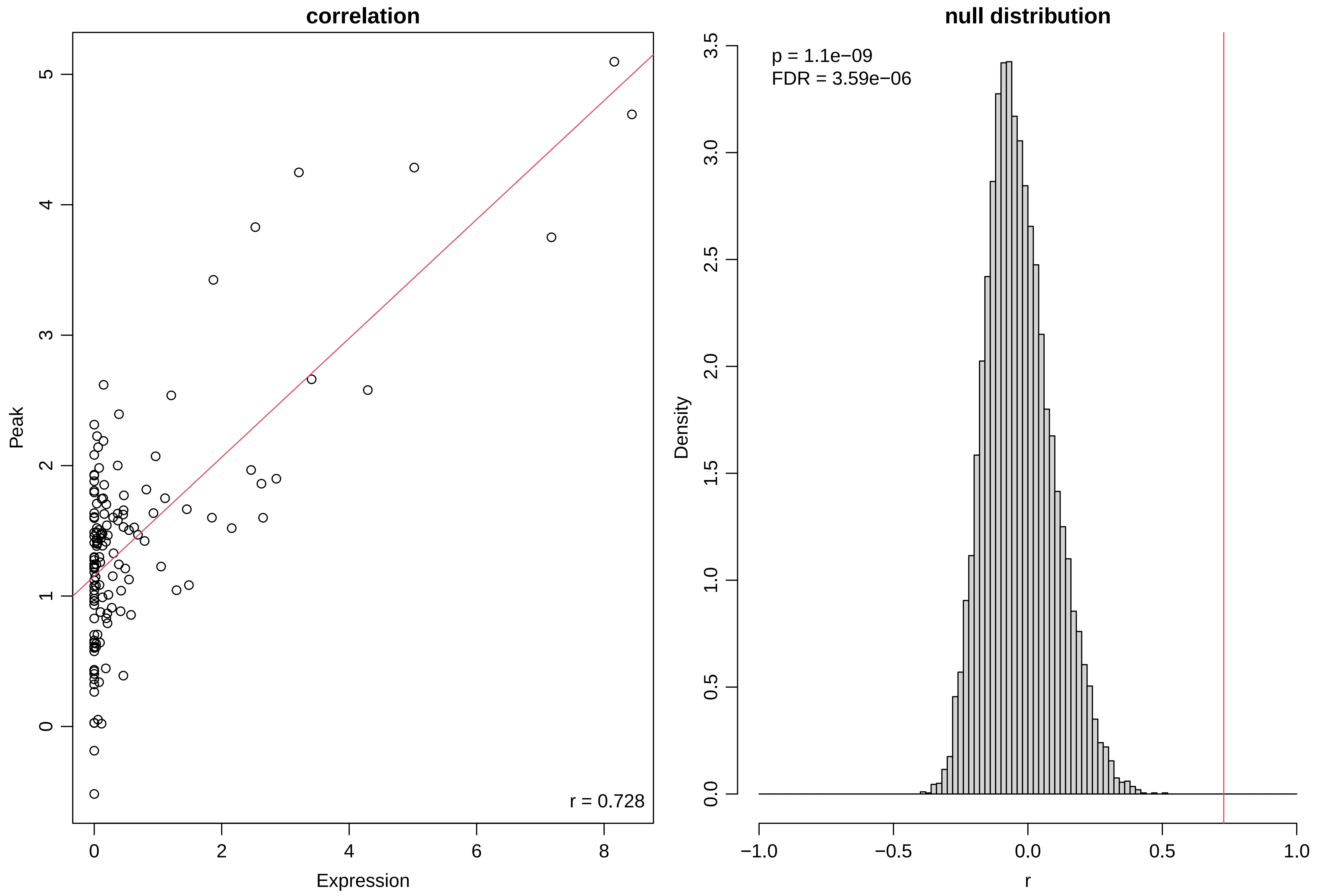

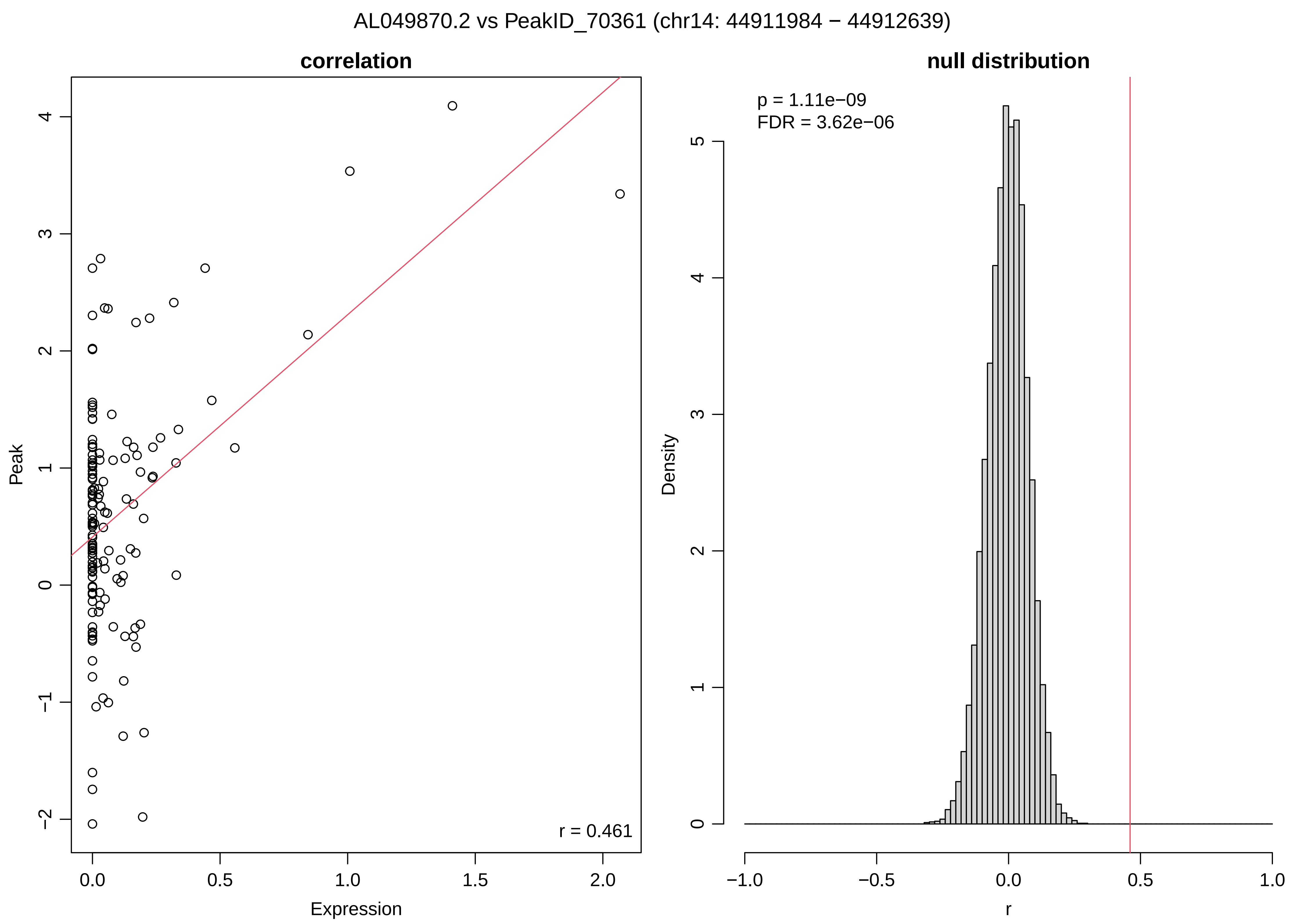
AL049870.2 vs PeakID_70361 (chr14: 44911984 − 44912639)
correlation
Peak
Expression
r = 0.461
null distribution
p = 1.11e−09
FDR = 3.62e−06
Density
r

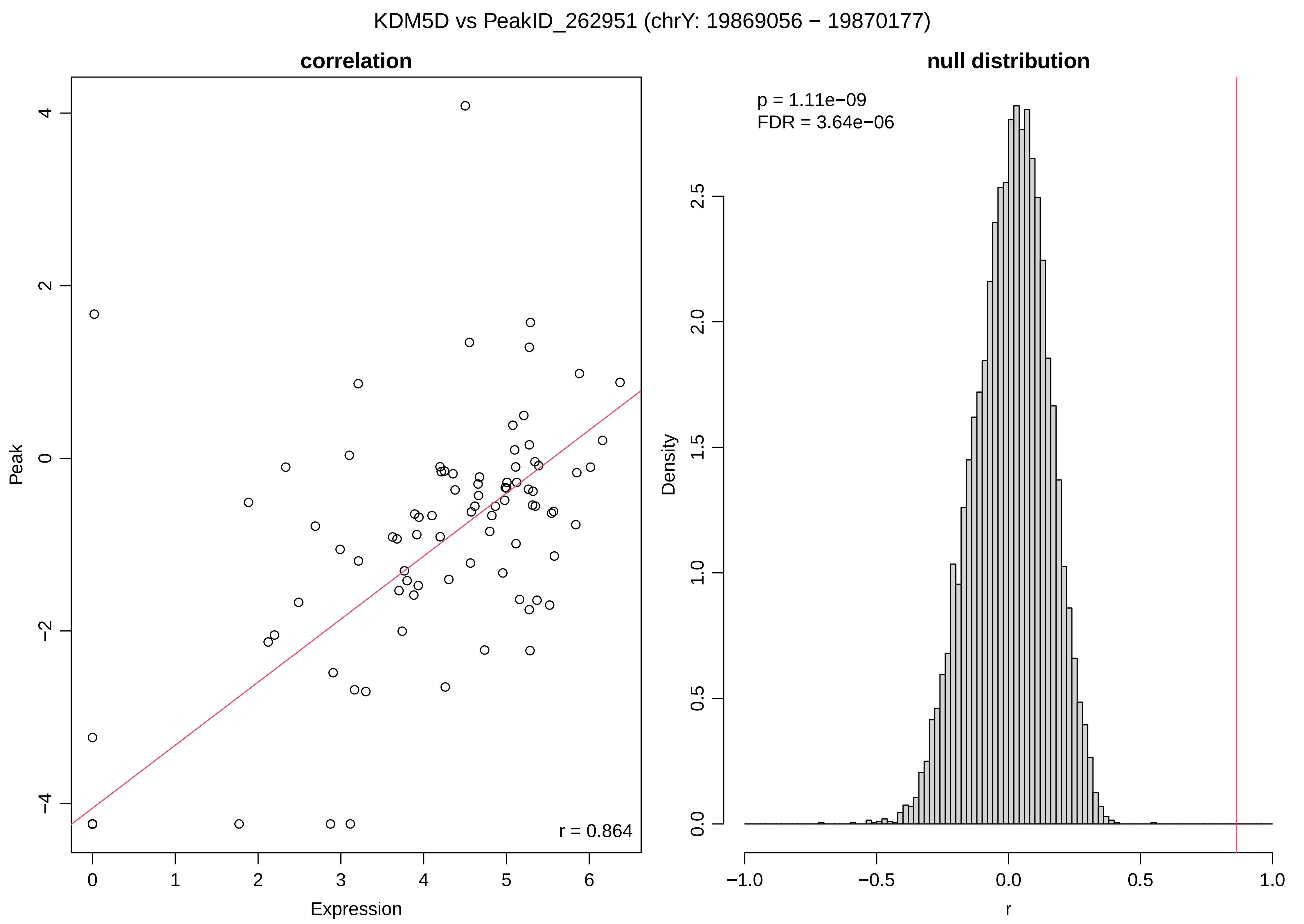
KDM5D vs PeakID_262951 (chrY: 19869056 − 19870177)
correlation
null distribution
p = 1.11e−09
FDR = 3.64e−06
Peak
Expression
r = 0.864
Density
r

PRSS3 vs PeakID_247072 (chr9: 33750219 − 33752314)

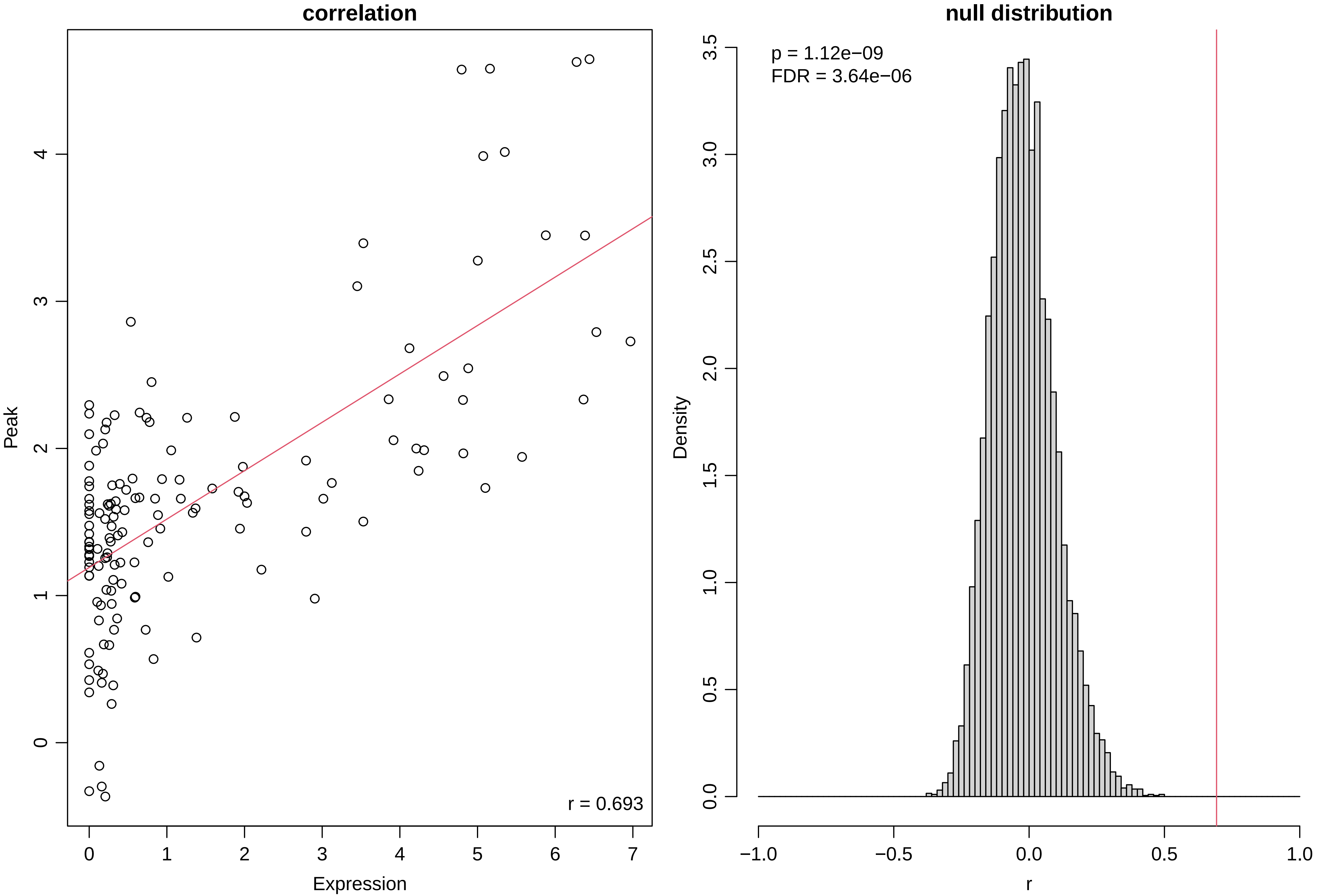

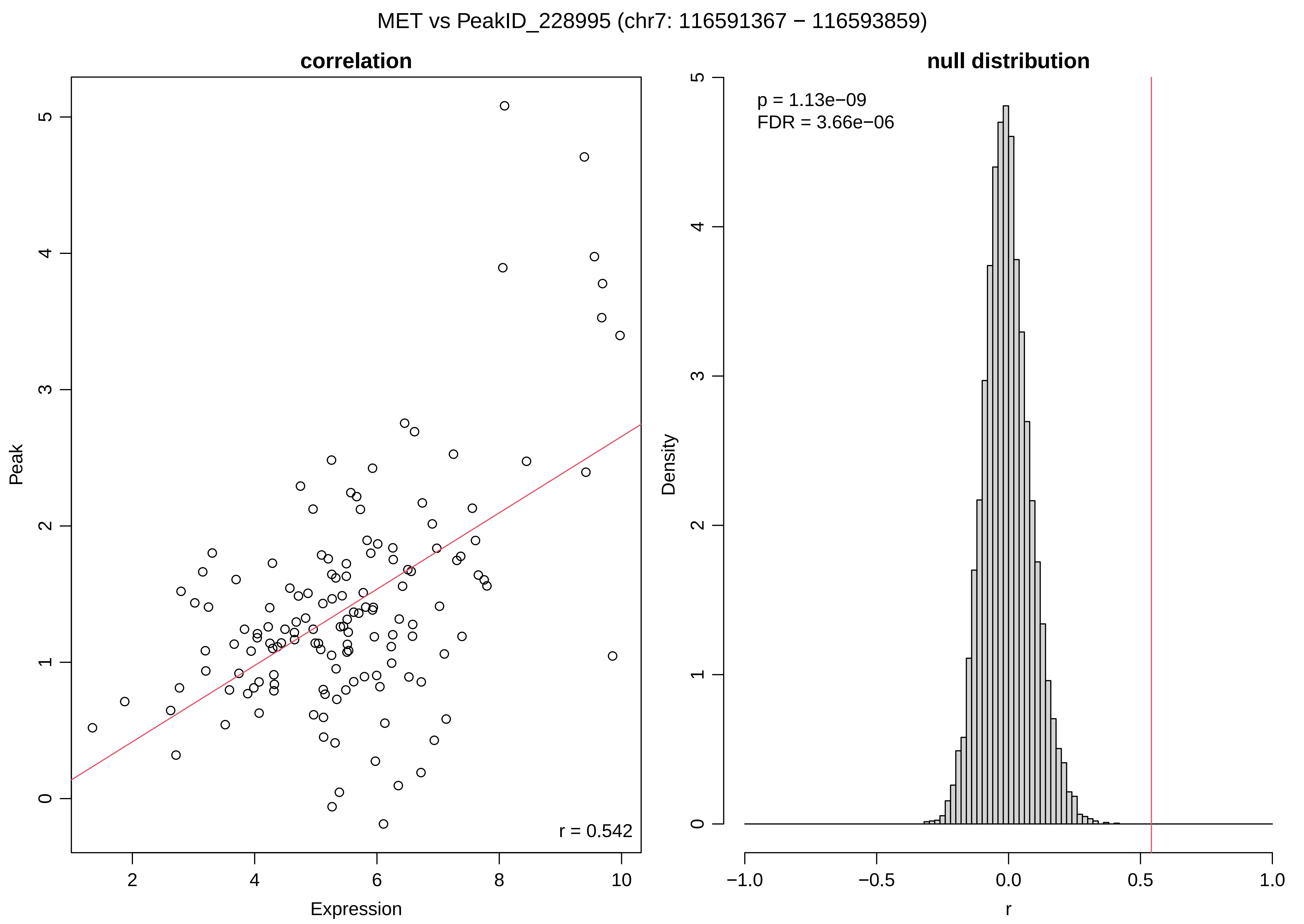

MET vs PeakID_228995 (chr7: 116591367 − 116593859)
correlation
Peak
Expression
r = 0.542
0
1
2
3
4
5
2
4
6
8
10
null distribution
p = 1.13e−09
FDR = 3.66e−06
Density
r
0
1
2
3
4
5
−1.0
−0.5
0.0
0.5
1.0

AC104088.1 vs PeakID_135802 (chr2: 172315047 − 172315716)

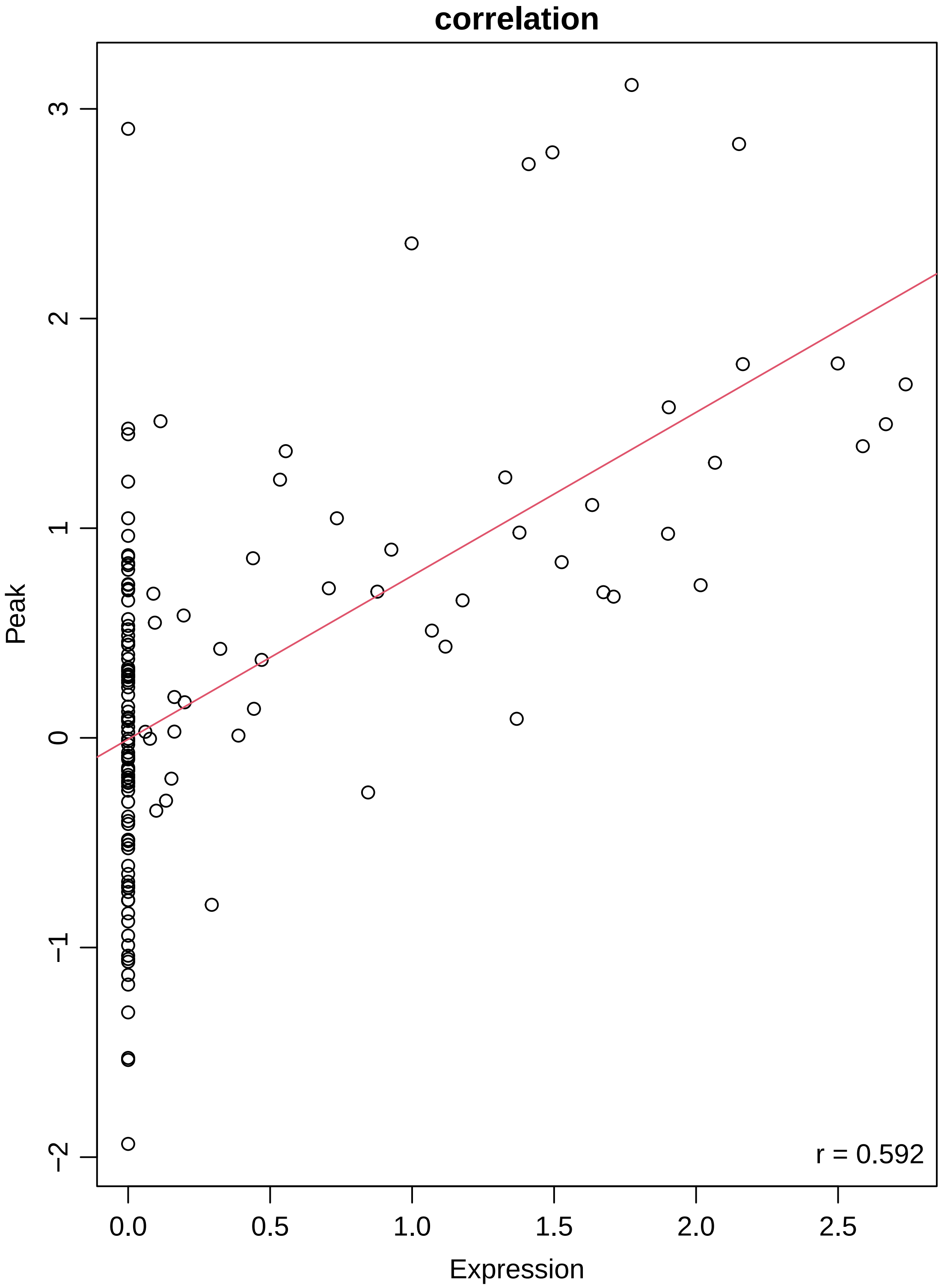

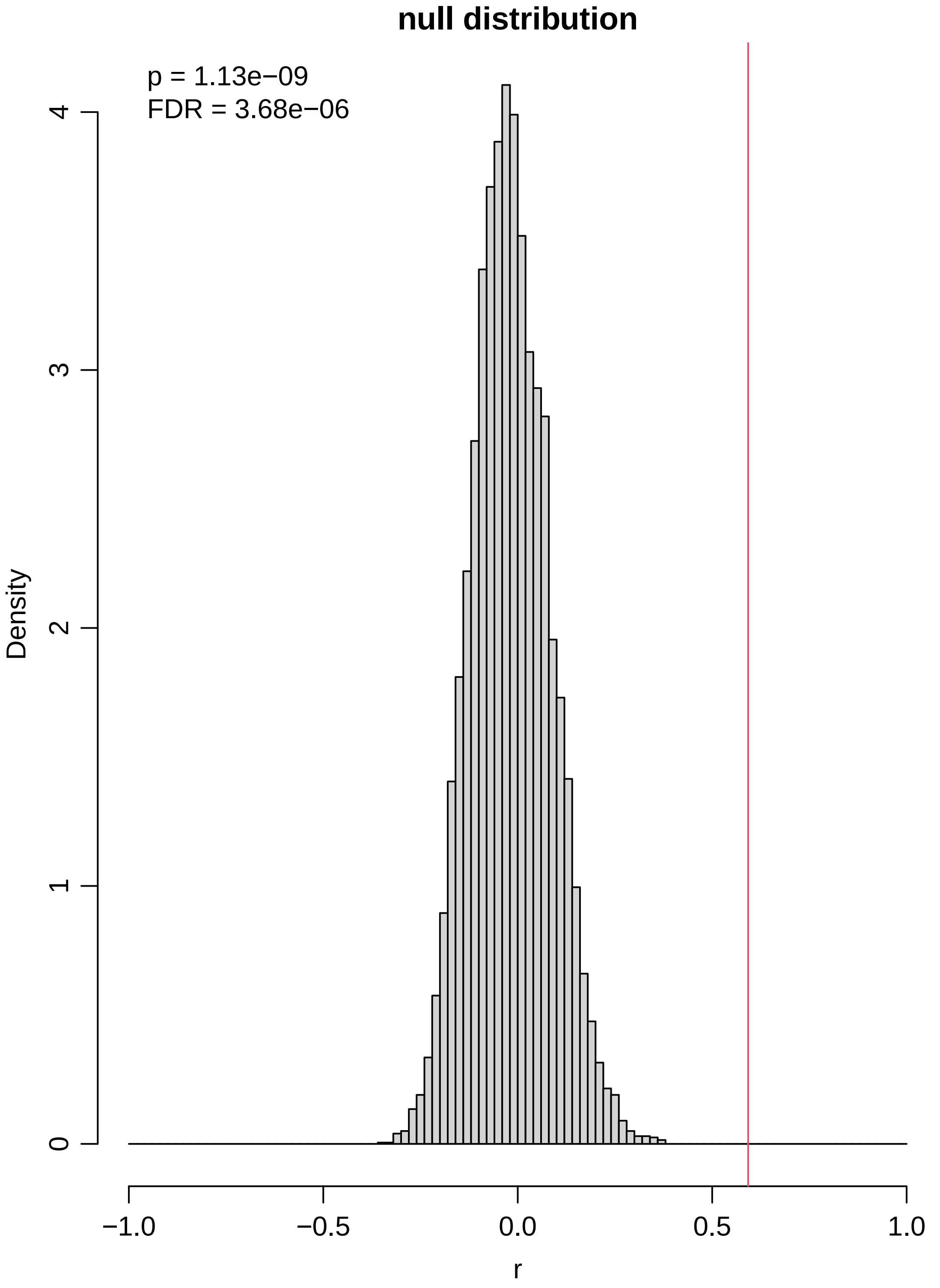

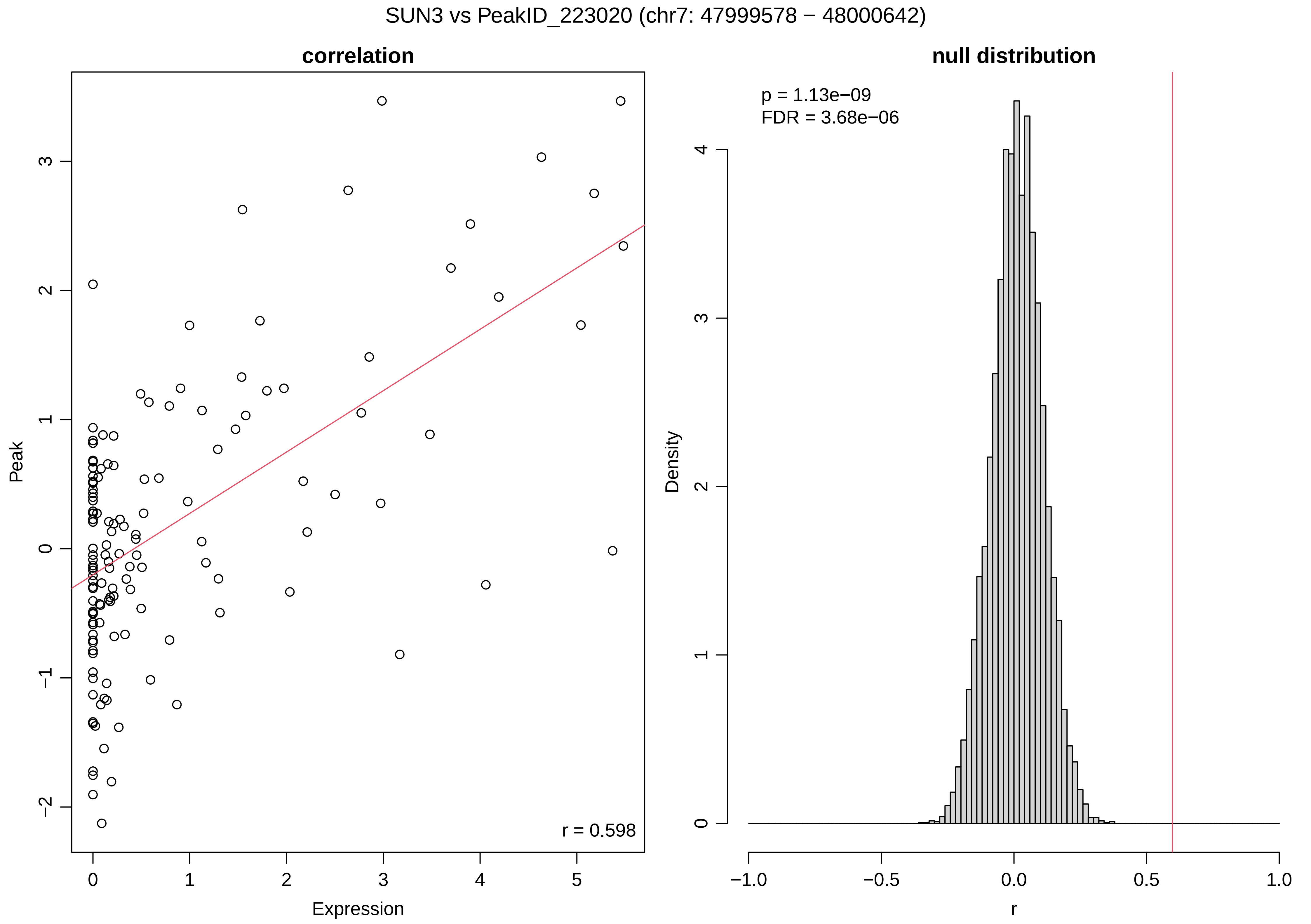

SUN3 vs PeakID_223020 (chr7: 47999578 − 48000642)
correlation
Peak
Expression
r = 0.598
null distribution
p = 1.13e−09
FDR = 3.68e−06
Density
r

GPR78 vs PeakID_176442 (chr4: 8557023 − 8559485)

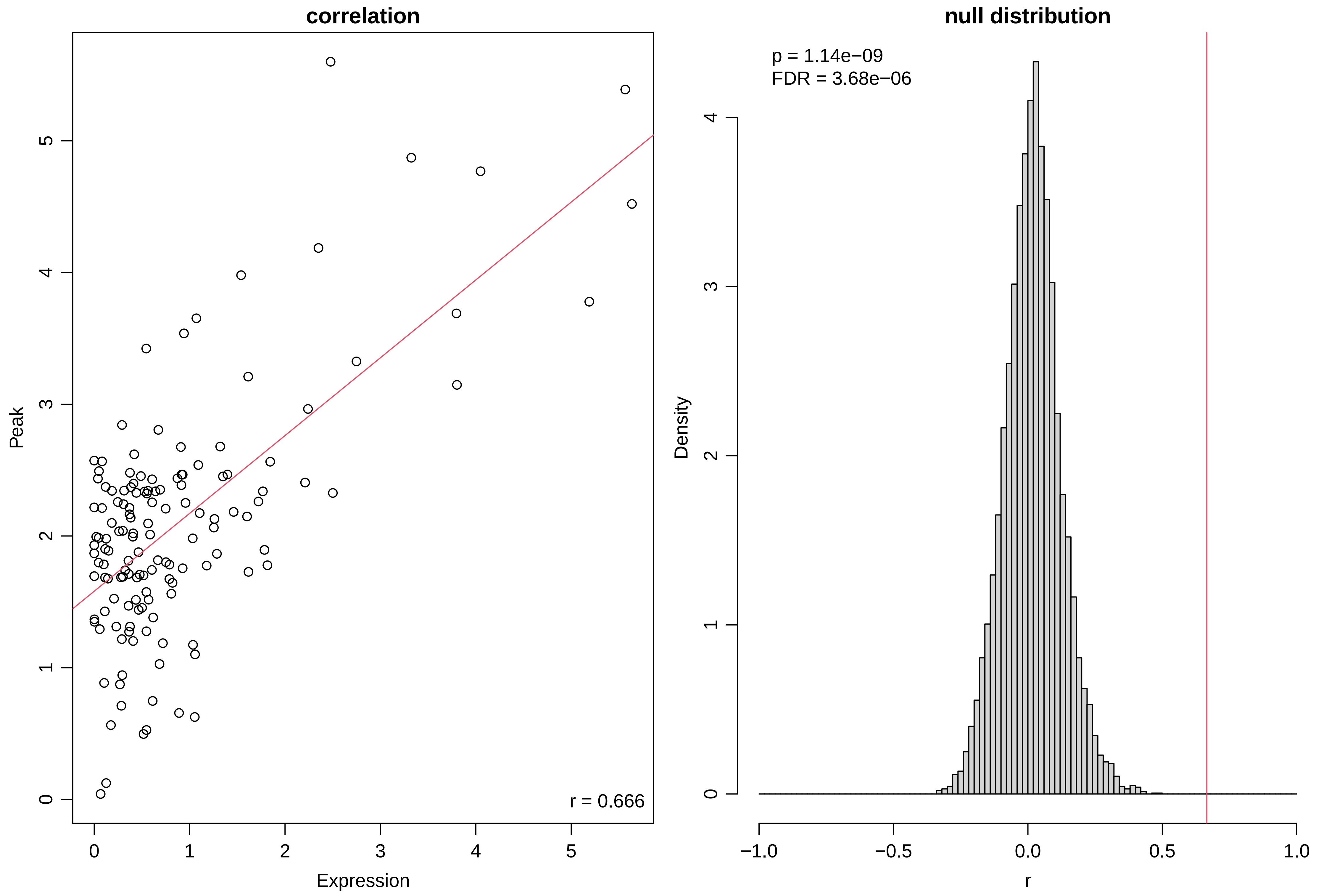

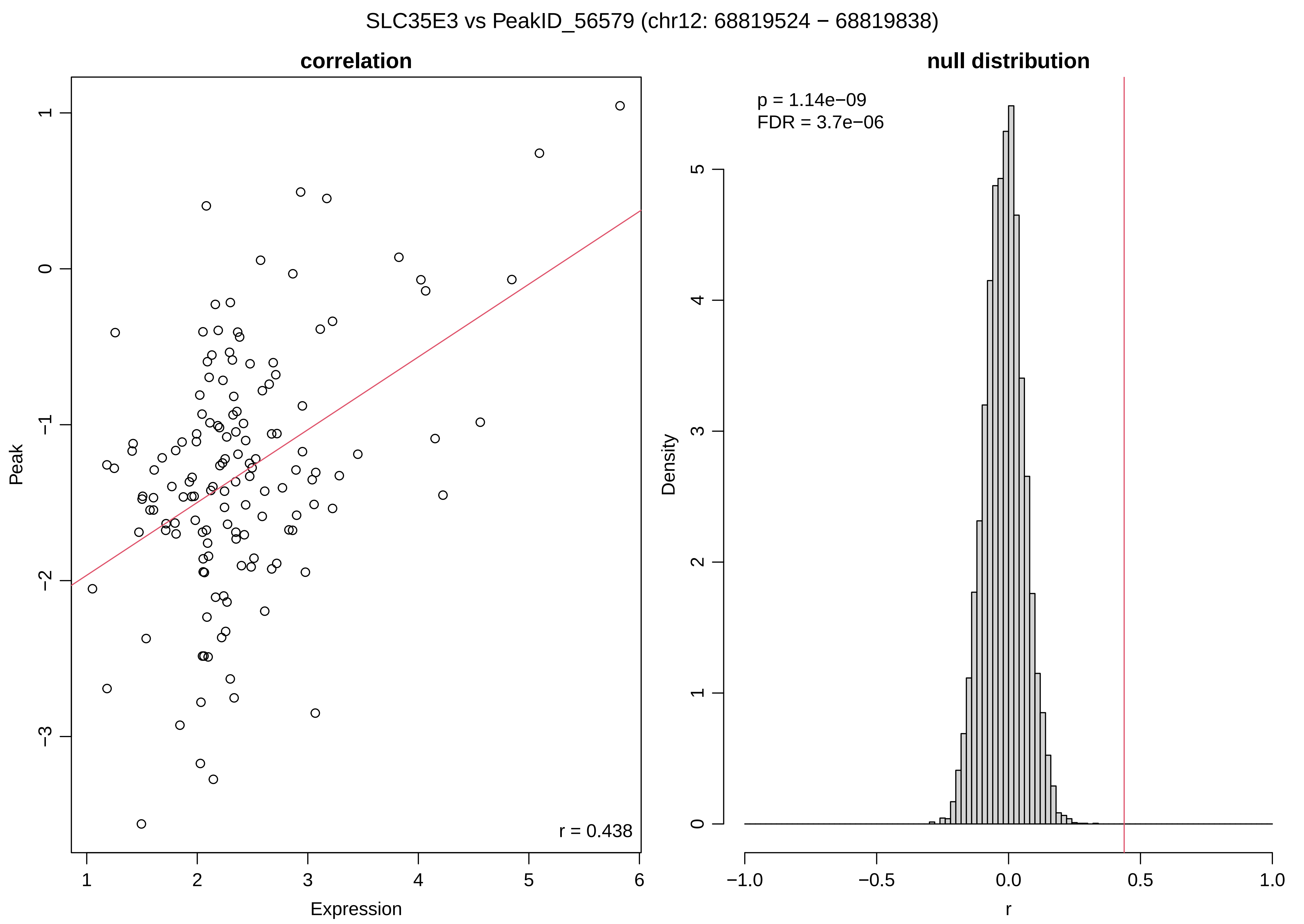
SLC35E3 vs PeakID_56579 (chr12: 68819524 − 68819838)
correlation
null distribution
Peak
Expression
r = 0.438
p = 1.14e−09
FDR = 3.7e−06
Density
r
1
0
−1
−2
−3
1
2
3
4
5
6
0
1
2
3
4
5
−1.0
−0.5
0.0
0.5
1.0

AL355796.1 vs PeakID_208955 (chr6: 79307149 − 79313380)

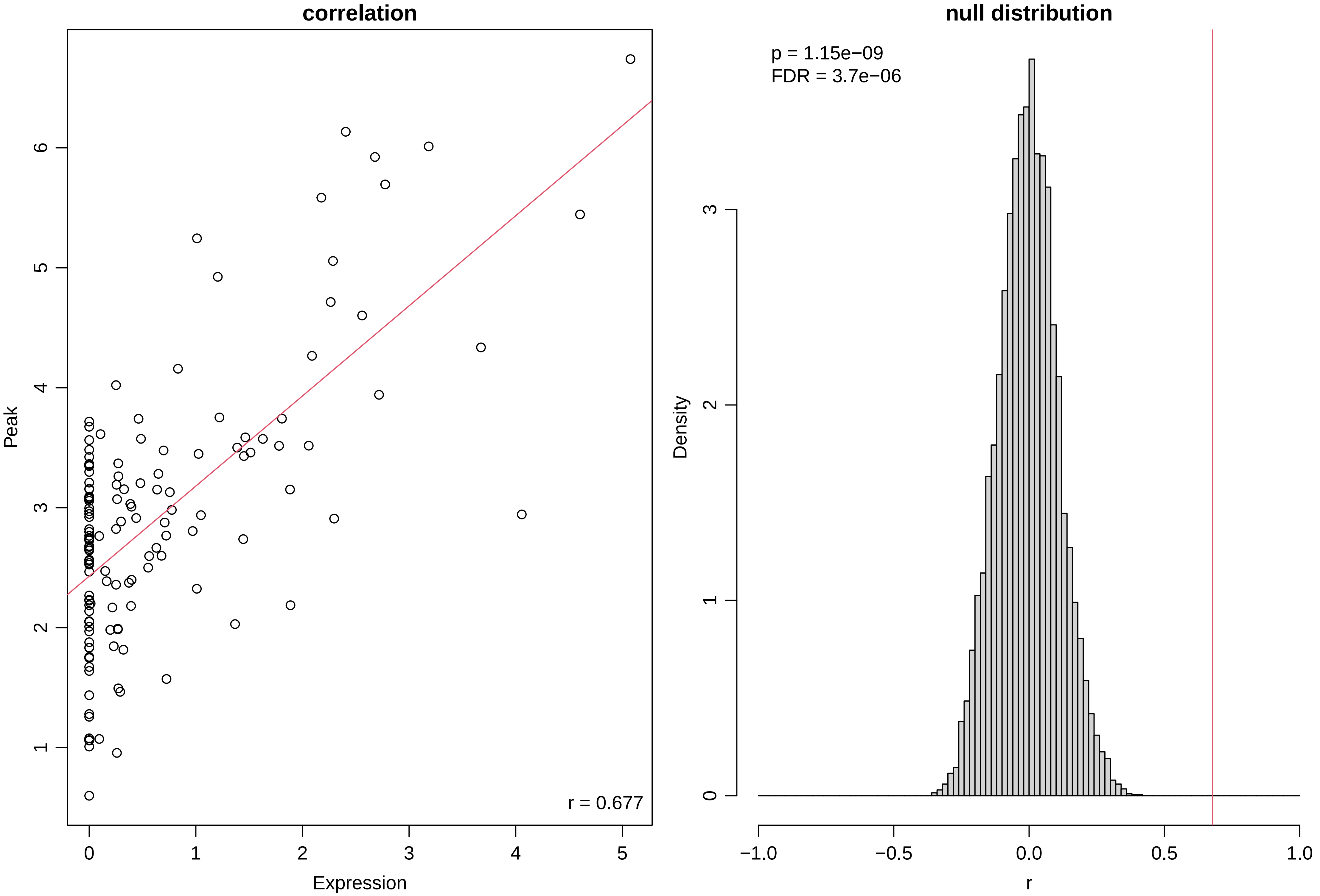

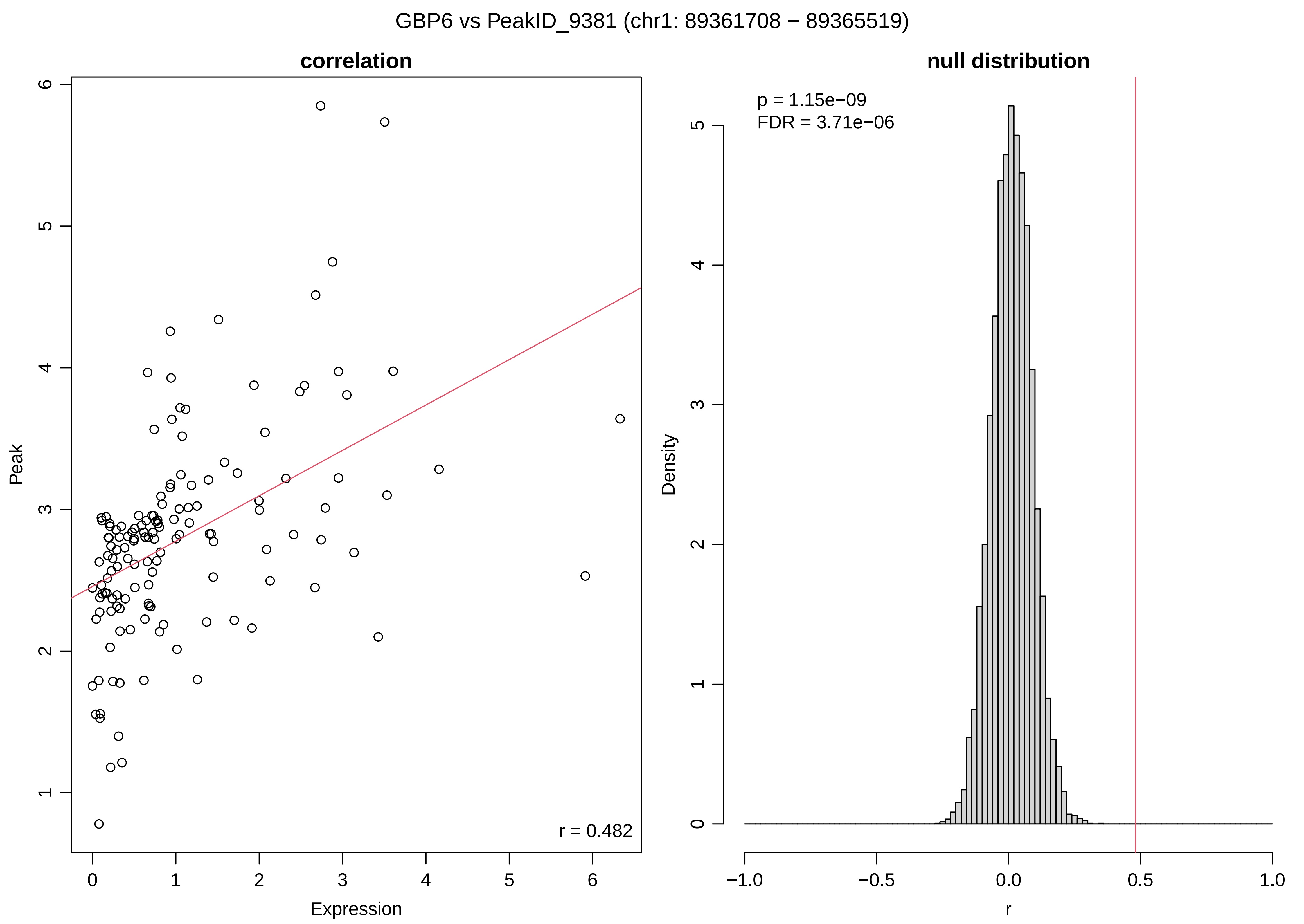

GBP6 vs PeakID_9381 (chr1: 89361708 − 89365519)
correlation
null distribution
p = 1.15e−09
FDR = 3.71e−06
Peak
Expression
r = 0.482
Density
r

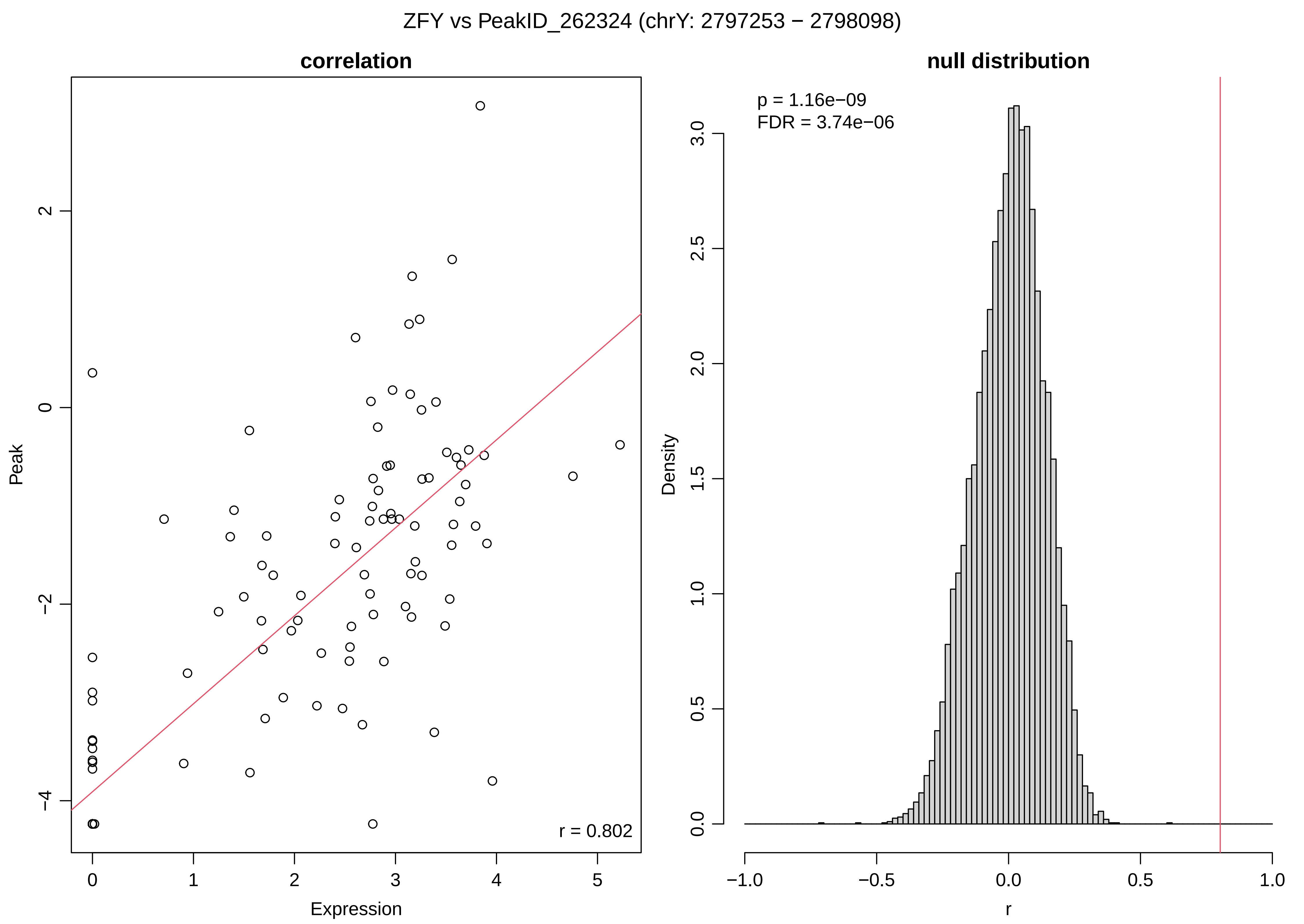
ZFY vs PeakID_262324 (chrY: 2797253 − 2798098)
correlation
Peak
Expression
r = 0.802
null distribution
p = 1.16e−09
FDR = 3.74e−06
Density
r

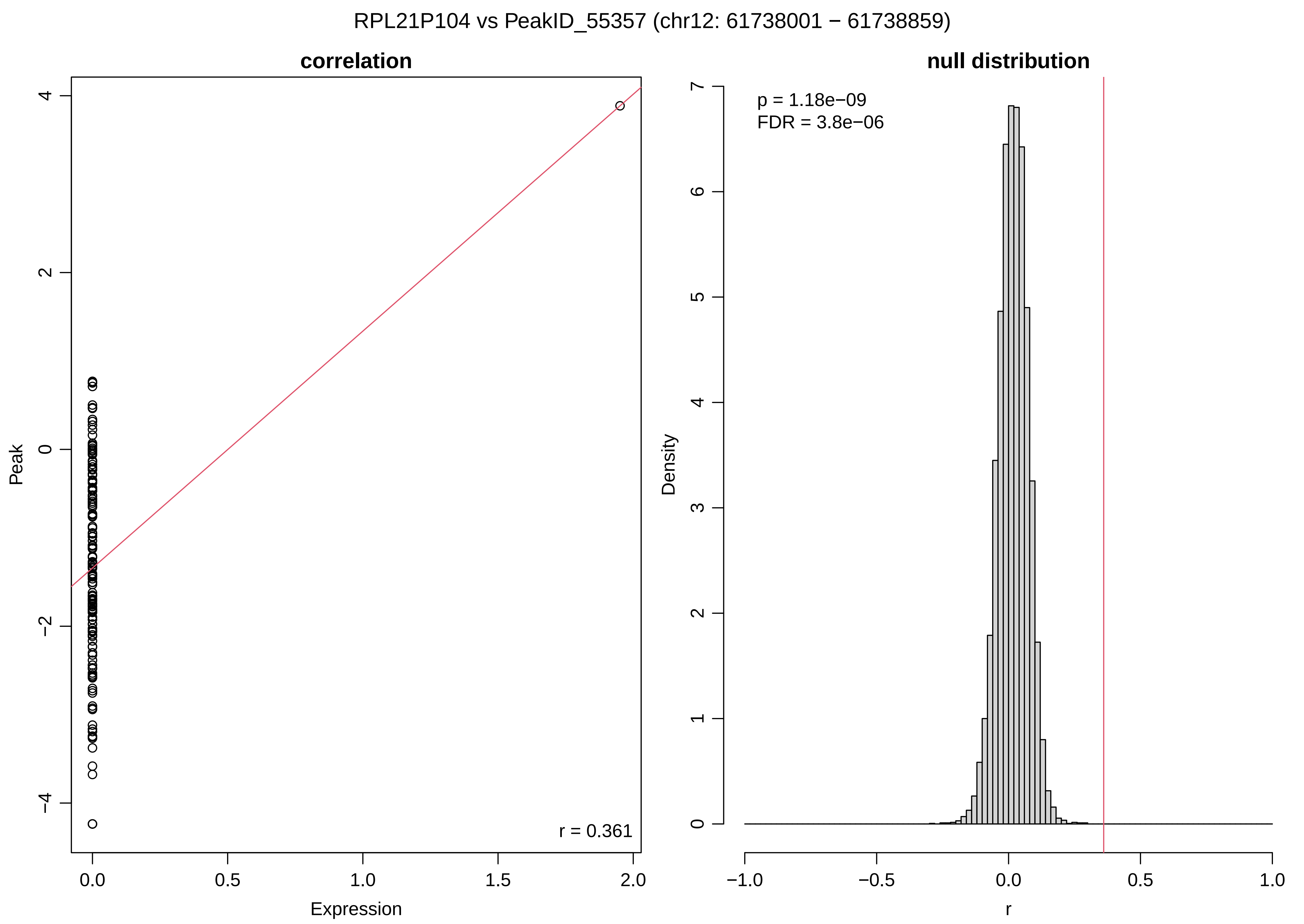

RPL21P104 vs PeakID_55357 (chr12: 61738001 − 61738859)
correlation
Peak
Expression
r = 0.361
null distribution
p = 1.18e−09
FDR = 3.8e−06
Density
r

CCT2 vs PeakID_56828 (chr12: 69434481 − 69443874)

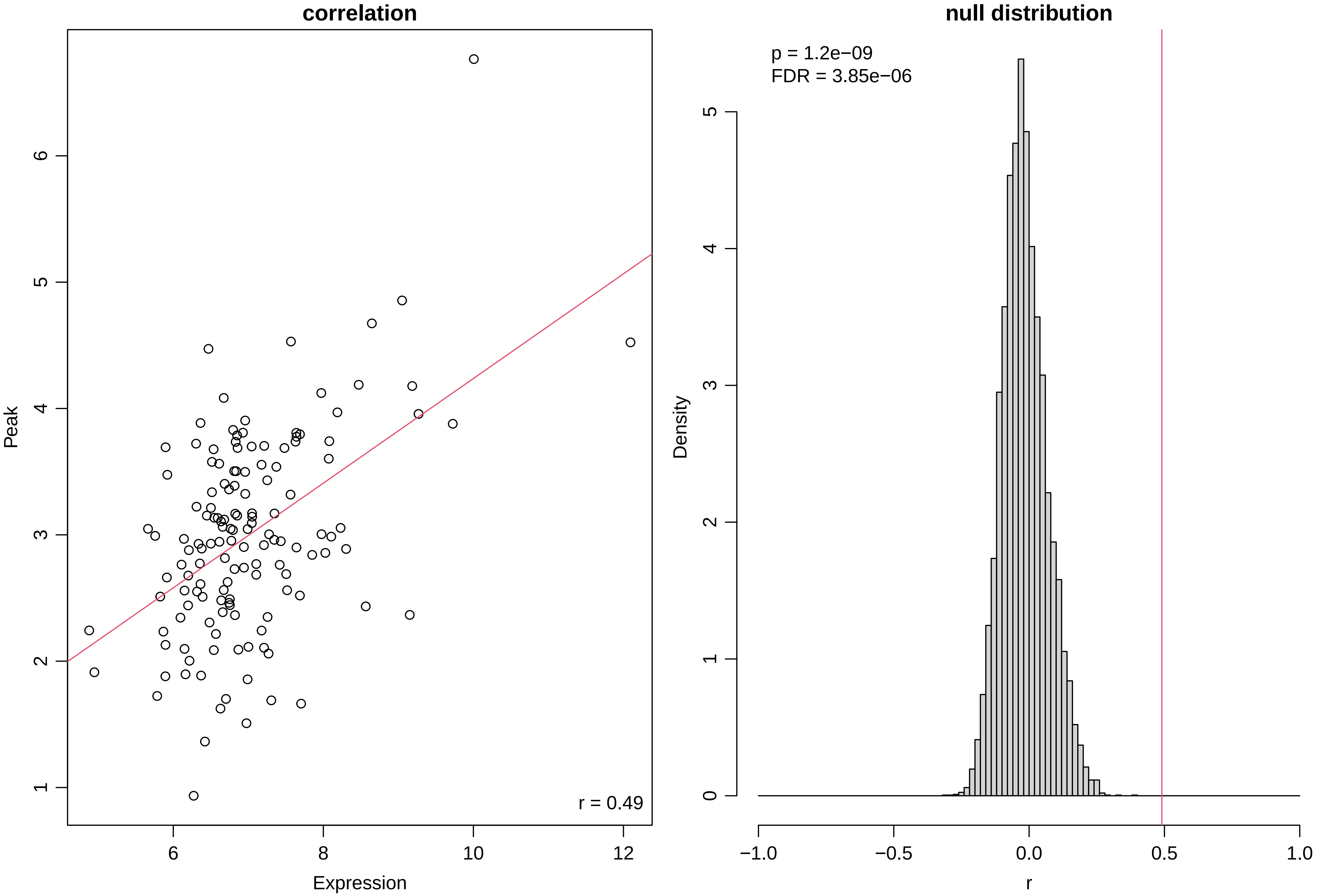

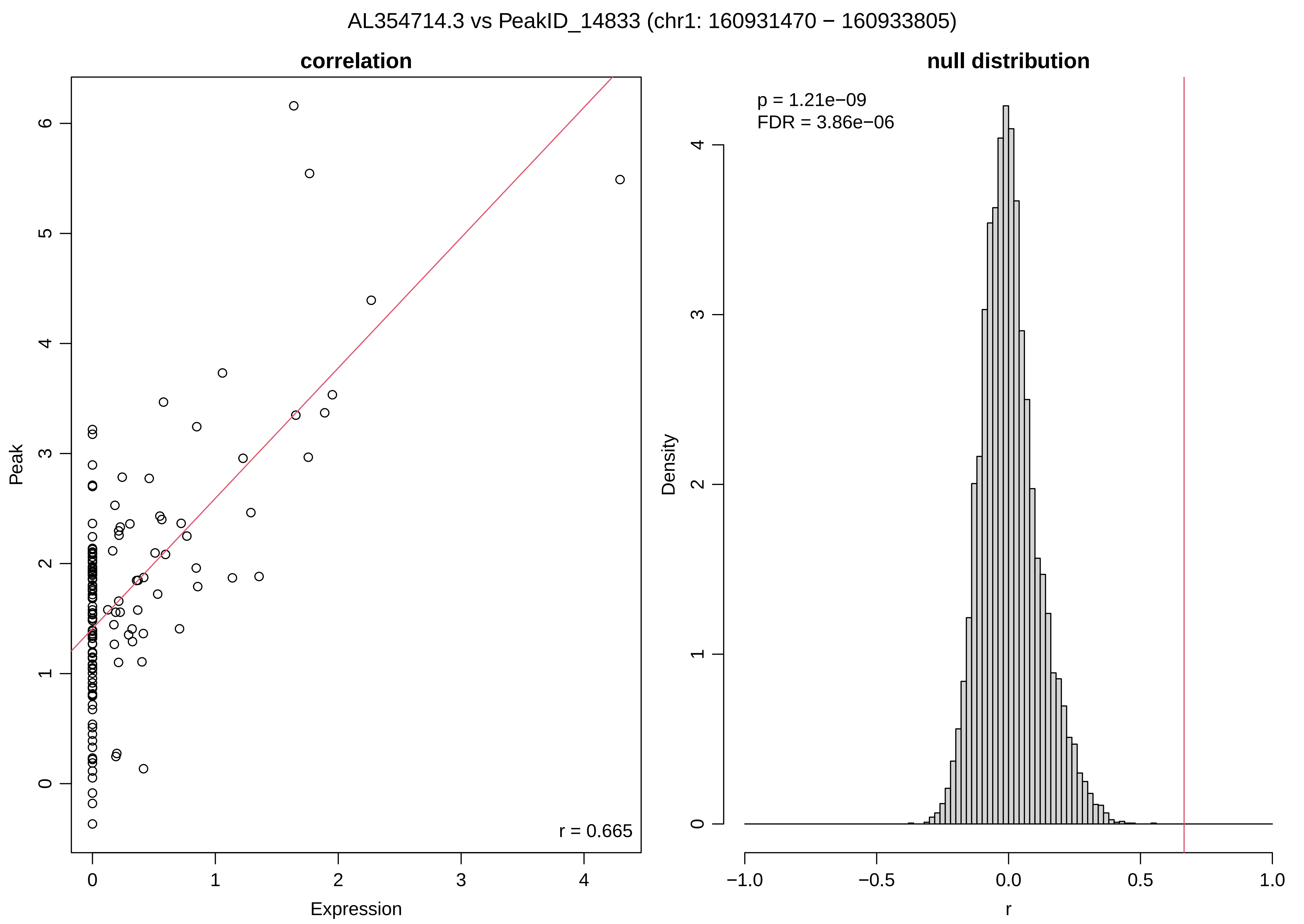

AL354714.3 vs PeakID_14833 (chr1: 160931470 − 160933805)
correlation
Peak
Expression
r = 0.665
null distribution
p = 1.21e−09
FDR = 3.86e−06
Density
r

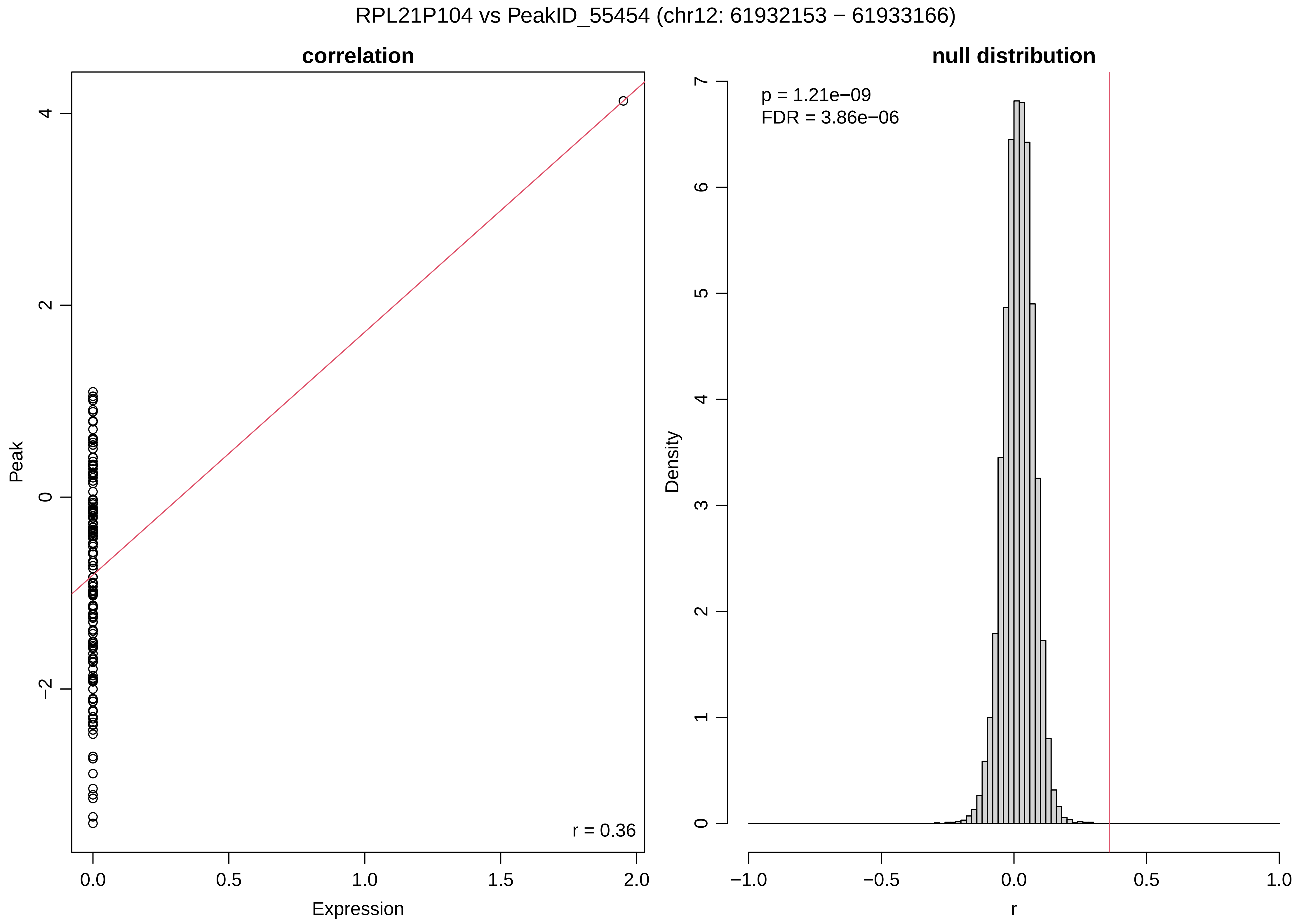

RPL21P104 vs PeakID_55454 (chr12: 61932153 − 61933166)
correlation
Peak
Expression
r = 0.36
4
2
0
−2
0.0
0.5
1.0
1.5
2.0
null distribution
p = 1.21e−09
FDR = 3.86e−06
Density
r
0
1
2
3
4
5
6
7
−1.0
−0.5
0.0
0.5
1.0

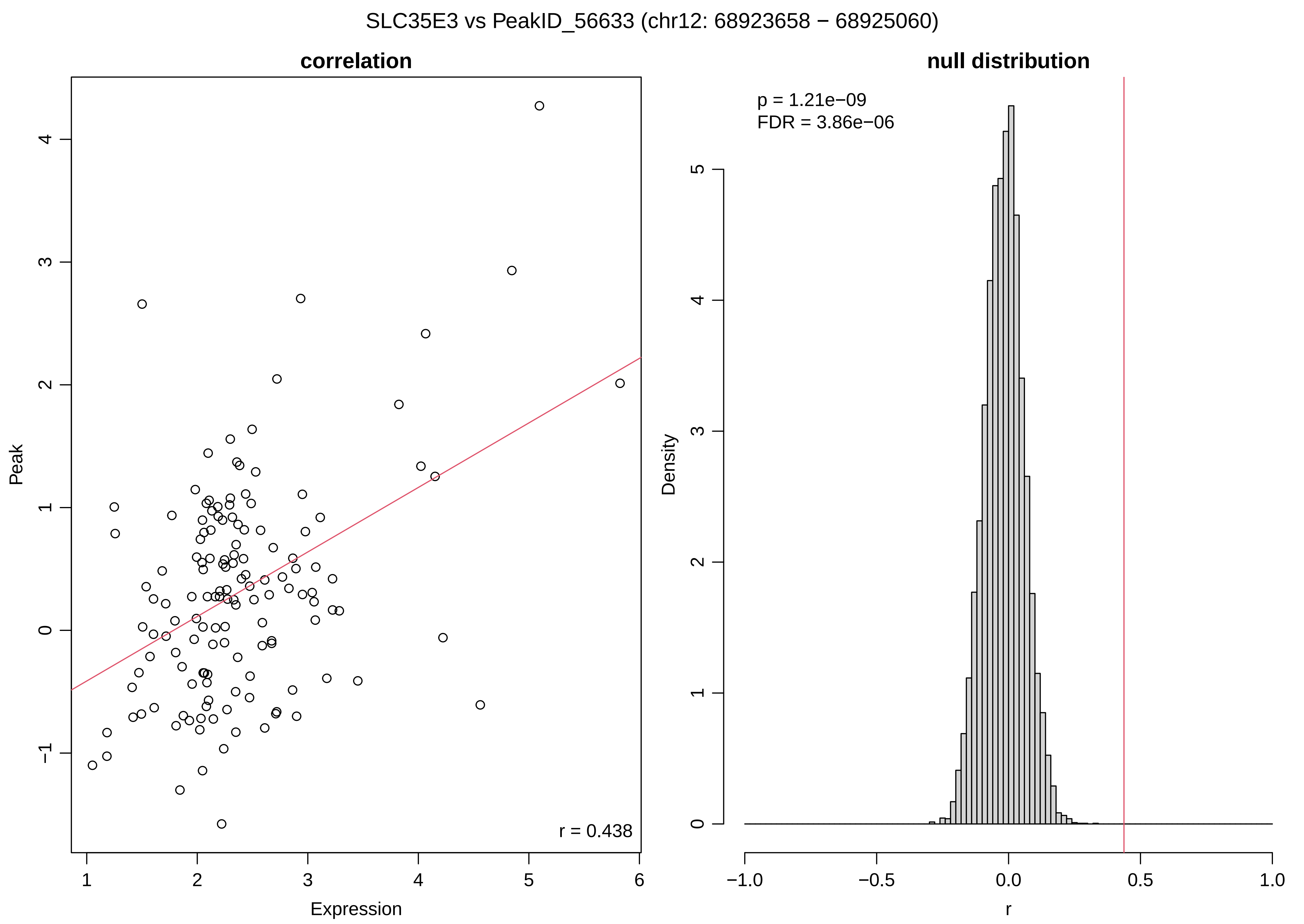

SLC35E3 vs PeakID_56633 (chr12: 68923658 − 68925060)
correlation
null distribution
Peak
Expression
r = 0.438
p = 1.21e−09
FDR = 3.86e−06
Density
r

TXLNGY vs PeakID_262931 (chrY: 19320986 − 19323286)

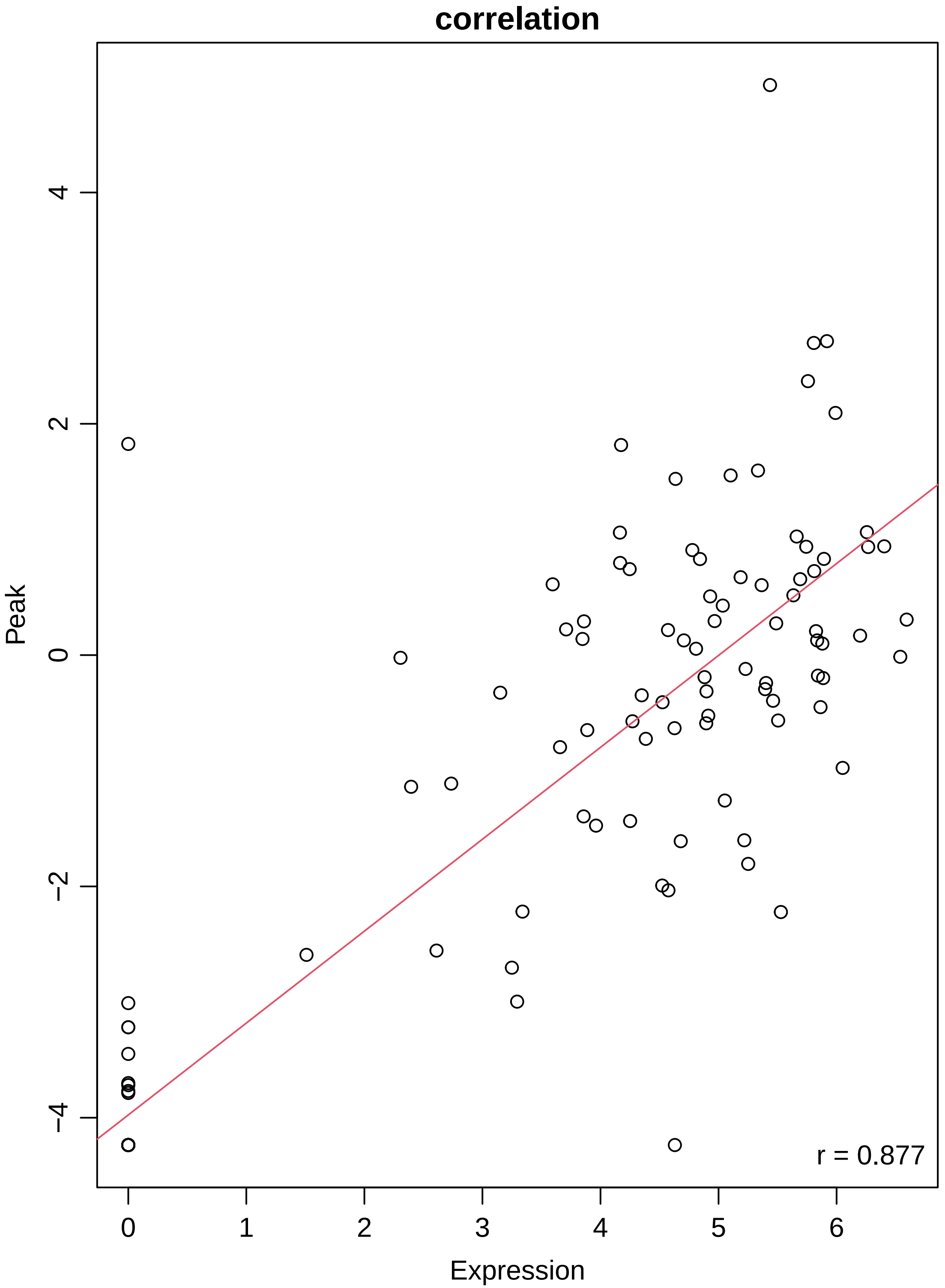

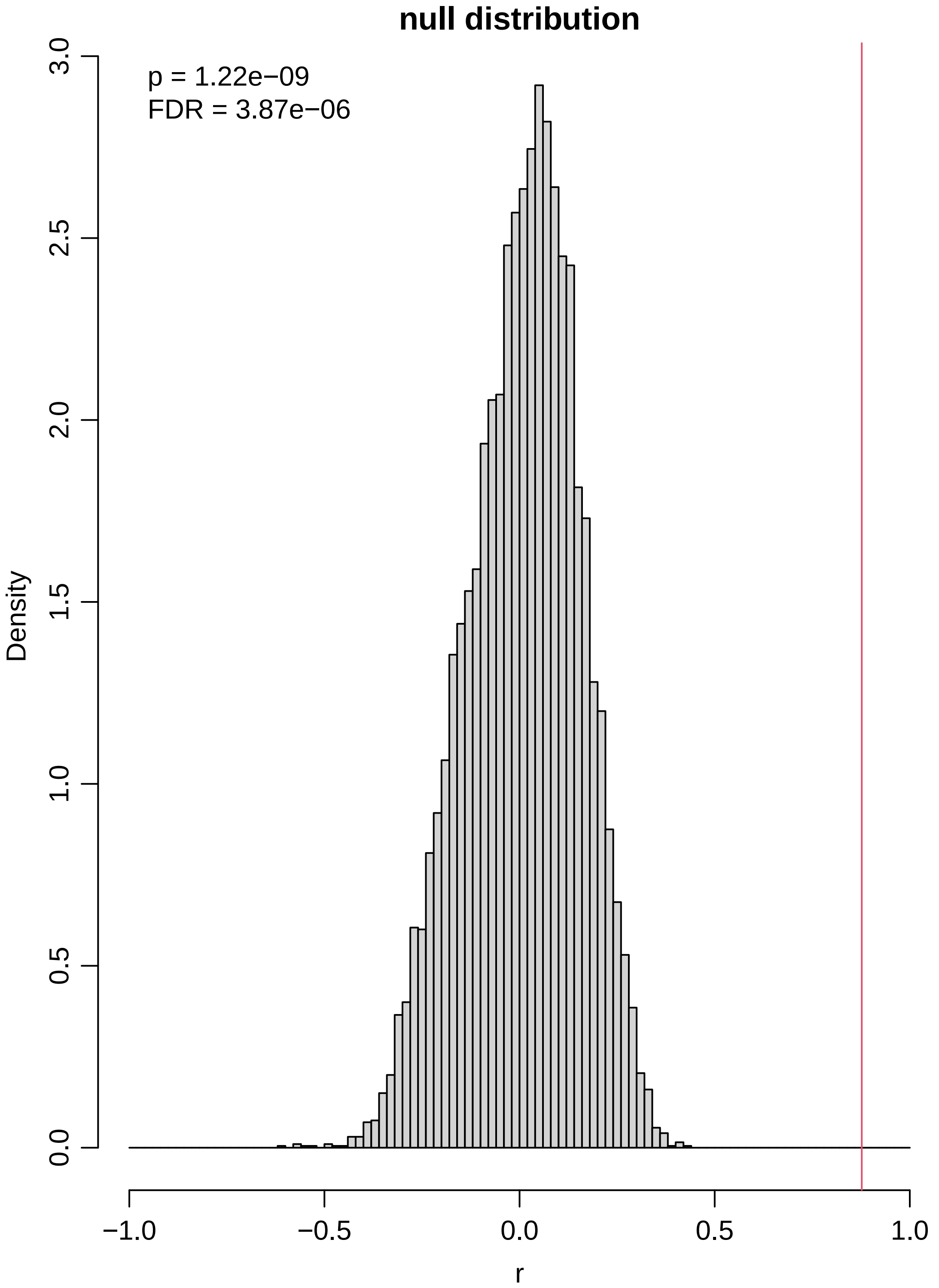

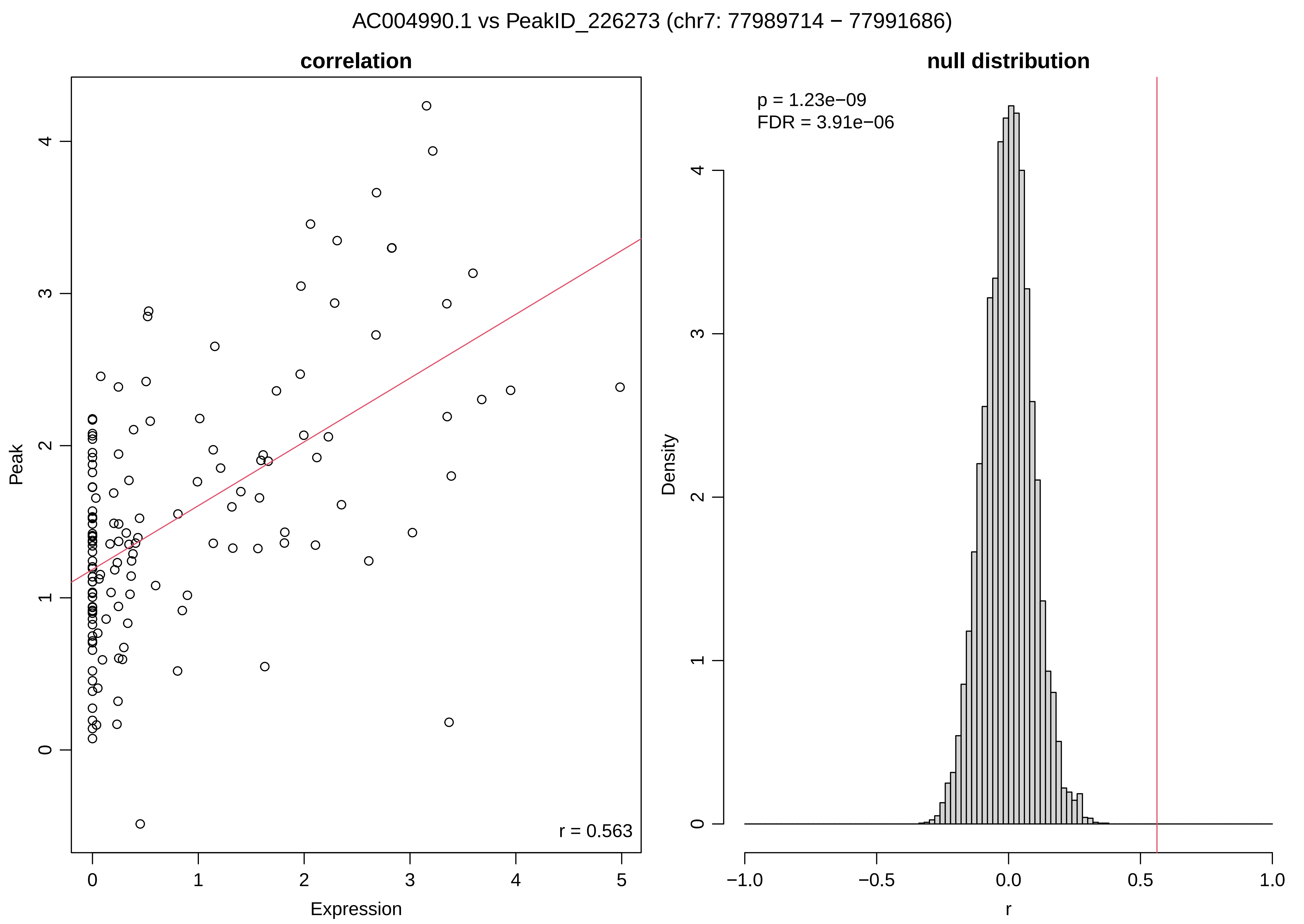
AC004990.1 vs PeakID_226273 (chr7: 77989714 − 77991686)
correlation
Peak
Expression
r = 0.563
0
1
2
3
4
5
null distribution
p = 1.23e−09
FDR = 3.91e−06
Density
r
−1.0
−0.5
0.0
0.5
1.0

FAM155B vs PeakID_259088 (chrX: 69503717 − 69505703)

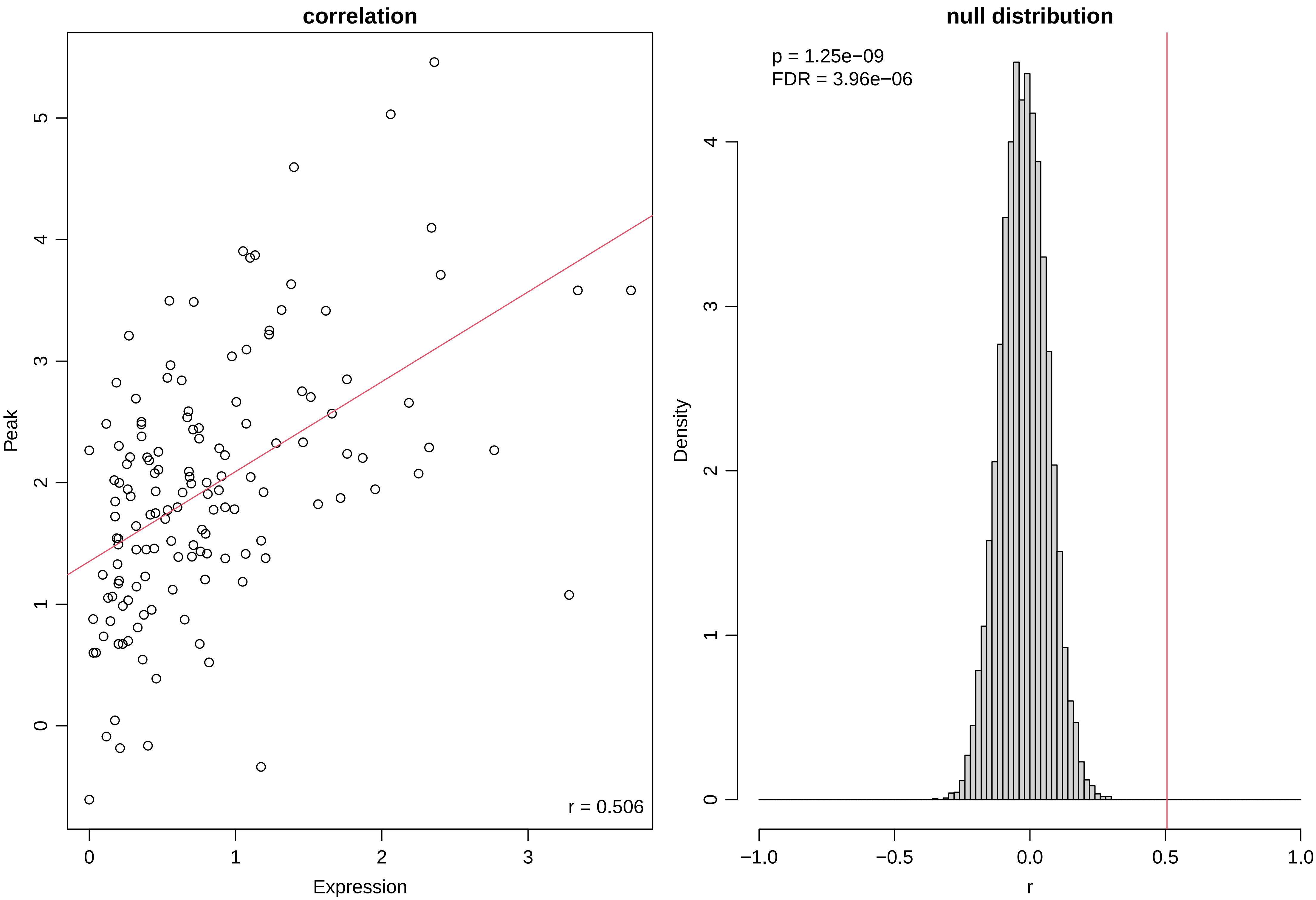

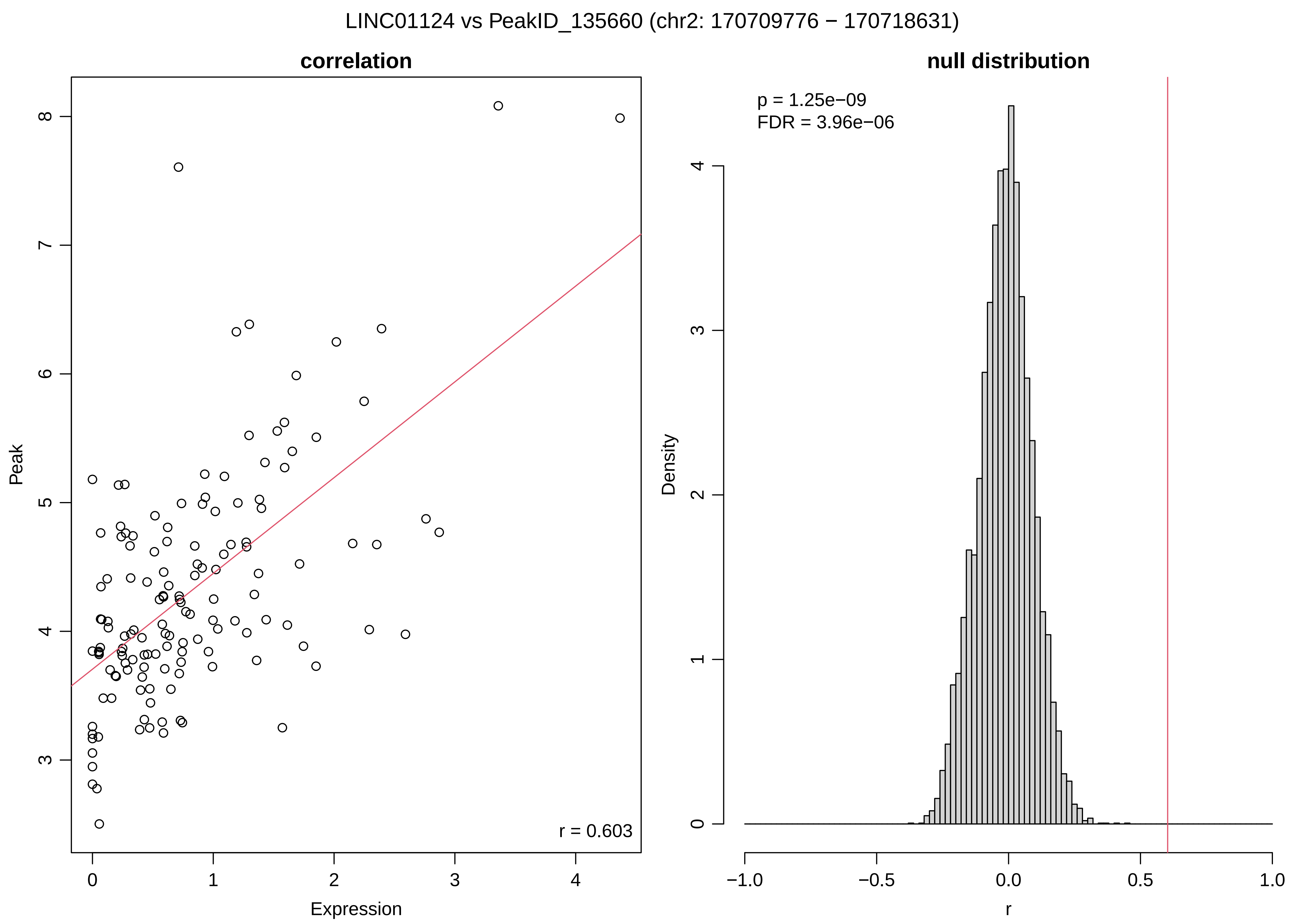

LINC01124 vs PeakID_135660 (chr2: 170709776 − 170718631)
correlation
Peak
Expression
r = 0.603
null distribution
p = 1.25e−09
FDR = 3.96e−06
Density
r

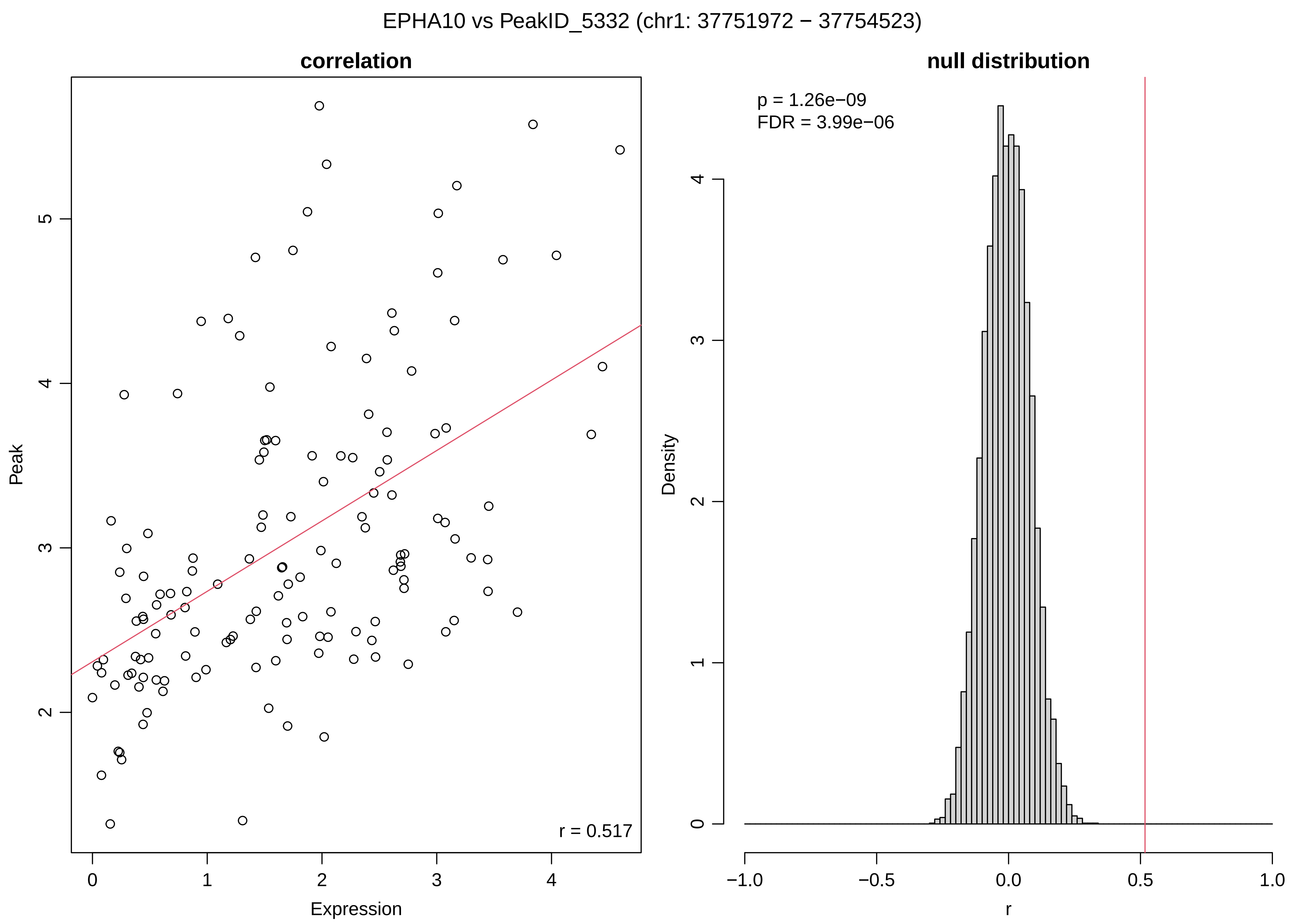

EPHA10 vs PeakID_5332 (chr1: 37751972 − 37754523)
correlation
Peak
Expression
r = 0.517
0
1
2
3
4
2
3
4
5
null distribution
p = 1.26e−09
FDR = 3.99e−06
Density
r
−1.0
−0.5
0.0
0.5
1.0
0
1
2
3
4

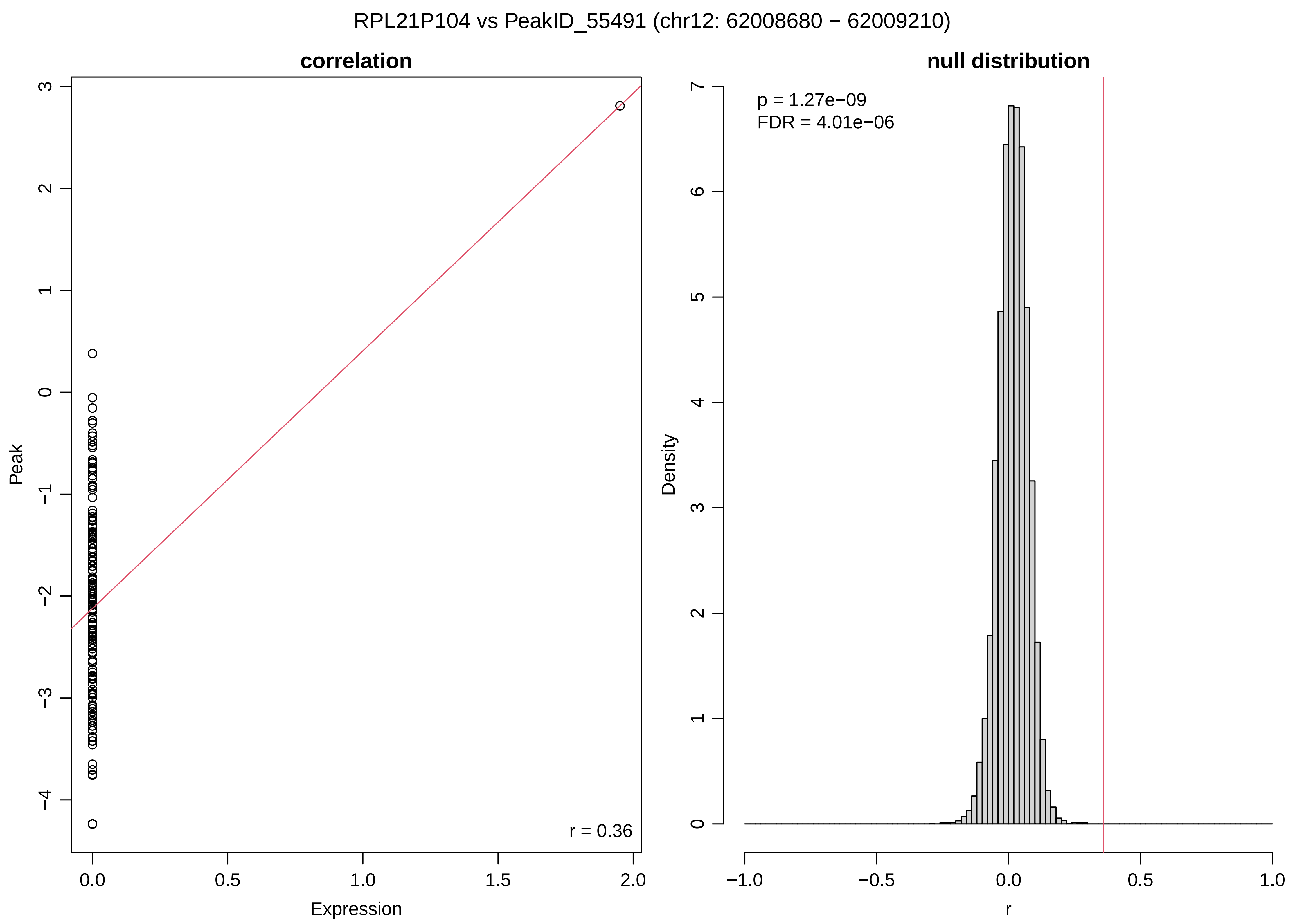

RPL21P104 vs PeakID_55491 (chr12: 62008680 − 62009210)
correlation
Peak
Expression
r = 0.36
null distribution
Density
r
p = 1.27e−09
FDR = 4.01e−06

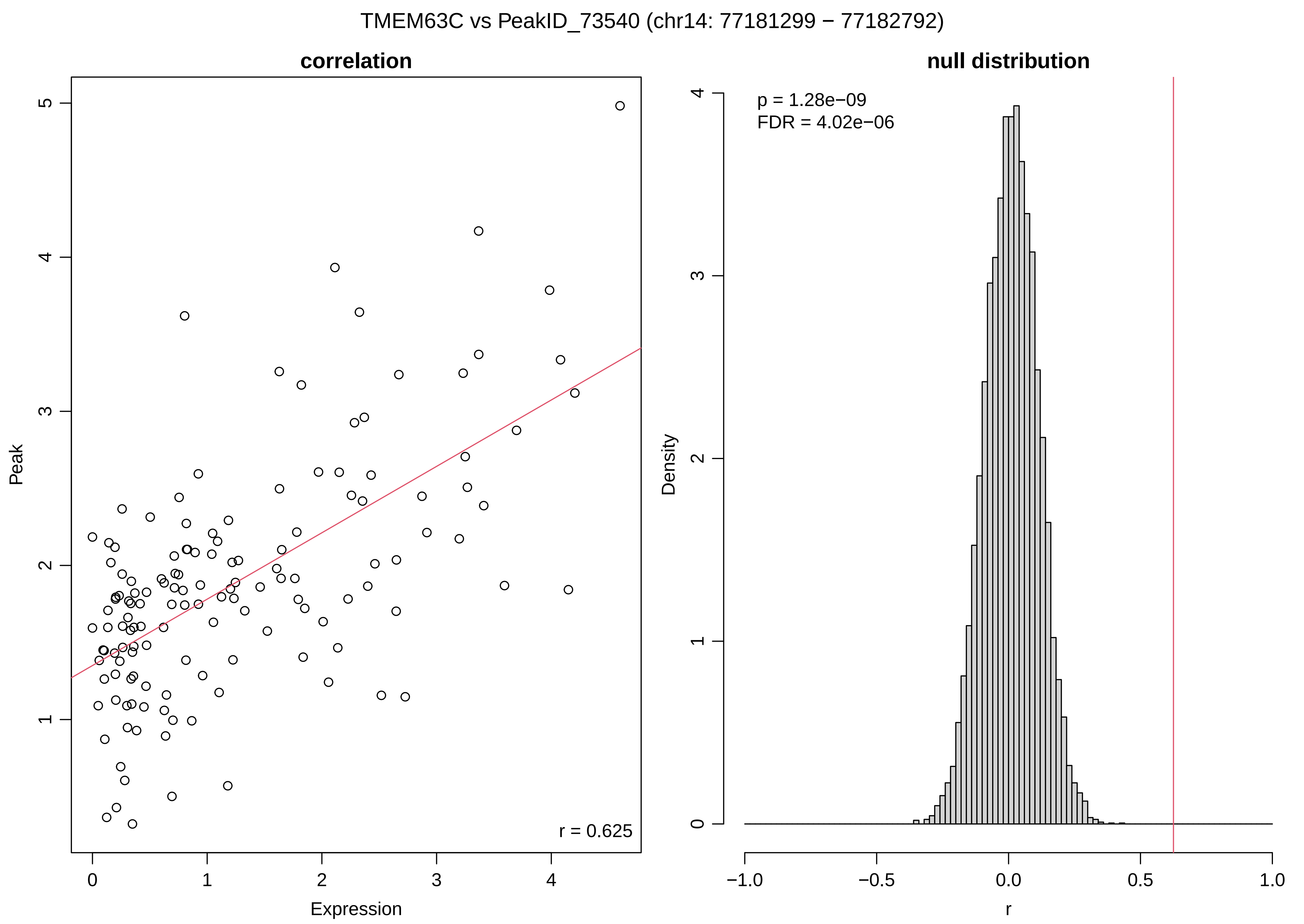

TMEM63C vs PeakID_73540 (chr14: 77181299 − 77182792)
correlation
Peak
Expression
r = 0.625
0
1
2
3
4
5
null distribution
p = 1.28e−09
FDR = 4.02e−06
Density
r
0
1
2
3
4
−1.0
−0.5
0.0
0.5
1.0

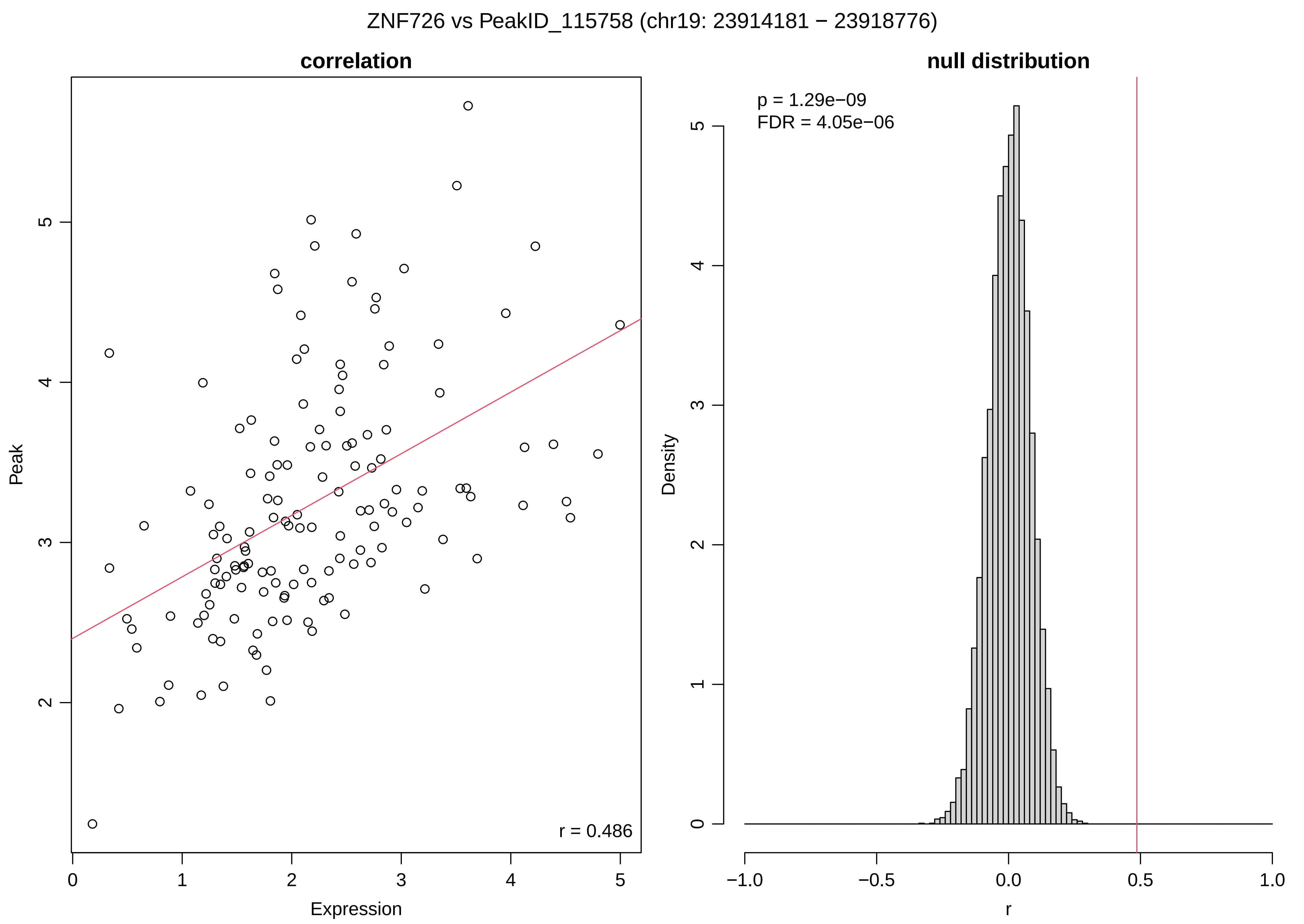

ZNF726 vs PeakID_115758 (chr19: 23914181 − 23918776)
correlation
null distribution
Peak
Expression
r = 0.486
p = 1.29e−09
FDR = 4.05e−06
Density
r

MSX2 vs PeakID_200105 (chr5: 174724504 − 174727452)

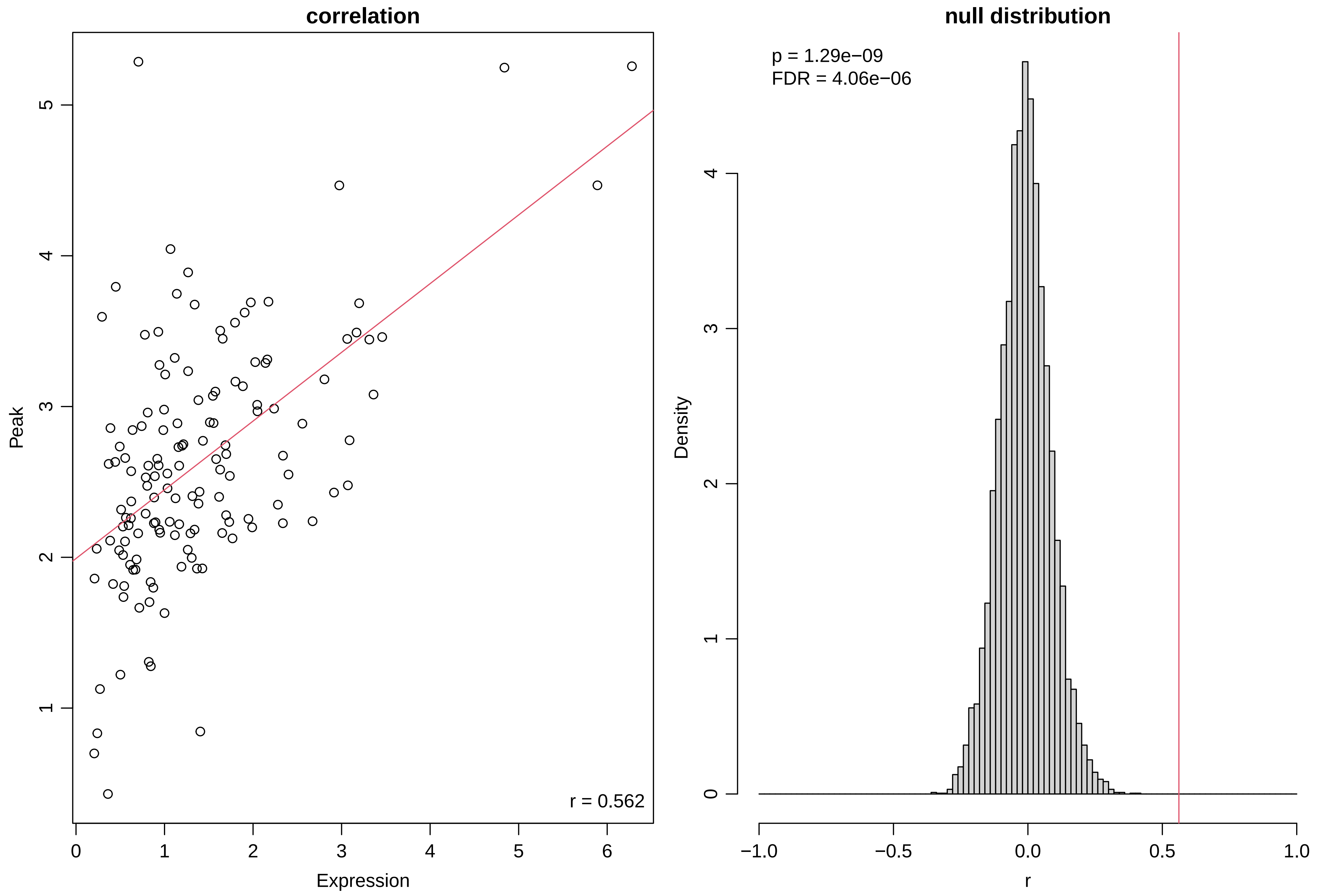

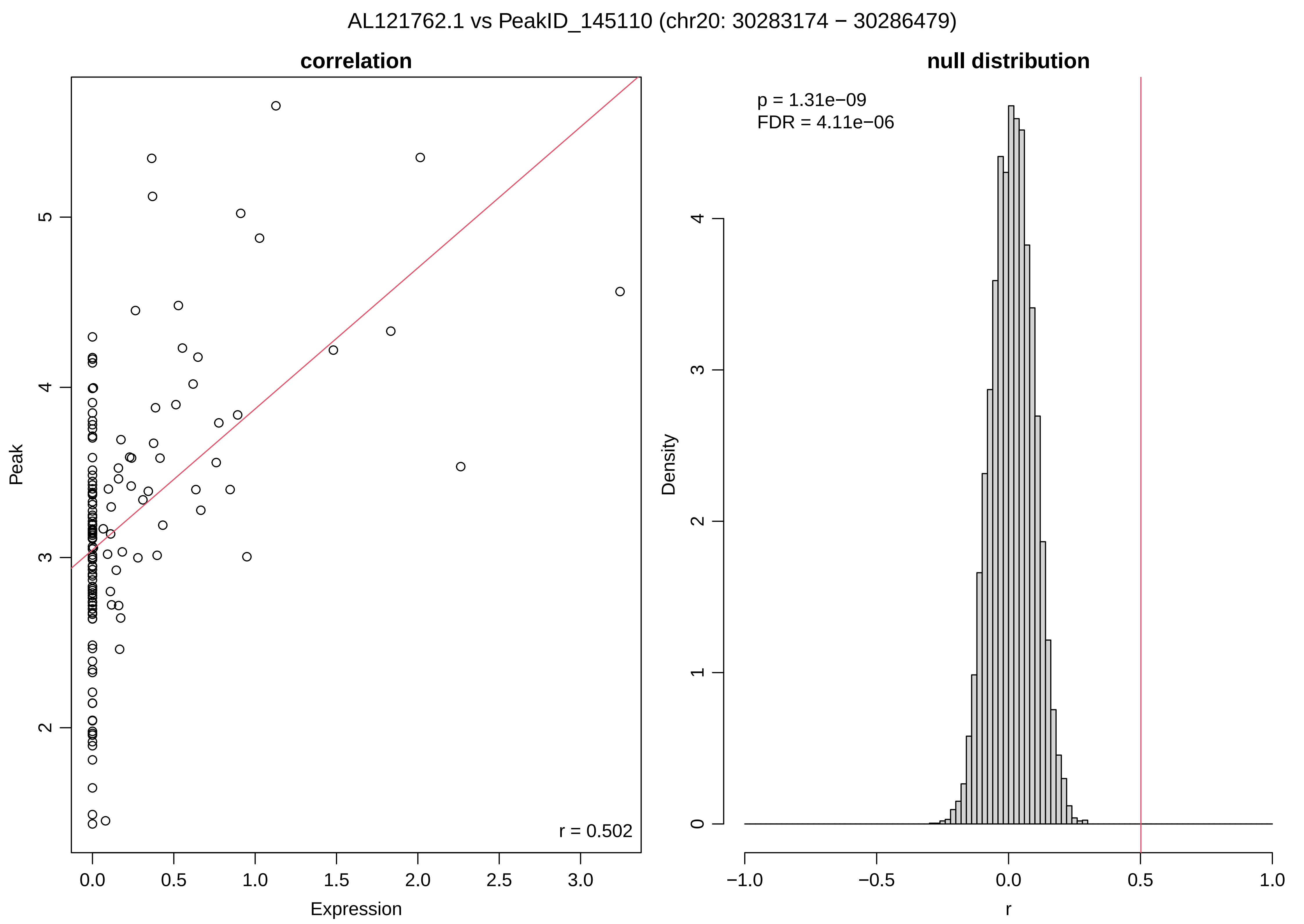
AL121762.1 vs PeakID_145110 (chr20: 30283174 − 30286479)
correlation
null distribution
p = 1.31e−09
FDR = 4.11e−06
r = 0.502
Peak
Expression
Density
r
0.0
0.5
1.0
1.5
2.0
2.5
3.0
2
3
4
5
−1.0
−0.5
0.0
0.5
1.0
0
1
2
3
4

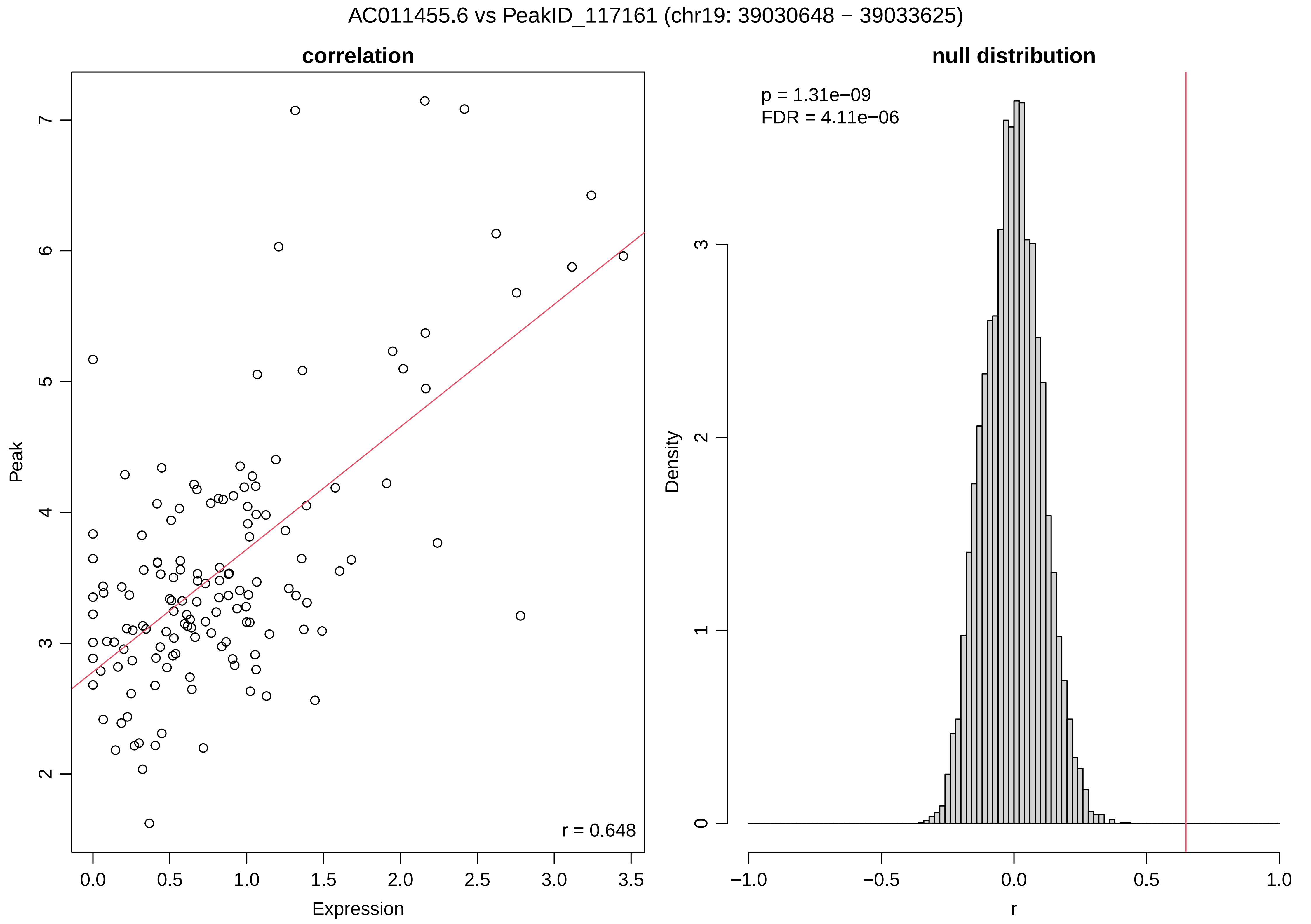

AC011455.6 vs PeakID_117161 (chr19: 39030648 − 39033625)
correlation
null distribution
p = 1.31e−09
FDR = 4.11e−06
r = 0.648
Peak
Expression
Density
r

PRKY vs PeakID_262393 (chrY: 7339414 − 7340329)

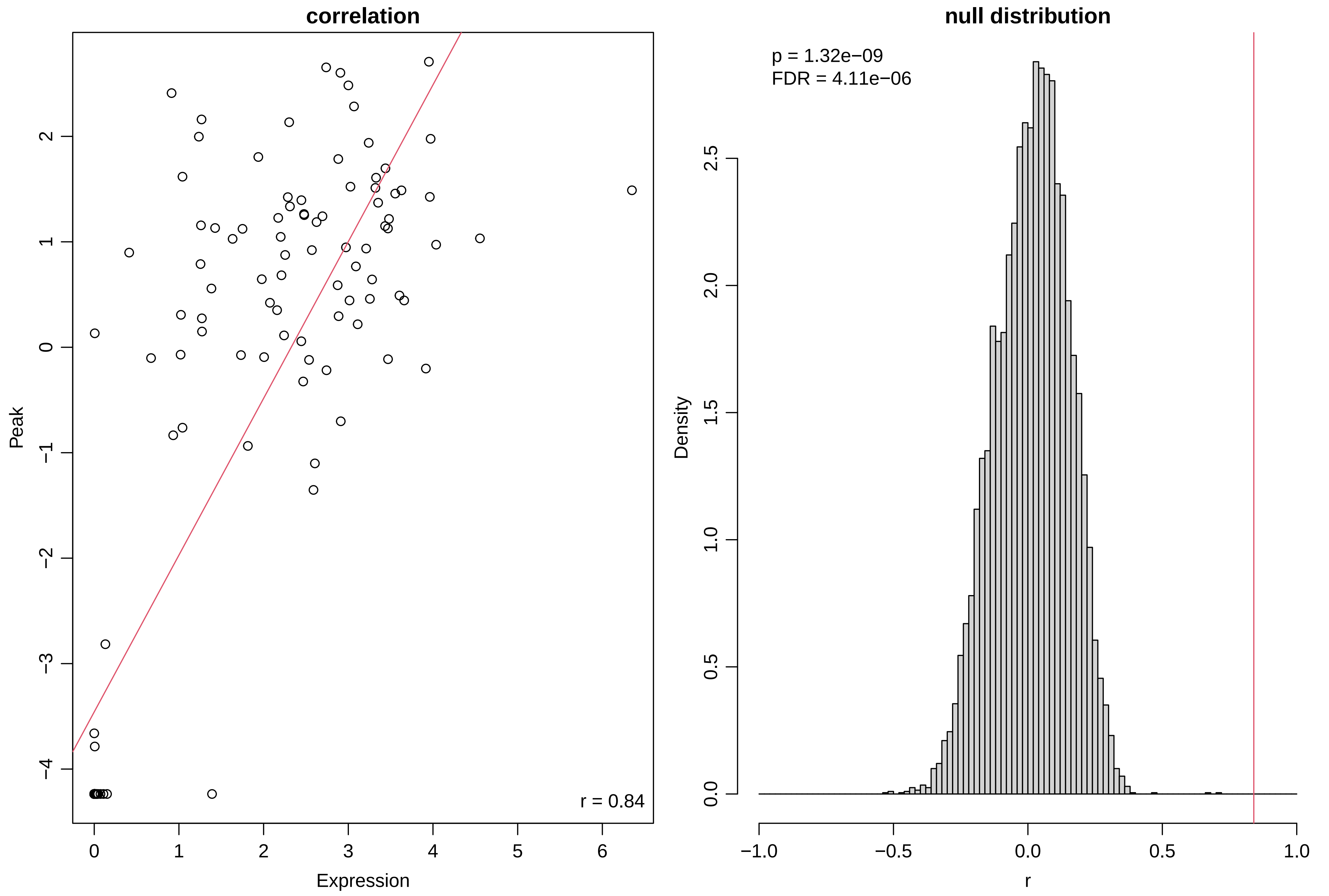

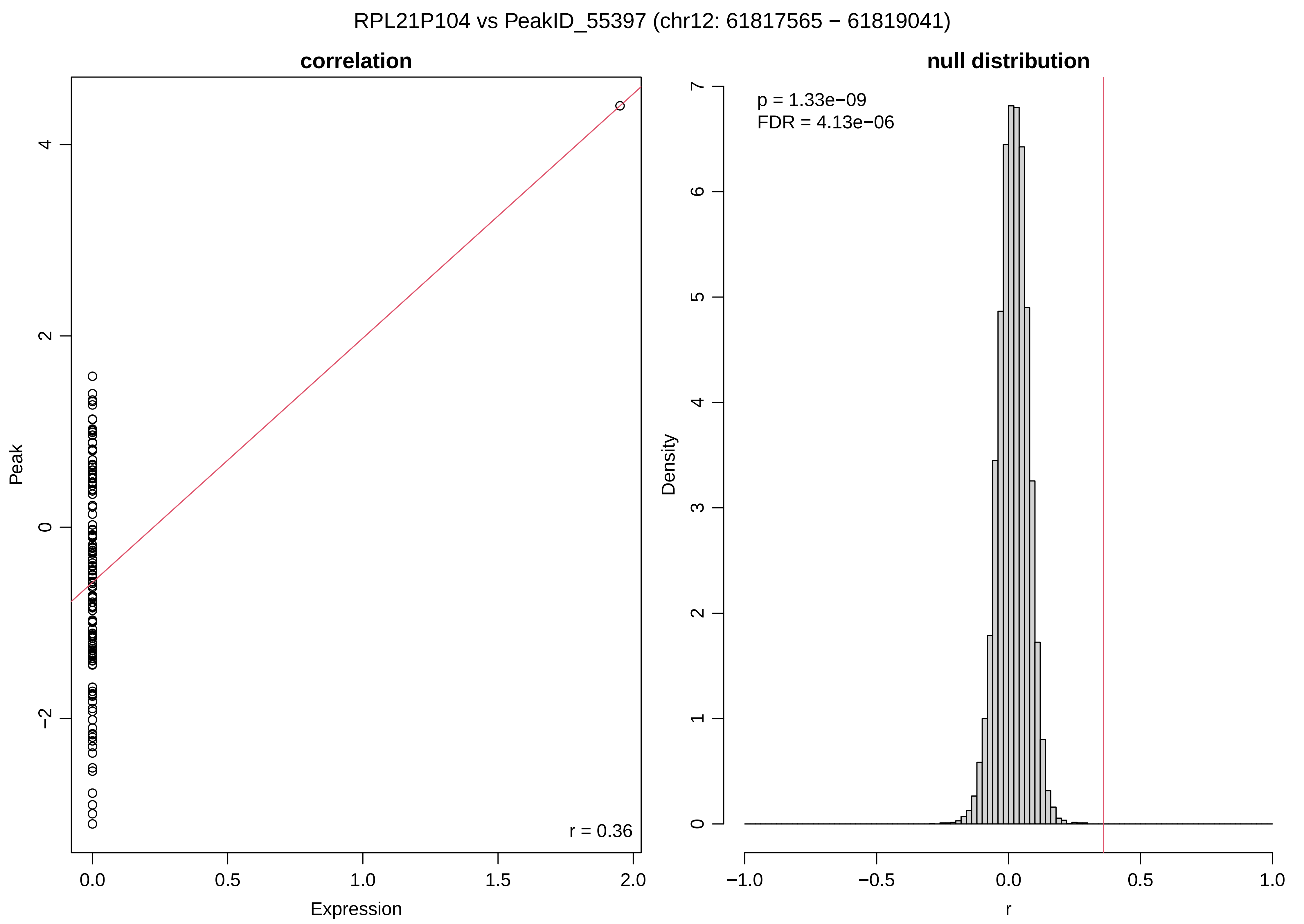

RPL21P104 vs PeakID_55397 (chr12: 61817565 − 61819041)
correlation
Peak
Expression
r = 0.36
null distribution
p = 1.33e−09
FDR = 4.13e−06
Density
r

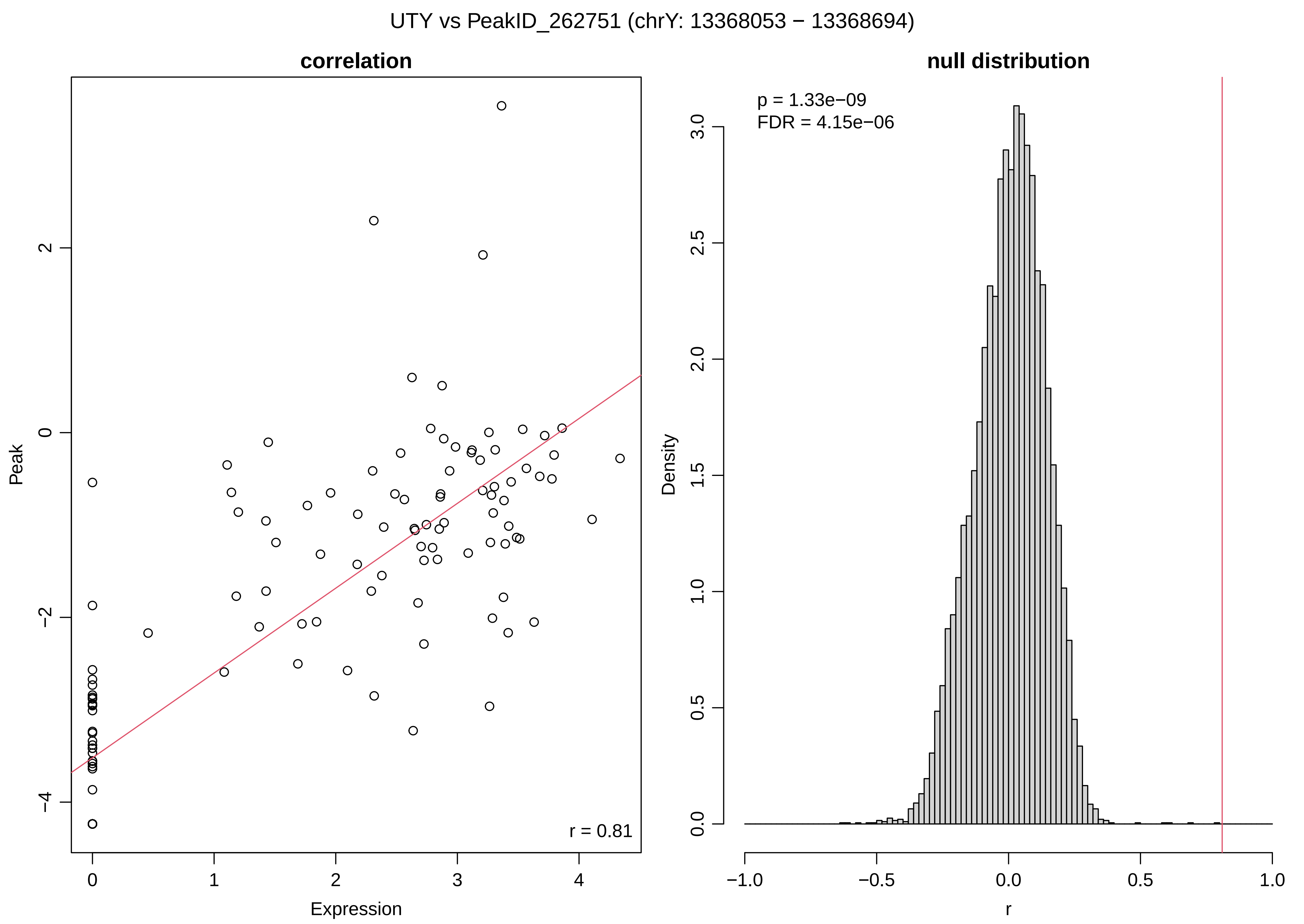
UTY vs PeakID_262751 (chrY: 13368053 − 13368694)
correlation
Peak
Expression
r = 0.81
null distribution
p = 1.33e−09
FDR = 4.15e−06
Density
r

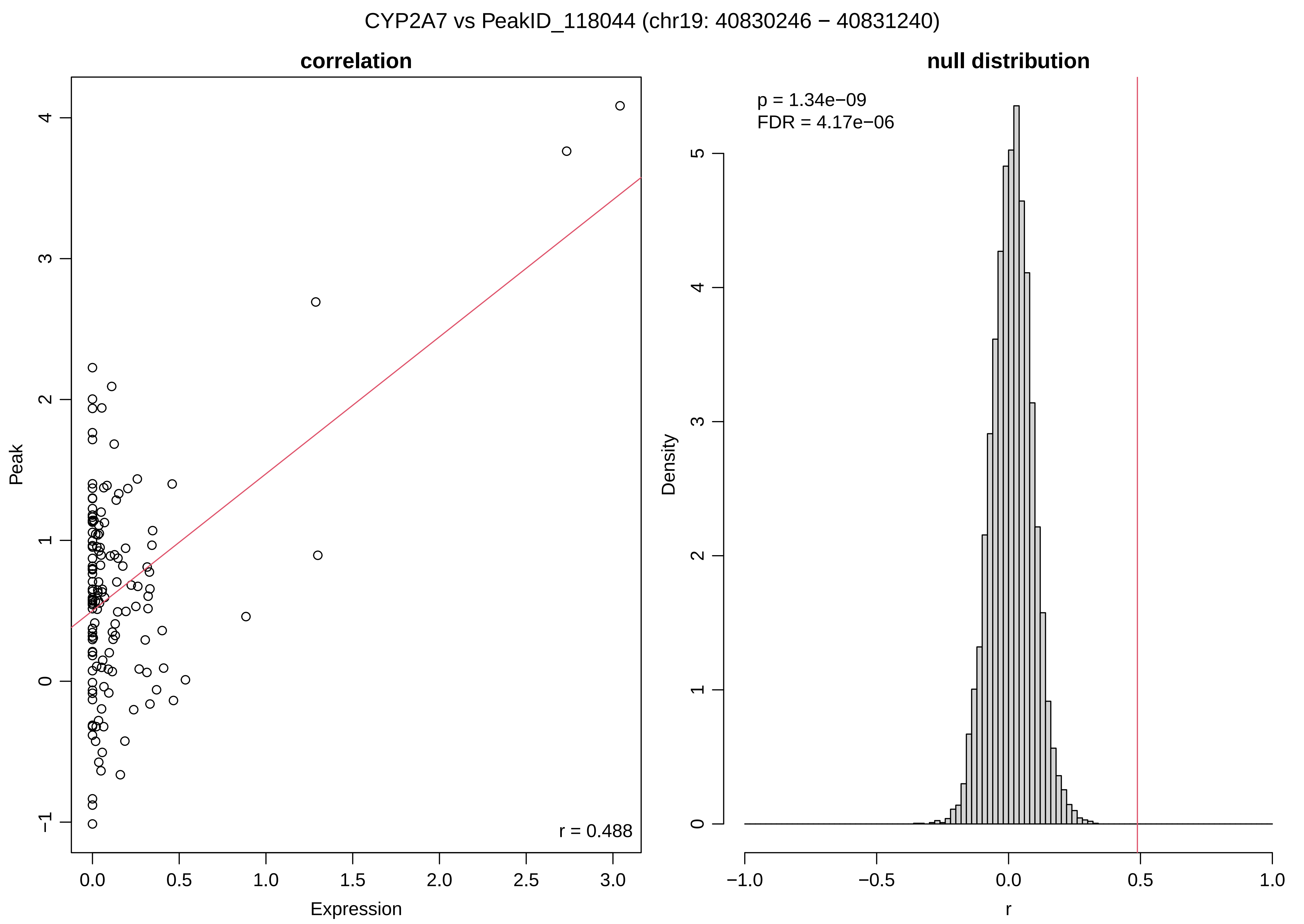
CYP2A7 vs PeakID_118044 (chr19: 40830246 − 40831240)
correlation
Peak
Expression
r = 0.488
null distribution
p = 1.34e−09
FDR = 4.17e−06
Density
r

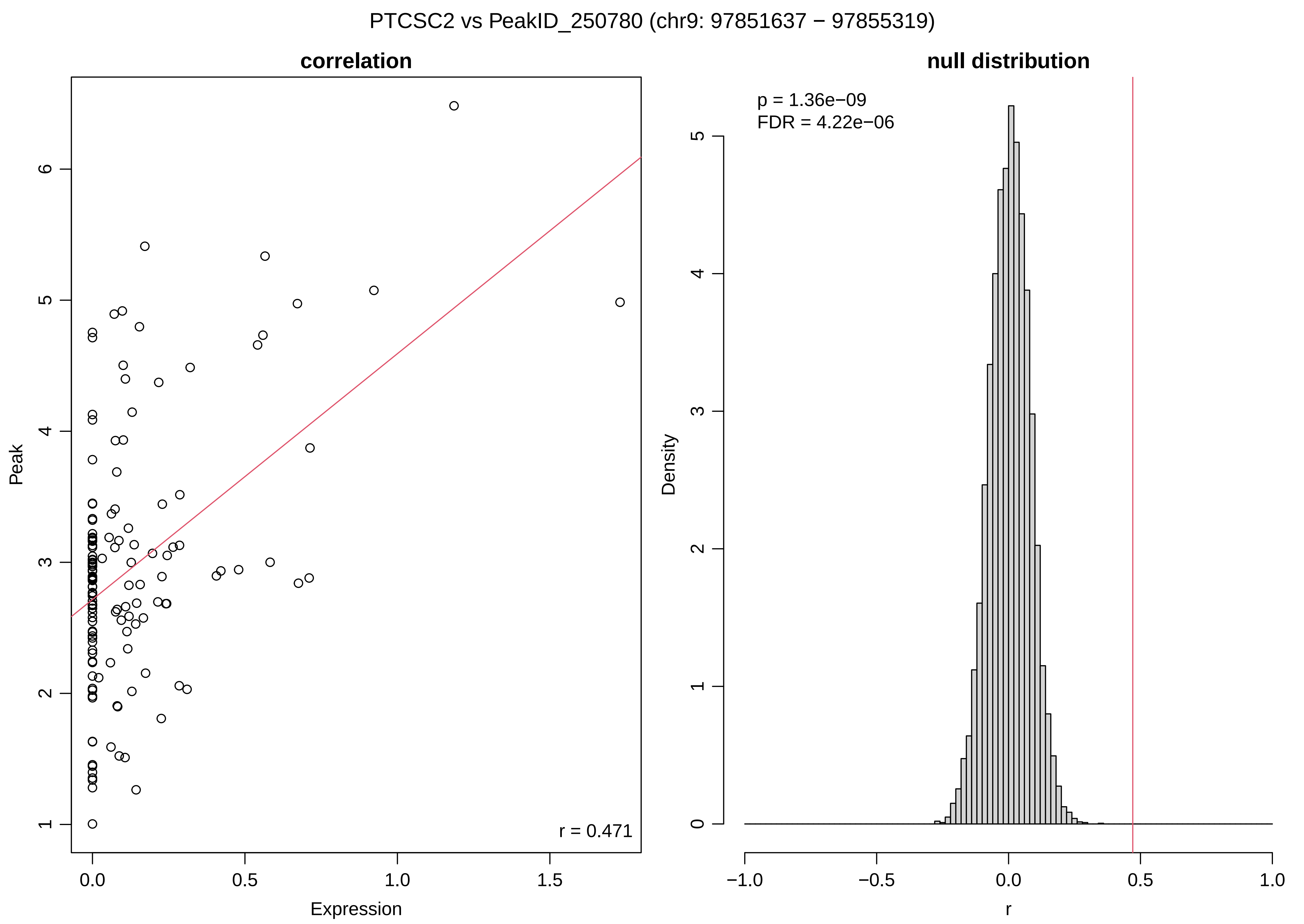
PTCSC2 vs PeakID_250780 (chr9: 97851637 − 97855319)
correlation
Peak
Expression
r = 0.471
null distribution
p = 1.36e−09
FDR = 4.22e−06
Density
r

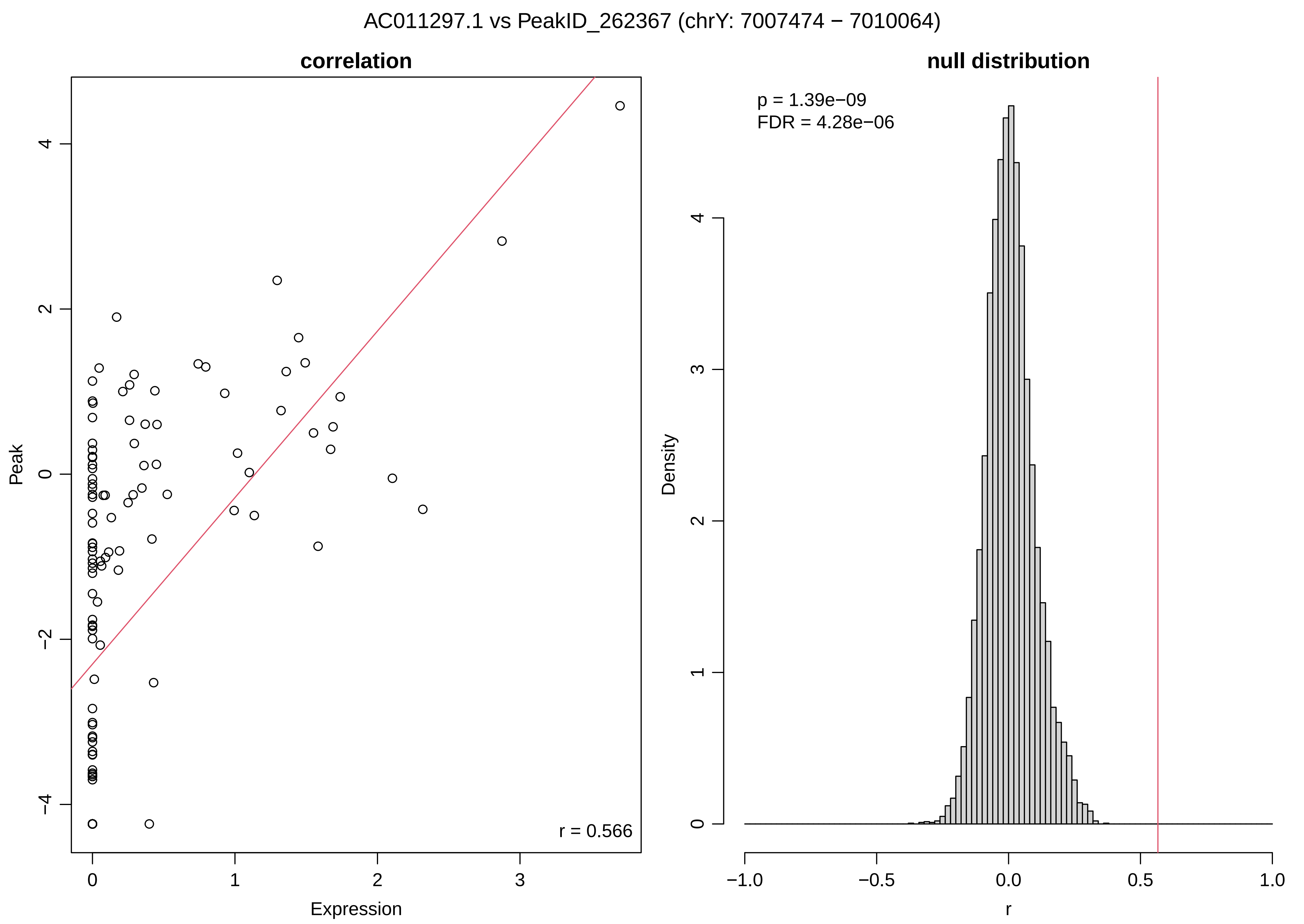
AC011297.1 vs PeakID_262367 (chrY: 7007474 − 7010064)
correlation
Peak
Expression
r = 0.566
null distribution
p = 1.39e−09
FDR = 4.28e−06
Density
r

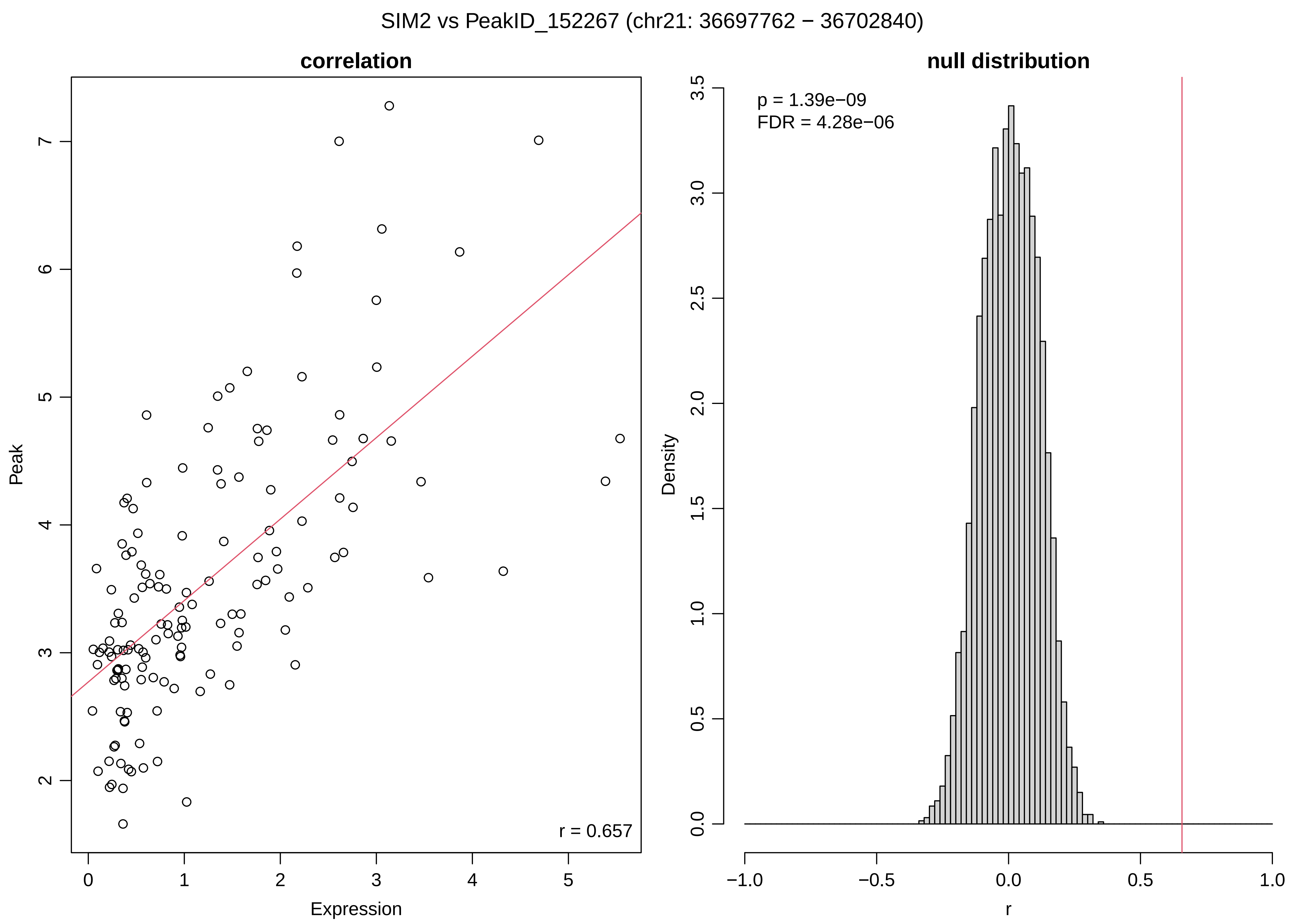
SIM2 vs PeakID_152267 (chr21: 36697762 − 36702840)
correlation
Peak
Expression
r = 0.657
null distribution
p = 1.39e−09
FDR = 4.28e−06
Density
r

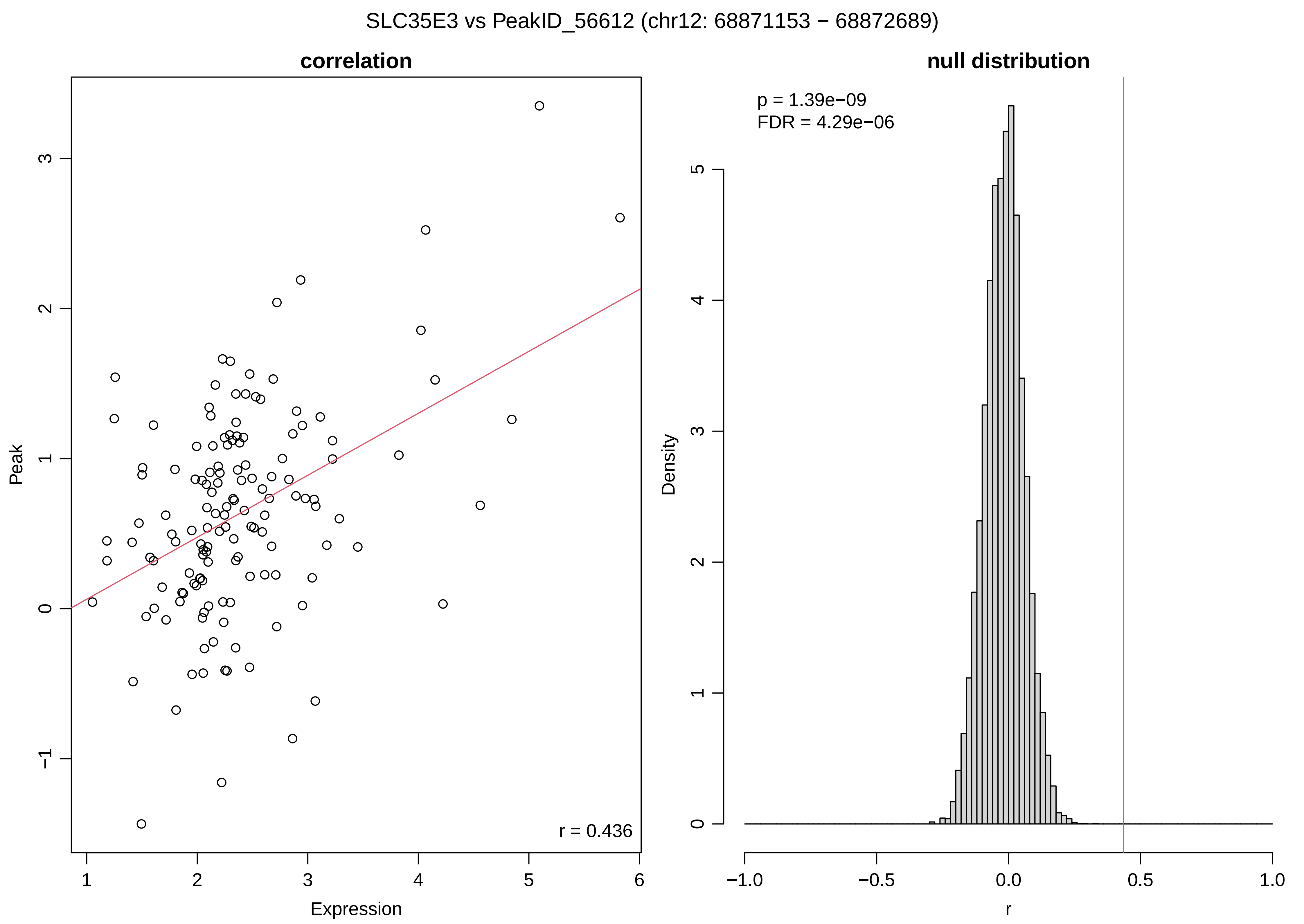

SLC35E3 vs PeakID_56612 (chr12: 68871153 − 68872689)
correlation
Peak
Expression
r = 0.436
null distribution
p = 1.39e−09
FDR = 4.29e−06
Density
r

GYG2P1 vs PeakID_262711 (chrY: 12303411 − 12304718)

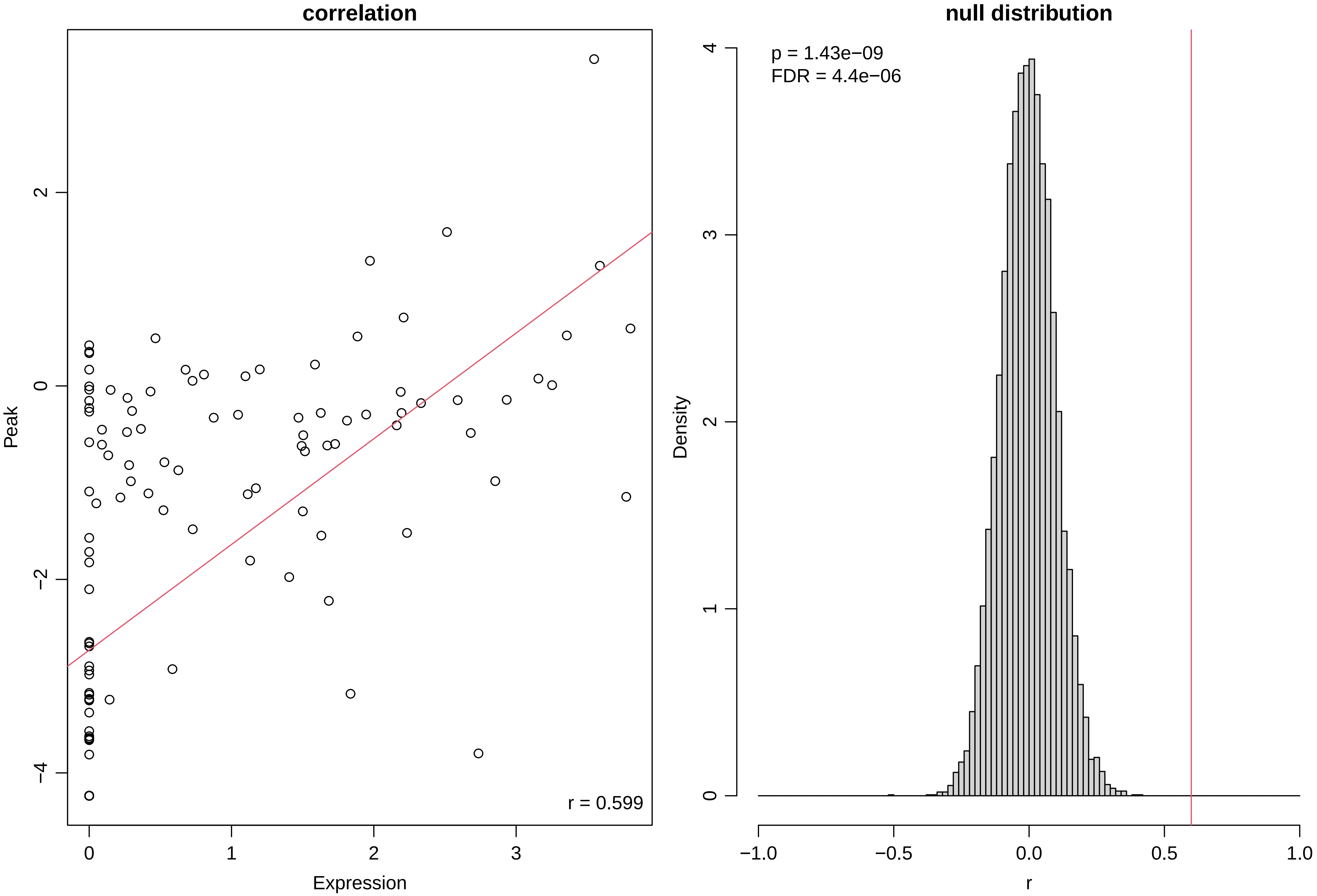

AC073324.1 vs PeakID_224252 (chr7: 55221408 − 55290386)

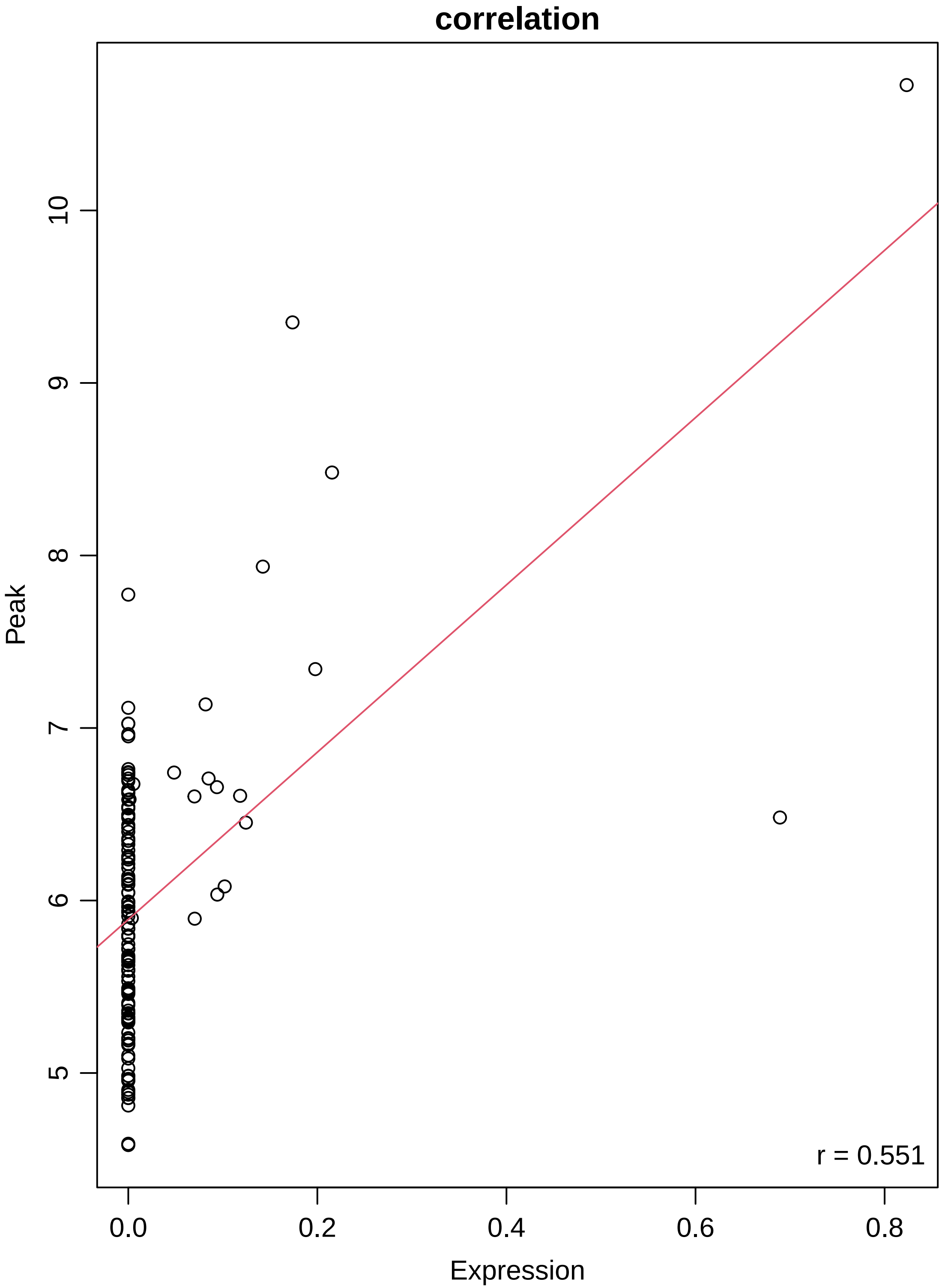

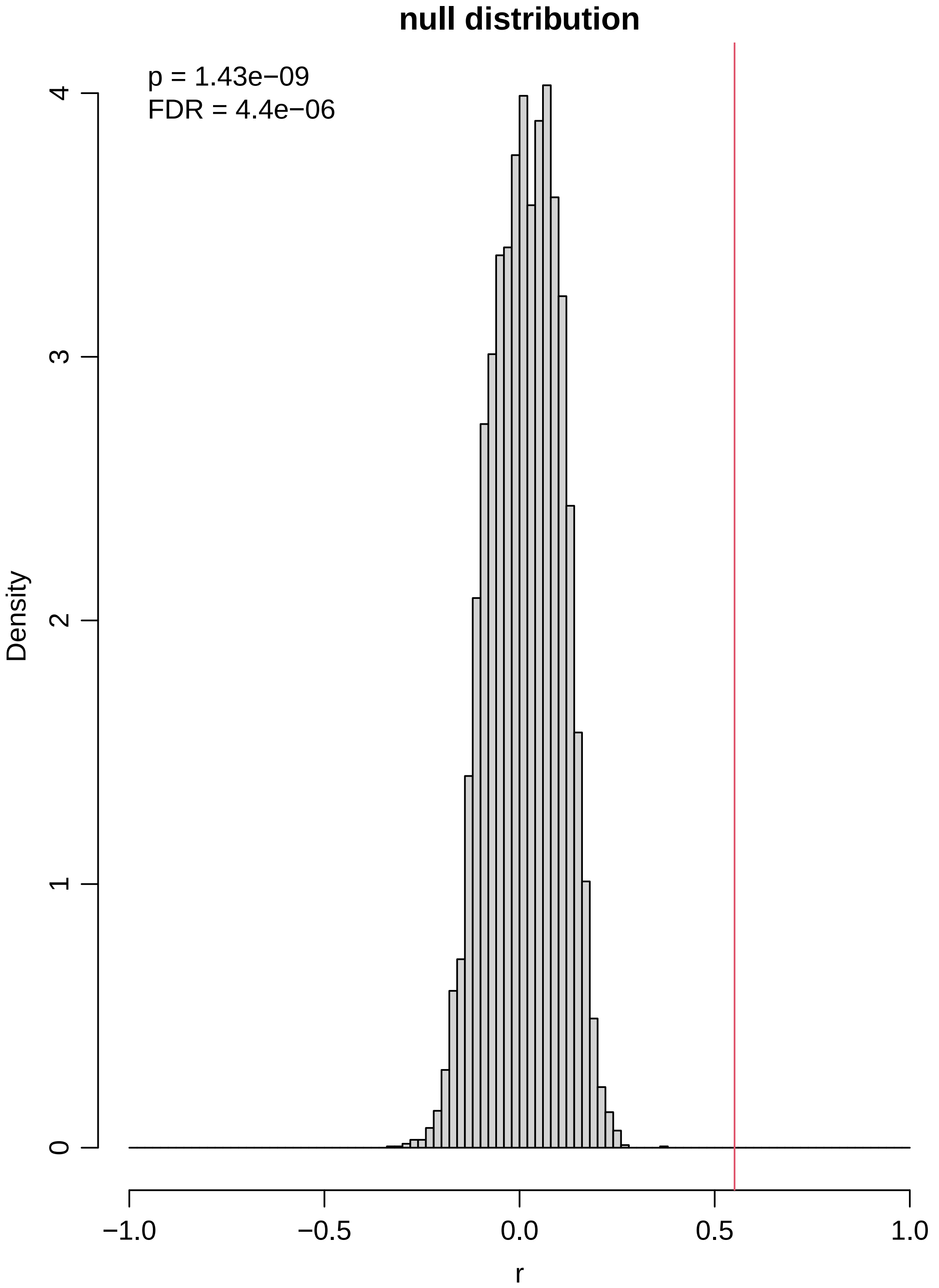

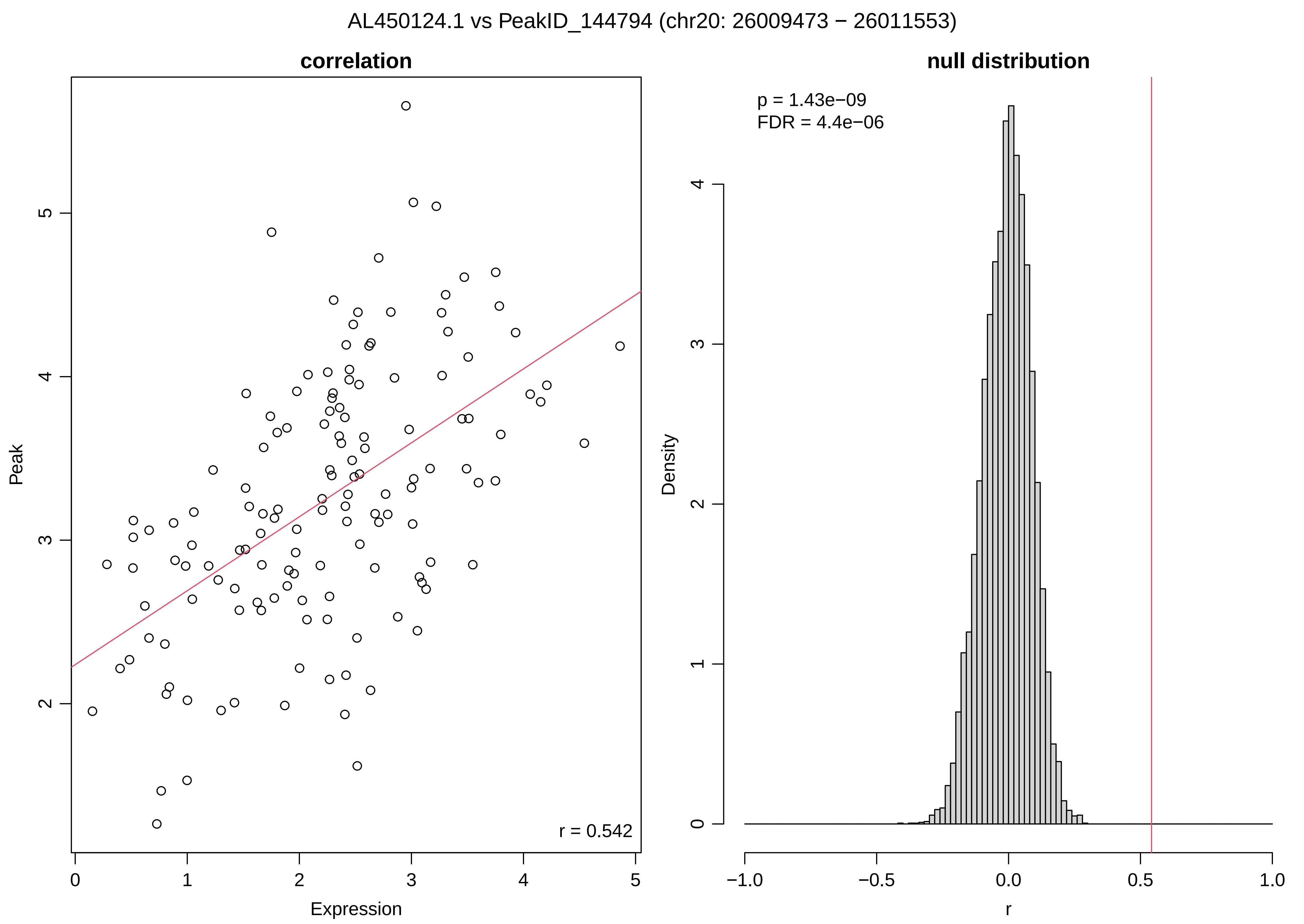

AL450124.1 vs PeakID_144794 (chr20: 26009473 − 26011553)
correlation
null distribution
p = 1.43e−09
FDR = 4.4e−06
r = 0.542
Peak
Expression
Density
r

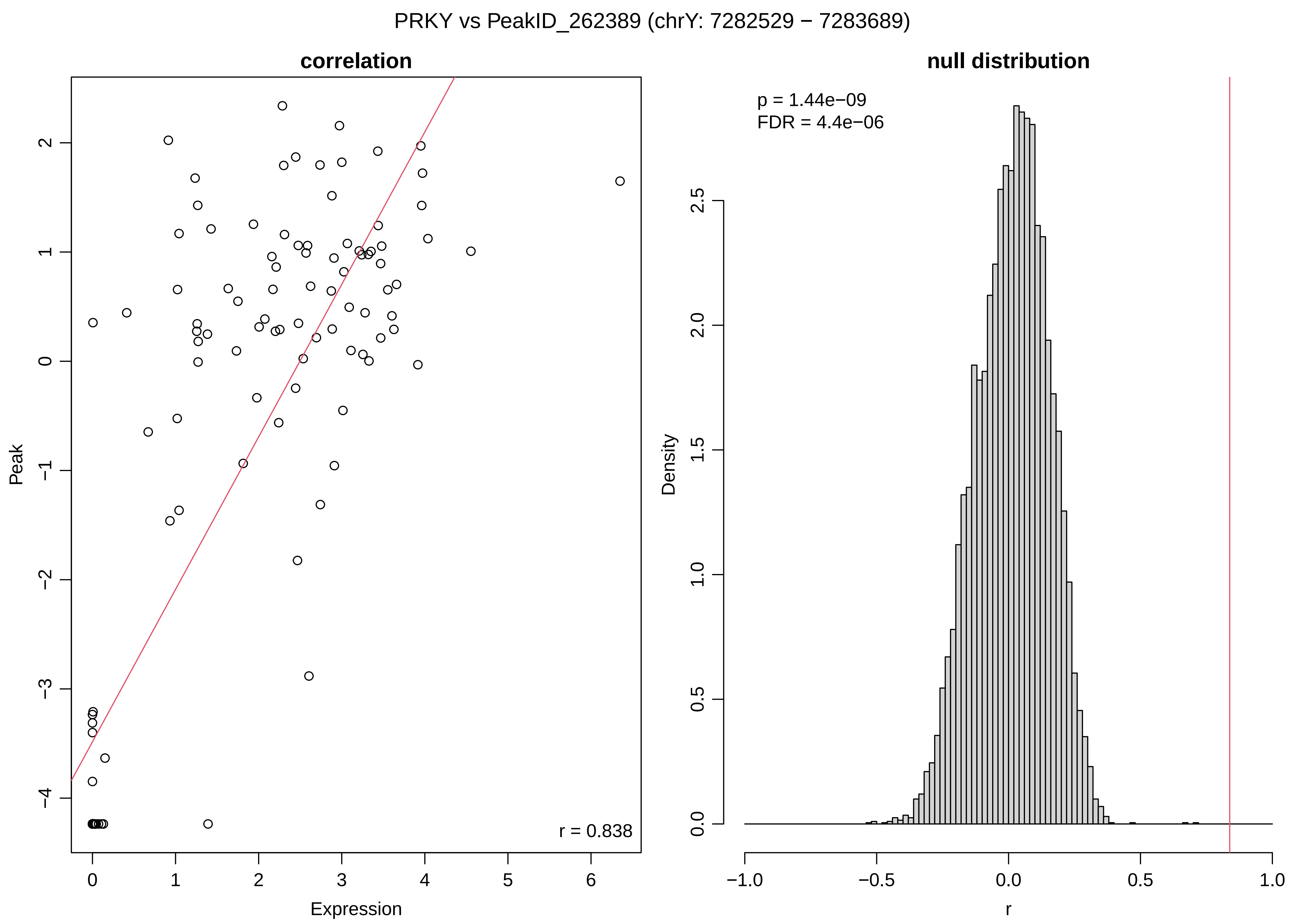
PRKY vs PeakID_262389 (chrY: 7282529 − 7283689)
correlation
Peak
Expression
r = 0.838
null distribution
p = 1.44e−09
FDR = 4.4e−06
Density
r

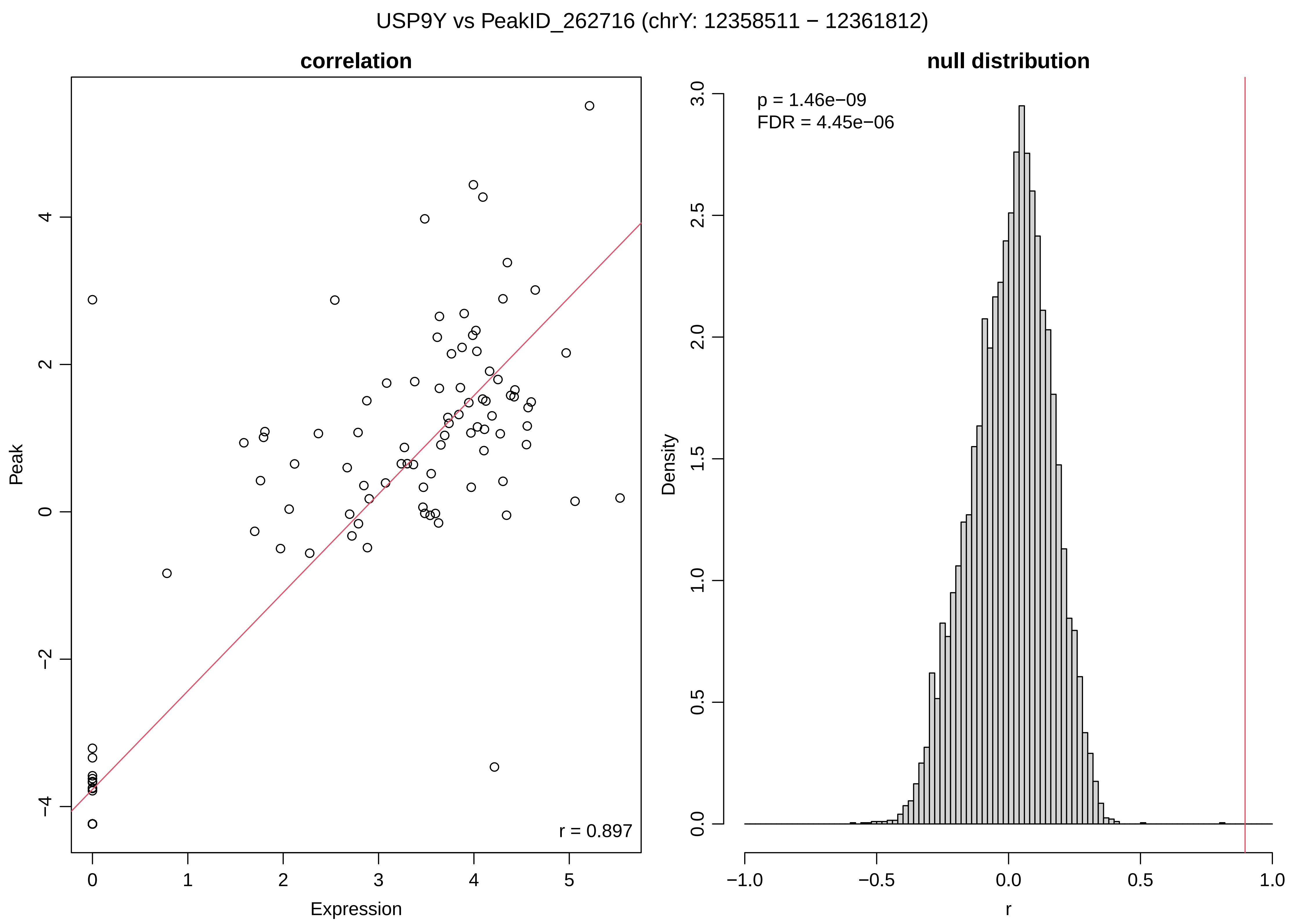

USP9Y vs PeakID_262716 (chrY: 12358511 − 12361812)
correlation
Peak
Expression
r = 0.897
null distribution
Density
r
p = 1.46e−09
FDR = 4.45e−06

IGLJCOR18 vs PeakID_106918 (chr18: 3383758 − 3384410)

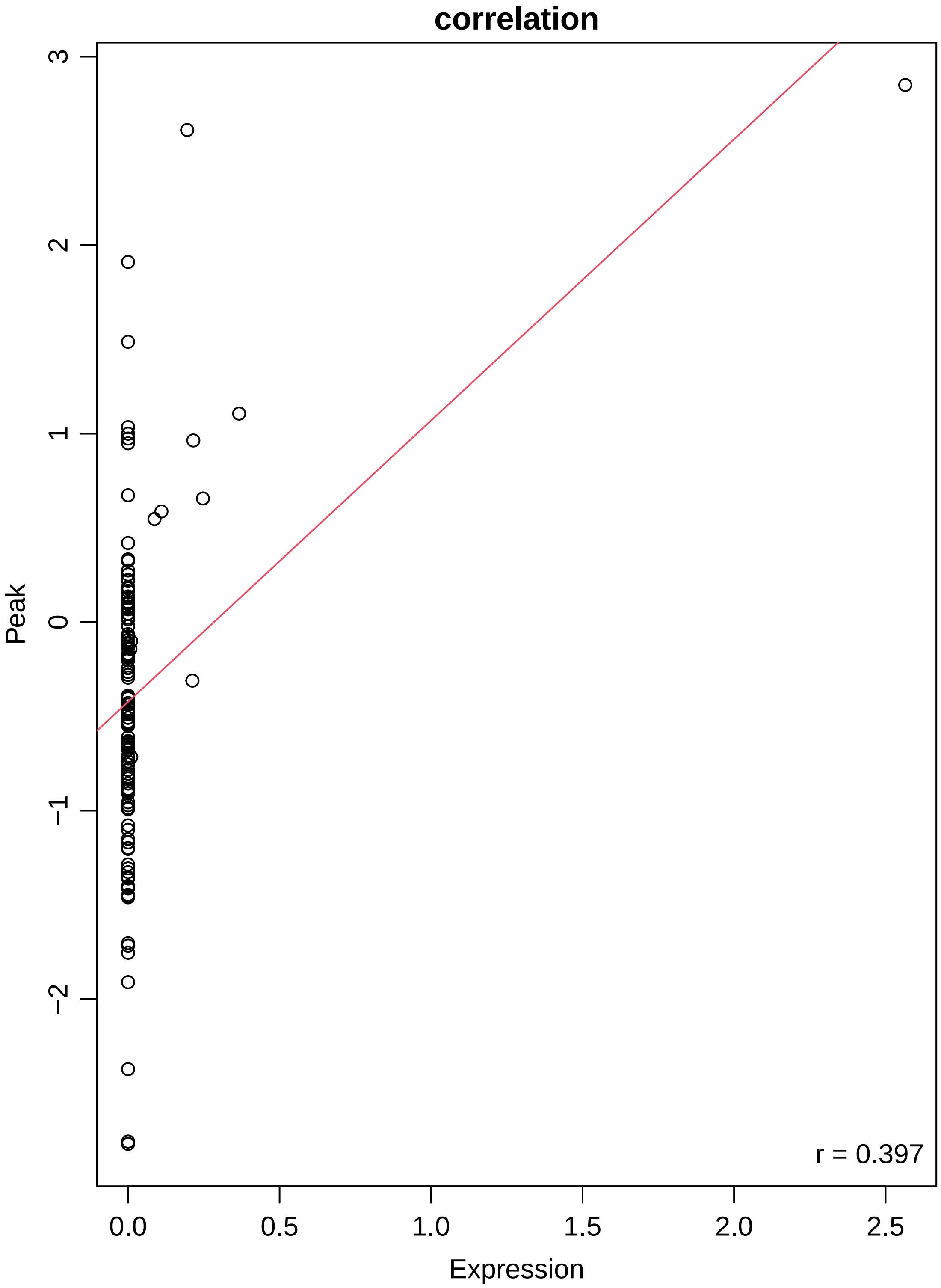

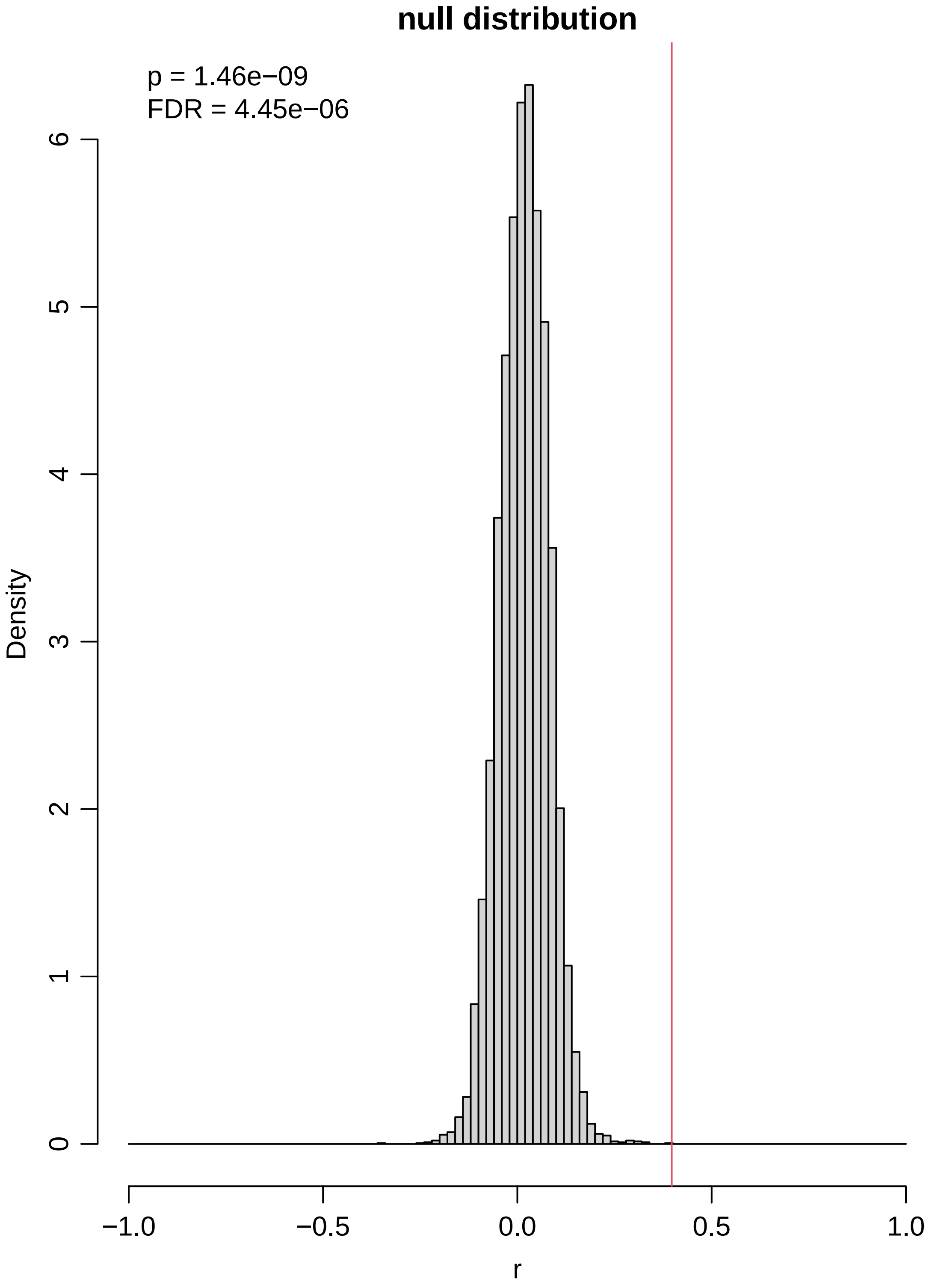

RNU4−20P vs PeakID_54834 (chr12: 59583037 − 59583559)

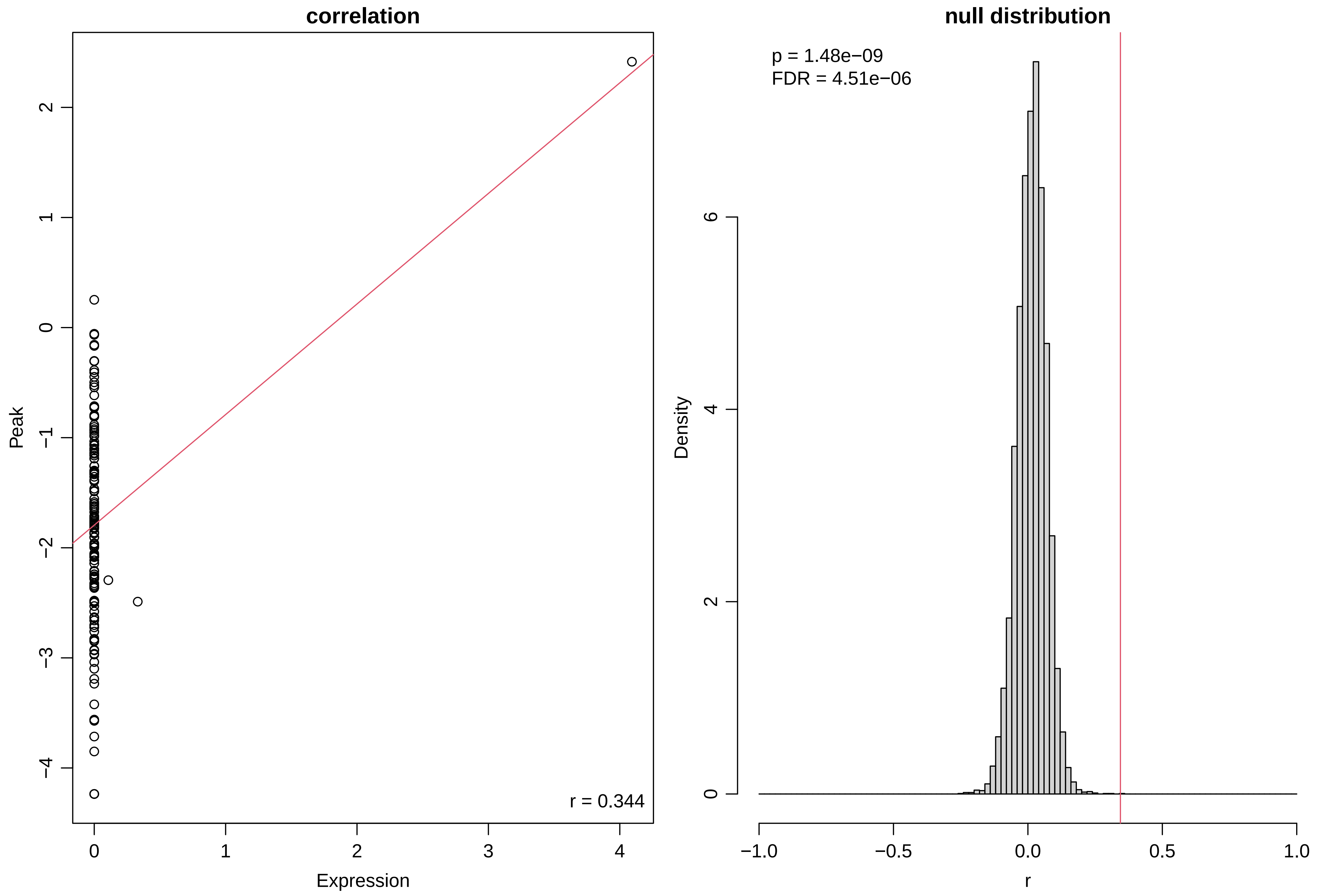

BEND3P1 vs PeakID_28919 (chr10: 50659922 − 50660307)

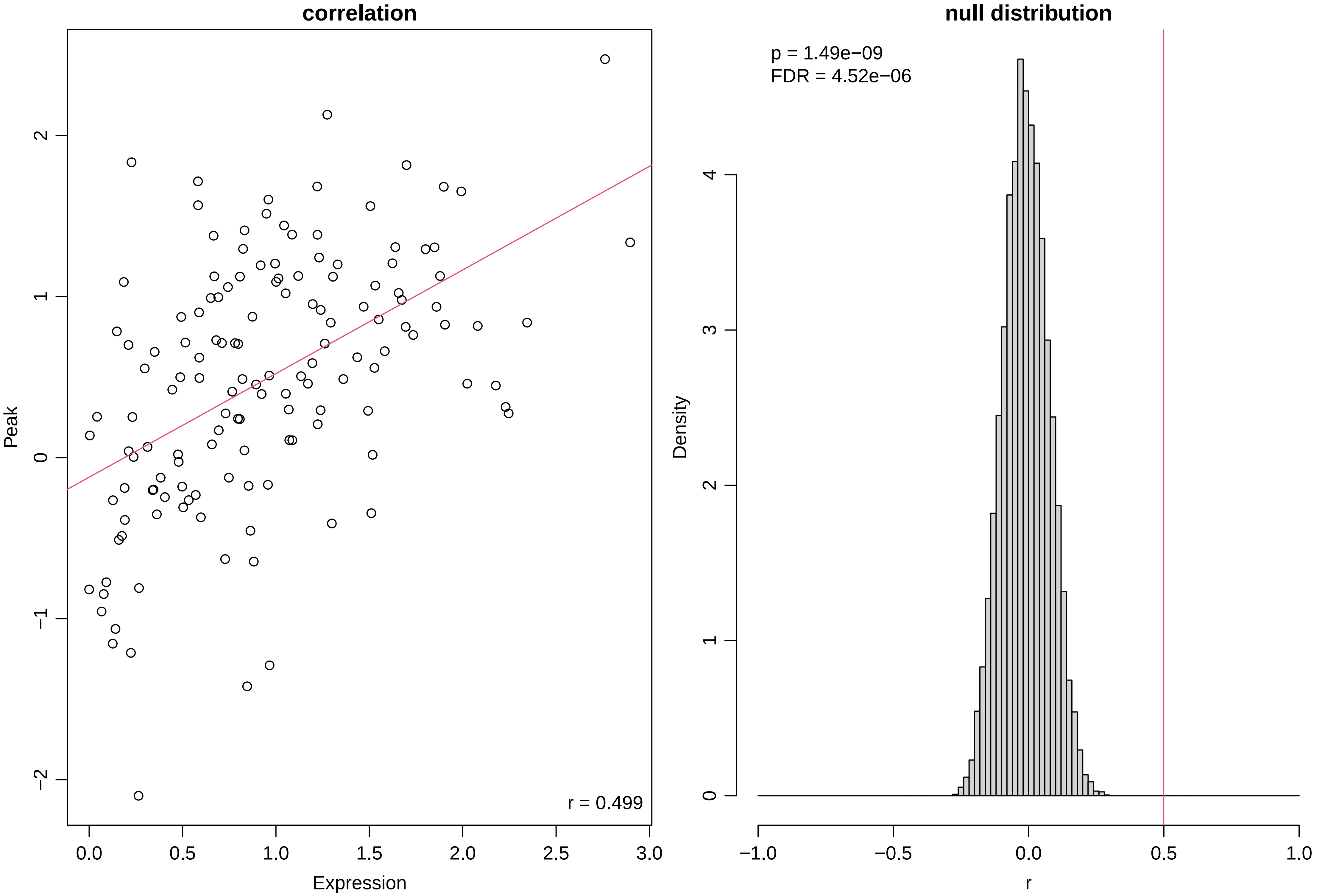

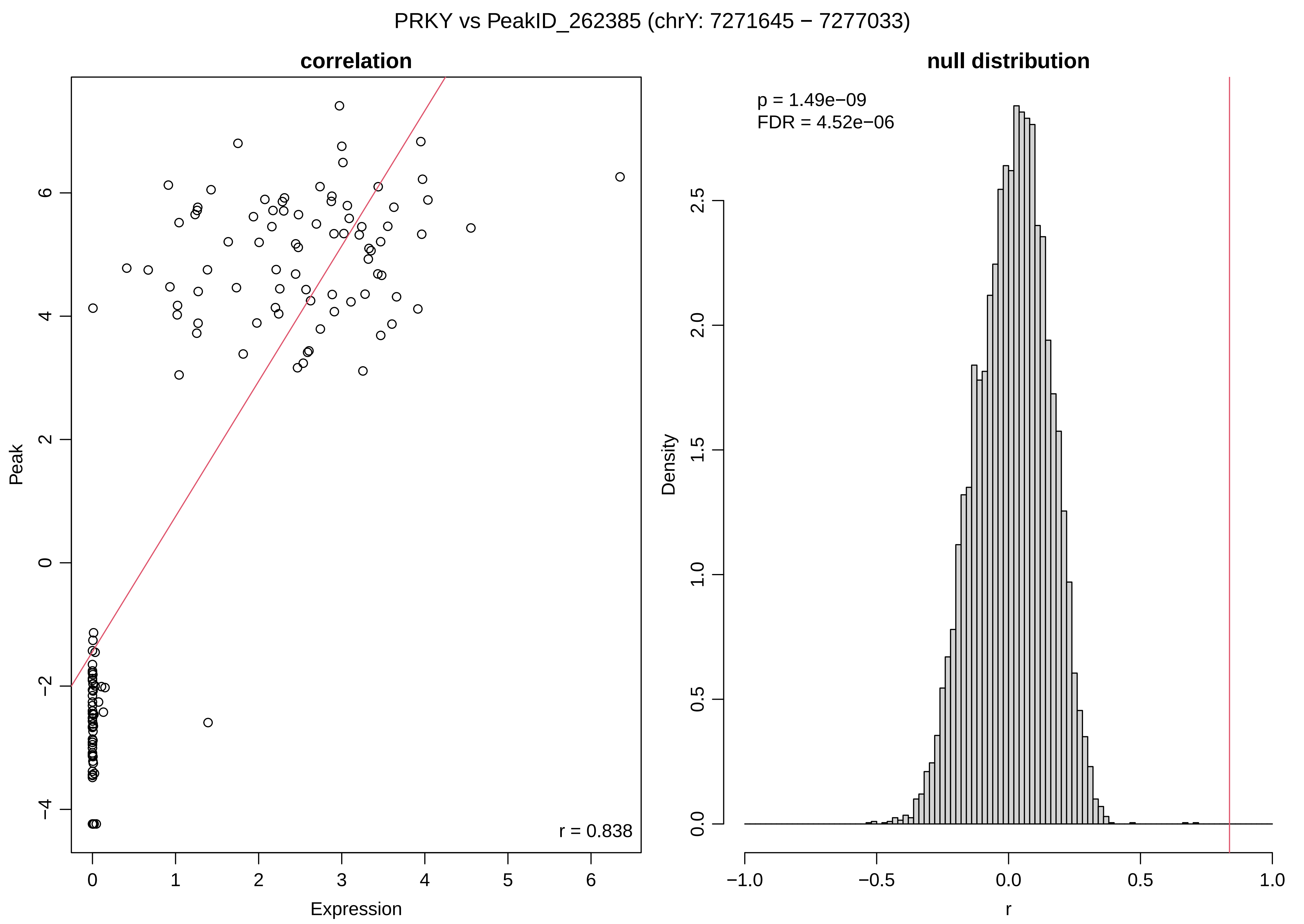
PRKY vs PeakID_262385 (chrY: 7271645 − 7277033)
correlation
null distribution
Peak
Expression
r = 0.838
Density
r
p = 1.49e−09
FDR = 4.52e−06

RNU4−20P vs PeakID_54818 (chr12: 59552464 − 59554117)

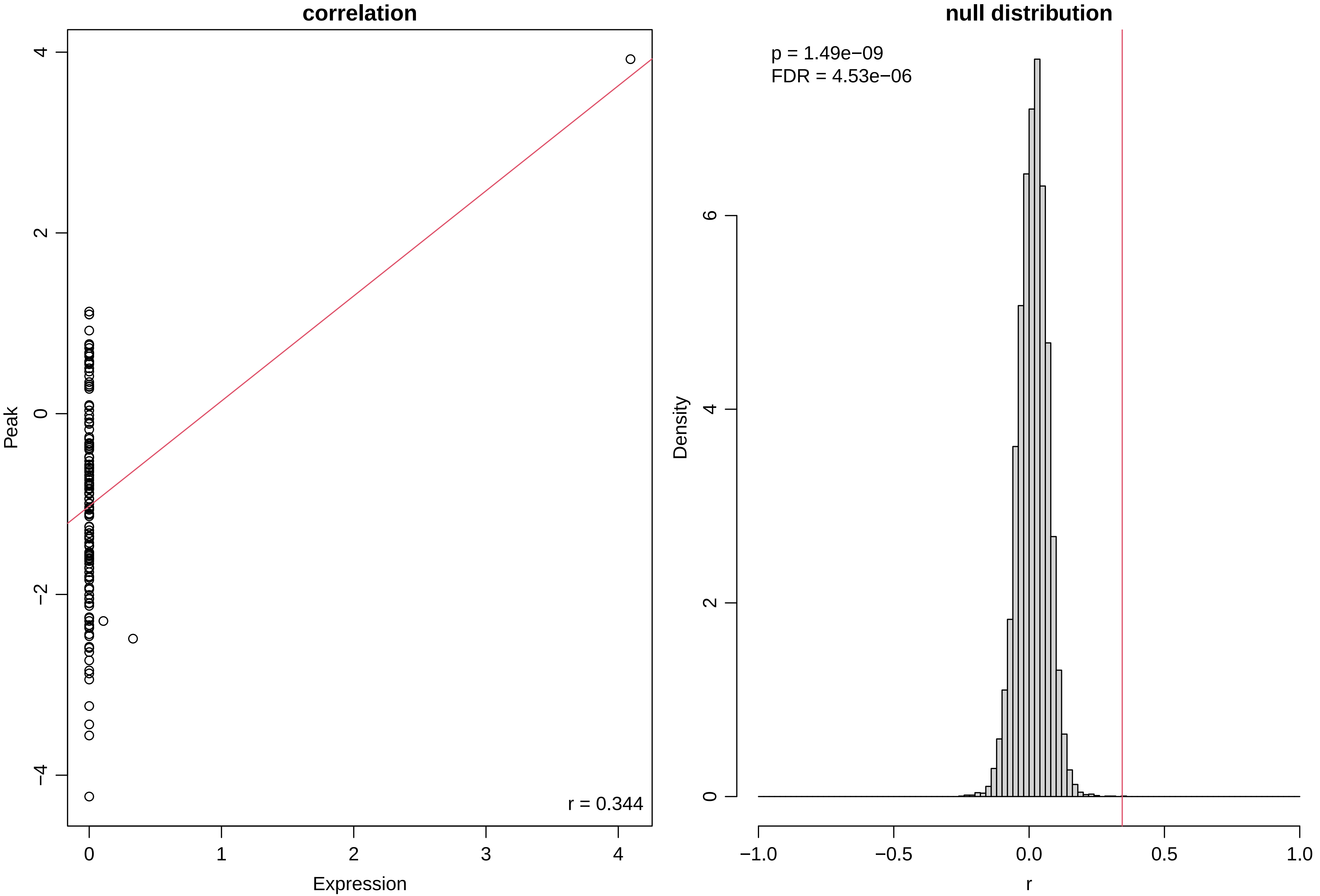

MIEN1 vs PeakID_99045 (chr17: 39692449 − 39763179)

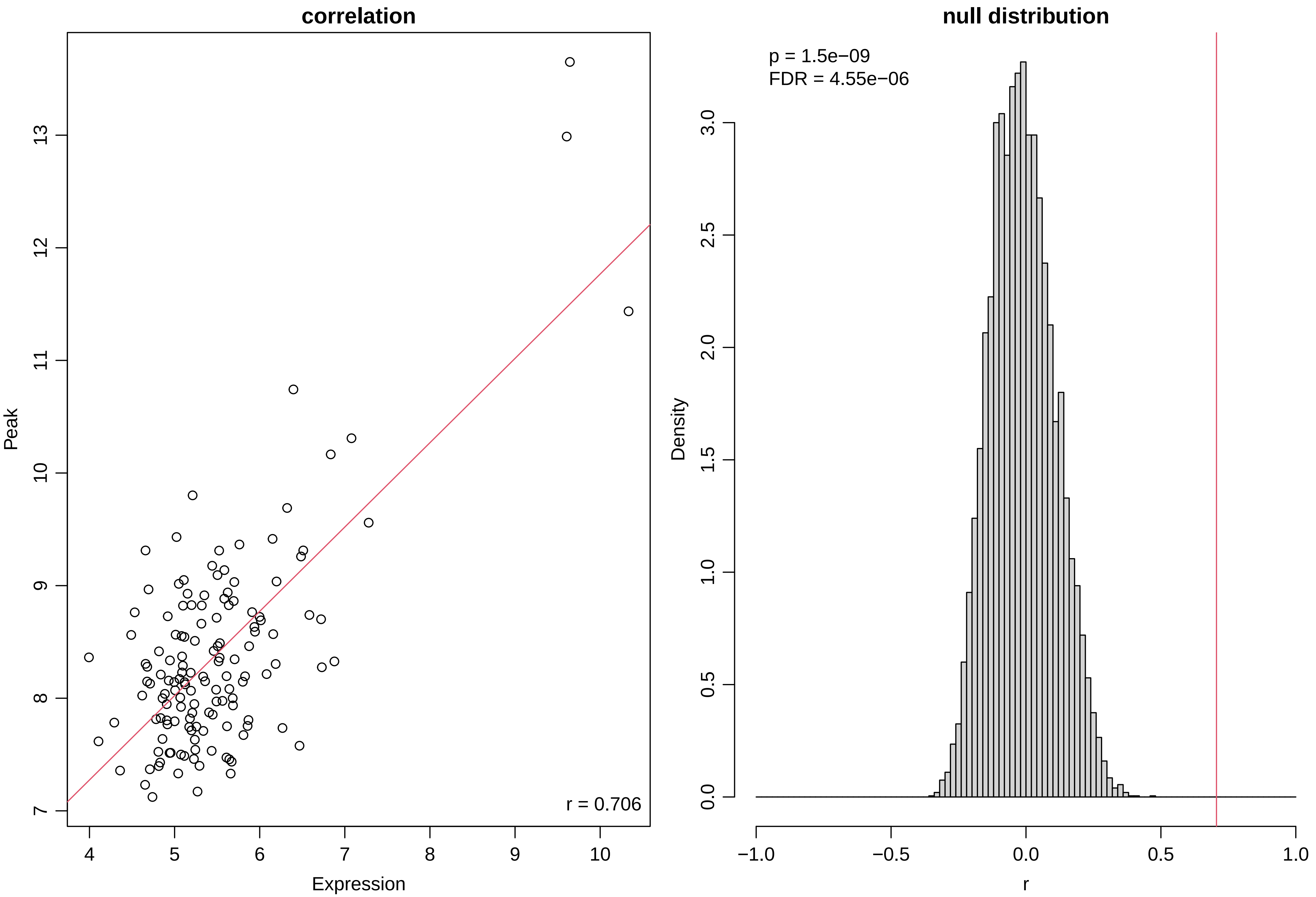

AC135776.1 vs PeakID_88803 (chr16: 35021769 − 35024090)

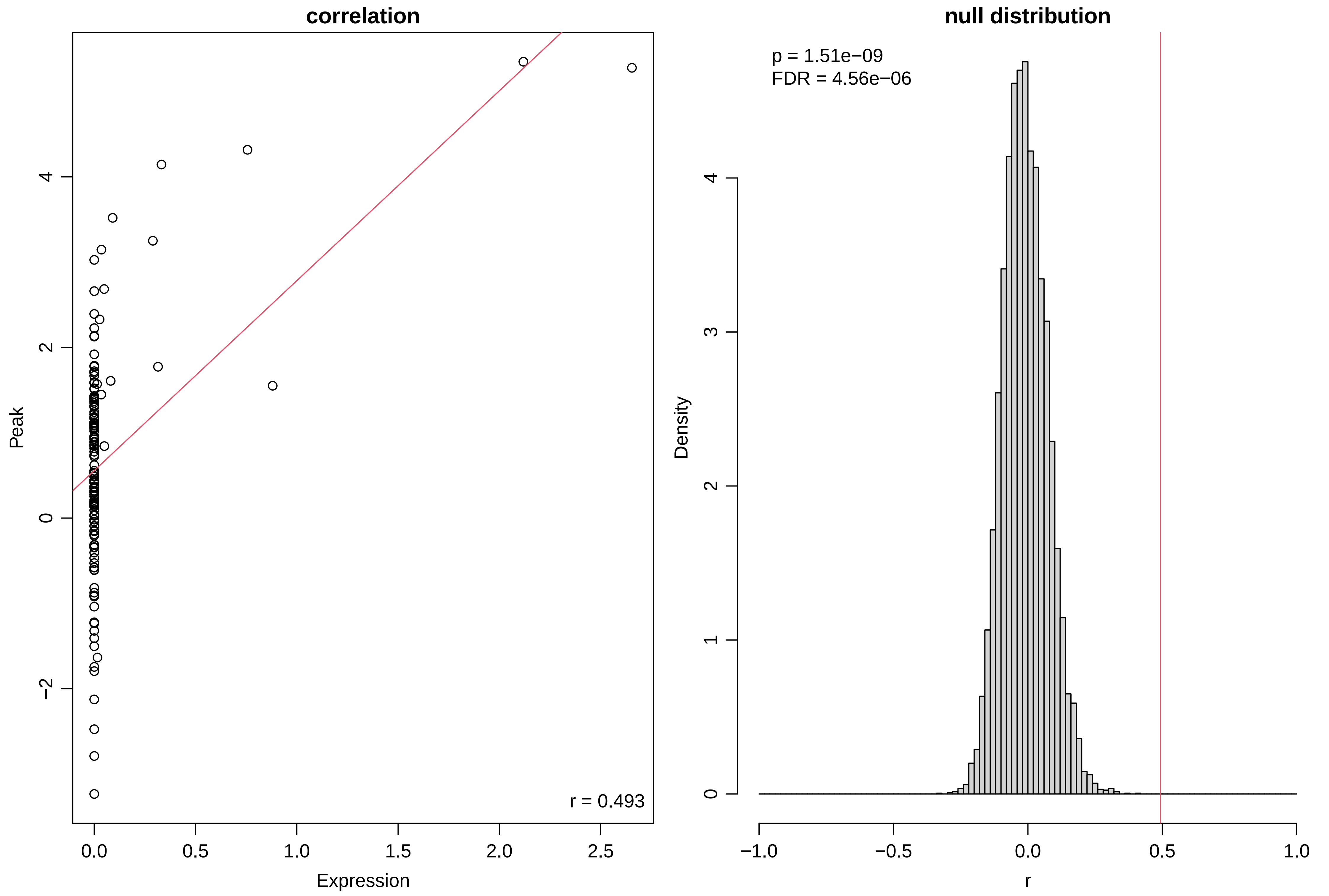

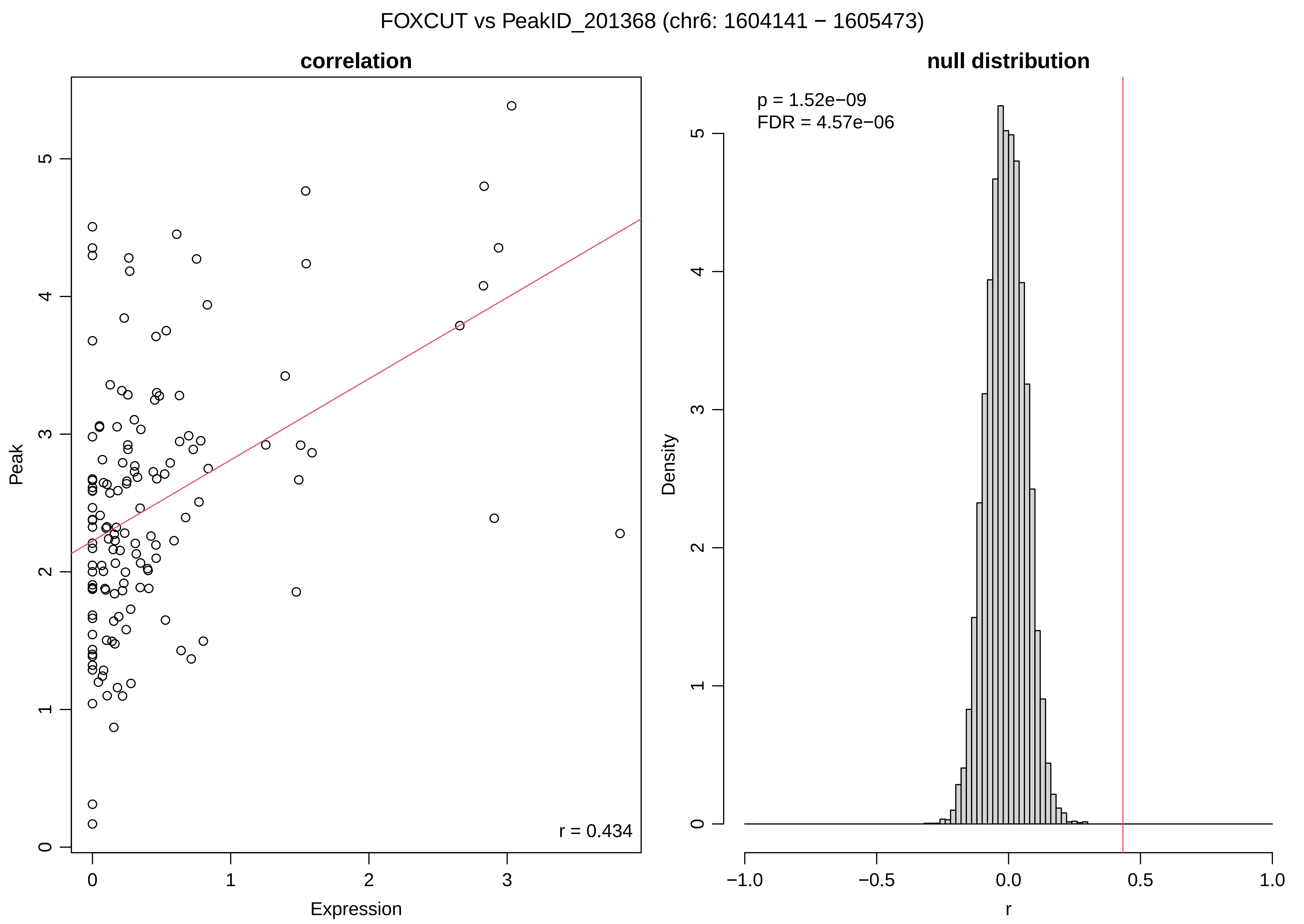

FOXCUT vs PeakID_201368 (chr6: 1604141 − 1605473)
correlation
Peak
Expression
r = 0.434
null distribution
p = 1.52e−09
FDR = 4.57e−06
Density
r

PRYP1 vs PeakID_262804 (chrY: 17729201 − 17729617)

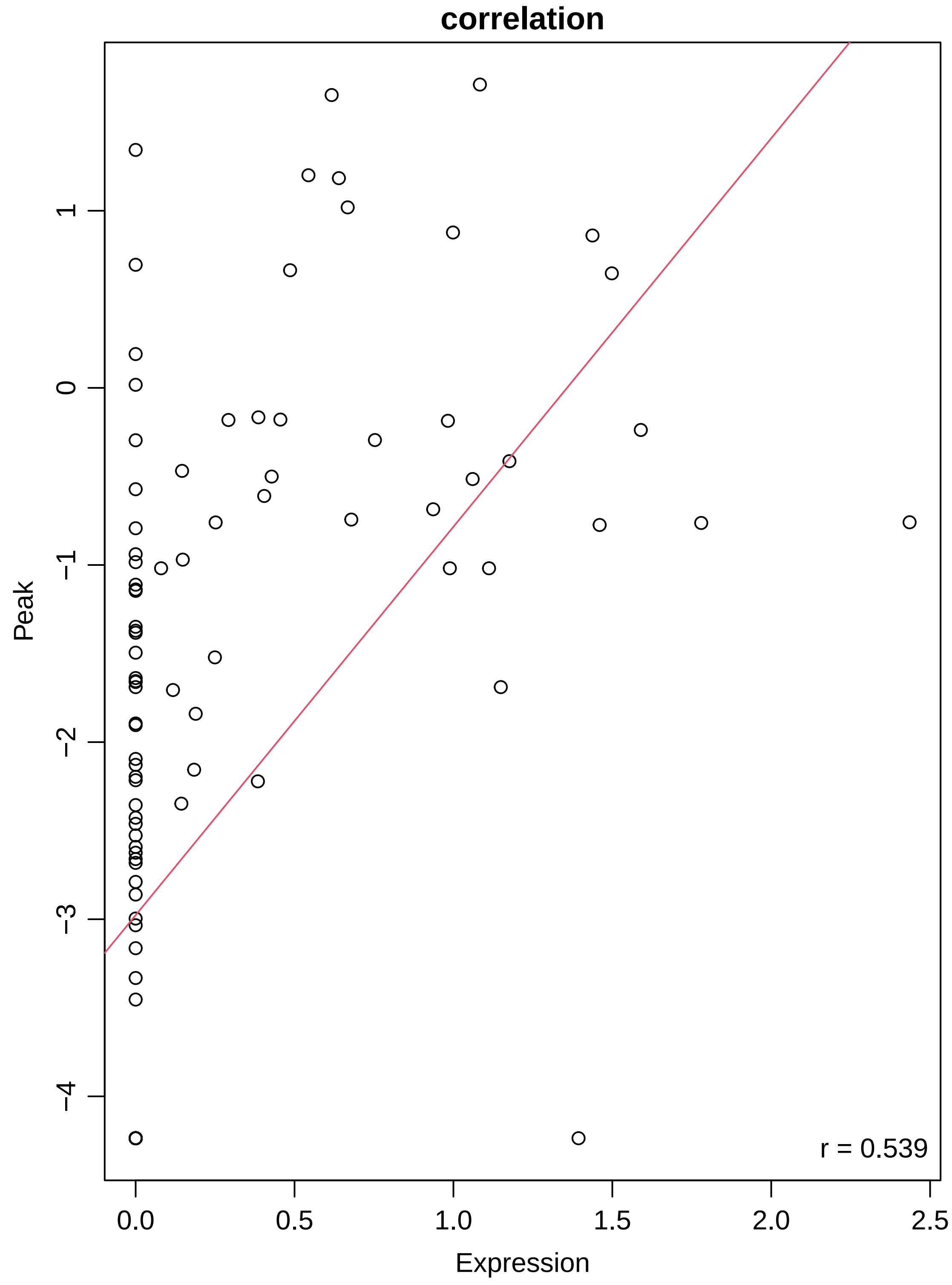

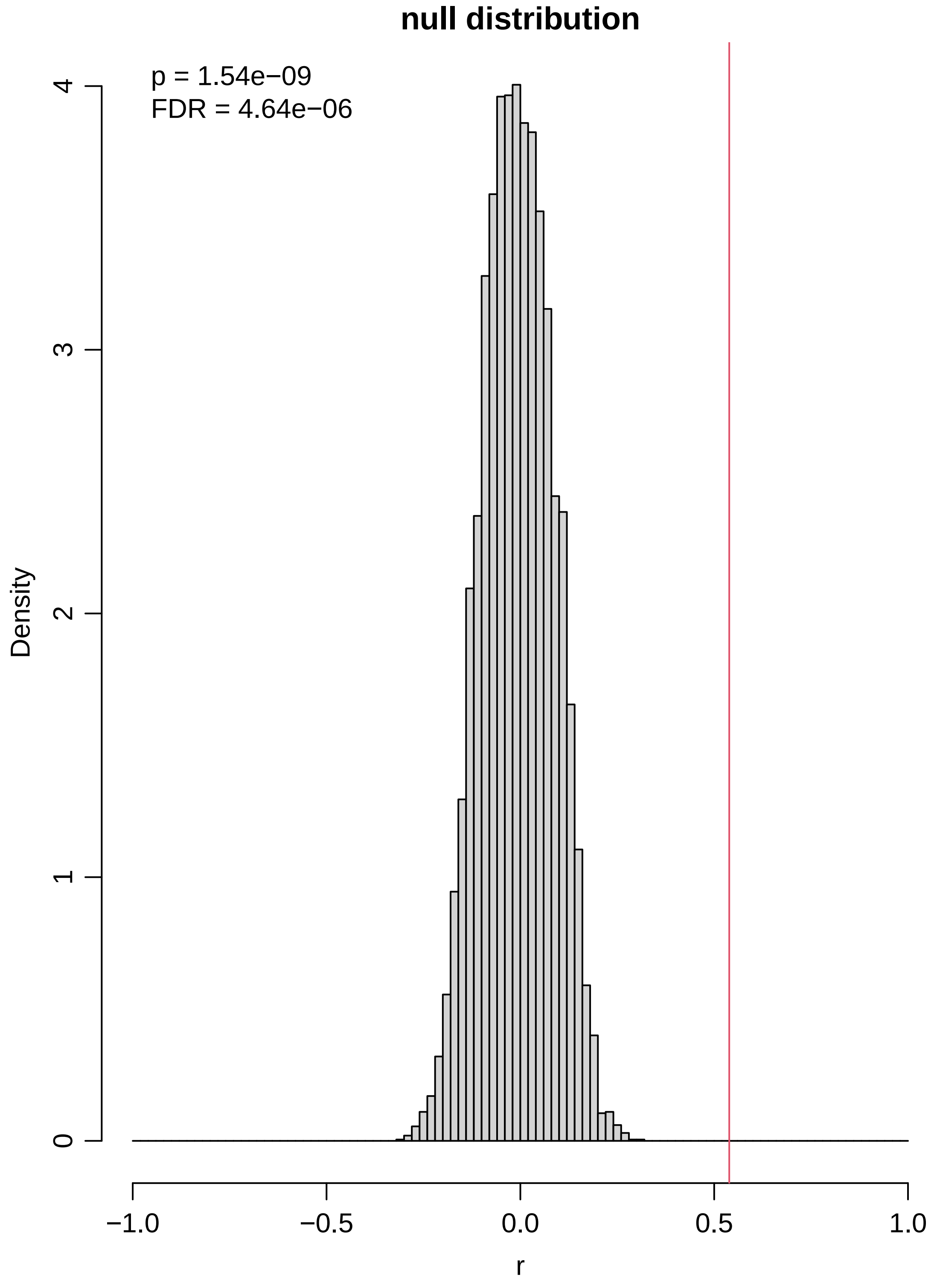

LINC00689 vs PeakID_232302 (chr7: 159011176 − 159019715)

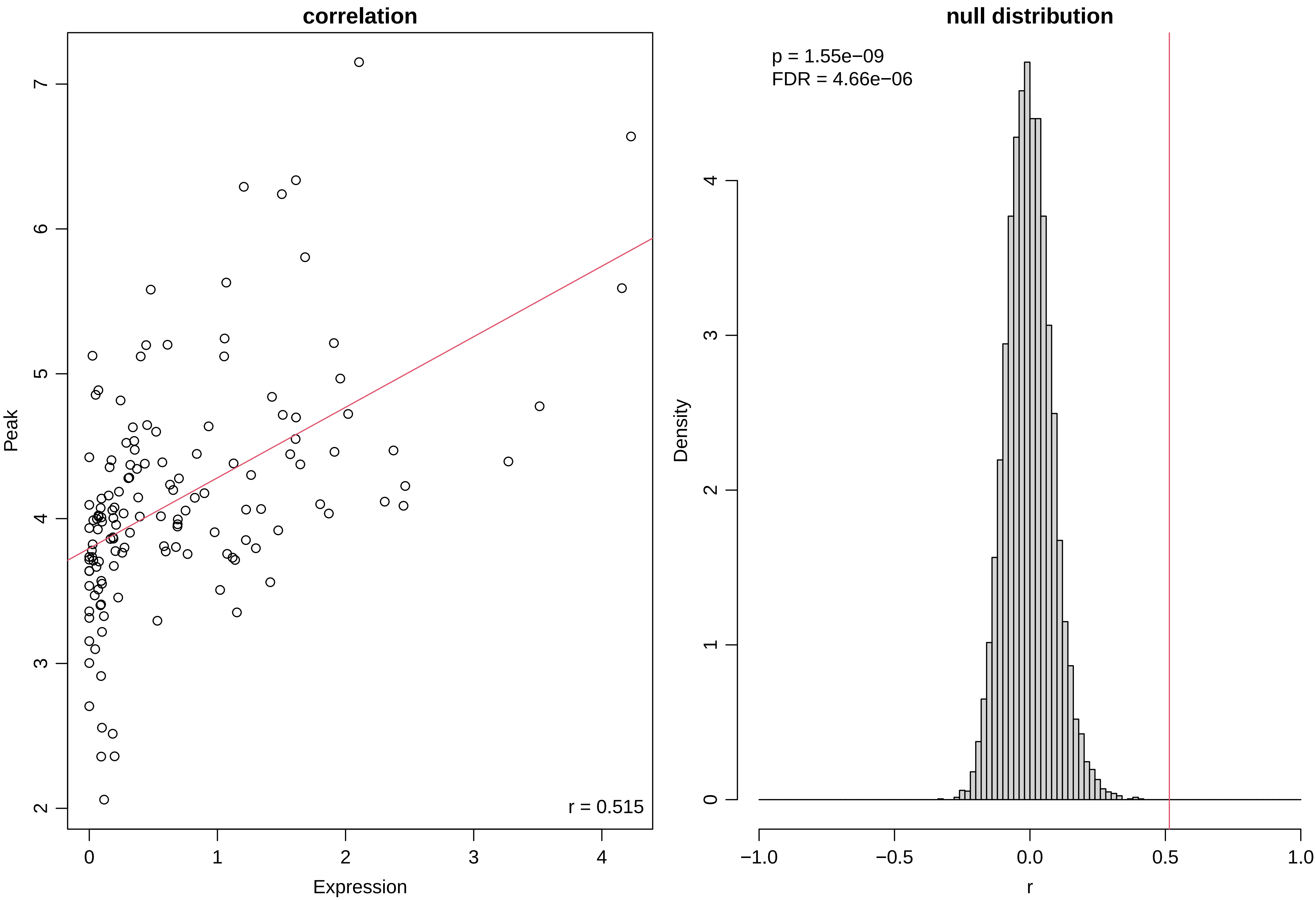

AC107881.1 vs PeakID_38028 (chr11: 12782726 − 12783958)

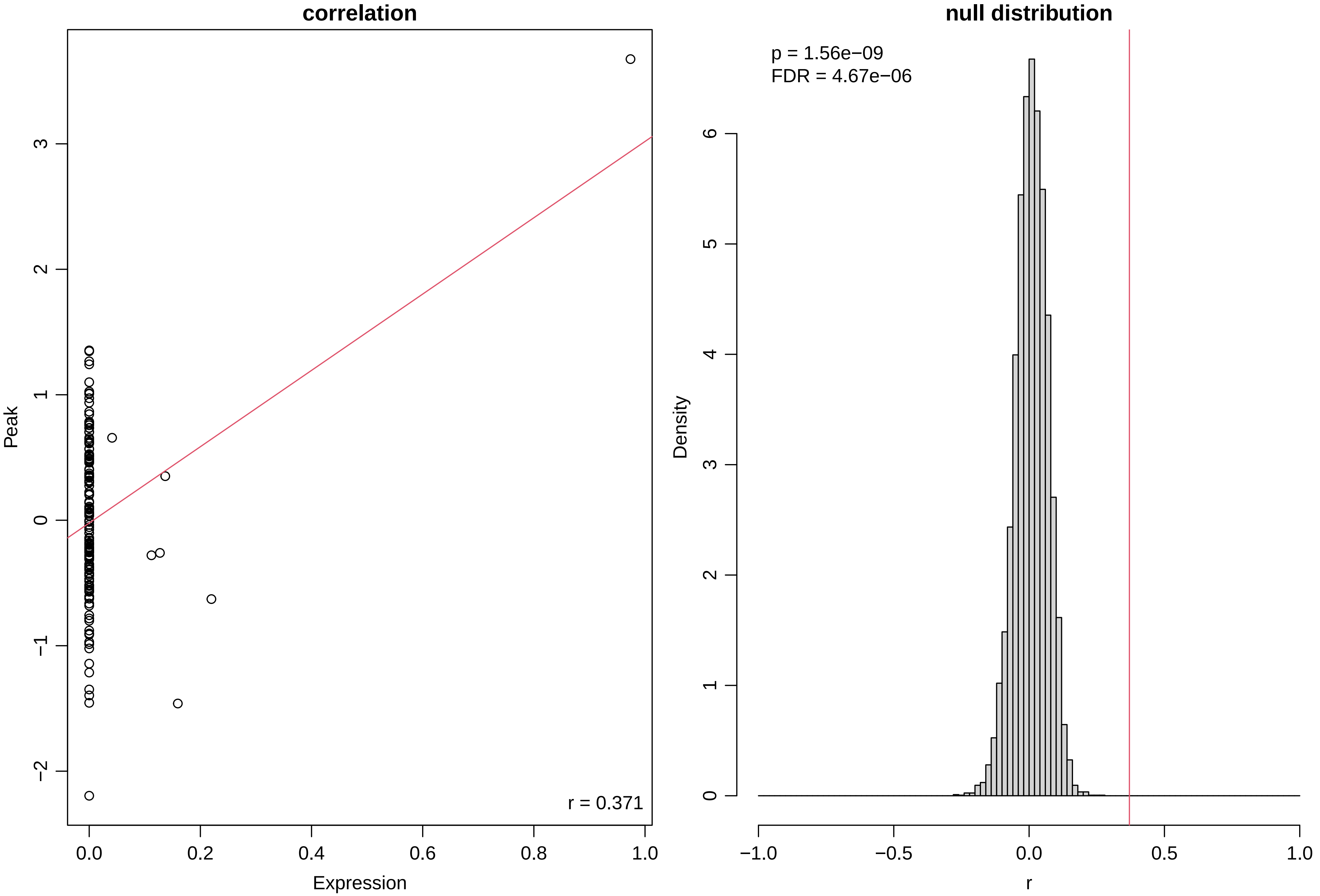

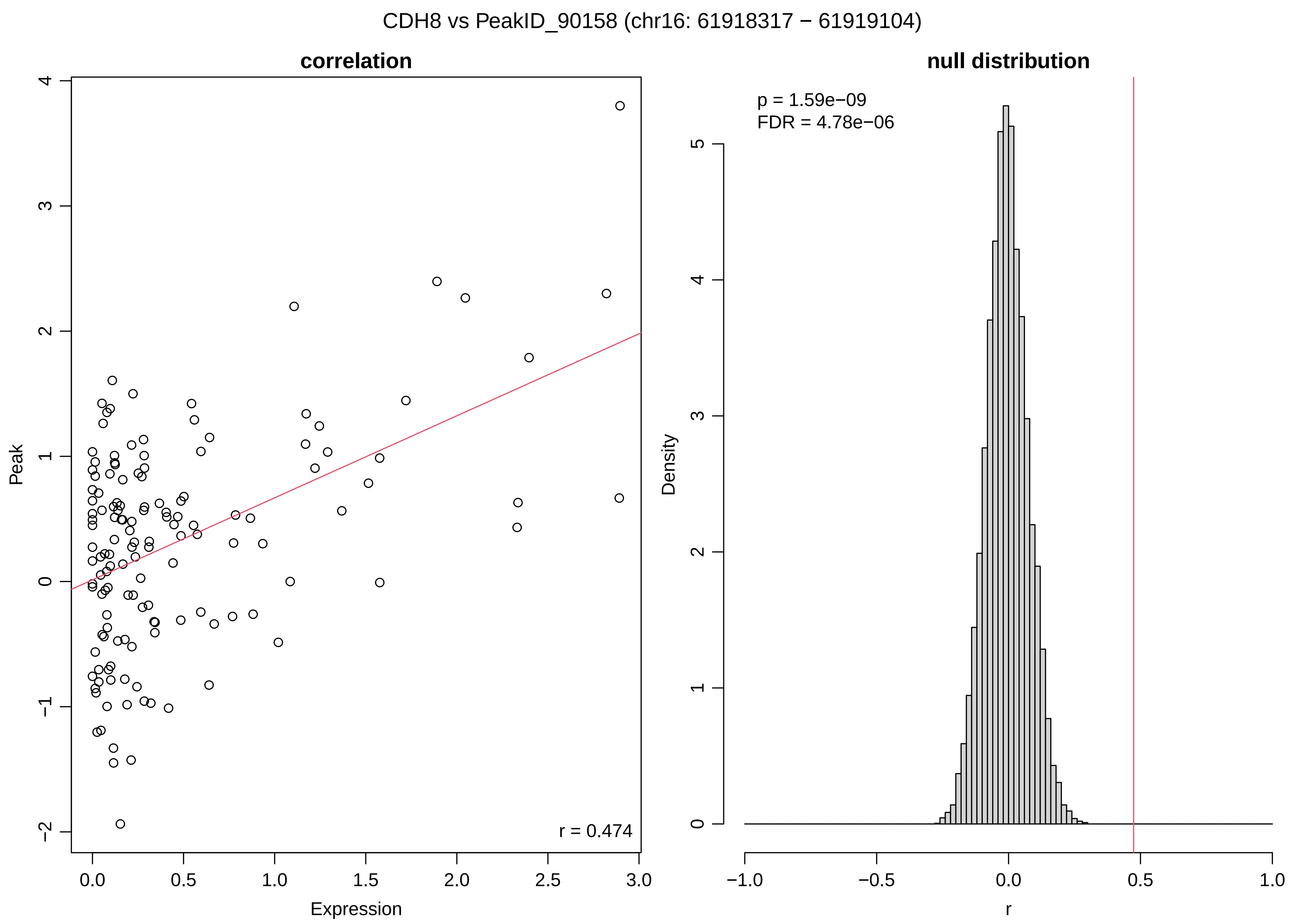

CDH8 vs PeakID_90158 (chr16: 61918317 − 61919104)
correlation
Peak
Expression
r = 0.474
null distribution
p = 1.59e−09
FDR = 4.78e−06
Density
r

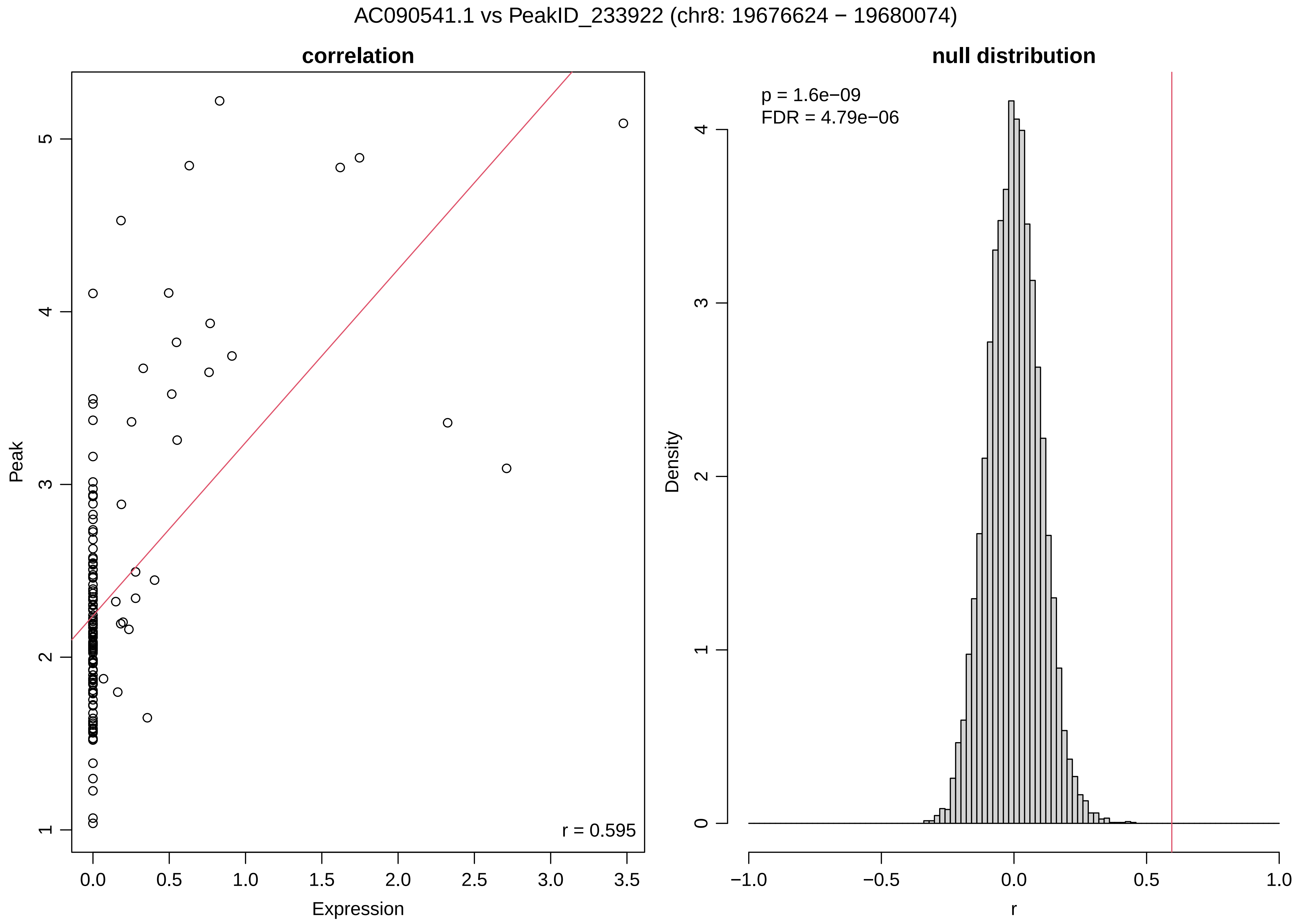
AC090541.1 vs PeakID_233922 (chr8: 19676624 − 19680074)
correlation
null distribution
Peak
Expression
r = 0.595
p = 1.6e−09
FDR = 4.79e−06
Density
r
1
2
3
4
5
0.0
0.5
1.0
1.5
2.0
2.5
3.0
3.5
0
−1.0
−0.5
1.0

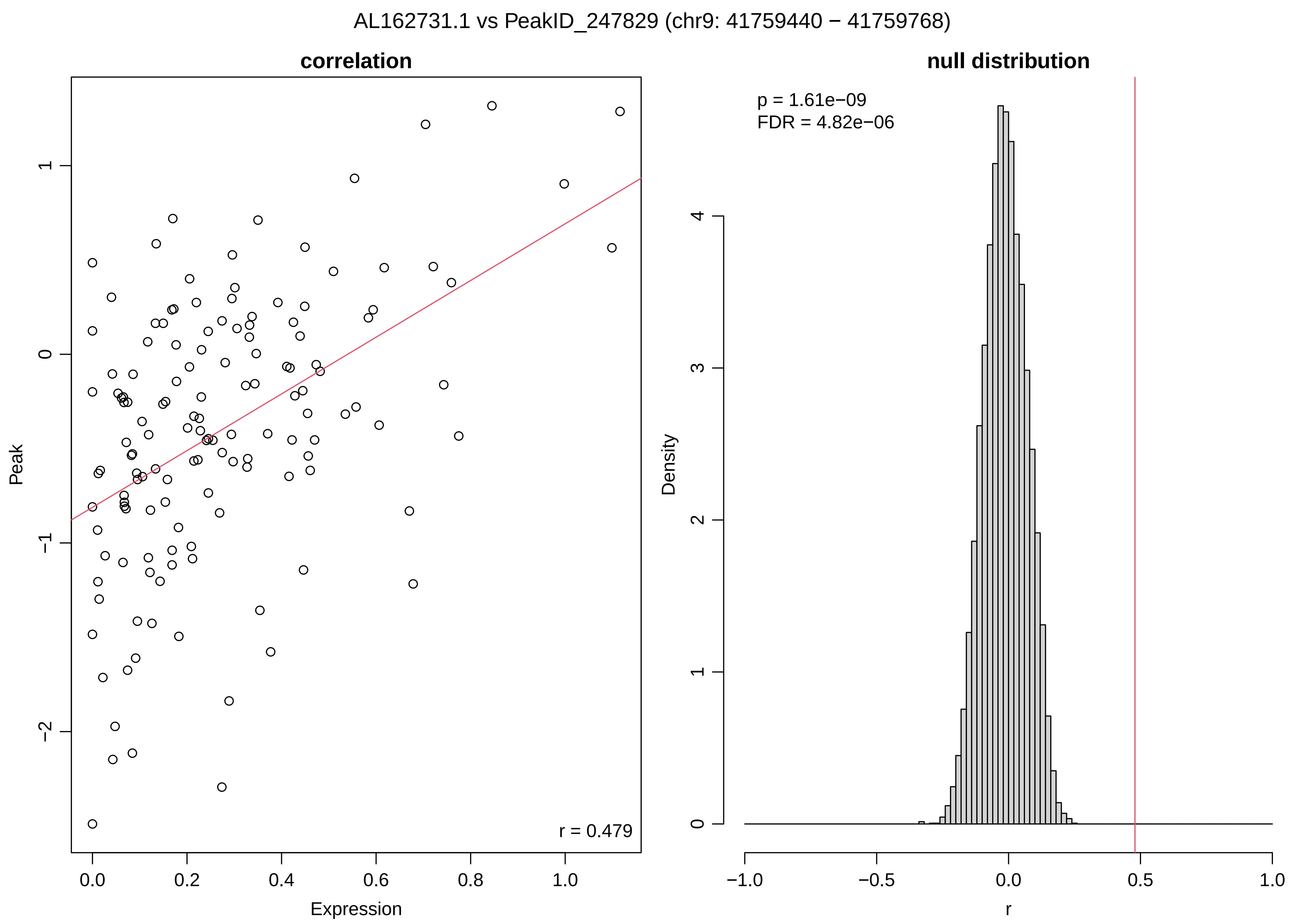
AL162731.1 vs PeakID_247829 (chr9: 41759440 − 41759768)
correlation
Peak
Expression
r = 0.479
null distribution
p = 1.61e−09
FDR = 4.82e−06
Density
r

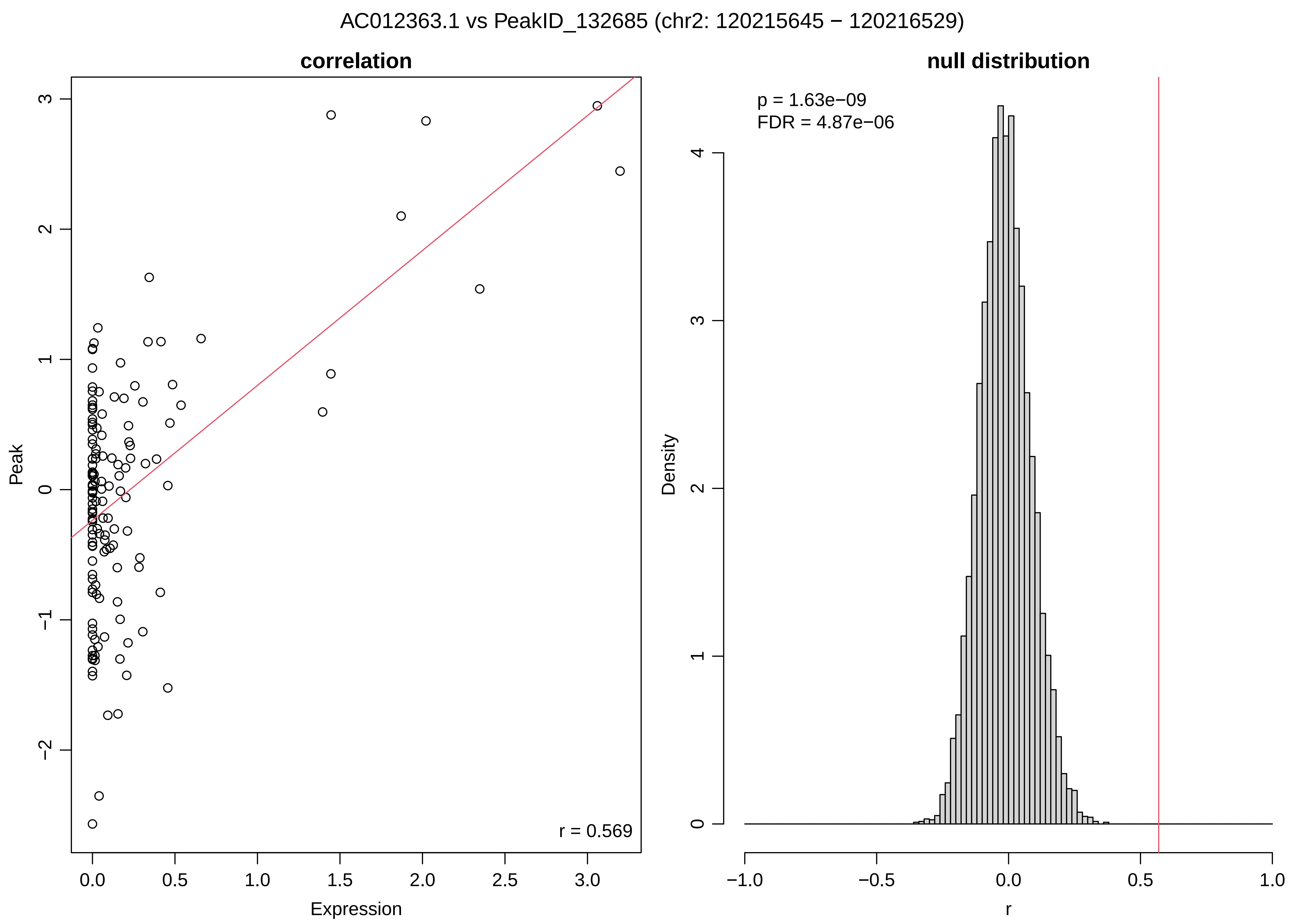
AC012363.1 vs PeakID_132685 (chr2: 120215645 − 120216529)
correlation
Peak
Expression
r = 0.569
null distribution
p = 1.63e−09
FDR = 4.87e−06
Density
r

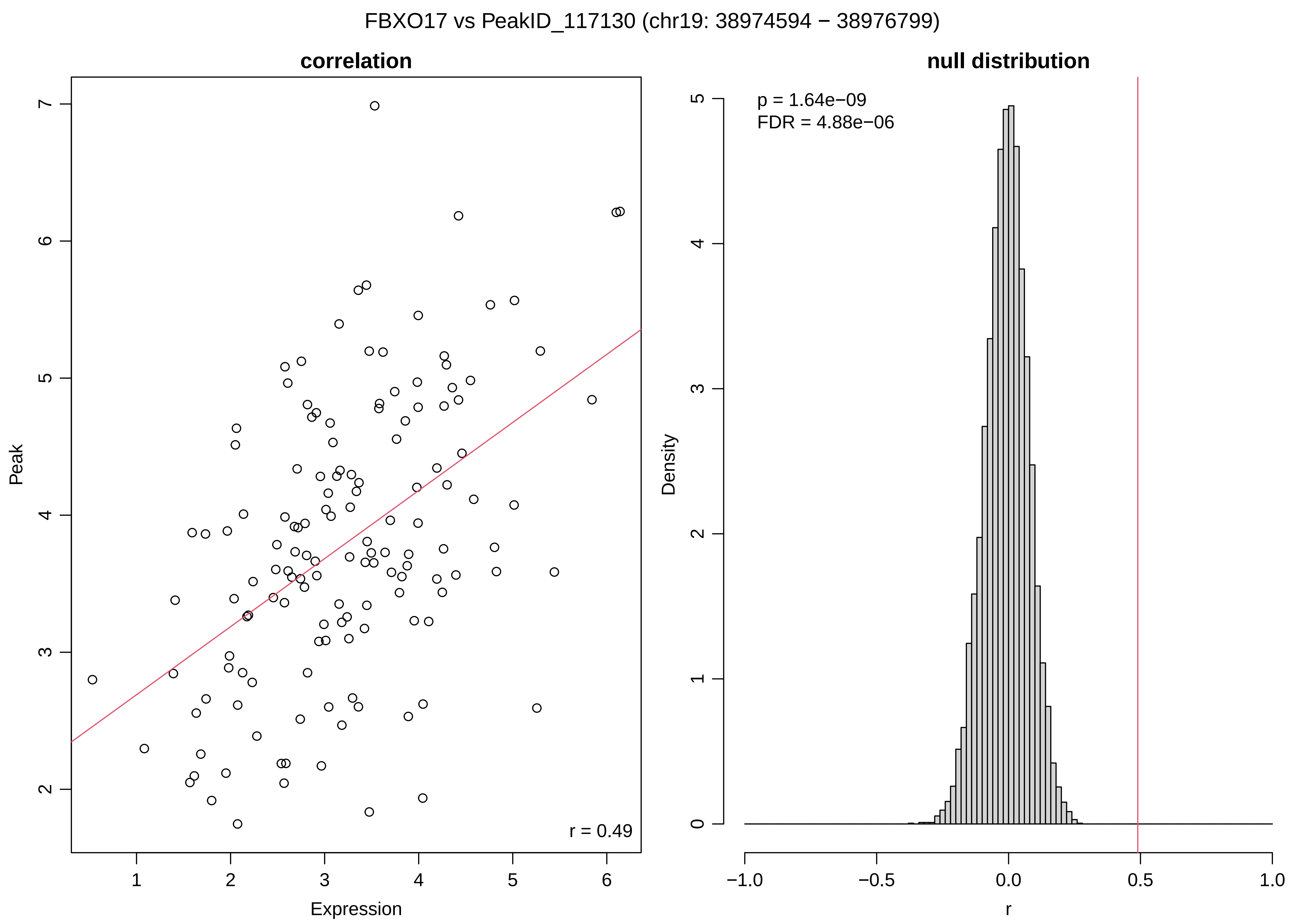

FBXO17 vs PeakID_117130 (chr19: 38974594 − 38976799)
correlation
Peak
Expression
r = 0.49
null distribution
p = 1.64e−09
FDR = 4.88e−06
Density
r

RPL21P104 vs PeakID_55473 (chr12: 61973520 − 61973908)

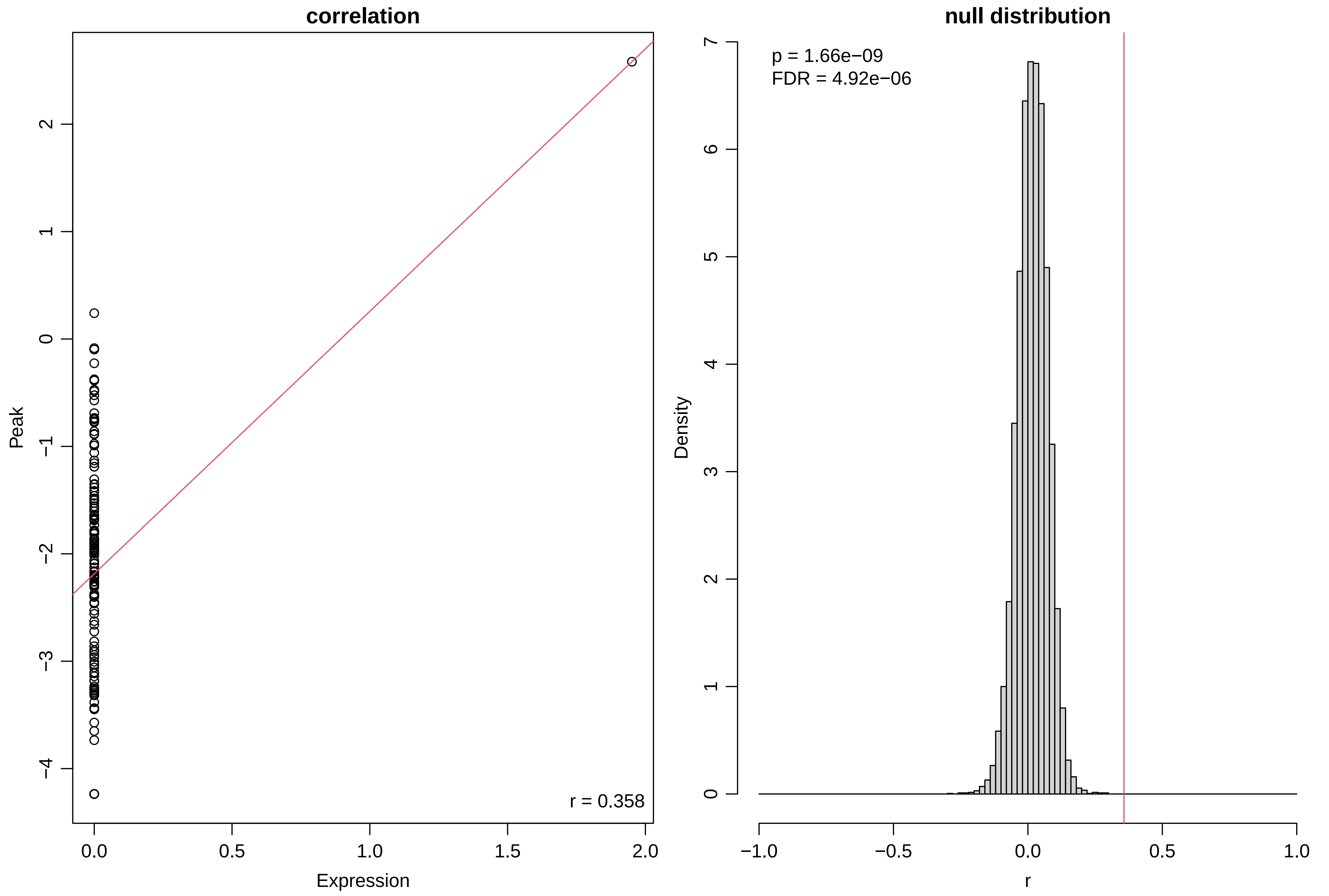

RPL21P104 vs PeakID_55414 (chr12: 61837582 − 61840331)

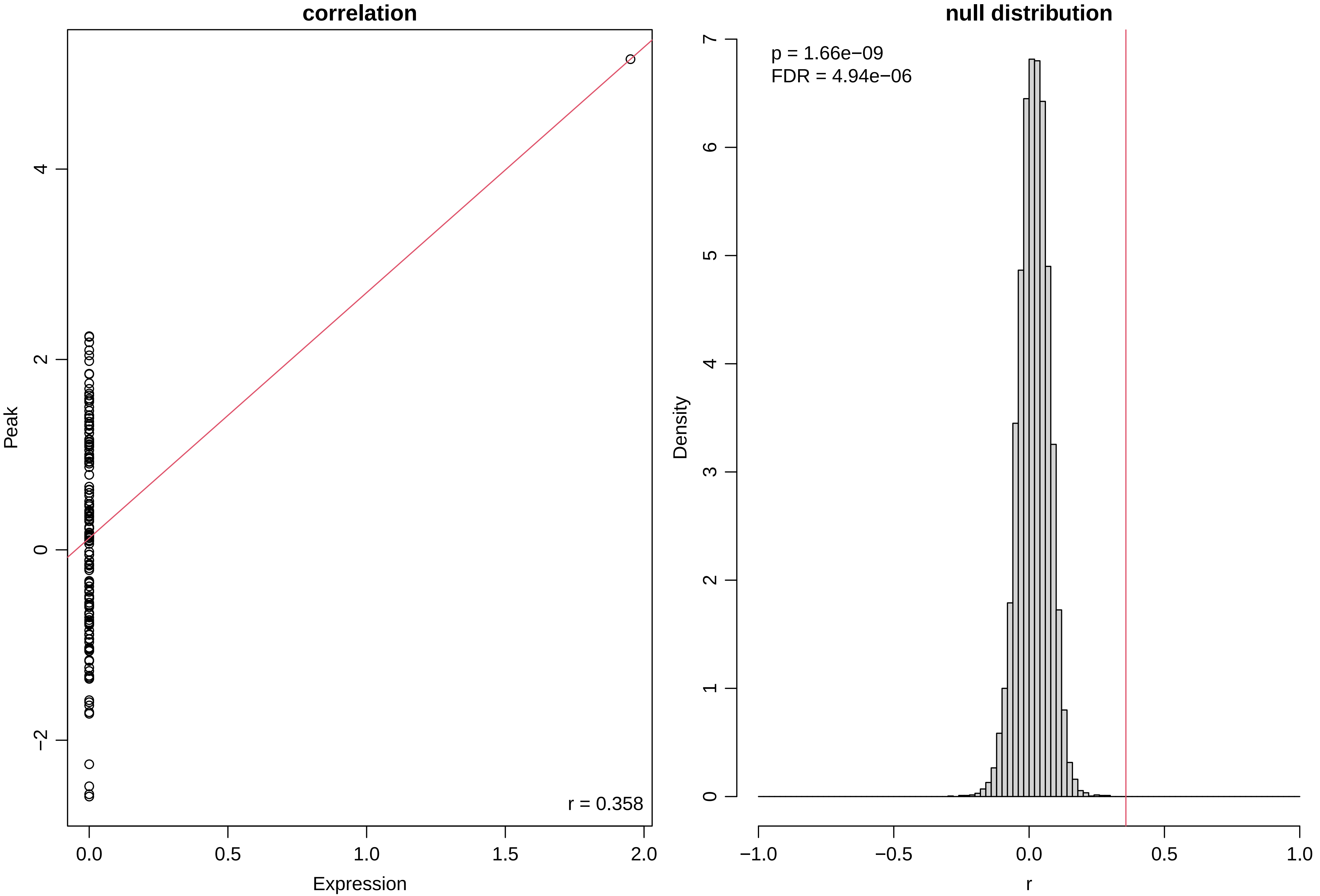

NOVA1 vs PeakID_69458 (chr14: 26596448 − 26599502)

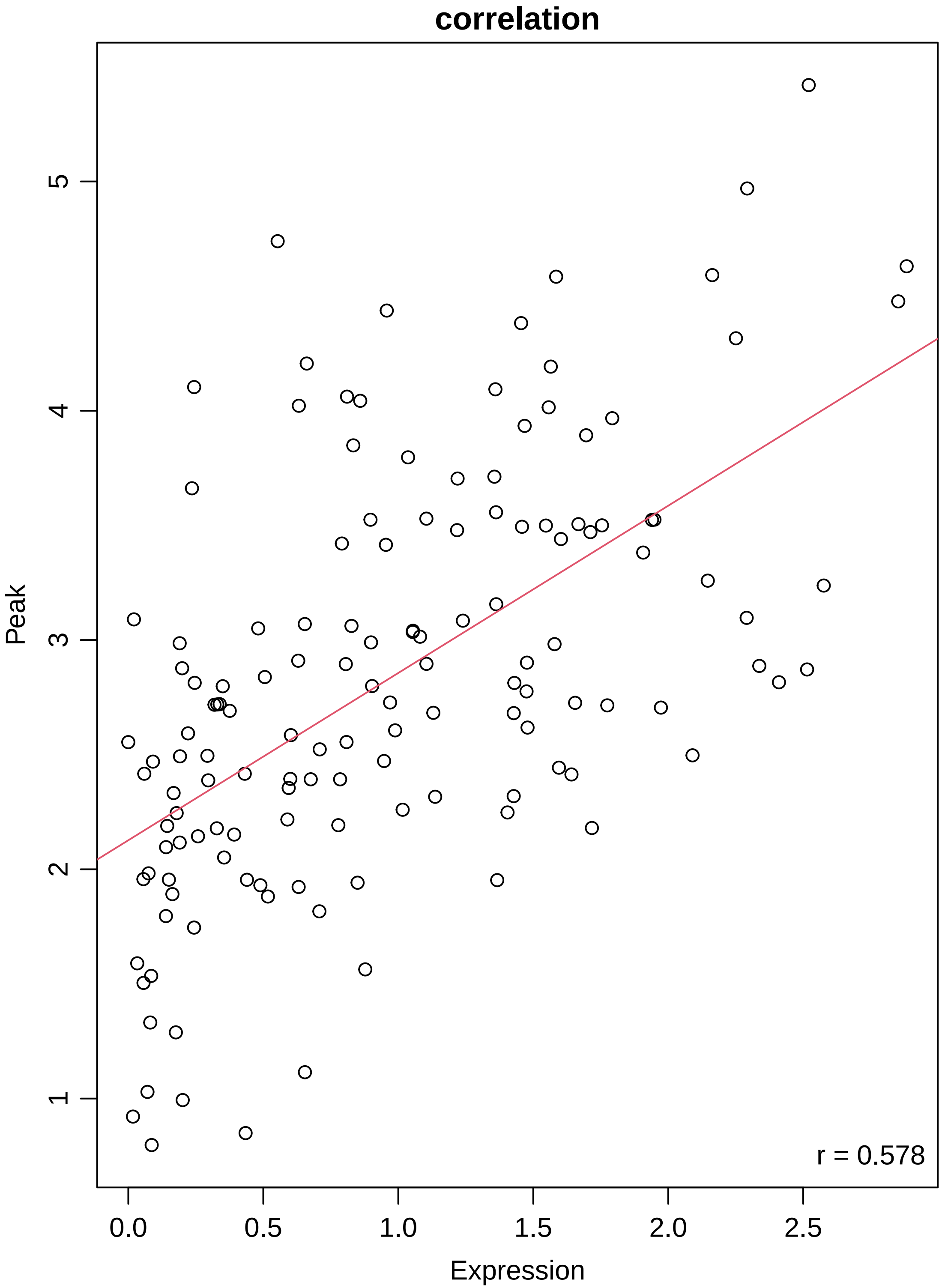

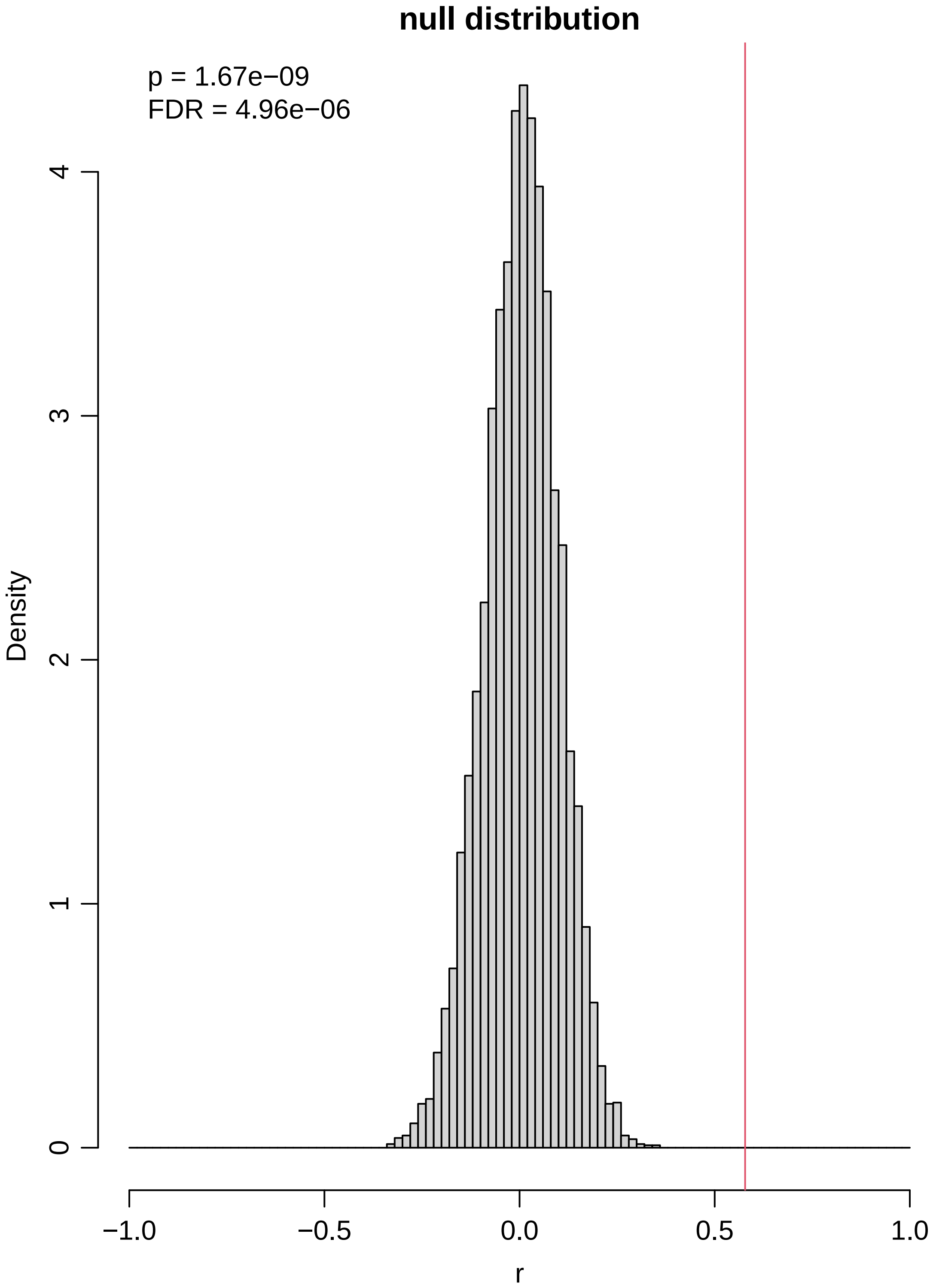

HOXC6 vs PeakID_53846 (chr12: 54015122 − 54022242)

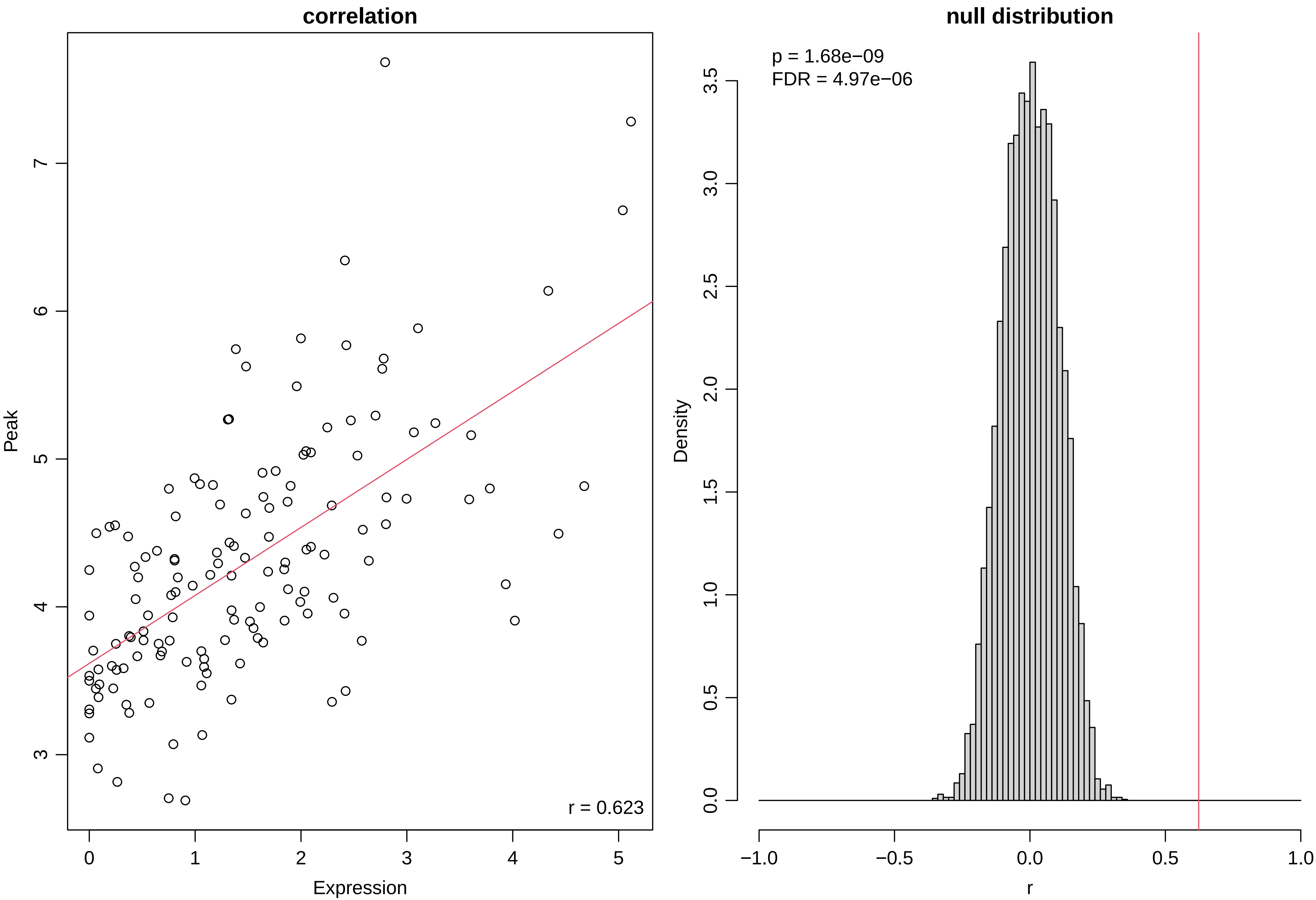

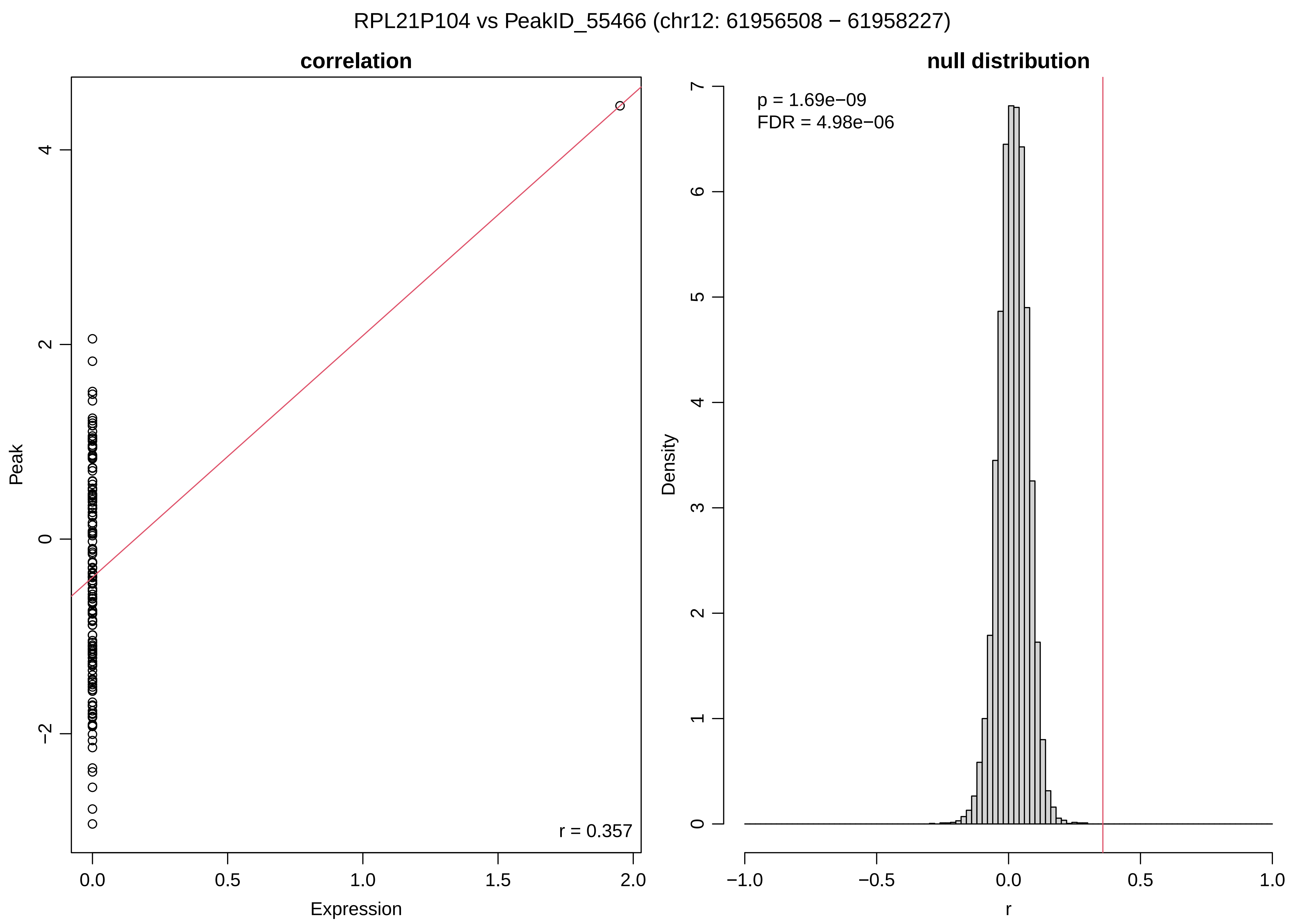
RPL21P104 vs PeakID_55466 (chr12: 61956508 − 61958227)
correlation
Peak
Expression
r = 0.357
null distribution
Density
r
p = 1.69e−09
FDR = 4.98e−06

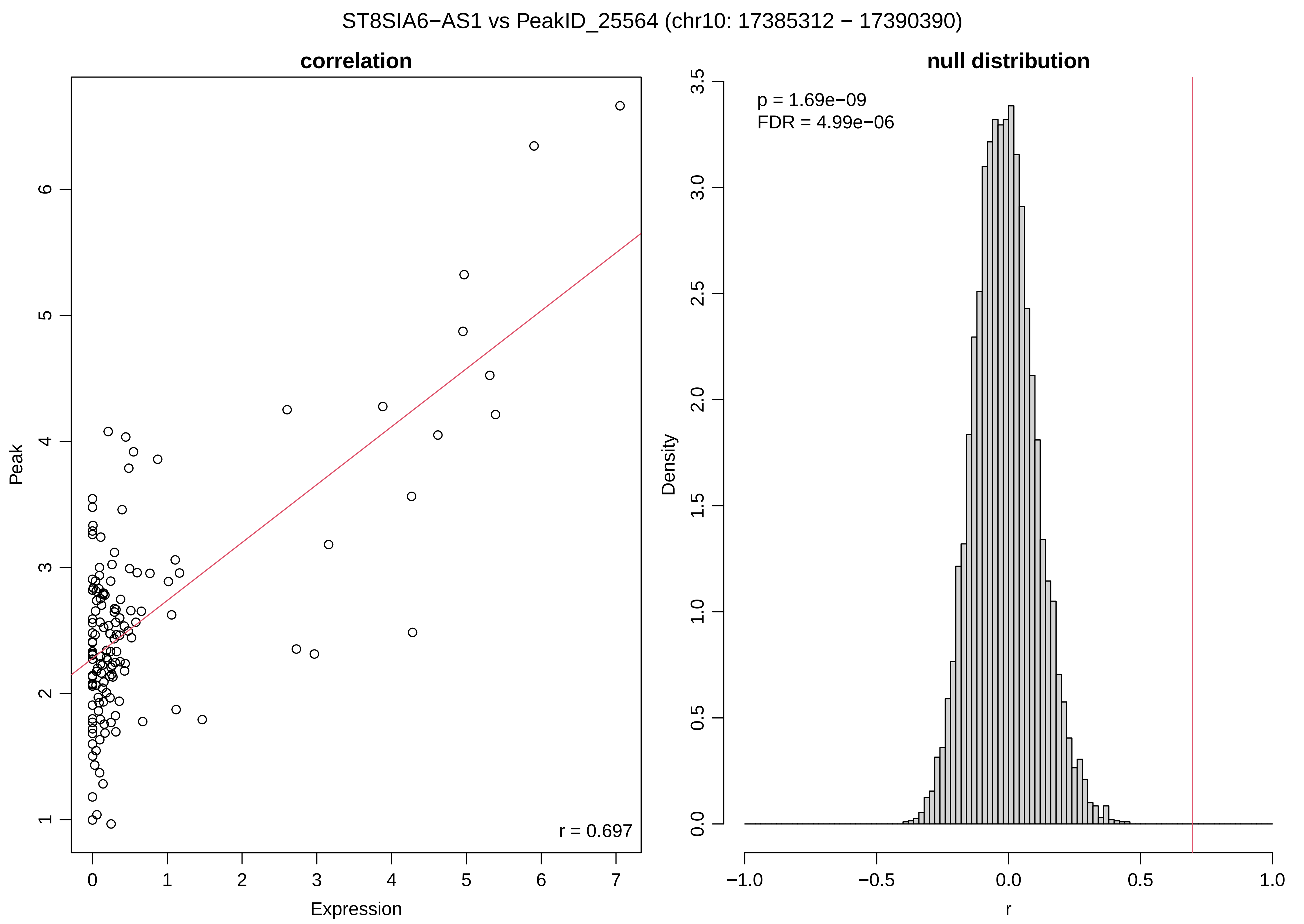
ST8SIA6−AS1 vs PeakID_25564 (chr10: 17385312 − 17390390)
correlation
Peak
Expression
r = 0.697
null distribution
p = 1.69e−09
FDR = 4.99e−06
Density
r

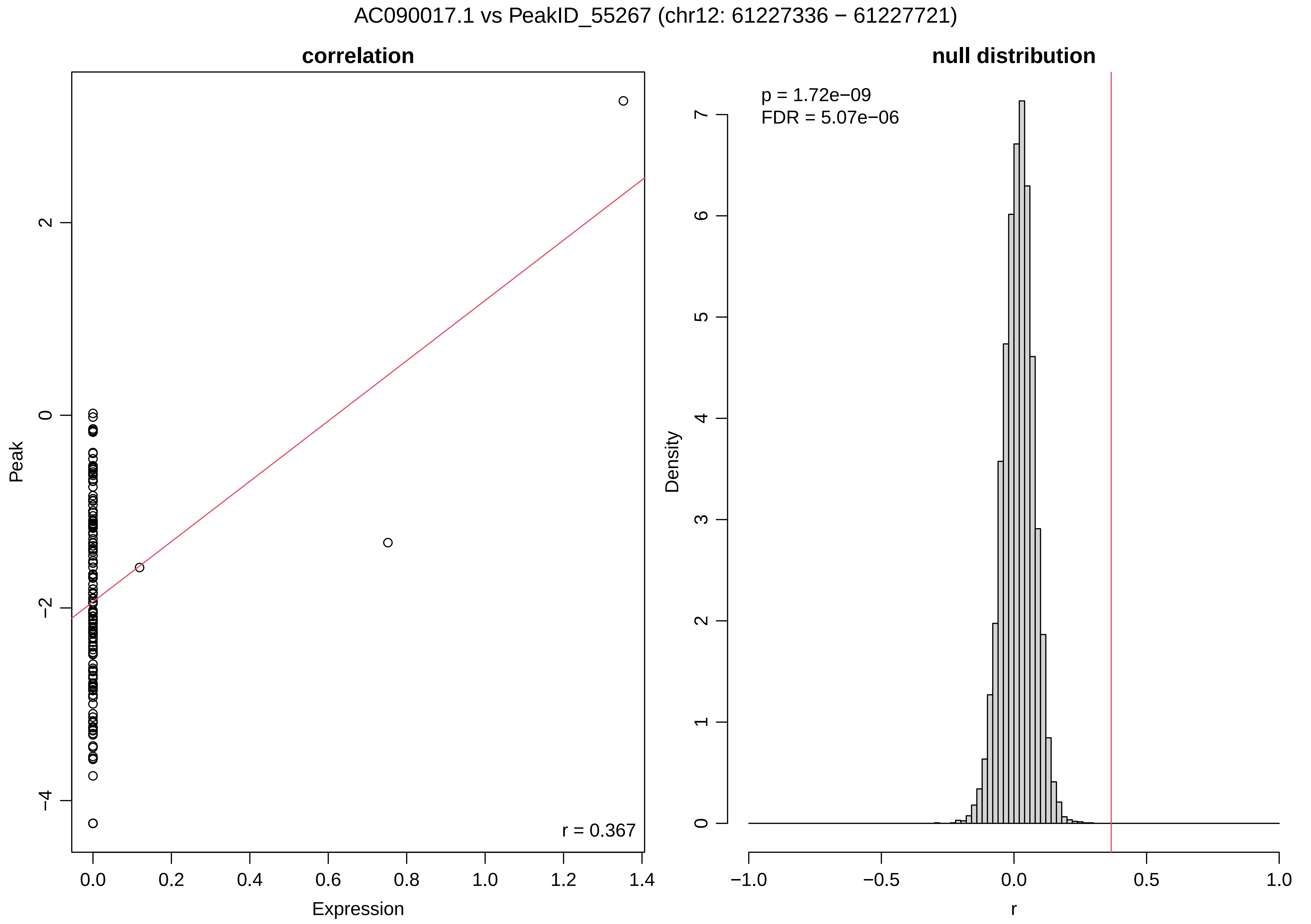
AC090017.1 vs PeakID_55267 (chr12: 61227336 − 61227721)
correlation
null distribution
Peak
Expression
r = 0.367
p = 1.72e−09
FDR = 5.07e−06
Density
r
0.0
0.2
0.4
0.6
0.8
1.0
1.2
1.4
2
0
−2
−4
−1.0
−0.5
0.0
0.5
1.0
0
1
2
3
4
5
6
7

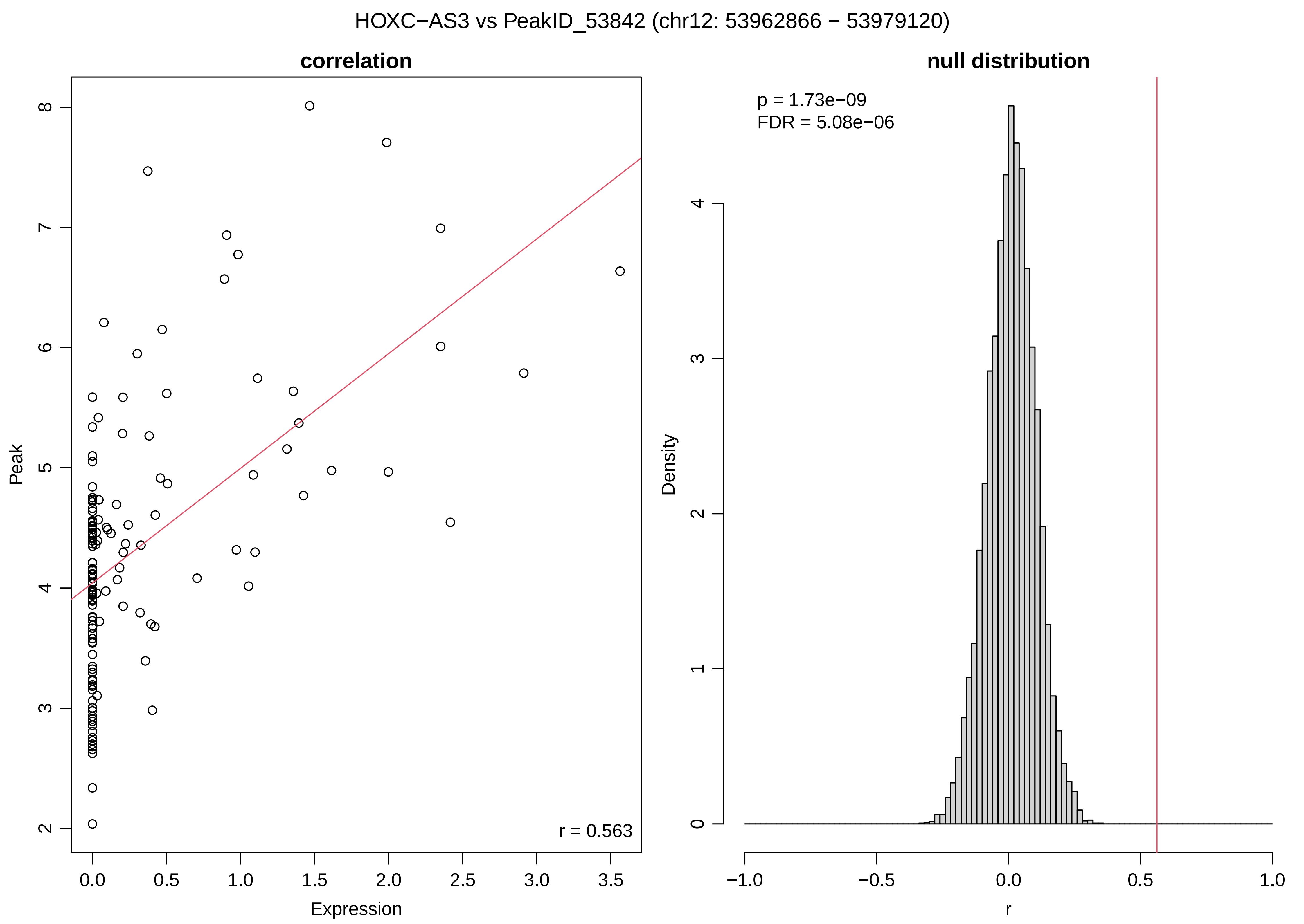

HOXC−AS3 vs PeakID_53842 (chr12: 53962866 − 53979120)
correlation
Peak
Expression
r = 0.563
null distribution
p = 1.73e−09
FDR = 5.08e−06
Density
r

FAR2P2 vs PeakID_133301 (chr2: 130427656 − 130429076)

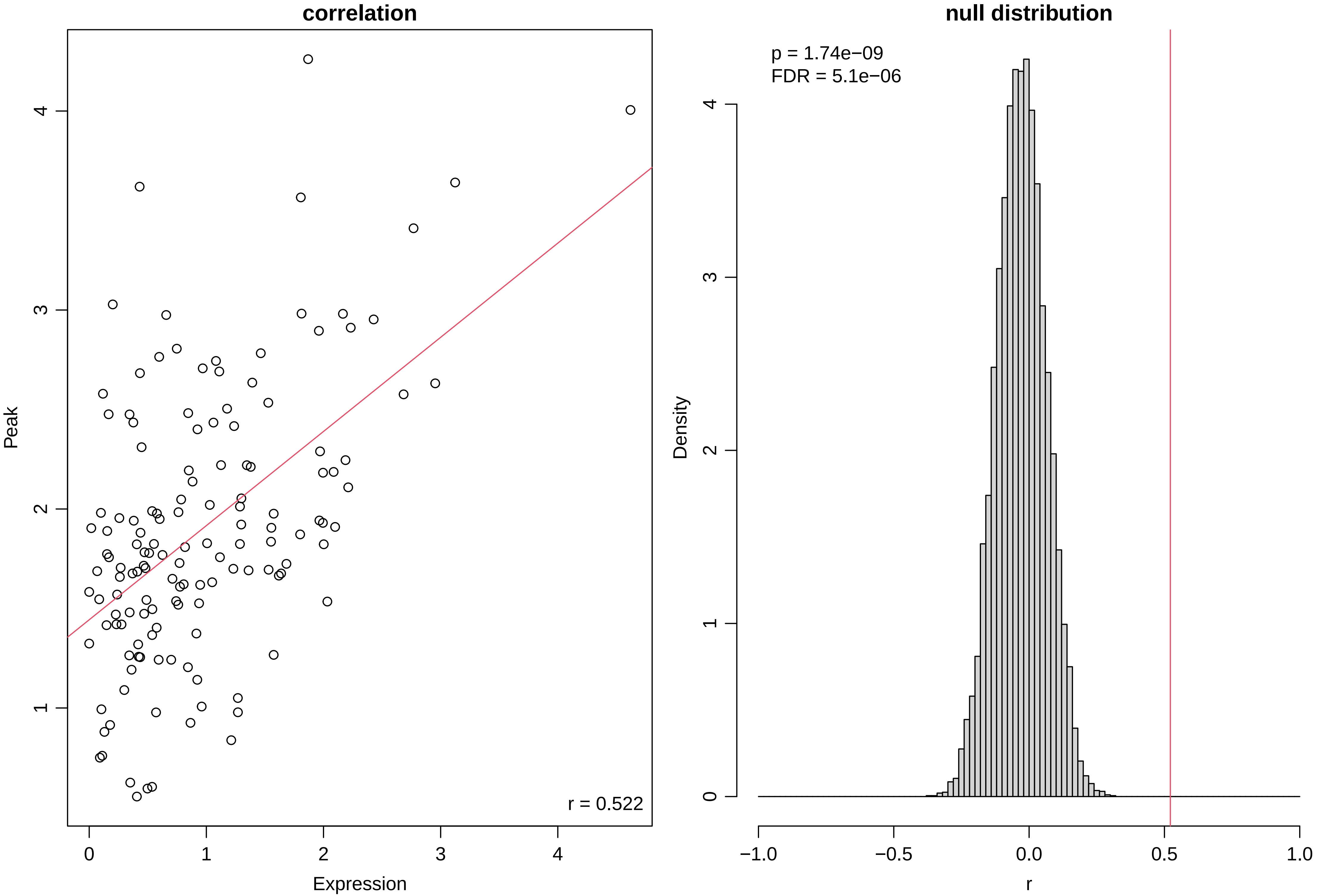

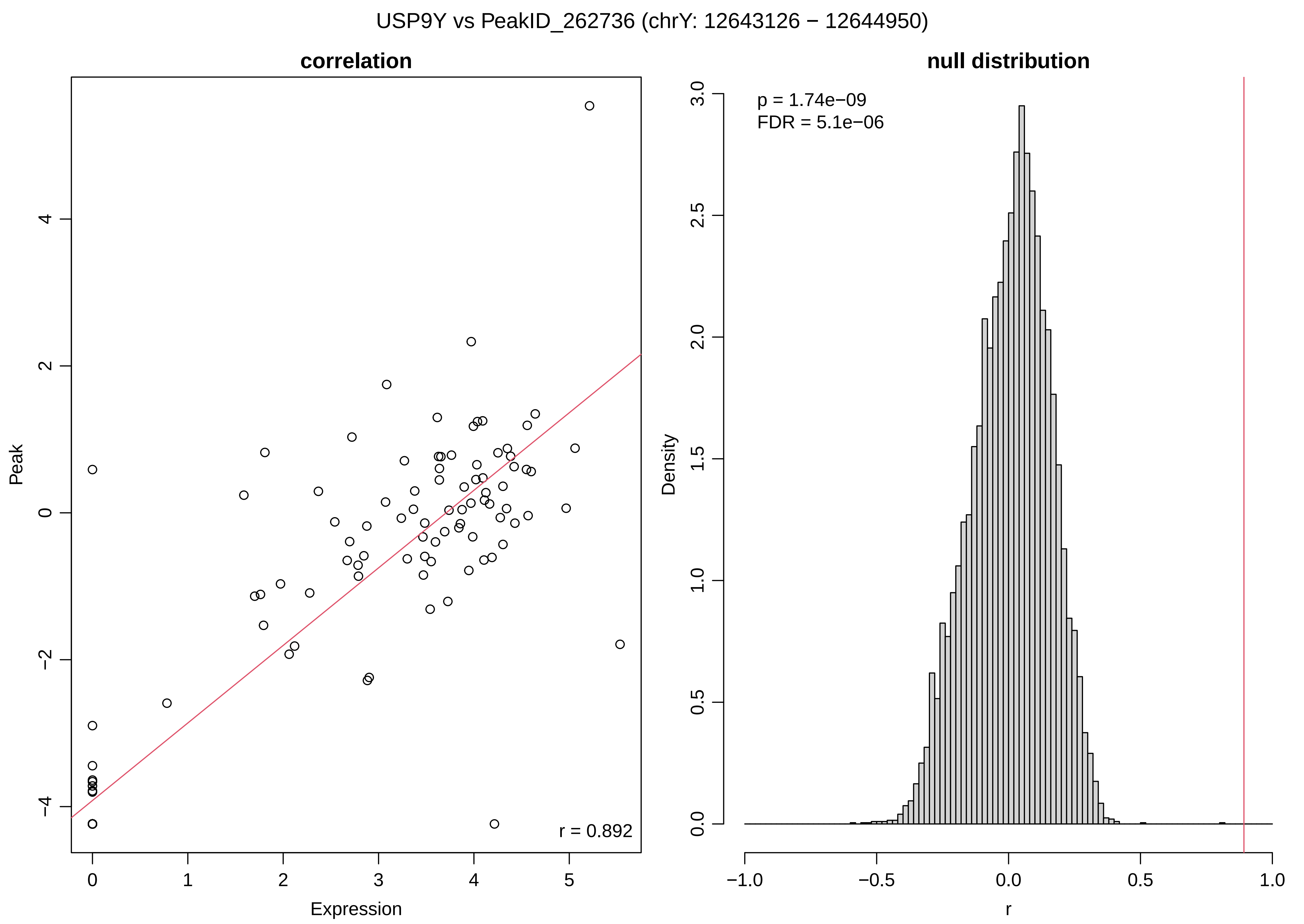

USP9Y vs PeakID_262736 (chrY: 12643126 − 12644950)
correlation
Peak
Expression
r = 0.892
null distribution
p = 1.74e−09
FDR = 5.1e−06
Density
r

RNF157 vs PeakID_103862 (chr17: 76237637 − 76238019)

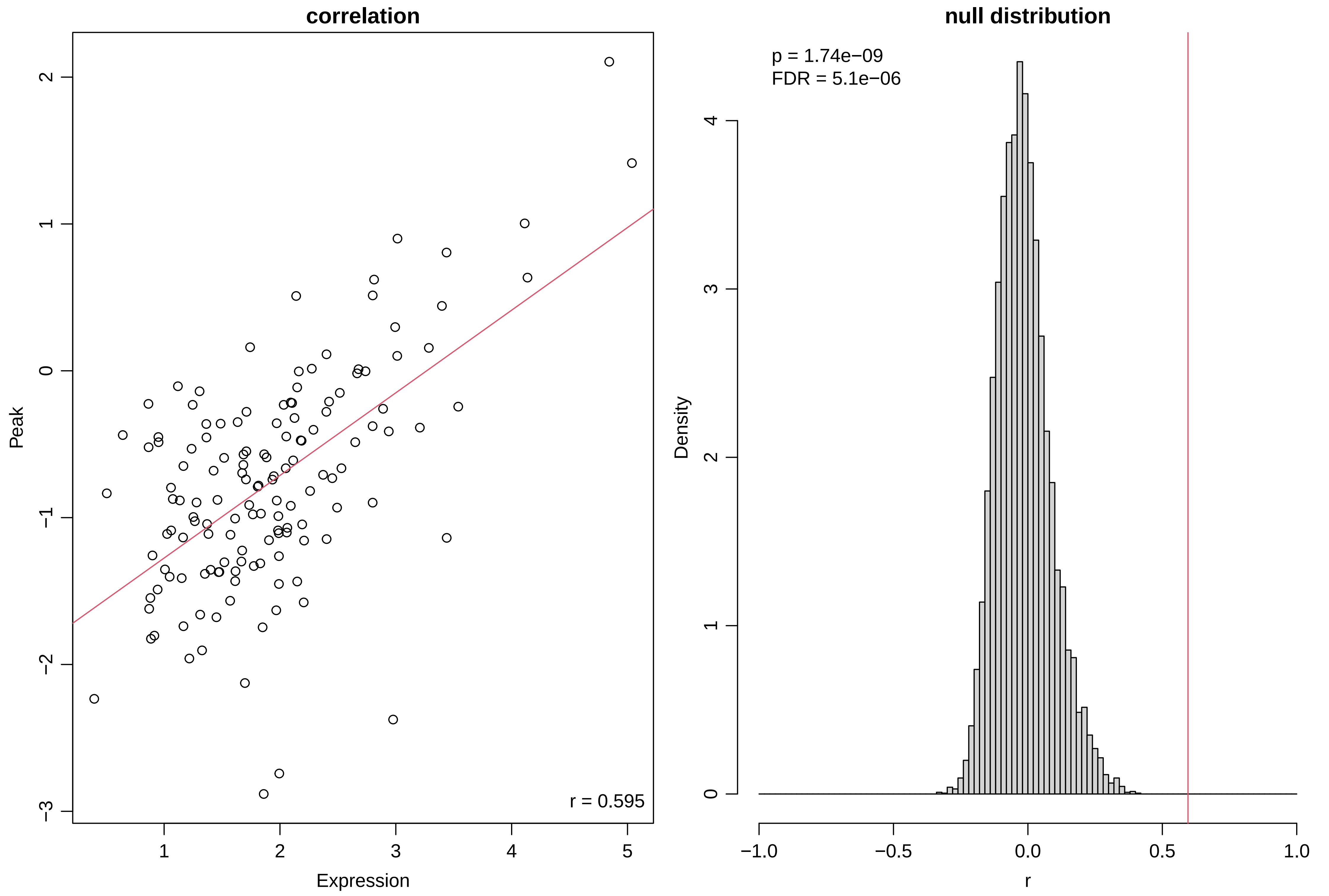

JAKMIP3 vs PeakID_35894 (chr10: 132093013 − 132096306)

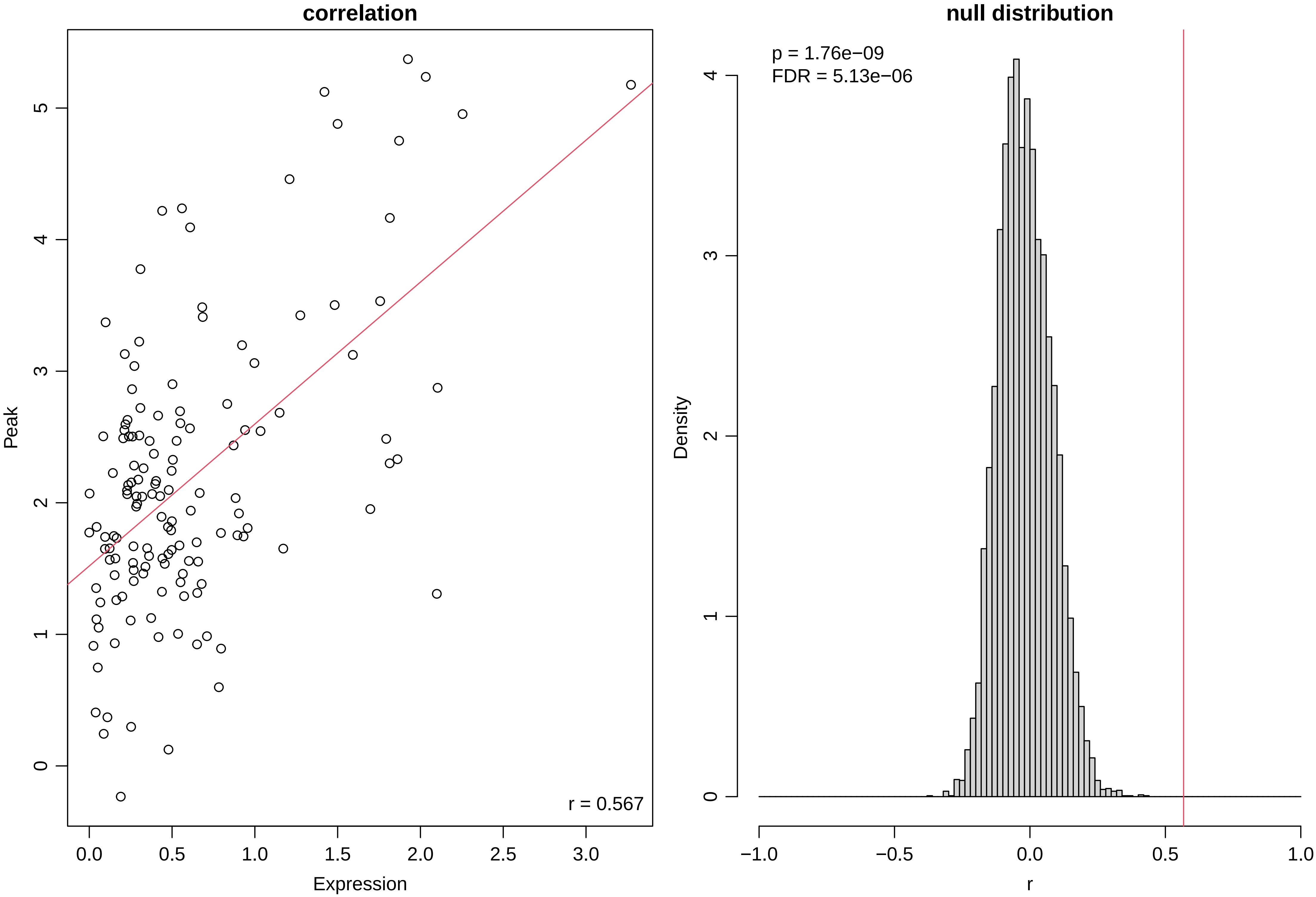

POU6F2 vs PeakID_222039 (chr7: 39013070 − 39015072)

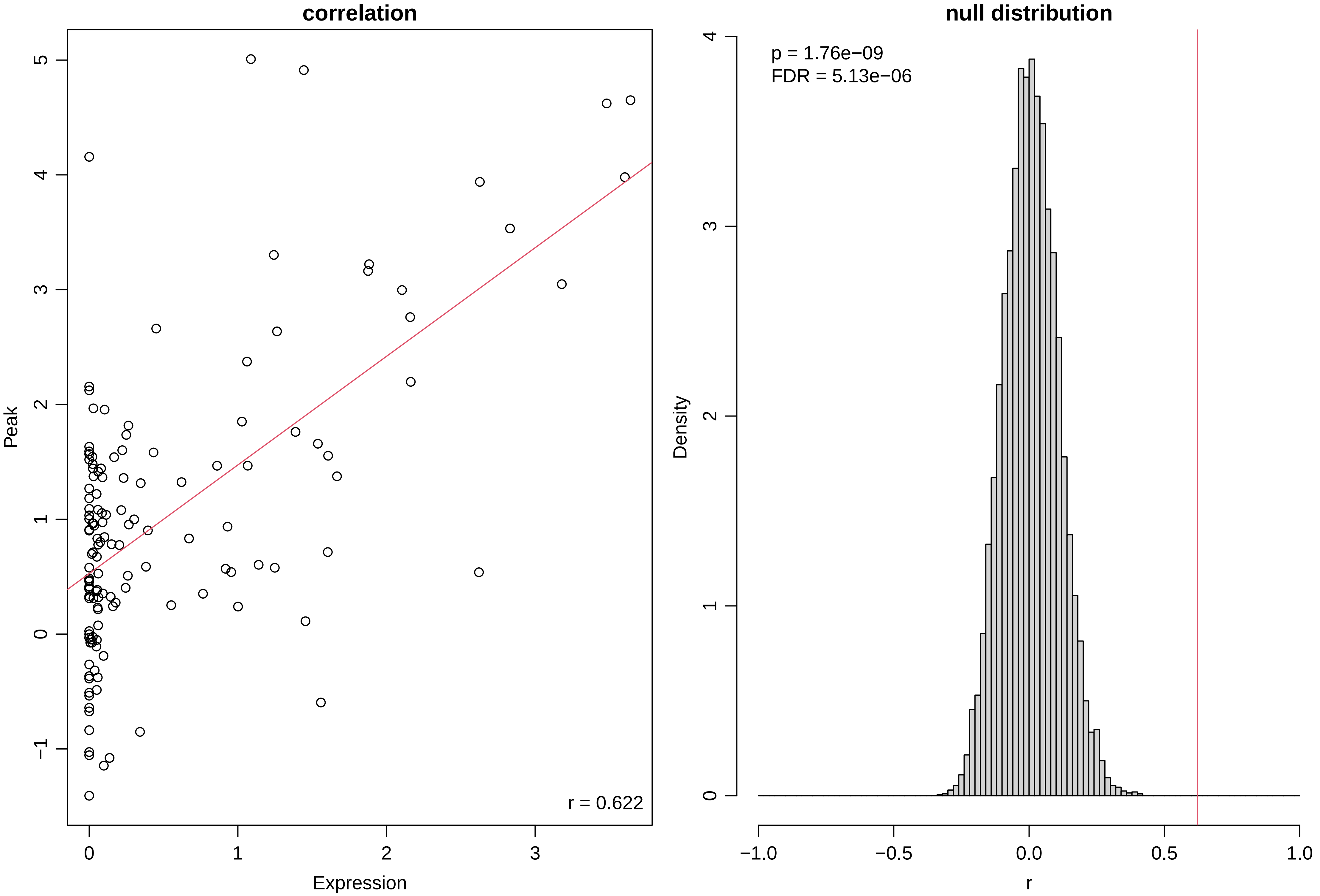

PRKY vs PeakID_262410 (chrY: 7516209 − 7516966)

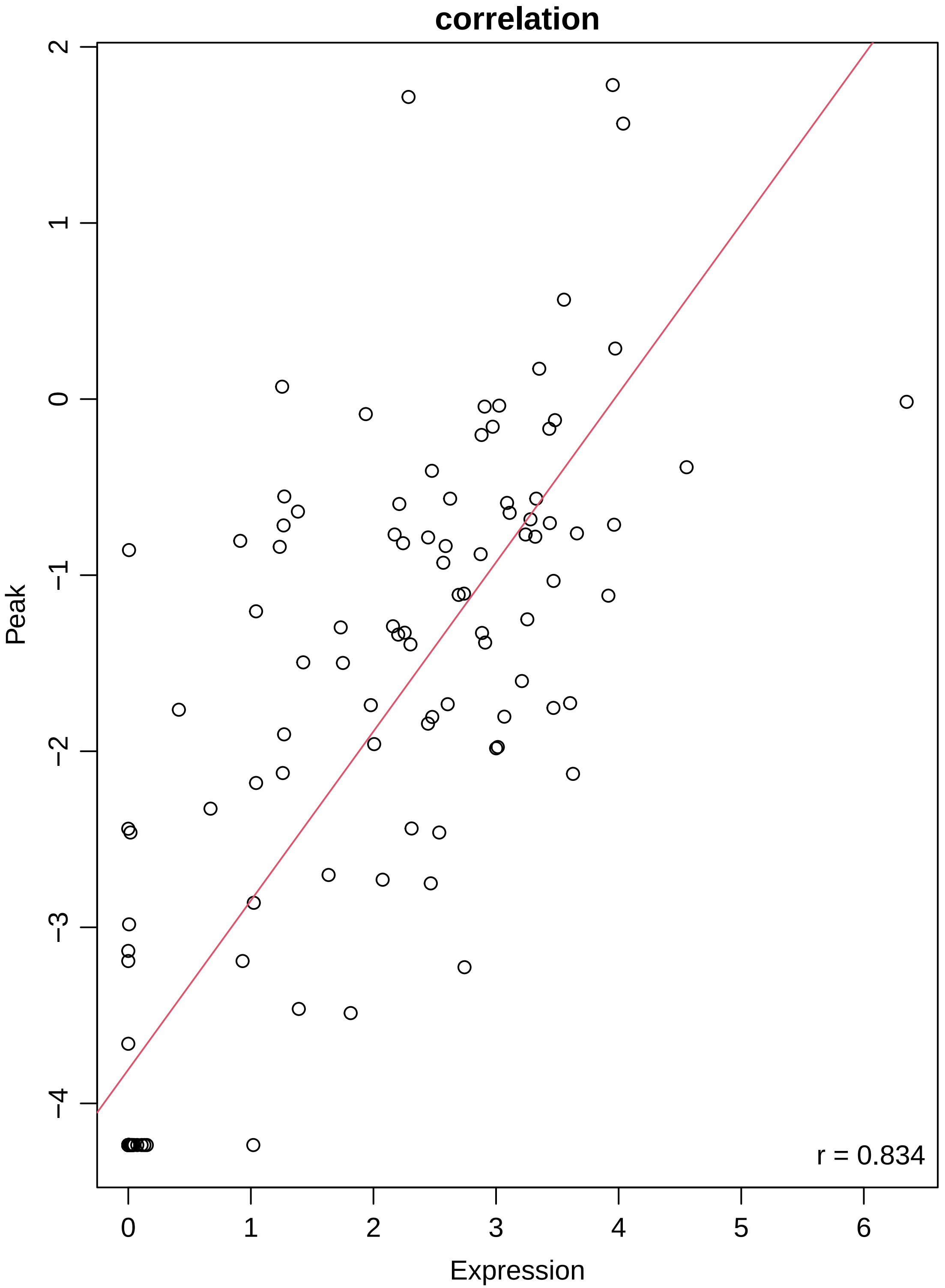

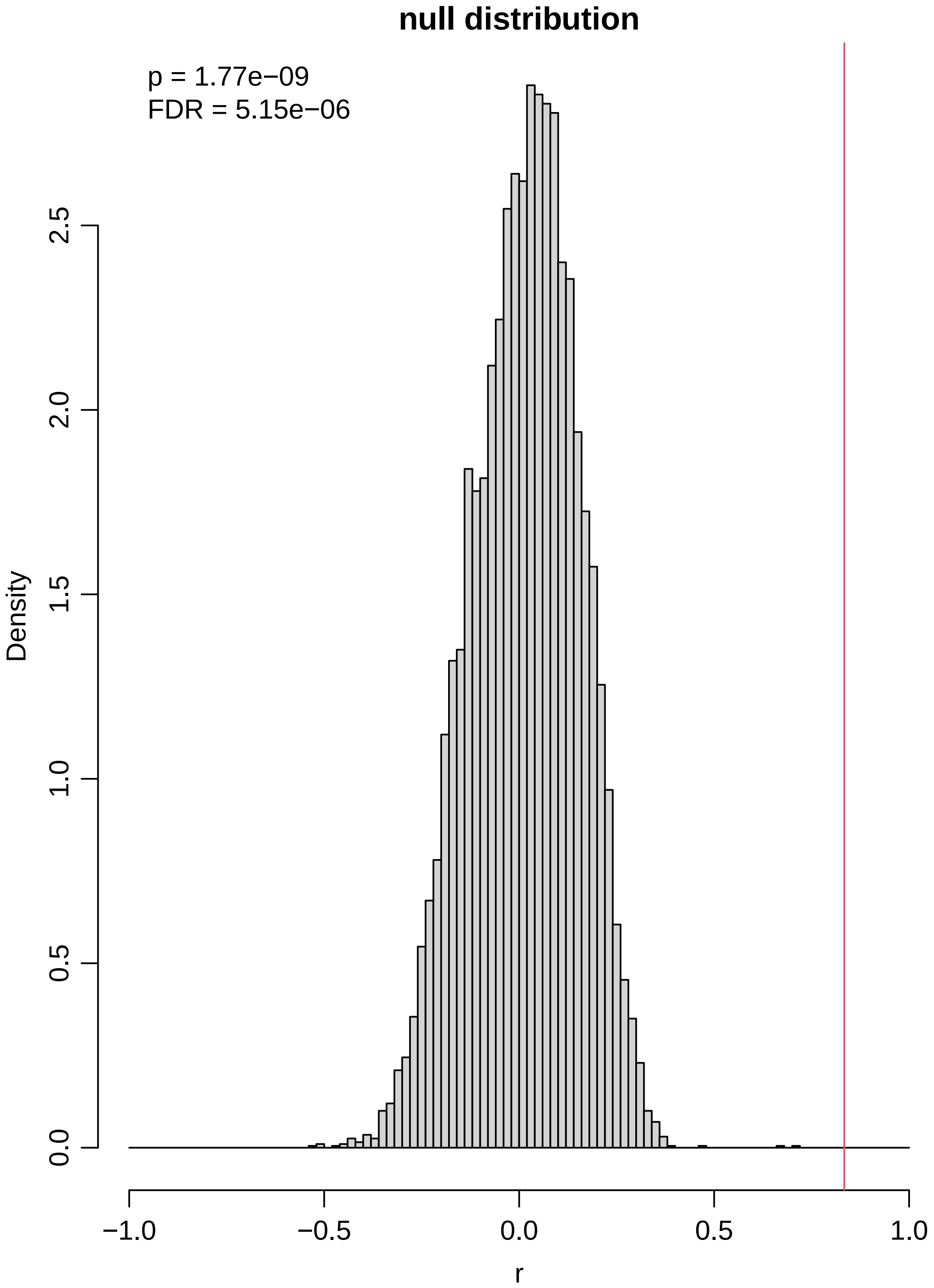

C12orf66 vs PeakID_55727 (chr12: 64078335 − 64084547)

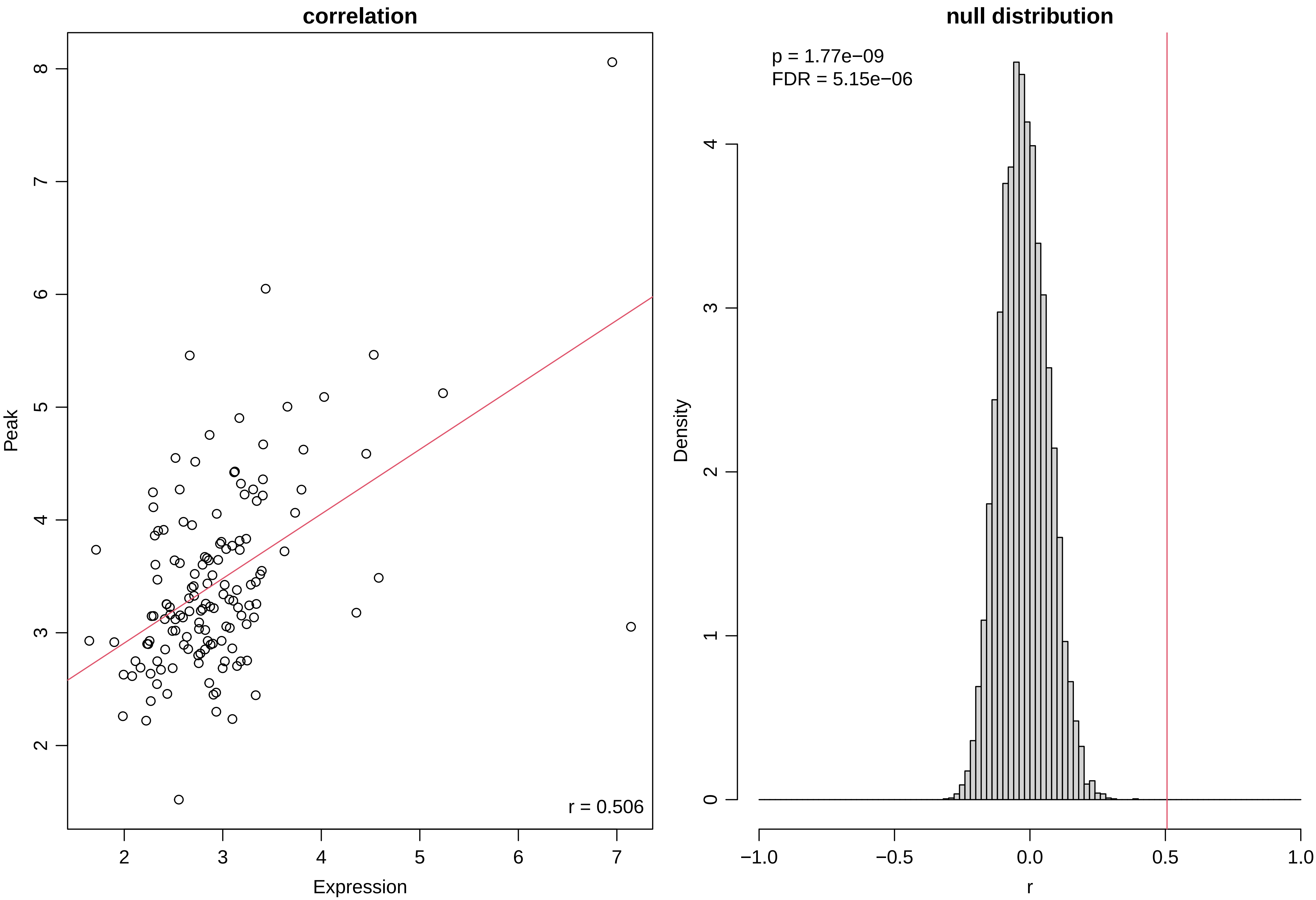

LINC00278 vs PeakID_262331 (chrY: 2887764 − 2888697)

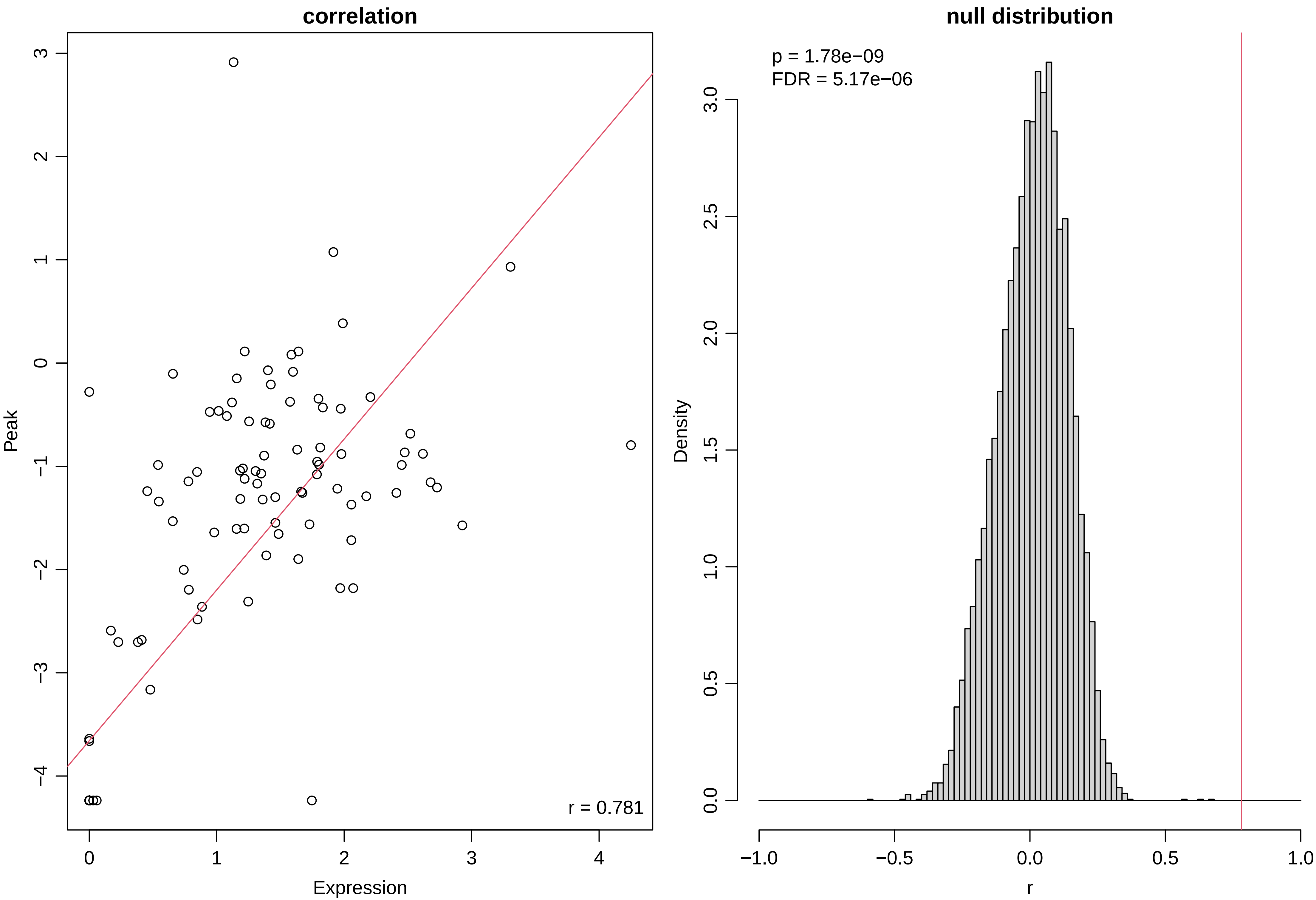

RFTN1P1 vs PeakID_262432 (chrY: 7880813 − 7883191)

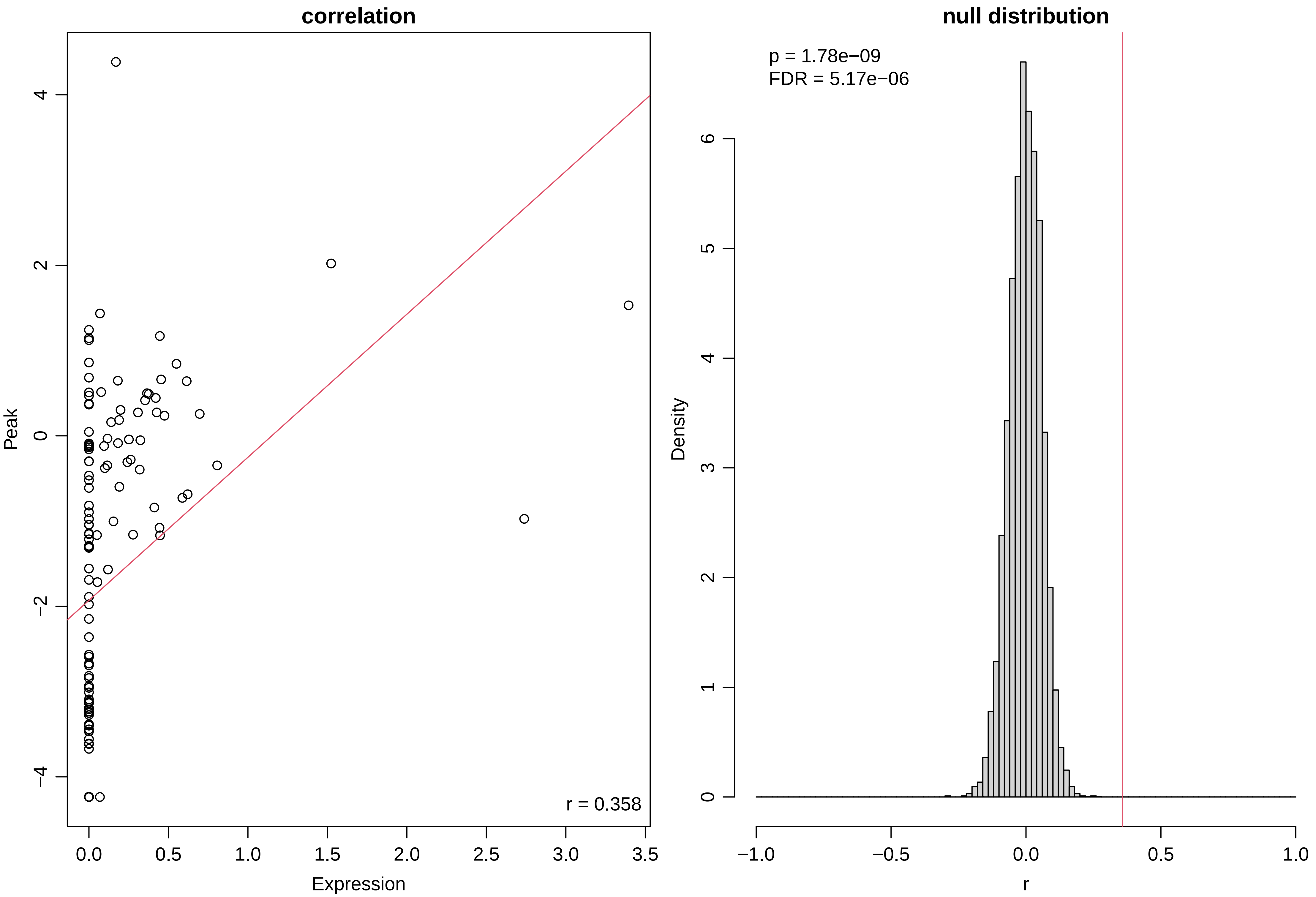

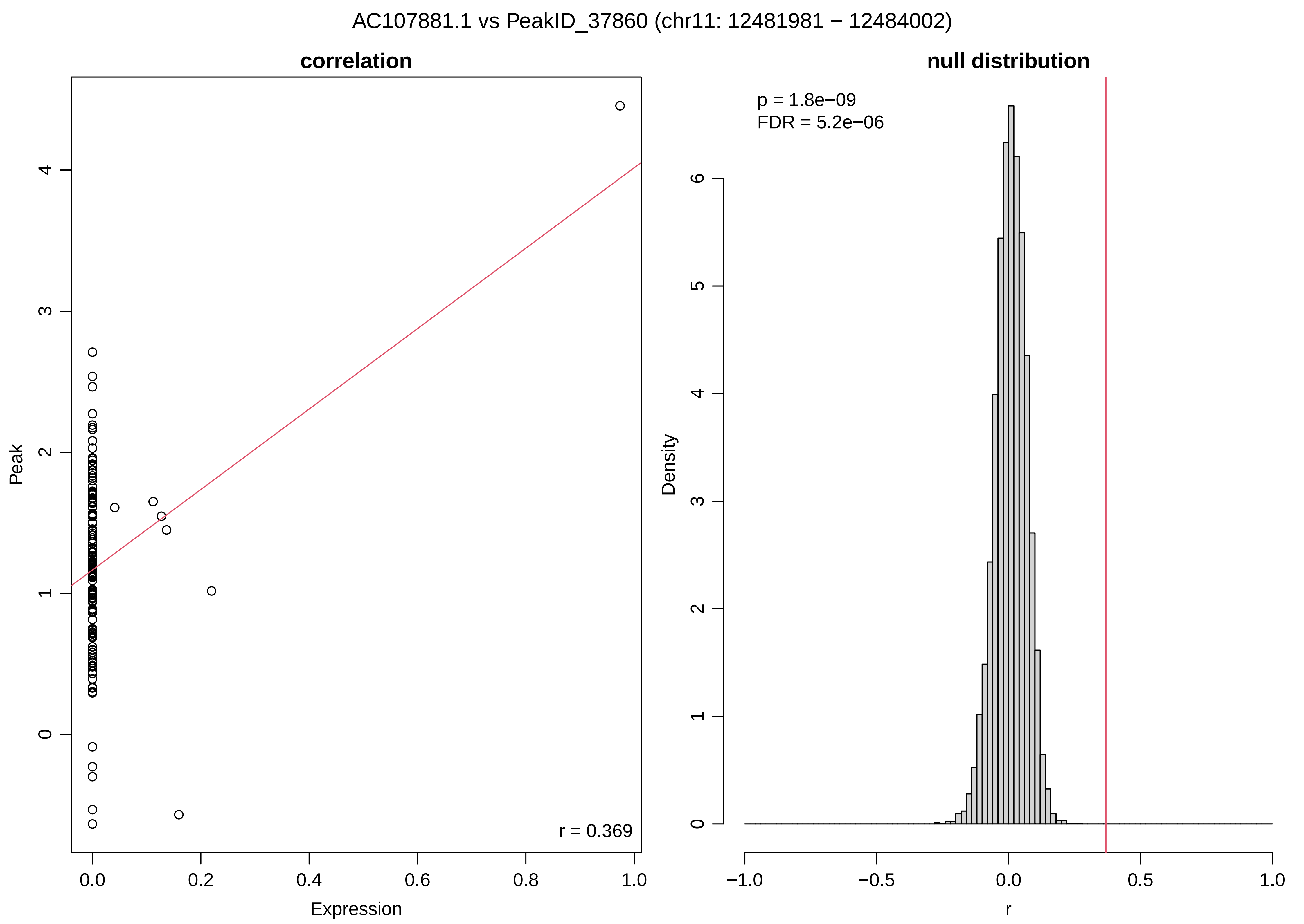

AC107881.1 vs PeakID_37860 (chr11: 12481981 − 12484002)
correlation
null distribution
Peak
Expression
r = 0.369
p = 1.8e−09
FDR = 5.2e−06
Density
r

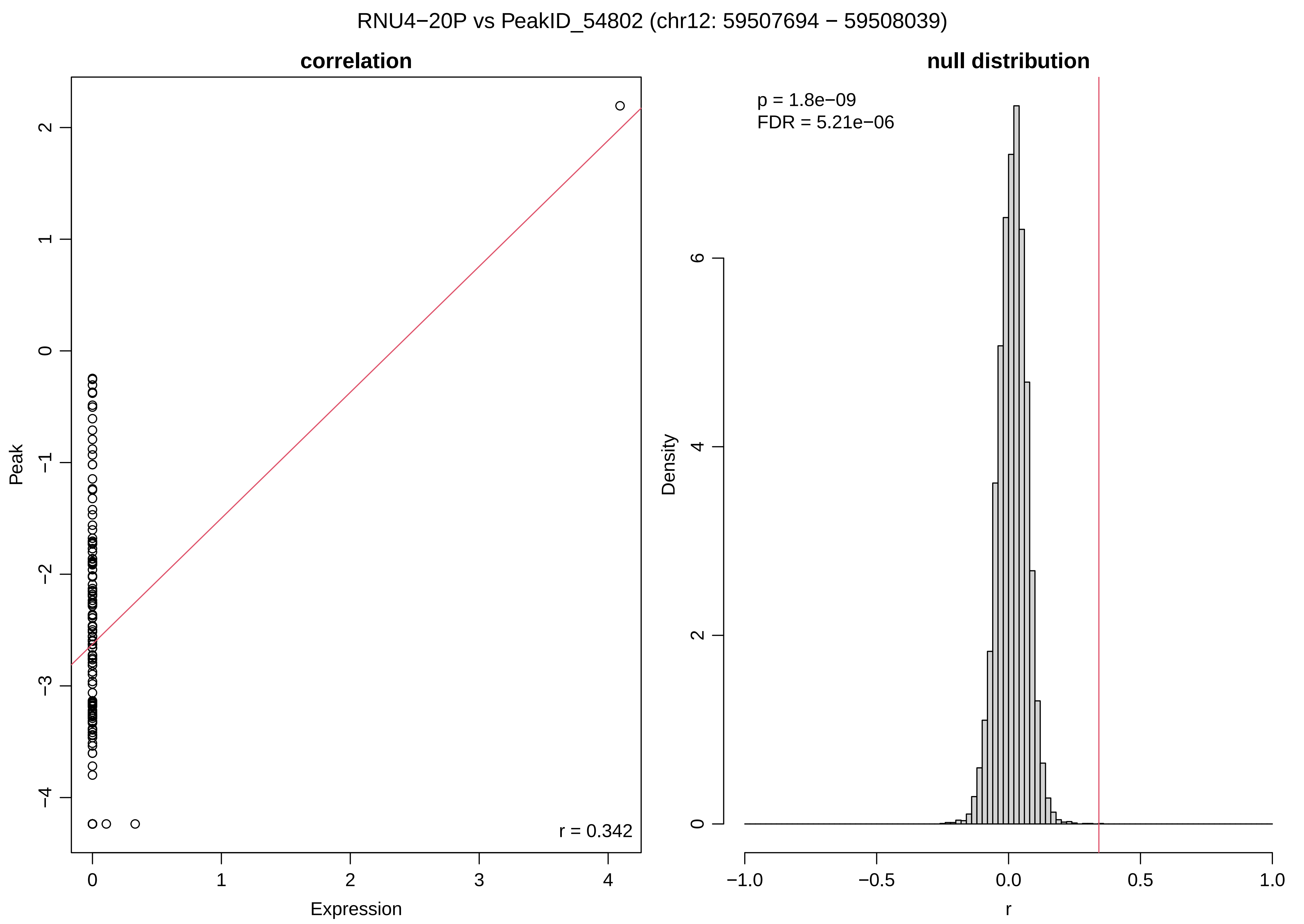

RNU4−20P vs PeakID_54802 (chr12: 59507694 − 59508039)
correlation
Peak
Expression
r = 0.342
null distribution
p = 1.8e−09
FDR = 5.21e−06
Density
r

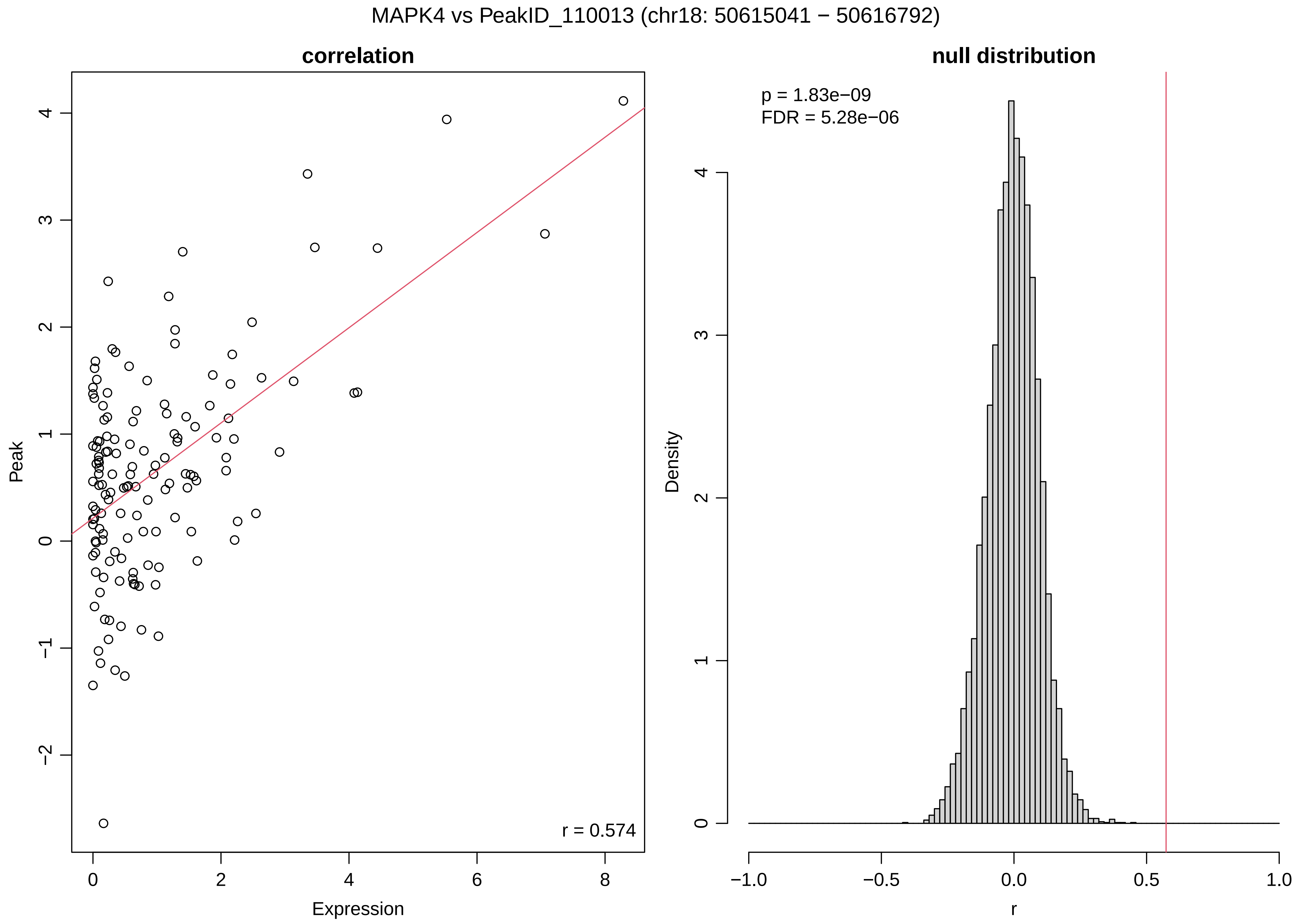
MAPK4 vs PeakID_110013 (chr18: 50615041 − 50616792)
correlation
null distribution
p = 1.83e−09
FDR = 5.28e−06
r = 0.574
Peak
Expression
Density
r
−2
−1
0
1
2
3
4
6
8
−1.0
−0.5
0.0
0.5
1.0

TBL1Y vs PeakID_262370 (chrY: 7056125 − 7057115)

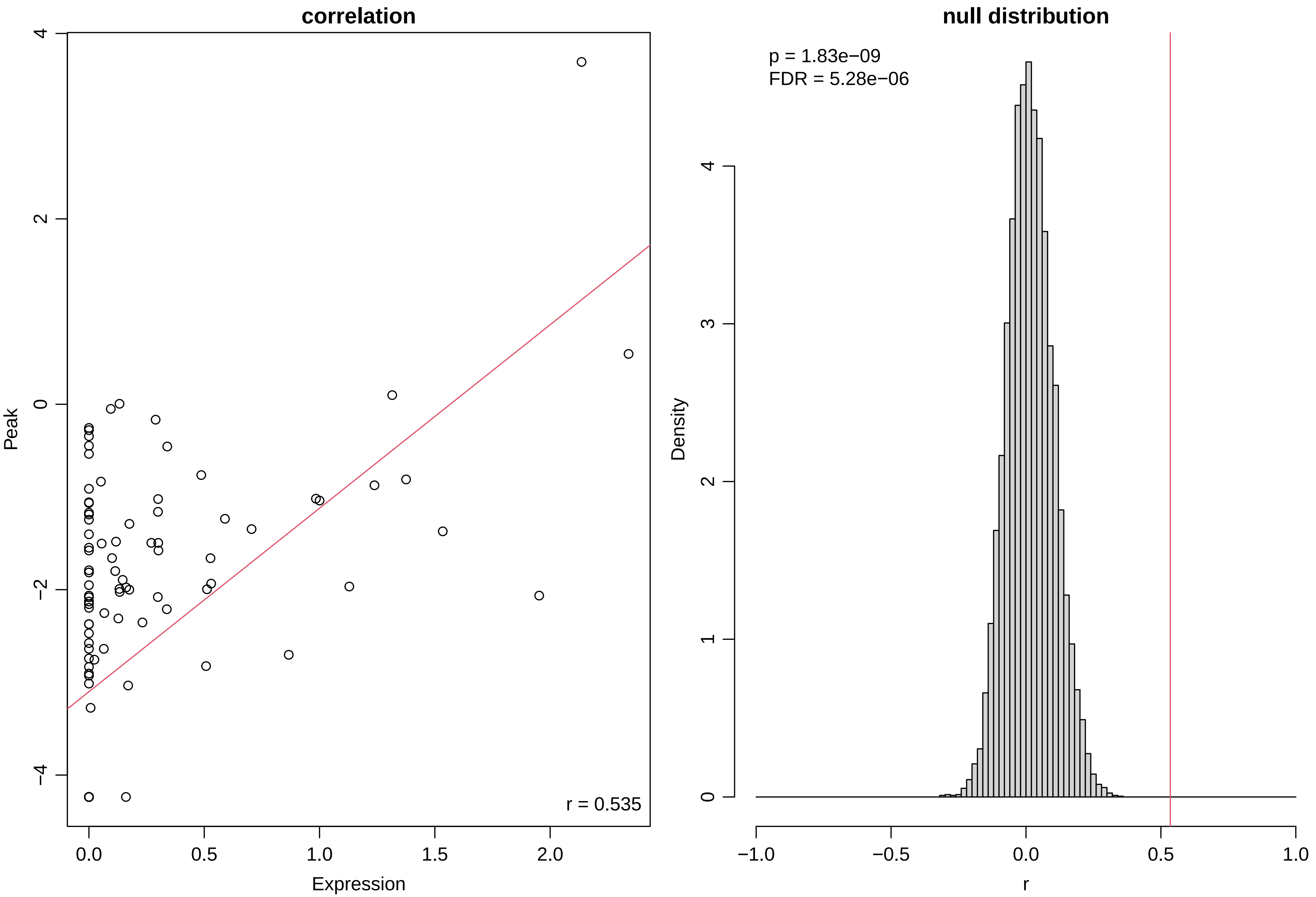

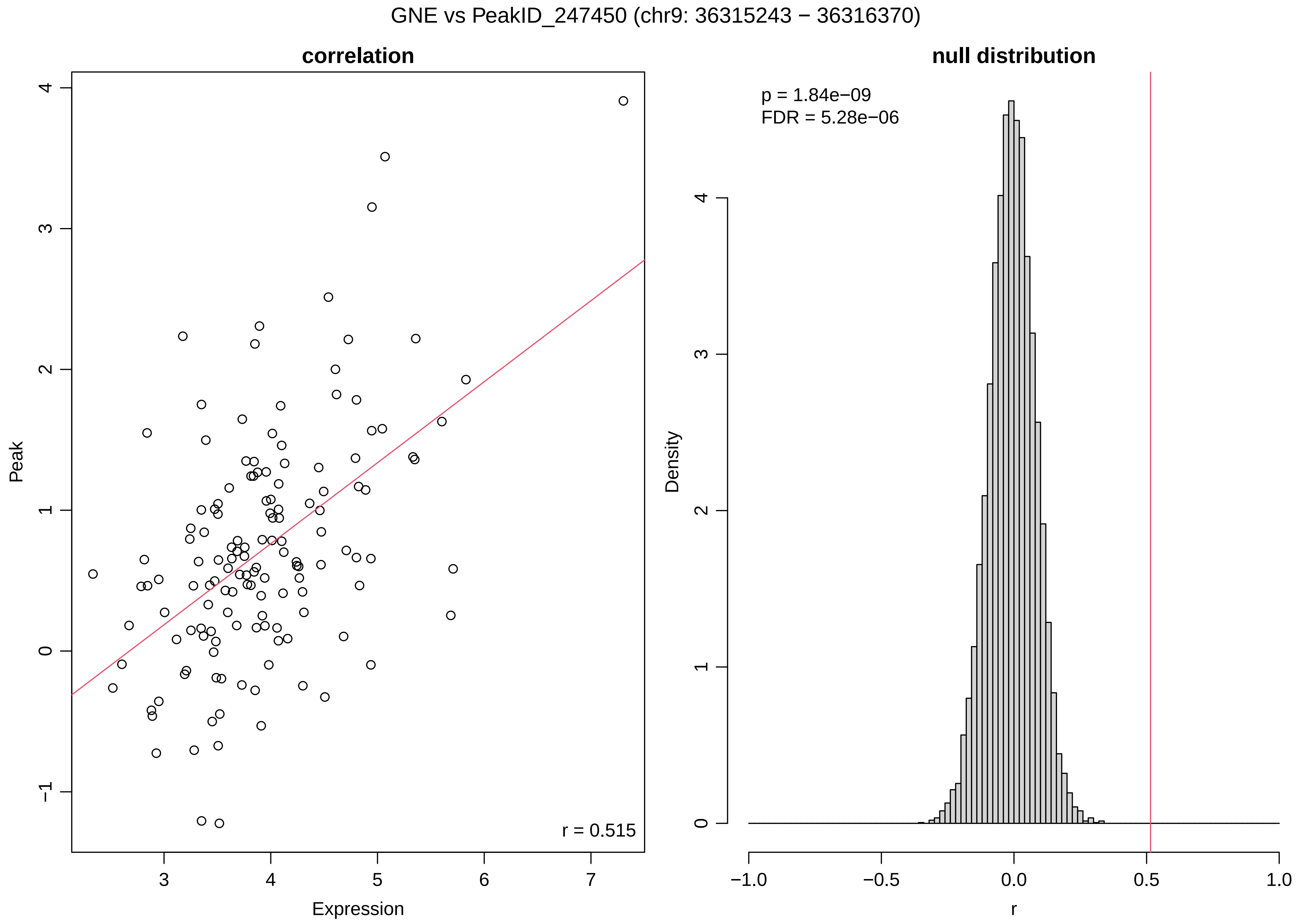
GNE vs PeakID_247450 (chr9: 36315243 − 36316370)
correlation
Peak
Expression
r = 0.515
null distribution
p = 1.84e−09
FDR = 5.28e−06
Density
r

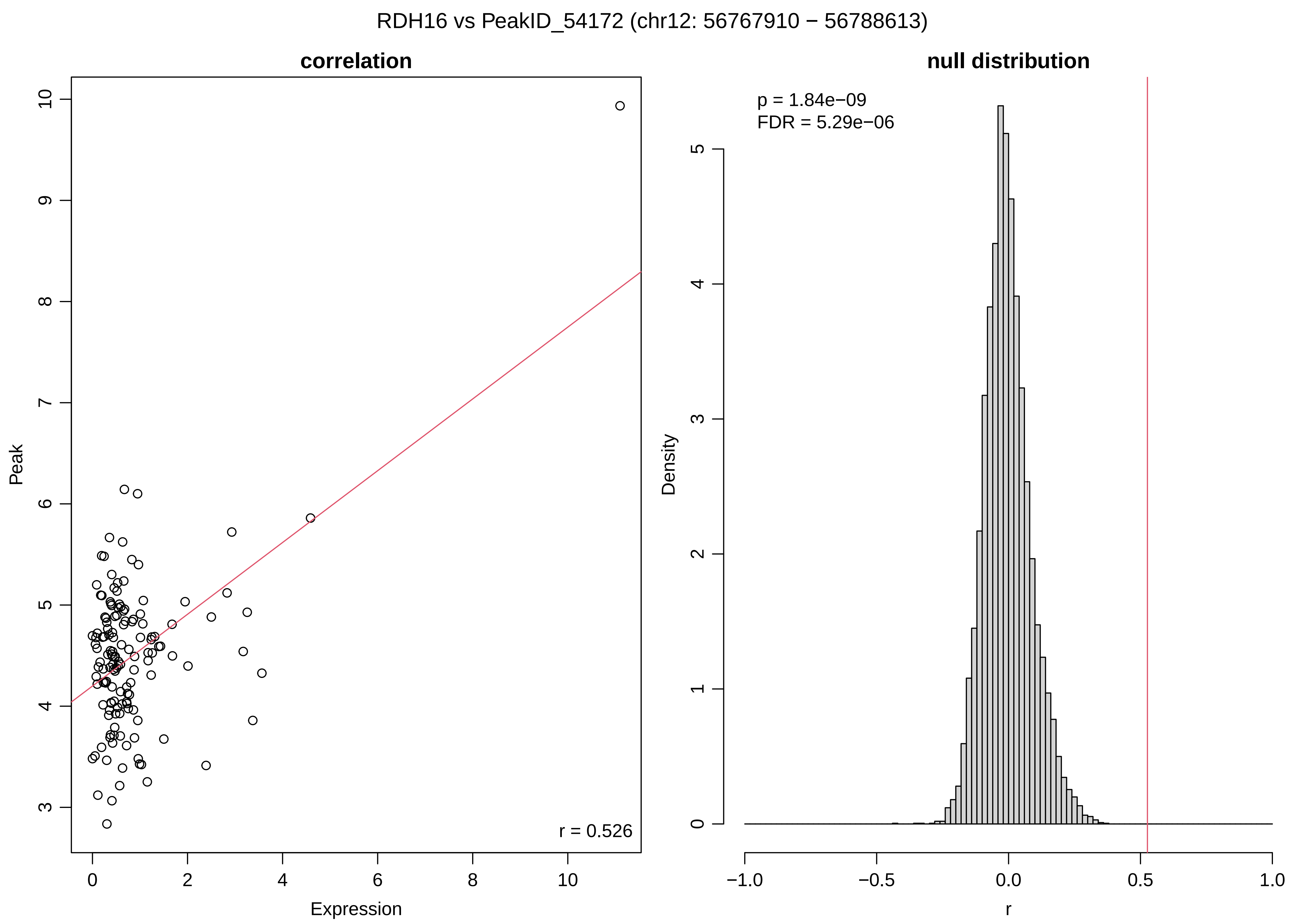

RDH16 vs PeakID_54172 (chr12: 56767910 − 56788613)
correlation
Peak
Expression
r = 0.526
null distribution
p = 1.84e−09
FDR = 5.29e−06
Density
r

RPS6KA6 vs PeakID_259511 (chrX: 84187270 − 84188542)

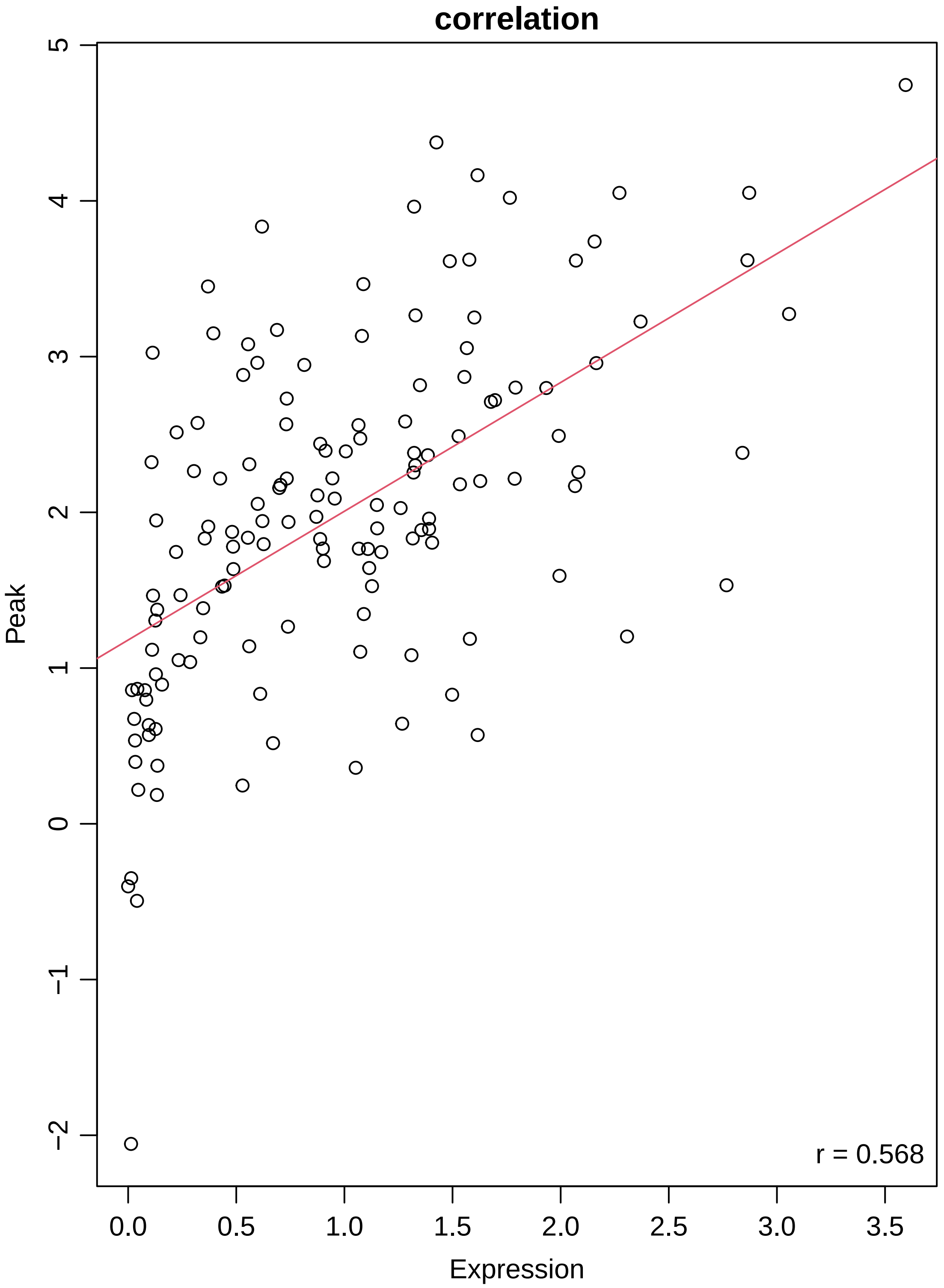

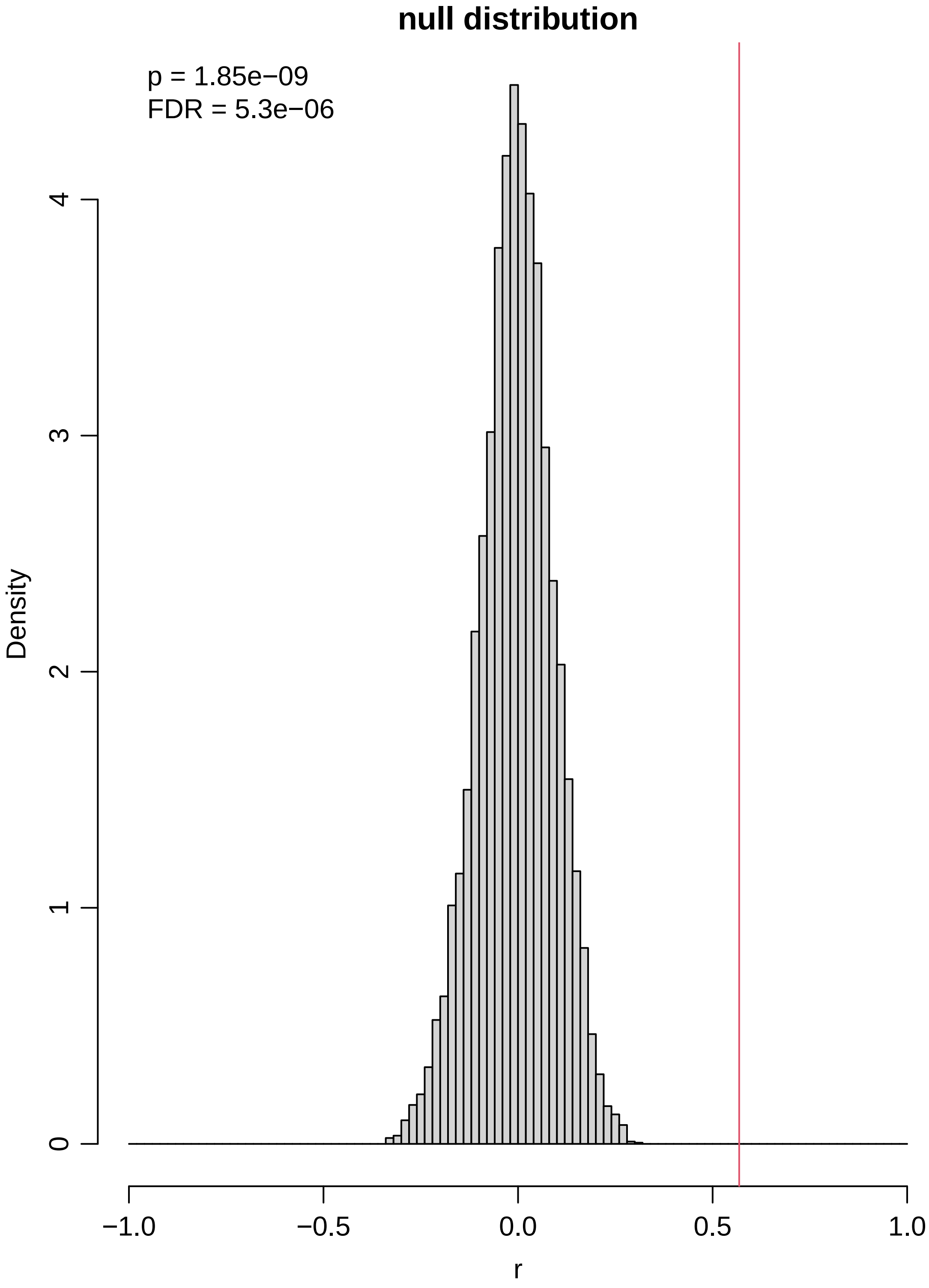

FAM224A vs PeakID_262828 (chrY: 18098233 − 18099203)

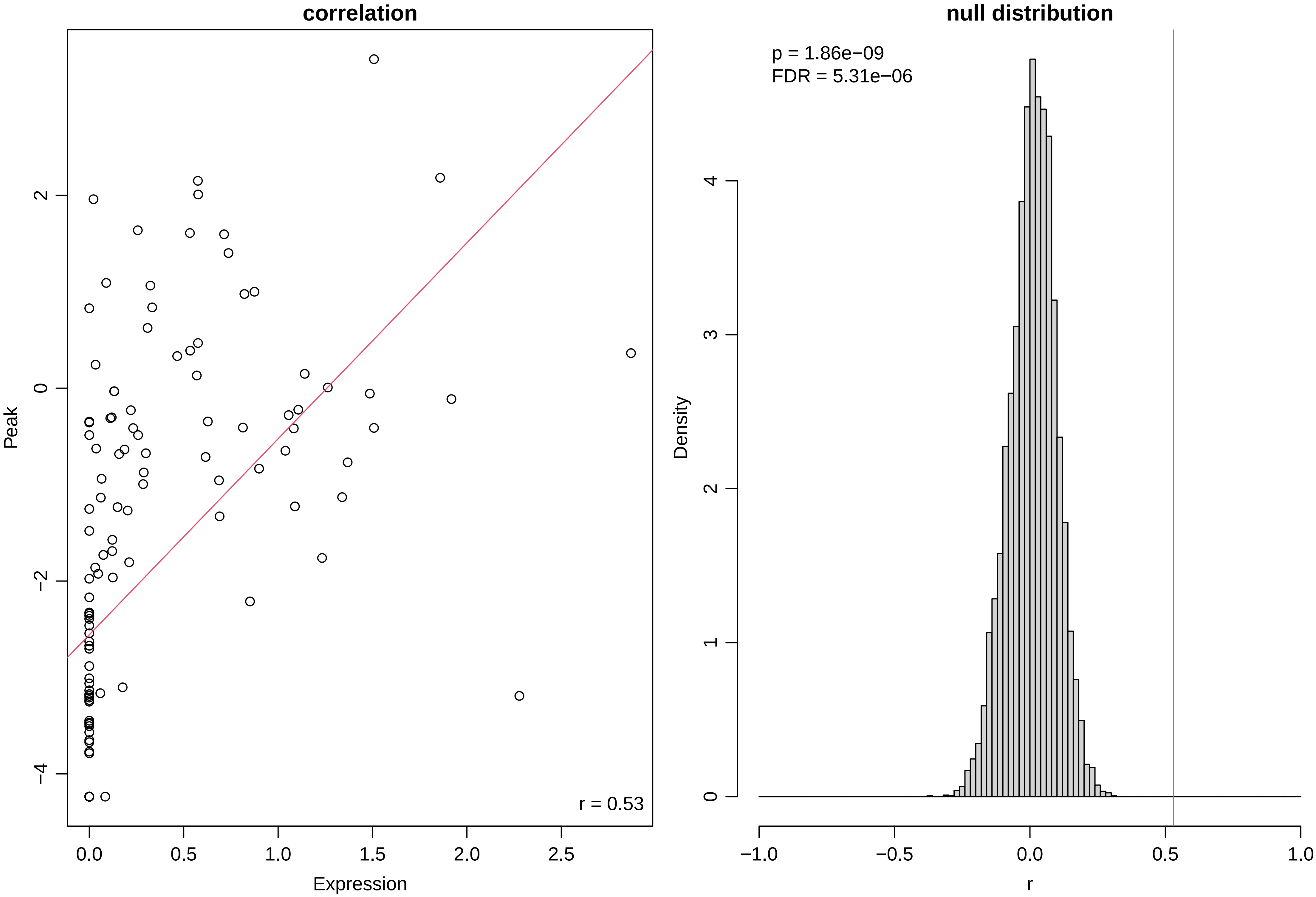

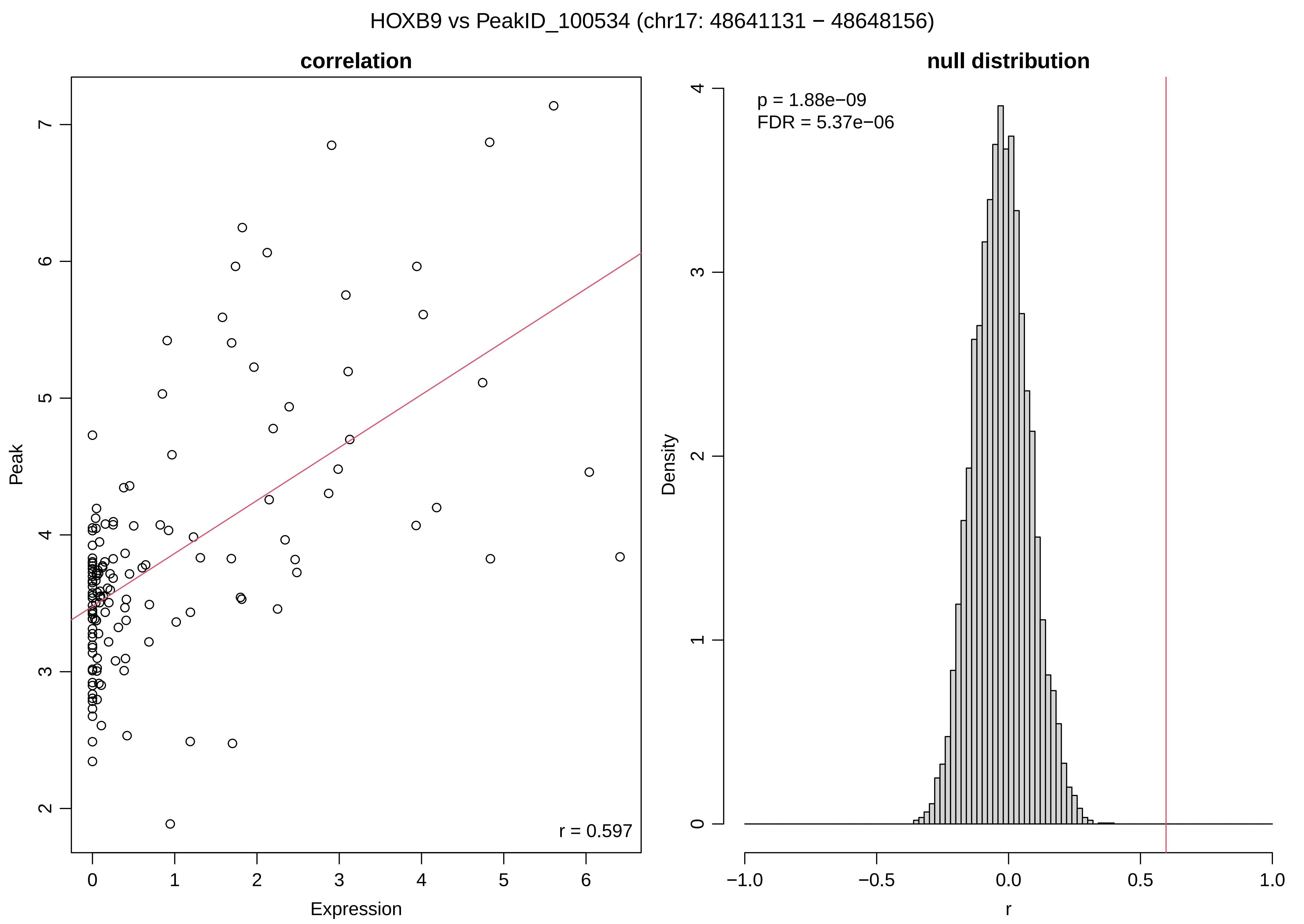
HOXB9 vs PeakID_100534 (chr17: 48641131 − 48648156)
correlation
null distribution
p = 1.88e−09
FDR = 5.37e−06
r = 0.597
Peak
Expression
Density
r

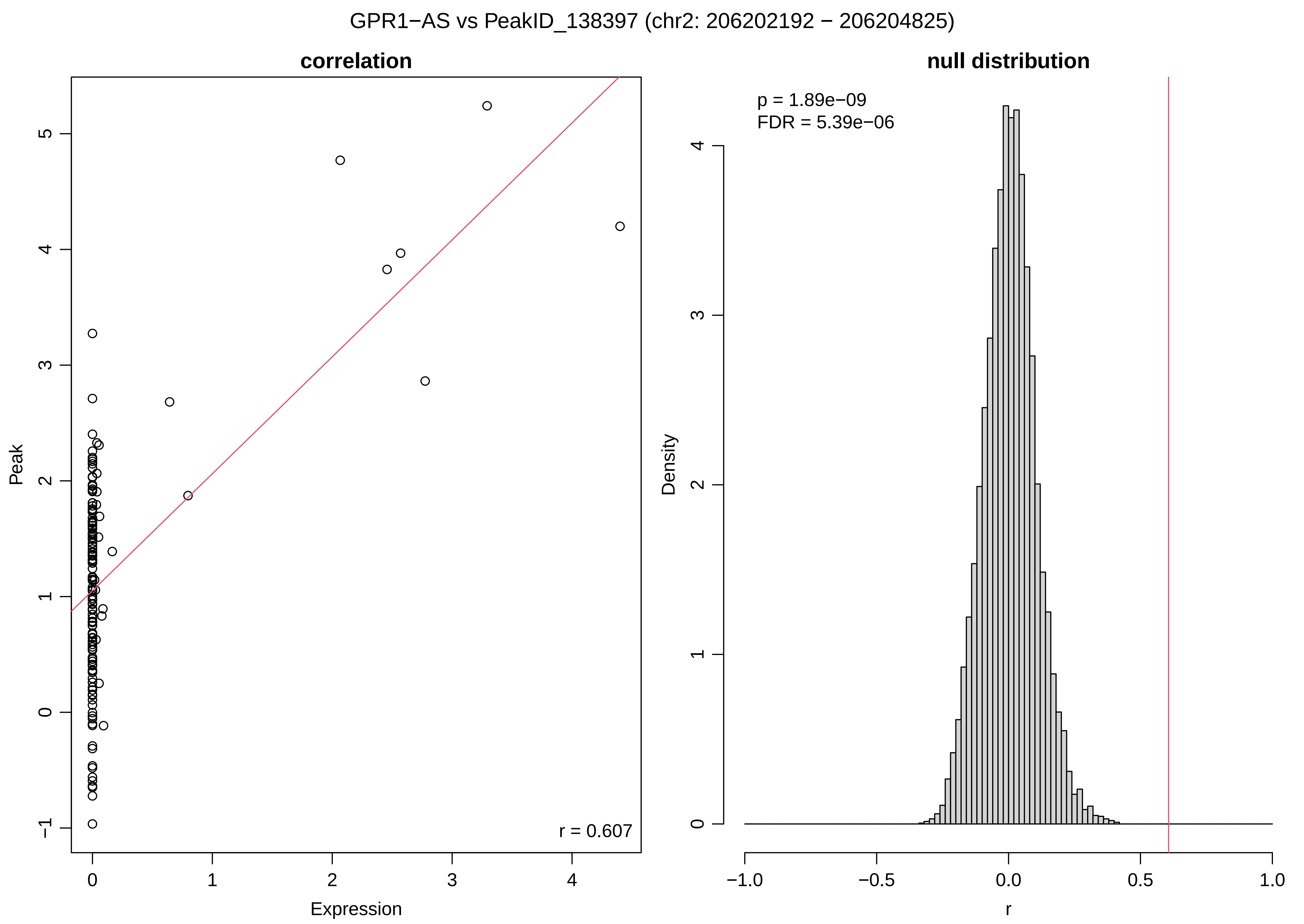

GPR1−AS vs PeakID_138397 (chr2: 206202192 − 206204825)
correlation
Peak
Expression
r = 0.607
null distribution
p = 1.89e−09
FDR = 5.39e−06
Density
r

CCT2 vs PeakID_56864 (chr12: 69526699 − 69530100)

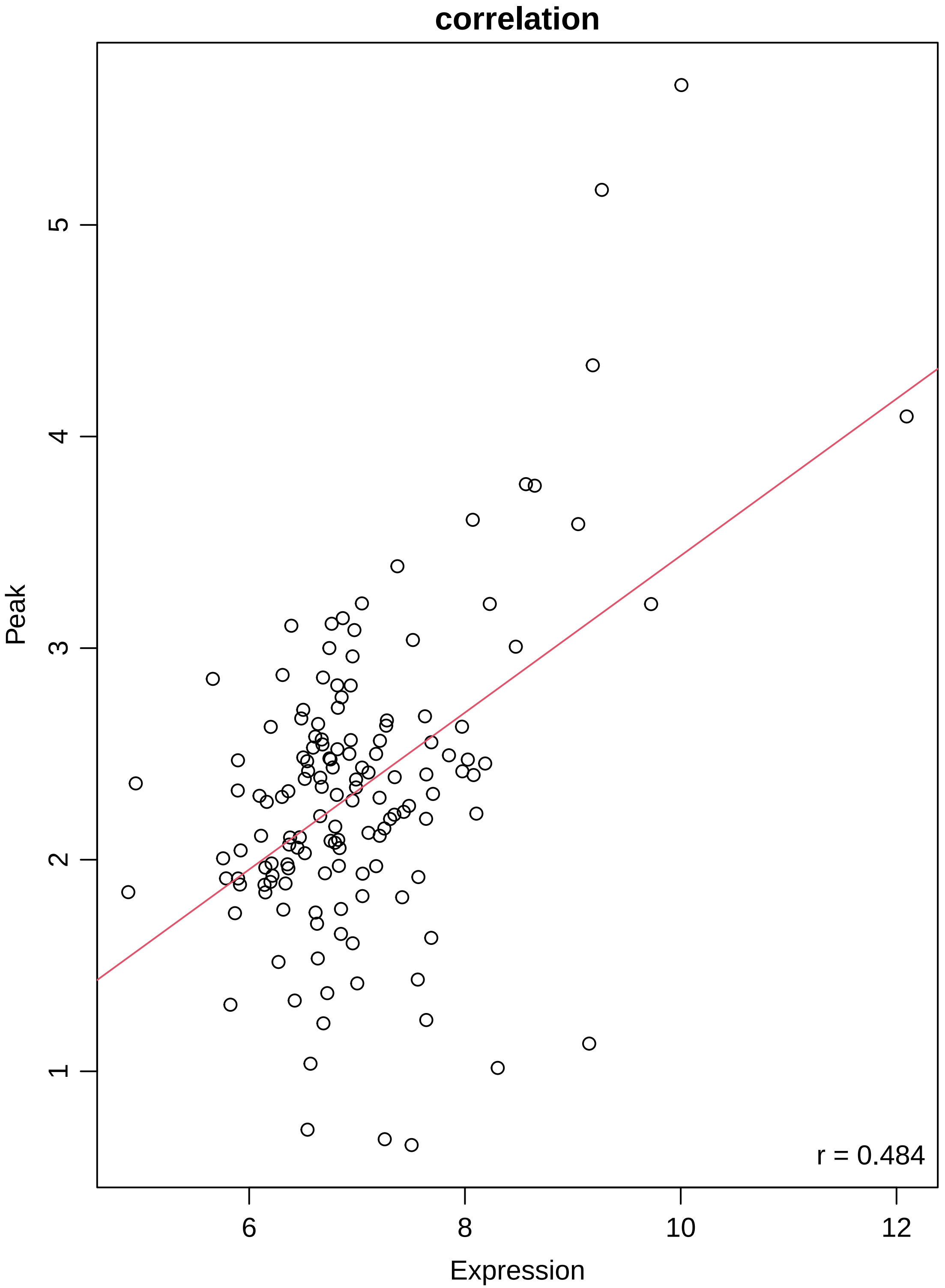

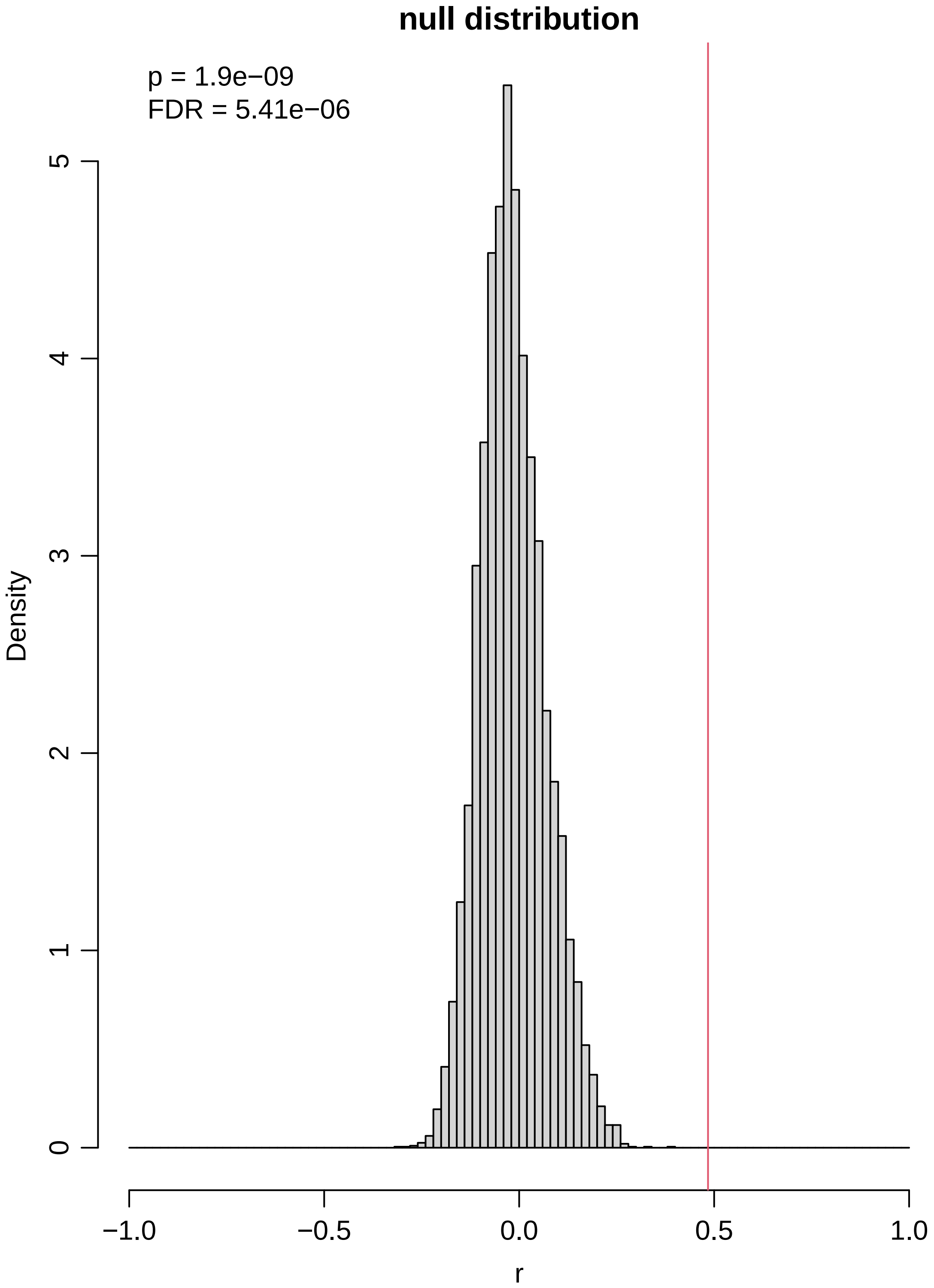

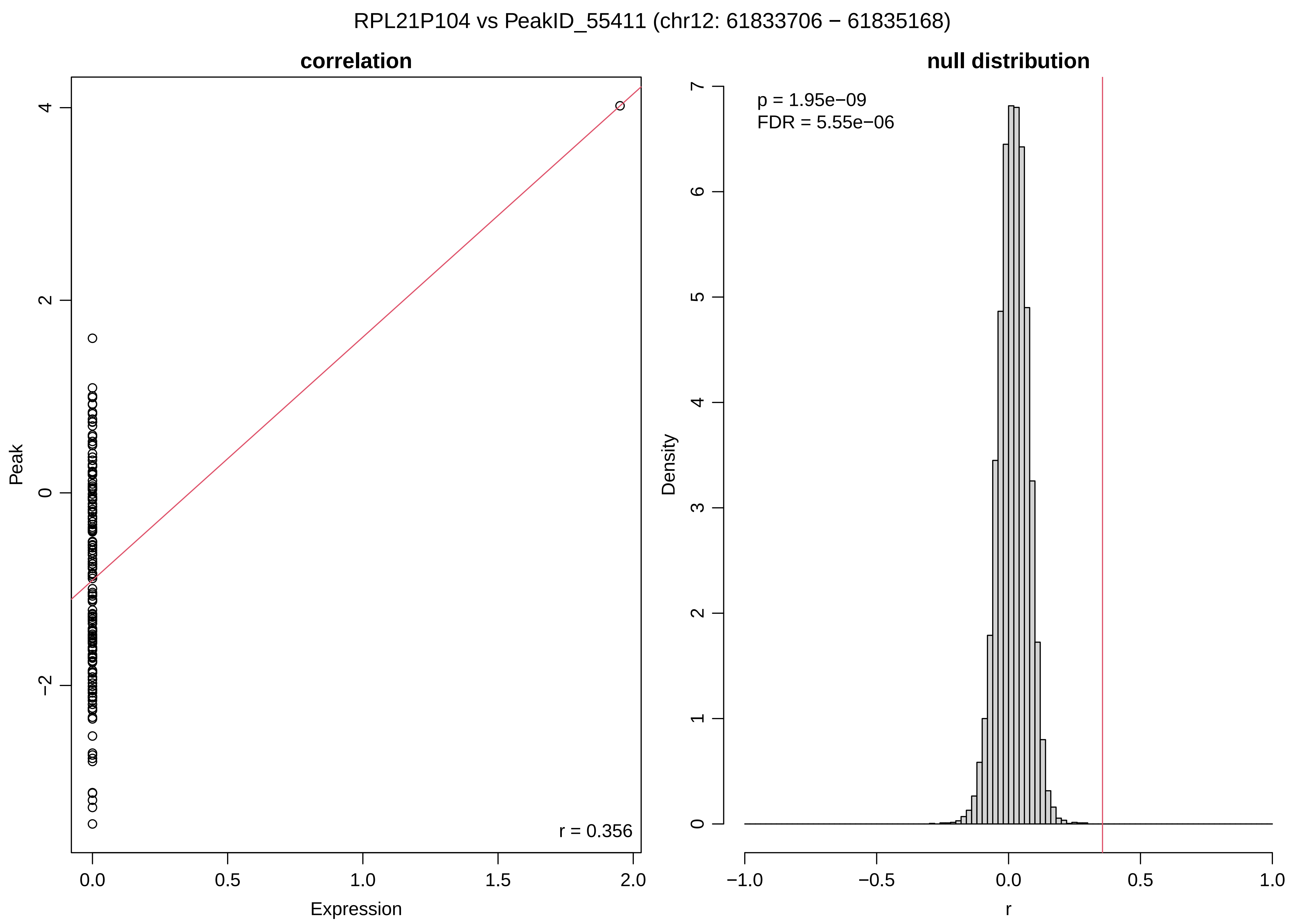

RPL21P104 vs PeakID_55411 (chr12: 61833706 − 61835168)
correlation
Peak
Expression
r = 0.356
0.0
0.5
1.0
1.5
2.0
4
2
0
−2
null distribution
p = 1.95e−09
FDR = 5.55e−06
Density
r
−1.0
−0.5
0.0
0.5
1.0
0
1
2
3
4
5
6
7

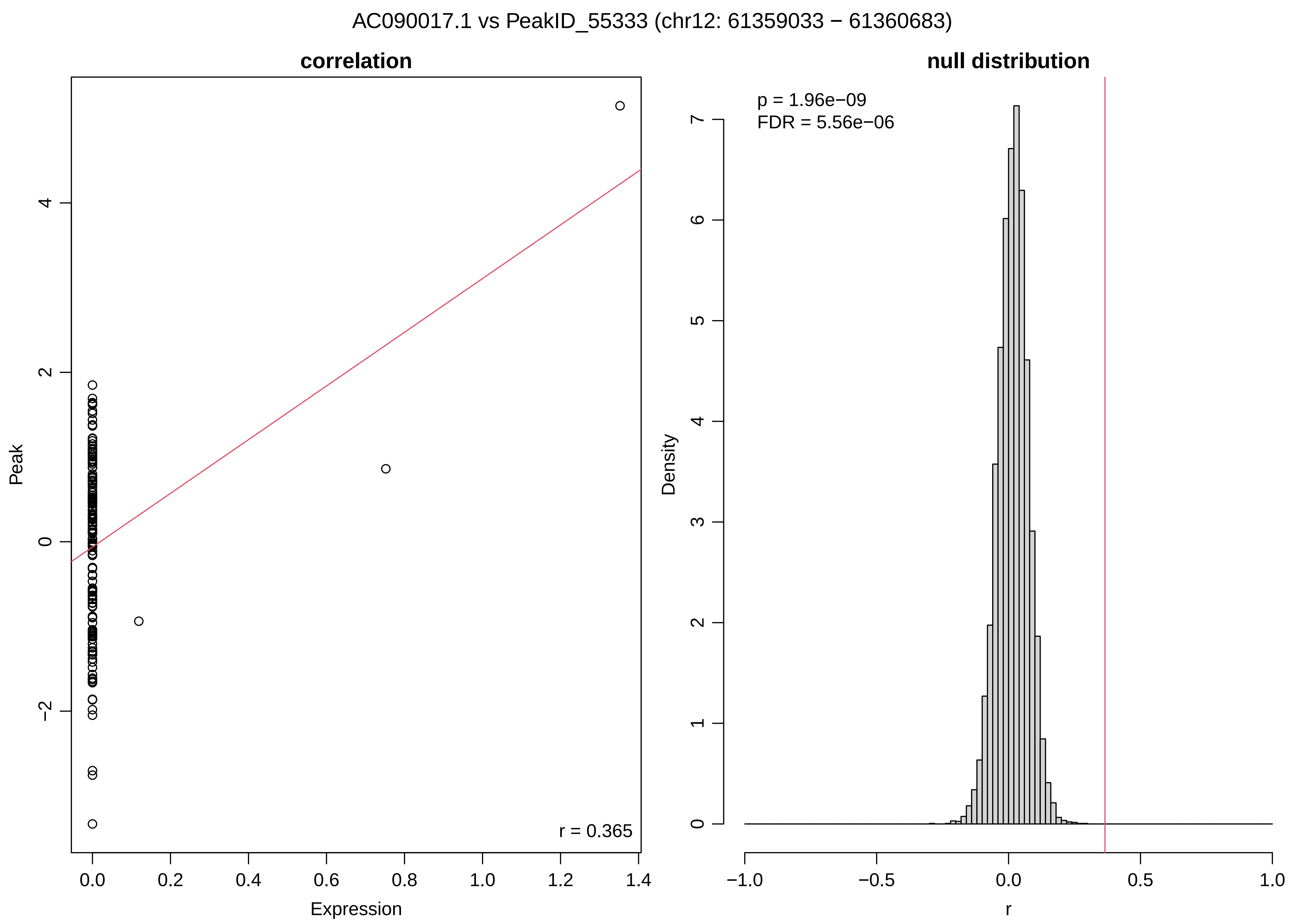
AC090017.1 vs PeakID_55333 (chr12: 61359033 − 61360683)
correlation
null distribution
Peak
Expression
r = 0.365
p = 1.96e−09
FDR = 5.56e−06
Density
r

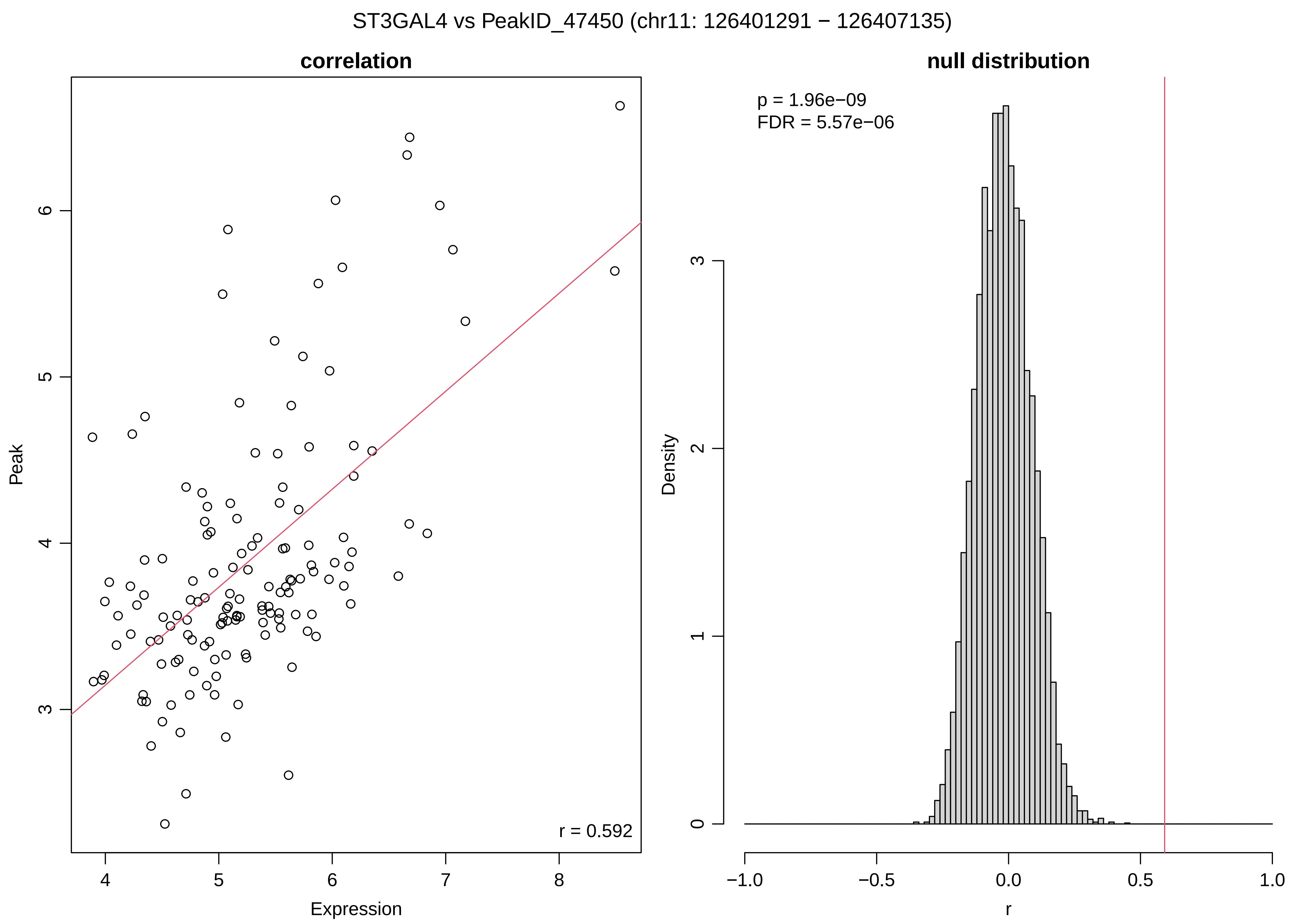

ST3GAL4 vs PeakID_47450 (chr11: 126401291 − 126407135)
correlation
Peak
Expression
r = 0.592
null distribution
p = 1.96e−09
FDR = 5.57e−06
Density
r

KDM5D vs PeakID_262943 (chrY: 19621358 − 19622712)

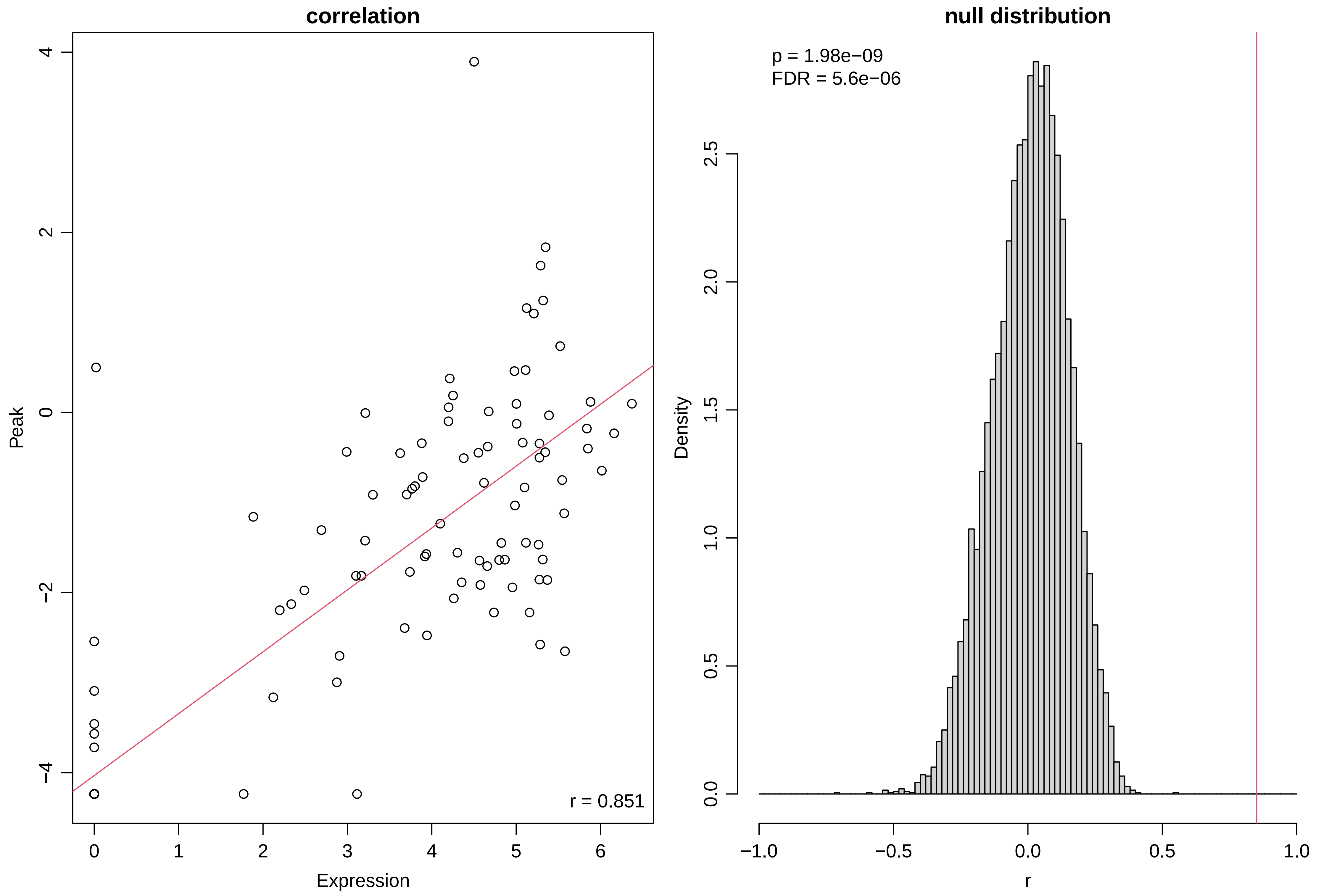

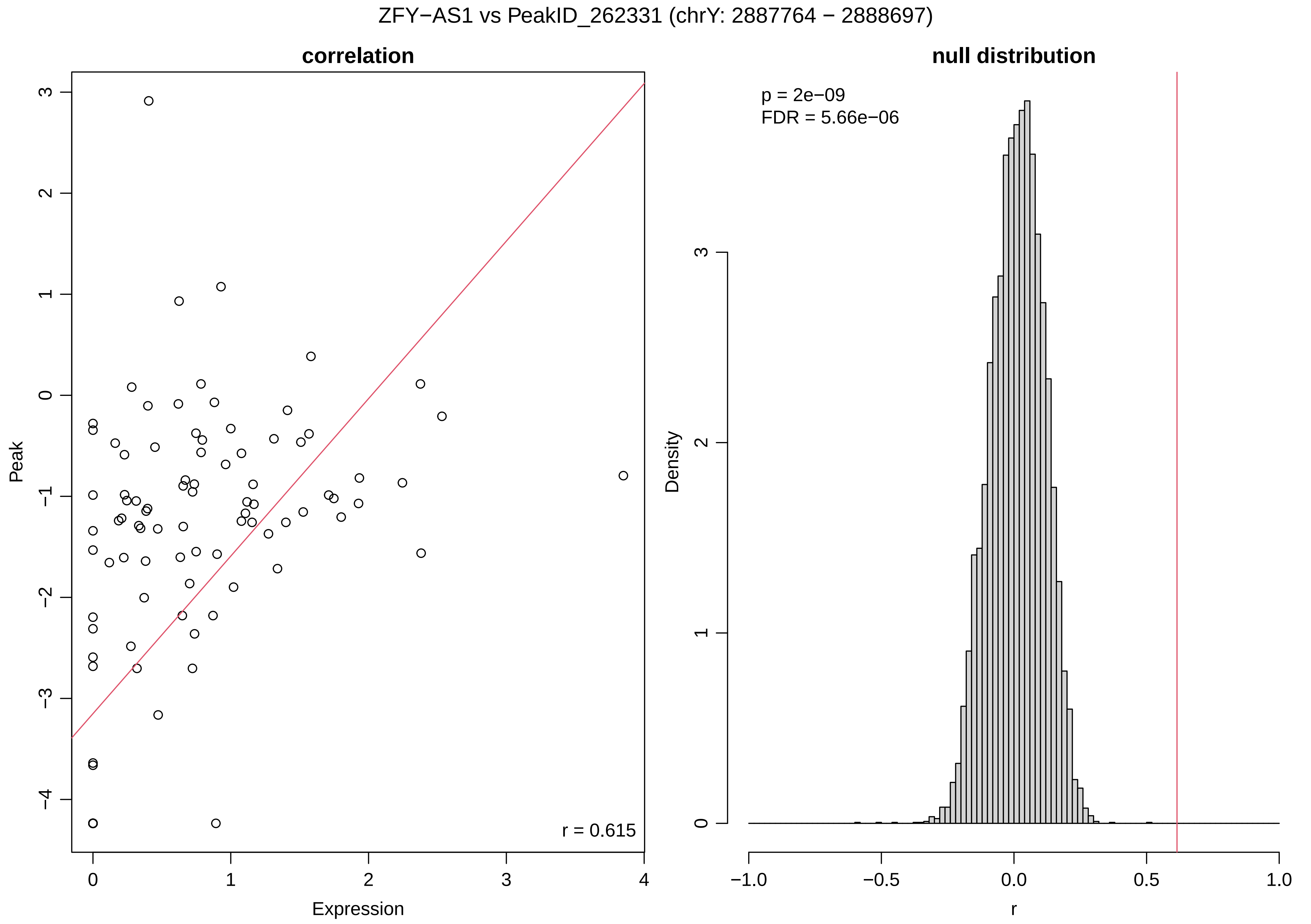

ZFY−AS1 vs PeakID_262331 (chrY: 2887764 − 2888697)
correlation
Peak
Expression
r = 0.615
null distribution
p = 2e−09
FDR = 5.66e−06
Density
r

RPL21P104 vs PeakID_55367 (chr12: 61761553 − 61762377)

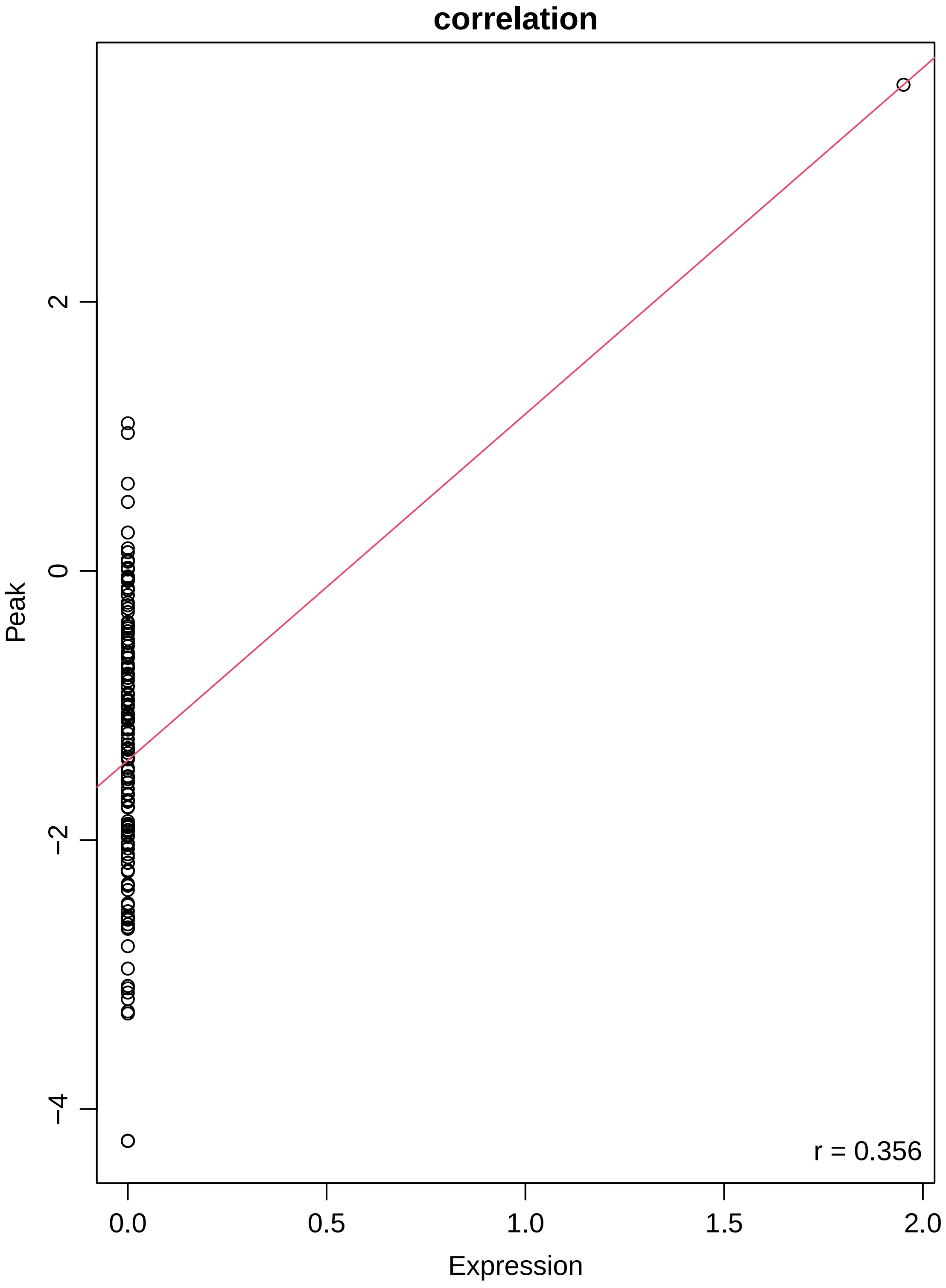

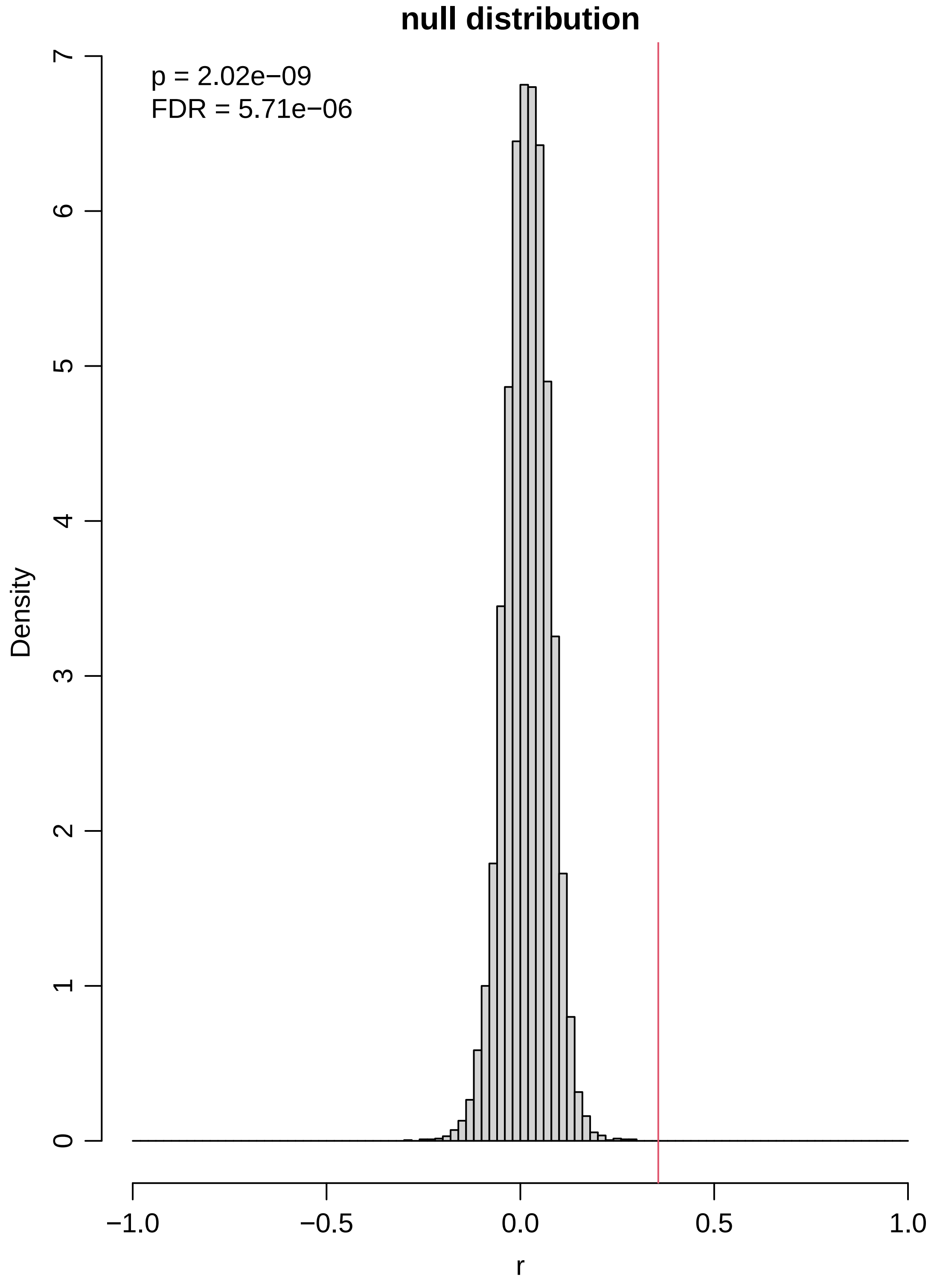

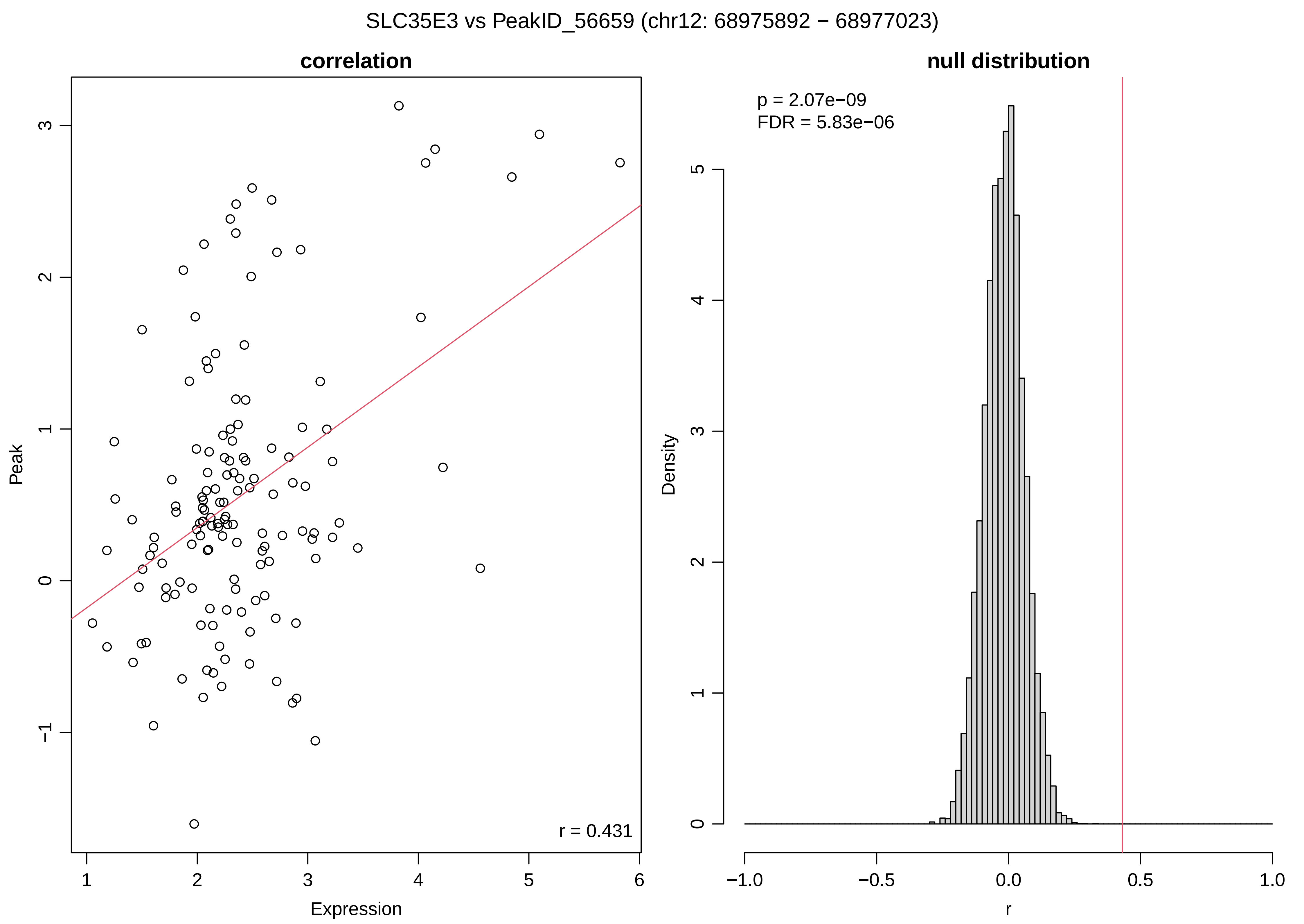
SLC35E3 vs PeakID_56659 (chr12: 68975892 − 68977023)
correlation
null distribution
p = 2.07e−09
FDR = 5.83e−06
r = 0.431
Peak
Expression
Density
r

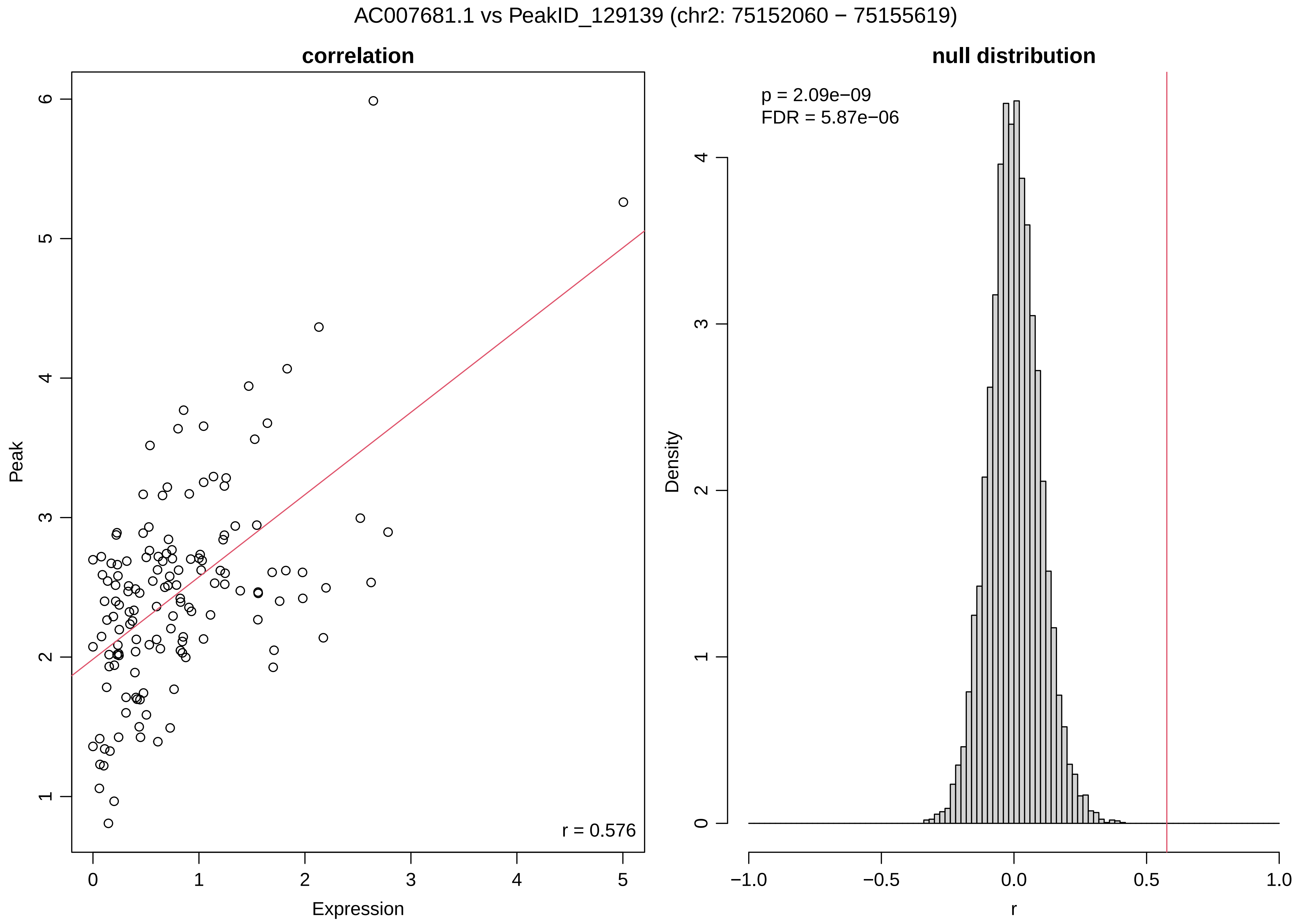
AC007681.1 vs PeakID_129139 (chr2: 75152060 − 75155619)
correlation
Peak
Expression
r = 0.576
0
1
2
3
4
5
6
null distribution
p = 2.09e−09
FDR = 5.87e−06
Density
r
−1.0
−0.5
0.0
0.5
1.0

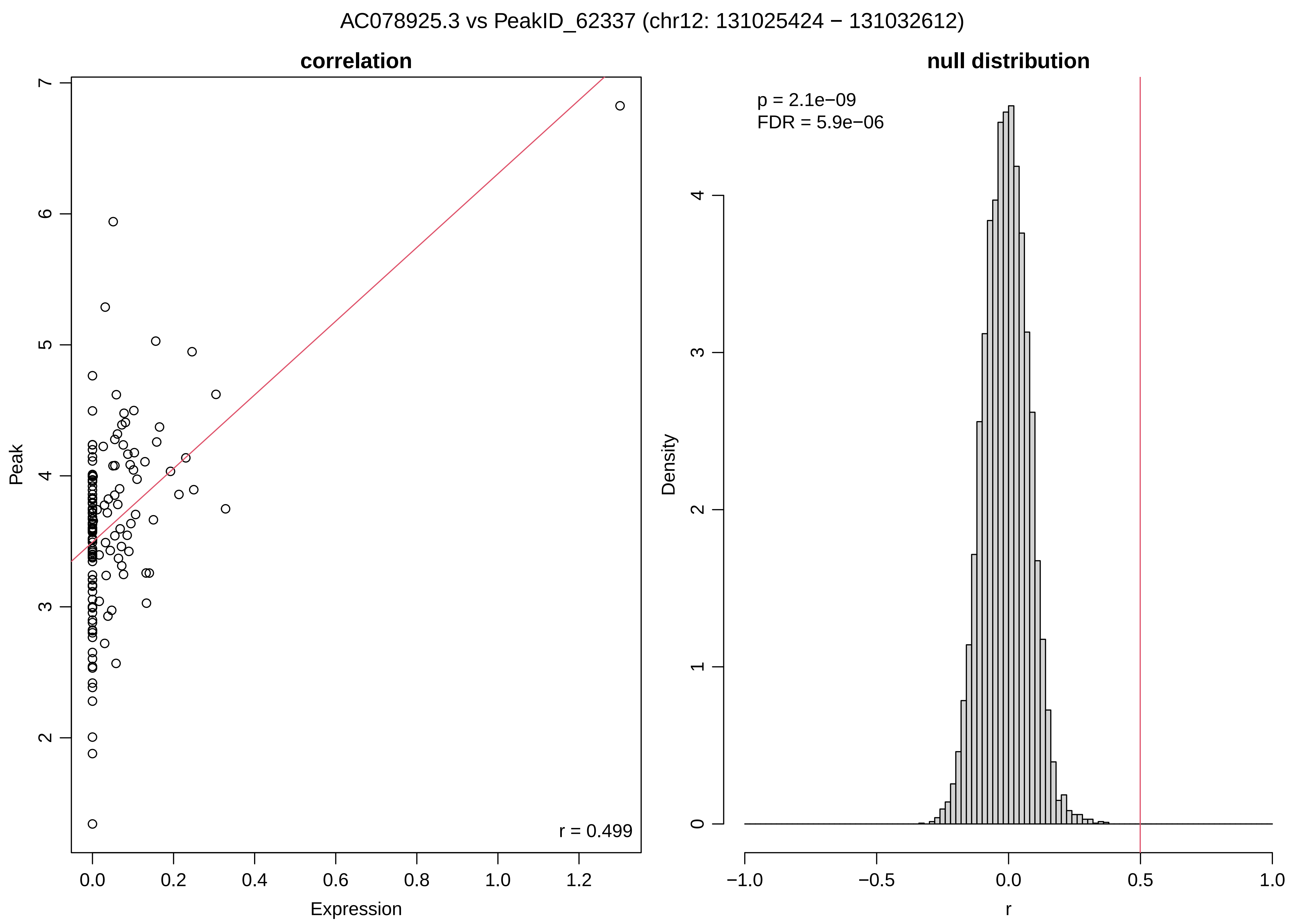

AC078925.3 vs PeakID_62337 (chr12: 131025424 − 131032612)
correlation
Peak
Expression
r = 0.499
0.0
0.2
0.4
0.6
0.8
1.0
1.2
2
3
4
5
6
7
null distribution
p = 2.1e−09
FDR = 5.9e−06
Density
r
−1.0
−0.5
0.0
0.5
1.0
0
1
2
3
4

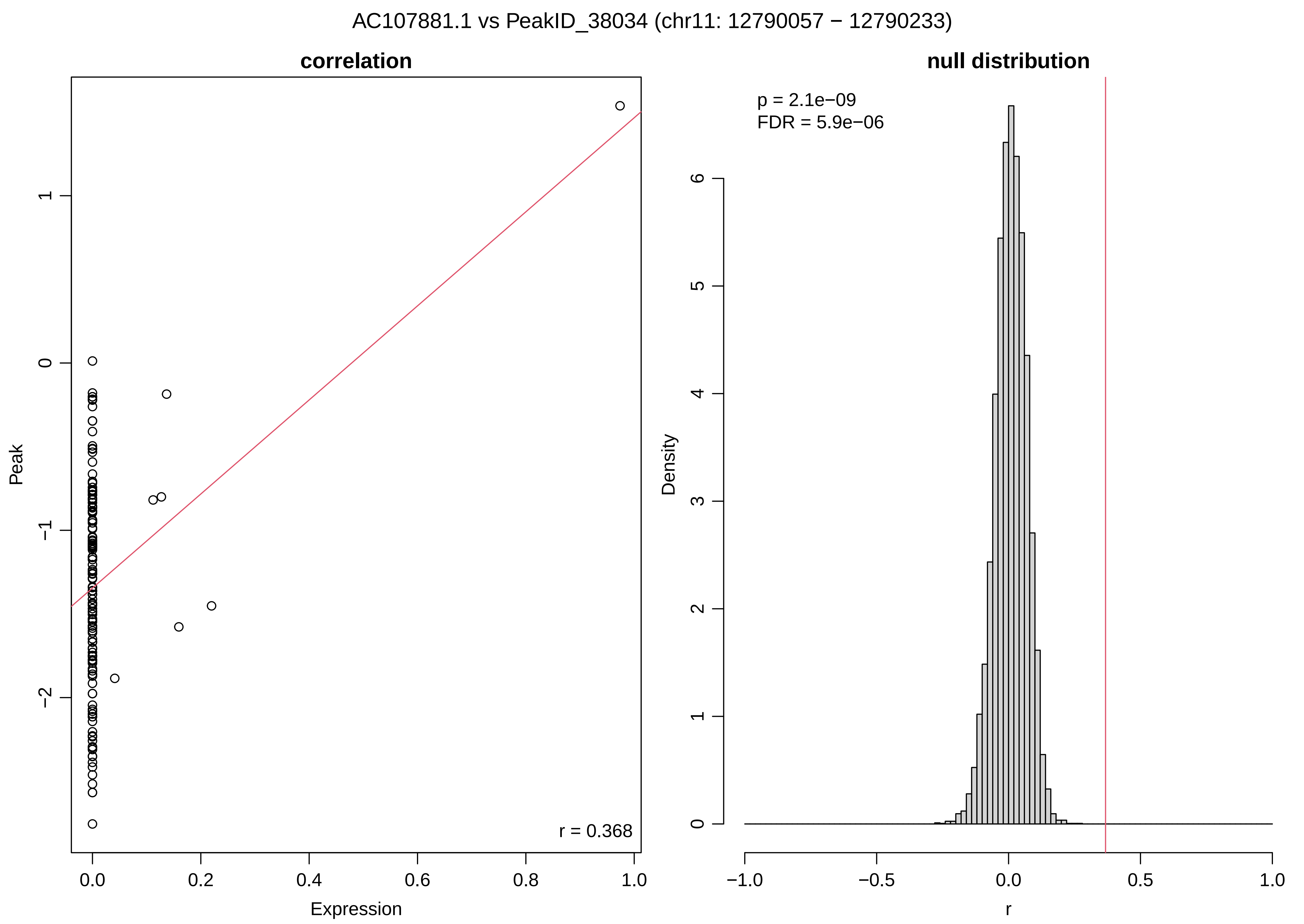

AC107881.1 vs PeakID_38034 (chr11: 12790057 − 12790233)
correlation
Peak
Expression
r = 0.368
null distribution
p = 2.1e−09
FDR = 5.9e−06
Density
r

AC107881.1 vs PeakID_37821 (chr11: 12416215 − 12416585)

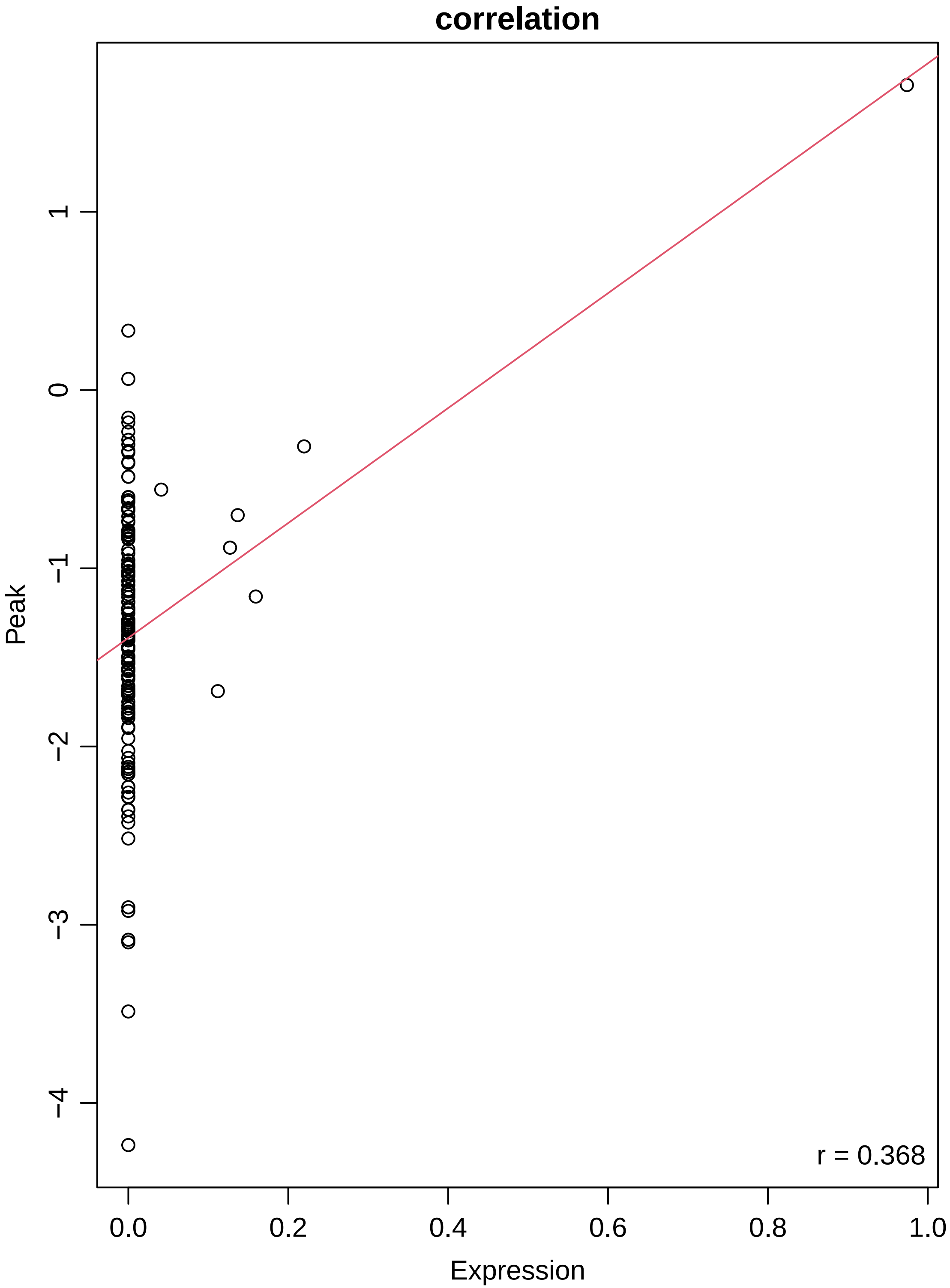

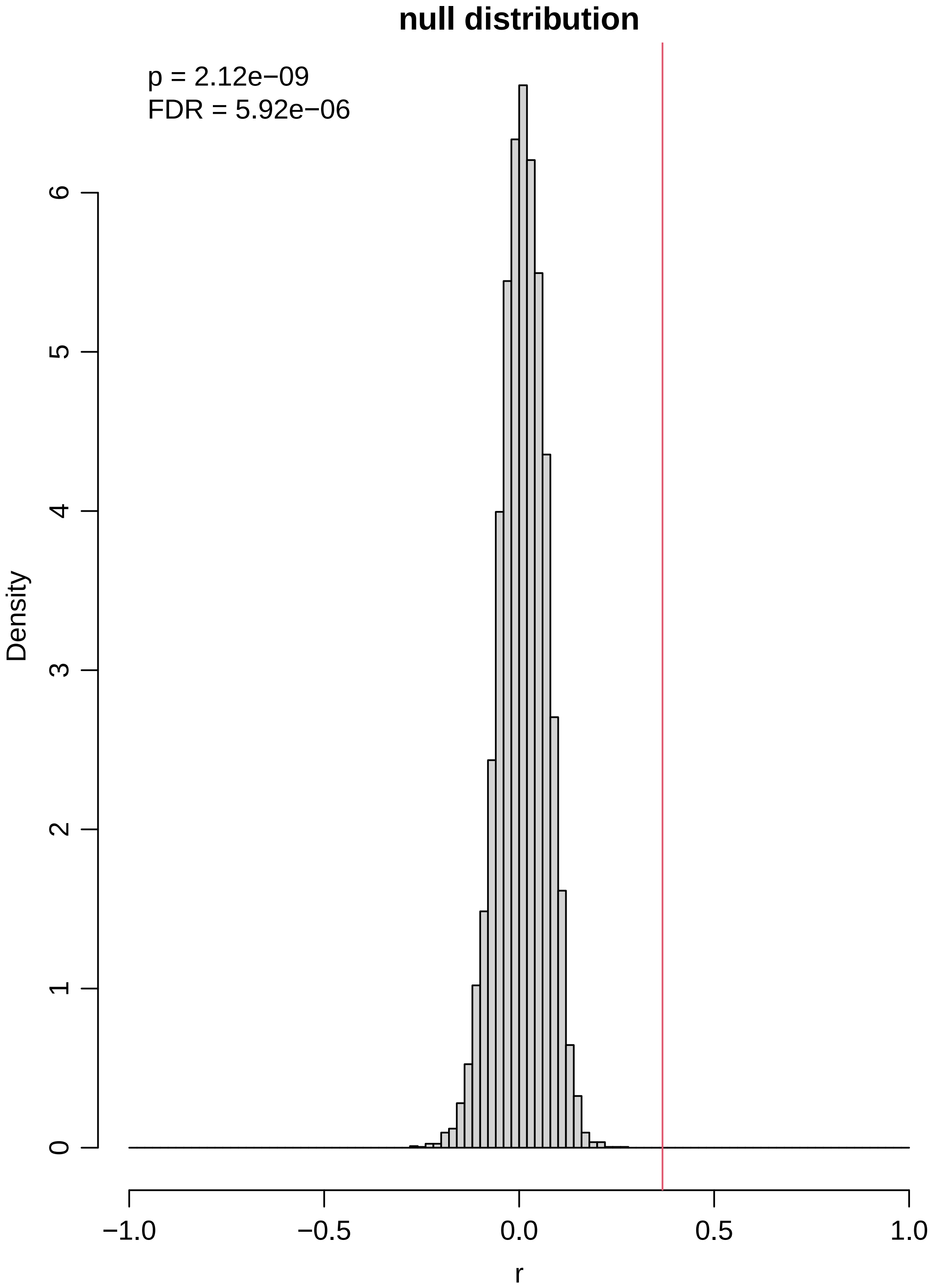

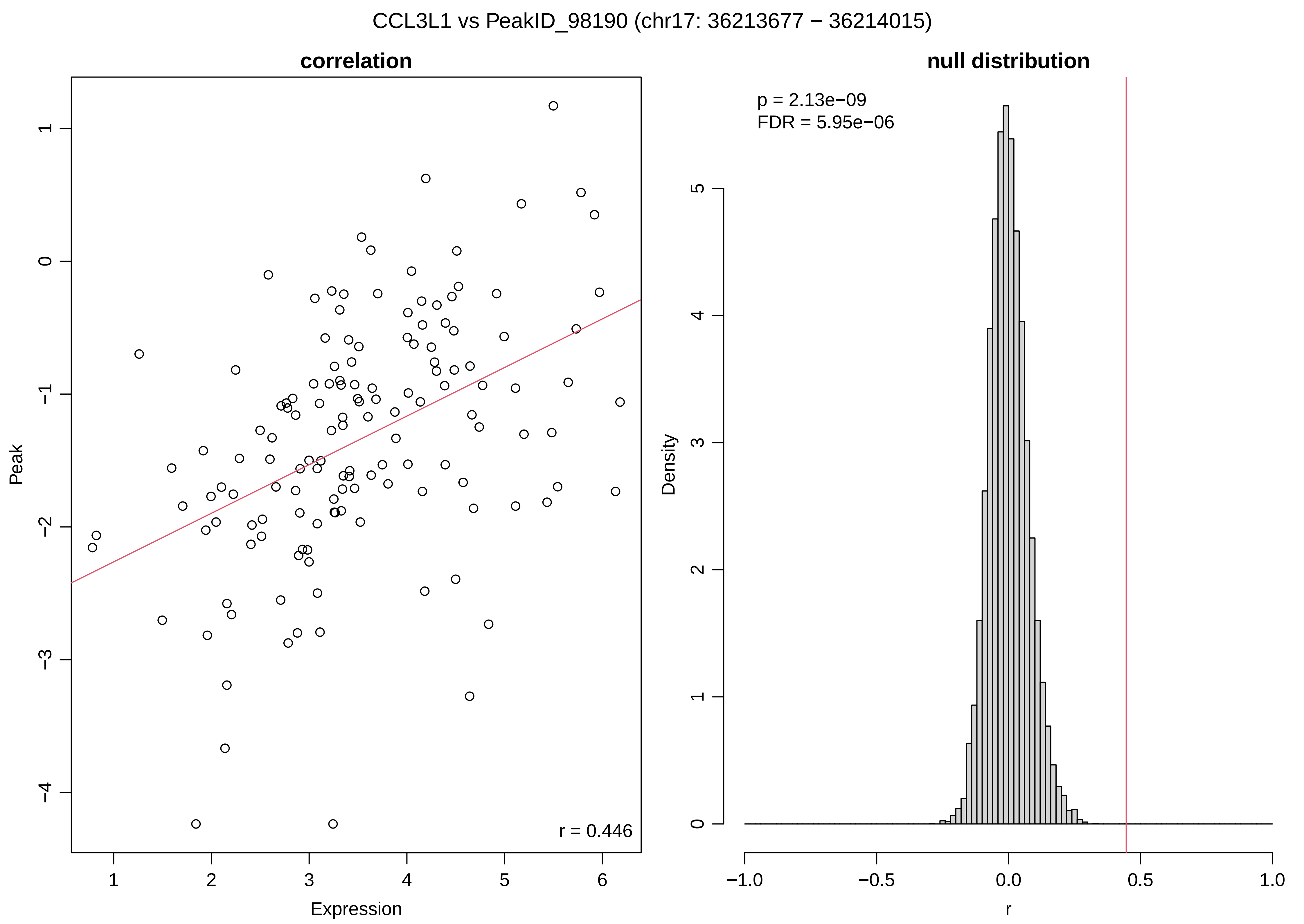

CCL3L1 vs PeakID_98190 (chr17: 36213677 − 36214015)
correlation
Peak
Expression
r = 0.446
null distribution
p = 2.13e−09
FDR = 5.95e−06
Density
r

KRT16P3 vs PeakID_96656 (chr17: 20504632 − 20505854)

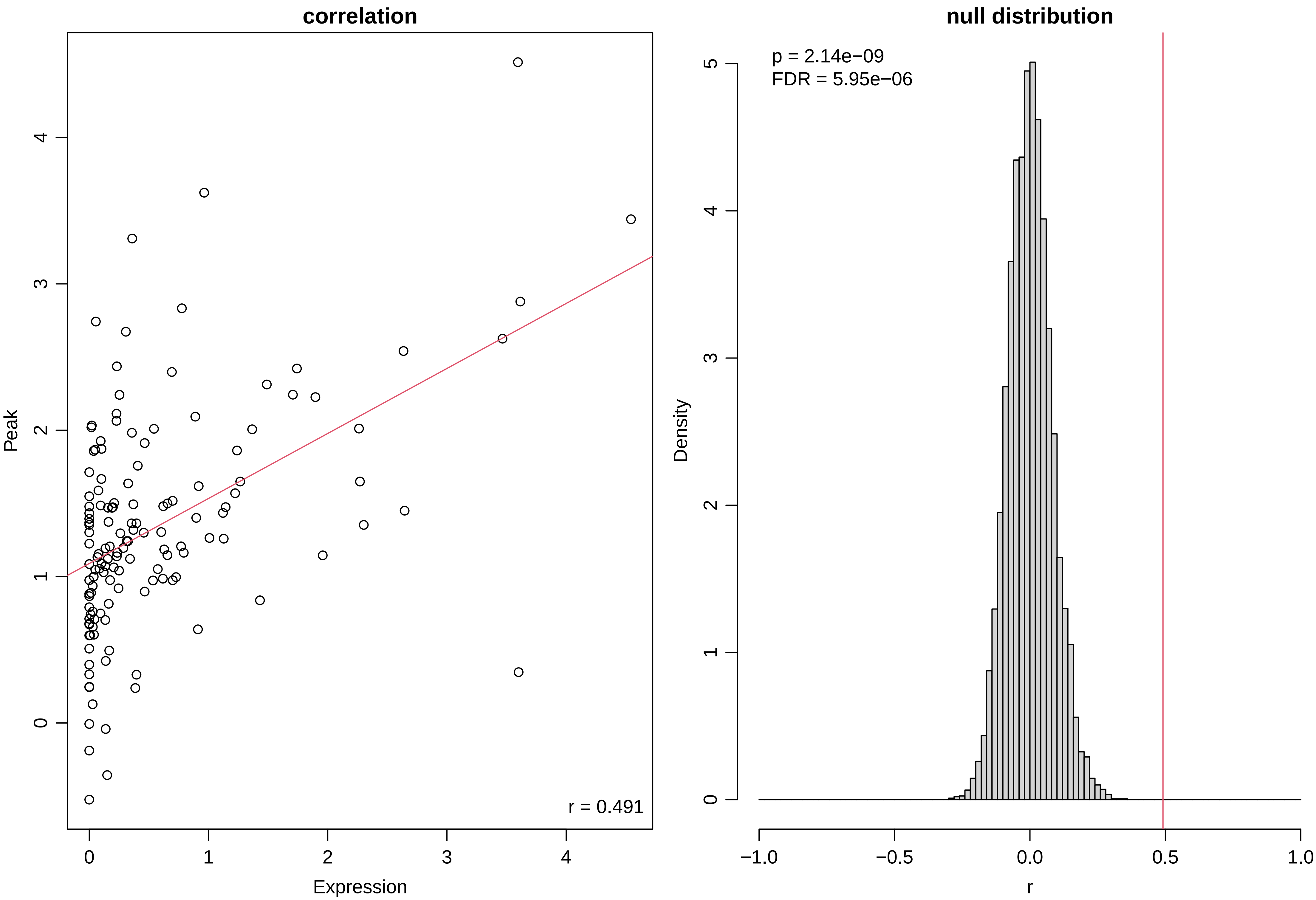

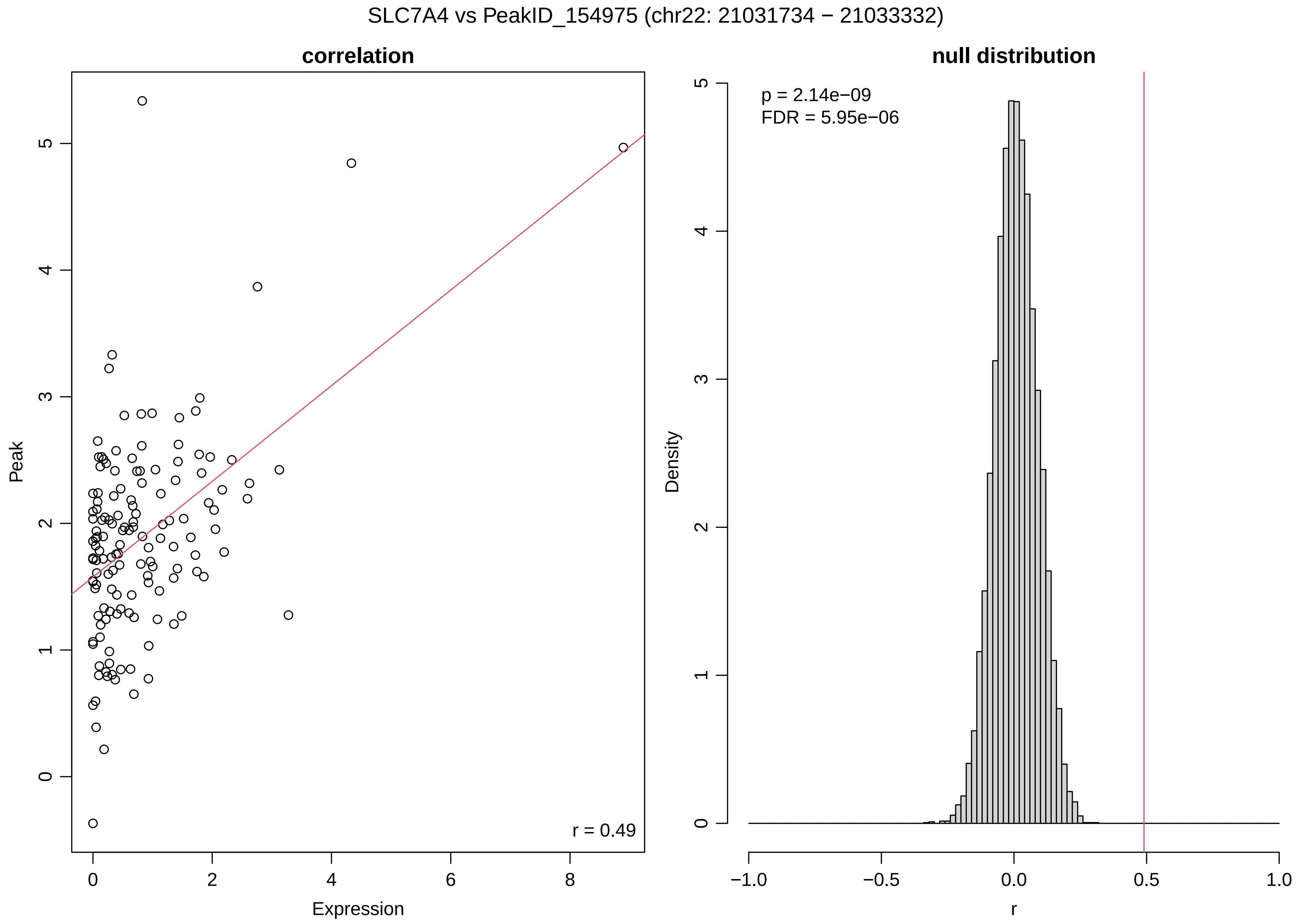
SLC7A4 vs PeakID_154975 (chr22: 21031734 − 21033332)
correlation
null distribution
Peak
Expression
r = 0.49
Density
r
p = 2.14e−09
FDR = 5.95e−06
0
1
2
3
4
5
0
2
4
6
8
−1.0
−0.5
0.0
0.5
1.0

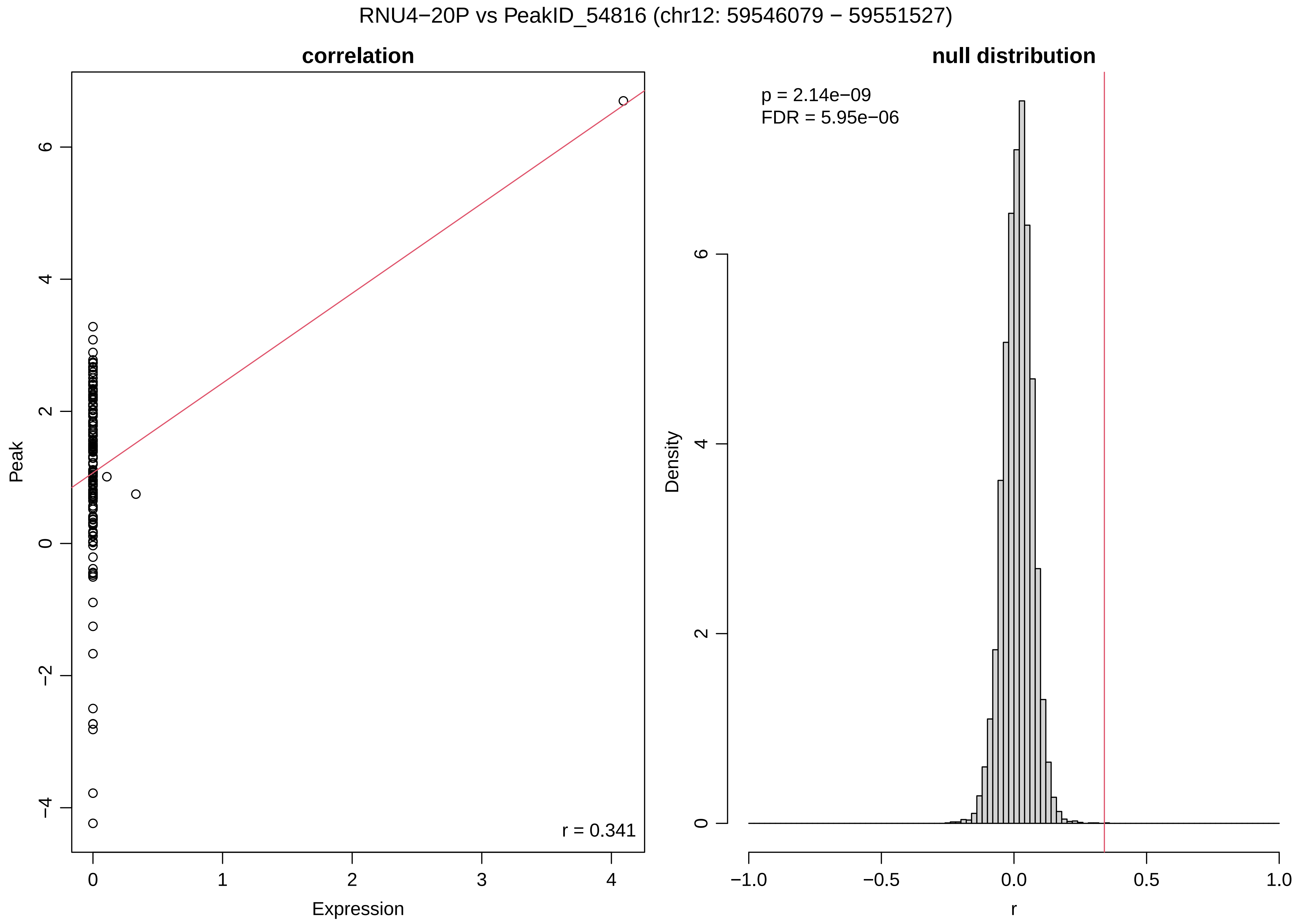

RNU4−20P vs PeakID_54816 (chr12: 59546079 − 59551527)
correlation
Peak
Expression
r = 0.341
null distribution
p = 2.14e−09
FDR = 5.95e−06
Density
r

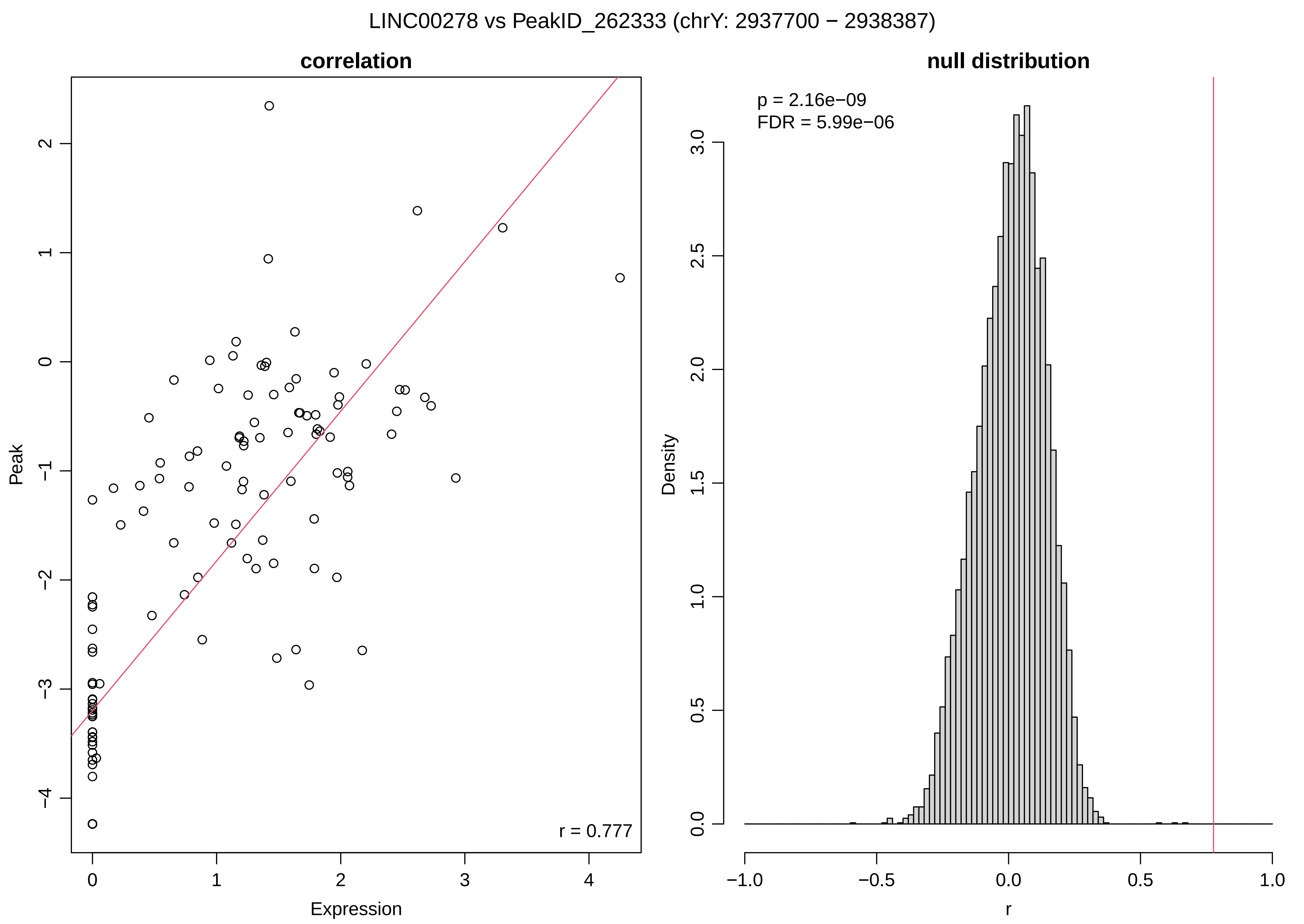
LINC00278 vs PeakID_262333 (chrY: 2937700 − 2938387)
correlation
Peak
Expression
r = 0.777
null distribution
p = 2.16e−09
FDR = 5.99e−06
Density
r

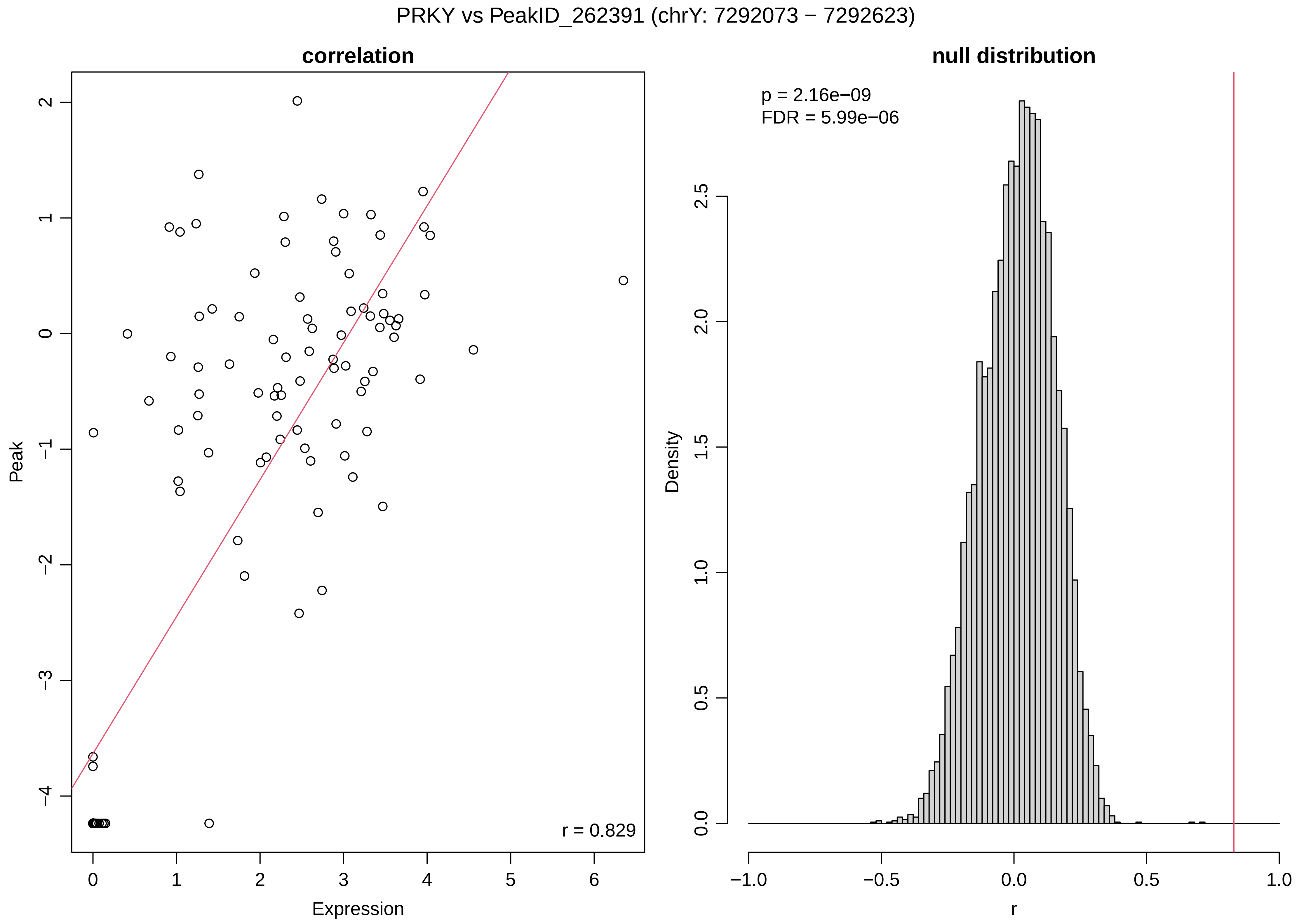
PRKY vs PeakID_262391 (chrY: 7292073 − 7292623)
correlation
Peak
Expression
r = 0.829
null distribution
p = 2.16e−09
FDR = 5.99e−06
Density
r

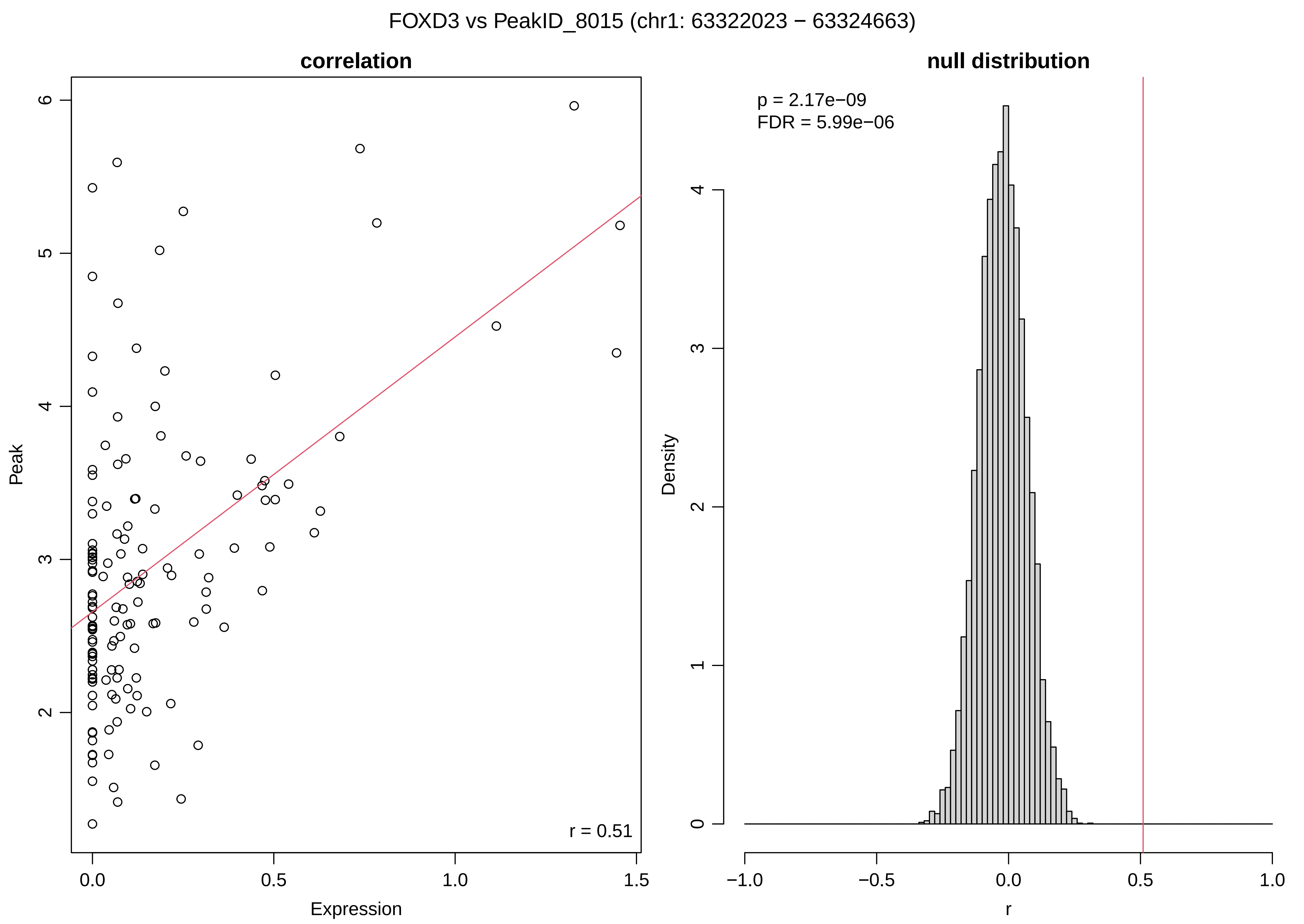

FOXD3 vs PeakID_8015 (chr1: 63322023 − 63324663)
correlation
null distribution
p = 2.17e−09
FDR = 5.99e−06
r = 0.51
Peak
Expression
Density
r

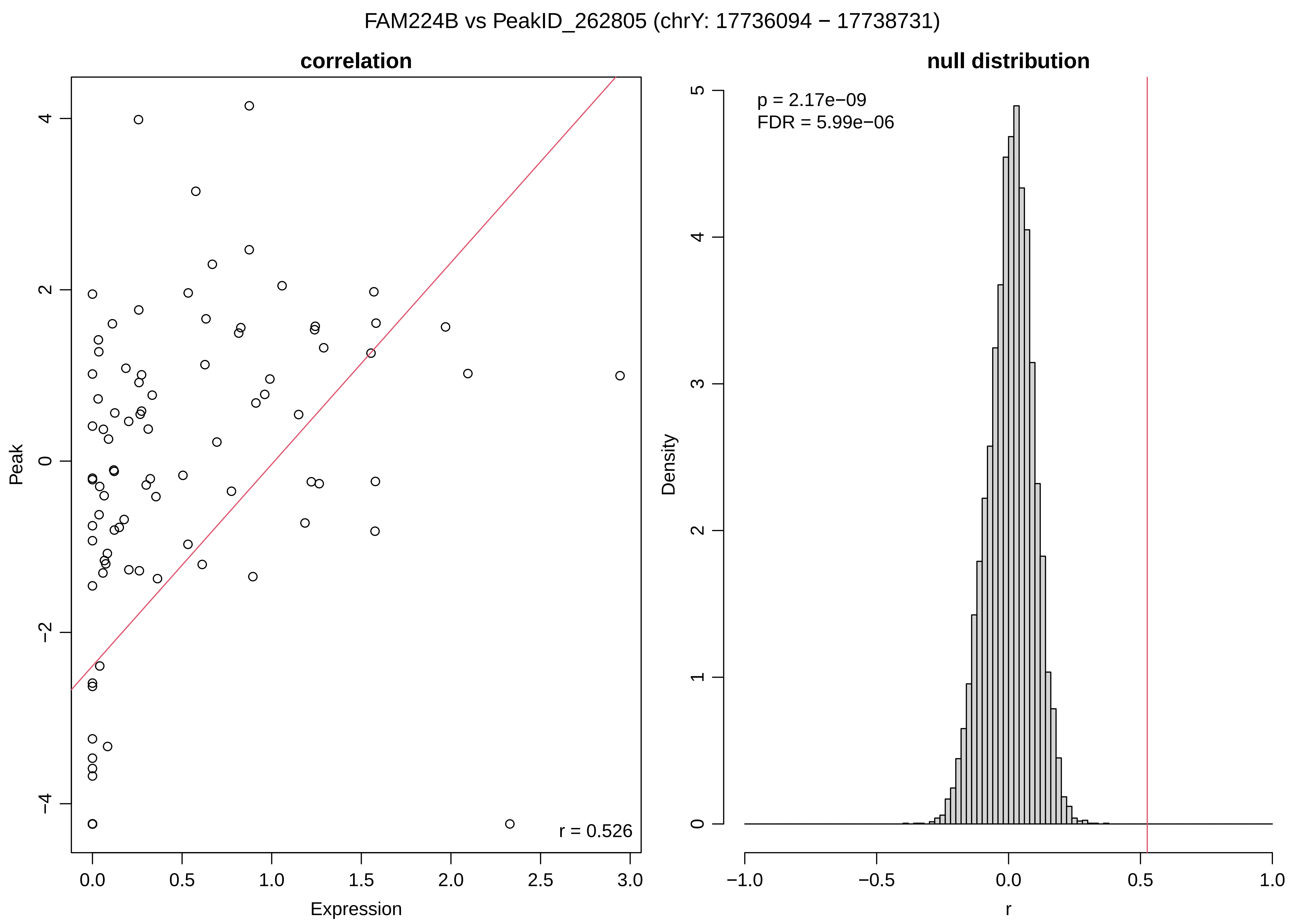

FAM224B vs PeakID_262805 (chrY: 17736094 − 17738731)
correlation
Peak
Expression
r = 0.526
null distribution
Density
r
p = 2.17e−09
FDR = 5.99e−06

RPL21P104 vs PeakID_55488 (chr12: 62004232 − 62005170)

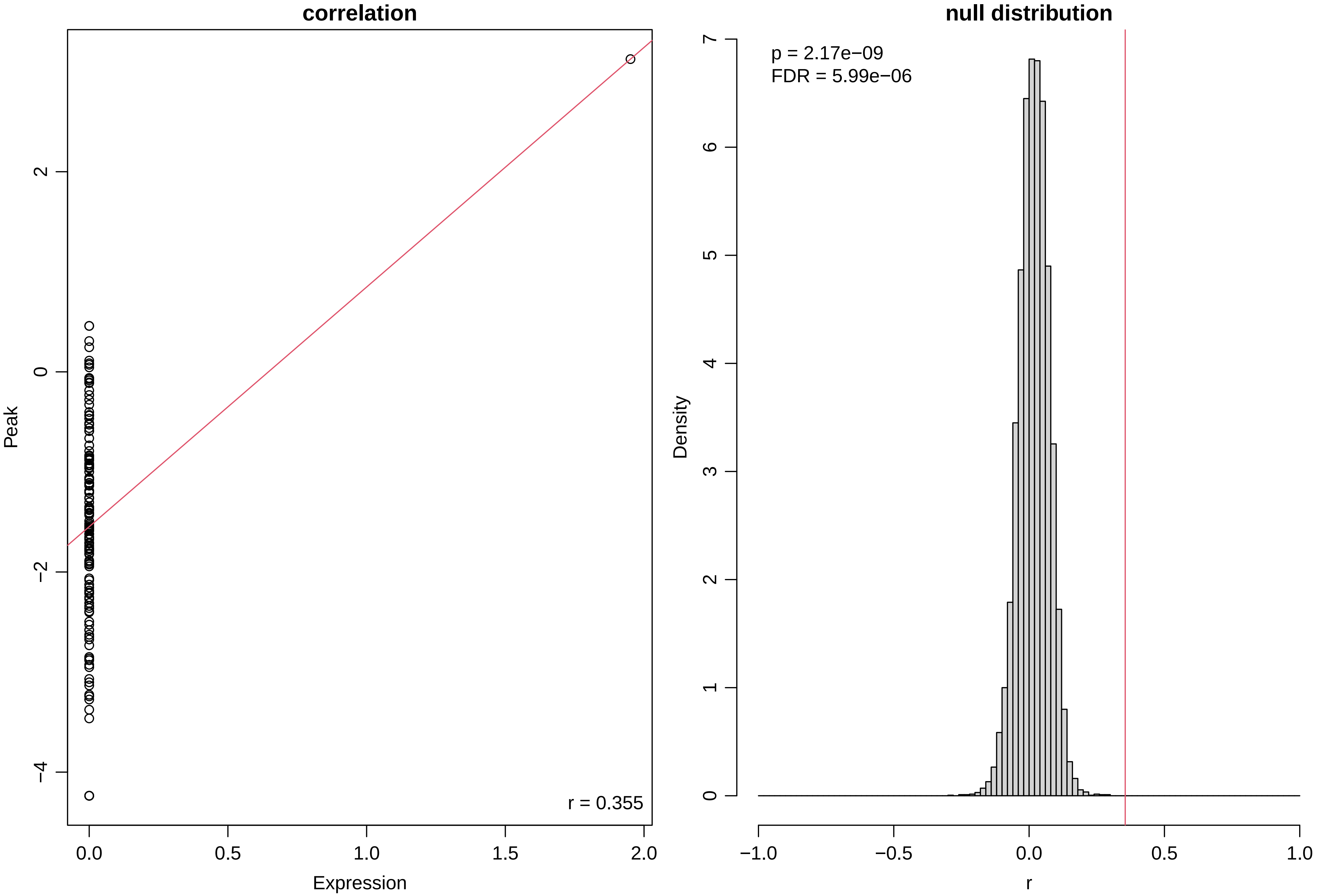

LANCL2 vs PeakID_224252 (chr7: 55221408 − 55290386)

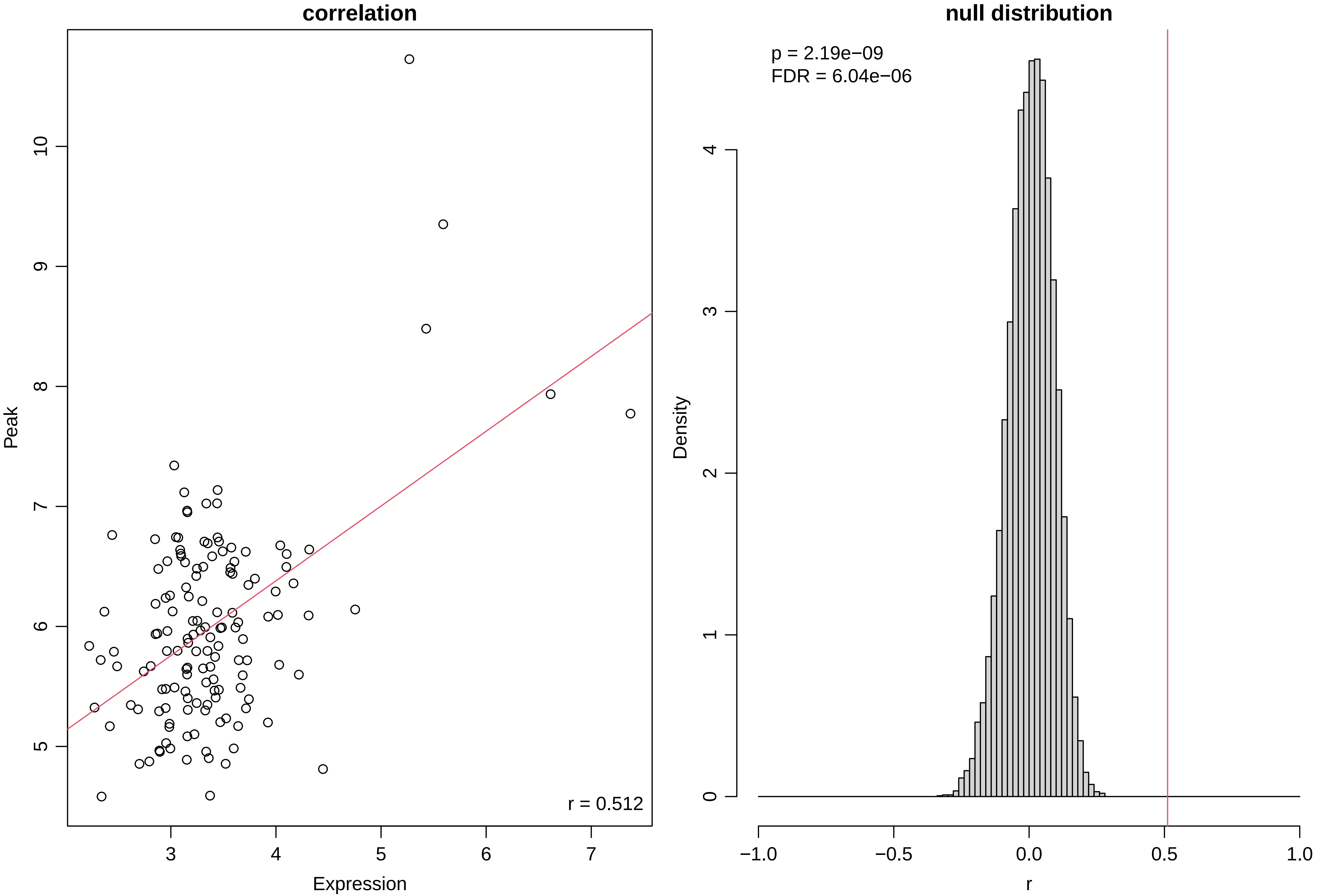

GPR37 vs PeakID_229493 (chr7: 124764207 − 124766478)

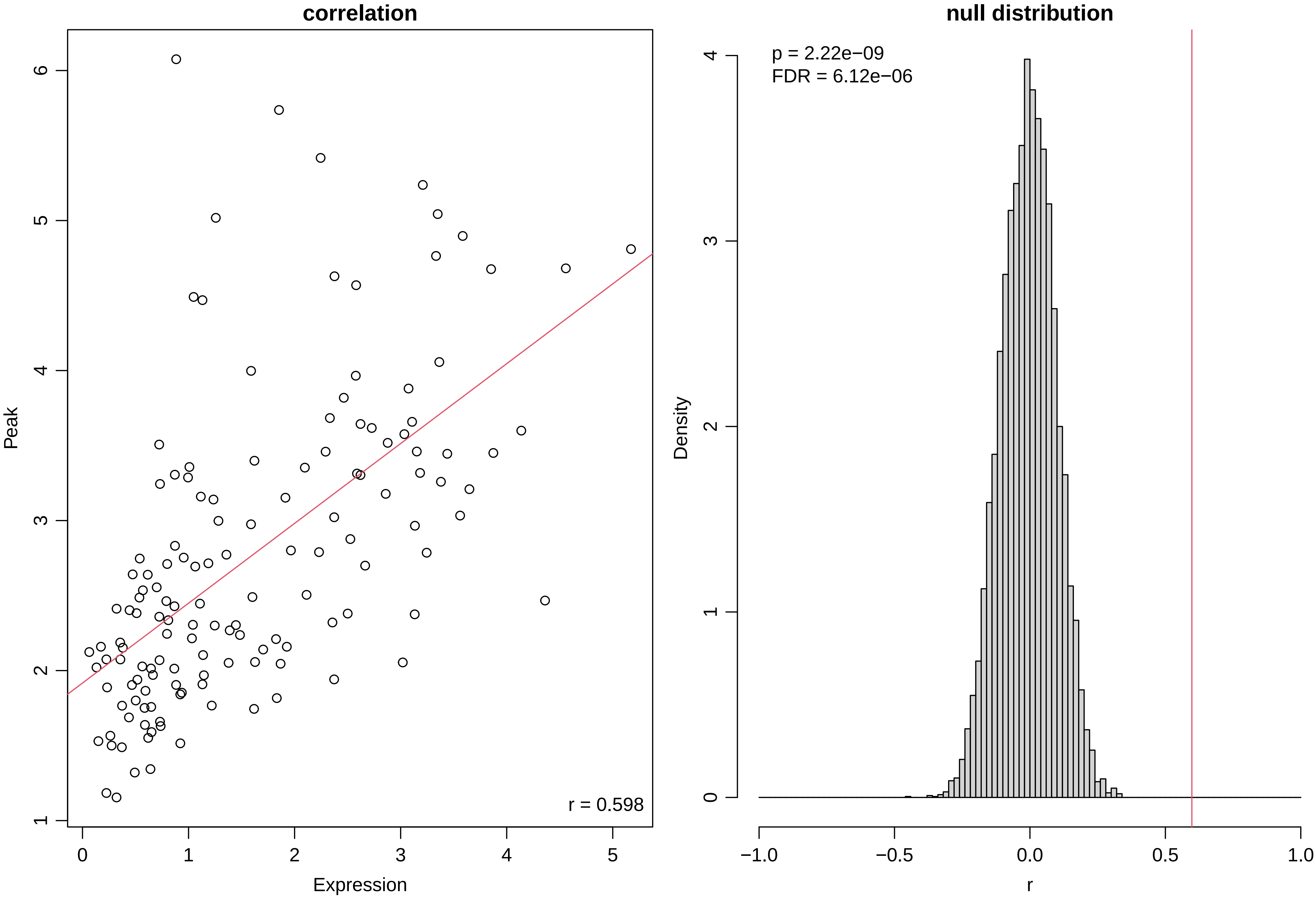

HS3ST5 vs PeakID_210853 (chr6: 114341260 − 114343842)

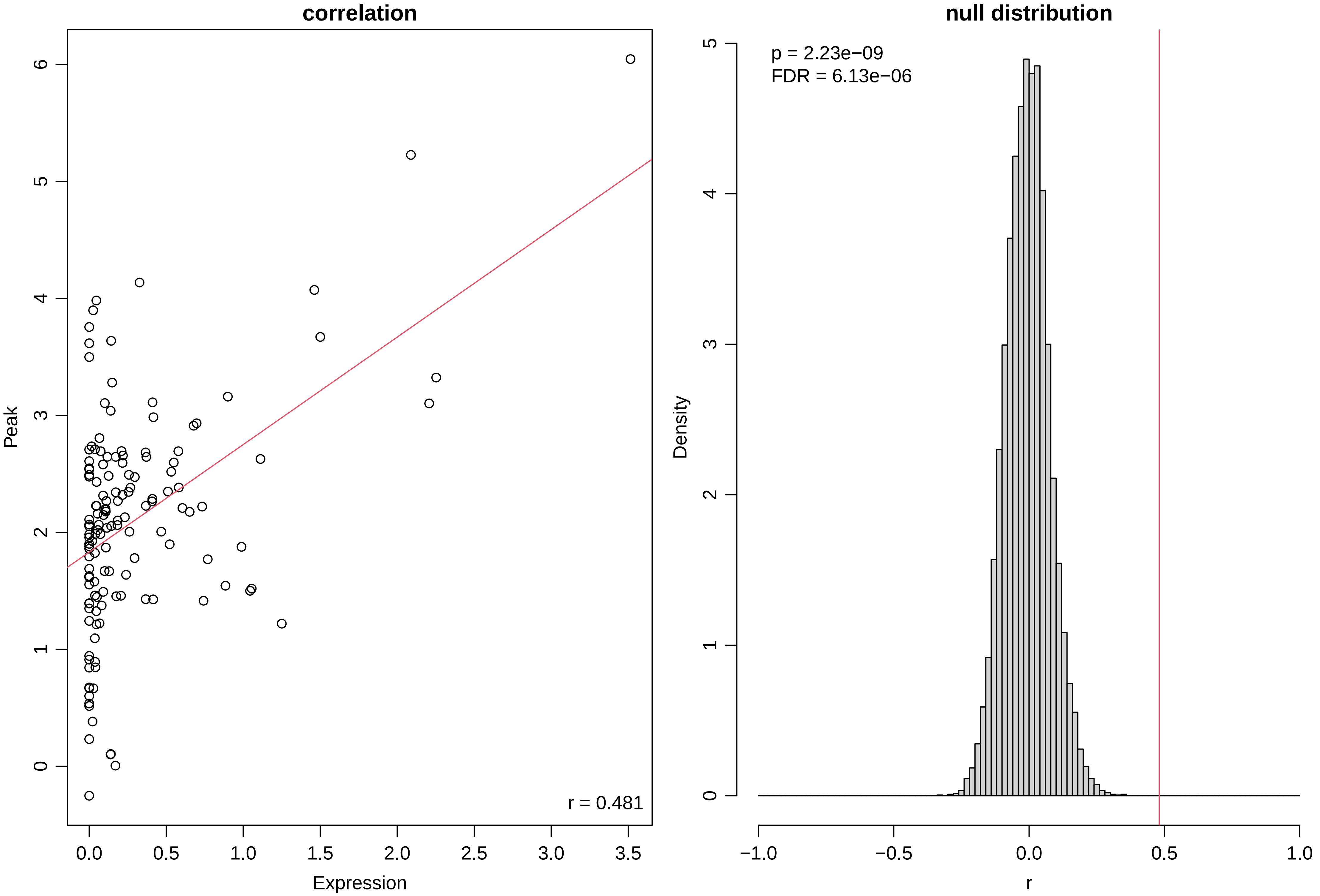

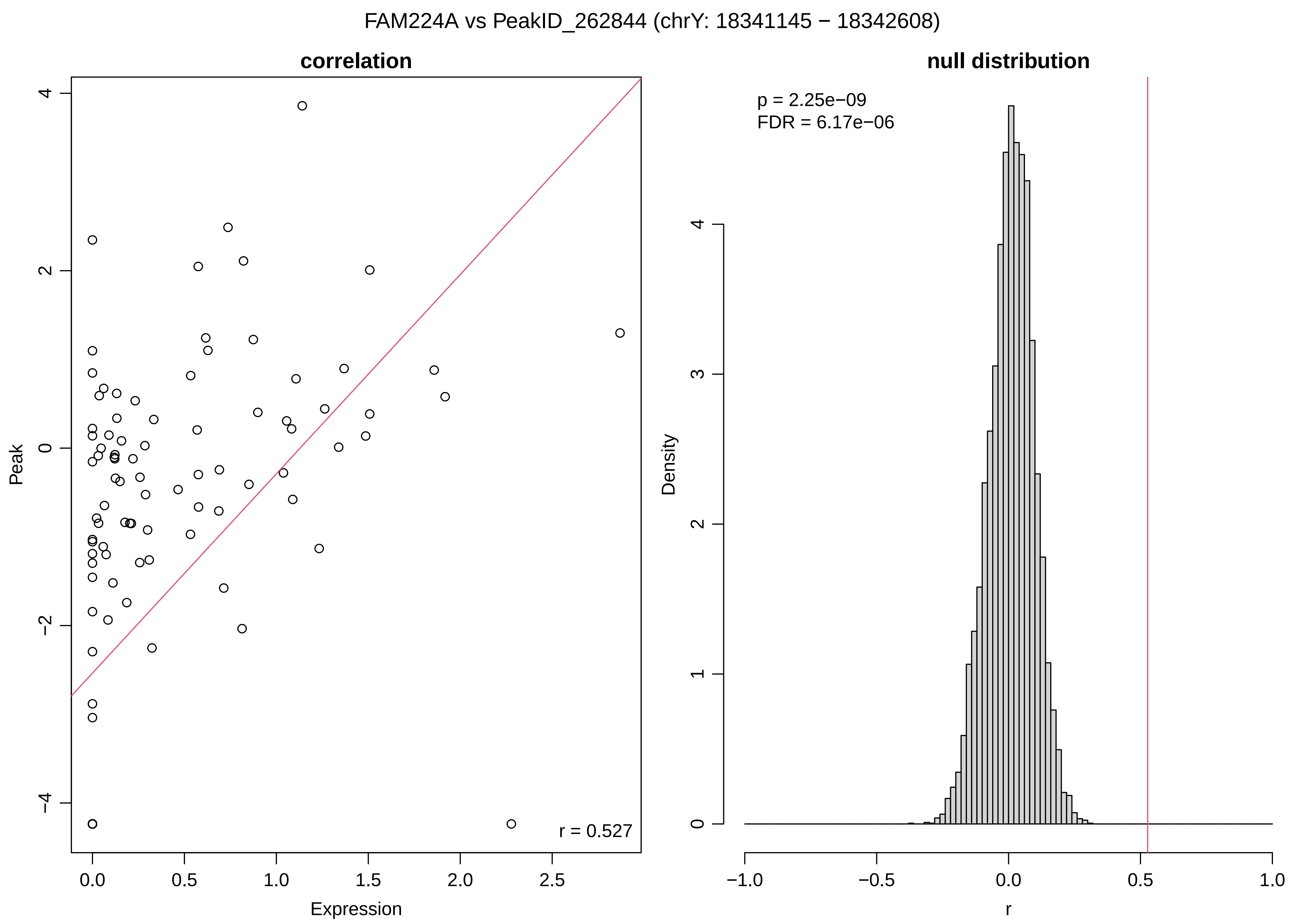
FAM224A vs PeakID_262844 (chrY: 18341145 − 18342608)
correlation
Peak
Expression
r = 0.527
null distribution
p = 2.25e−09
FDR = 6.17e−06
Density
r

PSMC1P7 vs PeakID_171805 (chr3: 161370790 − 161373956)

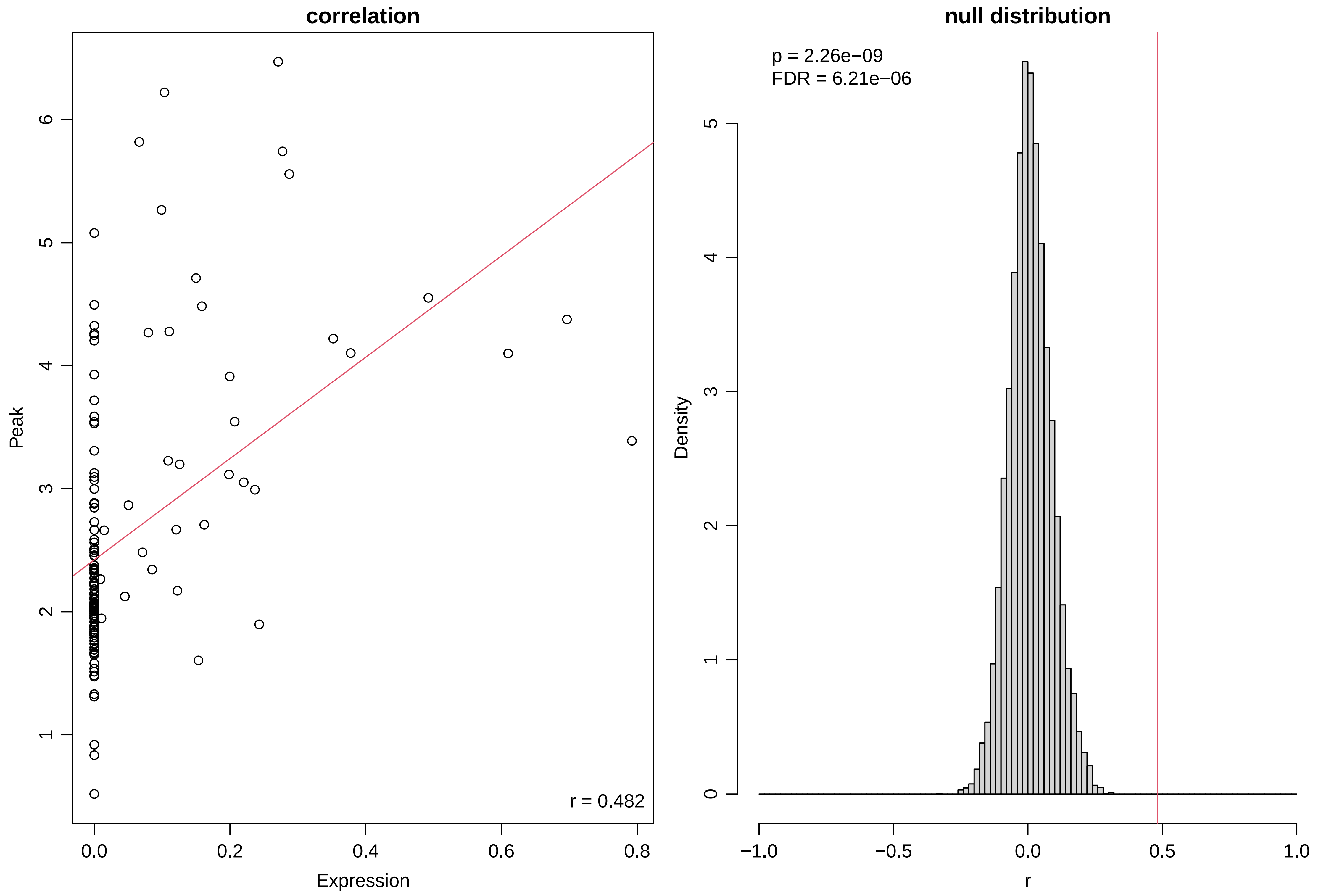

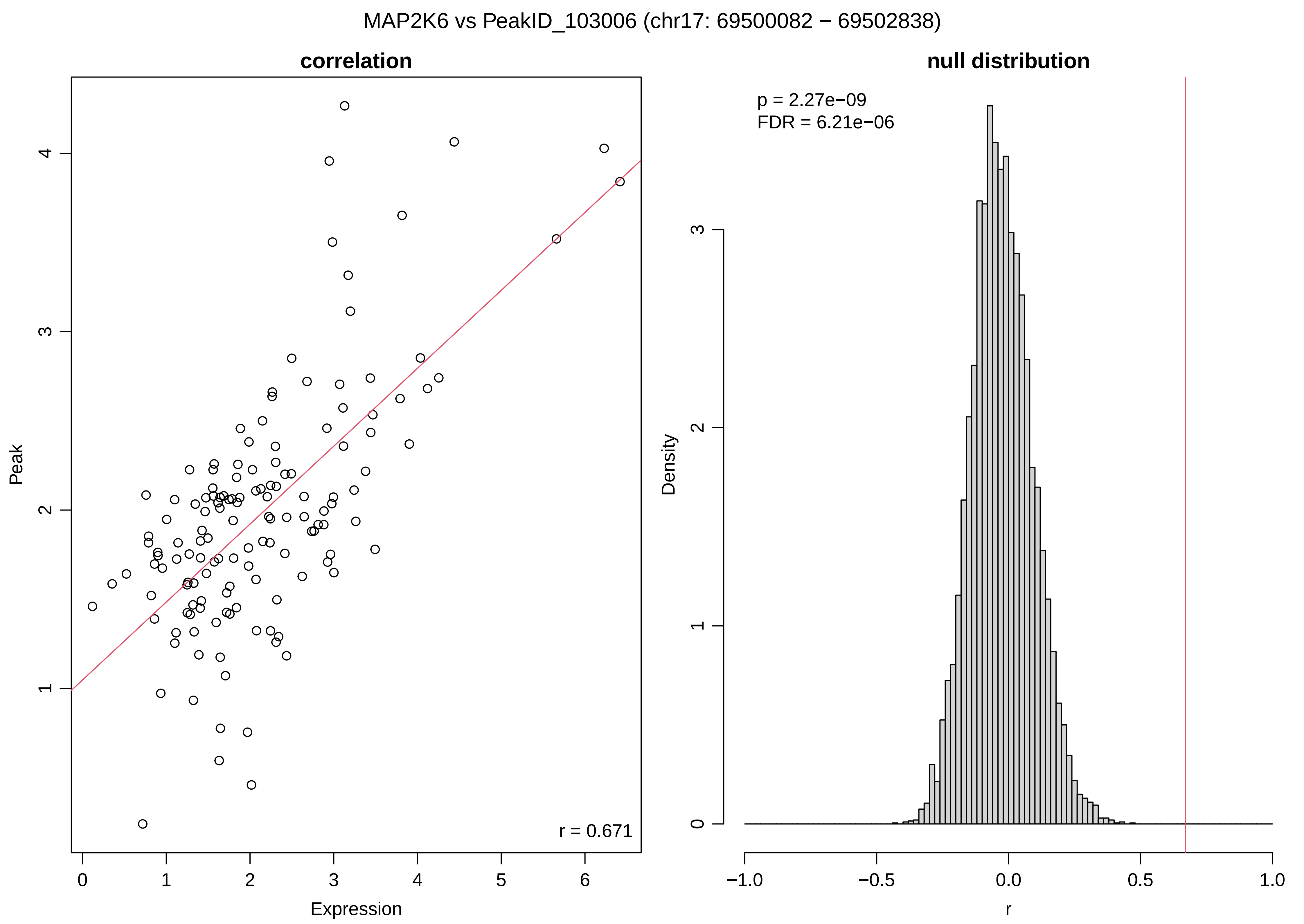

MAP2K6 vs PeakID_103006 (chr17: 69500082 − 69502838)
correlation
null distribution
Peak
Expression
r = 0.671
p = 2.27e−09
FDR = 6.21e−06
Density
r
0
1
2
3
4
5
6
−1.0
−0.5
0.0
0.5
1.0

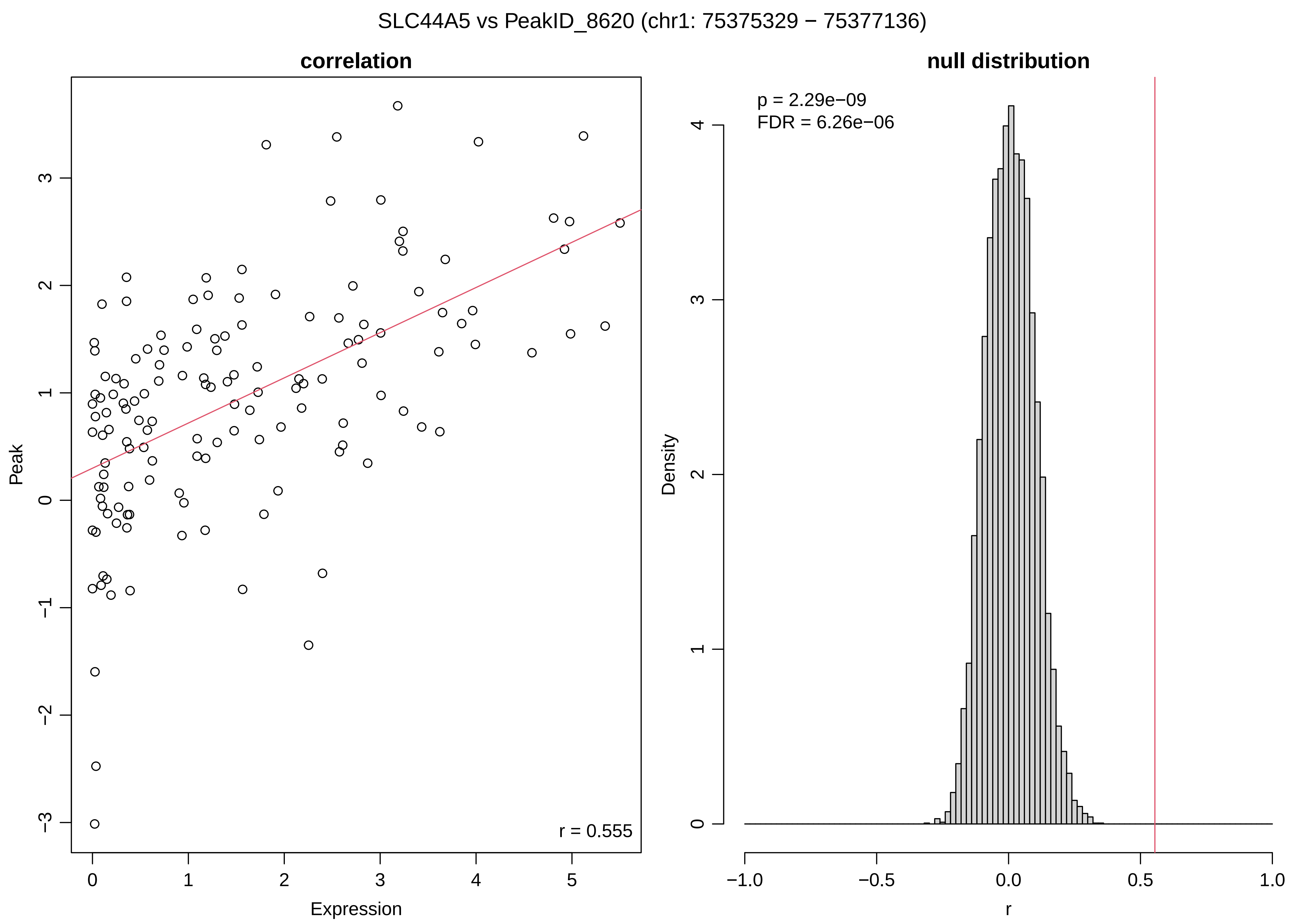

SLC44A5 vs PeakID_8620 (chr1: 75375329 − 75377136)
correlation
Peak
Expression
r = 0.555
null distribution
p = 2.29e−09
FDR = 6.26e−06
Density
r

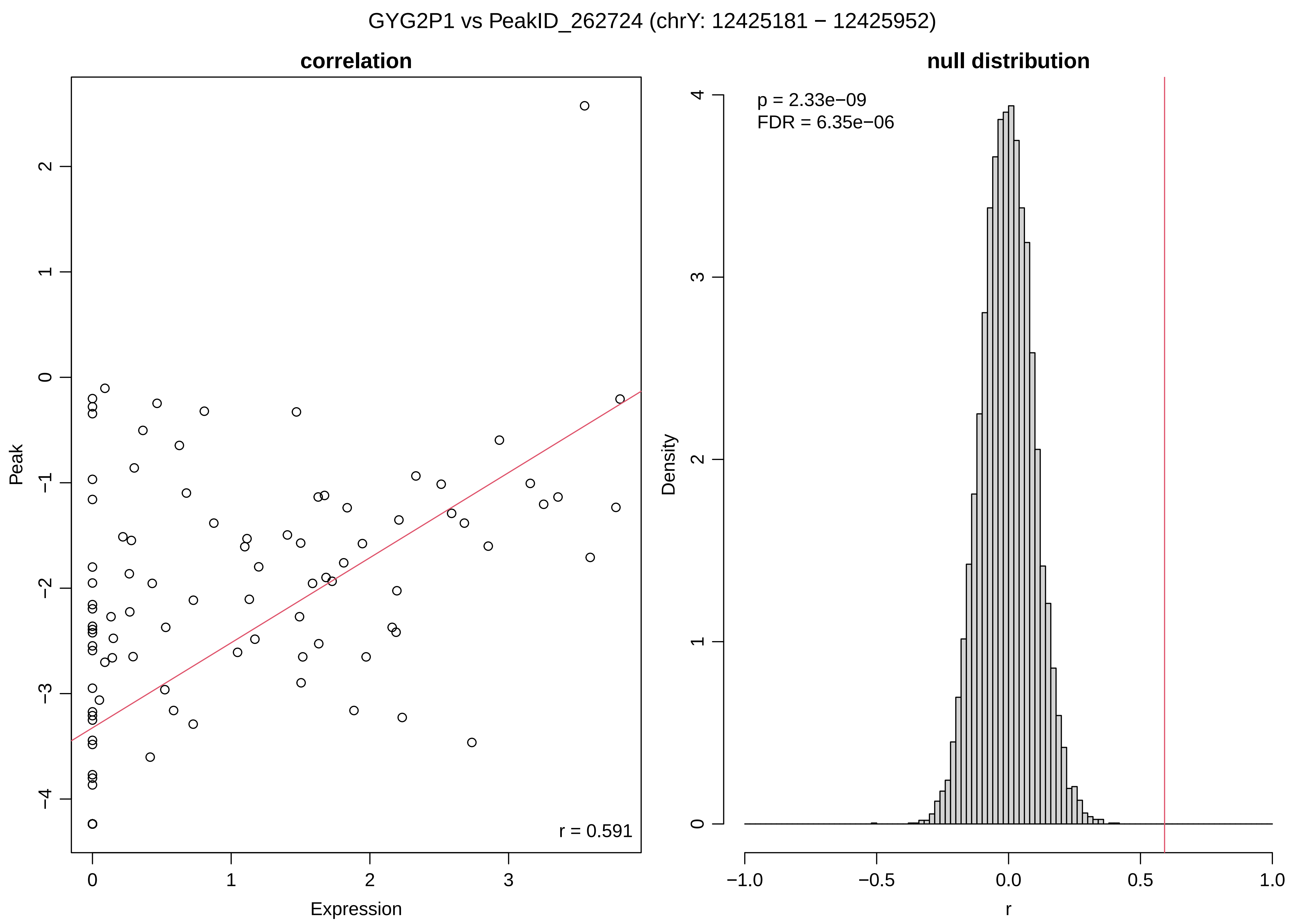
GYG2P1 vs PeakID_262724 (chrY: 12425181 − 12425952)
correlation
Peak
Expression
r = 0.591
null distribution
p = 2.33e−09
FDR = 6.35e−06
Density
r

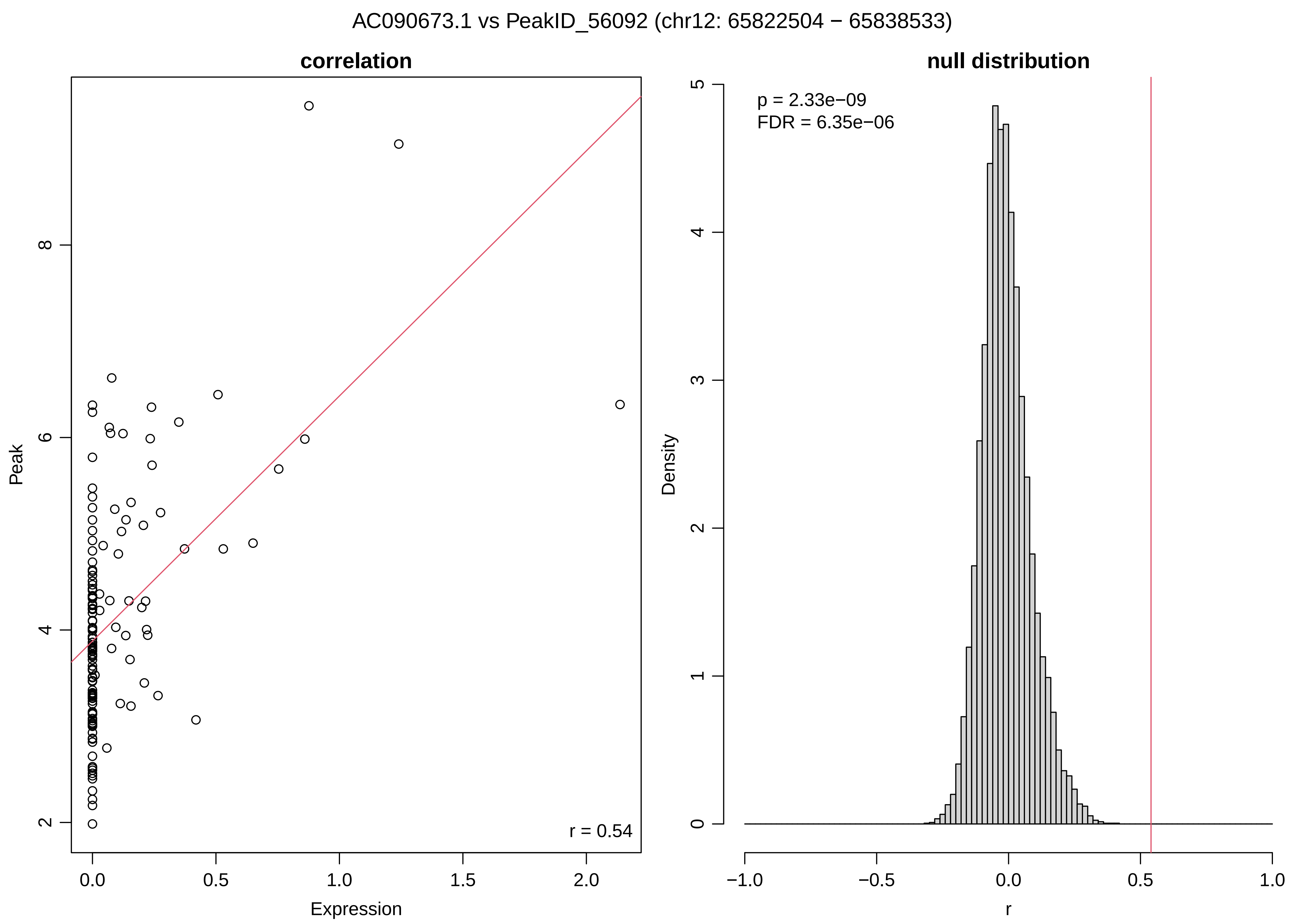
AC090673.1 vs PeakID_56092 (chr12: 65822504 − 65838533)
correlation
null distribution
Peak
Expression
r = 0.54
Density
r
p = 2.33e−09
FDR = 6.35e−06
0.0
0.5
1.0
1.5
2.0
2
4
6
8
−1.0
−0.5
0.0
0.5
1.0
0
1
2
3
4
5

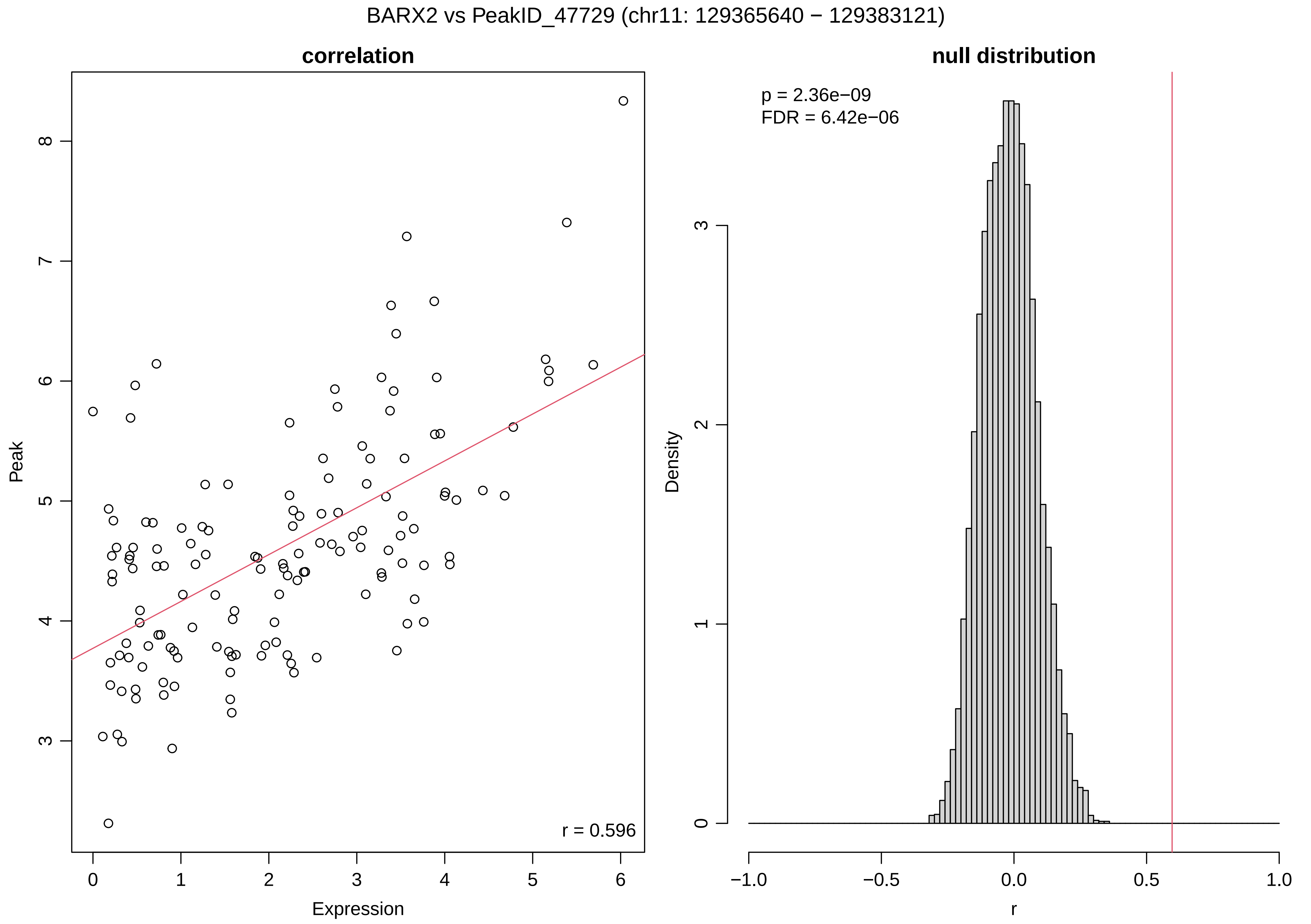
BARX2 vs PeakID_47729 (chr11: 129365640 − 129383121)
correlation
Peak
Expression
r = 0.596
null distribution
p = 2.36e−09
FDR = 6.42e−06
Density
r

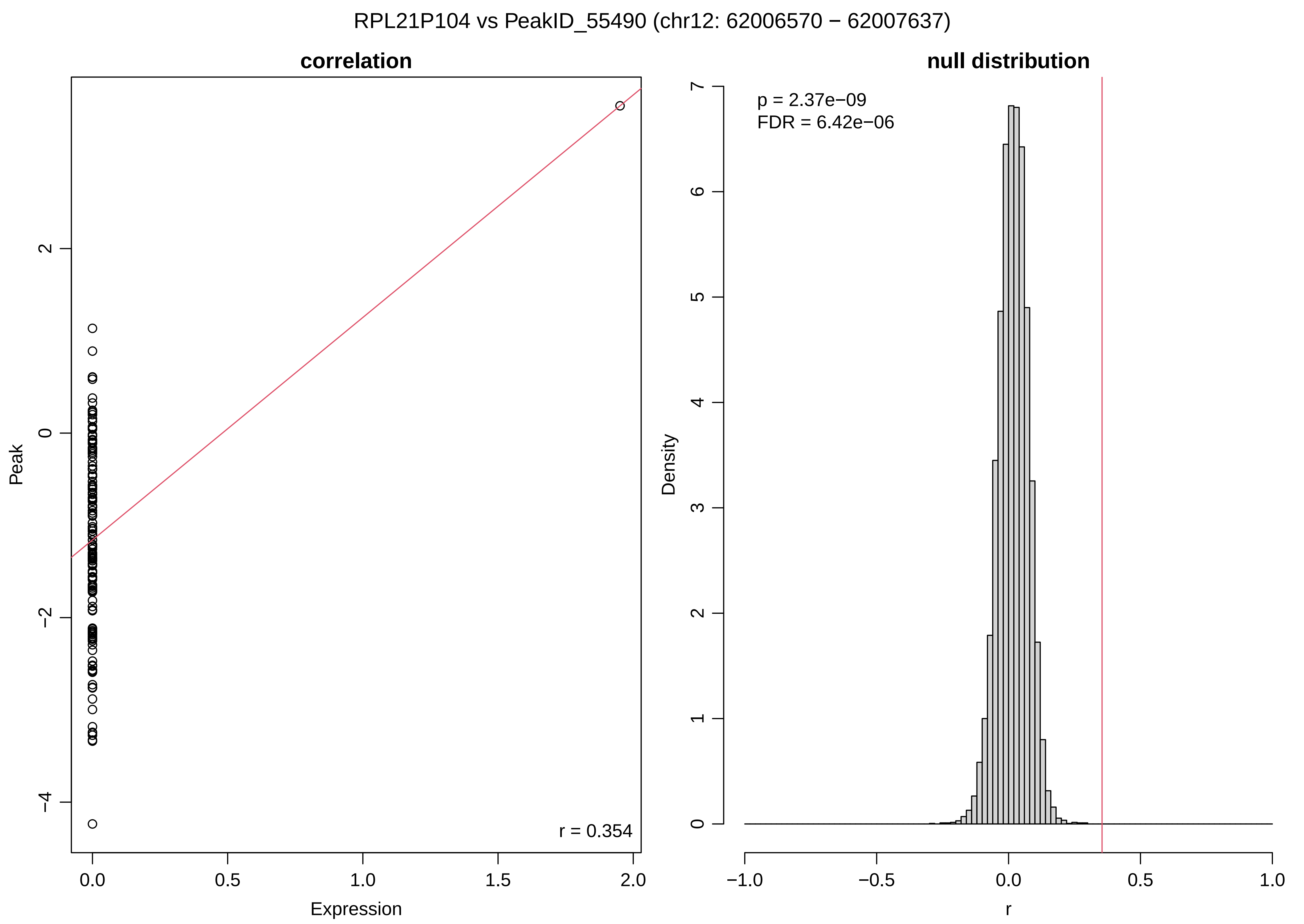
RPL21P104 vs PeakID_55490 (chr12: 62006570 − 62007637)
correlation
Peak
Expression
r = 0.354
2
0
−2
−4
0.0
0.5
1.0
1.5
2.0
null distribution
p = 2.37e−09
FDR = 6.42e−06
Density
r
0
1
2
3
4
5
6
7
−1.0
−0.5
0.0
0.5
1.0

PVT1 vs PeakID_242972 (chr8: 127806180 − 127812516)

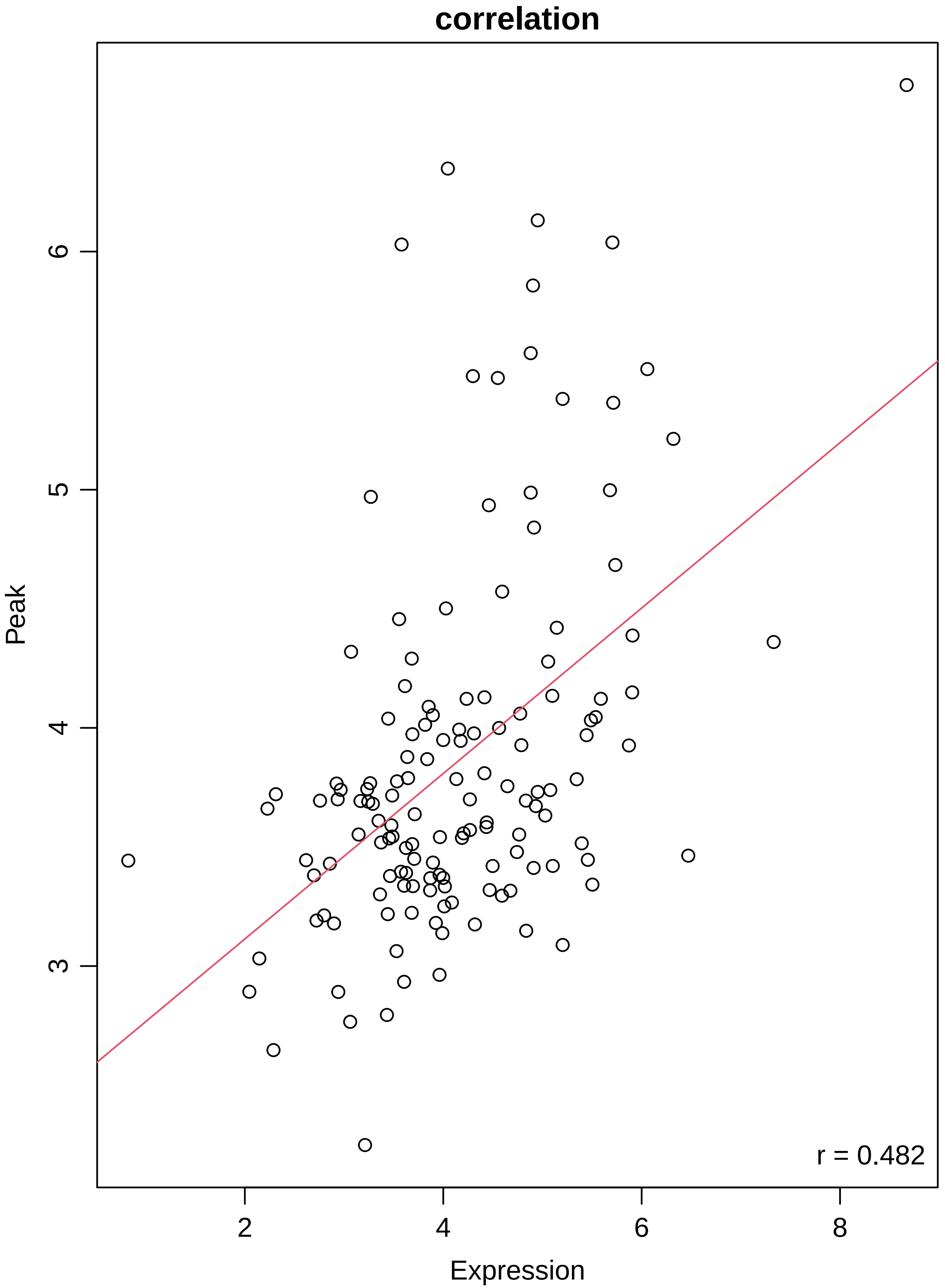

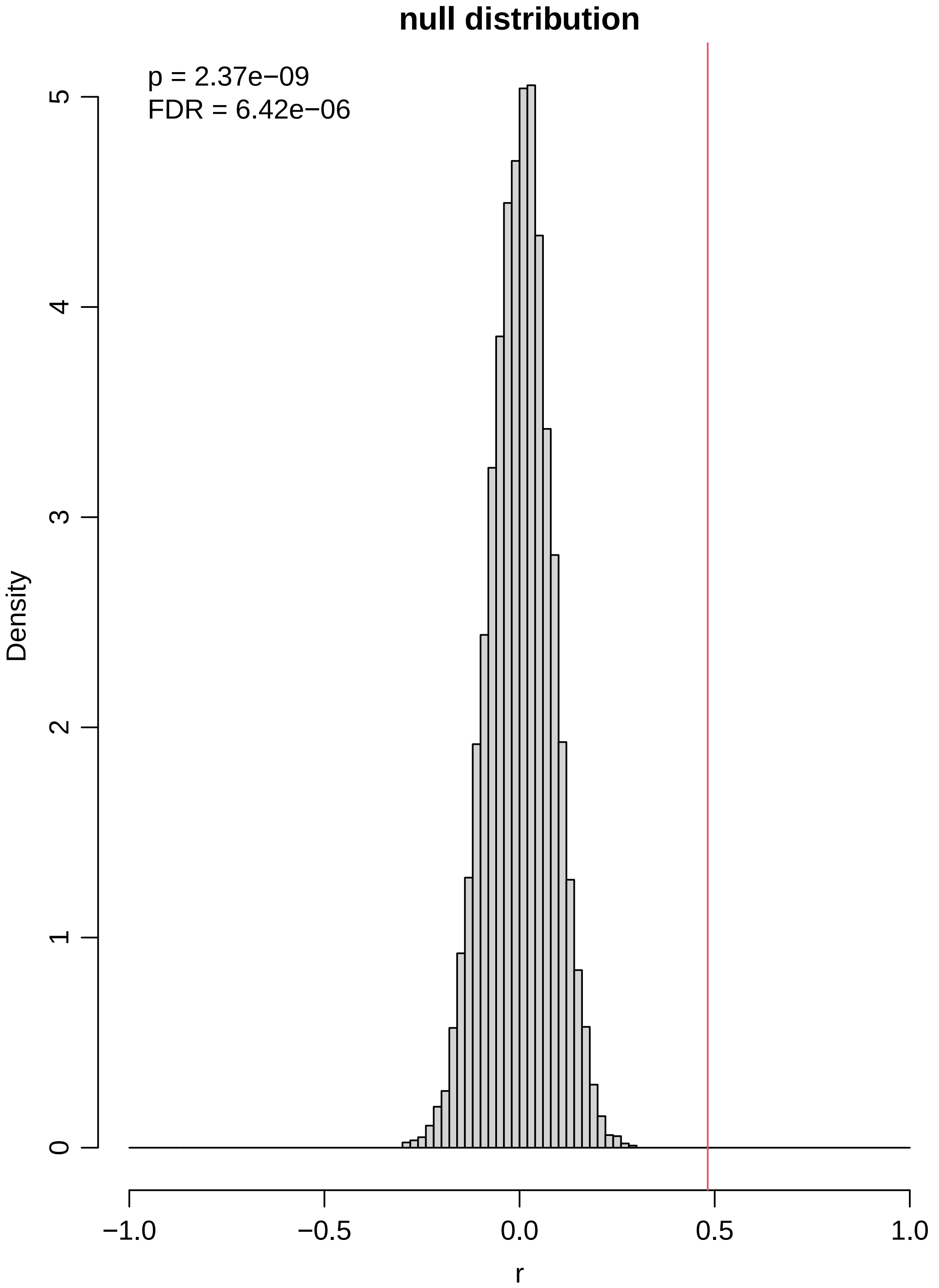

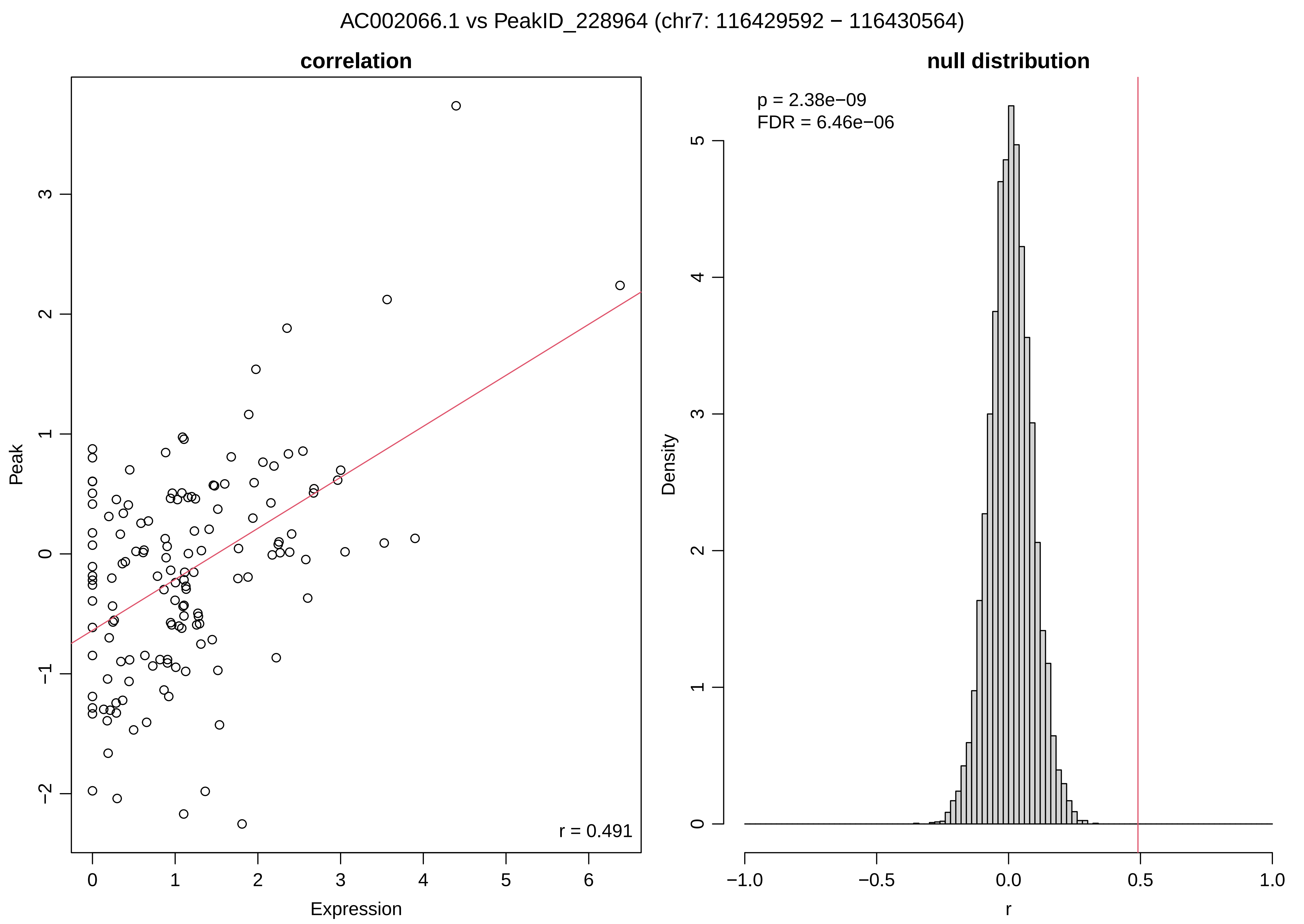
AC002066.1 vs PeakID_228964 (chr7: 116429592 − 116430564)
correlation
Peak
Expression
r = 0.491
null distribution
p = 2.38e−09
FDR = 6.46e−06
Density
r

LINC01269 vs PeakID_72669 (chr14: 70697312 − 70699460)

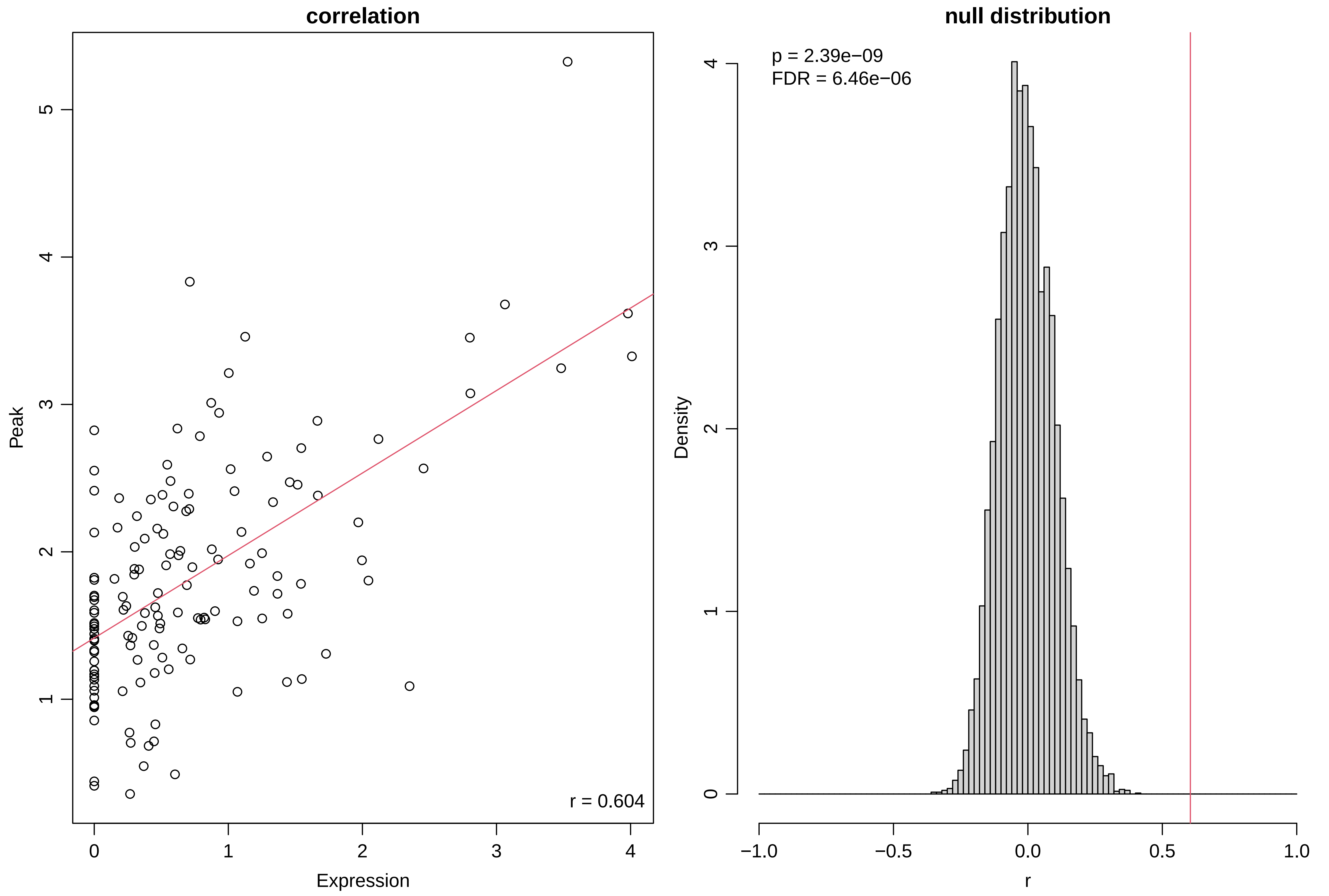

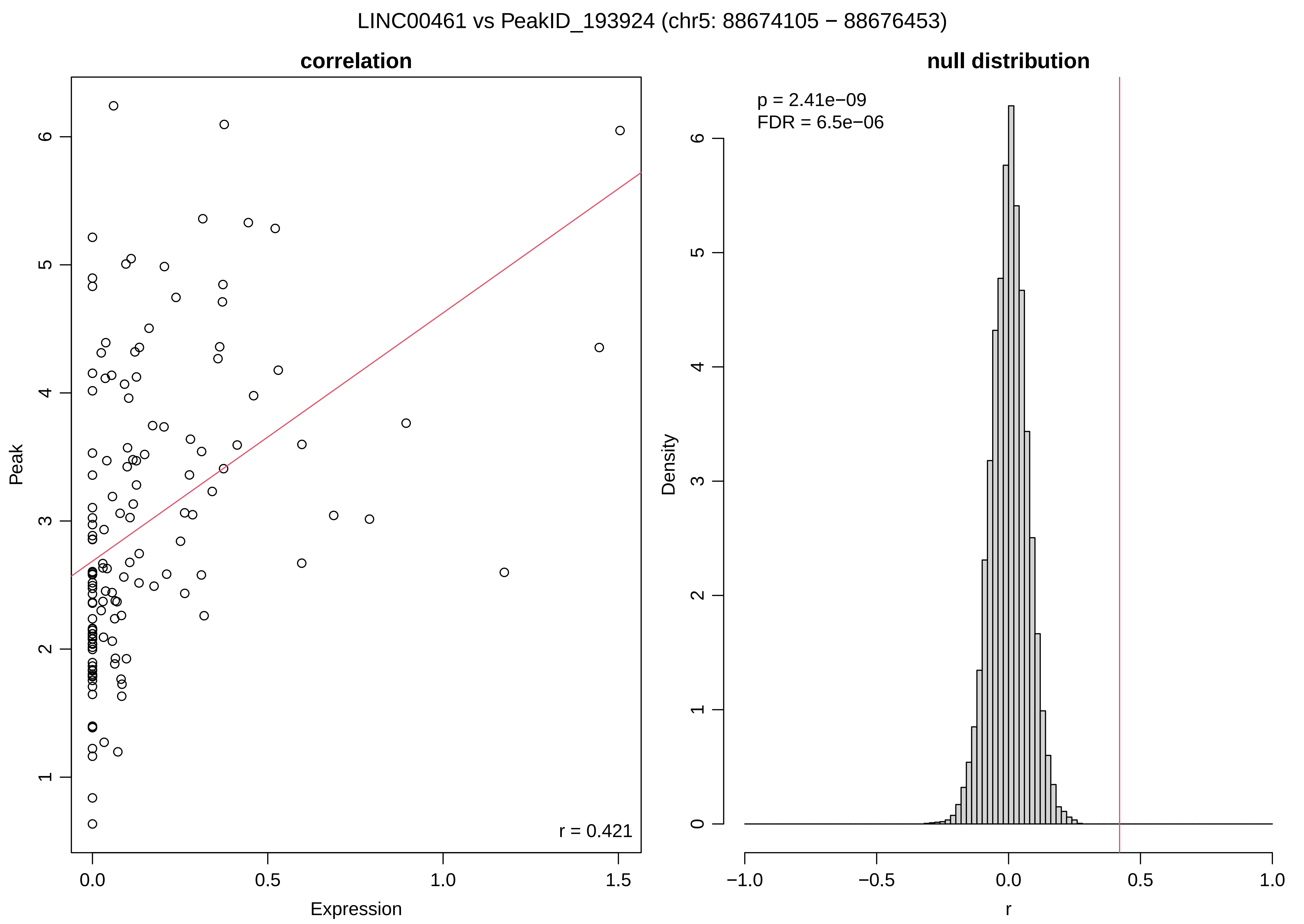
LINC00461 vs PeakID_193924 (chr5: 88674105 − 88676453)
correlation
Peak
Expression
r = 0.421
null distribution
p = 2.41e−09
FDR = 6.5e−06
Density
r
0.0
0.5
1.0
1.5
1
2
3
4
5
6
−1.0
−0.5
0

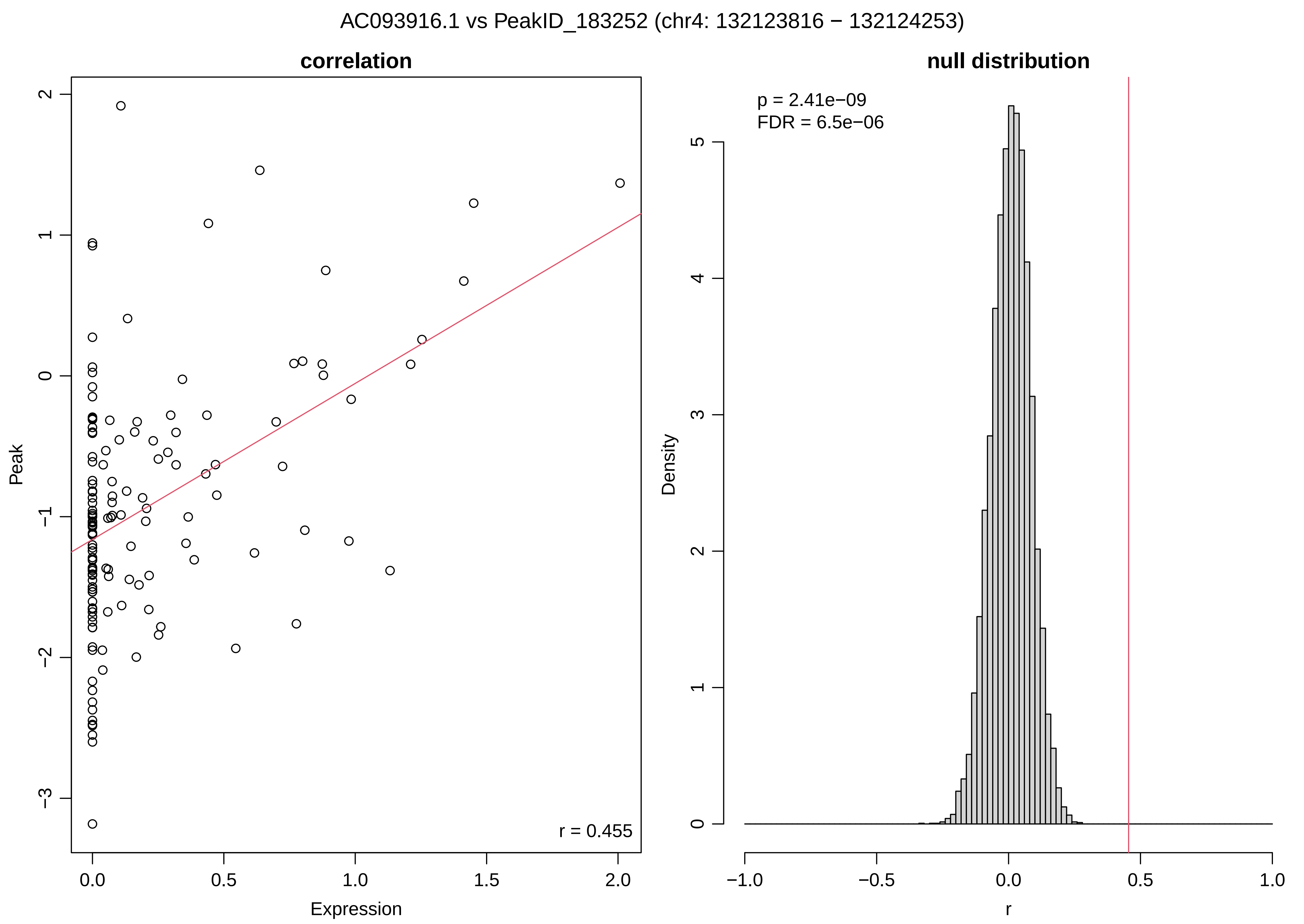
AC093916.1 vs PeakID_183252 (chr4: 132123816 − 132124253)
correlation
Peak
Expression
r = 0.455
null distribution
p = 2.41e−09
FDR = 6.5e−06
Density
r

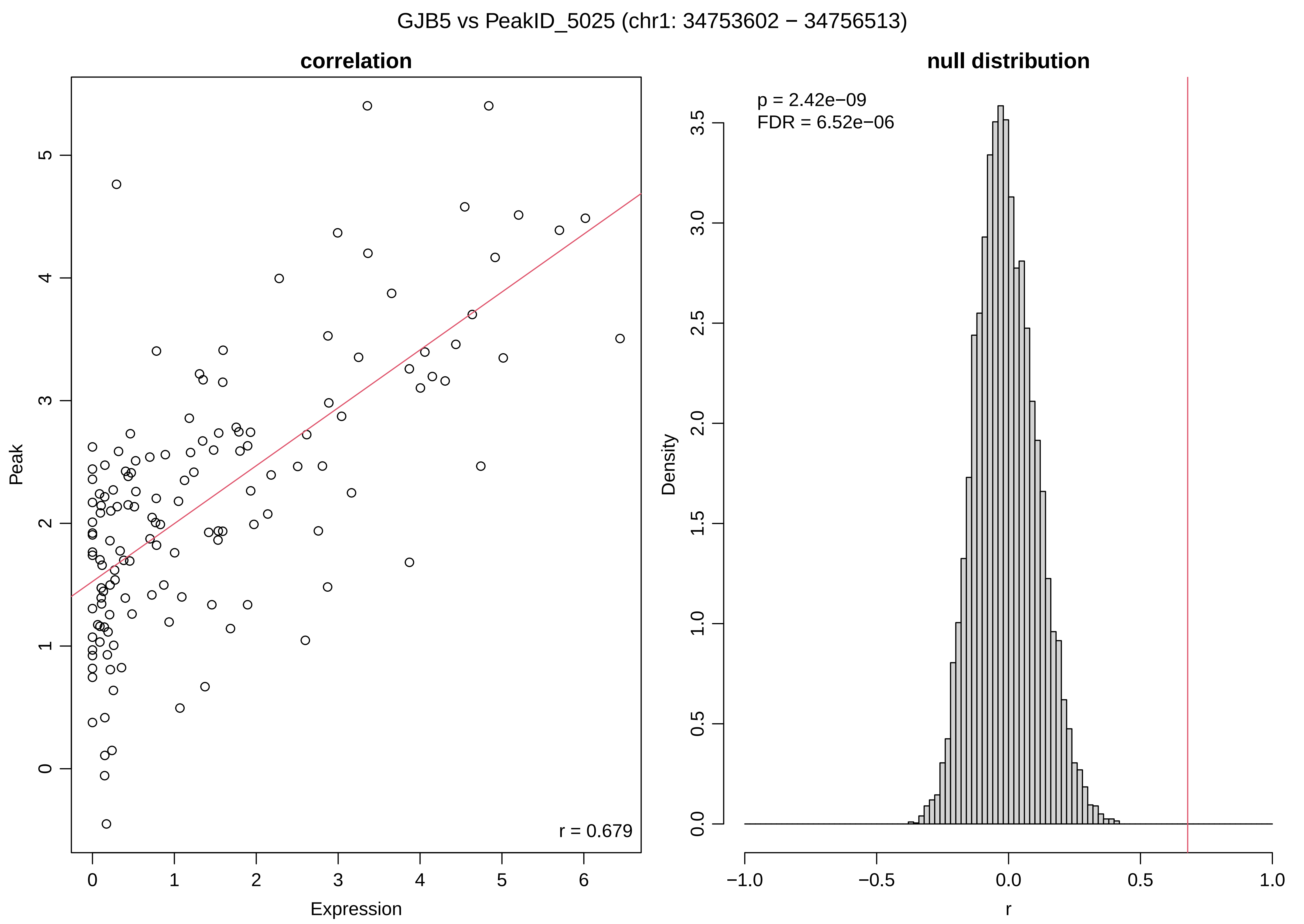

GJB5 vs PeakID_5025 (chr1: 34753602 − 34756513)
correlation
Peak
Expression
r = 0.679
null distribution
p = 2.42e−09
FDR = 6.52e−06
Density
r

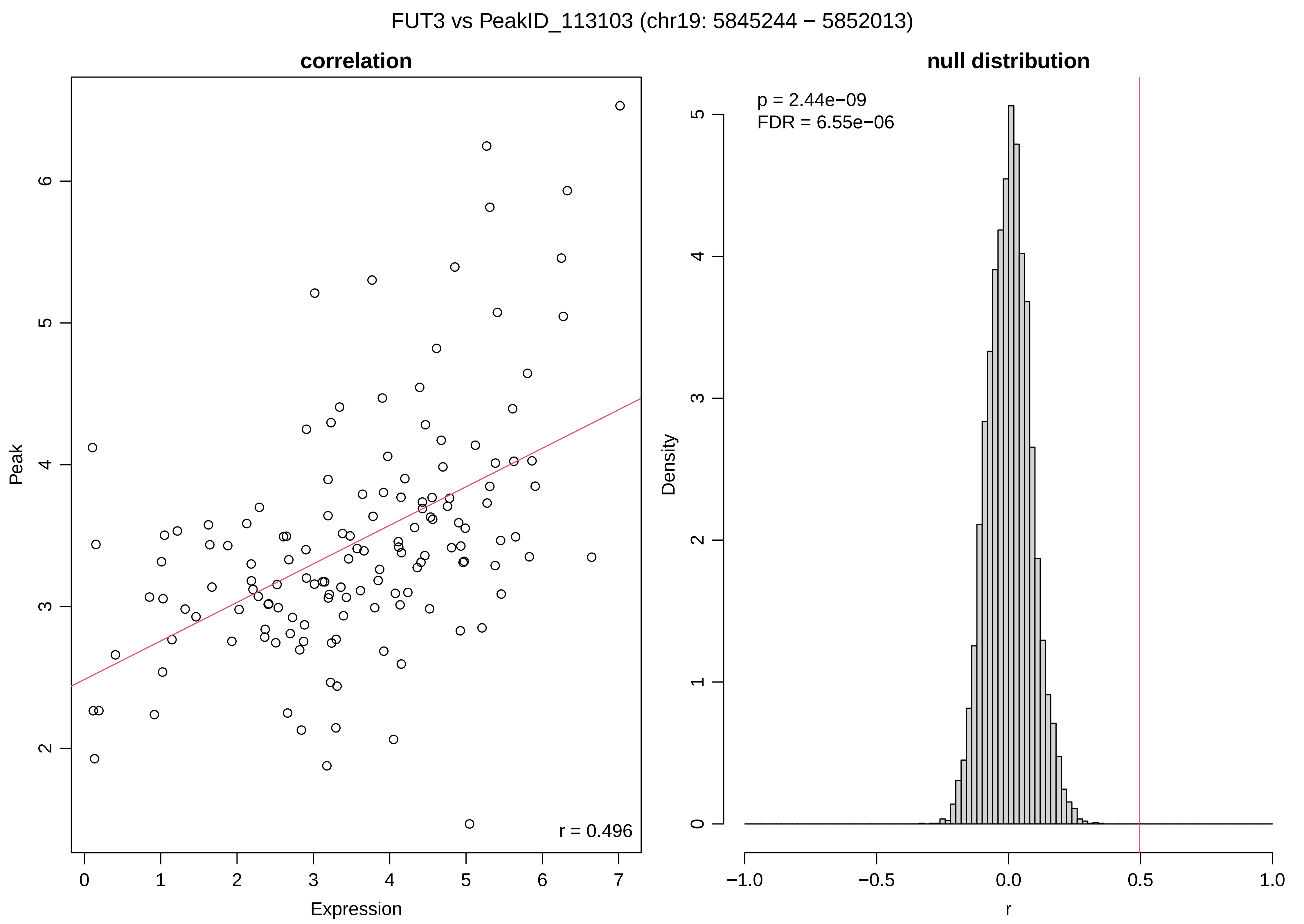

FUT3 vs PeakID_113103 (chr19: 5845244 − 5852013)
correlation
Peak
Expression
r = 0.496
null distribution
p = 2.44e−09
FDR = 6.55e−06
Density
r

LINC02403 vs PeakID_54454 (chr12: 58030288 − 58031350)

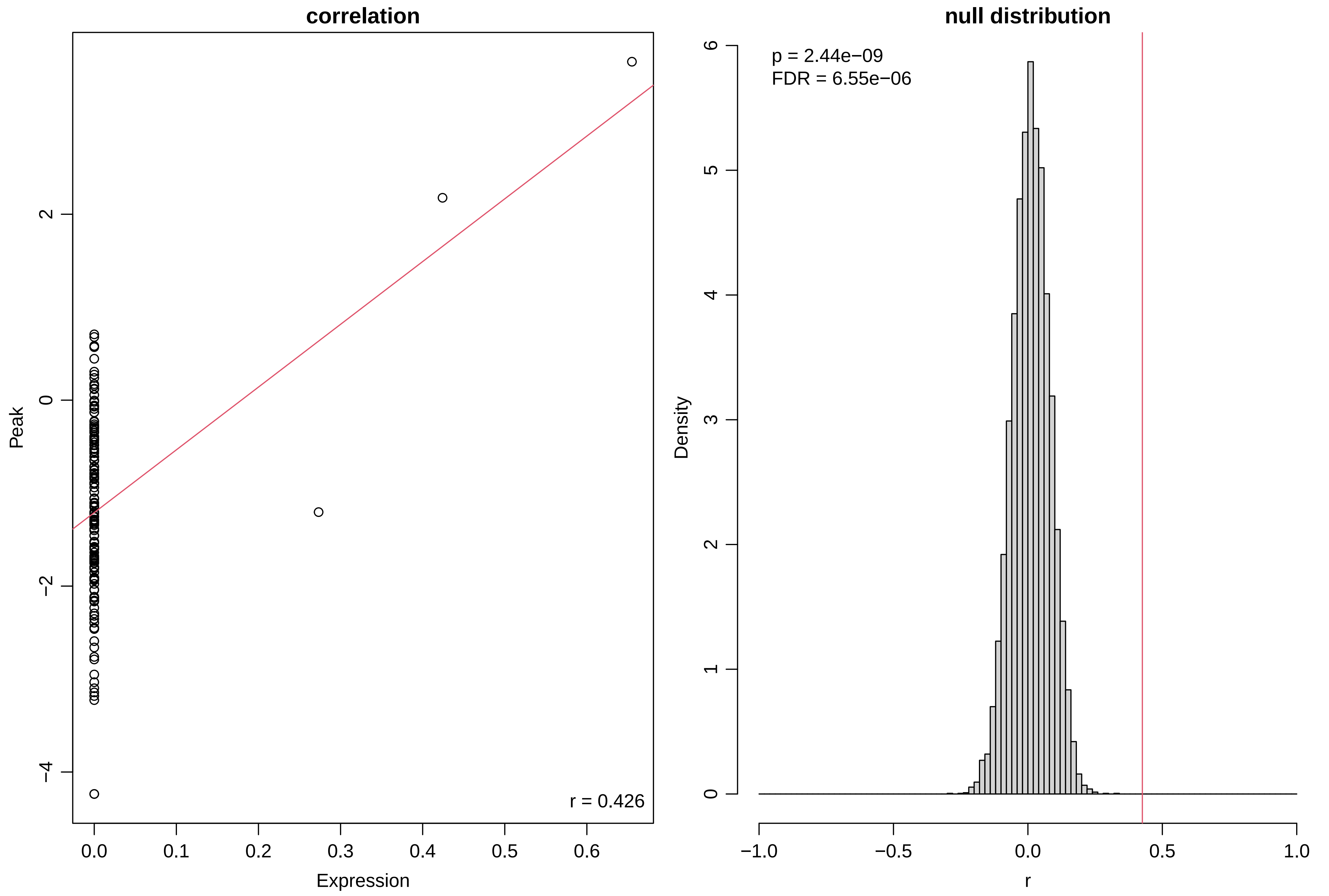

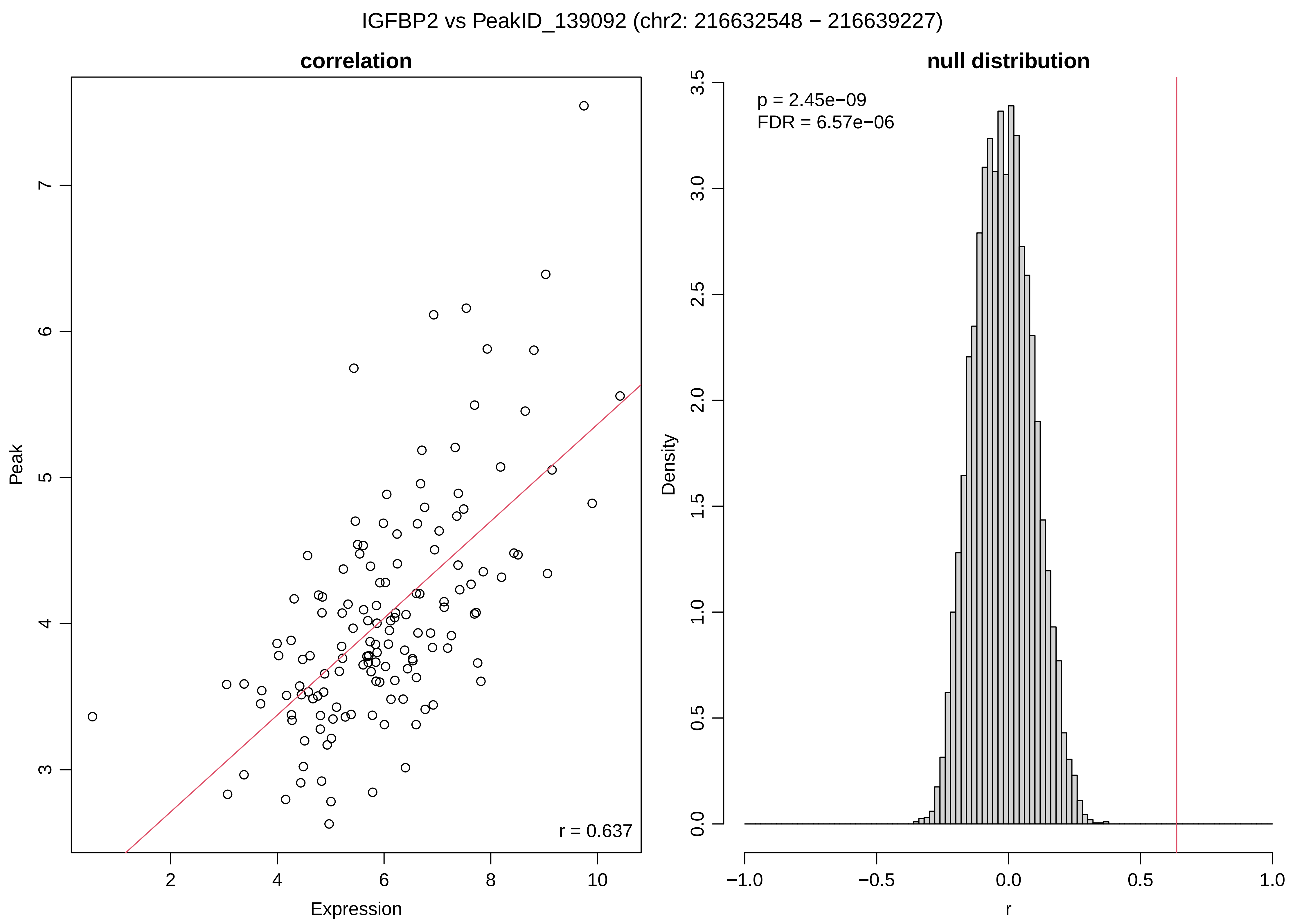
IGFBP2 vs PeakID_139092 (chr2: 216632548 − 216639227)
correlation
Peak
Expression
r = 0.637
null distribution
p = 2.45e−09
FDR = 6.57e−06
Density
r

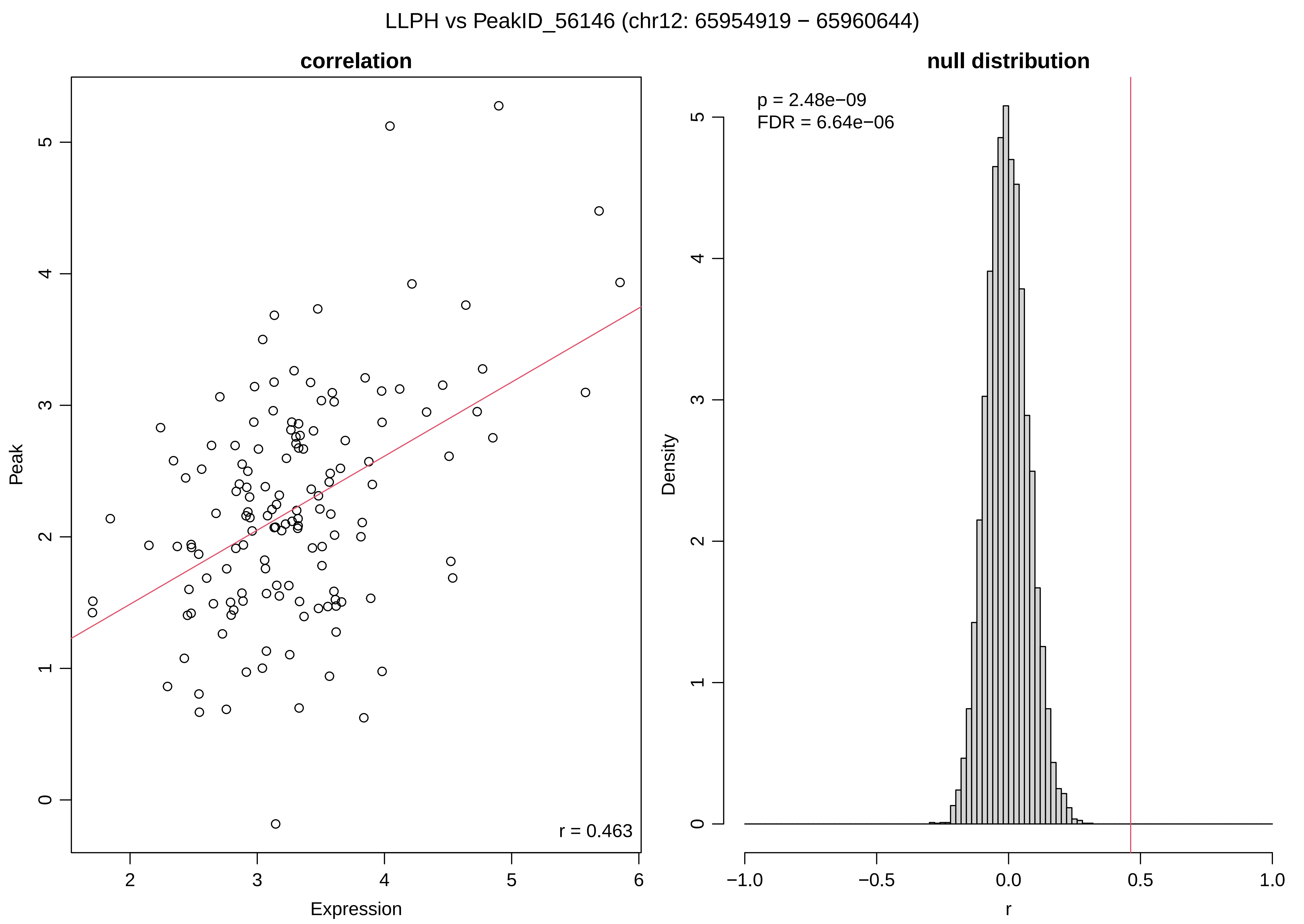

LLPH vs PeakID_56146 (chr12: 65954919 − 65960644)
correlation
Peak
Expression
r = 0.463
null distribution
p = 2.48e−09
FDR = 6.64e−06
Density
r

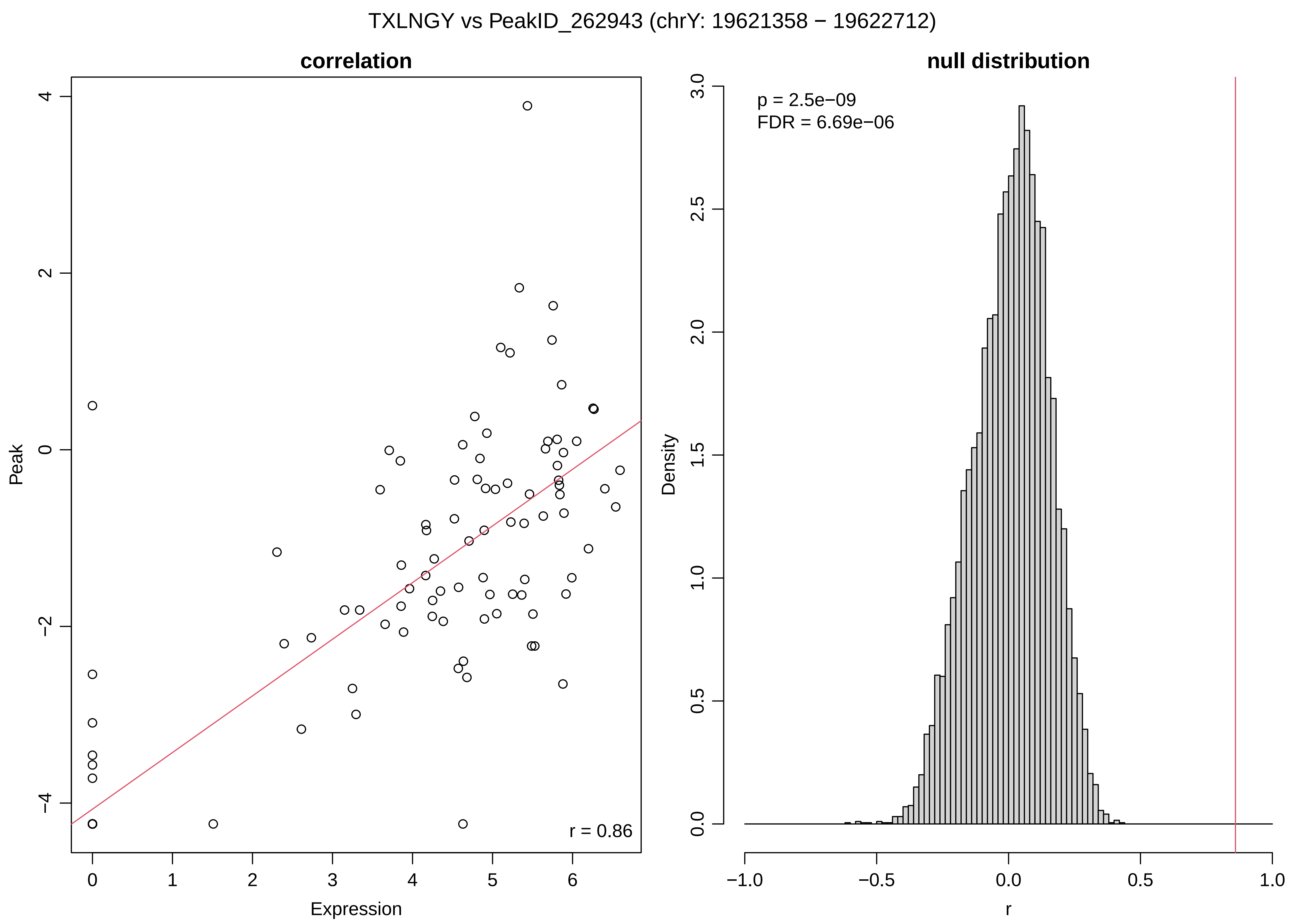
TXLNGY vs PeakID_262943 (chrY: 19621358 − 19622712)
correlation
Peak
Expression
r = 0.86
null distribution
Density
r
p = 2.5e−09
FDR = 6.69e−06

YEATS4 vs PeakID_56890 (chr12: 69580617 − 69581623)

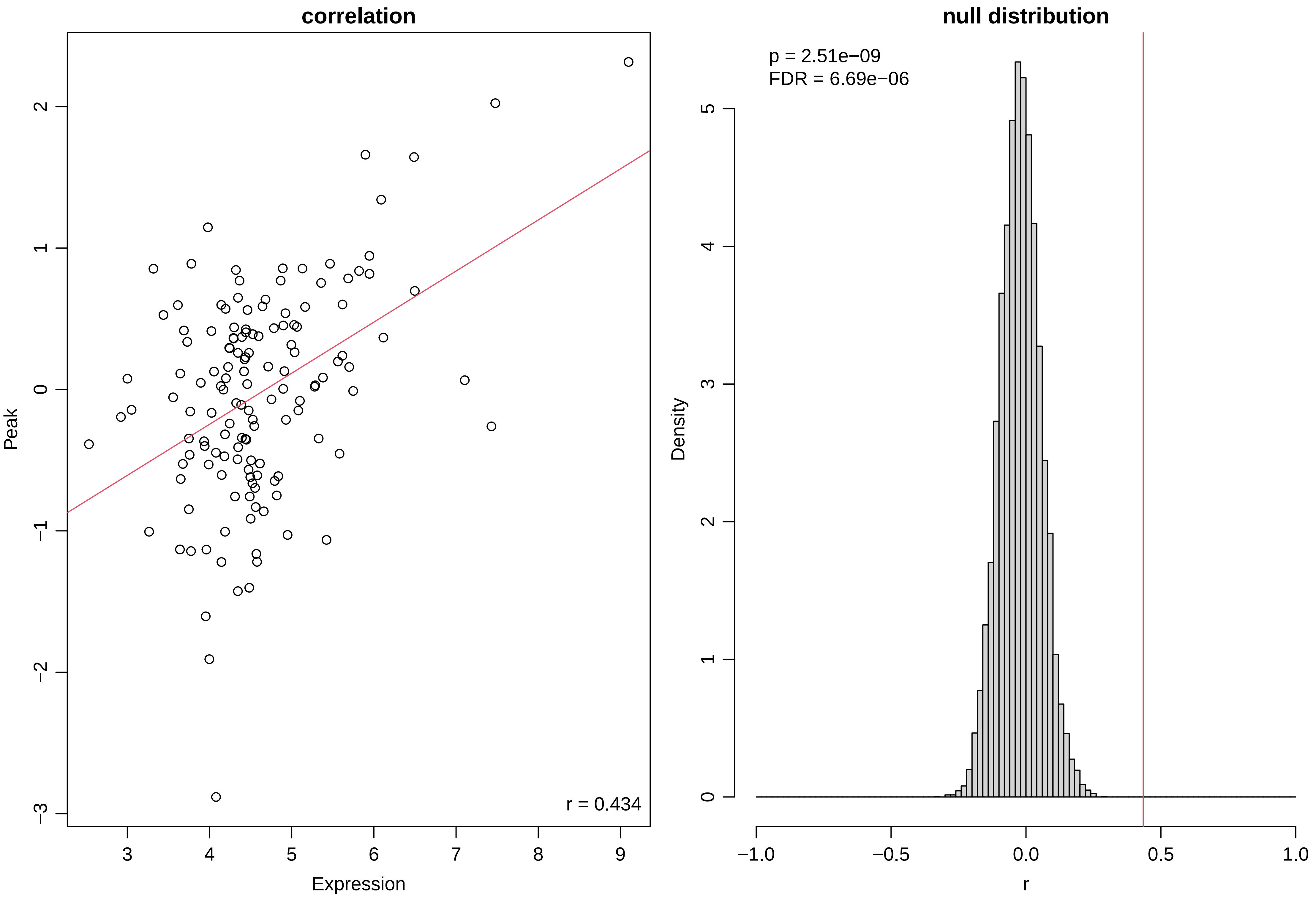

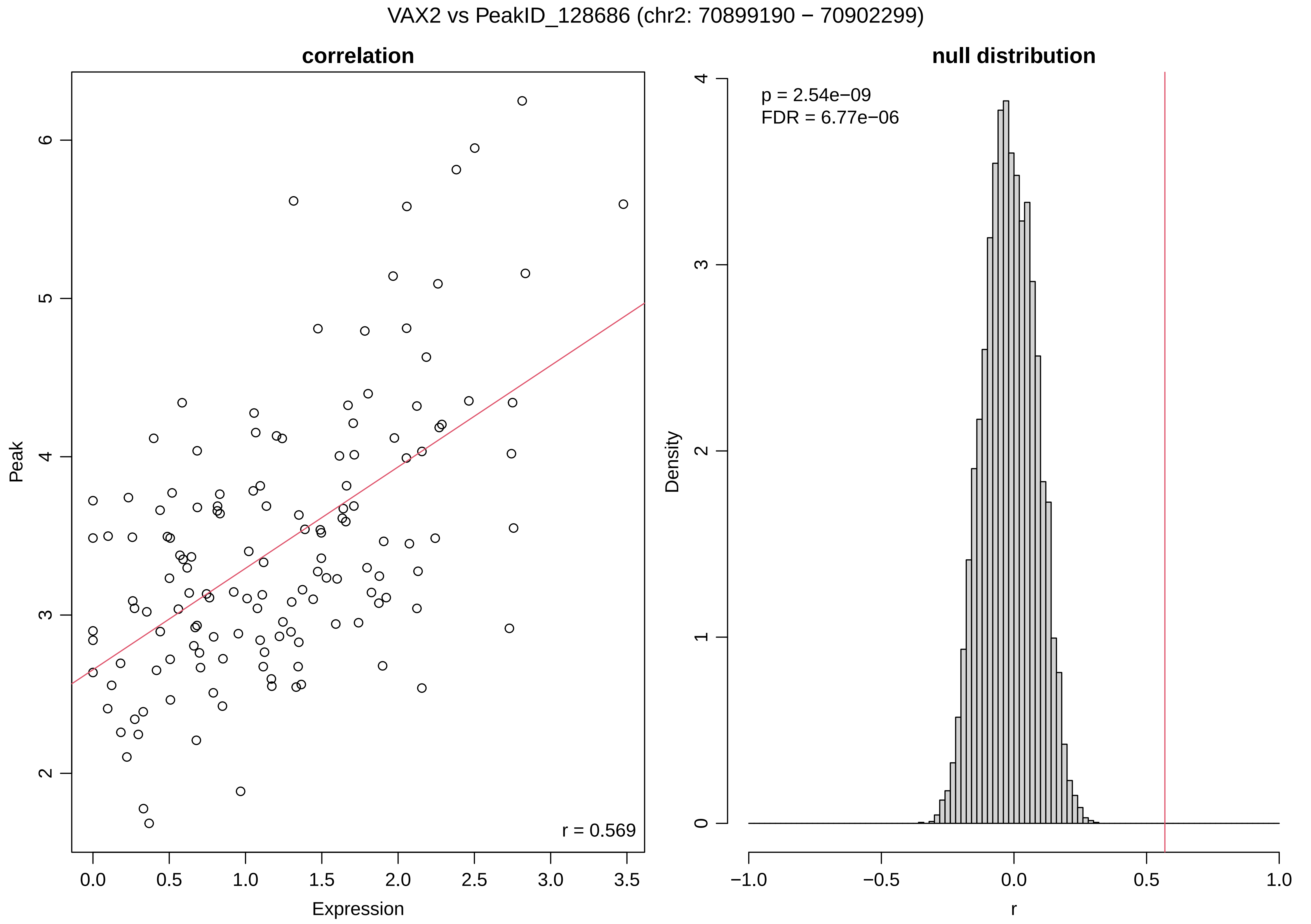

VAX2 vs PeakID_128686 (chr2: 70899190 − 70902299)
correlation
Peak
Expression
r = 0.569
null distribution
p = 2.54e−09
FDR = 6.77e−06
Density
r

AC011447.3 vs PeakID_115527 (chr19: 20234933 − 20238701)

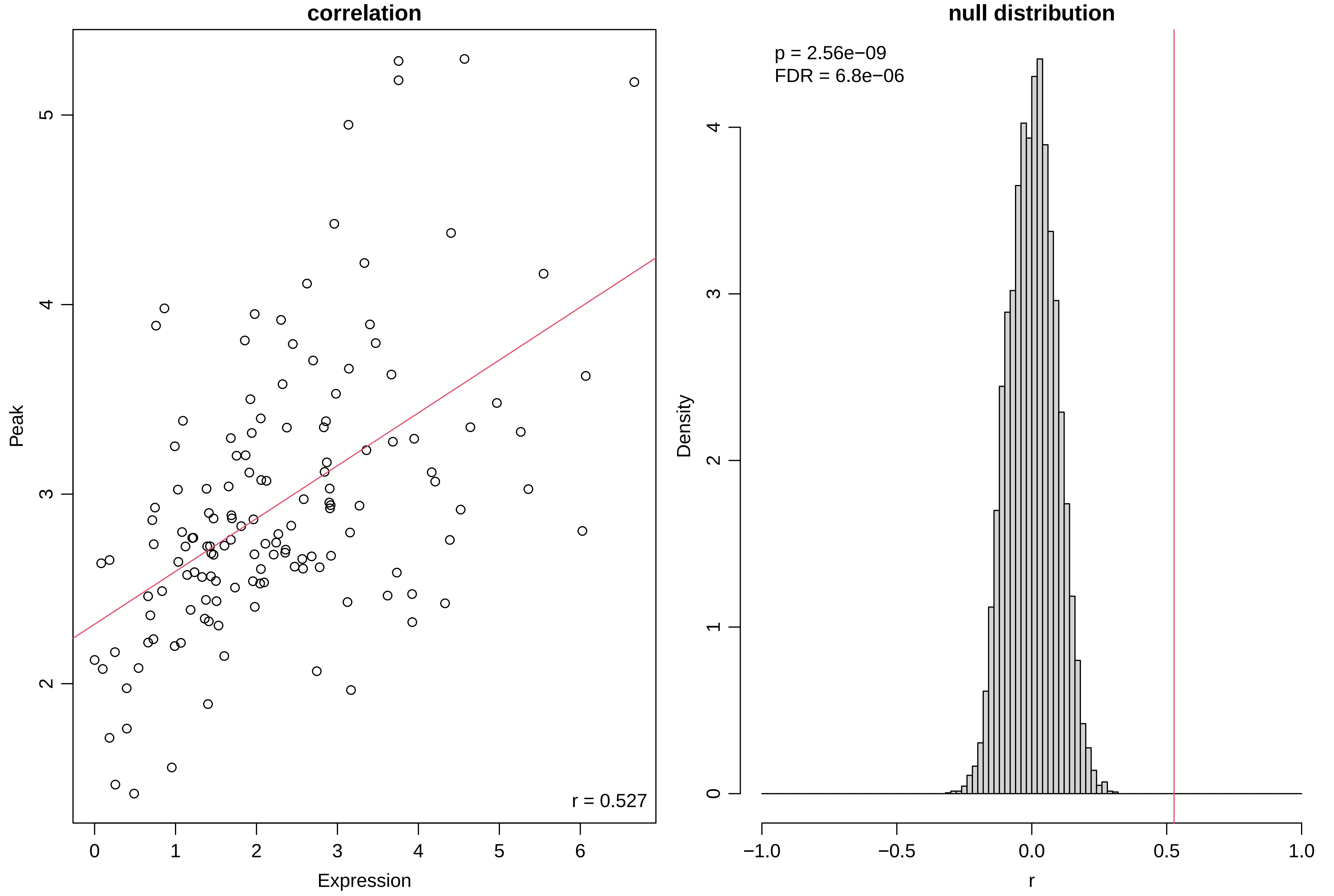

SOX21−AS1 vs PeakID_66826 (chr13: 94710543 − 94714127)

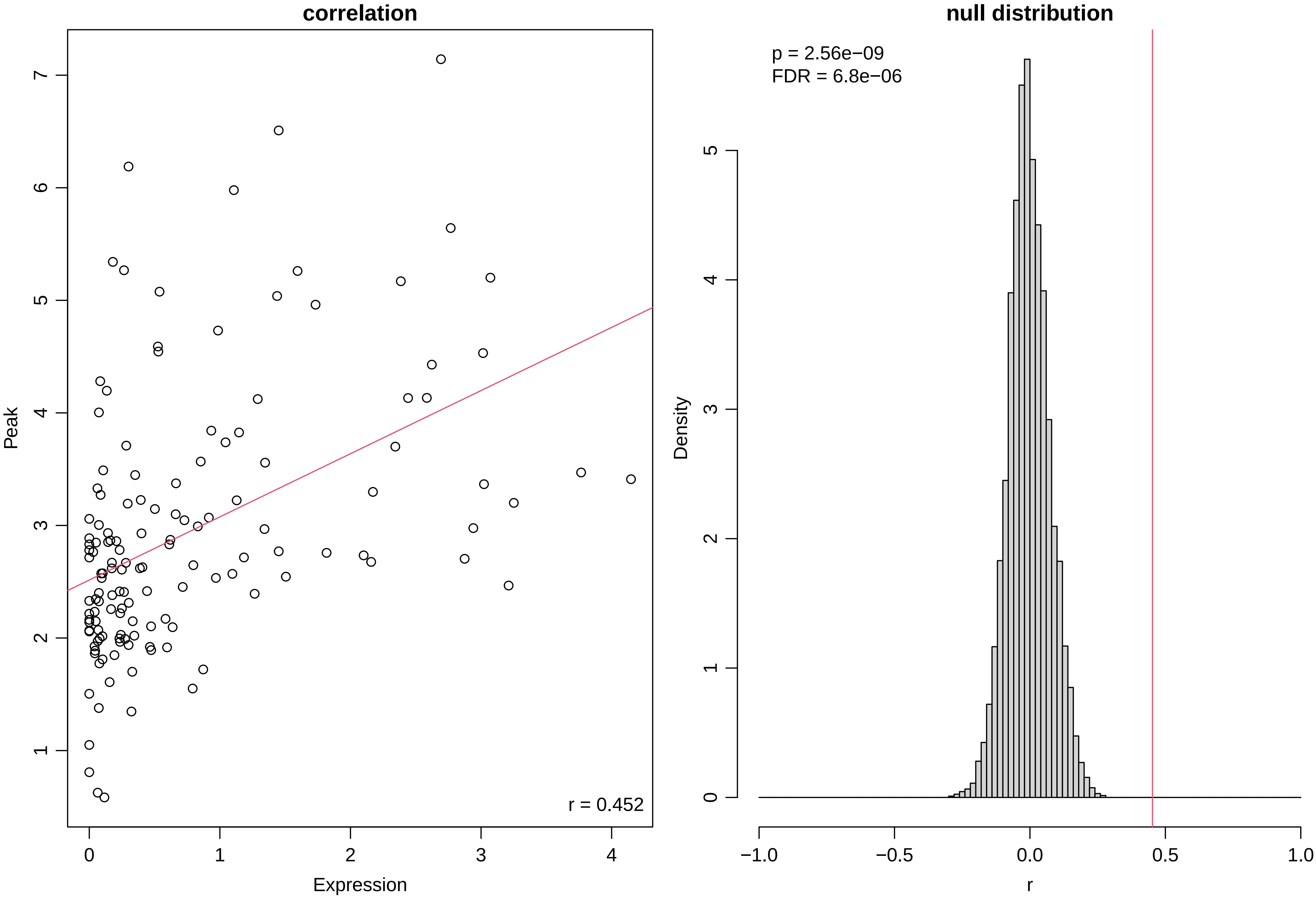

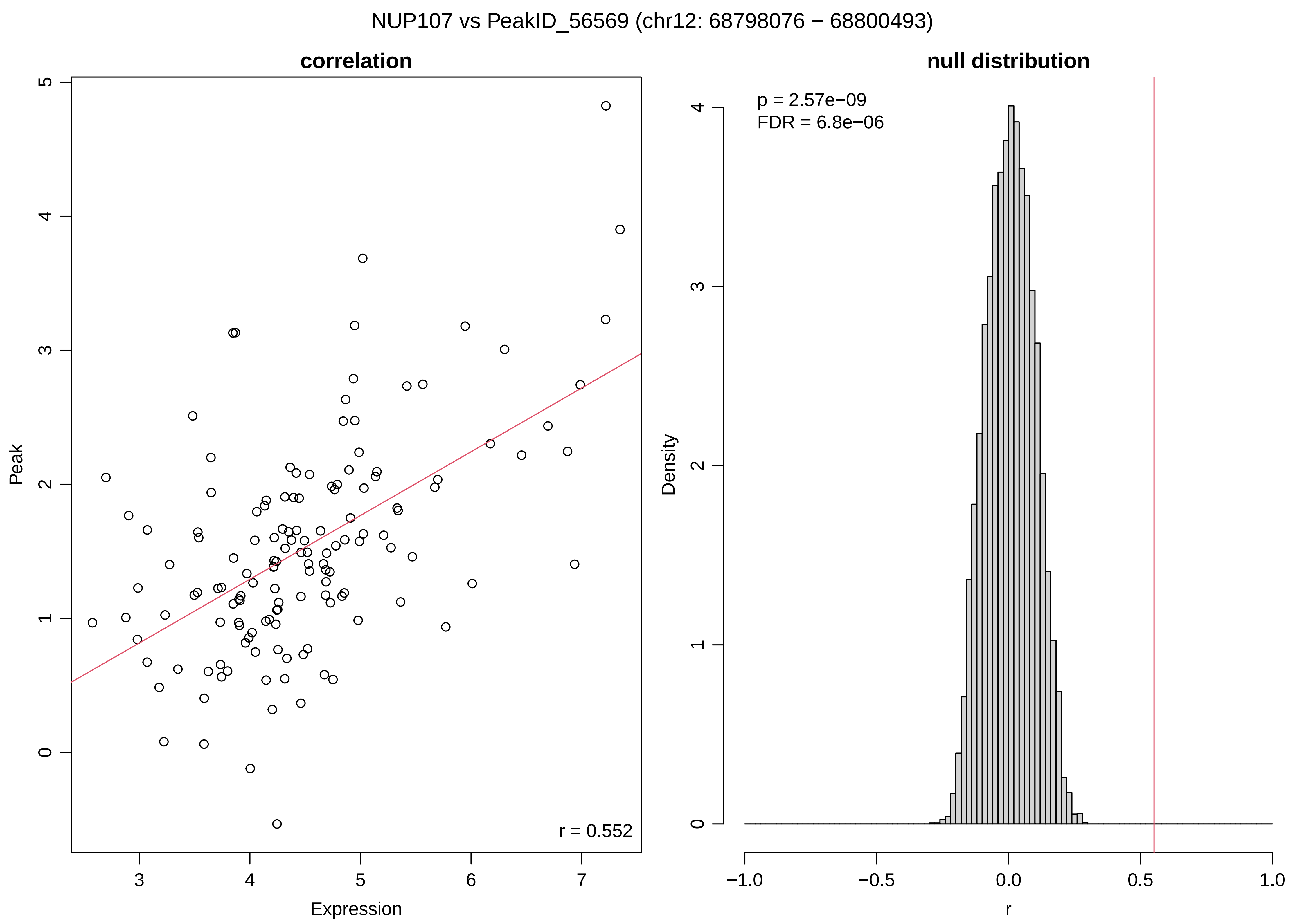

NUP107 vs PeakID_56569 (chr12: 68798076 − 68800493)
correlation
Peak
Expression
r = 0.552
null distribution
p = 2.57e−09
FDR = 6.8e−06
Density
r

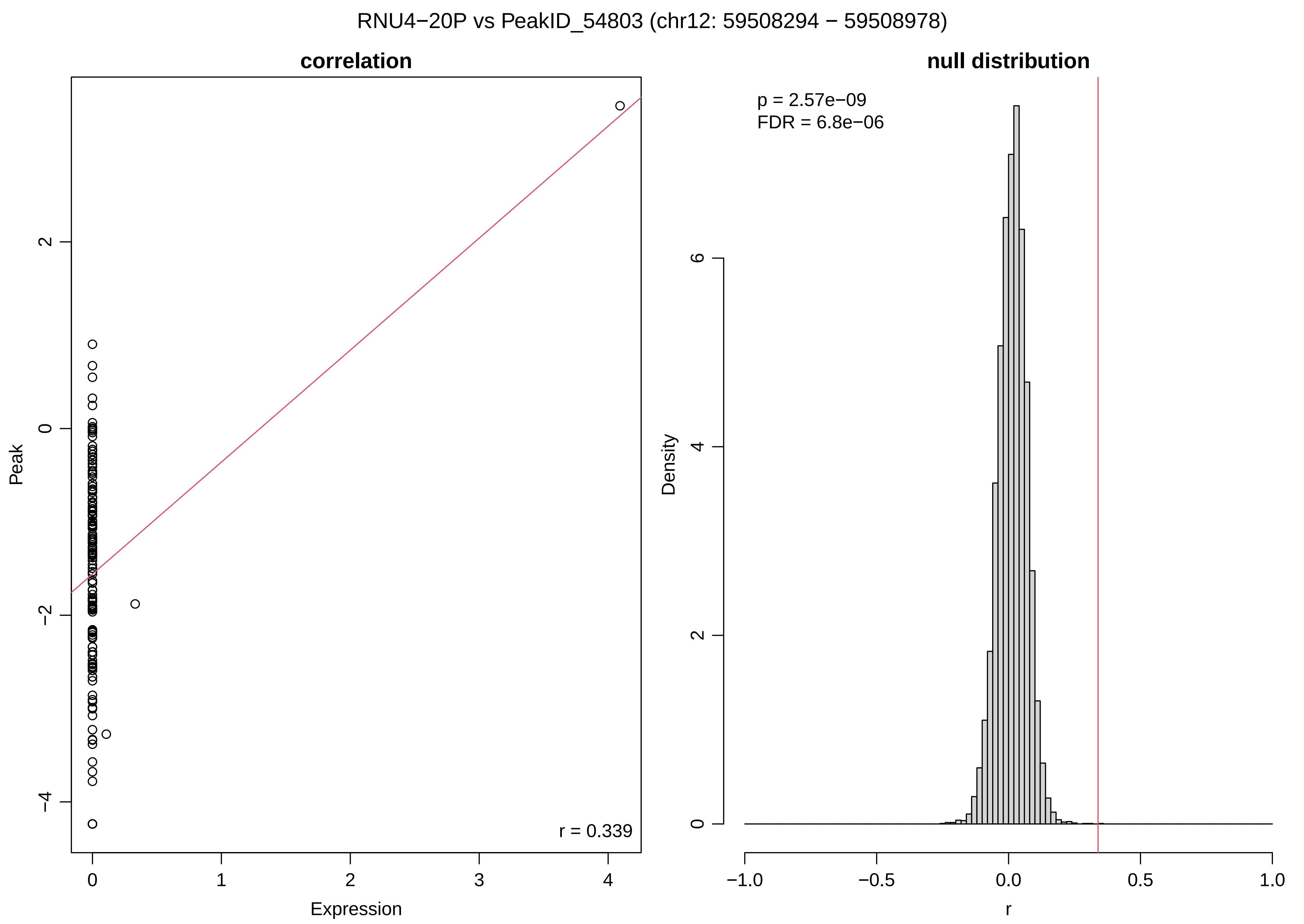

RNU4−20P vs PeakID_54803 (chr12: 59508294 − 59508978)
correlation
Peak
Expression
r = 0.339
null distribution
p = 2.57e−09
FDR = 6.8e−06
Density
r

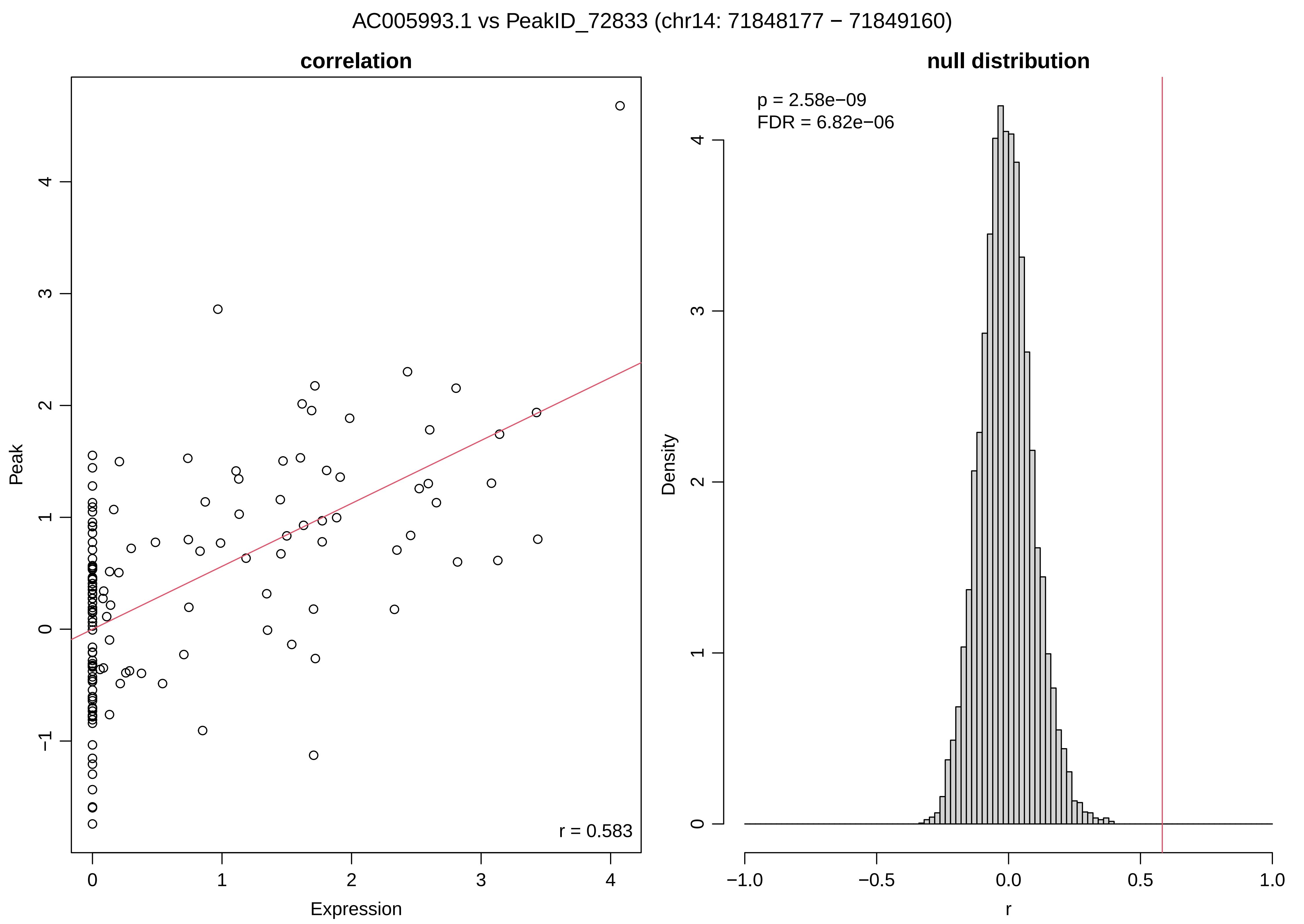
AC005993.1 vs PeakID_72833 (chr14: 71848177 − 71849160)
correlation
Peak
Expression
r = 0.583
null distribution
p = 2.58e−09
FDR = 6.82e−06
Density
r

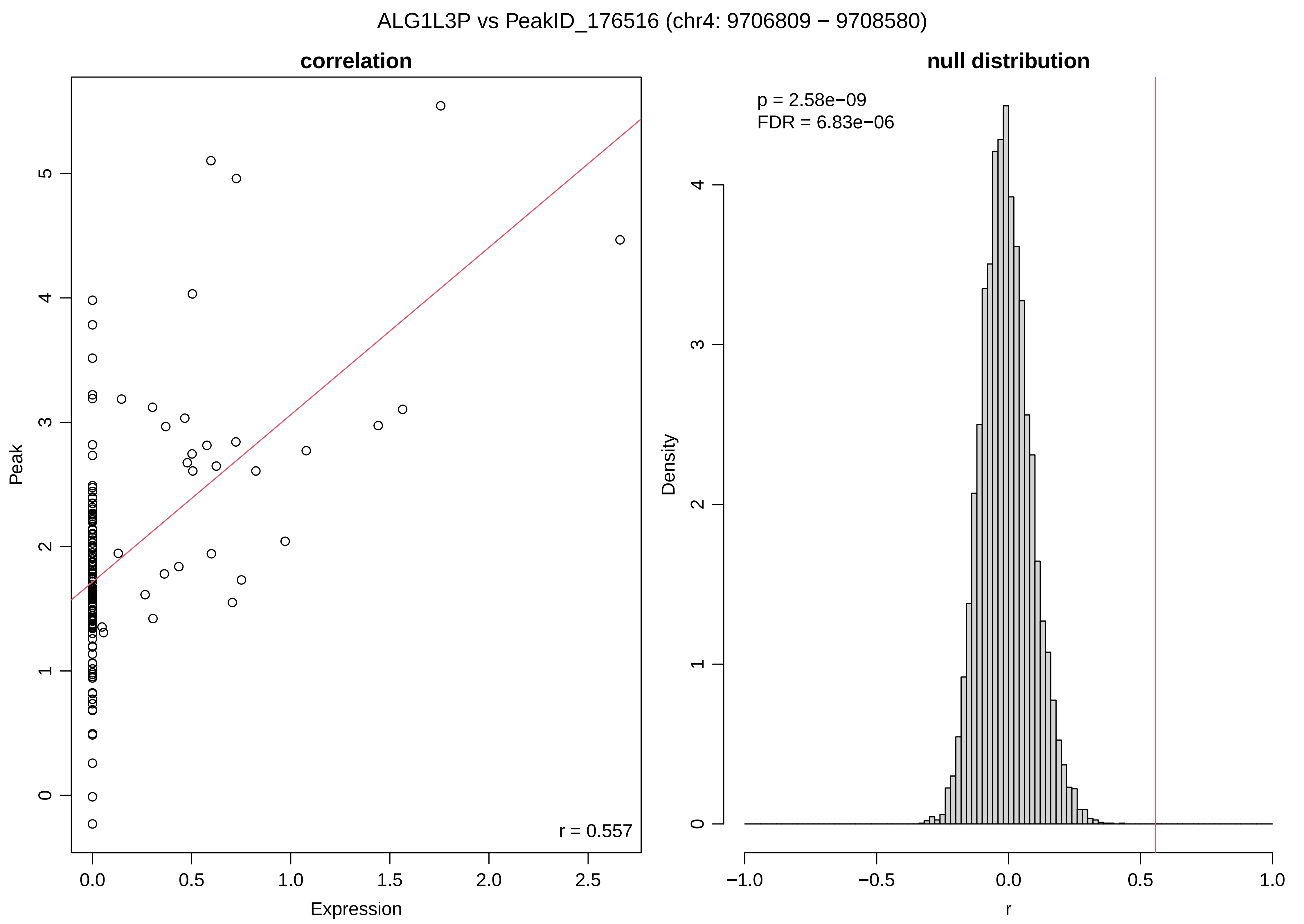
ALG1L3P vs PeakID_176516 (chr4: 9706809 − 9708580)
correlation
Peak
Expression
r = 0.557
null distribution
p = 2.58e−09
FDR = 6.83e−06
Density
r

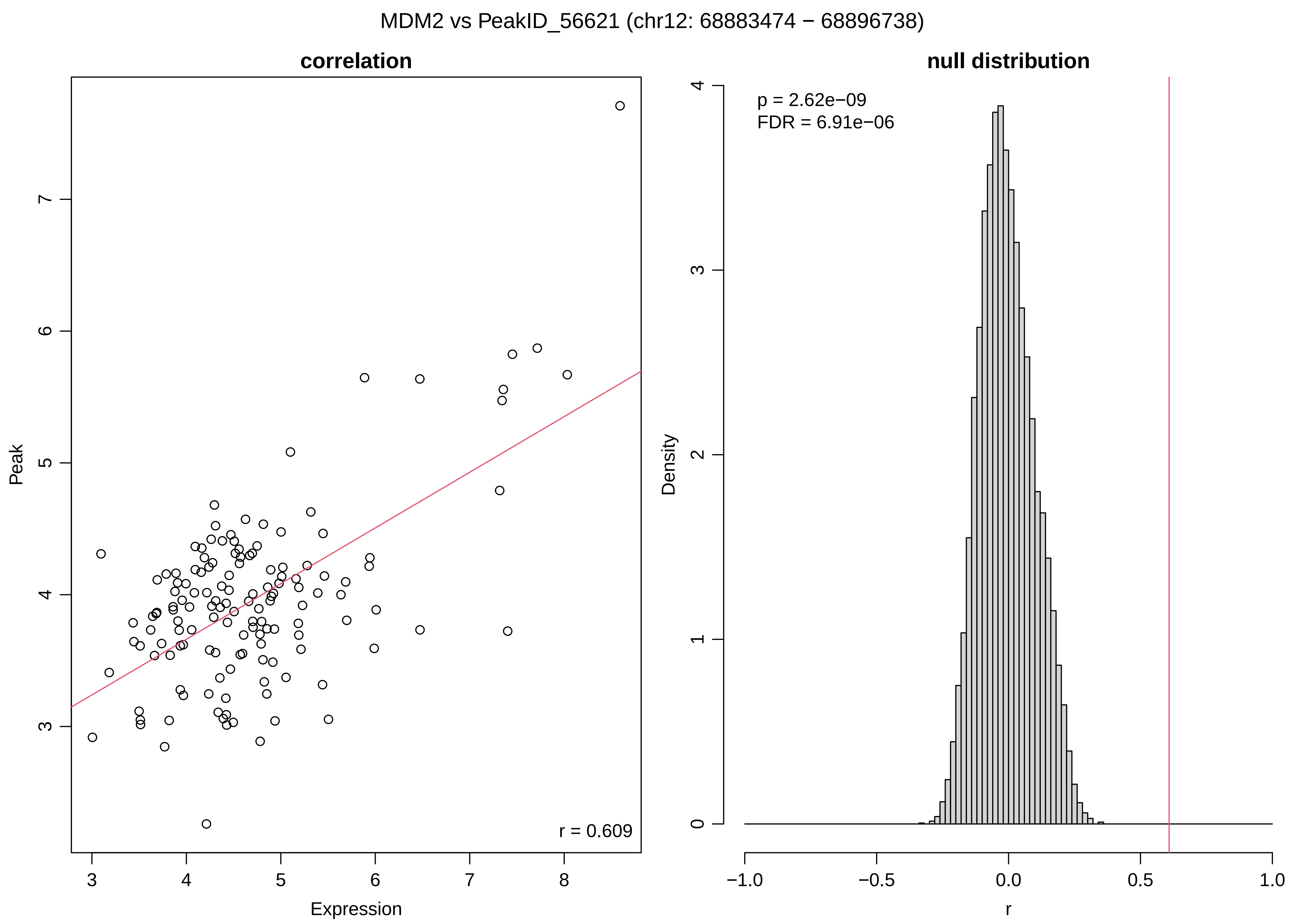

MDM2 vs PeakID_56621 (chr12: 68883474 − 68896738)
correlation
Peak
Expression
r = 0.609
null distribution
Density
r
p = 2.62e−09
FDR = 6.91e−06

AC010148.1 vs PeakID_141186 (chr2: 234887934 − 234888868)

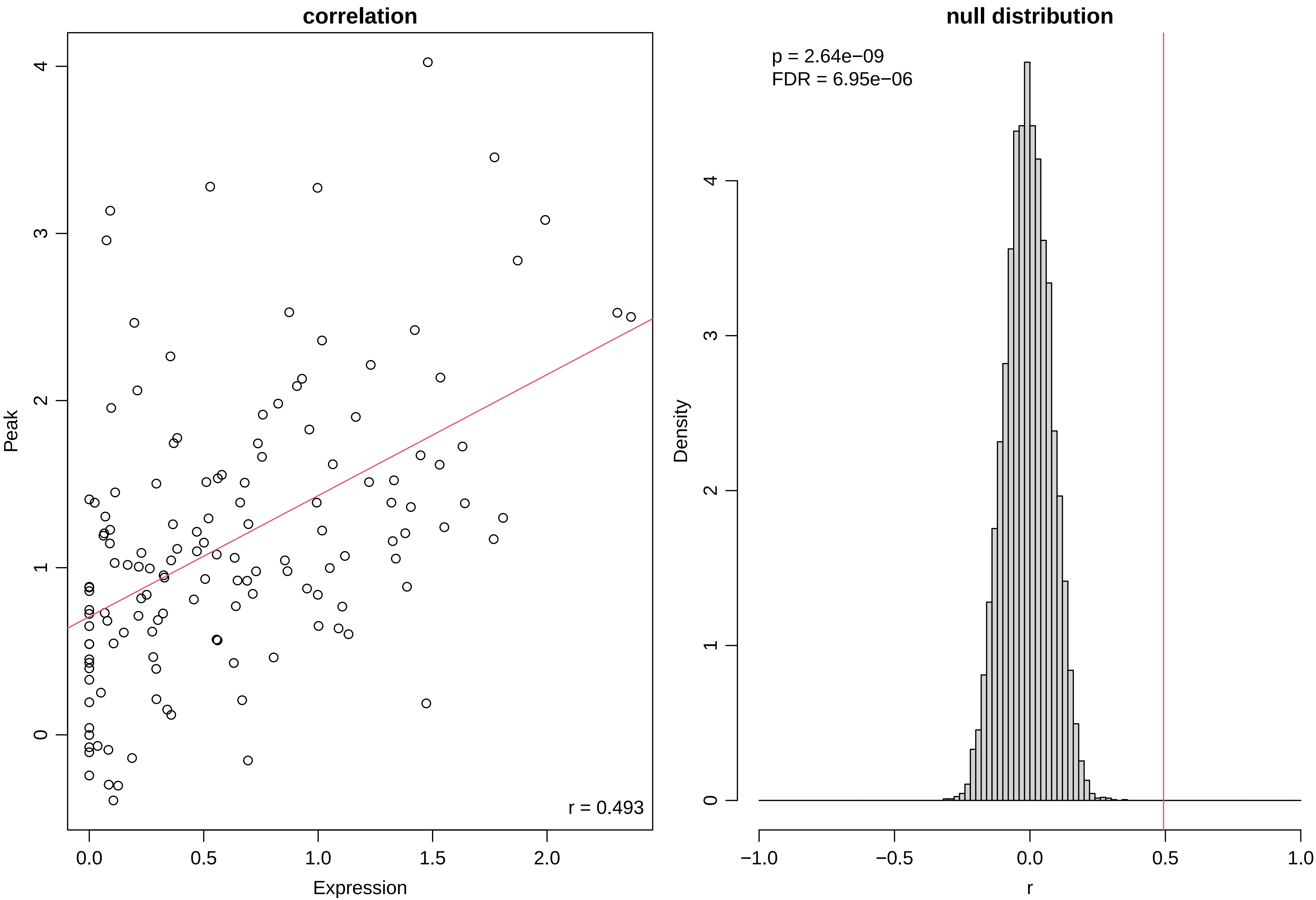

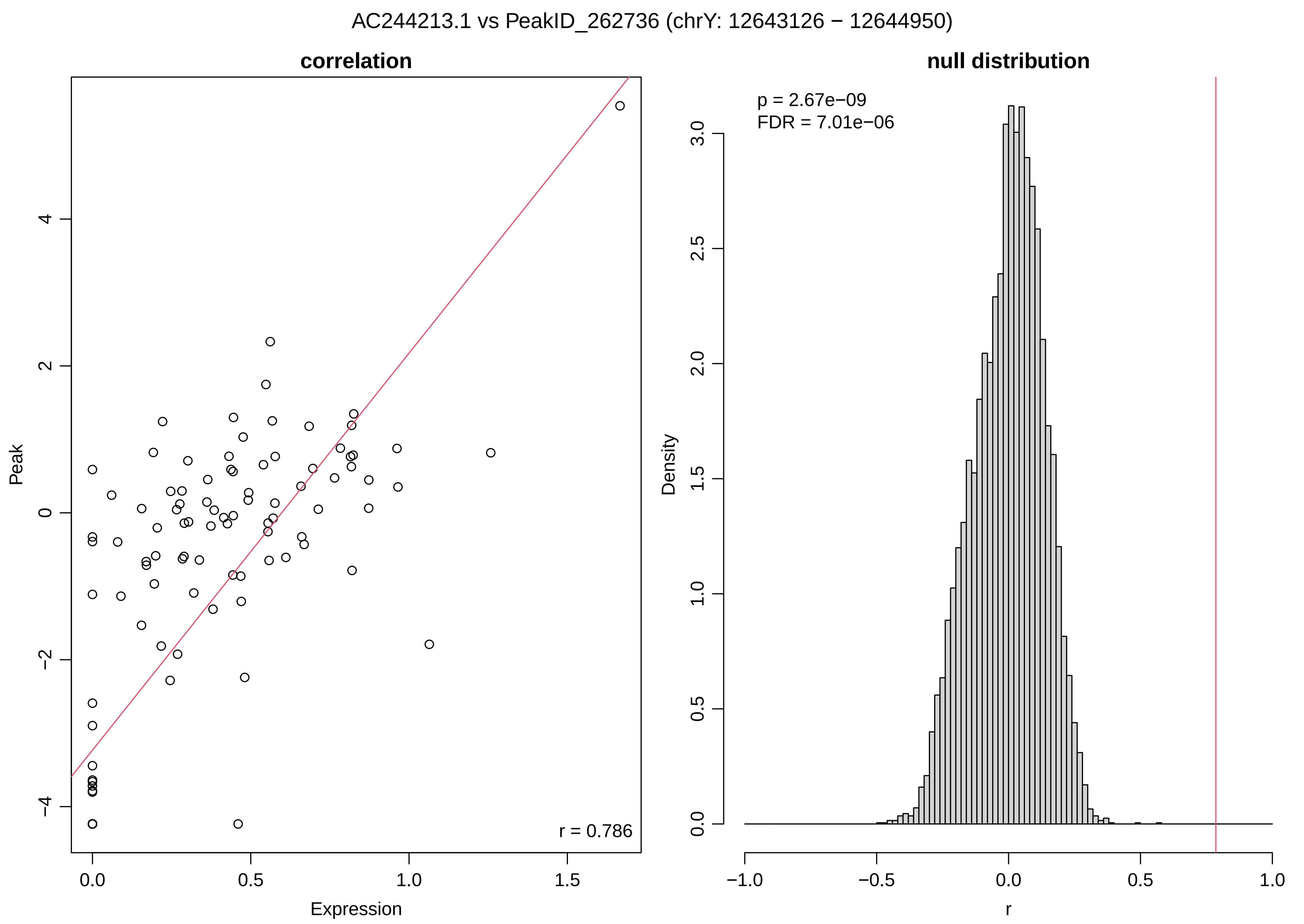
AC244213.1 vs PeakID_262736 (chrY: 12643126 − 12644950)
correlation
Peak
Expression
r = 0.786
−4
−2
0
2
4
0.0
0.5
1.0
1.5
null distribution
p = 2.67e−09
FDR = 7.01e−06
Density
r
0.0
0.5
1.0
1.5
2.0
2.5
3.0
−1.0
−0.5
0.0
0.5
1.0

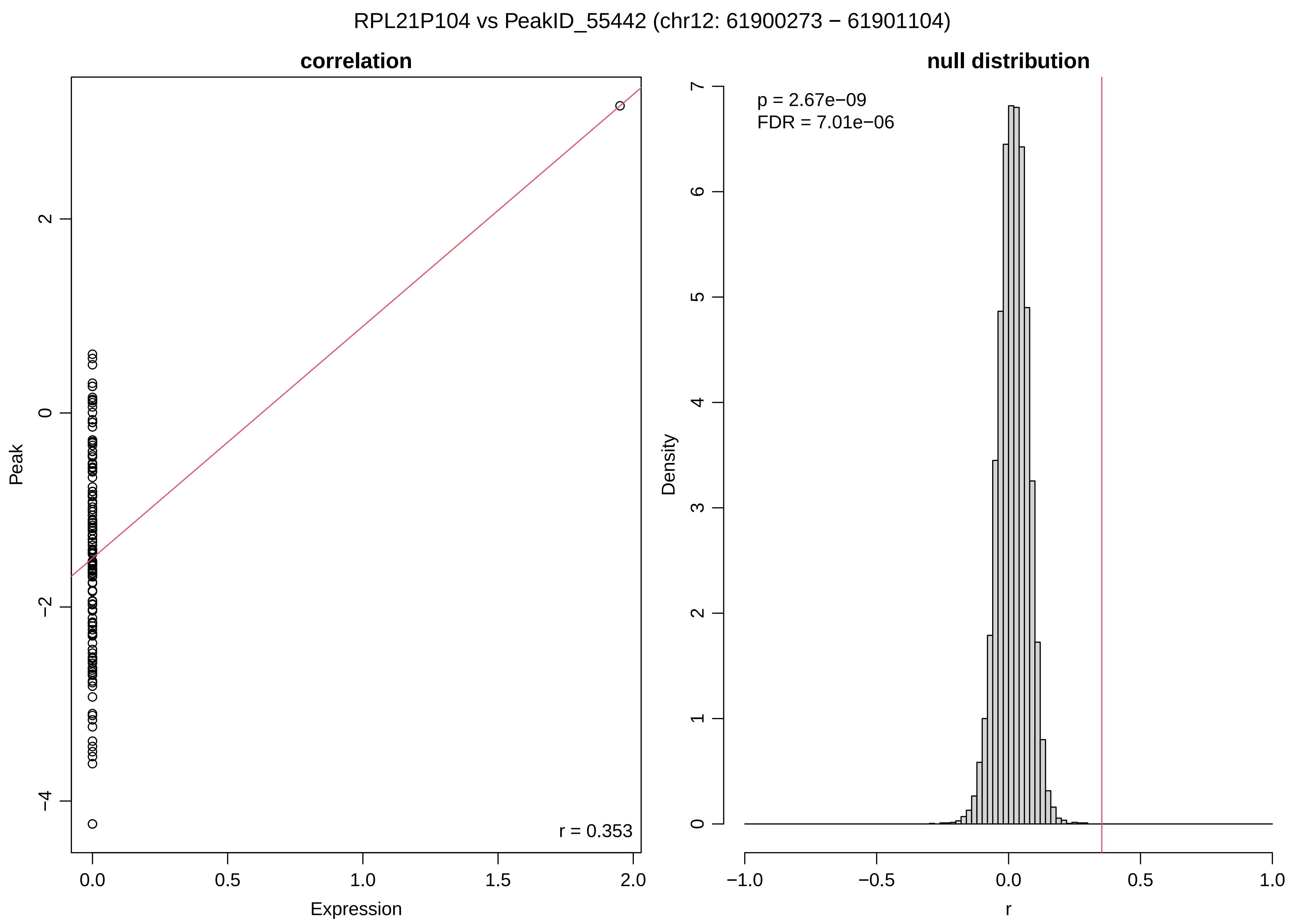
RPL21P104 vs PeakID_55442 (chr12: 61900273 − 61901104)
correlation
Peak
Expression
r = 0.353
null distribution
Density
r
p = 2.67e−09
FDR = 7.01e−06
2
0
−2
−4
0.0
0.5
1.0
1.5
2.0
0
1
2
3
4
5
6
7
−1.0
−0.5
0.0
0.5
1.0

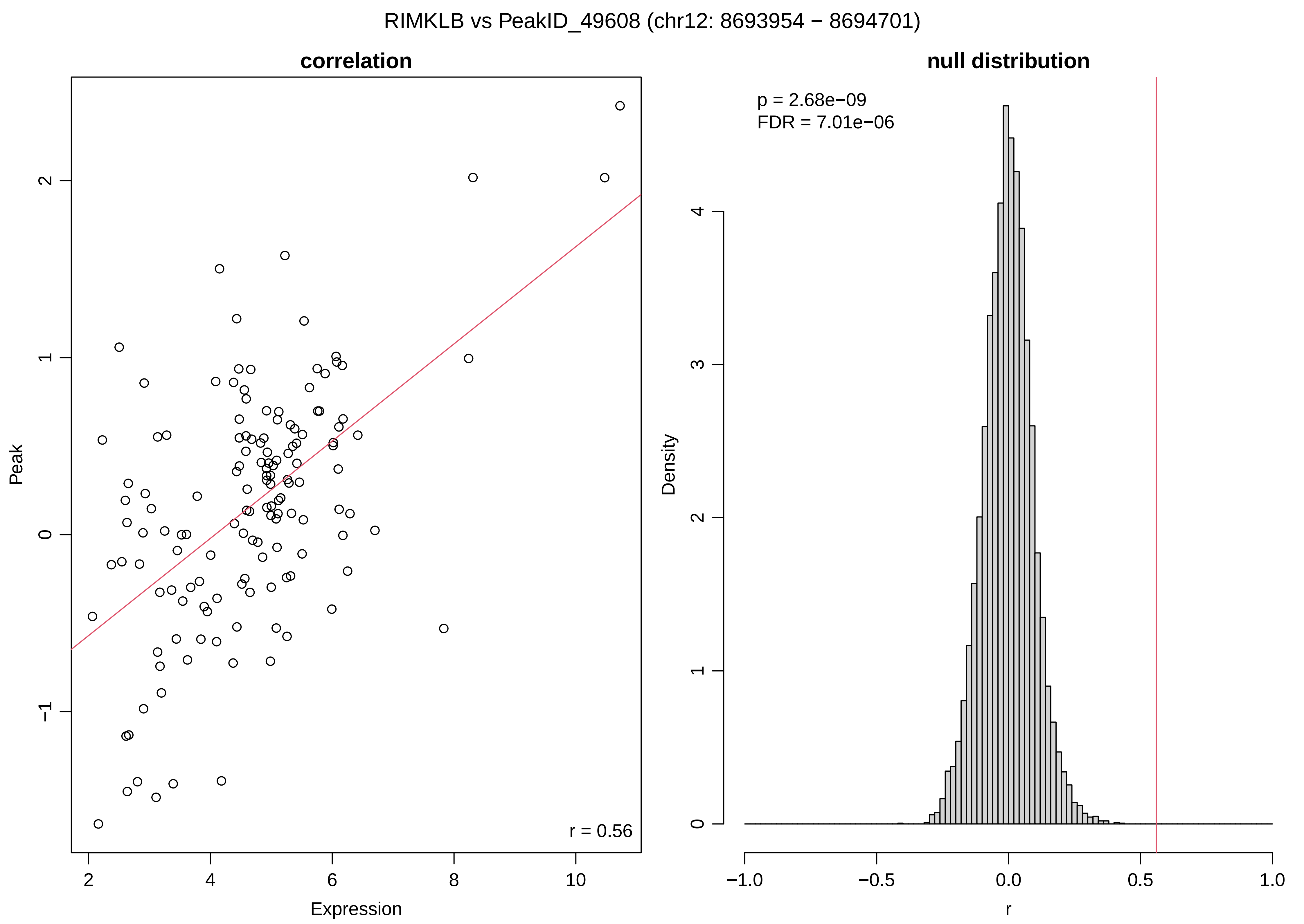
RIMKLB vs PeakID_49608 (chr12: 8693954 − 8694701)
correlation
Peak
Expression
r = 0.56
null distribution
p = 2.68e−09
FDR = 7.01e−06
Density
r

SLC7A11 vs PeakID_183322 (chr4: 138234518 − 138239966)

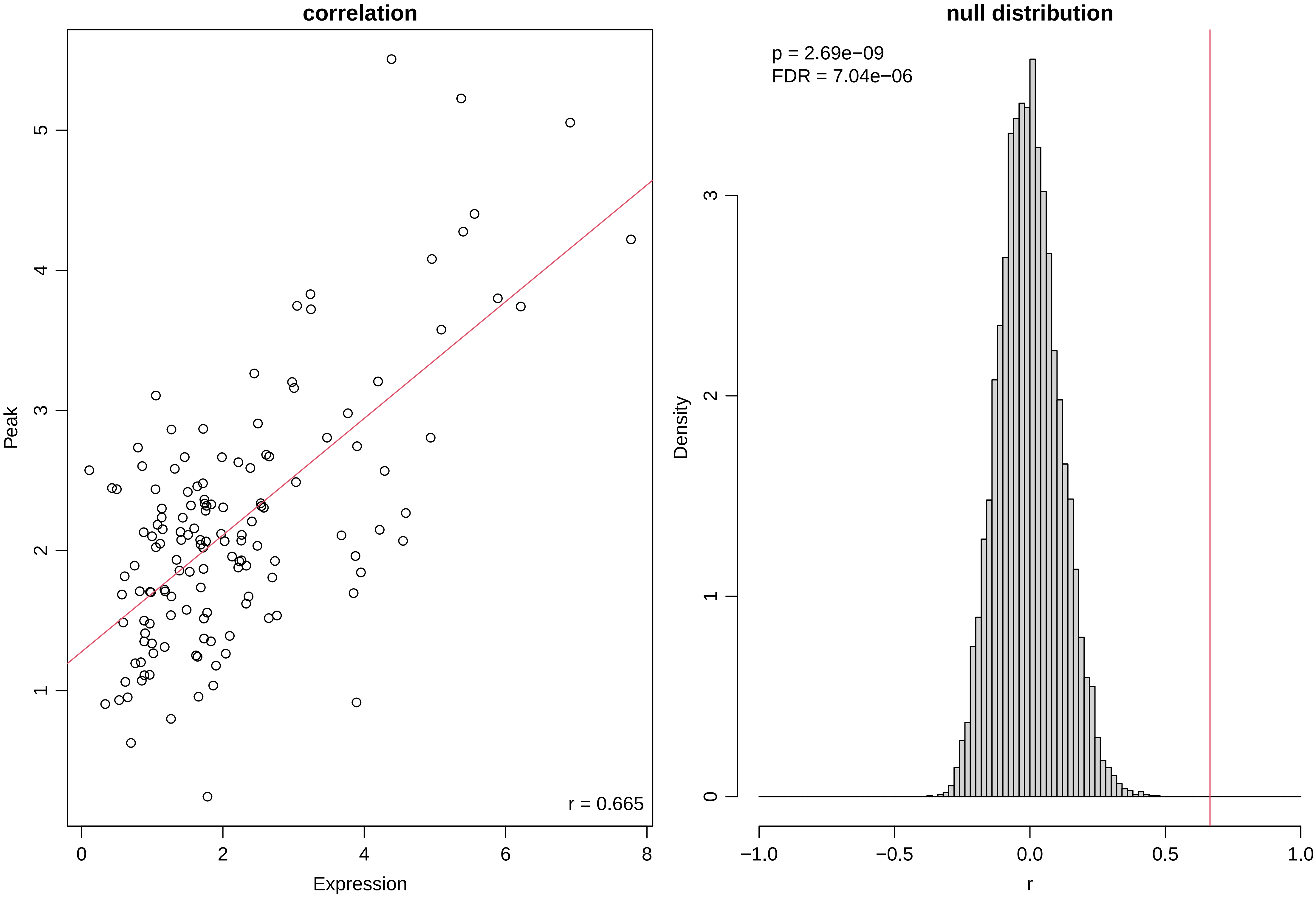

AC138649.4 vs PeakID_76722 (chr15: 22713992 − 22716520)

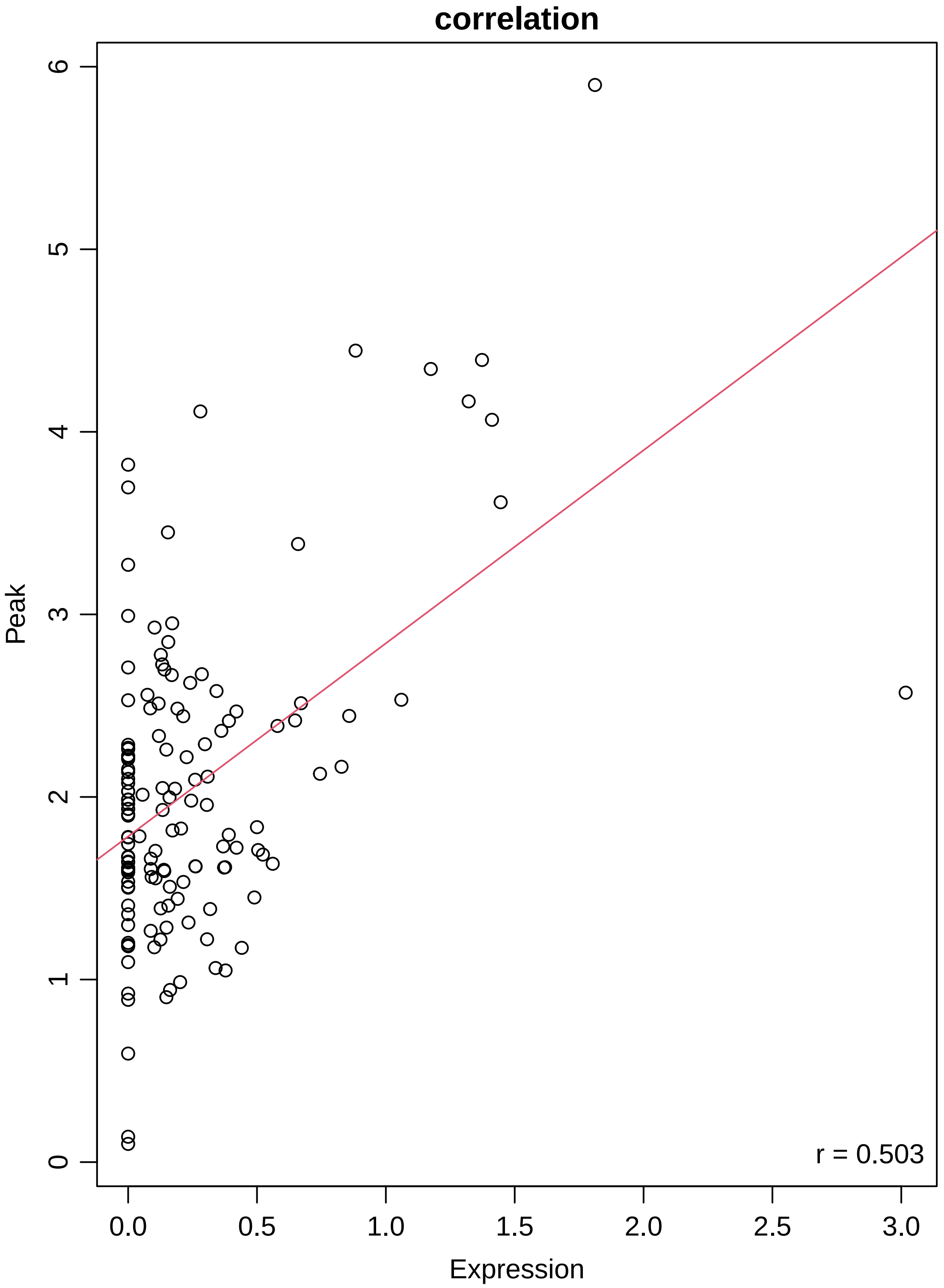

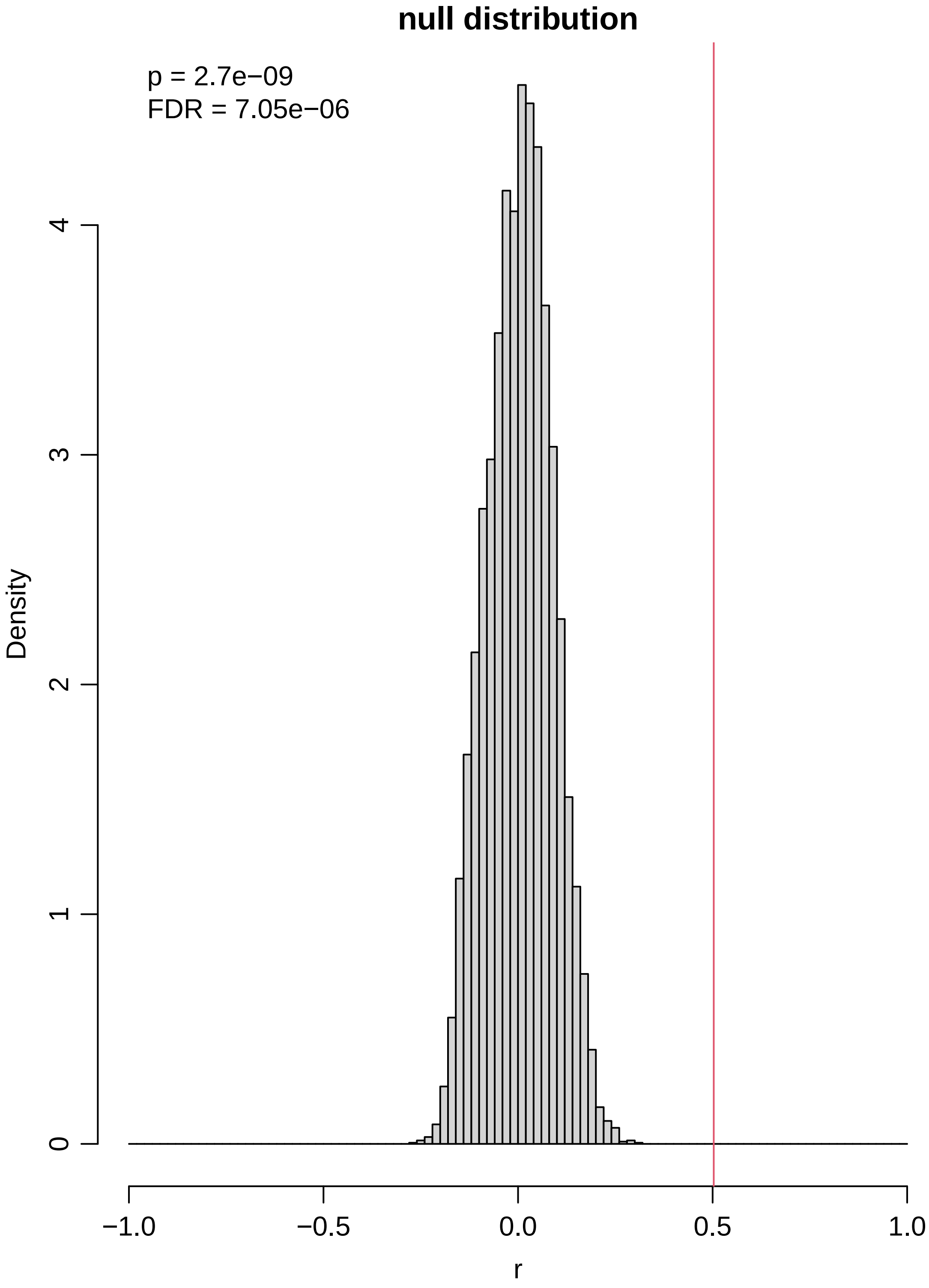

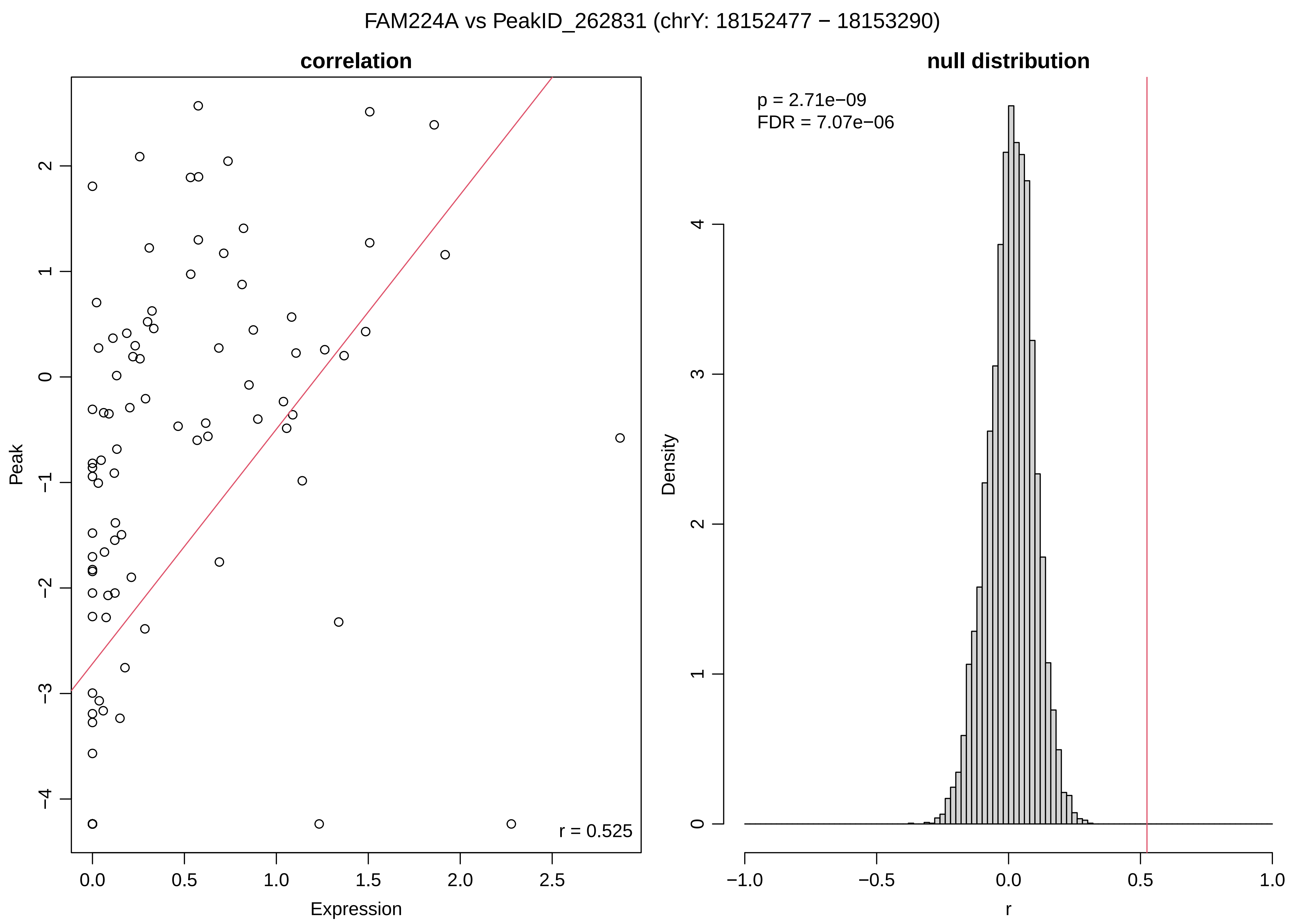

FAM224A vs PeakID_262831 (chrY: 18152477 − 18153290)
correlation
Peak
Expression
r = 0.525
null distribution
p = 2.71e−09
FDR = 7.07e−06
Density
r

RASAL1 vs PeakID_60445 (chr12: 113133644 − 113139934)

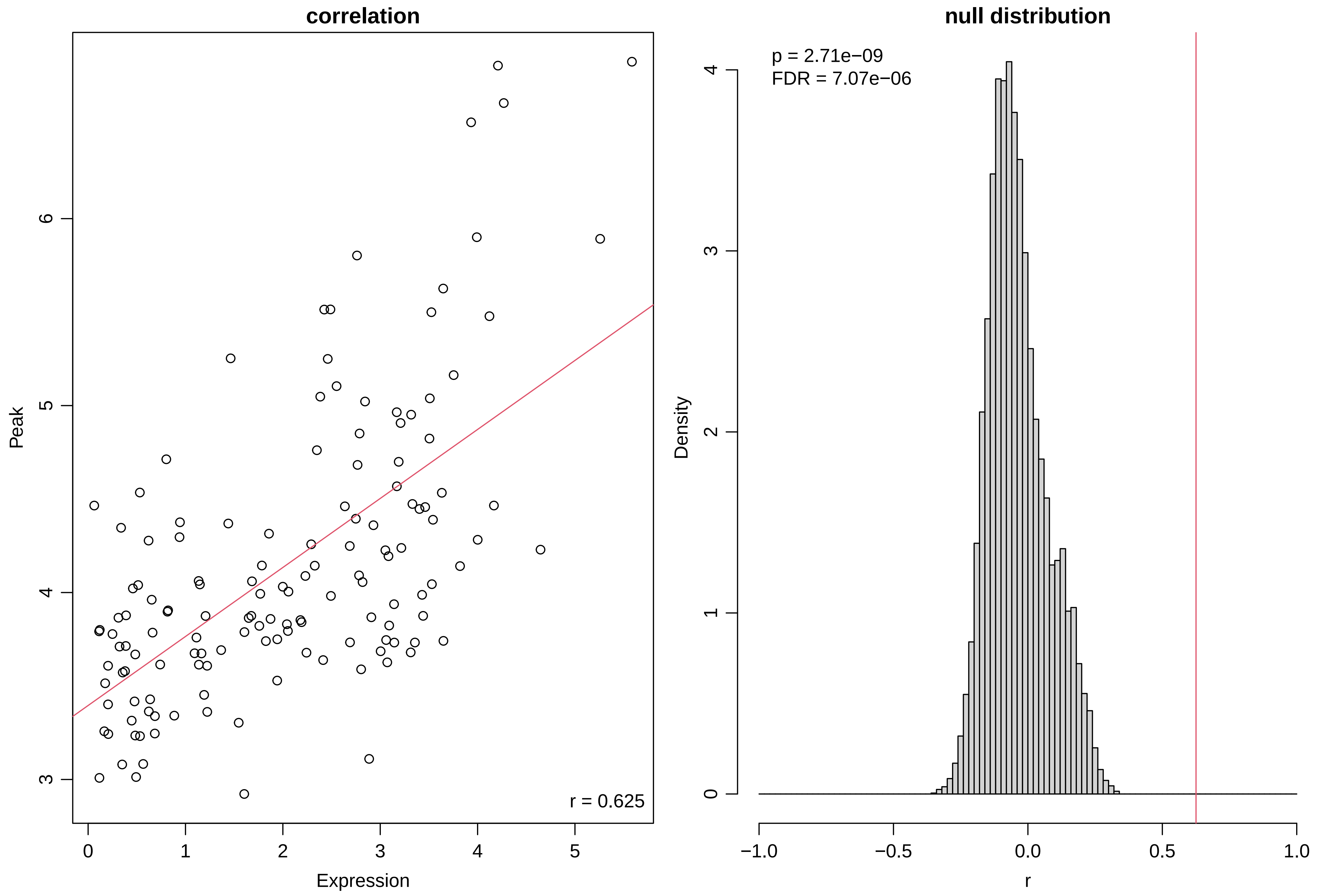

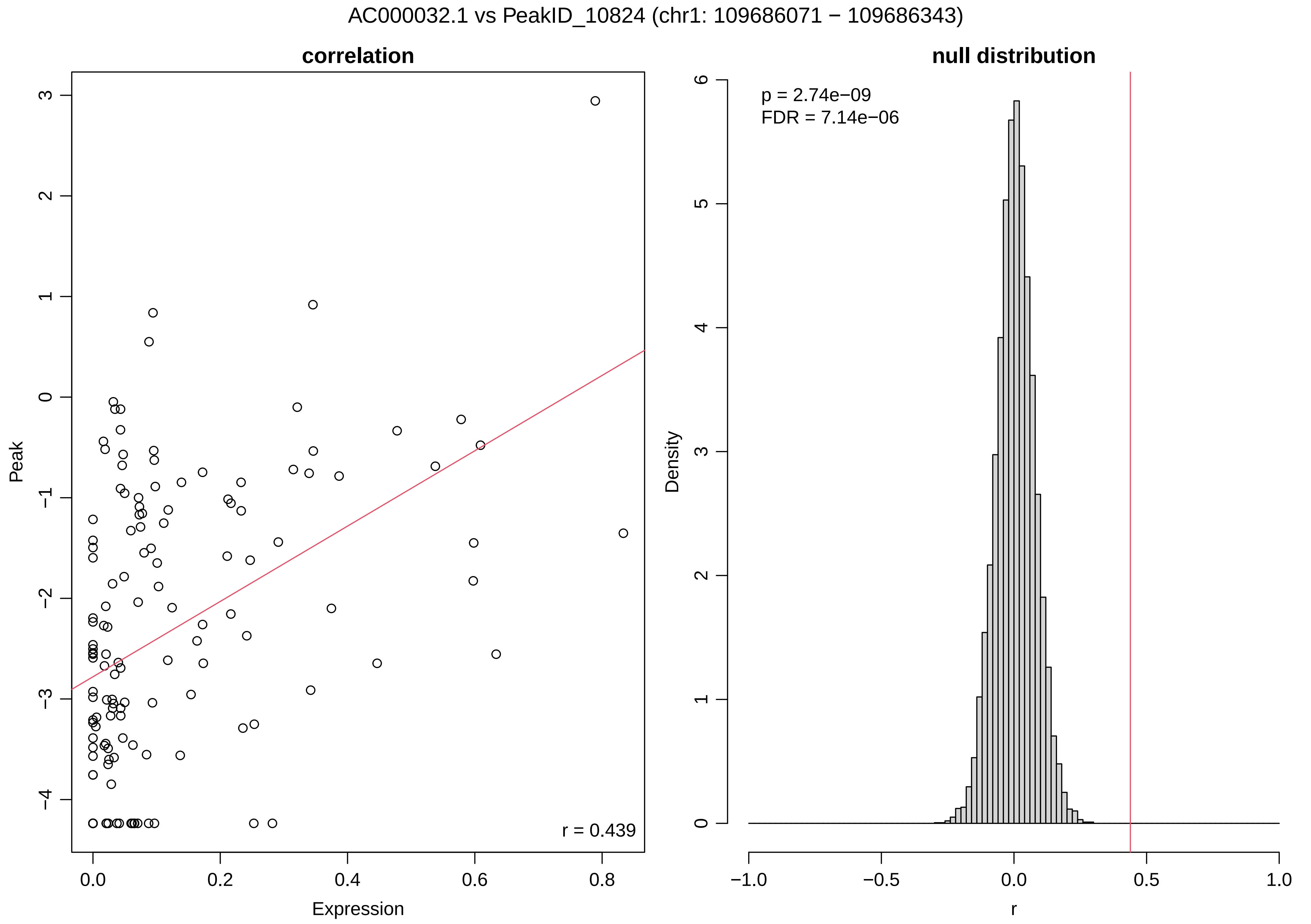
AC000032.1 vs PeakID_10824 (chr1: 109686071 − 109686343)
correlation
Peak
Expression
r = 0.439
null distribution
p = 2.74e−09
FDR = 7.14e−06
Density
r

USP9Y vs PeakID_262735 (chrY: 12641507 − 12642886)

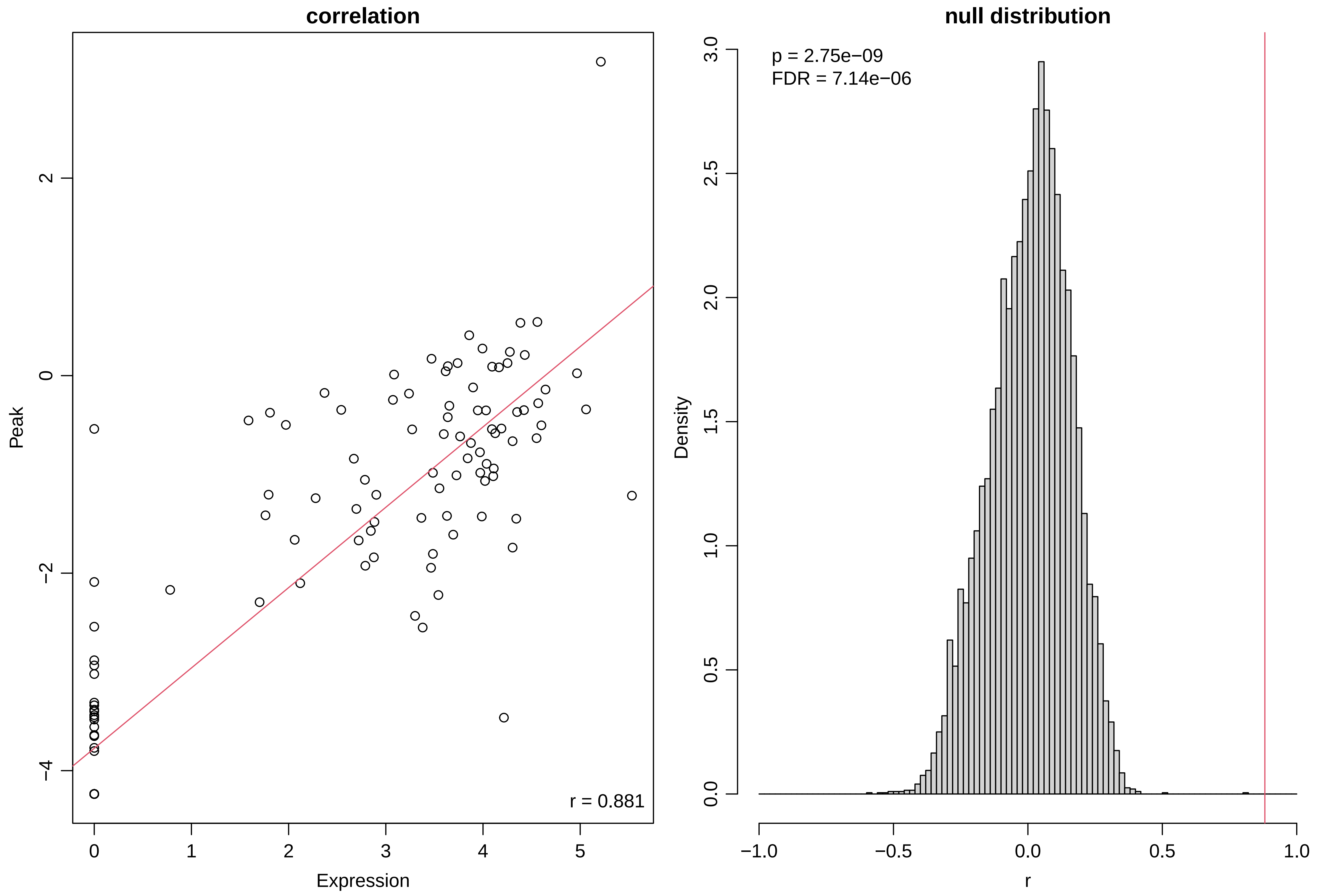

TMPRSS3 vs PeakID_153088 (chr21: 42394388 − 42397251)

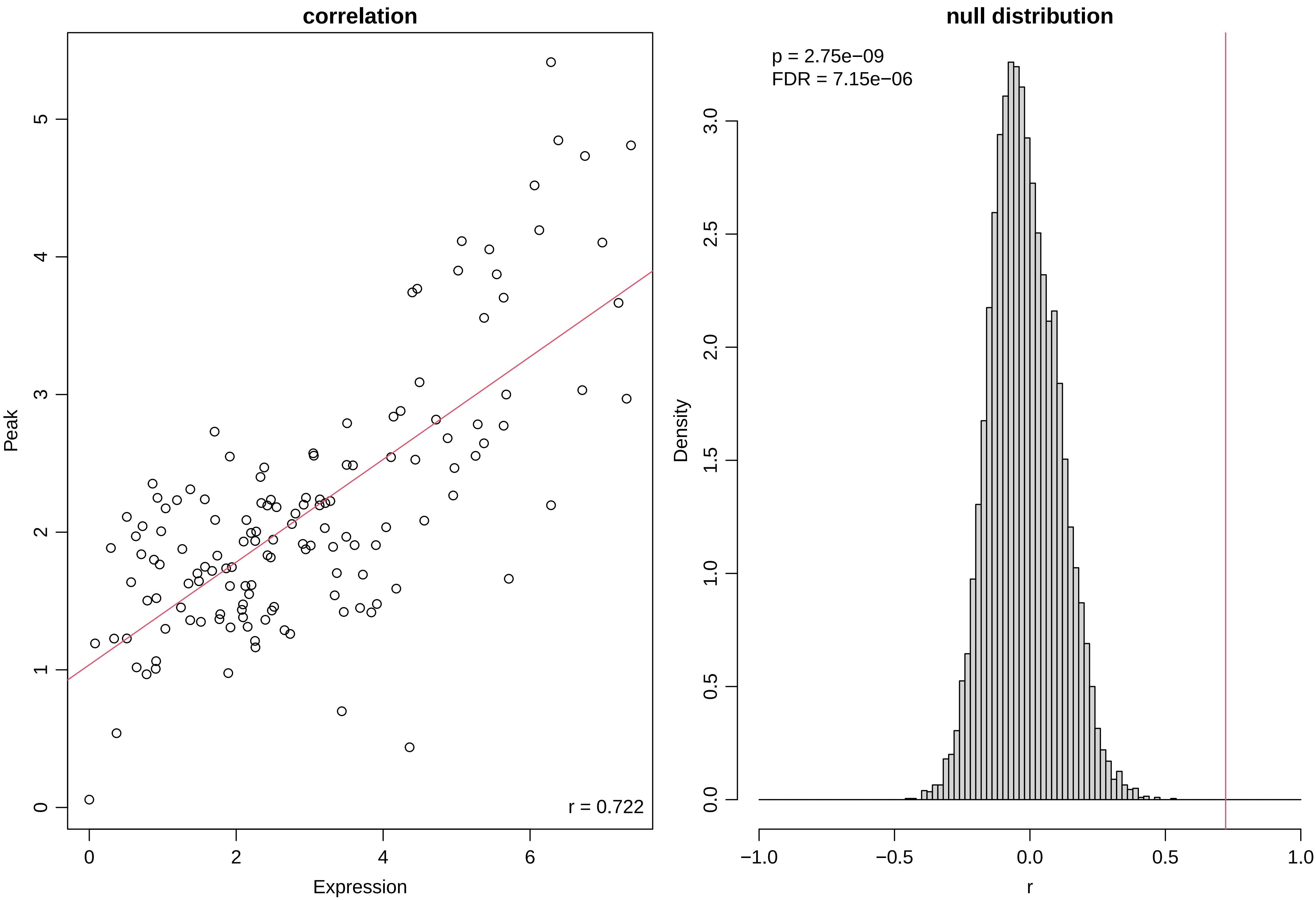

CYP2T3P vs PeakID_118165 (chr19: 41134383 − 41137473)

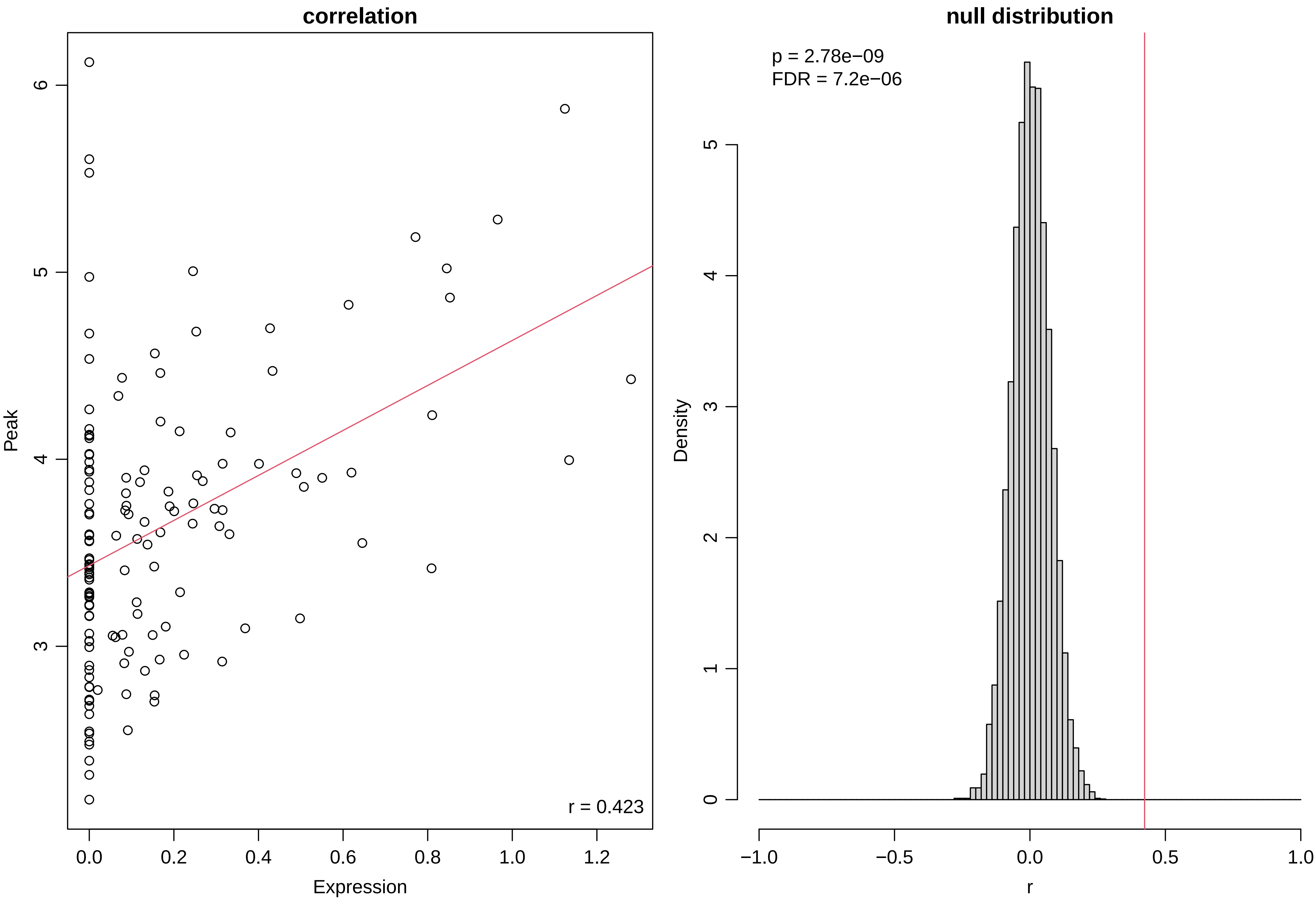

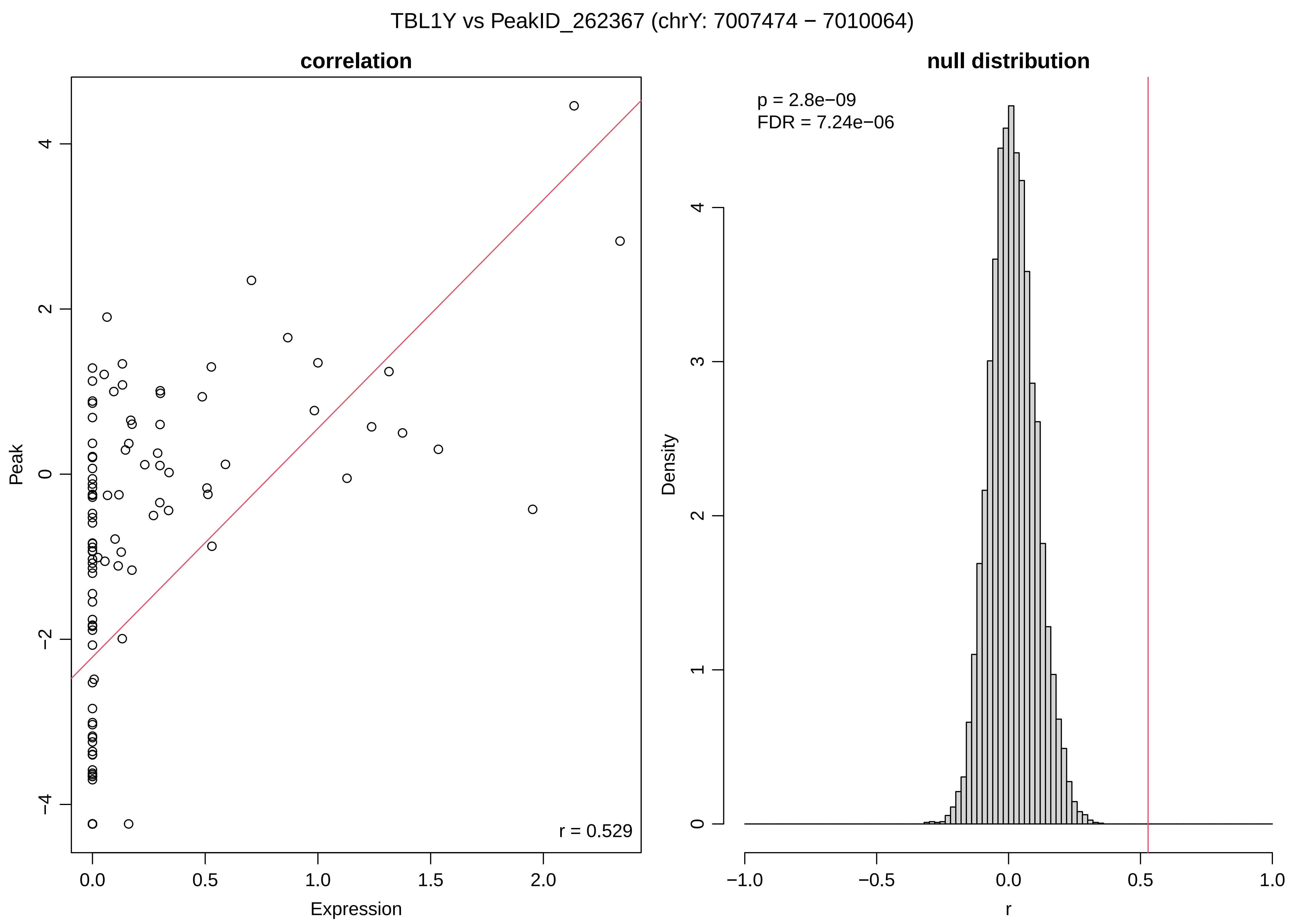
TBL1Y vs PeakID_262367 (chrY: 7007474 − 7010064)
correlation
Peak
Expression
r = 0.529
null distribution
p = 2.8e−09
FDR = 7.24e−06
Density
r

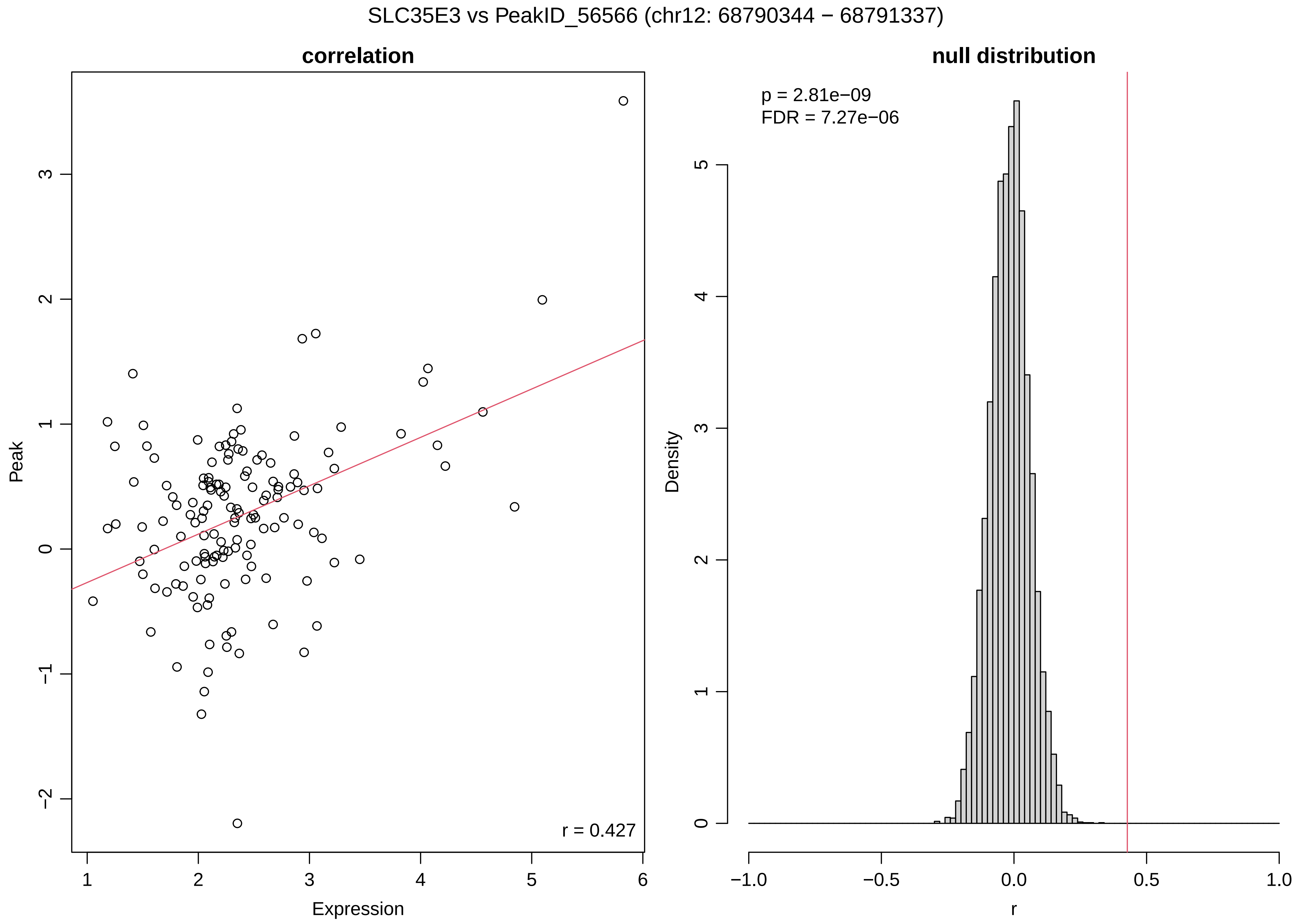
SLC35E3 vs PeakID_56566 (chr12: 68790344 − 68791337)
correlation
Peak
Expression
r = 0.427
1
2
3
4
5
6
−2
−1
0
1
2
3
null distribution
p = 2.81e−09
FDR = 7.27e−06
Density
r
−1.0
−0.5
0.0
0.5
1.0
0
1
2
3
4
5

HOXC5 vs PeakID_53853 (chr12: 54033832 − 54035170)

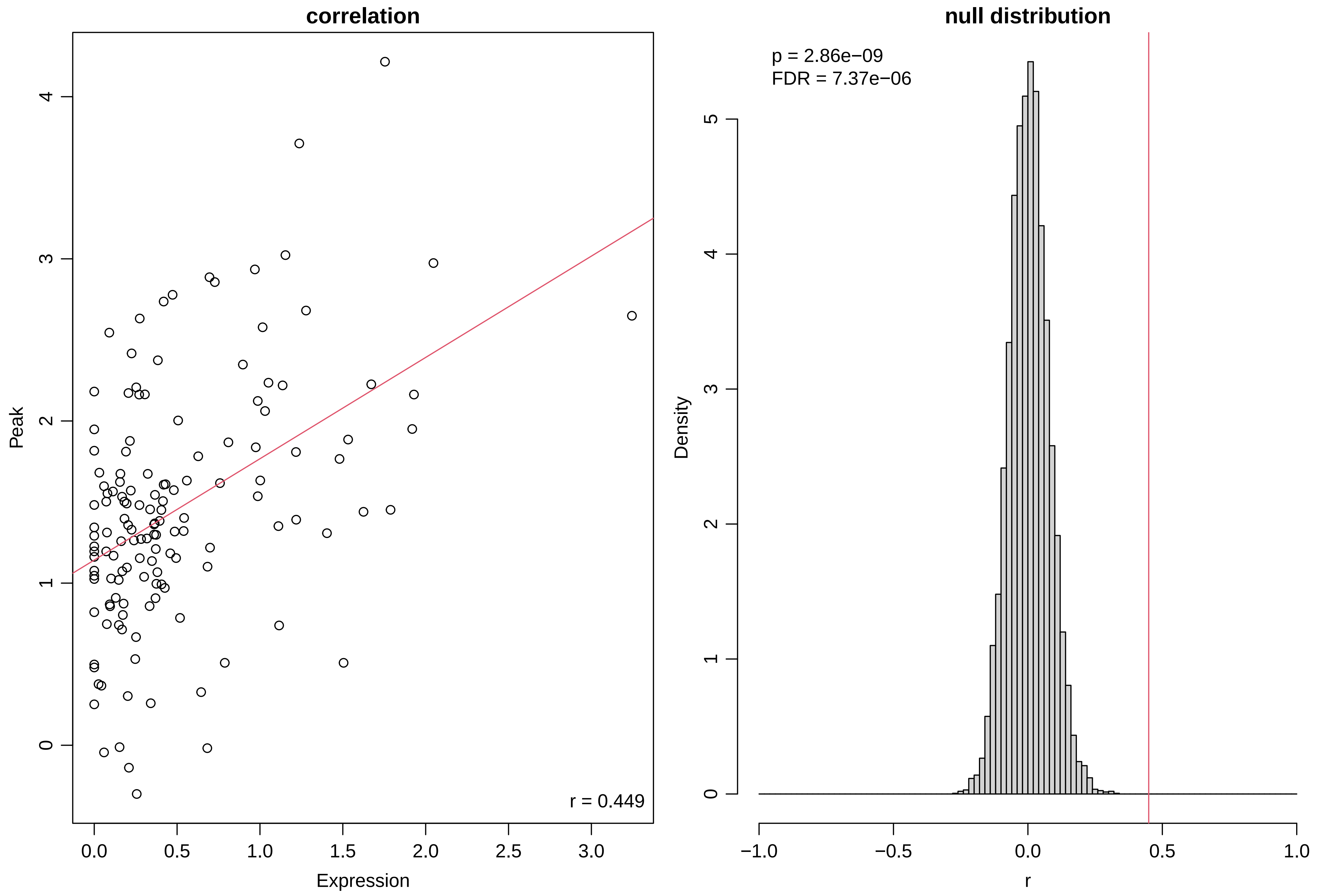

PRKY vs PeakID_262390 (chrY: 7287450 − 7291766)

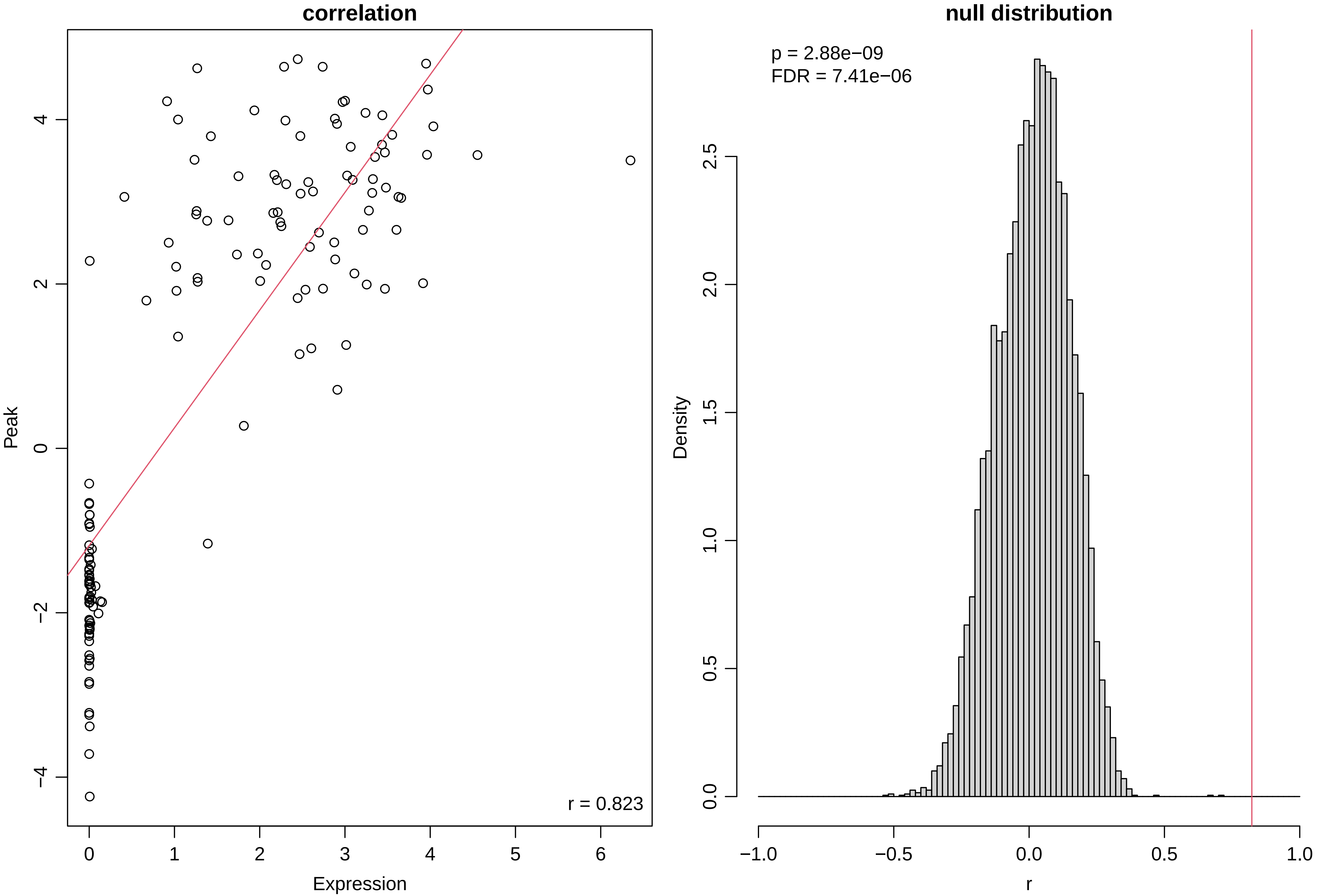

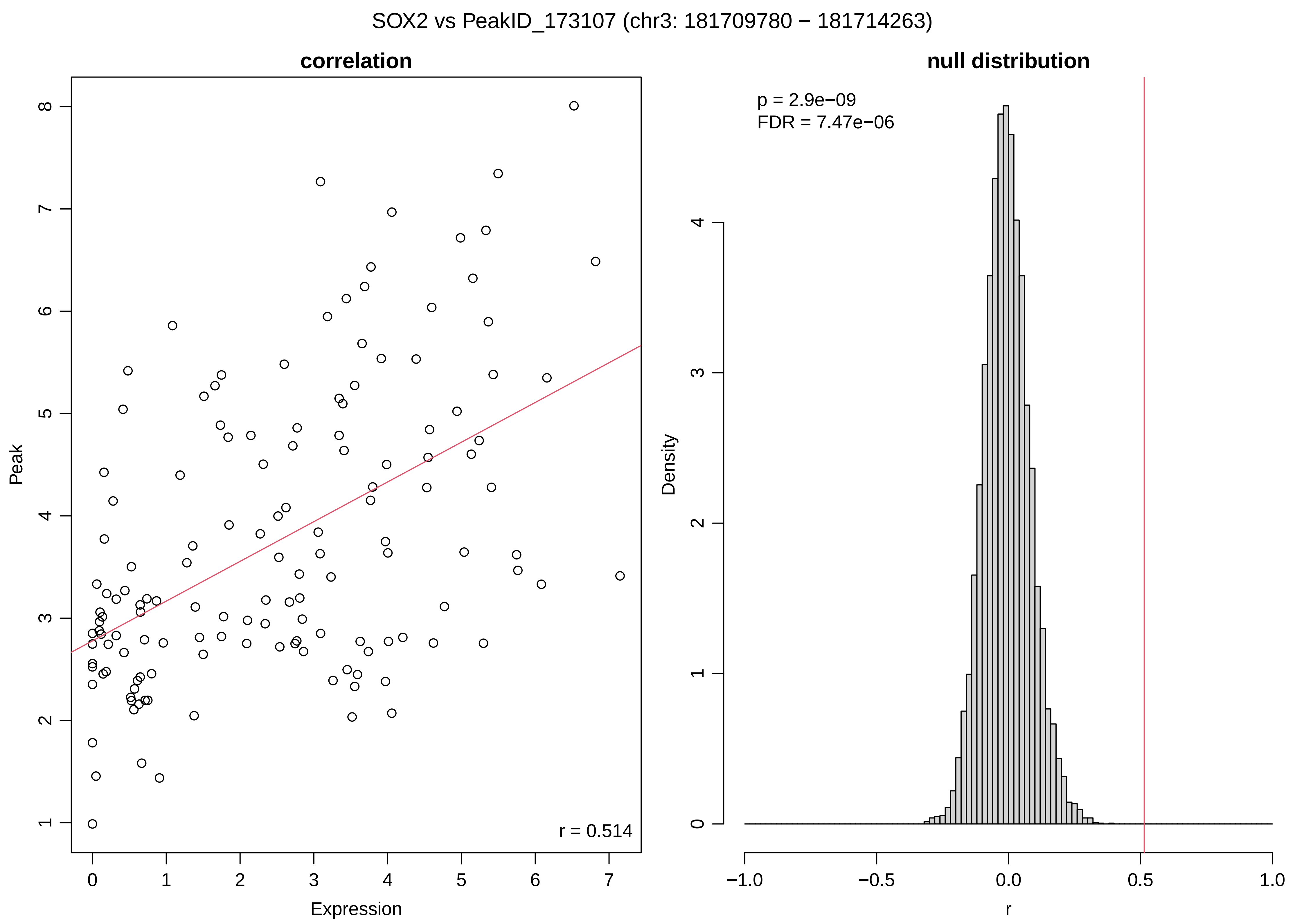
SOX2 vs PeakID_173107 (chr3: 181709780 − 181714263)
correlation
Peak
Expression
r = 0.514
null distribution
p = 2.9e−09
FDR = 7.47e−06
Density
r

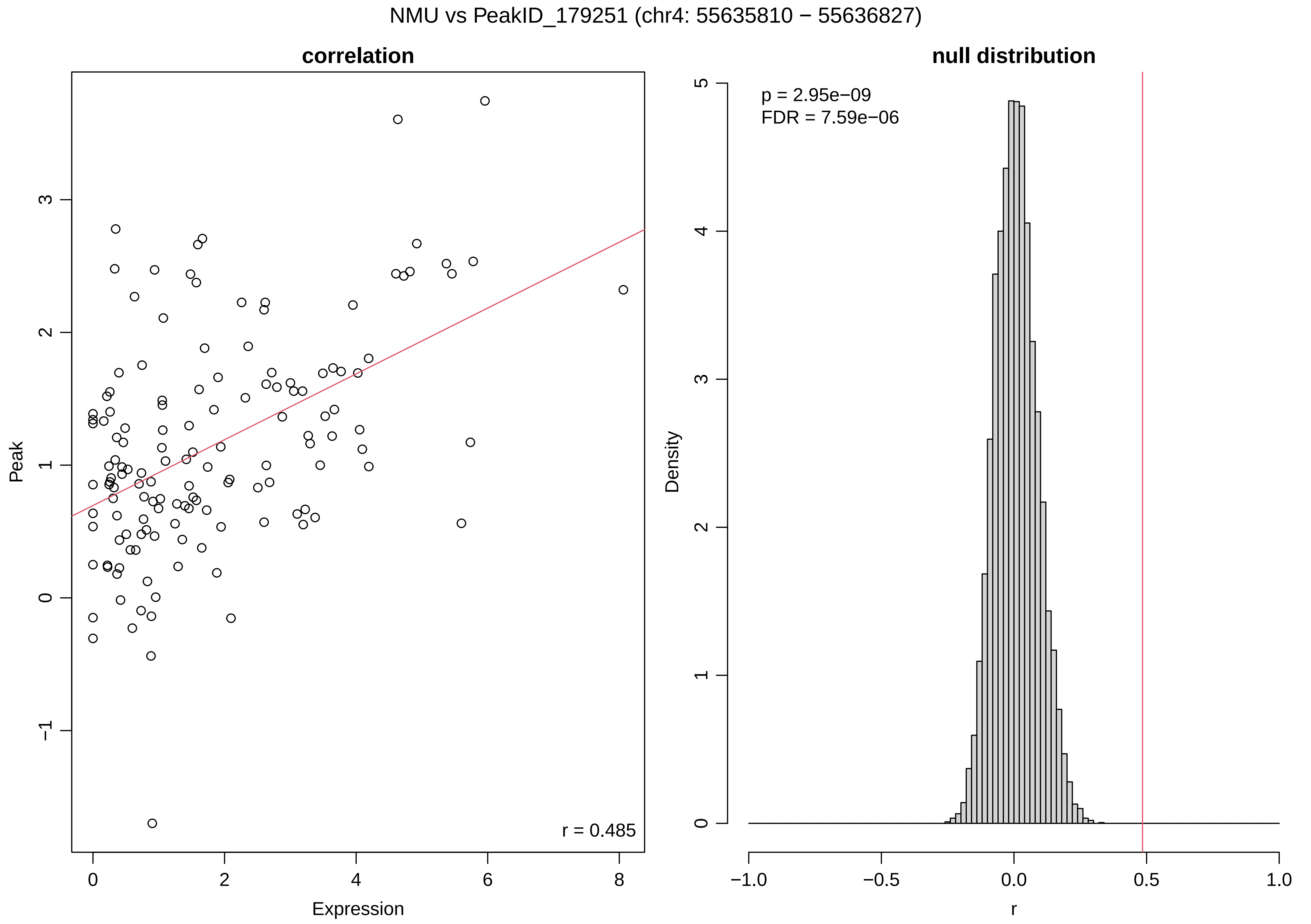
NMU vs PeakID_179251 (chr4: 55635810 − 55636827)
correlation
Peak
Expression
r = 0.485
null distribution
p = 2.95e−09
FDR = 7.59e−06
Density
r

RPL21P104 vs PeakID_55358 (chr12: 61739129 − 61740673)

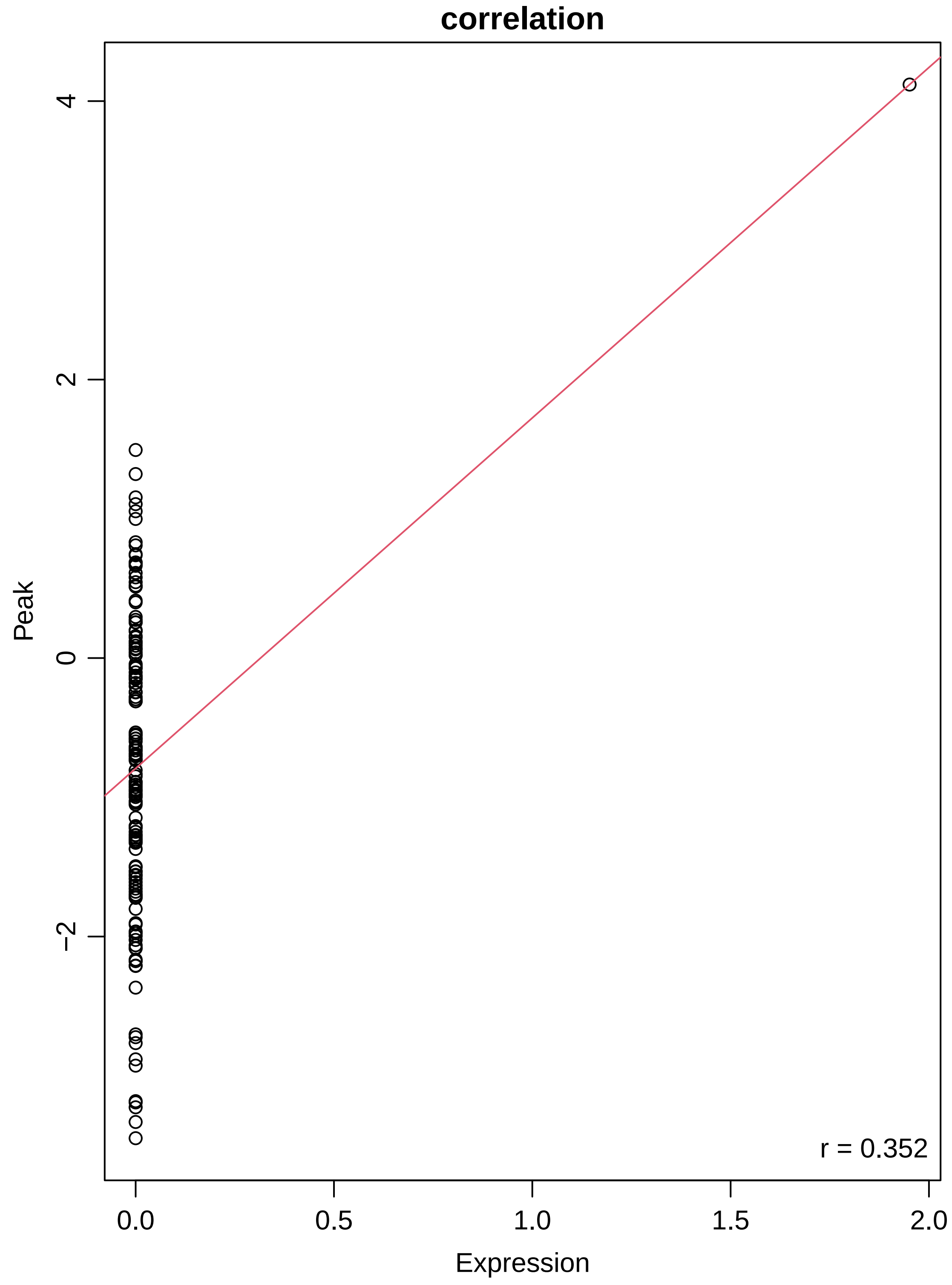

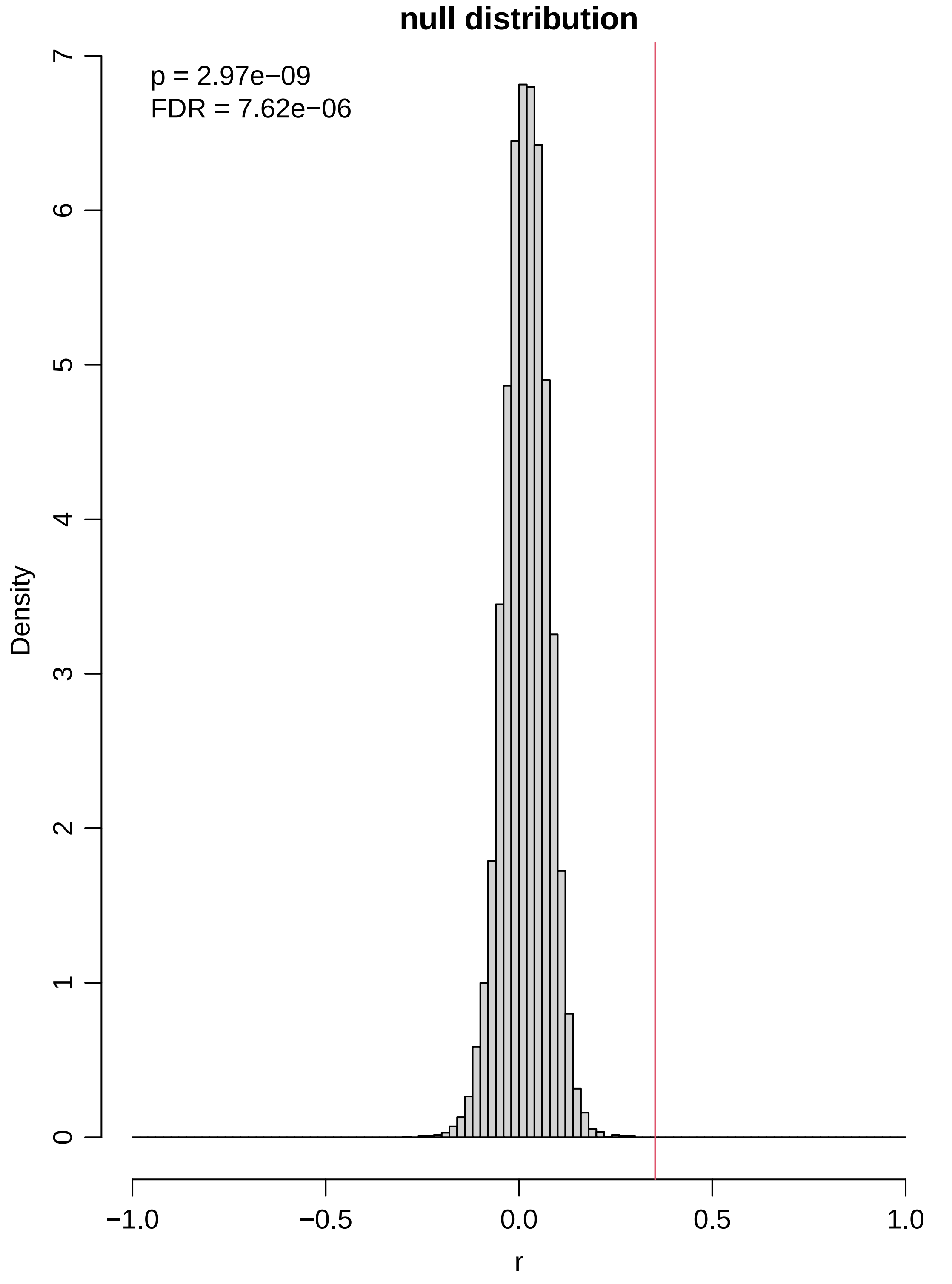

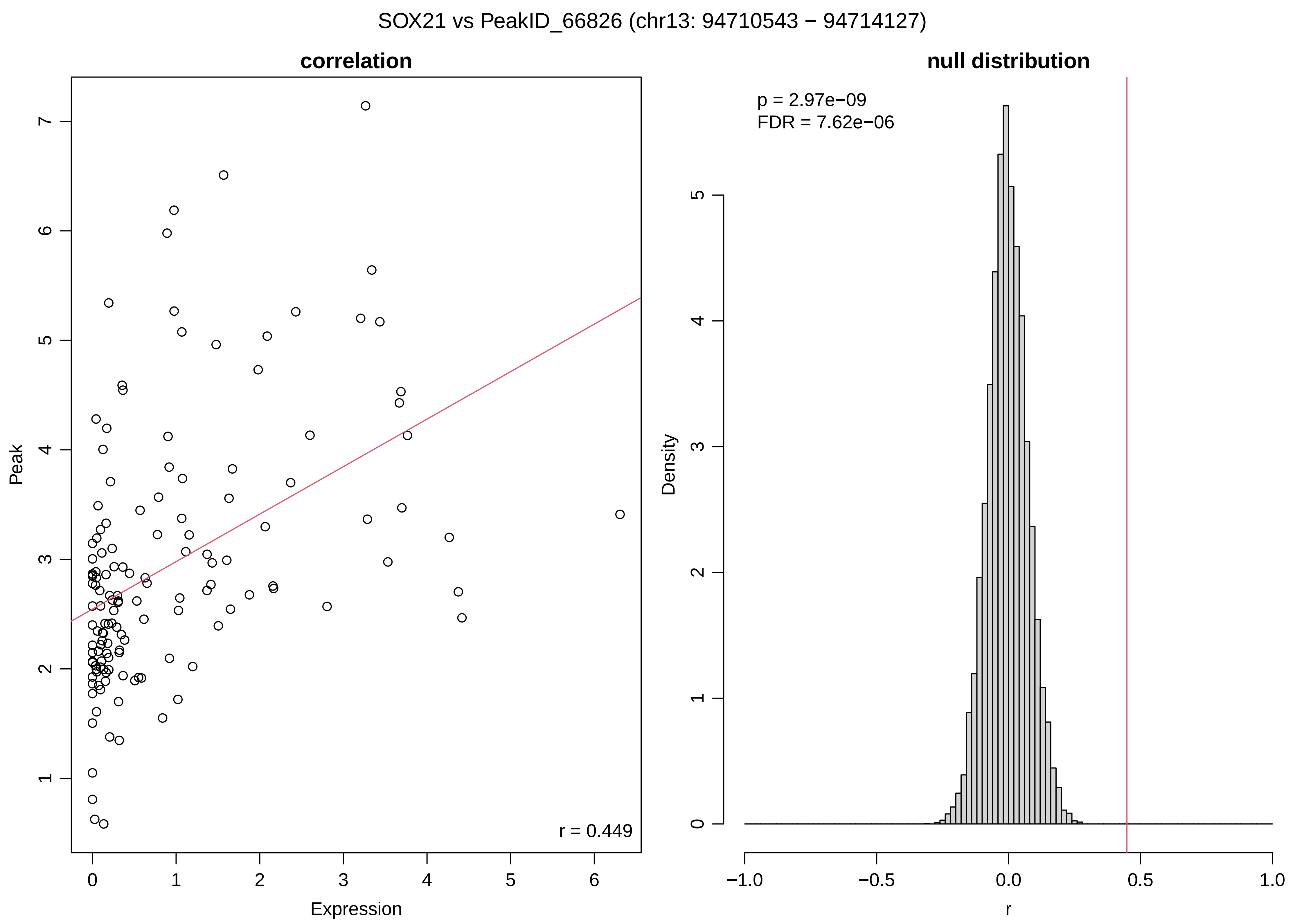
SOX21 vs PeakID_66826 (chr13: 94710543 − 94714127)
correlation
Peak
Expression
r = 0.449
null distribution
p = 2.97e−09
FDR = 7.62e−06
Density
r

YEATS4 vs PeakID_56814 (chr12: 69400863 − 69410150)

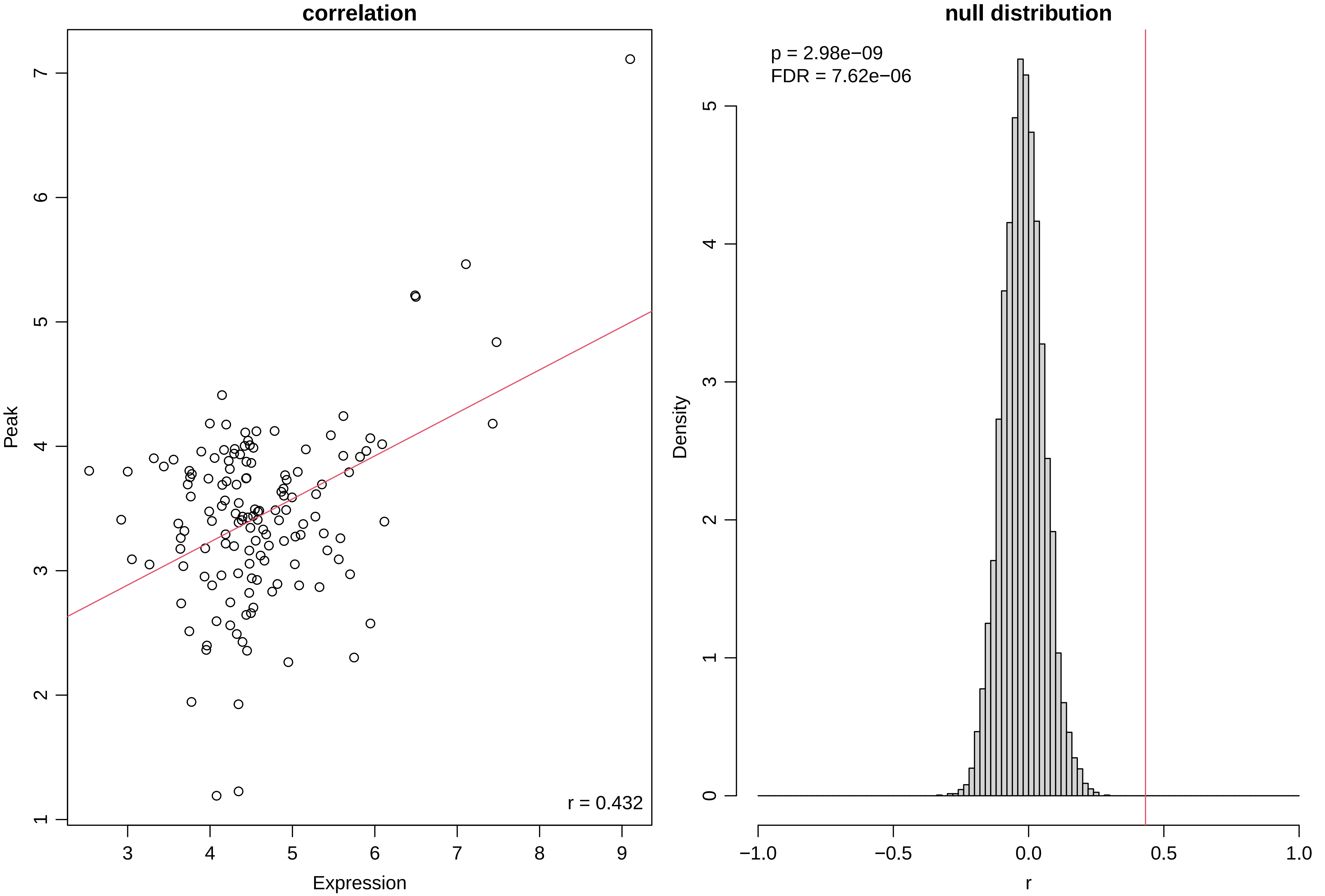

FAM177B vs PeakID_20687 (chr1: 222736154 − 222738975)

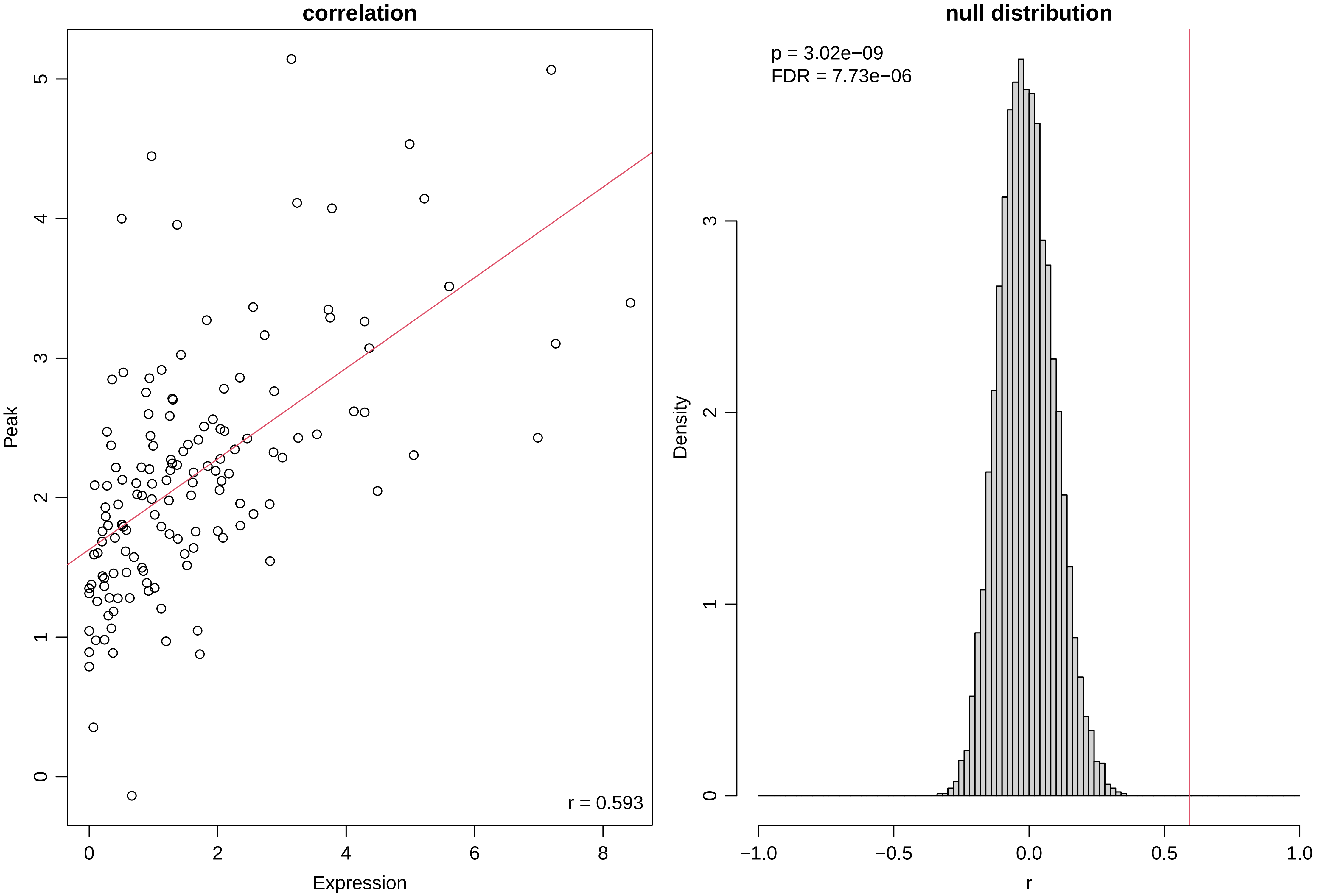

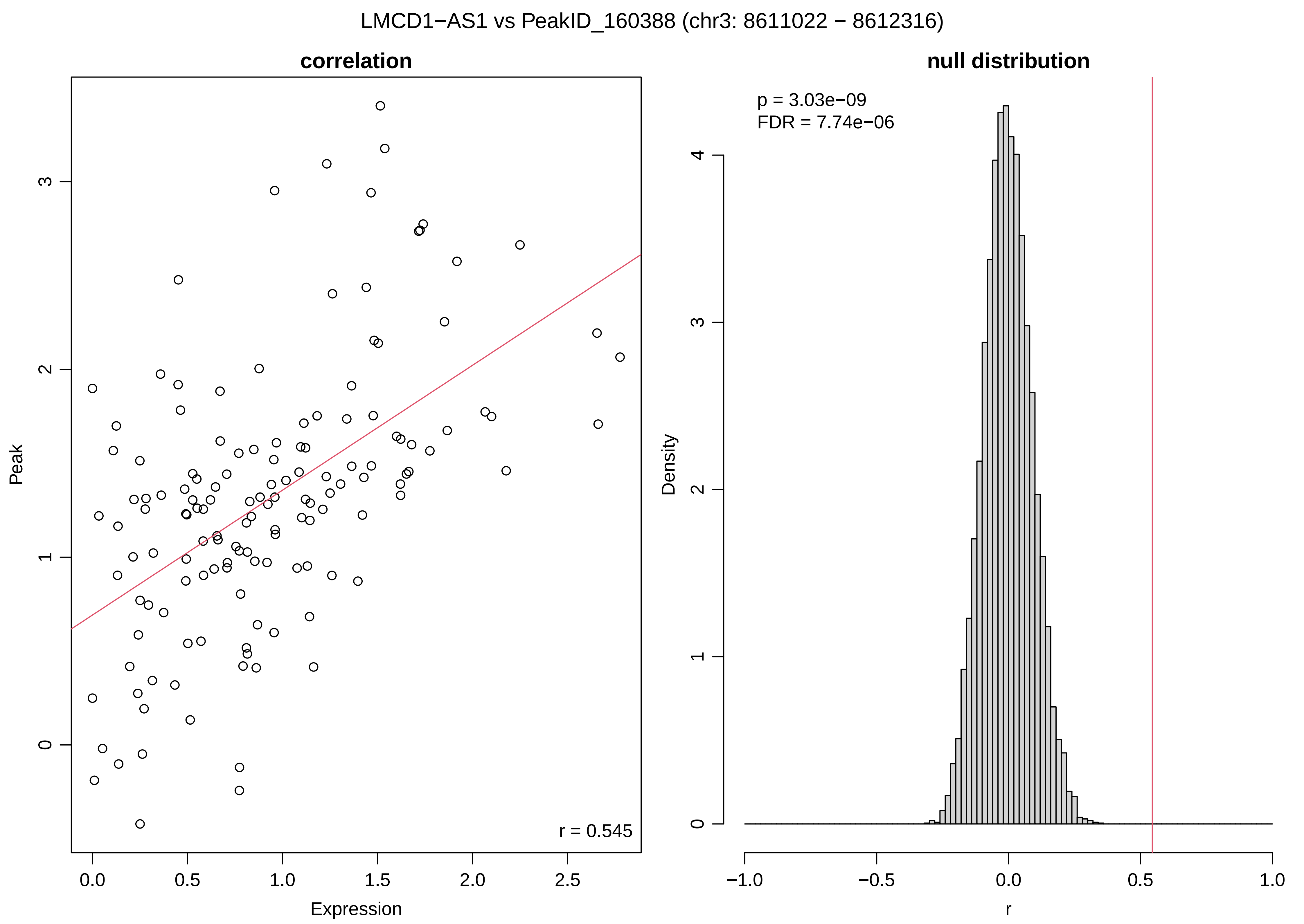

LMCD1−AS1 vs PeakID_160388 (chr3: 8611022 − 8612316)
correlation
Peak
Expression
r = 0.545
null distribution
p = 3.03e−09
FDR = 7.74e−06
Density
r

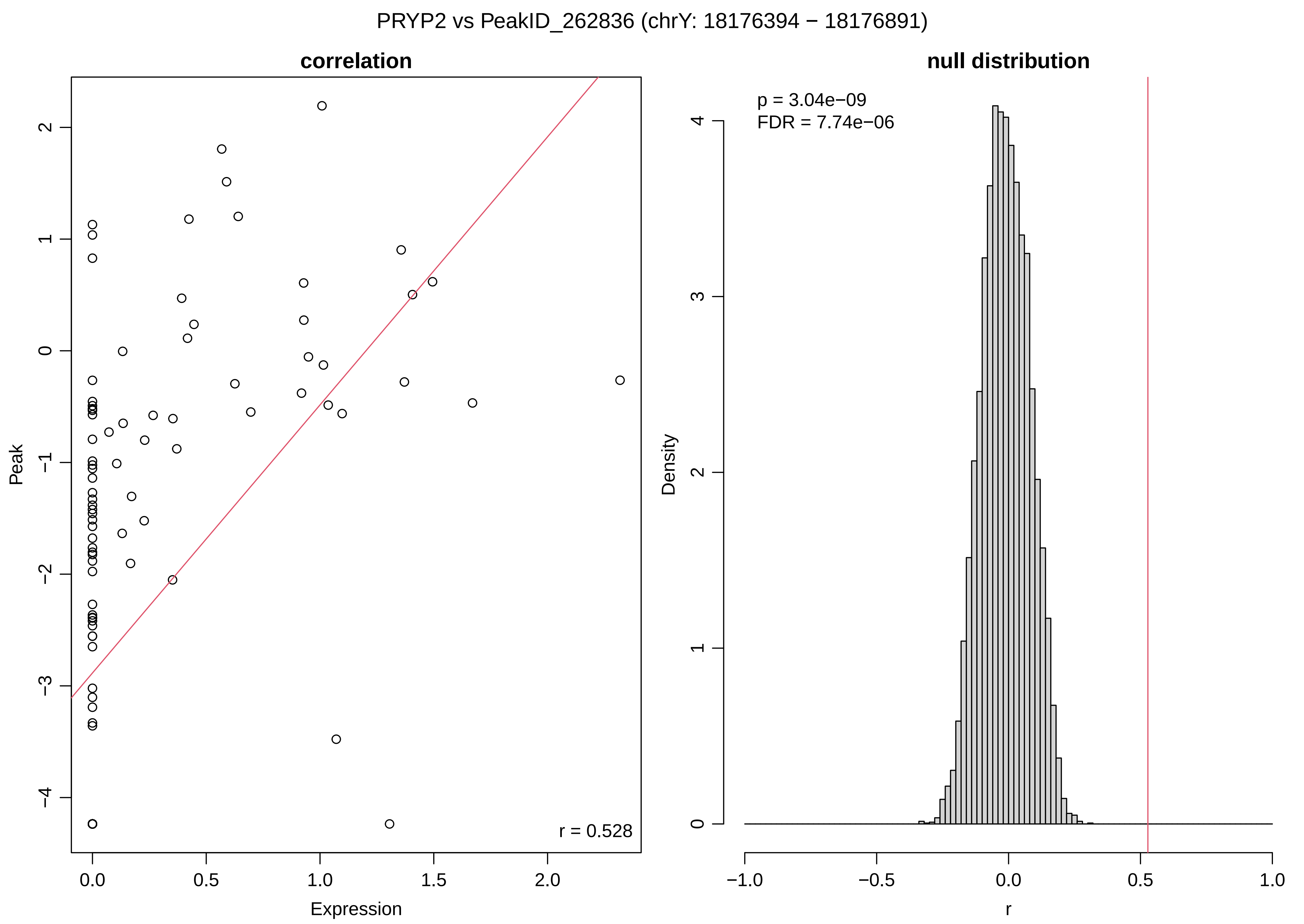

PRYP2 vs PeakID_262836 (chrY: 18176394 − 18176891)
correlation
Peak
Expression
r = 0.528
null distribution
p = 3.04e−09
FDR = 7.74e−06
Density
r

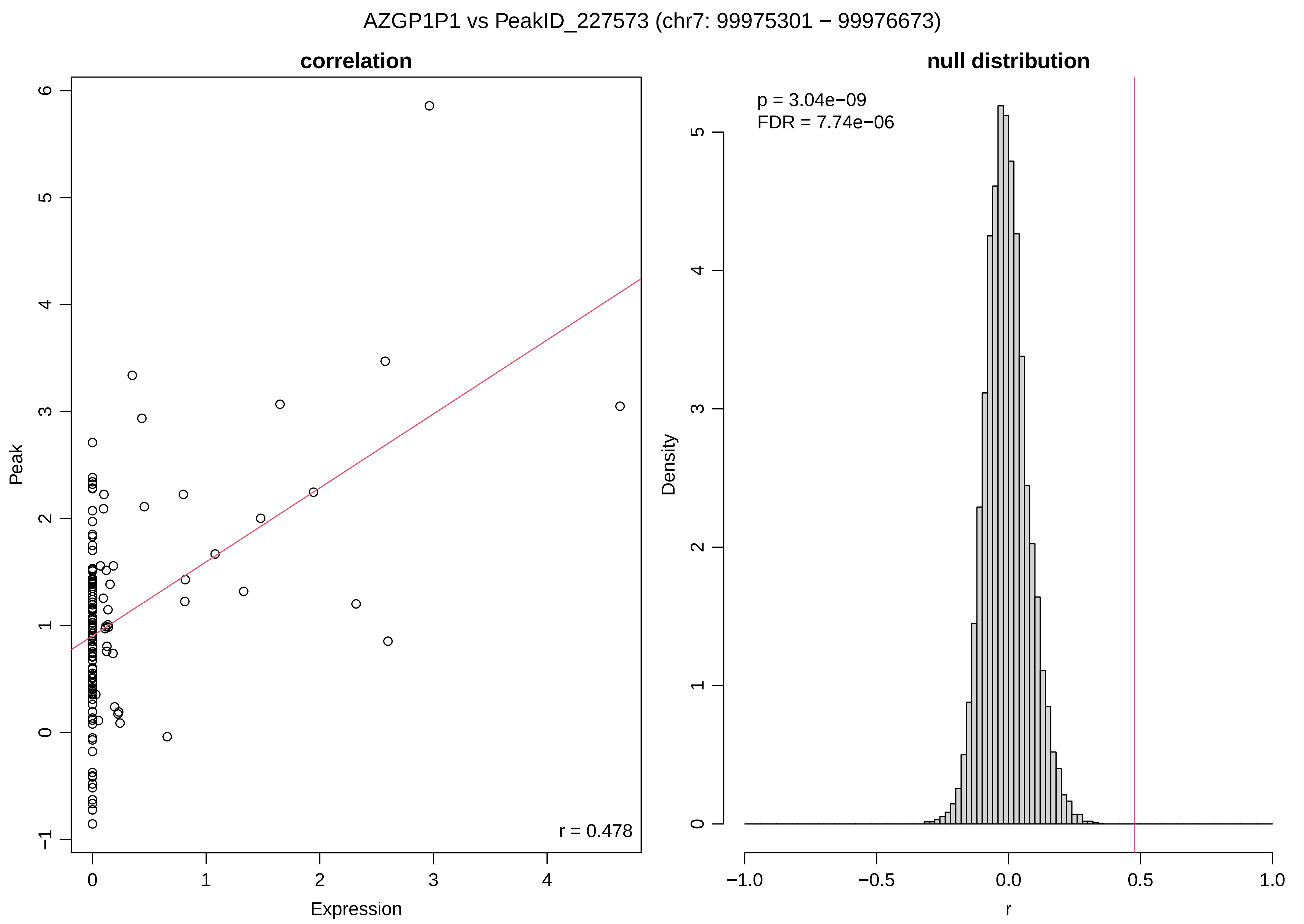
AZGP1P1 vs PeakID_227573 (chr7: 99975301 − 99976673)
correlation
Peak
Expression
r = 0.478
null distribution
p = 3.04e−09
FDR = 7.74e−06
Density
r

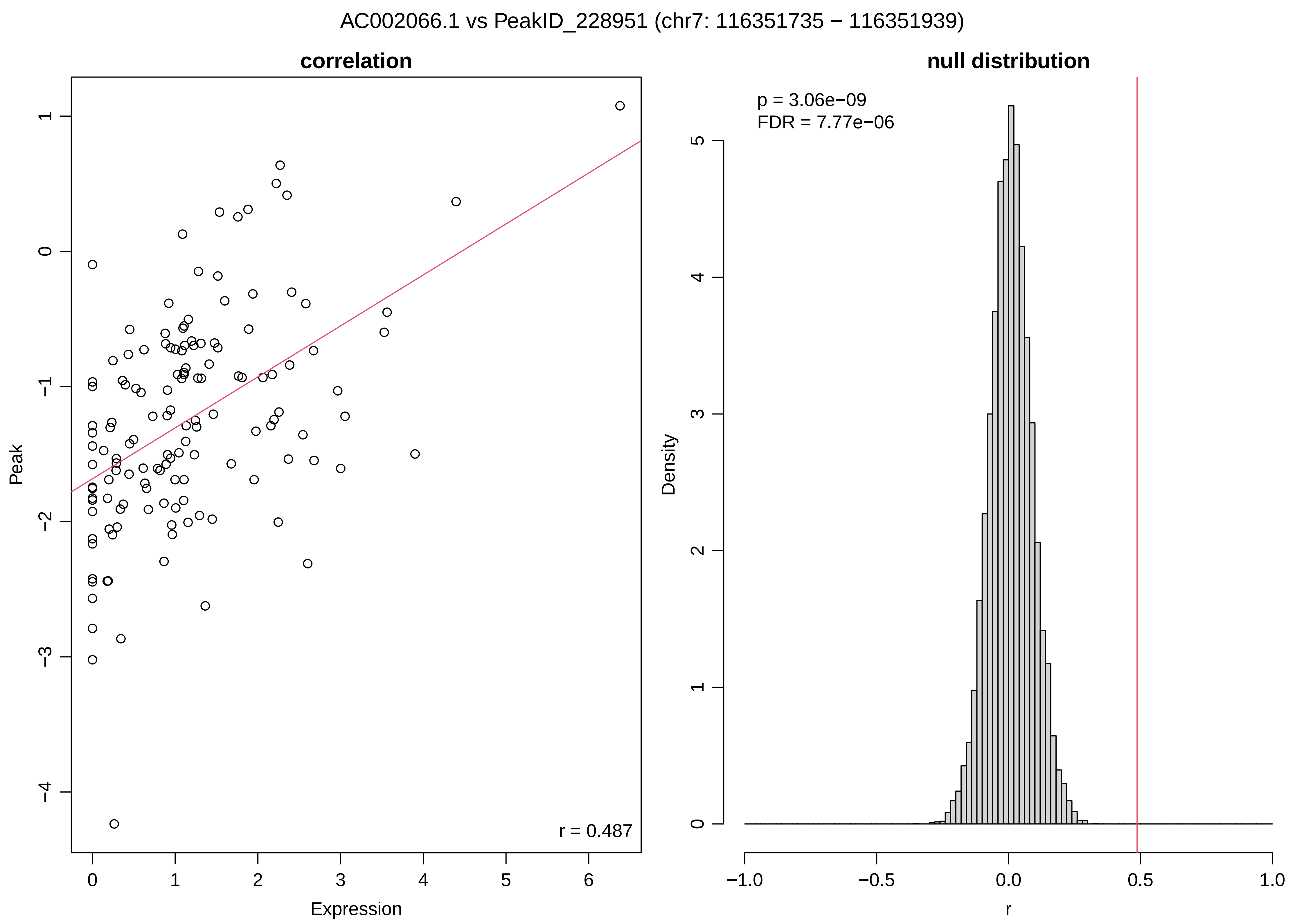
AC002066.1 vs PeakID_228951 (chr7: 116351735 − 116351939)
correlation
Peak
Expression
r = 0.487
null distribution
p = 3.06e−09
FDR = 7.77e−06
Density
r

TRIM71 vs PeakID_162412 (chr3: 32815757 − 32820967)

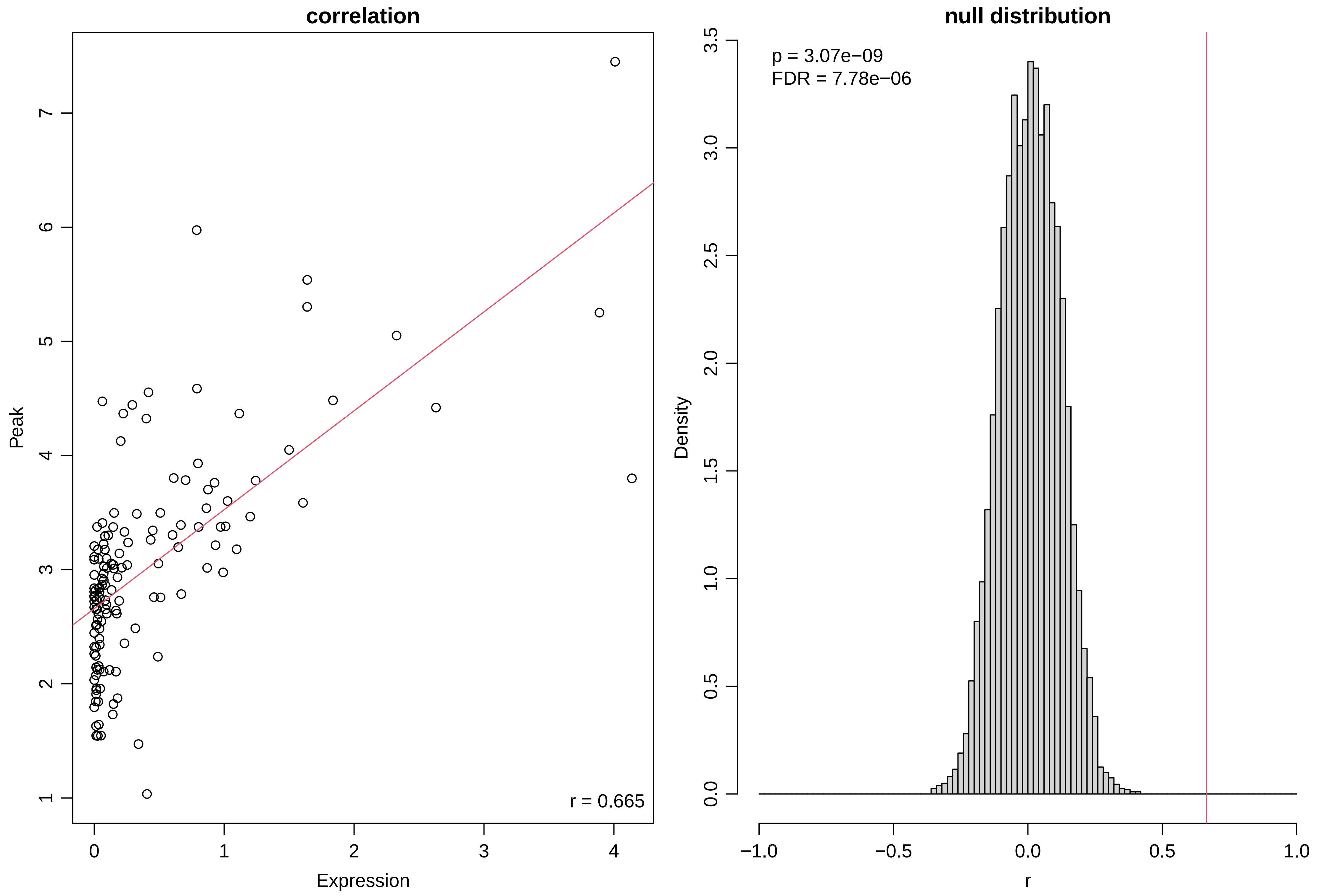

AC107881.1 vs PeakID_37882 (chr11: 12514476 − 12515115)

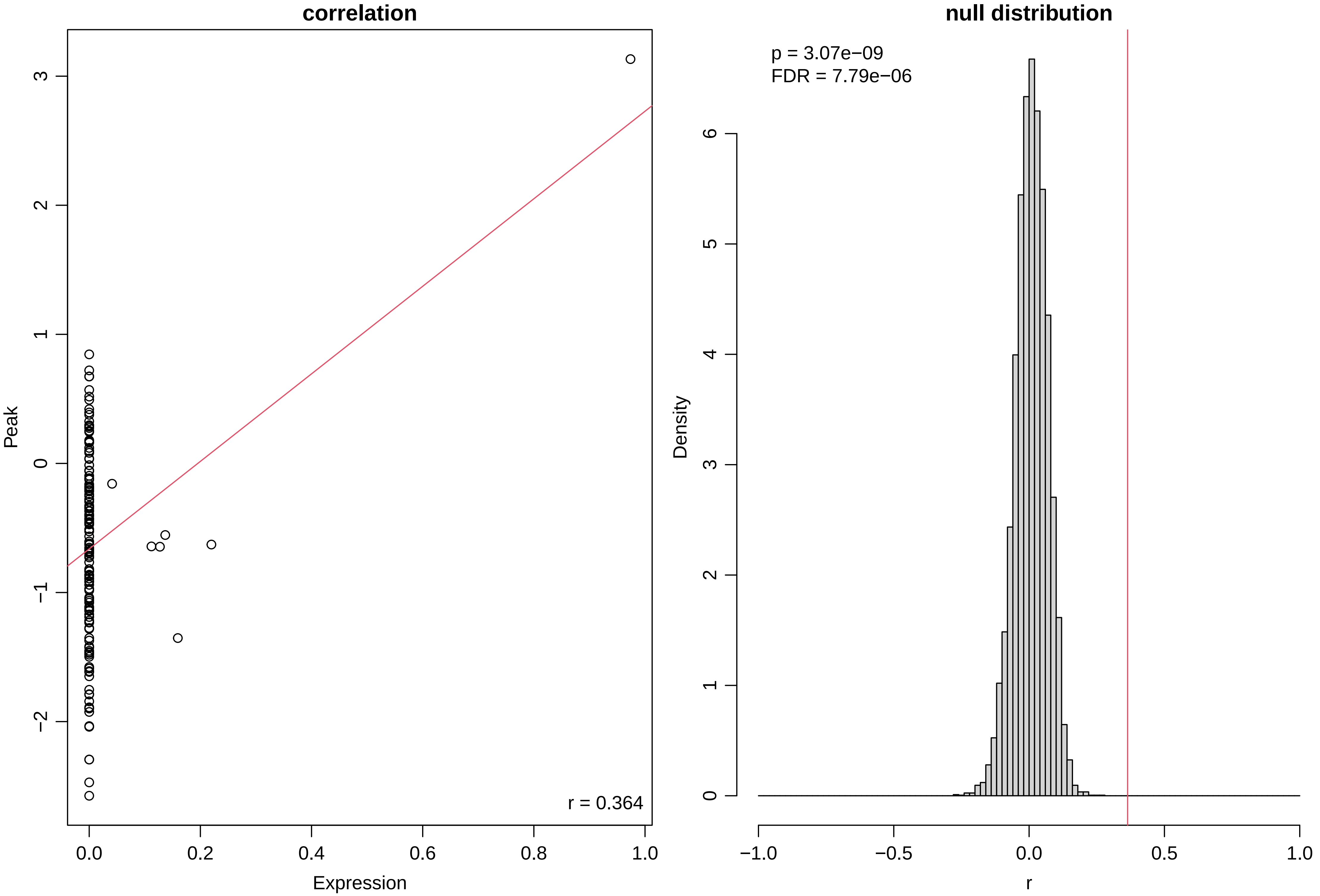

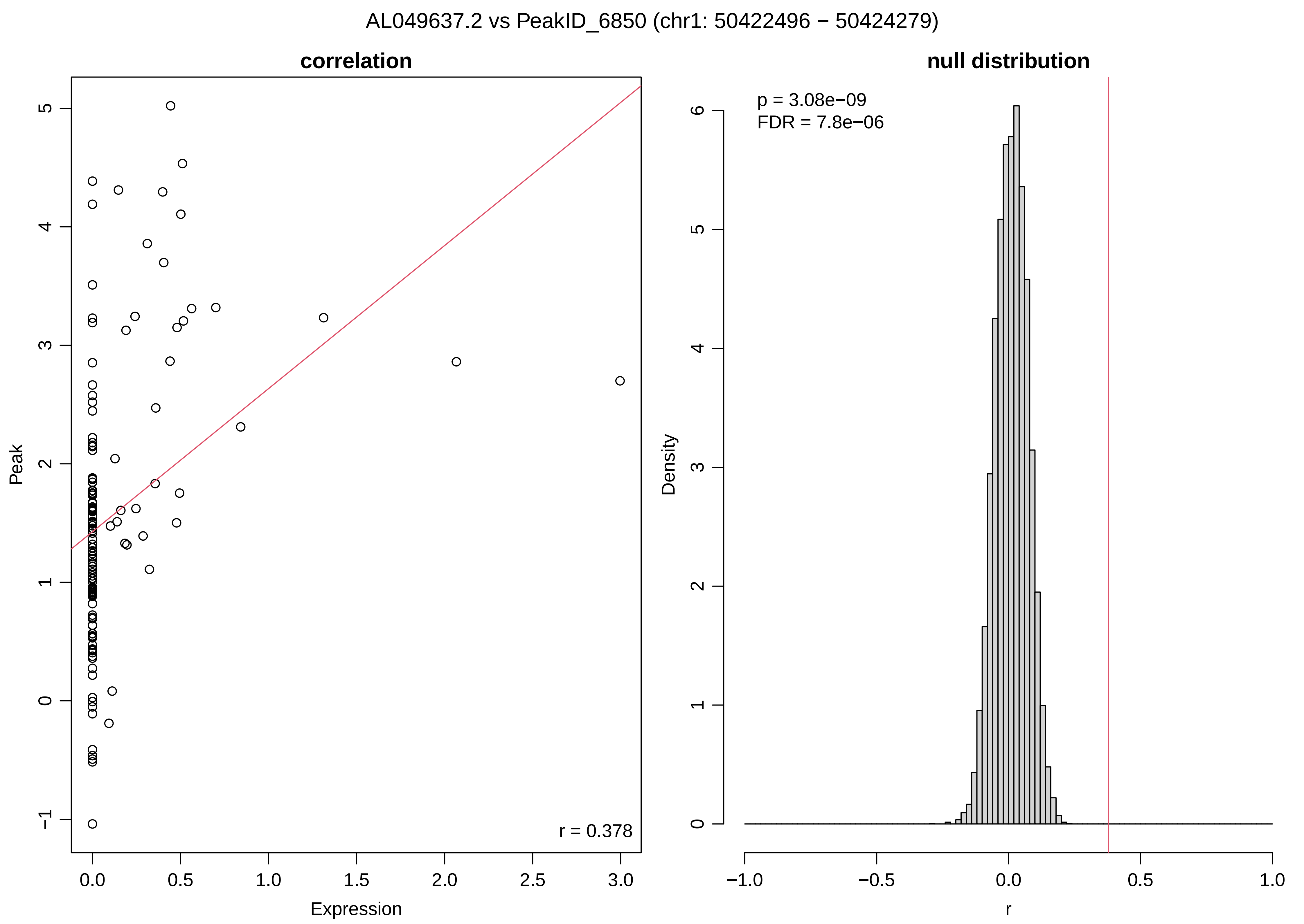
AL049637.2 vs PeakID_6850 (chr1: 50422496 − 50424279)
correlation
Peak
Expression
r = 0.378
null distribution
p = 3.08e−09
FDR = 7.8e−06
Density
r

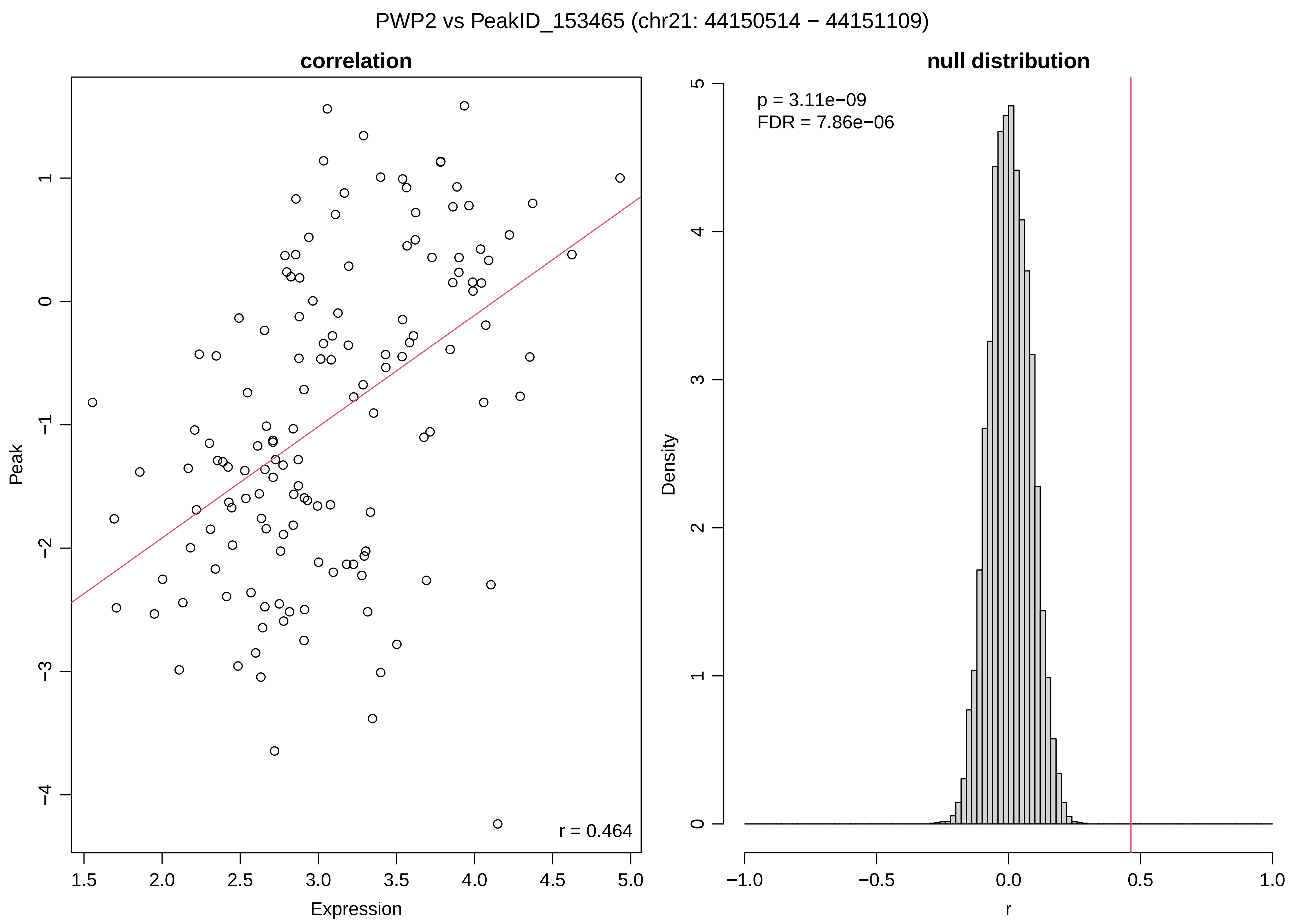

PWP2 vs PeakID_153465 (chr21: 44150514 − 44151109)
correlation
null distribution
p = 3.11e−09
FDR = 7.86e−06
r = 0.464
Peak
Expression
Density
r
1.5
2.0
2.5
3.0
3.5
4.0
4.5
5.0
1
0
−1
−2
−3
−4
5
4
3
2
1
0
−1.0
−0.5
0.0
0.5
1.0

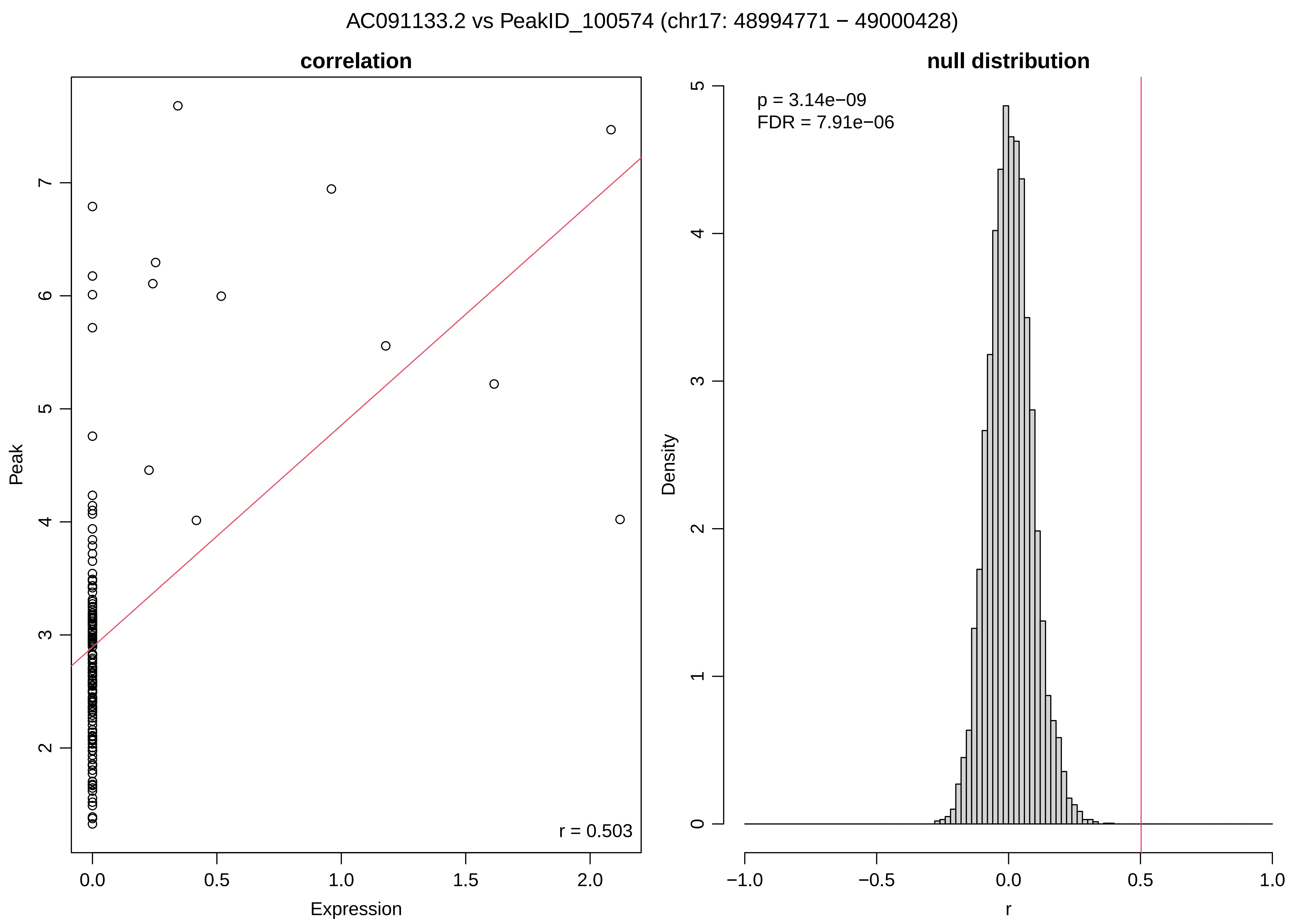

AC091133.2 vs PeakID_100574 (chr17: 48994771 − 49000428)
correlation
Peak
Expression
r = 0.503
null distribution
Density
r
p = 3.14e−09
FDR = 7.91e−06

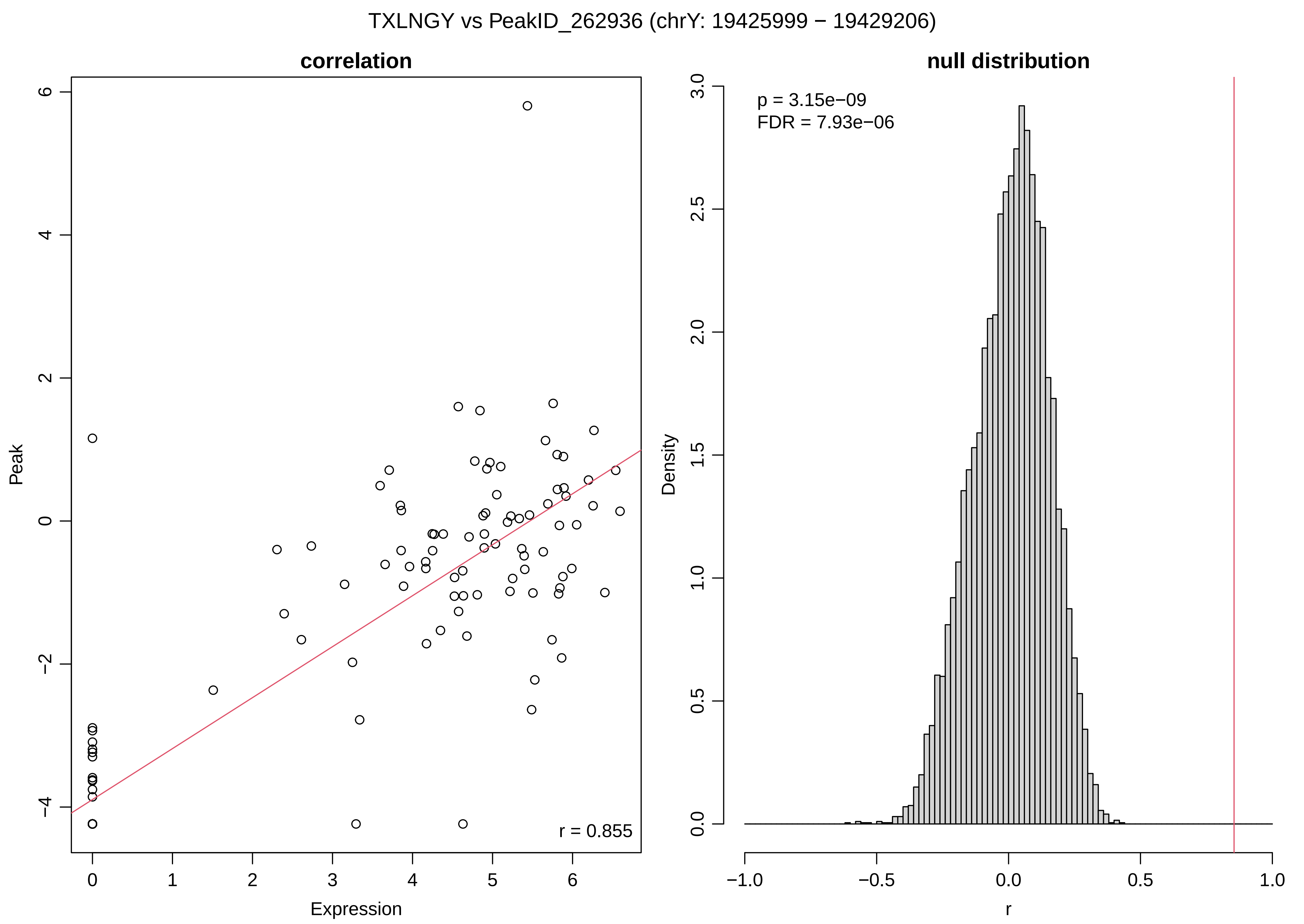
TXLNGY vs PeakID_262936 (chrY: 19425999 − 19429206)
correlation
Peak
Expression
r = 0.855
null distribution
Density
r
p = 3.15e−09
FDR = 7.93e−06

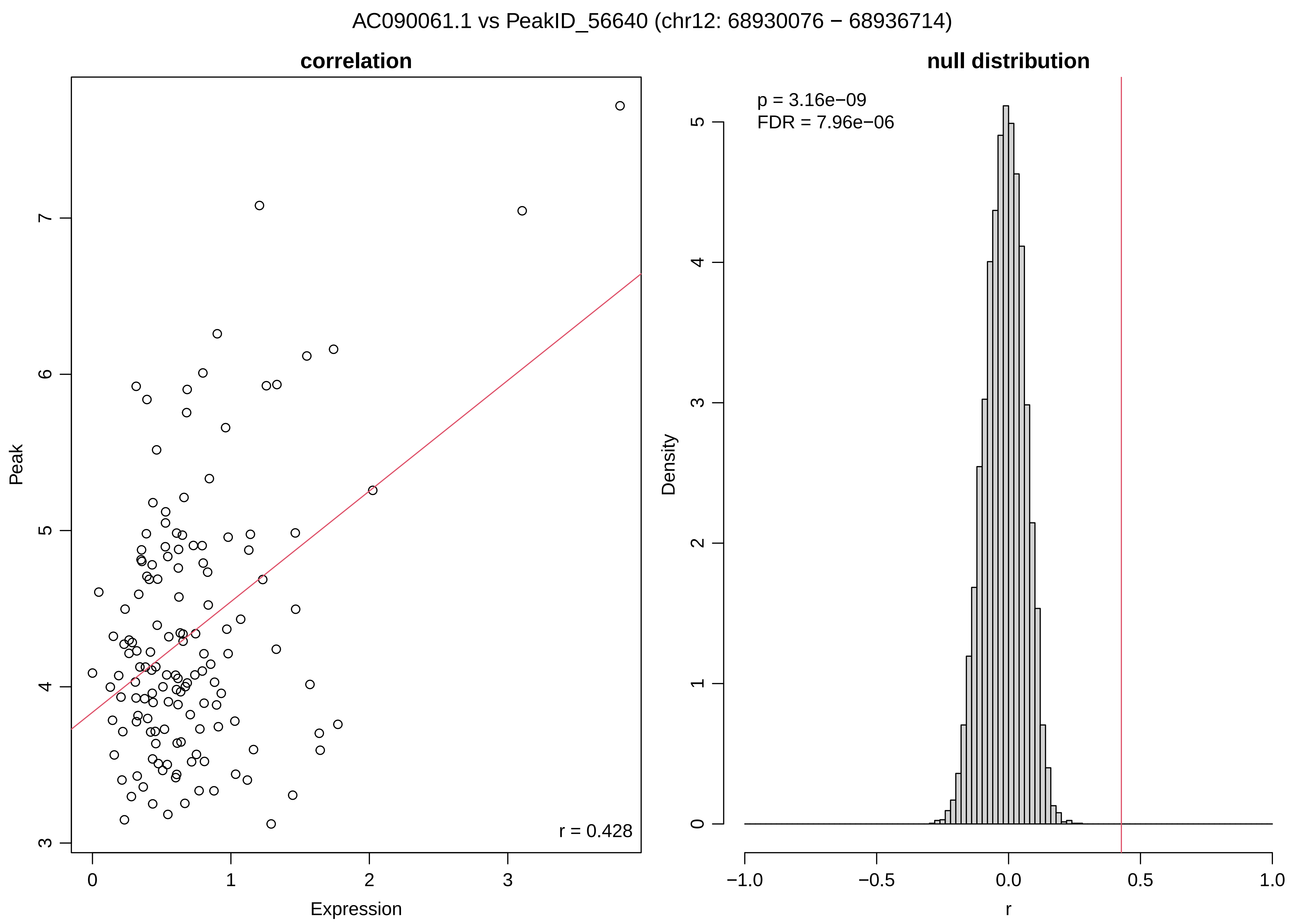
AC090061.1 vs PeakID_56640 (chr12: 68930076 − 68936714)
correlation
null distribution
Peak
Expression
r = 0.428
p = 3.16e−09
FDR = 7.96e−06
Density
r
3
4
5
6
7
0
1
2
3
−1.0
−0.5
0.0
0.5
1.0

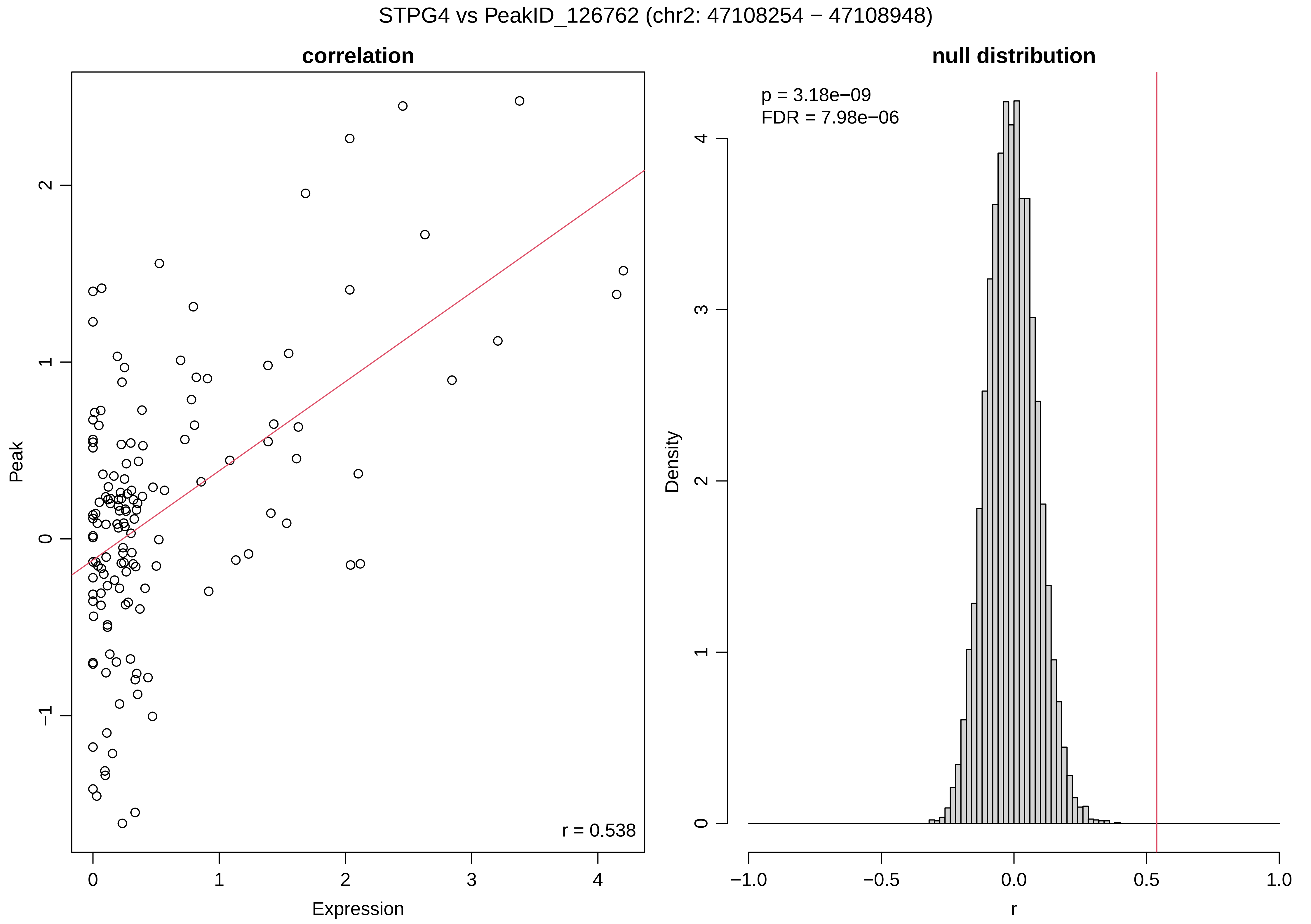

STPG4 vs PeakID_126762 (chr2: 47108254 − 47108948)
correlation
Peak
Expression
r = 0.538
null distribution
p = 3.18e−09
FDR = 7.98e−06
Density
r

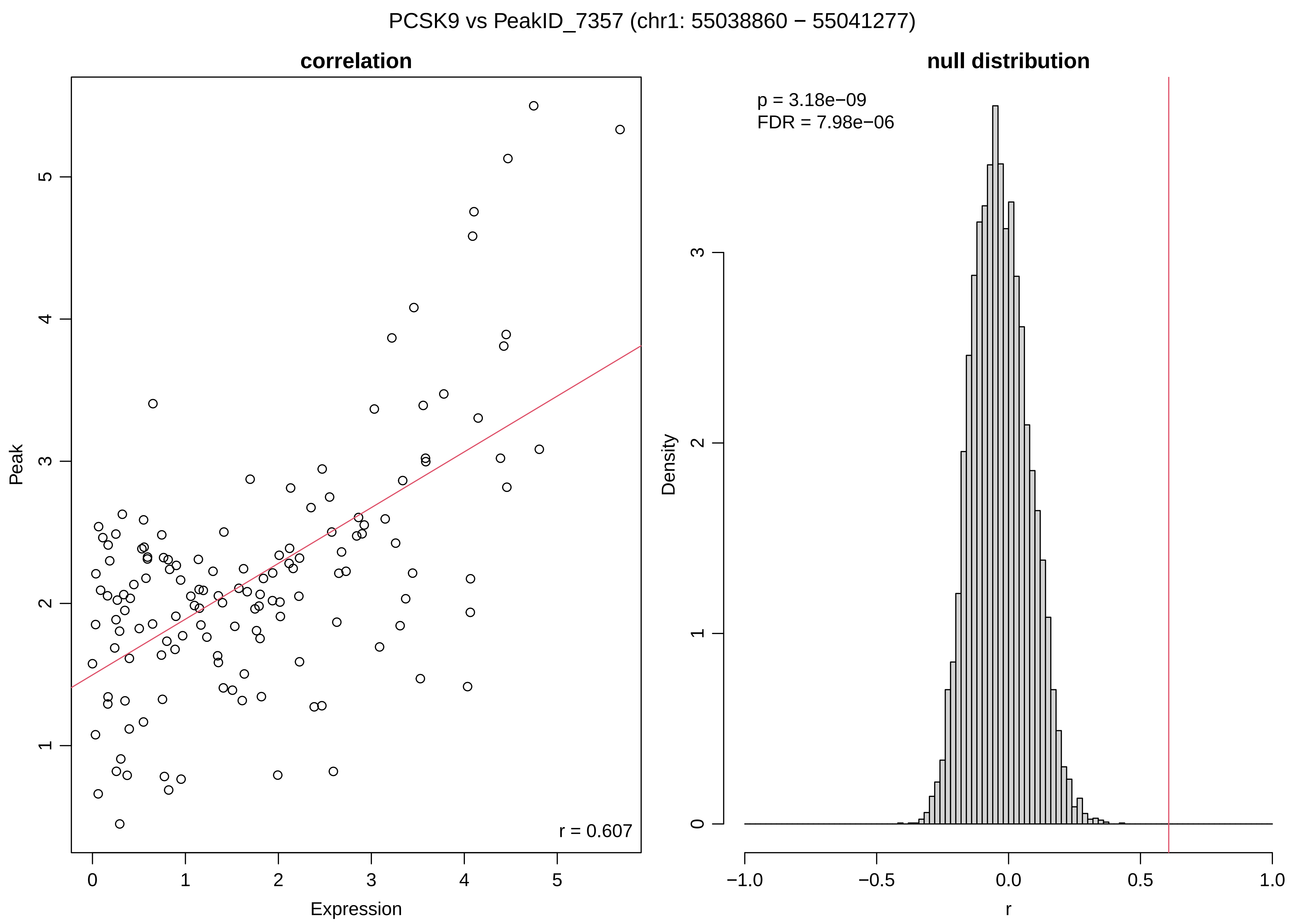
PCSK9 vs PeakID_7357 (chr1: 55038860 − 55041277)
correlation
Peak
Expression
r = 0.607
null distribution
p = 3.18e−09
FDR = 7.98e−06
Density
r

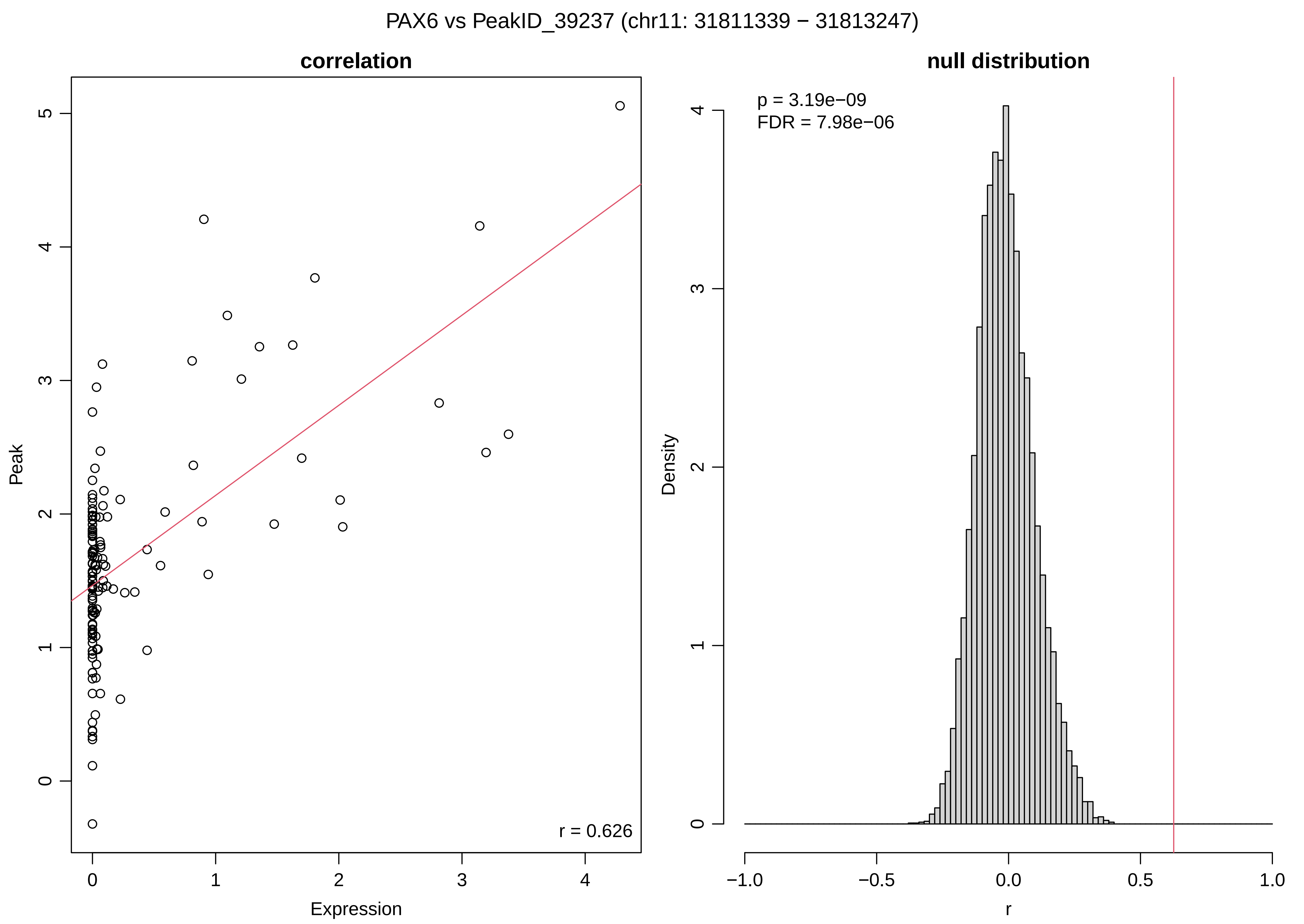
PAX6 vs PeakID_39237 (chr11: 31811339 − 31813247)
correlation
Peak
Expression
r = 0.626
0
1
2
3
4
5
null distribution
p = 3.19e−09
FDR = 7.98e−06
Density
r
−1.0
−0.5
0.0
0.5
1.0

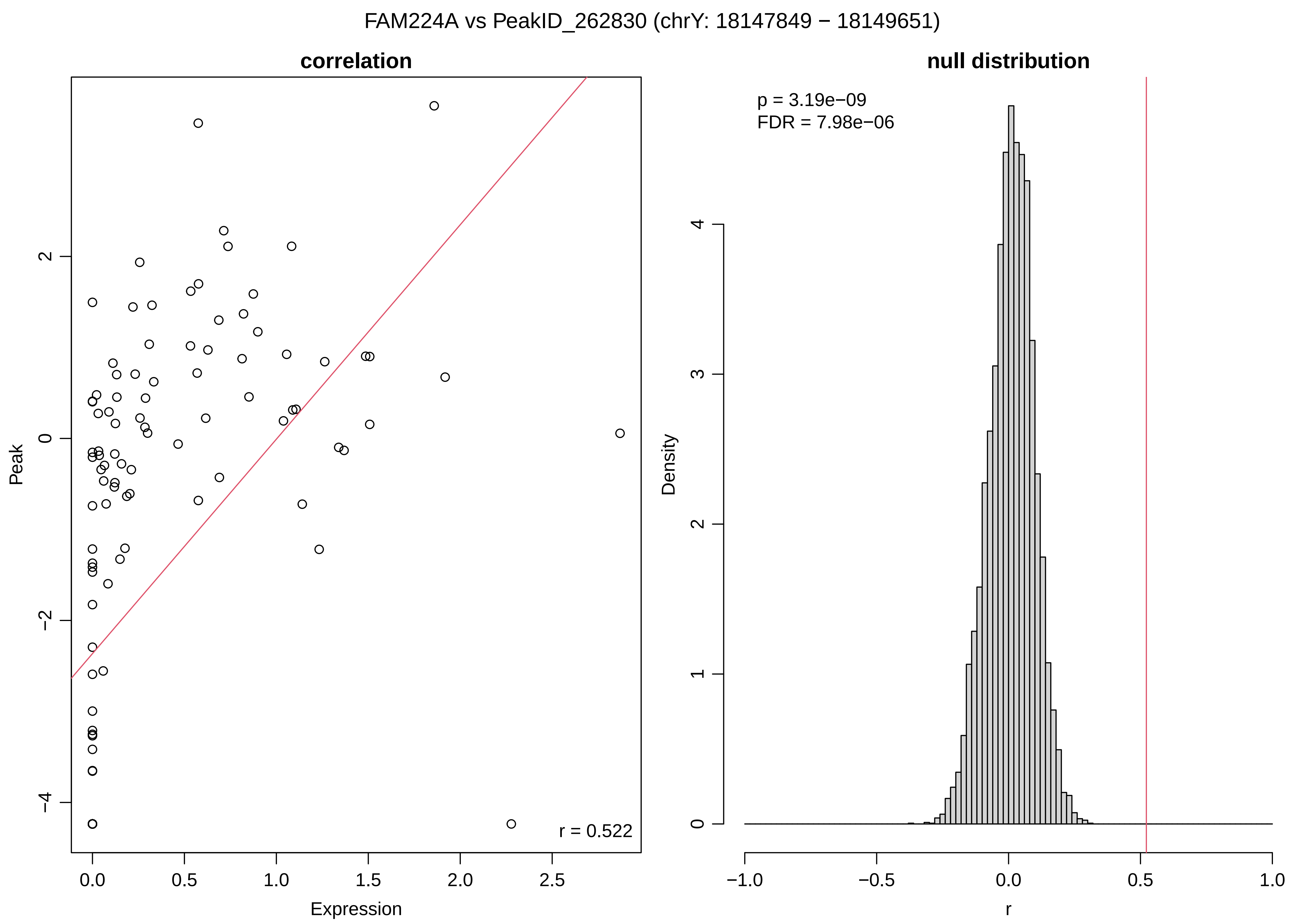

FAM224A vs PeakID_262830 (chrY: 18147849 − 18149651)
correlation
Peak
Expression
r = 0.522
null distribution
p = 3.19e−09
FDR = 7.98e−06
Density
r

APOBEC3B vs PeakID_157633 (chr22: 38963185 − 38969058)

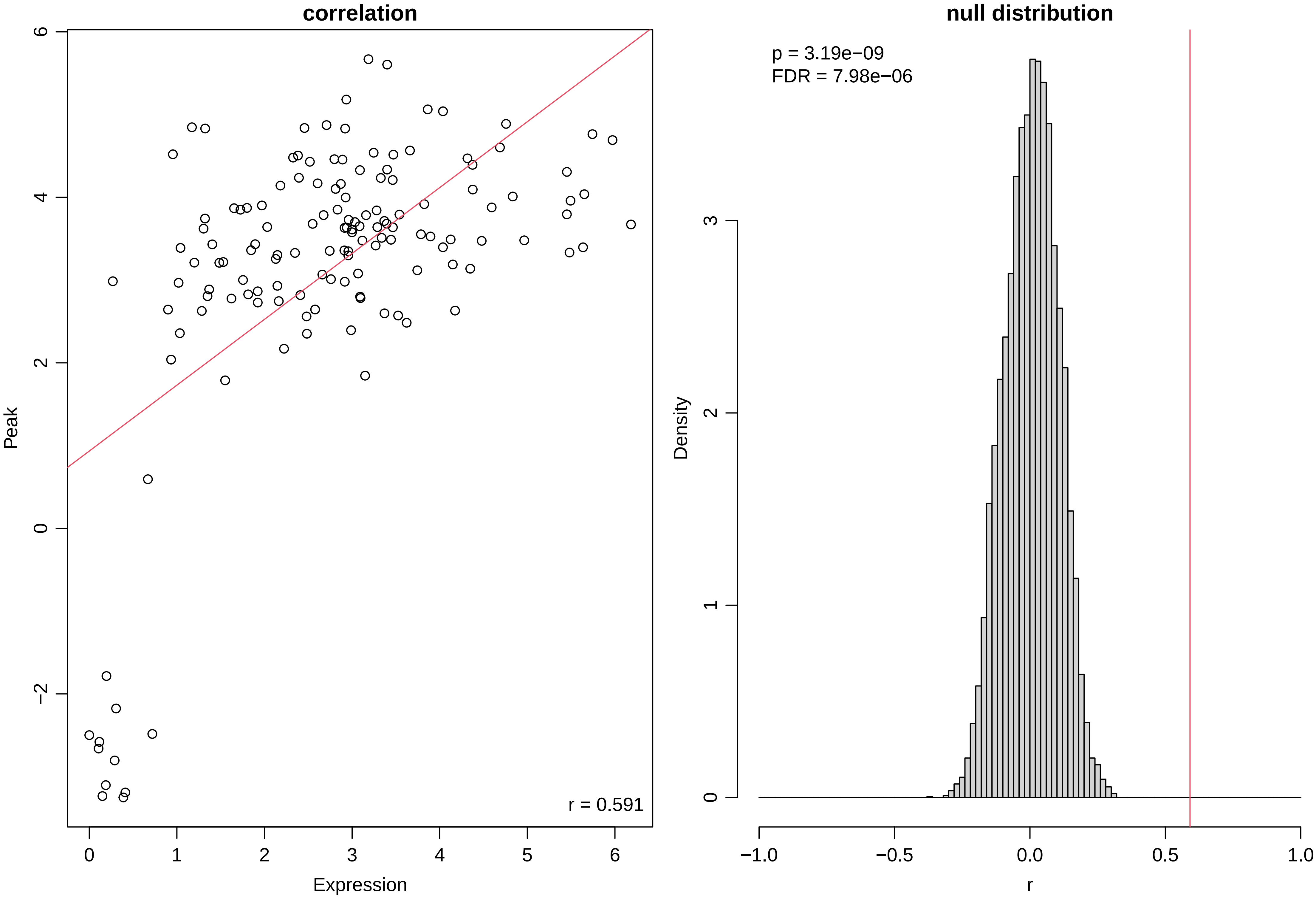

RPL21P104 vs PeakID_55360 (chr12: 61745206 − 61747249)

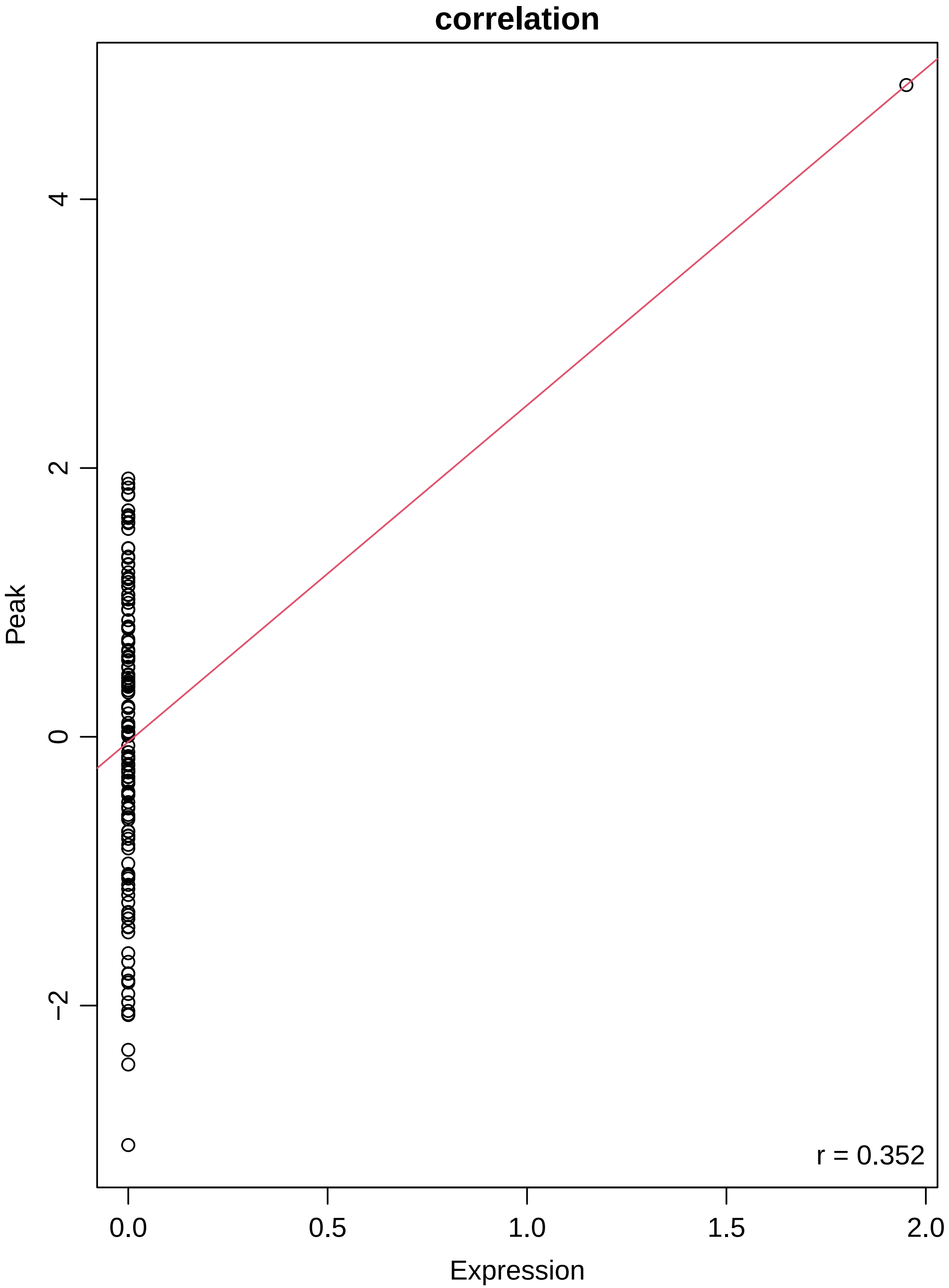

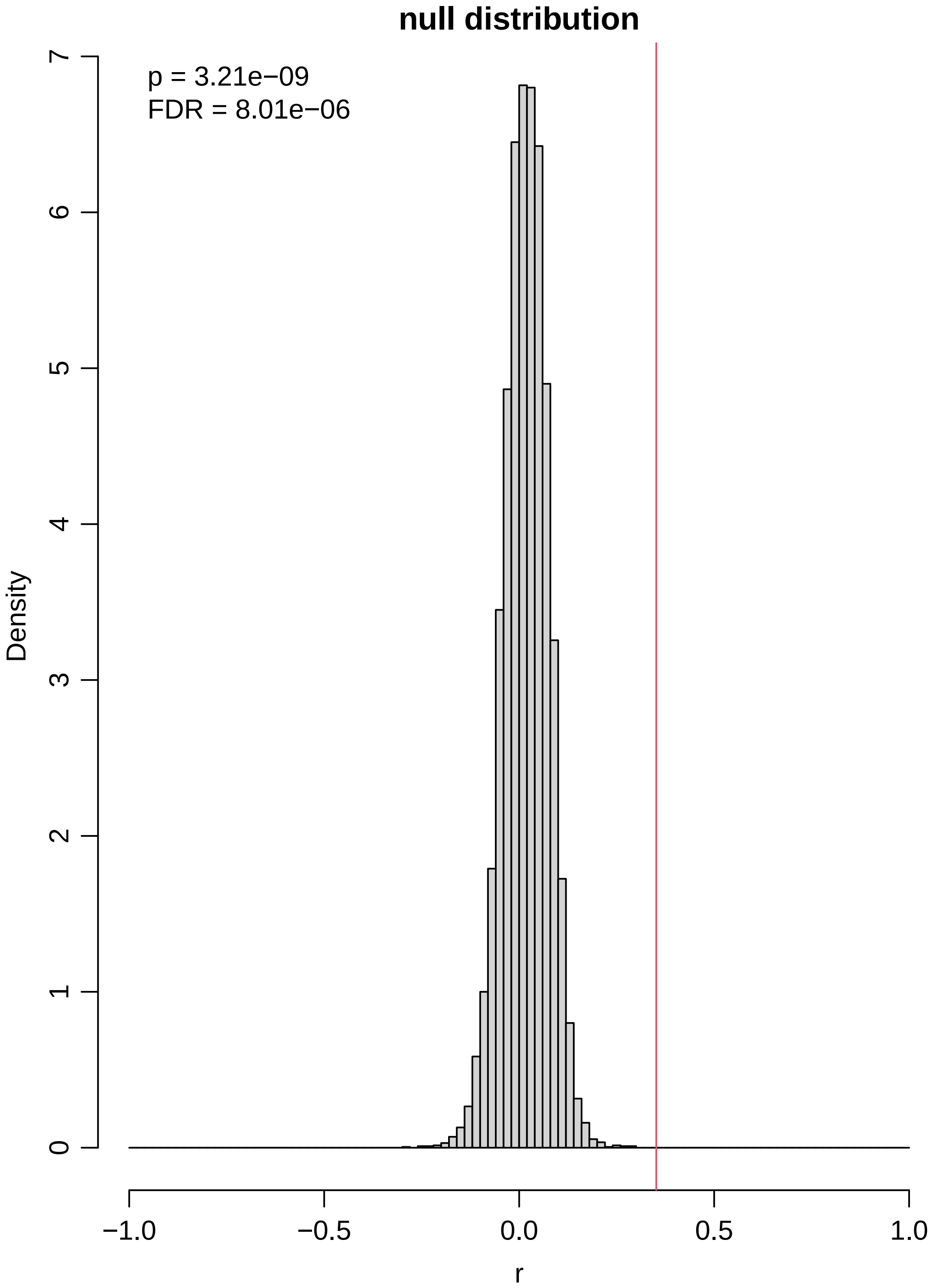

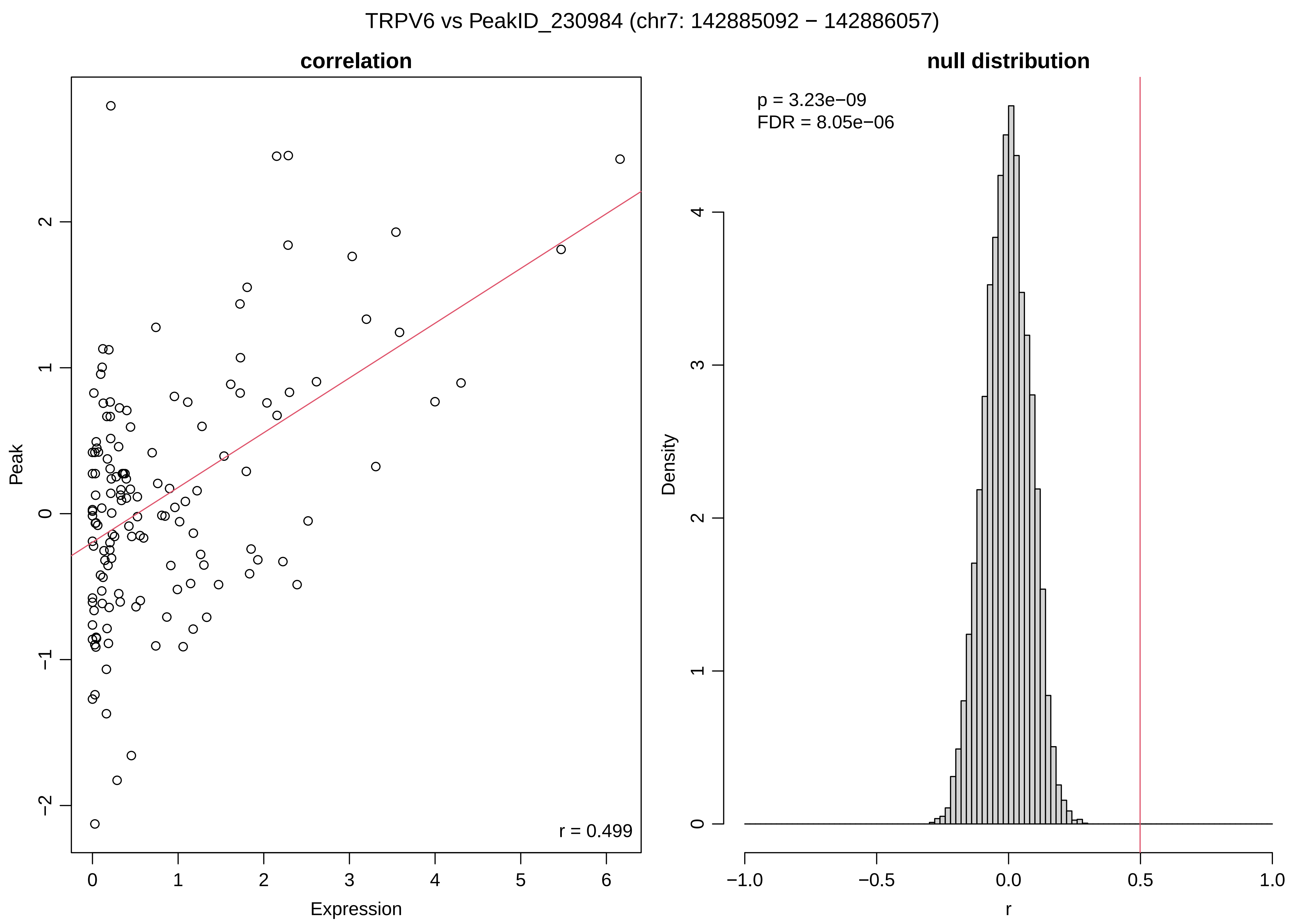
TRPV6 vs PeakID_230984 (chr7: 142885092 − 142886057)
correlation
Peak
Expression
r = 0.499
null distribution
p = 3.23e−09
FDR = 8.05e−06
Density
r

FLRT2 vs PeakID_73797 (chr14: 85524951 − 85536575)

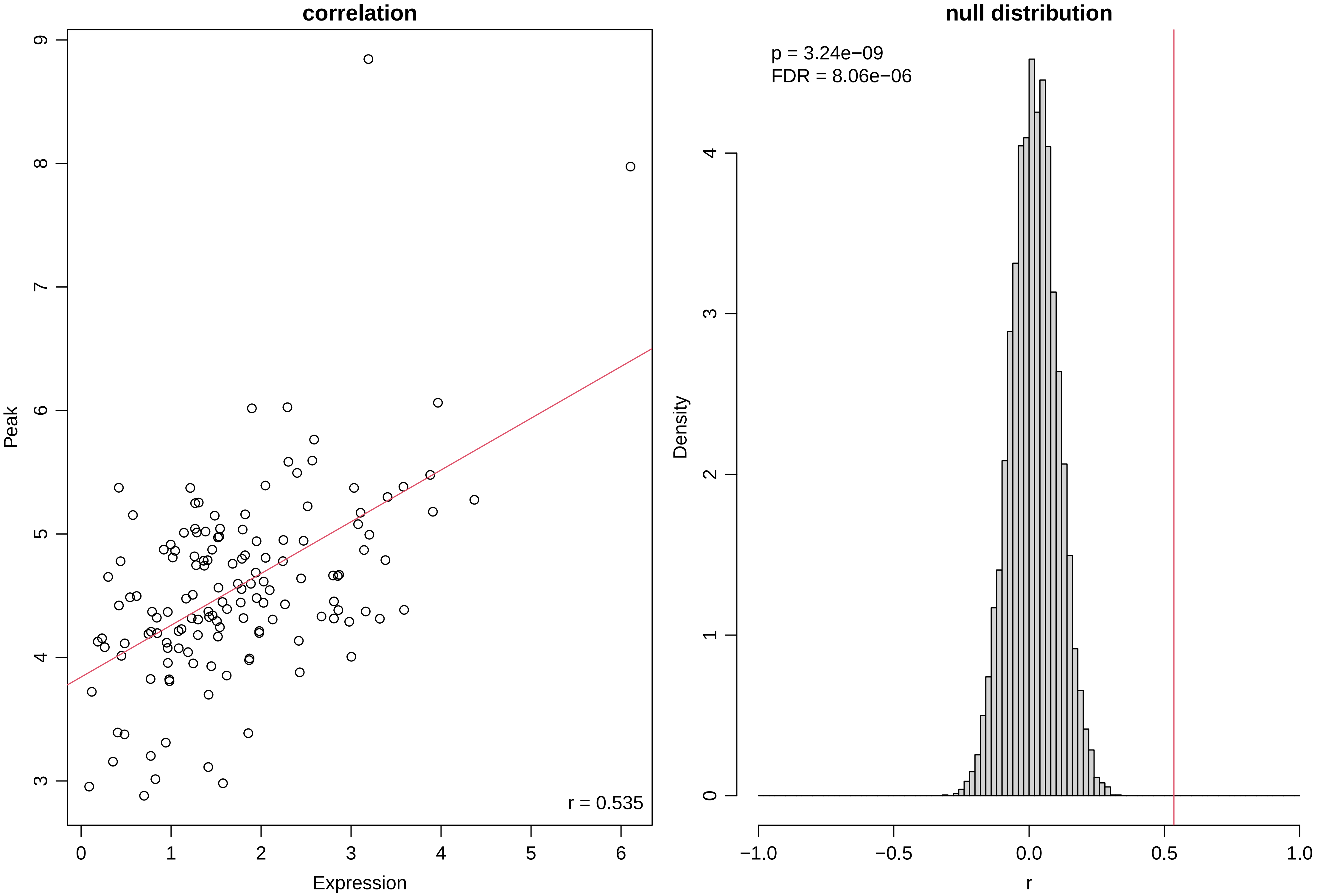

AC124293.1 vs PeakID_238059 (chr8: 72479317 − 72480036)

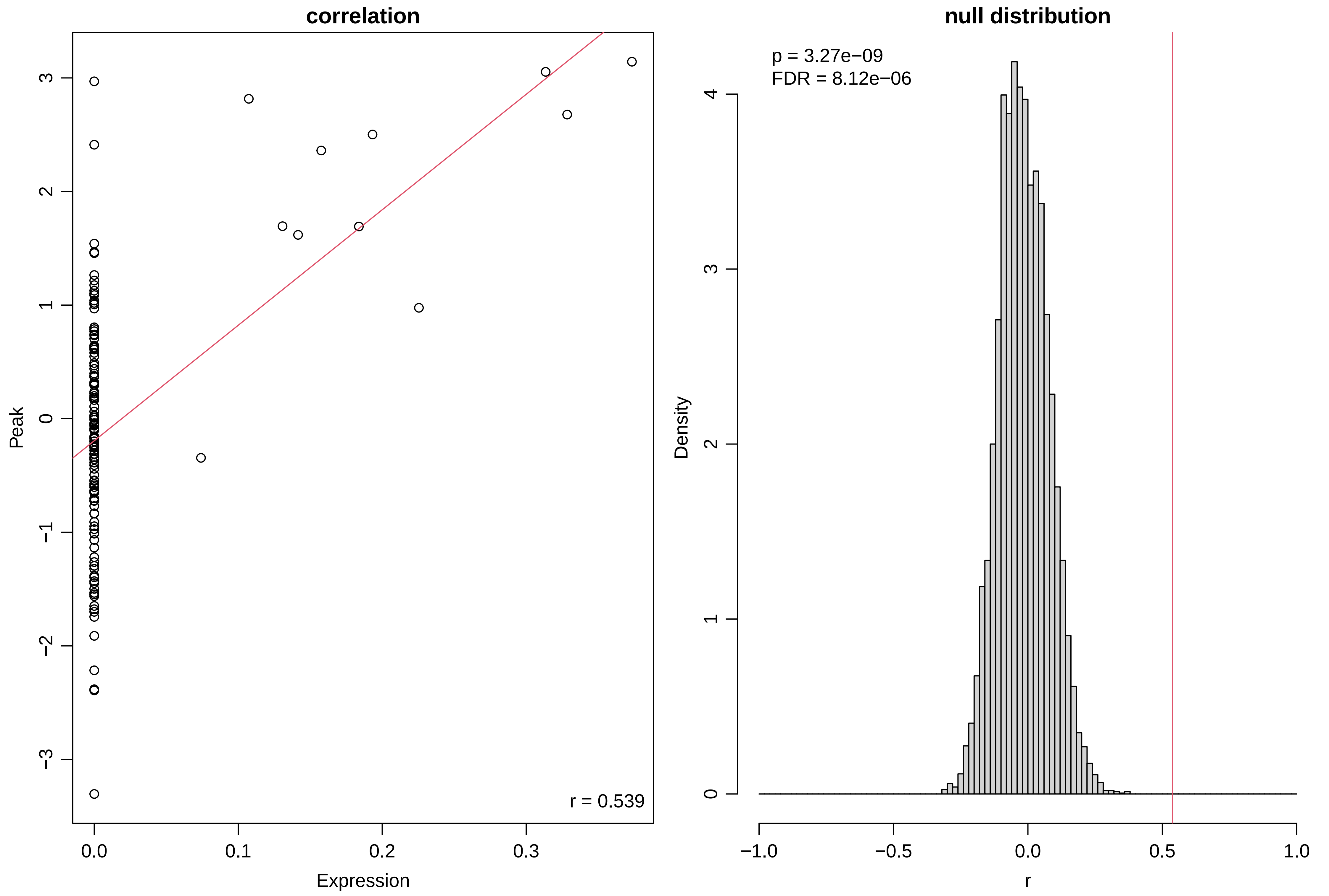

AC107881.1 vs PeakID_38071 (chr11: 12851503 − 12853456)

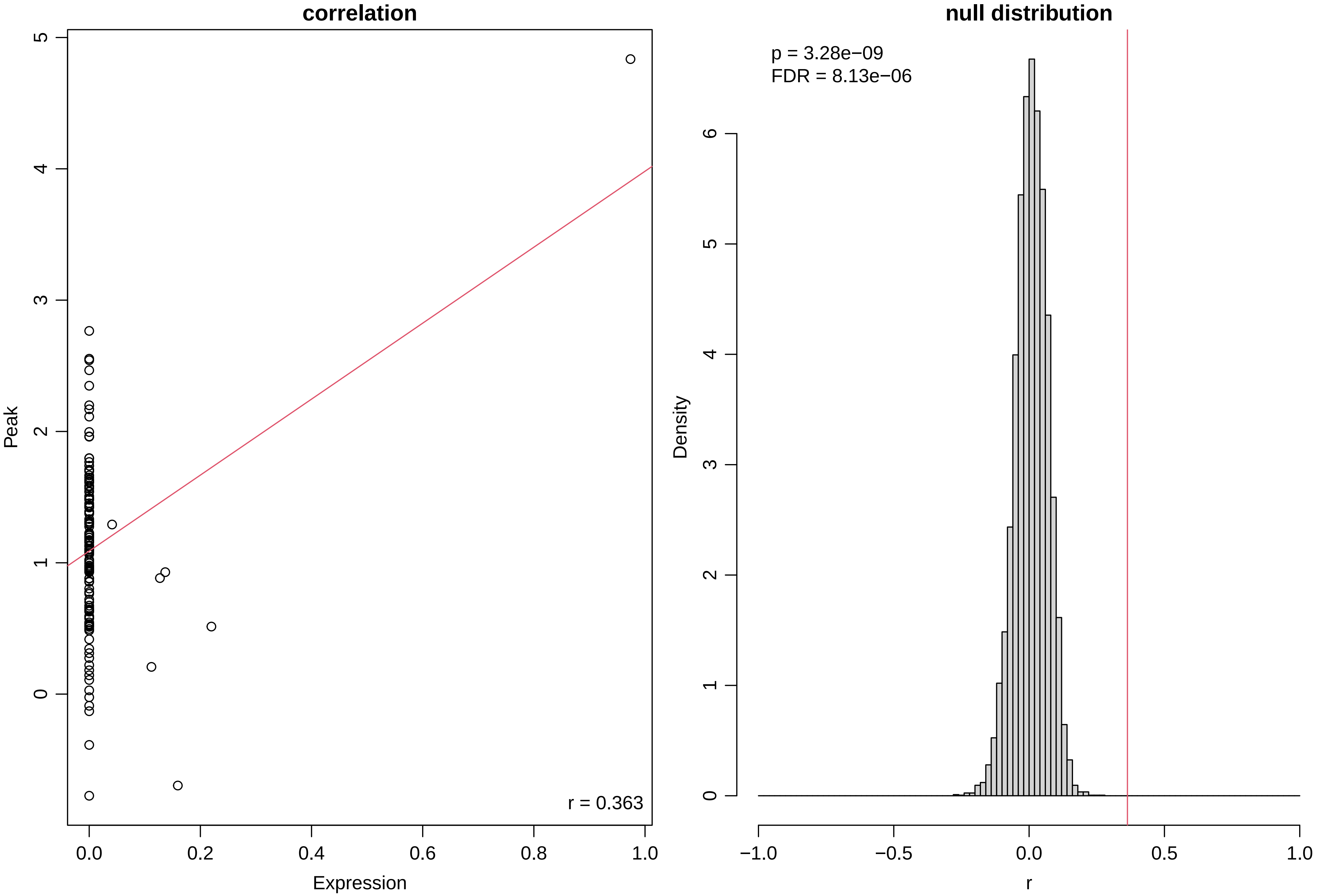

AL354824.1 vs PeakID_143369 (chr20: 10171869 − 10172782)

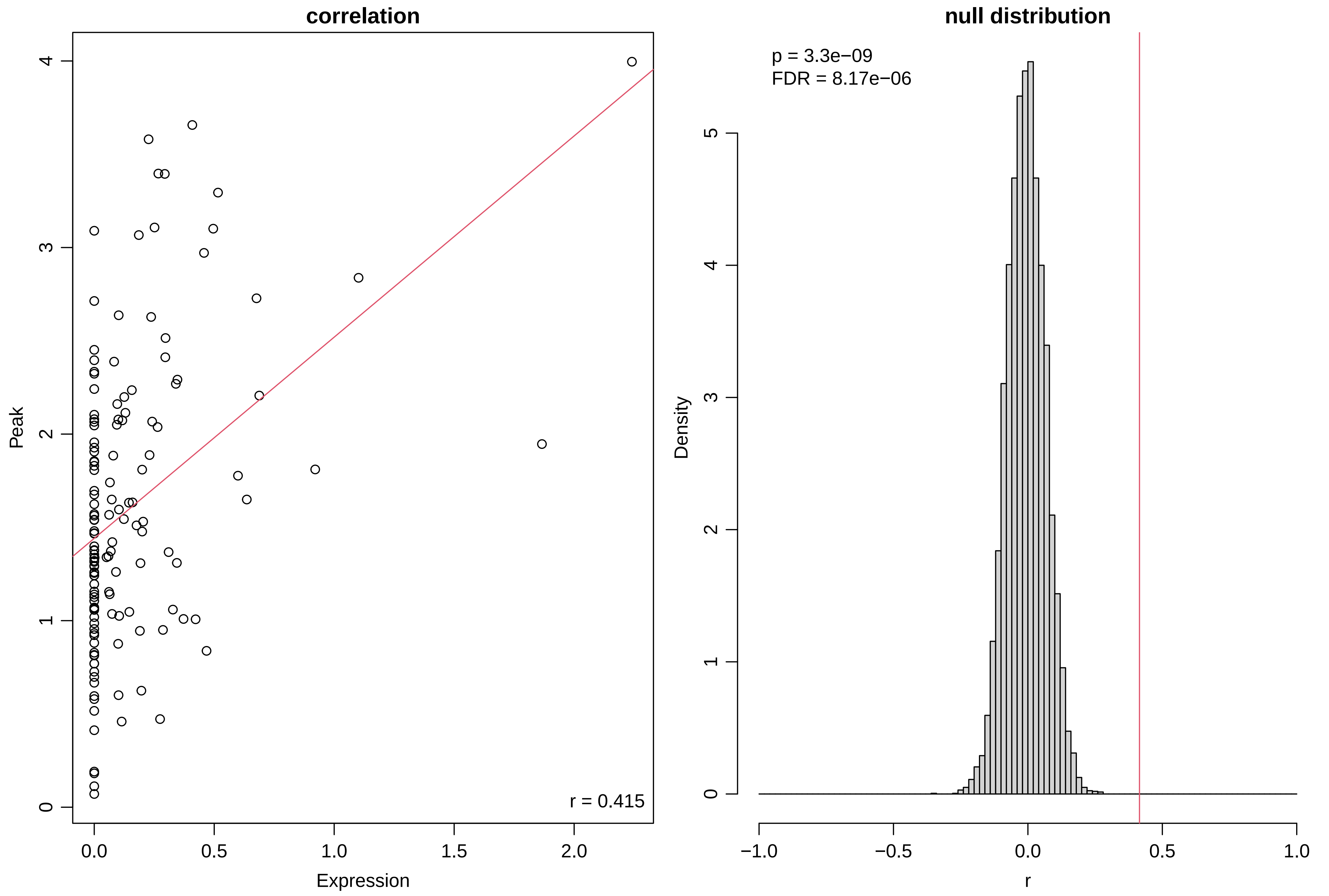

MYO3B vs PeakID_135625 (chr2: 170180158 − 170180565)

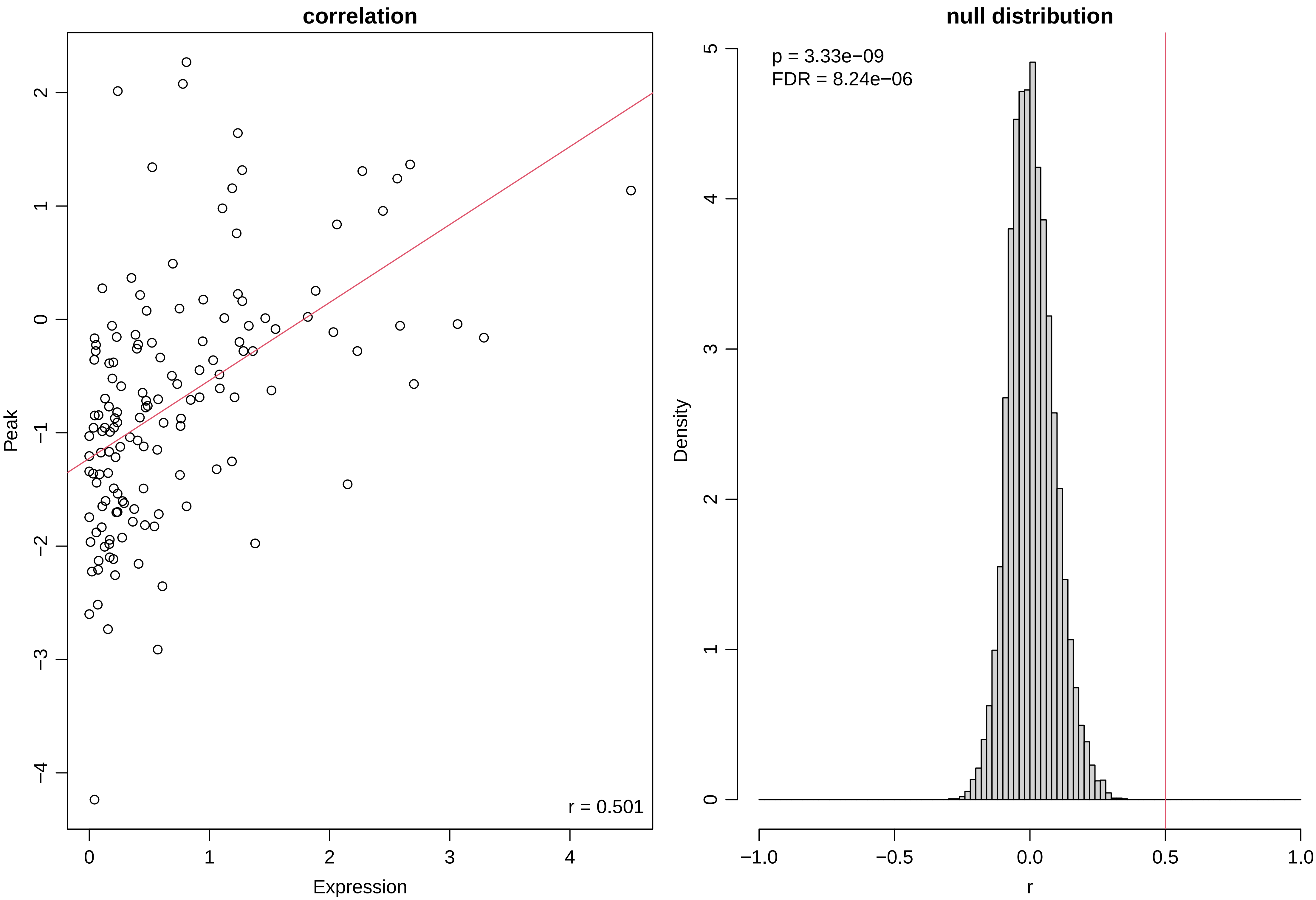

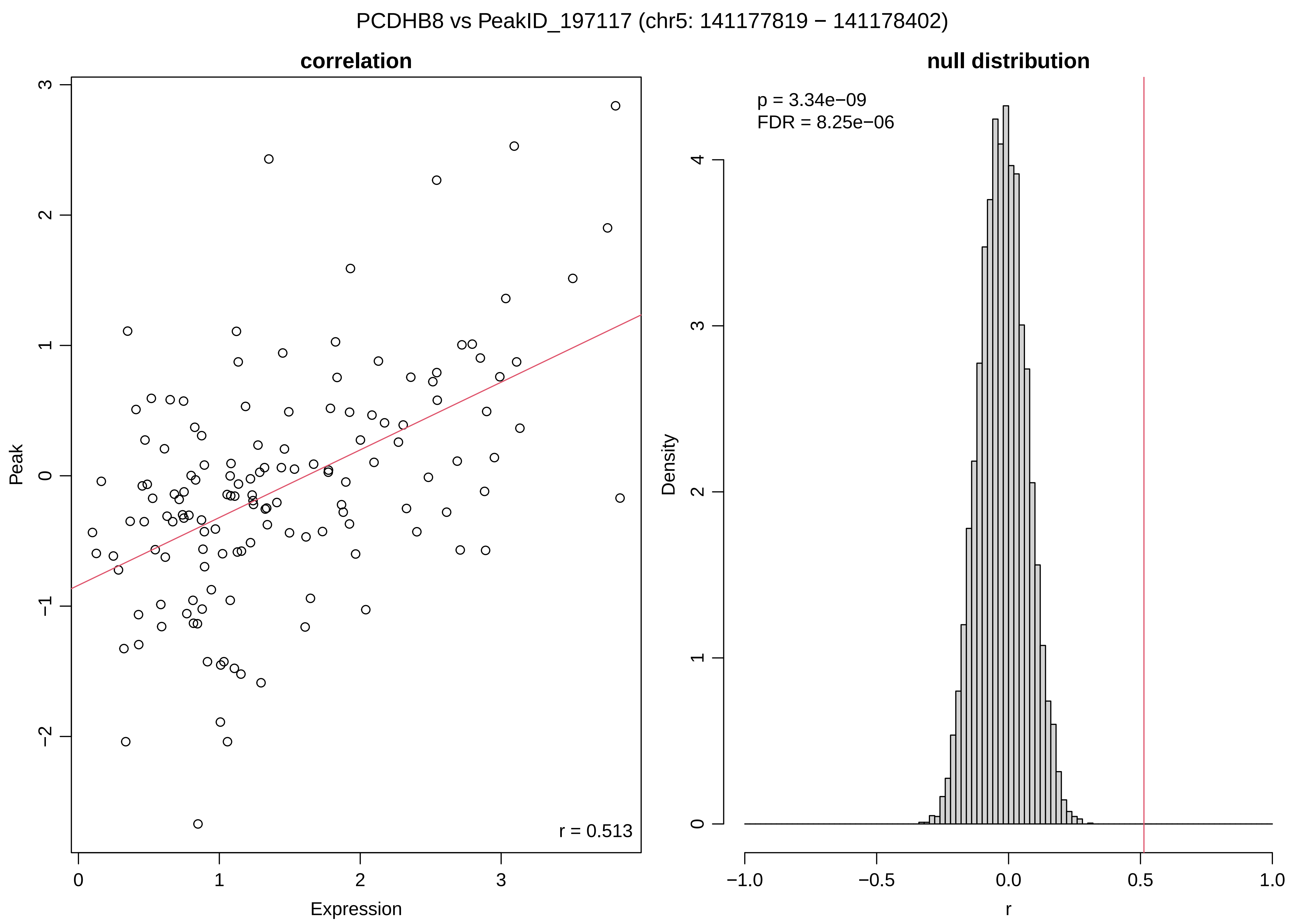
PCDHB8 vs PeakID_197117 (chr5: 141177819 − 141178402)
correlation
null distribution
Peak
Expression
r = 0.513
p = 3.34e−09
FDR = 8.25e−06
Density
r
3
2
1
0
−1
−2
0
1
2
3
4
−1.0
−0.5
0.0
0.5
1.0

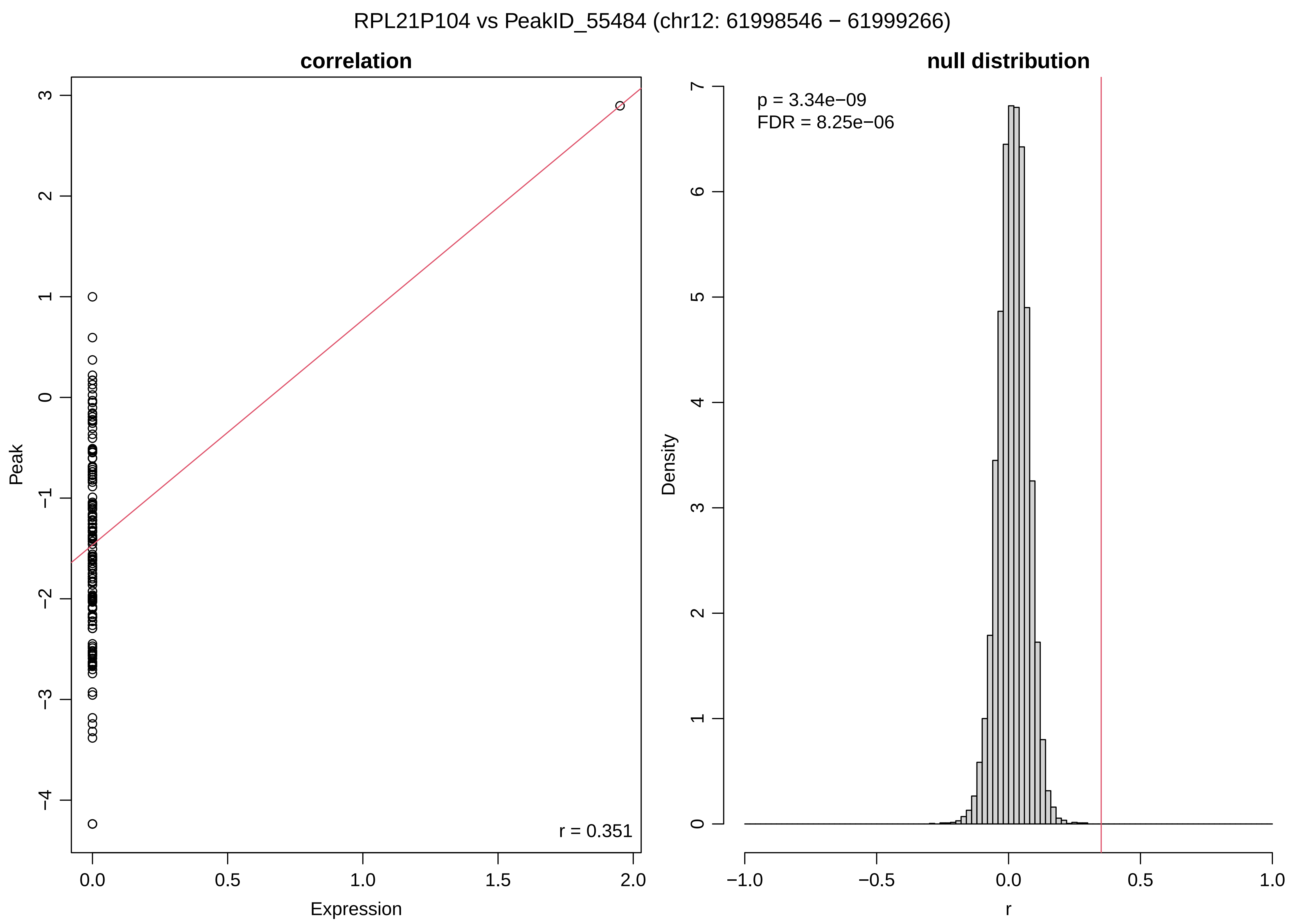

RPL21P104 vs PeakID_55484 (chr12: 61998546 − 61999266)
correlation
Peak
Expression
r = 0.351
null distribution
p = 3.34e−09
FDR = 8.25e−06
Density
r

GYG2P1 vs PeakID_262735 (chrY: 12641507 − 12642886)

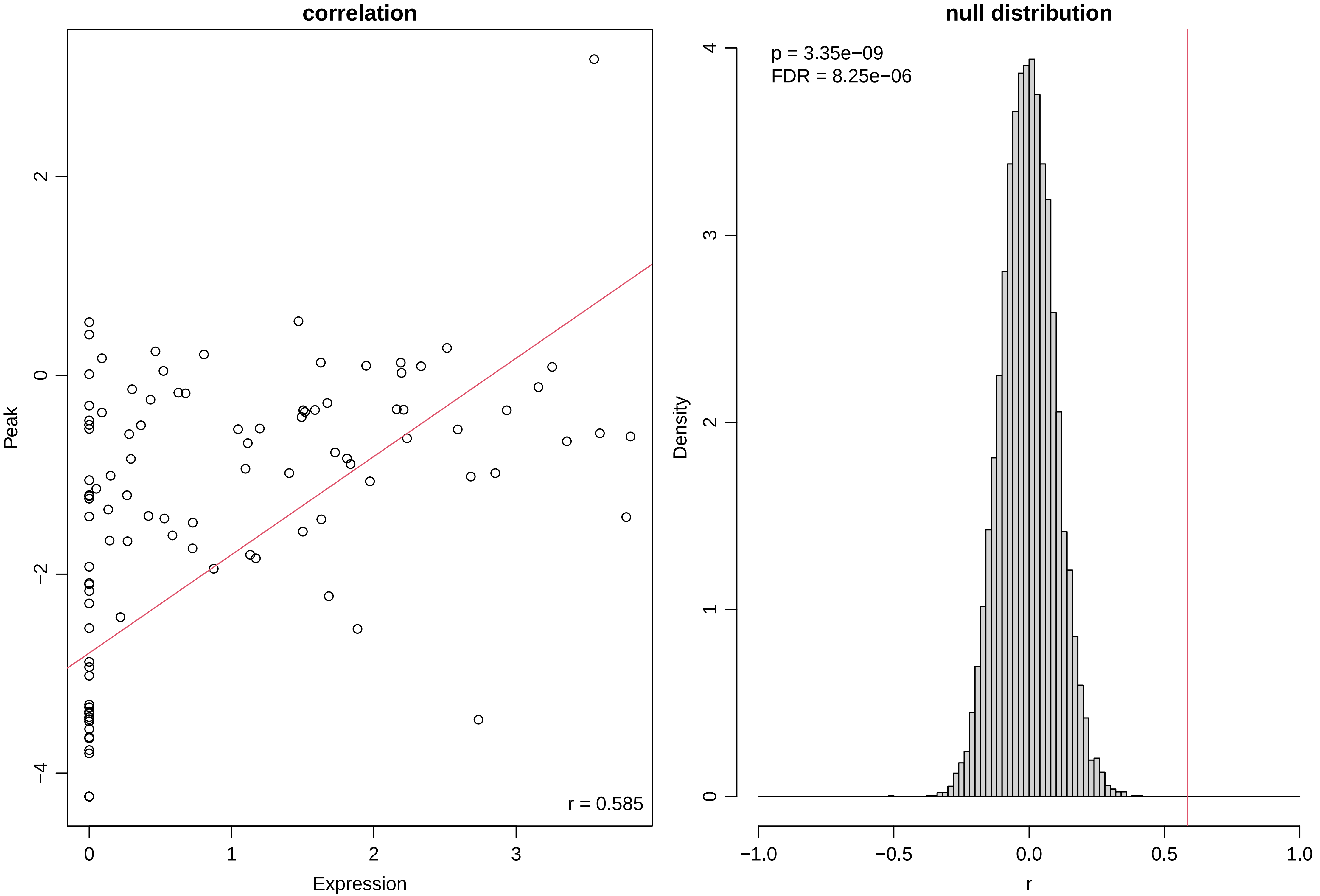

AC011370.1 vs PeakID_197774 (chr5: 147721387 − 147721932)

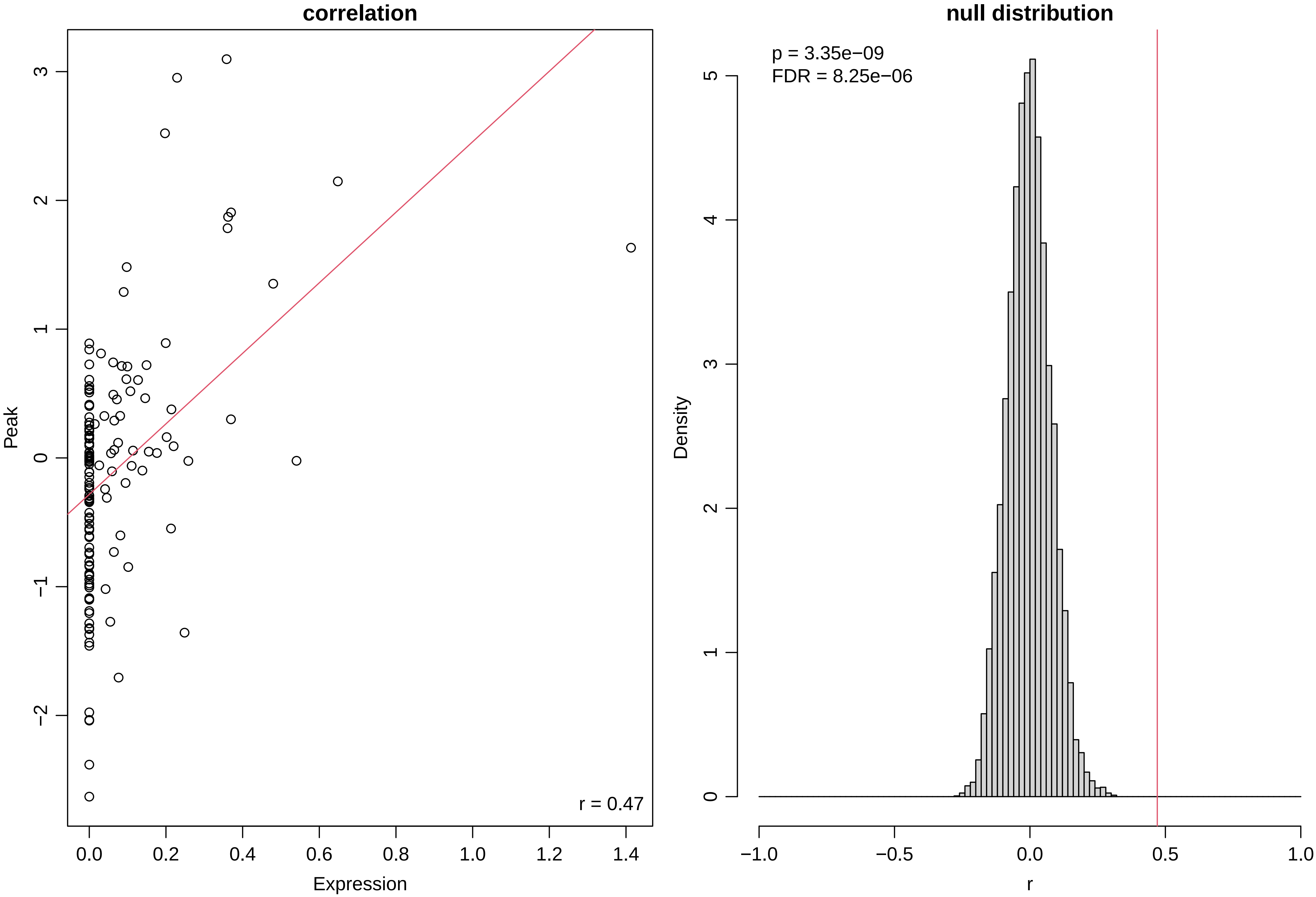

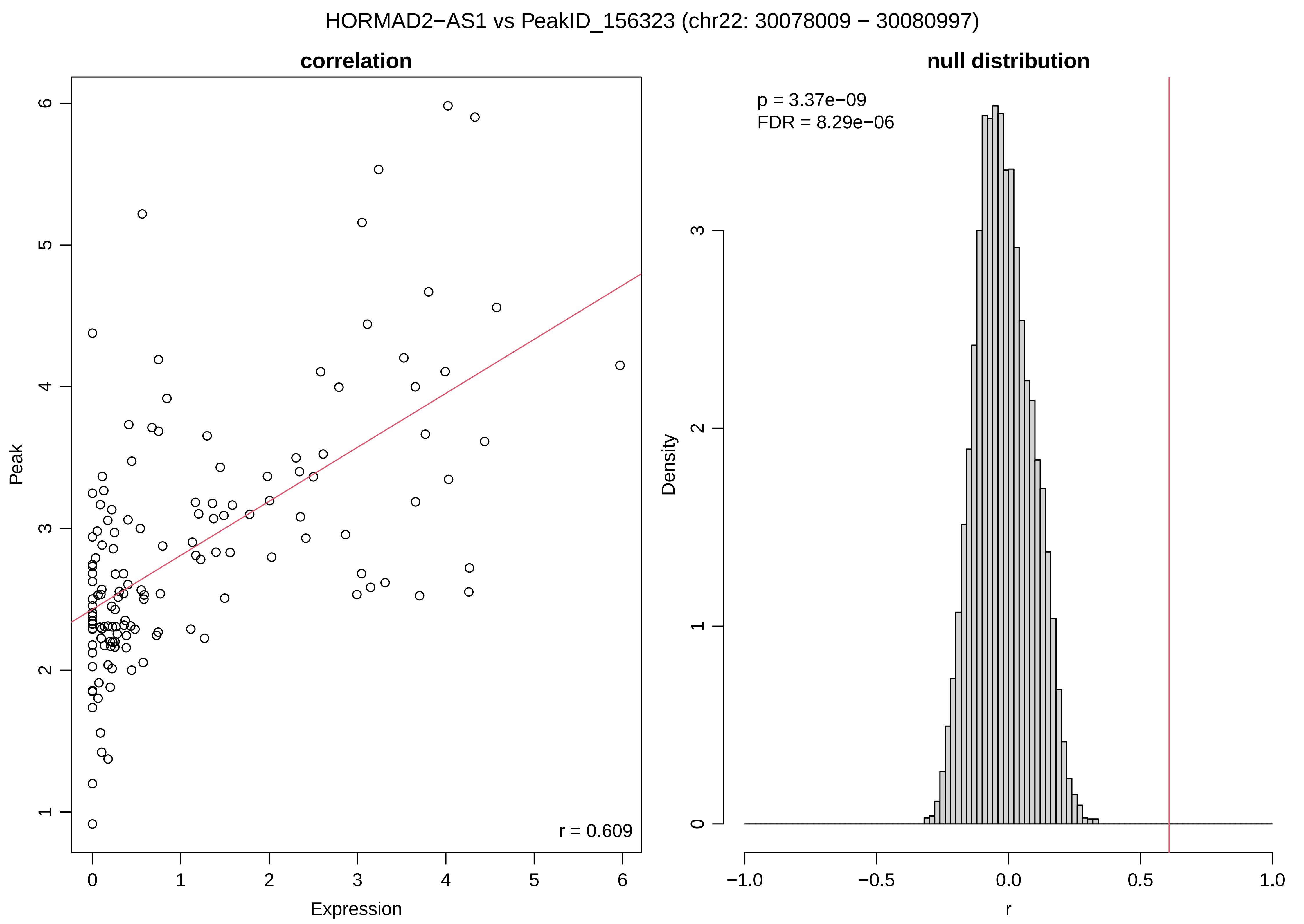
HORMAD2−AS1 vs PeakID_156323 (chr22: 30078009 − 30080997)
correlation
null distribution
p = 3.37e−09
FDR = 8.29e−06
r = 0.609
Peak
Expression
Density
r

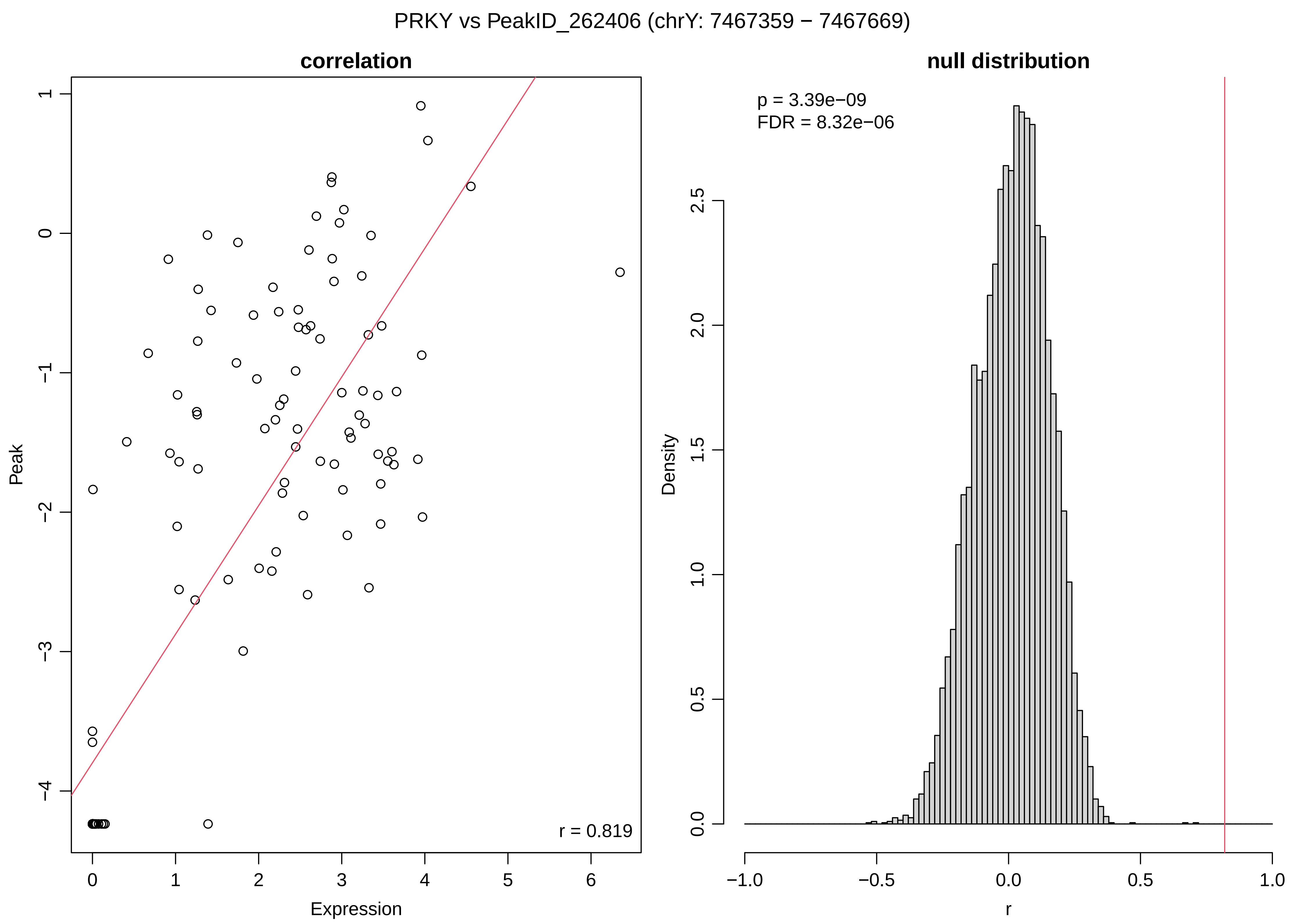

PRKY vs PeakID_262406 (chrY: 7467359 − 7467669)
correlation
Peak
Expression
r = 0.819
null distribution
p = 3.39e−09
FDR = 8.32e−06
Density
r

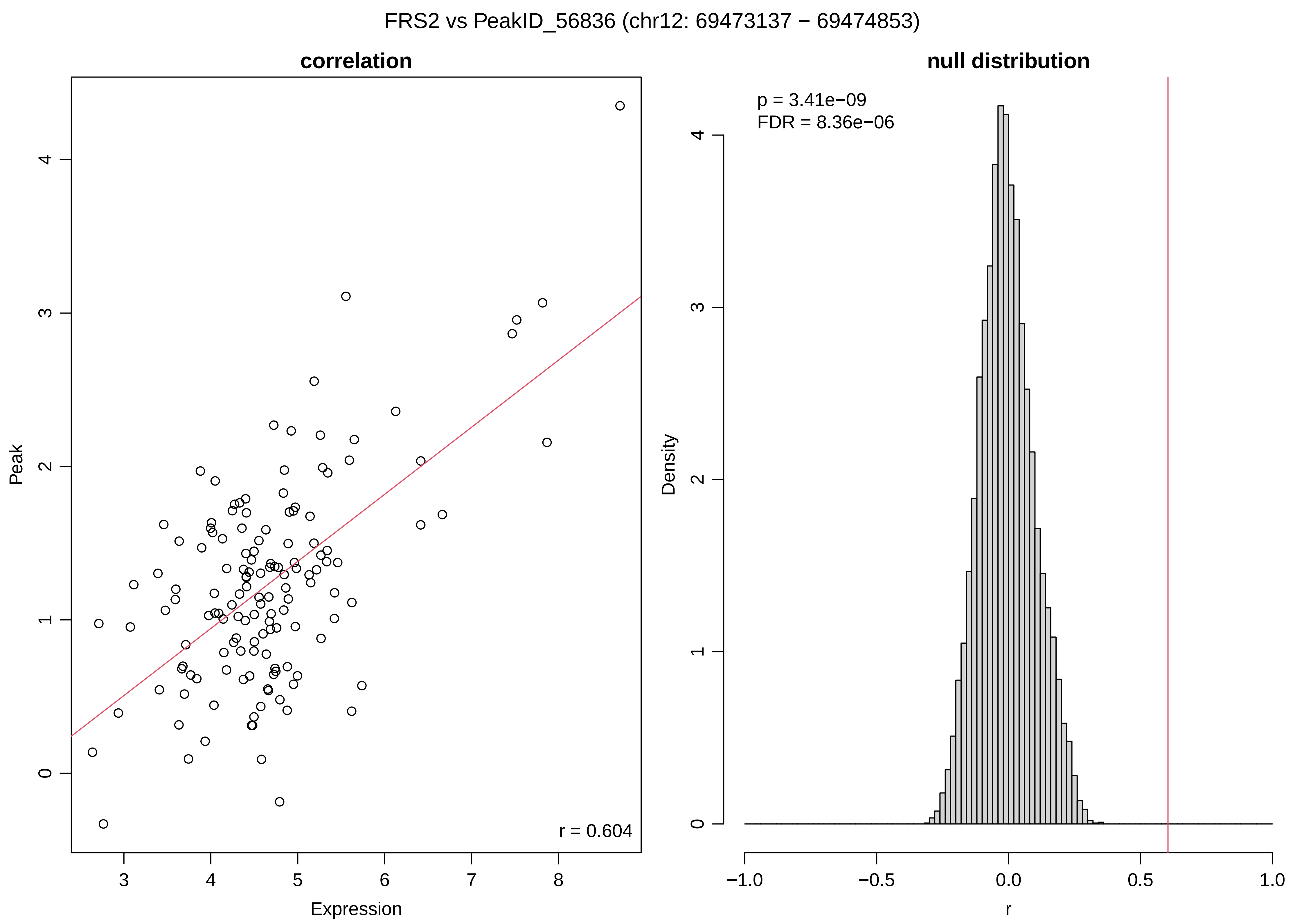
FRS2 vs PeakID_56836 (chr12: 69473137 − 69474853)
correlation
Peak
Expression
r = 0.604
null distribution
p = 3.41e−09
FDR = 8.36e−06
Density
r

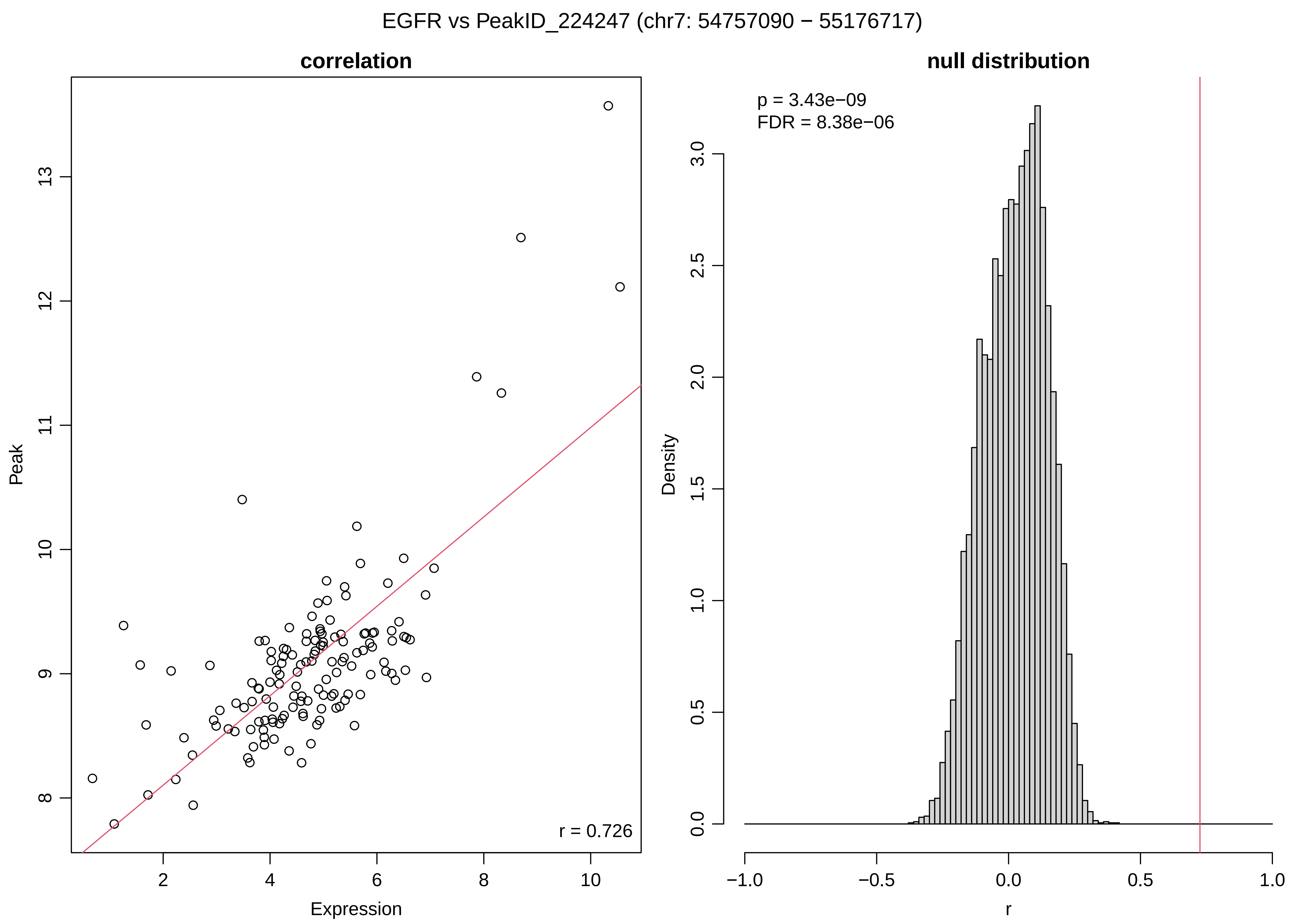
EGFR vs PeakID_224247 (chr7: 54757090 − 55176717)
correlation
Peak
Expression
r = 0.726
13
12
11
10
9
8
2
4
6
8
10
null distribution
p = 3.43e−09
FDR = 8.38e−06
Density
r
3.0
2.5
2.0
1.5
1.0
0.5
0.0
−1.0
−0.5
0.0
0.5
1.0

AP005482.1 vs PeakID_107860 (chr18: 12776762 − 12778140)

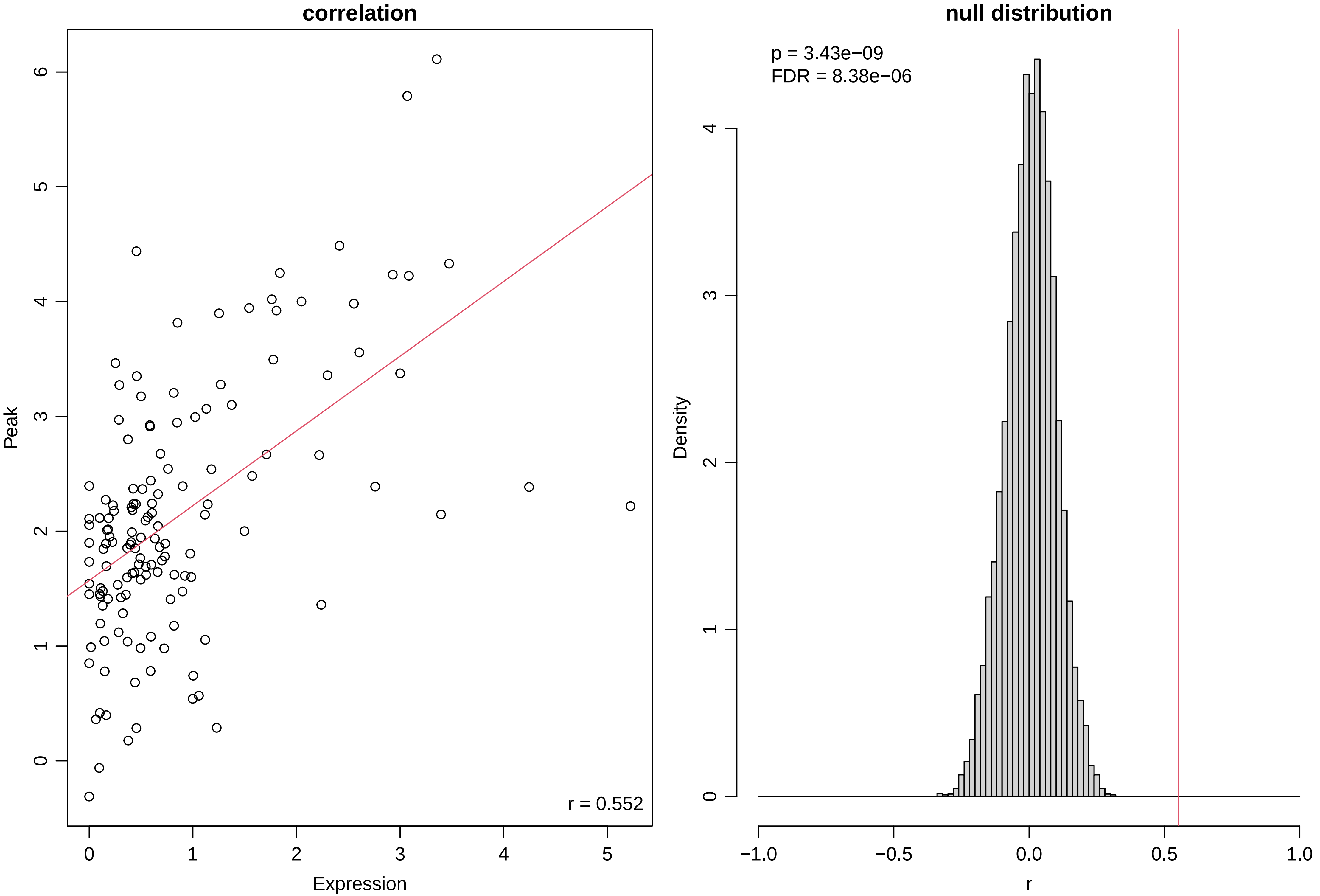

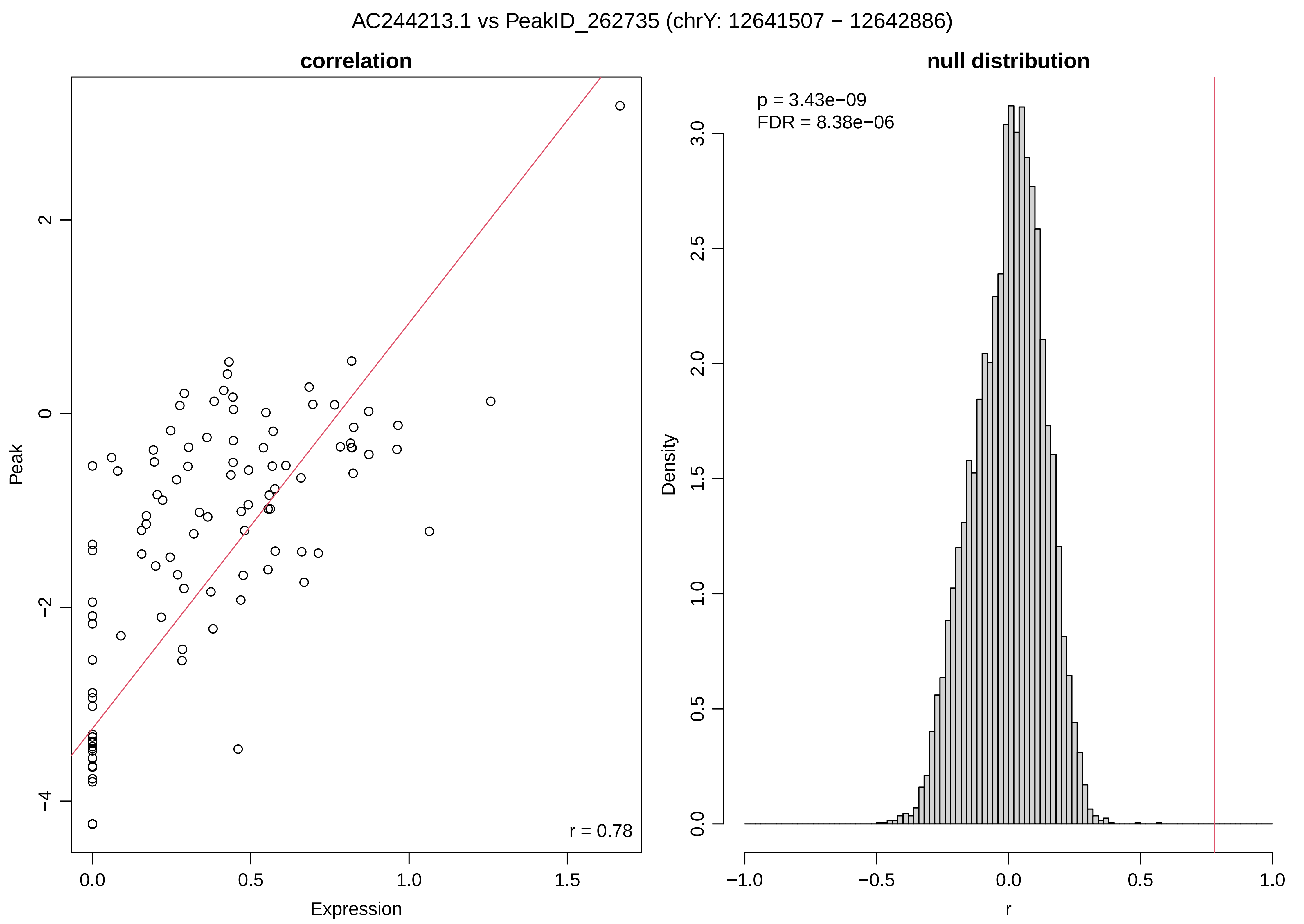

AC244213.1 vs PeakID_262735 (chrY: 12641507 − 12642886)
correlation
Peak
Expression
r = 0.78
null distribution
p = 3.43e−09
FDR = 8.38e−06
Density
r

AC107881.1 vs PeakID_37832 (chr11: 12436398 − 12437159)

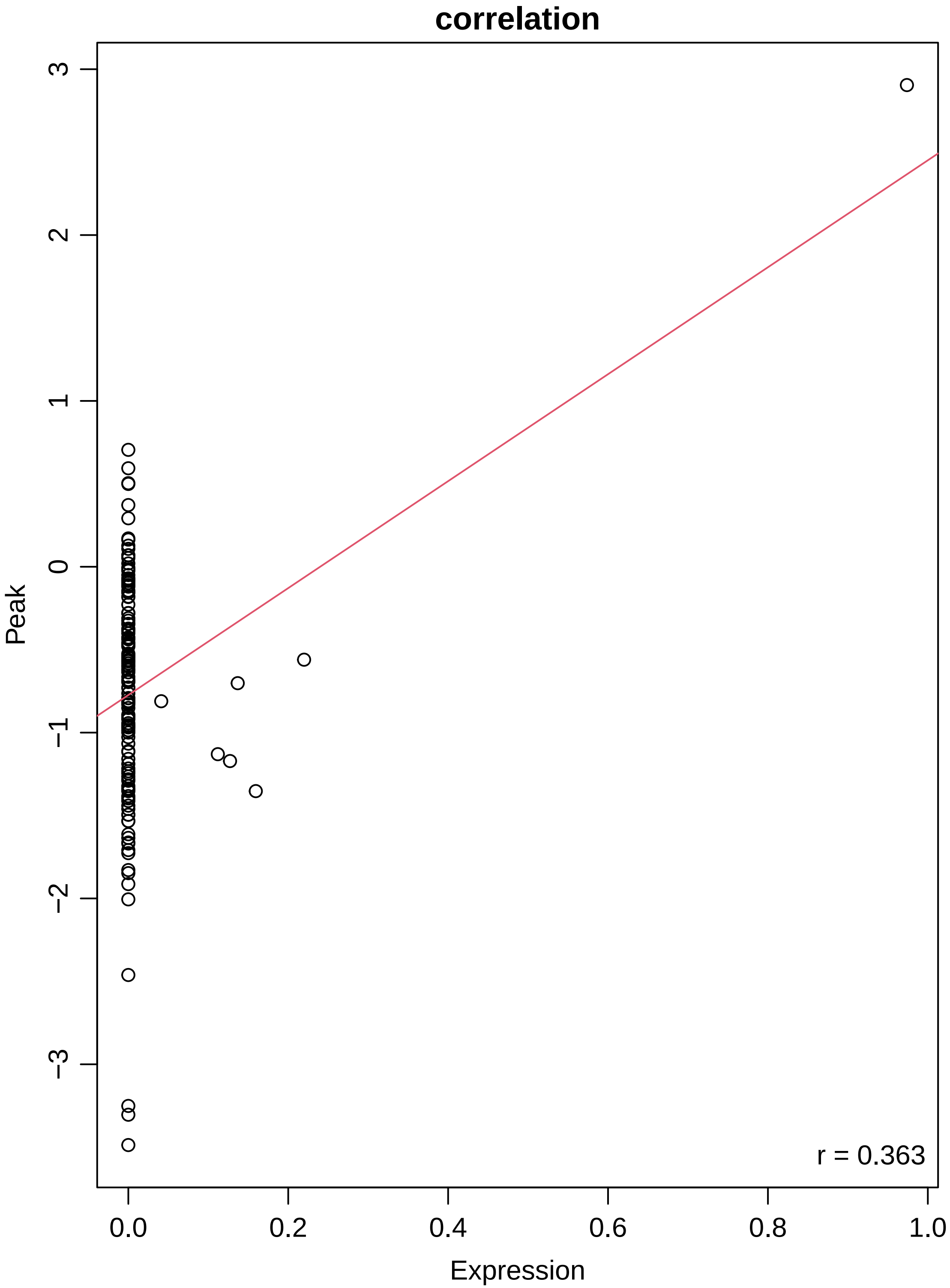

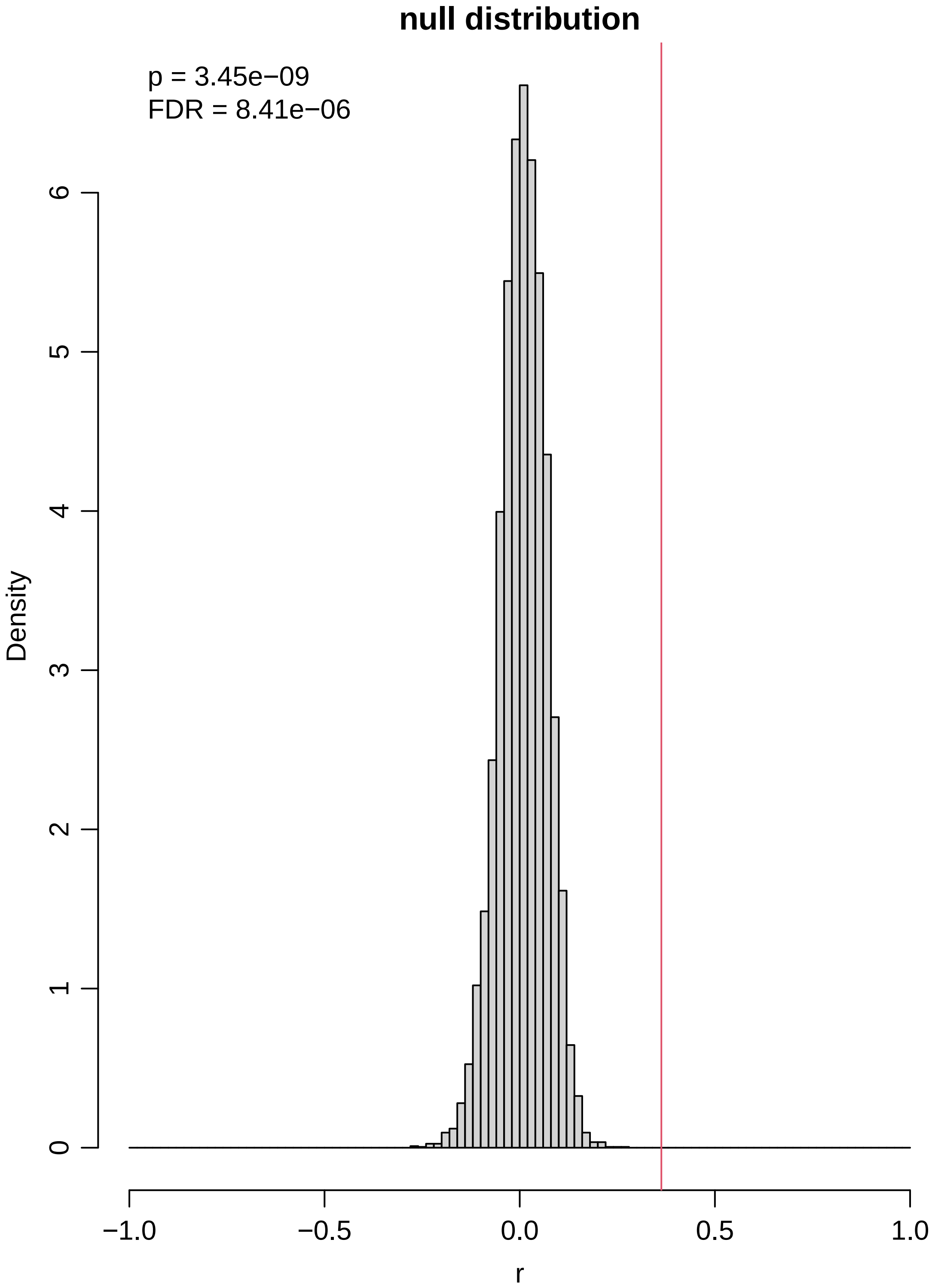

RBP1 vs PeakID_170090 (chr3: 139537295 − 139540512)

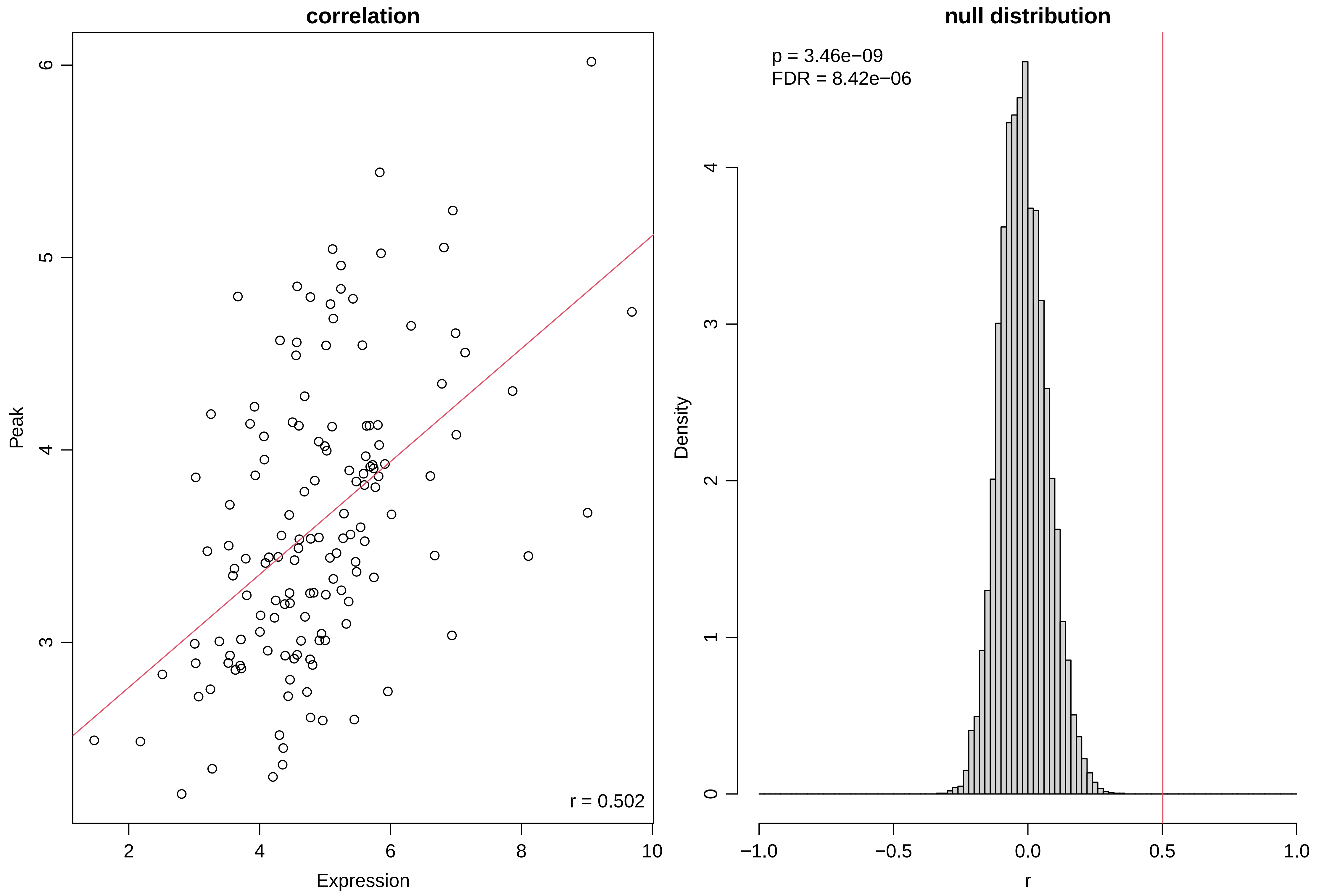

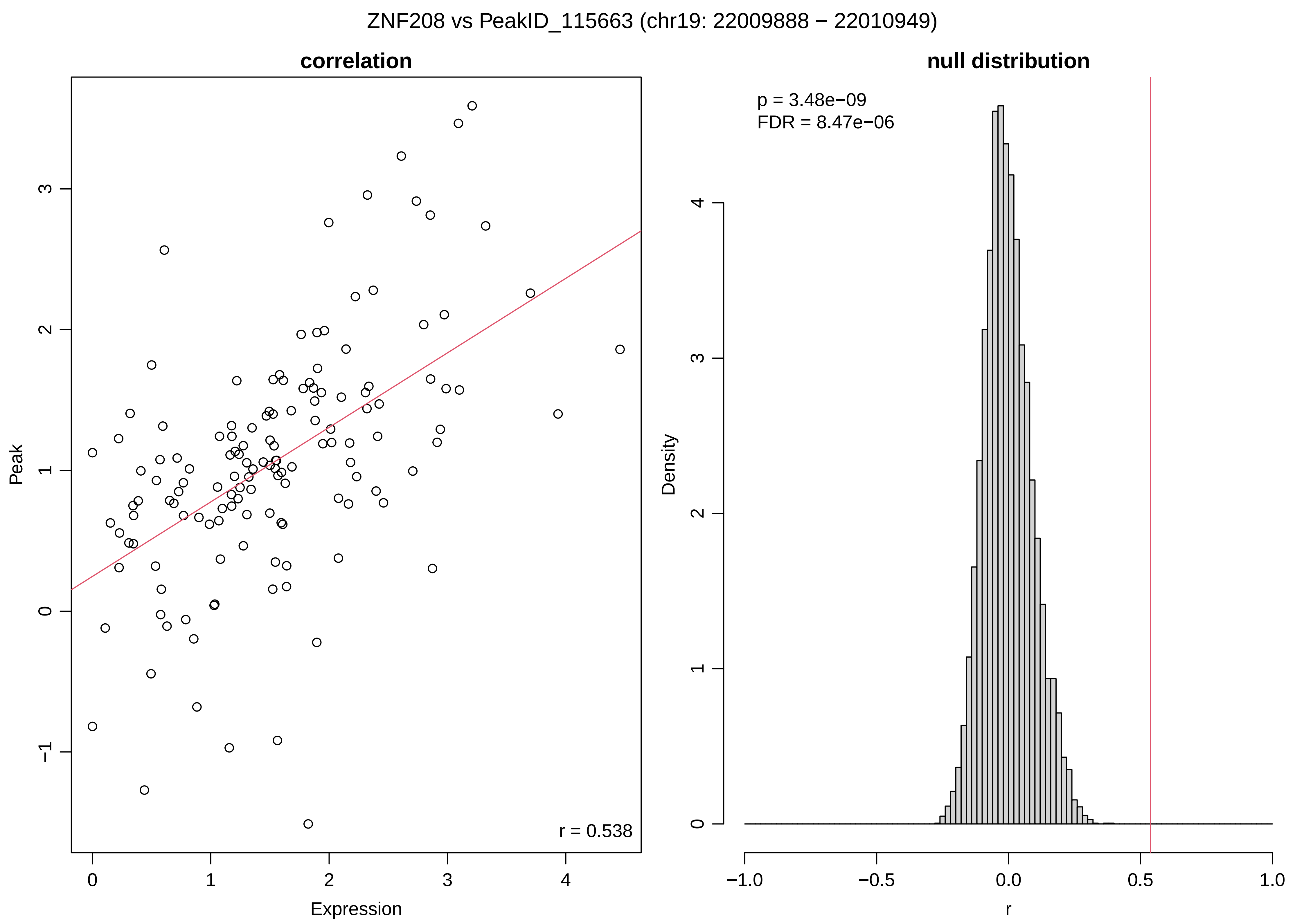
ZNF208 vs PeakID_115663 (chr19: 22009888 − 22010949)
correlation
null distribution
p = 3.48e−09
FDR = 8.47e−06
r = 0.538
Peak
Expression
Density
r
−1
0
1
2
3
4
−1.0
−0.5
0.0
0.5
1.0

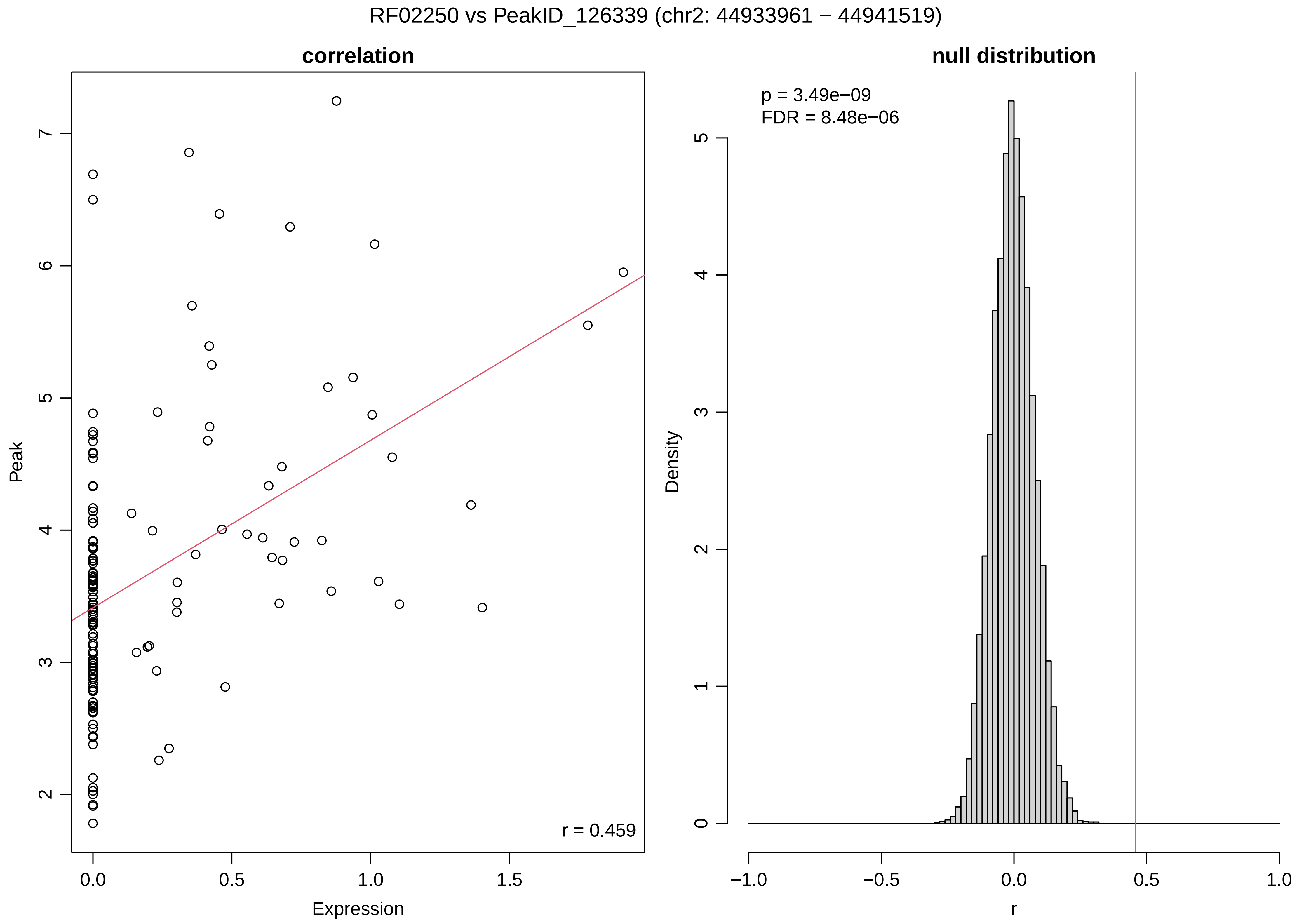
RF02250 vs PeakID_126339 (chr2: 44933961 − 44941519)
correlation
Peak
Expression
r = 0.459
null distribution
p = 3.49e−09
FDR = 8.48e−06
Density
r

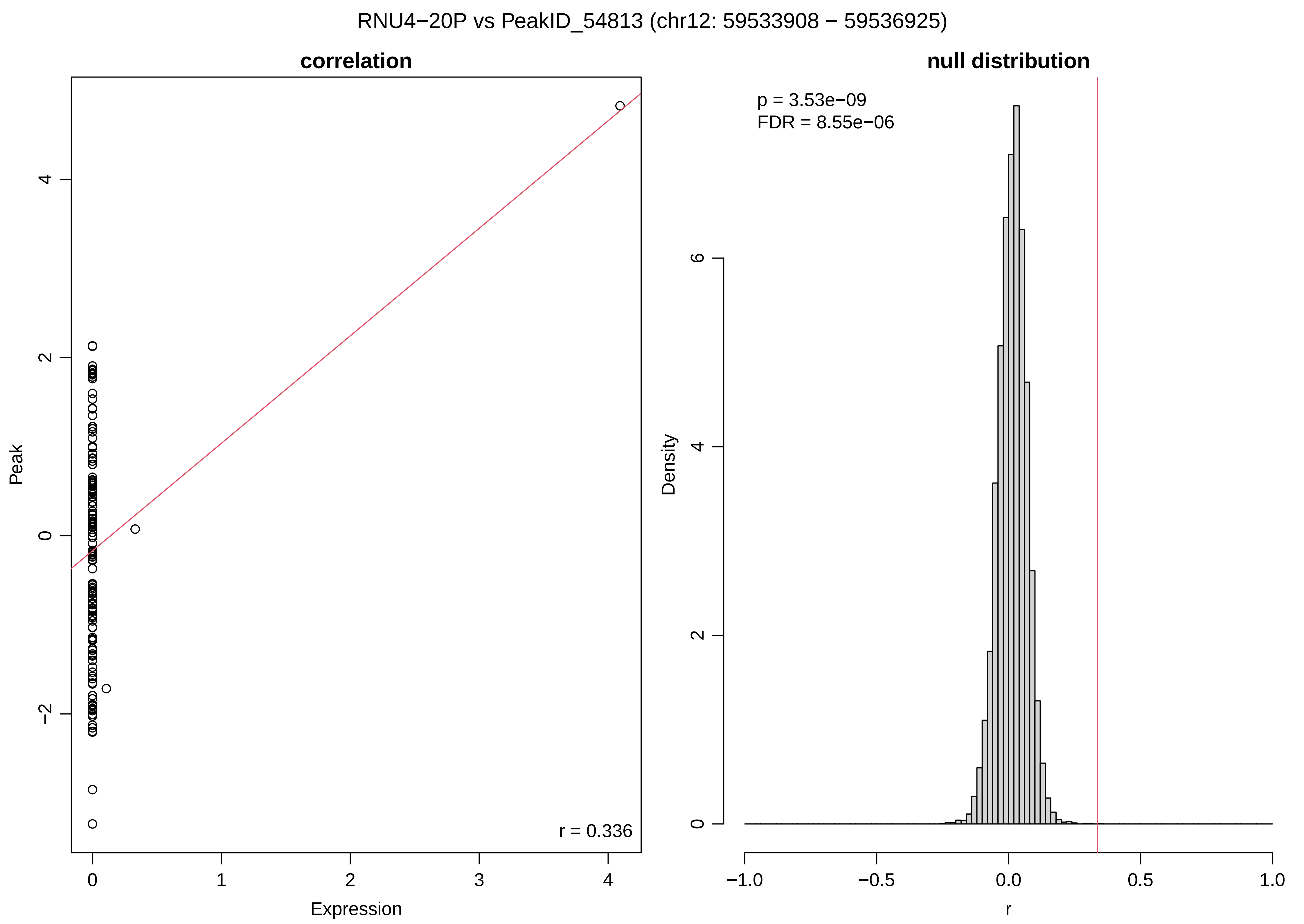

RNU4−20P vs PeakID_54813 (chr12: 59533908 − 59536925)
correlation
Peak
Expression
r = 0.336
null distribution
p = 3.53e−09
FDR = 8.55e−06
Density
r

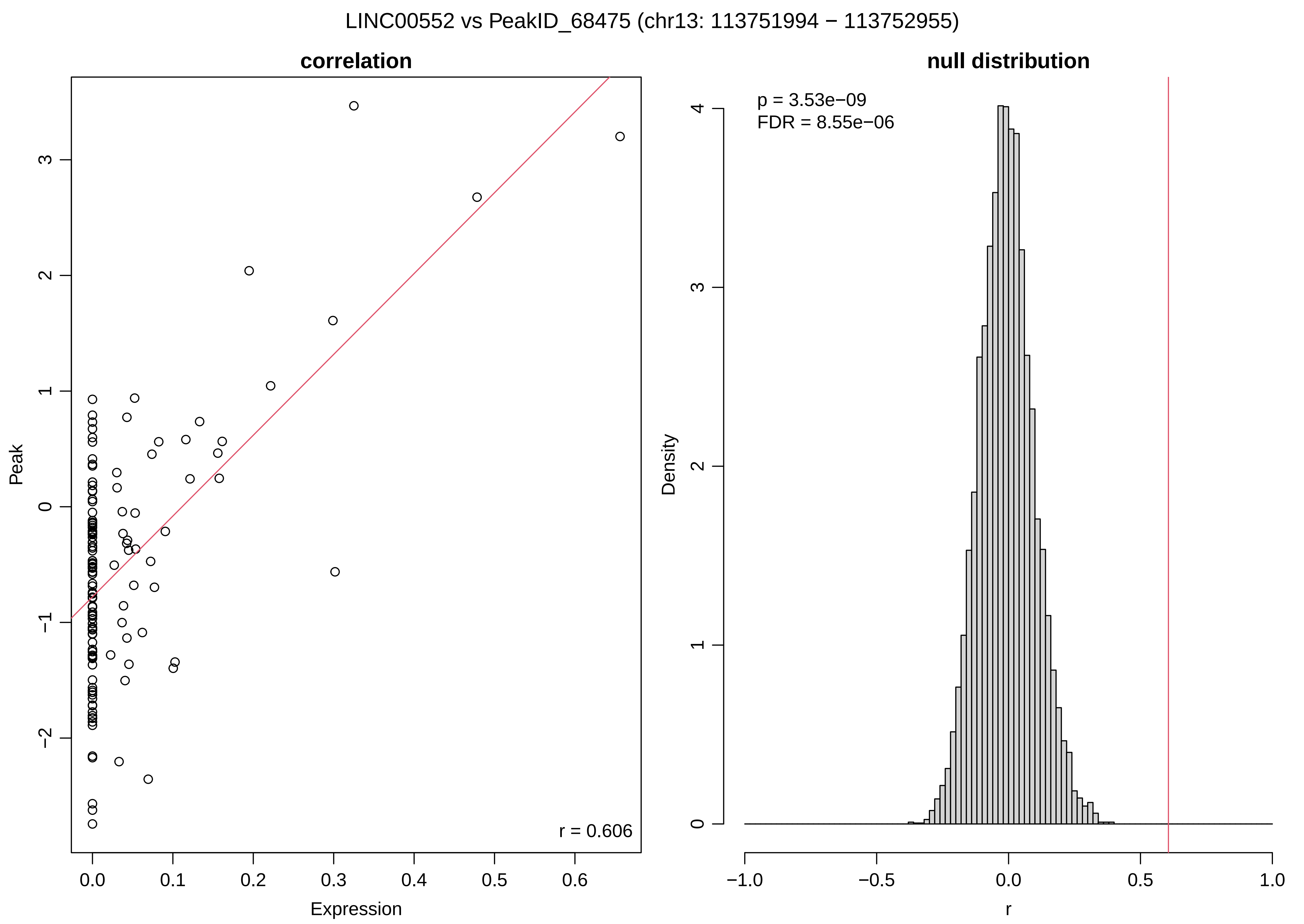
LINC00552 vs PeakID_68475 (chr13: 113751994 − 113752955)
correlation
null distribution
p = 3.53e−09
FDR = 8.55e−06
r = 0.606
Peak
Expression
Density
r
0.0
0.1
0.2
0.3
0.4
0.5
0.6
−2
−1
0
1
2
3
4
−1.0
−0.5
0.0
0.5
1.0

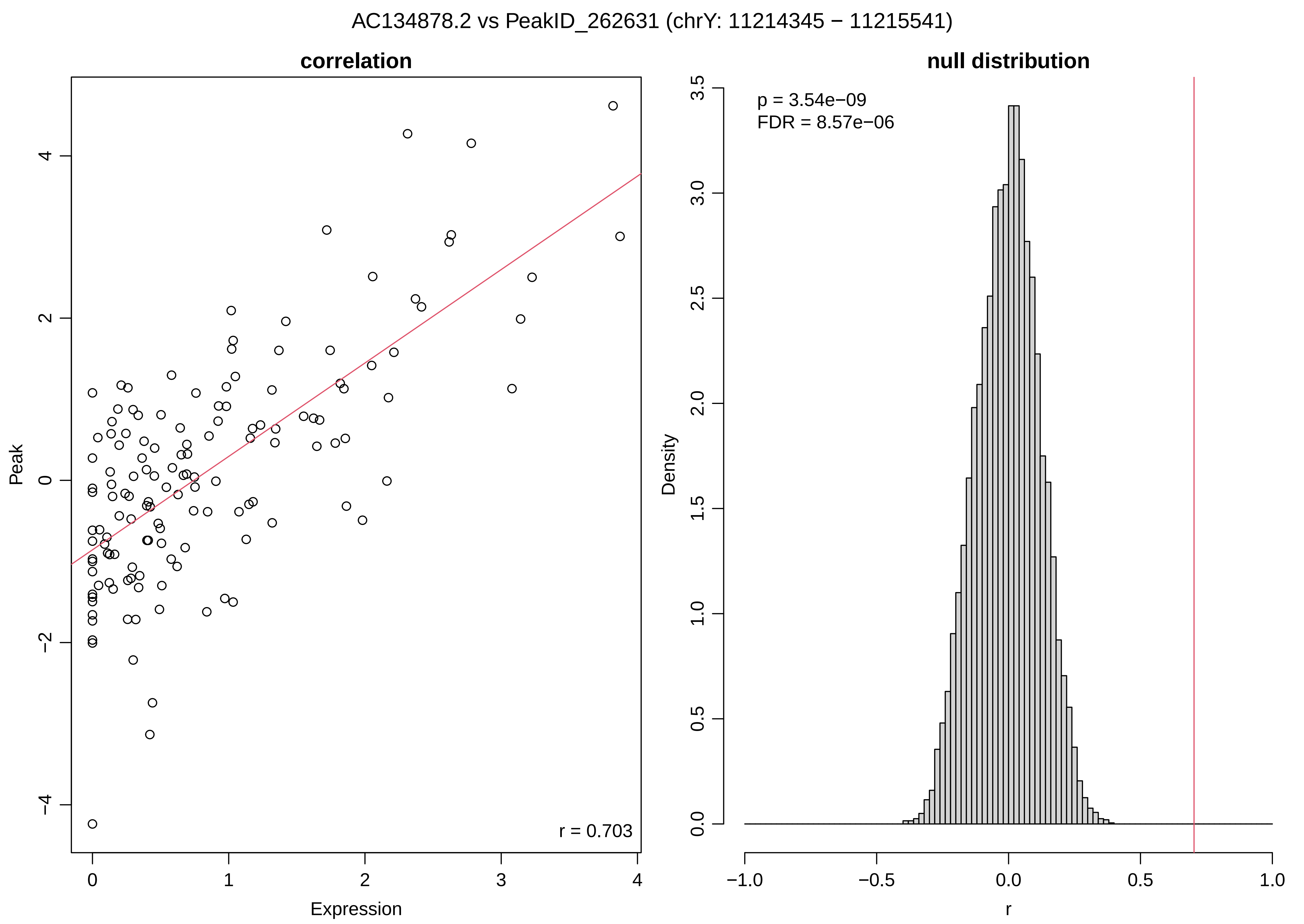

AC134878.2 vs PeakID_262631 (chrY: 11214345 − 11215541)
correlation
Peak
Expression
r = 0.703
null distribution
Density
r
p = 3.54e−09
FDR = 8.57e−06

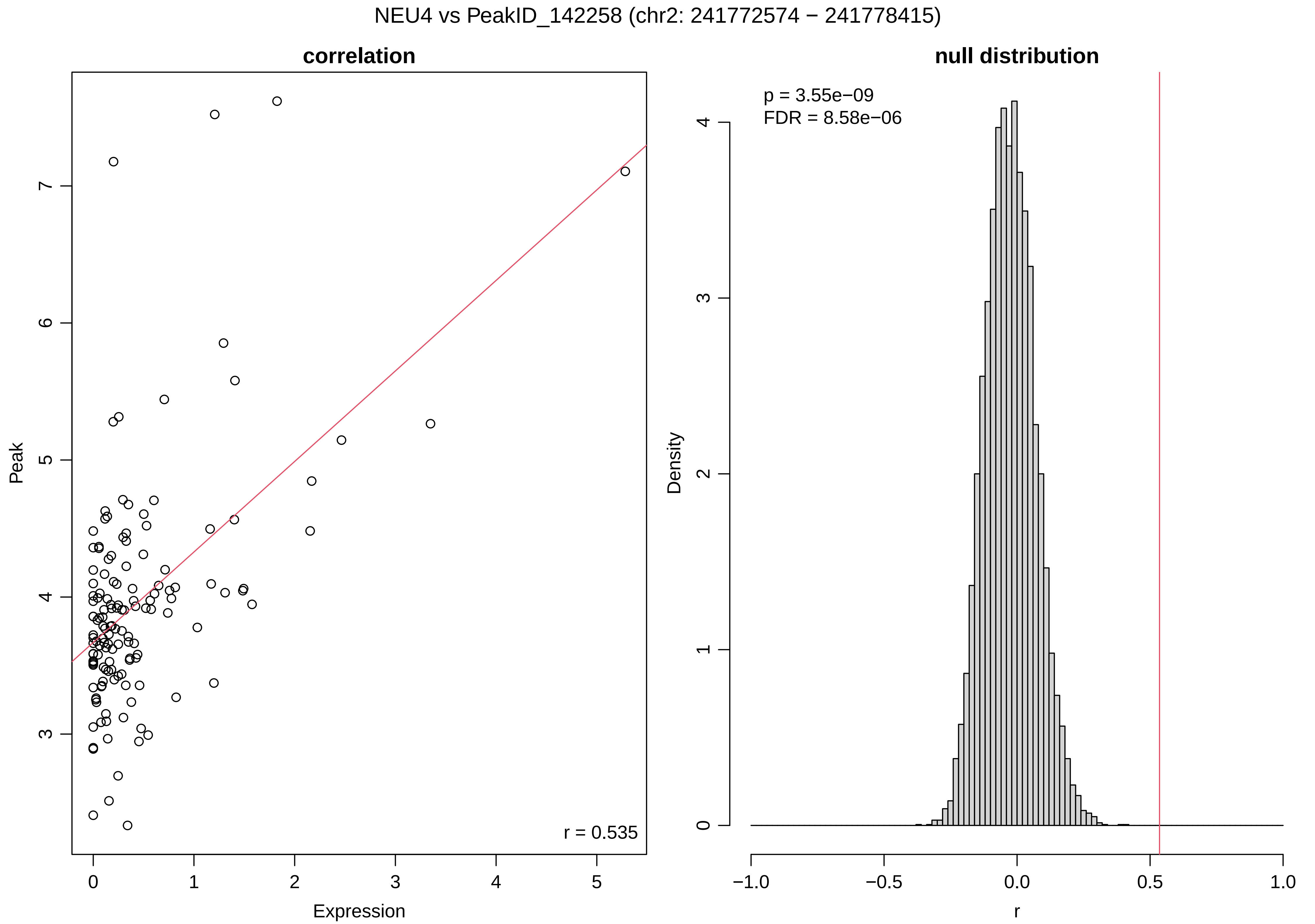

NEU4 vs PeakID_142258 (chr2: 241772574 − 241778415)
correlation
Peak
Expression
r = 0.535
null distribution
p = 3.55e−09
FDR = 8.58e−06
Density
r

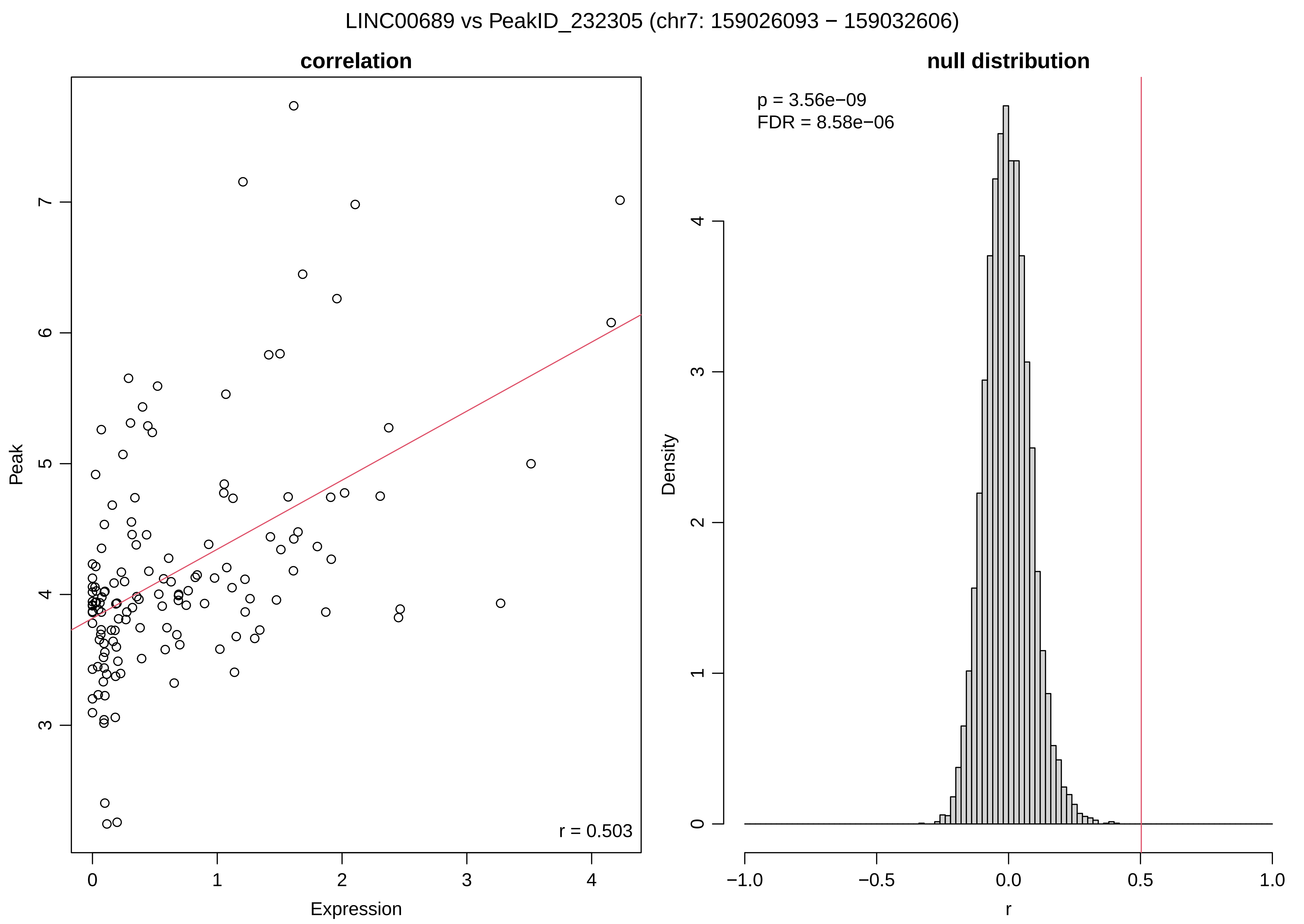
LINC00689 vs PeakID_232305 (chr7: 159026093 − 159032606)
correlation
Peak
Expression
r = 0.503
null distribution
p = 3.56e−09
FDR = 8.58e−06
Density
r

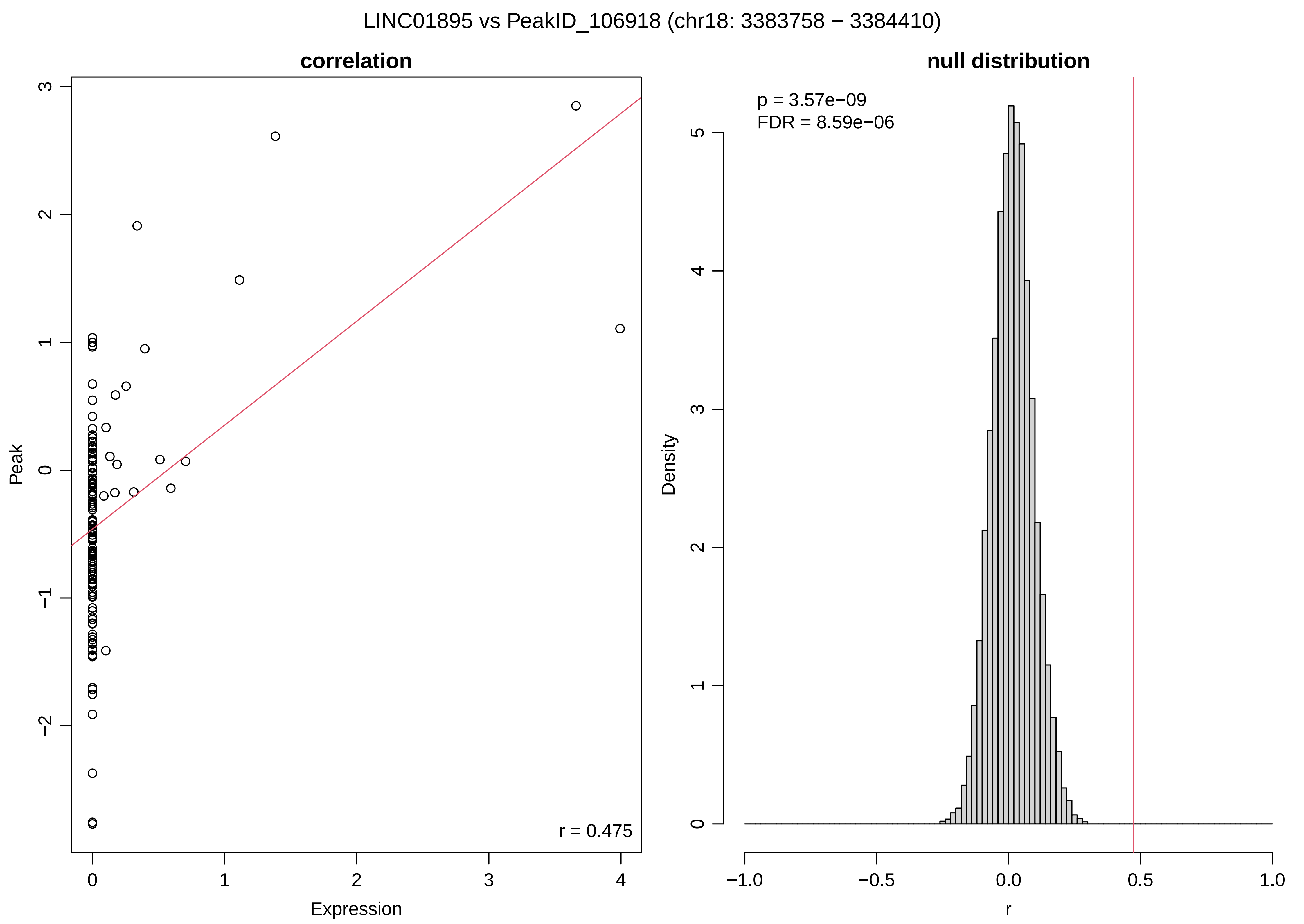
LINC01895 vs PeakID_106918 (chr18: 3383758 − 3384410)
correlation
Peak
Expression
r = 0.475
null distribution
p = 3.57e−09
FDR = 8.59e−06
Density
r

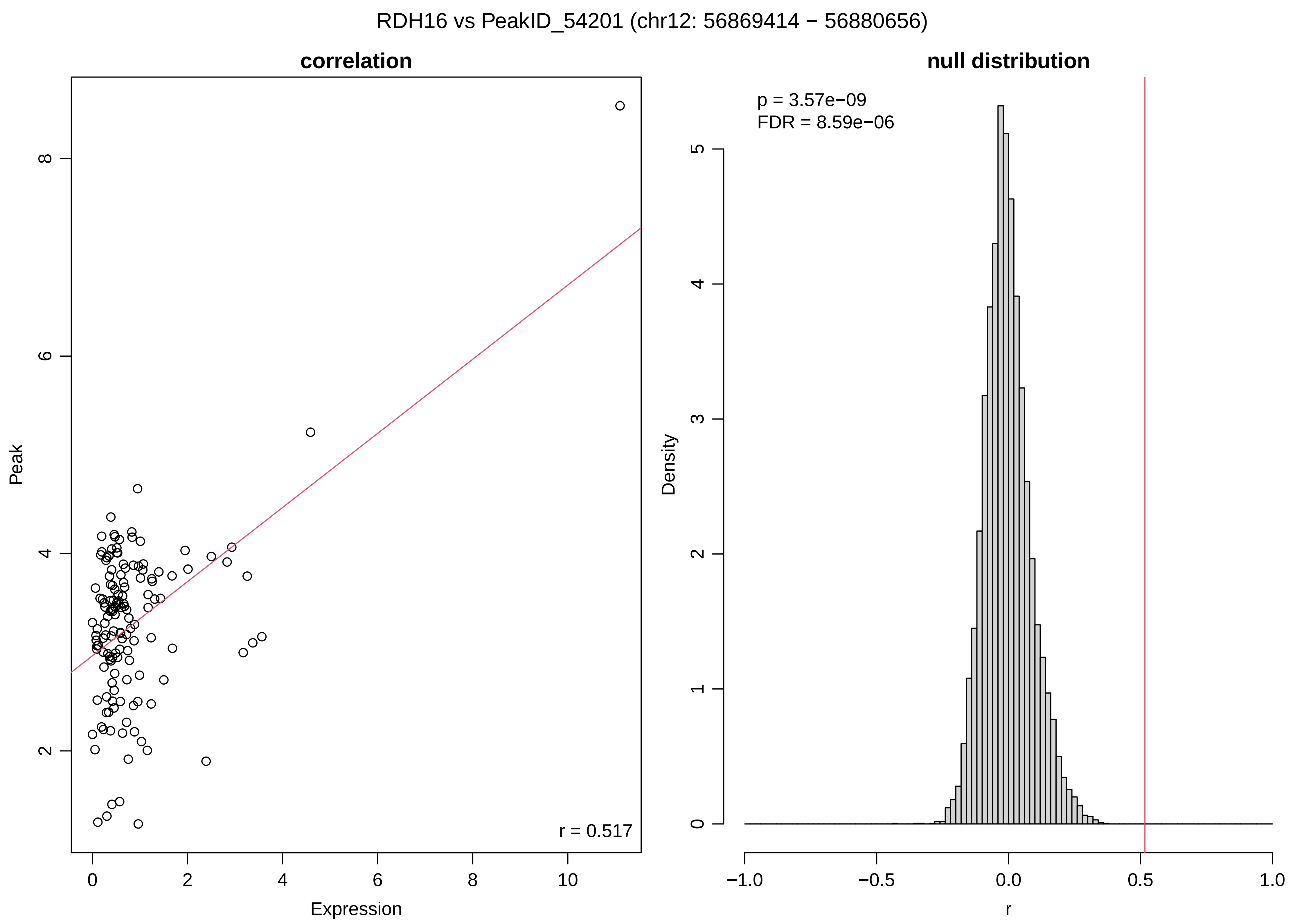

RDH16 vs PeakID_54201 (chr12: 56869414 − 56880656)
correlation
Peak
Expression
r = 0.517
null distribution
p = 3.57e−09
FDR = 8.59e−06
Density
r

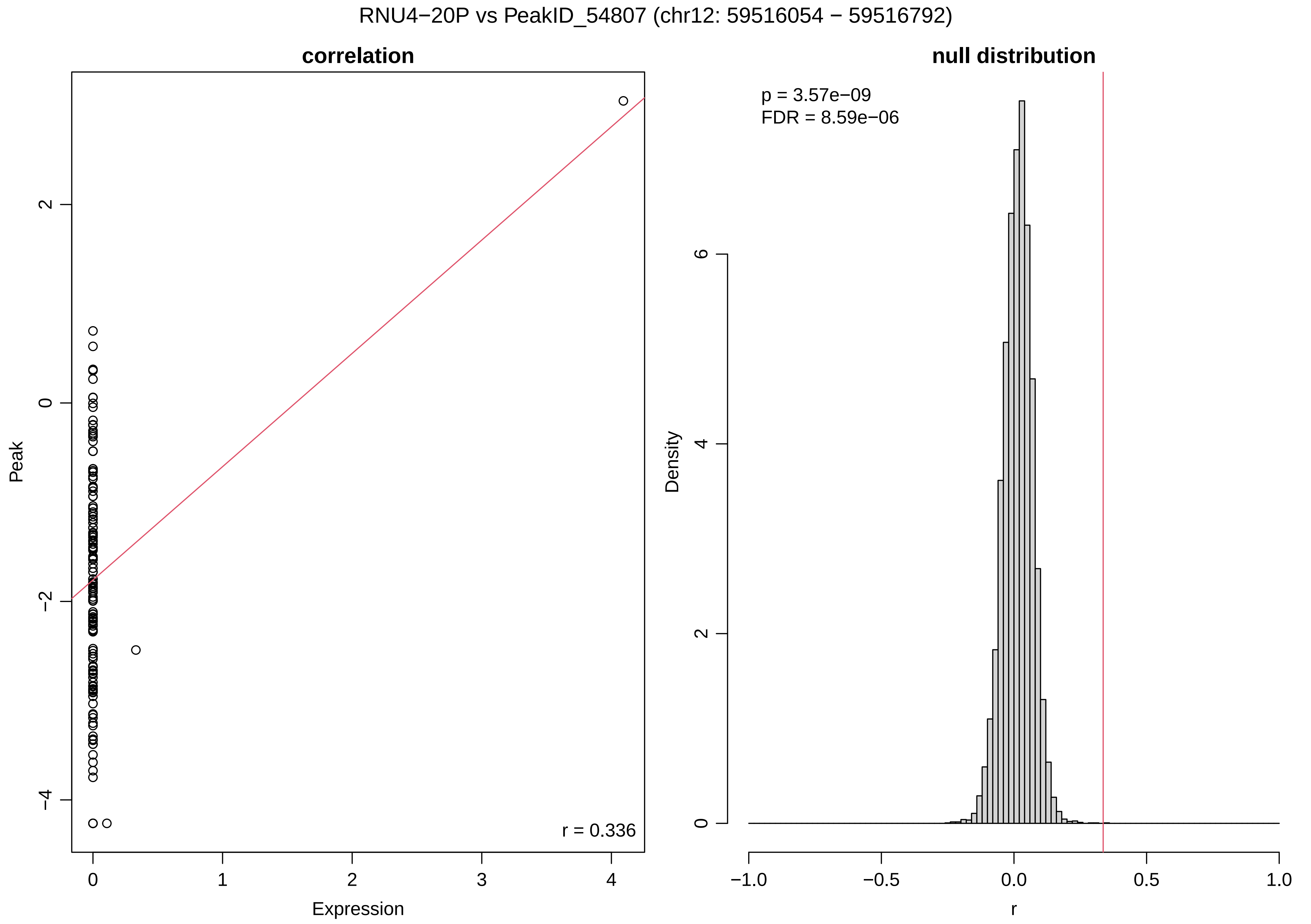

RNU4−20P vs PeakID_54807 (chr12: 59516054 − 59516792)
correlation
Peak
Expression
r = 0.336
null distribution
p = 3.57e−09
FDR = 8.59e−06
Density
r

AL391427.1 vs PeakID_24125 (chr10: 4971837 − 4973696)

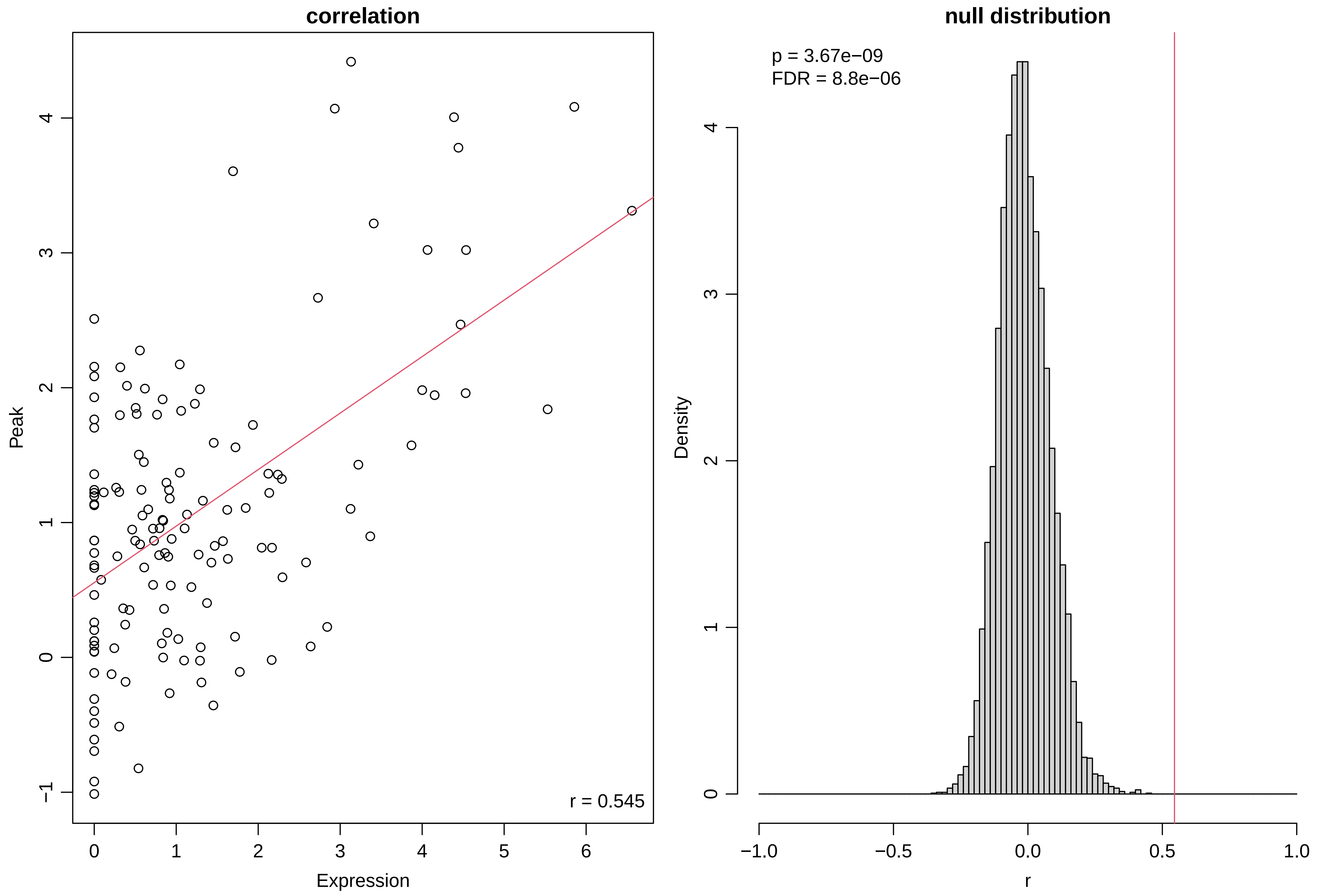

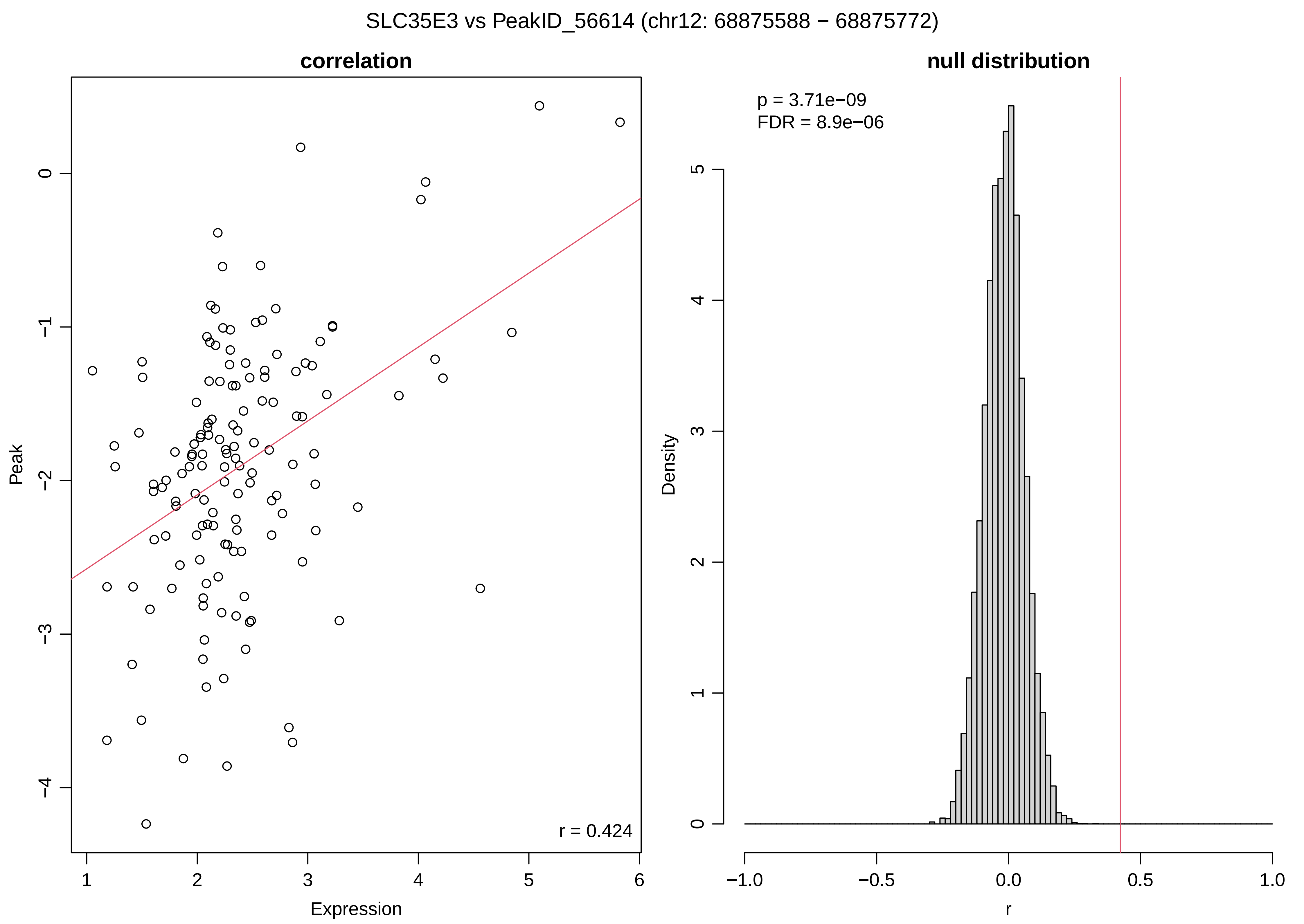
SLC35E3 vs PeakID_56614 (chr12: 68875588 − 68875772)
correlation
Peak
Expression
r = 0.424
null distribution
p = 3.71e−09
FDR = 8.9e−06
Density
r

DLX6−AS1 vs PeakID_227262 (chr7: 97005006 − 97007426)

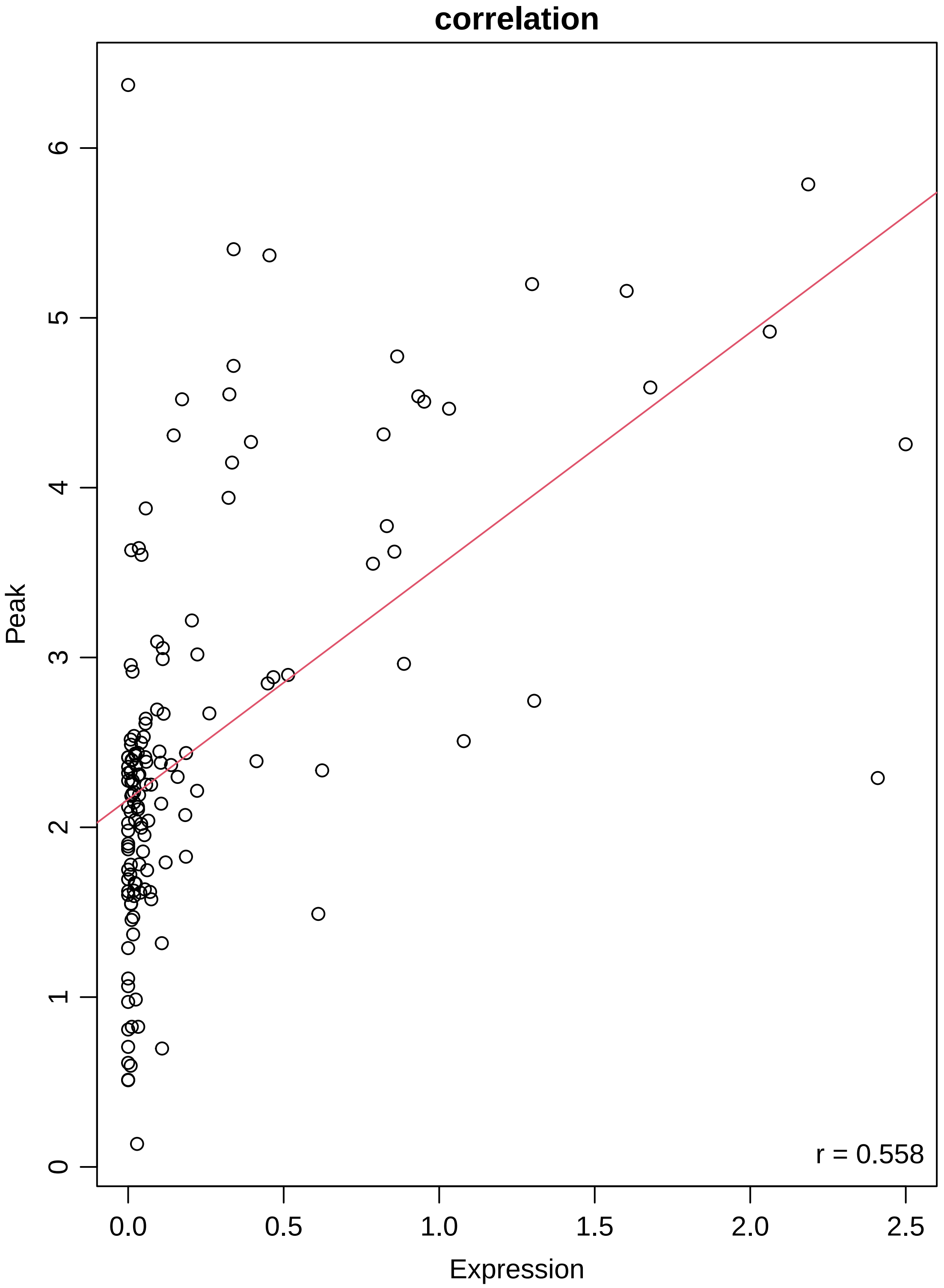

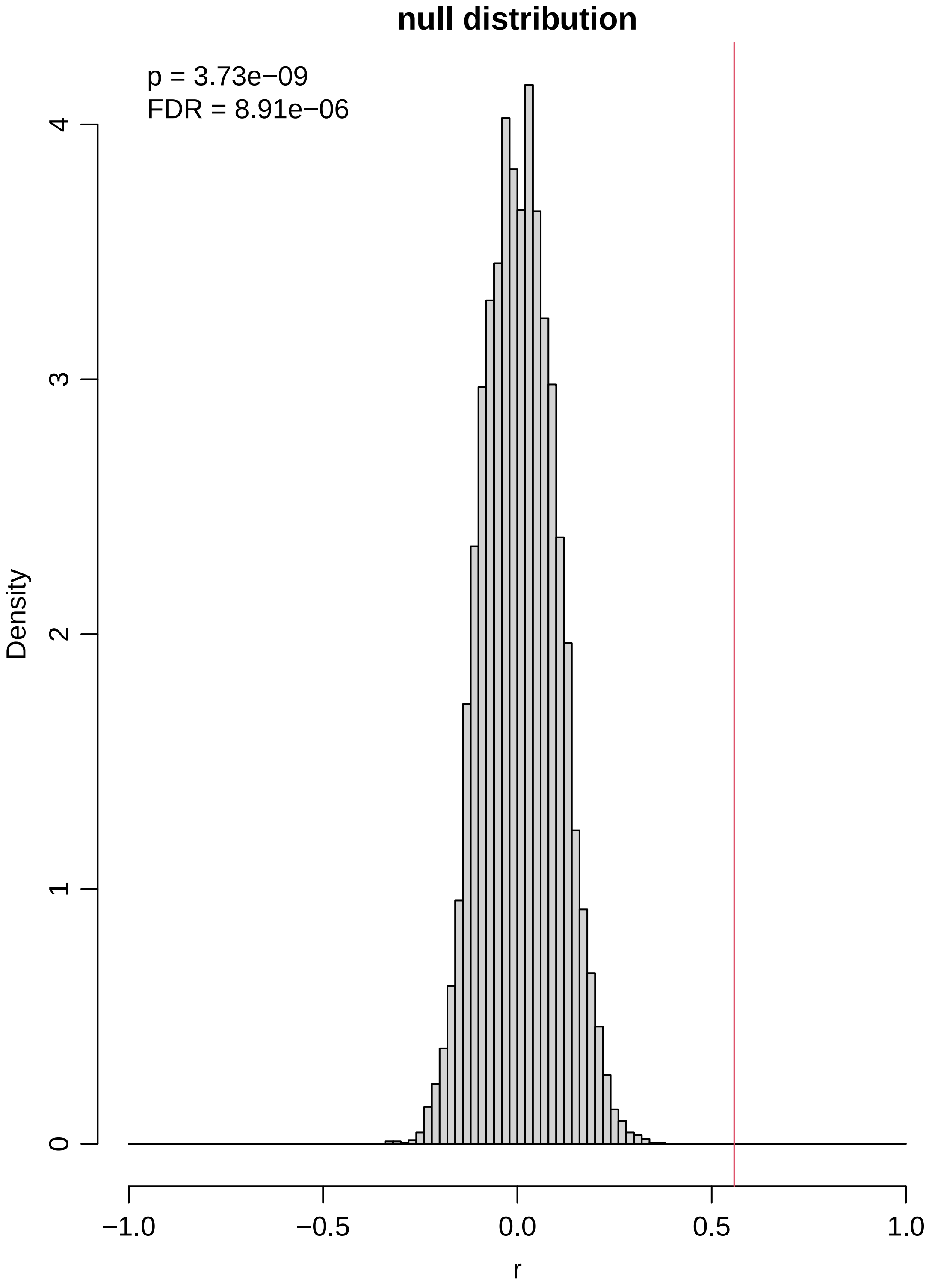

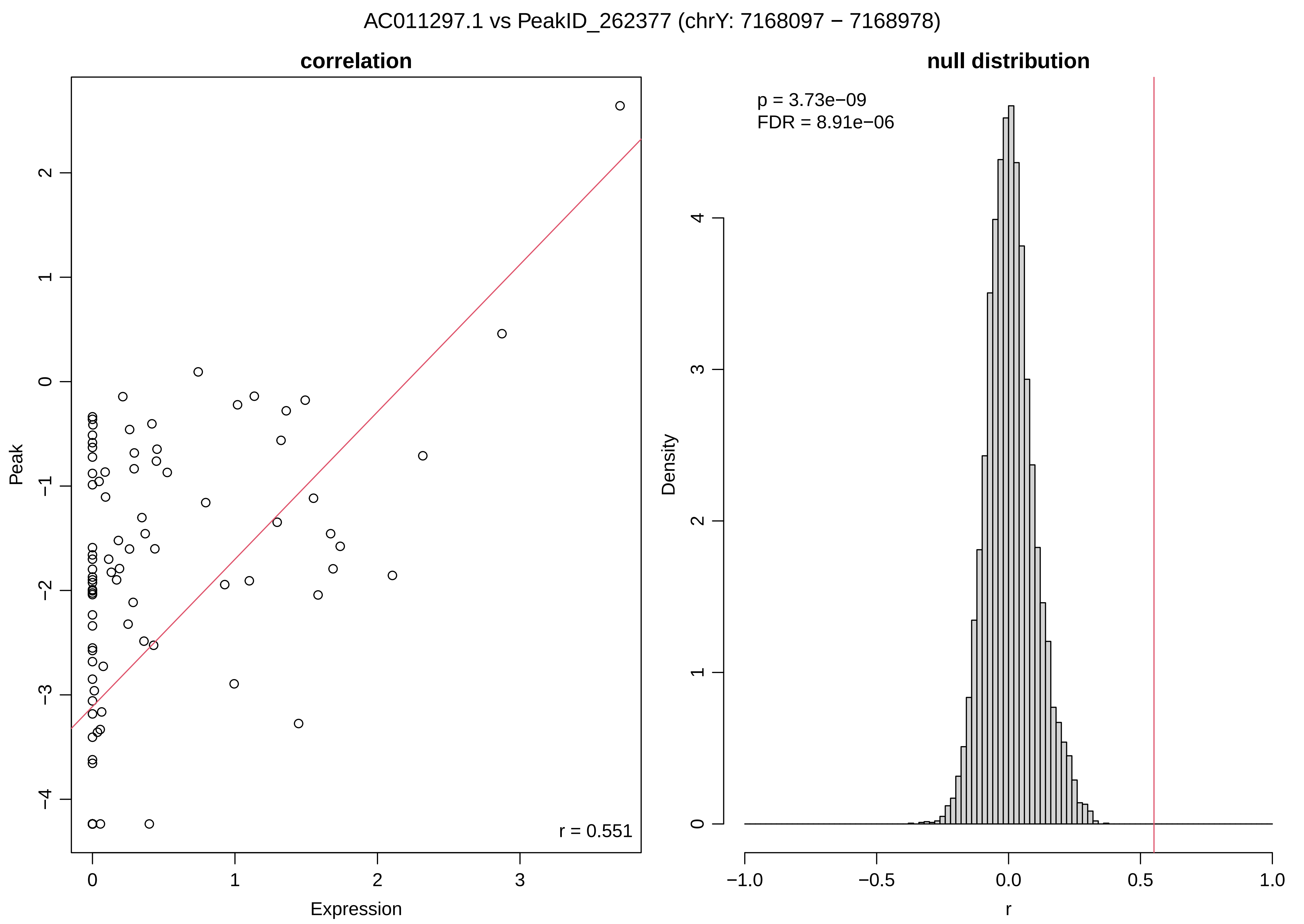
AC011297.1 vs PeakID_262377 (chrY: 7168097 − 7168978)
correlation
Peak
Expression
r = 0.551
null distribution
p = 3.73e−09
FDR = 8.91e−06
Density
r

AL391427.1 vs PeakID_24132 (chr10: 4992660 − 4992983)

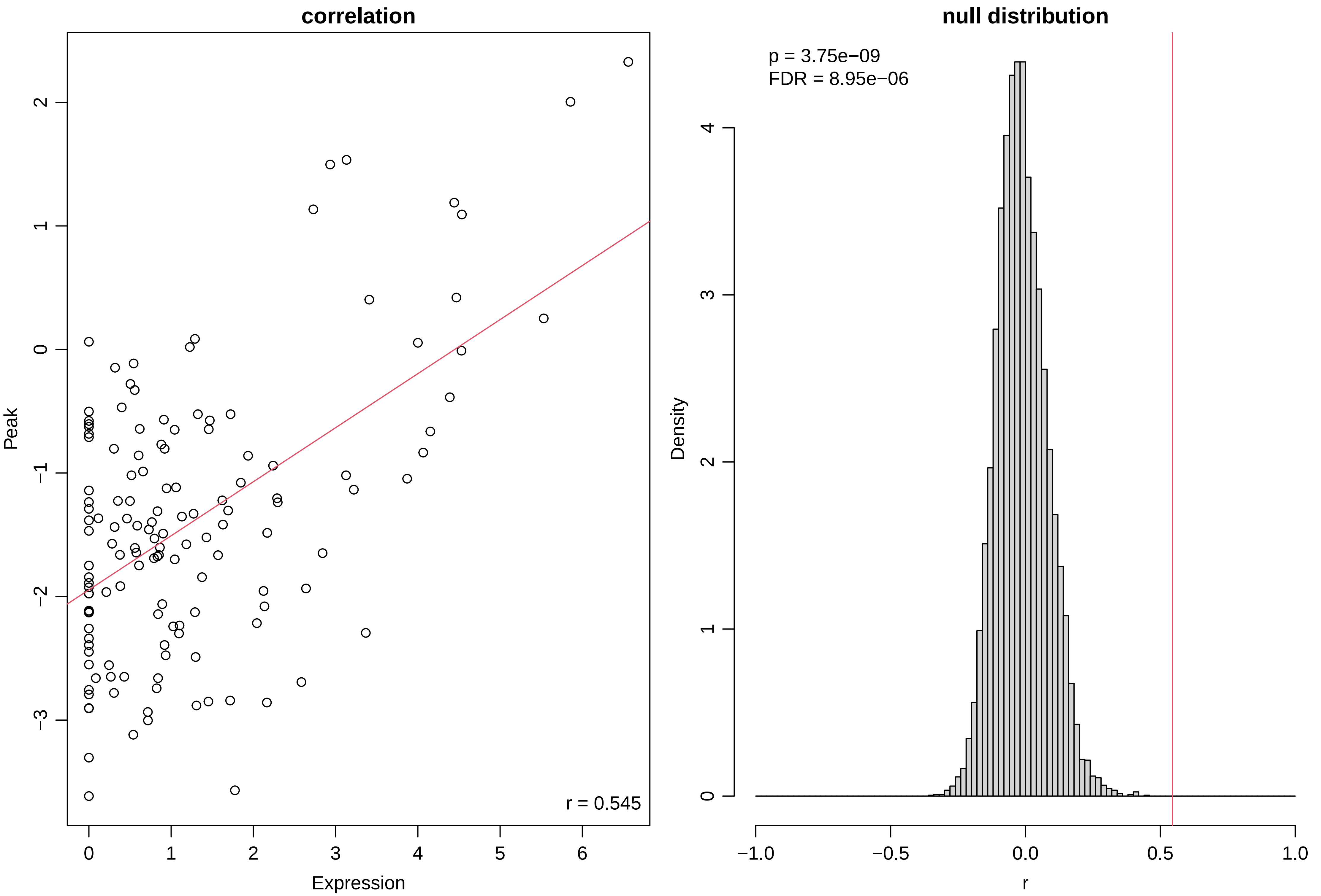

GJC3 vs PeakID_227573 (chr7: 99975301 − 99976673)

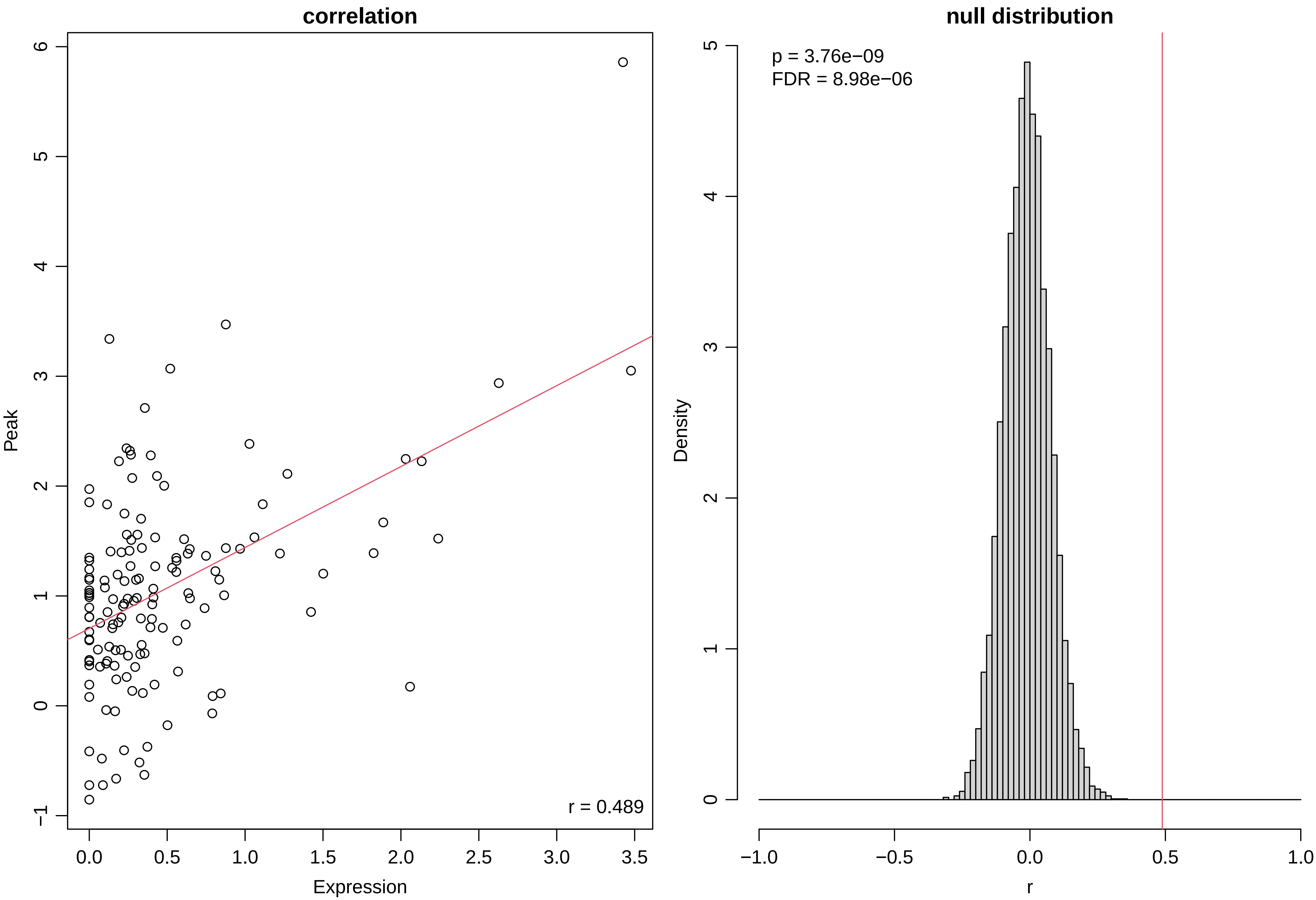

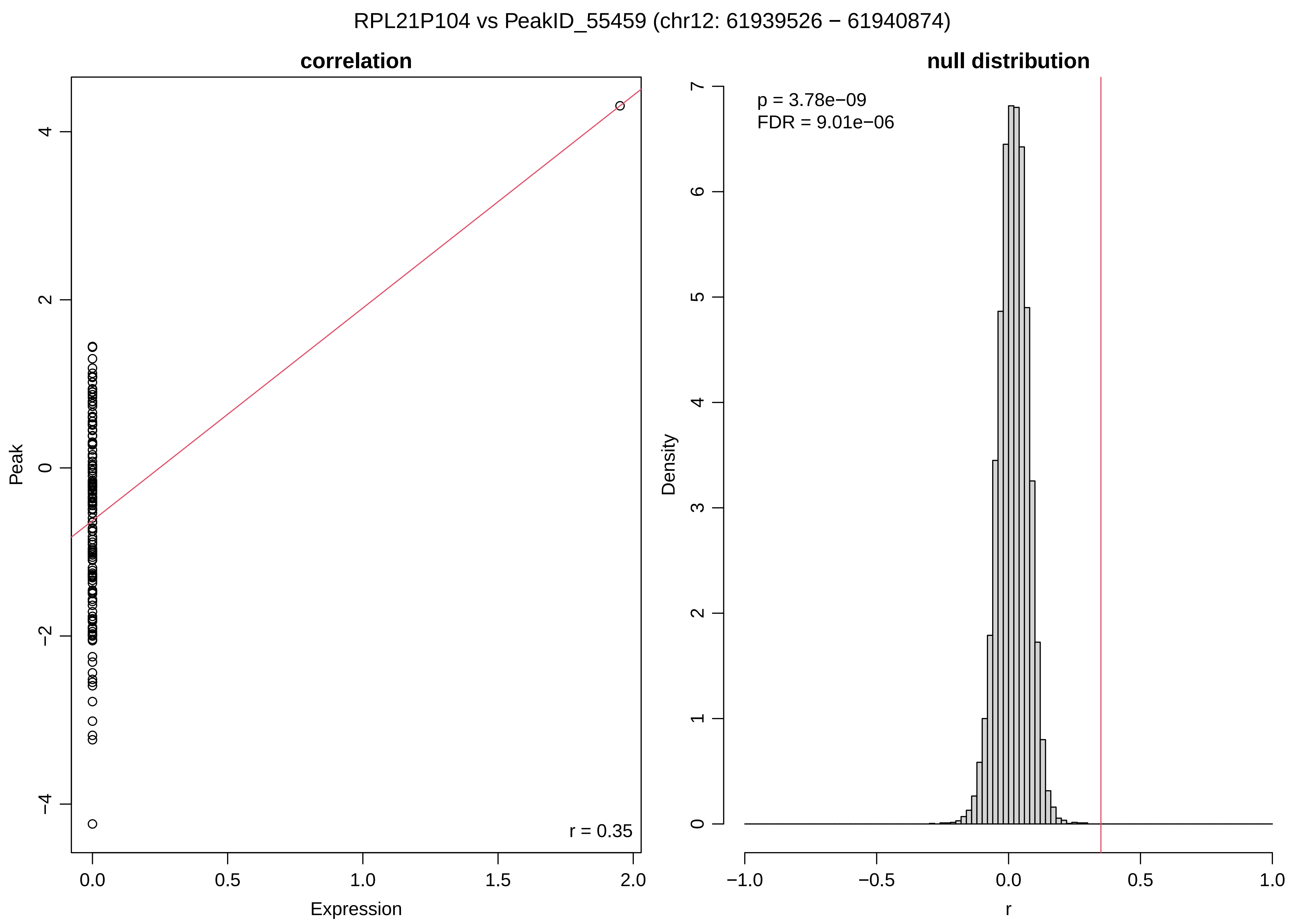
RPL21P104 vs PeakID_55459 (chr12: 61939526 − 61940874)
correlation
Peak
Expression
r = 0.35
null distribution
p = 3.78e−09
FDR = 9.01e−06
Density
r

LINC01518 vs PeakID_28081 (chr10: 42690071 − 42692152)

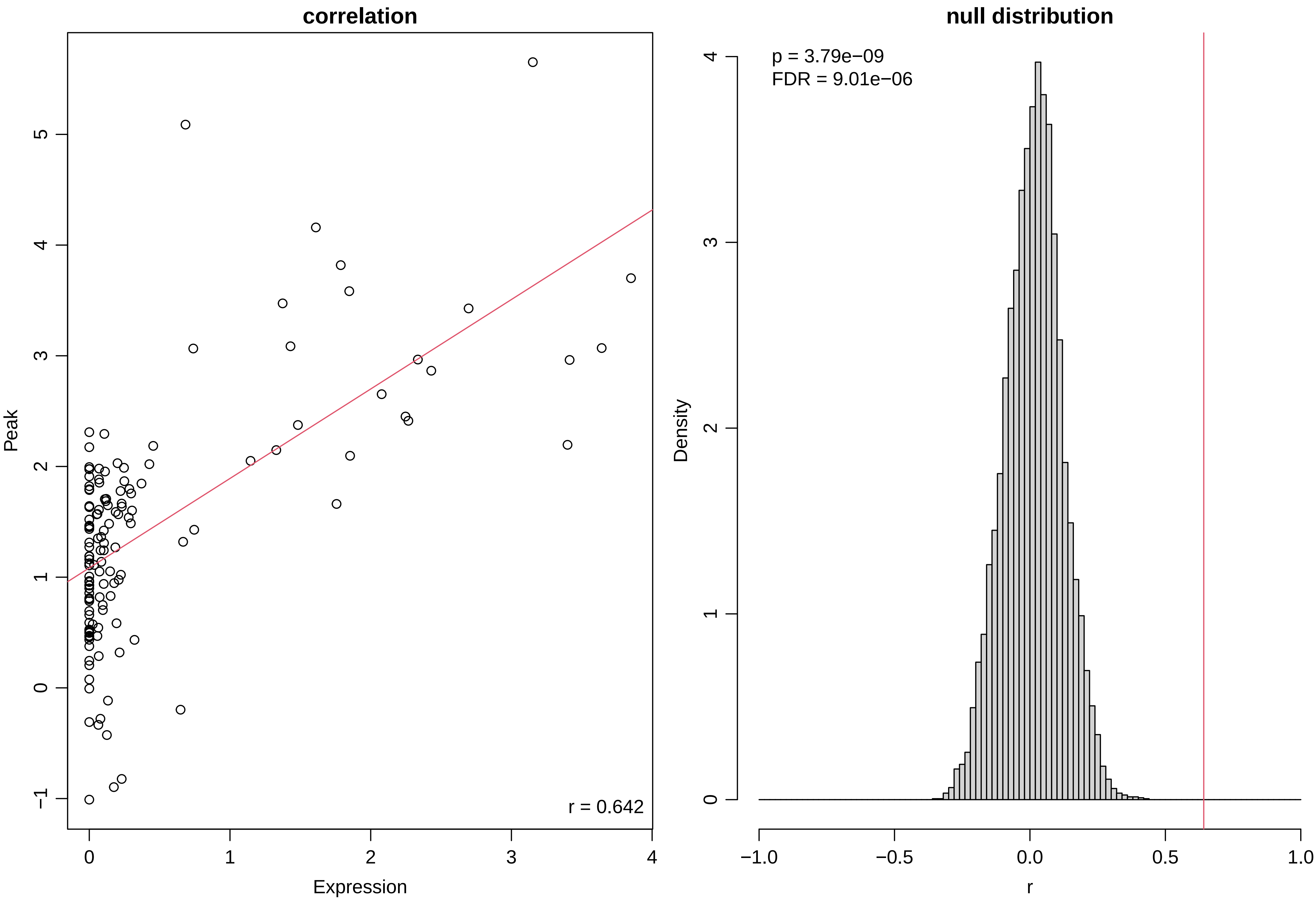

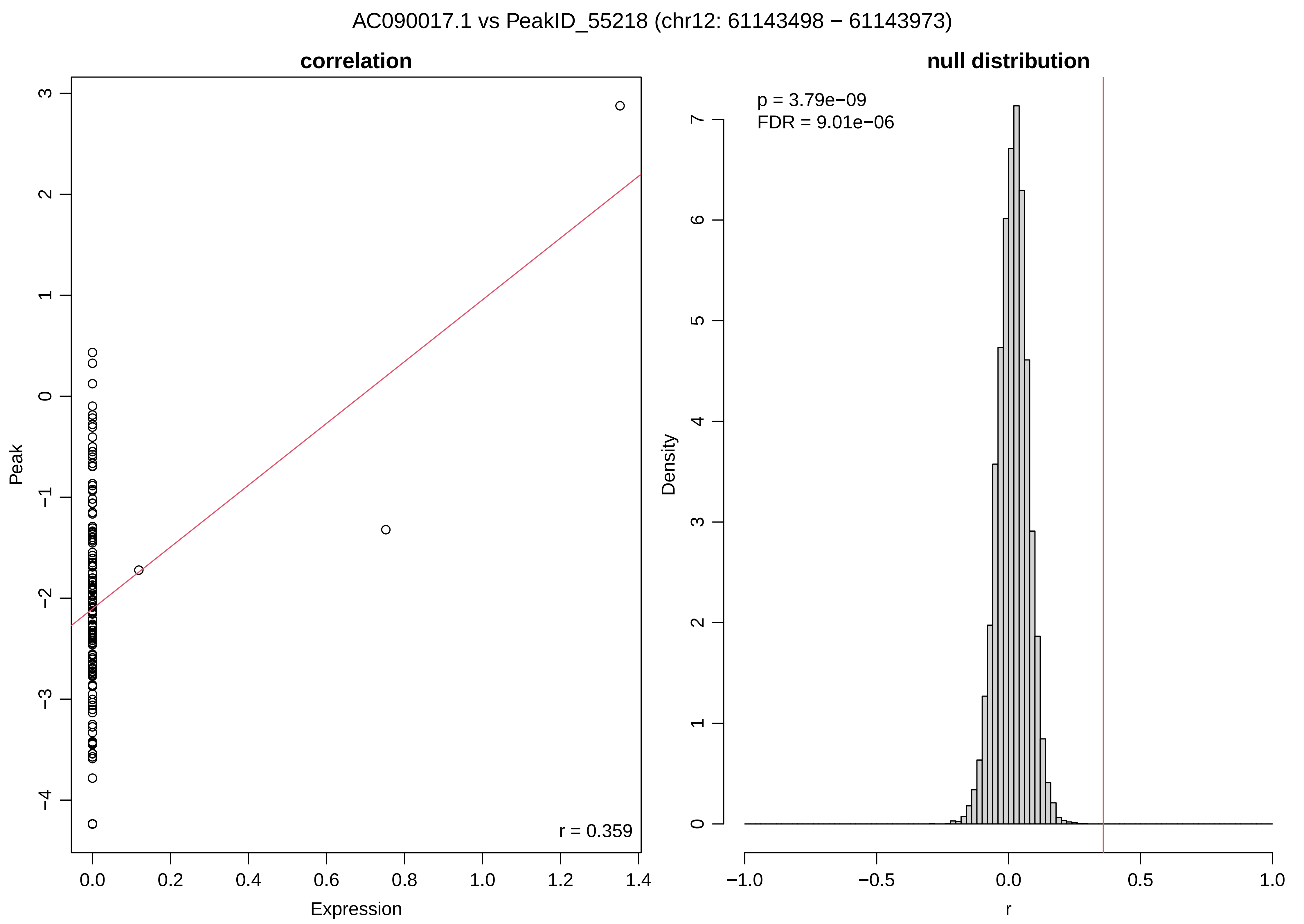

AC090017.1 vs PeakID_55218 (chr12: 61143498 − 61143973)
correlation
null distribution
p = 3.79e−09
FDR = 9.01e−06
Peak
Expression
r = 0.359
Density
r

CHL1−AS2 vs PeakID_159962 (chr3: 195587 − 200424)

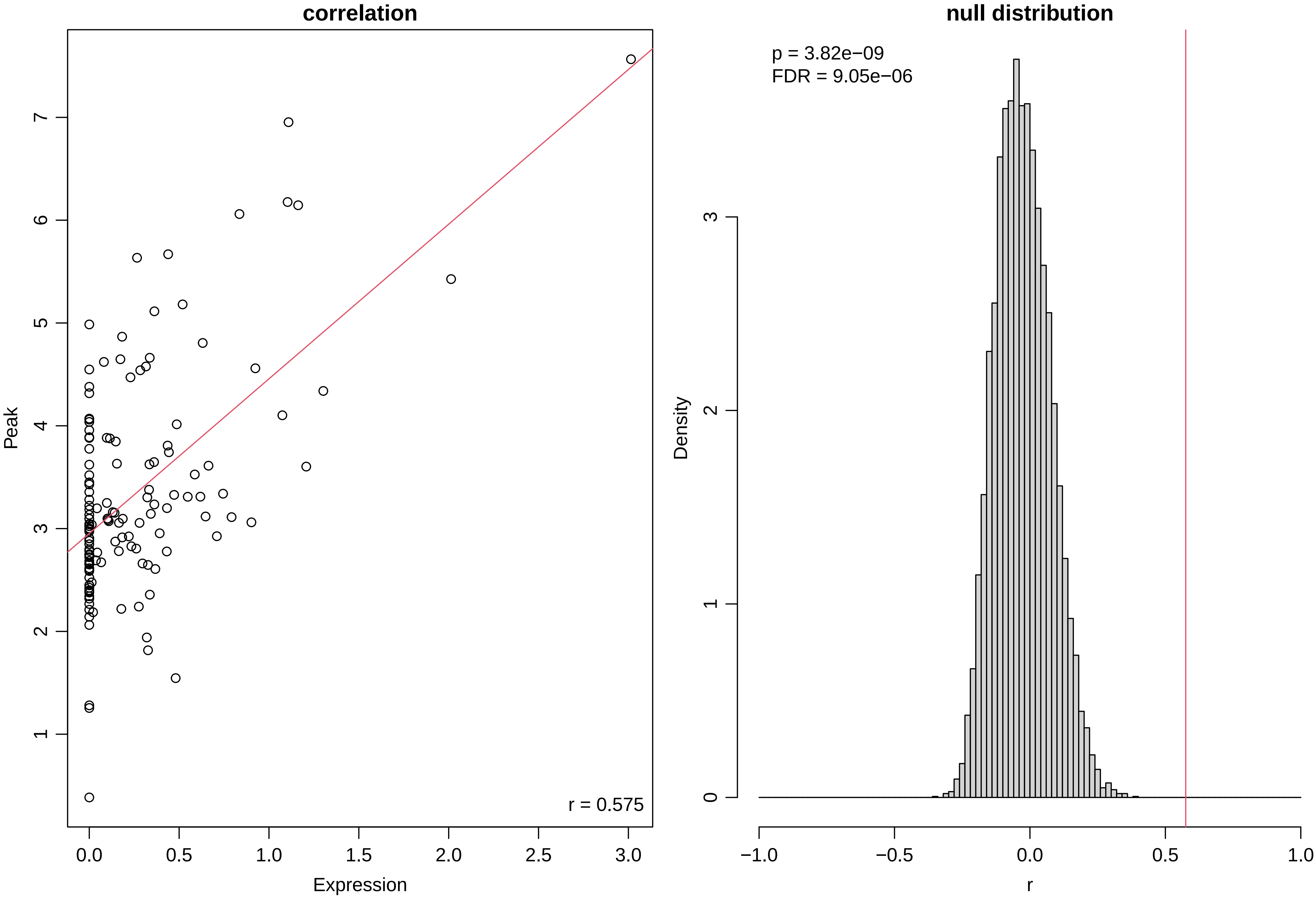

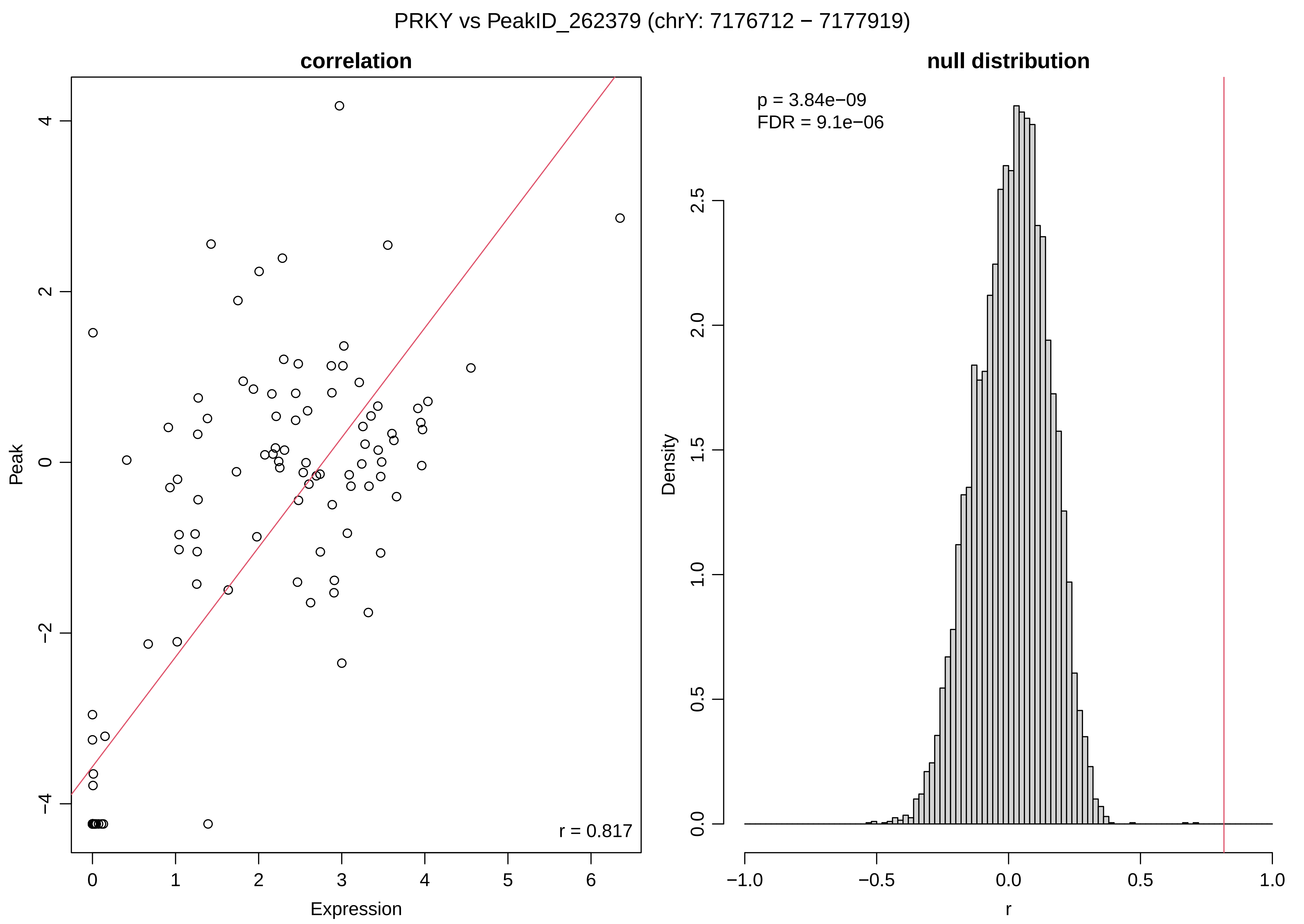
PRKY vs PeakID_262379 (chrY: 7176712 − 7177919)
correlation
Peak
Expression
r = 0.817
null distribution
p = 3.84e−09
FDR = 9.1e−06
Density
r

AC002066.1 vs PeakID_228935 (chr7: 116262652 − 116263048)

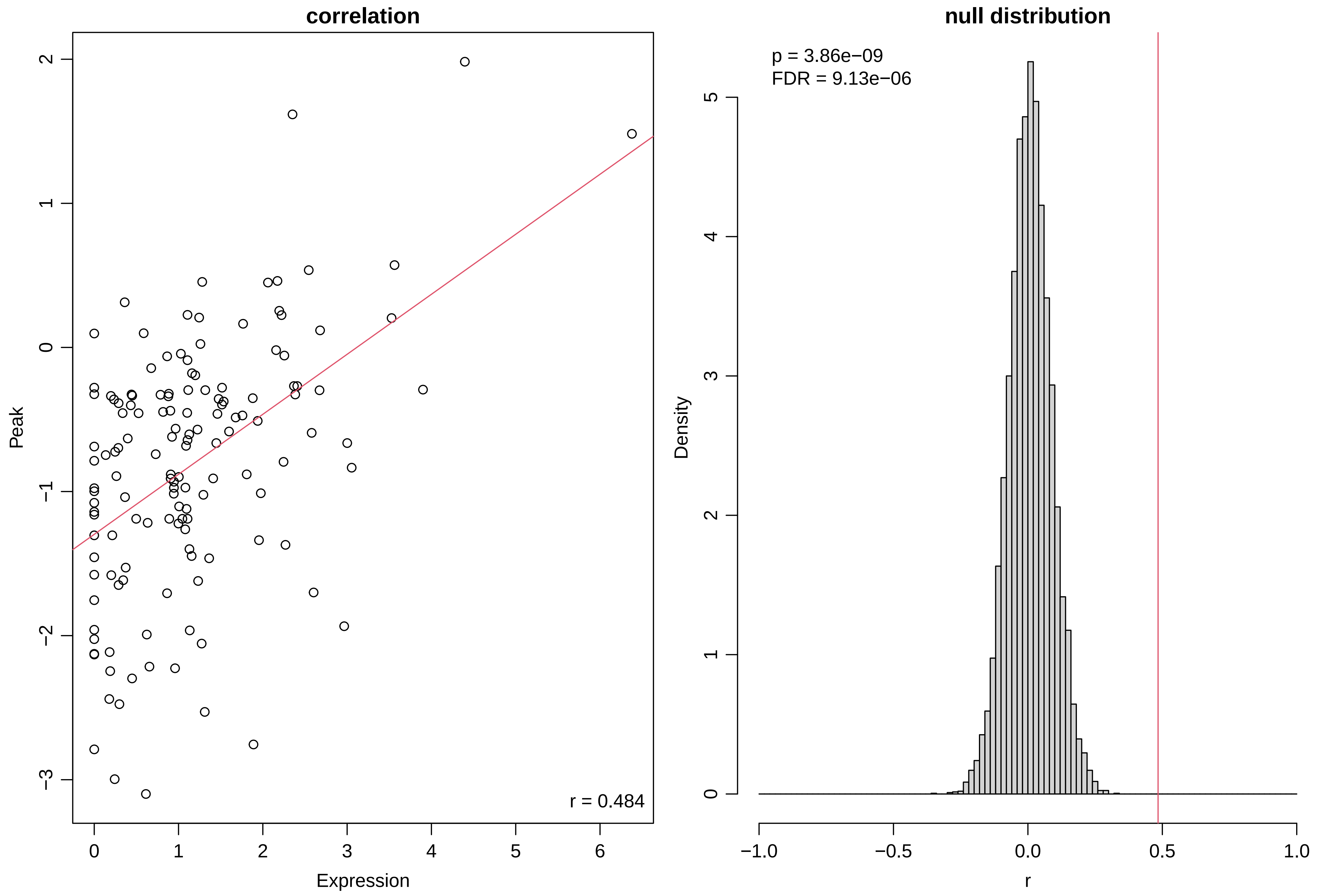

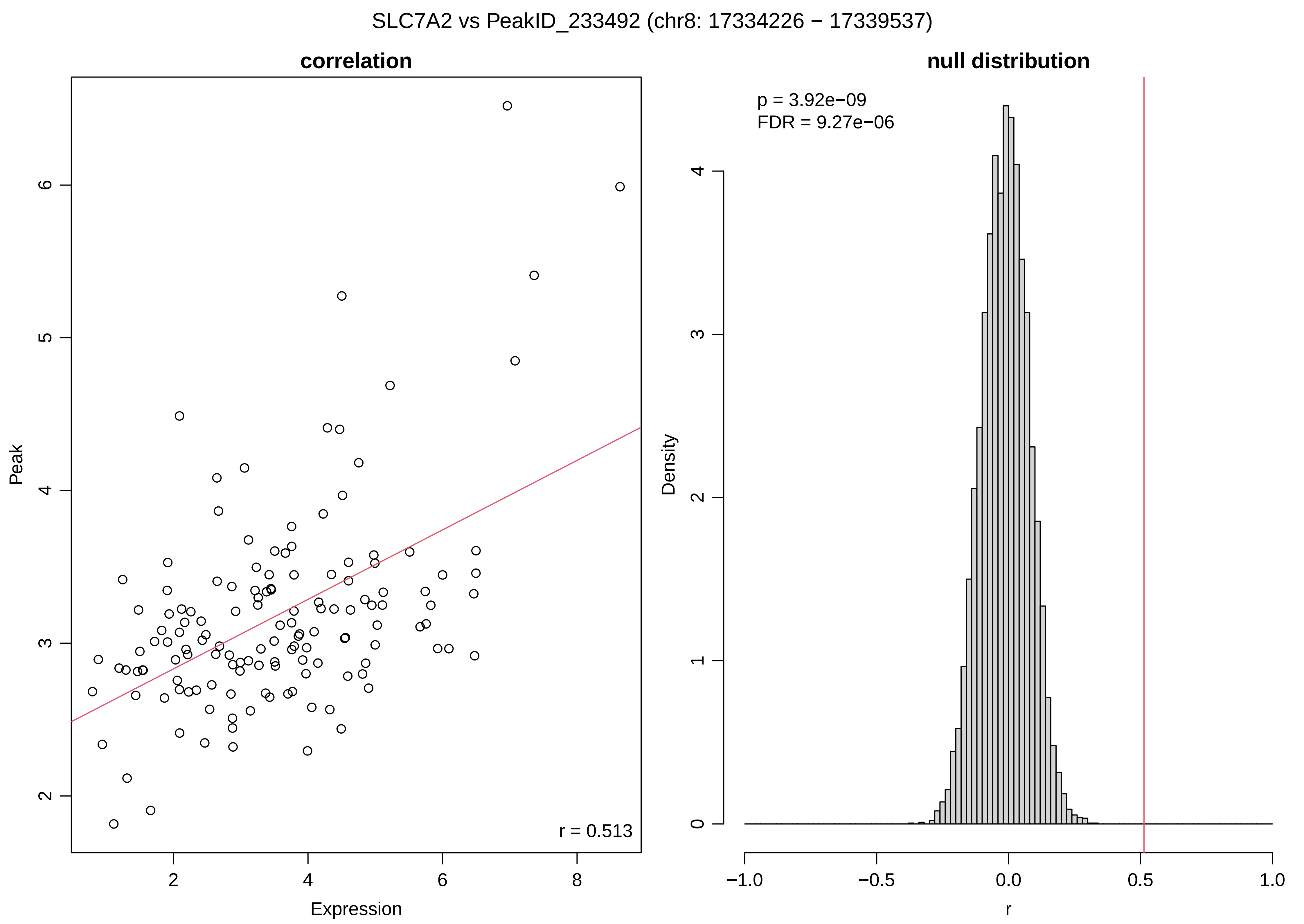
SLC7A2 vs PeakID_233492 (chr8: 17334226 − 17339537)
correlation
Peak
Expression
r = 0.513
null distribution
p = 3.92e−09
FDR = 9.27e−06
Density
r

LINC00842 vs PeakID_28454 (chr10: 46374736 − 46378659)

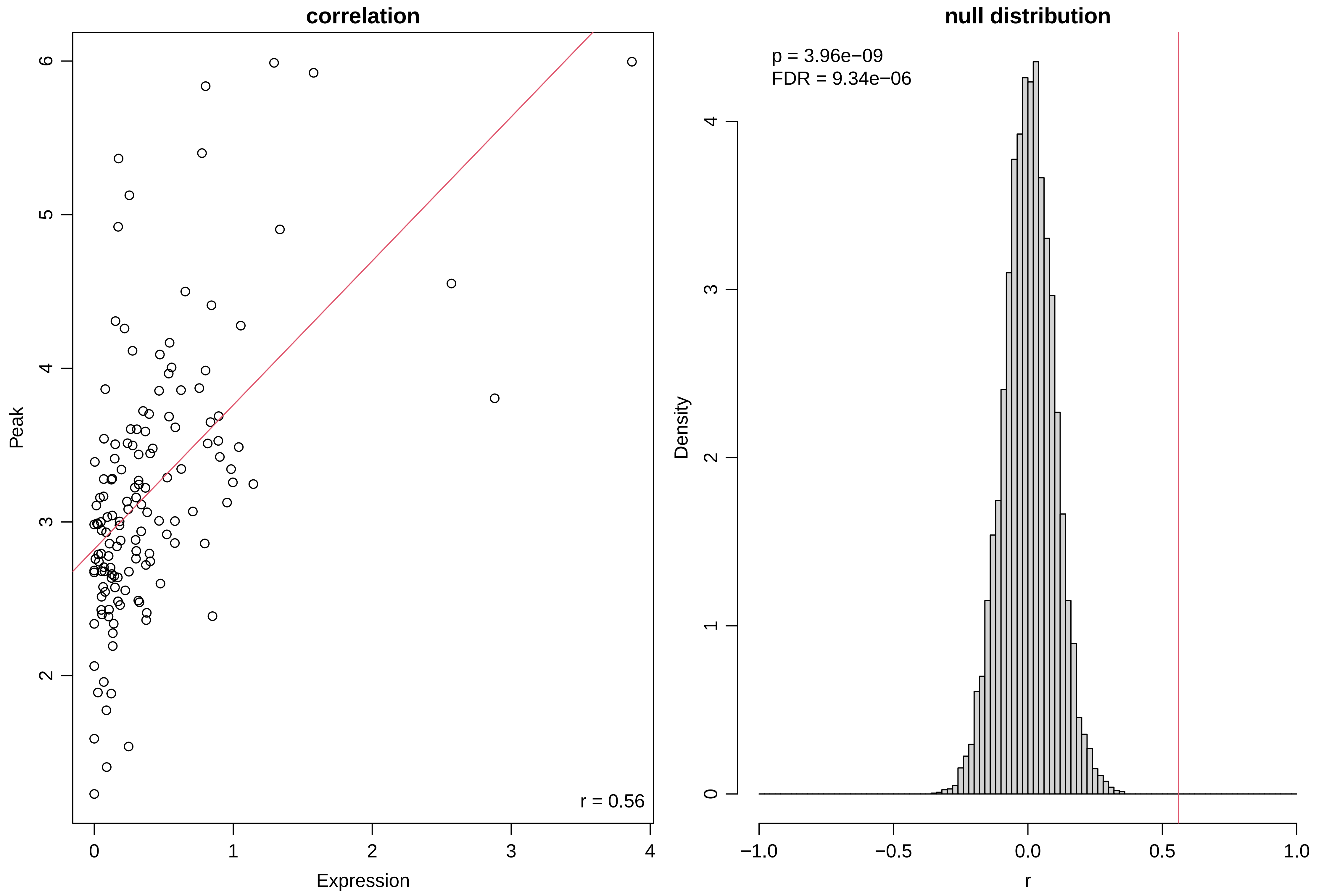

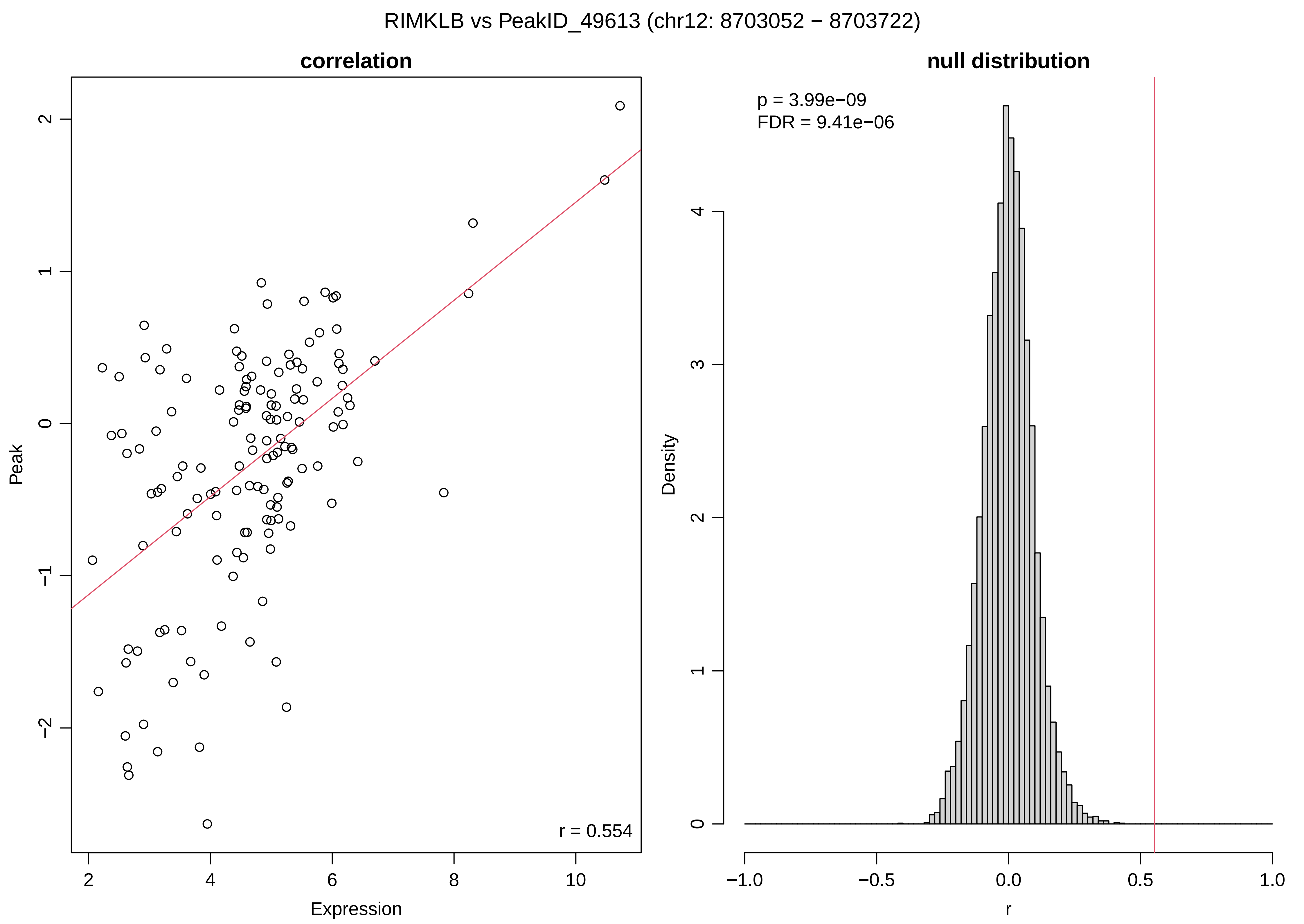
RIMKLB vs PeakID_49613 (chr12: 8703052 − 8703722)
correlation
Peak
Expression
r = 0.554
null distribution
p = 3.99e−09
FDR = 9.41e−06
Density
r

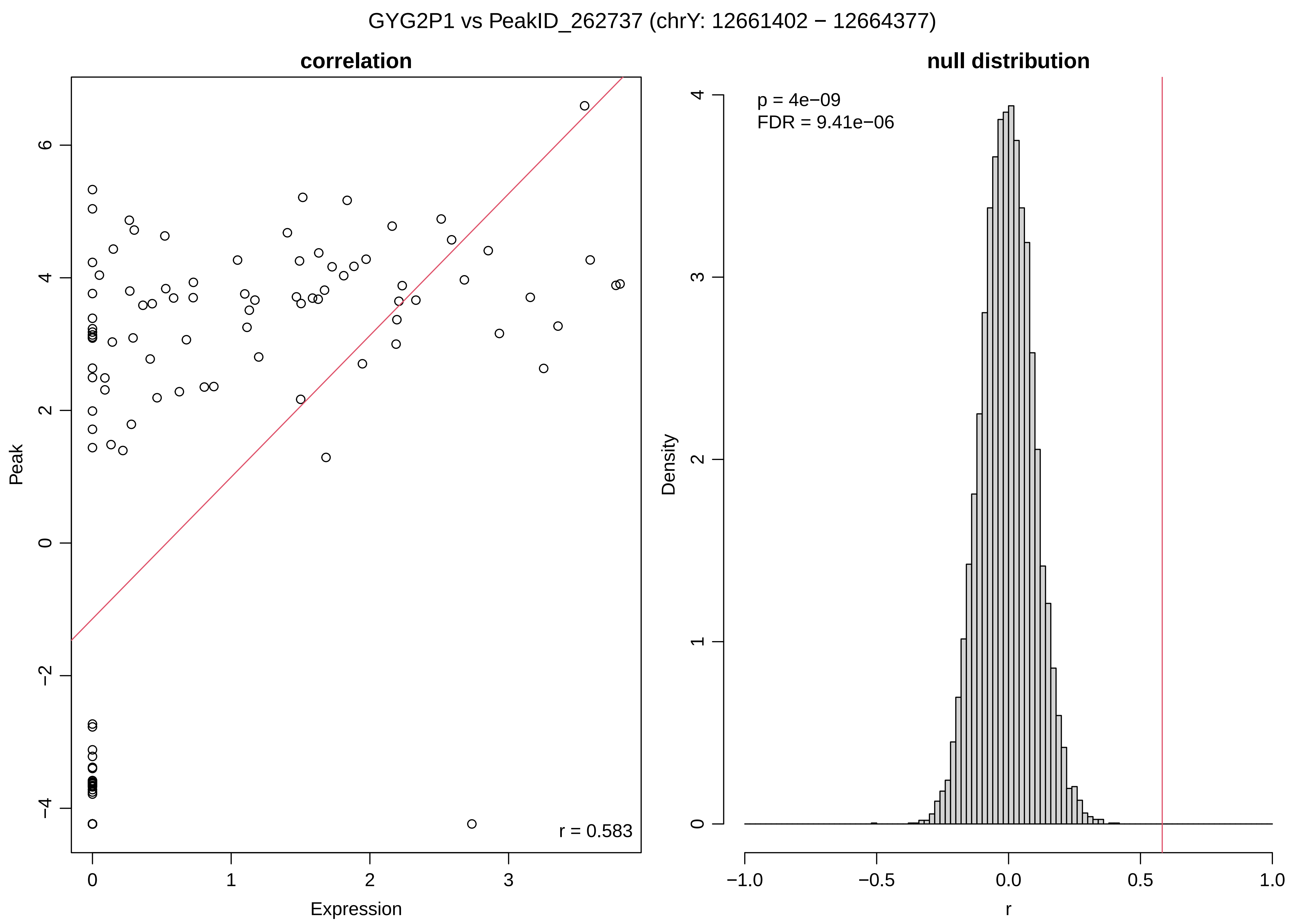
GYG2P1 vs PeakID_262737 (chrY: 12661402 − 12664377)
correlation
Peak
Expression
r = 0.583
null distribution
p = 4e−09
FDR = 9.41e−06
Density
r

FAM224A vs PeakID_262833 (chrY: 18167306 − 18168294)

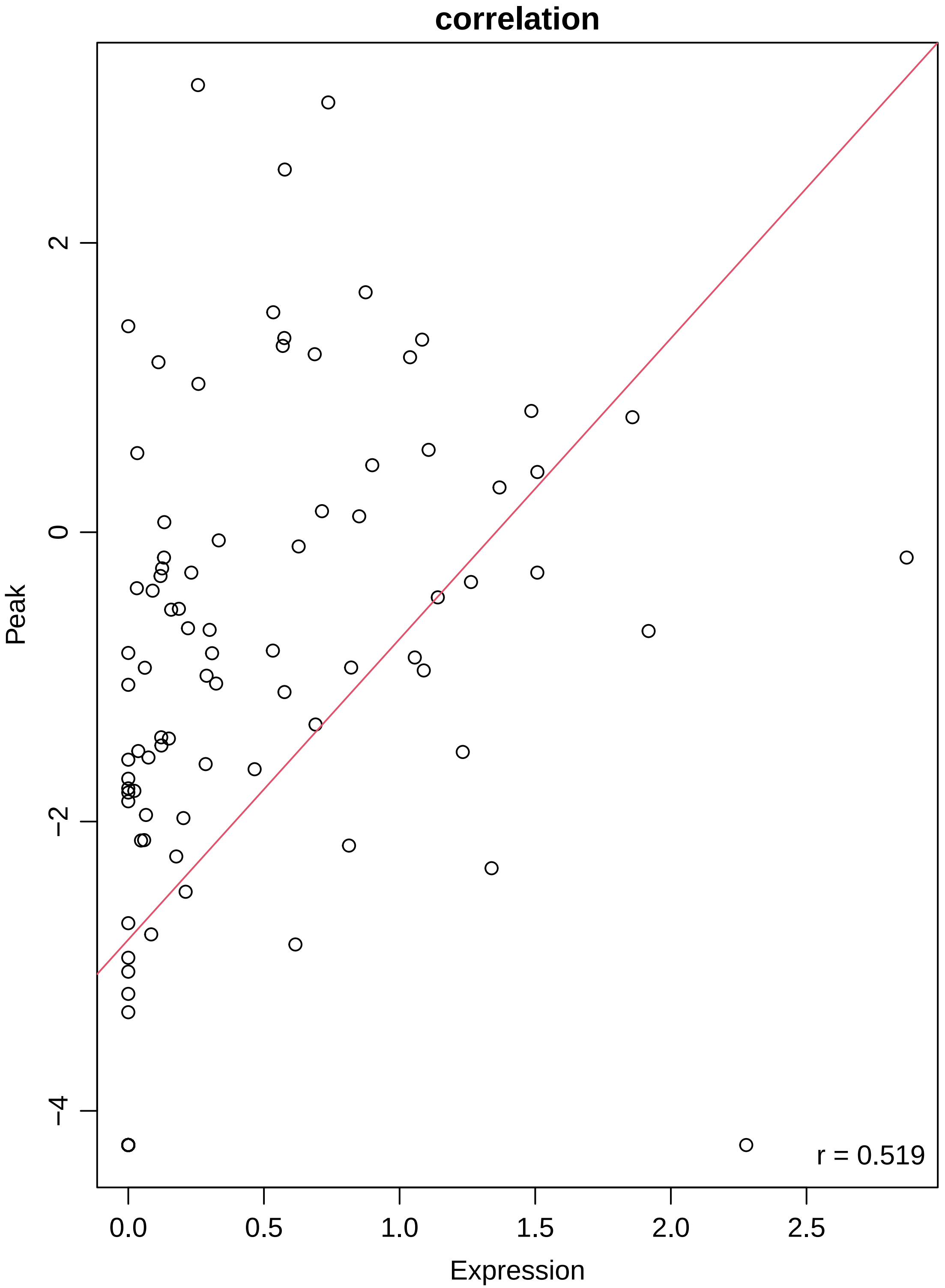

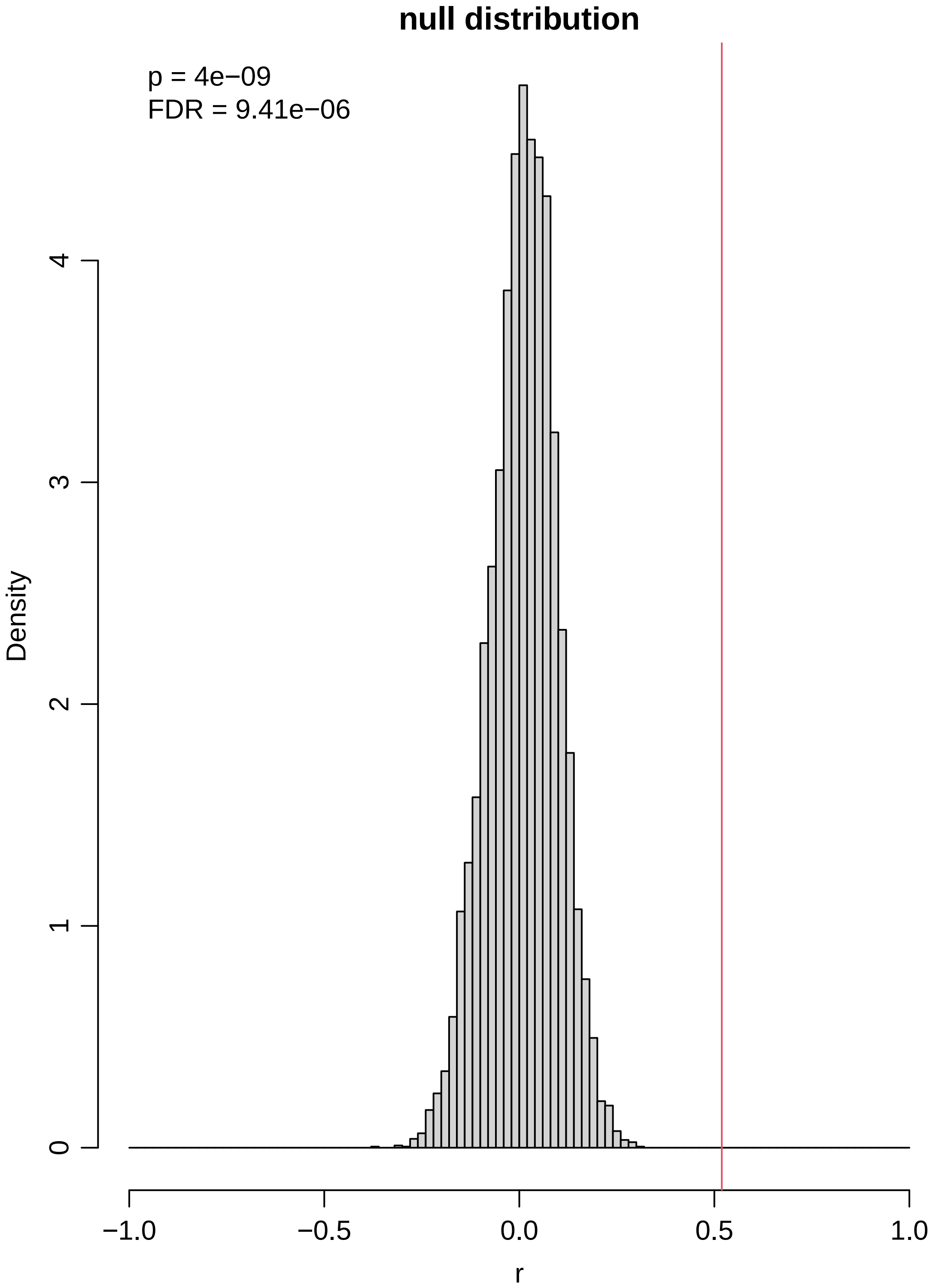

AC107881.1 vs PeakID_38082 (chr11: 12867200 − 12867494)

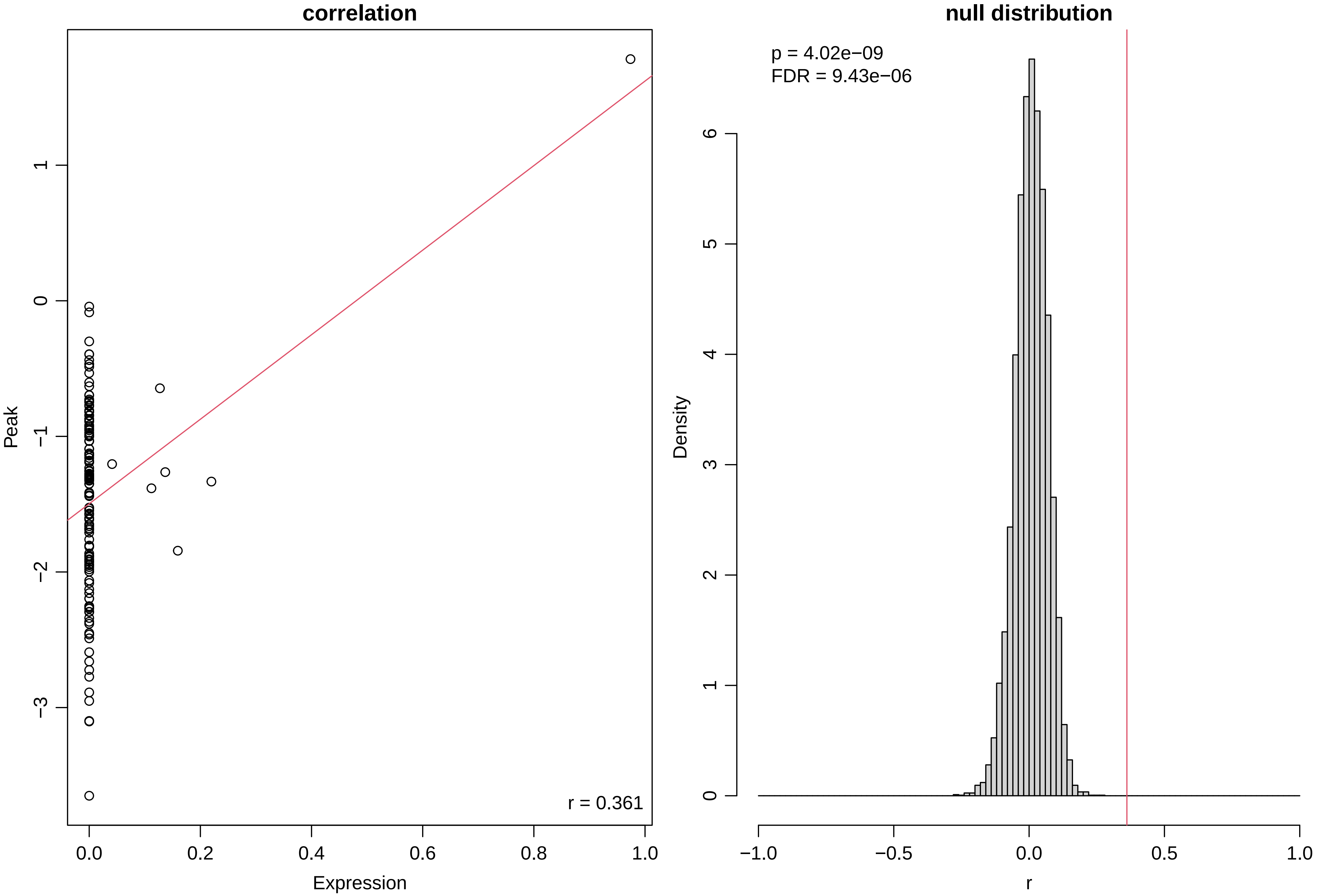

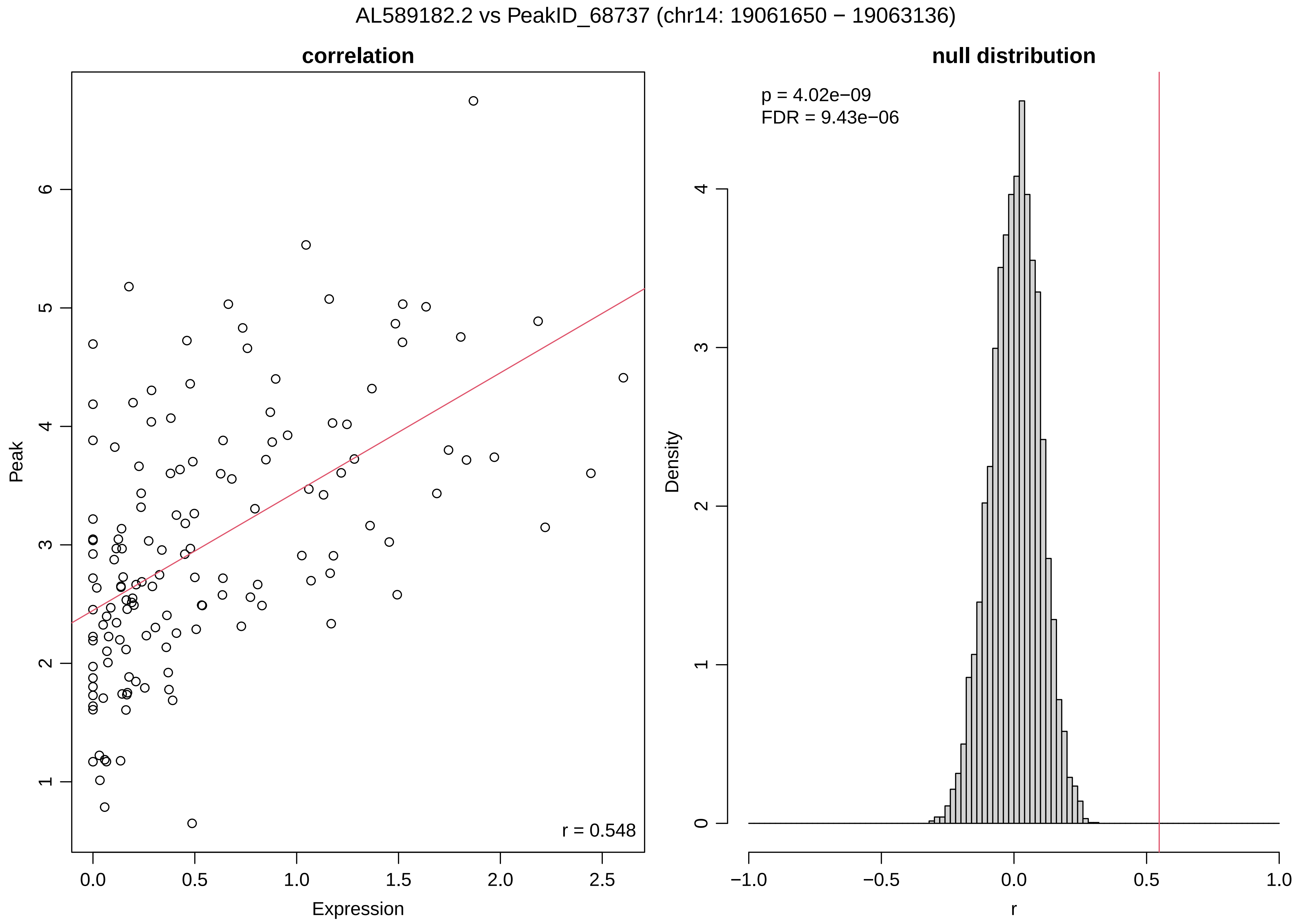
AL589182.2 vs PeakID_68737 (chr14: 19061650 − 19063136)
correlation
null distribution
Peak
Expression
r = 0.548
p = 4.02e−09
FDR = 9.43e−06
Density
r
0.0
0.5
1.0
1.5
2.0
2.5
1
2
3
4
5
6
−1.0
−0.5
0

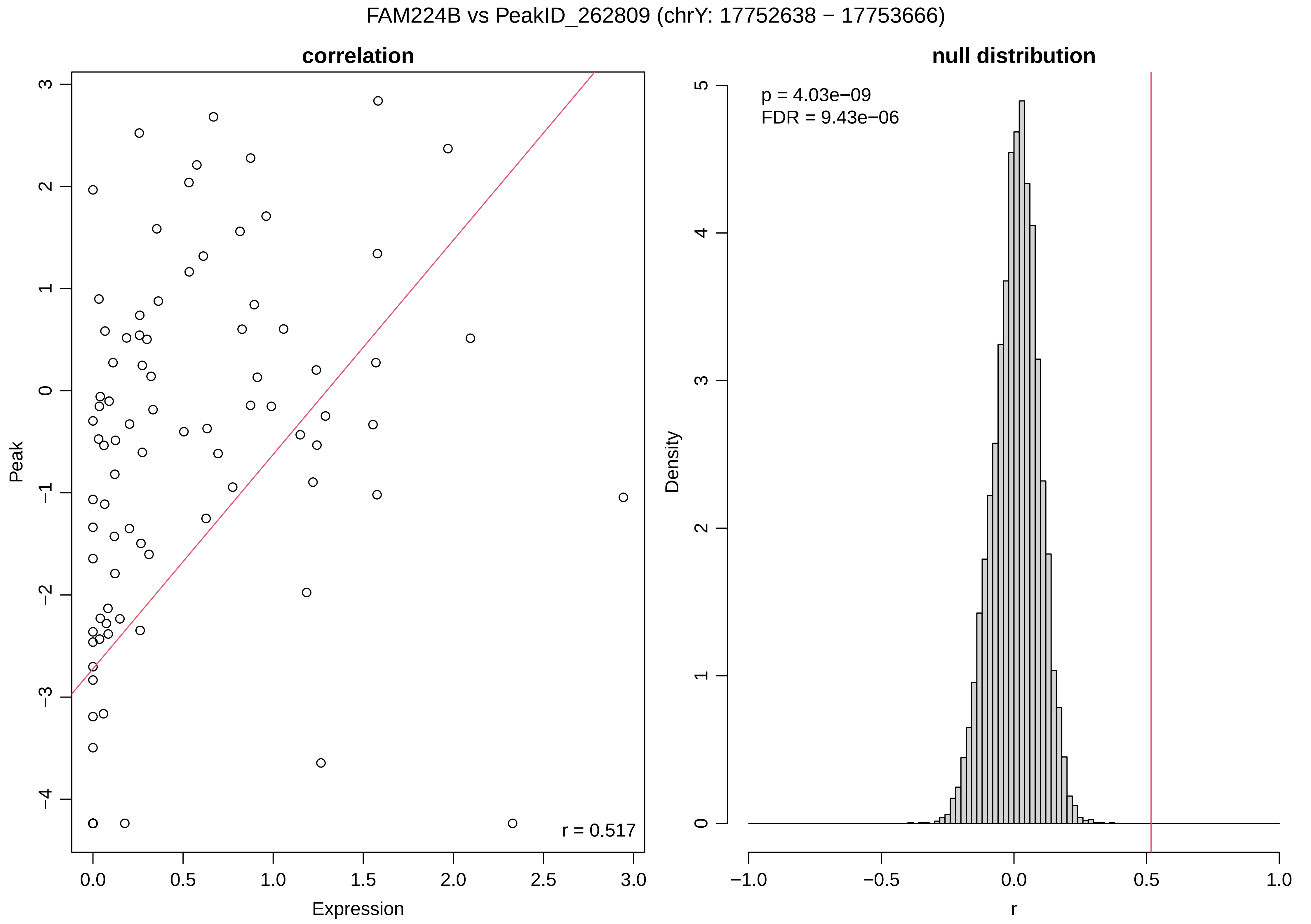

FAM224B vs PeakID_262809 (chrY: 17752638 − 17753666)
correlation
Peak
Expression
r = 0.517
null distribution
Density
r
p = 4.03e−09
FDR = 9.43e−06

PRKY vs PeakID_262408 (chrY: 7468549 − 7469073)

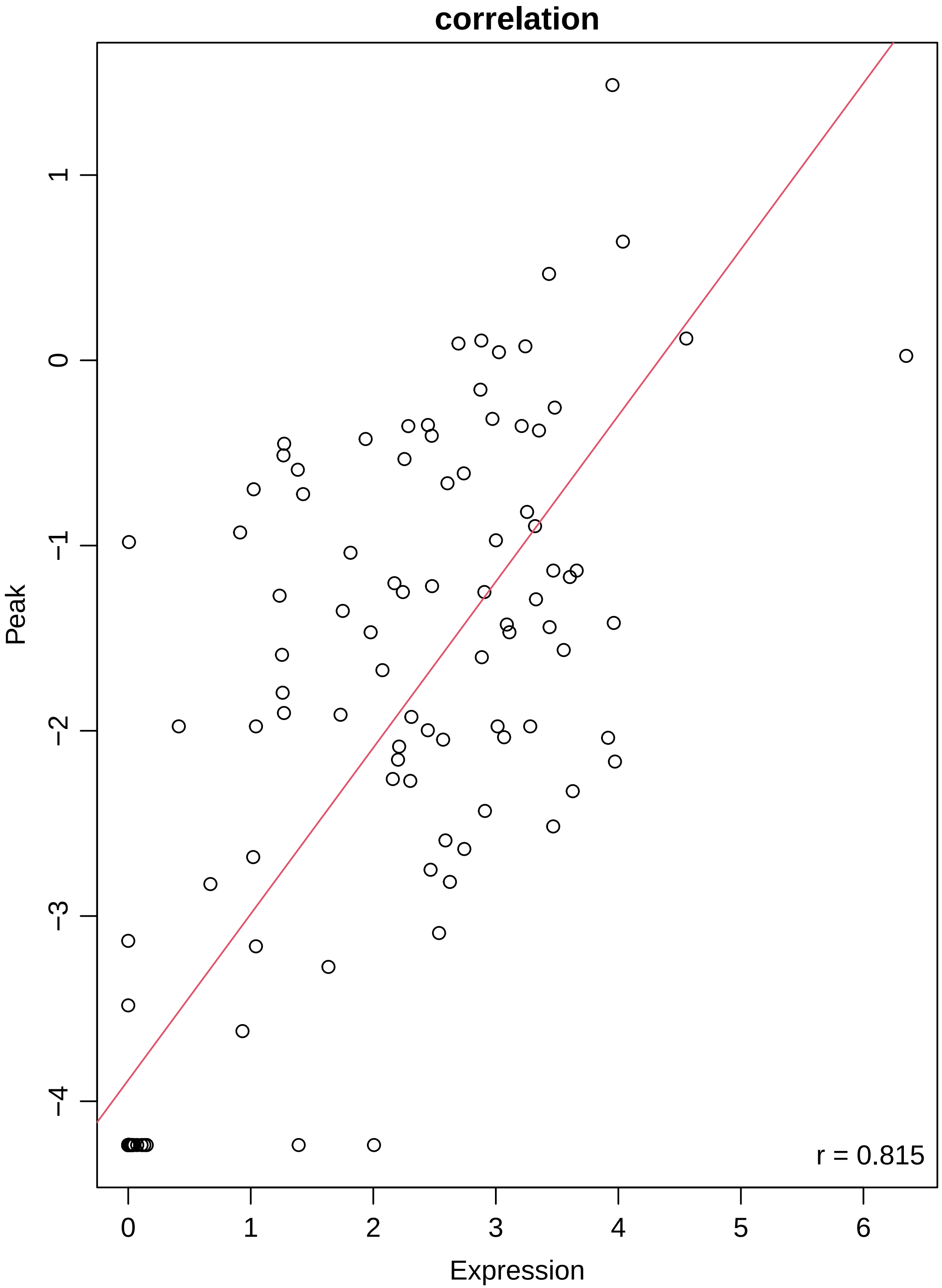

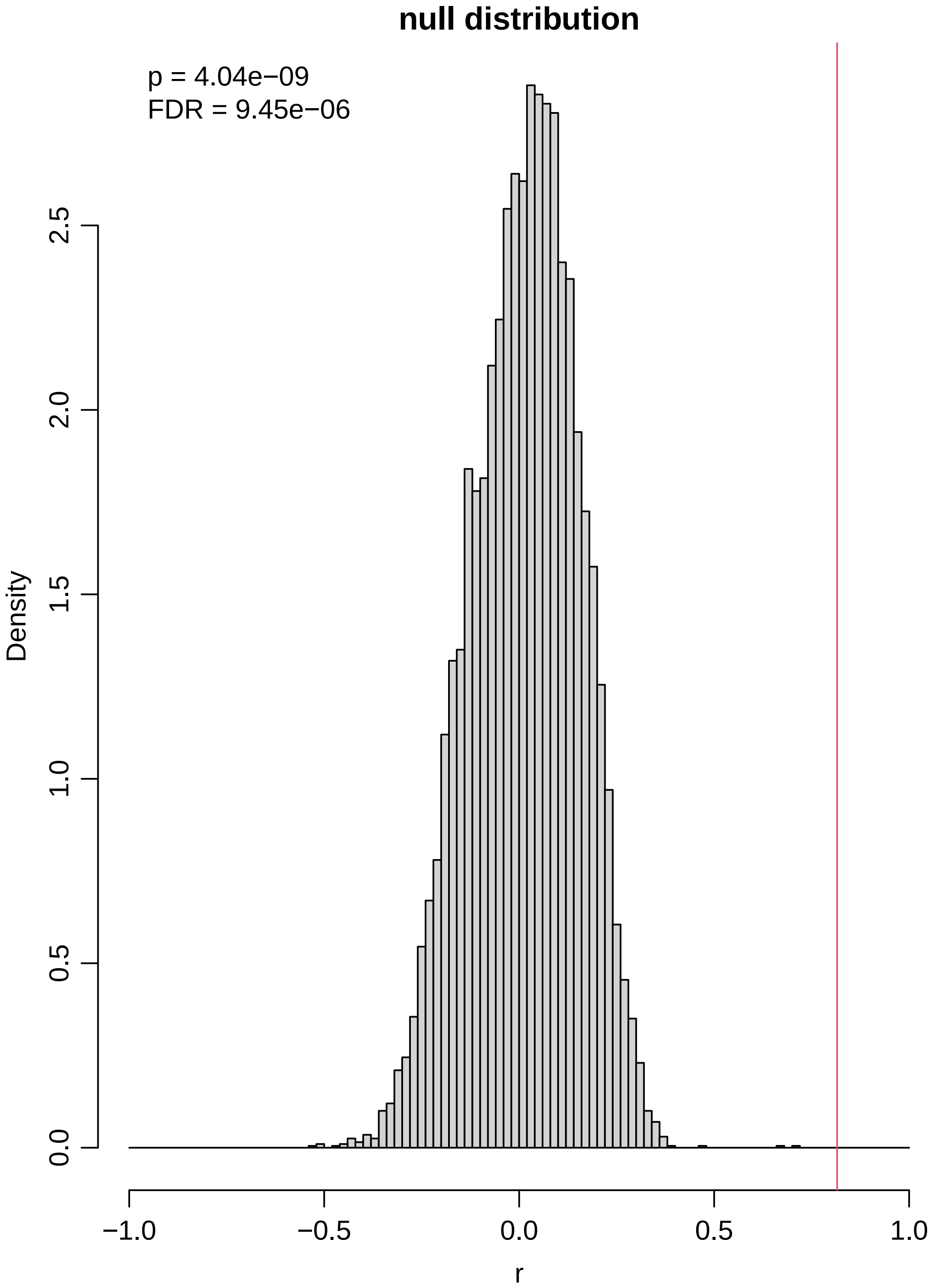

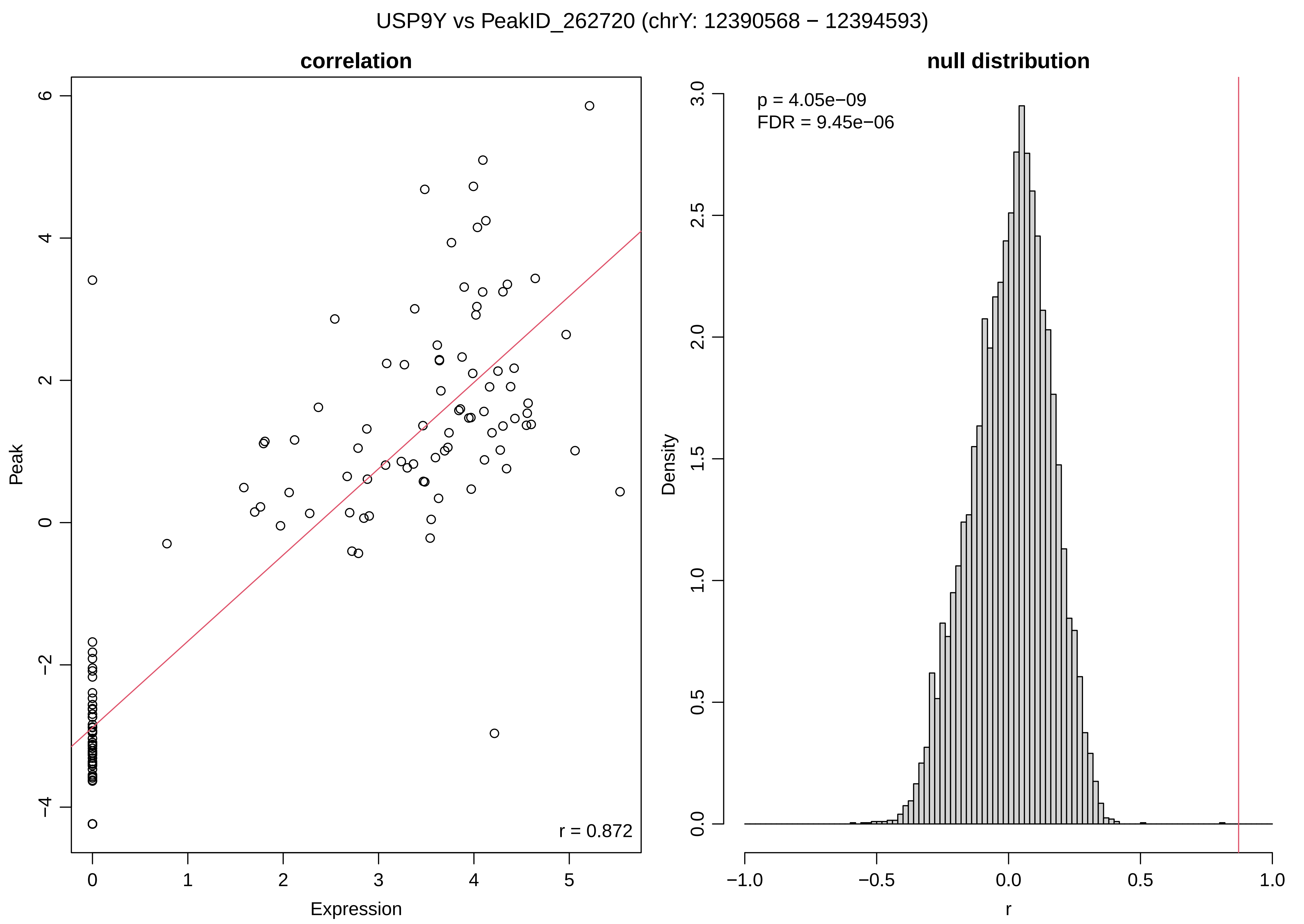

USP9Y vs PeakID_262720 (chrY: 12390568 − 12394593)
correlation
Peak
Expression
r = 0.872
null distribution
p = 4.05e−09
FDR = 9.45e−06
Density
r

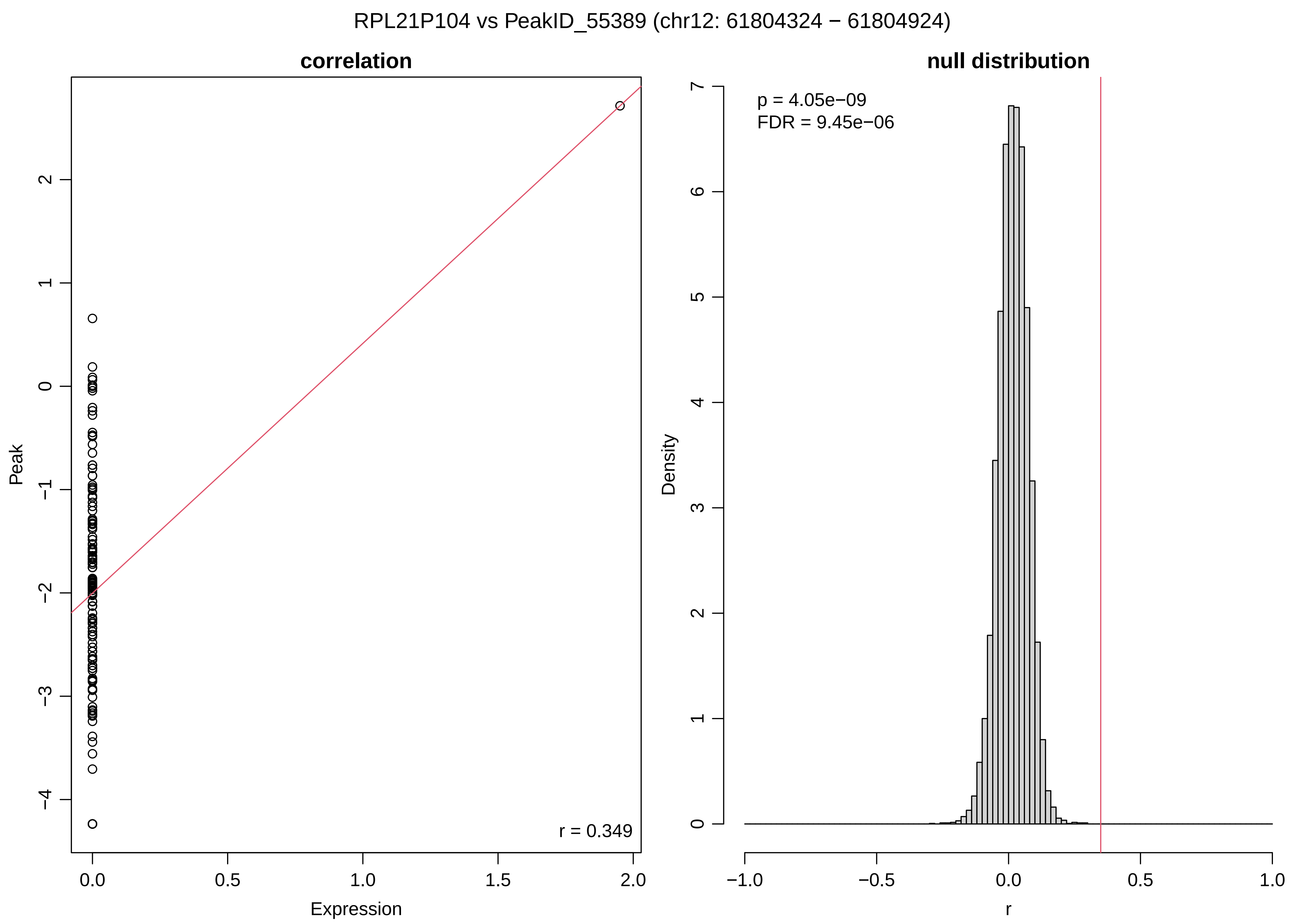
RPL21P104 vs PeakID_55389 (chr12: 61804324 − 61804924)
correlation
Peak
Expression
r = 0.349
null distribution
Density
r
p = 4.05e−09
FDR = 9.45e−06

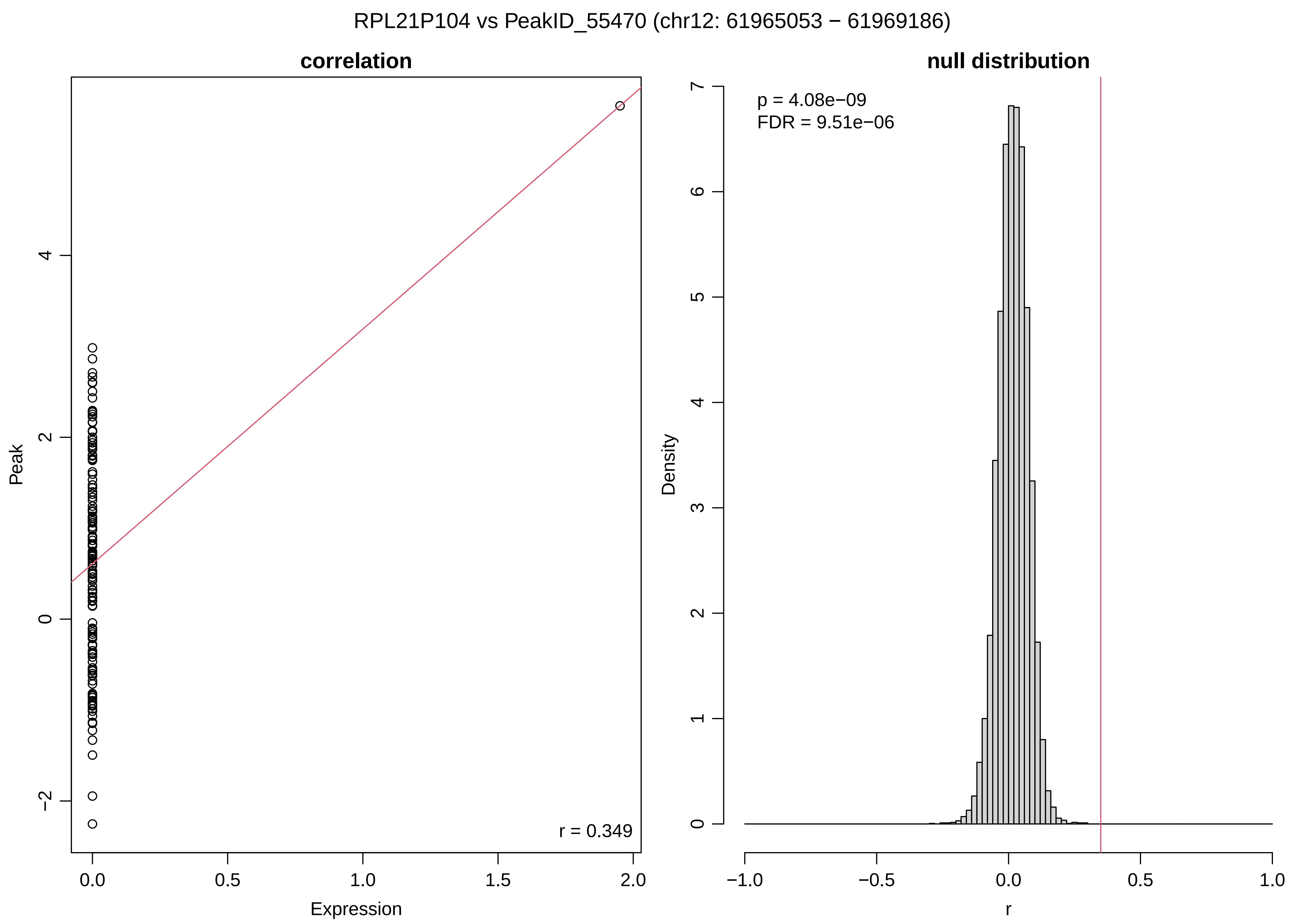
RPL21P104 vs PeakID_55470 (chr12: 61965053 − 61969186)
correlation
Peak
Expression
r = 0.349
null distribution
p = 4.08e−09
FDR = 9.51e−06
Density
r

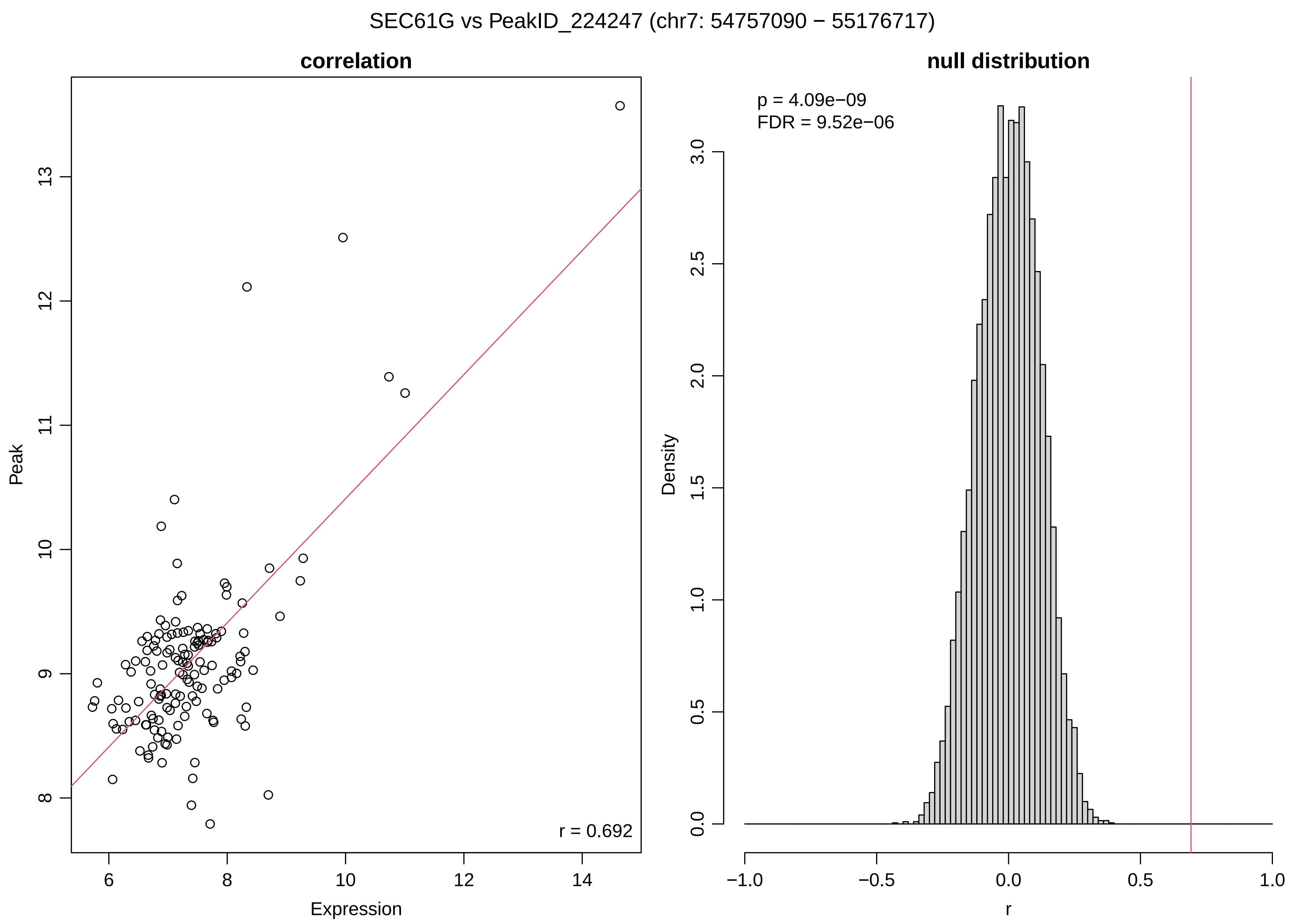

SEC61G vs PeakID_224247 (chr7: 54757090 − 55176717)
correlation
Peak
Expression
r = 0.692
8
9
10
11
12
13
6
8
10
12
14
null distribution
p = 4.09e−09
FDR = 9.52e−06
Density
r
0.0
0.5
1.0
1.5
2.0
2.5
3.0
−1.0
−0.5
0.0
0.5
1.0

SLC8A1−AS1 vs PeakID_125767 (chr2: 39838127 − 39840332)

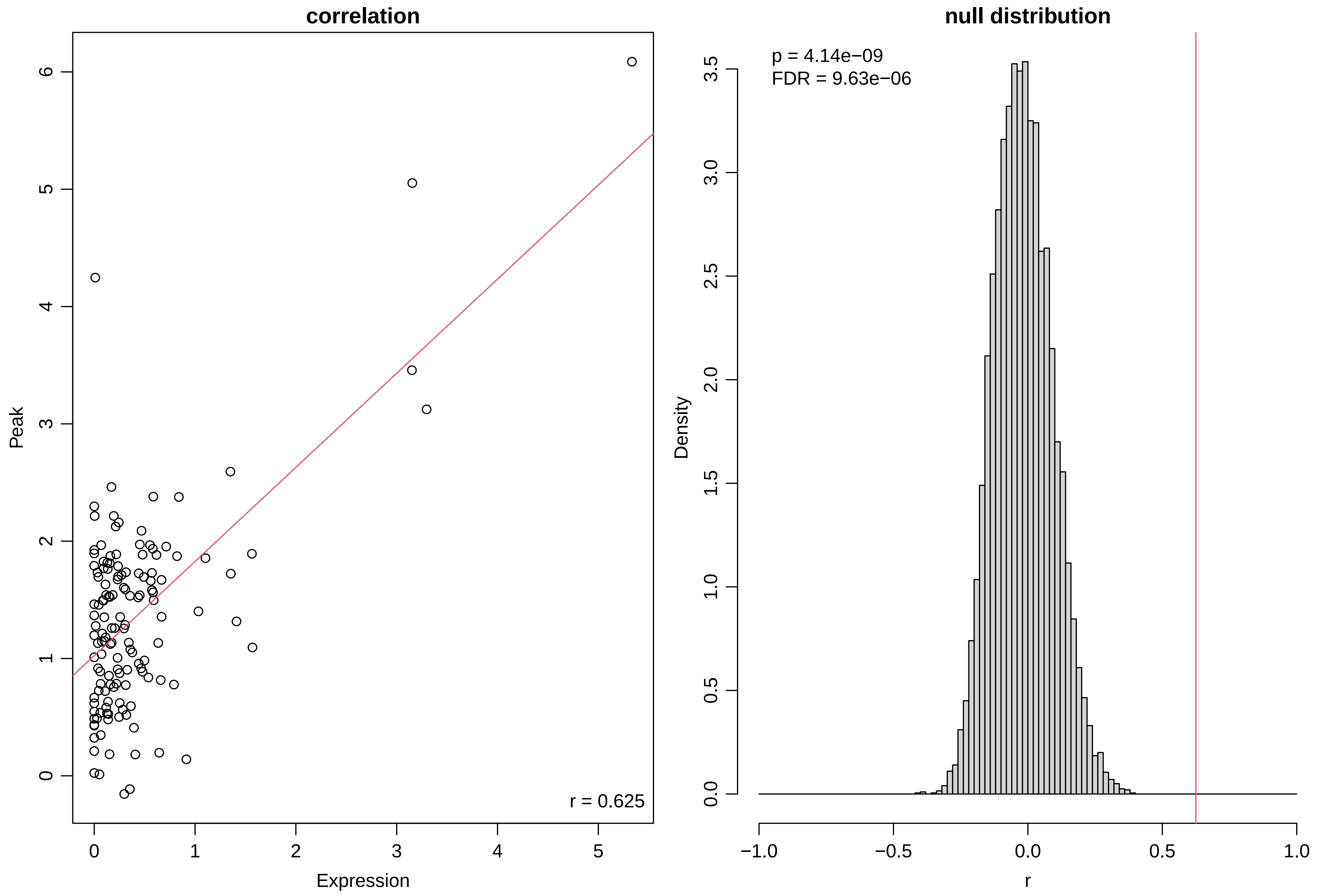

SLC6A11 vs PeakID_160647 (chr3: 10815753 − 10816916)

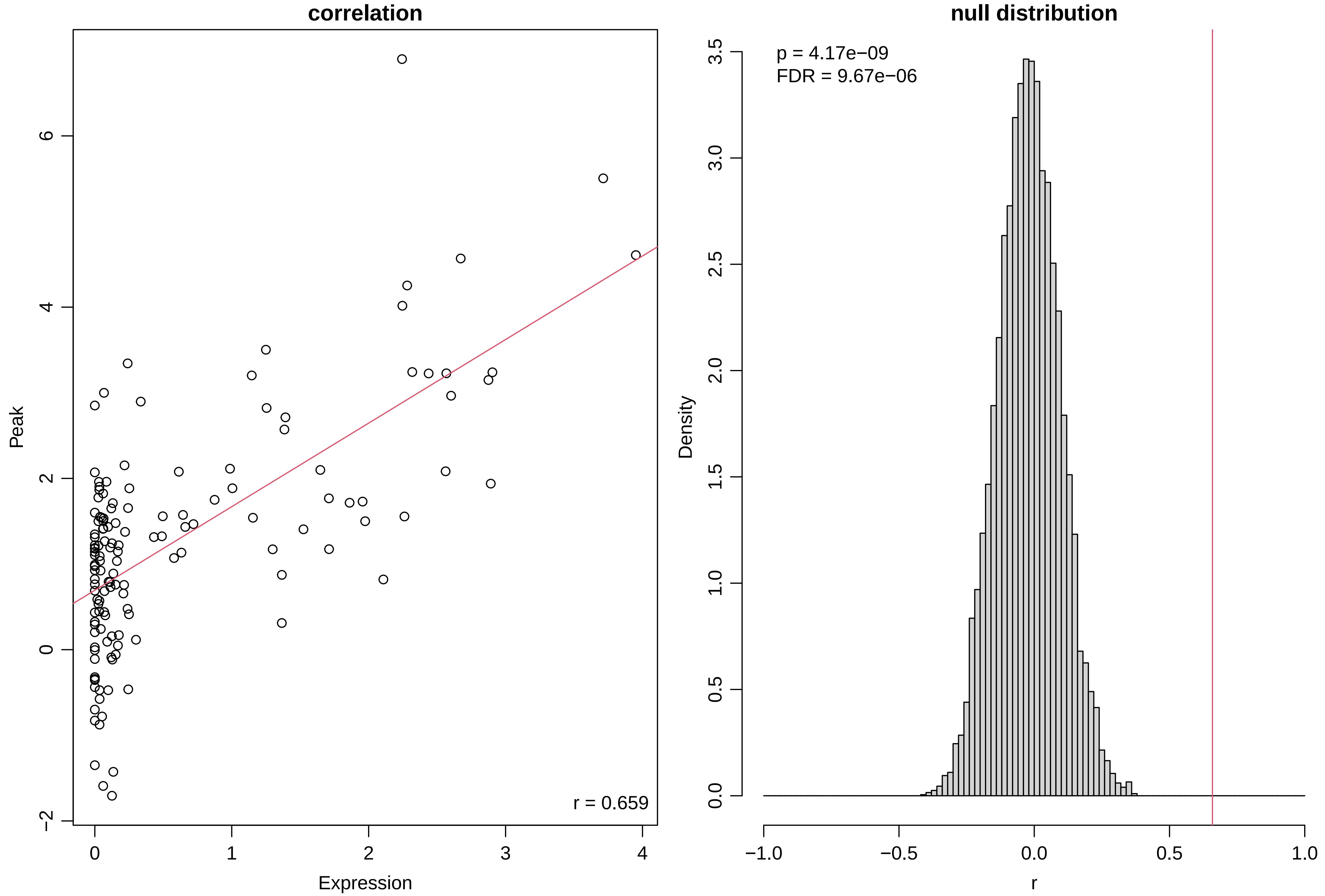

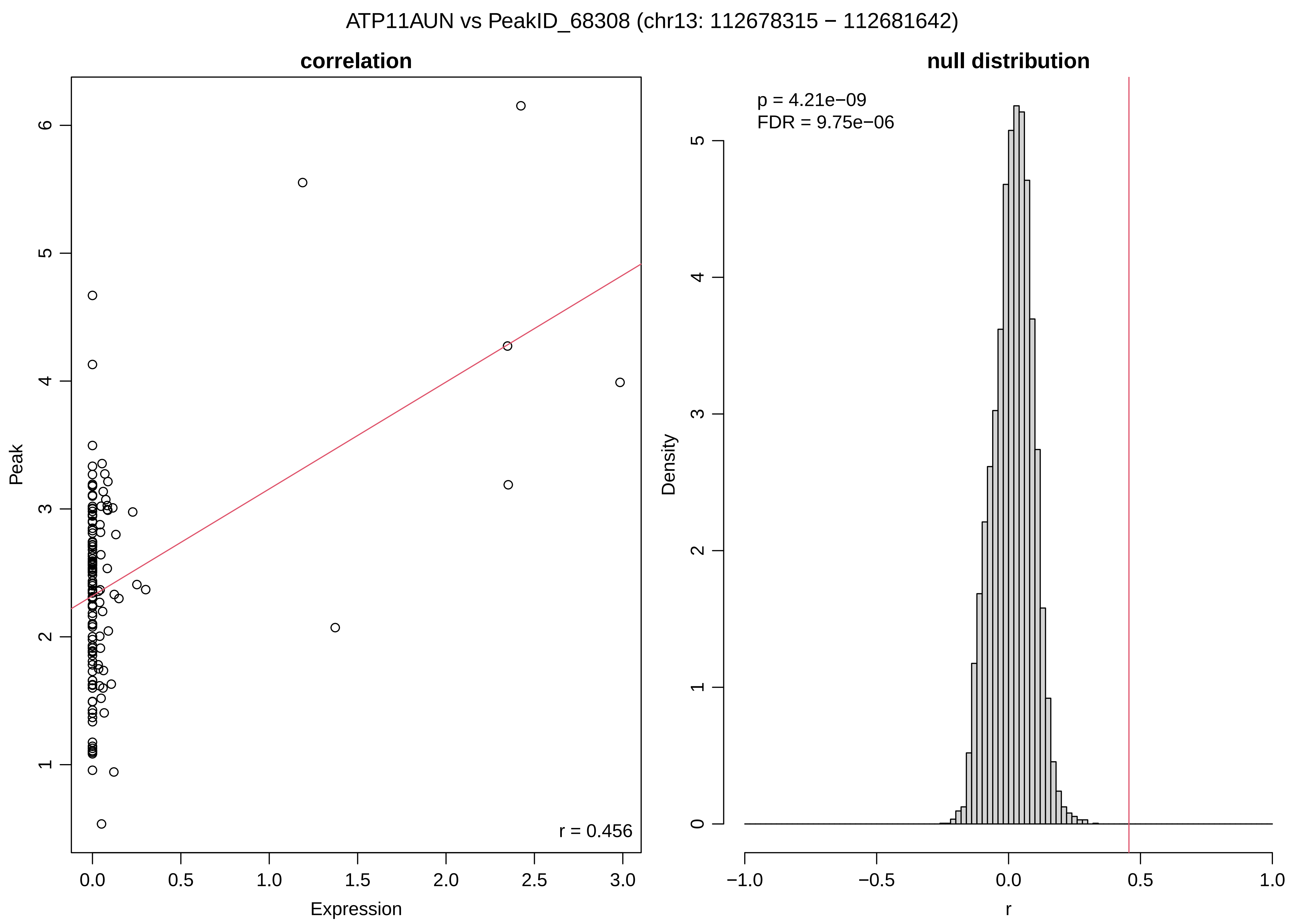

ATP11AUN vs PeakID_68308 (chr13: 112678315 − 112681642)
correlation
Peak
Expression
r = 0.456
null distribution
p = 4.21e−09
FDR = 9.75e−06
Density
r

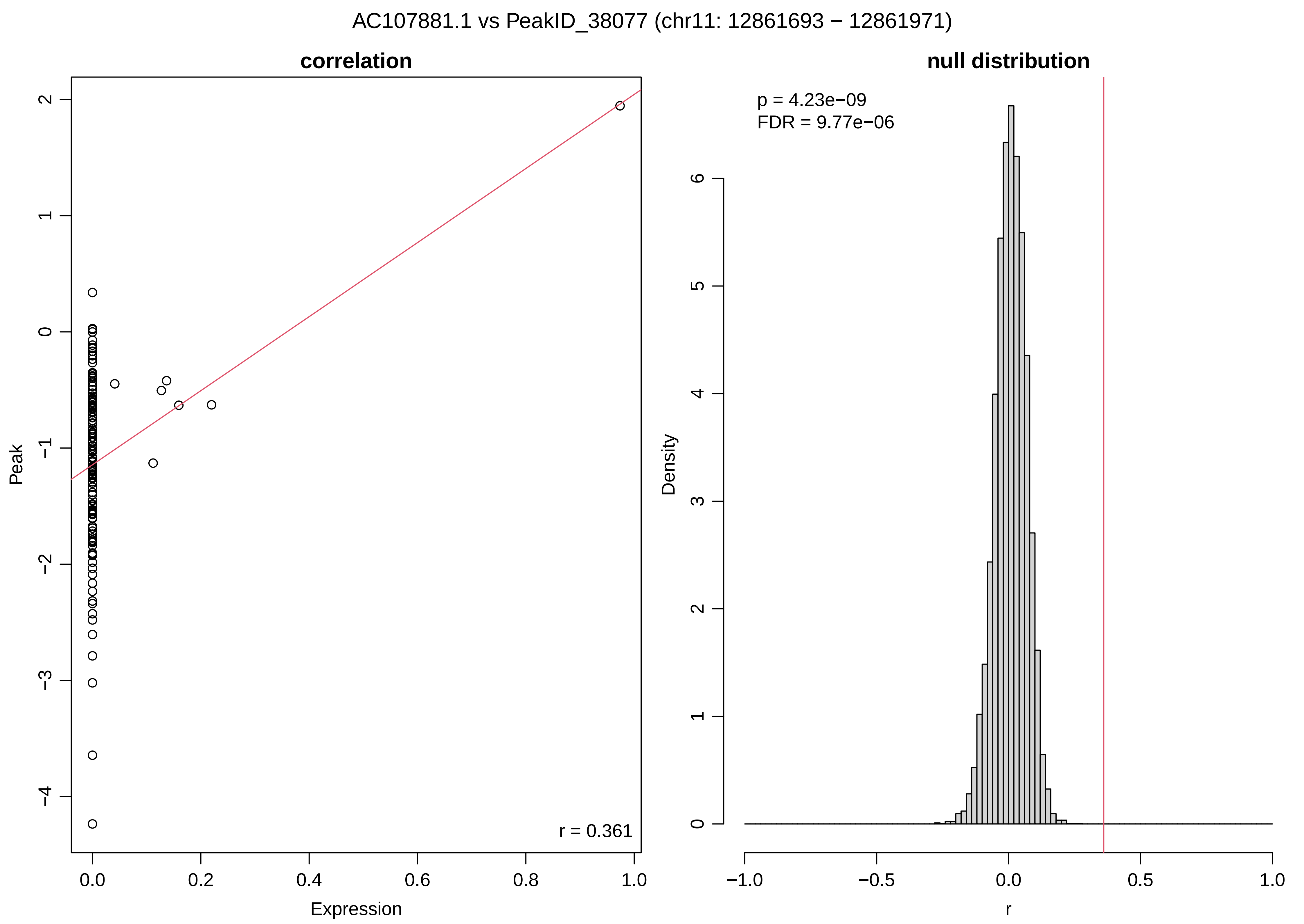

AC107881.1 vs PeakID_38077 (chr11: 12861693 − 12861971)
correlation
Peak
Expression
r = 0.361
null distribution
p = 4.23e−09
FDR = 9.77e−06
Density
r

LINC01833 vs PeakID_126339 (chr2: 44933961 − 44941519)

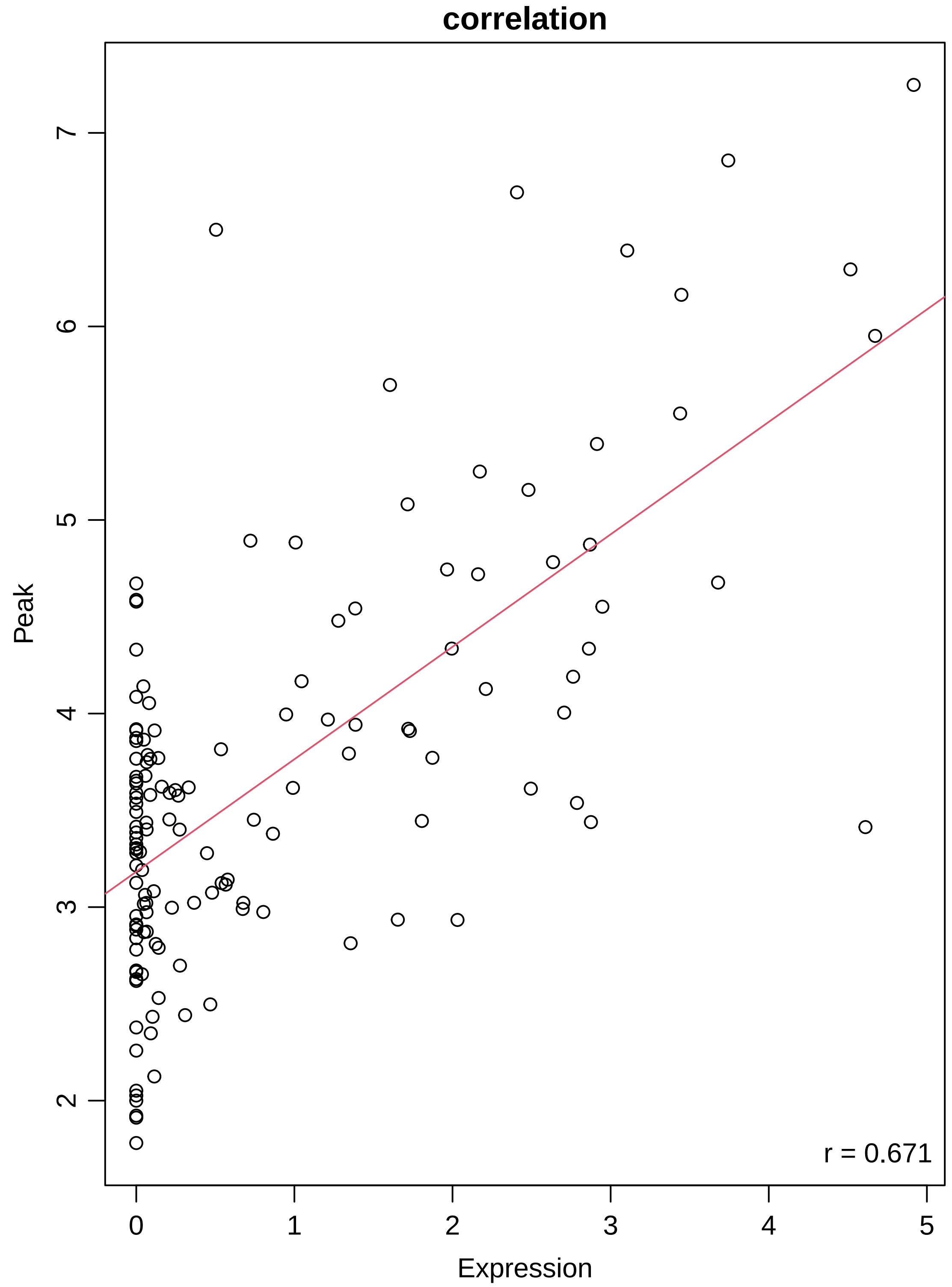

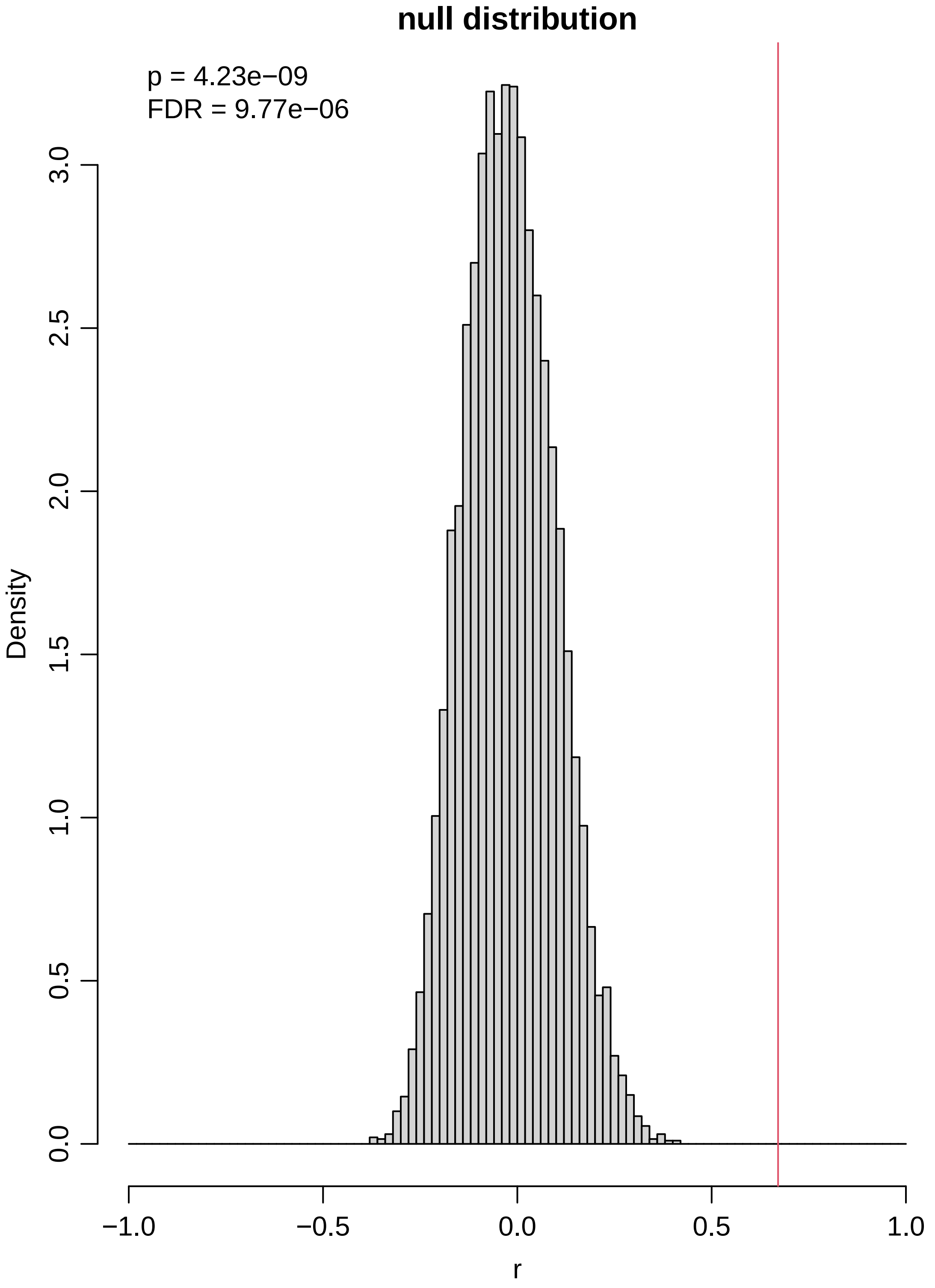

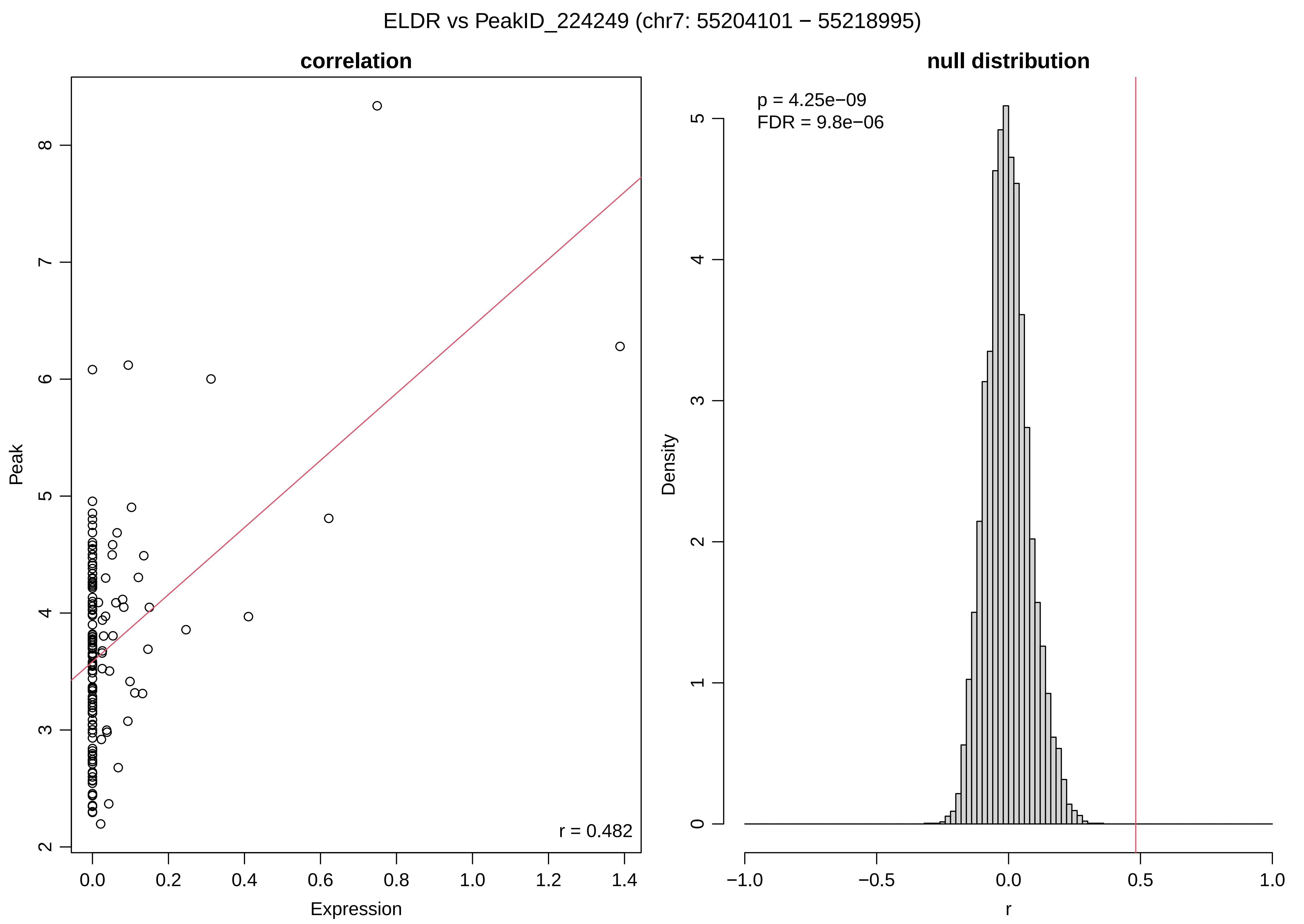
ELDR vs PeakID_224249 (chr7: 55204101 − 55218995)
correlation
Peak
Expression
r = 0.482
null distribution
p = 4.25e−09
FDR = 9.8e−06
Density
r

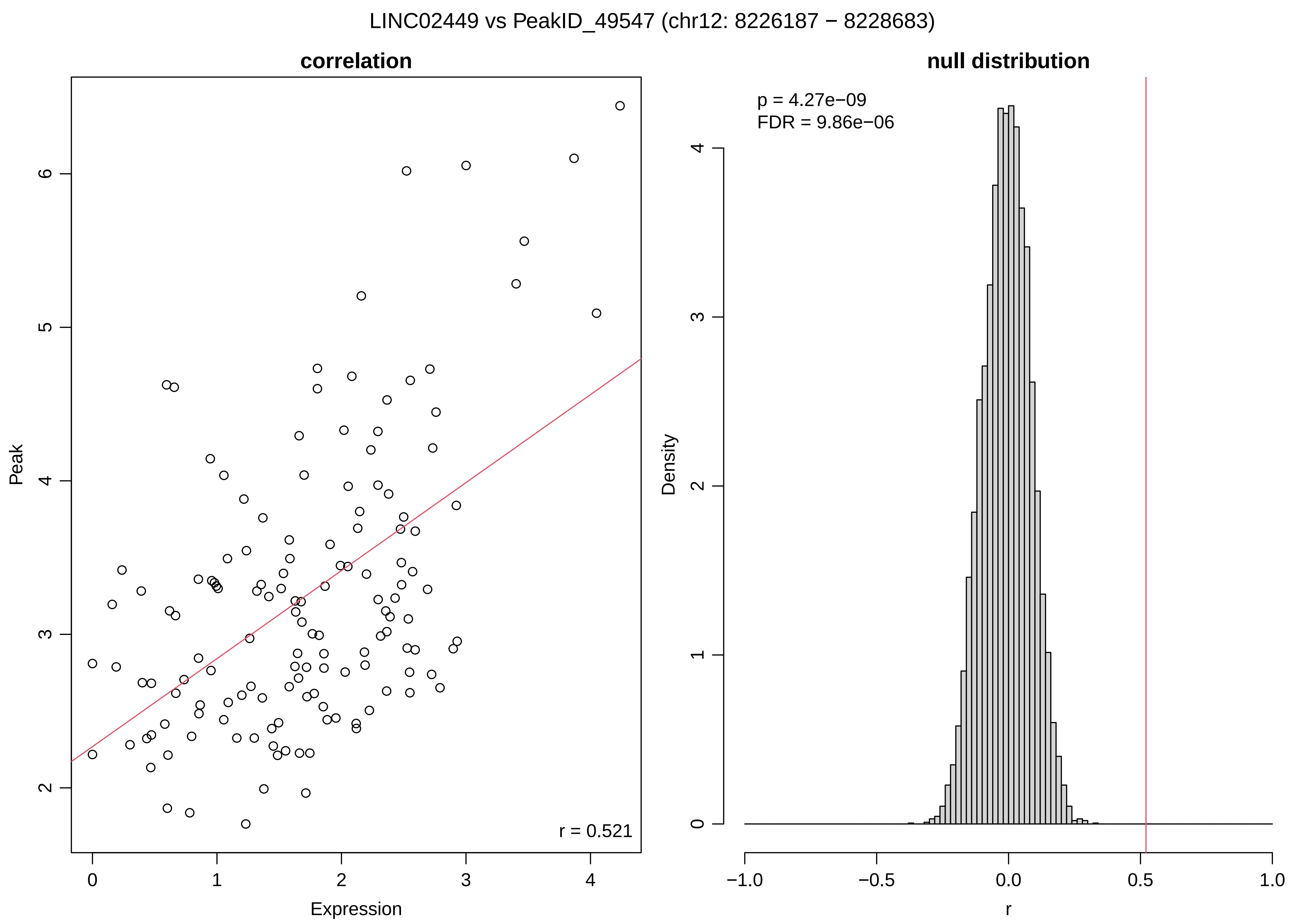
LINC02449 vs PeakID_49547 (chr12: 8226187 − 8228683)
correlation
null distribution
p = 4.27e−09
FDR = 9.86e−06
Peak
Expression
r = 0.521
Density
r

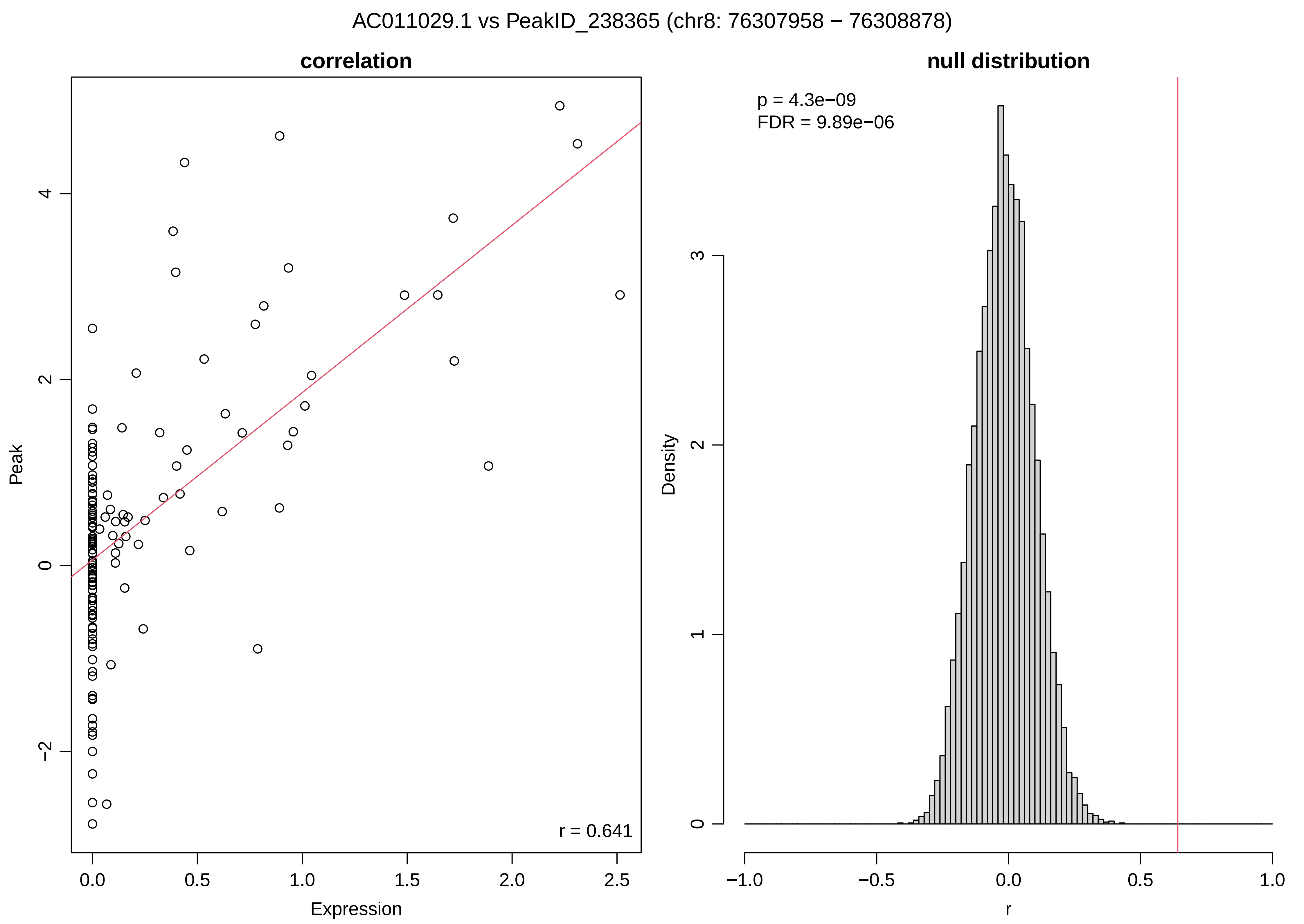
AC011029.1 vs PeakID_238365 (chr8: 76307958 − 76308878)
correlation
Peak
Expression
r = 0.641
null distribution
p = 4.3e−09
FDR = 9.89e−06
Density
r

RNU4−20P vs PeakID_54790 (chr12: 59482692 − 59484493)

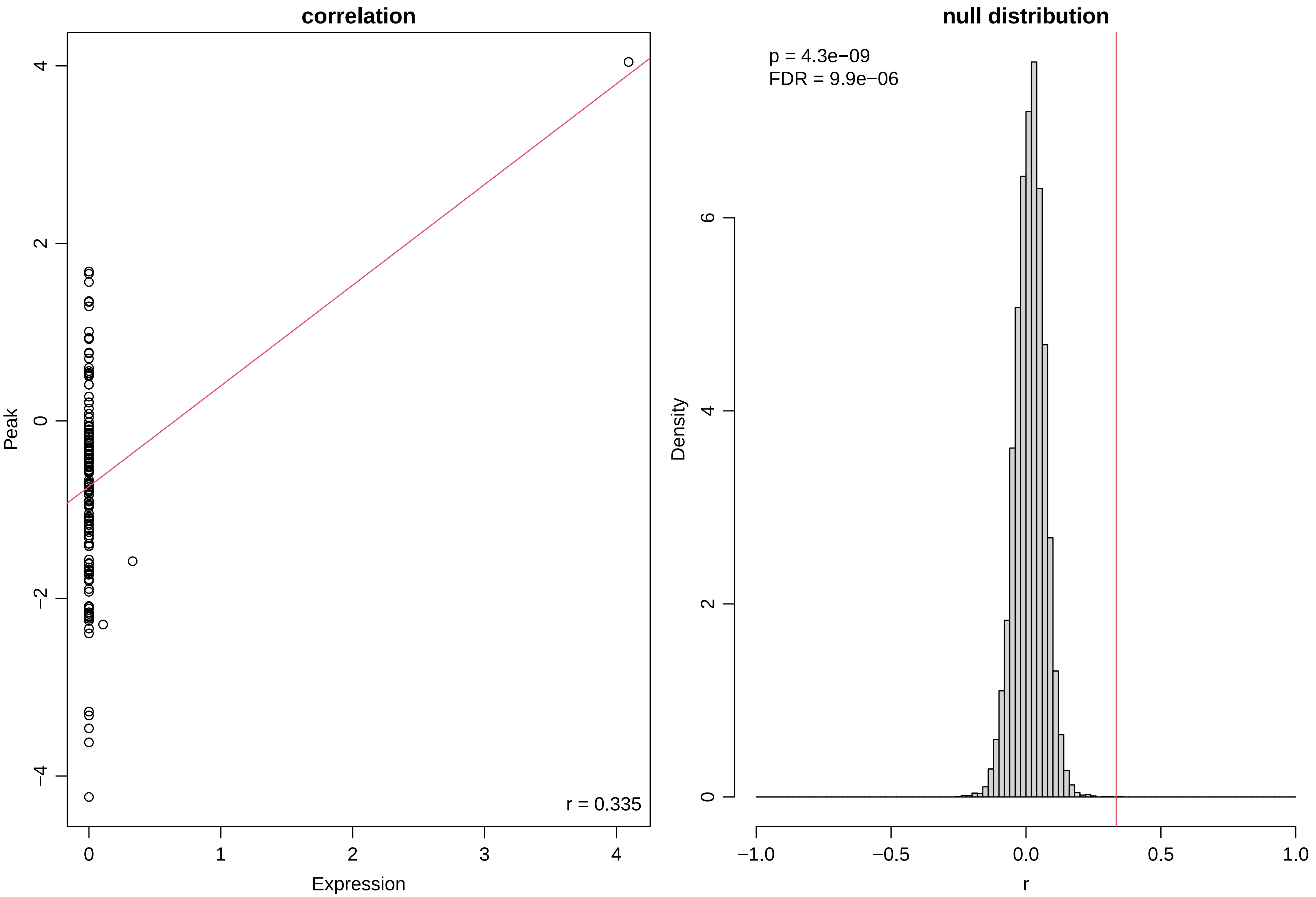

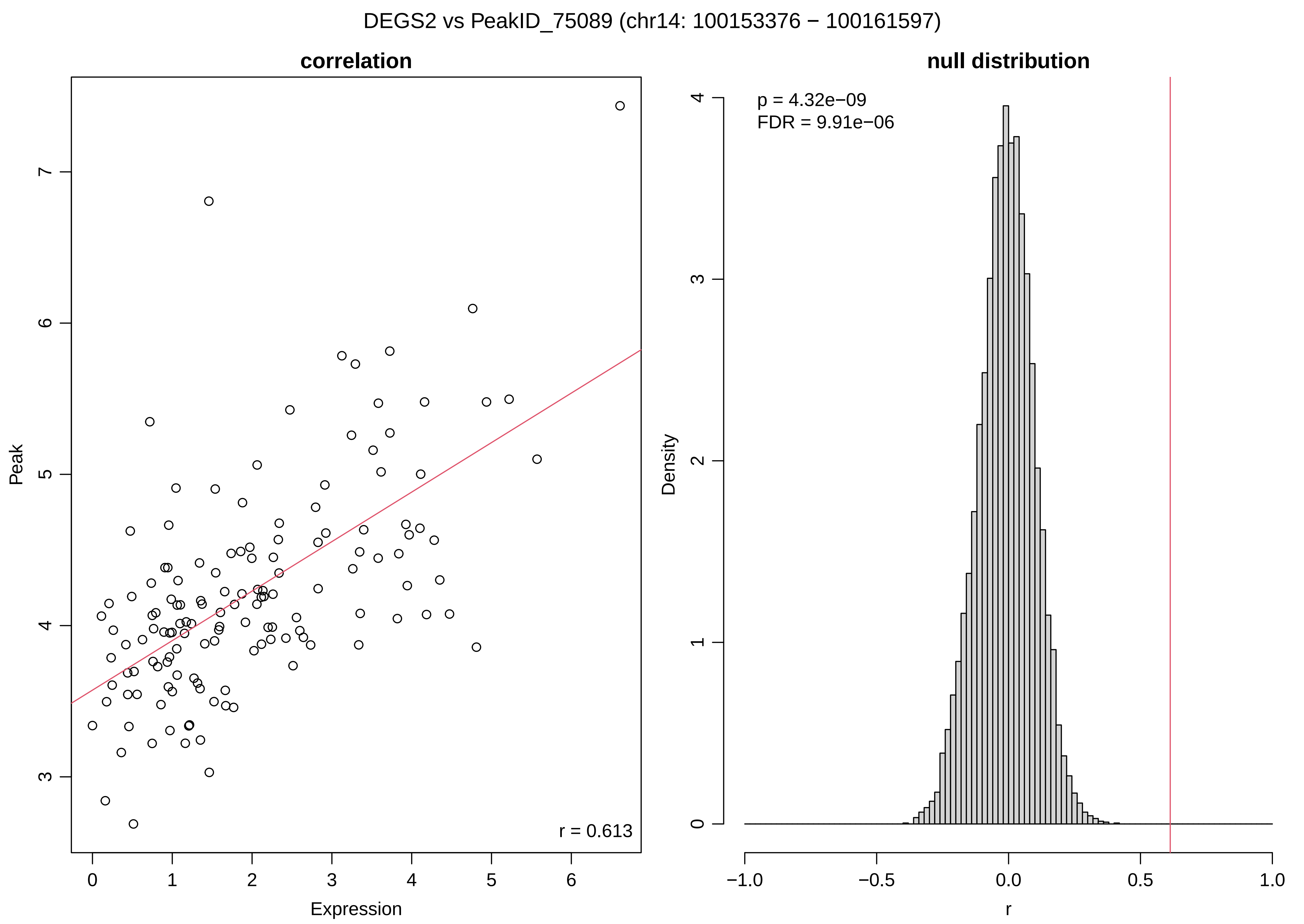
DEGS2 vs PeakID_75089 (chr14: 100153376 − 100161597)
correlation
Peak
Expression
r = 0.613
null distribution
Density
r
p = 4.32e−09
FDR = 9.91e−06

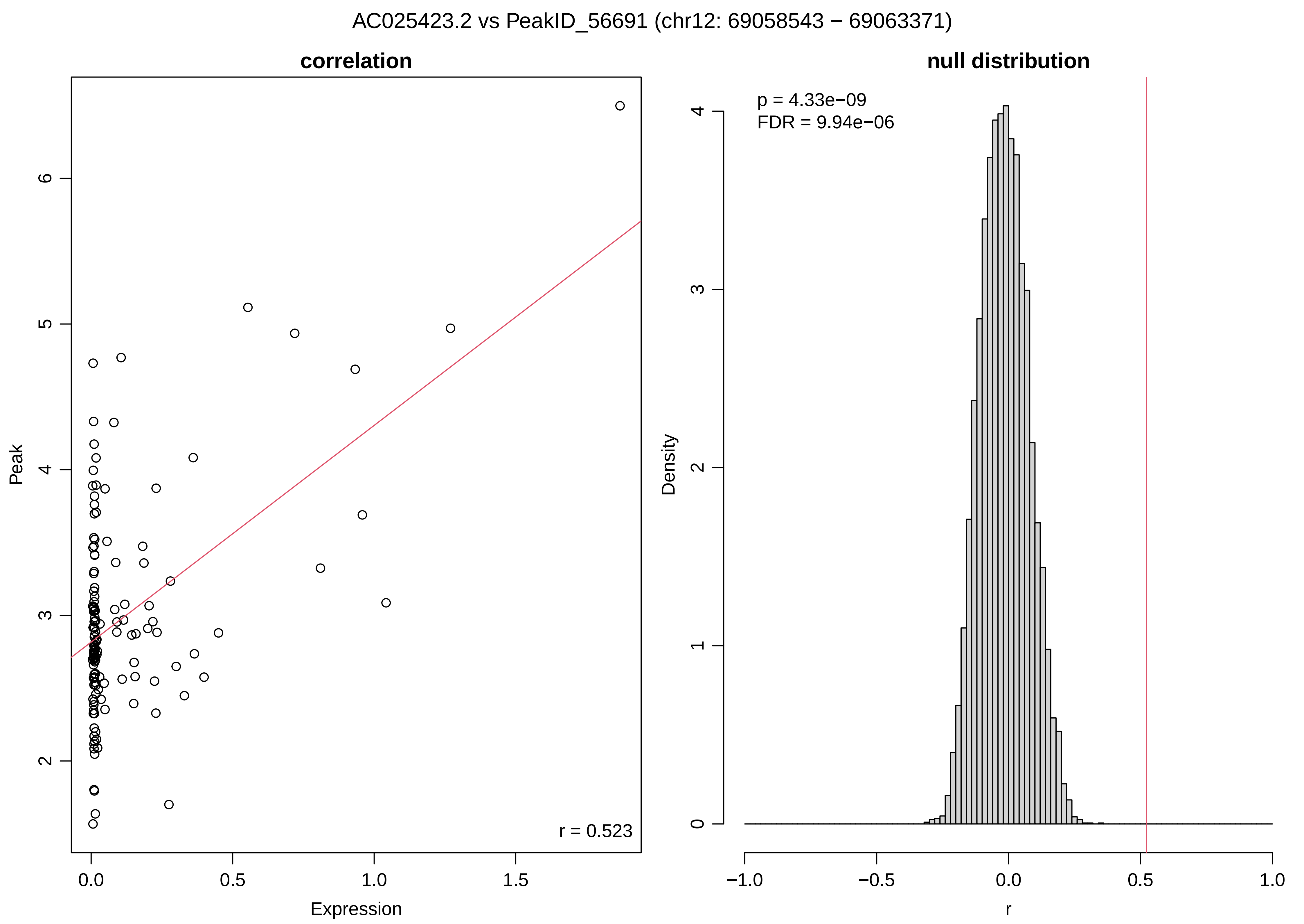

AC025423.2 vs PeakID_56691 (chr12: 69058543 − 69063371)
correlation
Peak
Expression
r = 0.523
0.0
0.5
1.0
1.5
2
3
4
5
6
null distribution
p = 4.33e−09
FDR = 9.94e−06
Density
r
−1.0
−0.5
0.0
0.5
1.0
0
1
2
3
4

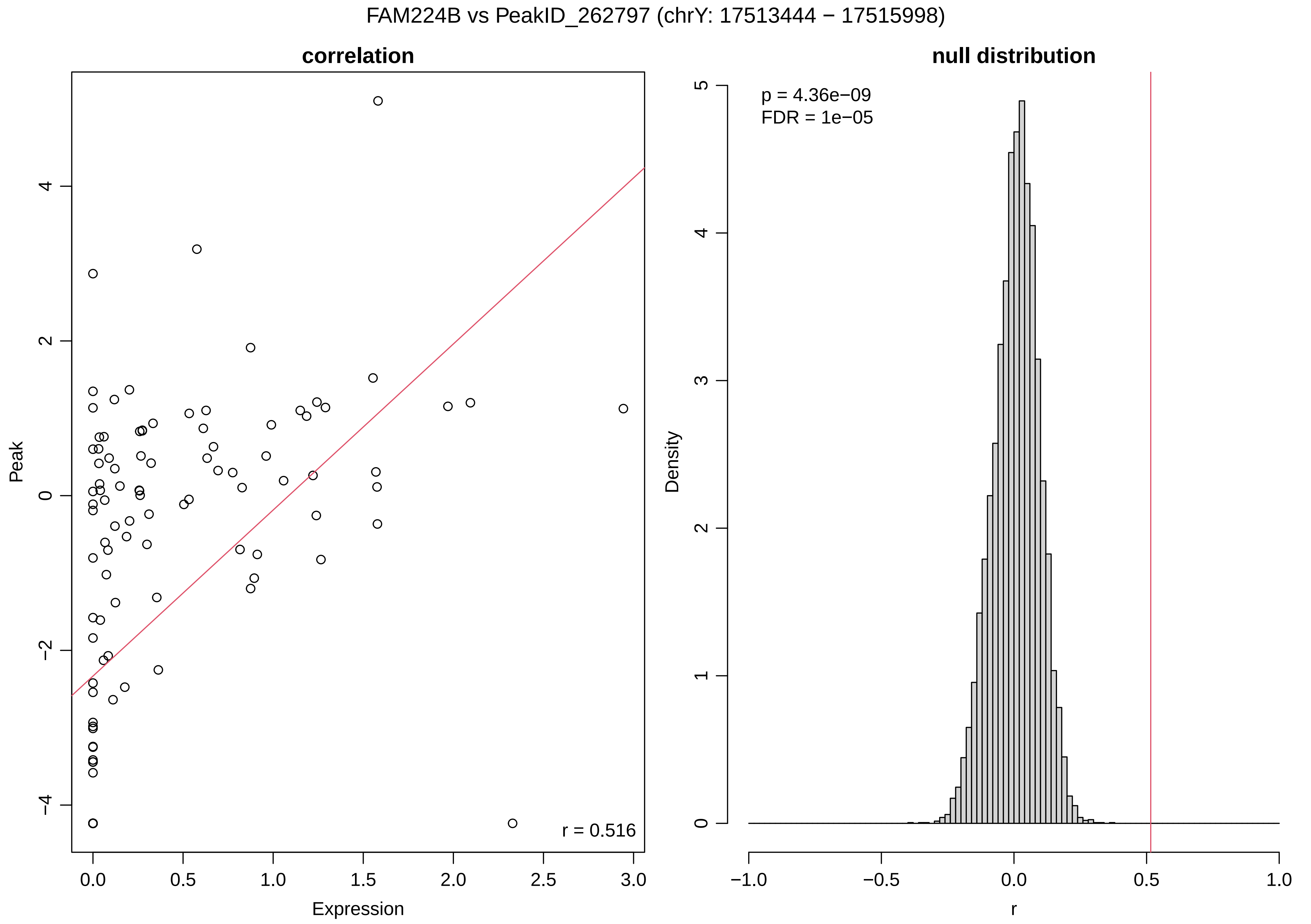

FAM224B vs PeakID_262797 (chrY: 17513444 − 17515998)
correlation
Peak
Expression
r = 0.516
null distribution
p = 4.36e−09
FDR = 1e−05
Density
r

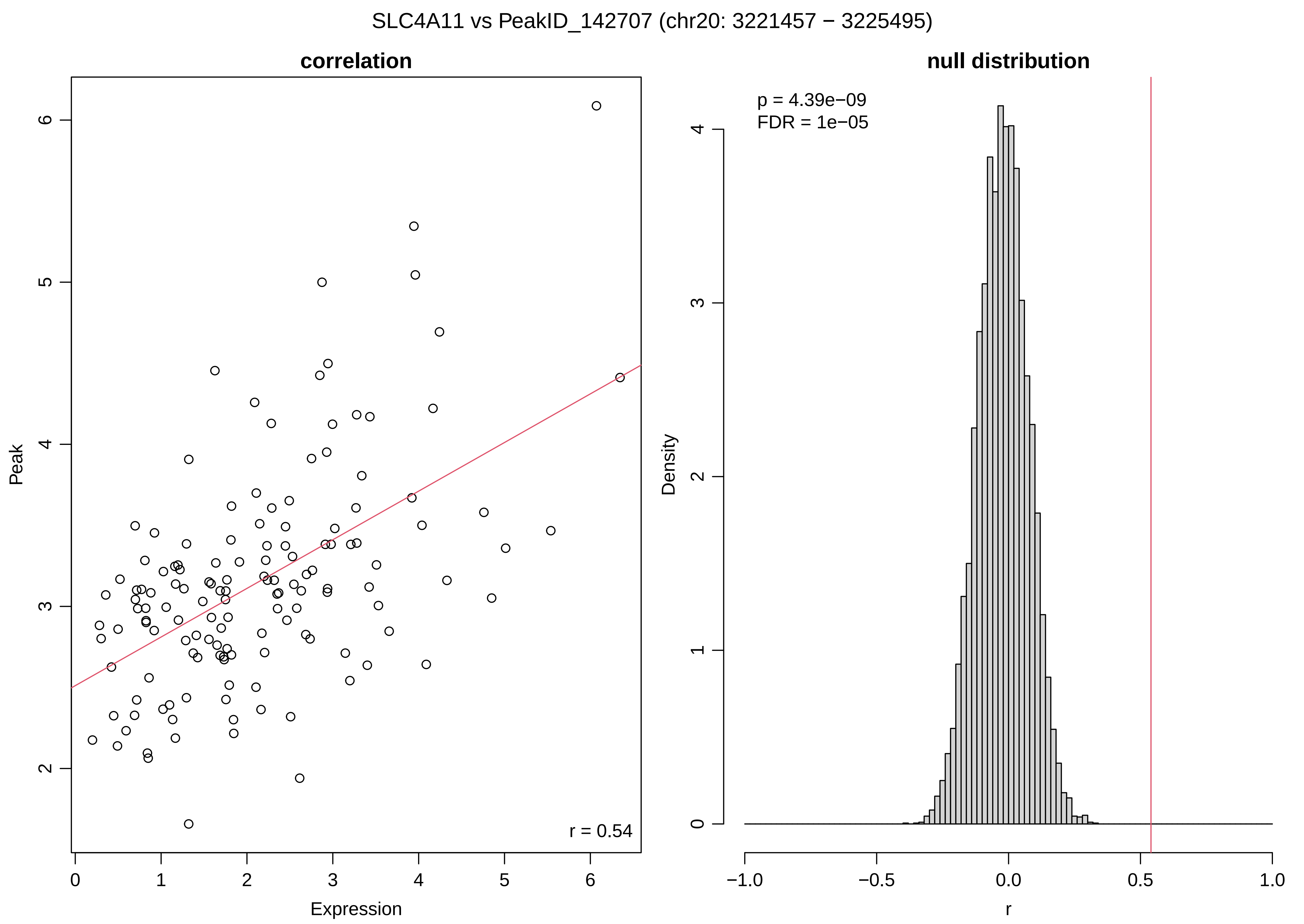
SLC4A11 vs PeakID_142707 (chr20: 3221457 − 3225495)
correlation
Peak
Expression
r = 0.54
null distribution
p = 4.39e−09
FDR = 1e−05
Density
r

EFNA5 vs PeakID_194764 (chr5: 107540886 − 107541068)

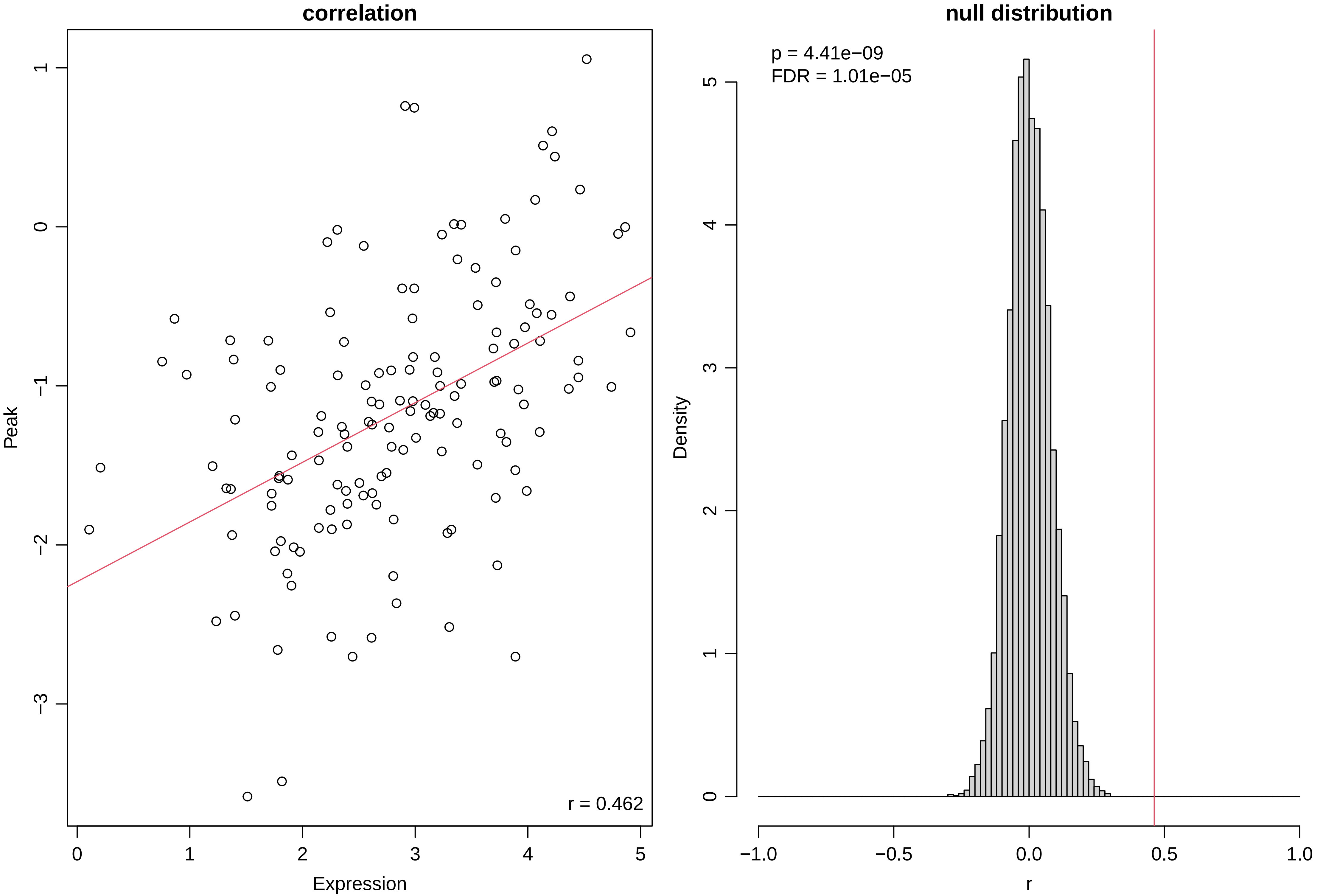

MUC20P1 vs PeakID_174761 (chr3: 195720404 − 195721979)

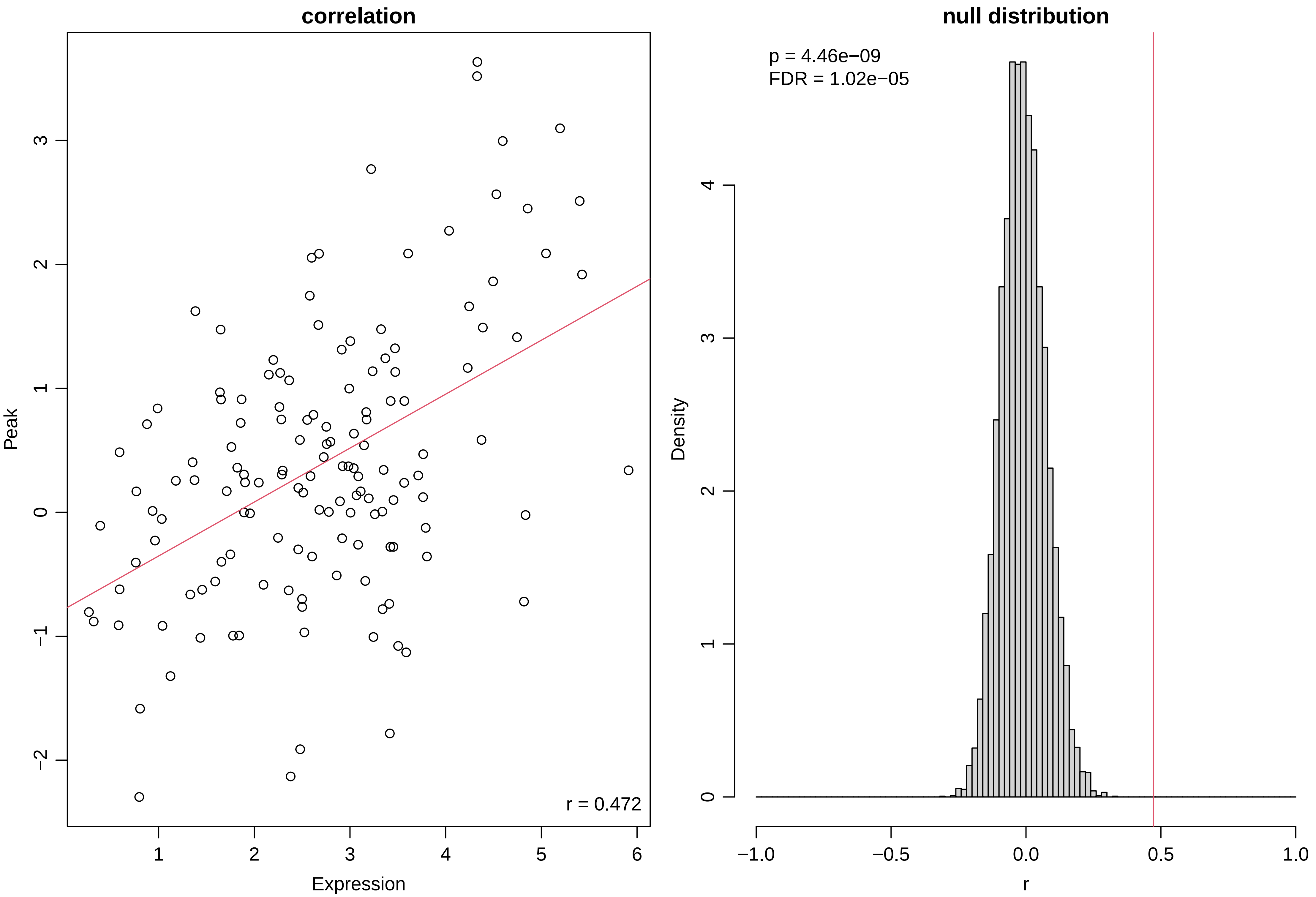

UGT1A12P vs PeakID_140961 (chr2: 233583042 − 233583884)

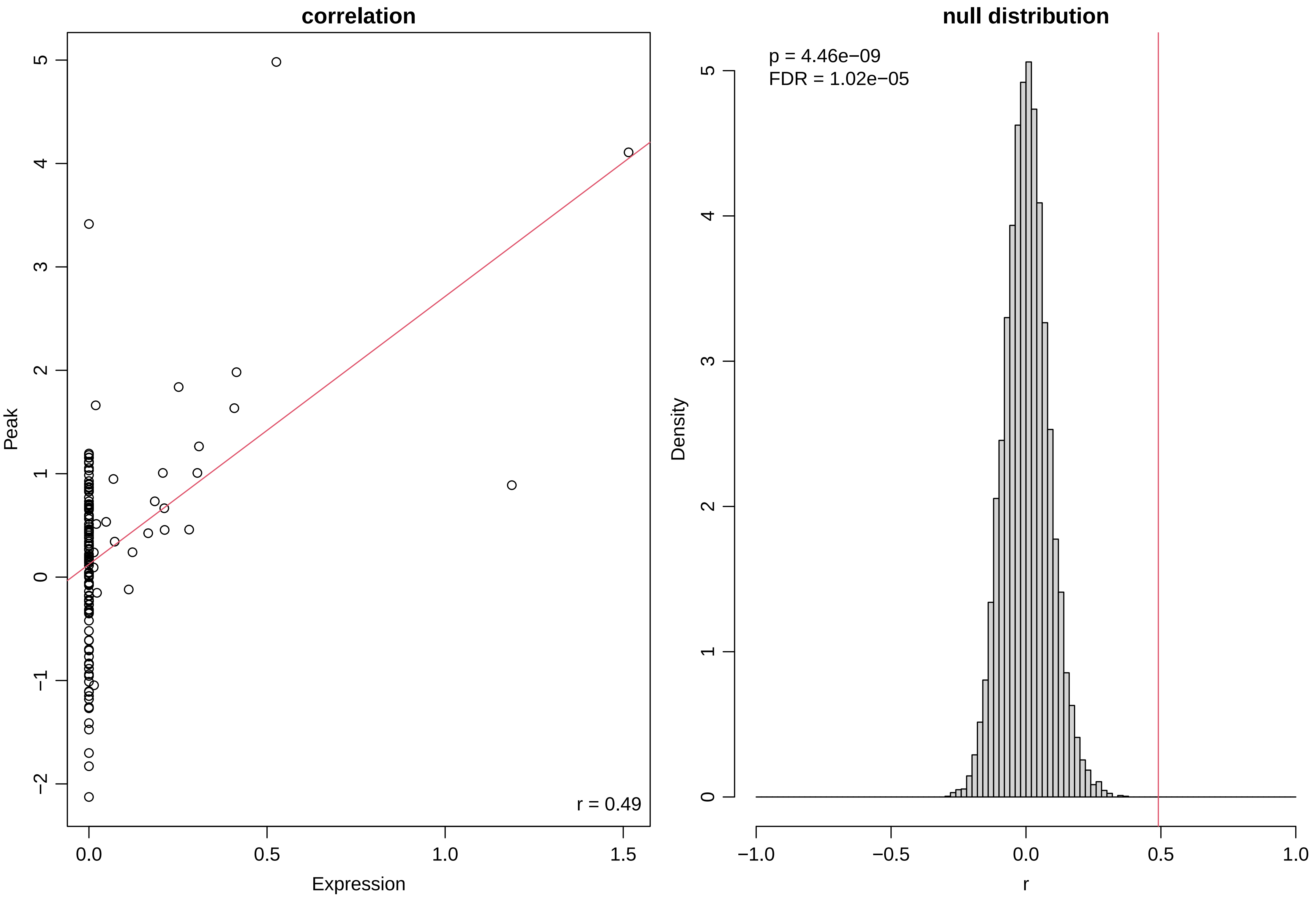

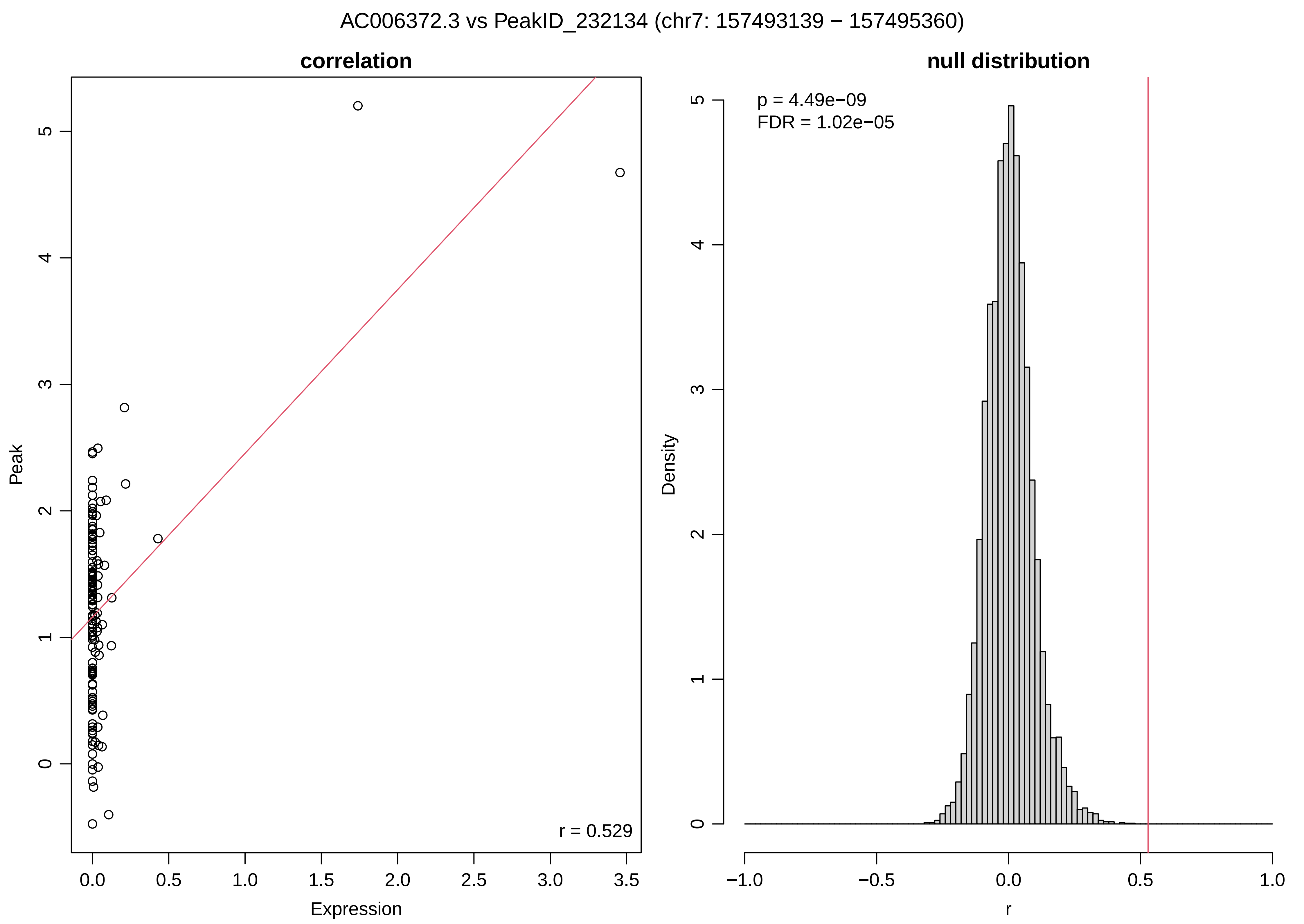
AC006372.3 vs PeakID_232134 (chr7: 157493139 − 157495360)
correlation
Peak
Expression
r = 0.529
0.0
0.5
1.0
1.5
2.0
2.5
3.0
3.5
0
1
2
3
4
5
null distribution
p = 4.49e−09
FDR = 1.02e−05
Density
r
−1.0
−0.5
0.0
0.5
1.0

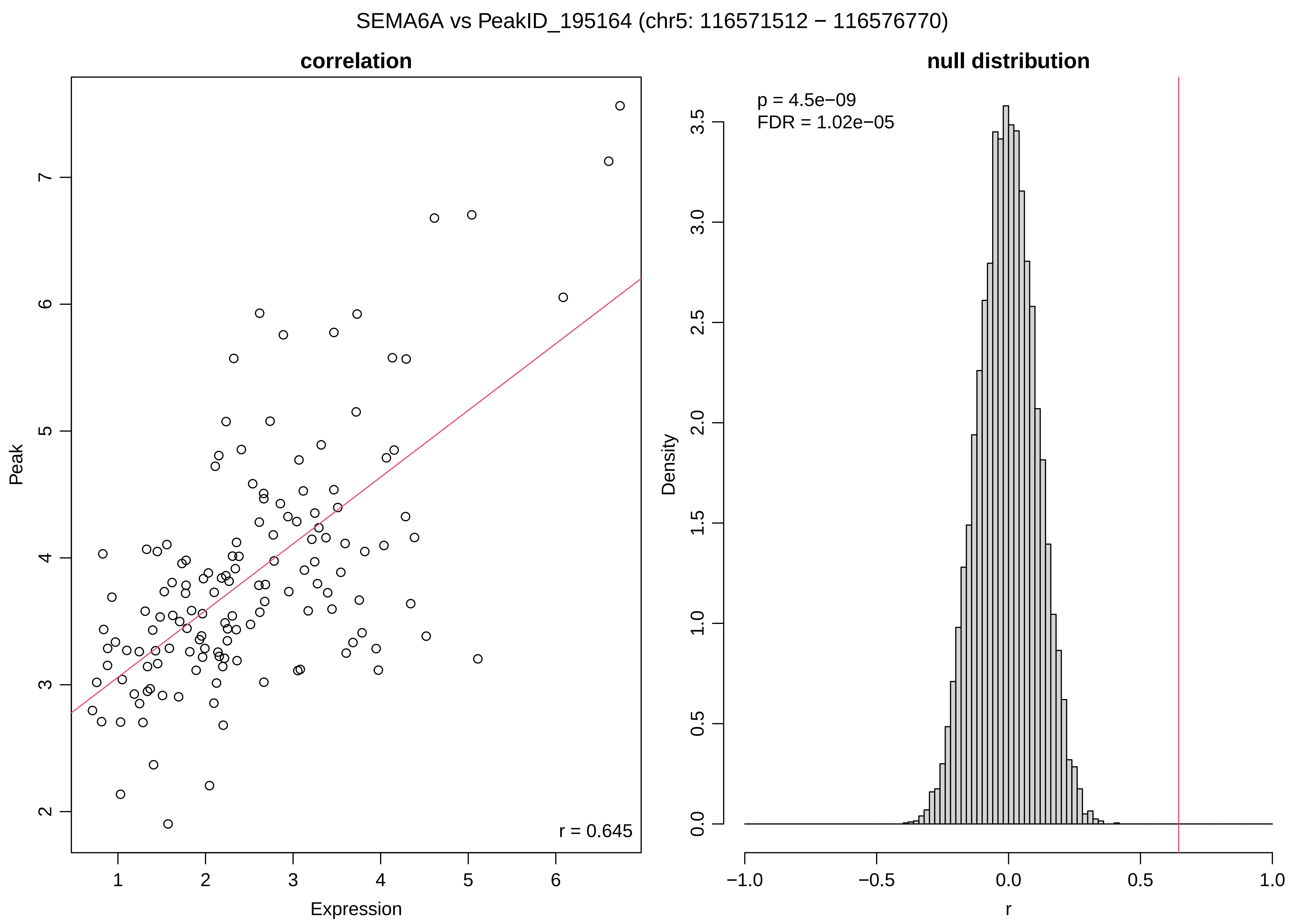

SEMA6A vs PeakID_195164 (chr5: 116571512 − 116576770)
correlation
Peak
Expression
r = 0.645
null distribution
p = 4.5e−09
FDR = 1.02e−05
Density
r

YEATS4 vs PeakID_56751 (chr12: 69249647 − 69250008)

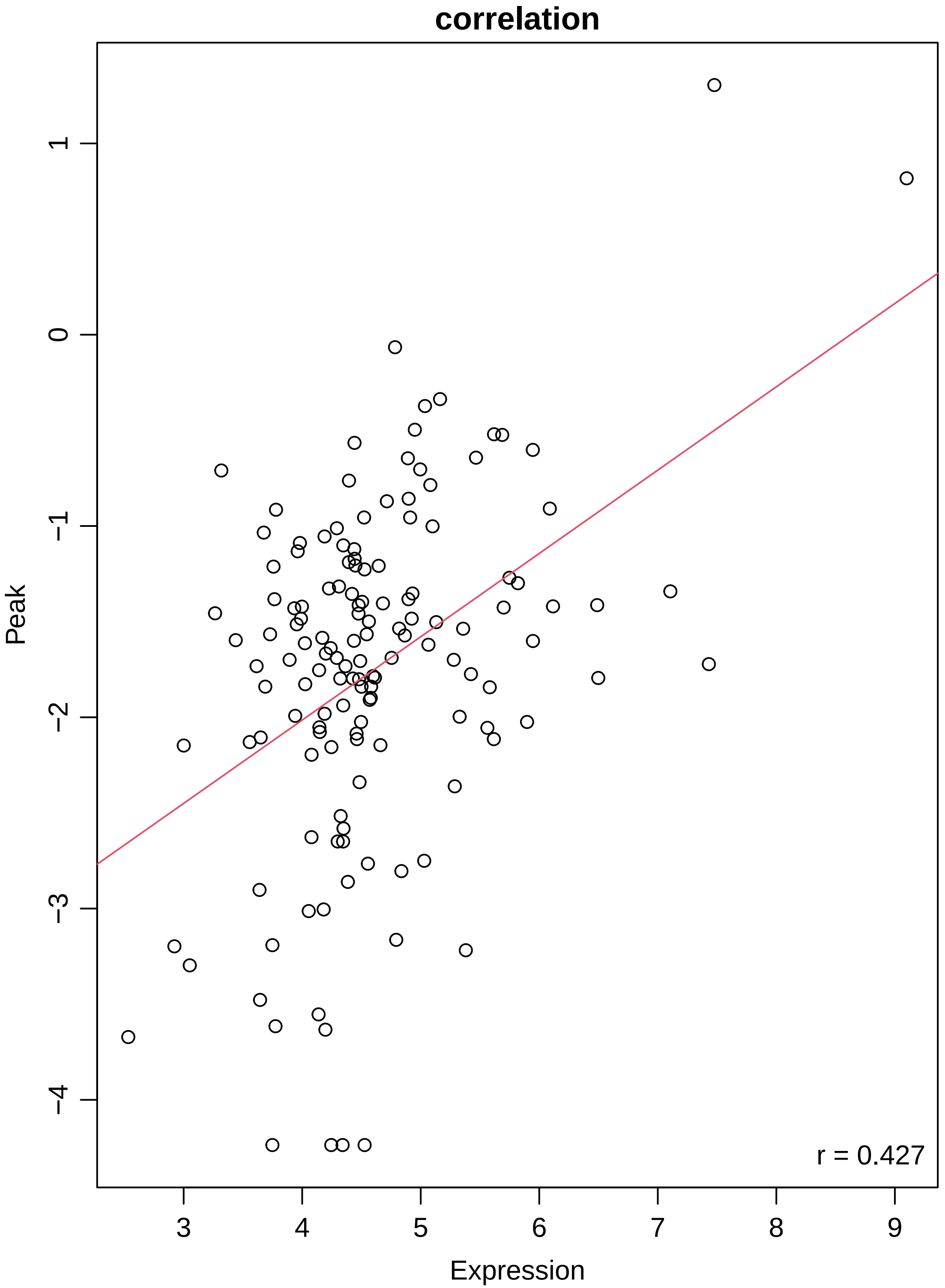

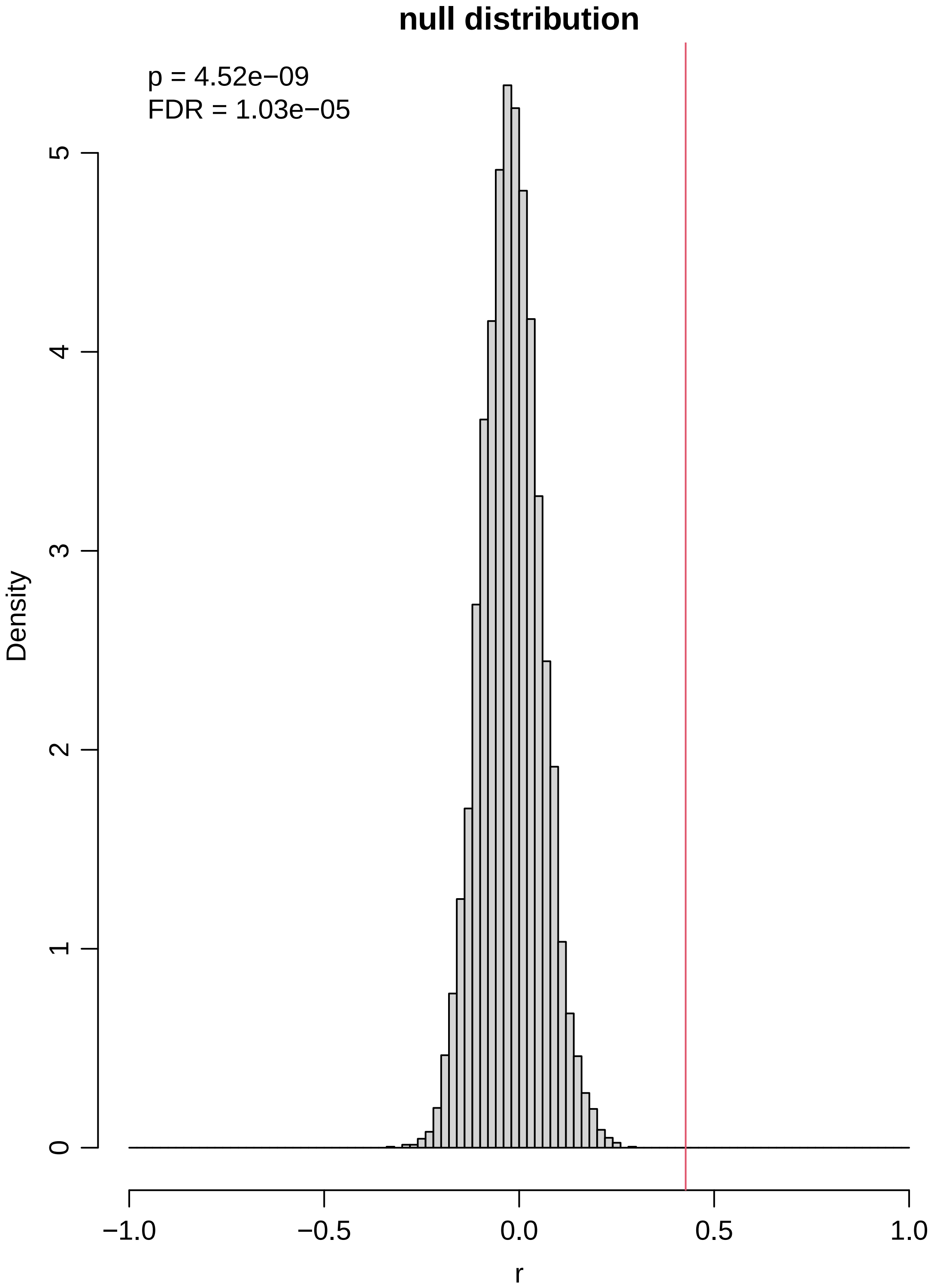

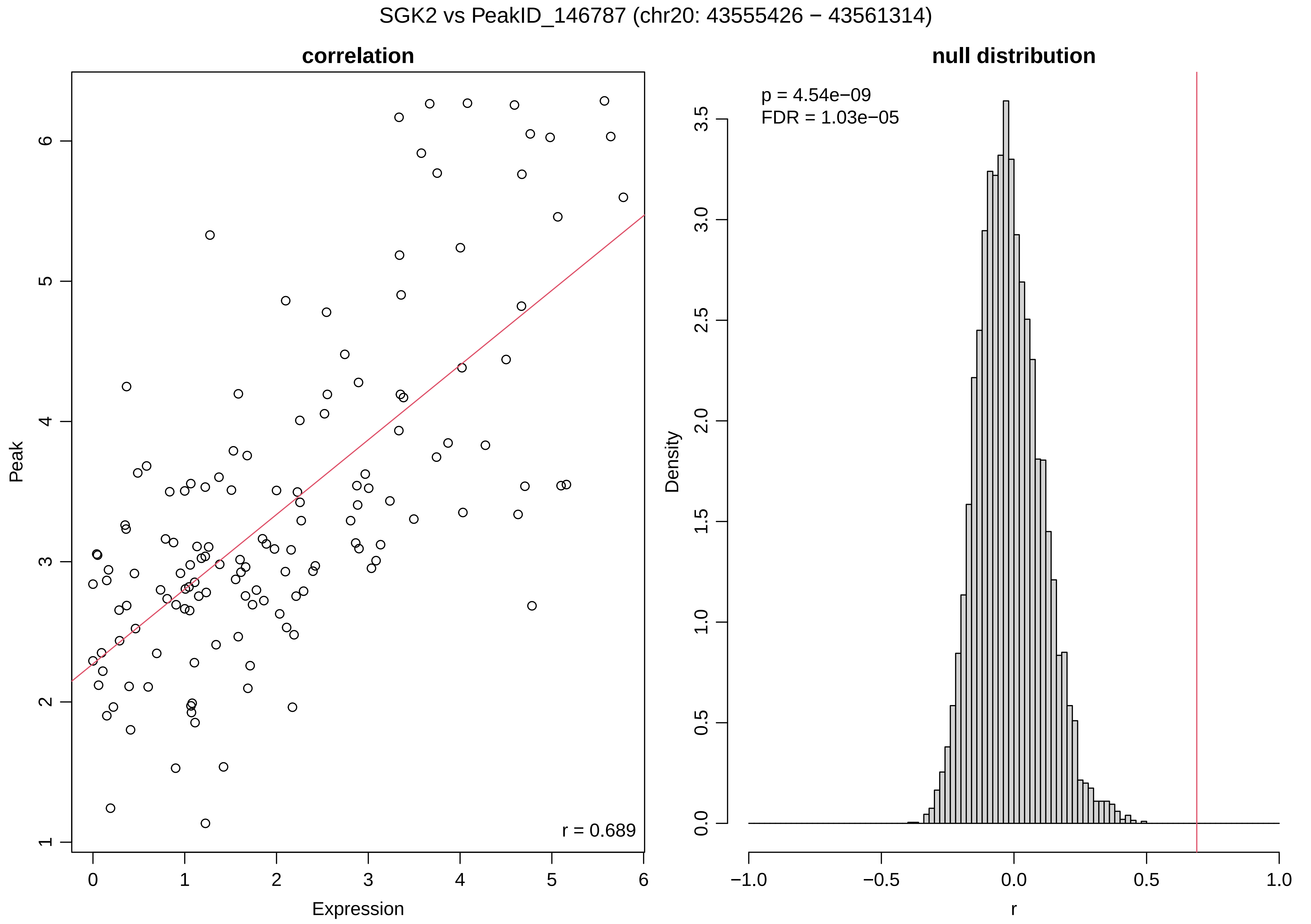
SGK2 vs PeakID_146787 (chr20: 43555426 − 43561314)
correlation
null distribution
Peak
Expression
r = 0.689
p = 4.54e−09
FDR = 1.03e−05
Density
r
0
1
2
3
4
5
6
0.0
0.5
1.0
1.5
2.0
2.5
3.0
3.5
−1.0
−0.5
0.0
0.5
1.0

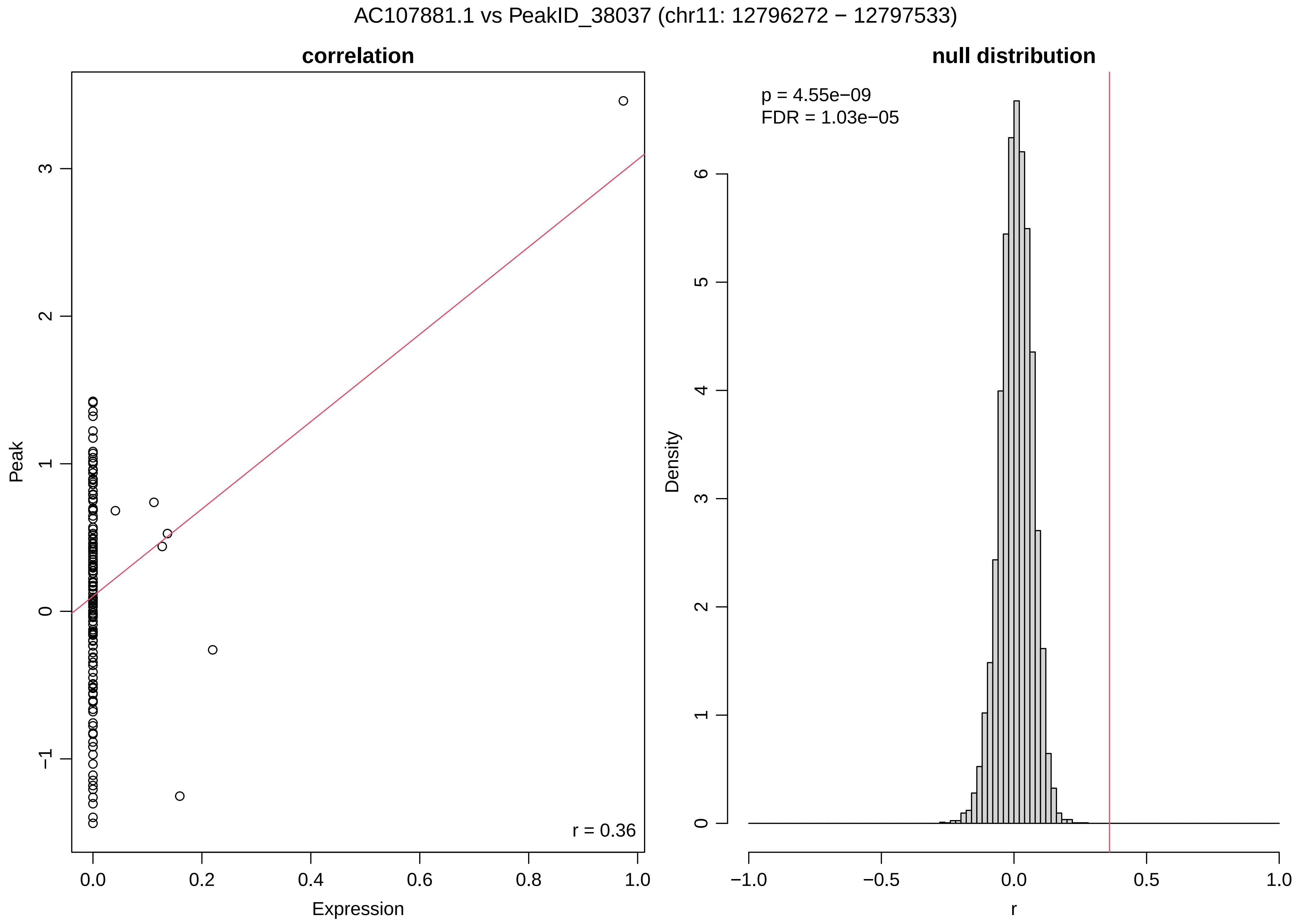
AC107881.1 vs PeakID_38037 (chr11: 12796272 − 12797533)
correlation
Peak
Expression
r = 0.36
null distribution
p = 4.55e−09
FDR = 1.03e−05
Density
r

YEATS4 vs PeakID_56789 (chr12: 69358490 − 69361674)

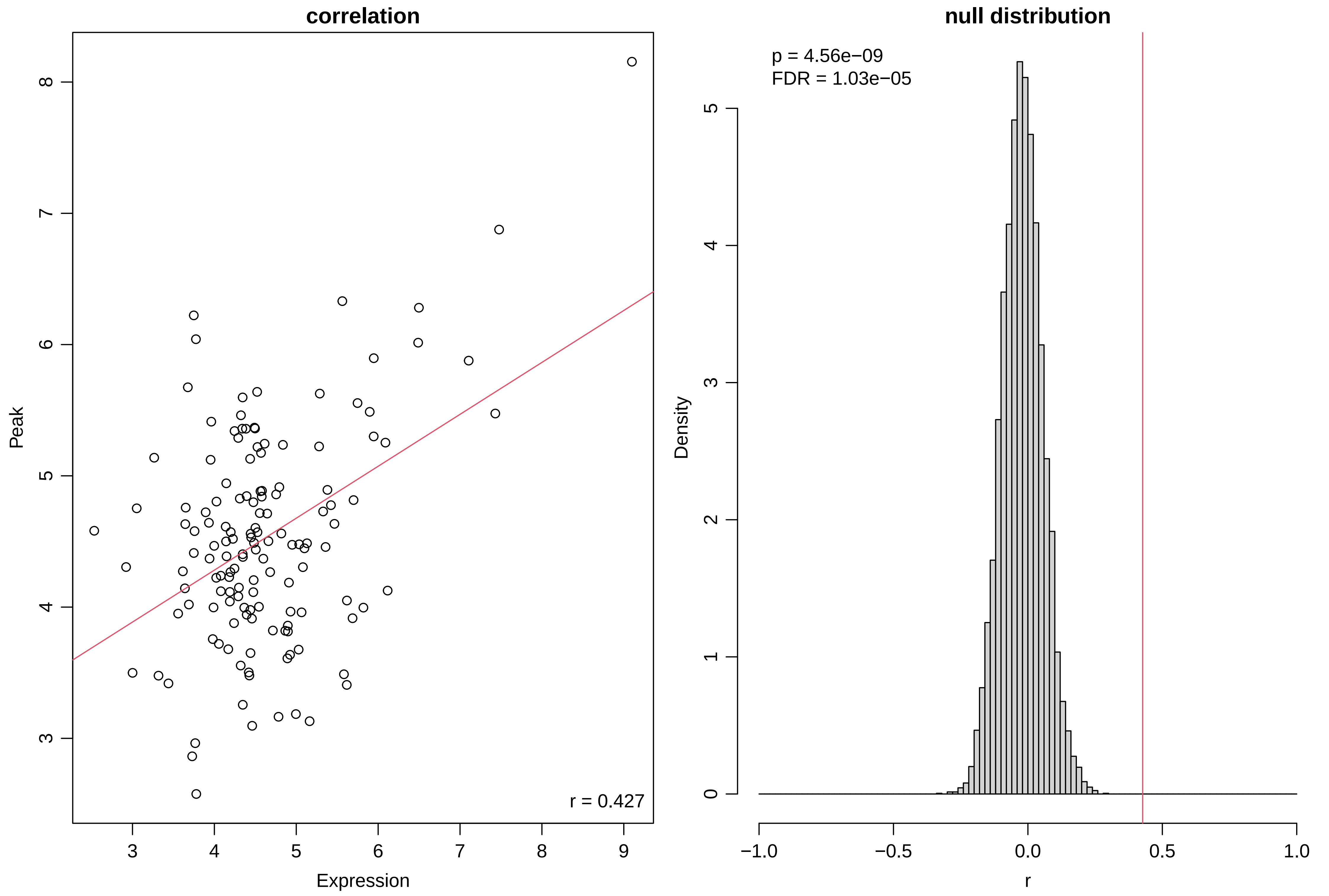

AC011632.1 vs PeakID_238263 (chr8: 74866502 − 74867296)

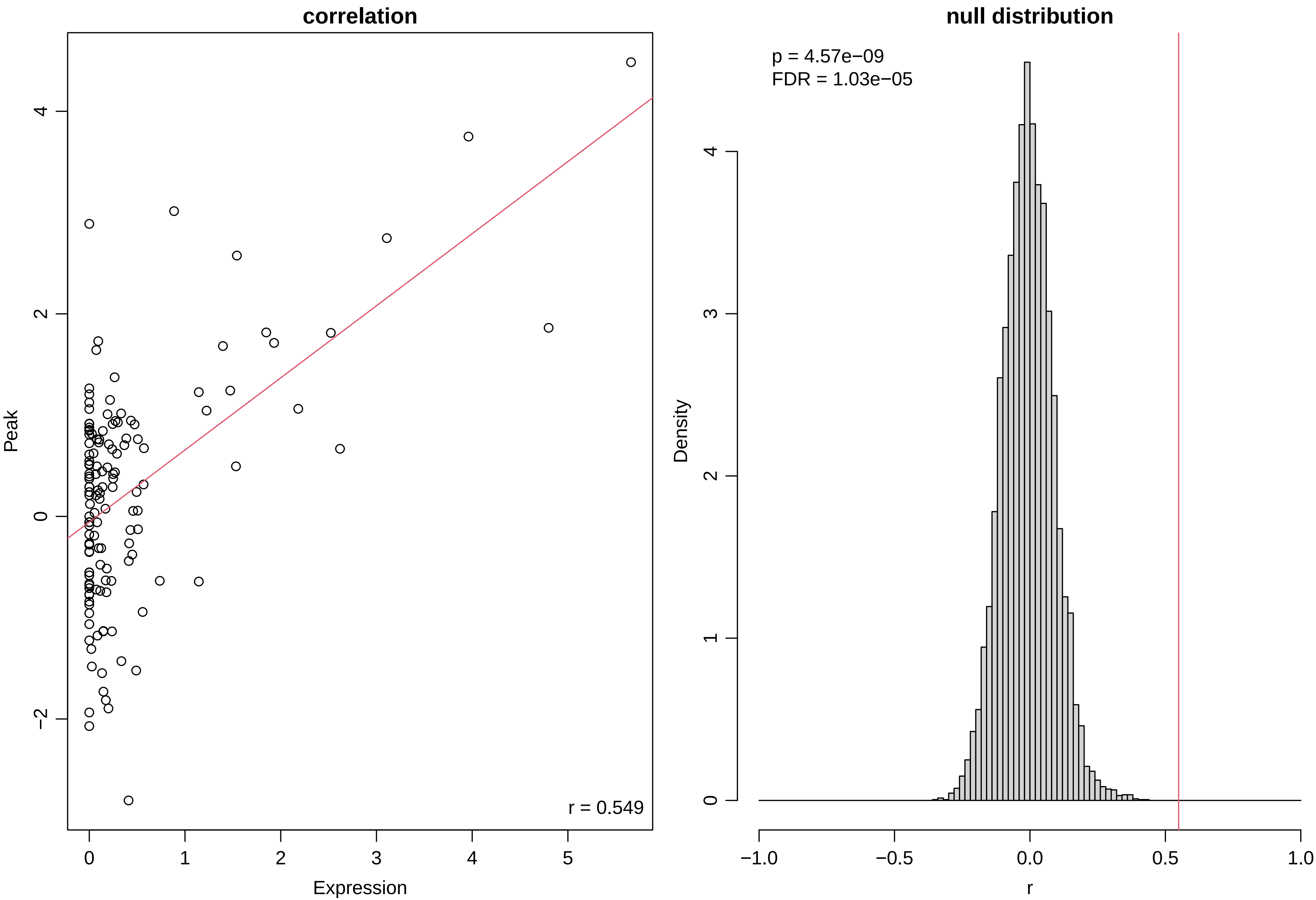

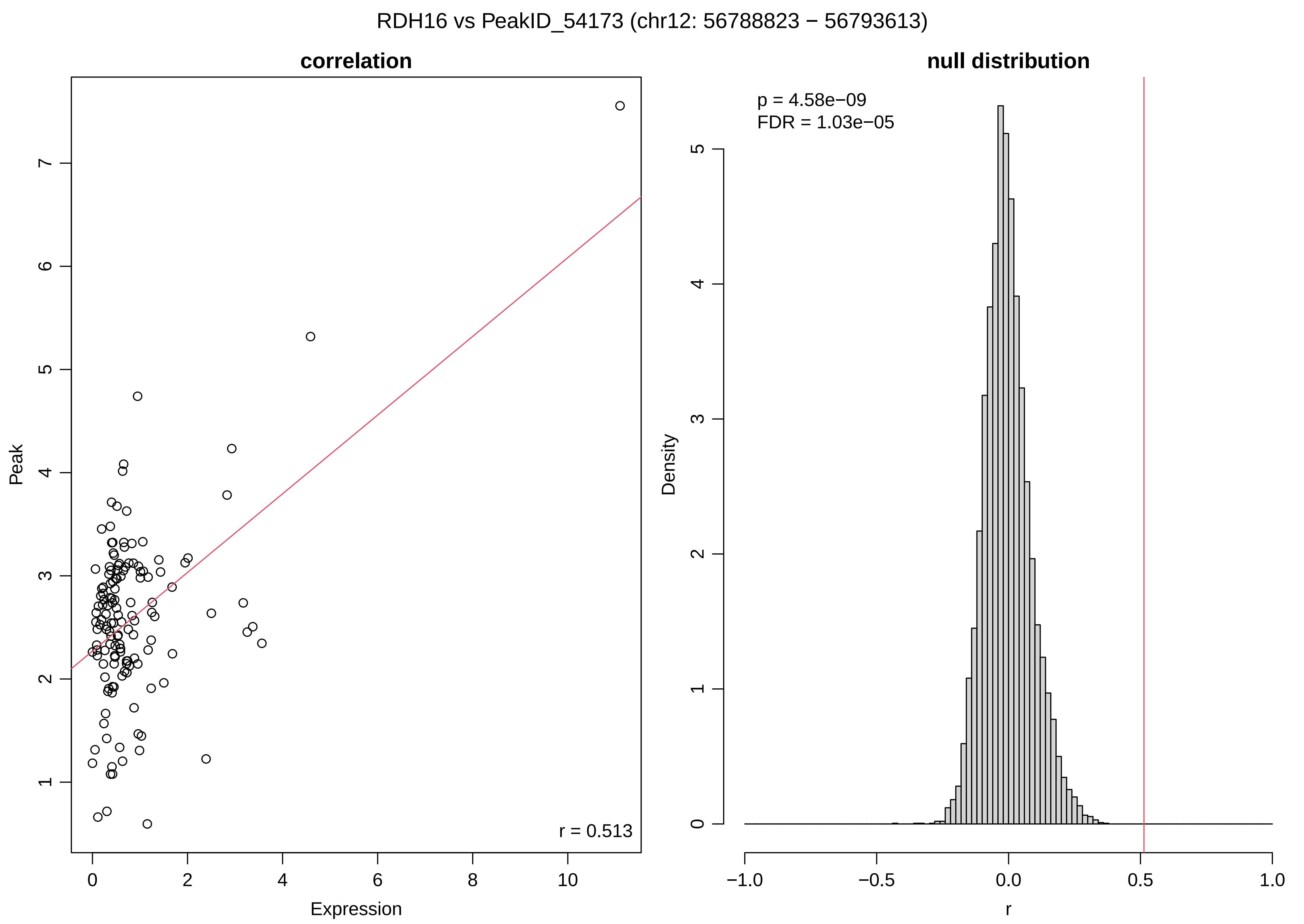

RDH16 vs PeakID_54173 (chr12: 56788823 − 56793613)
correlation
Peak
Expression
r = 0.513
null distribution
p = 4.58e−09
FDR = 1.03e−05
Density
r

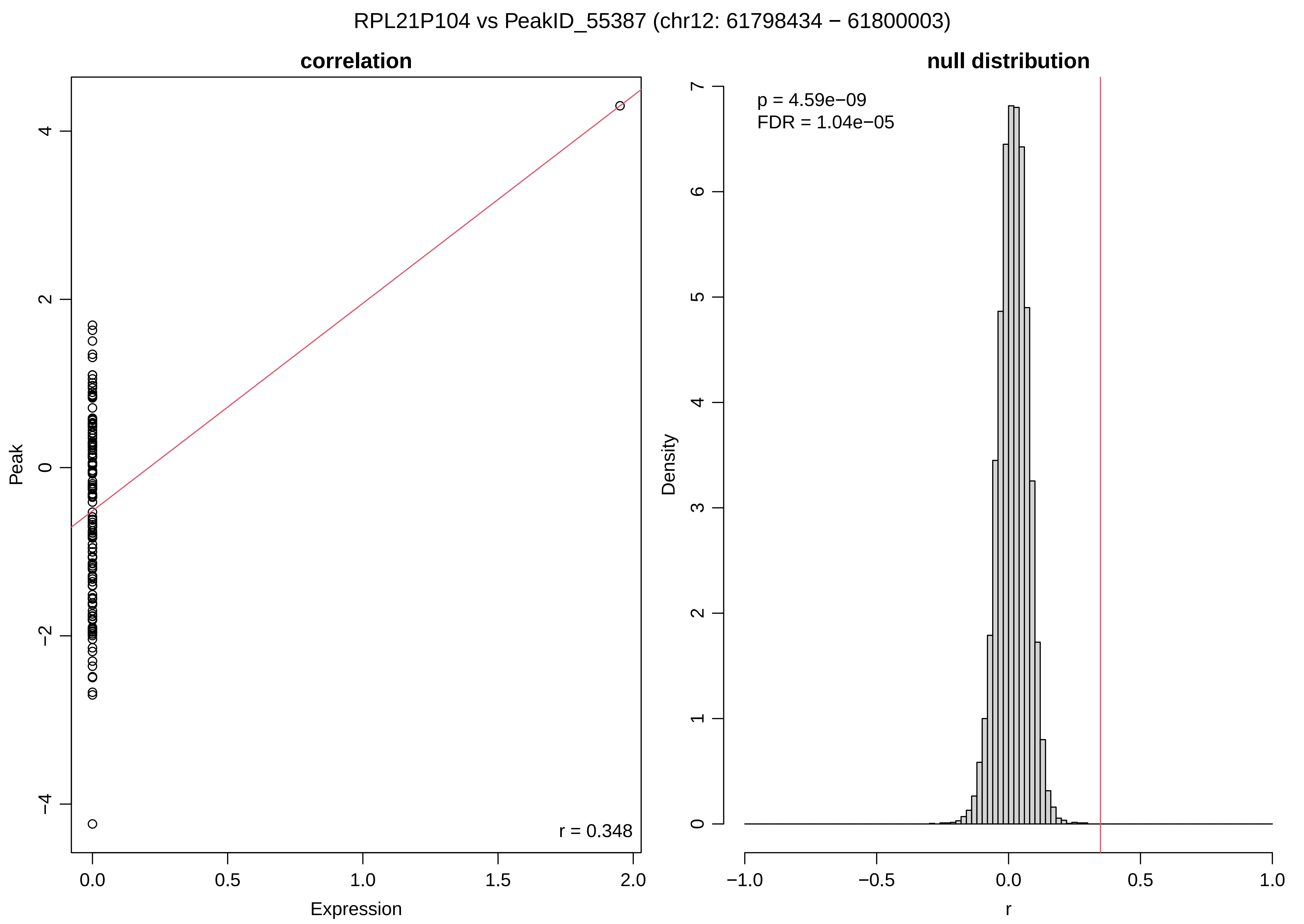
RPL21P104 vs PeakID_55387 (chr12: 61798434 − 61800003)
correlation
Peak
Expression
r = 0.348
null distribution
p = 4.59e−09
FDR = 1.04e−05
Density
r

RNU4−20P vs PeakID_54798 (chr12: 59504243 − 59504570)

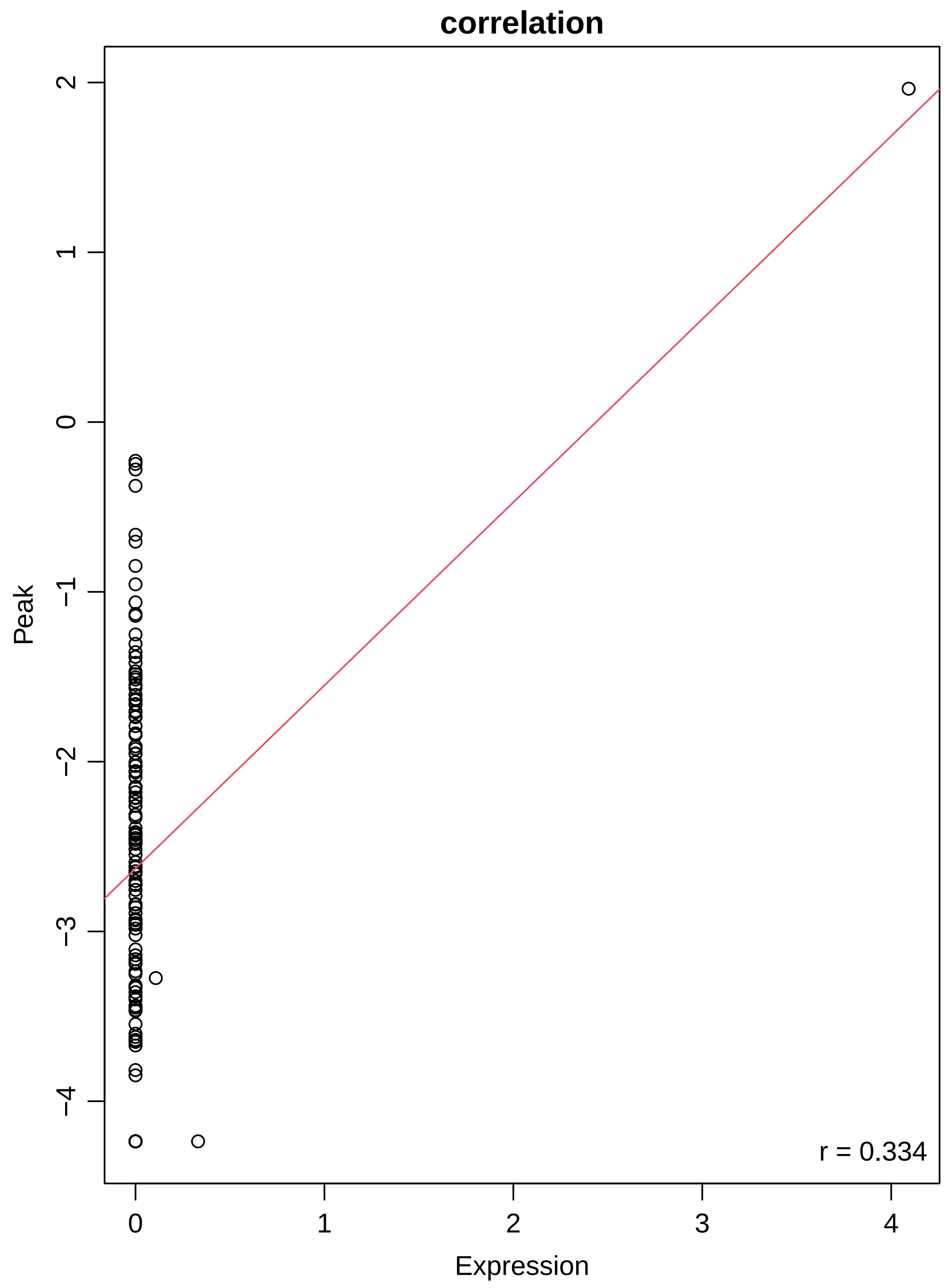

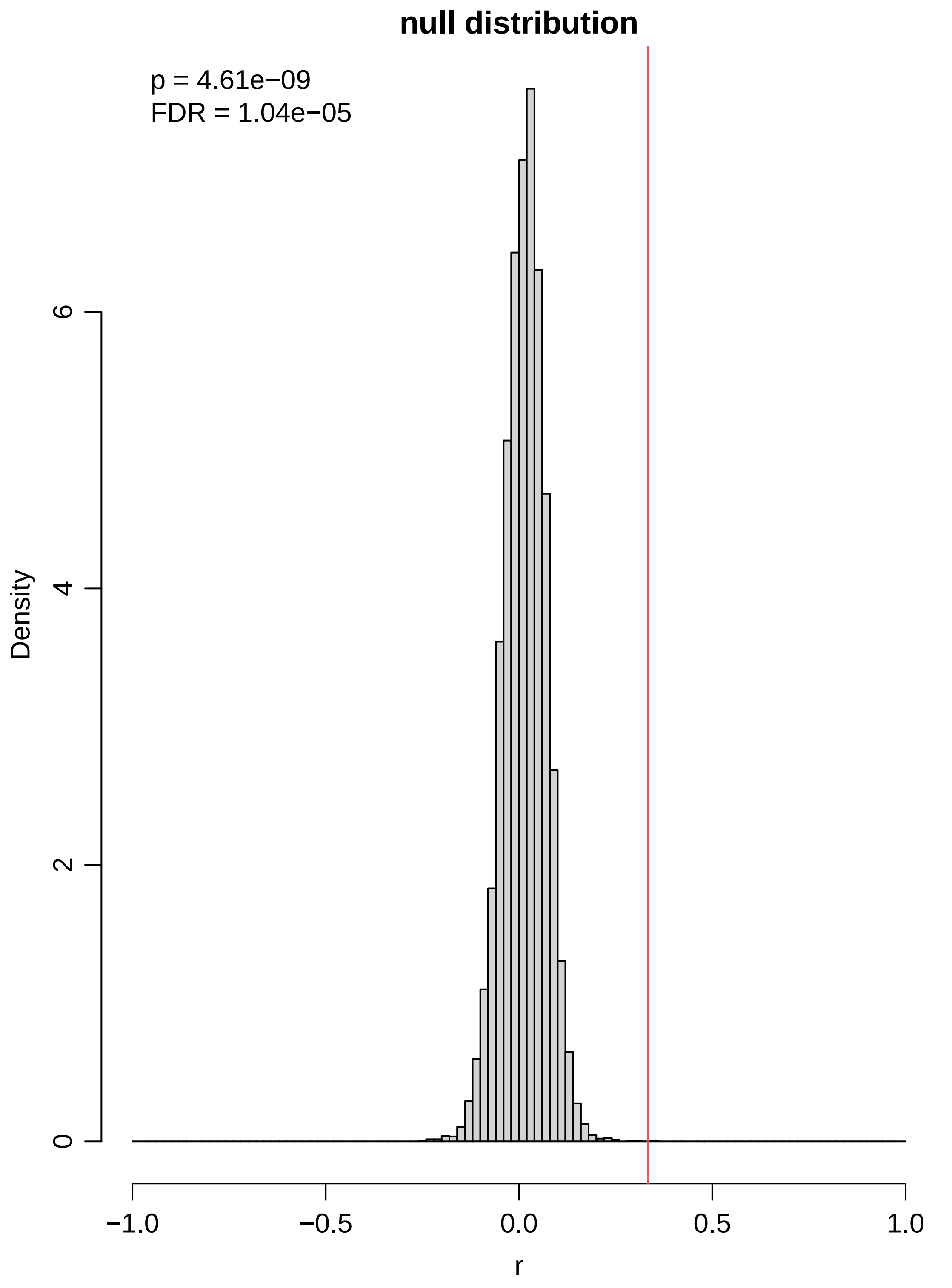

RPL21P104 vs PeakID_55461 (chr12: 61947549 − 61950471)

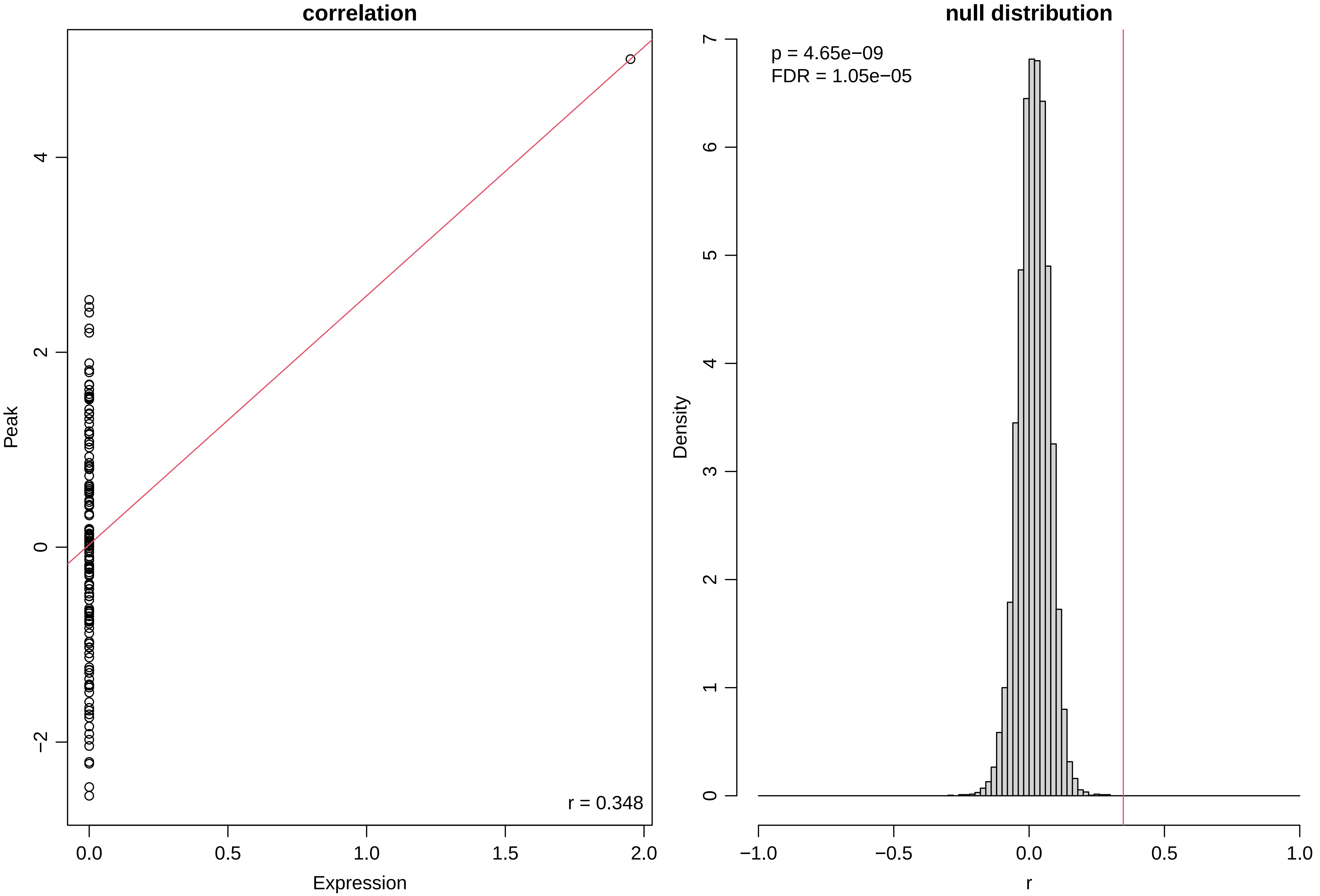

LINC01597 vs PeakID_145111 (chr20: 30287241 − 30287836)

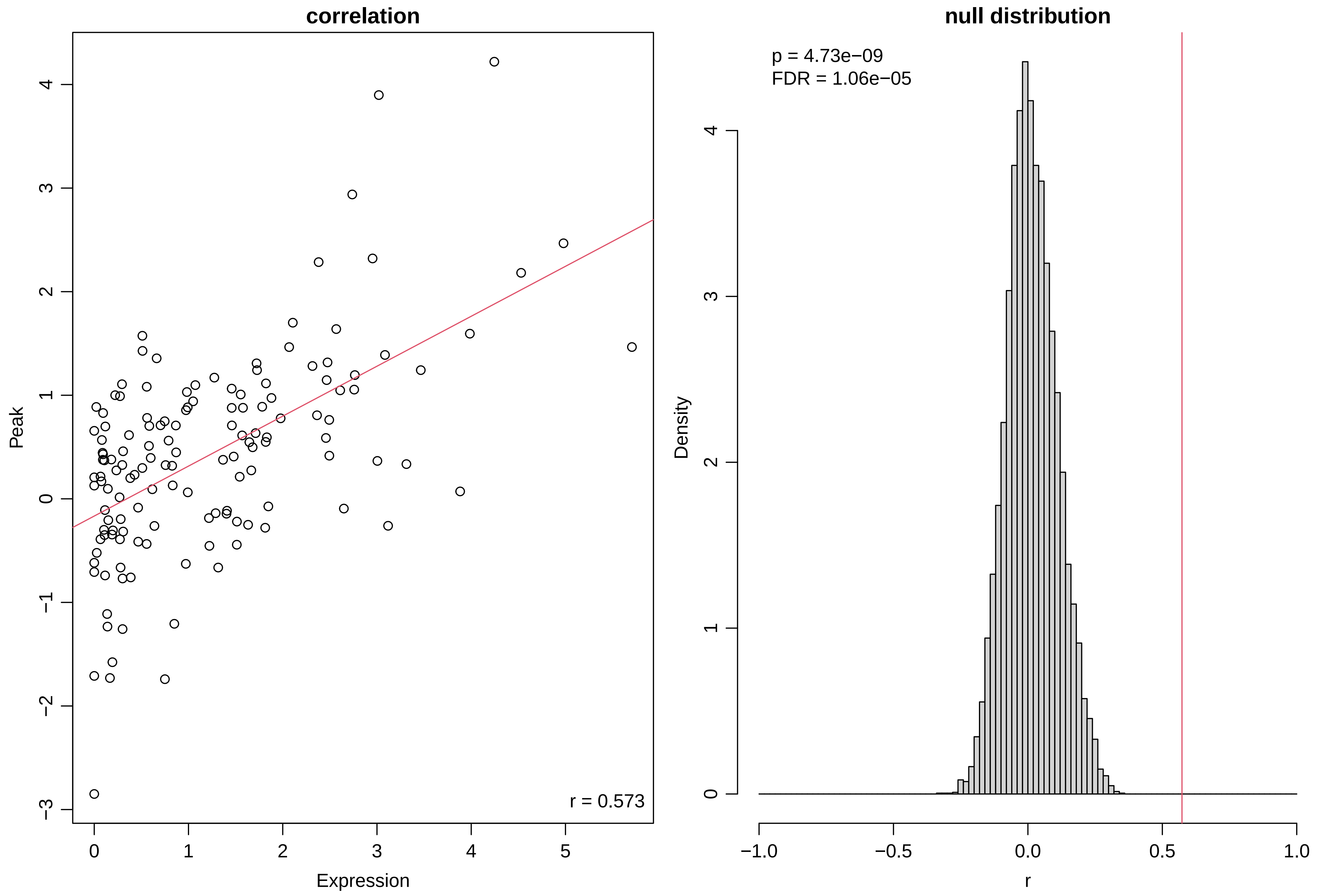

AC002066.1 vs PeakID_228975 (chr7: 116457729 − 116458093)

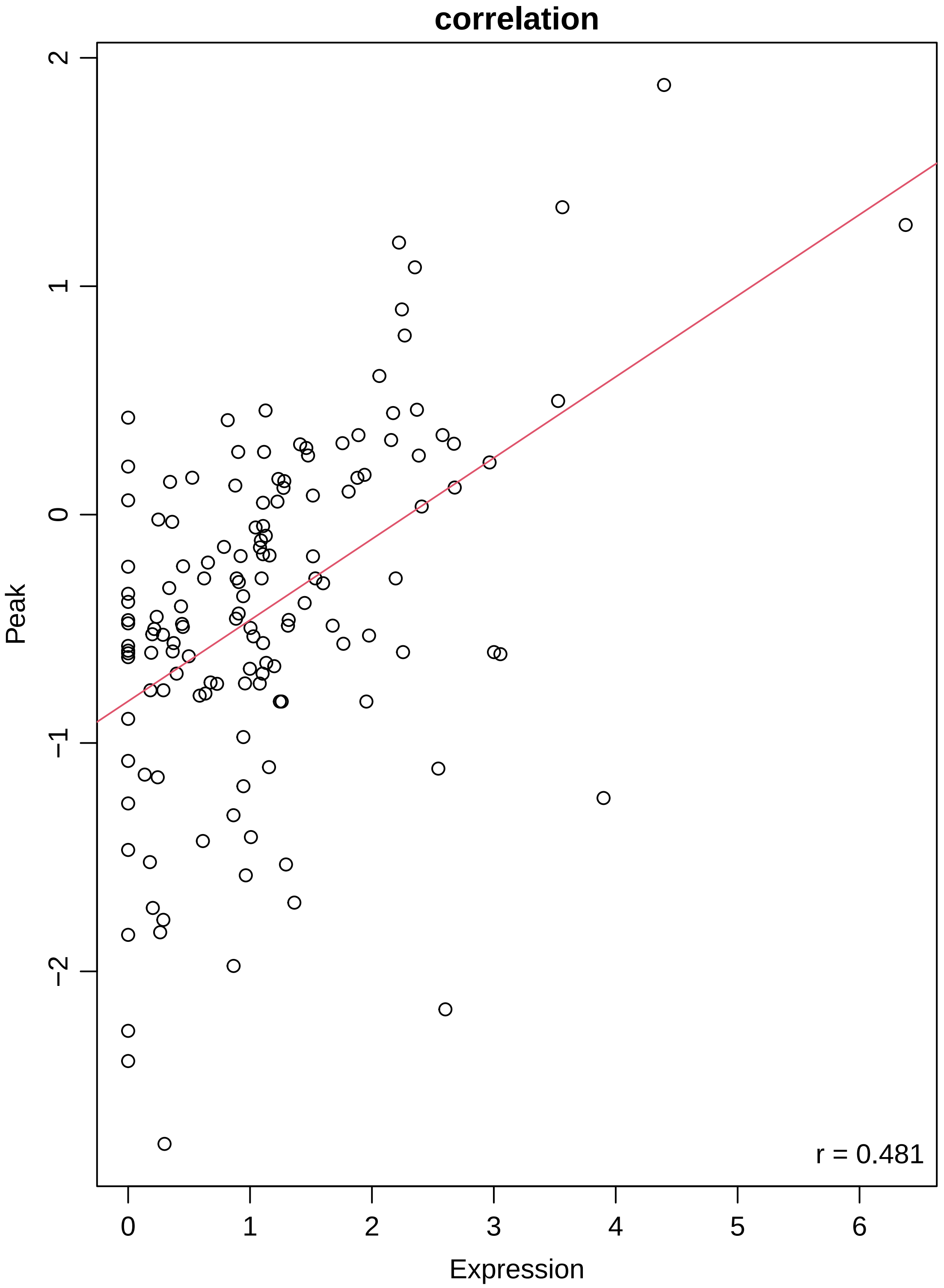

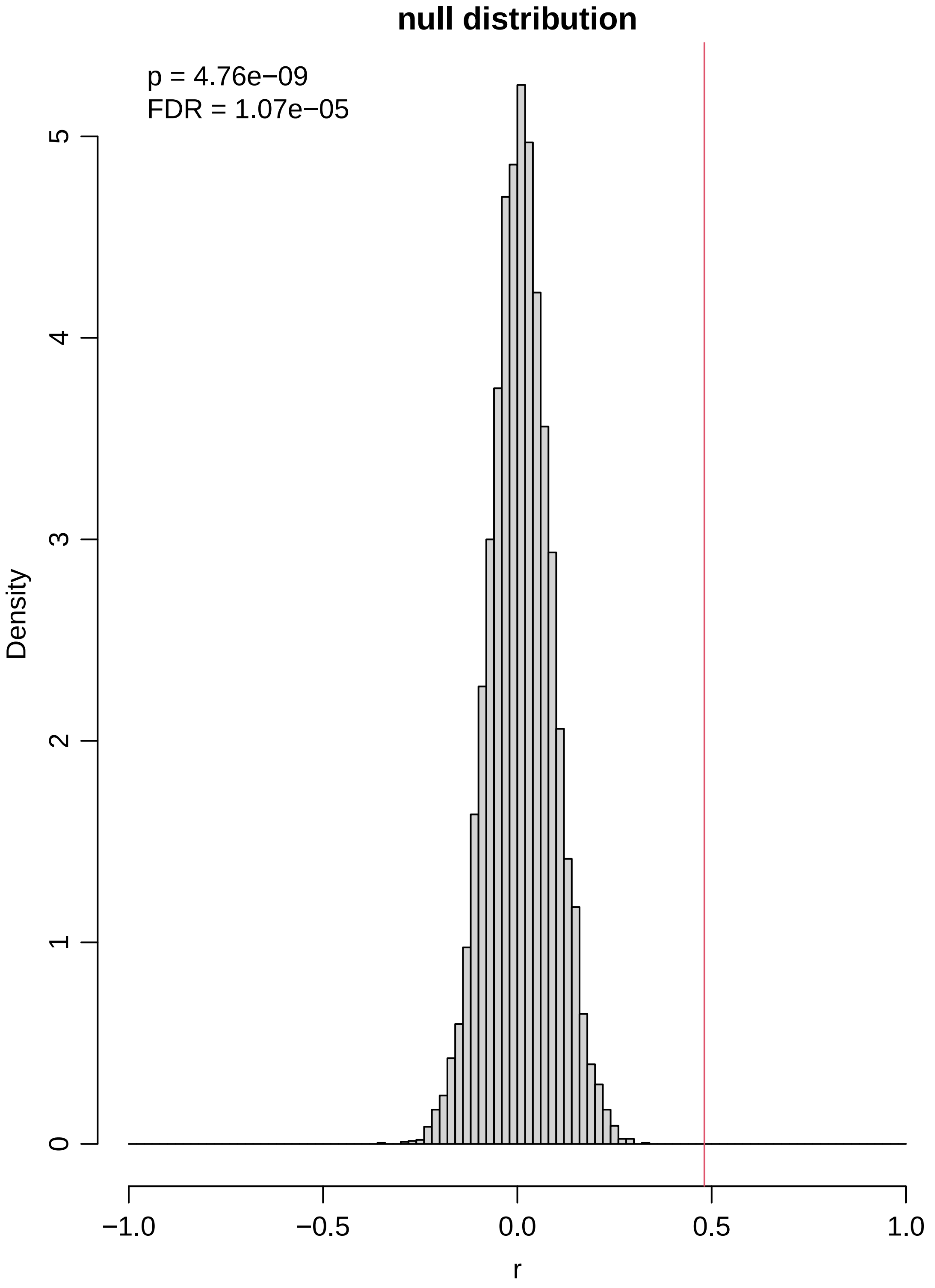

AC107881.1 vs PeakID_37802 (chr11: 12391463 − 12391791)

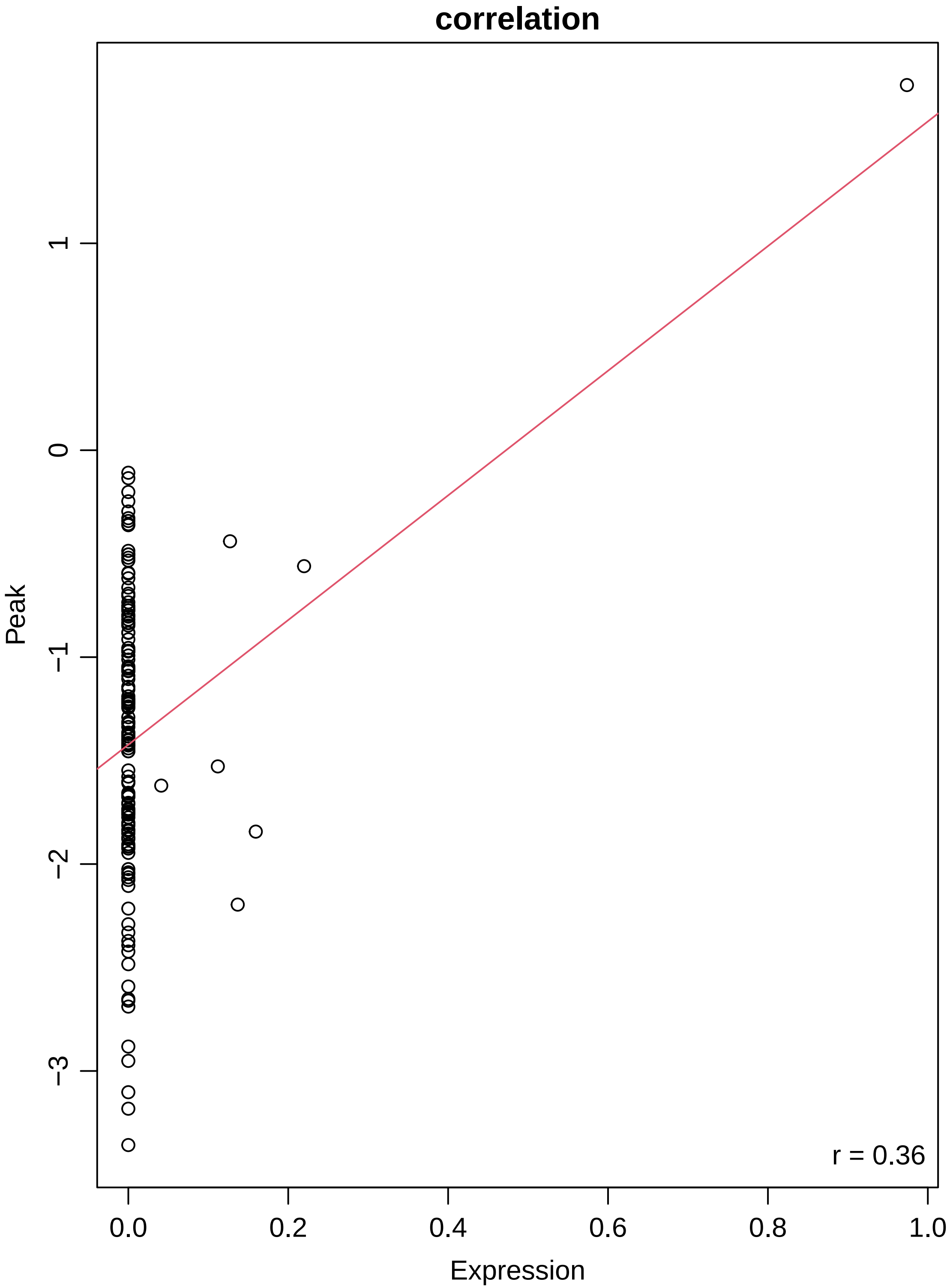

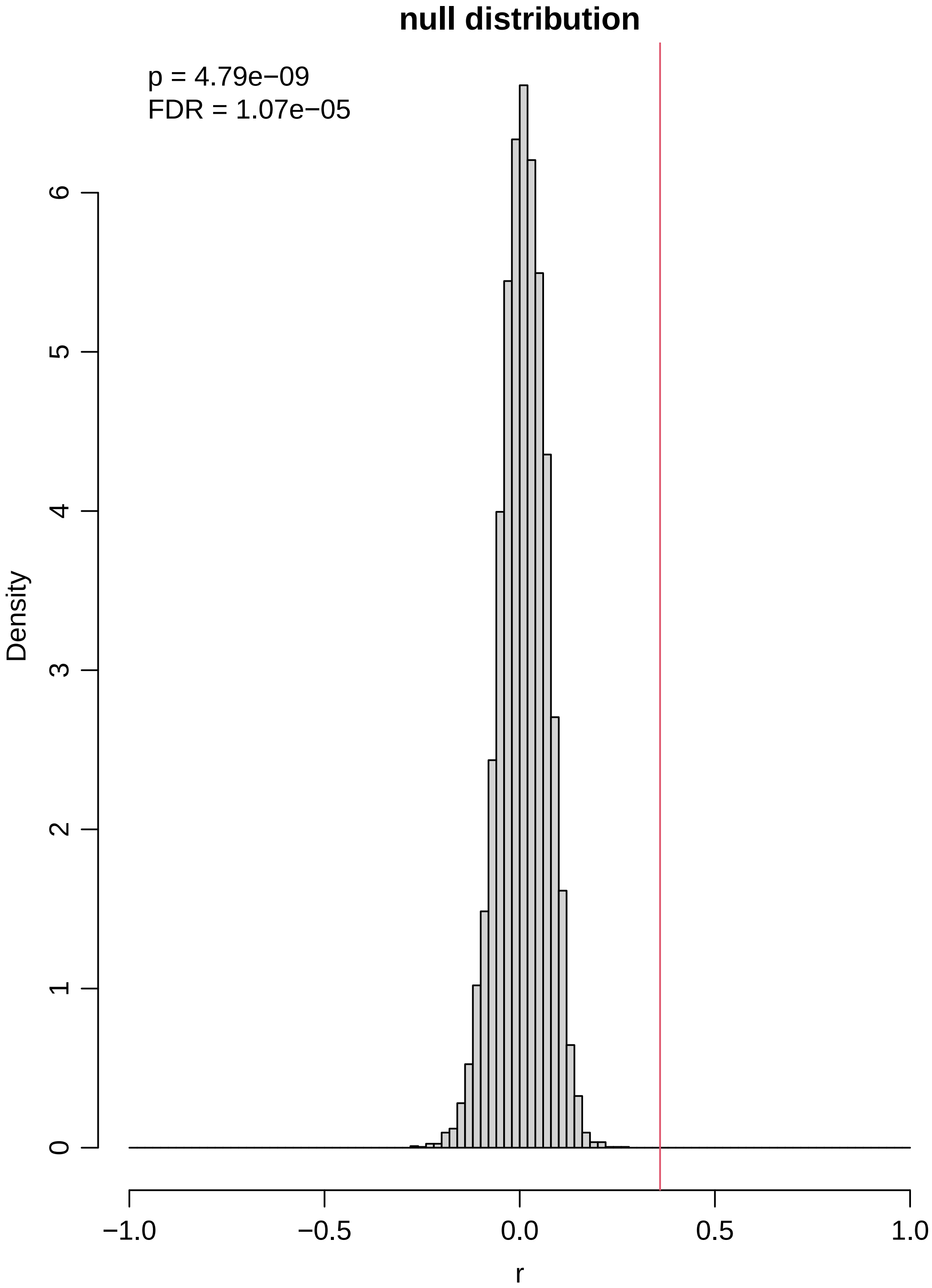

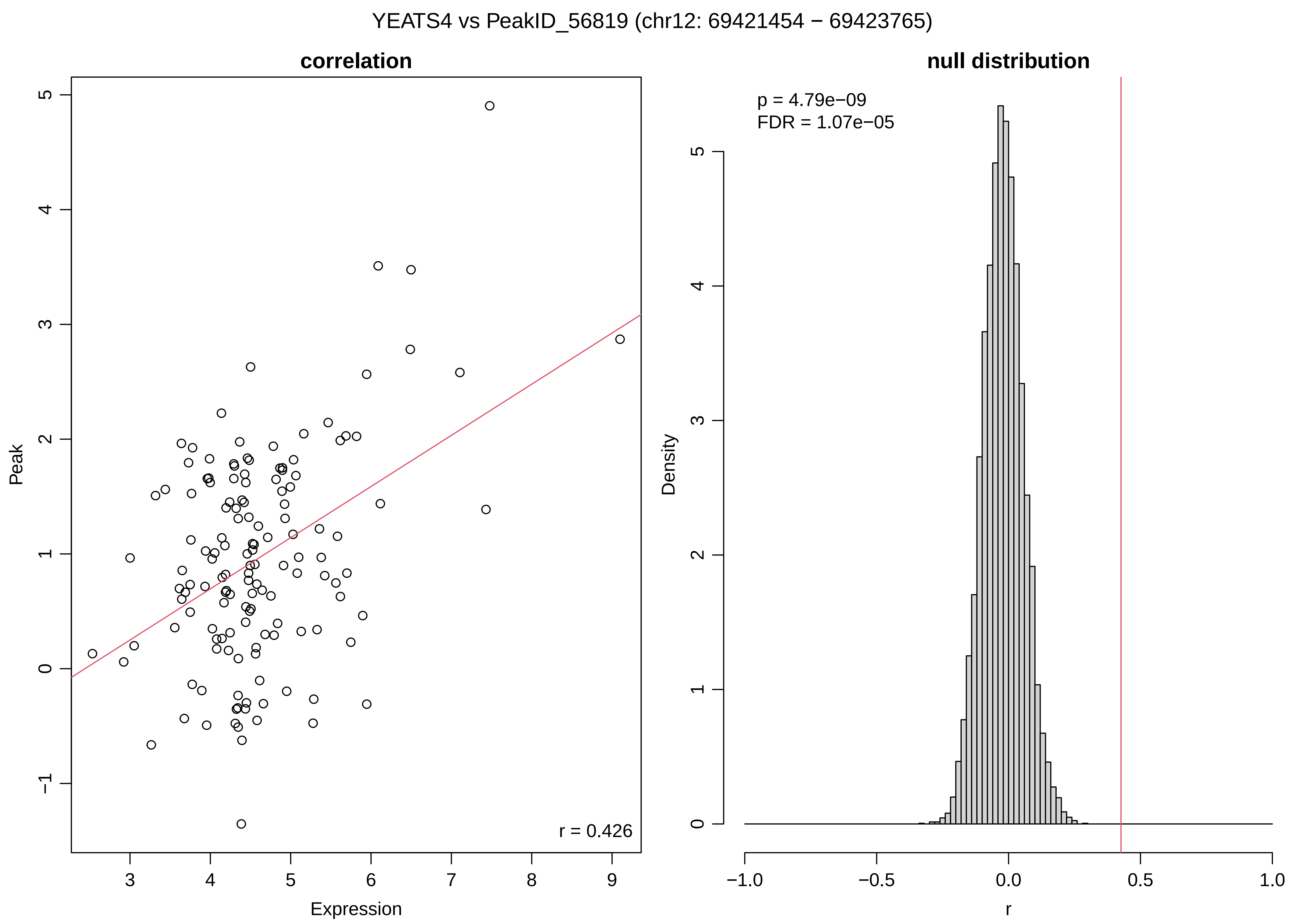

YEATS4 vs PeakID_56819 (chr12: 69421454 − 69423765)
correlation
Peak
Expression
r = 0.426
null distribution
p = 4.79e−09
FDR = 1.07e−05
Density
r

BCORP1 vs PeakID_262935 (chrY: 19410522 − 19412542)

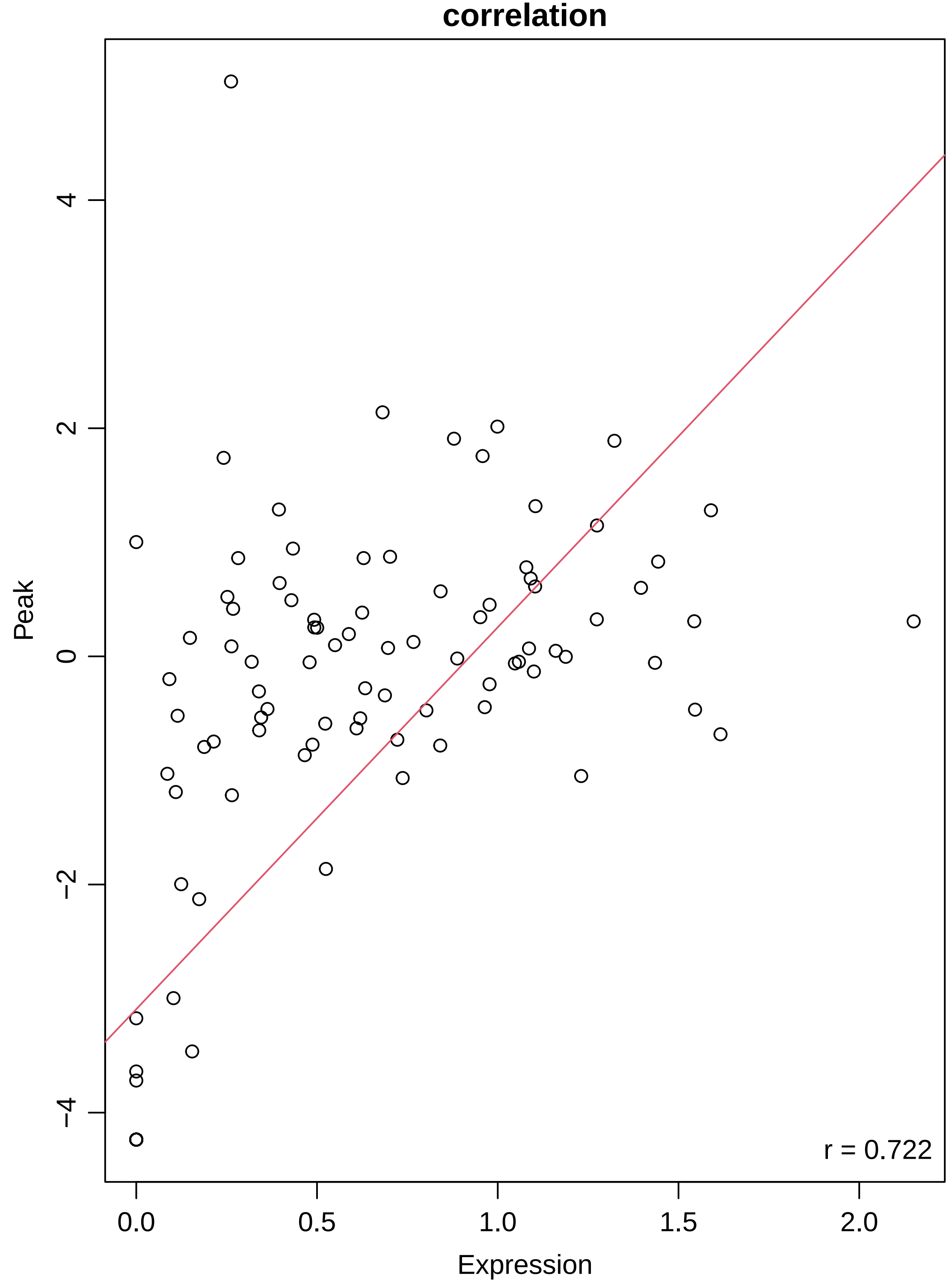

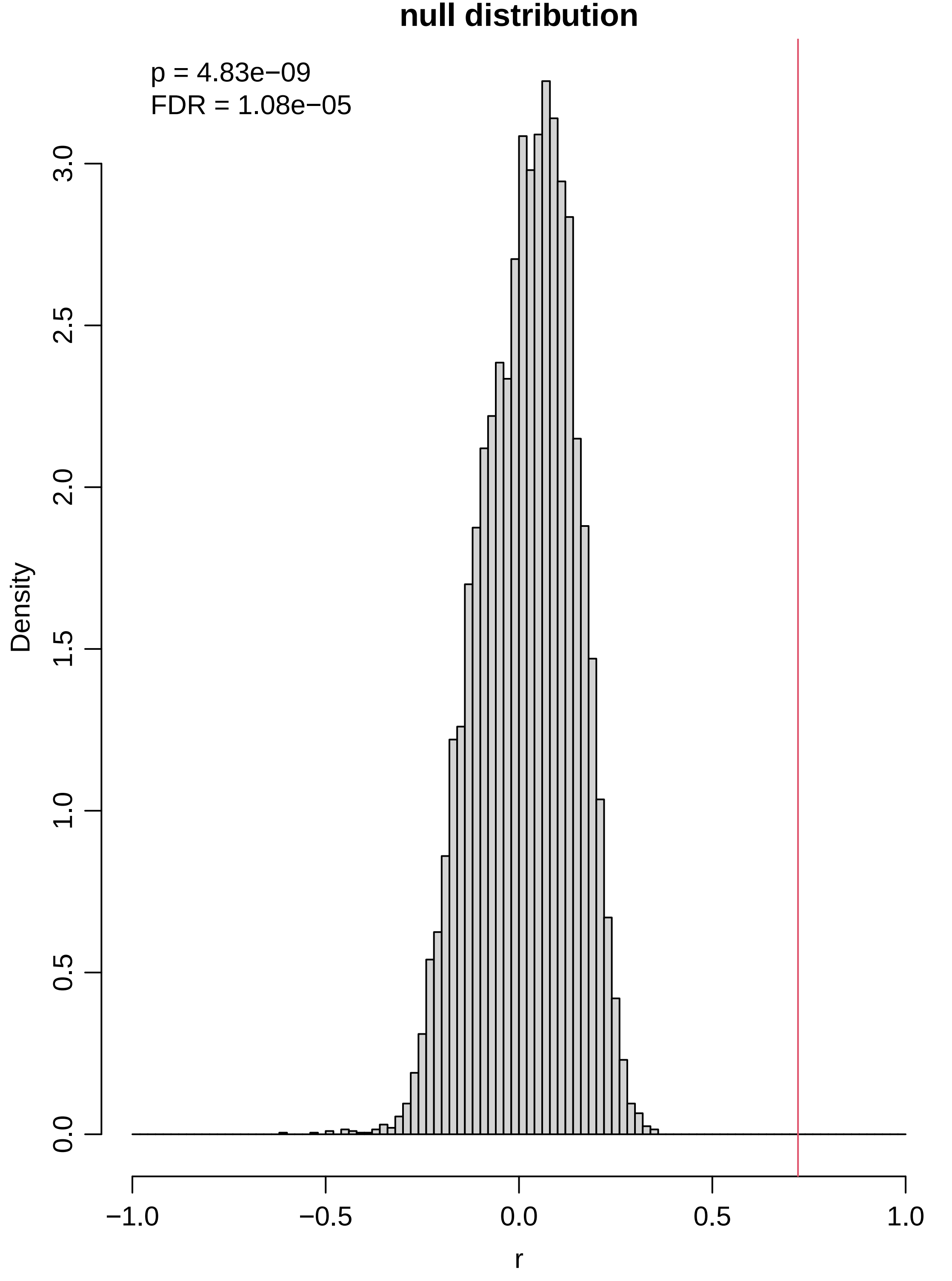

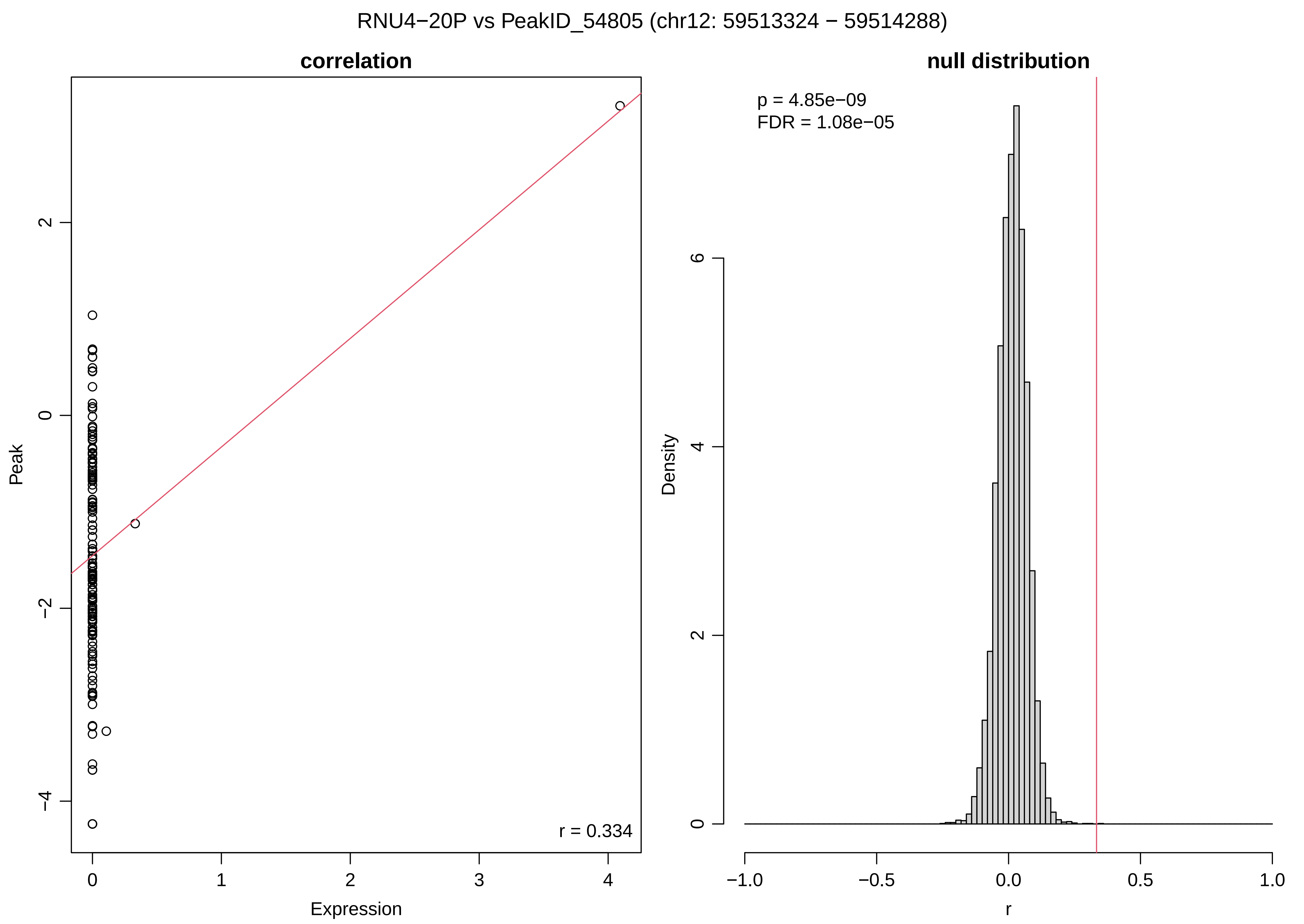
RNU4−20P vs PeakID_54805 (chr12: 59513324 − 59514288)
correlation
null distribution
p = 4.85e−09
FDR = 1.08e−05
r = 0.334
Peak
Expression
Density
r
2
0
−2
−4
0
1
2
3
4
6
4
2
0
−1.0
−0.5
0.0
0.5
1.0

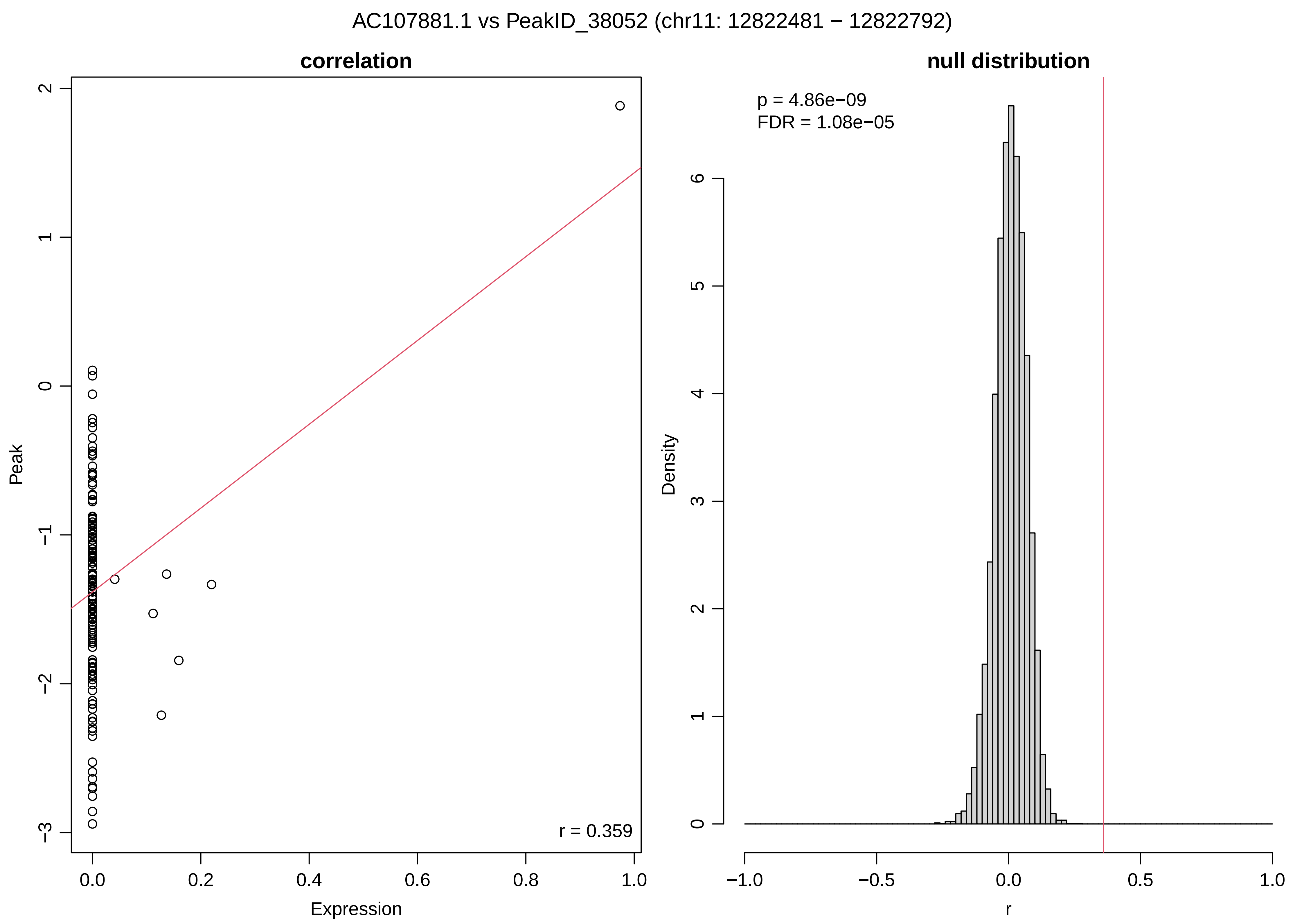
AC107881.1 vs PeakID_38052 (chr11: 12822481 − 12822792)
correlation
Peak
Expression
r = 0.359
null distribution
p = 4.86e−09
FDR = 1.08e−05
Density
r

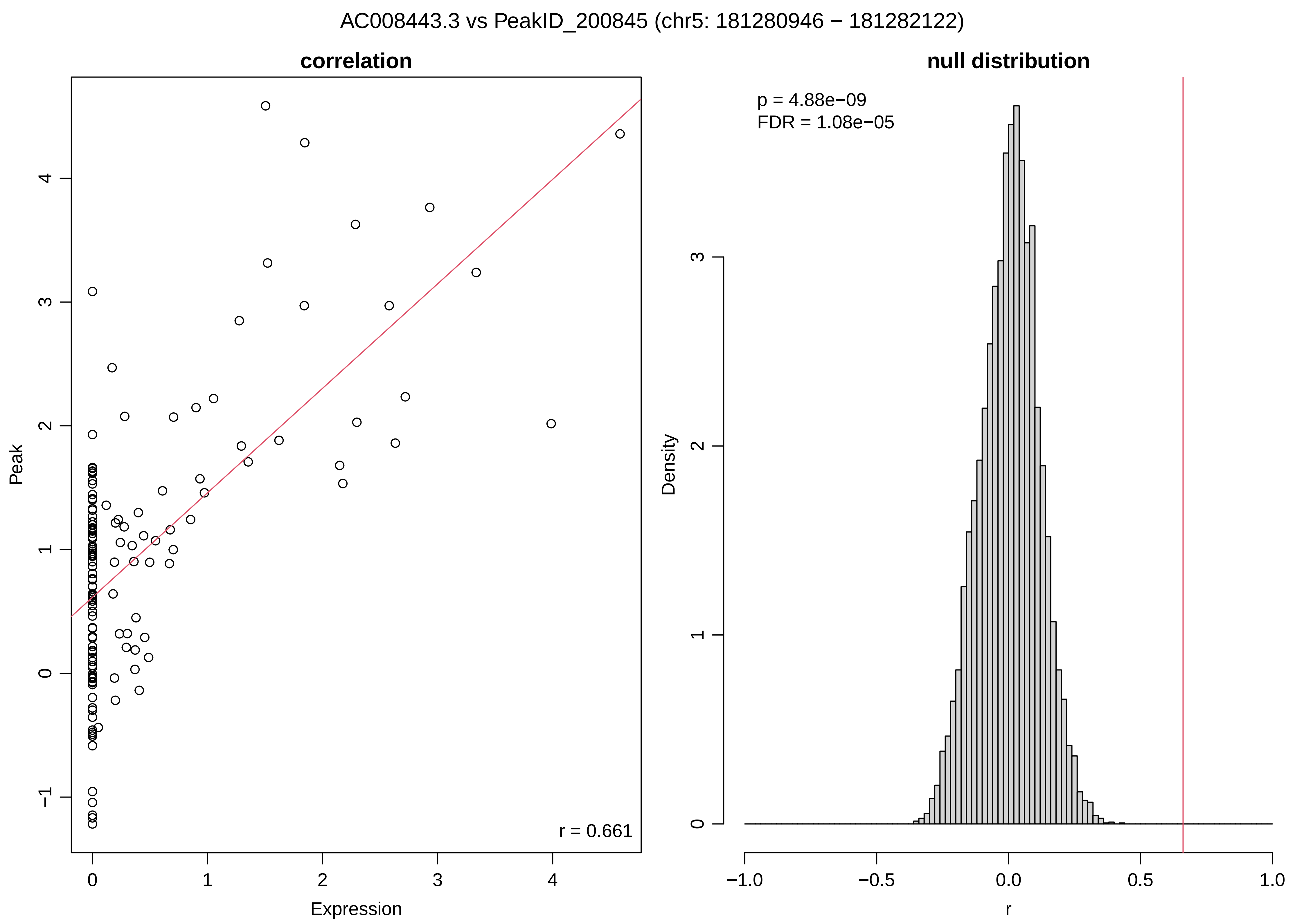
AC008443.3 vs PeakID_200845 (chr5: 181280946 − 181282122)
correlation
Peak
Expression
r = 0.661
null distribution
p = 4.88e−09
FDR = 1.08e−05
Density
r

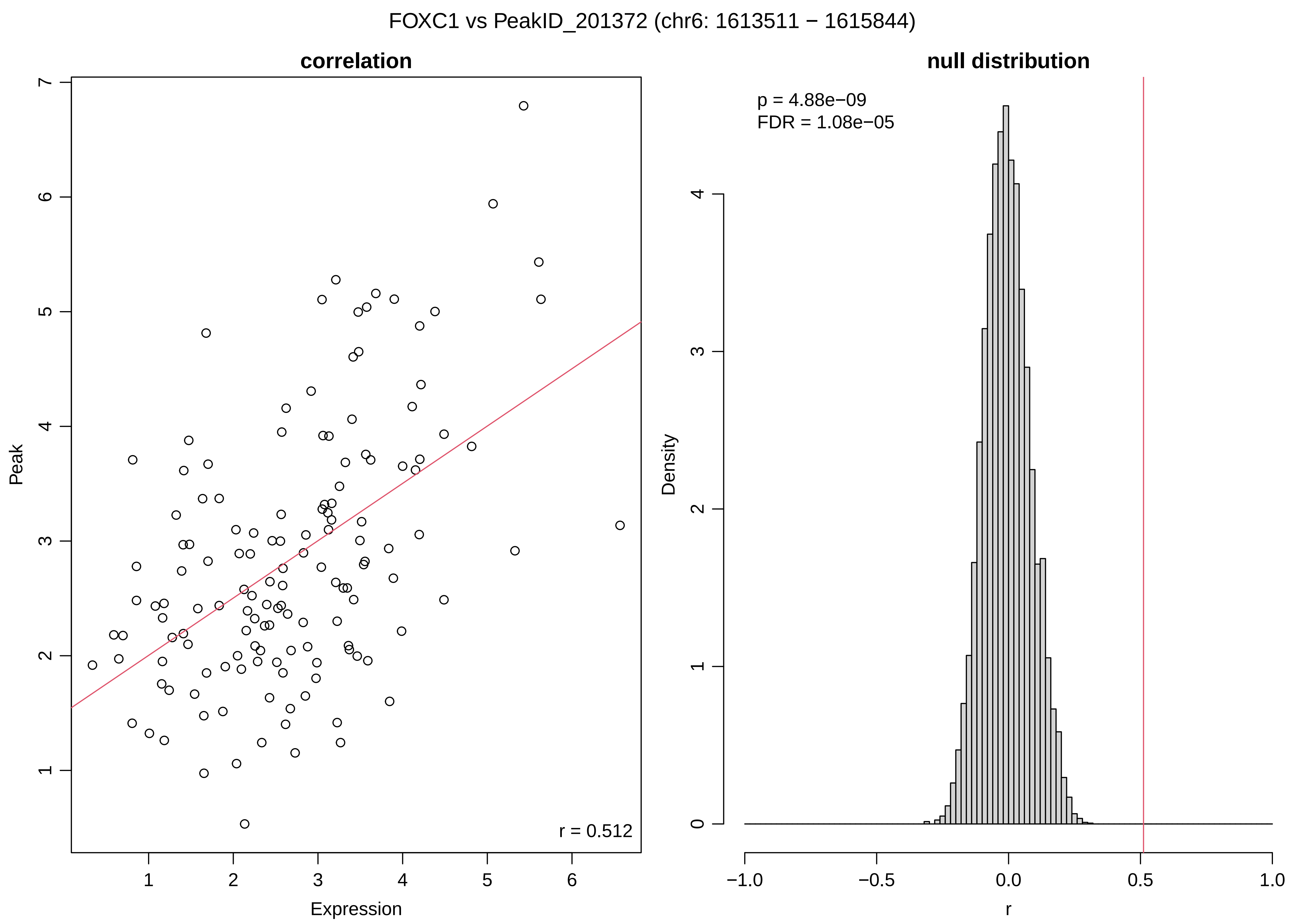
FOXC1 vs PeakID_201372 (chr6: 1613511 − 1615844)
correlation
Peak
Expression
r = 0.512
null distribution
p = 4.88e−09
FDR = 1.08e−05
Density
r

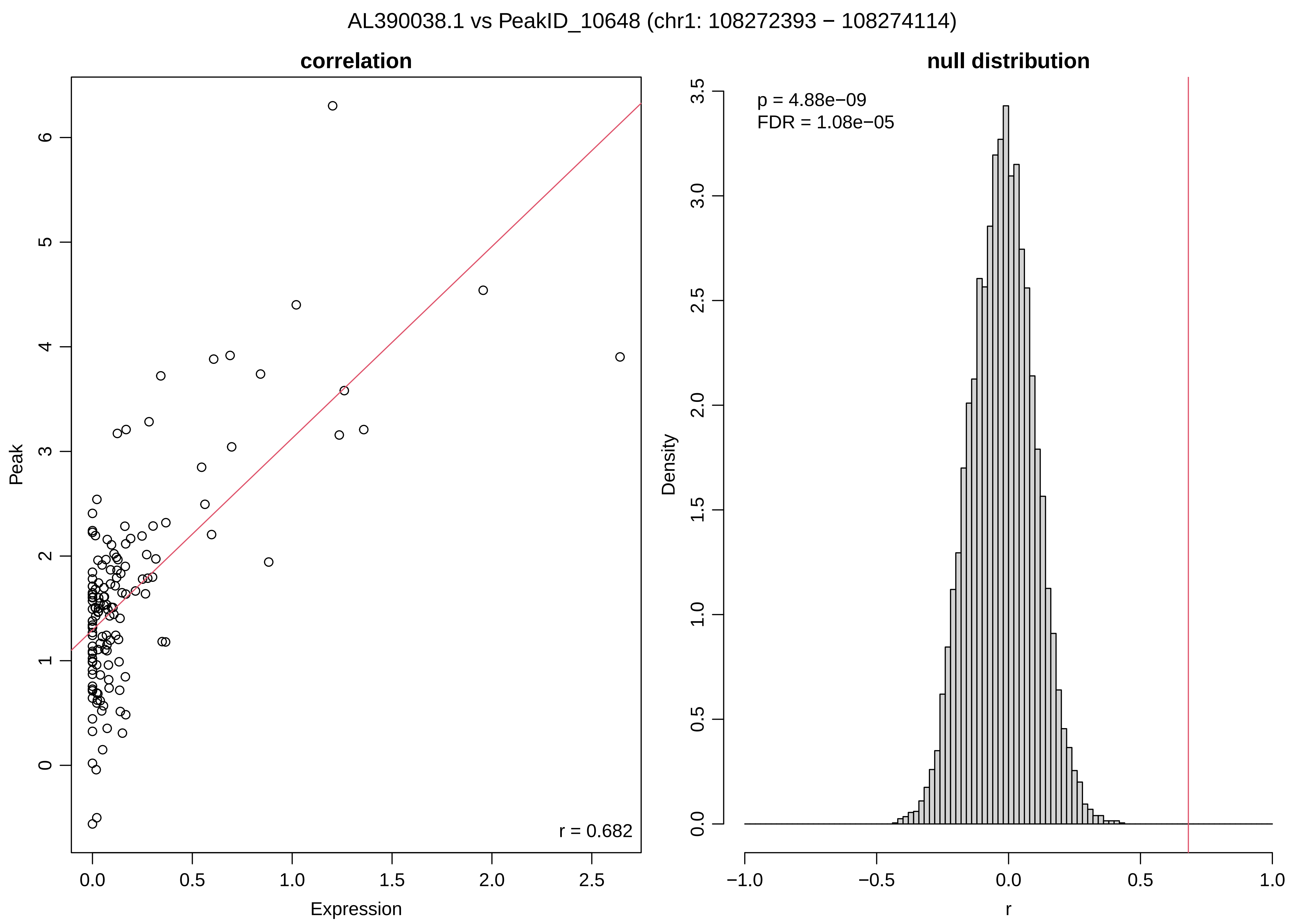
AL390038.1 vs PeakID_10648 (chr1: 108272393 − 108274114)
correlation
Peak
Expression
r = 0.682
0.0
0.5
1.0
1.5
2.0
2.5
0
1
2
3
4
5
6
null distribution
p = 4.88e−09
FDR = 1.08e−05
Density
r
−1.0
−0.5
0.0
0.5
1.0
0.0
0.5
1.0
1.5
2.0
2.5
3.0
3.5

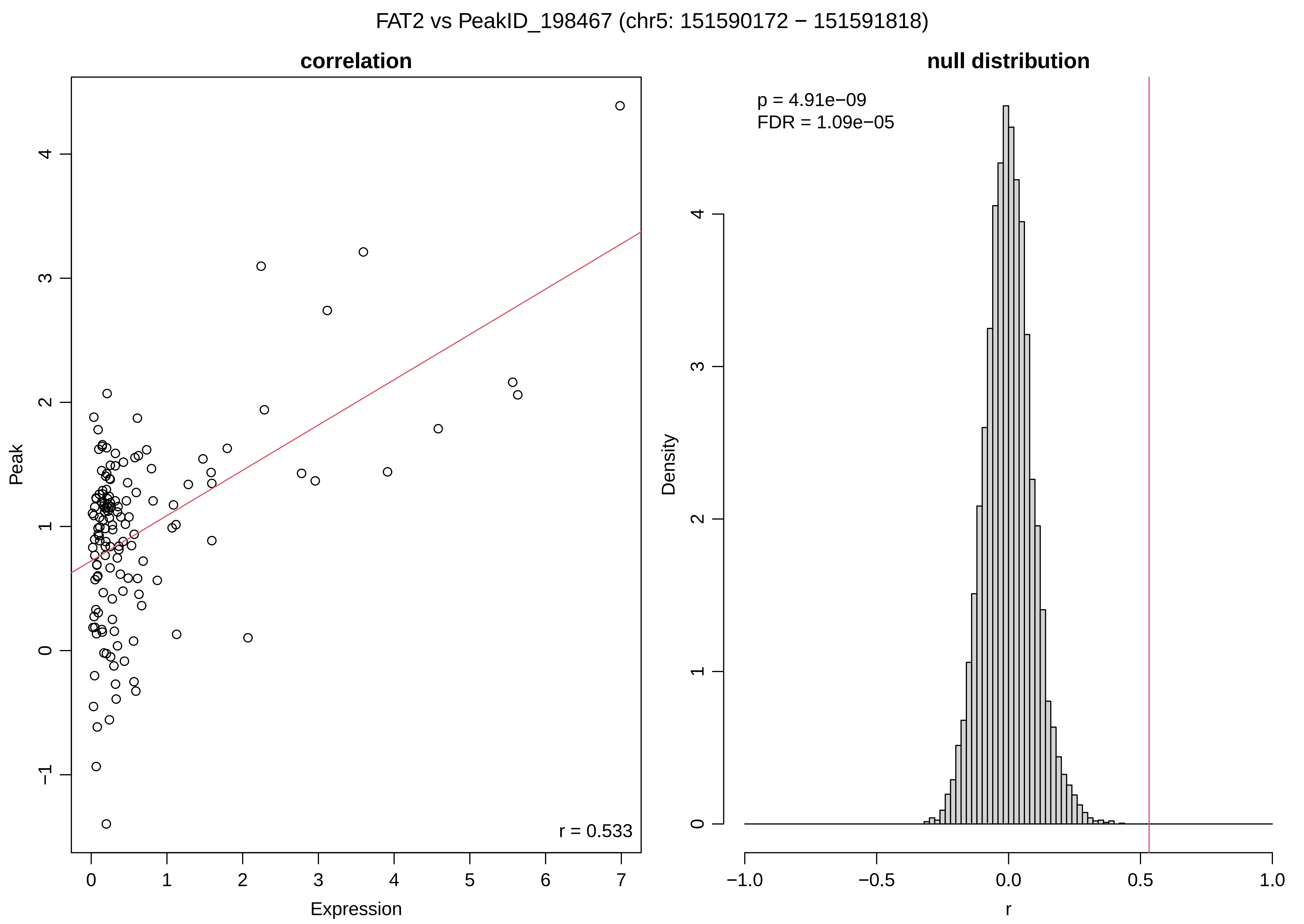
FAT2 vs PeakID_198467 (chr5: 151590172 − 151591818)
correlation
Peak
Expression
r = 0.533
null distribution
p = 4.91e−09
FDR = 1.09e−05
Density
r

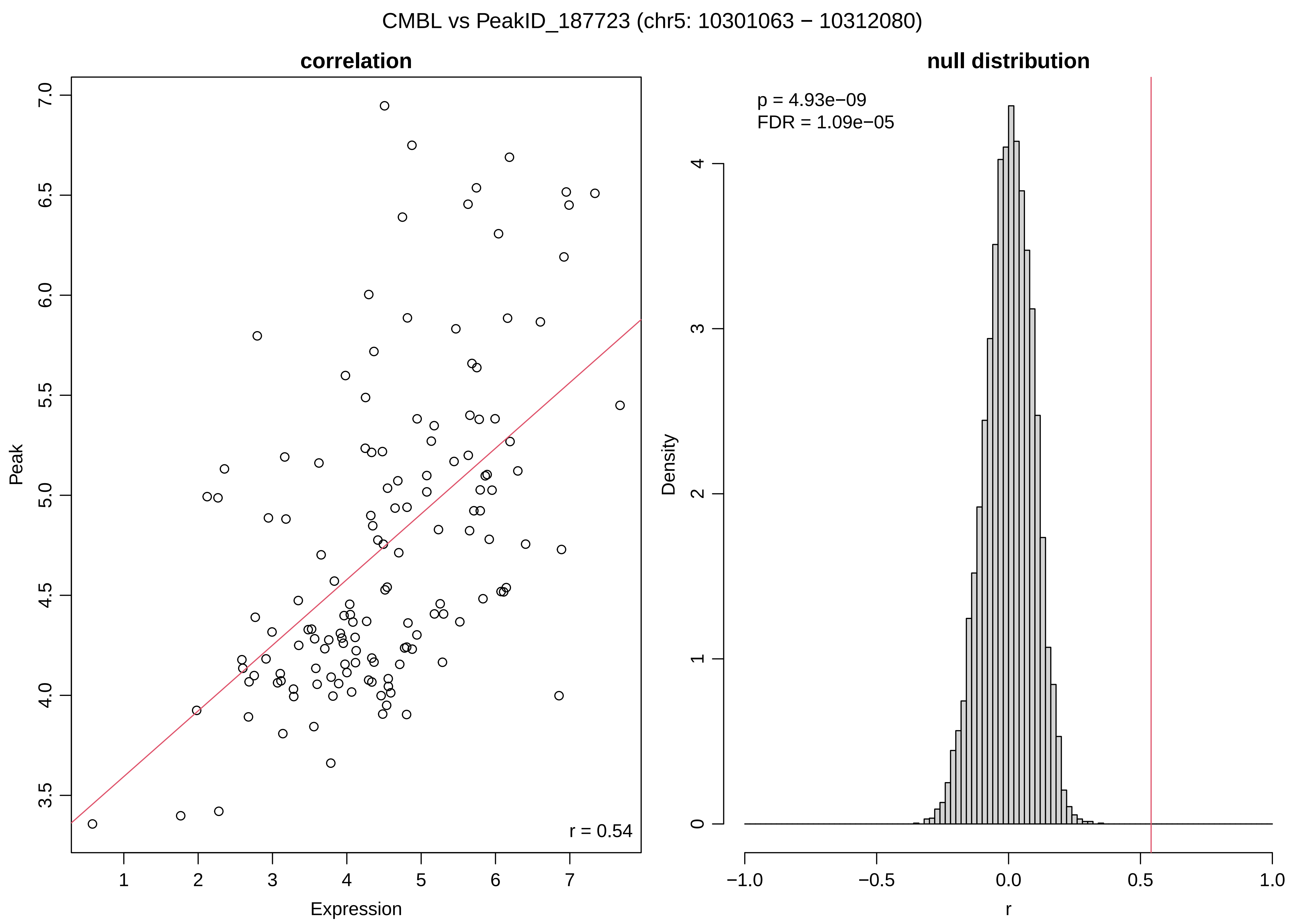
CMBL vs PeakID_187723 (chr5: 10301063 − 10312080)
correlation
null distribution
p = 4.93e−09
FDR = 1.09e−05
r = 0.54
Peak
Expression
Density
r

USP9Y vs PeakID_262723 (chrY: 12419450 − 12422048)

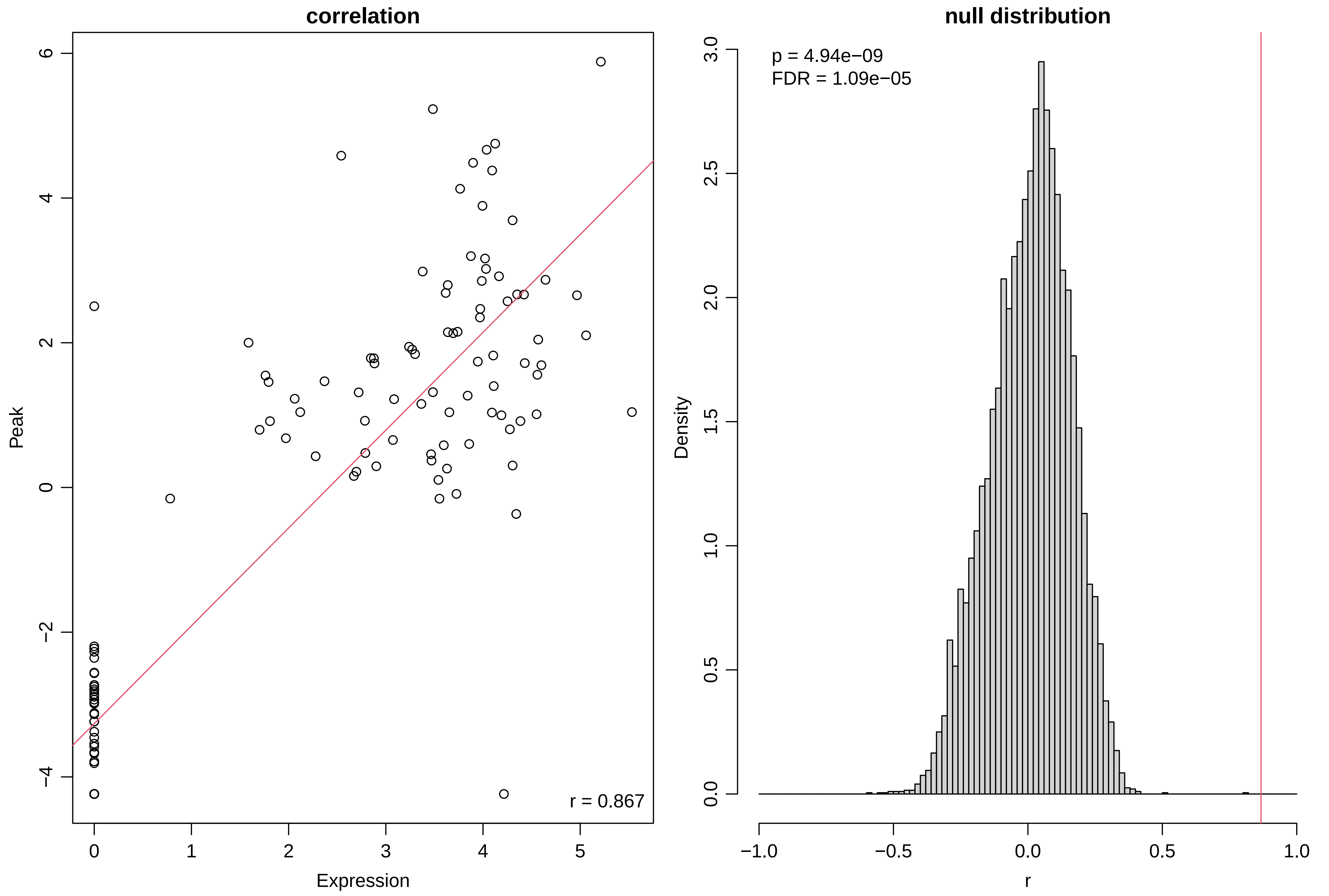

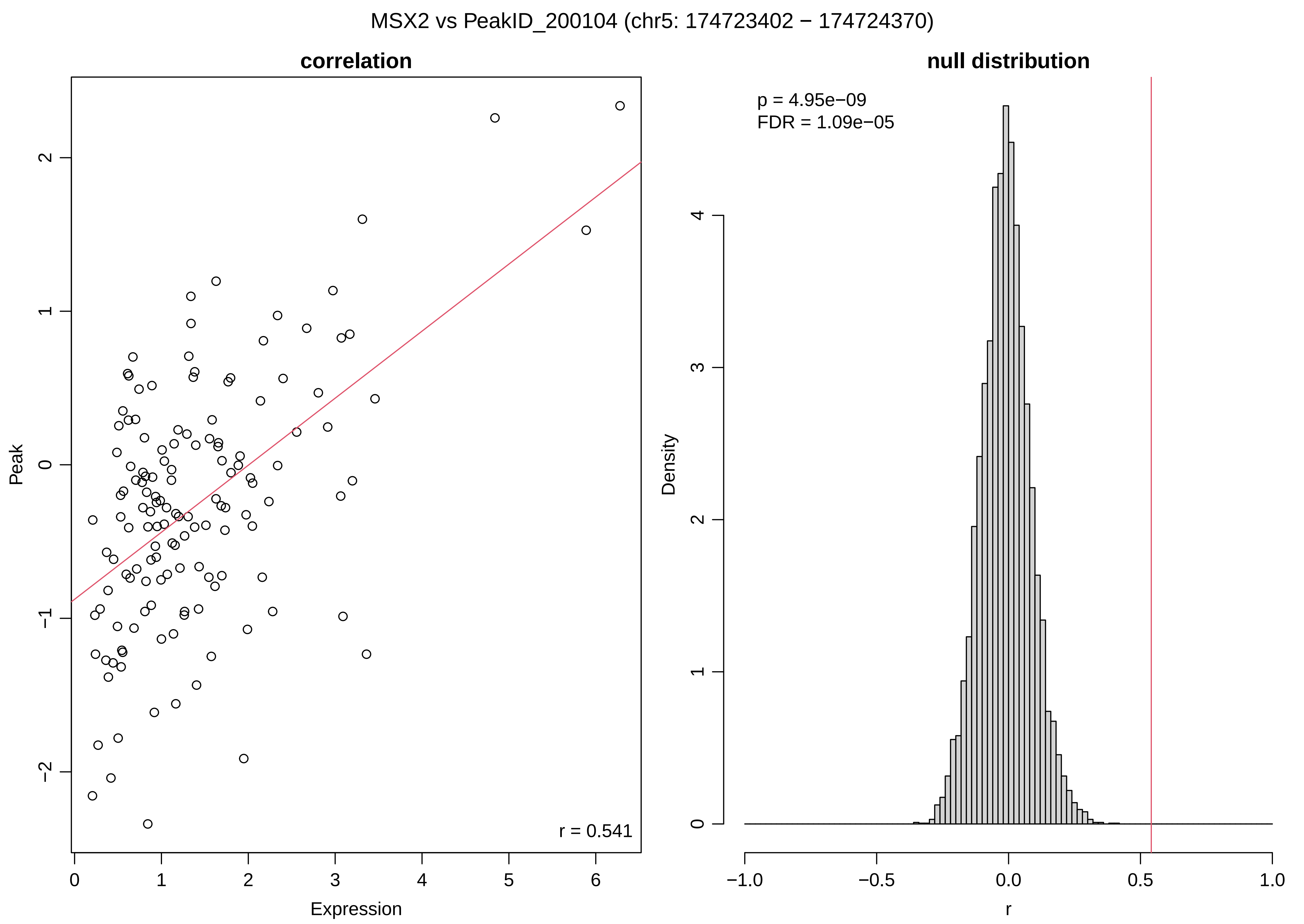

MSX2 vs PeakID_200104 (chr5: 174723402 − 174724370)
correlation
Peak
Expression
r = 0.541
null distribution
p = 4.95e−09
FDR = 1.09e−05
Density
r

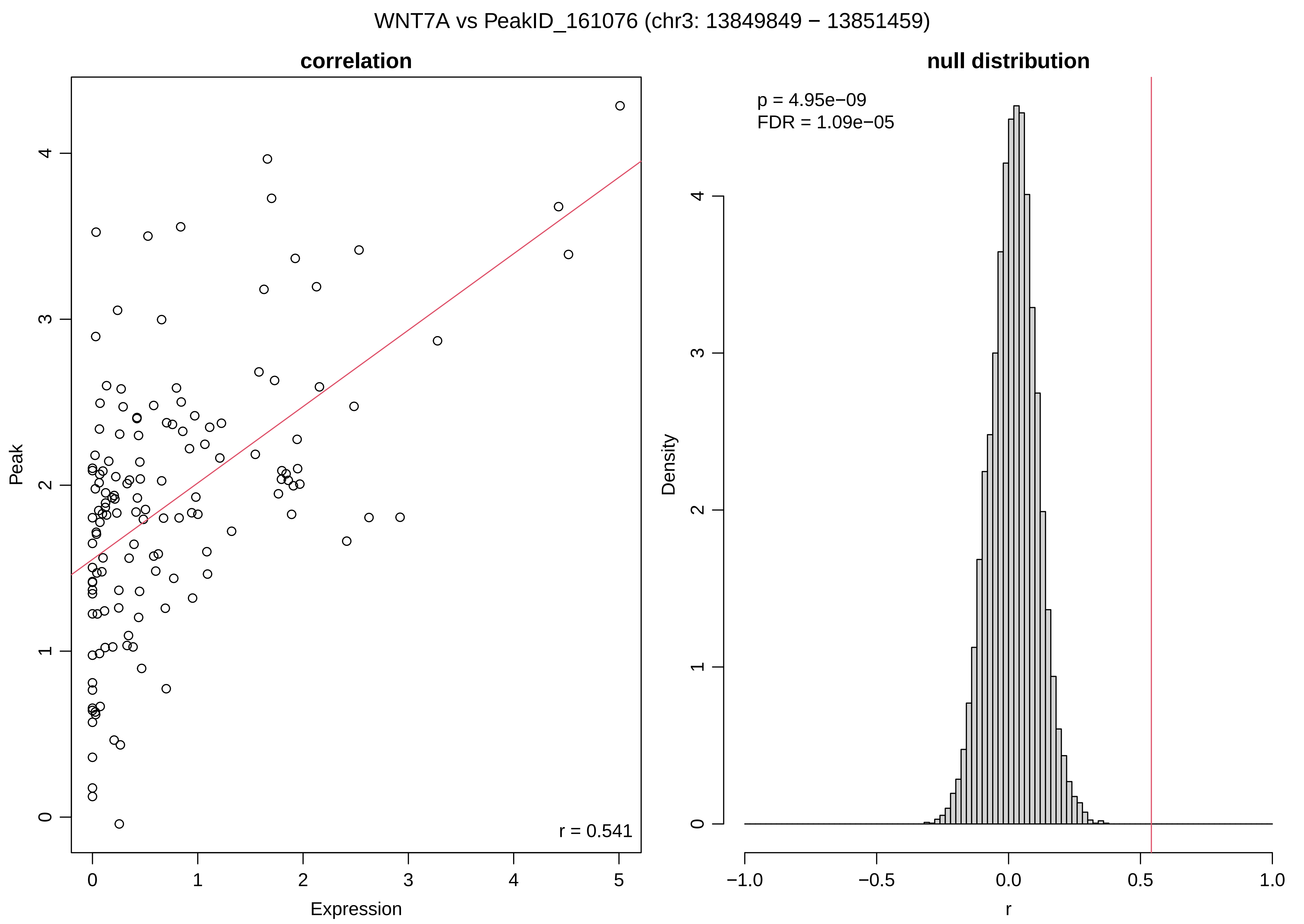
WNT7A vs PeakID_161076 (chr3: 13849849 − 13851459)
correlation
Peak
Expression
r = 0.541
null distribution
p = 4.95e−09
FDR = 1.09e−05
Density
r

RPL21P104 vs PeakID_55456 (chr12: 61935674 − 61937177)

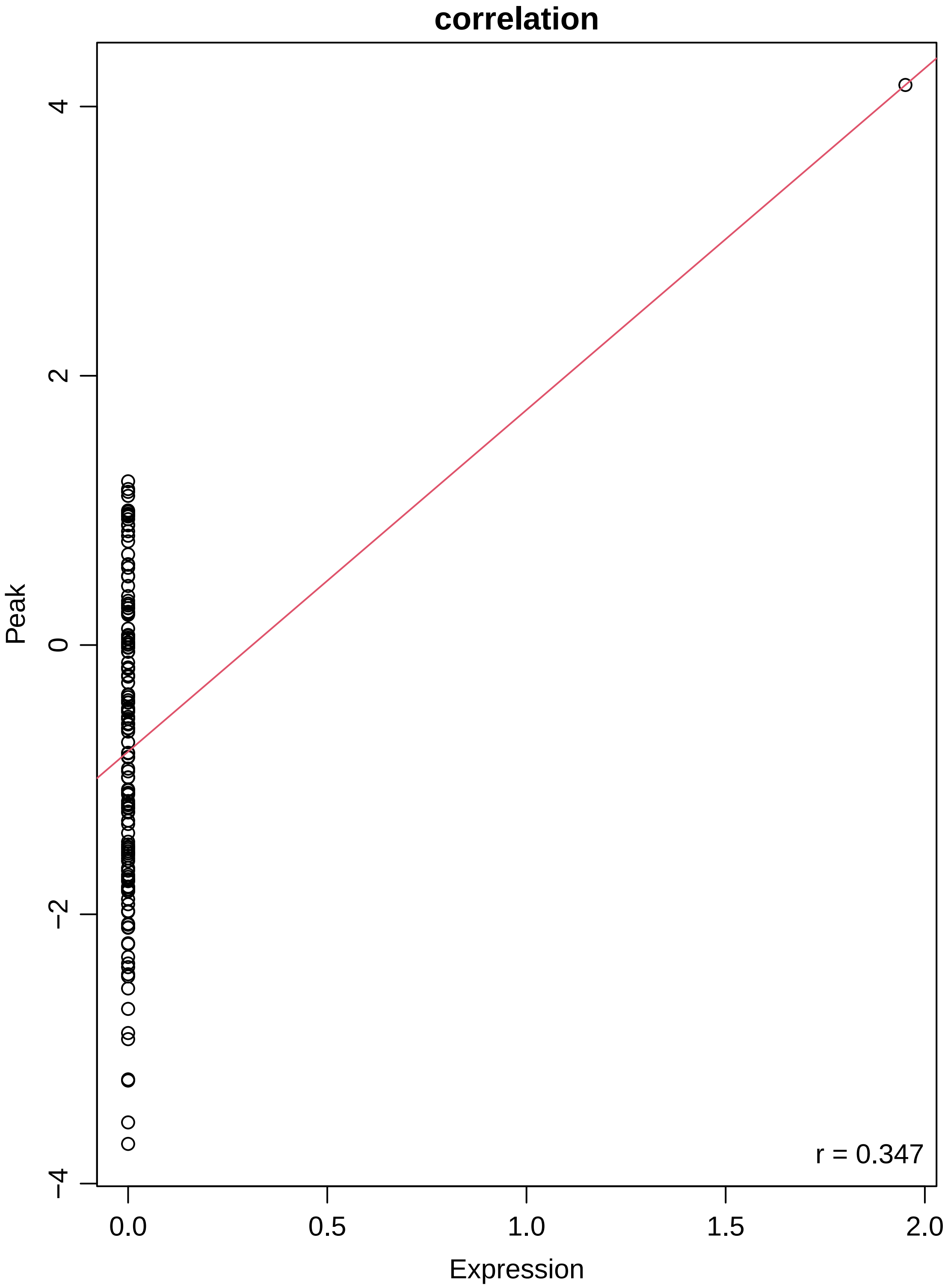

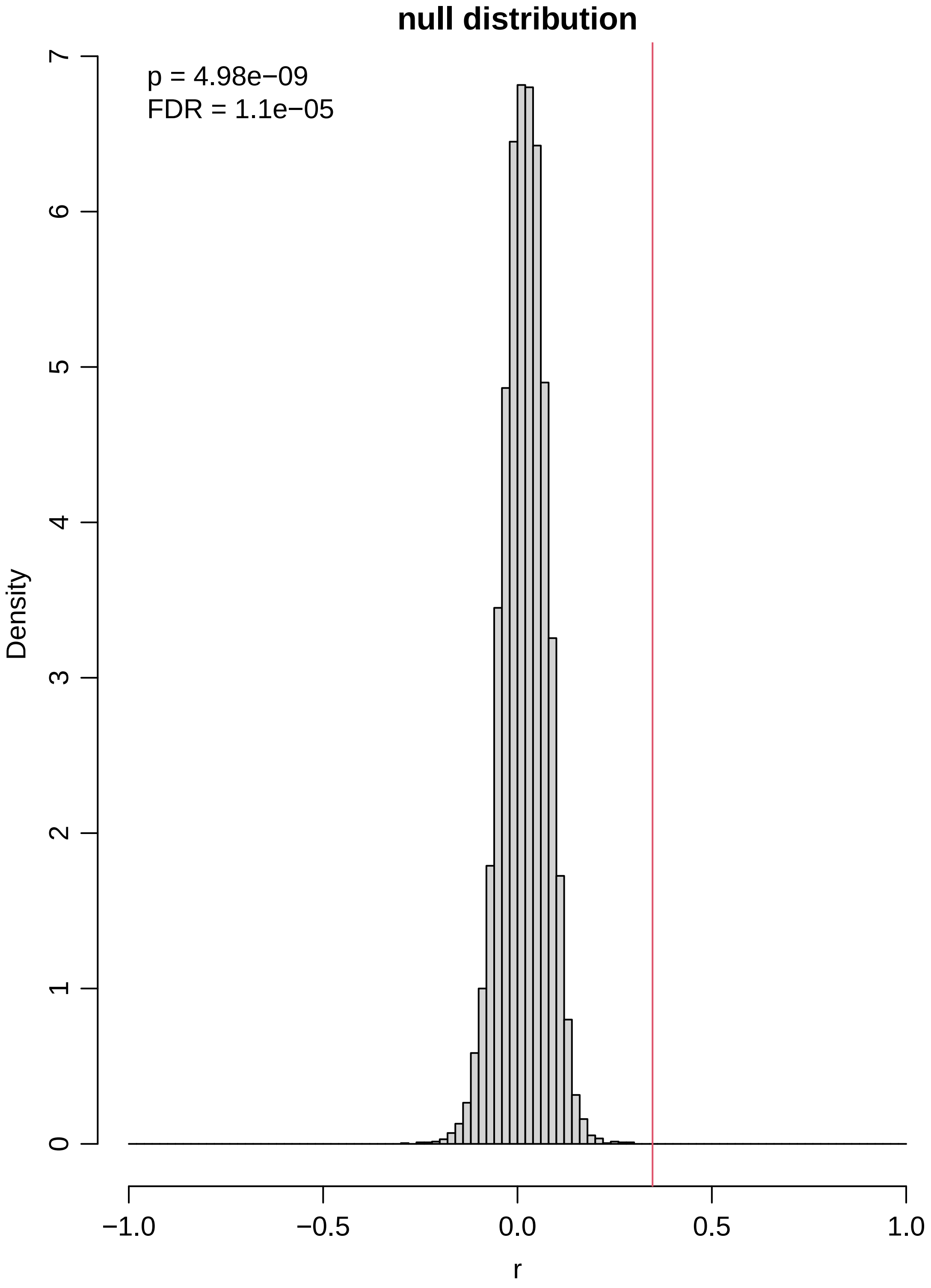

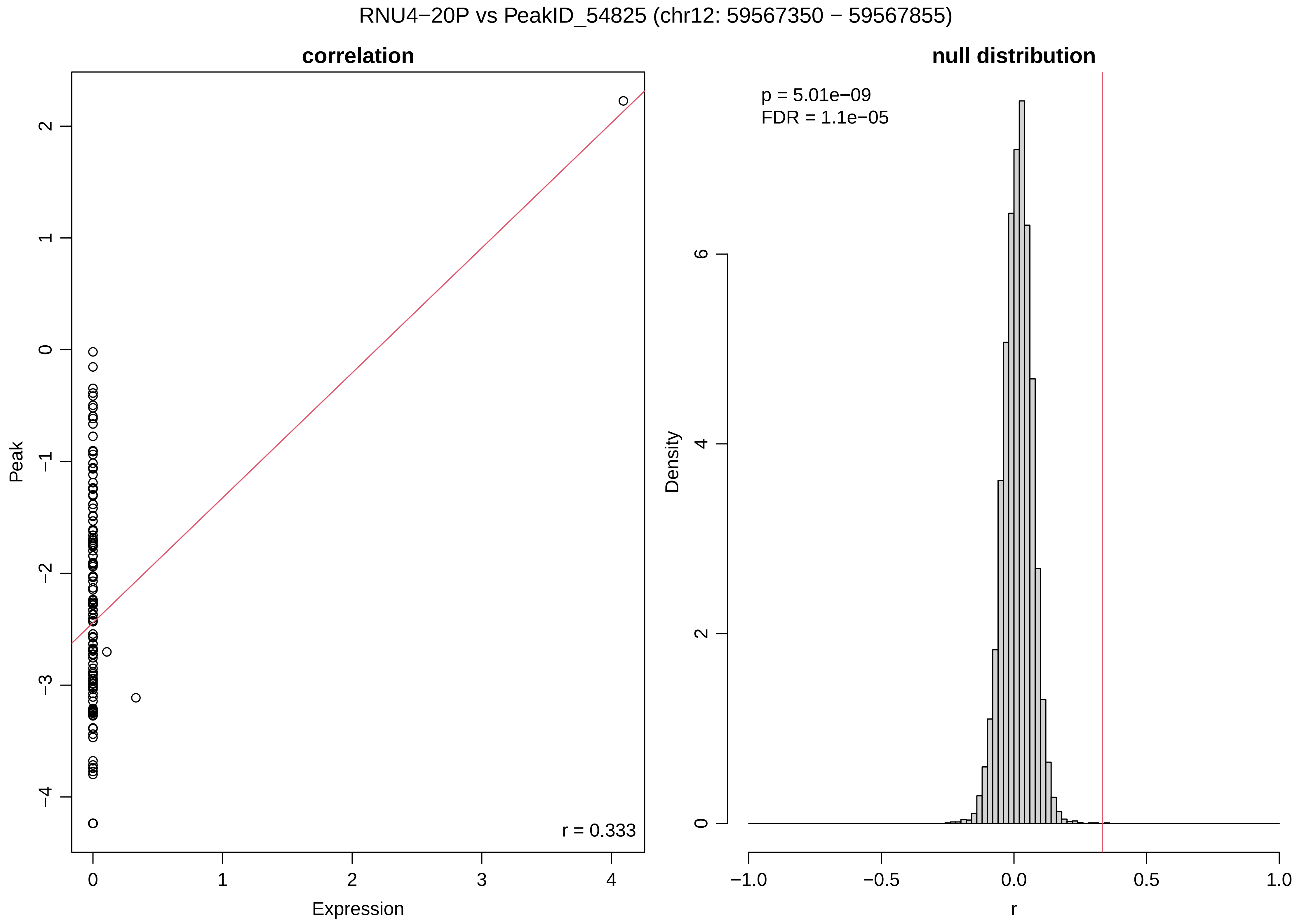

RNU4−20P vs PeakID_54825 (chr12: 59567350 − 59567855)
correlation
Peak
Expression
r = 0.333
null distribution
p = 5.01e−09
FDR = 1.1e−05
Density
r

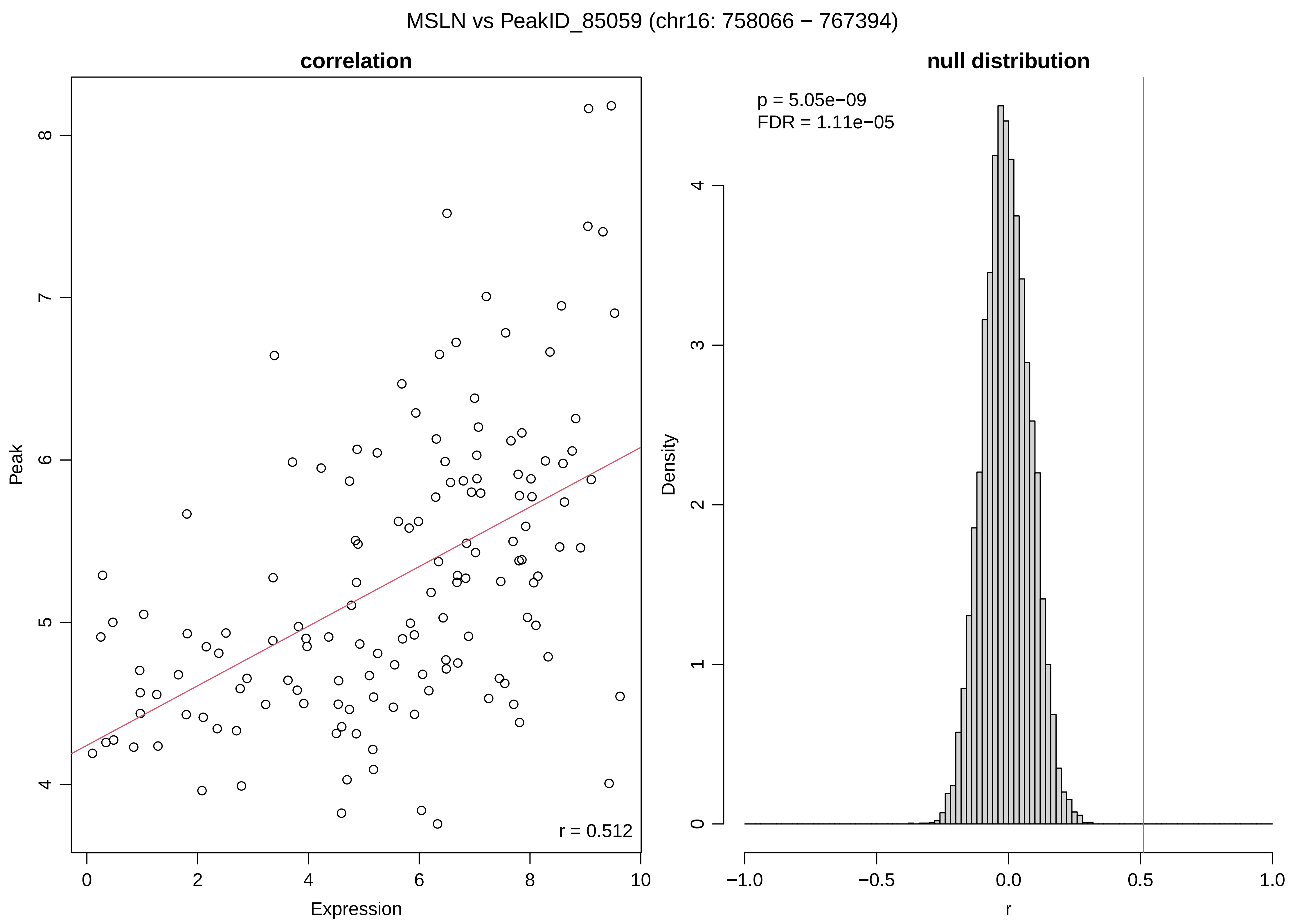
MSLN vs PeakID_85059 (chr16: 758066 − 767394)
correlation
Peak
Expression
r = 0.512
0
2
4
6
8
10
4
5
6
7
8
null distribution
p = 5.05e−09
FDR = 1.11e−05
Density
r
−1.0
−0.5
0.0
0.5
1.0
0
1
2
3
4

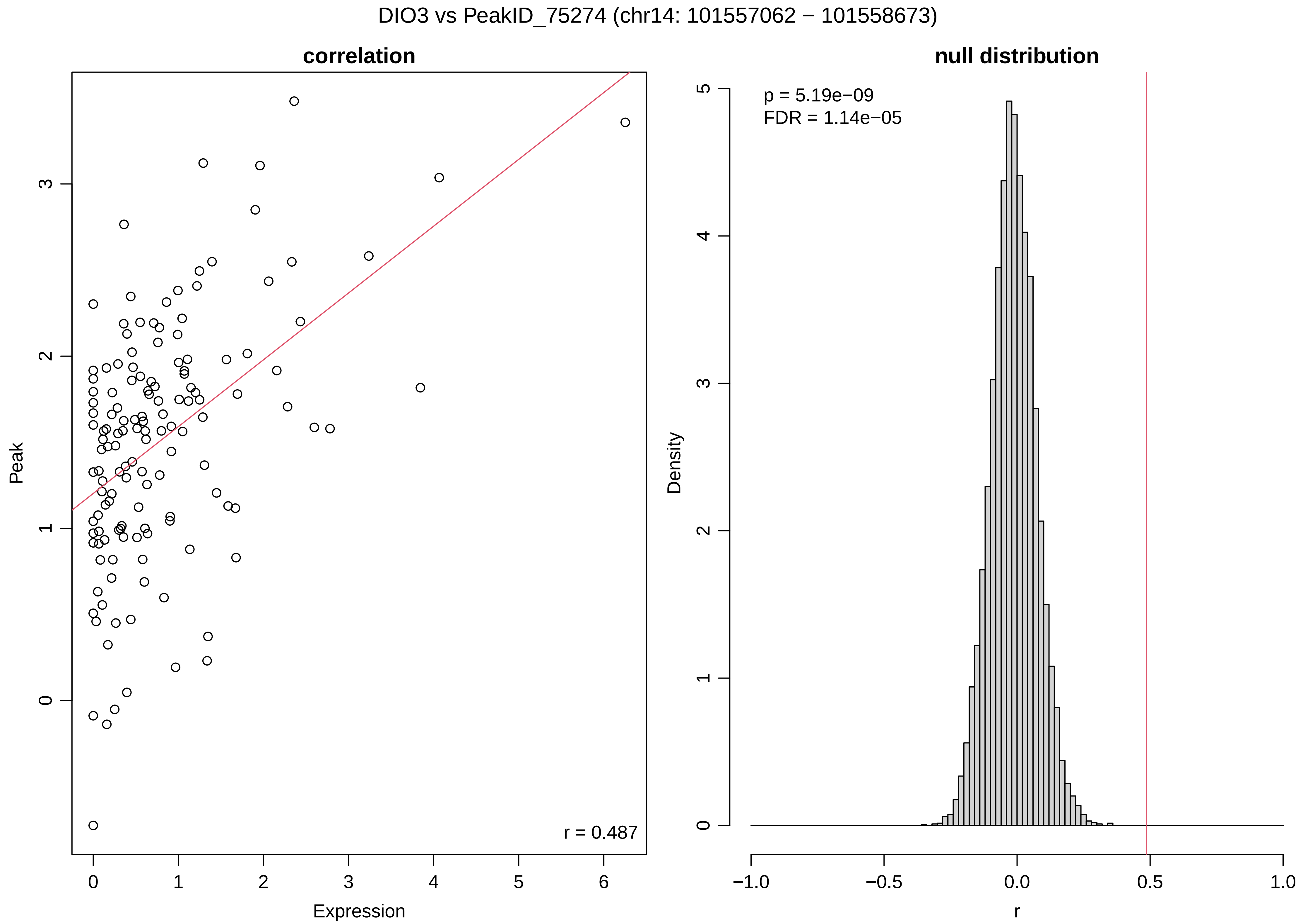
DIO3 vs PeakID_75274 (chr14: 101557062 − 101558673)
correlation
Peak
Expression
r = 0.487
null distribution
p = 5.19e−09
FDR = 1.14e−05
Density
r

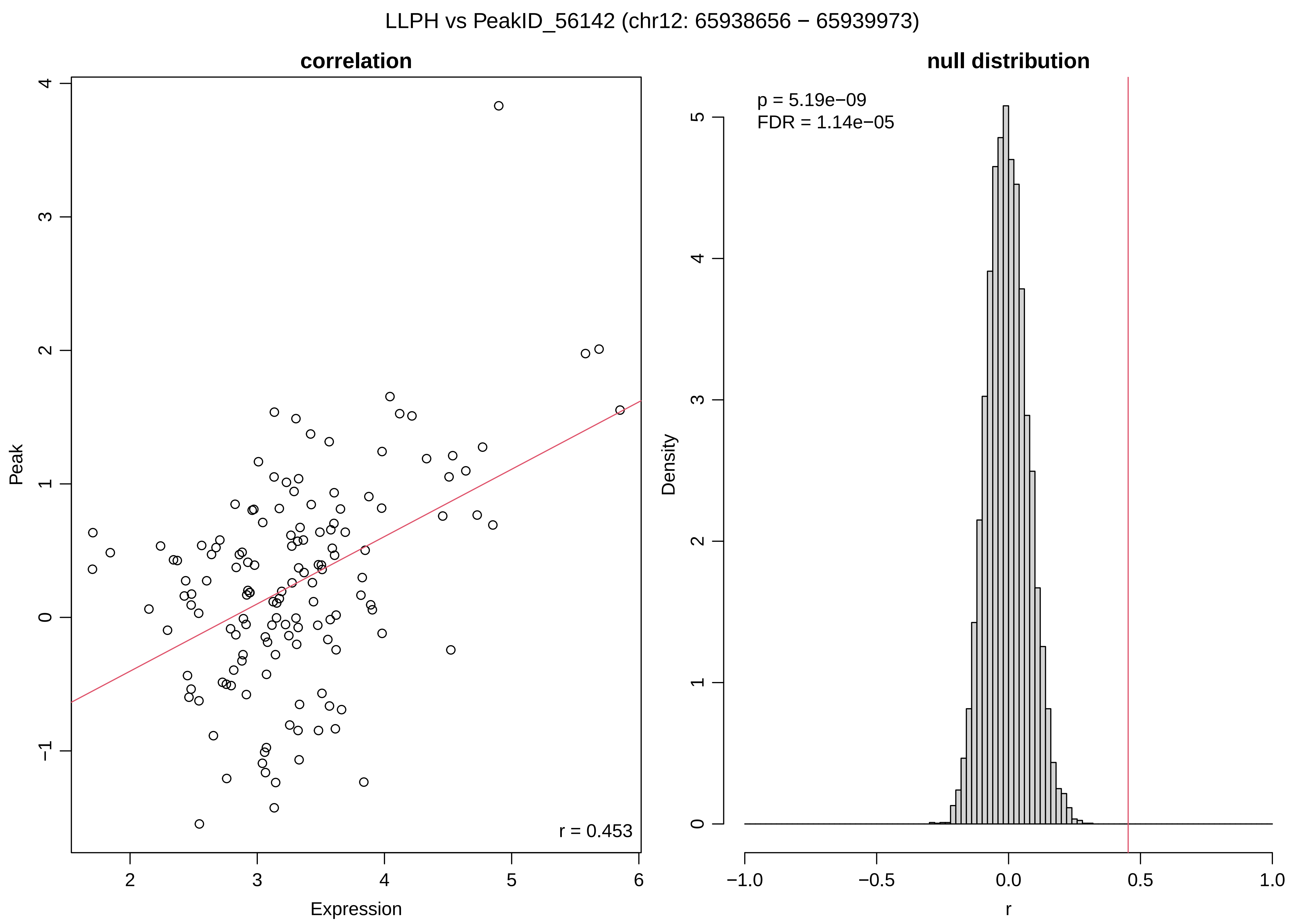
LLPH vs PeakID_56142 (chr12: 65938656 − 65939973)
correlation
null distribution
Peak
Expression
r = 0.453
p = 5.19e−09
FDR = 1.14e−05
Density
r

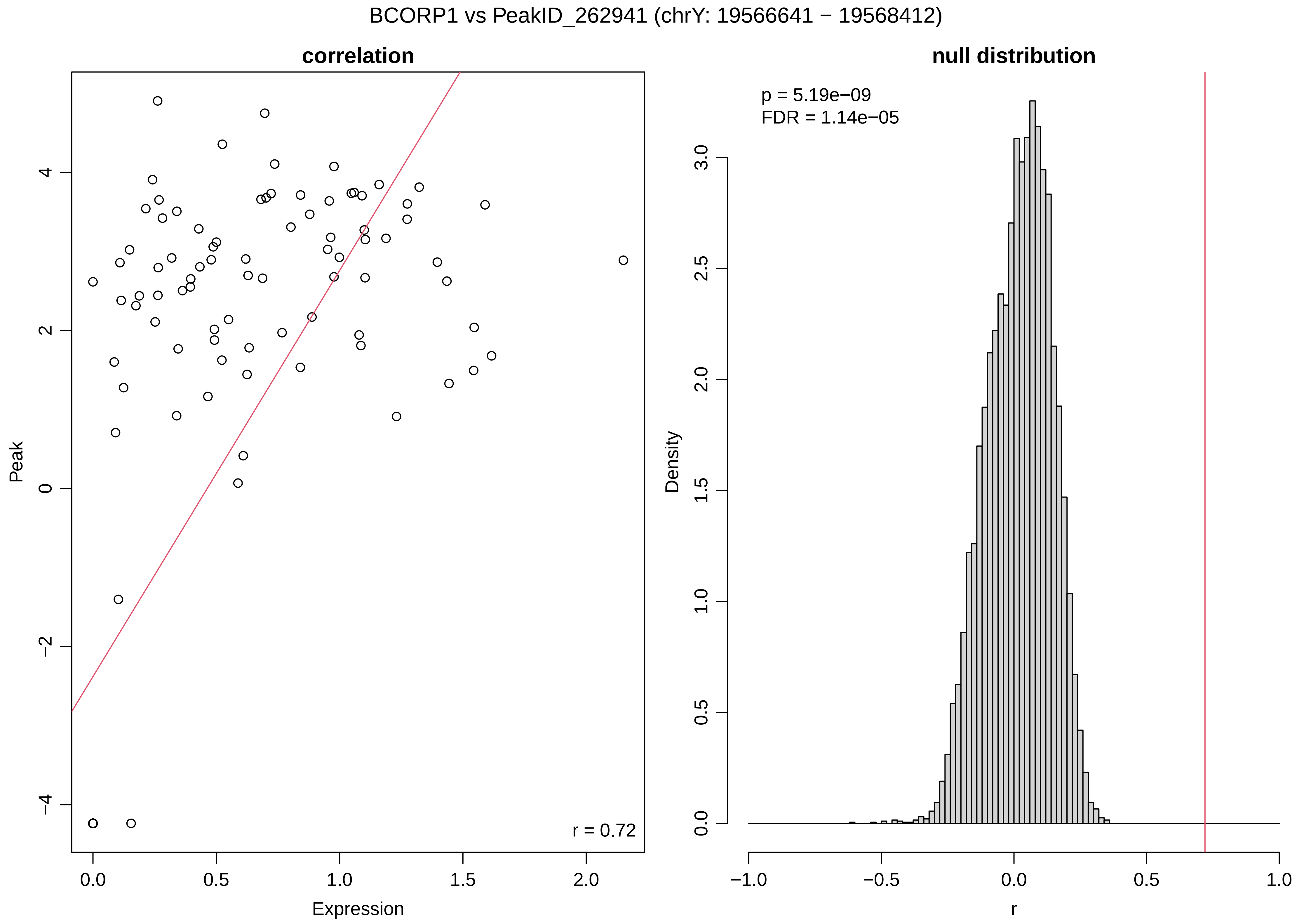

BCORP1 vs PeakID_262941 (chrY: 19566641 − 19568412)
correlation
Peak
Expression
r = 0.72
null distribution
p = 5.19e−09
FDR = 1.14e−05
Density
r

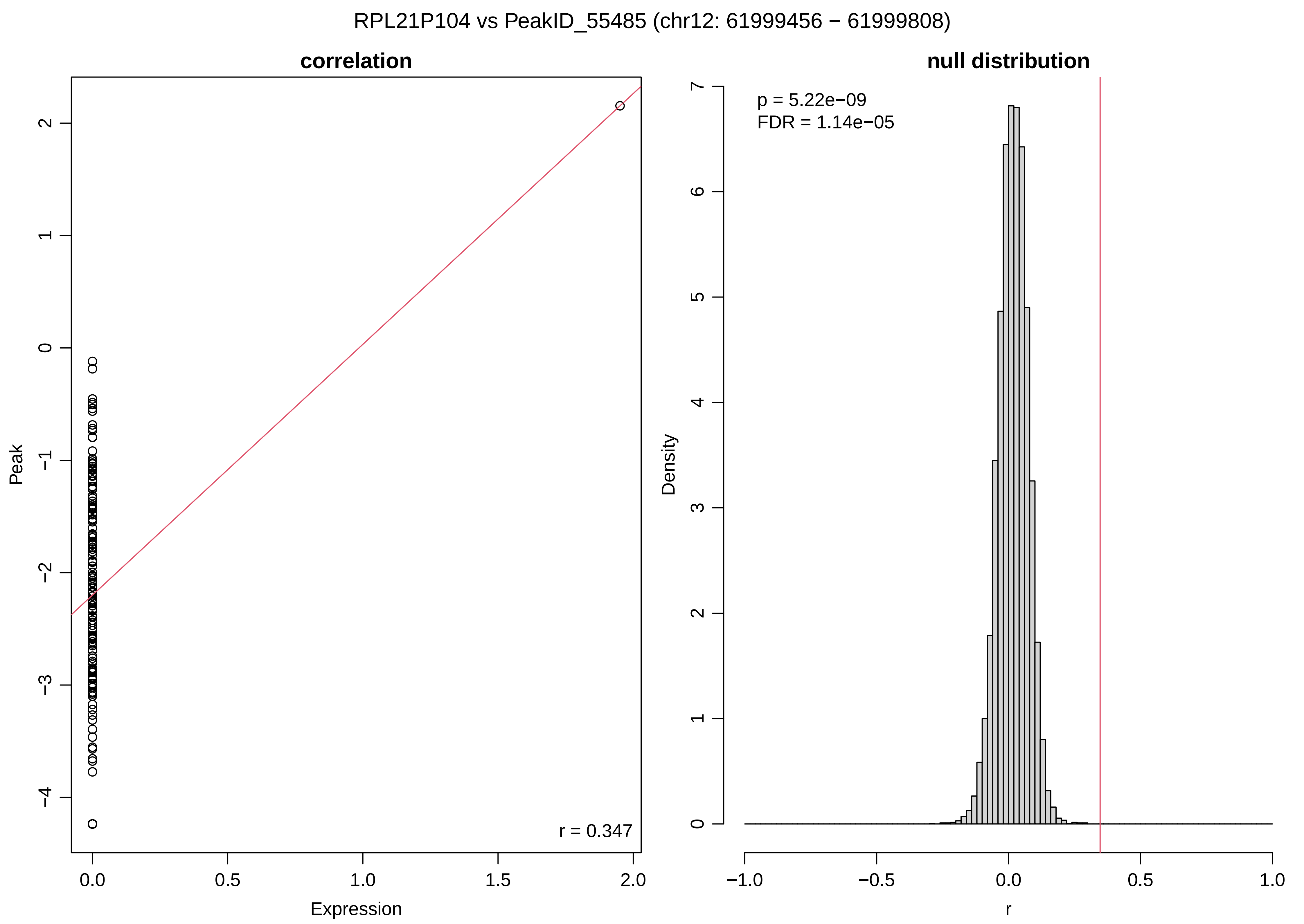

RPL21P104 vs PeakID_55485 (chr12: 61999456 − 61999808)
correlation
Peak
Expression
r = 0.347
0.0
0.5
1.0
1.5
2.0
−4
−3
−2
−1
0
1
2
null distribution
p = 5.22e−09
FDR = 1.14e−05
Density
r
−1.0
−0.5
0.0
0.5
1.0
0
1
2
3
4
5
6
7

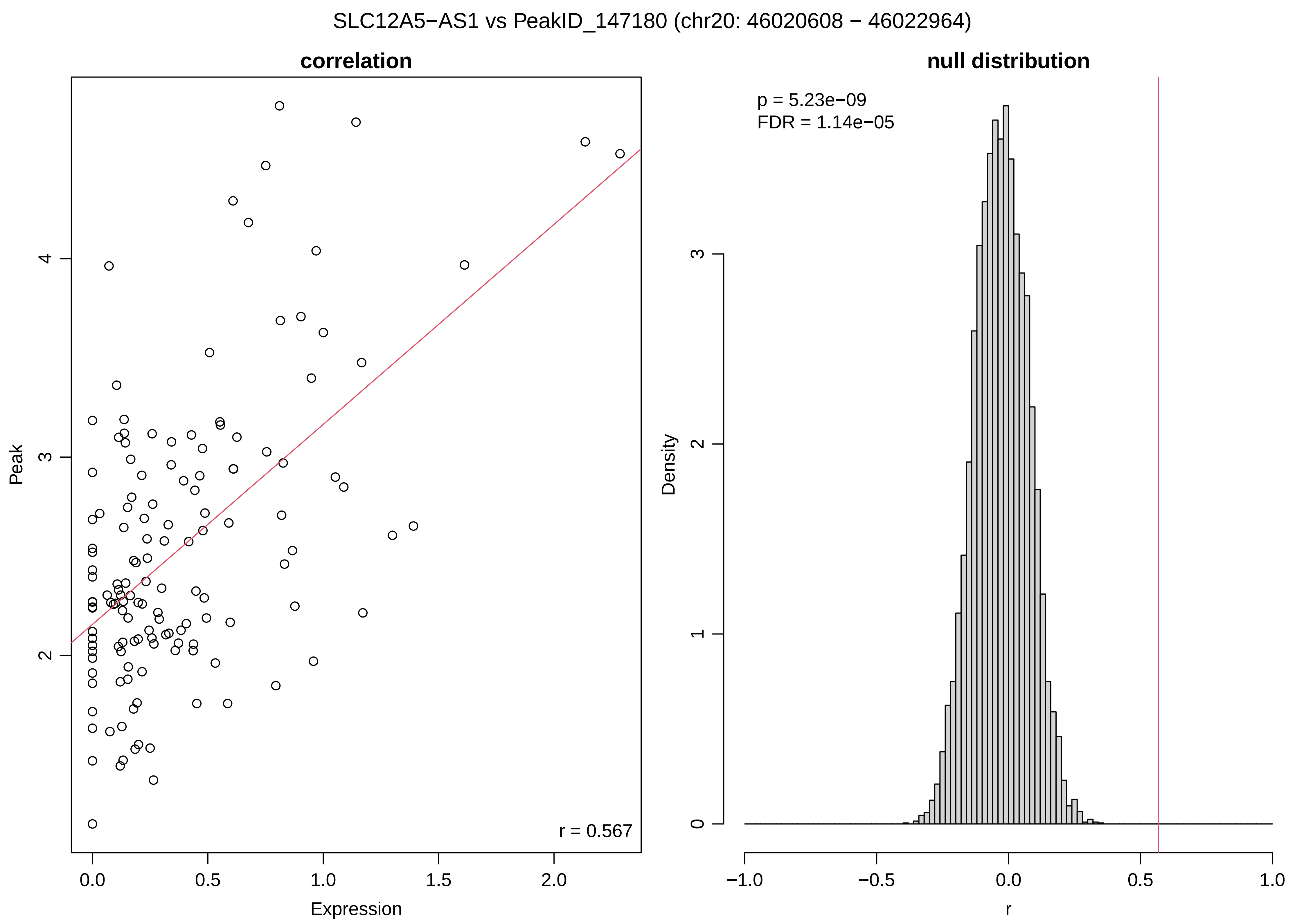
SLC12A5−AS1 vs PeakID_147180 (chr20: 46020608 − 46022964)
correlation
Peak
Expression
r = 0.567
null distribution
p = 5.23e−09
FDR = 1.14e−05
Density
r

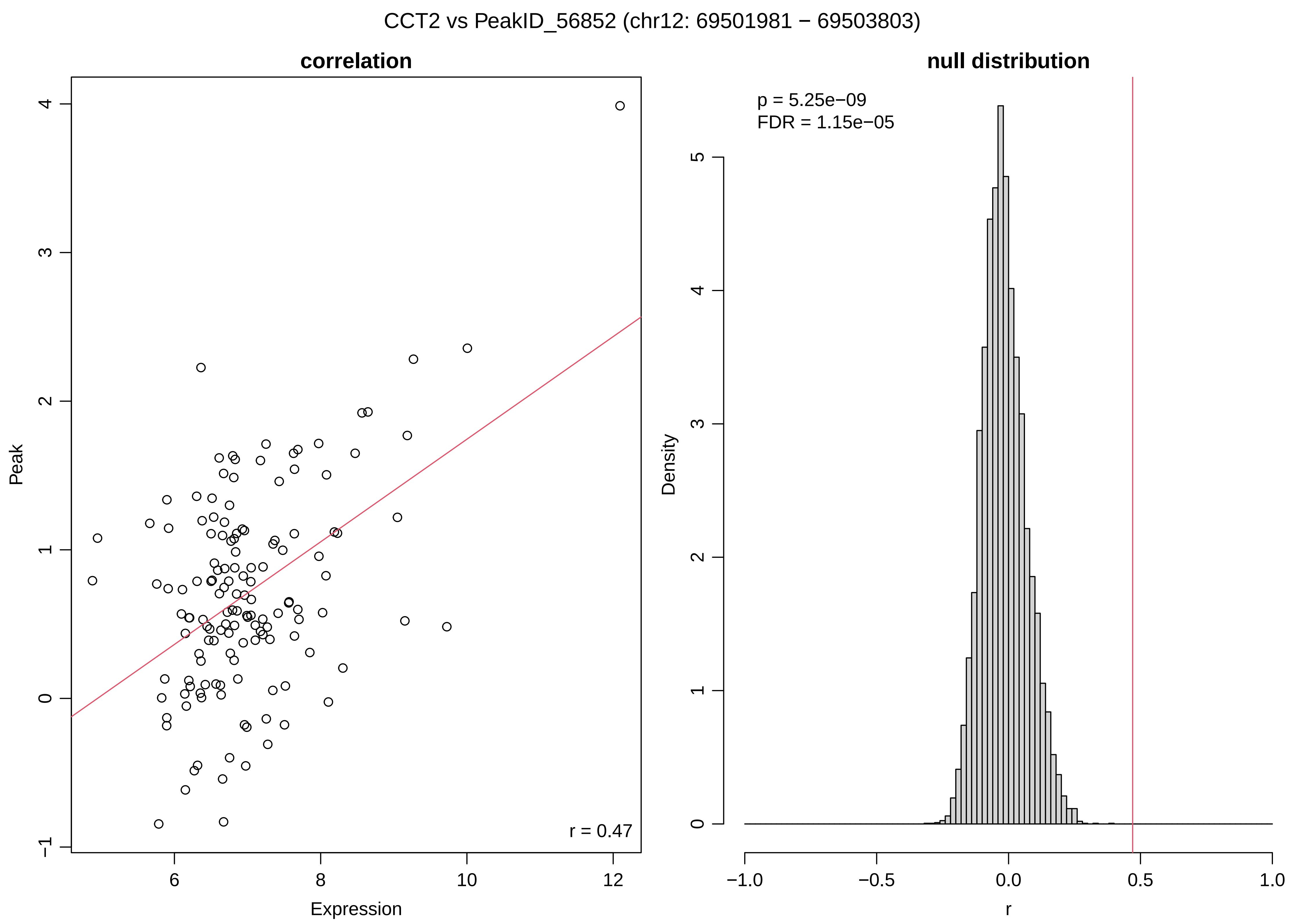
CCT2 vs PeakID_56852 (chr12: 69501981 − 69503803)
correlation
null distribution
p = 5.25e−09
FDR = 1.15e−05
r = 0.47
Peak
Expression
Density
r

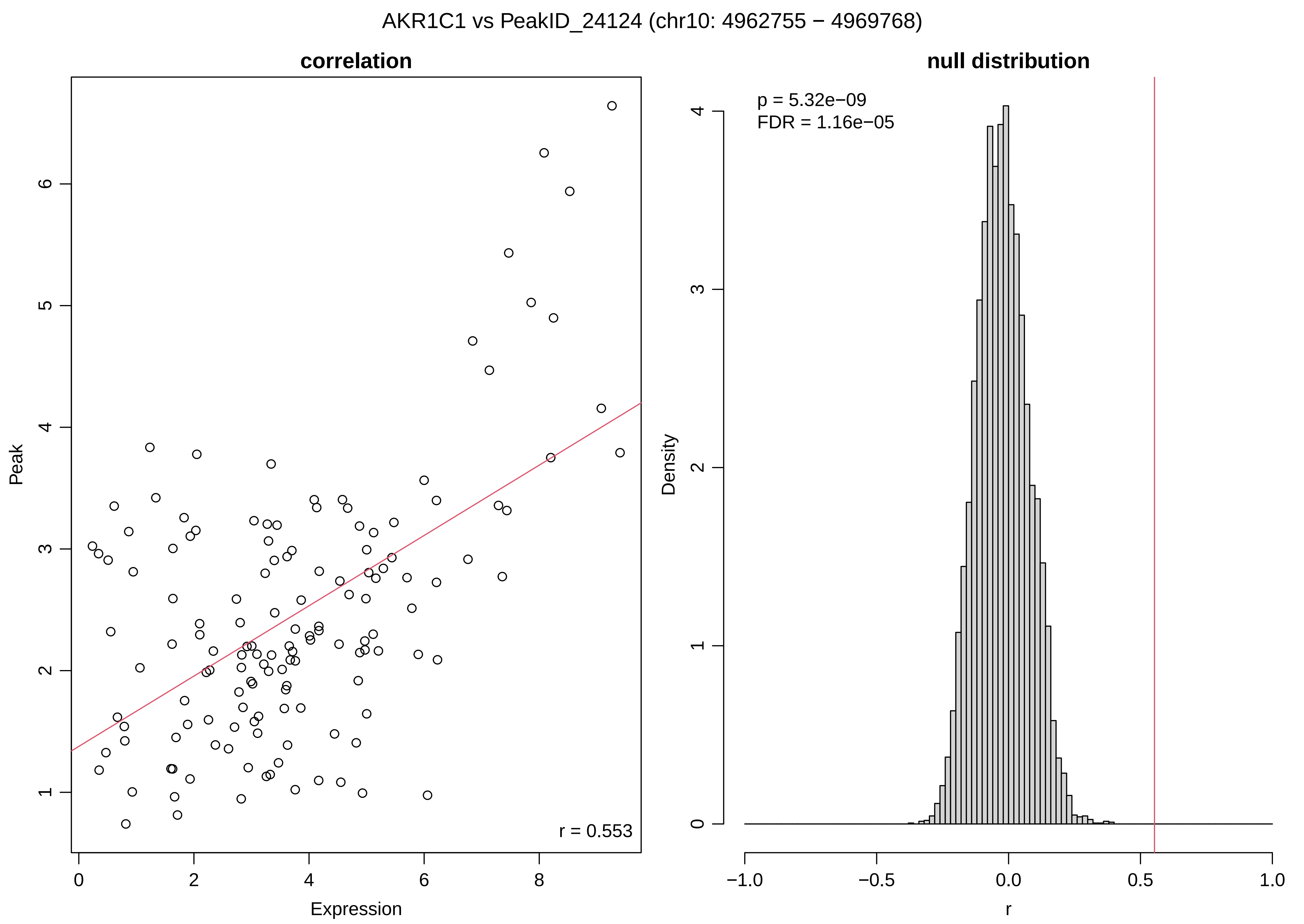
AKR1C1 vs PeakID_24124 (chr10: 4962755 − 4969768)
correlation
Peak
Expression
r = 0.553
null distribution
p = 5.32e−09
FDR = 1.16e−05
Density
r

AC002066.1 vs PeakID_229010 (chr7: 116670297 − 116718405)

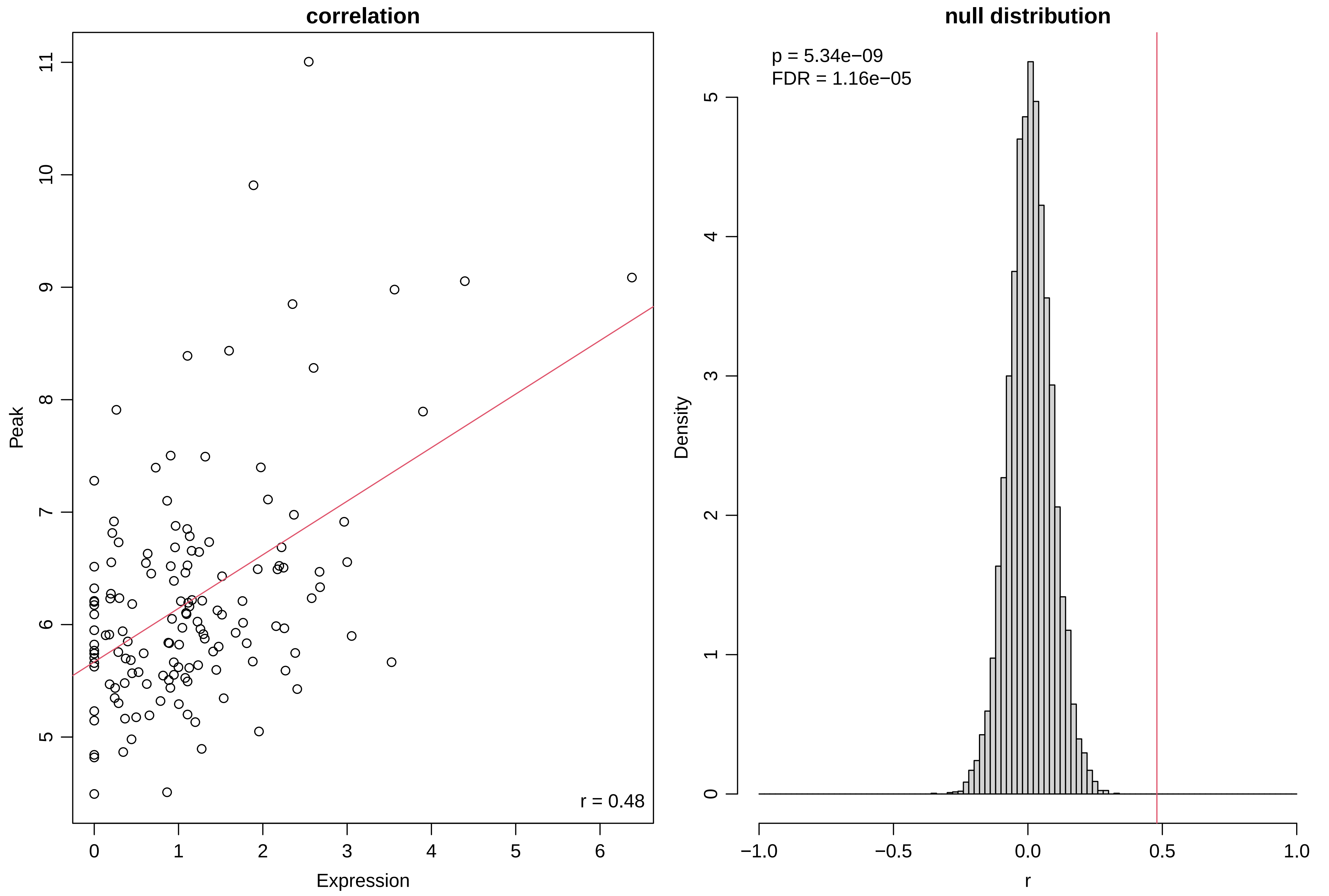

GATA3 vs PeakID_24670 (chr10: 8053280 − 8063277)

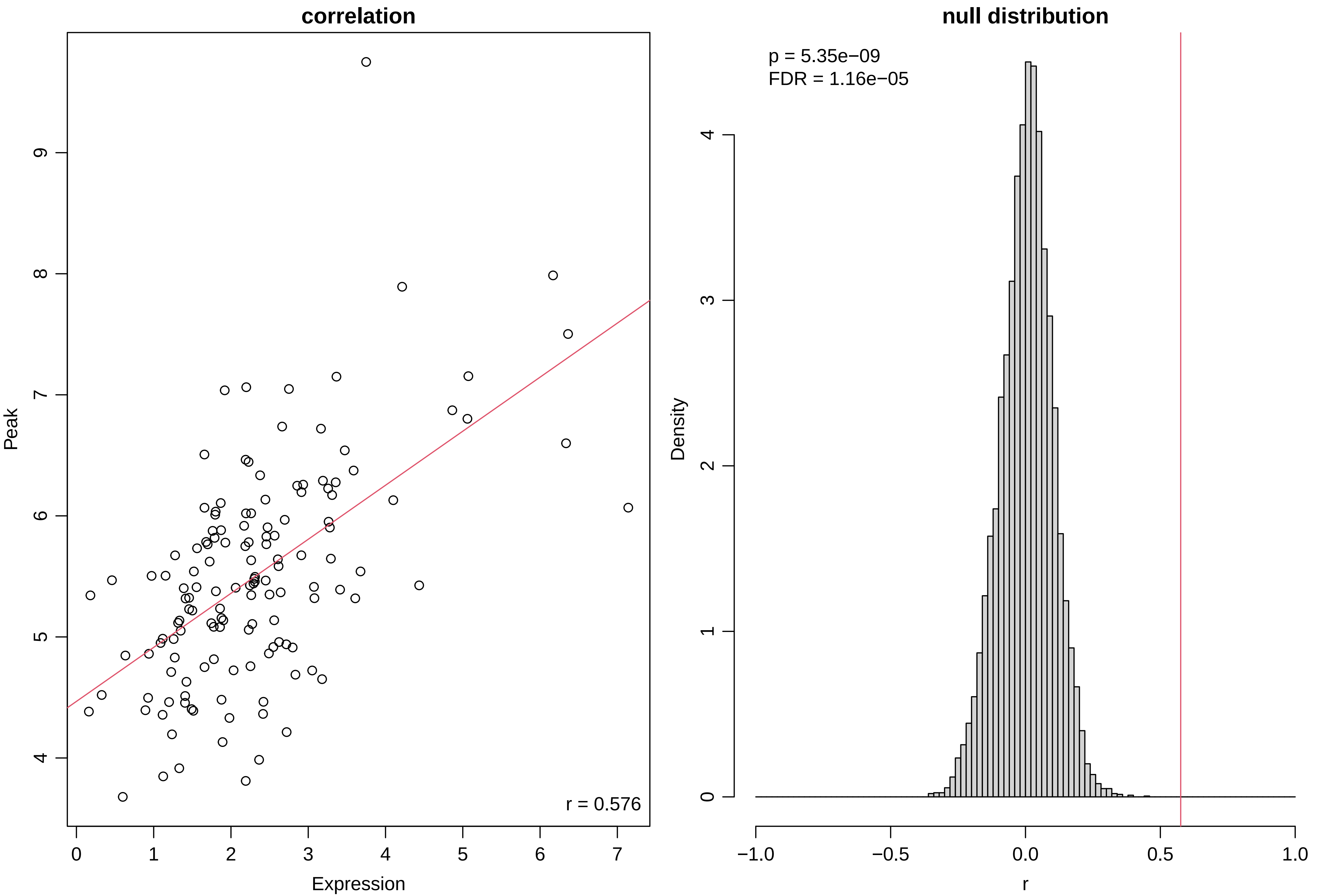

AC104958.2 vs PeakID_241724 (chr8: 120771646 − 120778730)

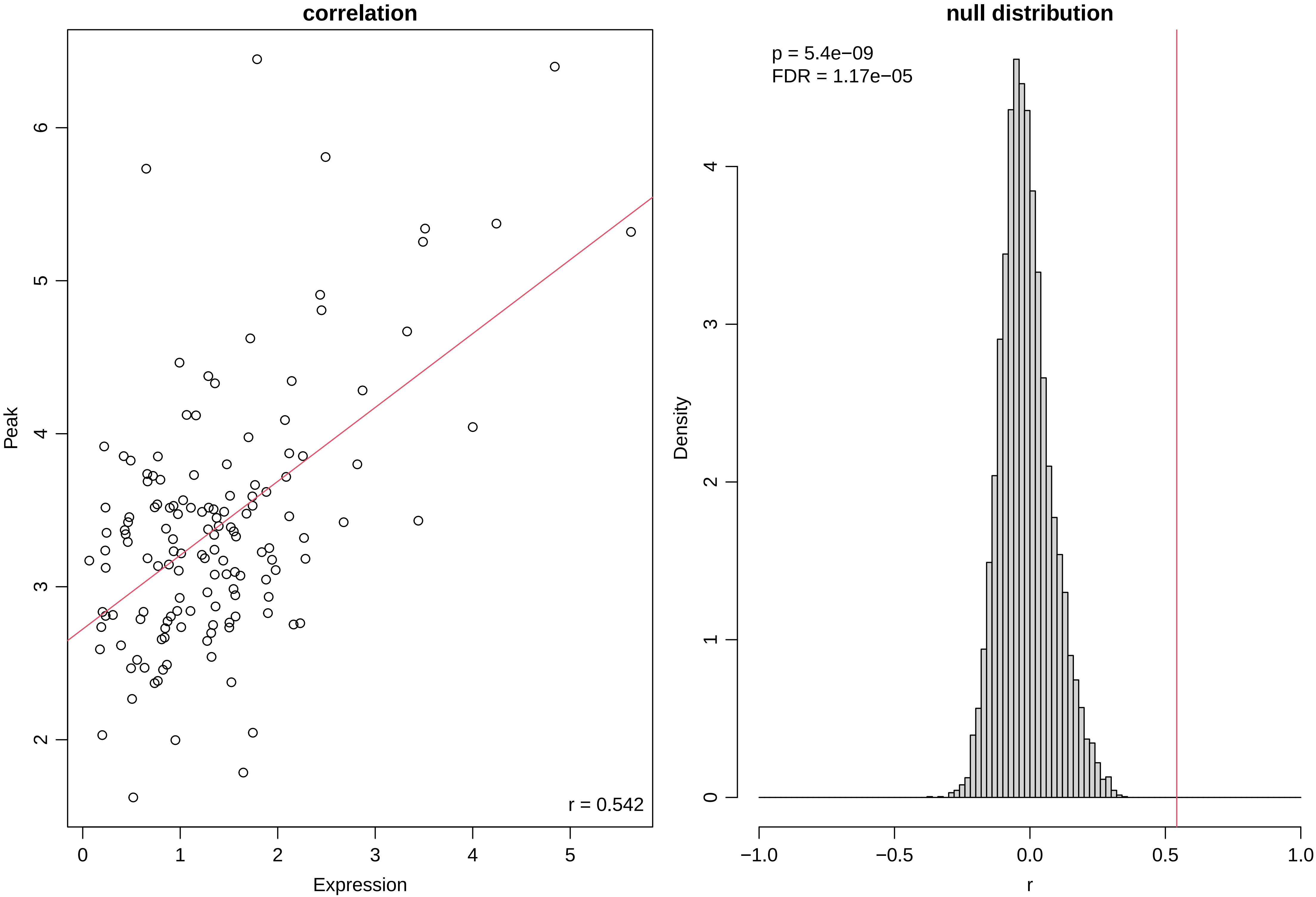

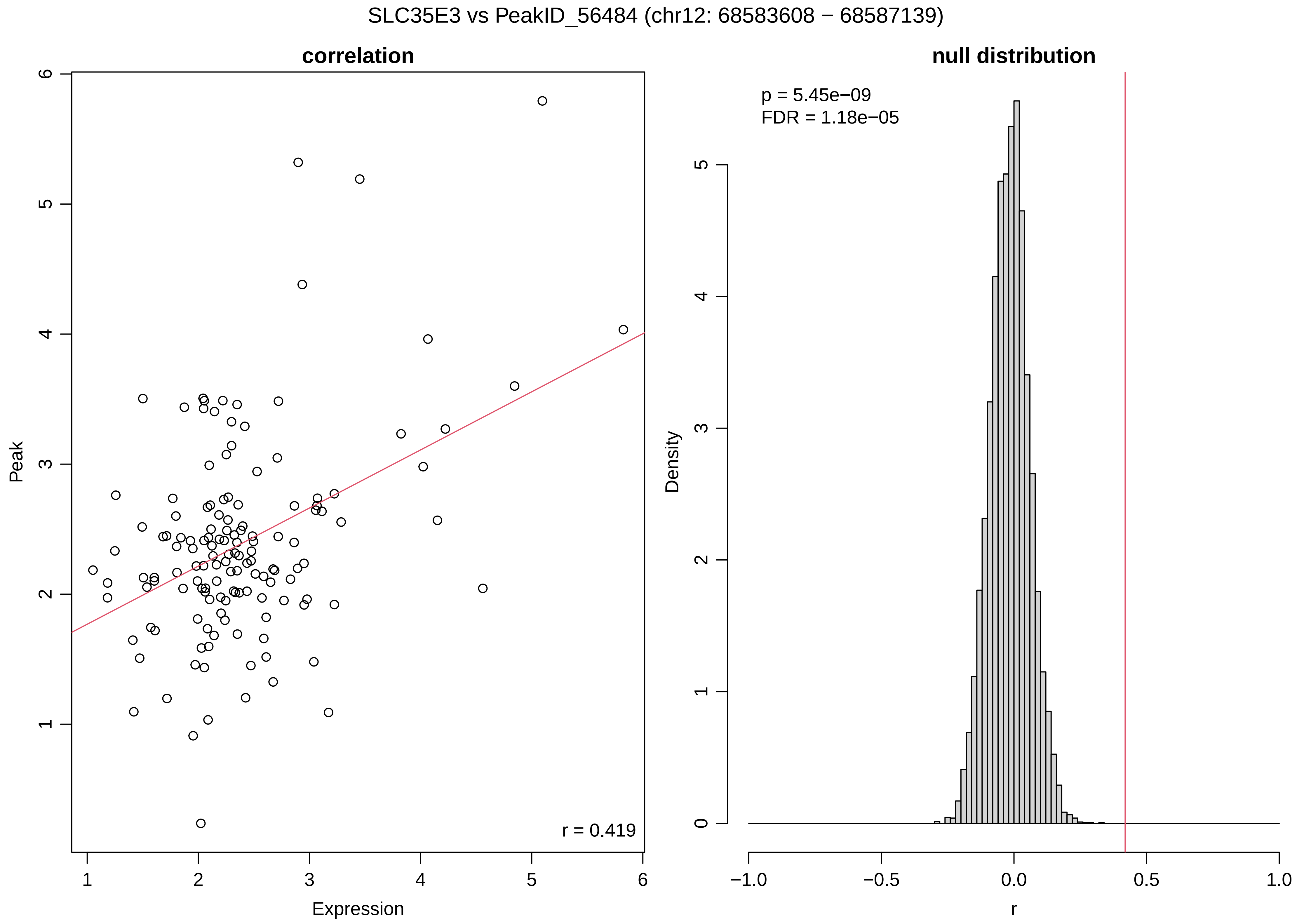

SLC35E3 vs PeakID_56484 (chr12: 68583608 − 68587139)
correlation
Peak
Expression
r = 0.419
null distribution
p = 5.45e−09
FDR = 1.18e−05
Density
r

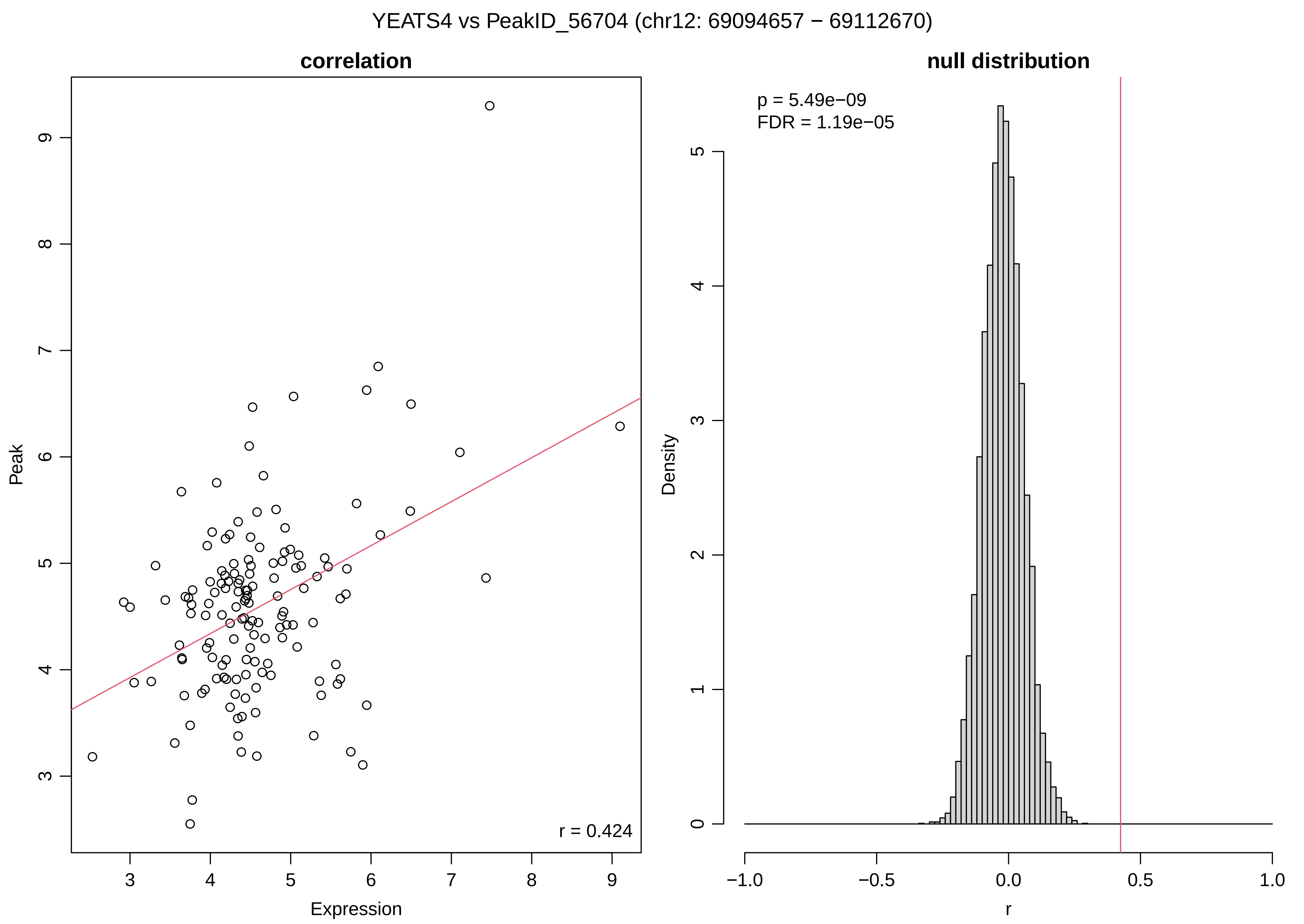
YEATS4 vs PeakID_56704 (chr12: 69094657 − 69112670)
correlation
null distribution
p = 5.49e−09
FDR = 1.19e−05
Peak
Expression
r = 0.424
Density
r

ANOS2P vs PeakID_262755 (chrY: 13564513 − 13565684)

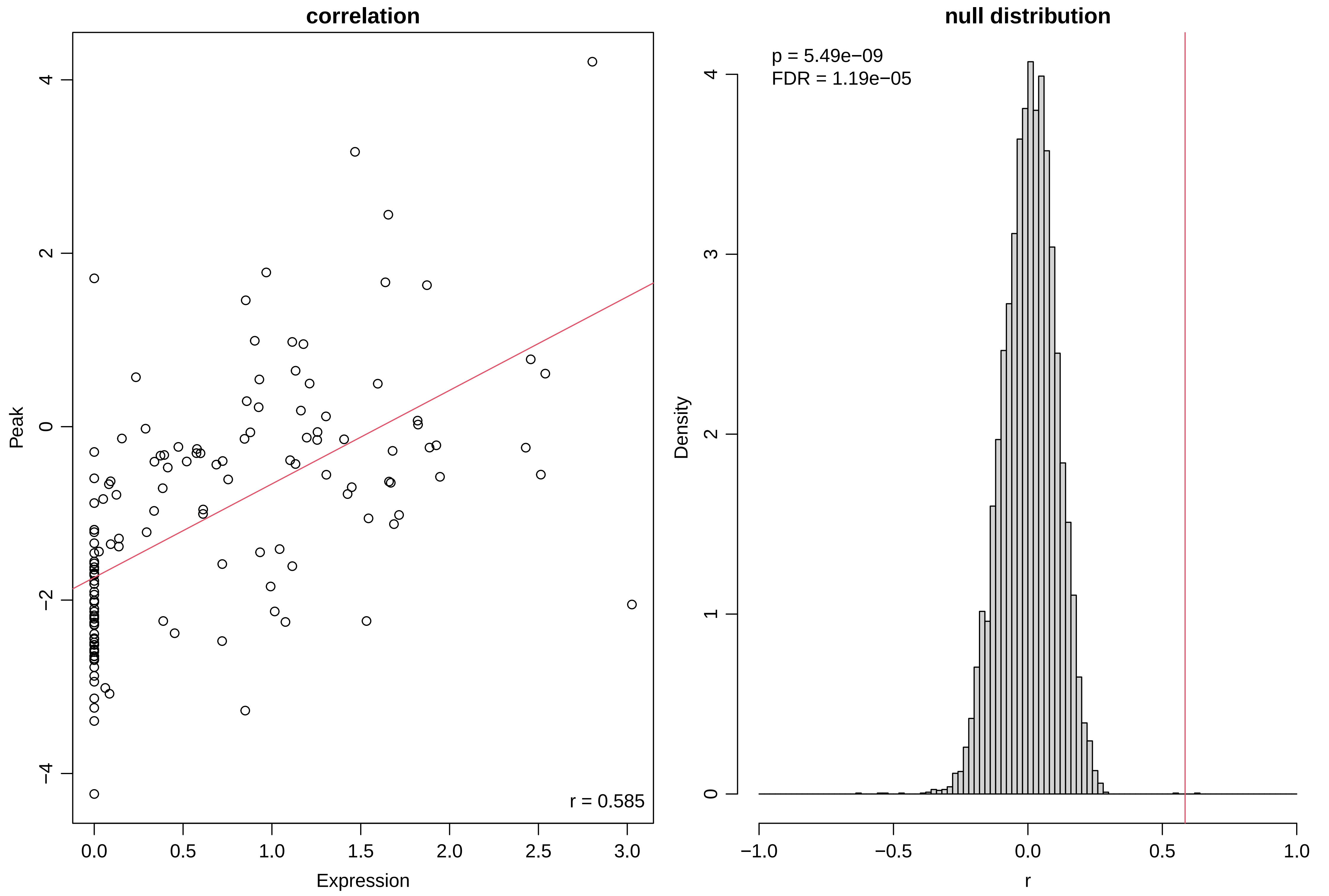

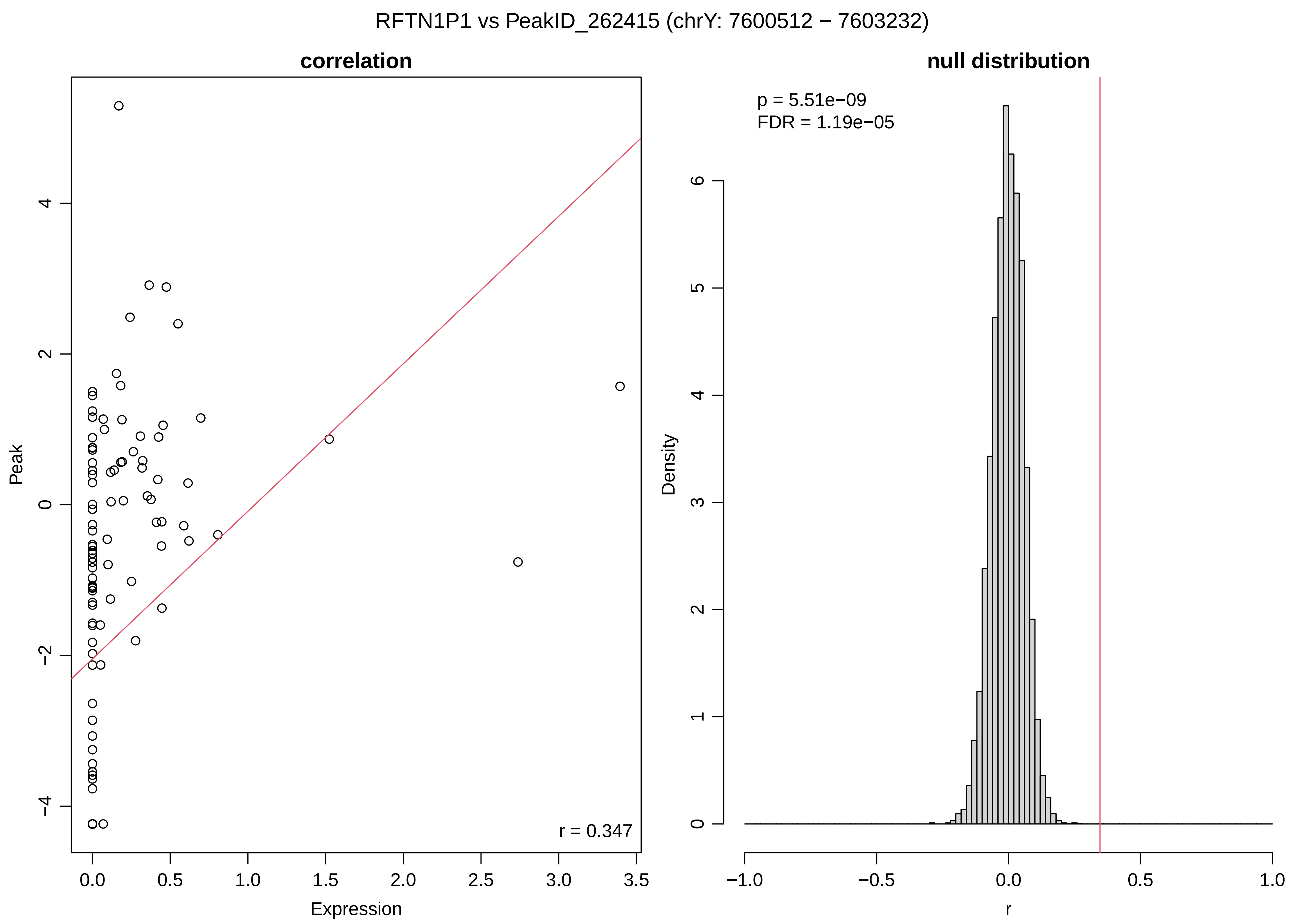
RFTN1P1 vs PeakID_262415 (chrY: 7600512 − 7603232)
correlation
null distribution
p = 5.51e−09
FDR = 1.19e−05
r = 0.347
Peak
Expression
Density
r
−4
−2
0
2
4
0.0
0.5
1.0
1.5
2.0
2.5
3.0
3.5
1
3
5
6
−1.0
−0.5
0.0
0.5
1.0

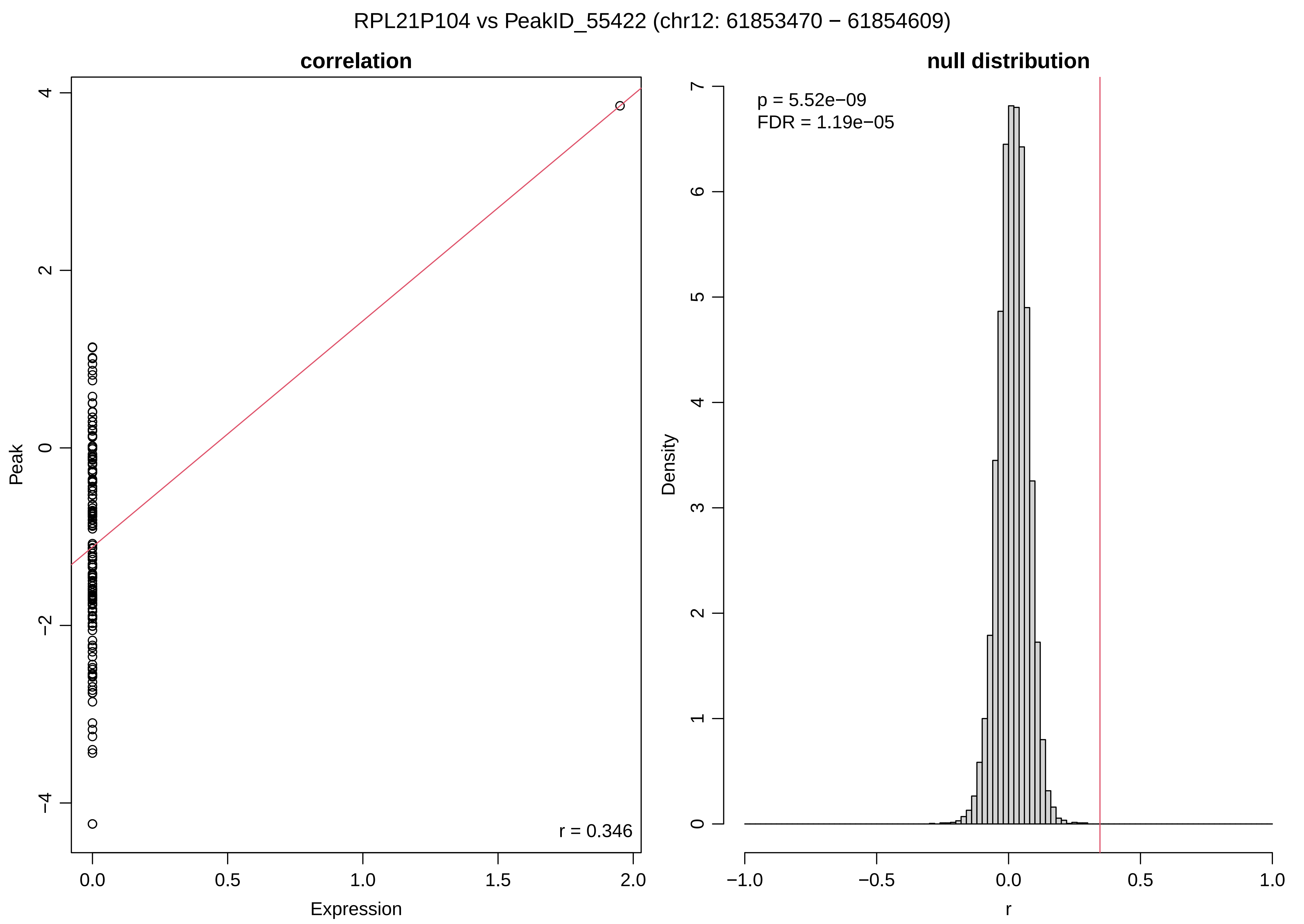
RPL21P104 vs PeakID_55422 (chr12: 61853470 − 61854609)
correlation
Peak
Expression
4
2
0
−2
−4
0.0
0.5
1.0
1.5
2.0
r = 0.346
null distribution
Density
r
p = 5.52e−09
FDR = 1.19e−05
0
1
2
3
4
5
6
7
−1.0
−0.5
0.0
0.5
1.0

AC092881.1 vs PeakID_56992 (chr12: 70001176 − 70001722)

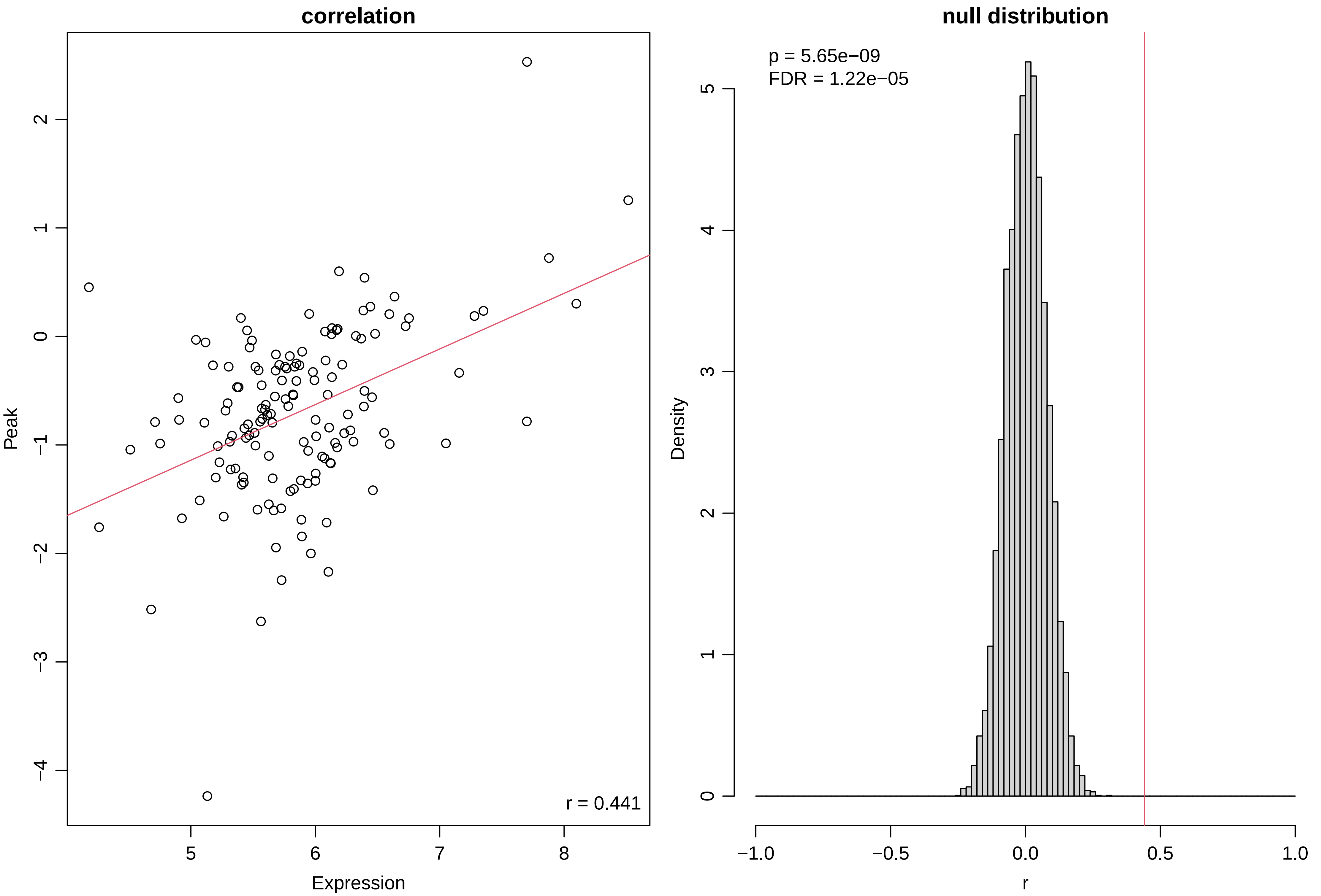

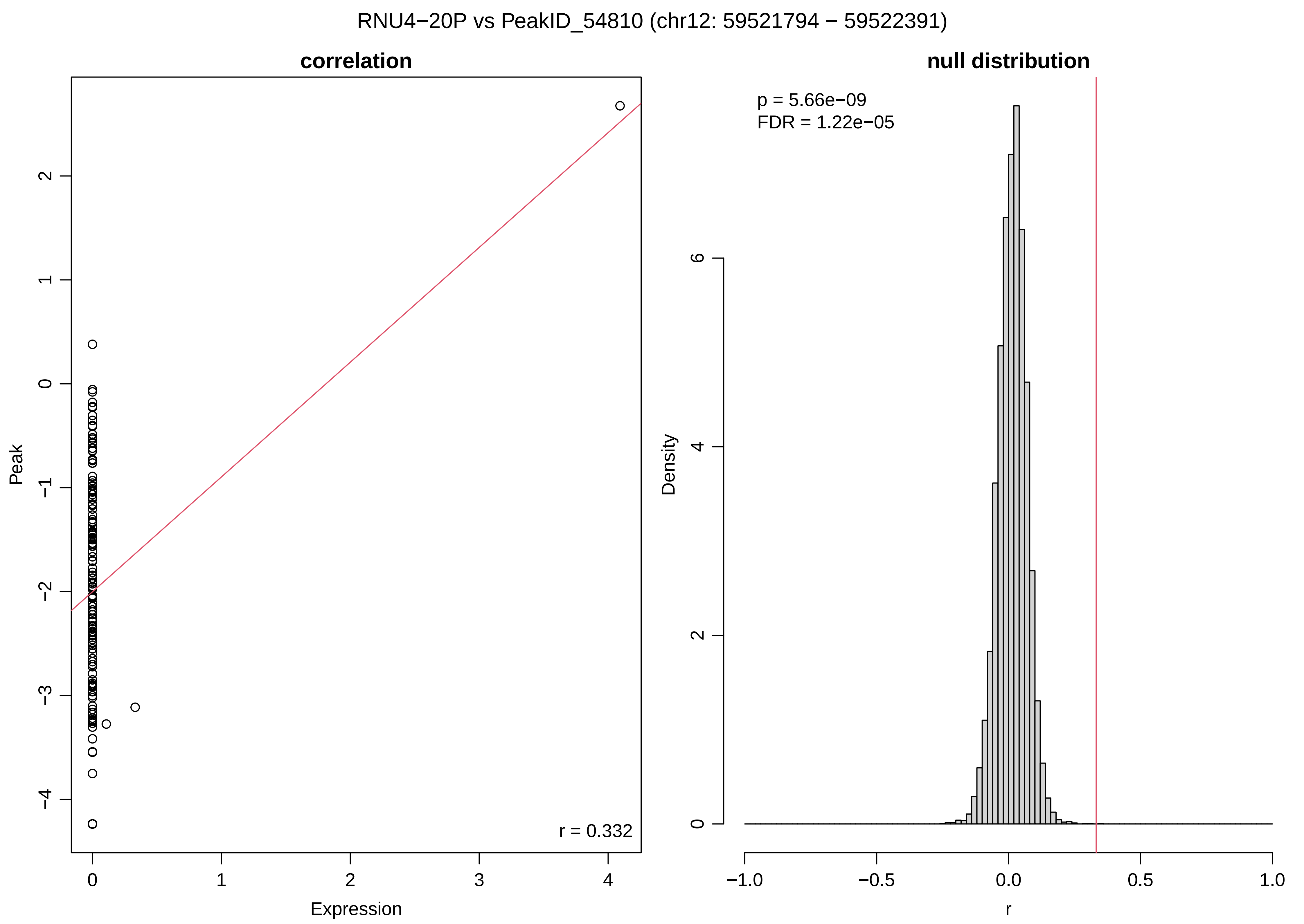

RNU4−20P vs PeakID_54810 (chr12: 59521794 − 59522391)
correlation
Peak
Expression
r = 0.332
null distribution
p = 5.66e−09
FDR = 1.22e−05
Density
r

RPL21P104 vs PeakID_55427 (chr12: 61861946 − 61862549)

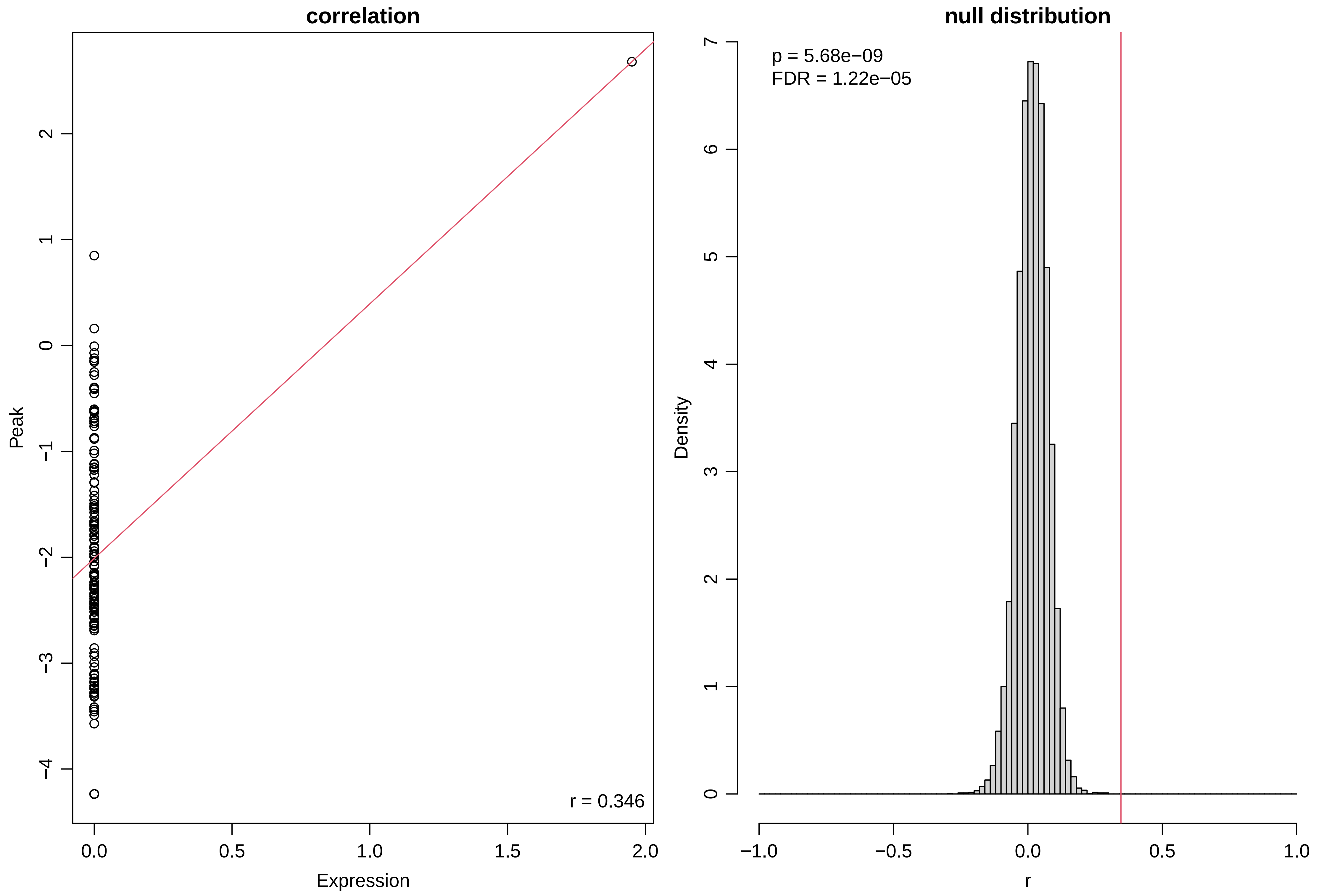

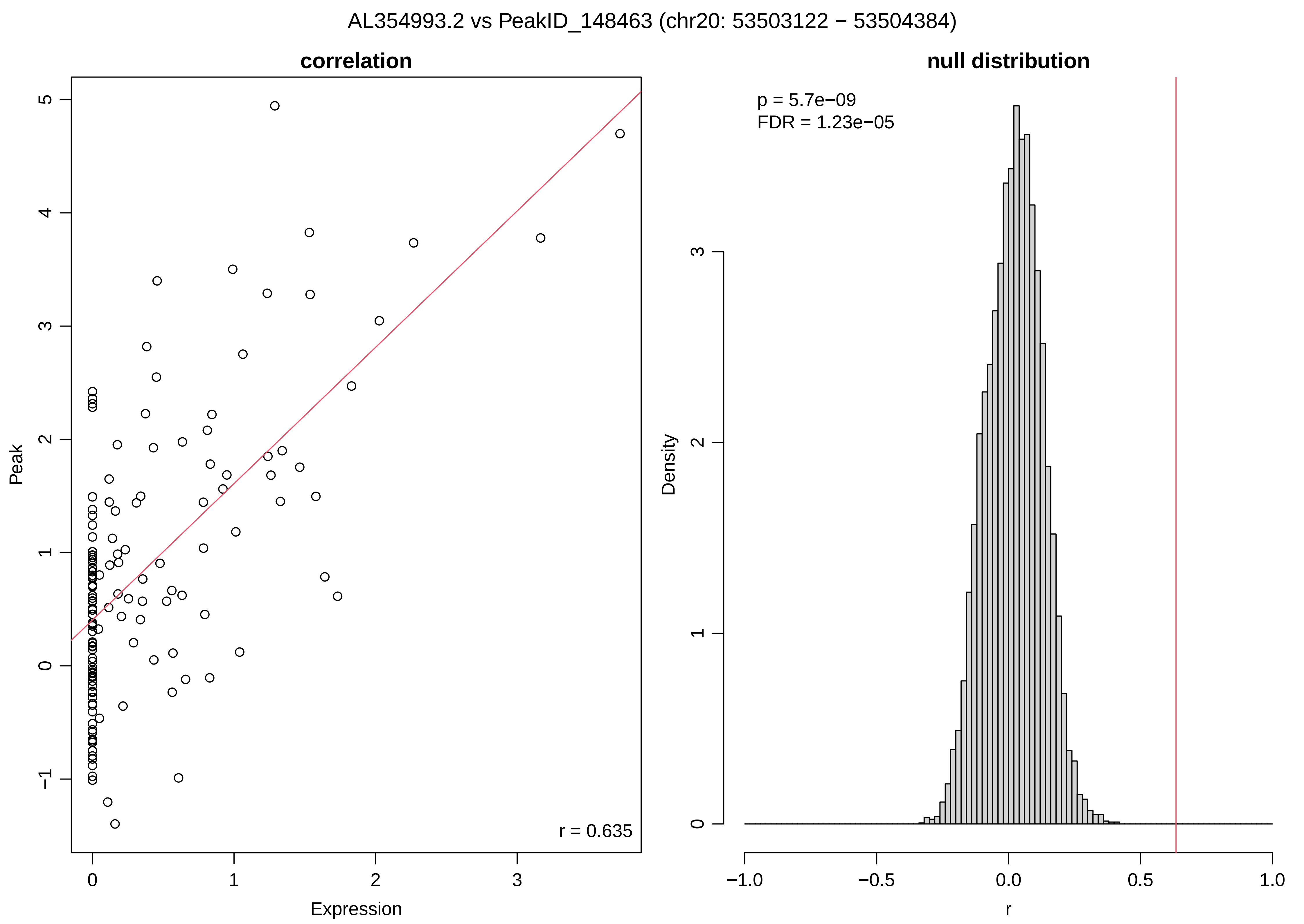
AL354993.2 vs PeakID_148463 (chr20: 53503122 − 53504384)
correlation
Peak
Expression
r = 0.635
null distribution
p = 5.7e−09
FDR = 1.23e−05
Density
r

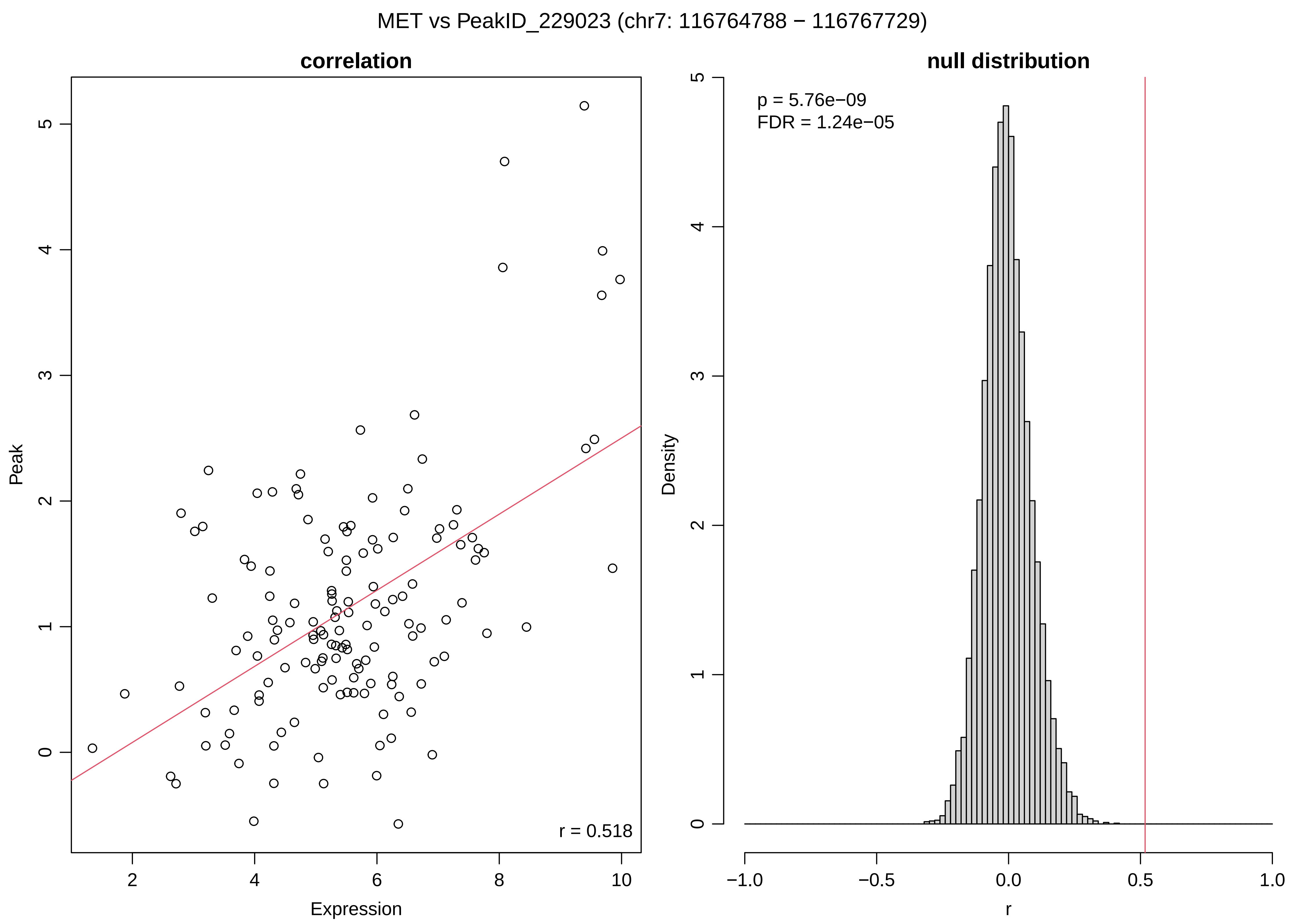
MET vs PeakID_229023 (chr7: 116764788 − 116767729)
correlation
null distribution
Peak
Expression
r = 0.518
p = 5.76e−09
FDR = 1.24e−05
Density
r

RNU4−20P vs PeakID_54797 (chr12: 59502325 − 59503522)

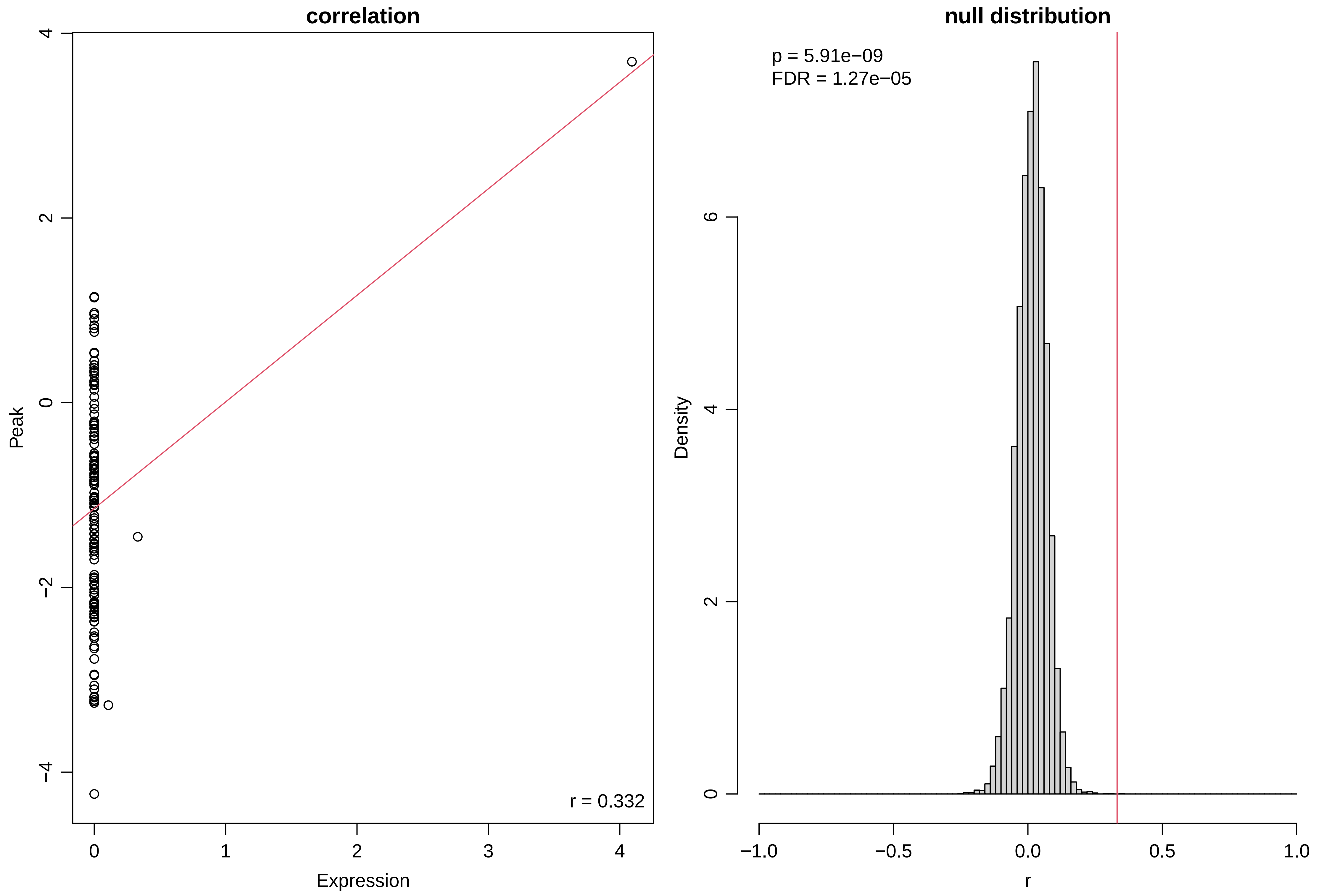

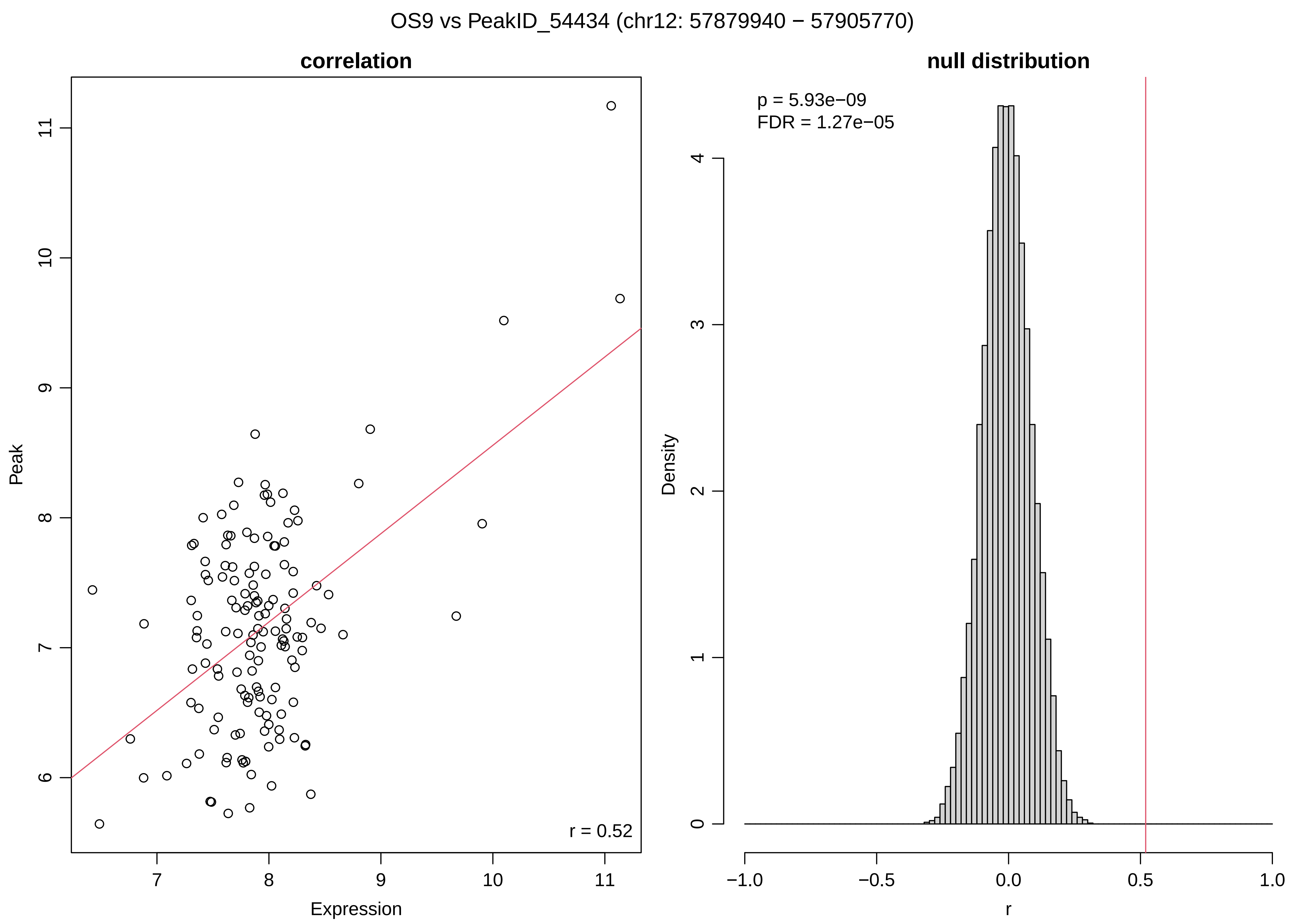
OS9 vs PeakID_54434 (chr12: 57879940 − 57905770)
correlation
Peak
Expression
r = 0.52
null distribution
p = 5.93e−09
FDR = 1.27e−05
Density
r

BMS1P13 vs PeakID_248110 (chr9: 65824697 − 65826029)

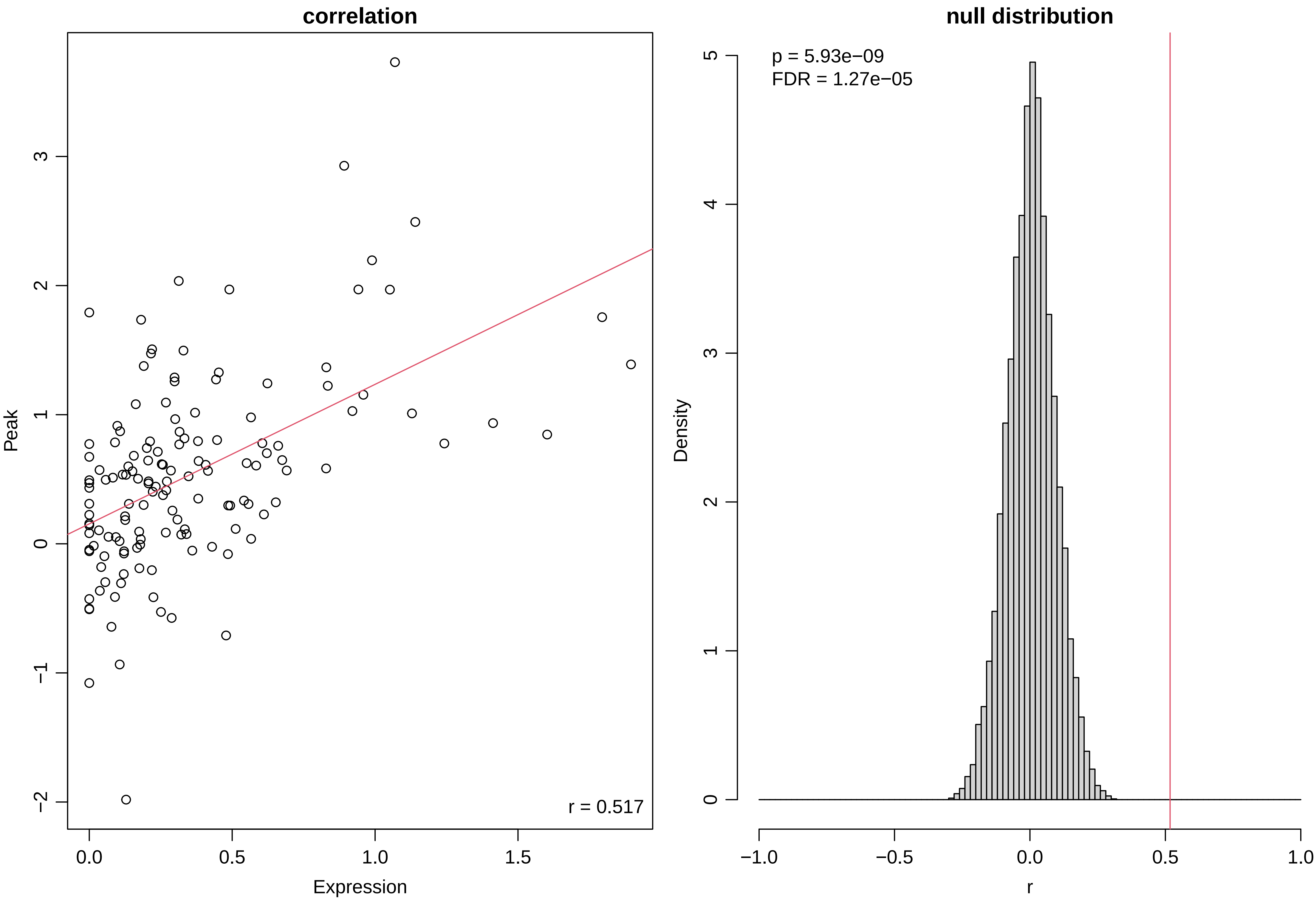

GYG2P1 vs PeakID_262728 (chrY: 12453057 − 12454068)

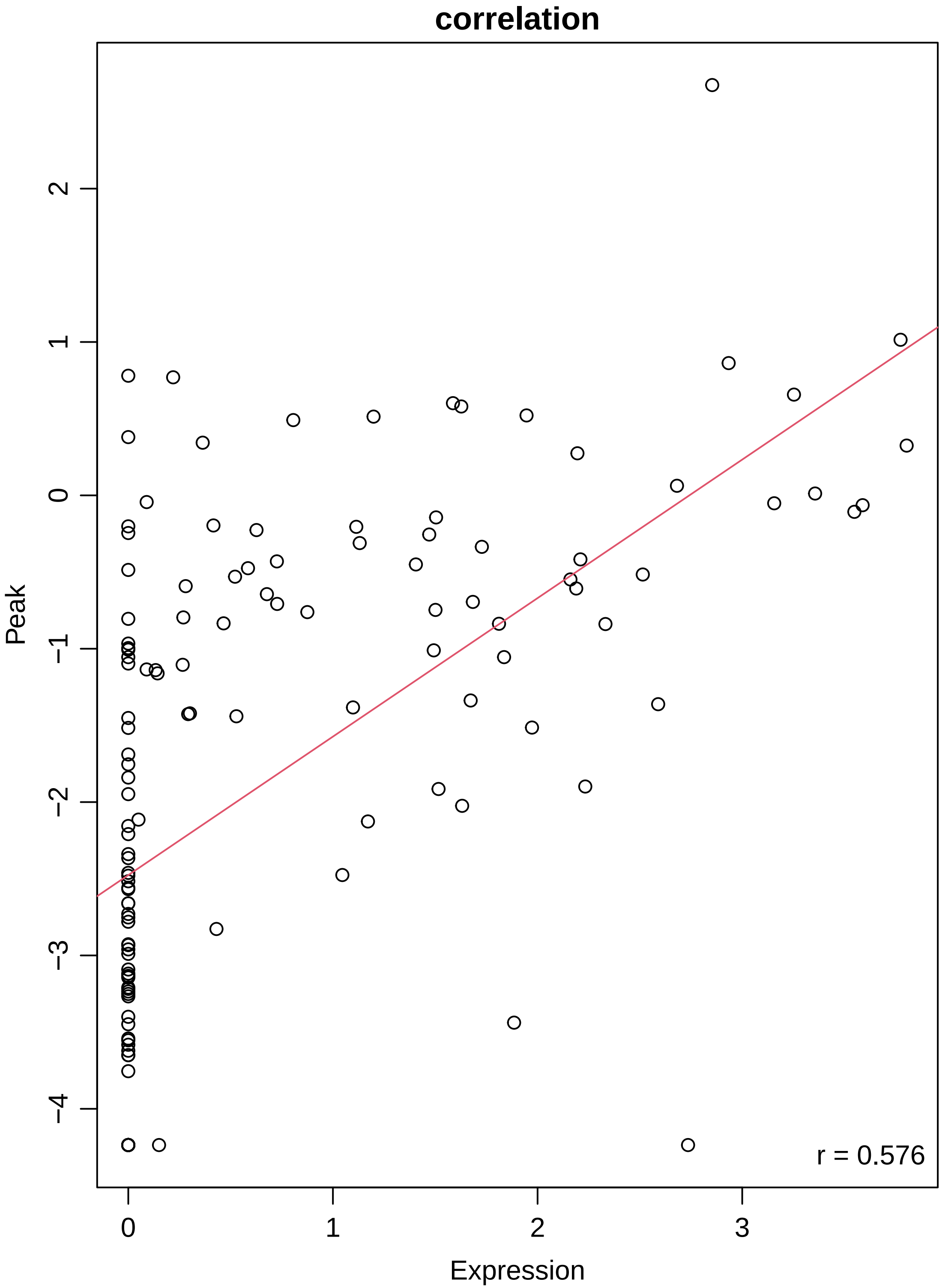

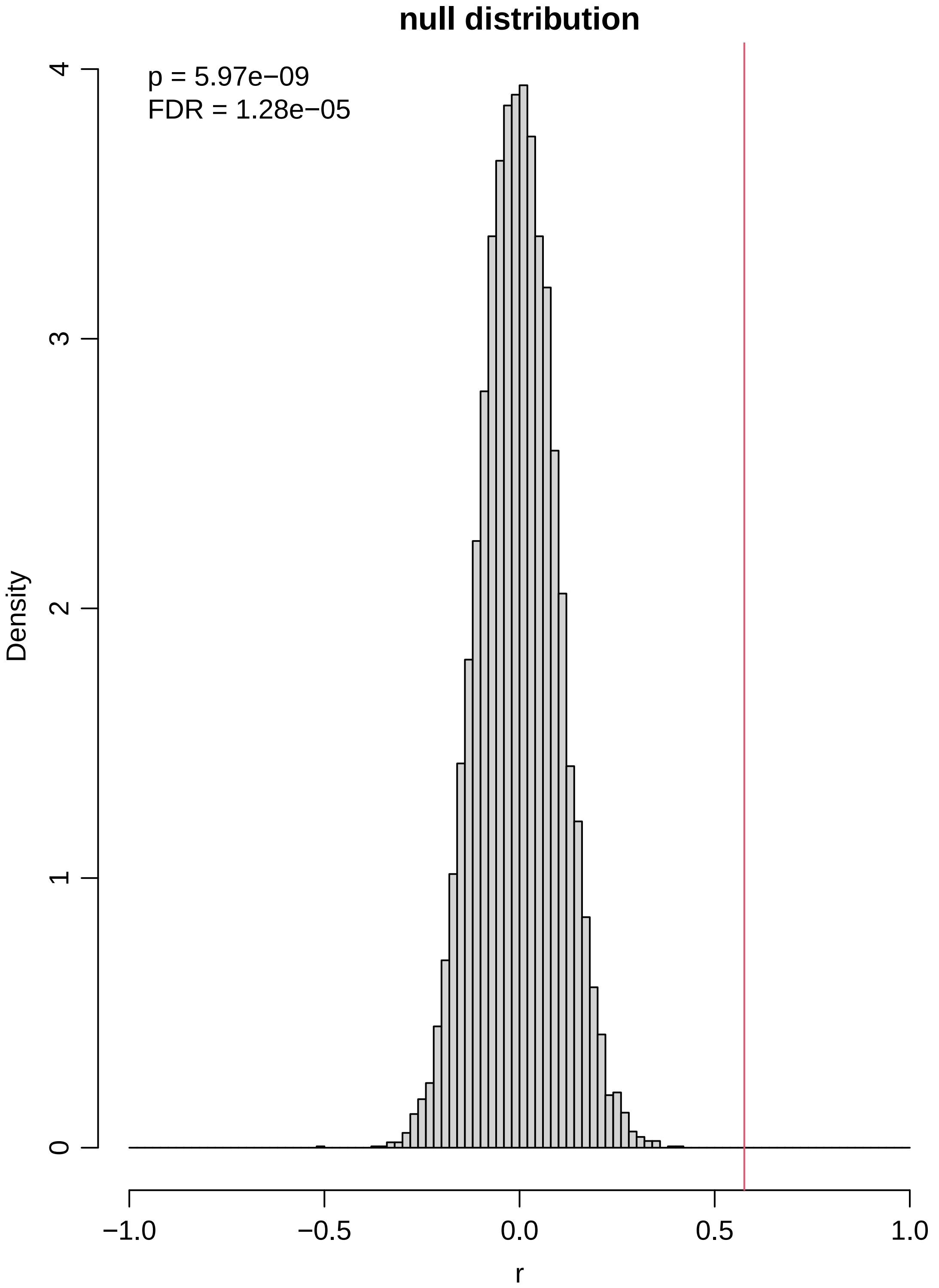

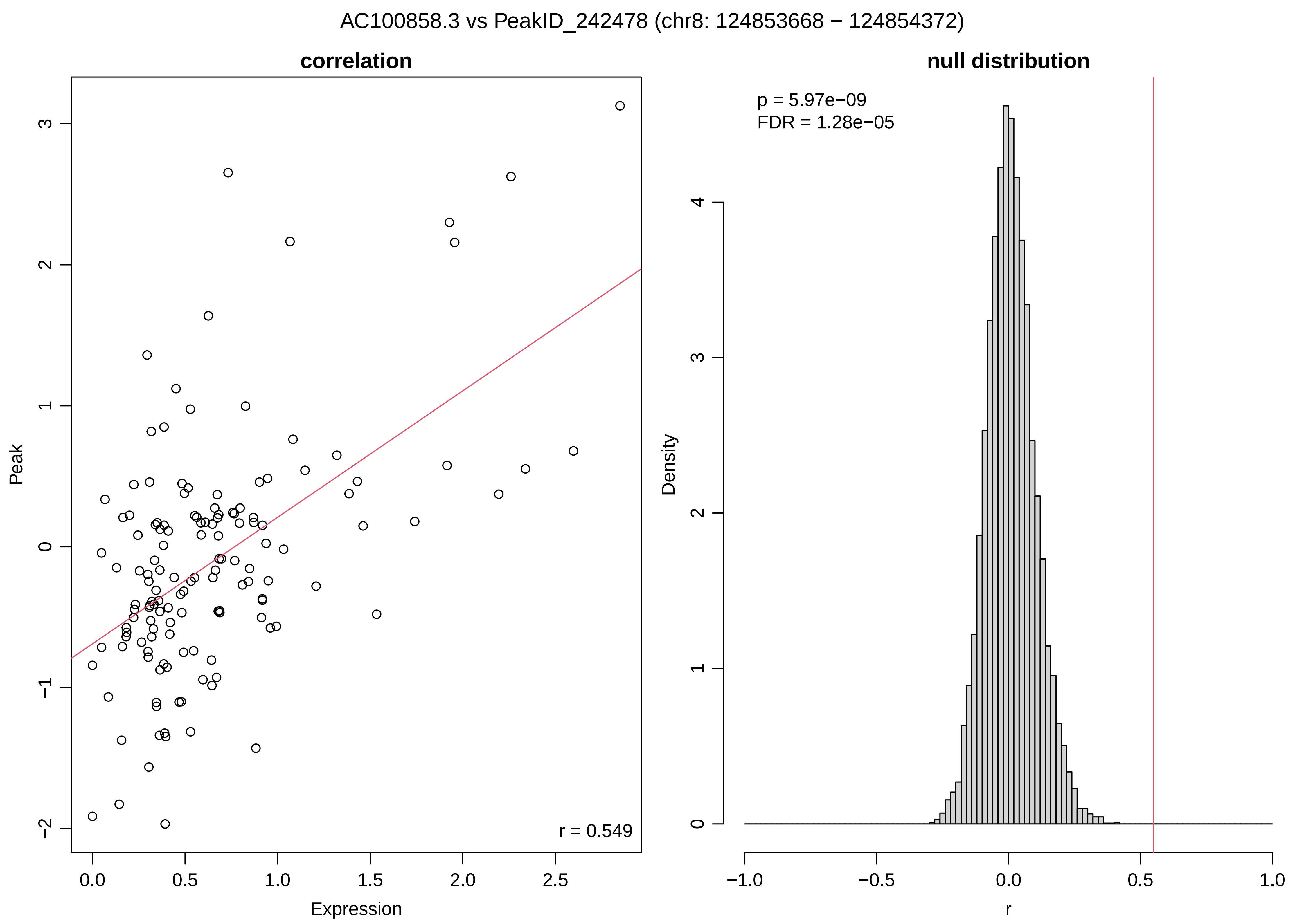
AC100858.3 vs PeakID_242478 (chr8: 124853668 − 124854372)
correlation
Peak
Expression
r = 0.549
null distribution
p = 5.97e−09
FDR = 1.28e−05
Density
r

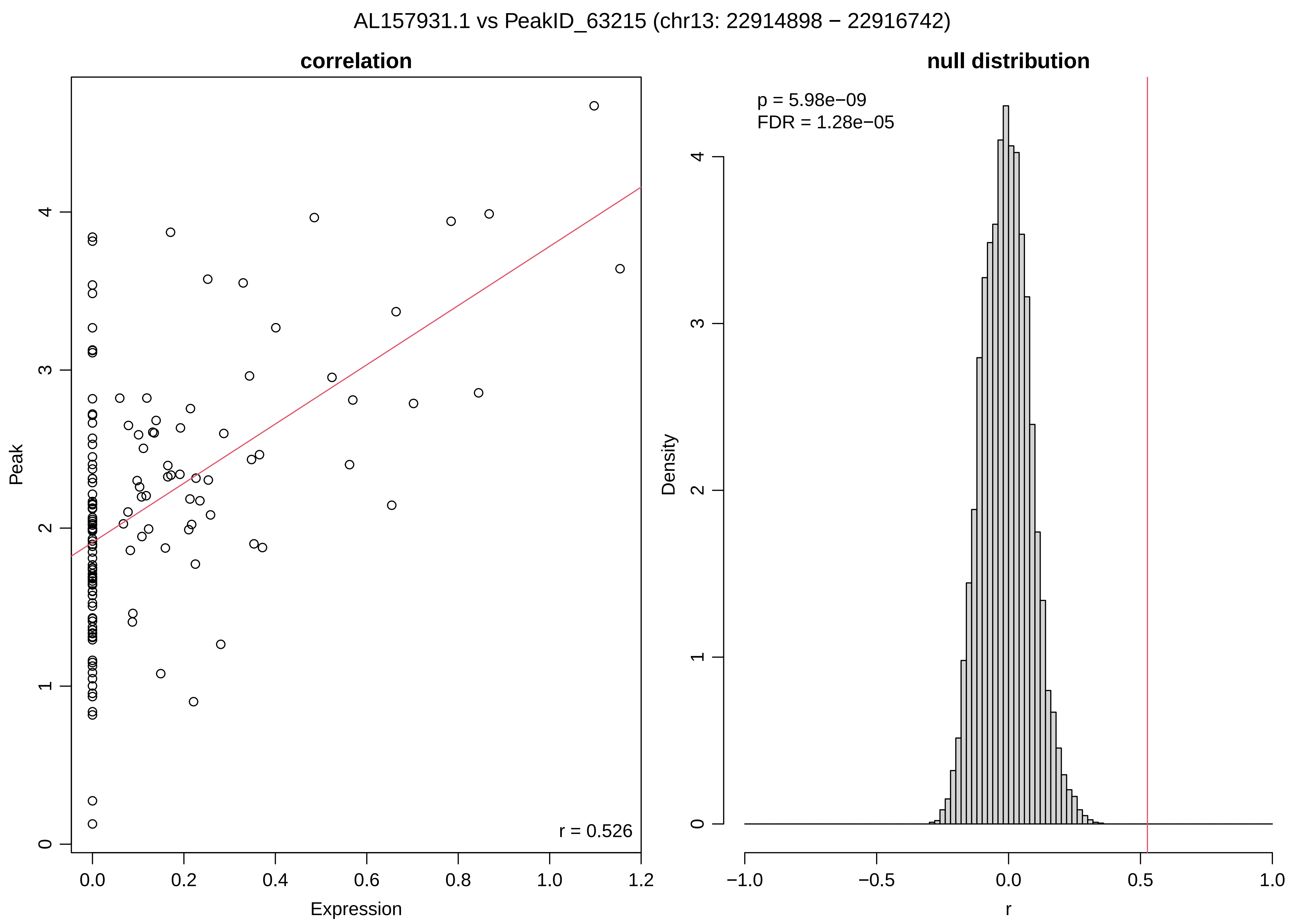

AL157931.1 vs PeakID_63215 (chr13: 22914898 − 22916742)
correlation
null distribution
Peak
Expression
r = 0.526
p = 5.98e−09
FDR = 1.28e−05
Density
r
0
1
2
3
4
0.0
0.2
0.4
0.6
0.8
1.0
1.2
−1.0
−0.5
0.0
0.5
1.0

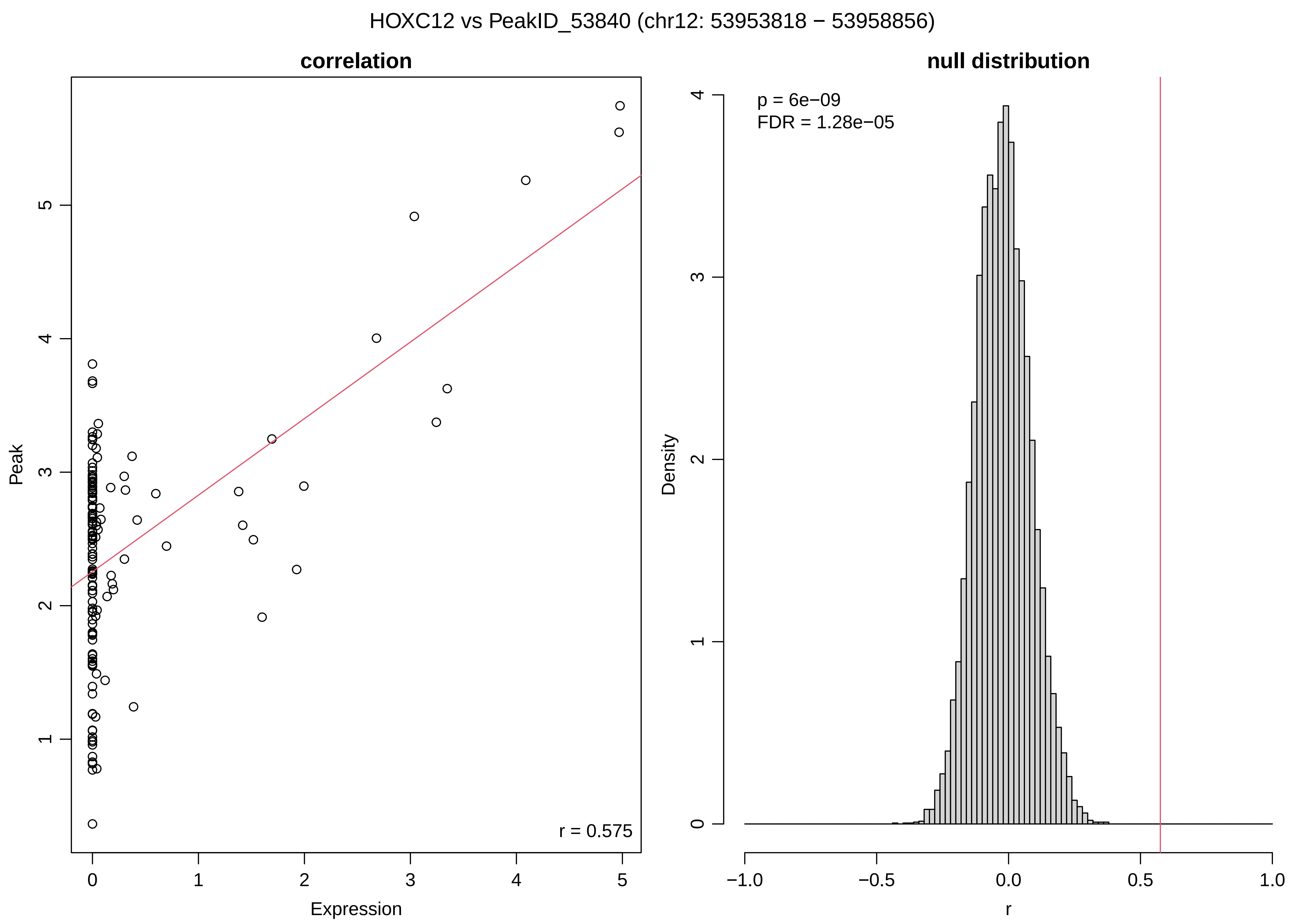
HOXC12 vs PeakID_53840 (chr12: 53953818 − 53958856)
correlation
null distribution
p = 6e−09
FDR = 1.28e−05
r = 0.575
Peak
Expression
Density
r
0
1
2
3
4
5
−1.0
−0.5
0.0
0.5
1.0

AC016582.1 vs PeakID_116818 (chr19: 37816688 − 37818104)

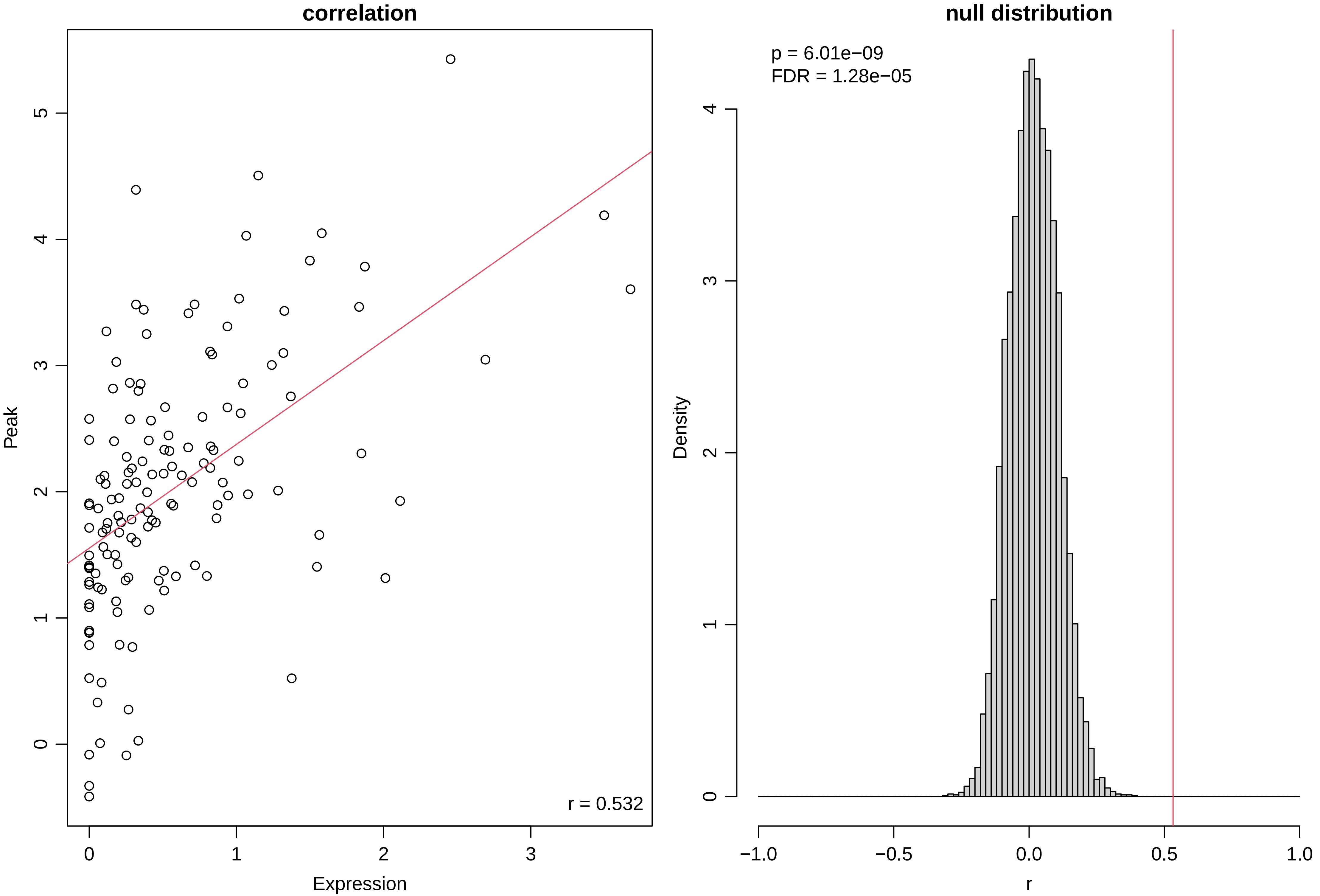

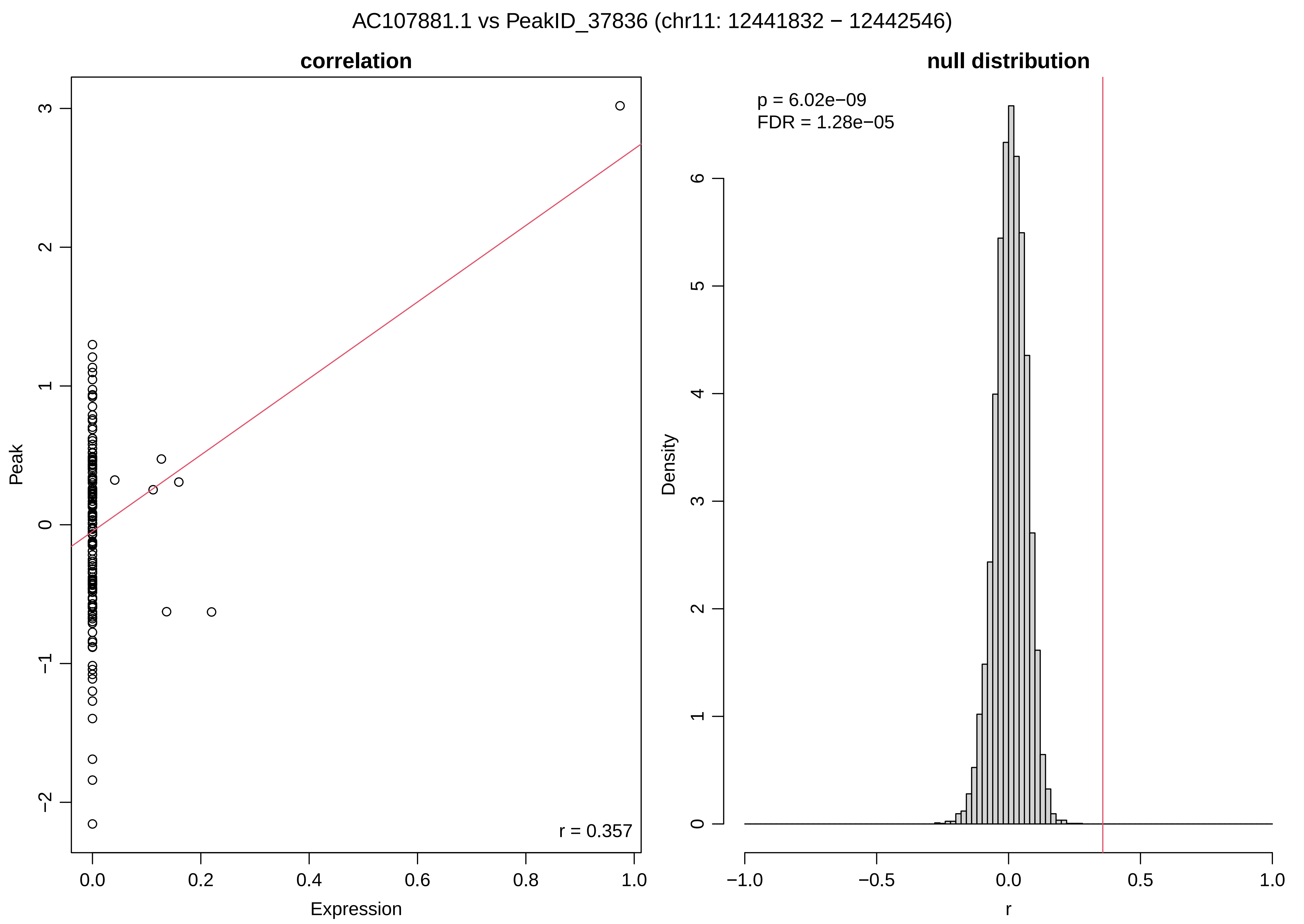
AC107881.1 vs PeakID_37836 (chr11: 12441832 − 12442546)
correlation
Peak
Expression
r = 0.357
0.0
0.2
0.4
0.6
0.8
1.0
−2
−1
0
1
2
3
null distribution
p = 6.02e−09
FDR = 1.28e−05
Density
r
−1.0
−0.5
0.0
0.5
1.0
0
1
2
3
4
5
6

RPL21P104 vs PeakID_55429 (chr12: 61864465 − 61866226)

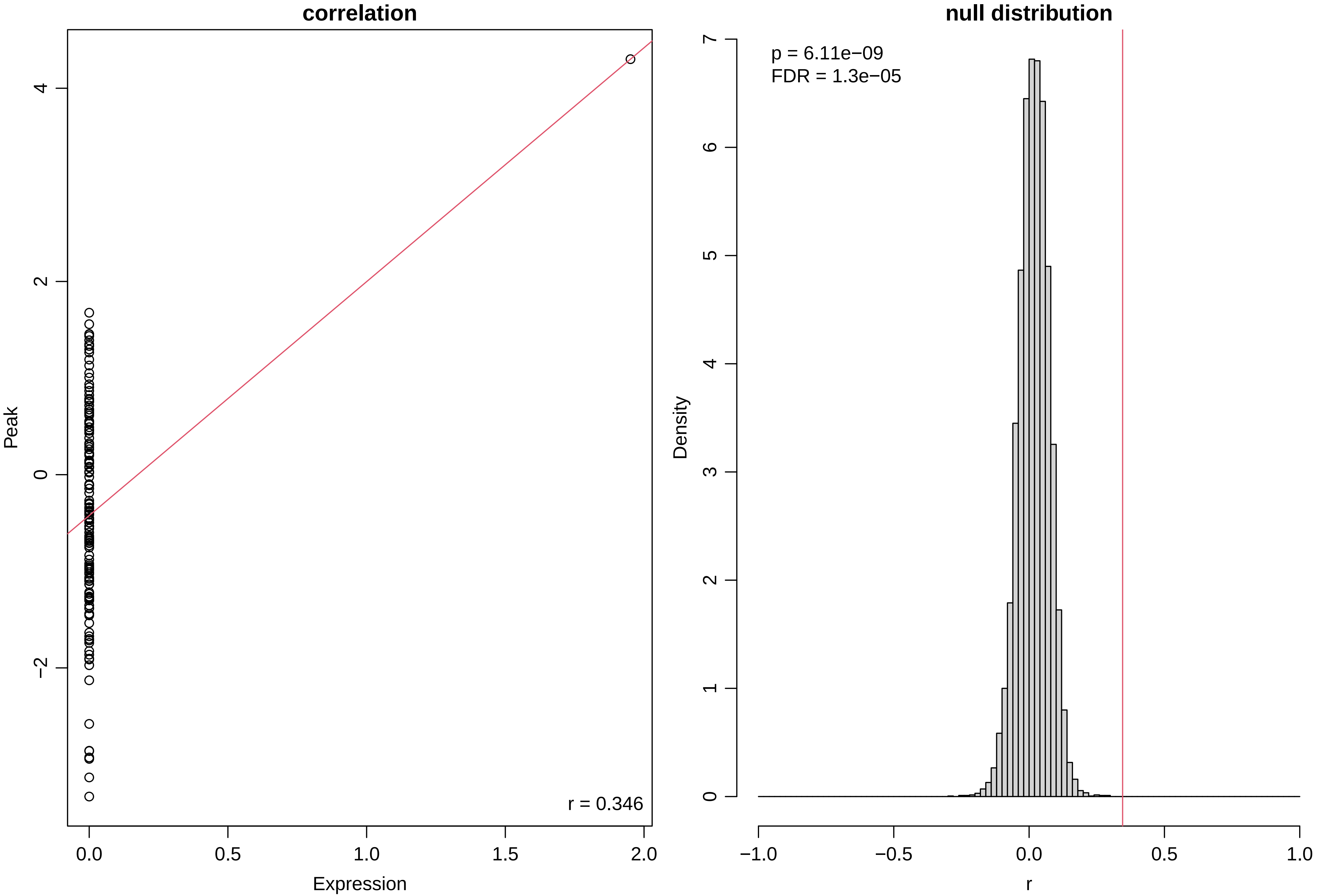

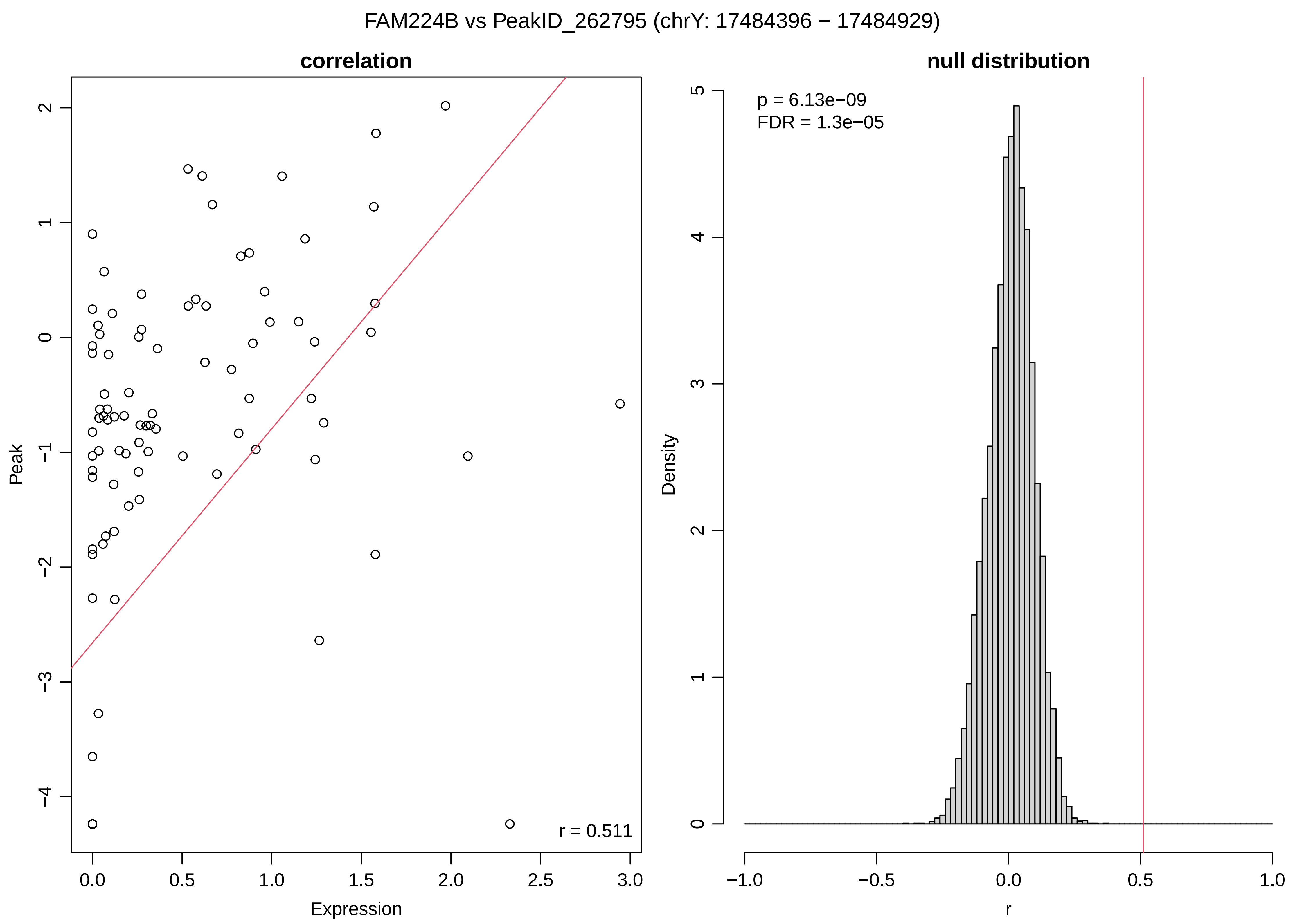
FAM224B vs PeakID_262795 (chrY: 17484396 − 17484929)
correlation
Peak
Expression
r = 0.511
null distribution
p = 6.13e−09
FDR = 1.3e−05
Density
r

MAEL vs PeakID_15454 (chr1: 166988839 − 166990022)

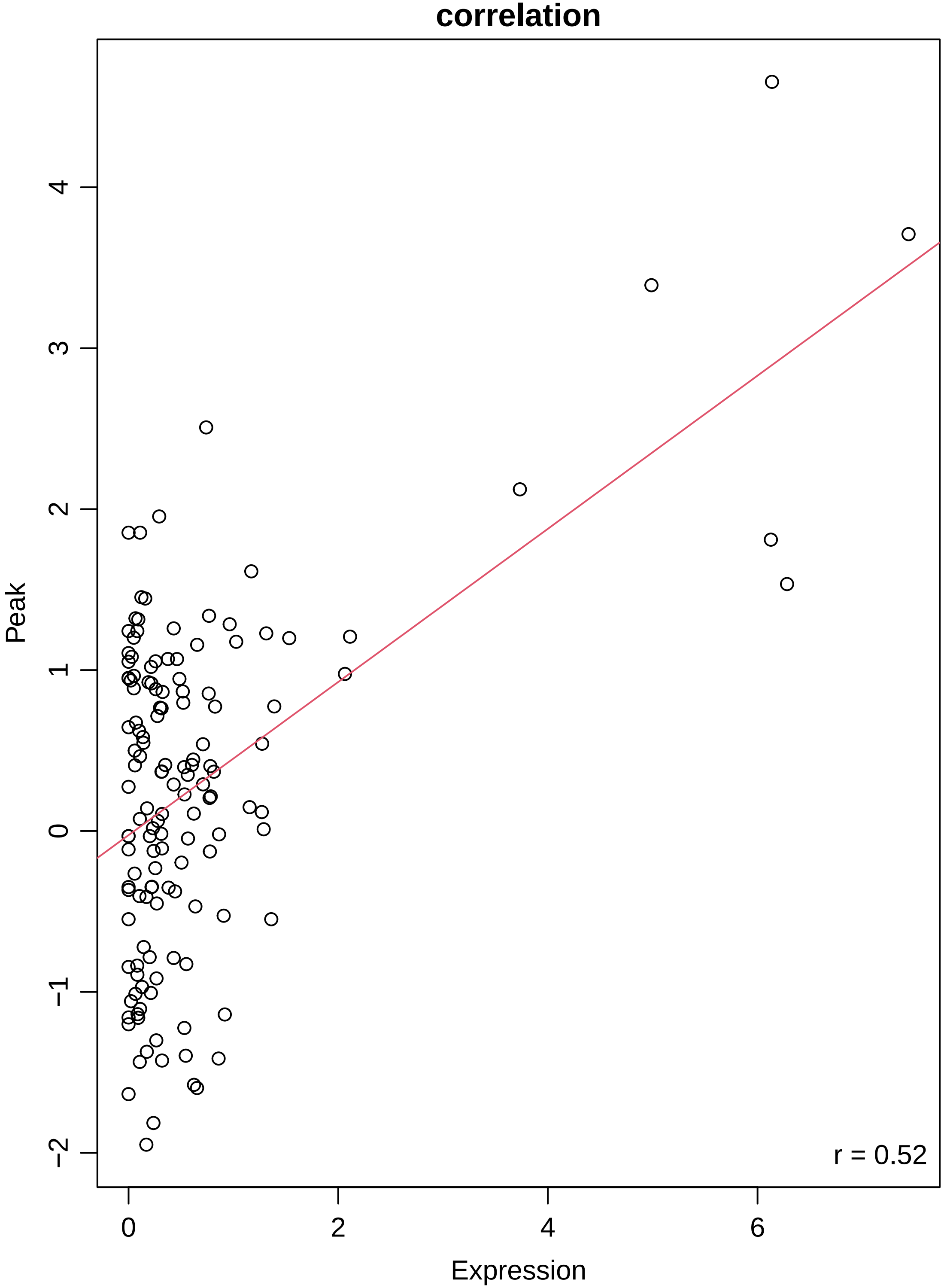

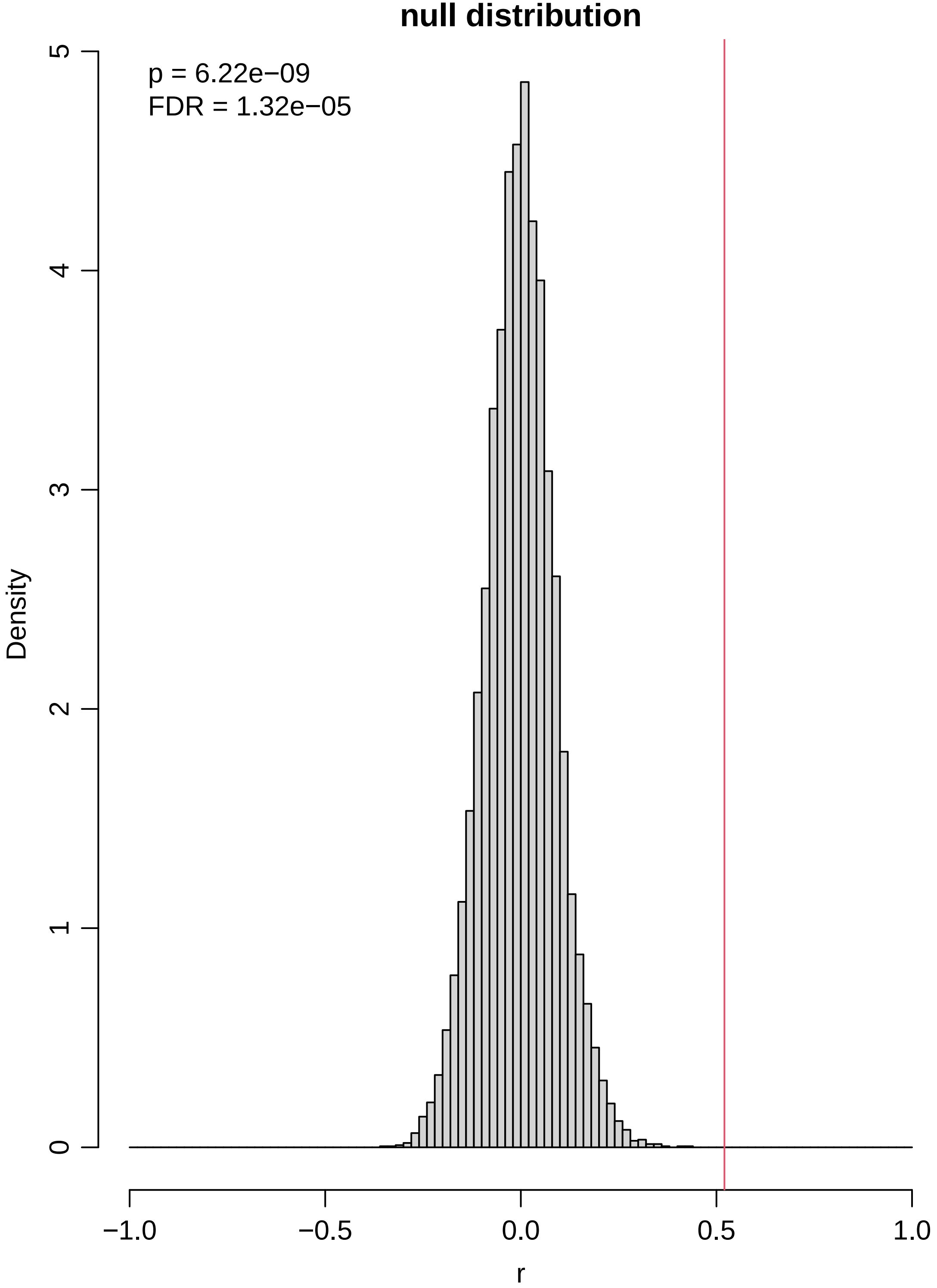

ZIC5 vs PeakID_67431 (chr13: 99978957 − 99987208)

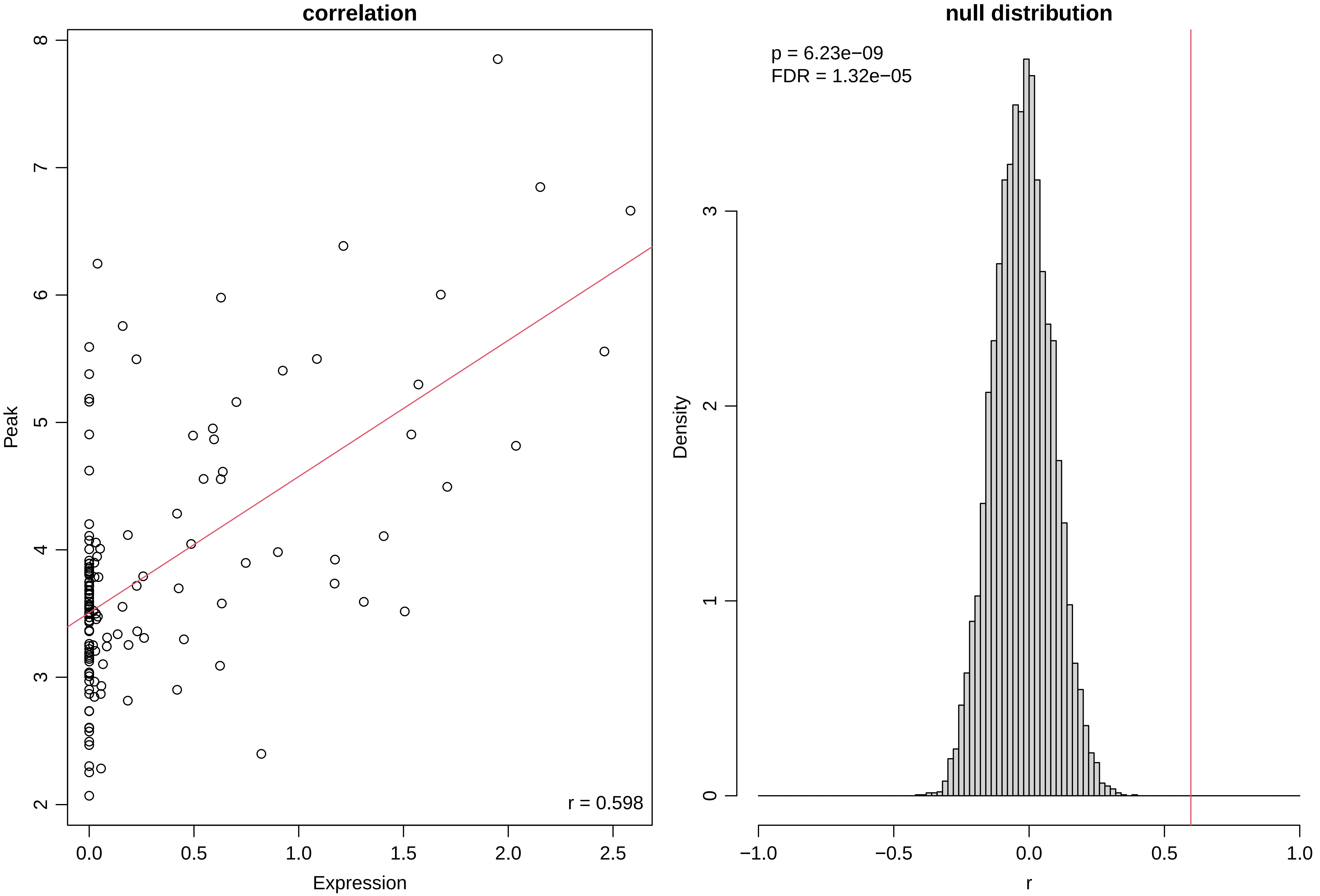

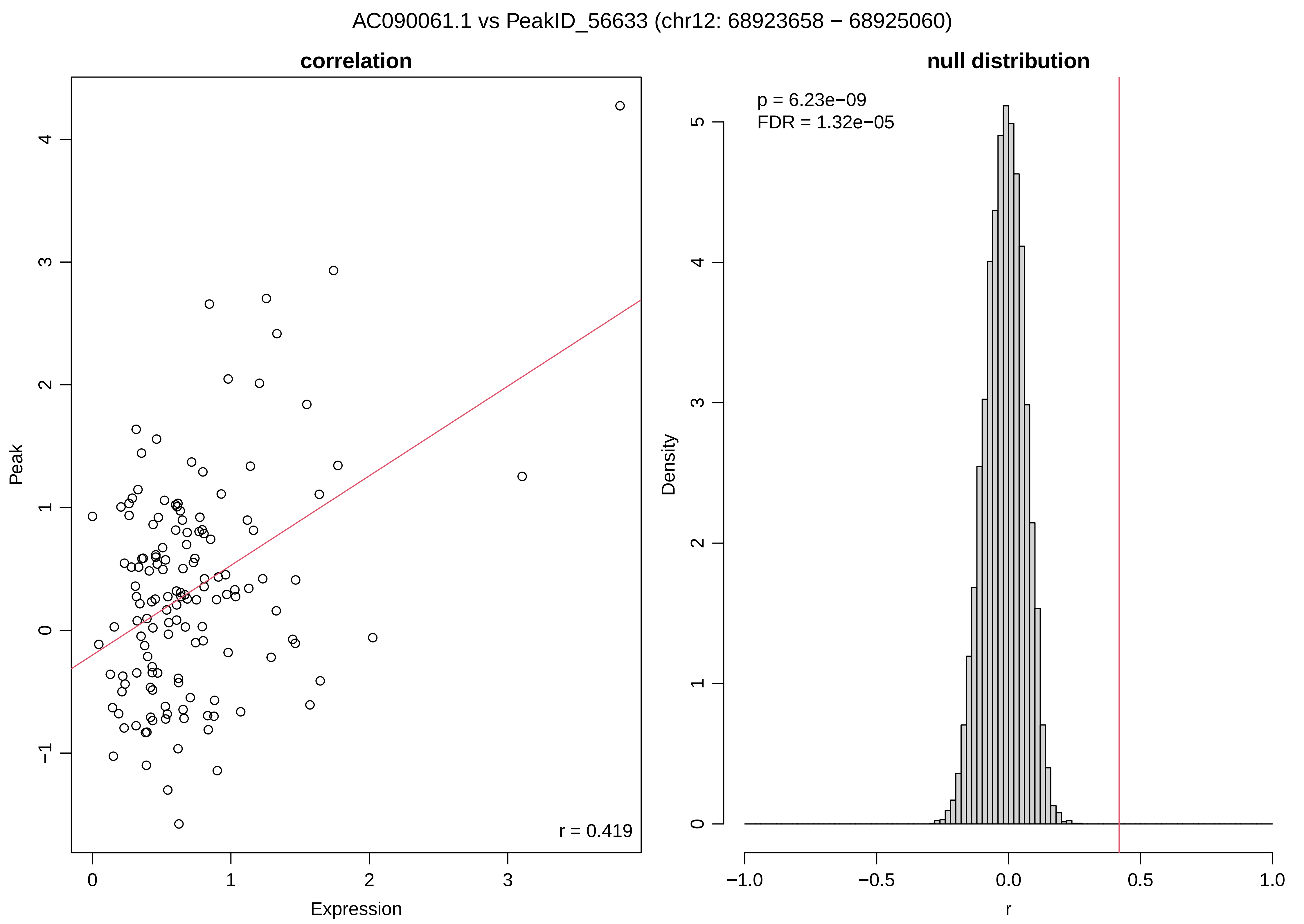
AC090061.1 vs PeakID_56633 (chr12: 68923658 − 68925060)
correlation
Peak
Expression
r = 0.419
null distribution
p = 6.23e−09
FDR = 1.32e−05
Density
r

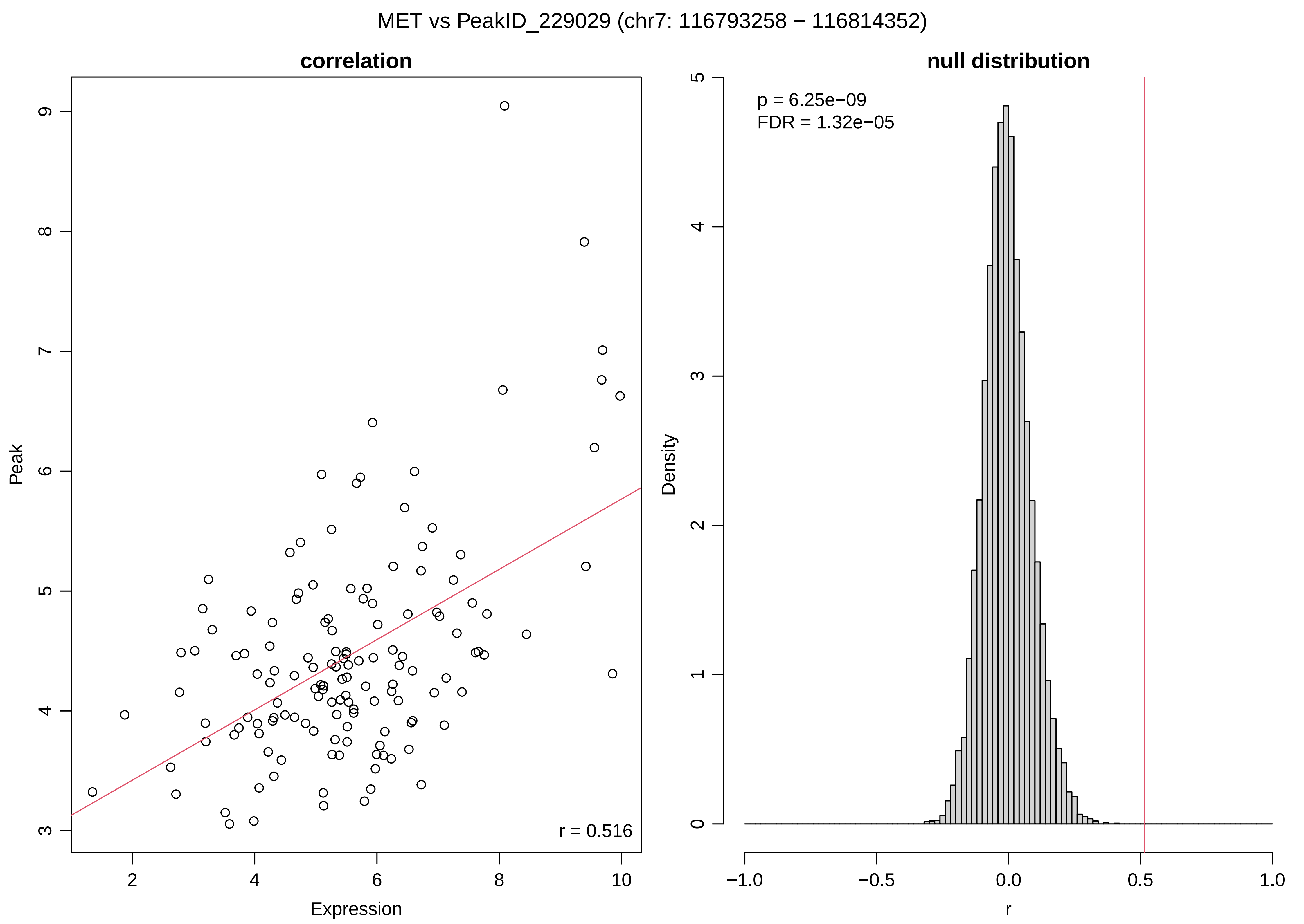
MET vs PeakID_229029 (chr7: 116793258 − 116814352)
correlation
Peak
Expression
r = 0.516
null distribution
Density
r
p = 6.25e−09
FDR = 1.32e−05

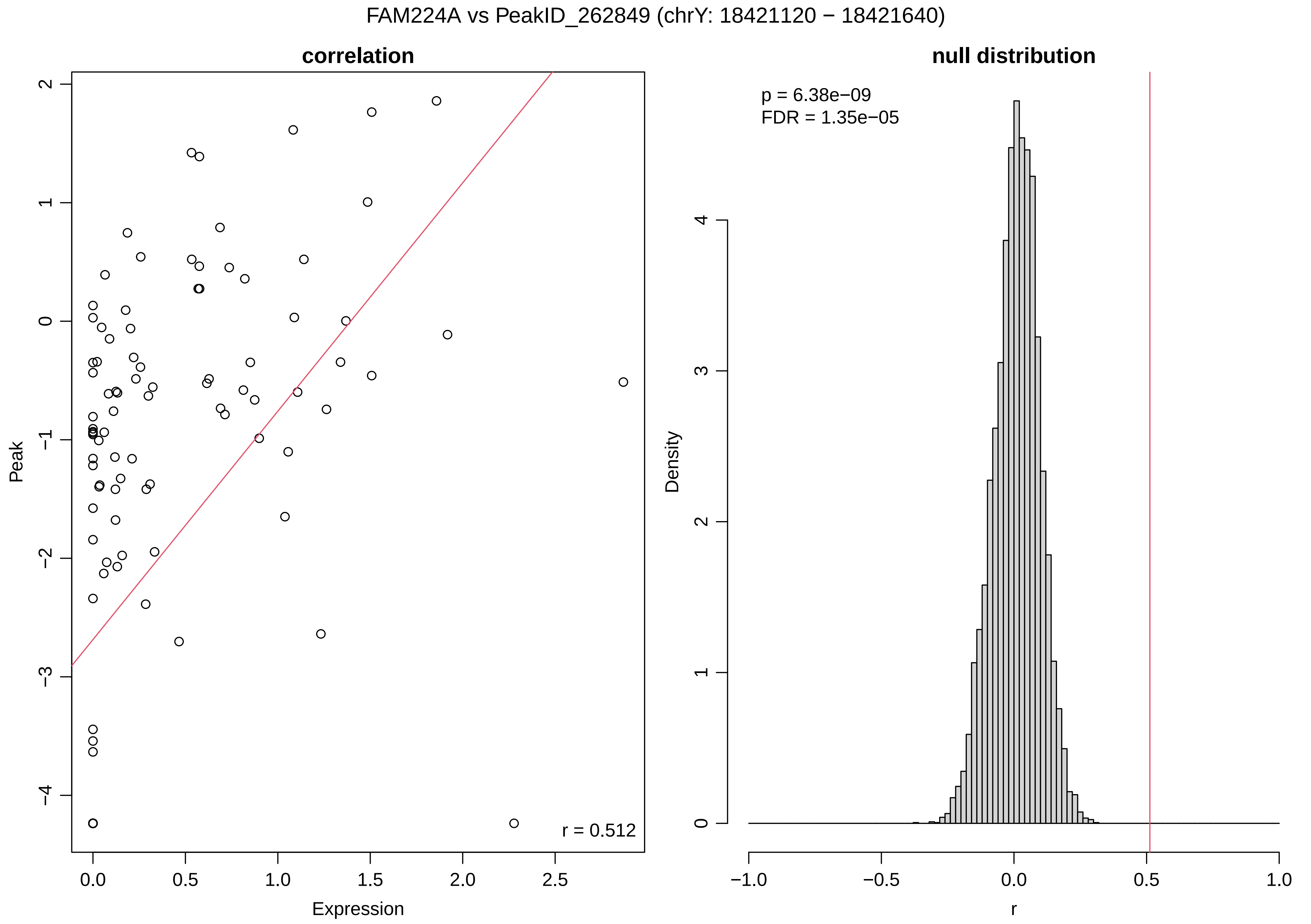
FAM224A vs PeakID_262849 (chrY: 18421120 − 18421640)
correlation
Peak
Expression
r = 0.512
null distribution
p = 6.38e−09
FDR = 1.35e−05
Density
r

EYA2 vs PeakID_147271 (chr20: 46893366 − 46902306)

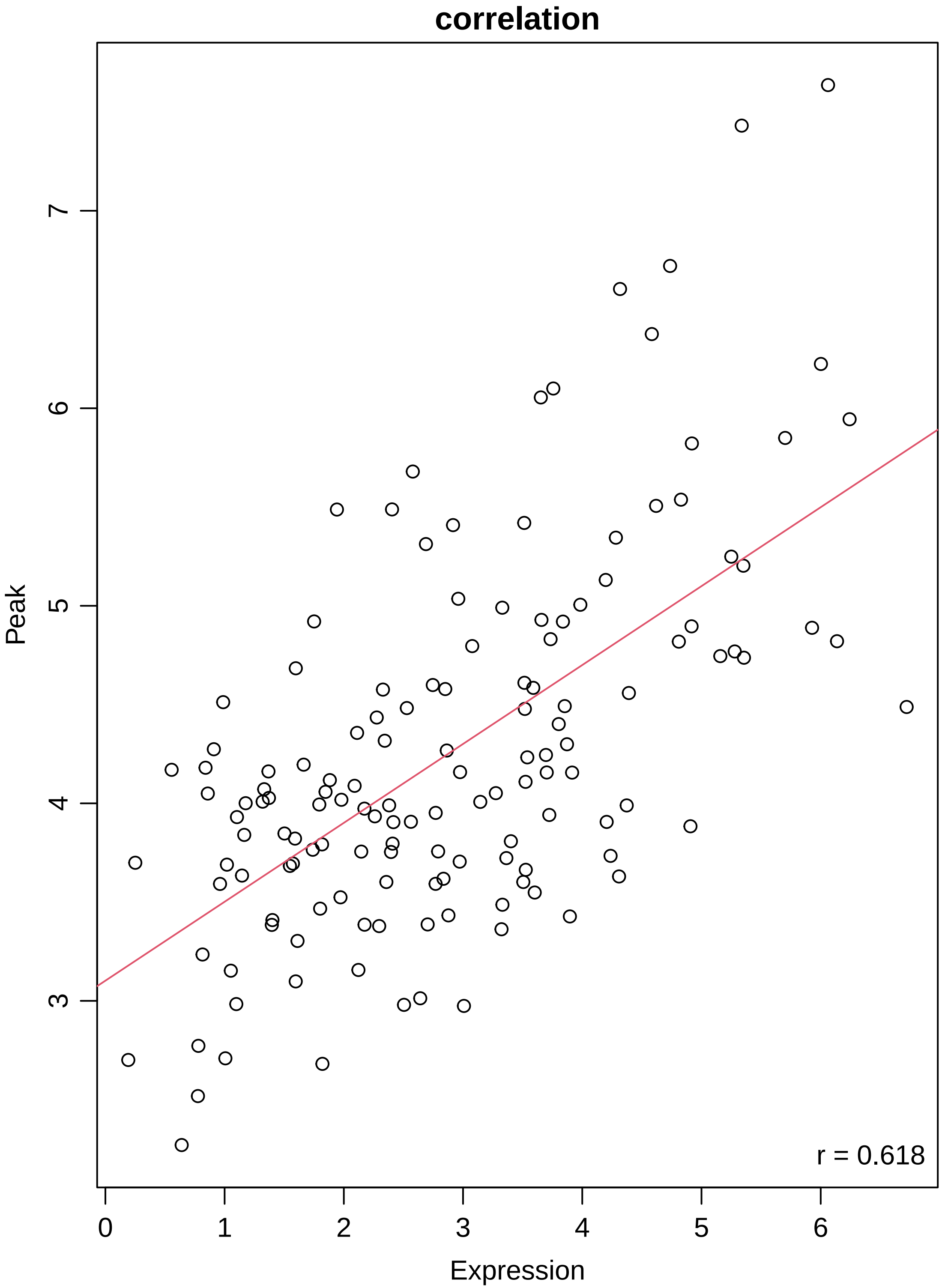

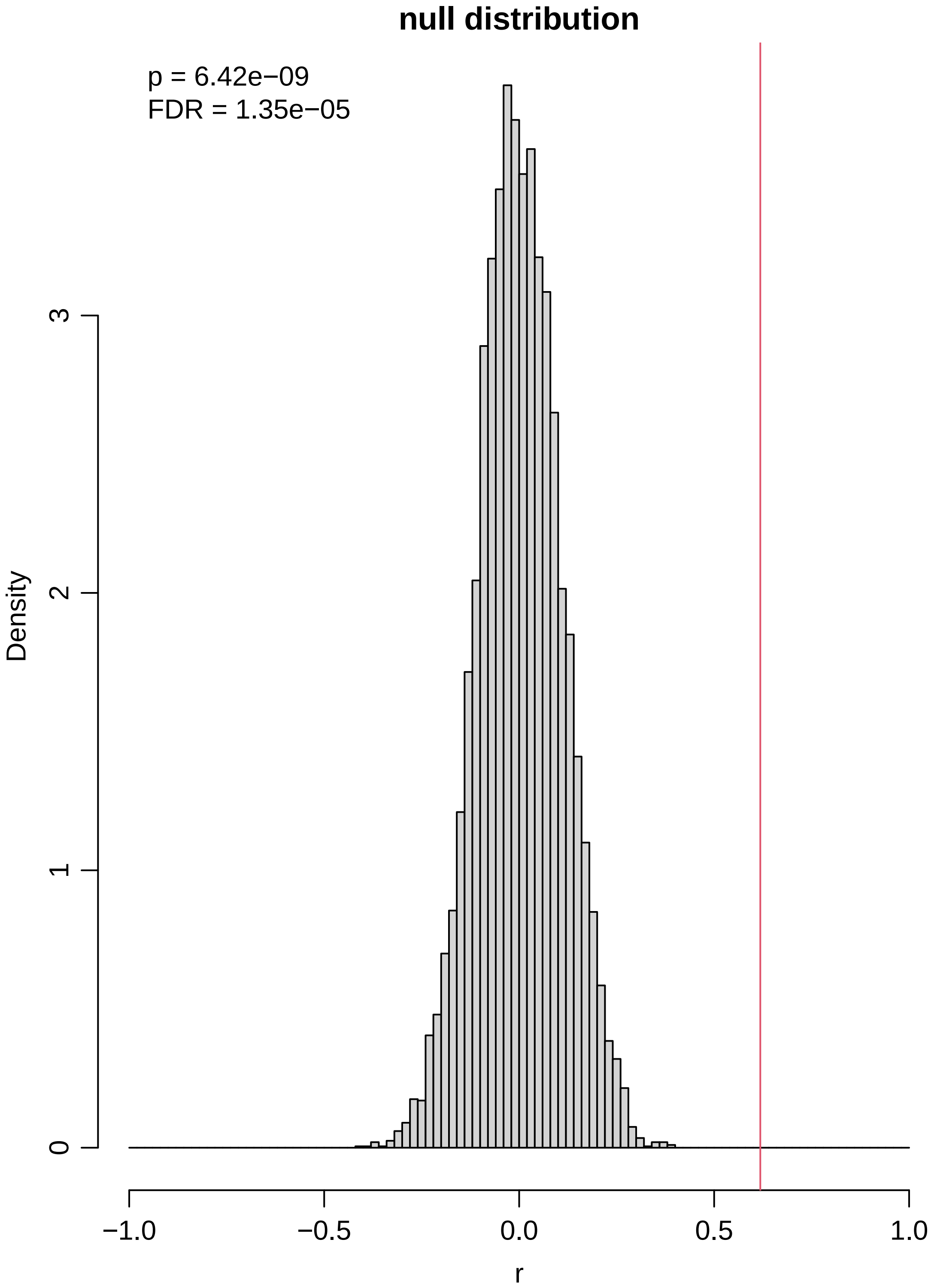

AC025423.2 vs PeakID_56674 (chr12: 69004581 − 69013537)

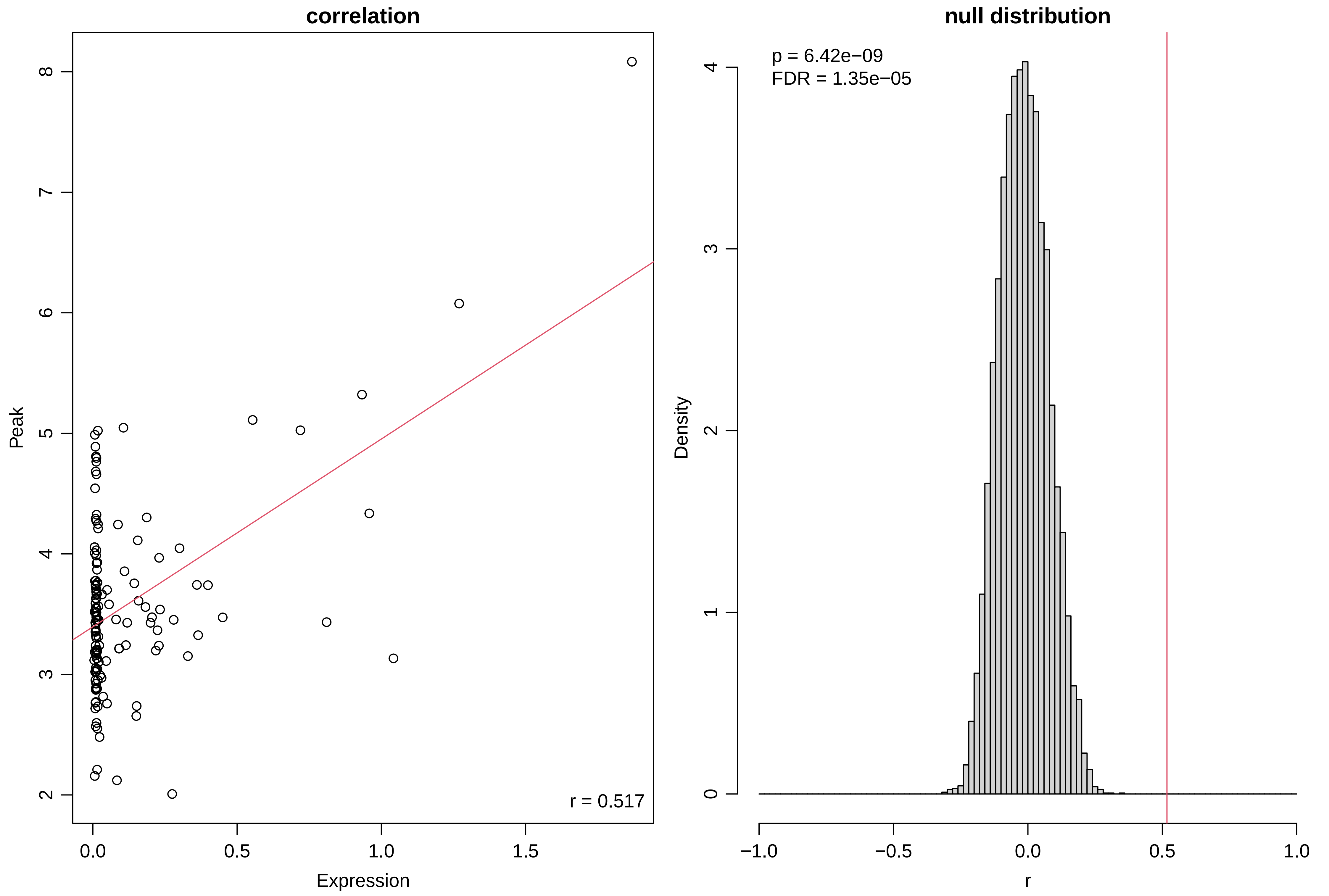

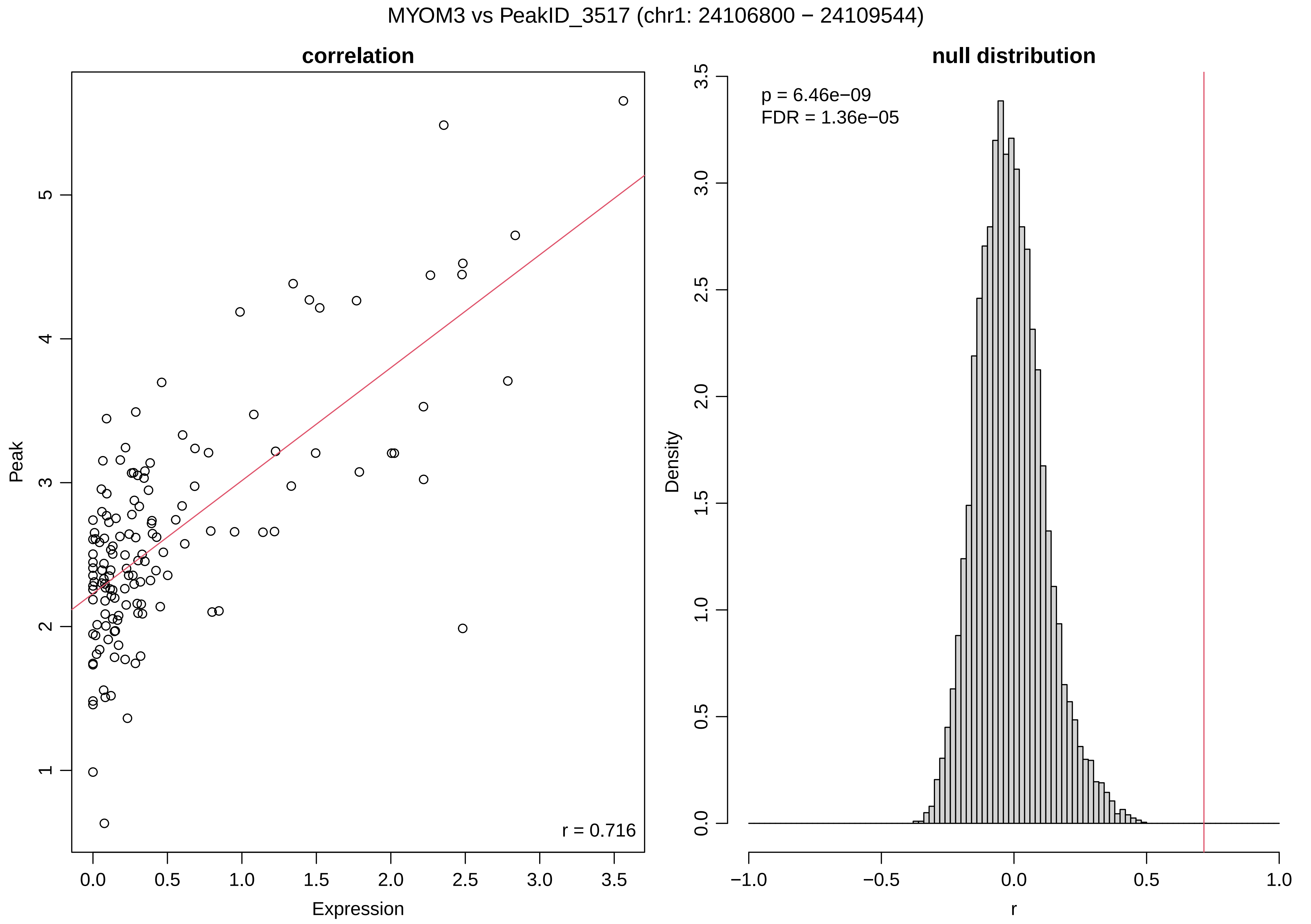
MYOM3 vs PeakID_3517 (chr1: 24106800 − 24109544)
correlation
Peak
Expression
r = 0.716
null distribution
p = 6.46e−09
FDR = 1.36e−05
Density
r

YEATS4 vs PeakID_56885 (chr12: 69572871 − 69573139)

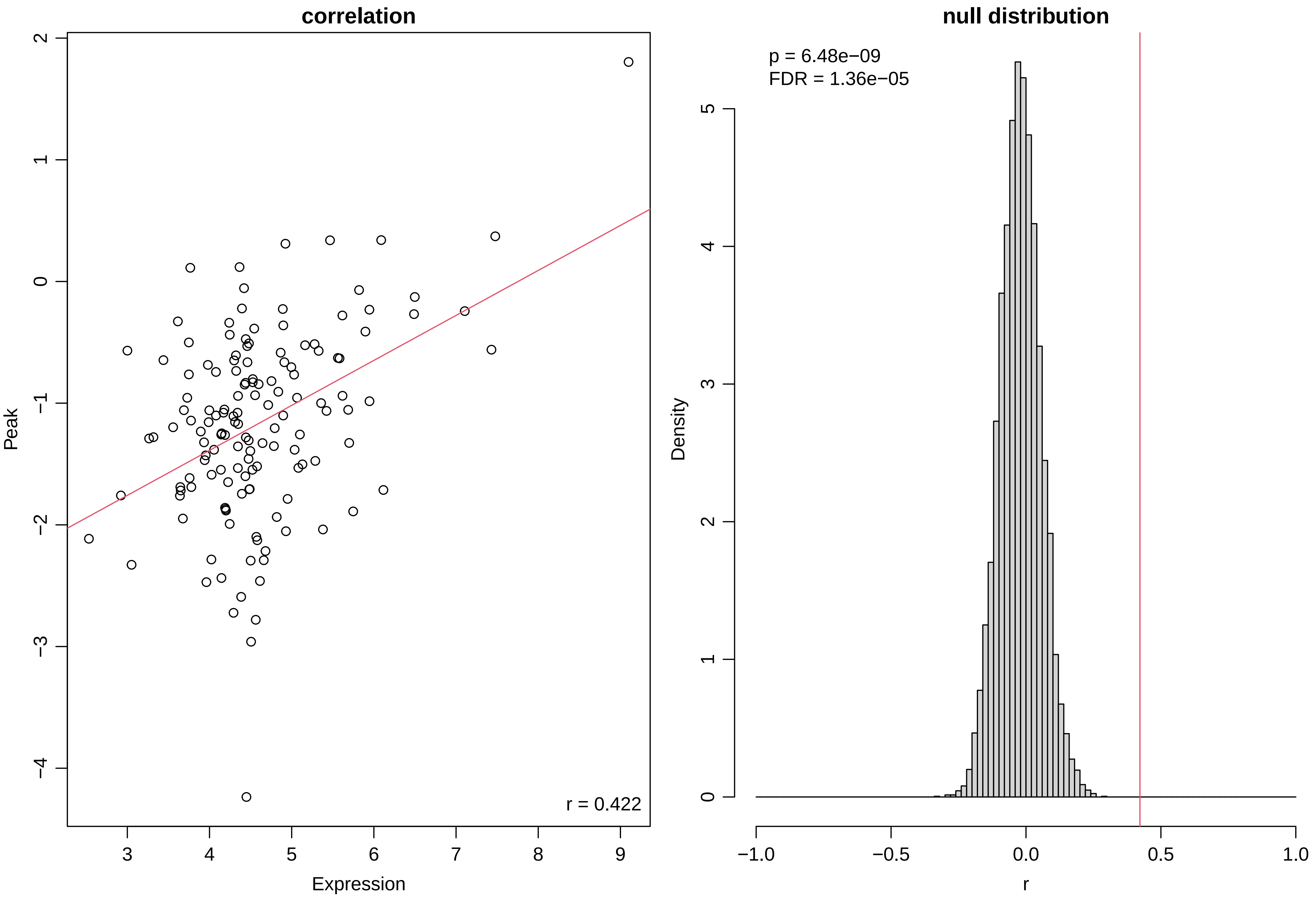

ASPH vs PeakID_237344 (chr8: 61778825 − 61784821)

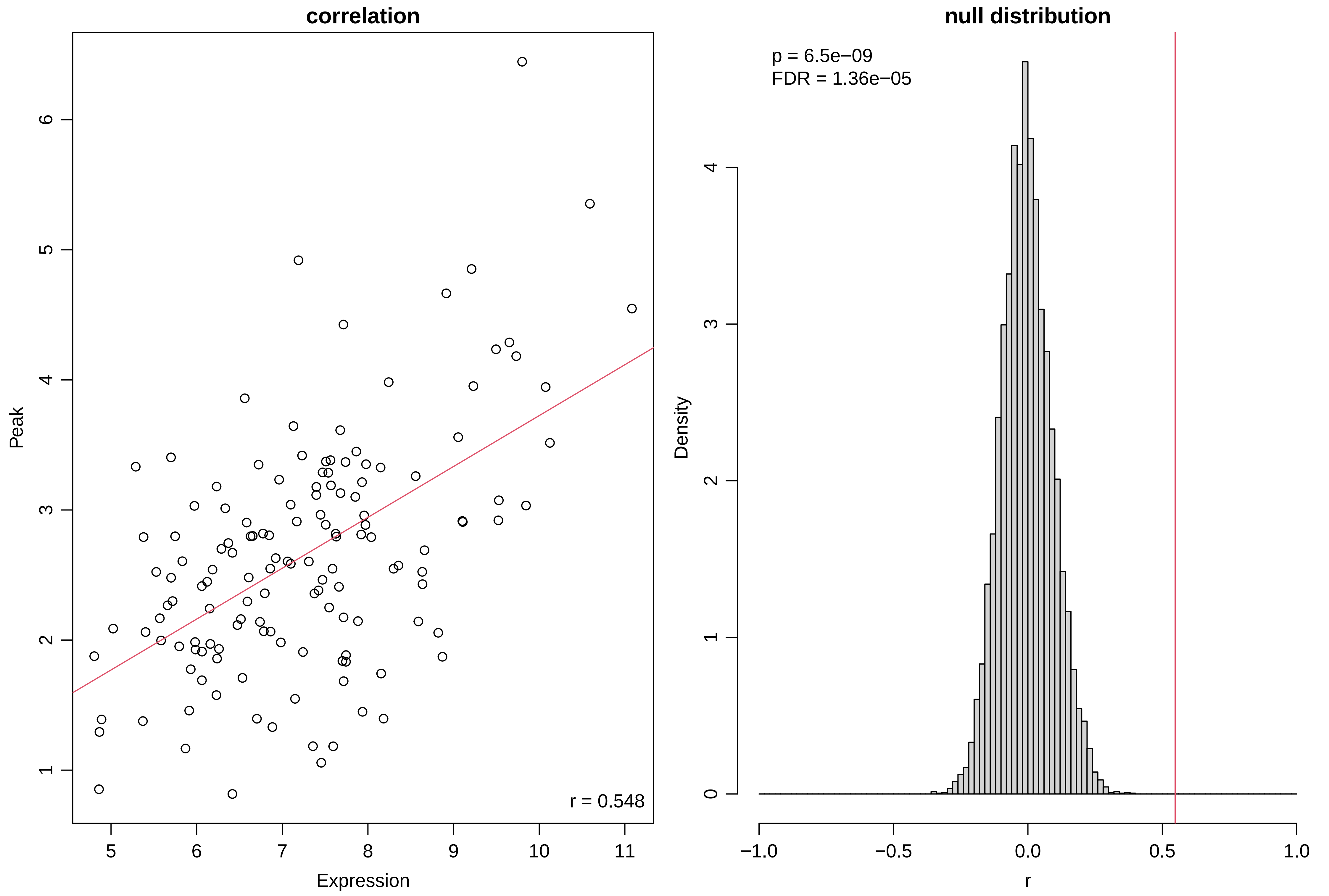

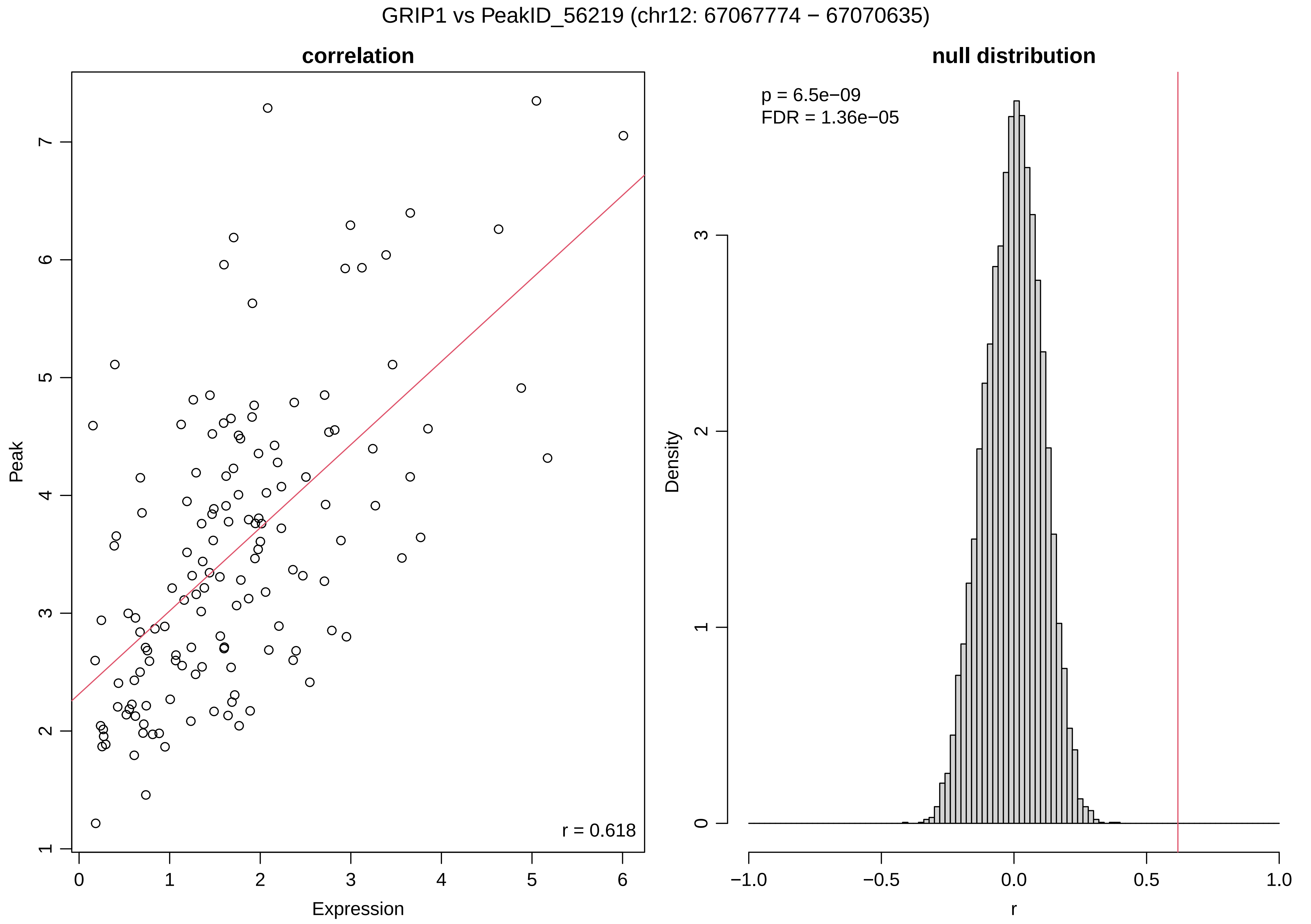

GRIP1 vs PeakID_56219 (chr12: 67067774 − 67070635)
correlation
Peak
Expression
r = 0.618
null distribution
p = 6.5e−09
FDR = 1.36e−05
Density
r

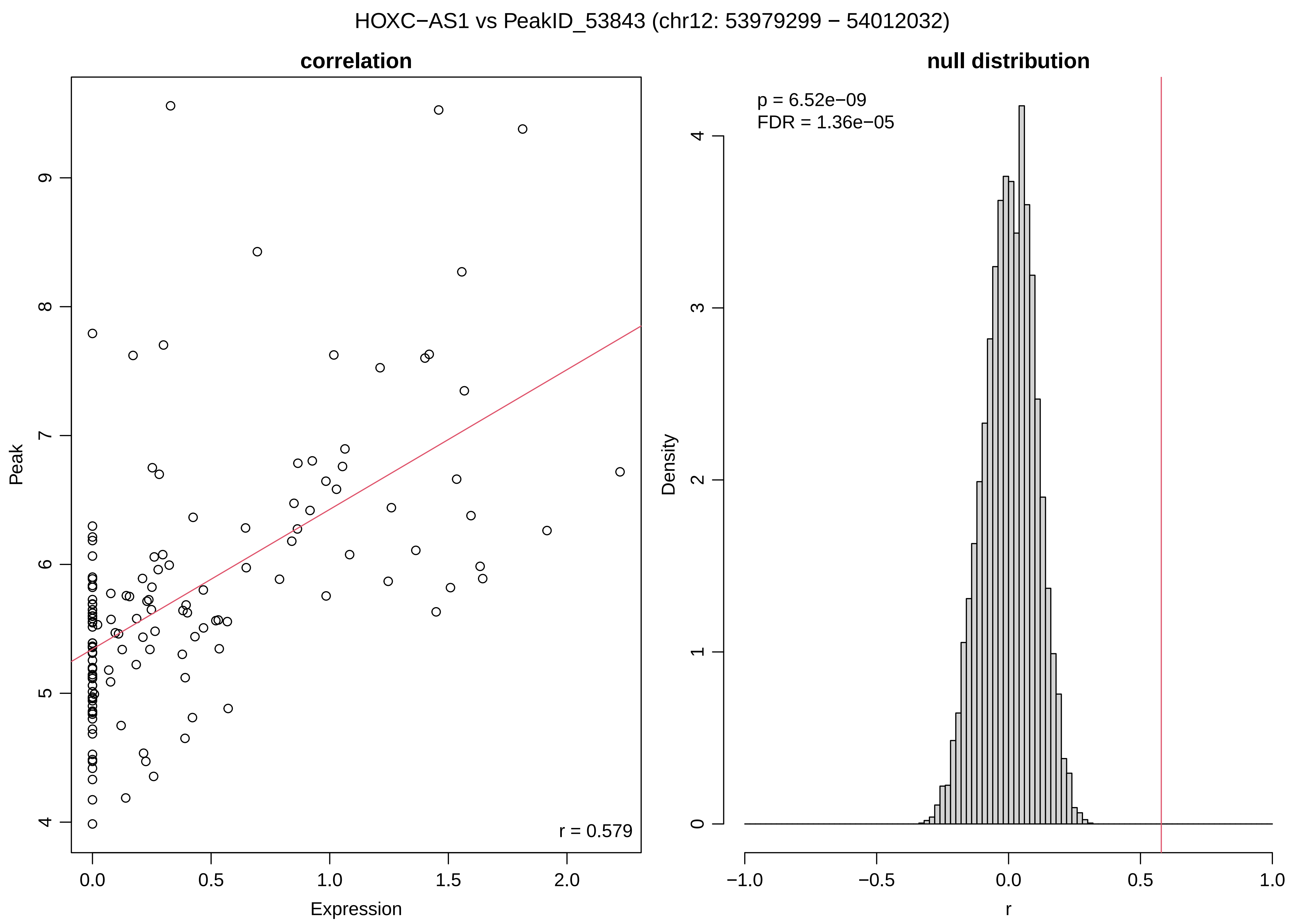

HOXC−AS1 vs PeakID_53843 (chr12: 53979299 − 54012032)
correlation
null distribution
p = 6.52e−09
FDR = 1.36e−05
r = 0.579
Peak
Expression
Density
r

RPL21P104 vs PeakID_55394 (chr12: 61811385 − 61813213)

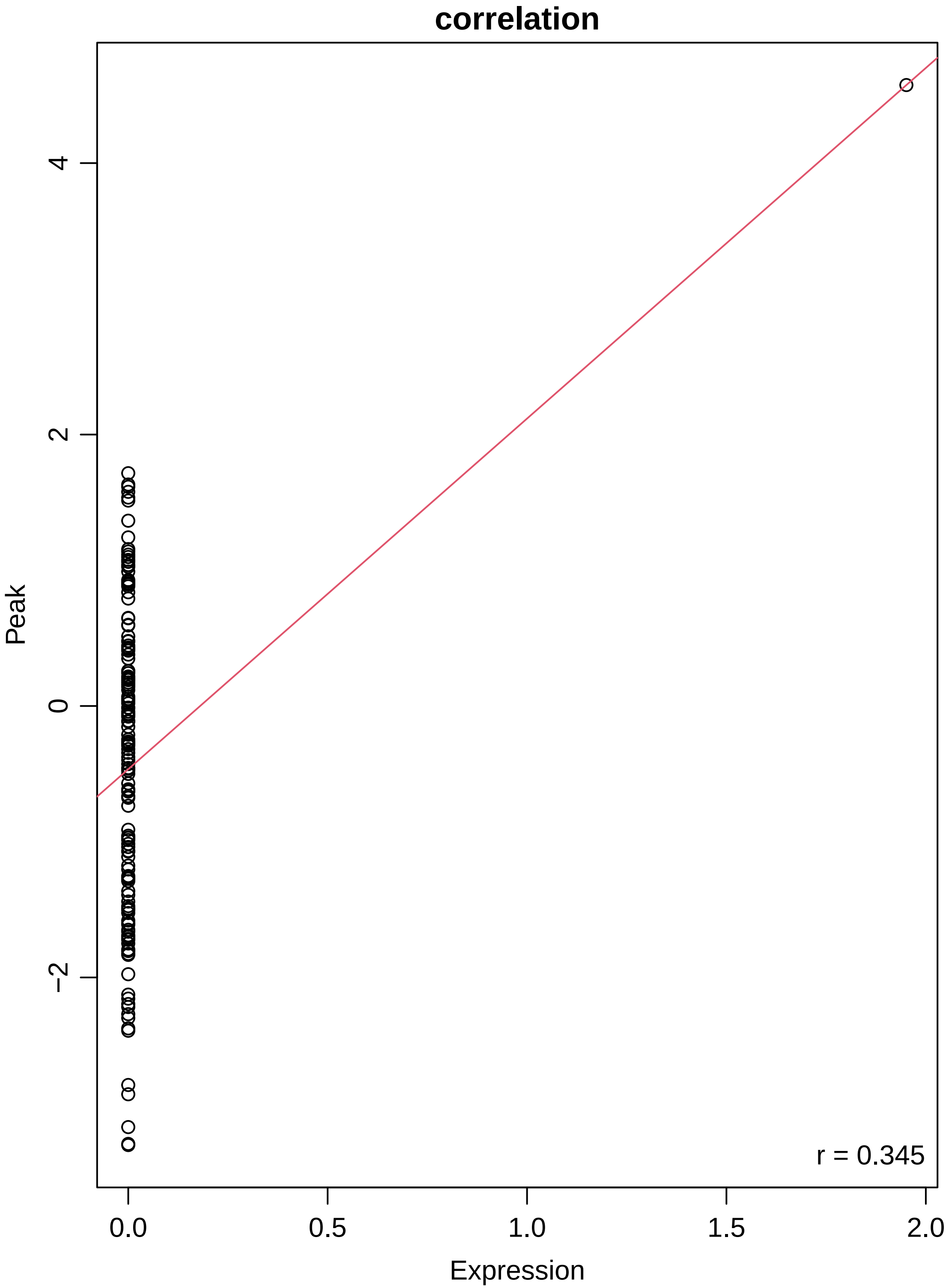

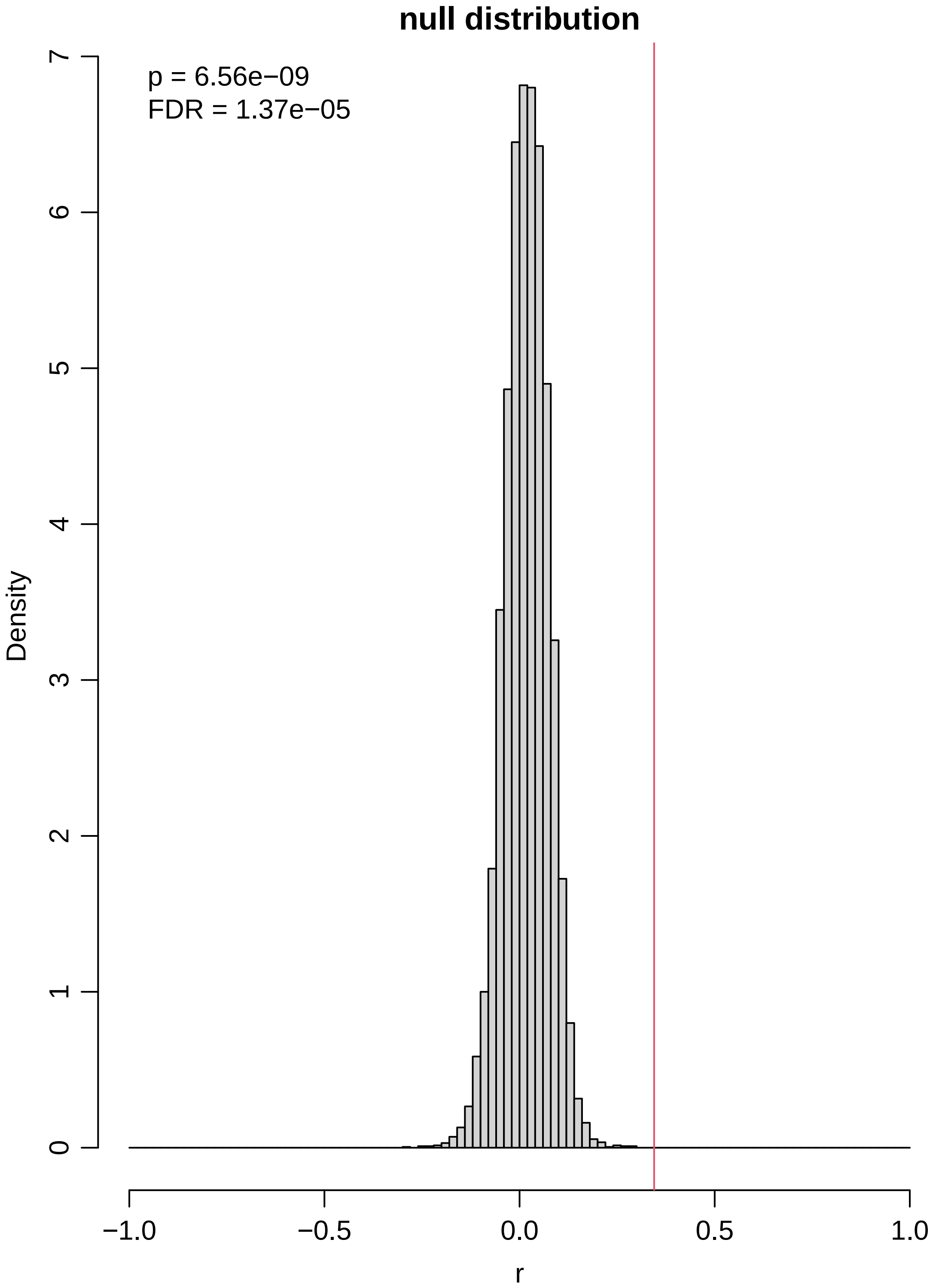

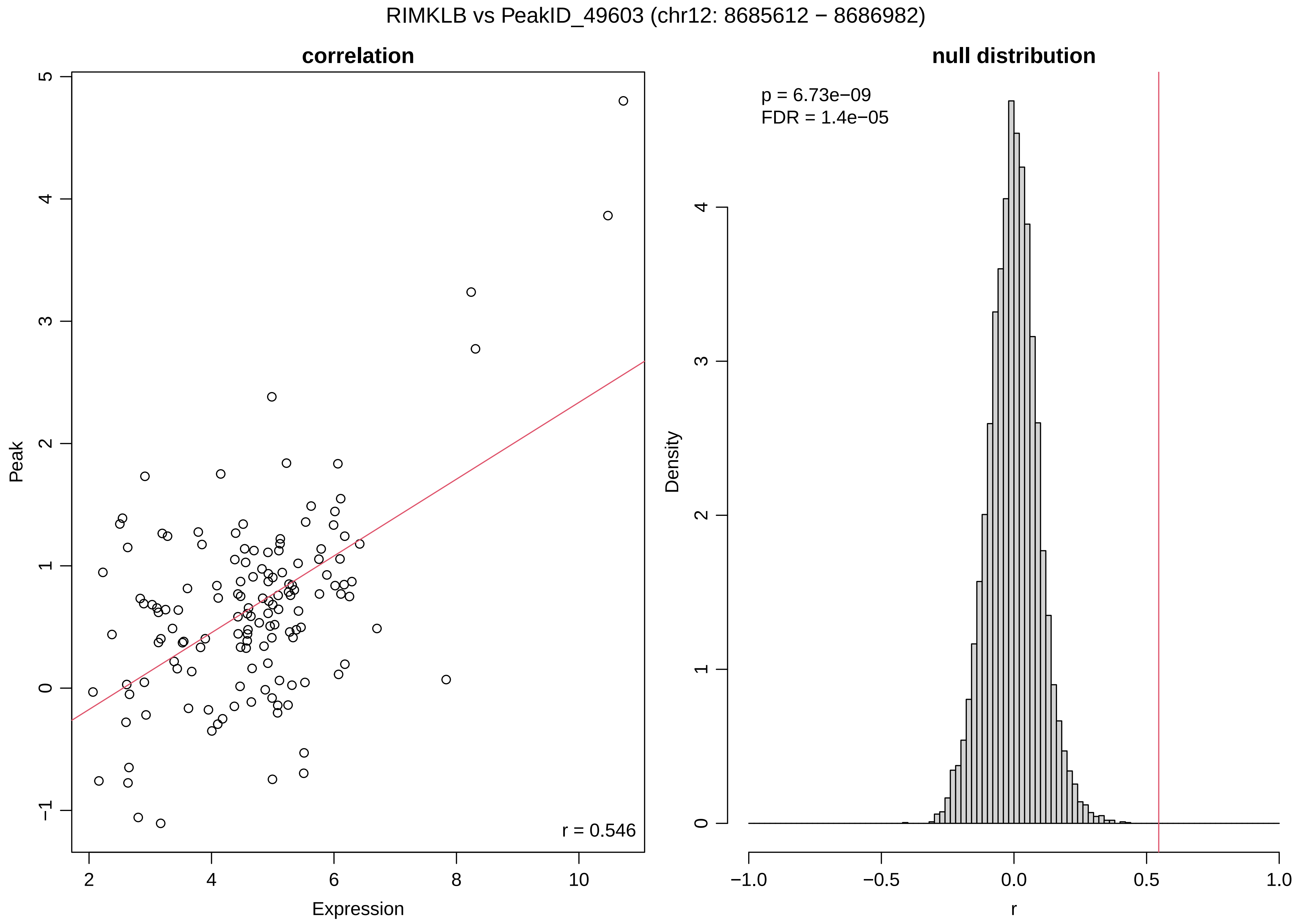
RIMKLB vs PeakID_49603 (chr12: 8685612 − 8686982)
correlation
Peak
Expression
r = 0.546
null distribution
p = 6.73e−09
FDR = 1.4e−05
Density
r

RNU4−20P vs PeakID_54804 (chr12: 59509230 − 59513021)

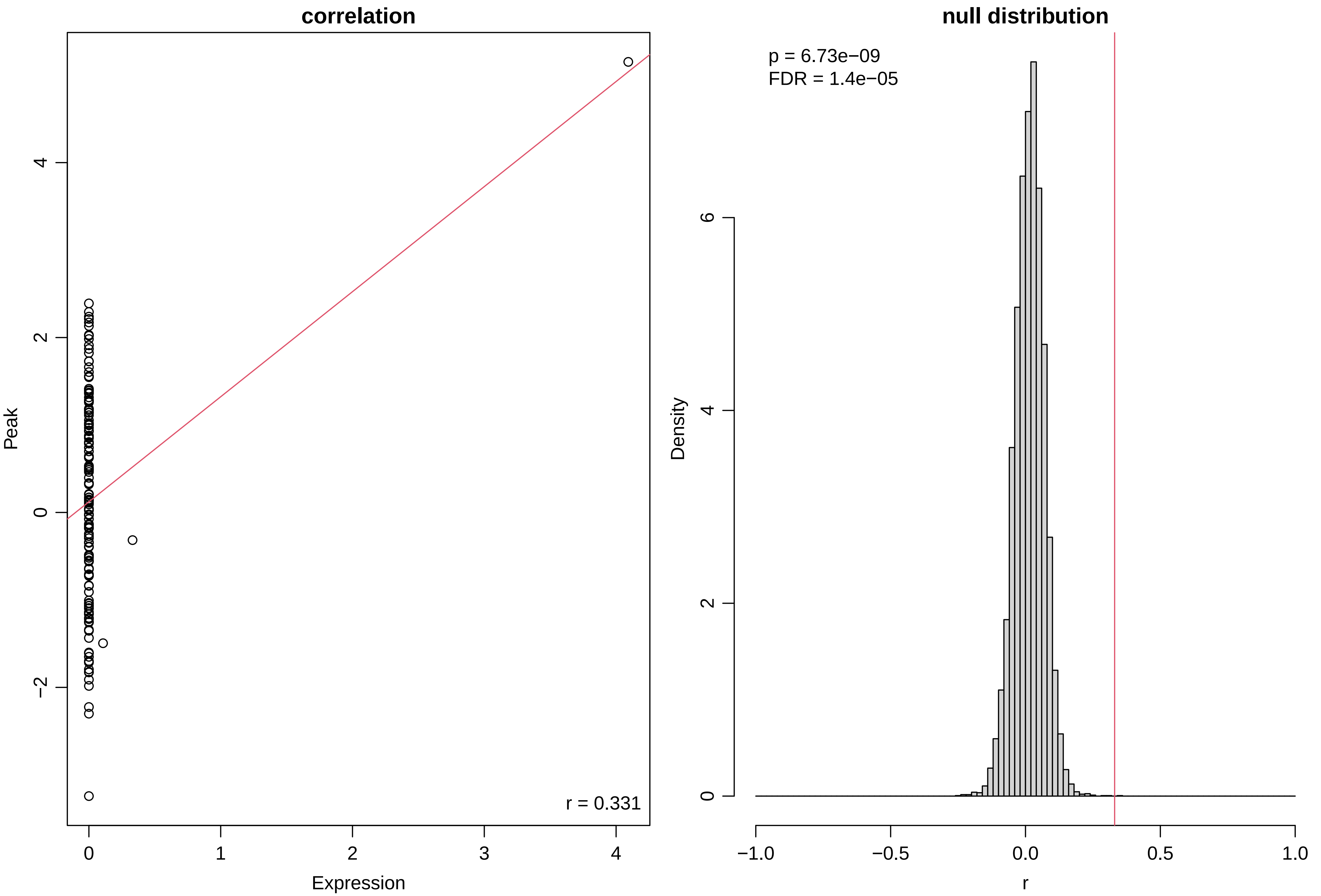

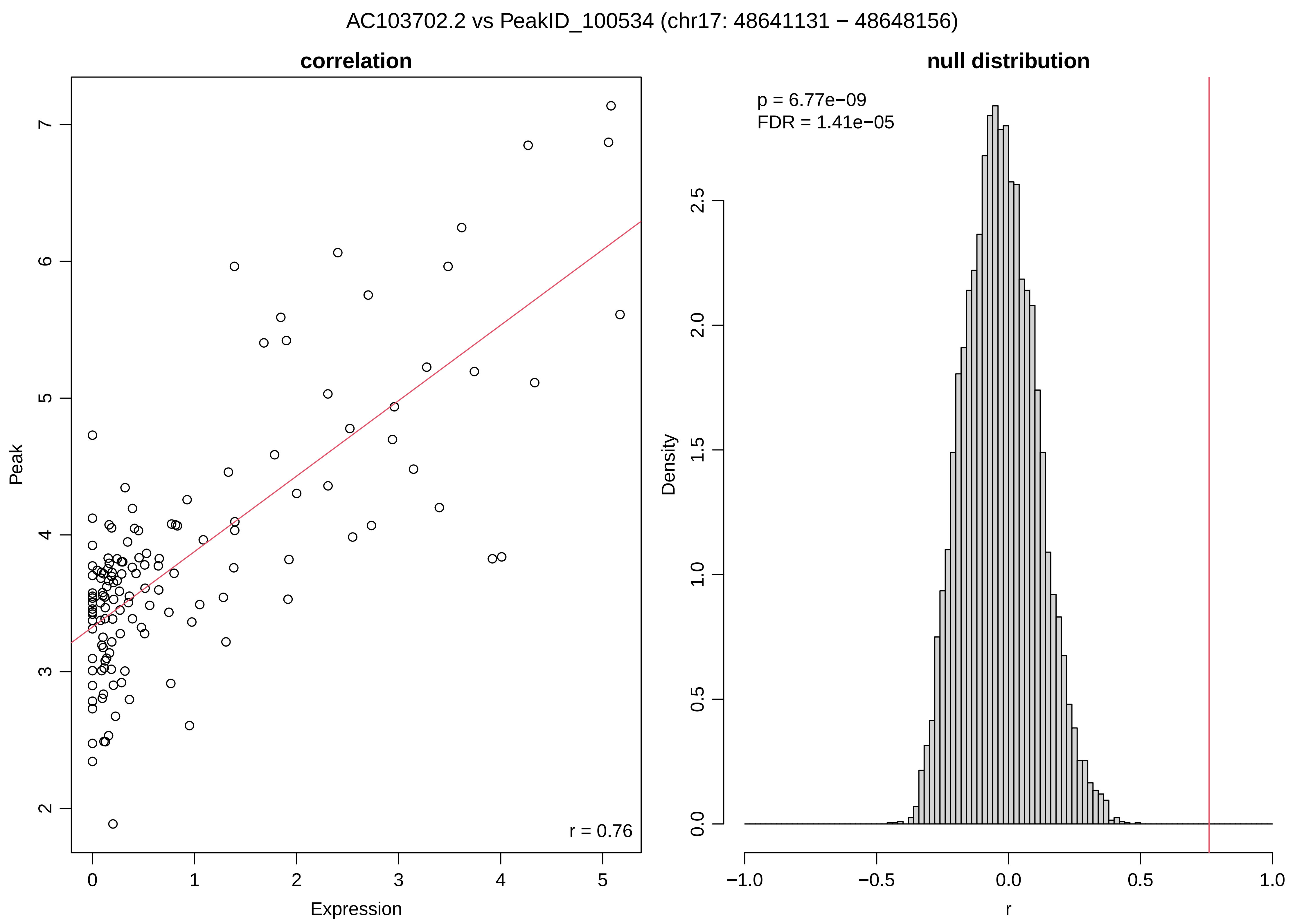

AC103702.2 vs PeakID_100534 (chr17: 48641131 − 48648156)
correlation
Peak
Expression
r = 0.76
null distribution
p = 6.77e−09
FDR = 1.41e−05
Density
r

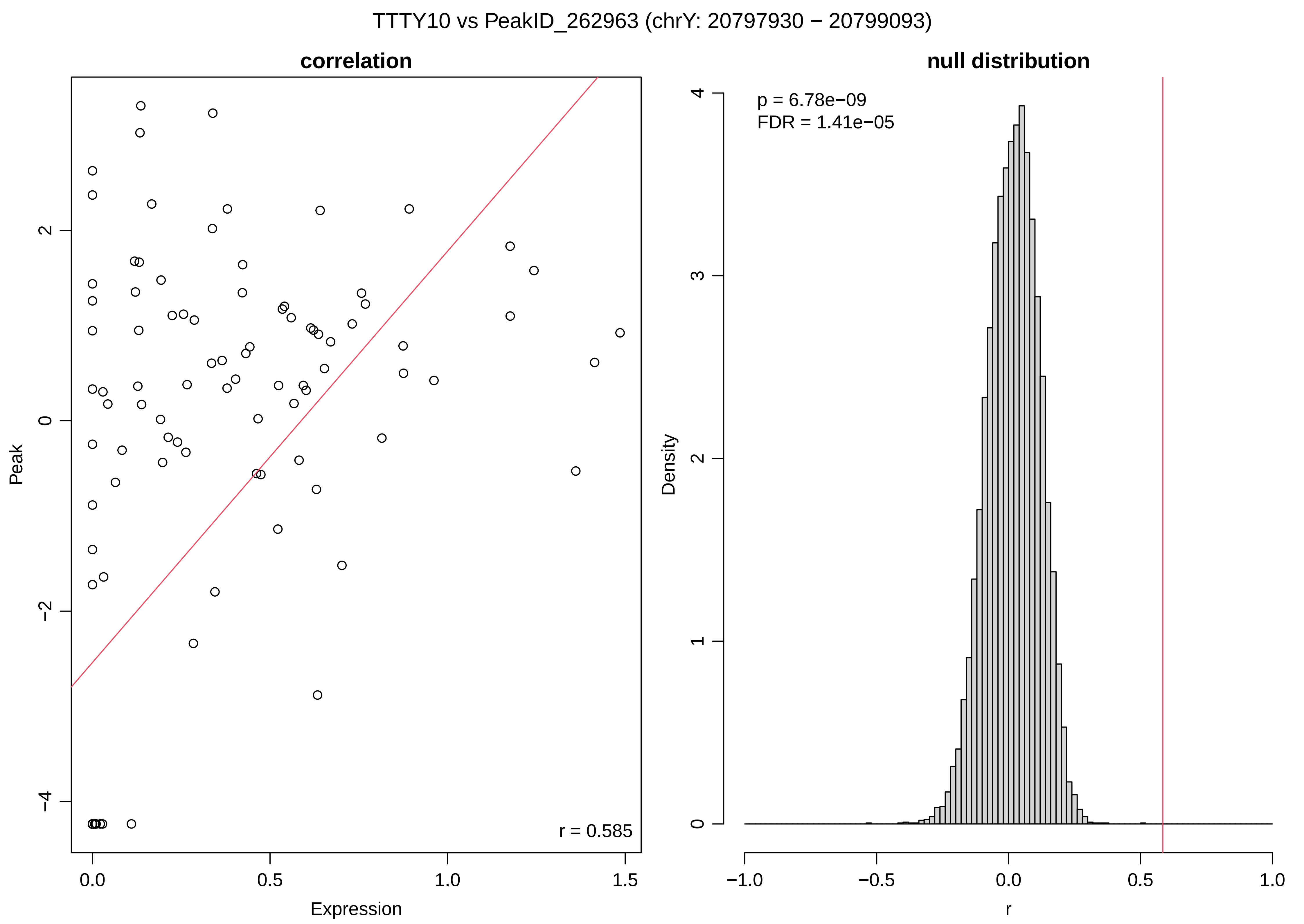

TTTY10 vs PeakID_262963 (chrY: 20797930 − 20799093)
correlation
Peak
Expression
r = 0.585
null distribution
p = 6.78e−09
FDR = 1.41e−05
Density
r

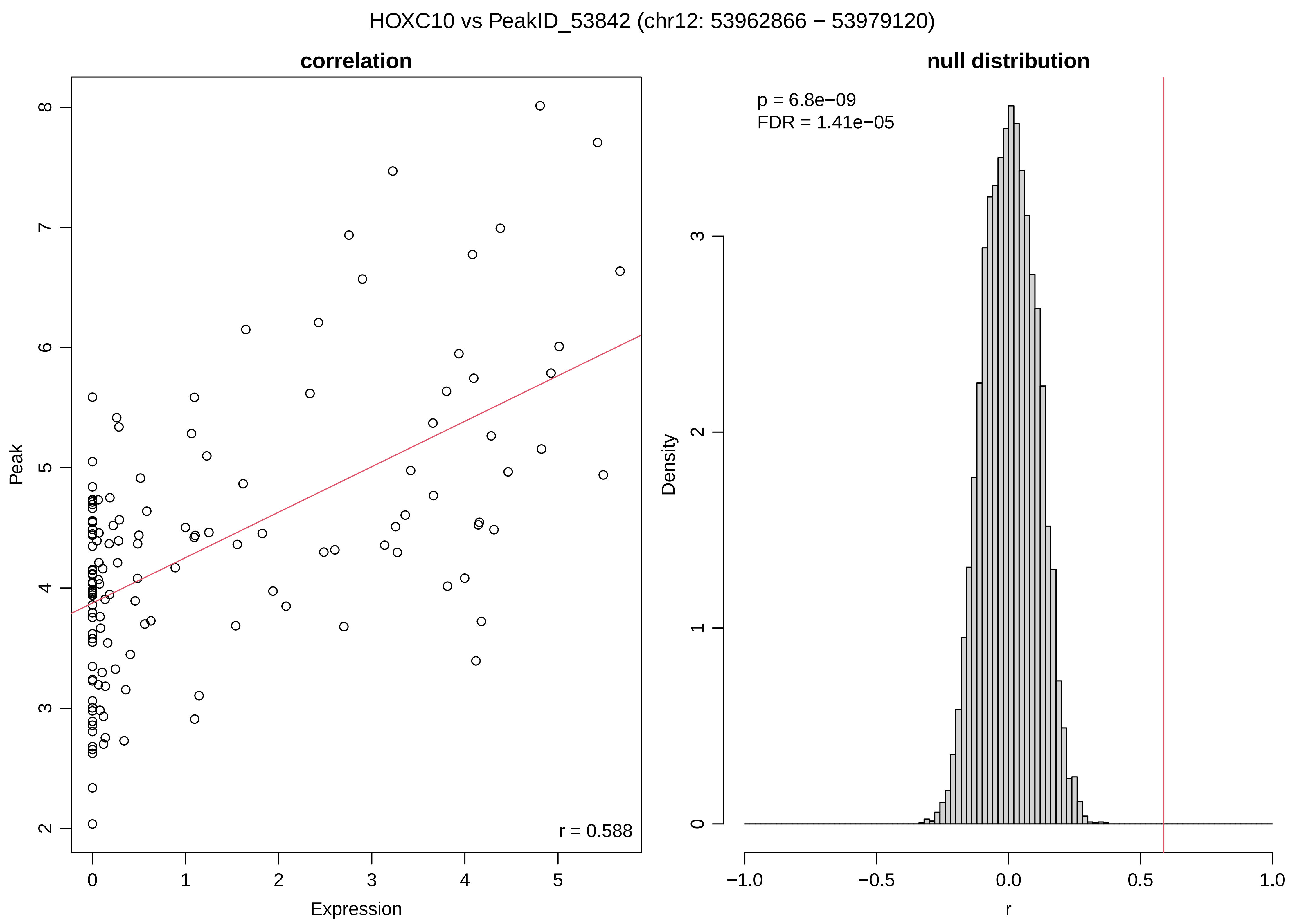
HOXC10 vs PeakID_53842 (chr12: 53962866 − 53979120)
correlation
null distribution
p = 6.8e−09
FDR = 1.41e−05
r = 0.588
Peak
Expression
Density
r
2
3
4
5
6
7
8
0
1
2
3
4
5
−1.0
−0.5
0.0
0.5
1.0

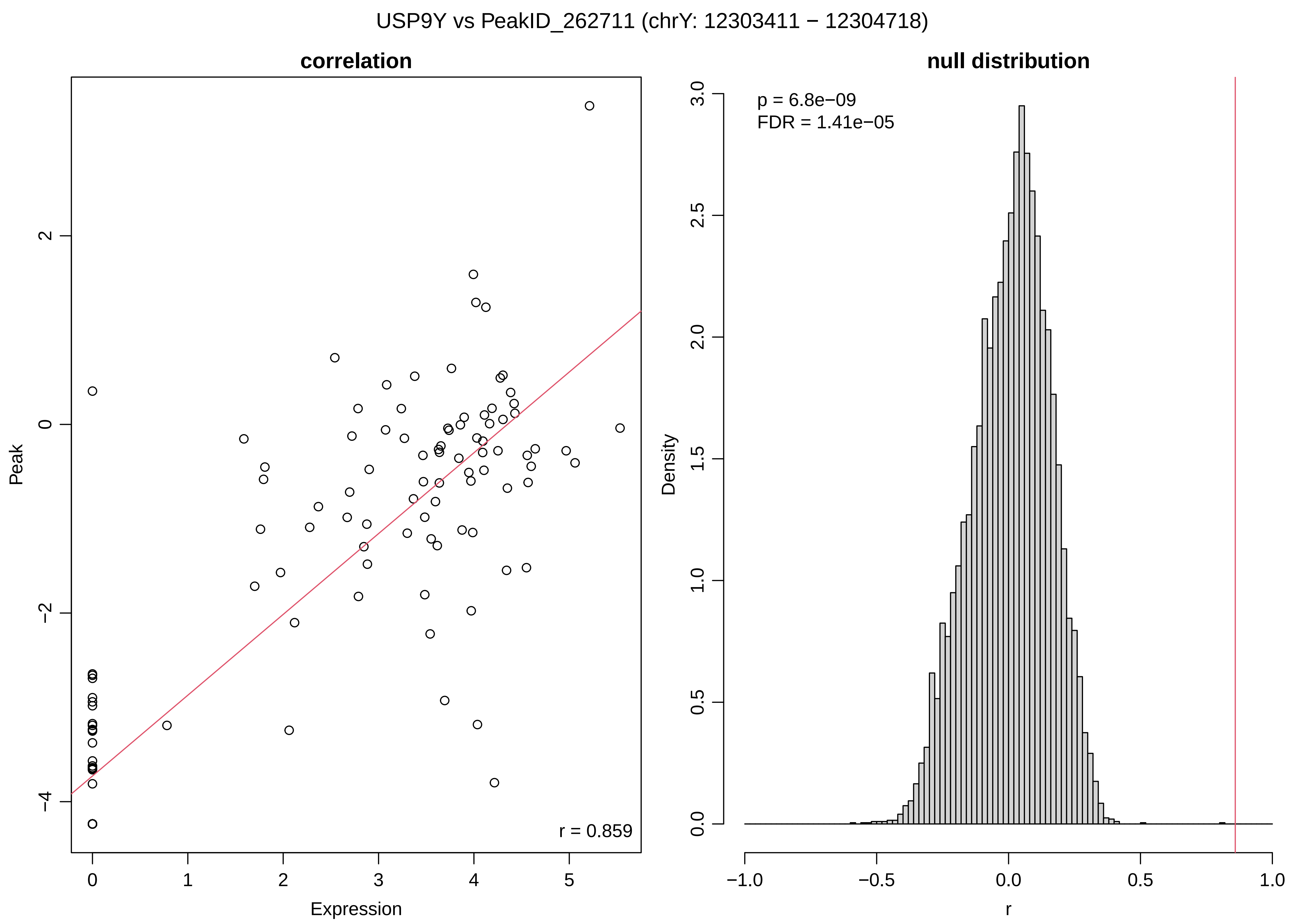

USP9Y vs PeakID_262711 (chrY: 12303411 − 12304718)
correlation
Peak
Expression
r = 0.859
null distribution
Density
r
p = 6.8e−09
FDR = 1.41e−05

CHMP1B2P vs PeakID_259478 (chrX: 80334864 − 80335685)

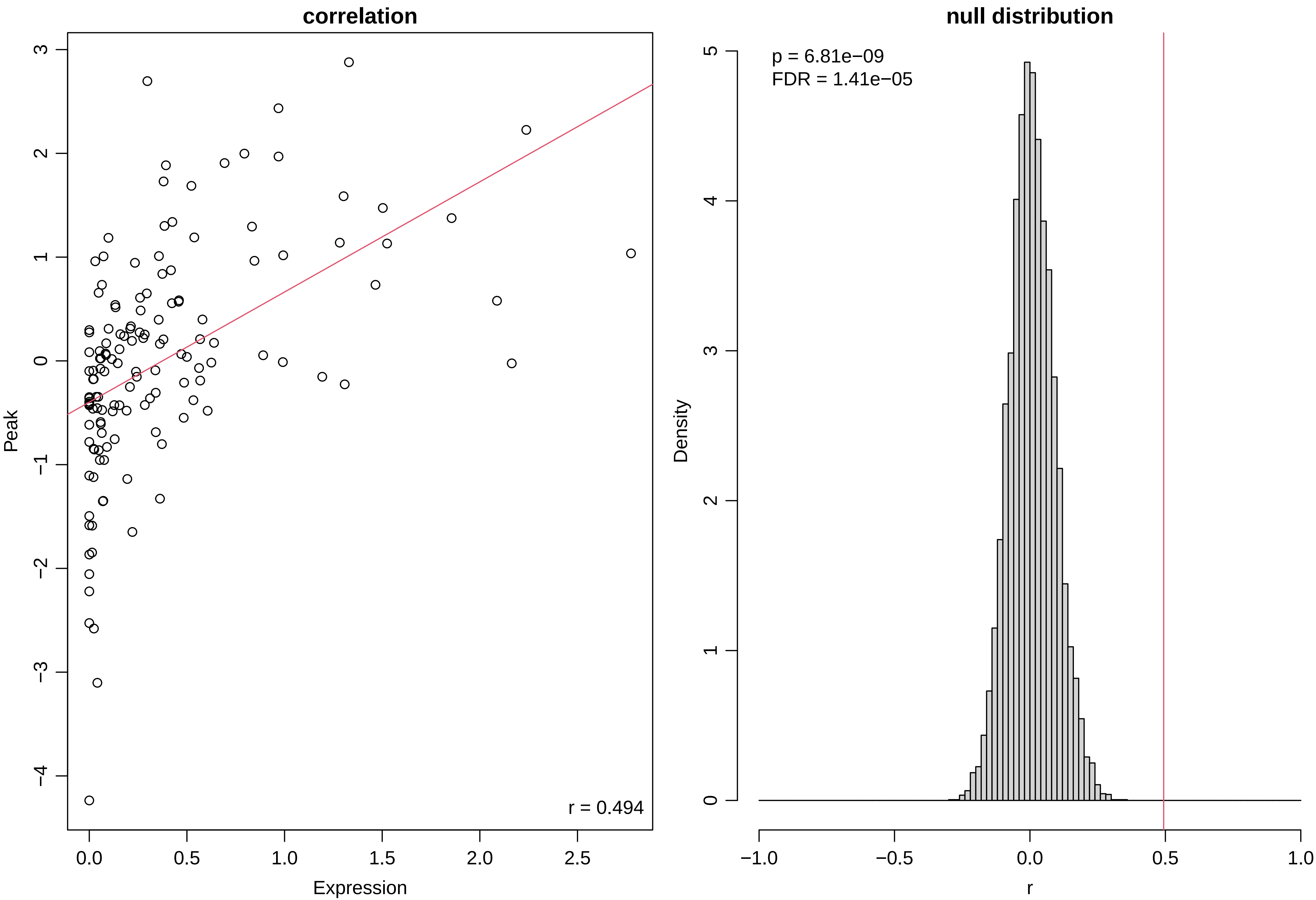

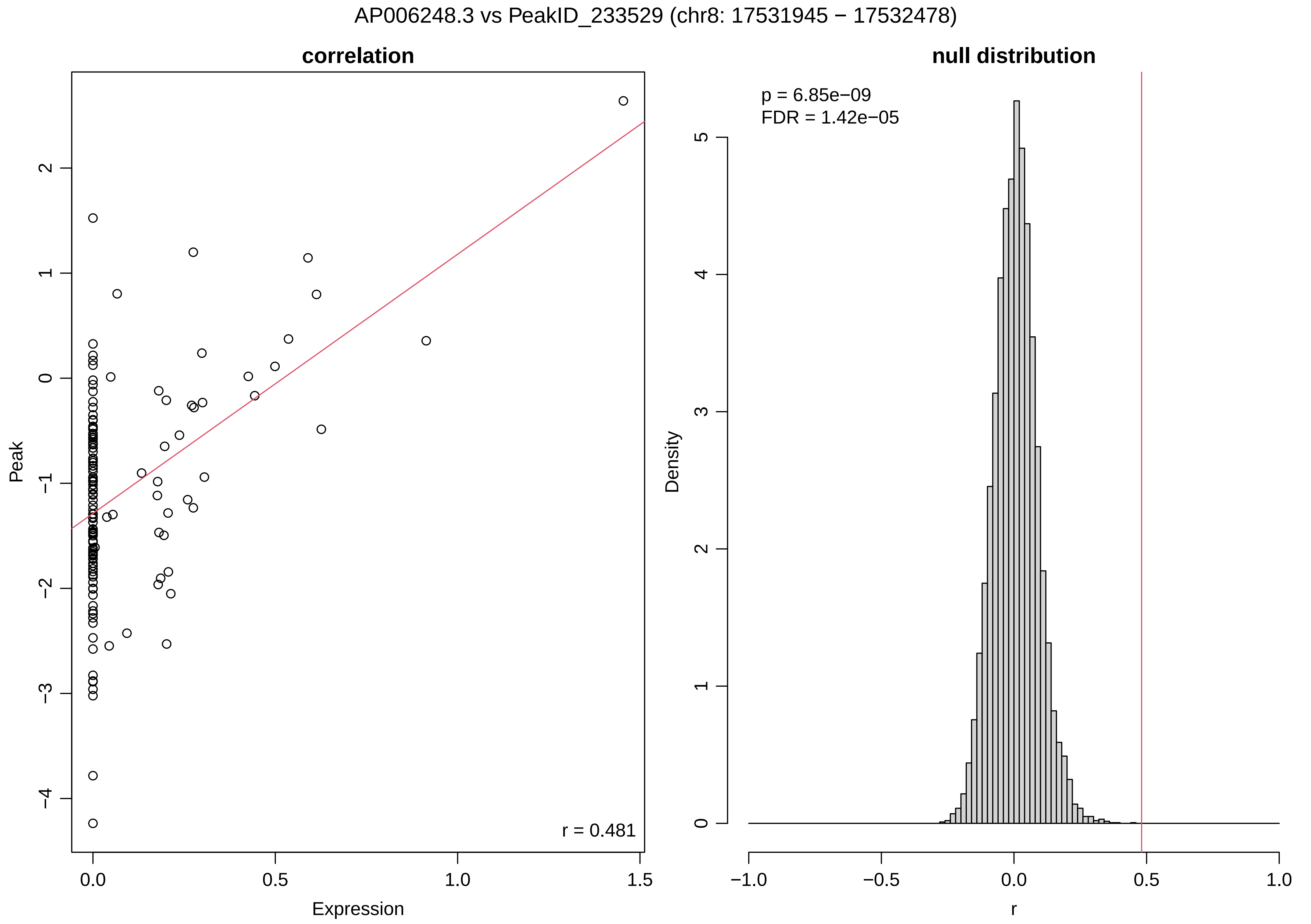
AP006248.3 vs PeakID_233529 (chr8: 17531945 − 17532478)
correlation
null distribution
p = 6.85e−09
FDR = 1.42e−05
r = 0.481
Peak
Expression
Density
r

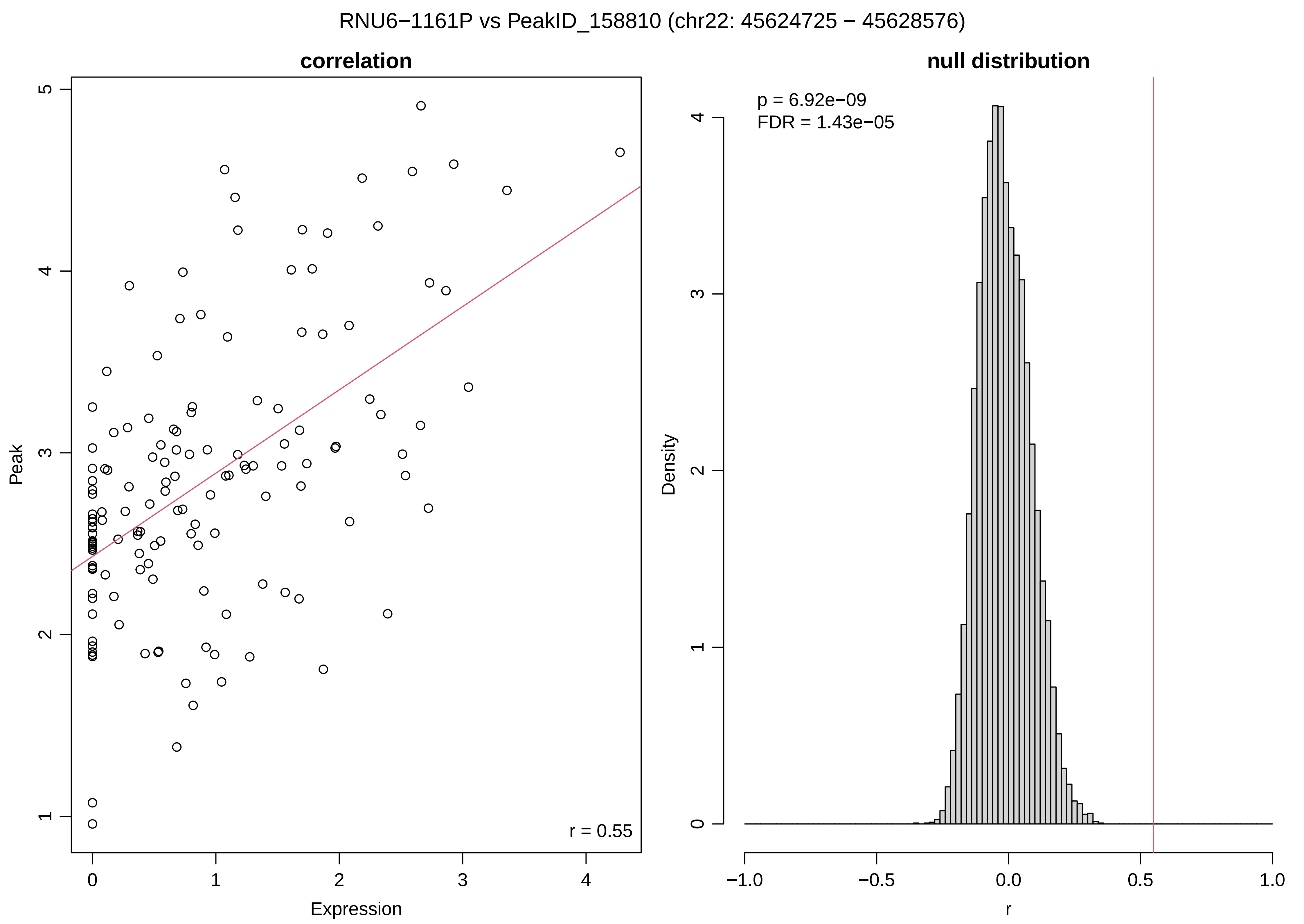

RNU6−1161P vs PeakID_158810 (chr22: 45624725 − 45628576)
correlation
Peak
Expression
r = 0.55
null distribution
p = 6.92e−09
FDR = 1.43e−05
Density
r

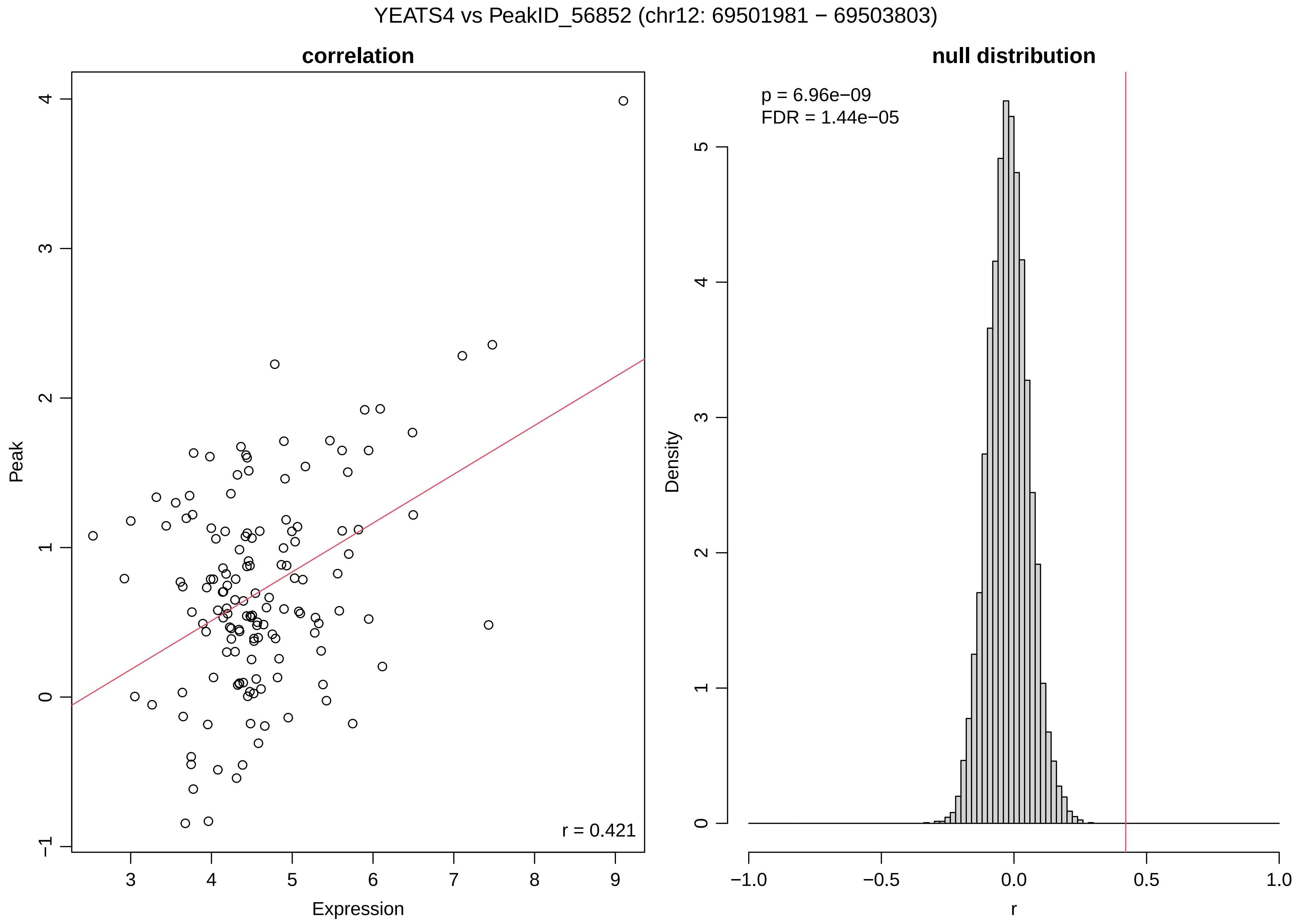
YEATS4 vs PeakID_56852 (chr12: 69501981 − 69503803)
correlation
Peak
Expression
r = 0.421
null distribution
p = 6.96e−09
FDR = 1.44e−05
Density
r

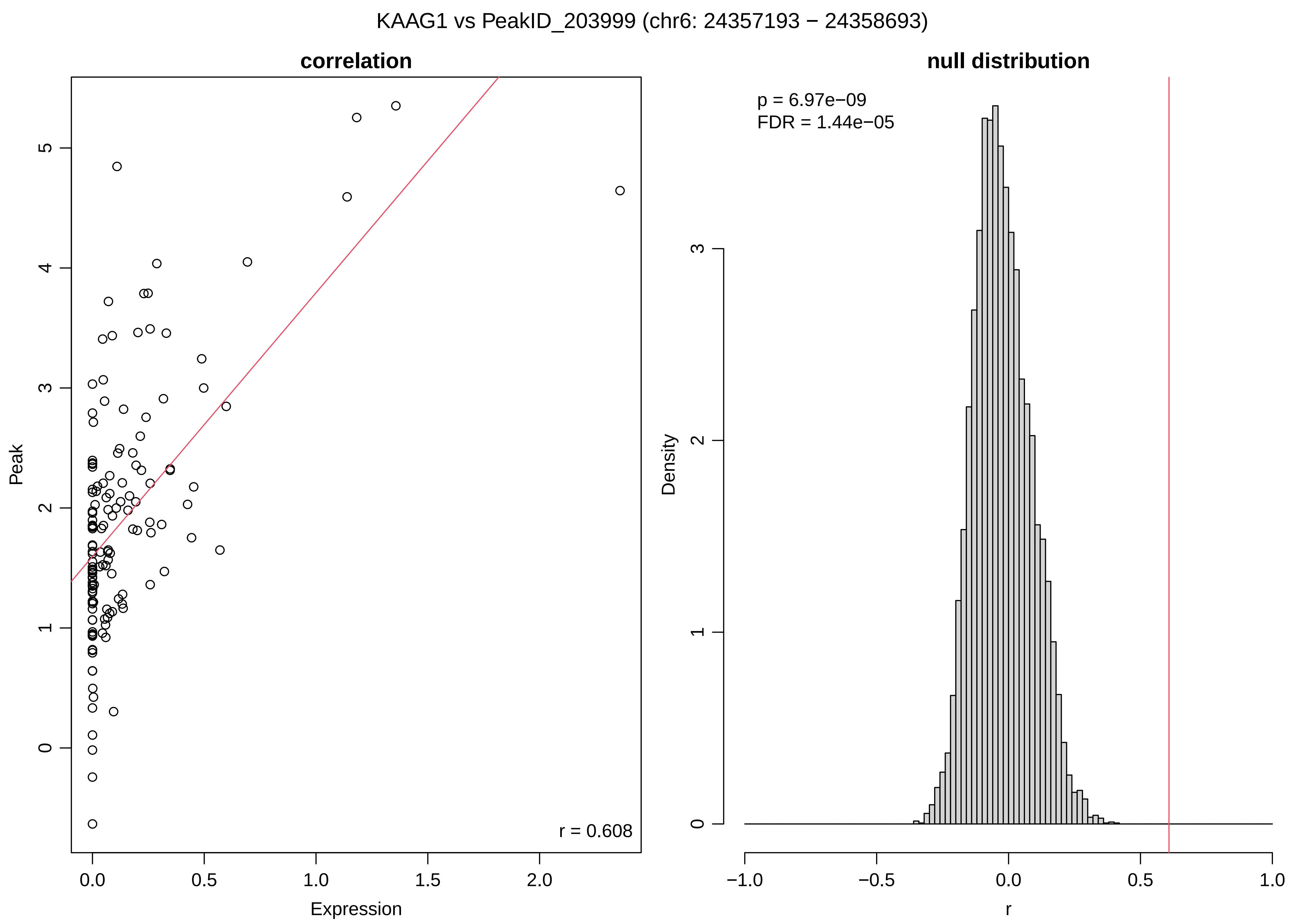
KAAG1 vs PeakID_203999 (chr6: 24357193 − 24358693)
correlation
Peak
Expression
r = 0.608
null distribution
p = 6.97e−09
FDR = 1.44e−05
Density
r

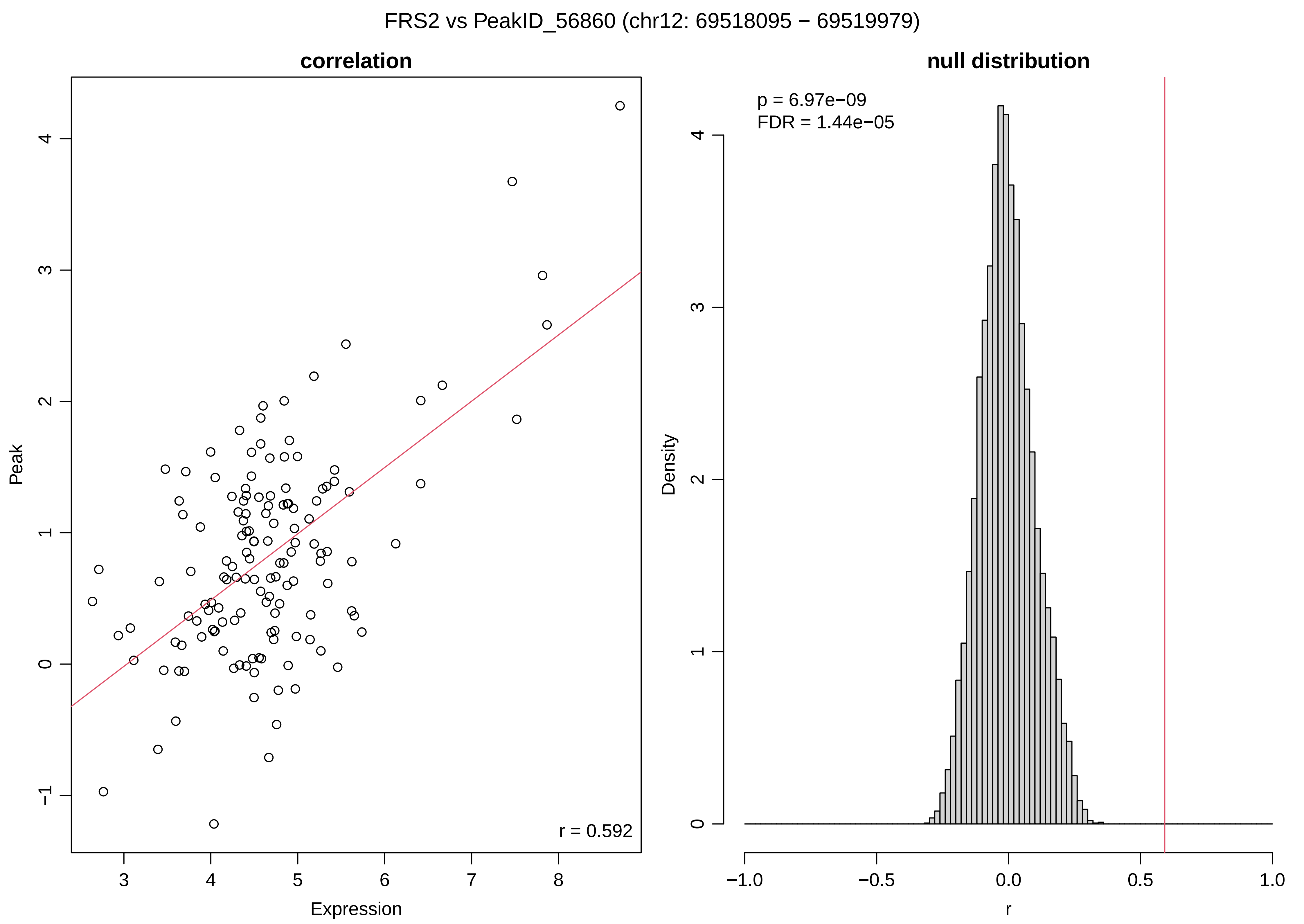
FRS2 vs PeakID_56860 (chr12: 69518095 − 69519979)
correlation
Peak
Expression
r = 0.592
null distribution
p = 6.97e−09
FDR = 1.44e−05
Density
r

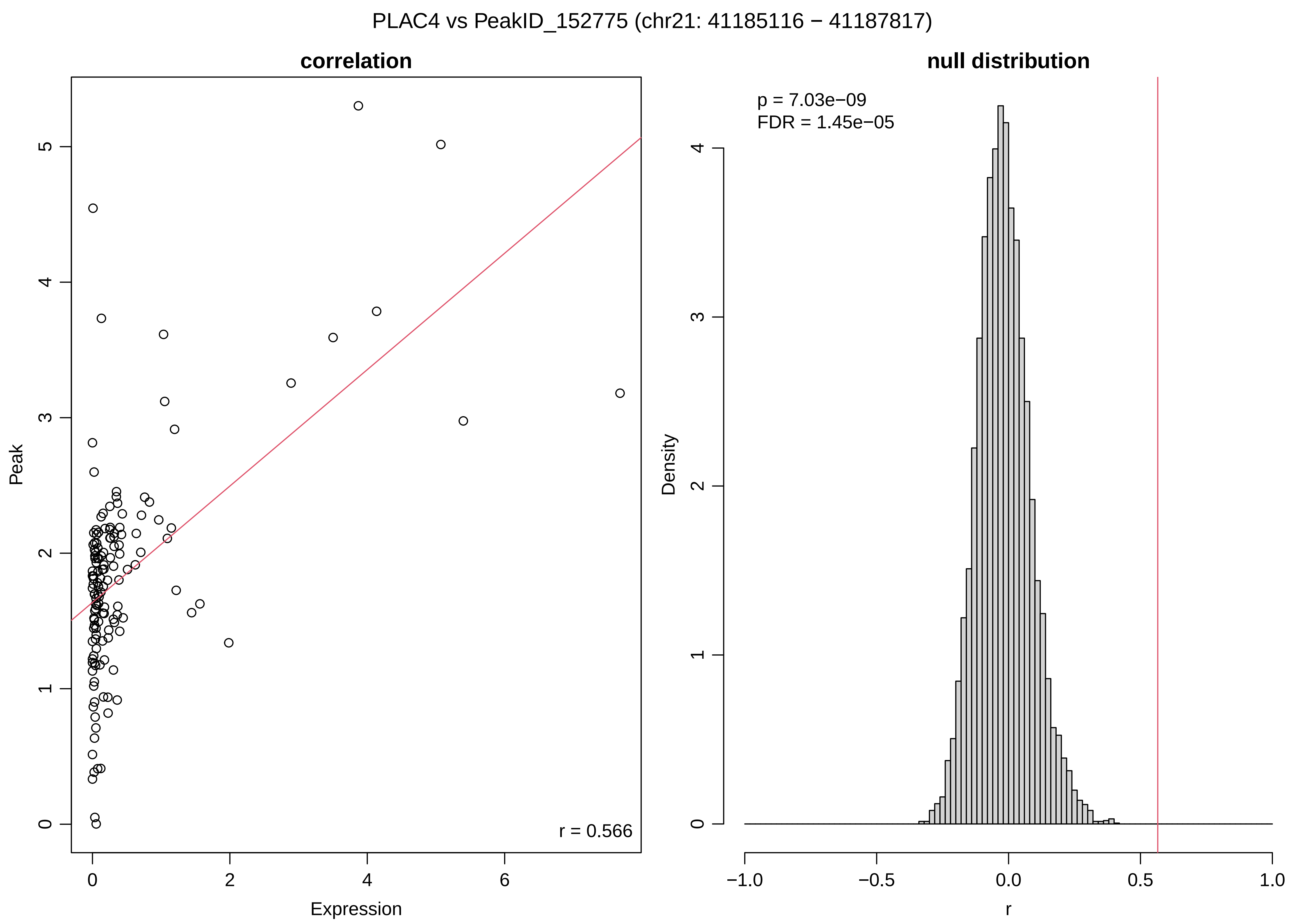

PLAC4 vs PeakID_152775 (chr21: 41185116 − 41187817)
correlation
Peak
Expression
r = 0.566
0
1
2
3
4
5
0
2
4
6
null distribution
p = 7.03e−09
FDR = 1.45e−05
Density
r
0
1
2
3
4
−1.0
−0.5
0.0
0.5
1.0

RPL21P104 vs PeakID_55405 (chr12: 61826059 − 61827359)

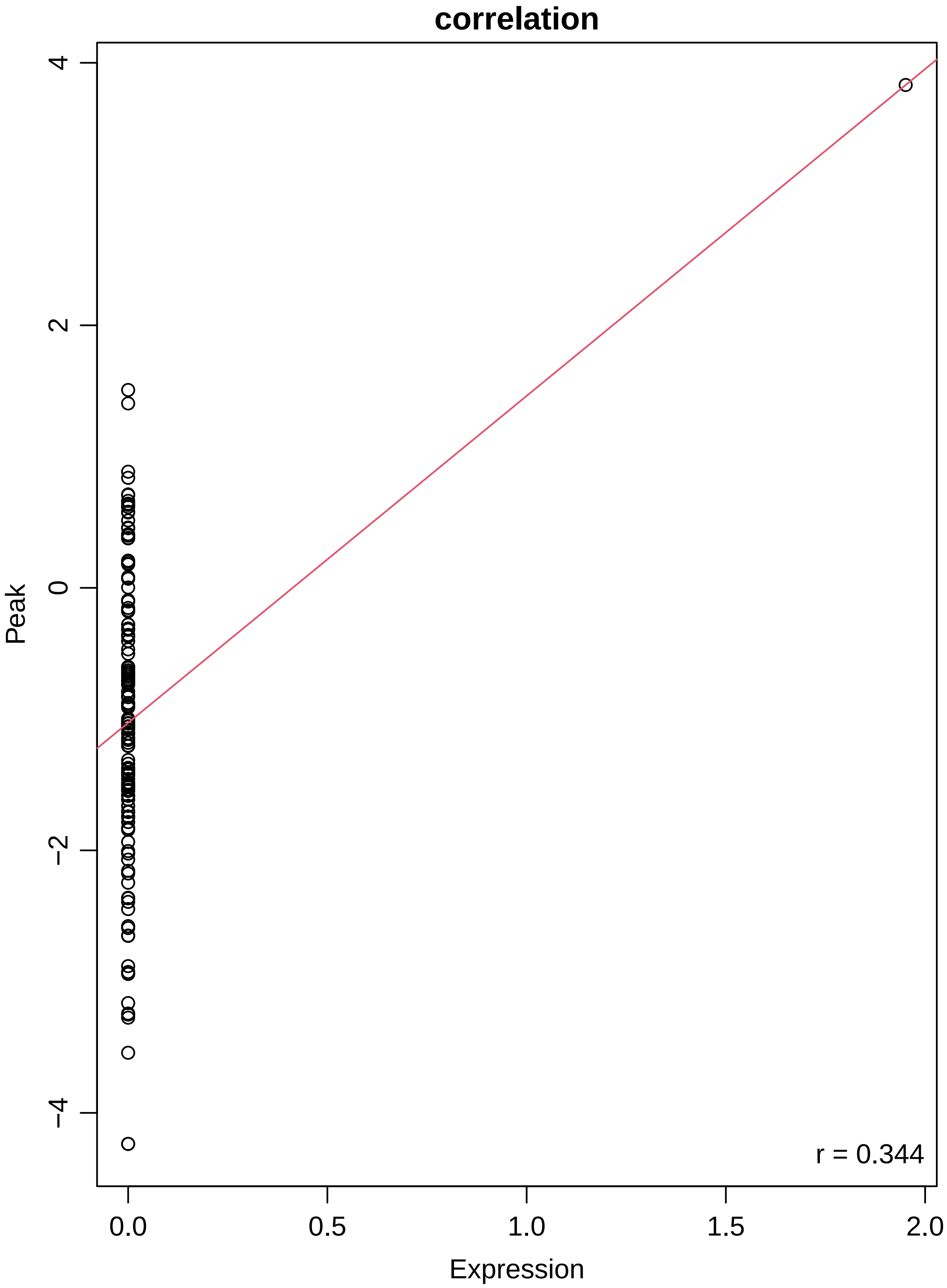

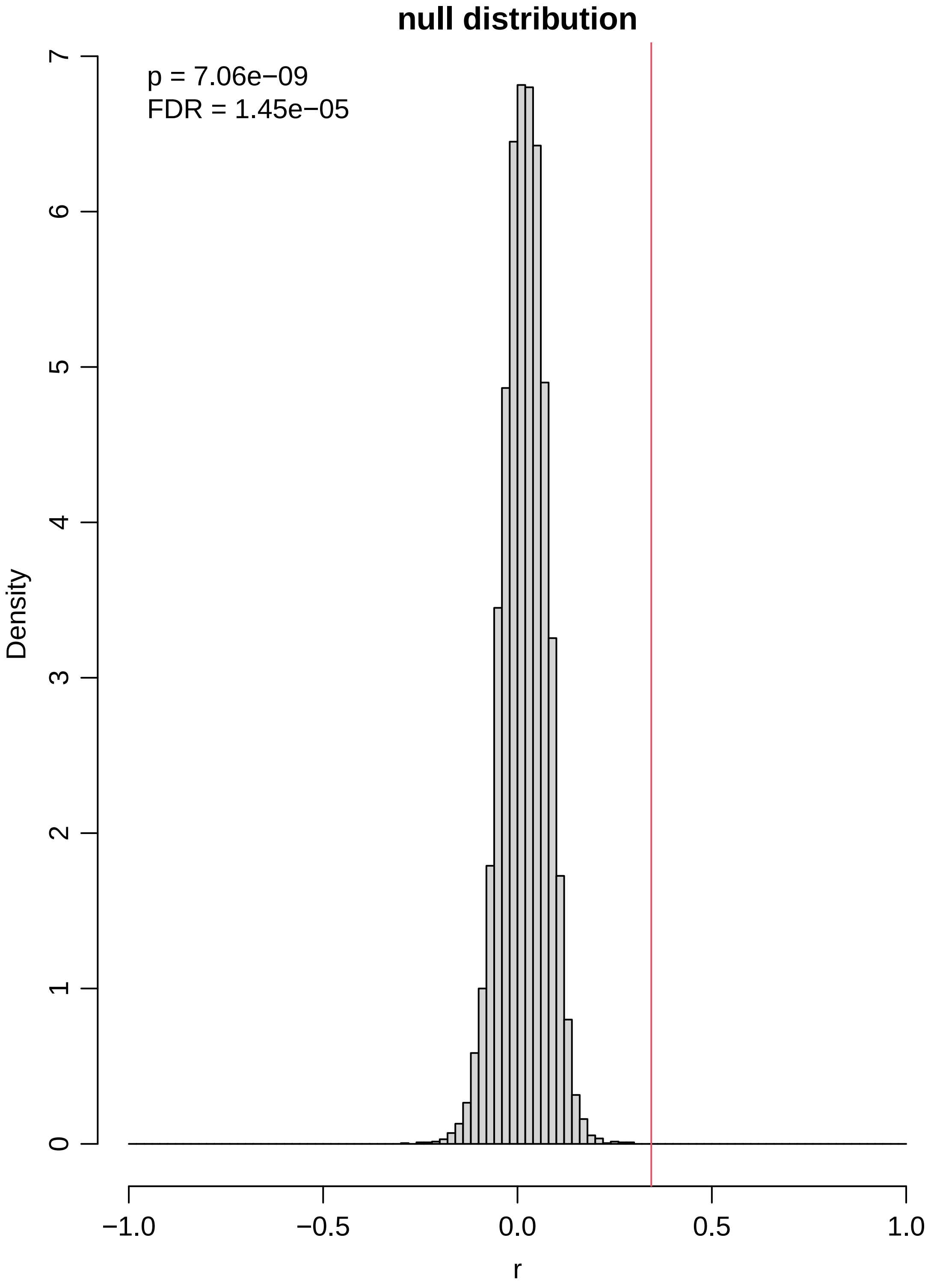

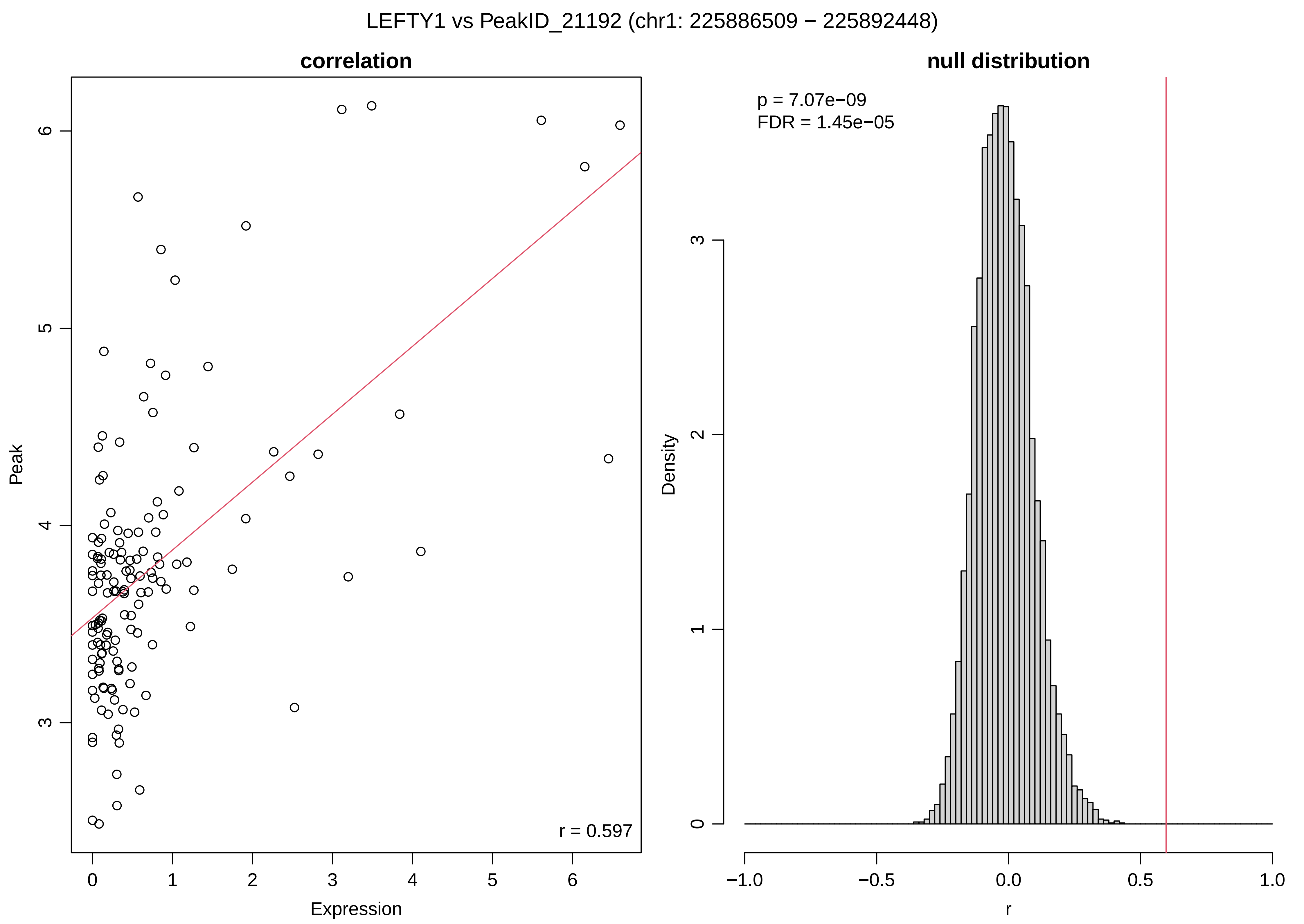

LEFTY1 vs PeakID_21192 (chr1: 225886509 − 225892448)
correlation
null distribution
p = 7.07e−09
FDR = 1.45e−05
r = 0.597
Peak
Expression
Density
r

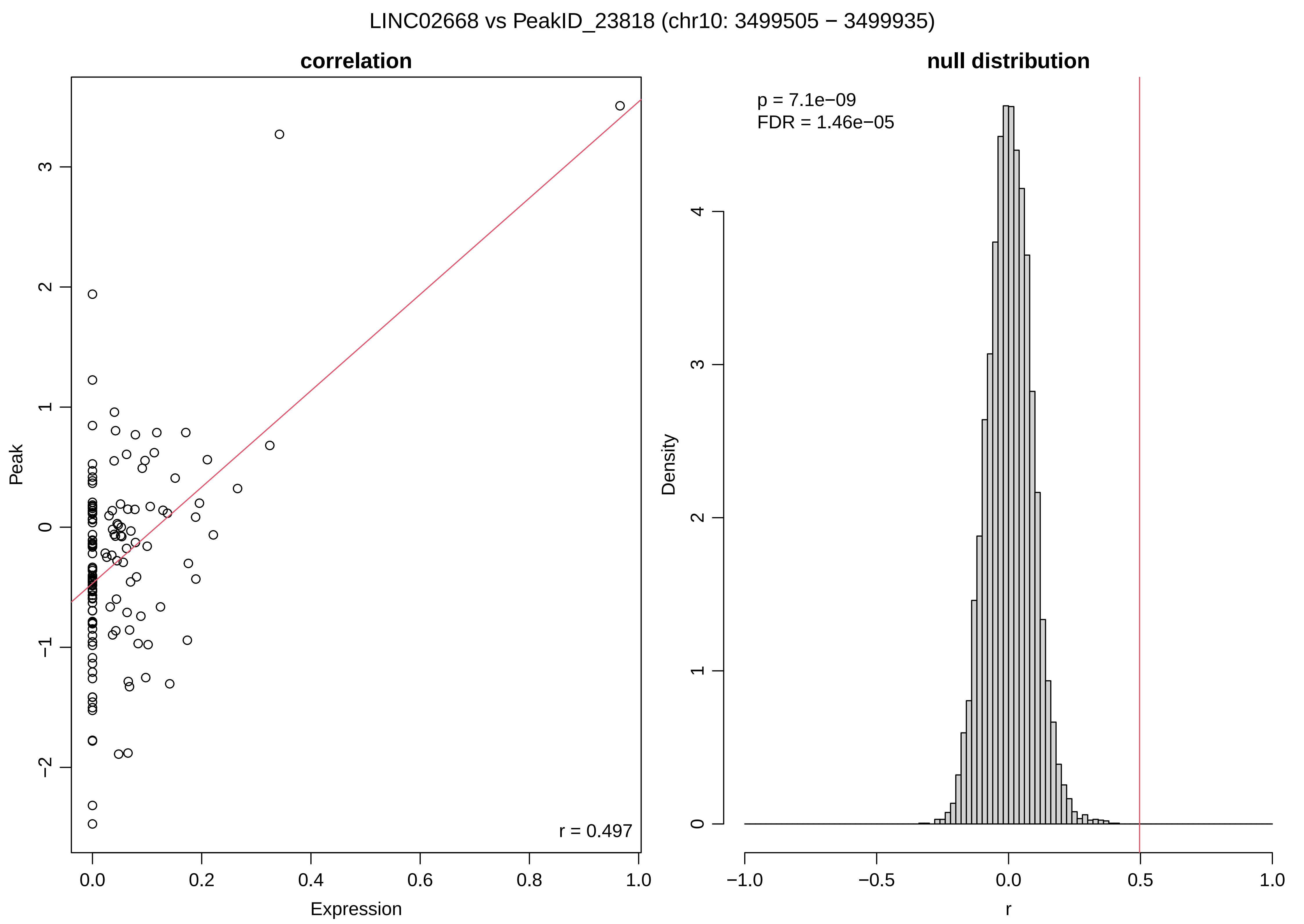
LINC02668 vs PeakID_23818 (chr10: 3499505 − 3499935)
correlation
Peak
Expression
r = 0.497
null distribution
p = 7.1e−09
FDR = 1.46e−05
Density
r

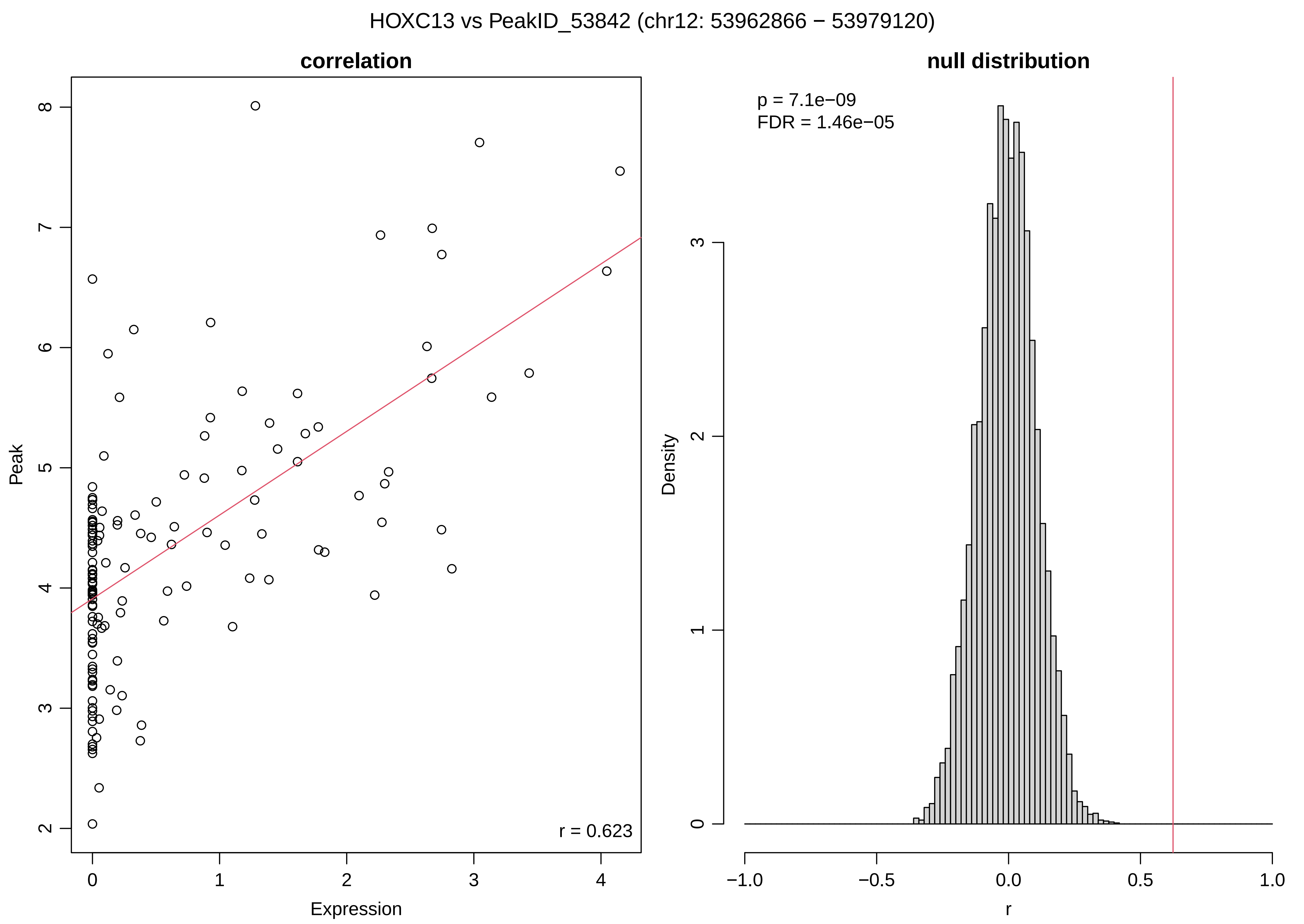

HOXC13 vs PeakID_53842 (chr12: 53962866 − 53979120)
correlation
null distribution
p = 7.1e−09
FDR = 1.46e−05
r = 0.623
Peak
Expression
Density
r

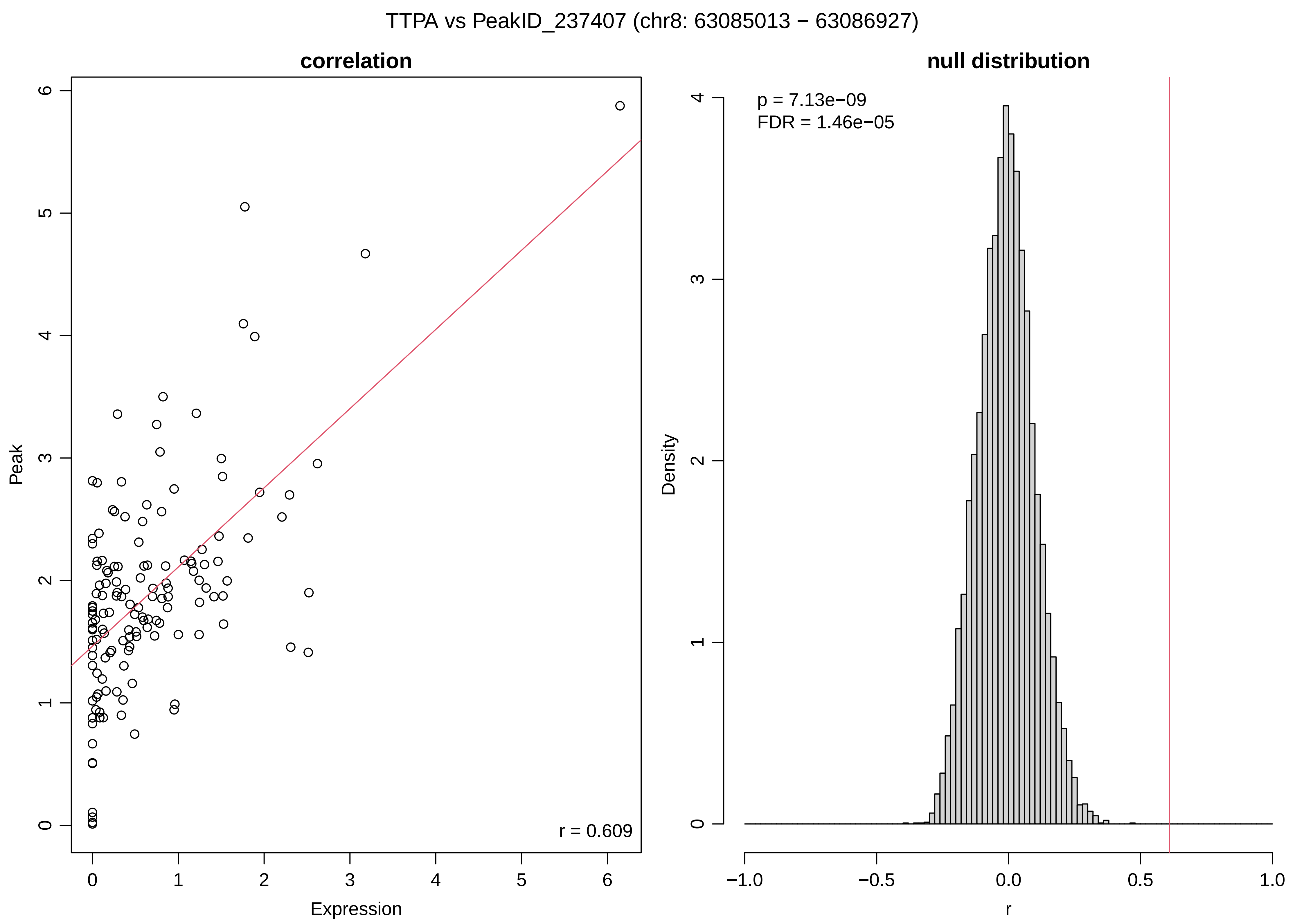

TTPA vs PeakID_237407 (chr8: 63085013 − 63086927)
correlation
null distribution
p = 7.13e−09
FDR = 1.46e−05
r = 0.609
Peak
Expression
Density
r

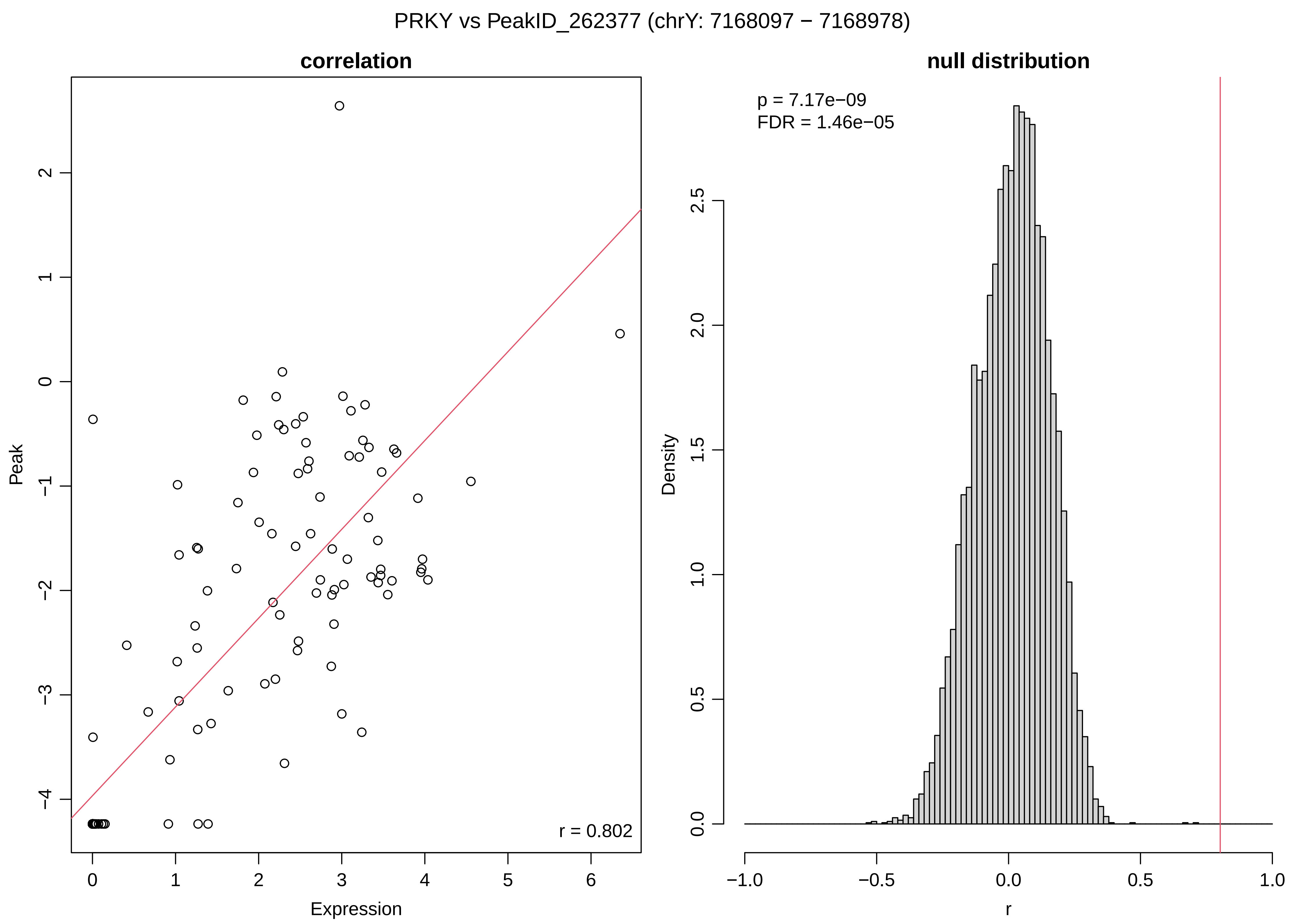
PRKY vs PeakID_262377 (chrY: 7168097 − 7168978)
correlation
Peak
Expression
r = 0.802
null distribution
p = 7.17e−09
FDR = 1.46e−05
Density
r

SLC16A9 vs PeakID_29259 (chr10: 59708728 − 59710379)

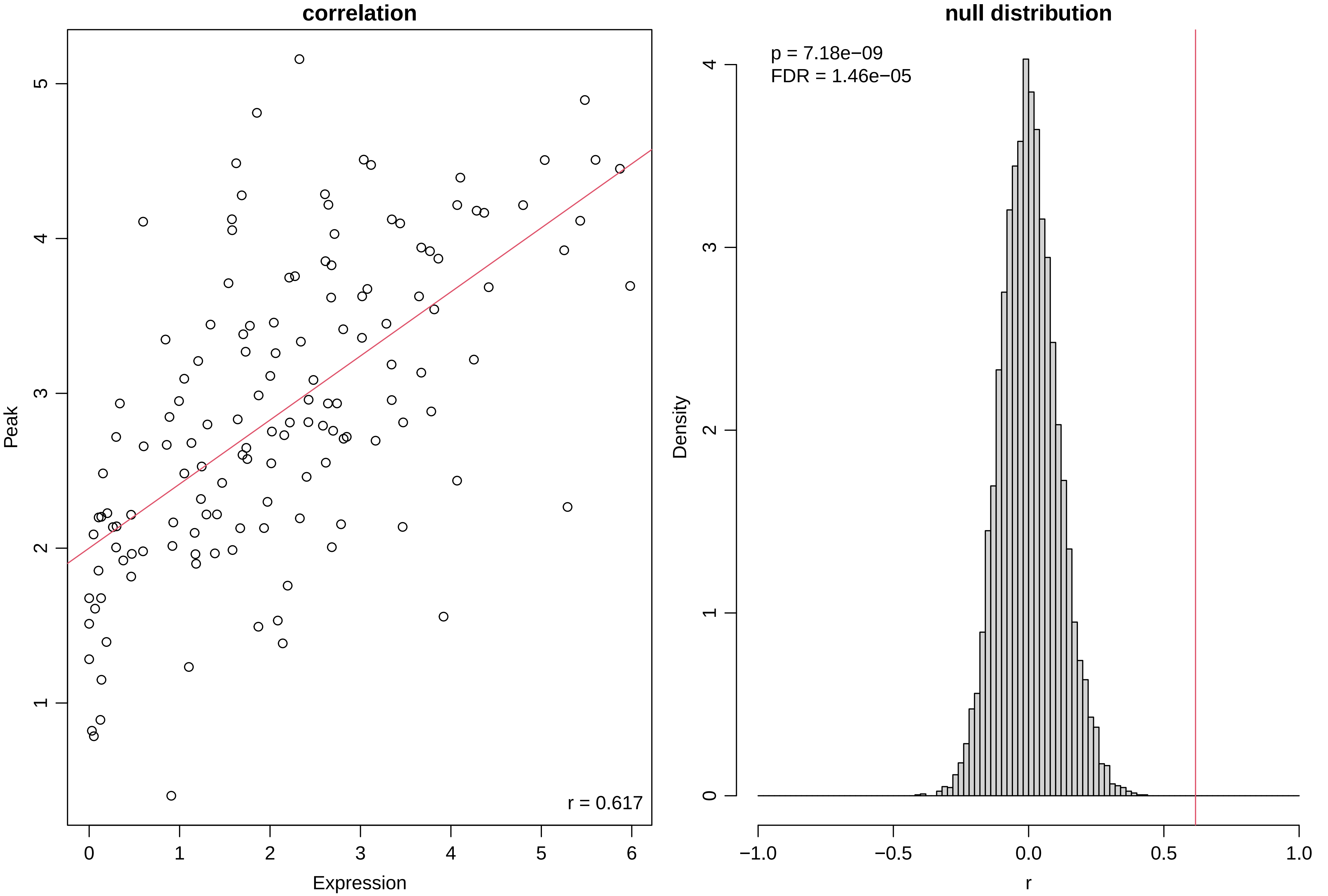

PRKY vs PeakID_262386 (chrY: 7278790 − 7280500)

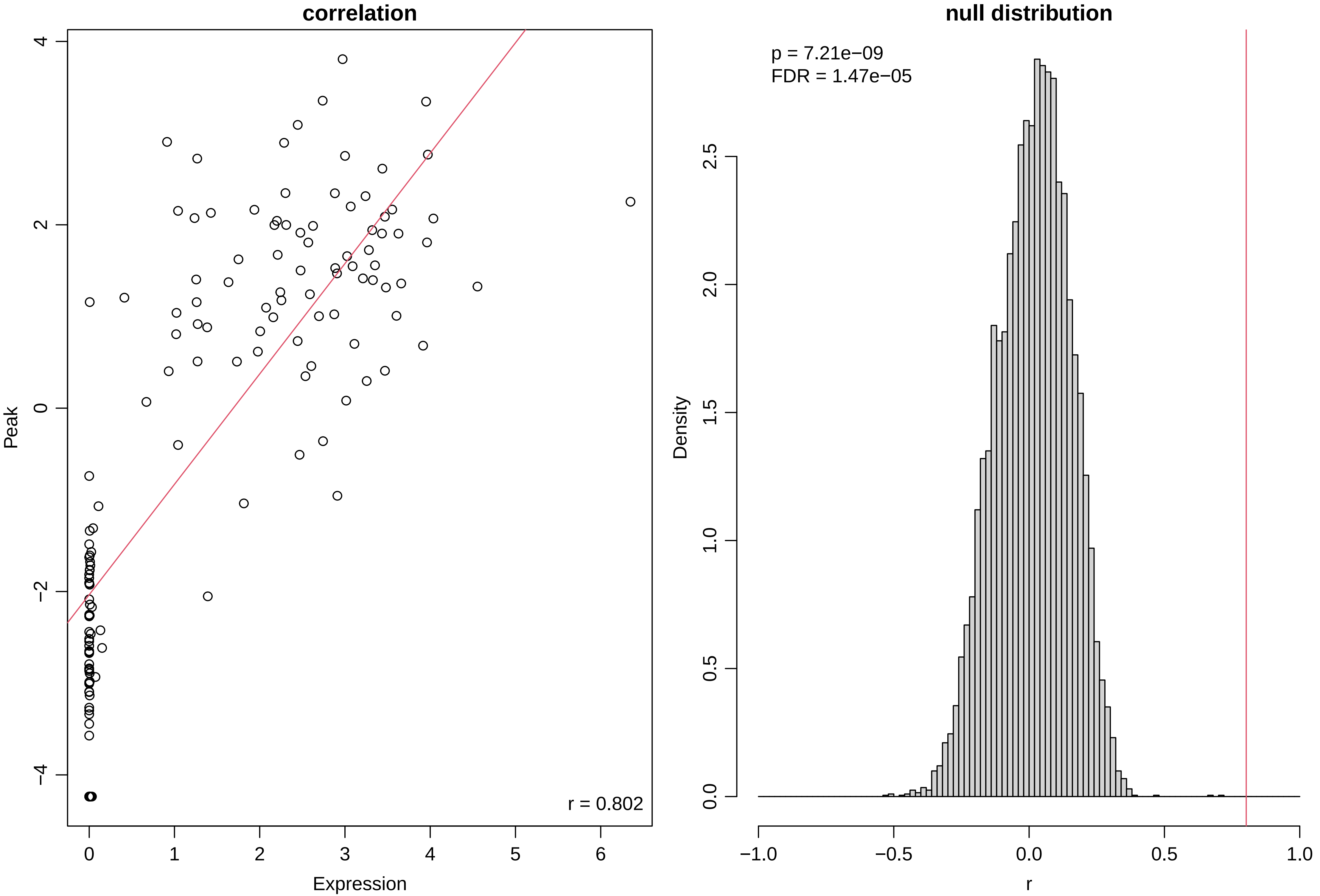

C4BPB vs PeakID_19422 (chr1: 207087867 − 207090612)

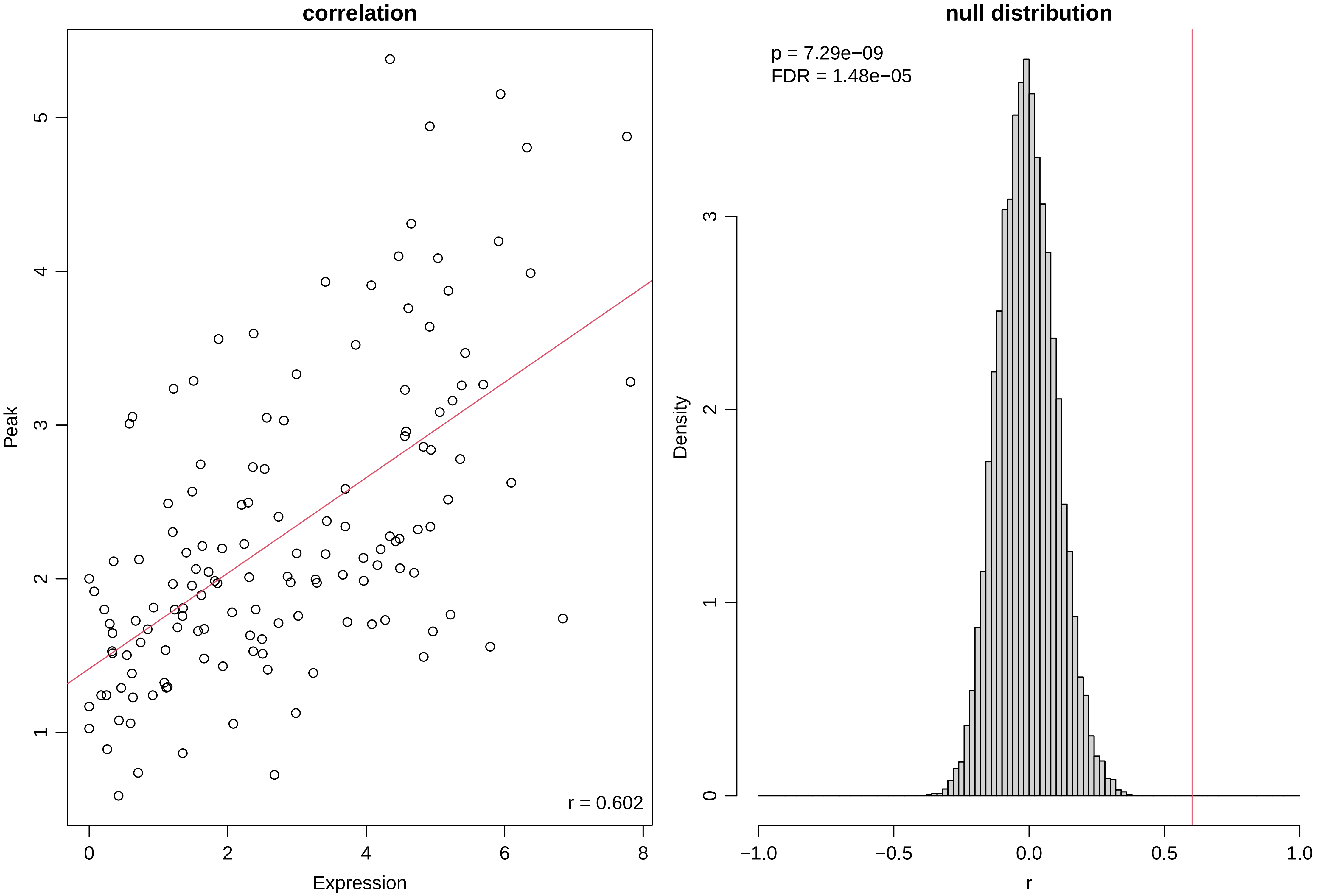

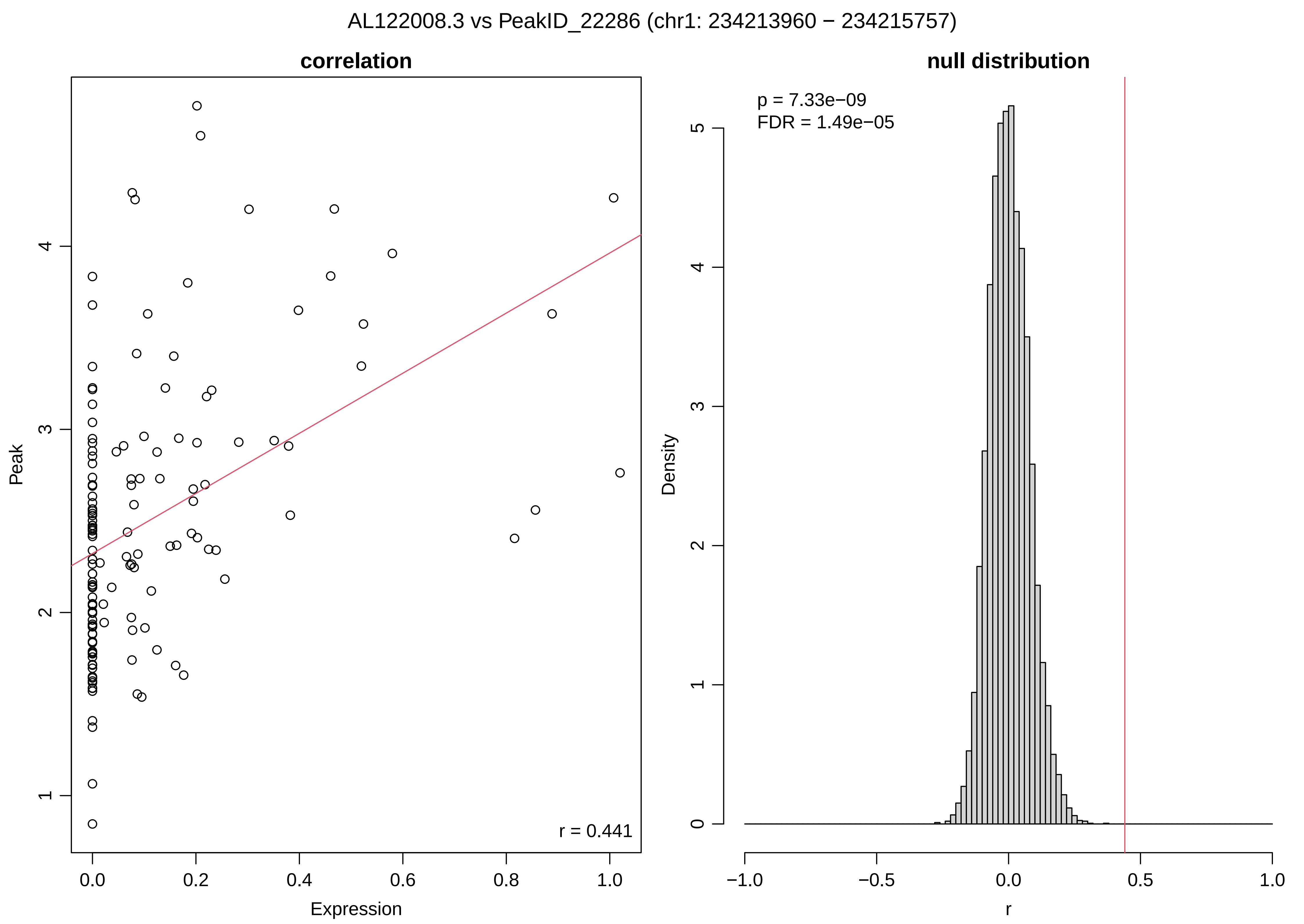
AL122008.3 vs PeakID_22286 (chr1: 234213960 − 234215757)
correlation
Peak
Expression
r = 0.441
0.0
0.2
0.4
0.6
0.8
1.0
1
2
3
4
null distribution
p = 7.33e−09
FDR = 1.49e−05
Density
r
−1.0
−0.5
0.0
0.5
1.0
0
1
2
3
4
5

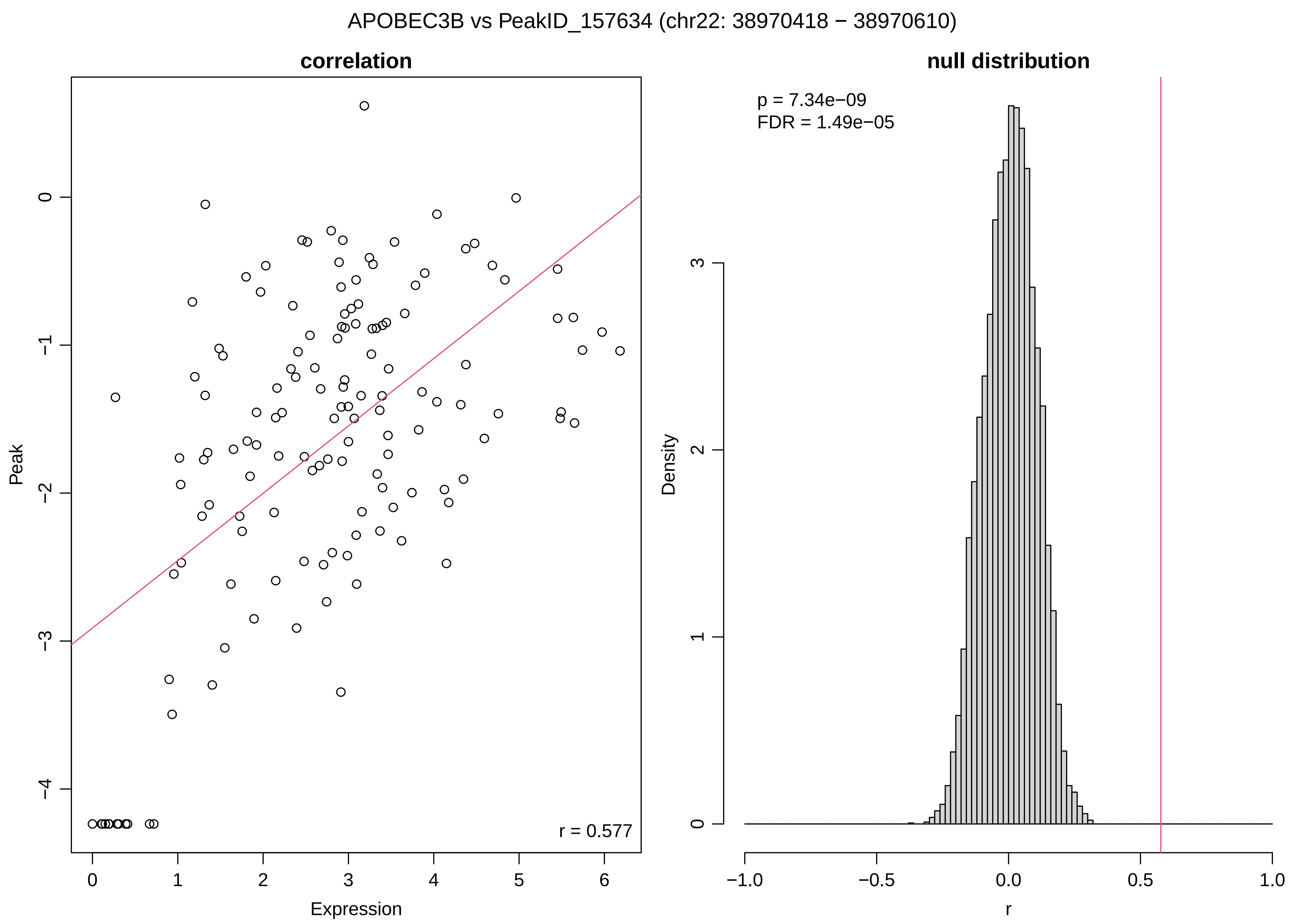
APOBEC3B vs PeakID_157634 (chr22: 38970418 − 38970610)
correlation
Peak
Expression
r = 0.577
null distribution
p = 7.34e−09
FDR = 1.49e−05
Density
r

FRS2 vs PeakID_56837 (chr12: 69475065 − 69478102)

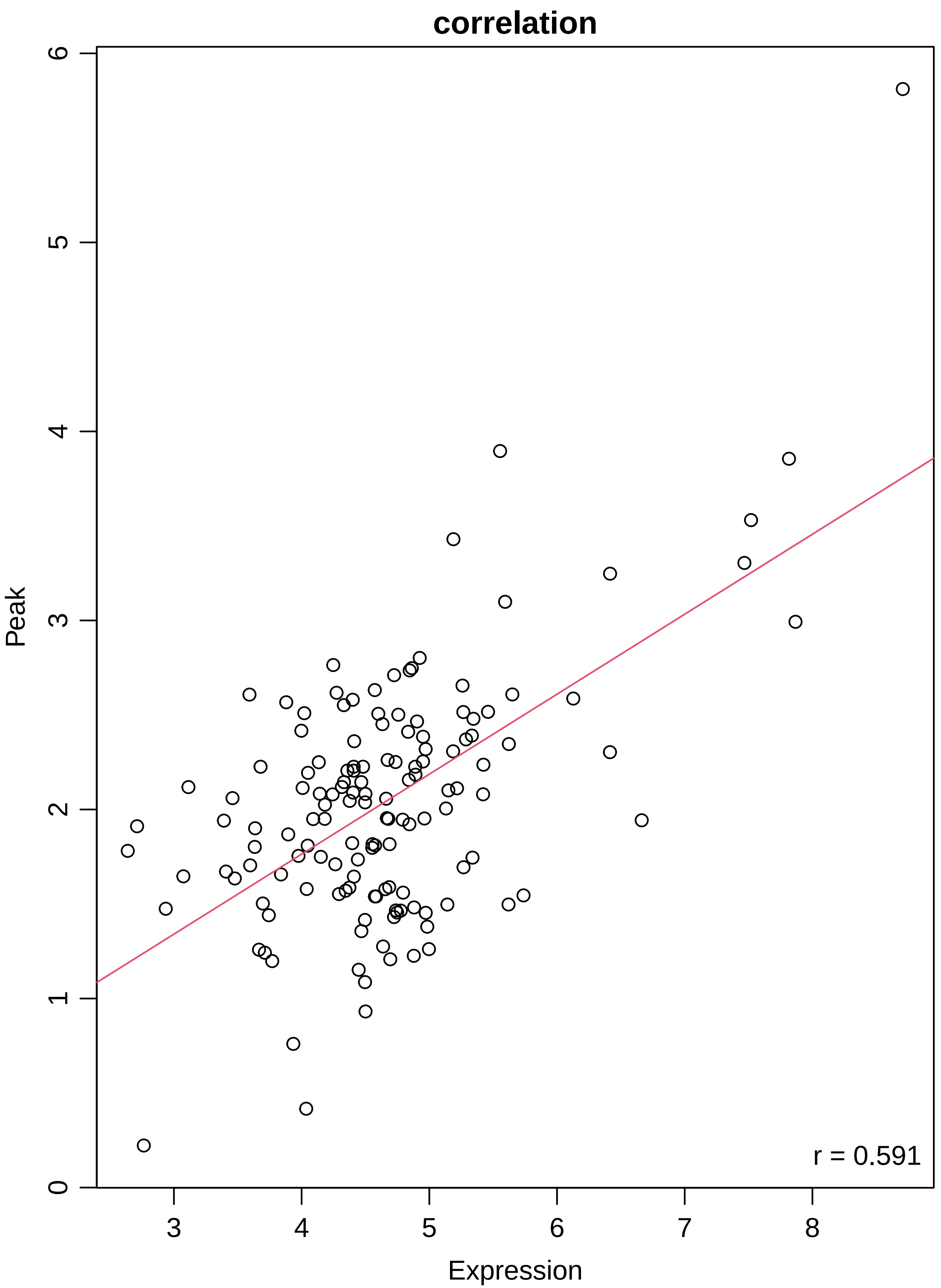

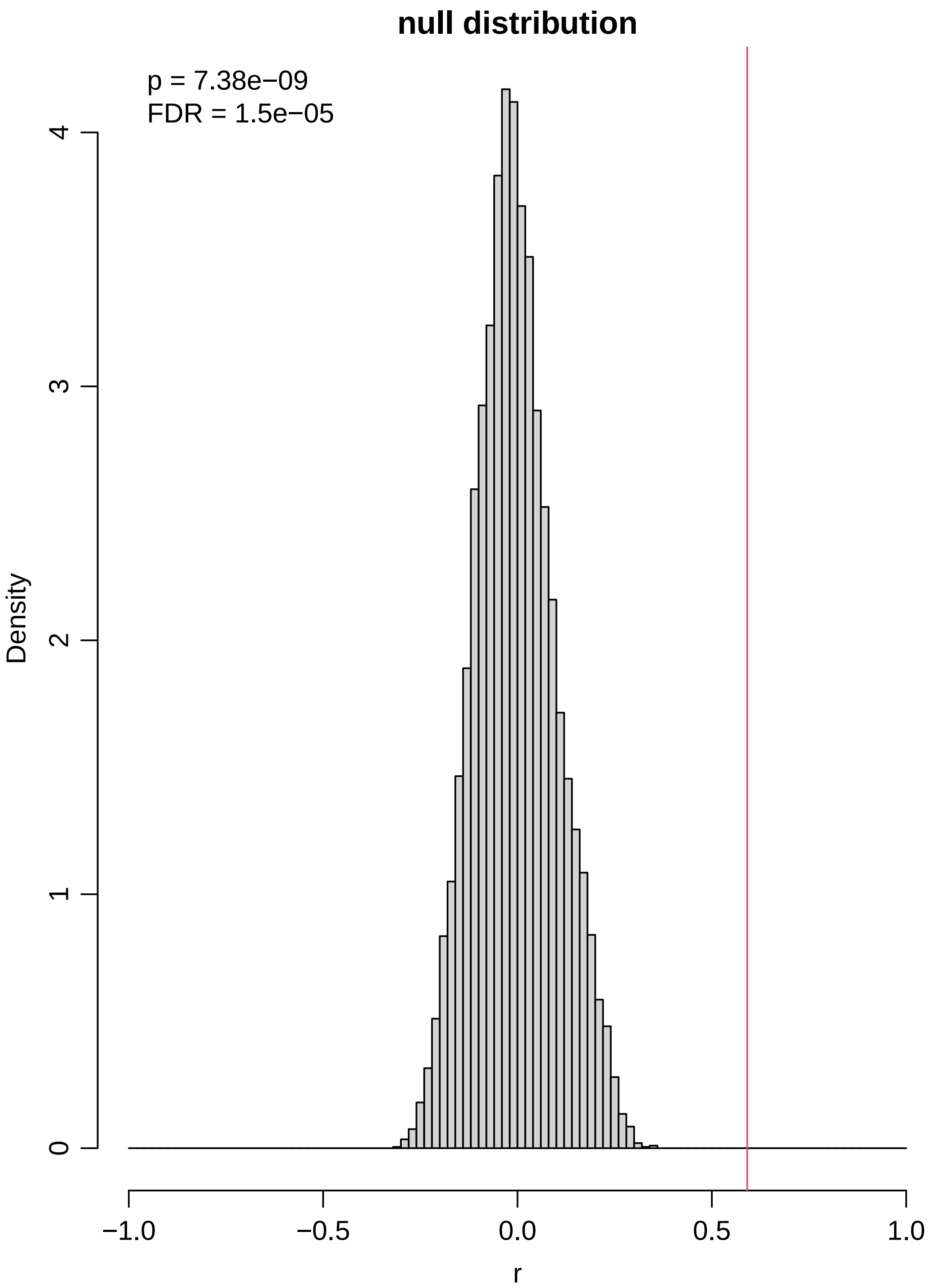

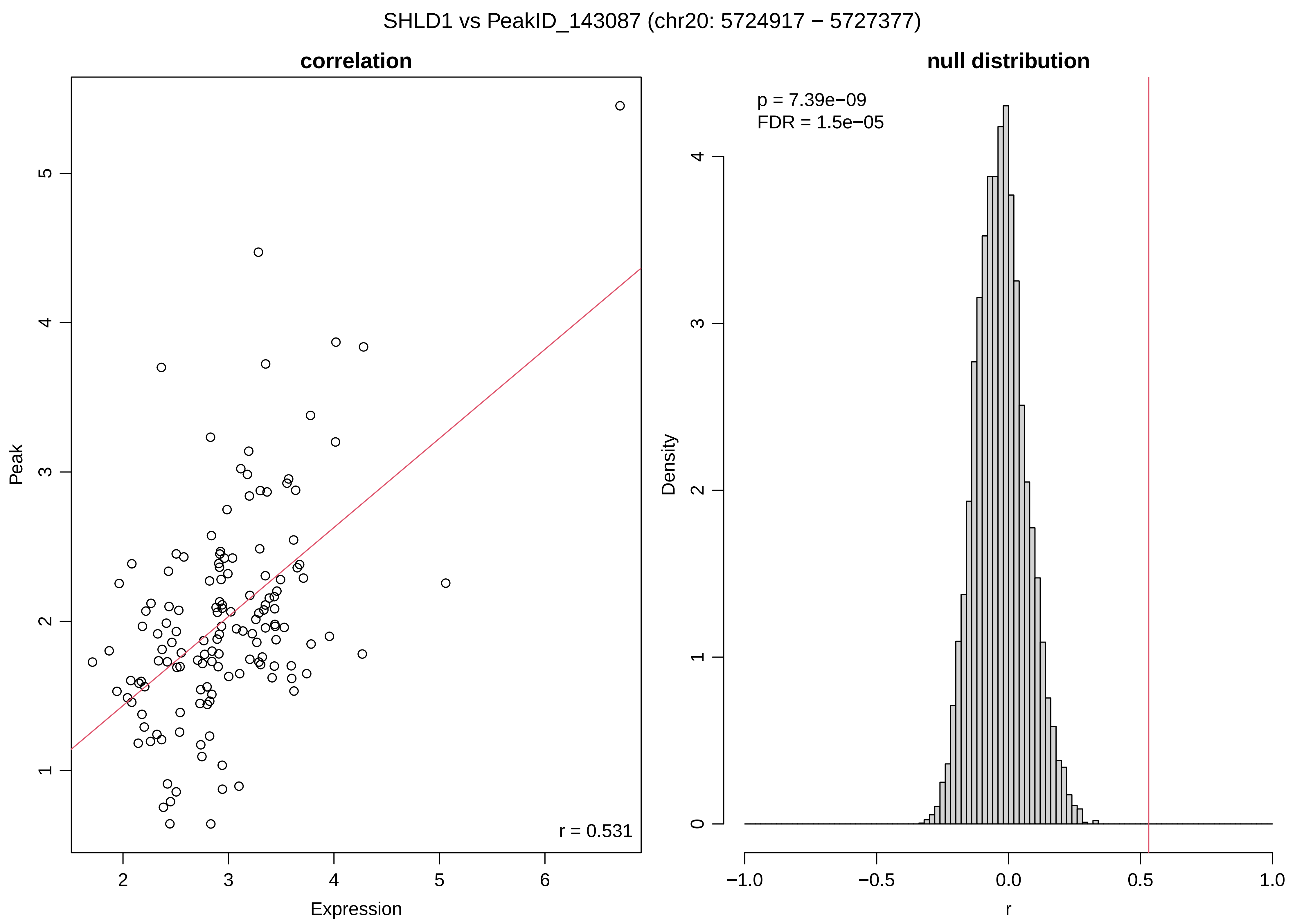
SHLD1 vs PeakID_143087 (chr20: 5724917 − 5727377)
correlation
Peak
Expression
r = 0.531
null distribution
p = 7.39e−09
FDR = 1.5e−05
Density
r

SLC35E3 vs PeakID_56610 (chr12: 68869972 − 68870302)

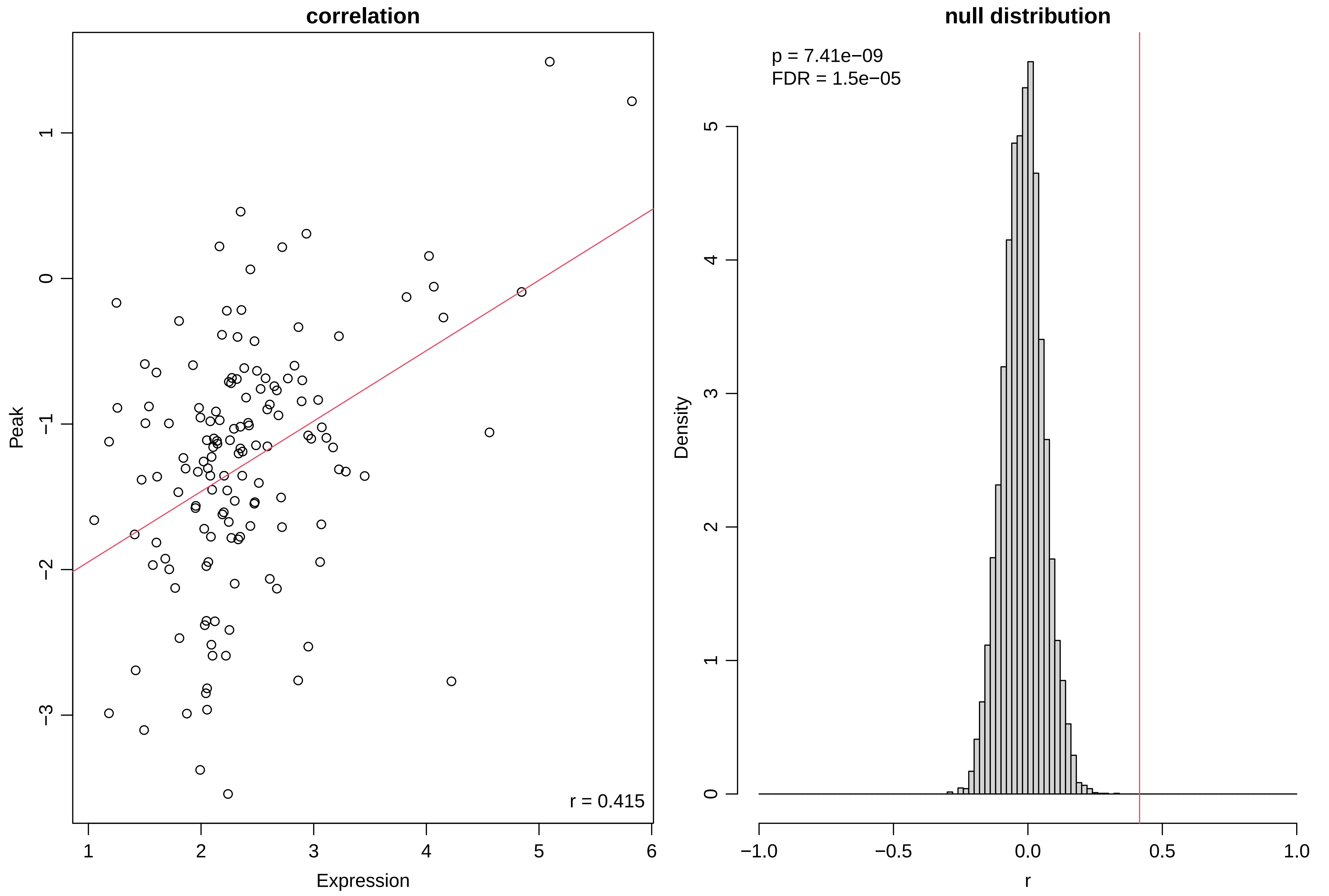

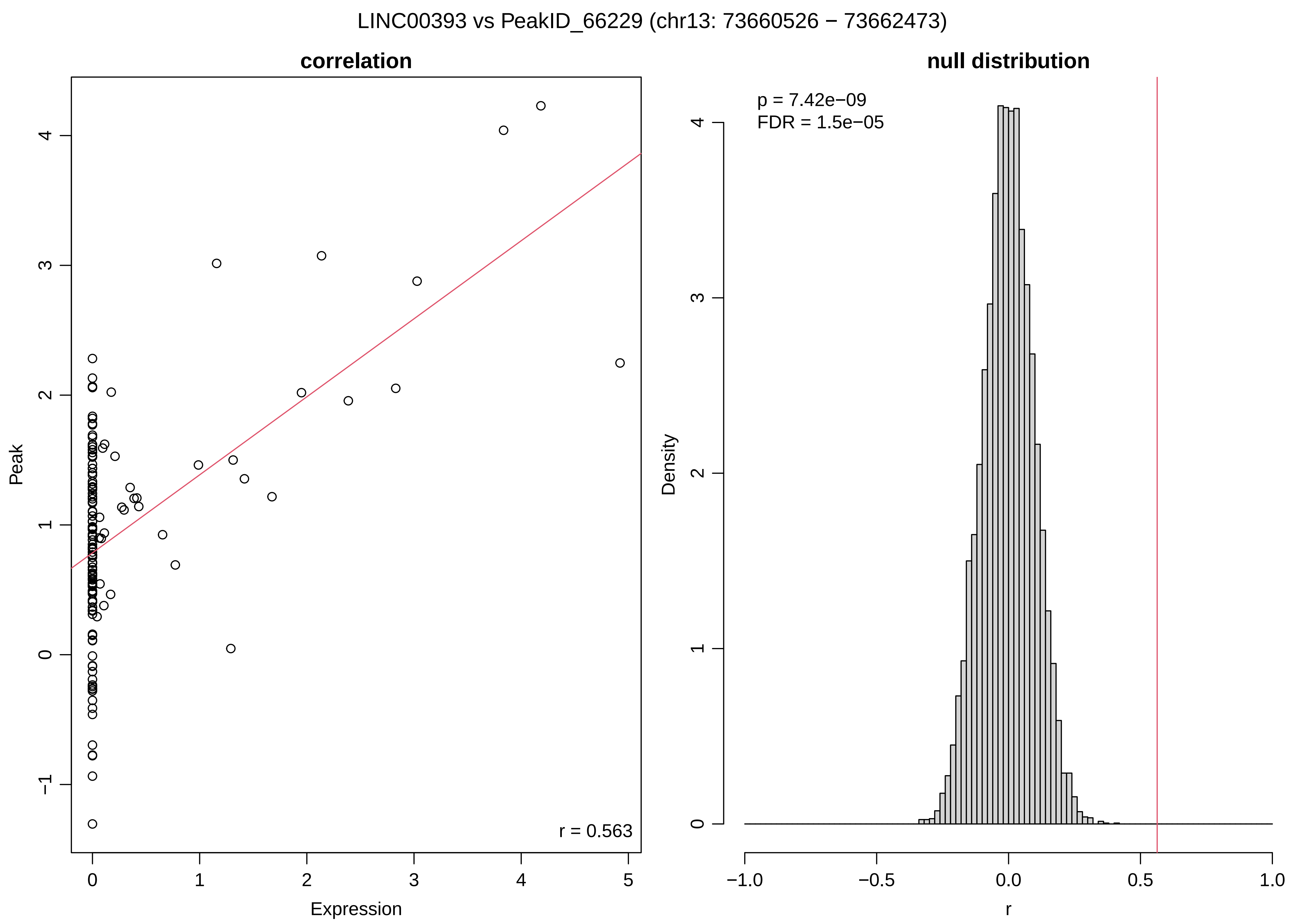
LINC00393 vs PeakID_66229 (chr13: 73660526 − 73662473)
correlation
null distribution
Peak
Expression
r = 0.563
Density
r
p = 7.42e−09
FDR = 1.5e−05
−1
0
1
2
3
4
5
−1.0
−0.5
0.0
0.5
1.0

YEATS4 vs PeakID_56755 (chr12: 69254154 − 69254543)

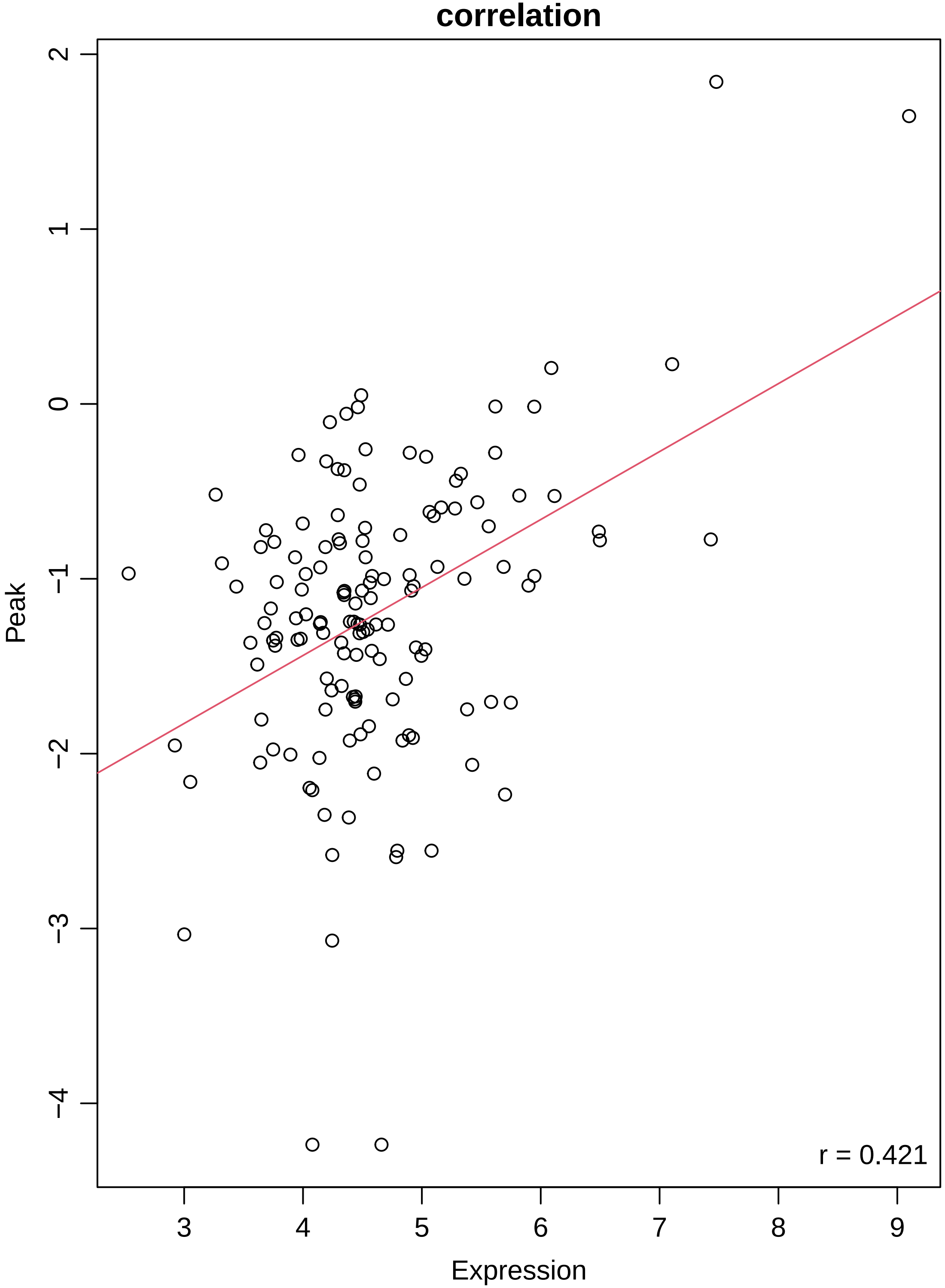

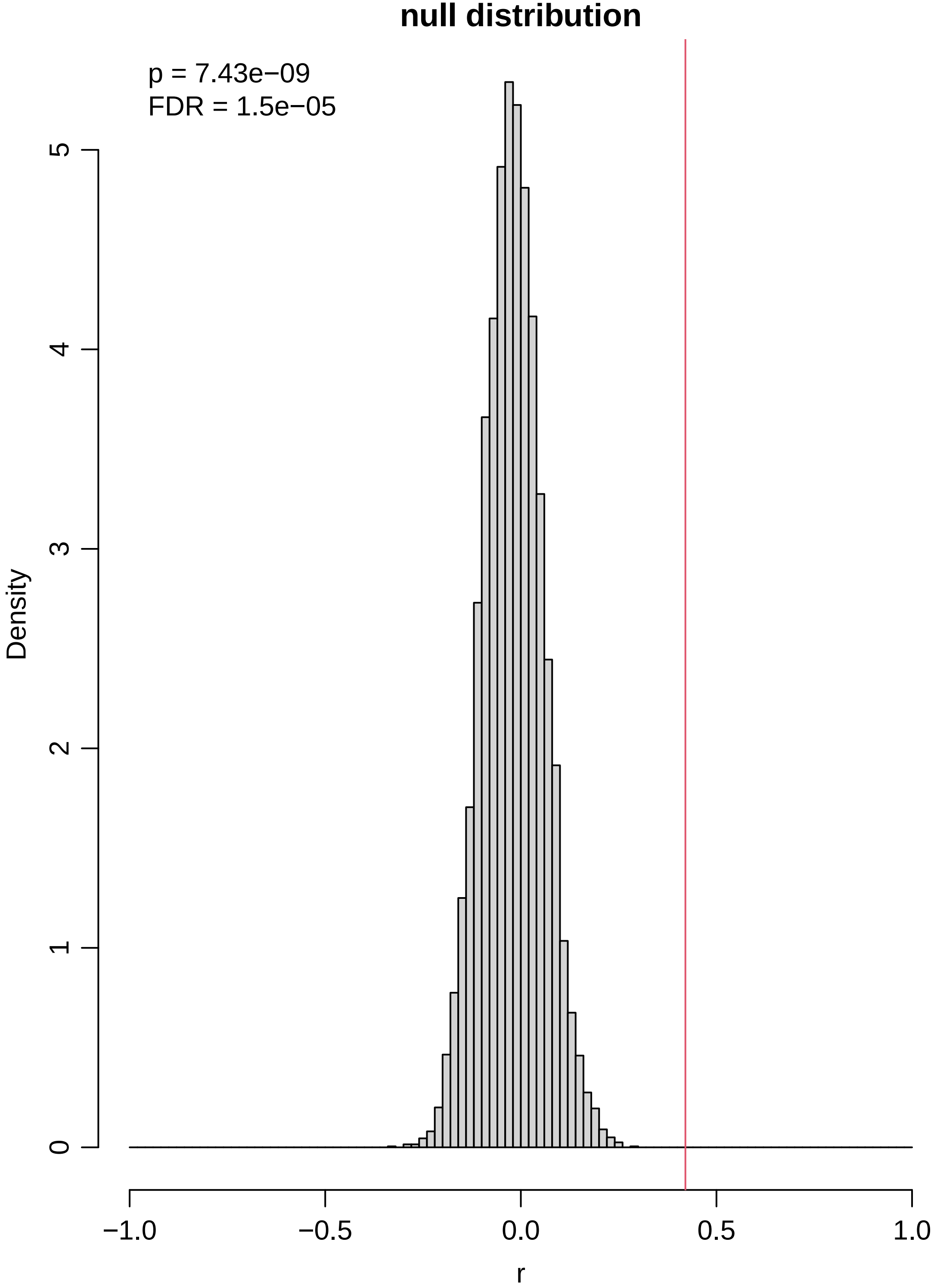

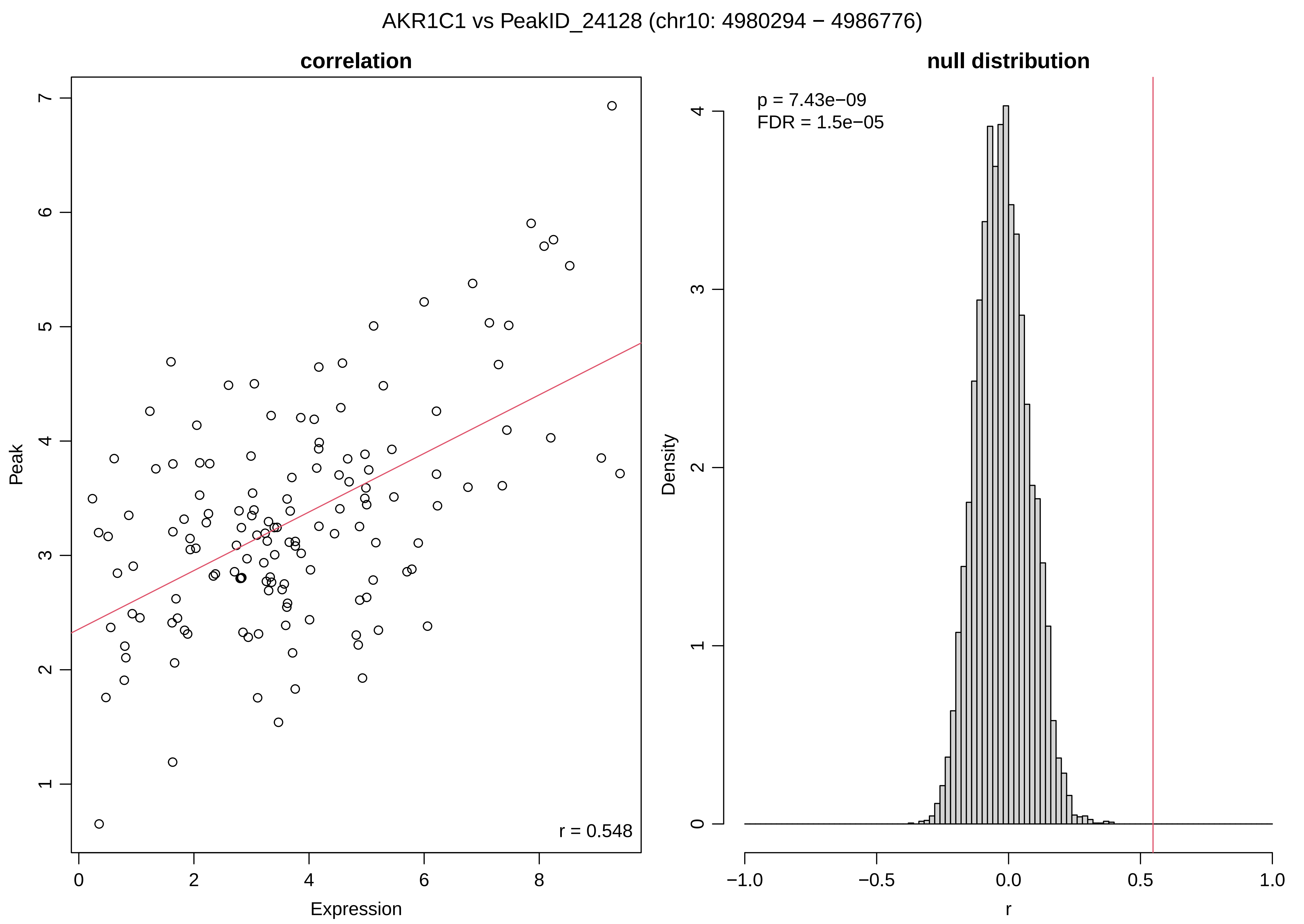

AKR1C1 vs PeakID_24128 (chr10: 4980294 − 4986776)
correlation
Peak
Expression
r = 0.548
null distribution
p = 7.43e−09
FDR = 1.5e−05
Density
r

AC079160.1 vs PeakID_180783 (chr4: 84298427 − 84299287)

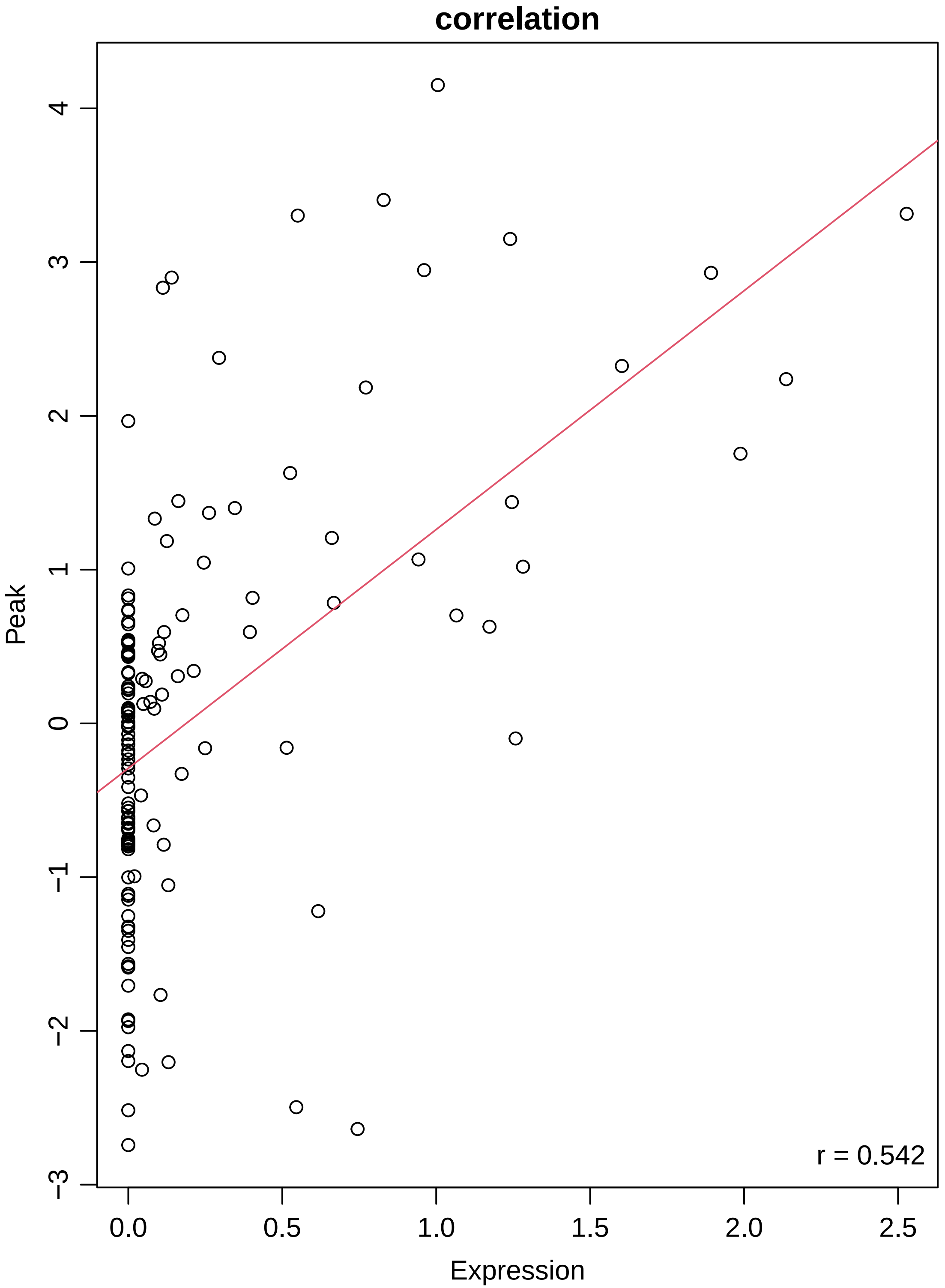

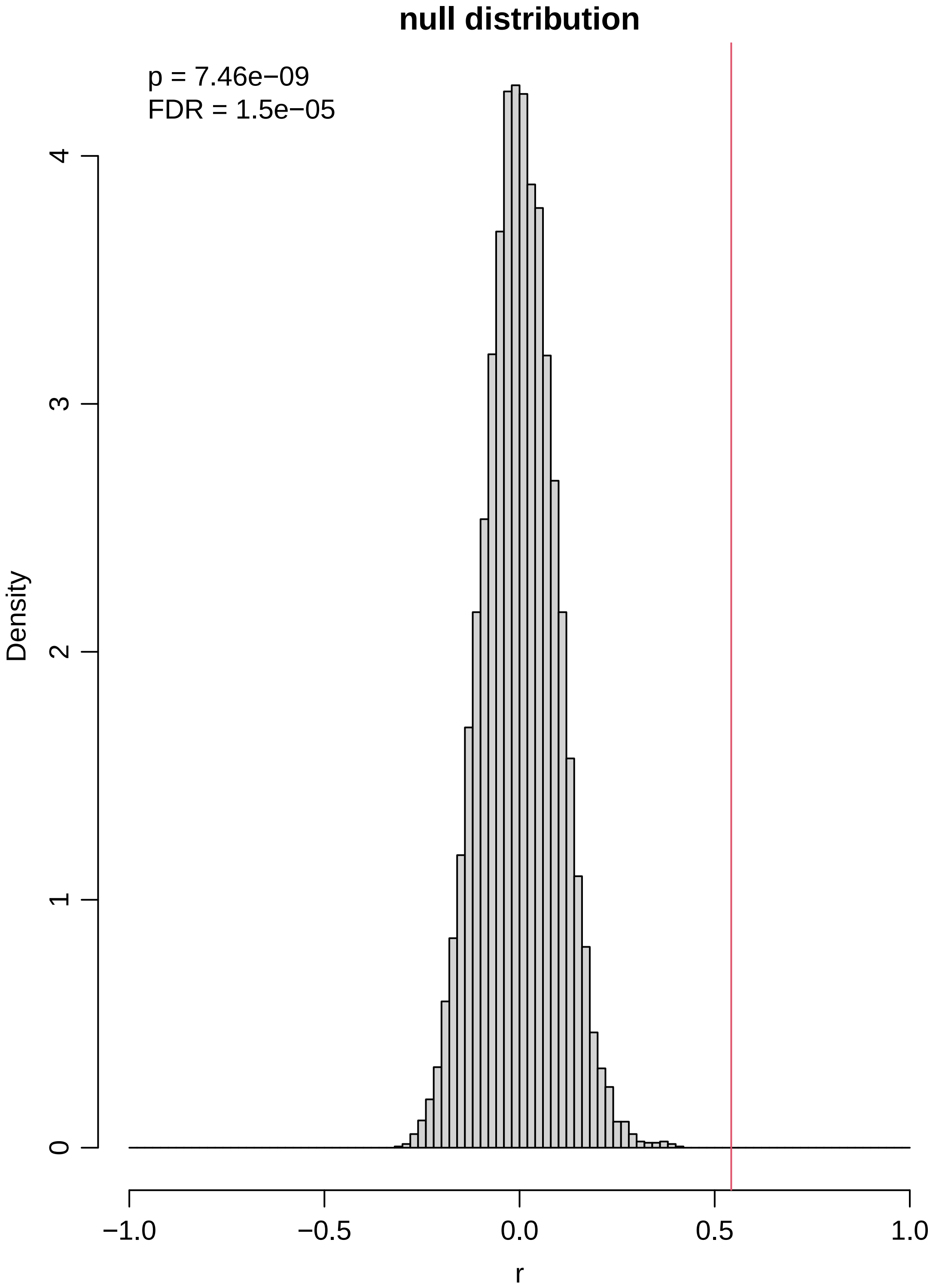

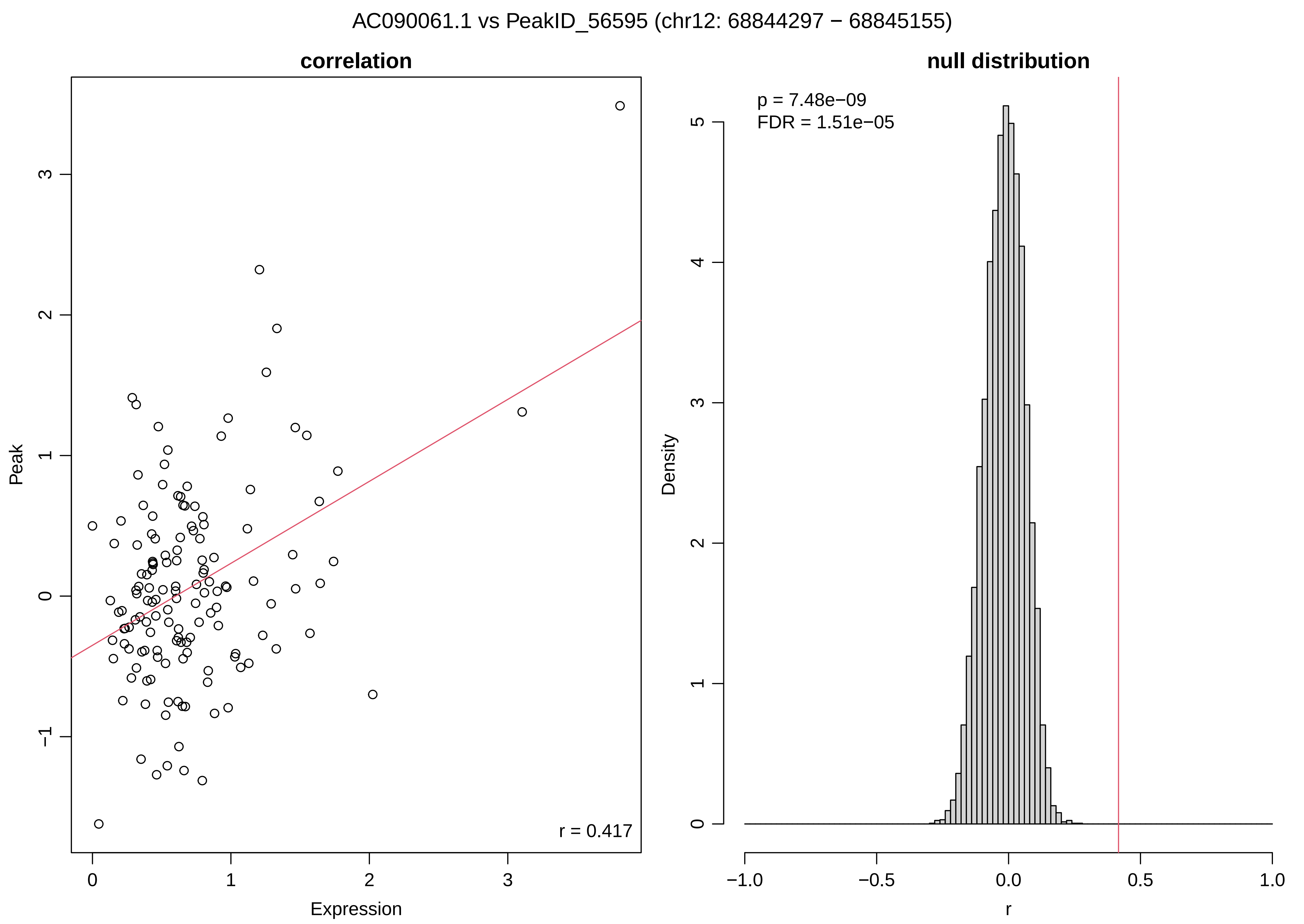
AC090061.1 vs PeakID_56595 (chr12: 68844297 − 68845155)
correlation
Peak
Expression
r = 0.417
null distribution
p = 7.48e−09
FDR = 1.51e−05
Density
r

GYG2P1 vs PeakID_262705 (chrY: 12243575 − 12248604)

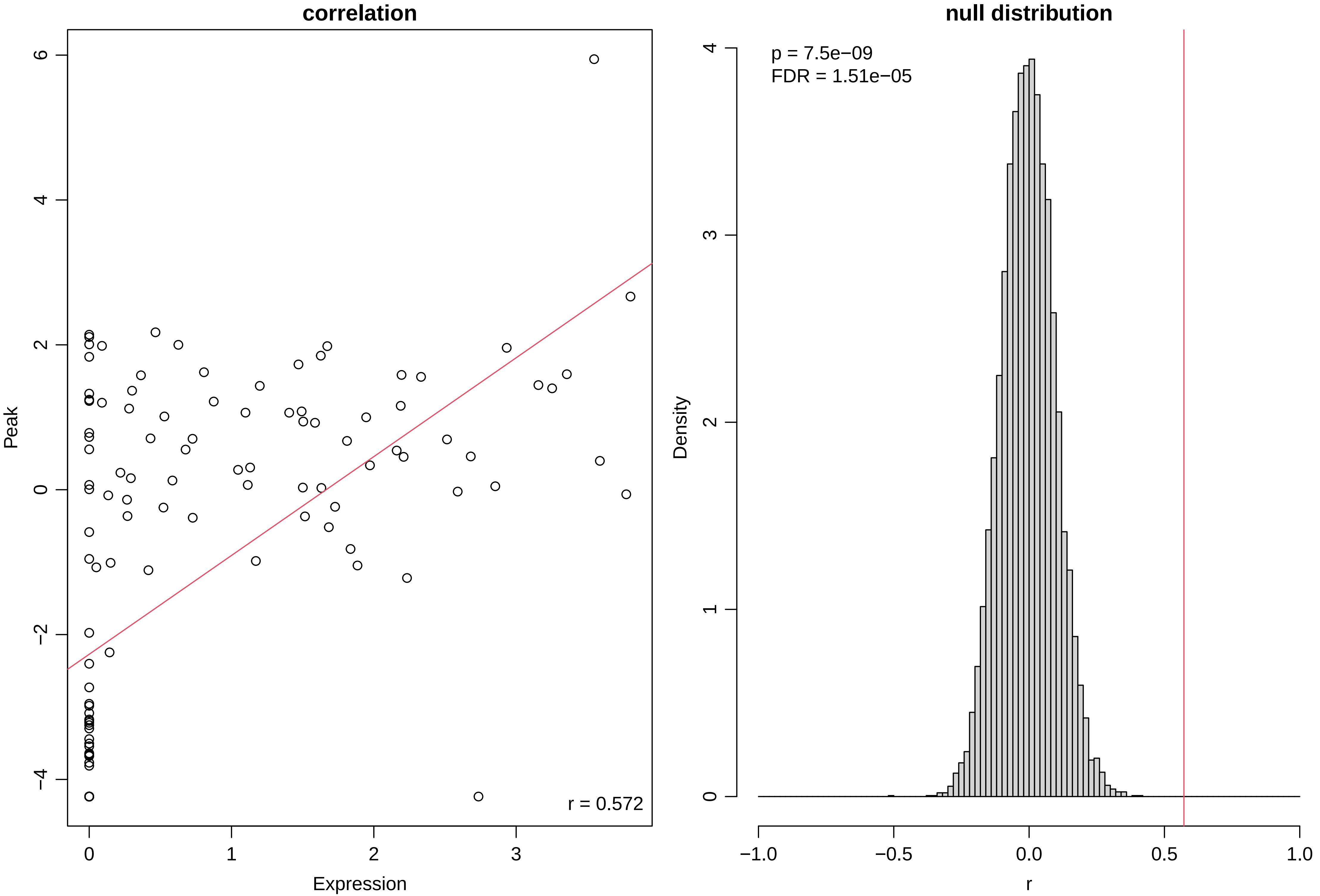

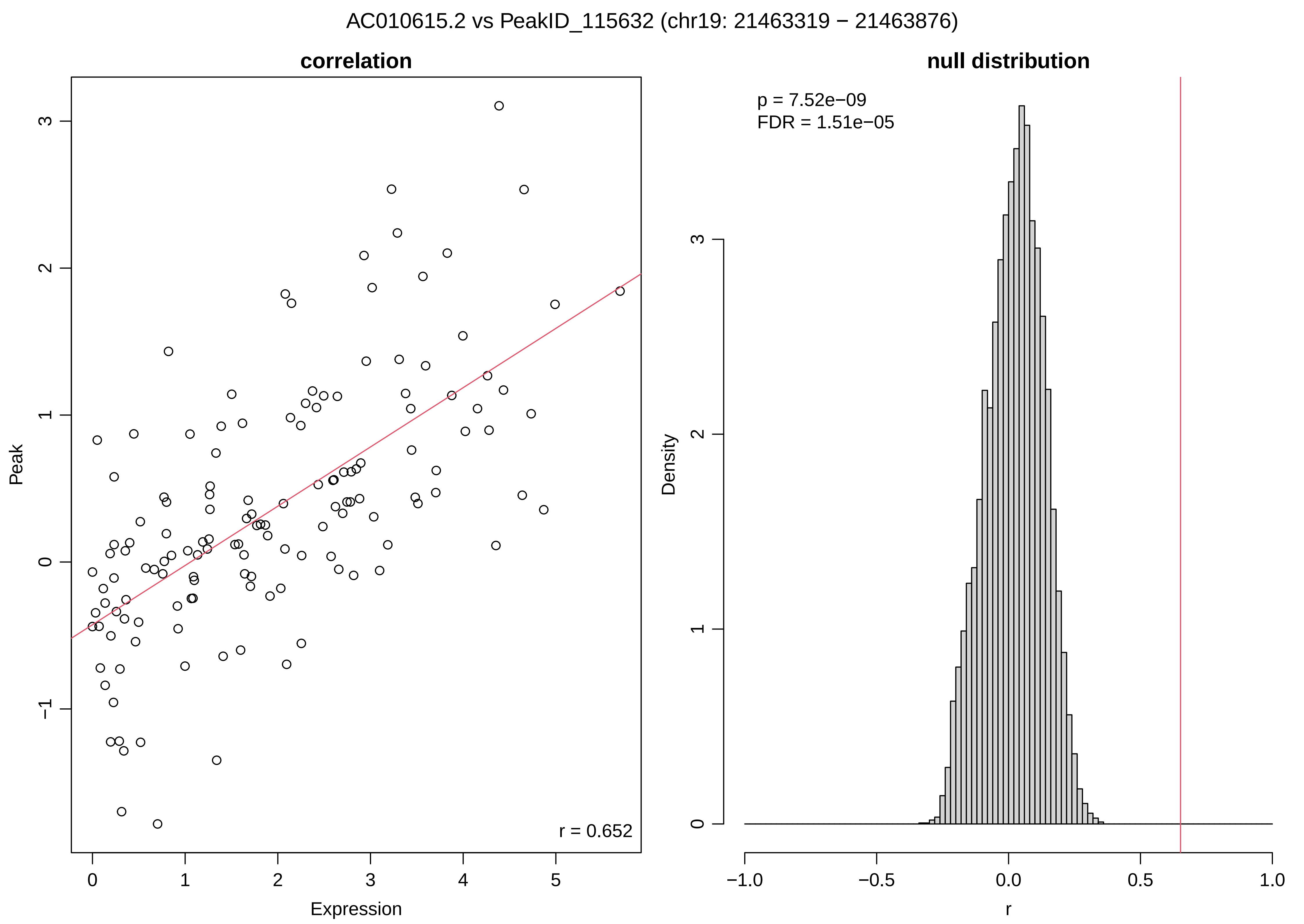

AC010615.2 vs PeakID_115632 (chr19: 21463319 − 21463876)
correlation
Peak
Expression
r = 0.652
null distribution
p = 7.52e−09
FDR = 1.51e−05
Density
r

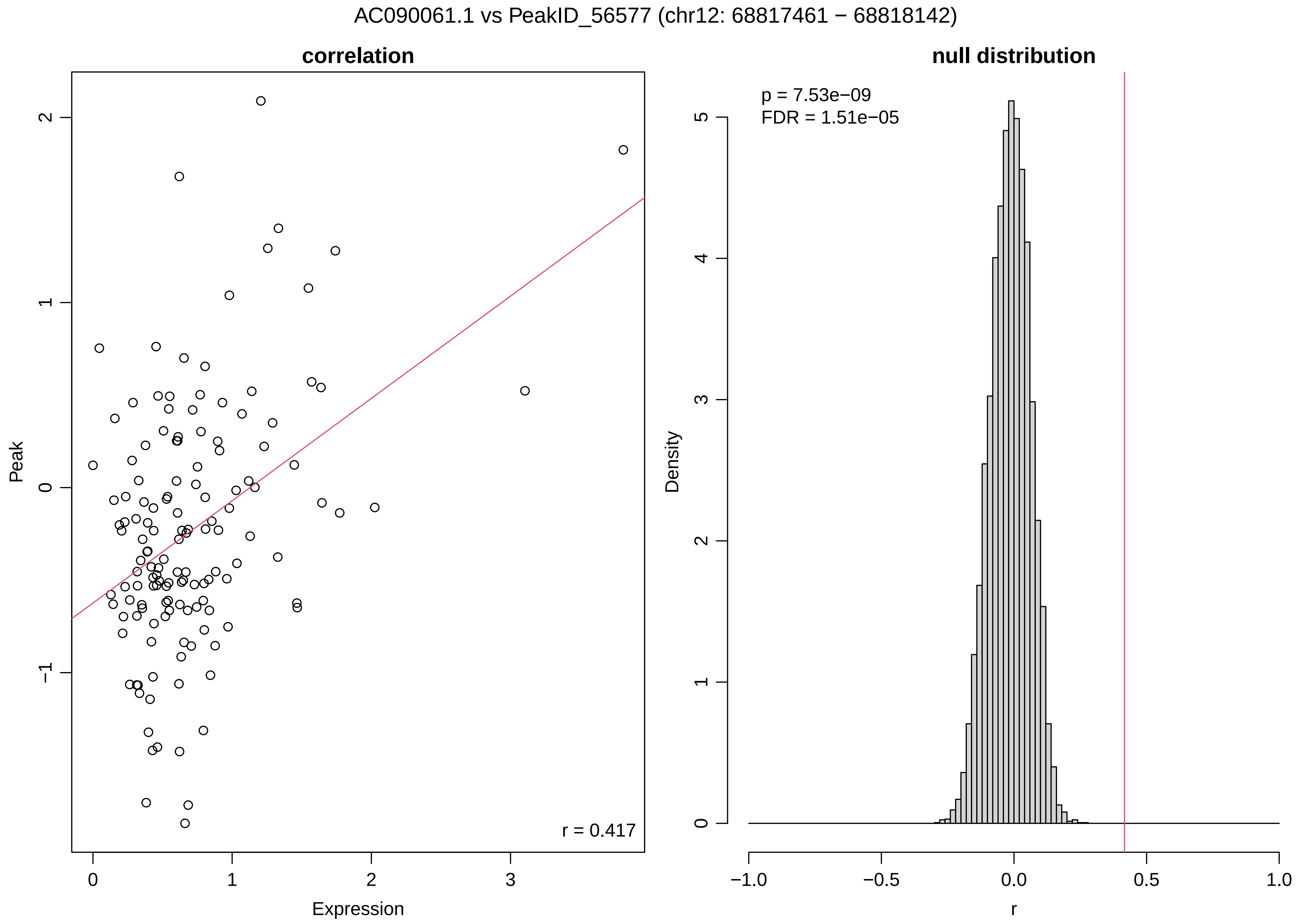

AC090061.1 vs PeakID_56577 (chr12: 68817461 − 68818142)
correlation
Peak
Expression
r = 0.417
null distribution
p = 7.53e−09
FDR = 1.51e−05
Density
r

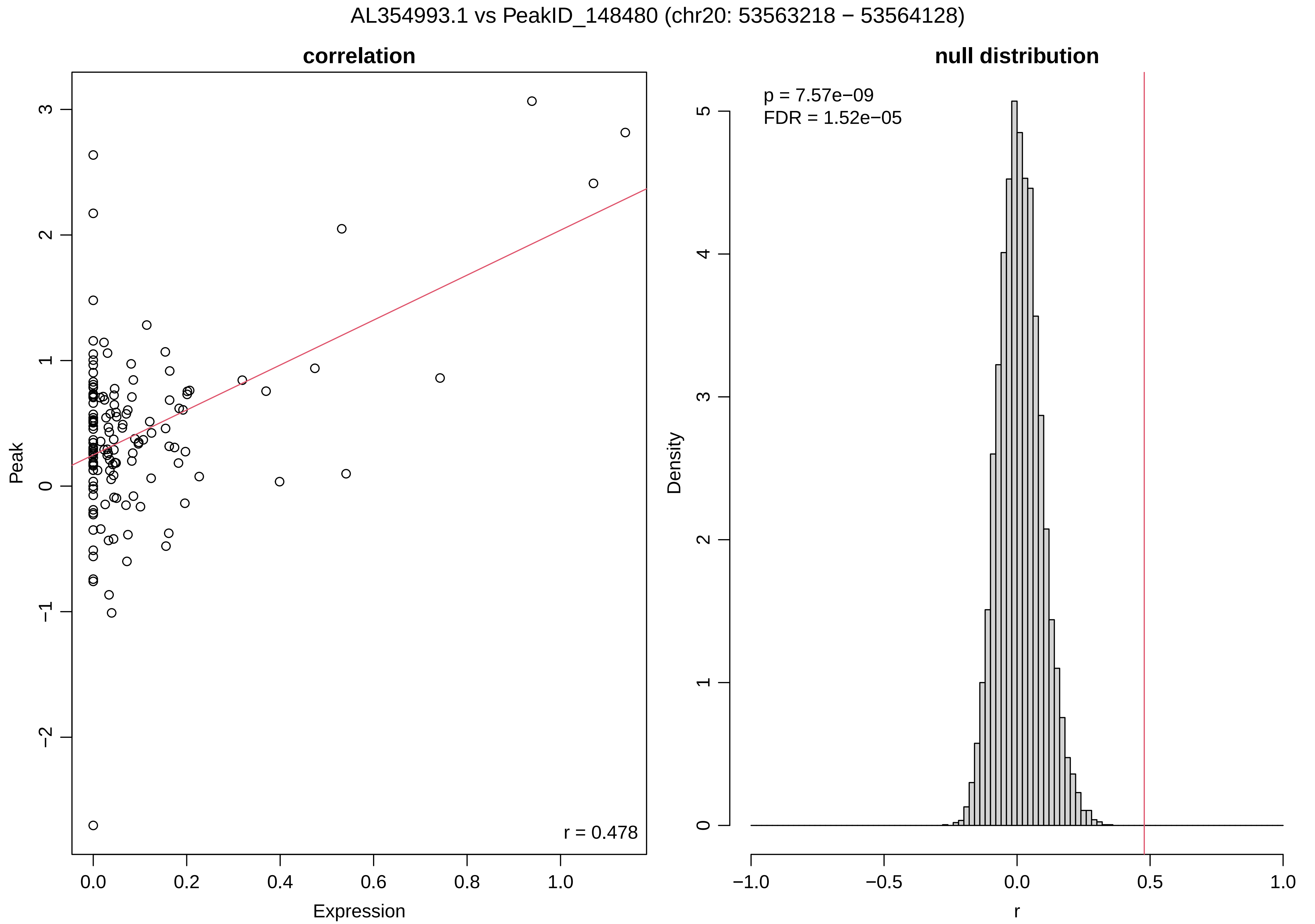
AL354993.1 vs PeakID_148480 (chr20: 53563218 − 53564128)
correlation
Peak
Expression
r = 0.478
null distribution
p = 7.57e−09
FDR = 1.52e−05
Density
r

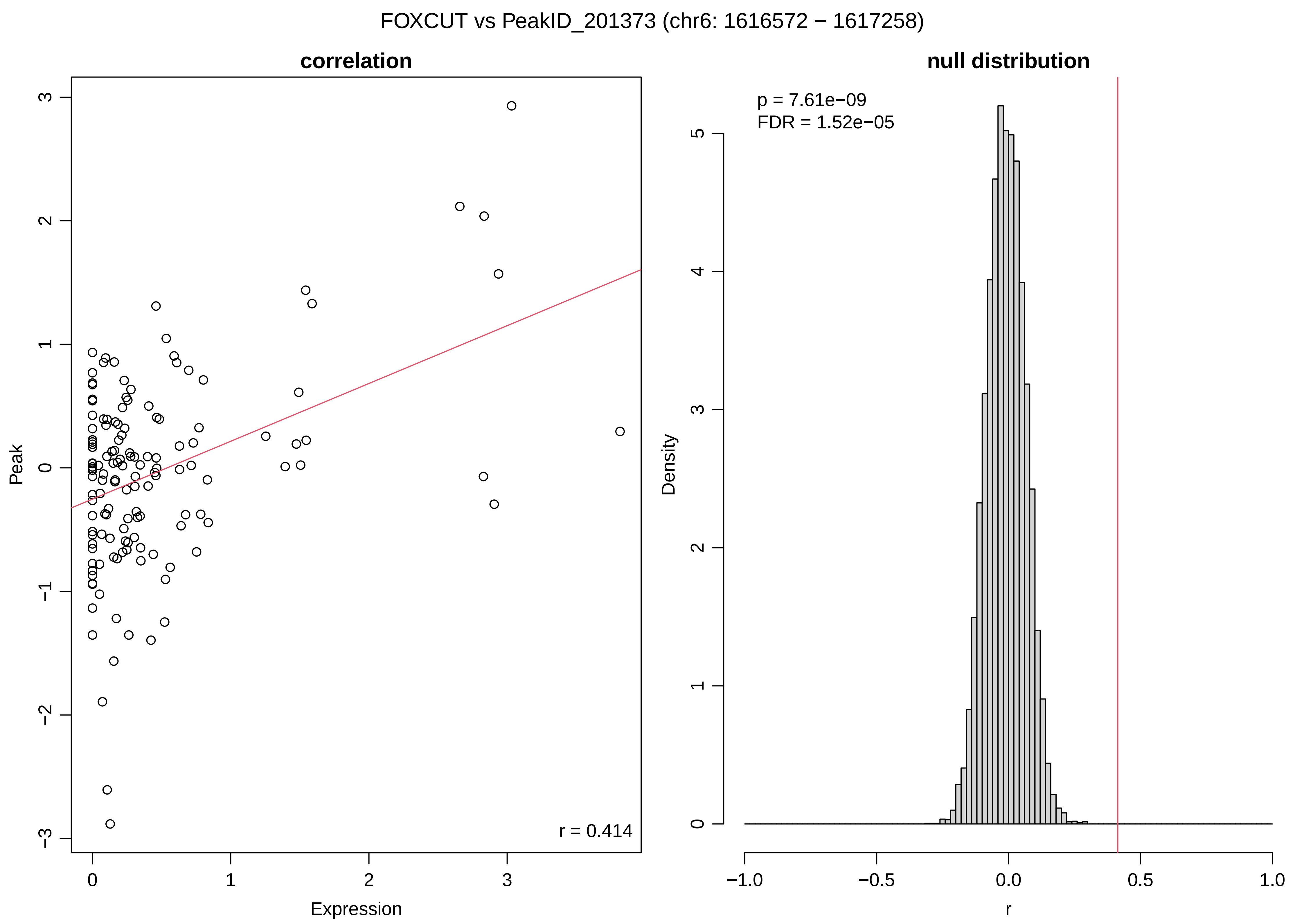

FOXCUT vs PeakID_201373 (chr6: 1616572 − 1617258)
correlation
null distribution
Peak
Expression
r = 0.414
p = 7.61e−09
FDR = 1.52e−05
Density
r

AC090017.1 vs PeakID_55231 (chr12: 61171320 − 61171926)

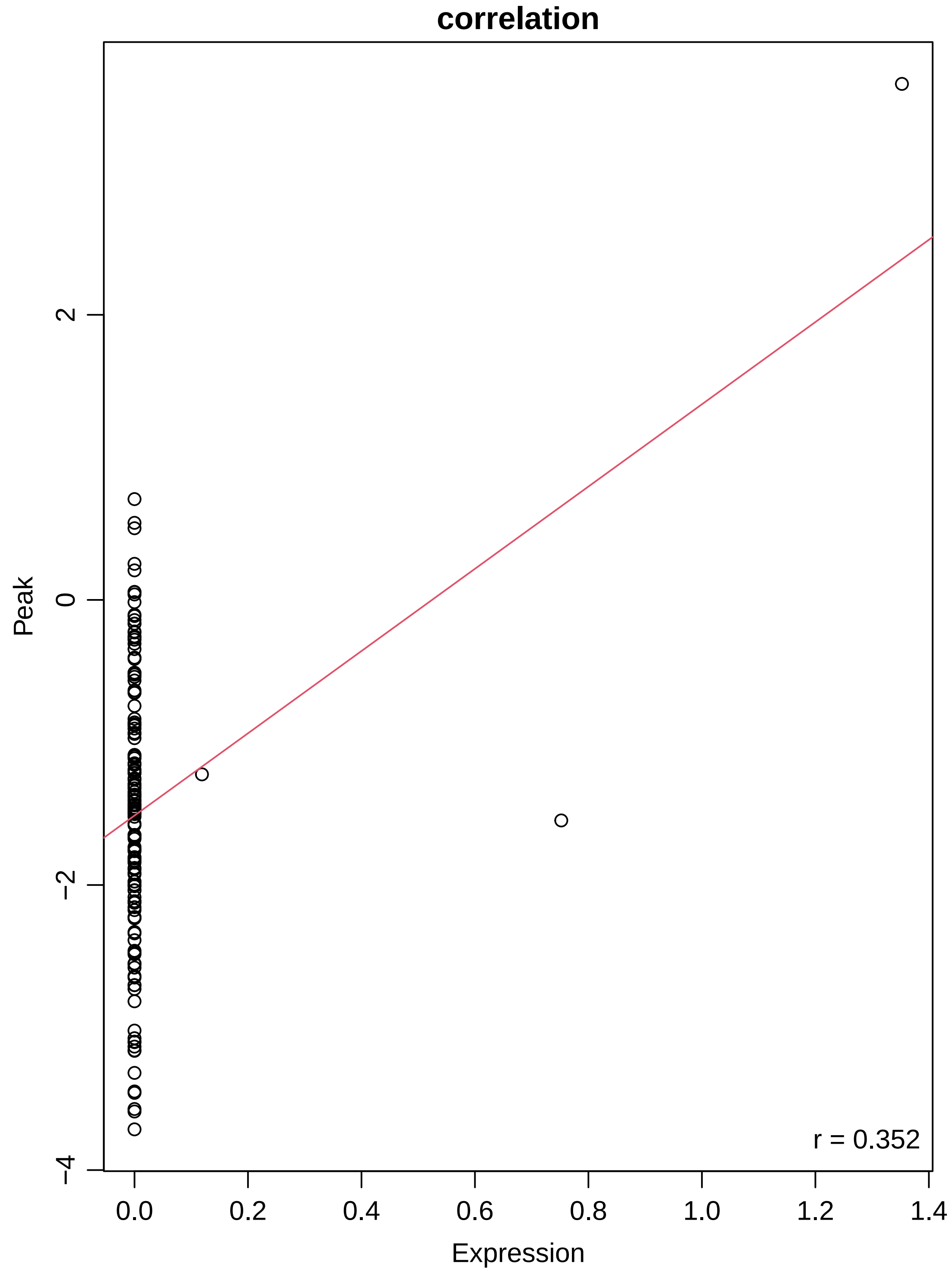

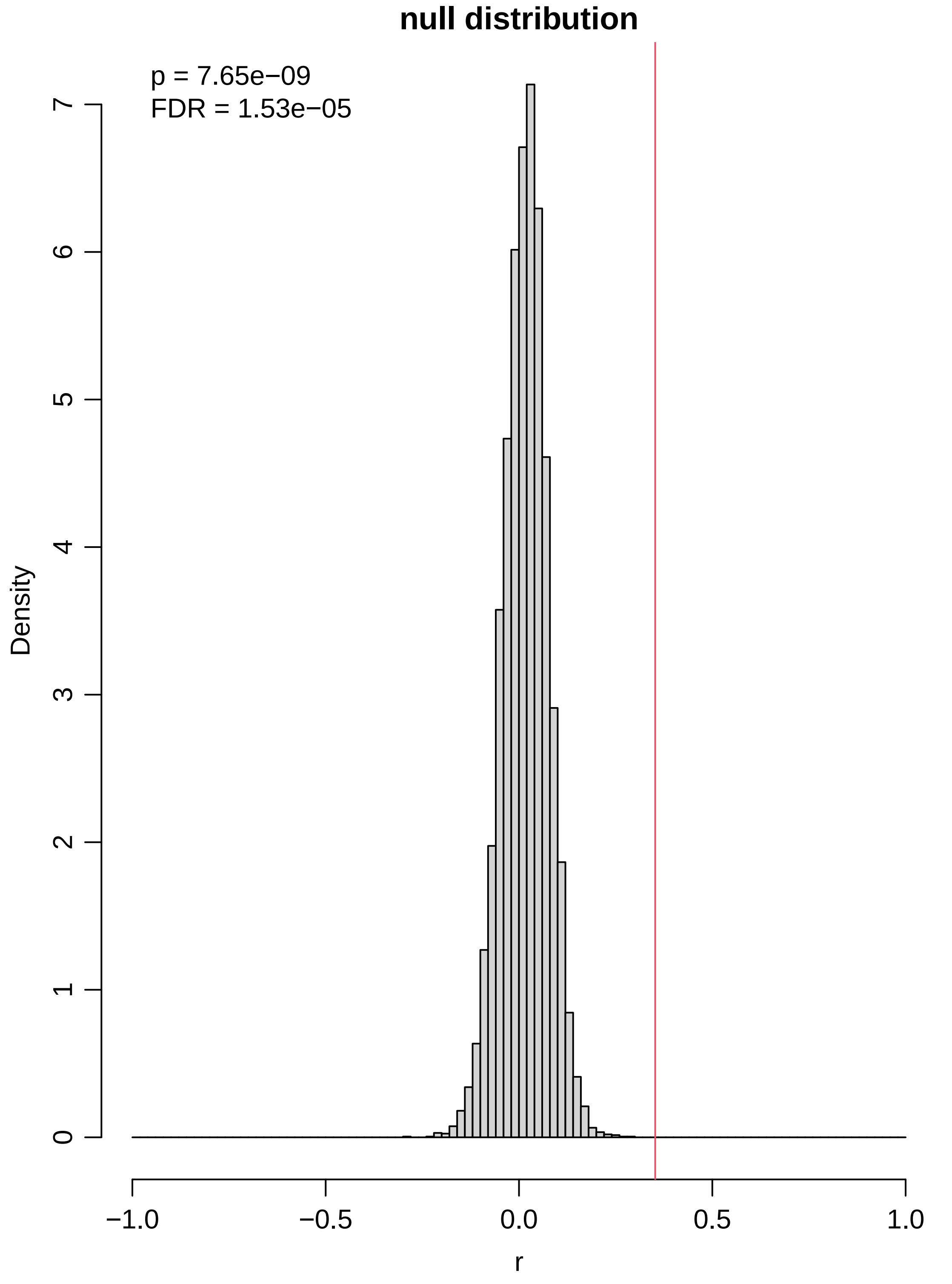

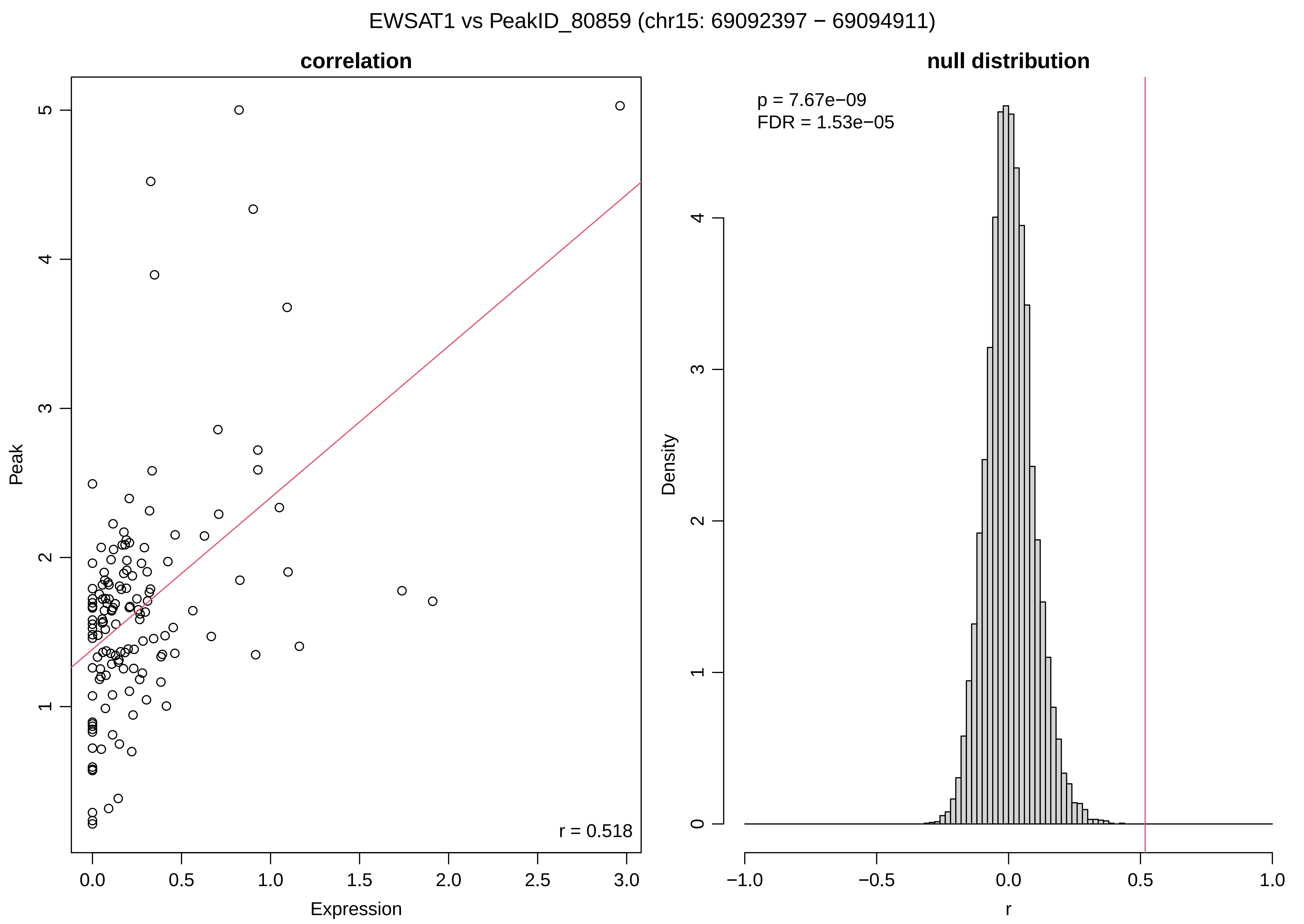
EWSAT1 vs PeakID_80859 (chr15: 69092397 − 69094911)
correlation
Peak
Expression
r = 0.518
null distribution
p = 7.67e−09
FDR = 1.53e−05
Density
r

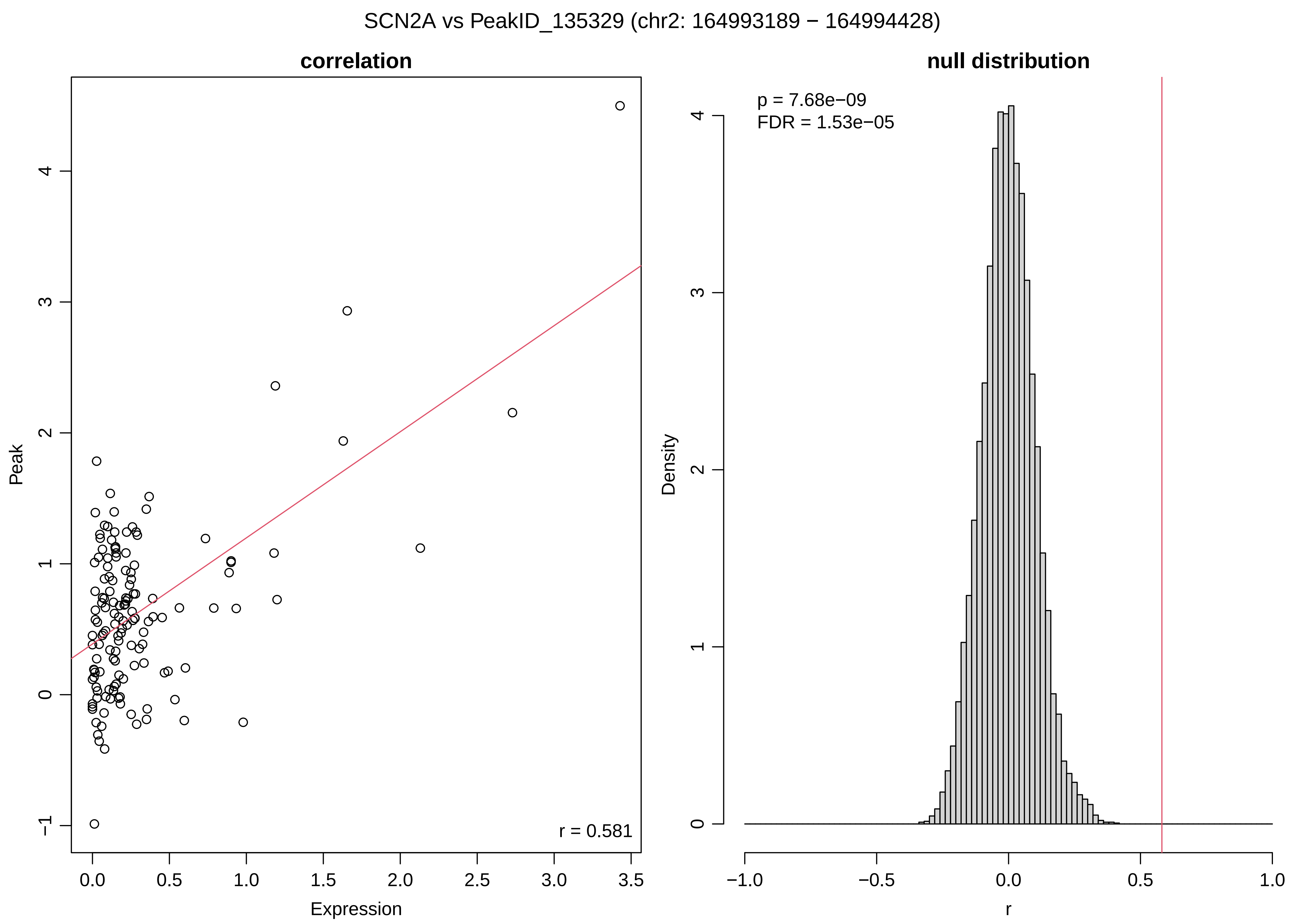
SCN2A vs PeakID_135329 (chr2: 164993189 − 164994428)
correlation
Peak
Expression
r = 0.581
null distribution
p = 7.68e−09
FDR = 1.53e−05
Density
r

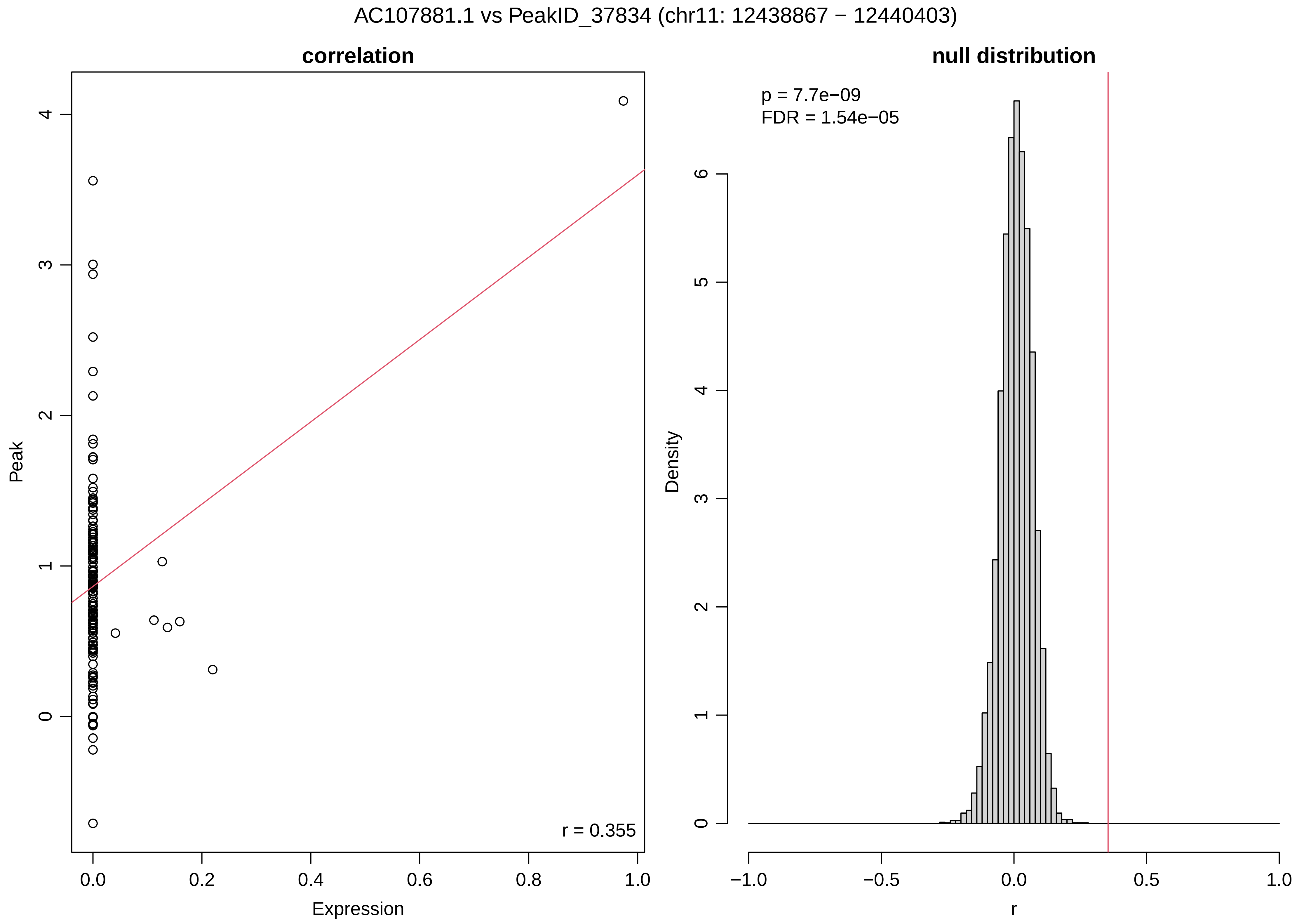
AC107881.1 vs PeakID_37834 (chr11: 12438867 − 12440403)
correlation
Peak
Expression
r = 0.355
null distribution
p = 7.7e−09
FDR = 1.54e−05
Density
r

AP005119.2 vs PeakID_106755 (chr18: 1406910 − 1407264)

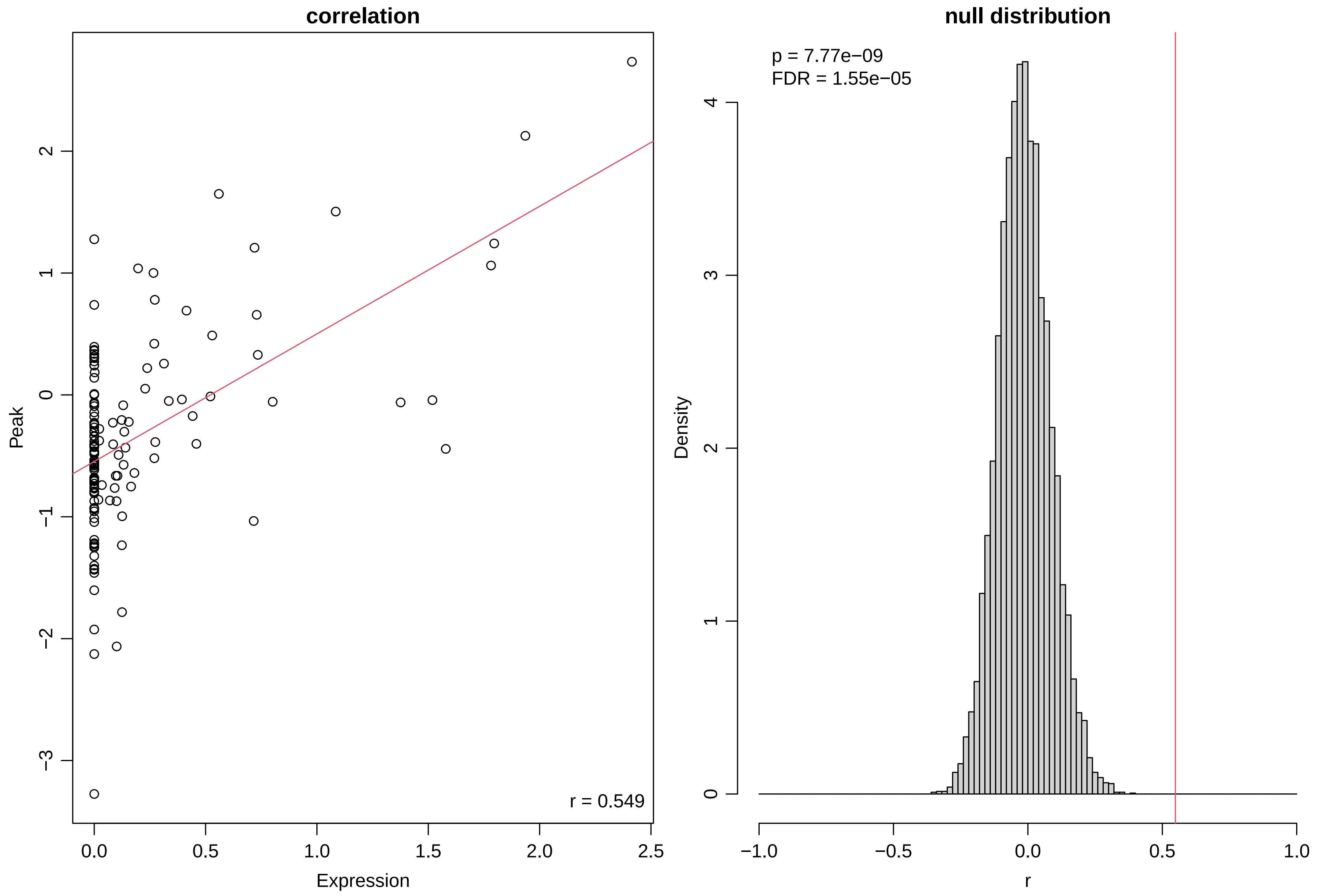

UGT1A4 vs PeakID_140976 (chr2: 233688739 − 233703275)

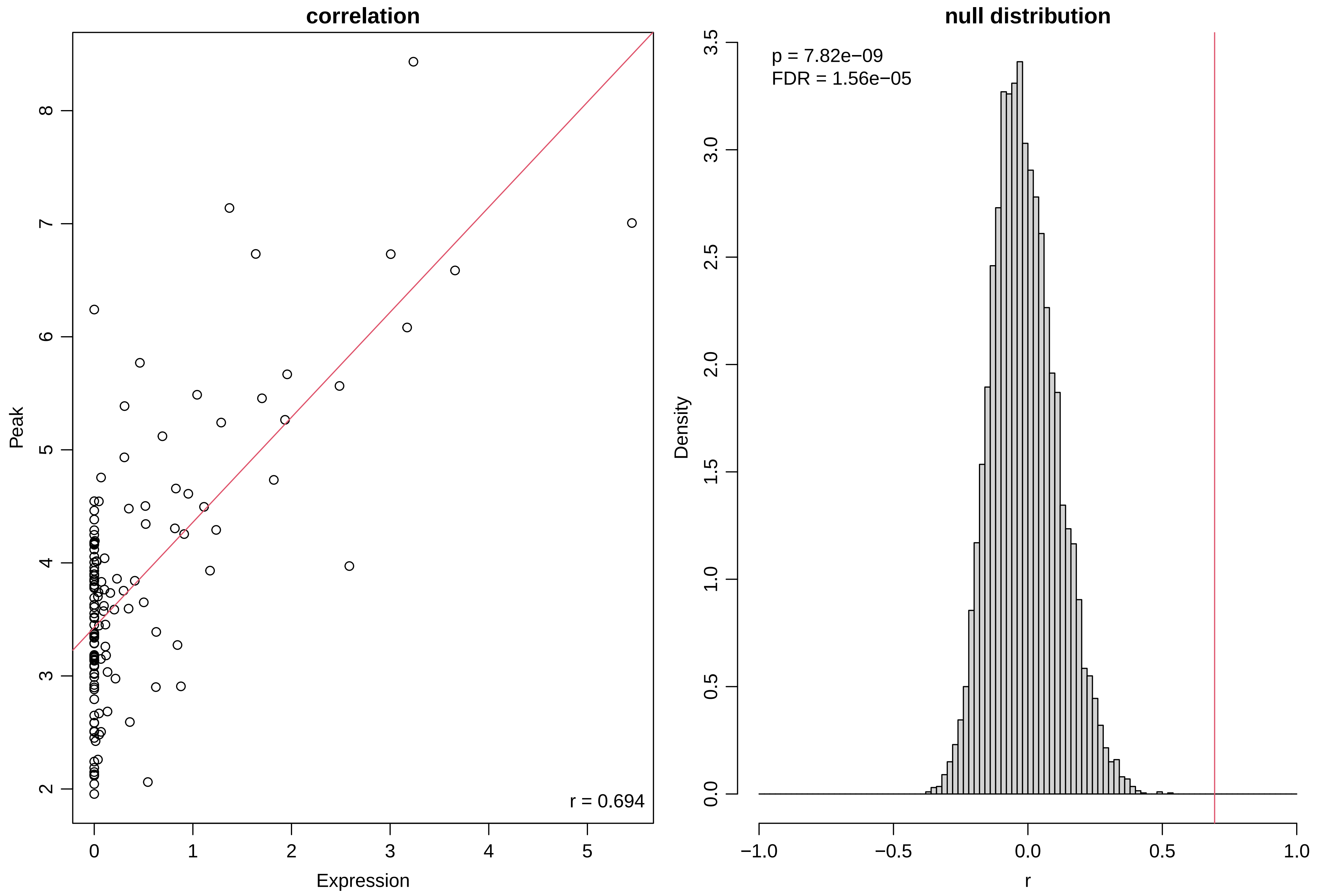

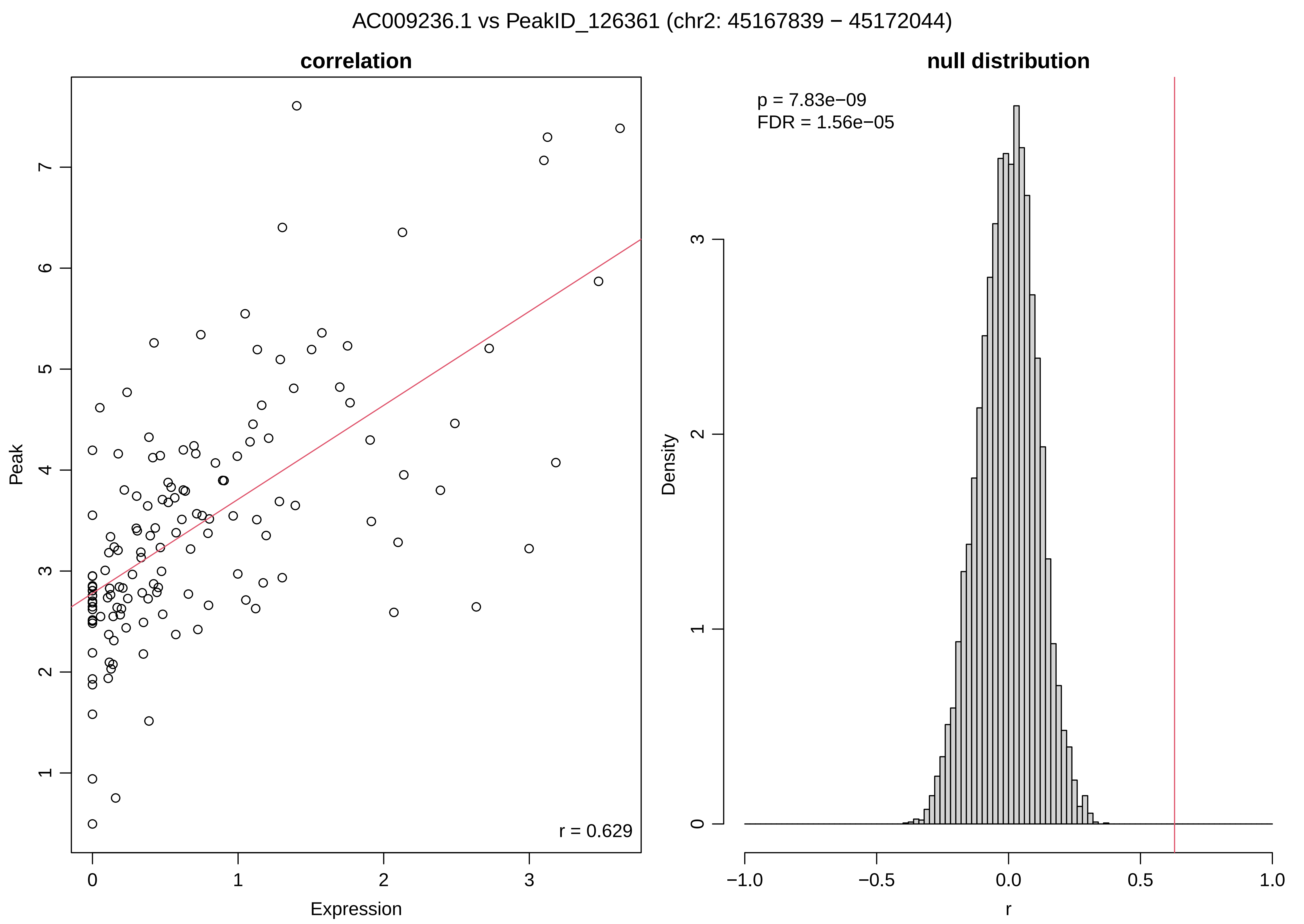
AC009236.1 vs PeakID_126361 (chr2: 45167839 − 45172044)
correlation
Peak
Expression
r = 0.629
null distribution
p = 7.83e−09
FDR = 1.56e−05
Density
r

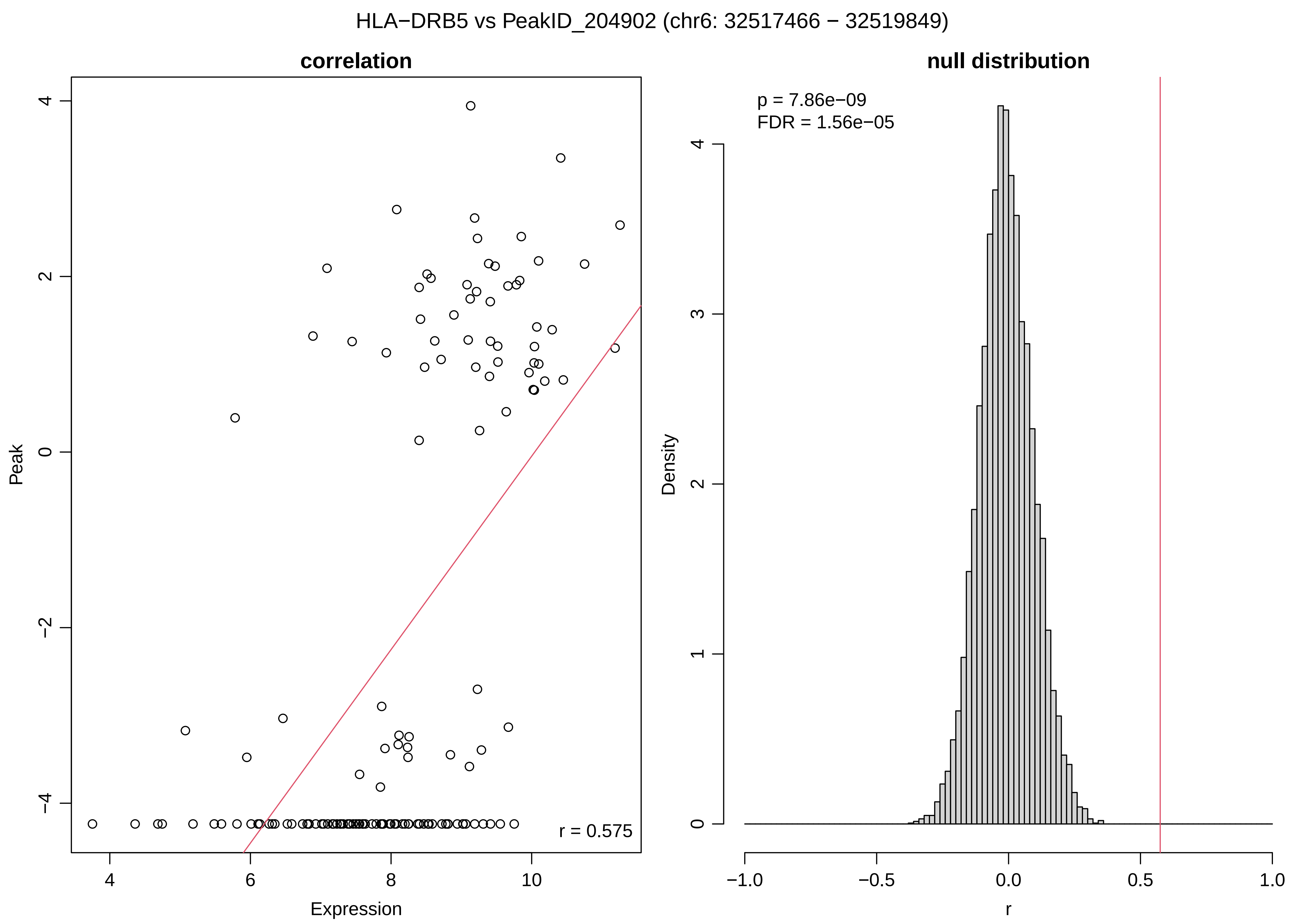
HLA−DRB5 vs PeakID_204902 (chr6: 32517466 − 32519849)
correlation
Peak
Expression
r = 0.575
4
2
0
−2
−4
4
6
8
10
null distribution
p = 7.86e−09
FDR = 1.56e−05
Density
r
0
1
2
3
4
−1.0
−0.5
0.0
0.5
1.0

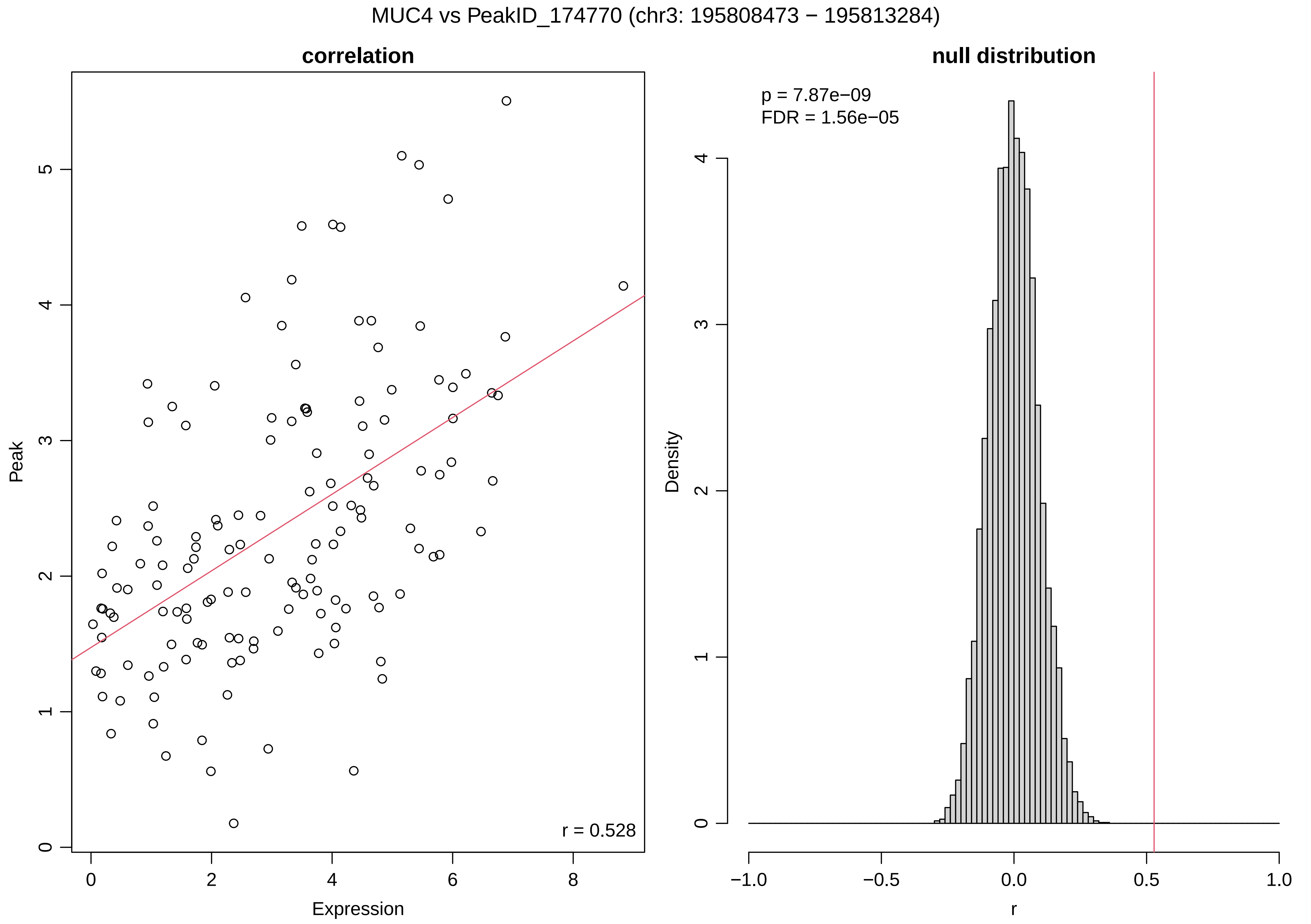
MUC4 vs PeakID_174770 (chr3: 195808473 − 195813284)
correlation
Peak
Expression
r = 0.528
null distribution
p = 7.87e−09
FDR = 1.56e−05
Density
r

WNT7A vs PeakID_161081 (chr3: 13878468 − 13880739)

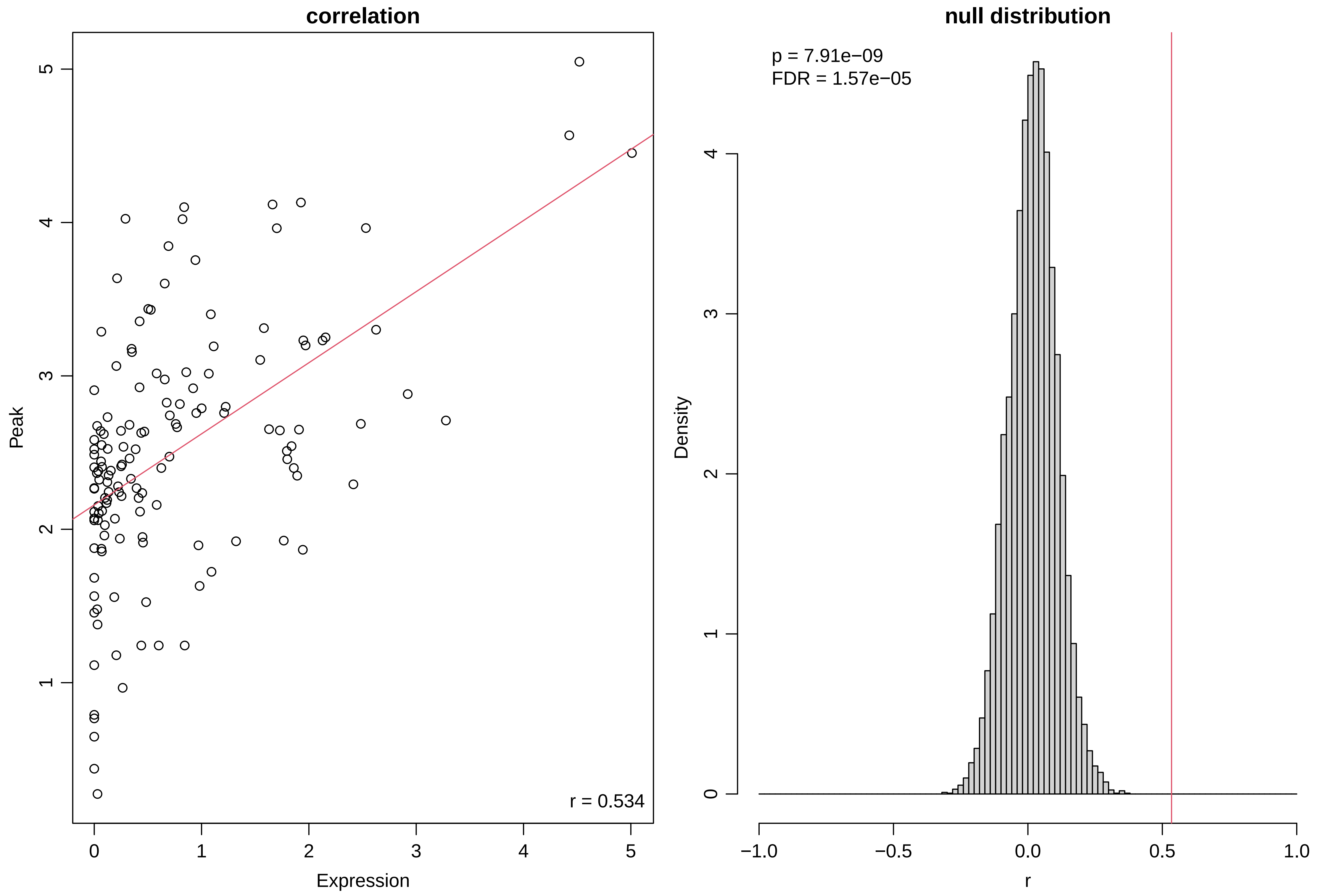

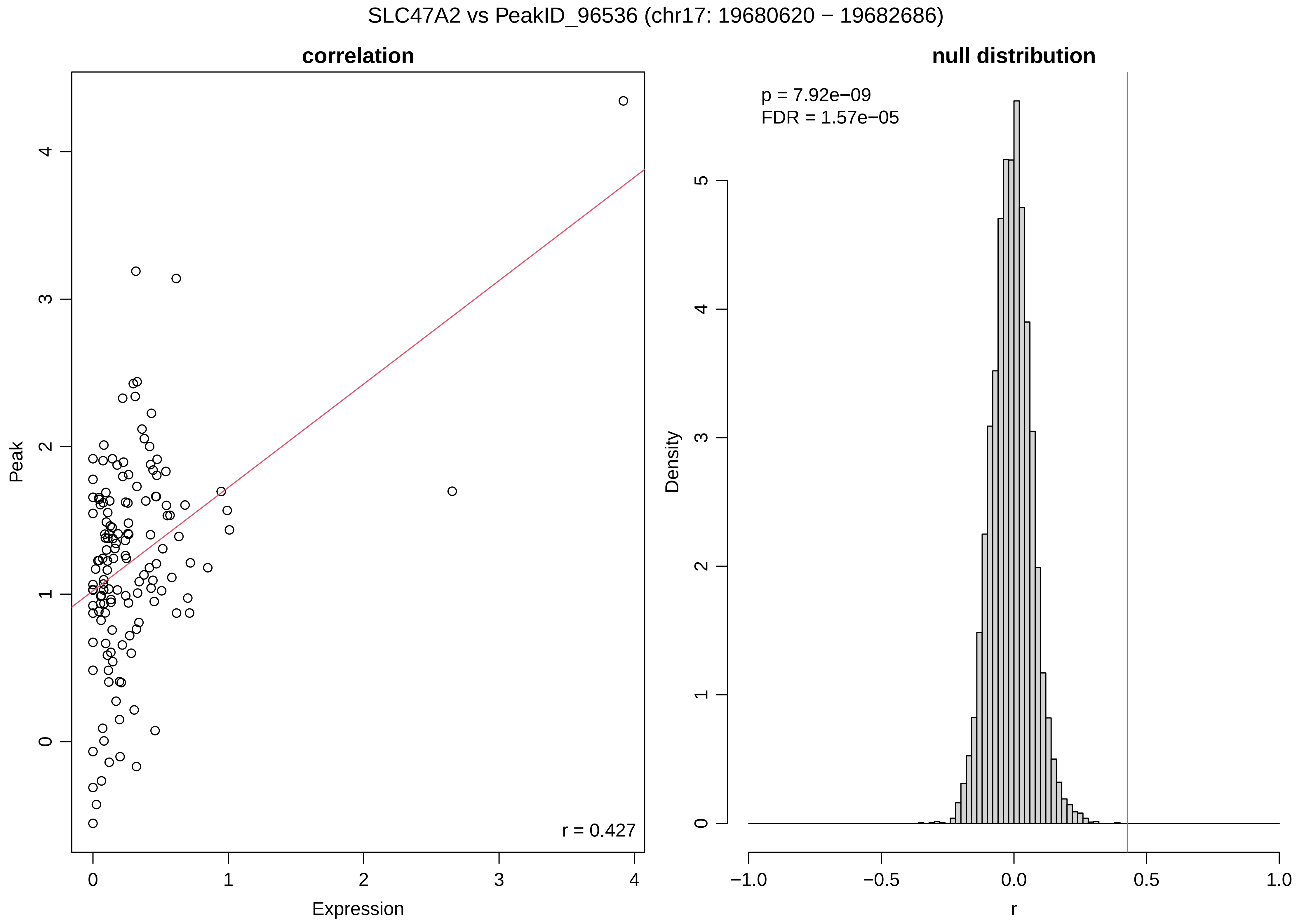

SLC47A2 vs PeakID_96536 (chr17: 19680620 − 19682686)
correlation
Peak
Expression
r = 0.427
0
1
2
3
4
null distribution
p = 7.92e−09
FDR = 1.57e−05
Density
r
−1.0
−0.5
0.0
0.5
1.0

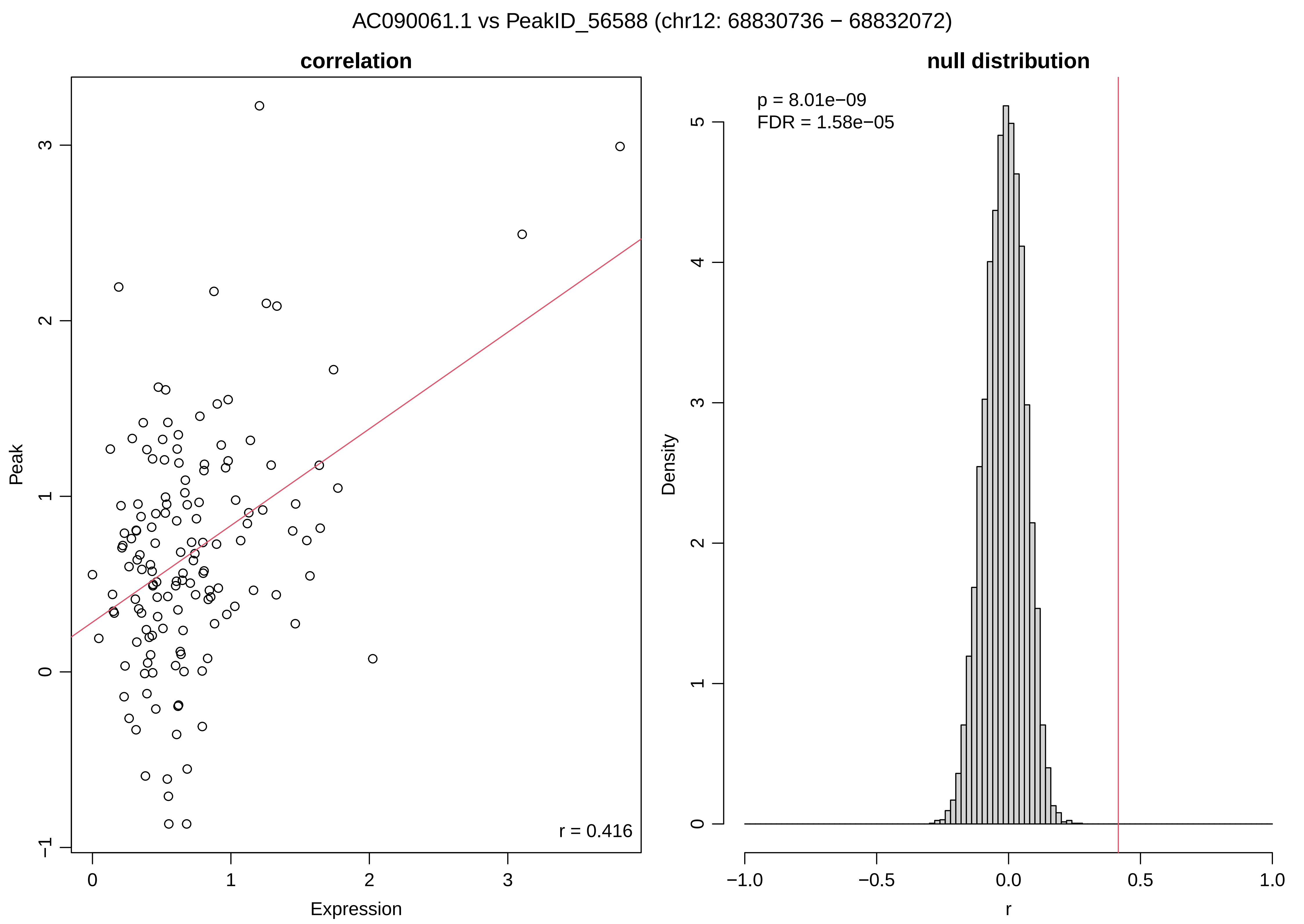

AC090061.1 vs PeakID_56588 (chr12: 68830736 − 68832072)
correlation
Peak
Expression
r = 0.416
null distribution
p = 8.01e−09
FDR = 1.58e−05
Density
r

TCERG1L−AS1 vs PeakID_35826 (chr10: 131093963 − 131095526)

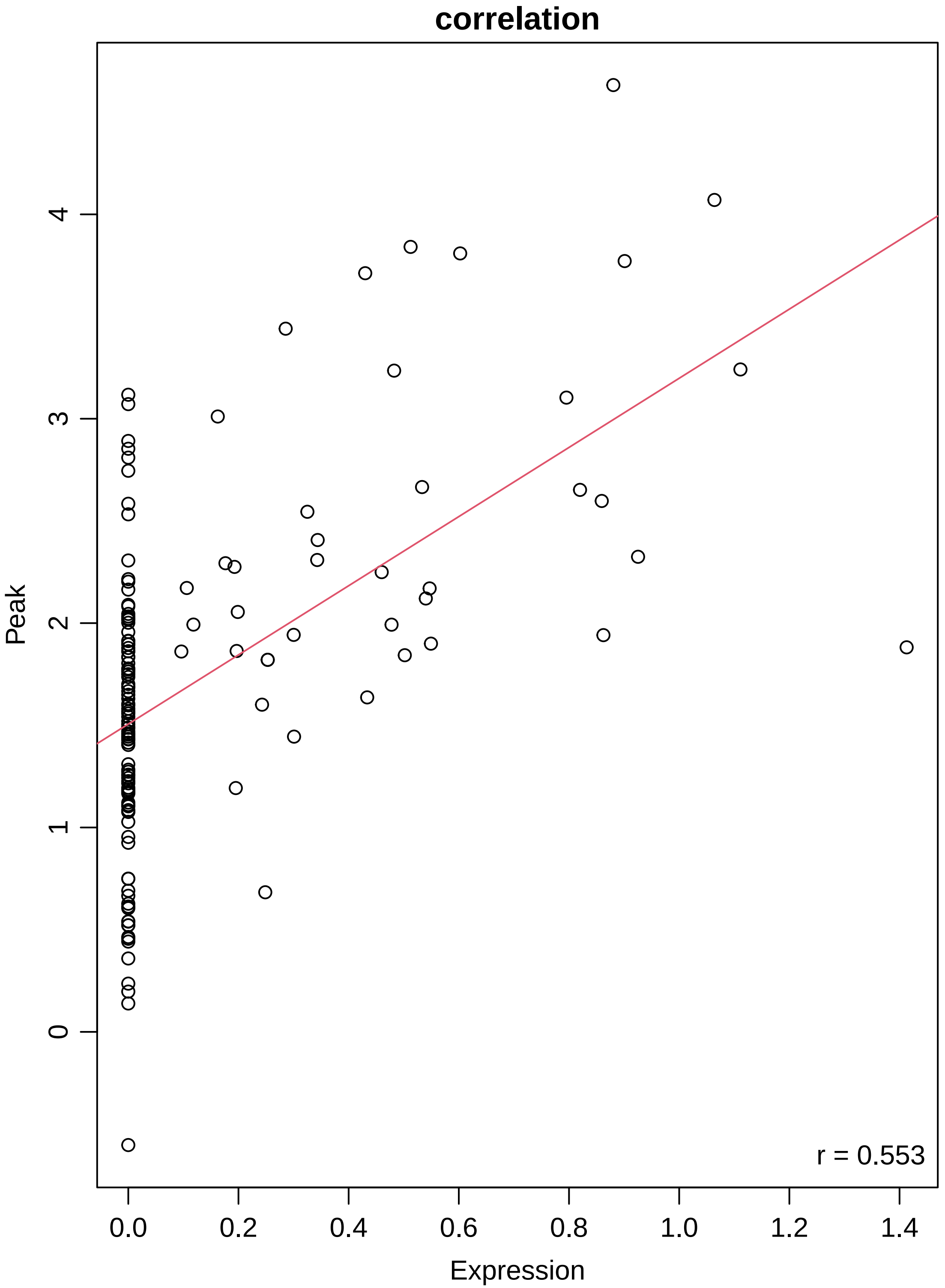

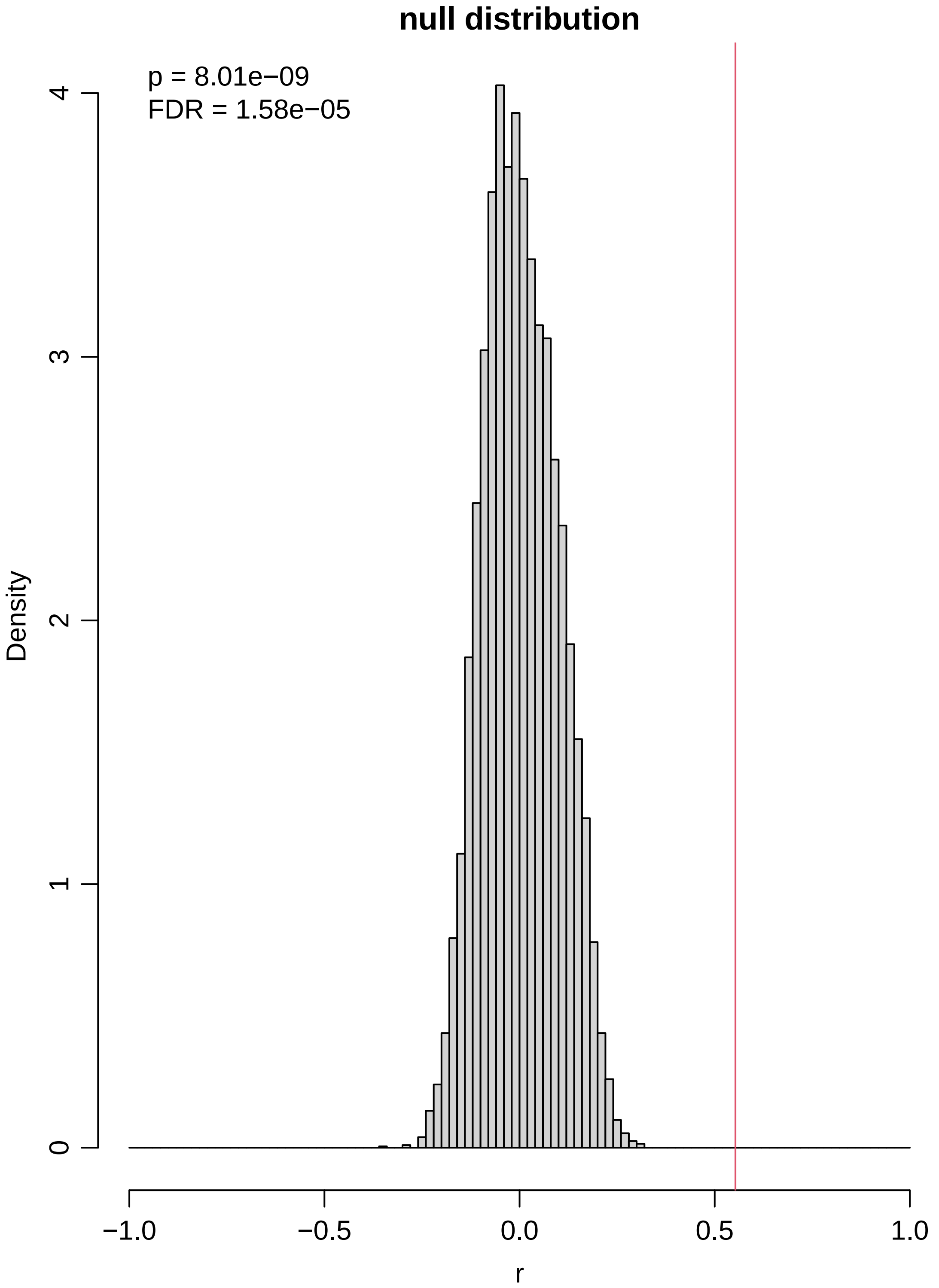

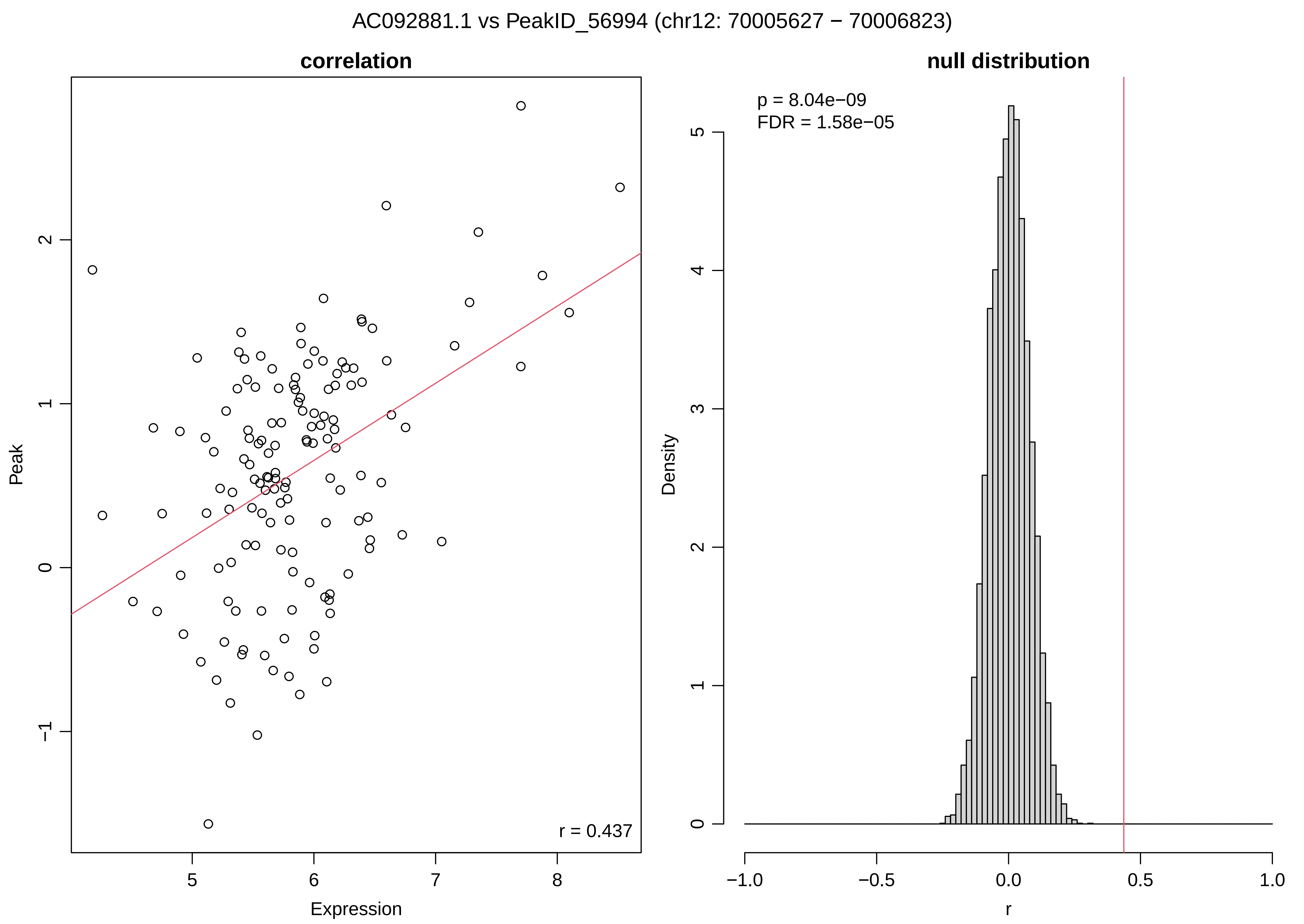

AC092881.1 vs PeakID_56994 (chr12: 70005627 − 70006823)
correlation
null distribution
p = 8.04e−09
FDR = 1.58e−05
r = 0.437
Peak
Expression
Density
r
−1
0
1
2
5
6
7
8
0
1
2
3
4
5
−1.0
−0.5
0.0
0.5
1.0

MNX1 vs PeakID_232059 (chr7: 157006286 − 157011807)

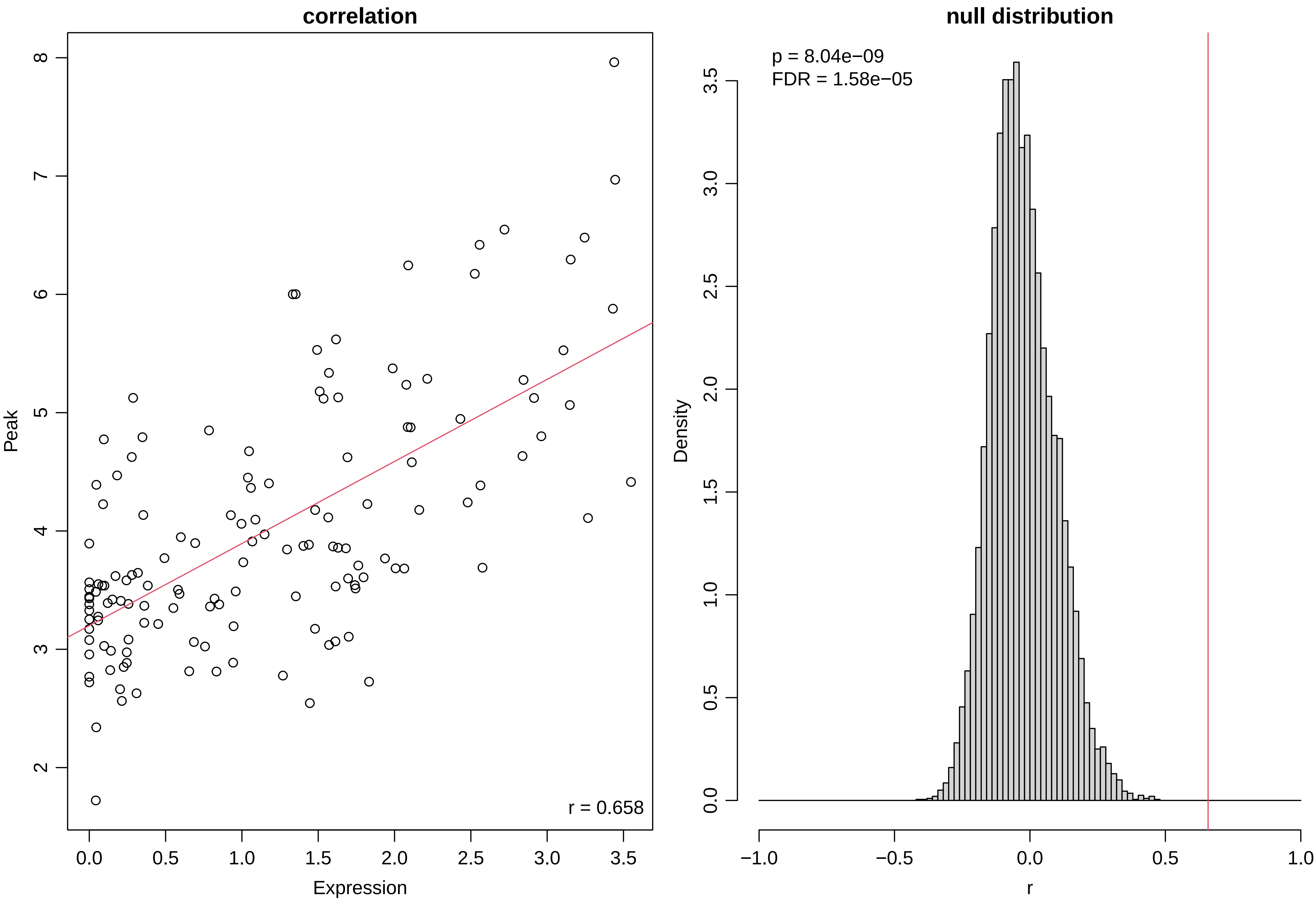

AC012512.1 vs PeakID_139836 (chr2: 223723553 − 223723960)

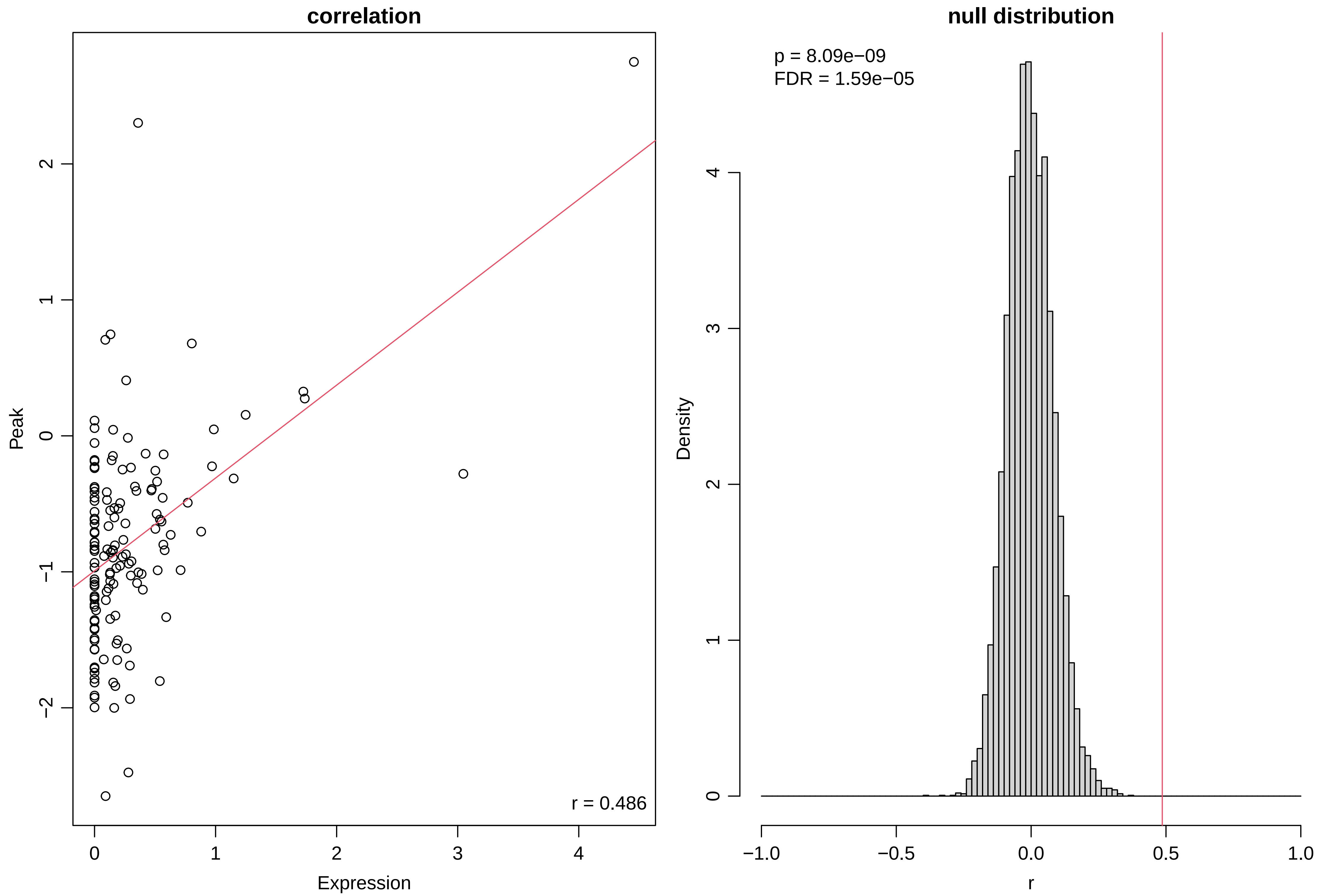

RPL31P59 vs PeakID_106918 (chr18: 3383758 − 3384410)

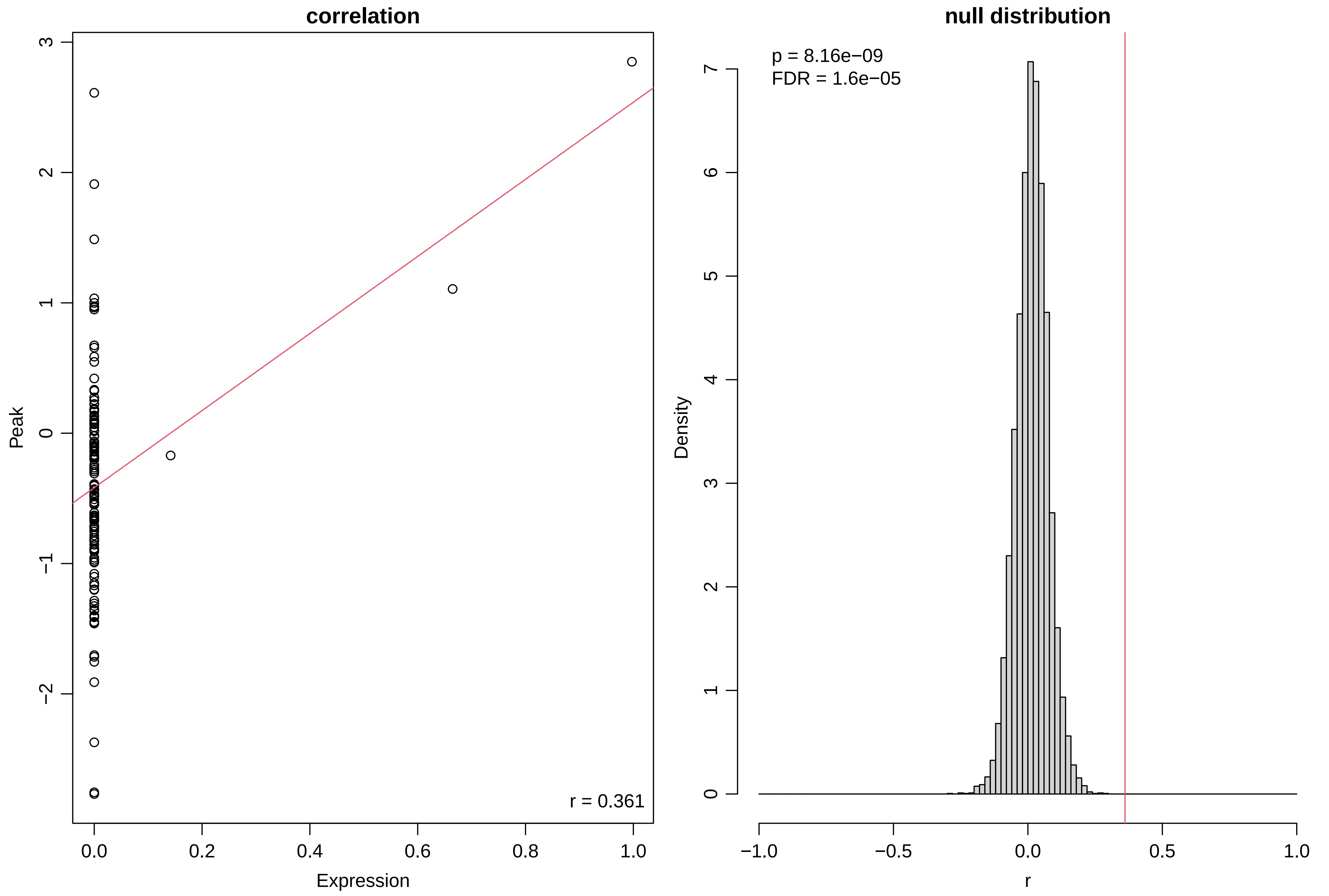

AL589182.2 vs PeakID_68765 (chr14: 19336812 − 19338377)

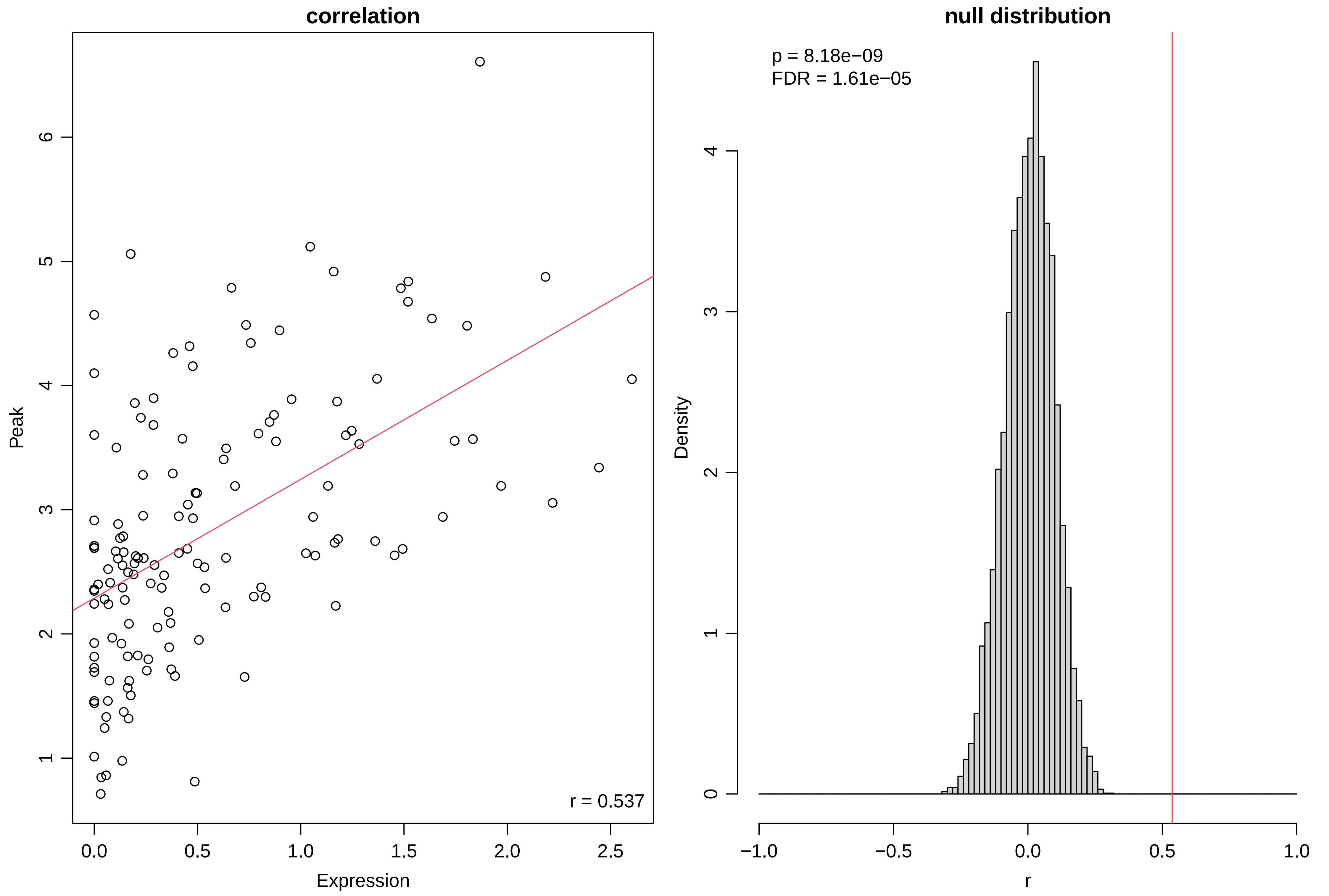

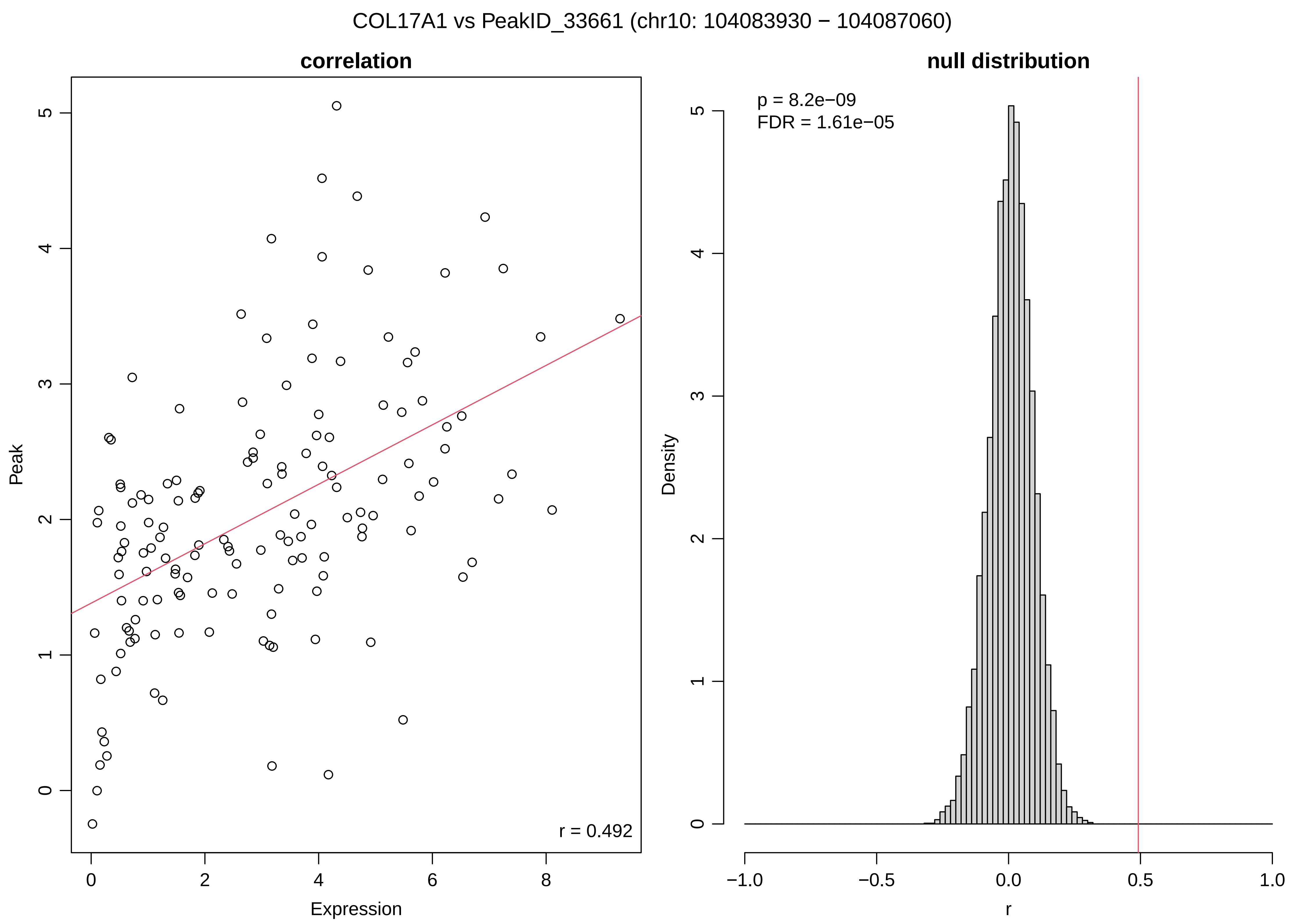

COL17A1 vs PeakID_33661 (chr10: 104083930 − 104087060)
correlation
Peak
Expression
r = 0.492
null distribution
p = 8.2e−09
FDR = 1.61e−05
Density
r

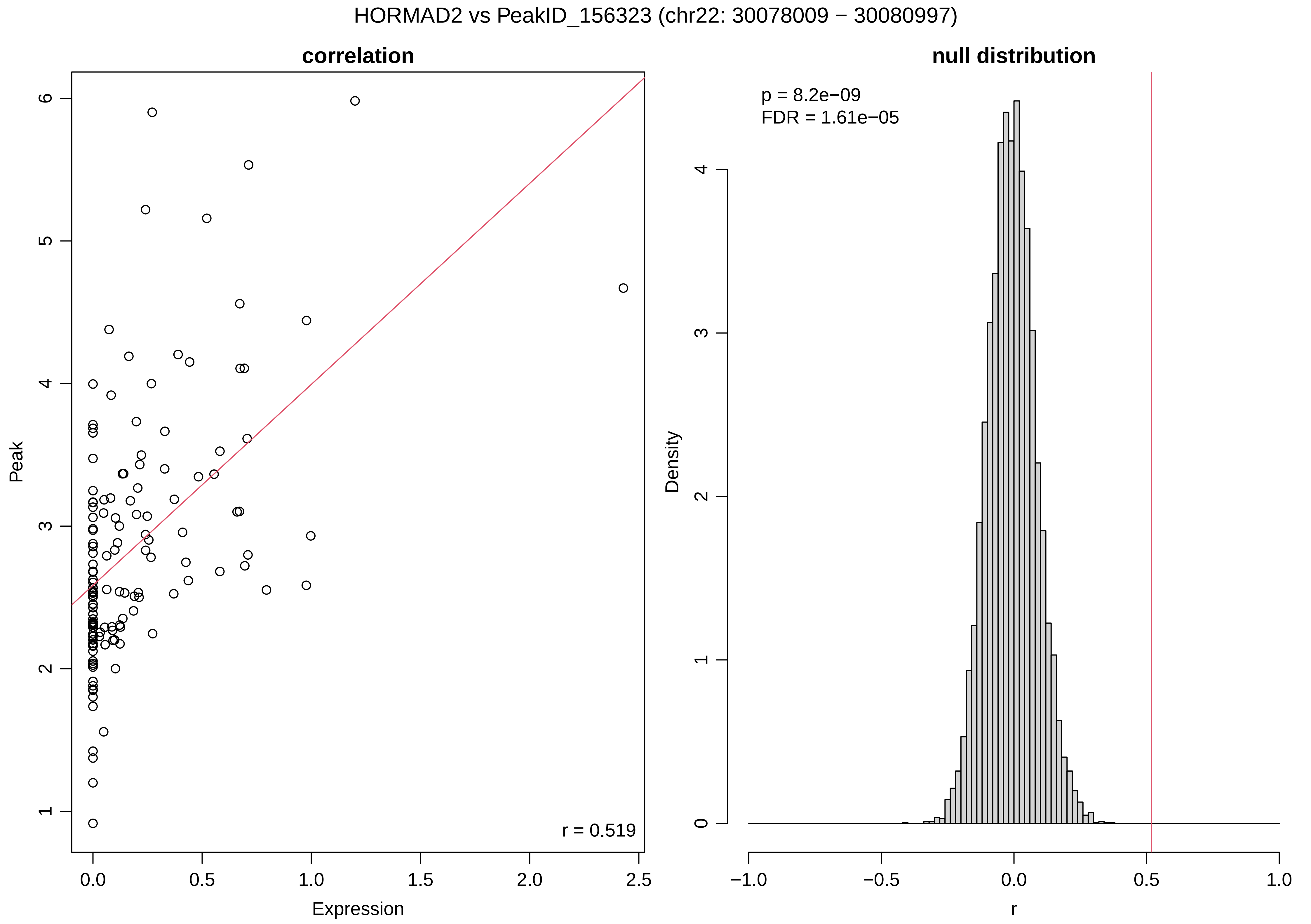
HORMAD2 vs PeakID_156323 (chr22: 30078009 − 30080997)
correlation
null distribution
Peak
Expression
r = 0.519
p = 8.2e−09
FDR = 1.61e−05
Density
r

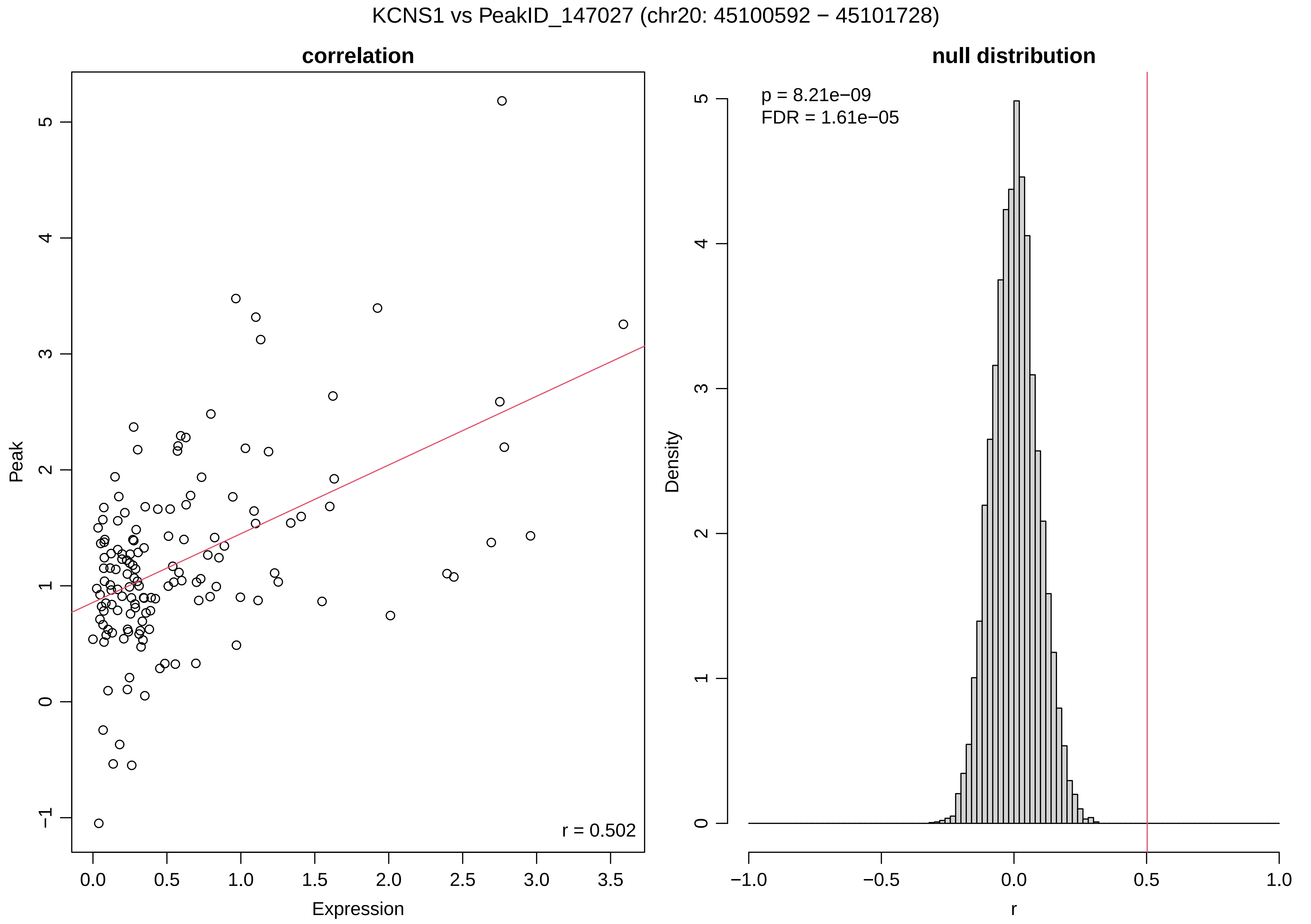
KCNS1 vs PeakID_147027 (chr20: 45100592 − 45101728)
correlation
Peak
Expression
r = 0.502
null distribution
p = 8.21e−09
FDR = 1.61e−05
Density
r

AC005722.3 vs PeakID_96552 (chr17: 19747211 − 19750615)

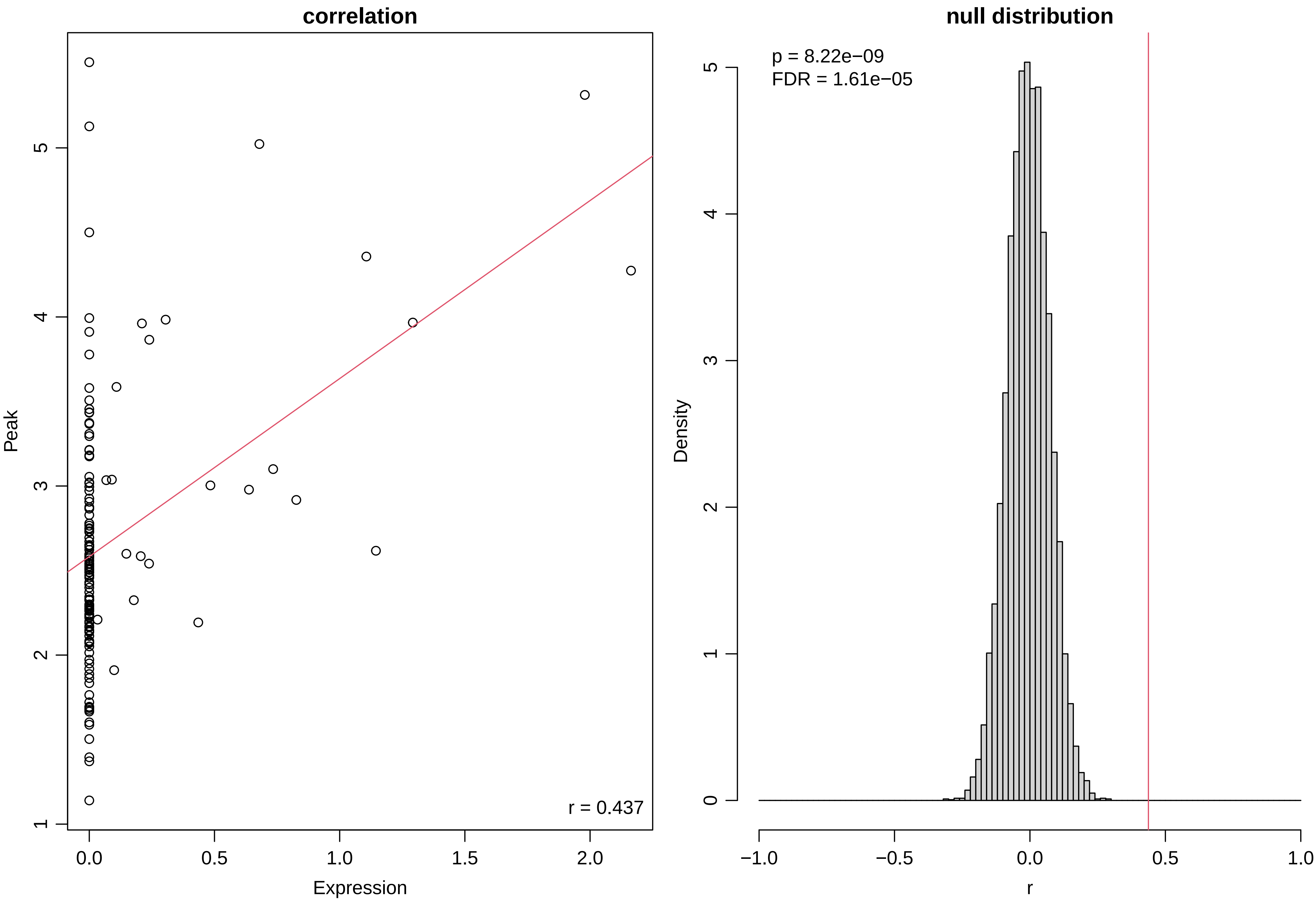

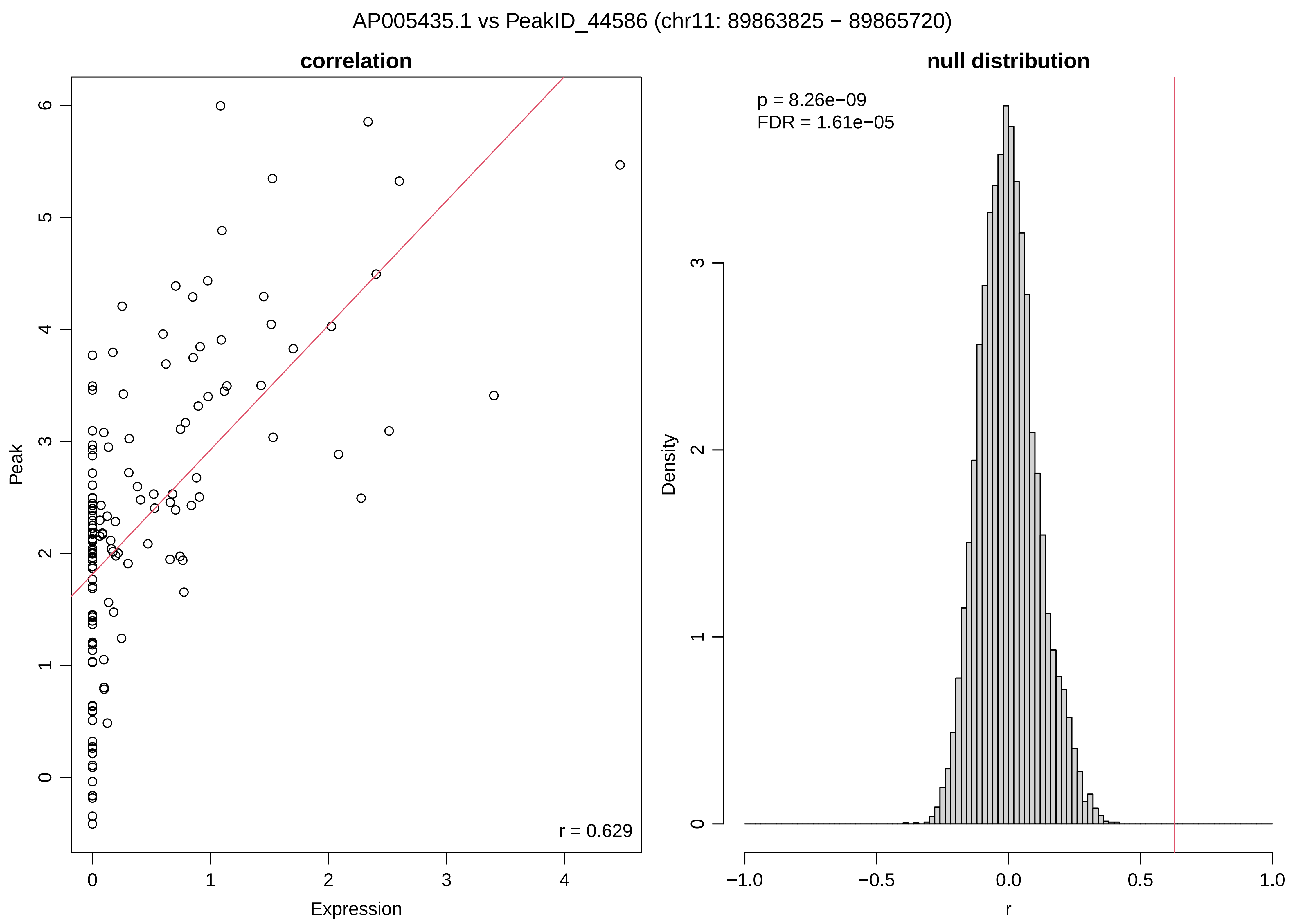

AP005435.1 vs PeakID_44586 (chr11: 89863825 − 89865720)
correlation
Peak
Expression
r = 0.629
null distribution
p = 8.26e−09
FDR = 1.61e−05
Density
r

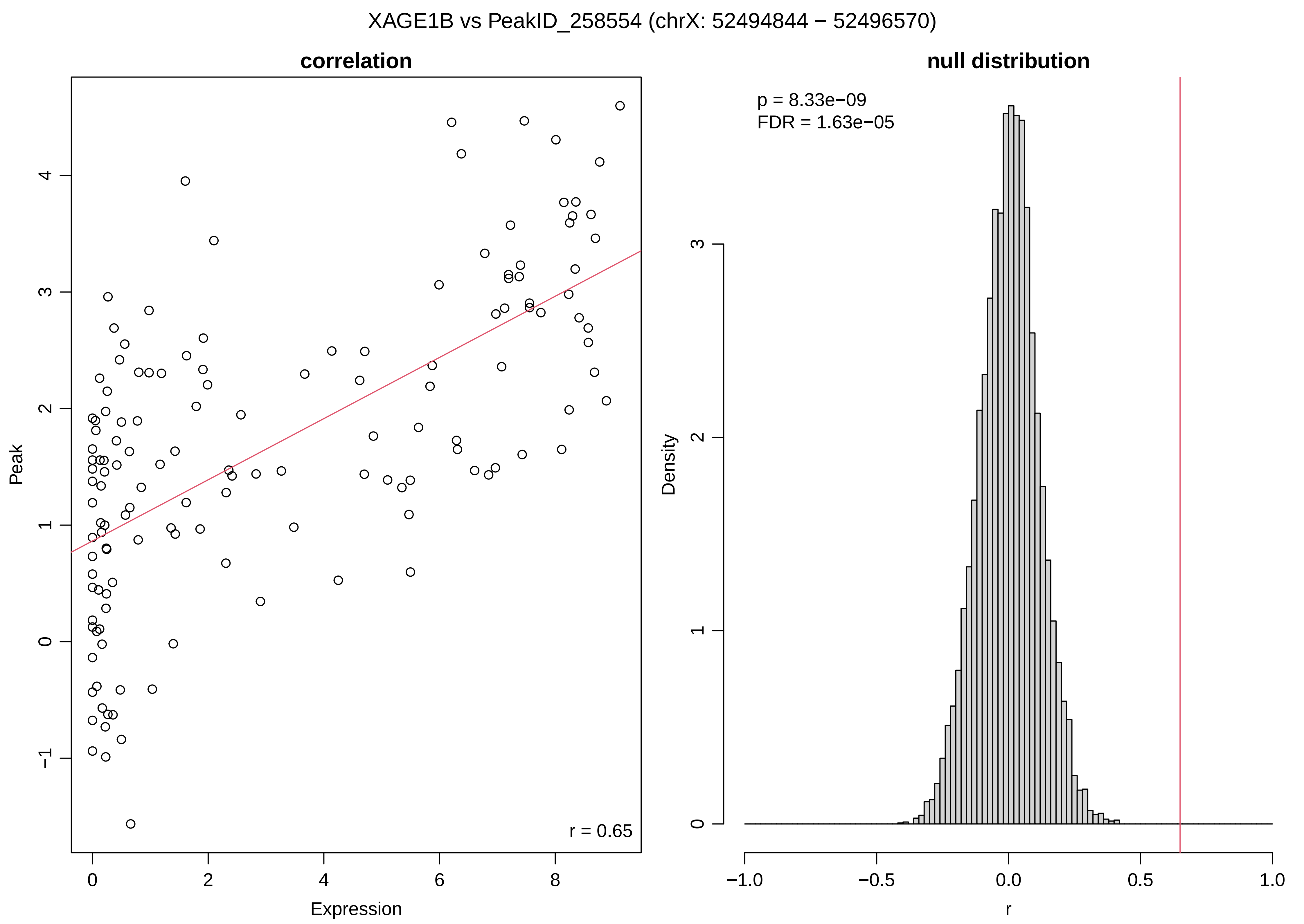
XAGE1B vs PeakID_258554 (chrX: 52494844 − 52496570)
correlation
null distribution
p = 8.33e−09
FDR = 1.63e−05
r = 0.65
Peak
Expression
Density
r

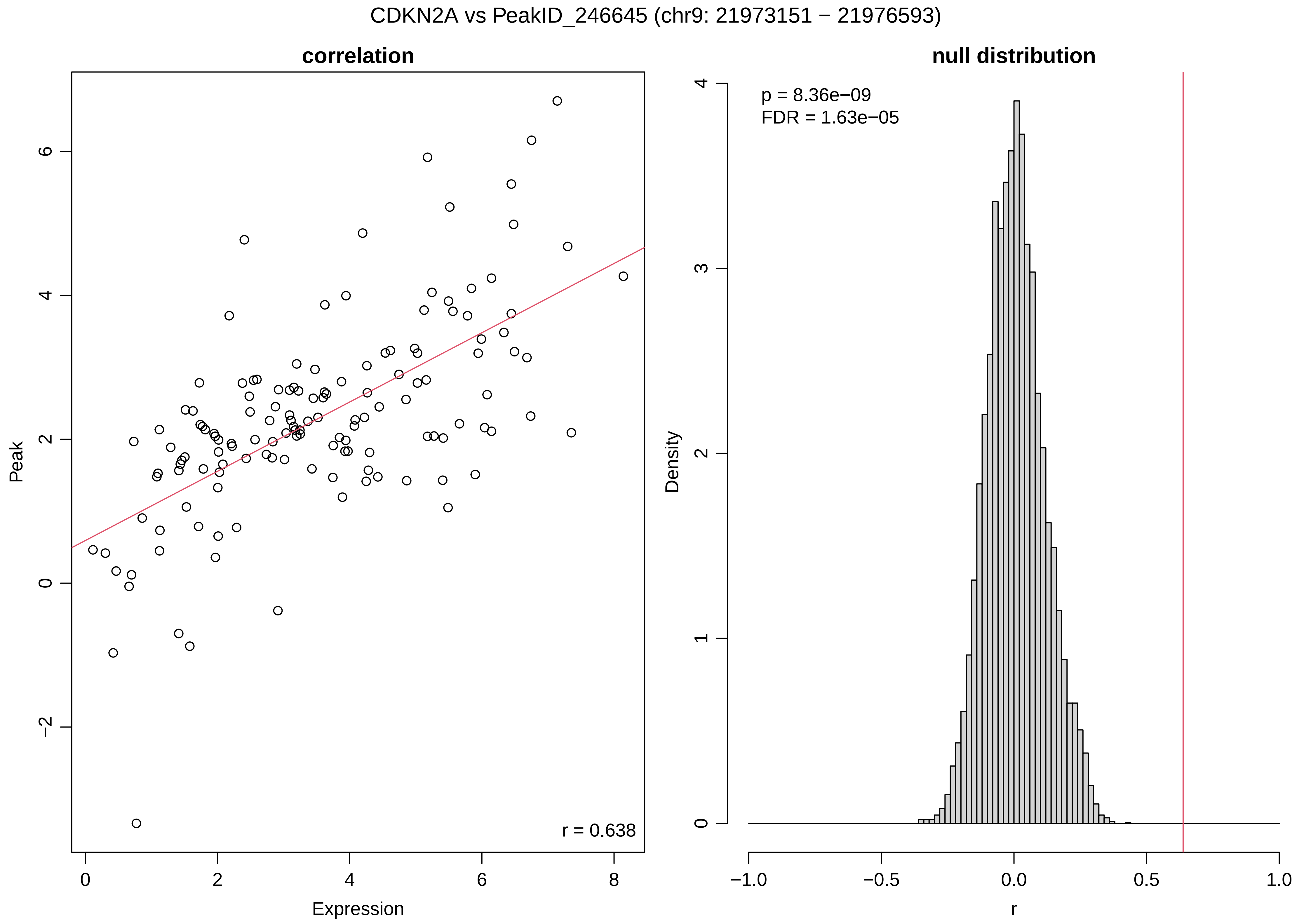

CDKN2A vs PeakID_246645 (chr9: 21973151 − 21976593)
correlation
null distribution
Peak
Expression
r = 0.638
Density
r
p = 8.36e−09
FDR = 1.63e−05

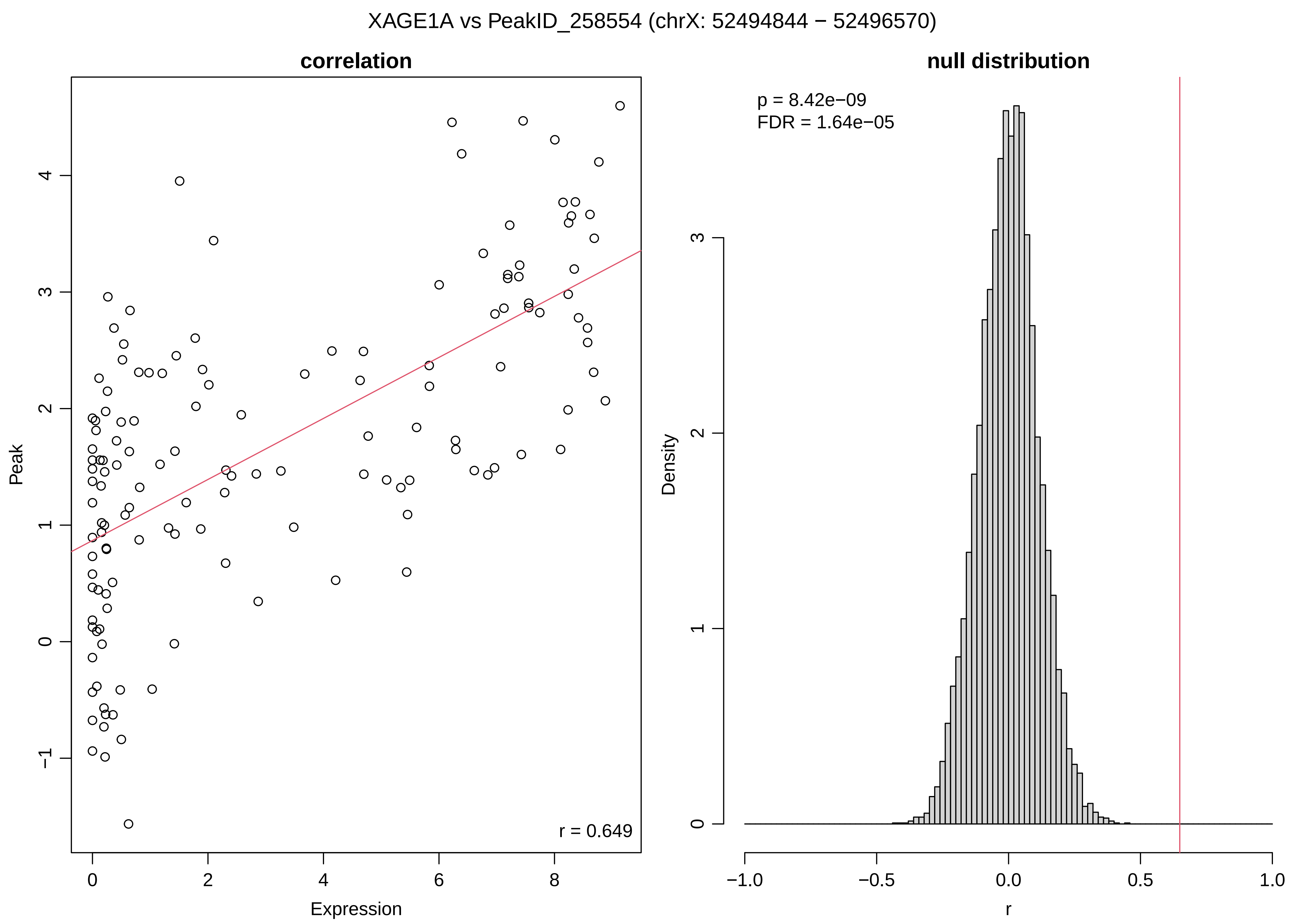
XAGE1A vs PeakID_258554 (chrX: 52494844 − 52496570)
correlation
null distribution
p = 8.42e−09
FDR = 1.64e−05
r = 0.649
Peak
Expression
Density
r

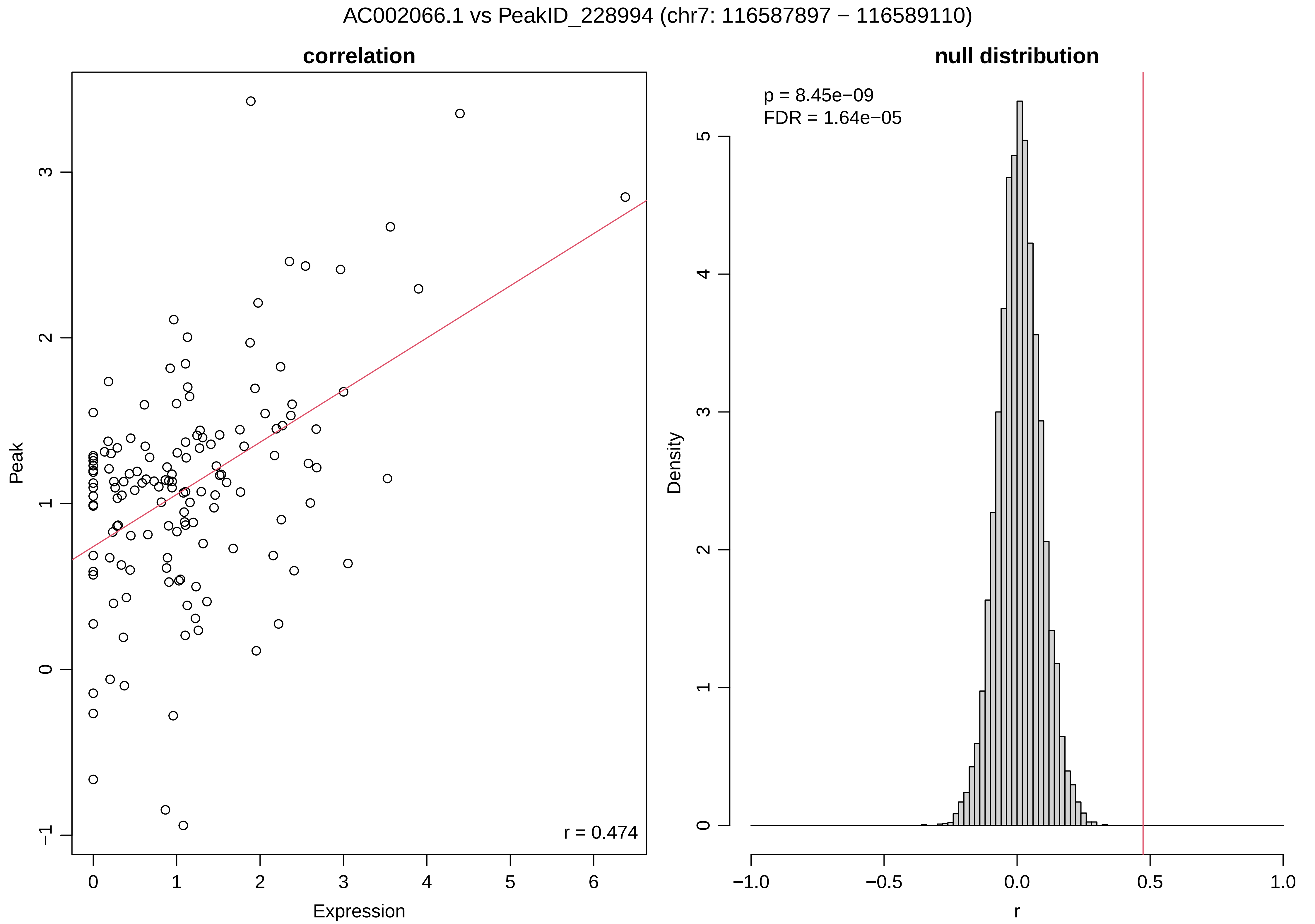
AC002066.1 vs PeakID_228994 (chr7: 116587897 − 116589110)
correlation
null distribution
p = 8.45e−09
FDR = 1.64e−05
Peak
Expression
r = 0.474
Density
r
−1
0
1
2
3
4
5
6
−1.0
−0.5
0.0
0.5
1.0

AC093817.2 vs PeakID_184643 (chr4: 157821809 − 157825358)

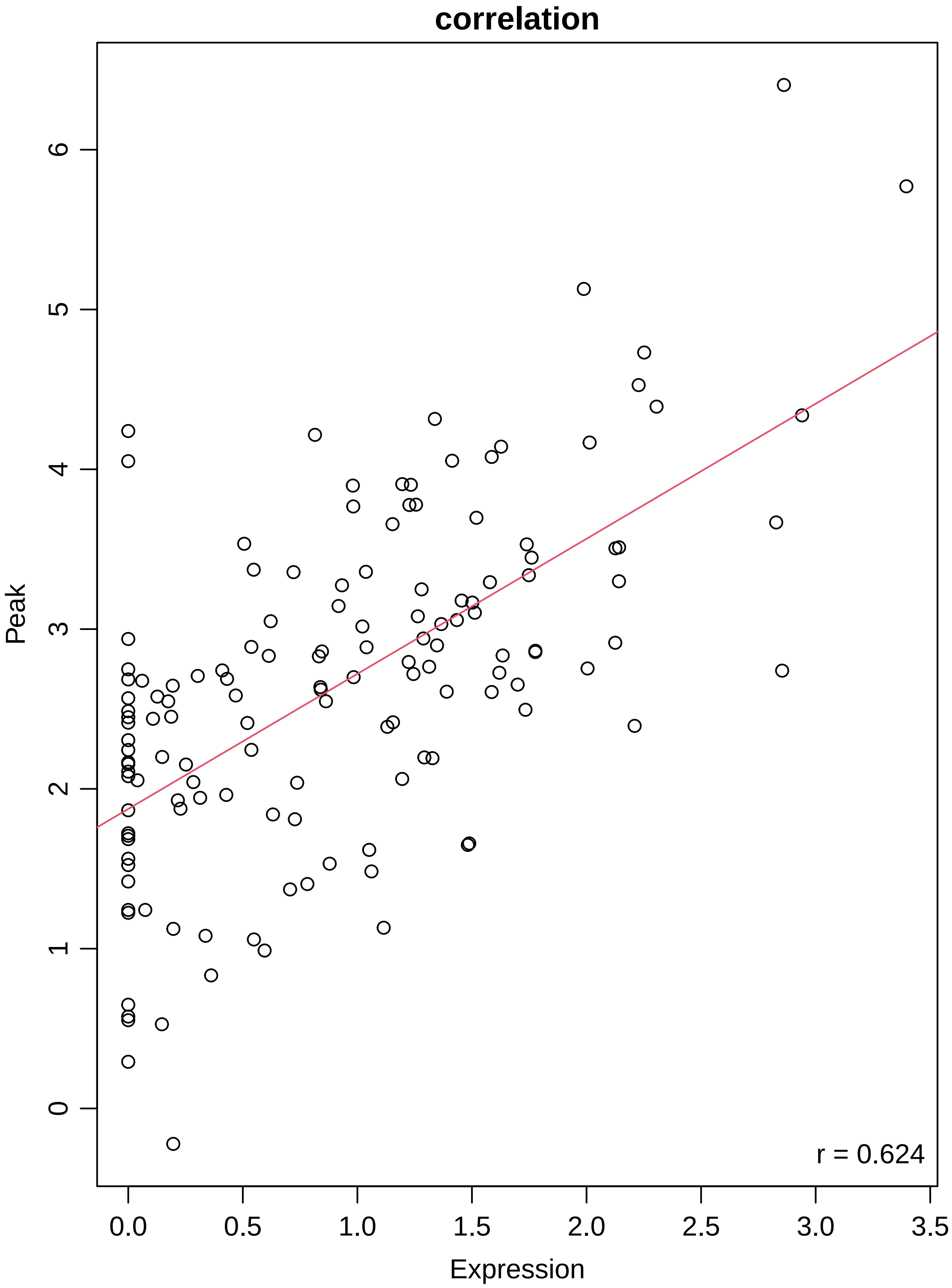

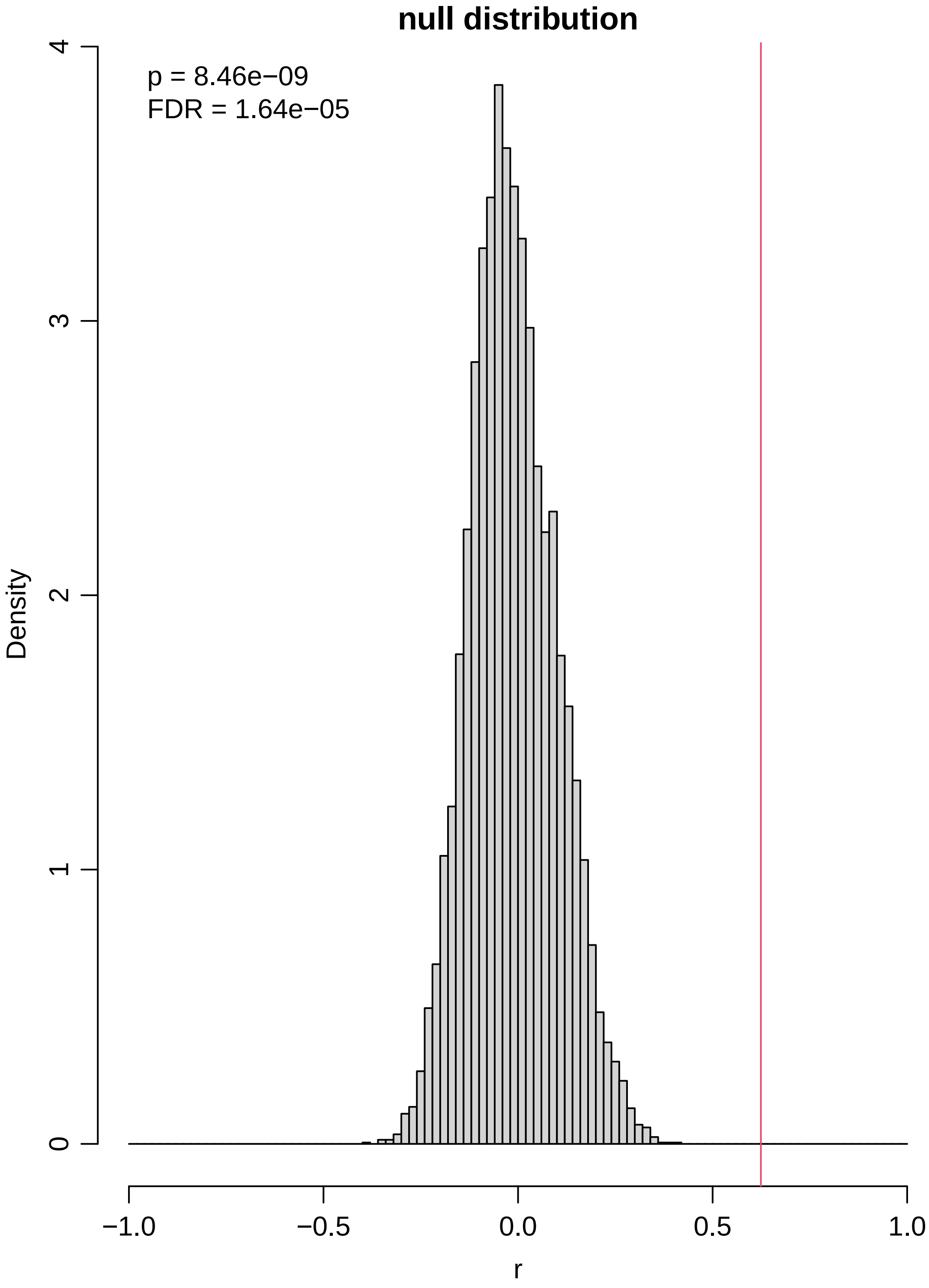

AC002066.1 vs PeakID_228950 (chr7: 116339392 − 116341777)

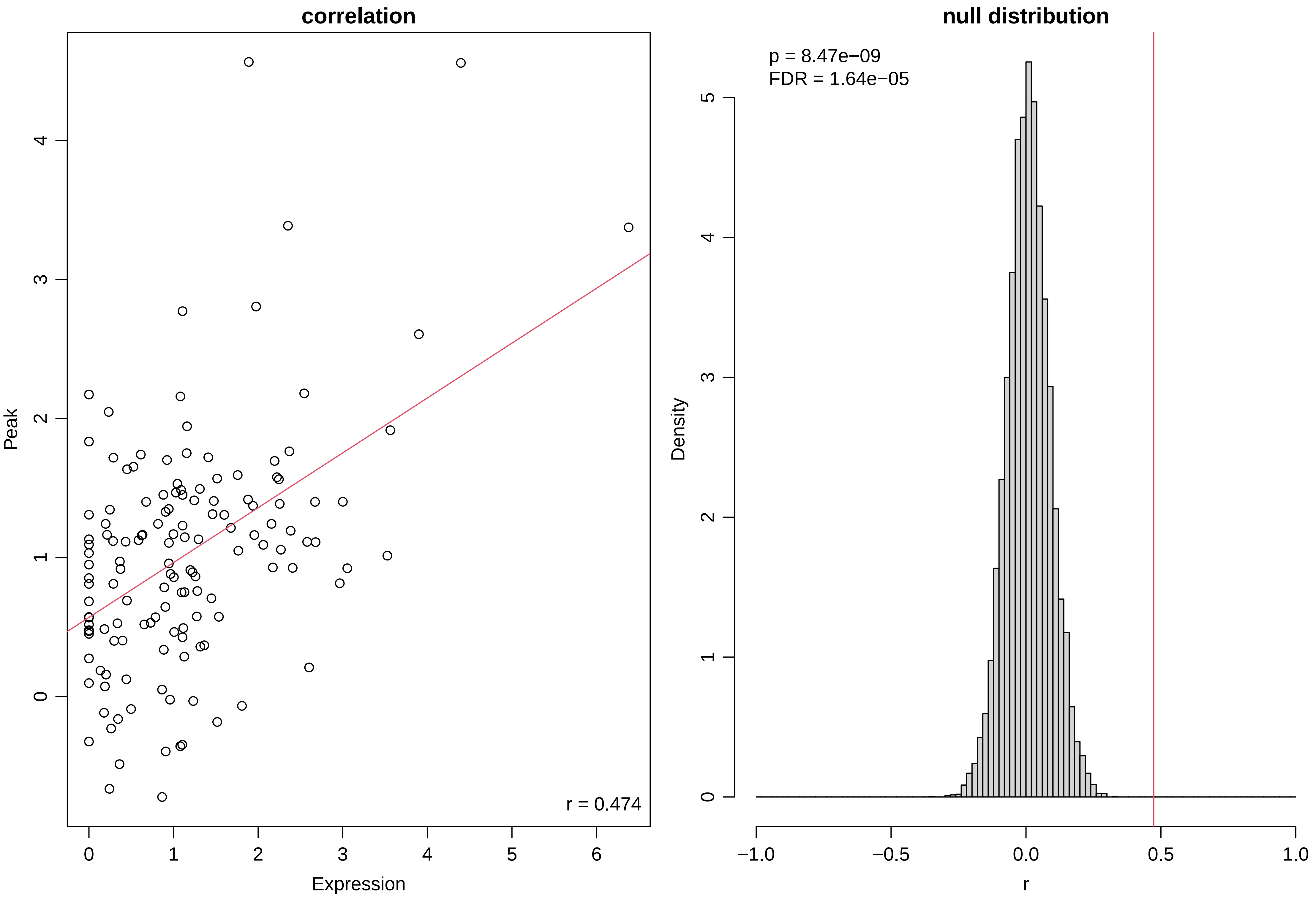

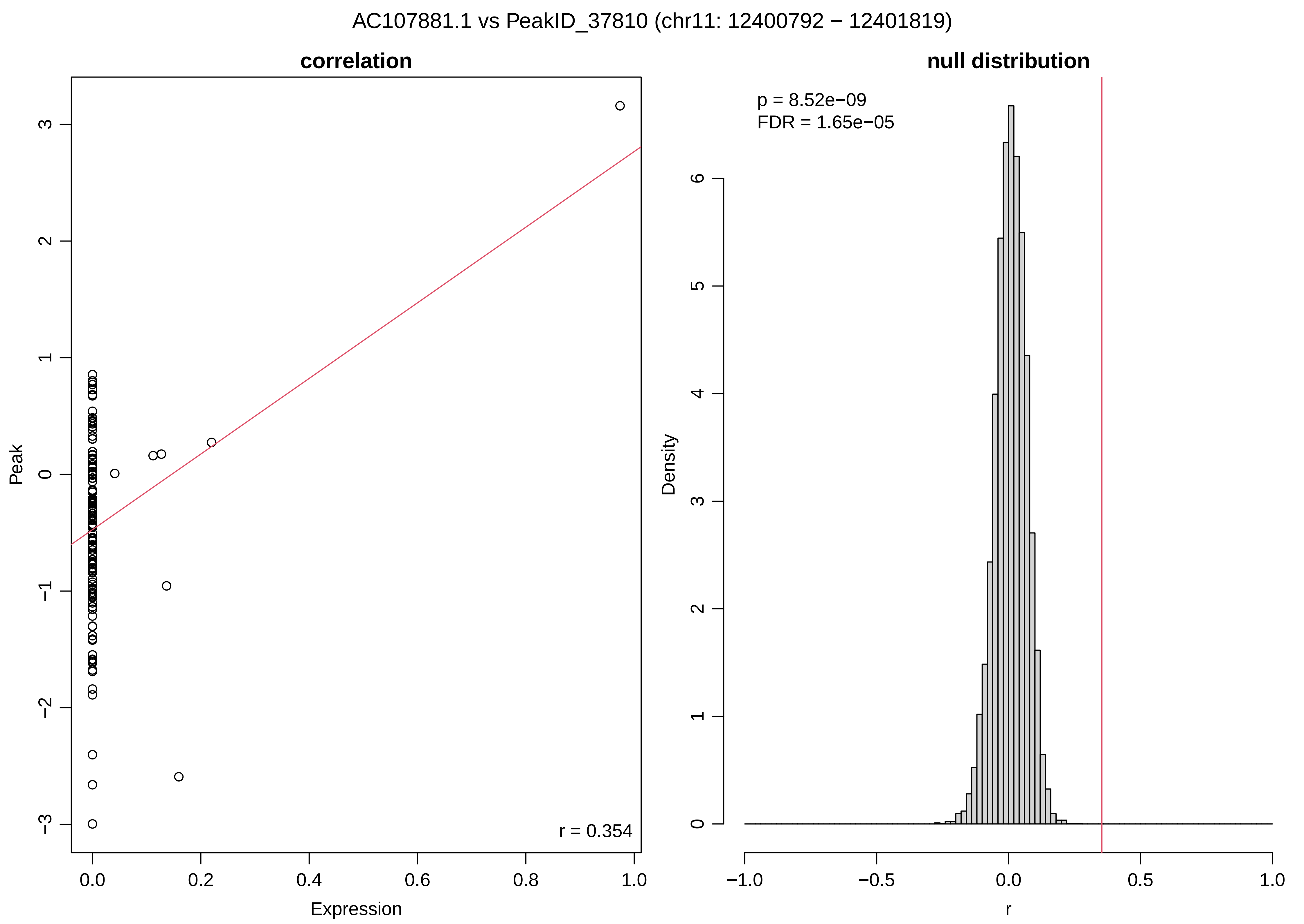
AC107881.1 vs PeakID_37810 (chr11: 12400792 − 12401819)
correlation
null distribution
Peak
Expression
r = 0.354
p = 8.52e−09
FDR = 1.65e−05
Density
r
0.0
0.2
0.4
0.6
0.8
1.0
−3
−2
−1
0
1
2
3
−1.0
−0.5
0.0
0.5
1.0
0
1
2
3
4
5
6

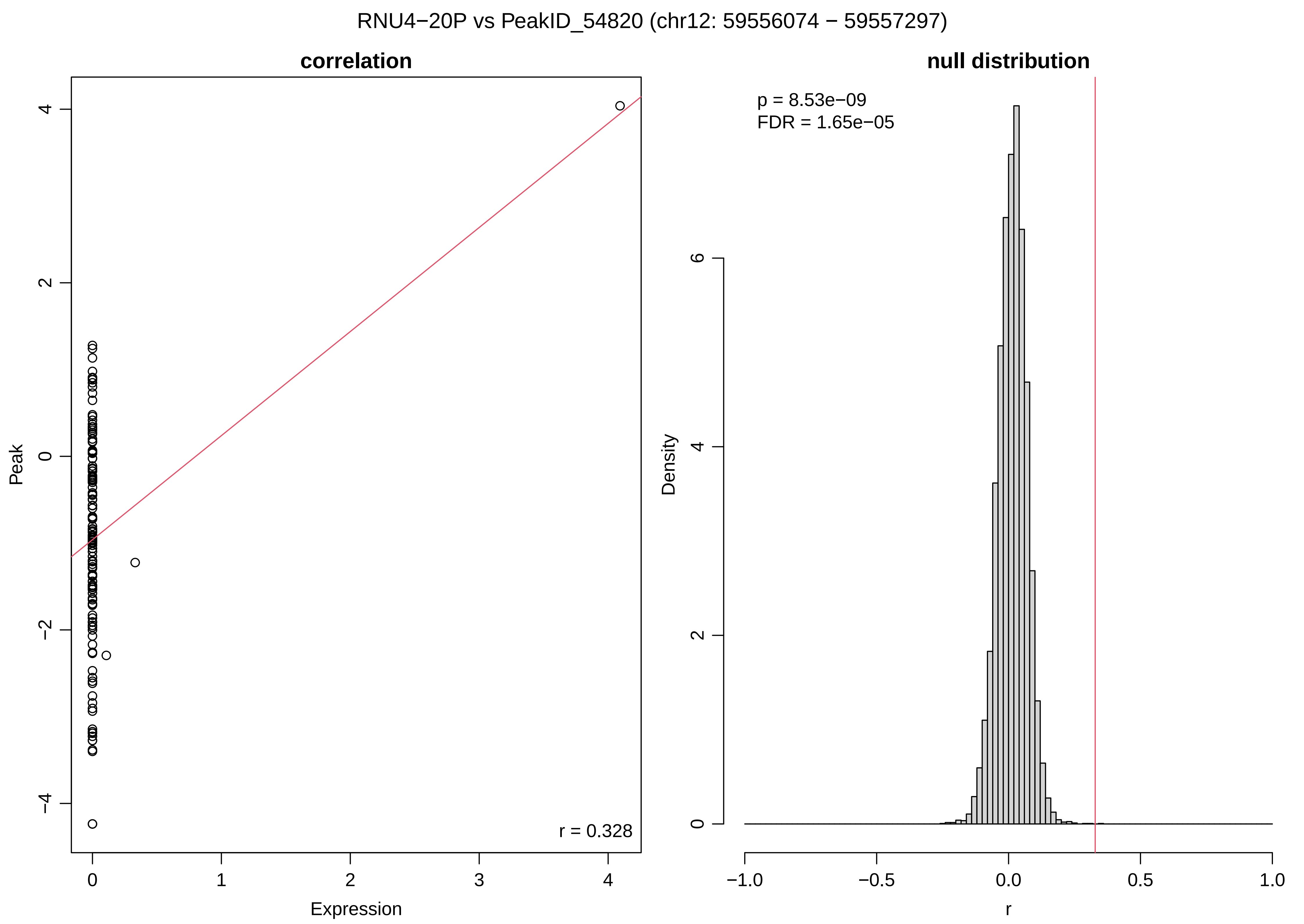
RNU4−20P vs PeakID_54820 (chr12: 59556074 − 59557297)
correlation
null distribution
p = 8.53e−09
FDR = 1.65e−05
r = 0.328
Peak
Expression
Density
r
−4
−2
0
2
4
0
1
2
3
4
0
2
4
6
−1.0
−0.5
0.0
0.5
1.0

ANXA8L1 vs PeakID_28454 (chr10: 46374736 − 46378659)

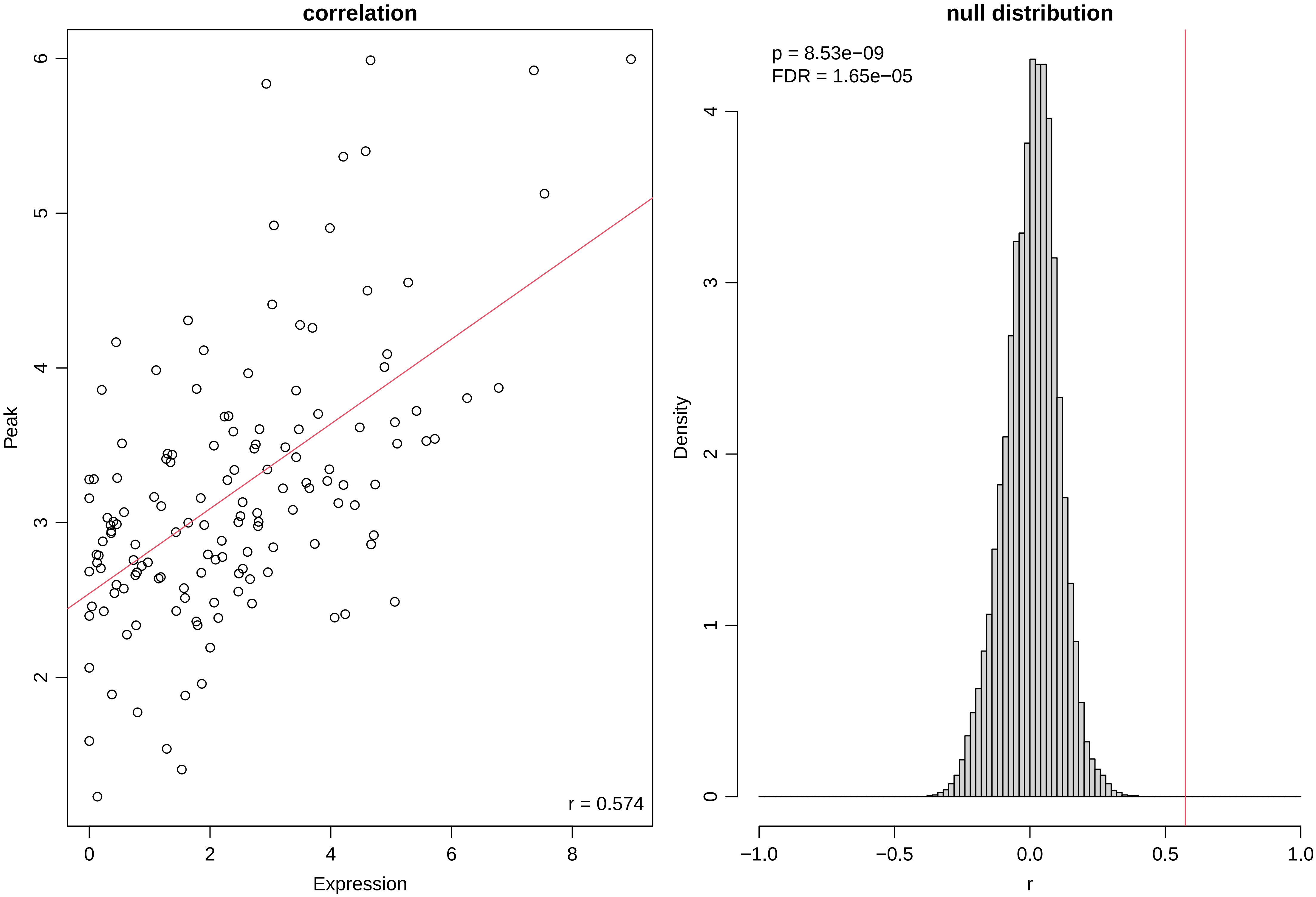

FAM224B vs PeakID_262814 (chrY: 17817662 − 17818947)

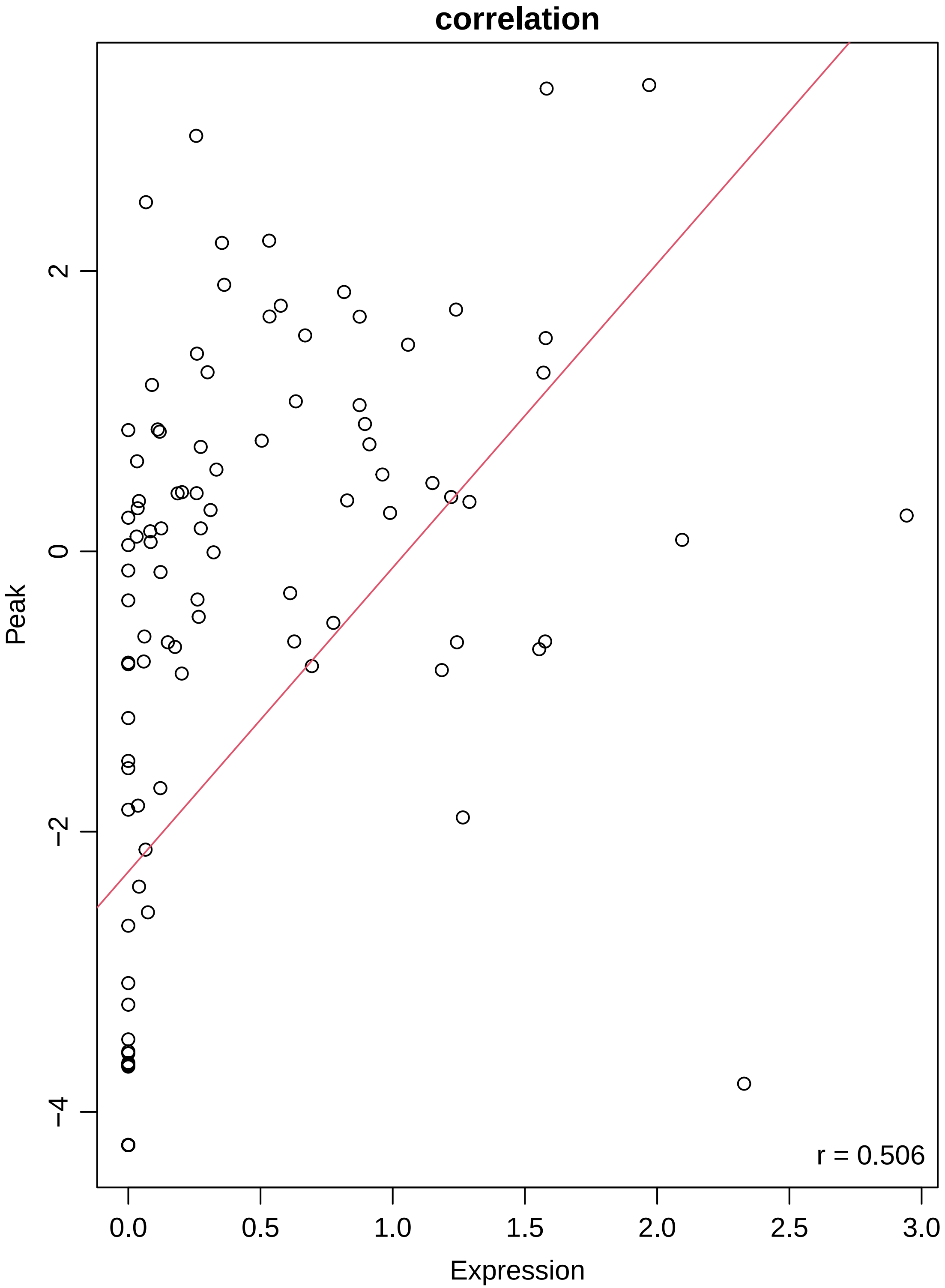

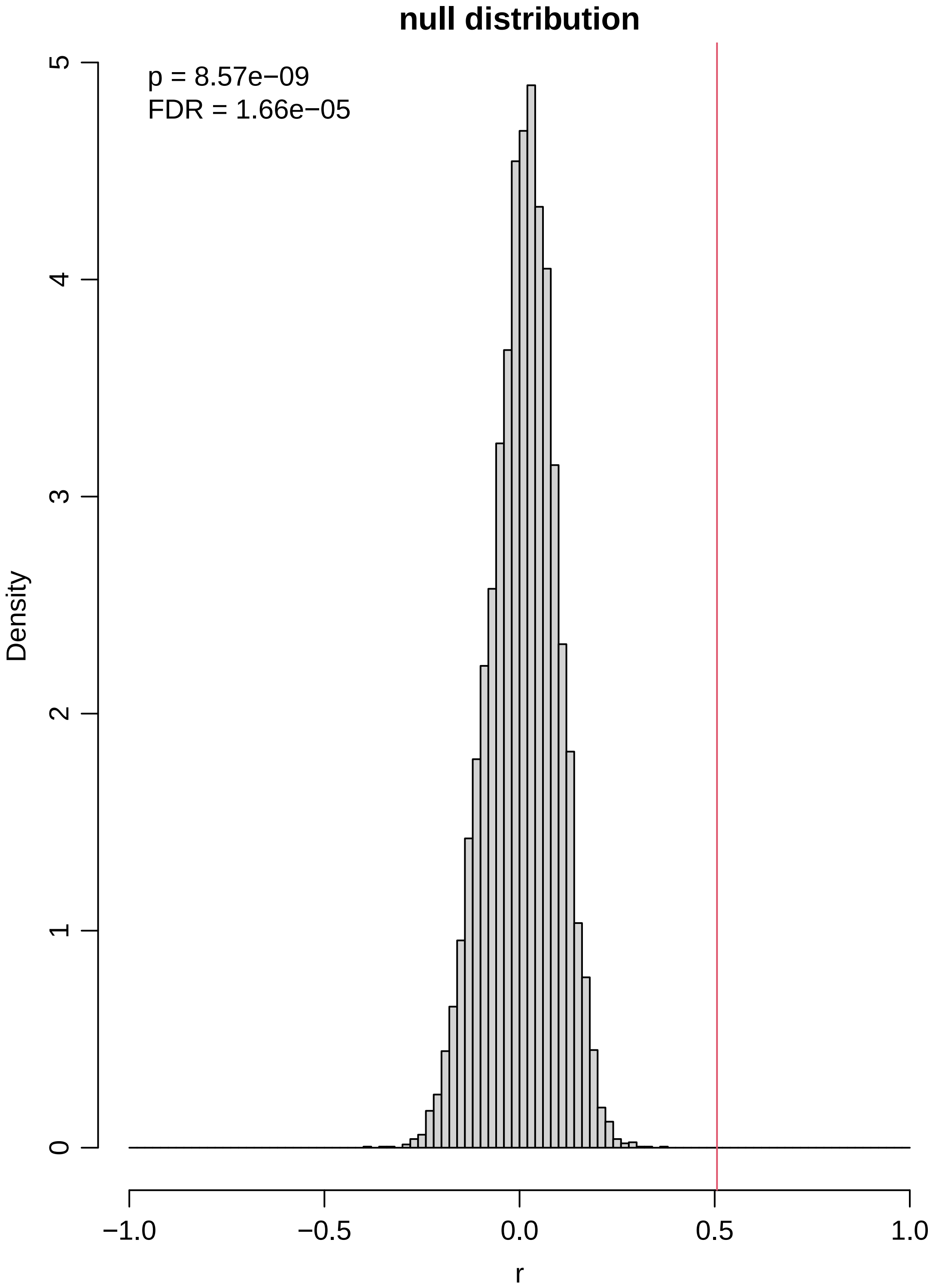

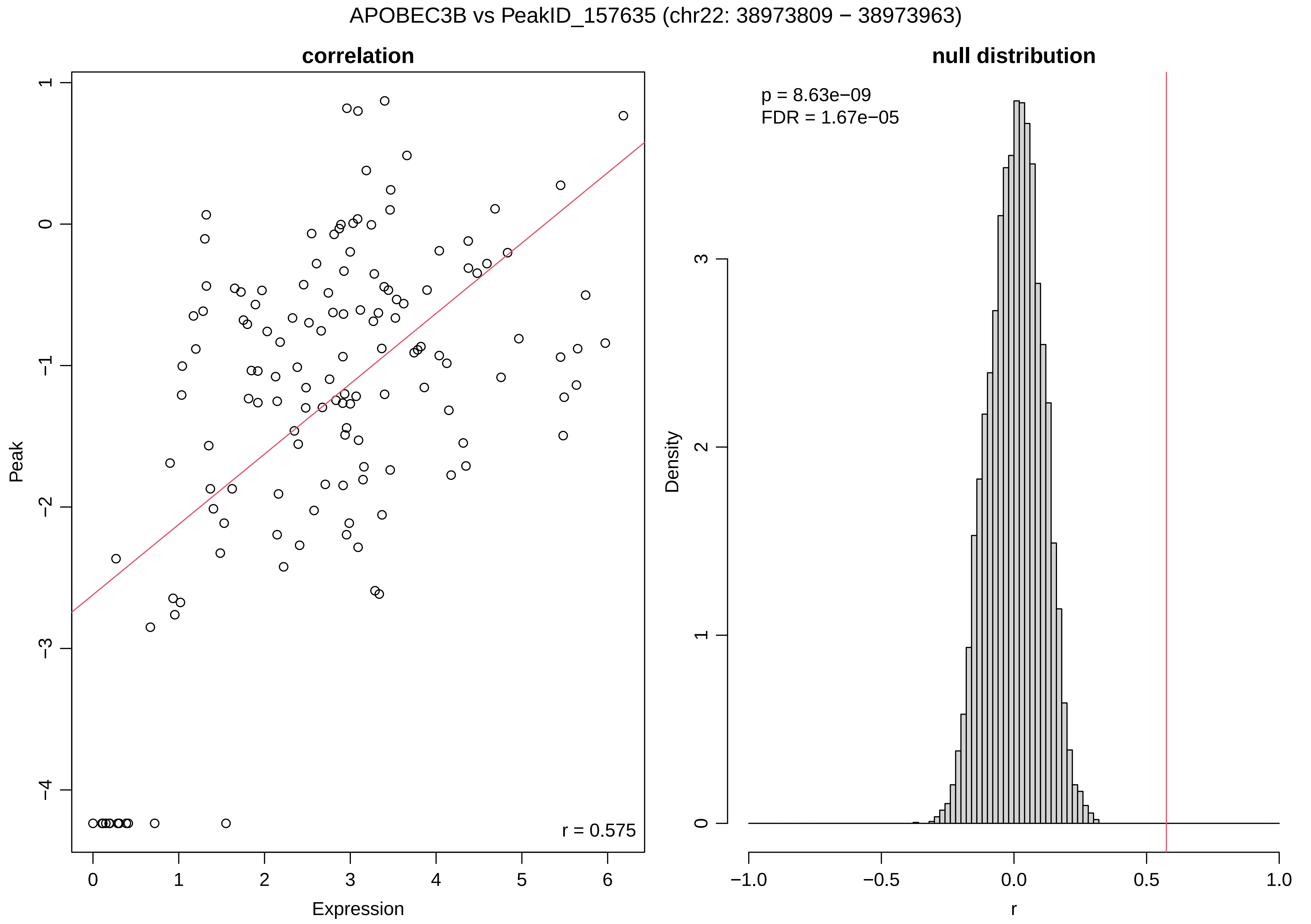
APOBEC3B vs PeakID_157635 (chr22: 38973809 − 38973963)
correlation
Peak
Expression
r = 0.575
null distribution
p = 8.63e−09
FDR = 1.67e−05
Density
r

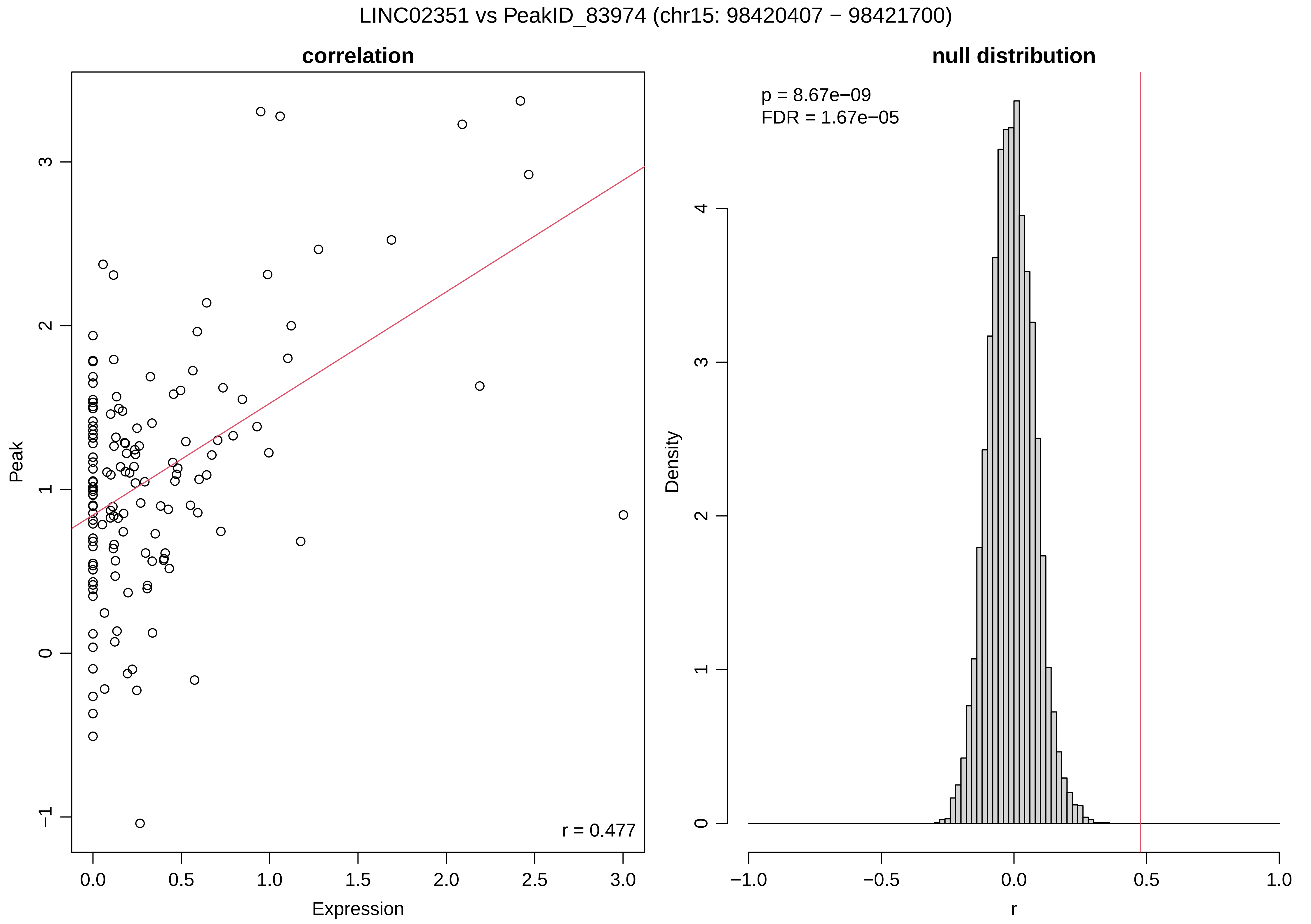

LINC02351 vs PeakID_83974 (chr15: 98420407 − 98421700)
correlation
null distribution
Peak
Expression
r = 0.477
Density
r
p = 8.67e−09
FDR = 1.67e−05

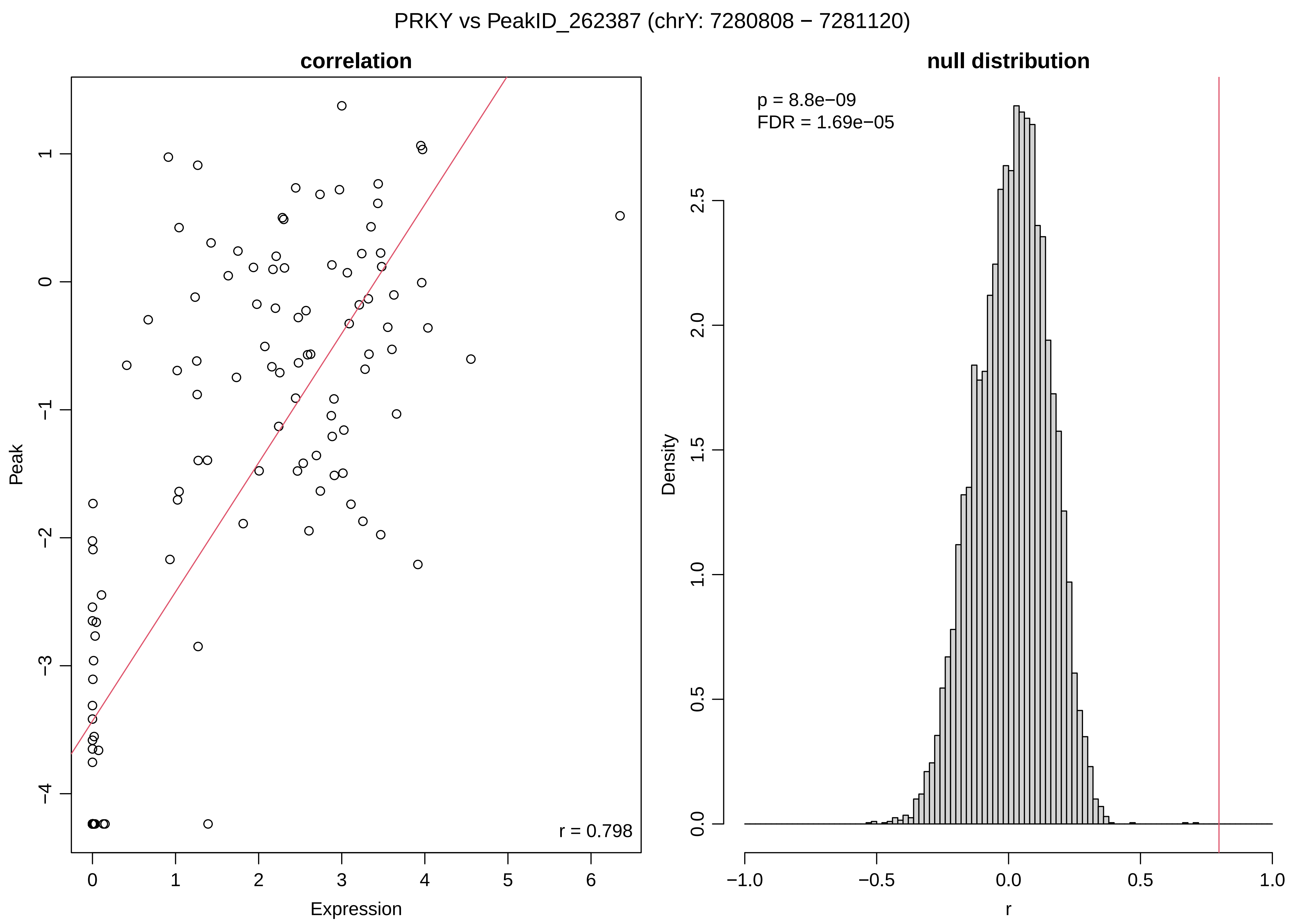
PRKY vs PeakID_262387 (chrY: 7280808 − 7281120)
correlation
Peak
Expression
r = 0.798
null distribution
p = 8.8e−09
FDR = 1.69e−05
Density
r

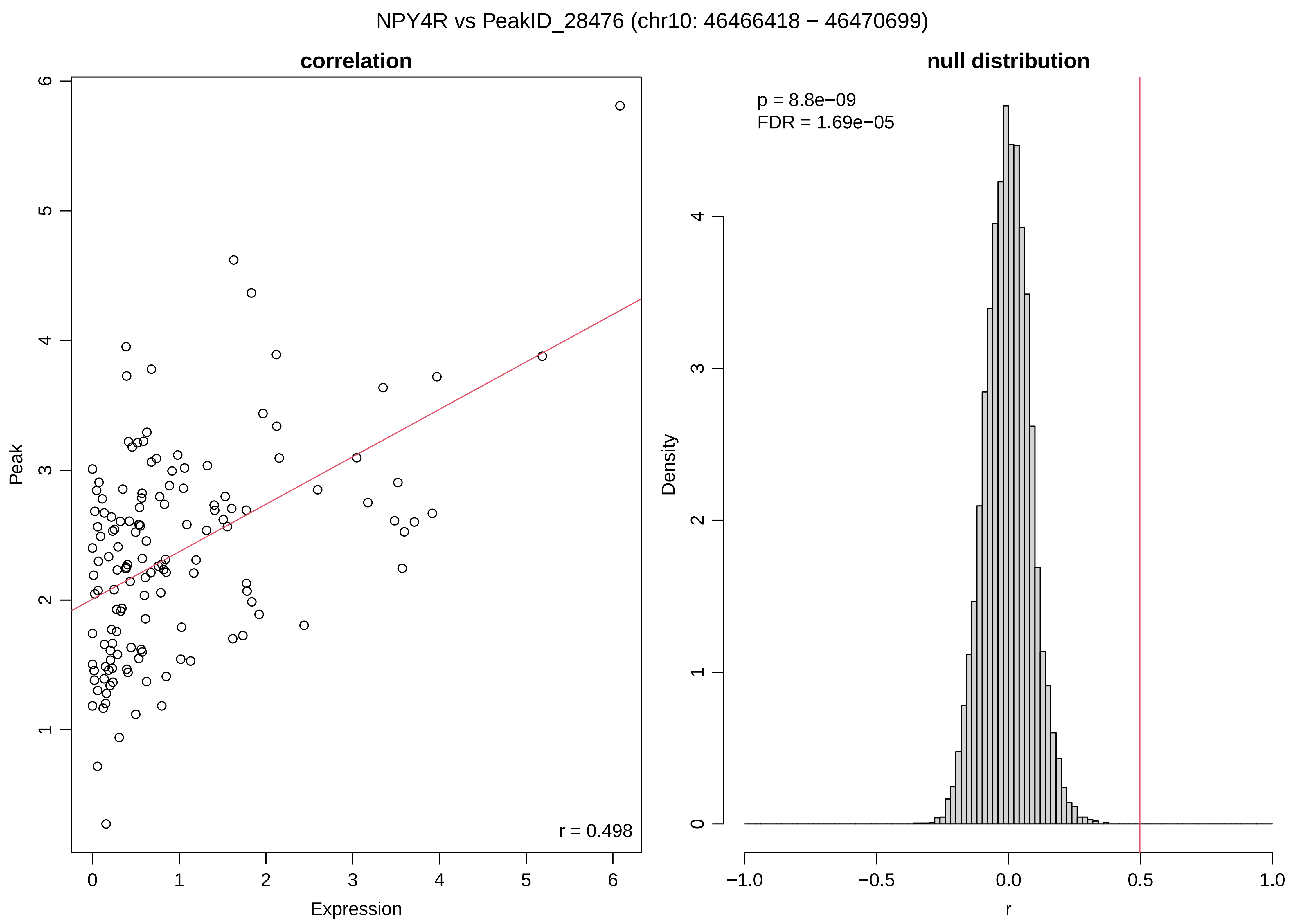
NPY4R vs PeakID_28476 (chr10: 46466418 − 46470699)
correlation
Peak
Expression
r = 0.498
null distribution
p = 8.8e−09
FDR = 1.69e−05
Density
r

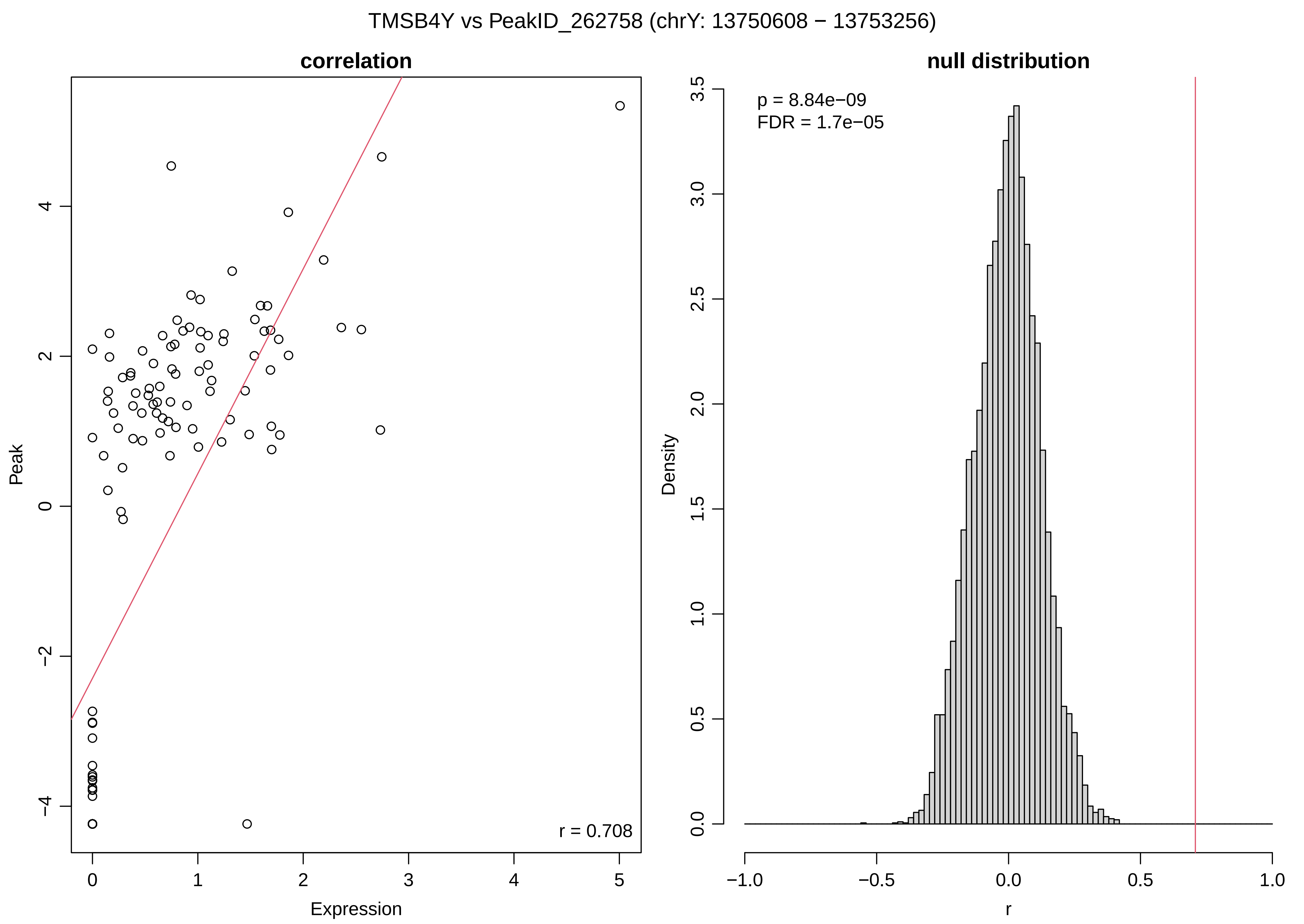
TMSB4Y vs PeakID_262758 (chrY: 13750608 − 13753256)
correlation
Peak
Expression
r = 0.708
null distribution
p = 8.84e−09
FDR = 1.7e−05
Density
r

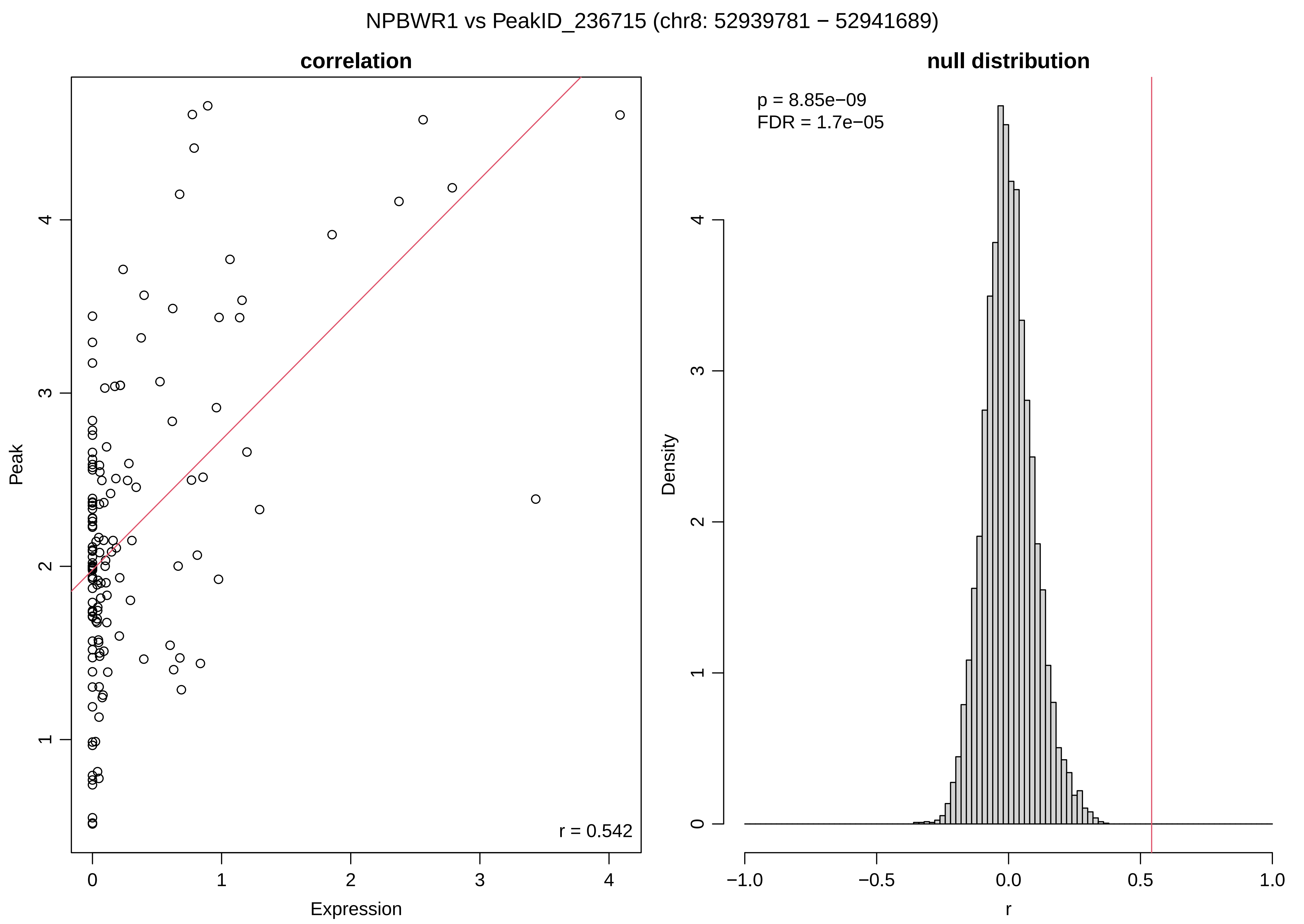
NPBWR1 vs PeakID_236715 (chr8: 52939781 − 52941689)
correlation
Peak
Expression
r = 0.542
null distribution
p = 8.85e−09
FDR = 1.7e−05
Density
r

LINC01060 vs PeakID_186373 (chr4: 188445910 − 188446572)

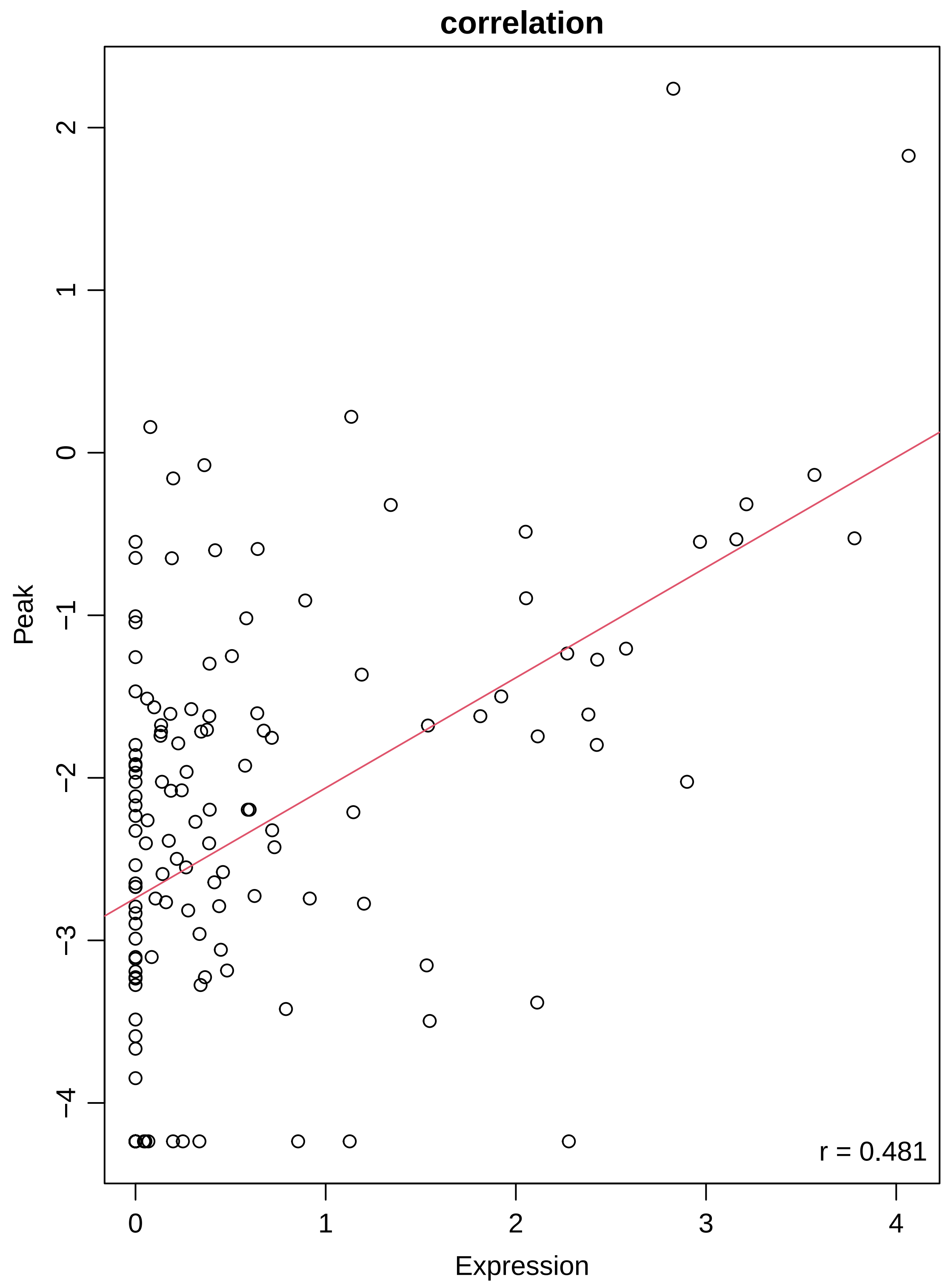

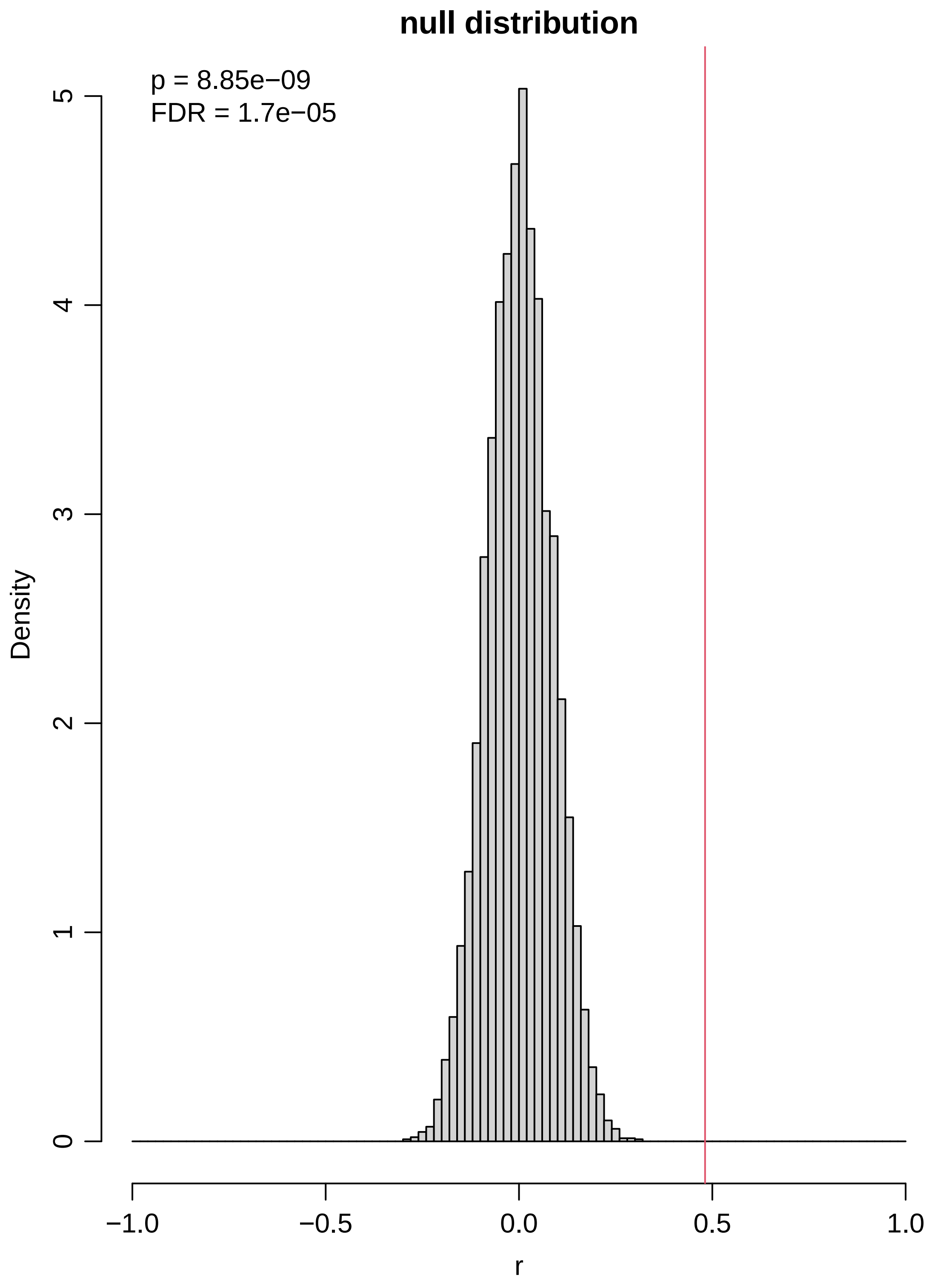

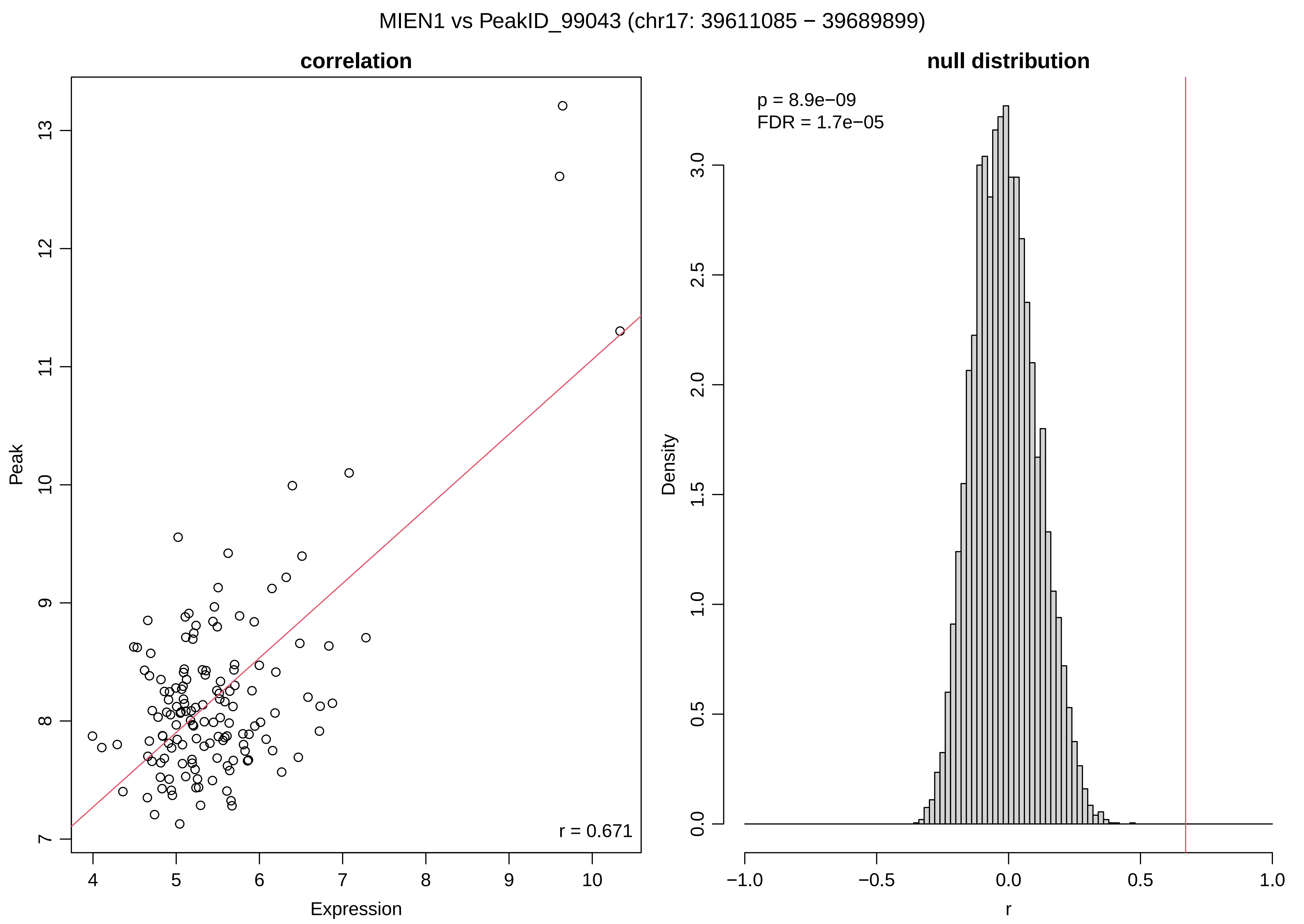
MIEN1 vs PeakID_99043 (chr17: 39611085 − 39689899)
correlation
Peak
Expression
r = 0.671
null distribution
p = 8.9e−09
FDR = 1.7e−05
Density
r

NOXO1 vs PeakID_85336 (chr16: 1977430 − 1987612)

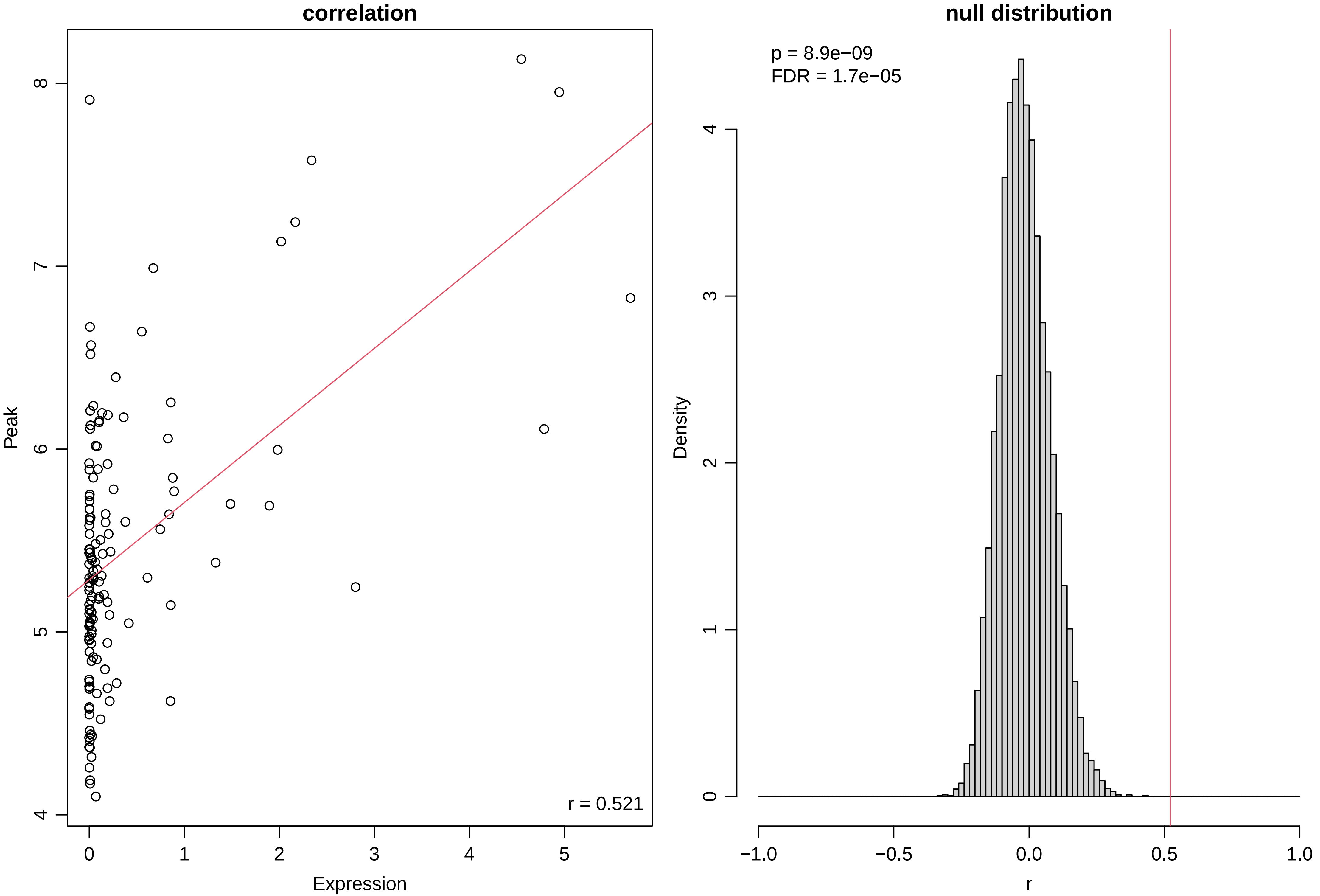

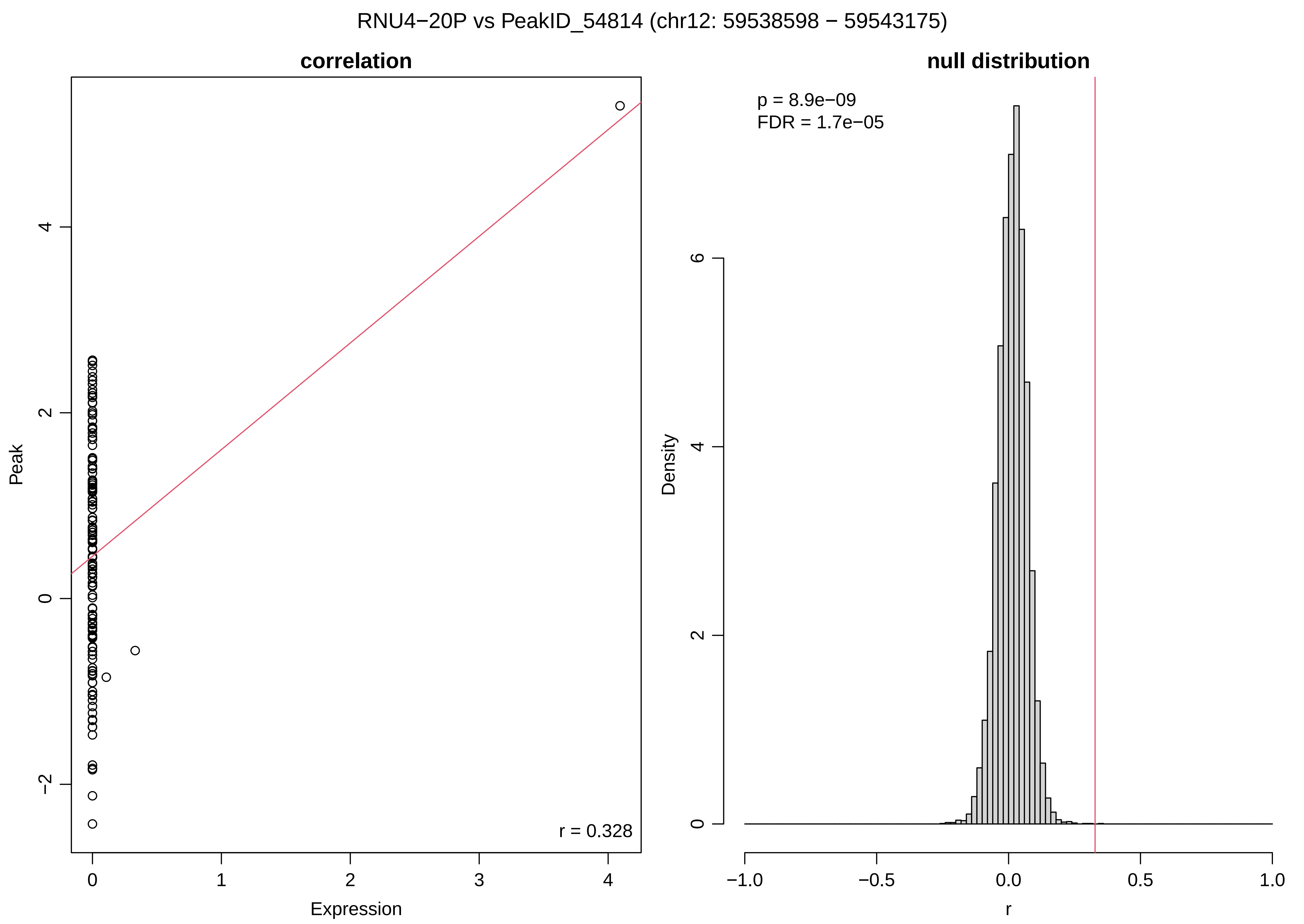
RNU4−20P vs PeakID_54814 (chr12: 59538598 − 59543175)
correlation
Peak
Expression
r = 0.328
null distribution
p = 8.9e−09
FDR = 1.7e−05
Density
r

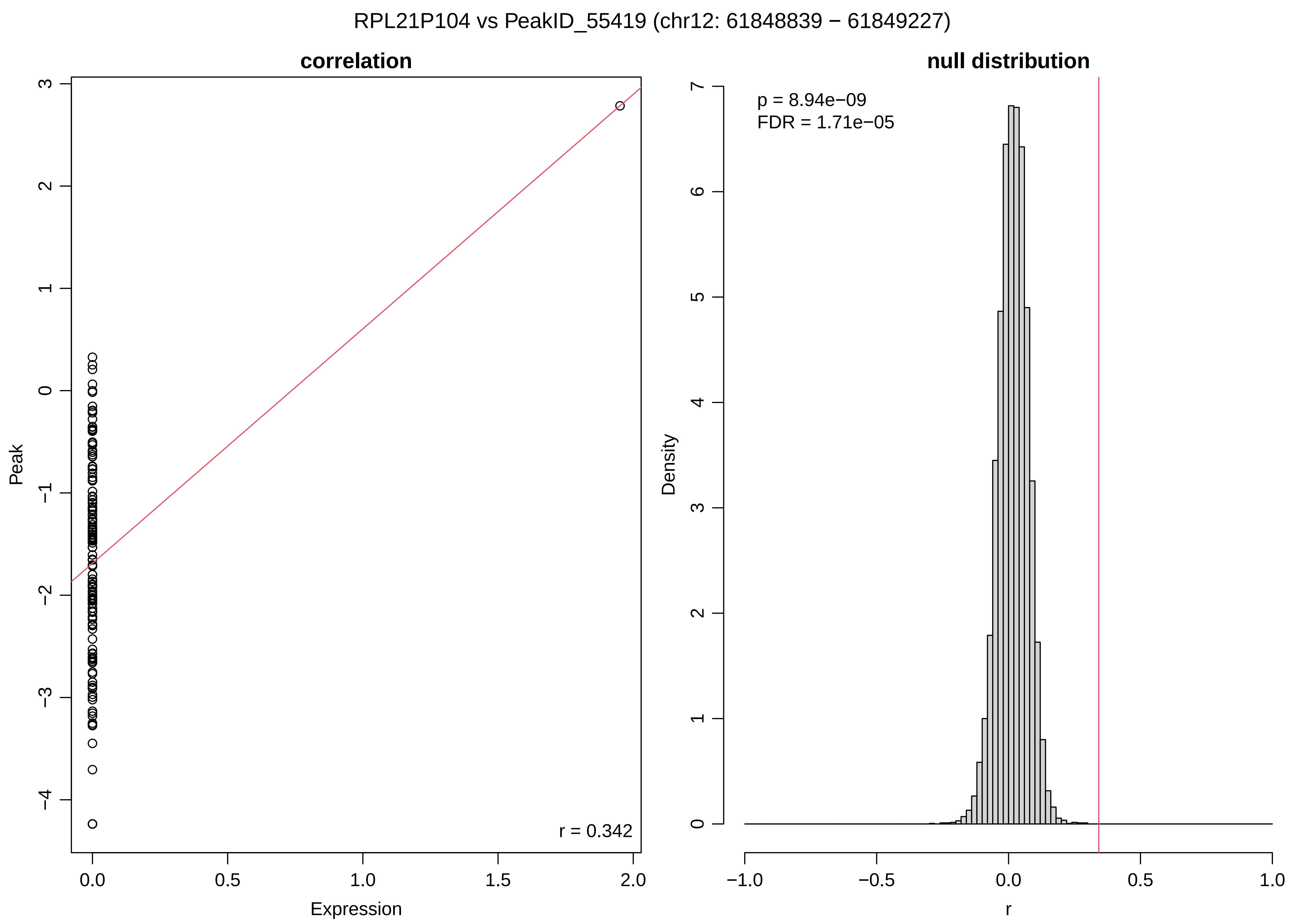

RPL21P104 vs PeakID_55419 (chr12: 61848839 − 61849227)
correlation
Peak
Expression
r = 0.342
3
2
1
0
−1
−2
−3
−4
0.0
0.5
1.0
1.5
2.0
null distribution
p = 8.94e−09
FDR = 1.71e−05
Density
r
7
6
5
4
3
2
1
0
−1.0
−0.5
0.0
0.5
1.0

DSP vs PeakID_202256 (chr6: 7540616 − 7544432)

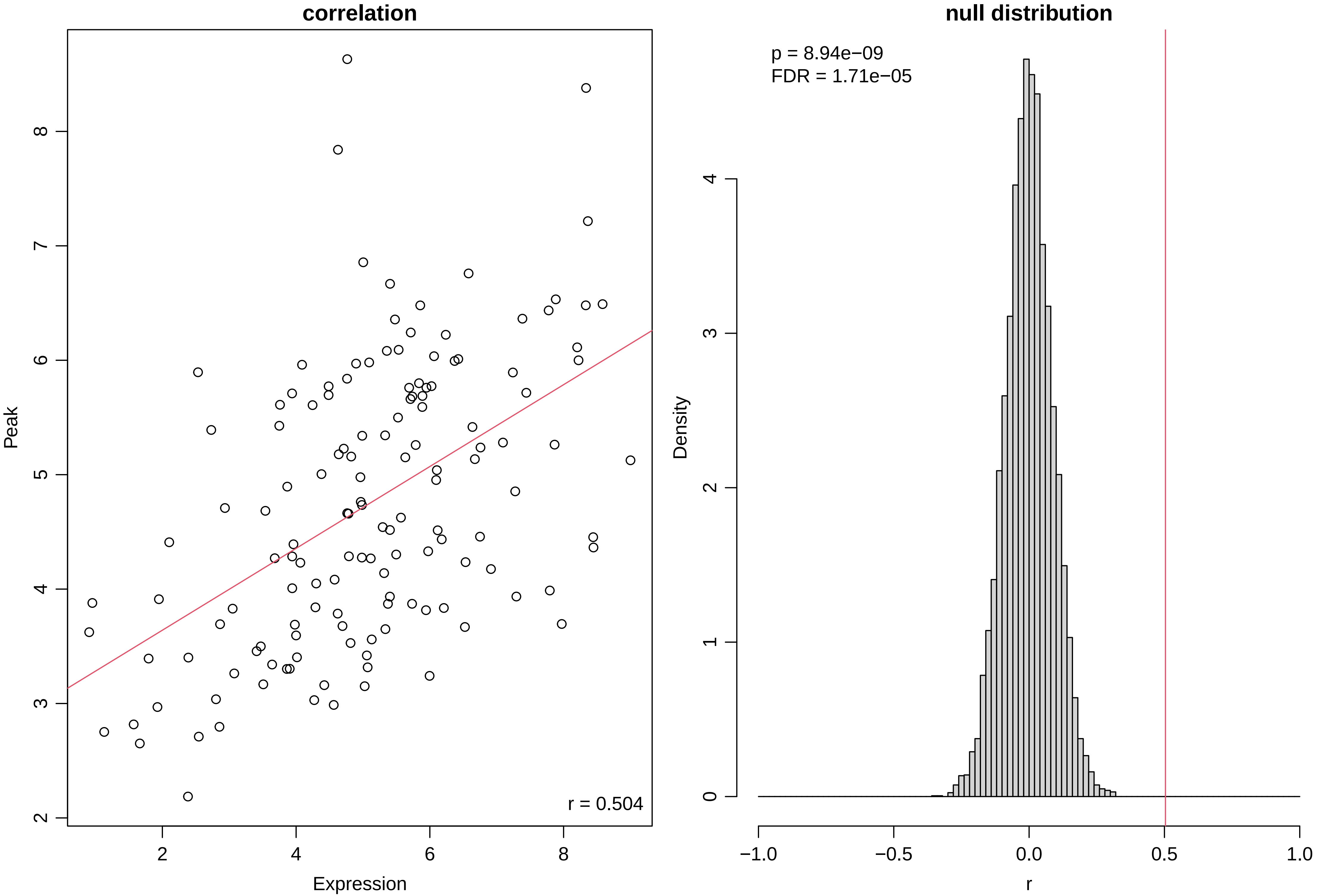

AC024901.1 vs PeakID_50932 (chr12: 19688014 − 19690584)

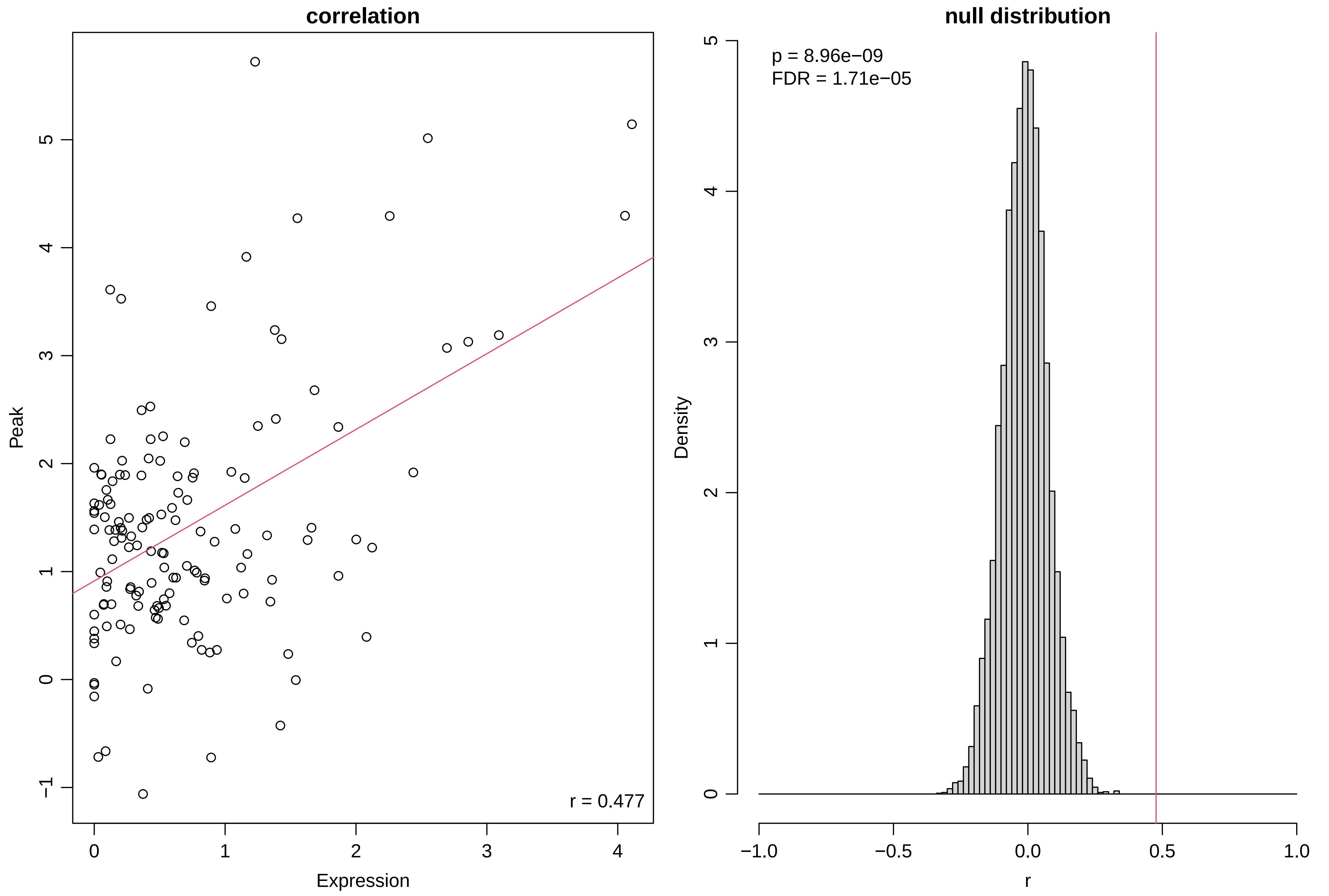

AC106771.1 vs PeakID_187926 (chr5: 12573853 − 12575398)

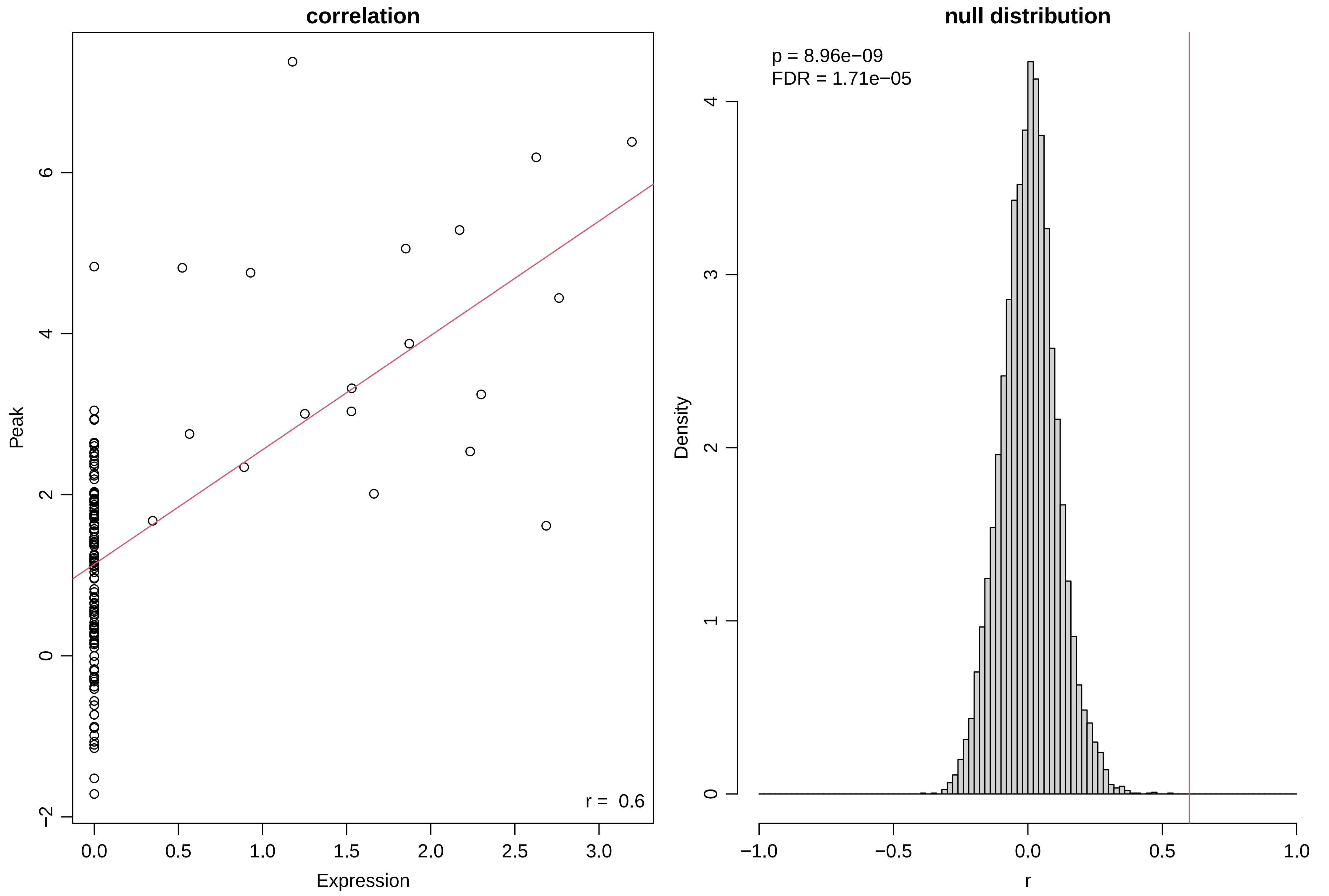

AC244213.1 vs PeakID_262731 (chrY: 12463377 − 12465370)

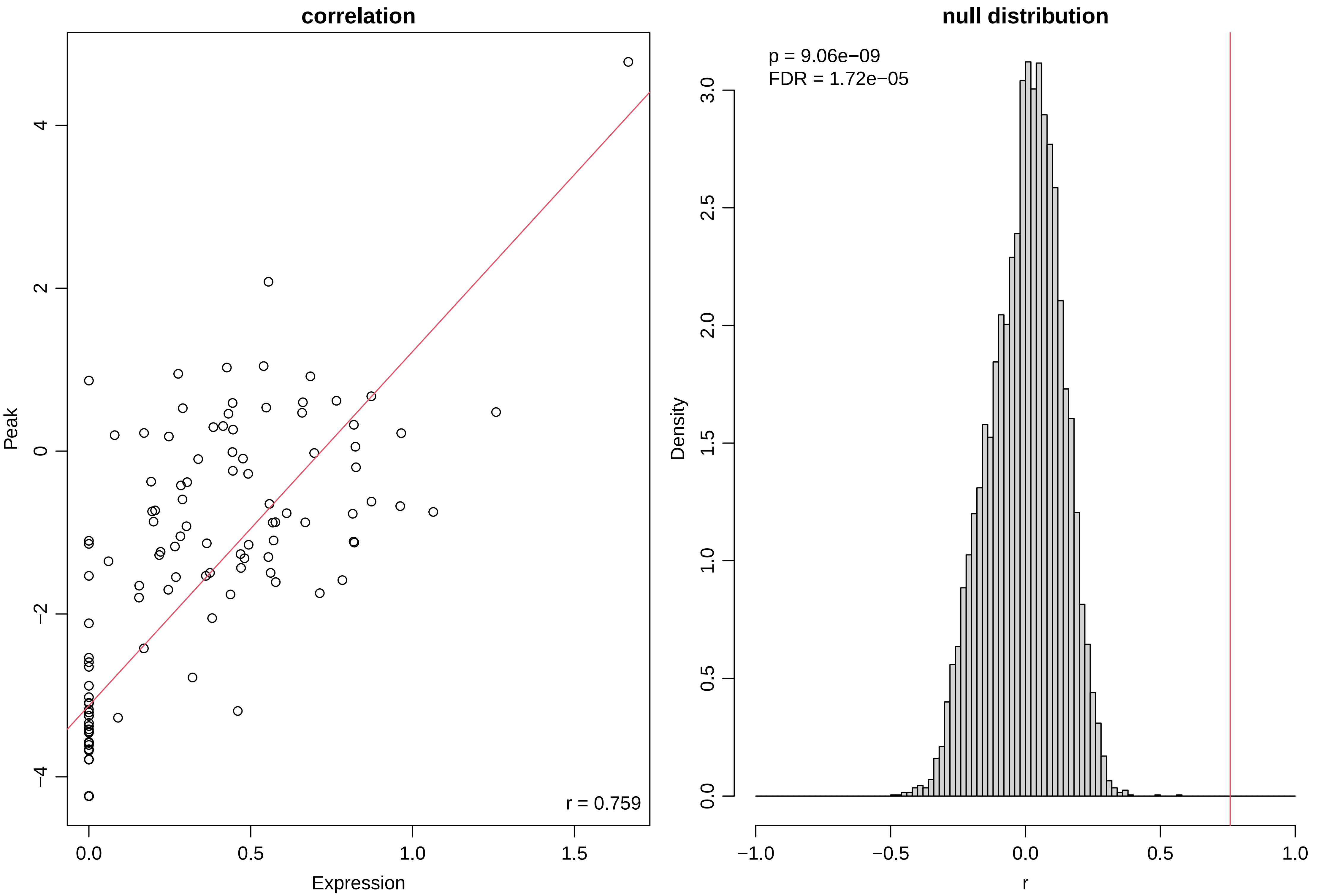

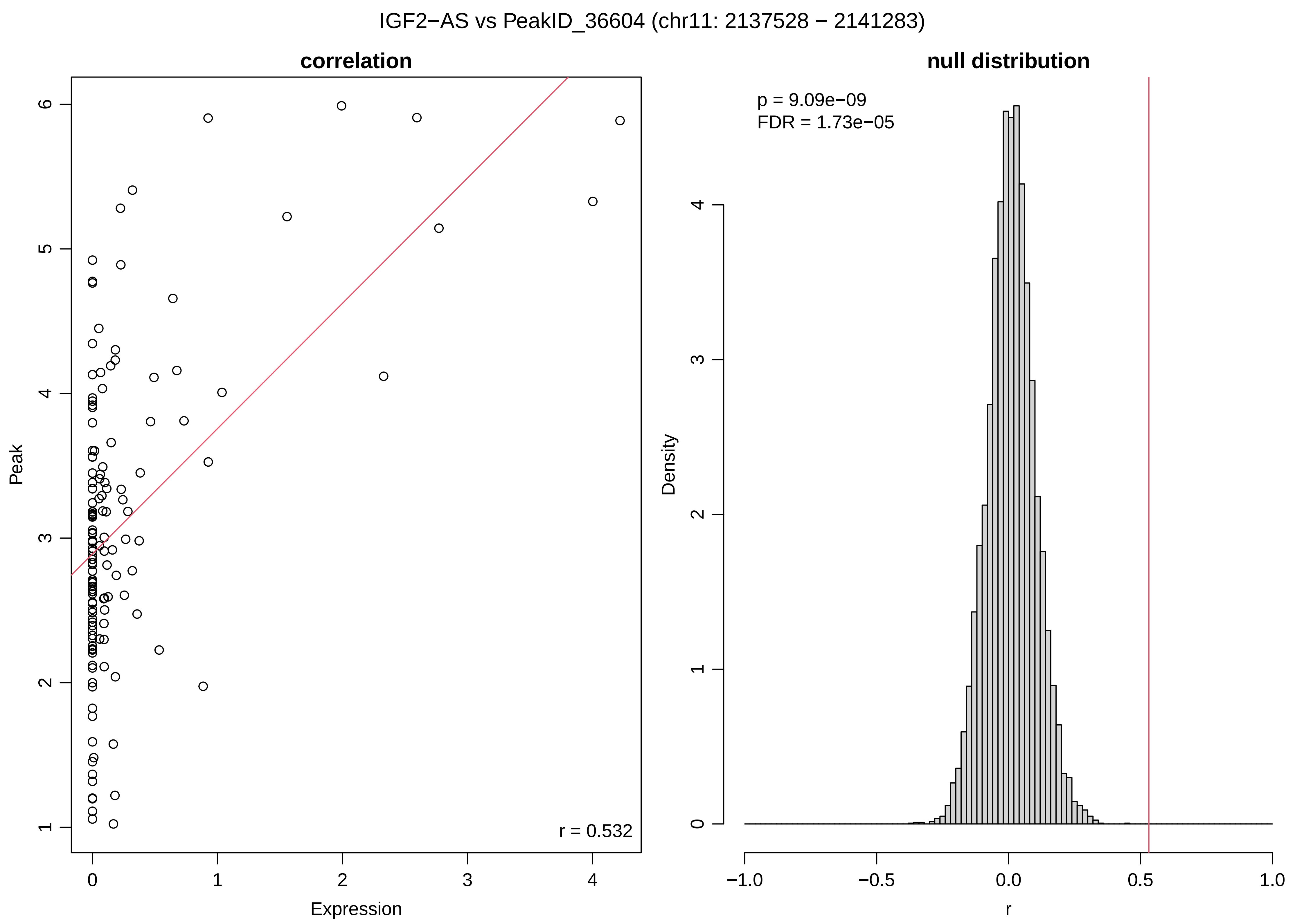
IGF2−AS vs PeakID_36604 (chr11: 2137528 − 2141283)
correlation
Peak
Expression
r = 0.532
null distribution
p = 9.09e−09
FDR = 1.73e−05
Density
r

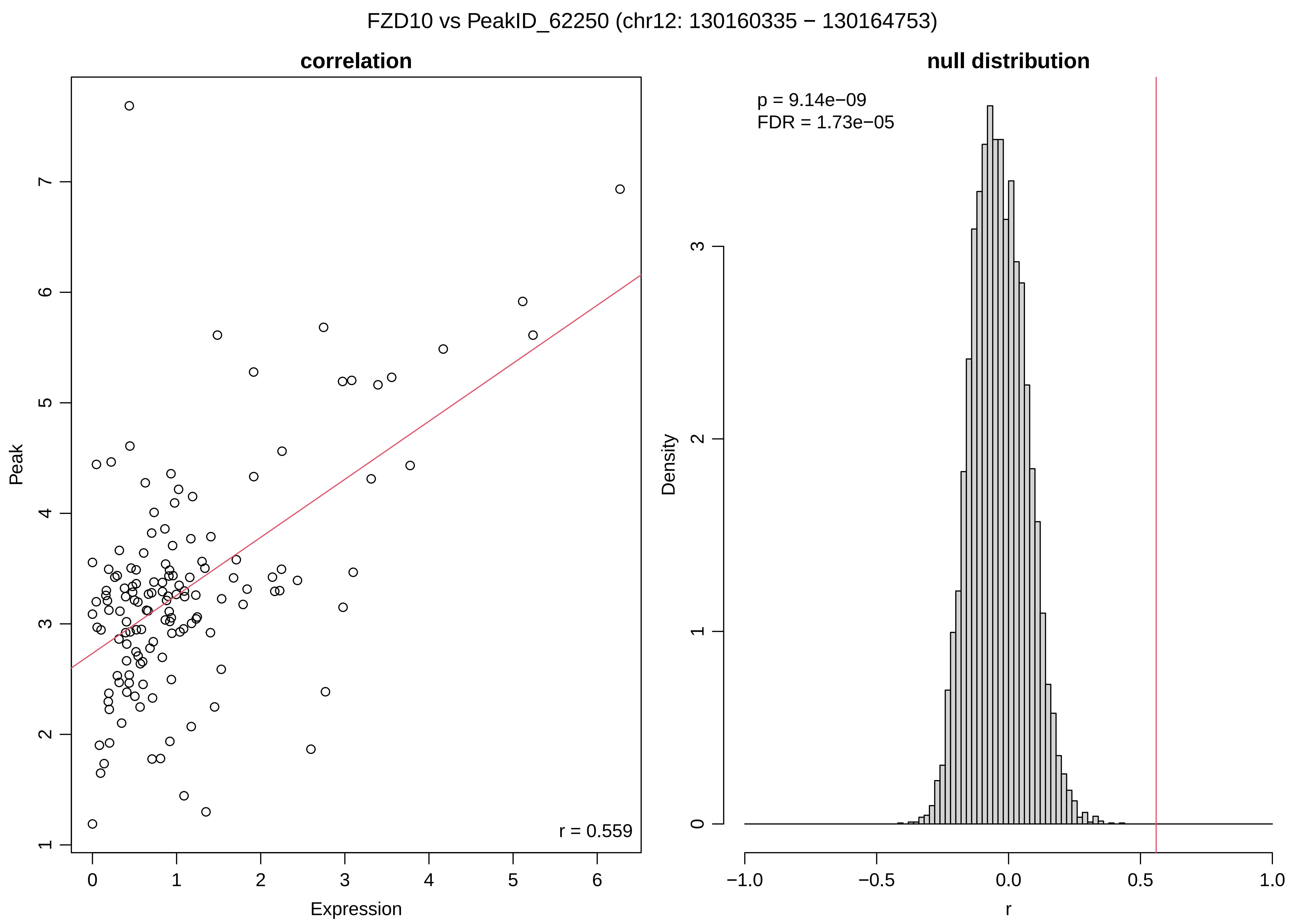
FZD10 vs PeakID_62250 (chr12: 130160335 − 130164753)
correlation
Peak
Expression
r = 0.559
null distribution
p = 9.14e−09
FDR = 1.73e−05
Density
r

XAGE1B vs PeakID_258557 (chrX: 52516275 − 52517982)

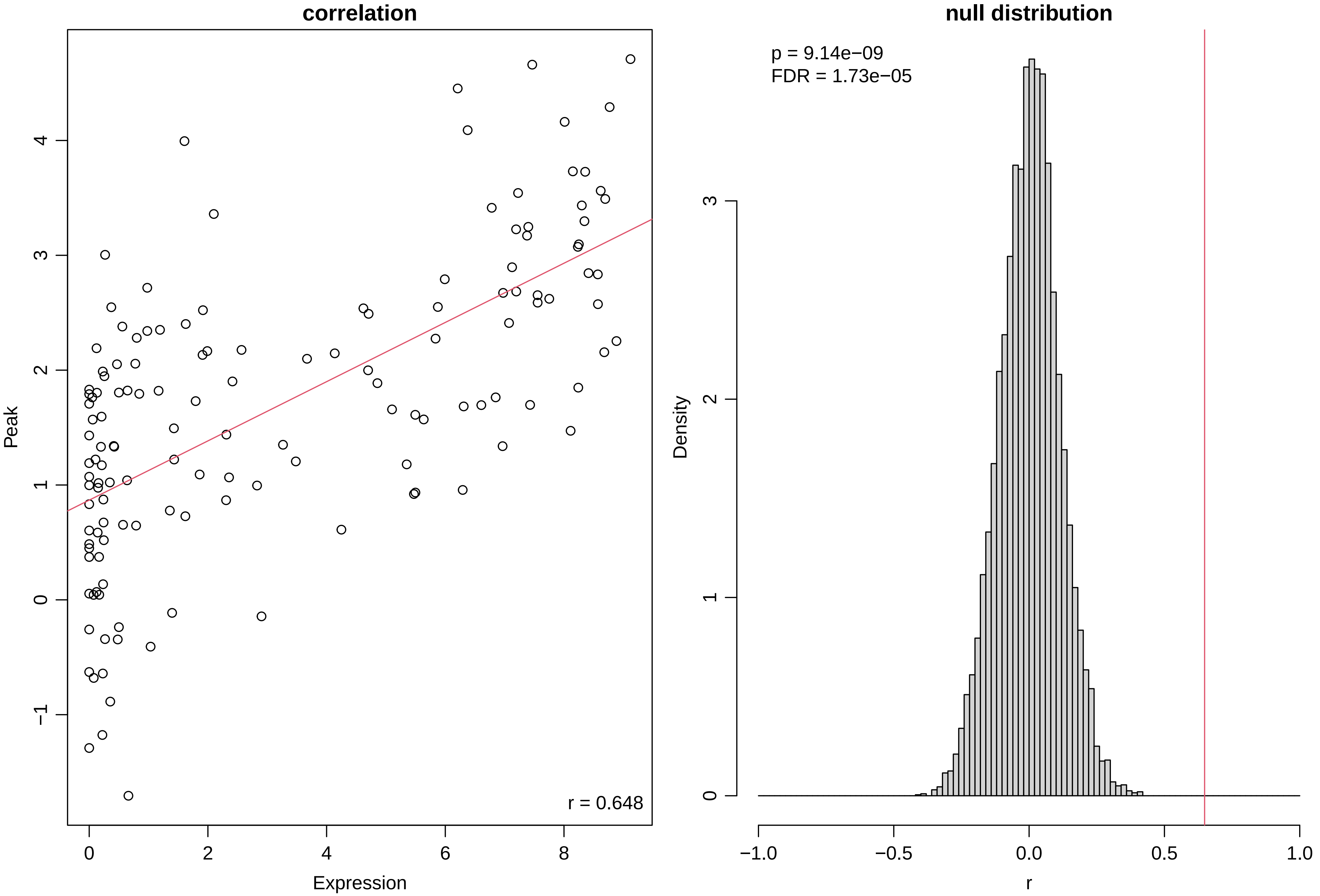

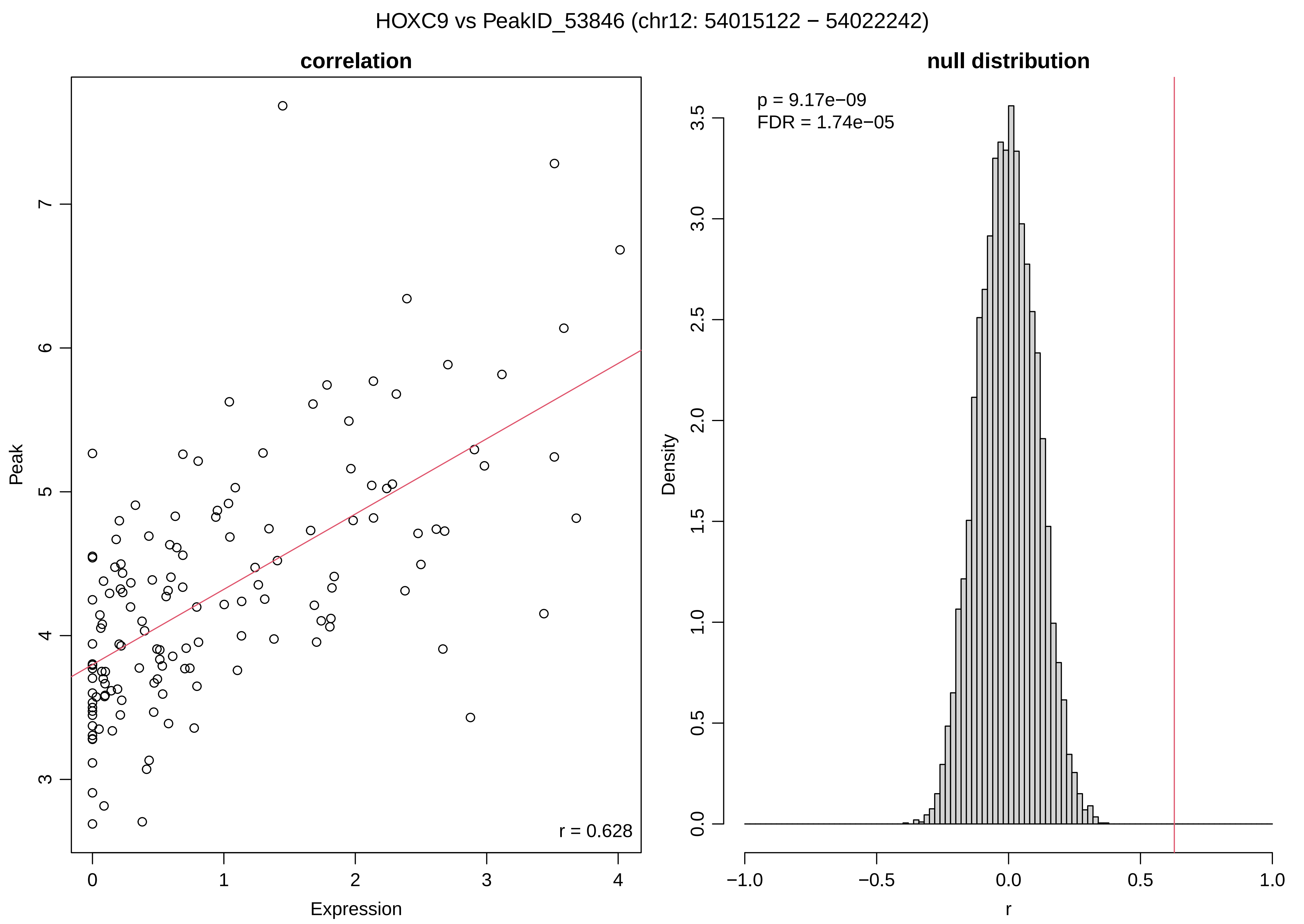
HOXC9 vs PeakID_53846 (chr12: 54015122 − 54022242)
correlation
null distribution
p = 9.17e−09
FDR = 1.74e−05
r = 0.628
Peak
Expression
Density
r

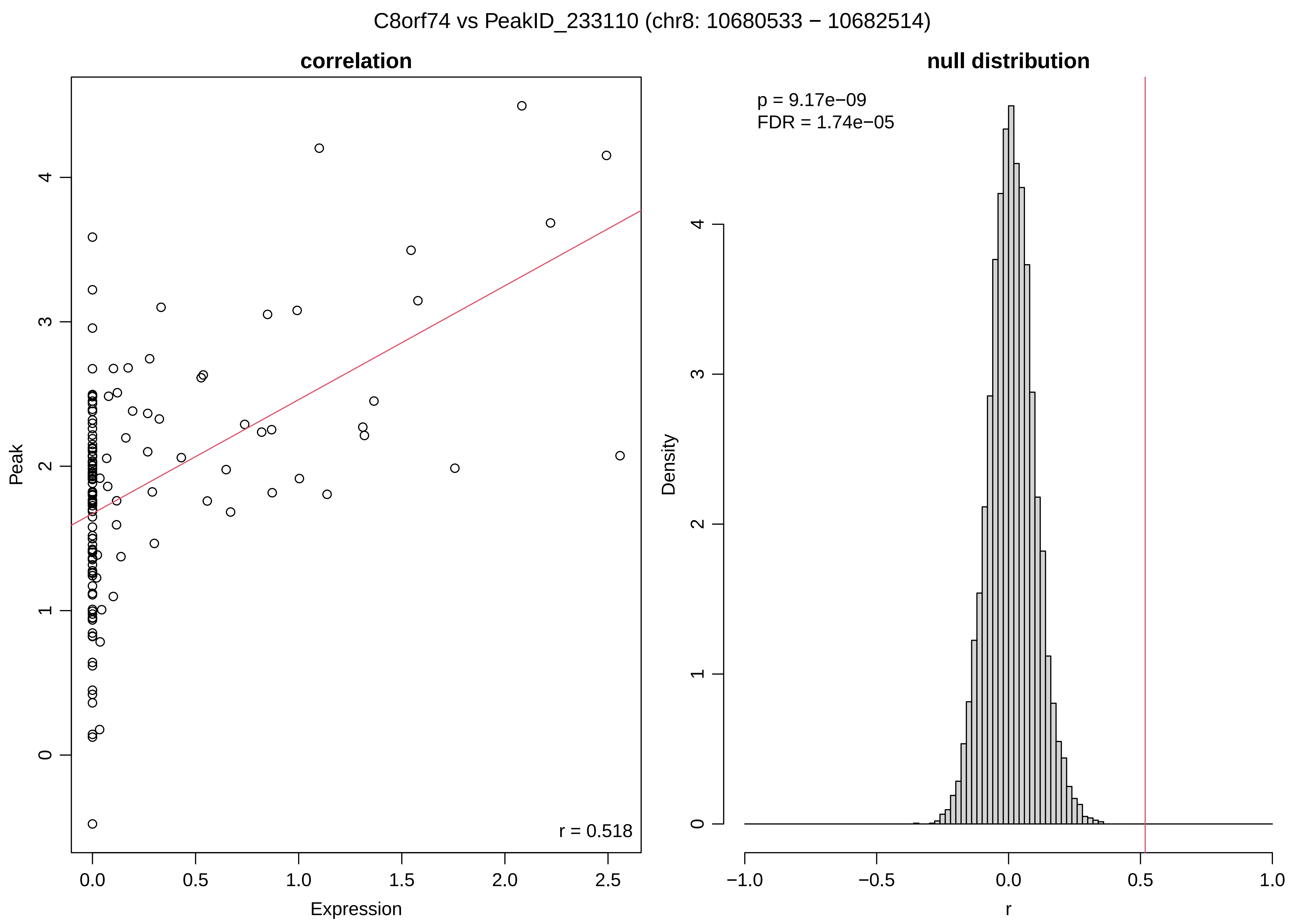
C8orf74 vs PeakID_233110 (chr8: 10680533 − 10682514)
correlation
null distribution
Peak
Expression
r = 0.518
p = 9.17e−09
FDR = 1.74e−05
Density
r

XAGE1A vs PeakID_258557 (chrX: 52516275 − 52517982)

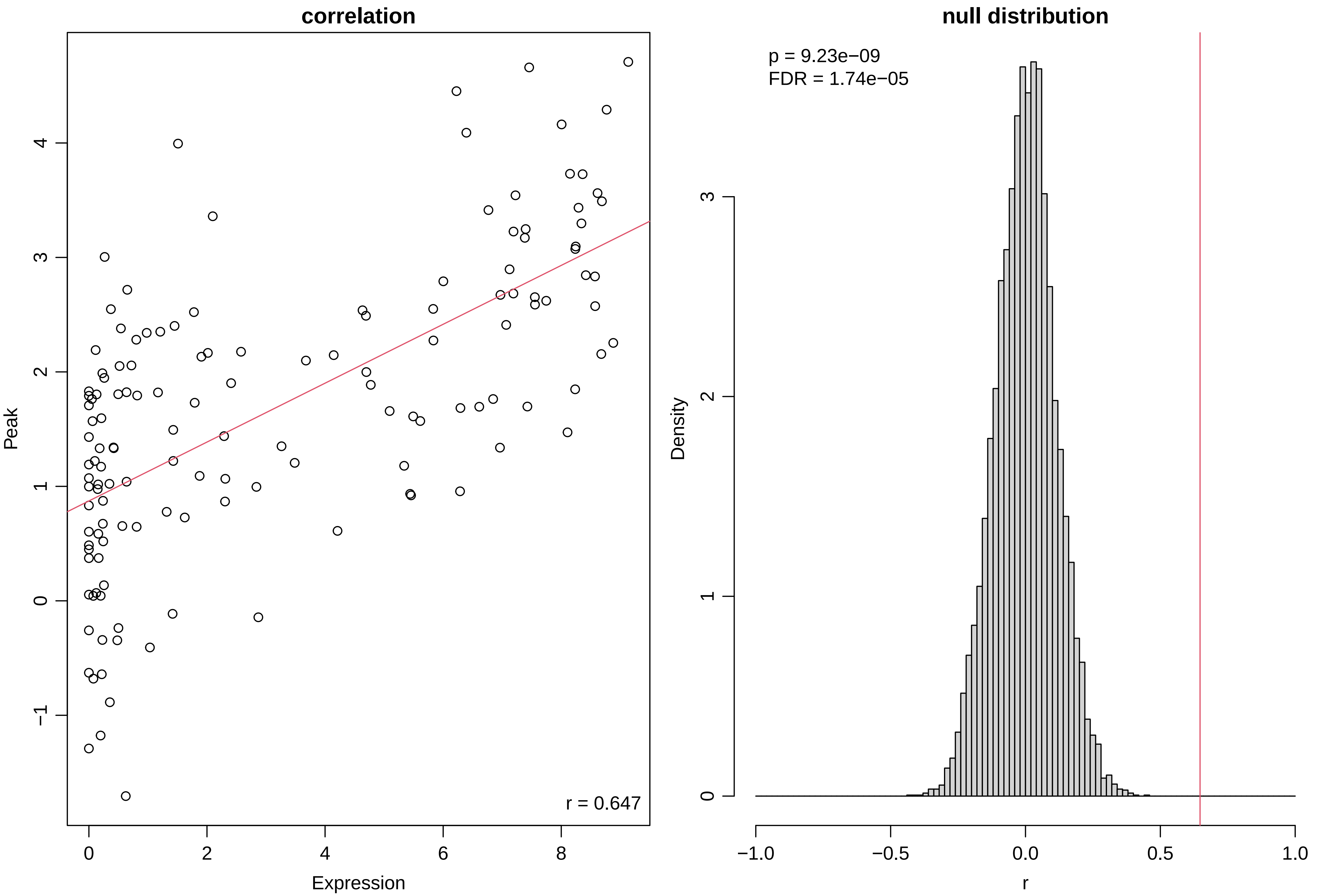

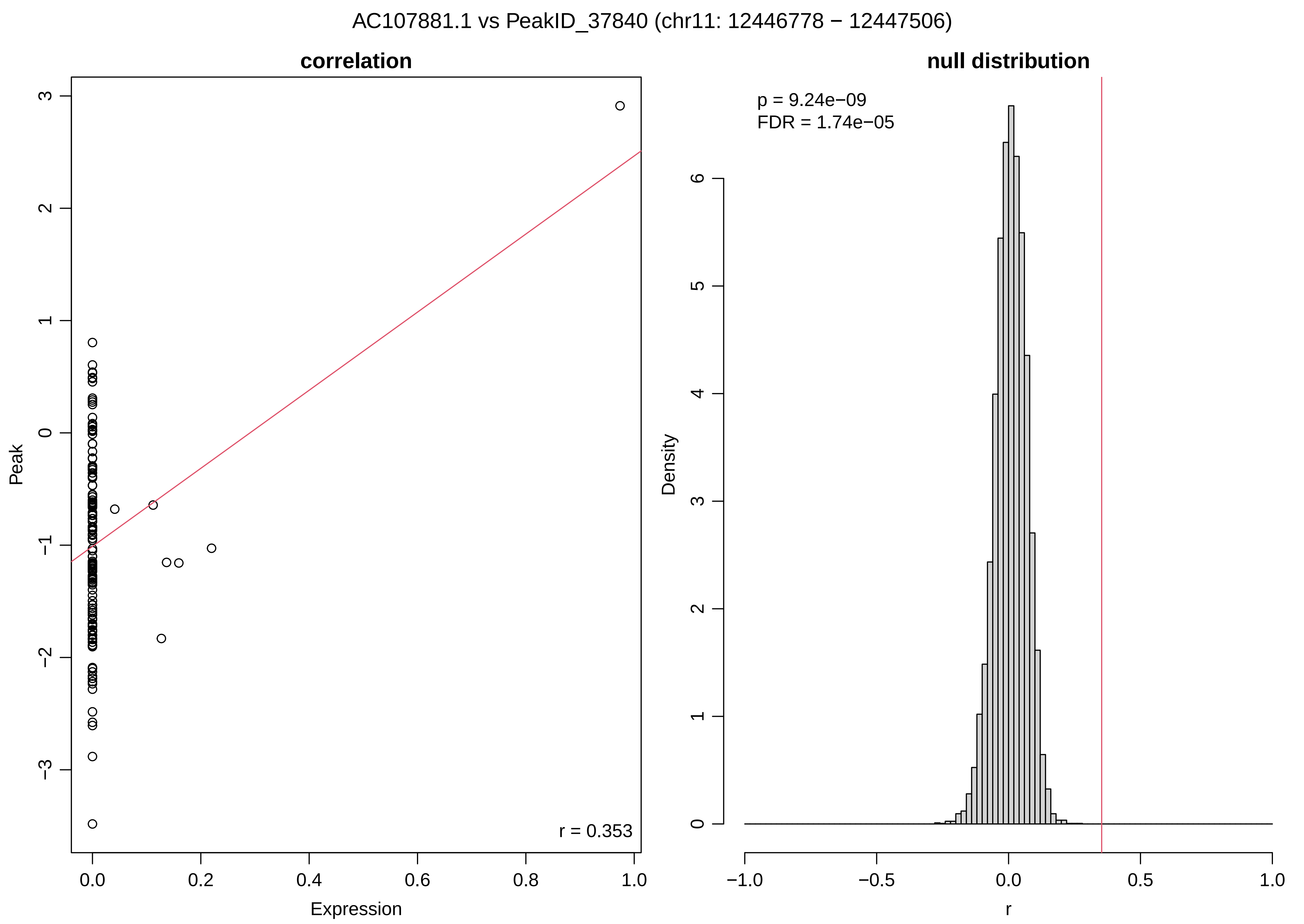
AC107881.1 vs PeakID_37840 (chr11: 12446778 − 12447506)
correlation
Peak
Expression
r = 0.353
null distribution
p = 9.24e−09
FDR = 1.74e−05
Density
r

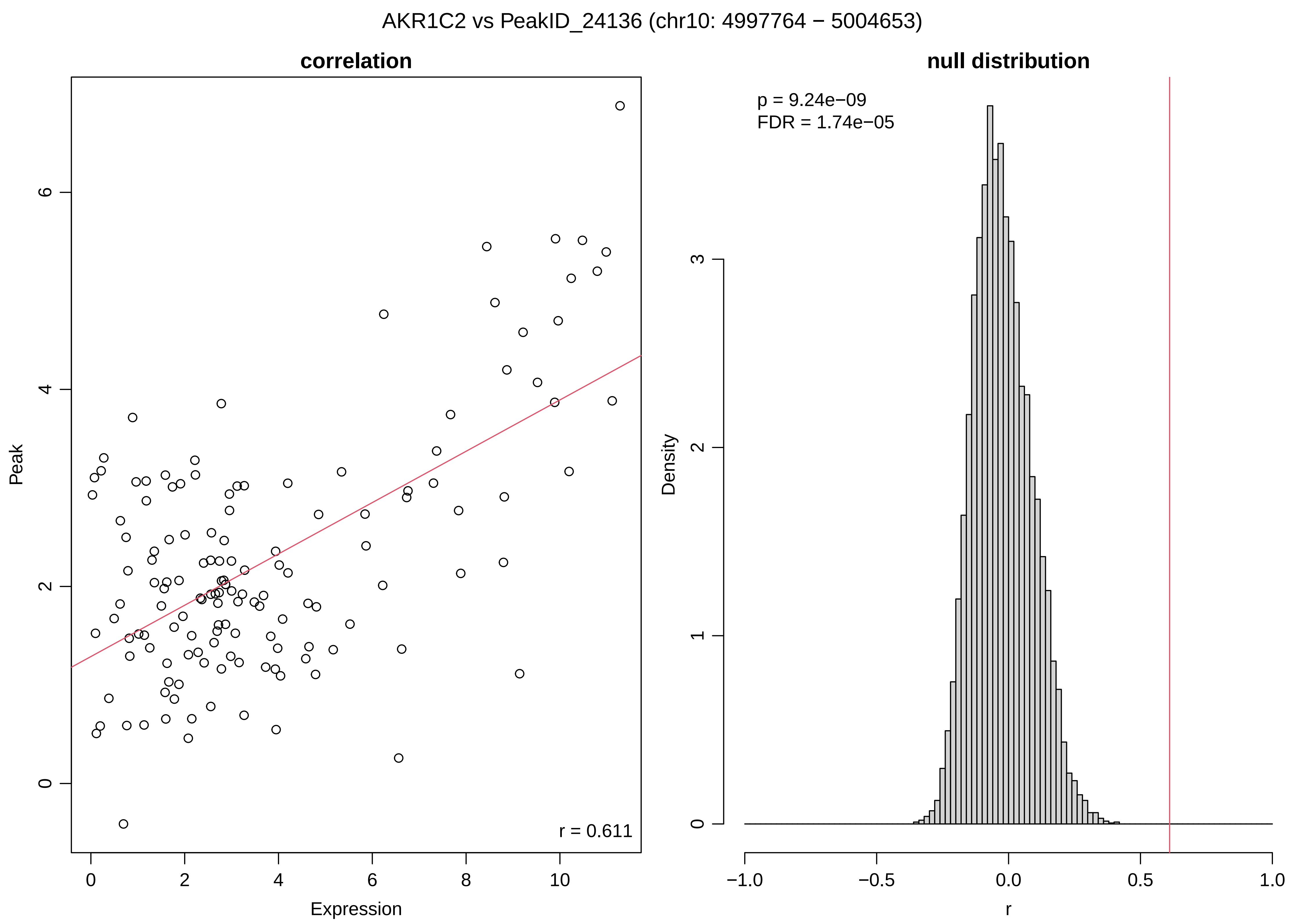

AKR1C2 vs PeakID_24136 (chr10: 4997764 − 5004653)
correlation
Peak
Expression
r = 0.611
0
2
4
6
0
2
4
6
8
10
null distribution
p = 9.24e−09
FDR = 1.74e−05
Density
r
0
1
2
3
−1.0
−0.5
0.0
0.5
1.0

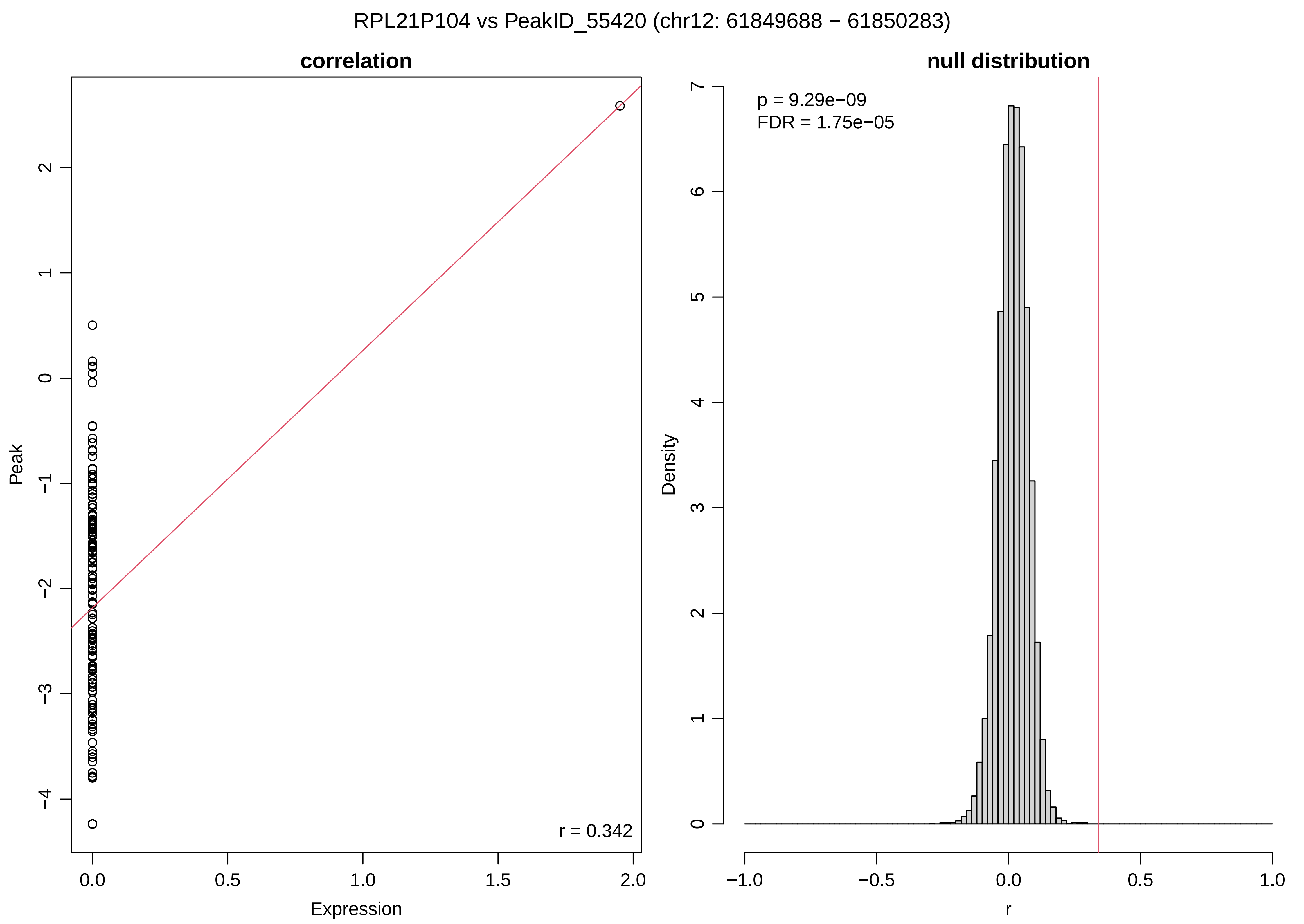
RPL21P104 vs PeakID_55420 (chr12: 61849688 − 61850283)
correlation
Peak
Expression
r = 0.342
null distribution
Density
r
p = 9.29e−09
FDR = 1.75e−05

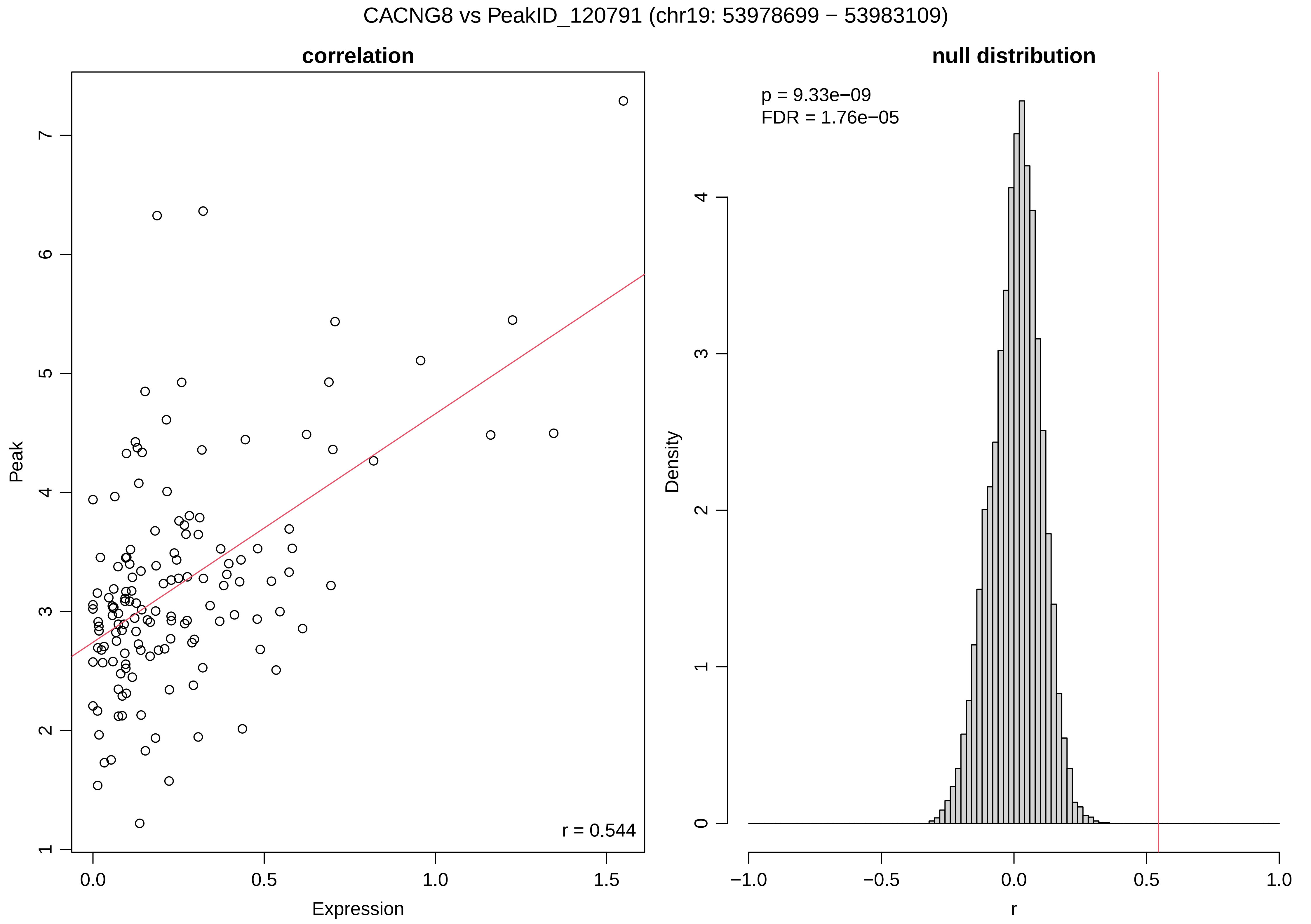

CACNG8 vs PeakID_120791 (chr19: 53978699 − 53983109)
correlation
Peak
Expression
r = 0.544
null distribution
p = 9.33e−09
FDR = 1.76e−05
Density
r

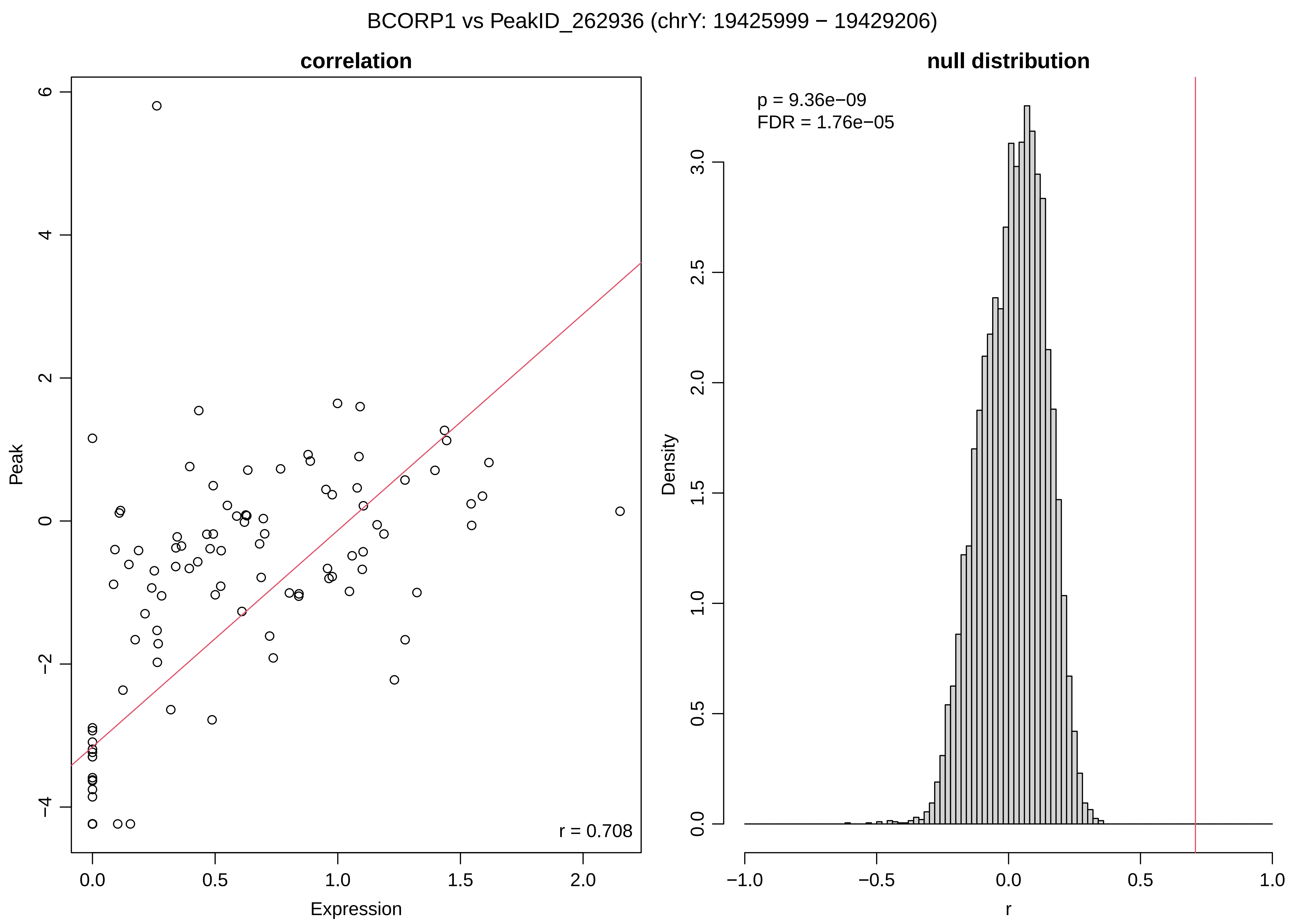

BCORP1 vs PeakID_262936 (chrY: 19425999 − 19429206)
correlation
Peak
Expression
r = 0.708
null distribution
p = 9.36e−09
FDR = 1.76e−05
Density
r

HOXB13 vs PeakID_100538 (chr17: 48725096 − 48730655)

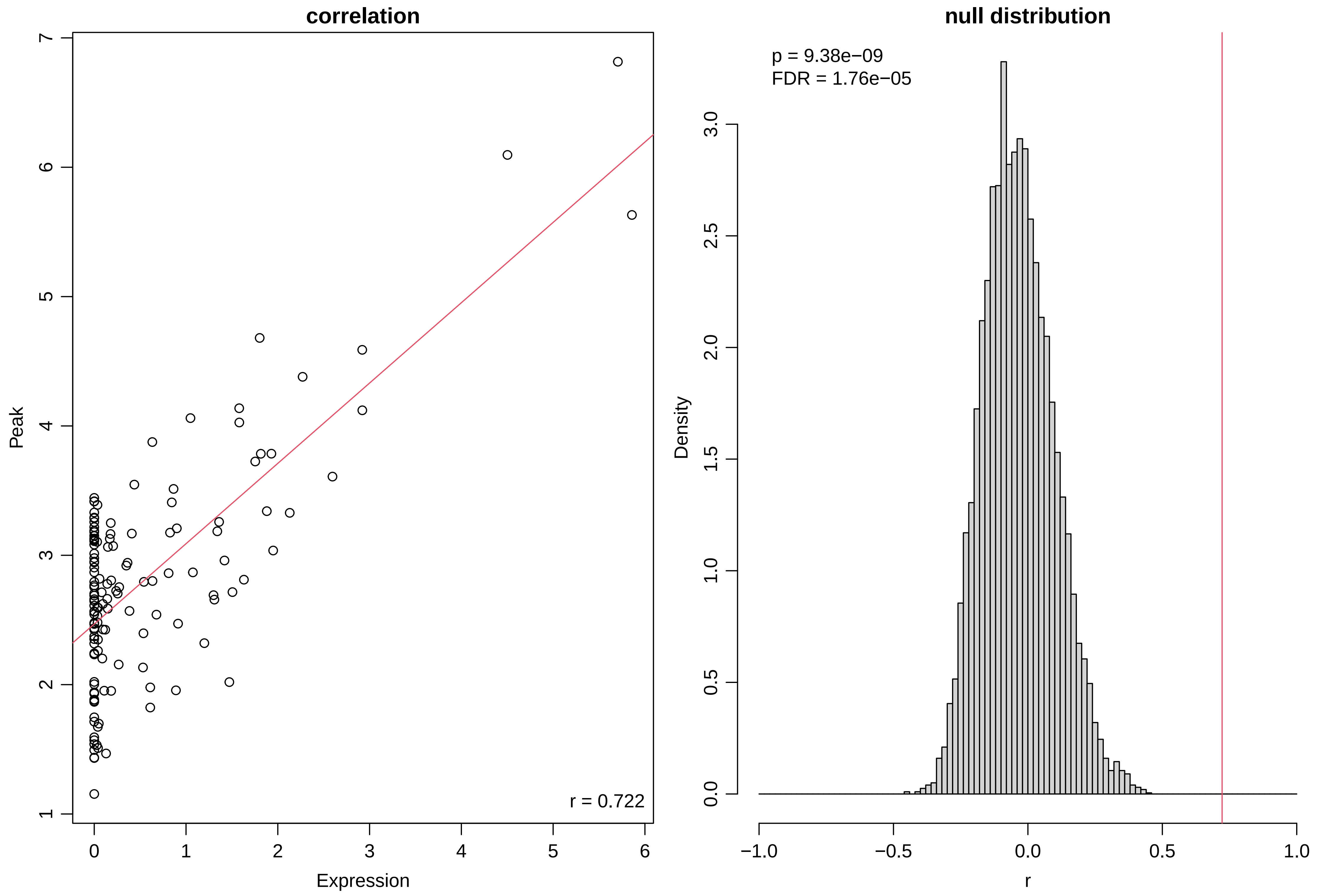

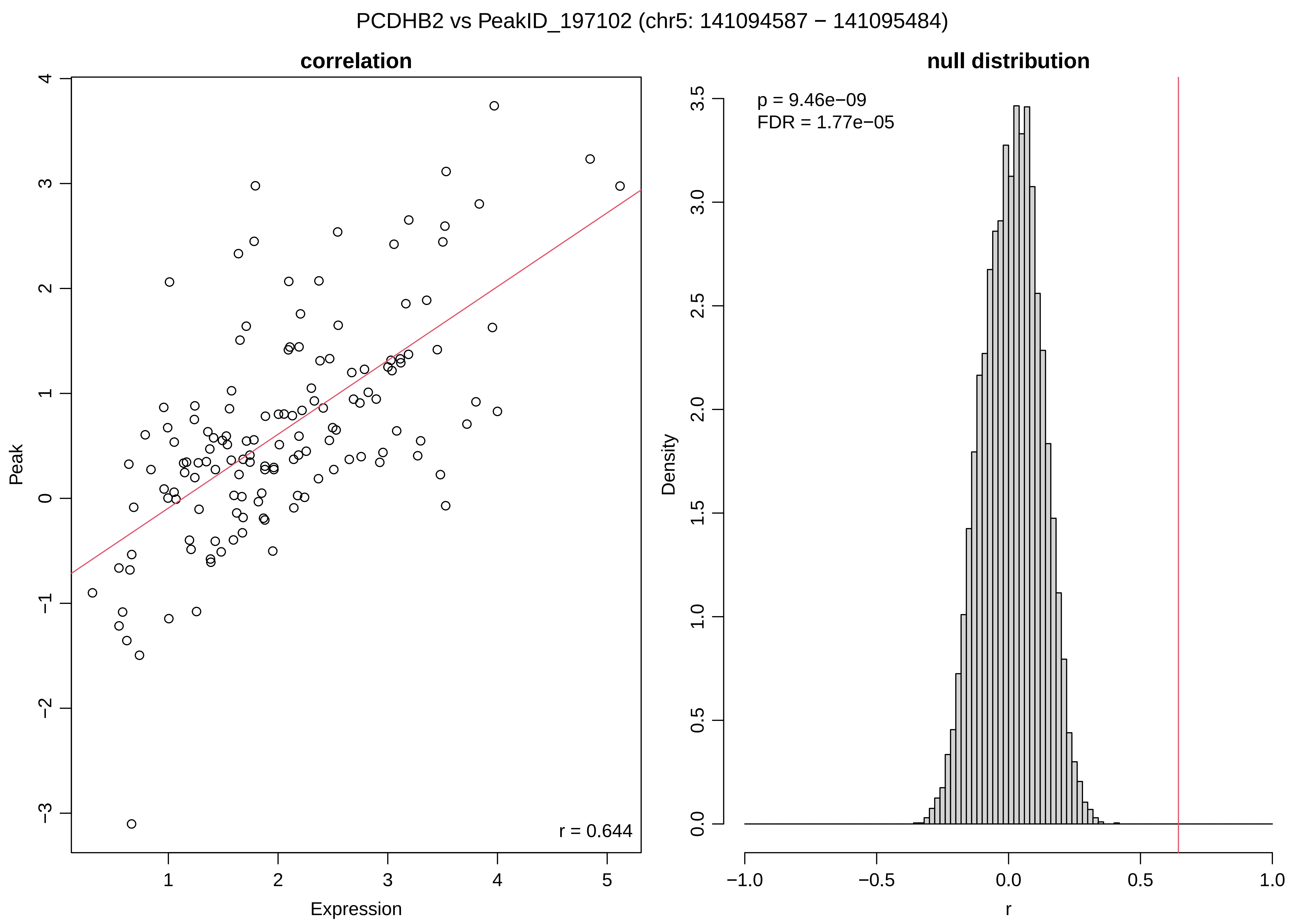
PCDHB2 vs PeakID_197102 (chr5: 141094587 − 141095484)
correlation
Peak
Expression
r = 0.644
null distribution
p = 9.46e−09
FDR = 1.77e−05
Density
r

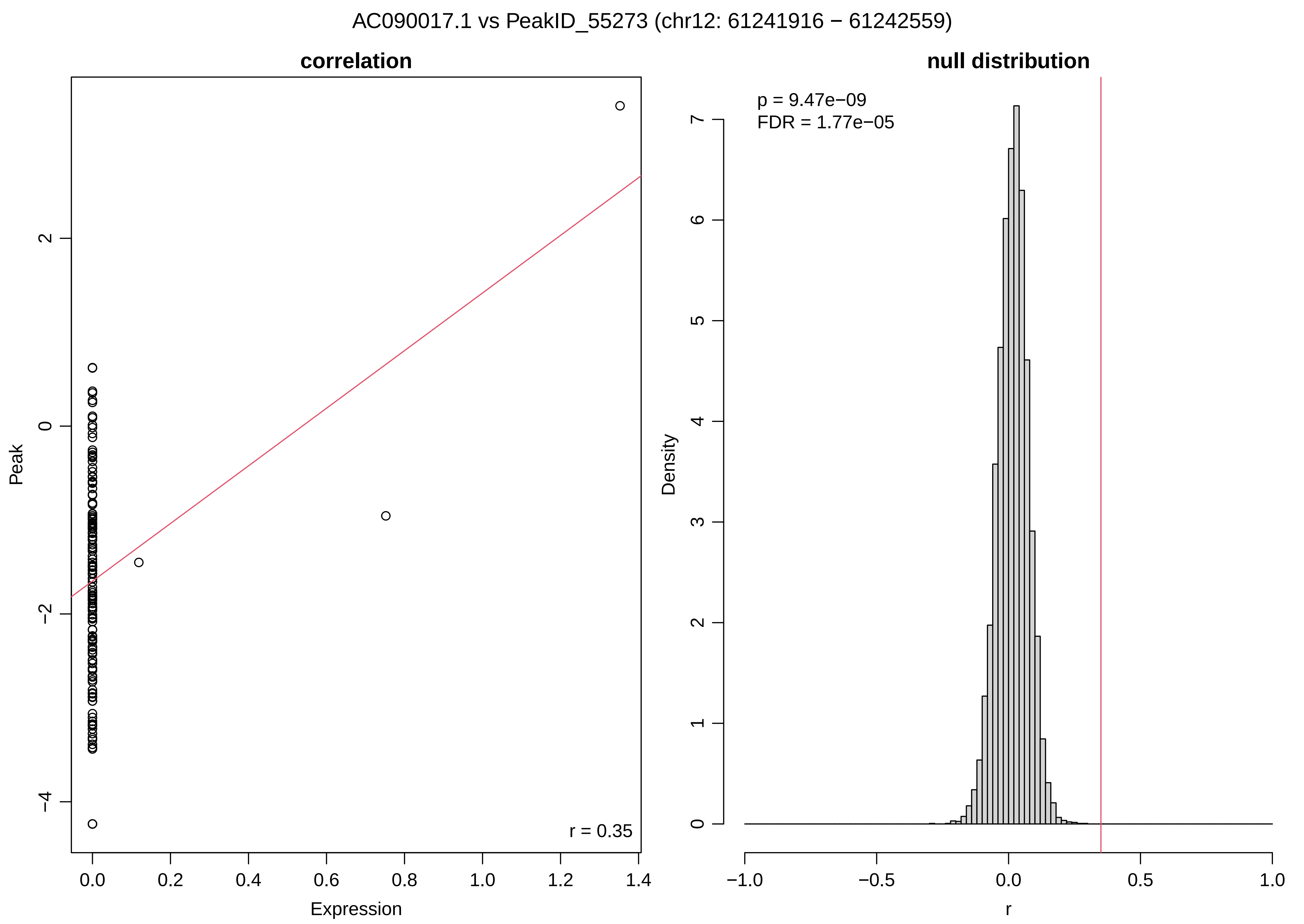

AC090017.1 vs PeakID_55273 (chr12: 61241916 − 61242559)
correlation
Peak
Expression
r = 0.35
null distribution
p = 9.47e−09
FDR = 1.77e−05
Density
r

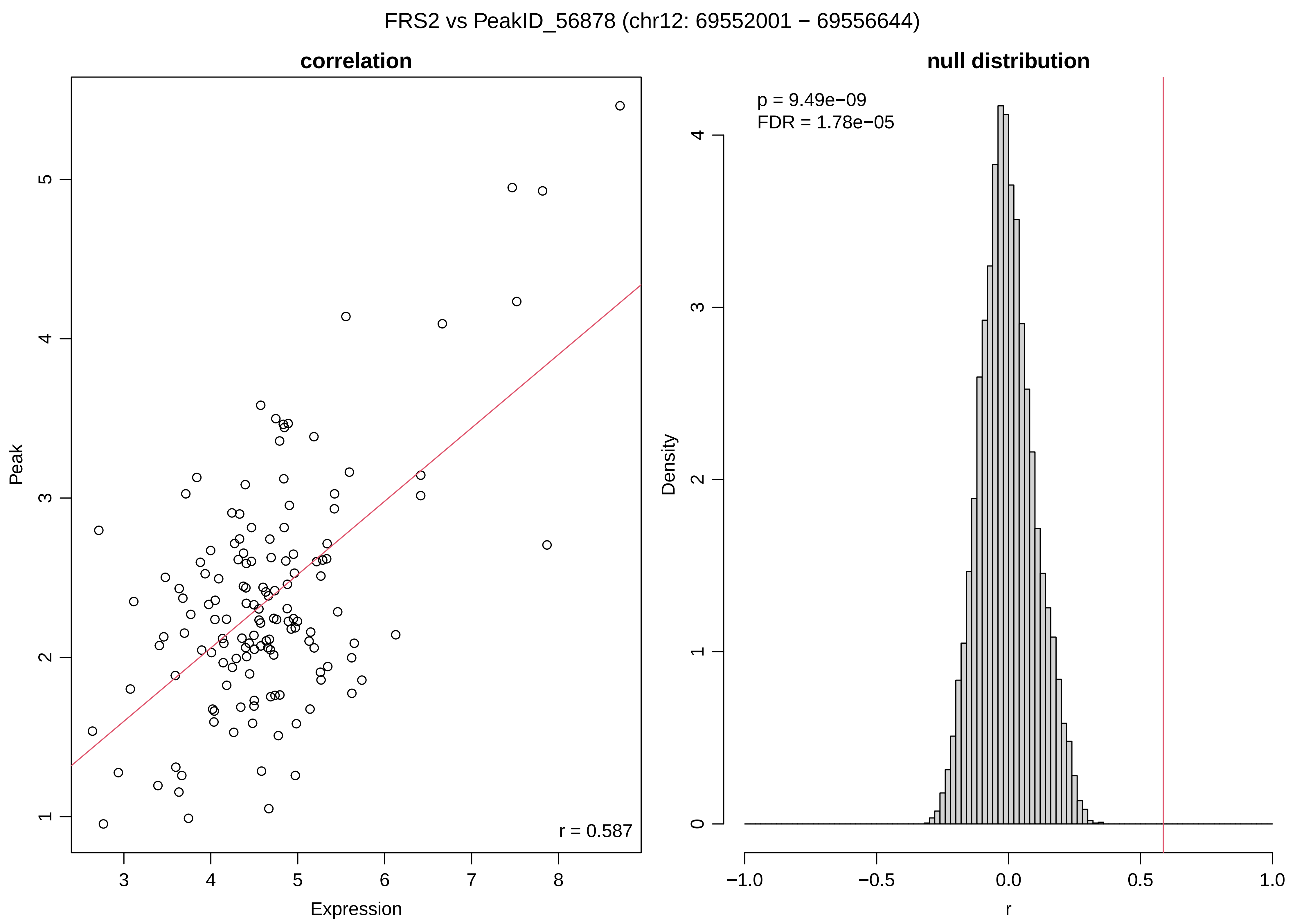
FRS2 vs PeakID_56878 (chr12: 69552001 − 69556644)
correlation
Peak
Expression
r = 0.587
null distribution
p = 9.49e−09
FDR = 1.78e−05
Density
r

HOXB6 vs PeakID_100531 (chr17: 48540951 − 48629745)

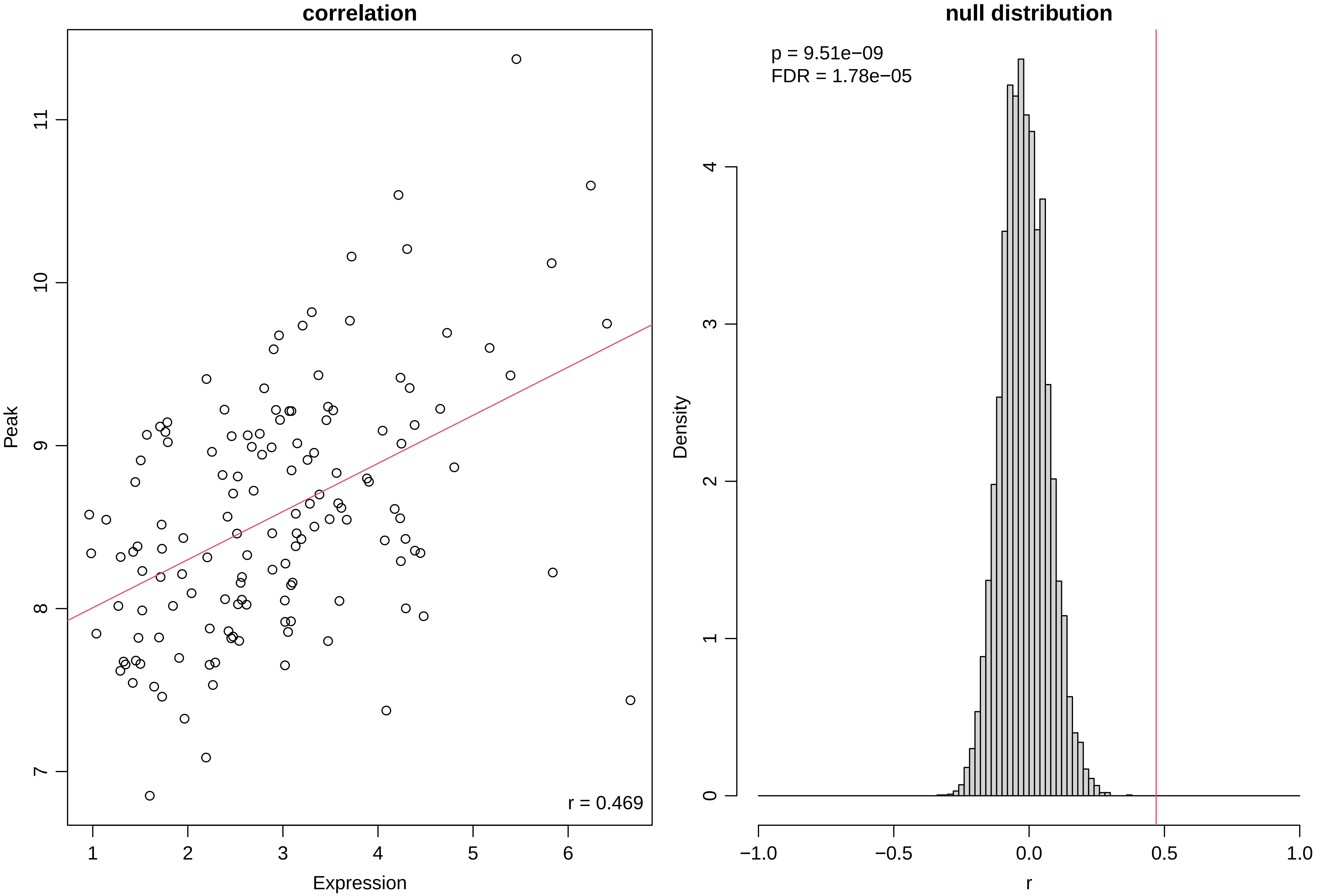

AL356740.3 vs PeakID_68375 (chr13: 113021338 − 113024137)

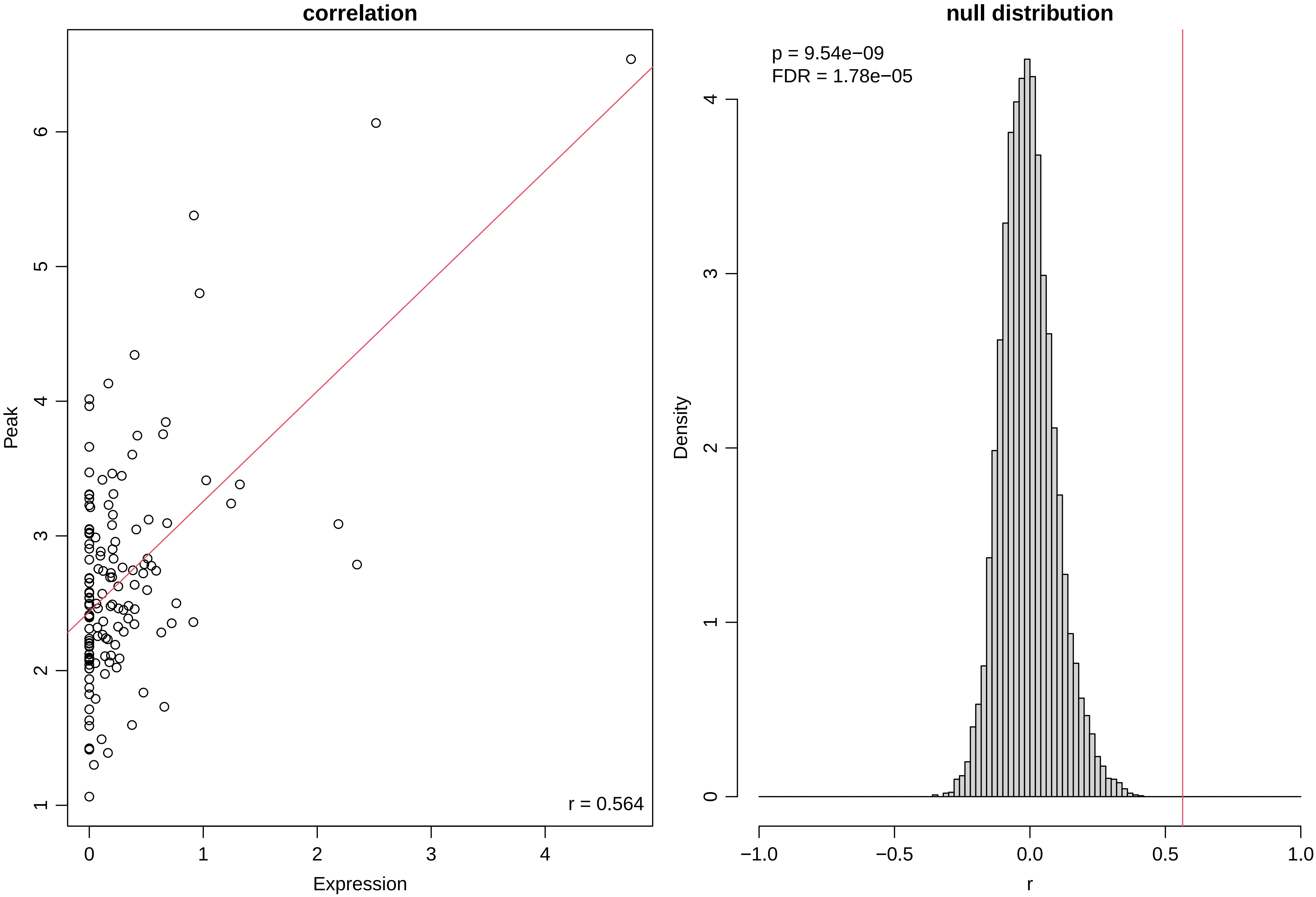

NKX2−3 vs PeakID_33013 (chr10: 99519154 − 99541543)

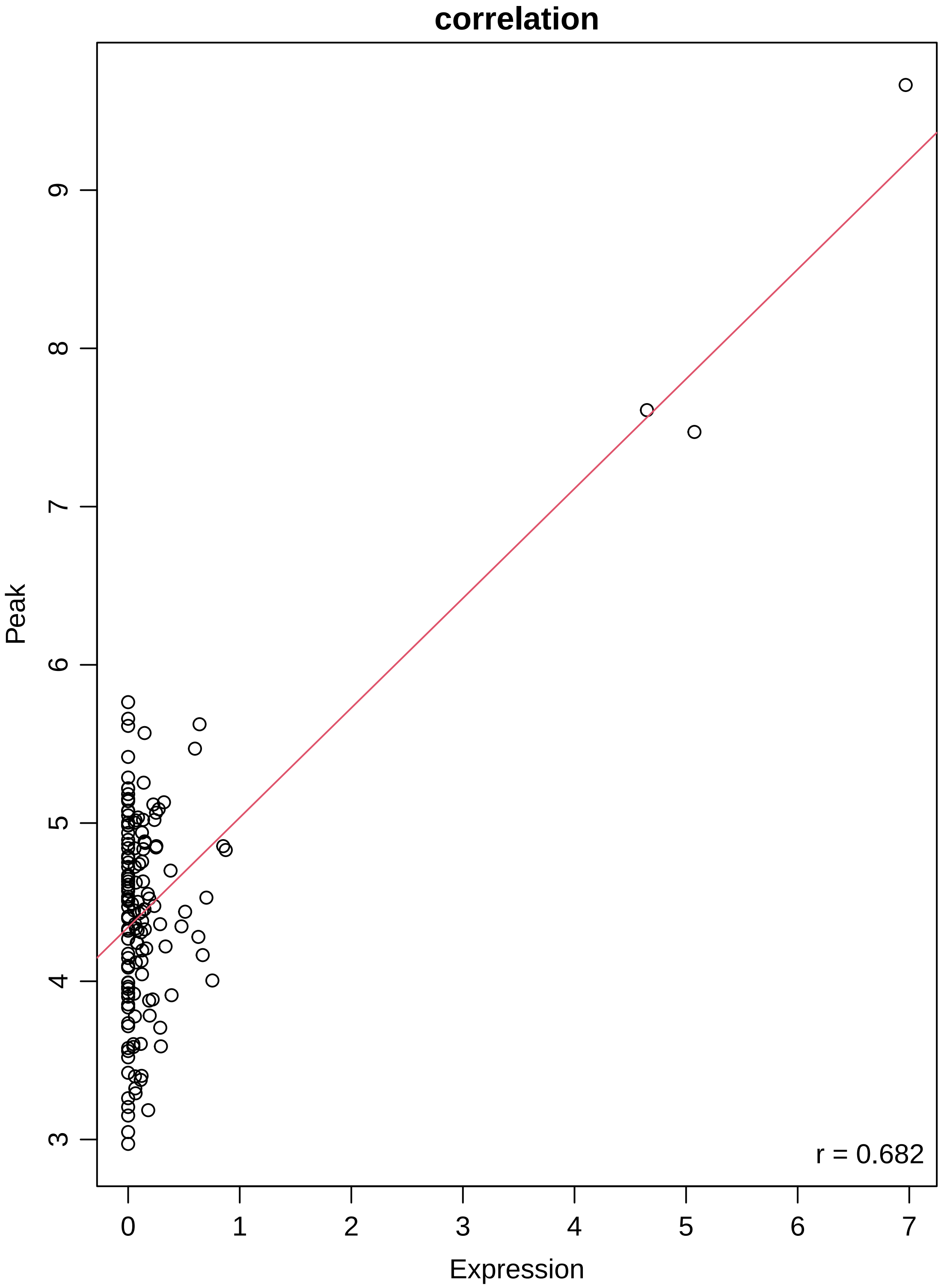

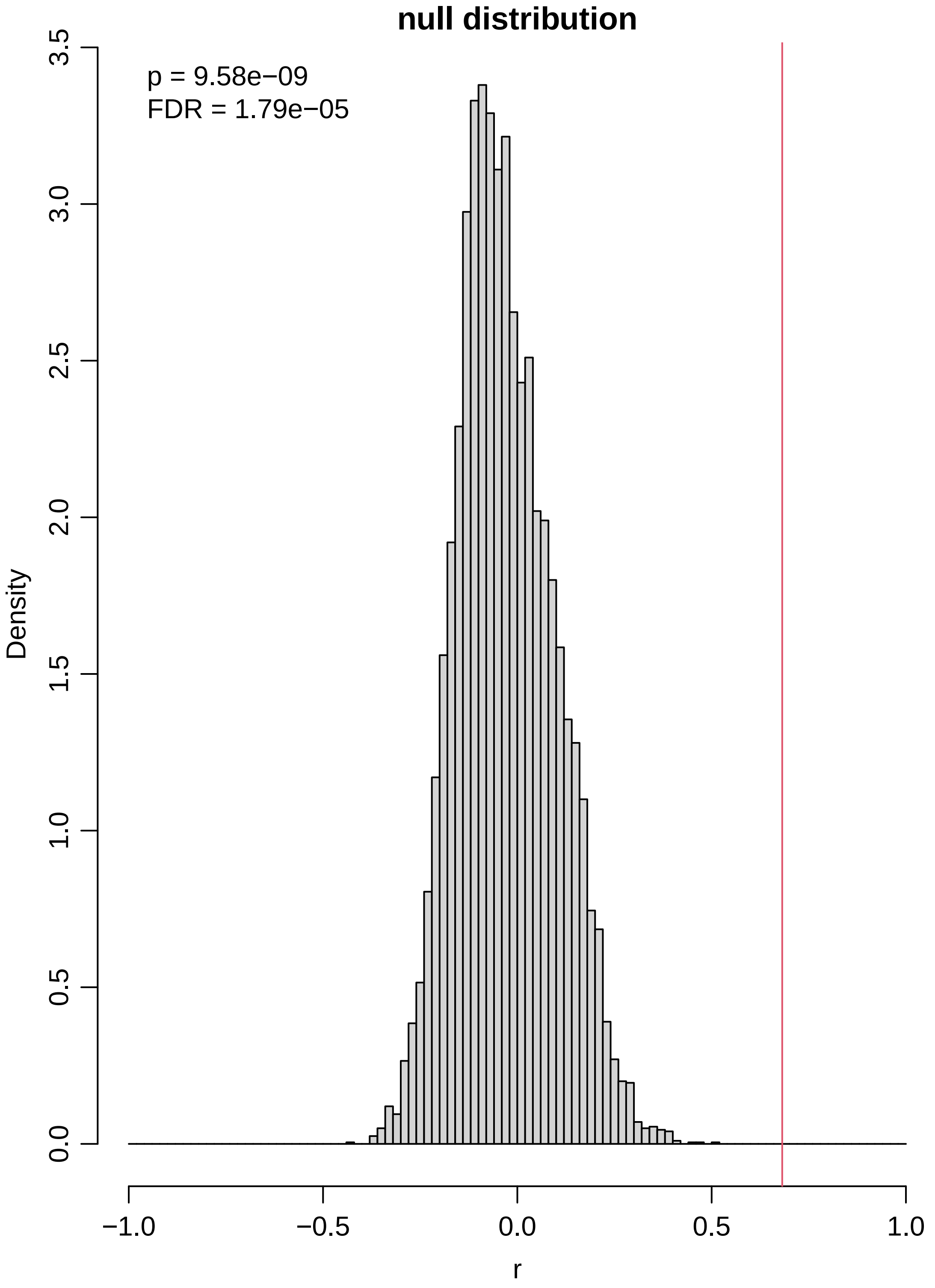

AC002066.1 vs PeakID_228972 (chr7: 116448089 − 116448294)

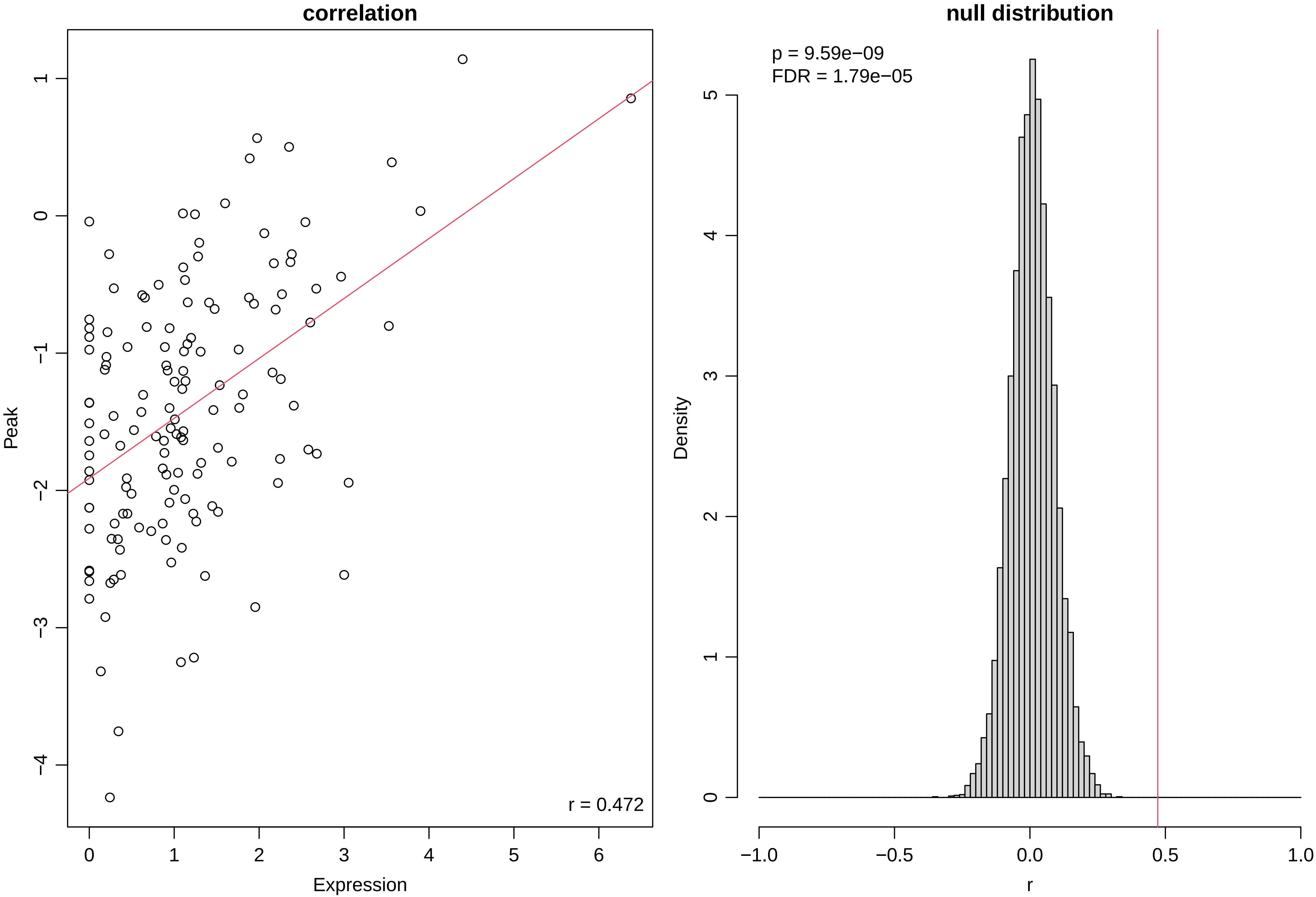

DSC3 vs PeakID_108823 (chr18: 31040463 − 31043262)

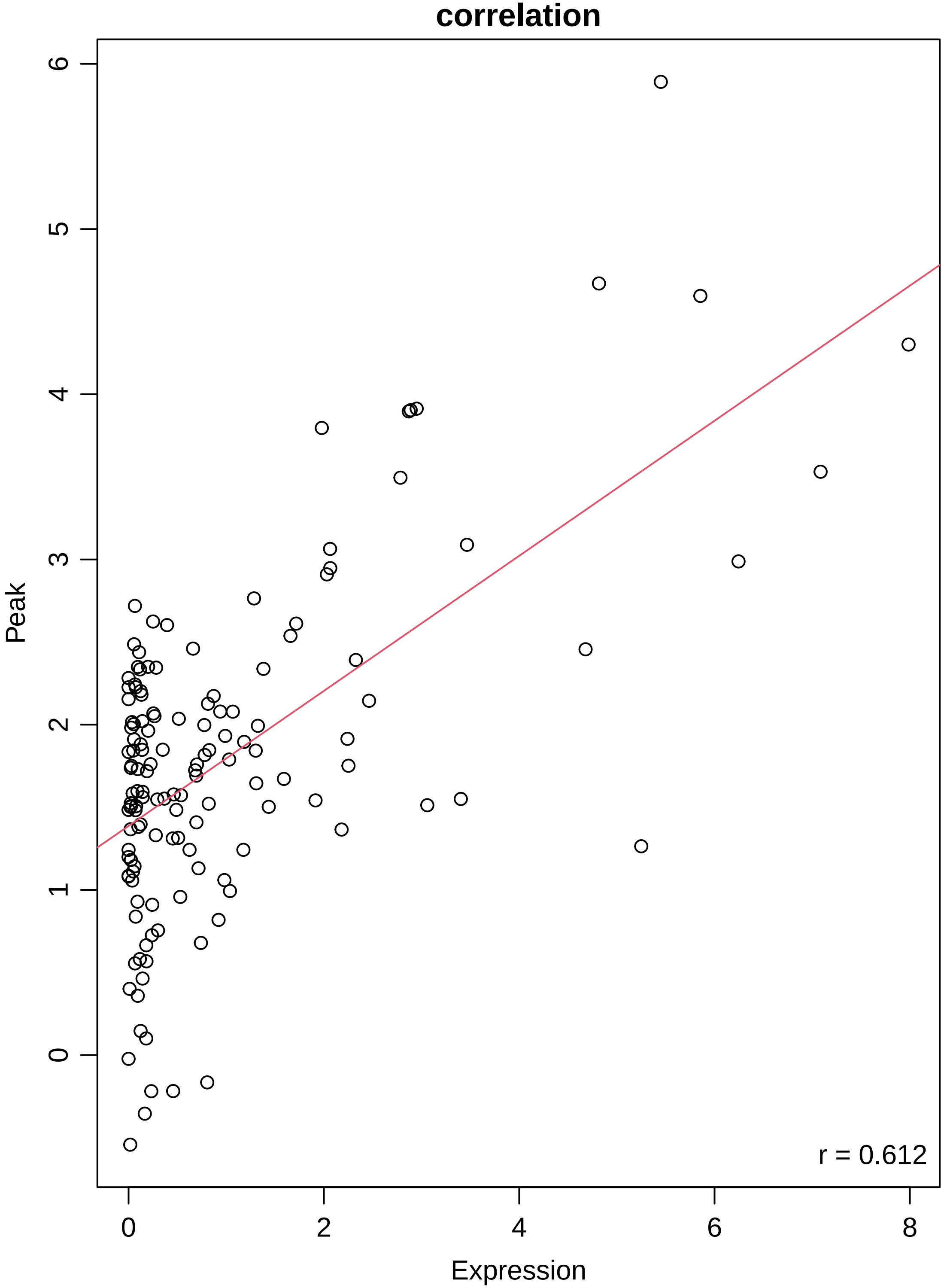

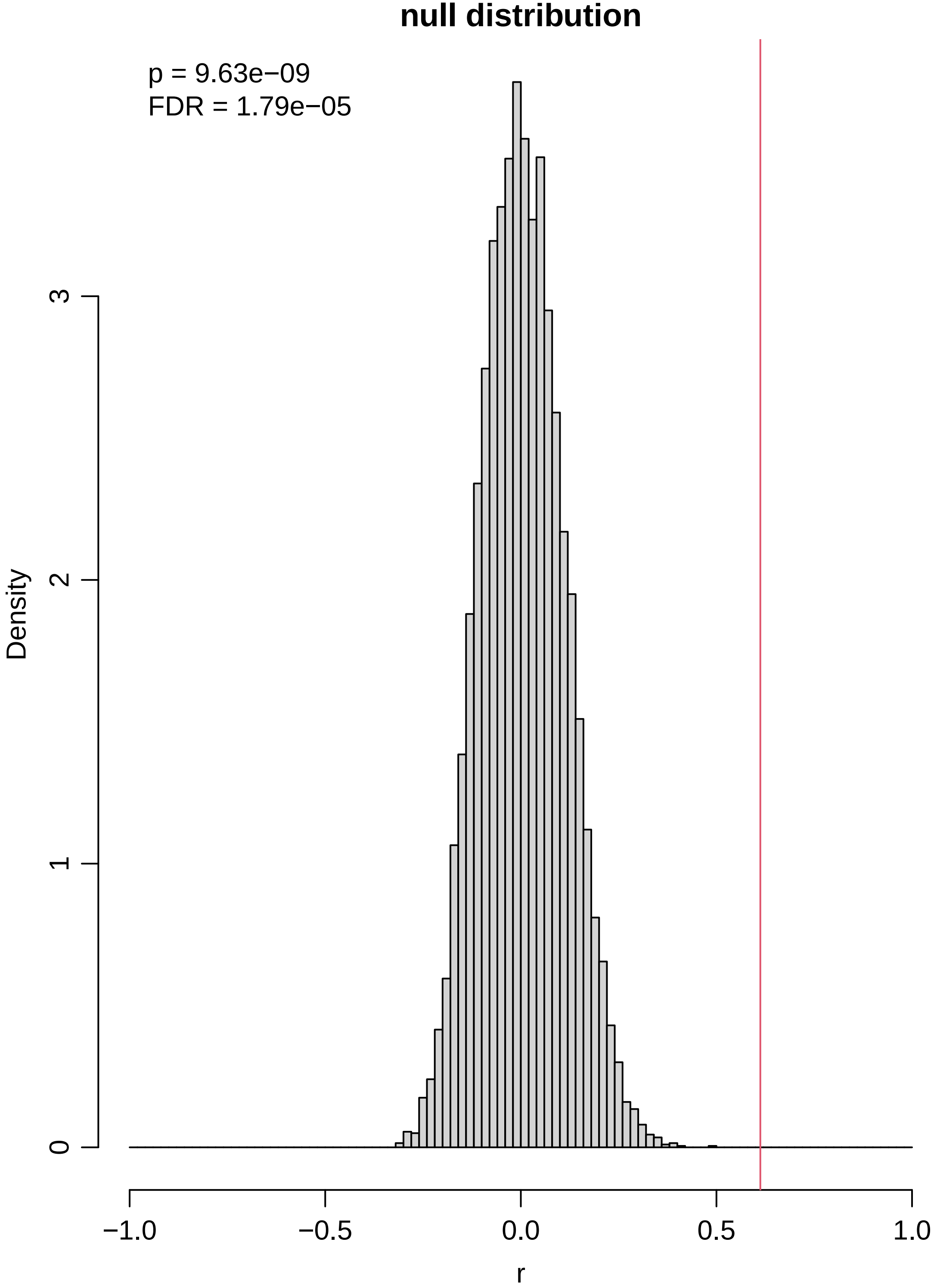

AC244213.1 vs PeakID_262737 (chrY: 12661402 − 12664377)

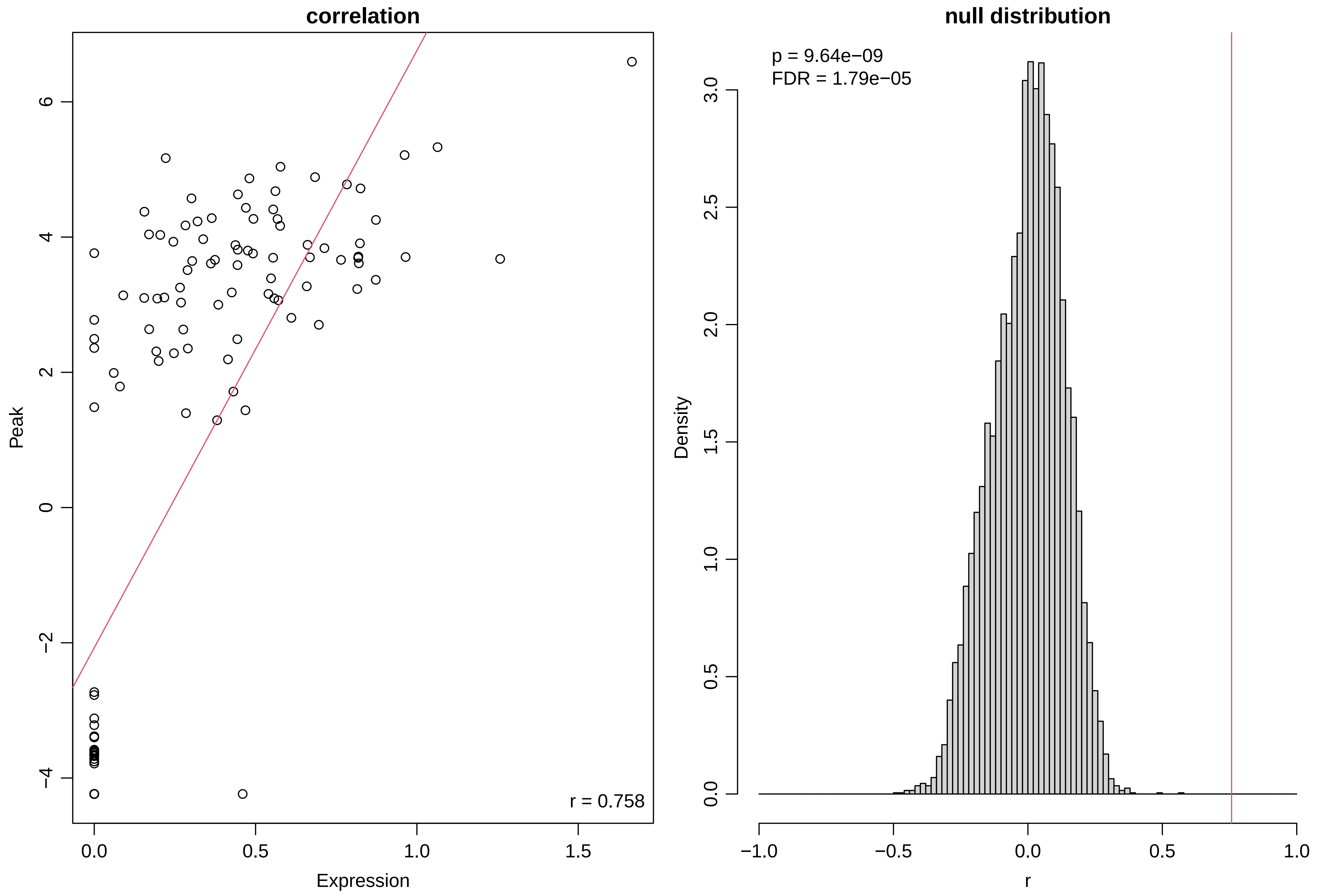

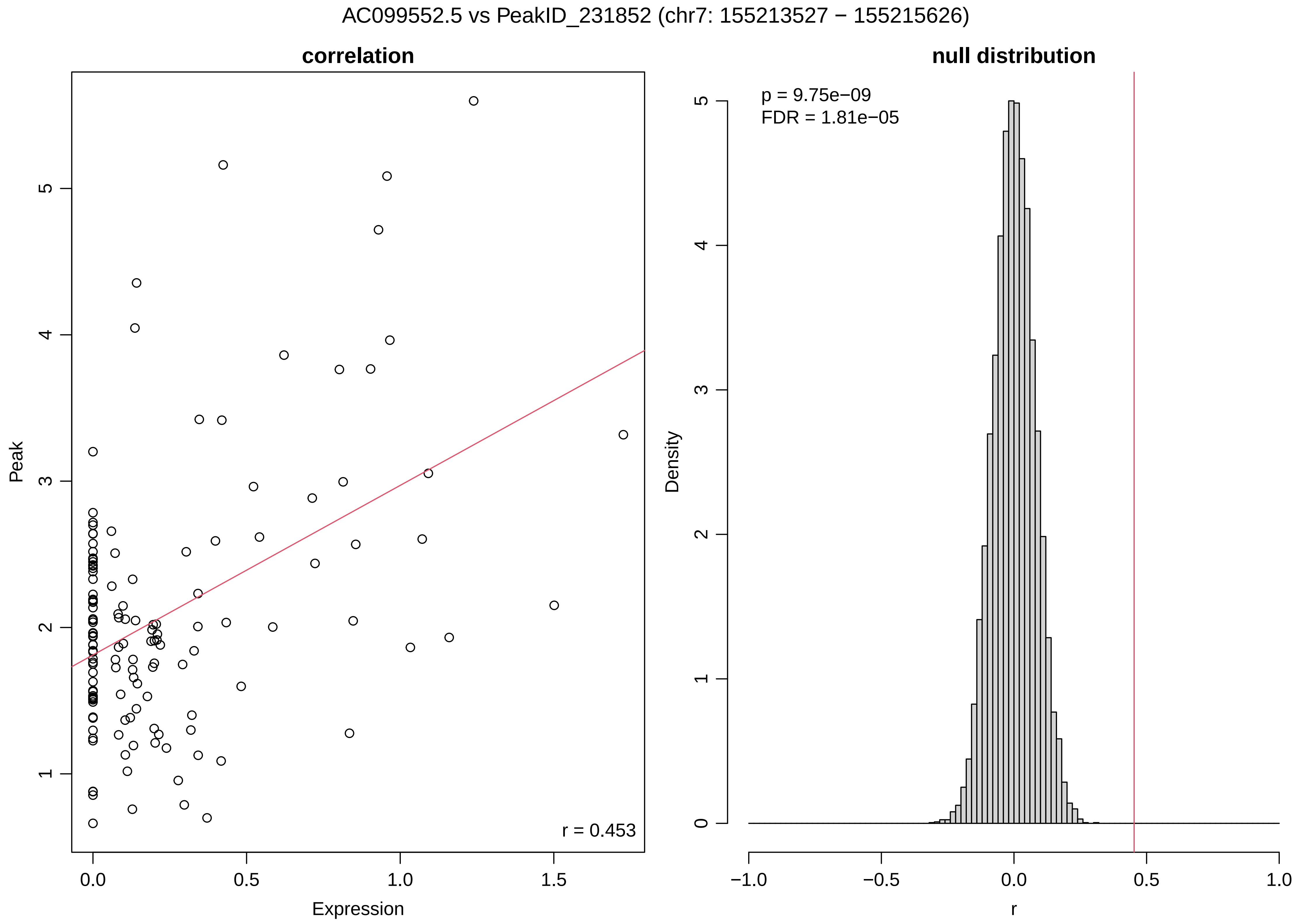
AC099552.5 vs PeakID_231852 (chr7: 155213527 − 155215626)
correlation
Peak
Expression
r = 0.453
null distribution
p = 9.75e−09
FDR = 1.81e−05
Density
r

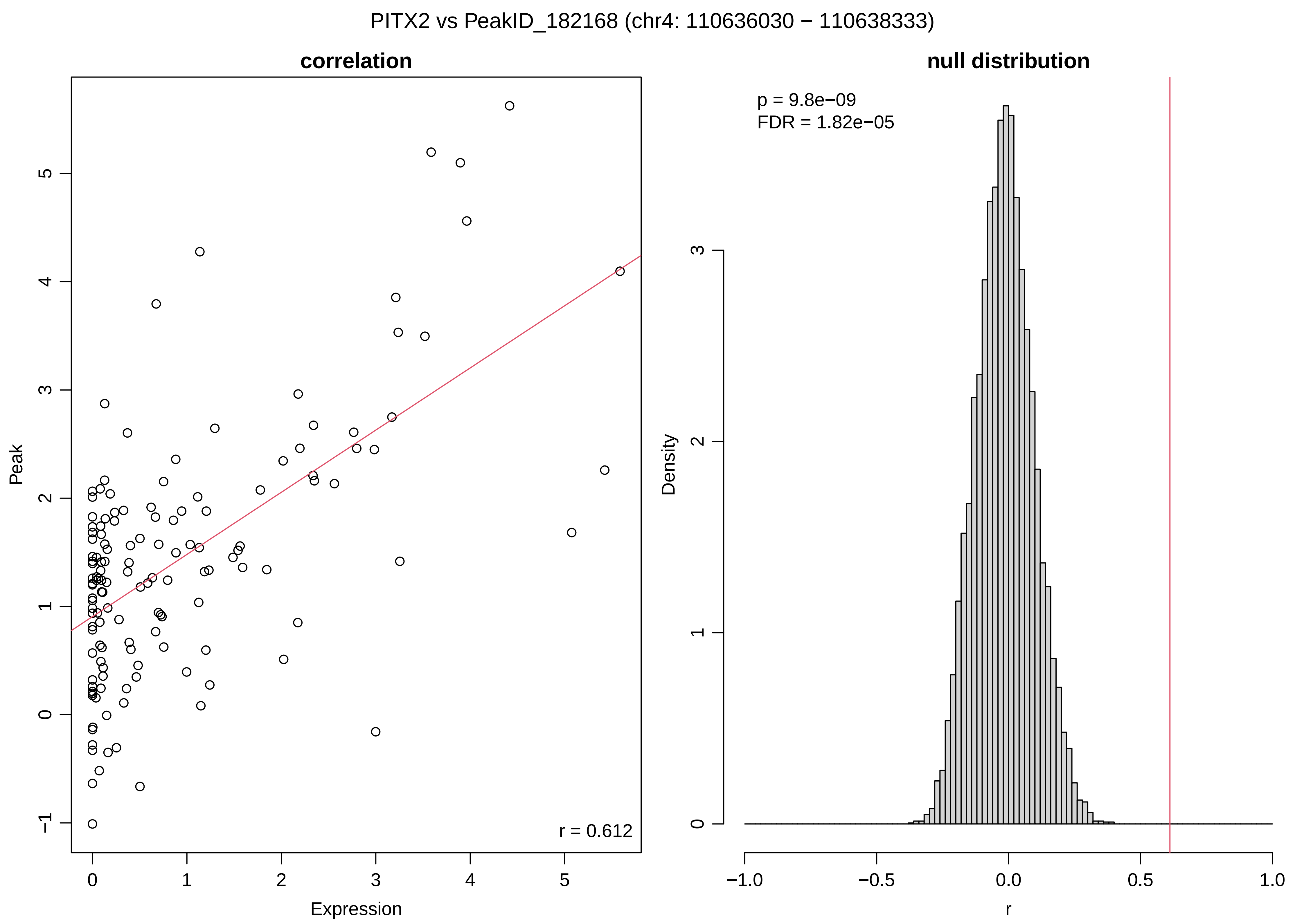
PITX2 vs PeakID_182168 (chr4: 110636030 − 110638333)
correlation
Peak
Expression
r = 0.612
null distribution
p = 9.8e−09
FDR = 1.82e−05
Density
r

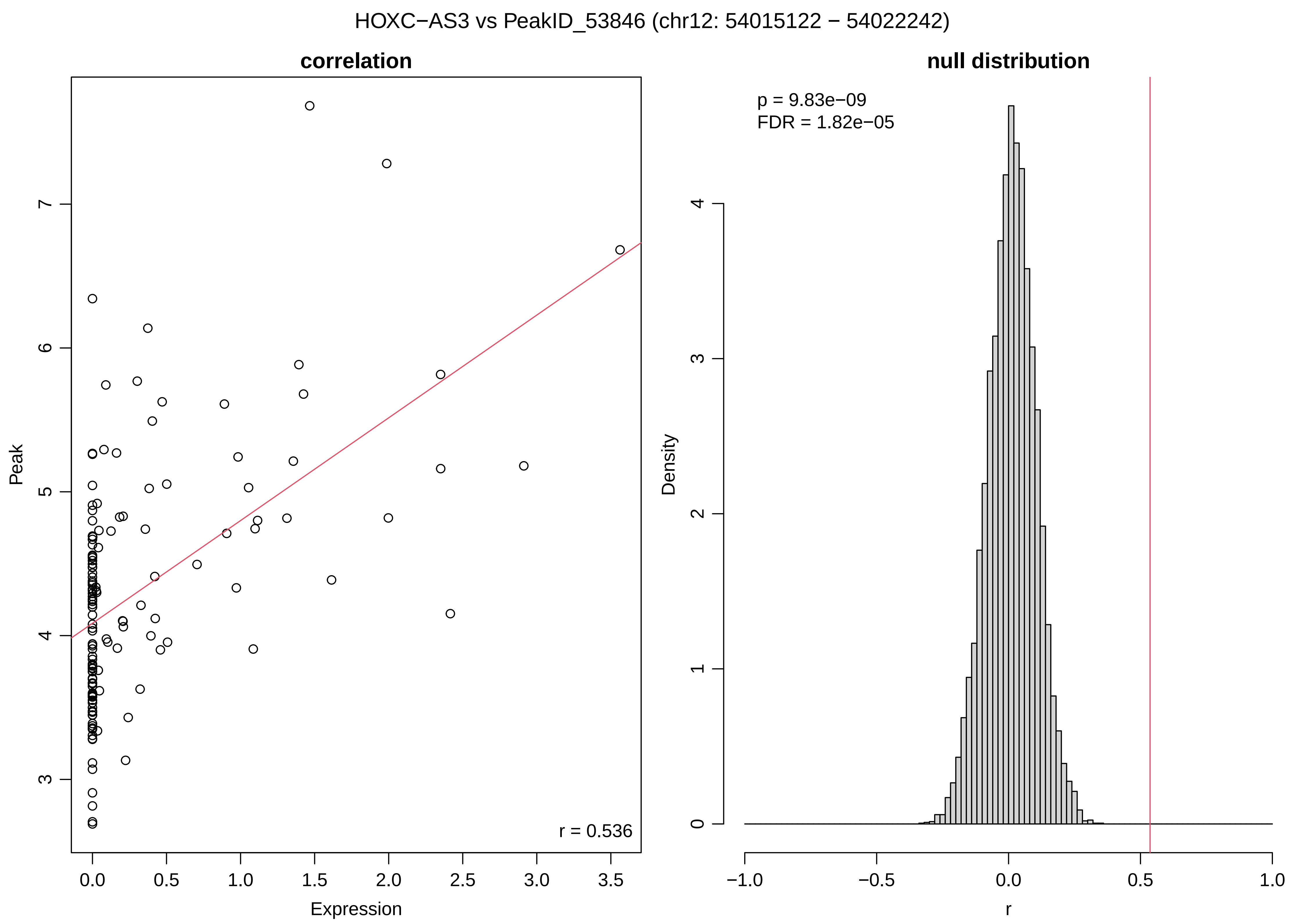
HOXC−AS3 vs PeakID_53846 (chr12: 54015122 − 54022242)
correlation
null distribution
p = 9.83e−09
FDR = 1.82e−05
r = 0.536
Peak
Expression
Density
r
0.0
0.5
1.0
1.5
2.0
2.5
3.0
3.5
3
4
5
6
7
−1.0
−0.5
0.0
0.5
1.0
0
1
2
3
4

BEGAIN vs PeakID_75162 (chr14: 100438834 − 100440181)

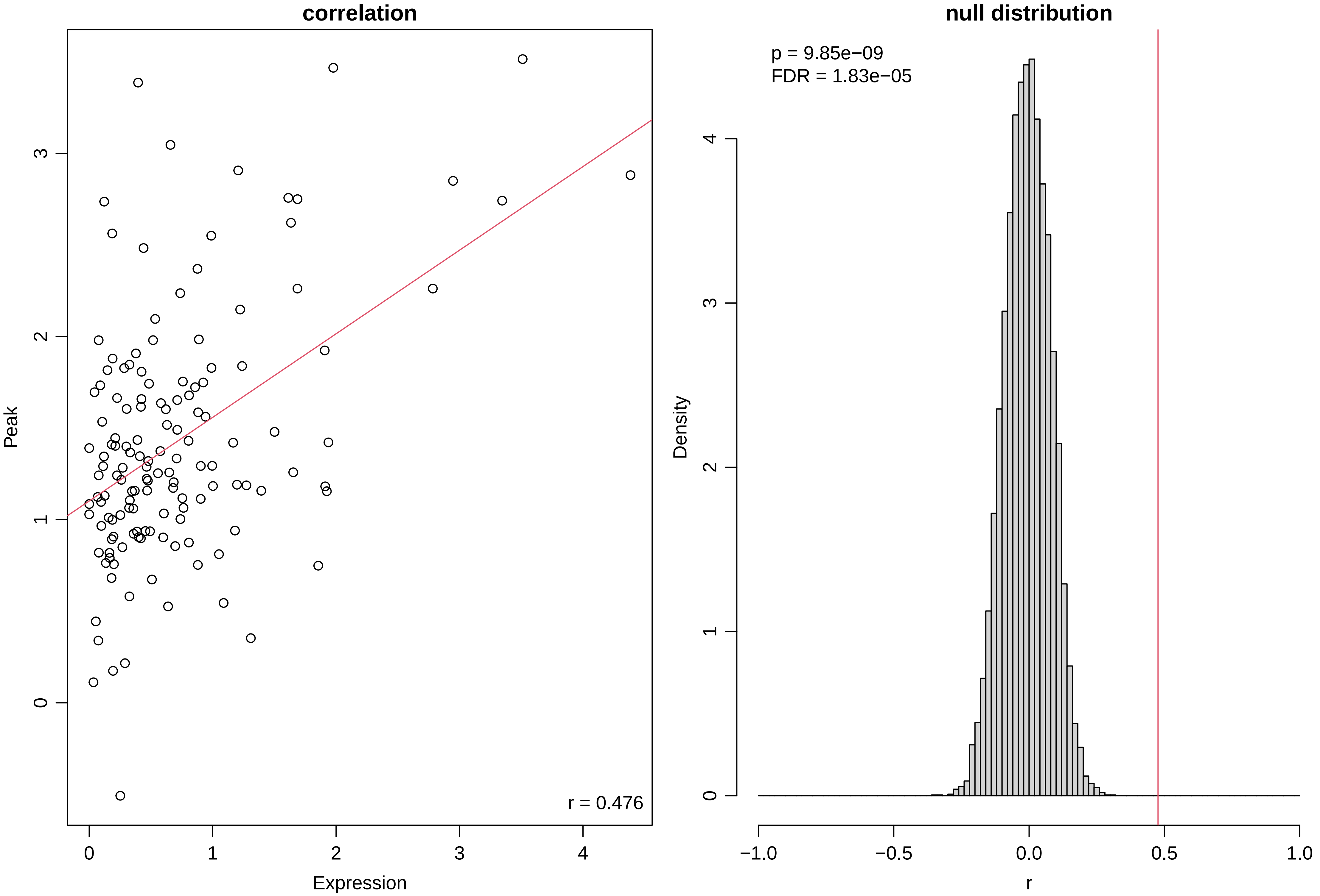

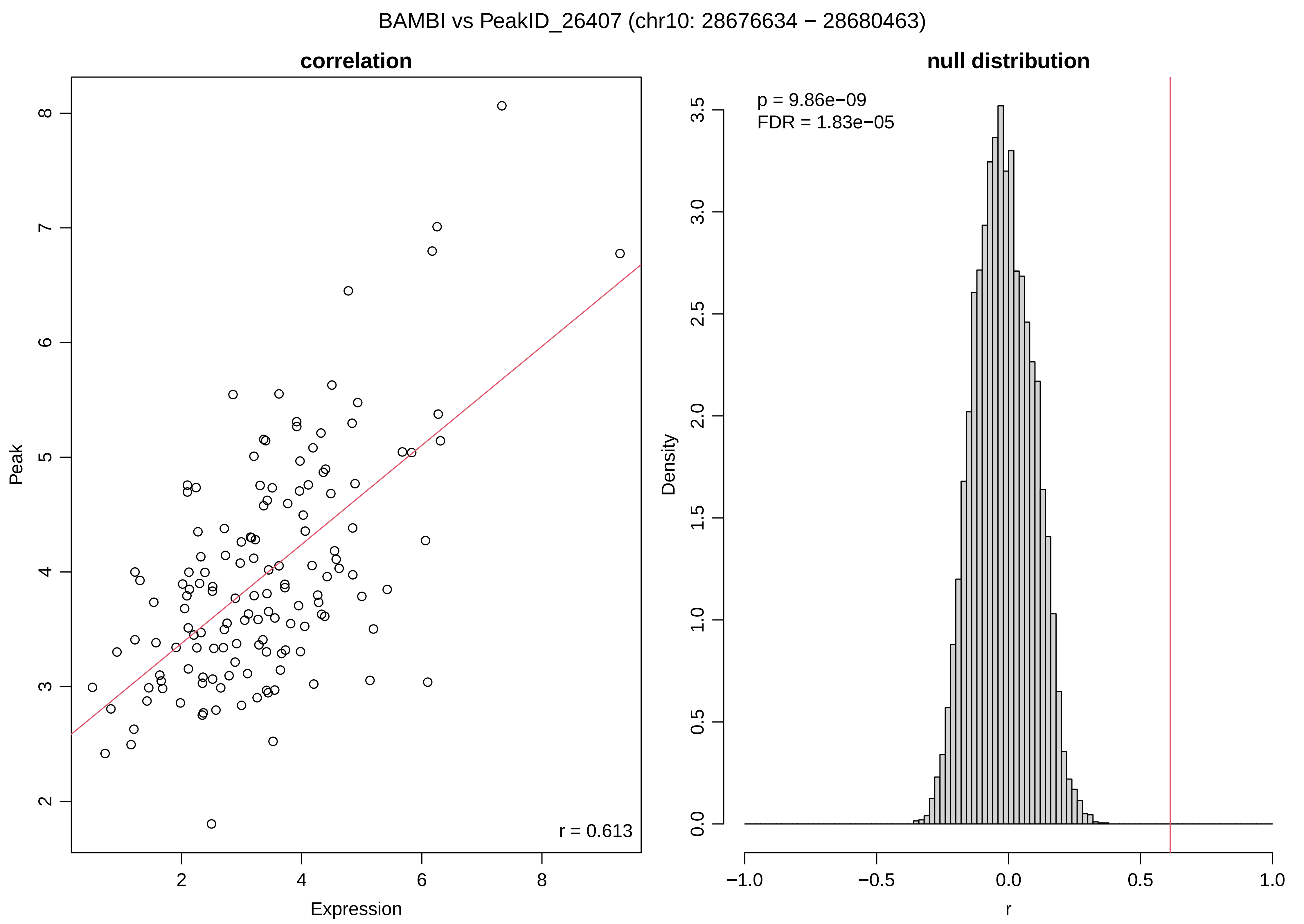
BAMBI vs PeakID_26407 (chr10: 28676634 − 28680463)
correlation
Peak
Expression
r = 0.613
null distribution
p = 9.86e−09
FDR = 1.83e−05
Density
r

CDX2 vs PeakID_63684 (chr13: 27959597 − 27969895)

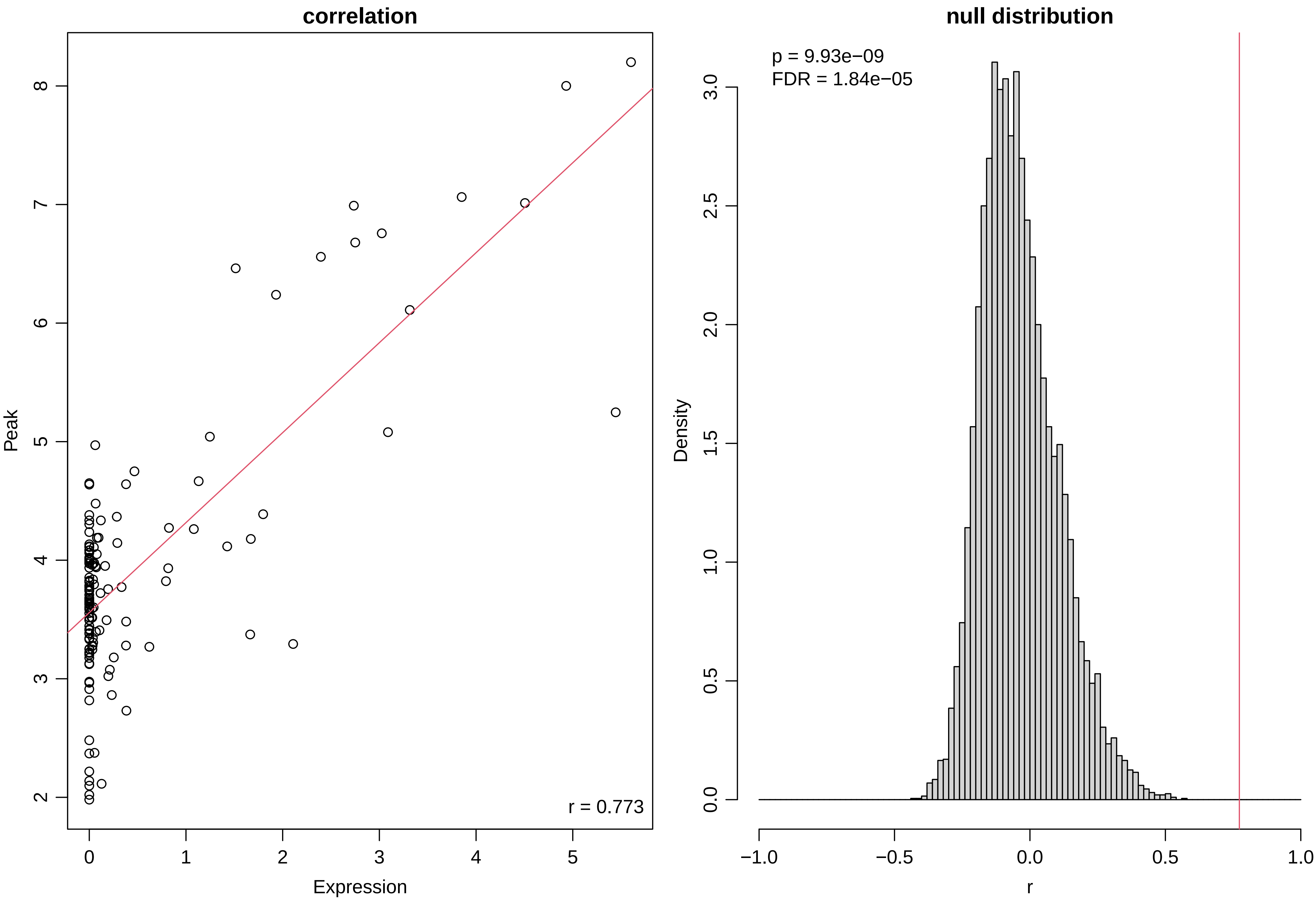

RPL21P104 vs PeakID_55447 (chr12: 61912553 − 61912944)

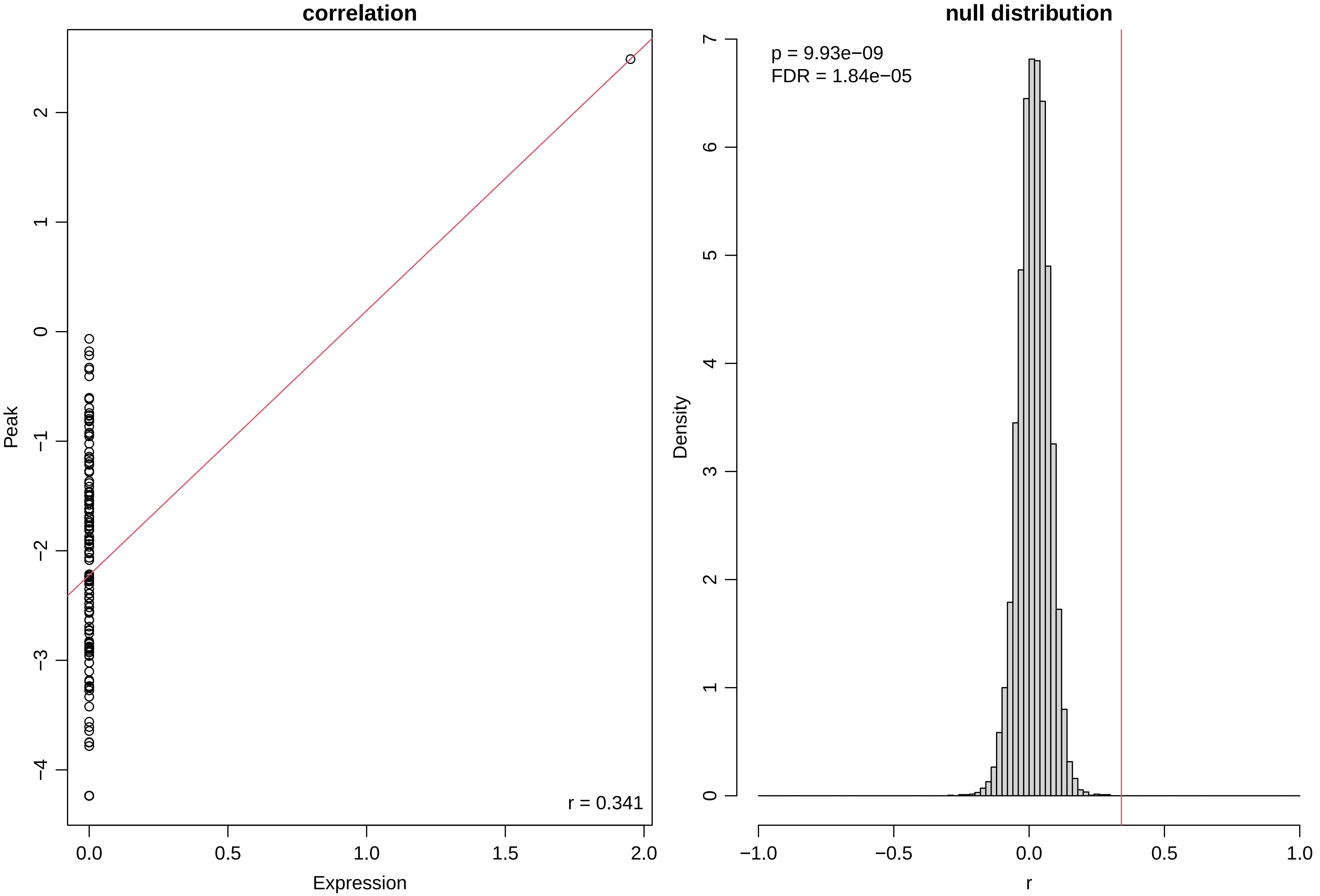

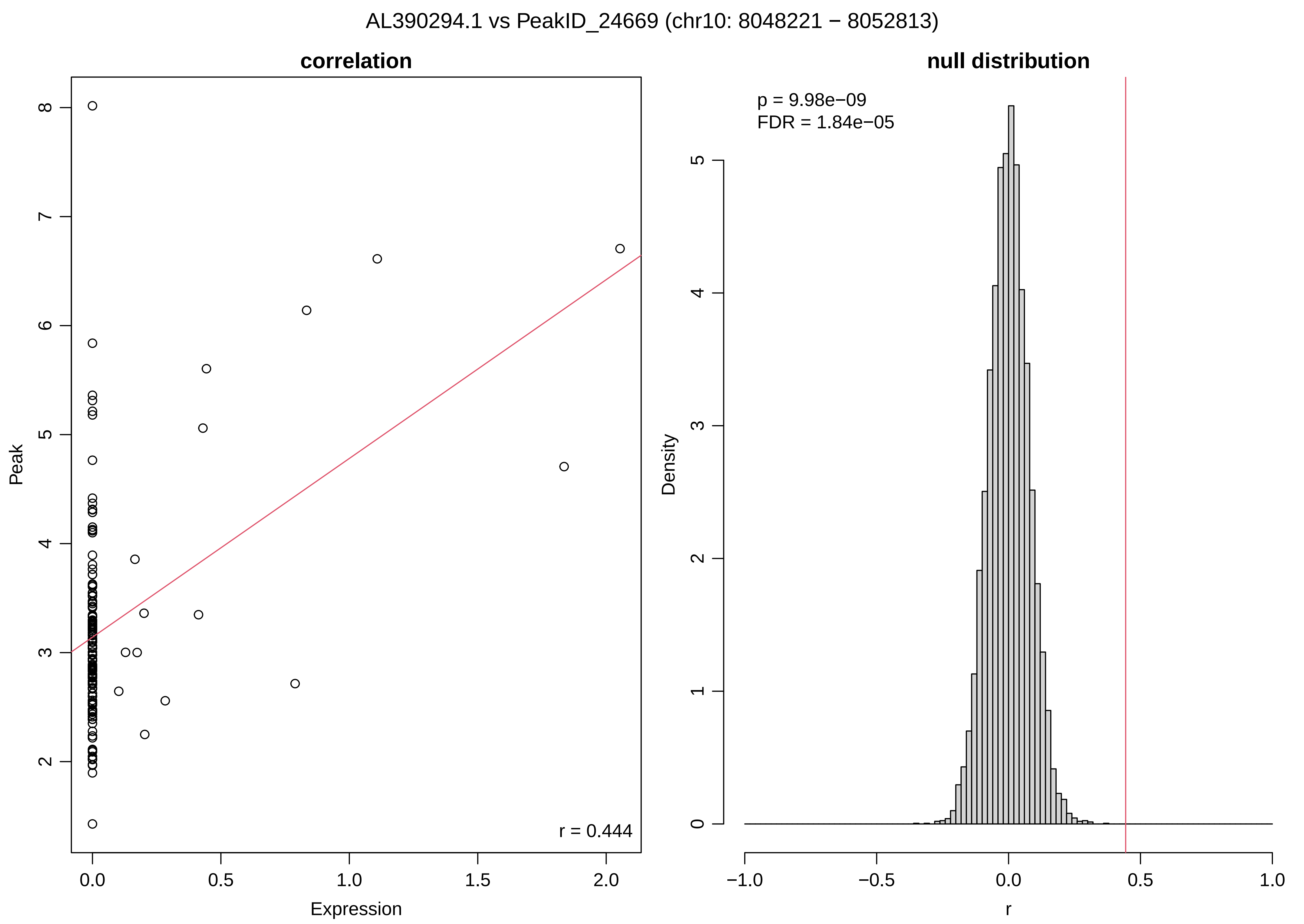
AL390294.1 vs PeakID_24669 (chr10: 8048221 − 8052813)
correlation
null distribution
p = 9.98e−09
FDR = 1.84e−05
r = 0.444
Peak
Expression
Density
r

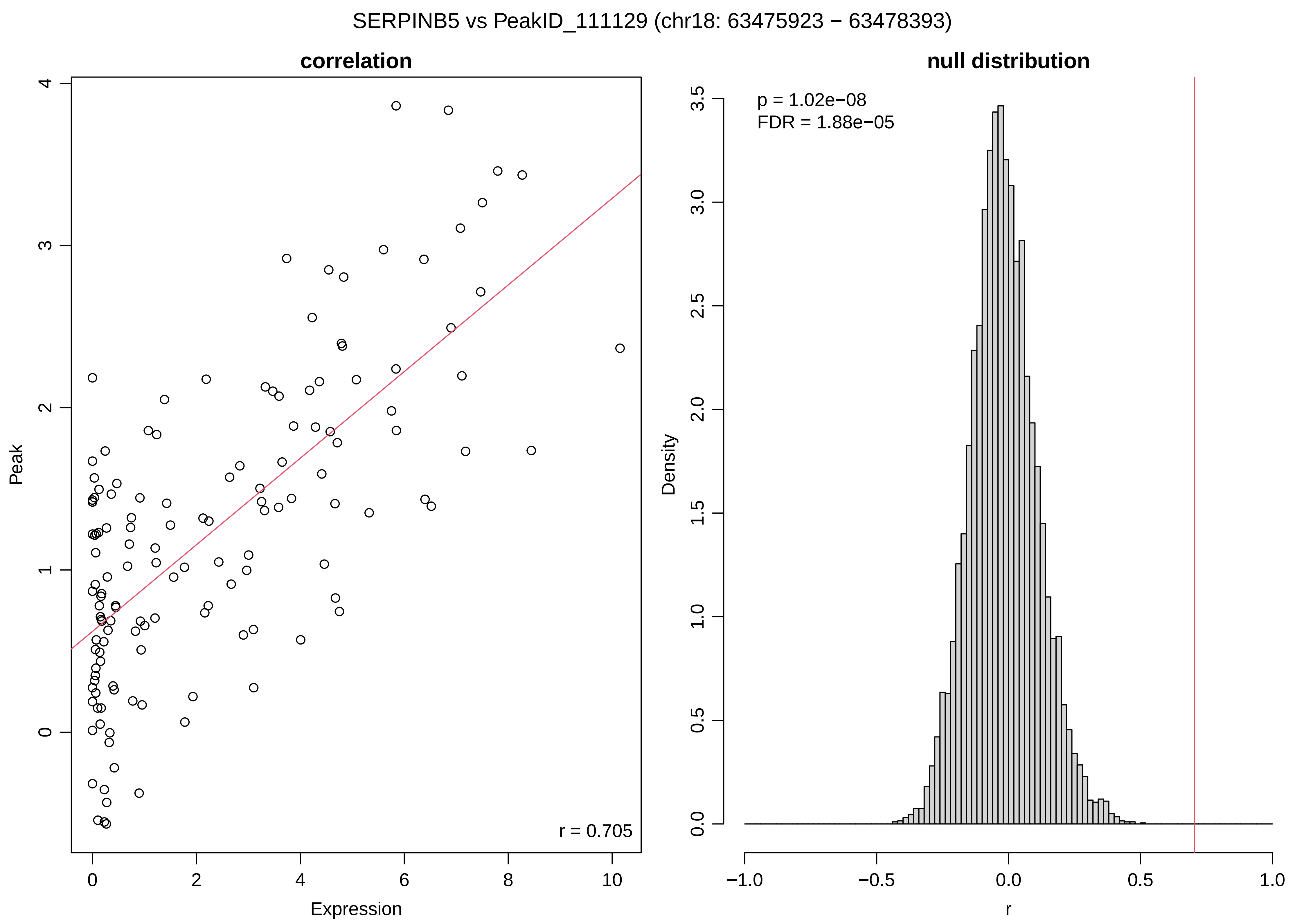
SERPINB5 vs PeakID_111129 (chr18: 63475923 − 63478393)
correlation
Peak
Expression
r = 0.705
0
1
2
3
4
0
2
4
6
8
10
null distribution
p = 1.02e−08
FDR = 1.88e−05
Density
r
0.0
0.5
1.0
1.5
2.0
2.5
3.0
3.5
−1.0
−0.5
0.0
0.5
1.0

RIMKLB vs PeakID_49609 (chr12: 8695727 − 8696027)

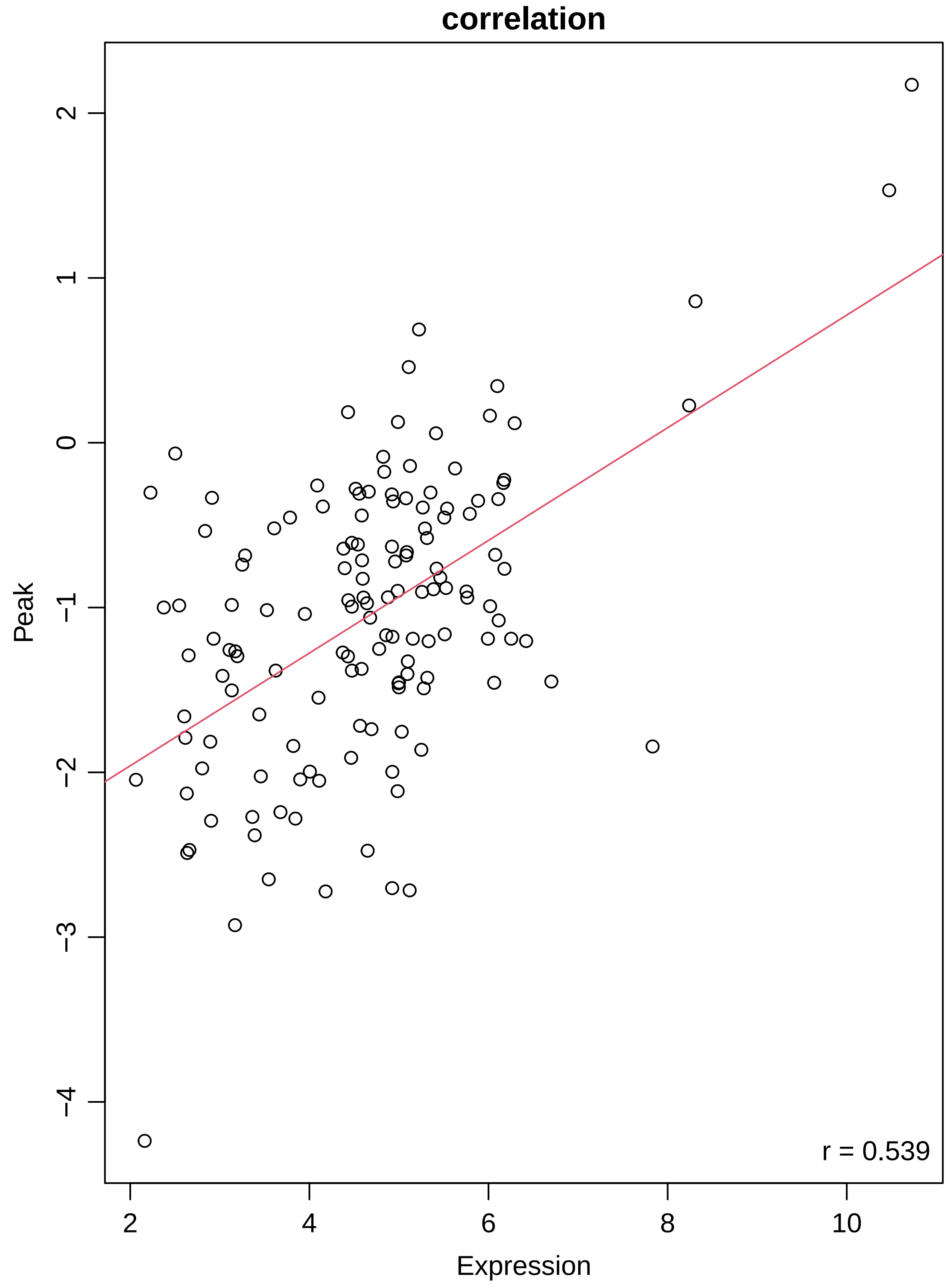

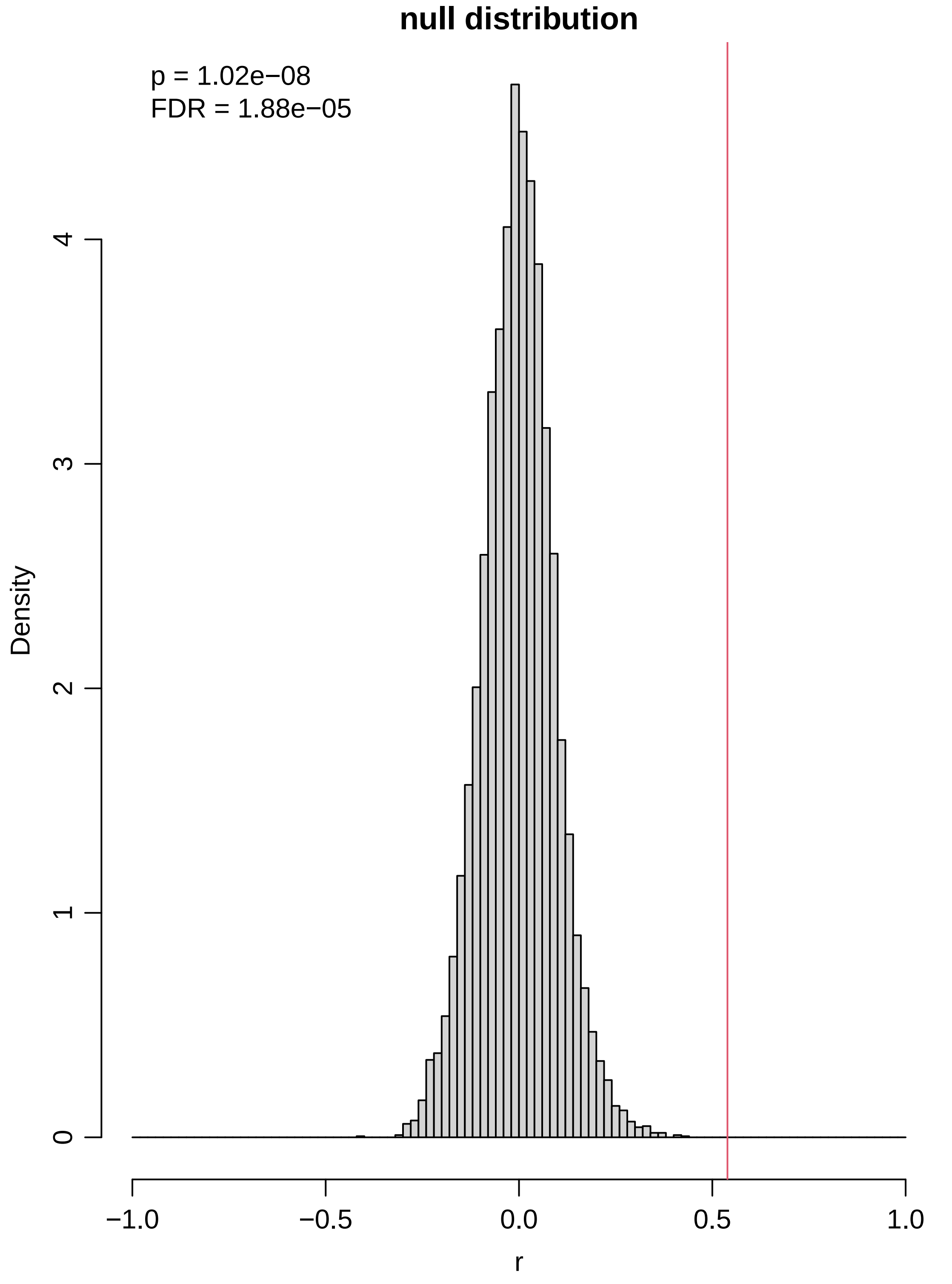

SLC5A5 vs PeakID_115125 (chr19: 17871725 − 17874815)

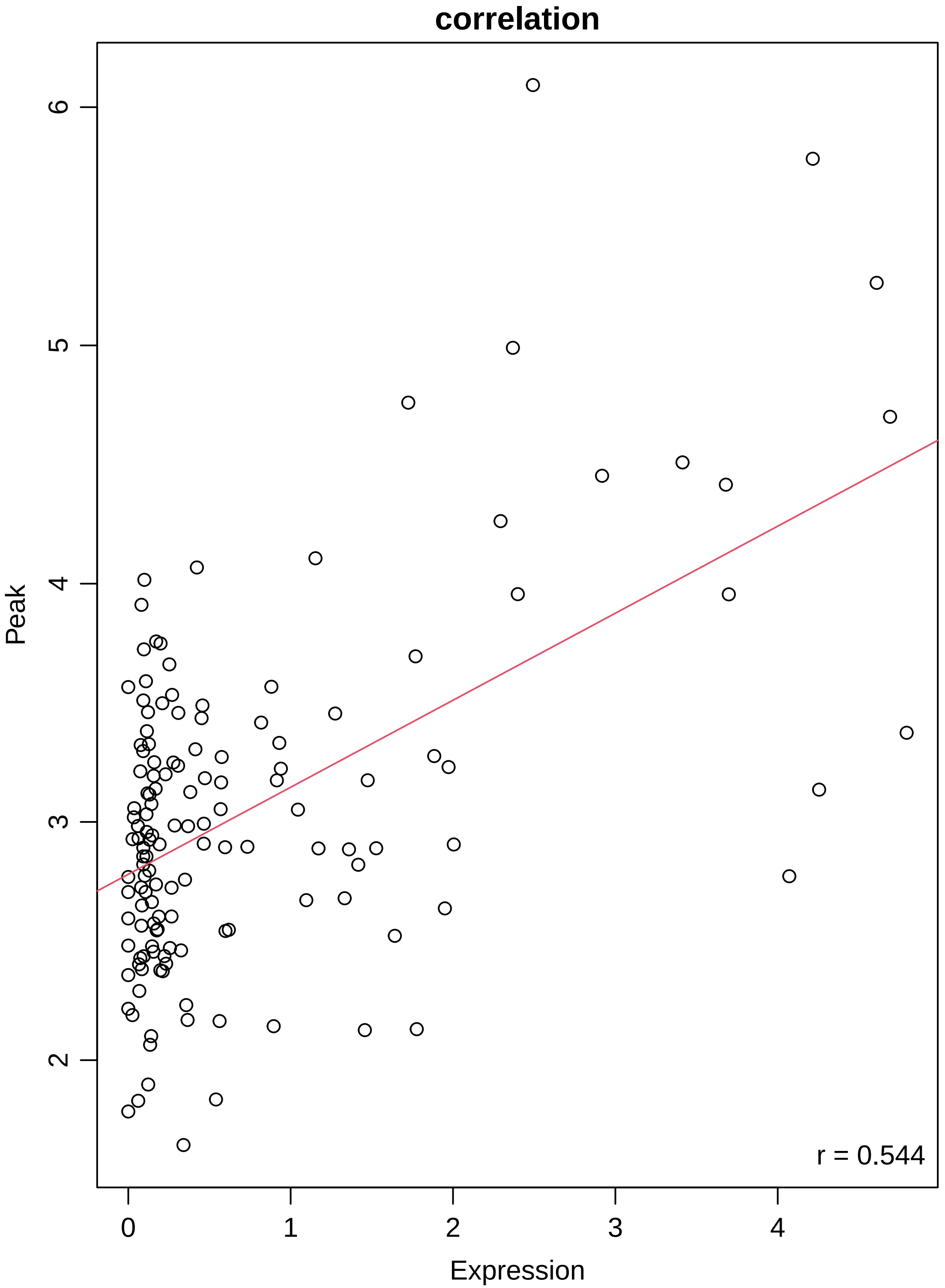

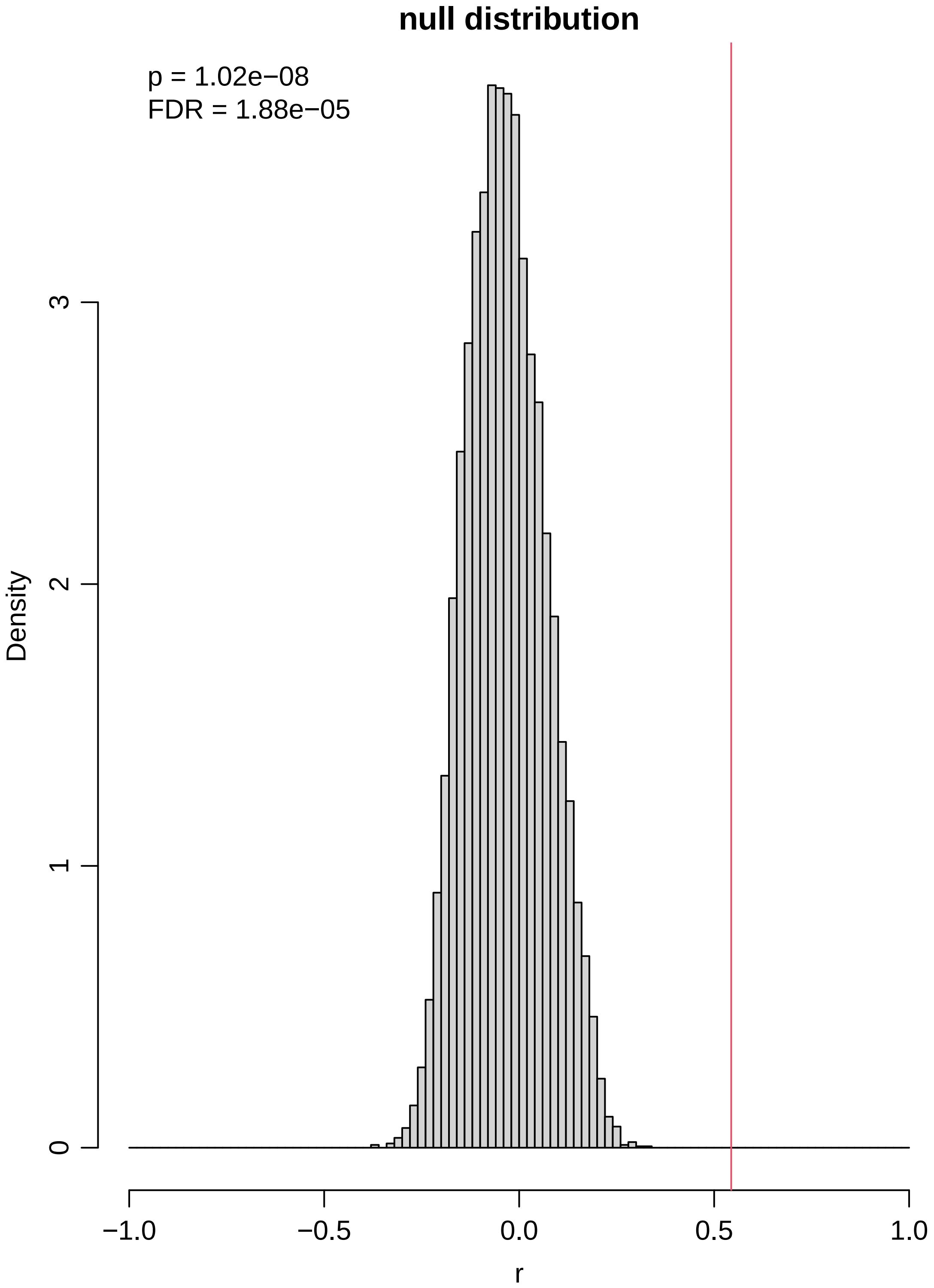

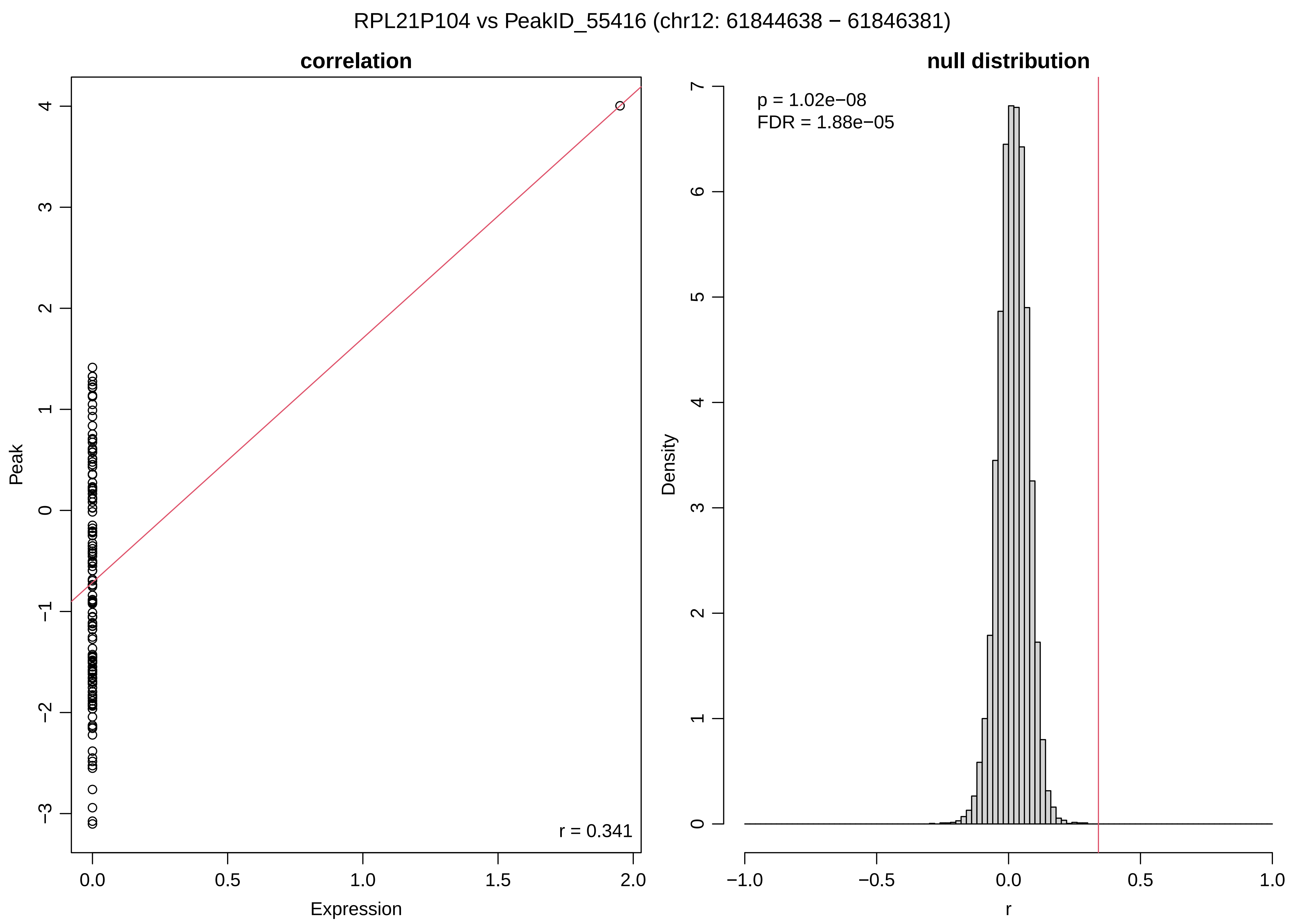
RPL21P104 vs PeakID_55416 (chr12: 61844638 − 61846381)
correlation
Peak
Expression
r = 0.341
null distribution
p = 1.02e−08
FDR = 1.88e−05
Density
r

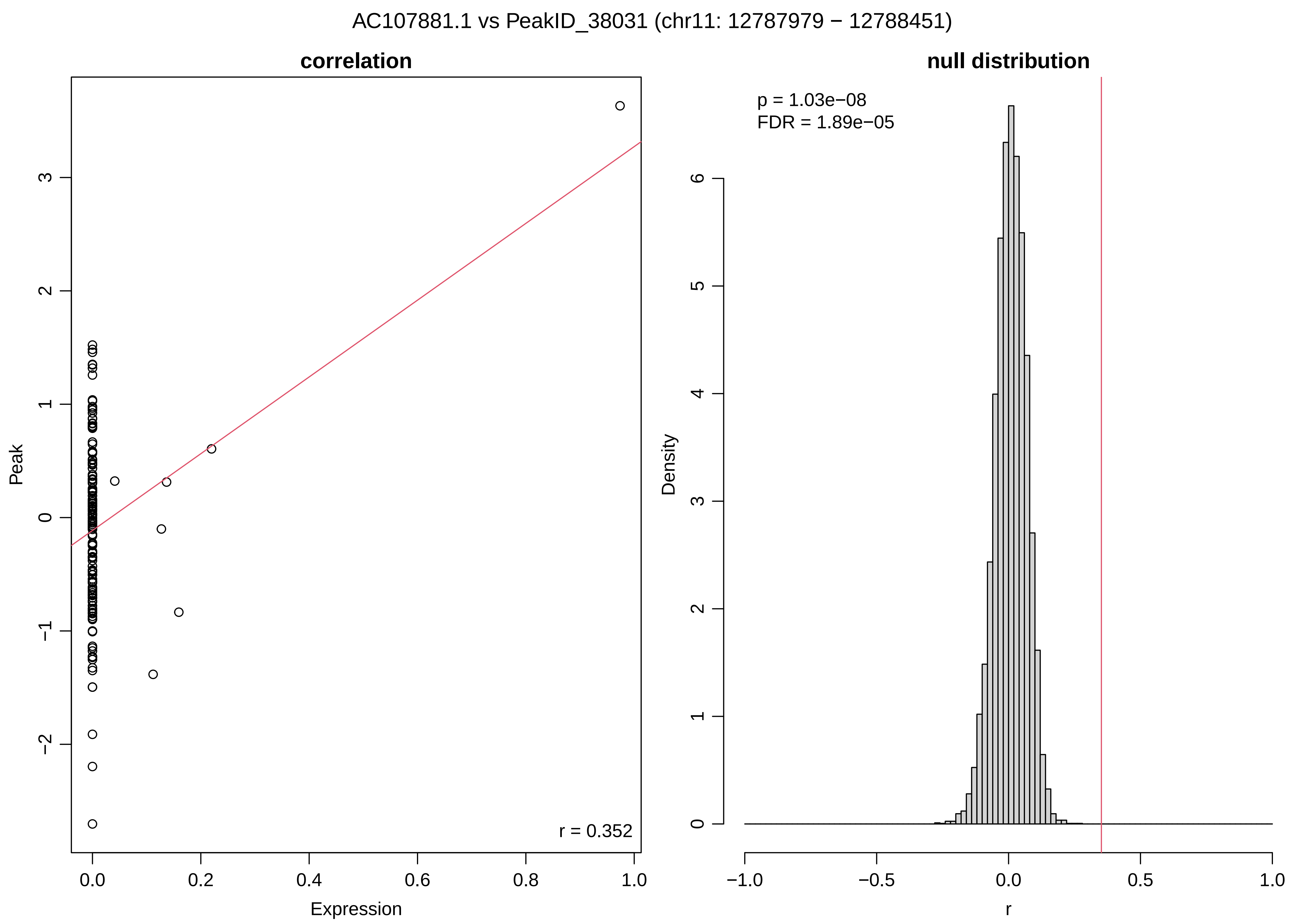
AC107881.1 vs PeakID_38031 (chr11: 12787979 − 12788451)
correlation
Peak
Expression
r = 0.352
null distribution
p = 1.03e−08
FDR = 1.89e−05
Density
r

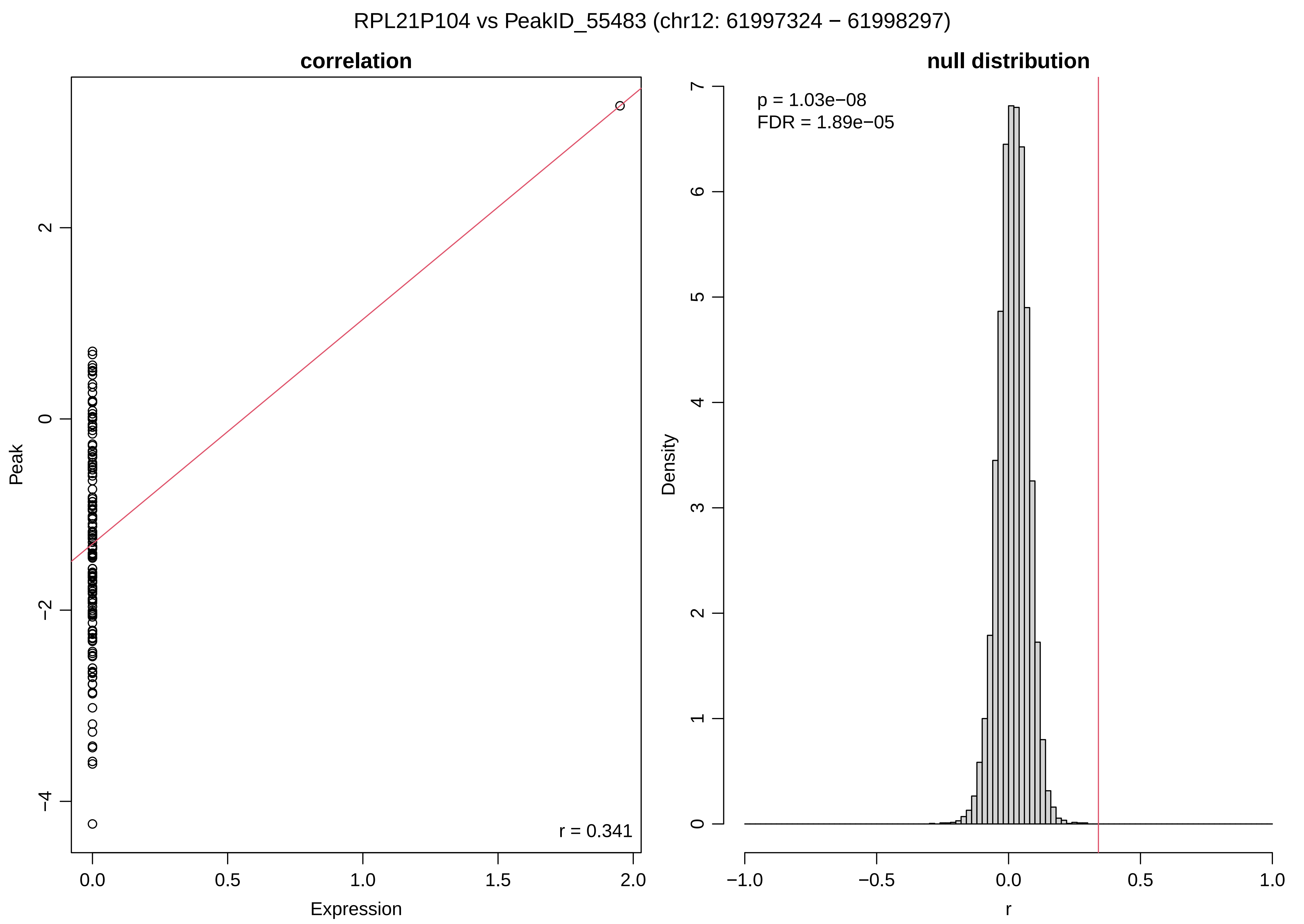

RPL21P104 vs PeakID_55483 (chr12: 61997324 − 61998297)
correlation
Peak
Expression
r = 0.341
null distribution
p = 1.03e−08
FDR = 1.89e−05
Density
r

RPS4Y1 vs PeakID_262324 (chrY: 2797253 − 2798098)

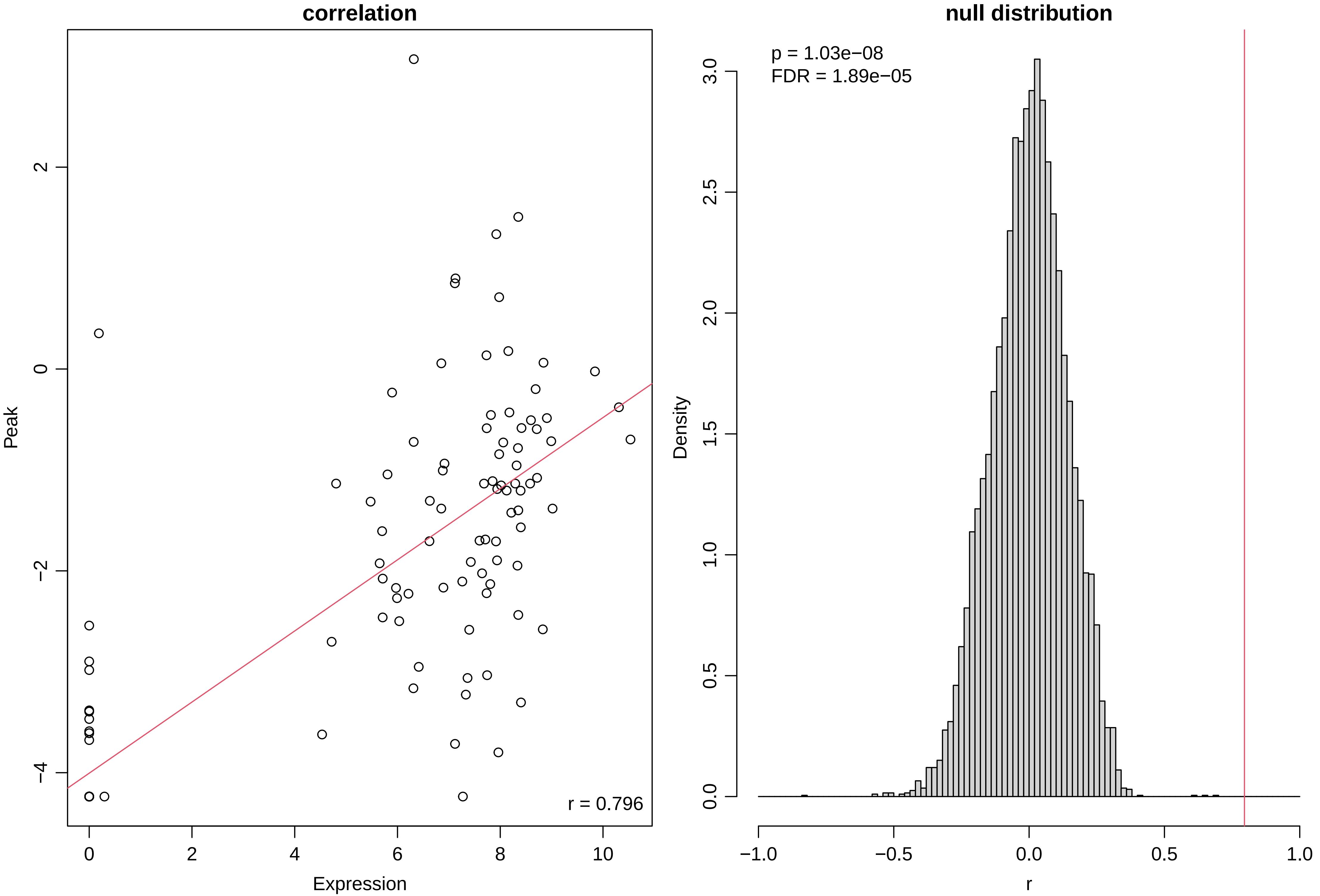

YEATS4 vs PeakID_56836 (chr12: 69473137 − 69474853)

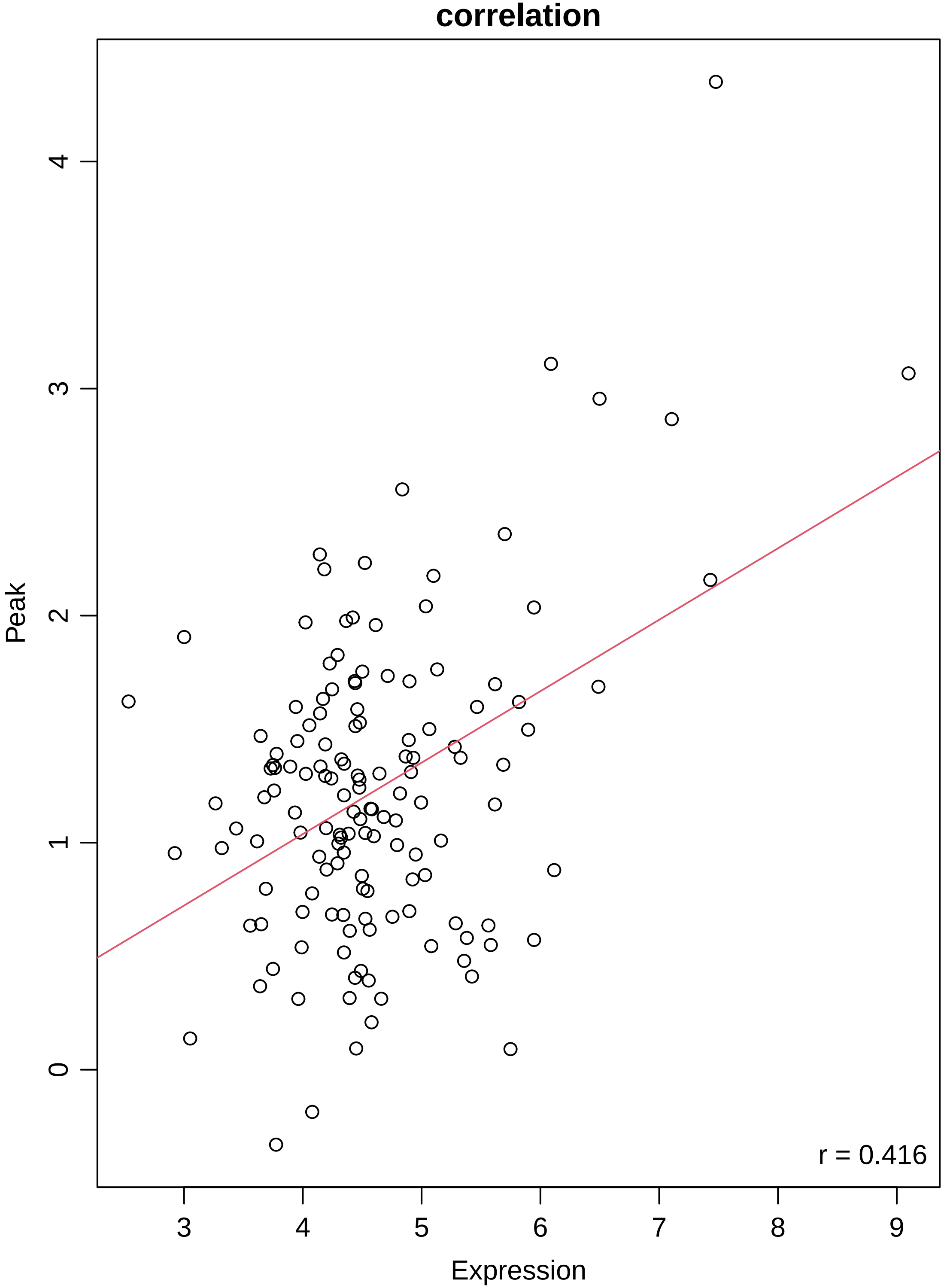

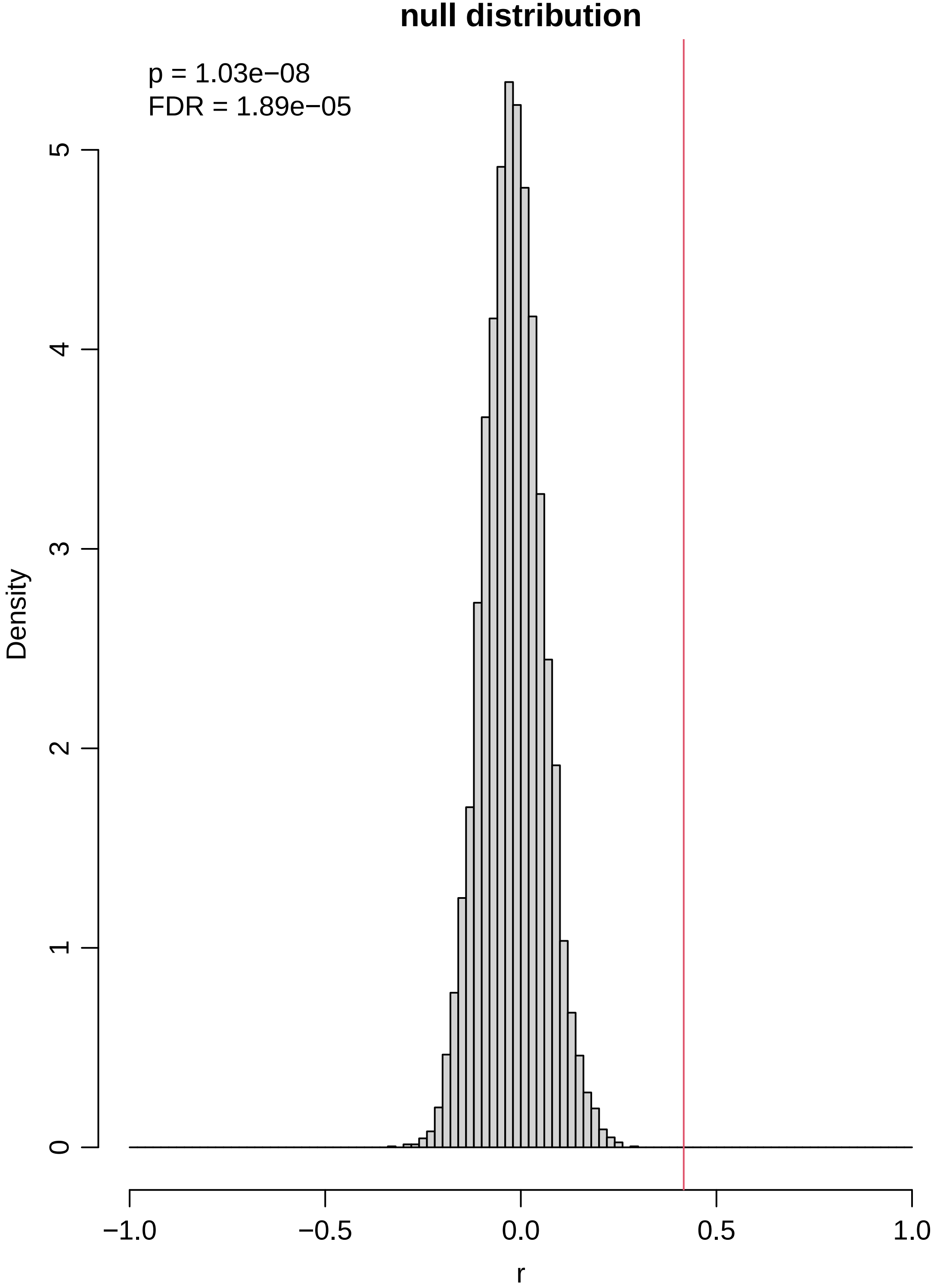

ALDH1B1 vs PeakID_247701 (chr9: 38380526 − 38383508)

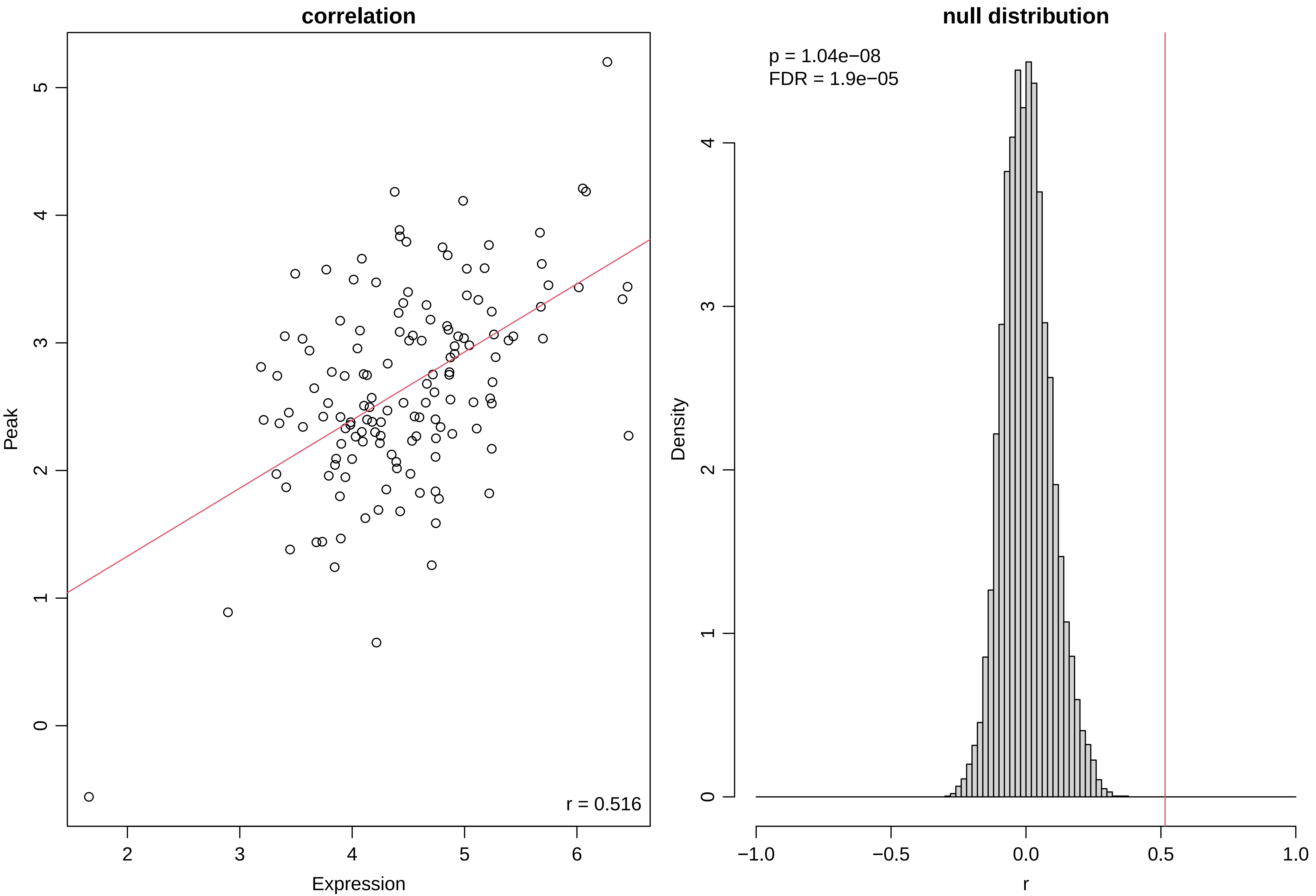

SNAP25−AS1 vs PeakID_143386 (chr20: 10366367 − 10371188)

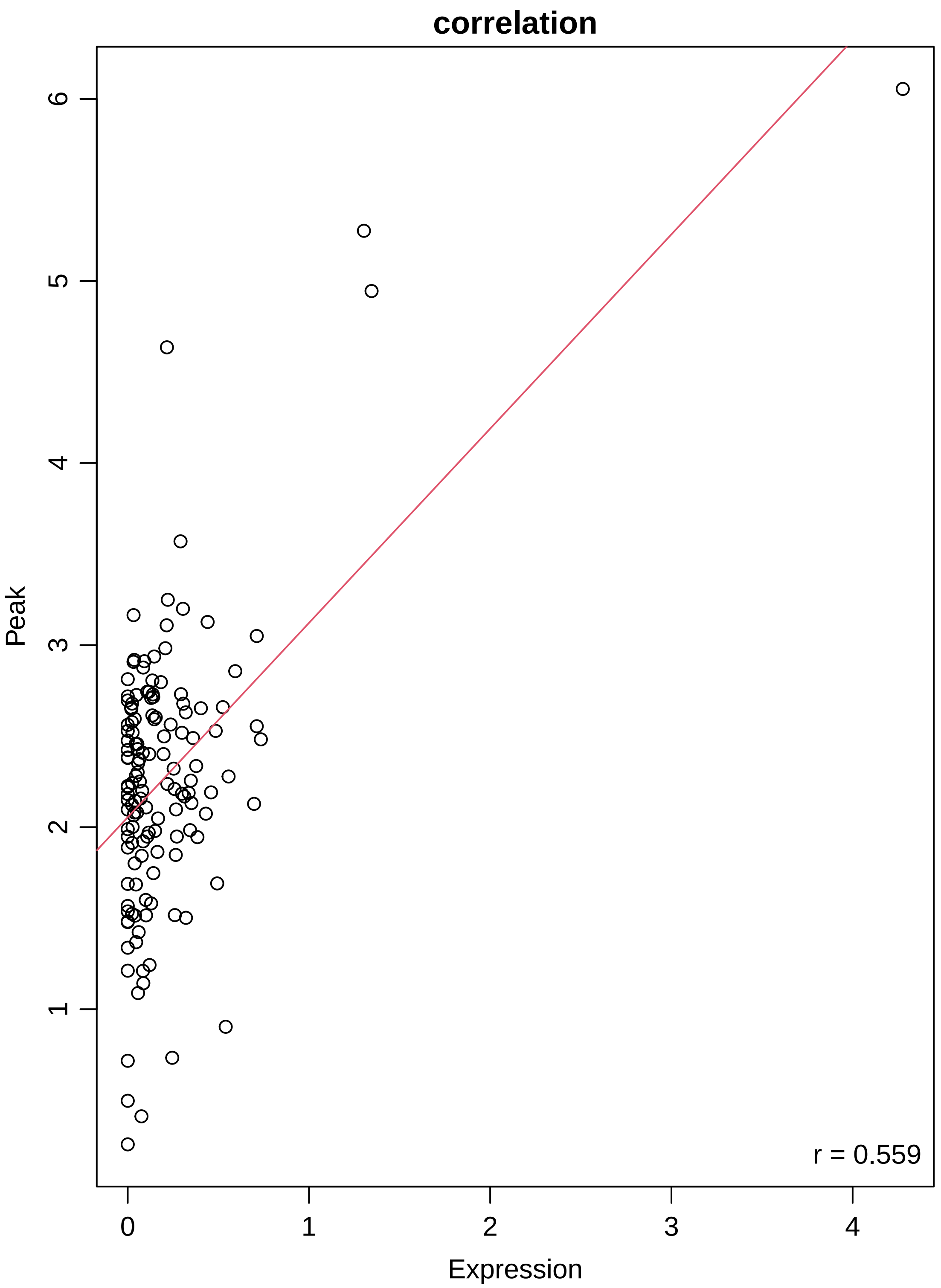

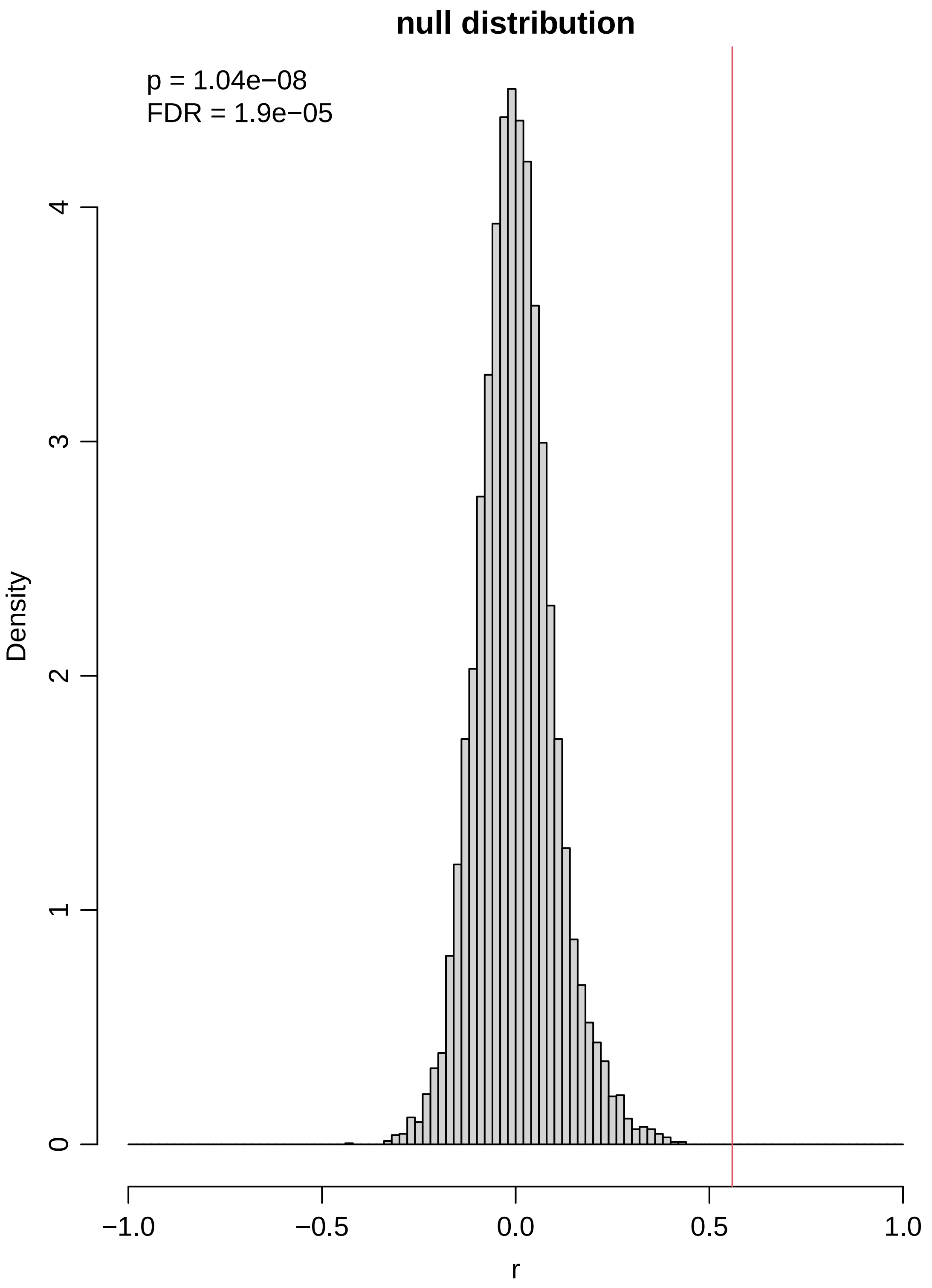

TMSB4Y vs PeakID_262757 (chrY: 13738852 − 13739204)

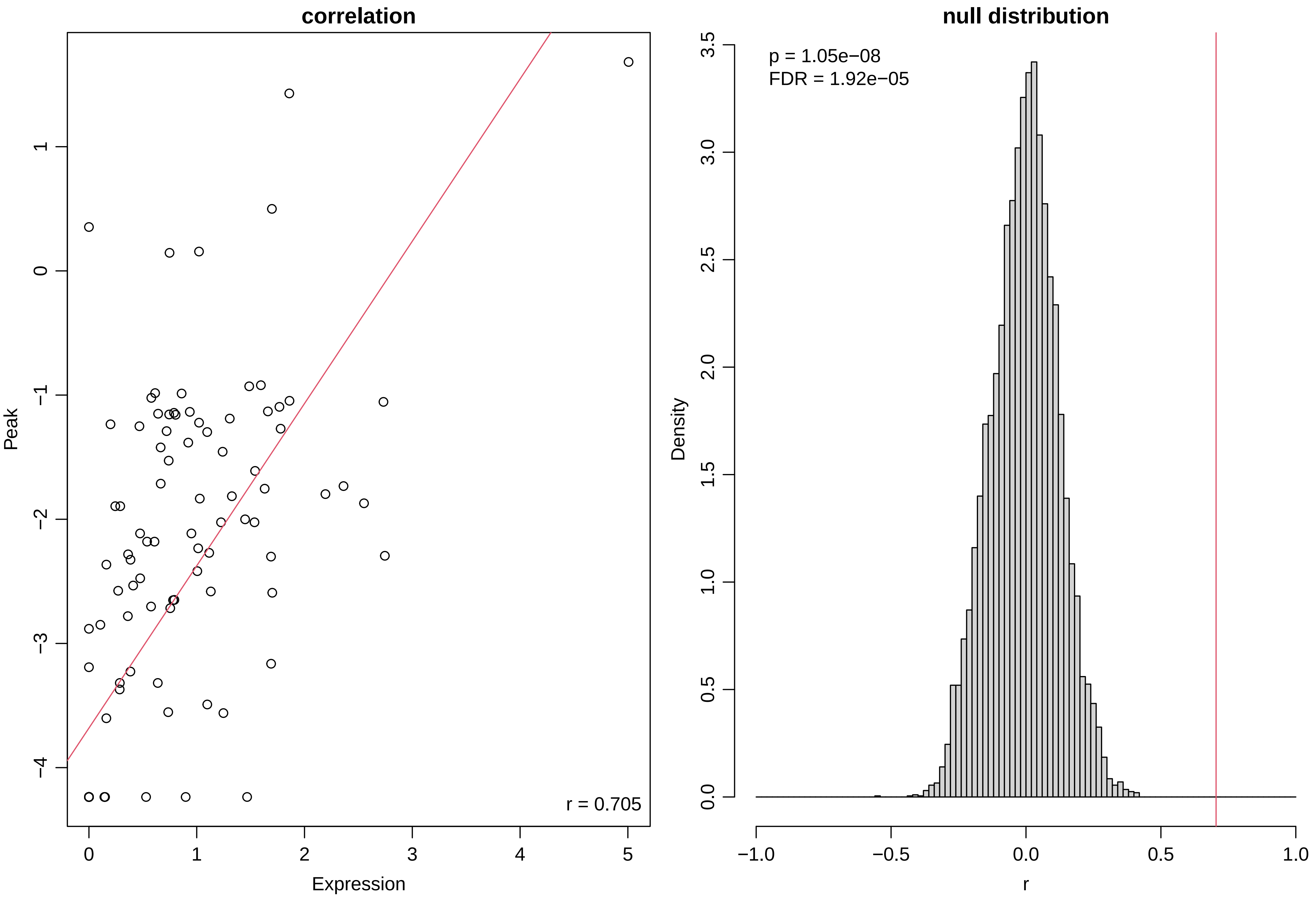

FOXCUT vs PeakID_201370 (chr6: 1607678 − 1612639)

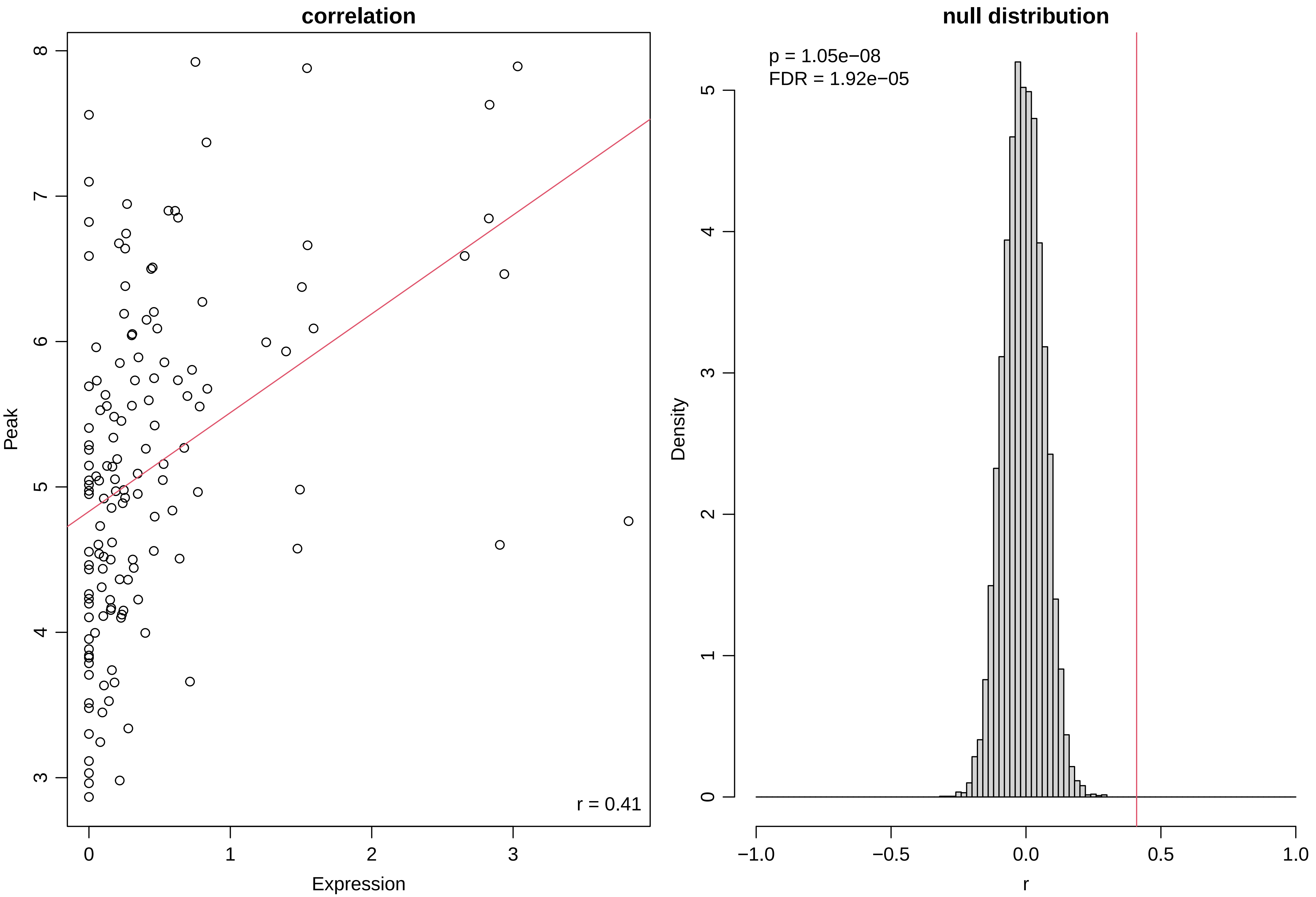

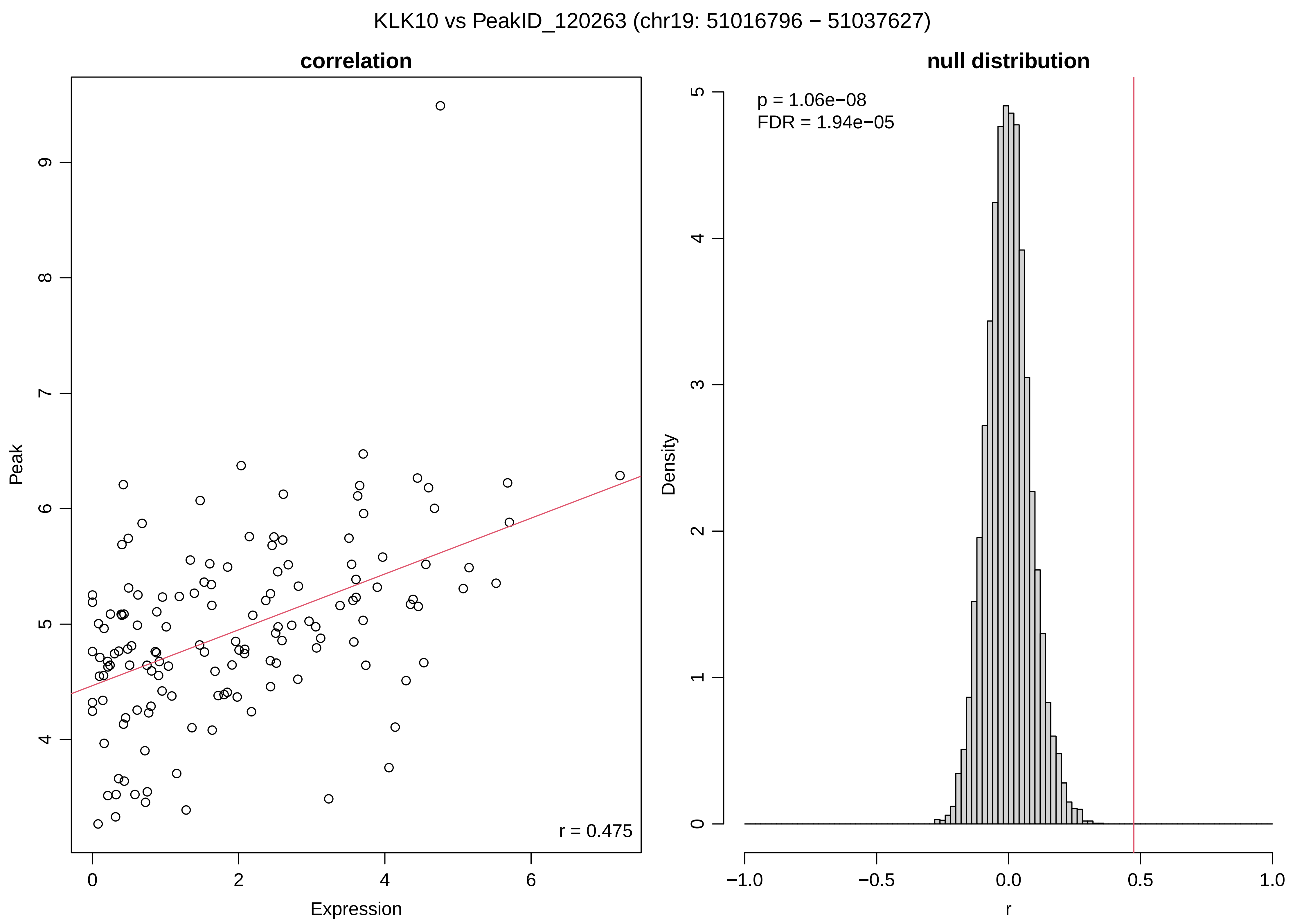
KLK10 vs PeakID_120263 (chr19: 51016796 − 51037627)
correlation
Peak
Expression
r = 0.475
null distribution
p = 1.06e−08
FDR = 1.94e−05
Density
r

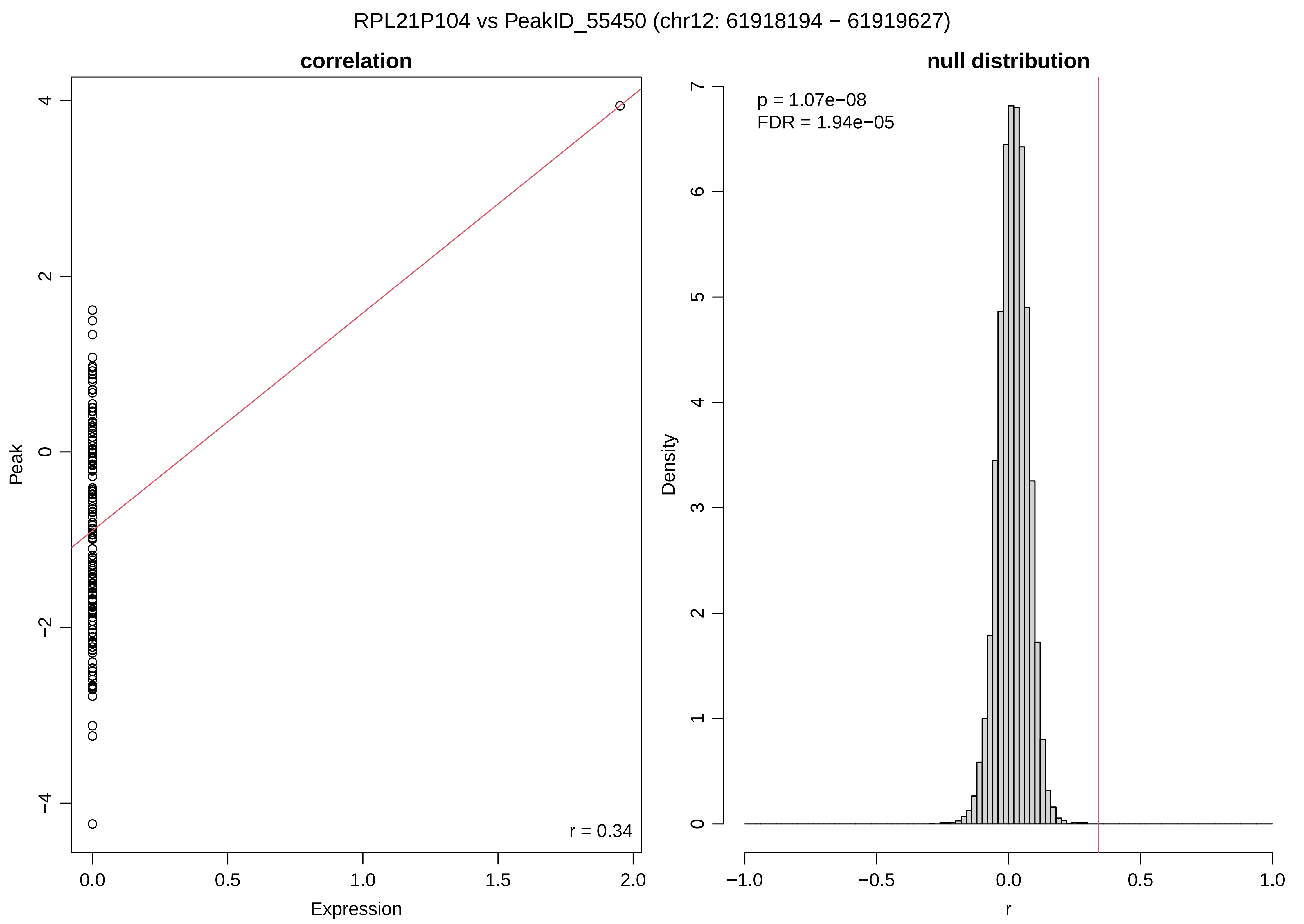
RPL21P104 vs PeakID_55450 (chr12: 61918194 − 61919627)
correlation
Peak
Expression
r = 0.34
null distribution
Density
r
p = 1.07e−08
FDR = 1.94e−05

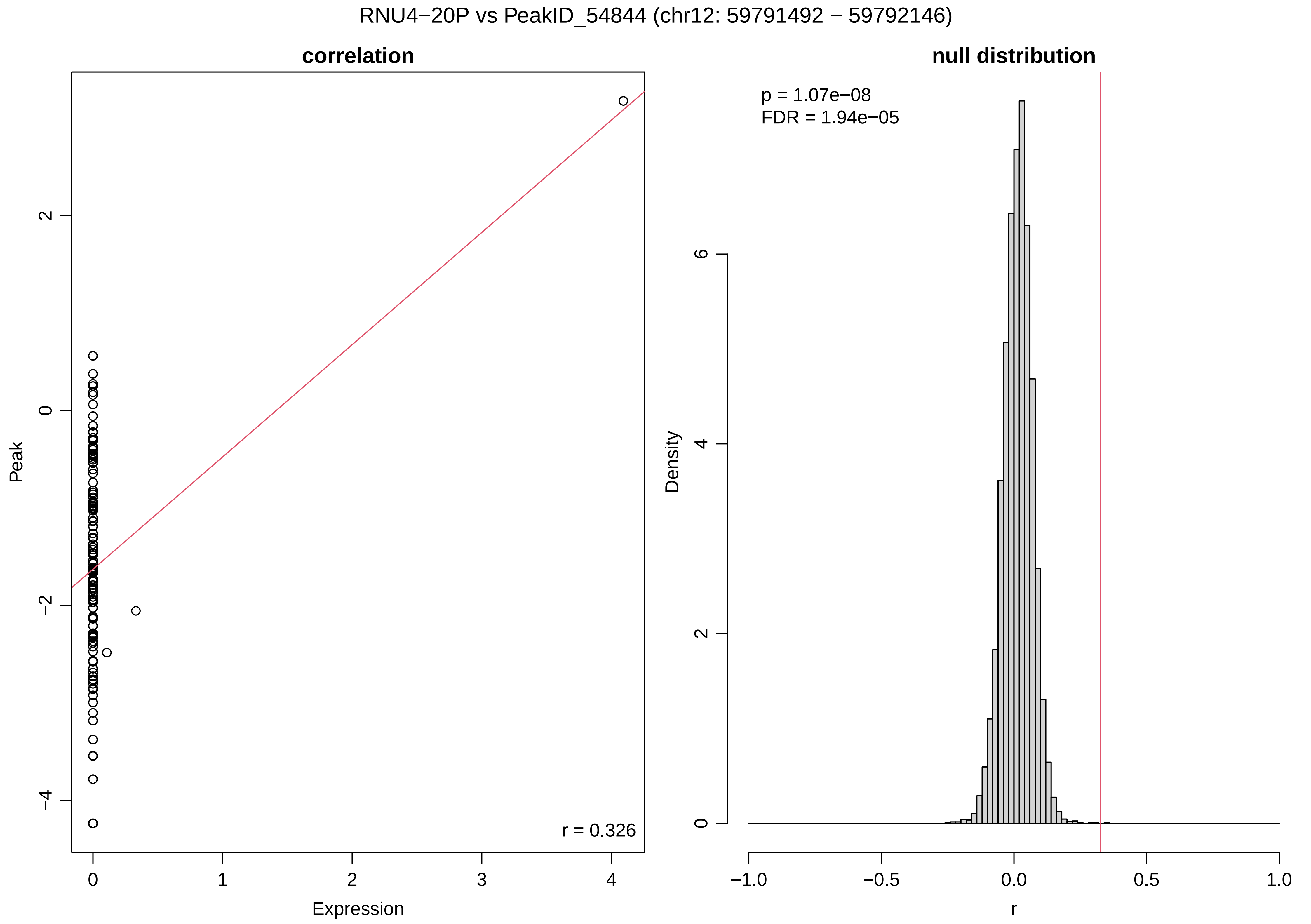

RNU4−20P vs PeakID_54844 (chr12: 59791492 − 59792146)
correlation
null distribution
p = 1.07e−08
FDR = 1.94e−05
r = 0.326
Peak
Expression
Density
r
2
0
−2
−4
0
1
2
3
4
6
4
2
0
−1.0
−0.5
0.0
0.5
1.0

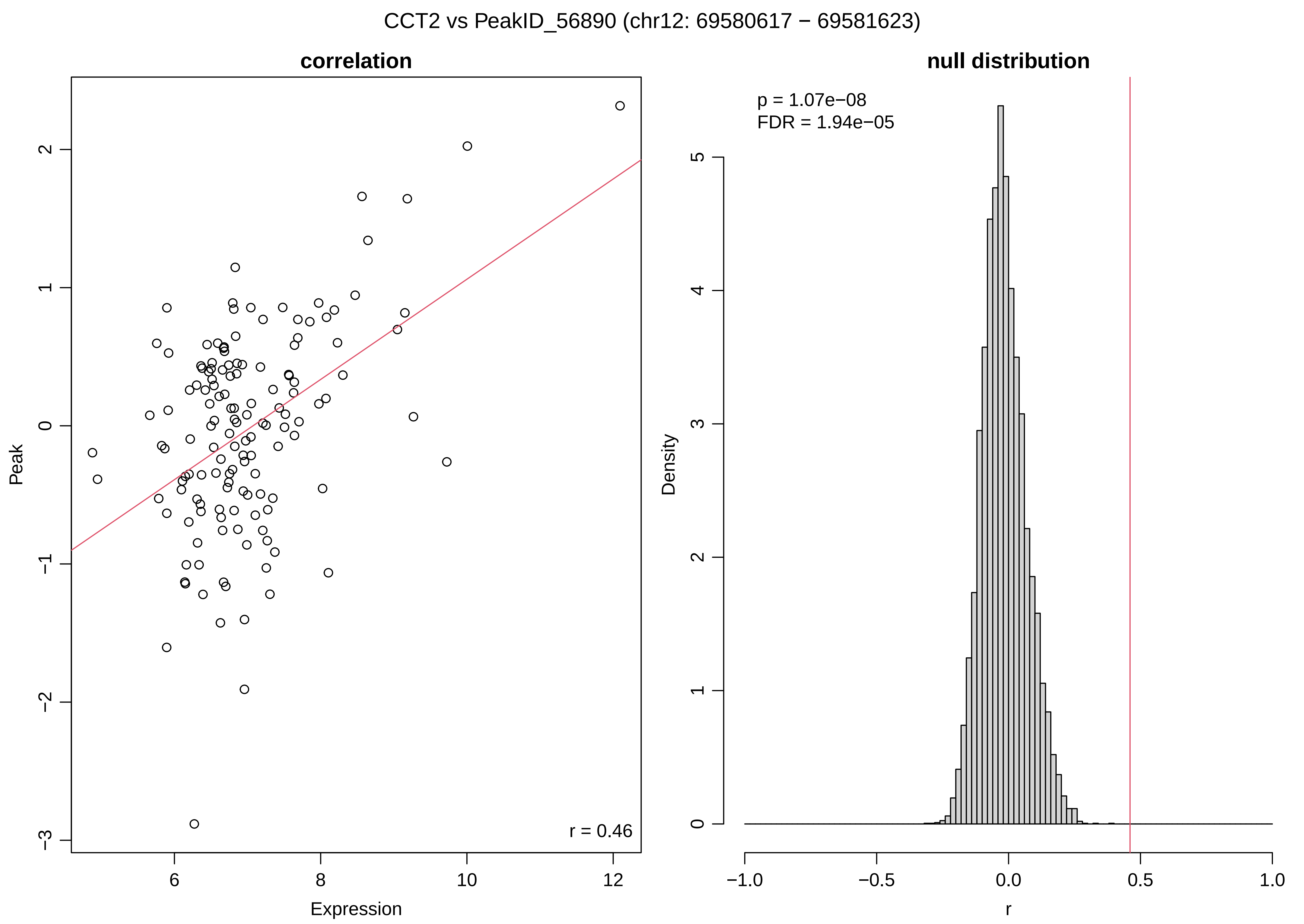
CCT2 vs PeakID_56890 (chr12: 69580617 − 69581623)
correlation
Peak
Expression
r = 0.46
null distribution
p = 1.07e−08
FDR = 1.94e−05
Density
r
